# Antiviral Mx proteins have an ancient origin and widespread distribution among eukaryotes

**DOI:** 10.1073/pnas.2416811122

**Published:** 2025-01-24

**Authors:** Caroline A. Langley, Peter A. Dietzen, Michael Emerman, Jeannette L. Tenthorey, Harmit S. Malik

**Affiliations:** ^a^Molecular and Cellular Biology Graduate Program, University of Washington, Seattle, WA 98195; ^b^Division of Human Biology, Fred Hutchinson Cancer Center, Seattle, WA 98109; ^c^Division of Basic Science, Fred Hutchinson Cancer Center, Seattle, WA 98109; ^d^Cellular Molecular Pharmacology Department, University of California San Francisco, San Francisco, CA 94143; ^e^HHMI, Fred Hutchinson Cancer Center, Seattle, WA 98109

**Keywords:** antiviral, phylogenomics, early eukaryotes, dynamin, gene turnover

## Abstract

Mx proteins, first identified in mammals, encode potent antiviral activity against a wide range of viruses. Mx proteins arose within the Dynamin superfamily of proteins (DSP), which mediate critical cellular processes, such as endocytosis and mitochondrial, plastid, and peroxisomal dynamics. Despite their crucial role, the evolutionary origins of Mx proteins are poorly understood. Through comprehensive phylogenomic analyses with progressively expanded taxonomic sampling, we demonstrate that Mx proteins predate the interferon signaling system in vertebrates. Our analyses find an ancient monophyletic DSP lineage in eukaryotes that groups vertebrate and invertebrate Mx proteins with fungal MxF proteins, the largely uncharacterized plant and algal Dynamin 4A/4C proteins, and representatives from several other eukaryotic lineages, suggesting that Mx-like proteins date back close to the origin of Eukarya. Our phylogenetic analyses also find host-encoded and nucleocytoplasmic large DNA viruses-encoded DSPs interspersed in four distinct DSP lineages, indicating recurrent viral theft of host DSPs. Our analyses thus reveal an ancient history of viral and antiviral functions encoded by the Dynamin superfamily in eukaryotes.

## Significance

Broadly antiviral Mx proteins were among the first innate antiviral genes to be discovered in mammals. They were found to be related to Dynamin proteins, which mediate critical cellular processes, such as endocytosis and organelle dynamics. Mx gene expression is triggered by interferon signaling. These findings led to the prevailing view that Mx proteins arose in bony vertebrates, coincident with interferon signaling. Using detailed phylogenomic analyses, we find that Mx proteins significantly predate interferon signaling. An ancient Mx clade includes representatives from animals, fungi, plants, and multiple other eukaryotic lineages. We also find evidence of recurrent gene transfers between eukaryote genomes and giant DNA viruses. Our findings suggest that Dynamin-related functions might be an ancient axis of host–virus arms races.

The vertebrate interferon (IFN) system acts as a first line of defense against viruses and other pathogens by inducing dozens of IFN-stimulated genes (ISGs) that create an antiviral environment. Functional IFN systems exist in many vertebrates, including bony fishes (1–3). Among the most rapidly and highly expressed ISGs upon IFN induction in human cells are Mx proteins, identified as antiviral proteins soon after the discovery of IFN (4–6). Although Mx proteins from different species have different antiviral specificities, they have exceptionally broad antiviral activity. For example, the human MxA protein restricts diverse viruses, including influenza A (7–9), vesicular stomatitis virus (8, 9), measles (10), and hepatitis B (11), whereas the human MxB paralog restricts retroviruses (12–14) and herpesviruses (15, 16). Most mammals have two *Mx* genes (17), although they were lost or pseudogenized in toothed whales (18). Birds have a single *Mx* gene, and fish encode up to seven *Mx* paralogs, which evolved by gene or genome duplication (1–3). The well-documented presence of Mx proteins in fish and mammalian lineages suggested that these proteins may have originated alongside the IFN system in the common ancestor of bony fish and mammals. However, recent findings have revealed that ISGs such as STING and cGAS predate vertebrates (19–22). In addition, two reports of Mx-like genes from invertebrate species (23, 24) suggested an earlier origin.

Mx proteins are a member of the Dynamin superfamily of proteins (DSP), multidomain GTPases that mediate many critical cellular processes within eukaryotic cells. Most DSPs localize to distinct cellular membranes, where they facilitate membrane remodeling. For example, Dynamin (or Dyn) proteins localize to the outer cellular membrane (25–33) and endosomes (34), whereas the Optic atrophy 1 (or Opa1) and Mitofusin (or Mfn) proteins act at mitochondrial membranes (35–41) alongside Dynamin-related proteins (or Drps) (42–46). In contrast to other studied DSPs, Mx proteins can function independently of membranes (6, 47). Although Mx antiviral mechanisms are still poorly understood, one model proposes that human MxA acts by binding viral ribonucleoproteins (RNPs) and exerting a GTP hydrolysis-dependent power stroke to restrict virus replication (48), analogous to the power stroke exerted by Dynamin proteins on cellular membranes in the final step of endocytosis (49, 50).

Previous studies on the evolution and diversification of DSPs (51–53) included few or no Mx protein sequences, leaving the evolutionary origins of Mx proteins unclear. Conversely, studies on Mx evolution focused exclusively on vertebrate or mammalian Mx sequences (17, 54). Here, we analyzed the deep phylogenetic history of Mx in the context of DSPs using stepwise increments of eukaryotic phylogenetic coverage. We find unambiguous evidence that Mx-like proteins predate the birth of IFN in animals and are present within plants, fungi, and most eukaryotic lineages. Expanding our analyses to all eukaryotic DSPs, we reveal an ancient and ongoing history of lateral transfer between host genomes and nucleocytoplasmic large DNA viruses (NCLDVs) in four DSP lineages. Our study suggests an understudied potential arms race for dynamin-related functions between host and viral genomes.

## Results

### Mx Predates the Birth of IFN.

**Fig. 1.**
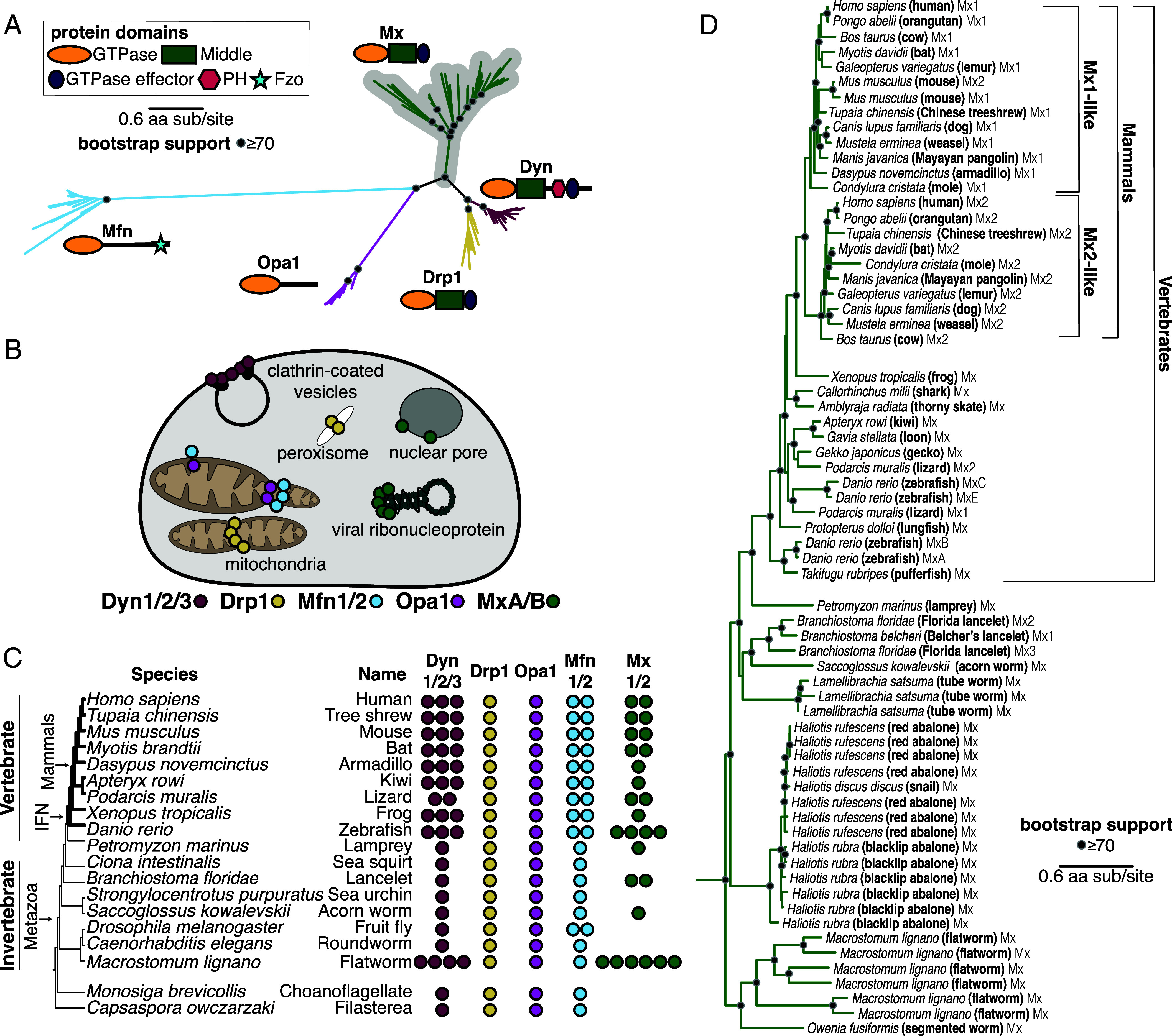
The evolutionary origin of Mx in animals predates the IFN signaling network. (*A*) Phylogenetic analyses of Dynamin-superfamily proteins (DSPs) based on their common GTPase domain in representative animals and outgroup species reveal five distinct DSP clades—Dyn, Drp1, Mfn, Opa1, and Mx (gray highlight). (*B*) Summary of localization of different DSPs in human cells. Dyn proteins localize to clathrin-coated pits on the plasma membrane, Drp proteins localize to mitochondria and peroxisomes, whereas Mfn and Opa1 proteins localize to mitochondrial membranes. In contrast, Mx proteins act independent of host membranes and localize to viral RNP complexes in the cytoplasm (MxA) or proximal to the nuclear pore (MxB) or in the nucleoplasm in other mammals (not shown). (*C*) Retention of different DSP clades in representative animals or their outgroup species (tree not drawn to scale). Drp1 and Opa1 are represented in a single copy in all representative animals and outgroup species. Mfn is also present in a single copy except in two cases. Mfn duplicated in bony vertebrates, giving rise to Mfn1 and Mfn2, coincident with the birth of the IFN system (IFN, bold lines). Mfn independently duplicated in the lineage leading to *Drosophila melanogaster*. Mx proteins are present in 1 to 4 copies in bony vertebrates and 1 to 6 copies in some invertebrate species but have also been independently lost in several lineages. (*D*) Phylogenetic analysis of Mx-like proteins reveals a phylogenetic split between Mx1-like and Mx2-like genes in mammals and independent duplications in several fish lineages. We also find unambiguous evidence of Mx-like genes in several invertebrate species, whose branching pattern suggests that Mx genes have been vertically inherited in animals, followed by frequent subsequent loss and duplication events. (*A*), (*D*) Black dots indicate nodes with bootstrap support greater than 70% based on FastTree analyses (*Materials and Methods*); a scale bar indicates the level of amino acid divergence.

To evaluate the evolutionary origins of Mx in the context of the broader Dynamin superfamily, we carried out BLAST searches on representative *Metazoa* (animal) species with fully sequenced genomes using different human DSPs as queries. Although many DSP genes undergo alternate splicing, we focused only on the longest isoform encoded by each DSP gene. We aligned all metazoan DSPs recovered with different query sequences using the MAFFT program (55). Additionally, we added hits from *Choanoflagellates* and *Filasterea* as outgroups to *Metazoa* to define DSP clades. Because only the GTPase domain is conserved across all DSPs, we manually extracted the GTPase domain from alignments of different DSPs. We used these sequences to generate a DSP GTPase alignment, which was further trimmed manually. We subsequently used these sequence alignments to create phylogenetic trees using FastTree (56, 57) (Fig. 1*A*) (*Materials and Methods*). The resulting phylogeny reveals five distinct clades of DSPs with high bootstrap support, indicating high confidence in their phylogenetic relatedness (Fig. 1*A*). These five clades are consistent with the five DSP groups previously established in animal cells: Dyn, Drp1, Opa1, Mfn, and Mx proteins. We obtained a nearly identical tree topology using the IQ-Tree (58, 59) method (*SI Appendix*, Fig. S1*A*), except for minor differences in branching topology within clades. The phylogenetic congruence of these trees supports the strength of our conclusions.

Next, we analyzed the domain organization of the different homologs assigned to each of the five *Metazoa* DSP clades using the NCBI conserved protein domain database (CDD) (60, 61). This analysis confirmed that all members of each DSP clade shared the characteristic domain differences previously used to distinguish DSPs (Fig. 1*A*). For instance, a hallmark of canonical Dyn proteins is the Pleckstrin homology domain (PH) domain, which facilitates the recruitment of these proteins to the outer cellular membrane (62). Our analyses show that all Dyn proteins encode a PH domain (Fig. 1*A*), which appears only in this clade. Similarly, Mfn proteins encode the Fzo (fuzzy onion) domain, which is restricted to this clade (Fig. 1*A*). In contrast, the GTPase effector domain (GED) and Middle domains (that separate the GTPase from GED domains) are found in Dyn, Drp, and Mx proteins but not in Opa1 and Mfn proteins. Thus, the GTPase domain is the only universal domain common to all five DSP clades.

Proteins from these five DSP clades localize to distinct cellular compartments in human cells (Fig. 1*B*). Dyn proteins, which localize to the outer cellular membrane and other internal cellular membranes (25–34), phylogenetically group with Drp1 proteins, which localize to mitochondria and peroxisomes and are critical for organelle fission/fusion (42–46). Mx antiviral proteins, which have been shown to localize to the cytoplasm, the nucleoplasm, or the nuclear pore, form an outgroup lineage to the Dyn and Drp sister clades (6, 47). Opa1 proteins localize to the inner mitochondrial membrane (35–37) and are an outgroup to the Mx, Drp, and Dyn clades. The most distally branching clade is the Mfn clade, which encodes proteins that localize to the outer mitochondrial membrane (38–41).

Based on their phylogenetic groupings and protein-domain analysis (Fig. 1*A*), we assigned all DSPs from representative *Metazoan* species to each of the five distinct DSP clades (Fig. 1*C*) to analyze gene loss or duplication. We often found several DSP paralogs from the same clade present within the same species. For example, the human genome encodes three Dyn paralogs (Dyn1, Dyn2, and Dyn3), two Mfn paralogs (Mfn1 and Mfn2), and two Mx paralogs (MxA and MxB) but only one Drp1 and one Opa1 (Fig. 1*C*). A Mfn duplication in bony vertebrates gave rise to Mfn2, which modulates antiviral immunity (63, 64), while an independent Mfn duplication gave rise to the Marf and Fzo proteins in the *Drosophila* species. In addition to Dyn duplications observed in bony vertebrates, independent Dyn duplications have also occurred in *Macrostomum lignano* (flatworm). In contrast, Drp1 and Opa1 are encoded by single-copy genes in all Metazoans and outgroups.

These analyses also confirm the presence of Mx proteins in the vertebrate lineage and its absence in well-studied invertebrate models like *Caenorhabditis elegans* and *D. melanogaster*. However, our findings provide unambiguous evidence, supported by bootstrap analysis and domain characterization, of Mx orthologs in several invertebrate species, including *Branchiostoma floridae* (lancelet), *Saccoglossus kowalevskii* (acorn worm), and *M. lignano* (flatworm), which encodes at least six distinct Mx proteins. These findings suggest that Mx proteins arose in animals well before the emergence of the IFN gene network in bony vertebrates. This ancient origin was followed by recurrent loss of Mx proteins from multiple invertebrate lineages and at least one lineage of mammals (18). This pattern of recurrent gene turnover-through loss and duplication is typical of evolutionary arms races between hosts and viruses, where viral adaptations can render particular host antiviral genes obsolete or, conversely, drive gene expansions in some host lineages to expand antiviral functions (65).

To rule out the alternative possibility that the invertebrate Mx homologs might have resulted from horizontal gene transfers (HGT) following their origins in vertebrates, we expanded our BLAST analyses to identify additional Mx proteins in animal genomes, using invertebrate Mx proteins as queries. We performed phylogenetic analyses using an alignment of the GTPase domain for all DSPs that unambiguously group within the Mx clade (Fig. 1*D*). Our analyses recapitulate and extend findings from previous studies of Mx proteins in mammals (66, 67). We found two lineages of Mx proteins (Mx1 and Mx2) in mammals as well as Mx representatives from bird, amphibian, shark, and fish lineages. In addition to previously identified invertebrate lineages, such as the lancelet, acorn worm, and flatworm, we identified Mx homologs in *Lamellibrachia satsuma* (tube worm), multiple *Haliotis* species (snail, abalone), and *Owenia fusiformis* (segmented worm) (Fig. 1 *B*–*D* and *SI Appendix*, Fig. S1*B*). Most importantly, the topology of the Mx tree (Fig. 1*D*) largely mirrors the species tree (Fig. 1*C*), consistent with the early origin of Mx proteins in the animal phylogeny. Together, these analyses show that animal Mx proteins are more ancient than the IFN system in vertebrates, have largely been subject to vertical inheritance, and have undergone several lineage-specific gene duplications and losses.

### Phylogeny of Animal, Fungal, and Plant DSPs Reveals Ancient Mx Orthologs.

**Fig. 2.**
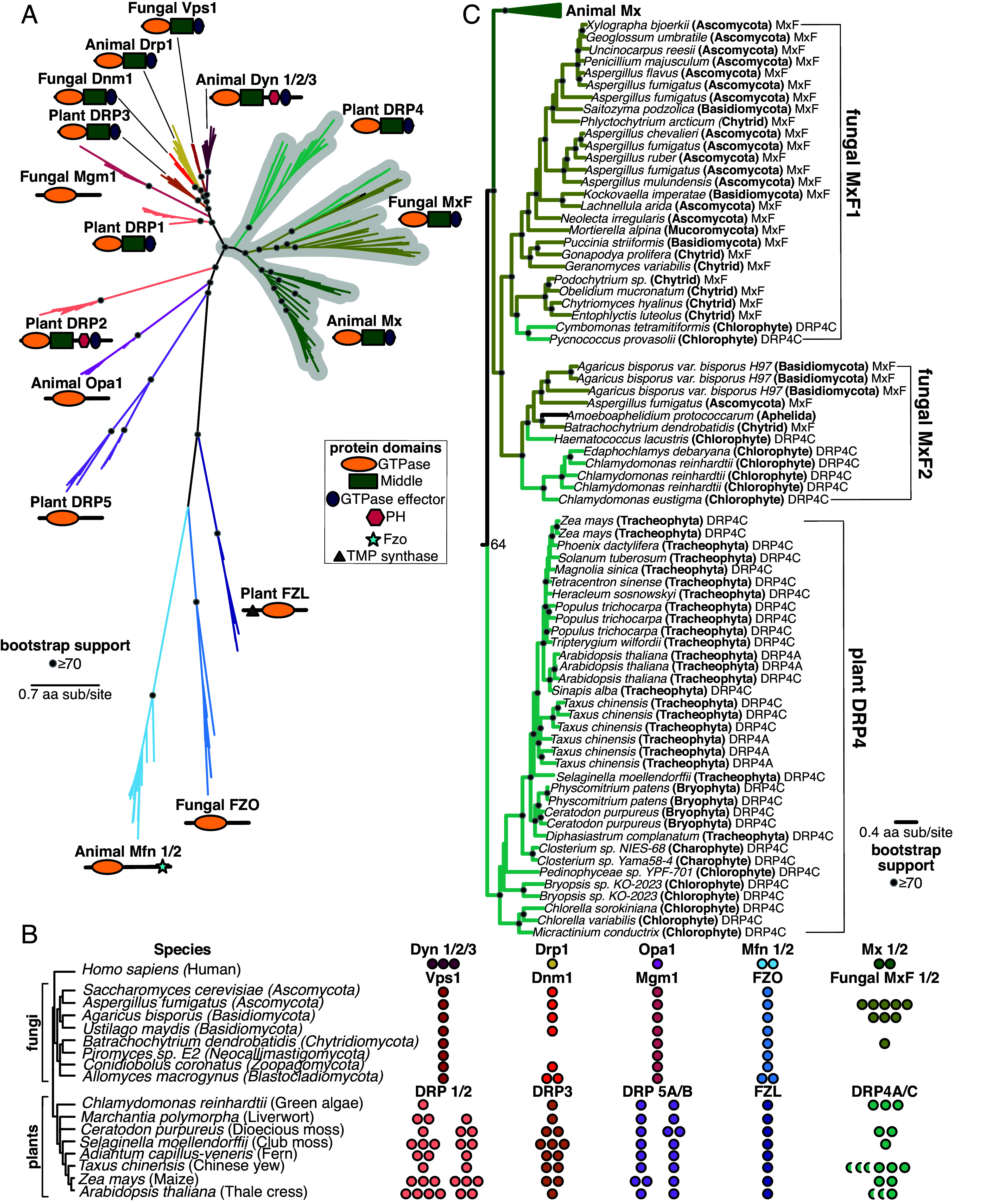
Mx and other DSP orthologs in fungal and plant genomes. (*A*) Phylogenetic analysis of animal, fungal, and plant DSPs based on their common GTPase domain reveals broad groupings. Animal Mx proteins appear orthologous to uncharacterized fungal MxF proteins and plant DRP4 proteins (gray highlight). Animal Dyn proteins group with Fungal Vps1 proteins but not with plant DRP1 or DRP2 proteins, even though only Animal Dyn and plant DRP2 proteins share a C-terminal PH domain. Animal Drp proteins group with Fungal Dnm1 and Plant DRP3 proteins. Fungal Mgm1 proteins are considered the functional equivalent of Animal Opa1 and Plant DRP5A/5B proteins but do not form a robust monophyletic group. Finally, the animal Mfn lineage groups with Fungal FZO and Plant FZL proteins; these proteins are significantly diverged from the rest of the DSPs. (*B*) Representation of various DSP classes in representative fungal and plant (and green algal) genomes. Among fungi, Vps1, Mgm1, and FZO are encoded by single-copy genes (except for an *FZO* duplication in *A. macrogynus*). *Mx*-like genes vary from zero to five copies (in *A. fumigatus*). Among plants, FZL is encoded by a single copy gene in all representative algae and plants. DRP5A and DRP5B are mostly also encoded by single-copy genes in plants (except for a *DRP5A* duplication in maize and a *DRP5B* duplication in a moss species). DRP1 varies from one to four copies in plants, whereas DRP2 is present in one to three copies (and absent in green algae). DRP3 varies from one to three copies in all algae and plants. Finally, DRP4C is present from zero to three copies. In addition to DRP4C (full circles), many plants also encode shorter DRP4A proteins (semicircles). (*C*) MxF proteins are found in a variety of fungal lineages, including *Ascomycota*, *Basidiomycota*, *Chytrids*, and *Mucoromycota*, as well as *Aphelia*, which are a pseudo-fungi-like sister lineage to *Fungi*. Fungal MxF forms two distinct clades, MxF1 and MxF2. Plant DRP4C proteins from various lineages of plants—*Chlorophytes, Charophytes, Bryophytes, and Tracheophytes* are primarily found in a single lineage, interspersed with shorter DRP4A proteins. However, some *Chlorophyte* DRP4C proteins group with fungal MxF proteins rather than other DRP4C, suggesting at least two MxF fungal-to-algal horizontal transfer events. (*A*), (*C*) Black dots indicate nodes with bootstrap support greater than 70% based on FastTree analyses (*Materials and Methods*); a scale bar indicates the level of amino acid divergence.

Given our finding that the Mx clade originated early in Animal evolution, we extended our analysis of potential Mx origins to two additional lineages where DSPs have also been extensively studied: fungi (a sister lineage to animals) and plants. Using the same approach of iterative BLAST searches of representative animal, fungal, and plant genomes using different DSP queries, we carried out phylogenetic analyses of all DSPs recovered from these genomes based on their common GTPase domain with FastTree (Fig. 2*A*) and IQ-Tree (*SI Appendix*, Fig. S2*A*). We also included outgroup species to fungi or plants to find clear delineations in DSP clades, and we analyzed their domain architecture using the CDD (Fig. 2*A*). These analyses revealed fungal and plant orthologs of animal Mx proteins. For example, animal Mx proteins unambiguously group with uncharacterized DSPs in some fungi, which we rename MxF (for Mx-like proteins from Fungi). *Aspergillus fumigatus* encodes five MxF proteins, *Agaricus bisporus* encodes three, and *Batrachochytrium dendrobatidis* encodes one (Fig. 2*B*). However, many different fungal lineages encode no MxF proteins at all *(e.g., Saccharomyces cerevisiae, Ustilago maydis, Piromyces sp. E2, Conidiobolus coronatus, and Allomyces macrogynus*) (Fig. 2*B*). This extreme dynamism in copy number is highly reminiscent of the gene loss and expansion seen in Mx genes in animal genomes. Phylogenetic analysis reveals the presence of two distinct clades of MxF, which we designate as MxF1 and MxF2 (Fig. 2 *C* and *D*). MxF1 and MxF2 are present broadly in fungal species, suggesting they likely represent ancient paralogs in fungi. The complete absence of MxF orthologs in some model fungal species, such as *S. cerevisiae,* may have obscured the discovery and led to the misannotation of MxF orthologs in other fungal species. Despite their heterogeneous presence, MxF proteins are found in most major clades of fungi (Fig. 2*C*), including *Ascomycota, Basidiomycota, Chytrid,* and *Mucoromycota,* as well as *Aphelida*—believed to be the sister lineage to true fungi (68, 69).

Consistent with a previous proposal (70), we find that plant DRP4 proteins are orthologous to animal Mx and fungal MxF proteins (Fig. 2 *A* and *C* and *SI Appendix*, Fig. S2 *A* and *B*). Plants encode full-length DRP4C proteins, which resemble animal Mx proteins in length and domain architecture, and much shorter DRP4A proteins, which often comprise only a GTPase domain with a truncated stalk domain or, in some cases, not even a full-length GTPase domain. We considered whether these two proteins represent an ancient specialization. If that were the case, phylogenetic analyses would reveal an ancient separation between the DRP4A and DRP4C clades. Contrary to this expectation, we found that DRP4A and DRP4C proteins encoded by the same species are often phylogenetically more closely related to each other than to homologs from different plant species (*SI Appendix*, Fig. S3). This suggests that *DRP4A* genes have independently arisen multiple times in plant evolution through partial gene duplication of full-length *DRP4C* genes. Given that no plant genome encodes *DRP4A* without also encoding *DRP4C*, we speculate that the shorter *DRP4A* genes may have recurrently evolved to regulate the activity of full-length DRP4C proteins. Alternatively, the DRP4A and DRP4C clades could result from ancient divergence, which has been obscured by recurrent gene conversion, a phenomenon documented in other multigene families (71–73).

Detailed phylogenetic analyses (Fig. 2 and *SI Appendix*, Fig. S2*B*) show that DRP4 proteins are widespread across green algal and plant lineages, being present in *Chlorophytes, Charophytes* (another green algal lineage)*, Bryophytes* (nonvascular plants)*, and Tracheophytes* (vascular plants, including ferns, gymnosperms, and angiosperms). Our analyses also reveal two instances of potential HGT from *Fungi* to *Chlorophytes*, where Chlorophyte proteins nestle within fungal clades (Fig. 2*C* and *SI Appendix*, Fig. S2*B*). The first instance occurred from a Chytrid MxF1 into the ancestor of two Chlorophytes, *Pycnococcus provasolii* and *Cymbomonas tetramitiformis*, whereas the second event occurred from an MxF2 into *Haematococcus lacustris, Edaphochlamys debaryana,* and *Chlamydomonas reinhardtii.* These are indicated as DRP4C proteins in our phylogeny (Fig. 2*C* and *SI Appendix*, Fig. S2*B*) but are more likely MxF proteins. Like animal Mx and fungal MxF proteins, we identify highly dynamic gene turnover within Plant DRP4 proteins (Fig. 2*B*), consistent with the possibility of their engagement in evolutionary arms races with viruses. We conclude that the Mx lineage is much more ancient than previously believed and includes animal, fungal, and plant representatives.

### Phylogenetic Relationships between Other Animal, Fungal, and Plant DSPs.

Our analyses also provide unique insights and clarify the phylogenetic relationships among other DSPs. For example, the grouping of animal Drp proteins, fungal Dnm1 (dynamin-related GTPase), and plant DRP3 (Fig. 2*A*) is consistent with their localization and function in mitochondria and peroxisomes (70, 74–78). Dnm1 is encoded by a single copy gene in representative fungi, except for a duplication in *A. macrogynus* and a loss in both *Batrachochytrium dendrobatidis* and *Piromyces sp. E2,* whereas *DRP3* genes are present in 1-3 copies in all representative plant species (Fig. 2*B*). The apparent loss of Dnm1 in some fungal species is unexpected, given its essential roles in many organisms, highlighting the possibility of functional redundancy among different DSP clades.

The “Dyn” grouping is more puzzling. Fungal Vps1 (vacuolar protein sorting) proteins, encoded by a single copy gene in most fungi, are closest in sequence (Fig. 2*A*) and considered the functional equivalent of animal Dyns (79, 80). Vps1 proteins are implicated in vacuolar fusion (81–83), membrane scission (82–85), and peroxisomal partitioning (76, 86–88). Most plants encode 1 to 4 copies of two Dyn-like proteins, DRP1 and DRP2, which play a role in clathrin-mediated endocytosis (70, 89–94) and at the cell plate during cytokinesis (95, 96). And yet, neither fungal Vps1 nor plant DRP1 proteins encode a PH domain, a defining characteristic of animal Dyn proteins. In contrast, plant DRP2 proteins (Fig. 2*A*) encode a PH domain despite being highly divergent from animal Dyn proteins. To address this apparent contradiction, we made a separate phylogenetic tree of all Dyn, Drp, and Mx proteins based either on their shared GTPase domains (as before, Fig. 2*A*) or on their shared Middle and GED domains (*SI Appendix*, Fig. S4*A*). Based on the Middle+GED domain phylogeny, we find that plant DRP1 and DRP2 proteins are sister lineages (*SI Appendix*, Fig. S4*B*), even though the DRP2 appears to be much more divergent than DRP1 in the GTPase phylogeny (Fig. 2*A*). We propose that an ancestral plant DRP1/2 protein originally encoded a PH domain. A subsequent duplication gave rise to DRP1, which subsequently lost the PH domain, and DRP2, which likely acquired a divergent GTPase domain through recombination (*SI Appendix*, Fig. S4*B*). An alternative possibility is that the ancestral plant DRP1 GTPase domains evolved more rapidly than other DSPs, leading to their (incorrect) divergent placement in the GTPase phylogeny. While the green algae *C. reinhardtii* encodes only DRP1, most other plants encode 1 to 4 copies of DRP1 and DRP2 (Fig. 2*B*).

Fungal Mgm1 has a similar domain architecture to Animal Opa1, maintains mitochondrial ultrastructure and morphology, and regulates mitochondrial fusion (97–99). However, the phylogenetic grouping of fungal Mgm1 and animal Opa1 proteins is weak (Fig. 2*A*). Like Opa1 in animals, Mgm1 is encoded by a single copy gene in most Fungi, while most plant genomes encode 1 to 2 copies of both *DRP5A* and *DRP5B* (Fig. 2*B*). Based on their similar structure, plant DRP5 proteins should be excellent candidates for functional equivalents of animal Opa1. However, unlike animal Opa1 proteins, which exclusively function in mitochondria, plant DRP5 proteins function instead in cytokinesis (78), chloroplast and peroxisome division (100, 101), and mitochondrial morphogenesis/division (102). Thus, the “Opa1” grouping of animal Opa1, fungal Mgm1, and plant DRP5 proteins shows neither strong evidence of monophyly (Fig. 2*A*) nor functional similarity.

The animal Mfn, fungal FZO, and plant FZL proteins are encoded mainly by single-copy genes (Fig. 2*B*). They group together to the exclusion of the rest of the DSPs (Fig. 2*A* and *SI Appendix*, Fig. S2*A*). Animal Mfn and fungal Fzo proteins mediate the interaction between mitochondrial outer membranes to drive mitochondrial fusion (103, 104), whereas plant FZL proteins localize to chloroplasts and function in thylakoid organization (105). Thus, it is unclear whether this grouping reflects true orthology or is simply a result of their high divergence from the rest of the DSPs.

### Deep Evolutionary Origins of the Mx-Like DSPs in Eukaryotes.

**Fig. 3.**
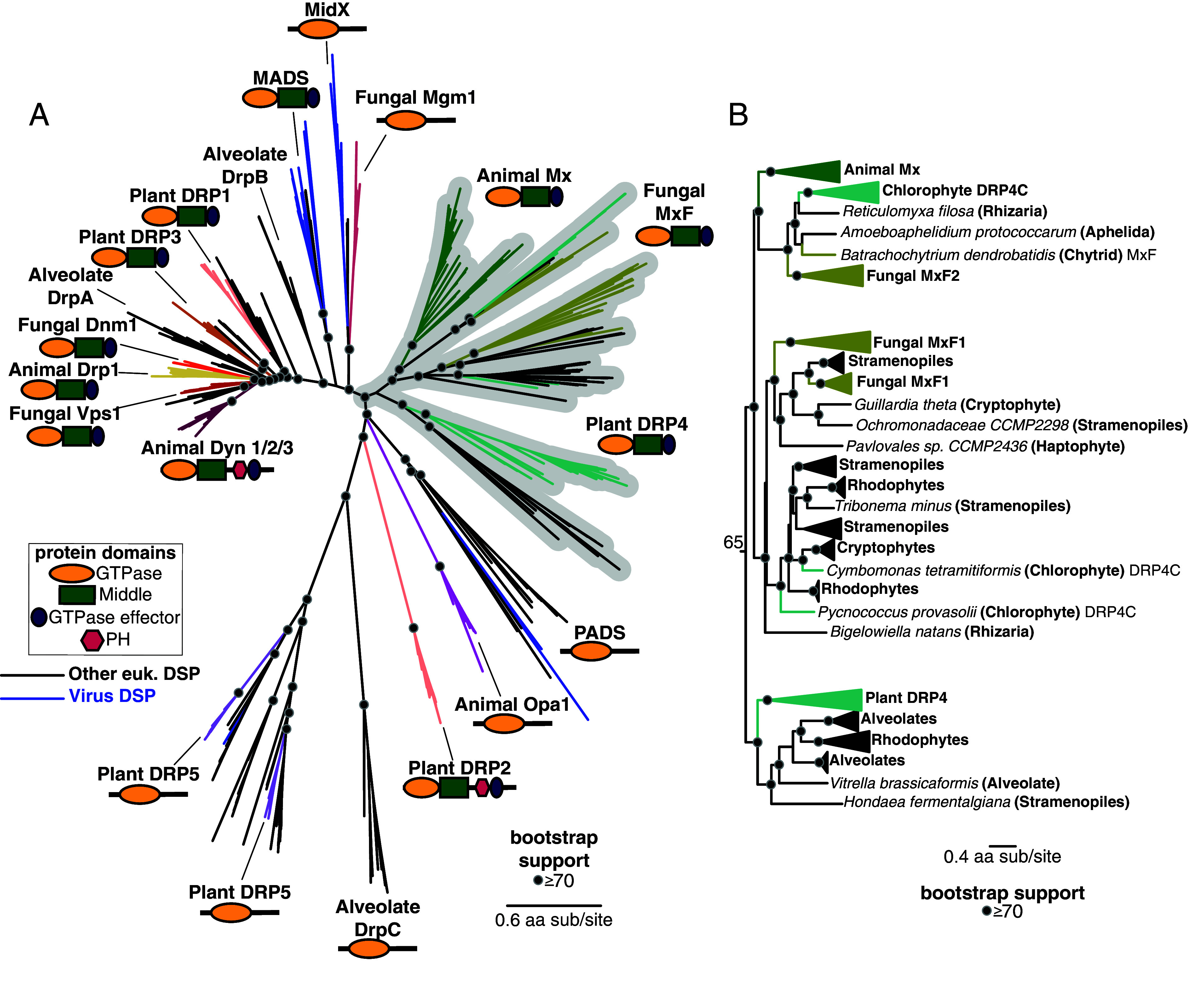
Mx proteins are ancient and widespread among eukaryotes. (*A*) Phylogenetic analysis of the GTPase domain from eukaryotic DSPs reveals an ancient Dyn/Drp clade that includes animal Drp, plant DRP3, and fungal Vsp1 along with fungal Dnm1, alveolate DrpA and DrpB, plant DRP1, and DSPs from several other eukaryotic lineages (eukaryotic DSPs from “other eukaryotes” excluding animals, fungi, and plants are indicated with black branches). Branching outside the Dyn/Drp cades are the MADS and MidX/ fungal Mgm1 clades (blue branches indicate DSPs encoded in viral genomes). Next is a single monophyletic clade of Mx-like DSPs and the newly discovered PADS lineage. Finally, we find a grouping of animal OPA1, plant DRP5 (which also contains DSPs from *Stramenopiles, Amoebozoa*, and a single virus), and alveolate DrpC. Although plant DRP2 also groups with this final grouping, we believe its correct phylogenetic position (based on the Middle and GED domains) is as a sister to plant DRP1 (*SI Appendix*, Fig. S4). (*B*) Our phylogenetic analysis delineates three lineages of Mx-like proteins in eukaryotes. The first consists of representatives from animals, fungi, algae, and *Rhizaria*. The second lineage consists of representatives from *Fungi*, *Stramenopiles, Haptophytes, Cryptophytes, Chlorophytes,* and *Rhizaria*. The third lineage consists of Mx-like sequences from plants, *Alveolates, Rhodophytes,* and *Stramenopiles*. (*A*), (*B*) Black dots indicate nodes with bootstrap support greater than 70% based on FastTree analyses (*Materials and Methods*); a scale bar indicates the level of amino acid divergence.

To pinpoint the evolutionary origin of Mx, we expanded our survey of Mx-like and other DSPs to include a broader range of eukaryotes and eukaryotic viruses (Fig. 3*A* and *SI Appendix*, Fig. S5*A*). In particular, we included representative genomes from *Rhodophytes* (red algae, an outgroup for plants and algae), *Cryptophytes* (a group of divergent plastid-bearing algae, also referred to as *Cryptomonads*), *Haptophytes* (a distinct divergent group of algae), *Amoebozoa* (an outgroup for animals and fungi), as well as Excavates (*Discoba*) and the TSAR (*Telonemia*–*Stramenopiles*–*Alveolates*–*Rhizaria*) supergroup of eukaryotes (106, 107). We excluded Mfn, FZO, and FZL proteins from this analysis due to their high divergence from other eukaryotic DSPs. We also omitted highly divergent dynamin-like GTPases, such as Sarcalumenins and EF-hand proteins, which appear to have been independently transferred from bacteria to eukaryotes (108, 109).

Our survey identified DSPs across well-established eukaryotic clades (Fig. 3*A*). Specifically, we found Dyn and Drp representatives in *Stramenopiles* (also referred to as *Heterokonts*)*, Alveolates, Rhizaria, Amoebozoa, Discoba,* and *Haptopytes*, indicating that the Dyn/Drp clade was already present as a fully specialized, distinct DSP clade in the last eukaryotic common ancestor (LECA). Additionally, we identified eukaryotic DSP lineages that were previously unidentified (e.g., PADS) or only recently discovered (e.g., MADS, MidX) (53), further clarifying the phylogenetic relationships among different DSPs. For instance, in line with recent findings (53), the fungal Mgm1 clade shows high bootstrap support for a closer relationship to the recently identified MidX rather than to other Dyn/Drp homologs. Thus, fungal Mgm1 proteins are not as closely related phylogenetically to animal OPA1, alveolate DrpC, or plant DRP5 proteins.

Our analyses also revealed Mx-like proteins beyond animals, fungi, and plants (Fig. 3 *A* and *B* and *SI Appendix*, Fig. S5 *A* and *B*). We identified Mx-like proteins in three lineages (Fig. 3*B* and *SI Appendix*, Fig. S5*B*. The first lineage comprises animal Mx proteins, fungal MxF2 proteins (including *Chytrids* and *Aphelida*), *Chlorophyte* (green algae) DRP4C, and a single Mx-like representative from *Rhizaria*. The second Mx-like lineage consists of fungal MxF1, *Chlorophyte* DRP4C, and proteins from *Stramenopiles*, *Cryptophytes*, *Haptophytes*, *Rhodophytes*, and *Rhizaria*. The third lineage of Mx-like proteins consists of plant DRP4 and Mx-like representatives from *Alveolates, Rhodophytes,* and *Stramenopiles*. Thus, Mx-like proteins are found in representatives of most of the extant eukaryotic supergroups, including TSAR (e.g., *Stramenopiles, Alveolates, Rhizaria*), *Haptists* (e.g., *Haptophytes*), *Archaeplastida* (e.g., red algae, green algae, land plants), *Cryptista* (e.g., *Cryptophytes*), and *Amorphea* (e.g., animals, fungi) (110). Given their presence across several eukaryotic supergroups, we infer that Mx proteins arose significantly earlier than previously suspected, near or shortly after LECA emerged. Including other eukaryotic groups adds a more nuanced perspective to our previous analysis of animal, fungal, and plant Mx-like representatives (Fig. 2*C*). Instead of their apparent orthology, the presence of multiple distinct lineages of Mx proteins (Fig. 3*B*) suggests multiple Mx-like paralogs existed in ancient eukaryotes, one of which gave rise to plant and algal DRP4s, whereas other(s) gave rise to animal and fungal Mx proteins.

**Fig. 4.**
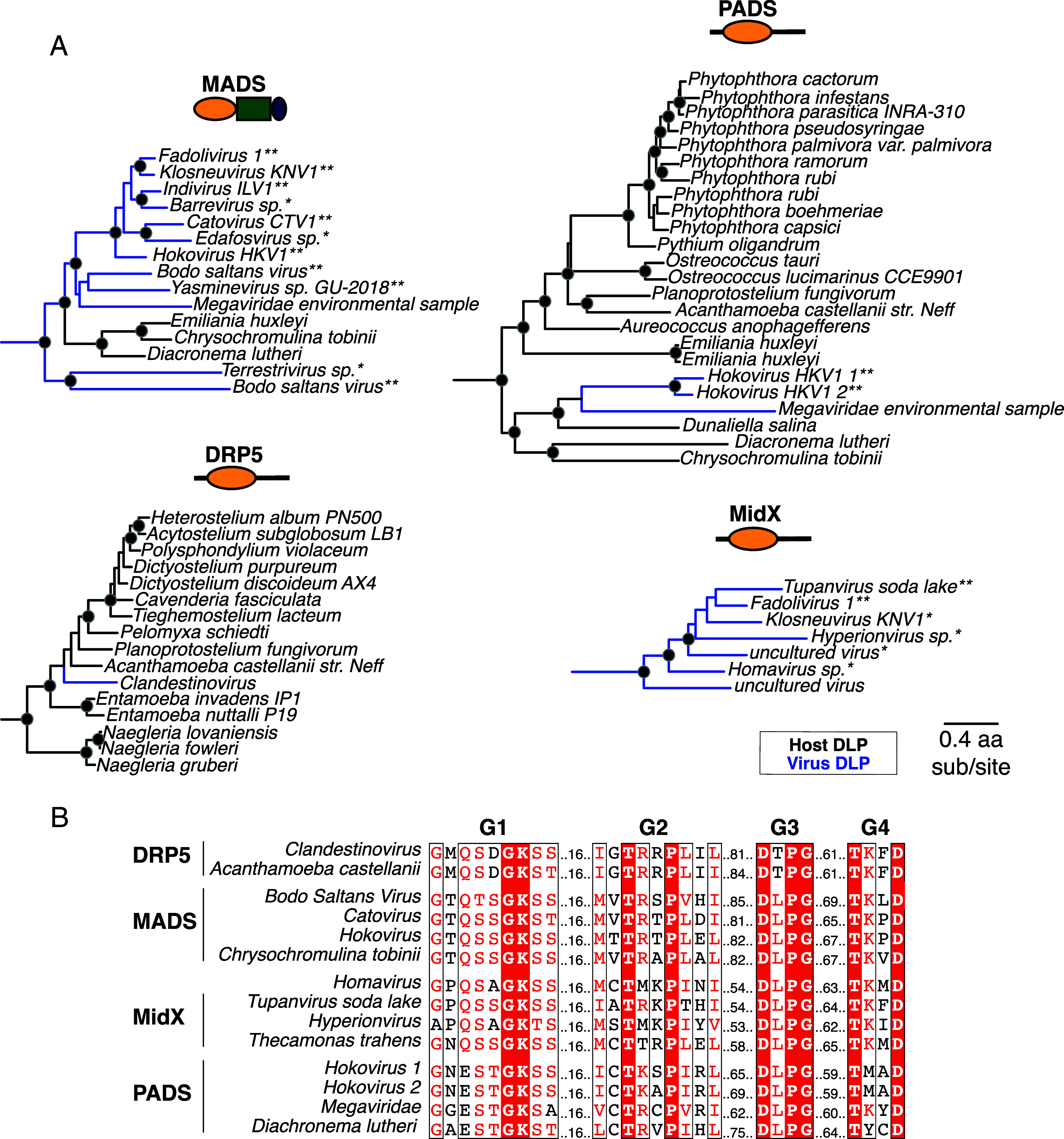
DSPs are encoded in giant viral genomes. (*A*) At least four major DSP clades contain viral DSP sequences, suggesting an ancient history of HGT between host and virus. MADS mainly contains NCLDV sequences, with three eukaryotic DSPs as outgroups. PADS consists of a few NCLDV sequences that serve as an outgroup to a diverse clade of eukaryotes. Our analysis only uncovered viral MidX DSPs. However, previous studies also uncovered eukaryotic representatives from metagenomic data (52). Finally, nestled among DRP5 sequences from *Amoebozoa* is a single NCLDV DSP. Black dots indicate nodes with bootstrap support greater than 70% based on FastTree analyses (*Materials and Methods*); the scale bar indicates the level of amino acid divergence for all four clades shown here. To confirm viral DSP sequences are from NCLDVs and not incorrect annotation or host contamination, we analyzed assemblies (*Materials and Methods*) to confirm sequences come from either fully sequenced viral genomes (**) or are of unambiguously viral origin (*). (*B*) Alignment of G Box motifs in viral DSPs indicates preservation of catalytic motifs associated with GTP hydrolysis in viral DSP sequences.

Finally, we identified four distinct DSP clades containing genes from both eukaryotes and viruses, strongly suggesting HGT (Fig. 4*A*). For example, the MADS clade [for Mimivirus-associated DRPs, previously described as “Clade D” (53)] appears in *Haptophytes*, such as *Emiliania huxleyi, Chrysochromulina tobinii*, and *Diacronema lutheri*, alongside interspersed lineages of *Nucleocytoviricota* (NCLDVs) [blue lineages, Fig. 4*A*; two asterisks denotes viral DSP sequences from full-length viral genome sequences, while one asterisk denotes viral DSP sequences from unambiguously viral contigs (*Materials and Methods*)]. Similarly, the PADS lineage (for *Phytopthora*-associated DRPs) includes DSPs from *Haptophytes, Chlorophytes* (green algae), *Oomycetes* (part of the *Stramenopiles*), and NCLDVs (Fig. 4*A*). Previous studies uncovered a large insertion from an ancient giant virus in oomycete genomes (111), but we found no evidence that the oomycete PADS sequence originates from this insertion. Our analyses also uncovered MidX sequences from NCLDVs (Fig. 4*A*). Although we did not recover any MidX sequences from eukaryotic host genomes based on our analyses of the well-curated nonredundant database in NCBI, metagenomic data have revealed additional host MidX sequences in a recent study (53). Finally, we recovered a DRP5-like DSP sequence from a *Clandestinovirus* NCLDV nestled within the DRP5 phylogeny from *Amoebozoa* (112). In nearly all cases, the viral DSPs have perfectly preserved all catalytic residues, suggesting they all have retained GTPase activity (Fig. 4*B*) (113, 114).

Thus, in addition to the deep evolutionary origins of Mx proteins in eukaryotes and their recurrent gene turnover—a characteristic of antiviral function—we find that giant DNA viruses have repeatedly co-opted DSP proteins. Our findings that Mx proteins are ancient and that giant viruses have repeatedly acquired DSPs suggest that the adaptable functionality of DSP proteins has positioned them centrally in the evolutionary arms race between hosts and viruses.

## Discussion

Dynamin-like proteins play essential roles in cellular remodeling, prompting significant interest in their evolutionary and functional diversification across eukaryotes. Previous studies have leveraged advances in cellular localization, structural insights, and expanding sequencing databases to propose models of the origin and diversification of this important gene family (17, 29, 53, 62). Building on these foundations, we investigated the origins of the distinctive Mx antiviral proteins in eukaryotes, whose evolutionary history remains poorly understood. Our findings indicate that the Mx lineage of DSPs is far more ancient than previously thought. While earlier reports suggested that its evolution coincided with the birth of the IFN system in bony vertebrates (17, 54), our analyses reveal distinct Mx-like lineages across animals (including invertebrates), fungi, plants, and most major eukaryotic supergroups.

Our analyses provide further insight into early events of Dynamin specialization across eukaryotes, uncovering novel DSP lineages with currently unknown cellular functions. For instance, a recent study showed that MidX proteins can remodel mitochondrial membrane topology from within the matrix (53), unlike Opa1 and Mfn, which remodel mitochondrial membranes from the outer membrane or intermembrane space, respectively. Beyond MidX and MADS, we identify a novel lineage, PADS (Fig. 3*A*). These findings suggest that substantial DSP diversification in extant eukaryotes likely occurred early in eukaryotic evolution, challenging prior classifications of “modern” versus “ancient” dynamins. In earlier analyses, such classifications may have been influenced by the overrepresentation of well-studied model systems, such as animals, fungi, and plants.

It remains unclear whether all Mx-like lineages perform antiviral roles, as they do in vertebrates, though some evidence suggests this may be the case. For example, specific Plant DRP4C proteins have been implicated in antiviral functions (62, 70, 78). If Mx-related proteins in nonanimal species were instead primarily involved in essential cellular membrane remodeling, we would expect less genetic turnover. However, our findings reveal widespread gene amplification and several instances of complete gene loss in animals, plants, and fungi. Such gene turnover aligns more closely with the inconsistent selective pressures typically imposed by viruses rather than with consistent selective pressures associated with cellular housekeeping functions. Our results suggest that Dynamin specialization for antiviral function emerged early in eukaryotic evolution.

While other IFN-induced antiviral genes, such as cGAS, STING, and Viperin, originate in bacteria (20, 21, 115), our analyses could not identify any bacterial or archaeal orthologs of eukaryotic Mx-like genes. Nonetheless, at least one bacterial Dynamin-like protein is known to participate in antiviral defense (116), while others have been proposed to act as antiviral effectors (117). These all weakly cluster with Mfn, Fzo, and Fzl (52, 116, 118, 119). Although these divergent prokaryotic DSPs are significantly divergent from Mx genes, they support our hypothesis that DSPs are primed to serve as antiviral factors.

A theme among DSPs is the recurrence of HGTs, including a fungal-to-algal transfer of Mx-like genes (Fig. 2*C*), which might represent the transfer of antiviral functionality. Understanding the functional implications of these inferred HGTs could shed light on the roles of Mx proteins beyond animals. Notably, the interspersion of eukaryotic and viral DSPs across at least four distinct lineages—MidX, MADS, PADS, and DRP5—suggests an ancient and ongoing history of DSP HGT events between various eukaryotic host lineages and NCLDV. NCLDV are a family of double-stranded DNA viruses, including *Mimiviridae*, *Poxviridae*, *Asfarviridae*, *Iridoviridae*, and *Phycodnaviridae*, typified by large genomes and viral particle sizes (120–122). These viruses infect many eukaryotes, including algae, amoeba, and animals (120–122). Only NCLDVs encode Dynamin-like proteins among viruses, suggesting some unique aspect of their cell biology may require Dynamin-like function. The discovery of complex membrane remodeling in *Molliviridae, Mimiviridae*, and *Poxviridae* highlights specific cell biological requirements for assembling large viral particles of NCLDVs (123–127). Although Dynamin proteins have not been directly implicated in these processes thus far, the cell biology of most NCLDVs remains largely unexplored. Our findings and previous studies (53) suggest that NCLDV assembly of new envelope-bound virions may require or benefit from Dynamin-like activity, potentially motivating some NCLDVs to acquire and repurpose host DSPs for their own functions. Alternatively, NCLDVs might acquire host DSPs to disrupt host DSP-mediated processes through a “dominant-negative” mechanism. However, if this were the case, we would not expect consistent retention of all features necessary for GTPase catalytic activity in viral DSPs (Fig. 4*B*).

Paralleling the bacterial specialization of dynamin-like proteins for antiviral defense (116, 117), our study highlights two ancient host–virus battles involving Dynamin-like functions across eukaryotic lineages. First, we demonstrate that the Mx clade of DSPs is ancient and may represent one of the earliest DSP specializations (for antiviral function) in eukaryotic evolution. Focused studies on Mx representatives in fungi, plants, and divergent eukaryotes could provide additional insights into Mx function and mechanisms. Second, we observe a recurring pattern of DSP acquisition by various NCLDV lineages, suggesting that both host and viral DSPs may play essential yet poorly understood roles in this widespread lineage of viruses. Investigating how these evolutionary arms races shape cellular and viral membrane remodeling functions may reveal unique aspects of viral biology and host defense. Thus, beyond their established roles in membrane remodeling, our findings suggest that DSPs have played crucial roles in antiviral and viral functions throughout most of eukaryotic evolution.

## Materials and Methods

### Phylogenetic Trees.

DSPs were identified via iterative BLAST searches on representative species with fully sequenced genomes using bona fide DSPs as queries. In cases with multiple hits from the same species, only the longest isoform encoded by each DSP gene was utilized in downstream analysis. We used Clustal Omega (128) or MAFFT (55) to align sequences obtained from the same query. We extracted and realigned the GTPase domains from these alignments using MAFFT to generate an alignment of GTPase domains from all DSPs. This alignment was then manually inspected to eliminate incomplete sequences and used to create phylogenetic trees using FastTree (56, 57) with default settings, which was also used to perform bootstrap analyses. FastTree combines the minimum evolution criterion with Nearest Neighbor Interchange (NNI) and Subtree Pruning and Regrafting rearrangements, followed by ML-based NNI rearrangements, to perform high-accuracy phylogenetic inference rapidly. As independent confirmation of our phylogenetic inferences, we also built trees using IQ-Tree under default settings with UltraFast Bootstraps (58) using the LG+I+G4 model. IQTree uses a more thorough but significantly slower stochastic algorithm for maximum likelihood tree estimation. The resulting trees were manually annotated to indicate the bootstrap support level and highlight groupings. We expanded our search space by increasing phylogenetic coverage, starting with animals, then plants and fungi, and finally all eukaryotes and eukaryotic viruses. More focused trees (Figs. 1 *D*, 2 *C*, 3 *B*, and 4 *A*) were excised from the larger trees (Figs. 1 *A*, 2 *A*, and 3 *A*, respectively).

To analyze Dynamin Middle Domains and GEDs, we aligned sequences using Clustal Omega (120), and manually extracted and realigned the Dynamin Middle and GEDs. This alignment was then manually inspected to eliminate incomplete sequences and used to generate phylogenetic trees using FastTree (56, 57) with default settings, also used to perform bootstrap analyses. To distinguish the evolutionary history of DRP4A and DRP4C, we took the plant and green algae sequences clustering with DRP4 from Fig. 2*A* and aligned the full-length sequences of the genes using MAFFT. This alignment included incomplete sequences, a signature of DRP4A, and was used to build a tree using FastTree.

### Protein Domain Analysis.

We uploaded full-length alignments of sequences retrieved from iterative Blast searches to the NCBI Conserved Domains tool using the Batch-CD search tool (60, 61). We present schematic versions of the domain analyses in our figures. We restrict our analyses only to domains reliably identified by the NCBI CD tool. However, in some instances, we additionally searched selected full-length DSP sequences using subsequent manual BLAST searches to confirm the absence of individual domains, such as the PH, Fuzzy onion (Fzo), or the TMP-synthase domains. We also visually inspected the GTPase domains identified to ensure that they bore all the hallmarks of catalytically active domains, including preservation of the previously identified catalytic residues.

### Cell Biological Localization.

We focused on previously published cell biological studies in human cells to represent the cytological location of different DSPs (Fig. 1*B*).

### DSP Repertoires in Representative Genomes.

To comprehensively identify all DSPs in representative genomes, we performed BLAST searches to specific fully sequenced genomes using the ref_seq database using representative DSPs from each identified clade, taking care to correctly and comprehensively identify true paralogs. We used phylogenetic analyses and (when possible) shared synteny analyses to distinguish between orthologs and paralogs. These analyses allowed us to identify cases of gene duplication within clades and confidently identify cases of specific gene losses of specific DSP clades for each representative genome sequenced.

### Analysis of Viral Assemblies.

To confirm whether a viral DSP sequence truly has a viral origin and is not a contaminating host sequence, we obtained the DNA assembles coding each viral DSP. We separated these into fully sequenced viral genomes and partial contigs. We used Geneious Prime to search the partial contigs for the viral DSP ORFs. We took 5kbp upstream and downstream of the viral DSP ORF and used Blastx searches to find the best matches to neighboring genes. We then identified the sequences with the highest e-value hit (after masking the query itself); if the neighboring hits were of viral origin, we denoted this as an unambiguous viral DSP sequence. This allowed us to assign most, but not all, DSPs analyzed to viral origin. Even in cases where DSPs did not meet this criterion, we did not find obvious host-origin homologs in the surrounding genes, suggesting that even most of these putative viral DSPs are not the result of host contamination.

### GTPase Catalytic Residue Alignment.

Representative sequences were selected from each viral DSP clade. These were aligned, and the G1, G2/Switch I, G3, and G4 boxes were manually extracted and realigned using MAFFT. This alignment was run on ESPript 3 (107) with default settings to display the different G Box motifs.

## Supplementary Material

Appendix 01 (PDF)

Dataset S01 (CSV)

Dataset S02 (PDF)

Dataset S03 (PDF)

Dataset S04 (CSV)

Dataset S05 (PDF)

Dataset S06 (PDF)

Dataset S07 (CSV)

Dataset S08 (PDF)

Dataset S09 (PDF)

Dataset S10 (PDF)

Dataset S11 (PDF)

Dataset S12 (PDF)

Dataset S13 (CSV)

Dataset S14 (PDF)

Dataset S15 (PDF)

Dataset S16 (PDF)

Dataset S17 (PDF)

Dataset S18 (XLSX)

## Data Availability

All study data are included in the article, *SI Appendix*, or supplementary datasets.
