# Supplementary material for: Antiviral Mx proteins have an ancient origin and widespread distribution among eukaryotes
Source: Proc Natl Acad Sci U S A. 2025 Jan 24;122(4):e2416811122. doi: 10.1073/pnas.2416811122 (PMC11789081; doi:10.1073/pnas.2416811122)
Supplement: Supplementary file 1 — Appendix 01 (PDF) [file pnas.2416811122.sapp.pdf]

Supporting Information for
Antiviral Mx proteins have an ancient origin and widespread distribution among eukaryotes

Caroline A. Langley, Peter A. Dietzen, Michael Emerman, Jeannette L. Tenthorey, and Harmit S. Malik

Corresponding author: Harmit S. Malik
Email: hsmalik@fhcrc.org

Supplementary Figures:

Supplementary Figure 1. The evolutionary origin of Mx in animals predates the interferon signaling network.
Supplementary Figure 2. Mx-like proteins in fungal and plant genomes.
Supplementary Figure 3. DRP4A arose multiple times in plant evolution.
Supplementary Figure 4. Evolutionary analyses of Plant DRP1 and DRP2 proteins.
Supplementary Figure 5. Phylogenetic analysis of DSPs in eukaryotes reveals an ancient Mx lineage.

Supporting datasets:
Dataset S1-S3: Figure 1: List of Accession numbers, MAFTT alignment, FastTree nexus file (3 files total)
Dataset S4-S6: Figure 2: List of Accession numbers, MAFTT alignment, FastTree nexus file (3 files total)
Dataset S7-S9: Figure 3: List of Accession numbers, MAFTT alignment, FastTree nexus file (3 files total)
Dataset S10: Supplementary Figure 1: IQTree nexus file (1 file total)
Dataset S11: Supplementary Figure 2: IQTree nexus file (1 file total)
Dataset S12-S14: Supplementary Figure 3: List of accession numbers, MAFTT alignment, FastTree nexus file (3 files total)
Dataset S15-S16: Supplementary Figure 4: CLUSTAL alignment file, FastTree nexus file (2 files total)
Dataset S17: Supplementary Figure 5: IQTree nexus file (1 file total)
Dataset S18: Viral DSPs genomic context

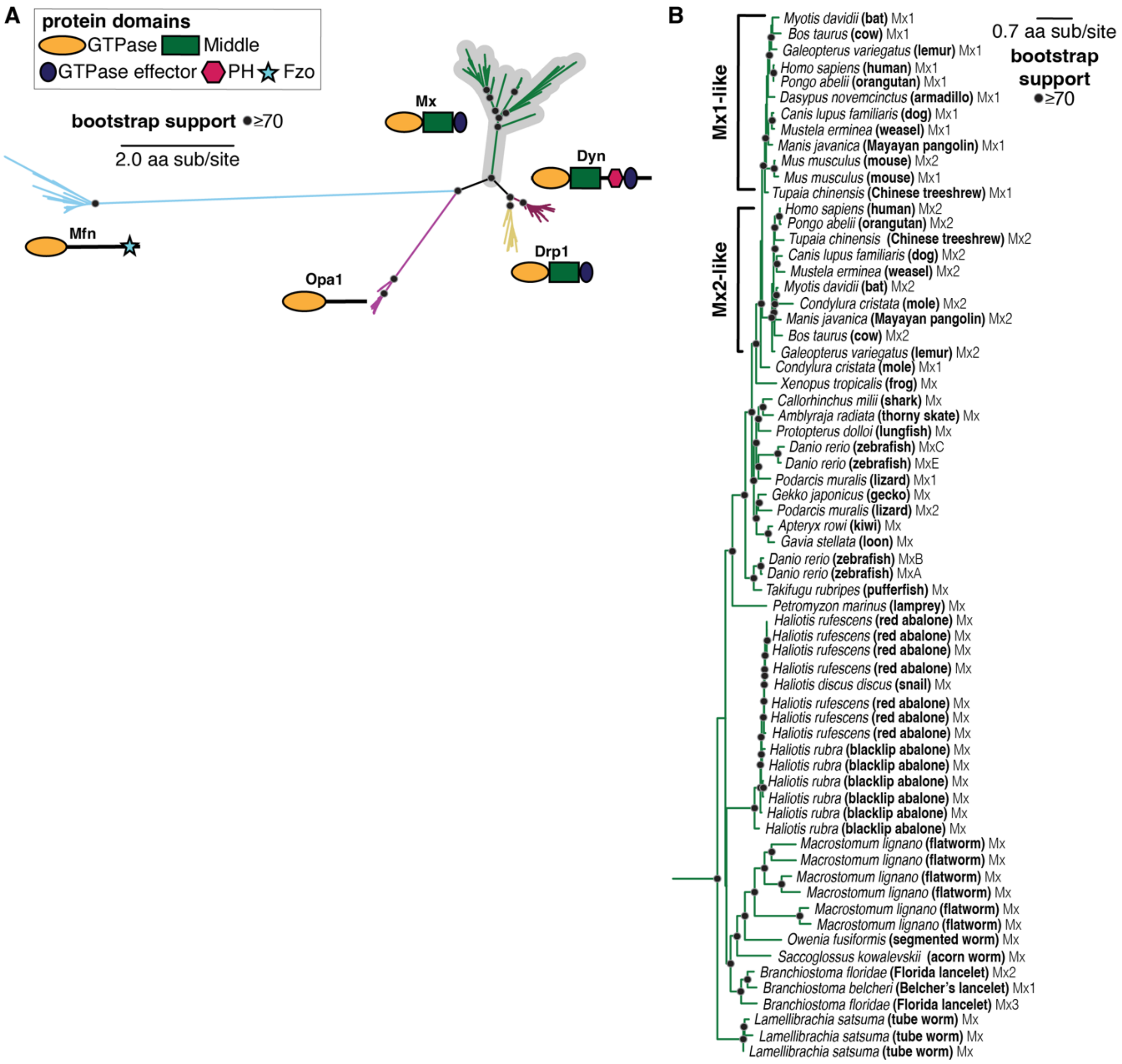

Supplementary Figure 1. The evolutionary origin of Mx in animals predates the interferon signaling network. (A) Phylogenetic analyses of Dynamin-superfamily proteins (DSPs) using IQ-Tree (112,113) based on their common GTPase domain in representative animals and outgroup species reveal five distinct DSP clades – Dyn, Drp1, Mfn, Opa1, and Mx (gray highlight**)**, consistent with FastTree analysis in Figure 1A. Black dots indicate nodes with UltraFast bootstrap support greater than 70% based on IQ-Tree analyses (Methods); a scale bar indicates the level of amino acid divergence. **(B**) Phylogenetic analysis of Mx-like proteins in animal species, consistent with Figure 1D.

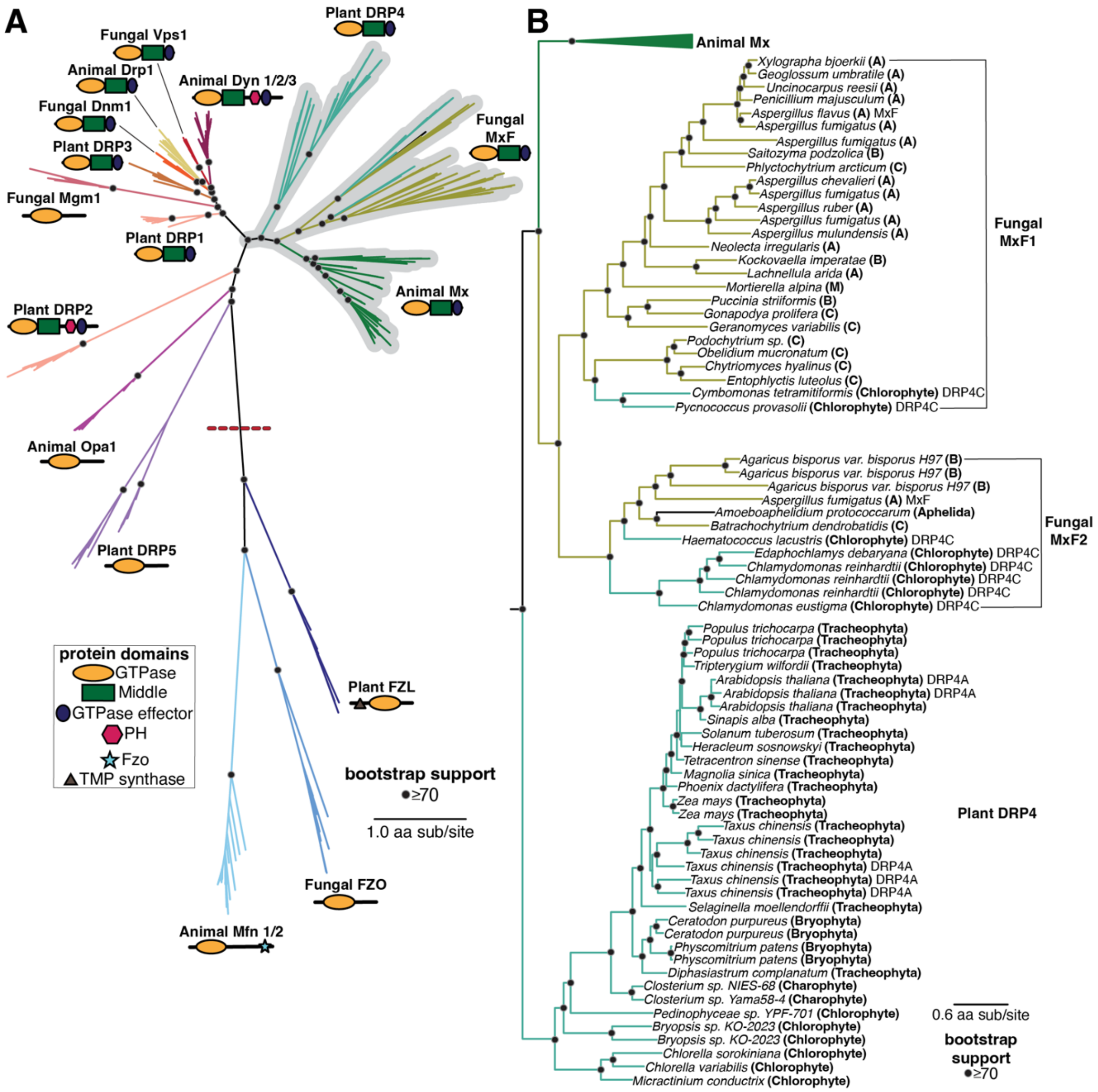

Supplementary Figure 2. Mx-like proteins in fungal and plant genomes. (A) Phylogenetic analysis using IQ-Tree (112,113) of animal, fungal, and plant DSPs based on their common GTPase domain reveals broad groupings (consistent with FastTree analysis in Figure 2A). Black dots indicate nodes with UltraFast bootstrap support greater than 70% based on IQ-Tree analyses (Methods); a scale bar indicates the level of amino acid divergence. **(B)** Phylogenetic analysis of animal Mx, fungal MxF, and plant DRP4 proteins using IQ-Tree, consistent with FastTree analysis in Figure 2C.

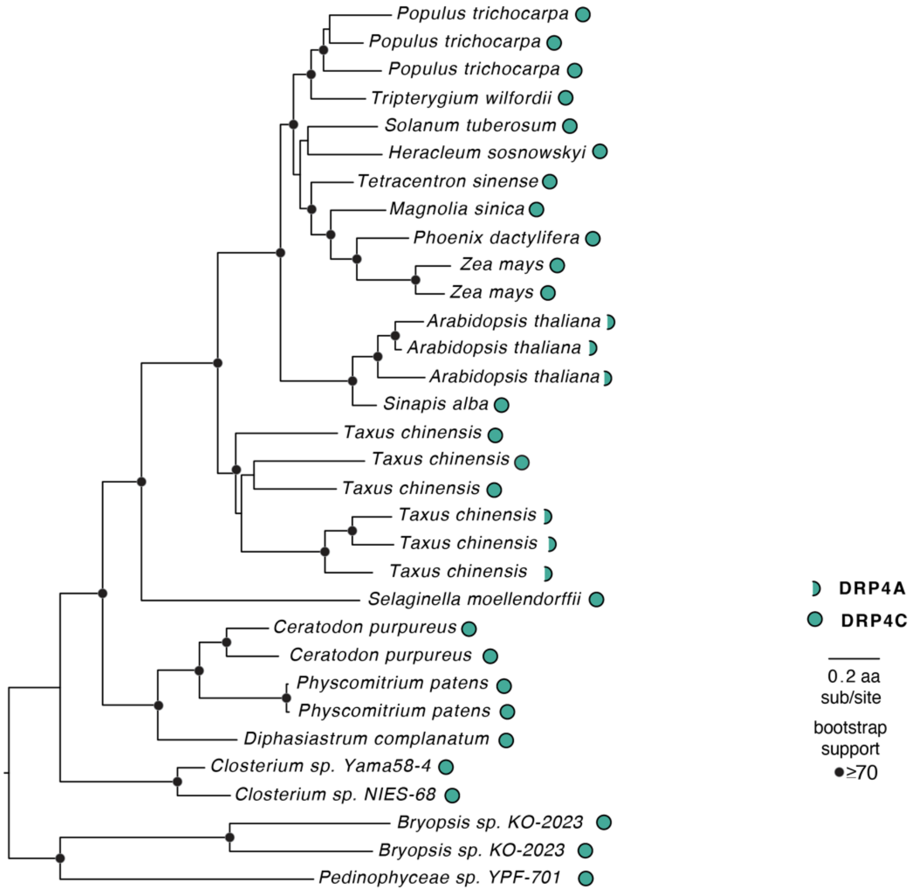

Supplementary Figure 3. DRP4A arose multiple times in plant evolution. Phylogenetic analysis of the DRP4A and DRP4C proteins using their common GTPase domains shows that DRP4A genes have independently arisen in plants multiple times. DRP4A genes often cluster with their DRP4C paralogs from the same species, indicating they have likely independently arisen via duplications of DRP4C. Full green circles indicate DRP4C proteins, while semicircles indicate the much shorter DRP4A proteins. Black dots indicate nodes with bootstrap support greater than 70% based on FastTree analyses (see Methods); a scale bar indicates the level of amino acid divergence.

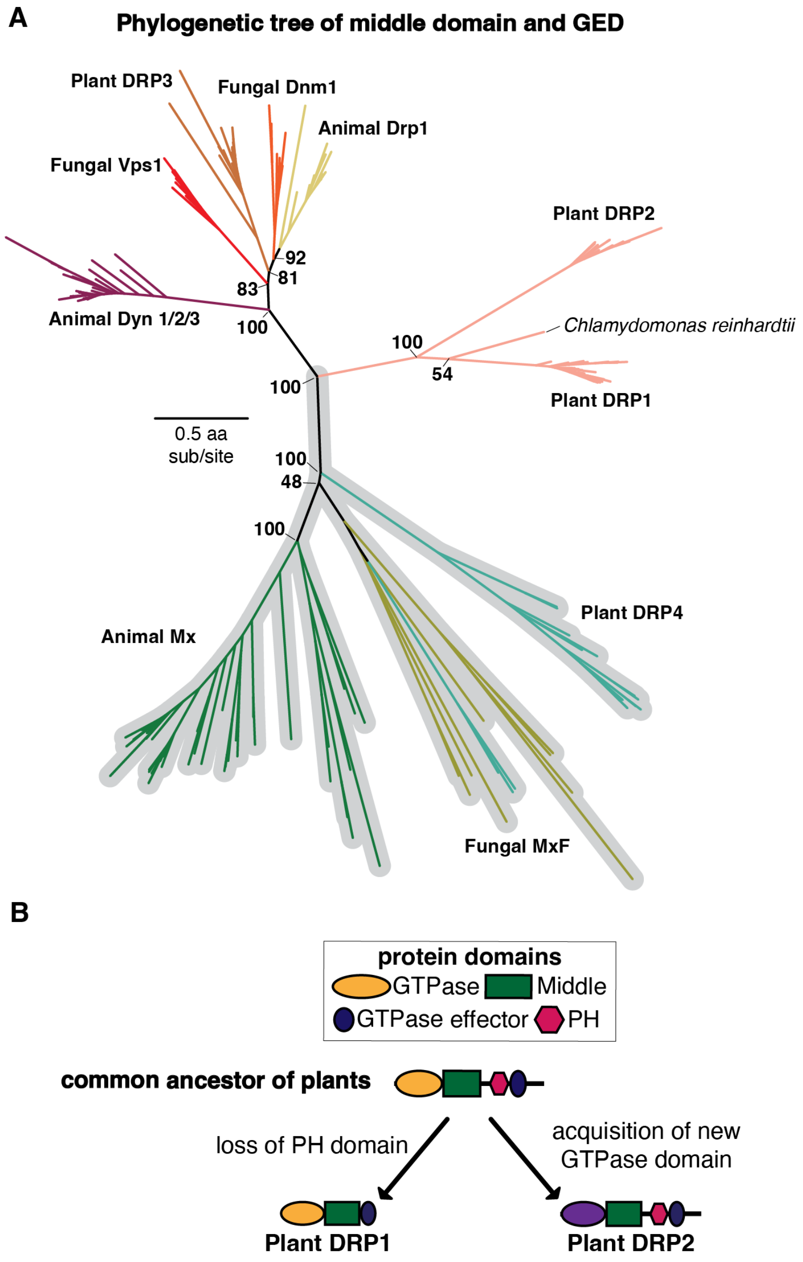

Supplementary Figure 4. Evolutionary analyses of Plant DRP1 and DRP2 proteins. (A) Phylogenetic analysis of plant, animal, and fungal DSPs based on an alignment of their Middle and GED domains reveals that Plant DRP1 and DRP2 are much more closely related in the Middle+GED phylogeny than in the GTPase phylogeny (Figure 2). Numbers indicate bootstrap support of specific nodes based on FastTree analyses (see Methods); a scale bar indicates the level of amino acid divergence. **(B**) A proposed model of plant DRP1 and DRP2 evolution, in which the plant DRP1/DRP2 ancestor encoded GTPase, Middle, GED, and PH domains. Gene duplication gave rise to DRP1, which lost its PH domains, and DRP2, which retained the PH domain but acquired a divergent GTPase domain via recombination.

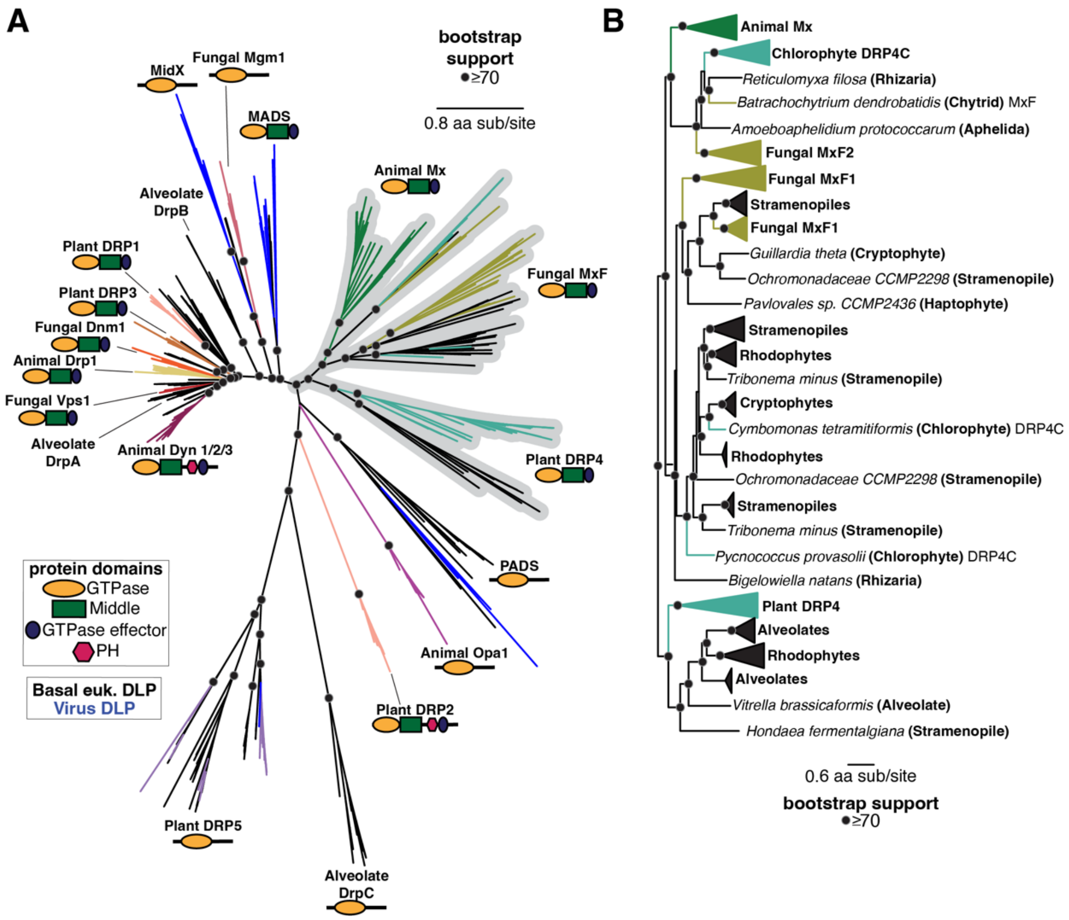

Supplementary Figure 5. Phylogenetic analysis of DSPs in eukaryotes reveals an ancient Mx lineage. (A) Phylogenetic analysis of all eukaryotic DSPs (excluding the Mfn, FZO, and FZL clades) using IQ-Tree (112,113) reveals an ancient Dyn/Drp clade that includes animal Drp, plant DRP3, and fungal Vsp1 along with fungal Dnm1, alveolate DrpA and DrpB, plant DRP1, and DSPs from several eukaryotic lineages (shown with black branches). The tree topology is consistent with FastTree analysis in Figure 3A. Black dots indicate nodes with UltraFast bootstrap support greater than 70% based on IQ-Tree analyses (see Methods); a scale bar indicates the level of amino acid divergence. **(B)** Phylogenetic analysis using IQ-Tree of three deeply branching lineages of Mx-like proteins in eukaryotes, compared to FastTree analysis in Figure 3B.
