# Supplementary material for: Antiviral Mx proteins have an ancient origin and widespread distribution among eukaryotes
Source: Proc Natl Acad Sci U S A. 2025 Jan 24;122(4):e2416811122. doi: 10.1073/pnas.2416811122 (PMC11789081; doi:10.1073/pnas.2416811122)
Supplement: Supplementary file 3 — Dataset S02 (PDF) [file pnas.2416811122.sd02.pdf]

Dataset S2. Figure_1_FastTree

#NEXUS
begin taxa;
dimensions ntax=180;
taxlabels
XP_006812840.1[&Organism="Saccoglossus kowalevskii",Description="PREDICTED: dynamin-1-like, partial [Saccoglossus kowalevskii]","Genetic Code"="Standard",Taxonomy="Eukaryota; Metazoa; Hemichordata; Enteropneusta; Harrimaniidae; Saccoglossus"]
XP_012378586.1[&Organism="Dasypus novemcinctus",Description="dynamin-1, partial [Dasypus novemcinctus]","Genetic Code"="Standard",Taxonomy="Eukaryota; Metazoa; Chordata; Craniata; Vertebrata; Euteleostomi; Mammalia; Eutheria; Xenarthra; Cingulata; Dasypodidae; Dasypus","Common Name"="nine-banded armadillo"]
EPQ17174.1[&Organism="Myotis brandtii",Description="PREDICTED: dynamin-1 [Myotis brandtii]","Genetic Code"="Standard",Taxonomy="Eukaryota; Metazoa; Chordata; Craniata; Vertebrata; Euteleostomi; Mammalia; Eutheria; Laurasiatheria; Chiroptera; Microchiroptera; Vespertilionidae; Myotis","Common Name"="Brandt's bat"]
ELW62001.1[&Organism="Tupaia chinensis",Description="dynamin-1 isoform X3 [Tupaia chinensis]","Genetic Code"="Standard",Taxonomy="Eukaryota; Metazoa; Chordata; Craniata; Vertebrata; Euteleostomi; Mammalia; Eutheria; Euarchontoglires; Scandentia; Tupaiidae; Tupaia","Common Name"="Chinese tree shrew"]
EAW87759.1[&Organism="Homo sapiens",Description="dynamin-1 isoform 2 [Homo sapiens]","Genetic Code"="Standard",Taxonomy="Eukaryota; Metazoa; Chordata; Craniata; Vertebrata; Euteleostomi; Mammalia; Eutheria; Euarchontoglires; Primates; Haplorrhini; Catarrhini; Hominidae; Homo","Common Name"="human"]
BAB27759.1[&Organism="Mus musculus",Description="dynamin-1 isoform X5 [Mus musculus]","Genetic Code"="Standard",Taxonomy="Eukaryota; Metazoa; Chordata; Craniata; Vertebrata; Euteleostomi; Mammalia; Eutheria; Euarchontoglires; Glires; Rodentia; Myomorpha; Muroidea; Muridae; Murinae; Mus; Mus","Common Name"="house mouse"]
XP_025915522.1[&Organism="Apteryx rowi",Description="dynamin-1 isoform X1 [Apteryx rowi]","Genetic Code"="Standard",Taxonomy="Eukaryota; Metazoa; Chordata; Craniata; Vertebrata; Euteleostomi; Archelosauria; Archosauria; Dinosauria; Saurischia; Theropoda; Coelurosauria; Aves; Palaeognathae; Apterygiformes; Apterygidae; Apteryx","Common Name"="Okarito brown kiwi"]
XP_028570166.1[&Organism="Podarcis muralis",Description="dynamin-1 isoform X14 [Podarcis muralis]","Genetic Code"="Standard",Taxonomy="Eukaryota; Metazoa; Chordata; Craniata; Vertebrata; Euteleostomi; Lepidosauria; Squamata; Bifurcata; Unidentata; Episquamata; Laterata; Lacertibaenia; Lacertidae; Podarcis","Common Name"="Common wall lizard"]
KAE8583055.1[&Organism="Xenopus tropicalis",Description="dynamin-1 isoform X18 [Xenopus tropicalis]","Genetic Code"="Standard",Taxonomy="Eukaryota; Metazoa;

Chordata; Craniata; Vertebrata; Euteleostomi; Amphibia; Batrachia; Anura; Pipoidea; Pipidae; Xenopodinae; Xenopus; Silurana","Common Name"="tropical clawed frog"]

XP_005165639.1[&Organism="Danio rerio",Description="dynamin-1 isoform X3 [Danio rerio]","Genetic Code"="Standard",Taxonomy="Eukaryota; Metazoa; Chordata; Craniata; Vertebrata; Euteleostomi; Actinopterygii; Neopterygii; Teleostei; Ostariophysi; Cypriniformes; Cyprinidae; Danio","Common Name"="zebrafish"]

XP_032814666.1[&Organism="Petromyzon marinus",Description="dynamin-1-like isoform X25 [Petromyzon marinus]","Genetic Code"="Standard",Taxonomy="Eukaryota; Metazoa; Chordata; Craniata; Vertebrata; Cyclostomata; Hyperoartia; Petromyzontiformes; Petromyzontidae; Petromyzon","Common Name"="sea lamprey"]

XP_012379251.1[&Organism="Dasypus novemcinctus",Description="dynamin-3 [Dasypus novemcinctus]","Genetic Code"="Standard",Taxonomy="Eukaryota; Metazoa; Chordata; Craniata; Vertebrata; Euteleostomi; Mammalia; Eutheria; Xenarthra; Cingulata; Dasypodidae; Dasypus","Common Name"="nine-banded armadillo"]

XP_006496668.1[&Organism="Mus musculus",Description="dynamin-3 isoform X6 [Mus musculus]","Genetic Code"="Standard",Taxonomy="Eukaryota; Metazoa; Chordata; Craniata; Vertebrata; Euteleostomi; Mammalia; Eutheria; Euarchontoglires; Glires; Rodentia; Myomorpha; Muroidea; Muridae; Murinae; Mus; Mus","Common Name"="house mouse"]

XP_016856477.1[&Organism="Homo sapiens",Description="dynamin-3 isoform d [Homo sapiens]","Genetic Code"="Standard",Taxonomy="Eukaryota; Metazoa; Chordata; Craniata; Vertebrata; Euteleostomi; Mammalia; Eutheria; Euarchontoglires; Primates; Haplorrhini; Catarrhini; Hominidae; Homo","Common Name"="human"]

XP_027623811.1[&Organism="Tupaia chinensis",Description="dynamin-3 isoform X3 [Tupaia chinensis]","Genetic Code"="Standard",Taxonomy="Eukaryota; Metazoa; Chordata; Craniata; Vertebrata; Euteleostomi; Mammalia; Eutheria; Euarchontoglires; Scandentia; Tupaiidae; Tupaia","Common Name"="Chinese tree shrew"]

EPQ08653.1[&Organism="Myotis brandtii",Description="PREDICTED: dynamin-3 isoform X1 [Myotis brandtii]","Genetic Code"="Standard",Taxonomy="Eukaryota; Metazoa; Chordata; Craniata; Vertebrata; Euteleostomi; Mammalia; Eutheria; Laurasiatheria; Chiroptera; Microchiroptera; Vespertilionidae; Myotis","Common Name"="Brandt's bat"]

XP_025944940.1[&Organism="Apteryx rowi",Description="dynamin-3 isoform X1 [Apteryx rowi]","Genetic Code"="Standard",Taxonomy="Eukaryota; Metazoa; Chordata; Craniata; Vertebrata; Euteleostomi; Archelosauria; Archosauria; Dinosauria; Saurischia; Theropoda; Coelurosauria; Aves; Palaeognathae; Apterygiformes; Apterygidae; Apteryx","Common Name"="Okarito brown kiwi"]

XP_014389433.1[&Organism="Myotis brandtii",Description="PREDICTED: dynamin-2 isoform X6 [Myotis brandtii]","Genetic Code"="Standard",Taxonomy="Eukaryota; Metazoa; Chordata; Craniata; Vertebrata; Euteleostomi; Mammalia; Eutheria; Laurasiatheria; Chiroptera; Microchiroptera; Vespertilionidae; Myotis","Common Name"="Brandt's bat"]

NP_001005360.1[&Organism="Homo sapiens",Description="dynamin-2 isoform 4 [Homo sapiens]","Genetic Code"="Standard",Taxonomy="Eukaryota; Metazoa; Chordata; Craniata; Vertebrata; Euteleostomi; Mammalia; Eutheria; Euarchontoglires; Primates; Haplorrhini; Catarrhini; Hominidae; Homo","Common Name"="human"]

XP_006510037.1[&Organism="Mus musculus",Description="dynamin-2 isoform X18 [Mus musculus]","Genetic Code"="Standard",Taxonomy="Eukaryota; Metazoa; Chordata; Craniata; Vertebrata; Euteleostomi; Mammalia; Eutheria; Euarchontoglires; Glires; Rodentia; Myomorpha; Muroidea; Muridae; Murinae; Mus; Mus","Common Name"="house mouse"]

XP_025920181.1[&Organism="Apteryx rowi",Description="dynamin-2 isoform X1 [Apteryx rowi]","Genetic Code"="Standard",Taxonomy="Eukaryota; Metazoa; Chordata; Craniata; Vertebrata; Euteleostomi; Archelosauria; Archosauria; Dinosauria; Saurischia; Theropoda; Coelurosauria; Aves; Palaeognathae; Apterygiformes; Apterygidae; Apteryx","Common Name"="Okarito brown kiwi"]

XP_028568434.1[&Organism="Podarcis muralis",Description="dynamin-2 isoform X7 [Podarcis muralis]","Genetic Code"="Standard",Taxonomy="Eukaryota; Metazoa; Chordata; Craniata; Vertebrata; Euteleostomi; Lepidosauria; Squamata; Bifurcata; Unidentata; Episquamata; Laterata; Lacertibaenia; Lacertidae; Podarcis","Common Name"="Common wall lizard"]

XP_012381548.1[&Organism="Dasypus novemcinctus",Description="dynamin-2 [Dasypus novemcinctus]","Genetic Code"="Standard",Taxonomy="Eukaryota; Metazoa; Chordata; Craniata; Vertebrata; Euteleostomi; Mammalia; Eutheria; Xenarthra; Cingulata; Dasypodidae; Dasypus","Common Name"="nine-banded armadillo"]

XP_006161648.2.2[&Organism="Tupaia chinensis",Description="dynamin-2 [Tupaia chinensis]","Genetic Code"="Standard",Taxonomy="Eukaryota; Metazoa; Chordata; Craniata; Vertebrata; Euteleostomi; Mammalia; Eutheria; Euarchontoglires; Scandentia; Tupaiidae; Tupaia","Common Name"="Chinese tree shrew"]

XP_031753735.1[&Organism="Xenopus tropicalis",Description="dynamin-2 isoform X2 [Xenopus tropicalis]","Genetic Code"="Standard",Taxonomy="Eukaryota; Metazoa; Chordata; Craniata; Vertebrata; Euteleostomi; Amphibia; Batrachia; Anura; Pipoidea; Pipidae; Xenopodinae; Xenopus; Silurana","Common Name"="tropical clawed frog"]

XP_021326548.1[&Organism="Danio rerio",Description="dynamin-2 isoform X5 [Danio rerio]","Genetic Code"="Standard",Taxonomy="Eukaryota; Metazoa; Chordata; Craniata; Vertebrata; Euteleostomi; Actinopterygii; Neopterygii; Teleostei; Ostariophysi; Cypriniformes; Cyprinidae; Danio","Common Name"="zebrafish"]

NP_001025299.1[&Organism="Danio rerio",Description="dynamin-3 [Danio rerio]","Genetic Code"="Standard",Taxonomy="Eukaryota; Metazoa; Chordata; Craniata; Vertebrata; Euteleostomi; Actinopterygii; Neopterygii; Teleostei; Ostariophysi; Cypriniformes; Cyprinidae; Danio","Common Name"="zebrafish"]

XP_035683496.1[&Organism="Branchiostoma floridae",Description="hypothetical protein BRAFLDRAFT_121263, partial [Branchiostoma floridae]","Genetic Code"="Standard",Taxonomy="Eukaryota; Metazoa; Chordata; Cephalochordata; Branchiostomidae; Branchiostoma","Common Name"="Florida lancelet"]

XP_030853442.1.2[&Organism="Strongylocentrotus purpuratus",Description="dynamin-1 isoform X1 [Strongylocentrotus purpuratus]","Genetic Code"="Standard",Taxonomy="Eukaryota; Metazoa; Echinodermata; Eleutherozoa; Echinozoa; Echinoidea; Euechinoidea; Echinacea; Echinoida; Strongylocentrotidae; Strongylocentrotus","Common Name"="purple sea urchin"]

XP_030853442.1[&Organism="Strongylocentrotus purpuratus",Description="dynamin-1 isoform X4 [Strongylocentrotus purpuratus]","Genetic Code"="Standard",Taxonomy="Eukaryota; Metazoa; Echinodermata; Eleutherozoa; Echinozoa; Echinoidea; Euechinoidea; Echinacea; Echinoida; Strongylocentrotidae; Strongylocentrotus","Common Name"="purple sea urchin"]

KMZ10000.1[&Organism="Drosophila melanogaster",Description="dynamin [Drosophila melanogaster]","Genetic Code"="Standard",Taxonomy="Eukaryota; Metazoa; Ecdysozoa; Arthropoda; Hexapoda; Insecta; Pterygota; Neoptera; Holometabola; Diptera; Brachycera; Muscomorpha; Ephydroidea; Drosophilidae; Drosophila; Sophophora","Common Name"="fruit fly"]

XP_026693152.1[&Organism="Ciona intestinalis",Description="dynamin-1 isoform X15 [Ciona intestinalis]","Genetic Code"="Standard",Taxonomy="Eukaryota; Metazoa; Chordata; Tunicata; Ascidiacea; Enterogona; Phlebobranchia; Cionidae; Ciona","Common Name"="vase tunicate"]

PAA65118.1[&Organism="Macrostomum lignano",Description="hypothetical protein BOX15_Mlig013747g2 [Macrostomum lignano]","Genetic Code"="Standard",Taxonomy="Eukaryota; Metazoa; Platyhelminthes; Rhabditophora; Macrostomorpha; Macrostomida; Macrostomidae; Macrostomum"]

PAA78248.1[&Organism="Macrostomum lignano",Description="hypothetical protein BOX15_Mlig010364g2 [Macrostomum lignano]","Genetic Code"="Standard",Taxonomy="Eukaryota; Metazoa; Platyhelminthes; Rhabditophora; Macrostomorpha; Macrostomida; Macrostomidae; Macrostomum"]

PAA59145.1[&Organism="Macrostomum lignano",Description="hypothetical protein BOX15_Mlig005677g1 [Macrostomum lignano]","Genetic Code"="Standard",Taxonomy="Eukaryota; Metazoa; Platyhelminthes; Rhabditophora; Macrostomorpha; Macrostomida; Macrostomidae; Macrostomum"]

PAA64382.1[&Organism="Macrostomum lignano",Description="hypothetical protein BOX15_Mlig016602g2 [Macrostomum lignano]","Genetic Code"="Standard",Taxonomy="Eukaryota; Metazoa; Platyhelminthes; Rhabditophora; Macrostomorpha; Macrostomida; Macrostomidae; Macrostomum"]

XP_001749319.1[&Organism="Monosiga brevicollis MX1",Description="uncharacterized protein MONBRDRAFT_28892 [Monosiga brevicollis MX1]","Genetic Code"="Standard",Taxonomy="Eukaryota; Choanoflagellata; Craspedida; Salpingoecidae; Monosiga"]

NP_001024332.1[&Organism="Caenorhabditis elegans",Description="Dynamin [Caenorhabditis elegans]","Genetic Code"="Standard",Taxonomy="Eukaryota; Metazoa; Ecdysozoa; Nematoda; Chromadorea; Rhabditida; Rhabditina; Rhabditomorpha; Rhabditoidea; Rhabditidae; Peloderinae; Caenorhabditis"]

XP_004347890.1[&Organism="Capsaspora owczarzaki ATCC 30864",Description="dynamin 1 [Capsaspora owczarzaki ATCC 30864]","Genetic Code"="Standard",Taxonomy="Eukaryota; Filasterea; Capsaspora"]

XP_031757197.1[&Organism="Xenopus tropicalis",Description="dynamin-3 [Xenopus tropicalis]","Genetic Code"="Standard",Taxonomy="Eukaryota; Metazoa;

Chordata; Craniata; Vertebrata; Euteleostomi; Amphibia; Batrachia; Anura; Pipoidea; Pipidae; Xenopodinae; Xenopus; Silurana","Common Name"="tropical clawed frog"]

XP_014153758.1[&Organism="Sphaeroforma arctica JP610",Description="dynamin-3 [Sphaeroforma arctica JP610]","Genetic Code"="Standard",Taxonomy="Eukaryota; Ichthyosporea; Ichthyophonida; Sphaeroforma"]

XP_014148725.1[&Organism="Sphaeroforma arctica JP610",Description="hypothetical protein SARC_12638, partial [Sphaeroforma arctica JP610]","Genetic Code"="Standard",Taxonomy="Eukaryota; Ichthyosporea; Ichthyophonida; Sphaeroforma"]

NP_741403.2[&Organism="Caenorhabditis elegans",Description="Dynamin GTPase [Caenorhabditis elegans]","Genetic Code"="Standard",Taxonomy="Eukaryota; Metazoa; Ecdysozoa; Nematoda; Chromadorea; Rhabditida; Rhabditina; Rhabditomorpha; Rhabditoidea; Rhabditidae; Peloderinae; Caenorhabditis"]

NP_957216.1[&Organism="Danio rerio",Description="dynamin-1-like protein [Danio rerio]","Genetic Code"="Standard",Taxonomy="Eukaryota; Metazoa; Chordata; Craniata; Vertebrata; Euteleostomi; Actinopterygii; Neopterygii; Teleostei; Ostariophysi; Cypriniformes; Danionidae; Danioninae; Danio","Common Name"="zebrafish"]

NP_001317309.1[&Organism="Homo sapiens",Description="dynamin-1-like protein isoform 8 [Homo sapiens]","Genetic Code"="Standard",Taxonomy="Eukaryota; Metazoa; Chordata; Craniata; Vertebrata; Euteleostomi; Mammalia; Eutheria; Euarchontoglires; Primates; Haplorrhini; Catarrhini; Hominidae; Homo","Common Name"="human"]

XP_006168142.1[&Organism="Tupaia chinensis",Description="dynamin-1-like protein isoform X1 [Tupaia chinensis]","Genetic Code"="Standard",Taxonomy="Eukaryota; Metazoa; Chordata; Craniata; Vertebrata; Euteleostomi; Mammalia; Eutheria; Euarchontoglires; Scandentia; Tupaiidae; Tupaia","Common Name"="Chinese tree shrew"]

NP_001392186.1[&Organism="Mus musculus",Description="dynamin-1-like protein isoform m [Mus musculus]","Genetic Code"="Standard",Taxonomy="Eukaryota; Metazoa; Chordata; Craniata; Vertebrata; Euteleostomi; Mammalia; Eutheria; Euarchontoglires; Glires; Rodentia; Myomorpha; Muroidea; Muridae; Murinae; Mus; Mus","Common Name"="house mouse"]

XP_014394711.1[&Organism="Myotis brandtii",Description="PREDICTED: dynamin-1-like protein isoform X6 [Myotis brandtii]","Genetic Code"="Standard",Taxonomy="Eukaryota; Metazoa; Chordata; Craniata; Vertebrata; Euteleostomi; Mammalia; Eutheria; Laurasiatheria; Chiroptera; Microchiroptera; Vespertilionidae; Myotis","Common Name"="Brandt's bat"]

XP_028602039.1[&Organism="Podarcis muralis",Description="dynamin-1-like protein isoform X3 [Podarcis muralis]","Genetic Code"="Standard",Taxonomy="Eukaryota; Metazoa; Chordata; Craniata; Vertebrata; Euteleostomi; Lepidosauria; Squamata; Bifurcata; Unidentata; Episquamata; Laterata; Lacertibaenia; Lacertidae; Podarcis","Common Name"="Common wall lizard"]

XP_025940269.1[&Organism="Apteryx rowi",Description="dynamin-1-like protein isoform X4 [Apteryx rowi]","Genetic Code"="Standard",Taxonomy="Eukaryota; Metazoa; Chordata; Craniata; Vertebrata; Euteleostomi; Archelosauria; Archosauria; Dinosauria;

Saurischia; Theropoda; Coelurosauria; Aves; Palaeognathae; Apterygiformes; Apterygidae; Apteryx","Common Name"="Okarito brown kiwi"]

XP_012382650.2[&Organism="Dasypus novemcinctus",Description="dynamin-1-like protein, partial [Dasypus novemcinctus]","Genetic Code"="Standard",Taxonomy="Eukaryota; Metazoa; Chordata; Craniata; Vertebrata; Euteleostomi; Mammalia; Eutheria; Xenarthra; Cingulata; Dasypodidae; Dasypus","Common Name"="nine-banded armadillo"]

XP_031753959.1[&Organism="Xenopus tropicalis",Description="dynamin-1-like protein [Xenopus tropicalis]","Genetic Code"="Standard",Taxonomy="Eukaryota; Metazoa; Chordata; Craniata; Vertebrata; Euteleostomi; Amphibia; Batrachia; Anura; Pipoidea; Pipidae; Xenopodinae; Xenopus; Silurana","Common Name"="tropical clawed frog"]

XP_032819300.1[&Organism="Petromyzon marinus",Description="dynamin-1-like protein isoform X2 [Petromyzon marinus]","Genetic Code"="Standard",Taxonomy="Eukaryota; Metazoa; Chordata; Craniata; Vertebrata; Cyclostomata; Hyperoartia; Petromyzontiformes; Petromyzontidae; Petromyzon","Common Name"="sea lamprey"]

XP_035676386.1[&Organism="Branchiostoma floridae",Description="dynamin-1-like protein isoform X5 [Branchiostoma floridae]","Genetic Code"="Standard",Taxonomy="Eukaryota; Metazoa; Chordata; Cephalochordata; Leptocardii; Amphioxiformes; Branchiostomatidae; Branchiostoma","Common Name"="Florida lancelet"]

XP_006821224.1[&Organism="Saccoglossus kowalevskii",Description="PREDICTED: dynamin-1-like protein-like [Saccoglossus kowalevskii]","Genetic Code"="Standard",Taxonomy="Eukaryota; Metazoa; Hemichordata; Enteropneusta; Harrimaniidae; Saccoglossus"]

XP_030827871.1[&Organism="Strongylocentrotus purpuratus",Description="dynamin-1-like protein isoform X2 [Strongylocentrotus purpuratus]","Genetic Code"="Standard",Taxonomy="Eukaryota; Metazoa; Echinodermata; Eleutherozoa; Echinozoa; Echinoidea; Euechinoidea; Echinacea; Camarodonta; Echinidea; Strongylocentrotidae; Strongylocentrotus","Common Name"="purple sea urchin"]

NP_001259946.1[&Organism="Drosophila melanogaster",Description="dynamin related protein 1, isoform B [Drosophila melanogaster]","Genetic Code"="Standard",Taxonomy="Eukaryota; Metazoa; Ecdysozoa; Arthropoda; Hexapoda; Insecta; Pterygota; Neoptera; Endopterygota; Diptera; Brachycera; Muscomorpha; Ephydroidea; Drosophilidae; Drosophila; Sophophora","Common Name"="fruit fly"]

PAA85687.1[&Organism="Macrostomum lignano",Description="hypothetical protein BOX15_Mlig022202g1 [Macrostomum lignano]","Genetic Code"="Standard",Taxonomy="Eukaryota; Metazoa; Spiralia; Lophotrochozoa; Platyhelminthes; Rhabditophora; Macrostomorpha; Macrostomida; Macrostomidae; Macrostomum"]

XP_002129967.2[&Organism="Ciona intestinalis",Description="dynamin-1-like protein [Ciona intestinalis]","Genetic Code"="Standard",Taxonomy="Eukaryota; Metazoa; Chordata; Tunicata; Ascidiacea; Phlebobranchia; Cionidae; Ciona","Common Name"="vase tunicate"]

XP_004348308.1[&Organism="Capsaspora owczarzaki ATCC 30864",Description="dynamin central region family protein [Capsaspora owczarzaki ATCC 30864]","Genetic Code"="Standard",Taxonomy="Eukaryota; Filasterea; Capsaspora"]

XP_014148015.1[&Organism="Sphaeroforma arctica JP610",Description="hypothetical protein SARC_13330, partial [Sphaeroforma arctica JP610]","Genetic Code"="Standard",Taxonomy="Eukaryota; Ichthyosporea; Ichthyophonida; Sphaeroforma"]

XP_001750431.1[&Organism="Monosiga brevicollis MX1",Description="uncharacterized protein MONBRDRAFT_34545 [Monosiga brevicollis MX1]","Genetic Code"="Standard",Taxonomy="Eukaryota; Choanoflagellata; Craspedida; Salpingoecidae; Monosiga"]

XP_004466363.1[&Organism="Dasypus novemcinctus",Description="interferon-induced GTP-binding protein Mx1 [Dasypus novemcinctus]","Genetic Code"="Standard",Taxonomy="Eukaryota; Metazoa; Chordata; Craniata; Vertebrata; Euteleostomi; Mammalia; Eutheria; Xenarthra; Cingulata; Dasypodidae; Dasypus","Common Name"="nine-banded armadillo"]

XP_006156437.1[&Organism="Tupaia chinensis",Description="interferon-induced GTP-binding protein Mx1 [Tupaia chinensis]","Genetic Code"="Standard",Taxonomy="Eukaryota; Metazoa; Chordata; Craniata; Vertebrata; Euteleostomi; Mammalia; Eutheria; Euarchontoglires; Scandentia; Tupaiidae; Tupaia","Common Name"="Chinese tree shrew"]

NP_002453.2.2[&Organism="Homo sapiens",Description="interferon-induced GTP-binding protein Mx1 isoform a [Homo sapiens]","Genetic Code"="Standard",Taxonomy="Eukaryota; Metazoa; Chordata; Craniata; Vertebrata; Euteleostomi; Mammalia; Eutheria; Euarchontoglires; Primates; Haplorrhini; Catarrhini; Hominidae; Homo","Common Name"="human"]

NP_001127618.1[&Organism="Pongo abelii",Description="interferon-induced GTP-binding protein Mx1 [Pongo abelii]","Genetic Code"="Standard",Taxonomy="Eukaryota; Metazoa; Chordata; Craniata; Vertebrata; Euteleostomi; Mammalia; Eutheria; Euarchontoglires; Primates; Haplorrhini; Catarrhini; Hominidae; Pongo","Common Name"="Sumatran orangutan"]

XP_017508130.1[&Organism="Manis javanica",Description="PREDICTED: interferon-induced GTP-binding protein Mx1 [Manis javanica]","Genetic Code"="Standard",Taxonomy="Eukaryota; Metazoa; Chordata; Craniata; Vertebrata; Euteleostomi; Mammalia; Eutheria; Laurasiatheria; Pholidota; Manidae; Manis","Common Name"="Malayan pangolin"]

NP_001003134.1[&Organism="Canis lupus familiaris",Description="interferon-induced GTP-binding protein Mx1 [Canis lupus familiaris]","Genetic Code"="Standard",Taxonomy="Eukaryota; Metazoa; Chordata; Craniata; Vertebrata; Euteleostomi; Mammalia; Eutheria; Laurasiatheria; Carnivora; Caniformia; Canidae; Canis","Common Name"="dog"]

XP_032211398.1[&Organism="Mustela erminea",Description="interferon-induced GTP-binding protein Mx1 isoform X1 [Mustela erminea]","Genetic Code"="Standard",Taxonomy="Eukaryota; Metazoa; Chordata; Craniata; Vertebrata;

Euteleostomi; Mammalia; Eutheria; Laurasiatheria; Carnivora; Caniformia; Mustelidae; Mustelinae; Mustela","Common Name"="ermine"]

XP_008569442.1[&Organism="Galeopterus variegatus",Description="PREDICTED: interferon-induced GTP-binding protein Mx1 [Galeopterus variegatus]","Genetic Code"="Standard",Taxonomy="Eukaryota; Metazoa; Chordata; Craniata; Vertebrata; Euteleostomi; Mammalia; Eutheria; Euarchontoglires; Dermoptera; Cynocephalidae; Galeopterus","Common Name"="Sunda flying lemur"]

XP_014388412.1[&Organism="Myotis brandtii",Description="PREDICTED: interferon-induced GTP-binding protein Mx1 isoform X1 [Myotis brandtii]","Genetic Code"="Standard",Taxonomy="Eukaryota; Metazoa; Chordata; Craniata; Vertebrata; Euteleostomi; Mammalia; Eutheria; Laurasiatheria; Chiroptera; Microchiroptera; Vespertilionidae; Myotis","Common Name"="Brandt's bat"]

XP_005202045.1[&Organism="Bos taurus",Description="interferon-induced GTP-binding protein Mx1 isoform X1 [Bos taurus]","Genetic Code"="Standard",Taxonomy="Eukaryota; Metazoa; Chordata; Craniata; Vertebrata; Euteleostomi; Mammalia; Eutheria; Laurasiatheria; Cetartiodactyla; Ruminantia; Pecora; Bovidae; Bovinae; Bos","Common Name"="cattle"]

NP_038634.1[&Organism="Mus musculus",Description="interferon-induced GTP-binding protein Mx2 [Mus musculus]","Genetic Code"="Standard",Taxonomy="Eukaryota; Metazoa; Chordata; Craniata; Vertebrata; Euteleostomi; Mammalia; Eutheria; Euarchontoglires; Glires; Rodentia; Myomorpha; Muroidea; Muridae; Murinae; Mus; Mus","Common Name"="house mouse"]

NP_034976.1[&Organism="Mus musculus",Description="interferon-induced GTP-binding protein Mx1 [Mus musculus]","Genetic Code"="Standard",Taxonomy="Eukaryota; Metazoa; Chordata; Craniata; Vertebrata; Euteleostomi; Mammalia; Eutheria; Euarchontoglires; Glires; Rodentia; Myomorpha; Muroidea; Muridae; Murinae; Mus; Mus","Common Name"="house mouse"]

XP_004675614.2.2[&Organism="Condylura cristata",Description="PREDICTED: interferon-induced GTP-binding protein Mx1 [Condylura cristata]","Genetic Code"="Standard",Taxonomy="Eukaryota; Metazoa; Chordata; Craniata; Vertebrata; Euteleostomi; Mammalia; Eutheria; Laurasiatheria; Insectivora; Talpidae; Condylura","Common Name"="star-nosed mole"]

XP_005885748.1[&Organism="Myotis brandtii",Description="PREDICTED: interferon-induced GTP-binding protein Mx2 [Myotis brandtii]","Genetic Code"="Standard",Taxonomy="Eukaryota; Metazoa; Chordata; Craniata; Vertebrata; Euteleostomi; Mammalia; Eutheria; Laurasiatheria; Chiroptera; Microchiroptera; Vespertilionidae; Myotis","Common Name"="Brandt's bat"]

XP_017508123.1[&Organism="Manis javanica",Description="PREDICTED: interferon-induced GTP-binding protein Mx2-like, partial [Manis javanica]","Genetic Code"="Standard",Taxonomy="Eukaryota; Metazoa; Chordata; Craniata; Vertebrata; Euteleostomi; Mammalia; Eutheria; Laurasiatheria; Pholidota; Manidae; Manis","Common Name"="Malayan pangolin"]

XP_008569440.1[&Organism="Galeopterus variegatus",Description="PREDICTED: interferon-induced GTP-binding protein Mx2 [Galeopterus variegatus]","Genetic

Code"="Standard",Taxonomy="Eukaryota; Metazoa; Chordata; Craniata; Vertebrata; Euteleostomi; Mammalia; Eutheria; Euarchontoglires; Dermoptera; Cynocephalidae; Galeopterus","Common Name"="Sunda flying lemur"]

NP_002454.1[&Organism="Homo sapiens",Description="interferon-induced GTP-binding protein Mx2 [Homo sapiens]","Genetic Code"="Standard",Taxonomy="Eukaryota; Metazoa; Chordata; Craniata; Vertebrata; Euteleostomi; Mammalia; Eutheria; Euarchontoglires; Primates; Haplorrhini; Catarrhini; Hominidae; Homo","Common Name"="human"]

XP_002830747.1[&Organism="Pongo abelii",Description="interferon-induced GTP-binding protein Mx2 [Pongo abelii]","Genetic Code"="Standard",Taxonomy="Eukaryota; Metazoa; Chordata; Craniata; Vertebrata; Euteleostomi; Mammalia; Eutheria; Euarchontoglires; Primates; Haplorrhini; Catarrhini; Hominidae; Pongo","Common Name"="Sumatran orangutan"]

NP_001003133.1[&Organism="Canis lupus familiaris",Description="interferon-induced GTP-binding protein Mx2 [Canis lupus familiaris]","Genetic Code"="Standard",Taxonomy="Eukaryota; Metazoa; Chordata; Craniata; Vertebrata; Euteleostomi; Mammalia; Eutheria; Laurasiatheria; Carnivora; Caniformia; Canidae; Canis","Common Name"="dog"]

NP_776366.1[&Organism="Bos taurus",Description="interferon-induced GTP-binding protein Mx2 [Bos taurus]","Genetic Code"="Standard",Taxonomy="Eukaryota; Metazoa; Chordata; Craniata; Vertebrata; Euteleostomi; Mammalia; Eutheria; Laurasiatheria; Artiodactyla; Ruminantia; Pecora; Bovidae; Bovinae; Bos","Common Name"="cattle"]

XP_032211320.1[&Organism="Mustela erminea",Description="interferon-induced GTP-binding protein Mx2 isoform X1 [Mustela erminea]","Genetic Code"="Standard",Taxonomy="Eukaryota; Metazoa; Chordata; Craniata; Vertebrata; Euteleostomi; Mammalia; Eutheria; Laurasiatheria; Carnivora; Caniformia; Mustelidae; Mustelinae; Mustela","Common Name"="ermine"]

XP_006156438.1[&Organism="Tupaia chinensis",Description="interferon-induced GTP-binding protein Mx2 [Tupaia chinensis]","Genetic Code"="Standard",Taxonomy="Eukaryota; Metazoa; Chordata; Craniata; Vertebrata; Euteleostomi; Mammalia; Eutheria; Euarchontoglires; Scandentia; Tupaiidae; Tupaia","Common Name"="Chinese tree shrew"]

XP_015269256.1[&Organism="Gekko japonicus",Description="PREDICTED: interferon-induced GTP-binding protein Mx1 [Gekko japonicus]","Genetic Code"="Standard",Taxonomy="Eukaryota; Metazoa; Chordata; Craniata; Vertebrata; Euteleostomi; Lepidosauria; Squamata; Bifurcata; Gekkota; Gekkonidae; Gekkoninae; Gekko"]

XP_028583072.1[&Organism="Podarcis muralis",Description="interferon-induced GTP-binding protein Mx2-like [Podarcis muralis]","Genetic Code"="Standard",Taxonomy="Eukaryota; Metazoa; Chordata; Craniata; Vertebrata; Euteleostomi; Lepidosauria; Squamata; Bifurcata; Unidentata; Episquamata; Laterata; Lacertibaenia; Lacertidae; Podarcis","Common Name"="Common wall lizard"]

XP_025933558.1[&Organism="Apteryx rowi",Description="interferon-induced GTP-binding protein Mx-like isoform X1 [Apteryx rowi]","Genetic Code"="Standard",Taxonomy="Eukaryota; Metazoa; Chordata; Craniata; Vertebrata; Euteleostomi; Archelosauria; Archosauria; Dinosauria; Saurischia; Theropoda; Coelurosauria; Aves; Palaeognathae; Apterygiformes; Apterygidae; Apteryx","Common Name"="Okarito brown kiwi"]

XP_009815891.1[&Organism="Gavia stellata",Description="PREDICTED: interferon-induced GTP-binding protein Mx-like [Gavia stellata]","Genetic Code"="Standard",Taxonomy="Eukaryota; Metazoa; Chordata; Craniata; Vertebrata; Euteleostomi; Archelosauria; Archosauria; Dinosauria; Saurischia; Theropoda; Coelurosauria; Aves; Neognathae; Gaviiformes; Gaviidae; Gavia","Common Name"="red-throated loon"]

AGU16245.1[&db_xref="taxon:27779",Organism="Protopterus dolloi",Description="Mx1 protein, partial [Protopterus dolloi]","Genetic Code"="Standard",Modified=Mon Mar 27 11:17:17 PDT 2023,Taxonomy="Eukaryota; Metazoa; Chordata; Craniata; Vertebrata; Euteleostomi; Dipnoi; Lepidosireniformes; Protopteridae; Protopterus",Accession="AGU16245.1","Common Name"="slender lungfish",Topology="linear","Molecule Type"="AA"]

XP_007904885.1[&Organism="Callorhinchus milii",Description="PREDICTED: interferon-induced GTP-binding protein Mx-like isoform X2 [Callorhinchus milii]","Genetic Code"="Standard",Taxonomy="Eukaryota; Metazoa; Chordata; Craniata; Vertebrata; Chondrichthyes; Holocephali; Chimaeriformes; Callorhinchidae; Callorhinchus","Common Name"="elephant shark"]

XP_032888405.1[&Organism="Amblyraja radiata",Description="interferon-induced GTP-binding protein Mx3-like isoform X1 [Amblyraja radiata]","Genetic Code"="Standard",Taxonomy="Eukaryota; Metazoa; Chordata; Craniata; Vertebrata; Chondrichthyes; Elasmobranchii; Batoidea; Rajiformes; Rajidae; Amblyraja","Common Name"="thorny skate"]

XP_028583068.1[&Organism="Podarcis muralis",Description="interferon-induced GTP-binding protein Mx1-like isoform X1 [Podarcis muralis]","Genetic Code"="Standard",Taxonomy="Eukaryota; Metazoa; Chordata; Craniata; Vertebrata; Euteleostomi; Lepidosauria; Squamata; Bifurcata; Unidentata; Episquamata; Laterata; Lacertibaenia; Lacertidae; Podarcis","Common Name"="Common wall lizard"]

XP_003973512.2.2[&Organism="Takifugu rubripes",Description="interferon-induced GTP-binding protein Mx [Takifugu rubripes]","Genetic Code"="Standard",Taxonomy="Eukaryota; Metazoa; Chordata; Craniata; Vertebrata; Euteleostomi; Actinopterygii; Neopterygii; Teleostei; Neoteleostei; Acanthomorphata; Eupercaria; Tetraodontiformes; Tetradontoidea; Tetraodontidae; Takifugu","Common Name"="torafugu"]

NP_891987.2.2[&Organism="Danio rerio",Description="interferon-induced GTP-binding protein MxA [Danio rerio]","Genetic Code"="Standard",Taxonomy="Eukaryota; Metazoa; Chordata; Craniata; Vertebrata; Euteleostomi; Actinopterygii; Neopterygii; Teleostei; Ostariophysi; Cypriniformes; Cyprinidae; Danio","Common Name"="zebrafish"]

XP_009304072.1[&Organism="Danio rerio",Description="interferon-induced GTP-binding protein MxB isoform X1 [Danio rerio]","Genetic Code"="Standard",Taxonomy="Eukaryota; Metazoa; Chordata; Craniata; Vertebrata; Euteleostomi; Actinopterygii; Neopterygii; Teleostei; Ostariophysi; Cypriniformes; Cyprinidae; Danio","Common Name"="zebrafish"]

XP_031752404.1[&Organism="Xenopus tropicalis",Description="interferon-induced GTP-binding protein Mx2 [Xenopus tropicalis]","Genetic Code"="Standard",Taxonomy="Eukaryota; Metazoa; Chordata; Craniata; Vertebrata; Euteleostomi; Amphibia; Batrachia; Anura; Pipoidea; Pipidae; Xenopodinae; Xenopus; Silurana","Common Name"="tropical clawed frog"]

NP_001007285.1[&Organism="Danio rerio",Description="interferon-induced GTP-binding protein MxC [Danio rerio]","Genetic Code"="Standard",Taxonomy="Eukaryota; Metazoa; Chordata; Craniata; Vertebrata; Euteleostomi; Actinopterygii; Neopterygii; Teleostei; Ostariophysi; Cypriniformes; Cyprinidae; Danio","Common Name"="zebrafish"]

XP_005167721.2.2[&Organism="Danio rerio",Description="interferon-induced GTP-binding protein MxE isoform X1 [Danio rerio]","Genetic Code"="Standard",Taxonomy="Eukaryota; Metazoa; Chordata; Craniata; Vertebrata; Euteleostomi; Actinopterygii; Neopterygii; Teleostei; Ostariophysi; Cypriniformes; Cyprinidae; Danio","Common Name"="zebrafish"]

XP_002608668.1[&Organism="Branchiostoma floridae",Description="hypothetical protein BRAFLDRAFT_58103 [Branchiostoma floridae]","Genetic Code"="Standard",Taxonomy="Eukaryota; Metazoa; Chordata; Cephalochordata; Branchiostomidae; Branchiostoma","Common Name"="Florida lancelet"]

XP_019617847.1[&Organism="Branchiostoma belcheri",Description="PREDICTED: interferon-induced GTP-binding protein Mx1-like [Branchiostoma belcheri]","Genetic Code"="Standard",Taxonomy="Eukaryota; Metazoa; Chordata; Cephalochordata; Branchiostomidae; Branchiostoma","Common Name"="Belcher's lancelet"]

XP_035690836.1[&Organism="Branchiostoma floridae",Description="interferon-induced GTP-binding protein Mx3-like [Branchiostoma floridae]","Genetic Code"="Standard",Taxonomy="Eukaryota; Metazoa; Chordata; Cephalochordata; Leptocardii; Amphioxiformes; Branchiostomidae; Branchiostoma","Common Name"="Florida lancelet"]

XP_012586448.1[&Organism="Condylura cristata",Description="PREDICTED: interferon-induced GTP-binding protein Mx2 [Condylura cristata]","Genetic Code"="Standard",Taxonomy="Eukaryota; Metazoa; Chordata; Craniata; Vertebrata; Euteleostomi; Mammalia; Eutheria; Laurasiatheria; Insectivora; Talpidae; Condylura","Common Name"="star-nosed mole"]

KAI0208044.1[&Organism="Lamellibrachia satsuma",Description="Interferon-induced GTP-binding protein Mx1 [Lamellibrachia satsuma]","Genetic Code"="Standard",Taxonomy="Eukaryota; Metazoa; Spiralia; Lophotrochozoa; Annelida; Polychaeta; Sedentaria; Canalipalpata; Sabellida; Siboglinidae; Lamellibrachia"]

KAI0213370.1[&Organism="Lamellibrachia satsuma",Description="Interferon-induced GTP-binding protein Mx2 [Lamellibrachia satsuma]","Genetic

Code"="Standard",Taxonomy="Eukaryota; Metazoa; Spiralia; Lophotrochozoa; Annelida; Polychaeta; Sedentaria; Canalipalpata; Sabellida; Siboglinidae; Lamellibrachia"]

KAI0218869.1[&Organism="Lamellibrachia satsuma",Description="hypothetical protein LSAT2_029455 [Lamellibrachia satsuma]","Genetic Code"="Standard",Taxonomy="Eukaryota; Metazoa; Spiralia; Lophotrochozoa; Annelida; Polychaeta; Sedentaria; Canalipalpata; Sabellida; Siboglinidae; Lamellibrachia"]

ABI53802.1[&Organism="Haliotis discus discus",Description="Mx [Haliotis discus discus]","Genetic Code"="Standard",Taxonomy="Eukaryota; Metazoa; Lophotrochozoa; Mollusca; Gastropoda; Vetigastropoda; Haliotoidea; Haliotidae; Haliotis","Common Name"="disc abalone"]

XP_046352531.2[&Organism="Haliotis rufescens",Description="interferon-induced GTP-binding protein Mx-like [Haliotis rufescens]","Genetic Code"="Standard",Taxonomy="Eukaryota; Metazoa; Spiralia; Lophotrochozoa; Mollusca; Gastropoda; Vetigastropoda; Lepetellida; Haliotoidea; Haliotidae; Haliotis","Common Name"="red abalone"]

XP_048258111.1[&Organism="Haliotis rufescens",Description="interferon-induced GTP-binding protein Mx-like [Haliotis rufescens]","Genetic Code"="Standard",Taxonomy="Eukaryota; Metazoa; Spiralia; Lophotrochozoa; Mollusca; Gastropoda; Vetigastropoda; Lepetellida; Haliotoidea; Haliotidae; Haliotis","Common Name"="red abalone"]

XP_048248472.1[&Organism="Haliotis rufescens",Description="LOW QUALITY PROTEIN: interferon-induced GTP-binding protein Mx-like [Haliotis rufescens]","Genetic Code"="Standard",Taxonomy="Eukaryota; Metazoa; Spiralia; Lophotrochozoa; Mollusca; Gastropoda; Vetigastropoda; Lepetellida; Haliotoidea; Haliotidae; Haliotis","Common Name"="red abalone"]

XP_048248473.1[&Organism="Haliotis rufescens",Description="interferon-induced GTP-binding protein Mx-like isoform X1 [Haliotis rufescens]","Genetic Code"="Standard",Taxonomy="Eukaryota; Metazoa; Spiralia; Lophotrochozoa; Mollusca; Gastropoda; Vetigastropoda; Lepetellida; Haliotoidea; Haliotidae; Haliotis","Common Name"="red abalone"]

XP_048248474.1[&Organism="Haliotis rufescens",Description="interferon-induced GTP-binding protein Mx-like isoform X2 [Haliotis rufescens]","Genetic Code"="Standard",Taxonomy="Eukaryota; Metazoa; Spiralia; Lophotrochozoa; Mollusca; Gastropoda; Vetigastropoda; Lepetellida; Haliotoidea; Haliotidae; Haliotis","Common Name"="red abalone"]

XP_048248476.1[&Organism="Haliotis rufescens",Description="interferon-induced GTP-binding protein Mx-like [Haliotis rufescens]","Genetic Code"="Standard",Taxonomy="Eukaryota; Metazoa; Spiralia; Lophotrochozoa; Mollusca; Gastropoda; Vetigastropoda; Lepetellida; Haliotoidea; Haliotidae; Haliotis","Common Name"="red abalone"]

XP_046352527.2[&Organism="Haliotis rufescens",Description="interferon-induced GTP-binding protein Mx1-like [Haliotis rufescens]","Genetic Code"="Standard",Taxonomy="Eukaryota; Metazoa; Spiralia; Lophotrochozoa; Mollusca;

Gastropoda; Vetigastropoda; Lepetellida; Haliotoidea; Haliotidae; Haliotis","Common Name"="red abalone"]

XP_046562919.1[&Organism="Haliotis rubra",Description="interferon-induced GTP-binding protein Mx-like [Haliotis rubra]","Genetic Code"="Standard",Taxonomy="Eukaryota; Metazoa; Spiralia; Lophotrochozoa; Mollusca; Gastropoda; Vetigastropoda; Lepetellida; Haliotoidea; Haliotidae; Haliotis","Common Name"="blacklip abalone"]

XP_046563124.1[&Organism="Haliotis rubra",Description="interferon-induced GTP-binding protein Mx-like [Haliotis rubra]","Genetic Code"="Standard",Taxonomy="Eukaryota; Metazoa; Spiralia; Lophotrochozoa; Mollusca; Gastropoda; Vetigastropoda; Lepetellida; Haliotoidea; Haliotidae; Haliotis","Common Name"="blacklip abalone"]

XP_046563126.1[&Organism="Haliotis rubra",Description="interferon-induced GTP-binding protein Mx-like [Haliotis rubra]","Genetic Code"="Standard",Taxonomy="Eukaryota; Metazoa; Spiralia; Lophotrochozoa; Mollusca; Gastropoda; Vetigastropoda; Lepetellida; Haliotoidea; Haliotidae; Haliotis","Common Name"="blacklip abalone"]

XP_046565195.1[&Organism="Haliotis rubra",Description="interferon-induced GTP-binding protein Mx-like [Haliotis rubra]","Genetic Code"="Standard",Taxonomy="Eukaryota; Metazoa; Spiralia; Lophotrochozoa; Mollusca; Gastropoda; Vetigastropoda; Lepetellida; Haliotoidea; Haliotidae; Haliotis","Common Name"="blacklip abalone"]

XP_046563125.1[&Organism="Haliotis rubra",Description="interferon-induced GTP-binding protein Mx-like [Haliotis rubra]","Genetic Code"="Standard",Taxonomy="Eukaryota; Metazoa; Spiralia; Lophotrochozoa; Mollusca; Gastropoda; Vetigastropoda; Lepetellida; Haliotoidea; Haliotidae; Haliotis","Common Name"="blacklip abalone"]

XP_046565196.1[&Organism="Haliotis rubra",Description="interferon-induced GTP-binding protein Mx-like [Haliotis rubra]","Genetic Code"="Standard",Taxonomy="Eukaryota; Metazoa; Spiralia; Lophotrochozoa; Mollusca; Gastropoda; Vetigastropoda; Lepetellida; Haliotoidea; Haliotidae; Haliotis","Common Name"="blacklip abalone"]

XP_032804093.1[&Organism="Petromyzon marinus",Description="interferon-induced GTP-binding protein Mx1-like isoform X2 [Petromyzon marinus]","Genetic Code"="Standard",Taxonomy="Eukaryota; Metazoa; Chordata; Craniata; Vertebrata; Cyclostomata; Hyperoartia; Petromyzontiformes; Petromyzontidae; Petromyzon","Common Name"="sea lamprey"]

XP_006815062.1[&Organism="Saccoglossus kowalevskii",Description="PREDICTED: interferon-induced GTP-binding protein Mx-like [Saccoglossus kowalevskii]","Genetic Code"="Standard",Taxonomy="Eukaryota; Metazoa; Hemichordata; Enteropneusta; Harrimaniidae; Saccoglossus"]

CAH1802128.1[&Organism="Owenia fusiformis",Description="unnamed protein product [Owenia fusiformis]","Genetic Code"="Standard",Taxonomy="Eukaryota; Metazoa; Spiralia; Lophotrochozoa; Annelida; Polychaeta; Sedentaria; Canalipalpata; Sabellida; Oweniida; Oweniidae; Owenia"]

PAA92268.1[&Organism="Macrostomum lignano",Description="hypothetical protein BOX15_Mlig009769g1 [Macrostomum lignano]","Genetic Code"="Standard",Taxonomy="Eukaryota; Metazoa; Platyhelminthes; Rhabditophora; Macrostomorpha; Macrostomida; Macrostomidae; Macrostomum"]

PAA74204.1[&Organism="Macrostomum lignano",Description="hypothetical protein BOX15_Mlig022940g2 [Macrostomum lignano]","Genetic Code"="Standard",Taxonomy="Eukaryota; Metazoa; Platyhelminthes; Rhabditophora; Macrostomorpha; Macrostomida; Macrostomidae; Macrostomum"]

PAA76532.1[&Organism="Macrostomum lignano",Description="hypothetical protein BOX15_Mlig002592g2 [Macrostomum lignano]","Genetic Code"="Standard",Taxonomy="Eukaryota; Metazoa; Platyhelminthes; Rhabditophora; Macrostomorpha; Macrostomida; Macrostomidae; Macrostomum"]

PAA69582.1[&Organism="Macrostomum lignano",Description="hypothetical protein BOX15_Mlig021727g2 [Macrostomum lignano]","Genetic Code"="Standard",Taxonomy="Eukaryota; Metazoa; Platyhelminthes; Rhabditophora; Macrostomorpha; Macrostomida; Macrostomidae; Macrostomum"]

PAA83069.1[&Organism="Macrostomum lignano",Description="hypothetical protein BOX15_Mlig009247g1 [Macrostomum lignano]","Genetic Code"="Standard",Taxonomy="Eukaryota; Metazoa; Platyhelminthes; Rhabditophora; Macrostomorpha; Macrostomida; Macrostomidae; Macrostomum"]

PAA94353.1[&Organism="Macrostomum lignano",Description="hypothetical protein BOX15_Mlig014920g1 [Macrostomum lignano]","Genetic Code"="Standard",Taxonomy="Eukaryota; Metazoa; Platyhelminthes; Rhabditophora; Macrostomorpha; Macrostomida; Macrostomidae; Macrostomum"]

XP_006813643.1[&Organism="Saccoglossus kowalevskii",Description="PREDICTED: dynamin-like 120 kDa protein, mitochondrial-like [Saccoglossus kowalevskii]","Genetic Code"="Standard",Taxonomy="Eukaryota; Metazoa; Hemichordata; Enteropneusta; Harrimaniidae; Saccoglossus"]

XP_030843280.1[&Organism="Strongylocentrotus purpuratus",Description="dynamin-like 120 kDa protein, mitochondrial [Strongylocentrotus purpuratus]","Genetic Code"="Standard",Taxonomy="Eukaryota; Metazoa; Echinodermata; Eleutherozoa; Echinozoa; Echinoidea; Euechinoidea; Echinacea; Echinoida; Strongylocentrotidae; Strongylocentrotus","Common Name"="purple sea urchin"]

XP_023440724.1[&Organism="Dasypus novemcinctus",Description="dynamin-like 120 kDa protein, mitochondrial [Dasypus novemcinctus]","Genetic Code"="Standard",Taxonomy="Eukaryota; Metazoa; Chordata; Craniata; Vertebrata; Euteleostomi; Mammalia; Eutheria; Xenarthra; Cingulata; Dasypodidae; Dasypus","Common Name"="nine-banded armadillo"]

XP_005873264.1[&Organism="Myotis brandtii",Description="PREDICTED: dynamin-like 120 kDa protein, mitochondrial isoform X5 [Myotis brandtii]","Genetic Code"="Standard",Taxonomy="Eukaryota; Metazoa; Chordata; Craniata; Vertebrata; Euteleostomi; Mammalia; Eutheria; Laurasiatheria; Chiroptera; Microchiroptera; Vespertilionidae; Myotis","Common Name"="Brandt's bat"]

XP_006163024.2.2[&Organism="Tupaia chinensis",Description="LOW QUALITY PROTEIN: dynamin-like 120 kDa protein, mitochondrial [Tupaia chinensis]","Genetic Code"="Standard",Taxonomy="Eukaryota; Metazoa; Chordata; Craniata; Vertebrata;

Euteleostomi; Mammalia; Eutheria; Euarchontoglires; Scandentia; Tupaiidae; Tupaia","Common Name"="Chinese tree shrew"]

NP_056375.2.2[&Organism="Homo sapiens",Description="dynamin-like 120 kDa protein, mitochondrial isoform 1 preproprotein [Homo sapiens]","Genetic Code"="Standard",Taxonomy="Eukaryota; Metazoa; Chordata; Craniata; Vertebrata; Euteleostomi; Mammalia; Eutheria; Euarchontoglires; Primates; Haplorrhini; Catarrhini; Hominidae; Homo","Common Name"="human"]

NP_598513.1[&Organism="Mus musculus",Description="dynamin-like 120 kDa protein, mitochondrial isoform 2 precursor [Mus musculus]","Genetic Code"="Standard",Taxonomy="Eukaryota; Metazoa; Chordata; Craniata; Vertebrata; Euteleostomi; Mammalia; Eutheria; Euarchontoglires; Glires; Rodentia; Myomorpha; Muroidea; Muridae; Murinae; Mus; Mus","Common Name"="house mouse"]

XP_028587646.1[&Organism="Podarcis muralis",Description="LOW QUALITY PROTEIN: dynamin-like 120 kDa protein, mitochondrial [Podarcis muralis]","Genetic Code"="Standard",Taxonomy="Eukaryota; Metazoa; Chordata; Craniata; Vertebrata; Euteleostomi; Lepidosauria; Squamata; Bifurcata; Unidentata; Episquamata; Laterata; Lacertibaenia; Lacertidae; Podarcis","Common Name"="Common wall lizard"]

XP_025913835.1[&Organism="Apteryx rowi",Description="dynamin-like 120 kDa protein, mitochondrial isoform X10 [Apteryx rowi]","Genetic Code"="Standard",Taxonomy="Eukaryota; Metazoa; Chordata; Craniata; Vertebrata; Euteleostomi; Archelosauria; Archosauria; Dinosauria; Saurischia; Theropoda; Coelurosauria; Aves; Palaeognathae; Apterygiformes; Apterygidae; Apteryx","Common Name"="Okarito brown kiwi"]

XP_021332524.1[&Organism="Danio rerio",Description="dynamin-like 120 kDa protein, mitochondrial isoform X5 [Danio rerio]","Genetic Code"="Standard",Taxonomy="Eukaryota; Metazoa; Chordata; Craniata; Vertebrata; Euteleostomi; Actinopterygii; Neopterygii; Teleostei; Ostariophysi; Cypriniformes; Cyprinidae; Danio","Common Name"="zebrafish"]

XP_031757388.1[&Organism="Xenopus tropicalis",Description="dynamin-like 120 kDa protein, mitochondrial isoform X3 [Xenopus tropicalis]","Genetic Code"="Standard",Taxonomy="Eukaryota; Metazoa; Chordata; Craniata; Vertebrata; Euteleostomi; Amphibia; Batrachia; Anura; Pipoidea; Pipidae; Xenopodinae; Xenopus; Silurana","Common Name"="tropical clawed frog"]

XP_032818114.1[&Organism="Petromyzon marinus",Description="dynamin-like 120 kDa protein, mitochondrial isoform X2 [Petromyzon marinus]","Genetic Code"="Standard",Taxonomy="Eukaryota; Metazoa; Chordata; Craniata; Vertebrata; Cyclostomata; Hyperoartia; Petromyzontiformes; Petromyzontidae; Petromyzon","Common Name"="sea lamprey"]

XP_018667792.1[&Organism="Ciona intestinalis",Description="dynamin-like 120 kDa protein, mitochondrial [Ciona intestinalis]","Genetic Code"="Standard",Taxonomy="Eukaryota; Metazoa; Chordata; Tunicata; Ascidiacea; Enterogona; Phlebobranchia; Cionidae; Ciona","Common Name"="vase tunicate"]

XP_002602331.1[&Organism="Branchiostoma floridae",Description="hypothetical protein BRAFLDRAFT_60684 [Branchiostoma floridae]","Genetic

Code"="Standard",Taxonomy="Eukaryota; Metazoa; Chordata; Cephalochordata; Branchiostomidae; Branchiostoma","Common Name"="Florida lancelet"]

XP_019637857.1[&Organism="Branchiostoma belcheri",Description="PREDICTED: dynamin-like 120 kDa protein, mitochondrial isoform X3 [Branchiostoma belcheri]","Genetic Code"="Standard",Taxonomy="Eukaryota; Metazoa; Chordata; Cephalochordata; Branchiostomidae; Branchiostoma","Common Name"="Belcher's lancelet"]

NP_495986.3.3[&Organism="Caenorhabditis elegans",Description="Dynamin-type G domain-containing protein [Caenorhabditis elegans]","Genetic Code"="Standard",Taxonomy="Eukaryota; Metazoa; Ecdysozoa; Nematoda; Chromadorea; Rhabditida; Rhabditina; Rhabditomorpha; Rhabditoidea; Rhabditidae; Peloderinae; Caenorhabditis"]

NP_610941.1[&Organism="Drosophila melanogaster",Description="optic atrophy 1, isoform B [Drosophila melanogaster]","Genetic Code"="Standard",Taxonomy="Eukaryota; Metazoa; Ecdysozoa; Arthropoda; Hexapoda; Insecta; Pterygota; Neoptera; Holometabola; Diptera; Brachycera; Muscomorpha; Ephydroidea; Drosophilidae; Drosophila; Sophophora","Common Name"="fruit fly"]

PAA68234.1[&Organism="Macrostomum lignano",Description="hypothetical protein BOX15_Mlig021919g1, partial [Macrostomum lignano]","Genetic Code"="Standard",Taxonomy="Eukaryota; Metazoa; Platyhelminthes; Rhabditophora; Macrostomorpha; Macrostomida; Macrostomidae; Macrostomum"]

PAA87312.1[&Organism="Macrostomum lignano",Description="hypothetical protein BOX15_Mlig004017g1, partial [Macrostomum lignano]","Genetic Code"="Standard",Taxonomy="Eukaryota; Metazoa; Platyhelminthes; Rhabditophora; Macrostomorpha; Macrostomida; Macrostomidae; Macrostomum"]

XP_004479029.1[&Organism="Dasypus novemcinctus",Description="mitofusin-1 isoform X2 [Dasypus novemcinctus]","Genetic Code"="Standard",Taxonomy="Eukaryota; Metazoa; Chordata; Craniata; Vertebrata; Euteleostomi; Mammalia; Eutheria; Xenarthra; Cingulata; Dasypodidae; Dasypus","Common Name"="nine-banded armadillo"]

NP_001193437.1[&Organism="Bos taurus",Description="mitofusin-1 [Bos taurus]","Genetic Code"="Standard",Taxonomy="Eukaryota; Metazoa; Chordata; Craniata; Vertebrata; Euteleostomi; Mammalia; Eutheria; Laurasiatheria; Artiodactyla; Ruminantia; Pecora; Bovidae; Bovinae; Bos","Common Name"="cattle"]

NP_077162.2.2[&Organism="Mus musculus",Description="mitofusin-1 [Mus musculus]","Genetic Code"="Standard",Taxonomy="Eukaryota; Metazoa; Chordata; Craniata; Vertebrata; Euteleostomi; Mammalia; Eutheria; Euarchontoglires; Glires; Rodentia; Myomorpha; Muroidea; Muridae; Murinae; Mus; Mus","Common Name"="house mouse"]

XP_006162789.1[&Organism="Tupaia chinensis",Description="mitofusin-1 [Tupaia chinensis]","Genetic Code"="Standard",Taxonomy="Eukaryota; Metazoa; Chordata; Craniata; Vertebrata; Euteleostomi; Mammalia; Eutheria; Euarchontoglires; Scandentia; Tupaiidae; Tupaia","Common Name"="Chinese tree shrew"]

XP_005883071.1[&Organism="Myotis brandtii",Description="PREDICTED: mitofusin-1 [Myotis brandtii]","Genetic Code"="Standard",Taxonomy="Eukaryota; Metazoa;

Chordata; Craniata; Vertebrata; Euteleostomi; Mammalia; Eutheria; Laurasiatheria; Chiroptera; Microchiroptera; Vespertilionidae; Myotis","Common Name"="Brandt's bat"]

XP_025917892.1[&Organism="Apteryx rowi",Description="mitofusin-1 isoform X1 [Apteryx rowi]","Genetic Code"="Standard",Taxonomy="Eukaryota; Metazoa; Chordata; Craniata; Vertebrata; Euteleostomi; Archelosauria; Archosauria; Dinosauria; Saurischia; Theropoda; Coelurosauria; Aves; Palaeognathae; Apterygiformes; Apterygidae; Apteryx","Common Name"="Okarito brown kiwi"]

NP_284941.2.2[&Organism="Homo sapiens",Description="mitofusin-1 [Homo sapiens]","Genetic Code"="Standard",Taxonomy="Eukaryota; Metazoa; Chordata; Craniata; Vertebrata; Euteleostomi; Mammalia; Eutheria; Euarchontoglires; Primates; Haplorrhini; Catarrhini; Hominidae; Homo","Common Name"="human"]

XP_028587453.1[&Organism="Podarcis muralis",Description="mitofusin-1 [Podarcis muralis]","Genetic Code"="Standard",Taxonomy="Eukaryota; Metazoa; Chordata; Craniata; Vertebrata; Euteleostomi; Lepidosauria; Squamata; Bifurcata; Unidentata; Episquamata; Laterata; Lacertibaenia; Lacertidae; Podarcis","Common Name"="Common wall lizard"]

NP_001016189.1[&Organism="Xenopus tropicalis",Description="mitofusin-1 [Xenopus tropicalis]","Genetic Code"="Standard",Taxonomy="Eukaryota; Metazoa; Chordata; Craniata; Vertebrata; Euteleostomi; Amphibia; Batrachia; Anura; Pipoidea; Pipidae; Xenopodinae; Xenopus; Silurana","Common Name"="tropical clawed frog"]

XP_017213868.2.2[&Organism="Danio rerio",Description="mitofusin-1 isoform X1 [Danio rerio]","Genetic Code"="Standard",Taxonomy="Eukaryota; Metazoa; Chordata; Craniata; Vertebrata; Euteleostomi; Actinopterygii; Neopterygii; Teleostei; Ostariophysi; Cypriniformes; Cyprinidae; Danio","Common Name"="zebrafish"]

XP_004482574.1[&Organism="Dasypus novemcinctus",Description="mitofusin-2 [Dasypus novemcinctus]","Genetic Code"="Standard",Taxonomy="Eukaryota; Metazoa; Chordata; Craniata; Vertebrata; Euteleostomi; Mammalia; Eutheria; Xenarthra; Cingulata; Dasypodidae; Dasypus","Common Name"="nine-banded armadillo"]

XP_006145367.1[&Organism="Tupaia chinensis",Description="mitofusin-2 isoform X2 [Tupaia chinensis]","Genetic Code"="Standard",Taxonomy="Eukaryota; Metazoa; Chordata; Craniata; Vertebrata; Euteleostomi; Mammalia; Eutheria; Euarchontoglires; Scandentia; Tupaiidae; Tupaia","Common Name"="Chinese tree shrew"]

XP_014400986.1[&Organism="Myotis brandtii",Description="PREDICTED: mitofusin-2 isoform X2 [Myotis brandtii]","Genetic Code"="Standard",Taxonomy="Eukaryota; Metazoa; Chordata; Craniata; Vertebrata; Euteleostomi; Mammalia; Eutheria; Laurasiatheria; Chiroptera; Microchiroptera; Vespertilionidae; Myotis","Common Name"="Brandt's bat"]

NP_001177198.1[&Organism="Bos taurus",Description="mitofusin-2 [Bos taurus]","Genetic Code"="Standard",Taxonomy="Eukaryota; Metazoa; Chordata; Craniata; Vertebrata; Euteleostomi; Mammalia; Eutheria; Laurasiatheria; Artiodactyla; Ruminantia; Pecora; Bovidae; Bovinae; Bos","Common Name"="cattle"]

NP_001272849.1[&Organism="Mus musculus",Description="mitofusin-2 [Mus musculus]","Genetic Code"="Standard",Taxonomy="Eukaryota; Metazoa; Chordata; Craniata; Vertebrata; Euteleostomi; Mammalia; Eutheria; Euarchontoglires; Glires;

Rodentia; Myomorpha; Muroidea; Muridae; Murinae; Mus; Mus","Common Name"="house mouse"]

NP_001121132.1[&Organism="Homo sapiens",Description="mitofusin-2 [Homo sapiens]","Genetic Code"="Standard",Taxonomy="Eukaryota; Metazoa; Chordata; Craniata; Vertebrata; Euteleostomi; Mammalia; Eutheria; Euarchontoglires; Primates; Haplorrhini; Catarrhini; Hominidae; Homo","Common Name"="human"]

XP_025929938.1[&Organism="Apteryx rowi",Description="mitofusin-2 [Apteryx rowi]","Genetic Code"="Standard",Taxonomy="Eukaryota; Metazoa; Chordata; Craniata; Vertebrata; Euteleostomi; Archelosauria; Archosauria; Dinosauria; Saurischia; Theropoda; Coelurosauria; Aves; Palaeognathae; Apterygiformes; Apterygidae; Apteryx","Common Name"="Okarito brown kiwi"]

XP_028597443.1[&Organism="Podarcis muralis",Description="LOW QUALITY PROTEIN: mitofusin-2 [Podarcis muralis]","Genetic Code"="Standard",Taxonomy="Eukaryota; Metazoa; Chordata; Craniata; Vertebrata; Euteleostomi; Lepidosauria; Squamata; Bifurcata; Unidentata; Episquamata; Laterata; Lacertibaenia; Lacertidae; Podarcis","Common Name"="Common wall lizard"]

XP_015268039.1[&Organism="Gekko japonicus",Description="PREDICTED: mitofusin-2 [Gekko japonicus]","Genetic Code"="Standard",Taxonomy="Eukaryota; Metazoa; Chordata; Craniata; Vertebrata; Euteleostomi; Lepidosauria; Squamata; Bifurcata; Gekkota; Gekkonidae; Gekkoninae; Gekko"]

NP_001121726.1[&Organism="Danio rerio",Description="mitofusin-2 [Danio rerio]","Genetic Code"="Standard",Taxonomy="Eukaryota; Metazoa; Chordata; Craniata; Vertebrata; Euteleostomi; Actinopterygii; Neopterygii; Teleostei; Ostariophysi; Cypriniformes; Danionidae; Danioninae; Danio","Common Name"="zebrafish"]

XP_002591612.1[&Organism="Branchiostoma floridae",Description="hypothetical protein BRAFLDRAFT_223384 [Branchiostoma floridae]","Genetic Code"="Standard",Taxonomy="Eukaryota; Metazoa; Chordata; Cephalochordata; Branchiostomidae; Branchiostoma","Common Name"="Florida lancelet"]

XP_019628129.1[&Organism="Branchiostoma belcheri",Description="PREDICTED: mitofusin-2-like [Branchiostoma belcheri]","Genetic Code"="Standard",Taxonomy="Eukaryota; Metazoa; Chordata; Cephalochordata; Branchiostomidae; Branchiostoma","Common Name"="Belcher's lancelet"]

XP_006819998.1[&Organism="Saccoglossus kowalevskii",Description="PREDICTED: mitofusin-2-like [Saccoglossus kowalevskii]","Genetic Code"="Standard",Taxonomy="Eukaryota; Metazoa; Hemichordata; Enteropneusta; Harrimaniidae; Saccoglossus"]

XP_030846906.1[&Organism="Strongylocentrotus purpuratus",Description="mitofusin-1-like [Strongylocentrotus purpuratus]","Genetic Code"="Standard",Taxonomy="Eukaryota; Metazoa; Echinodermata; Eleutherozoa; Echinozoa; Echinoidea; Euechinoidea; Echinacea; Echinoida; Strongylocentrotidae; Strongylocentrotus","Common Name"="purple sea urchin"]

XP_030847518.1[&Organism="Strongylocentrotus purpuratus",Description="mitofusin-2 [Strongylocentrotus purpuratus]","Genetic Code"="Standard",Taxonomy="Eukaryota; Metazoa; Echinodermata; Eleutherozoa;

Echinozoa; Echinoidea; Euechinoidea; Echinacea; Echinoida; Strongylocentrotidae; Strongylocentrotus","Common Name"="purple sea urchin"]
NP_996357.1[&Organism="Drosophila melanogaster",Description="mitochondrial assembly regulatory factor, isoform C [Drosophila melanogaster]","Genetic Code"="Standard",Taxonomy="Eukaryota; Metazoa; Ecdysozoa; Arthropoda; Hexapoda; Insecta; Pterygota; Neoptera; Holometabola; Diptera; Brachycera; Muscomorpha; Ephydroidea; Drosophilidae; Drosophila; Sophophora","Common Name"="fruit fly"]
XP_002126852.1[&Organism="Ciona intestinalis",Description="mitofusin-2 [Ciona intestinalis]","Genetic Code"="Standard",Taxonomy="Eukaryota; Metazoa; Chordata; Tunicata; Ascidiacea; Enterogona; Phlebobranchia; Cionidae; Ciona","Common Name"="vase tunicate"]
PAA75551.1[&Organism="Macrostomum lignano",Description="hypothetical protein BOX15_Mlig004622g1, partial [Macrostomum lignano]","Genetic Code"="Standard",Taxonomy="Eukaryota; Metazoa; Platyhelminthes; Rhabditophora; Macrostomorpha; Macrostomida; Macrostomidae; Macrostomum"]
PAA75258.1[&Organism="Macrostomum lignano",Description="hypothetical protein BOX15_Mlig033579g1, partial [Macrostomum lignano]","Genetic Code"="Standard",Taxonomy="Eukaryota; Metazoa; Platyhelminthes; Rhabditophora; Macrostomorpha; Macrostomida; Macrostomidae; Macrostomum"]
XP_004365821.1[&Organism="Capsaspora owczarzaki ATCC 30864",Description="transmembrane GTPase Marf [Capsaspora owczarzaki ATCC 30864]","Genetic Code"="Standard",Taxonomy="Eukaryota; Filasterea; Capsaspora"]
NP_495161.1[&Organism="Caenorhabditis elegans",Description="Transmembrane GTPase fzo-1 [Caenorhabditis elegans]","Genetic Code"="Standard",Taxonomy="Eukaryota; Metazoa; Ecdysozoa; Nematoda; Chromadorea; Rhabditida; Rhabditina; Rhabditomorpha; Rhabditoidea; Rhabditidae; Peloderinae; Caenorhabditis"]
XP_014153836.1[&Organism="Sphaeroforma arctica JP610",Description="hypothetical protein SARC_07694 [Sphaeroforma arctica JP610]","Genetic Code"="Standard",Taxonomy="Eukaryota; Ichthyosporea; Ichthyophonida; Sphaeroforma"]
XP_001745740.1[&Organism="Monosiga brevicollis MX1",Description="uncharacterized protein MONBRDRAFT_25320 [Monosiga brevicollis MX1]","Genetic Code"="Standard",Taxonomy="Eukaryota; Choanoflagellata; Craspedida; Salpingoecidae; Monosiga"]
;
end;

begin characters;
dimensions nchar=427;
format datatype=protein missing=? gap=-;
matrix
XP_006812840.1 ITKPSC-LILAVTP----------------GN---SDLANSDALK----VAKEVDPQGLRTIG-VITKLDLLDDGTDAREILENKLLP--------LRR-----------------------------------ASEWGEFLH----------------------------------CKGKKFT---NFDEIRMEIEAETDRLTGKN---

KGISPIPINLRVYSPHV----------LNLTLIDLPGMTKVPVGDQP--ADIEQQIR--------
SMLMEFITKPSCLILAVTPGNSDLANS-DALKVAKEVDPQGLRTIGVI-TKLDLLDDG-TD----
AREILENK-LLPLRR-GYIGVVNRGQKDIEGRKDIKSALASERKFFLSHPSY--RHM--ADR-LGT--
PYLQKALNQQLTNHIRDTL

XP_012378586.1 AGGPR----RRQSG---------------RAA----AAAEPEPERN--
FVGRDFLPRG---SG-IVTRRPLV-------------LQL--------VNA-----------------------------------
TTEYAEFLH----------------------------------CKGKKFT---DFEEVRLEIEAETDRVTGTN---
KGISPVPINLRVYSPHV----------LNLTLVDLPGMTKVPVGDQP--PDIEFQIR--------
DMLMQFVTKENCLILAVSPANSDLANS-DALKVAKEVDPQGQRTIGVI-TKLDLMDEG-TD----
ARDVLENK-LLPLRR-GYIGVVNRSQKDIDGKKDITAALAAERKFFLSHPSY--RHL--ADR-MGT--
PYLQKVLNQQLTNHIRDTL

EPQ17174.1 LDLPQ----IAVVG----------------GQ----SAGKSSVLEN--FVGRDFLPRG---
SG-IVTRRPLV-------------LQL--------VNA-----------------------------------STEYAEFLH----------------
------------------CKGKKFT---DFEEVRLEIEAETDRVTGTN---KGISPVPINLRVYSPHV----------
LNLTLVDLPGMTKVPVGDQP--ADIEFQIR--------DMLMQFVTKENCLILAVSPANSDLANS-
DALKIAKEVDPQGQRTIGVI-TKLDLMDEG-TD----ARDVLENK-LLPLRR-
GYIGVVNRSQKDIDGKKDITAALAAERKFFLSHPSY--RHL--ADR-MGT--
PYLQKVLNQQLTNHIRDTL

ELW62001.1 LDLPQ----IAVVG----------------GQ----SAGKSSVLEN--FVGRDFLPRG---
SG-IVTRRPLV-------------LQL--------VNA-----------------------------------TTEYAEFLH-----------------
-----------------CKGKKFT---DFEEVRLEIEAETDRVTGTN---KGISPVPINLRVYSPHV----------
LNLTLVDLPGMTKVPVGDQP--PDIEFQIR--------DMLMQFVTKENCLILAVSPANSDLANS-
DALKIAKEVDPQGQRTIGVI-TKLDLMDEG-TD----ARDVLENK-LLPLRR-
GYIGVVNRSQKDIDGKKDITAALAAERKFFLSHPSY--RHL--ADR-MGT--
PYLQKVLNQQLTNHIRDTL

EAW87759.1 LDLPQ----IAVVG----------------GQ----SAGKSSVLEN--FVGRDFLPRG---
SG-IVTRRPLV-------------LQL--------VNA-----------------------------------TTEYAEFLH-----------------
-----------------CKGKKFT---DFEEVRLEIEAETDRVTGTN---KGISPVPINLRVYSPHV----------
LNLTLVDLPGMTKVPVGDQP--PDIEFQIR--------DMLMQFVTKENCLILAVSPANSDLANS-
DALKVAKEVDPQGQRTIGVI-TKLDLMDEG-TD----ARDVLENK-LLPLRR-
GYIGVVNRSQKDIDGKKDITAALAAERKFFLSHPSY--RHL--ADR-MGT--
PYLQKVLNQQLTNHIRDTL

BAB27759.1 LDLPQ----IAVVG----------------GQ----SAGKSSVLEN--FVGRDFLPRG---
SG-IVTRRPLV-------------LQL--------VNS-----------------------------------TTEYAEFLH-----------------
-----------------CKGKKFT---DFEEVRLEIEAETDRVTGTN---KGISPVPINLRVYSPHV----------
LNLTLVDLPGMTKVPVGDQP--PDIEFQIR--------DMLMQFVTKENCLILAVSPANSDLANS-
DALKIAKEVDPQGQRTIGVI-TKLDLMDEG-TD----ARDVLENK-LLPLRR-
GYIGVVNRSQKDIDGKKDITAALAAERKFFLSHPSY--RHL--ADR-MGT--
PYLQKVLNQQLTNHIRDTL

XP_025915522.1 ---

---MTKVPVGDQP--PDIEFQIR--------
DMLMQFVTKENCLILAVSPANSDLANS-DALKIAKEVDPQGQRTIGVI-TKLDLMDEG-TD----

ARDVLENK-LLPLRR-GYIGVVNRSQKDIDGKKDIQAALAAERKFFLSHPAY--RHM--ADR-MGT--PYLQKVLNQQLTNHIRDTL

XP_028570166.1 LDLPQ----IAVVG----------------GQ----SAGKSSVLEN--FVGRDFLPRG---SG-IVTRRPLV-------------LQL--------VNS----------------------------------PTEYGEFLH----------------------------------CKGKKFT---DFDEIRQEIEAETDRITGSN---KGISPVPINLRVYSPHV----------LSLTLVDLPGMTKVPVGDQP--ADIEFQIR--------EMLMQFVTKENCLILAVSPANSDLANS-DALKIAKEVDPQGQRTIGVI-TKLDLMDEG-TD----ARDVLENK-LLPLRR-GYIGVVNRSQKDIDGKKDIQAALAAERKFFLTHPAY--RHM--ADR-MGT--PYLQKVLNQQLTNHIRDTL

KAE8583055.1 LDLPQ----IAVVG----------------GQ----SAGKSSVLEN--FVGKDFLPRG---SG-IVTRRPLV-------------LQL--------VNS----------------------------------STEYGEFLH----------------------------------CKGKKFT---DFDEIRLEIEAETDRATGTN---KGISPVPINLRVYSPNV----------LNLTLVDLPGMTKVPVGDQP--VDIEFQIR--------DMLMQFVTKENCLVLAVSPANSDLANS-DALKIAKEVDPKGLRTIGVI-TKLDLMDEG-TD----ARDVLENK-LLPLRR-GYIGVVNRSQKDIDGKKDIQAALAAERKFFLSHPSY--RHL--ADR-MGT--PYLQKALNQQLTNHIRDTL

XP_005165639.1 LDLPQ----IAVVG----------------GQ----SAGKSSVLEN--FVGKDFLPRG---SG-IVTRRPLV-------------LQL--------INC----------------------------------PTEYAEFLH----------------------------------CKGKKFT---DFDEVRQEIEAETDRITGQN---KGISPVPINLRVYSPNV----------LNLTLVDLPGMTKVPVGDQP--ADIEAQIR--------DMLMQFVTKENCLLLAVSPANSDLANS-DALKIAKEVDPQGMRTIGVI-TKLDLMDEG-TD----AREILENK-LLPLRR-GYIGVVNRSQKDIDGKKDITAAMSAERKFFLTHPSY--RHL--ADR-MGT--PYLQKALNQQLTNHIRDTL

XP_032814666.1 LDLPQ----IAVVG----------------GQ----SAGKSSVLEN--FVGRDFLPRG---SG-IVTRRPLI-------------LQL--------MFC----------------------------------KAEYAEFLH----------------------------------CKGKKFT---DFEEVRAEIEAETDRLTGSN---KGISPIPINLRVYSPHV----------LNLTLIDLPGMTKVPVGDQP--VDIEYQIR--------EMLMQFVTKENCLILAVSPANTDLANS-DALKIAKEVDPQGLRTIGVI-TKLDLMDDG-TD----ARDILENK-LLPLRR-GYIGVVNRSQKDIDGRKDINAAMAAERKFFLSHPSY--RHM--ADR-MGT--PYLQKTLNQQLTNHIRDTL

XP_012379251.1 ---VGRDFLPRG---SG-IVTRRPLV-------------LQL--------VTS----------------------------------KAEYAEFLH----------------------------------CKGKKFT---DFDEVRHEIEAETDRVTGMN---KGISSIPINLRVYSPHV----------LNLTLIDLPGITKVPVGDQP--PDIEYQIR--------EMIMQFITRENCLILAVTPANTDLANS-DALKLAKEVDPQGLRTIGVI-TKLDLMDEG-TD----ARDVLENK-LLPLRR-GYVGVVNRSQKDIDGKKDIKAAMLAERKFFLSHPAY--RHI--ADR-MGT--PHLQKVLNQQLTNHIRDTL

XP_006496668.1 LELPQ----IAVVG----------------GQ----SAGKSSVLEN--FVGRDFLPRG---SG-IVTRRPLV-------------LQL--------VTS----------------------------------KAEYAEFLH----------------------------------CKGKKFT---DFDEVRHEIEAETDRVTGMN---KGISSIPINLRVYSPHV----------LNLTLIDLPGITKVPVGDQP--PDIEYQIR--------DMIMQFITRENCLILAVTPANTDLANS-DALKLAKEVDPQGLRTIGVI-TKLDLMDEG-TD----ARDVLENK-LLPLRR-GYVGVVNRSQKDIDGKKDIKAAMLAERKFFLSHPAY--RHI--ADR-MGT--PHLQKVLNQQLTNHIRDTL

XP_016856477.1 LELPQ----IAVVG----------------GQ----SAGKSSVLEN--FVGRDFLPRG---SG-IVTRRPLV-------------LQL--------VTS----------------------------------KAEYAEFLH----------------------------------CKGKKFT---DFDEVRLEIEAETDRVTGMN---KGISSIPINLRVYSPHV----------LNLTLIDLPGITKVPVGDQP--PDIEYQIR--------EMIMQFITRENCLILAVTPANTDLANS-DALKLAKEVDPQGLRTIGVI-TKLDLMDEG-TD----ARDVLENK-LLPLRR-GYVGVVNRSQKDIDGKKDIKAAMLAERKFFLSHPAY--RHI--ADR-MGT--PHLQKVLNQQLTNHIRDTL

XP_027623811.1 LELPQ----IAVVG----------------GQ----SAGKSSVLEN--FVGRDFLPRG---SG-IVTRRPLV-------------LQL--------VTS----------------------------------KAEYGEFLH----------------------------------CKGKKFT---DFDEIRHEIEAETDRVTGVN---KGISSIPINLRVYSPHV----------LNLTLIDLPGITKVPVGDQP--PDIEYQIR--------EMIMQFITRENCLILAVTPANTDLANS-DALKLAKEVDPQGLRTIGVI-TKLDLMDEG-TD----ARDVLENK-LLPLRR-GYVGVVNRSQKDIDGKKDIKAAMLAERKFFLSHPAY--RHI--ADR-MGT--PHLQKVLNQQLTNHIRDTL

EPQ08653.1 LELPQ----IAVVG----------------GQ----SAGKSSVLEN--FVGRDFLPRG---SG-IVTRRPLV-------------LQL--------VTS----------------------------------KTEYAEFLH----------------------------------CKGKKFT---DFDEVRHEIEAETDRVTGMN---KGISSIPINLRVYSPHV----------LNLTLIDLPGITKVPVGDQP--VDIEHQIR--------EMIMQFITRENCLILAVTPANTDLANS-DALKLAKDVDPQGLRTIGVI-TKLDLMDEG-TD----ARDILENK-LLPLRR-GYVGVVNRSQKDIDGKKDIKAAMLAERKFFLSHPAY--RHI--ADR-MGT--PHLQKVLNQQLTNHIRDTL

XP_025944940.1 --MFFRDFLPRG---SG-IVTRRPLV-------------LQL--------VTA----------------------------------KTEYAEFLH----------------------------------CKGRKFT---DFDEVRQEIEVETDRITGVN---KGISSIPINLRIYSPHV----------LSLTLIDLPGITKVPVGDQP--PDIEQQIR--------DMIMQFISRENCLILAVTPANTDLANS-DALKLAKEVDPQGLRTIGVI-TKLDLMDEG-TD----AREILENK-LLPLRR-GYIGVVNRSQKDIDGKKDIKAALLAERKFFLSHPAY--RHM--ADR-MGT--PYLQKVLNQQLTNHIRDTL

XP_014389433.1 ------------------------------------AGKGQI------TAWDFLPRG---SG-IVTRRPLI-------------LQL--------IFS----------------------------------KTEYAEFLH----------------------------------CKSKKFT---DFDEVRQEIEAETDRVTGTN---KGISPVPINLRVYSPHV----------LNLTLIDLPGITKVPVGDQP--PDIEYQIK--------DMILQFISRESSLILAVTPANMDLANS-DALKMAKEVDPQGLRTIGVI-TKLDLMDEG-TD----ARDVLENK-LLPLRR-GYIGVVNRSQKDIEGKKDIRAALAAERKFFLSHPAY--RHM--ADR-MGT--PHLQKTLNQQLTNHIRESL

NP_001005360.1 LDLPQ----IAVVG----------------GQ----SAGKSSVLEN--FVGRDFLPRG---SG-IVTRRPLI-------------LQL--------IFS----------------------------------KTEHAEFLH----------------------------------CKSKKFT---DFDEVRQEIEAETDRVTGTN---KGISPVPINLRVYSPHV----------LNLTLIDLPGITKVPVGDQP--PDIEYQIK--------DMILQFISRESSLILAVTPANMDLANS-DALKLAKEVDPQGLRTIGVI-TKLDLMDEG-TD----ARDVLENK-LLPLRR-GYIGVVNRSQKDIEGKKDIRAALAAERKFFLSHPAY--RHM--ADR-MGT--PHLQKTLNQQLTNHIRESL

XP_006510037.1 LDLPQ----IAVVG----------------GQ----SAGKSSVLEN--FVGRDFLPRG---SG-IVTRRPLI-------------LQL--------IFS----------------------------------

KTEYAEFLH----------------------------------CKSKKFT---DFDEVRQEIEAETDRVTGTN---
KGISPVPINLRVYSPHV----------LNLTLIDLPGITKVPVGDQP--PDIEYQIK--------
DMILQFISRESSLILAVTPANMDLANS-DALKLAKEVDPQGLRTIGVI-TKLDLMDEG-TD----
ARDVLENK-LLPLRR-GYIGVVNRSQKDIEGKKDIRAALAAERKFFLSHPAY--RHM--ADR-MGT--
PHLQKTLNQQLTNHIRESL

XP_025920181.1 LDLPQ----IAVVG----------------GQ----SAGKSSVLEN--
FVGRDFLPRG---SG-IVTRRPLI-------------LQL--------IFS-----------------------------------
KTEYAEFLH----------------------------------CKSKKFT---DFDEVRQEIEAETDRVTGTN---
KGISPVPINLRVYSPHV----------LNLTLIDLPGITKVPVGDQP--QDIEYQIK--------
DMIMQFISRESSLILAVTPANMDLANS-DALKMAKEVDPQGLRTIGVI-TKLDLMDEG-TD----
ARDVLENK-LLPLRR-GYIGVVNRSQKDIDGKKDIRAALAAERKFFLSHPAY--RHM--ADR-MGT--
PHLQKVLNQQLTNHIRETL

XP_028568434.1 LDLPQ----IAVVG----------------GQ----SAGKSSVLEN--
FVGRDFLPRG---SG-IVTRRPLI-------------LQL--------IFS-----------------------------------
KTEYAEFLH----------------------------------CKSKKFT---DFDEVRQEIEAETDRVTGTN---
KGISPVPINLRVYSPHV----------LNLTLIDLPGITKVPVGDQP--QDIEYQIK--------
DMILQFISRESSLILAVTPANMDLANS-DALKMAKEVDPQGLRTIGVI-TKLDLMDEG-TD----
ARDVLENK-LLPLRR-GYIGVVNRSQKDIDGKKDIRAALAAERKFFLSHPAY--RHM--ADR-MGT--
PHLQKLLNQQLTNHIRETL

XP_012381548.1 ---

MILQFIGRESSLILAVTPANMDLANS-DALKLAKEVDPQGLRTIGVI-TKLDLMDEG-TD----
ARDVLENK-LLPLRR-GYIGVVNRSQKDIEGRKDIRSALAAERKFFFSHSAY--RHM--ADR-MGT--
LHLQKTLNQQLTNHIRESL

XP_006161648.2.2 ---PH----CTSVS----------------
RQAYSSPQGPRKVISVLFLPHRDFLPRG---SG-IVTRRPLI-------------LQL--------IFS-----------------
------------------KTEYAEFLH----------------------------------CKSKKFT---
DFDEVRQEIEAETDRVTGTN---KGISPVPINLRVYSPHV----------LNLTLIDLPGITKVPVGDQP--
PDIEYQIK--------DMILQFISRESSLILAVTPANMDLANS-DALKLAKEVDPQGLRTIGVI-
TKLDLMDEG-TD----ARDVLENK-LLPLRR-GYIGVVNRSQKDIEGKKDIRAALAAERKFFLSHPAY--
RHM--ADR-MGT--PHLQKTLNQQLTNHIRESL

XP_031753735.1 LDLPQ----IAVVG----------------GQ----SAGKSSVLEN--
FVGRDFLPRG---SG-IVTRRPLI-------------LQL--------IFS-----------------------------------
KTEYAEFLH----------------------------------CKSKKFT---DFDEVRQEIEAETDRVTGTN---
KGISPVPINLRVYSPNV----------LNLTLIDLPGITKVPVGDQP--HDIEYQIK--------
DMILQFISRDSCLILAVTPGNTDLANS-DALKMAKEVDPQGLRTIGVI-TKLDLMDEG-TD----
AKDILENK-LLPLRR-GYIGVVNRSQKDIDGKKDIKAALGAERKFFLSHPGY--RHI--AER-MGT--
PHLQKTLNQQLTNHIRETL

XP_021326548.1 LDLPQ----IAVVG----------------GQ----SAGKSSVLEN--
FVGRDFLPRG---SG-IVTRRPLI-------------LQL--------VNN-----------------------------------
KAEYAEFLH----------------------------------CKGRKFV---DFDEVRQEIEAETDRITGSN---
KGISPIPINLRVYSPNV----------LNLTLIDLPGMTKVAVGDQP--PDIEHQIR--------
DMIMQFITRESCLILAVTPANMDLANS-DALKVAKEVDPQGLRTIGVI-TKLDLMDEG-TD----

ARDILENK-LLPLRR-GYIGVVNRSQKDIDGRKDIRAALAAERKFFLSHPSY--RHM--AER-MGT--PHLQKALNQQLTNHIRDTL

NP_001025299.1 LDLPQ----IAVVG----------------GQ----SAGKSSVLEN--FVGRDFLPRG---SG-IVTRRPLI-------------LQL--------VNN-----------------------------------KAEYAEFLH----------------------------------CKGRKFV---DFDEVRQEIEAETDRITGSN---KGISPIPINLRVYSPNV----------LNLTLIDLPGMTKVAVGDQP--PDIEHQIR--------DMIMQFITRESCLILAVTPANMDLANS-DALKVAKEVDPQGLRTIGVI-TKLDLMDEG-TD----ARDILENK-LLPLRR-GYIGVVNRSQKDIDGRKDIRAALAAERKFFLSHPSY--RHM--AER-MGT--PHLQKALNQQLTNHIRDTL

XP_035683496.1 LDLPQ----IAVVG----------------GQ----SAGKSSVLEN--FVGRDFLPRG---SG-IVTRRPLV-------------LQL--------IHNP----------------------------------KAEYGEFLH----------------------------------AKGKMFS---DFHEIRAEIEAETDRMTGSN---KGISPVPINLRVYSPHV----------LNLTLIDLPGMTKVPVGDQP--PDIEQQIR--------DMLLQFITKDNCLILAVSPANQDLANS-DALKIAKEVDPQGMRTIGVI-TKLDLMDEG-TD----ARNILENR-TYPLRR-GYIGVVNRSQADIDGRKDIKAALAAERKFFLSHPAY--RHL--ADR-MGT--PYLQKTLNQQLTNHIRDTL

XP_030853442.1.2 LDLPQ----IAVVG----------------GQ----SAGKSSVLEN--FVGRDFLPRG---SG-IVTRRPLV-------------LQL--------NNS-----------------------------------KTEYGEFLH----------------------------------CKGKKFT---DFDEIRKEIEAETDRVTGSN---KGISNVPINLRVYSPNV----------LNLTLVDLPGMTKIAVGDQP--VDIEIQIR--------SMVMEFVTNESTLILAVSPANQDLANS-DALKVAKEVDPKGVRTIGVI-TKLDLMDDG-TD----AKDILENK-LLPLRR-GYVGVVNRSQRDIEGKKDIKAALAAERKFFLSHPSY--RHI--ADK-MGT--PWLQKILNQQLTNHIRDSL

XP_030853442.1 LDLPQ----IAVVG----------------GQ----SAGKSSVLEN--FVGRDFLPRG---SG-IVTRRPLV-------------LQL--------NNS-----------------------------------KTEYGEFLH----------------------------------CKGKKFT---DFDEIRKEIEAETDRVTGSN---KGISNVPINLRVYSPNV----------LNLTLVDLPGMTKIAVGDQP--VDIEIQIR--------SMVMEFVTNESTLILAVSPANQDLANS-DALKVAKEVDPKGVRTIGVI-TKLDLMDDG-TD----AKDILENK-LLPLRR-GYVGVVNRSQRDIEGKKDIKAALAAERKFFLSHPSY--RHI--ADK-MGT--PWLQKILNQQLTNHIRDSL

KMZ10000.1 LDLPQ----IAVVG----------------GQ----SAGKSSVLEN--FVGKDFLPRG---SG-IVTRRPLI-------------LQL--------ING-----------------------------------VTEYGEFLH----------------------------------IKGKKFS---SFDEIRKEIEDETDRVTGSN---KGISNIPINLRVYSPHV----------LNLTLIDLPGLTKVAIGDQP--VDIEQQIK--------QMIFQFIRKETCLILAVTPANTDLANS-DALKLAKEVDPQGVRTIGVI-TKLDLMDEG-TD----ARDILENK-LLPLRR-GYIGVVNRSQKDIEGRKDIHQALAAERKFFLSHPSY--RHM--ADR-LGT--PYLQRVLNQQLTNHIRDTL

XP_026693152.1 IDLPQ----IAVVG----------------GQ----SAGKSSVLEN--FVGKDFLPRG---SG-IVTRRPLV-------------LQL--------ITAK----------------------------------NGEWGEFLH----------------------------------CKGKKFT---DFNEIRKEIEEETDRMTGSN---KGISAIPINLRVHSPHV----------LNLTLVDLPGMTKVPVGDQP--ADIEQQIR--------DMIMQFVVKDNCLILAVSPANSDLANS-DALKIAKEFDPQGIRTIGVI-TKLDLMDEG-TD----AKHILENK-HLPLRR-GYVGVVNRSQKDIDGNKDIKAALSAERRFFLSHPAY--RHM--ADK-LGT--PYLQKILNQQLTNHIKETL

PAA65118.1 FDLPQ----IAVVG----------------SQ----SAGKSSVLEN--FVGKDFLPRG---SG-IVTRRPLI-------------LQL--------LYNP---------------------------------SAEYAEFGH----------------------------------QRGRKYT---NFEEVRQEIEAETDRLTGRN---KGISNVPIMLRVFSPHV----------LNLTLVDLPGMTKVAVGDQP--PDIEVQIR--------NMLLEFITKENCLILAVSPANSDLANS-DALKLAKEVDPAGTRTIGVI-TKLDLMDQG-TD----AREVLENK-LLPLRR-GYIGVVNRSQKDIEGRKDIKAAMAAERKFFLSHPSY--RHM--AER-MGT--PYLQRCLNQQLTNHIRETL

PAA78248.1 FDLPQ----IAVVG----------------SQ----SAGKSSVLEN--FVGKDFLPRG---SG-IVTRRPLV-------------LQL--------LTH----------------------------------PSEFAEFGH----------------------------------LRGKKFT---NFDEVRQEIENETDRLTGKN---KGISNVPITLRVFSPHV----------LNLTLVDLPGMTKVAVGDQP--PDIEQQIR--------AMLFEFISKENCLILAVSPANSDLANS-DALKIAKEVDPNGTRTIGVI-TKLDLMDQG-TD----AREVLENK-LLPLRR-GYIGVVNRSQKDIEGKKDIAAAMAAERKFFLSHPSY--RHM--AER-MGT--PYLQRCLNQQLTNHIRETL

PAA59145.1 FDLPQ----IAVVG----------------SQ----SAGKSSVLEN--FVGKDFLPRG---SG-IVTRRPLV-------------LQL--------INF----------------------------------HTEYAEFGH----------------------------------IRGKRFT---NFDEVRQEIENETDRVTGKN---KGISNVPIMLRVYSPQV----------LNLTLVDLPGLTKVAVGDQP--QDIELLIR--------AMILEFVSKDNCLILAVTPANSDLANS-DALKIAKEVDPSGTRTIGVI-TKLDLMDQG-TD----ARDVLENR-LLPLRR-GYIGVVNRSQKDIEGKKDIVAAMAAERKFFLSHPAY--RHM--AER-MGT--SYLQRCLNQQLTNHIRETL

PAA64382.1 IDLPQ----IAVVG----------------SQ----SAGKSSVLEN--FVGRDFLPRG---SG-IVTRRPLI-------------LQL--------MNY----------------------------------QTEYAEFGH----------------------------------IRGKKFV---NFDEVRREIEVETDRLTGQN---KGISNVPITLRVYSPQV----------LNLTLVDLPGMTKVAVGDQP--PDIEQQVR--------AMIWEFISKDNCLILAVSPANSDLANS-DALKLAKEADPSGSRTIGVL-TKLDLMDAG-TD----ARDVLENR-FLPLRR-GYVGVVNRSQKDIDGRKDISSAMAAERKFFLGHPAY--RHM--AER-MGT--AHLQRCLNQQLVGHIRDTL

XP_001749319.1 LDLPQ----IAVVG----------------GQ----SAGKSSVLEN--FVGKDFLPRG---SG-IVTRRPLV-------------LQL--------NYHP---------------------------------SAEWGEFLH----------------------------------ARGKKFT---DFNEIRQEIEAETDRMTGSN---KGISNIPINLRVYSPHV----------LNLTLVDLPGLTKVAVGDQP--ADIENQIR--------GMLMEFITKDNCIILAVTPANQDLANS-DALKLAKEVDPEGVRTIGVI-TKLDLMDSG-TD----ARAILTNE-FLPLRR-GYIGVVNRSQKDIDGRKDIRAALDAERKFFLMHPSY--KDI--ASK-NGT--PYLQKALNQQLTNHIRECL

NP_001024332.1 FELPQ----IAVVG----------------GQ----SAGKSSVLEN--FVGKDFLPRG---SG-IVTRRPLI-------------LQL--------IQD----------------------------------RNEYAEFLH----------------------------------KKGHRFV---DFDAVRKEIEDETDRVTGQN---KGISPHPINLRVFSPNV----------LNLTLIDLPGLTKVPVGDQP--ADIEQQIR--------DMILTFINRETCLILAVTPANSDLATS-DALKLAKEVDPQGLRTIGVL-TKLDLMDEG-TD----AREILENK-LFTLRR-GYVGVVNRGQKDIVGRKDIRAALDAERKFFISHPSY--RHM--ADR-LGT--SYLQHTLNQQLTNHIRDTL

XP_004347890.1 LDLPQ----IAVVG----------------SQ----SAGKSSVLEN--FVGKDFLPRG---SG-IVTRRPLV-------------LQL--------VNSK---------------------------------

GPEYGEFLH---------------------------------NKSKKFT---DFDEVRKEIEAETDRITGTN---
KGISPVPINLKVYSPNV----------LNLTLVDLPGITKVPIGDQP--TNIESLIR--------
EMIMQFIGRPNCLILAVSPANSDLANS-DALKLAREVDQQGIRTIGVI-TKLDLMDEG-TD----
AREVLENK-LIPLRR-GFIGVVNRSQKDIDGRKDIKAAMSAELRFFSTHPAY--RDL--ANK-NGT--
MYLQRVLNQQLTNHIRDTL

XP_031757197.1 VDLPQ----IAVVG----------------GQ----SAGKSSVLEN--LVGRWI----
-----------------------------H--------VLS---------------------------------STEYAEFLH--------------------
-------------CKGTKYT---DFSEVRQEIEEETERATGLN---KGISAIPISLRIYSPHV----------
LNLSLIDLPGVTKVPVGDQP--ADIETQIR--------DMIMNFISRENCLILAVTPANTDLANS-
DALKLAKEVDPQGLRTIGVI-TKLDLMDEG-TN----AQEILENK-LLPLRR-
GYVGVVNRSQKDIDGKKNINAALQAEQMFFLTHPAY--RHM--ADR-MGT--
SHLQKMLNQQLTNHIRETL

XP_014153758.1 N--
-------LYL--YIEWGEFLH---------------------------------
QPGRKYT---DFEEIMKEIEAETDRMTGSN---KGISNIPINLKVMSPHV----------
LDLTLVDLPGLTKVAVGDQP--ADIEQQIL--------GMIMEFITRPNCLILAVSPANADLANS-
DALKLAKEVDPQGLRTIGVI-TKLDLMDQG-TD----AREILENK-LLPLRR-
GYIGVVNRSQKDITGKKDIRAAQEAERRFFSTHPAY--RHL--AQN-MGT--
PKLQKVLNQQLTNHIRDSL

XP_014148725.1 ---
--TEYGEFLH---------------------------------
KPGRLFD---NFDEIRNEIEADTARITGAN---KGISHLPINLKVYSPHV----------
LDLTLVDLPGLTKVAVGDQP--ADIEMQIK--------NMIMEFITKPNCLILAVTPANSDLANS-
DALKLAKEVDPQGLRTIGVI-TKLDLMDAG-TD----ARDVLENK-LLPLRR-
GYVGVVNRSQKDIAGNKDIRAAQAAEKKFFKTHPAY--RHL--ADK-MGT--
PKLQQVLNQQLTDHIRQTL

NP_741403.2 IQLPQ----IVVVG----------------SQ----SAGKSSVLEN--LVGRDFLPRG---TG-
IVTRRPLI-------------LQL--------NHVALDDESKRRRSNG-------------------TLLTDDWAMFEH-------
---------------------------TGSKVFT---DFDAVRKEIEDETDRVTGVN---KGISLLPISLKIYSHRV----------
VSLSLVDLPGITKIPVGDQP--VNIEEQIR--------EMILLYISNPSSIILAVTPANQDFATS-
EPIKLAREVDAGGQRTLAVL-TKLDLMDQG-TD----AMDVLMGK-VIPVKL-
GIIGVVNRSQQNILDNKLIVDAVKDEQSFMQKK--Y--PTL--ASR-NGT--
PYLAKRLNMLLMHHIRNCL

NP_957216.1 IQLPQ----IAVVG----------------TQ----SSGKSSVLES--LVGRDLLPRG---TG-
IVTRRPLI-------------LQL--------VHVDPEDRRKTS-EEN-------------------GVDGEEWGKFLH--------
-------------------------TKNKIYT---DFDEIRQEIENETERVSGNN---KGISDEPIHLKIFSPHV----------
VNLTLVDLPGITKVPVGDQP--KDIELQIR--------ELILKYISNPNSIILAVTAANTDMATS-
EALKVAREVDPDGRRTLAVV-TKLDLMDAG-TD----AMDVLMGR-VIPVKL-
GLIGVVNRSQLDINNKKSVADSIRDEHGFLQKK--Y--PSL--ANR-NGT--
KYLARTLNRLLMHHIRDCL

NP_001317309.1 IQLPQ----IVVVG----------------TQ----SSGKSSVLES--
LVGRDLLPRG---TG-IVTRRPLI-------------LQL--------VHVSQEDKRKTTGEEN------
DPATWKNSRHLSKGVEAEEWGKFLH---------------------------------TKNKLYT---
DFDEIRQEIENETERISGNN---KGVSPEPIHLKIFSPNV----------VNLTLVDLPGMTKVPVGDQP--

KDIELQIR--------ELILRFISNPNSIILAVTAANTDMATS-EALKISREVDPDGRRTLAVI-TKLDLMDAG-TD----AMDVLMGR-VIPVKL-GIIGVVNRSQLDINNKKSVTDSIRDEYAFLQKK--Y--PSL--ANR-NGT--KYLARTLNRLLMHHIRDCL

XP_006168142.1 IQLPQ----IVVVG----------------TQ----SSGKSSVLES--LVGRDLLPRG---TG-IVTRRPLI-------------LQL--------VHVSPEDKRKTTGEEN------DPATWKNSRHLSKGVEAEEWGKFLH---------------------------------TKNKLYT---DFDEIRQEIENETERISGNN---KGVSPEPIHLKIFSPNV----------VNLTLVDLPGMTKVPVGDQP--KDIELQIR--------ELILRFISNPNSIILAVTAANTDMATS-EALKISREVDPDGRRTLAVI-TKLDLMDAG-TD----AMDVLMGR-VIPVKL-GIIGVVNRSQLDINNKKSVTDSIRDEYAFLQKK--Y--PSL--ANR-NGT--KYLARTLNRLLMHHIRDCL

NP_001392186.1 IQLPQ----IVVVG----------------TQ----SSGKSSVLES--LVGRDLLPRG---TG-VVTRRPLI-------------LQL--------VHVSPEDKRKTTGEENGKFQSWNPATWKNSRHLSKGVEAEEWGKFLH---------------------------------TKNKLYT---DFDEIRQEIENETERISGNN---KGVSPEPIHLKVFSPNV----------VNLTLVDLPGMTKVPVGDQP--KDIELQIR--------ELILRFISNPNSIILAVTAANTDMATS-EALKISREVDPDGRRTLAVI-TKLDLMDAG-TD----AMDVLMGR-VIPVKL-GIIGVVNRSQLDINNKKSVTDSIRDEYAFLQKK--Y--PSL--ANR-NGT--KYLARTLNRLLMHHIRDCL

XP_014394711.1 LLCPV----CVQIS----------------SL----SSGKSSVLES--LVGRDLLPRG---TG-IVTRRPLI-------------LQL--------VHVSPEDQRKTSGEEN------DPATWKNSRHLSKGVEAEEWGKFLH---------------------------------TKNKLYT---DFDEIRQEIENETERISGNN---KGVSPEPIHLKIFSPNV----------VNLTLVDLPGMTKVPVGDQP--KDIELQIR--------ELILRFISNPNSIILAVTAANTDMATS-EALKISREVDPDGRRTLAVI-TKLDLMDAG-TD----AMDVLMGR-VIPVKL-GIIGVVNRSQLDINNKKSVTDSIRDEYAFLQKK--Y--PSL--ANR-NGT--KYLARTLNRLLMHHIRDCL

XP_028602039.1 IQLPQ----IVVVG----------------TQ----SSGKSSVLES--LVGRDLLPRG---TG-IVTRRPLI-------------LQL--------VNVSAEDLRKKTGDEN------DPATWKHARHLTKGVDTEEWGKFLH---------------------------------TKNKLYS---DFDEIRQEIESETERISGNN---KGISPEPIHLKVFSPNV----------VNLTLVDLPGMTKVPVGDQP--KDIELQIR--------ELILRFISNPNSIILAVTAANTDMATS-EALKIAREVDPDGRRTLAVI-TKLDLMDAG-TD----AMDVLMGR-VIPVKL-GIIGVVNRSQLDINNKKSVADSIRDEYGFLQKK--Y--PSL--ANR-NGT--KYLARTLNRLLMHHIRDCL

XP_025940269.1 IQLPQ----IVVVG----------------TQ----SSGKSSVLES--LVGRDLLPRG---TG-VVTRRPLI-------------LQL--------VHVSPEDGRKTAGDEN-------------------EIDAEEWGKFLH---------------------------------TKNKVYT---DFDEIRQEIENETERISGNN---KGISPEPIHLKIFSSNV----------VNLTLVDLPGMTKVPVGDQP--KDIELQIR--------ELILQFISNPNSIILAVTAANTDMATS-EALKIAREVDPDGRRTLAVI-TKLDLMDAG-TD----AMDVLMGR-VIPVKL-GIIGVVNRSQLDINNKKSVADSIRDEYGFLQKK--Y--PSL--ANR-NGT--KYLARTLNRLLMHHIRDCL

XP_012382650.2 ---G---FK-LVISTKLI-------------LCC--------FSLTT-----------------DPATWKNSRHLSKGVEAEEWGKFLH---------------------------------TKNKLYT---DFDEIRQEIENETERISGNN---KGVSPEPIHLKIFSPNV----------VNLTLVDLPGMTKVPVGDQP--KDIELQIR--------ELILRFISNPNSIILAVTAANTDMATS-EALKISREVDPDGRRTLAVI-TKLDLMDAG-TD----AMDVLMGR-VIPVKL-GIIGIVNRSQLDINNKKSVTDSIRDEYAFLQKK--Y--PSL--ANR-NGT--KYLARTLNRLLMHHIRDCL

XP_031753959.1 IGFGP----VLRVP----------------LQ----PAKCWAAKES--MVLSASLLPV---PH-LYERLLLS-------------VVF--------FSLIP-----------------DPNAWKIPKHFSKGVETEEWGKFLH---------------------------------TKNKIYT---DFDEIRQEIENETERISGNN---KGISSEPIHLKIFSPNV----------VNLTLVDLPGMTKVPVGDQP--KDIEIQIR--------ELILRYISNPNSIILAVTAANTDMATS-EALKIARESDPDGRRTLAVI-TKLDLMDAG-TD----AMDVLLGR-VIPVKL-GIIGVVNRSQLDINNKKSVADSIRDEYGFLQKK--Y--PSL--ANR-NGT--KYLARTLNRLLMHHIRDCL

XP_032819300.1 IQLPQ----IVVVG----------------AQ----SSGKSSVLES--LVGRDFLPRG---TG-IVTRRPLV-------------LQL--------VHVIPDERIRPGGEEN-------------------GVEAEEWGKFLH---------------------------------TKNKVYS---DFNEIRQEIENETERITGTN---KGISSEAIHLKIFSPHV----------LNLTLVDLPGITKVPVGDQP--VDIEQQIR--------ELIIKFIGNPNSIILAVTAANTDLATS-EALKIAREVDTDGRRTLAVI-TKLDLMDAG-TD----AMDILTGR-VIPVKL-GIIGVVNRSQLDINTKKTILDAMQDEQSFMQKK--Y--PSL--ANR-NGT--KFLGKTLNRLLMHHIRDCL

XP_035676386.1 IQLPQ----IVVIG----------------TQ----SSGKSSVLES--LVGRDFLPRG---TG-IVTRRPLV-------------LQL--------VHVNSEEKKRPSEDED----------GGHKQDIKEHAHVEEWGKFLH---------------------------------TKNKIYT---DFDEIRQEIENETDRVTGTN---KGIIDDAIHLKIYSPKV----------LNLTLVDLPGITKVPVGDQP--PDIEVQIR--------EMCLKYIANPNSIILAVTSANTDMATS-EALKFAKEVDPDGRRTLAVI-TKLDLMDAG-TD----AHDVLMGR-VIPVKL-GIIGVVNRSQMDINKRKPIEEAIKDEAAFMQRK--Y--PSL--ASR-NGT--SHLARTLNRLLMHHIRDCL

XP_006821224.1 --MATS-EAIKLSREVDEDGRRTLAVI-TKLDLMDAG-TD----AVEIICGR-VIPVKL-GIIGVINRSQMDINNKKPIQESVKDEAAFLQRK--Y--PAL--ASR-NGT--PYLAKTLNRLLMHHIRDCL

XP_030827871.1 IQLPQ----IVVVG----------------NQ----SSGKSSVLEG--LVGKDFLPRG---NG-IVTRRPLV-------------LQM--------VHVDPEDKRGASGEGE------------------EEITADEWGKFLH---------------------------------TKNKVYT---DFEEIREEIQNETDRMAGTN---KGIVHDAIHLRIYSPKV----------LNLTLVDLPGLTKVPVGDQP--EDIESQIR--------EMLVKYIGNPNSIILAVTSANTDMATS-ESLKLAKEIDPDGRRTLAVI-TKLDLMDAG-TD----AVDVLCGR-VIPVKL-GIIGVVNRSQMDINNKKVIDDAVKDESAFLQRK--Y--PAL--ASR-NGT--AYLARTLNRLLMHHIRDCL

NP_001259946.1 IQLPQ----IVVLG----------------SQ----SSGKSSVIES--VVGRSFLPRG---TG-IVTRRPLV-------------LQL--------IYSPLDDRENRSAENG------------------TSNAEEWGRFLH---------------------------------TK-KCFT---DFDEIRKEIENETERAAGSN---KGICPEPINLKIFSTHV----------VNLTLVDLPGITKVPVGDQP--EDIEAQIK--------ELVLKYIENPNSIILAVTAANTDMATS-EALKLAKDVDPDGRRTLAVV-TKLDLMDAG-TD----AIDILCGR-VIPVKL-GIIGVMNRSQKDIMDQKHIDDQMKDEAAFLQRK--Y--PTL--ATR-NGT--PYLAKTLNRLLMHHIRDCL

PAA85687.1 IQLPQ----IVVIG----------------TQ----SSGKSSVLES--LVGRDFLPRG---TG-IVTRRPLV-------------LQL--------VHLEADEK-DEAGDRP-------------------AAEEEEWGKFLH---------------------------------TKGKIYT---DFNEIRDEIARETDRIAGSG---KCVSIDPINLKIYSPHV----------VSLTLVDLPGITKVPVADQP--EDIEVQIR--------ALCIEYIKNPNSIILAVTPANTDMATS-

ESLKLAKEVDPQGKRTLAVI-TKLDLMDAG-TD----AHDLLLGR-VIPVKL-
GIIGVVNRSQADIKNQKQVKEAVRDESSFLQRR--Y--PSL--ASR-NGT--PYLARTLNRLLMHHIRDCL
XP_002129967.2 LQLPQ----IVVVG----------------VQ----SSGKSSVLEN--
LVGRDFLPRG---TG-IVTRCPLV-------------LQM--------IHTTNEDTAQCSNEGS---
SGNNDSDSSGESFKETNEEVKEWVKFQH---------------------------------TKGKIFR---
SFKQVKKEIELETQRLSGNN---KGISSEAIRLKIFSPKV----------LNLTLVDLPGLMKIPVGDQP--
DDIEEQAR--------NLILRYISNPNSIILAVTPANVDFATS-EALQMARIVDPDGCRTLAVV-
TKLDLMDAG-TD----AIDVLCGR-IVPVKL-GIIGIVNRSQLDINKGKSVQDAIKDEQAFLQKK--Y--
PSF--ANR-SGS--RYLSITLNRLLMHHIRDCL
XP_004348308.1 IQLPQ----IAVVG----------------SQ----SSGKSSVLEN--
IVGKDFLPRG---HG-IVTRRPLI-------------LQL--------VHRKPGSPRPALPDDP-------
SSSGGHTDDGIDGEDVEEWGEFLH----------------------------------APGKRFI---
SFAEIRKEIEAETDRVTGSN---KGISSKPINLRIYSPNV----------LNLTLVDLPGITKVPVGDQP--
EDIEKQIR--------TLVRSYISNPNCIILAVTPANVDLANS-DALKLAKTIDPEGNRTIGVC-
TKIDLMDAG-TD----AMDILSGR-VVPVKL-GFIGVVNRSQADINTAKPIADSLKSEEQFFKSHPAY--
QAI--AHR-CGT--AYLSKALNKLLMHHIRDCL
XP_014148015.1 ---
------LQL--------VHHPVQR----------------------------GGPAAEWGEFLH--------------------------------
--QPGKIYT---DFSKIRDEIANETDRLTGTN---KGISHTPINLKLYSPNM----------
LDLTLVDLPGITKIAVGDQP--EDIEVQIH--------QLIESYINNPNCIILAVTAANTDIANS-
DALKMAKKADPKGLRTIGVA-TKLDLMDAG-TD----ALDILTGK-VVASKL-
GFIGVVNRSQADINQKVSIETAREAEQEYFRTHPAY--KSL--YKQ-SGT--EYLTRRLNQLLMTHIRRCL
XP_001750431.1 IQLPQ----IVVVGAQASSPADHPALRMSHEQ----SSGKSSILEN--
VVGKDFLPRG---TG-IVTRVPLV-------------LQL--------VQTA----------------------------------
DDEWATFQH----------------------------------AGGKVFR---DFEQVRQEIVDQTERITGPG---
KAVSNEPIHLRVHSPNV----------VNLTLVDLPGLTKVAVADQP--QDIGPQIR--------
RLVRHYIDNPNSLILAVSPANADIANS-DSLQIAKEVDPQGDRTLAIV-TKLDLMDRG-TD----
AKALLSGE-VLPVKL-GIIGIVNRSQNDINCKTSIQDSLDNEKRFFRTH--Y--PEM--ADR-CGC--
AFLADTLHHLLLQHIRACL
XP_004466363.1 LSLPA----IAVIG----------------DQ----SSGKSSVLEA--LSGVA-
LPRG---SG-IVTRCPLV-------------LKL--------KKLTN---------------------------------EEKWRGKVTYE-
-------------------------------DYEIDIS---DASEVEEEINKAQNVIAGEG---LGISQKLINLEVCSPDV-----
-----PDLTLIDLPGITRVAVGNQP--ADIGWQIK--------CLIKKYITRQETINLVVVPSNVDIATT-
EALSMAQEVDPNGDRTIGIL-TKPDLVDRG-TE--DKVVDVVRNL-VCHLKK-
GYMIVRCRGQQDIQDRLSLATALQKERAFFENHENF--RVLLEEGK-ATV--PHLAERLTTELITHISKTL
XP_006156437.1 LALPA----IAVIG----------------DQ----SSGKSSVLEA--LSGVA-
LPRG---SG-IVTRCPLV-------------LKL--------KKLIN--------------------------------
EDKWRGKVSYQ--------------------------------DIEVEIT---DPSKVEPEINKAQNVIAGEG---
MGISHELISLEVSSPHV----------PDLTLIDLPGITRVAVGNQP--ADIGRQIK--------
TLIKKYIHKQETINLVVVPSNVDIATT-EALSMAQEVDPDGDRTIGIL-TKPDLVDKG-TE--
EKVVDVVRNL-VCHLKK-GYMIVKCRGQQDIQDRLSLAEALQREKVFFEEHPYF--SFLLEEGK-ATI--
PCLAERLTTELIMHISKSL
NP_002453.2.2 LALPA----IAVIG----------------DQ----SSGKSSVLEA--LSGVA-
LPRG---SG-IVTRCPLV-------------LKL--------KKLVN--------------------------------

EDKWRGKVSYQ--------------------------------DYEIEIS---DASEVEKEINKAQNAIAGEG---
MGISHELITLEISSRDV----------PDLTLIDLPGITRVAVGNQP--ADIGYKIK--------
TLIKKYIQRQETISLVVVPSNVDIATT-EALSMAQEVDPEGDRTIGIL-TKPDLVDKG-TE--
DKVVDVVRNL-VFHLKK-GYMIVKCRGQQEIQDQLSLSEALQREKIFFENHPYF--RDLLEEGK-ATV--
PCLAEKLTSELITHICKSL

NP_001127618.1 LALPA----IAVIG----------------DQ----SSGKSSVLEA--LSGVA-
LPRG---SG-IVTRCPLV-------------LKL--------KKLVN--------------------------------
EDKWRGKVSYQ--------------------------------DYEIEIS---DASEVEKEINKAQNTIAGEG---
MGISHELITLEISSRDV----------PDLTLIDLPGITRVAVGNQP--ADIGYKIK--------
TLIKKYIQRQETISLVVVPSNVDIATT-EALSMAQEVDPEGDRTIGIL-TKPDLVDKG-TE--
DKVVDVVRNL-VFHLKK-GYMIVKCRGQQEIQDQLSLSEALQREKIFFEDHPYF--RDLLEEGK-ATV--
PCLAEKLTSELITHICKSL

XP_017508130.1 LALPA----IAVIG----------------DQ----SSGKSSVLEA--LSGVA-
LPRG---SG-IVTRCPLV-------------LKL--------KKLTN--------------------------------EETWRGKVSYQ-
-------------------------------DFEAELS---DPSEVEREINRAQNSIAGEG---TGISHELISLEISSPHV-----
-----PDLTLIDLPGITRVAVGNQP--ADIGRQIK--------ALIRKYIYKQETINLVVVPSNVDIATT-
EALSMAQEVDPDGDRTIGIL-TKPDLVDRG-TE--DKVVDVVRNL-VCHLKK-
GYMIVKCRGQQDIQDQLSLAEALKKERAFFEDNPYF--RDLLEEGR-ATV--
PCLADKLTVELITHICKSL

NP_001003134.1 LALPA----IAVIG----------------DQ----SSGKSSVLEA--LSGVA-
LPRG---SG-IVTRCPLV-------------LKL--------KKLIN--------------------------------
EDEWRGKVSYQ--------------------------------DTEMEIS---DPSEVEVEINKAQDAIAGEG---
QGISHELISLEVSSPHV----------PDLTLIDLPGITRVAVGNQP--ADIGRQTK--------
QLIRKYILKQETINLVVVPCNVDIATT-EALSMAQEVDPDGDRTIGIL-TKPDLVDRG-TE--
GKVVDVAQNL-VCHLKK-GYMIVKCRGQQDIQDQVSLAEALQKEKDFFEDHPHF--RVLLEEGR-
ATV--PNLAEKLTSELITHICKTL

XP_032211398.1 LALPA----IAVIG----------------DQ----SSGKSSVLEA--LSGVA-
LPRG---SG-IVTRCPLV-------------LKL--------KKVTN--------------------------------
QDEWRGKVSYQ--------------------------------DFEKEIS---DPSEVEAEINKAQNAVAGEG---
QGISHELISLEVSSSHV----------PDLTLIDLPGITRVAVGNQP--ADIGRQTK--------
QLIRKYILRQETINLVVVPCNVDIATT-EALSMAQEVDPDGDRTIGIL-TKPDLVDRG-TE--
SKVVDVAQNL-VCHLKK-GYMIVKCRGQQDIQDQVTLAEALQKERDFFEDHPHF--RVLLEEGR-
ATV--PCLADKLTSELIMHICKTL

XP_008569442.1 LALPA----IAVIG----------------DQ----SSGKSSVLEA--LSGVA-
LPRG---SG-IVTRCPLV-------------LKL--------KKLVH--------------------------------
GEEWKGKVSYR--------------------------------DLEIKIS---DALEVEEEVRKAQTIIAGEG---
MGISHELINLEISSPHV----------PDLTLIDLPGIARVAMGNQP--ADIGYQVK--------
XLIRKYIQRQETINLVVVPSNVDIATT-EALSMAQEVDPEGDRTIGIL-TKPDLVDKG-TE--
DKVVDVVRNL-VYHLKK-GYMIVKCRGQQDIQDQLSLATALQREKDFFEDHPQF--RDLLEEGR-
ATI--PCLAERLTTELITHICKSL

XP_014388412.1 LALPA----IAVIG----------------DQ----SSGKSSVLEA--LSGVS-
LPRG---SG-IVTRCPLV-------------LKL--------RKLRH--------------------------------
DDEWKGKVTYR--------------------------------DLEIDLS---AASEVEQEIRKAQNVIAGEG---
VGISQELINLEVSSPHV----------PDLTLIDLPGITRVAVGNQP--ADIGRQIT--------

ALIKKYILRQQTIMLVVVPSNVDIATT-EALSMAHEVDPDGDRTIGIL-TKPDLVDRG-TE--DKVVDVVRNL-VYHLKK-GYMIVKCRGQQDIQYQMSLSKALQRERAFFEDHPYF--RDLLEEGK-ATI--PCLAERLTNELIAHISKSL

XP_005202045.1 LALPA----IAVIG----------------DQ----SSGKSSVLEA--LSGVA-LPRG---SG-IVTRCPLV-------------LRL--------KKLGN--------------------------------EDEWKGKVSFL--------------------------------DKEIEIP---DASQVEKEISEAQIAIAGEG---TGISHELISLEVSSPHV----------PDLTLIDLPGITRVAVGNQP--PDIEYQIK--------SLIRKYILRQETINLVVVPANVDIATT-EALRMAQEVDPQGDRTIGIL-TKPDLVDKG-TE--DKVVDVVRNL-VFHLKK-GYMIVKCRGQQDIKHRMSLDKALQRERIFFEDHAHF--RDLLEEGK-ATI--PCLAERLTSELIMHICKTL

NP_038634.1 LALPA----IAVIG----------------DQ----SSGKSSVLEA--LSGVA-LPRG---SG-IVTRCPLV-------------LKL--------RKLNE--------------------------------GEEWRGKVSYD--------------------------------DIEVELS---DPSEVEEAINKGQNFIAGVG---LGISDKLISLDVSSPNV----------PDLTLIDLPGITRVAVGNQP--ADIGRQIK--------RLIKTYIQKQETINLVVVPSNVDIATT-EALSMAQEVDPEGDRTIGIL-TKPDLVDRG-TE--DKVVDVVRNL-VYHLKK-GYMIVKCRGQQDIQEQLSLTEALQNEQIFFKEHPHF--RVLLEDGK-ATV--PCLAERLTAELILHICKSL

NP_034976.1 LALPA----IAVIG----------------DQ----SSGKSSVLEA--LSGVA-LPRG---SG-IVTRCPLV-------------LKL--------RKLKE--------------------------------GEEWRGKVSYD--------------------------------DIEVELS---DPSEVEEAINKGQNFIAGVG---LGISDKLISLDVSSPNV----------PDLTLIDLPGITRVAVGNQP--ADIGRQIK--------RLIKTYIQKQETINLVVVPSNVDIATT-EALSMAQEVDPEGDRTIGVL-TKPDLVDRG-AE--GKVLDVMRNL-VYPLKK-GYMIVKCRGQQDIQEQLSLTEAFQKEQVFFKDHSYF--SILLEDGK-ATV--PCLAERLTEELTSHICKSL

XP_004675614.2.2 LALPA----IAVIG----------------DQ----SSGKSSVLEA--LSGVA-LPRG---SG-IVTRCPLV-------------LKL--------KKLMN--------------------------------EDSWKGKINYQ--------------------------------GVEVTIA---KASDVEQEVNKAQAVIAGDG---LGISHELITLEVSSPEV----------PDLTLIDLPGITRVAVGNQP--QDIGEQIK--------ALIRKYIQRQQTINLVVVPCNVDIATT-EALSMAREVDPDGDRTLGIL-TKPDLVDRG-TE--DRVVDVIRNF-ICPLKK-GYMIVKCRGQKDIQDRLSLAQALQKEQAFFEEHPHF--RQLLEEGR-ASI--PKLADRLTSELIRHISKSL

XP_005885748.1 LALPA----IAVIG----------------DQ----SSGKSSVLEA--LSGVA-LPRG---SG-IVTRCPLV-------------LKL--------KKQLAG--------------------------------ESLWTGKISYR--------------------------------STELQLQ---DPSQVEREIYKAQNTIAGNG---VGISHELINLEITSPEV----------PDLTLIDLPGIARVAVGNQP--QDIGLQIK--------ALIKKYIQRQQTINLVVVPCNVDIATT-EALSMAHEVDPDGDRTIGIL-TKPDLVDKG-AE--KNVVNVAQNL-TYRLKK-GYMIVKCRGQQEITDKLSLAEATKKEMMFFQTHPYF--RVLLEEGK-ATV--PRLAERLTTELIWHINKSL

XP_017508123.1 LALPA----IAVIG----------------DQ----SSGKSSVLEA--LSGVA-LPRG---SG-IVTRCPLV-------------LKL--------KKQLH--------------------------------EPAWTGRLSYQ--------------------------------TTELQLH---NPSQVEKEIQKAQNAIAGDG---VGISHELINLEITSPDV----------PDLTLIDLPGIARVAVGNQP--QDIGLQIK--------ALIKKYIQRQQTINLVVVPCNVDIATT-EALSMAQEVDPDGDRTIGIL-TKPDLVDKG-TE--

RVIVNVVQNL-TYHLKK-GYMIVKCRGQQEVTNKLSLAEATSKEMTFFQTHPYF--RILLEEGK-ATV--PRLAEKLTTELISHINKSL
XP_008569440.1 LALPA----IAVIG----------------DQ----SSGKSSVLEA--LSGVA-LPRG---SG-IVTRCPLV-------------LKL--------KKHLQ--------------------------------EDGWKGKISYR--------------------------------HTELLLQ---DPSQVEKEIHKAQNTIAGNG---VGISHELISLEITSPEV----------PDLTLIDLPGITRVAVGNQP--QDIGQQVK--------ALIKKYIQRQQTINLVVVPCNVDIATT-EALSMAQEVDPDGDRTIGIL-TKPDLVDKG-TE--KGVMNVARNL-TYHLKK-GYMIVKCRGQQDITNKLSLAEATKKEMAFFQTHPYF--RVLLEEGK-ATV--PCVAEKLTAELIVHINKSL
NP_002454.1 LALPA----IAVIG----------------DQ----SSGKSSVLEA--LSGVA-LPRG---SG-IVTRCPLV-------------LKL--------KKQPC--------------------------------E-AWAGRISYR--------------------------------NTELELQ---DPGQVEKEIHKAQNVMAGNG---RGISHELISLEITSPEV----------PDLTIIDLPGITRVAVDNQP--RDIGLQIK--------ALIKKYIQRQQTINLVVVPCNVDIATT-EALSMAHEVDPEGDRTIGIL-TKPDLMDRG-TE--KSVMNVVRNL-TYPLKK-GYMIVKCRGQQEITNRLSLAEATKKEITFFQTHPYF--RVLLEEGS-ATV--PRLAERLTTELIMHIQKSL
XP_002830747.1 LALPA----IAVIG----------------DQ----SSGKSSVLEA--LSGVA-LPRG---SG-IVTRCPLV-------------LKL--------KKQPC--------------------------------E-AWAGRISYR--------------------------------NTELELQ---DPGQVEKEIHKAQNIMAGNG---RGISHELISLEITSPEV----------PDLTIIDLPGITRVAVDNQP--RDIGLQIK--------ALIKKYIQRQQTINLVVVPCNVDIATT-EALSMAHEVDPEGDRTIGIL-TKPDLMDKG-TE--KSVMNVVRNL-TYPLKK-GYMIVRCRGQQELTNRLSLAEATKKEITFFQTHPYF--RVLLEEGS-ATV--PRLAERLTSELIMHIQKSL
NP_001003133.1 LALPA----IAVIG----------------DQ----SSGKSSVLEA--LSGVA-LPRG---SG-IVTRCPLV-------------LKL--------KRDPH--------------------------------K-AWRGRISYR--------------------------------KTELQFQ---DPSQVEKEIRQAQNIIAGQG---LGISHELISLEITSPEV----------PDLTLIDLPGITRVAVGNQP--QDIGVQIK--------ALIKNYIQKQETINLVVVPCNVDIATT-EALSMAQEVDPNGDRTIGVL-TKPDLVDRG-TE--KTVVNVAQNL-TYHLQK-GYMIVRCRGQEEITNQLSLAEATEKERMFFQTHPYF--RALLEEGK-ATV--PCLAERLTKELILHINKSL
NP_776366.1 LALPA----IAVIG----------------DQ----SSGKSSVLEA--LSGVA-LPRG---SG-IITRCPLV-------------LKL--------TKR----------------------------------ECEWTGKITYR--------------------------------NITQQLQ---NPSEVEWEIRRAQNIIAGNG---LGISHELINLEITSPEV----------PDLTLIDLPGITRVAVENQP--QDIGLQIK--------ALIKKYIQRQETINLVVVPCNVDIATT-EALSMAQEVDPDGDRTIGIL-TKPDLVDKG-TE--KGVLKVMQNL-TYHLKK-GYMIVKCRGQQDITNKLSLAEATRKETMFFETHPYF--RILLDEGK-ATV--PLLAERLTTELIWHINKSL
XP_032211320.1 LALPA----IAVIG----------------DQ----SSGKSSVLEA--LSGVA-LPRG---SG-IVTRCPLV-------------LKL--------KRQPQ--------------------------------ESAWKGRVIYG--------------------------------TREVRLQ---DPSQVEKEILKAQNTLAGDG---VSISHELISVDIISPEV----------PDLTLIDLPGITRVPVGNQP--QDIGLQIK--------ALIKKYIQRQETINLVVVPCNVDIATT-EALSMAQEVDPRGDRTIGIL-TKPDLVDKG-AE--PIVMKVAQNL-TYHLQK-GYMMVRCRGQEEITNRLSLAEATRKETMFFQKHPHF--RALLQEGK-ATV--PCLAERLTNELILHINKSL
XP_006156438.1 LALPA----IAVIG----------------DQ----SSGKSSVLEA--LSGVA-LPRG---SG-IVTRCPLV-------------LKL--------MKQSQ--------------------------------EPVWRGKIRYR--------------------------------NTEKKLG---DPTQVEAEICKAQNIIAGSG---VGISHELITLEITSPEV----------PDLTLIDLPGITRVALGNQP--QDISLQIK--------

ALIKKYIKRQQTINLVVVPCNVDIATT-EALSMAQEVDPEGDRTLGIL-TKPDLVDKG-SE--
KSVMNVLQNL-TFPLKK-GYMIVKCRGQQEIMNNLSLAEATRKELMFFQSHPHF--RVFLEEKK-ATV-
-PHLAERLTAELIAHIRKSL

XP_015269256.1 LALPA----IAVIG----------------DQ----SSGKSSVLEA--LSGVA-
LPRG---NG-IVTRCPLA-------------LKL--------KKTRQ--------------------------------
GCGWKGKISYR--------------------------------DINEELN---HPSEVEKEIRKAQISIAGEG---
VGISHELITLEIRSSEV----------PDLTLIDLPGIARVAVGNQP--QDIGHQIK--------
RLIKKIIAKDETINLVVVPCNVDIATT-EALKMAQEVDPDGERTLGIL-TKPDLVDKG-TE--
EAVVDIVRNL-IIHLKK-GYMIVKCRGQQDIQSNLDLASAIQKEKAFFEDNRHF--RILLAEKR-ATI--
PLLAEKLTSELVEHINKSL

XP_028583072.1 LALPA----IAVIG----------------DQ----SSGKSSVLEA--LSGVA-
LPRG---SG-IVTRCPLE-------------LRL--------KKLLP--------------------------------GEKWNGKISYL-
-------------------------------GKYMELA---NPSMVEIEIRKAQNIIAGDG---VAISDKLITLEIRSPEV------
----PDLTLIDLPGIARVAVGNQP--VNIGDQIK--------KLIKTFIDKQETINLVVVPSNVDIATT-
EALKMAQEVDPNGERTLGIV-TKPDLMDRG-TE--GTVVNIVRNQ-VIPLKK-
GYMIVKCRGQQDIQSNMTLASALKEERAFFEKHKCF--SILLQEKK-ATV--PLLAEKLTSELVEHISKSL

XP_025933558.1 LALPS----IAVIG----------------DQ----SSGKSSVLEA--LSGIA-LPRG-
--NG-IVTRCPLE-------------LKL--------KKTPA--------------------------------TQKWKGKISYH---------
-----------------------NTSEELK---NPSEVEKAIRGAQDVVAGTK---GAISRELISLEVWSPTV----------
PDLTLIDLPGIARVAVGDQP--EDIGEQIK--------KLLKNIIGNKETLNLVVVPCNVDIATT-
EALKMAQEVDPKGERTLGIL-TKPDLVDKG-TE--ESIVNIIRNL-TVPLKK-
GYMIVKCRGQQDIHNNLTLASAIQQEKEFFETHQHF--SILLNEGK-ATV--PLLAEKLTXELVGHIIKTL

XP_009815891.1 LALPA----IAVIG----------------DQ----SSGKSSVLEA--LSGIA-LPRG-
--NG-IVTRCPLE-------------LKL--------KRIPA--------------------------------TQAWKGKICYR---------
-----------------------NISSELQ---NASEVEKAIREAQDIVAGTR---GAISGELISLEIWSPDV----------
PDLTLIDLPGIARVAVGNQP--KDIGEQIK--------MLLKKIIGCKETLNLVVVPCNVDIATT-
EALKMAQEVDPSGERTLGIL-TKPDLVDRG-TE--ESIINIIRNL-VIPLKK-
GYMIVKCRGQQDIHNKLALAAAIQQERKFFETHEHF--SILLEEGK-ATV--PHLAEKLTNELVRHIIKTL

AGU16245.1 --LPA----IAVIG----------------DQ----SSGKSSVLEA--LSGVG-LPRG---SG-
IVTRCPLE-------------LKL--------KKAKK--------------------------------ETEWKATIRYE-----------------
---------------DEYKELT---SPSEVEKEIRTAQNAMAGSG---KGISDKLISLEIESDNV----------
PDLTLIDLPGIARVAVQGQP--YDIGEQIK--------KLIRKFIEKEETINLVVVPCNVDIATT-
EALKMAQDVDQSGERTLGIL-TKPDLVDKG-AE--QNIVDVVNNM-VIPLKK-
GYMIVKCRGQQDINENLTLAEAT--

XP_007904885.1 LSLPA----IAVIG----------------DQ----SSGKSSVLEA--LSGVS-
LPRG---TG-IVTRCPLE-------------LKL--------KKAKK--------------------------------ANVWKGAISFR-
-------------------------------EYSKEIT---NASEVEQEIRKAQNSMAGKE----GISHDLISLKIESSNV------
----PDLTLIDLPGIARVAVGNQP--LDIGDQIK--------KMIRSFINKQETINLVVVPCNVDIATT-
EALKMAQEVDPSGERTVGIL-TKPDLVDKG-TE--STIVDIVQNL-VVELKK-
GYMIVKCRGQKEINDKLTLQDAIARENRYFEEHEQF--RTLLDEKK-ASI--PHLAERLTNELVYHISKCL

XP_032888405.1 LGLPA----IAVIG----------------DQ----SSGKSSVLEA--LSGVA-
FPRG---SG-IVTRCPLE-------------LKL--------KNVKK--------------------------------
ANVWKGKISYK--------------------------------DYSNKLS---SAAEVEQAILKAQDSIAGKG---
VGISHELISLEIESTNV----------PDLTLIDLPGIARVAVGNQP--QDIGDQIK--------

RLIRLFIQKQETVNLVVVPCNVDIATT-EALKMAQEVDPTGDRTLGIL-TKPDLVDKG-TE--KNVVDIVKNL-TVELEK-GYMIVKCRGQNDINENISLVDAIAKEKEFFEDHEQF--RPLLEDGK-AGI--PNLAVRLTKELVNHINKSL

XP_028583068.1 LALPA----IAVIG----------------DQ----SSGKSSVLEA--LSGVA-LPRG---SG-IVTRCPLE-------------LKL--------KKTHN--------------------------------TKEWKGKISYL--------------------------------NTVEEMN---SSRQVEEQIIRAQNAMAGSG---SGISSELISLEISSSDV----------PDLTLIDLPGIARVAVGDQP--KDIGQQII--------KLIKKYINKQETINLVVVPSNVDIATT-EALKMAQEVDPTGERTLGIL-TKPDLVDKG-TE--AEVVDIIRNQ-RVPLRK-GYMIVKCRGQSDINDKVTLGDAIEKEREFFEEHDFF--RSLLEEGR-ATI--PLLAERLTQELIEHISKTL

XP_003973512.2.2 LALPA----IAVIG----------------DQ----SSGKSSVLEA--LSGVA-LPRG---SG-IVTRCPLE-------------LKM--------KRRKV--------------------------------GEPWYGNISYL--------------------------------DQEEVIE---DPADVEKKIQEAQNEMAGVG---VGISDDLISLEIASPEV----------PDLTLIDLPGIARVAVKGQP--ENIGEQIK--------RLIRKFITKQETISLVVVPCNVDIATT-EALKMAQEVDPDGERTLGIL-TKPDLVDKG-TE--ETVVDIIHNE-VIHLKK-GYMIVRCRGQKEIIDKVSLAEATETETAFFRDHAHF--QTLYDDGQ-ATI--LKLAEKLTLELVNHIEKSL

NP_891987.2.2 LALPA----IAVIG----------------DQ----SSGKSSVLEA--LSGVP-LPRG---SG-IVTRCPLE-------------LKM--------IRTKD--------------------------------QDKWHGRISYK--------------------------------TYEEDFD---DPAEVEKKIRQAQDEMAGAG---VGISEELISLQITSANV----------PDLTLIDLPGIARVAVKGQP--ENIGDQIK--------RLIRKFVTRQETINLVVVPCNVDIATT-EALQMAQAEDPDGERTLGIL-TKPDLVDKG-TE--GTVVDIVHNE-VIHLTK-GYMIVRCRGQKEIMDQVTLNEATETESAFFKDHPHF--SKLYEEGF-ATI--PKLAEKLTIELVHHIQKSL

XP_009304072.1 LALPA----IAVIG----------------DQ----SSGKSSVLEA--LSGVP-LPRG---SG-IVTRCPLE-------------LKM--------IRSKE--------------------------------DEKWHGRISYQ--------------------------------NHEEDFD---DPAEVEKKIREAQDEMAGAG---VGISEELISLQITSANV----------PDLTLIDLPGIARVAVKGQP--ENIGDQIK--------RLIRMFITKQETINLVVVPCNVDIATT-EALQMAQAEDPEGERTLGIL-TKPDLVDKG-TE--GTVVDIVHNE-VIHLTK-GYMIVRCRGQKEIMDQVTLNEATETESAFFKDHPHF--RKLYEEGF-ATI--PKLAEKLTIELVHHIQRSL

XP_031752404.1 LALPA----IAVIG----------------DQ----SSGKSSVLEA--LSGVT-LPRG---SG-IVTRCPLE-------------LKL--------KKAMK--------------------------------KTTWSGKISYR--------------------------------DHEIKIA---SAADVEEEVKRAQNLMAGSG---KGISDELISLEVISPDV----------PDLTLIDLPGITRVALPDQP--KDIEQQIK--------KMIRKYIQKQETINLVVVPSNVDIATT-EALEMAREVDPNGERTLGIL-TKPDLVDRG-AE--TDVISVVRNL-VYSLNK-GYMIVKCRGQQEIQENLSLKDALVNEQNFFKEHEHF--SVLLEEGY-ATI--ACLAGKLTNELVAHIVRNL

NP_001007285.1 LNLPA----IAVIG----------------DQ----SSGKSSVLEA--LSGVA-LPRG---IG-IVTRCPLI-------------LKL--------KKITR--------------------------------DKNWSGLLTYK--------------------------------DQTEILK---EPTGIENAVLKAQIALAGTG---EGISHEMITLEIQSCDV----------PDLTLIDLPGIARVATGNQP--EDIEKQIK--------DLIEKFIKRQETISLVVVPANIDIATT-EALKMASTVDPTGQRTLCIL-TKPDLVDRG-ME--DTVVRTVNNE-VIRLEK-GYMIVKCRGQQDINDKLNLVEALEKERRFFDEHPQF--SSLLEDGK-ATI--PLLGQRLTEELVEHIAKNV

XP_005167721.2.2 LNLPA----IAVIG----------------DQ----SSGKSSVLEA--LSGVA-LPRG---TG-IVTRCPLV-------------LKL--------KKITK--------------------------------DKSWHGLLTYN---------------------------------DKIRELK---DPAKIEKAVLNAQTALAGIG---EGISHEMITLEIQSCDV----------PDLTLIDLPGIARVATGNQP--EDIEKQIK--------SLIEKFIKRQETISLVVVPANIDIATT-EALKMASTVDPTGQRTLGIL-TKPDLVDRG-ME--DTVVRTVNNE-VIPLKK-GYMIVKCRGQQDINDKLGLVEALEKERRFFDENVHF--RSLLEDRK-ATI--PLLAERLTKELVEHIAKNL

XP_002608668.1 VTLPS----VVVIG----------------DQ----SAGKSSCLEA--MSGVQ-LPRG---SG-IVTRCPLE-------------LRL--------KKSQDP-------------------------------ESPWKGYIHYHF----------------------EGDR----DETGWKLT---DPSDVGEAVRKAQNNLAGDS---HGISPRLITLDVESPDI----------PDLTLIDLPGIARIAVDGQP--PDIGDQIK--------DLIKEYIQKDETIILAVVPCNVDIATT-EALQMAKDVDPTGSRTLGVL-TKPDLIDRG-TE--NTIVDIVNNQ-KYPLKK-GYTIIRCRGQEDINENVTLSEAMEKEERFFKTHEHF--KLPYHEKK-TGT--RTLAGKLTTELVEQIK---

XP_019617847.1 VTLPS----VVVIG----------------DQ----SAGKSSCLEA--ISGVQ-LPRG---SG-IVTRCPLE-------------LRL--------KKSPDP-------------------------------ESGWRGYIHFE-------------------------DK----GETRWELD---SPEDVGEAVKKAQNQLAGES---LCISPRLITLDVESPDI----------PDLTLIDLPGIARVPVGGQP--DDIGDQTK--------ALIREYIQMDETIILAVVPCNVDIATT-EALKMAKEVDPDGSRTLGVL-TKPDLIDRG-TE--NMTVDIVNNR-KYALKK-GYTIIKCRGQVDIENKVSLSDAMDKEEMFFQKHEHF--KILYEEKK-TGT--KTLAGKLTTELVEQIKKSI

XP_035690836.1 VSLPS----VVVIG----------------DQ----SAGKSSTLEA--ISGVQ-LPRG---SG-IVTRCPLE-------------LRL--------KKSQKK-------------------------------DAPWKGCIRYVK------------------------NK----KDVRFDVD---EPGNVGDAVKKAQNDLAGTT---NGISDSLITLDVESPDI----------PDLTLIDLPGIARIAAEGQP--TDIGQQIK--------DLISKYIQKKDTIILAVVPCNVDIATT-EALQMAQEVDADGSRTLGVL-TKPDLIDPG-TE--RGVLQILNNE-KYKLRK-GYTIIKCRGQMDIEKGMSLEEAMDKEQSYFKSHEHF--KSVYKEKK-AGV--RTLAGRLSTELVGQIKNSI

XP_012586448.1 LALPA----IAVIG----------------DQ----SSGKSSVLEA--LSGVA-LPRG---SG-IVTRCPLV-------------LKL--------KKQLQ--------------------------------GAPWTGTISYR--------------------------------GVTLGLQ---DPSAVEREIHVAQNVIAGHG---VGISHELITLEVSSPEV----------PDLTLIDLPGIARVAVGNQP--QDIGAQVSLSGAGRWGALVSGLPRSERC---------TGQETA-EALGGGRAAXXDGDRTLGIL-TKPDLVDKG-AE--KAVVNVAQNL-TYRLKK-GYMVVKCRGQQDIMDRLSLAQATEKEVAFFQTHPHF--RALLEEGK-ATV--PRLAEKLTSELILHINKSL

KAI0208044.1ISLPE----VAVIG----------------DQ----SAGKSSVLEA--ISGVQ-LPRG---SG-IVTRCPLA-------------LQL--------KSHDT--------------------------------PGYWNSVIKYKY---------------------GDEFDFEDIVVEKTIE---GPTKVDAGVREAQDAIAGKN---VGISDTLISLQITAYGV----------PDLTLIDLPGITRVAVQGQP--PDIGDQIK--------RLIGNYIKKEETIILAVVPANVDIATT-EALKMAKEVDPSGGRTLGVV-TKPDLIDIG-TE--KGLIDIINNE-TYPLEK-GYSCVRCRGQKAINEGQTLADAVQEDTDFFSSAPHF--SA-VDESI-LGV--KNLAMKLTFELVKQIKRAL

KAI0213370.1ISLPE----VAVIG----------------DQ----SAGKSSVLEA--ISGVQ-LPRG---SG-IVTRCPLA-------------LQL--------KSHDT--------------------------------PGYWNGVIKYET-------------

---------YNH------PVEKTIE---GPTEVGAEVREAQDVIAGKN---VGISDTLISLQITSHGV----------
PDLTLIDLPGITRVAVEGQP--KDIGDQIK--------RLIGHYIKKEETIILAVVPANVDIATT-
EALKMAKEVDPSGGRTLGVV-TKPDLIDIG-TE--KGLIDIINNE-TYPLEK-
GYSCVRCRGQKAINEGQTLAEAIQQDTDFFSSAPHF--SD-VDESI-LGV--
KNLAMKLTYELVKQIKRAL

KAI0218869.1IALPE----VAVIG----------------DQ----SAGKSSVLEA--ISGVQ-LPRG---SG-
IVTRCPLA-------------LQL--------KSDKT--------------------------------PGYWNGVIKYEI--------------
--------NER------LVEKTIV---GPAEVDAEVRNAQDVIAGKN---VGISSKLISLQITSYGI----------
PDLTLIDLPGITRVAVEGQP--QNIGEQIK--------RLIEKYIKKEETIILAVVPANVDIATT-
EALKMAKEVDPSGSRTLGVV-TKPDLIDAG-TE--KGLISIINNE-TYPLKK-
GYSCVRCRGQKAIDEGQTLAQAIQQDTDFFSIASHF--SD-VDQST-LGV--
KNLAMKLTYELVRQIKRAL

ABI53802.1 INLPA----VAVIG----------------DQ----SAGKSSVLEA--ISGVQ-LPRG---TG-
IVTRCPLE-------------MRM--------KHSEA--------------------------------EDMWEGKIMYKD----------
------------MYDV-----AHEEIIL---NRESVEELVRKAQKEMTDSA---KGISDELITLEVTSSDV----------
PDLTVIDLPGIARNAVEGQP--FDIEARIK--------NMIRRYIGRQETIILAVLQCNVDIATC-
EALKMAKEFDAEGGRTLGVL-TKPDLLDKG-AE--TGVVRILNNM-EFTLSK-
GYIIATCRGQEAISDGQSLTQALEVEEDFFKSHRYF--SS-LRPSQ-WGI--PNLSGRLSRELKKHIKKLL

XP_046352531.2 INLPS----VAVIG----------------DQ----SAGKSSVLEA--ISGVQ-
LPRG---TG-IVTRCPLE-------------MRM--------KHSED--------------------------------
EDMWEGKIMYKD----------------------MYDM-----AHEEIIL---NRESVGELVRKAQKEMTDSA---
KGISDELITLEVTSSDV----------PDLTVIDLPGIARNAVEGQP--FDIEARIK--------
NMIRKYIGRQETIILAVLQCNVDIATC-EALKMAKEFDAEGGRTLGVL-TKPDLLDKG-AE--
SGVVRILNNM-EFTLSK-GYIIVKCRGQEAISDGQTLKQALEVEEDFFKSHRHF--SS-LRPSQ-WGI--
PNLSGRLSRELKIHIKKLL

XP_048258111.1 INLPA----VAVIG----------------DQ----SAGKSSVLEA--ISGVQ-
LPRG---TG-IVTRCPLE-------------MRM--------KHSED--------------------------------
EDMWEGKIMYKD----------------------MHDM-----AHEEIIL---NRESVGELVRKAQKEMTDSA---
KGISDELITLEVTSSDV----------PDLTVIDLPGIARNAVEGQP--FDIEARIK--------
NMIRRYIGRQETIILAVLQCNVDIATC-EALKMAKEFDAEGGRTLGVL-TKPDLLDKG-AE--
AGVVRILNNM-EFTLSK-GYIIVKCRGQEAISYGQSLKQALEVEEDFFKSHRHF--SS-LRPSQ-WGI--
PNLSARLSRELKKHIKKLL

XP_048248472.1 INLPS----VAVIG----------------DQ----SAGKSSVLEA--ISGVQ-
LPRG---TG-IVTRCPLE-------------MRM--------KHSED--------------------------------
EDMWAGKIMYKD----------------------MYDM-----THEEIIL---NRESVGELVRKAQKEMTDSA---
KGISDELITLEVTSSDV----------PDLTVIDLPGIARNAVEGQP--LDIEARIK--------
NMIRRYIRRQETIILAVLQCNVDIATC-EALKMAKEFDAEGGRTLGVL-TKPDLLDKG-AE--
TGVVRILNNM-EFTLSK-GYIIVKCRGQEAISDGQSLKQALEVEEEFFKSHRHF--SS-LRPSQ-XGI--
PNLSGRLSRELKIHIKKRL

XP_048248473.1 INLPS----VAVIG----------------DQ----SAGKSSVLEA--ISGVQ-
LPRG---TG-IVTRCPLE-------------MRM--------KHSED--------------------------------
EDMWAGKIMYKD----------------------MYDM-----THEEIIL---NRESVGELVRKAQKEMTDSA---
KGISDELITLEVTSSDV----------PDLTVIDLPGIARNAVEGQP--LDIEARIK--------
NMIRRYIRRQETIILAVLQCNVDIATC-EALKMAKEFDAEGGRTLGVL-TKPDLLDKG-AE--

TGVVRILNNM-EFTLSK-GYIIVKCRGQEAISDGQSLKQALEVEEEFFKSHRHF--SS-LRPSQ-WGI--PNLSGRLSRELKIHIKKRL
XP_048248474.1 INLPS----VAVIG----------------DQ----SAGKSSVLEA--ISGVQ-LPRG---TG-IVTRCPLE-------------MRM--------KHSED--------------------------------EDMWAGKIMYKD----------------------MYDM-----THEEIIL---NRESVGELVRKAQKEMTDSA---KGISDELITLEVTSSDV----------PDLTVIDLPGIARNAVEGQP--LDIEARIK--------NMIRRYIRRQETIILAVLQCNVDIATC-EALKMAKEFDAEGGRTLGVL-TKPDLLDKG-AE--TGVVRILNNM-EFTLSK-GYIIVKCRGQEAISDGQSLKQALEVEEEFFKSHRHF--SS-LRPSQ-WGI--PNLSGRLSRELKIHIKKRL
XP_048248476.1 INLPA----VAVIG----------------DQ----SAGKSSVLEA--ISGVQ-LPRG---TG-IVTRCPLE-------------MRM--------KHSED--------------------------------GDMWEGKIMYKD----------------------MHDM-----AHEEIIL---NRESVGELVRKAQIEMTDSA---KGISDELITLEVTSSDV----------PDLTVIDLPGIARNAVEGQP--FDIEARIK--------NMIRRYIGRQETIILAVLQCNVDIATC-EALKMAKEFDTEGGRTLGVL-TKPDLLDKG-AE--SGVVRILNNK-EFTLSK-GYIIVKCRGQEAISDGQSLKQALEVEEDFFKSHRHF--SS-LRPSQ-WGI--PNLSMRLSRELKKHIKKLL
XP_046352527.2 INLPA----VAVIG----------------DQ----SAGKSSVLEA--ISGVQ-LPRG---TG-IVTRCPLE-------------MRM--------KHSED--------------------------------EDMWEGKIMYKD----------------------MHGE-----AHEEIIL---NRESVGELVRKAQKEMTDSA---KGISDELITLEVTSSDV----------PDLTVIDLPGIARNAVEGQP--VDIEARIK--------QMIRKYIGRQETIILAVLQCNVDIATC-EALKMAKEFDDEGGRTLGVL-TKPDLLDKG-AE--SGVVRILNNM-EFTLSK-GYIIVKCRGQEAISDGQSLKQALEVEEDFFKSHRHF--SS-LRPSQ-WGI--PNLSTRLSRELKKHIKKLL
XP_046562919.1 INLPA----VAVIG----------------DQ----SAGKSSVLEA--ISGVQ-LPRG---TG-IVTRCPLE-------------MRM--------KHSED--------------------------------EDKWEGKIMYKD----------------------KHDM-----RQEEVIL---NRESVGDLVRKAQKEMTDGA---KGISDELITLEVTSSDV----------PDLTVIDLPGITRNAVEGQP--FDIEARIK--------NMIRKYIKRQETIILAVLQCNVDIATC-EALKMAKEFDGEGGRTLGVL-TKPDLMDKG-AE--TGVIRILNNM-EFTLSK-GYIIVKCRGQEAISEGQSLKQALDIEEDFFKSHRHF--SS-LRPSQ-WGI--PDLSSRLSRELKRHIKKLL
XP_046563124.1 INLPA----VAVIG----------------DQ----SAGKSSVLEA--ISGVQ-LPRG---TG-IVTRCPLE-------------MRL--------KHSED--------------------------------EDKWEGKILYKD----------------------KHDM-----RQEEVIL---NRESVGDLVRKAQKEMTDSA---KGISDELITLEVTSSDV----------PDLTVIDLPGIARNAVEGQP--FDIEARIK--------NMIRRYIGRQETIILAVLQCNVDIATC-EALKMAKEFDDEGGRTLGVL-TKPDLLDKG-AE--TGVMRILNNM-EFTLSK-GYIIVKCRGQEAISEGQSLKHALDVEEDFFKSHRHF--SS-LRPSQ-WGI--PNLSARLSRELKKHIKKLL
XP_046563126.1 INLPA----VAVIG----------------DQ----SAGKSSVLEA--ISGVQ-LPRG---TG-IVTRCPLE-------------MRM--------KHSED--------------------------------EDKWEGKIMYKD----------------------KHDM-----PHEEVIL---NRESVGDLVRKAQTEMTDGA---TGISDELITLEVTSSDV----------PDLTVIDLPGIARNAVEGQP--FDIEARIK--------NMIRQYIERQETIILAVLQCNVDIATC-EALKMAKEFDDEGGRTLGVL-TKPDLLDKG-AE--TSVIRILNNM-EFTLSK-GYVIVKCRGQEAISEGQSLKHALDVEEDFFRSHRHF--SA-LRPSQ-WGI--PNLSERLSRELKKHIKKLL

XP_046565195.1 INLPA----VAVIG----------------DQ----SAGKSSVLEA--ISGVQ-LPRG---TG-IVTRCPLE-------------MRM--------KHSED--------------------------------EDKWEGKIMYKD----------------------KHDM-----PHEEVIL---NRESVGDLVRKAQKEMTDGA---TGISDELITLEVMSSDV----------PDLTVIDLPGIARNAVEGQP--FDIEARIK--------NMIRQYIQRQETIILAVLQCNVDIATC-EALKMAKEFDDEGGRTLGVL-TKPDLLDKG-AE--TGVIRILNNM-EFTLSK-GYVIVKCRGQEAISEGQSLKHALDVEEDFFRSHRHF--SA-MRPSQ-WGI--PNLSERLSRELKKHIKKLL

XP_046563125.1 INLPA----VAVIG----------------DQ----SAGKSSVLEA--ISGVQ-LPRG---TG-IVTRCPLE-------------MRM--------KHSED--------------------------------EDKWEGKIMYTD----------------------KHDE-----PHQEVIL---NRESVGDLVRKAQKEMTDSA---KGISDELITLEVTSSDV----------PDLTVIDLPGIARNAVEGQP--VDIEARIK--------NMIRQYIERQETIILAVLQCNVDIATC-EALKMAKEFDDEGGRTLGVL-TKPDLLDRG-AE--TGVMRILNNM-EFTLSK-GYIIVKCRGQEAISEGQSLKHALDVEEDFFKSHRHF--SS-LGPSQ-WGI--PNLSRRLSRELKKHIKKLL

XP_046565196.1 INLPA----VAVIG----------------DQ----SAGKSSVLEA--ISGVQ-LPRG---TG-IVTRCPLE-------------MRM--------KHSED--------------------------------EDKWEGKIMYKD----------------------KHDE-----LHKEDIQ---DRESVGDLVRKAQDEMTDCE---KGISDDLITLEVTSSDV----------PDLTLIDLPGIARNAVKGQP--VDIEKRIK--------DMIRKYIRRQETIILAVLQCNVDIATC-EALKMAKEFDEEGRRTLGVL-TKPDLLDKG-AE--NGVMRILNNM-EFSLSK-GYIIVKCRGQEAISKGQSLTEALGDEDNFFKDHSHF--RS-LKVSQ-WGI--LTLSSRLSLELQKHIK---

XP_032804093.1 VGLPA----VAVIG----------------DQ----SSGKSSVLEA--LSGVQ-LPRG---SG-IVTRCPLA-------------LKL--------KRA----------------------------------PGPWHGRIKYRV----------------------QGR------TVNTKLD---TPESVGDAVLQAQSELTGDD---LGVSKSLIELEVTSDSV----------PDLTLIDLPGIARVALAGQA--VDIETQIK--------DLIRDHIGRQETINLVVIPCNVDIATT-EALKMAQAVDPTGVRTLGVL-TKPDLMDEG-TE--RNALRILQNQ-VFPLSK-GYVLVKCRSQRDVEAHQTLAEASRVEAAFFKKHPVF--CHVHNGGKLTTT--TVLAAKLTEELVDNIKRTL

XP_006815062.1 VDLPA----VVVIG----------------DQ----SVGKSSVLEA--ISGVQ-LPRG---NE-IVTRCPIE-------------LRL--------KTLD---------------------------------NDEWCGKILYIN----------------------YSKE-----QVNKYID---SPDELGAAIRTAQQDITNSQ---KGISKTSITVEIQSAHV----------PNLTLIDLPGIARVPQEGQS--RNIADETK--------DLIKKYISKDDAIVLCVIPCNVDIATT-EAIKMAQEVDPTGSRTLGVL-TKPDLVDKG-SE--NVVVRIAENK-VINLKK-GYTIMKCRSQRNLEDAMSLEEAMDEEERFFREHKHY--SVL--SGQ-AGS--RLLAHRLTTELVEQILKSV

CAH1802128.1 VALPA----VVVIG----------------DQ----SVGKSSVLEA--MSGVQ-LPRG---TG-IVTRCPLE-------------LRM--------KQCD--------------------------------PGNFHAKISYDI----------------------QGGH----QPLEKTIT---DPSNIDFEIRQAQRALVGDS---GGVSDRLIRLEVQADYV----------PDLTLIDLPGIVRYSEGS----DTIVEETK--------NLIKTYVSRPETIILVVIPCNVDIDTV-EACNLAKQVDPNGDRTIGVL-TRPDLIDHG-VGPIKEVLDILENK-KMKLKK-GFYVVKCRSQKRIEEGQSLEQALAEEVQFFRSDERF--RV-INPSQ-CGV--KQLSSKLTNELFLHIKNCV

PAA92268.1 LKLPM----VAVVG----------------DQ----SVGKSSVLEA--ISGVE-FPRG---TG-MVTRCALQ-------------LSM--------QWNADP-------------------------------EAPWHGRISYKD--------

--------------VNGH-----KVDKELN---SPGEVDGAVREAQQRMTHGD---NEISSEQIDLAIKGPDV-----
-----PDLTLIDLPGIARYSATGG---SGIAQITK--------SLIAKYVSQPQVLILVVVPCHQDIETV-
EALSLAKEADPQGERTIGVL-TCPDMVNKG-AE--QETLKIANNE-KIPLKK-
GYVMVKCRSPEELNNGVTLSESVANEAAFFKTHRHF--SL-LPEQS-VGI--
RTLADKLTEELFESVKRNI

PAA74204.1 VRLPA----IAVVG----------------DQ----SVGKSSVLES--ISGID-LPRG---LG-
IVTRCPLM-------------LSM--------RNRE---------------------------------EAGWSARIKYKT-------------
---------KTGE-----GREKKLT---GASEVGQAIRDAQNEMTNSS---GEVSEQLIELWVESPES----------
PDLTLIDLPGIARYSIDGG---GAIAGLTK--------SLILSYIEKEEILILVVIPCHVDIETV-
EALSLAKEVDPESKRTIGVL-TCPDLVNPG-SE--SEVLALMQNR-KIPLKK-
GYVSVRCRTPQQLKDNMSLQQAAREEEVFFRTHPHF--RA-LDKFE-YGT--
KTLAVKLSSELYEAIKHNI

PAA76532.1 ISLPA----IAVVG----------------DQ----SVGKSSVLEA--ISGVE-FPRG---LG-
IVTRCPLM-------------LSM--------RGRE---------------------------------DSGWTARIRYET-------------
---------KSGQ-----ARDKPLS---TPAEIGQAIRDAQEEMTSSS---GEISEKLIELHIEGADT----------
PDLTLIDLPGIARFSIANA---GDIATVSK--------SLIMSYILKPEVLILVVIPCNVDVETV-
EAISLAREVDPECKRTLGVL-TCPDLVNPG-SE--TEVLAMMRNE-RLKLRK-
GFVTVRCRTPQQLKDNMGLREACKAEEEFFKLHPQF--CA-LGDYQ-RGC--
KTLANKLSVELYQAVKERI

PAA69582.1 LRLPT----VAVVG----------------DQ----SVGKSSVLEA--ISGVD-LPRG---TG-
IVTRCPLQ-------------LSM--------RSKP---------------------------------TGDWTGRISYQN-------------
---------RKGE-----HVEREIS---KKCEVDEMVRKVQNEITGDS---NGVSTEQIDLTIESADV----------
SDLTLVDLPGIARYSEKN----PKINEVTK--------QLILSYISQDQVIILVVVPCSVDIETV-
EAIALAKQVDPGGTRTIGVL-TCPDLTNPG-SE--EDIKAIVNNQGRVRLHK-
GFVMVKCRSPKELRNNISLSEVAKIEEDYFKNDPHF--SQ-LPKDI-VGT--KTLAEKLTNELFKAVAAGI

PAA83069.1 LKLPS----IVVIG----------------DQ----SSGKSSVLET--ISGVS-FPRG---NG-
VVTLCPLQ-------------LSM--------RSSD---------------------------------K-KWRGTVRYFD------------
----------AQGK-----EVHWDID---SPDDVENAIQNAQMRITGHK---KAISKNIIEMTLEAPDL----------
PNLTLVDLPGIARYSHSDGGS-VNLYKLTT--------DIIKEYIQREETIILTVIPLSADTATM-
EALQLAKDVDPYGLRTIGVL-TFPDLVNKG-AE--EEKLQIARNI-TFPLSK-
GYITVKCRNQEDIKSRKSLREAKVDEMRFFSNDPFF--SQ-LDPSQ-RGT--DTLAKRLSTELLTLIKKFI

PAA94353.1 LRLPS----IVVIG----------------DQ----STGKSSVLES--ISGVR-FPRG---NG-
VVTLCPLQ-------------LSM--------RTSD---------------------------------DGKWRGNIRYYD-----------
-----------TYGK-----LMKWDID---GPEDVEDAIQEAQMRITGNQ---RNVSKSIIEMTLESPEL----------
PNLTLIDLPGIARYNHNSAESGASLHQLTT--------DIIKEYIRREETIILVVIPLTSDTATM-
EALQLAKDADPYGMRTIGVL-TFPDLVNKG-AQ--EEKLMIARNI-TYPLSK-
GYVTVKCRNQEDIKNRKSLKDAKADEALFFNTDPFF--KQ-LDSMY-RGS--
DTLARRLSEELLYLVKKFI

XP_006813643.1 DHLPR----VVVVG----------------DQ----SSGKTSVLEM--
IAQARIFPRG---SGEMMTRTPVK-------------VTL--------SEGPY--------------------------------------
HVAQFKD----------------------S--------NKEYDLSKESELQSLRQEIELRMKNRVKKG---
QTVSNDTISLSVRGPGI----------QRMVLVDLPGMISTVTTGMA--ADTREAIH--------
NMSKSYMKNPNAIILCIQDGSVDAERS-IVTDLATTMDPEGKRTIFVL-TKVDLAEKNSANP-

SRIKQILDGK-LFPMKALGYFAVVTGRG---NTNESIEQIKNYEETFFRSSKLF-KTGTLKPSQ-MTT--QNLSFAVSDCFWKMVRESV

XP_030843280.1 DHLPR----VVVVG----------------DQ----SAGKTSVLEM--IAQARIFPRG---AGQMMTRAPVK-------------VTL--------SEGPN--------------------------------------HIAQFKD----------------------S--------GKEFDLTKESELKALRQEIEARMKGSVKEG---QTISPEVISLSVRGPGI----------QRMVLVDLPGMISTVTTGMA--ADTKTSIQ--------KMINGYMGNPNAIILCIQDGAIDAERS-IVTDLVNEIDPTGKRTIFVL-TKVDLAEKNHLNP-NRIRQILDGR-LFPMKALGYFAVVTGKG---NTSDSIDSIKQYEEQFFRHSALF-KSGVFKPSQ-LNT--QNLSFAVSDCFWKMVRESV

XP_023440724.1 DHLPR----VVVVG----------------DQ----SAGKTSVLEM--IAQARIFPRG---SGEMMTRSPVK-------------VTL--------SEGPH--------------------------------------HVALFKD----------------------S--------SREFDLTKEEDLAALRREIEIRMRKSVKEG---CTVSPETISLNVKGPGL----------QRMVLVDLPGVINTVTSGMA--PDTKETIF--------SISKAYMQNPNAIILCIQDGSVDAERS-IVTDLVSQMDPHGRRTIFVL-TKVDLAEKNVTSP-SRIQQIIEGK-LFPMKALGYFAVVTGKG---NSSESIEAIREYEEEFFQNSKLL-KTSMLKAHQ-VTT--RNLSLAVSDCFWKMVRESV

XP_005873264.1 DHLPR----VVVVG----------------DQ----SAGKTSVLEM--IAQARIFPRG---SGEMMTRSPVK-------------VTL--------SEGPH--------------------------------------HVALFKD----------------------S--------SREFDLTKEEDLAALRHEIELRMRKNVKEG---CTVSPETISLNVKGPGL----------QRMVLVDLPGVINTVTSGMA--PDTKETIF--------SMSKAYMQNPNAIILCIQDGSVDAERS-IVTDLVSQMDPHGRRTIFVL-TKVDLAEKNVASP-SRIQQIIEGK-LFPMKALGYFAVVTGKG---NSSESIEAIREYEEEFFQNSKLL-KASMLKAHQ-VTT--RNLSLAVSDCFWKMVRESV

XP_006163024.2.2 DHLPR----VVVVG----------------DQ----SAGKTSVLEM--IAQARIFPRG---SGEMMTRSPVK-------------VTL--------SEGPH--------------------------------------HVALFKD----------------------S--------SREFDLTKEEDLAALRHEIELRMRKNVKEG---CTVSPETISLNVKGPGL----------QRMVLVDLPGVINTVTSGMA--PDTKETIF--------SISKAYMQNPNAIILCIQDGSVDAERS-IVTDLVSQMDPHGRRTIFVL-TKVDLAEKNVASP-SRIQQIIEGK-LFPMKALGYFAVVTGKG---NSSESIEAIREYEEEFFQNSKLL-KTSMLKAHQ-VTT--RNLSLAVSDCFWKMVRESV

NP_056375.2.2 DHLPR----VVVVG----------------DQ----SAGKTSVLEM--IAQARIFPRG---SGEMMTRSPVK-------------VTL--------SEGPH--------------------------------------HVALFKD----------------------S--------SREFDLTKEEDLAALRHEIELRMRKNVKEG---CTVSPETISLNVKGPGL----------QRMVLVDLPGVINTVTSGMA--PDTKETIF--------SISKAYMQNPNAIILCIQDGSVDAERS-IVTDLVSQMDPHGRRTIFVL-TKVDLAEKNVASP-SRIQQIIEGK-LFPMKALGYFAVVTGKG---NSSESIEAIREYEEEFFQNSKLL-KTSMLKAHQ-VTT--RNLSLAVSDCFWKMVRESV

NP_598513.1 DHLPR----VVVVG----------------DQ----SAGKTSVLEM--IAQARIFPRG---SGEMMTRSPVK-------------VTL--------SEGPH--------------------------------------HVALFKD----------------------S--------SREFDLTKEEDLAALRHEIELRMRKNVKEG---CTVSPETISLNVKGPGL----------QRMVLVDLPGVINTVTSGMA--PDTKETIF--------SISKAYMQNPNAIILCIQDGSVDAERS-IVTDLVSQMDPHGRRTIFVL-TKVDLAEKNVASP-SRIQQIIEGK-LFPMKALGYFAVVTGKG---NSSESIEAIREYEEEFFQNSKLL-KTSMLKAHQ-VTT--RNLSLAVSDCFWKMVRESV

XP_028587646.1 DHLPR----VVVVG----------------DQ----SAGKTSVLEM--
IAQARIFPRG---SGEMMTRSPVK-------------VTL--------SEGPH-------------------------------------
HVAMFKD----------------------S--------SREFDLTKEEDLAALRNEIEIRMRKSVSDG---
CTVSTETISLSVKGPGL----------QRMVLVDLPGVISTVTSGMA--PDTKETIF--------
SISKAYMQNPNAIILCIQDGSVDAERS-IVTDMVSQMDPQGKRTIFVL-TKVDLAEKNVASP-
SRIQQIIEGK-LXPMKALGYFAVVTGKG---NSCESIESIKEYEEEFFQNSKLL-KNCMLKAHQ-VTT--
RNLSLAVSDCFWKMVRESV
XP_025913835.1 DHLPR----VVVVG----------------DQ----SAGKTSVLEM--
IAQARIFPRG---SGEMMTRSPVK-------------VTL--------SEGPH-------------------------------------
HVALFKD----------------------S--------SREFDLTKEEDLAALRNEIEIRMRNSVKEG---
CTVSTETISLSVKGPGL----------QRMVLVDLPGVISTVTSGMA--PDTKETIF--------
SISKAYMQNPNAIILCIQDGSVDAERS-IVTDLVSQMDPQGKRTIFVL-TKVDLAEKNVASP-
SRIQQIIEGK-LFPMKALGYFAVVTGKG---NSSESIDSIKEYEEEFFQNSKLL-KTCMLKAHQ-VTT--
KNLSLAVSDCFWKMVRESV
XP_021332524.1 DHLPR----VVVVG----------------DQ----SAGKTSVLEM--
IAQARIFPRG---SGEMMTRSPVK-------------VTL--------SEGPH-------------------------------------
HVAMFKD----------------------S--------SREFDLGKEEDLAALRHEIELRMRKSVKEG---
QTVSPETISLSVKGPGI----------QRMVLVDLPGVISTVTTGMA--ADTKETIF--------
SISKAYMQNPNAIILCIQDGSVDAERS-IVTDLVSQMDPQGKRTIFVL-TKVDLAEKNLASP-
SRIQQIVEGK-LFPMKALGYFAVVTGKG---SPNESIDSIKDYEEDFFQNSRLL-KDGMLKAHQ-VTT--
KNLSLAVSDCFWKMVRESV
XP_031757388.1 DHLPR----VVVVG----------------DQ----SAGKTSVLEM--
IAQARIFPRG---SGEMMTRSPVK-------------VTL--------SEGPH-------------------------------------
HVAMFKD----------------------S--------SREFDLSKETDLAALRNEIEVRMRKSVKNG---
QTVSPETISLSVKGPGI----------QRMVLVDLPGVINTVTSGMA--PDTKDTIF--------
NISKAYMLNPNAIILCIQDGSVDAERS-IVTDLVSQMDPQGRRTIFVL-TKVDLAEKNVASP-
NRIQQIIEGK-LFPMKALGYFAVVTGKG---NSNESIDSIKDYEEEFFQGSSLL-KKGMLKAHQ-VTT--
KNLSLAVSDCFWKMVRESI
XP_032818114.1 DQLPR----VVVVG----------------DQ----SSGKTSVLEM--
IAQARIFPRG---SGEMMTRSPVK-------------VTL--------SEGPH-------------------------------------
HVAIFKD----------------------S--------SREFDLTKEDDLAALRKEIEIRMKKSVKEG---
HTVSAETISLSVKGPGL----------QRMVLVDLPGVISTMTSGMA--PDTKDAIF--------
AMSKGYMQNPNAIILCIQDGSVDAERS-IVTDLVSNMDPQGKRTIFVL-TKVDLAEKNLASP-
NRIQQILDGK-LFPMKALGYFAVVTGKG---NRDESIESIKDYEEEFFQKSKLC-RSGMLKAHQ-VTT--
KNLSLAVSDCFWKMVRESV
XP_018667792.1 DHLPR----VVVVG----------------DQ----SAGKTSVLEM--
IAQARIFPRG---SGEMMTRAPVK-------------VTL--------SEGPN-------------------------------------
HVAQFRD----------------------S--------SREFDLSKEEELKSLRHEIELRMKSSCSDG---
KTVSNDTISLTVKGPGL----------QRMVLVDLPGMISTVTSGMA--PDTKDAIC--------
NMSKHYMENPNAIILCIQDGSVDAERS-IVTDLVSQMDPSGRRTIFVL-TKVDLAEKNITNP-
SRIQEILDGK-LFPMKALGYFAVVTGQG---SANSSITDIKEYEEEFFSNSKVF-KSGLLKASQ-LTT--
ANLSYAVSNCFWKMVRESV
XP_002602331.1 DELPR----VVVVG----------------DQ----SAGKTSVLEM--
VAQARIFPRG---AGEMMTRAPVK-------------VTL--------SEGPH-------------------------------------

HIAMFKD----------------------S--------DREFDLTKESELEALRREVEIRMKASVRPG---
QTVSMETIAMSVKGPGL----------QRMVLVDLPGIISTETQGMA--SATKESIK--------
MMCEHYMSNPNAIILCIQDGSVDAERS-NVTDLVSQMDPQGKRTIFVL-TKVDLAEKNITNP-
RRIKQILEGK-LFPMKALGYFAVVTGRG---NKDDSIDTIRGYEEEFFRNSQLF-RSGVLKASQ-MTT--
QNLSFAVSDCFWKMVKASV

XP_019637857.1 DELPR----VVVVG----------------DQ----SAGKTSVLEM--
VAQARIFPRG---AGEMMTRAPVK-------------VTL--------SEGPH---------------------------------------
HIAMFKD----------------------S--------DREFDLTKESELEALRREVEIRMKASVRPG---
QTVSMETISMSVKGPGL----------QRMVLVDLPGIISTETQGMA--SATKESIK--------
MMCEHYMSNPNAIILCIQDGSVDAERS-NVTDLVSQMDPQGKRTIFVL-TKVDLAEKNITNP-
HRIKQILEGK-LFPMKALGYFAVVMGRG---NKDDSIDTIRGYEEEFFRTSQLF-RSGVLKASQ-MTT--
QNLSFAVSDCFWKMVKASV

NP_495986.3.3 DNLPR----VVVVG----------------DQ----SAGKTSVLEM--
VAQARIFPRG---SGEMMTRAPVK-------------VTL--------SEGPY---------------------------------------
HVAQFRD----------------------S--------SREFDLTKETDLQQLRNETEVRMRNSVRDG---
KTVSNEVISLTVKGPNL----------PRMVLVDLPGVISTVTADMA--RETKDDII--------
RMSKAHMENPNAIILCIQDGSVDAERS-NVTDLVSSIDPSGKRTILVL-TKVDMAEKNLANP-
DRIKKILEGK-LFPMKALGYFGVVTGRG---NSSDSIDEIRKYEENFFSTSQLL-RDGVLKPSQ-MTT--
RNMSLAVSDCFWRMVRDSI

NP_610941.1 DHLPR----VVVVG----------------DQ----SSGKTSVLES--IAKARIFPRG---
SGEMMTRAPVK-------------VTL--------AEGPY---------------------------------------HVAQFRD---------
-------------S--------DREYDLTKESDLQDLRRDVEFRMKASVRGG---KTVSNEVIAMTVKGPGL-------
---QRMVLVDLPGIISTMTVDMA--SDTKDSIH--------QMTKHYMSNPNAIILCIQDGSVDAERS-
NVTDLVMQCDPLGRRTIFVL-TKVDLAEE-LADP-DRIRKILSGK-LFPMKALGYYAVVTGRG---
RKDDSIDAIRQYEEDFFKNSKLFHRRGVIMPHQ-VTS--RNLSLAVSDRFWKMVRETI

PAA68234.1 DHLPR----VVVIG----------------DQ----SSGKTSVLEA--VARARLFPRG---
AGEMMTRAPVQ-------------VTL--------ADGPY---------------------------------------HVARFKD---------
-------------DP-------DREFDLTKESELAALRDAIERRMRAAVRSSGPDAAVSTEAIPLSVQGPGL------
----PRMVLVDLPGIISTETAGMA--AQTRESIR--------QLARQYMRNPNAIILCVADACVDPERS-
NAFDLVAKHDPAGRRTIFVL-TKMDLAERDKVSP-DRVAKLLAGR-LLPLKALGYFAVVTGSG---
SQDESVEAIERHEAEYFASSRLF-KDGRLSPNQ-VTA--ANMARAVSRRFWALVRESV

PAA87312.1 DHLPR----VVVVG----------------DQ----SSGKTSVLEM--IAKARIFPRG---
AGEMMTRAPVQ-------------VTL--------AEGPY---------------------------------------HVARFKD---------
-------------NP-------SREYDLTQESELAALRDTIERRMRSVVQSG---GTVSAETISLSVQGPGL--------
--PRMVLVDLPGIISTETRGMA--SQTREAIR--------QLASQHMRNPNSIILCVADACVDPERS-
NAFDLVARHDPSGRRTIFVL-TKLDLAERDRISP-DRIGRLLAGR-LLPLKALGYFAVVTGSG---
GADESIPAIQRYEEQFFRNSQFF-KEGVLSVSQ-MTA--ANMAQAVSRRFWALVQESV

XP_004479029.1 EVLSRRHMKVAFFG----------------RT----SSGKSSVINA--
MLWDKVLPSG---IG-HTTNCFLS-------------VEG--------TDGDK---------------------------------------
AYLM-------------------------TEGS-----DEKKSVK---TVNQLAHALHMDN------------
DLKAGCLVHVFWPKA----KCALLRDDLVLVDSPGT------------DVTTELD--------
SWIDKFCLDADVFVLVANSESTLMNT--EKQFFHKVNERLSKPNIFILNNRWDASASE-PE----
YMEDVRRQ-HMERCL-NFLV---------EELKVVNPLEAQNRIFFVSAKEV-LSARKHKA--QGM--
PEGGGALADGF--------

NP_001193437.1 EVLSRRHMKVAFFG----------------RT----SSGKSSVINA--
MLWDKVLPSG---IG-HTTNCFLS-------------VEG--------TDGDK--------------------------------------
AYLM-------------------------TEGS-----DEKRSVK---TVNQLAHALHMDK------------
DLKAGCLVHVFWPKA----KCALLRDDLVLVDSPGT------------DVTTELD--------
SWIDKFCLDADVFVLVANSESTLMNT--EKQFFHKVNERLSKPNIFILNNRWDASASE-PE----
YMEDVRRQ-HMERCL-HFLV---------EELRVVDPLEARNRIFFVSAKEV-LSARKHKA--QGM--
PEGGGALAEGF--------
NP_077162.2.2 EVLSRRHMKVAFFG----------------RT----SSGKSSVINA--
MLWDKVLPSG---IG-HTTNCFLS-------------VEG--------TDGDK--------------------------------------
AYLM-------------------------TEGS-----DEKKSVK---TVNQLAHALHMDK------------
DLKAGCLVHVFWPKA----KCALLRDDLVLVDSPGT------------DVTTELD--------
IWIDKFCLDADVFVLVANSESTLMNT--EKHFFHKVNERLSKPNIFILNNRWDASASE-PE----
YMEDVRRQ-HMERCL-HFLV---------EELKVVSPSEARNRIFFVSAKEV-LNSRKHKA--QGM--
PEGGGALAEGF--------
XP_006162789.1 EVLSRRHMKVAFFG----------------RT----SSGKSSVINA--
MLWDKVLPSG---IG-HTTNCFLS-------------VEG--------TDGDK--------------------------------------
AYLM-------------------------TEGS-----DEKKSVK---TVNQLAHALHMDK------------
DLKAGCLVHVFWPKA----KCALLRDDLVLVDSPGT------------DVTTELD--------
SWIDKFCLDADVFVLVANSESTLMNT--EKHFFHKVNERLSKPNIFILNNRWDASASE-PE----
YMEDVRRQ-HMERCL-HFLV---------EELKVVGPSEARNRIFFVSAKEV-LSARKHRA--QGM--
PEGGGALAEGF--------
XP_005883071.1 EVLSRRHMKVAFFG----------------RT----SSGKSSVINA--
MLWDKVLPSG---IG-HTTNCLLS-------------VEG--------TDGDR--------------------------------------
AYLM-------------------------TEGS-----DEKKSVK---TVNQLAHALHMDK------------
DLKAGSLVHVFWPKA----KCALLRDDLVLVDSPGT------------DVTTELD--------
SWIDKFCLDADVFVLVANSESTLMNT--EKQFFHKVNERLSKPNIFILNNRWDASASE-PE----
YMEDVRRQ-HTERCL-HFLV---------EELKVVDPSEARNRIFFVSAKEV-LSARMHKA--QGM--
PEGGGALAEGF--------
XP_025917892.1 EVLSRRHMKVAFFG----------------RT----SSGKSSVINA--
MLWDKVLPSG---IG-HTTNCFLS-------------VEG--------TDGDK--------------------------------------
AYLM-------------------------TEGS-----DEKKSVK---TVNQLAHALHMDK------------
DLKAGCLVHVFWPKS----KCALLRDDLVLVDSPGT------------DVTTELD--------
SWIDKFCLDADVFVLVANSESTLMNT--EKHFFHKVNERLSKPNIFILNNRWDASASE-PE----
YMEDVRRQ-HMERCL-TFLV---------DELKVIDPIEARNRIFFVSAKEV-LSARRQKA--QGM--
PAGGEALAEGF--------
NP_284941.2.2 EVLSRRHMKVAFFG----------------RT----SSGKSSVINA--
MLWDKVLPSG---IG-HITNCFLS-------------VEG--------TDGDK--------------------------------------
AYLM-------------------------TEGS-----DEKKSVK---TVNQLAHALHMDK------------
DLKAGCLVRVFWPKA----KCALLRDDLVLVDSPGT------------DVTTELD--------
SWIDKFCLDADVFVLVANSESTLMNT--EKHFFHKVNERLSKPNIFILNNRWDASASE-PE----
YMEDVRRQ-HMERCL-HFLV---------EELKVVNALEAQNRIFFVSAKEV-LSARKQKA--QGM--
PESGVALAEGF--------
XP_028587453.1 GVLARRHMKVAFFG----------------RT----SSGKSSVINA--
MLWDRVLPSG---IG-HTTNCFLS-------------VEG--------TDGDK--------------------------------------

AYLM------------------------TEGS-----DEKKSVK---TVNQLAHALHMDK------------
DLEAGCLVHVFWPKA----KCALLRDDLVLVDSPGT------------DVTTELD--------
TWIDKFCLDADVFVLVANSESTLMNT--EKHFFHKVNEKLSKPNIFILNNRWDASASE-PE----
YMEHVRKQ-HMERCL-TFLV---------DELKVVDPSEAQNRIFFVSAKEV-LSARKQRA--QGM--
PEGGGALADGF--------
NP_001016189.1 EVLARRNMKVAFFG----------------RT----SSGKSTVINS--
MLWDKVLPSG---IG-HTTNCFLS-------------VEG--------TEGDK-------------------------------------
AYLM------------------------TEGS-----EEKKSVK---TVNQLAHALHMDK------------
DLGAGCLVHVFWPKA----KCALLRDDLVLVDSPGT------------DVTTELD--------
SWIDKFCLDADVFVLVANSESTLMNT--EKHFFYKVNEKLSKPNIFILNNRWDASASE-PE----
YMEDVRKQ-HMERCQ-SFLV---------DELKVVDSLEAQKRIFFVSAKEV-LNARMHKA--QGM--
PEAGAALAEGF--------
XP_017213868.2.2 EVLARRHMKVAFFG----------------RT----SNGKSTVINA--
MLRDRVLPSG---IG-HTTNCFLS-------------VEG--------TDEDK-------------------------------------
AFLK------------------------TEGS-----EEEKSIK---TVNQLAHALHMDE------------
SLDAGCLVKVFWPKT----KCALLRDDLVLVDSPGT------------DVTTELD--------
SWIDKFCLDADVFVLVANSESTLMNT--EKHFFHKVNEKLSKPNIFILNNRWDASAAE-PE----
YMEDVRKQ-HTDRCV-NFLV---------EELKVVDRAQAPNRIFFVSAKEV-LNSRMQRA--QGM--
PETGGALAEGF--------
XP_004482574.1 EVLARRHMKVAFFG----------------RT----SNGKSTVINA--
MLWDKVLPSG---IG-HTTNCFLR-------------VGG--------TDGHE-------------------------------------
AFLL------------------------TEGS-----EEKRSVK---TVNQLAHALHQDE------------
QLHAGSLVSVMWPNS----KCSLLKDDLVLMDSPGI------------DVTTELD--------
SWIDKFCLDADVFVLVANSESTLMQT--EKQFFHKVSERLSRPNIFILNNRWDASASE-PE----
YMEEVRRQ-HMERCT-SFLV---------DELGVVDRAQAGDRIFFVSAKEV-LNARIQKA--QGM--
PEGGGALAEGF--------
XP_006145367.1 EVLARRHMKVAFFG----------------RT----SNGKSTVINA--
MLWDKVLPSG---IG-HTTNCFLR-------------VEG--------TDGHE-------------------------------------
AFLL------------------------TEGS-----EEKRSVK---TVNQLAHALHQDE------------
QLHAGSLVSVMWPNS----KCPLLKDDLVLMDSPGI------------DVTTELD--------
SWIDKFCLDADVFVLVANSESTLMQT--EKQFFHKVSERLSRPNIFILNNRWDASASE-PE----
YMEEVRRQ-HMERCT-SFLV---------DELGVVDRAQAGDRIFFVSAKEV-LNARIQKA--QGM--
PEGGGALAEGF--------
XP_014400986.1 EVLARRHMKVAFFG----------------RT----SNGKSTVINA--
MLWDKVLPSG---IG-HTTNCFLR-------------VEG--------TDGHE-------------------------------------
AFLL------------------------TEGS-----EEKRSIK---TVNQLAHALHQDE------------
QLHAGSLVSVMWPNS----KCPLLKDDLVLMDSPGI------------DVTTELD--------
SWIDKFCLDADVFVLVANSESTLMQT--EKQFFHKVSERLSRPNIFILNNRWDASASE-PE----
YMEEVRRQ-HMERCT-SFLV---------DELGVVDRGQAGDRIFFVSAKEA-LNARIQKA--QGM--
PEGGGALAEGF--------
NP_001177198.1 EVLARRHMKVAFFG----------------RT----SNGKSTVINA--
MLWDKVLLSG---IG-HTTNCFLR-------------VEG--------TDGHE-------------------------------------
AFLL------------------------TEGS-----EEKRSVK---TVNQLAHALHQDE------------
QLHAGSLVSVMWPNS----KCPLLKDDLVLMDSPGI------------DVTTELD--------

SWIDKFCLDADVFVLVANSESTLMQT--EKQFFHKVSERLSRPNIFILNNRWDASASE-PE----
YMEEVRRQ-HMERCT-SFLV---------DELGVVDRGQAGDRIFFVSAKEV-LNARIQKA--QGM--
PEGGGALAEGF--------
NP_001272849.1 EVLARRHMKVAFFG----------------RT----SNGKSTVINA--
MLWDKVLPSG---IG-HTTNCFLR-------------VGG--------TDGHE-------------------------------------
AFLL-------------------------TEGS-----EEKKSVK---TVNQLAHALHQDE------------
QLHAGSMVSVMWPNS----KCPLLKDDLVLMDSPGI------------DVTTELD--------
SWIDKFCLDADVFVLVANSESTLMQT--EKQFFHKVSERLSRPNIFILNNRWDASASE-PE----
YMEEVRRQ-HMERCT-SFLV---------DELGVVDRAQAGDRIFFVSAKEV-LSARVQKA--QGM--
PEGGGALAEGF--------
NP_001121132.1 EVLARRHMKVAFFG----------------RT----SNGKSTVINA--
MLWDKVLPSG---IG-HTTNCFLR-------------VEG--------TDGHE-------------------------------------
AFLL-------------------------TEGS-----EEKRSAK---TVNQLAHALHQDK------------
QLHAGSLVSVMWPNS----KCPLLKDDLVLMDSPGI------------DVTTELD--------
SWIDKFCLDADVFVLVANSESTLMQT--EKHFFHKVSERLSRPNIFILNNRWDASASE-PE----
YMEEVRRQ-HMERCT-SFLV---------DELGVVDRSQAGDRIFFVSAKEV-LNARIQKA--QGM--
PEGGGALAEGF--------
XP_025929938.1 EVLARRHMKVAFFG----------------RT----SNGKSTVINA--
MLWDKVLPSG---IG-HTTNCFLR-------------VEG--------TDGHE-------------------------------------
AFLL-------------------------TEGS-----EEKKSVK---TVNQLAHALHQDE------------
LLDAGSLVSVMWPNS----KCPLLKDDLVLMDSPGI------------DVTTELD--------
SWIDKFCLDADVFVLVANSESTLMQT--EKQFFHKVNERLSRPNIFILNNRWDASASE-PE----
YMEEVRRQ-HMERCT-SFLV---------DELGVVDRAQAGDRIFFVSAKEV-LNARIQRA--QGM--
PEGGGALADGF--------
XP_028597443.1 QVLARRHMKVAFFG----------------RT----SNGKSTVINA--
MLWDKVLPSG---IG-HTTNCFLR-------------VEG--------TEGQD-------------------------------------
AFLL-------------------------TEGS-----EEKKSVK---TVNQLAHALHQDE------------
LLTAGGMVSVMWPNS----KCPLLKDDLVLMDSPGI------------DVTTELD--------
SWIDKFCLDADVFVLVANSESTLMQT--EKQFFHKVNTRLSRPNIFILNNRWDASASE-PE----
YMEEVRRQ-HMERCT-SFLV---------DELGVVDRAQAGDRIFFVSAKEV-LSARIQKA--QGM--
PEGGGALAEGF--------
XP_015268039.1 EVLARRHMKVAFFG----------------RT----SNGKSTVINA--
MLWDKVLPSG---IG-HTTNCFLR-------------VEG--------TDGHD-------------------------------------
AFLL-------------------------TEGS-----EXXXXXX---TVNQLAHALHQDE------------
LLTAGSLVSVMWPNS----KCPLLKDDLVLMDSPGI------------DVTTELD--------
SWIDKFCLDADVFVLVANSESTLMQT---VRRQ-HMERCT-
SFLV---------DELGVVDRAQAGDRIFFVSAKEV-LSARIQKA--QGM--PEGGGALAEGF--------
NP_001121726.1 EVLSRRHMKVVFFG----------------RT----SNGKSSVINA--
MLWDKVLPSG---IG-HTTNCFLR-------------VEG--------TDGNE-------------------------------------
SFLL-------------------------TEGS-----DERKSVK---TVNQLAHALHQDE------------
DLDAGSLVCVMWPKA----KCALLRDDLVLVDSPGI------------DVTTELD--------
SWIDKFCLDADVFVLVANSESTLMQT--EKSFFHKVNERLSSPNIFILNNRWDASANE-PE----
YMEEVRRQ-HMDRCT-SFLV---------DELRVVDRSHAGDRIFFVSAKEV-LQARVQKA--QGM--
PEAGGALAEGF--------

XP_002591612.1 QVISRDKMKVAFFG----------------RT----SNGKSTVVNA--MLRDKILPSG---IG-HTTNCFIN-------------VEG--------SDGYE------------------------------------AYLL-------------------------TPDS-----DDRKTVQ---SVGQLAHALCGER------------LEDSSILVKVFWPKG----RCALLRDDVVLLDSPGI------------DVTPDLD--------SWIDEHCLDADVFVLVANSESTLMRTAREKNFFHTVSERLSKPNIFILNNRWDASASE-PE----FMEAVKKQ-HLERCV-SFLV---------EELGVVDRLQAEDRVFFVSAKEA-LQSRLQKQ--QGM--PEEGGALAEGF--------

XP_019628129.1 QVISRDKMKVAFFG----------------RT----SNGKSTVVNA--MLRDKILPSG---IG-HTTNCFIN-------------VEG--------SDGFE------------------------------------AYLL-------------------------TPDS-----DDRKTVQ---SVGQLAHALCSER------------LEDSSVLVKVFWPKG----RCALLRDDVVLLDSPGI------------DVTPDLD--------SWIDEHCLDADVFVLVANSESTLMRT--EKNFFHTVSERLSKPNIFILNNRWDASASE-PE----FMEAVKKQ-HLERCV-SFLV---------EELGVVDRLQAEDRVFFVSAKEA-LQSRLQKQ--QGM--PEEGGALAEGF--------

XP_006819998.1 EVLARDHMKVAFFG----------------RT----SSGKSTVINA--MLKDKVLPTG---IG-HTTDCFLS-------------IEG--------SDTSE------------------------------------AYLI-------------------------IPQS-----NERRNVR---SVSQLAHALSNEK------------LADQSSLIHVFWPSS----RCALLKDDLVLVDSPGV------------DVTADLD--------SWIDDHCLDADVFVLVANAESTLMRT--EKSFFHKVAEKLSKPNIFILNNRWDASASE-PD----SMEDVKKQ-HLERSI-GFLV---------EELKVITKQQAEDRVFFVSAKEA-LCCRIQKV--QGM--PEAG---------------

XP_030846906.1 DVLERDHMKVAFFG----------------RT----SNGKSTVINA--MLRDKVLPSG---IG-HTTDCFLC-------------VEG--------CEGQE-------------------------------------GYMS-------------------------RQNS-----SEKISTT---SVSQLANALAGERDH---------EDFQQRSILHIFWPKT----QCHLLKNDVVLLDSPGI------------DVEHDMD--------EWIDDHYMDADVFVLVSNAESTLTRT--ETSFFLKVSAKLSKPNIFILNNRWDASANE-PE----NMEVVKRQ-HLEREI-KFLV---------EELKVMTEAQAKDRIFFVSAKEA-LNSRILQT--LST--PNAN-PIVEGY--------

XP_030847518.1 DVLERDHMKVAFFG----------------RT----SNGKSTVINA--MLRDKVLPSG---IG-HTTDCFLC-------------VEG--------CEGQE-------------------------------------GYMS-------------------------RQNS-----SEKMSIT---SVSQLAHALAGERDH---------EECQQSSILHIFWPKT----QCHLLMNDVVLLDSPGI------------DVEQDLD--------EWINTHCVDADVFVLVLNAESTLMRT--EKSFFHKVSEKLSKPNIFILNNRWDASANE-PE----FMEAVKRQ-HLERDV-KFLV---------EELKVMTEAQAKDRVFFVSAKEA-LNSRIPKT--LST--PDAN-PVVEGY--------

NP_996357.1 EVLQRDHMKVAFFG----------------RT----SNGKSSVINA--MLREKILPSG---IG-HTTNCFCQ-------------VEG--------SNGGE------------------------------------AYLM-------------------------TEGS-----EEKLNVV---NIKQLANALCQEK-------------LCESSLVRIFWPRE----RCSLLRDDVVFVDSPGV------------DVSANLD--------DWIDNHCLNADVFVLVLNAESTMTRA--EKQFFHTVSQKLSKPNIFILNNRWDASANE-PE----CQESVKSQ-HTERCI-DFLT---------KELKVSNEKEAAERVFFVSARET-LQARIEEA--KGN--PPHMGAIAEGF--------

XP_002126852.1 DMLTRNHMKVVFFG----------------RT----SNGKSSVVNA--MLWDRILPTG---IG-HTTNCFLS-------------VAGCSDEGSTSTDSDE-----------------------------------GAYLL-------------------------CNGS-----EEKRSIK---SVTQLSHALSEES------------

MSPDSLIQVFWPKS----KCALLKDDVVLVDSPGI------------DVSHDLD--------
QWIDKYCLDADVFILVANAESTLMQA--EKKFFHRVNEKLSKPNIFILNNRWDASASE-PE----
LMEQVRQQ-HLERGI-SFLA---------DELKVISKSQAKDRVFFVSAKET-LQSRMPKV--PGK--
ADSPVYMADGH--------
PAA75551.1 EVISRNQMKCAFFG----------------RT----SNGKSTVINA--MLGRKVLPSG---
IG-HTTNCFLQ-------------VEG--------TSKQS-------------------------------------AYLQ-------------------------TPNS-----SEEQPIE---SVSQLGSALSNEK-------------MDCESLVRVFWPKQ----
LCSLLREDVVLLDSPGV------------DVSPDLD--------TWIDQFCMDADVFILVCNSESTLMNT--
EKKFFHKVGSKLSKPNVFVLNNRWDCSDGE-LD----SAELVRKQ-HMDKSV-SFLA---------
DELKSCTRSEAESRVYFVSAKEA-LVNRLKETN-QGLESPSPAGSLADGW--------
PAA75258.1 EVISRNQMKCAFFG----------------RT----SNGKSTVINA--MLGRKVLPSG---
IG-HTTNCFLQ-------------VEG--------TSKES-------------------------------------AYLQ-------------------------TPNS-----SEERPIE---SVSQLGSALSNEK-------------LDCESLVRVFWPKQ----
LCSLLREDVVLLDSPGV------------DVSPDLD--------TWIDQFCMDADVFILVCNSESTLMNT--
EKKFFHKVGSKLSKPNVFVLNNRWDCSDGE-LD----SAELVRKQ-HMDKSV-SFLA---------
DELKSCTRSEAESRVYFVSAKEA-LVNRLKETN-QGLESPSPAGSLADGW--------
XP_004365821.1 KLFERDHMKVVFVG----------------QT----SNGKSTVVNA--
MLYNRILPSG---IG-HTTNCFVS-------------VSG--------SDANT-------------------------------------
PYII-------------------------DSLS-----SEQQPIS---NVLQLANALHPEG------------
SLNQSGLIRVFWPTT----KCRLLGDDVDLIDSPGL------------DLSNDIN--------
QWIDDYCMDADVFVLVANAEATLKVA--ERAFFFKVNEKLSKPNVFILNNRWDASDNEIDD----
SPERVREQ-HLEYAS-KFLA---------DELKVVSRSKILDRVFFVSARET-LLYRTTEN--WTR--
FKESQAV------------
NP_495161.1 DTFQRDNMKVVFFG----------------RT----SNGKSTTINA--MLHEKVLPQG--
-MG-HTTCCFLQ-------------VEG--------SEGEV-------------------------------------GHLQ-------------------------LDDN-----PQKIDMK---MLGKIGHALSDENSDL--------
PAMGQDSLLKVFHPKKSESGECRLLQNDVVILDSPGV------------DLSPEFD--------
SWIDKHCLDADVFVLVSNAESTLTQA--EKNFFLRVAKKLSKPNVFILNNRWDASAAE-TE----
NIEDVKKQ-HLTRFR-QFLV---------DELEVCSEREVNDRIFFVSSREV-LESRLKA---RGL--VQKA-
YQAEGH--------
XP_014153836.1 -MLRRDSMKVVFVG----------------HT----SNGKSTVINS--
MLGQKVLPMG---IG-HTTSCFCS-------------VTG--------TDEE--------------------------------------
PYIILGHEPKAKNSNSIRLNRVQRSDSPPSPPP-----EKRMAID---NVKTVANALCPES------------
DHDAYQFVRVFWDKR----KCNLLGDGVLFVDTPGL------------DIDENYD--------
NWIDKFCMDADVFVLVANGESTIKHT--EMNFFTKVAEKLSRPNVFILFNRWDGSDME-DD-----
VTPVQEQ-HKDRVR-SFFK---------KELQQ-DANIIDKRVFFVSGKEV-LTHRTKPD--KSV--
VKESNPSPMG---------
XP_001745740.1 RSVTRESMKVVFVG----------------RT----SNGKSTTINA--
MLHTRVLPAG---PG-HTTNCFVT-------------LQG--------SDQSK-------------------------------------
AYMQ-------------------------LPGD-----PTPRDLK---DVQSLTDALQQEH------------
VLPPGQSVEIHWPRD----QCHLLRDDVVILDSPGL------------DYDSDFD--------
AWIDETTRDADVFVLVVNAVSTLSGA--ESGFFHSVCKTVAKPNVFVIFNQWDNLDED-EA----
DVTGVRAQ-HMSKAR-DLLV---------RDLGICSEAELSSRVFFVSSKEV-LKSRAGSDS-
RTTSYTDPSSAVVPGLNTH-----

;
end;
begin trees;
tree tree_1 = [&U] [&branchAttributeNames={"FastTree support value"}](((XP_031753735.1[&Organism="Xenopus tropicalis","Genetic Code"="Standard",Taxonomy="Eukaryota; Metazoa; Chordata; Craniata; Vertebrata; Euteleostomi; Amphibia; Batrachia; Anura; Pipoidea; Pipidae; Xenopodinae; Xenopus; Silurana","Common Name"="tropical clawed frog"]:0.04274000000000022,(XP_025920181.1[&Organism="Apteryx rowi","Genetic Code"="Standard",Taxonomy="Eukaryota; Metazoa; Chordata; Craniata; Vertebrata; Euteleostomi; Archelosauria; Archosauria; Dinosauria; Saurischia; Theropoda; Coelurosauria; Aves; Palaeognathae; Apterygiformes; Apterygidae; Apteryx","Common Name"="Okarito brown kiwi"]:0.000540,((((NP_001005360.1[&Organism="Homo sapiens","Genetic Code"="Standard",Taxonomy="Eukaryota; Metazoa; Chordata; Craniata; Vertebrata; Euteleostomi; Mammalia; Eutheria; Euarchontoglires; Primates; Haplorrhini; Catarrhini; Hominidae; Homo","Common Name"="human"]:0.003670000000000506,(XP_012381548.1[&Organism="Dasypus novemcinctus","Genetic Code"="Standard",Taxonomy="Eukaryota; Metazoa; Chordata; Craniata; Vertebrata; Euteleostomi; Mammalia; Eutheria; Xenarthra; Cingulata; Dasypodidae; Dasypus","Common Name"="nine-banded armadillo"]:0.04507999999999565,XP_006161648.2.2[&Organism="Tupaia chinensis","Genetic Code"="Standard",Taxonomy="Eukaryota; Metazoa; Chordata; Craniata; Vertebrata; Euteleostomi; Mammalia; Eutheria; Euarchontoglires; Scandentia; Tupaiidae; Tupaia","Common Name"="Chinese tree shrew"]:0.0236799999999997)[&"FastTree support value"=1.0]:0.04271000000000047)[&"FastTree support value"=0.0]:0.000550,XP_006510037.1[&Organism="Mus musculus","Genetic Code"="Standard",Taxonomy="Eukaryota; Metazoa; Chordata; Craniata; Vertebrata; Euteleostomi; Mammalia; Eutheria; Euarchontoglires; Glires; Rodentia; Myomorpha; Muroidea; Muridae; Murinae; Mus; Mus","Common Name"="house mouse"]:0.000550)[&"FastTree support value"=0.768]:0.0037000000000002586,XP_014389433.1[&Organism="Myotis brandtii","Genetic Code"="Standard",Taxonomy="Eukaryota; Metazoa; Chordata; Craniata; Vertebrata; Euteleostomi; Mammalia; Eutheria; Laurasiatheria; Chiroptera; Microchiroptera; Vespertilionidae; Myotis","Common Name"="Brandt's bat"]:0.023229999999999862)[&"FastTree support value"=0.944]:0.014829999999999899,XP_028568434.1[&Organism="Podarcis muralis","Genetic Code"="Standard",Taxonomy="Eukaryota; Metazoa; Chordata; Craniata; Vertebrata; Euteleostomi; Lepidosauria; Squamata; Bifurcata; Unidentata; Episquamata; Laterata; Lacertibaenia; Lacertidae; Podarcis","Common Name"="Common wall lizard"]:0.0027099999999999546)[&"FastTree support value"=0.385]:0.004679999999999573)[&"FastTree support value"=0.595]:0.014630000000000365)[&"FastTree support

value"=0.977]:0.03807999999999989,((EPQ08653.1[&Organism="Myotis brandtii","Genetic Code"="Standard",Taxonomy="Eukaryota; Metazoa; Chordata; Craniata; Vertebrata; Euteleostomi; Mammalia; Eutheria; Laurasiatheria; Chiroptera; Microchiroptera; Vespertilionidae; Myotis","Common Name"="Brandt's bat"]:0.00786999999999996,(XP_006496668.1[&Organism="Mus musculus","Genetic Code"="Standard",Taxonomy="Eukaryota; Metazoa; Chordata; Craniata; Vertebrata; Euteleostomi; Mammalia; Eutheria; Euarchontoglires; Glires; Rodentia; Myomorpha; Muroidea; Muridae; Murinae; Mus; Mus","Common Name"="house mouse"]:0.0034799999999997056,(XP_027623811.1[&Organism="Tupaia chinensis","Genetic Code"="Standard",Taxonomy="Eukaryota; Metazoa; Chordata; Craniata; Vertebrata; Euteleostomi; Mammalia; Eutheria; Euarchontoglires; Scandentia; Tupaiidae; Tupaia","Common Name"="Chinese tree shrew"]:0.011240000000000805,(XP_012379251.1[&Organism="Dasypus novemcinctus","Genetic Code"="Standard",Taxonomy="Eukaryota; Metazoa; Chordata; Craniata; Vertebrata; Euteleostomi; Mammalia; Eutheria; Xenarthra; Cingulata; Dasypodidae; Dasypus","Common Name"="nine-banded armadillo"]:0.000550,XP_016856477.1[&Organism="Homo sapiens","Genetic Code"="Standard",Taxonomy="Eukaryota; Metazoa; Chordata; Craniata; Vertebrata; Euteleostomi; Mammalia; Eutheria; Euarchontoglires; Primates; Haplorrhini; Catarrhini; Hominidae; Homo","Common Name"="human"]:0.0037199999999995015)[&"FastTree support value"=0.0]:0.000550)[&"FastTree support value"=1.0]:0.000530)[&"FastTree support value"=0.769]:0.011009999999999742)[&"FastTree support value"=0.962]:0.024340000000000472,(XP_025944940.1[&Organism="Apteryx rowi","Genetic Code"="Standard",Taxonomy="Eukaryota; Metazoa; Chordata; Craniata; Vertebrata; Euteleostomi; Archelosauria; Archosauria; Dinosauria; Saurischia; Theropoda; Coelurosauria; Aves; Palaeognathae; Apterygiformes; Apterygidae; Apteryx","Common Name"="Okarito brown kiwi"]:0.04270999999999958,XP_031757197.1[&Organism="Xenopus tropicalis","Genetic Code"="Standard",Taxonomy="Eukaryota; Metazoa; Chordata; Craniata; Vertebrata; Euteleostomi; Amphibia; Batrachia; Anura; Pipoidea; Pipidae; Xenopodinae; Xenopus; Silurana","Common Name"="tropical clawed frog"]:0.14616000000000007)[&"FastTree support value"=0.767]:0.013470000000000076)[&"FastTree support value"=0.914]:0.024499999999999744)[&"FastTree support value"=0.801]:0.020240000000000258,(XP_021326548.1[&Organism="Danio rerio","Genetic Code"="Standard",Taxonomy="Eukaryota; Metazoa; Chordata; Craniata; Vertebrata; Euteleostomi; Actinopterygii; Neopterygii; Teleostei; Ostariophysi; Cypriniformes; Cyprinidae; Danio","Common Name"="zebrafish"]:0.0,NP_001025299.1[&Organism="Danio rerio","Genetic Code"="Standard",Taxonomy="Eukaryota; Metazoa; Chordata; Craniata; Vertebrata; Euteleostomi; Actinopterygii; Neopterygii; Teleostei; Ostariophysi; Cypriniformes; Cyprinidae; Danio","Common Name"="zebrafish"]:0.0):0.04976000000000026)[&"FastTree support value"=0.63]:0.013239999999999696,(((XP_035683496.1[&Organism="Branchiostoma floridae","Genetic Code"="Standard",Taxonomy="Eukaryota; Metazoa; Chordata;

Cephalochordata; Branchiostomidae; Branchiostoma","Common Name"="Florida lancelet"]:0.07881999999999945,(((((XP_014153758.1[&Organism="Sphaeroforma arctica JP610","Genetic Code"="Standard",Taxonomy="Eukaryota; Ichthyosporea; Ichthyophonida; Sphaeroforma"]:0.0874600000000001,XP_014148725.1[&Organism="Sphaeroforma arctica JP610","Genetic Code"="Standard",Taxonomy="Eukaryota; Ichthyosporea; Ichthyophonida; Sphaeroforma"]:0.12324999999999964)[&"FastTree support value"=0.922]:0.05618000000000034,(XP_001749319.1[&Organism="Monosiga brevicollis MX1","Genetic Code"="Standard",Taxonomy="Eukaryota; Choanoflagellata; Craspedida; Salpingoecidae; Monosiga"]:0.09123999999999999,(((XP_004348308.1[&Organism="Capsaspora owczarzaki ATCC 30864","Genetic Code"="Standard",Taxonomy="Eukaryota; Filasterea; Capsaspora"]:0.20411999999999964,XP_014148015.1[&Organism="Sphaeroforma arctica JP610","Genetic Code"="Standard",Taxonomy="Eukaryota; Ichthyosporea; Ichthyophonida; Sphaeroforma"]:0.30495999999999945)[&"FastTree support value"=0.041]:0.019569999999999865,(XP_001750431.1[&Organism="Monosiga brevicollis MX1","Genetic Code"="Standard",Taxonomy="Eukaryota; Choanoflagellata; Craspedida; Salpingoecidae; Monosiga"]:0.38597000000000037,((NP_001259946.1[&Organism="Drosophila melanogaster","Genetic Code"="Standard",Taxonomy="Eukaryota; Metazoa; Ecdysozoa; Arthropoda; Hexapoda; Insecta; Pterygota; Neoptera; Endopterygota; Diptera; Brachycera; Muscomorpha; Ephydroidea; Drosophilidae; Drosophila; Sophophora","Common Name"="fruit fly"]:0.15398000000000067,NP_741403.2[&Organism="Caenorhabditis elegans","Genetic Code"="Standard",Taxonomy="Eukaryota; Metazoa; Ecdysozoa; Nematoda; Chromadorea; Rhabditida; Rhabditina; Rhabditomorpha; Rhabditoidea; Rhabditidae; Peloderinae; Caenorhabditis"]:0.28471000000000046)[&"FastTree support value"=0.738]:0.039550000000000196,(((XP_002129967.2[&Organism="Ciona intestinalis","Genetic Code"="Standard",Taxonomy="Eukaryota; Metazoa; Chordata; Tunicata; Ascidiacea; Phlebobranchia; Cionidae; Ciona","Common Name"="vase tunicate"]:0.3687100000000001,XP_032819300.1[&Organism="Petromyzon marinus","Genetic Code"="Standard",Taxonomy="Eukaryota; Metazoa; Chordata; Craniata; Vertebrata; Cyclostomata; Hyperoartia; Petromyzontiformes; Petromyzontidae; Petromyzon","Common Name"="sea lamprey"]:0.08938999999999986)[&"FastTree support value"=0.199]:0.0316000000000007,(NP_957216.1[&Organism="Danio rerio","Genetic Code"="Standard",Taxonomy="Eukaryota; Metazoa; Chordata; Craniata; Vertebrata; Euteleostomi; Actinopterygii; Neopterygii; Teleostei; Ostariophysi; Cypriniformes; Danionidae; Danioninae; Danio","Common Name"="zebrafish"]:0.02429000000000059,(((XP_014394711.1[&Organism="Myotis brandtii","Genetic Code"="Standard",Taxonomy="Eukaryota; Metazoa; Chordata; Craniata; Vertebrata; Euteleostomi; Mammalia; Eutheria; Laurasiatheria; Chiroptera; Microchiroptera; Vespertilionidae; Myotis","Common Name"="Brandt's bat"]:0.028999999999999915,(XP_012382650.2[&Organism="Dasypus novemcinctus","Genetic Code"="Standard",Taxonomy="Eukaryota; Metazoa; Chordata; Craniata; Vertebrata; Euteleostomi; Mammalia; Eutheria; Xenarthra; Cingulata; Dasypodidae; Dasypus","Common Name"="nine-banded

armadillo"]:0.02557000000000093,XP_031753959.1[&Organism="Xenopus tropicalis","Genetic Code"="Standard",Taxonomy="Eukaryota; Metazoa; Chordata; Craniata; Vertebrata; Euteleostomi; Amphibia; Batrachia; Anura; Pipoidea; Pipidae; Xenopodinae; Xenopus; Silurana","Common Name"="tropical clawed frog"]:0.17985000000000007)[&"FastTree support value"=0.954]:0.05191000000000345)[&"FastTree support value"=0.806]:0.009539999999999438,(XP_006168142.1[&Organism="Tupaia chinensis","Genetic Code"="Standard",Taxonomy="Eukaryota; Metazoa; Chordata; Craniata; Vertebrata; Euteleostomi; Mammalia; Eutheria; Euarchontoglires; Scandentia; Tupaiidae; Tupaia","Common Name"="Chinese tree shrew"]:0.000550,(NP_001392186.1[&Organism="Mus musculus","Genetic Code"="Standard",Taxonomy="Eukaryota; Metazoa; Chordata; Craniata; Vertebrata; Euteleostomi; Mammalia; Eutheria; Euarchontoglires; Glires; Rodentia; Myomorpha; Muroidea; Muridae; Murinae; Mus; Mus","Common Name"="house mouse"]:0.009299999999999642,NP_001317309.1[&Organism="Homo sapiens","Genetic Code"="Standard",Taxonomy="Eukaryota; Metazoa; Chordata; Craniata; Vertebrata; Euteleostomi; Mammalia; Eutheria; Euarchontoglires; Primates; Haplorrhini; Catarrhini; Hominidae; Homo","Common Name"="human"]:0.003049999999999997)[&"FastTree support value"=0.749]:0.000510)[&"FastTree support value"=0.962]:0.000530)[&"FastTree support value"=0.978]:0.02646000000000015,(XP_025940269.1[&Organism="Apteryx rowi","Genetic Code"="Standard",Taxonomy="Eukaryota; Metazoa; Chordata; Craniata; Vertebrata; Euteleostomi; Archelosauria; Archosauria; Dinosauria; Saurischia; Theropoda; Coelurosauria; Aves; Palaeognathae; Apterygiformes; Apterygidae; Apteryx","Common Name"="Okarito brown kiwi"]:0.02644000000000907,XP_028602039.1[&Organism="Podarcis muralis","Genetic Code"="Standard",Taxonomy="Eukaryota; Metazoa; Chordata; Craniata; Vertebrata; Euteleostomi; Lepidosauria; Squamata; Bifurcata; Unidentata; Episquamata; Laterata; Lacertibaenia; Lacertidae; Podarcis","Common Name"="Common wall lizard"]:0.02876000000000012)[&"FastTree support value"=0.116]:0.0024999999999995026)[&"FastTree support value"=0.966]:0.03654000000000046)[&"FastTree support value"=0.971]:0.06032999999999955)[&"FastTree support value"=0.936]:0.05175000000000185,((XP_035676386.1[&Organism="Branchiostoma floridae","Genetic Code"="Standard",Taxonomy="Eukaryota; Metazoa; Chordata; Cephalochordata; Leptocardii; Amphioxiformes; Branchiostomatidae; Branchiostoma","Common Name"="Florida lancelet"]:0.07395999999999958,(XP_006821224.1[&Organism="Saccoglossus kowalevskii","Genetic Code"="Standard",Taxonomy="Eukaryota; Metazoa; Hemichordata; Enteropneusta; Harrimaniidae; Saccoglossus"]:0.10476000000000063,XP_030827871.1[&Organism="Strongylocentrotus purpuratus","Genetic Code"="Standard",Taxonomy="Eukaryota; Metazoa; Echinodermata; Eleutherozoa; Echinozoa; Echinoidea; Euechinoidea; Echinacea; Camarodonta; Echinidea; Strongylocentrotidae; Strongylocentrotus","Common Name"="purple sea urchin"]:0.08788000000000018)[&"FastTree support

value"=0.87]:0.04866999999999955)[&"FastTree support
value"=0.645]:0.040470000000000006,PAA85687.1[&Organism="Macrostomum
lignano","Genetic Code"="Standard",Taxonomy="Eukaryota; Metazoa; Spiralia;
Lophotrochozoa; Platyhelminthes; Rhabditophora; Macrostomorpha; Macrostomida;
Macrostomidae; Macrostomum"]:0.19941999999999993)[&"FastTree support
value"=0.686]:0.040770000000000195)[&"FastTree support
value"=0.857]:0.034880000000000244)[&"FastTree support
value"=0.992]:0.15169999999999995)[&"FastTree support
value"=0.949]:0.07288999999999923)[&"FastTree support
value"=0.877]:0.06943000000000055,(((((PAA75551.1[&Organism="Macrostomum
lignano","Genetic Code"="Standard",Taxonomy="Eukaryota; Metazoa; Platyhelminthes;
Rhabditophora; Macrostomorpha; Macrostomida; Macrostomidae;
Macrostomum"]:0.011420000000000208,PAA75258.1[&Organism="Macrostomum
lignano","Genetic Code"="Standard",Taxonomy="Eukaryota; Metazoa; Platyhelminthes;
Rhabditophora; Macrostomorpha; Macrostomida; Macrostomidae;
Macrostomum"]:0.000540)[&"FastTree support
value"=1.0]:0.3133999999999997,((XP_004365821.1[&Organism="Capsaspora owczarzaki
ATCC 30864","Genetic Code"="Standard",Taxonomy="Eukaryota; Filasterea;
Capsaspora"]:0.3587100000000003,XP_014153836.1[&Organism="Sphaeroforma arctica
JP610","Genetic Code"="Standard",Taxonomy="Eukaryota; Ichthyosporea; Ichthyophonida;
Sphaeroforma"]:0.5829699999999995)[&"FastTree support
value"=0.992]:0.24552999999999958,(XP_002126852.1[&Organism="Ciona
intestinalis","Genetic Code"="Standard",Taxonomy="Eukaryota; Metazoa; Chordata;
Tunicata; Ascidiacea; Enterogona; Phlebobranchia; Cionidae; Ciona","Common
Name"="vase tunicate"]:0.2565999999999997,(((NP_001121726.1[&Organism="Danio
rerio","Genetic Code"="Standard",Taxonomy="Eukaryota; Metazoa; Chordata; Craniata;
Vertebrata; Euteleostomi; Actinopterygii; Neopterygii; Teleostei; Ostariophysi;
Cypriniformes; Danionidae; Danioninae; Danio","Common
Name"="zebrafish"]:0.05816999999999961,((XP_025929938.1[&Organism="Apteryx
rowi","Genetic Code"="Standard",Taxonomy="Eukaryota; Metazoa; Chordata; Craniata;
Vertebrata; Euteleostomi; Archelosauria; Archosauria; Dinosauria; Saurischia; Theropoda;
Coelurosauria; Aves; Palaeognathae; Apterygiformes; Apterygidae; Apteryx","Common
Name"="Okarito brown
kiwi"]:0.008000000000000007,(XP_028597443.1[&Organism="Podarcis muralis","Genetic
Code"="Standard",Taxonomy="Eukaryota; Metazoa; Chordata; Craniata; Vertebrata;
Euteleostomi; Lepidosauria; Squamata; Bifurcata; Unidentata; Episquamata; Laterata;
Lacertibaenia; Lacertidae; Podarcis","Common Name"="Common wall
lizard"]:0.022389999999999688,XP_015268039.1[&Organism="Gekko japonicus","Genetic
Code"="Standard",Taxonomy="Eukaryota; Metazoa; Chordata; Craniata; Vertebrata;
Euteleostomi; Lepidosauria; Squamata; Bifurcata; Gekkota; Gekkonidae; Gekkoninae;
Gekko"]:0.000550)[&"FastTree support value"=0.946]:0.013679999999999914)[&"FastTree
support value"=0.682]:0.002200000000000202,((XP_006145367.1[&Organism="Tupaia
chinensis","Genetic Code"="Standard",Taxonomy="Eukaryota; Metazoa; Chordata;
Craniata; Vertebrata; Euteleostomi; Mammalia; Eutheria; Euarchontoglires; Scandentia;

Tupaiidae; Tupaia","Common Name"="Chinese tree
shrew"]:0.000540,(NP_001121132.1[&Organism="Homo sapiens","Genetic
Code"="Standard",Taxonomy="Eukaryota; Metazoa; Chordata; Craniata; Vertebrata;
Euteleostomi; Mammalia; Eutheria; Euarchontoglires; Primates; Haplorrhini; Catarrhini;
Hominidae; Homo","Common
Name"="human"]:0.015799999999999592,(XP_014400986.1[&Organism="Myotis
brandtii","Genetic Code"="Standard",Taxonomy="Eukaryota; Metazoa; Chordata; Craniata;
Vertebrata; Euteleostomi; Mammalia; Eutheria; Laurasiatheria; Chiroptera;
Microchiroptera; Vespertilionidae; Myotis","Common Name"="Brandt's
bat"]:0.007939999999999614,NP_001177198.1[&Organism="Bos taurus","Genetic
Code"="Standard",Taxonomy="Eukaryota; Metazoa; Chordata; Craniata; Vertebrata;
Euteleostomi; Mammalia; Eutheria; Laurasiatheria; Artiodactyla; Ruminantia; Pecora;
Bovidae; Bovinae; Bos","Common Name"="cattle"]:0.003999999999995595)[&"FastTree
support value"=0.934]:0.0037099999999988)[&"FastTree support
value"=0.996]:0.000550)[&"FastTree support
value"=0.144]:0.000550,(XP_004482574.1[&Organism="Dasypus novemcinctus","Genetic
Code"="Standard",Taxonomy="Eukaryota; Metazoa; Chordata; Craniata; Vertebrata;
Euteleostomi; Mammalia; Eutheria; Xenarthra; Cingulata; Dasypodidae;
Dasypus","Common Name"="nine-banded
armadillo"]:0.003989999999999938,NP_001272849.1[&Organism="Mus
musculus","Genetic Code"="Standard",Taxonomy="Eukaryota; Metazoa; Chordata;
Craniata; Vertebrata; Euteleostomi; Mammalia; Eutheria; Euarchontoglires; Glires;
Rodentia; Myomorpha; Muroidea; Muridae; Murinae; Mus; Mus","Common Name"="house
mouse"]:0.01590999999999987)[&"FastTree support
value"=0.673]:0.004010000000000069)[&"FastTree support
value"=0.914]:0.013889999999999958)[&"FastTree support
value"=0.975]:0.04731000000000041)[&"FastTree support
value"=0.971]:0.05208000000000126,(XP_017213868.2.2[&Organism="Danio
rerio","Genetic Code"="Standard",Taxonomy="Eukaryota; Metazoa; Chordata; Craniata;
Vertebrata; Euteleostomi; Actinopterygii; Neopterygii; Teleostei; Ostariophysi;
Cypriniformes; Cyprinidae; Danio","Common
Name"="zebrafish"]:0.08474000000000004,(NP_001016189.1[&Organism="Xenopus
tropicalis","Genetic Code"="Standard",Taxonomy="Eukaryota; Metazoa; Chordata;
Craniata; Vertebrata; Euteleostomi; Amphibia; Batrachia; Anura; Pipoidea; Pipidae;
Xenopodinae; Xenopus; Silurana","Common Name"="tropical clawed
frog"]:0.04126999999999992,(XP_028587453.1[&Organism="Podarcis muralis","Genetic
Code"="Standard",Taxonomy="Eukaryota; Metazoa; Chordata; Craniata; Vertebrata;
Euteleostomi; Lepidosauria; Squamata; Bifurcata; Unidentata; Episquamata; Laterata;
Lacertibaenia; Lacertidae; Podarcis","Common Name"="Common wall
lizard"]:0.0278299999999998,(XP_025917892.1[&Organism="Apteryx rowi","Genetic
Code"="Standard",Taxonomy="Eukaryota; Metazoa; Chordata; Craniata; Vertebrata;
Euteleostomi; Archelosauria; Archosauria; Dinosauria; Saurischia; Theropoda;
Coelurosauria; Aves; Palaeognathae; Apterygiformes; Apterygidae; Apteryx","Common
Name"="Okarito brown

kiwi"]:0.02836000000000163,(NP_284941.2.2[&Organism="Homo sapiens","Genetic Code"="Standard",Taxonomy="Eukaryota; Metazoa; Chordata; Craniata; Vertebrata; Euteleostomi; Mammalia; Eutheria; Euarchontoglires; Primates; Haplorrhini; Catarrhini; Hominidae; Homo","Common Name"="human"]:0.0239900000000004,((((NP_077162.2.2[&Organism="Mus musculus","Genetic Code"="Standard",Taxonomy="Eukaryota; Metazoa; Chordata; Craniata; Vertebrata; Euteleostomi; Mammalia; Eutheria; Euarchontoglires; Glires; Rodentia; Myomorpha; Muroidea; Muridae; Murinae; Mus; Mus","Common Name"="house mouse"]:0.01609000000000016,XP_006162789.1[&Organism="Tupaia chinensis","Genetic Code"="Standard",Taxonomy="Eukaryota; Metazoa; Chordata; Craniata; Vertebrata; Euteleostomi; Mammalia; Eutheria; Euarchontoglires; Scandentia; Tupaiidae; Tupaia","Common Name"="Chinese tree shrew"]:0.005049999999999777)[&"FastTree support value"=0.826]:0.007369999999999877,XP_005883071.1[&Organism="Myotis brandtii","Genetic Code"="Standard",Taxonomy="Eukaryota; Metazoa; Chordata; Craniata; Vertebrata; Euteleostomi; Mammalia; Eutheria; Laurasiatheria; Chiroptera; Microchiroptera; Vespertilionidae; Myotis","Common Name"="Brandt's bat"]:0.020220000000000127)[&"FastTree support value"=0.751]:0.004019999999996905,NP_001193437.1[&Organism="Bos taurus","Genetic Code"="Standard",Taxonomy="Eukaryota; Metazoa; Chordata; Craniata; Vertebrata; Euteleostomi; Mammalia; Eutheria; Laurasiatheria; Artiodactyla; Ruminantia; Pecora; Bovidae; Bovinae; Bos","Common Name"="cattle"]:0.008110000000000284)[&"FastTree support value"=0.725]:0.004459999999999085,XP_004479029.1[&Organism="Dasypus novemcinctus","Genetic Code"="Standard",Taxonomy="Eukaryota; Metazoa; Chordata; Craniata; Vertebrata; Euteleostomi; Mammalia; Eutheria; Xenarthra; Cingulata; Dasypodidae; Dasypus","Common Name"="nine-banded armadillo"]:0.015679999999999694)[&"FastTree support value"=0.668]:0.007950000000000124)[&"FastTree support value"=0.878]:0.008709999999999773)[&"FastTree support value"=0.92]:0.017509999999999692)[&"FastTree support value"=0.944]:0.02878999999999987)[&"FastTree support value"=0.908]:0.025859999999999772)[&"FastTree support value"=0.934]:0.040930000000000355)[&"FastTree support value"=0.99]:0.10615000000000041,((XP_006819998.1[&Organism="Saccoglossus kowalevskii","Genetic Code"="Standard",Taxonomy="Eukaryota; Metazoa; Hemichordata; Enteropneusta; Harrimaniidae; Saccoglossus"]:0.16448999999999998,(XP_002591612.1[&Organism="Branchiostoma floridae","Genetic Code"="Standard",Taxonomy="Eukaryota; Metazoa; Chordata; Cephalochordata; Branchiostomidae; Branchiostoma","Common Name"="Florida lancelet"]:0.002259999999997067,XP_019628129.1[&Organism="Branchiostoma belcheri","Genetic Code"="Standard",Taxonomy="Eukaryota; Metazoa; Chordata; Cephalochordata; Branchiostomidae; Branchiostoma","Common Name"="Belcher's lancelet"]:0.010030000000000427)[&"FastTree support value"=1.0]:0.16591000000000022)[&"FastTree support

value"=0.811]:0.017570000000000086,(NP_996357.1[&Organism="Drosophila melanogaster","Genetic Code"="Standard",Taxonomy="Eukaryota; Metazoa; Ecdysozoa; Arthropoda; Hexapoda; Insecta; Pterygota; Neoptera; Holometabola; Diptera; Brachycera; Muscomorpha; Ephydroidea; Drosophilidae; Drosophila; Sophophora","Common Name"="fruit fly"]:0.33619999999999983,(XP_030846906.1[&Organism="Strongylocentrotus purpuratus","Genetic Code"="Standard",Taxonomy="Eukaryota; Metazoa; Echinodermata; Eleutherozoa; Echinozoa; Echinoidea; Euechinoidea; Echinacea; Echinoida; Strongylocentrotidae; Strongylocentrotus","Common Name"="purple sea urchin"]:0.08598000000000017,XP_030847518.1[&Organism="Strongylocentrotus purpuratus","Genetic Code"="Standard",Taxonomy="Eukaryota; Metazoa; Echinodermata; Eleutherozoa; Echinozoa; Echinoidea; Euechinoidea; Echinacea; Echinoida; Strongylocentrotidae; Strongylocentrotus","Common Name"="purple sea urchin"]:0.028970000000000162)[&"FastTree support value"=1.0]:0.2647199999999996)[&"FastTree support value"=0.89]:0.05785000000000018)[&"FastTree support value"=0.958]:0.06245999999999974)[&"FastTree support value"=0.75]:0.04776000000000025)[&"FastTree support value"=0.454]:0.0664100000000003)[&"FastTree support value"=0.344]:0.07730000000000015)[&"FastTree support value"=0.0]:0.000540,(XP_001745740.1[&Organism="Monosiga brevicollis MX1","Genetic Code"="Standard",Taxonomy="Eukaryota; Choanoflagellata; Craspedida; Salpingoecidae; Monosiga"]:0.8712499999999999,NP_495161.1[&Organism="Caenorhabditis elegans","Genetic Code"="Standard",Taxonomy="Eukaryota; Metazoa; Ecdysozoa; Nematoda; Chromadorea; Rhabditida; Rhabditina; Rhabditomorpha; Rhabditoidea; Rhabditidae; Peloderinae; Caenorhabditis"]:0.31857999999999986)[&"FastTree support value"=0.859]:0.23867999999999956)[&"FastTree support value"=1.0]:3.4839200000000003,((PAA68234.1[&Organism="Macrostomum lignano","Genetic Code"="Standard",Taxonomy="Eukaryota; Metazoa; Platyhelminthes; Rhabditophora; Macrostomorpha; Macrostomida; Macrostomidae; Macrostomum"]:0.11982999999999988,PAA87312.1[&Organism="Macrostomum lignano","Genetic Code"="Standard",Taxonomy="Eukaryota; Metazoa; Platyhelminthes; Rhabditophora; Macrostomorpha; Macrostomida; Macrostomidae; Macrostomum"]:0.09414999999999996)[&"FastTree support value"=0.774]:0.13274000000000008,(NP_610941.1[&Organism="Drosophila melanogaster","Genetic Code"="Standard",Taxonomy="Eukaryota; Metazoa; Ecdysozoa; Arthropoda; Hexapoda; Insecta; Pterygota; Neoptera; Holometabola; Diptera; Brachycera; Muscomorpha; Ephydroidea; Drosophilidae; Drosophila; Sophophora","Common Name"="fruit fly"]:0.14365000000000006,(NP_495986.3.3[&Organism="Caenorhabditis elegans","Genetic Code"="Standard",Taxonomy="Eukaryota; Metazoa; Ecdysozoa; Nematoda; Chromadorea; Rhabditida; Rhabditina; Rhabditomorpha; Rhabditoidea; Rhabditidae; Peloderinae; Caenorhabditis"]:0.16979999999999995,((XP_002602331.1[&Organism="Branchiostoma floridae","Genetic Code"="Standard",Taxonomy="Eukaryota; Metazoa; Chordata;

Cephalochordata; Branchiostomidae; Branchiostoma","Common Name"="Florida
lancelet"]:0.00566000000000022,XP_019637857.1[&Organism="Branchiostoma
belcheri","Genetic Code"="Standard",Taxonomy="Eukaryota; Metazoa; Chordata;
Cephalochordata; Branchiostomidae; Branchiostoma","Common Name"="Belcher's
lancelet"]:0.00800000000000007)[&"FastTree support
value"=1.0]:0.11707000000000001,((XP_018667792.1[&Organism="Ciona
intestinalis","Genetic Code"="Standard",Taxonomy="Eukaryota; Metazoa; Chordata;
Tunicata; Ascidiacea; Enterogona; Phlebobranchia; Cionidae; Ciona","Common
Name"="vase
tunicate"]:0.12319000000000013,(XP_032818114.1[&Organism="Petromyzon
marinus","Genetic Code"="Standard",Taxonomy="Eukaryota; Metazoa; Chordata; Craniata;
Vertebrata; Cyclostomata; Hyperoartia; Petromyzontiformes; Petromyzontidae;
Petromyzon","Common Name"="sea
lamprey"]:0.06403999999999987,((XP_028587646.1[&Organism="Podarcis
muralis","Genetic Code"="Standard",Taxonomy="Eukaryota; Metazoa; Chordata; Craniata;
Vertebrata; Euteleostomi; Lepidosauria; Squamata; Bifurcata; Unidentata; Episquamata;
Laterata; Lacertibaenia; Lacertidae; Podarcis","Common Name"="Common wall
lizard"]:0.01989000000000185,((XP_023440724.1[&Organism="Dasypus
novemcinctus","Genetic Code"="Standard",Taxonomy="Eukaryota; Metazoa; Chordata;
Craniata; Vertebrata; Euteleostomi; Mammalia; Eutheria; Xenarthra; Cingulata;
Dasypodidae; Dasypus","Common Name"="nine-banded
armadillo"]:0.006409999999999805,(XP_005873264.1[&Organism="Myotis
brandtii","Genetic Code"="Standard",Taxonomy="Eukaryota; Metazoa; Chordata; Craniata;
Vertebrata; Euteleostomi; Mammalia; Eutheria; Laurasiatheria; Chiroptera;
Microchiroptera; Vespertilionidae; Myotis","Common Name"="Brandt's
bat"]:0.006780000000000008,(XP_006163024.2.2[&Organism="Tupaia chinensis","Genetic
Code"="Standard",Taxonomy="Eukaryota; Metazoa; Chordata; Craniata; Vertebrata;
Euteleostomi; Mammalia; Eutheria; Euarchontoglires; Scandentia; Tupaiidae;
Tupaia","Common Name"="Chinese tree shrew"]:0.0,NP_056375.2.2[&Organism="Homo
sapiens","Genetic Code"="Standard",Taxonomy="Eukaryota; Metazoa; Chordata; Craniata;
Vertebrata; Euteleostomi; Mammalia; Eutheria; Euarchontoglires; Primates; Haplorrhini;
Catarrhini; Hominidae; Homo","Common
Name"="human"]:0.0,NP_598513.1[&Organism="Mus musculus","Genetic
Code"="Standard",Taxonomy="Eukaryota; Metazoa; Chordata; Craniata; Vertebrata;
Euteleostomi; Mammalia; Eutheria; Euarchontoglires; Glires; Rodentia; Myomorpha;
Muroidea; Muridae; Murinae; Mus; Mus","Common Name"="house
mouse"]:0.0):0.000550)[&"FastTree support
value"=0.873]:0.00728000000000175)[&"FastTree support
value"=0.993]:0.036989999999999856,XP_025913835.1[&Organism="Apteryx
rowi","Genetic Code"="Standard",Taxonomy="Eukaryota; Metazoa; Chordata; Craniata;
Vertebrata; Euteleostomi; Archelosauria; Archosauria; Dinosauria; Saurischia; Theropoda;
Coelurosauria; Aves; Palaeognathae; Apterygiformes; Apterygidae; Apteryx","Common
Name"="Okarito brown kiwi"]:0.004589999999999872)[&"FastTree support
value"=0.372]:0.00680000000000139)[&"FastTree support

value"=0.909]:0.017290000000000028,(XP_021332524.1[&Organism="Danio rerio","Genetic Code"="Standard",Taxonomy="Eukaryota; Metazoa; Chordata; Craniata; Vertebrata; Euteleostomi; Actinopterygii; Neopterygii; Teleostei; Ostariophysi; Cypriniformes; Cyprinidae; Danio","Common Name"="zebrafish"]:0.04192999999999998,XP_031757388.1[&Organism="Xenopus tropicalis","Genetic Code"="Standard",Taxonomy="Eukaryota; Metazoa; Chordata; Craniata; Vertebrata; Euteleostomi; Amphibia; Batrachia; Anura; Pipoidea; Pipidae; Xenopodinae; Xenopus; Silurana","Common Name"="tropical clawed frog"]:0.04681999999999986)[&"FastTree support value"=0.792]:0.012370000000000214)[&"FastTree support value"=0.914]:0.03114999999999979)[&"FastTree support value"=0.994]:0.08445999999999998)[&"FastTree support value"=0.884]:0.03431000000000006,(XP_006813643.1[&Organism="Saccoglossus kowalevskii","Genetic Code"="Standard",Taxonomy="Eukaryota; Metazoa; Hemichordata; Enteropneusta; Harrimaniidae; Saccoglossus"]:0.10539999999999994,XP_030843280.1[&Organism="Strongylocentrotus purpuratus","Genetic Code"="Standard",Taxonomy="Eukaryota; Metazoa; Echinodermata; Eleutherozoa; Echinozoa; Echinoidea; Euechinoidea; Echinacea; Echinoida; Strongylocentrotidae; Strongylocentrotus","Common Name"="purple sea urchin"]:0.15691999999999995)[&"FastTree support value"=0.365]:0.02295999999999987)[&"FastTree support value"=0.922]:0.05369000000000015)[&"FastTree support value"=0.405]:0.05064000000000002)[&"FastTree support value"=0.265]:0.04186999999999985)[&"FastTree support value"=0.932]:0.1956199999999999)[&"FastTree support value"=0.983]:1.0973100000000002)[&"FastTree support value"=0.768]:0.3489299999999993,((CAH1802128.1[&Organism="Owenia fusiformis","Genetic Code"="Standard",Taxonomy="Eukaryota; Metazoa; Spiralia; Lophotrochozoa; Annelida; Polychaeta; Sedentaria; Canalipalpata; Sabellida; Oweniida; Oweniidae; Owenia"]:0.5098000000000003,((PAA83069.1[&Organism="Macrostomum lignano","Genetic Code"="Standard",Taxonomy="Eukaryota; Metazoa; Platyhelminthes; Rhabditophora; Macrostomorpha; Macrostomida; Macrostomidae; Macrostomum"]:0.11311999999999989,PAA94353.1[&Organism="Macrostomum lignano","Genetic Code"="Standard",Taxonomy="Eukaryota; Metazoa; Platyhelminthes; Rhabditophora; Macrostomorpha; Macrostomida; Macrostomidae; Macrostomum"]:0.16699999999999982)[&"FastTree support value"=1.0]:0.6317200000000001,((PAA74204.1[&Organism="Macrostomum lignano","Genetic Code"="Standard",Taxonomy="Eukaryota; Metazoa; Platyhelminthes; Rhabditophora; Macrostomorpha; Macrostomida; Macrostomidae; Macrostomum"]:0.14673999999999987,PAA76532.1[&Organism="Macrostomum lignano","Genetic Code"="Standard",Taxonomy="Eukaryota; Metazoa; Platyhelminthes; Rhabditophora; Macrostomorpha; Macrostomida; Macrostomidae; Macrostomum"]:0.2631700000000001)[&"FastTree support value"=0.998]:0.24018000000000006,(PAA92268.1[&Organism="Macrostomum

lignano","Genetic Code"="Standard",Taxonomy="Eukaryota; Metazoa; Platyhelminthes; Rhabditophora; Macrostomorpha; Macrostomida; Macrostomidae; Macrostomum"]:0.32726999999999995,PAA69582.1[&Organism="Macrostomum lignano","Genetic Code"="Standard",Taxonomy="Eukaryota; Metazoa; Platyhelminthes; Rhabditophora; Macrostomorpha; Macrostomida; Macrostomidae; Macrostomum"]:0.3430900000000001)[&"FastTree support value"=0.904]:0.12097999999999987)[&"FastTree support value"=0.951]:0.14803999999999995)[&"FastTree support value"=0.936]:0.12858999999999998)[&"FastTree support value"=0.95]:0.12808999999999937,((((((((XP_028583068.1[&Organism="Podarcis muralis","Genetic Code"="Standard",Taxonomy="Eukaryota; Metazoa; Chordata; Craniata; Vertebrata; Euteleostomi; Lepidosauria; Squamata; Bifurcata; Unidentata; Episquamata; Laterata; Lacertibaenia; Lacertidae; Podarcis","Common Name"="Common wall lizard"]:0.17128999999999994,(NP_001007285.1[&Organism="Danio rerio","Genetic Code"="Standard",Taxonomy="Eukaryota; Metazoa; Chordata; Craniata; Vertebrata; Euteleostomi; Actinopterygii; Neopterygii; Teleostei; Ostariophysi; Cypriniformes; Cyprinidae; Danio","Common Name"="zebrafish"]:0.08689999999999998,XP_005167721.2.2[&Organism="Danio rerio","Genetic Code"="Standard",Taxonomy="Eukaryota; Metazoa; Chordata; Craniata; Vertebrata; Euteleostomi; Actinopterygii; Neopterygii; Teleostei; Ostariophysi; Cypriniformes; Cyprinidae; Danio","Common Name"="zebrafish"]:0.03569999999999984)[&"FastTree support value"=1.0]:0.28037)[&"FastTree support value"=0.869]:0.04275999999999991,((XP_025933558.1[&Organism="Apteryx rowi","Genetic Code"="Standard",Taxonomy="Eukaryota; Metazoa; Chordata; Craniata; Vertebrata; Euteleostomi; Archelosauria; Archosauria; Dinosauria; Saurischia; Theropoda; Coelurosauria; Aves; Palaeognathae; Apterygiformes; Apterygidae; Apteryx","Common Name"="Okarito brown kiwi"]:0.05689000000000011,XP_009815891.1[&Organism="Gavia stellata","Genetic Code"="Standard",Taxonomy="Eukaryota; Metazoa; Chordata; Craniata; Vertebrata; Euteleostomi; Archelosauria; Archosauria; Dinosauria; Saurischia; Theropoda; Coelurosauria; Aves; Neognathae; Gaviiformes; Gaviidae; Gavia","Common Name"="red-throated loon"]:0.08173999999999992)[&"FastTree support value"=1.0]:0.1564000000000001,(XP_015269256.1[&Organism="Gekko japonicus","Genetic Code"="Standard",Taxonomy="Eukaryota; Metazoa; Chordata; Craniata; Vertebrata; Euteleostomi; Lepidosauria; Squamata; Bifurcata; Gekkota; Gekkonidae; Gekkoninae; Gekko"]:0.10316999999999998,XP_028583072.1[&Organism="Podarcis muralis","Genetic Code"="Standard",Taxonomy="Eukaryota; Metazoa; Chordata; Craniata; Vertebrata; Euteleostomi; Lepidosauria; Squamata; Bifurcata; Unidentata; Episquamata; Laterata; Lacertibaenia; Lacertidae; Podarcis","Common Name"="Common wall lizard"]:0.18415999999999988)[&"FastTree support value"=0.006]:0.031620000000000203)[&"FastTree support value"=0.918]:0.05170999999999992)[&"FastTree support value"=0.419]:0.02477000000000018,((XP_031752404.1[&Organism="Xenopus

tropicalis","Genetic Code"="Standard",Taxonomy="Eukaryota; Metazoa; Chordata; Craniata; Vertebrata; Euteleostomi; Amphibia; Batrachia; Anura; Pipoidea; Pipidae; Xenopodinae; Xenopus; Silurana","Common Name"="tropical clawed frog"]:0.26201000000000001,((((XP_008569440.1[&Organism="Galeopterus variegatus","Genetic Code"="Standard",Taxonomy="Eukaryota; Metazoa; Chordata; Craniata; Vertebrata; Euteleostomi; Mammalia; Eutheria; Euarchontoglires; Dermoptera; Cynocephalidae; Galeopterus","Common Name"="Sunda flying lemur"]:0.059330000000000105,((XP_017508123.1[&Organism="Manis javanica","Genetic Code"="Standard",Taxonomy="Eukaryota; Metazoa; Chordata; Craniata; Vertebrata; Euteleostomi; Mammalia; Eutheria; Laurasiatheria; Pholidota; Manidae; Manis","Common Name"="Malayan pangolin"]:0.077890000000000001,(XP_005885748.1[&Organism="Myotis brandtii","Genetic Code"="Standard",Taxonomy="Eukaryota; Metazoa; Chordata; Craniata; Vertebrata; Euteleostomi; Mammalia; Eutheria; Laurasiatheria; Chiroptera; Microchiroptera; Vespertilionidae; Myotis","Common Name"="Brandt's bat"]:0.020259999999999945,XP_012586448.1[&Organism="Condylura cristata","Genetic Code"="Standard",Taxonomy="Eukaryota; Metazoa; Chordata; Craniata; Vertebrata; Euteleostomi; Mammalia; Eutheria; Laurasiatheria; Insectivora; Talpidae; Condylura","Common Name"="star-nosed mole"]:0.24081999999999998)[&"FastTree support value"=0.869]:0.019979999999999887)[&"FastTree support value"=0.854]:0.018580000000000004,(XP_006156438.1[&Organism="Tupaia chinensis","Genetic Code"="Standard",Taxonomy="Eukaryota; Metazoa; Chordata; Craniata; Vertebrata; Euteleostomi; Mammalia; Eutheria; Euarchontoglires; Scandentia; Tupaiidae; Tupaia","Common Name"="Chinese tree shrew"]:0.12788999999999984,(NP_002454.1[&Organism="Homo sapiens","Genetic Code"="Standard",Taxonomy="Eukaryota; Metazoa; Chordata; Craniata; Vertebrata; Euteleostomi; Mammalia; Eutheria; Euarchontoglires; Primates; Haplorrhini; Catarrhini; Hominidae; Homo","Common Name"="human"]:0.0059900000000000162,XP_002830747.1[&Organism="Pongo abelii","Genetic Code"="Standard",Taxonomy="Eukaryota; Metazoa; Chordata; Craniata; Vertebrata; Euteleostomi; Mammalia; Eutheria; Euarchontoglires; Primates; Haplorrhini; Catarrhini; Hominidae; Pongo","Common Name"="Sumatran orangutan"]:0.011229999999999851)[&"FastTree support value"=0.995]:0.074219999999999995)[&"FastTree support value"=0.766]:0.014260000000000161)[&"FastTree support value"=0.182]:0.0055299999999999813)[&"FastTree support value"=0.566]:0.012239999999999807,(NP_001003133.1[&Organism="Canis lupus familiaris","Genetic Code"="Standard",Taxonomy="Eukaryota; Metazoa; Chordata; Craniata; Vertebrata; Euteleostomi; Mammalia; Eutheria; Laurasiatheria; Carnivora; Caniformia; Canidae; Canis","Common Name"="dog"]:0.072249999999999993,XP_032211320.1[&Organism="Mustela erminea","Genetic Code"="Standard",Taxonomy="Eukaryota; Metazoa; Chordata; Craniata; Vertebrata; Euteleostomi; Mammalia; Eutheria; Laurasiatheria; Carnivora; Caniformia; Mustelidae; Mustelinae; Mustela","Common Name"="ermine"]:0.10838999999999999)[&"FastTree support

value"=0.94]:0.03683000000000014)[&"FastTree support
value"=0.867]:0.024519999999999875,NP_776366.1[&Organism="Bos taurus","Genetic
Code"="Standard",Taxonomy="Eukaryota; Metazoa; Chordata; Craniata; Vertebrata;
Euteleostomi; Mammalia; Eutheria; Laurasiatheria; Artiodactyla; Ruminantia; Pecora;
Bovidae; Bovinae; Bos","Common Name"="cattle"]:0.08711000000000002)[&"FastTree
support value"=1.0]:0.13436000000000003,(XP_004675614.2.2[&Organism="Condylura
cristata","Genetic Code"="Standard",Taxonomy="Eukaryota; Metazoa; Chordata; Craniata;
Vertebrata; Euteleostomi; Mammalia; Eutheria; Laurasiatheria; Insectivora; Talpidae;
Condylura","Common Name"="star-nosed
mole"]:0.1381800000000002,((((XP_005202045.1[&Organism="Bos taurus","Genetic
Code"="Standard",Taxonomy="Eukaryota; Metazoa; Chordata; Craniata; Vertebrata;
Euteleostomi; Mammalia; Eutheria; Laurasiatheria; Cetartiodactyla; Ruminantia; Pecora;
Bovidae; Bovinae; Bos","Common
Name"="cattle"]:0.11654000000000009,(NP_002453.2.2[&Organism="Homo
sapiens","Genetic Code"="Standard",Taxonomy="Eukaryota; Metazoa; Chordata; Craniata;
Vertebrata; Euteleostomi; Mammalia; Eutheria; Euarchontoglires; Primates; Haplorrhini;
Catarrhini; Hominidae; Homo","Common
Name"="human"]:0.0038599999999999746,NP_001127618.1[&Organism="Pongo
abelii","Genetic Code"="Standard",Taxonomy="Eukaryota; Metazoa; Chordata; Craniata;
Vertebrata; Euteleostomi; Mammalia; Eutheria; Euarchontoglires; Primates; Haplorrhini;
Catarrhini; Hominidae; Pongo","Common Name"="Sumatran
orangutan"]:0.0029799999999999827)[&"FastTree support
value"=0.985]:0.05203999999999864)[&"FastTree support
value"=0.118]:0.019680000000000142,(XP_008569442.1[&Organism="Galeopterus
variegatus","Genetic Code"="Standard",Taxonomy="Eukaryota; Metazoa; Chordata;
Craniata; Vertebrata; Euteleostomi; Mammalia; Eutheria; Euarchontoglires; Dermoptera;
Cynocephalidae; Galeopterus","Common Name"="Sunda flying
lemur"]:0.07145000000000001,XP_014388412.1[&Organism="Myotis brandtii","Genetic
Code"="Standard",Taxonomy="Eukaryota; Metazoa; Chordata; Craniata; Vertebrata;
Euteleostomi; Mammalia; Eutheria; Laurasiatheria; Chiroptera; Microchiroptera;
Vespertilionidae; Myotis","Common Name"="Brandt's
bat"]:0.09780999999999995)[&"FastTree support
value"=0.516]:0.025040000000000173)[&"FastTree support
value"=0.881]:0.024480000000000057,((XP_006156437.1[&Organism="Tupaia
chinensis","Genetic Code"="Standard",Taxonomy="Eukaryota; Metazoa; Chordata;
Craniata; Vertebrata; Euteleostomi; Mammalia; Eutheria; Euarchontoglires; Scandentia;
Tupaiidae; Tupaia","Common Name"="Chinese tree
shrew"]:0.06058999999999992,(NP_038634.1[&Organism="Mus musculus","Genetic
Code"="Standard",Taxonomy="Eukaryota; Metazoa; Chordata; Craniata; Vertebrata;
Euteleostomi; Mammalia; Eutheria; Euarchontoglires; Glires; Rodentia; Myomorpha;
Muroidea; Muridae; Murinae; Mus; Mus","Common Name"="house
mouse"]:0.00843000000000016,NP_034976.1[&Organism="Mus musculus","Genetic
Code"="Standard",Taxonomy="Eukaryota; Metazoa; Chordata; Craniata; Vertebrata;
Euteleostomi; Mammalia; Eutheria; Euarchontoglires; Glires; Rodentia; Myomorpha;

Muroidea; Muridae; Murinae; Mus; Mus","Common Name"="house mouse"]:0.05713999999999997)[&"FastTree support value"=1.0]:0.11734)[&"FastTree support value"=0.659]:0.009539999999999882,(XP_017508130.1[&Organism="Manis javanica","Genetic Code"="Standard",Taxonomy="Eukaryota; Metazoa; Chordata; Craniata; Vertebrata; Euteleostomi; Mammalia; Eutheria; Laurasiatheria; Pholidota; Manidae; Manis","Common Name"="Malayan pangolin"]:0.05950999999999995,(NP_001003134.1[&Organism="Canis lupus familiaris","Genetic Code"="Standard",Taxonomy="Eukaryota; Metazoa; Chordata; Craniata; Vertebrata; Euteleostomi; Mammalia; Eutheria; Laurasiatheria; Carnivora; Caniformia; Canidae; Canis","Common Name"="dog"]:0.01980000000000004,XP_032211398.1[&Organism="Mustela erminea","Genetic Code"="Standard",Taxonomy="Eukaryota; Metazoa; Chordata; Craniata; Vertebrata; Euteleostomi; Mammalia; Eutheria; Laurasiatheria; Carnivora; Caniformia; Mustelidae; Mustelinae; Mustela","Common Name"="ermine"]:0.03765999999999805)[&"FastTree support value"=0.99]:0.05112000000000054)[&"FastTree support value"=0.87]:0.02208999999999943)[&"FastTree support value"=0.861]:0.01257000000000192)[&"FastTree support value"=0.81]:0.01319999999999878,XP_004466363.1[&Organism="Dasypus novemcinctus","Genetic Code"="Standard",Taxonomy="Eukaryota; Metazoa; Chordata; Craniata; Vertebrata; Euteleostomi; Mammalia; Eutheria; Xenarthra; Cingulata; Dasypodidae; Dasypus","Common Name"="nine-banded armadillo"]:0.09669999999999979)[&"FastTree support value"=0.886]:0.03132000000000014)[&"FastTree support value"=0.138]:0.04529000000000005)[&"FastTree support value"=0.928]:0.07262000000000013)[&"FastTree support value"=0.961]:0.0790700000000002,(XP_007904885.1[&Organism="Callorhinchus milii","Genetic Code"="Standard",Taxonomy="Eukaryota; Metazoa; Chordata; Craniata; Vertebrata; Chondrichthyes; Holocephali; Chimaeriformes; Callorhinchidae; Callorhinchus","Common Name"="elephant shark"]:0.13013999999999992,XP_032888405.1[&Organism="Amblyraja radiata","Genetic Code"="Standard",Taxonomy="Eukaryota; Metazoa; Chordata; Craniata; Vertebrata; Chondrichthyes; Elasmobranchii; Batoidea; Rajiformes; Rajidae; Amblyraja","Common Name"="thorny skate"]:0.14127)[&"FastTree support value"=0.961]:0.07933000000000012)[&"FastTree support value"=0.104]:0.01460000000000168)[&"FastTree support value"=0.843]:0.03808999999999951,AGU16245.1[&db_xref="taxon:27779",Organism="Protopterus dolloi","Genetic Code"="Standard",Modified=Mon Mar 27 11:17:17 PDT 2023,Taxonomy="Eukaryota; Metazoa; Chordata; Craniata; Vertebrata; Euteleostomi; Dipnoi; Lepidosireniformes; Protopteridae; Protopterus",Accession="AGU16245.1","Common Name"="slender lungfish",Topology="linear","Molecule Type"="AA"]:0.16343999999999959)[&"FastTree support value"=0.844]:0.08178000000000019,(XP_003973512.2.2[&Organism="Takifugu rubripes","Genetic Code"="Standard",Taxonomy="Eukaryota; Metazoa; Chordata; Craniata;

Vertebrata; Euteleostomi; Actinopterygii; Neopterygii; Teleostei; Neoteleostei; Acanthomorphata; Eupercaria; Tetraodontiformes; Tetradontoidea; Tetraodontidae; Takifugu","Common Name"="torafugu"]:0.10654999999999992,(NP_891987.2.2[&Organism="Danio rerio","Genetic Code"="Standard",Taxonomy="Eukaryota; Metazoa; Chordata; Craniata; Vertebrata; Euteleostomi; Actinopterygii; Neopterygii; Teleostei; Ostariophysi; Cypriniformes; Cyprinidae; Danio","Common Name"="zebrafish"]:0.03649000000000013,XP_009304072.1[&Organism="Danio rerio","Genetic Code"="Standard",Taxonomy="Eukaryota; Metazoa; Chordata; Craniata; Vertebrata; Euteleostomi; Actinopterygii; Neopterygii; Teleostei; Ostariophysi; Cypriniformes; Cyprinidae; Danio","Common Name"="zebrafish"]:0.01343000000000053)[&"FastTree support value"=0.996]:0.09981000000000018)[&"FastTree support value"=0.979]:0.11360999999999954)[&"FastTree support value"=0.986]:0.14922000000000057,XP_032804093.1[&Organism="Petromyzon marinus","Genetic Code"="Standard",Taxonomy="Eukaryota; Metazoa; Chordata; Craniata; Vertebrata; Cyclostomata; Hyperoartia; Petromyzontiformes; Petromyzontidae; Petromyzon","Common Name"="sea lamprey"]:0.4988999999999999)[&"FastTree support value"=0.922]:0.10626999999999942,((KAI0213370.1[&Organism="Lamellibrachia satsuma","Genetic Code"="Standard",Taxonomy="Eukaryota; Metazoa; Spiralia; Lophotrochozoa; Annelida; Polychaeta; Sedentaria; Canalipalpata; Sabellida; Siboglinidae; Lamellibrachia"]:0.000550,(KAI0208044.1[&Organism="Lamellibrachia satsuma","Genetic Code"="Standard",Taxonomy="Eukaryota; Metazoa; Spiralia; Lophotrochozoa; Annelida; Polychaeta; Sedentaria; Canalipalpata; Sabellida; Siboglinidae; Lamellibrachia"]:0.0630799999999998,KAI0218869.1[&Organism="Lamellibrachia satsuma","Genetic Code"="Standard",Taxonomy="Eukaryota; Metazoa; Spiralia; Lophotrochozoa; Annelida; Polychaeta; Sedentaria; Canalipalpata; Sabellida; Siboglinidae; Lamellibrachia"]:0.10996000000000006)[&"FastTree support value"=0.806]:0.01939000000000002)[&"FastTree support value"=1.0]:0.45840999999999976,(XP_006815062.1[&Organism="Saccoglossus kowalevskii","Genetic Code"="Standard",Taxonomy="Eukaryota; Metazoa; Hemichordata; Enteropneusta; Harrimaniidae; Saccoglossus"]:0.50379,(XP_035690836.1[&Organism="Branchiostoma floridae","Genetic Code"="Standard",Taxonomy="Eukaryota; Metazoa; Chordata; Cephalochordata; Leptocardii; Amphioxiformes; Branchiostomidae; Branchiostoma","Common Name"="Florida lancelet"]:0.21335000000000015,(XP_002608668.1[&Organism="Branchiostoma floridae","Genetic Code"="Standard",Taxonomy="Eukaryota; Metazoa; Chordata; Cephalochordata; Branchiostomidae; Branchiostoma","Common Name"="Florida lancelet"]:0.09283999999999937,XP_019617847.1[&Organism="Branchiostoma belcheri","Genetic Code"="Standard",Taxonomy="Eukaryota; Metazoa; Chordata; Cephalochordata; Branchiostomidae; Branchiostoma","Common Name"="Belcher's lancelet"]:0.12332999999999972)[&"FastTree support value"=0.923]:0.10554000000000041)[&"FastTree support

value"=0.967]:0.14315999999999995)[&"FastTree support
value"=0.852]:0.07362000000000002)[&"FastTree support
value"=0.179]:0.026869999999999727)[&"FastTree support
value"=0.863]:0.07502999999999993,(XP_046565196.1[&Organism="Haliotis
rubra","Genetic Code"="Standard",Taxonomy="Eukaryota; Metazoa; Spiralia;
Lophotrochozoa; Mollusca; Gastropoda; Vetigastropoda; Lepetellida; Haliotoidea;
Haliotidae; Haliotis","Common Name"="blacklip
abalone"]:0.0656200000000009,(((XP_046562919.1[&Organism="Haliotis rubra","Genetic
Code"="Standard",Taxonomy="Eukaryota; Metazoa; Spiralia; Lophotrochozoa; Mollusca;
Gastropoda; Vetigastropoda; Lepetellida; Haliotoidea; Haliotidae; Haliotis","Common
Name"="blacklip abalone"]:0.03966000000000003,XP_046563124.1[&Organism="Haliotis
rubra","Genetic Code"="Standard",Taxonomy="Eukaryota; Metazoa; Spiralia;
Lophotrochozoa; Mollusca; Gastropoda; Vetigastropoda; Lepetellida; Haliotoidea;
Haliotidae; Haliotis","Common Name"="blacklip
abalone"]:0.008340000000000458)[&"FastTree support
value"=0.286]:0.006270000000000664,(XP_046563125.1[&Organism="Haliotis
rubra","Genetic Code"="Standard",Taxonomy="Eukaryota; Metazoa; Spiralia;
Lophotrochozoa; Mollusca; Gastropoda; Vetigastropoda; Lepetellida; Haliotoidea;
Haliotidae; Haliotis","Common Name"="blacklip
abalone"]:0.0237999999999996,(XP_046563126.1[&Organism="Haliotis rubra","Genetic
Code"="Standard",Taxonomy="Eukaryota; Metazoa; Spiralia; Lophotrochozoa; Mollusca;
Gastropoda; Vetigastropoda; Lepetellida; Haliotoidea; Haliotidae; Haliotis","Common
Name"="blacklip
abalone"]:0.006709999999999994,XP_046565195.1[&Organism="Haliotis rubra","Genetic
Code"="Standard",Taxonomy="Eukaryota; Metazoa; Spiralia; Lophotrochozoa; Mollusca;
Gastropoda; Vetigastropoda; Lepetellida; Haliotoidea; Haliotidae; Haliotis","Common
Name"="blacklip abalone"]:0.0099800000000001)[&"FastTree support
value"=0.979]:0.02334999999999976)[&"FastTree support
value"=0.81]:0.00852000000000075)[&"FastTree support
value"=0.671]:0.0015999999999998238,(XP_046352527.2[&Organism="Haliotis
rufescens","Genetic Code"="Standard",Taxonomy="Eukaryota; Metazoa; Spiralia;
Lophotrochozoa; Mollusca; Gastropoda; Vetigastropoda; Lepetellida; Haliotoidea;
Haliotidae; Haliotis","Common Name"="red
abalone"]:0.020080000000000098,(XP_048248476.1[&Organism="Haliotis
rufescens","Genetic Code"="Standard",Taxonomy="Eukaryota; Metazoa; Spiralia;
Lophotrochozoa; Mollusca; Gastropoda; Vetigastropoda; Lepetellida; Haliotoidea;
Haliotidae; Haliotis","Common Name"="red
abalone"]:0.017389999999999795,(XP_048258111.1[&Organism="Haliotis
rufescens","Genetic Code"="Standard",Taxonomy="Eukaryota; Metazoa; Spiralia;
Lophotrochozoa; Mollusca; Gastropoda; Vetigastropoda; Lepetellida; Haliotoidea;
Haliotidae; Haliotis","Common Name"="red
abalone"]:0.006629999999999914,((XP_046352531.2[&Organism="Haliotis
rufescens","Genetic Code"="Standard",Taxonomy="Eukaryota; Metazoa; Spiralia;
Lophotrochozoa; Mollusca; Gastropoda; Vetigastropoda; Lepetellida; Haliotoidea;

Haliotidae; Haliotis","Common Name"="red
abalone"]:0.00735999999999811,(XP_048248472.1[&Organism="Haliotis
rufescens","Genetic Code"="Standard",Taxonomy="Eukaryota; Metazoa; Spiralia;
Lophotrochozoa; Mollusca; Gastropoda; Vetigastropoda; Lepetellida; Haliotoidea;
Haliotidae; Haliotis","Common Name"="red
abalone"]:0.000550,(XP_048248473.1[&Organism="Haliotis rufescens","Genetic
Code"="Standard",Taxonomy="Eukaryota; Metazoa; Spiralia; Lophotrochozoa; Mollusca;
Gastropoda; Vetigastropoda; Lepetellida; Haliotoidea; Haliotidae; Haliotis","Common
Name"="red abalone"]:0.0,XP_048248474.1[&Organism="Haliotis rufescens","Genetic
Code"="Standard",Taxonomy="Eukaryota; Metazoa; Spiralia; Lophotrochozoa; Mollusca;
Gastropoda; Vetigastropoda; Lepetellida; Haliotoidea; Haliotidae; Haliotis","Common
Name"="red abalone"]:0.0):0.000550)[&"FastTree support
value"=0.98]:0.0225499999999848)[&"FastTree support
value"=0.864]:0.00680000000000139,ABI53802.1[&Organism="Haliotis discus
discus","Genetic Code"="Standard",Taxonomy="Eukaryota; Metazoa; Lophotrochozoa;
Mollusca; Gastropoda; Vetigastropoda; Haliotoidea; Haliotidae; Haliotis","Common
Name"="disc abalone"]:0.0262199999999991)[&"FastTree support
value"=0.865]:0.00681000000000205)[&"FastTree support
value"=0.723]:0.000540)[&"FastTree support
value"=0.777]:0.00400000000000036)[&"FastTree support
value"=0.691]:0.03125000000000444)[&"FastTree support
value"=0.983]:0.08924000000000021)[&"FastTree support
value"=1.0]:0.32908999999999)[&"FastTree support
value"=0.736]:0.04049000000000014)[&"FastTree support
value"=0.99]:0.46616000000000035)[&"FastTree support
value"=0.964]:0.35960000000000036)[&"FastTree support
value"=0.993]:0.19428999999999963)[&"FastTree support
value"=0.904]:0.05298000000000069)[&"FastTree support
value"=0.206]:0.0329599999999921,XP_004347890.1[&Organism="Capsaspora
owczarzaki ATCC 30864","Genetic Code"="Standard",Taxonomy="Eukaryota; Filasterea;
Capsaspora"]:0.16350999999999996)[&"FastTree support
value"=0.863]:0.02360000000000065,XP_026693152.1[&Organism="Ciona
intestinalis","Genetic Code"="Standard",Taxonomy="Eukaryota; Metazoa; Chordata;
Tunicata; Ascidiacea; Enterogona; Phlebobranchia; Cionidae; Ciona","Common
Name"="vase tunicate"]:0.0978599999999984)[&"FastTree support
value"=0.262]:0.01787000000000275,XP_006812840.1[&Organism="Saccoglossus
kowalevskii","Genetic Code"="Standard",Taxonomy="Eukaryota; Metazoa; Hemichordata;
Enteropneusta; Harrimaniidae; Saccoglossus"]:0.2539299999999954)[&"FastTree
support value"=0.847]:0.0183699999999997)[&"FastTree support
value"=0.205]:0.01133000000000062,((XP_005165639.1[&Organism="Danio
rerio","Genetic Code"="Standard",Taxonomy="Eukaryota; Metazoa; Chordata; Craniata;
Vertebrata; Euteleostomi; Actinopterygii; Neopterygii; Teleostei; Ostariophysi;
Cypriniformes; Cyprinidae; Danio","Common
Name"="zebrafish"]:0.06090000000000176,(XP_028570166.1[&Organism="Podarcis

muralis","Genetic Code"="Standard",Taxonomy="Eukaryota; Metazoa; Chordata; Craniata; Vertebrata; Euteleostomi; Lepidosauria; Squamata; Bifurcata; Unidentata; Episquamata; Laterata; Lacertibaenia; Lacertidae; Podarcis","Common Name"="Common wall lizard"]:0.017699999999999605,(KAE8583055.1[&Organism="Xenopus tropicalis","Genetic Code"="Standard",Taxonomy="Eukaryota; Metazoa; Chordata; Craniata; Vertebrata; Euteleostomi; Amphibia; Batrachia; Anura; Pipoidea; Pipidae; Xenopodinae; Xenopus; Silurana","Common Name"="tropical clawed frog"]:0.030590000000000117,(EPQ17174.1[&Organism="Myotis brandtii","Genetic Code"="Standard",Taxonomy="Eukaryota; Metazoa; Chordata; Craniata; Vertebrata; Euteleostomi; Mammalia; Eutheria; Laurasiatheria; Chiroptera; Microchiroptera; Vespertilionidae; Myotis","Common Name"="Brandt's bat"]:0.000540,((ELW62001.1[&Organism="Tupaia chinensis","Genetic Code"="Standard",Taxonomy="Eukaryota; Metazoa; Chordata; Craniata; Vertebrata; Euteleostomi; Mammalia; Eutheria; Euarchontoglires; Scandentia; Tupaiidae; Tupaia","Common Name"="Chinese tree shrew"]:0.000550,(XP_012378586.1[&Organism="Dasypus novemcinctus","Genetic Code"="Standard",Taxonomy="Eukaryota; Metazoa; Chordata; Craniata; Vertebrata; Euteleostomi; Mammalia; Eutheria; Xenarthra; Cingulata; Dasypodidae; Dasypus","Common Name"="nine-banded armadillo"]:0.07051000000000052,EAW87759.1[&Organism="Homo sapiens","Genetic Code"="Standard",Taxonomy="Eukaryota; Metazoa; Chordata; Craniata; Vertebrata; Euteleostomi; Mammalia; Eutheria; Euarchontoglires; Primates; Haplorrhini; Catarrhini; Hominidae; Homo","Common Name"="human"]:0.000540)[&"FastTree support value"=0.841]:0.0036799999999992394)[&"FastTree support value"=0.0]:0.000540,(BAB27759.1[&Organism="Mus musculus","Genetic Code"="Standard",Taxonomy="Eukaryota; Metazoa; Chordata; Craniata; Vertebrata; Euteleostomi; Mammalia; Eutheria; Euarchontoglires; Glires; Rodentia; Myomorpha; Muroidea; Muridae; Murinae; Mus; Mus","Common Name"="house mouse"]:0.00264000000000042,XP_025915522.1[&Organism="Apteryx rowi","Genetic Code"="Standard",Taxonomy="Eukaryota; Metazoa; Chordata; Craniata; Vertebrata; Euteleostomi; Archelosauria; Archosauria; Dinosauria; Saurischia; Theropoda; Coelurosauria; Aves; Palaeognathae; Apterygiformes; Apterygidae; Apteryx","Common Name"="Okarito brown kiwi"]:0.019780000000000797)[&"FastTree support value"=0.938]:0.0010799999999999699)[&"FastTree support value"=0.582]:0.00741999999999976)[&"FastTree support value"=0.957]:0.019439999999999458)[&"FastTree support value"=0.831]:0.016329999999999956)[&"FastTree support value"=0.623]:0.016950000000000465)[&"FastTree support value"=0.678]:0.006109999999999616,(XP_030853442.1.2[&Organism="Strongylocentrotus purpuratus","Genetic Code"="Standard",Taxonomy="Eukaryota; Metazoa; Echinodermata; Eleutherozoa; Echinozoa; Echinoidea; Euechinoidea; Echinacea; Echinoida; Strongylocentrotidae; Strongylocentrotus","Common Name"="purple sea urchin"]:0.0,XP_030853442.1[&Organism="Strongylocentrotus purpuratus","Genetic Code"="Standard",Taxonomy="Eukaryota; Metazoa; Echinodermata; Eleutherozoa;

Echinozoa; Echinoidea; Euechinoidea; Echinacea; Echinoida; Strongylocentrotidae;
Strongylocentrotus","Common Name"="purple sea
urchin"]:0.0):0.11746999999999996)[&"FastTree support
value"=0.883]:0.017759999999999998)[&"FastTree support
value"=0.832]:0.02122000000000046,XP_032814666.1[&Organism="Petromyzon
marinus","Genetic Code"="Standard",Taxonomy="Eukaryota; Metazoa; Chordata; Craniata;
Vertebrata; Cyclostomata; Hyperoartia; Petromyzontiformes; Petromyzontidae;
Petromyzon","Common Name"="sea lamprey"]:0.05926000000000009)[&"FastTree support
value"=0.662]:0.012369999999999977,(((PAA59145.1[&Organism="Macrostomum
lignano","Genetic Code"="Standard",Taxonomy="Eukaryota; Metazoa; Platyhelminthes;
Rhabditophora; Macrostomorpha; Macrostomida; Macrostomidae;
Macrostomum"]:0.051569999999999894,PAA64382.1[&Organism="Macrostomum
lignano","Genetic Code"="Standard",Taxonomy="Eukaryota; Metazoa; Platyhelminthes;
Rhabditophora; Macrostomorpha; Macrostomida; Macrostomidae;
Macrostomum"]:0.11007999999999996)[&"FastTree support
value"=0.756]:0.020789999999999864,(PAA78248.1[&Organism="Macrostomum
lignano","Genetic Code"="Standard",Taxonomy="Eukaryota; Metazoa; Platyhelminthes;
Rhabditophora; Macrostomorpha; Macrostomida; Macrostomidae;
Macrostomum"]:0.028419999999999668,PAA65118.1[&Organism="Macrostomum
lignano","Genetic Code"="Standard",Taxonomy="Eukaryota; Metazoa; Platyhelminthes;
Rhabditophora; Macrostomorpha; Macrostomida; Macrostomidae;
Macrostomum"]:0.06540000000000035)[&"FastTree support
value"=0.726]:0.030259999999999287)[&"FastTree support
value"=0.999]:0.11606000000000094,(KMZ10000.1[&Organism="Drosophila
melanogaster","Genetic Code"="Standard",Taxonomy="Eukaryota; Metazoa; Ecdysozoa;
Arthropoda; Hexapoda; Insecta; Pterygota; Neoptera; Holometabola; Diptera; Brachycera;
Muscomorpha; Ephydroidea; Drosophilidae; Drosophila; Sophophora","Common
Name"="fruit fly"]:0.07181000000000015,NP_001024332.1[&Organism="Caenorhabditis
elegans","Genetic Code"="Standard",Taxonomy="Eukaryota; Metazoa; Ecdysozoa;
Nematoda; Chromadorea; Rhabditida; Rhabditina; Rhabditomorpha; Rhabditoidea;
Rhabditidae; Peloderinae; Caenorhabditis"]:0.1491100000000003)[&"FastTree support
value"=0.201]:0.025150000000000006)[&"FastTree support
value"=0.595]:0.0361899999999995);
end;
