# Supplementary material for: Antiviral Mx proteins have an ancient origin and widespread distribution among eukaryotes
Source: Proc Natl Acad Sci U S A. 2025 Jan 24;122(4):e2416811122. doi: 10.1073/pnas.2416811122 (PMC11789081; doi:10.1073/pnas.2416811122)
Supplement: Supplementary file 4 — Dataset S03 (PDF) [file pnas.2416811122.sd03.pdf]

Dataset S3. Figure_1_MAFFT

>XP_006812840.1
ITKPSC-LILAVTP----------------GN---SDLANSDALK----VAKEVDPQGLRTIG-
VITKLDLLDDGTDAREILENKLLP--------LRR-----------------------------------ASEWGEFLH-----------
-----------------------CKGKKFT---NFDEIRMEIEAETDRLTGKN---KGISPIPINLRVYSPHV----------
LNLTLIDLPGMTKVPVGDQP--ADIEQQIR--------SMLMEFITKPSCLILAVTPGNSDLANS-
DALKVAKEVDPQGLRTIGVI-TKLDLLDDG-TD----AREILENK-LLPLRR-
GYIGVVNRGQKDIEGRKDIKSALASERKFFLSHPSY--RHM--ADR-LGT--
PYLQKALNQQLTNHIRDTL
>XP_012378586.1
AGGPR----RRQSG---------------RAA----AAAEPEPERN--FVGRDFLPRG---SG-IVTRRPLV----------
---LQL--------VNA-----------------------------------TTEYAEFLH----------------------------------
CKGKKFT---DFEEVRLEIEAETDRVTGTN---KGISPVPINLRVYSPHV----------
LNLTLVDLPGMTKVPVGDQP--PDIEFQIR--------DMLMQFVTKENCLILAVSPANSDLANS-
DALKVAKEVDPQGQRTIGVI-TKLDLMDEG-TD----ARDVLENK-LLPLRR-
GYIGVVNRSQKDIDGKKDITAALAAERKFFLSHPSY--RHL--ADR-MGT--
PYLQKVLNQQLTNHIRDTL
>EPQ17174.1
LDLPQ----IAVVG----------------GQ----SAGKSSVLEN--FVGRDFLPRG---SG-IVTRRPLV------------
-LQL--------VNA-----------------------------------STEYAEFLH----------------------------------
CKGKKFT---DFEEVRLEIEAETDRVTGTN---KGISPVPINLRVYSPHV----------
LNLTLVDLPGMTKVPVGDQP--ADIEFQIR--------DMLMQFVTKENCLILAVSPANSDLANS-
DALKIAKEVDPQGQRTIGVI-TKLDLMDEG-TD----ARDVLENK-LLPLRR-
GYIGVVNRSQKDIDGKKDITAALAAERKFFLSHPSY--RHL--ADR-MGT--
PYLQKVLNQQLTNHIRDTL
>ELW62001.1
LDLPQ----IAVVG----------------GQ----SAGKSSVLEN--FVGRDFLPRG---SG-IVTRRPLV------------
-LQL--------VNA-----------------------------------TTEYAEFLH----------------------------------
CKGKKFT---DFEEVRLEIEAETDRVTGTN---KGISPVPINLRVYSPHV----------
LNLTLVDLPGMTKVPVGDQP--PDIEFQIR--------DMLMQFVTKENCLILAVSPANSDLANS-
DALKIAKEVDPQGQRTIGVI-TKLDLMDEG-TD----ARDVLENK-LLPLRR-
GYIGVVNRSQKDIDGKKDITAALAAERKFFLSHPSY--RHL--ADR-MGT--
PYLQKVLNQQLTNHIRDTL
>EAW87759.1
LDLPQ----IAVVG----------------GQ----SAGKSSVLEN--FVGRDFLPRG---SG-IVTRRPLV------------
-LQL--------VNA-----------------------------------TTEYAEFLH----------------------------------
CKGKKFT---DFEEVRLEIEAETDRVTGTN---KGISPVPINLRVYSPHV----------
LNLTLVDLPGMTKVPVGDQP--PDIEFQIR--------DMLMQFVTKENCLILAVSPANSDLANS-
DALKVAKEVDPQGQRTIGVI-TKLDLMDEG-TD----ARDVLENK-LLPLRR-
GYIGVVNRSQKDIDGKKDITAALAAERKFFLSHPSY--RHL--ADR-MGT--
PYLQKVLNQQLTNHIRDTL
>BAB27759.1

LDLPQ----IAVVG----------------GQ----SAGKSSVLEN--FVGRDFLPRG---SG-IVTRRPLV------------
-LQL--------VNS----------------------------------TTEYAEFLH---------------------------------
CKGKKFT---DFEEVRLEIEAETDRVTGTN---KGISPVPINLRVYSPHV----------
LNLTLVDLPGMTKVPVGDQP--PDIEFQIR--------DMLMQFVTKENCLILAVSPANSDLANS-
DALKIAKEVDPQGQRTIGVI-TKLDLMDEG-TD----ARDVLENK-LLPLRR-
GYIGVVNRSQKDIDGKKDITAALAAERKFFLSHPSY--RHL--ADR-MGT--
PYLQKVLNQQLTNHIRDTL
>XP_025915522.1

------------------MTKVPVGDQP--PDIEFQIR--------DMLMQFVTKENCLILAVSPANSDLANS-
DALKIAKEVDPQGQRTIGVI-TKLDLMDEG-TD----ARDVLENK-LLPLRR-
GYIGVVNRSQKDIDGKKDIQAALAAERKFFLSHPAY--RHM--ADR-MGT--
PYLQKVLNQQLTNHIRDTL
>XP_028570166.1
LDLPQ----IAVVG----------------GQ----SAGKSSVLEN--FVGRDFLPRG---SG-IVTRRPLV------------
-LQL--------VNS----------------------------------PTEYGEFLH---------------------------------
CKGKKFT---DFDEIRQEIEAETDRITGSN---KGISPVPINLRVYSPHV----------
LSLTLVDLPGMTKVPVGDQP--ADIEFQIR--------EMLMQFVTKENCLILAVSPANSDLANS-
DALKIAKEVDPQGQRTIGVI-TKLDLMDEG-TD----ARDVLENK-LLPLRR-
GYIGVVNRSQKDIDGKKDIQAALAAERKFFLTHPAY--RHM--ADR-MGT--
PYLQKVLNQQLTNHIRDTL
>KAE8583055.1
LDLPQ----IAVVG----------------GQ----SAGKSSVLEN--FVGKDFLPRG---SG-IVTRRPLV-------------
LQL--------VNS----------------------------------STEYGEFLH---------------------------------
CKGKKFT---DFDEIRLEIEAETDRATGTN---KGISPVPINLRVYSPNV----------
LNLTLVDLPGMTKVPVGDQP--VDIEFQIR--------DMLMQFVTKENCLVLAVSPANSDLANS-
DALKIAKEVDPKGLRTIGVI-TKLDLMDEG-TD----ARDVLENK-LLPLRR-
GYIGVVNRSQKDIDGKKDIQAALAAERKFFLSHPSY--RHL--ADR-MGT--
PYLQKALNQQLTNHIRDTL
>XP_005165639.1
LDLPQ----IAVVG----------------GQ----SAGKSSVLEN--FVGKDFLPRG---SG-IVTRRPLV-------------
LQL--------INC----------------------------------PTEYAEFLH---------------------------------CKGKKFT-
--DFDEVRQEIEAETDRITGQN---KGISPVPINLRVYSPNV----------LNLTLVDLPGMTKVPVGDQP--
ADIEAQIR--------DMLMQFVTKENCLLLAVSPANSDLANS-DALKIAKEVDPQGMRTIGVI-
TKLDLMDEG-TD----AREILENK-LLPLRR-GYIGVVNRSQKDIDGKKDITAAMSAERKFFLTHPSY--
RHL--ADR-MGT--PYLQKALNQQLTNHIRDTL
>XP_032814666.1
LDLPQ----IAVVG----------------GQ----SAGKSSVLEN--FVGRDFLPRG---SG-IVTRRPLI-------------
LQL--------MFC----------------------------------KAEYAEFLH---------------------------------
CKGKKFT---DFEEVRAEIEAETDRLTGSN---KGISPIPINLRVYSPHV----------
LNLTLIDLPGMTKVPVGDQP--VDIEYQIR--------EMLMQFVTKENCLILAVSPANTDLANS-
DALKIAKEVDPQGLRTIGVI-TKLDLMDDG-TD----ARDILENK-LLPLRR-

GYIGVVNRSQKDIDGRKDINAAMAAERKFFLSHPSY--RHM--ADR-MGT--
PYLQKTLNQQLTNHIRDTL
>XP_012379251.1
--VGRDFLPRG---SG-IVTRRPLV-------------LQL--------
VTS------------------------------------KAEYAEFLH-----------------------------------CKGKKFT---
DFDEVRHEIEAETDRVTGMN---KGISSIPINLRVYSPHV----------LNLTLIDLPGITKVPVGDQP--
PDIEYQIR--------EMIMQFITRENCLILAVTPANTDLANS-DALKLAKEVDPQGLRTIGVI-
TKLDLMDEG-TD----ARDVLENK-LLPLRR-GYVGVVNRSQKDIDGKKDIKAAMLAERKFFLSHPAY--
RHI--ADR-MGT--PHLQKVLNQQLTNHIRDTL
>XP_006496668.1
LELPQ----IAVVG----------------GQ----SAGKSSVLEN--FVGRDFLPRG---SG-IVTRRPLV-------------
LQL--------VTS------------------------------------KAEYAEFLH-----------------------------------CKGKKFT-
--DFDEVRHEIEAETDRVTGMN---KGISSIPINLRVYSPHV----------LNLTLIDLPGITKVPVGDQP--
PDIEYQIR--------DMIMQFITRENCLILAVTPANTDLANS-DALKLAKEVDPQGLRTIGVI-
TKLDLMDEG-TD----ARDVLENK-LLPLRR-GYVGVVNRSQKDIDGKKDIKAAMLAERKFFLSHPAY--
RHI--ADR-MGT--PHLQKVLNQQLTNHIRDTL
>XP_016856477.1
LELPQ----IAVVG----------------GQ----SAGKSSVLEN--FVGRDFLPRG---SG-IVTRRPLV-------------
LQL--------VTS------------------------------------KAEYAEFLH-----------------------------------CKGKKFT-
--DFDEVRLEIEAETDRVTGMN---KGISSIPINLRVYSPHV----------LNLTLIDLPGITKVPVGDQP--
PDIEYQIR--------EMIMQFITRENCLILAVTPANTDLANS-DALKLAKEVDPQGLRTIGVI-
TKLDLMDEG-TD----ARDVLENK-LLPLRR-GYVGVVNRSQKDIDGKKDIKAAMLAERKFFLSHPAY--
RHI--ADR-MGT--PHLQKVLNQQLTNHIRDTL
>XP_027623811.1
LELPQ----IAVVG----------------GQ----SAGKSSVLEN--FVGRDFLPRG---SG-IVTRRPLV-------------
LQL--------VTS------------------------------------KAEYGEFLH-----------------------------------
CKGKKFT---DFDEIRHEIEAETDRVTGVN---KGISSIPINLRVYSPHV----------
LNLTLIDLPGITKVPVGDQP--PDIEYQIR--------EMIMQFITRENCLILAVTPANTDLANS-
DALKLAKEVDPQGLRTIGVI-TKLDLMDEG-TD----ARDVLENK-LLPLRR-
GYVGVVNRSQKDIDGKKDIKAAMLAERKFFLSHPAY--RHI--ADR-MGT--
PHLQKVLNQQLTNHIRDTL
>EPQ08653.1
LELPQ----IAVVG----------------GQ----SAGKSSVLEN--FVGRDFLPRG---SG-IVTRRPLV-------------
LQL--------VTS------------------------------------KTEYAEFLH-----------------------------------CKGKKFT-
--DFDEVRHEIEAETDRVTGMN---KGISSIPINLRVYSPHV----------LNLTLIDLPGITKVPVGDQP--
VDIEHQIR--------EMIMQFITRENCLILAVTPANTDLANS-DALKLAKDVDPQGLRTIGVI-
TKLDLMDEG-TD----ARDILENK-LLPLRR-GYVGVVNRSQKDIDGKKDIKAAMLAERKFFLSHPAY--
RHI--ADR-MGT--PHLQKVLNQQLTNHIRDTL
>XP_025944940.1
--MFFRDFLPRG---SG-IVTRRPLV-------------LQL--------
VTA------------------------------------KTEYAEFLH-----------------------------------CKGRKFT---
DFDEVRQEIEVETDRITGVN---KGISSIPINLRIYSPHV----------LSLTLIDLPGITKVPVGDQP--
PDIEQQIR--------DMIMQFISRENCLILAVTPANTDLANS-DALKLAKEVDPQGLRTIGVI-

TKLDLMDEG-TD----AREILENK-LLPLRR-GYIGVVNRSQKDIDGKKDIKAALLAERKFFLSHPAY--
RHM--ADR-MGT--PYLQKVLNQQLTNHIRDTL
>XP_014389433.1
-------------------------------------AGKGQI------TAWDFLPRG---SG-IVTRRPLI------------LQL--------
IFS-----------------------------------KTEYAEFLH----------------------------------CKSKKFT---
DFDEVRQEIEAETDRVTGTN---KGISPVPINLRVYSPHV----------LNLTLIDLPGITKVPVGDQP--
PDIEYQIK--------DMILQFISRESSLILAVTPANMDLANS-DALKMAKEVDPQGLRTIGVI-
TKLDLMDEG-TD----ARDVLENK-LLPLRR-GYIGVVNRSQKDIEGKKDIRAALAAERKFFLSHPAY--
RHM--ADR-MGT--PHLQKTLNQQLTNHIRESL
>NP_001005360.1
LDLPQ----IAVVG----------------GQ----SAGKSSVLEN--FVGRDFLPRG---SG-IVTRRPLI------------
LQL--------IFS-----------------------------------KTEHAEFLH----------------------------------CKSKKFT-
--DFDEVRQEIEAETDRVTGTN---KGISPVPINLRVYSPHV----------LNLTLIDLPGITKVPVGDQP--
PDIEYQIK--------DMILQFISRESSLILAVTPANMDLANS-DALKLAKEVDPQGLRTIGVI-
TKLDLMDEG-TD----ARDVLENK-LLPLRR-GYIGVVNRSQKDIEGKKDIRAALAAERKFFLSHPAY--
RHM--ADR-MGT--PHLQKTLNQQLTNHIRESL
>XP_006510037.1
LDLPQ----IAVVG----------------GQ----SAGKSSVLEN--FVGRDFLPRG---SG-IVTRRPLI------------
LQL--------IFS-----------------------------------KTEYAEFLH----------------------------------CKSKKFT--
-DFDEVRQEIEAETDRVTGTN---KGISPVPINLRVYSPHV----------LNLTLIDLPGITKVPVGDQP--
PDIEYQIK--------DMILQFISRESSLILAVTPANMDLANS-DALKLAKEVDPQGLRTIGVI-
TKLDLMDEG-TD----ARDVLENK-LLPLRR-GYIGVVNRSQKDIEGKKDIRAALAAERKFFLSHPAY--
RHM--ADR-MGT--PHLQKTLNQQLTNHIRESL
>XP_025920181.1
LDLPQ----IAVVG----------------GQ----SAGKSSVLEN--FVGRDFLPRG---SG-IVTRRPLI------------
LQL--------IFS-----------------------------------KTEYAEFLH----------------------------------CKSKKFT--
-DFDEVRQEIEAETDRVTGTN---KGISPVPINLRVYSPHV----------LNLTLIDLPGITKVPVGDQP--
QDIEYQIK--------DMIMQFISRESSLILAVTPANMDLANS-DALKMAKEVDPQGLRTIGVI-
TKLDLMDEG-TD----ARDVLENK-LLPLRR-GYIGVVNRSQKDIDGKKDIRAALAAERKFFLSHPAY--
RHM--ADR-MGT--PHLQKVLNQQLTNHIRETL
>XP_028568434.1
LDLPQ----IAVVG----------------GQ----SAGKSSVLEN--FVGRDFLPRG---SG-IVTRRPLI------------
LQL--------IFS-----------------------------------KTEYAEFLH----------------------------------CKSKKFT--
-DFDEVRQEIEAETDRVTGTN---KGISPVPINLRVYSPHV----------LNLTLIDLPGITKVPVGDQP--
QDIEYQIK--------DMILQFISRESSLILAVTPANMDLANS-DALKMAKEVDPQGLRTIGVI-
TKLDLMDEG-TD----ARDVLENK-LLPLRR-GYIGVVNRSQKDIDGKKDIRAALAAERKFFLSHPAY--
RHM--ADR-MGT--PHLQKLLNQQLTNHIRETL
>XP_012381548.1

--MILQFIGRESSLILAVTPANMDLANS-
DALKLAKEVDPQGLRTIGVI-TKLDLMDEG-TD----ARDVLENK-LLPLRR-
GYIGVVNRSQKDIEGRKDIRSALAAERKFFFSHSAY--RHM--ADR-MGT--
LHLQKTLNQQLTNHIRESL

>XP_006161648.2.2
---PH----CTSVS----------------RQAYSSPQGPRKVISVLFLPHRDFLPRG---SG-IVTRRPLI-----------
-LQL--------IFS----------------------------------KTEYAEFLH---------------------------------CKSKKFT-
--DFDEVRQEIEAETDRVTGTN---KGISPVPINLRVYSPHV----------LNLTLIDLPGITKVPVGDQP--
PDIEYQIK--------DMILQFISRESSLILAVTPANMDLANS-DALKLAKEVDPQGLRTIGVI-
TKLDLMDEG-TD----ARDVLENK-LLPLRR-GYIGVVNRSQKDIEGKKDIRAALAAERKFFLSHPAY--
RHM--ADR-MGT--PHLQKTLNQQLTNHIRESL
>XP_031753735.1
LDLPQ----IAVVG----------------GQ----SAGKSSVLEN--FVGRDFLPRG---SG-IVTRRPLI------------
LQL--------IFS----------------------------------KTEYAEFLH---------------------------------CKSKKFT--
-DFDEVRQEIEAETDRVTGTN---KGISPVPINLRVYSPNV----------LNLTLIDLPGITKVPVGDQP--
HDIEYQIK--------DMILQFISRDSCLILAVTPGNTDLANS-DALKMAKEVDPQGLRTIGVI-
TKLDLMDEG-TD----AKDILENK-LLPLRR-GYIGVVNRSQKDIDGKKDIKAALGAERKFFLSHPGY--
RHI--AER-MGT--PHLQKTLNQQLTNHIRETL
>XP_021326548.1
LDLPQ----IAVVG----------------GQ----SAGKSSVLEN--FVGRDFLPRG---SG-IVTRRPLI------------
LQL--------VNN----------------------------------KAEYAEFLH---------------------------------
CKGRKFV---DFDEVRQEIEAETDRITGSN---KGISPIPINLRVYSPNV----------
LNLTLIDLPGMTKVAVGDQP--PDIEHQIR--------DMIMQFITRESCLILAVTPANMDLANS-
DALKVAKEVDPQGLRTIGVI-TKLDLMDEG-TD----ARDILENK-LLPLRR-
GYIGVVNRSQKDIDGRKDIRAALAAERKFFLSHPSY--RHM--AER-MGT--
PHLQKALNQQLTNHIRDTL
>NP_001025299.1
LDLPQ----IAVVG----------------GQ----SAGKSSVLEN--FVGRDFLPRG---SG-IVTRRPLI------------
LQL--------VNN----------------------------------KAEYAEFLH---------------------------------
CKGRKFV---DFDEVRQEIEAETDRITGSN---KGISPIPINLRVYSPNV----------
LNLTLIDLPGMTKVAVGDQP--PDIEHQIR--------DMIMQFITRESCLILAVTPANMDLANS-
DALKVAKEVDPQGLRTIGVI-TKLDLMDEG-TD----ARDILENK-LLPLRR-
GYIGVVNRSQKDIDGRKDIRAALAAERKFFLSHPSY--RHM--AER-MGT--
PHLQKALNQQLTNHIRDTL
>XP_035683496.1
LDLPQ----IAVVG----------------GQ----SAGKSSVLEN--FVGRDFLPRG---SG-IVTRRPLV-----------
-LQL--------IHNP---------------------------------KAEYGEFLH---------------------------------
AKGKMFS---DFHEIRAEIEAETDRMTGSN---KGISPVPINLRVYSPHV----------
LNLTLIDLPGMTKVPVGDQP--PDIEQQIR--------DMLLQFITKDNCLILAVSPANQDLANS-
DALKIAKEVDPQGMRTIGVI-TKLDLMDEG-TD----ARNILENR-TYPLRR-
GYIGVVNRSQADIDGRKDIKAALAAERKFFLSHPAY--RHL--ADR-MGT--
PYLQKTLNQQLTNHIRDTL
>XP_030853442.1.2
LDLPQ----IAVVG----------------GQ----SAGKSSVLEN--FVGRDFLPRG---SG-IVTRRPLV-----------
-LQL--------NNS----------------------------------KTEYGEFLH---------------------------------
CKGKKFT---DFDEIRKEIEAETDRVTGSN---KGISNVPINLRVYSPNV----------
LNLTLVDLPGMTKIAVGDQP--VDIEIQIR--------SMVMEFVTNESTLILAVSPANQDLANS-
DALKVAKEVDPKGVRTIGVI-TKLDLMDDG-TD----AKDILENK-LLPLRR-

GYVGVVNRSQRDIEGKKDIKAALAAERKFFLSHPSY--RHI--ADK-MGT--
PWLQKILNQQLTNHIRDSL
>XP_030853442.1
LDLPQ----IAVVG----------------GQ----SAGKSSVLEN--FVGRDFLPRG---SG-IVTRRPLV------------
-LQL--------NNS-----------------------------------KTEYGEFLH----------------------------------
CKGKKFT---DFDEIRKEIEAETDRVTGSN---KGISNVPINLRVYSPNV----------
LNLTLVDLPGMTKIAVGDQP--VDIEIQIR--------SMVMEFVTNESTLILAVSPANQDLANS-
DALKVAKEVDPKGVRTIGVI-TKLDLMDDG-TD----AKDILENK-LLPLRR-
GYVGVVNRSQRDIEGKKDIKAALAAERKFFLSHPSY--RHI--ADK-MGT--
PWLQKILNQQLTNHIRDSL
>KMZ10000.1
LDLPQ----IAVVG----------------GQ----SAGKSSVLEN--FVGKDFLPRG---SG-IVTRRPLI-------------
LQL--------ING-----------------------------------VTEYGEFLH----------------------------------IKGKKFS-
--SFDEIRKEIEDETDRVTGSN---KGISNIPINLRVYSPHV----------LNLTLIDLPGLTKVAIGDQP--
VDIEQQIK--------QMIFQFIRKETCLILAVTPANTDLANS-DALKLAKEVDPQGVRTIGVI-
TKLDLMDEG-TD----ARDILENK-LLPLRR-GYIGVVNRSQKDIEGRKDIHQALAAERKFFLSHPSY--
RHM--ADR-LGT--PYLQRVLNQQLTNHIRDTL
>XP_026693152.1
IDLPQ----IAVVG----------------GQ----SAGKSSVLEN--FVGKDFLPRG---SG-IVTRRPLV-------------
LQL--------ITAK----------------------------------NGEWGEFLH----------------------------------
CKGKKFT---DFNEIRKEIEEETDRMTGSN---KGISAIPINLRVHSPHV----------
LNLTLVDLPGMTKVPVGDQP--ADIEQQIR--------DMIMQFVVKDNCLILAVSPANSDLANS-
DALKIAKEFDPQGIRTIGVI-TKLDLMDEG-TD----AKHILENK-HLPLRR-
GYVGVVNRSQKDIDGNKDIKAALSAERRFFLSHPAY--RHM--ADK-LGT--
PYLQKILNQQLTNHIKETL
>PAA65118.1
FDLPQ----IAVVG----------------SQ----SAGKSSVLEN--FVGKDFLPRG---SG-IVTRRPLI-------------
LQL--------LYNP----------------------------------SAEYAEFGH----------------------------------
QRGRKYT---NFEEVRQEIEAETDRLTGRN---KGISNVPIMLRVFSPHV----------
LNLTLVDLPGMTKVAVGDQP--PDIEVQIR--------NMLLEFITKENCLILAVSPANSDLANS-
DALKLAKEVDPAGTRTIGVI-TKLDLMDQG-TD----AREVLENK-LLPLRR-
GYIGVVNRSQKDIEGRKDIKAAMAAERKFFLSHPSY--RHM--AER-MGT--
PYLQRCLNQQLTNHIRETL
>PAA78248.1
FDLPQ----IAVVG----------------SQ----SAGKSSVLEN--FVGKDFLPRG---SG-IVTRRPLV-------------
LQL--------LTH-----------------------------------PSEFAEFGH----------------------------------
LRGKKFT---NFDEVRQEIENETDRLTGKN---KGISNVPITLRVFSPHV----------
LNLTLVDLPGMTKVAVGDQP--PDIEQQIR--------AMLFEFISKENCLILAVSPANSDLANS-
DALKIAKEVDPNGTRTIGVI-TKLDLMDQG-TD----AREVLENK-LLPLRR-
GYIGVVNRSQKDIEGKKDIAAAMAAERKFFLSHPSY--RHM--AER-MGT--
PYLQRCLNQQLTNHIRETL
>PAA59145.1
FDLPQ----IAVVG----------------SQ----SAGKSSVLEN--FVGKDFLPRG---SG-IVTRRPLV-------------
LQL--------INF-----------------------------------HTEYAEFGH----------------------------------IRGKRFT-

--NFDEVRQEIENETDRVTGKN---KGISNVPIMLRVYSPQV----------LNLTLVDLPGLTKVAVGDQP--
QDIELLIR--------AMILEFVSKDNCLILAVTPANSDLANS-DALKIAKEVDPSGTRTIGVI-
TKLDLMDQG-TD----ARDVLENR-LLPLRR-GYIGVVNRSQKDIEGKKDIVAAMAAERKFFLSHPAY--
RHM--AER-MGT--SYLQRCLNQQLTNHIRETL
>PAA64382.1
IDLPQ----IAVVG----------------SQ----SAGKSSVLEN--FVGRDFLPRG---SG-IVTRRPLI------------
LQL--------MNY----------------------------------QTEYAEFGH---------------------------------
IRGKKFV---NFDEVRREIEVETDRLTGQN---KGISNVPITLRVYSPQV----------
LNLTLVDLPGMTKVAVGDQP--PDIEQQVR--------AMIWEFISKDNCLILAVSPANSDLANS-
DALKLAKEADPSGSRTIGVL-TKLDLMDAG-TD----ARDVLENR-FLPLRR-
GYVGVVNRSQKDIDGRKDISSAMAAERKFFLGHPAY--RHM--AER-MGT--
AHLQRCLNQQLVGHIRDTL
>XP_001749319.1
LDLPQ----IAVVG----------------GQ----SAGKSSVLEN--FVGKDFLPRG---SG-IVTRRPLV------------
LQL--------NYHP---------------------------------SAEWGEFLH---------------------------------
ARGKKFT---DFNEIRQEIEAETDRMTGSN---KGISNIPINLRVYSPHV----------
LNLTLVDLPGLTKVAVGDQP--ADIENQIR--------GMLMEFITKDNCIILAVTPANQDLANS-
DALKLAKEVDPEGVRTIGVI-TKLDLMDSG-TD----ARAILTNE-FLPLRR-
GYIGVVNRSQKDIDGRKDIRAALDAERKFFLMHPSY--KDI--ASK-NGT--
PYLQKALNQQLTNHIRECL
>NP_001024332.1
FELPQ----IAVVG----------------GQ----SAGKSSVLEN--FVGKDFLPRG---SG-IVTRRPLI------------
LQL--------IQD----------------------------------RNEYAEFLH---------------------------------
KKGHRFV---DFDAVRKEIEDETDRVTGQN---KGISPHPINLRVFSPNV----------
LNLTLIDLPGLTKVPVGDQP--ADIEQQIR--------DMILTFINRETCLILAVTPANSDLATS-
DALKLAKEVDPQGLRTIGVL-TKLDLMDEG-TD----AREILENK-LFTLRR-
GYVGVVNRGQKDIVGRKDIRAALDAERKFFISHPSY--RHM--ADR-LGT--
SYLQHTLNQQLTNHIRDTL
>XP_004347890.1
LDLPQ----IAVVG----------------SQ----SAGKSSVLEN--FVGKDFLPRG---SG-IVTRRPLV------------
LQL--------VNSK---------------------------------GPEYGEFLH---------------------------------
NKSKKFT---DFDEVRKEIEAETDRITGTN---KGISPVPINLKVYSPNV----------
LNLTLVDLPGITKVPIGDQP--TNIESLIR--------EMIMQFIGRPNCLILAVSPANSDLANS-
DALKLAREVDQQGIRTIGVI-TKLDLMDEG-TD----AREVLENK-LIPLRR-
GFIGVVNRSQKDIDGRKDIKAAMSAELRFFSTHPAY--RDL--ANK-NGT--
MYLQRVLNQQLTNHIRDTL
>XP_031757197.1
VDLPQ----IAVVG----------------GQ----SAGKSSVLEN--LVGRWI--------------------------------H----
----VLS---------------------------------STEYAEFLH---------------------------------CKGTKYT---
DFSEVRQEIEEETERATGLN---KGISAIPISLRIYSPHV----------LNLSLIDLPGVTKVPVGDQP--
ADIETQIR--------DMIMNFISRENCLILAVTPANTDLANS-DALKLAKEVDPQGLRTIGVI-
TKLDLMDEG-TN----AQEILENK-LLPLRR-GYVGVVNRSQKDIDGKKNINAALQAEQMFFLTHPAY--
RHM--ADR-MGT--SHLQKMLNQQLTNHIRETL
>XP_014153758.1

N--LYL------------------------
----------------------YIEWGEFLH----------------------------------QPGRKYT---
DFEEIMKEIEAETDRMTGSN---KGISNIPINLKVMSPHV----------LDLTLVDLPGLTKVAVGDQP--
ADIEQQIL--------GMIMEFITRPNCLILAVSPANADLANS-DALKLAKEVDPQGLRTIGVI-
TKLDLMDQG-TD----AREILENK-LLPLRR-GYIGVVNRSQKDITGKKDIRAAQEAERRFFSTHPAY--
RHL--AQN-MGT--PKLQKVLNQQLTNHIRDSL
>XP_014148725.1

---------------------TEYGEFLH----------------------------------KPGRLFD---
NFDEIRNEIEADTARITGAN---KGISHLPINLKVYSPHV----------LDLTLVDLPGLTKVAVGDQP--
ADIEMQIK--------NMIMEFITKPNCLILAVTPANSDLANS-DALKLAKEVDPQGLRTIGVI-
TKLDLMDAG-TD----ARDVLENK-LLPLRR-GYVGVVNRSQKDIAGNKDIRAAQAAEKKFFKTHPAY--
RHL--ADK-MGT--PKLQQVLNQQLTDHIRQTL
>NP_741403.2
IQLPQ----IVVVG----------------SQ----SAGKSSVLEN--LVGRDFLPRG---TG-IVTRRPLI-------------
LQL--------NHVALDDESKRRRSNG------------------TLLTDDWAMFEH-------------------------------
--TGSKVFT---DFDAVRKEIEDETDRVTGVN---KGISLLPISLKIYSHRV----------
VSLSLVDLPGITKIPVGDQP--VNIEEQIR--------EMILLYISNPSSIILAVTPANQDFATS-
EPIKLAREVDAGGQRTLAVL-TKLDLMDQG-TD----AMDVLMGK-VIPVKL-
GIIGVVNRSQQNILDNKLIVDAVKDEQSFMQKK--Y--PTL--ASR-NGT--
PYLAKRLNMLLMHHIRNCL
>NP_957216.1
IQLPQ----IAVVG----------------TQ----SSGKSSVLES--LVGRDLLPRG---TG-IVTRRPLI-------------
LQL--------VHVDPEDRRKTS-EEN-------------------GVDGEEWGKFLH-------------------------------
--TKNKIYT---DFDEIRQEIENETERVSGNN---KGISDEPIHLKIFSPHV----------
VNLTLVDLPGITKVPVGDQP--KDIELQIR--------ELILKYISNPNSIILAVTAANTDMATS-
EALKVAREVDPDGRRTLAVV-TKLDLMDAG-TD----AMDVLMGR-VIPVKL-
GLIGVVNRSQLDINNKKSVADSIRDEHGFLQKK--Y--PSL--ANR-NGT--
KYLARTLNRLLMHHIRDCL
>NP_001317309.1
IQLPQ----IVVVG----------------TQ----SSGKSSVLES--LVGRDLLPRG---TG-IVTRRPLI-------------
LQL--------VHVSQEDKRKTTGEEN------DPATWKNSRHLSKGVEAEEWGKFLH----------------------
------------TKNKLYT---DFDEIRQEIENETERISGNN---KGVSPEPIHLKIFSPNV----------
VNLTLVDLPGMTKVPVGDQP--KDIELQIR--------ELILRFISNPNSIILAVTAANTDMATS-
EALKISREVDPDGRRTLAVI-TKLDLMDAG-TD----AMDVLMGR-VIPVKL-
GIIGVVNRSQLDINNKKSVTDSIRDEYAFLQKK--Y--PSL--ANR-NGT--KYLARTLNRLLMHHIRDCL
>XP_006168142.1
IQLPQ----IVVVG----------------TQ----SSGKSSVLES--LVGRDLLPRG---TG-IVTRRPLI-------------
LQL--------VHVSPEDKRKTTGEEN------DPATWKNSRHLSKGVEAEEWGKFLH----------------------
------------TKNKLYT---DFDEIRQEIENETERISGNN---KGVSPEPIHLKIFSPNV----------
VNLTLVDLPGMTKVPVGDQP--KDIELQIR--------ELILRFISNPNSIILAVTAANTDMATS-
EALKISREVDPDGRRTLAVI-TKLDLMDAG-TD----AMDVLMGR-VIPVKL-
GIIGVVNRSQLDINNKKSVTDSIRDEYAFLQKK--Y--PSL--ANR-NGT--KYLARTLNRLLMHHIRDCL
>NP_001392186.1

IQLPQ----IVVVG----------------TQ----SSGKSSVLES--LVGRDLLPRG---TG-VVTRRPLI-------------
LQL--------VHVSPEDKRKTTGEENGKFQSWNPATWKNSRHLSKGVEAEEWGKFLH-----------------
-----------------TKNKLYT---DFDEIRQEIENETERISGNN---KGVSPEPIHLKVFSPNV----------
VNLTLVDLPGMTKVPVGDQP--KDIELQIR--------ELILRFISNPNSIILAVTAANTDMATS-
EALKISREVDPDGRRTLAVI-TKLDLMDAG-TD----AMDVLMGR-VIPVKL-
GIIGVVNRSQLDINNKKSVTDSIRDEYAFLQKK--Y--PSL--ANR-NGT--KYLARTLNRLLMHHIRDCL
>XP_014394711.1
LLCPV----CVQIS----------------SL----SSGKSSVLES--LVGRDLLPRG---TG-IVTRRPLI-------------
LQL--------VHVSPEDQRKTSGEEN------DPATWKNSRHLSKGVEAEEWGKFLH----------------------
------------TKNKLYT---DFDEIRQEIENETERISGNN---KGVSPEPIHLKIFSPNV----------
VNLTLVDLPGMTKVPVGDQP--KDIELQIR--------ELILRFISNPNSIILAVTAANTDMATS-
EALKISREVDPDGRRTLAVI-TKLDLMDAG-TD----AMDVLMGR-VIPVKL-
GIIGVVNRSQLDINNKKSVTDSIRDEYAFLQKK--Y--PSL--ANR-NGT--KYLARTLNRLLMHHIRDCL
>XP_028602039.1
IQLPQ----IVVVG----------------TQ----SSGKSSVLES--LVGRDLLPRG---TG-IVTRRPLI-------------
LQL--------VNVSAEDLRKKTGDEN------DPATWKHARHLTKGVDTEEWGKFLH----------------------
------------TKNKLYS---DFDEIRQEIESETERISGNN---KGISPEPIHLKVFSPNV----------
VNLTLVDLPGMTKVPVGDQP--KDIELQIR--------ELILRFISNPNSIILAVTAANTDMATS-
EALKIAREVDPDGRRTLAVI-TKLDLMDAG-TD----AMDVLMGR-VIPVKL-
GIIGVVNRSQLDINNKKSVADSIRDEYGFLQKK--Y--PSL--ANR-NGT--KYLARTLNRLLMHHIRDCL
>XP_025940269.1
IQLPQ----IVVVG----------------TQ----SSGKSSVLES--LVGRDLLPRG---TG-VVTRRPLI-------------
LQL--------VHVSPEDGRKTAGDEN-------------------EIDAEEWGKFLH---------------------------------
-TKNKVYT---DFDEIRQEIENETERISGNN---KGISPEPIHLKIFSSNV----------
VNLTLVDLPGMTKVPVGDQP--KDIELQIR--------ELILQFISNPNSIILAVTAANTDMATS-
EALKIAREVDPDGRRTLAVI-TKLDLMDAG-TD----AMDVLMGR-VIPVKL-
GIIGVVNRSQLDINNKKSVADSIRDEYGFLQKK--Y--PSL--ANR-NGT--KYLARTLNRLLMHHIRDCL
>XP_012382650.2
---G---FK-LVISTKLI-------------LCC--------FSLTT----
-------------DPATWKNSRHLSKGVEAEEWGKFLH----------------------------------TKNKLYT---
DFDEIRQEIENETERISGNN---KGVSPEPIHLKIFSPNV----------VNLTLVDLPGMTKVPVGDQP--
KDIELQIR--------ELILRFISNPNSIILAVTAANTDMATS-EALKISREVDPDGRRTLAVI-TKLDLMDAG-
TD----AMDVLMGR-VIPVKL-GIIGIVNRSQLDINNKKSVTDSIRDEYAFLQKK--Y--PSL--ANR-NGT--
KYLARTLNRLLMHHIRDCL
>XP_031753959.1
IGFGP----VLRVP----------------LQ----PAKCWAAKES--MVLSASLLPV---PH-LYERLLLS-------------
VVF--------FSLIP-----------------DPNAWKIPKHFSKGVETEEWGKFLH---------------------------------
-TKNKIYT---DFDEIRQEIENETERISGNN---KGISSEPIHLKIFSPNV----------
VNLTLVDLPGMTKVPVGDQP--KDIEIQIR--------ELILRYISNPNSIILAVTAANTDMATS-
EALKIARESDPDGRRTLAVI-TKLDLMDAG-TD----AMDVLLGR-VIPVKL-
GIIGVVNRSQLDINNKKSVADSIRDEYGFLQKK--Y--PSL--ANR-NGT--KYLARTLNRLLMHHIRDCL
>XP_032819300.1
IQLPQ----IVVVG----------------AQ----SSGKSSVLES--LVGRDFLPRG---TG-IVTRRPLV-------------
LQL--------VHVIPDERIRPGGEEN-------------------GVEAEEWGKFLH---------------------------------

-TKNKVYS---DFNEIRQEIENETERITGTN---KGISSEAIHLKIFSPHV----------
LNLTLVDLPGITKVPVGDQP--VDIEQQIR--------ELIIKFIGNPNSIILAVTAANTDLATS-
EALKIAREVDTDGRRTLAVI-TKLDLMDAG-TD----AMDILTGR-VIPVKL-
GIIGVVNRSQLDINTKKTILDAMQDEQSFMQKK--Y--PSL--ANR-NGT--
KFLGKTLNRLLMHHIRDCL
>XP_035676386.1
IQLPQ----IVVIG----------------TQ----SSGKSSVLES--LVGRDFLPRG---TG-IVTRRPLV-------------
LQL--------VHVNSEEKKRPSEDED----------GGHKQDIKEHAHVEEWGKFLH-------------------------
---------TKNKIYT---DFDEIRQEIENETDRVTGTN---KGIIDDAIHLKIYSPKV----------
LNLTLVDLPGITKVPVGDQP--PDIEVQIR--------EMCLKYIANPNSIILAVTSANTDMATS-
EALKFAKEVDPDGRRTLAVI-TKLDLMDAG-TD----AHDVLMGR-VIPVKL-
GIIGVVNRSQMDINKRKPIEEAIKDEAAFMQRK--Y--PSL--ASR-NGT--SHLARTLNRLLMHHIRDCL
>XP_006821224.1

---MATS-EAIKLSREVDEDGRRTLAVI-
TKLDLMDAG-TD----AVEIICGR-VIPVKL-GIIGVINRSQMDINNKKPIQESVKDEAAFLQRK--Y--PAL-
-ASR-NGT--PYLAKTLNRLLMHHIRDCL
>XP_030827871.1
IQLPQ----IVVVG----------------NQ----SSGKSSVLEG--LVGKDFLPRG---NG-IVTRRPLV-------------
LQM--------VHVDPEDKRGASGEGE------------------EEITADEWGKFLH-------------------------------
---TKNKVYT---DFEEIREEIQNETDRMAGTN---KGIVHDAIHLRIYSPKV----------
LNLTLVDLPGLTKVPVGDQP--EDIESQIR--------EMLVKYIGNPNSIILAVTSANTDMATS-
ESLKLAKEIDPDGRRTLAVI-TKLDLMDAG-TD----AVDVLCGR-VIPVKL-
GIIGVVNRSQMDINNKKVIDDAVKDESAFLQRK--Y--PAL--ASR-NGT--AYLARTLNRLLMHHIRDCL
>NP_001259946.1
IQLPQ----IVVLG----------------SQ----SSGKSSVIES--VVGRSFLPRG---TG-IVTRRPLV-------------
LQL--------IYSPLDDRENRSAENG-------------------TSNAEEWGRFLH---------------------------------
-TK-KCFT---DFDEIRKEIENETERAAGSN---KGICPEPINLKIFSTHV----------
VNLTLVDLPGITKVPVGDQP--EDIEAQIK--------ELVLKYIENPNSIILAVTAANTDMATS-
EALKLAKDVDPDGRRTLAVV-TKLDLMDAG-TD----AIDILCGR-VIPVKL-
GIIGVMNRSQKDIMDQKHIDDQMKDEAAFLQRK--Y--PTL--ATR-NGT--
PYLAKTLNRLLMHHIRDCL
>PAA85687.1
IQLPQ----IVVIG----------------TQ----SSGKSSVLES--LVGRDFLPRG---TG-IVTRRPLV-------------
LQL--------VHLEADEK-DEAGDRP-------------------AAEEEEWGKFLH---------------------------------
-TKGKIYT---DFNEIRDEIARETDRIAGSG---KCVSIDPINLKIYSPHV----------
VSLTLVDLPGITKVPVADQP--EDIEVQIR--------ALCIEYIKNPNSIILAVTPANTDMATS-
ESLKLAKEVDPQGKRTLAVI-TKLDLMDAG-TD----AHDLLLGR-VIPVKL-
GIIGVVNRSQADIKNQKQVKEAVRDESSFLQRR--Y--PSL--ASR-NGT--PYLARTLNRLLMHHIRDCL
>XP_002129967.2
LQLPQ----IVVVG----------------VQ----SSGKSSVLEN--LVGRDFLPRG---TG-IVTRCPLV-------------
LQM--------IHTTNEDTAQCSNEGS---SGNNDSDSSGESFKETNEEVKEWVKFQH-------------------
---------------TKGKIFR---SFKQVKKEIELETQRLSGNN---KGISSEAIRLKIFSPKV----------

LNLTLVDLPGLMKIPVGDQP--DDIEEQAR--------NLILRYISNPNSIILAVTPANVDFATS-
EALQMARIVDPDGCRTLAVV-TKLDLMDAG-TD----AIDVLCGR-IVPVKL-
GIIGIVNRSQLDINKGKSVQDAIKDEQAFLQKK--Y--PSF--ANR-SGS--RYLSITLNRLLMHHIRDCL
>XP_004348308.1
IQLPQ----IAVVG----------------SQ----SSGKSSVLEN--IVGKDFLPRG---HG-IVTRRPLI-------------
LQL--------VHRKPGSPRPALPDDP-------SSSGGHTDDGIDGEDVEEWGEFLH----------------------
------------APGKRFI---SFAEIRKEIEAETDRVTGSN---KGISSKPINLRIYSPNV----------
LNLTLVDLPGITKVPVGDQP--EDIEKQIR--------TLVRSYISNPNCIILAVTPANVDLANS-
DALKLAKTIDPEGNRTIGVC-TKIDLMDAG-TD----AMDILSGR-VVPVKL-
GFIGVVNRSQADINTAKPIADSLKSEEQFFKSHPAY--QAI--AHR-CGT--
AYLSKALNKLLMHHIRDCL
>XP_014148015.1
---LQL--------VHHPVQR---
-------------------------GGPAAEWGEFLH----------------------------------QPGKIYT---
DFSKIRDEIANETDRLTGTN---KGISHTPINLKLYSPNM----------LDLTLVDLPGITKIAVGDQP--
EDIEVQIH--------QLIESYINNPNCIILAVTAANTDIANS-DALKMAKKADPKGLRTIGVA-
TKLDLMDAG-TD----ALDILTGK-VVASKL-GFIGVVNRSQADINQKVSIETAREAEQEYFRTHPAY--
KSL--YKQ-SGT--EYLTRRLNQLLMTHIRRCL
>XP_001750431.1
IQLPQ----IVVVGAQASSPADHPALRMSHEQ----SSGKSSILEN--VVGKDFLPRG---TG-IVTRVPLV--
-----------LQL--------VQTA----------------------------------DDEWATFQH--------------------------------
--AGGKVFR---DFEQVRQEIVDQTERITGPG---KAVSNEPIHLRVHSPNV----------
VNLTLVDLPGLTKVAVADQP--QDIGPQIR--------RLVRHYIDNPNSLILAVSPANADIANS-
DSLQIAKEVDPQGDRTLAIV-TKLDLMDRG-TD----AKALLSGE-VLPVKL-
GIIGIVNRSQNDINCKTSIQDSLDNEKRFFRTH--Y--PEM--ADR-CGC--AFLADTLHHLLLQHIRACL
>XP_004466363.1
LSLPA----IAVIG----------------DQ----SSGKSSVLEA--LSGVA-LPRG---SG-IVTRCPLV-------------
LKL--------KKLTN---------------------------------EEKWRGKVTYE--------------------------------
DYEIDIS---DASEVEEEINKAQNVIAGEG---LGISQKLINLEVCSPDV----------
PDLTLIDLPGITRVAVGNQP--ADIGWQIK--------CLIKKYITRQETINLVVVPSNVDIATT-
EALSMAQEVDPNGDRTIGIL-TKPDLVDRG-TE--DKVVDVVRNL-VCHLKK-
GYMIVRCRGQQDIQDRLSLATALQKERAFFENHENF--RVLLEEGK-ATV--PHLAERLTTELITHISKTL
>XP_006156437.1
LALPA----IAVIG----------------DQ----SSGKSSVLEA--LSGVA-LPRG---SG-IVTRCPLV-------------
LKL--------KKLIN---------------------------------EDKWRGKVSYQ--------------------------------
DIEVEIT---DPSKVEPEINKAQNVIAGEG---MGISHELISLEVSSPHV----------
PDLTLIDLPGITRVAVGNQP--ADIGRQIK--------TLIKKYIHKQETINLVVVPSNVDIATT-
EALSMAQEVDPDGDRTIGIL-TKPDLVDKG-TE--EKVVDVVRNL-VCHLKK-
GYMIVKCRGQQDIQDRLSLAEALQREKVFFEEHPYF--SFLLEEGK-ATI--PCLAERLTTELIMHISKSL
>NP_002453.2.2
LALPA----IAVIG----------------DQ----SSGKSSVLEA--LSGVA-LPRG---SG-IVTRCPLV-------------
LKL--------KKLVN---------------------------------EDKWRGKVSYQ--------------------------------
DYEIEIS---DASEVEKEINKAQNAIAGEG---MGISHELITLEISSRDV----------
PDLTLIDLPGITRVAVGNQP--ADIGYKIK--------TLIKKYIQRQETISLVVVPSNVDIATT-

EALSMAQEVDPEGDRTIGIL-TKPDLVDKG-TE--DKVVDVVRNL-VFHLKK-
GYMIVKCRGQQEIQDQLSLSEALQREKIFFENHPYF--RDLLEEGK-ATV--PCLAEKLTSELITHICKSL
>NP_001127618.1
LALPA----IAVIG----------------DQ----SSGKSSVLEA--LSGVA-LPRG---SG-IVTRCPLV-------------
LKL--------KKLVN---------------------------------EDKWRGKVSYQ--------------------------------
DYEIEIS---DASEVEKEINKAQNTIAGEG---MGISHELITLEISSRDV----------
PDLTLIDLPGITRVAVGNQP--ADIGYKIK--------TLIKKYIQRQETISLVVVPSNVDIATT-
EALSMAQEVDPEGDRTIGIL-TKPDLVDKG-TE--DKVVDVVRNL-VFHLKK-
GYMIVKCRGQQEIQDQLSLSEALQREKIFFEDHPYF--RDLLEEGK-ATV--PCLAEKLTSELITHICKSL
>XP_017508130.1
LALPA----IAVIG----------------DQ----SSGKSSVLEA--LSGVA-LPRG---SG-IVTRCPLV-------------
LKL--------KKLTN---------------------------------EETWRGKVSYQ--------------------------------
DFEAELS---DPSEVEREINRAQNSIAGEG---TGISHELISLEISSPHV----------
PDLTLIDLPGITRVAVGNQP--ADIGRQIK--------ALIRKYIYKQETINLVVVPSNVDIATT-
EALSMAQEVDPDGDRTIGIL-TKPDLVDRG-TE--DKVVDVVRNL-VCHLKK-
GYMIVKCRGQQDIQDQLSLAEALKKERAFFEDNPYF--RDLLEEGR-ATV--
PCLADKLTVELITHICKSL
>NP_001003134.1
LALPA----IAVIG----------------DQ----SSGKSSVLEA--LSGVA-LPRG---SG-IVTRCPLV-------------
LKL--------KKLIN---------------------------------EDEWRGKVSYQ--------------------------------
DTEMEIS---DPSEVEVEINKAQDAIAGEG---QGISHELISLEVSSPHV----------
PDLTLIDLPGITRVAVGNQP--ADIGRQTK--------QLIRKYILKQETINLVVVPCNVDIATT-
EALSMAQEVDPDGDRTIGIL-TKPDLVDRG-TE--GKVVDVAQNL-VCHLKK-
GYMIVKCRGQQDIQDQVSLAEALQKEKDFFEDHPHF--RVLLEEGR-ATV--
PNLAEKLTSELITHICKTL
>XP_032211398.1
LALPA----IAVIG----------------DQ----SSGKSSVLEA--LSGVA-LPRG---SG-IVTRCPLV-------------
LKL--------KKVTN---------------------------------QDEWRGKVSYQ--------------------------------
DFEKEIS---DPSEVEAEINKAQNAVAGEG---QGISHELISLEVSSSHV----------
PDLTLIDLPGITRVAVGNQP--ADIGRQTK--------QLIRKYILRQETINLVVVPCNVDIATT-
EALSMAQEVDPDGDRTIGIL-TKPDLVDRG-TE--SKVVDVAQNL-VCHLKK-
GYMIVKCRGQQDIQDQVTLAEALQKERDFFEDHPHF--RVLLEEGR-ATV--
PCLADKLTSELIMHICKTL
>XP_008569442.1
LALPA----IAVIG----------------DQ----SSGKSSVLEA--LSGVA-LPRG---SG-IVTRCPLV-------------
LKL--------KKLVH---------------------------------GEEWKGKVSYR--------------------------------
DLEIKIS---DALEVEEEVRKAQTIIAGEG---MGISHELINLEISSPHV----------
PDLTLIDLPGIARVAMGNQP--ADIGYQVK--------XLIRKYIQRQETINLVVVPSNVDIATT-
EALSMAQEVDPEGDRTIGIL-TKPDLVDKG-TE--DKVVDVVRNL-VYHLKK-
GYMIVKCRGQQDIQDQLSLATALQREKDFFEDHPQF--RDLLEEGR-ATI--
PCLAERLTTELITHICKSL
>XP_014388412.1
LALPA----IAVIG----------------DQ----SSGKSSVLEA--LSGVS-LPRG---SG-IVTRCPLV-------------
LKL--------RKLRH---------------------------------DDEWKGKVTYR--------------------------------

DLEIDLS---AASEVEQEIRKAQNVIAGEG---VGISQELINLEVSSPHV----------
PDLTLIDLPGITRVAVGNQP--ADIGRQIT--------ALIKKYILRQQTIMLVVVPSNVDIATT-
EALSMAHEVDPDGDRTIGIL-TKPDLVDRG-TE--DKVVDVVRNL-VYHLKK-
GYMIVKCRGQQDIQYQMSLSKALQRERAFFEDHPYF--RDLLEEGK-ATI--
PCLAERLTNELIAHISKSL
>XP_005202045.1
LALPA----IAVIG----------------DQ----SSGKSSVLEA--LSGVA-LPRG---SG-IVTRCPLV------------
LRL--------KKLGN--------------------------------EDEWKGKVSFL-------------------------------
DKEIEIP---DASQVEKEISEAQIAIAGEG---TGISHELISLEVSSPHV----------
PDLTLIDLPGITRVAVGNQP--PDIEYQIK--------SLIRKYILRQETINLVVVPANVDIATT-
EALRMAQEVDPQGDRTIGIL-TKPDLVDKG-TE--DKVVDVVRNL-VFHLKK-
GYMIVKCRGQQDIKHRMSLDKALQRERIFFEDHAHF--RDLLEEGK-ATI--
PCLAERLTSELIMHICKTL
>NP_038634.1
LALPA----IAVIG----------------DQ----SSGKSSVLEA--LSGVA-LPRG---SG-IVTRCPLV------------
LKL--------RKLNE--------------------------------GEEWRGKVSYD-------------------------------
DIEVELS---DPSEVEEAINKGQNFIAGVG---LGISDKLISLDVSSPNV----------
PDLTLIDLPGITRVAVGNQP--ADIGRQIK--------RLIKTYIQKQETINLVVVPSNVDIATT-
EALSMAQEVDPEGDRTIGIL-TKPDLVDRG-TE--DKVVDVVRNL-VYHLKK-
GYMIVKCRGQQDIQEQLSLTEALQNEQIFFKEHPHF--RVLLEDGK-ATV--
PCLAERLTAELILHICKSL
>NP_034976.1
LALPA----IAVIG----------------DQ----SSGKSSVLEA--LSGVA-LPRG---SG-IVTRCPLV------------
LKL--------RKLKE--------------------------------GEEWRGKVSYD-------------------------------
DIEVELS---DPSEVEEAINKGQNFIAGVG---LGISDKLISLDVSSPNV----------
PDLTLIDLPGITRVAVGNQP--ADIGRQIK--------RLIKTYIQKQETINLVVVPSNVDIATT-
EALSMAQEVDPEGDRTIGVL-TKPDLVDRG-AE--GKVLDVMRNL-VYPLKK-
GYMIVKCRGQQDIQEQLSLTEAFQKEQVFFKDHSYF--SILLEDGK-ATV--
PCLAERLTEELTSHICKSL
>XP_004675614.2.2
LALPA----IAVIG----------------DQ----SSGKSSVLEA--LSGVA-LPRG---SG-IVTRCPLV------------
LKL--------KKLMN--------------------------------EDSWKGKINYQ-------------------------------
GVEVTIA---KASDVEQEVNKAQAVIAGDG---LGISHELITLEVSSPEV----------
PDLTLIDLPGITRVAVGNQP--QDIGEQIK--------ALIRKYIQRQQTINLVVVPCNVDIATT-
EALSMAREVDPDGDRTLGIL-TKPDLVDRG-TE--DRVVDVIRNF-ICPLKK-
GYMIVKCRGQKDIQDRLSLAQALQKEQAFFEEHPHF--RQLLEEGR-ASI--
PKLADRLTSELIRHISKSL
>XP_005885748.1
LALPA----IAVIG----------------DQ----SSGKSSVLEA--LSGVA-LPRG---SG-IVTRCPLV------------
LKL--------KKQLAG--------------------------------ESLWTGKISYR-------------------------------
STELQLQ---DPSQVEREIYKAQNTIAGNG---VGISHELINLEITSPEV----------
PDLTLIDLPGIARVAVGNQP--QDIGLQIK--------ALIKKYIQRQQTINLVVVPCNVDIATT-
EALSMAHEVDPDGDRTIGIL-TKPDLVDKG-AE--KNVVNVAQNL-TYRLKK-

GYMIVKCRGQQEITDKLSLAEATKKEMMFFQTHPYF--RVLLEEGK-ATV--
PRLAERLTTELIWHINKSL
>XP_017508123.1
LALPA----IAVIG----------------DQ----SSGKSSVLEA--LSGVA-LPRG---SG-IVTRCPLV-------------
LKL--------KKQLH---------------------------------EPAWTGRLSYQ--------------------------------
TTELQLH---NPSQVEKEIQKAQNAIAGDG---VGISHELINLEITSPDV----------
PDLTLIDLPGIARVAVGNQP--QDIGLQIK--------ALIKKYIQRQQTINLVVVPCNVDIATT-
EALSMAQEVDPDGDRTIGIL-TKPDLVDKG-TE--RVIVNVVQNL-TYHLKK-
GYMIVKCRGQQEVTNKLSLAEATSKEMTFFQTHPYF--RILLEEGK-ATV--PRLAEKLTTELISHINKSL
>XP_008569440.1
LALPA----IAVIG----------------DQ----SSGKSSVLEA--LSGVA-LPRG---SG-IVTRCPLV-------------
LKL--------KKHLQ---------------------------------EDGWKGKISYR--------------------------------
HTELLLQ---DPSQVEKEIHKAQNTIAGNG---VGISHELISLEITSPEV----------
PDLTLIDLPGITRVAVGNQP--QDIGQQVK--------ALIKKYIQRQQTINLVVVPCNVDIATT-
EALSMAQEVDPDGDRTIGIL-TKPDLVDKG-TE--KGVMNVARNL-TYHLKK-
GYMIVKCRGQQDITNKLSLAEATKKEMAFFQTHPYF--RVLLEEGK-ATV--PCVAEKLTAELIVHINKSL
>NP_002454.1
LALPA----IAVIG----------------DQ----SSGKSSVLEA--LSGVA-LPRG---SG-IVTRCPLV-------------
LKL--------KKQPC---------------------------------E-AWAGRISYR--------------------------------
NTELELQ---DPGQVEKEIHKAQNVMAGNG---RGISHELISLEITSPEV----------
PDLTIIDLPGITRVAVDNQP--RDIGLQIK--------ALIKKYIQRQQTINLVVVPCNVDIATT-
EALSMAHEVDPEGDRTIGIL-TKPDLMDRG-TE--KSVMNVVRNL-TYPLKK-
GYMIVKCRGQQEITNRLSLAEATKKEITFFQTHPYF--RVLLEEGS-ATV--PRLAERLTTELIMHIQKSL
>XP_002830747.1
LALPA----IAVIG----------------DQ----SSGKSSVLEA--LSGVA-LPRG---SG-IVTRCPLV-------------
LKL--------KKQPC---------------------------------E-AWAGRISYR--------------------------------
NTELELQ---DPGQVEKEIHKAQNIMAGNG---RGISHELISLEITSPEV----------
PDLTIIDLPGITRVAVDNQP--RDIGLQIK--------ALIKKYIQRQQTINLVVVPCNVDIATT-
EALSMAHEVDPEGDRTIGIL-TKPDLMDKG-TE--KSVMNVVRNL-TYPLKK-
GYMIVRCRGQQELTNRLSLAEATKKEITFFQTHPYF--RVLLEEGS-ATV--PRLAERLTSELIMHIQKSL
>NP_001003133.1
LALPA----IAVIG----------------DQ----SSGKSSVLEA--LSGVA-LPRG---SG-IVTRCPLV-------------
LKL--------KRDPH---------------------------------K-AWRGRISYR--------------------------------
KTELQFQ---DPSQVEKEIRQAQNIIAGQG---LGISHELISLEITSPEV----------
PDLTLIDLPGITRVAVGNQP--QDIGVQIK--------ALIKNYIQKQETINLVVVPCNVDIATT-
EALSMAQEVDPNGDRTIGVL-TKPDLVDRG-TE--KTVVNVAQNL-TYHLQK-
GYMIVRCRGQEEITNQLSLAEATEKERMFFQTHPYF--RALLEEGK-ATV--PCLAERLTKELILHINKSL
>NP_776366.1
LALPA----IAVIG----------------DQ----SSGKSSVLEA--LSGVA-LPRG---SG-IITRCPLV-------------
LKL--------TKR-----------------------------------ECEWTGKITYR--------------------------------
NITQQLQ---NPSEVEWEIRRAQNIIAGNG---LGISHELINLEITSPEV----------
PDLTLIDLPGITRVAVENQP--QDIGLQIK--------ALIKKYIQRQETINLVVVPCNVDIATT-
EALSMAQEVDPDGDRTIGIL-TKPDLVDKG-TE--KGVLKVMQNL-TYHLKK-
GYMIVKCRGQQDITNKLSLAEATRKETMFFETHPYF--RILLDEGK-ATV--PLLAERLTTELIWHINKSL

>XP_032211320.1
LALPA----IAVIG----------------DQ----SSGKSSVLEA--LSGVA-LPRG---SG-IVTRCPLV-------------
LKL--------KRQPQ--------------------------------ESAWKGRVIYG--------------------------------
TREVRLQ---DPSQVEKEILKAQNTLAGDG---VSISHELISVDIISPEV----------
PDLTLIDLPGITRVPVGNQP--QDIGLQIK--------ALIKKYIQRQETINLVVVPCNVDIATT-
EALSMAQEVDPRGDRTIGIL-TKPDLVDKG-AE--PIVMKVAQNL-TYHLQK-
GYMMVRCRGQEEITNRLSLAEATRKETMFFQKHPHF--RALLQEGK-ATV--
PCLAERLTNELILHINKSL
>XP_006156438.1
LALPA----IAVIG----------------DQ----SSGKSSVLEA--LSGVA-LPRG---SG-IVTRCPLV-------------
LKL--------MKQSQ--------------------------------EPVWRGKIRYR-------------------------------
NTEKKLG---DPTQVEAEICKAQNIIAGSG---VGISHELITLEITSPEV----------
PDLTLIDLPGITRVALGNQP--QDISLQIK--------ALIKKYIKRQQTINLVVVPCNVDIATT-
EALSMAQEVDPEGDRTLGIL-TKPDLVDKG-SE--KSVMNVLQNL-TFPLKK-
GYMIVKCRGQQEIMNNLSLAEATRKELMFFQSHPHF--RVFLEEKK-ATV--
PHLAERLTAELIAHIRKSL
>XP_015269256.1
LALPA----IAVIG----------------DQ----SSGKSSVLEA--LSGVA-LPRG---NG-IVTRCPLA-------------
LKL--------KKTRQ--------------------------------GCGWKGKISYR-------------------------------
DINEELN---HPSEVEKEIRKAQISIAGEG---VGISHELITLEIRSSEV----------
PDLTLIDLPGIARVAVGNQP--QDIGHQIK--------RLIKKIIAKDETINLVVVPCNVDIATT-
EALKMAQEVDPDGERTLGIL-TKPDLVDKG-TE--EAVVDIVRNL-IIHLKK-
GYMIVKCRGQQDIQSNLDLASAIQKEKAFFEDNRHF--RILLAEKR-ATI--PLLAEKLTSELVEHINKSL
>XP_028583072.1
LALPA----IAVIG----------------DQ----SSGKSSVLEA--LSGVA-LPRG---SG-IVTRCPLE-------------
LRL--------KKLLP--------------------------------GEKWNGKISYL-------------------------------
GKYMELA---NPSMVEIEIRKAQNIIAGDG---VAISDKLITLEIRSPEV----------
PDLTLIDLPGIARVAVGNQP--VNIGDQIK--------KLIKTFIDKQETINLVVVPSNVDIATT-
EALKMAQEVDPNGERTLGIV-TKPDLMDRG-TE--GTVVNIVRNQ-VIPLKK-
GYMIVKCRGQQDIQSNMTLASALKEERAFFEKHKCF--SILLQEKK-ATV--PLLAEKLTSELVEHISKSL
>XP_025933558.1
LALPS----IAVIG----------------DQ----SSGKSSVLEA--LSGIA-LPRG---NG-IVTRCPLE-------------
LKL--------KKTPA--------------------------------TQKWKGKISYH-------------------------------
NTSEELK---NPSEVEKAIRGAQDVVAGTK---GAISRELISLEVWSPTV----------
PDLTLIDLPGIARVAVGDQP--EDIGEQIK--------KLLKNIIGNKETLNLVVVPCNVDIATT-
EALKMAQEVDPKGERTLGIL-TKPDLVDKG-TE--ESIVNIIRNL-TVPLKK-
GYMIVKCRGQQDIHNNLTLASAIQQEKEFFETHQHF--SILLNEGK-ATV--PLLAEKLTXELVGHIIKTL
>XP_009815891.1
LALPA----IAVIG----------------DQ----SSGKSSVLEA--LSGIA-LPRG---NG-IVTRCPLE-------------
LKL--------KRIPA--------------------------------TQAWKGKICYR-------------------------------
NISSELQ---NASEVEKAIREAQDIVAGTR---GAISGELISLEIWSPDV----------
PDLTLIDLPGIARVAVGNQP--KDIGEQIK--------MLLKKIIGCKETLNLVVVPCNVDIATT-
EALKMAQEVDPSGERTLGIL-TKPDLVDRG-TE--ESIINIIRNL-VIPLKK-
GYMIVKCRGQQDIHNKLALAAAIQQERKFFETHEHF--SILLEEGK-ATV--PHLAEKLTNELVRHIIKTL

>AGU16245.1
--LPA----IAVIG----------------DQ----SSGKSSVLEA--LSGVG-LPRG---SG-IVTRCPLE-------------
LKL--------KKAKK---------------------------------ETEWKATIRYE--------------------------------
DEYKELT---SPSEVEKEIRTAQNAMAGSG---KGISDKLISLEIESDNV----------
PDLTLIDLPGIARVAVQGQP--YDIGEQIK--------KLIRKFIEKEETINLVVVPCNVDIATT-
EALKMAQDVDQSGERTLGIL-TKPDLVDKG-AE--QNIVDVVNNM-VIPLKK-
GYMIVKCRGQQDINENLTLAEAT--
>XP_007904885.1
LSLPA----IAVIG----------------DQ----SSGKSSVLEA--LSGVS-LPRG---TG-IVTRCPLE-------------
LKL--------KKAKK---------------------------------ANVWKGAISFR--------------------------------
EYSKEIT---NASEVEQEIRKAQNSMAGKE----GISHDLISLKIESSNV----------
PDLTLIDLPGIARVAVGNQP--LDIGDQIK--------KMIRSFINKQETINLVVVPCNVDIATT-
EALKMAQEVDPSGERTVGIL-TKPDLVDKG-TE--STIVDIVQNL-VVELKK-
GYMIVKCRGQKEINDKLTLQDAIARENRYFEEHEQF--RTLLDEKK-ASI--PHLAERLTNELVYHISKCL
>XP_032888405.1
LGLPA----IAVIG----------------DQ----SSGKSSVLEA--LSGVA-FPRG---SG-IVTRCPLE-------------
LKL--------KNVKK---------------------------------ANVWKGKISYK--------------------------------
DYSNKLS---SAAEVEQAILKAQDSIAGKG---VGISHELISLEIESTNV----------
PDLTLIDLPGIARVAVGNQP--QDIGDQIK--------RLIRLFIQKQETVNLVVVPCNVDIATT-
EALKMAQEVDPTGDRTLGIL-TKPDLVDKG-TE--KNVVDIVKNL-TVELEK-
GYMIVKCRGQNDINENISLVDAIAKEKEFFEDHEQF--RPLLEDGK-AGI--
PNLAVRLTKELVNHINKSL
>XP_028583068.1
LALPA----IAVIG----------------DQ----SSGKSSVLEA--LSGVA-LPRG---SG-IVTRCPLE-------------
LKL--------KKTHN---------------------------------TKEWKGKISYL--------------------------------
NTVEEMN---SSRQVEEQIIRAQNAMAGSG---SGISSELISLEISSSDV----------
PDLTLIDLPGIARVAVGDQP--KDIGQQII--------KLIKKYINKQETINLVVVPSNVDIATT-
EALKMAQEVDPTGERTLGIL-TKPDLVDKG-TE--AEVVDIIRNQ-RVPLRK-
GYMIVKCRGQSDINDKVTLGDAIEKEREFFEEHDFF--RSLLEEGR-ATI--PLLAERLTQELIEHISKTL
>XP_003973512.2.2
LALPA----IAVIG----------------DQ----SSGKSSVLEA--LSGVA-LPRG---SG-IVTRCPLE-------------
LKM--------KRRKV---------------------------------GEPWYGNISYL--------------------------------
DQEEVIE---DPADVEKKIQEAQNEMAGVG---VGISDDLISLEIASPEV----------
PDLTLIDLPGIARVAVKGQP--ENIGEQIK--------RLIRKFITKQETISLVVVPCNVDIATT-
EALKMAQEVDPDGERTLGIL-TKPDLVDKG-TE--ETVVDIIHNE-VIHLKK-
GYMIVRCRGQKEIIDKVSLAEATETETAFFRDHAHF--QTLYDDGQ-ATI--LKLAEKLTLELVNHIEKSL
>NP_891987.2.2
LALPA----IAVIG----------------DQ----SSGKSSVLEA--LSGVP-LPRG---SG-IVTRCPLE-------------
LKM--------IRTKD---------------------------------QDKWHGRISYK--------------------------------
TYEEDFD---DPAEVEKKIRQAQDEMAGAG---VGISEELISLQITSANV----------
PDLTLIDLPGIARVAVKGQP--ENIGDQIK--------RLIRKFVTRQETINLVVVPCNVDIATT-
EALQMAQAEDPDGERTLGIL-TKPDLVDKG-TE--GTVVDIVHNE-VIHLTK-
GYMIVRCRGQKEIMDQVTLNEATETESAFFKDHPHF--SKLYEEGF-ATI--PKLAEKLTIELVHHIQKSL
>XP_009304072.1

LALPA----IAVIG----------------DQ----SSGKSSVLEA--LSGVP-LPRG---SG-IVTRCPLE-------------
LKM--------IRSKE--------------------------------DEKWHGRISYQ-------------------------------
NHEEDFD---DPAEVEKKIREAQDEMAGAG---VGISEELISLQITSANV----------
PDLTLIDLPGIARVAVKGQP--ENIGDQIK--------RLIRMFITKQETINLVVVPCNVDIATT-
EALQMAQAEDPEGERTLGIL-TKPDLVDKG-TE--GTVVDIVHNE-VIHLTK-
GYMIVRCRGQKEIMDQVTLNEATETESAFFKDHPHF--RKLYEEGF-ATI--PKLAEKLTIELVHHIQRSL
>XP_031752404.1
LALPA----IAVIG----------------DQ----SSGKSSVLEA--LSGVT-LPRG---SG-IVTRCPLE-------------
LKL--------KKAMK--------------------------------KTTWSGKISYR-------------------------------
DHEIKIA---SAADVEEEVKRAQNLMAGSG---KGISDELISLEVISPDV----------
PDLTLIDLPGITRVALPDQP--KDIEQQIK--------KMIRKYIQKQETINLVVVPSNVDIATT-
EALEMAREVDPNGERTLGIL-TKPDLVDRG-AE--TDVISVVRNL-VYSLNK-
GYMIVKCRGQQEIQENLSLKDALVNEQNFFKEHEHF--SVLLEEGY-ATI--
ACLAGKLTNELVAHIVRNL
>NP_001007285.1
LNLPA----IAVIG----------------DQ----SSGKSSVLEA--LSGVA-LPRG---IG-IVTRCPLI-------------
LKL--------KKITR--------------------------------DKNWSGLLTYK-------------------------------
DQTEILK---EPTGIENAVLKAQIALAGTG---EGISHEMITLEIQSCDV----------
PDLTLIDLPGIARVATGNQP--EDIEKQIK--------DLIEKFIKRQETISLVVVPANIDIATT-
EALKMASTVDPTGQRTLCIL-TKPDLVDRG-ME--DTVVRTVNNE-VIRLEK-
GYMIVKCRGQQDINDKLNLVEALEKERRFFDEHPQF--SSLLEDGK-ATI--
PLLGQRLTEELVEHIAKNV
>XP_005167721.2.2
LNLPA----IAVIG----------------DQ----SSGKSSVLEA--LSGVA-LPRG---TG-IVTRCPLV-------------
LKL--------KKITK--------------------------------DKSWHGLLTYN-------------------------------
DKIRELK---DPAKIEKAVLNAQTALAGIG---EGISHEMITLEIQSCDV----------
PDLTLIDLPGIARVATGNQP--EDIEKQIK--------SLIEKFIKRQETISLVVVPANIDIATT-
EALKMASTVDPTGQRTLGIL-TKPDLVDRG-ME--DTVVRTVNNE-VIPLKK-
GYMIVKCRGQQDINDKLGLVEALEKERRFFDENVHF--RSLLEDRK-ATI--
PLLAERLTKELVEHIAKNL
>XP_002608668.1
VTLPS----VVVIG----------------DQ----SAGKSSCLEA--MSGVQ-LPRG---SG-IVTRCPLE-------------
LRL--------KKSQDP--------------------------------ESPWKGYIHYHF----------------------EGDR----
DETGWKLT---DPSDVGEAVRKAQNNLAGDS---HGISPRLITLDVESPDI----------
PDLTLIDLPGIARIAVDGQP--PDIGDQIK--------DLIKEYIQKDETIILAVVPCNVDIATT-
EALQMAKDVDPTGSRTLGVL-TKPDLIDRG-TE--NTIVDIVNNQ-KYPLKK-
GYTIIRCRGQEDINENVTLSEAMEKEERFFKTHEHF--KLPYHEKK-TGT--RTLAGKLTTELVEQIK---
>XP_019617847.1
VTLPS----VVVIG----------------DQ----SAGKSSCLEA--ISGVQ-LPRG---SG-IVTRCPLE-------------
LRL--------KKSPDP--------------------------------ESGWRGYIHFE-------------------------DK----
GETRWELD---SPEDVGEAVKKAQNQLAGES---LCISPRLITLDVESPDI----------
PDLTLIDLPGIARVPVGGQP--DDIGDQTK--------ALIREYIQMDETIILAVVPCNVDIATT-
EALKMAKEVDPDGSRTLGVL-TKPDLIDRG-TE--NMTVDIVNNR-KYALKK-
GYTIIKCRGQVDIENKVSLSDAMDKEEMFFQKHEHF--KILYEEKK-TGT--KTLAGKLTTELVEQIKKSI

>XP_035690836.1
VSLPS----VVVIG----------------DQ----SAGKSSTLEA--ISGVQ-LPRG---SG-IVTRCPLE------------
LRL--------KKSQKK-------------------------------DAPWKGCIRYVK-----------------------NK----
KDVRFDVD---EPGNVGDAVKKAQNDLAGTT---NGISDSLITLDVESPDI----------
PDLTLIDLPGIARIAAEGQP--TDIGQQIK--------DLISKYIQKKDTIILAVVPCNVDIATT-
EALQMAQEVDADGSRTLGVL-TKPDLIDPG-TE--RGVLQILNNE-KYKLRK-
GYTIIKCRGQMDIEKGMSLEEAMDKEQSYFKSHEHF--KSVYKEKK-AGV--
RTLAGRLSTELVGQIKNSI
>XP_012586448.1
LALPA----IAVIG----------------DQ----SSGKSSVLEA--LSGVA-LPRG---SG-IVTRCPLV------------
LKL--------KKQLQ--------------------------------GAPWTGTISYR-------------------------------
GVTLGLQ---DPSAVEREIHVAQNVIAGHG---VGISHELITLEVSSPEV----------
PDLTLIDLPGIARVAVGNQP--QDIGAQVSLSGAGRWGALVSGLPRSERC---------TGQETA-
EALGGGRAAXXDGDRTLGIL-TKPDLVDKG-AE--KAVVNVAQNL-TYRLKK-
GYMVVKCRGQQDIMDRLSLAQATEKEVAFFQTHPHF--RALLEEGK-ATV--
PRLAEKLTSELILHINKSL
>KAI0208044.1
ISLPE----VAVIG----------------DQ----SAGKSSVLEA--ISGVQ-LPRG---SG-IVTRCPLA------------
LQL--------KSHDT--------------------------------PGYWNSVIKYKY---------------------
GDEFDFEDIVVEKTIE---GPTKVDAGVREAQDAIAGKN---VGISDTLISLQITAYGV----------
PDLTLIDLPGITRVAVQGQP--PDIGDQIK--------RLIGNYIKKEETIILAVVPANVDIATT-
EALKMAKEVDPSGGRTLGVV-TKPDLIDIG-TE--KGLIDIINNE-TYPLEK-
GYSCVRCRGQKAINEGQTLADAVQEDTDFFSSAPHF--SA-VDESI-LGV--
KNLAMKLTFELVKQIKRAL
>KAI0213370.1
ISLPE----VAVIG----------------DQ----SAGKSSVLEA--ISGVQ-LPRG---SG-IVTRCPLA------------
LQL--------KSHDT--------------------------------PGYWNGVIKYET---------------------YNH------
PVEKTIE---GPTEVGAEVREAQDVIAGKN---VGISDTLISLQITSHGV----------
PDLTLIDLPGITRVAVEGQP--KDIGDQIK--------RLIGHYIKKEETIILAVVPANVDIATT-
EALKMAKEVDPSGGRTLGVV-TKPDLIDIG-TE--KGLIDIINNE-TYPLEK-
GYSCVRCRGQKAINEGQTLAEAIQQDTDFFSSAPHF--SD-VDESI-LGV--
KNLAMKLTYELVKQIKRAL
>KAI0218869.1
IALPE----VAVIG----------------DQ----SAGKSSVLEA--ISGVQ-LPRG---SG-IVTRCPLA------------
LQL--------KSDKT--------------------------------PGYWNGVIKYEI---------------------NER------
LVEKTIV---GPAEVDAEVRNAQDVIAGKN---VGISSKLISLQITSYGI----------
PDLTLIDLPGITRVAVEGQP--QNIGEQIK--------RLIEKYIKKEETIILAVVPANVDIATT-
EALKMAKEVDPSGSRTLGVV-TKPDLIDAG-TE--KGLISIINNE-TYPLKK-
GYSCVRCRGQKAIDEGQTLAQAIQQDTDFFSIASHF--SD-VDQST-LGV--
KNLAMKLTYELVRQIKRAL
>ABI53802.1
INLPA----VAVIG----------------DQ----SAGKSSVLEA--ISGVQ-LPRG---TG-IVTRCPLE------------
MRM--------KHSEA--------------------------------EDMWEGKIMYKD---------------------MYDV-----
AHEEIIL---NRESVEELVRKAQKEMTDSA---KGISDELITLEVTSSDV----------

PDLTVIDLPGIARNAVEGQP--FDIEARIK--------NMIRRYIGRQETIILAVLQCNVDIATC-
EALKMAKEFDAEGGRTLGVL-TKPDLLDKG-AE--TGVVRILNNM-EFTLSK-
GYIIATCRGQEAISDGQSLTQALEVEEDFFKSHRYF--SS-LRPSQ-WGI--PNLSGRLSRELKKHIKKLL
>XP_046352531.2
INLPS----VAVIG----------------DQ----SAGKSSVLEA--ISGVQ-LPRG---TG-IVTRCPLE------------
MRM--------KHSED--------------------------------EDMWEGKIMYKD---------------------MYDM-----
AHEEIIL---NRESVGELVRKAQKEMTDSA---KGISDELITLEVTSSDV----------
PDLTVIDLPGIARNAVEGQP--FDIEARIK--------NMIRKYIGRQETIILAVLQCNVDIATC-
EALKMAKEFDAEGGRTLGVL-TKPDLLDKG-AE--SGVVRILNNM-EFTLSK-
GYIIVKCRGQEAISDGQTLKQALEVEEDFFKSHRHF--SS-LRPSQ-WGI--PNLSGRLSRELKIHIKKLL
>XP_048258111.1
INLPA----VAVIG----------------DQ----SAGKSSVLEA--ISGVQ-LPRG---TG-IVTRCPLE------------
MRM--------KHSED--------------------------------EDMWEGKIMYKD---------------------MHDM-----
AHEEIIL---NRESVGELVRKAQKEMTDSA---KGISDELITLEVTSSDV----------
PDLTVIDLPGIARNAVEGQP--FDIEARIK--------NMIRRYIGRQETIILAVLQCNVDIATC-
EALKMAKEFDAEGGRTLGVL-TKPDLLDKG-AE--AGVVRILNNM-EFTLSK-
GYIIVKCRGQEAISYGQSLKQALEVEEDFFKSHRHF--SS-LRPSQ-WGI--
PNLSARLSRELKKHIKKLL
>XP_048248472.1
INLPS----VAVIG----------------DQ----SAGKSSVLEA--ISGVQ-LPRG---TG-IVTRCPLE------------
MRM--------KHSED--------------------------------EDMWAGKIMYKD---------------------MYDM-----
THEEIIL---NRESVGELVRKAQKEMTDSA---KGISDELITLEVTSSDV----------
PDLTVIDLPGIARNAVEGQP--LDIEARIK--------NMIRRYIRRQETIILAVLQCNVDIATC-
EALKMAKEFDAEGGRTLGVL-TKPDLLDKG-AE--TGVVRILNNM-EFTLSK-
GYIIVKCRGQEAISDGQSLKQALEVEEEFFKSHRHF--SS-LRPSQ-XGI--PNLSGRLSRELKIHIKKRL
>XP_048248473.1
INLPS----VAVIG----------------DQ----SAGKSSVLEA--ISGVQ-LPRG---TG-IVTRCPLE------------
MRM--------KHSED--------------------------------EDMWAGKIMYKD---------------------MYDM-----
THEEIIL---NRESVGELVRKAQKEMTDSA---KGISDELITLEVTSSDV----------
PDLTVIDLPGIARNAVEGQP--LDIEARIK--------NMIRRYIRRQETIILAVLQCNVDIATC-
EALKMAKEFDAEGGRTLGVL-TKPDLLDKG-AE--TGVVRILNNM-EFTLSK-
GYIIVKCRGQEAISDGQSLKQALEVEEEFFKSHRHF--SS-LRPSQ-WGI--
PNLSGRLSRELKIHIKKRL
>XP_048248474.1
INLPS----VAVIG----------------DQ----SAGKSSVLEA--ISGVQ-LPRG---TG-IVTRCPLE------------
MRM--------KHSED--------------------------------EDMWAGKIMYKD---------------------MYDM-----
THEEIIL---NRESVGELVRKAQKEMTDSA---KGISDELITLEVTSSDV----------
PDLTVIDLPGIARNAVEGQP--LDIEARIK--------NMIRRYIRRQETIILAVLQCNVDIATC-
EALKMAKEFDAEGGRTLGVL-TKPDLLDKG-AE--TGVVRILNNM-EFTLSK-
GYIIVKCRGQEAISDGQSLKQALEVEEEFFKSHRHF--SS-LRPSQ-WGI--
PNLSGRLSRELKIHIKKRL
>XP_048248476.1
INLPA----VAVIG----------------DQ----SAGKSSVLEA--ISGVQ-LPRG---TG-IVTRCPLE------------
MRM--------KHSED--------------------------------GDMWEGKIMYKD---------------------MHDM----

-AHEEIIL---NRESVGELVRKAQIEMTDSA---KGISDELITLEVTSSDV----------
PDLTVIDLPGIARNAVEGQP--FDIEARIK--------NMIRRYIGRQETIILAVLQCNVDIATC-
EALKMAKEFDTEGGRTLGVL-TKPDLLDKG-AE--SGVVRILNNK-EFTLSK-
GYIIVKCRGQEAISDGQSLKQALEVEEDFFKSHRHF--SS-LRPSQ-WGI--
PNLSMRLSRELKKHIKKLL
>XP_046352527.2
INLPA----VAVIG----------------DQ----SAGKSSVLEA--ISGVQ-LPRG---TG-IVTRCPLE-------------
MRM--------KHSED---------------------------------EDMWEGKIMYKD----------------------MHGE-----
AHEEIIL---NRESVGELVRKAQKEMTDSA---KGISDELITLEVTSSDV----------
PDLTVIDLPGIARNAVEGQP--VDIEARIK--------QMIRKYIGRQETIILAVLQCNVDIATC-
EALKMAKEFDDEGGRTLGVL-TKPDLLDKG-AE--SGVVRILNNM-EFTLSK-
GYIIVKCRGQEAISDGQSLKQALEVEEDFFKSHRHF--SS-LRPSQ-WGI--
PNLSTRLSRELKKHIKKLL
>XP_046562919.1
INLPA----VAVIG----------------DQ----SAGKSSVLEA--ISGVQ-LPRG---TG-IVTRCPLE-------------
MRM--------KHSED---------------------------------EDKWEGKIMYKD----------------------KHDM-----
RQEEVIL---NRESVGDLVRKAQKEMTDGA---KGISDELITLEVTSSDV----------
PDLTVIDLPGITRNAVEGQP--FDIEARIK--------NMIRKYIKRQETIILAVLQCNVDIATC-
EALKMAKEFDGEGGRTLGVL-TKPDLMDKG-AE--TGVIRILNNM-EFTLSK-
GYIIVKCRGQEAISEGQSLKQALDIEEDFFKSHRHF--SS-LRPSQ-WGI--PDLSSRLSRELKRHIKKLL
>XP_046563124.1
INLPA----VAVIG----------------DQ----SAGKSSVLEA--ISGVQ-LPRG---TG-IVTRCPLE-------------
MRL--------KHSED---------------------------------EDKWEGKILYKD----------------------KHDM-----
RQEEVIL---NRESVGDLVRKAQKEMTDSA---KGISDELITLEVTSSDV----------
PDLTVIDLPGIARNAVEGQP--FDIEARIK--------NMIRRYIGRQETIILAVLQCNVDIATC-
EALKMAKEFDDEGGRTLGVL-TKPDLLDKG-AE--TGVMRILNNM-EFTLSK-
GYIIVKCRGQEAISEGQSLKHALDVEEDFFKSHRHF--SS-LRPSQ-WGI--
PNLSARLSRELKKHIKKLL
>XP_046563126.1
INLPA----VAVIG----------------DQ----SAGKSSVLEA--ISGVQ-LPRG---TG-IVTRCPLE-------------
MRM--------KHSED---------------------------------EDKWEGKIMYKD----------------------KHDM-----
PHEEVIL---NRESVGDLVRKAQTEMTDGA---TGISDELITLEVTSSDV----------
PDLTVIDLPGIARNAVEGQP--FDIEARIK--------NMIRQYIERQETIILAVLQCNVDIATC-
EALKMAKEFDDEGGRTLGVL-TKPDLLDKG-AE--TSVIRILNNM-EFTLSK-
GYVIVKCRGQEAISEGQSLKHALDVEEDFFRSHRHF--SA-LRPSQ-WGI--
PNLSERLSRELKKHIKKLL
>XP_046565195.1
INLPA----VAVIG----------------DQ----SAGKSSVLEA--ISGVQ-LPRG---TG-IVTRCPLE-------------
MRM--------KHSED---------------------------------EDKWEGKIMYKD----------------------KHDM-----
PHEEVIL---NRESVGDLVRKAQKEMTDGA---TGISDELITLEVMSSDV----------
PDLTVIDLPGIARNAVEGQP--FDIEARIK--------NMIRQYIQRQETIILAVLQCNVDIATC-
EALKMAKEFDDEGGRTLGVL-TKPDLLDKG-AE--TGVIRILNNM-EFTLSK-
GYVIVKCRGQEAISEGQSLKHALDVEEDFFRSHRHF--SA-MRPSQ-WGI--
PNLSERLSRELKKHIKKLL

>XP_046563125.1
INLPA----VAVIG----------------DQ----SAGKSSVLEA--ISGVQ-LPRG---TG-IVTRCPLE------------
MRM--------KHSED--------------------------------EDKWEGKIMYTD---------------------KHDE-----
PHQEVIL---NRESVGDLVRKAQKEMTDSA---KGISDELITLEVTSSDV----------
PDLTVIDLPGIARNAVEGQP--VDIEARIK--------NMIRQYIERQETIILAVLQCNVDIATC-
EALKMAKEFDDEGGRTLGVL-TKPDLLDRG-AE--TGVMRILNNM-EFTLSK-
GYIIVKCRGQEAISEGQSLKHALDVEEDFFKSHRHF--SS-LGPSQ-WGI--
PNLSRRLSRELKKHIKKLL
>XP_046565196.1
INLPA----VAVIG----------------DQ----SAGKSSVLEA--ISGVQ-LPRG---TG-IVTRCPLE------------
MRM--------KHSED--------------------------------EDKWEGKIMYKD---------------------KHDE-----
LHKEDIQ---DRESVGDLVRKAQDEMTDCE---KGISDDLITLEVTSSDV----------
PDLTLIDLPGIARNAVKGQP--VDIEKRIK--------DMIRKYIRRQETIILAVLQCNVDIATC-
EALKMAKEFDEEGRRTLGVL-TKPDLLDKG-AE--NGVMRILNNM-EFSLSK-
GYIIVKCRGQEAISKGQSLTEALGDEDNFFKDHSHF--RS-LKVSQ-WGI--LTLSSRLSLELQKHIK---
>XP_032804093.1
VGLPA----VAVIG----------------DQ----SSGKSSVLEA--LSGVQ-LPRG---SG-IVTRCPLA------------
LKL--------KRA---------------------------------PGPWHGRIKYRV---------------------QGR------
TVNTKLD---TPESVGDAVLQAQSELTGDD---LGVSKSLIELEVTSDSV----------
PDLTLIDLPGIARVALAGQA--VDIETQIK--------DLIRDHIGRQETINLVVIPCNVDIATT-
EALKMAQAVDPTGVRTLGVL-TKPDLMDEG-TE--RNALRILQNQ-VFPLSK-
GYVLVKCRSQRDVEAHQTLAEASRVEAAFFKKHPVF--CHVHNGGKLTTT--
TVLAAKLTEELVDNIKRTL
>XP_006815062.1
VDLPA----VVVIG----------------DQ----SVGKSSVLEA--ISGVQ-LPRG---NE-IVTRCPIE------------
LRL--------KTLD---------------------------------NDEWCGKILYIN---------------------YSKE-----
QVNKYID---SPDELGAAIRTAQQDITNSQ---KGISKTSITVEIQSAHV----------
PNLTLIDLPGIARVPQEGQS--RNIADETK--------DLIKKYISKDDAIVLCVIPCNVDIATT-
EAIKMAQEVDPTGSRTLGVL-TKPDLVDKG-SE--NVVVRIAENK-VINLKK-
GYTIMKCRSQRNLEDAMSLEEAMDEEERFFREHKHY--SVL--SGQ-AGS--
RLLAHRLTTELVEQILKSV
>CAH1802128.1
VALPA----VVVIG----------------DQ----SVGKSSVLEA--MSGVQ-LPRG---TG-IVTRCPLE------------
LRM--------KQCD---------------------------------PGNFHAKISYDI---------------------QGGH----
QPLEKTIT---DPSNIDFEIRQAQRALVGDS---GGVSDRLIRLEVQADYV----------
PDLTLIDLPGIVRYSEGS----DTIVEETK--------NLIKTYVSRPETIILVVIPCNVDIDTV-
EACNLAKQVDPNGDRTIGVL-TRPDLIDHG-VGPIKEVLDILENK-KMKLKK-
GFYVVKCRSQKRIEEGQSLEQALAEEVQFFRSDERF--RV-INPSQ-CGV--
KQLSSKLTNELFLHIKNCV
>PAA92268.1
LKLPM----VAVVG----------------DQ----SVGKSSVLEA--ISGVE-FPRG---TG-MVTRCALQ------------
-LSM--------QWNADP------------------------------EAPWHGRISYKD---------------------VNGH----
-KVDKELN---SPGEVDGAVREAQQRMTHGD---NEISSEQIDLAIKGPDV----------
PDLTLIDLPGIARYSATGG---SGIAQITK--------SLIAKYVSQPQVLILVVVPCHQDIETV-

EALSLAKEADPQGERTIGVL-TCPDMVNKG-AE--QETLKIANNE-KIPLKK-
GYVMVKCRSPEELNNGVTLSESVANEAAFFKTHRHF--SL-LPEQS-VGI--
RTLADKLTEELFESVKRNI
>PAA74204.1
VRLPA----IAVVG----------------DQ----SVGKSSVLES--ISGID-LPRG---LG-IVTRCPLM------------
LSM--------RNRE---------------------------------EAGWSARIKYKT---------------------KTGE-----
GREKKLT---GASEVGQAIRDAQNEMTNSS---GEVSEQLIELWVESPES----------
PDLTLIDLPGIARYSIDGG---GAIAGLTK--------SLILSYIEKEEILILVVIPCHVDIETV-
EALSLAKEVDPESKRTIGVL-TCPDLVNPG-SE--SEVLALMQNR-KIPLKK-
GYVSVRCRTPQQLKDNMSLQQAAREEEVFFRTHPHF--RA-LDKFE-YGT--
KTLAVKLSSELYEAIKHNI
>PAA76532.1
ISLPA----IAVVG----------------DQ----SVGKSSVLEA--ISGVE-FPRG---LG-IVTRCPLM------------
LSM--------RGRE---------------------------------DSGWTARIRYET---------------------KSGQ-----
ARDKPLS---TPAEIGQAIRDAQEEMTSSS---GEISEKLIELHIEGADT----------
PDLTLIDLPGIARFSIANA---GDIATVSK--------SLIMSYILKPEVLILVVIPCNVDVETV-
EAISLAREVDPECKRTLGVL-TCPDLVNPG-SE--TEVLAMMRNE-RLKLRK-
GFVTVRCRTPQQLKDNMGLREACKAEEEFFKLHPQF--CA-LGDYQ-RGC--
KTLANKLSVELYQAVKERI
>PAA69582.1
LRLPT----VAVVG----------------DQ----SVGKSSVLEA--ISGVD-LPRG---TG-IVTRCPLQ------------
LSM--------RSKP---------------------------------TGDWTGRISYQN---------------------RKGE-----
HVEREIS---KKCEVDEMVRKVQNEITGDS---NGVSTEQIDLTIESADV----------
SDLTLVDLPGIARYSEKN----PKINEVTK--------QLILSYISQDQVIILVVVPCSVDIETV-
EAIALAKQVDPGGTRTIGVL-TCPDLTNPG-SE--EDIKAIVNNQGRVRLHK-
GFVMVKCRSPKELRNNISLSEVAKIEEDYFKNDPHF--SQ-LPKDI-VGT--KTLAEKLTNELFKAVAAGI
>PAA83069.1
LKLPS----IVVIG----------------DQ----SSGKSSVLET--ISGVS-FPRG---NG-VVTLCPLQ------------
LSM--------RSSD---------------------------------K-KWRGTVRYFD---------------------AQGK-----
EVHWDID---SPDDVENAIQNAQMRITGHK---KAISKNIIEMTLEAPDL----------
PNLTLVDLPGIARYSHSDGGS-VNLYKLTT--------DIIKEYIQREETIILTVIPLSADTATM-
EALQLAKDVDPYGLRTIGVL-TFPDLVNKG-AE--EEKLQIARNI-TFPLSK-
GYITVKCRNQEDIKSRKSLREAKVDEMRFFSNDPFF--SQ-LDPSQ-RGT--DTLAKRLSTELLTLIKKFI
>PAA94353.1
LRLPS----IVVIG----------------DQ----STGKSSVLES--ISGVR-FPRG---NG-VVTLCPLQ------------
LSM--------RTSD---------------------------------DGKWRGNIRYYD---------------------TYGK-----
LMKWDID---GPEDVEDAIQEAQMRITGNQ---RNVSKSIIEMTLESPEL----------
PNLTLIDLPGIARYNHNSAESGASLHQLTT--------DIIKEYIRREETIILVVIPLTSDTATM-
EALQLAKDADPYGMRTIGVL-TFPDLVNKG-AQ--EEKLMIARNI-TYPLSK-
GYVTVKCRNQEDIKNRKSLKDAKADEALFFNTDPFF--KQ-LDSMY-RGS--
DTLARRLSEELLYLVKKFI
>XP_006813643.1
DHLPR----VVVVG----------------DQ----SSGKTSVLEM--IAQARIFPRG---SGEMMTRTPVK----------
---VTL--------SEGPY-------------------------------------HVAQFKD---------------------S--------

NKEYDLSKESELQSLRQEIELRMKNRVKKG---QTVSNDTISLSVRGPGI----------
QRMVLVDLPGMISTVTTGMA--ADTREAIH--------NMSKSYMKNPNAIILCIQDGSVDAERS-
IVTDLATTMDPEGKRTIFVL-TKVDLAEKNSANP-SRIKQILDGK-LFPMKALGYFAVVTGRG---
NTNESIEQIKNYEETFFRSSKLF-KTGTLKPSQ-MTT--QNLSFAVSDCFWKMVRESV
>XP_030843280.1
DHLPR----VVVVG----------------DQ----SAGKTSVLEM--IAQARIFPRG---AGQMMTRAPVK----------
---VTL--------SEGPN--------------------------------------HIAQFKD----------------------S--------
GKEFDLTKESELKALRQEIEARMKGSVKEG---QTISPEVISLSVRGPGI----------
QRMVLVDLPGMISTVTTGMA--ADTKTSIQ--------KMINGYMGNPNAIILCIQDGAIDAERS-
IVTDLVNEIDPTGKRTIFVL-TKVDLAEKNHLNP-NRIRQILDGR-LFPMKALGYFAVVTGKG---
NTSDSIDSIKQYEEQFFRHSALF-KSGVFKPSQ-LNT--QNLSFAVSDCFWKMVRESV
>XP_023440724.1
DHLPR----VVVVG----------------DQ----SAGKTSVLEM--IAQARIFPRG---SGEMMTRSPVK----------
---VTL--------SEGPH--------------------------------------HVALFKD----------------------S--------
SREFDLTKEEDLAALRREIEIRMRKSVKEG---CTVSPETISLNVKGPGL----------
QRMVLVDLPGVINTVTSGMA--PDTKETIF--------SISKAYMQNPNAIILCIQDGSVDAERS-
IVTDLVSQMDPHGRRTIFVL-TKVDLAEKNVTSP-SRIQQIIEGK-LFPMKALGYFAVVTGKG---
NSSESIEAIREYEEEFFQNSKLL-KTSMLKAHQ-VTT--RNLSLAVSDCFWKMVRESV
>XP_005873264.1
DHLPR----VVVVG----------------DQ----SAGKTSVLEM--IAQARIFPRG---SGEMMTRSPVK----------
---VTL--------SEGPH--------------------------------------HVALFKD----------------------S--------
SREFDLTKEEDLAALRHEIELRMRKNVKEG---CTVSPETISLNVKGPGL----------
QRMVLVDLPGVINTVTSGMA--PDTKETIF--------SMSKAYMQNPNAIILCIQDGSVDAERS-
IVTDLVSQMDPHGRRTIFVL-TKVDLAEKNVASP-SRIQQIIEGK-LFPMKALGYFAVVTGKG---
NSSESIEAIREYEEEFFQNSKLL-KASMLKAHQ-VTT--RNLSLAVSDCFWKMVRESV
>XP_006163024.2.2
DHLPR----VVVVG----------------DQ----SAGKTSVLEM--IAQARIFPRG---SGEMMTRSPVK----------
---VTL--------SEGPH--------------------------------------HVALFKD----------------------S--------
SREFDLTKEEDLAALRHEIELRMRKNVKEG---CTVSPETISLNVKGPGL----------
QRMVLVDLPGVINTVTSGMA--PDTKETIF--------SISKAYMQNPNAIILCIQDGSVDAERS-
IVTDLVSQMDPHGRRTIFVL-TKVDLAEKNVASP-SRIQQIIEGK-LFPMKALGYFAVVTGKG---
NSSESIEAIREYEEEFFQNSKLL-KTSMLKAHQ-VTT--RNLSLAVSDCFWKMVRESV
>NP_056375.2.2
DHLPR----VVVVG----------------DQ----SAGKTSVLEM--IAQARIFPRG---SGEMMTRSPVK----------
---VTL--------SEGPH--------------------------------------HVALFKD----------------------S--------
SREFDLTKEEDLAALRHEIELRMRKNVKEG---CTVSPETISLNVKGPGL----------
QRMVLVDLPGVINTVTSGMA--PDTKETIF--------SISKAYMQNPNAIILCIQDGSVDAERS-
IVTDLVSQMDPHGRRTIFVL-TKVDLAEKNVASP-SRIQQIIEGK-LFPMKALGYFAVVTGKG---
NSSESIEAIREYEEEFFQNSKLL-KTSMLKAHQ-VTT--RNLSLAVSDCFWKMVRESV
>NP_598513.1
DHLPR----VVVVG----------------DQ----SAGKTSVLEM--IAQARIFPRG---SGEMMTRSPVK----------
---VTL--------SEGPH--------------------------------------HVALFKD----------------------S--------
SREFDLTKEEDLAALRHEIELRMRKNVKEG---CTVSPETISLNVKGPGL----------
QRMVLVDLPGVINTVTSGMA--PDTKETIF--------SISKAYMQNPNAIILCIQDGSVDAERS-

IVTDLVSQMDPHGRRTIFVL-TKVDLAEKNVASP-SRIQQIIEGK-LFPMKALGYFAVVTGKG---
NSSESIEAIREYEEEFFQNSKLL-KTSMLKAHQ-VTT--RNLSLAVSDCFWKMVRESV
>XP_028587646.1
DHLPR----VVVVG----------------DQ----SAGKTSVLEM--IAQARIFPRG---SGEMMTRSPVK----------
---VTL--------SEGPH--------------------------------------HVAMFKD----------------------S--------
SREFDLTKEEDLAALRNEIEIRMRKSVSDG---CTVSTETISLSVKGPGL----------
QRMVLVDLPGVISTVTSGMA--PDTKETIF--------SISKAYMQNPNAIILCIQDGSVDAERS-
IVTDMVSQMDPQGKRTIFVL-TKVDLAEKNVASP-SRIQQIIEGK-LXPMKALGYFAVVTGKG---
NSCESIESIKEYEEEFFQNSKLL-KNCMLKAHQ-VTT--RNLSLAVSDCFWKMVRESV
>XP_025913835.1
DHLPR----VVVVG----------------DQ----SAGKTSVLEM--IAQARIFPRG---SGEMMTRSPVK----------
---VTL--------SEGPH--------------------------------------HVALFKD----------------------S--------
SREFDLTKEEDLAALRNEIEIRMRNSVKEG---CTVSTETISLSVKGPGL----------
QRMVLVDLPGVISTVTSGMA--PDTKETIF--------SISKAYMQNPNAIILCIQDGSVDAERS-
IVTDLVSQMDPQGKRTIFVL-TKVDLAEKNVASP-SRIQQIIEGK-LFPMKALGYFAVVTGKG---
NSSESIDSIKEYEEEFFQNSKLL-KTCMLKAHQ-VTT--KNLSLAVSDCFWKMVRESV
>XP_021332524.1
DHLPR----VVVVG----------------DQ----SAGKTSVLEM--IAQARIFPRG---SGEMMTRSPVK----------
---VTL--------SEGPH--------------------------------------HVAMFKD----------------------S--------
SREFDLGKEEDLAALRHEIELRMRKSVKEG---QTVSPETISLSVKGPGI----------
QRMVLVDLPGVISTVTTGMA--ADTKETIF--------SISKAYMQNPNAIILCIQDGSVDAERS-
IVTDLVSQMDPQGKRTIFVL-TKVDLAEKNLASP-SRIQQIVEGK-LFPMKALGYFAVVTGKG---
SPNESIDSIKDYEEDFFQNSRLL-KDGMLKAHQ-VTT--KNLSLAVSDCFWKMVRESV
>XP_031757388.1
DHLPR----VVVVG----------------DQ----SAGKTSVLEM--IAQARIFPRG---SGEMMTRSPVK----------
---VTL--------SEGPH--------------------------------------HVAMFKD----------------------S--------
SREFDLSKETDLAALRNEIEVRMRKSVKNG---QTVSPETISLSVKGPGI----------
QRMVLVDLPGVINTVTSGMA--PDTKDTIF--------NISKAYMLNPNAIILCIQDGSVDAERS-
IVTDLVSQMDPQGRRTIFVL-TKVDLAEKNVASP-NRIQQIIEGK-LFPMKALGYFAVVTGKG---
NSNESIDSIKDYEEEFFQGSSLL-KKGMLKAHQ-VTT--KNLSLAVSDCFWKMVRESI
>XP_032818114.1
DQLPR----VVVVG----------------DQ----SSGKTSVLEM--IAQARIFPRG---SGEMMTRSPVK----------
---VTL--------SEGPH--------------------------------------HVAIFKD----------------------S--------
SREFDLTKEDDLAALRKEIEIRMKKSVKEG---HTVSAETISLSVKGPGL----------
QRMVLVDLPGVISTMTSGMA--PDTKDAIF--------AMSKGYMQNPNAIILCIQDGSVDAERS-
IVTDLVSNMDPQGKRTIFVL-TKVDLAEKNLASP-NRIQQILDGK-LFPMKALGYFAVVTGKG---
NRDESIESIKDYEEEFFQKSKLC-RSGMLKAHQ-VTT--KNLSLAVSDCFWKMVRESV
>XP_018667792.1
DHLPR----VVVVG----------------DQ----SAGKTSVLEM--IAQARIFPRG---SGEMMTRAPVK----------
---VTL--------SEGPN--------------------------------------HVAQFRD----------------------S--------
SREFDLSKEEELKSLRHEIELRMKSSCSDG---KTVSNDTISLTVKGPGL----------
QRMVLVDLPGMISTVTSGMA--PDTKDAIC--------NMSKHYMENPNAIILCIQDGSVDAERS-
IVTDLVSQMDPSGRRTIFVL-TKVDLAEKNITNP-SRIQEILDGK-LFPMKALGYFAVVTGQG---
SANSSITDIKEYEEEFFSNSKVF-KSGLLKASQ-LTT--ANLSYAVSNCFWKMVRESV

>XP_002602331.1
DELPR----VVVVG----------------DQ----SAGKTSVLEM--VAQARIFPRG---AGEMMTRAPVK----------
---VTL--------SEGPH--------------------------------------HIAMFKD----------------------S--------
DREFDLTKESELEALRREVEIRMKASVRPG---QTVSMETIAMSVKGPGL----------
QRMVLVDLPGIISTETQGMA--SATKESIK--------MMCEHYMSNPNAIILCIQDGSVDAERS-
NVTDLVSQMDPQGKRTIFVL-TKVDLAEKNITNP-RRIKQILEGK-LFPMKALGYFAVVTGRG---
NKDDSIDTIRGYEEEFFRNSQLF-RSGVLKASQ-MTT--QNLSFAVSDCFWKMVKASV
>XP_019637857.1
DELPR----VVVVG----------------DQ----SAGKTSVLEM--VAQARIFPRG---AGEMMTRAPVK----------
---VTL--------SEGPH--------------------------------------HIAMFKD----------------------S--------
DREFDLTKESELEALRREVEIRMKASVRPG---QTVSMETISMSVKGPGL----------
QRMVLVDLPGIISTETQGMA--SATKESIK--------MMCEHYMSNPNAIILCIQDGSVDAERS-
NVTDLVSQMDPQGKRTIFVL-TKVDLAEKNITNP-HRIKQILEGK-LFPMKALGYFAVVMGRG---
NKDDSIDTIRGYEEEFFRTSQLF-RSGVLKASQ-MTT--QNLSFAVSDCFWKMVKASV
>NP_495986.3.3
DNLPR----VVVVG----------------DQ----SAGKTSVLEM--VAQARIFPRG---SGEMMTRAPVK---------
----VTL--------SEGPY--------------------------------------HVAQFRD----------------------S--------
SREFDLTKETDLQQLRNETEVRMRNSVRDG---KTVSNEVISLTVKGPNL----------
PRMVLVDLPGVISTVTADMA--RETKDDII--------RMSKAHMENPNAIILCIQDGSVDAERS-
NVTDLVSSIDPSGKRTILVL-TKVDMAEKNLANP-DRIKKILEGK-LFPMKALGYFGVVTGRG---
NSSDSIDEIRKYEENFFSTSQLL-RDGVLKPSQ-MTT--RNMSLAVSDCFWRMVRDSI
>NP_610941.1
DHLPR----VVVVG----------------DQ----SSGKTSVLES--IAKARIFPRG---SGEMMTRAPVK-----------
--VTL--------AEGPY--------------------------------------HVAQFRD----------------------S--------
DREYDLTKESDLQDLRRDVEFRMKASVRGG---KTVSNEVIAMTVKGPGL----------
QRMVLVDLPGIISTMTVDMA--SDTKDSIH--------QMTKHYMSNPNAIILCIQDGSVDAERS-
NVTDLVMQCDPLGRRTIFVL-TKVDLAEE-LADP-DRIRKILSGK-LFPMKALGYYAVVTGRG---
RKDDSIDAIRQYEEDFFKNSKLFHRRGVIMPHQ-VTS--RNLSLAVSDRFWKMVRETI
>PAA68234.1
DHLPR----VVVIG----------------DQ----SSGKTSVLEA--VARARLFPRG---AGEMMTRAPVQ----------
---VTL--------ADGPY--------------------------------------HVARFKD----------------------DP-------
DREFDLTKESELAALRDAIERRMRAAVRSSGPDAAVSTEAIPLSVQGPGL----------
PRMVLVDLPGIISTETAGMA--AQTRESIR--------QLARQYMRNPNAIILCVADACVDPERS-
NAFDLVAKHDPAGRRTIFVL-TKMDLAERDKVSP-DRVAKLLAGR-LLPLKALGYFAVVTGSG---
SQDESVEAIERHEAEYFASSRLF-KDGRLSPNQ-VTA--ANMARAVSRRFWALVRESV
>PAA87312.1
DHLPR----VVVVG----------------DQ----SSGKTSVLEM--IAKARIFPRG---AGEMMTRAPVQ----------
---VTL--------AEGPY--------------------------------------HVARFKD----------------------NP-------
SREYDLTQESELAALRDTIERRMRSVVQSG---GTVSAETISLSVQGPGL----------
PRMVLVDLPGIISTETRGMA--SQTREAIR--------QLASQHMRNPNSIILCVADACVDPERS-
NAFDLVARHDPSGRRTIFVL-TKLDLAERDRISP-DRIGRLLAGR-LLPLKALGYFAVVTGSG---
GADESIPAIQRYEEQFFRNSQFF-KEGVLSVSQ-MTA--ANMAQAVSRRFWALVQESV
>XP_004479029.1

EVLSRRHMKVAFFG----------------RT----SSGKSSVINA--MLWDKVLPSG---IG-HTTNCFLS---------
----VEG--------TDGDK--------------------------------------AYLM-------------------------TEGS-----
DEKKSVK---TVNQLAHALHMDN------------DLKAGCLVHVFWPKA----KCALLRDDLVLVDSPGT---
---------DVTTELD--------SWIDKFCLDADVFVLVANSESTLMNT--
EKQFFHKVNERLSKPNIFILNNRWDASASE-PE----YMEDVRRQ-HMERCL-NFLV---------
EELKVVNPLEAQNRIFFVSAKEV-LSARKHKA--QGM--PEGGGALADGF--------
>NP_001193437.1
EVLSRRHMKVAFFG----------------RT----SSGKSSVINA--MLWDKVLPSG---IG-HTTNCFLS---------
----VEG--------TDGDK--------------------------------------AYLM-------------------------TEGS-----
DEKRSVK---TVNQLAHALHMDK------------DLKAGCLVHVFWPKA----KCALLRDDLVLVDSPGT----
--------DVTTELD--------SWIDKFCLDADVFVLVANSESTLMNT--
EKQFFHKVNERLSKPNIFILNNRWDASASE-PE----YMEDVRRQ-HMERCL-HFLV---------
EELRVVDPLEARNRIFFVSAKEV-LSARKHKA--QGM--PEGGGALAEGF--------
>NP_077162.2.2
EVLSRRHMKVAFFG----------------RT----SSGKSSVINA--MLWDKVLPSG---IG-HTTNCFLS---------
----VEG--------TDGDK--------------------------------------AYLM-------------------------TEGS-----
DEKKSVK---TVNQLAHALHMDK------------DLKAGCLVHVFWPKA----KCALLRDDLVLVDSPGT----
--------DVTTELD--------IWIDKFCLDADVFVLVANSESTLMNT--
EKHFFHKVNERLSKPNIFILNNRWDASASE-PE----YMEDVRRQ-HMERCL-HFLV---------
EELKVVSPSEARNRIFFVSAKEV-LNSRKHKA--QGM--PEGGGALAEGF--------
>XP_006162789.1
EVLSRRHMKVAFFG----------------RT----SSGKSSVINA--MLWDKVLPSG---IG-HTTNCFLS---------
----VEG--------TDGDK--------------------------------------AYLM-------------------------TEGS-----
DEKKSVK---TVNQLAHALHMDK------------DLKAGCLVHVFWPKA----KCALLRDDLVLVDSPGT----
--------DVTTELD--------SWIDKFCLDADVFVLVANSESTLMNT--
EKHFFHKVNERLSKPNIFILNNRWDASASE-PE----YMEDVRRQ-HMERCL-HFLV---------
EELKVVGPSEARNRIFFVSAKEV-LSARKHRA--QGM--PEGGGALAEGF--------
>XP_005883071.1
EVLSRRHMKVAFFG----------------RT----SSGKSSVINA--MLWDKVLPSG---IG-HTTNCLLS---------
----VEG--------TDGDR--------------------------------------AYLM-------------------------TEGS-----
DEKKSVK---TVNQLAHALHMDK------------DLKAGSLVHVFWPKA----KCALLRDDLVLVDSPGT----
--------DVTTELD--------SWIDKFCLDADVFVLVANSESTLMNT--
EKQFFHKVNERLSKPNIFILNNRWDASASE-PE----YMEDVRRQ-HTERCL-HFLV---------
EELKVVDPSEARNRIFFVSAKEV-LSARMHKA--QGM--PEGGGALAEGF--------
>XP_025917892.1
EVLSRRHMKVAFFG----------------RT----SSGKSSVINA--MLWDKVLPSG---IG-HTTNCFLS---------
----VEG--------TDGDK--------------------------------------AYLM-------------------------TEGS-----
DEKKSVK---TVNQLAHALHMDK------------DLKAGCLVHVFWPKS----KCALLRDDLVLVDSPGT----
--------DVTTELD--------SWIDKFCLDADVFVLVANSESTLMNT--
EKHFFHKVNERLSKPNIFILNNRWDASASE-PE----YMEDVRRQ-HMERCL-TFLV---------
DELKVIDPIEARNRIFFVSAKEV-LSARRQKA--QGM--PAGGEALAEGF--------
>NP_284941.2.2
EVLSRRHMKVAFFG----------------RT----SSGKSSVINA--MLWDKVLPSG---IG-HITNCFLS---------
----VEG--------TDGDK--------------------------------------AYLM-------------------------TEGS-----

DEKKSVK---TVNQLAHALHMDK------------DLKAGCLVRVFWPKA----KCALLRDDLVLVDSPGT----
--------DVTTELD--------SWIDKFCLDADVFVLVANSESTLMNT--
EKHFFHKVNERLSKPNIFILNNRWDASASE-PE----YMEDVRRQ-HMERCL-HFLV---------
EELKVVNALEAQNRIFFVSAKEV-LSARKQKA--QGM--PESGVALAEGF--------
>XP_028587453.1
GVLARRHMKVAFFG----------------RT----SSGKSSVINA--MLWDRVLPSG---IG-HTTNCFLS--------
-----VEG--------TDGDK--------------------------------------AYLM-------------------------TEGS-----
DEKKSVK---TVNQLAHALHMDK------------DLEAGCLVHVFWPKA----KCALLRDDLVLVDSPGT----
--------DVTTELD--------TWIDKFCLDADVFVLVANSESTLMNT--
EKHFFHKVNEKLSKPNIFILNNRWDASASE-PE----YMEHVRKQ-HMERCL-TFLV---------
DELKVVDPSEAQNRIFFVSAKEV-LSARKQRA--QGM--PEGGGALADGF--------
>NP_001016189.1
EVLARRNMKVAFFG----------------RT----SSGKSTVINS--MLWDKVLPSG---IG-HTTNCFLS---------
----VEG--------TEGDK--------------------------------------AYLM-------------------------TEGS-----
EEKKSVK---TVNQLAHALHMDK------------DLGAGCLVHVFWPKA----KCALLRDDLVLVDSPGT----
--------DVTTELD--------SWIDKFCLDADVFVLVANSESTLMNT--
EKHFFYKVNEKLSKPNIFILNNRWDASASE-PE----YMEDVRKQ-HMERCQ-SFLV---------
DELKVVDSLEAQKRIFFVSAKEV-LNARMHKA--QGM--PEAGAALAEGF--------
>XP_017213868.2.2
EVLARRHMKVAFFG----------------RT----SNGKSTVINA--MLRDRVLPSG---IG-HTTNCFLS---------
----VEG--------TDEDK--------------------------------------AFLK-------------------------TEGS-----
EEEKSIK---TVNQLAHALHMDE------------SLDAGCLVKVFWPKT----KCALLRDDLVLVDSPGT------
------DVTTELD--------SWIDKFCLDADVFVLVANSESTLMNT--
EKHFFHKVNEKLSKPNIFILNNRWDASAAE-PE----YMEDVRKQ-HTDRCV-NFLV---------
EELKVVDRAQAPNRIFFVSAKEV-LNSRMQRA--QGM--PETGGALAEGF--------
>XP_004482574.1
EVLARRHMKVAFFG----------------RT----SNGKSTVINA--MLWDKVLPSG---IG-HTTNCFLR--------
-----VGG--------TDGHE--------------------------------------AFLL-------------------------TEGS-----
EEKRSVK---TVNQLAHALHQDE------------QLHAGSLVSVMWPNS----KCSLLKDDLVLMDSPGI---
---------DVTTELD--------SWIDKFCLDADVFVLVANSESTLMQT--
EKQFFHKVSERLSRPNIFILNNRWDASASE-PE----YMEEVRRQ-HMERCT-SFLV---------
DELGVVDRAQAGDRIFFVSAKEV-LNARIQKA--QGM--PEGGGALAEGF--------
>XP_006145367.1
EVLARRHMKVAFFG----------------RT----SNGKSTVINA--MLWDKVLPSG---IG-HTTNCFLR--------
-----VEG--------TDGHE--------------------------------------AFLL-------------------------TEGS-----
EEKRSVK---TVNQLAHALHQDE------------QLHAGSLVSVMWPNS----KCPLLKDDLVLMDSPGI---
---------DVTTELD--------SWIDKFCLDADVFVLVANSESTLMQT--
EKQFFHKVSERLSRPNIFILNNRWDASASE-PE----YMEEVRRQ-HMERCT-SFLV---------
DELGVVDRAQAGDRIFFVSAKEV-LNARIQKA--QGM--PEGGGALAEGF--------
>XP_014400986.1
EVLARRHMKVAFFG----------------RT----SNGKSTVINA--MLWDKVLPSG---IG-HTTNCFLR--------
-----VEG--------TDGHE--------------------------------------AFLL-------------------------TEGS-----
EEKRSIK---TVNQLAHALHQDE------------QLHAGSLVSVMWPNS----KCPLLKDDLVLMDSPGI----
--------DVTTELD--------SWIDKFCLDADVFVLVANSESTLMQT--

EKQFFHKVSERLSRPNIFILNNRWDASASE-PE----YMEEVRRQ-HMERCT-SFLV---------
DELGVVDRGQAGDRIFFVSAKEA-LNARIQKA--QGM--PEGGGALAEGF--------
>NP_001177198.1
EVLARRHMKVAFFG----------------RT----SNGKSTVINA--MLWDKVLLSG---IG-HTTNCFLR---------
----VEG--------TDGHE-------------------------------------AFLL------------------------TEGS-----
EEKRSVK---TVNQLAHALHQDE------------QLHAGSLVSVMWPNS----KCPLLKDDLVLMDSPGI---
---------DVTTELD--------SWIDKFCLDADVFVLVANSESTLMQT--
EKQFFHKVSERLSRPNIFILNNRWDASASE-PE----YMEEVRRQ-HMERCT-SFLV---------
DELGVVDRGQAGDRIFFVSAKEV-LNARIQKA--QGM--PEGGGALAEGF--------
>NP_001272849.1
EVLARRHMKVAFFG----------------RT----SNGKSTVINA--MLWDKVLPSG---IG-HTTNCFLR--------
-----VGG--------TDGHE-------------------------------------AFLL------------------------TEGS-----
EEKKSVK---TVNQLAHALHQDE------------QLHAGSMVSVMWPNS----KCPLLKDDLVLMDSPGI--
----------DVTTELD--------SWIDKFCLDADVFVLVANSESTLMQT--
EKQFFHKVSERLSRPNIFILNNRWDASASE-PE----YMEEVRRQ-HMERCT-SFLV---------
DELGVVDRAQAGDRIFFVSAKEV-LSARVQKA--QGM--PEGGGALAEGF--------
>NP_001121132.1
EVLARRHMKVAFFG----------------RT----SNGKSTVINA--MLWDKVLPSG---IG-HTTNCFLR--------
-----VEG--------TDGHE-------------------------------------AFLL------------------------TEGS-----
EEKRSAK---TVNQLAHALHQDK------------QLHAGSLVSVMWPNS----KCPLLKDDLVLMDSPGI---
---------DVTTELD--------SWIDKFCLDADVFVLVANSESTLMQT--
EKHFFHKVSERLSRPNIFILNNRWDASASE-PE----YMEEVRRQ-HMERCT-SFLV---------
DELGVVDRSQAGDRIFFVSAKEV-LNARIQKA--QGM--PEGGGALAEGF--------
>XP_025929938.1
EVLARRHMKVAFFG----------------RT----SNGKSTVINA--MLWDKVLPSG---IG-HTTNCFLR--------
-----VEG--------TDGHE-------------------------------------AFLL------------------------TEGS-----
EEKKSVK---TVNQLAHALHQDE------------LLDAGSLVSVMWPNS----KCPLLKDDLVLMDSPGI----
--------DVTTELD--------SWIDKFCLDADVFVLVANSESTLMQT--
EKQFFHKVNERLSRPNIFILNNRWDASASE-PE----YMEEVRRQ-HMERCT-SFLV---------
DELGVVDRAQAGDRIFFVSAKEV-LNARIQRA--QGM--PEGGGALADGF--------
>XP_028597443.1
QVLARRHMKVAFFG----------------RT----SNGKSTVINA--MLWDKVLPSG---IG-HTTNCFLR--------
-----VEG--------TEGQD-------------------------------------AFLL------------------------TEGS-----
EEKKSVK---TVNQLAHALHQDE------------LLTAGGMVSVMWPNS----KCPLLKDDLVLMDSPGI---
---------DVTTELD--------SWIDKFCLDADVFVLVANSESTLMQT--
EKQFFHKVNTRLSRPNIFILNNRWDASASE-PE----YMEEVRRQ-HMERCT-SFLV---------
DELGVVDRAQAGDRIFFVSAKEV-LSARIQKA--QGM--PEGGGALAEGF--------
>XP_015268039.1
EVLARRHMKVAFFG----------------RT----SNGKSTVINA--MLWDKVLPSG---IG-HTTNCFLR--------
-----VEG--------TDGHD-------------------------------------AFLL------------------------TEGS-----
EXXXXXX---TVNQLAHALHQDE------------LLTAGSLVSVMWPNS----KCPLLKDDLVLMDSPGI-----
-------DVTTELD--------SWIDKFCLDADVFVLVANSESTLMQT--
VRRQ-HMERCT-SFLV---------DELGVVDRAQAGDRIFFVSAKEV-LSARIQKA--QGM--
PEGGGALAEGF--------

>NP_001121726.1
EVLSRRHMKVVFFG----------------RT----SNGKSSVINA--MLWDKVLPSG---IG-HTTNCFLR--------
-----VEG--------TDGNE--------------------------------------SFLL------------------------TEGS-----
DERKSVK---TVNQLAHALHQDE------------DLDAGSLVCVMWPKA----KCALLRDDLVLVDSPGI----
--------DVTTELD--------SWIDKFCLDADVFVLVANSESTLMQT--
EKSFFHKVNERLSSPNIFILNNRWDASANE-PE----YMEEVRRQ-HMDRCT-SFLV---------
DELRVVDRSHAGDRIFFVSAKEV-LQARVQKA--QGM--PEAGGALAEGF--------
>XP_002591612.1
QVISRDKMKVAFFG----------------RT----SNGKSTVVNA--MLRDKILPSG---IG-HTTNCFIN----------
---VEG--------SDGYE--------------------------------------AYLL------------------------TPDS-----
DDRKTVQ---SVGQLAHALCGER------------LEDSSILVKVFWPKG----RCALLRDDVVLLDSPGI-----
-------DVTPDLD--------
SWIDEHCLDADVFVLVANSESTLMRTAREKNFFHTVSERLSKPNIFILNNRWDASASE-PE----
FMEAVKKQ-HLERCV-SFLV---------EELGVVDRLQAEDRVFFVSAKEA-LQSRLQKQ--QGM--
PEEGGALAEGF--------
>XP_019628129.1
QVISRDKMKVAFFG----------------RT----SNGKSTVVNA--MLRDKILPSG---IG-HTTNCFIN----------
---VEG--------SDGFE--------------------------------------AYLL------------------------TPDS-----
DDRKTVQ---SVGQLAHALCSER------------LEDSSVLVKVFWPKG----RCALLRDDVVLLDSPGI-----
-------DVTPDLD--------SWIDEHCLDADVFVLVANSESTLMRT--
EKNFFHTVSERLSKPNIFILNNRWDASASE-PE----FMEAVKKQ-HLERCV-SFLV---------
EELGVVDRLQAEDRVFFVSAKEA-LQSRLQKQ--QGM--PEEGGALAEGF--------
>XP_006819998.1
EVLARDHMKVAFFG----------------RT----SSGKSTVINA--MLKDKVLPTG---IG-HTTDCFLS----------
---IEG--------SDTSE--------------------------------------AYLI------------------------IPQS-----
NERRNVR---SVSQLAHALSNEK------------LADQSSLIHVFWPSS----RCALLKDDLVLVDSPGV-----
-------DVTADLD--------SWIDDHCLDADVFVLVANAESTLMRT--
EKSFFHKVAEKLSKPNIFILNNRWDASASE-PD----SMEDVKKQ-HLERSI-GFLV---------
EELKVITKQQAEDRVFFVSAKEA-LCCRIQKV--QGM--PEAG---------------
>XP_030846906.1
DVLERDHMKVAFFG----------------RT----SNGKSTVINA--MLRDKVLPSG---IG-HTTDCFLC---------
----VEG--------CEGQE--------------------------------------GYMS------------------------RQNS-----
SEKISTT---SVSQLANALAGERDH---------EDFQQRSILHIFWPKT----QCHLLKNDVVLLDSPGI-----
-------DVEHDMD--------EWIDDHYMDADVFVLVSNAESTLTRT--
ETSFFLKVSAKLSKPNIFILNNRWDASANE-PE----NMEVVKRQ-HLEREI-KFLV---------
EELKVMTEAQAKDRIFFVSAKEA-LNSRILQT--LST--PNAN-PIVEGY--------
>XP_030847518.1
DVLERDHMKVAFFG----------------RT----SNGKSTVINA--MLRDKVLPSG---IG-HTTDCFLC---------
----VEG--------CEGQE--------------------------------------GYMS------------------------RQNS-----
SEKMSIT---SVSQLAHALAGERDH---------EECQQSSILHIFWPKT----QCHLLMNDVVLLDSPGI---
---------DVEQDLD--------EWINTHCVDADVFVLVLNAESTLMRT--
EKSFFHKVSEKLSKPNIFILNNRWDASANE-PE----FMEAVKRQ-HLERDV-KFLV---------
EELKVMTEAQAKDRVFFVSAKEA-LNSRIPKT--LST--PDAN-PVVEGY--------
>NP_996357.1

EVLQRDHMKVAFFG----------------RT----SNGKSSVINA--MLREKILPSG---IG-HTTNCFCQ---------
----VEG--------SNGGE--------------------------------------AYLM-------------------------TEGS-----
EEKLNVV---NIKQLANALCQEK-------------LCESSLVRIFWPRE----RCSLLRDDVVFVDSPGV-------
-----DVSANLD--------DWIDNHCLNADVFVLVLNAESTMTRA--
EKQFFHTVSQKLSKPNIFILNNRWDASANE-PE----CQESVKSQ-HTERCI-DFLT---------
KELKVSNEKEAAERVFFVSARET-LQARIEEA--KGN--PPHMGAIAEGF--------
>XP_002126852.1
DMLTRNHMKVVFFG----------------RT----SNGKSSVVNA--MLWDRILPTG---IG-HTTNCFLS--------
-----VAGCSDEGSTSTDSDE-------------------------------------GAYLL-------------------------CNGS---
--EEKRSIK---SVTQLSHALSEES-------------MSPDSLIQVFWPKS----KCALLKDDVVLVDSPGI------
------DVSHDLD--------QWIDKYCLDADVFILVANAESTLMQA--
EKKFFHRVNEKLSKPNIFILNNRWDASASE-PE----LMEQVRQQ-HLERGI-SFLA---------
DELKVISKSQAKDRVFFVSAKET-LQSRMPKV--PGK--ADSPVYMADGH--------
>PAA75551.1
EVISRNQMKCAFFG----------------RT----SNGKSTVINA--MLGRKVLPSG---IG-HTTNCFLQ---------
----VEG--------TSKQS--------------------------------------AYLQ-------------------------TPNS-----
SEEQPIE---SVSQLGSALSNEK-------------MDCESLVRVFWPKQ----LCSLLREDVVLLDSPGV------
------DVSPDLD--------TWIDQFCMDADVFILVCNSESTLMNT--
EKKFFHKVGSKLSKPNVFVLNNRWDCSDGE-LD----SAELVRKQ-HMDKSV-SFLA---------
DELKSCTRSEAESRVYFVSAKEA-LVNRLKETN-QGLESPSPAGSLADGW--------
>PAA75258.1
EVISRNQMKCAFFG----------------RT----SNGKSTVINA--MLGRKVLPSG---IG-HTTNCFLQ---------
----VEG--------TSKES--------------------------------------AYLQ-------------------------TPNS-----
SEERPIE---SVSQLGSALSNEK-------------LDCESLVRVFWPKQ----LCSLLREDVVLLDSPGV-------
-----DVSPDLD--------TWIDQFCMDADVFILVCNSESTLMNT--
EKKFFHKVGSKLSKPNVFVLNNRWDCSDGE-LD----SAELVRKQ-HMDKSV-SFLA---------
DELKSCTRSEAESRVYFVSAKEA-LVNRLKETN-QGLESPSPAGSLADGW--------
>XP_004365821.1
KLFERDHMKVVFVG----------------QT----SNGKSTVVNA--MLYNRILPSG---IG-HTTNCFVS---------
----VSG--------SDANT--------------------------------------PYII-------------------------DSLS-----
SEQQPIS---NVLQLANALHPEG------------SLNQSGLIRVFWPTT----KCRLLGDDVDLIDSPGL------
------DLSNDIN--------QWIDDYCMDADVFVLVANAEATLKVA--
ERAFFFKVNEKLSKPNVFILNNRWDASDNEIDD----SPERVREQ-HLEYAS-KFLA---------
DELKVVSRSKILDRVFFVSARET-LLYRTTEN--WTR--FKESQAV------------
>NP_495161.1
DTFQRDNMKVVFFG----------------RT----SNGKSTTINA--MLHEKVLPQG---MG-HTTCCFLQ-------
------VEG--------SEGEV--------------------------------------GHLQ-------------------------LDDN-----
PQKIDMK---MLGKIGHALSDENSDL--------
PAMGQDSLLKVFHPKKSESGECRLLQNDVVILDSPGV------------DLSPEFD--------
SWIDKHCLDADVFVLVSNAESTLTQA--EKNFFLRVAKKLSKPNVFILNNRWDASAAE-TE----
NIEDVKKQ-HLTRFR-QFLV---------DELEVCSEREVNDRIFFVSSREV-LESRLKA---RGL--VQKA-
YQAEGH--------
>XP_014153836.1

-MLRRDSMKVVFVG----------------HT----SNGKSTVINS--MLGQKVLPMG---IG-HTTSCFCS--------
-----VTG--------TDEE-------------------------------------
PYIILGHEPKAKNSNSIRLNRVQRSDSPPSPPP-----EKRMAID---NVKTVANALCPES------------
DHDAYQFVRVFWDKR----KCNLLGDGVLFVDTPGL------------DIDENYD--------
NWIDKFCMDADVFVLVANGESTIKHT--EMNFFTKVAEKLSRPNVFILFNRWDGSDME-DD-----
VTPVQEQ-HKDRVR-SFFK---------KELQQ-DANIIDKRVFFVSGKEV-LTHRTKPD--KSV--
VKESNPSPMG---------
>XP_001745740.1
RSVTRESMKVVFVG----------------RT----SNGKSTTINA--MLHTRVLPAG---PG-HTTNCFVT---------
----LQG--------SDQSK-------------------------------------AYMQ------------------------LPGD-----
PTPRDLK---DVQSLTDALQQEH------------VLPPGQSVEIHWPRD----QCHLLRDDVVILDSPGL----
--------DYDSDFD--------AWIDETTRDADVFVLVVNAVSTLSGA--
ESGFFHSVCKTVAKPNVFVIFNQWDNLDED-EA----DVTGVRAQ-HMSKAR-DLLV---------
RDLGICSEAELSSRVFFVSSKEV-LKSRAGSDS-RTTSYTDPSSAVVPGLNTH-----
