# Supplementary material for: Antiviral Mx proteins have an ancient origin and widespread distribution among eukaryotes
Source: Proc Natl Acad Sci U S A. 2025 Jan 24;122(4):e2416811122. doi: 10.1073/pnas.2416811122 (PMC11789081; doi:10.1073/pnas.2416811122)
Supplement: Supplementary file 6 — Dataset S05 (PDF) [file pnas.2416811122.sd05.pdf]

Dataset S5. Figure_2_FastTree

#NEXUS
begin taxa;
dimensions ntax=368;
taxlabels
XP_026693152.1[&Organism="Ciona intestinalis",Description="dynamin-1 isoform X15 [Ciona intestinalis]","Genetic Code"="Standard",Taxonomy="Eukaryota; Metazoa; Chordata; Tunicata; Ascidiacea; Enterogona; Phlebobranchia; Cionidae; Ciona","Common Name"="vase tunicate"]
XP_030853442.1.2[&Organism="Strongylocentrotus purpuratus",Description="dynamin-1 isoform X1 [Strongylocentrotus purpuratus]","Genetic Code"="Standard",Taxonomy="Eukaryota; Metazoa; Echinodermata; Eleutherozoa; Echinozoa; Echinoidea; Euechinoidea; Echinacea; Echinoida; Strongylocentrotidae; Strongylocentrotus","Common Name"="purple sea urchin"]
XP_030853442.1[&Organism="Strongylocentrotus purpuratus",Description="dynamin-1 isoform X4 [Strongylocentrotus purpuratus]","Genetic Code"="Standard",Taxonomy="Eukaryota; Metazoa; Echinodermata; Eleutherozoa; Echinozoa; Echinoidea; Euechinoidea; Echinacea; Echinoida; Strongylocentrotidae; Strongylocentrotus","Common Name"="purple sea urchin"]
XP_005165639.1[&Organism="Danio rerio",Description="dynamin-1 isoform X3 [Danio rerio]","Genetic Code"="Standard",Taxonomy="Eukaryota; Metazoa; Chordata; Craniata; Vertebrata; Euteleostomi; Actinopterygii; Neopterygii; Teleostei; Ostariophysi; Cypriniformes; Cyprinidae; Danio","Common Name"="zebrafish"]
XP_028570166.1[&Organism="Podarcis muralis",Description="dynamin-1 isoform X14 [Podarcis muralis]","Genetic Code"="Standard",Taxonomy="Eukaryota; Metazoa; Chordata; Craniata; Vertebrata; Euteleostomi; Lepidosauria; Squamata; Bifurcata; Unidentata; Episquamata; Laterata; Lacertibaenia; Lacertidae; Podarcis","Common Name"="Common wall lizard"]
EPQ17174.1[&Organism="Myotis brandtii",Description="PREDICTED: dynamin-1 [Myotis brandtii]","Genetic Code"="Standard",Taxonomy="Eukaryota; Metazoa; Chordata; Craniata; Vertebrata; Euteleostomi; Mammalia; Eutheria; Laurasiatheria; Chiroptera; Microchiroptera; Vespertilionidae; Myotis","Common Name"="Brandt's bat"]
BAB27759.1[&Organism="Mus musculus",Description="dynamin-1 isoform X5 [Mus musculus]","Genetic Code"="Standard",Taxonomy="Eukaryota; Metazoa; Chordata; Craniata; Vertebrata; Euteleostomi; Mammalia; Eutheria; Euarchontoglires; Glires; Rodentia; Myomorpha; Muroidea; Muridae; Murinae; Mus; Mus","Common Name"="house mouse"]
ELW62001.1[&Organism="Tupaia chinensis",Description="dynamin-1 isoform X3 [Tupaia chinensis]","Genetic Code"="Standard",Taxonomy="Eukaryota; Metazoa; Chordata; Craniata; Vertebrata; Euteleostomi; Mammalia; Eutheria; Euarchontoglires; Scandentia; Tupaiidae; Tupaia","Common Name"="Chinese tree shrew"]
EAW87759.1[&Organism="Homo sapiens",Description="dynamin-1 isoform 2 [Homo sapiens]","Genetic Code"="Standard",Taxonomy="Eukaryota; Metazoa; Chordata;

Craniata; Vertebrata; Euteleostomi; Mammalia; Eutheria; Euarchontoglires; Primates; Haplorrhini; Catarrhini; Hominidae; Homo","Common Name"="human"]

XP_025915522.1[&Organism="Apteryx rowi",Description="dynamin-1 isoform X1 [Apteryx rowi]","Genetic Code"="Standard",Taxonomy="Eukaryota; Metazoa; Chordata; Craniata; Vertebrata; Euteleostomi; Archelosauria; Archosauria; Dinosauria; Saurischia; Theropoda; Coelurosauria; Aves; Palaeognathae; Apterygiformes; Apterygidae; Apteryx","Common Name"="Okarito brown kiwi"]

XP_012378586.1[&Organism="Dasypus novemcinctus",Description="dynamin-1, partial [Dasypus novemcinctus]","Genetic Code"="Standard",Taxonomy="Eukaryota; Metazoa; Chordata; Craniata; Vertebrata; Euteleostomi; Mammalia; Eutheria; Xenarthra; Cingulata; Dasypodidae; Dasypus","Common Name"="nine-banded armadillo"]

KAE8583055.1[&Organism="Xenopus tropicalis",Description="dynamin-1 isoform X18 [Xenopus tropicalis]","Genetic Code"="Standard",Taxonomy="Eukaryota; Metazoa; Chordata; Craniata; Vertebrata; Euteleostomi; Amphibia; Batrachia; Anura; Pipoidea; Pipidae; Xenopodinae; Xenopus; Silurana","Common Name"="tropical clawed frog"]

XP_032814666.1[&Organism="Petromyzon marinus",Description="dynamin-1-like isoform X25 [Petromyzon marinus]","Genetic Code"="Standard",Taxonomy="Eukaryota; Metazoa; Chordata; Craniata; Vertebrata; Cyclostomata; Hyperoartia; Petromyzontiformes; Petromyzontidae; Petromyzon","Common Name"="sea lamprey"]

XP_025944940.1[&Organism="Apteryx rowi",Description="dynamin-3 isoform X1 [Apteryx rowi]","Genetic Code"="Standard",Taxonomy="Eukaryota; Metazoa; Chordata; Craniata; Vertebrata; Euteleostomi; Archelosauria; Archosauria; Dinosauria; Saurischia; Theropoda; Coelurosauria; Aves; Palaeognathae; Apterygiformes; Apterygidae; Apteryx","Common Name"="Okarito brown kiwi"]

EPQ08653.1[&Organism="Myotis brandtii",Description="PREDICTED: dynamin-3 isoform X1 [Myotis brandtii]","Genetic Code"="Standard",Taxonomy="Eukaryota; Metazoa; Chordata; Craniata; Vertebrata; Euteleostomi; Mammalia; Eutheria; Laurasiatheria; Chiroptera; Microchiroptera; Vespertilionidae; Myotis","Common Name"="Brandt's bat"]

XP_027623811.1[&Organism="Tupaia chinensis",Description="dynamin-3 isoform X3 [Tupaia chinensis]","Genetic Code"="Standard",Taxonomy="Eukaryota; Metazoa; Chordata; Craniata; Vertebrata; Euteleostomi; Mammalia; Eutheria; Euarchontoglires; Scandentia; Tupaiidae; Tupaia","Common Name"="Chinese tree shrew"]

XP_016856477.1[&Organism="Homo sapiens",Description="dynamin-3 isoform d [Homo sapiens]","Genetic Code"="Standard",Taxonomy="Eukaryota; Metazoa; Chordata; Craniata; Vertebrata; Euteleostomi; Mammalia; Eutheria; Euarchontoglires; Primates; Haplorrhini; Catarrhini; Hominidae; Homo","Common Name"="human"]

XP_006496668.1[&Organism="Mus musculus",Description="dynamin-3 isoform X6 [Mus musculus]","Genetic Code"="Standard",Taxonomy="Eukaryota; Metazoa; Chordata; Craniata; Vertebrata; Euteleostomi; Mammalia; Eutheria; Euarchontoglires; Glires; Rodentia; Myomorpha; Muroidea; Muridae; Murinae; Mus; Mus","Common Name"="house mouse"]

XP_012379251.1[&Organism="Dasypus novemcinctus",Description="dynamin-3 [Dasypus novemcinctus]","Genetic Code"="Standard",Taxonomy="Eukaryota; Metazoa;

Chordata; Craniata; Vertebrata; Euteleostomi; Mammalia; Eutheria; Xenarthra; Cingulata; Dasypodidae; Dasypus","Common Name"="nine-banded armadillo"]

XP_021326548.1[&Organism="Danio rerio",Description="dynamin-2 isoform X5 [Danio rerio]","Genetic Code"="Standard",Taxonomy="Eukaryota; Metazoa; Chordata; Craniata; Vertebrata; Euteleostomi; Actinopterygii; Neopterygii; Teleostei; Ostariophysi; Cypriniformes; Cyprinidae; Danio","Common Name"="zebrafish"]

NP_001025299.1[&Organism="Danio rerio",Description="dynamin-3 [Danio rerio]","Genetic Code"="Standard",Taxonomy="Eukaryota; Metazoa; Chordata; Craniata; Vertebrata; Euteleostomi; Actinopterygii; Neopterygii; Teleostei; Ostariophysi; Cypriniformes; Cyprinidae; Danio","Common Name"="zebrafish"]

XP_031753735.1[&Organism="Xenopus tropicalis",Description="dynamin-2 isoform X2 [Xenopus tropicalis]","Genetic Code"="Standard",Taxonomy="Eukaryota; Metazoa; Chordata; Craniata; Vertebrata; Euteleostomi; Amphibia; Batrachia; Anura; Pipoidea; Pipidae; Xenopodinae; Xenopus; Silurana","Common Name"="tropical clawed frog"]

XP_025920181.1[&Organism="Apteryx rowi",Description="dynamin-2 isoform X1 [Apteryx rowi]","Genetic Code"="Standard",Taxonomy="Eukaryota; Metazoa; Chordata; Craniata; Vertebrata; Euteleostomi; Archelosauria; Archosauria; Dinosauria; Saurischia; Theropoda; Coelurosauria; Aves; Palaeognathae; Apterygiformes; Apterygidae; Apteryx","Common Name"="Okarito brown kiwi"]

XP_028568434.1[&Organism="Podarcis muralis",Description="dynamin-2 isoform X7 [Podarcis muralis]","Genetic Code"="Standard",Taxonomy="Eukaryota; Metazoa; Chordata; Craniata; Vertebrata; Euteleostomi; Lepidosauria; Squamata; Bifurcata; Unidentata; Episquamata; Laterata; Lacertibaenia; Lacertidae; Podarcis","Common Name"="Common wall lizard"]

XP_006510037.1[&Organism="Mus musculus",Description="dynamin-2 isoform X18 [Mus musculus]","Genetic Code"="Standard",Taxonomy="Eukaryota; Metazoa; Chordata; Craniata; Vertebrata; Euteleostomi; Mammalia; Eutheria; Euarchontoglires; Glires; Rodentia; Myomorpha; Muroidea; Muridae; Murinae; Mus; Mus","Common Name"="house mouse"]

NP_001005360.1[&Organism="Homo sapiens",Description="dynamin-2 isoform 4 [Homo sapiens]","Genetic Code"="Standard",Taxonomy="Eukaryota; Metazoa; Chordata; Craniata; Vertebrata; Euteleostomi; Mammalia; Eutheria; Euarchontoglires; Primates; Haplorrhini; Catarrhini; Hominidae; Homo","Common Name"="human"]

XP_014389433.1[&Organism="Myotis brandtii",Description="PREDICTED: dynamin-2 isoform X6 [Myotis brandtii]","Genetic Code"="Standard",Taxonomy="Eukaryota; Metazoa; Chordata; Craniata; Vertebrata; Euteleostomi; Mammalia; Eutheria; Laurasiatheria; Chiroptera; Microchiroptera; Vespertilionidae; Myotis","Common Name"="Brandt's bat"]

XP_012381548.1[&Organism="Dasypus novemcinctus",Description="dynamin-2 [Dasypus novemcinctus]","Genetic Code"="Standard",Taxonomy="Eukaryota; Metazoa; Chordata; Craniata; Vertebrata; Euteleostomi; Mammalia; Eutheria; Xenarthra; Cingulata; Dasypodidae; Dasypus","Common Name"="nine-banded armadillo"]

XP_006161648.2.2[&Organism="Tupaia chinensis",Description="dynamin-2 [Tupaia chinensis]","Genetic Code"="Standard",Taxonomy="Eukaryota; Metazoa; Chordata;

Craniata; Vertebrata; Euteleostomi; Mammalia; Eutheria; Euarchontoglires; Scandentia; Tupaiidae; Tupaia","Common Name"="Chinese tree shrew"]

XP_035683496.1[&Organism="Branchiostoma floridae",Description="hypothetical protein BRAFLDRAFT_121263, partial [Branchiostoma floridae]","Genetic Code"="Standard",Taxonomy="Eukaryota; Metazoa; Chordata; Cephalochordata; Branchiostomidae; Branchiostoma","Common Name"="Florida lancelet"]

KMZ10000.1[&Organism="Drosophila melanogaster",Description="dynamin [Drosophila melanogaster]","Genetic Code"="Standard",Taxonomy="Eukaryota; Metazoa; Ecdysozoa; Arthropoda; Hexapoda; Insecta; Pterygota; Neoptera; Holometabola; Diptera; Brachycera; Muscomorpha; Ephydroidea; Drosophilidae; Drosophila; Sophophora","Common Name"="fruit fly"]

PAA78248.1[&Organism="Macrostomum lignano",Description="hypothetical protein BOX15_Mlig010364g2 [Macrostomum lignano]","Genetic Code"="Standard",Taxonomy="Eukaryota; Metazoa; Platyhelminthes; Rhabditophora; Macrostomorpha; Macrostomida; Macrostomidae; Macrostomum"]

PAA65118.1[&Organism="Macrostomum lignano",Description="hypothetical protein BOX15_Mlig013747g2 [Macrostomum lignano]","Genetic Code"="Standard",Taxonomy="Eukaryota; Metazoa; Platyhelminthes; Rhabditophora; Macrostomorpha; Macrostomida; Macrostomidae; Macrostomum"]

PAA59145.1[&Organism="Macrostomum lignano",Description="hypothetical protein BOX15_Mlig005677g1 [Macrostomum lignano]","Genetic Code"="Standard",Taxonomy="Eukaryota; Metazoa; Platyhelminthes; Rhabditophora; Macrostomorpha; Macrostomida; Macrostomidae; Macrostomum"]

PAA64382.1[&Organism="Macrostomum lignano",Description="hypothetical protein BOX15_Mlig016602g2 [Macrostomum lignano]","Genetic Code"="Standard",Taxonomy="Eukaryota; Metazoa; Platyhelminthes; Rhabditophora; Macrostomorpha; Macrostomida; Macrostomidae; Macrostomum"]

NP_001024332.1[&Organism="Caenorhabditis elegans",Description="Dynamin [Caenorhabditis elegans]","Genetic Code"="Standard",Taxonomy="Eukaryota; Metazoa; Ecdysozoa; Nematoda; Chromadorea; Rhabditida; Rhabditina; Rhabditomorpha; Rhabditoidea; Rhabditidae; Peloderinae; Caenorhabditis"]

XP_004347890.1[&Organism="Capsaspora owczarzaki ATCC 30864",Description="dynamin 1 [Capsaspora owczarzaki ATCC 30864]","Genetic Code"="Standard",Taxonomy="Eukaryota; Filasterea; Capsaspora"]

XP_001749319.1[&Organism="Monosiga brevicollis MX1",Description="uncharacterized protein MONBRDRAFT_28892 [Monosiga brevicollis MX1]","Genetic Code"="Standard",Taxonomy="Eukaryota; Choanoflagellata; Craspedida; Salpingoecidae; Monosiga"]

XP_031757197.1[&Organism="Xenopus tropicalis",Description="dynamin-3 [Xenopus tropicalis]","Genetic Code"="Standard",Taxonomy="Eukaryota; Metazoa; Chordata; Craniata; Vertebrata; Euteleostomi; Amphibia; Batrachia; Anura; Pipoidea; Pipidae; Xenopodinae; Xenopus; Silurana","Common Name"="tropical clawed frog"]

XP_014148725.1[&Organism="Sphaeroforma arctica JP610",Description="hypothetical protein SARC_12638, partial [Sphaeroforma arctica

JP610]","Genetic Code"="Standard",Taxonomy="Eukaryota; Ichthyosporea; Ichthyophonida; Sphaeroforma"]

XP_014153758.1[&Organism="Sphaeroforma arctica JP610",Description="dynamin-3 [Sphaeroforma arctica JP610]","Genetic Code"="Standard",Taxonomy="Eukaryota; Ichthyosporea; Ichthyophonida; Sphaeroforma"]

XP_006812840.1[&Organism="Saccoglossus kowalevskii",Description="PREDICTED: dynamin-1-like, partial [Saccoglossus kowalevskii]","Genetic Code"="Standard",Taxonomy="Eukaryota; Metazoa; Hemichordata; Enteropneusta; Harrimaniidae; Saccoglossus"]

XP_004348308.1[&Organism="Capsaspora owczarzaki ATCC 30864",Description="dynamin central region family protein [Capsaspora owczarzaki ATCC 30864]","Genetic Code"="Standard",Taxonomy="Eukaryota; Filasterea; Capsaspora"]

KNE67543.1[&Organism="Allomyces macrogynus ATCC 38327",Description="hypothetical protein AMAG_11997 [Allomyces macrogynus ATCC 38327]","Genetic Code"="Standard",Taxonomy="Eukaryota; Fungi; Fungi incertae sedis; Blastocladiomycota; Blastocladiomycota incertae sedis; Blastocladiomycetes; Blastocladiales; Blastocladiaceae; Allomyces"]

KNE61418.1[&Organism="Allomyces macrogynus ATCC 38327",Description="hypothetical protein AMAG_06247 [Allomyces macrogynus ATCC 38327]","Genetic Code"="Standard",Taxonomy="Eukaryota; Fungi; Fungi incertae sedis; Blastocladiomycota; Blastocladiomycota incertae sedis; Blastocladiomycetes; Blastocladiales; Blastocladiaceae; Allomyces"]

XP_011392073.1[&Organism="Ustilago maydis 521",Description="dynamin-related GTPase DNM1 [Ustilago maydis 521]","Genetic Code"="Standard",Taxonomy="Eukaryota; Fungi; Dikarya; Basidiomycota; Ustilaginomycotina; Ustilaginomycetes; Ustilaginales; Ustilaginaceae; Ustilago"]

XP_006461708.1[&Organism="Agaricus bisporus var. bisporus H97",Description="hypothetical protein AGABI2DRAFT_185821 [Agaricus bisporus var. bisporus H97]","Genetic Code"="Standard",Taxonomy="Eukaryota; Fungi; Dikarya; Basidiomycota; Agaricomycotina; Agaricomycetes; Agaricomycetidae; Agaricales; Agaricaceae; Agaricus"]

XP_746923.1[&Organism="Aspergillus fumigatus Af293",Description="dynamin-like GTPase Dnm1, putative [Aspergillus fumigatus Af293]","Genetic Code"="Standard",Taxonomy="Eukaryota; Fungi; Dikarya; Ascomycota; Pezizomycotina; Eurotiomycetes; Eurotiomycetidae; Eurotiales; Aspergillaceae; Aspergillus; Aspergillus subgen. Fumigati"]

KXN67416.1[&Organism="Conidiobolus coronatus NRRL 28638",Description="hypothetical protein CONCODRAFT_19627 [Conidiobolus coronatus NRRL 28638]","Genetic Code"="Standard",Taxonomy="Eukaryota; Fungi; Fungi incertae sedis; Zoopagomycota; Entomophthoromycotina; Entomophthoromycetes; Entomophthorales; Ancylistaceae; Conidiobolus"]

NP_013100.1[&Organism="Saccharomyces cerevisiae S288C",Description="dynamin-related GTPase DNM1 [Saccharomyces cerevisiae S288C]","Genetic Code"="Standard",Taxonomy="Eukaryota; Fungi; Dikarya; Ascomycota;

Saccharomycotina; Saccharomycetes; Saccharomycetales; Saccharomycetaceae; Saccharomyces"]

NP_012926.1[&Organism="Saccharomyces cerevisiae S288C",Description="dynamin-like GTPase VPS1 [Saccharomyces cerevisiae S288C]","Genetic Code"="Standard",Taxonomy="Eukaryota; Fungi; Dikarya; Ascomycota; Saccharomycotina; Saccharomycetes; Saccharomycetales; Saccharomycetaceae; Saccharomyces"]

OUM62108.1[&Organism="Piromyces sp. E2",Description="hypothetical protein PIROE2DRAFT_51763 [Piromyces sp. E2]","Genetic Code"="Standard",Taxonomy="Eukaryota; Fungi; Fungi incertae sedis; Chytridiomycota; Chytridiomycota incertae sedis; Neocallimastigomycetes; Neocallimastigales; Neocallimastigaceae; Piromyces; unclassified Piromyces"]

KXN66323.1[&Organism="Conidiobolus coronatus NRRL 28638",Description="vacuolar dynamin-like GTPase-like protein VpsA [Conidiobolus coronatus NRRL 28638]","Genetic Code"="Standard",Taxonomy="Eukaryota; Fungi; Fungi incertae sedis; Zoopagomycota; Entomophthoromycotina; Entomophthoromycetes; Entomophthorales; Ancylistaceae; Conidiobolus"]

XP_011389257.1[&Organism="Ustilago maydis 521",Description="putative dynamin-like GTPase VPS1 [Ustilago maydis 521]","Genetic Code"="Standard",Taxonomy="Eukaryota; Fungi; Dikarya; Basidiomycota; Ustilaginomycotina; Ustilaginomycetes; Ustilaginales; Ustilaginaceae; Ustilago"]

XP_006458578.1[&Organism="Agaricus bisporus var. bisporus H97",Description="hypothetical protein AGABI2DRAFT_190843 [Agaricus bisporus var. bisporus H97]","Genetic Code"="Standard",Taxonomy="Eukaryota; Fungi; Dikarya; Basidiomycota; Agaricomycotina; Agaricomycetes; Agaricomycetidae; Agaricales; Agaricaceae; Agaricus"]

KNE68830.1[&Organism="Allomyces macrogynus ATCC 38327",Description="hypothetical protein AMAG_13468 [Allomyces macrogynus ATCC 38327]","Genetic Code"="Standard",Taxonomy="Eukaryota; Fungi; Fungi incertae sedis; Blastocladiomycota; Blastocladiomycota incertae sedis; Blastocladiomycetes; Blastocladiales; Blastocladiaceae; Allomyces"]

XP_748106.1[&Organism="Aspergillus fumigatus Af293",Description="vacuolar dynamin-like GTPase VpsA, putative [Aspergillus fumigatus Af293]","Genetic Code"="Standard",Taxonomy="Eukaryota; Fungi; Dikarya; Ascomycota; Pezizomycotina; Eurotiomycetes; Eurotiomycetidae; Eurotiales; Aspergillaceae; Aspergillus; Aspergillus subgen. Fumigati"]

OAJ44422.1[&Organism="Batrachochytrium dendrobatidis JEL423",Description="hypothetical protein BDEG_27650 [Batrachochytrium dendrobatidis JEL423]","Genetic Code"="Standard",Taxonomy="Eukaryota; Fungi; Fungi incertae sedis; Chytridiomycota; Chytridiomycota incertae sedis; Chytridiomycetes; Rhizophydiales; Rhizophydiales incertae sedis; Batrachochytrium"]

XP_002129967.2[&Organism="Ciona intestinalis",Description="dynamin-1-like protein [Ciona intestinalis]","Genetic Code"="Standard",Taxonomy="Eukaryota; Metazoa;

Chordata; Tunicata; Ascidiacea; Phlebobranchia; Cionidae; Ciona","Common Name"="vase tunicate"]

NP_001259946.1[&Organism="Drosophila melanogaster",Description="dynamin related protein 1, isoform B [Drosophila melanogaster]","Genetic Code"="Standard",Taxonomy="Eukaryota; Metazoa; Ecdysozoa; Arthropoda; Hexapoda; Insecta; Pterygota; Neoptera; Endopterygota; Diptera; Brachycera; Muscomorpha; Ephydroidea; Drosophilidae; Drosophila; Sophophora","Common Name"="fruit fly"]

XP_035676386.1[&Organism="Branchiostoma floridae",Description="dynamin-1-like protein isoform X5 [Branchiostoma floridae]","Genetic Code"="Standard",Taxonomy="Eukaryota; Metazoa; Chordata; Cephalochordata; Leptocardii; Amphioxiformes; Branchiostomatidae; Branchiostoma","Common Name"="Florida lancelet"]

XP_006821224.1[&Organism="Saccoglossus kowalevskii",Description="PREDICTED: dynamin-1-like protein-like [Saccoglossus kowalevskii]","Genetic Code"="Standard",Taxonomy="Eukaryota; Metazoa; Hemichordata; Enteropneusta; Harrimaniidae; Saccoglossus"]

XP_030827871.1[&Organism="Strongylocentrotus purpuratus",Description="dynamin-1-like protein isoform X2 [Strongylocentrotus purpuratus]","Genetic Code"="Standard",Taxonomy="Eukaryota; Metazoa; Echinodermata; Eleutherozoa; Echinozoa; Echinoidea; Euechinoidea; Echinacea; Camarodonta; Echinidea; Strongylocentrotidae; Strongylocentrotus","Common Name"="purple sea urchin"]

XP_032819300.1[&Organism="Petromyzon marinus",Description="dynamin-1-like protein isoform X2 [Petromyzon marinus]","Genetic Code"="Standard",Taxonomy="Eukaryota; Metazoa; Chordata; Craniata; Vertebrata; Cyclostomata; Hyperoartia; Petromyzontiformes; Petromyzontidae; Petromyzon","Common Name"="sea lamprey"]

NP_957216.1[&Organism="Danio rerio",Description="dynamin-1-like protein [Danio rerio]","Genetic Code"="Standard",Taxonomy="Eukaryota; Metazoa; Chordata; Craniata; Vertebrata; Euteleostomi; Actinopterygii; Neopterygii; Teleostei; Ostariophysi; Cypriniformes; Danionidae; Danioninae; Danio","Common Name"="zebrafish"]

XP_025940269.1[&Organism="Apteryx rowi",Description="dynamin-1-like protein isoform X4 [Apteryx rowi]","Genetic Code"="Standard",Taxonomy="Eukaryota; Metazoa; Chordata; Craniata; Vertebrata; Euteleostomi; Archelosauria; Archosauria; Dinosauria; Saurischia; Theropoda; Coelurosauria; Aves; Palaeognathae; Apterygiformes; Apterygidae; Apteryx","Common Name"="Okarito brown kiwi"]

XP_006168142.1[&Organism="Tupaia chinensis",Description="dynamin-1-like protein isoform X1 [Tupaia chinensis]","Genetic Code"="Standard",Taxonomy="Eukaryota; Metazoa; Chordata; Craniata; Vertebrata; Euteleostomi; Mammalia; Eutheria; Euarchontoglires; Scandentia; Tupaiidae; Tupaia","Common Name"="Chinese tree shrew"]

NP_001317309.1[&Organism="Homo sapiens",Description="dynamin-1-like protein isoform 8 [Homo sapiens]","Genetic Code"="Standard",Taxonomy="Eukaryota; Metazoa; Chordata; Craniata; Vertebrata; Euteleostomi; Mammalia; Eutheria; Euarchontoglires; Primates; Haplorrhini; Catarrhini; Hominidae; Homo","Common Name"="human"]

NP_001392186.1[&Organism="Mus musculus",Description="dynamin-1-like protein isoform m [Mus musculus]","Genetic Code"="Standard",Taxonomy="Eukaryota; Metazoa; Chordata; Craniata; Vertebrata; Euteleostomi; Mammalia; Eutheria; Euarchontoglires; Glires; Rodentia; Myomorpha; Muroidea; Muridae; Murinae; Mus; Mus","Common Name"="house mouse"]

XP_014394711.1[&Organism="Myotis brandtii",Description="PREDICTED: dynamin-1-like protein isoform X6 [Myotis brandtii]","Genetic Code"="Standard",Taxonomy="Eukaryota; Metazoa; Chordata; Craniata; Vertebrata; Euteleostomi; Mammalia; Eutheria; Laurasiatheria; Chiroptera; Microchiroptera; Vespertilionidae; Myotis","Common Name"="Brandt's bat"]

XP_028602039.1[&Organism="Podarcis muralis",Description="dynamin-1-like protein isoform X3 [Podarcis muralis]","Genetic Code"="Standard",Taxonomy="Eukaryota; Metazoa; Chordata; Craniata; Vertebrata; Euteleostomi; Lepidosauria; Squamata; Bifurcata; Unidentata; Episquamata; Laterata; Lacertibaenia; Lacertidae; Podarcis","Common Name"="Common wall lizard"]

XP_012382650.2[&Organism="Dasypus novemcinctus",Description="dynamin-1-like protein, partial [Dasypus novemcinctus]","Genetic Code"="Standard",Taxonomy="Eukaryota; Metazoa; Chordata; Craniata; Vertebrata; Euteleostomi; Mammalia; Eutheria; Xenarthra; Cingulata; Dasypodidae; Dasypus","Common Name"="nine-banded armadillo"]

XP_031753959.1[&Organism="Xenopus tropicalis",Description="dynamin-1-like protein [Xenopus tropicalis]","Genetic Code"="Standard",Taxonomy="Eukaryota; Metazoa; Chordata; Craniata; Vertebrata; Euteleostomi; Amphibia; Batrachia; Anura; Pipoidea; Pipidae; Xenopodinae; Xenopus; Silurana","Common Name"="tropical clawed frog"]

PAA85687.1[&Organism="Macrostomum lignano",Description="hypothetical protein BOX15_Mlig022202g1 [Macrostomum lignano]","Genetic Code"="Standard",Taxonomy="Eukaryota; Metazoa; Spiralia; Lophotrochozoa; Platyhelminthes; Rhabditophora; Macrostomorpha; Macrostomida; Macrostomidae; Macrostomum"]

NP_741403.2[&Organism="Caenorhabditis elegans",Description="Dynamin GTPase [Caenorhabditis elegans]","Genetic Code"="Standard",Taxonomy="Eukaryota; Metazoa; Ecdysozoa; Nematoda; Chromadorea; Rhabditida; Rhabditina; Rhabditomorpha; Rhabditoidea; Rhabditidae; Peloderinae; Caenorhabditis"]

XP_042914770.1[&Organism="Chlamydomonas reinhardtii",Description="uncharacterized protein CHLRE_17g724150v5 [Chlamydomonas reinhardtii]","Genetic Code"="Standard",Taxonomy="Eukaryota; Viridiplantae; Chlorophyta; core chlorophytes; Chlorophyceae; CS clade; Chlamydomonadales; Chlamydomonadaceae; Chlamydomonas"]

PWZ09977.1[&Organism="Zea mays",Description="Dynamin-related protein 3A [Zea mays]","Genetic Code"="Standard",Taxonomy="Eukaryota; Viridiplantae; Streptophyta; Embryophyta; Tracheophyta; Spermatophyta; Magnoliopsida; Liliopsida; Poales; Poaceae; PACMAD clade; Panicoideae; Andropogonodae; Andropogoneae; Tripsacinae; Zea"]

KAH9304002.1[&Organism="Taxus chinensis",Description="hypothetical protein KI387_008406 [Taxus chinensis]","Genetic Code"="Standard",Taxonomy="Eukaryota;

Viridiplantae; Streptophyta; Embryophyta; Tracheophyta; Spermatophyta; Pinopsida; Pinidae; Conifers II; Cupressales; Taxaceae; Taxus"]

EFJ15047.1[&Organism="Selaginella moellendorffii",Description="hypothetical protein SELMODRAFT_119205 [Selaginella moellendorffii]","Genetic Code"="Standard",Taxonomy="Eukaryota; Viridiplantae; Streptophyta; Embryophyta; Tracheophyta; Lycopodiopsida; Selaginellales; Selaginellaceae; Selaginella"]

EFJ37641.1[&Organism="Selaginella moellendorffii",Description="hypothetical protein SELMODRAFT_437242 [Selaginella moellendorffii]","Genetic Code"="Standard",Taxonomy="Eukaryota; Viridiplantae; Streptophyta; Embryophyta; Tracheophyta; Lycopodiopsida; Selaginellales; Selaginellaceae; Selaginella"]

KAI5070335.1[&Organism="Adiantum capillus-veneris",Description="hypothetical protein GOP47_0014678 [Adiantum capillus-veneris]","Genetic Code"="Standard",Taxonomy="Eukaryota; Viridiplantae; Streptophyta; Embryophyta; Tracheophyta; Polypodiopsida; Polypodiidae; Polypodiales; Pteridineae; Pteridaceae; Vittarioideae; Adiantum"]

KAI5070758.1[&Organism="Adiantum capillus-veneris",Description="hypothetical protein GOP47_0015101 [Adiantum capillus-veneris]","Genetic Code"="Standard",Taxonomy="Eukaryota; Viridiplantae; Streptophyta; Embryophyta; Tracheophyta; Polypodiopsida; Polypodiidae; Polypodiales; Pteridineae; Pteridaceae; Vittarioideae; Adiantum"]

PTQ35749.1[&Organism="Marchantia polymorpha",Description="hypothetical protein MARPO_0069s0084 [Marchantia polymorpha]","Genetic Code"="Standard",Taxonomy="Eukaryota; Viridiplantae; Streptophyta; Embryophyta; Marchantiophyta; Marchantiopsida; Marchantiidae; Marchantiales; Marchantiaceae; Marchantia","Common Name"="liverwort"]

KAG0555682.1[&Organism="Ceratodon purpureus",Description="hypothetical protein KC19_12G188200 [Ceratodon purpureus]","Genetic Code"="Standard",Taxonomy="Eukaryota; Viridiplantae; Streptophyta; Embryophyta; Bryophyta; Bryophytina; Bryopsida; Dicranidae; Pseudoditrichales; Ditrichaceae; Ceratodon"]

KAG0554580.1[&Organism="Ceratodon purpureus",Description="hypothetical protein KC19_12G102200 [Ceratodon purpureus]","Genetic Code"="Standard",Taxonomy="Eukaryota; Viridiplantae; Streptophyta; Embryophyta; Bryophyta; Bryophytina; Bryopsida; Dicranidae; Pseudoditrichales; Ditrichaceae; Ceratodon"]

XP_024362051.1[&Organism="Physcomitrium patens",Description="dynamin-related protein 3A-like isoform X1 [Physcomitrium patens]","Genetic Code"="Standard",Taxonomy="Eukaryota; Viridiplantae; Streptophyta; Embryophyta; Bryophyta; Bryophytina; Bryopsida; Funariidae; Funariales; Funariaceae; Physcomitrium"]

AAC61784.1[&Organism="Arabidopsis thaliana",Description="similar to dynamin-like protein encoded by GenBank Accession Number X99669 [Arabidopsis thaliana]","Genetic Code"="Standard",Taxonomy="Eukaryota; Viridiplantae; Streptophyta; Embryophyta; Tracheophyta; Spermatophyta; Magnoliopsida; eudicotyledons;

Gunneridae; Pentapetalae; rosids; malvids; Brassicales; Brassicaceae; Camelineae; Arabidopsis","Common Name"="thale cress"]

XP_052310486.1[&Organism="Populus trichocarpa",Description="dynamin-related protein 3A [Populus trichocarpa]","Genetic Code"="Standard",Taxonomy="Eukaryota; Viridiplantae; Streptophyta; Embryophyta; Tracheophyta; Spermatophyta; Magnoliopsida; eudicotyledons; Gunneridae; Pentapetalae; rosids; fabids; Malpighiales; Salicaceae; Saliceae; Populus","Common Name"="Populus balsamifera subsp. trichocarpa"]

ONM18162.1[&Organism="Zea mays",Description="Dynamin-related protein 3A [Zea mays]","Genetic Code"="Standard",Taxonomy="Eukaryota; Viridiplantae; Streptophyta; Embryophyta; Tracheophyta; Spermatophyta; Magnoliopsida; Liliopsida; Poales; Poaceae; PACMAD clade; Panicoideae; Andropogonodae; Andropogoneae; Tripsacinae; Zea"]

KAH9327796.1[&Organism="Taxus chinensis",Description="hypothetical protein KI387_007974, partial [Taxus chinensis]","Genetic Code"="Standard",Taxonomy="Eukaryota; Viridiplantae; Streptophyta; Embryophyta; Tracheophyta; Spermatophyta; Pinopsida; Pinidae; Conifers II; Cupressales; Taxaceae; Taxus"]

XP_014148015.1[&Organism="Sphaeroforma arctica JP610",Description="hypothetical protein SARC_13330, partial [Sphaeroforma arctica JP610]","Genetic Code"="Standard",Taxonomy="Eukaryota; Ichthyosporea; Ichthyophonida; Sphaeroforma"]

XP_001750431.1[&Organism="Monosiga brevicollis MX1",Description="uncharacterized protein MONBRDRAFT_34545 [Monosiga brevicollis MX1]","Genetic Code"="Standard",Taxonomy="Eukaryota; Choanoflagellata; Craspedida; Salpingoecidae; Monosiga"]

XP_042924642.1[&Organism="Chlamydomonas reinhardtii",Description="uncharacterized protein CHLRE_05g245950v5 [Chlamydomonas reinhardtii]","Genetic Code"="Standard",Taxonomy="Eukaryota; Viridiplantae; Chlorophyta; core chlorophytes; Chlorophyceae; CS clade; Chlamydomonadales; Chlamydomonadaceae; Chlamydomonas"]

NP_001190448.1[&Organism="Arabidopsis thaliana",Description="dynamin-like protein [Arabidopsis thaliana]","Genetic Code"="Standard",Taxonomy="Eukaryota; Viridiplantae; Streptophyta; Embryophyta; Tracheophyta; Spermatophyta; Magnoliopsida; eudicotyledons; Gunneridae; Pentapetalae; rosids; malvids; Brassicales; Brassicaceae; Camelineae; Arabidopsis","Common Name"="thale cress"]

XP_002299468.1[&Organism="Populus trichocarpa",Description="dynamin-related protein 5A isoform X2 [Populus trichocarpa]","Genetic Code"="Standard",Taxonomy="Eukaryota; Viridiplantae; Streptophyta; Embryophyta; Tracheophyta; Spermatophyta; Magnoliopsida; eudicotyledons; Gunneridae; Pentapetalae; rosids; fabids; Malpighiales; Salicaceae; Saliceae; Populus","Common Name"="Populus balsamifera subsp. trichocarpa"]

XP_002302631.1[&Organism="Populus trichocarpa",Description="phragmoplastin DRP1B isoform X1 [Populus trichocarpa]","Genetic Code"="Standard",Taxonomy="Eukaryota; Viridiplantae; Streptophyta; Embryophyta; Tracheophyta; Spermatophyta; Magnoliopsida; eudicotyledons; Gunneridae;

Pentapetalae; rosids; fabids; Malpighiales; Salicaceae; Saliceae; Populus","Common Name"="Populus balsamifera subsp. trichocarpa"]

AQK88296.1[&Description="Dynamin-related protein 1A [Zea mays]"]

PTQ45603.1[&Organism="Marchantia polymorpha",Description="hypothetical protein MARPO_0014s0125 [Marchantia polymorpha]","Genetic Code"="Standard",Taxonomy="Eukaryota; Viridiplantae; Streptophyta; Embryophyta; Marchantiophyta; Marchantiopsida; Marchantiidae; Marchantiales; Marchantiaceae; Marchantia","Common Name"="liverwort"]

KAI5072318.1[&Organism="Adiantum capillus-veneris",Description="hypothetical protein GOP47_0012424 [Adiantum capillus-veneris]","Genetic Code"="Standard",Taxonomy="Eukaryota; Viridiplantae; Streptophyta; Embryophyta; Tracheophyta; Polypodiopsida; Polypodiidae; Polypodiales; Pteridineae; Pteridaceae; Vittarioideae; Adiantum"]

KAI5058380.1[&Organism="Adiantum capillus-veneris",Description="hypothetical protein GOP47_0026550 [Adiantum capillus-veneris]","Genetic Code"="Standard",Taxonomy="Eukaryota; Viridiplantae; Streptophyta; Embryophyta; Tracheophyta; Polypodiopsida; Polypodiidae; Polypodiales; Pteridineae; Pteridaceae; Vittarioideae; Adiantum"]

XP_002987566.1[&Organism="Selaginella moellendorffii",Description="dynamin-related protein 1E [Selaginella moellendorffii]","Genetic Code"="Standard",Taxonomy="Eukaryota; Viridiplantae; Streptophyta; Embryophyta; Tracheophyta; Lycopodiopsida; Selaginellales; Selaginellaceae; Selaginella"]

KAI5602084.1[&Organism="Populus trichocarpa",Description="hypothetical protein BDE02_01G133700 [Populus trichocarpa]","Genetic Code"="Standard",Taxonomy="Eukaryota; Viridiplantae; Streptophyta; Embryophyta; Tracheophyta; Spermatophyta; Magnoliopsida; eudicotyledons; Gunneridae; Pentapetalae; rosids; fabids; Malpighiales; Salicaceae; Saliceae; Populus","Common Name"="Populus balsamifera subsp. trichocarpa"]

XP_006375094.1[&Organism="Populus trichocarpa",Description="phragmoplastin DRP1E [Populus trichocarpa]","Genetic Code"="Standard",Taxonomy="Eukaryota; Viridiplantae; Streptophyta; Embryophyta; Tracheophyta; Spermatophyta; Magnoliopsida; eudicotyledons; Gunneridae; Pentapetalae; rosids; fabids; Malpighiales; Salicaceae; Saliceae; Populus","Common Name"="Populus balsamifera subsp. trichocarpa"]

XP_002315854.1[&Organism="Populus trichocarpa",Description="phragmoplastin DRP1C [Populus trichocarpa]","Genetic Code"="Standard",Taxonomy="Eukaryota; Viridiplantae; Streptophyta; Embryophyta; Tracheophyta; Spermatophyta; Magnoliopsida; eudicotyledons; Gunneridae; Pentapetalae; rosids; fabids; Malpighiales; Salicaceae; Saliceae; Populus","Common Name"="Populus balsamifera subsp. trichocarpa"]

NP_001147100.1[&Organism="Zea mays",Description="dynamin-related protein 1C [Zea mays]","Genetic Code"="Standard",Taxonomy="Eukaryota; Viridiplantae; Streptophyta; Embryophyta; Tracheophyta; Spermatophyta; Magnoliopsida; Liliopsida; Poales; Poaceae; PACMAD clade; Panicoideae; Andropogonodae; Andropogoneae; Tripsacinae; Zea"]

AAF22292.1[&Organism="Arabidopsis thaliana",Description="dynamin-like protein 4 [Arabidopsis thaliana]","Genetic Code"="Standard",Taxonomy="Eukaryota; Viridiplantae;

Streptophyta; Embryophyta; Tracheophyta; Spermatophyta; Magnoliopsida; eudicotyledons; Gunneridae; Pentapetalae; rosids; malvids; Brassicales; Brassicaceae; Camelineae; Arabidopsis","Common Name"="thale cress"]

KAG0556007.1[&Organism="Ceratodon purpureus",Description="hypothetical protein KC19_11G019300 [Ceratodon purpureus]","Genetic Code"="Standard",Taxonomy="Eukaryota; Viridiplantae; Streptophyta; Embryophyta; Bryophyta; Bryophytina; Bryopsida; Dicranidae; Pseudoditrichales; Ditrichaceae; Ceratodon"]

KAH9306600.1[&Organism="Taxus chinensis",Description="hypothetical protein KI387_011004, partial [Taxus chinensis]","Genetic Code"="Standard",Taxonomy="Eukaryota; Viridiplantae; Streptophyta; Embryophyta; Tracheophyta; Spermatophyta; Pinopsida; Pinidae; Conifers II; Cupressales; Taxaceae; Taxus"]

AAF79238.1[&Organism="Arabidopsis thaliana",Description="F10B6.23 [Arabidopsis thaliana]","Genetic Code"="Standard",Taxonomy="Eukaryota; Viridiplantae; Streptophyta; Embryophyta; Tracheophyta; Spermatophyta; Magnoliopsida; eudicotyledons; Gunneridae; Pentapetalae; rosids; malvids; Brassicales; Brassicaceae; Camelineae; Arabidopsis","Common Name"="thale cress"]

PWZ36850.1[&Organism="Zea mays",Description="Dynamin-related protein 1E [Zea mays]","Genetic Code"="Standard",Taxonomy="Eukaryota; Viridiplantae; Streptophyta; Embryophyta; Tracheophyta; Spermatophyta; Magnoliopsida; Liliopsida; Poales; Poaceae; PACMAD clade; Panicoideae; Andropogonodae; Andropogoneae; Tripsacinae; Zea"]

NP_850420.1[&Organism="Arabidopsis thaliana",Description="DYNAMIN-like 1D [Arabidopsis thaliana]","Genetic Code"="Standard",Taxonomy="Eukaryota; Viridiplantae; Streptophyta; Embryophyta; Tracheophyta; Spermatophyta; Magnoliopsida; eudicotyledons; Gunneridae; Pentapetalae; rosids; malvids; Brassicales; Brassicaceae; Camelineae; Arabidopsis","Common Name"="thale cress"]

EFJ15761.1[&Organism="Selaginella moellendorffii",Description="hypothetical protein SELMODRAFT_451592 [Selaginella moellendorffii]","Genetic Code"="Standard",Taxonomy="Eukaryota; Viridiplantae; Streptophyta; Embryophyta; Tracheophyta; Lycopodiopsida; Selaginellales; Selaginellaceae; Selaginella"]

PTQ29980.1[&Organism="Marchantia polymorpha",Description="hypothetical protein MARPO_0132s0051 [Marchantia polymorpha]","Genetic Code"="Standard",Taxonomy="Eukaryota; Viridiplantae; Streptophyta; Embryophyta; Marchantiophyta; Marchantiopsida; Marchantiidae; Marchantiales; Marchantiaceae; Marchantia","Common Name"="liverwort"]

EFJ23099.1[&Organism="Selaginella moellendorffii",Description="hypothetical protein SELMODRAFT_232702 [Selaginella moellendorffii]","Genetic Code"="Standard",Taxonomy="Eukaryota; Viridiplantae; Streptophyta; Embryophyta; Tracheophyta; Lycopodiopsida; Selaginellales; Selaginellaceae; Selaginella"]

EFJ35472.1[&Organism="Selaginella moellendorffii",Description="hypothetical protein SELMODRAFT_404911 [Selaginella moellendorffii]","Genetic Code"="Standard",Taxonomy="Eukaryota; Viridiplantae; Streptophyta; Embryophyta; Tracheophyta; Lycopodiopsida; Selaginellales; Selaginellaceae; Selaginella"]

KXN72852.1[&Organism="Conidiobolus coronatus NRRL 28638",Description="hypothetical protein CONCODRAFT_77587 [Conidiobolus coronatus NRRL 28638]","Genetic Code"="Standard",Taxonomy="Eukaryota; Fungi; Fungi incertae sedis; Zoopagomycota; Entomophthoromycotina; Entomophthoromycetes; Entomophthorales; Ancylistaceae; Conidiobolus"]

KNE54706.1[&Organism="Allomyces macrogynus ATCC 38327",Description="hypothetical protein AMAG_00665 [Allomyces macrogynus ATCC 38327]","Genetic Code"="Standard",Taxonomy="Eukaryota; Fungi; Fungi incertae sedis; Blastocladiomycota; Blastocladiomycota incertae sedis; Blastocladiomycetes; Blastocladiales; Blastocladiaceae; Allomyces"]

OAJ38404.1[&Organism="Batrachochytrium dendrobatidis JEL423",Description="hypothetical protein BDEG_22342 [Batrachochytrium dendrobatidis JEL423]","Genetic Code"="Standard",Taxonomy="Eukaryota; Fungi; Fungi incertae sedis; Chytridiomycota; Chytridiomycota incertae sedis; Chytridiomycetes; Rhizophydiales; Rhizophydiales incertae sedis; Batrachochytrium"]

XP_011389557.1[&Organism="Ustilago maydis 521",Description="putative dynamin-related GTPase [Ustilago maydis 521]","Genetic Code"="Standard",Taxonomy="Eukaryota; Fungi; Dikarya; Basidiomycota; Ustilaginomycotina; Ustilaginomycetes; Ustilaginales; Ustilaginaceae; Ustilago"]

XP_006459124.1[&Organism="Agaricus bisporus var. bisporus H97",Description="hypothetical protein AGABI2DRAFT_201103 [Agaricus bisporus var. bisporus H97]","Genetic Code"="Standard",Taxonomy="Eukaryota; Fungi; Dikarya; Basidiomycota; Agaricomycotina; Agaricomycetes; Agaricomycetidae; Agaricales; Agaricaceae; Agaricus"]

XP_752563.1[&Organism="Aspergillus fumigatus Af293",Description="mitochondrial dynamin GTPase (Msp1), putative [Aspergillus fumigatus Af293]","Genetic Code"="Standard",Taxonomy="Eukaryota; Fungi; Dikarya; Ascomycota; Pezizomycotina; Eurotiomycetes; Eurotiomycetidae; Eurotiales; Aspergillaceae; Aspergillus; Aspergillus subgen. Fumigati"]

OUM67143.1[&Organism="Piromyces sp. E2",Description="hypothetical protein PIROE2DRAFT_40291 [Piromyces sp. E2]","Genetic Code"="Standard",Taxonomy="Eukaryota; Fungi; Fungi incertae sedis; Chytridiomycota; Chytridiomycota incertae sedis; Neocallimastigomycetes; Neocallimastigales; Neocallimastigaceae; Piromyces; unclassified Piromyces"]

NP_014854.2[&Organism="Saccharomyces cerevisiae S288C",Description="dynamin-related GTPase MGM1 [Saccharomyces cerevisiae S288C]","Genetic Code"="Standard",Taxonomy="Eukaryota; Fungi; Dikarya; Ascomycota; Saccharomycotina; Saccharomycetes; Saccharomycetales; Saccharomycetaceae; Saccharomyces"]

PSC76263.1[&Organism="Micractinium conductrix",Description="dynamin-related 4C-like [Micractinium conductrix]","Genetic Code"="Standard",Taxonomy="Eukaryota; Viridiplantae; Chlorophyta; core chlorophytes; Trebouxiophyceae; Chlorellales; Chlorellaceae; Chlorella clade; Micractinium"]

XP_005849062.1[&Organism="Chlorella variabilis",Description="hypothetical protein CHLNCDRAFT_21811, partial [Chlorella variabilis]","Genetic Code"="Standard",Taxonomy="Eukaryota; Viridiplantae; Chlorophyta; core chlorophytes; Trebouxiophyceae; Chlorellales; Chlorellaceae; Chlorella clade; Chlorella"]

PRW56740.1[&Organism="Chlorella sorokiniana",Description="chromatin-remodeling complex ATPase chain [Chlorella sorokiniana]","Genetic Code"="Standard",Taxonomy="Eukaryota; Viridiplantae; Chlorophyta; core chlorophytes; Trebouxiophyceae; Chlorellales; Chlorellaceae; Chlorella clade; Chlorella"]

XP_006815062.1[&Organism="Saccoglossus kowalevskii",Description="PREDICTED: interferon-induced GTP-binding protein Mx-like [Saccoglossus kowalevskii]","Genetic Code"="Standard",Taxonomy="Eukaryota; Metazoa; Hemichordata; Enteropneusta; Harrimaniidae; Saccoglossus"]

XP_035690836.1[&Organism="Branchiostoma floridae",Description="interferon-induced GTP-binding protein Mx3-like [Branchiostoma floridae]","Genetic Code"="Standard",Taxonomy="Eukaryota; Metazoa; Chordata; Cephalochordata; Leptocardii; Amphioxiformes; Branchiostomidae; Branchiostoma","Common Name"="Florida lancelet"]

XP_002608668.1[&Organism="Branchiostoma floridae",Description="hypothetical protein BRAFLDRAFT_58103 [Branchiostoma floridae]","Genetic Code"="Standard",Taxonomy="Eukaryota; Metazoa; Chordata; Cephalochordata; Branchiostomidae; Branchiostoma","Common Name"="Florida lancelet"]

XP_019617847.1[&Organism="Branchiostoma belcheri",Description="PREDICTED: interferon-induced GTP-binding protein Mx1-like [Branchiostoma belcheri]","Genetic Code"="Standard",Taxonomy="Eukaryota; Metazoa; Chordata; Cephalochordata; Branchiostomidae; Branchiostoma","Common Name"="Belcher's lancelet"]

XP_003973512.2.2[&Organism="Takifugu rubripes",Description="interferon-induced GTP-binding protein Mx [Takifugu rubripes]","Genetic Code"="Standard",Taxonomy="Eukaryota; Metazoa; Chordata; Craniata; Vertebrata; Euteleostomi; Actinopterygii; Neopterygii; Teleostei; Neoteleostei; Acanthomorphata; Eupercaria; Tetraodontiformes; Tetradontoidea; Tetraodontidae; Takifugu","Common Name"="torafugu"]

NP_891987.2.2[&Organism="Danio rerio",Description="interferon-induced GTP-binding protein MxA [Danio rerio]","Genetic Code"="Standard",Taxonomy="Eukaryota; Metazoa; Chordata; Craniata; Vertebrata; Euteleostomi; Actinopterygii; Neopterygii; Teleostei; Ostariophysi; Cypriniformes; Cyprinidae; Danio","Common Name"="zebrafish"]

XP_009304072.1[&Organism="Danio rerio",Description="interferon-induced GTP-binding protein MxB isoform X1 [Danio rerio]","Genetic Code"="Standard",Taxonomy="Eukaryota; Metazoa; Chordata; Craniata; Vertebrata; Euteleostomi; Actinopterygii; Neopterygii; Teleostei; Ostariophysi; Cypriniformes; Cyprinidae; Danio","Common Name"="zebrafish"]

AGU16245.1[&db_xref="taxon:27779",Organism="Protopterus dolloi",Description="Mx1 protein, partial [Protopterus dolloi]","Genetic Code"="Standard",Modified=Mon Mar 27 11:17:17 PDT 2023,Taxonomy="Eukaryota; Metazoa; Chordata; Craniata; Vertebrata; Euteleostomi; Dipnoi; Lepidosireniformes;

Protopteridae; Protopterus",Accession="AGU16245.1","Common Name"="slender lungfish",Topology="linear","Molecule Type"="AA"]

XP_007904885.1[&Organism="Callorhinchus milii",Description="PREDICTED: interferon-induced GTP-binding protein Mx-like isoform X2 [Callorhinchus milii]","Genetic Code"="Standard",Taxonomy="Eukaryota; Metazoa; Chordata; Craniata; Vertebrata; Chondrichthyes; Holocephali; Chimaeriformes; Callorhinchidae; Callorhinchus","Common Name"="elephant shark"]

XP_032888405.1[&Organism="Amblyraja radiata",Description="interferon-induced GTP-binding protein Mx3-like isoform X1 [Amblyraja radiata]","Genetic Code"="Standard",Taxonomy="Eukaryota; Metazoa; Chordata; Craniata; Vertebrata; Chondrichthyes; Elasmobranchii; Batoidea; Rajiformes; Rajidae; Amblyraja","Common Name"="thorny skate"]

XP_025933558.1[&Organism="Apteryx rowi",Description="interferon-induced GTP-binding protein Mx-like isoform X1 [Apteryx rowi]","Genetic Code"="Standard",Taxonomy="Eukaryota; Metazoa; Chordata; Craniata; Vertebrata; Euteleostomi; Archelosauria; Archosauria; Dinosauria; Saurischia; Theropoda; Coelurosauria; Aves; Palaeognathae; Apterygiformes; Apterygidae; Apteryx","Common Name"="Okarito brown kiwi"]

XP_009815891.1[&Organism="Gavia stellata",Description="PREDICTED: interferon-induced GTP-binding protein Mx-like [Gavia stellata]","Genetic Code"="Standard",Taxonomy="Eukaryota; Metazoa; Chordata; Craniata; Vertebrata; Euteleostomi; Archelosauria; Archosauria; Dinosauria; Saurischia; Theropoda; Coelurosauria; Aves; Neognathae; Gaviiformes; Gaviidae; Gavia","Common Name"="red-throated loon"]

XP_015269256.1[&Organism="Gekko japonicus",Description="PREDICTED: interferon-induced GTP-binding protein Mx1 [Gekko japonicus]","Genetic Code"="Standard",Taxonomy="Eukaryota; Metazoa; Chordata; Craniata; Vertebrata; Euteleostomi; Lepidosauria; Squamata; Bifurcata; Gekkota; Gekkonidae; Gekkoninae; Gekko"]

XP_006156438.1[&Organism="Tupaia chinensis",Description="interferon-induced GTP-binding protein Mx2 [Tupaia chinensis]","Genetic Code"="Standard",Taxonomy="Eukaryota; Metazoa; Chordata; Craniata; Vertebrata; Euteleostomi; Mammalia; Eutheria; Euarchontoglires; Scandentia; Tupaiidae; Tupaia","Common Name"="Chinese tree shrew"]

NP_002454.1[&Organism="Homo sapiens",Description="interferon-induced GTP-binding protein Mx2 [Homo sapiens]","Genetic Code"="Standard",Taxonomy="Eukaryota; Metazoa; Chordata; Craniata; Vertebrata; Euteleostomi; Mammalia; Eutheria; Euarchontoglires; Primates; Haplorrhini; Catarrhini; Hominidae; Homo","Common Name"="human"]

XP_002830747.1[&Organism="Pongo abelii",Description="interferon-induced GTP-binding protein Mx2 [Pongo abelii]","Genetic Code"="Standard",Taxonomy="Eukaryota; Metazoa; Chordata; Craniata; Vertebrata; Euteleostomi; Mammalia; Eutheria; Euarchontoglires; Primates; Haplorrhini; Catarrhini; Hominidae; Pongo","Common Name"="Sumatran orangutan"]

XP_008569440.1[&Organism="Galeopterus variegatus",Description="PREDICTED: interferon-induced GTP-binding protein Mx2 [Galeopterus variegatus]","Genetic Code"="Standard",Taxonomy="Eukaryota; Metazoa; Chordata; Craniata; Vertebrata; Euteleostomi; Mammalia; Eutheria; Euarchontoglires; Dermoptera; Cynocephalidae; Galeopterus","Common Name"="Sunda flying lemur"]

XP_017508123.1[&Organism="Manis javanica",Description="PREDICTED: interferon-induced GTP-binding protein Mx2-like, partial [Manis javanica]","Genetic Code"="Standard",Taxonomy="Eukaryota; Metazoa; Chordata; Craniata; Vertebrata; Euteleostomi; Mammalia; Eutheria; Laurasiatheria; Pholidota; Manidae; Manis","Common Name"="Malayan pangolin"]

XP_005885748.1[&Organism="Myotis brandtii",Description="PREDICTED: interferon-induced GTP-binding protein Mx2 [Myotis brandtii]","Genetic Code"="Standard",Taxonomy="Eukaryota; Metazoa; Chordata; Craniata; Vertebrata; Euteleostomi; Mammalia; Eutheria; Laurasiatheria; Chiroptera; Microchiroptera; Vespertilionidae; Myotis","Common Name"="Brandt's bat"]

NP_001003133.1[&Organism="Canis lupus familiaris",Description="interferon-induced GTP-binding protein Mx2 [Canis lupus familiaris]","Genetic Code"="Standard",Taxonomy="Eukaryota; Metazoa; Chordata; Craniata; Vertebrata; Euteleostomi; Mammalia; Eutheria; Laurasiatheria; Carnivora; Caniformia; Canidae; Canis","Common Name"="dog"]

NP_776366.1[&Organism="Bos taurus",Description="interferon-induced GTP-binding protein Mx2 [Bos taurus]","Genetic Code"="Standard",Taxonomy="Eukaryota; Metazoa; Chordata; Craniata; Vertebrata; Euteleostomi; Mammalia; Eutheria; Laurasiatheria; Artiodactyla; Ruminantia; Pecora; Bovidae; Bovinae; Bos","Common Name"="cattle"]

XP_032211320.1[&Organism="Mustela erminea",Description="interferon-induced GTP-binding protein Mx2 isoform X1 [Mustela erminea]","Genetic Code"="Standard",Taxonomy="Eukaryota; Metazoa; Chordata; Craniata; Vertebrata; Euteleostomi; Mammalia; Eutheria; Laurasiatheria; Carnivora; Caniformia; Mustelidae; Mustelinae; Mustela","Common Name"="ermine"]

XP_004675614.2.2[&Organism="Condylura cristata",Description="PREDICTED: interferon-induced GTP-binding protein Mx1 [Condylura cristata]","Genetic Code"="Standard",Taxonomy="Eukaryota; Metazoa; Chordata; Craniata; Vertebrata; Euteleostomi; Mammalia; Eutheria; Laurasiatheria; Insectivora; Talpidae; Condylura","Common Name"="star-nosed mole"]

XP_004466363.1[&Organism="Dasypus novemcinctus",Description="interferon-induced GTP-binding protein Mx1 [Dasypus novemcinctus]","Genetic Code"="Standard",Taxonomy="Eukaryota; Metazoa; Chordata; Craniata; Vertebrata; Euteleostomi; Mammalia; Eutheria; Xenarthra; Cingulata; Dasypodidae; Dasypus","Common Name"="nine-banded armadillo"]

NP_002453.2.2[&Organism="Homo sapiens",Description="interferon-induced GTP-binding protein Mx1 isoform a [Homo sapiens]","Genetic Code"="Standard",Taxonomy="Eukaryota; Metazoa; Chordata; Craniata; Vertebrata;

Euteleostomi; Mammalia; Eutheria; Euarchontoglires; Primates; Haplorrhini; Catarrhini; Hominidae; Homo","Common Name"="human"]

NP_001127618.1[&Organism="Pongo abelii",Description="interferon-induced GTP-binding protein Mx1 [Pongo abelii]","Genetic Code"="Standard",Taxonomy="Eukaryota; Metazoa; Chordata; Craniata; Vertebrata; Euteleostomi; Mammalia; Eutheria; Euarchontoglires; Primates; Haplorrhini; Catarrhini; Hominidae; Pongo","Common Name"="Sumatran orangutan"]

XP_006156437.1[&Organism="Tupaia chinensis",Description="interferon-induced GTP-binding protein Mx1 [Tupaia chinensis]","Genetic Code"="Standard",Taxonomy="Eukaryota; Metazoa; Chordata; Craniata; Vertebrata; Euteleostomi; Mammalia; Eutheria; Euarchontoglires; Scandentia; Tupaiidae; Tupaia","Common Name"="Chinese tree shrew"]

XP_017508130.1[&Organism="Manis javanica",Description="PREDICTED: interferon-induced GTP-binding protein Mx1 [Manis javanica]","Genetic Code"="Standard",Taxonomy="Eukaryota; Metazoa; Chordata; Craniata; Vertebrata; Euteleostomi; Mammalia; Eutheria; Laurasiatheria; Pholidota; Manidae; Manis","Common Name"="Malayan pangolin"]

NP_001003134.1[&Organism="Canis lupus familiaris",Description="interferon-induced GTP-binding protein Mx1 [Canis lupus familiaris]","Genetic Code"="Standard",Taxonomy="Eukaryota; Metazoa; Chordata; Craniata; Vertebrata; Euteleostomi; Mammalia; Eutheria; Laurasiatheria; Carnivora; Caniformia; Canidae; Canis","Common Name"="dog"]

XP_032211398.1[&Organism="Mustela erminea",Description="interferon-induced GTP-binding protein Mx1 isoform X1 [Mustela erminea]","Genetic Code"="Standard",Taxonomy="Eukaryota; Metazoa; Chordata; Craniata; Vertebrata; Euteleostomi; Mammalia; Eutheria; Laurasiatheria; Carnivora; Caniformia; Mustelidae; Mustelinae; Mustela","Common Name"="ermine"]

XP_008569442.1[&Organism="Galeopterus variegatus",Description="PREDICTED: interferon-induced GTP-binding protein Mx1 [Galeopterus variegatus]","Genetic Code"="Standard",Taxonomy="Eukaryota; Metazoa; Chordata; Craniata; Vertebrata; Euteleostomi; Mammalia; Eutheria; Euarchontoglires; Dermoptera; Cynocephalidae; Galeopterus","Common Name"="Sunda flying lemur"]

XP_014388412.1[&Organism="Myotis brandtii",Description="PREDICTED: interferon-induced GTP-binding protein Mx1 isoform X1 [Myotis brandtii]","Genetic Code"="Standard",Taxonomy="Eukaryota; Metazoa; Chordata; Craniata; Vertebrata; Euteleostomi; Mammalia; Eutheria; Laurasiatheria; Chiroptera; Microchiroptera; Vespertilionidae; Myotis","Common Name"="Brandt's bat"]

XP_005202045.1[&Organism="Bos taurus",Description="interferon-induced GTP-binding protein Mx1 isoform X1 [Bos taurus]","Genetic Code"="Standard",Taxonomy="Eukaryota; Metazoa; Chordata; Craniata; Vertebrata; Euteleostomi; Mammalia; Eutheria; Laurasiatheria; Cetartiodactyla; Ruminantia; Pecora; Bovidae; Bovinae; Bos","Common Name"="cattle"]

NP_038634.1[&Organism="Mus musculus",Description="interferon-induced GTP-binding protein Mx2 [Mus musculus]","Genetic Code"="Standard",Taxonomy="Eukaryota;

Metazoa; Chordata; Craniata; Vertebrata; Euteleostomi; Mammalia; Eutheria; Euarchontoglires; Glires; Rodentia; Myomorpha; Muroidea; Muridae; Murinae; Mus; Mus","Common Name"="house mouse"]

NP_034976.1[&Organism="Mus musculus",Description="interferon-induced GTP-binding protein Mx1 [Mus musculus]","Genetic Code"="Standard",Taxonomy="Eukaryota; Metazoa; Chordata; Craniata; Vertebrata; Euteleostomi; Mammalia; Eutheria; Euarchontoglires; Glires; Rodentia; Myomorpha; Muroidea; Muridae; Murinae; Mus; Mus","Common Name"="house mouse"]

XP_028583072.1[&Organism="Podarcis muralis",Description="interferon-induced GTP-binding protein Mx2-like [Podarcis muralis]","Genetic Code"="Standard",Taxonomy="Eukaryota; Metazoa; Chordata; Craniata; Vertebrata; Euteleostomi; Lepidosauria; Squamata; Bifurcata; Unidentata; Episquamata; Laterata; Lacertibaenia; Lacertidae; Podarcis","Common Name"="Common wall lizard"]

XP_028583068.1[&Organism="Podarcis muralis",Description="interferon-induced GTP-binding protein Mx1-like isoform X1 [Podarcis muralis]","Genetic Code"="Standard",Taxonomy="Eukaryota; Metazoa; Chordata; Craniata; Vertebrata; Euteleostomi; Lepidosauria; Squamata; Bifurcata; Unidentata; Episquamata; Laterata; Lacertibaenia; Lacertidae; Podarcis","Common Name"="Common wall lizard"]

XP_031752404.1[&Organism="Xenopus tropicalis",Description="interferon-induced GTP-binding protein Mx2 [Xenopus tropicalis]","Genetic Code"="Standard",Taxonomy="Eukaryota; Metazoa; Chordata; Craniata; Vertebrata; Euteleostomi; Amphibia; Batrachia; Anura; Pipoidea; Pipidae; Xenopodinae; Xenopus; Silurana","Common Name"="tropical clawed frog"]

NP_001007285.1[&Organism="Danio rerio",Description="interferon-induced GTP-binding protein MxC [Danio rerio]","Genetic Code"="Standard",Taxonomy="Eukaryota; Metazoa; Chordata; Craniata; Vertebrata; Euteleostomi; Actinopterygii; Neopterygii; Teleostei; Ostariophysi; Cypriniformes; Cyprinidae; Danio","Common Name"="zebrafish"]

XP_005167721.2.2[&Organism="Danio rerio",Description="interferon-induced GTP-binding protein MxE isoform X1 [Danio rerio]","Genetic Code"="Standard",Taxonomy="Eukaryota; Metazoa; Chordata; Craniata; Vertebrata; Euteleostomi; Actinopterygii; Neopterygii; Teleostei; Ostariophysi; Cypriniformes; Cyprinidae; Danio","Common Name"="zebrafish"]

XP_012586448.1[&Organism="Condylura cristata",Description="PREDICTED: interferon-induced GTP-binding protein Mx2 [Condylura cristata]","Genetic Code"="Standard",Taxonomy="Eukaryota; Metazoa; Chordata; Craniata; Vertebrata; Euteleostomi; Mammalia; Eutheria; Laurasiatheria; Insectivora; Talpidae; Condylura","Common Name"="star-nosed mole"]

KAI0213370.1[&Organism="Lamellibrachia satsuma",Description="Interferon-induced GTP-binding protein Mx2 [Lamellibrachia satsuma]","Genetic Code"="Standard",Taxonomy="Eukaryota; Metazoa; Spiralia; Lophotrochozoa; Annelida; Polychaeta; Sedentaria; Canalipalpata; Sabellida; Siboglinidae; Lamellibrachia"]

KAI0208044.1[&Organism="Lamellibrachia satsuma",Description="Interferon-induced GTP-binding protein Mx1 [Lamellibrachia satsuma]","Genetic

Code"="Standard",Taxonomy="Eukaryota; Metazoa; Spiralia; Lophotrochozoa; Annelida; Polychaeta; Sedentaria; Canalipalpata; Sabellida; Siboglinidae; Lamellibrachia"]

KAI0218869.1[&Organism="Lamellibrachia satsuma",Description="hypothetical protein LSAT2_029455 [Lamellibrachia satsuma]","Genetic Code"="Standard",Taxonomy="Eukaryota; Metazoa; Spiralia; Lophotrochozoa; Annelida; Polychaeta; Sedentaria; Canalipalpata; Sabellida; Siboglinidae; Lamellibrachia"]

XP_032804093.1[&Organism="Petromyzon marinus",Description="interferon-induced GTP-binding protein Mx1-like isoform X2 [Petromyzon marinus]","Genetic Code"="Standard",Taxonomy="Eukaryota; Metazoa; Chordata; Craniata; Vertebrata; Cyclostomata; Hyperoartia; Petromyzontiformes; Petromyzontidae; Petromyzon","Common Name"="sea lamprey"]

XP_046565196.1[&Organism="Haliotis rubra",Description="interferon-induced GTP-binding protein Mx-like [Haliotis rubra]","Genetic Code"="Standard",Taxonomy="Eukaryota; Metazoa; Spiralia; Lophotrochozoa; Mollusca; Gastropoda; Vetigastropoda; Lepetellida; Haliotoidea; Haliotidae; Haliotis","Common Name"="blacklip abalone"]

XP_046562919.1[&Organism="Haliotis rubra",Description="interferon-induced GTP-binding protein Mx-like [Haliotis rubra]","Genetic Code"="Standard",Taxonomy="Eukaryota; Metazoa; Spiralia; Lophotrochozoa; Mollusca; Gastropoda; Vetigastropoda; Lepetellida; Haliotoidea; Haliotidae; Haliotis","Common Name"="blacklip abalone"]

XP_046563124.1[&Organism="Haliotis rubra",Description="interferon-induced GTP-binding protein Mx-like [Haliotis rubra]","Genetic Code"="Standard",Taxonomy="Eukaryota; Metazoa; Spiralia; Lophotrochozoa; Mollusca; Gastropoda; Vetigastropoda; Lepetellida; Haliotoidea; Haliotidae; Haliotis","Common Name"="blacklip abalone"]

XP_046563126.1[&Organism="Haliotis rubra",Description="interferon-induced GTP-binding protein Mx-like [Haliotis rubra]","Genetic Code"="Standard",Taxonomy="Eukaryota; Metazoa; Spiralia; Lophotrochozoa; Mollusca; Gastropoda; Vetigastropoda; Lepetellida; Haliotoidea; Haliotidae; Haliotis","Common Name"="blacklip abalone"]

XP_046565195.1[&Organism="Haliotis rubra",Description="interferon-induced GTP-binding protein Mx-like [Haliotis rubra]","Genetic Code"="Standard",Taxonomy="Eukaryota; Metazoa; Spiralia; Lophotrochozoa; Mollusca; Gastropoda; Vetigastropoda; Lepetellida; Haliotoidea; Haliotidae; Haliotis","Common Name"="blacklip abalone"]

XP_046563125.1[&Organism="Haliotis rubra",Description="interferon-induced GTP-binding protein Mx-like [Haliotis rubra]","Genetic Code"="Standard",Taxonomy="Eukaryota; Metazoa; Spiralia; Lophotrochozoa; Mollusca; Gastropoda; Vetigastropoda; Lepetellida; Haliotoidea; Haliotidae; Haliotis","Common Name"="blacklip abalone"]

XP_046352527.2[&Organism="Haliotis rufescens",Description="interferon-induced GTP-binding protein Mx1-like [Haliotis rufescens]","Genetic Code"="Standard",Taxonomy="Eukaryota; Metazoa; Spiralia; Lophotrochozoa; Mollusca; Gastropoda; Vetigastropoda; Lepetellida; Haliotoidea; Haliotidae; Haliotis","Common Name"="red abalone"]

XP_048248476.1[&Organism="Haliotis rufescens",Description="interferon-induced GTP-binding protein Mx-like [Haliotis rufescens]","Genetic Code"="Standard",Taxonomy="Eukaryota; Metazoa; Spiralia; Lophotrochozoa; Mollusca;

Gastropoda; Vetigastropoda; Lepetellida; Haliotoidea; Haliotidae; Haliotis","Common Name"="red abalone"]

XP_048258111.1[&Organism="Haliotis rufescens",Description="interferon-induced GTP-binding protein Mx-like [Haliotis rufescens]","Genetic Code"="Standard",Taxonomy="Eukaryota; Metazoa; Spiralia; Lophotrochozoa; Mollusca; Gastropoda; Vetigastropoda; Lepetellida; Haliotoidea; Haliotidae; Haliotis","Common Name"="red abalone"]

XP_046352531.2[&Organism="Haliotis rufescens",Description="interferon-induced GTP-binding protein Mx-like [Haliotis rufescens]","Genetic Code"="Standard",Taxonomy="Eukaryota; Metazoa; Spiralia; Lophotrochozoa; Mollusca; Gastropoda; Vetigastropoda; Lepetellida; Haliotoidea; Haliotidae; Haliotis","Common Name"="red abalone"]

XP_048248472.1[&Organism="Haliotis rufescens",Description="LOW QUALITY PROTEIN: interferon-induced GTP-binding protein Mx-like [Haliotis rufescens]","Genetic Code"="Standard",Taxonomy="Eukaryota; Metazoa; Spiralia; Lophotrochozoa; Mollusca; Gastropoda; Vetigastropoda; Lepetellida; Haliotoidea; Haliotidae; Haliotis","Common Name"="red abalone"]

XP_048248473.1[&Organism="Haliotis rufescens",Description="interferon-induced GTP-binding protein Mx-like isoform X1 [Haliotis rufescens]","Genetic Code"="Standard",Taxonomy="Eukaryota; Metazoa; Spiralia; Lophotrochozoa; Mollusca; Gastropoda; Vetigastropoda; Lepetellida; Haliotoidea; Haliotidae; Haliotis","Common Name"="red abalone"]

XP_048248474.1[&Organism="Haliotis rufescens",Description="interferon-induced GTP-binding protein Mx-like isoform X2 [Haliotis rufescens]","Genetic Code"="Standard",Taxonomy="Eukaryota; Metazoa; Spiralia; Lophotrochozoa; Mollusca; Gastropoda; Vetigastropoda; Lepetellida; Haliotoidea; Haliotidae; Haliotis","Common Name"="red abalone"]

ABI53802.1[&Organism="Haliotis discus discus",Description="Mx [Haliotis discus discus]","Genetic Code"="Standard",Taxonomy="Eukaryota; Metazoa; Lophotrochozoa; Mollusca; Gastropoda; Vetigastropoda; Haliotoidea; Haliotidae; Haliotis","Common Name"="disc abalone"]

CAH1802128.1[&Organism="Owenia fusiformis",Description="unnamed protein product [Owenia fusiformis]","Genetic Code"="Standard",Taxonomy="Eukaryota; Metazoa; Spiralia; Lophotrochozoa; Annelida; Polychaeta; Sedentaria; Canalipalpata; Sabellida; Oweniida; Oweniidae; Owenia"]

PAA74204.1[&Organism="Macrostomum lignano",Description="hypothetical protein BOX15_Mlig022940g2 [Macrostomum lignano]","Genetic Code"="Standard",Taxonomy="Eukaryota; Metazoa; Platyhelminthes; Rhabditophora; Macrostomorpha; Macrostomida; Macrostomidae; Macrostomum"]

PAA76532.1[&Organism="Macrostomum lignano",Description="hypothetical protein BOX15_Mlig002592g2 [Macrostomum lignano]","Genetic Code"="Standard",Taxonomy="Eukaryota; Metazoa; Platyhelminthes; Rhabditophora; Macrostomorpha; Macrostomida; Macrostomidae; Macrostomum"]

PAA92268.1[&Organism="Macrostomum lignano",Description="hypothetical protein BOX15_Mlig009769g1 [Macrostomum lignano]","Genetic Code"="Standard",Taxonomy="Eukaryota; Metazoa; Platyhelminthes; Rhabditophora; Macrostomorpha; Macrostomida; Macrostomidae; Macrostomum"]

PAA69582.1[&Organism="Macrostomum lignano",Description="hypothetical protein BOX15_Mlig021727g2 [Macrostomum lignano]","Genetic Code"="Standard",Taxonomy="Eukaryota; Metazoa; Platyhelminthes; Rhabditophora; Macrostomorpha; Macrostomida; Macrostomidae; Macrostomum"]

PAA83069.1[&Organism="Macrostomum lignano",Description="hypothetical protein BOX15_Mlig009247g1 [Macrostomum lignano]","Genetic Code"="Standard",Taxonomy="Eukaryota; Metazoa; Platyhelminthes; Rhabditophora; Macrostomorpha; Macrostomida; Macrostomidae; Macrostomum"]

PAA94353.1[&Organism="Macrostomum lignano",Description="hypothetical protein BOX15_Mlig014920g1 [Macrostomum lignano]","Genetic Code"="Standard",Taxonomy="Eukaryota; Metazoa; Platyhelminthes; Rhabditophora; Macrostomorpha; Macrostomida; Macrostomidae; Macrostomum"]

GMH43921.1[&Organism="Bryopsis sp. KO-2023",Description="hypothetical protein BSKO_11855 [Bryopsis sp. KO-2023]","Genetic Code"="Standard",Taxonomy="Eukaryota; Viridiplantae; Chlorophyta; Ulvophyceae; TCBD clade; Bryopsidales; Bryopsidineae; Bryopsidaceae; Bryopsis"]

GMH36208.1[&Organism="Bryopsis sp. KO-2023",Description="hypothetical protein BSKO_04076 [Bryopsis sp. KO-2023]","Genetic Code"="Standard",Taxonomy="Eukaryota; Viridiplantae; Chlorophyta; Ulvophyceae; TCBD clade; Bryopsidales; Bryopsidineae; Bryopsidaceae; Bryopsis"]

GJP35534.1[&Organism="Closterium sp. NIES-68",Description="hypothetical protein CLOM_g20043 [Closterium sp. NIES-68]","Genetic Code"="Standard",Taxonomy="Eukaryota; Viridiplantae; Streptophyta; Zygnemophyceae; Zygnematophycidae; Desmidiales; Closteriaceae; Closterium; Closterium peracerosum-strigosum-littorale complex"]

CAI5480041.1[&Organism="Closterium sp. Yama58-4",Description="unnamed protein product [Closterium sp. Yama58-4]","Genetic Code"="Standard",Taxonomy="Eukaryota; Viridiplantae; Streptophyta; Zygnemophyceae; Zygnematophycidae; Desmidiales; Closteriaceae; Closterium; Closterium peracerosum-strigosum-littorale complex"]

KAJ7294545.1[&Organism="Diphasiastrum complanatum",Description="hypothetical protein O6H91_Y251600 [Diphasiastrum complanatum]","Genetic Code"="Standard",Taxonomy="Eukaryota; Viridiplantae; Streptophyta; Embryophyta; Tracheophyta; Lycopodiopsida; Lycopodiales; Lycopodiaceae; Lycopodioideae; Diphasiastrum"]

XP_024380180.1[&Organism="Physcomitrium patens",Description="dynamin-related protein 4C-like [Physcomitrium patens]","Genetic Code"="Standard",Taxonomy="Eukaryota; Viridiplantae; Streptophyta; Embryophyta; Bryophyta; Bryophytina; Bryopsida; Funariidae; Funariales; Funariaceae; Physcomitrium"]

XP_024367947.1[&Organism="Physcomitrium patens",Description="dynamin-related protein 4C-like, partial [Physcomitrium patens]","Genetic Code"="Standard",Taxonomy="Eukaryota; Viridiplantae; Streptophyta; Embryophyta; Bryophyta; Bryophytina; Bryopsida; Funariidae; Funariales; Funariaceae; Physcomitrium"]

KAG0619429.1[&Organism="Ceratodon purpureus",Description="hypothetical protein M758_4G139100 [Ceratodon purpureus]","Genetic Code"="Standard",Taxonomy="Eukaryota; Viridiplantae; Streptophyta; Embryophyta; Bryophyta; Bryophytina; Bryopsida; Dicranidae; Pseudoditrichales; Ditrichaceae; Ceratodon"]

KAG0561847.1[&Organism="Ceratodon purpureus",Description="hypothetical protein KC19_9G097200 [Ceratodon purpureus]","Genetic Code"="Standard",Taxonomy="Eukaryota; Viridiplantae; Streptophyta; Embryophyta; Bryophyta; Bryophytina; Bryopsida; Dicranidae; Pseudoditrichales; Ditrichaceae; Ceratodon"]

KAH9290598.1[&Organism="Taxus chinensis",Description="hypothetical protein KI387_034715 [Taxus chinensis]","Genetic Code"="Standard",Taxonomy="Eukaryota; Viridiplantae; Streptophyta; Embryophyta; Tracheophyta; Spermatophyta; Pinopsida; Pinidae; Conifers II; Cupressales; Taxaceae; Taxus"]

KAH9320939.1[&Organism="Taxus chinensis",Description="hypothetical protein KI387_015578 [Taxus chinensis]","Genetic Code"="Standard",Taxonomy="Eukaryota; Viridiplantae; Streptophyta; Embryophyta; Tracheophyta; Spermatophyta; Pinopsida; Pinidae; Conifers II; Cupressales; Taxaceae; Taxus"]

KAF8079489.1[&Organism="Sinapis alba",Description="hypothetical protein N665_1024s0016 [Sinapis alba]","Genetic Code"="Standard",Taxonomy="Eukaryota; Viridiplantae; Streptophyta; Embryophyta; Tracheophyta; Spermatophyta; Magnoliopsida; eudicotyledons; Gunneridae; Pentapetalae; rosids; malvids; Brassicales; Brassicaceae; Brassiceae; Sinapis","Common Name"="white mustard"]

OAP13353.1[&Description="hypothetical protein AXX17_AT1G53610 [Arabidopsis thaliana]"]

OAP19580.1[&Description="DRP4A [Arabidopsis thaliana]"]

OAP13972.1[&Description="hypothetical protein AXX17_AT1G53540 [Arabidopsis thaliana]"]

XP_038984915.1[&Organism="Phoenix dactylifera",Description="dynamin-related protein 4C-like [Phoenix dactylifera]","Genetic Code"="Standard",Taxonomy="Eukaryota; Viridiplantae; Streptophyta; Embryophyta; Tracheophyta; Spermatophyta; Magnoliopsida; Liliopsida; Arecaceae; Coryphoideae; Phoeniceae; Phoenix","Common Name"="date palm"]

KAF8391993.1[&Organism="Tetracentron sinense",Description="hypothetical protein HHK36_022333 [Tetracentron sinense]","Genetic Code"="Standard",Taxonomy="Eukaryota; Viridiplantae; Streptophyta; Embryophyta; Tracheophyta; Spermatophyta; Magnoliopsida; Trochodendrales; Trochodendraceae; Tetracentron"]

XP_058079501.1[&Organism="Magnolia sinica",Description="dynamin-related protein 4C-like [Magnolia sinica]","Genetic Code"="Standard",Taxonomy="Eukaryota;

Viridiplantae; Streptophyta; Embryophyta; Tracheophyta; Spermatophyta; Magnoliopsida; Magnoliidae; Magnoliales; Magnoliaceae; Magnolia"]

KAF5727250.1[&Organism="Tripterygium wilfordii",Description="hypothetical protein HS088_TW22G00939 [Tripterygium wilfordii]","Genetic Code"="Standard",Taxonomy="Eukaryota; Viridiplantae; Streptophyta; Embryophyta; Tracheophyta; Spermatophyta; Magnoliopsida; eudicotyledons; Gunneridae; Pentapetalae; rosids; fabids; Celastrales; Celastraceae; Tripterygium"]

XP_002303204.3[&Organism="Populus trichocarpa",Description="dynamin-related protein 4C isoform X2 [Populus trichocarpa]","Genetic Code"="Standard",Taxonomy="Eukaryota; Viridiplantae; Streptophyta; Embryophyta; Tracheophyta; Spermatophyta; Magnoliopsida; eudicotyledons; Gunneridae; Pentapetalae; rosids; fabids; Malpighiales; Salicaceae; Saliceae; Populus","Common Name"="Populus balsamifera subsp. trichocarpa"]

KAK1401877.1[&Organism="Heracleum sosnowskyi",Description="Dynamin-related protein 4C [Heracleum sosnowskyi]","Genetic Code"="Standard",Taxonomy="Eukaryota; Viridiplantae; Streptophyta; Embryophyta; Tracheophyta; Spermatophyta; Magnoliopsida; eudicotyledons; Gunneridae; Pentapetalae; asterids; campanulids; Apiales; Apiaceae; Apioideae; apioid superclade; Tordylieae; Tordyliinae; Heracleum"]

XP_002297993.1[&Description="dynamin-related protein 4C [Populus trichocarpa]"]

XP_024439231.1[&Description="dynamin-related protein 4C [Populus trichocarpa]"]

KAH0683503.1[&Organism="Solanum tuberosum",Description="hypothetical protein KY289_021255 [Solanum tuberosum]","Genetic Code"="Standard",Taxonomy="Eukaryota; Viridiplantae; Streptophyta; Embryophyta; Tracheophyta; Spermatophyta; Magnoliopsida; eudicotyledons; Gunneridae; Pentapetalae; asterids; lamiids; Solanales; Solanaceae; Solanoideae; Solaneae; Solanum","Common Name"="potato"]

PWZ56863.1[&Organism="Zea mays",Description="Dynamin-related protein 4C [Zea mays]","Genetic Code"="Standard",Taxonomy="Eukaryota; Viridiplantae; Streptophyta; Embryophyta; Tracheophyta; Spermatophyta; Magnoliopsida; Liliopsida; Poales; Poaceae; PACMAD clade; Panicoideae; Andropogonodae; Andropogoneae; Tripsacinae; Zea"]

PWZ56864.1[&Organism="Zea mays",Description="Dynamin-related protein 4C [Zea mays]","Genetic Code"="Standard",Taxonomy="Eukaryota; Viridiplantae; Streptophyta; Embryophyta; Tracheophyta; Spermatophyta; Magnoliopsida; Liliopsida; Poales; Poaceae; PACMAD clade; Panicoideae; Andropogonodae; Andropogoneae; Tripsacinae; Zea"]

KAH9291961.1[&Organism="Taxus chinensis",Description="hypothetical protein KI387_042849 [Taxus chinensis]","Genetic Code"="Standard",Taxonomy="Eukaryota; Viridiplantae; Streptophyta; Embryophyta; Tracheophyta; Spermatophyta; Pinopsida; Pinidae; Conifers II; Cupressales; Taxaceae; Taxus"]

KAH9325151.1[&Description="hypothetical protein KI387_005329, partial [Taxus chinensis]"]

KAH9300179.1[&Description="hypothetical protein KI387_011762, partial [Taxus chinensis]"]

KAH9314974.1[&Description="hypothetical protein KI387_023601, partial [Taxus chinensis]"]

EFJ22917.1[&Description="hypothetical protein SELMODRAFT_104286 [Selaginella moellendorffii]"]

CAG9460856.1[&Organism="Pedinophyceae sp. YPF-701",Description="unnamed protein product [Pedinophyceae sp. YPF-701]","Genetic Code"="Standard",Taxonomy="Eukaryota; Viridiplantae; Chlorophyta; Pedinophyceae"]

KAK3283006.1[&Organism="Cymbomonas tetramitiformis",Description="hypothetical protein CYMTET_9279 [Cymbomonas tetramitiformis]","Genetic Code"="Standard",Taxonomy="Eukaryota; Viridiplantae; Chlorophyta; Pyramimonadophyceae; Pyramimonadales; Pyramimonadaceae; Cymbomonas"]

GHP04420.1[&Organism="Pycnococcus provasolii",Description="hypothetical protein PPROV_000317400 [Pycnococcus provasolii]","Genetic Code"="Standard",Taxonomy="Eukaryota; Viridiplantae; Chlorophyta; Pycnococcaceae; Pycnococcus"]

'KAJ3066410.1'[&"% Charged Amino Acids"=27.50%,"% Acidic Amino Acids"=15.00%,"% Hydrophobic Amino Acids"=47.14%,Description="JEL0797 KAJ3066410.1",Modified=Fri Jun 28 11:50:27 PDT 2024,"% GC-rich Amino Acids"=22.14%,"# Nucleotide Sequences With Quality"=0,"Extinction Coefficient"=13075.0,"Molecular Weight (kDa)"=30.847098080000013,Topology="linear","Alignment method"="MAFFT Alignment",Created=Fri Jun 28 11:46:17 PDT 2024,"Charge at pH 7"=-10.798238550692695,"Isoelectric Point"=4.645633697509766,"% Basic Amino Acids"=12.50%,"% AT-rich Amino Acids"=21.07%,"% Polar Uncharged Amino Acids"=25.71%,"Free end gaps"=true,"Molecule Type"="AA"]

KAI9324922.1[&"% Charged Amino Acids"=24.91%,"% Acidic Amino Acids"=13.17%,"% Hydrophobic Amino Acids"=46.26%,Description="KAI9324922.1",Modified=Fri Jun 28 11:50:37 PDT 2024,"% GC-rich Amino Acids"=21.71%,"# Nucleotide Sequences With Quality"=0,"Extinction Coefficient"=13075.0,"Molecular Weight (kDa)"=30.80993478000001,Topology="linear","Alignment method"="MAFFT Alignment",Created=Fri Jun 28 11:46:17 PDT 2024,"Charge at pH 7"=-7.798989763504893,"Isoelectric Point"=4.897228240966797,"% Basic Amino Acids"=11.74%,"% AT-rich Amino Acids"=21.71%,"% Polar Uncharged Amino Acids"=29.18%,"Free end gaps"=true,"Molecule Type"="AA"]

KAI8836453.1[&"% Charged Amino Acids"=29.14%,"% Acidic Amino Acids"=13.67%,"% Hydrophobic Amino Acids"=46.76%,Description="JEL632 KAI8836453.1",Modified=Fri Jun 28 11:50:45 PDT 2024,"% GC-rich Amino Acids"=22.30%,"# Nucleotide Sequences With Quality"=0,"Extinction Coefficient"=13200.0,"Molecular Weight (kDa)"=30.927449480000007,Topology="linear","Alignment method"="MAFFT Alignment",Created=Fri Jun 28 11:46:17 PDT 2024,"Charge at pH 7"=-3.4437602930013576,"Isoelectric Point"=6.172344207763672,"% Basic Amino Acids"=15.47%,"% AT-rich Amino Acids"=21.94%,"% Polar Uncharged Amino Acids"=24.46%,"Free end gaps"=true,"Molecule Type"="AA"]

KAJ3350919.1[&"% Charged Amino Acids"=24.29%,"% Acidic Amino Acids"=12.14%,"% Hydrophobic Amino Acids"=49.64%,Description="KAJ3350919.1",Modified=Fri Jun 28 11:50:59 PDT 2024,"% GC-rich Amino Acids"=20.71%,"# Nucleotide Sequences With Quality"=0,"Extinction Coefficient"=13200.0,"Molecular Weight (kDa)"=30.724561180000016,Topology="linear","Alignment method"="MAFFT Alignment",Created=Fri Jun 28 11:46:17 PDT 2024,"Charge at pH 7"=-4.908779490673721,"Isoelectric Point"=5.475826263427734,"% Basic Amino Acids"=12.14%,"% AT-rich Amino Acids"=24.64%,"% Polar Uncharged Amino Acids"=26.43%,"Free end gaps"=true,"Molecule Type"="AA"]
KAI8587516.1[&"% Charged Amino Acids"=22.91%,"% Acidic Amino Acids"=11.27%,"% Hydrophobic Amino Acids"=52.00%,Description="KAI8587516.1",Modified=Fri Jun 28 11:50:12 PDT 2024,"% GC-rich Amino Acids"=23.27%,"# Nucleotide Sequences With Quality"=0,"Extinction Coefficient"=24075.0,"Molecular Weight (kDa)"=30.21366338000001,Topology="linear","Alignment method"="MAFFT Alignment",Created=Fri Jun 28 11:46:17 PDT 2024,"Charge at pH 7"=-3.7092413986402963,"Isoelectric Point"=5.703121185302734,"% Basic Amino Acids"=11.64%,"% AT-rich Amino Acids"=21.82%,"% Polar Uncharged Amino Acids"=26.18%,"Free end gaps"=true,"Molecule Type"="AA"]
XP_047808890.1[&"% Charged Amino Acids"=27.17%,"% Acidic Amino Acids"=12.68%,"% Hydrophobic Amino Acids"=47.10%,Description="XP_047808890.1",Modified=Fri Jun 28 11:49:32 PDT 2024,"% GC-rich Amino Acids"=23.91%,"# Nucleotide Sequences With Quality"=0,"Extinction Coefficient"=13200.0,"Molecular Weight (kDa)"=30.56279258,Topology="linear","Alignment method"="MAFFT Alignment",Created=Fri Jun 28 11:46:17 PDT 2024,"Charge at pH 7"=-1.6440202409101712,"Isoelectric Point"=6.441722869873047,"% Basic Amino Acids"=14.49%,"% AT-rich Amino Acids"=22.83%,"% Polar Uncharged Amino Acids"=26.09%,"Free end gaps"=true,"Molecule Type"="AA"]
KXS17655.1[&"% Charged Amino Acids"=25.81%,"% Acidic Amino Acids"=13.26%,"% Hydrophobic Amino Acids"=53.05%,Description="KXS17655.1",Modified=Fri Jun 28 11:49:57 PDT 2024,"% GC-rich Amino Acids"=23.30%,"# Nucleotide Sequences With Quality"=0,"Extinction Coefficient"=14105.0,"Molecular Weight (kDa)"=30.090607580000007,Topology="linear","Alignment method"="MAFFT Alignment",Created=Fri Jun 28 11:46:17 PDT 2024,"Charge at pH 7"=-7.601807258076357,"Isoelectric Point"=5.110424041748047,"% Basic Amino Acids"=12.54%,"% AT-rich Amino Acids"=23.30%,"% Polar Uncharged Amino Acids"=21.86%,"Free end gaps"=true,"Molecule Type"="AA"]
XP_021869222.1[&"% Charged Amino Acids"=32.55%,"% Acidic Amino Acids"=15.77%,"% Hydrophobic Amino Acids"=44.30%,Description="XP_021869222.1",Modified=Fri Jun 28 11:49:08 PDT 2024,"% GC-rich Amino Acids"=21.48%,"# Nucleotide Sequences With Quality"=0,"Extinction Coefficient"=21095.0,"Molecular Weight

(kDa)"=33.45680067999999,Topology="linear","Alignment method"="MAFFT Alignment",Created=Fri Jun 28 11:46:17 PDT 2024,"Charge at pH 7"=-6.272273006910691,"Isoelectric Point"=5.793697357177734,"% Basic Amino Acids"=16.78%,"% AT-rich Amino Acids"=21.48%,"% Polar Uncharged Amino Acids"=24.16%,"Free end gaps"=true,"Molecule Type"="AA"]
TVY17522.1[&"% Charged Amino Acids"=29.25%,"% Acidic Amino Acids"=13.95%,"% Hydrophobic Amino Acids"=50.00%,Description="TVY17522.1",Modified=Fri Jun 28 11:46:17 PDT 2024,"% GC-rich Amino Acids"=22.79%,"# Nucleotide Sequences With Quality"=0,"Extinction Coefficient"=19605.0,"Molecular Weight (kDa)"=32.82586588,Topology="linear","Alignment method"="MAFFT Alignment",Created=Fri Jun 28 11:46:17 PDT 2024,"Charge at pH 7"=-3.4030920147103245,"Isoelectric Point"=6.098857879638672,"% Basic Amino Acids"=15.31%,"% AT-rich Amino Acids"=18.71%,"% Polar Uncharged Amino Acids"=21.77%,"Free end gaps"=true,"Molecule Type"="AA"]
OLL24579.1[&"% Charged Amino Acids"=26.39%,"% Acidic Amino Acids"=12.15%,"% Hydrophobic Amino Acids"=44.79%,Description="OLL24579.1",Modified=Fri Jun 28 11:46:17 PDT 2024,"% GC-rich Amino Acids"=20.49%,"# Nucleotide Sequences With Quality"=0,"Extinction Coefficient"=14565.0,"Molecular Weight (kDa)"=31.81140938000001,Topology="linear","Alignment method"="MAFFT Alignment",Created=Fri Jun 28 11:46:17 PDT 2024,"Charge at pH 7"=3.0255167076407066,"Isoelectric Point"=8.445613861083984,"% Basic Amino Acids"=14.24%,"% AT-rich Amino Acids"=25.69%,"% Polar Uncharged Amino Acids"=29.17%,"Free end gaps"=true,"Molecule Type"="AA"]
KAI9096888.1[&"% Charged Amino Acids"=30.14%,"% Acidic Amino Acids"=15.07%,"% Hydrophobic Amino Acids"=44.18%,Description="KAI9096888.1",Modified=Fri Jun 28 11:51:56 PDT 2024,"% GC-rich Amino Acids"=17.47%,"# Nucleotide Sequences With Quality"=0,"Extinction Coefficient"=20065.0,"Molecular Weight (kDa)"=33.101868080000024,Topology="linear","Alignment method"="MAFFT Alignment",Created=Fri Jun 28 11:46:17 PDT 2024,"Charge at pH 7"=-4.760784008823309,"Isoelectric Point"=5.568431854248047,"% Basic Amino Acids"=15.07%,"% AT-rich Amino Acids"=25.68%,"% Polar Uncharged Amino Acids"=26.37%,"Free end gaps"=true,"Molecule Type"="AA"]
RSH87279.1[&"% Charged Amino Acids"=29.37%,"% Acidic Amino Acids"=15.03%,"% Hydrophobic Amino Acids"=47.20%,Description="RSH87279.1",Modified=Fri Jun 28 11:48:55 PDT 2024,"% GC-rich Amino Acids"=24.13%,"# Nucleotide Sequences With Quality"=0,"Extinction Coefficient"=7115.0,"Molecular Weight (kDa)"=31.83822928,Topology="linear","Alignment method"="MAFFT Alignment",Created=Fri Jun 28 11:46:17 PDT 2024,"Charge at pH 7"=-5.858968677562601,"Isoelectric Point"=5.208797454833984,"% Basic Amino Acids"=14.34%,"% AT-rich Amino Acids"=20.63%,"% Polar Uncharged Amino Acids"=23.78%,"Free end gaps"=true,"Molecule Type"="AA"]

XP_041144356.1[&"% Charged Amino Acids"=26.92%,"% Acidic Amino Acids"=12.24%,"% Hydrophobic Amino Acids"=48.25%,Description="XM_041290714.1",Modified=Fri Jun 28 11:46:17 PDT 2024,"% GC-rich Amino Acids"=22.03%,"# Nucleotide Sequences With Quality"=0,"Extinction Coefficient"=17085.0,"Molecular Weight (kDa)"=31.70809888000001,Topology="linear","Alignment method"="MAFFT Alignment",Created=Fri Jun 28 11:46:17 PDT 2024,"Charge at pH 7"=-3.1075449530009323,"Isoelectric Point"=6.306392669677734,"% Basic Amino Acids"=14.69%,"% AT-rich Amino Acids"=19.58%,"% Polar Uncharged Amino Acids"=25.52%,"Free end gaps"=true,"Molecule Type"="AA"]
KAJ5704467.1[&"% Charged Amino Acids"=24.13%,"% Acidic Amino Acids"=11.89%,"% Hydrophobic Amino Acids"=49.30%,Description="KAJ5704467.1",Modified=Fri Jun 28 11:46:17 PDT 2024,"% GC-rich Amino Acids"=23.78%,"# Nucleotide Sequences With Quality"=0,"Extinction Coefficient"=19605.0,"Molecular Weight (kDa)"=31.15122537999999,Topology="linear","Alignment method"="MAFFT Alignment",Created=Fri Jun 28 11:46:17 PDT 2024,"Charge at pH 7"=-5.574711435859037,"Isoelectric Point"=5.575748443603516,"% Basic Amino Acids"=12.24%,"% AT-rich Amino Acids"=15.73%,"% Polar Uncharged Amino Acids"=27.62%,"Free end gaps"=true,"Molecule Type"="AA"]
MCJ1392161.1[&"% Charged Amino Acids"=26.22%,"% Acidic Amino Acids"=13.29%,"% Hydrophobic Amino Acids"=47.90%,Description="MCJ1392161.1",Modified=Fri Jun 28 11:46:17 PDT 2024,"% GC-rich Amino Acids"=21.68%,"# Nucleotide Sequences With Quality"=0,"Extinction Coefficient"=22585.0,"Molecular Weight (kDa)"=31.422763779999993,Topology="linear","Alignment method"="MAFFT Alignment",Created=Fri Jun 28 11:46:17 PDT 2024,"Charge at pH 7"=-6.599380872602497,"Isoelectric Point"=5.295635223388672,"% Basic Amino Acids"=12.94%,"% AT-rich Amino Acids"=17.83%,"% Polar Uncharged Amino Acids"=26.92%,"Free end gaps"=true,"Molecule Type"="AA"]
KAI9774215.1[&"% Charged Amino Acids"=25.87%,"% Acidic Amino Acids"=12.24%,"% Hydrophobic Amino Acids"=47.90%,Description="KAI9774215.1",Modified=Fri Jun 28 11:46:17 PDT 2024,"% GC-rich Amino Acids"=21.68%,"# Nucleotide Sequences With Quality"=0,"Extinction Coefficient"=21095.0,"Molecular Weight (kDa)"=31.32162668,Topology="linear","Alignment method"="MAFFT Alignment",Created=Fri Jun 28 11:46:17 PDT 2024,"Charge at pH 7"=-4.3741271905474495,"Isoelectric Point"=6.005184173583984,"% Basic Amino Acids"=13.64%,"% AT-rich Amino Acids"=18.53%,"% Polar Uncharged Amino Acids"=27.27%,"Free end gaps"=true,"Molecule Type"="AA"]
XP_002543522.1[&"% Charged Amino Acids"=26.92%,"% Acidic Amino Acids"=12.59%,"% Hydrophobic Amino Acids"=48.95%,Description="1704 XP_002543522.1",Modified=Fri Jun 28 11:46:17 PDT 2024,"% GC-rich Amino Acids"=23.78%,"# Nucleotide Sequences With Quality"=0,"Extinction Coefficient"=14105.0,"Molecular Weight

(kDa)"=31.483921279999993,Topology="linear","Alignment method"="MAFFT Alignment",Created=Fri Jun 28 11:46:17 PDT 2024,"Charge at pH 7"=-2.40438839054951,"Isoelectric Point"=6.292827606201172,"% Basic Amino Acids"=14.34%,"% AT-rich Amino Acids"=19.58%,"% Polar Uncharged Amino Acids"=24.83%,"Free end gaps"=true,"Molecule Type"="AA"]

XP_746402.1[&Organism="Aspergillus fumigatus Af293",Description="dynamin GTPase, putative [Aspergillus fumigatus Af293]","Genetic Code"="Standard",Taxonomy="Eukaryota; Fungi; Dikarya; Ascomycota; Pezizomycotina; Eurotiomycetes; Eurotiomycetidae; Eurotiales; Aspergillaceae; Aspergillus; Aspergillus subgen. Fumigati"]

XP_751069.1[&Organism="Aspergillus fumigatus Af293",Description="dynamin GTPase, putative [Aspergillus fumigatus Af293]","Genetic Code"="Standard",Taxonomy="Eukaryota; Fungi; Dikarya; Ascomycota; Pezizomycotina; Eurotiomycetes; Eurotiomycetidae; Eurotiales; Aspergillaceae; Aspergillus; Aspergillus subgen. Fumigati"]

XP_026607910.1[&"% Charged Amino Acids"=25.93%,"% Acidic Amino Acids"=11.11%,"% Hydrophobic Amino Acids"=48.82%,Description="XM_026742065.1",Modified=Fri Jun 28 11:46:17 PDT 2024,"% GC-rich Amino Acids"=24.58%,"# Nucleotide Sequences With Quality"=0,"Extinction Coefficient"=11710.0,"Molecular Weight (kDa)"=32.72361798000001,Topology="linear","Alignment method"="MAFFT Alignment",Created=Fri Jun 28 11:46:17 PDT 2024,"Charge at pH 7"=2.553564522492767,"Isoelectric Point"=7.953372955322266,"% Basic Amino Acids"=14.81%,"% AT-rich Amino Acids"=22.56%,"% Polar Uncharged Amino Acids"=25.59%,"Free end gaps"=true,"Molecule Type"="AA"]

XP_748757.2[&Organism="Aspergillus fumigatus Af293",Description="dynamin GTPase, putative [Aspergillus fumigatus Af293]","Genetic Code"="Standard",Taxonomy="Eukaryota; Fungi; Dikarya; Ascomycota; Pezizomycotina; Eurotiomycetes; Eurotiomycetidae; Eurotiales; Aspergillaceae; Aspergillus; Aspergillus subgen. Fumigati"]

XP_040633937.1[&"% Charged Amino Acids"=27.74%,"% Acidic Amino Acids"=14.04%,"% Hydrophobic Amino Acids"=51.71%,Description="XM_040785195.1",Modified=Fri Jun 28 11:46:17 PDT 2024,"% GC-rich Amino Acids"=27.40%,"# Nucleotide Sequences With Quality"=0,"Extinction Coefficient"=7115.0,"Molecular Weight (kDa)"=31.85812448,Topology="linear","Alignment method"="MAFFT Alignment",Created=Fri Jun 28 11:46:17 PDT 2024,"Charge at pH 7"=-11.101735485136608,"Isoelectric Point"=5.194911956787109,"% Basic Amino Acids"=13.70%,"% AT-rich Amino Acids"=17.12%,"% Polar Uncharged Amino Acids"=20.89%,"Free end gaps"=true,"Molecule Type"="AA"]

XP_043140374.1[&"% Charged Amino Acids"=27.74%,"% Acidic Amino Acids"=14.38%,"% Hydrophobic Amino Acids"=51.03%,Description="XM_043283056.1",Modified=Fri Jun 28 11:46:17 PDT 2024,"% GC-rich Amino Acids"=26.71%,"# Nucleotide Sequences With Quality"=0,"Extinction Coefficient"=7115.0,"Molecular Weight

(kDa)"=32.242680480000004,Topology="linear","Alignment method"="MAFFT Alignment",Created=Fri Jun 28 11:46:17 PDT 2024,"Charge at pH 7"=-8.599323914913342,"Isoelectric Point"=4.979366302490234,"% Basic Amino Acids"=13.36%,"% AT-rich Amino Acids"=20.21%,"% Polar Uncharged Amino Acids"=21.58%,"Free end gaps"=true,"Molecule Type"="AA"]

XP_754266.1[&Organism="Aspergillus fumigatus Af293",Description="dynamin GTPase, putative [Aspergillus fumigatus Af293]","Genetic Code"="Standard",Taxonomy="Eukaryota; Fungi; Dikarya; Ascomycota; Pezizomycotina; Eurotiomycetes; Eurotiomycetidae; Eurotiales; Aspergillaceae; Aspergillus; Aspergillus subgen. Fumigati"]

KAF9951223.1[&"% Charged Amino Acids"=29.71%,"% Acidic Amino Acids"=15.22%,"% Hydrophobic Amino Acids"=45.65%,Description="KAF9951223.1",Modified=Fri Jun 28 11:51:59 PDT 2024,"% GC-rich Amino Acids"=21.01%,"# Nucleotide Sequences With Quality"=0,"Extinction Coefficient"=27055.0,"Molecular Weight (kDa)"=31.347671079999994,Topology="linear","Alignment method"="MAFFT Alignment",Created=Fri Jun 28 11:46:17 PDT 2024,"Charge at pH 7"=-6.773009592825655,"Isoelectric Point"=5.118595123291016,"% Basic Amino Acids"=14.49%,"% AT-rich Amino Acids"=23.55%,"% Polar Uncharged Amino Acids"=25.72%,"Free end gaps"=true,"Molecule Type"="AA"]

GAX85982.1[&Description="GAX85982.1"]

XP_042923301.1[&Description="uncharacterized protein CHLRE_06g250650v5 [Chlamydomonas reinhardtii]"]

XP_042924848.1[&Description="uncharacterized protein CHLRE_05g237200v5 [Chlamydomonas reinhardtii]"]

KAG2488600.1[&Description="KAG2488600.1"]

XP_042924875.1[&Description="uncharacterized protein CHLRE_05g238290v5 [Chlamydomonas reinhardtii]"]

KAJ9515210.1[&Description="KAJ9515210.1"]

KAI3646081.1[&Organism="Amoeboaphelidium protococcarum",Description="hypothetical protein MP228_009009 [Amoeboaphelidium protococcarum]","Genetic Code"="Ciliate",Taxonomy="Eukaryota; Aphelida; Aphelidea; Amoeboaphelidium"]

OAJ38670.1[&Organism="Batrachochytrium dendrobatidis JEL423",Description="hypothetical protein BDEG_22578 [Batrachochytrium dendrobatidis JEL423]","Genetic Code"="Standard",Taxonomy="Eukaryota; Fungi; Fungi incertae sedis; Chytridiomycota; Chytridiomycota incertae sedis; Chytridiomycetes; Rhizophydiales; Rhizophydiales incertae sedis; Batrachochytrium"]

XP_006461472.1[&Organism="Agaricus bisporus var. bisporus H97",Description="hypothetical protein AGABI2DRAFT_222252 [Agaricus bisporus var. bisporus H97]","Genetic Code"="Standard",Taxonomy="Eukaryota; Fungi; Dikarya; Basidiomycota; Agaricomycotina; Agaricomycetes; Agaricomycetidae; Agaricales; Agaricaceae; Agaricus"]

XP_006461433.1[&Organism="Agaricus bisporus var. bisporus H97",Description="hypothetical protein AGABI2DRAFT_185678 [Agaricus bisporus var. bisporus H97]","Genetic Code"="Standard",Taxonomy="Eukaryota; Fungi; Dikarya; Basidiomycota; Agaricomycotina; Agaricomycetes; Agaricomycetidae; Agaricales; Agaricaceae; Agaricus"]

XP_006457072.1[&Organism="Agaricus bisporus var. bisporus H97",Description="hypothetical protein AGABI2DRAFT_139599 [Agaricus bisporus var. bisporus H97]","Genetic Code"="Standard",Taxonomy="Eukaryota; Fungi; Dikarya; Basidiomycota; Agaricomycotina; Agaricomycetes; Agaricomycetidae; Agaricales; Agaricaceae; Agaricus"]

XP_750654.1[&Organism="Aspergillus fumigatus Af293",Description="dynamin GTPase, putative [Aspergillus fumigatus Af293]","Genetic Code"="Standard",Taxonomy="Eukaryota; Fungi; Dikarya; Ascomycota; Pezizomycotina; Eurotiomycetes; Eurotiomycetidae; Eurotiales; Aspergillaceae; Aspergillus; Aspergillus subgen. Fumigati"]

PAA68234.1[&Organism="Macrostomum lignano",Description="hypothetical protein BOX15_Mlig021919g1, partial [Macrostomum lignano]","Genetic Code"="Standard",Taxonomy="Eukaryota; Metazoa; Platyhelminthes; Rhabditophora; Macrostomorpha; Macrostomida; Macrostomidae; Macrostomum"]

PAA87312.1[&Organism="Macrostomum lignano",Description="hypothetical protein BOX15_Mlig004017g1, partial [Macrostomum lignano]","Genetic Code"="Standard",Taxonomy="Eukaryota; Metazoa; Platyhelminthes; Rhabditophora; Macrostomorpha; Macrostomida; Macrostomidae; Macrostomum"]

XP_002602331.1[&Organism="Branchiostoma floridae",Description="hypothetical protein BRAFLDRAFT_60684 [Branchiostoma floridae]","Genetic Code"="Standard",Taxonomy="Eukaryota; Metazoa; Chordata; Cephalochordata; Branchiostomidae; Branchiostoma","Common Name"="Florida lancelet"]

XP_019637857.1[&Organism="Branchiostoma belcheri",Description="PREDICTED: dynamin-like 120 kDa protein, mitochondrial isoform X3 [Branchiostoma belcheri]","Genetic Code"="Standard",Taxonomy="Eukaryota; Metazoa; Chordata; Cephalochordata; Branchiostomidae; Branchiostoma","Common Name"="Belcher's lancelet"]

XP_006813643.1[&Organism="Saccoglossus kowalevskii",Description="PREDICTED: dynamin-like 120 kDa protein, mitochondrial-like [Saccoglossus kowalevskii]","Genetic Code"="Standard",Taxonomy="Eukaryota; Metazoa; Hemichordata; Enteropneusta; Harrimaniidae; Saccoglossus"]

XP_030843280.1[&Organism="Strongylocentrotus purpuratus",Description="dynamin-like 120 kDa protein, mitochondrial [Strongylocentrotus purpuratus]","Genetic Code"="Standard",Taxonomy="Eukaryota; Metazoa; Echinodermata; Eleutherozoa; Echinozoa; Echinoidea; Euechinoidea; Echinacea; Echinoida; Strongylocentrotidae; Strongylocentrotus","Common Name"="purple sea urchin"]

XP_018667792.1[&Organism="Ciona intestinalis",Description="dynamin-like 120 kDa protein, mitochondrial [Ciona intestinalis]","Genetic

Code"="Standard",Taxonomy="Eukaryota; Metazoa; Chordata; Tunicata; Ascidiacea; Enterogona; Phlebobranchia; Cionidae; Ciona","Common Name"="vase tunicate"]

XP_032818114.1[&Organism="Petromyzon marinus",Description="dynamin-like 120 kDa protein, mitochondrial isoform X2 [Petromyzon marinus]","Genetic Code"="Standard",Taxonomy="Eukaryota; Metazoa; Chordata; Craniata; Vertebrata; Cyclostomata; Hyperoartia; Petromyzontiformes; Petromyzontidae; Petromyzon","Common Name"="sea lamprey"]

XP_021332524.1[&Organism="Danio rerio",Description="dynamin-like 120 kDa protein, mitochondrial isoform X5 [Danio rerio]","Genetic Code"="Standard",Taxonomy="Eukaryota; Metazoa; Chordata; Craniata; Vertebrata; Euteleostomi; Actinopterygii; Neopterygii; Teleostei; Ostariophysi; Cypriniformes; Cyprinidae; Danio","Common Name"="zebrafish"]

XP_028587646.1[&Organism="Podarcis muralis",Description="LOW QUALITY PROTEIN: dynamin-like 120 kDa protein, mitochondrial [Podarcis muralis]","Genetic Code"="Standard",Taxonomy="Eukaryota; Metazoa; Chordata; Craniata; Vertebrata; Euteleostomi; Lepidosauria; Squamata; Bifurcata; Unidentata; Episquamata; Laterata; Lacertibaenia; Lacertidae; Podarcis","Common Name"="Common wall lizard"]

XP_025913835.1[&Organism="Apteryx rowi",Description="dynamin-like 120 kDa protein, mitochondrial isoform X10 [Apteryx rowi]","Genetic Code"="Standard",Taxonomy="Eukaryota; Metazoa; Chordata; Craniata; Vertebrata; Euteleostomi; Archelosauria; Archosauria; Dinosauria; Saurischia; Theropoda; Coelurosauria; Aves; Palaeognathae; Apterygiformes; Apterygidae; Apteryx","Common Name"="Okarito brown kiwi"]

XP_023440724.1[&Organism="Dasypus novemcinctus",Description="dynamin-like 120 kDa protein, mitochondrial [Dasypus novemcinctus]","Genetic Code"="Standard",Taxonomy="Eukaryota; Metazoa; Chordata; Craniata; Vertebrata; Euteleostomi; Mammalia; Eutheria; Xenarthra; Cingulata; Dasypodidae; Dasypus","Common Name"="nine-banded armadillo"]

XP_005873264.1[&Organism="Myotis brandtii",Description="PREDICTED: dynamin-like 120 kDa protein, mitochondrial isoform X5 [Myotis brandtii]","Genetic Code"="Standard",Taxonomy="Eukaryota; Metazoa; Chordata; Craniata; Vertebrata; Euteleostomi; Mammalia; Eutheria; Laurasiatheria; Chiroptera; Microchiroptera; Vespertilionidae; Myotis","Common Name"="Brandt's bat"]

XP_006163024.2.2[&Organism="Tupaia chinensis",Description="LOW QUALITY PROTEIN: dynamin-like 120 kDa protein, mitochondrial [Tupaia chinensis]","Genetic Code"="Standard",Taxonomy="Eukaryota; Metazoa; Chordata; Craniata; Vertebrata; Euteleostomi; Mammalia; Eutheria; Euarchontoglires; Scandentia; Tupaiidae; Tupaia","Common Name"="Chinese tree shrew"]

NP_056375.2.2[&Organism="Homo sapiens",Description="dynamin-like 120 kDa protein, mitochondrial isoform 1 preproprotein [Homo sapiens]","Genetic Code"="Standard",Taxonomy="Eukaryota; Metazoa; Chordata; Craniata; Vertebrata; Euteleostomi; Mammalia; Eutheria; Euarchontoglires; Primates; Haplorrhini; Catarrhini; Hominidae; Homo","Common Name"="human"]

NP_598513.1[&Organism="Mus musculus",Description="dynamin-like 120 kDa protein, mitochondrial isoform 2 precursor [Mus musculus]","Genetic Code"="Standard",Taxonomy="Eukaryota; Metazoa; Chordata; Craniata; Vertebrata; Euteleostomi; Mammalia; Eutheria; Euarchontoglires; Glires; Rodentia; Myomorpha; Muroidea; Muridae; Murinae; Mus; Mus","Common Name"="house mouse"]

XP_031757388.1[&Organism="Xenopus tropicalis",Description="dynamin-like 120 kDa protein, mitochondrial isoform X3 [Xenopus tropicalis]","Genetic Code"="Standard",Taxonomy="Eukaryota; Metazoa; Chordata; Craniata; Vertebrata; Euteleostomi; Amphibia; Batrachia; Anura; Pipoidea; Pipidae; Xenopodinae; Xenopus; Silurana","Common Name"="tropical clawed frog"]

NP_495986.3.3[&Organism="Caenorhabditis elegans",Description="Dynamin-type G domain-containing protein [Caenorhabditis elegans]","Genetic Code"="Standard",Taxonomy="Eukaryota; Metazoa; Ecdysozoa; Nematoda; Chromadorea; Rhabditida; Rhabditina; Rhabditomorpha; Rhabditoidea; Rhabditidae; Peloderinae; Caenorhabditis"]

NP_610941.1[&Organism="Drosophila melanogaster",Description="optic atrophy 1, isoform B [Drosophila melanogaster]","Genetic Code"="Standard",Taxonomy="Eukaryota; Metazoa; Ecdysozoa; Arthropoda; Hexapoda; Insecta; Pterygota; Neoptera; Holometabola; Diptera; Brachycera; Muscomorpha; Ephydroidea; Drosophilidae; Drosophila; Sophophora","Common Name"="fruit fly"]

EFJ33653.1[&Organism="Selaginella moellendorffii",Description="hypothetical protein SELMODRAFT_439053 [Selaginella moellendorffii]","Genetic Code"="Standard",Taxonomy="Eukaryota; Viridiplantae; Streptophyta; Embryophyta; Tracheophyta; Lycopodiopsida; Selaginellales; Selaginellaceae; Selaginella"]

EFJ28901.1[&Organism="Selaginella moellendorffii",Description="hypothetical protein SELMODRAFT_171046 [Selaginella moellendorffii]","Genetic Code"="Standard",Taxonomy="Eukaryota; Viridiplantae; Streptophyta; Embryophyta; Tracheophyta; Lycopodiopsida; Selaginellales; Selaginellaceae; Selaginella"]

KAG0632288.1[&Organism="Ceratodon purpureus",Description="hypothetical protein M758_1G317400 [Ceratodon purpureus]","Genetic Code"="Standard",Taxonomy="Eukaryota; Viridiplantae; Streptophyta; Embryophyta; Bryophyta; Bryophytina; Bryopsida; Dicranidae; Pseudoditrichales; Ditrichaceae; Ceratodon"]

'KAG0555995.1'[&Organism="Ceratodon purpureus",Description="hypothetical protein KC19_11G018700 [Ceratodon purpureus]","Genetic Code"="Standard",Taxonomy="Eukaryota; Viridiplantae; Streptophyta; Embryophyta; Bryophyta; Bryophytina; Bryopsida; Dicranidae; Pseudoditrichales; Ditrichaceae; Ceratodon"]

XP_024391061.1[&Organism="Physcomitrium patens",Description="dynamin-2B-like [Physcomitrium patens]","Genetic Code"="Standard",Taxonomy="Eukaryota; Viridiplantae; Streptophyta; Embryophyta; Bryophyta; Bryophytina; Bryopsida; Funariidae; Funariales; Funariaceae; Physcomitrium"]

XP_024368367.1[&Organism="Physcomitrium patens",Description="dynamin-2A-like [Physcomitrium patens]","Genetic Code"="Standard",Taxonomy="Eukaryota;

Viridiplantae; Streptophyta; Embryophyta; Bryophyta; Bryophytina; Bryopsida; Funariidae; Funariales; Funariaceae; Physcomitrium"]

OAE31801.1[&Organism="Marchantia polymorpha subsp. ruderalis",Description="hypothetical protein AXG93_1838s1110 [Marchantia polymorpha subsp. ruderalis]","Genetic Code"="Standard",Taxonomy="Eukaryota; Viridiplantae; Streptophyta; Embryophyta; Marchantiophyta; Marchantiopsida; Marchantiidae; Marchantiales; Marchantiaceae; Marchantia"]

KAI5073815.1[&Organism="Adiantum capillus-veneris",Description="hypothetical protein GOP47_0011828 [Adiantum capillus-veneris]","Genetic Code"="Standard",Taxonomy="Eukaryota; Viridiplantae; Streptophyta; Embryophyta; Tracheophyta; Polypodiopsida; Polypodiidae; Polypodiales; Pteridineae; Pteridaceae; Vittarioideae; Adiantum"]

XP_008646219.1[&Organism="Zea mays",Description="dynamin-2A [Zea mays]","Genetic Code"="Standard",Taxonomy="Eukaryota; Viridiplantae; Streptophyta; Embryophyta; Tracheophyta; Spermatophyta; Magnoliopsida; Liliopsida; Poales; Poaceae; PACMAD clade; Panicoideae; Andropogonodae; Andropogoneae; Tripsacinae; Zea"]

ACG47836.1[&Organism="Zea mays",Description="dynamin-2A [Zea mays]","Genetic Code"="Standard",Taxonomy="Eukaryota; Viridiplantae; Streptophyta; Embryophyta; Tracheophyta; Spermatophyta; Magnoliopsida; Liliopsida; Poales; Poaceae; PACMAD clade; Panicoideae; Andropogonodae; Andropogoneae; Tripsacinae; Zea"]

'KAG7649995.1'[&Organism="Arabidopsis thaliana",Description="DRP2B [Arabidopsis thaliana]","Genetic Code"="Standard",Taxonomy="Eukaryota; Viridiplantae; Streptophyta; Embryophyta; Tracheophyta; Spermatophyta; Magnoliopsida; eudicotyledons; Gunneridae; Pentapetalae; rosids; malvids; Brassicales; Brassicaceae; Camelineae; Arabidopsis","Common Name"="thale cress"]

'NP_172500.1'[&Organism="Arabidopsis thaliana",Description="unnamed protein product [Arabidopsis thaliana]","Genetic Code"="Standard",Taxonomy="Eukaryota; Viridiplantae; Streptophyta; Embryophyta; Tracheophyta; Spermatophyta; Magnoliopsida; eudicotyledons; Gunneridae; Pentapetalae; rosids; malvids; Brassicales; Brassicaceae; Camelineae; Arabidopsis","Common Name"="thale cress"]

XP_006385192.1[&Organism="Populus trichocarpa",Description="dynamin-2A [Populus trichocarpa]","Genetic Code"="Standard",Taxonomy="Eukaryota; Viridiplantae; Streptophyta; Embryophyta; Tracheophyta; Spermatophyta; Magnoliopsida; eudicotyledons; Gunneridae; Pentapetalae; rosids; fabids; Malpighiales; Salicaceae; Saliceae; Populus","Common Name"="Populus balsamifera subsp. trichocarpa"]

KAH9330549.1[&Organism="Taxus chinensis",Description="hypothetical protein KI387_002657 [Taxus chinensis]","Genetic Code"="Standard",Taxonomy="Eukaryota; Viridiplantae; Streptophyta; Embryophyta; Tracheophyta; Spermatophyta; Pinopsida; Pinidae; Conifers II; Cupressales; Taxaceae; Taxus"]

ONM04707.1[&Organism="Zea mays",Description="Dynamin-2A [Zea mays]","Genetic Code"="Standard",Taxonomy="Eukaryota; Viridiplantae; Streptophyta; Embryophyta; Tracheophyta; Spermatophyta; Magnoliopsida; Liliopsida; Poales; Poaceae; PACMAD clade; Panicoideae; Andropogonodae; Andropogoneae; Tripsacinae; Zea"]

XP_042918632.1[&Organism="Chlamydomonas reinhardtii",Description="uncharacterized protein CHLRE_12g529450v5 [Chlamydomonas reinhardtii]","Genetic Code"="Standard",Taxonomy="Eukaryota; Viridiplantae; Chlorophyta; core chlorophytes; Chlorophyceae; CS clade; Chlamydomonadales; Chlamydomonadaceae; Chlamydomonas"]

NP_001130364.1[&Organism="Zea mays",Description="Dynamin-related protein 5A-like [Zea mays]","Genetic Code"="Standard",Taxonomy="Eukaryota; Viridiplantae; Streptophyta; Embryophyta; Tracheophyta; Spermatophyta; Magnoliopsida; Liliopsida; Poales; Poaceae; PACMAD clade; Panicoideae; Andropogonodae; Andropogoneae; Tripsacinae; Zea"]

PWZ11893.1[&Organism="Zea mays",Description="Dynamin-related protein 5A [Zea mays]","Genetic Code"="Standard",Taxonomy="Eukaryota; Viridiplantae; Streptophyta; Embryophyta; Tracheophyta; Spermatophyta; Magnoliopsida; Liliopsida; Poales; Poaceae; PACMAD clade; Panicoideae; Andropogonodae; Andropogoneae; Tripsacinae; Zea"]

XP_002317496.2[&Organism="Populus trichocarpa",Description="dynamin-related protein 5A [Populus trichocarpa]","Genetic Code"="Standard",Taxonomy="Eukaryota; Viridiplantae; Streptophyta; Embryophyta; Tracheophyta; Spermatophyta; Magnoliopsida; eudicotyledons; Gunneridae; Pentapetalae; rosids; fabids; Malpighiales; Salicaceae; Saliceae; Populus","Common Name"="Populus balsamifera subsp. trichocarpa"]

AAF87857.1[&Organism="Arabidopsis thaliana",Description="Hypothetical protein [Arabidopsis thaliana]","Genetic Code"="Standard",Taxonomy="Eukaryota; Viridiplantae; Streptophyta; Embryophyta; Tracheophyta; Spermatophyta; Magnoliopsida; eudicotyledons; Gunneridae; Pentapetalae; rosids; malvids; Brassicales; Brassicaceae; Camelineae; Arabidopsis","Common Name"="thale cress"]

KAI5058044.1[&Organism="Adiantum capillus-veneris",Description="hypothetical protein GOP47_0026214 [Adiantum capillus-veneris]","Genetic Code"="Standard",Taxonomy="Eukaryota; Viridiplantae; Streptophyta; Embryophyta; Tracheophyta; Polypodiopsida; Polypodiidae; Polypodiales; Pteridineae; Pteridaceae; Vittarioideae; Adiantum"]

EFJ18064.1[&Organism="Selaginella moellendorffii",Description="hypothetical protein SELMODRAFT_113285 [Selaginella moellendorffii]","Genetic Code"="Standard",Taxonomy="Eukaryota; Viridiplantae; Streptophyta; Embryophyta; Tracheophyta; Lycopodiopsida; Selaginellales; Selaginellaceae; Selaginella"]

KAG0628798.1[&Organism="Ceratodon purpureus",Description="hypothetical protein M758_1G053700 [Ceratodon purpureus]","Genetic Code"="Standard",Taxonomy="Eukaryota; Viridiplantae; Streptophyta; Embryophyta; Bryophyta; Bryophytina; Bryopsida; Dicranidae; Pseudoditrichales; Ditrichaceae; Ceratodon"]

PTQ33908.1[&Organism="Marchantia polymorpha",Description="hypothetical protein MARPO_0084s0004 [Marchantia polymorpha]","Genetic Code"="Standard",Taxonomy="Eukaryota; Viridiplantae; Streptophyta; Embryophyta; Marchantiophyta; Marchantiopsida; Marchantiidae; Marchantiales; Marchantiaceae; Marchantia","Common Name"="liverwort"]

KAH9322298.1[&Organism="Taxus chinensis",Description="hypothetical protein KI387_016937, partial [Taxus chinensis]","Genetic Code"="Standard",Taxonomy="Eukaryota; Viridiplantae; Streptophyta; Embryophyta; Tracheophyta; Spermatophyta; Pinopsida; Pinidae; Conifers II; Cupressales; Taxaceae; Taxus"]

XP_009032466.1[&Organism="Aureococcus anophagefferens",Description="hypothetical protein AURANDRAFT_60924 [Aureococcus anophagefferens]","Genetic Code"="Standard",Taxonomy="Eukaryota; Sar; Stramenopiles; Ochrophyta; Pelagophyceae; Pelagomonadales; Aureococcus"]

XP_042920073.1[&Organism="Chlamydomonas reinhardtii",Description="uncharacterized protein CHLRE_10g433050v5 [Chlamydomonas reinhardtii]","Genetic Code"="Standard",Taxonomy="Eukaryota; Viridiplantae; Chlorophyta; core chlorophytes; Chlorophyceae; CS clade; Chlamydomonadales; Chlamydomonadaceae; Chlamydomonas"]

PWZ44616.1[&Organism="Zea mays",Description="Dynamin-like protein ARC5 [Zea mays]","Genetic Code"="Standard",Taxonomy="Eukaryota; Viridiplantae; Streptophyta; Embryophyta; Tracheophyta; Spermatophyta; Magnoliopsida; Liliopsida; Poales; Poaceae; PACMAD clade; Panicoideae; Andropogonodae; Andropogoneae; Tripsacinae; Zea"]

XP_002309632.3[&Organism="Populus trichocarpa",Description="dynamin-like protein ARC5 [Populus trichocarpa]","Genetic Code"="Standard",Taxonomy="Eukaryota; Viridiplantae; Streptophyta; Embryophyta; Tracheophyta; Spermatophyta; Magnoliopsida; eudicotyledons; Gunneridae; Pentapetalae; rosids; fabids; Malpighiales; Salicaceae; Saliceae; Populus","Common Name"="Populus balsamifera subsp. trichocarpa"]

NP_001189935.1[&Organism="Arabidopsis thaliana",Description="P-loop containing nucleoside triphosphate hydrolases superfamily protein [Arabidopsis thaliana]","Genetic Code"="Standard",Taxonomy="Eukaryota; Viridiplantae; Streptophyta; Embryophyta; Tracheophyta; Spermatophyta; Magnoliopsida; eudicotyledons; Gunneridae; Pentapetalae; rosids; malvids; Brassicales; Brassicaceae; Camelineae; Arabidopsis","Common Name"="thale cress"]

EFJ19523.1[&Organism="Selaginella moellendorffii",Description="hypothetical protein SELMODRAFT_110974 [Selaginella moellendorffii]","Genetic Code"="Standard",Taxonomy="Eukaryota; Viridiplantae; Streptophyta; Embryophyta; Tracheophyta; Lycopodiopsida; Selaginellales; Selaginellaceae; Selaginella"]

KAI5064281.1[&Organism="Adiantum capillus-veneris",Description="hypothetical protein GOP47_0020951 [Adiantum capillus-veneris]","Genetic Code"="Standard",Taxonomy="Eukaryota; Viridiplantae; Streptophyta; Embryophyta; Tracheophyta; Polypodiopsida; Polypodiidae; Polypodiales; Pteridineae; Pteridaceae; Vittarioideae; Adiantum"]

PTQ34556.1[&Organism="Marchantia polymorpha",Description="hypothetical protein MARPO_0079s0059 [Marchantia polymorpha]","Genetic Code"="Standard",Taxonomy="Eukaryota; Viridiplantae; Streptophyta; Embryophyta; Marchantiophyta; Marchantiopsida; Marchantiidae; Marchantiales; Marchantiaceae; Marchantia","Common Name"="liverwort"]

KAH9308354.1[&Organism="Taxus chinensis",Description="hypothetical protein KI387_036265, partial [Taxus chinensis]","Genetic Code"="Standard",Taxonomy="Eukaryota; Viridiplantae; Streptophyta; Embryophyta; Tracheophyta; Spermatophyta; Pinopsida; Pinidae; Conifers II; Cupressales; Taxaceae; Taxus"]

KAG0561482.1[&Organism="Ceratodon purpureus",Description="hypothetical protein KC19_9G067700 [Ceratodon purpureus]","Genetic Code"="Standard",Taxonomy="Eukaryota; Viridiplantae; Streptophyta; Embryophyta; Bryophyta; Bryophytina; Bryopsida; Dicranidae; Pseudoditrichales; Ditrichaceae; Ceratodon"]

KAG0605142.1[&Organism="Ceratodon purpureus",Description="hypothetical protein M758_9G034900 [Ceratodon purpureus]","Genetic Code"="Standard",Taxonomy="Eukaryota; Viridiplantae; Streptophyta; Embryophyta; Bryophyta; Bryophytina; Bryopsida; Dicranidae; Pseudoditrichales; Ditrichaceae; Ceratodon"]

XP_042916771.1[&Organism="Chlamydomonas reinhardtii",Description="uncharacterized protein CHLRE_14g616600v5 [Chlamydomonas reinhardtii]","Genetic Code"="Standard",Taxonomy="Eukaryota; Viridiplantae; Chlorophyta; core chlorophytes; Chlorophyceae; CS clade; Chlamydomonadales; Chlamydomonadaceae; Chlamydomonas"]

EFJ26018.1[&Organism="Selaginella moellendorffii",Description="hypothetical protein SELMODRAFT_413416 [Selaginella moellendorffii]","Genetic Code"="Standard",Taxonomy="Eukaryota; Viridiplantae; Streptophyta; Embryophyta; Tracheophyta; Lycopodiopsida; Selaginellales; Selaginellaceae; Selaginella"]

KAG0631008.1[&Organism="Ceratodon purpureus",Description="hypothetical protein M758_1G220200 [Ceratodon purpureus]","Genetic Code"="Standard",Taxonomy="Eukaryota; Viridiplantae; Streptophyta; Embryophyta; Bryophyta; Bryophytina; Bryopsida; Dicranidae; Pseudoditrichales; Ditrichaceae; Ceratodon"]

OAE29693.1[&Organism="Marchantia polymorpha subsp. ruderalis",Description="hypothetical protein AXG93_509s1460 [Marchantia polymorpha subsp. ruderalis]","Genetic Code"="Standard",Taxonomy="Eukaryota; Viridiplantae; Streptophyta; Embryophyta; Marchantiophyta; Marchantiopsida; Marchantiidae; Marchantiales; Marchantiaceae; Marchantia"]

KAH9315399.1[&Organism="Taxus chinensis",Description="hypothetical protein KI387_024026, partial [Taxus chinensis]","Genetic Code"="Standard",Taxonomy="Eukaryota; Viridiplantae; Streptophyta; Embryophyta; Tracheophyta; Spermatophyta; Pinopsida; Pinidae; Conifers II; Cupressales; Taxaceae; Taxus"]

KAI5059498.1[&Organism="Adiantum capillus-veneris",Description="hypothetical protein GOP47_0025817 [Adiantum capillus-veneris]","Genetic Code"="Standard",Taxonomy="Eukaryota; Viridiplantae; Streptophyta; Embryophyta; Tracheophyta; Polypodiopsida; Polypodiidae; Polypodiales; Pteridineae; Pteridaceae; Vittarioideae; Adiantum"]

CAD5311589.1[&Organism="Arabidopsis thaliana",Description="unnamed protein product [Arabidopsis thaliana]","Genetic Code"="Standard",Taxonomy="Eukaryota; Viridiplantae; Streptophyta; Embryophyta; Tracheophyta; Spermatophyta; Magnoliopsida; eudicotyledons; Gunneridae; Pentapetalae; rosids; malvids; Brassicales; Brassicaceae; Camelineae; Arabidopsis","Common Name"="thale cress"]

XP_008649599.1[&Organism="Zea mays",Description="probable transmembrane GTPase FZO-like, chloroplastic [Zea mays]","Genetic Code"="Standard",Taxonomy="Eukaryota; Viridiplantae; Streptophyta; Embryophyta; Tracheophyta; Spermatophyta; Magnoliopsida; Liliopsida; Poales; Poaceae; PACMAD clade; Panicoideae; Andropogonodae; Andropogoneae; Tripsacinae; Zea"]

OUM66167.1[&Organism="Piromyces sp. E2",Description="hypothetical protein PIROE2DRAFT_59551 [Piromyces sp. E2]","Genetic Code"="Standard",Taxonomy="Eukaryota; Fungi; Fungi incertae sedis; Chytridiomycota; Chytridiomycota incertae sedis; Neocallimastigomycetes; Neocallimastigales; Neocallimastigaceae; Piromyces"]

XP_001481516.1[&Organism="Aspergillus fumigatus Af293",Description="transmembrane GTPase Fzo1, putative [Aspergillus fumigatus Af293]","Genetic Code"="Standard",Taxonomy="Eukaryota; Fungi; Dikarya; Ascomycota; Pezizomycotina; Eurotiomycetes; Eurotiomycetidae; Eurotiales; Aspergillaceae; Aspergillus; Aspergillus subgen. Fumigati"]

XP_011392385.1[&Organism="Ustilago maydis 521",Description="GTP-binding protein [Ustilago maydis 521]","Genetic Code"="Standard",Taxonomy="Eukaryota; Fungi; Dikarya; Basidiomycota; Ustilaginomycotina; Ustilaginomycetes; Ustilaginales; Ustilaginaceae; Ustilago"]

XP_006462464.1[&Organism="Agaricus bisporus var. bisporus H97",Description="hypothetical protein AGABI2DRAFT_119324 [Agaricus bisporus var. bisporus H97]","Genetic Code"="Standard",Taxonomy="Eukaryota; Fungi; Dikarya; Basidiomycota; Agaricomycotina; Agaricomycetes; Agaricomycetidae; Agaricales; Agaricineae; Agaricaceae; Agaricus"]

XP_006676761.1[&Organism="Batrachochytrium dendrobatidis JAM81",Description="mitofusin [Batrachochytrium dendrobatidis JAM81]","Genetic Code"="Standard",Taxonomy="Eukaryota; Fungi; Fungi incertae sedis; Chytridiomycota; Chytridiomycota incertae sedis; Chytridiomycetes; Rhizophydiales; Rhizophydiales incertae sedis; Batrachochytrium"]

KXN69997.1[&Organism="Conidiobolus coronatus NRRL 28638",Description="hypothetical protein CONCODRAFT_79060 [Conidiobolus coronatus NRRL 28638]","Genetic Code"="Standard",Taxonomy="Eukaryota; Fungi; Fungi incertae sedis; Zoopagomycota; Entomophthoromycotina; Entomophthoromycetes; Entomophthorales; Ancylistaceae; Conidiobolus"]

NP_009738.1[&Organism="Saccharomyces cerevisiae S288C",Description="mitofusin [Saccharomyces cerevisiae S288C]","Genetic Code"="Standard",Taxonomy="Eukaryota; Fungi; Dikarya; Ascomycota; Saccharomycotina; Saccharomycetes; Saccharomycetales; Saccharomycetaceae; Saccharomyces"]

KNE73082.1[&Organism="Allomyces macrogynus ATCC 38327",Description="hypothetical protein AMAG_17236 [Allomyces macrogynus ATCC 38327]","Genetic Code"="Standard",Taxonomy="Eukaryota; Fungi; Fungi incertae sedis; Blastocladiomycota; Blastocladiomycota incertae sedis; Blastocladiomycetes; Blastocladiales; Blastocladiaceae; Allomyces"]

KNE65701.1[&Organism="Allomyces macrogynus ATCC 38327",Description="hypothetical protein AMAG_09684 [Allomyces macrogynus ATCC 38327]","Genetic Code"="Standard",Taxonomy="Eukaryota; Fungi; Fungi incertae sedis; Blastocladiomycota; Blastocladiomycota incertae sedis; Blastocladiomycetes; Blastocladiales; Blastocladiaceae; Allomyces"]

XP_001745740.1[&Organism="Monosiga brevicollis MX1",Description="uncharacterized protein MONBRDRAFT_25320 [Monosiga brevicollis MX1]","Genetic Code"="Standard",Taxonomy="Eukaryota; Choanoflagellata; Craspedida; Salpingoecidae; Monosiga"]

NP_495161.1[&Organism="Caenorhabditis elegans",Description="Transmembrane GTPase fzo-1 [Caenorhabditis elegans]","Genetic Code"="Standard",Taxonomy="Eukaryota; Metazoa; Ecdysozoa; Nematoda; Chromadorea; Rhabditida; Rhabditina; Rhabditomorpha; Rhabditoidea; Rhabditidae; Peloderinae; Caenorhabditis"]

PAA75551.1[&Organism="Macrostomum lignano",Description="hypothetical protein BOX15_Mlig004622g1, partial [Macrostomum lignano]","Genetic Code"="Standard",Taxonomy="Eukaryota; Metazoa; Platyhelminthes; Rhabditophora; Macrostomorpha; Macrostomida; Macrostomidae; Macrostomum"]

PAA75258.1[&Organism="Macrostomum lignano",Description="hypothetical protein BOX15_Mlig033579g1, partial [Macrostomum lignano]","Genetic Code"="Standard",Taxonomy="Eukaryota; Metazoa; Platyhelminthes; Rhabditophora; Macrostomorpha; Macrostomida; Macrostomidae; Macrostomum"]

XP_002126852.1[&Organism="Ciona intestinalis",Description="mitofusin-2 [Ciona intestinalis]","Genetic Code"="Standard",Taxonomy="Eukaryota; Metazoa; Chordata; Tunicata; Ascidiacea; Enterogona; Phlebobranchia; Cionidae; Ciona","Common Name"="vase tunicate"]

XP_006819998.1[&Organism="Saccoglossus kowalevskii",Description="PREDICTED: mitofusin-2-like [Saccoglossus kowalevskii]","Genetic Code"="Standard",Taxonomy="Eukaryota; Metazoa; Hemichordata; Enteropneusta; Harrimaniidae; Saccoglossus"]

XP_002591612.1[&Organism="Branchiostoma floridae",Description="hypothetical protein BRAFLDRAFT_223384 [Branchiostoma floridae]","Genetic Code"="Standard",Taxonomy="Eukaryota; Metazoa; Chordata; Cephalochordata; Branchiostomidae; Branchiostoma","Common Name"="Florida lancelet"]

XP_019628129.1[&Organism="Branchiostoma belcheri",Description="PREDICTED: mitofusin-2-like [Branchiostoma belcheri]","Genetic Code"="Standard",Taxonomy="Eukaryota; Metazoa; Chordata; Cephalochordata; Branchiostomidae; Branchiostoma","Common Name"="Belcher's lancelet"]

NP_001121726.1[&Organism="Danio rerio",Description="mitofusin-2 [Danio rerio]","Genetic Code"="Standard",Taxonomy="Eukaryota; Metazoa; Chordata; Craniata;

Vertebrata; Euteleostomi; Actinopterygii; Neopterygii; Teleostei; Ostariophysi; Cypriniformes; Danionidae; Danioninae; Danio","Common Name"="zebrafish"]

XP_025929938.1[&Organism="Apteryx rowi",Description="mitofusin-2 [Apteryx rowi]","Genetic Code"="Standard",Taxonomy="Eukaryota; Metazoa; Chordata; Craniata; Vertebrata; Euteleostomi; Archelosauria; Archosauria; Dinosauria; Saurischia; Theropoda; Coelurosauria; Aves; Palaeognathae; Apterygiformes; Apterygidae; Apteryx","Common Name"="Okarito brown kiwi"]

XP_004482574.1[&Organism="Dasypus novemcinctus",Description="mitofusin-2 [Dasypus novemcinctus]","Genetic Code"="Standard",Taxonomy="Eukaryota; Metazoa; Chordata; Craniata; Vertebrata; Euteleostomi; Mammalia; Eutheria; Xenarthra; Cingulata; Dasypodidae; Dasypus","Common Name"="nine-banded armadillo"]

XP_006145367.1[&Organism="Tupaia chinensis",Description="mitofusin-2 isoform X2 [Tupaia chinensis]","Genetic Code"="Standard",Taxonomy="Eukaryota; Metazoa; Chordata; Craniata; Vertebrata; Euteleostomi; Mammalia; Eutheria; Euarchontoglires; Scandentia; Tupaiidae; Tupaia","Common Name"="Chinese tree shrew"]

XP_014400986.1[&Organism="Myotis brandtii",Description="PREDICTED: mitofusin-2 isoform X2 [Myotis brandtii]","Genetic Code"="Standard",Taxonomy="Eukaryota; Metazoa; Chordata; Craniata; Vertebrata; Euteleostomi; Mammalia; Eutheria; Laurasiatheria; Chiroptera; Microchiroptera; Vespertilionidae; Myotis","Common Name"="Brandt's bat"]

NP_001177198.1[&Organism="Bos taurus",Description="mitofusin-2 [Bos taurus]","Genetic Code"="Standard",Taxonomy="Eukaryota; Metazoa; Chordata; Craniata; Vertebrata; Euteleostomi; Mammalia; Eutheria; Laurasiatheria; Artiodactyla; Ruminantia; Pecora; Bovidae; Bovinae; Bos","Common Name"="cattle"]

NP_001272849.1[&Organism="Mus musculus",Description="mitofusin-2 [Mus musculus]","Genetic Code"="Standard",Taxonomy="Eukaryota; Metazoa; Chordata; Craniata; Vertebrata; Euteleostomi; Mammalia; Eutheria; Euarchontoglires; Glires; Rodentia; Myomorpha; Muroidea; Muridae; Murinae; Mus; Mus","Common Name"="house mouse"]

NP_001121132.1[&Organism="Homo sapiens",Description="mitofusin-2 [Homo sapiens]","Genetic Code"="Standard",Taxonomy="Eukaryota; Metazoa; Chordata; Craniata; Vertebrata; Euteleostomi; Mammalia; Eutheria; Euarchontoglires; Primates; Haplorrhini; Catarrhini; Hominidae; Homo","Common Name"="human"]

XP_015268039.1[&Organism="Gekko japonicus",Description="PREDICTED: mitofusin-2 [Gekko japonicus]","Genetic Code"="Standard",Taxonomy="Eukaryota; Metazoa; Chordata; Craniata; Vertebrata; Euteleostomi; Lepidosauria; Squamata; Bifurcata; Gekkota; Gekkonidae; Gekkoninae; Gekko"]

XP_028597443.1[&Organism="Podarcis muralis",Description="LOW QUALITY PROTEIN: mitofusin-2 [Podarcis muralis]","Genetic Code"="Standard",Taxonomy="Eukaryota; Metazoa; Chordata; Craniata; Vertebrata; Euteleostomi; Lepidosauria; Squamata; Bifurcata; Unidentata; Episquamata; Laterata; Lacertibaenia; Lacertidae; Podarcis","Common Name"="Common wall lizard"]

XP_017213868.2.2[&Organism="Danio rerio",Description="mitofusin-1 isoform X1 [Danio rerio]","Genetic Code"="Standard",Taxonomy="Eukaryota; Metazoa; Chordata;

Craniata; Vertebrata; Euteleostomi; Actinopterygii; Neopterygii; Teleostei; Ostariophysi; Cypriniformes; Cyprinidae; Danio","Common Name"="zebrafish"]

NP_001016189.1[&Organism="Xenopus tropicalis",Description="mitofusin-1 [Xenopus tropicalis]","Genetic Code"="Standard",Taxonomy="Eukaryota; Metazoa; Chordata; Craniata; Vertebrata; Euteleostomi; Amphibia; Batrachia; Anura; Pipoidea; Pipidae; Xenopodinae; Xenopus; Silurana","Common Name"="tropical clawed frog"]

XP_028587453.1[&Organism="Podarcis muralis",Description="mitofusin-1 [Podarcis muralis]","Genetic Code"="Standard",Taxonomy="Eukaryota; Metazoa; Chordata; Craniata; Vertebrata; Euteleostomi; Lepidosauria; Squamata; Bifurcata; Unidentata; Episquamata; Laterata; Lacertibaenia; Lacertidae; Podarcis","Common Name"="Common wall lizard"]

XP_025917892.1[&Organism="Apteryx rowi",Description="mitofusin-1 isoform X1 [Apteryx rowi]","Genetic Code"="Standard",Taxonomy="Eukaryota; Metazoa; Chordata; Craniata; Vertebrata; Euteleostomi; Archelosauria; Archosauria; Dinosauria; Saurischia; Theropoda; Coelurosauria; Aves; Palaeognathae; Apterygiformes; Apterygidae; Apteryx","Common Name"="Okarito brown kiwi"]

XP_004479029.1[&Organism="Dasypus novemcinctus",Description="mitofusin-1 isoform X2 [Dasypus novemcinctus]","Genetic Code"="Standard",Taxonomy="Eukaryota; Metazoa; Chordata; Craniata; Vertebrata; Euteleostomi; Mammalia; Eutheria; Xenarthra; Cingulata; Dasypodidae; Dasypus","Common Name"="nine-banded armadillo"]

NP_001193437.1[&Organism="Bos taurus",Description="mitofusin-1 [Bos taurus]","Genetic Code"="Standard",Taxonomy="Eukaryota; Metazoa; Chordata; Craniata; Vertebrata; Euteleostomi; Mammalia; Eutheria; Laurasiatheria; Artiodactyla; Ruminantia; Pecora; Bovidae; Bovinae; Bos","Common Name"="cattle"]

NP_077162.2.2[&Organism="Mus musculus",Description="mitofusin-1 [Mus musculus]","Genetic Code"="Standard",Taxonomy="Eukaryota; Metazoa; Chordata; Craniata; Vertebrata; Euteleostomi; Mammalia; Eutheria; Euarchontoglires; Glires; Rodentia; Myomorpha; Muroidea; Muridae; Murinae; Mus; Mus","Common Name"="house mouse"]

XP_006162789.1[&Organism="Tupaia chinensis",Description="mitofusin-1 [Tupaia chinensis]","Genetic Code"="Standard",Taxonomy="Eukaryota; Metazoa; Chordata; Craniata; Vertebrata; Euteleostomi; Mammalia; Eutheria; Euarchontoglires; Scandentia; Tupaiidae; Tupaia","Common Name"="Chinese tree shrew"]

XP_005883071.1[&Organism="Myotis brandtii",Description="PREDICTED: mitofusin-1 [Myotis brandtii]","Genetic Code"="Standard",Taxonomy="Eukaryota; Metazoa; Chordata; Craniata; Vertebrata; Euteleostomi; Mammalia; Eutheria; Laurasiatheria; Chiroptera; Microchiroptera; Vespertilionidae; Myotis","Common Name"="Brandt's bat"]

NP_284941.2.2[&Organism="Homo sapiens",Description="mitofusin-1 [Homo sapiens]","Genetic Code"="Standard",Taxonomy="Eukaryota; Metazoa; Chordata; Craniata; Vertebrata; Euteleostomi; Mammalia; Eutheria; Euarchontoglires; Primates; Haplorrhini; Catarrhini; Hominidae; Homo","Common Name"="human"]

XP_030846906.1[&Organism="Strongylocentrotus purpuratus",Description="mitofusin-1-like [Strongylocentrotus purpuratus]","Genetic Code"="Standard",Taxonomy="Eukaryota; Metazoa; Echinodermata; Eleutherozoa;

Echinozoa; Echinoidea; Euechinoidea; Echinacea; Echinoida; Strongylocentrotidae; Strongylocentrotus","Common Name"="purple sea urchin"]
XP_030847518.1[&Organism="Strongylocentrotus purpuratus",Description="mitofusin-2 [Strongylocentrotus purpuratus]","Genetic Code"="Standard",Taxonomy="Eukaryota; Metazoa; Echinodermata; Eleutherozoa; Echinozoa; Echinoidea; Euechinoidea; Echinacea; Echinoida; Strongylocentrotidae; Strongylocentrotus","Common Name"="purple sea urchin"]
NP_996357.1[&Organism="Drosophila melanogaster",Description="mitochondrial assembly regulatory factor, isoform C [Drosophila melanogaster]","Genetic Code"="Standard",Taxonomy="Eukaryota; Metazoa; Ecdysozoa; Arthropoda; Hexapoda; Insecta; Pterygota; Neoptera; Holometabola; Diptera; Brachycera; Muscomorpha; Ephydroidea; Drosophilidae; Drosophila; Sophophora","Common Name"="fruit fly"]
XP_004365821.1[&Organism="Capsaspora owczarzaki ATCC 30864",Description="transmembrane GTPase Marf [Capsaspora owczarzaki ATCC 30864]","Genetic Code"="Standard",Taxonomy="Eukaryota; Filasterea; Capsaspora"]
XP_014153836.1[&Organism="Sphaeroforma arctica JP610",Description="hypothetical protein SARC_07694 [Sphaeroforma arctica JP610]","Genetic Code"="Standard",Taxonomy="Eukaryota; Ichthyosporea; Ichthyophonida; Sphaeroforma"]
;
end;

begin characters;
dimensions nchar=941;
format datatype=protein missing=? gap=-;
matrix
XP_026693152.1 ------------IDLPQIAVVGG----Q----------------SAGKSSVLE-----N---FV-GKDFLPRG---SGIVTRRPL---------VLQ---L-ITAK-NG---EW---G-------------------------------------E----------------FLH---CKG------KKF-----TDFN---EIRKEIEEETDR-----MTGSN-------------------KG-------------------------IS--AIPINLRVHSPH--VLNLTLVDLPGMTK--VP-------------------VGDQPA----------DI---EQQIR--------DMIMQFVVK------------DNCLILAVSPANS--DLA-NS--DALKIAKEFDPQGIRTIGVI-TKLDLMDEGT--------DAKHILENK---H---------------------LPLR-RGYVGV----------------------------------VNR-----------------------------------S-QKDID----G---------------------------NKDIK----------A----ALSA---ERRFF---------LS-------HP--------------------AY-----------------R-H------M--------------------------ADK-LGT-----PYLQKILNQQLTNHIKETL----
XP_030853442.1.2 ------------LDLPQIAVVGG----Q----------------SAGKSSVLE-----N---FV-GRDFLPRG---SGIVTRRPL---------VLQ---L-N-NS-KT---EY---G-------------------------------------E----------------FLH---CKG------KKF-----TDFD---EIRKEIEAETDR-----VTGSN-------------------KG-------------------------IS--NVPINLRVYSPN--VLNLTLVDLPGMTK--IA-------------------VGDQPV----------DI---EIQIR--------SMVMEFVTN------------ESTLILAVSPANQ--DLA-NS--DALKVAKEVDPKGVRTIGVI-TKLDLMDDGT--------

DAKDILENK---L---------------------LPLR-RGYVGV----------------------------------VNR----------------
-------------------S-QRDIE----G---------------------------KKDIK----------A----ALAA---ERKFF---------
LS-------HP--------------------SY------------------R-H------I--------------------------ADK-MGT-----
PWLQKILNQQLTNHIRDSL----
XP_030853442.1 ------------LDLPQIAVVGG----Q----------------SAGKSSVLE-----N---
FV-GRDFLPRG---SGIVTRRPL---------VLQ---L-N-NS-KT---
------------------------------EY---G-------------------------------------E----------------FLH-----------------
---------------------------CKG------KKF-----TDFD---EIRKEIEAETDR-----VTGSN-------------------KG--
------------------------IS--NVPINLRVYSPN---
---VLNLTLVDLPGMTK--IA--------------------VGDQPV----------DI---EIQIR--------SMVMEFVTN------
------ESTLILAVSPANQ--DLA-NS--DALKVAKEVDPKGVRTIGVI-TKLDLMDDGT--------
DAKDILENK---L---------------------LPLR-RGYVGV----------------------------------VNR----------------
-------------------S-QRDIE----G---------------------------KKDIK----------A----ALAA---ERKFF---------
LS-------HP--------------------SY------------------R-H------I--------------------------ADK-MGT-----
PWLQKILNQQLTNHIRDSL----
XP_005165639.1 ------------LDLPQIAVVGG----Q----------------SAGKSSVLE-----N---
FV-GKDFLPRG---SGIVTRRPL---------VLQ---L-I-NC-PT---
-----------------------------EY---A------------------------------------E----------------FLH-------------------
-------------------------CKG------KKF-----TDFD---EVRQEIEAETDR-----ITGQN-------------------KG---
-----------------------IS--PVPINLRVYSPN---
-VLNLTLVDLPGMTK--VP--------------------VGDQPA----------DI---EAQIR--------DMLMQFVTK------
------ENCLLLAVSPANS--DLA-NS--DALKIAKEVDPQGMRTIGVI-TKLDLMDEGT--------
DAREILENK---L---------------------LPLR-RGYIGV----------------------------------VNR------------------
------------------S-QKDID----G--------------------------KKDIT----------A----AMSA---ERKFF---------
LT-------HP--------------------SY------------------R-H------L--------------------------ADR-MGT-----
PYLQKALNQQLTNHIRDTL----
XP_028570166.1 ------------LDLPQIAVVGG----Q----------------SAGKSSVLE-----N---
FV-GRDFLPRG---SGIVTRRPL---------VLQ---L-V-NS-PT--
------------------------------EY---G-------------------------------------E----------------FLH-----------------
---------------------------CKG------KKF-----TDFD---EIRQEIEAETDR-----ITGSN-------------------KG--
------------------------IS--PVPINLRVYSPH---
--VLSLTLVDLPGMTK--VP--------------------VGDQPA----------DI---EFQIR--------EMLMQFVTK------
------ENCLILAVSPANS--DLA-NS--DALKIAKEVDPQGQRTIGVI-TKLDLMDEGT--------
DARDVLENK---L---------------------LPLR-RGYIGV----------------------------------VNR----------------
-------------------S-QKDID----G--------------------------KKDIQ----------A----ALAA---ERKFF---------
LT-------HP--------------------AY------------------R-H------M--------------------------ADR-MGT-----
PYLQKVLNQQLTNHIRDTL----
EPQ17174.1 ------------LDLPQIAVVGG----Q----------------SAGKSSVLE-----N---FV-
GRDFLPRG---SGIVTRRPL---------VLQ---L-V-NA-ST---
-------------------------EY---A-------------------------------------E----------------FLH----------------------
--------------------CKG------KKF-----TDFE---EVRLEIEAETDR-----VTGTN-------------------KG--------
------------------IS--PVPINLRVYSPH---
VLNLTLVDLPGMTK--VP--------------------VGDQPA----------DI---EFQIR--------DMLMQFVTK-------
-----ENCLILAVSPANS--DLA-NS--DALKIAKEVDPQGQRTIGVI-TKLDLMDEGT--------

DARDVLENK---L--------------------LPLR-RGYIGV--------------------------------VNR---------------
------------------S-QKDID----G-------------------------KKDIT---------A----ALAA---ERKFF---------
LS-------HP-------------------SY----------------R-H------L------------------------ADR-MGT-----
PYLQKVLNQQLTNHIRDTL----
BAB27759.1 ------------LDLPQIAVVGG----Q---------------SAGKSSVLE-----N---FV-
GRDFLPRG---SGIVTRRPL---------VLQ---L-V-NS-TT---
------------------------EY---A---------------------------------E---------------FLH---------------------
--------------------CKG------KKF-----TDFE---EVRLEIEAETDR-----VTGTN------------------KG--------
-----------------IS--PVPINLRVYSPH---
VLNLTLVDLPGMTK--VP------------------VGDQPP----------DI---EFQIR--------DMLMQFVTK-------
-----ENCLILAVSPANS--DLA-NS--DALKIAKEVDPQGQRTIGVI-TKLDLMDEGT--------
DARDVLENK---L--------------------LPLR-RGYIGV--------------------------------VNR---------------
------------------S-QKDID----G-------------------------KKDIT---------A----ALAA---ERKFF---------
LS-------HP-------------------SY----------------R-H------L------------------------ADR-MGT-----
PYLQKVLNQQLTNHIRDTL----
ELW62001.1 ------------LDLPQIAVVGG----Q---------------SAGKSSVLE-----N---FV-
GRDFLPRG---SGIVTRRPL---------VLQ---L-V-NA-TT---
------------------------EY---A---------------------------------E---------------FLH---------------------
--------------------CKG------KKF-----TDFE---EVRLEIEAETDR-----VTGTN------------------KG--------
-----------------IS--PVPINLRVYSPH---
VLNLTLVDLPGMTK--VP------------------VGDQPP----------DI---EFQIR--------DMLMQFVTK-------
-----ENCLILAVSPANS--DLA-NS--DALKIAKEVDPQGQRTIGVI-TKLDLMDEGT--------
DARDVLENK---L--------------------LPLR-RGYIGV--------------------------------VNR---------------
------------------S-QKDID----G-------------------------KKDIT---------A----ALAA---ERKFF---------
LS-------HP-------------------SY----------------R-H------L------------------------ADR-MGT-----
PYLQKVLNQQLTNHIRDTL----
EAW87759.1 ------------LDLPQIAVVGG----Q---------------SAGKSSVLE-----N---FV-
GRDFLPRG---SGIVTRRPL---------VLQ---L-V-NA-TT---
------------------------EY---A---------------------------------E---------------FLH---------------------
--------------------CKG------KKF-----TDFE---EVRLEIEAETDR-----VTGTN------------------KG--------
-----------------IS--PVPINLRVYSPH---
VLNLTLVDLPGMTK--VP------------------VGDQPP----------DI---EFQIR--------DMLMQFVTK-------
-----ENCLILAVSPANS--DLA-NS--DALKVAKEVDPQGQRTIGVI-TKLDLMDEGT--------
DARDVLENK---L--------------------LPLR-RGYIGV--------------------------------VNR---------------
------------------S-QKDID----G-------------------------KKDIT---------A----ALAA---ERKFF---------
LS-------HP-------------------SY----------------R-H------L------------------------ADR-MGT-----
PYLQKVLNQQLTNHIRDTL----
XP_025915522.1 --

--MTK--VP------------------VGDQPP----------DI--
-EFQIR--------DMLMQFVTK------------ENCLILAVSPANS--DLA-NS--
DALKIAKEVDPQGQRTIGVI-TKLDLMDEGT--------DARDVLENK---L--------------------LPLR-

RGYIGV--------------------------------VNR--------------------------------S-QKDID----G------------
--------------KKDIQ----------A----ALAA---ERKFF---------LS-------HP------------------AY------------
-----R-H------M-------------------------ADR-MGT-----PYLQKVLNQQLTNHIRDTL----

XP_012378586.1 -------------------AGGPRRRQ---------------SGRAAAAAEPEPERN--
-FV-GRDFLPRG---SGIVTRRPL---------VLQ---L-V-NA-TT--
-----------------------------EY---A----------------------------------E---------------FLH-----------------
------------------------CKG------KKF-----TDFE---EVRLEIEAETDR-----VTGTN------------------KG---
---------------------IS--PVPINLRVYSPH--
-VLNLTLVDLPGMTK--VP-------------------VGDQPP----------DI---EFQIR--------DMLMQFVTK------
------ENCLILAVSPANS--DLA-NS--DALKVAKEVDPQGQRTIGVI-TKLDLMDEGT--------
DARDVLENK---L--------------------LPLR-RGYIGV--------------------------------VNR----------------
-------------------S-QKDID----G--------------------------KKDIT----------A----ALAA---ERKFF---------
LS-------HP-------------------SY-----------------R-H------L-------------------------ADR-MGT-----
PYLQKVLNQQLTNHIRDTL----

KAE8583055.1 ------------LDLPQIAVVGG----Q---------------SAGKSSVLE-----N---
FV-GKDFLPRG---SGIVTRRPL---------VLQ---L-V-NS-ST--
-----------------------------EY---G----------------------------------E---------------FLH-----------------
------------------------CKG------KKF-----TDFD---EIRLEIEAETDR-----ATGTN------------------KG---
---------------------IS--PVPINLRVYSPN--
-VLNLTLVDLPGMTK--VP-------------------VGDQPV----------DI---EFQIR--------DMLMQFVTK------
------ENCLVLAVSPANS--DLA-NS--DALKIAKEVDPKGLRTIGVI-TKLDLMDEGT--------
DARDVLENK---L--------------------LPLR-RGYIGV--------------------------------VNR----------------
-------------------S-QKDID----G--------------------------KKDIQ----------A----ALAA---ERKFF---------
LS-------HP-------------------SY-----------------R-H------L-------------------------ADR-MGT-----
PYLQKALNQQLTNHIRDTL----

XP_032814666.1 ------------LDLPQIAVVGG----Q---------------SAGKSSVLE-----N---
FV-GRDFLPRG---SGIVTRRPL---------ILQ---L-M-FC-KA--
-----------------------------EY---A----------------------------------E---------------FLH-----------------
------------------------CKG------KKF-----TDFE---EVRAEIEAETDR-----LTGSN------------------KG---
---------------------IS--PIPINLRVYSPH--
VLNLTLIDLPGMTK--VP-------------------VGDQPV----------DI---EYQIR--------EMLMQFVTK--------
----ENCLILAVSPANT--DLA-NS--DALKIAKEVDPQGLRTIGVI-TKLDLMDDGT--------DARDILENK-
--L--------------------LPLR-RGYIGV--------------------------------VNR--------------------------------
--S-QKDID----G--------------------------RKDIN----------A----AMAA---ERKFF---------LS-------HP--
------------------SY-----------------R-H------M------------------------ADR-MGT-----
PYLQKTLNQQLTNHIRDTL----

XP_025944940.1 ---MF-
FRDFLPRG---SGIVTRRPL---------VLQ---L-V-TA-KT---
-----------------------EY---A----------------------------------E---------------FLH---------------------
-------------------CKG------RKF-----TDFD---EVRQEIEVETDR-----ITGVN------------------KG--------
-----------------IS--SIPINLRIYSPH--
VLSLTLIDLPGITK--VP-------------------VGDQPP----------DI---EQQIR--------DMIMQFISR-----------
-ENCLILAVTPANT--DLA-NS--DALKLAKEVDPQGLRTIGVI-TKLDLMDEGT--------DAREILENK---
L--------------------LPLR-RGYIGV--------------------------------VNR--------------------------------

S-QKDID----G----------------------------KKDIK----------A----ALLA---ERKFF---------LS-------HP------
--------------AY-----------------R-H------M--------------------------ADR-MGT-----
PYLQKVLNQQLTNHIRDTL----
EPQ08653.1 ------------LELPQIAVVGG----Q----------------SAGKSSVLE-----N---FV-
GRDFLPRG---SGIVTRRPL---------VLQ---L-V-TS-KT--
--------------------------EY---A-------------------------------------E----------------FLH-----------------------
--------------------CKG------KKF-----TDFD---EVRHEIEAETDR-----VTGMN-------------------KG------
-------------------IS--SIPINLRVYSPH--
VLNLTLIDLPGITK--VP-------------------VGDQPV----------DI---EHQIR--------EMIMQFITR-----------
-ENCLILAVTPANT--DLA-NS--DALKLAKDVDPQGLRTIGVI-TKLDLMDEGT--------DARDILENK---
L---------------------LPLR-RGYVGV-----------------------------------VNR-----------------------------------
-S-QKDID----G----------------------------KKDIK----------A----AMLA---ERKFF---------LS-------HP----
----------------AY-----------------R-H------I--------------------------ADR-MGT-----
PHLQKVLNQQLTNHIRDTL----
XP_027623811.1 ------------LELPQIAVVGG----Q----------------SAGKSSVLE-----N---
FV-GRDFLPRG---SGIVTRRPL---------VLQ---L-V-TS-KA--
------------------------------EY---G-------------------------------------E----------------FLH------------------
-------------------------CKG------KKF-----TDFD---EIRHEIEAETDR-----VTGVN-------------------KG--
-----------------------IS--SIPINLRVYSPH--
-VLNLTLIDLPGITK--VP-------------------VGDQPP----------DI---EYQIR--------EMIMQFITR-----------
-ENCLILAVTPANT--DLA-NS--DALKLAKEVDPQGLRTIGVI-TKLDLMDEGT--------DARDVLENK---
L---------------------LPLR-RGYVGV-----------------------------------VNR-----------------------------------
-S-QKDID----G----------------------------KKDIK----------A----AMLA---ERKFF---------LS-------HP----
----------------AY-----------------R-H------I--------------------------ADR-MGT-----
PHLQKVLNQQLTNHIRDTL----
XP_016856477.1 ------------LELPQIAVVGG----Q----------------SAGKSSVLE-----N---
FV-GRDFLPRG---SGIVTRRPL---------VLQ---L-V-TS-KA--
------------------------------EY---A-------------------------------------E----------------FLH-------------------
-------------------------CKG------KKF-----TDFD---EVRLEIEAETDR-----VTGMN-------------------KG--
-----------------------IS--SIPINLRVYSPH--
-VLNLTLIDLPGITK--VP-------------------VGDQPP----------DI---EYQIR--------EMIMQFITR-----------
-ENCLILAVTPANT--DLA-NS--DALKLAKEVDPQGLRTIGVI-TKLDLMDEGT--------DARDVLENK---
L---------------------LPLR-RGYVGV-----------------------------------VNR-----------------------------------
-S-QKDID----G----------------------------KKDIK----------A----AMLA---ERKFF---------LS-------HP----
----------------AY-----------------R-H------I--------------------------ADR-MGT-----
PHLQKVLNQQLTNHIRDTL----
XP_006496668.1 ------------LELPQIAVVGG----Q----------------SAGKSSVLE-----N---
FV-GRDFLPRG---SGIVTRRPL---------VLQ---L-V-TS-KA--
------------------------------EY---A-------------------------------------E----------------FLH-------------------
-------------------------CKG------KKF-----TDFD---EVRHEIEAETDR-----VTGMN-------------------KG--
-----------------------IS--SIPINLRVYSPH--
-VLNLTLIDLPGITK--VP-------------------VGDQPP----------DI---EYQIR--------DMIMQFITR----------
--ENCLILAVTPANT--DLA-NS--DALKLAKEVDPQGLRTIGVI-TKLDLMDEGT--------DARDVLENK--
-L---------------------LPLR-RGYVGV-----------------------------------VNR----------------------------------

--S-QKDID----G--------------------------KKDIK----------A----AMLA---ERKFF---------LS-------HP---
-----------------AY----------------R-H------I-------------------------ADR-MGT-----
PHLQKVLNQQLTNHIRDTL----
XP_012379251.1 --V-
GRDFLPRG---SGIVTRRPL---------VLQ---L-V-TS-KA--
------------------------EY---A-----------------------------------E----------------FLH-----------------------
--------------------CKG------KKF-----TDFD---EVRHEIEAETDR-----VTGMN------------------------KG------
------------------IS--SIPINLRVYSPH--
VLNLTLIDLPGITK--VP-------------------VGDQPP----------DI---EYQIR--------EMIMQFITR-----------
-ENCLILAVTPANT--DLA-NS--DALKLAKEVDPQGLRTIGVI-TKLDLMDEGT--------DARDVLENK---
L---------------------LPLR-RGYVGV---------------------------------VNR----------------------------------
-S-QKDID----G--------------------------KKDIK----------A----AMLA---ERKFF---------LS-------HP----
----------------AY----------------R-H------I-------------------------ADR-MGT-----
PHLQKVLNQQLTNHIRDTL----
XP_021326548.1 ------------LDLPQIAVVGG----Q----------------SAGKSSVLE-----N---
FV-GRDFLPRG---SGIVTRRPL---------ILQ---L-V-NN-KA---
-----------------------------EY---A-----------------------------------E----------------FLH------------------
------------------------CKG------RKF-----VDFD---EVRQEIEAETDR-----ITGSN------------------KG--
-----------------------IS--PIPINLRVYSPN---
-VLNLTLIDLPGMTK--VA-------------------VGDQPP----------DI---EHQIR--------DMIMQFITR--------
----ESCLILAVTPANM--DLA-NS--DALKVAKEVDPQGLRTIGVI-TKLDLMDEGT--------DARDILENK-
--L---------------------LPLR-RGYIGV---------------------------------VNR--------------------------------
--S-QKDID----G--------------------------RKDIR----------A----ALAA---ERKFF---------LS-------HP---
-----------------SY----------------R-H------M-------------------------AER-MGT-----
PHLQKALNQQLTNHIRDTL----
NP_001025299.1 ------------LDLPQIAVVGG----Q----------------SAGKSSVLE-----N---
FV-GRDFLPRG---SGIVTRRPL---------ILQ---L-V-NN-KA---
-----------------------------EY---A-----------------------------------E----------------FLH------------------
------------------------CKG------RKF-----VDFD---EVRQEIEAETDR-----ITGSN------------------KG--
-----------------------IS--PIPINLRVYSPN---
-VLNLTLIDLPGMTK--VA-------------------VGDQPP----------DI---EHQIR--------DMIMQFITR--------
----ESCLILAVTPANM--DLA-NS--DALKVAKEVDPQGLRTIGVI-TKLDLMDEGT--------DARDILENK-
--L---------------------LPLR-RGYIGV---------------------------------VNR--------------------------------
--S-QKDID----G--------------------------RKDIR----------A----ALAA---ERKFF---------LS-------HP---
-----------------SY----------------R-H------M-------------------------AER-MGT-----
PHLQKALNQQLTNHIRDTL----
XP_031753735.1 ------------LDLPQIAVVGG----Q----------------SAGKSSVLE-----N---
FV-GRDFLPRG---SGIVTRRPL---------ILQ---L-I-FS-KT---
---------------------------EY---A-----------------------------------E----------------FLH-------------------
-----------------------CKS------KKF-----TDFD---EVRQEIEAETDR-----VTGTN------------------KG----
---------------------IS--PVPINLRVYSPN--
VLNLTLIDLPGITK--VP-------------------VGDQPH----------DI---EYQIK--------DMILQFISR-----------
-DSCLILAVTPGNT--DLA-NS--DALKMAKEVDPQGLRTIGVI-TKLDLMDEGT--------DAKDILENK---
L---------------------LPLR-RGYIGV---------------------------------VNR----------------------------------

S-QKDID----G--------------------------KKDIK----------A----ALGA---ERKFF---------LS-------HP-----
---------------GY-----------------R-H------I-------------------------AER-MGT-----
PHLQKTLNQQLTNHIRETL----
XP_025920181.1 ------------LDLPQIAVVGG----Q---------------SAGKSSVLE-----N---
FV-GRDFLPRG---SGIVTRRPL---------ILQ---L-I-FS-KT---
---------------------------EY---A----------------------------------E---------------FLH------------------
----------------------CKS------KKF-----TDFD---EVRQEIEAETDR-----VTGTN------------------KG----
--------------------IS--PVPINLRVYSPH---
VLNLTLIDLPGITK--VP------------------VGDQPQ----------DI---EYQIK--------DMIMQFISR----------
--ESSLILAVTPANM--DLA-NS--DALKMAKEVDPQGLRTIGVI-TKLDLMDEGT--------DARDVLENK-
--L---------------------LPLR-RGYIGV----------------------------------VNR----------------------------------
--S-QKDID----G--------------------------KKDIR----------A----ALAA---ERKFF---------LS-------HP---
-----------------AY-----------------R-H------M-------------------------ADR-MGT-----
PHLQKVLNQQLTNHIRETL----
XP_028568434.1 ------------LDLPQIAVVGG----Q---------------SAGKSSVLE-----N---
FV-GRDFLPRG---SGIVTRRPL---------ILQ---L-I-FS-KT---
---------------------------EY---A----------------------------------E---------------FLH------------------
----------------------CKS------KKF-----TDFD---EVRQEIEAETDR-----VTGTN------------------KG----
--------------------IS--PVPINLRVYSPH---
VLNLTLIDLPGITK--VP------------------VGDQPQ----------DI---EYQIK--------DMILQFISR-----------
-ESSLILAVTPANM--DLA-NS--DALKMAKEVDPQGLRTIGVI-TKLDLMDEGT--------DARDVLENK--
-L---------------------LPLR-RGYIGV----------------------------------VNR-----------------------------------
-S-QKDID----G--------------------------KKDIR----------A----ALAA---ERKFF---------LS-------HP----
----------------AY-----------------R-H------M-------------------------ADR-MGT-----
PHLQKLLNQQLTNHIRETL----
XP_006510037.1 ------------LDLPQIAVVGG----Q---------------SAGKSSVLE-----N---
FV-GRDFLPRG---SGIVTRRPL---------ILQ---L-I-FS-KT---
---------------------------EY---A----------------------------------E---------------FLH------------------
----------------------CKS------KKF-----TDFD---EVRQEIEAETDR-----VTGTN------------------KG----
--------------------IS--PVPINLRVYSPH---
VLNLTLIDLPGITK--VP------------------VGDQPP----------DI---EYQIK--------DMILQFISR------------
ESSLILAVTPANM--DLA-NS--DALKLAKEVDPQGLRTIGVI-TKLDLMDEGT--------DARDVLENK---
L---------------------LPLR-RGYIGV----------------------------------VNR------------------------------------
S-QKDIE----G--------------------------KKDIR----------A----ALAA---ERKFF---------LS-------HP------
--------------AY-----------------R-H------M-------------------------ADR-MGT-----
PHLQKTLNQQLTNHIRESL----
NP_001005360.1 ------------LDLPQIAVVGG----Q---------------SAGKSSVLE-----N---
FV-GRDFLPRG---SGIVTRRPL---------ILQ---L-I-FS-KT---
---------------------------EH---A----------------------------------E---------------FLH-----------------
------------------------CKS------KKF-----TDFD---EVRQEIEAETDR-----VTGTN------------------KG---
----------------------IS--PVPINLRVYSPH---
-VLNLTLIDLPGITK--VP------------------VGDQPP----------DI---EYQIK--------DMILQFISR-----------
-ESSLILAVTPANM--DLA-NS--DALKLAKEVDPQGLRTIGVI-TKLDLMDEGT--------DARDVLENK---
L---------------------LPLR-RGYIGV----------------------------------VNR------------------------------------

S-QKDIE----G--------------------------KKDIR----------A----ALAA---ERKFF---------LS-------HP------
--------------AY-----------------R-H------M-------------------------ADR-MGT-----
PHLQKTLNQQLTNHIRESL----
XP_014389433.1 ---AGKGQI-----------T-
AWDFLPRG---SGIVTRRPL---------ILQ---L-I-FS-KT---
------------------------EY--A-----------------------------------E---------------FLH-----------------------
-------------------CKS------KKF-----TDFD---EVRQEIEAETDR-----VTGTN-------------------KG--------
-----------------IS--PVPINLRVYSPH---
VLNLTLIDLPGITK--VP-------------------VGDQPP----------DI---EYQIK--------DMILQFISR-----------
ESSLILAVTPANM--DLA-NS--DALKMAKEVDPQGLRTIGVI-TKLDLMDEGT--------DARDVLENK---
L---------------------LPLR-RGYIGV----------------------------------VNR------------------------------------
S-QKDIE----G--------------------------KKDIR----------A----ALAA---ERKFF---------LS-------HP------
--------------AY-----------------R-H------M-------------------------ADR-MGT-----
PHLQKTLNQQLTNHIRESL----
XP_012381548.1 --

------MILQFIGR------------ESSLILAVTPANM--DLA-NS--DALKLAKEVDPQGLRTIGVI-
TKLDLMDEGT--------DARDVLENK---L---------------------LPLR-RGYIGV------------------------------
----VNR------------------------------------S-QKDIE----G--------------------------RKDIR----------S----
ALAA---ERKFF---------FS-------HS--------------------AY-----------------R-H------M--------------------
------ADR-MGT-----LHLQKTLNQQLTNHIRESL----
XP_006161648.2.2 ----------------PHCTSVSR----QAYS------------SPQGPRKVIS-----VL-
FLP-HRDFLPRG---SGIVTRRPL---------ILQ---L-I-FS-KT---
-----------------------------EY--A-----------------------------------E---------------FLH-------------------
------------------------CKS------KKF-----TDFD---EVRQEIEAETDR-----VTGTN-------------------KG---
----------------------IS--PVPINLRVYSPH---
-VLNLTLIDLPGITK--VP-------------------VGDQPP----------DI---EYQIK--------DMILQFISR-----------
-ESSLILAVTPANM--DLA-NS--DALKLAKEVDPQGLRTIGVI-TKLDLMDEGT--------DARDVLENK---
L---------------------LPLR-RGYIGV----------------------------------VNR------------------------------------
S-QKDIE----G--------------------------KKDIR----------A----ALAA---ERKFF---------LS-------HP------
--------------AY-----------------R-H------M-------------------------ADR-MGT-----
PHLQKTLNQQLTNHIRESL----
XP_035683496.1 ------------LDLPQIAVVGG----Q----------------SAGKSSVLE-----N---
FV-GRDFLPRG---SGIVTRRPL---------VLQ---L-IHNP-KA---
------------------------------EY---G------------------------------------E----------------FLH-----------------
-------------------------AKG------KMF-----SDFH---EIRAEIEAETDR-----MTGSN------------------
KG-------------------------IS--PVPINLRVYSPH--
--------VLNLTLIDLPGMTK--VP-------------------VGDQPP----------DI---EQQIR--------DMLLQFITK-
-----------DNCLILAVSPANQ--DLA-NS--DALKIAKEVDPQGMRTIGVI-TKLDLMDEGT--------
DARNILENR---T--------------------YPLR-RGYIGV----------------------------------VNR-----------------
------------------S-QADID----G--------------------------RKDIK----------A----ALAA---ERKFF---------

LS-------HP-------------------AY----------------R-H------L------------------------ADR-MGT-----
PYLQKTLNQQLTNHIRDTL----
KMZ10000.1 ------------LDLPQIAVVGG----Q---------------SAGKSSVLE-----N---FV-
GKDFLPRG---SGIVTRRPL---------ILQ---L-I-NG-VT---
-----------------------EY---G---------------------------------E---------------FLH---------------------
-------------------IKG------KKF-----SSFD---EIRKEIEDETDR-----VTGSN------------------KG---------
---------------IS--NIPINLRVYSPH---
VLNLTLIDLPGLTK--VA------------------IGDQPV----------DI---EQQIK--------QMIFQFIRK-----------
-ETCLILAVTPANT--DLA-NS--DALKLAKEVDPQGVRTIGVI-TKLDLMDEGT--------DARDILENK---
L--------------------LPLR-RGYIGV--------------------------------VNR---------------------------------
S-QKDIE----G-------------------------RKDIH----------Q----ALAA---ERKFF---------LS-------HP-----
---------------SY----------------R-H------M------------------------ADR-LGT-----
PYLQRVLNQQLTNHIRDTL----
PAA78248.1 ------------FDLPQIAVVGS----Q---------------SAGKSSVLE-----N---FV-
GKDFLPRG---SGIVTRRPL---------VLQ---L-L-TH-PS--
------------------------EF---A---------------------------------E---------------FGH--------------------
--------------------LRG------KKF-----TNFD---EVRQEIENETDR-----LTGKN------------------KG------
------------------IS--NVPITLRVFSPH--
VLNLTLVDLPGMTK--VA------------------VGDQPP----------DI---EQQIR--------AMLFEFISK---------
---ENCLILAVSPANS--DLA-NS--DALKIAKEVDPNGTRTIGVI-TKLDLMDQGT--------DAREVLENK--
-L--------------------LPLR-RGYIGV--------------------------------VNR--------------------------------
-S-QKDIE----G--------------------------KKDIA----------A----AMAA---ERKFF---------LS-------HP----
----------------SY----------------R-H------M------------------------AER-MGT-----
PYLQRCLNQQLTNHIRETL----
PAA65118.1 ------------FDLPQIAVVGS----Q---------------SAGKSSVLE-----N---FV-
GKDFLPRG---SGIVTRRPL---------ILQ---L-L-YN-PS--
-------------------A---EY---A---------------------------------E---------------FGH-------------------
---------------------QRG------RKY-----TNFE---EVRQEIEAETDR-----LTGRN------------------KG-----
------------------IS--NVPIMLRVFSPH---
VLNLTLVDLPGMTK--VA------------------VGDQPP----------DI---EVQIR--------NMLLEFITK---------
---ENCLILAVSPANS--DLA-NS--DALKLAKEVDPAGTRTIGVI-TKLDLMDQGT--------DAREVLENK-
--L--------------------LPLR-RGYIGV--------------------------------VNR-------------------------------
--S-QKDIE----G--------------------------RKDIK----------A----AMAA---ERKFF---------LS-------HP---
-----------------SY----------------R-H------M------------------------AER-MGT-----
PYLQRCLNQQLTNHIRETL----
PAA59145.1 ------------FDLPQIAVVGS----Q---------------SAGKSSVLE-----N---FV-
GKDFLPRG---SGIVTRRPL---------VLQ---L-I-NF-HT--
------------------------EY---A---------------------------------E---------------FGH--------------------
--------------------IRG------KRF-----TNFD---EVRQEIENETDR-----VTGKN------------------KG------
------------------IS--NVPIMLRVYSPQ---
VLNLTLVDLPGLTK--VA------------------VGDQPQ----------DI---ELLIR--------AMILEFVSK-----------
-DNCLILAVTPANS--DLA-NS--DALKIAKEVDPSGTRTIGVI-TKLDLMDQGT--------DARDVLENR---
L--------------------LPLR-RGYIGV--------------------------------VNR---------------------------------
S-QKDIE----G-------------------------KKDIV----------A----AMAA---ERKFF---------LS-------HP-----

---------------AY-----------------R-H------M--------------------------AER-MGT-----
SYLQRCLNQQLTNHIRETL----
PAA64382.1 ------------IDLPQIAVVGS----Q----------------SAGKSSVLE-----N---FV-
GRDFLPRG---SGIVTRRPL---------ILQ---L-M-NY-QT--
-------------------------EY---A-----------------------------------E----------------FGH--------------------
----------------------IRG------KKF-----VNFD---EVRREIEVETDR-----LTGQN-------------------KG-----
--------------------IS--NVPITLRVYSPQ--
VLNLTLVDLPGMTK--VA-------------------VGDQPP----------DI---EQQVR--------AMIWEFISK--------
----DNCLILAVSPANS--DLA-NS--DALKLAKEADPSGSRTIGVL-TKLDLMDAGT--------
DARDVLENR---F---------------------LPLR-RGYVGV----------------------------------VNR---------------
--------------------S-QKDID----G--------------------------RKDIS----------S----AMAA---ERKFF-------
--LG-------HP--------------------AY-----------------R-H------M--------------------------AER-MGT-----
AHLQRCLNQQLVGHIRDTL----
NP_001024332.1 ------------FELPQIAVVGG----Q----------------SAGKSSVLE-----N---
FV-GKDFLPRG---SGIVTRRPL---------ILQ---L-I-QD-RN--
-----------------------------EY---A-----------------------------------E----------------FLH-------------------
------------------------KKG------HRF-----VDFD---AVRKEIEDETDR-----VTGQN-------------------KG--
-----------------------IS--PHPINLRVFSPN--
---VLNLTLIDLPGLTK--VP-------------------VGDQPA----------DI---EQQIR--------DMILTFINR--------
----ETCLILAVTPANS--DLA-TS--DALKLAKEVDPQGLRTIGVL-TKLDLMDEGT--------DAREILENK--
-L---------------------FTLR-RGYVGV----------------------------------VNR-----------------------------------
--G-QKDIV----G---------------------------RKDIR----------A----ALDA---ERKFF---------IS-------HP----
----------------SY-----------------R-H------M--------------------------ADR-LGT-----
SYLQHTLNQQLTNHIRDTL----
XP_004347890.1 ------------LDLPQIAVVGS----Q----------------SAGKSSVLE-----N---
FV-GKDFLPRG---SGIVTRRPL---------VLQ---L-V-NS-KG--
-------------------------P---EY---G-----------------------------------E----------------FLH-----------------
-------------------------NKS------KKF-----TDFD---EVRKEIEAETDR-----ITGTN-------------------KG--
-----------------------IS--PVPINLKVYSPN--
--VLNLTLVDLPGITK--VP-------------------IGDQPT----------NI---ESLIR--------EMIMQFIGR----------
--PNCLILAVSPANS--DLA-NS--DALKLAREVDQQGIRTIGVI-TKLDLMDEGT--------DAREVLENK--
-L---------------------IPLR-RGFIGV----------------------------------VNR-------------------------------------
S-QKDID----G---------------------------RKDIK----------A----AMSA---ELRFF---------ST-------HP-----
---------------AY-----------------R-D------L--------------------------ANK-NGT-----
MYLQRVLNQQLTNHIRDTL----
XP_001749319.1 ------------LDLPQIAVVGG----Q----------------SAGKSSVLE-----N---
FV-GKDFLPRG---SGIVTRRPL---------VLQ---L-N-YH-PS--
-------------------------A---EW---G-----------------------------------E----------------FLH---------------
----------------------------ARG------KKF-----TDFN---EIRQEIEAETDR-----MTGSN-------------------
KG-------------------------IS--NIPINLRVYSPH--
-------VLNLTLVDLPGLTK--VA-------------------VGDQPA----------DI---ENQIR--------GMLMEFITK--
----------DNCIILAVTPANQ--DLA-NS--DALKLAKEVDPEGVRTIGVI-TKLDLMDSGT--------
DARAILTNE---F---------------------LPLR-RGYIGV----------------------------------VNR------------------
-----------------S-QKDID----G--------------------------RKDIR----------A----ALDA---ERKFF---------

LM-------HP-------------------SY-----------------K-D------I-------------------------ASK-NGT-----
PYLQKALNQQLTNHIRECL----
XP_031757197.1 ------------VDLPQIAVVGG----Q---------------SAGKSSVLE-----N---
LV-GR---------------------------WIH---V-L--S-ST---
-------------------EY---A-----------------------------------E----------------FLH----------------------------
--------------CKG------TKY-----TDFS---EVRQEIEEETER-----ATGLN-------------------KG--------------
-----------IS--AIPISLRIYSPH---
VLNLSLIDLPGVTK--VP-------------------VGDQPA----------DI---ETQIR--------DMIMNFISR----------
--ENCLILAVTPANT--DLA-NS--DALKLAKEVDPQGLRTIGVI-TKLDLMDEGT--------NAQEILENK---
L---------------------LPLR-RGYVGV----------------------------------VNR------------------------------------
-S-QKDID----G---------------------------KKNIN----------A----ALQA---EQMFF---------LT-------HP---
-----------------AY-----------------R-H------M--------------------------ADR-MGT-----
SHLQKMLNQQLTNHIRETL----
XP_014148725.1 ---T-------------------
--EY---
G-------------------------------------E----------------FLH--KPG-----
-RLF-----DNFD---EIRNEIEADTAR-----ITGAN-------------------KG-------------------------IS--
HLPINLKVYSPH---VLDLTLVDLPGLTK--
VA-------------------VGDQPA----------DI---EMQIK--------NMIMEFITK------------PNCLILAVTPANS-
-DLA-NS--DALKLAKEVDPQGLRTIGVI-TKLDLMDAGT--------DARDVLENK---L---------------------
LPLR-RGYVGV----------------------------------VNR------------------------------------S-QKDIA----G----
-----------------------NKDIR----------A----AQAA---EKKFF---------KT-------HP--------------------AY---
--------------R-H------L--------------------------ADK-MGT-----PKLQQVLNQQLTDHIRQTL----
XP_014153758.1 ---N-------------------
-----------------LY---LYI---
EW---G-------------------------------------E----------------FLH--
QPG------RKY-----TDFE---EIMKEIEAETDR-----MTGSN-------------------KG-------------------------
IS--NIPINLKVMSPH---
VLDLTLVDLPGLTK--VA-------------------VGDQPA----------DI---EQQIL--------GMIMEFITR----------
--PNCLILAVSPANA--DLA-NS--DALKLAKEVDPQGLRTIGVI-TKLDLMDQGT--------DAREILENK--
-L---------------------LPLR-RGYIGV----------------------------------VNR------------------------------------
-S-QKDIT----G---------------------------KKDIR----------A----AQEA---ERRFF---------ST-------HP-----
---------------AY-----------------R-H------L--------------------------AQN-MGT-----
PKLQKVLNQQLTNHIRDSL----
XP_006812840.1 ------------ITKPSCLILAV--------------------TPGNSDLAN-----
SDALKV-AKEVDPQGLRTIGVITKLDLLDDGTDAREILENKLLPL-RR-AS--------------------------------
---EW---G-------------------------------------E---------------
FLH--CKG------KKF-----TNFD---EIRMEIEAETDR-----LTGKN--
-----------------KG-------------------------IS--PIPINLRVYSPH---
------------------------VLNLTLIDLPGMTK--VP-------------------VGDQPA----------DI---EQQIR--------
SMLMEFITK------------PSCLILAVTPGNS--DLA-NS--DALKVAKEVDPQGLRTIGVI-TKLDLLDDGT-
-------DAREILENK---L---------------------LPLR-RGYIGV----------------------------------VNR----------
------------------------G-QKDIE----G---------------------------RKDIK----------S----ALAS---ERKFF---

------LS-------HP-------------------SY----------------R-H------M------------------------ADR-LGT----
-PYLQKALNQQLTNHIRDTL----
XP_004348308.1 ------------IQLPQIAVVGS----Q---------------SSGKSSVLE-----N---
IV-GKDFLPRG---HGIVTRRPL---------ILQ---L-V-HRKPGS-----------------PRPALPDDPSSSG------
-----------------------GHTDDGIDGEDVE---EW---G----------------------------------E---------------
FLH---APG------KRF-----ISFA---EIRKEIEAETDR-----VTGSN----
---------------KG------------------------IS--SKPINLRIYSPN--
---------------------VLNLTLVDLPGITK--VP------------------VGDQPE----------DI---EKQIR--------
TLVRSYISN------------PNCIILAVTPANV--DLA-NS--DALKLAKTIDPEGNRTIGVC-TKIDLMDAGT---
-----DAMDILSGR---V--------------------VPVK-LGFIGV---------------------------------VNR-----------
----------------------S-QADIN----T--------------------------AKPIA----------D----SLKS---EEQFF------
---KS-------HP-------------------AY----------------Q-A------I------------------------AHR-CGT-----
AYLSKALNKLLMHHIRDCL----
KNE67543.1 ------------LDLPQIAVVGS----Q---------------SSGKSSVLE-----A---IV-
GKDFLPKG---AGIVTRRPL---------ILQ---L-N-YA-PV-------------------DPETPDE--------------------
------------------------PE---EW---A-----------------------------------E---------------FQH-------------
----------------------------LPG------QQF-----ADFG---EVKREIERETAR-----IAGDN------------------
KG------------------------IS--DEPITLRVHSPS--
--------VVDLTLVDLPGLTK--IP------------------VGDQPS----------DI---ELQIR--------DLIMKFILQ---
---------PNCIILAVSPANV--DLA-NS--DSLKLAREVDPQGLRTLGLL-TKVDLMETGS--------
HALDILGGR---V--------------------YPLR-LGFVAV---------------------------------VNR----------------
-----------------S-QRDIE----A--------------------------RRTLE----------W----SRKR---EQQFF---------
--SGR-CGT-----
AALARTLNSVLLDHIRAQLP---
KNE61418.1 ------------LDLPQIAVVGS----Q---------------SSGKSSVLE-----A---IV-
GKDFLPKG---NGIVTRRPL---------VLQ---L-R-NV-P---------------------PHAVPE--------------------
------------------------GE---EAVDVA-----------------------------------E---------------FSH------------
-----------------------------CPD------RVF-----TDFA---DVRREIEAETAR-----IAGDN------------------
KG------------------------IA--TDPIRLCVRSPN--
--------VVDLTLVDLPGMTK--IP------------------VGDQPS----------DI---ELQIR--------ELIMGFITK---
---------PNCLILAVSPANV--DLA-NS--DSLKLAREVDPTGDRTLGLL-TKVDLMEPGT--------
HALDILAGR---V--------------------YPLR-LGFVGV---------------------------------VNR---------------
------------------S-QRDID----Q--------------------------GKSLE----------F----ARKR---EQQFF-------
--AT-------HP-------------------VY----------------A-P------V------------------------ASR-CGT-----
TVLARTLNQVLLAHIRDRLP---
XP_011392073.1 ------------VDLPQIVVVGS----Q---------------SAGKSSVLE-----T---
IV-GRDFLPRG---SGIVTRRPL---------VLQ---L-I-HT-PS-------------------TKEQPRQPKQSSRPY-
DLSDGLA-----------SDMQRGGSHASSADTRSPTYE---EY---G-----------------------------------E----
------------FLH---L-D------KRF-----TDFN---EIRREIENETFR-----
VAGQN-------------------KG------------------------VS--KLPIHLKIYSPN--------------------------------
-----------------------------------VLNLTLVDLPGLTK--IP------------------VGDQPS----------DI---
ERQIR--------NLVTDYVSK------------PNCIILAVSPANV--DLA-NS--DSLKLARTVDPQGRRTIGVL-
TKLDLMDQGT--------HALDILTGR---V--------------------YPLK-LGFIGV-------------------------------
--VNR---------------------------------S-QQDIN----G--------------------------NVSML----------A----

ARRA---EEDFF---------RS-------HA-------------------AY-----------------K-N------I---------------------
-----AHR-CGT-----KYLAKTLNQVLMSHIRDKLP---
XP_006461708.1 ------------LDMPQIVVVGS----Q----------------SAGKSSVLE-----T---
IV-GKDFLPRG---SGIVTRRPL---------VLQ---L-I-HT-PV-----------------------PSEPSPNALP---------
------------------------------YT---EW---G-----------------------------------Q----------------FLH----------
----------------------------------I-D------KRF-----TDFN---DIRKEIEQETFR-----VAGQN-------------------
-KG------------------------IS--RLPISLRVYSPN--
--------VLDLTLVDLPGLTK--IP-------------------VGDQPS----------DI---EKQIR--------NLVVDYISK---
---------PNSVILAVSAANV--DLA-NS--ESLKLARSVDPQGRRTIGVL-TKLDLMDAGT--------
NALDILTGR---V---------------------YPLK-LGFIGI-----------------------------------VNR--------------------
----------------S-QQDIN----V---------------------------EKSLT----------D----AVES---EAEFF---------
RN-------HA-------------------VY-----------------R-N------I---------------------------AHK-NGT-----
RYLAKTLNQVLMNHIRDKLP---
XP_746923.1 ------------LDLPQIVVVGS----Q----------------SSGKSSVLE-----N---IV-
GRDFLPRG---SGIVTRRPL---------ILQ---L-I-NI-PS-------------------EHNDRPGDNDVLA-PH------
-------------------------TAASVAGQH---EW---A-----------------------------------E----------------FHH--
--LPG------RKF-----EDFA---LVKQEIEAETAR-----IAGNN---------
----------KG-------------------------IN--RQPINLKIFSPH---
------------------VLNLTMVDLPGLTK--VP-------------------IGDQPS----------DI---EKQTR--------
TLILEYIAK------------PNSIILAVSPANV--DLV-NS--EALKLARQVDPMGRRTIGVL-TKLDLMDHGT---
-----NAMDILSGR---V---------------------YPLK-LGFIGV-----------------------------------VNR------------
-----------------------S-QQDIQ----S---------------------------GKSLS----------E----ALQA---EAEFF----
-----RH-------HP-------------------AY-----------------R-N------M---------------------------ANR-CGT----
-QFLAKTLNTTLMAHIRDRLP---
KXN67416.1 ------------LDLPQIVVVGS----Q----------------SSGKSSVLE-----N---LV-
GRDFLPRG---NGIVTRRPL---------VLQ---L-V-NL-RE-------------------D-----ADLDLT----------------
-------------------------------ER---A-----------------------------------Q----------------FLH----------------
---------------------------NPT------KFY-----TDFS---EVRQEIEQETNR-----LAGEN-------------------
KG-------------------------IS--RNPIHLKIFSTQ--
-------VLNLTVVDLPGLTK--IP-------------------IGDQPT----------DI---EKQTK--------SLILDYISK------
------PNSIILAVSPANV--DLV-NS--ESLKLAKEVDPEGKRTIGII-TKIDLMDAGT--------NALDILTGR---
V---------------------LNLK-LGFIGV-----------------------------------INR------------------------------------
S-QQDTV----A---------------------------KKPIR----------E----SLEA---ELEFF---------RT-------HP------
--------------AY-----------------R-N------I---------------------------SQR-CGT-----
GHLSKTLNQVLVNHIRDRLP---
NP_013100.1 ------------LDLPILAVVGS----Q----------------SSGKSSILE-----T---LV-
GRDFLPRG---TGIVTRRPL---------VLQ---L-N-NI-SP-------------------NSPLIEEDDNSVNPH-
DEVTKI------------SGFEAGTKPLEYRGKERNHAD---EW---G------------------------------------E------
----------FLH---IPG------KRF-----YDFD---DIKREIENETAR-----
IAGKD-------------------KG-------------------------IS--KIPINLKVFSPH------------------------------------
----------------------------------VLNLTLVDLPGITK--VP-------------------IGEQPP----------DI---EKQIK-
-------NLILDYIAT------------PNCLILAVSPANV--DLV-NS--ESLKLAREVDPQGKRTIGVI-
TKLDLMDSGT--------NALDILSGK---M---------------------YPLK-LGFVGV------------------------------
----VNR-----------------------------------S-QQDIQ----L---------------------------NKTVE----------E----

SLDK---EEDYF---------RK-------HP--------------------VY-----------------R-T------I----------------------
----STK-CGT-----RYLAKLLNQTLLSHIRDKLP---

NP_012926.1 ------------IDLPQITVVGS----Q----------------SSGKSSVLE-----N---IV-
GRDFLPRG---TGIVTRRPL---------VLQ---L-I-NR-RP--------------------
KKSEHAKVNQTANELIDLNINDD-----------DKKKDESGKHQNEGQSEDNKE---EW---G-------------
-----------------------E----------------FLH--LPG------KKF-----
YNFD---EIRKEIVKETDK-----VTGAN-------------------SG-------------------------IS--SVPINLRIYSPH-
---VLTLTLVDLPGLTK--VP-------------------
VGDQPP----------DI---ERQIK--------DMLLKYISK------------PNAIILSVNAANT--DLA-NS--
DGLKLAREVDPEGTRTIGVL-TKVDLMDQGT--------DVIDILAGR---V---------------------IPLR-
YGYIPV-----------------------------------INR------------------------------------G-QKDIE----H--------------
-------------KKTIR----------E----ALEN---ERKFF---------EN-------HP--------------------SY--------------
---S-S------K--------------------------AHY-CGT-----PYLAKKLNSILLHHIRQTLP---

OUM62108.1 ------------IDLPQIVVIGS----Q----------------SSGKSSVLE-----N---IV-
GRDFLPRG---TGIVTRRPL---------ILQ---L-I-NK-RD--------------------GLTTNS------------------------
---------------APANSEQNMN---EW---G-------------------------------------E----------------FLH---------
-----------------------------------LPG------KKF-----YDFT---EIRKEIEKETEL-----KVGKN------------------
-AG--------------------------IS--DQPINLRIFSPK--
--------VLTLTLVDLPGLTK--VP-------------------VGDQPK----------DI---ERLIR--------DMILKYITK-----
-------PNAIILAVTAANT--DLA-NS--DGLKLAREVDPEGVRTIGVL-TKVDLMDAGT--------
DVIDILAGR---V---------------------IPLR-LGYVPV----------------------------------VNR------------------
-----------------G-QKDIE----T---------------------------NKSIS----------K----ALEA---EKQFF---------
EH-------HS--------------------SY-----------------K-S------K--------------------------AQY-CGT-----
PFLARKLNMILMHHIRNTLP---

KXN66323.1 ------------VDLPQIVVIGS----Q----------------SSGKSSVLE-----N---IV-
GRDFLPRG---TGIVTRRPL---------VLQ---L-F-NV-PQ--------------------TQ------------------------------
--------------ADSAKENEPA---EY---G-------------------------------------E----------------FLH-------------
------------------------------LPG------TKF-----TDFD---KIRKEIESETES-----KTGKN-------------------
AG--------------------------IS--PLPINLRIFSPK--
------VLTLTLVDLPGLTK--VP-------------------VGDQPK----------DI---EKQIR--------DMIMKYINK-----
-------PNAIILAVTAANT--DLA-NS--DGLKLAREVDPEGSRTIGVL-TKVDLMDHGT--------
DVVDILAGR---V---------------------IPLR-LGYVPV----------------------------------INR------------------
-----------------S-QRDIE----S---------------------------KKNIS----------A----ALDH---ERQFF---------
EK-------HP--------------------SY-----------------T-S------K--------------------------VQY-CGT-----
PFLARKLSMILMHHIRNTLP---

XP_011389257.1 ------------IDLPQITVLGS----Q----------------SSGKSSVLE-----N---
IV-GRDFLPRG---TGIVTRRPL---------VLQ---L-I-NR-PA--------------------TSKANDE------------------
----------------AAPAVSSKGANNPD---EW---G-------------------------------------E----------------FLH--
--LPG------EKF-----FDFD---KIREEIVRDTEL-----KTGRN----------
---------AG--------------------------IS--PQPINLRIYSPN--
----------------VLTLTLVDLPGLTK--VP-------------------VGDQPR----------DI---ERQIR--------
DTVLKFISK------------PNAVILAVTAANT--DLA-NS--DGLKLAREVDPEGTRTVGVL-TKVDLMDAGT-
-------DVVDILAGR---V---------------------IPLR-LGYVPV----------------------------------VNR----------
-------------------------G-QRDID----Q---------------------------KKLVS----------A----ALTA---EKEFF---

------EN-------HP--------------------SY-----------------R-S------K--------------------------AQY-CGT----
-PFLARKLNTILMHHIRNTLP---
XP_006458578.1 ------------IDLPQICVLGS----Q----------------SSGKSSVLE-----N---
IV-GRDFLPRG---TGIVTRRPL---------VLQ---L-I-NR-PA--------------------GTPG-----------------------
----------GPQINGTDKSSDKHANAD---EW---G------------------------------------E----------------FLH-
--LPG------EKF-----FDFN---KIRAEIVRDTEV-----KTGKN---------
----------AG-------------------------IS--PLPINLRVFSPN--
------------------VLTLTLVDLPGLTK--VP-------------------VGDQPR----------DI---EKQIR--------
DMLLKYISK------------SACIILAVTAGNT--DLA-NS--DGLKMAREVDPEGLRTIGVL-TKIDLMDKGT--
------DVVDILAGR---I---------------------IPLR-LGYVPV-----------------------------------VNR------------
-----------------------G-QRDIE----S---------------------------SKPIS----------A----ALEY---ERSFF------
---EN-------HA--------------------SY-----------------K-S------K--------------------------AQF-CGT-----
PFLARKLNMILMHHIKATIP---
KNE68830.1 ------------IDLPQITVIGS----Q----------------SSGKSSVLE-----N---IV-
GRDFLPRG---TGIVTRRPL---------VLQ---L-I-NR-PA--------------------TPAAPGS----------------------
-----PATDGEPAADGTSGDNQDNKN---EW---G------------------------------------E----------------
FLH--APN------KRF-----YNFD---EIRKEIIRDTDE-----KAGAN--
-----------------TG-------------------------IS--HAPINLRIYSPN---
------------------------VLTLTLVDLPGLTK--VP-------------------VGDQPK----------DI---EIQIR--------
EMLLKYITK------------PNAIILAVTAANT--DLA-NS--DGLKLAREVDPEGSRTIGVL-TKVDLMDPGT--
------DVVDILAGR---V---------------------IPLR-LGYVPV-----------------------------------VNR-----------
------------------------G-QKDID----K---------------------------RKSIA----------L----ALEH---ERSYF-----
----EE-------HP--------------------SY-----------------R-S------K--------------------------AQY-CGT-----
PFLARKLNMILLHHIKNTLP---
XP_748106.1 ------------IDLPQIVVVGS----Q----------------SSGKSSVLE-----N---IV-
GRDFLPRG---SGIVTRRPL---------ILQ---L-I-NR-PS--------------------RNSV--------------------------
------TNGVKEEKLETTDSEANVD---EY---G------------------------------------E----------------FLH------
------------------------------------IPG------QKF-----YDFN---KIREEIVRETEQ-----KVGRN-------------
------AG-------------------------IS--PAPINLRIYSPN---
-------------VLTLTLVDLPGLTK--VP-------------------VGDQPK----------DI---ERQIR--------
DMVLKYISK------------PNAIILAVTSANQ--DLA-NS--DGLKLAREVDPEGQRTIGVL-
TKVDLMDEGT--------DVVDILAGR---I---------------------IPLR-LGYVPV----------------------------------
--VNR-----------------------------------G-QRDIE----N----------------------------KRPIS----------Y----
ALEH---EKNFF---------ES-------HK--------------------AY-----------------R-N------K-------------------
------ASY-CGT-----PYLARKLNLILMMHIKQTLP---
OAJ44422.1 ------------IDLPQIAVVGS----Q----------------SSGKSSVLE-----N---IV-
GKDFLPRG---SGIVTRRPL---------VLQ---L-I-NR-PN--------------------SKGEIGGIAAGT----
DKPPSSP-----------STPGAKSATTSNAAVTVEEGD---EW---G------------------------------------E------
----------FLH--IPG------KRF-----TDFN---EIRDEIDRETEK-----
TTGKN-------------------AG-------------------------VS--SNPINLRIYSPN------------------------------------
-----------------------------------VLTLTLVDLPGLTK--VP-------------------VGDQPK----------DI---
EKLIK--------DMILKYISK------------SNAIILAVTSANT--DLA-NS--DGLKLAREVDPEGVRTIGVL-
TKIDLMDQGT--------DVIDILAGR---V--------------------IPLR-LGYVPV-----------------------------------
-VNR-----------------------------------G-QRDIE----N---------------------------KKKIS----------L----

ALEA---EKNYF---------EN-------HA-------------------SY----------------R-S------K-------------------
------AQY-CA----------------------------
XP_002129967.2 ------------LQLPQIVVVGV----Q---------------SSGKSSVLE-----N---
LV-GRDFLPRG---TGIVTRCPL---------VLQ---M-I-HT-TN------------------EDTAQCSNEGSSGNN--
--------------------DSDSSGESFK-ETNEEVK---EW---V----------------------------------K-------------
--FQH---TKG------KIF-----RSFK---QVKKEIELETQR-----
LSGNN------------------KG------------------------IS--SEAIRLKIFSPK--------------------------------
---------------------------------VLNLTLVDLPGLMK--IP------------------VGDQPD----------DI---
EEQAR--------NLILRYISN------------PNSIILAVTPANV--DFA-TS--EALQMARIVDPDGCRTLAVV-
TKLDLMDAGT--------DAIDVLCGR---I-------------------VPVK-LGIIGI--------------------------------
-VNR---------------------------------S-QLDIN----K-------------------------GKSVQ----------D----
AIKD---EQAFL---------QK-------K---------------------Y----------------P-S------F----------------------
---ANR-SGS-----RYLSITLNRLLMHHIRDCL----
NP_001259946.1 ------------IQLPQIVVLGS----Q---------------SSGKSSVIE-----S---
VV-GRSFLPRG---TGIVTRRPL---------VLQ---L-I-YS-PL------------------DDRENRSAENG-----------
----------------------------TSNAE---EW---G----------------------------------R---------------FLH------
-------------------------------------TK-------KCF-----TDFD---EIRKEIENETER-----AAGSN---------------
----KG------------------------IC--PEPINLKIFSTH---
-----------VVNLTLVDLPGITK--VP------------------VGDQPE----------DI---EAQIK--------
ELVLKYIEN------------PNSIILAVTAANT--DMA-TS--EALKLAKDVDPDGRRTLAVV-TKLDLMDAGT--
------DAIDILCGR---V-------------------IPVK-LGIIGV---------------------------------MNR------------
----------------------S-QKDIM----D---------------------------QKHID----------D----QMKD---EAAFL---
------QR-------K---------------------Y----------------P-T------L-------------------------ATR-NGT-----
PYLAKTLNRLLMHHIRDCL----
XP_035676386.1 ------------IQLPQIVVIGT----Q---------------SSGKSSVLE-----S---LV-
GRDFLPRG---TGIVTRRPL---------VLQ---L-V-HV-NS------------------EEKKRPSEDED-------------
-----------------GGHKQDIKEHAHVE---EW---G----------------------------------K---------------FLH-
--TKN------KIY-----TDFD---EIRQEIENETDR-----VTGTN----------
---------KG------------------------II--DDAIHLKIYSPK---
---------------VLNLTLVDLPGITK--VP------------------VGDQPP----------DI---EVQIR--------
EMCLKYIAN------------PNSIILAVTSANT--DMA-TS--EALKFAKEVDPDGRRTLAVI-TKLDLMDAGT--
------DAHDVLMGR---V-------------------IPVK-LGIIGV---------------------------------VNR-----------
-----------------------S-QMDIN----K-------------------------RKPIE----------E----AIKD---EAAFM----
-----QR-------K---------------------Y----------------P-S------L-------------------------ASR-NGT-----
SHLARTLNRLLMHHIRDCL----
XP_006821224.1 --

--MA-TS--EAIKLSREVDEDGRRTLAVI-TKLDLMDAGT--------
DAVEIICGR---V-------------------IPVK-LGIIGV---------------------------------INR-------------------
--------------S-QMDIN----N-------------------------KKPIQ----------E----SVKD---EAAFL---------

QR-------K---------------------Y----------------P-A------L-------------------------ASR-NGT-----
PYLAKTLNRLLMHHIRDCL----
XP_030827871.1 ------------IQLPQIVVVGN----Q---------------SSGKSSVLE-----G---
LV-GKDFLPRG---NGIVTRRPL---------VLQ---M-V-HV-DP------------------EDKRGASGEGE--------
------------------------------EEITAD---EW---G----------------------------------K---------------FLH---
--------------------------------------TKN------KVY-----TDFE---EIREEIQNETDR-----MAGTN----------
---------KG------------------------IV--HDAIHLRIYSPK---
----------------VLNLTLVDLPGLTK--VP------------------VGDQPE----------DI---ESQIR--------
EMLVKYIGN------------PNSIILAVTSANT--DMA-TS--ESLKLAKEIDPDGRRTLAVI-TKLDLMDAGT---
-----DAVDVLCGR---V--------------------IPVK-LGIIGV---------------------------------VNR------------
----------------------S-QMDIN----N--------------------------KKVID----------D----AVKD---ESAFL-----
----QR-------K---------------------Y----------------P-A------L-------------------------ASR-NGT-----
AYLARTLNRLLMHHIRDCL----
XP_032819300.1 ------------IQLPQIVVVGA----Q---------------SSGKSSVLE-----S---
LV-GRDFLPRG---TGIVTRRPL---------VLQ---L-V-HV-IP------------------DERIRPGGEEN------------
---------------------------GVEAE---EW---G---------------------------------K---------------FLH------
-----------------------------------TKN------KVY-----SDFN---EIRQEIENETER-----ITGTN---------------
----KG------------------------IS--SEAIHLKIFSPH---
-----------VLNLTLVDLPGITK--VP------------------VGDQPV----------DI---EQQIR--------ELIIKFIGN-
-----------PNSIILAVTAANT--DLA-TS--EALKIAREVDTDGRRTLAVI-TKLDLMDAGT--------
DAMDILTGR---V--------------------IPVK-LGIIGV---------------------------------VNR-------------------
----------------S-QLDIN----T--------------------------KKTIL----------D----AMQD---EQSFM---------
QK-------K---------------------Y----------------P-S------L-------------------------ANR-NGT-----
KFLGKTLNRLLMHHIRDCL----
NP_957216.1 ------------IQLPQIAVVGT----Q---------------SSGKSSVLE-----S---LV-
GRDLLPRG---TGIVTRRPL---------ILQ---L-V-HV-DP------------------EDRRKTS-EEN----------------
-----------------------GVDGE---EW---G----------------------------------K---------------FLH----------
--------------------------------TKN------KIY-----TDFD---EIRQEIENETER-----VSGNN------------------
-KG-------------------------IS--DEPIHLKIFSPH--
--------VVNLTLVDLPGITK--VP------------------VGDQPK----------DI---ELQIR--------ELILKYISN----
--------PNSIILAVTAANT--DMA-TS--EALKVAREVDPDGRRTLAVV-TKLDLMDAGT--------
DAMDVLMGR---V--------------------IPVK-LGLIGV---------------------------------VNR----------------
-------------------S-QLDIN----N--------------------------KKSVA----------D----SIRD---EHGFL--------
-QK-------K---------------------Y----------------P-S------L-------------------------ANR-NGT-----
KYLARTLNRLLMHHIRDCL----
XP_025940269.1 ------------IQLPQIVVVGT----Q---------------SSGKSSVLE-----S---
LV-GRDLLPRG---TGVVTRRPL---------ILQ---L-V-HV-SP------------------EDGRKTAGDEN----------
------------------------------EIDAE---EW---G----------------------------------K---------------FLH-----
--------------------------------------TKN------KVY-----TDFD---EIRQEIENETER-----ISGNN------------
------KG-------------------------IS--PEPIHLKIFSSN---
-------------VVNLTLVDLPGMTK--VP------------------VGDQPK----------DI---ELQIR--------
ELILQFISN------------PNSIILAVTAANT--DMA-TS--EALKIAREVDPDGRRTLAVI-TKLDLMDAGT----
----DAMDVLMGR---V--------------------IPVK-LGIIGV---------------------------------VNR------------
----------------------S-QLDIN----N--------------------------KKSVA----------D----SIRD---EYGFL------

---QK-------K---------------------Y----------------P-S------L-------------------------ANR-NGT-----
KYLARTLNRLLMHHIRDCL----
XP_006168142.1 ------------IQLPQIVVVGT----Q---------------SSGKSSVLE-----S---
LV-GRDLLPRG---TGIVTRRPL---------ILQ---L-V-HV-SP------------------EDKRKTTGEEN-----------
---------------DPATWKNSRHLSKGVEAE---EW---G---------------------------------K---------------
FLH---TKN------KLY-----TDFD---EIRQEIENETER-----ISGNN---
----------------KG------------------------VS--PEPIHLKIFSPN--
-----------------------VVNLTLVDLPGMTK--VP------------------VGDQPK----------DI---ELQIR--------
ELILRFISN------------PNSIILAVTAANT--DMA-TS--EALKISREVDPDGRRTLAVI-TKLDLMDAGT-----
---DAMDVLMGR---V--------------------IPVK-LGIIGV---------------------------------VNR-------------
--------------------S-QLDIN----N--------------------------KKSVT----------D----SIRD---EYAFL--------
-QK-------K---------------------Y----------------P-S------L-------------------------ANR-NGT-----
KYLARTLNRLLMHHIRDCL----
NP_001317309.1 ------------IQLPQIVVVGT----Q---------------SSGKSSVLE-----S---
LV-GRDLLPRG---TGIVTRRPL---------ILQ---L-V-HV-SQ------------------EDKRKTTGEEN-----------
----------------DPATWKNSRHLSKGVEAE---EW---G---------------------------------K---------------
FLH---TKN------KLY-----TDFD---EIRQEIENETER-----ISGNN---
----------------KG------------------------VS--PEPIHLKIFSPN--
-----------------------VVNLTLVDLPGMTK--VP------------------VGDQPK----------DI---ELQIR--------
ELILRFISN------------PNSIILAVTAANT--DMA-TS--EALKISREVDPDGRRTLAVI-TKLDLMDAGT-----
---DAMDVLMGR---V--------------------IPVK-LGIIGV---------------------------------VNR-------------
--------------------S-QLDIN----N--------------------------KKSVT----------D----SIRD---EYAFL--------
-QK-------K---------------------Y----------------P-S------L-------------------------ANR-NGT-----
KYLARTLNRLLMHHIRDCL----
NP_001392186.1 ------------IQLPQIVVVGT----Q---------------SSGKSSVLE-----S---
LV-GRDLLPRG---TGVVTRRPL---------ILQ---L-V-HV-SP------------------EDKRKTTGEENGKFQS--
-------------------WNPATWKNSRHLSKGVEAE---EW---G---------------------------------K-----------
-----FLH---TKN------KLY-----TDFD---EIRQEIENETER-----
ISGNN-------------------KG------------------------VS--PEPIHLKVFSPN---------------------------------
-----------------------------------VVNLTLVDLPGMTK--VP------------------VGDQPK----------DI---
ELQIR--------ELILRFISN------------PNSIILAVTAANT--DMA-TS--EALKISREVDPDGRRTLAVI-
TKLDLMDAGT--------DAMDVLMGR---V--------------------IPVK-LGIIGV------------------------------
---VNR----------------------------------S-QLDIN----N--------------------------KKSVT----------D----
SIRD---EYAFL---------QK-------K---------------------Y----------------P-S------L----------------------
--ANR-NGT-----KYLARTLNRLLMHHIRDCL----
XP_014394711.1 ------------LLCPVCVQISS----L---------------SSGKSSVLE-----S---
LV-GRDLLPRG---TGIVTRRPL---------ILQ---L-V-HV-SP------------------EDQRKTSGEEN-----------
----------------DPATWKNSRHLSKGVEAE---EW---G---------------------------------K---------------
FLH---TKN------KLY-----TDFD---EIRQEIENETER-----ISGNN---
----------------KG------------------------VS--PEPIHLKIFSPN--
-----------------------VVNLTLVDLPGMTK--VP------------------VGDQPK----------DI---ELQIR--------
ELILRFISN------------PNSIILAVTAANT--DMA-TS--EALKISREVDPDGRRTLAVI-TKLDLMDAGT-----
---DAMDVLMGR---V--------------------IPVK-LGIIGV---------------------------------VNR-------------
--------------------S-QLDIN----N--------------------------KKSVT----------D----SIRD---EYAFL--------

-QK-------K---------------------Y-----------------P-S------L-------------------------ANR-NGT-----
KYLARTLNRLLMHHIRDCL----

XP_028602039.1 ------------IQLPQIVVVGT----Q----------------SSGKSSVLE-----S---
LV-GRDLLPRG---TGIVTRRPL---------ILQ---L-V-NV-SA-------------------EDLRKKTGDEN-----------
----------------DPATWKHARHLTKGVDTE---EW---G-----------------------------------K----------------
FLH---TKN------KLY-----SDFD---EIRQEIESETER-----ISGNN---
----------------KG------------------------IS--PEPIHLKVFSPN---
-----------------------VVNLTLVDLPGMTK--VP-------------------VGDQPK----------DI---ELQIR--------
ELILRFISN------------PNSIILAVTAANT--DMA-TS--EALKIAREVDPDGRRTLAVI-TKLDLMDAGT-----
---DAMDVLMGR---V---------------------IPVK-LGIIGV----------------------------------VNR--------------
---------------------S-QLDIN----N--------------------------KKSVA----------D----SIRD---EYGFL-------
--QK-------K---------------------Y-----------------P-S------L-------------------------ANR-NGT-----
KYLARTLNRLLMHHIRDCL----

XP_012382650.2 ---G---
FKLVISTKL---------ILC---C-F-SL-TT---
DPATWKNSRHLSKGVEAE---EW---G-----------------------------------K----------------FLH-----------
--------------------------------TKN------KLY-----TDFD---EIRQEIENETER-----ISGNN-------------------
KG------------------------VS--PEPIHLKIFSPN---
--------VVNLTLVDLPGMTK--VP-------------------VGDQPK----------DI---ELQIR--------ELILRFISN---
---------PNSIILAVTAANT--DMA-TS--EALKISREVDPDGRRTLAVI-TKLDLMDAGT--------
DAMDVLMGR---V---------------------IPVK-LGIIGI----------------------------------VNR-----------------
-------------------S-QLDIN----N--------------------------KKSVT----------D----SIRD---EYAFL---------
QK-------K---------------------Y-----------------P-S------L-------------------------ANR-NGT-----
KYLARTLNRLLMHHIRDCL----

XP_031753959.1 ------------IGFGPVLRVPL----Q----------------PAKCWAAKE-----S---
MVLSASLLP-V---PHLYERLLL---------SVV---F-F-SL-IP--
------DPNAWKIPKHFSKGVETE---EW---G-----------------------------------K----------------FLH------
-------------------------------------TKN------KIY-----TDFD---EIRQEIENETER-----ISGNN---------------
----KG------------------------IS--SEPIHLKIFSPN--
-----------VVNLTLVDLPGMTK--VP-------------------VGDQPK----------DI---EIQIR--------
ELILRYISN------------PNSIILAVTAANT--DMA-TS--EALKIARESDPDGRRTLAVI-TKLDLMDAGT-----
---DAMDVLLGR---V---------------------IPVK-LGIIGV----------------------------------VNR--------------
---------------------S-QLDIN----N--------------------------KKSVA----------D----SIRD---EYGFL-------
--QK-------K---------------------Y-----------------P-S------L-------------------------ANR-NGT-----
KYLARTLNRLLMHHIRDCL----

PAA85687.1 ------------IQLPQIVVIGT----Q----------------SSGKSSVLE-----S---LV-
GRDFLPRG---TGIVTRRPL---------VLQ---L-V-HL-EA-------------------DEKDEAGDRPA--------------
---------------------------AEEE---EW---G-----------------------------------K----------------FLH---------
----------------------------------TKG------KIY-----TDFN---EIRDEIARETDR-----IAGSG------------------
-KC------------------------VS--IDPINLKIYSPH---
--------VVSLTLVDLPGITK--VP-------------------VADQPE----------DI---EVQIR--------ALCIEYIKN----
--------PNSIILAVTPANT--DMA-TS--ESLKLAKEVDPQGKRTLAVI-TKLDLMDAGT--------
DAHDLLLGR---V---------------------IPVK-LGIIGV----------------------------------VNR------------------
-----------------S-QADIK----N--------------------------QKQVK----------E----AVRD---ESSFL---------

QR-------R---------------------Y-----------------P-S------L-------------------------ASR-NGT-----
PYLARTLNRLLMHHIRDCL----
NP_741403.2 ------------IQLPQIVVVGS----Q----------------SAGKSSVLE-----N---LV-
GRDFLPRG---TGIVTRRPL---------ILQ---L-N-HV-AL-------------------DDESKRRRSNG--------------
-------------------------TLLTD---DW---A-----------------------------------M----------------FEH---------
---------------------------------TGS------KVF-----TDFD---AVRKEIEDETDR-----VTGVN----------------
---KG-------------------------IS--LLPISLKIYSHR---
---------VVSLSLVDLPGITK--IP-------------------VGDQPV----------NI---EEQIR--------EMILLYISN---
---------PSSIILAVTPANQ--DFA-TS--EPIKLAREVDAGGQRTLAVL-TKLDLMDQGT--------
DAMDVLMGK---V--------------------IPVK-LGIIGV---------------------------------VNR----------------
-----------------S-QQNIL----D--------------------------NKLIV----------D----AVKD---EQSFM---------
QK-------K---------------------Y-----------------P-T------L-------------------------ASR-NGT-----
PYLAKRLNMLLMHHIRNCL----
XP_042914770.1 ------------LDLPQVAVVGS----Q----------------SSGKSSVLE-----A---
LV-GRDFLPRG---NDIVTRRPL---------LLQ---L-V-KT-TP--
-----------------GPT--GRPS---EW---G-----------------------------------E----------------FLH------------
------------------------------APG------KMF-----YDFD---RIRDEIHQETER-----LVGYN-----------------
-KN-------------------------VS--DKPIRLKIFSPR--
--------VLTMTLVDLPGLTR--VP------------------VGDQPG----------DI---EQRIR--------EMALEYIRR-
-----------PNCIILAVSPANV--DLA-TS--DALQLSQVADPEGVRTIGVL-TKLDIMDRGT--------
DAAHILRNA---H--------------------IPLR-LGYIGV---------------------------------VLR-----------------
-----------------A-QADIA----A--------------------------KLPMS----------E----CRKR---EESFF---------
AS-------RA-------------------EY-----------------R-D------V-------------------------AAH-CGV-----
PTLARRLNVILVEHIR-------
PWZ09977.1 ------------IDLPQVAVVGS----Q----------------SSGKSSVLE-----A---LV-
GRDFLPRG---SDICTRRPL---------VLQ---L-V-HQ-PR--
---------------RPAD-AEAD---EW---G-----------------------------------E----------------FLH-------------
-----------------------------LSG------RRF-----YDFR---EIRREIQAETDR-----EAGGN------------------
KG-------------------------VS--DRQIRLKIFSPN--
--------VLNITLVDLPGITK--VP------------------VGDQPT----------DI---EARIR--------TMIMSYIKH----
--------KTCIILAVSPANA--DLA-NS--DALQMARVADPDGSRTIGVI-TKLDIMDRGT--------
DARNFLLGN---V--------------------IPLK-LGYVGV---------------------------------VNR----------------
------------------S-QQDIN----S--------------------------DLSIK----------D----ALAR---EEKFF---------
RT-------QP-------------------AY-----------------H-G------L-------------------------AQY-CGI-----
PQLAKKLNQILVQHIK-------
KAH9304002.1 ---

-------------MVQAETER-----EVGVN-------------------KG------------------------VS--DKQIRLKIYSPN-
--VLNITLVDLPGLTK--VP------------------
VGDQPT----------DI---EARIR--------TMIMSYIKH------------ETCIILAVSPANA--DLA-NS--
DALQMARVADADGHRTIGVI-TKLDIMDRGT--------DATNFLLGN---V--------------------IPLR-
LGYVGV---------------------------------VNR----------------------------------S-QADIN----G------------

---------------NKSIQ----------D----ALAY---EERFF---------RS-------RP-------------------VY------------
-----H-R------L-------------------------ADR-CGI-----PQLAKKLNYILVQHIR-------
EFJ15047.1 --------------LPQVAVVGS----Q----------------SSGKSSVLE-----A---LV-
GRDFLPRG---CDICTRRPL---------VLQ---L-V-QT-TR--
--------------RGD---EVV---EW---G----------------------------------E----------------FLH---------------
-----------------------------LPN------RRF-----TDFS---AIRKEIQAETDR-----ELGSN-------------------
KG------------------------IS--DKQIRLKIFSPK--
-------VLNITLVDLPGITK--VP-------------------VGDQPT----------DI---EARIR--------TMILSYIKH------
------ETCIILAVSPANA--DLA-NS--DALQMARIADPDGSRTIGVI-TKLDIMDRGT--------
DARNFLLGS---V---------------------IPLR-LGYIGV---------------------------------VNR-----------------
------------------S-QEDIT----S--------------------------NRSIQ----------D----ALMY---EEQFF---------
RS-------RP-------------------VY-----------------H-S------L-------------------------SDR-CGI-----
PQLAKKLNQILVQHIRTILP---
EFJ37641.1 ------------IDLPQVAVVGS----Q----------------SSGKSSVLE-----A---LV-
GRDFLPRG---CDICTRRPL---------VLQ---L-V-QT-TR--
--------------RGD---EVV---EW---G----------------------------------E----------------FLH---------------
-----------------------------LPN------RRF-----TDFT---AIRKEIQAETDR-----ELGSN-------------------
KG------------------------IS--DKQIRLKIFSPK--
-------VLNITLVDLPGITK--VP-------------------VGDQPT----------DI---EARIR--------TMILSYIKH------
------ETCIILAVSPANA--DLA-NS--DALQMARIADPDGSRTIGVI-TKLDIMDRGT--------
DARNFLLGS---V---------------------IPLR-LGYIGV---------------------------------VNR-----------------
------------------S-QEDIT----S--------------------------NRSIQ----------D----ALMY---EEQFF---------
RS-------RP-------------------VY-----------------H-S------L-------------------------SDR-CGI-----
PQLAKKLNQILVQHIR-------
KAI5070335.1------------IDLPQVAVIGS----Q----------------SSGKSSVLE-----A---LV-
GRDFLPRG---SDICTRRPL---------VLQ---L-V-QL-VR---
--------------RPDDKSDVM---EW---G----------------------------------E----------------FLH------------
--------------------------------TPG------KRY-----TDFS---LIRKEIQAETER-----ELGSN-------------------
RG------------------------IS--DKQIRLKIFSPS---
-------VLNITLVDLPGITK--VP-------------------VGDQPS----------DI---EARVR--------TMILSYIKH-----
-------ETCIILAVSPANA--DLA-NS--DALQMARMADPDGSRTIGVI-TKLDIMDRGT--------
DARNFLLGS---A---------------------IPLR-LGYIGV---------------------------------VNR-----------------
------------------S-QADIL----G--------------------------NKSIR----------E----ALAY---EDNFF---------
RS-------LP-------------------VY-----------------H-S------L-------------------------ADR-CGI-----
PQLAKKLNNILVQHIK-------
KAI5070758.1------------VNLPQVAVVGS----Q----------------SSGKSSVLE-----G---LV-
GRDFLPRG---SDICTRRPL---------VLQ---L-I-QT-TR--
-------------RPDEKSEVV---EW---G----------------------------------E----------------FLH---------------
-----------------------------APG------KRF-----TDFS---LIRKEIEAETER-----ELGSN-------------------KG-
------------------------IS--DKQIRLKIFSPN--
--VLTITLVDLPGITK--VP-------------------VGDQPS----------DI---ESRVR--------TMILSYIKH-----------
-ETCIILAVSPANA--DLA-NS--DALQMARLADPDGSRTIGVI-TKLDIMDRGT--------DARNLLLGN---
V--------------------IPLR-LGYIGV---------------------------------VNR----------------------------------
S-QEDIL----G--------------------------NKSVV----------D----ALLY---EENFF---------RS-------RP-----

----------------VY------------------H-N------L----------------------------ADR-CGI-----
AQLAKKLNTILVQHIK-------
PTQ35749.1 ------------IDLPQVAVVGS----Q----------------SSGKSSVLE-----A---MV-
GRDFLPRG---ADICTRRPL---------VLQ---L-V-QT-VR--
---------------RPEDRSELV---EW---G---------------------------------------E----------------FLH--------------
-------------------------------IPG------RRF-----TDFT---AIRKEIQAETDR-----ELGTN-------------------
KG---------------------------IS--EKQIRLKIFSPN---
-------VLNITLVDLPGITK--VP------------------VGDQPS----------DI---EARVR--------TMILNYIKH----
--------ETCIILAVSPANA--DLA-NS--DALQMARIADPDGSRTIGVI-TKLDIMDRGT--------
DARNFLLGN---V----------------------VPLR-LGYIGV-------------------------------------VNR----------------
-------------------S-QEDIQ----A----------------------------NKTIR----------E----ALGY---EENFF---------
RS-------RP--------------------VY------------------H-S------L----------------------------SER-CGI-----
PQLAKKLNSILVQHIR-------
KAG0555682.1 ------------IDLPQVAVVGS----Q----------------SSGKSSVLE-----A---
LV-GRDFLPRG---SDICTRRPL---------VLQ---L-V-QT-SR--
--------------------RPEDRTELV---EW---G---------------------------------------E----------------FLH-----------
------------------------------------IPG------RRF-----TDFA---AIRKEIQAETDR-----ELGTN-------------------
KG---------------------------IS--EKQIRLKIFSPN---
-------VLNITLVDLPGITK--VP------------------VGDQPN----------DI---EARVR--------TMILSYIKH----
--------ETCIILAVSPANA--DLA-NS--DALQMARIADPDGSRTIGVI-TKLDIMDRGT--------
DARNFLLGN---V----------------------IPLR-LGYIGV-------------------------------------VNR-----------------
-------------------S-QEDII----A----------------------------NKSIR----------D----ALVY---EEGFF---------
RS-------KP--------------------VY------------------H-N------L----------------------------ADR-CGV-----
PQLAIRLNTILVQHIK-------
KAG0554580.1 ------------IDLPQVAVVGS----Q----------------SSGKSSVLE-----A---
LV-GRDFLPRG---SDICTRRPL---------VLQ---L-V-QT-LR--
------------------RPDEKSEPV---EW---G---------------------------------------E----------------FLH-----------
-----------------------------------IPG------RRF-----TDFS---AIRKEIQLETER-----ELGTN-------------------
KG---------------------------IS--EKQIRLKIFSPN---
-------VLNITLVDLPGITK--VP------------------VGDQPT----------DI---EARVR--------TMILSYIKH-----
-------ETCIILAVSPANA--DLA-NS--DALQMARIADPDGSRTIGVI-SKLDIMDRGT--------
DARSFLLGN---I----------------------IPLR-LGYVGV-------------------------------------VNR-----------------
-------------------S-QEDIS----A----------------------------NKSIR----------D----ALTY---EENFF---------
RS-------RP--------------------VY------------------H-N------L----------------------------SDR-CGV-----
PQLAKKLNTILVQHIK-------
XP_024362051.1 ------------IDLPQVAVVGS----Q----------------SSGKSSVLE-----A---
LV-GKDFLPRS---SDICTRRPL---------VLQ---L-V-QT-LR---
------------------RSDEKSELV---EW---G---------------------------------------E----------------FLH-----------
-----------------------------------IPG------RQF-----TNFS---AIRKEIQLETER-----DMGTN------------------
-KG---------------------------IS--EKQIRLKIFSPD--
--------VLNITLVDLPGITK--VP------------------VGDQPT----------DI---EARVR--------TMILSYIKH----
--------KTCIILAVSPANA--DLA-NS--DALQMARIADPDGSRTIGVI-SKLDIMDRGT--------
DARSLLLGN---V----------------------IPLR-LGYVGV-------------------------------------VNR-----------------
-------------------S-QEDIS----R----------------------------NRSIR----------D----ALTN---EENFF---------

RS-------RP-------------------VY-----------------H-N------L-------------------------SDR-CGV-----
PQLAKKLNTILVQHIK-------
AAC61784.1 ------------IALPQVVVVGS----Q----------------SSGKSSVLE-----A---LV-
GRDFLPRG---NDICTRRPL---------VLQ---L-L-QT-KS---
--------------RANG-GSDD---EW---G-----------------------------------E----------------FRH------------
-------------------------------LPE------TRF-----YDFS---EIRREIEAETNR-----LVGEN-------------------
KG-------------------------VA--DTQIRLKISSPN--
--------VLNITLVDLPGITK--VP-------------------VGDQPS----------DI---EARIR--------TMILSYIKQ-----
-------DTCLILAVTPANT--DLA-NS--DALQIASIVDPDGHRTIGVI-TKLDIMDKGT--------
DARKLLLGN---V---------------------VPLR-LGYVGV-----------------------------------VNR----------------
-------------------C-QEDIL----L---------------------------NRTVK----------E----ALLA---EEKFF---------
RS-------HP-------------------VY-----------------H-G------L-------------------------ADR-LGV-----
PQLAKKLNQILVQHIK-------
XP_052310486.1 ------------IELPQVAVVGS----Q----------------SSGKSSVLE-----A---
LV-GRDFLPRG---NEICTRRPL---------VLQ---L-L-QT-KR---
------------------KGDG-SGED---EW---G-----------------------------------E----------------FLH---------
-----------------------------------LPG------KRF-----YDFS---EIRSEIQAETAK-----EAGGN----------------
---KG-------------------------VS--DKQIRLKIFSPN---
-----------VLDITLVDLPGITK--VP-------------------VGDQPS----------DI---EARIR--------TMIMSYIKK-
-----------PSCLILAVTAANS--DLA-NS--DALQIAGNADPDGYRTIGII-TKLDIMDRGT--------
DARNLLLGK---V---------------------IPLR-LGYVGV-----------------------------------VNR-----------------
-------------------S-QEDII----L---------------------------NRSIK----------D----ALAA---EEKFF---------
RS-------RP-------------------VY-----------------N-G------L-------------------------ADR-CGV-----
PQLAKKLNQILVQHIK-------
ONM18162.1 ------------LELPQVAAIGG----Q----------------SSGKSSVLE-----A---LV-
GRDFLPRG---PDICTRRPL---------VLQ---L-V-RH---
------------------AAPE---EW---G-----------------------------------E----------------FLH------------------
--------------------------VPG------RQF-----HDFE---QIKREIQLETDK-----EAGDN-------------------KG-
-------------------------VS--EKQIRLKIFSPN---
---VLDITLVDLPGITR--VP-------------------VGDQPS----------DI---ESRIR--------AMIMQYIKH--------
----PSCIILAVSPANA--DLA-NS--DALQLARLADPDGSRTIGVI-TKLDIMDRGT--------DARNFLLGN-
--V---------------------IPLK-FGYVGV-----------------------------------VNR-----------------------------------
--S-QEDIN----F---------------------------NRSVK----------D----ALAF---EEKYF---------LT-------LP----
----------------AY-----------------H-G------L-------------------------AHC-CGV-----
PQLAKKLNMILLKHVT-------
KAH9327796.1 --MYP-----W---LV-VKEF---
-----------PD---------AIA---L---

--------------------------QVETER-----EAGGN-------------------KG-------------------------IS--
AKQIRLKIFSPF--VLNINLVDLPGIMK--
VP-------------------VGDQPI----------DI---EARIR--------TMIMSYIKH------------PSCIILAVSPANS--
DLA-NS--DALQIARVADTDGSRTIGVI-TKLDIMDRGT--------DARNFLLGN---V---------------------
IPLQ-LGYIGV-----------------------------------VNR-----------------------------------S-QEDII----A-------

-------------------NQSIR----------D----ALAY---EENFF---------RG-------HP-------------------VY------
-----------C-S------L-------------------------ADQ-CGI-----PQLARKLNQILVKHIR-------

XP_014148015.1 --LQ---------LV--
HHPVQRGG---------PA----------------A--
----------------EW---G------------------------------------E---------------FLH-----------------------------
-------------QPG------KIY-----TDFS---KIRDEIANETDR-----LTGTN-------------------KG----------------
---------IS--HTPINLKLYSPN---
MLDLTLVDLPGITK--IA-------------------VGDQPE----------DI---EVQIH--------QLIESYINN-----------
-PNCIILAVTAANT--DIA-NS--DALKMAKKADPKGLRTIGVA-TKLDLMDAGT--------DALDILTGK---V-
--------------------VASK-LGFIGV----------------------------------VNR-----------------------------------S-
QADIN----Q--------------------------KVSIE---------T----AREA---EQEYF---------RT-------HP---------
-----------AY-----------------K-S------L-------------------------YKQ-SGT-----
EYLTRRLNQLLMTHIRRCL----

XP_001750431.1 ------------IQLPQIVVVGA----
QASSPADHPALRMSHEQSSGKSSILE-----N---VV-GKDFLPRG---TGIVTRVPL---------VLQ---L-V-
QT-AD--D---EW---A------------------
------------------T---------------FQH--AGG------KVF-----RDFE--
-QVRQEIVDQTER-----ITGPG-------------------KA------------------------VS--NEPIHLRVHSPN------
---VVNLTLVDLPGLTK--VA-------------------
VADQPQ----------DI---GPQIR--------RLVRHYIDN------------PNSLILAVSPANA--DIA-NS--
DSLQIAKEVDPQGDRTLAIV-TKLDLMDRGT--------DAKALLSGE---V---------------------LPVK-
LGIIGI-----------------------------------VNR-----------------------------------S-QNDIN----C--------------
-------------KTSIQ----------D----SLDN---EKRFF---------RT-------H---------------------Y---------------
--P-E------M-------------------------ADR-CGC-----AFLADTLHHLLLQHIRACL----

XP_042924642.1 --------------LPTIVVVGG----Q----------------SSGKSSVLE-----A---
VV-GRDFLPRG---TGIVTRRPL---------VLQ---L---VK-TD--
---------------------DPNAV---DY---G-----------------------------------E---------------FAH--------------
----------------------------APG------RKF-----TNFD---DITTEIEDETTRH--LQRQGGT-------------------
KV------------------------VS--PDPIYLTVYSVN--
-------VPNLTLVDMPGLTK--VP-------------------IDGQPA----------SI---VQELD--------
DMARQYVKS------------DNAIILAVTPANA--DLA-TS--DALRMARDVDPSGDRTIGVL-
TKVDIMDRGT--------DCRDVLLGK---T---------------------LKLK-HGWVAV-----------------------------
-----VNR-----------------------------------G-QADLN----S---------------------------KVTMK----------D---
-ARAR---EQEFF---------KG-------KP-------------------EY----------------Q-D------L------------------
-------QN--TGT-----TFLAEKLSNHLINEIMKSLP---

NP_001190448.1 --------------LPAIAVVGG----Q----------------SSGKSSVLE-----S---IV-
GKDFLPRG---SGIVTRRPL---------VLQ---L---QK-ID---
-------------------DGTR---EY---A-----------------------------------E---------------FLH-------------------
-------------------------LPR------KKF-----TDFA---AVRKEIQDETDR-----ETGRS-------------------KA----
---------------------IS--SVPIHLSIYSPN--
VVNLTLIDLPGLTK--VA-------------------VDGQSD----------SI---VKDIE--------NMVRSYIEK----------
--PNCIILAISPANQ--DLA-TS--DAIKISREVDPSGDRTFGVL-TKIDLMDKGT--------DAVEILEGR---S-
--------------------FKLK-YPWVGV---------------------------------VNR-----------------------------------
S-QADIN----K--------------------------NVDMI---------A----ARKR---EREYF---------SN-------TT-----

---------------EY-----------------R-H------L--------------------------ANK-MGS-----
EHLAKMLSKHLERVIKSRIP---
XP_002299468.1 --------------LPSIAVVGG----Q----------------SSGKSSVLE-----S---
VV-GKDFLPRG---SGIVTRRPL---------VLQ---L---HK-ID---
----------------------EGSR---EY---A-----------------------------------E----------------FLH--------------
----------------------------LPR------KRF-----TDFA---AVRREIQDETDR-----ETGRS------------------
KQ------------------------IS--SVPIHLSIYSPN--
-------VVNLTLVDLPGLTK--VA-------------------VEGQPD----------SI---VQDIE--------NMVRAYIEK---
---------PNCIILAISPANQ--DLA-TS--DAIKISREVDPTGERTLGVL-TKIDLMDKGT--------
DAVDMLEGK---S---------------------YRLK-FPWVGV-----------------------------------VNR--------------
---------------------S-QADIN----K--------------------------NVDMI----------A----ARRR---EREYF------
---SS-------TP--------------------EY-----------------K-H------L-------------------------AHR-MGS-----
EHLAKMLSKHLEVVIKSKIP---
XP_002302631.1 --------------LPSIAVVGG----Q----------------SSGKSSVLE-----S---IV-
GKDFLPRG---AGIVTRRPL---------VLQ---L---HK-ID---
------------------EG-K---EY---A-----------------------------------E----------------FMH-------------------
------------------------LPR------KKF-----TDFA---AVRKEIADETDR-----ETGRS-------------------KQ----
---------------------IS--SVPIHLSIFSPN---
VVNLTLVDLPGLTK--VA-------------------IDGQPE----------SI---VHDIE--------NMVRSYIEK----------
--PNCIILAISPANQ--DLA-TS--DAIKISREVDPRGERTFGVL-TKIDLMDKGT--------DAVDILEGK---S-
--------------------YKLQ-FPWIGV-----------------------------------VNR------------------------------------
S-QADIN----K---------------------------SVDMI----------A----ARRR---EREYF---------QS-------SP-----
---------------EY-----------------G-H------L--------------------------ASR-MGS-----
EHLGKMLSKHLEQVIKSRIP---
AQK88296.1 --------------ISRVAGGTA----R----------------SSGKSSVLE-----S---VV-
GKDFLPRG---SGIVTRRPL---------VLQ---L---HR-ID--
-------------------GDR---EY---A-----------------------------------E----------------FMH-------------------
------------------------LPR------KRF-----TDFA---AVRKEIADETDR-----ETGRS-------------------KQ----
---------------------IS--TVPIHLSIFSPHGKMQTLEGYIVTVALSHLNCPLP-------------------------------
---------------IVNLTLIDLPGLTK--VA-------------------VDGQPE----------SI---VHDIE--------
NMVRSYIEK------------PNCIILAVSPANQ--DLA-TS--DAIKISREVDPKGERTFGVL-TKIDLMDKGT---
-----DAVDILEGR---S---------------------YRLQ-TPWVGV-----------------------------------VNR----------
-------------------------S-QQDIN----K---------------------------NVDMI----------A----ARRR---EREYF--
-------AS-------TP--------------------EY-----------------K-H------M-------------------------ASR-MGS---
--EYLGKMLSKHLEQVIKSRIP---
PTQ45603.1 --------------LPSVVVVGG----Q----------------SSGKSSVLE-----S---IV-
GRDFLPRG---SGIVTRRPL---------VLQ---L---HK-T---
-----------------DDGS---DY---A------------------------------------E----------------FLH-------------------
------------------------HPR------RRF-----ADFA---AVRKEIADETDR-----VTGRS-------------------KM--
-----------------------IS--PVPIHLSIYSPN---
-VVNLTLIDLPGLTK--VA-------------------VEGQPD----------SI---VHDIE--------NMVRSYVEK--------
----PNSIILAISPANQ--DIA-TS--DAIKLAREVDPAGERTWGVL-TKLDLMDRGT--------NALDVLEGR-
--S---------------------YRLQ-LPWIGV----------------------------------VNR--------------------------------
---S-QADIN----K--------------------------NVDMI----------A----ARRR---EREYF---------QS-------SQ-

-------------------DY-----------------G-H------L--------------------------AGK-MGS-----
EYLAKMLSKHLEAVIKSRIP---
KAI5072318.1--------------LPSVAVVGG----Q----------------SSGKSSVLE-----S---IV-
GRDFLPRG---SGIVTRRPL---------VLQ---L---YR-T---
-----------------DKGP---EY---A-----------------------------------E----------------FLH-------------------
-----------------------APK------KRF-----TDFA---AVRKEISDETDR-----ITGRS-------------------KQ------
-------------------IS--PVPIHLSIYSPN--
VVNLTLIDLPGLTK--VA-------------------VEGQPD----------SI---VADIE--------NMVRSYVEK----------
--PNSLILAISPANQ--DIA-TS--DAIKLAREVDPSGERTFGVL-TKLDLMDKGT--------NALDTLEGR---
A---------------------YRLQ-HPWVGV----------------------------------VNR---------------------------------
---S-QADIN----K---------------------------SVDMM----------A----ARRR---EREYF---------AT-------SP-
-------------------DY-----------------K-H------L--------------------------ASR-MGS-----
EYLGQMLSKHLESVIKSRIP---
KAI5058380.1--------------LPSVAVVGG----Q----------------SSGKSSVLE-----S---IV-
GRDFLPRG---SGIVTRRPL---------VLQ---L---HK-L--
-----------------DEGS---EY---A-----------------------------------E----------------FMH-------------------
------------------------LPK------RRF-----TDFA---AVRKEIQDETDR-----VTGRS-------------------KQ---
---------------------IS--PVPIHLSIYSPH--
VVNLTLIDLPGLTK--VA-------------------VEGQPE----------SI---VADIE--------NMVRLYVDK----------
--PNTIILAISPANQ--DIA-TS--DAIKLAREVDPTGERTWGVL-TKLDLMDKGT--------NAIDVLEGR---
A---------------------YHLK-NPWIGV----------------------------------VNR------------------------------------
-S-QADIN----K---------------------------NVDMM----------A----ARRR---EREYF---------AT-------SS---
-----------------DY-----------------S-H------L--------------------------TSR-MGS-----
EYLGKMLSKHLEAVIKARIP---
XP_002987566.1 --------------LPSVVVVGG----Q----------------SSGKSSVLE-----S---
IV-GRDFLPRG---SGIVTRRPL---------VLQ---L---HK-T---
--------------------EGGA---EY---A-----------------------------------E----------------FLH-----------------
--------------------------IPK------KRF-----TDFS---LVRKEIQDETDR-----VTGRS-------------------KQ---
---------------------IS--PIPIQLSIYSPN--
VVNLTLIDLPGLTK--IA-------------------IEGQPD----------SI---VADIE--------NMVRSYVEK-----------
-QNSVILAISPANQ--DIA-TS--DAMKLAREVDPTGERTFGVL-TKLDLMDKGT--------NALDVLEGR---
S---------------------YKLQ-HPWVGV----------------------------------VNR------------------------------------
--S-QADIN----R---------------------------SVDMV----------A----ARRR---EREYF---------SS-------SA--
------------------DY-----------------G-H------L--------------------------TSR-MGS-----
EYLAKILSKHLEAFIKARIP---
KAI5602084.1--------------LPSVAVVGG----Q----------------SSGKSSVLE-----S---IV-
GRDFLPRG---SGIVTRRPL---------VLQ---L---HK-TE---
------------------PGIT---EY---A-----------------------------------E----------------FLH--------------------
---------------------KQR------ERF-----TDFA---MVRKEIQDETDK-----ITGKS------------------KQ-----
--------------------IS--PVPIHLSIYSPN---
VVNLTLIDLPGLTK--VA-------------------VEGQPE----------SI---VKDIE--------NMVRLYVEK----------
--PNCIILAITPANQ--DIA-TS--DAIKLAREVDPAGERTFGVL-TKLDLMDKGT--------NAQDVLEGR---
A---------------------YPLQ-HPWVGI----------------------------------VNR---------------------------------
--S-QADIN----K---------------------------NVDMI----------A----ARRR---EREFF---------ST-------SP---

-----------------DY-----------------G-H------L-------------------------AGR-MGS-----
EYLAKLLSKHLESVIKTRIP---
XP_006375094.1 --------------LPSVAVVGG----Q----------------SSGKSSVLE-----S---
IV-GRDFLPRG---SGIVTRRPL---------VLQ---L---HK-TE---
--------------------DGSQ---EY---A-----------------------------------E----------------FLH----------------
--------------------------LPK------RRF-----SDFA---VVRKEIQDETDR-----ITGKT-------------------KQ-
----------------------IS--PVPIHLSIYSPN---
--VVNLTLIDLPGLTK--VA-------------------VEGQPE----------SI---VQDIE--------TMVRTYVEK---------
---PNCIILAISPANQ--DIA-TS--DAIKLAREVDPSGERTFGVL-TKLDLMDKGT--------NALDVIEGR---
S---------------------YRLQ-HPWVGI-----------------------------------VNR------------------------------------
--S-QADIN----K--------------------------NVDMI----------V----ARRK---EREYF---------AT-------SP----
----------------DY-----------------G-H------L-------------------------ANK-MGS-----
EYLAKLLSKHLESAIRARIP---
XP_002315854.1 --------------LPSVAVVGG----Q----------------SSGKSSVLE-----S---
VV-GRDFLPRG---SGIVTRRPL---------VLQ---L---HK-I---
--------------------DGGS---DY---A-----------------------------------E----------------FLH---------------
---------------------------APR------KKF-----TDFA---SVRKEIADETDR-----ITGKS-------------------KQ-
----------------------IS--NVPIHLSIYSPN--
---VVNLTLIDLPGLTK--VA-------------------VEGQPE----------SI---VEDIE--------NMVRSYVEK-------
-----PNSIILAISPANQ--DIA-TS--DAIKLAREVDPSGERTFGVL-TKLDLMDKGT--------NALDVIEGR--
-S---------------------YRLQ-HPWVGI-----------------------------------VNR-----------------------------------
---S-QADIN----K---------------------------NVDMI----------A----ARRK---EREYF---------ET-------SP--
------------------EY-----------------G-H------L-------------------------SSK-MGA-----
EYLAKLLSKHLETVIRQRIP---
NP_001147100.1 --------------LPSVAVVGG----Q----------------SSGKSSVLE-----S---
IV-GRDFLPRG---SGIVTRRPL---------VLQ---L---HK-T--
-------------------DGGH---EY---A-----------------------------------E----------------FLH----------------
--------------------------APR------KRF-----TDFA---AVRKEIADETDR-----ITGKT-------------------KA--
----------------------IS--NVPIHLSIYSPH--
--VVNLTLIDLPGLTK--VA-------------------VEGQPE----------SI---VQDIE--------NMVRAYVDK--------
----PNCIILAISPANQ--DIA-TS--DAIKLARDVDPSGDRTFGVL-TKLDLMDKGT--------NAVDVLEGR-
--Q---------------------YRLQ-HPWVGI-----------------------------------VNR----------------------------------
----S-QADIN----K---------------------------NVDML----------S----ARRK---EKEYF---------ES-------
SP--------------------EY-----------------G-H------L-------------------------AHK-MGA-----
EYLAKLLSQHLEAVIRAKIP---
AAF22292.1 --------------LPTVAVVGG----Q----------------SSGKSSVLE-----S---IV-
GRDFLPRG---SGIVTRRPL---------VLQ---L---HK-TD--
------------------DGTE---EY---A-----------------------------------E----------------FLH--------------------
----------------------LPK------KQF-----TDFA---LVRREIQDETDR-----ITGKN-------------------KQ-----
--------------------IS--PVPIHLSIYSPN---
VVNLTLIDLPGLTK--VA-------------------VEGQPE----------TI---AEDIE--------SMVRTYVDK-----------
-PNCIILAISPANQ--DIA-TS--DAIKLAKDVDPTGERTFGVL-TKLDLMDKGT--------NALEVLEGR---S-
--------------------YRLQ-HPWVGI-----------------------------------SEPF-----------------------------------
N-KQDIN----K--------------------------NVDMM----------L----ARRK---EREYF---------DT-------SP---

-----------------DY-----------------G-H------L---------------------------ASK-MGS-----
EYLAKLLSKHLESVIRTRIP---
KAG0556007.1 --------------LPSVAVVGG----Q---------------SSGKSSVLE-----S---
IV-GRDFLPRG---SGIVTRRPL---------VLQ---L---HK-T--
--------------------EDKY---EY---A-------------------------------------E---------------FLH------------------
-------------------------MPK------RRF-----TDFA---AVRKEISDETDR-----ITGRS-------------------KQ---
----------------------IS--PVPIHLSVYSPN---
-VVNLTLIDLPGLTK--IA-------------------VEGQSD----------SI---VGDIE--------NMVRSYIEK----------
--PNCIILAVSPANQ--DIA-TS--DAIKIAREVDPNGERTFGVL-TKLDLMDKGT--------NAIDVLEGR---
S--------------------YKLI-QPWIGV-----------------------------------VNR-----------------------------------
S-QQDIN----K--------------------------NVDMI----------A----ARRR---EREYF---------QT-------SP----
----------------DY----------------S-H------L-------------------------QSK-MGS-----
EYLGRVLSKHLEAVIRSRIP---
KAH9306600.1 --------------LPSVAVVGG----Q---------------SSGKSSVLE-----S---
IV-GRDFLPRG---SGIVTRRPL---------VLQ---L---HK-TD--
----------------------EGTP---EY---G-------------------------------------E---------------FLH----------------
---------------------------RPN------KRI-----TDFA---KVRSEIQEETDR-----ITGRT-------------------KM--
----------------------IS--PVPIHLSIYSPN---
-VVNLTLIDLPGLTK--VA-------------------VEGQPE----------SI---VGDIE--------
NMVRSYVEKLLKLVVTADFLLPNSIILAISPANQ--DIA-TS--DAIKLAREVDPTGERTFGVL-
TKLDLMDKGT--------NALDVLDGR---S--------------------YRLQ-HPWVGV----------------------------
-------VNR-----------------------------------S-QADIN----K--------------------------SVDMI----------A---
-ARRR---EREYF---------ST-------ST-------------------DY-----------------G-H------L-------------------
------SSR-MGS-----EYLAKLLSKHLENVIKARIP---
AAF79238.1 --------------LPTVAVVGG----Q---------------SSGKSSVLE-----S---VV-
GRDFLPRG---SGIVTRRPL---------VLQ---L---HK-TE--
------------------DGTT---EY---A-------------------------------------E---------------FLH--------------------
----------------------APK------KRF-----ADFA---AVRKEIEDETDR-----ITGKS-------------------KQ-----
--------------------IS--NIPIQLSIYSPNG----------
LCLRPHFLLCIPIVVSSETNTFDTSCNEITSSTIRFEDSNFANLFHVTLISHSTLFSTVVNLTLIDLPGLTK
--VAVVTDMNLVLKLVTDMNIIRVDGQPE----------SI---VQDIE--------NMVRSYVEK-----------
PNCIILAISPANQ--DIA-TS--DAIKLAREVDPTGERTFGVA-TKLDIMDKGT--------DCLDVLEGR---S--
-------------------YRLQ-HPWVGI-----------------------------------VNR-----------------------------------
S-QADIN----K--------------------------RVDMI----------A----ARRK---EQEYF---------ET-------SP-----
---------------EY-----------------G-H------L-------------------------ASR-MGS-----
EYLAKLLSQHLETVIRQKIP---
PWZ36850.1 --------------LPTIAVVGG----Q---------------SSGKSSVLE-----S---IV-
GTDFLPRG---SGIVTRRPL---------VLQ---L---QQ-TE--
------------------DGSQ---EY---A-------------------------------------E---------------FLH------------------
-----------------------MPK------RRF-----SDFA---LVRQEIADETDR-----LTGKT-------------------KQ---
----------------------IS--PVPIHLSIYSPK--
VVNLTMIDLPGLTK--VA-------------------VEGQSE----------SI---VQDIE--------NMVRSYVDK--------
----PNCIILAISPANQ--DIA-TS--DAIKLSKEVDPTGERTFGVL-TKLDLMDKGT--------NALDVLEGR--
-A--------------------YRLQ-NPWVGI-----------------------------------VNR-------------------------------

---S-QADIN----R---------------------------KVDMI----------S----AREK---EREYF---------ET-------SP---
-----------------DY-----------------A-H------L---------------------------SSR-MGS-----
GYLAKLLSQHLESVIKVRIP---
NP_850420.1 --------------LPSVAVVGG----Q----------------SSGKSSVLE-----S---IV-
GRDFLPRG---SGIVTRRPL---------VLQ---L---HK-TE--
------------------NGTE---DN---A-------------------------------------E----------------FLH------------------
-------------------------LTN------KKF-----TNFS---LVRKEIEDETDR-----ITGKN-------------------KQ----
---------------------IS--SIPIHLSIFSPN--
VVNLTLIDLPGLTK--VA-------------------VEGQPE----------TI---VEDIE--------SMVRSYVEK-----------
-PNCLILAISPANQ--DIA-TS--DAMKLAKEVDPIGDRTFGVL-TKLDLMDKGT--------NALDVINGR---
S---------------------YKLK-YPWVGI----------------------------------VNR-----------------------------------
S-QADIN----K----------------------------NVDMM----------V----ARRK---EREYF---------ET-------SP----
----------------DY-----------------G-H------L---------------------------ATR-MGS-----
EYLAKLLSKLLESVIRSRIP---
EFJ15761.1 --------------LPSVVVVGG----Q----------------SSGKSSVLE-----S---IV-
GRDFLPRG---SGIVTRRPL---------VLQ---L---HR-T---
-----------------EDGP---DY---A-------------------------------------E-----------------FLH--------------------
-----------------------LPK------KKF-----TDFA---LVRKEIQDETDR-----ITGRS-------------------KQ------
-------------------IS--PVPIHLSIYSRN---
VVNLTMIDLPGLTK--IA-------------------VDGQPE----------SI---VGDIE--------NMVRSYVEK---------
---ENTIILAISPANQ--DIA-TS--DAMKLARENIALGDRTFGVL-TKLDLMDKGT--------NAIDVLEGH---
S---------------------YRLQ-RPWIGV----------------------------------VNR----------------------------------
-S-QADIN----K---------------------------SVDMI----------V----ARRR---EREYF---------SS-------SP----
----------------DY-----------------R-H------L---------------------------ASR-MGS-----
EYLGRVLSKHLEAVIKARIP---
PTQ29980.1 --------------LPSVVVVGG----Q----------------SSGKSSVLE-----S---VV-
GKDFLPRG---SGIVTRRPL---------VLQ---L---QK-TE--
------------------DGTR---EW---A-------------------------------------E----------------FLH------------------
-------------------------APR------KRF-----EDFA---LVRKEISDETDR-----VGGR--------------------KG---
----------------------IS--KIPIHLTVYSPN--
VVNLTLVDLPGLTK--VA-------------------VEGQSD----------SI---VADIE--------DMVRSYVEK---------
---PNSIILAVSPANQ--DIA-TS--DAIKIAREVDPNGERTFGVA-TKLDLMDKGT--------NALDVLEGR---
T---------------------YRLQ-LGWVGV----------------------------------VNR---------------------------------
--S-QQDIN----K---------------------------NTDML----------A----ARRR---EREYF---------QT-------SQ-
-------------------DY-----------------G-H------L--------------------------ASR-MGS-----
EYLGKLLSKHLEQVIKARIP---
EFJ23099.1 --------------LPSVAVVGG----Q----------------SSGKSSVLE-----S---VV-
GRDFLPRG---SGIVTRRPL---------VLQ---L---HK-T--
-----------------EGGQ---EY---A-------------------------------------E-----------------FLH--------------------
-----------------------NPK------TKF-----SDFS---LVRKEIEDETDR-----MTGHT-------------------KQ----
---------------------IS--PVPIHLSIYSPNG---------TCLSHPVKFSMPWFVYS----------------------------
-------------VVNLTLIDLPGLTK--IA-------------------VGKCISFSK---MMCLLI---LADIE--------
NMVRSYVEK------------QNSIILAISPANQ--DIA-TS--DAMKLAKEVDPTGERTFGVL-TKLDLMDKGT-
-------NALEVLEGR---A---------------------YRLQ-FQWVGV----------------------------------VNR-------

---------------------------S-QADIN----K--------------------------SVDMI----------A----ARKK---
EREFF---------AS-------SP-------------------DY-----------------G-H------L-------------------------
ANR-MGS-----EYLAKMLSKHLETVIKTRLP---
EFJ35472.1 ------------VDLPQLVHVQR----K----------------------MD-----------DREF----
--S--
-EW---G-----------------------------------E---------------FLH--
LPG------RRF-----TDFS---HIRKEIQAETER-----ELGDK-------------------KG------------------------
VS--DKQIRLKIFSPN---
VLNITLVDLPGLTR--VP-------------------VGDQPS----------DI---ESRIR--------AMILSYIKH-----------
-ATCIILAVSPANT--DLS-NS--DSLQMAKLVDPDGSRTIGVV-TKLDIMDRGT--------DARSILLGT---V-
--------------------IPLR-LGYVGV----------------------------------VNR----------------------------------S-
QEVFR----SPFFCLKILMKHGCRIFMPTNRSRMLLLRKSIF----------S----EVER---CTKAF---------
WI-------D----------------------V------------------A-S------L--------------------------NSR-KSL-----
IKYELEPVKILVQHIK-------
KXN72852.1 ------------LNLPSIVVVGS----Q----------------SSGKSSVLE-----A---IV-
GKEFLPKG---DNMVTRRPI---------ELT---L-I-HS-P--
-----------------NLTQ---EF---G-----------------------------------V----------------FPQ--------------------
-----------------------LGP------SKI-----TDFN---LITSQLT-ELNL-----QVSDK-------------------EC-------
------------------IS--EEPVQLHIYSPS--
VPDLTLIDLPGYIQ--IH-------------------TKDQPS----------DL---KPKIR--------ALCRKYLAP-----------
-PN-LILAVCPADV--DLA-NS--EALLESRRADPSGQRTLGVV-TKLDLVDPAL---------GASILLNH---
D---------------------YPLN-LGYVGV----------------------------------ICR----------------------------------
KNK-PSAVS----F---------------------------SRDPL----------Q-----------------------------------HP--------
------------NY------------------Q-S------P--------------------------DIR-VGI-----
QTLQSTLIRVLEQRMLKSLH---
KNE54706.1 ------------LVLPSIVVIGS----Q----------------SSGKSSVLE-----A---IV-
GREFLPKG---TNMVTRRPL---------ELT---L-I-HT-P--
-----------------DSTE---EY---G-----------------------------------E----------------FPQ--------------------
-----------------------LGF------GKV-----TNFQ---QIQRTLY-DLNM-----AVQD--------------------EC----
--------------------IS--DKPIELRIYSPN---
VPDLTLVDLPGYIQ--IH-------------------SKNQPR----------DL---KEKIA--------DLCEKYIQE----------
--PN-VILAVCAADV--DLA-NS--EALRASRKADPLGLRTIGVL-TKLDLVSPEM---------GEQLIAHN---
D---------------------YPLH-LGYVGV----------------------------------VCG----------------------------------
----GKNPQ----A---------------------------ALVPF----------Q----K------R----------------------------------
--DLT-VGV-----
PYLRQLLMRTLEERMGSRLS---
OAJ38404.1 ------------LNLPSIVVVGS----Q----------------SSGKSSVLE-----A---VV-
GHEFLPKG---ANMVTRRPI---------ELT---L-I-HT-P--
-----------------DSKE---EY---S-----------------------------------E----------------FPQ--------------------
-----------------------LGL------GKI-----KDFS---QVRRTLT-DLNL-----AVSDA-------------------EC-----
--------------------VS--EIPIELRVYSPN---
IPDLTLVDLPGYIQ--IH-------------------TKDQPP----------IL---KEKIA--------ALCQKYIQE-----------
PN-IILAVCAADV--DLA-NS--EALRASRKIDPLGLRTIGVI-TKMDLVEPQA---------AVNILENK---S----
-----------------YPLA-LGYIGV----------------------------------VNK-------------------------------------

TSSRS----F---------------------------SQALT----------R----Q------E-SYF---------RS-------HP-----------
---------EF-----------------N----------------------------------NAM-VGT-----
ATLRRRLVEVLEEHMGRSLH---
XP_011389557.1 ------------LTLPSIVVIGS----Q---------------SSGKSSVLE-----A---IV-
GHEFLPKG---NNMVTRRPI---------ELT---L-I-HT-TT--
------------QPGR-PTKDTVVEY---A-----------------------------------E---------------FPG--------------
----------------------------LGL------GRI-----TDFS---HVQKTLY-DLNM-----AVPAS------------------
EC------------------------VS--DEPIELRIHSPH---
--------VPDLTLIDLPGYVQ--IA-------------------SMDQPD----------EL---REKIQ--------KLCHKYIQE-
-----------PN-IILAVCAADV--DLA-NS--PALRASRQVDPLGLRTIGVV-TKMDLVPPEV---------
GAGILSNN---K--------------------YPLA-LGYVGV----------------------------------VCKNNLGVFQS---
------------------SKGHDRA-TGEGR----M--------------------------SSLVM----------K----Q------
ESDYF---------ST-------NR--------------------EH-----------------FCA------PTRGRNA------------------
GVQPM-TGT-----DTLRRRLMSVLEESMGSSLH---
XP_006459124.1 ------------LKLPSIVVIGS----Q---------------SSGKSSVLE-----A---IV-
GHEFLPKG---NNMVTRRPI---------ELT---L-I-HT-PT---
------------KDGQLPT-----EY---G-----------------------------------E---------------FPG-----------------
-------------------------LGM------GKI-----TNFA---DIQRTLT-DLNL-----AVPAS-------------------DC---
----------------------VN--NDPIDLRIYSPN---
--VPDLTLIDLPGYVQ--IS-------------------SLDQPE----------SL---KEKIA--------SLCEKYIRE---------
---PN-IILAVCAADV--DLA-NS--PALRASRKVDPLGLRTIGVI-TKMDLVPPER---------GAQILAGN---
R---------------------YPLH-LGYVGV----------------------------------VAK------------------------------------
KS-PVEPS----S---------------------------ALVTQ----------R----A------EDSYF---------HQ-------NR----
----------------DF-----------------F-----------GNSS--------------------QLL-VGT-----
GTLRRRLMEVLESSMASSLH---
XP_752563.1 ------------LTLPSIVVIGS----Q---------------SSGKSSVLE-----A---IV-
GHEFLPKG---TNMVTRRPI---------ELT---L-V-NT-P---
-----------------NAQS---EY---G-----------------------------------E---------------FPA--------------------
-----------------------LGL------GKI-----TDFS---QIQRTLT-DLNL-----AVPER-------------------DC------
-------------------VS--DDPIKLTIYSPN---
VPDLSLIDLPGYIQ--VA-------------------GKDQPP----------EL---KQKIA--------DLCDKYIQP---------
---PN-VILAISAADV--DLA-NS--TALRASRRVDPRGERTIGVI-TKMDLVDPER---------GFSILSDQ---
K---------------------YPLR-LGYVGV----------------------------------VSR----VPQT---------------------
TAL--FS-RGSGN----I---------------------------TSAIL----------K----N------ENAYF---------SA-------
HP--------------------SE-----------------F-----------GPQS-------------------GVS-VGV-----
STLRGKLMHVLEQTMAASLA---
OUM67143.1 ------------LQLPSIVVIGS----Q---------------SSGKSSVLE-----A---IV-
GHEFLPK-----------PI---------ELT---L-V-HT-P---
----------NAEE---EY---G-----------------------------------E---------------FPE--------------------------
-----------------IGM------KNI-----TDFG---HIQKTLS-ELNM-----SVSES-------------------EC-------------
--------------IS--TDPIELKIFSPN---
VPDLTLIDLPGYIQ--VI-------------------NRKQPP----------VL---KRKIV--------ELCDRYIVE------------
PN-IILAISSADV--DLA-NS--EALTHSRKVDPYGQRTIGVI-TKMDLVEPEK---------GVDLLINN---D---
------------------YPLE-LGYVGM----------------------------------VCK-----------------------------------

PPG-KSGFS----R--------------------------QLSLT----------Q----K------SDEYF---------RK-------NP---
----------------IF----------------H-Q------P-------------------------DVQ-VGL-----
SALKKRLTTILEENMGQNLY---

NP_014854.2 ------------LTLPSIVVIGS----Q---------------SSGKSSVLE-----S---IV-
GREFLPKG---SNMVTRRPI---------ELT---L-V-NT-P--
----------------NSNN---VT---A----------------------------------D---------------FPS-------------------
----------------------MRL------YNI-----KDFK---EVKRMLM-ELNM-----AVPTS------------------EA----
--------------------VS--EEPIQLTIKSSR---
VPDLSLVDLPGYIQ--VE------------------AADQPI----------EL---KTKIR--------DLCEKYLTA----------
-PN-IILAISAADV--DLA-NS--SALKASKAADPKGLRTIGVI-TKLDLVDPEK---------ARSILNNK---K----
----------------YPLS-MGYVGV--------------------------------ITK----
TPSSINRKHLGLFGEAPSSSLSGIFSKG-QHG-QSSGE----E--------------------------NTNGL---------
-K----QIVSHQFEKAYF---------KE-------NK-------------------KY----------------F----------------------
-----------TNCQ-VST-----KKLREKLIKILEISMSNALE---

PSC76263.1   RLLGGIESG---IDLPKIAVVGD----Q---------------SSGKSSVIE-----S---IF-
GIS-LPRG---SGIVTRTPI---------QVE---H---RY-T---
---------------EG---QA---Y-AEL------------------------------S---------------YKT-------------------
SPDD----------------EEWTK------RVV-----EDLD---GIEQAVEEATDA-----VTGTT------------------
KG--
-----LLDLPGIMR--IA------------------VDDQPK----------NI---EEIVE--------NQIRRHIEG-----------
DNVVILCVLHGTS--DPS-TA--SAIKLAQEYDEDGDQTMGVV-TKPDRCEKAQ----V--
EDLIGSVLGAG-SS--------------------IKLK-LGFIPV--------------------------------RNR--------------
-------------------T-TSELT----D--------------------------GTSLD----------Q----VREN---EAAFF---------
KC-------HP-------------------LL----------------S-Q------L---A---------------------KDR-RGI-----
PALVDQLAHVQMQRVHKALPELK

XP_005849062.1 ------------LQIPKIAVVGD----Q---------------SSGKSSLIE-----S---IF-
GIS-LPRG---EDIVTRTPI---------QVE---H---RY-SD----------------------G----------------------------
-----------------------EA---Y-AEL------------------------------S---------------FRS-------------------
SPDSE----------------ELIK------KRI-----ADLS---TIDDEVREATRR-----VAGSG------------------KG--
----------------------VV--DSPIYLRVYTNK---
--LPDLTVLDLPGITR--NA------------------VEGQPE----------DI---EEIN--------TMIESHIAG---------
---ETTVVLAIVQANV--DFS-TA--AAIKLAKKFDPNRDRTM--

PRW56740.1 ---SGMPSAKRSKDMYRVVVVGD----Q---------------SSGKSSLIE-----S---
MF-SFE-LPRG---QDIVTRTPI---------QVK---H---RF-E--
----------------------KG---PP---R-AEI------------------------------S---------------FR-----------------
---KPGE----------------SDLTV------VKL-----DSLD---EIEEAVIQATDI-----VAGEG------------------
KD------------------------IV--DSTIFLRVFSDA---
-------LPDLTLTDLPGITR--NA------------------TQGQPE----------NI---EEIVT--------SVIENHIKG----
--------EMVVILCVVPANV--DFS-TA--GGIKLARRHDADGVRTMGVV-TKIDLTESGQ--------
EMLDRLSGAG-KN--------------------LKLK-LGFIPV--------------------------------RNR-------------
--------------------T-AQELK---D--------------------------GTPLE----------K----VCKA---EGDFF-------

--SS-------HP--------------------QL-----------------S-K------V---N----------------------PDR-RGI-----
SALVTELARLQMQRVTKSLPGVK
XP_006815062.1 ------------VDLPAVVVIGD----Q----------------SVGKSSVLE-----A---
IS-GVQ-LPRG---NEIVTRCPI---------ELR---L---KT-LD---
--------------------N--D---EW---C-GKI--------------------------------L----------------YIN----------------
----YSKE-------------------QVN------KYI-----DSPD---ELGAAIRTAQQD-----ITNSQ-------------------
KG-------------------------IS--KTSITVEIQSAH---
-------VPNLTLIDLPGIAR--VP-------------------QEGQSR----------NI---ADETK--------DLIKKYISK----
--------DDAIVLCVIPCNV--DIA-TT--EAIKMAQEVDPTGSRTLGVL-TKPDLVDKGS----E--
NVVVRIAENK---V---------------------INLK-KGYTIM------------------------------------KCR----------------
-------------------S-QRNLE----D---------------------------AMSLE----------E----AMDE---EERFF-------
--RE-------HK--------------------HY-----------------S-V------L-------------------------SGQ-AGS-----
RLLAHRLTTELVEQILKSV----
XP_035690836.1 ------------VSLPSVVVIGD----Q----------------SAGKSSTLE-----A---
IS-GVQ-LPRG---SGIVTRCPL---------ELR---L---KK-SQ--
---------------------KKDA---PW---K-GCI--------------------------------R----------------YV----------------
-----KNKK-------------------DVR------FDV-----DEPG---NVGDAVKKAQND-----LAGTT-----------------
---NG-------------------------IS--DSLITLDVESPD---
-----------IPDLTLIDLPGIAR--IA-------------------AEGQPT----------DI---GQQIK--------DLISKYIQK--
----------KDTIILAVVPCNV--DIA-TT--EALQMAQEVDADGSRTLGVL-TKPDLIDPGT----E--
RGVLQILNNE---K---------------------YKLR-KGYTII------------------------------------KCR-----------------
------------------G-QMDIE----K---------------------------GMSLE----------E----AMDK---EQSYF--------
-KS-------HE--------------------HF-----------------K-S------V--YK----------------------EKK-AGV-----
RTLAGRLSTELVGQIKNSI----
XP_002608668.1 ------------VTLPSVVVIGD----Q----------------SAGKSSCLE-----A---
MS-GVQ-LPRG---SGIVTRCPL---------ELR---L---KK-SQ---
----------------------DPES---PW---K-GYI--------------------------------H----------------YHF------------
--------EGDR------------------DETG------WKL-----TDPS---DVGEAVRKAQNN-----LAGDS------------
--------HG-------------------------IS--PRLITLDVESPD--
----------------IPDLTLIDLPGIAR--IA-------------------VDGQPP----------DI---GDQIK--------
DLIKEYIQK------------DETIILAVVPCNV--DIA-TT--EALQMAKDVDPTGSRTLGVL-TKPDLIDRGT----
E--NTIVDIVNNQ---K--------------------YPLK-KGYTII------------------------------------RCR-------------
----------------------G-QEDIN----E---------------------------NVTLS----------E----AMEK---EERFF-----
----KT-------HE--------------------HF-----------------K-L------P--YH----------------------EKK-TGT-----
RTLAGKLTTELVEQIK-------
XP_019617847.1 ------------VTLPSVVVIGD----Q----------------SAGKSSCLE-----A---
IS-GVQ-LPRG---SGIVTRCPL---------ELR---L---KK-SP---
---------------------DPES---GW---R-GYI--------------------------------H----------------FE---------------
--------DK------------------GETR------WEL-----DSPE---DVGEAVKKAQNQ-----LAGES---------------
----LC-------------------------IS--PRLITLDVESPD---
-----------IPDLTLIDLPGIAR--VP-------------------VGGQPD----------DI---GDQTK--------
ALIREYIQM------------DETIILAVVPCNV--DIA-TT--EALKMAKEVDPDGSRTLGVL-TKPDLIDRGT----
E--NMTVDIVNNR---K--------------------YALK-KGYTII------------------------------------KCR------------
----------------------G-QVDIE----N---------------------------KVSLS----------D----AMDK---EEMFF---

------QK-------HE-------------------HF-----------------K-I------L--YE---------------------EKK-TGT-----
KTLAGKLTTELVEQIKKSI----

XP_003973512.2.2 ------------LALPAIAVIGD----Q----------------SSGKSSVLE-----A---
LS-GVA-LPRG---SGIVTRCPL---------ELK---M---KR-RK--
----------------------V-GE---PW---Y-GNI-------------------------------S---------------YL---------------
----------------------------DQE------EVI-----EDPA---DVEKKIQEAQNE-----MAGVG-------------------
VG------------------------IS--DDLISLEIASPE---
-------VPDLTLIDLPGIAR--VA-------------------VKGQPE----------NI---GEQIK--------RLIRKFITK------
------QETISLVVVPCNV--DIA-TT--EALKMAQEVDPDGERTLGIL-TKPDLVDKGT----E--
ETVVDIIHNE---V---------------------IHLK-KGYMIV---------------------------------RCR-----------------
------------------G-QKEII----D--------------------------KVSLA----------E----ATET---ETAFF---------
RD-------HA--------------------HF-----------------Q-T------L--YD---------------------DGQ-ATI-----
LKLAEKLTLELVNHIEKSL----

NP_891987.2.2 ------------LALPAIAVIGD----Q----------------SSGKSSVLE-----A---
LS-GVP-LPRG---SGIVTRCPL---------ELK---M---IR-TK---
--------------------D-QD---KW---H-GRI-------------------------------S---------------YK---------------
----------------------------TYE------EDF-----DDPA---EVEKKIRQAQDE-----MAGAG-------------------
VG------------------------IS--EELISLQITSAN---
------VPDLTLIDLPGIAR--VA-------------------VKGQPE----------NI---GDQIK--------RLIRKFVTR-----
-------QETINLVVVPCNV--DIA-TT--EALQMAQAEDPDGERTLGIL-TKPDLVDKGT----E--
GTVVDIVHNE---V---------------------IHLT-KGYMIV---------------------------------RCR----------------
-------------------G-QKEIM----D--------------------------QVTLN----------E----ATET---ESAFF---------
KD-------HP--------------------HF-----------------S-K------L--YE---------------------EGF-ATI-----
PKLAEKLTIELVHHIQKSL----

XP_009304072.1 ------------LALPAIAVIGD----Q----------------SSGKSSVLE-----A---
LS-GVP-LPRG---SGIVTRCPL---------ELK---M---IR-SK--
---------------------E-DE---KW---H-GRI-------------------------------S---------------YQ---------------
----------------------------NHE------EDF-----DDPA---EVEKKIREAQDE-----MAGAG-------------------
VG------------------------IS--EELISLQITSAN---
------VPDLTLIDLPGIAR--VA-------------------VKGQPE----------NI---GDQIK--------RLIRMFITK------
------QETINLVVVPCNV--DIA-TT--EALQMAQAEDPEGERTLGIL-TKPDLVDKGT----E--
GTVVDIVHNE---V---------------------IHLT-KGYMIV---------------------------------RCR----------------
-------------------G-QKEIM----D--------------------------QVTLN----------E----ATET---ESAFF---------
KD-------HP--------------------HF-----------------R-K------L--YE---------------------EGF-ATI-----
PKLAEKLTIELVHHIQRSL----

AGU16245.1 --------------LPAIAVIGD----Q----------------SSGKSSVLE-----A---LS-GVG-
LPRG---SGIVTRCPL---------ELK---L---KK-AK---
----------K-ET---EW---K-ATI--------------------------------R----------------YE----------------------------
---------------DEY------KEL-----TSPS---EVEKEIRTAQNA-----MAGSG-------------------KG------------
-------------IS--DKLISLEIESDN---
VPDLTLIDLPGIAR--VA-------------------VQGQPY----------DI---GEQIK--------KLIRKFIEK------------
EETINLVVVPCNV--DIA-TT--EALKMAQDVDQSGERTLGIL-TKPDLVDKGA----E--QNIVDVVNNM--
-V--------------------IPLK-KGYMIV---------------------------------KCR----------------------------------

G-QQDIN----E--------------------------NLTLA----------E----AT--
----
XP_007904885.1 ------------LSLPAIAVIGD----Q---------------SSGKSSVLE-----A---
LS-GVS-LPRG---TGIVTRCPL---------ELK---L---KK-AK--
------------------K-AN---VW---K-GAI-------------------------------S---------------FR----------------
--------------------------EYS------KEI-----TNAS---EVEQEIRKAQNS-----MAGK-------------------EG-
----------------------IS--HDLISLKIESSN--
--VPDLTLIDLPGIAR--VA------------------VGNQPL----------DI---GDQIK--------KMIRSFINK---------
---QETINLVVVPCNV--DIA-TT--EALKMAQEVDPSGERTVGIL-TKPDLVDKGT----E--STIVDIVQNL--
-V--------------------VELK-KGYMIV--------------------------------KCR--------------------------------
-G-QKEIN----D--------------------------KLTLQ----------D----AIAR---ENRYF---------EE-------HE----
---------------QF----------------R-T------L--LD---------------------EKK-ASI-----
PHLAERLTNELVYHISKCL----
XP_032888405.1 ------------LGLPAIAVIGD----Q---------------SSGKSSVLE-----A---
LS-GVA-FPRG---SGIVTRCPL---------ELK---L---KN-VK--
-------------------K-AN---VW---K-GKI------------------------------S---------------YK----------------
--------------------------DYS------NKL-----SSAA---EVEQAILKAQDS-----IAGKG------------------
VG------------------------IS--HELISLEIESTN---
------VPDLTLIDLPGIAR--VA------------------VGNQPQ----------DI---GDQIK--------RLIRLFIQK-----
-------QETVNLVVVPCNV--DIA-TT--EALKMAQEVDPTGDRTLGIL-TKPDLVDKGT----E--
KNVVDIVKNL---T--------------------VELE-KGYMIV--------------------------------KCR---------------
-------------------G-QNDIN----E--------------------------NISLV----------D----AIAK---EKEFF---------
ED-------HE-------------------QF----------------R-P------L--LE---------------------DGK-AGI-----
PNLAVRLTKELVNHINKSL----
XP_025933558.1 ------------LALPSIAVIGD----Q---------------SSGKSSVLE-----A---
LS-GIA-LPRG---NGIVTRCPL---------ELK---L---KK-TP--
------------------A-TQ---KW---K-GKI-------------------------------S---------------YH-----------------
-------------------------NTS------EEL-----KNPS---EVEKAIRGAQDV-----VAGTK------------------GA-
----------------------IS--RELISLEVWSPT---
---VPDLTLIDLPGIAR--VA------------------VGDQPE----------DI---GEQIK--------KLLKNIIGN--------
----KETLNLVVVPCNV--DIA-TT--EALKMAQEVDPKGERTLGIL-TKPDLVDKGT----E--ESIVNIIRNL--
-T--------------------VPLK-KGYMIV--------------------------------KCR---------------------------------
G-QQDIH----N--------------------------NLTLA----------S----AIQQ---EKEFF---------ET-------HQ----
---------------HF----------------S-I------L--LN---------------------EGK-ATV-----
PLLAEKLTXELVGHIIKTL----
XP_009815891.1 ------------LALPAIAVIGD----Q---------------SSGKSSVLE-----A---
LS-GIA-LPRG---NGIVTRCPL---------ELK---L---KR-IP--
------------------A-TQ---AW---K-GKI-------------------------------C---------------YR----------------
--------------------------NIS------SEL-----QNAS---EVEKAIREAQDI-----VAGTR------------------GA-
----------------------IS--GELISLEIWSPD---
---VPDLTLIDLPGIAR--VA------------------VGNQPK----------DI---GEQIK--------MLLKKIIGC--------
----KETLNLVVVPCNV--DIA-TT--EALKMAQEVDPSGERTLGIL-TKPDLVDRGT----E--ESIINIIRNL---
V--------------------IPLK-KGYMIV--------------------------------KCR--------------------------------
G-QQDIH----N--------------------------KLALA----------A----AIQQ---ERKFF---------ET-------HE----

----------------HF-----------------S-I------L--LE---------------------EGK-ATV-----
PHLAEKLTNELVRHIIKTL----
XP_015269256.1 ------------LALPAIAVIGD----Q----------------SSGKSSVLE-----A---
LS-GVA-LPRG---NGIVTRCPL---------ALK---L---KK-TR--
---------------------Q-GC---GW---K-GKI------------------------------S----------------YR---------------
-----------------------------DIN------EEL-----NHPS---EVEKEIRKAQIS-----IAGEG-------------------
VG------------------------IS--HELITLEIRSSE--
------VPDLTLIDLPGIAR--VA-------------------VGNQPQ----------DI---GHQIK--------RLIKKIIAK------
------DETINLVVVPCNV--DIA-TT--EALKMAQEVDPDGERTLGIL-TKPDLVDKGT----E--
EAVVDIVRNL---I---------------------IHLK-KGYMIV-----------------------------------KCR-----------------
------------------G-QQDIQ----S---------------------------NLDLA----------S----AIQK---EKAFF---------
ED-------NR--------------------HF-----------------R-I------L--LA---------------------EKR-ATI-----
PLLAEKLTSELVEHINKSL----
XP_006156438.1 ------------LALPAIAVIGD----Q----------------SSGKSSVLE-----A---
LS-GVA-LPRG---SGIVTRCPL---------VLK---L---MK-QS---
----------------------Q-EP---VW---R-GKI------------------------------R----------------YR---------------
-----------------------------NTE------KKL-----GDPT---QVEAEICKAQNI-----IAGSG-------------------
VG------------------------IS--HELITLEITSPE--
------VPDLTLIDLPGITR--VA-------------------LGNQPQ----------DI---SLQIK--------ALIKKYIKR-------
-----QQTINLVVVPCNV--DIA-TT--EALSMAQEVDPEGDRTLGIL-TKPDLVDKGS----E--
KSVMNVLQNL---T---------------------FPLK-KGYMIV-----------------------------------KCR--------------
---------------------G-QQEIM----N----------------------------NLSLA----------E----ATRK---ELMFF------
---QS-------HP--------------------HF-----------------R-V------F--LE---------------------EKK-ATV-----
PHLAERLTAELIAHIRKSL----
NP_002454.1 ------------LALPAIAVIGD----Q----------------SSGKSSVLE-----A---LS-GVA-
LPRG---SGIVTRCPL---------VLK---L---KK-QP--
-----------C--E---AW---A-GRI------------------------------S----------------YR-------------------------
-----------------NTE------LEL-----QDPG---QVEKEIHKAQNV-----MAGNG-------------------RG-------
------------------IS--HELISLEITSPE--
VPDLTIIDLPGITR--VA-------------------VDNQPR----------DI---GLQIK--------ALIKKYIQR------------
QQTINLVVVPCNV--DIA-TT--EALSMAHEVDPEGDRTIGIL-TKPDLMDRGT----E--KSVMNVVRNL--
-T---------------------YPLK-KGYMIV-----------------------------------KCR-------------------------------------
G-QQEIT----N----------------------------RLSLA----------E----ATKK---EITFF---------QT-------HP-------
-------------YF-----------------R-V------L--LE---------------------EGS-ATV-----
PRLAERLTTELIMHIQKSL----
XP_002830747.1 ------------LALPAIAVIGD----Q----------------SSGKSSVLE-----A---
LS-GVA-LPRG---SGIVTRCPL---------VLK---L---KK-QP---
---------------------C--E---AW---A-GRI------------------------------S----------------YR-----------------
---------------------------NTE------LEL-----QDPG---QVEKEIHKAQNI-----MAGNG------------------
RG------------------------IS--HELISLEITSPE--
------VPDLTIIDLPGITR--VA-------------------VDNQPR----------DI---GLQIK--------ALIKKYIQR-------
-----QQTINLVVVPCNV--DIA-TT--EALSMAHEVDPEGDRTIGIL-TKPDLMDKGT----E--
KSVMNVVRNL---T---------------------YPLK-KGYMIV-----------------------------------RCR--------------
---------------------G-QQELT----N----------------------------RLSLA----------E----ATKK---EITFF---------

QT-------HP------------------YF----------------R-V------L--LE---------------------EGS-ATV-----
PRLAERLTSELIMHIQKSL----
XP_008569440.1 ------------LALPAIAVIGD----Q---------------SSGKSSVLE-----A---
LS-GVA-LPRG---SGIVTRCPL---------VLK---L---KK-HL--
--------------------Q-ED---GW---K-GKI------------------------------S---------------YR---------------
---------------------------HTE------LLL-----QDPS---QVEKEIHKAQNT-----IAGNG------------------
VG------------------------IS--HELISLEITSPE---
------VPDLTLIDLPGITR--VA------------------VGNQPQ----------DI---GQQVK--------ALIKKYIQR----
--------QQTINLVVVPCNV--DIA-TT--EALSMAQEVDPDGDRTIGIL-TKPDLVDKGT----E--
KGVMNVARNL---T--------------------YHLK-KGYMIV--------------------------------KCR-------------
--------------------G-QQDIT----N-------------------------KLSLA----------E----ATKK---EMAFF-------
--QT-------HP------------------YF----------------R-V------L--LE---------------------EGK-ATV-----
PCVAEKLTAELIVHINKSL----
XP_017508123.1 ------------LALPAIAVIGD----Q---------------SSGKSSVLE-----A---
LS-GVA-LPRG---SGIVTRCPL---------VLK---L---KK-QL--
--------------------H-EP---AW---T-GRL------------------------------S---------------YQ---------------
---------------------------TTE------LQL-----HNPS---QVEKEIQKAQNA-----IAGDG------------------
VG------------------------IS--HELINLEITSPD---
-------VPDLTLIDLPGIAR--VA------------------VGNQPQ----------DI---GLQIK--------ALIKKYIQR----
--------QQTINLVVVPCNV--DIA-TT--EALSMAQEVDPDGDRTIGIL-TKPDLVDKGT----E--
RVIVNVVQNL---T--------------------YHLK-KGYMIV--------------------------------KCR--------------
--------------------G-QQEVT----N-------------------------KLSLA----------E----ATSK---EMTFF--------
-QT-------HP------------------YF----------------R-I------L--LE---------------------EGK-ATV-----
PRLAEKLTTELISHINKSL----
XP_005885748.1 ------------LALPAIAVIGD----Q---------------SSGKSSVLE-----A---
LS-GVA-LPRG---SGIVTRCPL---------VLK---L---KK-QL--
--------------------AGES---LW---T-GKI------------------------------S---------------YR-----------------
--------------------------STE------LQL-----QDPS---QVEREIYKAQNT-----IAGNG------------------
VG------------------------IS--HELINLEITSPE---
------VPDLTLIDLPGIAR--VA------------------VGNQPQ----------DI---GLQIK--------ALIKKYIQR-----
-------QQTINLVVVPCNV--DIA-TT--EALSMAHEVDPDGDRTIGIL-TKPDLVDKGA----E--
KNVVNVAQNL---T--------------------YRLK-KGYMIV--------------------------------KCR-------------
--------------------G-QQEIT----D-------------------------KLSLA----------E----ATKK---EMMFF-------
--QT-------HP------------------YF----------------R-V------L--LE---------------------EGK-ATV-----
PRLAERLTTELIWHINKSL----
NP_001003133.1 ------------LALPAIAVIGD----Q---------------SSGKSSVLE-----A---
LS-GVA-LPRG---SGIVTRCPL---------VLK---L---KR-DP--
--------------------H--K---AW---R-GRI------------------------------S---------------YR----------------
--------------------------KTE------LQF-----QDPS---QVEKEIRQAQNI-----IAGQG------------------
LG------------------------IS--HELISLEITSPE---
------VPDLTLIDLPGITR--VA------------------VGNQPQ----------DI---GVQIK--------ALIKNYIQK-----
-------QETINLVVVPCNV--DIA-TT--EALSMAQEVDPNGDRTIGVL-TKPDLVDRGT----E--
KTVVNVAQNL---T--------------------YHLQ-KGYMIV--------------------------------RCR-------------
--------------------G-QEEIT----N-------------------------QLSLA----------E----ATEK---ERMFF-------

--QT-------HP-------------------YF-----------------R-A------L--LE---------------------EGK-ATV-----
PCLAERLTKELILHINKSL----
NP_776366.1 ------------LALPAIAVIGD----Q----------------SSGKSSVLE-----A---LS-GVA-
LPRG---SGIITRCPL---------VLK---L---TK-R--
----------EC---EW---T-GKI-------------------------------T----------------YR------------------------------
-------------NIT------QQL-----QNPS---EVEWEIRRAQNI-----IAGNG-------------------LG-------------
------------IS--HELINLEITSPE--
VPDLTLIDLPGITR--VA-------------------VENQPQ----------DI---GLQIK--------ALIKKYIQR-----------
QETINLVVVPCNV--DIA-TT--EALSMAQEVDPDGDRTIGIL-TKPDLVDKGT----E--KGVLKVMQNL---
T---------------------YHLK-KGYMIV-----------------------------------KCR------------------------------------
G-QQDIT----N---------------------------KLSLA----------E----ATRK---ETMFF---------ET-------HP------
--------------YF-----------------R-I------L--LD---------------------EGK-ATV-----
PLLAERLTTELIWHINKSL----
XP_032211320.1 ------------LALPAIAVIGD----Q----------------SSGKSSVLE-----A---
LS-GVA-LPRG---SGIVTRCPL---------VLK---L---KR-QP---
---------------------Q-ES---AW---K-GRV-------------------------------I----------------YG----------------
----------------------------TRE------VRL-----QDPS---QVEKEILKAQNT-----LAGDG-------------------
VS-------------------------IS--HELISVDIISPE---
------VPDLTLIDLPGITR--VP-------------------VGNQPQ----------DI---GLQIK--------ALIKKYIQR------
------QETINLVVVPCNV--DIA-TT--EALSMAQEVDPRGDRTIGIL-TKPDLVDKGA----E--
PIVMKVAQNL---T---------------------YHLQ-KGYMMV-----------------------------------RCR-------------
----------------------G-QEEIT----N---------------------------RLSLA----------E----ATRK---ETMFF-------
--QK-------HP-------------------HF-----------------R-A------L--LQ---------------------EGK-ATV-----
PCLAERLTNELILHINKSL----
XP_004675614.2.2 ------------LALPAIAVIGD----Q----------------SSGKSSVLE-----A---
LS-GVA-LPRG---SGIVTRCPL---------VLK---L---KK-LM--
---------------------N-ED---SW---K-GKI-------------------------------N----------------YQ---------------
-----------------------------GVE------VTI-----AKAS---DVEQEVNKAQAV-----IAGDG-------------------
LG-------------------------IS--HELITLEVSSPE---
-------VPDLTLIDLPGITR--VA-------------------VGNQPQ----------DI---GEQIK--------ALIRKYIQR----
--------QQTINLVVVPCNV--DIA-TT--EALSMAREVDPDGDRTLGIL-TKPDLVDRGT----E--
DRVVDVIRNF---I---------------------CPLK-KGYMIV-----------------------------------KCR---------------
--------------------G-QKDIQ----D---------------------------RLSLA----------Q----ALQK---EQAFF------
---EE-------HP-------------------HF-----------------R-Q------L--LE---------------------EGR-ASI-----
PKLADRLTSELIRHISKSL----
XP_004466363.1 ------------LSLPAIAVIGD----Q----------------SSGKSSVLE-----A---
LS-GVA-LPRG---SGIVTRCPL---------VLK---L---KK-LT--
--------------------N-EE---KW---R-GKV-------------------------------T----------------YE-----------------
---------------------------DYE------IDI-----SDAS---EVEEEINKAQNV-----IAGEG-------------------LG-
-------------------------IS--QKLINLEVCSPD---
----VPDLTLIDLPGITR--VA-------------------VGNQPA----------DI---GWQIK--------CLIKKYITR-------
-----QETINLVVVPSNV--DIA-TT--EALSMAQEVDPNGDRTIGIL-TKPDLVDRGT----E--
DKVVDVVRNL---V---------------------CHLK-KGYMIV-----------------------------------RCR-------------
----------------------G-QQDIQ----D---------------------------RLSLA----------T----ALQK---ERAFF-----

----EN-------HE--------------------NF-----------------R-V------L--LE----------------------EGK-ATV-----
PHLAERLTTELITHISKTL----
NP_002453.2.2 ------------LALPAIAVIGD----Q----------------SSGKSSVLE-----A---
LS-GVA-LPRG---SGIVTRCPL---------VLK---L---KK-LV--
--------------------N-ED---KW---R-GKV---------------------------------S----------------YQ----------------
-----------------------------DYE------IEI-----SDAS---EVEKEINKAQNA-----IAGEG-------------------
MG--------------------------IS--HELITLEISSRD---
-------VPDLTLIDLPGITR--VA-------------------VGNQPA----------DI---GYKIK--------TLIKKYIQR------
------QETISLVVVPSNV--DIA-TT--EALSMAQEVDPEGDRTIGIL-TKPDLVDKGT----E--
DKVVDVVRNL---V---------------------FHLK-KGYMIV-----------------------------------KCR--------------
---------------------G-QQEIQ----D----------------------------QLSLS----------E----ALQR---EKIFF-------
--EN-------HP--------------------YF-----------------R-D------L--LE----------------------EGK-ATV-----
PCLAEKLTSELITHICKSL----
NP_001127618.1 ------------LALPAIAVIGD----Q----------------SSGKSSVLE-----A---
LS-GVA-LPRG---SGIVTRCPL---------VLK---L---KK-LV--
--------------------N-ED---KW---R-GKV---------------------------------S----------------YQ----------------
-----------------------------DYE------IEI-----SDAS---EVEKEINKAQNT-----IAGEG-------------------
MG--------------------------IS--HELITLEISSRD---
-------VPDLTLIDLPGITR--VA-------------------VGNQPA----------DI---GYKIK--------TLIKKYIQR------
------QETISLVVVPSNV--DIA-TT--EALSMAQEVDPEGDRTIGIL-TKPDLVDKGT----E--
DKVVDVVRNL---V---------------------FHLK-KGYMIV-----------------------------------KCR--------------
---------------------G-QQEIQ----D----------------------------QLSLS----------E----ALQR---EKIFF-------
--ED-------HP--------------------YF-----------------R-D------L--LE----------------------EGK-ATV-----
PCLAEKLTSELITHICKSL----
XP_006156437.1 ------------LALPAIAVIGD----Q----------------SSGKSSVLE-----A---
LS-GVA-LPRG---SGIVTRCPL---------VLK---L---KK-LI--
--------------------N-ED---KW---R-GKV---------------------------------S----------------YQ----------------
-----------------------------DIE------VEI-----TDPS---KVEPEINKAQNV-----IAGEG-------------------
MG--------------------------IS--HELISLEVSSPH--
--------VPDLTLIDLPGITR--VA-------------------VGNQPA----------DI---GRQIK--------TLIKKYIHK-----
-------QETINLVVVPSNV--DIA-TT--EALSMAQEVDPDGDRTIGIL-TKPDLVDKGT----E--
EKVVDVVRNL---V---------------------CHLK-KGYMIV-----------------------------------KCR--------------
---------------------G-QQDIQ----D----------------------------RLSLA----------E----ALQR---EKVFF------
---EE-------HP--------------------YF-----------------S-F------L--LE----------------------EGK-ATI-----
PCLAERLTTELIMHISKSL----
XP_017508130.1 ------------LALPAIAVIGD----Q----------------SSGKSSVLE-----A---
LS-GVA-LPRG---SGIVTRCPL---------VLK---L---KK-LT--
--------------------N-EE---TW---R-GKV---------------------------------S----------------YQ-----------------
----------------------------DFE------AEL-----SDPS---EVEREINRAQNS-----IAGEG-------------------
TG--------------------------IS--HELISLEISSPH--
------VPDLTLIDLPGITR--VA-------------------VGNQPA----------DI---GRQIK--------ALIRKYIYK------
------QETINLVVVPSNV--DIA-TT--EALSMAQEVDPDGDRTIGIL-TKPDLVDRGT----E--
DKVVDVVRNL---V---------------------CHLK-KGYMIV-----------------------------------KCR-------------
----------------------G-QQDIQ----D----------------------------QLSLA----------E----ALKK---ERAFF-----

```
----ED-------NP-------------------YF-----------------R-D------L--LE---------------------EGR-ATV-----
PCLADKLTVELITHICKSL----
        NP_001003134.1      ------------LALPAIAVIGD----Q----------------SSGKSSVLE-----A---
LS-GVA-LPRG---SGIVTRCPL---------VLK---L---KK-LI---------------------------------------------------
--------------------N-ED---EW---R-GKV-------------------------------S----------------YQ---------------
----------------------------DTE------MEI-----SDPS---EVEVEINKAQDA-----IAGEG-------------------
QG------------------------IS--HELISLEVSSPH------------------------------------------------------------
--------VPDLTLIDLPGITR--VA-------------------VGNQPA----------DI---GRQTK--------QLIRKYILK----
--------QETINLVVVPCNV--DIA-TT--EALSMAQEVDPDGDRTIGIL-TKPDLVDRGT----E--
GKVVDVAQNL---V---------------------CHLK-KGYMIV----------------------------------KCR-------------
----------------------G-QQDIQ----D---------------------------QVSLA----------E----ALQK---EKDFF----
-----ED-------HP-------------------HF-----------------R-V------L--LE---------------------EGR-ATV----
-PNLAEKLTSELITHICKTL----
        XP_032211398.1      ------------LALPAIAVIGD----Q----------------SSGKSSVLE-----A---
LS-GVA-LPRG---SGIVTRCPL---------VLK---L---KK-VT------------------------------------------------------
--------------------N-QD---EW---R-GKV-------------------------------S----------------YQ---------------
-----------------------------DFE------KEI-----SDPS---EVEAEINKAQNA-----VAGEG-------------------
QG------------------------IS--HELISLEVSSSH------------------------------------------------------------
--------VPDLTLIDLPGITR--VA-------------------VGNQPA----------DI---GRQTK--------QLIRKYILR---
---------QETINLVVVPCNV--DIA-TT--EALSMAQEVDPDGDRTIGIL-TKPDLVDRGT----E--
SKVVDVAQNL---V---------------------CHLK-KGYMIV----------------------------------KCR--------------
---------------------G-QQDIQ----D----------------------------QVTLA----------E----ALQK---ERDFF-----
----ED-------HP-------------------HF-----------------R-V------L--LE---------------------EGR-ATV-----
PCLADKLTSELIMHICKTL----
        XP_008569442.1      ------------LALPAIAVIGD----Q----------------SSGKSSVLE-----A---
LS-GVA-LPRG---SGIVTRCPL---------VLK---L---KK-LV------------------------------------------------------
--------------------H-GE---EW---K-GKV-------------------------------S----------------YR----------------
----------------------------DLE------IKI-----SDAL---EVEEEVRKAQTI-----IAGEG-------------------MG-
------------------------IS--HELINLEISSPH------------------------------------------------------------------
--VPDLTLIDLPGIAR--VA-------------------MGNQPA----------DI---GYQVK--------XLIRKYIQR--------
----QETINLVVVPSNV--DIA-TT--EALSMAQEVDPEGDRTIGIL-TKPDLVDKGT----E--
DKVVDVVRNL---V---------------------YHLK-KGYMIV----------------------------------KCR--------------
---------------------G-QQDIQ----D----------------------------QLSLA----------T----ALQR---EKDFF------
---ED-------HP-------------------QF-----------------R-D------L--LE---------------------EGR-ATI-----
PCLAERLTTELITHICKSL----
        XP_014388412.1      ------------LALPAIAVIGD----Q----------------SSGKSSVLE-----A---
LS-GVS-LPRG---SGIVTRCPL---------VLK---L---RK-LR--------------------------------------------------
---------------------H-DD---EW---K-GKV-------------------------------T----------------YR----------------
----------------------------DLE------IDL-----SAAS---EVEQEIRKAQNV-----IAGEG------------------
VG------------------------IS--QELINLEVSSPH------------------------------------------------------------
--------VPDLTLIDLPGITR--VA-------------------VGNQPA----------DI---GRQIT--------ALIKKYILR-----
-------QQTIMLVVVPSNV--DIA-TT--EALSMAHEVDPDGDRTIGIL-TKPDLVDRGT----E--
DKVVDVVRNL---V--------------------YHLK-KGYMIV----------------------------------KCR--------------
---------------------G-QQDIQ----Y---------------------------QMSLS----------K----ALQR---ERAFF-----
```

----ED-------HP-------------------YF-----------------R-D------L--LE---------------------EGK-ATI-----
PCLAERLTNELIAHISKSL----

XP_005202045.1 ------------LALPAIAVIGD----Q---------------SSGKSSVLE-----A---
LS-GVA-LPRG---SGIVTRCPL---------VLR---L---KK-LG---
---------------------N-ED---EW---K-GKV------------------------------S---------------FL---------------
---------------------------DKE------IEI-----PDAS---QVEKEISEAQIA-----IAGEG------------------TG--
----------------------IS--HELISLEVSSPH---
--VPDLTLIDLPGITR--VA------------------VGNQPP----------DI---EYQIK--------SLIRKYILR-----------
-QETINLVVVPANV--DIA-TT--EALRMAQEVDPQGDRTIGIL-TKPDLVDKGT----E--DKVVDVVRNL--
-V--------------------FHLK-KGYMIV---------------------------------KCR---------------------------------
-G-QQDIK----H--------------------------RMSLD----------K----ALQR---ERIFF---------ED-------HA---
-----------------HF-----------------R-D------L--LE---------------------EGK-ATI-----
PCLAERLTSELIMHICKTL----

NP_038634.1 ------------LALPAIAVIGD----Q---------------SSGKSSVLE-----A---LS-GVA-
LPRG---SGIVTRCPL---------VLK---L---RK-LN---
----------E-GE---EW---R-GKV------------------------------S---------------YD-------------------------
------------------DIE------VEL-----SDPS---EVEEAINKGQNF-----IAGVG------------------LG----------
---------------IS--DKLISLDVSSPN---
VPDLTLIDLPGITR--VA------------------VGNQPA----------DI---GRQIK--------RLIKTYIQK-----------
QETINLVVVPSNV--DIA-TT--EALSMAQEVDPEGDRTIGIL-TKPDLVDRGT----E--DKVVDVVRNL---
V---------------------YHLK-KGYMIV---------------------------------KCR---------------------------------
G-QQDIQ----E--------------------------QLSLT----------E----ALQN---EQIFF---------KE-------HP-----
---------------HF-----------------R-V------L--LE---------------------DGK-ATV-----
PCLAERLTAELILHICKSL----

NP_034976.1 ------------LALPAIAVIGD----Q---------------SSGKSSVLE-----A---LS-GVA-
LPRG---SGIVTRCPL---------VLK---L---RK-LK--
----------E-GE---EW---R-GKV------------------------------S---------------YD-------------------------
------------------DIE------VEL-----SDPS---EVEEAINKGQNF-----IAGVG------------------LG----------
---------------IS--DKLISLDVSSPN---
VPDLTLIDLPGITR--VA------------------VGNQPA----------DI---GRQIK--------RLIKTYIQK-----------
QETINLVVVPSNV--DIA-TT--EALSMAQEVDPEGDRTIGVL-TKPDLVDRGA----E--GKVLDVMRNL--
-V--------------------YPLK-KGYMIV---------------------------------KCR--------------------------------
-G-QQDIQ----E--------------------------QLSLT----------E----AFQK---EQVFF---------KD-------HS---
-----------------YF-----------------S-I------L--LE---------------------DGK-ATV-----
PCLAERLTEELTSHICKSL----

XP_028583072.1 ------------LALPAIAVIGD----Q---------------SSGKSSVLE-----A---
LS-GVA-LPRG---SGIVTRCPL---------ELR---L---KK-LL--
---------------------P-GE---KW---N-GKI------------------------------S---------------YL---------------
---------------------------GKY------MEL-----ANPS---MVEIEIRKAQNI-----IAGDG------------------
VA-------------------------IS--DKLITLEIRSPE--
------VPDLTLIDLPGIAR--VA------------------VGNQPV----------NI---GDQIK--------KLIKTFIDK------
------QETINLVVVPSNV--DIA-TT--EALKMAQEVDPNGERTLGIV-TKPDLMDRGT----E--
GTVVNIVRNQ---V--------------------IPLK-KGYMIV---------------------------------KCR---------------
-------------------G-QQDIQ----S--------------------------NMTLA----------S----ALKE---ERAFF--------

-EK-------HK-------------------CF----------------S-I------L--LQ--------------------EKK-ATV-----
PLLAEKLTSELVEHISKSL----
XP_028583068.1 ------------LALPAIAVIGD----Q---------------SSGKSSVLE-----A---
LS-GVA-LPRG---SGIVTRCPL---------ELK---L---KK-TH--
--------------------N-TK---EW---K-GKI------------------------------S---------------YL----------------
--------------------------NTV------EEM-----NSSR---QVEEQIIRAQNA-----MAGSG------------------
SG-----------------------IS--SELISLEISSSD---
------VPDLTLIDLPGIAR--VA------------------VGDQPK----------DI---GQQII--------KLIKKYINK------
------QETINLVVVPSNV--DIA-TT--EALKMAQEVDPTGERTLGIL-TKPDLVDKGT----E--
AEVVDIIRNQ---R--------------------VPLR-KGYMIV--------------------------------KCR--------------
-------------------G-QSDIN----D-------------------------KVTLG----------D----AIEK---EREFF--------
-EE-------HD-------------------FF----------------R-S------L--LE--------------------EGR-ATI-----
PLLAERLTQELIEHISKTL----
XP_031752404.1 ------------LALPAIAVIGD----Q---------------SSGKSSVLE-----A---
LS-GVT-LPRG---SGIVTRCPL---------ELK---L---KK-AM--
--------------------K-KT---TW---S-GKI------------------------------S---------------YR----------------
-------------------------DHE------IKI-----ASAA---DVEEEVKRAQNL-----MAGSG------------------
KG-----------------------IS--DELISLEVISPD---
-------VPDLTLIDLPGITR--VA------------------LPDQPK----------DI---EQQIK--------KMIRKYIQK-----
-------QETINLVVVPSNV--DIA-TT--EALEMAREVDPNGERTLGIL-TKPDLVDRGA----E--
TDVISVVRNL---V--------------------YSLN-KGYMIV--------------------------------KCR---------------
------------------G-QQEIQ----E-------------------------NLSLK----------D----ALVN---EQNFF-------
--KE-------HE-------------------HF----------------S-V------L--LE--------------------EGY-ATI-----
ACLAGKLTNELVAHIVRNL----
NP_001007285.1 ------------LNLPAIAVIGD----Q---------------SSGKSSVLE-----A---
LS-GVA-LPRG---IGIVTRCPL---------ILK---L---KK-IT---
-----------------R-DK---NW---S-GLL------------------------------T---------------YK-------------------
------------------------DQT------EIL-----KEPT---GIENAVLKAQIA-----LAGTG------------------EG----
--------------------IS--HEMITLEIQSCD---
VPDLTLIDLPGIAR--VA------------------TGNQPE----------DI---EKQIK--------DLIEKFIKR-----------
QETISLVVVPANI--DIA-TT--EALKMASTVDPTGQRTLCIL-TKPDLVDRGM----E--DTVVRTVNNE---
V--------------------IRLE-KGYMIV--------------------------------KCR---------------------------------
G-QQDIN----D-------------------------KLNLV----------E----ALEK---ERRFF---------DE-------HP----
----------------QF----------------S-S------L--LE--------------------DGK-ATI-----
PLLGQRLTEELVEHIAKNV----
XP_005167721.2.2 ------------LNLPAIAVIGD----Q---------------SSGKSSVLE-----A---
LS-GVA-LPRG---TGIVTRCPL---------VLK---L---KK-IT--
-------------------K-DK---SW---H-GLL-------------------------------T---------------YN-----------------
-------------------------DKI------REL-----KDPA---KIEKAVLNAQTA-----LAGIG------------------EG---
----------------------IS--HEMITLEIQSCD---
-VPDLTLIDLPGIAR--VA------------------TGNQPE----------DI---EKQIK--------SLIEKFIKR-----------
QETISLVVVPANI--DIA-TT--EALKMASTVDPTGQRTLGIL-TKPDLVDRGM----E--DTVVRTVNNE---
V--------------------IPLK-KGYMIV--------------------------------KCR---------------------------------
G-QQDIN----D-------------------------KLGLV----------E----ALEK---ERRFF---------DE-------NV----

----------------HF-----------------R-S------L--LE---------------------DRK-ATI-----
PLLAERLTKELVEHIAKNL----
XP_012586448.1 ------------LALPAIAVIGD----Q----------------SSGKSSVLE-----A---
LS-GVA-LPRG---SGIVTRCPL---------VLK---L---KK-QL---
---------------------Q-GA---PW---T-GTI-------------------------------S---------------YR----------------
---------------------------GVT------LGL-----QDPS---AVEREIHVAQNV-----IAGHG-------------------
VG-------------------------IS--HELITLEVSSPE---
-------VPDLTLIDLPGIAR--VA-------------------VGNQPQ----------DI---
GAQVSLSGAGRWGALVSGLPRS------------ERCTGQ-----------E-TA--
EALGGGRAAXXDGDRTLGIL-TKPDLVDKGA----E--KAVVNVAQNL---T--------------------YRLK-
KGYMVV---------------------------------KCR---------------------------------G-QQDIM----D---------
-----------------RLSLA----------Q----ATEK---EVAFF---------QT-------HP-------------------HF---------
--------R-A------L--LE---------------------EGK-ATV-----PRLAEKLTSELILHINKSL----
KAI0213370.1------------ISLPEVAVIGD----Q----------------SAGKSSVLE-----A---IS-GVQ-
LPRG---SGIVTRCPL---------ALQ---L---KS-HD---
------------T-PG---YW---N-GVI-------------------------------K---------------YE--------------------
TYNH-------------------PVE------KTI-----EGPT---EVGAEVREAQDV-----IAGKN------------------VG-
-------------------------IS--DTLISLQITSHG--
--VPDLTLIDLPGITR--VA-------------------VEGQPK----------DI---GDQIK--------RLIGHYIKK----------
--EETIILAVVPANV--DIA-TT--EALKMAKEVDPSGGRTLGVV-TKPDLIDIGT----E--KGLIDIINNE---T---
-------------------YPLE-KGYSCV---------------------------------RCR---------------------------------G-
QKAIN----E--------------------------GQTLA----------E----AIQQ---DTDFF---------SS-------AP--------
------------HF-----------------S-D------V---D---------------------ESI-LGV-----
KNLAMKLTYELVKQIKRAL----
KAI0208044.1------------ISLPEVAVIGD----Q----------------SAGKSSVLE-----A---IS-GVQ-
LPRG---SGIVTRCPL---------ALQ---L---KS-HD---
------------T-PG---YW---N-SVI-------------------------------K---------------YK--------------------
YGDEFD-------------FEDIVVE------KTI-----EGPT---KVDAGVREAQDA-----IAGKN------------------
VG-------------------------IS--DTLISLQITAYG--
------VPDLTLIDLPGITR--VA-------------------VQGQPP----------DI---GDQIK--------RLIGNYIKK-----
-------EETIILAVVPANV--DIA-TT--EALKMAKEVDPSGGRTLGVV-TKPDLIDIGT----E--KGLIDIINNE--
-T--------------------YPLE-KGYSCV---------------------------------RCR---------------------------------
-G-QKAIN----E--------------------------GQTLA----------D----AVQE---DTDFF---------SS-------AP---
-----------------HF-----------------S-A------V---D---------------------ESI-LGV-----
KNLAMKLTFELVKQIKRAL----
KAI0218869.1------------IALPEVAVIGD----Q----------------SAGKSSVLE-----A---IS-GVQ-
LPRG---SGIVTRCPL---------ALQ---L---KS-DK---
-----------T-PG---YW---N-GVI-------------------------------K---------------YE--------------------
INER-------------------LVE------KTI-----VGPA---EVDAEVRNAQDV-----IAGKN------------------VG--
-----------------------IS--SKLISLQITSYG---
IPDLTLIDLPGITR--VA-------------------VEGQPQ----------NI---GEQIK--------RLIEKYIKK-----------
EETIILAVVPANV--DIA-TT--EALKMAKEVDPSGSRTLGVV-TKPDLIDAGT----E--KGLISIINNE---T-----
----------------YPLK-KGYSCV---------------------------------RCR---------------------------------G-
QKAID----E--------------------------GQTLA----------Q----AIQQ---DTDFF---------SI-------AS--------

------------HF-----------------S-D------V---D---------------------QST-LGV-----
KNLAMKLTYELVRQIKRAL----
XP_032804093.1 ------------VGLPAVAVIGD----Q----------------SSGKSSVLE-----A---
LS-GVQ-LPRG---SGIVTRCPL---------ALK---L---KR-A--
----------------------PG---PW---H-GRI-------------------------------K----------------YR-----------------
----VQGR-------------------TVN------TKL-----DTPE---SVGDAVLQAQSE-----LTGDD------------------
-LG------------------------VS--KSLIELEVTSDS---
--------VPDLTLIDLPGIAR--VA-------------------LAGQAV----------DI---ETQIK--------DLIRDHIGR----
--------QETINLVVIPCNV--DIA-TT--EALKMAQAVDPTGVRTLGVL-TKPDLMDEGT----E--
RNALRILQNQ---V--------------------FPLS-KGYVLV---------------------------------KCR---------------
--------------------S-QRDVE----A--------------------------HQTLA----------E----ASRV---EAAFF-------
--KK-------HP-------------------VF-----------------C-H------V--HN---------------------GGKLTTT-----
TVLAAKLTEELVDNIKRTL----
XP_046565196.1 ------------INLPAVAVIGD----Q----------------SAGKSSVLE-----A---
IS-GVQ-LPRG---TGIVTRCPL---------EMR---M---KH-SE--
----------------------D-ED---KW---E-GKI-------------------------------M----------------YKD-------------
-------KHDE-------------------LHK------EDI-----QDRE---SVGDLVRKAQDE-----MTDCE--------------
-----KG------------------------IS--DDLITLEVTSSD---
------------VPDLTLIDLPGIAR--NA-------------------VKGQPV----------DI---EKRIK--------
DMIRKYIRR------------QETIILAVLQCNV--DIA-TC--EALKMAKEFDEEGRRTLGVL-TKPDLLDKGA--
--E--NGVMRILNNM---E--------------------FSLS-KGYIIV---------------------------------KCR---------
--------------------------G-QEAIS----K--------------------------GQSLT----------E----ALGD---EDNFF-
--------KD-------HS--------------------HF-----------------R-S------L---K---------------------VSQ-WGI-
----LTLSSRLSLELQKHIK-------
XP_046562919.1 ------------INLPAVAVIGD----Q----------------SAGKSSVLE-----A---
IS-GVQ-LPRG---TGIVTRCPL---------EMR---M---KH-SE--
----------------------D-ED---KW---E-GKI-------------------------------M----------------YKD-------------
-------KHDM-------------------RQE------EVI-----LNRE---SVGDLVRKAQKE-----MTDGA--------------
-----KG------------------------IS--DELITLEVTSSD---
------------VPDLTVIDLPGITR--NA-------------------VEGQPF----------DI---EARIK--------
NMIRKYIKR------------QETIILAVLQCNV--DIA-TC--EALKMAKEFDGEGGRTLGVL-
TKPDLMDKGA----E--TGVIRILNNM---E--------------------FTLS-KGYIIV-------------------------------
---KCR----------------------------------G-QEAIS----E--------------------------GQSLK----------Q----
ALDI---EEDFF---------KS-------HR-------------------HF-----------------S-S------L---R----------------
------PSQ-WGI-----PDLSSRLSRELKRHIKKLL----
XP_046563124.1 ------------INLPAVAVIGD----Q----------------SAGKSSVLE-----A---
IS-GVQ-LPRG---TGIVTRCPL---------EMR---L---KH-SE--
----------------------D-ED---KW---E-GKI-------------------------------L----------------YKD-------------
-------KHDM-------------------RQE------EVI-----LNRE---SVGDLVRKAQKE-----MTDSA--------------
-----KG------------------------IS--DELITLEVTSSD---
------------VPDLTVIDLPGIAR--NA-------------------VEGQPF----------DI---EARIK--------
NMIRRYIGR------------QETIILAVLQCNV--DIA-TC--EALKMAKEFDDEGGRTLGVL-TKPDLLDKGA-
---E--TGVMRILNNM---E--------------------FTLS-KGYIIV---------------------------------KCR---------
--------------------------G-QEAIS----E--------------------------GQSLK----------H----ALDV---EEDFF-

--------KS-------HR-------------------HF----------------S-S------L---R---------------------PSQ-WGI-
----PNLSARLSRELKKHIKKLL----
XP_046563126.1 ------------INLPAVAVIGD----Q---------------SAGKSSVLE-----A---
IS-GVQ-LPRG---TGIVTRCPL---------EMR---M---KH-SE---
---------------------D-ED---KW---E-GKI-------------------------------M---------------YKD------------
-------KHDM-------------------PHE------EVI-----LNRE---SVGDLVRKAQTE-----MTDGA-------------
-----TG------------------------IS--DELITLEVTSSD---
-----------VPDLTVIDLPGIAR--NA------------------VEGQPF----------DI---EARIK--------
NMIRQYIER------------QETIILAVLQCNV--DIA-TC--EALKMAKEFDDEGGRTLGVL-TKPDLLDKGA-
---E--TSVIRILNNM---E--------------------FTLS-KGYVIV--------------------------------KCR----------
------------------------G-QEAIS----E-------------------------GQSLK----------H----ALDV---EEDFF--
-------RS-------HR-------------------HF----------------S-A------L---R---------------------PSQ-WGI--
---PNLSERLSRELKKHIKKLL----
XP_046565195.1 ------------INLPAVAVIGD----Q---------------SAGKSSVLE-----A---
IS-GVQ-LPRG---TGIVTRCPL---------EMR---M---KH-SE---
---------------------D-ED---KW---E-GKI-------------------------------M---------------YKD------------
-------KHDM-------------------PHE------EVI-----LNRE---SVGDLVRKAQKE-----MTDGA-------------
-----TG------------------------IS--DELITLEVMSSD---
------------VPDLTVIDLPGIAR--NA------------------VEGQPF----------DI---EARIK--------
NMIRQYIQR------------QETIILAVLQCNV--DIA-TC--EALKMAKEFDDEGGRTLGVL-
TKPDLLDKGA----E--TGVIRILNNM---E--------------------FTLS-KGYVIV------------------------------
---KCR----------------------------------G-QEAIS----E-------------------------GQSLK----------H----
ALDV---EEDFF---------RS-------HR-------------------HF----------------S-A------M---R--------------
-------PSQ-WGI-----PNLSERLSRELKKHIKKLL----
XP_046563125.1 ------------INLPAVAVIGD----Q---------------SAGKSSVLE-----A---
IS-GVQ-LPRG---TGIVTRCPL---------EMR---M---KH-SE---
---------------------D-ED---KW---E-GKI-------------------------------M---------------YTD------------
-------KHDE-------------------PHQ------EVI-----LNRE---SVGDLVRKAQKE-----MTDSA---------------
----KG------------------------IS--DELITLEVTSSD---
-----------VPDLTVIDLPGIAR--NA------------------VEGQPV----------DI---EARIK--------
NMIRQYIER------------QETIILAVLQCNV--DIA-TC--EALKMAKEFDDEGGRTLGVL-TKPDLLDRGA-
---E--TGVMRILNNM---E--------------------FTLS-KGYIIV--------------------------------KCR---------
-------------------------G-QEAIS----E-------------------------GQSLK----------H----ALDV---EEDFF-
--------KS-------HR-------------------HF----------------S-S------L---G---------------------PSQ-
WGI-----PNLSRRLSRELKKHIKKLL----
XP_046352527.2 ------------INLPAVAVIGD----Q---------------SAGKSSVLE-----A---
IS-GVQ-LPRG---TGIVTRCPL---------EMR---M---KH-SE---
---------------------D-ED---MW---E-GKI-------------------------------M---------------YKD-----------
--------MHGE-------------------AHE------EII-----LNRE---SVGELVRKAQKE-----MTDSA--------------
----KG------------------------IS--DELITLEVTSSD---
-----------VPDLTVIDLPGIAR--NA------------------VEGQPV----------DI---EARIK--------
QMIRKYIGR------------QETIILAVLQCNV--DIA-TC--EALKMAKEFDDEGGRTLGVL-TKPDLLDKGA-
---E--SGVVRILNNM---E--------------------FTLS-KGYIIV--------------------------------KCR---------
-------------------------G-QEAIS----D-------------------------GQSLK----------Q----ALEV---EEDFF-

--------KS-------HR-------------------HF----------------S-S------L---R---------------------PSQ-WGI-
----PNLSTRLSRELKKHIKKLL----
XP_048248476.1 ------------INLPAVAVIGD----Q---------------SAGKSSVLE-----A---
IS-GVQ-LPRG---TGIVTRCPL---------EMR---M---KH-SE---
----------------------D-GD---MW---E-GKI------------------------------M---------------YKD----------
---------MHDM------------------AHE------EII-----LNRE---SVGELVRKAQIE-----MTDSA-------------
-----KG-----------------------IS--DELITLEVTSSD---
------------VPDLTVIDLPGIAR--NA------------------VEGQPF----------DI---EARIK--------
NMIRRYIGR------------QETIILAVLQCNV--DIA-TC--EALKMAKEFDTEGGRTLGVL-TKPDLLDKGA--
--E--SGVVRILNNK---E--------------------FTLS-KGYIIV---------------------------------KCR----------
------------------------G-QEAIS----D-------------------------GQSLK----------Q----ALEV---EEDFF---
------KS-------HR-------------------HF----------------S-S------L---R---------------------PSQ-WGI---
--PNLSMRLSRELKKHIKKLL----
XP_048258111.1 ------------INLPAVAVIGD----Q---------------SAGKSSVLE-----A---
IS-GVQ-LPRG---TGIVTRCPL---------EMR---M---KH-SE---
----------------------D-ED---MW---E-GKI------------------------------M---------------YKD-----------
--------MHDM------------------AHE------EII-----LNRE---SVGELVRKAQKE-----MTDSA-------------
-----KG-----------------------IS--DELITLEVTSSD---
------------VPDLTVIDLPGIAR--NA------------------VEGQPF----------DI---EARIK--------
NMIRRYIGR------------QETIILAVLQCNV--DIA-TC--EALKMAKEFDAEGGRTLGVL-TKPDLLDKGA-
---E--AGVVRILNNM---E--------------------FTLS-KGYIIV---------------------------------KCR---------
--------------------------G-QEAIS----Y--------------------------GQSLK----------Q----ALEV---EEDFF--
-------KS-------HR-------------------HF----------------S-S------L---R---------------------PSQ-WGI--
---PNLSARLSRELKKHIKKLL----
XP_046352531.2 ------------INLPSVAVIGD----Q---------------SAGKSSVLE-----A---
IS-GVQ-LPRG---TGIVTRCPL---------EMR---M---KH-SE---
----------------------D-ED---MW---E-GKI------------------------------M---------------YKD-----------
--------MYDM------------------AHE------EII-----LNRE---SVGELVRKAQKE-----MTDSA--------------
----KG-----------------------IS--DELITLEVTSSD---
-----------VPDLTVIDLPGIAR--NA------------------VEGQPF----------DI---EARIK--------
NMIRKYIGR------------QETIILAVLQCNV--DIA-TC--EALKMAKEFDAEGGRTLGVL-TKPDLLDKGA-
---E--SGVVRILNNM---E--------------------FTLS-KGYIIV---------------------------------KCR---------
--------------------------G-QEAIS----D--------------------------GQTLK----------Q----ALEV---EEDFF-
--------KS-------HR-------------------HF----------------S-S------L---R---------------------PSQ-WGI-
----PNLSGRLSRELKIHIKKLL----
XP_048248472.1 ------------INLPSVAVIGD----Q---------------SAGKSSVLE-----A---
IS-GVQ-LPRG---TGIVTRCPL---------EMR---M---KH-SE---
----------------------D-ED---MW---A-GKI------------------------------M---------------YKD-----------
--------MYDM------------------THE------EII-----LNRE---SVGELVRKAQKE-----MTDSA--------------
----KG-----------------------IS--DELITLEVTSSD---
-----------VPDLTVIDLPGIAR--NA------------------VEGQPL----------DI---EARIK--------
NMIRRYIRR------------QETIILAVLQCNV--DIA-TC--EALKMAKEFDAEGGRTLGVL-TKPDLLDKGA-
---E--TGVVRILNNM---E--------------------FTLS-KGYIIV---------------------------------KCR---------
------------------------G-QEAIS----D--------------------------GQSLK----------Q----ALEV---EEEFF--

-------KS-------HR-------------------HF-----------------S-S------L---R---------------------PSQ-XGI---
--PNLSGRLSRELKIHIKKRL----
XP_048248473.1 ------------INLPSVAVIGD----Q----------------SAGKSSVLE-----A---
IS-GVQ-LPRG---TGIVTRCPL---------EMR---M---KH-SE---
----------------------D-ED---MW---A-GKI--------------------------------M----------------YKD------------
--------MYDM-------------------THE------EII-----LNRE---SVGELVRKAQKE-----MTDSA---------------
----KG-------------------------IS--DELITLEVTSSD--
-----------VPDLTVIDLPGIAR--NA-------------------VEGQPL----------DI---EARIK--------
NMIRRYIRR------------QETIILAVLQCNV--DIA-TC--EALKMAKEFDAEGGRTLGVL-TKPDLLDKGA-
---E--TGVVRILNNM---E---------------------FTLS-KGYIIV---------------------------------KCR----------
-------------------------G-QEAIS----D--------------------------GQSLK----------Q----ALEV---EEEFF--
-------KS-------HR-------------------HF-----------------S-S------L---R---------------------PSQ-WGI--
---PNLSGRLSRELKIHIKKRL----
XP_048248474.1 ------------INLPSVAVIGD----Q----------------SAGKSSVLE-----A---
IS-GVQ-LPRG---TGIVTRCPL---------EMR---M---KH-SE---
----------------------D-ED---MW---A-GKI--------------------------------M----------------YKD------------
--------MYDM-------------------THE------EII-----LNRE---SVGELVRKAQKE-----MTDSA---------------
----KG-------------------------IS--DELITLEVTSSD--
-----------VPDLTVIDLPGIAR--NA-------------------VEGQPL----------DI---EARIK--------
NMIRRYIRR------------QETIILAVLQCNV--DIA-TC--EALKMAKEFDAEGGRTLGVL-TKPDLLDKGA-
---E--TGVVRILNNM---E---------------------FTLS-KGYIIV---------------------------------KCR----------
-------------------------G-QEAIS----D--------------------------GQSLK----------Q----ALEV---EEEFF--
-------KS-------HR-------------------HF-----------------S-S------L---R---------------------PSQ-WGI--
---PNLSGRLSRELKIHIKKRL----
ABI53802.1 ------------INLPAVAVIGD----Q----------------SAGKSSVLE-----A---IS-GVQ-
LPRG---TGIVTRCPL---------EMR---M---KH-SE--
------------A-ED---MW---E-GKI--------------------------------M----------------YKD--------------------
MYDV-------------------AHE------EII-----LNRE---SVEELVRKAQKE-----MTDSA-------------------KG-
------------------------IS--DELITLEVTSSD---
--VPDLTVIDLPGIAR--NA-------------------VEGQPF----------DI---EARIK--------NMIRRYIGR---------
---QETIILAVLQCNV--DIA-TC--EALKMAKEFDAEGGRTLGVL-TKPDLLDKGA----E--
TGVVRILNNM---E---------------------FTLS-KGYIIA---------------------------------TCR-----------------
------------------G-QEAIS----D--------------------------GQSLT----------Q----ALEV---EEDFF---------
KS-------HR-------------------YF-----------------S-S------L---R---------------------PSQ-WGI-----
PNLSGRLSRELKKHIKKLL----
CAH1802128.1 ------------VALPAVVVIGD----Q----------------SVGKSSVLE-----A---
MS-GVQ-LPRG---TGIVTRCPL---------ELR---M---KQ-CD--
-------------------------PG---NF---H-AKI--------------------------------S----------------YDI--------------
------QGGH------------------QPLE------KTI-----TDPS---NIDFEIRQAQRA-----LVGDS-----------------
--GG-------------------------VS--DRLIRLEVQADY--
-----------VPDLTLIDLPGIVR--YS-------------------EGSD-----------TI---VEETK--------NLIKTYVSR---
---------PETIILVVIPCNV--DID-TV--EACNLAKQVDPNGDRTIGVL-TRPDLIDHGV----
GPIKEVLDILENK---K---------------------MKLK-KGFYVV---------------------------------KCR-----------
-----------------------S-QKRIE----E--------------------------GQSLE----------Q----ALAE---EVQFF---

------RS-------DE--------------------RF-----------------R-V------I---N----------------------PSQ-CGV---
--KQLSSKLTNELFLHIKNCV----
PAA74204.1 ------------VRLPAIAVVGD----Q----------------SVGKSSVLE-----S---IS-GID-
LPRG---LGIVTRCPL---------MLS---M---RN--
-----------REEA---GW---S-ARI--------------------------------K----------------YKT--------------------
KTGE-------------------GRE------KKL-----TGAS---EVGQAIRDAQNE-----MTNSS-------------------
GE-------------------------VS--EQLIELWVESPE--
---------SPDLTLIDLPGIAR--YS-------------------IDGGG-----------AI---AGLTK--------SLILSYIEK-----
-------EEILILVVIPCHV--DIE-TV--EALSLAKEVDPESKRTIGVL-TCPDLVNPGS----E--
SEVLALMQNR---K---------------------IPLK-KGYVSV----------------------------------RCR--------------
---------------------T-PQQLK----D---------------------------NMSLQ----------Q----AARE---EEVFF-----
----RT-------HP--------------------HF-----------------R-A------L---D----------------------KFE-YGT-----
KTLAVKLSSELYEAIKHNI----
PAA76532.1 ------------ISLPAIAVVGD----Q----------------SVGKSSVLE-----A---IS-GVE-
FPRG---LGIVTRCPL---------MLS---M---RG--
-----------REDS---GW---T-ARI--------------------------------R----------------YET--------------------
KSGQ-------------------ARD------KPL-----STPA---EIGQAIRDAQEE-----MTSSS-------------------GE-
------------------------IS--EKLIELHIEGAD---
--TPDLTLIDLPGIAR--FS-------------------IANAG-----------DI---ATVSK--------SLIMSYILK------------
PEVLILVVIPCNV--DVE-TV--EAISLAREVDPECKRTLGVL-TCPDLVNPGS----E--TEVLAMMRNE---
R---------------------LKLR-KGFVTV----------------------------------RCR----------------------------------
-T-PQQLK----D---------------------------NMGLR----------E----ACKA---EEEFF---------KL-------HP---
-----------------QF-----------------C-A------L---G----------------------DYQ-RGC-----
KTLANKLSVELYQAVKERI----
PAA92268.1 ------------LKLPMVAVVGD----Q----------------SVGKSSVLE-----A---IS-
GVE-FPRG---TGMVTRCAL---------QLS---M---QW-NA--
---------------------DPEA---PW---H-GRI--------------------------------S----------------YKD-------------
-------VNGH-------------------KVD------KEL-----NSPG---EVDGAVREAQQR-----MTHGD------------
-------NE-------------------------IS--SEQIDLAIKGPD---
---------------VPDLTLIDLPGIAR--YS-------------------ATGGS-----------GI---AQITK--------
SLIAKYVSQ------------PQVLILVVVPCHQ--DIE-TV--EALSLAKEADPQGERTIGVL-TCPDMVNKGA-
---E--QETLKIANNE---K---------------------IPLK-KGYVMV----------------------------------KCR---------
--------------------------S-PEELN----N---------------------------GVTLS----------E----SVAN---EAAFF--
-------KT-------HR--------------------HF-----------------S-L------L---P----------------------EQS-VGI----
-RTLADKLTEELFESVKRNI----
PAA69582.1 ------------LRLPTVAVVGD----Q----------------SVGKSSVLE-----A---IS-GVD-
LPRG---TGIVTRCPL---------QLS---M---R---S---
----------KPTG---DW---T-GRI--------------------------------S----------------YQN--------------------
RKGE-------------------HVE------REI-----SKKC---EVDEMVRKVQNE-----ITGDS-------------------
NG-------------------------VS--TEQIDLTIESAD--
--------VSDLTLVDLPGIAR--YS-------------------EKN-P-----------KI---NEVTK--------QLILSYISQ-----
-------DQVIILVVVPCSV--DIE-TV--EAIALAKQVDPGGTRTIGVL-TCPDLTNPGS----E--
EDIKAIVNNQG--R---------------------VRLH-KGFVMV----------------------------------KCR-------------
----------------------S-PKELR----N---------------------------NISLS----------E----VAKI---EEDYF--------

-KN-------DP--------------------HF-----------------S-Q------L---P---------------------KDI-VGT-----
KTLAEKLTNELFKAVAAGI----
PAA83069.1 ------------LKLPSIVVIGD----Q----------------SSGKSSVLE-----T---IS-GVS-
FPRG---NGVVTLCPL---------QLS---M---RS-SD--
-------------K------KW---R-GTV-------------------------------R----------------YFD-------------------
AQGK-------------------EVH------WDI-----DSPD---DVENAIQNAQMR-----ITGHK-------------------
KA------------------------IS--KNIIEMTLEAPD--
-------LPNLTLVDLPGIAR--YS-------------------HSDGGS---------VNL---YKLTT--------DIIKEYIQR----
--------EETIILTVIPLSA--DTA-TM--EALQLAKDVDPYGLRTIGVL-TFPDLVNKGA----E--
EEKLQIARNI---T---------------------FPLS-KGYITV---------------------------------KCR------------------
-----------------N-QEDIK----S--------------------------RKSLR----------E----AKVD---EMRFF---------
SN-------DP--------------------FF-----------------S-Q------L---D---------------------PSQ-RGT-----
DTLAKRLSTELLTLIKKFI----
PAA94353.1 ------------LRLPSIVVIGD----Q----------------STGKSSVLE-----S---IS-GVR-
FPRG---NGVVTLCPL---------QLS---M---RT-SD--
-------------D--G---KW---R-GNI-------------------------------R----------------YYD-------------------
TYGK-------------------LMK------WDI-----DGPE---DVEDAIQEAQMR-----ITGNQ-------------------
RN------------------------VS--KSIIEMTLESPE--
-------LPNLTLIDLPGIAR--YN-------------------HNSAESG--------ASL---HQLTT--------DIIKEYIRR---
---------EETIILVVIPLTS--DTA-TM--EALQLAKDADPYGMRTIGVL-TFPDLVNKGA----Q--
EEKLMIARNI---T---------------------YPLS-KGYVTV---------------------------------KCR------------------
------------------N-QEDIK----N--------------------------RKSLK----------D----AKAD---EALFF---------
NT-------DP--------------------FF-----------------K-Q------L---D---------------------SMY-RGS-----
DTLARRLSEELLYLVKKFI----
GMH43921.1 S-----------QMLTAIVVVGD----Q----------------SSGKSSTLE-----R---IA-GID-
LPRG---QGICTRVPL---------EMQ---M---RK-GS--
---------------------KF---S-ATL-------------------------------E----------------YQQ-------------------
EKGGS------------------KQS------VEI-----KDAS---KISDAIQAATRD-----IVGNS-------------------KN--
-----------------------VE--DLPLVLRISSPI---
-YQDLTLIDLPGIAR--AP-------------------LPGQRS----------DI---EEQTL--------EMMRRYITG--------
----EAKVILCALPATN--DFV-TS--AALKLALQLDPDGERTLGAV-TKIDQARKGI----A--KKLEGTDASE-
-----------------------ITLH-LGFAGV---------------------------------RCR-----------------------------------
-T-ENETD----A--------------------------GITLE----------Q----VRQA---EEELF---------RT-------HD-----
---------------EL-----------------K-H------V---D---------------------DSC-KGV-----
SALLQKLVAVQRGRLISHLPKVL
GMH36208.1 -------------MLPAIVVVGD----Q----------------SSGKSSLLE-----I---LS-GVT-
LPRG---EGICTRVPL---------ELQ---L---RN-GT--
-----------EV----------S-AQI-------------------------------E----------------YQT-------------------
DLDAP------------------RVS------KHI-----M-VE---EVKNEILLATKR-----IAGME-------------------LN--
-----------------------IK--DLPIVLRMTGPT---
--YQDLTLIDLPGIAR--MP-------------------LRGQPD----------NI---EELTM--------EMIQKYING-------
-----DSKVILCAVPANN--EFV-TS--AALKLASNVDPLGLRTLGVV-TKADQFSRGM----R--
RRLEGLDDT----D--------------------VKLK-LGFVAV--------------------------------RCR---------------
--------------------T-QKELE----E--------------------------GISLQ----------D----VRMR---EELLF--------

-ET-------DP-------------------EL-----------------R-D------V---Q---------------------PHC-RGI-----
STLVDKLVDIQKERLIEQLPRIV
GJP35534.1 -------EG---IELPTIVVGD----Q----------------SSGKSSVLE-----N---LS-GIS-
LPRG---KGIVTRVPL---------ILR---L---QS-CV--
----------K--G---KD---M-ITI--------------------------------E---------------Y---------------------TPVTG-
-----------------KVS------KVL-----SDEE---MIEEEISEATIA-----LAGSR-------------------KG------------
-------------VM--NCPITLQVQRPD---
LPDLTLVDLPGITR--VP-------------------IEDQPK----------DI---YNQVK--------NMIMHYITP----------
--KESVILNVLAAEV--DFS-TC--ESIVMSQEVDSDGDRTLAVV-TKVDRAPDGL----Y--EKIQGN-------
S---------------------VRIG-LGYVCV-----------------------------------RNK-----------------------------------
TDADASH----D--D----ARRA---EAAFF---------NS-------HP------
--------------EL-----------------S-Q------I---E---------------------SHC-LGI-----
PALAQRLTEIQAKRVADSIPRIR
CAI5480041.1 -------EG---IELPTIVVGD----Q----------------SSGKSSVLE-----N---
LS-GIS-LPRG---NGIVTRVPL---------ILR---L---QS-CT--
--------------------S--K---DG---E-ITI--------------------------------E----------------YN-------------------
-NPSSG------------------KIF------KIL-----PDEE---SIQEEISKATVT-----LAGSR-------------------KG---
----------------------VM--DRPITLQVKRSG---
---LPDLTLVDLPGITR--VP-------------------VDDQPK----------DI---YNQVK--------KMIMQYITP-------
-----EESVILNVLAAEV--DFS-TC--ESIVMSQEVDQDGDRTLAVV-TKVDRAPDGL----Y--EKIQGN----
---S--------------------VRIG-LGYVCV-----------------------------------RNK---------------------------------
---TDADASH----A--A----ARLA---ETDFF---------DR-------HP----
----------------EL-----------------S-R------I---E---------------------TDS-RGI-----
PALAQRLSEIQAKRVAESIPRIR
KAJ7294545.1 --------G---IQLPTIVVGD----Q----------------SHGKSSVLE-----S---
LA-EIT-LPRR---QGIATRVPL---------ILR---L---QS-CK--
-------------------VA-S---EQ---S-ITI--------------------------------E----------------Y-----------------------
-LNV------------------KD--------EI-----KSEE---LIEAAIDEATNV-----LAGPR-------------------KD------
-------------------VR--DTPISLHVRKLG---
APDLTMVDLPGITR--VP-------------------VHGQPE----------NI---YEQIA--------AMIQKYINP---------
---PESIILNVISATV--DFP-TC--ESIRMSQLADKEGKRTLAVV-TKVDKAPEGL----Y--EKVTSD-------A-
--------------------VNIG-LGYICV-----------------------------------RNR-------------------------------------
TEKENSN----E--Q----ARFV---EKHLF---------DT-------HP-------
-------------SL-----------------C-K------L---D---------------------KSM-VGI-----
PMLAYRLTCIQAQMIQGCLPGL-
XP_024380180.1 -------EN---VNIPTIVVGD----Q----------------SSGKSSVLE-----S---
LA-GIT-LPRG---QGIATRVPL---------ILR---L---QS-CL--
--------------------SE-Q---DS---K-ILM--------------------------------E----------------Y--------------------
----GSV------------------KE-------MRI-----NSED---DIEAAINAATDD-----LAGSN------------------
KN-------------------------IR--DTPILLHIRKPD--
-------APDLTMVDLPGITR--VP-------------------VHGQPE----------NI---YEQVR--------DMIMHYIKP-
-----------EESIILNVLPAEV--DFS-TC--ESIRLSQTVDKKGVRTLAVV-TKVDKAPEGL----F--EKVTSD--
-----A--------------------VSIG-LGYVCV-----------------------------------RNR-----------------------------
------TPADDSI----A--V----ARCR---ELELF---------ND-------HP--

------------------DL-----------------R-N------I---D----------------------RSM-VGI-----
PTLGRRLVKIQSDMVRGCLPRIR
XP_024367947.1 -------EN---VNIPTIVVGD----Q----------------SSGKSSVLE-----S---
LA-GIT-LPRG---QGIATRVPL---------ILR---L---QS-CL---
--------------------SE-Q---DS---K-ILM--------------------------------E----------------Y--------------------
----ENV------------------KE-------MRI-----NSED---DIEAAINAATDD-----LAGSN-------------------
KN-------------------------IR--NTPISLHIRKPD--
-------APDLTMVDLPGITR--VP-------------------VHGQPE----------NI---YEQVR--------DMIMHYIKP-
-----------EESIILNVLPAEV--DFS-TC--ESIRLSQTVDKKGVRTLAVV-TKVDKAPEGL----F--EKVTSD--
-----A---------------------VSIG-LGYVCV----------------------------------RNR-----------------------------
------TPADDSI----A--V----ARCR---ELELF---------ND-------HP--
------------------DL-----------------R-N------I---D----------------------RSM-VGI-----
PTLARRLVKIQSDMVRGCLPRIR
KAG0619429.1 --------N---VQLPTIVVGD----Q----------------SSGKSSVLE-----S---
LA-GIT-LPRG---QGIATRVPL---------ILR---L---QS-CD---
--------------------ST-E---ES---L-IRM--------------------------------D----------------Y---------------------
---GNV------------------KD-------REI-----DGEE---QIEAAINEATNV-----LAGGN-------------------KD-
------------------------VK--DTPISLHIRKPH--
---APDLTMVDLPGITR--VP-------------------VHGQPQ----------NI---YEQIQ--------AMIMKHISP-----
-------EESIILNVLSAQV--DFP-TC--ESIRMSQQVDKDGKRTLAVV-TKVDKAPEGL----L--EKVTTD---
----A---------------------VNIG-LGYVCV----------------------------------RNR------------------------------
-----TDDDDTI----S--V----ARIR---EQRLF---------ES-------HP----
----------------AL-----------------K-D------L---D----------------------RSM-VGI-----
PALARKLTKIQSDMVKGCLPRI-
KAG0561847.1 --------N---IQLPTIVVGD----Q----------------SSGKSSVLE-----S---
LA-GIT-LPRG---QGIATRVPL---------VLR---L---QS-C--
--------------------QL-E---ES---I-IKM--------------------------------D----------------Y---------------------
---GNV------------------KD-------QEI-----SGEE---QIEAAINAATNA-----LAGSG-------------------KG-
------------------------VK--DSPIQLLIRKPN--
---SPDLTMVDLPGITR--VP-------------------VHGQPK----------NI---YEQIR--------GMIMRYITP------
------EESIILNVLSAQV--DFP-TC--ESIRMSQQVDKEGNRTLAVV-TKVDKAPEGL----L--EKVTTD----
---A---------------------VNIG-LGYICV----------------------------------RNR--------------------------------
---IDVDDSI----A--I----ARQR---ERELF---------ES-------HP------
--------------AL-----------------K-E------L---D----------------------GSM-VGI-----
PALARKLTKIQSDMVKECLPRI-
KAH9290598.1 --------G---IHLPTIVVGD----Q----------------SSGKSSVLE-----S---
LS-GID-LPRG---QGICTRVPL---------IMR---L---QN-ST--
---------------------E--E---YS---V-ISV--------------------------------E----------------Y---------------------
----KD------------------RK--------LS-----INEH---QIVDTINLVTEE-----IAGRN-------------------KG-----
--------------------IS--DDPITLHVRKKN--
VPDLTLVDLPGITR--VP-------------------VYGQPK----------DI---YEQVY--------KIIMKYISP------------
RDSIILNVLSATV--DFP-TC--ESIRMSQKVDEDGERTLAVV-TKVDKAPEGL----R--EKVAED-------A--
-------------------MNIG-LGYVCV----------------------------------RNR-----------------------------------V-
EGESI----V--K----ARKK---ENELF---------KT-------HP-------------

-------LL-----------------S-G------I---D---------------------KSI-VGI-----
PILAHKLMKIQAAGITNSLPRI-
KAH9320939.1 --------G---IQLPSIVVGD----Q----------------SSGKSSVLE-----S---
LA-GIK-LPRG---QGICTRVPL---------VMR---L---QS-CA--
----------------------EE-S---EE---E-ISI-------------------------------E---------------F-------------------
-----NG------------------VE--------KF-----IQES---DITSSIDTATQE-----IAGNG-------------------KG----
---------------------IS--HTPITLHVTKVG--
APDLTMVDLPGITR--VP-------------------VGGQPG----------DI---FEQIC--------EIIKEYITP-----------
-KESIILNVLAANV--DFP-TC--ESIRMSQKVDELGERTLAVV-TKSDRAPDGL----K--EKVTTD-------A-
--------------------VNIG-LGYVCV---------------------------------RNG-----------------------------------I-
GDESN----A---E----AREK---EKNLF---------DF-------HP---------
----------LL-----------------K-D------L---D---------------------KSM-VGI-----
PTLAKKLMQIQATTISATLPQI-
KAF8079489.1 -------EG---IQLPTIVVGD----Q----------------SSGKSSVLE-----S---
LA-GIS-LPRG---QGICTRVPL---------VMR---L---QG-SA--
-----------------------S--S---EP---E-IWL-------------------------------E----------------Y------------------
-------SD------------------NV--------VP-----TDEE---HIAEAISAATDV-----IAGSH-------------------KG-
-----------------------VS--DAPLTLHVKKAG--
-----APDLTMVDLPGITR--VP-------------------VKGQPE----------NI---YEQIS--------GMIMKYIKP-----
-------QESIILNVLSATV--DFT-TC--ESIRMSRQVDKTGERTLAVV-TKADMAPEGL----L--QKVTSD----
---D---------------------VSIG-LGYVCV---------------------------------RNR-------------------------------
----V-GEETY----E--E----ARKQ---EELLF---------ET-------HP------
--------------TL-----------------S-M------I---D---------------------ENI-VGV-----
PVLAQKLIQIQTTMIARCLPKIV
OAP13353.1 ------------IQLPTIVVGD----Q----------------SSGKSSVLE-----S---LA-GIS-
LPRG---QGICTRVPL---------VMR---L---QR-RR---
-------------S--P---EP---E-IWL-------------------------------E----------------Y------------------------
GD------------------KI--------VP-----TDEE---HIAQTICAATDV-----IAGM--------------------------------
----
---F--------------------

-
OAP19580.1 ------------IQLPTIVVGD----Q----------------SSGKSSVLE-----S---LA-GIN-
LPRG---QGICTRVPL---------VMR---L---QR-SS---
------------S--P---EP---E-IWL-------------------------------E----------------Y------------------------
SD------------------KV--------VP-----TDEE---HVAEAICAATDV-----IAGK--------------------------------
----
--FS-LS--PS--------
--Q-CSVKCV-----------------------------------

--LLQK----

OAP13972.1 ------------IQLPTIVVVGD----Q---------------SSGKSSVLD-----S---LA-GIS-
LPRG---QGICTRVPL---------VMR---L---QR-SS--
------------S--P---VP---E-IWL-------------------------------E---------------Y-----------------------SD-
-----------------KI--------VP-----TDEE---HIAEAICAATDV-----IAGK--------------------------------------
----
---FT-L--------------------
---Y-LGIKCV---

XP_038984915.1 -------EG---IELPTIVVVGD----Q---------------SSGKSSVLE-----S---
LA-GIS-LPRG---QGICTRVPL---------IMR---L---QD-DP--
---------------------SL-S---QP---Q-LQL-------------------------------E---------------Y----------------
--------KD------------------KA--------IH-----TSED---GIADAINSATDD-----IAGSG------------------
KG------------------------IS--NAPLTLVVRKRG--
--------VPDLTMVDLPGITR--VP-------------------VHGQPD----------NI---YEQIS--------NIIMEYIAP--
----------KASIILNVLSATV--DFP-TC--ESIRMSQSVDRTGERTLAVV-TKADKAPEGL----L--EKVTAD--
-----D--------------------VNIG-LGYVCV--------------------------------RNR---------------------------
------I-GDESY----E---E----ARAE---ERNLF---------KR-------HP---
-----------------LL-----------------S-R------I---D---------------------KSI-VGI-----
PVLAQRLMQIQAASIAKSLPDIV
KAF8391993.1 -------EG---IELPTIVVVGD----Q---------------SSGKSSVLE-----S---
LA-GIS-LPRG---QGICTRVPL---------IMR---L---QH-HS--
---------------------T--P---SP---E-LHL-------------------------------E---------------Y-------------------
-----HN------------------KI--------IP-----TNET---HVAEAINMATNE-----IAGNG------------------KG---
---------------------IS--NTPLTLVVKKKG---
-VPDLTMVDLPGITR--VP-------------------VHGQPE----------DI---YEQIS--------SIIMEYIKP----------
--KESTILNVLSATV--DFP-TC--ESIRMSQRVDKTGERTLAVV-TKCDKAPEGL----L--EKVTAD-------
D--------------------VNIG-LGYVCV--------------------------------RNR---------------------------------
-I-GEELY----E---E----ARME---EATLF---------ES-------HQ--------
------------LL-----------------S-K------I---D---------------------KSI-VGV-----
PILAQKLVQIQASIIAKCLPDIV
XP_058079501.1 -------EG---IELPTIVVVGD----Q---------------SSGKSSVIE-----S---
LA-GIS-LPRG---QGICTRVPL---------IMC---L---QN-VP--
---------------------T--D---KP---Q-MHL-------------------------------E---------------Y-----------------
-------QG------------------KI--------VL-----TSEN---QISDSISMATDE-----IAGNG------------------KG-
-----------------------IS--NIPLTLVVKKKG--
--VPDLTIVDLPGITR--VP-------------------VHGQPE----------DI---YEQIS--------NIIMDYIKP----------
--KESIILNVLSATV--DFP-TC--ESIRMSQHVDKTGERTLAVV-TKADKAPEGL----L--EKVTAD-------D-
-------------------VNIG-LGYVCV--------------------------------RNR----------------------------------I-
GDETY----E---E----ARIE---EATLF---------KS-------HP-------------
-------LL-----------------S-K------I---D---------------------KSI-VGI-----
PVLAQKLFQIQANSLSQCLPDIV
KAF5727250.1 -------EG---IQLPTIVVVGD----Q---------------SSGKSSVLE-----S---
LA-GIS-LPRG---QGICTRVPL---------VMR---L---QH-HH---

----------------------I--P---EP---E-LYL-------------------------------E---------------F------------------
------NG------------------KT--------VQ-----TTES---RISEAINLATDE-----IAGNG-------------------KG--
----------------------VS--NTPLTLVVKKHG--
---VPDLTMVDLPGITR--VP-------------------VHDQPE----------NI---YEQIA--------GIIMEYIKP--------
----DESIILNVLSATV--DFS-TC--ESIRMSQQVDKTGERTLAVV-TKVDKAPEGL----L--EKVTAD-------
D---------------------VNVG-LGYVCV---------------------------------RNR---------------------------------
--I-GDETY----E--E----ARRK---EAALF---------ES-------HP--------
------------LL-----------------C-L------I---D---------------------KSI-VGV-----
PVLAQKLVQIQAAIIMKCLPEIV
XP_002303204.3 --------G---IQLPTIVVVGD----Q----------------SSGKSSVLE-----S---
LA-GIS-LPRG---QGICTRVPL---------IMR---L---QH-HT--
---------------------A--P---EP---E-LSL-------------------------------E---------------F------------------
------NG------------------KT--------VP-----TSEA---KIANAISLATDE-----IAGNA-------------------KG---
---------------------IS--NTPLTLVVKKNG--
-VPDLTMVDLPGITR--VP-------------------VHGQPE----------NI---YEQIA--------DIIMEYIRP----------
--EESIILNVLSATV--DFT-TC--ESIRMSQKVDKNGERTLAVV-TKADRAPEGL----L--EKVTAD-------D-
--------------------VNIG-LGYVCV---------------------------------RNR-----------------------------------I-
GDESY----K--E----ARKE---EADLF---------EN-------HP-----------
---------LL-----------------S-K------I---D---------------------KSM-VGI-----
PVLAQKLVQIQATIIARCLPEI-
KAK1401877.1 -------EG---IQLPTIVVVGD----Q----------------SSGKSSVLE-----S---
LA-GIS-LPRG---QGICTRVPL---------IMR---L---HH-HS--
----------------------K--P---ES---E-LHL-------------------------------E---------------Y------------------
------CG------------------KV--------VS-----TDEL---KIAESINMATAE-----IAGDG-------------------KG--
----------------------IS--HTPLTLVVKKNG--
--VPDLTMVDLPGITR--VP-------------------VHGQPE----------NI---YEQIS--------GIIMEYIKP---------
---EESIILNVLSATV--DFP-TC--ESIRMSQSVDKTGDRTLAVV-TKSDKSPEGL----R--DKVMAD-------
D---------------------VNIG-LGYVCV---------------------------------RNR---------------------------------
-I-GDESY----E--E----ARMA---ESMLF---------ES-------HP-------
-------------LL-----------------S-K------I---D---------------------KSM-VSV-----
PVLARKLVQIQARIIAKCLPEIV
XP_002297993.1 ------------IQLPTIVVVGD----Q----------------SSGKSSVLE-----S---
LA-GIS-LPRG---QGICTRVPL---------IMR---L---QH-HT--
---------------------S--L---IP---E-MFL-------------------------------E---------------F------------------
------NG------------------KT--------TQ-----TDEA---NVADDINIATEE-----IAGSG-------------------KG--
----------------------IS--DAPLTLVIKKNG---
--VPDLTMVDLPGITR--VP-------------------VHGQPD----------NI---YEQIA--------GIVMQYIQP------
------EESIILNVLPASV--DFT-TC--ESIRMSRQVDKTGERTLAVV-TKADKAPEGL----L--EKVTAD------
-D---------------------VNIG-LGYVCV---------------------------------RNR--------------------------------
--I-GDESY----D--N----ARME---EANLF---------AT-------HP------
--------------LL-----------------S-R------I---D---------------------KSI-VGI-----
PVLAKKLMQVQATIMAKCWP---
XP_024439231.1 ------------IQLPTIVVVGD----Q----------------SSGKSSVLE-----S---
LA-CIN-LPRG---DGICTRVPL---------IVR---L---KH-HP--

---------------------S--L---VP---E-IFL-------------------------------Q----------------F---------------------
----NG------------------KT--------VP-----TDEA---HVADAINLVTDE-----IAGNG-------------------KG---
----------------------IS--NTELTLVVKKNG---
-VPDLTLVDLPGITR--VP-------------------VHGQPE----------NI---YEQIA--------YIIMKYISP-----------
-DESVILNVLSASV--DFS-TC--ESIRMSQKVDKNGQRTLAVV-TKVDKSPEGL----L--EKVTRN-------
D---------------------VNIG-LGYVCV----------------------------------RNR-----------------------------------
-I-GNESY----E--D----ARKE---EAALF---------AT-------HQ--------
------------LL-----------------S-K------I---D---------------------KST-VGI-----
QVLAQKLVQIQANIIAKCLP---

KAH0683503.1 -------EG---IQLPTIVVVGD----Q----------------SSGKSSVLE-----S---
LA-GIS-LPRG---QGICTRVPL---------VMR---L---KN-DP---
----------------------NI-T---AP---N-LQL--------------------------------E---------------Y-------------------
------NN------------------KS--------LP-----VDEI---GIADAIILATDE-----IAGHG-------------------KG---
----------------------IS--NNPLTLVVKKNG---
--VPDLTMVDLPGITR--VA-------------------VQGQPE----------DI---YEQVY--------DIIMKYIVP--------
----EESIILNVLSATV--DFP-TC--ESIRMSQKVDKTGERTLAVV-TKADKAPEGL----L--EKVTAD-------
E---------------------VNIG-LGYVCV----------------------------------RNR-----------------------------------
-I-GNESY----E--E----ARSD---EQRLF---------ST-------HP--------
------------LL-----------------S-K------I---D---------------------KSM-VSV-----
PILAQKLVRIQASIISKCLPEIV

PWZ56863.1 --------G---IQLPTIVVVGD----Q----------------SSGKSSVLE-----S---LA-GIS-
LPRG---QGICTRVPL---------VMR---L---QD-DP---
-------------SA-D---SP---K-LQL--------------------------------E---------------Y-----------------------
SNG------------------RV--------VT-----TTEA---DVADAINAATAE-----IAGSG-------------------KG------
-------------------IS--DAPITLVVRKRG--
VPDLTLVDLPGITR--VP-------------------VHGQPE----------DI---YDQVA--------KIIKEYIAP------------
KESIILNVLSATV--DFP-TC--ESIRMSQQVDRSGERTLAVV-TKVDKNPEGL----L--EKVTMD-------D-
---------------------VNIG-LGYVCV----------------------------------RNR-----------------------------------I-
GDETY----D--Q----ARVE---EERLF---------KY-------HP----------
---------LL-----------------S-K------I---D---------------------KDM-VGI-----
PVLANRLMQIQSTIIAKCLPDI-

PWZ56864.1 --------G---IQLPTIVVVGD----Q----------------SSGKSSVLE-----S---LA-GIS-
LPRG---QGICTRVPL---------VMR---L---QG-DP---
-------------ST-D---SP---K-LQL--------------------------------E---------------Y-----------------------
SNG------------------RV--------VT-----TTEA---KVADAINAATAE-----IAGSG-------------------KG-------
------------------IS--DAPITLVVRKSG--
VPDLTLVDLPGITR--VP-------------------VQGQPE----------DI---YDQIA--------NIIKEYITP------------
KESIILNVLSATV--DFP-TC--ESIRMSQQVDRTGERTLAVV-TKVDKAPEGL----L--EKVTMD-------D--
-------------------VHIG-LGYVCV----------------------------------RNR-----------------------------------V-
GEETY----D--Q----ARVA---EAQLF---------KN-------HP----------
----------LL-----------------S-Q------I---D---------------------KSM-VGI-----
PVLAQRLMQIQASIIAKCLPDI-

KAH9291961.1 --------G---IQLPYIVVVGD----Q----------------SSGKSSVLE-----C---
LT-GIS-LPRG---VGICTRVPL---------IMR---L---QN-SS---

--------------------E--Q---DS---E-IVV------------------------------E---------------Y--------------------
----ND-----------------TV--------EH-----IIES---QITERIDSITKE-----IAGTN------------------KG-------
------------------IS--HVPIRLNVKKMN---
APDLTLVDLPGIAR--VS------------------LNGNPD----------D----HELIS--------KIVMEYISP----------
--ADSIILNVLSATV--NFR-TC--ESIRMSQRVDVHGERTLGVV-TKVDIAPEGL----L--EKVALD-------
D--------------------VNTG-LGYVCV---------------------------------RNR----------------------------------
--V-GDECN----E---E----AREA---EAELF---------RS-------HT------
--------------QL-----------------N-K------F---D---------------------EAM-VGI-----
PMLARRLMQIQTKRISKCFPDI-

KAH9325151.1 ------------IQLLSIVVVGD----Q----------------SSGKSSMLR-----S---LA-GIN-LPKG---QWICTRVPL---------IMR---L---QI-SK--
---------------------S--Q---ET---E-ITI-------------------------------E---------------Y---------------------
---SG-----------------VK--------NN-----IFEY---KIIEALNAATDE-----IAGVG------------------KG------
-------------------IS--DTPITLNVSKSN--
IPNITMVDFPCITK--MP------------------VHDQPQ----------DI---YDQIS--------QVIKQYITP----------
--KESIILNVLFTSV--DFP-TC--ESIRMSQLVDVKGEKTLAVV-TKVDKAVEGL----F--EKVTVDV-V----S-
--------------------VNIG-LGYVCV----------------------------------RNK------------------------------------I-
GNESN----A--

KAH9300179.1 -------------NCLVLLL--
---WETSRLE--

-------------------------------------PG------------------RS------------------------IN--
DTPITLNVGKSN--APDLTMVDFPSITR--
VP------------------VHGQPQ----------DI---YDQIS--------QVIKQYITP------------KESIIMNVLSASV--
DFP-TC--ESIRMSQLVDEKGERTLAVF-TKVNKAAEVL----F--EKVTVD-------A--------------------
VNIG-LGYVCV---------------------------------RNR---------------------------------I-GNESN----A-----
----

KAH9314974.1 --

-------------ALNAATNE-----FAGAG------------------KS-----------------------IS--DTPITLNVSKSN--
--APNLTMVDLPGITW--VP-----------------
-VHGQPQ----------DI---YDQIS--------QVIKQYITP-----------KESIIMNVLSASV--DFP-TC--
ESIRMSQLVDEKGERTLAVV-TKVDNAAEGL----F--EKVTVD-------V--------------------VNIG-
LGYTCV---------------------------------YSF---------------------------------A-KRSRN----LA-V---------
-----------QACDYLDRI----------I----CRVI---DPQLQ---------ASS-----RRA-------------------FQ------
-----------A-L------I---D---------------------RKR-DKC-----IQYVEDAMEMQKSIVYTENP---

EFJ22917.1 ------------IKLPTIVVVGD----Q----------------SSGKSSVLE-----S---LA-QVD-LPRG---QGVVTRVPL---------VLR---L---QN-TS---
------------VTDQ---SH---Q-VVI-----------------------------Q---------------Y-----------------------
GG------------------KK--------RV-----IEEA---EISAAVVEATIE-----LAGD------------------KH----------
---------------IV--NKPISLHITKPG---

APDLTMIDLPGITR--VP-------------------VHGQPE----------DI---EEQIK--------KIIQEYISP-----------
KETIILNVICSTV--DFP-TC--ESILMSRQVDREGERTMAVV-TKVDMSPKDL----K--EKVMAD-------V--
-------------------VGIG-LGYICV---------------------------------RNR----------------------------------I-
GDETH----E--E----GRDR---EAELF---------RT-------DP----------
----------HL-----------------R-D------L---P---------------------ESM-LGI-----
RQLAKRLTEFQADSLRKNLP---

CAG9460856.1 ------------DKLPTIVVTGD----Q---------------SAGKSSVLE-----S---
LS-GIA-FPVG---DGIVTRLPC---------QVA---L---RE-GP--
----------------------AF-R---AV---C-TPP------------------------------E---------------G----------------
--------HG------------------EA-------VTL-----TDPK---AVTKWIEDTTAA-----VAGDK-------------------
KG------------------------VL--DKPLSIKVEREG---
--------SADLTLVDLPGITR--VA-------------------VDGQAD----------DI---EEQVK--------RMIQRYISR-
-----------EAAVVLCVLPANV--DFS-TA--ECIKMARAVDPGGERTLGVV-TKVDRAERGI----V--
TRLNAFGTT----G---------------------WALR-LGYVAV---------------------------------KNL---------------
--------------------S-QDERA----K-------------------------HGVSTT----------K----VLEL---EDAFF-------
--DDGVGRPAHLA--------------------EL-----------------A-D------L---D---------------------ADM-
RGL-----RTLVQKLVQVQGERIEAFMPSLV

KAK3283006.1 --------QD--IPIPQIAVMGD----Q---------------SSGKSSVLE-----A---
LS-GVH-FPRG---TGLVTKCAV---------EVR---M---KR-LQP--
-------------------------NE---DW---N-ASV--------SLSW-------------------------------------D----------
----------RP-QP------------------SEA------GVA-----TTPN---EVGEKISKLTEV---LLRARGN-------------
------RA------------------------TFEP-EHRIQVELKSPD---
----------------VSDLTIIDLPGIVR--TN-------------------VAGQCK----------KV---IAEVD--------
ALLDKYLRQ------------ERTIILAVIPSTV--DIA-TV--DVIERAEKVDPHGLRTIGVL-TKADQISSDD----
E--AERVAVLRGV---R---------------------KPLK-LGYFMV---------------------------------KNR----------
-------------------------T-QTELE----A---------------------------GVTLA----------E----ARLA---EARYF----
-----SA-------HK-------------------TF-----------------G-K------L---H---------------------PGL-FGS-----
QNLAERLSDVLATRIRDDLPLLV

GHP04420.1 --------QDA-IAIPQIAVMGD----Q---------------SSGKSSVLE-----A---IS-
GVP-FPRG---KGLVTKCAT---------QVI---M---RT-APK--
--------------------GS---PW---S-AVT--------SVRWADG-----------------T---------------TEH-----------
--------DQ-QP------------------EEA------GVI-----ASPE---EVAGVIERLTQV---LLKKSGH--------------
----QK-----------------------SFS--EHSIIIKLSSPE---
-----------HPNLSMVDLPGLVR--TV-------------------TEDQDD----------RD---IETVS--------
ELITRFMKQ------------ERTIILGVIPVNA--DIA-TS--EVLQRAKHYDPSGMRTLAVL-TEPDLVDPGS---
-E--NEMIEVLMNR---R--------------------VNLM-LGFCMV---------------------------------KLR--------
---------------------------G-QKELDECEGK--------------------------DVNST----------ELTRRAREA---
EEKFF---------RE-------NE--------------------AL-----------------A-R------L---GDE-------------------
VDGQ-LGI-----PNLITRLSDTLSNRIREQFPTIK

'KAJ3066410.1' --------DKY-VELPQIAVMGD----T---------------SSGKSSVLS-----A---
IS-GIT-FPSS---SELTTRCPT---------QLI---L---SE-AE--
-------------------------EF---S-GTV--------RLM--------------------R---------------FKP------------------
--QEGEI------------------LES------TTL-----SSPA---DITGEIERLTKQ-----IVSEQ-------------------QL--
----------------------IS--DDAIIIEVRGPG--

-YPNLTLTDLPGLIR--TV-------------------EDHEDK----------DI---IRRVR--------GLVDRYLVQ---------
---NRTVILAVVPANV--DVH-NT--EILQAAQDADPEGIRTISII-TKPDRIDPGA----E--SQVVDLLMNR--
-K---------------------KKLK-LGYHAV----------------------------------RCR----------------------------------
--G-QQDLD----D---------------------------GVTIA----------D----GIVN---ETKFF---------SE-------HK--
-------------------AW-----------------S-D------V---D---------------------PSY-VGI-----
NRLTEKLVKILQSIIASSL----

KAI9324922.1--------DRY-VELPQIATMGD----T----------------SSGKSSVLS-----A---IS-GIT-FPSS---SELTTRCPT---------QLI---L---SQ-SE---
-----------------TF---S-GSV--------RLQ--------------------R----------------FKPQ-------------------
QNEEP-------------------PET------KTL-----TNAN---EIEHEIERLTRQ-----LVQEN--------------------QS--
------------------------IS--DDSIIINVSGPN---
--YPNLTLTDLPGLIR--TV-------------------EDSEDP----------AI---IGRVR--------ALVDRYLVQ---------
---SRTVILAVVPANV--DVH-NT--EILQAAEAADPQGVRTISII-TKPDLIDSGA----E--SQVVELLLNR---
K---------------------KMLK-LGYHAV----------------------------------KCR----------------------------------
-G-QKDLN----N---------------------------GVSIA----------D----GIAK---EAEFF---------ET-------HA----
-----------------VW-----------------R-K------V---D---------------------SSY-FGI-----
SKLTEKLVKILETVIGGSL----

KAI8836453.1--------DKY-IELPQIAVMGD----T----------------SSGKSSVLS-----A---IS-GIE-FPSS---DTLTTRCPT---------QIV---L---SE-AD---
-----------------KF---S-GTV--------CLV--------------------R----------------FGSG-------------------IS---
-------------------SHL------THL-----KNRN---EITTEIARLTQV-----IRDEG-------------------QT-----------
---------------IS--DDAIVIEVRGPE--
YPNLTLTDLPGIIR--TV-------------------QDNEDP----------AM---IPRVR--------QLVDRYLVQ---------
---KRTVILAVVPANV--DFH-NS--EILQAAEKVDPKGERTIAII-TKPDAIDPGA----E--QSVLDLLMNK--
-K---------------------KALR-LGYHAV----------------------------------RCR----------------------------------
--G-KQHHD----D---------------------------KMTIP----------E----GLEM---EHDFF---------HN-------HK--------------------VW-----------------K-S------V---A---------------------PSY-VGC-----
ERLTEKLVKVLRNIITDSL----

KAJ3350919.1 --------DQY-VELPQIAVMGD----T----------------SSGKSSVLS-----A---IS-GIE-FPSH---AELTTRCPT---------QLV---L---ST-AE--
---------------------------AF---T-CTV--------CII--------------------R----------------VKN------------------
--SKASS------------------PPS------LKL-----KEPG---EIRGAITELTKI-----IIDDG-------------------PA---
-----------------------IS--EDMISIEVSGPK---
-YPNLTLIDLPGIVR--TV-------------------ADNEDP----------SM---ILNIR--------DLVTSYLQK----------
--KRTVILAVIPANV--DMH-NV--EILQLAESVDPSGDRTIAII-TKPDAVDCGA----E--KQIVDLLLNR---
K---------------------KFLK-LGYHAM----------------------------------RCR----------------------------------
-G-QQDLN----E---------------------------KMSIH----------E----GVIK---ESKFF---------YN-------HP---
-----------------VW-----------------R-N------I---S---------------------PEL-LGV-----
ESLVPKLVNTLQNVINQSL----

KAI8587516.1--------GQD-VSLPQLVVAGD----Q----------------SSGKSSLLE-----S---LS-GIS-FPKA---AELCTTFPT---------QIV---M---RT-K--
-----------------K---TW---E-ARV--------YTVPE--
IAN------------------FPA------TNC-----ASKV---AVQNIIQFIKRD---VLKATAG--------------------------
-------------------VS--ETVLVIELGSPE--

LPNLTIIDLPGYVH--TL------------------VKGQSE----------NF---KQKID--------NMVDKFIQD---------
---RRSIILAVIPANK--DFA-TN--VVLQRAQEWDPEGDRTIGVV-TKPDLVDQGT----E--
AAVIRMIQGH---Y--------------------KELK-LGYYMV---------------------------------HNR-------------
---------------------S-HMDLQ----N--------------------------GVDLA----------A----AVAK---EAEFF-----
----S--------QP-------------------AW----------------D-A------L---N---------------------ARQ-LGT-----
QQLQTAVVEVLAAHVVKEF----

XP_047808890.1 --------QDM-VSLPQIAVVGD----Q----------------SSGKSTLLE-----Y--
-IS-GVT-FPKD---AGMCTCFAT---------EVS---M---RP-S--
------------------------T---QF---S-ARVF------INHQPD-SRI---
---------------------------------------KQP-----RSPE---DVAGVIQQAKKL---FVEASGQ------------------
-KA------------------------IY--DDILTVELNGPG--
---------LPILTLVDLPGYIH--TH------------------ATGQPE----------SI---VKDIE--------QLVERYLNS---
---------PRTVIMAVIPVNR--DFE-TN--VAIKHIRRFDPSGKRTLCVL-TKPDQVDAGT----E--
RNVLDVLAGK---K--------------------MHLD-RGYHII---------------------------------KNK-------------
---------------------N-FEECQ----A--------------------------GDNRE----------A----TSKK---EGHFF-----
----A--------RS-------------------PW----------------S-S------I---S---------------------PTE-KGI-----
ASLVDRLSDTLNAQVEKEF----

KXS17655.1 --------NHL-ISLPQIAVVGD----Q----------------SAGKSSLLE-----A---IS-GIS-
FPKD---KEMCTTFAT---------QIV---M---AK-G---
--------------A---SF---A-AKV--------TIDPDPSNI---
S-----------------VGL------PVP-----KSPL---DVAAVIEEAKNL---MSEGNSN-------------------LI-------
------------------IA--DKILTIELTGPN--
YPRLTLVDLPGYVQ--SV------------------IKGQSE----------TI---IEDIA--------DIVDRHLKD-----------
-ERTITLAVIPANK--DLA-TN--VVVGKVDKLGSNGARTLGVI-TKVDVIDAGE----E--EAVLEILHGR---
R---------------------CDFG-LGFHAV---------------------------------RNR---------------------------------
--N-WAEVN----G---------------------------SLSTE----------E----LLVK---EAQFF---------A--------RA---
-----------------PW-----------------S-Q------L---D---------------------KSM-KGI-----
VSLRSKLVEILHNHVEKEL----

XP_021869222.1 --------GAE-VELPQLVVVGN----Q----------------SSGKSSVLE-----A-
--LT-GIP-FPRD---DGLCTRFAT---------RIT---F---RR-A---
-----------------------L---ET---R-YQA--------KIVPDKLSS----------------K----------------EHQ------------
--------DKCQQ------------------WGQ-------EL-----ESFDL-FQIADLMKKVRTV---MGVSDKT----------
---------SDSTYPAG-----------------SAFS--NDVLSLEITGPG--
-----------------------EEHFSIVDVPGTFK--VE------------------AEGVTT----------KE--DIKLVD--------
DMVKRYMTN------------SRSIMLTVVNCND--DIS-SH--DIIQKARDIDPHGERTLGIL-
TKPDLADEGA----E--QKIIDILDGK---Q-------------------HRLF-HGWHIL-----------------------------
-----RNR-----------------------------------G-QKDLR----D--------------------------ATSLS----------D---
-RHAT---ERKFF---------TD-------KD--------------------PW-----------------N-K------L---D--------------
--------KSL-VGI-----DALNHRLHAVLATQLNKEF----

TVY17522.1 --------GDY-IDLPQVVVVGD----Q----------------SSGKSSVLE-----G---LT-
NLP-FPRD---SGLCTKFVT---------KIT---F---RR-S---
------------------P---MT---R-IAI--------TIIPAKNSS--------------T---------------EHV-------------------
EHVRG------------------WAK------ADL-----RSLDE-KTFADIMKEVHTV---MGLSEQV------------------
-DGMTPP-------------------TFS--DDVLSLEVCGPK--

--------------QEHLSIIDVPGIFK--KR-------------------TQGLTS----------KA--DIQMVK--------
SMVLGYMKN------------PRSVMLTVIPANV--DIV-TQ--EILEMAEEVDPDGQRTLGVL-
TKPDLVDKGA----E--KTIVDLIEGR---R---------------------HRLA-LGWLLV------------------------------
----RNP-----------------------------------G-QQELT----D-----------------------------PMT----------D----
RHAL---EKSFF---------SH-------EQ--------------------PW-----------------N-S------L---D--------------
--------KEK-VGI-----PALQVRVRVILAEHIRRDF----
OLL24579.1 --------NSV-ISLPQIVVCGD----Q----------------SSGKSSVLE-----A---LT-
QIP-FPRS---DGLCTKFAT---------QVI---L---RR-A---
------------------S---KT---S-VRV--------QIIPDCKRP---------------E----------------AEQ------------------
---RALQS------------------VD-------IKL-----KKLE---DMTILIEEASKH---MGVQSSS-------------------
STS-----------------------TFS--SDILSIEVSGPK--
--------QPHLTVVDLPGYIR--TT-------------------SGNQTK----------KD---ITLIY--------DLVKDYISD---
---------KRSIILAVIPANV--DVA-NA--EILEKASEADPNKTRTLGVI-TKPDLVDNGA----E--
NQVLDLAANV---T---------------------KPLK-LGYFIV-----------------------------------RNR---------------
--------------------N-YNELK----S---------------------------ASDSK----------A----RNKS---EAAFF-------
--A--------QS---------------------PW-----------------S-E------I---N---------------------KTR-IGI-----
DRLRLYLSDLLQEHIFKEL----
KAI9096888.1--------SEY-VSLPQLVVCGD----Q----------------SAGKSSVLE-----A---IT-
EVP-FPQN---QGTCTRFAT---------QIV---L---RR-S--
-------------------V---VT---E-AIV--------TIIPESRRT---------------D-----------------NEV-------------------
-AKFAQ------------------FK-------KKI-----EDLK---DLPSIITEAEAL---ILFSNNV-------------------RTK--
----------------------FS--KDVLNVEISGPK---
--QPHLTVVDLPGIIHTSSS-------------------TTEDEE----------GDEFEVEVVK--------ELVKGYMKE----
--------DRTIILAIVAGNY--DYN-NQ--IILQMAKELDQDRTRTLGIV-TKPDLQEVGSD--YE--
KTLVKMVKNE---V---------------------KHLS-LGWHVL-----------------------------------KNR-------------
----------------------G-FKE------R---------------------------ECSIE----------Q----RNIA---EEKFF--------
-N--------QG--------------------VW-----------------T-S------L---P---------------------RKD-VGV-----
ESLRIKLSNLLYQHIKREL----
RSH87279.1 --------DSV-LSLPQIVVCGD----Q----------------SSGKSSLLE-----A---LT-
EIP-FPRN---DNLCTRFAT---------EIS---L---RR-E---
------------------S---ES---S-LTI--------RIIPAHSRP---------------Q----------------EEQ--------------------
-KRLER------------------FS-------EVI-----TDFE---DLPAVMDKAMQE---LGISEGT------------------
GA-------------------------FA--KDTLSVEIQGPD---
---------RPQLTLVDIPGLIQ--TS------------------TRGVSE----------AD---VALVA--------EITDSYIKQ---
---------PRTICLAVVSATN--DAA-NQ--PILSRVRKFDPHGKRTLGVI-TKPDRLPSGSA--SE--
SKFLELARNE---D--------------------VFFK-LGWHVI-----------------------------------KNR--------------
---------------------K-FEE------T--------------------------AFSID----------E----RNLV---ERTFF---------
A--------TS---------------------NF-----------------K-A------L---P---------------------RED-VGI-----
DALRVKLSTLLFDHVKKEL----
XP_041144356.1 --------SHY-ISLPQIIVCGD----Q----------------SSGKSSVLE-----A---
IS-GVS-FPVK---SNLCTRFPT---------ELV---L---RK-S---
----------------------S---HI---G-VKV--------SIVPHRSRS---------------H----------------VEQ------------
--------DALSR------------------FH-------EEL-----ESFE---GLPTLIENAKAA---MGIFTHG---------------
----KA-------------------------FS--NDLLRVEVSGPD---

--------------RPHLTIVDLPGLIH--SE-------------------TKLQSA----------AD---VALVQ--------
DVVQSYMKE------------PRSIILAVVSAKN--DFA-NQ--IVLRLAREADSFGHRTLGVI-
TKPDTLVEGSE--SE--YQFVSLAKNQ---E---------------------VTFR-LGWHVL--------------------------
-------KNM-----------------------------------D-TEK------G---------------------------NYNLS----------V---
-RGQE---EAEFF---------S--------RG-------------------IW-----------------E-D------L---P--------------
-------RSH-VGI-----DTLRQRLSKLLLGQIATEL----
KAJ5704467.1 --------SHY-VSLPQIIVCGD----Q----------------SSGKSSVLE-----A---
IS-GVS-FPVK---SSLCTRFPT---------ELV---L---RK-N---
----------------------S---QV---G-VRV--------SIVPHQSRS---------------D----------------AEQ-----------
---------HSLGS------------------FC-------EQL-----DGFD---GLANLIENAKAA---MGISTHG-----------
--------KA-------------------------FS--NDLLRVEVSGPD---
------------------RPHLTIVDLPGLIH--SE-------------------TRQQSA----------AD---VQLVQ--------
DVVQSYMRE------------PRSVILAVVSAKN--DFA-NQ--IVLRLARDADPSGNRTLGVI-
SKPDTLVPGSE--SE--ASFVSLAKNQ---D---------------------VEFR-LGWHVL-------------------------
--------MNM-----------------------------------D-SEK------G---------------------------QWSLS----------
D----RDIQ---ERKFF---------S--------GG-------------------IW-----------------E-D------L---P---------
------------RSL-VGV-----DSLRTRMSSLLLGQIAGEL----
MCJ1392161.1 --------SHY-VSLPQIIVCGD----Q----------------SSGKSSVLE-----A---
IS-GVS-FPVK---SNLSTRFPT---------ELV---L---RK-T---
---------------------S---QI---S-VSV--------SIVPHQARS---------------E----------------SER--------------
------LILSS------------------FH-------EEL-----EGFE---GLPSLIENAKSA---MGISTLG-------------------
KA-------------------------FS--KDLLRVEISGPD--
---------RPHLTIVDLPGLIH--SE-------------------TKQQSA----------SD---VELVQ--------
DVVQSYMKE------------PRCIILAVVSAKN--DYA-NQ--IVLKLARAADKKGTRTLGVI-TKPDTLIAGSE-
-SE--AMYVSLARNQ---D---------------------VEFR-LGWHAL----------------------------------KNM-----
-------------------------------D-SET------G---------------------------EWSLA----------E----RDVE---
EQEFF---------S--------QG-------------------IW-----------------R-D------M---S---------------------
RSL-LGV-----DDLRSRLSKVLLGQIAAEL----
KAI9774215.1--------SHY-VSLPQIIVCGD----Q----------------SSGKSSVLE-----A---IS-
GVS-FPIK---SNLCTRFPT---------ELV---L---RK-T---
-----------------S---QI---G-VSV--------SIVPHQSRS---------------E----------------SEQ-----------------
---HTLSS------------------FH-------EEL-----DGFE---GLPALIENAKAV---MGISTHG-------------------
KA-------------------------FS--KDLLRVEVSGPD---
---------RPHLTIVDLPGLIH--SE-------------------TKHQSA----------SD---VELVQ--------
DVVQAYMKE------------PRSIILAVVSAKN--DAA-NQ--IVLKLARAADKKGNRTLGVI-
TKPDVLIPGSE--SE--ASYICLARNQ---N--------------------VEFR-LGWHVL--------------------------
-------RNT-----------------------------------D-SEM------G--------------------------DWSLA----------D--
--RDAQ---EKEFF---------S--------QG--------------------IW-----------------E-Q------M---S-----------
----------QSL-LGV-----DKLRSRLSKVLLGQIATEL----
XP_002543522.1 --------SHY-ISLPQIIVCGD----Q----------------SSGKSSVLE-----A---
IS-GVA-FPVK---SNLCTRFPT---------ELV---L---RK-S---
----------------------P---EV---H-VSV--------SIVPHRSRS---------------E---------------SEQ-------------
-------HSLES------------------FR-------EDL-----DSFE---GLPELIENVKTA---LGISTHG-----------------
--RA-------------------------FS--NDILRIEISGPD---

---------RPHLTIVDLPGLIH--SE-------------------TKQQSA----------SD---VDLVQ--------
DVVQAYMRE------------PRSIILAVISAKN--DIA-NQ--IVLKLARGADRFGRRTLGVI-TKPDTLIPGSA--
TE--SIFVSLAKNQ---E---------------------VDFR-LGWHVL-----------------------------------KNM--------
---------------------------D-SEK------G----------------------------ISTLA----------D----RDVE---ERQFF--
-------S--------QG--------------------VW-----------------K-D------I---A----------------------PST-MGI---
--ARLRGRLSKVLLGQIATEL----
XP_746402.1 ------------ISLPQIIVCGD----Q----------------SSGKSSVLE-----A---IS-GVS-
FPIR---SSLCTRFPT---------ELV---L---RK-S---
-----------S---QV---G-VCV--------SIVPHRSRS---------------E----------------SER--------------------
EALAQ------------------FH-------EEL-----DSFE---GLPQLIENAKSA---MGIYTNA--------------------
KS-------------------------FS--NDLLRVEVSGPD--
----------RPHLTIVDLPGLIH--SE-------------------TKQQSA----------AD---VELVH--------
DVVKSYMEE------------PRSIILAVVSAKN--DVP-NQ--IVLKLARAADPHGTRTLGVI-
TKPDTLVRGSD--SE--AQFVSLAKNQ---E---------------------VEFR-LGWHAL---------------------------
--------KNM------------------------------------D-TDK------G----------------------------AWTLA----------E-
---RDKE---EHAFF---------A--------SG--------------------VW-----------------E-A------L---P------------
----------RSH-VGI-----DQLRKRLSKLLLAQIATELPSL-
XP_751069.1 ------------VELPQLIVCGN----Q----------------SSGKSSVLE-----A---IS-RVR-
FPAK---SNVCTRFAT---------EVI---L---RR-N--
------------A---AF---SKIKV--------SIEPGPSRT--------------DE----------------DER--------------------
RRLRS------------------FTY------EDF-----SNGD---DLPPLIEKAKVH---MGITESV-------------------
NTG------------------------FS--DDVLKVEISGPD---
----------KPELTLVDLPGLYY--ST-------------------SQEQDL----------QG---ILIVR--------
KLTERYMSN------------PRSIILAVISAKT--DYH-LQ--EVLNIAEQFDPKRERTLGII-TQPDILEANSE--
EE--DTYLHFVKNE---K---------------------IPLE-LGWHVL-----------------------------------RNR---------
--------------------------S-FET------R----------------------------DISDD----------A----RDEM---EKAFF---
------N--------QG--------------------RW-----------------A-S------L---S----------------------REC-VGI---
--ESLRRRLSGVLLRLIRRNLPGL-
XP_026607910.1 --------GEV-VALPQLAVCGD----Q----------------SAGKSSVLE-----A-
--IT-GIP-FPQQ---DGLCTRFPT---------EIT---L---RH-S---
------------------------E---AT---QSITIF------ASIRPHSVRS---------------R----------------KEK-----------
---------DYLAS------------------YQ-------KTL-----GAIS---ELPSIIADASKL---MGIRGYG----------------
---GQKNGP--------------------AFA--ADVLRIEITGPI---
--------------GLQLSVVDLPGLIS--VV-------------------SEEQNE----------ND---VVMIH--------
DMVTSYLQS------------SRTIILAVVQASN--DFA-NQ--CIIRMARKHDPEGQRTVGII-TKPDLINQGT-
---E--SKIARIAKNL---D--------------------TIKLK-LGFFLL-----------------------------------KNP-----------
------------------------S-PMERK----D---------------------------CHSMT----------A----RSAL---EDRFF--
-------S--------RP--------------------SW-----------------A-IH----HL---D----------------------KKR-IGS--
---ESLRTFLQKLLDSHIEHEL----
XP_748757.2 ------------ISLPQLVVSGD----Q----------------SSGKSSVLT-----A---VT-GFS-
FPRR---EGTCTRFAT---------EII---L---RH-S--
-----------K---ET---ETIIT-------ASIIPSLSRH--------------D----------------GSE--------------------
EALKR------------------FK-------KVL-----KSTE---ELPSVIHEASVA---MGIRGYS-------------------
DSDDSP--------------------AFT--ADVLRIEVVGDT---

------------GLCLTIVDLPGLIS--VS-------------------DYDEGE----------AD---VQLVN--------
TLIDSYLAN------------TRSIILAVVQASN--DIQ-NQ--NIIQRARRFDKLGERTVGII-TKPDLVNKGT----
E--SHIVRLANNL---D--------------------IVRLK-LGFFLM----------------------------------KNP----------
------------------------S-PEQLK----N---------------------------NISMF----------E----WKQK---ELEFF---
------N--------SP--------------------PW-----------------K-DL----ML---D---------------------HNR-
VGA-----ECLRSFLEKILEEHIERELP---
XP_040633937.1 --------GDH-VALPQLVVCGD----Q----------------SAGKSSVLE-----
G---IS-GIP-FPRQ---DGLCTRFAT---------EII---L---RH--
------------------------E---PG---EQRAT-------AMIIPHVSRT---------------D----------------EEK---------
-----------SSLGA-------------------FH-------RNI-----SDFT---ELPIIVEEARSI---MDIHGHG-------------
------IGSNAS--------------------AFS--ADVLRLELVGST--
-----------------GLHLTMVDLPGLIS--VS----------------------ENE----------HD---VQLVR--------
DLVDSYLEN------------SRTIIMAVVPASS--DVD-TQ--GILQRARHFDKTGLRTVGVI-TKPDLINAGT--
--E--PRVARLARNL---D--------------------GAKLN-LGFFLL----------------------------------KNP-------
----------------------------S-PAELE----A---------------------------GTTLP----------E----RRKV---ELEFF--
-------S--------SG--------------------AW-----------------K-GQ----GL---D---------------------PSR-IGI-
----DNLRSFLQDLLDHHIEREL----
XP_043140374.1 --------GDH-IALPQLVVCGD----Q----------------SAGKSSVLE-----G-
--IS-GIP-FPRQ---DGVCTRFAT---------EII---L---RH---
----------------------E---PN---DQRNT-------ATIIPHMSRA---------------E----------------DEK----------
----------VRLSA-------------------FH-------RDI-----NDLV---NLPGIVDEAARL---MGVQGAN-----------
--------DSTDAP--------------------TFA--ADVLRLEVVGDT---
--------------------GLHLTLVDLPGLIS--VS----------------------ENE----------ED---VQLVG--------
NLVNSYLEN------------SRTIILAVVPASS--DVD-TQ--SIIQRARRFDKDGLRTVGII-TKPDLINDGT----
E--SRVAKLANNA---D--------------------RTKLK-LGFFLL----------------------------------KNP----------
-------------------------R-PIDLE----K---------------------------GMTMV----------E----RRKM---EAQFF--
-------A--------NP--------------------PW-----------------N-NL----GL---N---------------------PSR-VGI-
----DNLRVFMQDLLDRHIEREL----
XP_754266.1 ------------IALPQLVVCGD----Q----------------SAGKSSVLE-----G---IT-GIP-
FPRQ---DGVCTRFAT---------EII---L---RH--
------------E---PN---HRRNT-------ATILPHISRT---------------E----------------EEK-------------------
AKLSA-------------------FR-------REV-----SDLA---QLPGIIEEAARL---MGVQGMN-------------------
DLADAP--------------------TFA--ADVLRLEIVGDT---
-----------GLHLTLVDLPGLIS--VS----------------------END----------DD---VQLVG--------
DLVNSYLEN------------SRSIILAVVPASS--DVD-TQ--SIIQRARRFDKDGFRTVGII-TKPDLINDGT----
E--GRIAKLANNA---D--------------------RTKLR-LGFFLV----------------------------------KNP----------
-------------------------R-PIDLE----K---------------------------GMTTA----------E----RRKV---EAEFF----
-----A--------HP--------------------PW-----------------N-KL----GL---D---------------------PSR-VGI---
--DNLRIFMQDLLDRHIERELPKV-
KAF9951223.1 --------NHV-LSVPQIAIVGD----Q----------------SSGKSSVLE-----A--
-FT-QLS-FPRD---KGMCTRFAT---------QVN---L---CR-DL--
----T-------------------LDKD---TL---S-ARI-------------------------------D---------------DE-------------
-----------DS------------------FN-------ERW-----KTFQV-DQFYAVIKEAVSL---LCGTS------------------
----------------------------DIS--DKVLELTLSGPH---

-----------QSPLTVVDLPGFIN--TT-------------------LDGQDK----------NI---PHTIR--------
DINERYMKD------------PRTIILAVIPANV--DLN-NS--YVLARAEEHDPKNERTVPIV-TKPDMIDKGT--
--L--SELIDMVLNN---R---------------------KKMP-LGYLVM---------------------------------RNT--------
--------------------------G-YAD------R--------------------------DLSWE----------E----AQRA---
EEDYF---------AQ-------DK-------------------AW----------------E-A------V---P---------------------
RSN-RGR-----VMVKKFLGDLLYFHIKKEL----

GAX85982.1 ---RQLGVSND-IKLPTLVTAGN----Q----------------SSGKSSVVE-----A---IA-GIP-LPRS---SGTCTRCPT---------EVR---M---RS--
----
---VHQVVRLTLTDVDQK---------------KP--KEPEHSRS------
FLEL--PTEYQLEFT--RNSVVLEIEGAD--
-ADLTIIDLPGIIQ-----------------------SHHKGP----------HY---VEMIK--------SMVLNSIES-----------
DHVIIVMVITAMD--DVE-NQ--AINLEARNVDPEGQRTIGVI-TKPDNIPKGEH------
DKWVALASNRR--P--------------------GQELS-LGYYVV----------------------------------RNP-----------
-----------------------G-QNELD----E---------------------------SIRFE----------D----ARVK---ESEYF----
-----ET-------SP--------------------YW---------------------PSNGEL-------------------------QGR-LGT---
--TFLRNALSESLVQGIKKGLPGMQ

XP_042923301.1 ------------LKVPALVIAGD----Q----------------SSGKSSVVE-----A---IA-GVP-LPRS---DGTCTRCPT---------EVR---M---RT-HG-----------------------AP----------------------
----------------------GEGGSA--VW---Q-CRI--------KVVR--------------------N----------------FDS-----
---------------TGKPLAP-----------GEAHEKLF------CTV-----TDKA---
HITACISAAQAVLLNPTVVGDAVADGAERFVPLLSAAEPGGRAPEASSA------
MRGLGDAAGYELQFT--ANKVVLEIVGAE---
----ADLTIIDLPGIIH-----------------------SHPKDP----------SL---IDVVK--------SLVKCYLAP-----------
-AHHIIVMTLPAGM--DAE-TQ--AILQFAREADPEGRRSIGII-TKPDKIGTDER--TE-
WGKLCNLVAGAR--A-PTGVPAAGGSRAAAAPN---PHLQ-LGYYVV----------------------------------
KNP-----------------------------------G-QEQLA----A---------------------------GISFE----------Q----
ARAA---EERYF---------AD-------HP--------------------LW-----------------ASAMKANSLL--------------
------------SQR-LGT-----NALRDGLSALLVDKIGEHMP---

XP_042924848.1 ------------LQVPTLVIAGD----Q----------------SSGKSSVVE-----A---SA-GVP-LPRS---DGTCTRCPT---------EVR---M---RT-
TQQLPAATGVCGGSALSAAAASSPPGPHSGEDGEDDEDDEDEEDEE-------DD-
AVVNNAAGLPPGTTRGGGGGSSASAW---Q-CRI--------KLCR--------------------E----------------
FDS--------------------DGVRLAT------------KPPEQPF------CVV-----RDKA---
HIATCVLAAQAVLLNPRAVEDT-PGGAGAFVPLLSSAQP-GRQPAAAAK------LLALRDASHYELPFT-
-PNKVVLEIDGAE---ADLTIIDLPGIIH----
-------------------SH-EDP----------RL---IELVK--------DMVKANLAP------------EHHIIAMALPAGQ--
DAE-TQ--AIRLMTREVDPDGRRSIGII-TKPDRVPEHEA--GE-TLKLIRLVGACG--APPAGAGAAGGS-
-ARVAH---PQHP-LGHYVV----------------------------------KNP-----------------------------------S-
QDGLA----M----------------------------NITFE----------Q----ARAD---EAAYF---------AG-------HK------
--------------HW-----------------AAALRRQPEL--------------------------QRR-MGA-----
AALRRGLSGLLVELVIAQLP---

KAG2488600.1 --LRALGVGSA-LQLPALVIAGD----Q----------------SSGKSSVVE-----A---IA-GVS-LPRS---DGTCTRCPT---------EVR---L---RT-HA-----------------------GP-----------------

---------------DANGDSP-----MPDGDV--PW---T-CRI--------KLHR--------------------E----------------
YDS--------------------DGHPLTE------------LPPEELF------ATL-----TNKA---
HIAAFVTAAQAVLLNPRAADAA-PGGARAFVPDVSGDRP--RDPQPLRA---------LGHPTTYELSFT--
ANKVVLEVDGAD---ADLTIIDLPGIIH----
-------------------DHPKGK----------QY---VDMVE--------RMTKAQLRP------------EHHIIAMALPAGL-
-DPE-TQ--AIRLWVREVDPSGSRSIGII-TKPDTIADDAH--IT-YGKLVKLVGGST--M-
AGGAAGAAASAGPAAGHDESHQLT-LGYYVV----------------------------------RNP---------------------
--------------G-QEQLE----D----------------------------CIGFA----------E----ARAA---EQRYF---------AT-
------NT--------------------HW-----------------VQAVAALPSL--------------------------KQR-LGA-----
NHLRSGLSALLVERIETQLPHMR
XP_042924875.1 --MR--------TQAANADLASD---ME----------------SSGGGEDGS-----
-------GME-----E--DGEGAANGA---------DGK-------RRP---
-------------------------------AW---R-CRI--------KLCR--------------------D----------------YDS----------
----------EDKPLAE------------KPPEQPF------CVV-----RDKA---HIAACVSAAQAVLLNPRAVEAA-
AGGPQAFVPELSSA----HSPSH--------------------------PNKVVLEIDGAE------------------------------
--ADLTIIDLPGIIH-----------------------DHPKGR----------HL---
VEVVE--------RMTKTNLAP------------AHHIIAMALPAGL--DPE-TQ--AIRLWAREVDPDGHRSIGII-
TKPDMIAEEAH--IV-CNKLVKLVGARG-ELGPGGTRSQPD-----------GHLR-LGYYVV-----------------
-----------------KNP-----------------------------------S-QEQLV----E----------------------------GITFE-----
-----K----AREI---EARYF---------AN-------HV--------------------HW-----------------RPAMATSPGL----
----------------------VQR-LGA-----NALRSGLSLLLVERIEEQMP---
KAJ9515210.1 --LADFGAAHE-ISYPTIVVCGD----Q----------------SAGKSSIIQ-----
R---IS-GID-LPRS---SGTCTRCPM---------EVRM-TL---SE--
----------------------------GGV--PW---S-CKI--------KI----------------------R----------------REW--------
------------DDGKRKTLS---------KVSWEDFG------APL-----LDKE---AVGPAVSRAQKA-----VLNPG--
-----------------KGYASFVD------PTSPILADADELGFS--RNVVVLEIQGAD------------------------------
--IS-LSLIDLPGIIN-----------------------STEKKE----------DQ-
YLVNMIK--------DMVKQYIEA------------SQTIIVLAVHALS--DIQ-NQ--
VVYQMAREADPHQQRTLGVI-TKVDVIPPGS----H--SMWIRMMRGE---L---------------------FPLD-
LGYYMV----------------------------------VNP------------------------------------N-QVDLD----Q-----------
----------------GTSHEVGHTGRMWKDD----AVDK---EMRFFETDANLGVLAQ-------SV---------------
-----VW-----------------S-S------------------------------------H-LGL-----
SNLTAALSKQLVDRTMAELPHMR
KAI3646081.1------------LNLPGIVVCGN----Q----------------SSGKSSLIE-----A---IS-RVP-
LPRA---AGTCTRCPF---------ECR---L---SE---
------------SKN--PW---S-CKV--------ML----------------------R----------------YEV--------------------
DA-KGQAL---TLKA-----VLNPG-------------------LEAQQFLT-
------DTNWEKIEDQQKFT--ENVVCLTIEGEG---
-------VGYLTLTDLPGIIQ-----------------------STNDAA----------DE-HFVELIK--------NLVEKYVSC---
---------PTNIIVEVITCKD--DME-NQ--IVHTLARKADPSGLRTVGVL-TKPDMIEEGC----T--
DDWLNVLRNN---T---------------------CPLK-HGYYMV----------------------------------KNP-----------
------------------------A-TKDLQ----A----------------------------NITFS----------Q----ARQK---EVAFF----
-----SQ--------K--------------------PW-----------------S-T------E---RAL--------------------SKR-FGI-----
YNLKEFLSNLLSNLLKQNLPQL-

OAJ38670.1 ------------LSLPTLVVCGN----Q---------------SVGKSSLVE-----A---IC-GIT-LPKA---AGTCTRCVT---------EVR---L---SE-YSDV-------------------GPVDSVRKKSVAGDRNSTESTITQGVYIDALDTVSSGSVPSAFHIDGHEKESS--TW---S-CTI--------TL---------------------R----------------FEY-------------------DE-AGIPLR---------SIREVLFG------PPL-----VEKS---LVALAVRRAQKA-----LLNPT------------------LDPSVFLTHIFTDNDQSNSDTKSNQLKFT--KNIVCLDIQGAG---IN-LALVDLPGIIR----------------------NVEHPD----------DA-MFIPMIE--------DLVKSYIQK------------ERTIIVATITCKD--EME-NQ--AIVHLAREVDPTGIRTIGVL-TKPDTIESGT----A--ARWADILMGN---L--------------------YPLK-LGYFMV--------------------------------RCL---------------------------------S-KAELA----A--------------------------GNTLQ----------D----AQKL---ENAFF---------AQ-------SQ-------------------PW----------------S-T------L---RRK-------------------SAR-FGA-----PALRFELSRLLINLVDMSLP---

XP_006461472.1 ------------IDLPQIAVVGN----Q---------------SAGKSSLIE-----S---IS-GIT-LPRA---SGTCTRCPT---------ECR---L---SY---SSQ--PW---K-CVV--------SL---------------------R---------------ITT--------------------DR-SGQPLG---------QSRNETFG------STI-----YDKK---EVDDRIRRAQLA-----ILNPD------------------KPAKSFLN------DDEPSLMEGNFLTFS--KNCVSLAISGPD---VADLSFVDLPGLIA----------------------SVGRGG----------NA-GDIKLVE--------GLVTTYIHK------------TNCIILLTVACET--DFE-NQ--GAHQLAKQYDPEGKRTIGVL-TKPDRIPAGE----E--QNWLKFIRNE---K--------------------EPLQ-NNWFCV---------------------------------KQP----------------------------------A-SSDLK----N--------------------------NWTWQ----------Q----ARQK---EDEFF---------TA-------TS-------------------PW----------------N-E------L---EAM-------------------YVRY-LRT-----KNLVERLSQVLSDLIAKTLP---

XP_006461433.1 ------------IDLPQIAVIGS----Q---------------SAGKSSLIE-----S---IS-GIT-LPRA---AGTCTRCPT---------ECR---L---SY---SPL--PW---K-CTV--------HL---------------------R---------------FTT-------------------DG-NGTPLG---------QSRNEIFG------PTI-----YEKS---EVEERIRRAQRA-----ILNPG------------------KPTKLFLE------DDDEMSGEAE-LSFS--NNCVSLQISGPD--VADLSFCDLPGLIA----------------------SVGRGG----------NT-NDIKLVE--------SLVTSYIKK------------PSCIILLTVACET--DFE-NQ--GAHQISKAYDPEGKRTIGVL-TKPDRIPLGE----E--LNWLKFLKNE---R--------------------EPLE-NNWYCV---------------------------------KQP----------------------------------S-SNDLK----N--------------------------NWTWQ----------E----AREK---EQQFF---------AA-------TA-------------------PW----------------C-E------L---EGM-------------------YQKF-LRT-----TNLVERLSGVLSDLIAKRLP---

XP_006457072.1 ------------FDLPKIVVIGN----Q---------------SAGKSSLIE-----A---VT-GIN-VPRD---SGTCTRCPM---------ECS---M---SS-DTR--SW---S-CTISLRSGTSTSIPPTSPRVL-----RSTRGTSIAS---------------ASS--------------------TASAGVTPTPARASTGVRNITTQSFG------PTI-----TDKS---QVELWLRRAQGA-----ILSTD-----------ADKSQWLNKSAEEIRQAI---------QNKTGMRDFT--EDTIVVDIQDPT---ATDLSFVDLPGLIS-----------------------NADPGS---------------IDLIK--------NLVRQHVAG------------ENTLILVTIPASD--DIQ-NH--GAVVLAKEADGNGDRTIVVL-TKPDSLGPGDT-GLQ--ETWRQTFKNPN--V-PENQ----------------NYLR-HGYYCV--------------------------------QLP---------------------------------NDQQRQQ--------------------------------GLTAH----------T-----------LPNYL---------GV-------TW-----

---------------PW-----------------S-E------F---AG--------------------QGR-FGV-----
TNLVKNVSALLVQMIEANLP---

XP_750654.1 ------------IALPKICVIGD----Q---------------STGKSSLIE-----G---MS-QIK-VPRS---AGTCTRCPM---------EIN---L---SE-GE--------------------P---GQ---DW---N-CRI--------FLSR-------------------K---------------YIF--------------------DGSRKVTKLPKKSQPLGPWIEQDQED------EHF-----TDVRDKDGVQAAIKWAQLA-----ILNPGRPS---------TDYQPGHNGDTDESYC---------------QVKFS--PNVVRLDISAPN---FPNLSFYDLPGVIS--QA-------------------EHDHER----------YL---VSLVE--------NLVREYISQ------------ENCIVLLALPMTD--DAT-NS--SAAKIMRDVPGAKERTLGVL-TKPDRIQTGE----S-YDQWVEILEGD---K---------------------FALG-HGYYIV---------------------------------RNN----------------------------------P-NPAIEH---S--R----AREE---EAVFF-----------------AKS--------------------PW-----------------ATD------L---SAY--------------------QNR-FGT-----RNLQSALSSLLLEQIQGCLP---

PAA68234.1 ------------DHLPRVVVIGD----Q---------------SSGKTSVLE-----A---VA-RARLFPRGA--GEMMTRAPV---------QVT---L--ADG--PY---HVARF--------------------------------K---------------DDP---DRE------FDL-----TKESELAALRDAIERRMRA-----AVRSS-------------------GP---------------------DAAVS--TEAIPLSVQGPG--LPRMVLVDLPGIIS--TE-------------------TAGMAA----------QT---RESIR--------QLARQYMRN------------PNAIILCVADACV--DPE-RS--NAFDLVAKHDPAGRRTIFVL-TKMDLAERDK---VS-PDRVAKLLAGR---L--------------------LPLKALGYFAV---------------------------------VTGS----------------------------------G---------SQ---------------------------DESVE---------------AIERH--EAEYF---------AS-------SR-------------------LF-----------------K-D----GRL---S---------------------PNQ-VTA-----ANMARAVSRRFWALVRESV----

PAA87312.1 ------------DHLPRVVVVGD----Q---------------SSGKTSVLE-----M---IA-KARIFPRGA--GEMMTRAPV---------QVT---L---AEG--PY---HVARF--------------------------------K---------------DNP---SRE------YDL-----TQESELAALRDTIERRMRS-----VVQSG-------------------G------------------------TVS--AETISLSVQGPG---LPRMVLVDLPGIIS--TE-------------------TRGMAS----------QT---REAIR--------QLASQHMRN------------PNSIILCVADACV--DPE-RS--NAFDLVARHDPSGRRTIFVL-TKLDLAERDR---IS-PDRIGRLLAGR---L--------------------LPLKALGYFAV---------------------------------VTGS-----------------------------------G---------GA---------------------------DESIP---------------AIQRY--EEQFF---------RN-------SQ-------------------FF-----------------K-E----GVL---S---------------------VSQ-MTA-----ANMAQAVSRRFWALVQESV----

XP_002602331.1 ------------DELPRVVVVGD----Q---------------SAGKTSVLE-----M---VA-QARIFPRGA--GEMMTRAPV---------KVT---L---SEG--PH---HIAMF--------------------------------K---------------DS---DRE------FDL-----TKESELEALRREVEIRMKA-----SVRPG-------------------Q------------------------TVS--METIAMSVKGPG--LQRMVLVDLPGIIS--TE-------------------TQGMAS----------AT---KESIK--------MMCEHYMSN------------PNAIILCIQDGSV--DAE-RS--NVTDLVSQMDPQGKRTIFVL-TKVDLAEKNI---TN-PRRIKQILEGK---L--------------------FPMKALGYFAV---------------------------------VTGR---------------------------------G---------NK---------------------------DDSID---------------

TIRGY--EEEFF---------RN-------SQ--------------------LF-----------------R-S----GVL---K---------------
-------ASQ-MTT-----QNLSFAVSDCFWKMVKASV----

XP_019637857.1 ------------DELPRVVVVGD----Q----------------SAGKTSVLE-----M---VA-QARIFPRGA--GEMMTRAPV---------KVT---L--
-----------------------SEG--PH---HIAMF--------------------------------K----------------DS---------------
------------------------------DRE------FDL-----TKESELEALRREVEIRMKA-----SVRPG-------------------
Q--------------------------TVS--METISMSVKGPG--
----------LQRMVLVDLPGIIS--TE-------------------TQGMAS----------AT---KESIK--------
MMCEHYMSN------------PNAIILCIQDGSV--DAE-RS--NVTDLVSQMDPQGKRTIFVL-
TKVDLAEKNI---TN-PHRIKQILEGK---L--------------------FPMKALGYFAV------------------------------
-----VMGR-----------------------------------G---------NK---------------------------DDSID---------------
TIRGY--EEEFF---------RT-------SQ--------------------LF-----------------R-S----GVL---K----------------
------ASQ-MTT-----QNLSFAVSDCFWKMVKASV----

XP_006813643.1 ------------DHLPRVVVVGD----Q----------------SSGKTSVLE-----M---IA-QARIFPRGS--GEMMTRTPV---------KVT---L--
-----------------------SEG--PY---HVAQF--------------------------------K----------------DS---------------
------------------------------NKE------YDL-----SKESELQSLRQEIELRMKN-----RVKKG------------------
-Q-------------------------TVS--NDTISLSVRGPG--
----------IQRMVLVDLPGMIS--TV-------------------TTGMAA----------DT---REAIH--------
NMSKSYMKN------------PNAIILCIQDGSV--DAE-RS--IVTDLATTMDPEGKRTIFVL-TKVDLAEKNS-
--AN-PSRIKQILDGK---L---------------------FPMKALGYFAV-----------------------------------VTGR-----
------------------------------G---------NT--------------------------NESIE---------------QIKNY--EETFF---
------RS-------SK-------------------LF------------------K-T----GTL---K-----------------------PSQ-MTT----
-QNLSFAVSDCFWKMVRESV----

XP_030843280.1 ------------DHLPRVVVVGD----Q----------------SAGKTSVLE-----M---IA-QARIFPRGA--GQMMTRAPV---------KVT---L--
------------------------SEG--PN---HIAQF--------------------------------K----------------DS--------------
-------------------------------GKE------FDL-----TKESELKALRQEIEARMKG-----SVKEG-----------------
--Q-------------------------TIS--PEVISLSVRGPG--
----------IQRMVLVDLPGMIS--TV-------------------TTGMAA----------DT---KTSIQ--------
KMINGYMGN------------PNAIILCIQDGAI--DAE-RS--IVTDLVNEIDPTGKRTIFVL-TKVDLAEKNH--
-LN-PNRIRQILDGR---L---------------------FPMKALGYFAV-----------------------------------VTGK------
-----------------------------G---------NT--------------------------SDSID---------------SIKQY--EEQFF----
-----RH-------SA-------------------LF------------------K-S----GVF---K----------------------PSQ-LNT---
--QNLSFAVSDCFWKMVRESV----

XP_018667792.1 ------------DHLPRVVVVGD----Q----------------SAGKTSVLE-----M---IA-QARIFPRGS--GEMMTRAPV---------KVT---L--
-----------------------SEG--PN---HVAQF--------------------------------R----------------DS--------------
-------------------------------SRE------FDL-----SKEEELKSLRHEIELRMKS-----SCSDG-----------------
--K-------------------------TVS--NDTISLTVKGPG--
----------LQRMVLVDLPGMIS--TV-------------------TSGMAP----------DT---KDAIC--------
NMSKHYMEN------------PNAIILCIQDGSV--DAE-RS--IVTDLVSQMDPSGRRTIFVL-
TKVDLAEKNI---TN-PSRIQEILDGK---L--------------------FPMKALGYFAV-----------------------------
-----VTGQ-----------------------------------G---------SA---------------------------NSSIT---------------

DIKEY--EEEFF---------SN-------SK-------------------VF-----------------K-S----GLL---K----------------
------ASQ-LTT-----ANLSYAVSNCFWKMVRESV----

XP_032818114.1 ------------DQLPRVVVVGD----Q----------------SSGKTSVLE-----M--
-IA-QARIFPRGS--GEMMTRSPV---------KVT---L--
-----------------------SEG--PH---HVAIF--------------------------------K----------------DS----------------
----------------------------SRE------FDL-----TKEDDLAALRKEIEIRMKK-----SVKEG-------------------
H------------------------TVS--AETISLSVKGPG--
--------LQRMVLVDLPGVIS--TM-------------------TSGMAP----------DT---KDAIF--------
AMSKGYMQN------------PNAIILCIQDGSV--DAE-RS--IVTDLVSNMDPQGKRTIFVL-
TKVDLAEKNL---AS-PNRIQQILDGK---L---------------------FPMKALGYFAV---------------------------
-------VTGK----------------------------------G----------NR-------------------------DESIE---------------
SIKDY--EEEFF---------QK-------SK-------------------LC-----------------R-S----GML---K--------------
--------AHQ-VTT-----KNLSLAVSDCFWKMVRESV----

XP_021332524.1 ------------DHLPRVVVVGD----Q----------------SAGKTSVLE-----M--
-IA-QARIFPRGS--GEMMTRSPV---------KVT---L--
-----------------------SEG--PH---HVAMF--------------------------------K----------------DS--------------
------------------------------SRE------FDL-----GKEEDLAALRHEIELRMRK-----SVKEG------------------
--Q------------------------TVS--PETISLSVKGPG---
----------IQRMVLVDLPGVIS--TV-------------------TTGMAA----------DT---KETIF--------
SISKAYMQN------------PNAIILCIQDGSV--DAE-RS--IVTDLVSQMDPQGKRTIFVL-TKVDLAEKNL-
--AS-PSRIQQIVEGK---L---------------------FPMKALGYFAV----------------------------------VTGK-----
-----------------------------G----------SP-------------------------NESID---------------SIKDY--EEDFF---
------QN-------SR-------------------LL-----------------K-D----GML---K---------------------AHQ-VTT-
----KNLSLAVSDCFWKMVRESV----

XP_028587646.1 ------------DHLPRVVVVGD----Q----------------SAGKTSVLE-----M--
-IA-QARIFPRGS--GEMMTRSPV---------KVT---L--
-----------------------SEG--PH---HVAMF--------------------------------K----------------DS--------------
------------------------------SRE------FDL-----TKEEDLAALRNEIEIRMRK-----SVSDG------------------
-C-------------------------TVS--TETISLSVKGPG--
---------LQRMVLVDLPGVIS--TV-------------------TSGMAP----------DT---KETIF--------
SISKAYMQN------------PNAIILCIQDGSV--DAE-RS--IVTDMVSQMDPQGKRTIFVL-
TKVDLAEKNV---AS-PSRIQQIIEGK---L---------------------XPMKALGYFAV-----------------------------
-----VTGK----------------------------------G----------NS-------------------------CESIE---------------
SIKEY--EEEFF---------QN-------SK-------------------LL-----------------K-N----CML---K--------------
--------AHQ-VTT-----RNLSLAVSDCFWKMVRESV----

XP_025913835.1 ------------DHLPRVVVVGD----Q----------------SAGKTSVLE-----M--
-IA-QARIFPRGS--GEMMTRSPV---------KVT---L--
-----------------------SEG--PH---HVALF--------------------------------K----------------DS---------------
-----------------------------SRE------FDL-----TKEEDLAALRNEIEIRMRN-----SVKEG-------------------
C-------------------------TVS--TETISLSVKGPG---
-------LQRMVLVDLPGVIS--TV-------------------TSGMAP----------DT---KETIF--------SISKAYMQN--
----------PNAIILCIQDGSV--DAE-RS--IVTDLVSQMDPQGKRTIFVL-TKVDLAEKNV---AS-
PSRIQQIIEGK---L---------------------FPMKALGYFAV---------------------------------VTGK------------
----------------------G----------NS-------------------------SESID---------------SIKEY--EEEFF---------

QN-------SK-------------------LL-----------------K-T----CML---K---------------------AHQ-VTT-----
KNLSLAVSDCFWKMVRESV----
XP_023440724.1 ------------DHLPRVVVVGD----Q----------------SAGKTSVLE-----M--
-IA-QARIFPRGS--GEMMTRSPV---------KVT---L--
-----------------------SEG--PH---HVALF--------------------------------K----------------DS---------------
-----------------------------SRE------FDL-----TKEEDLAALRREIEIRMRK-----SVKEG-------------------
C------------------------TVS--PETISLNVKGPG---
--------LQRMVLVDLPGVIN--TV-------------------TSGMAP----------DT---KETIF--------SISKAYMQN-
-----------PNAIILCIQDGSV--DAE-RS--IVTDLVSQMDPHGRRTIFVL-TKVDLAEKNV---TS-
PSRIQQIIEGK---L---------------------FPMKALGYFAV---------------------------------VTGK------------
----------------------G----------NS-------------------------SESIE---------------AIREY--EEEFF---------
QN-------SK-------------------LL-----------------K-T----SML---K---------------------AHQ-VTT-----
RNLSLAVSDCFWKMVRESV----
XP_005873264.1 ------------DHLPRVVVVGD----Q----------------SAGKTSVLE-----M--
-IA-QARIFPRGS--GEMMTRSPV---------KVT---L--
-----------------------SEG--PH---HVALF--------------------------------K----------------DS---------------
-----------------------------SRE------FDL-----TKEEDLAALRHEIELRMRK-----NVKEG------------------
-C------------------------TVS--PETISLNVKGPG---
---------LQRMVLVDLPGVIN--TV-------------------TSGMAP----------DT---KETIF--------
SMSKAYMQN------------PNAIILCIQDGSV--DAE-RS--IVTDLVSQMDPHGRRTIFVL-
TKVDLAEKNV---AS-PSRIQQIIEGK---L---------------------FPMKALGYFAV----------------------------
-----VTGK----------------------------------G----------NS-------------------------SESIE---------------
AIREY--EEEFF---------QN-------SK-------------------LL-----------------K-A----SML---K---------------
-------AHQ-VTT-----RNLSLAVSDCFWKMVRESV----
XP_006163024.2.2 ------------DHLPRVVVVGD----Q----------------SAGKTSVLE-----M--
-IA-QARIFPRGS--GEMMTRSPV---------KVT---L--
-----------------------SEG--PH---HVALF--------------------------------K----------------DS---------------
-----------------------------SRE------FDL-----TKEEDLAALRHEIELRMRK-----NVKEG------------------
-C------------------------TVS--PETISLNVKGPG---
---------LQRMVLVDLPGVIN--TV-------------------TSGMAP----------DT---KETIF--------
SISKAYMQN------------PNAIILCIQDGSV--DAE-RS--IVTDLVSQMDPHGRRTIFVL-TKVDLAEKNV-
--AS-PSRIQQIIEGK---L---------------------FPMKALGYFAV---------------------------------VTGK------
----------------------------G----------NS-------------------------SESIE---------------AIREY--EEEFF-----
----QN-------SK-------------------LL-----------------K-T----SML---K---------------------AHQ-VTT-----
RNLSLAVSDCFWKMVRESV----
NP_056375.2.2 ------------DHLPRVVVVGD----Q----------------SAGKTSVLE-----M--
-IA-QARIFPRGS--GEMMTRSPV---------KVT---L--
-----------------------SEG--PH---HVALF--------------------------------K----------------DS---------------
-----------------------------SRE------FDL-----TKEEDLAALRHEIELRMRK-----NVKEG------------------
-C------------------------TVS--PETISLNVKGPG---
---------LQRMVLVDLPGVIN--TV-------------------TSGMAP----------DT---KETIF--------
SISKAYMQN------------PNAIILCIQDGSV--DAE-RS--IVTDLVSQMDPHGRRTIFVL-TKVDLAEKNV-
--AS-PSRIQQIIEGK---L---------------------FPMKALGYFAV---------------------------------VTGK------
----------------------------G----------NS-------------------------SESIE---------------AIREY--EEEFF-----

----QN-------SK-------------------LL-----------------K-T----SML---K---------------------AHQ-VTT-----
RNLSLAVSDCFWKMVRESV----
NP_598513.1 ------------DHLPRVVVVGD----Q----------------SAGKTSVLE-----M---IA-
QARIFPRGS--GEMMTRSPV---------KVT---L--
-------------------SEG--PH---HVALF-------------------------------K----------------DS-------------------
-------------------------SRE------FDL-----TKEEDLAALRHEIELRMRK-----NVKEG-------------------C-
-----------------------TVS--PETISLNVKGPG---
-----LQRMVLVDLPGVIN--TV-------------------TSGMAP----------DT---KETIF--------SISKAYMQN----
--------PNAIILCIQDGSV--DAE-RS--IVTDLVSQMDPHGRRTIFVL-TKVDLAEKNV---AS-
PSRIQQIIEGK---L---------------------FPMKALGYFAV----------------------------------VTGK------------
----------------------G----------NS-------------------------SESIE---------------AIREY--EEEFF---------
QN-------SK-------------------LL-----------------K-T----SML---K---------------------AHQ-VTT-----
RNLSLAVSDCFWKMVRESV----
XP_031757388.1 ------------DHLPRVVVVGD----Q----------------SAGKTSVLE-----M--
-IA-QARIFPRGS--GEMMTRSPV---------KVT---L---
-----------------------SEG--PH---HVAMF-------------------------------K----------------DS--------------
-----------------------------SRE------FDL-----SKETDLAALRNEIEVRMRK-----SVKNG-----------------
--Q------------------------TVS--PETISLSVKGPG---
----------IQRMVLVDLPGVIN--TV-------------------TSGMAP----------DT---KDTIF--------
NISKAYMLN------------PNAIILCIQDGSV--DAE-RS--IVTDLVSQMDPQGRRTIFVL-TKVDLAEKNV-
--AS-PNRIQQIIEGK---L---------------------FPMKALGYFAV----------------------------------VTGK------
-----------------------------G----------NS-------------------------NESID---------------SIKDY--EEEFF----
-----QG-------SS-------------------LL-----------------K-K----GML---K---------------------AHQ-VTT---
--KNLSLAVSDCFWKMVRESI----
NP_495986.3.3 ------------DNLPRVVVVGD----Q----------------SAGKTSVLE-----M--
-VA-QARIFPRGS--GEMMTRAPV---------KVT---L---
------------------------SEG--PY---HVAQF-------------------------------R----------------DS--------------
-----------------------------SRE------FDL-----TKETDLQQLRNETEVRMRN-----SVRDG---------------
----K------------------------TVS--NEVISLTVKGPN--
------------LPRMVLVDLPGVIS--TV-------------------TADMAR----------ET---KDDII--------
RMSKAHMEN------------PNAIILCIQDGSV--DAE-RS--NVTDLVSSIDPSGKRTILVL-
TKVDMAEKNL---AN-PDRIKKILEGK---L--------------------FPMKALGYFGV--------------------------
-------VTGR-----------------------------------G----------NS-------------------------SDSID---------------
EIRKY--EENFF---------ST-------SQ-------------------LL-----------------R-D----GVL---K--------------
-------PSQ-MTT-----RNMSLAVSDCFWRMVRDSI----
NP_610941.1 ------------DHLPRVVVVGD----Q----------------SSGKTSVLE-----S---IA-
KARIFPRGS--GEMMTRAPV---------KVT---L--
------------------AEG--PY---HVAQF--------------------------------R----------------DS-------------------
------------------------DRE------YDL-----TKESDLQDLRRDVEFRMKA-----SVRGG-------------------K-
-----------------------TVS--NEVIAMTVKGPG---
------LQRMVLVDLPGIIS--TM-------------------TVDMAS----------DT---KDSIH--------
QMTKHYMSN------------PNAIILCIQDGSV--DAE-RS--NVTDLVMQCDPLGRRTIFVL-TKVDLAEE-
L---AD-PDRIRKILSGK---L--------------------FPMKALGYYAV----------------------------------VTGR---
------------------------------G----------RK-------------------------DDSID---------------AIRQY--

EEDFF---------KN-------SK-------------------LF----------------HR-R----GVI---M----------------------
PHQ-VTS-----RNLSLAVSDRFWKMVRETI----
EFJ33653.1 -----------------VVTIGN---T-----------------GAGKSAVLN-----S---II-
GYTVMPTG---ENGATRAPI---------VVE---L---ER-DQ--
-------------------SEGK---GL---A-VMT---
------------------------EGR------ARP-----SSAN---EIRLSLQSRISR-----IASSR-------------------SG-----
-----------------R------PEEIRLRLRSSA--
APPLTLIDLPGLA--SLD-D------QFVNEYGSH------------
NDAVLLVVVPATSVRDIT-GS--QALKMARELDPEFSRTVGVI-SKVDQSASDPK---S-
LAAVQAVLSGQG-PS---------------------ASAD-ITWVAL----------------------------------IGQS---------
-----------------------V-S-IAAAH----AGS---------------------VGTDDSLE----------T----AWKA---
ETETL---------RS------I--L---TAA-------------------PSTR-
LGR-----AALVDVISKQIRKRIRQRLP---
EFJ28901.1 -----------------VVALGN---T-----------------GAGKSAVLN-----S---LI-
GYAVLPTG---ENGATRAPV---------TID---L---EA-DD--
------------------SGNKR--GL---T-VQM---
-------------------------EGK------SMQ-----VSAT---DIRHSLQNKFGR-----M--ST-------------------GA--
--------------------V------KENIHLKLKCSS---
--APPLKLVDLPGLES---RSVS-D------SLVREYIDS------------
NDALLLVVIPATSVRDIT-GS--QALKIAQDIDHEGSRTVGVI-SKIDQAASDPK---S-
LAAVQAVLSGQG-PS---------------------ITSK-FTWIAL----------------------------------IGQS-----------
---------------------V-S-IAGAH----S-------------------------KDDSLE----------T----AWKA---EMESL---
------KS------I--L---GGA------------------SSSR-LGR-----
SSLVEAIAKQIRQRMQQRLP---
KAG0632288.1 -----------------VVTLGS---V-----------------GAGKSAVLN-----S---LM-
GYPVLPTG---ENGATRAPI---------IIQ---M---QR-GS--
------------------SSTNR--GL---Y-VVL---
-----------------------DGR------TSN-----VTAS---DVRHSLQGRLKSW----TPNAR-------------------SG-
---------------------R------TEGIQLTLQSSA--
---APPLKLFDLPGLDT--RATSD-D------SLVQEFAEH-----------
-SDAILLVVVPAASVREVG-TS--KALKLAQELDSDATRTVGVI-SKVDQAASDRR---S-
LDAVAALLSGNG-PA---------------------ITQE-IPWVAM----------------------------------IGQS----------
----------------------V-S-IAAAH----GS-------------------------EDSLD----------T----AWKA---EAESL--
-------KS------L--L---TQA------------------APTK-LGR-----
VALVEAIAKQIRKRLKQRIP---
'KAG0555995.1' -----------------VVALGS---V-----------------SAGKSAVLN-----S---LI-
GHPVLPTG---ENGATRAPI---------ILD---M---ER-DK--
------------------SSSSR--GL---A-VVL---
-----------------------EGR------TQN-----VSAS---DVRHSLQGRLKN------ASSS------------------KG--
--------------------R------TEGIRLTLRSAS---
-TPPLKLIDLPGVS---GSID-D------SPAHDLAAN------------
NDTILLIVIPATSCRDVA-AS--KALKLAQELDSDGTRTVGVI-SKVDQAASDPR---S-
LAAVNALISGQG-PP--------------------STAD-IPWVAL---------------------------------IGQS---------
---------------------V-S-IAAAH----SSG------------------------EDSLD----------T----AWKA---EMESL-

--------KS------I---L---NGA-------------------PSAK-LGR----
-IALVETLSHQIRTRLKQRLP---
XP_024391061.1 -----------------AIALGN---V-----------------SAGKSAVLN-----S---LI-
GHPVLPTG---ENGATRAPI---------IID---M---ER-DK--
------------------SGRPG--GL---A-VVL---
------------------------EGR------TQN-----VSAS---DIRHSLQGRLKS------VSSS-------------------KG---
-------------------R------GDGIRLTLRSNS--
--GPPLKLIDLPGIDS--RGSID-D------SPAHDLAAN------------
NDTILLVVIAATSCRDVA-VN--RALKLAQELDSDGSRTIGVI-SKVDQAASDPR---S-
LAAVNALISGQG-PS---------------------NTQD-MPWVAL-------------------------------------IGQS-------
-------------------------V-S-IAAAH----SSS-------------------------EDPLD----------T----AWKA---
EMESL---------NS------I---L---KGA-------------------PPAK-
LGR-----IALLETLASKIQSRLKQRIP---
XP_024368367.1 -----------------AIALGS---V-----------------SAGKSAVLN-----T---II-
GHPVLPTG---ENGATRAPI---------IID---M---ER-DK--
------------------SGRPG--GL---A-VVL---
------------------------EGR------TQN-----VSAS---DVRHSLQGRLKS------VSSS-------------------KS--
--------------------R------GDGIRLTLRSNS---
---GPPLKLIDLPGIDS--RGSLD-D------SPAHDLAAN----------
--NDTILLVVIAATSCRDVA-VN--RALKLAQELDSDGSRTVGVI-SKVDQAASDPR---S-
LAAVNALISGQG-PS---------------------NTQD-IPWVAL-------------------------------------IGQS---------
-----------------------V-S-IAAAH----SSP-------------------------EDSLD----------T----AWKA---EMESL-
--------KT------I---L---KGA-------------------PSAK-LGR-----
IALLETLASKIQSRLKQRIP---
OAE31801.1 -----------------VVAVGH---I-----------------SAGKSAVLN-----S---LV-
GYPVLPTG---ENGATRVPI---------IIE---M---KR-DE---
-----------------SGNRK--GL---A-IQV--
-----------------------EGG----R-IQS-----VSAS---DLRHNLQGRLQK-----WTPNA-------------------
KG----------------------R------PDEIKLRLVSSA---
-------APPLKLIDLPGMEV--RAPIE-D------SIVRDYAEH--------
----NDAVLLLVIPATQAANIL-GA--RALKLVQDLDGEGTRTVGVI-SKVDQAAADPR---S-
LAAVQALLSGQG-PS---------------------STAE-FPWVAL-------------------------------------IGQS---------
-----------------------V-S-IAAAH----AGG-----------------------GAEDSLE----------T----AWRA---
EAESL---------KQ------I--L---PQA-------------------SPSK-
LGR-----VALVETLSSQIRKRLKNRLP---
KAI5073815.1-----------------VVALGN---V-----------------GAGKSAVLN-----S---LT-
GHPVLPTG---ENGATRVPI---------VID---M---ER-DE--
------------------GLNSK--QL---V-LQI---
-----------------------ESK------TQQ-----VSAS---SIRRSLQEKLTS-------ASE-------------------RG------
----------------R------LDEIYLKLRSST--
APPLKLVDLPGLDQ--RGSD-E------STVSAYTDQ------------
GDAILLVVVPAFQTSEIS-NS--RALRLAHDLDPDGSRTVGVI-SKVDQAASDSR---N-
LAAVQALLVGQG-PS---------------------ITLD-VPWVAL------------------------------------IGQS----------
----------------------A-S-IATAH----SSS---------------------TGGDNSLE----------T----AWRA---

EMENL---------KS------V--L---GSA-----------------
PQSK-LGR-----IALVDTLSKQIRKRLKLRLP---
XP_008646219.1 -----------------AVALGN---V-----------------GAGKSAVLN-----S---LI-
GHPVLPTG---ENGATRAPI---------VVD---L---AR-DP---
-------------------GLSSK--SI---V-LQI--
-----------------------DSK------SQQ-----VSAS---ALRHSLQDRLSK---GASSGSG-------------------
RS----------------------R------SDEIYLKLRTST--
------APPLKLIDLPGIDQ---RVMD-D------STISEYAGH---------
---NDALLIVVIPAMQAADVA-SS--RALRLAKDIDPDGTRTIGVL-SKIDQAAADAK---T-
VSCVQSILSNKGAPR------------------AAAD-IEWVAL-------------------------------IGQS-------------
----------------------V-S-IASAQ----SGS----------------------VGSDNSLE----------T----AWRA---
EAETL---------KS------I--L---TGA-------------------PQSK-
LGR-----IALVDTIAKQIRKRMKVRLP---
ACG47836.1 -----------------AVALGN---V-----------------GAGKSAVLN-----S---LI-
GHPVLPTG---ENGATRAPI---------VVD---L---QR-EP---
-------------------GLSSK--SI---V-LQI--
-----------------------DSK------SQQ-----VSAS---ALRHSLQDRLSR---GASGGSG-------------------
RG----------------------R------VDEIYLKLRTST--
-------APSLKLIDLPGIDQ---RAVD-D------SMINEYAGH--------
----NDAILLIVIPAMQAADVA-SS--RALRLAKDIDADGTRTVGVI-SKVDQANGDAK---T-
IACVQALLSNKG-PK-------------------NLPD-IEWVAL---------------------------------IGQS----------
---------------------V-A-IASAQ----S------------------------VGSENSLE----------T----AWRA---EAESL-
--------KN------I--L---TGS-------------------PQNK-LGR---
--IALVDTIAKQIRKRMKVRVP---
'KAG7649995.1' -----------------VVALGN---V-----------------GAGKSAVLN-----S---LI-
GHPVLPTG---ENGATRAPI---------IID---L---SR-EE---
----------------SLSSK--AI---I-LQI--
------------------DNK------NQQ-----VSAS---ALRHSLQDRLSK------GASG-------------------RG------
-----------------------RDEIYLKLRTST--
APPLKLIDLPGLDQ--RIVD-E------SMIGEHAQH------------
NDAILLVVVPASQASEIS-SS--RALKIAKEYDPDSTRTVGII-SKIDQAAENPK---S-LAAVQALLSNQG-
PP---------------------KTTD-IPWVAL----------------------------------IGQS--------------------------------
V-S-IASAQ----SG------------------------GSENSLE----------T----AWRA---ESESL---------KS------I----
---L---TGA-------------------PQSK-LGR-----
IALVDTLASQIRSRMKLRLP---
'NP_172500.1' -----------------VVALGN---V-----------------GAGKSAVLN-----S---LI-
GHPVLPTG---ENGATRAPI---------IIE---L---SR-ES---
----------------SLSSK--AI---I-LQI--
------------------DNK------SQQ-----VSAS---ALRHSLQDRLSK------GASG-------------------KN-------
----------------------RDEINLKLRTST--
APPLKLVDLPGLDQ--RIVD-E------SMIAEYAQH------------
NDAILLVIVPASQASEIS-SS--RALKIAKEYDPESTRTIGII-GKIDQAAENSK---A-LAAVQALLSNQG-
PP---------------------KTTD-IPWVAV--------------------------------IGQS--------------------------------
V-S-IASAQ----SG------------------------SGENSLE----------T----AWRA---ESESL---------KS------I----

--L---TGA-------------------PQSK-LGR-----
IALVDTLASQIRSRMKLRLP---
XP_006385192.1 -----------------VVALGN---V-----------------GAGKSAVLN-----S---LI-
GHPVLPTG---ENGATRAPI---------SID---L---SR-DS--
------------------SVSSK--SI---I-LQI---
---------------------DSK------NQQ-----VSAS---ALRHSLQERLSK------VSSG-------------------RS-----
------------------------RDEIYLKLRTST--
APPLKLIDLPGVDQ--RIVD-D------SMISEYVQH---------
NDAILLVVIPAIQAPEIS-SS--RALRIAKEYDAESTRTVGII-SKIDQAATESK---A-IAAVQALLLNQG-PP-
--------------------KTSD-IPWVAL-----------------------------------IGQS--------------------------------V-
S-IASVQ----SGS---------------------ASSESSLE---------T----AWRA---ESESL---------KS------I------
--L---TGA-------------------PQSK-LGR-----
VALVDVLAGQIRSRMKLRLP---
KAH9330549.1 -----------------VVALGN---V-----------------GAGKSAVLN-----S---LI-
GHPLLPTG---ENGATRAPI---------IID---L---QR-DS---
-----------------SVSSR--AI---F-LQI--
---------------------ENK------TQQ-----VSAS---ALRHSLQDKLSK---GL------------------------------------
---------------------GIPPMKLRTST--
SPPLKLIDLPGLDQ--RVAD-D------SMISNVAEH---------
NDAILLVVVPASQAPEIS-SS--RALKLALEFDPD--
--A-IASAQ----SGS---------
-------------VGGESSLE----------T----AWRA---ESESL---------KA------I------------------------------------
---------------L---TGA-------------------PQTK-LGR-----IALVETLARQIRKRIKIRLP---
ONM04707.1 -----------------VVAIGN---V-----------------GAGKSAVLN-----G---LI-
GHPVLPTG---ENGATRAPI---------CVE---L---QR-DA--
-------------------SLSSK--AI---V-LQI--
----------------------DSK------SQQ-----VSAS---SIRHSLQDRLSK---V----SG-------------------KG-----
-----------------R------PDEITVKLCTST---
APPLKLIDIPGVDQ---RSTD--------ESISNYAAR-----------
NDAILLVIIPALQAPDVA-SS--RALRIARELDSEGTRTIGVL-SKIDQASGEQK---A-LGAVQALLVNQG-
PR--------------------TAAD-IQWVAT-----------------------------------IGHS--------------------------------
-V-P-TASAQ----SEA-----------------------GSETPPE----------A----YWQA---EVKTL---------VS------T--
--L---GGA-------------------PESK-LGR-----
VALVDSLSKQIKARIKARLP---
XP_042918632.1 ------------LAIPEIVAIGG----Q----------------SDGKSSLLE-----A---
FL-GFRFNVRE---VEMGTRRPL---------IVQ---M---VH-DP--
------------------------TAQE---PR-----CRL---------------------------------Q---------------EED-----------
-------------------------------SDEYG------PPI-----VPET---AVADAIQRRTEE-----HLRKM------------------
--GG-----------------------IAVS--SKPIVMRAEYAY---
-----------CPNLTIIDTPGFIL--KA--------------------KTGELD----------NT---PDEIM--------
SMVKAQASP------------PHRMILFLQQSSV--EWA-SS--LWLRVVQEVDPYFQRTVIVA-
SKFDNRLKEF---AE-RWEVDKYLSATG-YL-PPN-----VRP-------FFVALP---------------------------------
---------KDRV---------------------------------IQS-SAEWR--R--

--SMTEV--DTAIY---------KH------MRD-------------------GI------------------K----GGF---DEE--------
----------RFASR-IGF-----SNLKKFLEEELSRRYREAAP---
NP_001130364.1 ------------LPIPEIVVIGG----Q----------------SDGKSSLLE-----A---
LL-GFRFNVRE---VEMGTRRPL---------VLQ---M---VH-DP--
-----------------------TALE---PR-----CRF-------------------------------Q----------------EED------------
-----------------------------SEEYG------SPM-----VVAT---AIADLIKQRTES-----HLRKI-------------------
QA-----------------------AVS--SKPIVMRAEYAH--
----------CPNLTIIDTPGFVL--KA-------------------KRGEPE----------ST---PDEIR--------
SMVKSLATP------------PHRLVLFLQQSSV--EWC-SS--IWLDTLKEIDPTFRRTMIVI-SKFDNRLKEF-
--TE-RWEVDAFLSASG-YL-GDN-----IHP-------FFVALP---KDRG-
-------------------------------TIS-NEEFR--R----QICHV--
DIDVL---------RH------LRD-------------------NV-------------------K----GGF---NEE------------------
KYGSH-IGF-----SCLRKYLESELQKRYKEAAP---
PWZ11893.1 ------------LPIPEIVAIGG----Q----------------SDGKSSLLE-----A---LL-
GFRFNVRE---VEMGTRRPL---------VLQ---M---VH-DP--
--------------------TALE---PR-----CRF-------------------------------Q----------------EED----------------
-------------------------SEEYG------SPM-----VLAT---AIADLIKQRTES-----HLRKI-------------------QA-
----------------------AVS--SKPIVMRAEYAH---
-----CPNLTIIDTPGFVL--KA-------------------KRGEPG----------ST---PDEIR---------------------------
--------LQQSSV--EWC-SS--IWLDTLKEIDPTFRRTIIVI-SKFDNRLKEF---TE-RWEVDTFLSASG-YL-
GDN-----IHP-------FFVALP---KDHG-------------------------------
TIS-NEEFR--R----QICQV--DIDVL---------RH------LRE-----
---------------NV-------------------K----GGF---NEE------------------KYVSC-IGF-----
SCLKKYLESELQKRYKEAAP---
XP_002317496.2 ------------LPIPEIVALGG----Q----------------SDGKSSLLE-----A---
LL-GFRFNVRE---VEMGTRRPL---------ILQ---M---VH-DP---
-----------------------SALE---PR-----CRF-------------------------------Q----------------EED-----------
------------------------------SEEYG------SSV-----VSST---TIADIIKSRTEV-----LLKRT------------------
KT------------------------AVS--SKPIVMRAEYAH--
---------CPNLTIIDTPGFVL--KA-------------------RKGEPE----------NT---PDEIL--------SMVKSLASP-
-----------PHRILLFLQQSSV--EWC-SS--LWLDAIKDIDPNFRRTVIVV-SKFDNRLKEF---SD-
RWEVDRYLSASG-YL-GEN-----TRP-------FFVALP---KDKN-------
-------------------------TIT-NDEFR--R----QISQV--DSEIL-----
----HH------LRD-------------------GV-------------------K----GGF---DEE------------------KFRPY-IGF-
----STLRDYLESELQKRYKEAAP---
AAF87857.1 ------------LPIPEIVAIGG----Q----------------SDGKSSLLE-----A---LL-
GFRFNVRE---VEMGTRRPL---------ILQ---M---VH-DL---
--------------------SALE---PR-----CRF--------------------------------QISRIFFVELAILITDLDED--------
---------------------------------SEEYG------SPI-----VSAT---AVADVIRSRTEA-----LLKKT-----------------
--KT------------------------AVS--PKPIVMRAEYAH--
-----------CPNLTIIDTPGFVL--KA-------------------KKGEPE----------TT---PDEIL--------
SMVKSLASP------------PHRILLFLQQSSV--EWC-SS--LWLDAVREIDSSFRRTIVVV-
SKFDNRLKEF---SD-RGEVDRYLSASG-YL-GEN-----TRP-------YFVALP--------------------------------
---------KDRS--------------------------------TIS-NDEFR--R---

-QISQV--DTEVI---------RH------LRE--------------------GV-------------------K----GGF---DEE---------
---------KFRSC-IGF-----GSLRDFLESELQKRYKEAAP---

KAI5058044.1------------LPIPEIVALGG----Q----------------SDGKSSLLE-----A---LL-
GFRFNIRE---VEMGTRRPL---------MIQ---M---VH-DA---
--------------------SALE---PL-----CRL--------------------------------Q----------------DED---------------
--------------------------SDDYG------PVI-----APAS---AVAEAIKLRTEE-----HLKKE-------------------
RT------------------------AVS--SKPIVMRVEYAY--
--------CPNLTIIDTPGFVL--KA-------------------KKGEPE----------NT---PEDIM--------SMVRTLAAP-
-----------QHRLLLFLQQSSV--EWC-SS--LWLDSIRTVDPSLRRTIIVI-SKFDNRLKEF---GE-
RWEVDRYLSAGG-YL-GDT-----AHP-------FFVALP---KDRT-------
-------------------------MTS-NEEFR---R----QIGAV--DSDVH--
-------RY------LRN--------------------NI-------------------K----GGF---DEE------------------KFGDF-
IGF-----LNLKQYLELELQRRYRDAAP---

EFJ18064.1   ------------LSIPEIVAVGG----Q----------------SDGKSSLLE-----A---LL-
GFRFNVRE---VEMGTRRPL---------VLQ---M---IH-EP--
-------------------AAVD---PR-----CRL--------------------------------Q----------------HED---------------
--------------------------DEEYG------PVI-----VPHY---AVAEAIKLRTEE-----HLKKI-------------------
RA------------------------AVS--SKPIVMRVEYAY---
---------CPNLTIIDTPGFIL--KA-------------------KKGEPE----------ST---PDDIL--------QMVRALALP--
----------PNRLLLFLQQSSV--EWC-SS--LWLDTVRSIDPGFHRTVVVV-SKFDNRLGEF---AE-
KWEVDRYLSAGG-YL-GDH-----VRP-------FFVALP---KDRG------
--------------------------SVT-NEEFR---S----QIASV--DAEVL---
------KH------LRE--------------------RI-------------------S----GGF---SED------------------KYSGS-
IGF-----GNLRNYLEAELQRRYREAAP---

KAG0628798.1   ------------LPIPEIVAVGG----Q----------------SDGKSSLLE-----A---
LL-GFRFNVKE---VEMGTRRPL---------MLQ---M---IH-DP---
------------------------EALE---PR-----CRL--------------------------------Q----------------DED-----------
------------------------------ADDYG------PVI-----TPVS---SVADHIRIRTEG-----FLKKL------------------
-GT------------------------AVS--AKPIVMRAEYAY---
----------CPNLTIIDTPGFIL--KA-------------------KKGEPD----------ST---PDEIE--------AMVRELAAP-
-----------QHRLLLFLQQSSV--EWC-SS--LWLDVVKSIDPSLQRTMVVV-SKFDNRLKEF---TE-
RWEVDRYLSTGG-YL-GEN-----ARP-------FFVALP---KDRG-------
-------------------------TTT-NDDYR---H----QISVV--DIDIL-----
----KQ------LRE--------------------NV-------------------A----GGF---DEE------------------RFGNY-
VGF-----GKLRQFLEAELQRRYRDAAP---

PTQ33908.1   ------------LPIPEIVAVGG----Q----------------SDGKSSLLE-----A---LL-
GFRFNVRE---VEMGTRRPL---------MLQ---M---IH-DP---
--------------------GALE---PR-----CRL--------------------------------Q----------------DED---------------
--------------------------SDEYG------PAI-----VPAS---AVAEAIRSRTEL-----FLKRT-------------------GT-
-----------------------AVA--SKPIVMRAEYAF--
-----CSNLTIIDTPGFIL--KA-------------------KKGESE----------ST---PDDIV--------AMVRELAAP------
------PNRILVFLQQSSV--EWC-SS--LWLDTVRAIDPALRRTIVVV-SKFDNRLKEF---AE-
KWEVDRYLSAGG-YL-GDS-----TRP-------FFVALP---KERS--------
------------------------SVS-NEEFR---R----SIARV--DNEVV----

-----RH------LRE-------------------NV------------------S----GGF---DED-----------------QFGDR-IGF-----SNLRRFLEAELQRRYRQSAP---
KAH9322298.1 --DED---------------------------------------SDDYG------APV-----ALAS---AVADAIKSRTEE-----HLKKI------------------RA-----------------------AVS--AKPIIMRAEYAH--CPNLTIIDTPGFVL--KA------------------KKGEPD----------ST---PEDIL--------SMVKALAAP------------PNRLLLFLQQSSV--EWC-SS--LWLDTIRAVDPTLRRTIVVV-SKFDNRLKEF---GE-RWEVDRYLSASG-YL-GEN-----TRP-------FFVALP---------------------------------------KDRG-------------------------------AIT-NEEFR--T----QIAQV--DAEVN---------CY------LKE-------------------SV------------------I----GGY---DEE-----------------KFGAY-VGF-----GNLRGYLENELQRRYRDAAP---
XP_009032466.1 ------------FDVPSVVVIGA----Q---------------SSGKSALVE-----A---LM-GFQFNEVG---GGTRTRRPI---------ALQ---M---HY-NA---ACDE---PA-----CYI-------------------------------M---------------DERFS-------------GGEPVDGGAP----------------FERRA-------TL-----AEAR---RFIEEENRRLER-----DQHR--SFE--AREIVMRVEYRH---CPNLVLVDTPGLVG-GGGDVFG--------------DDFGEESHESPHARGMKRQ---AREAY--------ELALGKARA------------RNAVLLCVDDGN---DWKLGS--IARRLCADADPTLSRTVVVS-TKLDTKLVQF---GS-GRDVASFLRAKV--L-HDLHPRLLAGP-------FFTSVP-CGRVAGAISPGGDAWDPQGGAPENQPWDLDDEGFYEDDGVAFR----------------------------------G-DAEFR--A----ATARA--SRADR---------SL------VKS--------------------KV----------------GF-E------F--FDKA--------------------APQ-LGV-----GALRQFLERHVELQYRSNVA---
XP_042920073.1 ------------FDAPAILVVGH----Q---------------TDGKSALVE-----G---LM-GFQFNSVG---GGTKTRRPI---------AIN---M---KY-NG---ACST---PA-----CFL-------------------------------K---------------LE--------------------DGVS-----------------EQEMS--------L-----AELQ---AYIDADNAALER-----E--Q---RFA--AKEIVVRMEYKH---CPNLTIIDTPGLIS-PAP---------------------GKKN------CALQNC---AAQVE--------EIVRAKAQV------------PEYVILCLEDCS---DWS-NA--TTRRLVMQVDPNLVRTVLVS-TKFDTRIPQF---AR-AADCEMFLRPSA----LDSMGMLGDGP-------FFTSVP-SGRVGS----GADC--------------------------VFP---------------------------------S-HDVFR--E----RLADR--EATDV---------AE------LES-------------------KL----------------AR-K------L--SRGE-------------------RDH-IGV-----GALRRYLEQLLQKRYLDAVP---
PWZ44616.1 ------------AAAPAVVVVGH----Q---------------TDGKSALVE-----A---LM-GFQFNHVG---GGTKTRRPV---------ALH---L---RF-NP---RCDE---PQ-----CRL-------------------------------L---------------SA--------------------TGDAE----------------EHDEAGVAARPMPL-----ADIQ---AYIEAENLRLEN-----DPCQ---FS--EKEIIIKVEYKH---SPNLTIIDTPGLIL-PAP--------------------GRKN------RVLQSQ---ASAVE--------SLVRAKIQH------------KETIILCLEDCS---DWS-NA--TTRRVVMQVDPDLARTVLVS-TKLDTKIPQF---AR-PSDVEVFLHPPN-CV-LDV-SLLGDSP-------FFTSVP-SGRVGS----CHEA-----------------------

--VFR----------------------------------S-NGEFK--K----AILSR-
-ELEDI---------AS------IED-------------------KL---------------GR-S------L--TTME------------------
KDR-IGV-----GNLRLYLEELLQKRYVKSVP---
XP_002309632.3 ------------FDAPAVLVVGH----Q---------------TDGKSALVE-----G---
LM-GFQFNHVG---GGTKTRRPI---------TLH---M---KY-DP--
-----------------------ECEV---PT-----CHL-------------------------------V---------------SD-------------
-------DDPSF---------------AQEKS--------L-----HEIQ---AYIEYENMRLEK-----ESFQ-----------------
----------------------------FS--AKEIIIRVEYKH--
-------CPNLTIIDTPGLIA-PAP--------------------GRKN------QALQSQ---AHAVE--------
SLVRAKMQH------------KEFIILCLEDCS---DWS-NA--TTRRVVMQIDPELSRTIVVS-TKLDTRIPQF--
-AR-ASDVEVFLSPPA-HT-LDG-FILGDSP-------FFTSVP-SGRVGS----GHDS-----------------------
-VYS----------------------------------S-NDEFK---Q----AISLR-
-EVEDI---------AS------LEE-------------------KL----------------CR-P------L--SMQE------------------
-RNR-IGV-----SKLRSFLEELLQKRYMDSVP---
NP_001189935.1 ------------FEAPAVLVVGQ----Q----------------TDGKSALVE-----A---
LM-GFQFNHVG---GGTKTRRPI---------TLH---M---KY-DP--
-----------------------QCQF---PL-----CHL--------------------------------G---------------SD-----------
---------DDPSV----------------SLPKS--------L-----SQIQ---AYIEAENMRLEQ-----EPCS--------------
------------------------------PFS--AKEIIVKVQYKY--
-----------CPNLTIIDTPGLIA-PAP--------------------GLKN------RALQVQ---ARAVE--------
ALVRAKMQH------------KEFIILCLEDSS---DWS-IA--TTRRIVMQVDPELSRTIVVS-TKLDTKIPQF---
SC-SSDVEVFLSPPA-SA-LDS-SLLGDSP-------FFTSVP-SGRVGY----GQDS------------------------
VYK----------------------------------S-NDEFK---Q----AVSLR-
-EMEDI---------AS------LEK-------------------KL----------------GR-L------L--TKQE------------------
KSR-IGI-----SKLRLFLEELLWKRYKESVP---
EFJ19523.1 ------------FEAPAILLVGH----Q----------------TDGKSALIE-----A---LM-
GFQFNHVG---GGTKTRRPI---------TLH---M---KY-NA---
--------------------ACSE---PL-----CYL-------------------------------M---------------TE-----------------
---DGLPR----------------EEERS--------L-----DDIQ---AHIESENLRLER-----DTHQ--------------------
-------------------------FW--AKEIIIKIEYKY---
---CPNLTIIDTPGLIA-PPP--------------------QSNAT------TALQAQ---AKAVE--------ALVRSKMQH---
---------KEFIILCLEDCS---DWS-NA--TTRRVVMQVDPELSRTIVVS-TKLDTRIPQF---AR-
AADVELFLRPPP-RL-LDG-DILGGTP-------FFTSVP-SGRVGS----GRDA-------------------------
VYK----------------------------------S-NEQFR---E----ASPAR-
-ELEDV---------SS------LEE-------------------KL----------------GR-P------L--LREE-------------------
RNH-VGV-----SRLRWFLEQILQRKYMESVP---
KAI5064281.1------------FDAPAILVVGH----Q----------------TDGKSALVE-----A---LM-
GFQFNHVG---GGTKTRRPI---------TLH---M---KY-NA---
--------------------GCSE---PV-----CYL-------------------------------M---------------TD----------------
----DKPPI----------------EEERS--------L-----EELQ---AFIEAENMRLEQ-----EACQ--------------------
-------------------------FW--AKEIVVKIEYKY---
-----CPNLTIIDTPGLIA-AAP--------------------GRKN------HLLQAQ---ARAVE--------ALVRTKMQQ-
-----------KEFIILCLEDCS---DWS-NA--TTRRVVMQMDPELSRTVVVS-TKLDTKIPQF---AR-
AADVELFLRPPP-RL-LDG-NILGETP-------FFTSVP-SGRVGT----SRDS-------------------------VFR-

---------------------------------S-NEQFR---E----AVAAR--
EAQDL---------SA------LEE--------------------KM----------------DR-H------L--LPDE-------------------
-RAR-VGV-----SRLRWFLEQLLQQRYMESVP---
PTQ34556.1 ------------FDAPAILVVGH----Q----------------TDGKSALVE-----A---LM-
GFQFNHVG---GGTKTRRPI---------TLH---M---TY-NA---
-------------------ECTE---PR-----CFL--------------------------------L----------------SE-------------------
--DAPPK----------------EEEKS--------L-----DDLQ---AYIESENMRLEL-----EPSQ----------------------
------------------------FW--AKEIVVKIEYKF--
----CPNLTIIDTPGLIS-AAP--------------------GRKN------ISLQSQ---ARAVE--------ALVRSKMQQ---
---------KEFIILCLEDCS---DWT-NA--TTRRFVMQMDPELTRTVIVS-TKLDTRIPQF---AR-
PADVELFLRPPS-RL-LDG-NILGDTP-------FFTSVP-SGRVGS----GRDS-------------------------
VYR-----------------------------------T-NESFR--E----AVAMR--
EALDV---------AM------LEE--------------------KM----------------DR-P------L--LNEE-------------------
-RNR-VGI-----SRLRCFLEQLLQRRYMDSVP---
KAH9308354.1 --

---AQ---
ARAVE--------MLVRTKMQH------------KEYIILCLEDCS---DWS-NA--
TTRRVVMQVDPELSRTVMVA-TKLDTKIPQF---GR-SSDVELFLRPPT-RL-LDG-SILGETP-------
FFTSVP-SGRVGT----NRDA--------------------------VYR-----------------------------------S-NDQFK----
---E----AVASR--EAQDV---------AA------LED--------------------
KL----------------GR-S------L--LKEE--------------------RAR-VGV-----SRLGCFLEELLQRRYIDSVP---
KAG0561482.1 ------------FDAPAIVVVGH----Q----------------TDGKSALVE-----A---
LM-GFQFNHVG---GGTKTRRPI---------TLH---M---KY-NA--
-----------------------MCAE---PR-----CYL--------------------------------I----------------TE--------------
-------DRPPR----------------EEEKS--------L-----EELQ---AYIEAENLRLER-----EVCQ------------------
-----------------------------FW--EKEIILKIEYKF--
-------CPNLTIIDTPGLIS-AAP---------------------GRKN------QSLQSQ---ARSVE--------
VLVRTKMQH------------KEFIILCLEDSS---DWS-NA--TTRSIVMQIDPDLSRTVVVS-TKLDTRIPQF--
-AC-RADVELFLRPSQ-RL-LEG-NILSGSP-------FFTSVP-SGRVGV----TRDS--------------------------
VHR-----------------------------------S-NDHFR--E----AIALR-
-EAQDI---------AL------LEE--------------------KL----------------DR-Q------L--TKEE--------------------
LAH-VGV-----SRLRLFLEQLLQRQYMDSVP---
KAG0605142.1 ------------FDAPAILVVGH----Q----------------TDGKSALVE-----A---
LM-GFQFNHVG---GGTKTRRPI---------TLH---M---KY-NA--
-----------------------NCAQ---PR-----CFL--------------------------------I----------------SE--------------
-------DQPHR----------------EKEQS--------L-----EEIQ---AYIEAENKRLER-----EACQ-----------------
------------------------------FS--AKEIILKIEYKF---
-------CPNLTIIDTPGLIS-AAP---------------------GRKN------QSLQSQ---GGAVE--------
ALVQLKMQQ------------KEFIILCLEDSS---DWS-NA--TTRRVVMQADPELRRTVLVS-TKLDTRIPQF-
--AR-PDDVELFLKPPS-CL-LDG-IILGGSP-------FFTSVP-SGRVGS----SKDS-------------------------
-VFR-----------------------------------S-NTDFQ--E----

AVAAR--QIQDL---------AL------LEE-------------------KL---------------NR-P------L--TRDE----------
----------CSR-IGI-----SRLRWFLEQLLQCRYMDSVP---
XP_042916771.1 ---------------FLVAVVGE---F-----------------NSGKSSVIN-----A---LL-GRRYLAEGI--------LPT----TN---EIS--IL---
-----------------KY---S---
-----------------------------------DTAPATSNPSQV----QLVQQ---
------SDGLYVRYLPAK---L---
LQDLNIVDTPGTNV----------------------ILERQQ---------------------------RLTEEYVPR-------------
ADLVLFVMSA----DRPFSE--SEVRFLEYIRQWQKKVVFVV-NKSDILESSD----E-
VDAVKEFVAANAQRI---------------------LRLDRPSVIAV---------------------------------SSRS---------
-------------------------ALRAKLT-------------------------------ASNLP----------I----TASF---DSDLP-----
----SA-------SPTAPLSNVDPEAMEAALSNSRDW-----------------A--------V----------------------------
-SNF-----SELERNVSNFL------------
EFJ26018.1 ---------------FLLVVVGE---F-----------------NSGKSSVIN-----A---LL-GDRFLKQGV--------LPT----TN---EIT--LL---
-------------------KY---S---
-----------------------------------DESYE--ERPA--------RH---
--PDGHLMRYLSAG--L---
LKQMNLVDTPGTNV----------------------ILQRQQ---------------------------RLTEEFVPR-------------
ADLVLFVIGA----ERPLTE--SEAS---------SHTSFCI-SRFDVIRLLSGFFFT-VEEVRRFVADNVRQL----
-----------------LNIEAAMIFPI---------------------------------SARK---------------------------------
ALHAKVK--AKQL---ESKNL---------ER-------DP---------
-----------LW-----------------T--------A----------------------------SGF-----DKLEQYVLDFL------------
KAG0631008.1 ---------------FLSVVVGE---F-----------------NSGKSSIIN-----A---LL-GKRFLKEGV--------LPT----TN---EIT--LL---
-------------------RH---A---
-----------------------------------NDGGNTEEREE--------RH--
-----PDGHFLRFLPAS---L---
LKQMNLVDTPGTNV----------------------ILQRQQ---------------------------RLTEEFVPR-------------
ADLVLFVLSA----DRPLTE--SEVTFLRYIRQWGKKVVFIL-NKSDVLSTYS----E-
VEEVRNFVRDNAQRL---------------------LTVDQALVYPV---------------------------------SARQ-------
--------------------------ALQAKLS-------------------------------ASL------------E----DGTV---DTARL---
------SE-------DP-------------------LW-----------------T-------T----------------------------SGF-----
KDLEEFIFSFM------------
OAE29693.1 ---------------FLLVIVGE---F-----------------NSGKSSVIN-----A---LL-GERFLKEGV--------LPT----TN---EIT--VL---
-------------------RH---T---
-----------------------------------GEGDEGKERSE--------KH--
-----PDGHFLRYLPAE---L---
LKQMNLVDTPGTNV----------------------ILQRQQ---------------------------RLTEEFVPR-------------
ADLVLFVLSV----DRPLTE--SEVTFLRYIRQWGKKIIFIL-NKSDVLADRK----E-LEEVLKFVKENAQSL-
-------------------LSVEEASVYPV---------------------------------SARR---------------------------------
ALLAKQA-------------------------------AVN-----------E----DGVV---DRELL---------MQ-------NS----

-----------------SW------------------K--------S--------------------------------SGF-----DELEDFIFCFL--------
----
KAH9315399.1 ---------------FLLVIVGE---F-----------------NSGKSTVIN-----A---ML-
GKRYLKEGV--------VPT----TN---EIS--VL---
-------------------CY---S---
--------------------------------------GEGHDEEERSE--------RH---
-----PNGYFIQYLPAS---L---
LKQMSLVDTPGTNV-----------------------ILQRQ---------------------------RLTEEFLPR-------------
ADLVLFVISA----DRPLTE--SEVTFLRYIRQWGKKVIFIL-NKSDIFKDVK----E-
LDEAVTFVKDNAQQL---------------------LSTEQIILYPV---------------------------------SSRS----------
-----------------------ALEAKIA-------------------------------ATT-----------G----DGGV---DLEIL-------
--SK-------DP--------------------NW-----------------I--------I-----------------------------SGF-----
SALEDFIFDFL------------
KAI5059498.1---------------FLLMVVGE---F-----------------NSGKSSVIN-----A---LL-
GKRYLPEGV--------LPT----TN---EIA--LL---
-------------------KH---A---
--------------------------------------GNGFNDKERSE--------RH---
-----PDGHFMYYLPAE---L---
LKEINLVDTPGTNV-----------------------ILKRQQ---------------------------RLTEEFVPR-------------
ADLVLFVLSA----DRPFTE--SEMTFLKYIMQWDKRIVFLL-NKSDIFSDQK----E-
LEEVVKFVKDNAQQL---------------------LSVEKATIYAV---------------------------------SARK----------
-----------------------AFQAKNE-------------------------------VGV-----------D----SGNL---DVEEL----
-----LQ-------NS--------------------AW-----------------N--------V-----------------------------SGF-----
RDLEKFIADFL------------
CAD5311589.1 ---------------FLMVIVGE---F-----------------NSGKSTVIN-----A---LL-
GKRYLKEGV--------VPT----TN---EIT--FL---
-------------------CY---S---
--------------------------------------DLESEEQQRCQ--------TH--
----PDGQYVCYLPAP---I---
LKDINIVDTPGTNV-----------------------ILQRQQ---------------------------RLTEEFVPR-------------
ADLLVFVLSA----DRPLTE--SEVAFLRYTQQWKKKFVFIL-NKSDIYRDAR----E-
LEEAISFVKENTRKL---------------------LNTENVILYPV---------------------------------SARS-----------
-----------------------ALEAKLS-------------------------------TAS-----------L----VGRD---DLEV---------
--S-------DPG------------------SNW-----------------R--------V-----------------------------QSF-----
NELEKFLYSFL------------
XP_008649599.1 ---------------FLLVIVGE---F-----------------NSGKSTFIN-----A---LL-
GRQYLQEGV--------VPT----TN---EIT--LL---
--------------------SY---S---
---------------------------------------EVESENFERCE--------RH---
-----PDGQFMCYLSVP---I---
LKEMNLVDTPGTNV-----------------------ILQRQQ---------------------------RLTEEYVPR-------------
ADLVLFVLSS----DRPLTE--SEVGFLQYVQQWKKKVVFIL-NKLDLYRNSN----E-
LEEATAFVKENAMKL---------------------LTAEDVTLFPV---------------------------------SSRS----------
-----------------------ALEAKLSY-------------------------------SKN-----------S----DGKH---STEAM----

-----YN-------DP-------------------RW----------------R--------S----------------------------SKF-----
FELEDYLLSFL------------
OUM66167.1 ----------------KVLVMGE----T----------------NSGKTTFIN-----A---IV-
GGEILFEN---RFPCKAPLC---------EIK--HS---SQ-NN---
--------------------DKE---EV---H-CFI---NER-----------------
--------------------------------CDI-----KSID---EFKTLIKDIEKK---
-------------YEEKMPYDFYRIFYNDGV------------EQ--PKSLI--------------------------------------
NNN----LVNIILLDSPGLNI------------------------------------DQ---------K----KTKIVEEALED--------
-----IDAVIFVCDA----TYTIKE--IEYNYLKNNLGKSRDYVFIVVNKMDIITDDED-----KKECKDRINSK--
----
IEKVL--P-K---------------------------------------TF---EERD---LLIHY--------VSA-------RN------------
-------------------------AK-T----KEI--T----------------------QEDIQNF-----NNLKNALQTFL------------
XP_001481516.1 ----------------KVLITGD---L-----------------NAGKSTFCN-----A---LL-
RRKVLPED---QQPCTSIFC---------EVL--DA---RE-NS--
---------------------GVE---EV---HAVHK--------DK---------------------Q---------------YDR-----------
----------------------------NDEST------YDV-----YTLP---ELENIVIDNS--------------------------------------
--------------------------KYMQCKVYVK-DV------------RTID-ESLL---
NNG----VVDIALIDAPGLNS-------------------------------------DS---------------LKTTAVFARQE------
-----EIDVVVFVVSAAN--HFT---L-SAKEFILNAAHEKAYIFIVV-NGFDQIRDKQ--------RCERMILDQ-
----
IGKLS--P-R---TY---KEAA---ELVHF--------VSS-------NA----------
---------VPVAPPVQMEQSGGGSGGGS-D----PHG--DDDDDHSDNRG-KGKG------KEREKIRDF--
---ENLEGSLRRFV------------
XP_011392385.1 ----------------KVLVTGD---L-----------------NAGKSTFVN-----A---LL-
RRPLMPTD---QQPCTTVFC---------EVL--DA---SHLDS---
-----------------------SAE---EV---HMLKP--------GF---------------------K---------------YNC----------
------------------------------NDDSS------FTR-----HSLA---EIEQIVAEAEQV-----------------------------
--------------------------SP-EDAPILKCYAH-DT------------RAAQ-DSLL----------------------------------
-----KNG----IVDIALIDAPGLNR--------------------------------------DS---------------LKTTALFARQE--
---------EIDVVVFVVSAEN--HFT---L-SAKEFLWNASHDKAFVFIVV-NKFDSIKNKD--------
KCRKLVLDQ--
------------IRQLS--P-R---TY---DDAA---NLVHF--------VDS-------
QT--------------------V----------FGADVDAVV-D----SSV--
AAPDTPSEELGCKVEGAQLVQRRDSESLKAF-----ARLEAALRDFV------------
XP_006462464.1 ----------------KVLVTGD---L-----------------NAGKSTFVN-----A---LL-
RREIMPVD---QQPCTTAFC---------EVH--DA---AE-NQ--
----------------------AKE---EV---HFLKE--------GV---------------------E---------------YNI-------------
--------------------------NDEST------FTR-----GTIA---QLEEFVADNE-----------------------------------
-------------------------DTQQMIKVYLA-DT------------RAPS-ESLL--------------------------------------
NNG----VVDISLIDAPGLNR---------------------------------------DS---------------LKTTAVFARQE------
-----EIDVVVFVVSAEN--HFT---L-SAKEFLWNASNEKAYLFIVV-NKFDQIKNKE--------
KCRRLVLDQ--
------------IRQLS--P-R--TH---DDAE---DLVHF--------VDS-------

AT-------------------------------------AL-Q----PFT--ANP-------------------------SF-----
DDLESALRSFV------------
XP_006676761.1 ----------------RVLVTGD---V-----------------NAGKSTFVN-----T---LL-
RRQIVPDD---QEPCTALFV---------EVM--DP---QQ-NG--
----------------------GVE---EF---HAIKE--------NV---------------------D----------------YHP-----------
----------------------------SDRSS------FTR-----FDLH---ELRSVVEINE----------------------------------
---------------------------PEFSMIKVYCM-DN-----------RDSV-SSLL-----------------------------------
---HNG----VVDISFIDSPGLNI--------------------------------------DS--------------VKTTSLFTKQE----
-------EIDVIVFVVRAEN--HFT---Q-SSCDFLATACKEKASVFIVV-NRFDQIRRKD--------
RCRREILDQ--
-----------IQGIS--Q-H--TF---ADAA---NLVHF--------VSA-------
RM-------------------VL----------------ES-D----LKL--AE-------------------RNEEAEVSF-----
KHLEQSLRSFV------------
KXN69997.1 ----------------KVLVTGD---L-----------------NSGKSTFVN-----A---LL-
KRRVMPTD---QQPCTMLFV---------ETL--NV---KL-ND--
---------------------GVE---EA---HAIPV--------AA---------------------L----------------YDR------------
---------------------------TDPST------FVV-----IPME---DLERTVAEDY----------------------------------
-------------------------KEYELVKVYAH-DP-----------ED-N-ESML--------------------------------------
YNG----ILDIALIDSPGLNR--------------------------------------DS---------------IKTTQLFARQE-------
----EIDVVVFCVHAEN--QFT---L-SGQEFLQSAGREKAYIFIVV-NRFDTIRDKN--------RCKRQILDQ-
----
IKNLS--P-H--TY---QDAD---DLVHF--------VSA-------DH--------
-----------CF----------------PE-D----EDI--PDA------------------LPSDEIPEDF-----GRLENNLRGFI---
----
NP_009738.1 ----------------KVFITGD---V-----------------NTGKSALCN-----S---LL-
KQRLLPED---QLPCTNVFS---------EIL--EA---RE-ND---
--------------------GIE---EV---HAIPL--------NIA-------------PTLKEAIDM----------------YSI------------
---------------------------QNPKT------YEI-----HTLK---ELPDLVPQNG----------------------------------
---------------------------KYALLKIYIKDDK-----------RPAS-TSLL--------------------------------------
RNG----TVDISLIDSPGLNM--------------------------------------DS--------------LQTAEVMSRQE----
-------EIDLVIFVVNAEN--QLT---L-SAKEFISLASREKKLMFFVV-KKFDKIRDKQ--------RCKELILKQ-
----
IRDLS--P-E--TY---KRAA---DFVHF--------VSK-------NG---------
---------------------------DE-L----PHY--HNENDNEDHGDRKPDDDP---YSSSDPDPDF-----
DSLEDSLRNFV------------
KNE73082.1 ----------------RILVTGD---L-----------------NSGKSSFVN-----A---LL-
RRNVVPVD---QQPLTNVFV---------EVL--DA---RH-NTA--
--------------------RGQRE---EV---HLVRD--------GATILSSSQGGDDKSKRAVQPAAQK---------------
-RHP--KHKAA------AHVDEADVYPLD---QLAHLVADPN---------
---GYEMIKCFISEDP-----------AATSTSVLV-------------
-------------------------GNE----SVDVHMIDSPGLNR--------------------------------------DV-----------
----WQTMALFSQEK-----------EIDVIVFVVSAPD--HFT---L-SSREFLTKAGQEKAYIFVVI-
NKFDSIRDKE--------RCKRTILRQ--
---------------------------------IQQLS--P-H--TF---EYRD---

KLVHF--------VSA-------EA--------------------ML-------RDVQAAQ---R-A----GAV--AVSE-------------
-------TPRIAEF-----LHVERALKEFT------------
KNE65701.1 ----------------RVLVGGD---L-----------------NAGKSTFVN-----A---LL-
RRDVVPVD---QQPLTNVFV---------EVL--DA---RH-NQL--
---------------------HPGVE---EV---H--
-------------------------AHKEV------GSV-------------IAHSSRRPD--
---------------------DCELVKVYCNEEQ------------AERS-SVLV--
GNE----HVDVRVIDSPGLNR--DM---------------WQTMALFAQQK-
----------EIDVIVFL------------------------AGQEKAYIFIVV-NKFDAIKDKE--------RCKKIILQQ-----------
--IEELS--P-H-
--TY---RYKE---KLVHF--------VSA-------ER--------------------MM--
-----RDVAAIRTLAE-Q----GQL--ADDEE----------------ETMTTAMAEF-----VRVEEALKEFV------------
XP_001745740.1 --------RSVTRESMKVVFVGR----T----------------SNGKSTTIN-----A--
-ML-HTRVLPAG---PGHTTNCFV---------TLQ--G--
----------------------SDQS---K------AYM---------------------------------Q-----------------------------------
-----LPGDP-------------------TP------RDL-----KDVQ---SLTDALQQEHV----------L----------------------
------------------------------PPGQSVEIHWPRD-------------Q---CHL--------------------------------------
------L---RDDVVILDSPGL--DY---DSDFD--------AWIDETTRD---
---------ADVFVLVVNAVST---LS-GA--ESGFFHSVCKTVAKPNVFVIFNQWDNLDED--------
EADVTGVRAQH--M---------------------------------S----------------------------------KARD----------------
----------------LL--VRDLGIC--S---E----AELS---SRVFF--------
VSS-------KE--------------------VL----------------KS-R------A---G-----------------------SDS-RTT---
SYTDPSSAVVPGLNTH---------
NP_495161.1 --------DTFQRDNMKVVFFGR----T----------------SNGKSTTIN-----A---ML-
HEKVLPQG---MGHTTCCFL---------QVE--G--
------------------SEGE---V------GHL---------------------------------Q--
LDDNP-------------------QK------IDM-----KMLG---KIGHALSDENSD-LPA-----M----------------------
-----------------------------GQDSLLKVFHPKKSE---------SGE----CRL-------------------------------------
-------L---QNDVVILDSPGV--DL---SPEFD--------SWIDKHCLD-
-----------ADVFVLVSNAEST---LT-QA--EKNFFLRVAKKLSKPNVFILNNRWDASAAE--------
TENIEDVKKQH--L---------------------------------T----------------------------------RFRQ-------------------
-------------FL--VDELEVC--S---E----REVN---DRIFF--------VSS--
-----RE--------------------VL----------------ES-R------L---K----------------------A---RGLV------
QKAYQAEGH------------
PAA75551.1 --------EVISRNQMKCAFFGR----T----------------SNGKSTVIN-----A---ML-
GRKVLPSG---IGHTTNCFL---------QVE--G---
-----------------TSKQ---S------AYL---------------------------------Q--
TPNSS-------------------EE------QPI-----ESVS---QLGSALSNEK-----------M----------------------------
-----------------------DCESLVRVFWPKQ-------------L----CSL--
L---REDVVLLDSPGV--DV---SPDLD--------TWIDQFCMD--------
----ADVFILVCNSEST---LM-NT--EKKFFHKVGSKLSKPNVFVLNNRWDCSDGE--------
LDSAELVRKQH--M---------------------------------D----------------------------------KSVS-----------------
---------------FL--ADELKSC--T---R----SEAE---SRVYF--------

VSA-------KE--------------------AL----------------VN-R------L---K---------------------ETN-QGLE---
SPSPAGSLADGW------------
PAA75258.1 --------EVISRNQMKCAFFGR----T---------------SNGKSTVIN-----A---ML-
GRKVLPSG---IGHTTNCFL---------QVE--G--
-----------------TSKE---S------AYL---------------------------------Q--
TPNSS-------------------EE------RPI-----ESVS---QLGSALSNEK-----------L------------------------------
---------------------DCESLVRVFWPKQ-----------L----CSL--L--
-REDVVLLDSPGV---DV---SPDLD--------TWIDQFCMD-----------
-ADVFILVCNSEST---LM-NT--EKKFFHKVGSKLSKPNVFVLNNRWDCSDGE--------
LDSAELVRKQH--M--------------------------------D---------------------------------KSVS-----------------
---------------FL--ADELKSC--T---R----SEAE---SRVYF--------
VSA-------KE--------------------AL----------------VN-R------L---K---------------------ETN-QGLE---
SPSPAGSLADGW------------
XP_002126852.1 --------DMLTRNHMKVVFFGR----T---------------SNGKSSVVN-----
A---ML-WDRILPTG---IGHTTNCFL---------SVA--GC-S-DEGST--
------------------------------STDSD---EG-----AYL---------------------------------L---------------------------
------------CNGSE-------------------EK------RSI-----KSVT---QLSHALSEES-----------M---------------
------------------------------------SPDSLIQVFWPKS-------------K----CAL----------------------------------
----------L---KDDVVLVDSPGI--DV---SHDLD--------
QWIDKYCLD------------ADVFILVANAEST---LM-QA--EKKFFHRVNEKLSKPNIFILNNRWDASASE-
-------PELMEQVRQQH--L----------------------------------E----------------------------------RGIS----------
----------------------FL--ADELKVI--S---K----SQAK---DRVFF------
--VSA-------KE--------------------TL----------------QS-R------M---------------------------PKV-PGK-----
ADSPVYMADGH------------
XP_006819998.1 --------EVLARDHMKVAFFGR----T---------------SSGKSTVIN-----A--
-ML-KDKVLPTG---IGHTTDCFL---------SIE--G--
--------------------SDTS---E------AYL---------------------------------I--
IPQSN-------------------ER------RNV-----RSVS---QLAHALSNEKL----------A---------------------------
------------------------DQSSLIHVFWPSS-------------R----CAL--
L---KDDLVLVDSPGV--DV---TADLD--------SWIDDHCLD--------
----ADVFVLVANAEST---LM-RT--EKSFFHKVAEKLSKPNIFILNNRWDASASE--------
PDSMEDVKKQH--L---------------------------------E----------------------------------RSIG------------------
---------------FL--VEELKVI--T---K----QQAE---DRVFF--------VSA-
------KE--------------------AL----------------CC-R------I---------------------------QKV-QGM-----PEAG-
----
XP_002591612.1 --------QVISRDKMKVAFFGR----T---------------SNGKSTVVN-----A-
--ML-RDKILPSG---IGHTTNCFI---------NVE--G---
----------------------SDGY---E------AYL---------------------------------L-------------------------------------
---TPDSD-------------------DR------KTV-----QSVG---QLAHALCGERL-----------E-----------------------
-----------------------------DSSILVKVFWPKG-------------R----CAL---
---L---RDDVVLLDSPGI--DV---TPDLD--------SWIDEHCLD------
------ADVFVLVANSEST---LM-RTAREKNFFHTVSERLSKPNIFILNNRWDASASE--------
PEFMEAVKKQH--L----------------------------------E----------------------------------RCVS------------------
---------------FL--VEELGVV--D---R----LQAE---DRVFF--------

VSA-------KE--------------------AL----------------QS-R------L-------------------------QKQ-QGM-----
PEEGGALAEGF------------
XP_019628129.1 --------QVISRDKMKVAFFGR----T---------------SNGKSTVVN-----A-
--ML-RDKILPSG---IGHTTNCFI---------NVE--G--
----------------------SDGF---E------AYL---------------------------------L------------------------------------
---TPDSD-------------------DR------KTV-----QSVG---QLAHALCSERL----------E-----------------------
---------------------------DSSVLVKVFWPKG-------------R----CAL--
---L---RDDVVLLDSPGI---------------------------------------DV---TPDLD--------SWIDEHCLD------
------ADVFVLVANSEST---LM-RT--EKNFFHTVSERLSKPNIFILNNRWDASASE--------
PEFMEAVKKQH--L--------------------------------E---------------------------------RCVS-----------------
---------------FL--VEELGVV--D--R----LQAE---DRVFF--------
VSA-------KE--------------------AL----------------QS-R------L-------------------------QKQ-QGM-----
PEEGGALAEGF------------
NP_001121726.1 --------EVLSRRHMKVVFFGR----T---------------SNGKSSVIN-----A-
--ML-WDKVLPSG---IGHTTNCFL---------RVE--G--
------------------------TDGN---E------SFL--------------------------------L----------------------------------
-----TEGSD-------------------ER------KSV-----KTVN---QLAHALHQDED----------L--------------------
------------------------------DAGSLVCVMWPKA-------------K----CAL------------------------------------
-------L---RDDLVLVDSPGI---DV---TTELD--------SWIDKFCLD---
---------ADVFVLVANSEST---LM-QT--EKSFFHKVNERLSSPNIFILNNRWDASANE--------
PEYMEEVRRQH--M--------------------------------D---------------------------------RCTS----------------
----------------FL--VDELRVV--D--R----SHAG---DRIFF--------
VSA-------KE--------------------VL----------------QA-R------V-------------------------QKA-QGM-----
PEAGGALAEGF------------
XP_025929938.1 --------EVLARRHMKVAFFGR----T---------------SNGKSTVIN-----A--
-ML-WDKVLPSG---IGHTTNCFL---------RVE--G---
-----------------------TDGH---E------AFL--------------------------------L-----------------------------------
----TEGSE-------------------EK------KSV-----KTVN---QLAHALHQDEL----------L----------------------
----------------------------DAGSLVSVMWPNS-------------K----CPL--------------------------------------
-----L---KDDLVLMDSPGI---DV---TTELD--------SWIDKFCLD-----
-------ADVFVLVANSEST---LM-QT--EKQFFHKVNERLSRPNIFILNNRWDASASE--------
PEYMEEVRRQH--M--------------------------------E---------------------------------RCTS----------------
----------------FL--VDELGVV--D--R----AQAG---DRIFF--------
VSA-------KE--------------------VL----------------NA-R------I-------------------------QRA-QGM-----
PEGGGALADGF------------
XP_004482574.1 --------EVLARRHMKVAFFGR----T---------------SNGKSTVIN-----A--
-ML-WDKVLPSG---IGHTTNCFL---------RVG--G---
-----------------------TDGH---E------AFL--------------------------------L-----------------------------------
----TEGSE-------------------EK------RSV-----KTVN---QLAHALHQDEQ----------L----------------------
----------------------------HAGSLVSVMWPNS-------------K----CSL--------------------------------------
-----L---KDDLVLMDSPGI---DV---TTELD--------SWIDKFCLD-----
-------ADVFVLVANSEST---LM-QT--EKQFFHKVSERLSRPNIFILNNRWDASASE--------
PEYMEEVRRQH--M--------------------------------E---------------------------------RCTS----------------
----------------FL--VDELGVV--D--R----AQAG---DRIFF--------

VSA-------KE------------------VL---------------NA-R------I------------------------QKA-QGM-----
PEGGGALAEGF------------
XP_006145367.1 --------EVLARRHMKVAFFGR----T---------------SNGKSTVIN-----A--
-ML-WDKVLPSG---IGHTTNCFL---------RVE--G--
----------------------TDGH---E------AFL------------------------------L---------------------------------
----TEGSE------------------EK------RSV-----KTVN---QLAHALHQDEQ---------L--------------------
---------------------------HAGSLVSVMWPNS------------K----CPL-------------------------------------
-----L---KDDLVLMDSPGI---------------------------------------DV---TTELD--------SWIDKFCLD-----
-------ADVFVLVANSEST---LM-QT--EKQFFHKVSERLSRPNIFILNNRWDASASE--------
PEYMEEVRRQH--M------------------------------E-------------------------------RCTS---------------
---------------FL--VDELGVV--D--R----AQAG---DRIFF--------
VSA-------KE------------------VL---------------NA-R------I------------------------QKA-QGM-----
PEGGGALAEGF------------
XP_014400986.1 --------EVLARRHMKVAFFGR----T---------------SNGKSTVIN-----A--
-ML-WDKVLPSG---IGHTTNCFL---------RVE--G--
----------------------TDGH---E------AFL------------------------------L---------------------------------
----TEGSE------------------EK------RSI-----KTVN---QLAHALHQDEQ---------L--------------------
---------------------------HAGSLVSVMWPNS------------K----CPL-------------------------------------
-----L---KDDLVLMDSPGI---------------------------------------DV---TTELD--------SWIDKFCLD-----
-------ADVFVLVANSEST---LM-QT--EKQFFHKVSERLSRPNIFILNNRWDASASE--------
PEYMEEVRRQH--M------------------------------E-------------------------------RCTS---------------
---------------FL--VDELGVV--D--R----GQAG---DRIFF--------
VSA-------KE------------------AL---------------NA-R------I------------------------QKA-QGM-----
PEGGGALAEGF------------
NP_001177198.1 --------EVLARRHMKVAFFGR----T---------------SNGKSTVIN-----A--
-ML-WDKVLLSG---IGHTTNCFL---------RVE--G--
----------------------TDGH---E------AFL------------------------------L---------------------------------
----TEGSE------------------EK------RSV-----KTVN---QLAHALHQDEQ---------L--------------------
---------------------------HAGSLVSVMWPNS------------K----CPL-------------------------------------
-----L---KDDLVLMDSPGI---------------------------------------DV---TTELD--------SWIDKFCLD-----
-------ADVFVLVANSEST---LM-QT--EKQFFHKVSERLSRPNIFILNNRWDASASE--------
PEYMEEVRRQH--M------------------------------E-------------------------------RCTS---------------
---------------FL--VDELGVV--D--R----GQAG---DRIFF--------
VSA-------KE------------------VL---------------NA-R------I------------------------QKA-QGM-----
PEGGGALAEGF------------
NP_001272849.1 --------EVLARRHMKVAFFGR----T---------------SNGKSTVIN-----A--
-ML-WDKVLPSG---IGHTTNCFL---------RVG--G--
----------------------TDGH---E------AFL------------------------------L---------------------------------
----TEGSE------------------EK------KSV-----KTVN---QLAHALHQDEQ---------L--------------------
---------------------------HAGSMVSVMWPNS------------K----CPL-------------------------------------
------L---KDDLVLMDSPGI---------------------------------------DV---TTELD--------SWIDKFCLD----
--------ADVFVLVANSEST---LM-QT--EKQFFHKVSERLSRPNIFILNNRWDASASE--------
PEYMEEVRRQH--M------------------------------E-------------------------------RCTS---------------
---------------FL--VDELGVV--D--R----AQAG---DRIFF--------

VSA-------KE--------------------VL----------------SA-R------V--------------------------QKA-QGM-----
PEGGGALAEGF------------
NP_001121132.1 --------EVLARRHMKVAFFGR----T----------------SNGKSTVIN-----A--
-ML-WDKVLPSG---IGHTTNCFL---------RVE--G---
-----------------------TDGH---E------AFL-------------------------------L-----------------------------------
----TEGSE-------------------EK------RSA-----KTVN---QLAHALHQDKQ----------L---------------------
---------------------------HAGSLVSVMWPNS-------------K----CPL--------------------------------------
-----L---KDDLVLMDSPGI---DV---TTELD--------SWIDKFCLD-----
-------ADVFVLVANSEST---LM-QT--EKHFFHKVSERLSRPNIFILNNRWDASASE--------
PEYMEEVRRQH--M--------------------------------E---------------------------------RCTS----------------
----------------FL--VDELGVV--D---R----SQAG---DRIFF--------
VSA-------KE--------------------VL----------------NA-R------I--------------------------QKA-QGM-----
PEGGGALAEGF------------
XP_015268039.1 --------EVLARRHMKVAFFGR----T----------------SNGKSTVIN-----A--
-ML-WDKVLPSG---IGHTTNCFL---------RVE--G---
-----------------------TDGH---D------AFL-------------------------------L----------------------------------
-----TEGSE-------------------XX------XXX-----XTVN---QLAHALHQDEL----------L---------------------
---------------------------TAGSLVSVMWPNS-------------K----CPL--------------------------------------
----L---KDDLVLMDSPGI---DV---TTELD--------SWIDKFCLD------
------ADVFVLVANSEST---LM-QT--VRRQH--M----------------
----------------E---------------------------------RCTS---------------------------------FL--VDELGVV--D----
---------------------------------------R----AQAG---DRIFF--------VSA-------KE--------------------VL------
----------SA-R------I--------------------------QKA-QGM-----PEGGGALAEGF------------
XP_028597443.1 --------QVLARRHMKVAFFGR----T----------------SNGKSTVIN-----A-
--ML-WDKVLPSG---IGHTTNCFL---------RVE--G--
------------------------TEGQ---D------AFL-------------------------------L-----------------------------------
-----TEGSE-------------------EK------KSV-----KTVN---QLAHALHQDEL----------L--------------------
----------------------------TAGGMVSVMWPNS-------------K----CPL-------------------------------------
------L---KDDLVLMDSPGI---DV---TTELD--------SWIDKFCLD----
--------ADVFVLVANSEST---LM-QT--EKQFFHKVNTRLSRPNIFILNNRWDASASE--------
PEYMEEVRRQH--M--------------------------------E---------------------------------RCTS----------------
----------------FL--VDELGVV--D---R----AQAG---DRIFF--------
VSA-------KE--------------------VL----------------SA-R------I--------------------------QKA-QGM-----
PEGGGALAEGF------------
XP_017213868.2.2 --------EVLARRHMKVAFFGR----T----------------SNGKSTVIN-----A--
-ML-RDRVLPSG---IGHTTNCFL---------SVE--G---
----------------------TDED---K------AFL-------------------------------K-----------------------------------
---TEGSE-------------------EE------KSI-----KTVN---QLAHALHMDES----------L-----------------------
-------------------------DAGCLVKVFWPKT-------------K----CAL---------------------------------------
--L---RDDLVLVDSPGT---DV---TTELD--------SWIDKFCLD--------
----ADVFVLVANSEST---LM-NT--EKHFFHKVNEKLSKPNIFILNNRWDASAAE--------
PEYMEDVRKQH--T--------------------------------D---------------------------------RCVN----------------
----------------FL--VEELKVV--D---R----AQAP---NRIFF--------

VSA-------KE-------------------VL---------------NS-R------M------------------------QRA-QGM-----
PETGGALAEGF------------
NP_001016189.1 --------EVLARRNMKVAFFGR----T---------------SSGKSTVIN-----S--
-ML-WDKVLPSG---IGHTTNCFL---------SVE--G---
---------------------TEGD---K------AYL------------------------------M---------------------------------
----TEGSE-------------------EK------KSV-----KTVN---QLAHALHMDKD---------L---------------------
---------------------------GAGCLVHVFWPKA------------K----CAL-----------------------------------
-----L---RDDLVLVDSPGT-------------------------------------DV---TTELD--------SWIDKFCLD-----
-------ADVFVLVANSEST---LM-NT--EKHFFYKVNEKLSKPNIFILNNRWDASASE--------
PEYMEDVRKQH--M------------------------------E------------------------------RCQS---------------
-----------------FL--VDELKVV--D---S----LEAQ---KRIFF--------
VSA-------KE-------------------VL---------------NA-R------M------------------------HKA-QGM-----
PEAGAALAEGF------------
XP_028587453.1 --------GVLARRHMKVAFFGR----T---------------SSGKSSVIN-----A-
--ML-WDRVLPSG---IGHTTNCFL---------SVE--G---
----------------------TDGD---K------AYL------------------------------M-------------------------------
------TEGSD-------------------EK------KSV-----KTVN---QLAHALHMDKD---------L-------------------
------------------------------EAGCLVHVFWPKA------------K----CAL-----------------------------------
-------L---RDDLVLVDSPGT-------------------------------------DV---TTELD--------TWIDKFCLD---
---------ADVFVLVANSEST---LM-NT--EKHFFHKVNEKLSKPNIFILNNRWDASASE--------
PEYMEHVRKQH--M------------------------------E------------------------------RCLT-----------------
---------------FL--VDELKVV--D---P----SEAQ---NRIFF--------
VSA-------KE-------------------VL---------------SA-R------K------------------------QRA-QGM-----
PEGGGALADGF------------
XP_025917892.1 --------EVLSRRHMKVAFFGR----T---------------SSGKSSVIN-----A--
-ML-WDKVLPSG---IGHTTNCFL---------SVE--G---
---------------------TDGD---K------AYL------------------------------M---------------------------------
-----TEGSD-------------------EK------KSV-----KTVN---QLAHALHMDKD---------L-------------------
------------------------------KAGCLVHVFWPKS------------K----CAL-----------------------------------
------L---RDDLVLVDSPGT-------------------------------------DV---TTELD--------SWIDKFCLD----
--------ADVFVLVANSEST---LM-NT--EKHFFHKVNERLSKPNIFILNNRWDASASE--------
PEYMEDVRRQH--M------------------------------E------------------------------RCLT-----------------
---------------FL--VDELKVI--D---P----IEAR---NRIFF--------VSA--
-----KE-------------------VL---------------SA-R------R------------------------QKA-QGM-----
PAGGEALAEGF------------
XP_004479029.1 --------EVLSRRHMKVAFFGR----T---------------SSGKSSVIN-----A--
-ML-WDKVLPSG---IGHTTNCFL---------SVE--G---
---------------------TDGD---K------AYL------------------------------M---------------------------------
-----TEGSD-------------------EK------KSV-----KTVN---QLAHALHMDND---------L-------------------
------------------------------KAGCLVHVFWPKA------------K----CAL-----------------------------------
------L---RDDLVLVDSPGT-------------------------------------DV---TTELD--------SWIDKFCLD----
--------ADVFVLVANSEST---LM-NT--EKQFFHKVNERLSKPNIFILNNRWDASASE--------
PEYMEDVRRQH--M------------------------------E------------------------------RCLN---------------
-----------------FL--VEELKVV--N---P----LEAQ---NRIFF--------

VSA-------KE--------------------VL----------------SA-R------K--------------------------HKA-QGM-----
PEGGGALADGF------------
NP_001193437.1 --------EVLSRRHMKVAFFGR----T----------------SSGKSSVIN-----A--
-ML-WDKVLPSG---IGHTTNCFL---------SVE--G---
-----------------------TDGD---K------AYL--------------------------------M----------------------------------
-----TEGSD-------------------EK------RSV-----KTVN---QLAHALHMDKD----------L--------------------
------------------------------KAGCLVHVFWPKA-------------K----CAL-------------------------------------
------L---RDDLVLVDSPGT---DV---TTELD--------SWIDKFCLD----
--------ADVFVLVANSEST---LM-NT--EKQFFHKVNERLSKPNIFILNNRWDASASE--------
PEYMEDVRRQH--M--------------------------------E---------------------------------RCLH---------------
-----------------FL--VEELRVV--D--P----LEAR---NRIFF--------
VSA-------KE--------------------VL----------------SA-R------K--------------------------HKA-QGM-----
PEGGGALAEGF------------
NP_077162.2.2 --------EVLSRRHMKVAFFGR----T----------------SSGKSSVIN-----A--
-ML-WDKVLPSG---IGHTTNCFL---------SVE--G---
-----------------------TDGD---K------AYL--------------------------------M----------------------------------
-----TEGSD-------------------EK------KSV-----KTVN---QLAHALHMDKD----------L--------------------
------------------------------KAGCLVHVFWPKA-------------K----CAL-------------------------------------
------L---RDDLVLVDSPGT---DV---TTELD--------IWIDKFCLD-----
-------ADVFVLVANSEST---LM-NT--EKHFFHKVNERLSKPNIFILNNRWDASASE--------
PEYMEDVRRQH--M--------------------------------E---------------------------------RCLH---------------
-----------------FL--VEELKVV--S--P----SEAR---NRIFF--------
VSA-------KE--------------------VL----------------NS-R------K--------------------------HKA-QGM-----
PEGGGALAEGF------------
XP_006162789.1 --------EVLSRRHMKVAFFGR----T----------------SSGKSSVIN-----A--
-ML-WDKVLPSG---IGHTTNCFL---------SVE--G---
-----------------------TDGD---K------AYL--------------------------------M----------------------------------
-----TEGSD-------------------EK------KSV-----KTVN---QLAHALHMDKD----------L--------------------
------------------------------KAGCLVHVFWPKA-------------K----CAL-------------------------------------
------L---RDDLVLVDSPGT---DV---TTELD--------SWIDKFCLD----
--------ADVFVLVANSEST---LM-NT--EKHFFHKVNERLSKPNIFILNNRWDASASE--------
PEYMEDVRRQH--M--------------------------------E---------------------------------RCLH---------------
-----------------FL--VEELKVV--G--P----SEAR---NRIFF--------
VSA-------KE--------------------VL----------------SA-R------K--------------------------HRA-QGM-----
PEGGGALAEGF------------
XP_005883071.1 --------EVLSRRHMKVAFFGR----T----------------SSGKSSVIN-----A--
-ML-WDKVLPSG---IGHTTNCLL---------SVE--G---
-----------------------TDGD---R------AYL--------------------------------M----------------------------------
-----TEGSD-------------------EK------KSV-----KTVN---QLAHALHMDKD----------L--------------------
------------------------------KAGSLVHVFWPKA-------------K----CAL-------------------------------------
------L---RDDLVLVDSPGT---DV---TTELD--------SWIDKFCLD----
--------ADVFVLVANSEST---LM-NT--EKQFFHKVNERLSKPNIFILNNRWDASASE--------
PEYMEDVRRQH--T--------------------------------E---------------------------------RCLH-----------------
---------------FL--VEELKVV--D--P----SEAR---NRIFF--------VSA-

------KE-------------------VL----------------SA-R------M-------------------------HKA-QGM-----
PEGGGALAEGF------------
NP_284941.2.2 --------EVLSRRHMKVAFFGR----T----------------SSGKSSVIN-----A--
-ML-WDKVLPSG---IGHITNCFL---------SVE--G--
----------------------TDGD---K------AYL-------------------------------M-----------------------------------
----TEGSD-------------------EK------KSV-----KTVN---QLAHALHMDKD----------L--------------------
----------------------------KAGCLVRVFWPKA-------------K----CAL-------------------------------------
-----L---RDDLVLVDSPGT---------------------------------------DV---TTELD--------SWIDKFCLD-----
-------ADVFVLVANSEST---LM-NT--EKHFFHKVNERLSKPNIFILNNRWDASASE--------
PEYMEDVRRQH--M-------------------------------E--------------------------------RCLH---------------
-----------------FL--VEELKVV--N---A----LEAQ---NRIFF--------
VSA-------KE-------------------VL----------------SA-R------K-------------------------QKA-QGM-----
PESGVALAEGF------------
XP_030846906.1 --------DVLERDHMKVAFFGR----T----------------SNGKSTVIN-----A-
--ML-RDKVLPSG---IGHTTDCFL---------CVE--G---
-----------------------CEGQ---E------GYM-------------------------------S-----------------------------------
------RQNSS-------------------EK------IST-----TSVS---QLANALAGERDH-------EDF-------------------
------------------------------QQRSILHIFWPKT-------------Q----CHL-------------------------------------
------L---KNDVVLLDSPGI---DV---EHDMD--------EWIDDHYMD-
-----------ADVFVLVSNAEST---LT-RT--ETSFFLKVSAKLSKPNIFILNNRWDASANE--------
PENMEVVKRQH--L-------------------------------E--------------------------------REIK------------------
--------------FL--VEELKVM--T---E----AQAK---DRIFF--------VSA--
-----KE--------------------AL----------------NS-R------I-------------------------LQT-LST-----PNAN-
PIVEGY------------
XP_030847518.1 --------DVLERDHMKVAFFGR----T----------------SNGKSTVIN-----A-
--ML-RDKVLPSG---IGHTTDCFL---------CVE--G---
-----------------------CEGQ---E------GYM-------------------------------S-----------------------------------
------RQNSS-------------------EK------MSI-----TSVS---QLAHALAGERDH-------EEC-----------------
---------------------------------QQSSILHIFWPKT-------------Q----CHL----------------------------------
--------L---MNDVVLLDSPGI---DV---EQDLD--------
EWINTHCVD------------ADVFVLVLNAEST---LM-RT--EKSFFHKVSEKLSKPNIFILNNRWDASANE-
-------PEFMEAVKRQH--L-------------------------------E--------------------------------RDVK----------
----------------------FL--VEELKVM--T---E----AQAK---DRVFF------
--VSA-------KE-------------------AL----------------NS-R------I-------------------------PKT-LST-----
PDAN-PVVEGY------------
NP_996357.1 --------EVLQRDHMKVAFFGR----T----------------SNGKSSVIN-----A---ML-
REKILPSG---IGHTTNCFC---------QVE--G--
----------------SNGG---E------AYL-------------------------------M--
TEGSE-------------------EK------LNV-----VNIK---QLANALCQEK-----------L---------------------------
----------------------CESSLVRIFWPRE-------------R----CSL--L--
-RDDVVFVDSPGV---DV---SANLD--------DWIDNHCLN----------
--ADVFVLVLNAEST---MT-RA--EKQFFHTVSQKLSKPNIFILNNRWDASANE--------
PECQESVKSQH--T-------------------------------E--------------------------------RCID------------------
--------------FL--TKELKVS--N---E----KEAA---ERVFF--------VSA-

------RE--------------------TL----------------QA-R------I--------------------------EEA-KGN-----
PPHMGAIAEGF------------
XP_004365821.1 --------KLFERDHMKVVFVGQ----T----------------SNGKSTVVN-----
A---ML-YNRILPSG---IGHTTNCFV---------SVS--G---
-------------------------SDAN---T------PYI-------------------------------I----------------------------------
----DSLSS-------------------EQ------QPI-----SNVL---QLANALHPEGS----------L---------------------
-----------------------------NQSGLIRVFWPTT-------------K----CRL---
---L---GDDVDLIDSPGL--DL---SNDIN--------QWIDDYCMD----
--------ADVFVLVANAEAT---LK-VA--ERAFFFKVNEKLSKPNVFILNNRWDASDNEI-------
DDSPERVREQH--L--------------------------------E---------------------------------YASK------------------
--------------FL--ADELKVV--S---R----SKIL---DRVFF--------VSA--
-----RE--------------------TL----------------LY-R------T---T---------------------ENW-TRF-----KESQAV-
----
XP_014153836.1 ---------MLRRDSMKVVFVGH----T----------------SNGKSTVIN-----S-
--ML-GQKVLPMG---IGHTTSCFC---------SVT--G---
------------------------TDEE----------PYI--------------------------------ILG-------------
HEPKAKNSNSIRLNRVQRSDSPPSPPPE-------------------KR------MAI-----DNVK---
TVANALCPESD----------H---DAYQFVRVFWDKR---------
----K----CNL--L---GDGVLFVDTPGL------------------------------
-----------DI---DENYD--------NWIDKFCMD------------ADVFVLVANGEST---IK-HT--
EMNFFTKVAEKLSRPNVFILFNRWDGSDME---------DDVTPVQEQH--K------------------------------
--D---------------------------------RVRS----------------------------------FF--KKELQQ---D-----------------
-------------------------A----NIID---KRVFF--------VSG-------KE--------------------VL----------------
TH-R------T---K---------------------PD--KSVVKESNPSPMG------------------

;
end;
begin trees;
tree tree_1 = [&U] [&branchAttributeNames={"FastTree support value"}](XP_026693152.1[&Organism="Ciona intestinalis","Genetic Code"="Standard",Taxonomy="Eukaryota; Metazoa; Chordata; Tunicata; Ascidiacea; Enterogona; Phlebobranchia; Cionidae; Ciona","Common Name"="vase tunicate"]:0.09567000000000014,XP_004347890.1[&Organism="Capsaspora owczarzaki ATCC 30864","Genetic Code"="Standard",Taxonomy="Eukaryota; Filasterea; Capsaspora"]:0.1577700000000002,(((XP_005165639.1[&Organism="Danio rerio","Genetic Code"="Standard",Taxonomy="Eukaryota; Metazoa; Chordata; Craniata; Vertebrata; Euteleostomi; Actinopterygii; Neopterygii; Teleostei; Ostariophysi; Cypriniformes; Cyprinidae; Danio","Common Name"="zebrafish"]:0.05093999999999976,((KAE8583055.1[&Organism="Xenopus tropicalis","Genetic Code"="Standard",Taxonomy="Eukaryota; Metazoa; Chordata; Craniata; Vertebrata; Euteleostomi; Amphibia; Batrachia; Anura; Pipoidea; Pipidae; Xenopodinae; Xenopus; Silurana","Common Name"="tropical clawed frog"]:0.02737999999999996,((((XP_012378586.1[&Organism="Dasypus novemcinctus","Genetic Code"="Standard",Taxonomy="Eukaryota; Metazoa; Chordata;

Craniata; Vertebrata; Euteleostomi; Mammalia; Eutheria; Xenarthra; Cingulata;
Dasypodidae; Dasypus","Common Name"="nine-banded
armadillo"]:0.02828999999999926,EAW87759.1[&Organism="Homo sapiens","Genetic
Code"="Standard",Taxonomy="Eukaryota; Metazoa; Chordata; Craniata; Vertebrata;
Euteleostomi; Mammalia; Eutheria; Euarchontoglires; Primates; Haplorrhini; Catarrhini;
Hominidae; Homo","Common Name"="human"]:0.000540)[&"FastTree support
value"=0.823]:0.003440000000003317,ELW62001.1[&Organism="Tupaia
chinensis","Genetic Code"="Standard",Taxonomy="Eukaryota; Metazoa; Chordata;
Craniata; Vertebrata; Euteleostomi; Mammalia; Eutheria; Euarchontoglires; Scandentia;
Tupaiidae; Tupaia","Common Name"="Chinese tree shrew"]:0.000550)[&"FastTree support
value"=0.345]:0.000530,BAB27759.1[&Organism="Mus musculus","Genetic
Code"="Standard",Taxonomy="Eukaryota; Metazoa; Chordata; Craniata; Vertebrata;
Euteleostomi; Mammalia; Eutheria; Euarchontoglires; Glires; Rodentia; Myomorpha;
Muroidea; Muridae; Murinae; Mus; Mus","Common Name"="house
mouse"]:0.003439999999994435)[&"FastTree support
value"=0.439]:0.006910000000000416,EPQ17174.1[&Organism="Myotis brandtii","Genetic
Code"="Standard",Taxonomy="Eukaryota; Metazoa; Chordata; Craniata; Vertebrata;
Euteleostomi; Mammalia; Eutheria; Laurasiatheria; Chiroptera; Microchiroptera;
Vespertilionidae; Myotis","Common Name"="Brandt's bat"]:0.000550)[&"FastTree support
value"=0.948]:0.01919000000000004)[&"FastTree support
value"=0.73]:0.01684000000000188,(XP_025915522.1[&Organism="Apteryx
rowi","Genetic Code"="Standard",Taxonomy="Eukaryota; Metazoa; Chordata; Craniata;
Vertebrata; Euteleostomi; Archelosauria; Archosauria; Dinosauria; Saurischia; Theropoda;
Coelurosauria; Aves; Palaeognathae; Apterygiformes; Apterygidae; Apteryx","Common
Name"="Okarito brown
kiwi"]:0.005709999999999966,XP_028570166.1[&Organism="Podarcis muralis","Genetic
Code"="Standard",Taxonomy="Eukaryota; Metazoa; Chordata; Craniata; Vertebrata;
Euteleostomi; Lepidosauria; Squamata; Bifurcata; Unidentata; Episquamata; Laterata;
Lacertibaenia; Lacertidae; Podarcis","Common Name"="Common wall
lizard"]:0.01010000000000442)[&"FastTree support
value"=0.757]:0.005060000000002865)[&"FastTree support
value"=0.884]:0.01895999999999866)[&"FastTree support
value"=0.793]:0.01313999999999993,(XP_006812840.1[&Organism="Saccoglossus
kowalevskii","Genetic Code"="Standard",Taxonomy="Eukaryota; Metazoa; Hemichordata;
Enteropneusta; Harrimaniidae;
Saccoglossus"]:0.2605700000000004,(XP_035683496.1[&Organism="Branchiostoma
floridae","Genetic Code"="Standard",Taxonomy="Eukaryota; Metazoa; Chordata;
Cephalochordata; Branchiostomidae; Branchiostoma","Common Name"="Florida
lancelet"]:0.07038000000000011,(XP_032814666.1[&Organism="Petromyzon
marinus","Genetic Code"="Standard",Taxonomy="Eukaryota; Metazoa; Chordata; Craniata;
Vertebrata; Cyclostomata; Hyperoartia; Petromyzontiformes; Petromyzontidae;
Petromyzon","Common Name"="sea
lamprey"]:0.0482800000000001,((XP_021326548.1[&Organism="Danio rerio","Genetic
Code"="Standard",Taxonomy="Eukaryota; Metazoa; Chordata; Craniata; Vertebrata;

Euteleostomi; Actinopterygii; Neopterygii; Teleostei; Ostariophysi; Cypriniformes; Cyprinidae; Danio","Common Name"="zebrafish"]:0.0,NP_001025299.1[&Organism="Danio rerio","Genetic Code"="Standard",Taxonomy="Eukaryota; Metazoa; Chordata; Craniata; Vertebrata; Euteleostomi; Actinopterygii; Neopterygii; Teleostei; Ostariophysi; Cypriniformes; Cyprinidae; Danio","Common Name"="zebrafish"]:0.0):0.04132000000000069,(((EPQ08653.1[&Organism="Myotis brandtii","Genetic Code"="Standard",Taxonomy="Eukaryota; Metazoa; Chordata; Craniata; Vertebrata; Euteleostomi; Mammalia; Eutheria; Laurasiatheria; Chiroptera; Microchiroptera; Vespertilionidae; Myotis","Common Name"="Brandt's bat"]:0.007520000000000415,(XP_006496668.1[&Organism="Mus musculus","Genetic Code"="Standard",Taxonomy="Eukaryota; Metazoa; Chordata; Craniata; Vertebrata; Euteleostomi; Mammalia; Eutheria; Euarchontoglires; Glires; Rodentia; Myomorpha; Muroidea; Muridae; Murinae; Mus; Mus","Common Name"="house mouse"]:0.0032399999999999096,(XP_027623811.1[&Organism="Tupaia chinensis","Genetic Code"="Standard",Taxonomy="Eukaryota; Metazoa; Chordata; Craniata; Vertebrata; Euteleostomi; Mammalia; Eutheria; Euarchontoglires; Scandentia; Tupaiidae; Tupaia","Common Name"="Chinese tree shrew"]:0.010480000000000267,(XP_016856477.1[&Organism="Homo sapiens","Genetic Code"="Standard",Taxonomy="Eukaryota; Metazoa; Chordata; Craniata; Vertebrata; Euteleostomi; Mammalia; Eutheria; Euarchontoglires; Primates; Haplorrhini; Catarrhini; Hominidae; Homo","Common Name"="human"]:0.003490000000000215,XP_012379251.1[&Organism="Dasypus novemcinctus","Genetic Code"="Standard",Taxonomy="Eukaryota; Metazoa; Chordata; Craniata; Vertebrata; Euteleostomi; Mammalia; Eutheria; Xenarthra; Cingulata; Dasypodidae; Dasypus","Common Name"="nine-banded armadillo"]:0.000550)[&"FastTree support value"=0.0]:0.000540)[&"FastTree support value"=1.0]:0.000550)[&"FastTree support value"=0.473]:0.010120000000000573)[&"FastTree support value"=0.954]:0.023109999999999076,(XP_025944940.1[&Organism="Apteryx rowi","Genetic Code"="Standard",Taxonomy="Eukaryota; Metazoa; Chordata; Craniata; Vertebrata; Euteleostomi; Archelosauria; Archosauria; Dinosauria; Saurischia; Theropoda; Coelurosauria; Aves; Palaeognathae; Apterygiformes; Apterygidae; Apteryx","Common Name"="Okarito brown kiwi"]:0.03845000000000095,XP_031757197.1[&Organism="Xenopus tropicalis","Genetic Code"="Standard",Taxonomy="Eukaryota; Metazoa; Chordata; Craniata; Vertebrata; Euteleostomi; Amphibia; Batrachia; Anura; Pipoidea; Pipidae; Xenopodinae; Xenopus; Silurana","Common Name"="tropical clawed frog"]:0.13576999999999995)[&"FastTree support value"=0.785]:0.012659999999999945)[&"FastTree support value"=0.94]:0.026060000000000194,(XP_031753735.1[&Organism="Xenopus tropicalis","Genetic Code"="Standard",Taxonomy="Eukaryota; Metazoa; Chordata; Craniata; Vertebrata; Euteleostomi; Amphibia; Batrachia; Anura; Pipoidea; Pipidae; Xenopodinae; Xenopus; Silurana","Common Name"="tropical clawed frog"]:0.03249999999999975,(((((XP_012381548.1[&Organism="Dasypus novemcinctus","Genetic Code"="Standard",Taxonomy="Eukaryota; Metazoa; Chordata;

Craniata; Vertebrata; Euteleostomi; Mammalia; Eutheria; Xenarthra; Cingulata; Dasypodidae; Dasypus","Common Name"="nine-banded armadillo"]:0.03878999999999966,XP_006161648.2.2[&Organism="Tupaia chinensis","Genetic Code"="Standard",Taxonomy="Eukaryota; Metazoa; Chordata; Craniata; Vertebrata; Euteleostomi; Mammalia; Eutheria; Euarchontoglires; Scandentia; Tupaiidae; Tupaia","Common Name"="Chinese tree shrew"]:0.03232999999999997)[&"FastTree support value"=1.0]:0.030070000000000263,NP_001005360.1[&Organism="Homo sapiens","Genetic Code"="Standard",Taxonomy="Eukaryota; Metazoa; Chordata; Craniata; Vertebrata; Euteleostomi; Mammalia; Eutheria; Euarchontoglires; Primates; Haplorrhini; Catarrhini; Hominidae; Homo","Common Name"="human"]:0.003429999999999822)[&"FastTree support value"=0.0]:0.000550,XP_006510037.1[&Organism="Mus musculus","Genetic Code"="Standard",Taxonomy="Eukaryota; Metazoa; Chordata; Craniata; Vertebrata; Euteleostomi; Mammalia; Eutheria; Euarchontoglires; Glires; Rodentia; Myomorpha; Muroidea; Muridae; Murinae; Mus; Mus","Common Name"="house mouse"]:0.000550)[&"FastTree support value"=0.788]:0.0035199999999999676,XP_014389433.1[&Organism="Myotis brandtii","Genetic Code"="Standard",Taxonomy="Eukaryota; Metazoa; Chordata; Craniata; Vertebrata; Euteleostomi; Mammalia; Eutheria; Laurasiatheria; Chiroptera; Microchiroptera; Vespertilionidae; Myotis","Common Name"="Brandt's bat"]:0.021420000000000883)[&"FastTree support value"=0.884]:0.007279999999999731,(XP_025920181.1[&Organism="Apteryx rowi","Genetic Code"="Standard",Taxonomy="Eukaryota; Metazoa; Chordata; Craniata; Vertebrata; Euteleostomi; Archelosauria; Archosauria; Dinosauria; Saurischia; Theropoda; Coelurosauria; Aves; Palaeognathae; Apterygiformes; Apterygidae; Apteryx","Common Name"="Okarito brown kiwi"]:0.004520000000003016,XP_028568434.1[&Organism="Podarcis muralis","Genetic Code"="Standard",Taxonomy="Eukaryota; Metazoa; Chordata; Craniata; Vertebrata; Euteleostomi; Lepidosauria; Squamata; Bifurcata; Unidentata; Episquamata; Laterata; Lacertibaenia; Lacertidae; Podarcis","Common Name"="Common wall lizard"]:0.002580000000000267)[&"FastTree support value"=0.818]:0.006470000000000198)[&"FastTree support value"=0.91]:0.01391999999999971)[&"FastTree support value"=0.971]:0.03678999999999988)[&"FastTree support value"=0.828]:0.02370000000000072)[&"FastTree support value"=0.929]:0.019989999999999952)[&"FastTree support value"=0.881]:0.02100999999999953)[&"FastTree support value"=0.158]:0.011630000000000251)[&"FastTree support value"=0.802]:0.011079999999999757)[&"FastTree support value"=0.667]:0.014249999999999652,(((KMZ10000.1[&Organism="Drosophila melanogaster","Genetic Code"="Standard",Taxonomy="Eukaryota; Metazoa; Ecdysozoa; Arthropoda; Hexapoda; Insecta; Pterygota; Neoptera; Holometabola; Diptera; Brachycera; Muscomorpha; Ephydroidea; Drosophilidae; Drosophila; Sophophora","Common

Name"="fruit fly"]:0.04943000000000097,NP_001024332.1[&Organism="Caenorhabditis elegans","Genetic Code"="Standard",Taxonomy="Eukaryota; Metazoa; Ecdysozoa; Nematoda; Chromadorea; Rhabditida; Rhabditina; Rhabditomorpha; Rhabditoidea; Rhabditidae; Peloderinae; Caenorhabditis"]:0.1514300000000004)[&"FastTree support value"=0.919]:0.03333999999999904,(((PAA78248.1[&Organism="Macrostomum lignano","Genetic Code"="Standard",Taxonomy="Eukaryota; Metazoa; Platyhelminthes; Rhabditophora; Macrostomorpha; Macrostomida; Macrostomidae; Macrostomum"]:0.018240000000000478,PAA65118.1[&Organism="Macrostomum lignano","Genetic Code"="Standard",Taxonomy="Eukaryota; Metazoa; Platyhelminthes; Rhabditophora; Macrostomorpha; Macrostomida; Macrostomidae; Macrostomum"]:0.05787000000000031)[&"FastTree support value"=0.869]:0.027060000000000528,(PAA59145.1[&Organism="Macrostomum lignano","Genetic Code"="Standard",Taxonomy="Eukaryota; Metazoa; Platyhelminthes; Rhabditophora; Macrostomorpha; Macrostomida; Macrostomidae; Macrostomum"]:0.044929999999999914,PAA64382.1[&Organism="Macrostomum lignano","Genetic Code"="Standard",Taxonomy="Eukaryota; Metazoa; Platyhelminthes; Rhabditophora; Macrostomorpha; Macrostomida; Macrostomidae; Macrostomum"]:0.10461000000000009)[&"FastTree support value"=0.621]:0.020800000000000374)[&"FastTree support value"=0.99]:0.07676999999999978,((OAJ44422.1[&Organism="Batrachochytrium dendrobatidis JEL423","Genetic Code"="Standard",Taxonomy="Eukaryota; Fungi; Fungi incertae sedis; Chytridiomycota; Chytridiomycota incertae sedis; Chytridiomycetes; Rhizophydiales; Rhizophydiales incertae sedis; Batrachochytrium"]:0.14408999999999939,((((XP_011389257.1[&Organism="Ustilago maydis 521","Genetic Code"="Standard",Taxonomy="Eukaryota; Fungi; Dikarya; Basidiomycota; Ustilaginomycotina; Ustilaginomycetes; Ustilaginales; Ustilaginaceae; Ustilago"]:0.08176999999999968,XP_006458578.1[&Organism="Agaricus bisporus var. bisporus H97","Genetic Code"="Standard",Taxonomy="Eukaryota; Fungi; Dikarya; Basidiomycota; Agaricomycotina; Agaricomycetes; Agaricomycetidae; Agaricales; Agaricaceae; Agaricus"]:0.15010999999999974)[&"FastTree support value"=0.834]:0.027140000000000164,(KXN66323.1[&Organism="Conidiobolus coronatus NRRL 28638","Genetic Code"="Standard",Taxonomy="Eukaryota; Fungi; Fungi incertae sedis; Zoopagomycota; Entomophthoromycotina; Entomophthoromycetes; Entomophthorales; Ancylistaceae; Conidiobolus"]:0.10740000000000016,OUM62108.1[&Organism="Piromyces sp. E2","Genetic Code"="Standard",Taxonomy="Eukaryota; Fungi; Fungi incertae sedis; Chytridiomycota; Chytridiomycota incertae sedis; Neocallimastigomycetes; Neocallimastigales; Neocallimastigaceae; Piromyces; unclassified Piromyces"]:0.10905999999999949)[&"FastTree support value"=0.666]:0.019610000000000127)[&"FastTree support value"=0.389]:0.030730000000000146,(KNE68830.1[&Organism="Allomyces macrogynus ATCC 38327","Genetic Code"="Standard",Taxonomy="Eukaryota; Fungi; Fungi incertae sedis; Blastocladiomycota; Blastocladiomycota incertae sedis; Blastocladiomycetes; Blastocladiales; Blastocladiaceae;

Allomyces"]:0.11237999999999992,NP_012926.1[&Organism="Saccharomyces cerevisiae S288C","Genetic Code"="Standard",Taxonomy="Eukaryota; Fungi; Dikarya; Ascomycota; Saccharomycotina; Saccharomycetes; Saccharomycetales; Saccharomycetaceae; Saccharomyces"]:0.17493999999999943)[&"FastTree support value"=0.662]:0.04081000000000046)[&"FastTree support value"=0.498]:0.02202999999999994,XP_748106.1[&Organism="Aspergillus fumigatus Af293","Genetic Code"="Standard",Taxonomy="Eukaryota; Fungi; Dikarya; Ascomycota; Pezizomycotina; Eurotiomycetes; Eurotiomycetidae; Eurotiales; Aspergillaceae; Aspergillus; Aspergillus subgen. Fumigati"]:0.13966000000000012)[&"FastTree support value"=0.859]:0.04193999999999942)[&"FastTree support value"=0.999]:0.15477000000000096,((((NP_013100.1[&Organism="Saccharomyces cerevisiae S288C","Genetic Code"="Standard",Taxonomy="Eukaryota; Fungi; Dikarya; Ascomycota; Saccharomycotina; Saccharomycetes; Saccharomycetales; Saccharomycetaceae; Saccharomyces"]:0.2662199999999997,(XP_746923.1[&Organism="Aspergillus fumigatus Af293","Genetic Code"="Standard",Taxonomy="Eukaryota; Fungi; Dikarya; Ascomycota; Pezizomycotina; Eurotiomycetes; Eurotiomycetidae; Eurotiales; Aspergillaceae; Aspergillus; Aspergillus subgen. Fumigati"]:0.1616200000000001,KXN67416.1[&Organism="Conidiobolus coronatus NRRL 28638","Genetic Code"="Standard",Taxonomy="Eukaryota; Fungi; Fungi incertae sedis; Zoopagomycota; Entomophthoromycotina; Entomophthoromycetes; Entomophthorales; Ancylistaceae; Conidiobolus"]:0.22487999999999975)[&"FastTree support value"=0.578]:0.0366200000000001)[&"FastTree support value"=0.931]:0.05796000000000456,((XP_011392073.1[&Organism="Ustilago maydis 521","Genetic Code"="Standard",Taxonomy="Eukaryota; Fungi; Dikarya; Basidiomycota; Ustilaginomycotina; Ustilaginomycetes; Ustilaginales; Ustilaginaceae; Ustilago"]:0.12134999999999962,XP_006461708.1[&Organism="Agaricus bisporus var. bisporus H97","Genetic Code"="Standard",Taxonomy="Eukaryota; Fungi; Dikarya; Basidiomycota; Agaricomycotina; Agaricomycetes; Agaricomycetidae; Agaricales; Agaricaceae; Agaricus"]:0.1093699999999993)[&"FastTree support value"=0.952]:0.08072000000000035,(KNE67543.1[&Organism="Allomyces macrogynus ATCC 38327","Genetic Code"="Standard",Taxonomy="Eukaryota; Fungi; Fungi incertae sedis; Blastocladiomycota; Blastocladiomycota incertae sedis; Blastocladiomycetes; Blastocladiales; Blastocladiaceae; Allomyces"]:0.13511999999999968,KNE61418.1[&Organism="Allomyces macrogynus ATCC 38327","Genetic Code"="Standard",Taxonomy="Eukaryota; Fungi; Fungi incertae sedis; Blastocladiomycota; Blastocladiomycota incertae sedis; Blastocladiomycetes; Blastocladiales; Blastocladiaceae; Allomyces"]:0.14499999999999957)[&"FastTree support value"=0.999]:0.2384000000000004)[&"FastTree support value"=0.472]:0.03465000000000007)[&"FastTree support value"=0.987]:0.0906899999999995,(XP_004348308.1[&Organism="Capsaspora owczarzaki ATCC 30864","Genetic Code"="Standard",Taxonomy="Eukaryota; Filasterea; Capsaspora"]:0.18308999999999997,((XP_001750431.1[&Organism="Monosiga brevicollis MX1","Genetic Code"="Standard",Taxonomy="Eukaryota; Choanoflagellata; Craspedida;

Salpingoecidae; Monosiga"]:0.37582000000000093,(XP_002129967.2[&Organism="Ciona intestinalis","Genetic Code"="Standard",Taxonomy="Eukaryota; Metazoa; Chordata; Tunicata; Ascidiacea; Phlebobranchia; Cionidae; Ciona","Common Name"="vase tunicate"]:0.24733999999999945,(((PAA85687.1[&Organism="Macrostomum lignano","Genetic Code"="Standard",Taxonomy="Eukaryota; Metazoa; Spiralia; Lophotrochozoa; Platyhelminthes; Rhabditophora; Macrostomorpha; Macrostomida; Macrostomidae; Macrostomum"]:0.18630999999999975,(XP_035676386.1[&Organism="Branchiostoma floridae","Genetic Code"="Standard",Taxonomy="Eukaryota; Metazoa; Chordata; Cephalochordata; Leptocardii; Amphioxiformes; Branchiostomatidae; Branchiostoma","Common Name"="Florida lancelet"]:0.06264000000000003,(XP_006821224.1[&Organism="Saccoglossus kowalevskii","Genetic Code"="Standard",Taxonomy="Eukaryota; Metazoa; Hemichordata; Enteropneusta; Harrimaniidae; Saccoglossus"]:0.08640000000000025,XP_030827871.1[&Organism="Strongylocentrotus purpuratus","Genetic Code"="Standard",Taxonomy="Eukaryota; Metazoa; Echinodermata; Eleutherozoa; Echinozoa; Echinoidea; Euechinoidea; Echinacea; Camarodonta; Echinidea; Strongylocentrotidae; Strongylocentrotus","Common Name"="purple sea urchin"]:0.08265000000000011)[&"FastTree support value"=0.859]:0.04560999999999993)[&"FastTree support value"=0.568]:0.037039999999999296)[&"FastTree support value"=0.807]:0.04875000000000007,(XP_032819300.1[&Organism="Petromyzon marinus","Genetic Code"="Standard",Taxonomy="Eukaryota; Metazoa; Chordata; Craniata; Vertebrata; Cyclostomata; Hyperoartia; Petromyzontiformes; Petromyzontidae; Petromyzon","Common Name"="sea lamprey"]:0.09692999999999952,(((((XP_006168142.1[&Organism="Tupaia chinensis","Genetic Code"="Standard",Taxonomy="Eukaryota; Metazoa; Chordata; Craniata; Vertebrata; Euteleostomi; Mammalia; Eutheria; Euarchontoglires; Scandentia; Tupaiidae; Tupaia","Common Name"="Chinese tree shrew"]:0.000550,(NP_001392186.1[&Organism="Mus musculus","Genetic Code"="Standard",Taxonomy="Eukaryota; Metazoa; Chordata; Craniata; Vertebrata; Euteleostomi; Mammalia; Eutheria; Euarchontoglires; Glires; Rodentia; Myomorpha; Muroidea; Muridae; Murinae; Mus; Mus","Common Name"="house mouse"]:0.00849000000000109,NP_001317309.1[&Organism="Homo sapiens","Genetic Code"="Standard",Taxonomy="Eukaryota; Metazoa; Chordata; Craniata; Vertebrata; Euteleostomi; Mammalia; Eutheria; Euarchontoglires; Primates; Haplorrhini; Catarrhini; Hominidae; Homo","Common Name"="human"]:0.0027100000000004343)[&"FastTree support value"=0.825]:0.000550)[&"FastTree support value"=0.954]:0.000530,(XP_014394711.1[&Organism="Myotis brandtii","Genetic Code"="Standard",Taxonomy="Eukaryota; Metazoa; Chordata; Craniata; Vertebrata; Euteleostomi; Mammalia; Eutheria; Laurasiatheria; Chiroptera; Microchiroptera; Vespertilionidae; Myotis","Common Name"="Brandt's bat"]:0.02563000000000486,(XP_012382650.2[&Organism="Dasypus novemcinctus","Genetic Code"="Standard",Taxonomy="Eukaryota; Metazoa; Chordata;

Craniata; Vertebrata; Euteleostomi; Mammalia; Eutheria; Xenarthra; Cingulata; Dasypodidae; Dasypus","Common Name"="nine-banded armadillo"]:0.02286000000000547,XP_031753959.1[&Organism="Xenopus tropicalis","Genetic Code"="Standard",Taxonomy="Eukaryota; Metazoa; Chordata; Craniata; Vertebrata; Euteleostomi; Amphibia; Batrachia; Anura; Pipoidea; Pipidae; Xenopodinae; Xenopus; Silurana","Common Name"="tropical clawed frog"]:0.14993000000000034)[&"FastTree support value"=0.97]:0.04391999999999996)[&"FastTree support value"=0.81]:0.008659999999999989)[&"FastTree support value"=0.988]:0.02367000000000008,XP_028602039.1[&Organism="Podarcis muralis","Genetic Code"="Standard",Taxonomy="Eukaryota; Metazoa; Chordata; Craniata; Vertebrata; Euteleostomi; Lepidosauria; Squamata; Bifurcata; Unidentata; Episquamata; Laterata; Lacertibaenia; Lacertidae; Podarcis","Common Name"="Common wall lizard"]:0.027619999999999756)[&"FastTree support value"=0.229]:0.003369999999999429,XP_025940269.1[&Organism="Apteryx rowi","Genetic Code"="Standard",Taxonomy="Eukaryota; Metazoa; Chordata; Craniata; Vertebrata; Euteleostomi; Archelosauria; Archosauria; Dinosauria; Saurischia; Theropoda; Coelurosauria; Aves; Palaeognathae; Apterygiformes; Apterygidae; Apteryx","Common Name"="Okarito brown kiwi"]:0.023439999999999017)[&"FastTree support value"=0.954]:0.030440000000000467,NP_957216.1[&Organism="Danio rerio","Genetic Code"="Standard",Taxonomy="Eukaryota; Metazoa; Chordata; Craniata; Vertebrata; Euteleostomi; Actinopterygii; Neopterygii; Teleostei; Ostariophysi; Cypriniformes; Danionidae; Danioninae; Danio","Common Name"="zebrafish"]:0.023229999999999862)[&"FastTree support value"=0.953]:0.05426999999999982)[&"FastTree support value"=0.863]:0.04103999999999974)[&"FastTree support value"=0.909]:0.04446000000000083,(NP_001259946.1[&Organism="Drosophila melanogaster","Genetic Code"="Standard",Taxonomy="Eukaryota; Metazoa; Ecdysozoa; Arthropoda; Hexapoda; Insecta; Pterygota; Neoptera; Endopterygota; Diptera; Brachycera; Muscomorpha; Ephydroidea; Drosophilidae; Drosophila; Sophophora","Common Name"="fruit fly"]:0.14022999999999985,NP_741403.2[&Organism="Caenorhabditis elegans","Genetic Code"="Standard",Taxonomy="Eukaryota; Metazoa; Ecdysozoa; Nematoda; Chromadorea; Rhabditida; Rhabditina; Rhabditomorpha; Rhabditoidea; Rhabditidae; Peloderinae; Caenorhabditis"]:0.25253999999999976)[&"FastTree support value"=0.184]:0.03633000000000042)[&"FastTree support value"=0.829]:0.04223999999999961)[&"FastTree support value"=0.929]:0.08811000000000035)[&"FastTree support value"=0.926]:0.05433999999999983,XP_014148015.1[&Organism="Sphaeroforma arctica JP610","Genetic Code"="Standard",Taxonomy="Eukaryota; Ichthyosporea; Ichthyophonida; Sphaeroforma"]:0.3359300000000003)[&"FastTree support value"=0.096]:0.013009999999999522)[&"FastTree support value"=0.968]:0.07150999999999996)[&"FastTree support value"=0.872]:0.03753999999999991,((((XP_042924642.1[&Organism="Chlamydomonas reinhardtii","Genetic Code"="Standard",Taxonomy="Eukaryota; Viridiplantae; Chlorophyta;

core chlorophytes; Chlorophyceae; CS clade; Chlamydomonadales; Chlamydomonadaceae; Chlamydomonas"]:0.33921000000000046,(NP_001190448.1[&Organism="Arabidopsis thaliana","Genetic Code"="Standard",Taxonomy="Eukaryota; Viridiplantae; Streptophyta; Embryophyta; Tracheophyta; Spermatophyta; Magnoliopsida; eudicotyledons; Gunneridae; Pentapetalae; rosids; malvids; Brassicales; Brassicaceae; Camelineae; Arabidopsis","Common Name"="thale cress"]:0.05184000000000033,(((XP_002302631.1[&Organism="Populus trichocarpa","Genetic Code"="Standard",Taxonomy="Eukaryota; Viridiplantae; Streptophyta; Embryophyta; Tracheophyta; Spermatophyta; Magnoliopsida; eudicotyledons; Gunneridae; Pentapetalae; rosids; fabids; Malpighiales; Salicaceae; Saliceae; Populus","Common Name"="Populus balsamifera subsp. trichocarpa"]:0.03230000000000022,AQK88296.1:0.0906400000000005)[&"FastTree support value"=0.044]:0.015569999999999418,(((KAG0556007.1[&Organism="Ceratodon purpureus","Genetic Code"="Standard",Taxonomy="Eukaryota; Viridiplantae; Streptophyta; Embryophyta; Bryophyta; Bryophytina; Bryopsida; Dicranidae; Pseudoditrichales; Ditrichaceae; Ceratodon"]:0.09192,(((((KAI5602084.1[&Organism="Populus trichocarpa","Genetic Code"="Standard",Taxonomy="Eukaryota; Viridiplantae; Streptophyta; Embryophyta; Tracheophyta; Spermatophyta; Magnoliopsida; eudicotyledons; Gunneridae; Pentapetalae; rosids; fabids; Malpighiales; Salicaceae; Saliceae; Populus","Common Name"="Populus balsamifera subsp. trichocarpa"]:0.06795999999999935,(((AAF79238.1[&Organism="Arabidopsis thaliana","Genetic Code"="Standard",Taxonomy="Eukaryota; Viridiplantae; Streptophyta; Embryophyta; Tracheophyta; Spermatophyta; Magnoliopsida; eudicotyledons; Gunneridae; Pentapetalae; rosids; malvids; Brassicales; Brassicaceae; Camelineae; Arabidopsis","Common Name"="thale cress"]:0.06214999999999993,(XP_002315854.1[&Organism="Populus trichocarpa","Genetic Code"="Standard",Taxonomy="Eukaryota; Viridiplantae; Streptophyta; Embryophyta; Tracheophyta; Spermatophyta; Magnoliopsida; eudicotyledons; Gunneridae; Pentapetalae; rosids; fabids; Malpighiales; Salicaceae; Saliceae; Populus","Common Name"="Populus balsamifera subsp. trichocarpa"]:0.0371100000000002,NP_001147100.1[&Organism="Zea mays","Genetic Code"="Standard",Taxonomy="Eukaryota; Viridiplantae; Streptophyta; Embryophyta; Tracheophyta; Spermatophyta; Magnoliopsida; Liliopsida; Poales; Poaceae; PACMAD clade; Panicoideae; Andropogonodae; Andropogoneae; Tripsacinae; Zea"]:0.06409999999999982)[&"FastTree support value"=0.308]:0.017059999999999853)[&"FastTree support value"=0.931]:0.03016000000000041,PWZ36850.1[&Organism="Zea mays","Genetic Code"="Standard",Taxonomy="Eukaryota; Viridiplantae; Streptophyta; Embryophyta; Tracheophyta; Spermatophyta; Magnoliopsida; Liliopsida; Poales; Poaceae; PACMAD clade; Panicoideae; Andropogonodae; Andropogoneae; Tripsacinae; Zea"]:0.10221000000000036)[&"FastTree support value"=0.297]:0.007389999999991195,(XP_006375094.1[&Organism="Populus trichocarpa","Genetic Code"="Standard",Taxonomy="Eukaryota; Viridiplantae;

Streptophyta; Embryophyta; Tracheophyta; Spermatophyta; Magnoliopsida; eudicotyledons; Gunneridae; Pentapetalae; rosids; fabids; Malpighiales; Salicaceae; Saliceae; Populus","Common Name"="Populus balsamifera subsp. trichocarpa"]:0.03990999999999989,(AAF22292.1[&Organism="Arabidopsis thaliana","Genetic Code"="Standard",Taxonomy="Eukaryota; Viridiplantae; Streptophyta; Embryophyta; Tracheophyta; Spermatophyta; Magnoliopsida; eudicotyledons; Gunneridae; Pentapetalae; rosids; malvids; Brassicales; Brassicaceae; Camelineae; Arabidopsis","Common Name"="thale cress"]:0.06524999999999981,NP_850420.1[&Organism="Arabidopsis thaliana","Genetic Code"="Standard",Taxonomy="Eukaryota; Viridiplantae; Streptophyta; Embryophyta; Tracheophyta; Spermatophyta; Magnoliopsida; eudicotyledons; Gunneridae; Pentapetalae; rosids; malvids; Brassicales; Brassicaceae; Camelineae; Arabidopsis","Common Name"="thale cress"]:0.08664000000000005)[&"FastTree support value"=0.873]:0.02472999999999992)[&"FastTree support value"=0.644]:0.008359999999999701)[&"FastTree support value"=0.877]:0.0164200000000001)[&"FastTree support value"=0.895]:0.020820000000000505,KAH9306600.1[&Organism="Taxus chinensis","Genetic Code"="Standard",Taxonomy="Eukaryota; Viridiplantae; Streptophyta; Embryophyta; Tracheophyta; Spermatophyta; Pinopsida; Pinidae; Conifers II; Cupressales; Taxaceae; Taxus"]:0.05635999999999744)[&"FastTree support value"=0.764]:0.014039999999999608,(EFJ23099.1[&Organism="Selaginella moellendorffii","Genetic Code"="Standard",Taxonomy="Eukaryota; Viridiplantae; Streptophyta; Embryophyta; Tracheophyta; Lycopodiopsida; Selaginellales; Selaginellaceae; Selaginella"]:0.1290899999999997,(XP_002987566.1[&Organism="Selaginella moellendorffii","Genetic Code"="Standard",Taxonomy="Eukaryota; Viridiplantae; Streptophyta; Embryophyta; Tracheophyta; Lycopodiopsida; Selaginellales; Selaginellaceae; Selaginella"]:0.05194999999999972,EFJ15761.1[&Organism="Selaginella moellendorffii","Genetic Code"="Standard",Taxonomy="Eukaryota; Viridiplantae; Streptophyta; Embryophyta; Tracheophyta; Lycopodiopsida; Selaginellales; Selaginellaceae; Selaginella"]:0.08901999999999965)[&"FastTree support value"=0.751]:0.0044100000000000025)[&"FastTree support value"=0.948]:0.022819999999999396)[&"FastTree support value"=0.874]:0.016810000000000436,KAI5058380.1[&Organism="Adiantum capillus-veneris","Genetic Code"="Standard",Taxonomy="Eukaryota; Viridiplantae; Streptophyta; Embryophyta; Tracheophyta; Polypodiopsida; Polypodiidae; Polypodiales; Pteridineae; Pteridaceae; Vittarioideae; Adiantum"]:0.0730499999999994)[&"FastTree support value"=0.391]:0.010969999999999948,KAI5072318.1[&Organism="Adiantum capillus-veneris","Genetic Code"="Standard",Taxonomy="Eukaryota; Viridiplantae; Streptophyta; Embryophyta; Tracheophyta; Polypodiopsida; Polypodiidae; Polypodiales; Pteridineae; Pteridaceae; Vittarioideae; Adiantum"]:0.04598999999999975)[&"FastTree support value"=0.759]:0.009669999999999845)[&"FastTree support value"=0.178]:0.015210000000000612,PTQ45603.1[&Organism="Marchantia polymorpha","Genetic Code"="Standard",Taxonomy="Eukaryota; Viridiplantae;

Streptophyta; Embryophyta; Marchantiophyta; Marchantiopsida; Marchantiidae; Marchantiales; Marchantiaceae; Marchantia","Common Name"="liverwort"]:0.05877999999999961)[&"FastTree support value"=0.44]:0.02381999999999973,PTQ29980.1[&Organism="Marchantia polymorpha","Genetic Code"="Standard",Taxonomy="Eukaryota; Viridiplantae; Streptophyta; Embryophyta; Marchantiophyta; Marchantiopsida; Marchantiidae; Marchantiales; Marchantiaceae; Marchantia","Common Name"="liverwort"]:0.10687999999999942)[&"FastTree support value"=0.999]:0.08049000000000017)[&"FastTree support value"=0.945]:0.0248999999999997,XP_002299468.1[&Organism="Populus trichocarpa","Genetic Code"="Standard",Taxonomy="Eukaryota; Viridiplantae; Streptophyta; Embryophyta; Tracheophyta; Spermatophyta; Magnoliopsida; eudicotyledons; Gunneridae; Pentapetalae; rosids; fabids; Malpighiales; Salicaceae; Saliceae; Populus","Common Name"="Populus balsamifera subsp. trichocarpa"]:0.03997999999999905)[&"FastTree support value"=0.713]:0.01294000000000396)[&"FastTree support value"=1.0]:0.26898)[&"FastTree support value"=0.932]:0.11783000000000055,((((((OAE31801.1[&Organism="Marchantia polymorpha subsp. ruderalis","Genetic Code"="Standard",Taxonomy="Eukaryota; Viridiplantae; Streptophyta; Embryophyta; Marchantiophyta; Marchantiopsida; Marchantiidae; Marchantiales; Marchantiaceae; Marchantia"]:0.22481000000000018,(KAI5073815.1[&Organism="Adiantum capillus-veneris","Genetic Code"="Standard",Taxonomy="Eukaryota; Viridiplantae; Streptophyta; Embryophyta; Tracheophyta; Polypodiopsida; Polypodiidae; Polypodiales; Pteridineae; Pteridaceae; Vittarioideae; Adiantum"]:0.16583000000000014,((KAH9330549.1[&Organism="Taxus chinensis","Genetic Code"="Standard",Taxonomy="Eukaryota; Viridiplantae; Streptophyta; Embryophyta; Tracheophyta; Spermatophyta; Pinopsida; Pinidae; Conifers II; Cupressales; Taxaceae; Taxus"]:0.11452000000000018,(XP_006385192.1[&Organism="Populus trichocarpa","Genetic Code"="Standard",Taxonomy="Eukaryota; Viridiplantae; Streptophyta; Embryophyta; Tracheophyta; Spermatophyta; Magnoliopsida; eudicotyledons; Gunneridae; Pentapetalae; rosids; fabids; Malpighiales; Salicaceae; Saliceae; Populus","Common Name"="Populus balsamifera subsp. trichocarpa"]:0.07947000000000015,('KAG7649995.1'[&Organism="Arabidopsis thaliana","Genetic Code"="Standard",Taxonomy="Eukaryota; Viridiplantae; Streptophyta; Embryophyta; Tracheophyta; Spermatophyta; Magnoliopsida; eudicotyledons; Gunneridae; Pentapetalae; rosids; malvids; Brassicales; Brassicaceae; Camelineae; Arabidopsis","Common Name"="thale cress"]:0.01541000000000146,'NP_172500.1'[&Organism="Arabidopsis thaliana","Genetic Code"="Standard",Taxonomy="Eukaryota; Viridiplantae; Streptophyta; Embryophyta; Tracheophyta; Spermatophyta; Magnoliopsida; eudicotyledons; Gunneridae; Pentapetalae; rosids; malvids; Brassicales; Brassicaceae; Camelineae; Arabidopsis","Common Name"="thale cress"]:0.04222999999999999)[&"FastTree support value"=0.355]:0.02140999999999993)[&"FastTree support

value"=0.888]:0.04172000000000002)[&"FastTree support value"=0.843]:0.028939999999999966,((XP_008646219.1[&Organism="Zea mays","Genetic Code"="Standard",Taxonomy="Eukaryota; Viridiplantae; Streptophyta; Embryophyta; Tracheophyta; Spermatophyta; Magnoliopsida; Liliopsida; Poales; Poaceae; PACMAD clade; Panicoideae; Andropogonodae; Andropogoneae; Tripsacinae; Zea"]:0.05121000000000002,ACG47836.1[&Organism="Zea mays","Genetic Code"="Standard",Taxonomy="Eukaryota; Viridiplantae; Streptophyta; Embryophyta; Tracheophyta; Spermatophyta; Magnoliopsida; Liliopsida; Poales; Poaceae; PACMAD clade; Panicoideae; Andropogonodae; Andropogoneae; Tripsacinae; Zea"]:0.06483000000000017)[&"FastTree support value"=0.925]:0.05170999999999992,ONM04707.1[&Organism="Zea mays","Genetic Code"="Standard",Taxonomy="Eukaryota; Viridiplantae; Streptophyta; Embryophyta; Tracheophyta; Spermatophyta; Magnoliopsida; Liliopsida; Poales; Poaceae; PACMAD clade; Panicoideae; Andropogonodae; Andropogoneae; Tripsacinae; Zea"]:0.2387999999999999)[&"FastTree support value"=0.82]:0.04901)[&"FastTree support value"=0.973]:0.08176000000000005)[&"FastTree support value"=0.994]:0.11328000000000005)[&"FastTree support value"=0.608]:0.025040000000000173,(KAG0632288.1[&Organism="Ceratodon purpureus","Genetic Code"="Standard",Taxonomy="Eukaryota; Viridiplantae; Streptophyta; Embryophyta; Bryophyta; Bryophytina; Bryopsida; Dicranidae; Pseudoditrichales; Ditrichaceae; Ceratodon"]:0.19998000000000005,('KAG0555995.1'[&Organism="Ceratodon purpureus","Genetic Code"="Standard",Taxonomy="Eukaryota; Viridiplantae; Streptophyta; Embryophyta; Bryophyta; Bryophytina; Bryopsida; Dicranidae; Pseudoditrichales; Ditrichaceae; Ceratodon"]:0.034170000000000034,(XP_024391061.1[&Organism="Physcomitrium patens","Genetic Code"="Standard",Taxonomy="Eukaryota; Viridiplantae; Streptophyta; Embryophyta; Bryophyta; Bryophytina; Bryopsida; Funariidae; Funariales; Funariaceae; Physcomitrium"]:0.020989999999999842,XP_024368367.1[&Organism="Physcomitrium patens","Genetic Code"="Standard",Taxonomy="Eukaryota; Viridiplantae; Streptophyta; Embryophyta; Bryophyta; Bryophytina; Bryopsida; Funariidae; Funariales; Funariaceae; Physcomitrium"]:0.019489999999999785)[&"FastTree support value"=0.996]:0.08272000000000013)[&"FastTree support value"=0.986]:0.0771399999999999)[&"FastTree support value"=0.988]:0.08056999999999981)[&"FastTree support value"=0.975]:0.07965,EFJ28901.1[&Organism="Selaginella moellendorffii","Genetic Code"="Standard",Taxonomy="Eukaryota; Viridiplantae; Streptophyta; Embryophyta; Tracheophyta; Lycopodiopsida; Selaginellales; Selaginellaceae; Selaginella"]:0.20156000000000018)[&"FastTree support value"=0.83]:0.07520000000000016,EFJ33653.1[&Organism="Selaginella moellendorffii","Genetic Code"="Standard",Taxonomy="Eukaryota; Viridiplantae; Streptophyta; Embryophyta; Tracheophyta; Lycopodiopsida; Selaginellales; Selaginellaceae; Selaginella"]:0.11682000000000015)[&"FastTree support value"=1.0]:1.2805900000000001,((((((NP_495986.3.3[&Organism="Caenorhabditis

elegans","Genetic Code"="Standard",Taxonomy="Eukaryota; Metazoa; Ecdysozoa; Nematoda; Chromadorea; Rhabditida; Rhabditina; Rhabditomorpha; Rhabditoidea; Rhabditidae; Peloderinae; Caenorhabditis"]:0.1495099999999998,NP_610941.1[&Organism="Drosophila melanogaster","Genetic Code"="Standard",Taxonomy="Eukaryota; Metazoa; Ecdysozoa; Arthropoda; Hexapoda; Insecta; Pterygota; Neoptera; Holometabola; Diptera; Brachycera; Muscomorpha; Ephydroidea; Drosophilidae; Drosophila; Sophophora","Common Name"="fruit fly"]:0.14554)[&"FastTree support value"=0.857]:0.04992999999999981,((XP_018667792.1[&Organism="Ciona intestinalis","Genetic Code"="Standard",Taxonomy="Eukaryota; Metazoa; Chordata; Tunicata; Ascidiacea; Enterogona; Phlebobranchia; Cionidae; Ciona","Common Name"="vase tunicate"]:0.11273,(XP_032818114.1[&Organism="Petromyzon marinus","Genetic Code"="Standard",Taxonomy="Eukaryota; Metazoa; Chordata; Craniata; Vertebrata; Cyclostomata; Hyperoartia; Petromyzontiformes; Petromyzontidae; Petromyzon","Common Name"="sea lamprey"]:0.05867000000000022,((XP_028587646.1[&Organism="Podarcis muralis","Genetic Code"="Standard",Taxonomy="Eukaryota; Metazoa; Chordata; Craniata; Vertebrata; Euteleostomi; Lepidosauria; Squamata; Bifurcata; Unidentata; Episquamata; Laterata; Lacertibaenia; Lacertidae; Podarcis","Common Name"="Common wall lizard"]:0.018530000000000157,((XP_023440724.1[&Organism="Dasypus novemcinctus","Genetic Code"="Standard",Taxonomy="Eukaryota; Metazoa; Chordata; Craniata; Vertebrata; Euteleostomi; Mammalia; Eutheria; Xenarthra; Cingulata; Dasypodidae; Dasypus","Common Name"="nine-banded armadillo"]:0.005900000000000016,(XP_005873264.1[&Organism="Myotis brandtii","Genetic Code"="Standard",Taxonomy="Eukaryota; Metazoa; Chordata; Craniata; Vertebrata; Euteleostomi; Mammalia; Eutheria; Laurasiatheria; Chiroptera; Microchiroptera; Vespertilionidae; Myotis","Common Name"="Brandt's bat"]:0.006330000000000169,(XP_006163024.2.2[&Organism="Tupaia chinensis","Genetic Code"="Standard",Taxonomy="Eukaryota; Metazoa; Chordata; Craniata; Vertebrata; Euteleostomi; Mammalia; Eutheria; Euarchontoglires; Scandentia; Tupaiidae; Tupaia","Common Name"="Chinese tree shrew"]:0.0,NP_056375.2.2[&Organism="Homo sapiens","Genetic Code"="Standard",Taxonomy="Eukaryota; Metazoa; Chordata; Craniata; Vertebrata; Euteleostomi; Mammalia; Eutheria; Euarchontoglires; Primates; Haplorrhini; Catarrhini; Hominidae; Homo","Common Name"="human"]:0.0,NP_598513.1[&Organism="Mus musculus","Genetic Code"="Standard",Taxonomy="Eukaryota; Metazoa; Chordata; Craniata; Vertebrata; Euteleostomi; Mammalia; Eutheria; Euarchontoglires; Glires; Rodentia; Myomorpha; Muroidea; Muridae; Murinae; Mus; Mus","Common Name"="house mouse"]:0.0):0.000550)[&"FastTree support value"=0.873]:0.006860000000000088)[&"FastTree support value"=0.997]:0.03437000000000001,XP_025913835.1[&Organism="Apteryx rowi","Genetic Code"="Standard",Taxonomy="Eukaryota; Metazoa; Chordata; Craniata; Vertebrata; Euteleostomi; Archelosauria; Archosauria; Dinosauria; Saurischia; Theropoda; Coelurosauria; Aves; Palaeognathae; Apterygiformes; Apterygidae; Apteryx","Common

Name"="Okarito brown kiwi"]:0.004290000000000127)[&"FastTree support
value"=0.231]:0.006219999999999892)[&"FastTree support
value"=0.91]:0.015589999999999993,(XP_021332524.1[&Organism="Danio rerio","Genetic
Code"="Standard",Taxonomy="Eukaryota; Metazoa; Chordata; Craniata; Vertebrata;
Euteleostomi; Actinopterygii; Neopterygii; Teleostei; Ostariophysi; Cypriniformes;
Cyprinidae; Danio","Common
Name"="zebrafish"]:0.038450000000000095,XP_031757388.1[&Organism="Xenopus
tropicalis","Genetic Code"="Standard",Taxonomy="Eukaryota; Metazoa; Chordata;
Craniata; Vertebrata; Euteleostomi; Amphibia; Batrachia; Anura; Pipoidea; Pipidae;
Xenopodinae; Xenopus; Silurana","Common Name"="tropical clawed
frog"]:0.043829999999999814)[&"FastTree support
value"=0.566]:0.01161000000000012)[&"FastTree support
value"=0.921]:0.029520000000000213)[&"FastTree support
value"=0.991]:0.07799000000000023)[&"FastTree support
value"=0.896]:0.0347599999999999,(XP_006813643.1[&Organism="Saccoglossus
kowalevskii","Genetic Code"="Standard",Taxonomy="Eukaryota; Metazoa; Hemichordata;
Enteropneusta; Harrimaniidae;
Saccoglossus"]:0.09380999999999995,XP_030843280.1[&Organism="Strongylocentrotus
purpuratus","Genetic Code"="Standard",Taxonomy="Eukaryota; Metazoa; Echinodermata;
Eleutherozoa; Echinozoa; Echinoidea; Euechinoidea; Echinacea; Echinoida;
Strongylocentrotidae; Strongylocentrotus","Common Name"="purple sea
urchin"]:0.14175000000000004)[&"FastTree support
value"=0.29]:0.020389999999999908)[&"FastTree support
value"=0.945]:0.055320000000000036)[&"FastTree support
value"=0.374]:0.019280000000000186,(XP_002602331.1[&Organism="Branchiostoma
floridae","Genetic Code"="Standard",Taxonomy="Eukaryota; Metazoa; Chordata;
Cephalochordata; Branchiostomidae; Branchiostoma","Common Name"="Florida
lancelet"]:0.005560000000000009,XP_019637857.1[&Organism="Branchiostoma
belcheri","Genetic Code"="Standard",Taxonomy="Eukaryota; Metazoa; Chordata;
Cephalochordata; Branchiostomidae; Branchiostoma","Common Name"="Belcher's
lancelet"]:0.007109999999999995)[&"FastTree support
value"=0.992]:0.09193000000000007)[&"FastTree support
value"=1.0]:0.3084699999999998,PAA87312.1[&Organism="Macrostomum
lignano","Genetic Code"="Standard",Taxonomy="Eukaryota; Metazoa; Platyhelminthes;
Rhabditophora; Macrostomorpha; Macrostomida; Macrostomidae;
Macrostomum"]:0.08456000000000019)[&"FastTree support
value"=0.177]:0.07329000000000008,PAA68234.1[&Organism="Macrostomum
lignano","Genetic Code"="Standard",Taxonomy="Eukaryota; Metazoa; Platyhelminthes;
Rhabditophora; Macrostomorpha; Macrostomida; Macrostomidae;
Macrostomum"]:0.03765000000000018)[&"FastTree support
value"=1.0]:0.9271000000000003,(((XP_042918632.1[&Organism="Chlamydomonas
reinhardtii","Genetic Code"="Standard",Taxonomy="Eukaryota; Viridiplantae; Chlorophyta;
core chlorophytes; Chlorophyceae; CS clade; Chlamydomonadales;
Chlamydomonadaceae;

Chlamydomonas"]:0.1155400000000002,(((NP_001130364.1[&Organism="Zea mays","Genetic Code"="Standard",Taxonomy="Eukaryota; Viridiplantae; Streptophyta; Embryophyta; Tracheophyta; Spermatophyta; Magnoliopsida; Liliopsida; Poales; Poaceae; PACMAD clade; Panicoideae; Andropogonodae; Andropogoneae; Tripsacinae; Zea"]:0.0189499999999998,PWZ11893.1[&Organism="Zea mays","Genetic Code"="Standard",Taxonomy="Eukaryota; Viridiplantae; Streptophyta; Embryophyta; Tracheophyta; Spermatophyta; Magnoliopsida; Liliopsida; Poales; Poaceae; PACMAD clade; Panicoideae; Andropogonodae; Andropogoneae; Tripsacinae; Zea"]:0.017549999999999955)[&"FastTree support value"=1.0]:0.10488000000000008,(XP_002317496.2[&Organism="Populus trichocarpa","Genetic Code"="Standard",Taxonomy="Eukaryota; Viridiplantae; Streptophyta; Embryophyta; Tracheophyta; Spermatophyta; Magnoliopsida; eudicotyledons; Gunneridae; Pentapetalae; rosids; fabids; Malpighiales; Salicaceae; Saliceae; Populus","Common Name"="Populus balsamifera subsp. trichocarpa"]:0.0786699999999998,AAF87857.1[&Organism="Arabidopsis thaliana","Genetic Code"="Standard",Taxonomy="Eukaryota; Viridiplantae; Streptophyta; Embryophyta; Tracheophyta; Spermatophyta; Magnoliopsida; eudicotyledons; Gunneridae; Pentapetalae; rosids; malvids; Brassicales; Brassicaceae; Camelineae; Arabidopsis","Common Name"="thale cress"]:0.04041000000000006)[&"FastTree support value"=0.96]:0.04977000000000009)[&"FastTree support value"=0.792]:0.037840000000000096,((KAI5058044.1[&Organism="Adiantum capillus-veneris","Genetic Code"="Standard",Taxonomy="Eukaryota; Viridiplantae; Streptophyta; Embryophyta; Tracheophyta; Polypodiopsida; Polypodiidae; Polypodiales; Pteridineae; Pteridaceae; Vittarioideae; Adiantum"]:0.13915999999999995,(EFJ18064.1[&Organism="Selaginella moellendorffii","Genetic Code"="Standard",Taxonomy="Eukaryota; Viridiplantae; Streptophyta; Embryophyta; Tracheophyta; Lycopodiopsida; Selaginellales; Selaginellaceae; Selaginella"]:0.1477599999999999,(KAG0628798.1[&Organism="Ceratodon purpureus","Genetic Code"="Standard",Taxonomy="Eukaryota; Viridiplantae; Streptophyta; Embryophyta; Bryophyta; Bryophytina; Bryopsida; Dicranidae; Pseudoditrichales; Ditrichaceae; Ceratodon"]:0.15864999999999974,PTQ33908.1[&Organism="Marchantia polymorpha","Genetic Code"="Standard",Taxonomy="Eukaryota; Viridiplantae; Streptophyta; Embryophyta; Marchantiophyta; Marchantiopsida; Marchantiidae; Marchantiales; Marchantiaceae; Marchantia","Common Name"="liverwort"]:0.10671000000000008)[&"FastTree support value"=0.545]:0.02369000000000021)[&"FastTree support value"=0.67]:0.02466999999999997)[&"FastTree support value"=0.108]:0.03381999999999996,KAH9322298.1[&Organism="Taxus chinensis","Genetic Code"="Standard",Taxonomy="Eukaryota; Viridiplantae; Streptophyta; Embryophyta; Tracheophyta; Spermatophyta; Pinopsida; Pinidae; Conifers II; Cupressales; Taxaceae; Taxus"]:0.12297000000000002)[&"FastTree support value"=0.899]:0.03964999999999996)[&"FastTree support value"=0.951]:0.12089000000000016)[&"FastTree support

value"=0.987]:0.4891200000000002,(XP_009032466.1[&Organism="Aureococcus anophagefferens","Genetic Code"="Standard",Taxonomy="Eukaryota; Sar; Stramenopiles; Ochrophyta; Pelagophyceae; Pelagomonadales; Aureococcus"]:0.5847800000000003,(XP_042920073.1[&Organism="Chlamydomonas reinhardtii","Genetic Code"="Standard",Taxonomy="Eukaryota; Viridiplantae; Chlorophyta; core chlorophytes; Chlorophyceae; CS clade; Chlamydomonadales; Chlamydomonadaceae; Chlamydomonas"]:0.22039000000000009,((EFJ19523.1[&Organism="Selaginella moellendorffii","Genetic Code"="Standard",Taxonomy="Eukaryota; Viridiplantae; Streptophyta; Embryophyta; Tracheophyta; Lycopodiopsida; Selaginellales; Selaginellaceae; Selaginella"]:0.12516999999999978,((PTQ34556.1[&Organism="Marchantia polymorpha","Genetic Code"="Standard",Taxonomy="Eukaryota; Viridiplantae; Streptophyta; Embryophyta; Marchantiophyta; Marchantiopsida; Marchantiidae; Marchantiales; Marchantiaceae; Marchantia","Common Name"="liverwort"]:0.10168999999999961,(KAG0561482.1[&Organism="Ceratodon purpureus","Genetic Code"="Standard",Taxonomy="Eukaryota; Viridiplantae; Streptophyta; Embryophyta; Bryophyta; Bryophytina; Bryopsida; Dicranidae; Pseudoditrichales; Ditrichaceae; Ceratodon"]:0.10144999999999982,KAG0605142.1[&Organism="Ceratodon purpureus","Genetic Code"="Standard",Taxonomy="Eukaryota; Viridiplantae; Streptophyta; Embryophyta; Bryophyta; Bryophytina; Bryopsida; Dicranidae; Pseudoditrichales; Ditrichaceae; Ceratodon"]:0.12924000000000024)[&"FastTree support value"=0.879]:0.029600000000000293)[&"FastTree support value"=0.518]:0.029819999999999958,(KAI5064281.1[&Organism="Adiantum capillus-veneris","Genetic Code"="Standard",Taxonomy="Eukaryota; Viridiplantae; Streptophyta; Embryophyta; Tracheophyta; Polypodiopsida; Polypodiidae; Polypodiales; Pteridineae; Pteridaceae; Vittarioideae; Adiantum"]:0.06024000000000029,KAH9308354.1[&Organism="Taxus chinensis","Genetic Code"="Standard",Taxonomy="Eukaryota; Viridiplantae; Streptophyta; Embryophyta; Tracheophyta; Spermatophyta; Pinopsida; Pinidae; Conifers II; Cupressales; Taxaceae; Taxus"]:0.11746999999999996)[&"FastTree support value"=0.397]:0.026830000000000354)[&"FastTree support value"=0.458]:0.026650000000000063)[&"FastTree support value"=0.952]:0.06829000000000018,((XP_002309632.3[&Organism="Populus trichocarpa","Genetic Code"="Standard",Taxonomy="Eukaryota; Viridiplantae; Streptophyta; Embryophyta; Tracheophyta; Spermatophyta; Magnoliopsida; eudicotyledons; Gunneridae; Pentapetalae; rosids; fabids; Malpighiales; Salicaceae; Saliceae; Populus","Common Name"="Populus balsamifera subsp. trichocarpa"]:0.06339000000000006,NP_001189935.1[&Organism="Arabidopsis thaliana","Genetic Code"="Standard",Taxonomy="Eukaryota; Viridiplantae; Streptophyta; Embryophyta; Tracheophyta; Spermatophyta; Magnoliopsida; eudicotyledons; Gunneridae; Pentapetalae; rosids; malvids; Brassicales; Brassicaceae; Camelineae; Arabidopsis","Common Name"="thale cress"]:0.1436099999999998)[&"FastTree support value"=0.708]:0.03143000000000029,PWZ44616.1[&Organism="Zea mays","Genetic

Code"="Standard",Taxonomy="Eukaryota; Viridiplantae; Streptophyta; Embryophyta; Tracheophyta; Spermatophyta; Magnoliopsida; Liliopsida; Poales; Poaceae; PACMAD clade; Panicoideae; Andropogonodae; Andropogoneae; Tripsacinae; Zea"]:0.24495999999999984)[&"FastTree support value"=0.9]:0.07598000000000038)[&"FastTree support value"=0.904]:0.09060999999999986)[&"FastTree support value"=0.983]:0.2915000000000001)[&"FastTree support value"=1.0]:0.5779200000000002)[&"FastTree support value"=1.0]:0.96393,((XP_042916771.1[&Organism="Chlamydomonas reinhardtii","Genetic Code"="Standard",Taxonomy="Eukaryota; Viridiplantae; Chlorophyta; core chlorophytes; Chlorophyceae; CS clade; Chlamydomonadales; Chlamydomonadaceae; Chlamydomonas"]:0.21752000000000038,((KAG0631008.1[&Organism="Ceratodon purpureus","Genetic Code"="Standard",Taxonomy="Eukaryota; Viridiplantae; Streptophyta; Embryophyta; Bryophyta; Bryophytina; Bryopsida; Dicranidae; Pseudoditrichales; Ditrichaceae; Ceratodon"]:0.14400000000000013,EFJ26018.1[&Organism="Selaginella moellendorffii","Genetic Code"="Standard",Taxonomy="Eukaryota; Viridiplantae; Streptophyta; Embryophyta; Tracheophyta; Lycopodiopsida; Selaginellales; Selaginellaceae; Selaginella"]:0.45999999999999996)[&"FastTree support value"=0.607]:0.030219999999999914,((KAH9315399.1[&Organism="Taxus chinensis","Genetic Code"="Standard",Taxonomy="Eukaryota; Viridiplantae; Streptophyta; Embryophyta; Tracheophyta; Spermatophyta; Pinopsida; Pinidae; Conifers II; Cupressales; Taxaceae; Taxus"]:0.13780000000000037,(CAD5311589.1[&Organism="Arabidopsis thaliana","Genetic Code"="Standard",Taxonomy="Eukaryota; Viridiplantae; Streptophyta; Embryophyta; Tracheophyta; Spermatophyta; Magnoliopsida; eudicotyledons; Gunneridae; Pentapetalae; rosids; malvids; Brassicales; Brassicaceae; Camelineae; Arabidopsis","Common Name"="thale cress"]:0.17558000000000007,XP_008649599.1[&Organism="Zea mays","Genetic Code"="Standard",Taxonomy="Eukaryota; Viridiplantae; Streptophyta; Embryophyta; Tracheophyta; Spermatophyta; Magnoliopsida; Liliopsida; Poales; Poaceae; PACMAD clade; Panicoideae; Andropogonodae; Andropogoneae; Tripsacinae; Zea"]:0.2576400000000003)[&"FastTree support value"=0.994]:0.18527999999999967)[&"FastTree support value"=0.991]:0.12425999999999959,(KAI5059498.1[&Organism="Adiantum capillus-veneris","Genetic Code"="Standard",Taxonomy="Eukaryota; Viridiplantae; Streptophyta; Embryophyta; Tracheophyta; Polypodiopsida; Polypodiidae; Polypodiales; Pteridineae; Pteridaceae; Vittarioideae; Adiantum"]:0.28415000000000035,OAE29693.1[&Organism="Marchantia polymorpha subsp. ruderalis","Genetic Code"="Standard",Taxonomy="Eukaryota; Viridiplantae; Streptophyta; Embryophyta; Marchantiophyta; Marchantiopsida; Marchantiidae; Marchantiales; Marchantiaceae; Marchantia"]:0.1492199999999997)[&"FastTree support value"=0.687]:0.05869000000000035)[&"FastTree support value"=0.81]:0.05295000000000005)[&"FastTree support value"=0.983]:0.3449)[&"FastTree support value"=0.978]:0.84002,((XP_001745740.1[&Organism="Monosiga brevicollis MX1","Genetic

Code"="Standard",Taxonomy="Eukaryota; Choanoflagellata; Craspedida; Salpingoecidae; Monosiga"]:0.53092999999999997,(((PAA75551.1[&Organism="Macrostomum lignano","Genetic Code"="Standard",Taxonomy="Eukaryota; Metazoa; Platyhelminthes; Rhabditophora; Macrostomorpha; Macrostomida; Macrostomidae; Macrostomum"]:0.010239999999999583,PAA75258.1[&Organism="Macrostomum lignano","Genetic Code"="Standard",Taxonomy="Eukaryota; Metazoa; Platyhelminthes; Rhabditophora; Macrostomorpha; Macrostomida; Macrostomidae; Macrostomum"]:0.000540)[&"FastTree support value"=0.999]:0.2871100000000002,((XP_004365821.1[&Organism="Capsaspora owczarzaki ATCC 30864","Genetic Code"="Standard",Taxonomy="Eukaryota; Filasterea; Capsaspora"]:0.36488999999999994,XP_014153836.1[&Organism="Sphaeroforma arctica JP610","Genetic Code"="Standard",Taxonomy="Eukaryota; Ichthyosporea; Ichthyophonida; Sphaeroforma"]:0.51381999999999999)[&"FastTree support value"=0.94]:0.15245999999999996,(XP_002126852.1[&Organism="Ciona intestinalis","Genetic Code"="Standard",Taxonomy="Eukaryota; Metazoa; Chordata; Tunicata; Ascidiacea; Enterogona; Phlebobranchia; Cionidae; Ciona","Common Name"="vase tunicate"]:0.21591999999999967,(((NP_001121726.1[&Organism="Danio rerio","Genetic Code"="Standard",Taxonomy="Eukaryota; Metazoa; Chordata; Craniata; Vertebrata; Euteleostomi; Actinopterygii; Neopterygii; Teleostei; Ostariophysi; Cypriniformes; Danionidae; Danioninae; Danio","Common Name"="zebrafish"]:0.053139999999999965,((XP_025929938.1[&Organism="Apteryx rowi","Genetic Code"="Standard",Taxonomy="Eukaryota; Metazoa; Chordata; Craniata; Vertebrata; Euteleostomi; Archelosauria; Archosauria; Dinosauria; Saurischia; Theropoda; Coelurosauria; Aves; Palaeognathae; Apterygiformes; Apterygidae; Apteryx","Common Name"="Okarito brown kiwi"]:0.0072299999999999848,(XP_028597443.1[&Organism="Podarcis muralis","Genetic Code"="Standard",Taxonomy="Eukaryota; Metazoa; Chordata; Craniata; Vertebrata; Euteleostomi; Lepidosauria; Squamata; Bifurcata; Unidentata; Episquamata; Laterata; Lacertibaenia; Lacertidae; Podarcis","Common Name"="Common wall lizard"]:0.020209999999999617,XP_015268039.1[&Organism="Gekko japonicus","Genetic Code"="Standard",Taxonomy="Eukaryota; Metazoa; Chordata; Craniata; Vertebrata; Euteleostomi; Lepidosauria; Squamata; Bifurcata; Gekkota; Gekkonidae; Gekkoninae; Gekko"]:0.000540)[&"FastTree support value"=0.94]:0.012360000000000149)[&"FastTree support value"=0.686]:0.0020300000000004204,((XP_006145367.1[&Organism="Tupaia chinensis","Genetic Code"="Standard",Taxonomy="Eukaryota; Metazoa; Chordata; Craniata; Vertebrata; Euteleostomi; Mammalia; Eutheria; Euarchontoglires; Scandentia; Tupaiidae; Tupaia","Common Name"="Chinese tree shrew"]:0.000540,(NP_001121132.1[&Organism="Homo sapiens","Genetic Code"="Standard",Taxonomy="Eukaryota; Metazoa; Chordata; Craniata; Vertebrata; Euteleostomi; Mammalia; Eutheria; Euarchontoglires; Primates; Haplorrhini; Catarrhini; Hominidae; Homo","Common Name"="human"]:0.014280000000000292,(XP_014400986.1[&Organism="Myotis brandtii","Genetic Code"="Standard",Taxonomy="Eukaryota; Metazoa; Chordata; Craniata; Vertebrata; Euteleostomi; Mammalia; Eutheria; Laurasiatheria; Chiroptera;

Microchiroptera; Vespertilionidae; Myotis","Common Name"="Brandt's bat"]:0.007189999999999586,NP_001177198.1[&Organism="Bos taurus","Genetic Code"="Standard",Taxonomy="Eukaryota; Metazoa; Chordata; Craniata; Vertebrata; Euteleostomi; Mammalia; Eutheria; Laurasiatheria; Artiodactyla; Ruminantia; Pecora; Bovidae; Bovinae; Bos","Common Name"="cattle"]:0.003610000000000113)[&"FastTree support value"=0.924]:0.0033200000000004337)[&"FastTree support value"=0.993]:0.000550)[&"FastTree support value"=0.545]:0.000550,(XP_004482574.1[&Organism="Dasypus novemcinctus","Genetic Code"="Standard",Taxonomy="Eukaryota; Metazoa; Chordata; Craniata; Vertebrata; Euteleostomi; Mammalia; Eutheria; Xenarthra; Cingulata; Dasypodidae; Dasypus","Common Name"="nine-banded armadillo"]:0.0036199999999997345,NP_001272849.1[&Organism="Mus musculus","Genetic Code"="Standard",Taxonomy="Eukaryota; Metazoa; Chordata; Craniata; Vertebrata; Euteleostomi; Mammalia; Eutheria; Euarchontoglires; Glires; Rodentia; Myomorpha; Muroidea; Muridae; Murinae; Mus; Mus","Common Name"="house mouse"]:0.01438999999999968)[&"FastTree support value"=0.692]:0.003569999999999851)[&"FastTree support value"=0.925]:0.012500000000000178)[&"FastTree support value"=0.984]:0.04263999999999957)[&"FastTree support value"=0.965]:0.046020000000000394,(XP_017213868.2.2[&Organism="Danio rerio","Genetic Code"="Standard",Taxonomy="Eukaryota; Metazoa; Chordata; Craniata; Vertebrata; Euteleostomi; Actinopterygii; Neopterygii; Teleostei; Ostariophysi; Cypriniformes; Cyprinidae; Danio","Common Name"="zebrafish"]:0.07589999999999986,(NP_001016189.1[&Organism="Xenopus tropicalis","Genetic Code"="Standard",Taxonomy="Eukaryota; Metazoa; Chordata; Craniata; Vertebrata; Euteleostomi; Amphibia; Batrachia; Anura; Pipoidea; Pipidae; Xenopodinae; Xenopus; Silurana","Common Name"="tropical clawed frog"]:0.03692999999999991,(XP_028587453.1[&Organism="Podarcis muralis","Genetic Code"="Standard",Taxonomy="Eukaryota; Metazoa; Chordata; Craniata; Vertebrata; Euteleostomi; Lepidosauria; Squamata; Bifurcata; Unidentata; Episquamata; Laterata; Lacertibaenia; Lacertidae; Podarcis","Common Name"="Common wall lizard"]:0.025330000000000297,(XP_025917892.1[&Organism="Apteryx rowi","Genetic Code"="Standard",Taxonomy="Eukaryota; Metazoa; Chordata; Craniata; Vertebrata; Euteleostomi; Archelosauria; Archosauria; Dinosauria; Saurischia; Theropoda; Coelurosauria; Aves; Palaeognathae; Apterygiformes; Apterygidae; Apteryx","Common Name"="Okarito brown kiwi"]:0.025660000000000238,(NP_284941.2.2[&Organism="Homo sapiens","Genetic Code"="Standard",Taxonomy="Eukaryota; Metazoa; Chordata; Craniata; Vertebrata; Euteleostomi; Mammalia; Eutheria; Euarchontoglires; Primates; Haplorrhini; Catarrhini; Hominidae; Homo","Common Name"="human"]:0.02165999999999979,(((XP_005883071.1[&Organism="Myotis brandtii","Genetic Code"="Standard",Taxonomy="Eukaryota; Metazoa; Chordata; Craniata; Vertebrata; Euteleostomi; Mammalia; Eutheria; Laurasiatheria; Chiroptera; Microchiroptera; Vespertilionidae; Myotis","Common Name"="Brandt's

bat"]:0.01822999999999997,(NP_077162.2.2[&Organism="Mus musculus","Genetic
Code"="Standard",Taxonomy="Eukaryota; Metazoa; Chordata; Craniata; Vertebrata;
Euteleostomi; Mammalia; Eutheria; Euarchontoglires; Glires; Rodentia; Myomorpha;
Muroidea; Muridae; Murinae; Mus; Mus","Common Name"="house
mouse"]:0.014429999999999943,XP_006162789.1[&Organism="Tupaia
chinensis","Genetic Code"="Standard",Taxonomy="Eukaryota; Metazoa; Chordata;
Craniata; Vertebrata; Euteleostomi; Mammalia; Eutheria; Euarchontoglires; Scandentia;
Tupaiidae; Tupaia","Common Name"="Chinese tree
shrew"]:0.004509999999999792)[&"FastTree support
value"=0.841]:0.006719999999999615)[&"FastTree support
value"=0.767]:0.003650000000000375,NP_001193437.1[&Organism="Bos
taurus","Genetic Code"="Standard",Taxonomy="Eukaryota; Metazoa; Chordata; Craniata;
Vertebrata; Euteleostomi; Mammalia; Eutheria; Laurasiatheria; Artiodactyla; Ruminantia;
Pecora; Bovidae; Bovinae; Bos","Common
Name"="cattle"]:0.007299999999999862)[&"FastTree support
value"=0.763]:0.004019999999996905,XP_004479029.1[&Organism="Dasypus
novemcinctus","Genetic Code"="Standard",Taxonomy="Eukaryota; Metazoa; Chordata;
Craniata; Vertebrata; Euteleostomi; Mammalia; Eutheria; Xenarthra; Cingulata;
Dasypodidae; Dasypus","Common Name"="nine-banded
armadillo"]:0.014149999999999885)[&"FastTree support
value"=0.676]:0.007229999999999848)[&"FastTree support
value"=0.865]:0.007939999999999614)[&"FastTree support
value"=0.917]:0.015719999999999956)[&"FastTree support
value"=0.933]:0.026239999999999597)[&"FastTree support
value"=0.895]:0.023589999999999556)[&"FastTree support
value"=0.946]:0.03683000000000014)[&"FastTree support
value"=0.986]:0.09438999999999975,((XP_006819998.1[&Organism="Saccoglossus
kowalevskii","Genetic Code"="Standard",Taxonomy="Eukaryota; Metazoa; Hemichordata;
Enteropneusta; Harrimaniidae;
Saccoglossus"]:0.14865999999999957,(XP_002591612.1[&Organism="Branchiostoma
floridae","Genetic Code"="Standard",Taxonomy="Eukaryota; Metazoa; Chordata;
Cephalochordata; Branchiostomidae; Branchiostoma","Common Name"="Florida
lancelet"]:0.003199999999996476,XP_019628129.1[&Organism="Branchiostoma
belcheri","Genetic Code"="Standard",Taxonomy="Eukaryota; Metazoa; Chordata;
Cephalochordata; Branchiostomidae; Branchiostoma","Common Name"="Belcher's
lancelet"]:0.007819999999999716)[&"FastTree support
value"=1.0]:0.14876000000000023)[&"FastTree support
value"=0.784]:0.016799999999999926,((XP_030846906.1[&Organism="Strongylocentrotu
s purpuratus","Genetic Code"="Standard",Taxonomy="Eukaryota; Metazoa; Echinodermata;
Eleutherozoa; Echinozoa; Echinoidea; Euechinoidea; Echinacea; Echinoida;
Strongylocentrotidae; Strongylocentrotus","Common Name"="purple sea
urchin"]:0.08117000000000019,XP_030847518.1[&Organism="Strongylocentrotus
purpuratus","Genetic Code"="Standard",Taxonomy="Eukaryota; Metazoa; Echinodermata;
Eleutherozoa; Echinozoa; Echinoidea; Euechinoidea; Echinacea; Echinoida;

Strongylocentrotidae; Strongylocentrotus","Common Name"="purple sea
urchin"]:0.022739999999999976)[&"FastTree support
value"=0.999]:0.23312000000000044,NP_996357.1[&Organism="Drosophila
melanogaster","Genetic Code"="Standard",Taxonomy="Eukaryota; Metazoa; Ecdysozoa;
Arthropoda; Hexapoda; Insecta; Pterygota; Neoptera; Holometabola; Diptera; Brachycera;
Muscomorpha; Ephydroidea; Drosophilidae; Drosophila; Sophophora","Common
Name"="fruit fly"]:0.30025000000000013)[&"FastTree support
value"=0.88]:0.05137999999999998)[&"FastTree support
value"=0.968]:0.05449000000000037)[&"FastTree support
value"=0.9]:0.05738999999999983)[&"FastTree support
value"=0.263]:0.06784999999999997)[&"FastTree support
value"=0.597]:0.06519999999999992)[&"FastTree support
value"=0.95]:0.1806700000000001,NP_495161.1[&Organism="Caenorhabditis
elegans","Genetic Code"="Standard",Taxonomy="Eukaryota; Metazoa; Ecdysozoa;
Nematoda; Chromadorea; Rhabditida; Rhabditina; Rhabditomorpha; Rhabditoidea;
Rhabditidae; Peloderinae; Caenorhabditis"]:0.28514000000000017)[&"FastTree support
value"=0.172]:0.26342)[&"FastTree support
value"=0.999]:1.5830599999999997,(OUM66167.1[&Organism="Piromyces sp.
E2","Genetic Code"="Standard",Taxonomy="Eukaryota; Fungi; Fungi incertae sedis;
Chytridiomycota; Chytridiomycota incertae sedis; Neocallimastigomycetes;
Neocallimastigales; Neocallimastigaceae;
Piromyces"]:1.0772599999999999,(KXN69997.1[&Organism="Conidiobolus coronatus
NRRL 28638","Genetic Code"="Standard",Taxonomy="Eukaryota; Fungi; Fungi incertae
sedis; Zoopagomycota; Entomophthoromycotina; Entomophthoromycetes;
Entomophthorales; Ancylistaceae;
Conidiobolus"]:0.31515999999999966,(((KNE73082.1[&Organism="Allomyces
macrogynus ATCC 38327","Genetic Code"="Standard",Taxonomy="Eukaryota; Fungi; Fungi
incertae sedis; Blastocladiomycota; Blastocladiomycota incertae sedis;
Blastocladiomycetes; Blastocladiales; Blastocladiaceae;
Allomyces"]:0.20805999999999997,KNE65701.1[&Organism="Allomyces macrogynus ATCC
38327","Genetic Code"="Standard",Taxonomy="Eukaryota; Fungi; Fungi incertae sedis;
Blastocladiomycota; Blastocladiomycota incertae sedis; Blastocladiomycetes;
Blastocladiales; Blastocladiaceae; Allomyces"]:0.21272000000000002)[&"FastTree
support
value"=1.0]:0.4261400000000002,XP_006676761.1[&Organism="Batrachochytrium
dendrobatidis JAM81","Genetic Code"="Standard",Taxonomy="Eukaryota; Fungi; Fungi
incertae sedis; Chytridiomycota; Chytridiomycota incertae sedis; Chytridiomycetes;
Rhizophydiales; Rhizophydiales incertae sedis;
Batrachochytrium"]:0.39963000000000015)[&"FastTree support
value"=0.582]:0.07868999999999993,((XP_001481516.1[&Organism="Aspergillus
fumigatus Af293","Genetic Code"="Standard",Taxonomy="Eukaryota; Fungi; Dikarya;
Ascomycota; Pezizomycotina; Eurotiomycetes; Eurotiomycetidae; Eurotiales;
Aspergillaceae; Aspergillus; Aspergillus subgen.
Fumigati"]:0.1630200000000004,NP_009738.1[&Organism="Saccharomyces cerevisiae

S288C","Genetic Code"="Standard",Taxonomy="Eukaryota; Fungi; Dikarya; Ascomycota; Saccharomycotina; Saccharomycetes; Saccharomycetales; Saccharomycetaceae; Saccharomyces"]:0.6015199999999998)[&"FastTree support value"=0.961]:0.1544800000000004,(XP_011392385.1[&Organism="Ustilago maydis 521","Genetic Code"="Standard",Taxonomy="Eukaryota; Fungi; Dikarya; Basidiomycota; Ustilaginomycotina; Ustilaginomycetes; Ustilaginales; Ustilaginaceae; Ustilago"]:0.21060000000000034,XP_006462464.1[&Organism="Agaricus bisporus var. bisporus H97","Genetic Code"="Standard",Taxonomy="Eukaryota; Fungi; Dikarya; Basidiomycota; Agaricomycotina; Agaricomycetes; Agaricomycetidae; Agaricales; Agaricineae; Agaricaceae; Agaricus"]:0.15355000000000008)[&"FastTree support value"=0.984]:0.15714000000000006)[&"FastTree support value"=0.893]:0.09755999999999965)[&"FastTree support value"=0.46]:0.08450000000000024)[&"FastTree support value"=0.958]:0.6098099999999995)[&"FastTree support value"=0.959]:0.78458)[&"FastTree support value"=0.636]:0.6055099999999998)[&"FastTree support value"=0.991]:1.1366100000000001)[&"FastTree support value"=0.733]:0.08201999999999998)[&"FastTree support value"=0.837]:0.13472000000000062)[&"FastTree support value"=0.862]:0.19455999999999918,(((PSC76263.1[&Organism="Micractinium conductrix","Genetic Code"="Standard",Taxonomy="Eukaryota; Viridiplantae; Chlorophyta; core chlorophytes; Trebouxiophyceae; Chlorellales; Chlorellaceae; Chlorella clade; Micractinium"]:0.2499800000000003,(PRW56740.1[&Organism="Chlorella sorokiniana","Genetic Code"="Standard",Taxonomy="Eukaryota; Viridiplantae; Chlorophyta; core chlorophytes; Trebouxiophyceae; Chlorellales; Chlorellaceae; Chlorella clade; Chlorella"]:0.3840900000000005,XP_005849062.1[&Organism="Chlorella variabilis","Genetic Code"="Standard",Taxonomy="Eukaryota; Viridiplantae; Chlorophyta; core chlorophytes; Trebouxiophyceae; Chlorellales; Chlorellaceae; Chlorella clade; Chlorella"]:0.25588000000000033)[&"FastTree support value"=0.868]:0.08830999999999989)[&"FastTree support value"=0.999]:0.3711500000000001,((GMH43921.1[&Organism="Bryopsis sp. KO-2023","Genetic Code"="Standard",Taxonomy="Eukaryota; Viridiplantae; Chlorophyta; Ulvophyceae; TCBD clade; Bryopsidales; Bryopsidineae; Bryopsidaceae; Bryopsis"]:0.3886500000000006,GMH36208.1[&Organism="Bryopsis sp. KO-2023","Genetic Code"="Standard",Taxonomy="Eukaryota; Viridiplantae; Chlorophyta; Ulvophyceae; TCBD clade; Bryopsidales; Bryopsidineae; Bryopsidaceae; Bryopsis"]:0.2993800000000002)[&"FastTree support value"=1.0]:0.3853799999999996,(CAG9460856.1[&Organism="Pedinophyceae sp. YPF-701","Genetic Code"="Standard",Taxonomy="Eukaryota; Viridiplantae; Chlorophyta; Pedinophyceae"]:0.6261900000000002,((GJP35534.1[&Organism="Closterium sp. NIES-68","Genetic Code"="Standard",Taxonomy="Eukaryota; Viridiplantae; Streptophyta; Zygnemophyceae; Zygnematophycidae; Desmidiales; Closteriaceae; Closterium; Closterium peracerosum-strigosum-littorale complex"]:0.0976300000000001,CAI5480041.1[&Organism="Closterium sp. Yama58-

4","Genetic Code"="Standard",Taxonomy="Eukaryota; Viridiplantae; Streptophyta; Zygnemophyceae; Zygnematophycidae; Desmidiales; Closteriaceae; Closterium; Closterium peracerosum-strigosum-littorale complex"]:0.07383000000000006)[&"FastTree support value"=0.996]:0.19755000000000056,((KAJ7294545.1[&Organism="Diphasiastrum complanatum","Genetic Code"="Standard",Taxonomy="Eukaryota; Viridiplantae; Streptophyta; Embryophyta; Tracheophyta; Lycopodiopsida; Lycopodiales; Lycopodiaceae; Lycopodioideae; Diphasiastrum"]:0.20131999999999994,((XP_024380180.1[&Organism="Physcomitrium patens","Genetic Code"="Standard",Taxonomy="Eukaryota; Viridiplantae; Streptophyta; Embryophyta; Bryophyta; Bryophytina; Bryopsida; Funariidae; Funariales; Funariaceae; Physcomitrium"]:0.010050000000000114,XP_024367947.1[&Organism="Physcomitrium patens","Genetic Code"="Standard",Taxonomy="Eukaryota; Viridiplantae; Streptophyta; Embryophyta; Bryophyta; Bryophytina; Bryopsida; Funariidae; Funariales; Funariaceae; Physcomitrium"]:0.005840000000000067)[&"FastTree support value"=1.0]:0.1691600000000002,(KAG0619429.1[&Organism="Ceratodon purpureus","Genetic Code"="Standard",Taxonomy="Eukaryota; Viridiplantae; Streptophyta; Embryophyta; Bryophyta; Bryophytina; Bryopsida; Dicranidae; Pseudoditrichales; Ditrichaceae; Ceratodon"]:0.05590999999999904,KAG0561847.1[&Organism="Ceratodon purpureus","Genetic Code"="Standard",Taxonomy="Eukaryota; Viridiplantae; Streptophyta; Embryophyta; Bryophyta; Bryophytina; Bryopsida; Dicranidae; Pseudoditrichales; Ditrichaceae; Ceratodon"]:0.0956100000000002)[&"FastTree support value"=0.891]:0.04309999999999916)[&"FastTree support value"=0.951]:0.06732000000000005)[&"FastTree support value"=0.961]:0.08641999999999994,(((((KAK1401877.1[&Organism="Heracleum sosnowskyi","Genetic Code"="Standard",Taxonomy="Eukaryota; Viridiplantae; Streptophyta; Embryophyta; Tracheophyta; Spermatophyta; Magnoliopsida; eudicotyledons; Gunneridae; Pentapetalae; asterids; campanulids; Apiales; Apiaceae; Apioideae; apioid superclade; Tordylieae; Tordyliinae; Heracleum"]:0.09690999999999983,(KAF8391993.1[&Organism="Tetracentron sinense","Genetic Code"="Standard",Taxonomy="Eukaryota; Viridiplantae; Streptophyta; Embryophyta; Tracheophyta; Spermatophyta; Magnoliopsida; Trochodendrales; Trochodendraceae; Tetracentron"]:0.05697000000000019,((KAH0683503.1[&Organism="Solanum tuberosum","Genetic Code"="Standard",Taxonomy="Eukaryota; Viridiplantae; Streptophyta; Embryophyta; Tracheophyta; Spermatophyta; Magnoliopsida; eudicotyledons; Gunneridae; Pentapetalae; asterids; lamiids; Solanales; Solanaceae; Solanoideae; Solaneae; Solanum","Common Name"="potato"]:0.15664999999999996,(XP_038984915.1[&Organism="Phoenix dactylifera","Genetic Code"="Standard",Taxonomy="Eukaryota; Viridiplantae; Streptophyta; Embryophyta; Tracheophyta; Spermatophyta; Magnoliopsida; Liliopsida; Arecaceae; Coryphoideae; Phoeniceae; Phoenix","Common Name"="date palm"]:0.08503000000000016,(PWZ56863.1[&Organism="Zea mays","Genetic

Code"="Standard",Taxonomy="Eukaryota; Viridiplantae; Streptophyta; Embryophyta; Tracheophyta; Spermatophyta; Magnoliopsida; Liliopsida; Poales; Poaceae; PACMAD clade; Panicoideae; Andropogonodae; Andropogoneae; Tripsacinae; Zea"]:0.0396399999999999,PWZ56864.1[&Organism="Zea mays","Genetic Code"="Standard",Taxonomy="Eukaryota; Viridiplantae; Streptophyta; Embryophyta; Tracheophyta; Spermatophyta; Magnoliopsida; Liliopsida; Poales; Poaceae; PACMAD clade; Panicoideae; Andropogonodae; Andropogoneae; Tripsacinae; Zea"]:0.03794999999999993)[&"FastTree support value"=0.998]:0.08816999999999986)[&"FastTree support value"=0.945]:0.04065999999999992)[&"FastTree support value"=0.351]:0.020300000000000207,XP_058079501.1[&Organism="Magnolia sinica","Genetic Code"="Standard",Taxonomy="Eukaryota; Viridiplantae; Streptophyta; Embryophyta; Tracheophyta; Spermatophyta; Magnoliopsida; Magnoliidae; Magnoliales; Magnoliaceae; Magnolia"]:0.09799999999999986)[&"FastTree support value"=0.654]:0.029370000000000118)[&"FastTree support value"=0.917]:0.02707000000000015)[&"FastTree support value"=0.643]:0.00981999999999994,(((XP_002297993.1:0.10568,XP_024439231.1:0.11258999999999997)[&"FastTree support value"=0.907]:0.03385000000000016,XP_002303204.3[&Organism="Populus trichocarpa","Genetic Code"="Standard",Taxonomy="Eukaryota; Viridiplantae; Streptophyta; Embryophyta; Tracheophyta; Spermatophyta; Magnoliopsida; eudicotyledons; Gunneridae; Pentapetalae; rosids; fabids; Malpighiales; Salicaceae; Saliceae; Populus","Common Name"="Populus balsamifera subsp. trichocarpa"]:0.05905000000000005)[&"FastTree support value"=0.289]:0.005889999999999951,KAF5727250.1[&Organism="Tripterygium wilfordii","Genetic Code"="Standard",Taxonomy="Eukaryota; Viridiplantae; Streptophyta; Embryophyta; Tracheophyta; Spermatophyta; Magnoliopsida; eudicotyledons; Gunneridae; Pentapetalae; rosids; fabids; Celastrales; Celastraceae; Tripterygium"]:0.08562000000000003)[&"FastTree support value"=0.899]:0.021079999999999988)[&"FastTree support value"=0.883]:0.041180000000000216,(KAF8079489.1[&Organism="Sinapis alba","Genetic Code"="Standard",Taxonomy="Eukaryota; Viridiplantae; Streptophyta; Embryophyta; Tracheophyta; Spermatophyta; Magnoliopsida; eudicotyledons; Gunneridae; Pentapetalae; rosids; malvids; Brassicales; Brassicaceae; Brassiceae; Sinapis","Common Name"="white mustard"]:0.04037999999999986,(OAP13353.1:0.06733000000000011,(OAP19580.1:0.08377999999999997,OAP13972.1:0.02854000000000001)[&"FastTree support value"=0.062]:0.000550)[&"FastTree support value"=0.925]:0.08951999999999982)[&"FastTree support value"=0.976]:0.12331000000000003)[&"FastTree support value"=0.992]:0.11549999999999994,((KAH9320939.1[&Organism="Taxus chinensis","Genetic Code"="Standard",Taxonomy="Eukaryota; Viridiplantae; Streptophyta; Embryophyta; Tracheophyta; Spermatophyta; Pinopsida; Pinidae; Conifers II; Cupressales; Taxaceae;

Taxus"]:0.20414999999999983,(KAH9325151.1:0.09968000000000021,(KAH9300179.1:0.11534999999999984,KAH9314974.1:0.21309999999999985)[&"FastTree support value"=0.813]:0.09315000000000007)[&"FastTree support value"=0.997]:0.24404000000000003)[&"FastTree support value"=0.723]:0.03672999999999993,(KAH9290598.1[&Organism="Taxus chinensis","Genetic Code"="Standard",Taxonomy="Eukaryota; Viridiplantae; Streptophyta; Embryophyta; Tracheophyta; Spermatophyta; Pinopsida; Pinidae; Conifers II; Cupressales; Taxaceae; Taxus"]:0.2029399999999999,KAH9291961.1[&Organism="Taxus chinensis","Genetic Code"="Standard",Taxonomy="Eukaryota; Viridiplantae; Streptophyta; Embryophyta; Tracheophyta; Spermatophyta; Pinopsida; Pinidae; Conifers II; Cupressales; Taxaceae; Taxus"]:0.26704000000000017)[&"FastTree support value"=0.867]:0.05946000000000007)[&"FastTree support value"=0.824]:0.02132999999999985)[&"FastTree support value"=0.933]:0.07356000000000007,EFJ22917.1:0.38363000000000014)[&"FastTree support value"=0.905]:0.0607000000000002)[&"FastTree support value"=0.982]:0.15807000000000038)[&"FastTree support value"=1.0]:0.31837999999999944)[&"FastTree support value"=0.517]:0.04588999999999986)[&"FastTree support value"=0.476]:0.07417000000000051)[&"FastTree support value"=0.991]:0.24476999999999904,((((GAX85982.1:0.27283999999999997,(XP_042923301.1:0.21519000000000021,(XP_042924848.1:0.22973,(KAG2488600.1:0.24070999999999998,XP_042924875.1:0.27707000000000015)[&"FastTree support value"=0.981]:0.1080899999999998)[&"FastTree support value"=0.383]:0.05425000000000013)[&"FastTree support value"=0.998]:0.3535300000000001)[&"FastTree support value"=0.999]:0.41606999999999994,((((XP_006457072.1[&Organism="Agaricus bisporus var. bisporus H97","Genetic Code"="Standard",Taxonomy="Eukaryota; Fungi; Dikarya; Basidiomycota; Agaricomycotina; Agaricomycetes; Agaricomycetidae; Agaricales; Agaricaceae; Agaricus"]:0.78864,(XP_006461472.1[&Organism="Agaricus bisporus var. bisporus H97","Genetic Code"="Standard",Taxonomy="Eukaryota; Fungi; Dikarya; Basidiomycota; Agaricomycotina; Agaricomycetes; Agaricomycetidae; Agaricales; Agaricaceae; Agaricus"]:0.12299000000000015,XP_006461433.1[&Organism="Agaricus bisporus var. bisporus H97","Genetic Code"="Standard",Taxonomy="Eukaryota; Fungi; Dikarya; Basidiomycota; Agaricomycotina; Agaricomycetes; Agaricomycetidae; Agaricales; Agaricaceae; Agaricus"]:0.11629000000000023)[&"FastTree support value"=1.0]:0.44593000000000016)[&"FastTree support value"=0.882]:0.12266000000000021,XP_750654.1[&Organism="Aspergillus fumigatus Af293","Genetic Code"="Standard",Taxonomy="Eukaryota; Fungi; Dikarya; Ascomycota; Pezizomycotina; Eurotiomycetes; Eurotiomycetidae; Eurotiales; Aspergillaceae; Aspergillus; Aspergillus subgen. Fumigati"]:0.87981)[&"FastTree support value"=0.85]:0.14701999999999993,(KAI3646081.1[&Organism="Amoeboaphelidium protococcarum","Genetic Code"="Ciliate",Taxonomy="Eukaryota; Aphelida; Aphelidea; Amoeboaphelidium"]:0.48175999999999997,OAJ38670.1[&Organism="Batrachochytrium dendrobatidis JEL423","Genetic Code"="Standard",Taxonomy="Eukaryota; Fungi; Fungi

incertae sedis; Chytridiomycota; Chytridiomycota incertae sedis; Chytridiomycetes; Rhizophydiales; Rhizophydiales incertae sedis; Batrachochytrium"]:0.4142800000000002)[&"FastTree support value"=0.851]:0.1600999999999999)[&"FastTree support value"=0.633]:0.11839999999999984,KAJ9515210.1:0.4977900000000002)[&"FastTree support value"=0.716]:0.10646999999999984)[&"FastTree support value"=1.0]:0.40085000000000015,(((KAF9951223.1[&"% Charged Amino Acids"=29.71%,"% Acidic Amino Acids"=15.22%,"% Hydrophobic Amino Acids"=45.65%,Modified=Fri Jun 28 11:51:59 PDT 2024,"% GC-rich Amino Acids"=21.01%,"Molecular Weight (kDa)"=31.347671079999994,"Extinction Coefficient"=27055.0,"# Nucleotide Sequences With Quality"=0,Topology="linear","Alignment method"="MAFFT Alignment",Created=Fri Jun 28 11:46:17 PDT 2024,"Charge at pH 7"=-6.773009592825655,"Isoelectric Point"=5.118595123291016,"% Basic Amino Acids"=14.49%,"% AT-rich Amino Acids"=23.55%,"% Polar Uncharged Amino Acids"=25.72%,"Free end gaps"=true,"Molecule Type"="AA"]:0.78444,(OLL24579.1[&"% Charged Amino Acids"=26.39%,"% Acidic Amino Acids"=12.15%,"% Hydrophobic Amino Acids"=44.79%,Modified=Fri Jun 28 11:46:17 PDT 2024,"% GC-rich Amino Acids"=20.49%,"Molecular Weight (kDa)"=31.81140938000001,"Extinction Coefficient"=14565.0,"# Nucleotide Sequences With Quality"=0,Topology="linear","Alignment method"="MAFFT Alignment",Created=Fri Jun 28 11:46:17 PDT 2024,"Charge at pH 7"=3.0255167076407066,"Isoelectric Point"=8.445613861083984,"% Basic Amino Acids"=14.24%,"% AT-rich Amino Acids"=25.69%,"% Polar Uncharged Amino Acids"=29.17%,"Free end gaps"=true,"Molecule Type"="AA"]:0.45570999999999984,((XP_021869222.1[&"% Charged Amino Acids"=32.55%,"% Acidic Amino Acids"=15.77%,"% Hydrophobic Amino Acids"=44.30%,Modified=Fri Jun 28 11:49:08 PDT 2024,"% GC-rich Amino Acids"=21.48%,"Molecular Weight (kDa)"=33.45680067999999,"Extinction Coefficient"=21095.0,"# Nucleotide Sequences With Quality"=0,Topology="linear","Alignment method"="MAFFT Alignment",Created=Fri Jun 28 11:46:17 PDT 2024,"Charge at pH 7"=-6.272273006910691,"Isoelectric Point"=5.793697357177734,"% Basic Amino Acids"=16.78%,"% AT-rich Amino Acids"=21.48%,"% Polar Uncharged Amino Acids"=24.16%,"Free end gaps"=true,"Molecule Type"="AA"]:0.4044500000000002,TVY17522.1[&"% Charged Amino Acids"=29.25%,"% Acidic Amino Acids"=13.95%,"% Hydrophobic Amino Acids"=50.00%,Modified=Fri Jun 28 11:46:17 PDT 2024,"% GC-rich Amino Acids"=22.79%,"Molecular Weight (kDa)"=32.82586588,"Extinction Coefficient"=19605.0,"# Nucleotide Sequences With Quality"=0,Topology="linear","Alignment method"="MAFFT Alignment",Created=Fri Jun 28 11:46:17 PDT 2024,"Charge at pH 7"=-3.4030920147103245,"Isoelectric Point"=6.098857879638672,"% Basic Amino Acids"=15.31%,"% AT-rich Amino Acids"=18.71%,"% Polar Uncharged Amino Acids"=21.77%,"Free end gaps"=true,"Molecule Type"="AA"]:0.33618000000000015)[&"FastTree support value"=1.0]:0.4367000000000001,((XP_026607910.1[&"% Charged Amino Acids"=25.93%,"% Acidic Amino Acids"=11.11%,"% Hydrophobic Amino Acids"=48.82%,Modified=Fri Jun 28 11:46:17 PDT 2024,"% GC-rich Amino

Acids"=24.58%,"Molecular Weight (kDa)"=32.72361798000001,"Extinction Coefficient"=11710.0,"# Nucleotide Sequences With Quality"=0,Topology="linear","Alignment method"="MAFFT Alignment",Created=Fri Jun 28 11:46:17 PDT 2024,"Charge at pH 7"=2.553564522492767,"Isoelectric Point"=7.953372955322266,"% Basic Amino Acids"=14.81%,"% AT-rich Amino Acids"=22.56%,"% Polar Uncharged Amino Acids"=25.59%,"Free end gaps"=true,"Molecule Type"="AA"]:0.33679000000000014,(XP_748757.2[&Organism="Aspergillus fumigatus Af293","Genetic Code"="Standard",Taxonomy="Eukaryota; Fungi; Dikarya; Ascomycota; Pezizomycotina; Eurotiomycetes; Eurotiomycetidae; Eurotiales; Aspergillaceae; Aspergillus; Aspergillus subgen. Fumigati"]:0.35790999999999995,(XP_040633937.1[&"% Charged Amino Acids"=27.74%,"% Acidic Amino Acids"=14.04%,"% Hydrophobic Amino Acids"=51.71%,Modified=Fri Jun 28 11:46:17 PDT 2024,"% GC-rich Amino Acids"=27.40%,"Molecular Weight (kDa)"=31.85812448,"Extinction Coefficient"=7115.0,"# Nucleotide Sequences With Quality"=0,Topology="linear","Alignment method"="MAFFT Alignment",Created=Fri Jun 28 11:46:17 PDT 2024,"Charge at pH 7"=-11.101735485136608,"Isoelectric Point"=5.194911956787109,"% Basic Amino Acids"=13.70%,"% AT-rich Amino Acids"=17.12%,"% Polar Uncharged Amino Acids"=20.89%,"Free end gaps"=true,"Molecule Type"="AA"]:0.18232999999999988,(XP_043140374.1[&"% Charged Amino Acids"=27.74%,"% Acidic Amino Acids"=14.38%,"% Hydrophobic Amino Acids"=51.03%,Modified=Fri Jun 28 11:46:17 PDT 2024,"% GC-rich Amino Acids"=26.71%,"Molecular Weight (kDa)"=32.242680480000004,"Extinction Coefficient"=7115.0,"# Nucleotide Sequences With Quality"=0,Topology="linear","Alignment method"="MAFFT Alignment",Created=Fri Jun 28 11:46:17 PDT 2024,"Charge at pH 7"=-8.599323914913342,"Isoelectric Point"=4.979366302490234,"% Basic Amino Acids"=13.36%,"% AT-rich Amino Acids"=20.21%,"% Polar Uncharged Amino Acids"=21.58%,"Free end gaps"=true,"Molecule Type"="AA"]:0.04653999999999804,XP_754266.1[&Organism="Aspergillus fumigatus Af293","Genetic Code"="Standard",Taxonomy="Eukaryota; Fungi; Dikarya; Ascomycota; Pezizomycotina; Eurotiomycetes; Eurotiomycetidae; Eurotiales; Aspergillaceae; Aspergillus; Aspergillus subgen. Fumigati"]:0.07710999999999979)[&"FastTree support value"=0.968]:0.11267000000000005)[&"FastTree support value"=0.992]:0.1656200000000001)[&"FastTree support value"=0.832]:0.05567000000000011)[&"FastTree support value"=1.0]:0.35279000000000016,(KAI9096888.1[&"% Charged Amino Acids"=30.14%,"% Acidic Amino Acids"=15.07%,"% Hydrophobic Amino Acids"=44.18%,Modified=Fri Jun 28 11:51:56 PDT 2024,"% GC-rich Amino Acids"=17.47%,"Molecular Weight (kDa)"=33.101868080000024,"Extinction Coefficient"=20065.0,"# Nucleotide Sequences With Quality"=0,Topology="linear","Alignment method"="MAFFT Alignment",Created=Fri Jun 28 11:46:17 PDT 2024,"Charge at pH 7"=-4.760784008823309,"Isoelectric Point"=5.568431854248047,"% Basic Amino Acids"=15.07%,"% AT-rich Amino Acids"=25.68%,"% Polar Uncharged Amino Acids"=26.37%,"Free end gaps"=true,"Molecule Type"="AA"]:0.4982099999999998,((((XP_041144356.1[&"% Charged Amino Acids"=26.92%,"% Acidic Amino Acids"=12.24%,"% Hydrophobic Amino

Acids"=48.25%,Modified=Fri Jun 28 11:46:17 PDT 2024,"% GC-rich Amino Acids"=22.03%,"Molecular Weight (kDa)"=31.70809888000001,"Extinction Coefficient"=17085.0,"# Nucleotide Sequences With Quality"=0,Topology="linear","Alignment method"="MAFFT Alignment",Created=Fri Jun 28 11:46:17 PDT 2024,"Charge at pH 7"=-3.1075449530009323,"Isoelectric Point"=6.306392669677734,"% Basic Amino Acids"=14.69%,"% AT-rich Amino Acids"=19.58%,"% Polar Uncharged Amino Acids"=25.52%,"Free end gaps"=true,"Molecule Type"="AA"]:0.08279000000000014,XP_746402.1[&Organism="Aspergillus fumigatus Af293","Genetic Code"="Standard",Taxonomy="Eukaryota; Fungi; Dikarya; Ascomycota; Pezizomycotina; Eurotiomycetes; Eurotiomycetidae; Eurotiales; Aspergillaceae; Aspergillus; Aspergillus subgen. Fumigati"]:0.12986999999999993)[&"FastTree support value"=0.843]:0.03230000000000022,(KAJ5704467.1[&"% Charged Amino Acids"=24.13%,"% Acidic Amino Acids"=11.89%,"% Hydrophobic Amino Acids"=49.30%,Modified=Fri Jun 28 11:46:17 PDT 2024,"% GC-rich Amino Acids"=23.78%,"Molecular Weight (kDa)"=31.15122537999999,"Extinction Coefficient"=19605.0,"# Nucleotide Sequences With Quality"=0,Topology="linear","Alignment method"="MAFFT Alignment",Created=Fri Jun 28 11:46:17 PDT 2024,"Charge at pH 7"=-5.574711435859037,"Isoelectric Point"=5.575748443603516,"% Basic Amino Acids"=12.24%,"% AT-rich Amino Acids"=15.73%,"% Polar Uncharged Amino Acids"=27.62%,"Free end gaps"=true,"Molecule Type"="AA"]:0.09892000000000012,(XP_002543522.1[&"% Charged Amino Acids"=26.92%,"% Acidic Amino Acids"=12.59%,"% Hydrophobic Amino Acids"=48.95%,Modified=Fri Jun 28 11:46:17 PDT 2024,"% GC-rich Amino Acids"=23.78%,"Molecular Weight (kDa)"=31.483921279999993,"Extinction Coefficient"=14105.0,"# Nucleotide Sequences With Quality"=0,Topology="linear","Alignment method"="MAFFT Alignment",Created=Fri Jun 28 11:46:17 PDT 2024,"Charge at pH 7"=-2.40438839054951,"Isoelectric Point"=6.292827606201172,"% Basic Amino Acids"=14.34%,"% AT-rich Amino Acids"=19.58%,"% Polar Uncharged Amino Acids"=24.83%,"Free end gaps"=true,"Molecule Type"="AA"]:0.1602800000000002,(MCJ1392161.1[&"% Charged Amino Acids"=26.22%,"% Acidic Amino Acids"=13.29%,"% Hydrophobic Amino Acids"=47.90%,Modified=Fri Jun 28 11:46:17 PDT 2024,"% GC-rich Amino Acids"=21.68%,"Molecular Weight (kDa)"=31.422763779999993,"Extinction Coefficient"=22585.0,"# Nucleotide Sequences With Quality"=0,Topology="linear","Alignment method"="MAFFT Alignment",Created=Fri Jun 28 11:46:17 PDT 2024,"Charge at pH 7"=-6.599380872602497,"Isoelectric Point"=5.295635223388672,"% Basic Amino Acids"=12.94%,"% AT-rich Amino Acids"=17.83%,"% Polar Uncharged Amino Acids"=26.92%,"Free end gaps"=true,"Molecule Type"="AA"]:0.07203999999999988,KAI9774215.1[&"% Charged Amino Acids"=25.87%,"% Acidic Amino Acids"=12.24%,"% Hydrophobic Amino Acids"=47.90%,Modified=Fri Jun 28 11:46:17 PDT 2024,"% GC-rich Amino Acids"=21.68%,"Molecular Weight (kDa)"=31.32162668,"Extinction Coefficient"=21095.0,"# Nucleotide Sequences With Quality"=0,Topology="linear","Alignment method"="MAFFT Alignment",Created=Fri Jun 28 11:46:17 PDT 2024,"Charge at pH 7"=-4.3741271905474495,"Isoelectric Point"=6.005184173583984,"% Basic Amino Acids"=13.64%,"% AT-rich Amino

Acids"=18.53%,"% Polar Uncharged Amino Acids"=27.27%,"Free end gaps"=true,"Molecule Type"="AA"]:0.05787999999999993)[&"FastTree support value"=0.923]:0.040939999999999976)[&"FastTree support value"=0.576]:0.03372000000000194)[&"FastTree support value"=0.734]:0.015690000000000204)[&"FastTree support value"=0.998]:0.2950499999999998,XP_751069.1[&Organism="Aspergillus fumigatus Af293","Genetic Code"="Standard",Taxonomy="Eukaryota; Fungi; Dikarya; Ascomycota; Pezizomycotina; Eurotiomycetes; Eurotiomycetidae; Eurotiales; Aspergillaceae; Aspergillus; Aspergillus subgen. Fumigati"]:0.5620600000000002)[&"FastTree support value"=0.913]:0.11726999999999999,RSH87279.1[&"% Charged Amino Acids"=29.37%,"% Acidic Amino Acids"=15.03%,"% Hydrophobic Amino Acids"=47.20%,Modified=Fri Jun 28 11:48:55 PDT 2024,"% GC-rich Amino Acids"=24.13%,"Molecular Weight (kDa)"=31.83822928,"Extinction Coefficient"=7115.0,"# Nucleotide Sequences With Quality"=0,Topology="linear","Alignment method"="MAFFT Alignment",Created=Fri Jun 28 11:46:17 PDT 2024,"Charge at pH 7"=-5.858968677562601,"Isoelectric Point"=5.208797454833984,"% Basic Amino Acids"=14.34%,"% AT-rich Amino Acids"=20.63%,"% Polar Uncharged Amino Acids"=23.78%,"Free end gaps"=true,"Molecule Type"="AA"]:0.48841)[&"FastTree support value"=0.249]:0.06601999999999997)[&"FastTree support value"=0.822]:0.13842)[&"FastTree support value"=0.835]:0.11562999999999999)[&"FastTree support value"=0.2]:0.07139000000000006)[&"FastTree support value"=0.928]:0.15485000000000015)[&"FastTree support value"=0.926]:0.12632000000000065,((XP_047808890.1[&"% Charged Amino Acids"=27.17%,"% Acidic Amino Acids"=12.68%,"% Hydrophobic Amino Acids"=47.10%,Modified=Fri Jun 28 11:49:32 PDT 2024,"% GC-rich Amino Acids"=23.91%,"Molecular Weight (kDa)"=30.56279258,"Extinction Coefficient"=13200.0,"# Nucleotide Sequences With Quality"=0,Topology="linear","Alignment method"="MAFFT Alignment",Created=Fri Jun 28 11:46:17 PDT 2024,"Charge at pH 7"=-1.6440202409101712,"Isoelectric Point"=6.441722869873047,"% Basic Amino Acids"=14.49%,"% AT-rich Amino Acids"=22.83%,"% Polar Uncharged Amino Acids"=26.09%,"Free end gaps"=true,"Molecule Type"="AA"]:0.5031499999999998,KXS17655.1[&"% Charged Amino Acids"=25.81%,"% Acidic Amino Acids"=13.26%,"% Hydrophobic Amino Acids"=53.05%,Modified=Fri Jun 28 11:49:57 PDT 2024,"% GC-rich Amino Acids"=23.30%,"Molecular Weight (kDa)"=30.090607580000007,"Extinction Coefficient"=14105.0,"# Nucleotide Sequences With Quality"=0,Topology="linear","Alignment method"="MAFFT Alignment",Created=Fri Jun 28 11:46:17 PDT 2024,"Charge at pH 7"=-7.601807258076357,"Isoelectric Point"=5.110424041748047,"% Basic Amino Acids"=12.54%,"% AT-rich Amino Acids"=23.30%,"% Polar Uncharged Amino Acids"=21.86%,"Free end gaps"=true,"Molecule Type"="AA"]:0.45002999999999993)[&"FastTree support value"=0.889]:0.1417600000000001,KAI8587516.1[&"% Charged Amino Acids"=22.91%,"% Acidic Amino Acids"=11.27%,"% Hydrophobic Amino Acids"=52.00%,Modified=Fri Jun 28 11:50:12 PDT 2024,"% GC-rich Amino Acids"=23.27%,"Molecular Weight

(kDa)"=30.21366338000001,"Extinction Coefficient"=24075.0,"# Nucleotide Sequences With Quality"=0,Topology="linear","Alignment method"="MAFFT Alignment",Created=Fri Jun 28 11:46:17 PDT 2024,"Charge at pH 7"=-3.7092413986402963,"Isoelectric Point"=5.703121185302734,"% Basic Amino Acids"=11.64%,"% AT-rich Amino Acids"=21.82%,"% Polar Uncharged Amino Acids"=26.18%,"Free end gaps"=true,"Molecule Type"="AA"]:0.6553999999999998)[&"FastTree support value"=0.913]:0.1619000000000006)[&"FastTree support value"=0.973]:0.1963399999999993,((('KAJ3066410.1'[&"% Charged Amino Acids"=27.50%,"% Acidic Amino Acids"=15.00%,"% Hydrophobic Amino Acids"=47.14%,Modified=Fri Jun 28 11:50:27 PDT 2024,"% GC-rich Amino Acids"=22.14%,"Molecular Weight (kDa)"=30.847098080000013,"Extinction Coefficient"=13075.0,"# Nucleotide Sequences With Quality"=0,Topology="linear","Alignment method"="MAFFT Alignment",Created=Fri Jun 28 11:46:17 PDT 2024,"Charge at pH 7"=-10.798238550692695,"Isoelectric Point"=4.645633697509766,"% Basic Amino Acids"=12.50%,"% AT-rich Amino Acids"=21.07%,"% Polar Uncharged Amino Acids"=25.71%,"Free end gaps"=true,"Molecule Type"="AA"]:0.09438000000000013,KAI9324922.1[&"% Charged Amino Acids"=24.91%,"% Acidic Amino Acids"=13.17%,"% Hydrophobic Amino Acids"=46.26%,Modified=Fri Jun 28 11:50:37 PDT 2024,"% GC-rich Amino Acids"=21.71%,"Molecular Weight (kDa)"=30.80993478000001,"Extinction Coefficient"=13075.0,"# Nucleotide Sequences With Quality"=0,Topology="linear","Alignment method"="MAFFT Alignment",Created=Fri Jun 28 11:46:17 PDT 2024,"Charge at pH 7"=-7.798989763504893,"Isoelectric Point"=4.897228240966797,"% Basic Amino Acids"=11.74%,"% AT-rich Amino Acids"=21.71%,"% Polar Uncharged Amino Acids"=29.18%,"Free end gaps"=true,"Molecule Type"="AA"]:0.18867000000000012)[&"FastTree support value"=0.771]:0.059719999999999995,(KAI8836453.1[&"% Charged Amino Acids"=29.14%,"% Acidic Amino Acids"=13.67%,"% Hydrophobic Amino Acids"=46.76%,Modified=Fri Jun 28 11:50:45 PDT 2024,"% GC-rich Amino Acids"=22.30%,"Molecular Weight (kDa)"=30.927449480000007,"Extinction Coefficient"=13200.0,"# Nucleotide Sequences With Quality"=0,Topology="linear","Alignment method"="MAFFT Alignment",Created=Fri Jun 28 11:46:17 PDT 2024,"Charge at pH 7"=-3.4437602930013576,"Isoelectric Point"=6.172344207763672,"% Basic Amino Acids"=15.47%,"% AT-rich Amino Acids"=21.94%,"% Polar Uncharged Amino Acids"=24.46%,"Free end gaps"=true,"Molecule Type"="AA"]:0.17702000000000018,KAJ3350919.1[&"% Charged Amino Acids"=24.29%,"% Acidic Amino Acids"=12.14%,"% Hydrophobic Amino Acids"=49.64%,Modified=Fri Jun 28 11:50:59 PDT 2024,"% GC-rich Amino Acids"=20.71%,"Molecular Weight (kDa)"=30.724561180000016,"Extinction Coefficient"=13200.0,"# Nucleotide Sequences With Quality"=0,Topology="linear","Alignment method"="MAFFT Alignment",Created=Fri Jun 28 11:46:17 PDT 2024,"Charge at pH 7"=-4.908779490673721,"Isoelectric Point"=5.475826263427734,"% Basic Amino Acids"=12.14%,"% AT-rich Amino Acids"=24.64%,"% Polar Uncharged Amino Acids"=26.43%,"Free end gaps"=true,"Molecule Type"="AA"]:0.3658800000000002)[&"FastTree support value"=0.763]:0.11802000000000001)[&"FastTree support

value"=1.0]:0.5563099999999999,(KAK3283006.1[&Organism="Cymbomonas tetramitiformis","Genetic Code"="Standard",Taxonomy="Eukaryota; Viridiplantae; Chlorophyta; Pyramimonadophyceae; Pyramimonadales; Pyramimonadaceae; Cymbomonas"]:0.5175000000000001,GHP04420.1[&Organism="Pycnococcus provasolii","Genetic Code"="Standard",Taxonomy="Eukaryota; Viridiplantae; Chlorophyta; Pycnococcaceae; Pycnococcus"]:0.40458000000000016)[&"FastTree support value"=0.975]:0.22318999999999978)[&"FastTree support value"=0.696]:0.10013999999999967)[&"FastTree support value"=0.948]:0.19572000000000056)[&"FastTree support value"=0.977]:0.1541800000000002,((XP_006815062.1[&Organism="Saccoglossus kowalevskii","Genetic Code"="Standard",Taxonomy="Eukaryota; Metazoa; Hemichordata; Enteropneusta; Harrimaniidae; Saccoglossus"]:0.39963000000000015,(CAH1802128.1[&Organism="Owenia fusiformis","Genetic Code"="Standard",Taxonomy="Eukaryota; Metazoa; Spiralia; Lophotrochozoa; Annelida; Polychaeta; Sedentaria; Canalipalpata; Sabellida; Oweniida; Oweniidae; Owenia"]:0.49373999999999985,((PAA83069.1[&Organism="Macrostomum lignano","Genetic Code"="Standard",Taxonomy="Eukaryota; Metazoa; Platyhelminthes; Rhabditophora; Macrostomorpha; Macrostomida; Macrostomidae; Macrostomum"]:0.09462999999999999,PAA94353.1[&Organism="Macrostomum lignano","Genetic Code"="Standard",Taxonomy="Eukaryota; Metazoa; Platyhelminthes; Rhabditophora; Macrostomorpha; Macrostomida; Macrostomidae; Macrostomum"]:0.16556000000000015)[&"FastTree support value"=1.0]:0.5450200000000001,((PAA74204.1[&Organism="Macrostomum lignano","Genetic Code"="Standard",Taxonomy="Eukaryota; Metazoa; Platyhelminthes; Rhabditophora; Macrostomorpha; Macrostomida; Macrostomidae; Macrostomum"]:0.12822999999999984,PAA76532.1[&Organism="Macrostomum lignano","Genetic Code"="Standard",Taxonomy="Eukaryota; Metazoa; Platyhelminthes; Rhabditophora; Macrostomorpha; Macrostomida; Macrostomidae; Macrostomum"]:0.2512599999999998)[&"FastTree support value"=0.998]:0.24244999999999983,(PAA92268.1[&Organism="Macrostomum lignano","Genetic Code"="Standard",Taxonomy="Eukaryota; Metazoa; Platyhelminthes; Rhabditophora; Macrostomorpha; Macrostomida; Macrostomidae; Macrostomum"]:0.3074699999999999,PAA69582.1[&Organism="Macrostomum lignano","Genetic Code"="Standard",Taxonomy="Eukaryota; Metazoa; Platyhelminthes; Rhabditophora; Macrostomorpha; Macrostomida; Macrostomidae; Macrostomum"]:0.3029799999999998)[&"FastTree support value"=0.917]:0.11024000000000012)[&"FastTree support value"=0.942]:0.11650999999999989)[&"FastTree support value"=0.948]:0.1136499999999998)[&"FastTree support value"=0.933]:0.08865000000000034)[&"FastTree support value"=0.855]:0.062129999999999797,((((XP_032804093.1[&Organism="Petromyzon marinus","Genetic Code"="Standard",Taxonomy="Eukaryota; Metazoa; Chordata; Craniata; Vertebrata; Cyclostomata; Hyperoartia; Petromyzontiformes; Petromyzontidae; Petromyzon","Common Name"="sea

lamprey"]:0.44564999999999966,((XP_003973512.2.2[&Organism="Takifugu rubripes","Genetic Code"="Standard",Taxonomy="Eukaryota; Metazoa; Chordata; Craniata; Vertebrata; Euteleostomi; Actinopterygii; Neopterygii; Teleostei; Neoteleostei; Acanthomorphata; Eupercaria; Tetraodontiformes; Tetradontoidea; Tetraodontidae; Takifugu","Common Name"="torafugu"]:0.10187999999999997,(NP_891987.2.2[&Organism="Danio rerio","Genetic Code"="Standard",Taxonomy="Eukaryota; Metazoa; Chordata; Craniata; Vertebrata; Euteleostomi; Actinopterygii; Neopterygii; Teleostei; Ostariophysi; Cypriniformes; Cyprinidae; Danio","Common Name"="zebrafish"]:0.03521999999999981,XP_009304072.1[&Organism="Danio rerio","Genetic Code"="Standard",Taxonomy="Eukaryota; Metazoa; Chordata; Craniata; Vertebrata; Euteleostomi; Actinopterygii; Neopterygii; Teleostei; Ostariophysi; Cypriniformes; Cyprinidae; Danio","Common Name"="zebrafish"]:0.011730000000000018)[&"FastTree support value"=0.997]:0.09100999999999981)[&"FastTree support value"=0.984]:0.09715000000000007,(AGU16245.1[&db_xref="taxon:27779",Organism="Protopterus dolloi","Genetic Code"="Standard",Modified=Mon Mar 27 11:17:17 PDT 2023,Taxonomy="Eukaryota; Metazoa; Chordata; Craniata; Vertebrata; Euteleostomi; Dipnoi; Lepidosireniformes; Protopteridae; Protopterus",Accession="AGU16245.1","Common Name"="slender lungfish",Topology="linear","Molecule Type"="AA"]:0.14762999999999948,((XP_007904885.1[&Organism="Callorhinchus milii","Genetic Code"="Standard",Taxonomy="Eukaryota; Metazoa; Chordata; Craniata; Vertebrata; Chondrichthyes; Holocephali; Chimaeriformes; Callorhinchidae; Callorhinchus","Common Name"="elephant shark"]:0.11882999999999999,XP_032888405.1[&Organism="Amblyraja radiata","Genetic Code"="Standard",Taxonomy="Eukaryota; Metazoa; Chordata; Craniata; Vertebrata; Chondrichthyes; Elasmobranchii; Batoidea; Rajiformes; Rajidae; Amblyraja","Common Name"="thorny skate"]:0.13264999999999993)[&"FastTree support value"=0.979]:0.07698999999999989,(((((((((NP_776366.1[&Organism="Bos taurus","Genetic Code"="Standard",Taxonomy="Eukaryota; Metazoa; Chordata; Craniata; Vertebrata; Euteleostomi; Mammalia; Eutheria; Laurasiatheria; Artiodactyla; Ruminantia; Pecora; Bovidae; Bovinae; Bos","Common Name"="cattle"]:0.1025999999999998,((XP_012586448.1[&Organism="Condylura cristata","Genetic Code"="Standard",Taxonomy="Eukaryota; Metazoa; Chordata; Craniata; Vertebrata; Euteleostomi; Mammalia; Eutheria; Laurasiatheria; Insectivora; Talpidae; Condylura","Common Name"="star-nosed mole"]:0.22780999999999985,XP_005885748.1[&Organism="Myotis brandtii","Genetic Code"="Standard",Taxonomy="Eukaryota; Metazoa; Chordata; Craniata; Vertebrata; Euteleostomi; Mammalia; Eutheria; Laurasiatheria; Chiroptera; Microchiroptera; Vespertilionidae; Myotis","Common Name"="Brandt's bat"]:0.015330000000000066)[&"FastTree support value"=0.856]:0.017549999999999955,XP_017508123.1[&Organism="Manis javanica","Genetic Code"="Standard",Taxonomy="Eukaryota; Metazoa; Chordata; Craniata;

Vertebrata; Euteleostomi; Mammalia; Eutheria; Laurasiatheria; Pholidota; Manidae; Manis","Common Name"="Malayan pangolin"]:0.07516999999999996)[&"FastTree support value"=0.706]:0.008430000000000016)[&"FastTree support value"=0.727]:0.016950000000000002,(NP_001003133.1[&Organism="Canis lupus familiaris","Genetic Code"="Standard",Taxonomy="Eukaryota; Metazoa; Chordata; Craniata; Vertebrata; Euteleostomi; Mammalia; Eutheria; Laurasiatheria; Carnivora; Caniformia; Canidae; Canis","Common Name"="dog"]:0.07214000000000009,XP_032211320.1[&Organism="Mustela erminea","Genetic Code"="Standard",Taxonomy="Eukaryota; Metazoa; Chordata; Craniata; Vertebrata; Euteleostomi; Mammalia; Eutheria; Laurasiatheria; Carnivora; Caniformia; Mustelidae; Mustelinae; Mustela","Common Name"="ermine"]:0.1015600000000001)[&"FastTree support value"=0.964]:0.04037999999999986)[&"FastTree support value"=0.245]:0.008220000000000116,XP_008569440.1[&Organism="Galeopterus variegatus","Genetic Code"="Standard",Taxonomy="Eukaryota; Metazoa; Chordata; Craniata; Vertebrata; Euteleostomi; Mammalia; Eutheria; Euarchontoglires; Dermoptera; Cynocephalidae; Galeopterus","Common Name"="Sunda flying lemur"]:0.04604000000000008)[&"FastTree support value"=0.861]:0.014339999999999797,(NP_002454.1[&Organism="Homo sapiens","Genetic Code"="Standard",Taxonomy="Eukaryota; Metazoa; Chordata; Craniata; Vertebrata; Euteleostomi; Mammalia; Eutheria; Euarchontoglires; Primates; Haplorrhini; Catarrhini; Hominidae; Homo","Common Name"="human"]:0.005320000000002134,XP_002830747.1[&Organism="Pongo abelii","Genetic Code"="Standard",Taxonomy="Eukaryota; Metazoa; Chordata; Craniata; Vertebrata; Euteleostomi; Mammalia; Eutheria; Euarchontoglires; Primates; Haplorrhini; Catarrhini; Hominidae; Pongo","Common Name"="Sumatran orangutan"]:0.011060000000000007)[&"FastTree support value"=0.999]:0.07412999999999981)[&"FastTree support value"=0.812]:0.014339999999999797,XP_006156438.1[&Organism="Tupaia chinensis","Genetic Code"="Standard",Taxonomy="Eukaryota; Metazoa; Chordata; Craniata; Vertebrata; Euteleostomi; Mammalia; Eutheria; Euarchontoglires; Scandentia; Tupaiidae; Tupaia","Common Name"="Chinese tree shrew"]:0.11734)[&"FastTree support value"=1.0]:0.10684000000000005,(XP_004675614.2.2[&Organism="Condylura cristata","Genetic Code"="Standard",Taxonomy="Eukaryota; Metazoa; Chordata; Craniata; Vertebrata; Euteleostomi; Mammalia; Eutheria; Laurasiatheria; Insectivora; Talpidae; Condylura","Common Name"="star-nosed mole"]:0.12102000000000013,((((XP_006156437.1[&Organism="Tupaia chinensis","Genetic Code"="Standard",Taxonomy="Eukaryota; Metazoa; Chordata; Craniata; Vertebrata; Euteleostomi; Mammalia; Eutheria; Euarchontoglires; Scandentia; Tupaiidae; Tupaia","Common Name"="Chinese tree shrew"]:0.05886000000000134,(NP_038634.1[&Organism="Mus musculus","Genetic Code"="Standard",Taxonomy="Eukaryota; Metazoa; Chordata; Craniata; Vertebrata; Euteleostomi; Mammalia; Eutheria; Euarchontoglires; Glires; Rodentia; Myomorpha; Muroidea; Muridae; Murinae; Mus; Mus","Common Name"="house

mouse"]:0.007699999999999818,NP_034976.1[&Organism="Mus musculus","Genetic Code"="Standard",Taxonomy="Eukaryota; Metazoa; Chordata; Craniata; Vertebrata; Euteleostomi; Mammalia; Eutheria; Euarchontoglires; Glires; Rodentia; Myomorpha; Muroidea; Muridae; Murinae; Mus; Mus","Common Name"="house mouse"]:0.05378999999999978)[&"FastTree support value"=1.0]:0.10926999999999998)[&"FastTree support value"=0.703]:0.007099999999999884,(XP_017508130.1[&Organism="Manis javanica","Genetic Code"="Standard",Taxonomy="Eukaryota; Metazoa; Chordata; Craniata; Vertebrata; Euteleostomi; Mammalia; Eutheria; Laurasiatheria; Pholidota; Manidae; Manis","Common Name"="Malayan pangolin"]:0.05543999999999934,(NP_001003134.1[&Organism="Canis lupus familiaris","Genetic Code"="Standard",Taxonomy="Eukaryota; Metazoa; Chordata; Craniata; Vertebrata; Euteleostomi; Mammalia; Eutheria; Laurasiatheria; Carnivora; Caniformia; Canidae; Canis","Common Name"="dog"]:0.018920000000000048,XP_032211398.1[&Organism="Mustela erminea","Genetic Code"="Standard",Taxonomy="Eukaryota; Metazoa; Chordata; Craniata; Vertebrata; Euteleostomi; Mammalia; Eutheria; Laurasiatheria; Carnivora; Caniformia; Mustelidae; Mustelinae; Mustela","Common Name"="ermine"]:0.03506000000000009)[&"FastTree support value"=0.989]:0.04818999999999955)[&"FastTree support value"=0.869]:0.020979999999999777)[&"FastTree support value"=0.441]:0.010110000000000063,((XP_005202045.1[&Organism="Bos taurus","Genetic Code"="Standard",Taxonomy="Eukaryota; Metazoa; Chordata; Craniata; Vertebrata; Euteleostomi; Mammalia; Eutheria; Laurasiatheria; Cetartiodactyla; Ruminantia; Pecora; Bovidae; Bovinae; Bos","Common Name"="cattle"]:0.10773000000000001,(XP_008569442.1[&Organism="Galeopterus variegatus","Genetic Code"="Standard",Taxonomy="Eukaryota; Metazoa; Chordata; Craniata; Vertebrata; Euteleostomi; Mammalia; Eutheria; Euarchontoglires; Dermoptera; Cynocephalidae; Galeopterus","Common Name"="Sunda flying lemur"]:0.06667999999999985,XP_014388412.1[&Organism="Myotis brandtii","Genetic Code"="Standard",Taxonomy="Eukaryota; Metazoa; Chordata; Craniata; Vertebrata; Euteleostomi; Mammalia; Eutheria; Laurasiatheria; Chiroptera; Microchiroptera; Vespertilionidae; Myotis","Common Name"="Brandt's bat"]:0.09224999999999994)[&"FastTree support value"=0.093]:0.01922999999999986)[&"FastTree support value"=0.355]:0.018650000000000055,(NP_002453.2.2[&Organism="Homo sapiens","Genetic Code"="Standard",Taxonomy="Eukaryota; Metazoa; Chordata; Craniata; Vertebrata; Euteleostomi; Mammalia; Eutheria; Euarchontoglires; Primates; Haplorrhini; Catarrhini; Hominidae; Homo","Common Name"="human"]:0.005079999999999973,NP_001127618.1[&Organism="Pongo abelii","Genetic Code"="Standard",Taxonomy="Eukaryota; Metazoa; Chordata; Craniata; Vertebrata; Euteleostomi; Mammalia; Eutheria; Euarchontoglires; Primates; Haplorrhini; Catarrhini; Hominidae; Pongo","Common Name"="Sumatran orangutan"]:0.0013600000000000279)[&"FastTree support

value"=0.992]:0.05180000000000007)[&"FastTree support
value"=0.851]:0.027179999999999982)[&"FastTree support
value"=0.56]:0.012480000000000047,XP_004466363.1[&Organism="Dasypus
novemcinctus","Genetic Code"="Standard",Taxonomy="Eukaryota; Metazoa; Chordata;
Craniata; Vertebrata; Euteleostomi; Mammalia; Eutheria; Xenarthra; Cingulata;
Dasypodidae; Dasypus","Common Name"="nine-banded
armadillo"]:0.08752000000000004)[&"FastTree support
value"=0.942]:0.04219000000000017)[&"FastTree support
value"=0.927]:0.05169999999999986)[&"FastTree support
value"=0.789]:0.055019999999999985,XP_031752404.1[&Organism="Xenopus
tropicalis","Genetic Code"="Standard",Taxonomy="Eukaryota; Metazoa; Chordata;
Craniata; Vertebrata; Euteleostomi; Amphibia; Batrachia; Anura; Pipoidea; Pipidae;
Xenopodinae; Xenopus; Silurana","Common Name"="tropical clawed
frog"]:0.2599800000000001)[&"FastTree support
value"=0.95]:0.0663800000000001,((XP_025933558.1[&Organism="Apteryx rowi","Genetic
Code"="Standard",Taxonomy="Eukaryota; Metazoa; Chordata; Craniata; Vertebrata;
Euteleostomi; Archelosauria; Archosauria; Dinosauria; Saurischia; Theropoda;
Coelurosauria; Aves; Palaeognathae; Apterygiformes; Apterygidae; Apteryx","Common
Name"="Okarito brown kiwi"]:0.05458000000000007,XP_009815891.1[&Organism="Gavia
stellata","Genetic Code"="Standard",Taxonomy="Eukaryota; Metazoa; Chordata; Craniata;
Vertebrata; Euteleostomi; Archelosauria; Archosauria; Dinosauria; Saurischia; Theropoda;
Coelurosauria; Aves; Neognathae; Gaviiformes; Gaviidae; Gavia","Common Name"="red-
throated loon"]:0.0751400000000002)[&"FastTree support
value"=1.0]:0.1471300000000002,(XP_015269256.1[&Organism="Gekko
japonicus","Genetic Code"="Standard",Taxonomy="Eukaryota; Metazoa; Chordata;
Craniata; Vertebrata; Euteleostomi; Lepidosauria; Squamata; Bifurcata; Gekkota;
Gekkonidae; Gekkoninae;
Gekko"]:0.10082000000000013,XP_028583072.1[&Organism="Podarcis muralis","Genetic
Code"="Standard",Taxonomy="Eukaryota; Metazoa; Chordata; Craniata; Vertebrata;
Euteleostomi; Lepidosauria; Squamata; Bifurcata; Unidentata; Episquamata; Laterata;
Lacertibaenia; Lacertidae; Podarcis","Common Name"="Common wall
lizard"]:0.16557999999999984)[&"FastTree support
value"=0.175]:0.026250000000000107)[&"FastTree support
value"=0.838]:0.027820000000000178)[&"FastTree support
value"=0.9]:0.033970000000000056,((NP_001007285.1[&Organism="Danio rerio","Genetic
Code"="Standard",Taxonomy="Eukaryota; Metazoa; Chordata; Craniata; Vertebrata;
Euteleostomi; Actinopterygii; Neopterygii; Teleostei; Ostariophysi; Cypriniformes;
Cyprinidae; Danio","Common
Name"="zebrafish"]:0.0823100000000001,XP_005167721.2.2[&Organism="Danio
rerio","Genetic Code"="Standard",Taxonomy="Eukaryota; Metazoa; Chordata; Craniata;
Vertebrata; Euteleostomi; Actinopterygii; Neopterygii; Teleostei; Ostariophysi;
Cypriniformes; Cyprinidae; Danio","Common
Name"="zebrafish"]:0.03374999999999995)[&"FastTree support
value"=1.0]:0.25863000000000014,XP_028583068.1[&Organism="Podarcis

muralis","Genetic Code"="Standard",Taxonomy="Eukaryota; Metazoa; Chordata; Craniata; Vertebrata; Euteleostomi; Lepidosauria; Squamata; Bifurcata; Unidentata; Episquamata; Laterata; Lacertibaenia; Lacertidae; Podarcis","Common Name"="Common wall lizard"]:0.16215000000000002)[&"FastTree support value"=0.773]:0.02970000000000006)[&"FastTree support value"=0.421]:0.025290000000000035)[&"FastTree support value"=0.542]:0.036809999999999565)[&"FastTree support value"=0.905]:0.08468000000000053)[&"FastTree support value"=0.987]:0.1315799999999996)[&"FastTree support value"=0.962]:0.10011000000000081,(KAI0213370.1[&Organism="Lamellibrachia satsuma","Genetic Code"="Standard",Taxonomy="Eukaryota; Metazoa; Spiralia; Lophotrochozoa; Annelida; Polychaeta; Sedentaria; Canalipalpata; Sabellida; Siboglinidae; Lamellibrachia"]:0.007039999999999935,(KAI0218869.1[&Organism="Lamellibrachia satsuma","Genetic Code"="Standard",Taxonomy="Eukaryota; Metazoa; Spiralia; Lophotrochozoa; Annelida; Polychaeta; Sedentaria; Canalipalpata; Sabellida; Siboglinidae; Lamellibrachia"]:0.09967000000000015,KAI0208044.1[&Organism="Lamellibrachia satsuma","Genetic Code"="Standard",Taxonomy="Eukaryota; Metazoa; Spiralia; Lophotrochozoa; Annelida; Polychaeta; Sedentaria; Canalipalpata; Sabellida; Siboglinidae; Lamellibrachia"]:0.058399999999999785)[&"FastTree support value"=0.349]:0.012960000000000083)[&"FastTree support value"=1.0]:0.37754000000000065)[&"FastTree support value"=0.439]:0.024639999999999773,(XP_046565196.1[&Organism="Haliotis rubra","Genetic Code"="Standard",Taxonomy="Eukaryota; Metazoa; Spiralia; Lophotrochozoa; Mollusca; Gastropoda; Vetigastropoda; Lepetellida; Haliotoidea; Haliotidae; Haliotis","Common Name"="blacklip abalone"]:0.05237999999999987,(((XP_046562919.1[&Organism="Haliotis rubra","Genetic Code"="Standard",Taxonomy="Eukaryota; Metazoa; Spiralia; Lophotrochozoa; Mollusca; Gastropoda; Vetigastropoda; Lepetellida; Haliotoidea; Haliotidae; Haliotis","Common Name"="blacklip abalone"]:0.03655000000000008,XP_046563124.1[&Organism="Haliotis rubra","Genetic Code"="Standard",Taxonomy="Eukaryota; Metazoa; Spiralia; Lophotrochozoa; Mollusca; Gastropoda; Vetigastropoda; Lepetellida; Haliotoidea; Haliotidae; Haliotis","Common Name"="blacklip abalone"]:0.008100000000000218)[&"FastTree support value"=0.214]:0.005739999999999856,((XP_046563126.1[&Organism="Haliotis rubra","Genetic Code"="Standard",Taxonomy="Eukaryota; Metazoa; Spiralia; Lophotrochozoa; Mollusca; Gastropoda; Vetigastropoda; Lepetellida; Haliotoidea; Haliotidae; Haliotis","Common Name"="blacklip abalone"]:0.00627000000000022,XP_046565195.1[&Organism="Haliotis rubra","Genetic Code"="Standard",Taxonomy="Eukaryota; Metazoa; Spiralia; Lophotrochozoa; Mollusca; Gastropoda; Vetigastropoda; Lepetellida; Haliotoidea; Haliotidae; Haliotis","Common Name"="blacklip abalone"]:0.009329999999999838)[&"FastTree support value"=0.981]:0.021720000000000184,XP_046563125.1[&Organism="Haliotis rubra","Genetic Code"="Standard",Taxonomy="Eukaryota; Metazoa; Spiralia; Lophotrochozoa; Mollusca; Gastropoda; Vetigastropoda; Lepetellida; Haliotoidea;

Haliotidae; Haliotis","Common Name"="blacklip
abalone"]:0.02224000000000038)[&"FastTree support
value"=0.854]:0.00828000000000065)[&"FastTree support
value"=0.689]:0.00137000000000934,(XP_046352527.2[&Organism="Haliotis
rufescens","Genetic Code"="Standard",Taxonomy="Eukaryota; Metazoa; Spiralia;
Lophotrochozoa; Mollusca; Gastropoda; Vetigastropoda; Lepetellida; Haliotoidea;
Haliotidae; Haliotis","Common Name"="red
abalone"]:0.018429999999999946,((XP_048258111.1[&Organism="Haliotis
rufescens","Genetic Code"="Standard",Taxonomy="Eukaryota; Metazoa; Spiralia;
Lophotrochozoa; Mollusca; Gastropoda; Vetigastropoda; Lepetellida; Haliotoidea;
Haliotidae; Haliotis","Common Name"="red
abalone"]:0.00619000000000014,((XP_046352531.2[&Organism="Haliotis
rufescens","Genetic Code"="Standard",Taxonomy="Eukaryota; Metazoa; Spiralia;
Lophotrochozoa; Mollusca; Gastropoda; Vetigastropoda; Lepetellida; Haliotoidea;
Haliotidae; Haliotis","Common Name"="red
abalone"]:0.00692000000000037,(XP_048248472.1[&Organism="Haliotis
rufescens","Genetic Code"="Standard",Taxonomy="Eukaryota; Metazoa; Spiralia;
Lophotrochozoa; Mollusca; Gastropoda; Vetigastropoda; Lepetellida; Haliotoidea;
Haliotidae; Haliotis","Common Name"="red
abalone"]:0.000550,(XP_048248473.1[&Organism="Haliotis rufescens","Genetic
Code"="Standard",Taxonomy="Eukaryota; Metazoa; Spiralia; Lophotrochozoa; Mollusca;
Gastropoda; Vetigastropoda; Lepetellida; Haliotoidea; Haliotidae; Haliotis","Common
Name"="red abalone"]:0.0,XP_048248474.1[&Organism="Haliotis rufescens","Genetic
Code"="Standard",Taxonomy="Eukaryota; Metazoa; Spiralia; Lophotrochozoa; Mollusca;
Gastropoda; Vetigastropoda; Lepetellida; Haliotoidea; Haliotidae; Haliotis","Common
Name"="red abalone"]:0.0):0.000550)[&"FastTree support
value"=0.979]:0.02112999999999987)[&"FastTree support
value"=0.879]:0.00633000000000169,ABI53802.1[&Organism="Haliotis discus
discus","Genetic Code"="Standard",Taxonomy="Eukaryota; Metazoa; Lophotrochozoa;
Mollusca; Gastropoda; Vetigastropoda; Haliotoidea; Haliotidae; Haliotis","Common
Name"="disc abalone"]:0.02457000000000203)[&"FastTree support
value"=0.879]:0.006369999999999987)[&"FastTree support
value"=0.638]:0.000540,XP_048248476.1[&Organism="Haliotis rufescens","Genetic
Code"="Standard",Taxonomy="Eukaryota; Metazoa; Spiralia; Lophotrochozoa; Mollusca;
Gastropoda; Vetigastropoda; Lepetellida; Haliotoidea; Haliotidae; Haliotis","Common
Name"="red abalone"]:0.01621000000000058)[&"FastTree support
value"=0.726]:0.004119999999999015)[&"FastTree support
value"=0.589]:0.02927999999999973)[&"FastTree support
value"=0.983]:0.09292999999999996)[&"FastTree support
value"=1.0]:0.34098000000000006)[&"FastTree support
value"=0.585]:0.03621000000000052,(XP_035690836.1[&Organism="Branchiostoma
floridae","Genetic Code"="Standard",Taxonomy="Eukaryota; Metazoa; Chordata;
Cephalochordata; Leptocardii; Amphioxiformes; Branchiostomidae;
Branchiostoma","Common Name"="Florida

lancelet"]:0.18857999999999997,(XP_002608668.1[&Organism="Branchiostoma floridae","Genetic Code"="Standard",Taxonomy="Eukaryota; Metazoa; Chordata; Cephalochordata; Branchiostomidae; Branchiostoma","Common Name"="Florida lancelet"]:0.08423999999999943,XP_019617847.1[&Organism="Branchiostoma belcheri","Genetic Code"="Standard",Taxonomy="Eukaryota; Metazoa; Chordata; Cephalochordata; Branchiostomidae; Branchiostoma","Common Name"="Belcher's lancelet"]:0.11602999999999941)[&"FastTree support value"=0.97]:0.1039500000000011)[&"FastTree support value"=0.995]:0.1633300000000002)[&"FastTree support value"=0.353]:0.05320999999999998)[&"FastTree support value"=0.996]:0.22586999999999957)[&"FastTree support value"=0.934]:0.12231999999999932)[&"FastTree support value"=0.637]:0.09736000000000011)[&"FastTree support value"=0.976]:0.2421400000000009)[&"FastTree support value"=0.105]:0.04995999999999956,((((OAJ38404.1[&Organism="Batrachochytrium dendrobatidis JEL423","Genetic Code"="Standard",Taxonomy="Eukaryota; Fungi; Fungi incertae sedis; Chytridiomycota; Chytridiomycota incertae sedis; Chytridiomycetes; Rhizophydiales; Rhizophydiales incertae sedis; Batrachochytrium"]:0.1757099999999996,((NP_014854.2[&Organism="Saccharomyces cerevisiae S288C","Genetic Code"="Standard",Taxonomy="Eukaryota; Fungi; Dikarya; Ascomycota; Saccharomycotina; Saccharomycetes; Saccharomycetales; Saccharomycetaceae; Saccharomyces"]:0.45218000000000025,XP_752563.1[&Organism="Aspergillus fumigatus Af293","Genetic Code"="Standard",Taxonomy="Eukaryota; Fungi; Dikarya; Ascomycota; Pezizomycotina; Eurotiomycetes; Eurotiomycetidae; Eurotiales; Aspergillaceae; Aspergillus; Aspergillus subgen. Fumigati"]:0.1670600000000002)[&"FastTree support value"=0.969]:0.11093000000000064,(XP_011389557.1[&Organism="Ustilago maydis 521","Genetic Code"="Standard",Taxonomy="Eukaryota; Fungi; Dikarya; Basidiomycota; Ustilaginomycotina; Ustilaginomycetes; Ustilaginales; Ustilaginaceae; Ustilago"]:0.15533000000000108,XP_006459124.1[&Organism="Agaricus bisporus var. bisporus H97","Genetic Code"="Standard",Taxonomy="Eukaryota; Fungi; Dikarya; Basidiomycota; Agaricomycotina; Agaricomycetes; Agaricomycetidae; Agaricales; Agaricaceae; Agaricus"]:0.17001000000000044)[&"FastTree support value"=0.966]:0.08633999999999986)[&"FastTree support value"=0.94]:0.06895999999999969)[&"FastTree support value"=0.39]:0.031010000000000204,OUM67143.1[&Organism="Piromyces sp. E2","Genetic Code"="Standard",Taxonomy="Eukaryota; Fungi; Fungi incertae sedis; Chytridiomycota; Chytridiomycota incertae sedis; Neocallimastigomycetes; Neocallimastigales; Neocallimastigaceae; Piromyces; unclassified Piromyces"]:0.36372999999999944)[&"FastTree support value"=0.355]:0.04178000000000015,KNE54706.1[&Organism="Allomyces macrogynus ATCC 38327","Genetic Code"="Standard",Taxonomy="Eukaryota; Fungi; Fungi incertae sedis; Blastocladiomycota; Blastocladiomycota incertae sedis; Blastocladiomycetes; Blastocladiales; Blastocladiaceae; Allomyces"]:0.22668)[&"FastTree support

value"=0.832]:0.09450999999999965,KXN72852.1[&Organism="Conidiobolus coronatus NRRL 28638","Genetic Code"="Standard",Taxonomy="Eukaryota; Fungi; Fungi incertae sedis; Zoopagomycota; Entomophthoromycotina; Entomophthoromycetes; Entomophthorales; Ancylistaceae; Conidiobolus"]:0.28243999999999936)[&"FastTree support value"=1.0]:0.7017300000000004)[&"FastTree support value"=0.845]:0.06686000000000014,(XP_042914770.1[&Organism="Chlamydomonas reinhardtii","Genetic Code"="Standard",Taxonomy="Eukaryota; Viridiplantae; Chlorophyta; core chlorophytes; Chlorophyceae; CS clade; Chlamydomonadales; Chlamydomonadaceae; Chlamydomonas"]:0.33641000000000076,(EFJ35472.1[&Organism="Selaginella moellendorffii","Genetic Code"="Standard",Taxonomy="Eukaryota; Viridiplantae; Streptophyta; Embryophyta; Tracheophyta; Lycopodiopsida; Selaginellales; Selaginellaceae; Selaginella"]:0.33237000000000005,((KAH9304002.1[&Organism="Taxus chinensis","Genetic Code"="Standard",Taxonomy="Eukaryota; Viridiplantae; Streptophyta; Embryophyta; Tracheophyta; Spermatophyta; Pinopsida; Pinidae; Conifers II; Cupressales; Taxaceae; Taxus"]:0.07298999999999989,(((KAI5070335.1[&Organism="Adiantum capillus-veneris","Genetic Code"="Standard",Taxonomy="Eukaryota; Viridiplantae; Streptophyta; Embryophyta; Tracheophyta; Polypodiopsida; Polypodiidae; Polypodiales; Pteridineae; Pteridaceae; Vittarioideae; Adiantum"]:0.05642000000000014,KAI5070758.1[&Organism="Adiantum capillus-veneris","Genetic Code"="Standard",Taxonomy="Eukaryota; Viridiplantae; Streptophyta; Embryophyta; Tracheophyta; Polypodiopsida; Polypodiidae; Polypodiales; Pteridineae; Pteridaceae; Vittarioideae; Adiantum"]:0.05089000000000077)[&"FastTree support value"=0.89]:0.019549999999999734,((KAG0554580.1[&Organism="Ceratodon purpureus","Genetic Code"="Standard",Taxonomy="Eukaryota; Viridiplantae; Streptophyta; Embryophyta; Bryophyta; Bryophytina; Bryopsida; Dicranidae; Pseudoditrichales; Ditrichaceae; Ceratodon"]:0.005469999999999864,XP_024362051.1[&Organism="Physcomitrium patens","Genetic Code"="Standard",Taxonomy="Eukaryota; Viridiplantae; Streptophyta; Embryophyta; Bryophyta; Bryophytina; Bryopsida; Funariidae; Funariales; Funariaceae; Physcomitrium"]:0.04241999999999999)[&"FastTree support value"=0.969]:0.03190999999999988,(PTQ35749.1[&Organism="Marchantia polymorpha","Genetic Code"="Standard",Taxonomy="Eukaryota; Viridiplantae; Streptophyta; Embryophyta; Marchantiophyta; Marchantiopsida; Marchantiidae; Marchantiales; Marchantiaceae; Marchantia","Common Name"="liverwort"]:0.04879999999999955,KAG0555682.1[&Organism="Ceratodon purpureus","Genetic Code"="Standard",Taxonomy="Eukaryota; Viridiplantae; Streptophyta; Embryophyta; Bryophyta; Bryophytina; Bryopsida; Dicranidae; Pseudoditrichales; Ditrichaceae; Ceratodon"]:0.03536999999999946)[&"FastTree support value"=0.003]:0.009599999999999831)[&"FastTree support value"=0.864]:0.015690000000000204)[&"FastTree support value"=0.898]:0.02414000000000005,(EFJ15047.1[&Organism="Selaginella moellendorffii","Genetic Code"="Standard",Taxonomy="Eukaryota; Viridiplantae; Streptophyta; Embryophyta; Tracheophyta; Lycopodiopsida; Selaginellales;

Selaginellaceae; Selaginella"]:0.000540,EFJ37641.1[&Organism="Selaginella
moellendorffii","Genetic Code"="Standard",Taxonomy="Eukaryota; Viridiplantae;
Streptophyta; Embryophyta; Tracheophyta; Lycopodiopsida; Selaginellales;
Selaginellaceae; Selaginella"]:0.0031699999999999007)[&"FastTree support
value"=0.977]:0.036540000000000046)[&"FastTree support
value"=0.931]:0.032169999999999981)[&"FastTree support
value"=0.537]:0.012970000000000148,(((AAC61784.1[&Organism="Arabidopsis
thaliana","Genetic Code"="Standard",Taxonomy="Eukaryota; Viridiplantae; Streptophyta;
Embryophyta; Tracheophyta; Spermatophyta; Magnoliopsida; eudicotyledons;
Gunneridae; Pentapetalae; rosids; malvids; Brassicales; Brassicaceae; Camelineae;
Arabidopsis","Common Name"="thale
cress"]:0.12905999999999995,XP_052310486.1[&Organism="Populus
trichocarpa","Genetic Code"="Standard",Taxonomy="Eukaryota; Viridiplantae;
Streptophyta; Embryophyta; Tracheophyta; Spermatophyta; Magnoliopsida;
eudicotyledons; Gunneridae; Pentapetalae; rosids; fabids; Malpighiales; Salicaceae;
Saliceae; Populus","Common Name"="Populus balsamifera subsp.
trichocarpa"]:0.057210000000000043)[&"FastTree support
value"=0.981]:0.063930000000000004,KAH9327796.1[&Organism="Taxus
chinensis","Genetic Code"="Standard",Taxonomy="Eukaryota; Viridiplantae; Streptophyta;
Embryophyta; Tracheophyta; Spermatophyta; Pinopsida; Pinidae; Conifers II; Cupressales;
Taxaceae; Taxus"]:0.16982000000000053)[&"FastTree support
value"=0.577]:0.016689999999999965,(PWZ09977.1[&Organism="Zea mays","Genetic
Code"="Standard",Taxonomy="Eukaryota; Viridiplantae; Streptophyta; Embryophyta;
Tracheophyta; Spermatophyta; Magnoliopsida; Liliopsida; Poales; Poaceae; PACMAD
clade; Panicoideae; Andropogonodae; Andropogoneae; Tripsacinae;
Zea"]:0.039069999999999716,ONM18162.1[&Organism="Zea mays","Genetic
Code"="Standard",Taxonomy="Eukaryota; Viridiplantae; Streptophyta; Embryophyta;
Tracheophyta; Spermatophyta; Magnoliopsida; Liliopsida; Poales; Poaceae; PACMAD
clade; Panicoideae; Andropogonodae; Andropogoneae; Tripsacinae;
Zea"]:0.17797000000000018)[&"FastTree support
value"=0.928]:0.033619999999999998)[&"FastTree support
value"=0.611]:0.019770000000000287)[&"FastTree support
value"=0.854]:0.033260000000000029)[&"FastTree support
value"=0.96]:0.099140000000000023)[&"FastTree support
value"=0.979]:0.13506999999999998)[&"FastTree support
value"=0.9]:0.060469999999999958)[&"FastTree support
value"=0.892]:0.051880000000000059)[&"FastTree support
value"=1.0]:0.20129999999999998)[&"FastTree support
value"=0.802]:0.023759999999999337)[&"FastTree support
value"=0.845]:0.022680000000000256,((XP_014148725.1[&Organism="Sphaeroforma
arctica JP610","Genetic Code"="Standard",Taxonomy="Eukaryota; Ichthyosporea;
Ichthyophonida;
Sphaeroforma"]:0.10878000000000032,XP_014153758.1[&Organism="Sphaeroforma
arctica JP610","Genetic Code"="Standard",Taxonomy="Eukaryota; Ichthyosporea;

Ichthyophonida; Sphaeroforma"]:0.07723000000000013)[&"FastTree support value"=0.99]:0.07193000000000005,((XP_030853442.1.2[&Organism="Strongylocentrotus purpuratus","Genetic Code"="Standard",Taxonomy="Eukaryota; Metazoa; Echinodermata; Eleutherozoa; Echinozoa; Echinoidea; Euechinoidea; Echinacea; Echinoida; Strongylocentrotidae; Strongylocentrotus","Common Name"="purple sea urchin"]:0.0,XP_030853442.1[&Organism="Strongylocentrotus purpuratus","Genetic Code"="Standard",Taxonomy="Eukaryota; Metazoa; Echinodermata; Eleutherozoa; Echinozoa; Echinoidea; Euechinoidea; Echinacea; Echinoida; Strongylocentrotidae; Strongylocentrotus","Common Name"="purple sea urchin"]:0.0):0.08388000000000062,XP_001749319.1[&Organism="Monosiga brevicollis MX1","Genetic Code"="Standard",Taxonomy="Eukaryota; Choanoflagellata; Craspedida; Salpingoecidae; Monosiga"]:0.12720999999999982)[&"FastTree support value"=0.767]:0.016960000000000086)[&"FastTree support value"=0.65]:0.006249999999999645)[&"FastTree support value"=0.834]:0.021239999999999704)[&"FastTree support value"=0.23]:0.013710000000000555);
end;
