# Supplementary material for: Antiviral Mx proteins have an ancient origin and widespread distribution among eukaryotes
Source: Proc Natl Acad Sci U S A. 2025 Jan 24;122(4):e2416811122. doi: 10.1073/pnas.2416811122 (PMC11789081; doi:10.1073/pnas.2416811122)
Supplement: Supplementary file 7 — Dataset S06 (PDF) [file pnas.2416811122.sd06.pdf]

Dataset S6. Figure_2_MAFFT

>XP_026693152.1
------------IDLPQIAVVGG----Q----------------SAGKSSVLE-----N---FV-GKDFLPRG---SGIVTRRPL-
--------VLQ---L-ITAK-NG---EW---
G-----------------------------------E----------------FLH---CKG-----
-KKF-----TDFN---EIRKEIEEETDR-----MTGSN-------------------KG------------------------IS--
AIPINLRVHSPH--VLNLTLVDLPGMTK--
VP-------------------VGDQPA----------DI---EQQIR--------DMIMQFVVK------------
DNCLILAVSPANS--DLA-NS--DALKIAKEFDPQGIRTIGVI-TKLDLMDEGT--------DAKHILENK---H-
--------------------LPLR-RGYVGV---------------------------------VNR-----------------------------------
S-QKDID----G---------------------------NKDIK----------A----ALSA---ERRFF---------LS-------HP-----
---------------AY-----------------R-H------M--------------------------ADK-LGT-----
PYLQKILNQQLTNHIKETL----
>XP_030853442.1.2
------------LDLPQIAVVGG----Q----------------SAGKSSVLE-----N---FV-GRDFLPRG---
SGIVTRRPL---------VLQ---L-N-NS-KT---
---------EY---G-----------------------------------E----------------FLH---------------------------------------
-----CKG------KKF-----TDFD---EIRKEIEAETDR-----VTGSN-------------------KG-----------------------
--IS--NVPINLRVYSPN--
VLNLTLVDLPGMTK--IA-------------------VGDQPV----------DI---EIQIR--------SMVMEFVTN---------
---ESTLILAVSPANQ--DLA-NS--DALKVAKEVDPKGVRTIGVI-TKLDLMDDGT--------DAKDILENK--
-L---------------------LPLR-RGYVGV---------------------------------VNR----------------------------------
--S-QRDIE----G---------------------------KKDIK----------A----ALAA---ERKFF---------LS-------HP----
----------------SY-----------------R-H------I--------------------------ADK-MGT-----
PWLQKILNQQLTNHIRDSL----
>XP_030853442.1
------------LDLPQIAVVGG----Q----------------SAGKSSVLE-----N---FV-GRDFLPRG---
SGIVTRRPL---------VLQ---L-N-NS-KT---
---------EY---G-----------------------------------E----------------FLH---------------------------------------
-----CKG------KKF-----TDFD---EIRKEIEAETDR-----VTGSN-------------------KG-----------------------
--IS--NVPINLRVYSPN--
VLNLTLVDLPGMTK--IA-------------------VGDQPV----------DI---EIQIR--------SMVMEFVTN---------
---ESTLILAVSPANQ--DLA-NS--DALKVAKEVDPKGVRTIGVI-TKLDLMDDGT--------DAKDILENK--
-L---------------------LPLR-RGYVGV---------------------------------VNR----------------------------------
--S-QRDIE----G---------------------------KKDIK----------A----ALAA---ERKFF---------LS-------HP----
----------------SY-----------------R-H------I--------------------------ADK-MGT-----
PWLQKILNQQLTNHIRDSL----
>XP_005165639.1
------------LDLPQIAVVGG----Q----------------SAGKSSVLE-----N---FV-GKDFLPRG---
SGIVTRRPL---------VLQ---L-I-NC-PT---
--------EY---A-----------------------------------E----------------FLH--
---CKG------KKF-----TDFD---EVRQEIEAETDR-----ITGQN-------------------KG-----------------------
-IS--PVPINLRVYSPN--

VLNLTLVDLPGMTK--VP-------------------VGDQPA----------DI---EAQIR--------DMLMQFVTK-------
-----ENCLLLAVSPANS--DLA-NS--DALKIAKEVDPQGMRTIGVI-TKLDLMDEGT--------
DAREILENK---L---------------------LPLR-RGYIGV----------------------------------VNR-----------------
------------------S-QKDID----G---------------------------KKDIT----------A----AMSA---ERKFF---------
LT-------HP--------------------SY-----------------R-H------L--------------------------ADR-MGT-----
PYLQKALNQQLTNHIRDTL----
>XP_028570166.1
------------LDLPQIAVVGG----Q----------------SAGKSSVLE-----N---FV-GRDFLPRG---
SGIVTRRPL---------VLQ---L-V-NS-PT--
--------EY---G-------------------------------------E----------------FLH---------------------------------------
----CKG------KKF-----TDFD---EIRQEIEAETDR-----ITGSN-------------------KG------------------------
-IS--PVPINLRVYSPH--
VLSLTLVDLPGMTK--VP-------------------VGDQPA----------DI---EFQIR--------EMLMQFVTK--------
----ENCLILAVSPANS--DLA-NS--DALKIAKEVDPQGQRTIGVI-TKLDLMDEGT--------
DARDVLENK---L---------------------LPLR-RGYIGV----------------------------------VNR----------------
-------------------S-QKDID----G---------------------------KKDIQ----------A----ALAA---ERKFF---------
LT-------HP--------------------AY-----------------R-H------M--------------------------ADR-MGT-----
PYLQKVLNQQLTNHIRDTL----
>EPQ17174.1
------------LDLPQIAVVGG----Q----------------SAGKSSVLE-----N---FV-GRDFLPRG---
SGIVTRRPL---------VLQ---L-V-NA-ST--
--------EY---A------------------------------------E----------------FLH--
---CKG------KKF-----TDFE---EVRLEIEAETDR-----VTGTN-------------------KG-------------------------
IS--PVPINLRVYSPH--
VLNLTLVDLPGMTK--VP-------------------VGDQPA----------DI---EFQIR--------DMLMQFVTK-------
-----ENCLILAVSPANS--DLA-NS--DALKIAKEVDPQGQRTIGVI-TKLDLMDEGT--------
DARDVLENK---L---------------------LPLR-RGYIGV----------------------------------VNR----------------
-------------------S-QKDID----G---------------------------KKDIT----------A----ALAA---ERKFF---------
LS-------HP--------------------SY-----------------R-H------L--------------------------ADR-MGT-----
PYLQKVLNQQLTNHIRDTL----
>BAB27759.1
------------LDLPQIAVVGG----Q----------------SAGKSSVLE-----N---FV-GRDFLPRG---
SGIVTRRPL---------VLQ---L-V-NS-TT--
--------EY---A------------------------------------E----------------FLH--
---CKG------KKF-----TDFE---EVRLEIEAETDR-----VTGTN-------------------KG-------------------------
IS--PVPINLRVYSPH--
VLNLTLVDLPGMTK--VP-------------------VGDQPP----------DI---EFQIR--------DMLMQFVTK-------
-----ENCLILAVSPANS--DLA-NS--DALKIAKEVDPQGQRTIGVI-TKLDLMDEGT--------
DARDVLENK---L---------------------LPLR-RGYIGV----------------------------------VNR----------------
-------------------S-QKDID----G---------------------------KKDIT----------A----ALAA---ERKFF---------
LS-------HP--------------------SY-----------------R-H------L--------------------------ADR-MGT-----
PYLQKVLNQQLTNHIRDTL----
>ELW62001.1

------------LDLPQIAVVGG----Q----------------SAGKSSVLE-----N---FV-GRDFLPRG---
SGIVTRRPL---------VLQ---L-V-NA-TT--
--------EY---A------------------------------------E----------------FLH--
---CKG------KKF-----TDFE---EVRLEIEAETDR-----VTGTN-------------------KG-------------------------
IS--PVPINLRVYSPH---
VLNLTLVDLPGMTK--VP-------------------VGDQPP----------DI---EFQIR--------DMLMQFVTK-------
-----ENCLILAVSPANS--DLA-NS--DALKIAKEVDPQGQRTIGVI-TKLDLMDEGT--------
DARDVLENK---L---------------------LPLR-RGYIGV----------------------------------VNR----------------
-------------------S-QKDID----G---------------------------KKDIT----------A----ALAA---ERKFF---------
LS-------HP--------------------SY-----------------R-H------L--------------------------ADR-MGT-----
PYLQKVLNQQLTNHIRDTL----
>EAW87759.1
------------LDLPQIAVVGG----Q----------------SAGKSSVLE-----N---FV-GRDFLPRG---
SGIVTRRPL---------VLQ---L-V-NA-TT--
--------EY---A------------------------------------E----------------FLH--
---CKG------KKF-----TDFE---EVRLEIEAETDR-----VTGTN-------------------KG-------------------------
IS--PVPINLRVYSPH---
VLNLTLVDLPGMTK--VP-------------------VGDQPP----------DI---EFQIR--------DMLMQFVTK-------
-----ENCLILAVSPANS--DLA-NS--DALKVAKEVDPQGQRTIGVI-TKLDLMDEGT--------
DARDVLENK---L---------------------LPLR-RGYIGV----------------------------------VNR----------------
-------------------S-QKDID----G---------------------------KKDIT----------A----ALAA---ERKFF---------
LS-------HP--------------------SY-----------------R-H------L--------------------------ADR-MGT-----
PYLQKVLNQQLTNHIRDTL----
>XP_025915522.1

------------------------MTK--VP-------------------VGDQPP----------DI---EFQIR--------DMLMQFVTK-
-----------ENCLILAVSPANS--DLA-NS--DALKIAKEVDPQGQRTIGVI-TKLDLMDEGT--------
DARDVLENK---L---------------------LPLR-RGYIGV----------------------------------VNR----------------
-------------------S-QKDID----G---------------------------KKDIQ----------A----ALAA---ERKFF---------
LS-------HP--------------------AY-----------------R-H------M--------------------------ADR-MGT-----
PYLQKVLNQQLTNHIRDTL----
>XP_012378586.1
--------------------AGGPRRRQ----------------SGRAAAAAEPEPERN---FV-GRDFLPRG---
SGIVTRRPL---------VLQ---L-V-NA-TT--
--------EY---A------------------------------------E----------------FLH--
---CKG------KKF-----TDFE---EVRLEIEAETDR-----VTGTN-------------------KG-------------------------
IS--PVPINLRVYSPH---
VLNLTLVDLPGMTK--VP-------------------VGDQPP----------DI---EFQIR--------DMLMQFVTK-------
-----ENCLILAVSPANS--DLA-NS--DALKVAKEVDPQGQRTIGVI-TKLDLMDEGT--------
DARDVLENK---L---------------------LPLR-RGYIGV----------------------------------VNR----------------
-------------------S-QKDID----G---------------------------KKDIT----------A----ALAA---ERKFF---------

LS-------HP--------------------SY-----------------R-H------L--------------------------ADR-MGT-----
PYLQKVLNQQLTNHIRDTL----
>KAE8583055.1
------------LDLPQIAVVGG----Q----------------SAGKSSVLE-----N---FV-GKDFLPRG---
SGIVTRRPL---------VLQ---L-V-NS-ST--
--------EY---G------------------------------------E----------------FLH---------------------------------------
----CKG------KKF-----TDFD---EIRLEIEAETDR-----ATGTN-------------------KG------------------------
-IS--PVPINLRVYSPN---
VLNLTLVDLPGMTK--VP-------------------VGDQPV----------DI---EFQIR--------DMLMQFVTK-------
-----ENCLVLAVSPANS--DLA-NS--DALKIAKEVDPKGLRTIGVI-TKLDLMDEGT--------
DARDVLENK---L---------------------LPLR-RGYIGV----------------------------------VNR----------------
-------------------S-QKDID----G---------------------------KKDIQ----------A----ALAA---ERKFF---------
LS-------HP--------------------SY-----------------R-H------L--------------------------ADR-MGT-----
PYLQKALNQQLTNHIRDTL----
>XP_032814666.1
------------LDLPQIAVVGG----Q----------------SAGKSSVLE-----N---FV-GRDFLPRG---
SGIVTRRPL---------ILQ---L-M-FC-KA--
--------EY---A------------------------------------E----------------FLH--
---CKG------KKF-----TDFE---EVRAEIEAETDR-----LTGSN-------------------KG-------------------------
IS--PIPINLRVYSPH---
VLNLTLIDLPGMTK--VP-------------------VGDQPV----------DI---EYQIR--------EMLMQFVTK--------
----ENCLILAVSPANT--DLA-NS--DALKIAKEVDPQGLRTIGVI-TKLDLMDDGT--------DARDILENK-
--L---------------------LPLR-RGYIGV----------------------------------VNR---------------------------------
--S-QKDID----G---------------------------RKDIN----------A----AMAA---ERKFF---------LS-------HP--
------------------SY-----------------R-H------M--------------------------ADR-MGT-----
PYLQKTLNQQLTNHIRDTL----
>XP_025944940.1
--MF-FRDFLPRG---SGIVTRRPL---------VLQ--
-L-V-TA-KT--EY---A----------------
--------------------E----------------FLH---CKG------RKF-----
TDFD---EVRQEIEVETDR-----ITGVN-------------------KG-------------------------IS--SIPINLRIYSPH-
--VLSLTLIDLPGITK--VP-------------------
VGDQPP----------DI---EQQIR--------DMIMQFISR------------ENCLILAVTPANT--DLA-NS--
DALKLAKEVDPQGLRTIGVI-TKLDLMDEGT--------DAREILENK---L---------------------LPLR-
RGYIGV----------------------------------VNR-----------------------------------S-QKDID----G-------------
--------------KKDIK----------A----ALLA---ERKFF---------LS-------HP--------------------AY-------------
----R-H------M--------------------------ADR-MGT-----PYLQKVLNQQLTNHIRDTL----
>EPQ08653.1
------------LELPQIAVVGG----Q----------------SAGKSSVLE-----N---FV-GRDFLPRG---
SGIVTRRPL---------VLQ---L-V-TS-KT--
-------EY---A------------------------------------E----------------FLH---
--CKG------KKF-----TDFD---EVRHEIEAETDR-----VTGMN-------------------KG------------------------
-IS--SIPINLRVYSPH---
VLNLTLIDLPGITK--VP-------------------VGDQPV----------DI---EHQIR--------EMIMQFITR-----------

-ENCLILAVTPANT--DLA-NS--DALKLAKDVDPQGLRTIGVI-TKLDLMDEGT--------DARDILENK---
L--------------------LPLR-RGYVGV--------------------------------VNR--------------------------------
-S-QKDID----G-------------------------KKDIK----------A----AMLA---ERKFF---------LS-------HP----
----------------AY-----------------R-H------I-------------------------ADR-MGT-----
PHLQKVLNQQLTNHIRDTL----
>XP_027623811.1
------------LELPQIAVVGG----Q----------------SAGKSSVLE-----N---FV-GRDFLPRG---
SGIVTRRPL---------VLQ---L-V-TS-KA--
--------EY---G-----------------------------------E---------------FLH------------------------------------
----CKG------KKF-----TDFD---EIRHEIEAETDR-----VTGVN-------------------KG----------------------
--IS--SIPINLRVYSPH--
VLNLTLIDLPGITK--VP-------------------VGDQPP----------DI---EYQIR--------EMIMQFITR-----------
-ENCLILAVTPANT--DLA-NS--DALKLAKEVDPQGLRTIGVI-TKLDLMDEGT--------DARDVLENK---
L--------------------LPLR-RGYVGV--------------------------------VNR--------------------------------
-S-QKDID----G-------------------------KKDIK----------A----AMLA---ERKFF---------LS-------HP----
----------------AY-----------------R-H------I-------------------------ADR-MGT-----
PHLQKVLNQQLTNHIRDTL----
>XP_016856477.1
------------LELPQIAVVGG----Q----------------SAGKSSVLE-----N---FV-GRDFLPRG---
SGIVTRRPL---------VLQ---L-V-TS-KA--
--------EY---A-----------------------------------E---------------FLH-------------------------------------
---CKG------KKF-----TDFD---EVRLEIEAETDR-----VTGMN-------------------KG----------------------
--IS--SIPINLRVYSPH--
VLNLTLIDLPGITK--VP-------------------VGDQPP----------DI---EYQIR--------EMIMQFITR-----------
-ENCLILAVTPANT--DLA-NS--DALKLAKEVDPQGLRTIGVI-TKLDLMDEGT--------DARDVLENK---
L--------------------LPLR-RGYVGV--------------------------------VNR--------------------------------
-S-QKDID----G-------------------------KKDIK----------A----AMLA---ERKFF---------LS-------HP----
----------------AY-----------------R-H------I-------------------------ADR-MGT-----
PHLQKVLNQQLTNHIRDTL----
>XP_006496668.1
------------LELPQIAVVGG----Q----------------SAGKSSVLE-----N---FV-GRDFLPRG---
SGIVTRRPL---------VLQ---L-V-TS-KA--
--------EY---A-----------------------------------E---------------FLH-------------------------------------
---CKG------KKF-----TDFD---EVRHEIEAETDR-----VTGMN-------------------KG----------------------
--IS--SIPINLRVYSPH--
VLNLTLIDLPGITK--VP-------------------VGDQPP----------DI---EYQIR--------DMIMQFITR-----------
-ENCLILAVTPANT--DLA-NS--DALKLAKEVDPQGLRTIGVI-TKLDLMDEGT--------DARDVLENK---
L--------------------LPLR-RGYVGV--------------------------------VNR--------------------------------
-S-QKDID----G-------------------------KKDIK----------A----AMLA---ERKFF---------LS-------HP----
----------------AY-----------------R-H------I-------------------------ADR-MGT-----
PHLQKVLNQQLTNHIRDTL----
>XP_012379251.1
---V-GRDFLPRG---SGIVTRRPL---------VLQ---
L-V-TS-KA--EY---A----------------

--------------------E----------------FLH--CKG------KKF-----
TDFD---EVRHEIEAETDR-----VTGMN-------------------KG-------------------------IS--
SIPINLRVYSPH--VLNLTLIDLPGITK--
VP-------------------VGDQPP----------DI---EYQIR--------EMIMQFITR------------ENCLILAVTPANT--
DLA-NS--DALKLAKEVDPQGLRTIGVI-TKLDLMDEGT--------DARDVLENK---L---------------------
LPLR-RGYVGV-----------------------------------VNR-----------------------------------S-QKDID----G----
-----------------------KKDIK----------A----AMLA---ERKFF---------LS-------HP--------------------AY---
--------------R-H------I--------------------------ADR-MGT-----PHLQKVLNQQLTNHIRDTL----
>XP_021326548.1
------------LDLPQIAVVGG----Q----------------SAGKSSVLE-----N---FV-GRDFLPRG---
SGIVTRRPL---------ILQ---L-V-NN-KA---
--------EY---A------------------------------------E----------------FLH--
---CKG------RKF-----VDFD---EVRQEIEAETDR-----ITGSN-------------------KG-------------------------
-IS--PIPINLRVYSPN---
VLNLTLIDLPGMTK--VA-------------------VGDQPP----------DI---EHQIR--------DMIMQFITR---------
---ESCLILAVTPANM--DLA-NS--DALKVAKEVDPQGLRTIGVI-TKLDLMDEGT--------DARDILENK--
-L---------------------LPLR-RGYIGV-----------------------------------VNR-----------------------------------
-S-QKDID----G---------------------------RKDIR----------A----ALAA---ERKFF---------LS-------HP----
----------------SY-----------------R-H------M--------------------------AER-MGT-----
PHLQKALNQQLTNHIRDTL----
>NP_001025299.1
------------LDLPQIAVVGG----Q----------------SAGKSSVLE-----N---FV-GRDFLPRG---
SGIVTRRPL---------ILQ---L-V-NN-KA---
--------EY---A------------------------------------E----------------FLH--
---CKG------RKF-----VDFD---EVRQEIEAETDR-----ITGSN-------------------KG-------------------------
-IS--PIPINLRVYSPN---
VLNLTLIDLPGMTK--VA-------------------VGDQPP----------DI---EHQIR--------DMIMQFITR---------
---ESCLILAVTPANM--DLA-NS--DALKVAKEVDPQGLRTIGVI-TKLDLMDEGT--------DARDILENK--
-L---------------------LPLR-RGYIGV-----------------------------------VNR-----------------------------------
-S-QKDID----G---------------------------RKDIR----------A----ALAA---ERKFF---------LS-------HP----
----------------SY-----------------R-H------M--------------------------AER-MGT-----
PHLQKALNQQLTNHIRDTL----
>XP_031753735.1
------------LDLPQIAVVGG----Q----------------SAGKSSVLE-----N---FV-GRDFLPRG---
SGIVTRRPL---------ILQ---L-I-FS-KT---
------EY---A------------------------------------E----------------FLH--
-CKS------KKF-----TDFD---EVRQEIEAETDR-----VTGTN-------------------KG-------------------------
IS--PVPINLRVYSPN---
VLNLTLIDLPGITK--VP-------------------VGDQPH----------DI---EYQIK--------DMILQFISR-----------
-DSCLILAVTPGNT--DLA-NS--DALKMAKEVDPQGLRTIGVI-TKLDLMDEGT--------DAKDILENK---
L---------------------LPLR-RGYIGV-----------------------------------VNR------------------------------------
S-QKDID----G---------------------------KKDIK----------A----ALGA---ERKFF---------LS-------HP-----
---------------GY-----------------R-H------I--------------------------AER-MGT-----
PHLQKTLNQQLTNHIRETL----

>XP_025920181.1
------------LDLPQIAVVGG----Q----------------SAGKSSVLE-----N---FV-GRDFLPRG---
SGIVTRRPL---------ILQ---L-I-FS-KT--
------EY---A------------------------------------E----------------FLH--
-CKS------KKF-----TDFD---EVRQEIEAETDR-----VTGTN-------------------KG-------------------------
IS--PVPINLRVYSPH--
VLNLTLIDLPGITK--VP-------------------VGDQPQ----------DI---EYQIK--------DMIMQFISR----------
--ESSLILAVTPANM--DLA-NS--DALKMAKEVDPQGLRTIGVI-TKLDLMDEGT--------DARDVLENK-
--L---------------------LPLR-RGYIGV----------------------------------VNR---------------------------------
--S-QKDID----G---------------------------KKDIR----------A----ALAA---ERKFF---------LS-------HP---
-----------------AY-----------------R-H------M--------------------------ADR-MGT-----
PHLQKVLNQQLTNHIRETL----
>XP_028568434.1
------------LDLPQIAVVGG----Q----------------SAGKSSVLE-----N---FV-GRDFLPRG---
SGIVTRRPL---------ILQ---L-I-FS-KT--
------EY---A------------------------------------E----------------FLH--
-CKS------KKF-----TDFD---EVRQEIEAETDR-----VTGTN-------------------KG-------------------------
IS--PVPINLRVYSPH--
VLNLTLIDLPGITK--VP-------------------VGDQPQ----------DI---EYQIK--------DMILQFISR-----------
-ESSLILAVTPANM--DLA-NS--DALKMAKEVDPQGLRTIGVI-TKLDLMDEGT--------DARDVLENK--
-L---------------------LPLR-RGYIGV----------------------------------VNR----------------------------------
-S-QKDID----G---------------------------KKDIR----------A----ALAA---ERKFF---------LS-------HP----
----------------AY-----------------R-H------M--------------------------ADR-MGT-----
PHLQKLLNQQLTNHIRETL----
>XP_006510037.1
------------LDLPQIAVVGG----Q----------------SAGKSSVLE-----N---FV-GRDFLPRG---
SGIVTRRPL---------ILQ---L-I-FS-KT--
------EY---A------------------------------------E----------------FLH--
-CKS------KKF-----TDFD---EVRQEIEAETDR-----VTGTN-------------------KG-------------------------
IS--PVPINLRVYSPH--
VLNLTLIDLPGITK--VP-------------------VGDQPP----------DI---EYQIK--------DMILQFISR------------
ESSLILAVTPANM--DLA-NS--DALKLAKEVDPQGLRTIGVI-TKLDLMDEGT--------DARDVLENK---
L---------------------LPLR-RGYIGV----------------------------------VNR-----------------------------------
S-QKDIE----G---------------------------KKDIR----------A----ALAA---ERKFF---------LS-------HP------
--------------AY-----------------R-H------M--------------------------ADR-MGT-----
PHLQKTLNQQLTNHIRESL----
>NP_001005360.1
------------LDLPQIAVVGG----Q----------------SAGKSSVLE-----N---FV-GRDFLPRG---
SGIVTRRPL---------ILQ---L-I-FS-KT--
------EH---A------------------------------------E----------------FLH---
--CKS------KKF-----TDFD---EVRQEIEAETDR-----VTGTN-------------------KG-------------------------
IS--PVPINLRVYSPH--
VLNLTLIDLPGITK--VP-------------------VGDQPP----------DI---EYQIK--------DMILQFISR------------
ESSLILAVTPANM--DLA-NS--DALKLAKEVDPQGLRTIGVI-TKLDLMDEGT--------DARDVLENK---

L---------------------LPLR-RGYIGV----------------------------------VNR-----------------------------------
S-QKDIE----G---------------------------KKDIR----------A----ALAA---ERKFF---------LS-------HP------
--------------AY-----------------R-H------M--------------------------ADR-MGT-----
PHLQKTLNQQLTNHIRESL----
>XP_014389433.1
---AGKGQI------------T-AWDFLPRG---SGIVTRRPL---------
ILQ---L-I-FS-KT--EY---A----------
--------------------------E----------------FLH---CKS------KKF-----
TDFD---EVRQEIEAETDR-----VTGTN-------------------KG-------------------------IS--
PVPINLRVYSPH---VLNLTLIDLPGITK--
VP-------------------VGDQPP----------DI---EYQIK--------DMILQFISR------------ESSLILAVTPANM--
DLA-NS--DALKMAKEVDPQGLRTIGVI-TKLDLMDEGT--------DARDVLENK---L---------------------
LPLR-RGYIGV----------------------------------VNR-----------------------------------S-QKDIE----G-----
----------------------KKDIR----------A----ALAA---ERKFF---------LS-------HP--------------------AY-----
------------R-H------M--------------------------ADR-MGT-----PHLQKTLNQQLTNHIRESL----
>XP_012381548.1

---MILQFIGR-----------
ESSLILAVTPANM--DLA-NS--DALKLAKEVDPQGLRTIGVI-TKLDLMDEGT--------DARDVLENK---
L---------------------LPLR-RGYIGV----------------------------------VNR-----------------------------------
S-QKDIE----G---------------------------RKDIR----------S----ALAA---ERKFF---------FS-------HS------
--------------AY-----------------R-H------M--------------------------ADR-MGT-----
LHLQKTLNQQLTNHIRESL----
>XP_006161648.2.2
---------------PHCTSVSR----QAYS------------SPQGPRKVIS-----VL-FLP-HRDFLPRG---
SGIVTRRPL---------ILQ---L-I-FS-KT--
------EY---A------------------------------------E----------------FLH--
-CKS------KKF-----TDFD---EVRQEIEAETDR-----VTGTN-------------------KG-------------------------
IS--PVPINLRVYSPH---
VLNLTLIDLPGITK--VP-------------------VGDQPP----------DI---EYQIK--------DMILQFISR------------
ESSLILAVTPANM--DLA-NS--DALKLAKEVDPQGLRTIGVI-TKLDLMDEGT--------DARDVLENK---
L---------------------LPLR-RGYIGV----------------------------------VNR-----------------------------------
S-QKDIE----G---------------------------KKDIR----------A----ALAA---ERKFF---------LS-------HP------
--------------AY-----------------R-H------M--------------------------ADR-MGT-----
PHLQKTLNQQLTNHIRESL----
>XP_035683496.1
------------LDLPQIAVVGG----Q----------------SAGKSSVLE-----N---FV-GRDFLPRG---
SGIVTRRPL---------VLQ---L-IHNP-KA---
---------EY---G------------------------------------E----------------FLH--------------------------------------
-----AKG------KMF-----SDFH---EIRAEIEAETDR-----MTGSN-------------------KG---------------------
----IS--PVPINLRVYSPH---

VLNLTLIDLPGMTK--VP-------------------VGDQPP----------DI---EQQIR--------DMLLQFITK---------
---DNCLILAVSPANQ--DLA-NS--DALKIAKEVDPQGMRTIGVI-TKLDLMDEGT--------
DARNILENR---T---------------------YPLR-RGYIGV----------------------------------VNR-----------------
------------------S-QADID----G----------------------------RKDIK----------A----ALAA---ERKFF---------
LS-------HP--------------------AY-----------------R-H------L--------------------------ADR-MGT-----
PYLQKTLNQQLTNHIRDTL----
>KMZ10000.1
------------LDLPQIAVVGG----Q----------------SAGKSSVLE-----N---FV-GKDFLPRG---
SGIVTRRPL---------ILQ---L-I-NG-VT--
-------EY---G-------------------------------------E----------------FLH---
---IKG------KKF-----SSFD---EIRKEIEDETDR-----VTGSN-------------------KG-------------------------
IS--NIPINLRVYSPH--
VLNLTLIDLPGLTK--VA-------------------IGDQPV----------DI---EQQIK--------QMIFQFIRK-----------
-ETCLILAVTPANT--DLA-NS--DALKLAKEVDPQGVRTIGVI-TKLDLMDEGT--------DARDILENK---
L---------------------LPLR-RGYIGV----------------------------------VNR-----------------------------------
S-QKDIE----G----------------------------RKDIH----------Q----ALAA---ERKFF---------LS-------HP-----
---------------SY-----------------R-H------M--------------------------ADR-LGT-----
PYLQRVLNQQLTNHIRDTL----
>PAA78248.1
------------FDLPQIAVVGS----Q----------------SAGKSSVLE-----N---FV-GKDFLPRG---
SGIVTRRPL---------VLQ---L-L-TH-PS---
--------EF---A-------------------------------------E----------------FGH--
----LRG------KKF-----TNFD---EVRQEIENETDR-----LTGKN-------------------KG-----------------------
--IS--NVPITLRVFSPH--
VLNLTLVDLPGMTK--VA-------------------VGDQPP----------DI---EQQIR--------AMLFEFISK---------
---ENCLILAVSPANS--DLA-NS--DALKIAKEVDPNGTRTIGVI-TKLDLMDQGT--------DAREVLENK--
-L---------------------LPLR-RGYIGV----------------------------------VNR----------------------------------
-S-QKDIE----G----------------------------KKDIA----------A----AMAA---ERKFF---------LS-------HP----
----------------SY-----------------R-H------M--------------------------AER-MGT-----
PYLQRCLNQQLTNHIRETL----
>PAA65118.1
------------FDLPQIAVVGS----Q----------------SAGKSSVLE-----N---FV-GKDFLPRG---
SGIVTRRPL---------ILQ---L-L-YN-PS--
---A---EY---A-------------------------------------E----------------FGH--
----QRG------RKY-----TNFE---EVRQEIEAETDR-----LTGRN-------------------KG-----------------------
--IS--NVPIMLRVFSPH--
VLNLTLVDLPGMTK--VA-------------------VGDQPP----------DI---EVQIR--------NMLLEFITK---------
---ENCLILAVSPANS--DLA-NS--DALKLAKEVDPAGTRTIGVI-TKLDLMDQGT--------DAREVLENK-
--L---------------------LPLR-RGYIGV----------------------------------VNR---------------------------------
--S-QKDIE----G----------------------------RKDIK----------A----AMAA---ERKFF---------LS-------HP---
-----------------SY-----------------R-H------M--------------------------AER-MGT-----
PYLQRCLNQQLTNHIRETL----
>PAA59145.1

------------FDLPQIAVVGS----Q----------------SAGKSSVLE-----N---FV-GKDFLPRG---
SGIVTRRPL---------VLQ---L-I-NF-HT--
--------EY---A-----------------------------------E---------------FGH---------------------------------------
----IRG------KRF-----TNFD---EVRQEIENETDR-----VTGKN-------------------KG----------------------
--IS--NVPIMLRVYSPQ---
VLNLTLVDLPGLTK--VA-------------------VGDQPQ----------DI---ELLIR--------AMILEFVSK----------
-DNCLILAVTPANS--DLA-NS--DALKIAKEVDPSGTRTIGVI-TKLDLMDQGT--------DARDVLENR---
L--------------------LPLR-RGYIGV--------------------------------VNR----------------------------------
S-QKDIE----G--------------------------KKDIV----------A----AMAA---ERKFF---------LS-------HP-----
---------------AY-----------------R-H------M-------------------------AER-MGT-----
SYLQRCLNQQLTNHIRETL----
>PAA64382.1
------------IDLPQIAVVGS----Q----------------SAGKSSVLE-----N---FV-GRDFLPRG---SGIVTRRPL-
--------ILQ---L-M-NY-QT--EY---
A-----------------------------------E---------------FGH---IRG-----
-KKF-----VNFD---EVRREIEVETDR-----LTGQN-------------------KG------------------------IS--
NVPITLRVYSPQ---VLNLTLVDLPGMTK--
VA-------------------VGDQPP----------DI---EQQVR--------AMIWEFISK------------
DNCLILAVSPANS--DLA-NS--DALKLAKEADPSGSRTIGVL-TKLDLMDAGT--------DARDVLENR---
F--------------------LPLR-RGYVGV--------------------------------VNR--------------------------------
-S-QKDID----G---------------------------RKDIS----------S----AMAA---ERKFF---------LG-------HP---
-----------------AY-----------------R-H------M-------------------------AER-MGT-----
AHLQRCLNQQLVGHIRDTL----
>NP_001024332.1
------------FELPQIAVVGG----Q----------------SAGKSSVLE-----N---FV-GKDFLPRG---
SGIVTRRPL---------ILQ---L-I-QD-RN--
--------EY---A-----------------------------------E---------------FLH---
---KKG------HRF-----VDFD---AVRKEIEDETDR-----VTGQN-------------------KG----------------------
--IS--PHPINLRVFSPN---
VLNLTLIDLPGLTK--VP-------------------VGDQPA----------DI---EQQIR--------DMILTFINR----------
-ETCLILAVTPANS--DLA-TS--DALKLAKEVDPQGLRTIGVL-TKLDLMDEGT--------DAREILENK---L-
--------------------FTLR-RGYVGV--------------------------------VNR---------------------------------
G-QKDIV----G---------------------------RKDIR----------A----ALDA---ERKFF---------IS-------HP------
--------------SY-----------------R-H------M-------------------------ADR-LGT-----
SYLQHTLNQQLTNHIRDTL----
>XP_004347890.1
------------LDLPQIAVVGS----Q----------------SAGKSSVLE-----N---FV-GKDFLPRG---SGIVTRRPL-
--------VLQ---L-V-NS-KG--P---EY---
G-----------------------------------E---------------FLH---NKS-----
-KKF-----TDFD---EVRKEIEAETDR-----ITGTN-------------------KG------------------------IS--
PVPINLKVYSPN---VLNLTLVDLPGITK--
VP-------------------IGDQPT----------NI---ESLIR--------EMIMQFIGR------------PNCLILAVSPANS--
DLA-NS--DALKLAREVDQQGIRTIGVI-TKLDLMDEGT--------DAREVLENK---L--------------------
IPLR-RGFIGV--------------------------------VNR----------------------------------S-QKDID----G------

--------------------RKDIK----------A----AMSA---ELRFF---------ST-------HP-------------------AY------
-----------R-D------L-------------------------ANK-NGT-----MYLQRVLNQQLTNHIRDTL----
>XP_001749319.1
------------LDLPQIAVVGG----Q----------------SAGKSSVLE-----N---FV-GKDFLPRG---
SGIVTRRPL---------VLQ---L-N-YH-PS---
-----A---EW---G------------------------------------E----------------FLH------------------------------------
-------ARG------KKF-----TDFN---EIRQEIEAETDR-----MTGSN-------------------KG-------------------
-----IS--NIPINLRVYSPH---
VLNLTLVDLPGLTK--VA-------------------VGDQPA----------DI---ENQIR--------GMLMEFITK---------
---DNCIILAVTPANQ--DLA-NS--DALKLAKEVDPEGVRTIGVI-TKLDLMDSGT--------DARAILTNE---
F--------------------LPLR-RGYIGV---------------------------------VNR-----------------------------------
S-QKDID----G---------------------------RKDIR----------A----ALDA---ERKFF---------LM-------HP----
----------------SY-----------------K-D------I-------------------------ASK-NGT-----
PYLQKALNQQLTNHIRECL----
>XP_031757197.1
------------VDLPQIAVVGG----Q----------------SAGKSSVLE-----N---LV-GR--------------------------
WIH---V-L--S-ST--EY---A--------
---------------------------E----------------FLH--CKG------TKY----
-TDFS---EVRQEIEEETER-----ATGLN-------------------KG-------------------------IS--AIPISLRIYSPH-
--VLNLSLIDLPGVTK--VP-------------------
VGDQPA----------DI---ETQIR--------DMIMNFISR------------ENCLILAVTPANT--DLA-NS--
DALKLAKEVDPQGLRTIGVI-TKLDLMDEGT--------NAQEILENK---L--------------------LPLR-
RGYVGV---------------------------------VNR-----------------------------------S-QKDID----G------------
---------------KKNIN----------A----ALQA---EQMFF---------LT-------HP-------------------AY-----------
------R-H------M-------------------------ADR-MGT-----SHLQKMLNQQLTNHIRETL----
>XP_014148725.1
--T---
--EY---G---------------------------------
---E----------------FLH--KPG------RLF-----DNFD---
EIRNEIEADTAR-----ITGAN-------------------KG-------------------------IS--HLPINLKVYSPH----------
---VLDLTLVDLPGLTK--VA-------------------
VGDQPA----------DI---EMQIK--------NMIMEFITK------------PNCLILAVTPANS--DLA-NS--
DALKLAKEVDPQGLRTIGVI-TKLDLMDAGT--------DARDVLENK---L--------------------LPLR-
RGYVGV---------------------------------VNR-----------------------------------S-QKDIA----G------------
---------------NKDIR----------A----AQAA---EKKFF---------KT-------HP-------------------AY-----------
------R-H------L-------------------------ADK-MGT-----PKLQQVLNQQLTDHIRQTL----
>XP_014153758.1
--N-----------------------------------LY---LYI---------
---EW---G------------------------------
------E----------------FLH---QPG------RKY-----TDFE---
EIMKEIEAETDR-----MTGSN-------------------KG-------------------------IS--NIPINLKVMSPH---------
---VLDLTLVDLPGLTK--VA-------------------
VGDQPA----------DI---EQQIL--------GMIMEFITR------------PNCLILAVSPANA--DLA-NS--
DALKLAKEVDPQGLRTIGVI-TKLDLMDQGT--------DAREILENK---L--------------------LPLR-

RGYIGV-----------------------------------VNR-------------------------------------S-QKDIT----G---------------
---------------KKDIR-----------A----AQEA---ERRFF---------ST-------HP--------------------AY-------------
-----R-H------L----------------------------AQN-MGT-----PKLQKVLNQQLTNHIRDSL----
>XP_006812840.1
-------------ITKPSCLILAV-------------------------TPGNSDLAN-----SDALKV-
AKEVDPQGLRTIGVITKLDLLDDGTDAREILENKLLPL-RR-AS--
-------------------------------------EW--G---------------------------------------E------------------FLH------------
----------------------------------CKG------KKF-----TNFD---EIRMEIEAETDR-----LTGKN-------------------
-KG---------------------------IS--PIPINLRVYSPH---
--------VLNLTLIDLPGMTK--VP-------------------VGDQPA----------DI---EQQIR--------SMLMEFITK-
-----------PSCLILAVTPGNS--DLA-NS--DALKVAKEVDPQGLRTIGVI-TKLDLLDDGT--------
DAREILENK---L-----------------------LPLR-RGYIGV------------------------------------VNR-------------------
-------------------G-QKDIE----G-----------------------------RKDIK----------S----ALAS---ERKFF-----------
LS-------HP--------------------SY------------------R-H------M----------------------------ADR-LGT-----
PYLQKALNQQLTNHIRDTL----
>XP_004348308.1
------------IQLPQIAVVGS----Q------------------SSGKSSVLE-----N---IV-GKDFLPRG---HGIVTRRPL-
--------ILQ---L-V-HRKPGS-------------------PRPALPDDPSSSG-------------------------------
GHTDDGIDGEDVE---EW---G---------------------------------------E-----------------FLH---------------------
------------------------APG------KRF-----ISFA---EIRKEIEAETDR-----VTGSN--------------------KG------
--------------------IS--SKPINLRIYSPN--
VLNLTLVDLPGITK--VP-------------------VGDQPE----------DI---EKQIR--------TLVRSYISN-------------
PNCIILAVTPANV--DLA-NS--DALKLAKTIDPEGNRTIGVC-TKIDLMDAGT--------DAMDILSGR---V-
---------------------VPVK-LGFIGV---------------------------------------VNR-----------------------------------S-
QADIN----T-----------------------------AKPIA----------D----SLKS---EEQFF---------KS-------HP-------
-----------AY------------------Q-A------I----------------------------AHR-CGT-----
AYLSKALNKLLMHHIRDCL----
>KNE67543.1
------------LDLPQIAVVGS----Q------------------SSGKSSVLE-----A---IV-GKDFLPKG---AGIVTRRPL--
-------ILQ---L-N-YA-PV---------------------DPETPDE--PE---
EW---A---------------------------------------E------------------FQH--
LPG------QQF-----ADFG---EVKREIERETAR-----IAGDN--------------------KG---------------------------
IS--DEPITLRVHSPS--
VVDLTLVDLPGLTK--IP--------------------VGDQPS----------DI---ELQIR--------DLIMKFILQ------------
-PNCIILAVSPANV--DLA-NS--DSLKLAREVDPQGLRTLGLL-TKVDLMETGS--------HALDILGGR---
V-----------------------YPLR-LGFVAV--------------------------------------VNR-------------------------------------
S-QRDIE----A-----------------------------RRTLE---------W----SRKR---EQQFF-----------------------------
---SGR-CGT-----
AALARTLNSVLLDHIRAQLP---
>KNE61418.1
------------LDLPQIAVVGS----Q------------------SSGKSSVLE-----A---IV-GKDFLPKG---NGIVTRRPL-
--------VLQ---L-R-NV-P------------------------PHAVPE---GE---
EAVDVA---------------------------------------E------------------FSH--
CPD------RVF-----TDFA---DVRREIEAETAR-----IAGDN--------------------KG---------------------------

IA--TDPIRLCVRSPN--
VVDLTLVDLPGMTK--IP-------------------VGDQPS----------DI---ELQIR--------ELIMGFITK-----------
-PNCLILAVSPANV--DLA-NS--DSLKLAREVDPTGDRTLGLL-TKVDLMEPGT--------HALDILAGR---
V--------------------YPLR-LGFVGV---------------------------------VNR---------------------------------
-S-QRDID----Q---------------------------GKSLE----------F----ARKR---EQQFF---------AT-------HP---
-----------------VY-----------------A-P------V-------------------------ASR-CGT-----
TVLARTLNQVLLAHIRDRLP---
>XP_011392073.1
------------VDLPQIVVVGS----Q----------------SAGKSSVLE-----T---IV-GRDFLPRG---SGIVTRRPL--
-------VLQ---L-I-HT-PS--------------------TKEQPRQPKQSSRPY-DLSDGLA-----------
SDMQRGGSHASSADTRSPTYE---EY---G------------------------------------E----------------FLH-------
------------------------------------L-D------KRF-----TDFN---EIRREIENETFR-----VAGQN---------------
----KG-------------------------VS--KLPIHLKIYSPN--
-----------VLNLTLVDLPGLTK--IP------------------VGDQPS----------DI---ERQIR--------
NLVTDYVSK------------PNCIILAVSPANV--DLA-NS--DSLKLARTVDPQGRRTIGVL-
TKLDLMDQGT--------HALDILTGR---V--------------------YPLK-LGFIGV--------------------------------
--VNR---------------------------------S-QQDIN----G--------------------------NVSML----------A----
ARRA---EEDFF---------RS-------HA--------------------AY----------------K-N------I--------------------
-----AHR-CGT-----KYLAKTLNQVLMSHIRDKLP---
>XP_006461708.1
------------LDMPQIVVVGS----Q----------------SAGKSSVLE-----T---IV-GKDFLPRG---SGIVTRRPL-
--------VLQ---L-I-HT-PV-----------------------PSEPSPNALP-------------------------------------YT---
EW---G-----------------------------------Q----------------FLH--I-
D------KRF-----TDFN---DIRKEIEQETFR-----VAGQN------------------KG------------------------IS--
RLPISLRVYSPN--VLDLTLVDLPGLTK--
IP------------------VGDQPS----------DI---EKQIR--------NLVVDYISK-----------PNSVILAVSAANV--
DLA-NS--ESLKLARSVDPQGRRTIGVL-TKLDLMDAGT--------NALDILTGR---V--------------------
YPLK-LGFIGI---------------------------------VNR---------------------------------S-QQDIN----V------
--------------------EKSLT----------D----AVES---EAEFF---------RN-------HA-------------------VY-----
------------R-N------I-------------------------AHK-NGT-----RYLAKTLNQVLMNHIRDKLP---
>XP_746923.1
------------LDLPQIVVVGS----Q----------------SSGKSSVLE-----N---IV-GRDFLPRG---SGIVTRRPL-
--------ILQ---L-I-NI-PS-------------------EHNDRPGDNDVLA-PH------------------------------
TAASVAGQH---EW---A-----------------------------------E----------------FHH---------------------------
----------------LPG------RKF-----EDFA---LVKQEIEAETAR-----IAGNN------------------KG------------
-------------IN--RQPINLKIFSPH--
VLNLTMVDLPGLTK--VP------------------IGDQPS----------DI---EKQTR--------TLILEYIAK-----------
PNSIILAVSPANV--DLV-NS--EALKLARQVDPMGRRTIGVL-TKLDLMDHGT--------NAMDILSGR---
V--------------------YPLK-LGFIGV---------------------------------VNR---------------------------------
S-QQDIQ----S---------------------------GKSLS----------E----ALQA---EAEFF---------RH-------HP----
----------------AY-----------------R-N------M-------------------------ANR-CGT-----
QFLAKTLNTTLMAHIRDRLP---
>KXN67416.1

------------LDLPQIVVVGS----Q---------------SSGKSSVLE-----N---LV-GRDFLPRG---
NGIVTRRPL---------VLQ---L-V-NL-RE------------------D-----ADLDLT--------------------------------
-------------ER---A----------------------------------Q---------------FLH--------------------------------
---------NPT------KFY-----TDFS---EVRQEIEQETNR-----LAGEN------------------KG-----------------
-------IS--RNPIHLKIFSTQ--
VLNLTVVDLPGLTK--IP------------------IGDQPT----------DI---EKQTK--------SLILDYISK-----------
PNSIILAVSPANV--DLV-NS--ESLKLAKEVDPEGKRTIGII-TKIDLMDAGT--------NALDILTGR---V-----
----------------LNLK-LGFIGV---------------------------------INR---------------------------------S-
QQDTV----A--------------------------KKPIR----------E----SLEA---ELEFF---------RT-------HP---------
-----------AY----------------R-N------I------------------------SQR-CGT-----
GHLSKTLNQVLVNHIRDRLP---
>NP_013100.1
------------LDLPILAVVGS----Q---------------SSGKSSILE-----T---LV-GRDFLPRG---TGIVTRRPL----
-----VLQ---L-N-NI-SP-------------------NSPLIEEDDNSVNPH-DEVTKI------------
SGFEAGTKPLEYRGKERNHAD---EW---G----------------------------------E---------------FLH------
------------------------------------IPG------KRF-----YDFD---DIKREIENETAR-----IAGKD---------------
----KG-----------------------IS--KIPINLKVFSPH---
-----------VLNLTLVDLPGITK--VP------------------IGEQPP----------DI---EKQIK--------NLILDYIAT---
---------PNCLILAVSPANV--DLV-NS--ESLKLAREVDPQGKRTIGVI-TKLDLMDSGT--------
NALDILSGK---M-------------------YPLK-LGFVGV---------------------------------VNR----------------
-------------------S-QQDIQ----L--------------------------NKTVE----------E----SLDK---EEDYF---------
RK-------HP--------------------VY----------------R-T------I------------------------STK-CGT-----
RYLAKLLNQTLLSHIRDKLP---
>NP_012926.1
------------IDLPQITVVGS----Q---------------SSGKSSVLE-----N---IV-GRDFLPRG---TGIVTRRPL---
------VLQ---L-I-NR-RP--------------------KKSEHAKVNQTANELIDLNINDD-----------
DKKKDESGKHQNEGQSEDNKE---EW---G----------------------------------E---------------FLH----
--------------------------------------LPG------KKF-----YNFD---EIRKEIVKETDK-----VTGAN------------
-------SG------------------------IS--SVPINLRIYSPH---
--------------VLTLTLVDLPGLTK--VP------------------VGDQPP----------DI---ERQIK--------
DMLLKYISK-----------PNAIILSVNAANT--DLA-NS--DGLKLAREVDPEGTRTIGVL-TKVDLMDQGT-
-------DVIDILAGR---V--------------------IPLR-YGYIPV---------------------------------INR-------------
----------------------G-QKDIE----H--------------------------KKTIR----------E----ALEN---ERKFF-------
--EN-------HP------------------SY----------------S-S------K------------------------AHY-CGT-----
PYLAKKLNSILLHHIRQTLP---
>OUM62108.1
------------IDLPQIVVIGS----Q---------------SSGKSSVLE-----N---IV-GRDFLPRG---TGIVTRRPL---
------ILQ---L-I-NK-RD--------------------GLTTNS-------------------------------------
APANSEQNMN---EW---G----------------------------------E---------------FLH-----------------------
-------------------LPG------KKF-----YDFT---EIRKEIEKETEL-----KVGKN------------------AG----------
---------------IS--DQPINLRIFSPK---
VLTLTLVDLPGLTK--VP------------------VGDQPK----------DI---ERLIR--------DMILKYITK-----------
PNAIILAVTAANT--DLA-NS--DGLKLAREVDPEGVRTIGVL-TKVDLMDAGT--------DVIDILAGR---V-
--------------------IPLR-LGYVPV---------------------------------VNR---------------------------------G-

QKDIE----T---------------------------NKSIS----------K----ALEA---EKQFF---------EH-------HS---------
-----------SY-----------------K-S------K-------------------------AQY-CGT-----
PFLARKLNMILMHHIRNTLP---
>KXN66323.1
------------VDLPQIVVIGS----Q----------------SSGKSSVLE-----N---IV-GRDFLPRG---TGIVTRRPL--
-------VLQ---L-F-NV-PQ-------------------TQ--ADSAKENEPA--
-EY---G-----------------------------------E----------------FLH---
LPG------TKF-----TDFD---KIRKEIESETES-----KTGKN-------------------AG------------------------IS--
PLPINLRIFSPK--VLTLTLVDLPGLTK--
VP-------------------VGDQPK----------DI---EKQIR--------DMIMKYINK-----------PNAIILAVTAANT--
DLA-NS--DGLKLAREVDPEGSRTIGVL-TKVDLMDHGT--------DVVDILAGR---V--------------------
IPLR-LGYVPV---------------------------------INR---------------------------------S-QRDIE----S-------
--------------------KKNIS----------A----ALDH---ERQFF---------EK-------HP--------------------SY------
-----------T-S------K-------------------------VQY-CGT-----PFLARKLSMILMHHIRNTLP---
>XP_011389257.1
------------IDLPQITVLGS----Q----------------SSGKSSVLE-----N---IV-GRDFLPRG---TGIVTRRPL---
------VLQ---L-I-NR-PA--------------------TSKANDE----------------------------------
AAPAVSSKGANNPD---EW---G-----------------------------------E----------------FLH------------------
-------------------------LPG------EKF-----FDFD---KIREEIVRDTEL-----KTGRN-------------------AG---
----------------------IS--PQPINLRIYSPN---
-VLTLTLVDLPGLTK--VP-------------------VGDQPR----------DI---ERQIR--------DTVLKFISK-----------
-PNAVILAVTAANT--DLA-NS--DGLKLAREVDPEGTRTVGVL-TKVDLMDAGT--------DVVDILAGR--
-V---------------------IPLR-LGYVPV----------------------------------VNR-----------------------------------
G-QRDID----Q---------------------------KKLVS----------A----ALTA---EKEFF---------EN-------HP-----
---------------SY-----------------R-S------K-------------------------AQY-CGT-----
PFLARKLNTILMHHIRNTLP---
>XP_006458578.1
------------IDLPQICVLGS----Q----------------SSGKSSVLE-----N---IV-GRDFLPRG---TGIVTRRPL--
-------VLQ---L-I-NR-PA--------------------GTPG---------------------------------
GPQINGTDKSSDKHANAD---EW---G-----------------------------------E----------------FLH-----------
---------------------------------LPG------EKF-----FDFN---KIRAEIVRDTEV-----KTGKN-------------------
AG------------------------IS--PLPINLRVFSPN--
--------VLTLTLVDLPGLTK--VP-------------------VGDQPR----------DI---EKQIR--------DMLLKYISK---
---------SACIILAVTAGNT--DLA-NS--DGLKMAREVDPEGLRTIGVL-TKIDLMDKGT--------
DVVDILAGR---I---------------------IPLR-LGYVPV-----------------------------------VNR------------------
-----------------G-QRDIE----S---------------------------SKPIS----------A----ALEY---ERSFF---------
EN-------HA--------------------SY-----------------K-S------K-------------------------AQF-CGT-----
PFLARKLNMILMHHIKATIP---
>KNE68830.1
------------IDLPQITVIGS----Q----------------SSGKSSVLE-----N---IV-GRDFLPRG---TGIVTRRPL----
-----VLQ---L-I-NR-PA--------------------TPAAPGS---------------------------
PATDGEPAADGTSGDNQDNKN---EW---G-----------------------------------E----------------FLH-----
-------------------------------------APN------KRF-----YNFD---EIRKEIIRDTDE-----KAGAN-------------
------TG------------------------IS--HAPINLRIYSPN--

-------------VLTLTLVDLPGLTK--VP-------------------VGDQPK----------DI---EIQIR--------
EMLLKYITK------------PNAIILAVTAANT--DLA-NS--DGLKLAREVDPEGSRTIGVL-TKVDLMDPGT--
------DVVDILAGR---V---------------------IPLR-LGYVPV----------------------------------VNR-----------
------------------------G-QKDID----K---------------------------RKSIA----------L----ALEH---ERSYF-----
----EE-------HP--------------------SY-----------------R-S------K--------------------------AQY-CGT-----
PFLARKLNMILLHHIKNTLP---
>XP_748106.1
------------IDLPQIVVVGS----Q----------------SSGKSSVLE-----N---IV-GRDFLPRG---SGIVTRRPL--
-------ILQ---L-I-NR-PS-------------------RNSV-------------------------------
TNGVKEEKLETTDSEANVD---EY---G------------------------------------E----------------FLH------------
-------------------------------IPG------QKF-----YDFN---KIREEIVRETEQ-----KVGRN-------------------
AG-------------------------IS--PAPINLRIYSPN--
-------VLTLTLVDLPGLTK--VP-------------------VGDQPK----------DI---ERQIR--------DMVLKYISK----
--------PNAIILAVTSANQ--DLA-NS--DGLKLAREVDPEGQRTIGVL-TKVDLMDEGT--------
DVVDILAGR---I---------------------IPLR-LGYVPV----------------------------------VNR------------------
-----------------G-QRDIE----N---------------------------KRPIS----------Y----ALEH---EKNFF---------
ES-------HK--------------------AY-----------------R-N------K--------------------------ASY-CGT-----
PYLARKLNLILMMHIKQTLP---
>OAJ44422.1
------------IDLPQIAVVGS----Q----------------SSGKSSVLE-----N---IV-GKDFLPRG---SGIVTRRPL--
-------VLQ---L-I-NR-PN--------------------SKGEIGGIAAGT----DKPPSSP-----------
STPGAKSATTSNAAVTVEEGD---EW---G------------------------------------E----------------FLH--------
-----------------------------------IPG------KRF-----TDFN---EIRDEIDRETEK-----TTGKN-----------------
--AG-------------------------VS--SNPINLRIYSPN--
----------VLTLTLVDLPGLTK--VP-------------------VGDQPK----------DI---EKLIK--------DMILKYISK---
---------SNAIILAVTSANT--DLA-NS--DGLKLAREVDPEGVRTIGVL-TKIDLMDQGT--------
DVIDILAGR---V---------------------IPLR-LGYVPV----------------------------------VNR------------------
-----------------G-QRDIE----N---------------------------KKKIS----------L----ALEA---EKNYF---------
EN-------HA--------------------SY-----------------R-S------K--------------------------AQY-CA------------
----
>XP_002129967.2
------------LQLPQIVVVGV----Q----------------SSGKSSVLE-----N---LV-GRDFLPRG---TGIVTRCPL-
--------VLQ---M-I-HT-TN-------------------EDTAQCSNEGSSGNN----------------------
DSDSSGESFK-ETNEEVK---EW---V-----------------------------------K---------------FQH-------------
-----------------------------TKG------KIF-----RSFK---QVKKEIELETQR-----LSGNN-------------------
KG------------------------IS--SEAIRLKIFSPK---
------VLNLTLVDLPGLMK--IP-------------------VGDQPD----------DI---EEQAR--------NLILRYISN----
--------PNSIILAVTPANV--DFA-TS--EALQMARIVDPDGCRTLAVV-TKLDLMDAGT--------
DAIDVLCGR---I---------------------VPVK-LGIIGI----------------------------------VNR-------------------
----------------S-QLDIN----K----------------------------GKSVQ----------D----AIKD---EQAFL---------
QK-------K---------------------Y-----------------P-S------F-------------------------ANR-SGS-----
RYLSITLNRLLMHHIRDCL----
>NP_001259946.1

```
------------IQLPQIVVLGS----Q----------------SSGKSSVIE-----S---VV-GRSFLPRG---TGIVTRRPL---
------VLQ---L-I-YS-PL-------------------DDRENRSAENG----------------------------------------TSNAE-
--EW---G------------------------------------R----------------FLH-------------------------------------------
TK-------KCF-----TDFD---EIRKEIENETER-----AAGSN-------------------KG-------------------------IC-
-PEPINLKIFSTH--------------------------------------------------------------------VVNLTLVDLPGITK--
VP-------------------VGDQPE----------DI---EAQIK--------ELVLKYIEN------------PNSIILAVTAANT--
DMA-TS--EALKLAKDVDPDGRRTLAVV-TKLDLMDAGT--------DAIDILCGR---V--------------------
IPVK-LGIIGV----------------------------------MNR-----------------------------------S-QKDIM----D------
---------------------QKHID----------D----QMKD---EAAFL---------QR-------K----------------------Y----
-------------P-T------L--------------------------ATR-NGT-----PYLAKTLNRLLMHHIRDCL----
>XP_035676386.1
------------IQLPQIVVIGT----Q----------------SSGKSSVLE-----S---LV-GRDFLPRG---TGIVTRRPL---
------VLQ---L-V-HV-NS-------------------EEKKRPSEDED-------------------------------
GGHKQDIKEHAHVE---EW---G------------------------------------K----------------FLH------------------
-------------------------TKN------KIY-----TDFD---EIRQEIENETDR-----VTGTN-------------------KG----
---------------------II--DDAIHLKIYSPK--------------------------------------------------------------------
VLNLTLVDLPGITK--VP-------------------VGDQPP----------DI---EVQIR--------EMCLKYIAN----------
--PNSIILAVTSANT--DMA-TS--EALKFAKEVDPDGRRTLAVI-TKLDLMDAGT--------DAHDVLMGR--
-V--------------------IPVK-LGIIGV----------------------------------VNR-----------------------------------
S-QMDIN----K---------------------------RKPIE----------E----AIKD---EAAFM---------QR-------K-------
---------------Y-----------------P-S------L--------------------------ASR-NGT-----
SHLARTLNRLLMHHIRDCL----
>XP_006821224.1
-----------------------------------------------------------------------------------------------------------------
-----------------------------------------------------------------------------------------------------------------
-----------------------------------------------------------------------------------------------------------------
-----------------------------------------------------------------------------------------------------------------
-----------------------------------------------------------------------------------------------------------------
-------MA-TS--EAIKLSREVDEDGRRTLAVI-TKLDLMDAGT--------DAVEIICGR---V-------------------
-IPVK-LGIIGV----------------------------------INR-----------------------------------S-QMDIN----N------
---------------------KKPIQ----------E----SVKD---EAAFL---------QR-------K----------------------Y-------
----------P-A------L--------------------------ASR-NGT-----PYLAKTLNRLLMHHIRDCL----
>XP_030827871.1
------------IQLPQIVVVGN----Q----------------SSGKSSVLE-----G---LV-GKDFLPRG---
NGIVTRRPL---------VLQ---M-V-HV-DP-------------------EDKRGASGEGE-----------------------------
----------EEITAD---EW---G------------------------------------K----------------FLH------------------------
-------------------TKN------KVY-----TDFE---EIREEIQNETDR-----MAGTN-------------------KG--------
-----------------IV--HDAIHLRIYSPK--------------------------------------------------------------------
VLNLTLVDLPGLTK--VP-------------------VGDQPE----------DI---ESQIR--------EMLVKYIGN----------
--PNSIILAVTSANT--DMA-TS--ESLKLAKEIDPDGRRTLAVI-TKLDLMDAGT--------DAVDVLCGR---
V--------------------IPVK-LGIIGV----------------------------------VNR-----------------------------------
S-QMDIN----N---------------------------KKVID----------D----AVKD---ESAFL---------QR-------K------
----------------Y-----------------P-A------L--------------------------ASR-NGT-----
AYLARTLNRLLMHHIRDCL----
```

>XP_032819300.1
------------IQLPQIVVVGA----Q----------------SSGKSSVLE-----S---LV-GRDFLPRG---TGIVTRRPL--
-------VLQ---L-V-HV-IP-------------------DERIRPGGEEN--
GVEAE---EW---G------------------------------------K----------------FLH----------------------------------
---------TKN------KVY-----SDFN---EIRQEIENETER-----ITGTN-------------------KG-------------------
-----IS--SEAIHLKIFSPH---
VLNLTLVDLPGITK--VP-------------------VGDQPV----------DI---EQQIR--------ELIIKFIGN------------
PNSIILAVTAANT--DLA-TS--EALKIAREVDTDGRRTLAVI-TKLDLMDAGT--------DAMDILTGR---V---
------------------IPVK-LGIIGV---------------------------------VNR----------------------------------S-
QLDIN----T---------------------------KKTIL----------D----AMQD---EQSFM---------QK-------K---------
-------------Y-----------------P-S------L--------------------------ANR-NGT-----
KFLGKTLNRLLMHHIRDCL----
>NP_957216.1
------------IQLPQIAVVGT----Q----------------SSGKSSVLE-----S---LV-GRDLLPRG---TGIVTRRPL---
------ILQ---L-V-HV-DP-------------------EDRRKTS-EEN--GVDGE-
--EW---G------------------------------------K----------------FLH---
TKN------KIY-----TDFD---EIRQEIENETER-----VSGNN-------------------KG-------------------------IS-
-DEPIHLKIFSPH---VVNLTLVDLPGITK--
VP-------------------VGDQPK----------DI---ELQIR--------ELILKYISN------------PNSIILAVTAANT--
DMA-TS--EALKVAREVDPDGRRTLAVV-TKLDLMDAGT--------DAMDVLMGR---V--------------------
-IPVK-LGLIGV---------------------------------VNR----------------------------------S-QLDIN----N-----
----------------------KKSVA----------D----SIRD---EHGFL---------QK-------K---------------------Y-----
------------P-S------L--------------------------ANR-NGT-----KYLARTLNRLLMHHIRDCL----
>XP_025940269.1
------------IQLPQIVVVGT----Q----------------SSGKSSVLE-----S---LV-GRDLLPRG---TGVVTRRPL--
-------ILQ---L-V-HV-SP-------------------EDGRKTAGDEN--
EIDAE---EW---G------------------------------------K----------------FLH----------------------------------
--------TKN------KVY-----TDFD---EIRQEIENETER-----ISGNN-------------------KG-------------------
-----IS--PEPIHLKIFSSN---
VVNLTLVDLPGMTK--VP-------------------VGDQPK----------DI---ELQIR--------ELILQFISN----------
--PNSIILAVTAANT--DMA-TS--EALKIAREVDPDGRRTLAVI-TKLDLMDAGT--------DAMDVLMGR--
-V---------------------IPVK-LGIIGV---------------------------------VNR----------------------------------
S-QLDIN----N---------------------------KKSVA----------D----SIRD---EYGFL---------QK-------K-------
---------------Y-----------------P-S------L--------------------------ANR-NGT-----
KYLARTLNRLLMHHIRDCL----
>XP_006168142.1
------------IQLPQIVVVGT----Q----------------SSGKSSVLE-----S---LV-GRDLLPRG---TGIVTRRPL---
------ILQ---L-V-HV-SP-------------------EDKRKTTGEEN---------------------------
DPATWKNSRHLSKGVEAE---EW---G------------------------------------K----------------FLH-----------
---------------------------------TKN------KLY-----TDFD---EIRQEIENETER-----ISGNN-------------------
KG-------------------------VS--PEPIHLKIFSPN---
--------VVNLTLVDLPGMTK--VP-------------------VGDQPK----------DI---ELQIR--------ELILRFISN---
---------PNSIILAVTAANT--DMA-TS--EALKISREVDPDGRRTLAVI-TKLDLMDAGT--------
DAMDVLMGR---V---------------------IPVK-LGIIGV---------------------------------VNR-----------------

------------------S-QLDIN----N---------------------------KKSVT----------D----SIRD---EYAFL---------
QK-------K----------------------Y-----------------P-S------L--------------------------ANR-NGT-----
KYLARTLNRLLMHHIRDCL----
>NP_001317309.1
------------IQLPQIVVVGT----Q----------------SSGKSSVLE-----S---LV-GRDLLPRG---TGIVTRRPL---
------ILQ---L-V-HV-SQ-------------------EDKRKTTGEEN---------------------------
DPATWKNSRHLSKGVEAE---EW---G------------------------------------K----------------FLH-----------
--------------------------------TKN------KLY-----TDFD---EIRQEIENETER-----ISGNN-------------------
KG-------------------------VS--PEPIHLKIFSPN---
--------VVNLTLVDLPGMTK--VP----------------VGDQPK----------DI---ELQIR--------ELILRFISN---
---------PNSIILAVTAANT--DMA-TS--EALKISREVDPDGRRTLAVI-TKLDLMDAGT--------
DAMDVLMGR---V---------------------IPVK-LGIIGV----------------------------------VNR-----------------
------------------S-QLDIN----N---------------------------KKSVT----------D----SIRD---EYAFL---------
QK-------K----------------------Y-----------------P-S------L--------------------------ANR-NGT-----
KYLARTLNRLLMHHIRDCL----
>NP_001392186.1
------------IQLPQIVVVGT----Q----------------SSGKSSVLE-----S---LV-GRDLLPRG---TGVVTRRPL--
-------ILQ---L-V-HV-SP-------------------EDKRKTTGEENGKFQS---------------------
WNPATWKNSRHLSKGVEAE---EW---G------------------------------------K----------------FLH--------
-----------------------------------TKN------KLY-----TDFD---EIRQEIENETER-----ISGNN----------------
---KG-------------------------VS--PEPIHLKVFSPN--
-----------VVNLTLVDLPGMTK--VP----------------VGDQPK----------DI---ELQIR--------
ELILRFISN------------PNSIILAVTAANT--DMA-TS--EALKISREVDPDGRRTLAVI-TKLDLMDAGT-----
---DAMDVLMGR---V---------------------IPVK-LGIIGV----------------------------------VNR--------------
---------------------S-QLDIN----N---------------------------KKSVT----------D----SIRD---EYAFL--------
-QK-------K----------------------Y-----------------P-S------L--------------------------ANR-NGT-----
KYLARTLNRLLMHHIRDCL----
>XP_014394711.1
------------LLCPVCVQISS----L----------------SSGKSSVLE-----S---LV-GRDLLPRG---TGIVTRRPL---
------ILQ---L-V-HV-SP-------------------EDQRKTSGEEN---------------------------
DPATWKNSRHLSKGVEAE---EW---G------------------------------------K----------------FLH-----------
--------------------------------TKN------KLY-----TDFD---EIRQEIENETER-----ISGNN-------------------
KG-------------------------VS--PEPIHLKIFSPN---
--------VVNLTLVDLPGMTK--VP----------------VGDQPK----------DI---ELQIR--------ELILRFISN---
---------PNSIILAVTAANT--DMA-TS--EALKISREVDPDGRRTLAVI-TKLDLMDAGT--------
DAMDVLMGR---V---------------------IPVK-LGIIGV----------------------------------VNR-----------------
------------------S-QLDIN----N---------------------------KKSVT----------D----SIRD---EYAFL---------
QK-------K----------------------Y-----------------P-S------L--------------------------ANR-NGT-----
KYLARTLNRLLMHHIRDCL----
>XP_028602039.1
------------IQLPQIVVVGT----Q----------------SSGKSSVLE-----S---LV-GRDLLPRG---TGIVTRRPL---
------ILQ---L-V-NV-SA-------------------EDLRKKTGDEN---------------------------
DPATWKHARHLTKGVDTE---EW---G------------------------------------K----------------FLH------------
-------------------------------TKN------KLY-----SDFD---EIRQEIESETER-----ISGNN-------------------

KG-------------------------IS--PEPIHLKVFSPN--
--------VVNLTLVDLPGMTK--VP-------------------VGDQPK----------DI---ELQIR--------ELILRFISN---
---------PNSIILAVTAANT--DMA-TS--EALKIAREVDPDGRRTLAVI-TKLDLMDAGT--------
DAMDVLMGR---V---------------------IPVK-LGIIGV----------------------------------VNR-----------------
------------------S-QLDIN----N---------------------------KKSVA----------D----SIRD---EYGFL---------
QK-------K----------------------Y-----------------P-S------L--------------------------ANR-NGT-----
KYLARTLNRLLMHHIRDCL----
>XP_012382650.2
---G---FKLVISTKL---------ILC---C-F-
SL-TT--DPATWKNSRHLSKGVEAE---EW---G------
------------------------------K----------------FLH--TKN------KLY---
--TDFD---EIRQEIENETER-----ISGNN-------------------KG-------------------------VS--
PEPIHLKIFSPN--VVNLTLVDLPGMTK--
VP-------------------VGDQPK----------DI---ELQIR--------ELILRFISN------------PNSIILAVTAANT--
DMA-TS--EALKISREVDPDGRRTLAVI-TKLDLMDAGT--------DAMDVLMGR---V---------------------
IPVK-LGIIGI----------------------------------VNR-----------------------------------S-QLDIN----N--------
-------------------KKSVT----------D----SIRD---EYAFL---------QK-------K----------------------Y-----------
------P-S------L--------------------------ANR-NGT-----KYLARTLNRLLMHHIRDCL----
>XP_031753959.1
------------IGFGPVLRVPL----Q----------------PAKCWAAKE-----S---MVLSASLLP-V---PHLYERLLL-
--------SVV---F-F-SL-IP--
DPNAWKIPKHFSKGVETE---EW---G-------------------------------------K----------------FLH------------
--------------------------------TKN------KIY-----TDFD---EIRQEIENETER-----ISGNN-------------------
KG-------------------------IS--SEPIHLKIFSPN---
-------VVNLTLVDLPGMTK--VP-------------------VGDQPK----------DI---EIQIR--------ELILRYISN----
--------PNSIILAVTAANT--DMA-TS--EALKIARESDPDGRRTLAVI-TKLDLMDAGT--------
DAMDVLLGR---V---------------------IPVK-LGIIGV----------------------------------VNR-----------------
------------------S-QLDIN----N---------------------------KKSVA----------D----SIRD---EYGFL---------
QK-------K----------------------Y-----------------P-S------L--------------------------ANR-NGT-----
KYLARTLNRLLMHHIRDCL----
>PAA85687.1
------------IQLPQIVVIGT----Q----------------SSGKSSVLE-----S---LV-GRDFLPRG---TGIVTRRPL---
------VLQ---L-V-HL-EA-------------------DEKDEAGDRPA--AEEE-
--EW---G-------------------------------------K----------------FLH--
TKG------KIY-----TDFN---EIRDEIARETDR-----IAGSG-------------------KC-------------------------VS-
-IDPINLKIYSPH--VVSLTLVDLPGITK--
VP-------------------VADQPE----------DI---EVQIR--------ALCIEYIKN------------PNSIILAVTPANT--
DMA-TS--ESLKLAKEVDPQGKRTLAVI-TKLDLMDAGT--------DAHDLLLGR---V---------------------
IPVK-LGIIGV----------------------------------VNR-----------------------------------S-QADIK----N-------
--------------------QKQVK----------E----AVRD---ESSFL---------QR-------R----------------------Y------
-----------P-S------L--------------------------ASR-NGT-----PYLARTLNRLLMHHIRDCL----
>NP_741403.2
------------IQLPQIVVVGS----Q----------------SAGKSSVLE-----N---LV-GRDFLPRG---TGIVTRRPL--
-------ILQ---L-N-HV-AL-------------------DDESKRRRSNG---

TLLTD---DW---A-------------------------------------M-----------------FEH------------------------------------
--------TGS------KVF-----TDFD---AVRKEIEDETDR-----VTGVN-------------------KG--------------------
------IS--LLPISLKIYSHR---
VVSLSLVDLPGITK--IP-------------------VGDQPV----------NI---EEQIR--------EMILLYISN------------
PSSIILAVTPANQ--DFA-TS--EPIKLAREVDAGGQRTLAVL-TKLDLMDQGT--------DAMDVLMGK---
V---------------------IPVK-LGIIGV-----------------------------------VNR------------------------------------
S-QQNIL----D----------------------------NKLIV----------D----AVKD---EQSFM---------QK-------K------
----------------Y-----------------P-T------L---------------------------ASR-NGT-----
PYLAKRLNMLLMHHIRNCL----
>XP_042914770.1
------------LDLPQVAVVGS----Q----------------SSGKSSVLE-----A---LV-GRDFLPRG---
NDIVTRRPL---------LLQ---L-V-KT-TP--
GPT--GRPS---EW---G-------------------------------------E-----------------FLH------------------------------
--------------APG------KMF-----YDFD---RIRDEIHQETER-----LVGYN--------------------KN-------------
-------------VS--DKPIRLKIFSPR---
VLTMTLVDLPGLTR--VP-------------------VGDQPG----------DI---EQRIR--------EMALEYIRR--------
---PNCIILAVSPANV--DLA-TS--DALQLSQVADPEGVRTIGVL-TKLDIMDRGT--------DAAHILRNA--
-H---------------------IPLR-LGYIGV-----------------------------------VLR------------------------------------
A-QADIA----A----------------------------KLPMS----------E----CRKR---EESFF---------AS-------RA-----
---------------EY-----------------R-D------V---------------------------AAH-CGV-----
PTLARRLNVILVEHIR-------
>PWZ09977.1
------------IDLPQVAVVGS----Q----------------SSGKSSVLE-----A---LV-GRDFLPRG---SDICTRRPL-
--------VLQ---L-V-HQ-PR--RPAD-AEAD---
EW---G-------------------------------------E-----------------FLH--
LSG------RRF-----YDFR---EIRREIQAETDR-----EAGGN-------------------KG--------------------------
VS--DRQIRLKIFSPN---
VLNITLVDLPGITK--VP-------------------VGDQPT----------DI---EARIR--------TMIMSYIKH------------
KTCIILAVSPANA--DLA-NS--DALQMARVADPDGSRTIGVI-TKLDIMDRGT--------DARNFLLGN---
V---------------------IPLK-LGYVGV-----------------------------------VNR------------------------------------
S-QQDIN----S----------------------------DLSIK----------D----ALAR---EEKFF---------RT-------QP------
--------------AY-----------------H-G------L---------------------------AQY-CGI-----
PQLAKKLNQILVQHIK-------
>KAH9304002.1

---MVQAETER-----
EVGVN-------------------KG--------------------------VS--DKQIRLKIYSPN----------------------------------
-------------------------------------VLNITLVDLPGLTK--VP-------------------VGDQPT----------DI---
EARIR--------TMIMSYIKH------------ETCIILAVSPANA--DLA-NS--DALQMARVADADGHRTIGVI-
TKLDIMDRGT--------DATNFLLGN---V---------------------IPLR-LGYVGV--------------------------------
---VNR------------------------------------S-QADIN----G----------------------------NKSIQ----------D----
ALAY---EERFF---------RS-------RP--------------------VY-----------------H-R------L-----------------------
----ADR-CGI-----PQLAKKLNYILVQHIR-------

>EFJ15047.1
-------------LPQVAVVGS----Q---------------SSGKSSVLE-----A---LV-GRDFLPRG---CDICTRRPL-
--------VLQ---L-V-QT-TR--RGD---EVV---
EW---G-----------------------------------E---------------FLH---
LPN------RRF-----TDFS---AIRKEIQAETDR-----ELGSN------------------KG------------------------
IS--DKQIRLKIFSPK---VLNITLVDLPGITK-
-VP------------------VGDQPT----------DI---EARIR--------TMILSYIKH-----------ETCIILAVSPANA--
DLA-NS--DALQMARIADPDGSRTIGVI-TKLDIMDRGT--------DARNFLLGS---V--------------------
IPLR-LGYIGV---------------------------------VNR---------------------------------S-QEDIT----S-------
--------------------NRSIQ----------D----ALMY---EEQFF---------RS-------RP------------------VY-----
------------H-S------L-------------------------SDR-CGI-----PQLAKKLNQILVQHIRTILP---
>EFJ37641.1
------------IDLPQVAVVGS----Q---------------SSGKSSVLE-----A---LV-GRDFLPRG---
CDICTRRPL---------VLQ---L-V-QT-TR--
RGD---EVV---EW---G-----------------------------------E---------------FLH----------------------------
--------------LPN------RRF-----TDFT---AIRKEIQAETDR-----ELGSN------------------KG-------------
-----------IS--DKQIRLKIFSPK--
VLNITLVDLPGITK--VP------------------VGDQPT----------DI---EARIR--------TMILSYIKH-----------
ETCIILAVSPANA--DLA-NS--DALQMARIADPDGSRTIGVI-TKLDIMDRGT--------DARNFLLGS---V-
--------------------IPLR-LGYIGV---------------------------------VNR---------------------------------S-
QEDIT----S--------------------------NRSIQ----------D----ALMY---EEQFF---------RS-------RP-------
------------VY-----------------H-S------L-------------------------SDR-CGI-----PQLAKKLNQILVQHIR-
------
>KAI5070335.1
------------IDLPQVAVIGS----Q---------------SSGKSSVLE-----A---LV-GRDFLPRG---SDICTRRPL--
-------VLQ---L-V-QL-VR--RPDDKSDVM---
EW---G-----------------------------------E---------------FLH---
TPG------KRY-----TDFS---LIRKEIQAETER-----ELGSN------------------RG------------------------IS-
-DKQIRLKIFSPS---VLNITLVDLPGITK--
VP------------------VGDQPS----------DI---EARVR--------TMILSYIKH-----------ETCIILAVSPANA--
DLA-NS--DALQMARMADPDGSRTIGVI-TKLDIMDRGT--------DARNFLLGS---A--------------------
IPLR-LGYIGV---------------------------------VNR---------------------------------S-QADIL----G-------
--------------------NKSIR----------E----ALAY---EDNFF---------RS-------LP------------------VY-------
----------H-S------L-------------------------ADR-CGI-----PQLAKKLNNILVQHIK-------
>KAI5070758.1
------------VNLPQVAVVGS----Q---------------SSGKSSVLE-----G---LV-GRDFLPRG---
SDICTRRPL---------VLQ---L-I-QT-TR--
RPDEKSEVV---EW---G-----------------------------------E---------------FLH----------------------------
---------------APG------KRF-----TDFS---LIRKEIEAETER-----ELGSN------------------KG-------------
-----------IS--DKQIRLKIFSPN---
VLTITLVDLPGITK--VP------------------VGDQPS----------DI---ESRVR--------TMILSYIKH-----------
ETCIILAVSPANA--DLA-NS--DALQMARLADPDGSRTIGVI-TKLDIMDRGT--------DARNLLLGN---
V--------------------IPLR-LGYIGV---------------------------------VNR---------------------------------
S-QEDIL----G--------------------------NKSVV----------D----ALLY---EENFF---------RS-------RP-----

---------------VY-----------------H-N------L-------------------------ADR-CGI-----
AQLAKKLNTILVQHIK-------
>PTQ35749.1
------------IDLPQVAVVGS----Q----------------SSGKSSVLE-----A---MV-GRDFLPRG---
ADICTRRPL---------VLQ---L-V-QT-VR--
RPEDRSELV---EW---G-----------------------------------E----------------FLH----------------------------
---------------IPG------RRF-----TDFT---AIRKEIQAETDR-----ELGTN-------------------KG--------------
-----------IS--EKQIRLKIFSPN---
VLNITLVDLPGITK--VP-------------------VGDQPS----------DI---EARVR--------TMILNYIKH-----------
-ETCIILAVSPANA--DLA-NS--DALQMARIADPDGSRTIGVI-TKLDIMDRGT--------DARNFLLGN---
V--------------------VPLR-LGYIGV---------------------------------VNR----------------------------------
S-QEDIQ----A---------------------------NKTIR----------E----ALGY---EENFF---------RS-------RP------
--------------VY-----------------H-S------L-------------------------SER-CGI-----
PQLAKKLNSILVQHIR-------
>KAG0555682.1
------------IDLPQVAVVGS----Q----------------SSGKSSVLE-----A---LV-GRDFLPRG---SDICTRRPL-
--------VLQ---L-V-QT-SR---RPEDRTELV---
EW---G-----------------------------------E----------------FLH--
IPG------RRF-----TDFA---AIRKEIQAETDR-----ELGTN-------------------KG-------------------------IS-
-EKQIRLKIFSPN---VLNITLVDLPGITK--
VP-------------------VGDQPN----------DI---EARVR--------TMILSYIKH------------ETCIILAVSPANA--
DLA-NS--DALQMARIADPDGSRTIGVI-TKLDIMDRGT--------DARNFLLGN---V---------------------
IPLR-LGYIGV---------------------------------VNR----------------------------------S-QEDII----A--------
-------------------NKSIR----------D----ALVY---EEGFF---------RS-------KP-------------------VY--------
---------H-N------L-------------------------ADR-CGV-----PQLAIRLNTILVQHIK-------
>KAG0554580.1
------------IDLPQVAVVGS----Q----------------SSGKSSVLE-----A---LV-GRDFLPRG---SDICTRRPL-
--------VLQ---L-V-QT-LR---RPDEKSEPV---
EW---G-----------------------------------E----------------FLH--
IPG------RRF-----TDFS---AIRKEIQLETER-----ELGTN-------------------KG-------------------------IS--
EKQIRLKIFSPN---VLNITLVDLPGITK--
VP-------------------VGDQPT----------DI---EARVR--------TMILSYIKH------------ETCIILAVSPANA--
DLA-NS--DALQMARIADPDGSRTIGVI-SKLDIMDRGT--------DARSFLLGN---I---------------------
IPLR-LGYVGV---------------------------------VNR----------------------------------S-QEDIS----A------
---------------------NKSIR----------D----ALTY---EENFF---------RS-------RP-------------------VY------
-----------H-N------L-------------------------SDR-CGV-----PQLAKKLNTILVQHIK-------
>XP_024362051.1
------------IDLPQVAVVGS----Q----------------SSGKSSVLE-----A---LV-GKDFLPRS---SDICTRRPL-
--------VLQ---L-V-QT-LR---RSDEKSELV---
EW---G-----------------------------------E----------------FLH--
IPG------RQF-----TNFS---AIRKEIQLETER-----DMGTN-------------------KG------------------------
IS--EKQIRLKIFSPD---VLNITLVDLPGITK-
-VP-------------------VGDQPT----------DI---EARVR--------TMILSYIKH------------KTCIILAVSPANA--
DLA-NS--DALQMARIADPDGSRTIGVI-SKLDIMDRGT--------DARSLLLGN---V---------------------

IPLR-LGYVGV----------------------------------VNR-----------------------------------S-QEDIS----R------
---------------------NRSIR----------D----ALTN---EENFF---------RS-------RP-------------------VY-----
------------H-N------L--------------------------SDR-CGV-----PQLAKKLNTILVQHIK-------
>AAC61784.1
------------IALPQVVVVGS----Q----------------SSGKSSVLE-----A---LV-GRDFLPRG---
NDICTRRPL---------VLQ---L-L-QT-KS---
RANG-GSDD---EW---G------------------------------------E----------------FRH--------------------------
-----------------LPE------TRF-----YDFS---EIRREIEAETNR-----LVGEN-------------------KG------------
-------------VA--DTQIRLKISSPN--
VLNITLVDLPGITK--VP-------------------VGDQPS----------DI---EARIR--------TMILSYIKQ------------
DTCLILAVTPANT--DLA-NS--DALQIASIVDPDGHRTIGVI-TKLDIMDKGT--------DARKLLLGN---V--
-------------------VPLR-LGYVGV-----------------------------------VNR-------------------------------------C-
QEDIL----L---------------------------NRTVK----------E----ALLA---EEKFF---------RS-------HP---------
-----------VY-----------------H-G------L--------------------------ADR-LGV-----PQLAKKLNQILVQHIK--
-----
>XP_052310486.1
------------IELPQVAVVGS----Q----------------SSGKSSVLE-----A---LV-GRDFLPRG---NEICTRRPL-
--------VLQ---L-L-QT-KR--KGDG-SGED---
EW---G------------------------------------E----------------FLH--
LPG------KRF-----YDFS---EIRSEIQAETAK-----EAGGN-------------------KG-------------------------
VS--DKQIRLKIFSPN---
VLDITLVDLPGITK--VP-------------------VGDQPS----------DI---EARIR--------TMIMSYIKK------------
PSCLILAVTAANS--DLA-NS--DALQIAGNADPDGYRTIGII-TKLDIMDRGT--------DARNLLLGK---V-
--------------------IPLR-LGYVGV-----------------------------------VNR-------------------------------------S-
QEDII----L---------------------------NRSIK----------D----ALAA---EEKFF---------RS-------RP----------
----------VY-----------------N-G------L--------------------------ADR-CGV-----PQLAKKLNQILVQHIK--
-----
>ONM18162.1
------------LELPQVAAIGG----Q----------------SSGKSSVLE-----A---LV-GRDFLPRG---PDICTRRPL-
--------VLQ---L-V-RH---AAPE---EW-
--G------------------------------------E----------------FLH--VPG---
---RQF-----HDFE---QIKREIQLETDK-----EAGDN-------------------KG-------------------------VS--
EKQIRLKIFSPN---VLDITLVDLPGITR--
VP-------------------VGDQPS----------DI---ESRIR--------AMIMQYIKH------------PSCIILAVSPANA-
-DLA-NS--DALQLARLADPDGSRTIGVI-TKLDIMDRGT--------DARNFLLGN---V--------------------
IPLK-FGYVGV----------------------------------VNR-----------------------------------S-QEDIN----F------
---------------------NRSVK----------D----ALAF---EEKYF---------LT-------LP--------------------AY------
-----------H-G------L--------------------------AHC-CGV-----PQLAKKLNMILLKHVT-------
>KAH9327796.1
--MYP-----W---LV-VKEF---------------PD---------AIA---L--

QVETER-----EAGGN-------------------KG-------------------------IS--AKQIRLKIFSPF-------------------
--VLNINLVDLPGIMK--VP-------------------VGDQPI-------

---DI---EARIR--------TMIMSYIKH------------PSCIILAVSPANS--DLA-NS--
DALQIARVADTDGSRTIGVI-TKLDIMDRGT--------DARNFLLGN---V--------------------IPLQ-
LGYIGV----------------------------------VNR-----------------------------------S-QEDII----A---------------
------------NQSIR----------D----ALAY---EENFF---------RG-------HP--------------------VY-------------
----C-S------L--------------------------ADQ-CGI-----PQLARKLNQILVKHIR-------
>XP_014148015.1
---LQ---------LV--HHPVQRGG---------PA-----------------
A---EW---G-------------------
-----------------E----------------FLH---QPG------KIY-----TDFS---
KIRDEIANETDR-----LTGTN-------------------KG-------------------------IS--HTPINLKLYSPN----------
---MLDLTLVDLPGITK--IA-------------------
VGDQPE----------DI---EVQIH--------QLIESYINN------------PNCIILAVTAANT--DIA-NS--
DALKMAKKADPKGLRTIGVA-TKLDLMDAGT--------DALDILTGK---V---------------------VASK-
LGFIGV----------------------------------VNR-----------------------------------S-QADIN----Q-------------
--------------KVSIE----------T----AREA---EQEYF---------RT-------HP--------------------AY--------------
---K-S------L--------------------------YKQ-SGT-----EYLTRRLNQLLMTHIRRCL----
>XP_001750431.1
------------IQLPQIVVVGA----QASSPADHPALRMSHEQSSGKSSILE-----N---VV-GKDFLPRG---
TGIVTRVPL---------VLQ---L-V-QT-AD--
----D---EW---A------------------------------------T----------------FQH-------------------------------------
------AGG------KVF-----RDFE---QVRQEIVDQTER-----ITGPG-------------------KA--------------------
-----VS--NEPIHLRVHSPN--
VVNLTLVDLPGLTK--VA-------------------VADQPQ----------DI---GPQIR--------RLVRHYIDN---------
---PNSLILAVSPANA--DIA-NS--DSLQIAKEVDPQGDRTLAIV-TKLDLMDRGT--------DAKALLSGE--
-V---------------------LPVK-LGIIGI----------------------------------VNR-----------------------------------
S-QNDIN----C---------------------------KTSIQ----------D----SLDN---EKRFF---------RT-------H------
----------------Y-----------------P-E------M--------------------------ADR-CGC-----
AFLADTLHHLLLQHIRACL----
>XP_042924642.1
--------------LPTIVVVGG----Q----------------SSGKSSVLE-----A---VV-GRDFLPRG---TGIVTRRPL---
------VLQ---L---VK-TD--DPNAV---DY---
G------------------------------------E----------------FAH---APG-----
-RKF-----TNFD---DITTEIEDETTRH--LQRQGGT-------------------KV-------------------------VS--
PDPIYLTVYSVN---VPNLTLVDMPGLTK--
VP-------------------IDGQPA----------SI---VQELD--------DMARQYVKS------------
DNAIILAVTPANA--DLA-TS--DALRMARDVDPSGDRTIGVL-TKVDIMDRGT--------DCRDVLLGK---
T---------------------LKLK-HGWVAV----------------------------------VNR----------------------------------
-G-QADLN----S---------------------------KVTMK----------D----ARAR---EQEFF---------KG-------KP--
------------------EY-----------------Q-D------L--------------------------QN--TGT-----
TFLAEKLSNHLINEIMKSLP---
>NP_001190448.1
--------------LPAIAVVGG----Q----------------SSGKSSVLE-----S---IV-GKDFLPRG---SGIVTRRPL---
------VLQ---L---QK-ID---DGTR---EY---
A------------------------------------E----------------FLH---LPR------

KKF-----TDFA---AVRKEIQDETDR-----ETGRS--------------------KA--------------------------IS--
SVPIHLSIYSPN--VVNLTLIDLPGLTK--
VA--------------------VDGQSD----------SI---VKDIE--------NMVRSYIEK------------PNCIILAISPANQ--
DLA-TS--DAIKISREVDPSGDRTFGVL-TKIDLMDKGT--------DAVEILEGR---S----------------------
FKLK-YPWVGV-----------------------------------VNR--------------------------------------S-QADIN----K----
------------------------NVDMI----------A----ARKR---EREYF---------SN-------TT---------------------EY---
--------------R-H------L---------------------------ANK-MGS-----EHLAKMLSKHLERVIKSRIP---
>XP_002299468.1
--------------LPSIAVVGG----Q-----------------SSGKSSVLE-----S---VV-GKDFLPRG---SGIVTRRPL--
-------VLQ---L---HK-ID---EGSR---EY---
A--------------------------------------E-----------------FLH---LPR------
KRF-----TDFA---AVRREIQDETDR-----ETGRS--------------------KQ--------------------------IS--
SVPIHLSIYSPN--VVNLTLVDLPGLTK--
VA--------------------VEGQPD----------SI---VQDIE--------NMVRAYIEK------------PNCIILAISPANQ--
DLA-TS--DAIKISREVDPTGERTLGVL-TKIDLMDKGT--------DAVDMLEGK---S----------------------
YRLK-FPWVGV-----------------------------------VNR---------------------------------------S-QADIN----K----
------------------------NVDMI----------A----ARRR---EREYF---------SS-------TP---------------------EY---
--------------K-H------L---------------------------AHR-MGS-----EHLAKMLSKHLEVVIKSKIP---
>XP_002302631.1
--------------LPSIAVVGG----Q-----------------SSGKSSVLE-----S---IV-GKDFLPRG---AGIVTRRPL---
------VLQ---L---HK-ID---EG-K---EY---
A--------------------------------------E-----------------FMH---LPR-----
-KKF-----TDFA---AVRKEIADETDR-----ETGRS--------------------KQ--------------------------IS--
SVPIHLSIFSPN--VVNLTLVDLPGLTK--
VA--------------------IDGQPE----------SI---VHDIE--------NMVRSYIEK------------PNCIILAISPANQ--
DLA-TS--DAIKISREVDPRGERTFGVL-TKIDLMDKGT--------DAVDILEGK---S----------------------
YKLQ-FPWIGV-----------------------------------VNR-------------------------------------S-QADIN----K-----
-----------------------SVDMI----------A----ARRR---EREYF-------QS-------SP---------------------EY---
--------------G-H------L---------------------------ASR-MGS-----EHLGKMLSKHLEQVIKSRIP---
>AQK88296.1
--------------ISRVAGGTA----R-----------------SSGKSSVLE-----S---VV-GKDFLPRG---SGIVTRRPL---
------VLQ---L---HR-ID---GDR---EY---
A--------------------------------------E-----------------FMH---LPR-----
-KRF-----TDFA---AVRKEIADETDR-----ETGRS--------------------KQ--------------------------IS--
TVPIHLSIFSPHGKMQTLEGYIVTVALSHLNCPLP--
IVNLTLIDLPGLTK--VA--------------------VDGQPE----------SI---VHDIE--------NMVRSYIEK------------
-PNCIILAVSPANQ--DLA-TS--DAIKISREVDPKGERTFGVL-TKIDLMDKGT--------DAVDILEGR---S-
--------------------YRLQ-TPWVGV-----------------------------------VNR-------------------------------------
S-QQDIN----K--------------------------NVDMI----------A----ARRR---EREYF--------AS-------TP-----
---------------EY-----------------K-H------M---------------------------ASR-MGS-----
EYLGKMLSKHLEQVIKSRIP---
>PTQ45603.1
--------------LPSVVVVGG----Q-----------------SSGKSSVLE-----S---IV-GRDFLPRG---SGIVTRRPL--
-------VLQ---L---HK-T---DDGS---DY---

A----------------------------------E----------------FLH--HPR-----
-RRF-----ADFA---AVRKEIADETDR-----VTGRS------------------KM------------------------IS--
PVPIHLSIYSPN--VVNLTLIDLPGLTK--
VA-------------------VEGQPD----------SI---VHDIE--------NMVRSYVEK------------PNSIILAISPANQ-
-DIA-TS--DAIKLAREVDPAGERTWGVL-TKLDLMDRGT--------NALDVLEGR---S--------------------
YRLQ-LPWIGV---------------------------------VNR---------------------------------S-QADIN----K-----
----------------------NVDMI----------A----ARRR---EREYF---------QS-------SQ--------------------DY--
---------------G-H------L-------------------------AGK-MGS-----EYLAKMLSKHLEAVIKSRIP---
>KAI5072318.1
--------------LPSVAVVGG----Q----------------SSGKSSVLE-----S---IV-GRDFLPRG---SGIVTRRPL--
-------VLQ---L---YR-T---DKGP---EY---
A----------------------------------E----------------FLH--APK------
KRF-----TDFA---AVRKEISDETDR-----ITGRS------------------KQ------------------------IS--
PVPIHLSIYSPN--VVNLTLIDLPGLTK--
VA-------------------VEGQPD----------SI---VADIE--------NMVRSYVEK------------PNSLILAISPANQ-
-DIA-TS--DAIKLAREVDPSGERTFGVL-TKLDLMDKGT--------NALDTLEGR---A--------------------
YRLQ-HPWVGV---------------------------------VNR---------------------------------S-QADIN----K---
------------------------SVDMM----------A----ARRR---EREYF---------AT-------SP--------------------DY-
----------------K-H------L-------------------------ASR-MGS-----EYLGQMLSKHLESVIKSRIP---
>KAI5058380.1
--------------LPSVAVVGG----Q----------------SSGKSSVLE-----S---IV-GRDFLPRG---SGIVTRRPL--
-------VLQ---L---HK-L---DEGS---EY---
A----------------------------------E----------------FMH--LPK-----
-RRF-----TDFA---AVRKEIQDETDR-----VTGRS------------------KQ------------------------IS--
PVPIHLSIYSPH--VVNLTLIDLPGLTK--
VA-------------------VEGQPE----------SI---VADIE--------NMVRLYVDK------------PNTIILAISPANQ--
DIA-TS--DAIKLAREVDPTGERTWGVL-TKLDLMDKGT--------NAIDVLEGR---A--------------------
YHLK-NPWIGV---------------------------------VNR---------------------------------S-QADIN----K----
-----------------------NVDMM----------A----ARRR---EREYF---------AT-------SS--------------------DY-
----------------S-H------L-------------------------TSR-MGS-----EYLGKMLSKHLEAVIKARIP---
>XP_002987566.1
--------------LPSVVVVGG----Q----------------SSGKSSVLE-----S---IV-GRDFLPRG---SGIVTRRPL--
-------VLQ---L---HK-T---EGGA---EY---
A----------------------------------E----------------FLH--IPK------
KRF-----TDFS---LVRKEIQDETDR-----VTGRS------------------KQ------------------------IS--
PIPIQLSIYSPN--VVNLTLIDLPGLTK--IA-
------------------IEGQPD----------SI---VADIE--------NMVRSYVEK------------QNSVILAISPANQ--
DIA-TS--DAMKLAREVDPTGERTFGVL-TKLDLMDKGT--------NALDVLEGR---S--------------------
YKLQ-HPWVGV---------------------------------VNR---------------------------------S-QADIN----R---
------------------------SVDMV----------A----ARRR---EREYF---------SS-------SA--------------------DY-
----------------G-H------L-------------------------TSR-MGS-----EYLAKILSKHLEAFIKARIP---
>KAI5602084.1
--------------LPSVAVVGG----Q----------------SSGKSSVLE-----S---IV-GRDFLPRG---SGIVTRRPL--
-------VLQ---L---HK-TE---PGIT---EY---

A-------------------------------------E----------------FLH--KQR-----
-ERF-----TDFA---MVRKEIQDETDK-----ITGKS-------------------KQ-------------------------IS--
PVPIHLSIYSPN--VVNLTLIDLPGLTK--
VA-------------------VEGQPE----------SI---VKDIE--------NMVRLYVEK------------PNCIILAITPANQ--
DIA-TS--DAIKLAREVDPAGERTFGVL-TKLDLMDKGT--------NAQDVLEGR---A---------------------
YPLQ-HPWVGI-----------------------------------VNR------------------------------------S-QADIN----K----
-----------------------NVDMI----------A----ARRR---EREFF---------ST-------SP---------------------DY---
--------------G-H------L--------------------------AGR-MGS-----EYLAKLLSKHLESVIKTRIP---
>XP_006375094.1
--------------LPSVAVVGG----Q----------------SSGKSSVLE-----S---IV-GRDFLPRG---SGIVTRRPL--
-------VLQ---L---HK-TE--DGSQ---EY--
-A------------------------------------E----------------FLH---LPK-----
-RRF-----SDFA---VVRKEIQDETDR-----ITGKT-------------------KQ-------------------------IS--
PVPIHLSIYSPN--VVNLTLIDLPGLTK--
VA-------------------VEGQPE----------SI---VQDIE--------TMVRTYVEK------------PNCIILAISPANQ--
DIA-TS--DAIKLAREVDPSGERTFGVL-TKLDLMDKGT--------NALDVIEGR---S--------------------
YRLQ-HPWVGI-----------------------------------VNR------------------------------------S-QADIN----K----
-----------------------NVDMI----------V----ARRK---EREYF---------AT-------SP---------------------DY---
--------------G-H------L--------------------------ANK-MGS-----EYLAKLLSKHLESAIRARIP---
>XP_002315854.1
--------------LPSVAVVGG----Q----------------SSGKSSVLE-----S---VV-GRDFLPRG---SGIVTRRPL-
--------VLQ---L---HK-I---DGGS---DY--
-A------------------------------------E----------------FLH---APR----
--KKF-----TDFA---SVRKEIADETDR-----ITGKS-------------------KQ-------------------------IS--
NVPIHLSIYSPN--VVNLTLIDLPGLTK--
VA-------------------VEGQPE----------SI---VEDIE--------NMVRSYVEK------------PNSIILAISPANQ--
DIA-TS--DAIKLAREVDPSGERTFGVL-TKLDLMDKGT--------NALDVIEGR---S--------------------
YRLQ-HPWVGI-----------------------------------VNR------------------------------------S-QADIN----K----
-----------------------NVDMI----------A----ARRK---EREYF---------ET-------SP---------------------EY---
--------------G-H------L--------------------------SSK-MGA-----EYLAKLLSKHLETVIRQRIP---
>NP_001147100.1
--------------LPSVAVVGG----Q----------------SSGKSSVLE-----S---IV-GRDFLPRG---SGIVTRRPL--
-------VLQ---L---HK-T--DGGH---EY--
-A------------------------------------E----------------FLH---APR----
--KRF-----TDFA---AVRKEIADETDR-----ITGKT-------------------KA-------------------------IS--
NVPIHLSIYSPH--VVNLTLIDLPGLTK--
VA-------------------VEGQPE----------SI---VQDIE--------NMVRAYVDK------------PNCIILAISPANQ-
-DIA-TS--DAIKLARDVDPSGDRTFGVL-TKLDLMDKGT--------NAVDVLEGR---Q---------------------
YRLQ-HPWVGI-----------------------------------VNR------------------------------------S-QADIN----K----
-----------------------NVDML----------S----ARRK---EKEYF---------ES-------SP---------------------EY--
---------------G-H------L--------------------------AHK-MGA-----EYLAKLLSQHLEAVIRAKIP---
>AAF22292.1
-------------LPTVAVVGG----Q----------------SSGKSSVLE-----S---IV-GRDFLPRG---SGIVTRRPL---
------VLQ---L---HK-TD--DGTE---EY---

A-------------------------------------E----------------FLH--LPK------
KQF-----TDFA---LVRREIQDETDR-----ITGKN-------------------KQ-------------------------IS--
PVPIHLSIYSPN--VVNLTLIDLPGLTK--
VA-------------------VEGQPE----------TI---AEDIE--------SMVRTYVDK------------PNCIILAISPANQ--
DIA-TS--DAIKLAKDVDPTGERTFGVL-TKLDLMDKGT--------NALEVLEGR---S---------------------
YRLQ-HPWVGI----------------------------------SEPF----------------------------------N-KQDIN----K----
-----------------------NVDMM----------L----ARRK---EREYF---------DT-------SP--------------------DY-
----------------G-H------L--------------------------ASK-MGS-----EYLAKLLSKHLESVIRTRIP---
>KAG0556007.1
--------------LPSVAVVGG----Q----------------SSGKSSVLE-----S---IV-GRDFLPRG---SGIVTRRPL--
-------VLQ---L---HK-T---EDKY---EY---
A-------------------------------------E----------------FLH--MPK-----
-RRF-----TDFA---AVRKEISDETDR-----ITGRS-------------------KQ-------------------------IS--
PVPIHLSVYSPN--VVNLTLIDLPGLTK--
IA-------------------VEGQSD----------SI---VGDIE--------NMVRSYIEK------------PNCIILAVSPANQ--
DIA-TS--DAIKIAREVDPNGERTFGVL-TKLDLMDKGT--------NAIDVLEGR---S---------------------
YKLI-QPWIGV----------------------------------VNR-----------------------------------S-QQDIN----K-----
----------------------NVDMI----------A----ARRR---EREYF---------QT-------SP--------------------DY---
--------------S-H------L--------------------------QSK-MGS-----EYLGRVLSKHLEAVIRSRIP---
>KAH9306600.1
--------------LPSVAVVGG----Q----------------SSGKSSVLE-----S---IV-GRDFLPRG---SGIVTRRPL--
-------VLQ---L---HK-TD--EGTP---EY---
G-------------------------------------E----------------FLH--RPN-----
-KRI-----TDFA---KVRSEIQEETDR-----ITGRT-------------------KM-------------------------IS--
PVPIHLSIYSPN--VVNLTLIDLPGLTK--
VA-------------------VEGQPE----------SI---VGDIE--------
NMVRSYVEKLLKLVVTADFLLPNSIILAISPANQ--DIA-TS--DAIKLAREVDPTGERTFGVL-
TKLDLMDKGT--------NALDVLDGR---S---------------------YRLQ-HPWVGV---------------------------
-------VNR-----------------------------------S-QADIN----K---------------------------SVDMI----------A---
-ARRR---EREYF---------ST-------ST--------------------DY-----------------G-H------L--------------------
------SSR-MGS-----EYLAKLLSKHLENVIKARIP---
>AAF79238.1
--------------LPTVAVVGG----Q----------------SSGKSSVLE-----S---VV-GRDFLPRG---SGIVTRRPL--
-------VLQ---L---HK-TE--DGTT---EY---
A-------------------------------------E----------------FLH--APK------
KRF-----ADFA---AVRKEIEDETDR-----ITGKS-------------------KQ-------------------------IS--
NIPIQLSIYSPNG----------
LCLRPHFLLCIPIVVSSETNTFDTSCNEITSSTIRFEDSNFANLFHVTLISHSTLFSTVVNLTLIDLPGLTK
--VAVVTDMNLVLKLVTDMNIIRVDGQPE----------SI---VQDIE--------NMVRSYVEK------------
PNCIILAISPANQ--DIA-TS--DAIKLAREVDPTGERTFGVA-TKLDIMDKGT--------DCLDVLEGR---S--
-------------------YRLQ-HPWVGI----------------------------------VNR-----------------------------------
S-QADIN----K---------------------------RVDMI----------A----ARRK---EQEYF---------ET-------SP-----
---------------EY-----------------G-H------L--------------------------ASR-MGS-----
EYLAKLLSQHLETVIRQKIP---

>PWZ36850.1
--------------LPTIAVVGG----Q----------------SSGKSSVLE-----S---IV-GTDFLPRG---SGIVTRRPL----
-----VLQ---L---QQ-TE--DGSQ---EY---
A-------------------------------------E-----------------FLH--MPK-----
-RRF-----SDFA---LVRQEIADETDR-----LTGKT-------------------KQ--------------------------IS--
PVPIHLSIYSPK--VVNLTMIDLPGLTK--
VA-------------------VEGQSE----------SI---VQDIE--------NMVRSYVDK-----------PNCIILAISPANQ-
-DIA-TS--DAIKLSKEVDPTGERTFGVL-TKLDLMDKGT--------NALDVLEGR---A--------------------
YRLQ-NPWVGI----------------------------------VNR------------------------------------S-QADIN----R----
-----------------------KVDMI----------S----AREK---EREYF---------ET-------SP--------------------DY----
-------------A-H------L--------------------------SSR-MGS-----GYLAKLLSQHLESVIKVRIP---
>NP_850420.1
--------------LPSVAVVGG----Q----------------SSGKSSVLE-----S---IV-GRDFLPRG---SGIVTRRPL--
-------VLQ---L---HK-TE--NGTE---DN--
-A-------------------------------------E-----------------FLH--LTN-----
-KKF-----TNFS---LVRKEIEDETDR-----ITGKN-------------------KQ--------------------------IS--
SIPIHLSIFSPN--VVNLTLIDLPGLTK--
VA-------------------VEGQPE----------TI---VEDIE--------SMVRSYVEK-----------PNCLILAISPANQ--
DIA-TS--DAMKLAKEVDPIGDRTFGVL-TKLDLMDKGT--------NALDVINGR---S--------------------
YKLK-YPWVGI----------------------------------VNR------------------------------------S-QADIN----K-----
----------------------NVDMM----------V----ARRK---EREYF---------ET-------SP--------------------DY---
--------------G-H------L--------------------------ATR-MGS-----EYLAKLLSKLLESVIRSRIP---
>EFJ15761.1
--------------LPSVVVVGG----Q----------------SSGKSSVLE-----S---IV-GRDFLPRG---SGIVTRRPL--
-------VLQ---L---HR-T---EDGP---DY---
A-------------------------------------E-----------------FLH--LPK------
KKF-----TDFA---LVRKEIQDETDR-----ITGRS-------------------KQ--------------------------IS--
PVPIHLSIYSRN--VVNLTMIDLPGLTK--
IA-------------------VDGQPE----------SI---VGDIE--------NMVRSYVEK-----------ENTIILAISPANQ--
DIA-TS--DAMKLARENIALGDRTFGVL-TKLDLMDKGT--------NAIDVLEGH---S--------------------
YRLQ-RPWIGV----------------------------------VNR------------------------------------S-QADIN----K----
-----------------------SVDMI----------V----ARRR---EREYF---------SS-------SP--------------------DY---
--------------R-H------L--------------------------ASR-MGS-----EYLGRVLSKHLEAVIKARIP---
>PTQ29980.1
--------------LPSVVVVGG----Q----------------SSGKSSVLE-----S---VV-GKDFLPRG---SGIVTRRPL-
--------VLQ---L---QK-TE--DGTR---
EW---A-------------------------------------E-----------------FLH--
APR------KRF-----EDFA---LVRKEISDETDR-----VGGR--------------------KG--------------------------
IS--KIPIHLTVYSPN--
VVNLTLVDLPGLTK--VA-------------------VEGQSD----------SI---VADIE--------DMVRSYVEK--------
---PNSIILAVSPANQ--DIA-TS--DAIKIAREVDPNGERTFGVA-TKLDLMDKGT--------NALDVLEGR---
T--------------------YRLQ-LGWVGV----------------------------------VNR----------------------------------
--S-QQDIN----K---------------------------NTDML----------A----ARRR---EREYF---------QT-------SQ-

-------------------DY-----------------G-H------L---------------------------ASR-MGS-----
EYLGKLLSKHLEQVIKARIP---
>EFJ23099.1
--------------LPSVAVVGG----Q----------------SSGKSSVLE-----S---VV-GRDFLPRG---SGIVTRRPL-
--------VLQ---L---HK-T---EGGQ---EY--
-A-------------------------------------E----------------FLH--NPK----
--TKF-----SDFS---LVRKEIEDETDR-----MTGHT-------------------KQ---------------------------IS--
PVPIHLSIYSPNG----------TCLSHPVKFSMPWFVYS--
VVNLTLIDLPGLTK--IA-------------------VGKCISFSK---MMCLLI---LADIE--------NMVRSYVEK------
------QNSIILAISPANQ--DIA-TS--DAMKLAKEVDPTGERTFGVL-TKLDLMDKGT--------
NALEVLEGR---A---------------------YRLQ-FQWVGV-----------------------------------VNR--------------
---------------------S-QADIN----K---------------------------SVDMI----------A----ARKK---EREFF-------
--AS-------SP--------------------DY-----------------G-H------L---------------------------ANR-MGS-----
EYLAKMLSKHLETVIKTRLP---
>EFJ35472.1
------------VDLPQLVHVQR----K-----------------------MD------------DREF-------------------------------
---S---EW---G-----------------
-------------------E----------------FLH--LPG------RRF-----TDFS--
-HIRKEIQAETER-----ELGDK-------------------KG-------------------------VS--DKQIRLKIFSPN--------
--VLNITLVDLPGLTR--VP-------------------
VGDQPS----------DI---ESRIR--------AMILSYIKH-----------ATCIILAVSPANT--DLS-NS--
DSLQMAKLVDPDGSRTIGVV-TKLDIMDRGT--------DARSILLGT---V---------------------IPLR-
LGYVGV----------------------------------VNR-----------------------------------S-QEVFR----
SPFFCLKILMKHGCRIFMPTNRSRMLLLRKSIF----------S----EVER---CTKAF---------WI-------D----
------------------V-----------------A-S------L---------------------------NSR-KSL-----
IKYELEPVKILVQHIK-------
>KXN72852.1
------------LNLPSIVVVGS----Q----------------SSGKSSVLE-----A---IV-GKEFLPKG---DNMVTRRPI-
--------ELT---L-I-HS-P--NLTQ---EF---
G-------------------------------------V----------------FPQ--LGP-----
-SKI-----TDFN---LITSQLT-ELNL-----QVSDK-------------------EC-------------------------IS--
EEPVQLHIYSPS--VPDLTLIDLPGYIQ--
IH-------------------TKDQPS----------DL---KPKIR--------ALCRKYLAP-----------PN-LILAVCPADV--
DLA-NS--EALLESRRADPSGQRTLGVV-TKLDLVDPAL---------GASILLNH---D---------------------
YPLN-LGYVGV----------------------------------ICR---------------------------------KNK-PSAVS----F----
-----------------------SRDPL----------Q-----------------------------------HP---------------------NY---------
--------Q-S------P---------------------------DIR-VGI-----QTLQSTLIRVLEQRMLKSLH---
>KNE54706.1
------------LVLPSIVVIGS----Q----------------SSGKSSVLE-----A---IV-GREFLPKG---TNMVTRRPL---
------ELT---L-I-HT-P--DSTE---EY---G-
------------------------------------E----------------FPQ--LGF------
GKV-----TNFQ---QIQRTLY-DLNM-----AVQD--------------------EC-------------------------IS--
DKPIELRIYSPN--VPDLTLVDLPGYIQ--
IH-------------------SKNQPR----------DL---KEKIA--------DLCEKYIQE-----------PN-VILAVCAADV-

-DLA-NS--EALRASRKADPLGLRTIGVL-TKLDLVSPEM---------GEQLIAHN---D--------------------
YPLH-LGYVGV----------------------------------VCG--GKNPQ----A----
-----------------------ALVPF----------Q----K------R--
---DLT-VGV-----PYLRQLLMRTLEERMGSRLS---
>OAJ38404.1
------------LNLPSIVVVGS----Q----------------SSGKSSVLE-----A---VV-GHEFLPKG---
ANMVTRRPI---------ELT---L-I-HT-P---
DSKE---EY---S--E----------------FPQ---
------LGL------GKI-----KDFS---QVRRTLT-DLNL-----AVSDA-------------------EC----------------------
---VS--EIPIELRVYSPN---
IPDLTLVDLPGYIQ--IH-------------------TKDQPP----------IL---KEKIA--------ALCQKYIQE-----------
PN-IILAVCAADV--DLA-NS--EALRASRKIDPLGLRTIGVI-TKMDLVEPQA---------AVNILENK---S----
-----------------YPLA-LGYIGV-------------------------------------VNK---
TSSRS----F------------------------------SQALT----------R----Q------E-SYF---------RS-------HP----------
---------EF-----------------N--------------------------------------NAM-VGT-----
ATLRRRLVEVLEEHMGRSLH---
>XP_011389557.1
------------LTLPSIVVIGS----Q----------------SSGKSSVLE-----A---IV-GHEFLPKG---NNMVTRRPI---
------ELT---L-I-HT-TT---QPGR-PTKDTVVEY-
--A--E----------------FPG---LGL---
---GRI-----TDFS---HVQKTLY-DLNM-----AVPAS-------------------EC------------------------VS--
DEPIELRIHSPH--VPDLTLIDLPGYVQ--
IA-------------------SMDQPD----------EL---REKIQ--------KLCHKYIQE------------PN-IILAVCAADV--
DLA-NS--PALRASRQVDPLGLRTIGVV-TKMDLVPPEV---------GAGILSNN---K--------------------
YPLA-LGYVGV------------------------------------VCKNNLGVFQS---------------------SKGHDRA-
TGEGR----M------------------------------SSLVM----------K----Q------ESDYF---------ST-------NR--------
------------EH------------------FCA------PTRGRNA------------------GVQPM-TGT-----
DTLRRRLMSVLEESMGSSLH---
>XP_006459124.1
------------LKLPSIVVIGS----Q----------------SSGKSSVLE-----A---IV-GHEFLPKG---NNMVTRRPI--
-------ELT---L-I-HT-PT---KDGQLPT-----EY--
-G--E----------------FPG---LGM---
---GKI-----TNFA---DIQRTLT-DLNL-----AVPAS-------------------DC------------------------VN--
NDPIDLRIYSPN---VPDLTLIDLPGYVQ--
IS-------------------SLDQPE----------SL---KEKIA--------SLCEKYIRE------------PN-IILAVCAADV--
DLA-NS--PALRASRKVDPLGLRTIGVI-TKMDLVPPER---------GAQILAGN---R--------------------
YPLH-LGYVGV-------------------------------------VAK------------------------------------KS-PVEPS----S-----
----------------------ALVTQ----------R----A------EDSYF---------HQ-------NR--------------------DF----
-------------F-----------GNSS--------------------QLL-VGT-----GTLRRRLMEVLESSMASSLH---
>XP_752563.1
------------LTLPSIVVIGS----Q----------------SSGKSSVLE-----A---IV-GHEFLPKG---TNMVTRRPI---
------ELT---L-V-NT-P--NAQS---EY---
G--E----------------FPA---LGL------
GKI-----TDFS---QIQRTLT-DLNL-----AVPER-------------------DC------------------------VS--

DDPIKLTIYSPN---VPDLSLIDLPGYIQ--
VA-------------------GKDQPP----------EL---KQKIA--------DLCDKYIQP------------PN-VILAISAADV--
DLA-NS--TALRASRRVDPRGERTIGVI-TKMDLVDPER---------GFSILSDQ---K----------------------
YPLR-LGYVGV---------------------------------VSR----VPQT--------------------TAL--FS-RGSGN----I-
--------------------------TSAIL----------K----N------ENAYF---------SA-------HP--------------------SE---
--------------F-----------GPQS--------------------GVS-VGV-----STLRGKLMHVLEQTMAASLA---
>OUM67143.1
------------LQLPSIVVIGS----Q----------------SSGKSSVLE-----A---IV-GHEFLPK-----------PI---------
ELT---L-V-HT-P---NAEE---EY---G------
------------------------------E----------------FPE---IGM------KNI---
--TDFG---HIQKTLS-ELNM-----SVSES-------------------EC-------------------------IS--
TDPIELKIFSPN---VPDLTLIDLPGYIQ--
VI-------------------NRKQPP----------VL---KRKIV--------ELCDRYIVE------------PN-IILAISSADV--
DLA-NS--EALTHSRKVDPYGQRTIGVI-TKMDLVEPEK---------GVDLLINN---D---------------------
YPLE-LGYVGM---------------------------------VCK---------------------------------PPG-KSGFS----R---
------------------------QLSLT----------Q----K------SDEYF---------RK-------NP--------------------IF-----
------------H-Q------P--------------------------DVQ-VGL-----SALKKRLTTILEENMGQNLY---
>NP_014854.2
------------LTLPSIVVIGS----Q----------------SSGKSSVLE-----S---IV-GREFLPKG---SNMVTRRPI---
------ELT---L-V-NT-P--NSNN---VT---
A------------------------------------D-----------------FPS---MRL-----
-YNI-----KDFK---EVKRMLM-ELNM-----AVPTS-------------------EA-------------------------VS--
EEPIQLTIKSSR---VPDLSLVDLPGYIQ--
VE-------------------AADQPI----------EL---KTKIR--------DLCEKYLTA------------PN-IILAISAADV--
DLA-NS--SALKASKAADPKGLRTIGVI-TKLDLVDPEK---------ARSILNNK---K---------------------
YPLS-MGYVGV----------------------------------ITK----TPSSINRKHLGLFGEAPSSSLSGIFSKG-
QHG-QSSGE----E----------------------------NTNGL----------K----QIVSHQFEKAYF---------KE-------
NK---------------------KY-----------------F----------------------------------TNCQ-VST-----
KKLREKLIKILEISMSNALE---
>PSC76263.1
RLLGGIESG---IDLPKIAVVGD----Q----------------SSGKSSVIE-----S---IF-GIS-LPRG---
SGIVTRTPI---------QVE---H---RY-T---
-EG---QA---Y-AEL--------------------------------S----------------YKT--------------------SPDD-----------
------EEWTK------RVV-----EDLD---GIEQAVEEATDA-----VTGTT------------------KG------------------
---LLDLPGIMR--
IA-------------------VDDQPK----------NI---EEIVE--------NQIRRHIEG------------DNVVILCVLHGTS-
-DPS-TA--SAIKLAQEYDEDGDQTMGVV-TKPDRCEKAQ----V--EDLIGSVLGAG-SS------------------
---IKLK-LGFIPV----------------------------------RNR-----------------------------------T-TSELT----D------
---------------------GTSLD----------Q----VREN---EAAFF---------KC-------HP--------------------LL----
-------------S-Q------L---A----------------------KDR-RGI-----PALVDQLAHVQMQRVHKALPELK
>XP_005849062.1
------------LQIPKIAVVGD----Q----------------SSGKSSLIE-----S---IF-GIS-LPRG---EDIVTRTPI------
---QVE---H---RY-SD-----------------------G--EA---Y-
AEL--------------------------------S----------------FRS--------------------SPDSE-----------------ELIK---

---KRI-----ADLS---TIDDEVREATRR-----VAGSG-------------------KG------------------------VV--
DSPIYLRVYTNK---LPDLTVLDLPGITR--
NA-------------------VEGQPE----------DI---EEIIN--------TMIESHIAG------------ETTVVLAIVQANV--
DFS-TA--AAIKLAKKFDPNRDRTM---

>PRW56740.1
---SGMPSAKRSKDMYRVVVVGD----Q----------------SSGKSSLIE-----S---MF-SFE-LPRG---
QDIVTRTPI---------QVK---H---RF-E---
--KG---PP---R-AEI-------------------------------S----------------FR--------------------KPGE----------
------SDLTV------VKL-----DSLD---EIEEAVIQATDI-----VAGEG-------------------KD-------------------
-----IV--DSTIFLRVFSDA--
LPDLTLTDLPGITR--NA-------------------TQGQPE----------NI---EEIVT--------SVIENHIKG----------
-EMVVILCVVPANV--DFS-TA--GGIKLARRHDADGVRTMGVV-TKIDLTESGQ--------
EMLDRLSGAG-KN---------------------LKLK-LGFIPV----------------------------------RNR-------------
---------------------T-AQELK----D---------------------------GTPLE----------K----VCKA---EGDFF-------
--SS-------HP--------------------QL------------------S-K------V---N----------------------PDR-RGI-----
SALVTELARLQMQRVTKSLPGVK
>XP_006815062.1
------------VDLPAVVVIGD----Q----------------SVGKSSVLE-----A---IS-GVQ-LPRG---NEIVTRCPI---
------ELR---L---KT-LD---N--D---EW---
C-GKI--------------------------------L----------------YIN-------------------YSKE-------------------QVN-
-----KYI-----DSPD---ELGAAIRTAQQD-----ITNSQ-------------------KG------------------------IS--
KTSITVEIQSAH---VPNLTLIDLPGIAR--
VP-------------------QEGQSR----------NI---ADETK--------DLIKKYISK------------DDAIVLCVIPCNV--
DIA-TT--EAIKMAQEVDPTGSRTLGVL-TKPDLVDKGS----E--NVVVRIAENK---V---------------------
INLK-KGYTIM----------------------------------KCR----------------------------------S-QRNLE----D-----
----------------------AMSLE----------E----AMDE---EERFF---------RE-------HK--------------------HY--
---------------S-V------L--------------------------SGQ-AGS-----RLLAHRLTTELVEQILKSV----
>XP_035690836.1
------------VSLPSVVVIGD----Q----------------SAGKSSTLE-----A---IS-GVQ-LPRG---SGIVTRCPL--
-------ELR---L---KK-SQ---KKDA---PW-
--K-GCI--------------------------------R----------------YV--------------------KNKK-------------------
DVR------FDV-----DEPG---NVGDAVKKAQND-----LAGTT-------------------NG-----------------------
-IS--DSLITLDVESPD--
IPDLTLIDLPGIAR--IA-------------------AEGQPT----------DI---GQQIK--------DLISKYIQK------------
KDTIILAVVPCNV--DIA-TT--EALQMAQEVDADGSRTLGVL-TKPDLIDPGT----E--RGVLQILNNE---
K--------------------YKLR-KGYTII----------------------------------KCR----------------------------------
G-QMDIE----K---------------------------GMSLE----------E----AMDK---EQSYF---------KS-------HE---
-----------------HF-----------------K-S------V--YK----------------------EKK-AGV-----
RTLAGRLSTELVGQIKNSI----
>XP_002608668.1

------------VTLPSVVVIGD----Q----------------SAGKSSCLE-----A---MS-GVQ-LPRG---
SGIVTRCPL---------ELR---L---KK-SQ--
-DPES---PW---K-GYI---------------------------------H----------------YHF--------------------EGDR------
------------DETG------WKL-----TDPS---DVGEAVRKAQNN-----LAGDS-------------------HG---------
----------------IS--PRLITLDVESPD---
IPDLTLIDLPGIAR--IA-------------------VDGQPP----------DI---GDQIK--------DLIKEYIQK------------
DETIILAVVPCNV--DIA-TT--EALQMAKDVDPTGSRTLGVL-TKPDLIDRGT----E--NTIVDIVNNQ---K-
--------------------YPLK-KGYTII-----------------------------------RCR------------------------------------G-
QEDIN----E----------------------------NVTLS----------E----AMEK---EERFF---------KT-------HE--------
------------HF-----------------K-L------P--YH----------------------EKK-TGT-----RTLAGKLTTELVEQIK--
-----
>XP_019617847.1
------------VTLPSVVVIGD----Q----------------SAGKSSCLE-----A---IS-GVQ-LPRG---SGIVTRCPL--
-------ELR---L---KK-SP---DPES---GW--
-R-GYI---------------------------------H----------------FE------------------------DK------------------GETR-
-----WEL-----DSPE---DVGEAVKKAQNQ-----LAGES-------------------LC-------------------------IS--
PRLITLDVESPD---IPDLTLIDLPGIAR--
VP-------------------VGGQPD----------DI---GDQTK--------ALIREYIQM------------DETIILAVVPCNV-
-DIA-TT--EALKMAKEVDPDGSRTLGVL-TKPDLIDRGT----E--NMTVDIVNNR---K---------------------
YALK-KGYTII-----------------------------------KCR------------------------------------G-QVDIE----N-------
--------------------KVSLS----------D----AMDK---EEMFF---------QK-------HE--------------------HF----
-------------K-I------L--YE----------------------EKK-TGT-----KTLAGKLTTELVEQIKKSI----
>XP_003973512.2.2
------------LALPAIAVIGD----Q----------------SSGKSSVLE-----A---LS-GVA-LPRG---SGIVTRCPL---
------ELK---M---KR-RK---V-GE---PW---
Y-GNI---------------------------------S----------------YL---DQE----
--EVI-----EDPA---DVEKKIQEAQNE-----MAGVG-------------------VG-------------------------IS--
DDLISLEIASPE---VPDLTLIDLPGIAR--
VA-------------------VKGQPE----------NI---GEQIK--------RLIRKFITK------------QETISLVVVPCNV--
DIA-TT--EALKMAQEVDPDGERTLGIL-TKPDLVDKGT----E--ETVVDIIHNE---V---------------------
IHLK-KGYMIV-----------------------------------RCR------------------------------------G-QKEII---D-------
--------------------KVSLA----------E----ATET---ETAFF---------RD-------HA--------------------HF------
-----------Q-T------L--YD----------------------DGQ-ATI-----LKLAEKLTLELVNHIEKSL----
>NP_891987.2.2
------------LALPAIAVIGD----Q----------------SSGKSSVLE-----A---LS-GVP-LPRG---SGIVTRCPL---
------ELK---M---IR-TK---D-QD---KW---
H-GRI---------------------------------S----------------YK---TYE-----
-EDF-----DDPA---EVEKKIRQAQDE-----MAGAG-------------------VG-------------------------IS--
EELISLQITSAN---VPDLTLIDLPGIAR--
VA-------------------VKGQPE----------NI---GDQIK--------RLIRKFVTR------------QETINLVVVPCNV-
-DIA-TT--EALQMAQAEDPDGERTLGIL-TKPDLVDKGT----E--GTVVDIVHNE---V---------------------
IHLT-KGYMIV-----------------------------------RCR------------------------------------G-QKEIM----D------
---------------------QVTLN----------E----ATET---ESAFF---------KD-------HP--------------------HF-----
------------S-K------L--YE----------------------EGF-ATI-----PKLAEKLTIELVHHIQKSL----

>XP_009304072.1
------------LALPAIAVIGD----Q---------------SSGKSSVLE-----A---LS-GVP-LPRG---SGIVTRCPL---
------ELK---M---IR-SK---E-DE---KW---
H-GRI-------------------------------S---------------YQ---NHE---
---EDF-----DDPA---EVEKKIREAQDE-----MAGAG------------------VG-----------------------IS--
EELISLQITSAN---VPDLTLIDLPGIAR--
VA-----------------VKGQPE----------NI---GDQIK--------RLIRMFITK-----------QETINLVVVPCNV-
-DIA-TT--EALQMAQAEDPEGERTLGIL-TKPDLVDKGT----E--GTVVDIVHNE---V-------------------
IHLT-KGYMIV--------------------------------RCR--------------------------------G-QKEIM----D------
--------------------QVTLN----------E----ATET---ESAFF---------KD-------HP------------------HF-----
------------R-K------L--YE--------------------EGF-ATI-----PKLAEKLTIELVHHIQRSL----
>AGU16245.1
-------------LPAIAVIGD----Q---------------SSGKSSVLE-----A---LS-GVG-LPRG---SGIVTRCPL----
-----ELK---L---KK-AK--K-ET---EW---K-
ATI--------------------------------R---------------YE--DEY------
KEL-----TSPS---EVEKEIRTAQNA-----MAGSG------------------KG-----------------------IS--
DKLISLEIESDN---VPDLTLIDLPGIAR--
VA-----------------VQGQPY----------DI---GEQIK--------KLIRKFIEK-----------EETINLVVVPCNV--
DIA-TT--EALKMAQDVDQSGERTLGIL-TKPDLVDKGA----E--QNIVDVVNNM---V-------------------
IPLK-KGYMIV--------------------------------KCR--------------------------------G-QQDIN----E-----
---------------------NLTLA----------E----AT---

>XP_007904885.1
------------LSLPAIAVIGD----Q---------------SSGKSSVLE-----A---LS-GVS-LPRG---TGIVTRCPL----
-----ELK---L---KK-AK--K-AN---VW---
K-GAI-------------------------------S---------------FR---EYS-----
-KEI-----TNAS---EVEQEIRKAQNS-----MAGK------------------EG-----------------------IS--
HDLISLKIESSN---VPDLTLIDLPGIAR--
VA-----------------VGNQPL----------DI---GDQIK--------KMIRSFINK-----------
QETINLVVVPCNV--DIA-TT--EALKMAQEVDPSGERTVGIL-TKPDLVDKGT----E--STIVDIVQNL---V-
-------------------VELK-KGYMIV--------------------------------KCR--------------------------------G-
QKEIN----D--------------------------KLTLQ----------D----AIAR---ENRYF---------EE-------HE--------
------------QF-----------------R-T------L--LD--------------------EKK-ASI-----
PHLAERLTNELVYHISKCL----
>XP_032888405.1
------------LGLPAIAVIGD----Q---------------SSGKSSVLE-----A---LS-GVA-FPRG---SGIVTRCPL---
------ELK---L---KN-VK--K-AN---VW---
K-GKI-------------------------------S---------------YK---DYS-----
-NKL-----SSAA---EVEQAILKAQDS-----IAGKG------------------VG-----------------------IS--
HELISLEIESTN---VPDLTLIDLPGIAR--
VA-----------------VGNQPQ----------DI---GDQIK--------RLIRLFIQK-----------
QETVNLVVVPCNV--DIA-TT--EALKMAQEVDPTGDRTLGIL-TKPDLVDKGT----E--KNVVDIVKNL---
T-------------------VELE-KGYMIV--------------------------------KCR--------------------------------
G-QNDIN----E--------------------------NISLV---------D----AIAK---EKEFF---------ED-------HE------

--------------QF-----------------R-P------L--LE---------------------DGK-AGI-----
PNLAVRLTKELVNHINKSL----
>XP_025933558.1
------------LALPSIAVIGD----Q---------------SSGKSSVLE-----A---LS-GIA-LPRG---NGIVTRCPL---
------ELK---L---KK-TP---A-TQ---KW---
K-GKI------------------------------S---------------YH--NTS-----
-EEL-----KNPS---EVEKAIRGAQDV-----VAGTK------------------GA-----------------------IS--
RELISLEVWSPT--VPDLTLIDLPGIAR--
VA------------------VGDQPE----------DI---GEQIK--------KLLKNIIGN------------KETLNLVVVPCNV-
-DIA-TT--EALKMAQEVDPKGERTLGIL-TKPDLVDKGT----E--ESIVNIIRNL---T--------------------
VPLK-KGYMIV-----------------------------------KCR-----------------------------------G-QQDIH----N----
----------------------NLTLA----------S----AIQQ---EKEFF---------ET-------HQ-------------------HF---
--------------S-I------L--LN---------------------EGK-ATV-----PLLAEKLTXELVGHIIKTL----
>XP_009815891.1
------------LALPAIAVIGD----Q---------------SSGKSSVLE-----A---LS-GIA-LPRG---NGIVTRCPL---
------ELK---L---KR-IP---A-TQ---AW---
K-GKI------------------------------C---------------YR--NIS-----
-SEL-----QNAS---EVEKAIREAQDI-----VAGTR------------------GA-----------------------IS--
GELISLEIWSPD--VPDLTLIDLPGIAR--
VA------------------VGNQPK----------DI---GEQIK--------MLLKKIIGC------------
KETLNLVVVPCNV--DIA-TT--EALKMAQEVDPSGERTLGIL-TKPDLVDRGT----E--ESIINIIRNL---V---
------------------IPLK-KGYMIV-----------------------------------KCR-----------------------------------G-
QQDIH----N---------------------------KLALA----------A----AIQQ---ERKFF---------ET-------HE-------
-------------HF-----------------S-I------L--LE---------------------EGK-ATV-----
PHLAEKLTNELVRHIIKTL----
>XP_015269256.1
------------LALPAIAVIGD----Q---------------SSGKSSVLE-----A---LS-GVA-LPRG---NGIVTRCPL--
-------ALK---L---KK-TR---Q-GC---GW--
-K-GKI------------------------------S---------------YR--DIN----
--EEL-----NHPS---EVEKEIRKAQIS-----IAGEG------------------VG-----------------------IS--
HELITLEIRSSE--VPDLTLIDLPGIAR--
VA------------------VGNQPQ----------DI---GHQIK--------RLIKKIIAK------------DETINLVVVPCNV--
DIA-TT--EALKMAQEVDPDGERTLGIL-TKPDLVDKGT----E--EAVVDIVRNL---I--------------------
IHLK-KGYMIV-----------------------------------KCR-----------------------------------G-QQDIQ----S-----
---------------------NLDLA----------S----AIQK---EKAFF---------ED-------NR-------------------HF---
--------------R-I------L--LA---------------------EKR-ATI-----PLLAEKLTSELVEHINKSL----
>XP_006156438.1
------------LALPAIAVIGD----Q---------------SSGKSSVLE-----A---LS-GVA-LPRG---SGIVTRCPL---
------VLK---L---MK-QS---Q-EP---VW---
R-GKI------------------------------R---------------YR--NTE-----
-KKL-----GDPT---QVEAEICKAQNI-----IAGSG------------------VG-----------------------IS--
HELITLEITSPE--VPDLTLIDLPGITR--VA-
------------------LGNQPQ----------DI---SLQIK--------ALIKKYIKR------------QQTINLVVVPCNV--
DIA-TT--EALSMAQEVDPEGDRTLGIL-TKPDLVDKGS----E--KSVMNVLQNL---T--------------------

FPLK-KGYMIV----------------------------------KCR------------------------------------G-QQEIM----N----
-----------------------NLSLA----------E----ATRK---ELMFF---------QS-------HP--------------------HF--
---------------R-V------F--LE----------------------EKK-ATV-----PHLAERLTAELIAHIRKSL----
>NP_002454.1
------------LALPAIAVIGD----Q----------------SSGKSSVLE-----A---LS-GVA-LPRG---SGIVTRCPL---
------VLK---L---KK-QP--C--E---AW---
A-GRI---------------------------------S----------------YR---NTE-----
-LEL-----QDPG---QVEKEIHKAQNV-----MAGNG-------------------RG--------------------------IS--
HELISLEITSPE--VPDLTIIDLPGITR--VA-
------------------VDNQPR----------DI---GLQIK--------ALIKKYIQR------------QQTINLVVVPCNV--
DIA-TT--EALSMAHEVDPEGDRTIGIL-TKPDLMDRGT----E--KSVMNVVRNL---T---------------------
YPLK-KGYMIV----------------------------------KCR------------------------------------G-QQEIT----N-----
----------------------RLSLA----------E----ATKK---EITFF---------QT-------HP--------------------YF------
-----------R-V------L--LE----------------------EGS-ATV-----PRLAERLTTELIMHIQKSL----
>XP_002830747.1
------------LALPAIAVIGD----Q----------------SSGKSSVLE-----A---LS-GVA-LPRG---SGIVTRCPL---
------VLK---L---KK-QP--C--E---AW---
A-GRI---------------------------------S----------------YR---NTE-----
-LEL-----QDPG---QVEKEIHKAQNI-----MAGNG-------------------RG--------------------------IS--
HELISLEITSPE--VPDLTIIDLPGITR--VA-
------------------VDNQPR----------DI---GLQIK--------ALIKKYIQR------------QQTINLVVVPCNV--
DIA-TT--EALSMAHEVDPEGDRTIGIL-TKPDLMDKGT----E--KSVMNVVRNL---T---------------------
YPLK-KGYMIV----------------------------------RCR------------------------------------G-QQELT----N-----
----------------------RLSLA----------E----ATKK---EITFF---------QT-------HP--------------------YF------
-----------R-V------L--LE----------------------EGS-ATV-----PRLAERLTSELIMHIQKSL----
>XP_008569440.1
------------LALPAIAVIGD----Q----------------SSGKSSVLE-----A---LS-GVA-LPRG---SGIVTRCPL---
------VLK---L---KK-HL--Q-ED---GW---
K-GKI---------------------------------S----------------YR---HTE-----
-LLL-----QDPS---QVEKEIHKAQNT-----IAGNG-------------------VG--------------------------IS--
HELISLEITSPE--VPDLTLIDLPGITR--VA-
------------------VGNQPQ----------DI---GQQVK--------ALIKKYIQR------------QQTINLVVVPCNV--
DIA-TT--EALSMAQEVDPDGDRTIGIL-TKPDLVDKGT----E--KGVMNVARNL---T---------------------
YHLK-KGYMIV----------------------------------KCR------------------------------------G-QQDIT----N-----
----------------------KLSLA----------E----ATKK---EMAFF---------QT-------HP--------------------YF----
-------------R-V------L--LE----------------------EGK-ATV-----PCVAEKLTAELIVHINKSL----
>XP_017508123.1
------------LALPAIAVIGD----Q----------------SSGKSSVLE-----A---LS-GVA-LPRG---SGIVTRCPL---
------VLK---L---KK-QL--H-EP---AW---
T-GRL---------------------------------S----------------YQ---TTE-----
-LQL-----HNPS---QVEKEIQKAQNA-----IAGDG-------------------VG--------------------------IS--
HELINLEITSPD--VPDLTLIDLPGIAR--
VA-------------------VGNQPQ----------DI---GLQIK--------ALIKKYIQR------------
QQTINLVVVPCNV--DIA-TT--EALSMAQEVDPDGDRTIGIL-TKPDLVDKGT----E--RVIVNVVQNL---

T--------------------YHLK-KGYMIV----------------------------------KCR-----------------------------------
G-QQEVT----N---------------------------KLSLA----------E----ATSK---EMTFF---------QT-------HP-----
---------------YF-----------------R-I------L--LE---------------------EGK-ATV-----
PRLAEKLTTELISHINKSL----
>XP_005885748.1
------------LALPAIAVIGD----Q----------------SSGKSSVLE-----A---LS-GVA-LPRG---SGIVTRCPL---
------VLK---L---KK-QL--AGES---LW---
T-GKI------------------------------S----------------YR--STE------
LQL-----QDPS---QVEREIYKAQNT-----IAGNG-------------------VG-------------------------IS--
HELINLEITSPE---VPDLTLIDLPGIAR--
VA-------------------VGNQPQ----------DI---GLQIK--------ALIKKYIQR-----------
QQTINLVVVPCNV--DIA-TT--EALSMAHEVDPDGDRTIGIL-TKPDLVDKGA----E--KNVVNVAQNL--
-T--------------------YRLK-KGYMIV----------------------------------KCR-----------------------------------
G-QQEIT----D---------------------------KLSLA----------E----ATKK---EMMFF---------QT-------HP-----
---------------YF-----------------R-V------L--LE---------------------EGK-ATV-----
PRLAERLTTELIWHINKSL----
>NP_001003133.1
------------LALPAIAVIGD----Q----------------SSGKSSVLE-----A---LS-GVA-LPRG---SGIVTRCPL---
------VLK---L---KR-DP---H--K---AW---
R-GRI--------------------------------S----------------YR--KTE-----
-LQF-----QDPS---QVEKEIRQAQNI-----IAGQG-------------------LG-------------------------IS--
HELISLEITSPE---VPDLTLIDLPGITR--VA-
------------------VGNQPQ----------DI---GVQIK--------ALIKNYIQK-----------QETINLVVVPCNV--
DIA-TT--EALSMAQEVDPNGDRTIGVL-TKPDLVDRGT----E--KTVVNVAQNL---T--------------------
YHLQ-KGYMIV----------------------------------RCR-----------------------------------G-QEEIT----N-----
----------------------QLSLA----------E----ATEK---ERMFF---------QT-------HP-------------------YF----
-------------R-A------L--LE---------------------EGK-ATV-----PCLAERLTKELILHINKSL----
>NP_776366.1
------------LALPAIAVIGD----Q----------------SSGKSSVLE-----A---LS-GVA-LPRG---SGIITRCPL----
-----VLK---L---TK-R---EC---EW---T-
GKI--------------------------------T----------------YR--NIT------
QQL-----QNPS---EVEWEIRRAQNI-----IAGNG-------------------LG-------------------------IS--
HELINLEITSPE---VPDLTLIDLPGITR--
VA-------------------VENQPQ----------DI---GLQIK--------ALIKKYIQR-----------QETINLVVVPCNV-
-DIA-TT--EALSMAQEVDPDGDRTIGIL-TKPDLVDKGT----E--KGVLKVMQNL---T--------------------
YHLK-KGYMIV----------------------------------KCR-----------------------------------G-QQDIT----N-----
----------------------KLSLA----------E----ATRK---ETMFF---------ET-------HP-------------------YF-----
------------R-I------L--LD---------------------EGK-ATV-----PLLAERLTTELIWHINKSL----
>XP_032211320.1
------------LALPAIAVIGD----Q----------------SSGKSSVLE-----A---LS-GVA-LPRG---SGIVTRCPL---
------VLK---L---KR-QP---Q-ES---AW---
K-GRV--------------------------------I----------------YG--TRE-----
-VRL-----QDPS---QVEKEILKAQNT-----LAGDG-------------------VS-------------------------IS--
HELISVDIISPE---VPDLTLIDLPGITR--VP-

------------------VGNQPQ----------DI---GLQIK--------ALIKKYIQR------------QETINLVVVPCNV--
DIA-TT--EALSMAQEVDPRGDRTIGIL-TKPDLVDKGA----E--PIVMKVAQNL---T--------------------
YHLQ-KGYMMV----------------------------------RCR-----------------------------------G-QEEIT----N---
------------------------RLSLA----------E----ATRK---ETMFF---------QK-------HP--------------------HF--
---------------R-A------L--LQ----------------------EGK-ATV-----PCLAERLTNELILHINKSL----
>XP_004675614.2.2
------------LALPAIAVIGD----Q----------------SSGKSSVLE-----A---LS-GVA-LPRG---SGIVTRCPL---
------VLK---L---KK-LM--N-ED---SW---
K-GKI--------------------------------N----------------YQ--GVE----
--VTI-----AKAS---DVEQEVNKAQAV-----IAGDG-------------------LG-------------------------IS--
HELITLEVSSPE---VPDLTLIDLPGITR--
VA-------------------VGNQPQ----------DI---GEQIK--------ALIRKYIQR------------
QQTINLVVVPCNV--DIA-TT--EALSMAREVDPDGDRTLGIL-TKPDLVDRGT----E--DRVVDVIRNF---
I---------------------CPLK-KGYMIV-----------------------------------KCR-----------------------------------
G-QKDIQ----D---------------------------RLSLA----------Q----ALQK---EQAFF---------EE-------HP----
----------------HF-----------------R-Q------L--LE----------------------EGR-ASI-----
PKLADRLTSELIRHISKSL----
>XP_004466363.1
------------LSLPAIAVIGD----Q----------------SSGKSSVLE-----A---LS-GVA-LPRG---SGIVTRCPL---
------VLK---L---KK-LT--N-EE---KW---
R-GKV--------------------------------T----------------YE--DYE-----
-IDI-----SDAS---EVEEEINKAQNV-----IAGEG-------------------LG-------------------------IS--
QKLINLEVCSPD---VPDLTLIDLPGITR--
VA-------------------VGNQPA----------DI---GWQIK--------CLIKKYITR------------QETINLVVVPSNV-
-DIA-TT--EALSMAQEVDPNGDRTIGIL-TKPDLVDRGT----E--DKVVDVVRNL---V--------------------
CHLK-KGYMIV-----------------------------------RCR-----------------------------------G-QQDIQ----D---
------------------------RLSLA----------T----ALQK---ERAFF---------EN-------HE--------------------NF--
---------------R-V------L--LE----------------------EGK-ATV-----PHLAERLTTELITHISKTL----
>NP_002453.2.2
------------LALPAIAVIGD----Q----------------SSGKSSVLE-----A---LS-GVA-LPRG---SGIVTRCPL---
------VLK---L---KK-LV--N-ED---KW---
R-GKV--------------------------------S----------------YQ--DYE----
--IEI-----SDAS---EVEKEINKAQNA-----IAGEG-------------------MG-------------------------IS--
HELITLEISSRD---VPDLTLIDLPGITR--
VA-------------------VGNQPA----------DI---GYKIK--------TLIKKYIQR------------QETISLVVVPSNV--
DIA-TT--EALSMAQEVDPEGDRTIGIL-TKPDLVDKGT----E--DKVVDVVRNL---V--------------------
FHLK-KGYMIV-----------------------------------KCR-----------------------------------G-QQEIQ----D----
-----------------------QLSLS----------E----ALQR---EKIFF---------EN-------HP--------------------YF---
--------------R-D------L--LE----------------------EGK-ATV-----PCLAEKLTSELITHICKSL----
>NP_001127618.1
------------LALPAIAVIGD----Q----------------SSGKSSVLE-----A---LS-GVA-LPRG---SGIVTRCPL---
------VLK---L---KK-LV--N-ED---KW---
R-GKV--------------------------------S----------------YQ--DYE----
--IEI-----SDAS---EVEKEINKAQNT-----IAGEG-------------------MG-------------------------IS--

HELITLEISSRD--VPDLTLIDLPGITR--
VA-------------------VGNQPA----------DI---GYKIK--------TLIKKYIQR------------QETISLVVVPSNV--
DIA-TT--EALSMAQEVDPEGDRTIGIL-TKPDLVDKGT----E--DKVVDVVRNL---V---------------------
FHLK-KGYMIV-----------------------------------KCR------------------------------------G-QQEIQ----D----
-----------------------QLSLS----------E----ALQR---EKIFF---------ED-------HP--------------------YF----
-------------R-D------L--LE----------------------EGK-ATV-----PCLAEKLTSELITHICKSL----
>XP_006156437.1
------------LALPAIAVIGD----Q----------------SSGKSSVLE-----A---LS-GVA-LPRG---SGIVTRCPL---
------VLK---L---KK-LI--N-ED---KW---
R-GKV---------------------------------S----------------YQ---DIE----
--VEI-----TDPS---KVEPEINKAQNV-----IAGEG-------------------MG-------------------------IS--
HELISLEVSSPH--VPDLTLIDLPGITR--
VA-------------------VGNQPA----------DI---GRQIK--------TLIKKYIHK------------QETINLVVVPSNV--
DIA-TT--EALSMAQEVDPDGDRTIGIL-TKPDLVDKGT----E--EKVVDVVRNL---V---------------------
CHLK-KGYMIV-----------------------------------KCR------------------------------------G-QQDIQ----D---
------------------------RLSLA----------E----ALQR---EKVFF---------EE-------HP--------------------YF--
---------------S-F------L--LE----------------------EGK-ATI-----PCLAERLTTELIMHISKSL----
>XP_017508130.1
------------LALPAIAVIGD----Q----------------SSGKSSVLE-----A---LS-GVA-LPRG---SGIVTRCPL---
------VLK---L---KK-LT--N-EE---TW---
R-GKV---------------------------------S----------------YQ---DFE----
--AEL-----SDPS---EVEREINRAQNS-----IAGEG-------------------TG-------------------------IS--
HELISLEISSPH--VPDLTLIDLPGITR--
VA-------------------VGNQPA----------DI---GRQIK--------ALIRKYIYK------------QETINLVVVPSNV--
DIA-TT--EALSMAQEVDPDGDRTIGIL-TKPDLVDRGT----E--DKVVDVVRNL---V---------------------
CHLK-KGYMIV-----------------------------------KCR------------------------------------G-QQDIQ----D---
------------------------QLSLA----------E----ALKK---ERAFF---------ED-------NP--------------------YF--
---------------R-D------L--LE----------------------EGR-ATV-----PCLADKLTVELITHICKSL----
>NP_001003134.1
------------LALPAIAVIGD----Q----------------SSGKSSVLE-----A---LS-GVA-LPRG---SGIVTRCPL---
------VLK---L---KK-LI--N-ED---EW---
R-GKV---------------------------------S----------------YQ---DTE----
--MEI-----SDPS---EVEVEINKAQDA-----IAGEG-------------------QG-------------------------IS--
HELISLEVSSPH--VPDLTLIDLPGITR--
VA-------------------VGNQPA----------DI---GRQTK--------QLIRKYILK------------QETINLVVVPCNV-
-DIA-TT--EALSMAQEVDPDGDRTIGIL-TKPDLVDRGT----E--GKVVDVAQNL---V---------------------
CHLK-KGYMIV-----------------------------------KCR------------------------------------G-QQDIQ----D---
------------------------QVSLA----------E----ALQK---EKDFF---------ED-------HP--------------------HF-
----------------R-V------L--LE----------------------EGR-ATV-----PNLAEKLTSELITHICKTL----
>XP_032211398.1
------------LALPAIAVIGD----Q----------------SSGKSSVLE-----A---LS-GVA-LPRG---SGIVTRCPL---
------VLK---L---KK-VT--N-QD---EW---
R-GKV---------------------------------S----------------YQ---DFE----
--KEI-----SDPS---EVEAEINKAQNA-----VAGEG-------------------QG-------------------------IS--

HELISLEVSSSH---VPDLTLIDLPGITR--
VA-------------------VGNQPA----------DI---GRQTK--------QLIRKYILR------------QETINLVVVPCNV-
-DIA-TT--EALSMAQEVDPDGDRTIGIL-TKPDLVDRGT----E--SKVVDVAQNL---V--------------------
CHLK-KGYMIV---------------------------------KCR----------------------------------G-QQDIQ----D---
------------------------QVTLA----------E----ALQK---ERDFF---------ED-------HP-------------------HF-
----------------R-V------L--LE---------------------EGR-ATV-----PCLADKLTSELIMHICKTL----
>XP_008569442.1
------------LALPAIAVIGD----Q----------------SSGKSSVLE-----A---LS-GVA-LPRG---SGIVTRCPL---
------VLK---L---KK-LV---H-GE---EW---
K-GKV--------------------------------S---------------YR---DLE----
--IKI-----SDAL---EVEEEVRKAQTI-----IAGEG-------------------MG------------------------IS--
HELINLEISSPH---VPDLTLIDLPGIAR--
VA-------------------MGNQPA----------DI---GYQVK--------XLIRKYIQR------------
QETINLVVVPSNV--DIA-TT--EALSMAQEVDPEGDRTIGIL-TKPDLVDKGT----E--DKVVDVVRNL---
V--------------------YHLK-KGYMIV---------------------------------KCR-----------------------------------
G-QQDIQ----D---------------------------QLSLA---------T----ALQR---EKDFF---------ED-------HP---
-----------------QF-----------------R-D------L--LE---------------------EGR-ATI-----
PCLAERLTTELITHICKSL----
>XP_014388412.1
------------LALPAIAVIGD----Q----------------SSGKSSVLE-----A---LS-GVS-LPRG---SGIVTRCPL---
------VLK---L---RK-LR---H-DD---EW---
K-GKV--------------------------------T---------------YR---DLE-----
-IDL-----SAAS---EVEQEIRKAQNV-----IAGEG-------------------VG------------------------IS--
QELINLEVSSPH---VPDLTLIDLPGITR--
VA-------------------VGNQPA----------DI---GRQIT--------ALIKKYILR------------QQTIMLVVVPSNV--
DIA-TT--EALSMAHEVDPDGDRTIGIL-TKPDLVDRGT----E--DKVVDVVRNL---V--------------------
YHLK-KGYMIV---------------------------------KCR----------------------------------G-QQDIQ----Y-----
----------------------QMSLS----------K----ALQR---ERAFF---------ED-------HP-------------------YF---
--------------R-D------L--LE---------------------EGK-ATI-----PCLAERLTNELIAHISKSL----
>XP_005202045.1
------------LALPAIAVIGD----Q----------------SSGKSSVLE-----A---LS-GVA-LPRG---SGIVTRCPL---
------VLR---L---KK-LG---N-ED---EW---
K-GKV--------------------------------S---------------FL---DKE----
--IEI-----PDAS---QVEKEISEAQIA-----IAGEG-------------------TG------------------------IS--
HELISLEVSSPH---VPDLTLIDLPGITR--
VA-------------------VGNQPP----------DI---EYQIK--------SLIRKYILR------------QETINLVVVPANV--
DIA-TT--EALRMAQEVDPQGDRTIGIL-TKPDLVDKGT----E--DKVVDVVRNL---V--------------------
FHLK-KGYMIV---------------------------------KCR----------------------------------G-QQDIK----H----
----------------------RMSLD----------K----ALQR---ERIFF---------ED-------HA-------------------HF--
---------------R-D------L--LE---------------------EGK-ATI-----PCLAERLTSELIMHICKTL----
>NP_038634.1
------------LALPAIAVIGD----Q----------------SSGKSSVLE-----A---LS-GVA-LPRG---SGIVTRCPL---
------VLK---L---RK-LN---E-GE---EW---
R-GKV--------------------------------S---------------YD---DIE----

--VEL-----SDPS---EVEEAINKGQNF-----IAGVG-------------------LG-------------------------IS--
DKLISLDVSSPN--VPDLTLIDLPGITR--
VA-------------------VGNQPA----------DI---GRQIK--------RLIKTYIQK------------QETINLVVVPSNV--
DIA-TT--EALSMAQEVDPEGDRTIGIL-TKPDLVDRGT----E--DKVVDVVRNL---V---------------------
YHLK-KGYMIV----------------------------------KCR-----------------------------------G-QQDIQ----E----
-----------------------QLSLT----------E----ALQN---EQIFF---------KE-------HP--------------------HF---
--------------R-V------L--LE----------------------DGK-ATV-----PCLAERLTAELILHICKSL----
>NP_034976.1
------------LALPAIAVIGD----Q----------------SSGKSSVLE-----A---LS-GVA-LPRG---SGIVTRCPL---
------VLK---L---RK-LK--E-GE---EW---
R-GKV--------------------------------S----------------YD---DIE----
--VEL-----SDPS---EVEEAINKGQNF-----IAGVG-------------------LG-------------------------IS--
DKLISLDVSSPN--VPDLTLIDLPGITR--
VA-------------------VGNQPA----------DI---GRQIK--------RLIKTYIQK------------QETINLVVVPSNV--
DIA-TT--EALSMAQEVDPEGDRTIGVL-TKPDLVDRGA----E--GKVLDVMRNL---V---------------------
YPLK-KGYMIV----------------------------------KCR-----------------------------------G-QQDIQ----E-----
----------------------QLSLT----------E----AFQK---EQVFF---------KD-------HS--------------------YF----
-------------S-I------L--LE----------------------DGK-ATV-----PCLAERLTEELTSHICKSL----
>XP_028583072.1
------------LALPAIAVIGD----Q----------------SSGKSSVLE-----A---LS-GVA-LPRG---SGIVTRCPL---
------ELR---L---KK-LL--P-GE---KW---
N-GKI--------------------------------S----------------YL---GKY-----
-MEL-----ANPS---MVEIEIRKAQNI-----IAGDG-------------------VA-------------------------IS--
DKLITLEIRSPE--VPDLTLIDLPGIAR--
VA-------------------VGNQPV----------NI---GDQIK--------KLIKTFIDK------------QETINLVVVPSNV--
DIA-TT--EALKMAQEVDPNGERTLGIV-TKPDLMDRGT----E--GTVVNIVRNQ---V---------------------
IPLK-KGYMIV----------------------------------KCR-----------------------------------G-QQDIQ----S-----
----------------------NMTLA----------S----ALKE---ERAFF---------EK-------HK--------------------CF---
--------------S-I------L--LQ----------------------EKK-ATV-----PLLAEKLTSELVEHISKSL----
>XP_028583068.1
------------LALPAIAVIGD----Q----------------SSGKSSVLE-----A---LS-GVA-LPRG---SGIVTRCPL---
------ELK---L---KK-TH--N-TK---EW---
K-GKI--------------------------------S----------------YL---NTV-----
-EEM-----NSSR---QVEEQIIRAQNA-----MAGSG-------------------SG-------------------------IS--
SELISLEISSSD--VPDLTLIDLPGIAR--
VA-------------------VGDQPK----------DI---GQQII--------KLIKKYINK------------QETINLVVVPSNV--
DIA-TT--EALKMAQEVDPTGERTLGIL-TKPDLVDKGT----E--AEVVDIIRNQ---R---------------------
VPLR-KGYMIV----------------------------------KCR-----------------------------------G-QSDIN----D----
-----------------------KVTLG----------D----AIEK---EREFF---------EE-------HD--------------------FF----
-------------R-S------L--LE----------------------EGR-ATI-----PLLAERLTQELIEHISKTL----
>XP_031752404.1
------------LALPAIAVIGD----Q----------------SSGKSSVLE-----A---LS-GVT-LPRG---SGIVTRCPL---
------ELK---L---KK-AM--K-KT---TW---
S-GKI--------------------------------S----------------YR---DHE----

--IKI-----ASAA---DVEEEVKRAQNL-----MAGSG-------------------KG-------------------------IS--
DELISLEVISPD---VPDLTLIDLPGITR--
VA-------------------LPDQPK----------DI---EQQIK--------KMIRKYIQK------------QETINLVVVPSNV-
-DIA-TT--EALEMAREVDPNGERTLGIL-TKPDLVDRGA----E--TDVISVVRNL---V--------------------
YSLN-KGYMIV-----------------------------------KCR------------------------------------G-QQEIQ----E-----
----------------------NLSLK----------D----ALVN---EQNFF---------KE-------HE--------------------HF--
---------------S-V------L--LE----------------------EGY-ATI-----ACLAGKLTNELVAHIVRNL----
>NP_001007285.1
------------LNLPAIAVIGD----Q----------------SSGKSSVLE-----A---LS-GVA-LPRG---IGIVTRCPL---
------ILK---L---KK-IT--R-DK---NW---S-
GLL-------------------------------T---------------YK---DQT------
EIL-----KEPT---GIENAVLKAQIA-----LAGTG-------------------EG-------------------------IS--
HEMITLEIQSCD---VPDLTLIDLPGIAR--
VA-------------------TGNQPE----------DI---EKQIK--------DLIEKFIKR------------QETISLVVVPANI--
DIA-TT--EALKMASTVDPTGQRTLCIL-TKPDLVDRGM----E--DTVVRTVNNE---V--------------------
IRLE-KGYMIV-----------------------------------KCR------------------------------------G-QQDIN----D-----
----------------------KLNLV----------E----ALEK---ERRFF---------DE-------HP--------------------QF----
-------------S-S------L--LE----------------------DGK-ATI-----PLLGQRLTEELVEHIAKNV----
>XP_005167721.2.2
------------LNLPAIAVIGD----Q----------------SSGKSSVLE-----A---LS-GVA-LPRG---TGIVTRCPL---
------VLK---L---KK-IT--K-DK---SW---
H-GLL-------------------------------T---------------YN---DKI-----
-REL-----KDPA---KIEKAVLNAQTA-----LAGIG-------------------EG-------------------------IS--
HEMITLEIQSCD---VPDLTLIDLPGIAR--
VA-------------------TGNQPE----------DI---EKQIK--------SLIEKFIKR------------QETISLVVVPANI--
DIA-TT--EALKMASTVDPTGQRTLGIL-TKPDLVDRGM----E--DTVVRTVNNE---V--------------------
IPLK-KGYMIV-----------------------------------KCR------------------------------------G-QQDIN----D-----
----------------------KLGLV----------E----ALEK---ERRFF---------DE-------NV--------------------HF----
-------------R-S------L--LE----------------------DRK-ATI-----PLLAERLTKELVEHIAKNL----
>XP_012586448.1
------------LALPAIAVIGD----Q----------------SSGKSSVLE-----A---LS-GVA-LPRG---SGIVTRCPL---
------VLK---L---KK-QL--Q-GA---PW---
T-GTI-------------------------------S---------------YR---GVT------
LGL-----QDPS---AVEREIHVAQNV-----IAGHG-------------------VG-------------------------IS--
HELITLEVSSPE---VPDLTLIDLPGIAR--
VA-------------------VGNQPQ----------DI---GAQVSLSGAGRWGALVSGLPRS------------ERCTGQ--
---------E-TA--EALGGGRAAXXDGDRTLGIL-TKPDLVDKGA----E--KAVVNVAQNL---T----------------
-----YRLK-KGYMVV-----------------------------------KCR------------------------------------G-QQDIM----
D--------------------------RLSLA----------Q----ATEK---EVAFF---------QT-------HP--------------------
HF-----------------R-A------L--LE----------------------EGK-ATV-----PRLAEKLTSELILHINKSL----
>KAI0213370.1
------------ISLPEVAVIGD----Q----------------SAGKSSVLE-----A---IS-GVQ-LPRG---SGIVTRCPL---
------ALQ---L---KS-HD--T-PG---YW---
N-GVI-------------------------------K---------------YE--------------------TYNH-------------------PVE--

----KTI-----EGPT---EVGAEVREAQDV-----IAGKN-------------------VG-------------------------IS--
DTLISLQITSHG---VPDLTLIDLPGITR--
VA-------------------VEGQPK----------DI---GDQIK--------RLIGHYIKK------------EETIILAVVPANV--
DIA-TT--EALKMAKEVDPSGGRTLGVV-TKPDLIDIGT----E--KGLIDIINNE---T---------------------
YPLE-KGYSCV----------------------------------RCR-----------------------------------G-QKAIN----E-----
----------------------GQTLA----------E----AIQQ---DTDFF---------SS-------AP--------------------HF---
--------------S-D------V---D----------------------ESI-LGV-----KNLAMKLTYELVKQIKRAL----
>KAI0208044.1
------------ISLPEVAVIGD----Q----------------SAGKSSVLE-----A---IS-GVQ-LPRG---SGIVTRCPL---
------ALQ---L---KS-HD---T-PG---YW---
N-SVI--------------------------------K----------------YK---------------------YGDEFD-------------
FEDIVVE------KTI-----EGPT---KVDAGVREAQDA-----IAGKN-------------------VG---------------------
----IS--DTLISLQITAYG---
VPDLTLIDLPGITR--VA-------------------VQGQPP----------DI---GDQIK--------RLIGNYIKK-----------
-EETIILAVVPANV--DIA-TT--EALKMAKEVDPSGGRTLGVV-TKPDLIDIGT----E--KGLIDIINNE---T----
-----------------YPLE-KGYSCV----------------------------------RCR-----------------------------------G-
QKAIN----E---------------------------GQTLA----------D----AVQE---DTDFF---------SS-------AP-------
-------------HF-----------------S-A------V---D----------------------ESI-LGV-----
KNLAMKLTFELVKQIKRAL----
>KAI0218869.1
------------IALPEVAVIGD----Q----------------SAGKSSVLE-----A---IS-GVQ-LPRG---SGIVTRCPL---
------ALQ---L---KS-DK---T-PG---YW---
N-GVI--------------------------------K----------------YE---------------------INER-------------------LVE---
---KTI-----VGPA---EVDAEVRNAQDV-----IAGKN-------------------VG-------------------------IS--
SKLISLQITSYG---IPDLTLIDLPGITR--VA-
------------------VEGQPQ----------NI---GEQIK--------RLIEKYIKK------------EETIILAVVPANV--DIA-
TT--EALKMAKEVDPSGSRTLGVV-TKPDLIDAGT----E--KGLISIINNE---T---------------------YPLK-
KGYSCV----------------------------------RCR-----------------------------------G-QKAID----E------------
---------------GQTLA----------Q----AIQQ---DTDFF---------SI-------AS--------------------HF----------
-------S-D------V---D----------------------QST-LGV-----KNLAMKLTYELVRQIKRAL----
>XP_032804093.1
------------VGLPAVAVIGD----Q----------------SSGKSSVLE-----A---LS-GVQ-LPRG---SGIVTRCPL-
--------ALK---L---KR-A--PG---PW---
H-GRI--------------------------------K----------------YR---------------------VQGR-------------------TVN-
-----TKL-----DTPE---SVGDAVLQAQSE-----LTGDD-------------------LG-------------------------VS--
KSLIELEVTSDS---VPDLTLIDLPGIAR--
VA-------------------LAGQAV----------DI---ETQIK--------DLIRDHIGR------------QETINLVVIPCNV--
DIA-TT--EALKMAQAVDPTGVRTLGVL-TKPDLMDEGT----E--RNALRILQNQ---V---------------------
FPLS-KGYVLV----------------------------------KCR-----------------------------------S-QRDVE----A-----
----------------------HQTLA----------E----ASRV---EAAFF---------KK-------HP--------------------VF----
-------------C-H------V--HN----------------------GGKLTTT-----TVLAAKLTEELVDNIKRTL----
>XP_046565196.1
------------INLPAVAVIGD----Q----------------SAGKSSVLE-----A---IS-GVQ-LPRG---TGIVTRCPL---
------EMR---M---KH-SE---D-ED---KW-

--E-GKI--------------------------------M----------------YKD--------------------KHDE-------------------
LHK------EDI-----QDRE---SVGDLVRKAQDE-----MTDCE-------------------KG-------------------------
IS--DDLITLEVTSSD---
VPDLTLIDLPGIAR--NA-------------------VKGQPV----------DI---EKRIK--------DMIRKYIRR-----------
-QETIILAVLQCNV--DIA-TC--EALKMAKEFDEEGRRTLGVL-TKPDLLDKGA----E--NGVMRILNNM-
--E---------------------FSLS-KGYIIV----------------------------------KCR----------------------------------
-G-QEAIS----K---------------------------GQSLT----------E----ALGD---EDNFF---------KD-------HS---
-----------------HF-----------------R-S------L---K----------------------VSQ-WGI-----
LTLSSRLSLELQKHIK-------
>XP_046562919.1
------------INLPAVAVIGD----Q----------------SAGKSSVLE-----A---IS-GVQ-LPRG---TGIVTRCPL---
------EMR---M---KH-SE---D-ED---KW-
--E-GKI--------------------------------M----------------YKD--------------------KHDM-------------------
RQE------EVI-----LNRE---SVGDLVRKAQKE-----MTDGA-------------------KG-------------------------
IS--DELITLEVTSSD---
VPDLTVIDLPGITR--NA-------------------VEGQPF----------DI---EARIK--------NMIRKYIKR-----------
-QETIILAVLQCNV--DIA-TC--EALKMAKEFDGEGGRTLGVL-TKPDLMDKGA----E--TGVIRILNNM--
-E---------------------FTLS-KGYIIV----------------------------------KCR-----------------------------------
G-QEAIS----E---------------------------GQSLK----------Q----ALDI---EEDFF---------KS-------HR-----
---------------HF-----------------S-S------L---R----------------------PSQ-WGI-----
PDLSSRLSRELKRHIKKLL----
>XP_046563124.1
------------INLPAVAVIGD----Q----------------SAGKSSVLE-----A---IS-GVQ-LPRG---TGIVTRCPL---
------EMR---L---KH-SE---D-ED---KW--
-E-GKI--------------------------------L----------------YKD--------------------KHDM-------------------
RQE------EVI-----LNRE---SVGDLVRKAQKE-----MTDSA-------------------KG-------------------------
IS--DELITLEVTSSD---
VPDLTVIDLPGIAR--NA-------------------VEGQPF----------DI---EARIK--------NMIRRYIGR----------
--QETIILAVLQCNV--DIA-TC--EALKMAKEFDDEGGRTLGVL-TKPDLLDKGA----E--
TGVMRILNNM---E---------------------FTLS-KGYIIV----------------------------------KCR----------------
-------------------G-QEAIS----E---------------------------GQSLK----------H----ALDV---EEDFF--------
-KS-------HR--------------------HF-----------------S-S------L---R----------------------PSQ-WGI-----
PNLSARLSRELKKHIKKLL----
>XP_046563126.1
------------INLPAVAVIGD----Q----------------SAGKSSVLE-----A---IS-GVQ-LPRG---TGIVTRCPL---
------EMR---M---KH-SE---D-ED---KW-
--E-GKI--------------------------------M----------------YKD--------------------KHDM-------------------
PHE------EVI-----LNRE---SVGDLVRKAQTE-----MTDGA-------------------TG-------------------------
IS--DELITLEVTSSD---
VPDLTVIDLPGIAR--NA-------------------VEGQPF----------DI---EARIK--------NMIRQYIER-----------
-QETIILAVLQCNV--DIA-TC--EALKMAKEFDDEGGRTLGVL-TKPDLLDKGA----E--TSVIRILNNM---
E---------------------FTLS-KGYVIV----------------------------------KCR-----------------------------------
G-QEAIS----E---------------------------GQSLK----------H----ALDV---EEDFF---------RS-------HR----

----------------HF-----------------S-A------L---R----------------------PSQ-WGI-----
PNLSERLSRELKKHIKKLL----
>XP_046565195.1
------------INLPAVAVIGD----Q----------------SAGKSSVLE-----A---IS-GVQ-LPRG---TGIVTRCPL---
------EMR---M---KH-SE---D-ED---KW-
--E-GKI---------------------------------M----------------YKD--------------------KHDM-------------------
PHE------EVI-----LNRE---SVGDLVRKAQKE-----MTDGA--------------------TG-------------------------
IS--DELITLEVMSSD---
VPDLTVIDLPGIAR--NA-------------------VEGQPF----------DI---EARIK--------NMIRQYIQR----------
--QETIILAVLQCNV--DIA-TC--EALKMAKEFDDEGGRTLGVL-TKPDLLDKGA----E--TGVIRILNNM--
-E---------------------FTLS-KGYVIV-----------------------------------KCR------------------------------------
G-QEAIS----E---------------------------GQSLK----------H----ALDV---EEDFF---------RS-------HR----
----------------HF-----------------S-A------M---R----------------------PSQ-WGI-----
PNLSERLSRELKKHIKKLL----
>XP_046563125.1
------------INLPAVAVIGD----Q----------------SAGKSSVLE-----A---IS-GVQ-LPRG---TGIVTRCPL---
------EMR---M---KH-SE---D-ED---KW-
--E-GKI---------------------------------M----------------YTD--------------------KHDE-------------------
PHQ------EVI-----LNRE---SVGDLVRKAQKE-----MTDSA-------------------KG-------------------------
IS--DELITLEVTSSD---
VPDLTVIDLPGIAR--NA-------------------VEGQPV----------DI---EARIK--------NMIRQYIER----------
-QETIILAVLQCNV--DIA-TC--EALKMAKEFDDEGGRTLGVL-TKPDLLDRGA----E--TGVMRILNNM-
--E---------------------FTLS-KGYIIV-----------------------------------KCR------------------------------------
G-QEAIS----E---------------------------GQSLK----------H----ALDV---EEDFF---------KS-------HR----
----------------HF-----------------S-S------L---G----------------------PSQ-WGI-----
PNLSRRLSRELKKHIKKLL----
>XP_046352527.2
------------INLPAVAVIGD----Q----------------SAGKSSVLE-----A---IS-GVQ-LPRG---TGIVTRCPL---
------EMR---M---KH-SE---D-ED---
MW---E-GKI---------------------------------M----------------YKD--------------------MHGE---------------
----AHE------EII-----LNRE---SVGELVRKAQKE-----MTDSA-------------------KG------------------------
--IS--DELITLEVTSSD---
VPDLTVIDLPGIAR--NA-------------------VEGQPV----------DI---EARIK--------QMIRKYIGR----------
--QETIILAVLQCNV--DIA-TC--EALKMAKEFDDEGGRTLGVL-TKPDLLDKGA----E--
SGVVRILNNM---E---------------------FTLS-KGYIIV-----------------------------------KCR----------------
-------------------G-QEAIS----D---------------------------GQSLK----------Q----ALEV---EEDFF--------
-KS-------HR--------------------HF-----------------S-S------L---R----------------------PSQ-WGI-----
PNLSTRLSRELKKHIKKLL----
>XP_048248476.1
------------INLPAVAVIGD----Q----------------SAGKSSVLE-----A---IS-GVQ-LPRG---TGIVTRCPL---
------EMR---M---KH-SE---D-GD---
MW---E-GKI---------------------------------M----------------YKD--------------------MHDM---------------
----AHE------EII-----LNRE---SVGELVRKAQIE-----MTDSA-------------------KG------------------------
-IS--DELITLEVTSSD---

VPDLTVIDLPGIAR--NA-------------------VEGQPF----------DI---EARIK--------NMIRRYIGR----------
--QETIILAVLQCNV--DIA-TC--EALKMAKEFDTEGGRTLGVL-TKPDLLDKGA----E--SGVVRILNNK--
-E---------------------FTLS-KGYIIV----------------------------------KCR-----------------------------------
G-QEAIS----D---------------------------GQSLK----------Q----ALEV---EEDFF---------KS-------HR----
----------------HF-----------------S-S------L---R----------------------PSQ-WGI-----
PNLSMRLSRELKKHIKKLL----
>XP_048258111.1
------------INLPAVAVIGD----Q----------------SAGKSSVLE-----A---IS-GVQ-LPRG---TGIVTRCPL---
------EMR---M---KH-SE---D-ED---
MW---E-GKI--------------------------------M----------------YKD--------------------MHDM---------------
----AHE------EII-----LNRE---SVGELVRKAQKE-----MTDSA-------------------KG-----------------------
--IS--DELITLEVTSSD---
VPDLTVIDLPGIAR--NA-------------------VEGQPF----------DI---EARIK--------NMIRRYIGR----------
--QETIILAVLQCNV--DIA-TC--EALKMAKEFDAEGGRTLGVL-TKPDLLDKGA----E--AGVVRILNNM-
--E---------------------FTLS-KGYIIV----------------------------------KCR-----------------------------------
G-QEAIS----Y---------------------------GQSLK----------Q----ALEV---EEDFF---------KS-------HR-----
---------------HF-----------------S-S------L---R----------------------PSQ-WGI-----
PNLSARLSRELKKHIKKLL----
>XP_046352531.2
------------INLPSVAVIGD----Q----------------SAGKSSVLE-----A---IS-GVQ-LPRG---TGIVTRCPL---
------EMR---M---KH-SE---D-ED---
MW---E-GKI--------------------------------M----------------YKD--------------------MYDM---------------
----AHE------EII-----LNRE---SVGELVRKAQKE-----MTDSA-------------------KG-----------------------
--IS--DELITLEVTSSD---
VPDLTVIDLPGIAR--NA-------------------VEGQPF----------DI---EARIK--------NMIRKYIGR----------
--QETIILAVLQCNV--DIA-TC--EALKMAKEFDAEGGRTLGVL-TKPDLLDKGA----E--SGVVRILNNM-
--E---------------------FTLS-KGYIIV----------------------------------KCR-----------------------------------
G-QEAIS----D---------------------------GQTLK----------Q----ALEV---EEDFF---------KS-------HR----
----------------HF-----------------S-S------L---R----------------------PSQ-WGI-----
PNLSGRLSRELKIHIKKLL----
>XP_048248472.1
------------INLPSVAVIGD----Q----------------SAGKSSVLE-----A---IS-GVQ-LPRG---TGIVTRCPL---
------EMR---M---KH-SE---D-ED---
MW---A-GKI--------------------------------M----------------YKD--------------------MYDM---------------
----THE------EII-----LNRE---SVGELVRKAQKE-----MTDSA-------------------KG------------------------
-IS--DELITLEVTSSD---
VPDLTVIDLPGIAR--NA-------------------VEGQPL----------DI---EARIK--------NMIRRYIRR-----------
-QETIILAVLQCNV--DIA-TC--EALKMAKEFDAEGGRTLGVL-TKPDLLDKGA----E--TGVVRILNNM--
-E---------------------FTLS-KGYIIV----------------------------------KCR-----------------------------------
G-QEAIS----D---------------------------GQSLK----------Q----ALEV---EEEFF---------KS-------HR----
----------------HF-----------------S-S------L---R----------------------PSQ-XGI-----
PNLSGRLSRELKIHIKKRL----
>XP_048248473.1

------------INLPSVAVIGD----Q----------------SAGKSSVLE-----A---IS-GVQ-LPRG---TGIVTRCPL---
------EMR---M---KH-SE---D-ED---
MW---A-GKI--------------------------------M----------------YKD--------------------MYDM---------------
----THE------EII-----LNRE---SVGELVRKAQKE-----MTDSA-------------------KG------------------------
-IS--DELITLEVTSSD--
VPDLTVIDLPGIAR--NA-------------------VEGQPL----------DI---EARIK--------NMIRRYIRR-----------
-QETIILAVLQCNV--DIA-TC--EALKMAKEFDAEGGRTLGVL-TKPDLLDKGA----E--TGVVRILNNM--
-E---------------------FTLS-KGYIIV----------------------------------KCR-----------------------------------
G-QEAIS----D---------------------------GQSLK----------Q----ALEV---EEEFF--------KS-------HR----
----------------HF-----------------S-S------L---R----------------------PSQ-WGI-----
PNLSGRLSRELKIHIKKRL----
>XP_048248474.1
------------INLPSVAVIGD----Q----------------SAGKSSVLE-----A---IS-GVQ-LPRG---TGIVTRCPL---
------EMR---M---KH-SE---D-ED---
MW---A-GKI--------------------------------M----------------YKD--------------------MYDM---------------
----THE------EII-----LNRE---SVGELVRKAQKE-----MTDSA-------------------KG------------------------
-IS--DELITLEVTSSD--
VPDLTVIDLPGIAR--NA-------------------VEGQPL----------DI---EARIK--------NMIRRYIRR-----------
-QETIILAVLQCNV--DIA-TC--EALKMAKEFDAEGGRTLGVL-TKPDLLDKGA----E--TGVVRILNNM--
-E---------------------FTLS-KGYIIV----------------------------------KCR-----------------------------------
G-QEAIS----D---------------------------GQSLK----------Q----ALEV---EEEFF--------KS-------HR----
----------------HF-----------------S-S------L---R----------------------PSQ-WGI-----
PNLSGRLSRELKIHIKKRL----
>ABI53802.1
------------INLPAVAVIGD----Q----------------SAGKSSVLE-----A---IS-GVQ-LPRG---TGIVTRCPL---
------EMR---M---KH-SE---A-ED---MW-
--E-GKI--------------------------------M----------------YKD--------------------MYDV------------------
AHE------EII-----LNRE---SVEELVRKAQKE-----MTDSA-------------------KG-------------------------
IS--DELITLEVTSSD---
VPDLTVIDLPGIAR--NA-------------------VEGQPF----------DI---EARIK--------NMIRRYIGR-----------
--QETIILAVLQCNV--DIA-TC--EALKMAKEFDAEGGRTLGVL-TKPDLLDKGA----E--TGVVRILNNM-
--E---------------------FTLS-KGYIIA----------------------------------TCR-----------------------------------
G-QEAIS----D---------------------------GQSLT----------Q----ALEV---EEDFF--------KS-------HR----
----------------YF-----------------S-S------L---R----------------------PSQ-WGI-----
PNLSGRLSRELKKHIKKLL----
>CAH1802128.1
------------VALPAVVVIGD----Q----------------SVGKSSVLE-----A---MS-GVQ-LPRG---TGIVTRCPL-
--------ELR---M---KQ-CD---PG---NF-
--H-AKI--------------------------------S----------------YDI--------------------QGGH------------------
QPLE------KTI-----TDPS---NIDFEIRQAQRA-----LVGDS-------------------GG-------------------------
VS--DRLIRLEVQADY---
VPDLTLIDLPGIVR--YS-------------------EGSD------------TI---VEETK--------NLIKTYVSR-----------
PETIILVVIPCNV--DID-TV--EACNLAKQVDPNGDRTIGVL-TRPDLIDHGV----GPIKEVLDILENK---K-
--------------------MKLK-KGFYVV----------------------------------KCR-----------------------------------

S-QKRIE----E---------------------------GQSLE----------Q----ALAE---EVQFF---------RS-------DE----
----------------RF-----------------R-V------I---N----------------------PSQ-CGV-----
KQLSSKLTNELFLHIKNCV----
>PAA74204.1
------------VRLPAIAVVGD----Q----------------SVGKSSVLE-----S---IS-GID-LPRG---LGIVTRCPL---
------MLS---M---RN---REEA---GW--
-S-ARI--------------------------------K----------------YKT--------------------KTGE-------------------GRE-
-----KKL-----TGAS---EVGQAIRDAQNE-----MTNSS-------------------GE-------------------------VS--
EQLIELWVESPE--SPDLTLIDLPGIAR--
YS-------------------IDGGG-----------AI---AGLTK--------SLILSYIEK------------EEILILVVIPCHV--
DIE-TV--EALSLAKEVDPESKRTIGVL-TCPDLVNPGS----E--SEVLALMQNR---K---------------------
IPLK-KGYVSV----------------------------------RCR-----------------------------------T-PQQLK----D------
---------------------NMSLQ----------Q----AARE---EEVFF---------RT-------HP--------------------HF---
--------------R-A------L---D----------------------KFE-YGT-----KTLAVKLSSELYEAIKHNI----
>PAA76532.1
------------ISLPAIAVVGD----Q----------------SVGKSSVLE-----A---IS-GVE-FPRG---LGIVTRCPL----
-----MLS---M---RG---REDS---GW---
T-ARI--------------------------------R----------------YET--------------------KSGQ-------------------ARD-
-----KPL-----STPA---EIGQAIRDAQEE-----MTSSS-------------------GE-------------------------IS--
EKLIELHIEGAD--TPDLTLIDLPGIAR--
FS-------------------IANAG-----------DI---ATVSK--------SLIMSYILK------------PEVLILVVIPCNV--
DVE-TV--EAISLAREVDPECKRTLGVL-TCPDLVNPGS----E--TEVLAMMRNE---R---------------------
LKLR-KGFVTV----------------------------------RCR-----------------------------------T-PQQLK----D-----
----------------------NMGLR----------E----ACKA---EEEFF---------KL-------HP--------------------QF--
---------------C-A------L---G----------------------DYQ-RGC-----KTLANKLSVELYQAVKERI----
>PAA92268.1
------------LKLPMVAVVGD----Q----------------SVGKSSVLE-----A---IS-GVE-FPRG---
TGMVTRCAL---------QLS---M---QW-NA--
-----DPEA---PW---H-GRI--------------------------------S----------------YKD--------------------VNGH-
------------------KVD------KEL-----NSPG---EVDGAVREAQQR-----MTHGD-------------------NE-----
--------------------IS--SEQIDLAIKGPD---
VPDLTLIDLPGIAR--YS-------------------ATGGS-----------GI---AQITK--------SLIAKYVSQ------------
PQVLILVVVPCHQ--DIE-TV--EALSLAKEADPQGERTIGVL-TCPDMVNKGA----E--QETLKIANNE---
K---------------------IPLK-KGYVMV----------------------------------KCR-----------------------------------
S-PEELN----N---------------------------GVTLS----------E----SVAN---EAAFF---------KT-------HR-----
---------------HF-----------------S-L------L---P----------------------EQS-VGI-----
RTLADKLTEELFESVKRNI----
>PAA69582.1
------------LRLPTVAVVGD----Q----------------SVGKSSVLE-----A---IS-GVD-LPRG---TGIVTRCPL--
-------QLS---M---R---S---KPTG---DW--
-T-GRI--------------------------------S----------------YQN--------------------RKGE-------------------
HVE------REI-----SKKC---EVDEMVRKVQNE-----ITGDS-------------------NG-------------------------
VS--TEQIDLTIESAD---
VSDLTLVDLPGIAR--YS-------------------EKN-P-----------KI---NEVTK--------QLILSYISQ------------

DQVIILVVVPCSV--DIE-TV--EAIALAKQVDPGGTRTIGVL-TCPDLTNPGS----E--EDIKAIVNNQG--
R---------------------VRLH-KGFVMV-----------------------------------KCR----------------------------------
--S-PKELR----N----------------------------NISLS----------E----VAKI---EEDYF----------KN-------DP----
----------------HF------------------S-Q------L---P----------------------KDI-VGT-----
KTLAEKLTNELFKAVAAGI----
>PAA83069.1
------------LKLPSIVVIGD----Q----------------SSGKSSVLE-----T---IS-GVS-FPRG---NGVVTLCPL---
------QLS---M---RS-SD--K------KW---
R-GTV------------------------------R----------------YFD--------------------AQGK-------------------
EVH------WDI-----DSPD---DVENAIQNAQMR-----ITGHK-------------------KA-------------------------
IS--KNIIEMTLEAPD--
LPNLTLVDLPGIAR--YS-------------------HSDGGS----------VNL---YKLTT--------DIIKEYIQR-----------
-EETIILTVIPLSA--DTA-TM--EALQLAKDVDPYGLRTIGVL-TFPDLVNKGA----E--EEKLQIARNI---T---
------------------FPLS-KGYITV-----------------------------------KCR------------------------------------N-
QEDIK----S---------------------------RKSLR----------E----AKVD---EMRFF----------SN-------DP-------
-------------FF------------------S-Q------L---D-----------------------PSQ-RGT-----
DTLAKRLSTELLTLIKKFI----
>PAA94353.1
------------LRLPSIVVIGD----Q----------------STGKSSVLE-----S---IS-GVR-FPRG---NGVVTLCPL---
------QLS---M---RT-SD---D--G---KW--
-R-GNI------------------------------R----------------YYD--------------------TYGK-------------------
LMK------WDI-----DGPE---DVEDAIQEAQMR-----ITGNQ-------------------RN-------------------------
-VS--KSIIEMTLESPE--
LPNLTLIDLPGIAR--YN-------------------HNSAESG---------ASL---HQLTT--------DIIKEYIRR------------
--EETIILVVIPLTS--DTA-TM--EALQLAKDADPYGMRTIGVL-TFPDLVNKGA----Q--EEKLMIARNI---T-
--------------------YPLS-KGYVTV-----------------------------------KCR------------------------------------N-
QEDIK----N---------------------------RKSLK----------D----AKAD---EALFF----------NT-------DP--------
------------FF------------------K-Q------L---D----------------------SMY-RGS-----
DTLARRLSEELLYLVKKFI----
>GMH43921.1
S-----------QMLTAIVVVGD----Q----------------SSGKSSTLE-----R---IA-GID-LPRG---QGICTRVPL-
--------EMQ---M---RK-GS---KF--
-S-ATL--------------------------------E----------------YQQ--------------------EKGGS------------------
KQS------VEI-----KDAS---KISDAIQAATRD-----IVGNS-------------------KN-------------------------
VE--DLPLVLRISSPI--
YQDLTLIDLPGIAR--AP-------------------LPGQRS----------DI---EEQTL--------EMMRRYITG---------
---EAKVILCALPATN--DFV-TS--AALKLALQLDPDGERTLGAV-TKIDQARKGI----A--KKLEGTDASE--
-----------------------ITLH-LGFAGV-----------------------------------RCR------------------------------------
T-ENETD----A----------------------------GITLE----------Q----VRQA---EEELF----------RT-------HD------
--------------EL-----------------K-H------V---D-----------------------DSC-KGV-----
SALLQKLVAVQRGRLISHLPKVL
>GMH36208.1
-------------MLPAIVVGD----Q-----------------SSGKSSLLE-----I---LS-GVT-LPRG---EGICTRVPL---
------ELQ---L---RN-GT---EV----------S-

AQI--------------------------------E----------------YQT--------------------DLDAP------------------RVS---
---KHI-----M-VE---EVKNEILLATKR-----IAGME-------------------LN-------------------------IK--
DLPIVLRMTGPT--YQDLTLIDLPGIAR--
MP-------------------LRGQPD----------NI---EELTM--------EMIQKYING------------
DSKVILCAVPANN--EFV-TS--AALKLASNVDPLGLRTLGVV-TKADQFSRGM----R--RRLEGLDDT---
-D---------------------VKLK-LGFVAV----------------------------------RCR---------------------------------
--T-QKELE----E---------------------------GISLQ----------D----VRMR---EELLF---------ET-------DP----
----------------EL-----------------R-D------V---Q----------------------PHC-RGI-----
STLVDKLVDIQKERLIEQLPRIV
>GJP35534.1
-------EG---IELPTIVVGD----Q----------------SSGKSSVLE-----N---LS-GIS-LPRG---KGIVTRVPL--
-------ILR---L---QS-CV---K--G---KD---
M-ITI--------------------------------E----------------Y----------------------TPVTG------------------KVS----
--KVL-----SDEE---MIEEEISEATIA-----LAGSR-------------------KG-------------------------VM--
NCPITLQVQRPD--LPDLTLVDLPGITR--
VP-------------------IEDQPK----------DI---YNQVK--------NMIMHYITP------------KESVILNVLAAEV--
DFS-TC--ESIVMSQEVDSDGDRTLAVV-TKVDRAPDGL----Y--EKIQGN-------S---------------------
VRIG-LGYVCV----------------------------------RNK----------------------------------TDADASH----D----
--------------------------------------D----ARRA---EAAFF---------NS-------HP--------------------EL-----
------------S-Q------I---E----------------------SHC-LGI-----PALAQRLTEIQAKRVADSIPRIR
>CAI5480041.1
-------EG---IELPTIVVGD----Q----------------SSGKSSVLE-----N---LS-GIS-LPRG---NGIVTRVPL--
-------ILR---L---QS-CT---S--K---DG---
E-ITI--------------------------------E----------------YN---------------------NPSSG------------------KIF---
---KIL-----PDEE---SIQEEISKATVT-----LAGSR-------------------KG-------------------------VM--
DRPITLQVKRSG--LPDLTLVDLPGITR--
VP-------------------VDDQPK----------DI---YNQVK--------KMIMQYITP------------EESVILNVLAAEV-
-DFS-TC--ESIVMSQEVDQDGDRTLAVV-TKVDRAPDGL----Y--EKIQGN-------S---------------------
VRIG-LGYVCV----------------------------------RNK----------------------------------TDADASH----A----
--------------------------------------A----ARLA---ETDFF---------DR-------HP--------------------EL------
-----------S-R------I---E----------------------TDS-RGI-----PALAQRLSEIQAKRVAESIPRIR
>KAJ7294545.1
--------G---IQLPTIVVGD----Q----------------SHGKSSVLE-----S---LA-EIT-LPRR---QGIATRVPL---
------ILR---L---QS-CK---VA-S---EQ---
S-ITI--------------------------------E----------------Y------------------------LNV------------------KD-------
-EI-----KSEE---LIEAAIDEATNV-----LAGPR-------------------KD-------------------------VR--
DTPISLHVRKLG--APDLTMVDLPGITR--
VP-------------------VHGQPE----------NI---YEQIA--------AMIQKYINP------------PESIILNVISATV--
DFP-TC--ESIRMSQLADKEGKRTLAVV-TKVDKAPEGL----Y--EKVTSD-------A---------------------
VNIG-LGYICV----------------------------------RNR----------------------------------TEKENSN----E-----
-------------------------------------Q----ARFV---EKHLF---------DT-------HP--------------------SL-------
----------C-K------L---D----------------------KSM-VGI-----PMLAYRLTCIQAQMIQGCLPGL-
>XP_024380180.1

-------EN---VNIPTIVVGD----Q----------------SSGKSSVLE-----S---LA-GIT-LPRG---QGIATRVPL--
-------ILR---L---QS-CL--SE-Q---DS---
K-ILM--------------------------------E----------------Y------------------------GSV------------------KE-----
--MRI-----NSED---DIEAAINAATDD-----LAGSN-------------------KN-------------------------IR--
DTPILLHIRKPD---APDLTMVDLPGITR--
VP-------------------VHGQPE----------NI---YEQVR--------DMIMHYIKP------------EESIILNVLPAEV-
-DFS-TC--ESIRLSQTVDKKGVRTLAVV-TKVDKAPEGL----F--EKVTSD-------A---------------------
VSIG-LGYVCV----------------------------------RNR-----------------------------------TPADDSI----A-----
-------------------------------------V----ARCR---ELELF---------ND-------HP--------------------DL------
-----------R-N------I---D----------------------RSM-VGI-----PTLGRRLVKIQSDMVRGCLPRIR
>XP_024367947.1
-------EN---VNIPTIVVGD----Q----------------SSGKSSVLE-----S---LA-GIT-LPRG---QGIATRVPL--
-------ILR---L---QS-CL--SE-Q---DS---
K-ILM--------------------------------E----------------Y------------------------ENV------------------KE-----
--MRI-----NSED---DIEAAINAATDD-----LAGSN-------------------KN-------------------------IR--
NTPISLHIRKPD---APDLTMVDLPGITR--
VP-------------------VHGQPE----------NI---YEQVR--------DMIMHYIKP------------EESIILNVLPAEV-
-DFS-TC--ESIRLSQTVDKKGVRTLAVV-TKVDKAPEGL----F--EKVTSD-------A---------------------
VSIG-LGYVCV----------------------------------RNR-----------------------------------TPADDSI----A-----
-------------------------------------V----ARCR---ELELF---------ND-------HP--------------------DL------
-----------R-N------I---D----------------------RSM-VGI-----PTLARRLVKIQSDMVRGCLPRIR
>KAG0619429.1
--------N---VQLPTIVVGD----Q----------------SSGKSSVLE-----S---LA-GIT-LPRG---QGIATRVPL--
-------ILR---L---QS-CD--ST-E---ES---
L-IRM--------------------------------D----------------Y------------------------GNV------------------KD----
---REI-----DGEE---QIEAAINEATNV-----LAGGN-------------------KD-------------------------VK--
DTPISLHIRKPH---APDLTMVDLPGITR--
VP-------------------VHGQPQ----------NI---YEQIQ--------AMIMKHISP------------EESIILNVLSAQV-
-DFP-TC--ESIRMSQQVDKDGKRTLAVV-TKVDKAPEGL----L--EKVTTD-------A---------------------
VNIG-LGYVCV----------------------------------RNR-----------------------------------TDDDDTI----S----
--------------------------------------V----ARIR---EQRLF---------ES-------HP--------------------AL-------
----------K-D------L---D----------------------RSM-VGI-----PALARKLTKIQSDMVKGCLPRI-
>KAG0561847.1
--------N---IQLPTIVVGD----Q----------------SSGKSSVLE-----S---LA-GIT-LPRG---QGIATRVPL--
-------VLR---L---QS-C---QL-E---ES---
I-IKM--------------------------------D----------------Y------------------------GNV------------------KD-----
--QEI-----SGEE---QIEAAINAATNA-----LAGSG-------------------KG-------------------------VK--
DSPIQLLIRKPN---SPDLTMVDLPGITR--
VP-------------------VHGQPK----------NI---YEQIR--------GMIMRYITP------------EESIILNVLSAQV--
DFP-TC--ESIRMSQQVDKEGNRTLAVV-TKVDKAPEGL----L--EKVTTD-------A---------------------
VNIG-LGYICV----------------------------------RNR-----------------------------------IDVDDSI----A------
------------------------------------I----ARQR---ERELF---------ES-------HP--------------------AL---------
--------K-E------L---D----------------------GSM-VGI-----PALARKLTKIQSDMVKECLPRI-
>KAH9290598.1

--------G---IHLPTIVVVGD----Q----------------SSGKSSVLE-----S---LS-GID-LPRG---QGICTRVPL-
--------IMR---L---QN-ST--E--E---YS---
V-ISV--------------------------------E----------------Y-------------------------KD------------------RK-------
-LS-----INEH---QIVDTINLVTEE-----IAGRN--------------------KG--------------------------IS--
DDPITLHVRKKN--VPDLTLVDLPGITR--
VP-------------------VYGQPK----------DI---YEQVY--------KIIMKYISP------------RDSIILNVLSATV--
DFP-TC--ESIRMSQKVDEDGERTLAVV-TKVDKAPEGL----R--EKVAED-------A---------------------
MNIG-LGYVCV-----------------------------------RNR------------------------------------V-EGESI----V-----
--------------------------------------K----ARKK---ENELF------KT-------HP---------------------LL--------
---------S-G------I---D----------------------KSI-VGI-----PILAHKLMKIQAAGITNSLPRI-
>KAH9320939.1
--------G---IQLPSIVVVGD----Q----------------SSGKSSVLE-----S---LA-GIK-LPRG---QGICTRVPL-
--------VMR---L---QS-CA---EE-S---EE-
--E-ISI--------------------------------E----------------F--------------------------NG------------------VE-----
---KF-----IQES---DITSSIDTATQE-----IAGNG--------------------KG--------------------------IS--
HTPITLHVTKVG--APDLTMVDLPGITR--
VP-------------------VGGQPG----------DI---FEQIC--------EIIKEYITP------------KESIILNVLAANV--
DFP-TC--ESIRMSQKVDELGERTLAVV-TKSDRAPDGL----K--EKVTTD-------A---------------------
VNIG-LGYVCV-----------------------------------RNG------------------------------------I-GDESN----A----
---------------------------------------E----AREK---EKNLF------DF-------HP---------------------LL------
----------K-D------L---D----------------------KSM-VGI-----PTLAKKLMQIQATTISATLPQI-
>KAF8079489.1
-------EG---IQLPTIVVVGD----Q----------------SSGKSSVLE-----S---LA-GIS-LPRG---QGICTRVPL-
--------VMR---L---QG-SA---S--S---EP--
-E-IWL--------------------------------E----------------Y-------------------------SD------------------NV----
----VP-----TDEE---HIAEAISAATDV-----IAGSH-------------------KG-------------------------VS--
DAPLTLHVKKAG--APDLTMVDLPGITR-
-VP-------------------VKGQPE----------NI---YEQIS--------GMIMKYIKP------------QESIILNVLSATV--
DFT-TC--ESIRMSRQVDKTGERTLAVV-TKADMAPEGL----L--QKVTSD-------D---------------------
VSIG-LGYVCV-----------------------------------RNR------------------------------------V-GEETY----E------
-------------------------------------E----ARKQ---EELLF------ET-------HP--------------------TL---------
--------S-M------I---D----------------------ENI-VGV-----PVLAQKLIQIQTTMIARCLPKIV
>OAP13353.1
------------IQLPTIVVVGD----Q----------------SSGKSSVLE-----S---LA-GIS-LPRG---QGICTRVPL---
------VMR---L---QR-RR---S--P---EP---
E-IWL--------------------------------E----------------Y-------------------------GD------------------KI------
--VP-----TDEE---HIAQTICAATDV-----IAGM--

---F---

>OAP19580.1

------------IQLPTIVVVGD----Q---------------SSGKSSVLE-----S---LA-GIN-LPRG---QGICTRVPL--
-------VMR---L---QR-SS--S--P---EP---
E-IWL--------------------------------E----------------Y-------------------------SD------------------KV------
--VP-----TDEE---HVAEAICAATDV-----IAGK--

--FS-LS--PS---------------------------------------
-------------------------------------Q-CSVKCV---

--LLQK-----------------
>OAP13972.1
------------IQLPTIVVVGD----Q---------------SSGKSSVLD-----S---LA-GIS-LPRG---QGICTRVPL--
-------VMR---L---QR-SS--S--P---VP---
E-IWL--------------------------------E----------------Y-------------------------SD------------------KI-------
-VP-----TDEE---HIAEAICAATDV-----IAGK--

--FT-L---
-------------------------------Y-LGIKCV---

>XP_038984915.1
-------EG---IELPTIVVVGD----Q---------------SSGKSSVLE-----S---LA-GIS-LPRG---QGICTRVPL--
-------IMR---L---QD-DP--SL-S---QP--
-Q-LQL--------------------------------E----------------Y-------------------------KD------------------KA----
----IH-----TSED---GIADAINSATDD-----IAGSG-------------------KG-------------------------IS--
NAPLTLVVRKRG--VPDLTMVDLPGITR--
VP--------------------VHGQPD----------NI---YEQIS--------NIIMEYIAP------------KASIILNVLSATV--
DFP-TC--ESIRMSQSVDRTGERTLAVV-TKADKAPEGL----L--EKVTAD-------D---------------------
VNIG-LGYVCV----------------------------------RNR-----------------------------------I-GDESY----E-----
-------------------------------------E----ARAE---ERNLF---------KR-------HP-------------------LL-------
----------S-R------I---D----------------------KSI-VGI-----PVLAQRLMQIQAASIAKSLPDIV
>KAF8391993.1
-------EG---IELPTIVVVGD----Q---------------SSGKSSVLE-----S---LA-GIS-LPRG---QGICTRVPL--
-------IMR---L---QH-HS--T--P---SP---
E-LHL--------------------------------E----------------Y-------------------------HN------------------KI------
--IP-----TNET---HVAEAINMATNE-----IAGNG-------------------KG-------------------------IS--
NTPLTLVVKKKG--VPDLTMVDLPGITR--
VP--------------------VHGQPE----------DI---YEQIS--------SIIMEYIKP------------KESTILNVLSATV--
DFP-TC--ESIRMSQRVDKTGERTLAVV-TKCDKAPEGL----L--EKVTAD-------D---------------------
VNIG-LGYVCV----------------------------------RNR-----------------------------------I-GEELY----E------
------------------------------------E----ARME---EATLF---------ES-------HQ--------------------LL--------
---------S-K------I---D----------------------KSI-VGV-----PILAQKLVQIQASIIAKCLPDIV
>XP_058079501.1
-------EG---IELPTIVVVGD----Q---------------SSGKSSVIE-----S---LA-GIS-LPRG---QGICTRVPL--
-------IMC---L---QN-VP--T--D---KP---

Q-MHL---------------------------------E----------------Y-------------------------QG------------------KI----
----VL-----TSEN---QISDSISMATDE-----IAGNG-------------------KG-------------------------IS--
NIPLTLVVKKKG--VPDLTIVDLPGITR--
VP-------------------VHGQPE----------DI---YEQIS--------NIIMDYIKP------------KESIILNVLSATV--
DFP-TC--ESIRMSQHVDKTGERTLAVV-TKADKAPEGL----L--EKVTAD-------D---------------------
VNIG-LGYVCV-----------------------------------RNR------------------------------------I-GDETY----E------
-------------------------------------E----ARIE---EATLF---------KS-------HP--------------------LL----------
-------S-K------I---D----------------------KSI-VGI-----PVLAQKLFQIQANSLSQCLPDIV
>KAF5727250.1
-------EG---IQLPTIVVVGD----Q----------------SSGKSSVLE-----S---LA-GIS-LPRG---QGICTRVPL-
--------VMR---L---QH-HH---I--P---EP--
-E-LYL---------------------------------E----------------F-------------------------NG------------------KT-----
---VQ-----TTES---RISEAINLATDE-----IAGNG-------------------KG-------------------------VS--
NTPLTLVVKKHG--VPDLTMVDLPGITR--
VP-------------------VHDQPE----------NI---YEQIA--------GIIMEYIKP------------DESIILNVLSATV--
DFS-TC--ESIRMSQQVDKTGERTLAVV-TKVDKAPEGL----L--EKVTAD-------D---------------------
VNVG-LGYVCV-----------------------------------RNR------------------------------------I-GDETY----E-----
--------------------------------------E----ARRK---EAALF---------ES-------HP--------------------LL--------
---------C-L------I---D----------------------KSI-VGV-----PVLAQKLVQIQAAIIMKCLPEIV
>XP_002303204.3
--------G---IQLPTIVVVGD----Q----------------SSGKSSVLE-----S---LA-GIS-LPRG---QGICTRVPL--
-------IMR---L---QH-HT---A--P---EP---
E-LSL---------------------------------E----------------F-------------------------NG------------------KT------
--VP-----TSEA---KIANAISLATDE-----IAGNA-------------------KG-------------------------IS--
NTPLTLVVKKNG--VPDLTMVDLPGITR--
VP-------------------VHGQPE----------NI---YEQIA--------DIIMEYIRP------------EESIILNVLSATV--
DFT-TC--ESIRMSQKVDKNGERTLAVV-TKADRAPEGL----L--EKVTAD-------D---------------------
VNIG-LGYVCV-----------------------------------RNR------------------------------------I-GDESY----K----
--------------------------------------E----ARKE---EADLF---------EN-------HP--------------------LL-------
----------S-K------I---D----------------------KSM-VGI-----PVLAQKLVQIQATIIARCLPEI-
>KAK1401877.1
-------EG---IQLPTIVVVGD----Q----------------SSGKSSVLE-----S---LA-GIS-LPRG---QGICTRVPL-
--------IMR---L---HH-HS---K--P---ES--
-E-LHL---------------------------------E----------------Y-------------------------CG------------------KV----
----VS-----TDEL---KIAESINMATAE-----IAGDG-------------------KG-------------------------IS--
HTPLTLVVKKNG--VPDLTMVDLPGITR--
VP-------------------VHGQPE----------NI---YEQIS--------GIIMEYIKP------------EESIILNVLSATV--
DFP-TC--ESIRMSQSVDKTGDRTLAVV-TKSDKSPEGL----R--DKVMAD-------D---------------------
VNIG-LGYVCV-----------------------------------RNR------------------------------------I-GDESY----E-----
--------------------------------------E----ARMA---ESMLF---------ES-------HP--------------------LL------
-----------S-K------I---D----------------------KSM-VSV-----PVLARKLVQIQARIIAKCLPEIV
>XP_002297993.1
------------IQLPTIVVVGD----Q----------------SSGKSSVLE-----S---LA-GIS-LPRG---QGICTRVPL---
------IMR---L---QH-HT---S--L---IP---E-

MFL---------------------------------E-----------------F---------------------------NG-------------------KT--------
TQ-----TDEA---NVADDINIATEE-----IAGSG--------------------KG--------------------------IS--
DAPLTLVIKKNG---VPDLTMVDLPGITR--
VP-------------------VHGQPD----------NI---YEQIA--------GIVMQYIQP-----------EESIILNVLPASV--
DFT-TC--ESIRMSRQVDKTGERTLAVV-TKADKAPEGL----L--EKVTAD-------D--------------------
VNIG-LGYVCV------------------------------------RNR--------------------------------------I-GDESY----D-----
--N----ARME---EANLF---------AT-------HP--------------------LL-------
----------S-R------I---D-----------------------KSI-VGI-----PVLAKKLMQVQATIMAKCWP---
>XP_024439231.1
------------IQLPTIVVVGD----Q----------------SSGKSSVLE-----S---LA-CIN-LPRG---DGICTRVPL---
------IVR---L---KH-HP--S--L---VP---E-
IFL---------------------------------Q-----------------F---------------------------NG-------------------KT--------
VP-----TDEA---HVADAINLVTDE-----IAGNG--------------------KG--------------------------IS--
NTELTLVVKKNG---VPDLTLVDLPGITR--
VP-------------------VHGQPE----------NI---YEQIA--------YIIMKYISP-----------DESVILNVLSASV--
DFS-TC--ESIRMSQKVDKNGQRTLAVV-TKVDKSPEGL----L--EKVTRN-------D--------------------
VNIG-LGYVCV------------------------------------RNR--------------------------------------I-GNESY----E-----
--D----ARKE---EAALF---------AT-------HQ--------------------LL-------
----------S-K------I---D-----------------------KST-VGI-----QVLAQKLVQIQANIIAKCLP---
>KAH0683503.1
-------EG---IQLPTIVVVGD----Q----------------SSGKSSVLE-----S---LA-GIS-LPRG---QGICTRVPL-
--------VMR---L---KN-DP---NI-T---AP--
-N-LQL----------------------------------E-----------------Y---------------------------NN-------------------KS----
----LP-----VDEI---GIADAIILATDE-----IAGHG--------------------KG---------------------------IS--
NNPLTLVVKKNG---VPDLTMVDLPGITR-
-VA-------------------VQGQPE----------DI---YEQVY--------DIIMKYIVP-----------EESIILNVLSATV--
DFP-TC--ESIRMSQKVDKTGERTLAVV-TKADKAPEGL----L--EKVTAD-------E--------------------
VNIG-LGYVCV------------------------------------RNR--------------------------------------I-GNESY----E-----
--E----ARSD---EQRLF---------ST-------HP--------------------LL-------
----------S-K------I---D-----------------------KSM-VSV-----PILAQKLVRIQASIISKCLPEIV
>PWZ56863.1
--------G---IQLPTIVVVGD----Q----------------SSGKSSVLE-----S---LA-GIS-LPRG---QGICTRVPL--
-------VMR---L---QD-DP---SA-D---SP-
--K-LQL----------------------------------E-----------------Y-------------------------SNG-------------------RV--
------VT-----TTEA---DVADAINAATAE-----IAGSG--------------------KG---------------------------IS--
DAPITLVVRKRG---VPDLTLVDLPGITR--
VP-------------------VHGQPE----------DI---YDQVA--------KIIKEYIAP-----------KESIILNVLSATV--
DFP-TC--ESIRMSQQVDRSGERTLAVV-TKVDKNPEGL----L--EKVTMD-------D--------------------
VNIG-LGYVCV------------------------------------RNR--------------------------------------I-GDETY----D-----
--Q----ARVE---EERLF---------KY-------HP--------------------LL-------
----------S-K------I---D-----------------------KDM-VGI-----PVLANRLMQIQSTIIAKCLPDI-
>PWZ56864.1
--------G---IQLPTIVVVGD----Q----------------SSGKSSVLE-----S---LA-GIS-LPRG---QGICTRVPL--
-------VMR---L---QG-DP---ST-D---SP--

-K-LQL-------------------------------E-----------------Y-------------------------SNG-------------------RV---
-----VT-----TTEA---KVADAINAATAE-----IAGSG--------------------KG--------------------------IS--
DAPITLVVRKSG--VPDLTLVDLPGITR--
VP-------------------VQGQPE----------DI---YDQIA--------NIIKEYITP------------KESIILNVLSATV--
DFP-TC--ESIRMSQQVDRTGERTLAVV-TKVDKAPEGL----L--EKVTMD-------D----------------------
VHIG-LGYVCV-----------------------------------RNR------------------------------------V-GEETY----D-----
---------------------------------------Q----ARVA---EAQLF---------KN-------HP---------------------LL------
-----------S-Q------I---D-----------------------KSM-VGI-----PVLAQRLMQIQASIIAKCLPDI-
>KAH9291961.1
--------G---IQLPYIVVVGD----Q-----------------SSGKSSVLE-----C---LT-GIS-LPRG---VGICTRVPL--
-------IMR---L---QN-SS---E--Q---DS---
E-IVV---------------------------------E-----------------Y--------------------------ND------------------TV-------
-EH-----IIES---QITERIDSITKE-----IAGTN--------------------KG--------------------------IS--
HVPIRLNVKKMN--APDLTLVDLPGIAR-
-VS-------------------LNGNPD----------D----HELIS--------KIVMEYISP------------ADSIILNVLSATV--
NFR-TC--ESIRMSQRVDVHGERTLGVV-TKVDIAPEGL----L--EKVALD-------D----------------------
VNTG-LGYVCV-----------------------------------RNR------------------------------------V-GDECN----E---
---E----AREA---EAELF---------RS-------HT---------------------QL-----
------------N-K------F---D-----------------------EAM-VGI-----PMLARRLMQIQTKRISKCFPDI-
>KAH9325151.1
------------IQLLSIVVVGD----Q-----------------SSGKSSMLR-----S---LA-GIN-LPKG---QWICTRVPL-
--------IMR---L---QI-SK---S--Q---ET---
E-ITI---------------------------------E-----------------Y--------------------------SG-------------------VK-------
NN-----IFEY---KIIEALNAATDE-----IAGVG--------------------KG--------------------------IS--
DTPITLNVSKSN--IPNITMVDFPCITK--
MP-------------------VHDQPQ----------DI---YDQIS--------QVIKQYITP------------KESIILNVLFTSV--
DFP-TC--ESIRMSQLVDVKGEKTLAVV-TKVDKAVEGL----F--EKVTVDV-V----S----------------------
VNIG-LGYVCV-----------------------------------RNK------------------------------------I-GNESN----A-----
----

>KAH9300179.1
-------------NCLVLLL--WETSRLE---------------------

----PG--------------------RS---------------------------IN--DTPITLNVGKSN------------------------------------
-------------------------------------APDLTMVDFPSITR--VP--------------------VHGQPQ----------DI---
YDQIS--------QVIKQYITP------------KESIIMNVLSASV--DFP-TC--ESIRMSQLVDEKGERTLAVF-
TKVNKAAEVL----F--EKVTVD-------A---------------------VNIG-LGYVCV---------------------------------
--RNR------------------------------------I-GNESN----A---

>KAH9314974.1

--ALNAATNE-----
FAGAG-------------------KS--------------------------IS--DTPITLNVSKSN------------------------------------
-----------------------------------APNLTMVDLPGITW--VP-------------------VHGQPQ----------DI---
YDQIS--------QVIKQYITP------------KESIIMNVLSASV--DFP-TC--ESIRMSQLVDEKGERTLAVV-
TKVDNAAEGL----F--EKVTVD-------V---------------------VNIG-LGYTCV--------------------------------
--YSF-------------------------------------A-KRSRN----LA-V---------------------QACDYLDRI----------I----
CRVI---DPQLQ---------ASS-----RRA-------------------FQ-----------------A-L------I---D--------------
--------RKR-DKC-----IQYVEDAMEMQKSIVYTENP---
>EFJ22917.1
------------IKLPTIVVVGD----Q----------------SSGKSSVLE-----S---LA-QVD-LPRG---QGVVTRVPL-
--------VLR---L---QN-TS---VTDQ---SH-
--Q-VVI---------------------------------Q----------------Y-------------------------GG------------------KK---
-----RV-----IEEA---EISAAVVEATIE-----LAGD---------------------KH--------------------------IV--
NKPISLHITKPG---APDLTMIDLPGITR--
VP-------------------VHGQPE----------DI---EEQIK--------KIIQEYISP------------KETIILNVICSTV--
DFP-TC--ESILMSRQVDREGERTMAVV-TKVDMSPKDL----K--EKVMAD-------V---------------------
VGIG-LGYICV-----------------------------------RNR--I-GDETH----E------
------------------------------------E----GRDR---EAELF---------RT-------DP-------------------HL--------
---------R-D------L---P---------------------ESM-LGI-----RQLAKRLTEFQADSLRKNLP---
>CAG9460856.1
------------DKLPTIVVTGD----Q----------------SAGKSSVLE-----S---LS-GIA-FPVG---DGIVTRLPC---
------QVA---L---RE-GP---AF-R---AV---
C-TPP--------------------------------E----------------G-------------------------HG------------------EA----
---VTL-----TDPK---AVTKWIEDTTAA-----VAGDK-------------------KG-------------------------VL--
DKPLSIKVEREG--SADLTLVDLPGITR--
VA-------------------VDGQAD----------DI---EEQVK--------RMIQRYISR------------
EAAVVLCVLPANV--DFS-TA--ECIKMARAVDPGGERTLGVV-TKVDRAERGI----V--TRLNAFGTT----
G---------------------WALR-LGYVAV-----------------------------------KNL------------------------------------
--S-QDERA----K--------------------------HGVSTT----------K----VLEL---EDAFF---------
DDGVGRPAHLA---------------------EL-----------------A-D------L---D----------------------ADM-RGL--
---RTLVQKLVQVQGERIEAFMPSLV
>KAK3283006.1
--------QD--IPIPQIAVMGD----Q----------------SSGKSSVLE-----A---LS-GVH-FPRG---
TGLVTKCAV---------EVR---M---KR-LQP---
-----NE---DW---N-ASV--------SLSW--D--------------------RP-QP--
----------------SEA------GVA-----TTPN---EVGEKISKLTEV---LLRARGN-------------------RA-----------
------------TFEP-EHRIQVELKSPD--
VSDLTIIDLPGIVR--TN-------------------VAGQCK----------KV---IAEVD--------ALLDKYLRQ----------
--ERTIILAVIPSTV--DIA-TV--DVIERAEKVDPHGLRTIGVL-TKADQISSDD----E--AERVAVLRGV---R-
--------------------KPLK-LGYFMV-------------------------------------KNR------------------------------------T-
QTELE----A--------------------------GVTLA----------E----ARLA---EARYF---------SA-------HK--------
------------TF-----------------G-K------L---H---------------------PGL-FGS-----
QNLAERLSDVLATRIRDDLPLLV
>GHP04420.1

--------QDA-IAIPQIAVMGD----Q---------------SSGKSSVLE-----A---IS-GVP-FPRG---
KGLVTKCAT---------QVI---M---RT-APK--
---GS---PW---S-AVT--------SVRWADG-----------------T----------------TEH--------------------DQ-
QP------------------EEA------GVI-----ASPE---EVAGVIERLTQV---LLKKSGH-------------------QK-----
-------------------SFS--EHSIIIKLSSPE--
HPNLSMVDLPGLVR--TV-------------------TEDQDD----------RD---IETVS--------ELITRFMKQ--------
----ERTIILGVIPVNA--DIA-TS--EVLQRAKHYDPSGMRTLAVL-TEPDLVDPGS----E--
NEMIEVLMNR---R---------------------VNLM-LGFCMV----------------------------------KLR-------------
----------------------G-QKELDECEGK--------------------------DVNST----------ELTRRAREA---
EEKFF---------RE-------NE------------------AL-----------------A-R------L---GDE------------------
VDGQ-LGI-----PNLITRLSDTLSNRIREQFPTIK
>KAJ3066410.1
--------DKY-VELPQIAVMGD----T---------------SSGKSSVLS-----A---IS-GIT-FPSS---SELTTRCPT---
------QLI---L---SE-AE---EF---S-
GTV--------RLM---------------------R----------------FKP--------------------QEGEI------------------LES-
-----TTL-----SSPA---DITGEIERLTKQ-----IVSEQ-------------------QL-------------------------IS--
DDAIIIEVRGPG--YPNLTLTDLPGLIR--
TV-------------------EDHEDK----------DI---IRRVR--------GLVDRYLVQ-----------NRTVILAVVPANV-
-DVH-NT--EILQAAQDADPEGIRTISII-TKPDRIDPGA----E--SQVVDLLMNR---K---------------------
KKLK-LGYHAV---------------------------------RCR----------------------------------G-QQDLD----D---
-----------------------GVTIA----------D----GIVN---ETKFF---------SE-------HK-------------------AW--
--------------S-D------V---D---------------------PSY-VGI-----NRLTEKLVKILQSIIASSL----
>KAI9324922.1
--------DRY-VELPQIATMGD----T---------------SSGKSSVLS-----A---IS-GIT-FPSS---SELTTRCPT---
------QLI---L---SQ-SE---TF---S-
GSV--------RLQ---------------------R----------------FKPQ-------------------QNEEP-----------------
PET------KTL-----TNAN---EIEHEIERLTRQ-----LVQEN-------------------QS-------------------------IS-
-DDSIIINVSGPN--YPNLTLTDLPGLIR--
TV-------------------EDSEDP----------AI---IGRVR--------ALVDRYLVQ-----------SRTVILAVVPANV--
DVH-NT--EILQAAEAADPQGVRTISII-TKPDLIDSGA----E--SQVVELLLNR---K---------------------
KMLK-LGYHAV---------------------------------KCR----------------------------------G-QKDLN----N---
-----------------------GVSIA----------D----GIAK---EAEFF---------ET-------HA-------------------VW--
--------------R-K------V---D---------------------SSY-FGI-----SKLTEKLVKILETVIGGSL----
>KAI8836453.1
--------DKY-IELPQIAVMGD----T---------------SSGKSSVLS-----A---IS-GIE-FPSS---DTLTTRCPT---
------QIV---L---SE-AD---KF---S-
GTV--------CLV--------------------R----------------FGSG-------------------IS-------------------SHL--
----THL-----KNRN---EITTEIARLTQV-----IRDEG-------------------QT-------------------------IS--
DDAIVIEVRGPE--YPNLTLTDLPGIIR--
TV-------------------QDNEDP----------AM---IPRVR--------QLVDRYLVQ-----------
KRTVILAVVPANV--DFH-NS--EILQAAEKVDPKGERTIAII-TKPDAIDPGA----E--QSVLDLLMNK---K-
-------------------KALR-LGYHAV---------------------------------RCR----------------------------------
G-KQHHD----D---------------------------KMTIP----------E----GLEM---EHDFF---------HN-------HK--

-----------------VW-----------------K-S------V---A---------------------PSY-VGC-----
ERLTEKLVKVLRNIITDSL----
>KAJ3350919.1
--------DQY-VELPQIAVMGD----T----------------SSGKSSVLS-----A---IS-GIE-FPSH---AELTTRCPT-
--------QLV---L---ST-AE---AF---T-
CTV--------CII---------------------R----------------VKN-------------------SKASS------------------PPS--
----LKL-----KEPG---EIRGAITELTKI-----IIDDG------------------PA------------------------IS--
EDMISIEVSGPK--YPNLTLIDLPGIVR--
TV-------------------ADNEDP----------SM---ILNIR--------DLVTSYLQK------------KRTVILAVIPANV--
DMH-NV--EILQLAESVDPSGDRTIAII-TKPDAVDCGA----E--KQIVDLLLNR---K--------------------
KFLK-LGYHAM---------------------------------RCR---------------------------------G-QQDLN----E---
------------------------KMSIH----------E----GVIK---ESKFF---------YN-------HP-------------------VW-
----------------R-N------I---S---------------------PEL-LGV-----ESLVPKLVNTLQNVINQSL----
>KAI8587516.1
--------GQD-VSLPQLVVAGD----Q----------------SSGKSSLLE-----S---LS-GIS-FPKA---
AELCTTFPT---------QIV---M---RT-K--
--K---TW---E-ARV--------YTVPE---IAN-----------
-------FPA------TNC-----ASKV---AVQNIIQFIKRD---VLKATAG---
---VS--ETVLVIELGSPE--
LPNLTIIDLPGYVH--TL-------------------VKGQSE----------NF---KQKID--------NMVDKFIQD---------
---RRSIILAVIPANK--DFA-TN--VVLQRAQEWDPEGDRTIGVV-TKPDLVDQGT----E--
AAVIRMIQGH---Y--------------------KELK-LGYYMV-----------------------------------HNR--------------
---------------------S-HMDLQ----N--------------------------GVDLA----------A----AVAK---EAEFF-----
----S--------QP-------------------AW-----------------D-A------L---N---------------------ARQ-LGT-----
QQLQTAVVEVLAAHVVKEF----
>XP_047808890.1
--------QDM-VSLPQIAVVGD----Q----------------SSGKSTLLE-----Y---IS-GVT-FPKD---
AGMCTCFAT---------EVS---M---RP-S--
-----T---QF---S-ARVF------INHQPD-SRI--
--------------------KQP-----RSPE---DVAGVIQQAKKL---FVEASGQ-------------------KA----------------
---------IY--DDILTVELNGPG--
LPILTLVDLPGYIH--TH-------------------ATGQPE----------SI---VKDIE--------QLVERYLNS------------
PRTVIMAVIPVNR--DFE-TN--VAIKHIRRFDPSGKRTLCVL-TKPDQVDAGT----E--RNVLDVLAGK---
K--------------------MHLD-RGYHII---------------------------------KNK---------------------------------
-N-FEECQ----A--------------------------GDNRE----------A----TSKK---EGHFF---------A--------RS---
-----------------PW-----------------S-S------I---S---------------------PTE-KGI-----
ASLVDRLSDTLNAQVEKEF----
>KXS17655.1
--------NHL-ISLPQIAVVGD----Q----------------SAGKSSLLE-----A---IS-GIS-FPKD---KEMCTTFAT-
--------QIV---M---AK-G--A---SF---
A-AKV--------TIDPDPSNI--S------------------VGL-
-----PVP-----KSPL---DVAAVIEEAKNL---MSEGNSN------------------LI------------------------IA--
DKILTIELTGPN--YPRLTLVDLPGYVQ--
SV-------------------IKGQSE----------TI---IEDIA--------DIVDRHLKD------------ERTITLAVIPANK--

DLA-TN--VVVGKVDKLGSNGARTLGVI-TKVDVIDAGE----E--EAVLEILHGR---R---------------------
CDFG-LGFHAV----------------------------------RNR-----------------------------------N-WAEVN----G--
-------------------------SLSTE----------E----LLVK---EAQFF---------A-------RA--------------------PW--
---------------S-Q------L---D----------------------KSM-KGI-----VSLRSKLVEILHNHVEKEL----
>XP_021869222.1
--------GAE-VELPQLVVVGN----Q----------------SSGKSSVLE-----A---LT-GIP-FPRD---
DGLCTRFAT---------RIT---F---RR-A---
--L---ET---R-YQA--------KIVPDKLSS---------------K----------------EHQ--------------------DKCQQ---
---------------WGQ-------EL-----ESFDL-FQIADLMKKVRTV---MGVSDKT-------------------
SDSTYPAG-----------------SAFS--NDVLSLEITGPG---
--------------EEHFSIVDVPGTFK--VE-------------------AEGVTT----------KE--DIKLVD--------
DMVKRYMTN------------SRSIMLTVVNCND--DIS-SH--DIIQKARDIDPHGERTLGIL-
TKPDLADEGA----E--QKIIDILDGK---Q---------------------HRLF-HGWHIL-----------------------------
-----RNR-----------------------------------G-QKDLR----D---------------------------ATSLS----------D---
-RHAT---ERKFF---------TD-------KD--------------------PW-----------------N-K------L---D--------------
--------KSL-VGI-----DALNHRLHAVLATQLNKEF----
>TVY17522.1
--------GDY-IDLPQVVVVGD----Q----------------SSGKSSVLE-----G---LT-NLP-FPRD---
SGLCTKFVT---------KIT---F---RR-S---
--P---MT---R-IAI--------TIIPAKNSS---------------T----------------EHV--------------------EHVRG-------
-----------WAK------ADL-----RSLDE-KTFADIMKEVHTV---MGLSEQV-------------------DGMTPP----
----------------TFS--DDVLSLEVCGPK---
QEHLSIIDVPGIFK--KR-------------------TQGLTS----------KA--DIQMVK--------SMVLGYMKN-------
-----PRSVMLTVIPANV--DIV-TQ--EILEMAEEVDPDGQRTLGVL-TKPDLVDKGA----E--
KTIVDLIEGR---R---------------------HRLA-LGWLLV----------------------------------RNP---------------
--------------------G-QQELT----D-----------------------------PMT----------D----RHAL---EKSFF--------
-SH-------EQ--------------------PW-----------------N-S------L---D----------------------KEK-VGI-----
PALQVRVRVILAEHIRRDF----
>OLL24579.1
--------NSV-ISLPQIVVCGD----Q----------------SSGKSSVLE-----A---LT-QIP-FPRS---DGLCTKFAT-
--------QVI---L---RR-A---S---KT---
S-VRV--------QIIPDCKRP---------------E----------------AEQ--------------------RALQS-----------------
-VD-------IKL-----KKLE---DMTILIEEASKH---MGVQSSS-------------------STS-----------------------
TFS--SDILSIEVSGPK---
QPHLTVVDLPGYIR--TT-------------------SGNQTK----------KD---ITLIY--------DLVKDYISD-----------
-KRSIILAVIPANV--DVA-NA--EILEKASEADPNKTRTLGVI-TKPDLVDNGA----E--NQVLDLAANV---
T---------------------KPLK-LGYFIV----------------------------------RNR-----------------------------------
N-YNELK----S---------------------------ASDSK----------A----RNKS---EAAFF---------A--------QS-----
---------------PW-----------------S-E------I---N----------------------KTR-IGI-----
DRLRLYLSDLLQEHIFKEL----
>KAI9096888.1
--------SEY-VSLPQLVVCGD----Q----------------SAGKSSVLE-----A---IT-EVP-FPQN---
QGTCTRFAT---------QIV---L---RR-S--
---V---VT---E-AIV--------TIIPESRRT---------------D----------------NEV--------------------AKFAQ-------

-----------FK-------KKI-----EDLK---DLPSIITEAEAL---ILFSNNV------------------RTK-----------------
------FS--KDVLNVEISGPK---
QPHLTVVDLPGIIHTSSS------------------TTEDEE----------GDEFEVEVVK--------ELVKGYMKE------
------DRTIILAIVAGNY--DYN-NQ--IILQMAKELDQDRTRTLGIV-TKPDLQEVGSD--YE--
KTLVKMVKNE---V-------------------KHLS-LGWHVL--------------------------------KNR------------
---------------------G-FKE------R--------------------------ECSIE----------Q----RNIA---EEKFF--------
-N--------QG-------------------VW----------------T-S------L---P--------------------RKD-VGV-----
ESLRIKLSNLLYQHIKREL----
>RSH87279.1
--------DSV-LSLPQIVVCGD----Q----------------SSGKSSLLE-----A---LT-EIP-FPRN---DNLCTRFAT-
--------EIS---L---RR-E---S---ES---S-
LTI--------RIIPAHSRP---------------Q----------------EEQ--------------------KRLER------------------FS-
------EVI-----TDFE---DLPAVMDKAMQE---LGISEGT------------------GA-------------------------FA--
KDTLSVEIQGPD---RPQLTLVDIPGLIQ--
TS------------------TRGVSE----------AD---VALVA--------EITDSYIKQ------------PRTICLAVVSATN--
DAA-NQ--PILSRVRKFDPHGKRTLGVI-TKPDRLPSGSA--SE--SKFLELARNE---D--------------------
-VFFK-LGWHVI---------------------------------KNR-----------------------------------K-FEE------T------
---------------------AFSID----------E----RNLV---ERTFF---------A--------TS-------------------NF-------
----------K-A------L---P---------------------RED-VGI-----DALRVKLSTLLFDHVKKEL----
>XP_041144356.1
--------SHY-ISLPQIIVCGD----Q----------------SSGKSSVLE-----A---IS-GVS-FPVK---SNLCTRFPT--
-------ELV---L---RK-S--S---HI---G-
VKV--------SIVPHRSRS---------------H----------------VEQ--------------------DALSR------------------
FH-------EEL-----ESFE---GLPTLIENAKAA---MGIFTHG-------------------KA-------------------------
FS--NDLLRVEVSGPD---
RPHLTIVDLPGLIH--SE-------------------TKLQSA----------AD---VALVQ--------DVVQSYMKE--------
----PRSIILAVVSAKN--DFA-NQ--IVLRLAREADSFGHRTLGVI-TKPDTLVEGSE--SE--
YQFVSLAKNQ---E--------------------VTFR-LGWHVL---------------------------------KNM------------
-----------------------D-TEK------G---------------------------NYNLS----------V----RGQE---EAEFF------
---S--------RG--------------------IW-----------------E-D------L---P---------------------RSH-VGI-----
DTLRQRLSKLLLGQIATEL----
>KAJ5704467.1
--------SHY-VSLPQIIVCGD----Q----------------SSGKSSVLE-----A---IS-GVS-FPVK---SSLCTRFPT-
--------ELV---L---RK-N--S---QV---
G-VRV--------SIVPHQSRS---------------D----------------AEQ-------------------HSLGS---------------
---FC-------EQL-----DGFD---GLANLIENAKAA---MGISTHG-------------------KA---------------------
---FS--NDLLRVEVSGPD---
RPHLTIVDLPGLIH--SE-------------------TRQQSA----------AD---VQLVQ--------DVVQSYMRE-------
-----PRSVILAVVSAKN--DFA-NQ--IVLRLARDADPSGNRTLGVI-SKPDTLVPGSE--SE--
ASFVSLAKNQ---D---------------------VEFR-LGWHVL----------------------------------MNM-----------
------------------------D-SEK------G--------------------------QWSLS----------D----RDIQ---ERKFF----
-----S--------GG-------------------IW----------------E-D------L---P---------------------RSL-VGV-----
DSLRTRMSSLLLGQIAGEL----
>MCJ1392161.1

--------SHY-VSLPQIIVCGD----Q---------------SSGKSSVLE-----A---IS-GVS-FPVK---SNLSTRFPT-
--------ELV---L---RK-T--S---QI---S-
VSV--------SIVPHQARS--------------E---------------SER------------------LILSS-----------------
FH-------EEL-----EGFE---GLPSLIENAKSA---MGISTLG------------------KA-----------------------
FS--KDLLRVEISGPD---
RPHLTIVDLPGLIH--SE------------------TKQQSA----------SD---VELVQ--------DVVQSYMKE--------
----PRCIILAVVSAKN--DYA-NQ--IVLKLARAADKKGTRTLGVI-TKPDTLIAGSE--SE--
AMYVSLARNQ---D--------------------VEFR-LGWHAL--------------------------------KNM-----------
------------------------D-SET------G-------------------------EWSLA----------E----RDVE---EQEFF----
-----S--------QG-------------------IW----------------R-D------M---S--------------------RSL-LGV----
-DDLRSRLSKVLLGQIAAEL----
>KAI9774215.1
--------SHY-VSLPQIIVCGD----Q---------------SSGKSSVLE-----A---IS-GVS-FPIK---SNLCTRFPT--
-------ELV---L---RK-T---S---QI---G-
VSV--------SIVPHQSRS--------------E---------------SEQ-------------------HTLSS-----------------
FH-------EEL-----DGFE---GLPALIENAKAV---MGISTHG------------------KA-----------------------
FS--KDLLRVEVSGPD---
RPHLTIVDLPGLIH--SE------------------TKHQSA----------SD---VELVQ--------DVVQAYMKE--------
----PRSIILAVVSAKN--DAA-NQ--IVLKLARAADKKGNRTLGVI-TKPDVLIPGSE--SE--
ASYICLARNQ---N--------------------VEFR-LGWHVL--------------------------------RNT------------
----------------------D-SEM------G-------------------------DWSLA----------D----RDAQ---EKEFF----
-----S--------QG-------------------IW----------------E-Q------M---S--------------------QSL-LGV----
-DKLRSRLSKVLLGQIATEL----
>XP_002543522.1
--------SHY-ISLPQIIVCGD----Q---------------SSGKSSVLE-----A---IS-GVA-FPVK---SNLCTRFPT--
-------ELV---L---RK-S---P---EV---H-
VSV--------SIVPHRSRS--------------E---------------SEQ-------------------HSLES-----------------
FR-------EDL-----DSFE---GLPELIENVKTA---LGISTHG------------------RA-----------------------
FS--NDILRIEISGPD---
RPHLTIVDLPGLIH--SE------------------TKQQSA----------SD---VDLVQ--------DVVQAYMRE-------
-----PRSIILAVISAKN--DIA-NQ--IVLKLARGADRFGRRTLGVI-TKPDTLIPGSA--TE--SIFVSLAKNQ--
-E--------------------VDFR-LGWHVL--------------------------------KNM------------------------------
----D-SEK------G-------------------------ISTLA----------D----RDVE---ERQFF---------S--------QG--
-----------------VW-----------------K-D------I---A--------------------PST-MGI-----
ARLRGRLSKVLLGQIATEL----
>XP_746402.1
------------ISLPQIIVCGD----Q---------------SSGKSSVLE-----A---IS-GVS-FPIR---SSLCTRFPT-----
----ELV---L---RK-S---S---QV---G-
VCV--------SIVPHRSRS--------------E---------------SER-------------------EALAQ-----------------
FH-------EEL-----DSFE---GLPQLIENAKSA---MGIYTNA------------------KS-----------------------
FS--NDLLRVEVSGPD---
RPHLTIVDLPGLIH--SE------------------TKQQSA----------AD---VELVH--------DVVKSYMEE--------
----PRSIILAVVSAKN--DVP-NQ--IVLKLARAADPHGTRTLGVI-TKPDTLVRGSD--SE--
AQFVSLAKNQ---E--------------------VEFR-LGWHAL--------------------------------KNM-----------

----------------------D-TDK------G--------------------------AWTLA----------E----RDKE---EHAFF-----
----A--------SG-------------------VW----------------E-A------L---P---------------------RSH-VGI-----
DQLRKRLSKLLLAQIATELPSL-
>XP_751069.1
------------VELPQLIVCGN----Q---------------SSGKSSVLE-----A---IS-RVR-FPAK---SNVCTRFAT--
-------EVI---L---RR-N---A---AF---
SKIKV--------SIEPGPSRT-------------DE---------------DER-------------------RRLRS----------------
-FTY------EDF-----SNGD---DLPPLIEKAKVH---MGITESV------------------NTG----------------------
-FS--DDVLKVEISGPD---
KPELTLVDLPGLYY--ST------------------SQEQDL----------QG---ILIVR--------KLTERYMSN----------
--PRSIILAVISAKT--DYH-LQ--EVLNIAEQFDPKRERTLGII-TQPDILEANSE--EE--DTYLHFVKNE---
K--------------------IPLE-LGWHVL--------------------------------RNR--------------------------------
-S-FET------R--------------------------DISDD----------A----RDEM---EKAFF---------N--------QG-----
--------------RW----------------A-S------L---S---------------------REC-VGI-----
ESLRRRLSGVLLRLIRRNLPGL-
>XP_026607910.1
--------GEV-VALPQLAVCGD----Q---------------SAGKSSVLE-----A---IT-GIP-FPQQ---
DGLCTRFPT---------EIT---L---RH-S--
---E---AT---QSITIF------ASIRPHSVRS--------------R---------------KEK-------------------DYLAS----
-------------YQ-------KTL-----GAIS---ELPSIIADASKL---MGIRGYG------------------GQKNGP------
-------------AFA--ADVLRIEITGPI---
GLQLSVVDLPGLIS--VV------------------SEEQNE----------ND---VVMIH--------DMVTSYLQS------
------SRTIILAVVQASN--DFA-NQ--CIIRMARKHDPEGQRTVGII-TKPDLINQGT----E--
SKIARIAKNL---D-------------------TIKLK-LGFFLL--------------------------------KNP----------------
-----------------S-PMERK----D--------------------------CHSMT----------A----RSAL---EDRFF--------
-S--------RP-------------------SW----------------A-IH----HL---D---------------------KKR-IGS-----
ESLRTFLQKLLDSHIEHEL----
>XP_748757.2
------------ISLPQLVVSGD----Q---------------SSGKSSVLT-----A---VT-GFS-FPRR---EGTCTRFAT---
------EII---L---RH-S---K---ET---
ETIIT-------ASIIPSLSRH--------------D---------------GSE-------------------EALKR-----------------
FK-------KVL-----KSTE---ELPSVIHEASVA---MGIRGYS------------------DSDDSP-------------------
AFT--ADVLRIEVVGDT---
GLCLTIVDLPGLIS--VS------------------DYDEGE----------AD---VQLVN--------TLIDSYLAN----------
--TRSIILAVVQASN--DIQ-NQ--NIIQRARRFDKLGERTVGII-TKPDLVNKGT----E--SHIVRLANNL---
D-------------------IVRLK-LGFFLM--------------------------------KNP--------------------------------
-S-PEQLK----N--------------------------NISMF----------E----WKQK---ELEFF---------N--------SP----
---------------PW----------------K-DL----ML---D---------------------HNR-VGA-----
ECLRSFLEKILEEHIERELP---
>XP_040633937.1
--------GDH-VALPQLVVCGD----Q---------------SAGKSSVLE-----G---IS-GIP-FPRQ---
DGLCTRFAT---------EII---L---RH--
-E---PG---EQRAT-------AMIIPHVSRT--------------D---------------EEK-------------------SSLGA----
-------------FH-------RNI-----SDFT---ELPIIVEEARSI---MDIHGHG------------------IGSNAS--------

------------AFS--ADVLRLELVGST--
GLHLTMVDLPGLIS--VS----------------------ENE----------HD---VQLVR--------DLVDSYLEN---------
---SRTIIMAVVPASS--DVD-TQ--GILQRARHFDKTGLRTVGVI-TKPDLINAGT----E--PRVARLARNL--
-D--------------------GAKLN-LGFFLL----------------------------------KNP--------------------------------
---S-PAELE----A---------------------------GTTLP----------E----RRKV---ELEFF---------S--------SG----
----------------AW-----------------K-GQ----GL---D----------------------PSR-IGI-----
DNLRSFLQDLLDHHIEREL----
>XP_043140374.1
--------GDH-IALPQLVVCGD----Q----------------SAGKSSVLE-----G---IS-GIP-FPRQ---
DGVCTRFAT---------EII---L---RH--
--E---PN---DQRNT-------ATIIPHMSRA---------------E----------------DEK---------------------VRLSA---
---------------FH-------RDI-----NDLV---NLPGIVDEAARL---MGVQGAN-------------------DSTDAP--
------------------TFA--ADVLRLEVVGDT---
GLHLTLVDLPGLIS--VS----------------------ENE----------ED---VQLVG--------NLVNSYLEN----------
--SRTIILAVVPASS--DVD-TQ--SIIQRARRFDKDGLRTVGII-TKPDLINDGT----E--SRVAKLANNA---
D---------------------RTKLK-LGFFLL----------------------------------KNP----------------------------------
-R-PIDLE----K---------------------------GMTMV----------E----RRKM---EAQFF--------A--------NP---
-----------------PW-----------------N-NL----GL---N----------------------PSR-VGI-----
DNLRVFMQDLLDRHIEREL----
>XP_754266.1
------------IALPQLVVCGD----Q----------------SAGKSSVLE-----G---IT-GIP-FPRQ---DGVCTRFAT--
-------EII---L---RH---E---PN---
HRRNT-------ATILPHISRT--------------E----------------EEK--------------------AKLSA-------------------
FR-------REV-----SDLA---QLPGIIEEAARL---MGVQGMN-------------------DLADAP------------------
--TFA--ADVLRLEIVGDT--
GLHLTLVDLPGLIS--VS----------------------END----------DD---VQLVG--------DLVNSYLEN--------
---SRSIILAVVPASS--DVD-TQ--SIIQRARRFDKDGFRTVGII-TKPDLINDGT----E--GRIAKLANNA---
D---------------------RTKLR-LGFFLV----------------------------------KNP----------------------------------
-R-PIDLE----K---------------------------GMTTA----------E----RRKV---EAEFF--------A--------HP-----
---------------PW-----------------N-KL----GL---D----------------------PSR-VGI-----
DNLRIFMQDLLDRHIERELPKV-
>KAF9951223.1
--------NHV-LSVPQIAIVGD----Q----------------SSGKSSVLE-----A---FT-QLS-FPRD---
KGMCTRFAT---------QVN---L---CR-DL--T----------------
---LDKD---TL---S-ARI-----------------------------D-----------------DE------------------------DS------
-----------FN-------ERW-----KTFQV-DQFYAVIKEAVSL---LCGTS--
-------DIS--DKVLELTLSGPH---
QSPLTVVDLPGFIN--TT-------------------LDGQDK----------NI---PHTIR--------DINERYMKD---------
---PRTIILAVIPANV--DLN-NS--YVLARAEEHDPKNERTVPIV-TKPDMIDKGT----L--SELIDMVLNN--
-R---------------------KKMP-LGYLVM----------------------------------RNT--------------------------------
--G-YAD------R---------------------------DLSWE----------E----AQRA---EEDYF---------AQ-------DK--
------------------AW-----------------E-A------V---P----------------------RSN-RGR-----
VMVKKFLGDLLYFHIKKEL----
>GAX85982.1

---RQLGVSND-IKLPTLVTAGN----Q----------------SSGKSSVVE-----A---IA-GIP-LPRS---
SGTCTRCPT---------EVR---M---RS--

------------------------------VHQVVRLTLTDVDQK---------------KP--KEPEHSRS------FLEL--
PTEYQLEFT--RNSVVLEIEGAD---
ADLTIIDLPGIIQ-----------------------SHHKGP----------HY---VEMIK--------SMVLNSIES------------
DHVIIVMVITAMD--DVE-NQ--AINLEARNVDPEGQRTIGVI-TKPDNIPKGEH------
DKWVALASNRR--P--------------------GQELS-LGYYVV-----------------------------------RNP-----------
------------------------G-QNELD----E--------------------------SIRFE----------D----ARVK---ESEYF----
-----ET-------SP-------------------YW---------------------PSNGEL-------------------------QGR-LGT---
--TFLRNALSESLVQGIKKGLPGMQ
>XP_042923301.1
------------LKVPALVIAGD----Q----------------SSGKSSVVE-----A---IA-GVP-LPRS---DGTCTRCPT--
-------EVR---M---RT-HG-----------------------AP--GEGGSA--
VW---Q-CRI--------KVVR--------------------N----------------FDS-------------------TGKPLAP---------
--GEAHEKLF------CTV-----TDKA---
HITACISAAQAVLLNPTVVGDAVADGAERFVPLLSAAEPGGRAPEASSA------
MRGLGDAAGYELQFT--ANKVVLEIVGAE---
----ADLTIIDLPGIIH-----------------------SHPKDP----------SL---IDVVK--------SLVKCYLAP-----------
-AHHIIVMTLPAGM--DAE-TQ--AILQFAREADPEGRRSIGII-TKPDKIGTDER--TE-
WGKLCNLVAGAR--A-PTGVPAAGGSRAAAAPN---PHLQ-LGYYVV---------------------------------
KNP-----------------------------------G-QEQLA----A---------------------------GISFE----------Q----
ARAA---EERYF---------AD-------HP-------------------LW-----------------ASAMKANSLL--------------
------------SQR-LGT-----NALRDGLSALLVDKIGEHMP---
>XP_042924848.1
------------LQVPTLVIAGD----Q----------------SSGKSSVVE-----A---SA-GVP-LPRS---DGTCTRCPT-
--------EVR---M---RT-TQQLPAATGVCGGSALSAAAASSPPGPHSGEDGEDDEDDEDEEDEE-------
DD-AVVNNAAGLPPGTTRGGGGGSSASAW---Q-CRI--------KLCR-------------------E--------------
--FDS--------------------DGVRLAT------------KPPEQPF------CVV-----RDKA---
HIATCVLAAQAVLLNPRAVEDT-PGGAGAFVPLLSSAQP-GRQPAAAAK------LLALRDASHYELPFT-
-PNKVVLEIDGAE---ADLTIIDLPGIIH----
-------------------SH-EDP----------RL---IELVK--------DMVKANLAP------------EHHIIAMALPAGQ--
DAE-TQ--AIRLMTREVDPDGRRSIGII-TKPDRVPEHEA--GE-TLKLIRLVGACG--APPAGAGAAGGS-
-ARVAH---PQHP-LGHYVV---------------------------------KNP----------------------------------S-
QDGLA----M--------------------------NITFE----------Q----ARAD---EAAYF---------AG-------HK------
--------------HW-----------------AAALRRQPEL-------------------------QRR-MGA-----
AALRRGLSGLLVELVIAQLP---
>KAG2488600.1
--LRALGVGSA-LQLPALVIAGD----Q----------------SSGKSSVVE-----A---IA-GVS-LPRS---
DGTCTRCPT---------EVR---L---RT-HA-----------------------GP--------------------------------
DANGDSP-----MPDGDV--PW---T-CRI--------KLHR-------------------E----------------YDS-----------
---------DGHPLTE------------LPPEELF------ATL-----TNKA---HIAAFVTAAQAVLLNPRAADAA-
PGGARAFVPDVSGDRP--RDPQPLRA---------LGHPTTYELSFT--ANKVVLEVDGAD-----------------
--ADLTIIDLPGIIH-----------------------DHPKGK--------

--QY---VDMVE--------RMTKAQLRP------------EHHIIAMALPAGL--DPE-TQ--
AIRLWVREVDPSGSRSIGII-TKPDTIADDAH--IT-YGKLVKLVGGST--M-
AGGAAGAAASAGPAAGHDESHQLT-LGYYVV---------------------------------RNP--------------------
--------------G-QEQLE----D--------------------------CIGFA----------E----ARAA---EQRYF---------AT-
------NT-------------------HW-----------------VQAVAALPSL-------------------------KQR-LGA-----
NHLRSGLSALLVERIETQLPHMR
>XP_042924875.1
--MR--------TQAANADLASD---ME----------------SSGGGEDGS------------GME-----E--
DGEGAANGA---------DGK-------RRP---
----------AW---R-CRI--------KLCR--------------------D---------------YDS-------------------
EDKPLAE------------KPPEQPF------CVV-----RDKA---HIAACVSAAQAVLLNPRAVEAA-
AGGPQAFVPELSSA----HSPSH-------------------------PNKVVLEIDGAE-----------------------------
---------------------------------------ADLTIIDLPGIIH----------------------DHPKGR----------HL---
VEVVE--------RMTKTNLAP------------AHHIIAMALPAGL--DPE-TQ--AIRLWAREVDPDGHRSIGII-
TKPDMIAEEAH--IV-CNKLVKLVGARG-ELGPGGTRSQPD-----------GHLR-LGYYVV-----------------
-----------------KNP---------------------------------S-QEQLV----E--------------------------GITFE-----
-----K----AREI---EARYF---------AN-------HV-------------------HW-----------------RPAMATSPGL----
---------------------VQR-LGA-----NALRSGLSLLLVERIEEQMP---
>KAJ9515210.1
--LADFGAAHE-ISYPTIVVCGD----Q----------------SAGKSSIIQ-----R---IS-GID-LPRS---
SGTCTRCPM---------EVRM-TL---SE---
-----GGV--PW---S-CKI--------KI----------------------R----------------REW-------------------
DDGKRKTLS---------KVSWEDFG------APL-----LDKE---AVGPAVSRAQKA-----VLNPG-------------
-----KGYASFVD------PTSPILADADELGFS--RNVVVLEIQGAD---
--------------------------IS-LSLIDLPGIIN----------------------STEKKE----------DQ-YLVNMIK--------
DMVKQYIEA------------SQTIIVLAVHALS--DIQ-NQ--VVYQMAREADPHQQRTLGVI-
TKVDVIPPGS----H--SMWIRMMRGE---L--------------------FPLD-LGYYMV--------------------------
-------VNP----------------------------------N-QVDLD----Q--------------------------
GTSHEVGHTGRMWKDD----AVDK---EMRFFETDANLGVLAQ-------SV-------------------VW-------
----------S-S----------------------------------H-LGL-----SNLTAALSKQLVDRTMAELPHMR
>KAI3646081.1
------------LNLPGIVVCGN----Q----------------SSGKSSLIE-----A---IS-RVP-LPRA---AGTCTRCPF--
-------ECR---L---SE---SKN--PW---
S-CKV--------ML----------------------R----------------YEV--------------------DA-KGQAL----------------
-------------------------------TLKA-----VLNPG-------------------LEAQQFLT-------
DTNWEKIEDQQKFT--ENVVCLTIEGEG---
-VGYLTLTDLPGIIQ----------------------STNDAA----------DE-HFVELIK--------NLVEKYVSC---------
---PTNIIVEVITCKD--DME-NQ--IVHTLARKADPSGLRTVGVL-TKPDMIEEGC----T--
DDWLNVLRNN---T--------------------CPLK-HGYYMV---------------------------------KNP-----------
------------------------A-TKDLQ----A---------------------------NITFS----------Q----ARQK---EVAFF----
-----SQ--------K-------------------PW-----------------S-T------E---RAL-------------------SKR-FGI-----
YNLKEFLSNLLSNLLKQNLPQL-
>OAJ38670.1

------------LSLPTLVVCGN----Q---------------SVGKSSLVE-----A---IC-GIT-LPKA---AGTCTRCVT---
------EVR---L---SE-YSDV--------------------
GPVDSVRKKSVAGDRNSTESTITQGVYIDALDTVSSGSVPSAFHIDGHEKESS--TW---S-CTI--------
TL---------------------R---------------FEY------------------DE-AGIPLR---------SIREVLFG------
PPL-----VEKS---LVALAVRRAQKA-----LLNPT------------------
LDPSVFLTHIFTDNDQSNSDTKSNQLKFT--KNIVCLDIQGAG--
-------------------------IN-LALVDLPGIIR----------------------NVEHPD----------DA-MFIPMIE--------
DLVKSYIQK------------ERTIIVATITCKD--EME-NQ--AIVHLAREVDPTGIRTIGVL-TKPDTIESGT----A-
-ARWADILMGN---L--------------------YPLK-LGYFMV--------------------------------RCL------------
----------------------S-KAELA----A--------------------------GNTLQ----------D----AQKL---ENAFF----
-----AQ-------SQ-------------------PW----------------S-T------L---RRK-------------------SAR-FGA--
---PALRFELSRLLINLVDMSLP---
>XP_006461472.1
------------IDLPQIAVVGN----Q---------------SAGKSSLIE-----S---IS-GIT-LPRA---SGTCTRCPT-----
----ECR---L---SY---SSQ--PW---K-
CVV--------SL---------------------R---------------ITT-------------------DR-SGQPLG---------
QSRNETFG------STI-----YDKK---EVDDRIRRAQLA-----ILNPD-------------------KPAKSFLN------
DDEPSLMEGNFLTFS--KNCVSLAISGPD--
---VADLSFVDLPGLIA----------------------SVGRGG----------NA-GDIKLVE--------GLVTTYIHK------
------TNCIILLTVACET--DFE-NQ--GAHQLAKQYDPEGKRTIGVL-TKPDRIPAGE----E--
QNWLKFIRNE---K--------------------EPLQ-NNWFCV--------------------------------KQP------------
----------------------A-SSDLK----N--------------------------NWTWQ----------Q----ARQK---EDEFF-
--------TA-------TS-------------------PW----------------N-E------L---EAM------------------YVRY-
LRT-----KNLVERLSQVLSDLIAKTLP---
>XP_006461433.1
------------IDLPQIAVIGS----Q---------------SAGKSSLIE-----S---IS-GIT-LPRA---AGTCTRCPT------
---ECR---L---SY---SPL--PW---K-
CTV--------HL---------------------R---------------FTT-------------------DG-NGTPLG---------
QSRNEIFG------PTI-----YEKS---EVEERIRRAQRA-----ILNPG-------------------KPTKLFLE------
DDDEMSGEAE-LSFS--NNCVSLQISGPD---
----VADLSFCDLPGLIA----------------------SVGRGG----------NT-NDIKLVE--------SLVTSYIKK------
------PSCIILLTVACET--DFE-NQ--GAHQISKAYDPEGKRTIGVL-TKPDRIPLGE----E--
LNWLKFLKNE---R--------------------EPLE-NNWYCV--------------------------------KQP------------
----------------------S-SNDLK----N--------------------------NWTWQ----------E----AREK---EQQFF-
--------AA-------TA-------------------PW----------------C-E------L---EGM------------------YQKF-
LRT-----TNLVERLSGVLSDLIAKRLP---
>XP_006457072.1
------------FDLPKIVVIGN----Q---------------SAGKSSLIE-----A---VT-GIN-VPRD---SGTCTRCPM--
-------ECS---M---SS-DTR--SW---
S-CTISLRSGTSTSIPPTSPRVL-----RSTRGTSIAS----------------ASS--------------------
TASAGVTPTPARASTGVRNITTQSFG------PTI-----TDKS---QVELWLRRAQGA-----ILSTD-----------
ADKSQWLNKSAEEIRQAI---------QNKTGMRDFT--EDTIVVDIQDPT----------------------------------
--------------------------------ATDLSFVDLPGLIS----------------------NADPGS---------------IDLIK--
------NLVRQHVAG------------ENTLILVTIPASD--DIQ-NH--GAVVLAKEADGNGDRTIVVL-

TKPDSLGPGDT-GLQ--ETWRQTFKNPN--V-PENQ----------------NYLR-HGYYCV--------------------
--------------QLP-----------------------------------NDQQRQQ--------------------------------GLTAH-----
-----T-----------LPNYL---------GV-------TW--------------------PW-----------------S-E------F---AG-----
----------------QGR-FGV-----TNLVKNVSALLVQMIEANLP---
>XP_750654.1
------------IALPKICVIGD----Q----------------STGKSSLIE-----G---MS-QIK-VPRS---AGTCTRCPM---
------EIN---L---SE-GE---------------------P---GQ---DW---
N-CRI--------FLSR--------------------K---------------YIF--------------------
DGSRKVTKLPKKSQPLGPWIEQDQED------EHF-----TDVRDKDGVQAAIKWAQLA-----
ILNPGRPS---------TDYQPGHNGDTDESYC---------------QVKFS--PNVVRLDISAPN-----------------
--FPNLSFYDLPGVIS--QA-------------------EHDHER---
-------YL---VSLVE--------NLVREYISQ------------ENCIVLLALPMTD--DAT-NS--
SAAKIMRDVPGAKERTLGVL-TKPDRIQTGE----S-YDQWVEILEGD---K---------------------FALG-
HGYYIV----------------------------------RNN-----------------------------------P-NPAIEH---S------------
------------------------------R----AREE---EAVFF-----------------AKS--------------------PW--------------
---ATD------L---SAY--------------------QNR-FGT-----RNLQSALSSLLLEQIQGCLP---
>PAA68234.1
------------DHLPRVVVIGD----Q----------------SSGKTSVLE-----A---VA-RARLFPRGA--
GEMMTRAPV---------QVT---L---
--ADG--PY---HVARF--------------------------------K----------------DDP----------------------------------
---------DRE------FDL-----TKESELAALRDAIERRMRA-----AVRSS-------------------GP----------------
------DAAVS--TEAIPLSVQGPG---
LPRMVLVDLPGIIS--TE-------------------TAGMAA----------QT---RESIR--------QLARQYMRN--------
----PNAIILCVADACV--DPE-RS--NAFDLVAKHDPAGRRTIFVL-TKMDLAERDK---VS-
PDRVAKLLAGR---L---------------------LPLKALGYFAV----------------------------------VTGS-----------
-----------------------G----------SQ--------------------------DESVE---------------AIERH--EAEYF-------
--AS-------SR--------------------LF-----------------K-D----GRL---S----------------------PNQ-VTA-----
ANMARAVSRRFWALVRESV----
>PAA87312.1
------------DHLPRVVVVGD----Q----------------SSGKTSVLE-----M---IA-KARIFPRGA--
GEMMTRAPV---------QVT---L---
--AEG--PY---HVARF--------------------------------K----------------DNP-----------------------------------
--------SRE------YDL-----TQESELAALRDTIERRMRS-----VVQSG-------------------G------------------
-------TVS--AETISLSVQGPG---
LPRMVLVDLPGIIS--TE-------------------TRGMAS----------QT---REAIR--------QLASQHMRN--------
----PNSIILCVADACV--DPE-RS--NAFDLVARHDPSGRRTIFVL-TKLDLAERDR---IS-
PDRIGRLLAGR---L---------------------LPLKALGYFAV----------------------------------VTGS------------
----------------------G----------GA--------------------------DESIP---------------AIQRY--EEQFF---------
RN-------SQ--------------------FF-----------------K-E----GVL---S----------------------VSQ-MTA-----
ANMAQAVSRRFWALVQESV----
>XP_002602331.1
------------DELPRVVVVGD----Q----------------SAGKTSVLE-----M---VA-QARIFPRGA--
GEMMTRAPV---------KVT---L---
--SEG--PH---HIAMF--------------------------------K----------------DS------------------------------------

--------DRE------FDL-----TKESELEALRREVEIRMKA-----SVRPG-------------------Q-------------------
------TVS--METIAMSVKGPG--
LQRMVLVDLPGIIS--TE-------------------TQGMAS----------AT---KESIK--------MMCEHYMSN-------
-----PNAIILCIQDGSV--DAE-RS--NVTDLVSQMDPQGKRTIFVL-TKVDLAEKNI---TN-
PRRIKQILEGK---L---------------------FPMKALGYFAV----------------------------------VTGR------------
----------------------G----------NK--------------------------DDSID---------------TIRGY--EEEFF---------
RN-------SQ--------------------LF-----------------R-S----GVL---K----------------------ASQ-MTT-----
QNLSFAVSDCFWKMVKASV----
>XP_019637857.1
------------DELPRVVVVGD----Q----------------SAGKTSVLE-----M---VA-QARIFPRGA--
GEMMTRAPV---------KVT---L--
--SEG--PH---HIAMF--------------------------------K----------------DS------------------------------------
--------DRE------FDL-----TKESELEALRREVEIRMKA-----SVRPG-------------------Q-------------------
------TVS--METISMSVKGPG--
LQRMVLVDLPGIIS--TE-------------------TQGMAS----------AT---KESIK--------MMCEHYMSN-------
-----PNAIILCIQDGSV--DAE-RS--NVTDLVSQMDPQGKRTIFVL-TKVDLAEKNI---TN-
PHRIKQILEGK---L---------------------FPMKALGYFAV----------------------------------VMGR-----------
-----------------------G----------NK--------------------------DDSID---------------TIRGY--EEEFF---------
RT-------SQ--------------------LF-----------------R-S----GVL---K----------------------ASQ-MTT-----
QNLSFAVSDCFWKMVKASV----
>XP_006813643.1
------------DHLPRVVVVGD----Q----------------SSGKTSVLE-----M---IA-QARIFPRGS--
GEMMTRTPV---------KVT---L---
-SEG--PY---HVAQF--------------------------------K----------------DS-------------------------------------
-------NKE------YDL-----SKESELQSLRQEIELRMKN-----RVKKG-------------------Q-------------------
------TVS--NDTISLSVRGPG--
IQRMVLVDLPGMIS--TV-------------------TTGMAA----------DT---REAIH--------NMSKSYMKN-------
-----PNAIILCIQDGSV--DAE-RS--IVTDLATTMDPEGKRTIFVL-TKVDLAEKNS---AN-
PSRIKQILDGK---L---------------------FPMKALGYFAV----------------------------------VTGR------------
----------------------G----------NT--------------------------NESIE---------------QIKNY--EETFF---------
RS-------SK--------------------LF-----------------K-T----GTL---K----------------------PSQ-MTT-----
QNLSFAVSDCFWKMVRESV----
>XP_030843280.1
------------DHLPRVVVVGD----Q----------------SAGKTSVLE-----M---IA-QARIFPRGA--
GQMMTRAPV---------KVT---L--
--SEG--PN---HIAQF--------------------------------K----------------DS------------------------------------
--------GKE------FDL-----TKESELKALRQEIEARMKG-----SVKEG-------------------Q------------------
-------TIS--PEVISLSVRGPG---
IQRMVLVDLPGMIS--TV-------------------TTGMAA----------DT---KTSIQ--------KMINGYMGN-------
-----PNAIILCIQDGAI--DAE-RS--IVTDLVNEIDPTGKRTIFVL-TKVDLAEKNH---LN-
PNRIRQILDGR---L---------------------FPMKALGYFAV----------------------------------VTGK-----------
-----------------------G----------NT--------------------------SDSID---------------SIKQY--EEQFF---------
RH-------SA--------------------LF-----------------K-S----GVF---K----------------------PSQ-LNT-----
QNLSFAVSDCFWKMVRESV----

>XP_018667792.1
------------DHLPRVVVVGD----Q---------------SAGKTSVLE-----M---IA-QARIFPRGS--
GEMMTRAPV---------KVT---L---
--SEG--PN---HVAQF-------------------------------R---------------DS-----------------------------------
---------SRE------FDL-----SKEEELKSLRHEIELRMKS-----SCSDG-------------------K-----------------
-------TVS--NDTISLTVKGPG---
LQRMVLVDLPGMIS--TV-------------------TSGMAP----------DT---KDAIC--------NMSKHYMEN------
------PNAIILCIQDGSV--DAE-RS--IVTDLVSQMDPSGRRTIFVL-TKVDLAEKNI---TN-
PSRIQEILDGK---L---------------------FPMKALGYFAV---------------------------------VTGQ-----------
-----------------------G----------SA-------------------------NSSIT---------------DIKEY--EEEFF---------
SN-------SK--------------------VF-----------------K-S----GLL---K---------------------ASQ-LTT-----
ANLSYAVSNCFWKMVRESV----
>XP_032818114.1
------------DQLPRVVVVGD----Q---------------SSGKTSVLE-----M---IA-QARIFPRGS--
GEMMTRSPV---------KVT---L---
-SEG--PH---HVAIF-------------------------------K---------------DS--------------------------------------
------SRE------FDL-----TKEDDLAALRKEIEIRMKK-----SVKEG-------------------H---------------------
---TVS--AETISLSVKGPG---
LQRMVLVDLPGVIS--TM-------------------TSGMAP----------DT---KDAIF--------AMSKGYMQN------
------PNAIILCIQDGSV--DAE-RS--IVTDLVSNMDPQGKRTIFVL-TKVDLAEKNL---AS-
PNRIQQILDGK---L---------------------FPMKALGYFAV---------------------------------VTGK-----------
-----------------------G----------NR-------------------------DESIE---------------SIKDY--EEEFF---------
QK-------SK--------------------LC-----------------R-S----GML---K---------------------AHQ-VTT-----
KNLSLAVSDCFWKMVRESV----
>XP_021332524.1
------------DHLPRVVVVGD----Q---------------SAGKTSVLE-----M---IA-QARIFPRGS--
GEMMTRSPV---------KVT---L---
-SEG--PH---HVAMF-------------------------------K---------------DS-----------------------------------
--------SRE------FDL-----GKEEDLAALRHEIELRMRK-----SVKEG-------------------Q------------------
-------TVS--PETISLSVKGPG---
IQRMVLVDLPGVIS--TV-------------------TTGMAA----------DT---KETIF--------SISKAYMQN----------
--PNAIILCIQDGSV--DAE-RS--IVTDLVSQMDPQGKRTIFVL-TKVDLAEKNL---AS-PSRIQQIVEGK-
--L---------------------FPMKALGYFAV---------------------------------VTGK-----------------------------
----G----------SP-------------------------NESID---------------SIKDY--EEDFF---------QN-------SR----
----------------LL-----------------K-D----GML---K---------------------AHQ-VTT-----
KNLSLAVSDCFWKMVRESV----
>XP_028587646.1
------------DHLPRVVVVGD----Q---------------SAGKTSVLE-----M---IA-QARIFPRGS--
GEMMTRSPV---------KVT---L---
-SEG--PH---HVAMF-------------------------------K---------------DS-----------------------------------
--------SRE------FDL-----TKEEDLAALRNEIEIRMRK-----SVSDG-------------------C-------------------
------TVS--TETISLSVKGPG---
LQRMVLVDLPGVIS--TV-------------------TSGMAP----------DT---KETIF--------SISKAYMQN---------
---PNAIILCIQDGSV--DAE-RS--IVTDMVSQMDPQGKRTIFVL-TKVDLAEKNV---AS-

PSRIQQIIEGK---L---------------------XPMKALGYFAV----------------------------------VTGK------------
----------------------G----------NS--------------------------CESIE---------------SIKEY--EEEFF---------
QN-------SK--------------------LL-----------------K-N----CML---K----------------------AHQ-VTT-----
RNLSLAVSDCFWKMVRESV----
>XP_025913835.1
------------DHLPRVVVVGD----Q----------------SAGKTSVLE-----M---IA-QARIFPRGS--
GEMMTRSPV---------KVT---L--
-SEG--PH---HVALF--------------------------------K----------------DS-------------------------------------
-------SRE------FDL-----TKEEDLAALRNEIEIRMRN-----SVKEG-------------------C--------------------
-----TVS--TETISLSVKGPG---
LQRMVLVDLPGVIS--TV-------------------TSGMAP----------DT---KETIF--------SISKAYMQN---------
---PNAIILCIQDGSV--DAE-RS--IVTDLVSQMDPQGKRTIFVL-TKVDLAEKNV---AS-PSRIQQIIEGK-
--L---------------------FPMKALGYFAV----------------------------------VTGK------------------------------
----G----------NS--------------------------SESID---------------SIKEY--EEEFF---------QN-------SK-----
---------------LL-----------------K-T----CML---K----------------------AHQ-VTT-----
KNLSLAVSDCFWKMVRESV----
>XP_023440724.1
------------DHLPRVVVVGD----Q----------------SAGKTSVLE-----M---IA-QARIFPRGS--
GEMMTRSPV---------KVT---L--
-SEG--PH---HVALF--------------------------------K----------------DS-------------------------------------
-------SRE------FDL-----TKEEDLAALRREIEIRMRK-----SVKEG-------------------C---------------------
----TVS--PETISLNVKGPG---
LQRMVLVDLPGVIN--TV-------------------TSGMAP----------DT---KETIF--------SISKAYMQN---------
---PNAIILCIQDGSV--DAE-RS--IVTDLVSQMDPHGRRTIFVL-TKVDLAEKNV---TS-PSRIQQIIEGK--
-L---------------------FPMKALGYFAV----------------------------------VTGK-------------------------------
---G----------NS--------------------------SESIE---------------AIREY--EEEFF---------QN-------SK------
--------------LL-----------------K-T----SML---K----------------------AHQ-VTT-----
RNLSLAVSDCFWKMVRESV----
>XP_005873264.1
------------DHLPRVVVVGD----Q----------------SAGKTSVLE-----M---IA-QARIFPRGS--
GEMMTRSPV---------KVT---L--
-SEG--PH---HVALF--------------------------------K----------------DS-------------------------------------
-------SRE------FDL-----TKEEDLAALRHEIELRMRK-----NVKEG-------------------C-------------------
------TVS--PETISLNVKGPG---
LQRMVLVDLPGVIN--TV-------------------TSGMAP----------DT---KETIF--------SMSKAYMQN-------
-----PNAIILCIQDGSV--DAE-RS--IVTDLVSQMDPHGRRTIFVL-TKVDLAEKNV---AS-
PSRIQQIIEGK---L---------------------FPMKALGYFAV----------------------------------VTGK------------
----------------------G----------NS--------------------------SESIE---------------AIREY--EEEFF---------
QN-------SK--------------------LL-----------------K-A----SML---K----------------------AHQ-VTT-----
RNLSLAVSDCFWKMVRESV----
>XP_006163024.2.2
------------DHLPRVVVVGD----Q----------------SAGKTSVLE-----M---IA-QARIFPRGS--
GEMMTRSPV---------KVT---L--
-SEG--PH---HVALF--------------------------------K----------------DS-------------------------------------

-------SRE------FDL-----TKEEDLAALRHEIELRMRK-----NVKEG-------------------C-------------------
------TVS--PETISLNVKGPG---
LQRMVLVDLPGVIN--TV-------------------TSGMAP----------DT---KETIF--------SISKAYMQN---------
---PNAIILCIQDGSV--DAE-RS--IVTDLVSQMDPHGRRTIFVL-TKVDLAEKNV---AS-PSRIQQIIEGK-
--L--------------------FPMKALGYFAV---------------------------------VTGK-----------------------------
----G----------NS-------------------------SESIE---------------AIREY--EEEFF---------QN-------SK-----
---------------LL-----------------K-T----SML---K---------------------AHQ-VTT-----
RNLSLAVSDCFWKMVRESV----
>NP_056375.2.2
------------DHLPRVVVVGD----Q----------------SAGKTSVLE-----M---IA-QARIFPRGS--
GEMMTRSPV---------KVT---L---
-SEG--PH---HVALF-------------------------------K---------------DS-------------------------------------
-------SRE------FDL-----TKEEDLAALRHEIELRMRK-----NVKEG-------------------C-------------------
------TVS--PETISLNVKGPG---
LQRMVLVDLPGVIN--TV-------------------TSGMAP----------DT---KETIF--------SISKAYMQN---------
---PNAIILCIQDGSV--DAE-RS--IVTDLVSQMDPHGRRTIFVL-TKVDLAEKNV---AS-PSRIQQIIEGK-
--L--------------------FPMKALGYFAV---------------------------------VTGK-----------------------------
----G----------NS-------------------------SESIE---------------AIREY--EEEFF---------QN-------SK-----
---------------LL-----------------K-T----SML---K---------------------AHQ-VTT-----
RNLSLAVSDCFWKMVRESV----
>NP_598513.1
------------DHLPRVVVVGD----Q----------------SAGKTSVLE-----M---IA-QARIFPRGS--
GEMMTRSPV---------KVT---L---
-SEG--PH---HVALF-------------------------------K---------------DS-------------------------------------
-------SRE------FDL-----TKEEDLAALRHEIELRMRK-----NVKEG-------------------C-------------------
------TVS--PETISLNVKGPG---
LQRMVLVDLPGVIN--TV-------------------TSGMAP----------DT---KETIF--------SISKAYMQN---------
---PNAIILCIQDGSV--DAE-RS--IVTDLVSQMDPHGRRTIFVL-TKVDLAEKNV---AS-PSRIQQIIEGK-
--L--------------------FPMKALGYFAV---------------------------------VTGK-----------------------------
----G----------NS-------------------------SESIE---------------AIREY--EEEFF---------QN-------SK-----
---------------LL-----------------K-T----SML---K---------------------AHQ-VTT-----
RNLSLAVSDCFWKMVRESV----
>XP_031757388.1
------------DHLPRVVVVGD----Q----------------SAGKTSVLE-----M---IA-QARIFPRGS--
GEMMTRSPV---------KVT---L---
-SEG--PH---HVAMF-------------------------------K---------------DS-----------------------------------
--------SRE------FDL-----SKETDLAALRNEIEVRMRK-----SVKNG------------------Q------------------
-------TVS--PETISLSVKGPG--
IQRMVLVDLPGVIN--TV-------------------TSGMAP----------DT---KDTIF--------NISKAYMLN----------
--PNAIILCIQDGSV--DAE-RS--IVTDLVSQMDPQGRRTIFVL-TKVDLAEKNV---AS-PNRIQQIIEGK--
-L--------------------FPMKALGYFAV---------------------------------VTGK------------------------------
---G----------NS-------------------------NESID---------------SIKDY--EEEFF---------QG-------SS-----
---------------LL-----------------K-K----GML---K---------------------AHQ-VTT-----
KNLSLAVSDCFWKMVRESI----

>NP_495986.3.3
------------DNLPRVVVVGD----Q---------------SAGKTSVLE-----M---VA-QARIFPRGS--
GEMMTRAPV---------KVT---L---
--SEG--PY---HVAQF--------------------------------R---------------DS-----------------------------------
--------SRE------FDL-----TKETDLQQLRNETEVRMRN-----SVRDG------------------K----------------
--------TVS--NEVISLTVKGPN---
LPRMVLVDLPGVIS--TV-------------------TADMAR----------ET---KDDII--------RMSKAHMEN--------
----PNAIILCIQDGSV--DAE-RS--NVTDLVSSIDPSGKRTILVL-TKVDMAEKNL---AN-
PDRIKKILEGK---L---------------------FPMKALGYFGV---------------------------------VTGR------------
----------------------G----------NS-------------------------SDSID--------------EIRKY--EENFF---------
ST-------SQ--------------------LL-----------------R-D----GVL---K---------------------PSQ-MTT-----
RNMSLAVSDCFWRMVRDSI----
>NP_610941.1
------------DHLPRVVVVGD----Q---------------SSGKTSVLE-----S---IA-KARIFPRGS--
GEMMTRAPV---------KVT---L---
--AEG--PY---HVAQF--------------------------------R---------------DS-----------------------------------
--------DRE------YDL-----TKESDLQDLRRDVEFRMKA-----SVRGG------------------K----------------
--------TVS--NEVIAMTVKGPG---
LQRMVLVDLPGIIS--TM-------------------TVDMAS----------DT---KDSIH--------QMTKHYMSN------
------PNAIILCIQDGSV--DAE-RS--NVTDLVMQCDPLGRRTIFVL-TKVDLAEE-L---AD-
PDRIRKILSGK---L---------------------FPMKALGYYAV---------------------------------VTGR------------
----------------------G----------RK-------------------------DDSID--------------AIRQY--EEDFF---------
KN-------SK--------------------LF----------------HR-R----GVI---M---------------------PHQ-VTS-----
RNLSLAVSDRFWKMVRETI----
>EFJ33653.1
-----------------VVTIGN---T-----------------GAGKSAVLN-----S---II-GYTVMPTG---ENGATRAPI------
---VVE---L---ER-DQ---SEGK---GL---A-
VMT---EGR------
ARP-----SSAN---EIRLSLQSRISR-----IASSR-------------------SG---------------------R------
PEEIRLRLRSSA--APPLTLIDLPGLA------
---------------------------------------SLD-D------QFVNEYGSH-----------NDAVLLVVVPATSVRDIT-
GS--QALKMARELDPEFSRTVGVI-SKVDQSASDPK---S-LAAVQAVLSGQG-PS---------------------
ASAD-ITWVAL----------------------------------IGQS---------------------------------V-S-IAAAH----AGS-
---------------------VGTDDSLE----------T----AWKA---ETETL---------RS------I--------------------------
-----------------------L---TAA-------------------PSTR-LGR-----AALVDVISKQIRKRIRQRLP---
>EFJ28901.1
-----------------VVALGN---T-----------------GAGKSAVLN-----S---LI-GYAVLPTG---ENGATRAPV-----
----TID---L---EA-DD---SGNKR--GL---
T-VQM---EGK------
SMQ-----VSAT---DIRHSLQNKFGR-----M--ST-------------------GA---------------------V------
KENIHLKLKCSS--APPLKLVDLPGLES--
---RSVS-D------SLVREYIDS-----------NDALLLVVIPATSVRDIT-
GS--QALKIAQDIDHEGSRTVGVI-SKIDQAASDPK---S-LAAVQAVLSGQG-PS--------------------
ITSK-FTWIAL----------------------------------IGQS---------------------------------V-S-IAGAH----S------

-------------------KDDSLE----------T----AWKA---EMESL---------KS------I-----------------------------
-------------------L---GGA------------------SSSR-LGR-----SSLVEAIAKQIRQRMQQRLP---
>KAG0632288.1
----------------VVTLGS---V----------------GAGKSAVLN-----S---LM-GYPVLPTG---ENGATRAPI----
-----IIQ---M---QR-GS---SSTNR--GL---
Y-VVL---DGR------
TSN-----VTAS---DVRHSLQGRLKSW----TPNAR------------------SG--------------------R------
TEGIQLTLQSSA--APPLKLFDLPGLDT---
---------------------------------------RATSD-D------SLVQEFAEH------------
SDAILLVVVPAASVREVG-TS--KALKLAQELDSDATRTVGVI-SKVDQAASDRR---S-
LDAVAALLSGNG-PA--------------------ITQE-IPWVAM--------------------------------IGQS---------
---------------------V-S-IAAAH----GS-------------------------EDSLD---------T----AWKA---EAESL--
-------KS------L---L---TQA------------------APTK-LGR-----
VALVEAIAKQIRKRLKQRIP---
>KAG0555995.1
----------------VVALGS---V----------------SAGKSAVLN-----S---LI-GHPVLPTG---ENGATRAPI-----
----ILD---M---ER-DK---SSSSR--GL---
A-VVL---EGR------
TQN-----VSAS---DVRHSLQGRLKN------ASSS------------------KG--------------------R------
TEGIRLTLRSAS---TPPLKLIDLPGVS------
---------------------------------------GSID-D------SPAHDLAAN------------NDTILLIVIPATSCRDVA-
AS--KALKLAQELDSDGTRTVGVI-SKVDQAASDPR---S-LAAVNALISGQG-PP--------------------
STAD-IPWVAL--------------------------------IGQS-----------------------------V-S-IAAAH----SSG-
-----------------------EDSLD---------T----AWKA---EMESL---------KS------I---------------------------
---------------------L---NGA------------------PSAK-LGR-----IALVETLSHQIRTRLKQRLP---
>XP_024391061.1
----------------AIALGN---V----------------SAGKSAVLN-----S---LI-GHPVLPTG---ENGATRAPI------
---IID---M---ER-DK--SGRPG--GL---A-
VVL---EGR------
TQN-----VSAS---DIRHSLQGRLKS------VSSS------------------KG--------------------R------
GDGIRLTLRSNS---GPPLKLIDLPGIDS--
---RGSID-D------SPAHDLAAN------------
NDTILLVVIAATSCRDVA-VN--RALKLAQELDSDGSRTIGVI-SKVDQAASDPR---S-
LAAVNALISGQG-PS--------------------NTQD-MPWVAL--------------------------------IGQS-------
-----------------------V-S-IAAAH----SSS-----------------------EDPLD---------T----AWKA---
EMESL---------NS------I---L---KGA------------------PPAK-
LGR-----IALLETLASKIQSRLKQRIP---
>XP_024368367.1
----------------AIALGS---V----------------SAGKSAVLN-----T---II-GHPVLPTG---ENGATRAPI-------
--IID---M---ER-DK--SGRPG--GL---A-
VVL---EGR------
TQN-----VSAS---DVRHSLQGRLKS------VSSS------------------KS--------------------R------
GDGIRLTLRSNS---GPPLKLIDLPGIDS--
---RGSLD-D------SPAHDLAAN------------

NDTILLVVIAATSCRDVA-VN--RALKLAQELDSDGSRTVGVI-SKVDQAASDPR---S-
LAAVNALISGQG-PS--------------------NTQD-IPWVAL--------------------------------IGQS--------
---------------------V-S-IAAAH----SSP------------------------EDSLD---------T----AWKA---EMESL-
-------KT-----I---L---KGA------------------PSAK-LGR-----
IALLETLASKIQSRLKQRIP---
>OAE31801.1
----------------VVAVGH---I----------------SAGKSAVLN-----S---LV-GYPVLPTG---ENGATRVPI-----
----IIE---M---KR-DE---SGNRK--GL---
A-IQV---EGG----
R-IQS-----VSAS---DLRHNLQGRLQK-----WTPNA------------------KG-------------------R------
PDEIKLRLVSSA---APPLKLIDLPGMEV---
--------------------------------------RAPIE-D------SIVRDYAEH---------NDAVLLLVIPATQAANIL-
GA--RALKLVQDLDGEGTRTVGVI-SKVDQAAADPR---S-LAAVQALLSGQG-PS-------------------
STAE-FPWVAL-------------------------------IGQS-----------------------------V-S-IAAAH----
AGG---------------------GAEDSLE---------T----AWRA---EAESL--------KQ------I-------------------
----------------------------L---PQA-----------------SPSK-LGR-----VALVETLSSQIRKRLKNRLP---
>KAI5073815.1
----------------VVALGN---V---------------GAGKSAVLN-----S---LT-GHPVLPTG---ENGATRVPI----
-----VID---M---ER-DE---GLNSK--QL---
V-LQI---ESK------
TQQ-----VSAS---SIRRSLQEKLTS-------ASE------------------RG--------------------R------
LDEIYLKLRSST--APPLKLVDLPGLDQ---
---------------------------------------RGSD-E------STVSAYTDQ------------
GDAILLVVVPAFQTSEIS-NS--RALRLAHDLDPDGSRTVGVI-SKVDQAASDSR---N-
LAAVQALLVGQG-PS--------------------ITLD-VPWVAL--------------------------------IGQS---------
--------------------A-S-IATAH----SSS---------------------TGGDNSLE---------T----AWRA---
EMENL---------KS------V--L---GSA------------------
PQSK-LGR-----IALVDTLSKQIRKRLKLRLP---
>XP_008646219.1
----------------AVALGN---V-----------------GAGKSAVLN-----S---LI-GHPVLPTG---ENGATRAPI----
-----VVD---L---AR-DP---GLSSK--SI---
V-LQI---DSK------
SQQ-----VSAS---ALRHSLQDRLSK---GASSGSG------------------RS--------------------R------
SDEIYLKLRTST--APPLKLIDLPGIDQ----
--------------------------------------RVMD-D------STISEYAGH------------
NDALLIVVIPAMQAADVA-SS--RALRLAKDIDPDGTRTIGVL-SKIDQAAADAK---T-
VSCVQSILSNKGAPR--------------------AAAD-IEWVAL--------------------------------IGQS--------
---------------------V-S-IASAQ----SGS---------------------VGSDNSLE---------T----AWRA---
EAETL---------KS------I--L---TGA------------------PQSK-
LGR-----IALVDTIAKQIRKRMKVRLP---
>ACG47836.1
----------------AVALGN---V-----------------GAGKSAVLN-----S---LI-GHPVLPTG---ENGATRAPI----
-----VVD---L---QR-EP---GLSSK--SI---
V-LQI---DSK------

SQQ-----VSAS---ALRHSLQDRLSR---GASGGSG------------------RG---------------------R------
VDEIYLKLRTST---APSLKLIDLPGIDQ----
--RAVD-D------SMINEYAGH------------
NDAILLIVIPAMQAADVA-SS--RALRLAKDIDADGTRTVGVI-SKVDQANGDAK---T-
IACVQALLSNKG-PK--------------------NLPD-IEWVAL---------------------------------IGQS---------
---------------------V-A-IASAQ----S-----------------------VGSENSLE----------T----AWRA---EAESL-
--------KN------I---L---TGS------------------PQNK-LGR---
--IALVDTIAKQIRKRMKVRVP---
>KAG7649995.1
----------------VVALGN---V----------------GAGKSAVLN-----S---LI-GHPVLPTG---ENGATRAPI----
-----IID---L---SR-EE---SLSSK--AI---I-
LQI---DNK------
NQQ-----VSAS---ALRHSLQDRLSK------GASG------------------RG----------------------------
RDEIYLKLRTST---APPLKLIDLPGLDQ---
--RIVD-E------SMIGEHAQH------------
NDAILLVVVPASQASEIS-SS--RALKIAKEYDPDSTRTVGII-SKIDQAAENPK---S-LAAVQALLSNQG-
PP--------------------KTTD-IPWVAL---------------------------------IGQS--------------------------------
V-S-IASAQ----SG-----------------------GSENSLE----------T----AWRA---ESESL---------KS------I----
--L---TGA------------------PQSK-LGR-----
IALVDTLASQIRSRMKLRLP---
>NP_172500.1
----------------VVALGN---V----------------GAGKSAVLN-----S---LI-GHPVLPTG---ENGATRAPI----
-----IIE---L---SR-ES--SLSSK--AI---I-
LQI---DNK------
SQQ-----VSAS---ALRHSLQDRLSK------GASG------------------KN----------------------------
RDEINLKLRTST---APPLKLVDLPGLDQ--
---RIVD-E------SMIAEYAQH------------NDAILLVIVPASQASEIS-
SS--RALKIAKEYDPESTRTIGII-GKIDQAAENSK---A-LAAVQALLSNQG-PP--------------------
KTTD-IPWVAV---------------------------------IGQS--------------------------------V-S-IASAQ----SG---
---------------------SGENSLE----------T----AWRA---ESESL---------KS------I---------------------------
---------------------L---TGA------------------PQSK-LGR-----IALVDTLASQIRSRMKLRLP---
>XP_006385192.1
----------------VVALGN---V----------------GAGKSAVLN-----S---LI-GHPVLPTG---ENGATRAPI----
-----SID---L---SR-DS--SVSSK--SI---I-
LQI---DSK------
NQQ-----VSAS---ALRHSLQERLSK------VSSG------------------RS----------------------------
RDEIYLKLRTST---APPLKLIDLPGVDQ---
--RIVD-D------SMISEYVQH------------NDAILLVVIPAIQAPEIS-
SS--RALRIAKEYDAESTRTVGII-SKIDQAATESK---A-IAAVQALLLNQG-PP--------------------KTSD-
IPWVAL---------------------------------IGQS--------------------------------V-S-IASVQ----SGS---------
-------------ASSESSLE----------T----AWRA---ESESL---------KS------I----------------------------------
---------------L---TGA------------------PQSK-LGR-----VALVDVLAGQIRSRMKLRLP---
>KAH9330549.1

-----------------VVALGN---V-----------------GAGKSAVLN-----S---LI-GHPLLPTG---ENGATRAPI-----
----IID---L---QR-DS--SVSSR--AI---F-
LQI---ENK------
TQQ-----VSAS---ALRHSLQDKLSK---GL---
GIPPMKLRTST--SPPLKLIDLPGLDQ----
--RVAD-D------SMISNVAEH------------
NDAILLVVVPASQAPEIS-SS--RALKLALEFDPD--
---A-IASAQ----SGS---------
--------------VGGESSLE----------T----AWRA---ESESL---------KA------I--------------------------------------
------------------L---TGA---------------------PQTK-LGR-----IALVETLARQIRKRIKIRLP---
>ONM04707.1
-----------------VVAIGN---V-----------------GAGKSAVLN-----G---LI-GHPVLPTG---ENGATRAPI-----
----CVE---L---QR-DA--SLSSK--AI---V-
LQI---DSK------
SQQ-----VSAS---SIRHSLQDRLSK---V----SG---------------------KG-------------------------R------
PDEITVKLCTST--APPLKLIDIPGVDQ----
--RSTD--------ESISNYAAR-----------NDAILLVIIPALQAPDVA-
SS--RALRIARELDSEGTRTIGVL-SKIDQASGEQK---A-LGAVQALLVNQG-PR-----------------------
TAAD-IQWVAT--------------------------------------IGHS------------------------------------V-P-TASAQ----SEA-
-------------------------GSETPPE-----------A----YWQA---EVKTL-------------VS------T--------------------------------
--------------------------L---GGA---------------------PESK-LGR-----VALVDSLSKQIKARIKARLP---
>XP_042918632.1
-------------LAIPEIVAIGG----Q------------------SDGKSSLLE-----A---FL-GFRFNVRE---VEMGTRRPL--
-------IVQ---M---VH-DP--TAQE---PR--
---CRL------------------------------------Q--------------------EED--
SDEYG------PPI-----VPET---AVADAIQRRTEE-----HLRKM---------------------GG--------------------------
IAVS--SKPIVMRAEYAY---
CPNLTIIDTPGFIL--KA-----------------------KTGELD-----------NT---PDEIM--------SMVKAQASP-----------
--PHRMILFLQQSSV--EWA-SS--LWLRVVQEVDPYFQRTVIVA-SKFDNRLKEF---AE-
RWEVDKYLSATG-YL-PPN-----VRP-------FFVALP--KDRV--------
---------------------------IQS-SAEWR---R----SMTEV--DTAIY-----
----KH------MRD----------------------GI----------------------K----GGF---DEE--------------------RFASR-IGF-
----SNLKKFLEEELSRRYREAAP---
>NP_001130364.1
-------------LPIPEIVVIGG----Q------------------SDGKSSLLE-----A---LL-GFRFNVRE---VEMGTRRPL--
-------VLQ---M---VH-DP--TALE---PR--
---CRF------------------------------------Q--------------------EED--SEEYG-
-----SPM-----VVAT---AIADLIKQRTES-----HLRKI---------------------QA---------------------------AVS--
SKPIVMRAEYAH--CPNLTIIDTPGFVL--
KA---------------------KRGEPE-----------ST---PDEIR--------SMVKSLATP-------------
PHRLVLFLQQSSV--EWC-SS--IWLDTLKEIDPTFRRTMIVI-SKFDNRLKEF---TE-
RWEVDAFLSASG-YL-GDN-----IHP-------FFVALP--KDRG-------
----------------------------TIS-NEEFR---R----QICHV--DIDVL----

-----RH------LRD--------------------NV-------------------K----GGF---NEE------------------KYGSH-
IGF-----SCLRKYLESELQKRYKEAAP---
>PWZ11893.1
------------LPIPEIVAIGG----Q----------------SDGKSSLLE-----A---LL-GFRFNVRE---VEMGTRRPL--
-------VLQ---M---VH-DP---TALE---PR--
---CRF--------------------------------Q----------------EED---SEEYG-
-----SPM-----VLAT---AIADLIKQRTES-----HLRKI-------------------QA------------------------AVS--
SKPIVMRAEYAH---CPNLTIIDTPGFVL--
KA-------------------KRGEPG----------ST---PDEIR------------------------------------LQQSSV--EWC-
SS--IWLDTLKEIDPTFRRTIIVI-SKFDNRLKEF---TE-RWEVDTFLSASG-YL-GDN-----IHP-------
FFVALP---KDHG--------------------------------TIS-NEEFR----------
-------------------------------------R----QICQV--DIDVL---------RH------LRE--------------------NV-----
--------------K----GGF---NEE------------------KYVSC-IGF-----SCLKKYLESELQKRYKEAAP---
>XP_002317496.2
------------LPIPEIVALGG----Q----------------SDGKSSLLE-----A---LL-GFRFNVRE---VEMGTRRPL-
--------ILQ---M---VH-DP---SALE---PR-
----CRF--------------------------------Q----------------EED---
SEEYG------SSV-----VSST---TIADIIKSRTEV-----LLKRT-------------------KT------------------------
AVS--SKPIVMRAEYAH---
CPNLTIIDTPGFVL--KA-------------------RKGEPE----------NT---PDEIL--------SMVKSLASP----------
--PHRILLFLQQSSV--EWC-SS--LWLDAIKDIDPNFRRTVIVV-SKFDNRLKEF---SD-
RWEVDRYLSASG-YL-GEN-----TRP-------FFVALP---KDKN-------
-------------------------TIT-NDEFR---R----QISQV--DSEIL-----
----HH------LRD--------------------GV-------------------K----GGF---DEE------------------KFRPY-IGF-
----STLRDYLESELQKRYKEAAP---
>AAF87857.1
------------LPIPEIVAIGG----Q----------------SDGKSSLLE-----A---LL-GFRFNVRE---VEMGTRRPL--
-------ILQ---M---VH-DL---SALE---PR--
---CRF--------------------------------QISRIFFVELAILITDLDED---
SEEYG------SPI-----VSAT---AVADVIRSRTEA-----LLKKT-------------------KT------------------------
AVS--PKPIVMRAEYAH---
CPNLTIIDTPGFVL--KA-------------------KKGEPE----------TT---PDEIL--------SMVKSLASP-----------
-PHRILLFLQQSSV--EWC-SS--LWLDAVREIDSSFRRTIVVV-SKFDNRLKEF---SD-
RGEVDRYLSASG-YL-GEN-----TRP-------YFVALP---KDRS--------
------------------------TIS-NDEFR---R----QISQV--DTEVI------
---RH------LRE--------------------GV-------------------K----GGF---DEE------------------KFRSC-IGF--
---GSLRDFLESELQKRYKEAAP---
>KAI5058044.1
------------LPIPEIVALGG----Q----------------SDGKSSLLE-----A---LL-GFRFNIRE---VEMGTRRPL--
-------MIQ---M---VH-DA---SALE---PL--
---CRL--------------------------------Q----------------DED---
SDDYG------PVI-----APAS---AVAEAIKLRTEE-----HLKKE-------------------RT------------------------
AVS--SKPIVMRVEYAY---
CPNLTIIDTPGFVL--KA-------------------KKGEPE----------NT---PEDIM--------SMVRTLAAP---------

---QHRLLLFLQQSSV--EWC-SS--LWLDSIRTVDPSLRRTIIVI-SKFDNRLKEF---GE-
RWEVDRYLSAGG-YL-GDT-----AHP-------FFVALP---KDRT-------
-------------------------MTS-NEEFR---R----QIGAV--DSDVH--
-------RY------LRN--------------------NI-------------------K----GGF---DEE------------------KFGDF-
IGF-----LNLKQYLELELQRRYRDAAP---
>EFJ18064.1
------------LSIPEIVAVGG----Q----------------SDGKSSLLE-----A---LL-GFRFNVRE---VEMGTRRPL-
--------VLQ---M---IH-EP---AAVD---PR-
----CRL--------------------------------Q----------------HED---
DEEYG------PVI-----VPHY---AVAEAIKLRTEE-----HLKKI-------------------RA------------------------
AVS--SKPIVMRVEYAY---
CPNLTIIDTPGFIL--KA-------------------KKGEPE----------ST---PDDIL--------QMVRALALP-----------
-PNRLLLFLQQSSV--EWC-SS--LWLDTVRSIDPGFHRTVVVV-SKFDNRLGEF---AE-
KWEVDRYLSAGG-YL-GDH-----VRP-------FFVALP---KDRG------
--------------------------SVT-NEEFR---S----QIASV--DAEVL---
------KH------LRE--------------------RI-------------------S----GGF---SED------------------KYSGS-
IGF-----GNLRNYLEAELQRRYREAAP---
>KAG0628798.1
------------LPIPEIVAVGG----Q----------------SDGKSSLLE-----A---LL-GFRFNVKE---VEMGTRRPL-
--------MLQ---M---IH-DP---EALE---PR-
----CRL--------------------------------Q----------------DED---
ADDYG------PVI-----TPVS---SVADHIRIRTEG-----FLKKL-------------------GT------------------------
AVS--AKPIVMRAEYAY---
CPNLTIIDTPGFIL--KA-------------------KKGEPD----------ST---PDEIE--------AMVRELAAP-----------
-QHRLLLFLQQSSV--EWC-SS--LWLDVVKSIDPSLQRTMVVV-SKFDNRLKEF---TE-
RWEVDRYLSTGG-YL-GEN-----ARP-------FFVALP---KDRG-------
-------------------------TTT-NDDYR---H----QISVV--DIDIL-----
----KQ------LRE--------------------NV-------------------A----GGF---DEE------------------RFGNY-
VGF-----GKLRQFLEAELQRRYRDAAP---
>PTQ33908.1
------------LPIPEIVAVGG----Q----------------SDGKSSLLE-----A---LL-GFRFNVRE---VEMGTRRPL-
--------MLQ---M---IH-DP---GALE---
PR-----CRL--------------------------------Q----------------DED---
SDEYG------PAI-----VPAS---AVAEAIRSRTEL-----FLKRT-------------------GT------------------------
AVA--SKPIVMRAEYAF---
CSNLTIIDTPGFIL--KA-------------------KKGESE----------ST---PDDIV--------AMVRELAAP-----------
-PNRILVFLQQSSV--EWC-SS--LWLDTVRAIDPALRRTIVVV-SKFDNRLKEF---AE-
KWEVDRYLSAGG-YL-GDS-----TRP-------FFVALP---KERS--------
------------------------SVS-NEEFR---R----SIARV--DNEVV----
-----RH------LRE--------------------NV-------------------S----GGF---DED------------------QFGDR-
IGF-----SNLRRFLEAELQRRYRQSAP---
>KAH9322298.1

------------------DED---SDDYG------APV-----ALAS---
AVADAIKSRTEE-----HLKKI-------------------RA------------------------AVS--AKPIIMRAEYAH----------
---CPNLTIIDTPGFVL--KA-------------------
KKGEPD----------ST---PEDIL--------SMVKALAAP------------PNRLLLFLQQSSV--EWC-SS--
LWLDTIRAVDPTLRRTIVVV-SKFDNRLKEF---GE-RWEVDRYLSASG-YL-GEN-----TRP-------
FFVALP---KDRG--------------------------------AIT-NEEFR-----------
-----------------------------------T----QIAQV--DAEVN---------CY------LKE--------------------SV-------
------------I----GGY---DEE------------------KFGAY-VGF-----GNLRGYLENELQRRYRDAAP---
>XP_009032466.1
------------FDVPSVVVIGA----Q----------------SSGKSALVE-----A---LM-GFQFNEVG---
GGTRTRRPI---------ALQ---M---HY-NA---
--ACDE---PA-----CYI--------------------------------M----------------DERFS-------------
GGEPVDGGAP----------------FERRA-------TL-----AEAR---RFIEEENRRLER-----DQHR--------------
--------------------------------SFE--AREIVMRVEYRH---
--------------CPNLVLVDTPGLVG-GGGDVFG--------------DDFGEESHESPHARGMKRQ---AREAY-
-------ELALGKARA------------RNAVLLCVDDGN---DWKLGS--IARRLCADADPTLSRTVVVS-
TKLDTKLVQF---GS-GRDVASFLRAKV--L-HDLHPRLLAGP-------FFTSVP-
CGRVAGAISPGGDAWDPQGGAPENQPWDLDDEGFYEDDGVAFR----------------------------------
-G-DAEFR---A----ATARA--SRADR---------SL------VKS-------
--------------KV----------------GF-E------F--FDKA--------------------APQ-LGV-----
GALRQFLERHVELQYRSNVA---
>XP_042920073.1
------------FDAPAILVVGH----Q----------------TDGKSALVE-----G---LM-GFQFNSVG---
GGTKTRRPI---------AIN---M---KY-NG---
--ACST---PA-----CFL--------------------------------K----------------LE---------------------DGVS--------
---------EQEMS--------L-----AELQ---AYIDADNAALER-----E--Q--
------RFA--AKEIVVRMEYKH---
CPNLTIIDTPGLIS-PAP---------------------GKKN------CALQNC---AAQVE--------EIVRAKAQV-------
-----PEYVILCLEDCS---DWS-NA--TTRRLVMQVDPNLVRTVLVS-TKFDTRIPQF---AR-
AADCEMFLRPSA----LDSMGMLGDGP-------FFTSVP-SGRVGS----GADC--------------------------
VFP-----------------------------------S-HDVFR---E----RLADR-
-EATDV---------AE------LES--------------------KL----------------AR-K------L--SRGE--------------------
RDH-IGV-----GALRRYLEQLLQKRYLDAVP---
>PWZ44616.1
------------AAAPAVVVVGH----Q----------------TDGKSALVE-----A---LM-GFQFNHVG---
GGTKTRRPV---------ALH---L---RF-NP---
--RCDE---PQ-----CRL--------------------------------L----------------SA---------------------TGDAE-----
-----------EHDEAGVAARPMPL-----ADIQ---AYIEAENLRLEN-----DPCQ-------------------------------
----------------FS--EKEIIIKVEYKH---
SPNLTIIDTPGLIL-PAP---------------------GRKN------RVLQSQ---ASAVE--------SLVRAKIQH--------
----KETIILCLEDCS---DWS-NA--TTRRVVMQVDPDLARTVLVS-TKLDTKIPQF---AR-
PSDVEVFLHPPN-CV-LDV-SLLGDSP-------FFTSVP-SGRVGS----CHEA--------------------------
VFR-----------------------------------S-NGEFK---K----AILSR--

ELEDI---------AS------IED--------------------KL----------------GR-S------L--TTME--------------------
KDR-IGV-----GNLRLYLEELLQKRYVKSVP---
>XP_002309632.3
------------FDAPAVLVVGH----Q----------------TDGKSALVE-----G---LM-GFQFNHVG---
GGTKTRRPI---------TLH---M---KY-DP--
-ECEV---PT-----CHL--------------------------------V----------------SD---------------------DDPSF-------
---------AQEKS--------L-----HEIQ---AYIEYENMRLEK-----ESFQ---
------FS--AKEIIIRVEYKH---
CPNLTIIDTPGLIA-PAP---------------------GRKN------QALQSQ---AHAVE--------SLVRAKMQH-----
-------KEFIILCLEDCS---DWS-NA--TTRRVVMQIDPELSRTIVVS-TKLDTRIPQF---AR-
ASDVEVFLSPPA-HT-LDG-FILGDSP-------FFTSVP-SGRVGS----GHDS--------------------------
VYS-----------------------------------S-NDEFK---Q----AISLR--
EVEDI---------AS------LEE--------------------KL----------------CR-P------L--SMQE--------------------
RNR-IGV-----SKLRSFLEELLQKRYMDSVP---
>NP_001189935.1
------------FEAPAVLVVGQ----Q----------------TDGKSALVE-----A---LM-GFQFNHVG---
GGTKTRRPI---------TLH---M---KY-DP--
-QCQF---PL-----CHL--------------------------------G----------------SD---------------------DDPSV-----
-----------SLPKS--------L-----SQIQ---AYIEAENMRLEQ-----EPCS--------------------------------------
--------PFS--AKEIIVKVQYKY---
CPNLTIIDTPGLIA-PAP---------------------GLKN------RALQVQ---ARAVE--------ALVRAKMQH------
------KEFIILCLEDSS---DWS-IA--TTRRIVMQVDPELSRTIVVS-TKLDTKIPQF---SC-
SSDVEVFLSPPA-SA-LDS-SLLGDSP-------FFTSVP-SGRVGY----GQDS--------------------------
VYK-----------------------------------S-NDEFK---Q----AVSLR-
-EMEDI---------AS------LEK--------------------KL----------------GR-L------L--TKQE--------------------
KSR-IGI-----SKLRLFLEELLWKRYKESVP---
>EFJ19523.1
------------FEAPAILLVGH----Q----------------TDGKSALIE-----A---LM-GFQFNHVG---GGTKTRRPI-
--------TLH---M---KY-NA---ACSE---PL-
----CYL--------------------------------M----------------TE---------------------DGLPR----------------
EEERS--------L-----DDIQ---AHIESENLRLER-----DTHQ---
FW--AKEIIIKIEYKY---CPNLTIIDTPGLIA-
PPP--------------------QSNAT------TALQAQ---AKAVE--------ALVRSKMQH------------
KEFIILCLEDCS---DWS-NA--TTRRVVMQVDPELSRTIVVS-TKLDTRIPQF---AR-AADVELFLRPPP-
RL-LDG-DILGGTP-------FFTSVP-SGRVGS----GRDA--------------------------VYK---------------------
--------------S-NEQFR---E---ASPAR--ELEDV---------SS-----
-LEE--------------------KL----------------GR-P------L--LREE--------------------RNH-VGV-----
SRLRWFLEQILQRKYMESVP---
>KAI5064281.1
------------FDAPAILVVGH----Q----------------TDGKSALVE-----A---LM-GFQFNHVG---
GGTKTRRPI---------TLH---M---KY-NA--
-GCSE---PV-----CYL--------------------------------M----------------TD---------------------DKPPI-------
---------EEERS--------L-----EELQ---AFIEAENMRLEQ-----EACQ---------------------------------------
--------FW--AKEIVVKIEYKY---

CPNLTIIDTPGLIA-AAP--------------------GRKN------HLLQAQ---ARAVE--------ALVRTKMQQ------
------KEFIILCLEDCS---DWS-NA--TTRRVVMQMDPELSRTVVVS-TKLDTKIPQF---AR-
AADVELFLRPPP-RL-LDG-NILGETP-------FFTSVP-SGRVGT----SRDS-------------------------VFR-
--------------------------------S-NEQFR--E----AVAAR--
EAQDL---------SA------LEE-------------------KM---------------DR-H------L--LPDE------------------
-RAR-VGV-----SRLRWFLEQLLQQRYMESVP---
>PTQ34556.1
------------FDAPAILVVGH----Q---------------TDGKSALVE-----A---LM-GFQFNHVG---
GGTKTRRPI---------TLH---M---TY-NA---
-ECTE---PR-----CFL------------------------------L---------------SE--------------------DAPPK-------
---------EEEKS--------L-----DDLQ---AYIESENMRLEL-----EPSQ--
-------FW--AKEIVVKIEYKF--
CPNLTIIDTPGLIS-AAP--------------------GRKN------ISLQSQ---ARAVE--------ALVRSKMQQ-------
-----KEFIILCLEDCS---DWT-NA--TTRRFVMQMDPELTRTVIVS-TKLDTRIPQF---AR-
PADVELFLRPPS-RL-LDG-NILGDTP-------FFTSVP-SGRVGS----GRDS-------------------------
VYR----------------------------------T-NESFR--E----AVAMR--
EALDV---------AM------LEE-------------------KM---------------DR-P------L--LNEE-------------------
-RNR-VGI-----SRLRCFLEQLLQRRYMDSVP---
>KAH9308354.1

---AQ---ARAVE--------MLVRTKMQH--------
----KEYIILCLEDCS---DWS-NA--TTRRVVMQVDPELSRTVMVA-TKLDTKIPQF---GR-
SSDVELFLRPPT-RL-LDG-SILGETP-------FFTSVP-SGRVGT----NRDA-------------------------VYR--
--------------------------------S-NDQFK--E----AVASR--
EAQDV---------AA------LED-------------------KL---------------GR-S------L--LKEE--------------------
RAR-VGV-----SRLGCFLEELLQRRYIDSVP---
>KAG0561482.1
------------FDAPAIVVVGH----Q---------------TDGKSALVE-----A---LM-GFQFNHVG---
GGTKTRRPI---------TLH---M---KY-NA--
-MCAE---PR-----CYL-------------------------------I---------------TE--------------------DRPPR-------
---------EEEKS--------L-----EELQ---AYIEAENLRLER-----EVCQ--
------FW--EKEIILKIEYKF---
CPNLTIIDTPGLIS-AAP--------------------GRKN------QSLQSQ---ARSVE--------VLVRTKMQH------
------KEFIILCLEDSS---DWS-NA--TTRSIVMQIDPDLSRTVVVS-TKLDTRIPQF---AC-
RADVELFLRPSQ-RL-LEG-NILSGSP-------FFTSVP-SGRVGV----TRDS-------------------------
VHR----------------------------------S-NDHFR--E----AIALR-
-EAQDI---------AL------LEE-------------------KL---------------DR-Q------L--TKEE--------------------
LAH-VGV-----SRLRLFLEQLLQRQYMDSVP---
>KAG0605142.1
------------FDAPAILVVGH----Q---------------TDGKSALVE-----A---LM-GFQFNHVG---
GGTKTRRPI---------TLH---M---KY-NA--

-NCAQ---PR-----CFL--------------------------------I----------------SE---------------------DQPHR------
----------EKEQS--------L-----EEIQ---AYIEAENKRLER-----EACQ--
-------FS--AKEIILKIEYKF---
CPNLTIIDTPGLIS-AAP---------------------GRKN------QSLQSQ---GGAVE--------ALVQLKMQQ----
--------KEFIILCLEDSS---DWS-NA--TTRRVVMQADPELRRTVLVS-TKLDTRIPQF---AR-
PDDVELFLKPPS-CL-LDG-IILGGSP-------FFTSVP-SGRVGS----SKDS--------------------------VFR-
----------------------------------S-NTDFQ---E----AVAAR--
QIQDL---------AL------LEE-------------------KL----------------NR-P------L--TRDE--------------------
CSR-IGI-----SRLRWFLEQLLQCRYMDSVP---
>XP_042916771.1
---------------FLVAVVGE---F-----------------NSGKSSVIN-----A---LL-GRRYLAEGI--------LPT----TN-
--EIS--IL--KY---S----------

DTAPATSNPSQV----QLVQQ---SDGLYVRYLPAK----------
---L---LQDLNIVDTPGTNV-----------------------
ILERQQ---------------------------RLTEEYVPR-------------ADLVLFVMSA----DRPFSE--
SEVRFLEYIRQWQKKVVFVV-NKSDILESSD----E-VDAVKEFVAANAQRI---------------------
LRLDRPSVIAV---------------------------------SSRS----------------------------------ALRAKLT-----------
---------------------ASNLP----------I----TASF---DSDLP---------SA-------
SPTAPLSNVDPEAMEAALSNSRDW-----------------A--------V-----------------------------SNF-----
SELERNVSNFL------------
>EFJ26018.1
---------------FLLVVVGE---F-----------------NSGKSSVIN-----A---LL-GDRFLKQGV--------LPT----
TN---EIT--LL--KY---S-----

----DESYE--ERPA--------RH---PDGHLMRYLSAG--------
---L---LKQMNLVDTPGTNV-----------------------
ILQRQQ----------------------------RLTEEFVPR------------ADLVLFVIGA----ERPLTE--SEAS---------
SHTSFCI-SRFDVIRLLSGFFFT-VEEVRRFVADNVRQL---------------------LNIEAAMIFPI----------
--------------------SARK----------------------------------ALHAKVK--
-----------AKQL---ESKNL---------ER-------DP--------------------LW-----------------T--------A----------
--------------------SGF-----DKLEQYVLDFL------------
>KAG0631008.1
---------------FLSVVVGE---F-----------------NSGKSSIIN-----A---LL-GKRFLKEGV--------LPT----TN-
--EIT--LL--RH---A--------

NDGGNTEEREE--------RH---PDGHFLRFLPAS----------
--L---LKQMNLVDTPGTNV-----------------------
ILQRQQ----------------------------RLTEEFVPR------------ADLVLFVLSA----DRPLTE--
SEVTFLRYIRQWGKKVVFIL-NKSDVLSTYS----E-VEEVRNFVRDNAQRL---------------------
LTVDQALVYPV---------------------------------SARQ----------------------------------ALQAKLS---------
-----------------------ASL-----------E----DGTV---DTARL---------SE-------DP--------------------LW----
-------------T--------T------------------------------SGF-----KDLEEFIFSFM-----------
>OAE29693.1

---------------FLLVIVGE---F-----------------NSGKSSVIN-----A---LL-GERFLKEGV--------LPT----TN-
--EIT--VL--RH---T---------

GEGDEGKERSE--------KH--PDGHFLRYLPAE-----------
--L---LKQMNLVDTPGTNV----------------------
ILQRQQ----------------------------RLTEEFVPR-------------ADLVLFVLSV----DRPLTE--
SEVTFLRYIRQWGKKIIFIL-NKSDVLADRK----E-LEEVLKFVKENAQSL--------------------
LSVEEASVYPV----------------------------------SARR----------------------------------ALLAKQA---------
----------------------AVN------------E----DGVV---DRELL---------MQ-------NS-------------------SW-
----------------K--------S-----------------------------SGF-----DELEDFIFCFL------------
>KAH9315399.1
---------------FLLVIVGE---F-----------------NSGKSTVIN-----A---ML-GKRYLKEGV--------VPT----TN-
--EIS--VL---CY---S---------

GEGHDEEERSE--------RH--PNGYFIQYLPAS------------
---L---LKQMSLVDTPGTNV----------------------
ILQRQQ----------------------------RLTEEFLPR-------------ADLVLFVISA----DRPLTE--
SEVTFLRYIRQWGKKVIFIL-NKSDIFKDVK----E-LDEAVTFVKDNAQQL--------------------
LSTEQIILYPV----------------------------------SSRS----------------------------------ALEAKIA-------------
-------------------ATT------------G----DGGV---DLEIL---------SK-------DP-------------------NW-------
----------I--------I-----------------------------SGF-----SALEDFIFDFL------------
>KAI5059498.1
---------------FLLMVVGE---F-----------------NSGKSSVIN-----A---LL-GKRYLPEGV--------LPT----
TN---EIA--LL---KH---A----

-----GNGFNDKERSE--------RH--PDGHFMYYLPAE-----
--L---LKEINLVDTPGTNV----------------------
ILKRQQ----------------------------RLTEEFVPR-------------ADLVLFVLSA----DRPFTE--
SEMTFLKYIMQWDKRIVFLL-NKSDIFSDQK----E-LEEVVKFVKDNAQQL--------------------
LSVEKATIYAV----------------------------------SARK----------------------------------AFQAKNE----------
---------------------VGV------------D----SGNL---DVEEL---------LQ-------NS-------------------AW---
--------------N--------V-----------------------------SGF-----RDLEKFIADFL------------
>CAD5311589.1
---------------FLMVIVGE---F-----------------NSGKSTVIN-----A---LL-GKRYLKEGV--------VPT----
TN---EIT--FL---CY---S-----

----DLESEEQQRCQ--------TH--PDGQYVCYLPAP------
--I---LKDINIVDTPGTNV----------------------
ILQRQQ----------------------------RLTEEFVPR-------------ADLLVFVLSA----DRPLTE--
SEVAFLRYTQQWKKKFVFIL-NKSDIYRDAR----E-LEEAISFVKENTRKL--------------------
LNTENVILYPV----------------------------------SARS----------------------------------ALEAKLS-----------
---------------------TAS------------L----VGRD---DLEV----------S-------DPG------------------SNW----
-------------R--------V-----------------------------QSF-----NELEKFLYSFL------------
>XP_008649599.1

---------------FLLVIVGE---F-----------------NSGKSTFIN-----A---LL-GRQYLQEGV--------VPT----TN-
--EIT--LL---SY---S---------

EVESENFERCE--------RH--PDGQFMCYLSVP----------
---I---LKEMNLVDTPGTNV----------------------
ILQRQQ----------------------------RLTEEYVPR-------------ADLVLFVLSS----DRPLTE--
SEVGFLQYVQQWKKKVVFIL-NKLDLYRNSN----E-LEEATAFVKENAMKL--------------------
LTAEDVTLFPV----------------------------------SSRS-----------------------------------ALEAKLSY---------
----------------------SKN------------S----DGKH---STEAM---------YN-------DP--------------------RW--
---------------R--------S-----------------------------SKF-----FELEDYLLSFL------------
>OUM66167.1
----------------KVLVMGE----T----------------NSGKTTFIN-----A---IV-GGEILFEN---RFPCKAPLC-----
----EIK--HS---SQ-NN---DKE---EV---
H-CFI---NER---
CDI-----KSID---EFKTLIKDIEKK---
YEEKMPYDFYRIFYNDGV------------EQ--PKSLI--NNN----
LVNIILLDSPGLNI---DQ---------K----KTKIVEEALED------------
IDAVIFVCDA----TYTIKE--IEYNYLKNNLGKSRDYVFIVVNKMDIITDDED-----KKECKDRINSK-------
---IEKVL--
P-K--TF---EERD---LLIHY--------VSA-------RN--------------------
-----------------AK-T----KEI--T----------------------QEDIQNF-----NNLKNALQTFL------------
>XP_001481516.1
----------------KVLITGD---L-----------------NAGKSTFCN-----A---LL-RRKVLPED---QQPCTSIFC----
-----EVL--DA---RE-NS---GVE---EV---
HAVHK--------DK---------------------Q---------------YDR---
NDEST------YDV-----YTLP---ELENIVIDNS---
KYMQCKVYVK-DV------------RTID-ESLL--NNG----
VVDIALIDAPGLNS--DS--------------LKTTAVFARQE-----------
EIDVVVFVVSAAN--HFT---L-SAKEFILNAAHEKAYIFIVV-NGFDQIRDKQ--------RCERMILDQ------
--IGKLS--
P-R---TY---KEAA---ELVHF--------VSS-------NA-------------------
VPVAPPVQMEQSGGGSGGGS-D----PHG--DDDDDHSDNRG-KGKG------KEREKIRDF-----
ENLEGSLRRFV------------
>XP_011392385.1
----------------KVLVTGD---L-----------------NAGKSTFVN-----A---LL-RRPLMPTD---QQPCTTVFC--
-------EVL--DA---SHLDS--SAE---EV-
--HMLKP--------GF---------------------K---------------YNC--
NDDSS------FTR-----HSLA---EIEQIVAEAEQV---
SP-EDAPILKCYAH-DT------------RAAQ-DSLL---KNG----
IVDIALIDAPGLNR---DS---------------LKTTALFARQE-----------
EIDVVVFVVSAEN--HFT---L-SAKEFLWNASHDKAFVFIVV-NKFDSIKNKD--------KCRKLVLDQ----
----
IRQLS--P-R---------------------------------------TY---DDAA---NLVHF--------VDS-------QT---------

----------V----------FGADVDAVV-D----SSV--AAPDTPSEELGCKVEGAQLVQRRDSESLKAF-----
ARLEAALRDFV------------
>XP_006462464.1
----------------KVLVTGD---L-----------------NAGKSTFVN-----A---LL-RREIMPVD---QQPCTTAFC---
------EVH--DA---AE-NQ---AKE---EV--
-HFLKE--------GV----------------------E----------------YNI--
NDEST------FTR-----GTIA---QLEEFVADNE---
DTQQMIKVYLA-DT------------RAPS-ESLL--NNG----
VVDISLIDAPGLNR--DS---------------LKTTAVFARQE-----------
EIDVVVFVVSAEN--HFT---L-SAKEFLWNASNEKAYLFIVV-NKFDQIKNKE--------KCRRLVLDQ-----
---IRQLS-
-P-R---TH---DDAE---DLVHF--------VDS-------AT------------------
--------------------AL-Q----PFT--ANP--------------------------SF-----DDLESALRSFV------------
>XP_006676761.1
----------------RVLVTGD---V-----------------NAGKSTFVN-----T---LL-RRQIVPDD---QEPCTALFV----
-----EVM--DP---QQ-NG---GVE---EF--
-HAIKE--------NV----------------------D----------------YHP--
SDRSS------FTR-----FDLH---ELRSVVEINE---
PEFSMIKVYCM-DN------------RDSV-SSLL--HNG----
VVDISFIDSPGLNI--DS---------------VKTTSLFTKQE-----------
EIDVIVFVVRAEN--HFT---Q-SSCDFLATACKEKASVFIVV-NRFDQIRRKD--------RCRREILDQ------
--IQGIS--
Q-H---TF---ADAA---NLVHF--------VSA-------RM------------------
--VL----------------ES-D----LKL--AE--------------------RNEEAEVSF-----KHLEQSLRSFV------------
>KXN69997.1
----------------KVLVTGD---L-----------------NSGKSTFVN-----A---LL-KRRVMPTD---QQPCTMLFV--
-------ETL--NV---KL-ND---GVE---EA--
-HAIPV--------AA----------------------L----------------YDR--
TDPST------FVV-----IPME---DLERTVAEDY---
KEYELVKVYAH-DP------------ED-N-ESML--YNG----
ILDIALIDSPGLNR--DS---------------IKTTQLFARQE-----------
EIDVVVFCVHAEN--QFT---L-SGQEFLQSAGREKAYIFIVV-NRFDTIRDKN--------RCKRQILDQ-----
---IKNLS-
-P-H---TY---QDAD---DLVHF--------VSA-------DH-----------------
---CF----------------PE-D----EDI--PDA------------------LPSDEIPEDF-----GRLENNLRGFI-----------
-
>NP_009738.1
----------------KVFITGD---V-----------------NTGKSALCN-----S---LL-KQRLLPED---QLPCTNVFS---
------EIL--EA---RE-ND---GIE---EV---
HAIPL--------NIA-------------PTLKEAIDM----------------YSI--
QNPKT------YEI-----HTLK---ELPDLVPQNG--
KYALLKIYIKDDK------------RPAS-TSLL--RNG----
TVDISLIDSPGLNM--DS---------------LQTAEVMSRQE-----------
EIDLVIFVVNAEN--QLT---L-SAKEFISLASREKKLMFFVV-KKFDKIRDKQ--------RCKELILKQ-------

---IRDLS--P-
E--TY---KRAA---DFVHF--------VSK-------NG----------------------
----------------DE-L----PHY--HNENDNEDHGDRKPDDDP---YSSSDPDPDF-----DSLEDSLRNFV--
----
>KNE73082.1
----------------RILVTGD---L-----------------NSGKSSFVN-----A---LL-RRNVVPVD---QQPLTNVFV---
------EVL--DA---RH-NTA---RGQRE---
EV---HLVRD--------GATILSSSQGGDDKSKRAVQPAAQK----------------RHP--------------------------
---------------KHKAA------AHVDEADVYPLD---QLAHLVADPN--
---------------------GYEMIKCFISEDP------------AATSTSVLV---------------------------------------GNE-
---SVDVHMIDSPGLNR---------------------------------------DV-------------WQTMALFSQEK--------
---EIDVIVFVVSAPD--HFT---L-SSREFLTKAGQEKAYIFVVI-NKFDSIRDKE--------RCKRTILRQ------
---IQQLS--
P-H---TF---EYRD---KLVHF--------VSA-------EA--------------------
ML-------RDVQAAQ---R-A----GAV--AVSE--------------------TPRIAEF-----LHVERALKEFT-----------
-
>KNE65701.1
----------------RVLVGGD---L-----------------NAGKSTFVN-----A---LL-RRDVVPVD---QQPLTNVFV-
--------EVL--DA---RH-NQL---HPGVE---
EV---H---
AHKEV------GSV------------IAHSSRRPD---
DCELVKVYCNEEQ------------AERS-SVLV---------------------------------------GNE----
HVDVRVIDSPGLNR---------------------------------------DM-------------WQTMALFAQQK----------
EIDVIVFL-----------------------AGQEKAYIFIVV-NKFDAIKDKE--------RCKKIILQQ---------------------
--IEELS--P-H-----------
-------------------------------TY---RYKE---KLVHF--------VSA-------ER--------------------MM-------
RDVAAIRTLAE-Q----GQL--ADDEE--------------ETMTTAMAEF-----VRVEEALKEFV------------
>XP_001745740.1
--------RSVTRESMKVVFVGR----T---------------SNGKSTTIN-----A---ML-HTRVLPAG---
PGHTTNCFV---------TLQ--G---
SDQS---K------AYM--------------------------------Q---------------------------------------LPGDP---------
----------TP------RDL-----KDVQ---SLTDALQQEHV----------L--
------PPGQSVEIHWPRD-------------Q----CHL---L---
RDDVVILDSPGL---DY---DSDFD--------AWIDETTRD------------
ADVFVLVVNAVST---LS-GA--ESGFFHSVCKTVAKPNVFVIFNQWDNLDED--------
EADVTGVRAQH--M--------------------------------S---------------------------------KARD----------------
----------------LL--VRDLGIC--S--E----AELS---SRVFF--------
VSS-------KE--------------------VL-----------------KS-R------A---G----------------------SDS-RTT---
SYTDPSSAVVPGLNTH---------
>NP_495161.1
--------DTFQRDNMKVVFFGR----T----------------SNGKSTTIN-----A---ML-HEKVLPQG---
MGHTTCCFL---------QVE--G---
-SEGE---V------GHL--------------------------------Q---------------------------------------LDDNP--------
-----------QK------IDM-----KMLG---KIGHALSDENSD-LPA-----M---------------------------------------

-----------GQDSLLKVFHPKKSE---------SGE----CRL--L---
QNDVVILDSPGV--DL---SPEFD--------SWIDKHCLD-----------
ADVFVLVSNAEST---LT-QA--EKNFFLRVAKKLSKPNVFILNNRWDASAAE--------TENIEDVKKQH--
L-------------------------------T--------------------------------RFRQ-------------------------------FL--
VDELEVC--S--E----REVN---DRIFF--------VSS-------RE---------
----------VL---------------ES-R------L---K---------------------A---RGLV------QKAYQAEGH-----------
-
>PAA75551.1
--------EVISRNQMKCAFFGR----T---------------SNGKSTVIN-----A---ML-GRKVLPSG---
IGHTTNCFL---------QVE--G---
TSKQ---S------AYL-------------------------------Q-------------------------------------TPNSS----------
--------EE------QPI-----ESVS---QLGSALSNEK----------M--
---DCESLVRVFWPKQ-------------L----CSL---L---
REDVVLLDSPGV--DV---SPDLD--------TWIDQFCMD-----------
ADVFILVCNSEST---LM-NT--EKKFFHKVGSKLSKPNVFVLNNRWDCSDGE--------
LDSAELVRKQH--M-------------------------------D--------------------------------KSVS----------------
---------------FL--ADELKSC--T---R----SEAE---SRVYF--------
VSA-------KE--------------------AL----------------VN-R------L---K---------------------ETN-QGLE---
SPSPAGSLADGW-----------
>PAA75258.1
--------EVISRNQMKCAFFGR----T---------------SNGKSTVIN-----A---ML-GRKVLPSG---
IGHTTNCFL---------QVE--G---
TSKE---S------AYL-------------------------------Q-------------------------------------TPNSS----------
--------EE------RPI-----ESVS---QLGSALSNEK----------L--
--DCESLVRVFWPKQ-------------L----CSL---L---
REDVVLLDSPGV--DV---SPDLD--------TWIDQFCMD-----------
ADVFILVCNSEST---LM-NT--EKKFFHKVGSKLSKPNVFVLNNRWDCSDGE--------
LDSAELVRKQH--M-------------------------------D--------------------------------KSVS----------------
---------------FL--ADELKSC--T---R----SEAE---SRVYF--------
VSA-------KE--------------------AL----------------VN-R------L---K---------------------ETN-QGLE---
SPSPAGSLADGW-----------
>XP_002126852.1
--------DMLTRNHMKVVFFGR----T----------------SNGKSSVVN-----A---ML-WDRILPTG---
IGHTTNCFL---------SVA--GC-S-DEGST--
---STDSD---EG-----AYL-------------------------------L-------------------------------------CNGSE----
---------------EK------RSI-----KSVT---QLSHALSEES----------M---------------------------------------
---------SPDSLIQVFWPKS-------------K----CAL---L---
KDDVVLVDSPGI--DV---SHDLD--------QWIDKYCLD-----------
ADVFILVANAEST---LM-QA--EKKFFHRVNEKLSKPNIFILNNRWDASASE--------
PELMEQVRQQH--L-------------------------------E--------------------------------RGIS----------------
---------------FL--ADELKVI--S---K----SQAK---DRVFF--------
VSA-------KE--------------------TL----------------QS-R------M-------------------------PKV-PGK-----
ADSPVYMADGH-----------
>XP_006819998.1

--------EVLARDHMKVAFFGR----T----------------SSGKSTVIN-----A---ML-KDKVLPTG---
IGHTTDCFL---------SIE--G---
SDTS---E------AYL--------------------------------I--------------------------------------IPQSN------------
-------ER------RNV-----RSVS---QLAHALSNEKL----------A--
---DQSSLIHVFWPSS-------------R----CAL--L---
KDDLVLVDSPGV---DV---TADLD--------SWIDDHCLD------------
ADVFVLVANAEST---LM-RT--EKSFFHKVAEKLSKPNIFILNNRWDASASE--------PDSMEDVKKQH-
-L--------------------------------E--------------------------------RSIG--------------------------------FL--
VEELKVI--T---K----QQAE---DRVFF--------VSA-------KE----------
----------AL----------------CC-R------I--------------------------QKV-QGM-----PEAG-------------------
>XP_002591612.1
--------QVISRDKMKVAFFGR----T----------------SNGKSTVVN-----A---ML-RDKILPSG---
IGHTTNCFI---------NVE--G---
SDGY---E------AYL--------------------------------L--------------------------------------TPDSD-----------
--------DR------KTV-----QSVG---QLAHALCGERL----------E--
-----DSSILVKVFWPKG-------------R----CAL--L---
RDDVVLLDSPGI---DV---TPDLD--------SWIDEHCLD------------
ADVFVLVANSEST---LM-RTAREKNFFHTVSERLSKPNIFILNNRWDASASE--------
PEFMEAVKKQH--L--------------------------------E---------------------------------RCVS------------------
---------------FL--VEELGVV--D---R----LQAE---DRVFF--------
VSA-------KE--------------------AL----------------QS-R------L--------------------------QKQ-QGM-----
PEEGGALAEGF------------
>XP_019628129.1
--------QVISRDKMKVAFFGR----T----------------SNGKSTVVN-----A---ML-RDKILPSG---
IGHTTNCFI---------NVE--G---
SDGF---E------AYL--------------------------------L--------------------------------------TPDSD----------
---------DR------KTV-----QSVG---QLAHALCSERL----------E--
------DSSVLVKVFWPKG-------------R----CAL--L---
RDDVVLLDSPGI---DV---TPDLD--------SWIDEHCLD------------
ADVFVLVANSEST---LM-RT--EKNFFHTVSERLSKPNIFILNNRWDASASE--------PEFMEAVKKQH-
-L--------------------------------E--------------------------------RCVS--------------------------------FL--
VEELGVV--D---R----LQAE---DRVFF--------VSA-------KE---------
-----------AL----------------QS-R------L--------------------------QKQ-QGM-----PEEGGALAEGF------
------
>NP_001121726.1
--------EVLSRRHMKVVFFGR----T----------------SNGKSSVIN-----A---ML-WDKVLPSG---
IGHTTNCFL---------RVE--G---
TDGN---E------SFL--------------------------------L--------------------------------------TEGSD----------
---------ER------KSV-----KTVN---QLAHALHQDED----------L--
------DAGSLVCVMWPKA-------------K----CAL--L---
RDDLVLVDSPGI---DV---TTELD--------SWIDKFCLD------------
ADVFVLVANSEST---LM-QT--EKSFFHKVNERLSSPNIFILNNRWDASANE--------
PEYMEEVRRQH--M--------------------------------D--------------------------------RCTS-----------------
----------------FL--VDELRVV--D---R----SHAG---DRIFF--------

VSA-------KE--------------------VL----------------QA-R------V--------------------------QKA-QGM-----
PEAGGALAEGF------------
>XP_025929938.1
--------EVLARRHMKVAFFGR----T----------------SNGKSTVIN-----A---ML-WDKVLPSG---
IGHTTNCFL---------RVE--G--
TDGH---E------AFL---------------------------------L--TEGSE----------
---------EK------KSV-----KTVN---QLAHALHQDEL---------L--
-----DAGSLVSVMWPNS------------K----CPL--L---
KDDLVLMDSPGI--DV---TTELD--------SWIDKFCLD------------
ADVFVLVANSEST---LM-QT--EKQFFHKVNERLSRPNIFILNNRWDASASE--------
PEYMEEVRRQH--M---------------------------------E----------------------------------RCTS----------------
----------------FL--VDELGVV--D---R----AQAG---DRIFF--------
VSA-------KE--------------------VL----------------NA-R------I--------------------------QRA-QGM-----
PEGGGALADGF------------
>XP_004482574.1
--------EVLARRHMKVAFFGR----T----------------SNGKSTVIN-----A---ML-WDKVLPSG---
IGHTTNCFL---------RVG--G--
TDGH---E------AFL---------------------------------L--TEGSE----------
---------EK------RSV-----KTVN---QLAHALHQDEQ---------L--
------HAGSLVSVMWPNS------------K----CSL--L---
KDDLVLMDSPGI--DV---TTELD--------SWIDKFCLD------------
ADVFVLVANSEST---LM-QT--EKQFFHKVSERLSRPNIFILNNRWDASASE--------
PEYMEEVRRQH--M---------------------------------E----------------------------------RCTS----------------
----------------FL--VDELGVV--D---R----AQAG---DRIFF--------
VSA-------KE--------------------VL----------------NA-R------I--------------------------QKA-QGM-----
PEGGGALAEGF------------
>XP_006145367.1
--------EVLARRHMKVAFFGR----T----------------SNGKSTVIN-----A---ML-WDKVLPSG---
IGHTTNCFL---------RVE--G--
TDGH---E------AFL---------------------------------L--TEGSE----------
---------EK------RSV-----KTVN---QLAHALHQDEQ---------L--
------HAGSLVSVMWPNS------------K----CPL--L---
KDDLVLMDSPGI--DV---TTELD--------SWIDKFCLD------------
ADVFVLVANSEST---LM-QT--EKQFFHKVSERLSRPNIFILNNRWDASASE--------
PEYMEEVRRQH--M---------------------------------E----------------------------------RCTS----------------
----------------FL--VDELGVV--D---R----AQAG---DRIFF--------
VSA-------KE--------------------VL----------------NA-R------I--------------------------QKA-QGM-----
PEGGGALAEGF------------
>XP_014400986.1
--------EVLARRHMKVAFFGR----T----------------SNGKSTVIN-----A---ML-WDKVLPSG---
IGHTTNCFL---------RVE--G--
TDGH---E------AFL---------------------------------L--TEGSE----------
---------EK------RSI-----KTVN---QLAHALHQDEQ---------L--
-----HAGSLVSVMWPNS------------K----CPL--L---

KDDLVLMDSPGI---DV---TTELD--------SWIDKFCLD------------
ADVFVLVANSEST---LM-QT--EKQFFHKVSERLSRPNIFILNNRWDASASE--------
PEYMEEVRRQH--M--------------------------------E---------------------------------RCTS----------------
----------------FL--VDELGVV--D--R----GQAG---DRIFF--------
VSA-------KE--------------------AL----------------NA-R------I--------------------------QKA-QGM-----
PEGGGALAEGF------------
>NP_001177198.1
--------EVLARRHMKVAFFGR----T----------------SNGKSTVIN-----A---ML-WDKVLLSG---
IGHTTNCFL---------RVE--G--
TDGH---E------AFL--------------------------------L---------------------------------------TEGSE----------
---------EK------RSV-----KTVN---QLAHALHQDEQ----------L--
------HAGSLVSVMWPNS-------------K----CPL---L---
KDDLVLMDSPGI---DV---TTELD--------SWIDKFCLD------------
ADVFVLVANSEST---LM-QT--EKQFFHKVSERLSRPNIFILNNRWDASASE--------
PEYMEEVRRQH--M--------------------------------E---------------------------------RCTS----------------
----------------FL--VDELGVV--D--R----GQAG---DRIFF--------
VSA-------KE--------------------VL----------------NA-R------I--------------------------QKA-QGM-----
PEGGGALAEGF------------
>NP_001272849.1
--------EVLARRHMKVAFFGR----T----------------SNGKSTVIN-----A---ML-WDKVLPSG---
IGHTTNCFL---------RVG--G--
TDGH---E------AFL--------------------------------L---------------------------------------TEGSE----------
---------EK------KSV-----KTVN---QLAHALHQDEQ----------L--
------HAGSMVSVMWPNS-------------K----CPL---L---
KDDLVLMDSPGI---DV---TTELD--------SWIDKFCLD------------
ADVFVLVANSEST---LM-QT--EKQFFHKVSERLSRPNIFILNNRWDASASE--------
PEYMEEVRRQH--M--------------------------------E---------------------------------RCTS----------------
----------------FL--VDELGVV--D--R----AQAG---DRIFF--------
VSA-------KE--------------------VL----------------SA-R------V--------------------------QKA-QGM-----
PEGGGALAEGF------------
>NP_001121132.1
--------EVLARRHMKVAFFGR----T----------------SNGKSTVIN-----A---ML-WDKVLPSG---
IGHTTNCFL---------RVE--G--
TDGH---E------AFL--------------------------------L---------------------------------------TEGSE----------
---------EK------RSA-----KTVN---QLAHALHQDKQ----------L--
------HAGSLVSVMWPNS-------------K----CPL---L---
KDDLVLMDSPGI---DV---TTELD--------SWIDKFCLD------------
ADVFVLVANSEST---LM-QT--EKHFFHKVSERLSRPNIFILNNRWDASASE--------
PEYMEEVRRQH--M--------------------------------E---------------------------------RCTS----------------
----------------FL--VDELGVV--D--R----SQAG---DRIFF--------
VSA-------KE--------------------VL----------------NA-R------I--------------------------QKA-QGM-----
PEGGGALAEGF------------
>XP_015268039.1

--------EVLARRHMKVAFFGR----T---------------SNGKSTVIN-----A---ML-WDKVLPSG---
IGHTTNCFL---------RVE--G--
TDGH---D------AFL--------------------------------L---TEGSE----------
---------XX------XXX-----XTVN---QLAHALHQDEL----------L--
-----TAGSLVSVMWPNS-------------K----CPL---L---
KDDLVLMDSPGI---DV---TTELD--------SWIDKFCLD------------
ADVFVLVANSEST---LM-QT--VRRQH--M--------------------
----------E-------------------------------RCTS--------------------------------FL--VDELGVV--D----------
-------------------------------R----AQAG---DRIFF--------VSA-------KE-------------------VL------------
----SA-R------I--------------------------QKA-QGM-----PEGGGALAEGF------------
>XP_028597443.1
--------QVLARRHMKVAFFGR----T---------------SNGKSTVIN-----A---ML-WDKVLPSG---
IGHTTNCFL---------RVE--G--
TEGQ---D------AFL--------------------------------L---TEGSE----------
---------EK------KSV-----KTVN---QLAHALHQDEL----------L--
-----TAGGMVSVMWPNS-------------K----CPL---L---
KDDLVLMDSPGI---DV---TTELD--------SWIDKFCLD------------
ADVFVLVANSEST---LM-QT--EKQFFHKVNTRLSRPNIFILNNRWDASASE--------
PEYMEEVRRQH--M--------------------------------E---------------------------------RCTS----------------
----------------FL--VDELGVV--D---R----AQAG---DRIFF--------
VSA-------KE-------------------VL----------------SA-R------I--------------------------QKA-QGM-----
PEGGGALAEGF------------
>XP_017213868.2.2
--------EVLARRHMKVAFFGR----T---------------SNGKSTVIN-----A---ML-RDRVLPSG---
IGHTTNCFL---------SVE--G--
TDED---K------AFL--------------------------------K---TEGSE-----------
--------EE------KSI-----KTVN---QLAHALHMDES----------L--
----DAGCLVKVFWPKT-------------K----CAL---L---
RDDLVLVDSPGT---DV---TTELD--------SWIDKFCLD------------
ADVFVLVANSEST---LM-NT--EKHFFHKVNEKLSKPNIFILNNRWDASAAE--------
PEYMEDVRKQH--T--------------------------------D---------------------------------RCVN----------------
----------------FL--VEELKVV--D---R----AQAP---NRIFF--------
VSA-------KE-------------------VL----------------NS-R------M--------------------------QRA-QGM-----
PETGGALAEGF------------
>NP_001016189.1
--------EVLARRNMKVAFFGR----T---------------SSGKSTVIN-----S---ML-WDKVLPSG---
IGHTTNCFL---------SVE--G--
TEGD---K------AYL--------------------------------M---TEGSE----------
---------EK------KSV-----KTVN---QLAHALHMDKD----------L--
------GAGCLVHVFWPKA-------------K----CAL---L---
RDDLVLVDSPGT---DV---TTELD--------SWIDKFCLD------------
ADVFVLVANSEST---LM-NT--EKHFFYKVNEKLSKPNIFILNNRWDASASE--------
PEYMEDVRKQH--M--------------------------------E---------------------------------RCQS---------------
-----------------FL--VDELKVV--D---S----LEAQ---KRIFF--------

VSA-------KE-------------------VL---------------NA-R------M------------------------HKA-QGM-----
PEAGAALAEGF------------
>XP_028587453.1
--------GVLARRHMKVAFFGR----T---------------SSGKSSVIN-----A---ML-WDRVLPSG---
IGHTTNCFL---------SVE--G---
TDGD---K------AYL-------------------------------M--------------------------------------TEGSD---------
----------EK------KSV-----KTVN---QLAHALHMDKD----------L---
-------EAGCLVHVFWPKA------------K----CAL---L---
RDDLVLVDSPGT--DV---TTELD--------TWIDKFCLD------------
ADVFVLVANSEST---LM-NT--EKHFFHKVNEKLSKPNIFILNNRWDASASE--------
PEYMEHVRKQH--M------------------------------E--------------------------------RCLT----------------
---------------FL--VDELKVV--D---P----SEAQ---NRIFF--------
VSA-------KE-------------------VL---------------SA-R------K------------------------QRA-QGM-----
PEGGGALADGF------------
>XP_025917892.1
--------EVLSRRHMKVAFFGR----T---------------SSGKSSVIN-----A---ML-WDKVLPSG---
IGHTTNCFL---------SVE--G---
TDGD---K------AYL-------------------------------M--------------------------------------TEGSD---------
----------EK------KSV-----KTVN---QLAHALHMDKD----------L---
-------KAGCLVHVFWPKS------------K----CAL---L---
RDDLVLVDSPGT--DV---TTELD--------SWIDKFCLD------------
ADVFVLVANSEST---LM-NT--EKHFFHKVNERLSKPNIFILNNRWDASASE--------
PEYMEDVRRQH--M------------------------------E--------------------------------RCLT----------------
---------------FL--VDELKVI--D---P----IEAR---NRIFF--------VSA--
-----KE-------------------VL---------------SA-R------R------------------------QKA-QGM-----
PAGGEALAEGF------------
>XP_004479029.1
--------EVLSRRHMKVAFFGR----T---------------SSGKSSVIN-----A---ML-WDKVLPSG---
IGHTTNCFL---------SVE--G---
TDGD---K------AYL-------------------------------M--------------------------------------TEGSD---------
----------EK------KSV-----KTVN---QLAHALHMDND----------L---
-------KAGCLVHVFWPKA------------K----CAL---L---
RDDLVLVDSPGT--DV---TTELD--------SWIDKFCLD------------
ADVFVLVANSEST---LM-NT--EKQFFHKVNERLSKPNIFILNNRWDASASE--------
PEYMEDVRRQH--M------------------------------E-------------------------------RCLN--------------
-----------------FL--VEELKVV--N--P----LEAQ---NRIFF--------
VSA-------KE-------------------VL---------------SA-R------K------------------------HKA-QGM-----
PEGGGALADGF------------
>NP_001193437.1
--------EVLSRRHMKVAFFGR----T---------------SSGKSSVIN-----A---ML-WDKVLPSG---
IGHTTNCFL---------SVE--G---
TDGD---K------AYL-------------------------------M--------------------------------------TEGSD---------
----------EK------RSV-----KTVN---QLAHALHMDKD----------L---
-------KAGCLVHVFWPKA------------K----CAL---L---

RDDLVLVDSPGT---DV---TTELD--------SWIDKFCLD------------
ADVFVLVANSEST---LM-NT--EKQFFHKVNERLSKPNIFILNNRWDASASE--------
PEYMEDVRRQH--M--------------------------------E---------------------------------RCLH---------------
-----------------FL--VEELRVV--D--P----LEAR---NRIFF--------
VSA-------KE--------------------VL----------------SA-R------K--------------------------HKA-QGM-----
PEGGGALAEGF------------
>NP_077162.2.2
--------EVLSRRHMKVAFFGR----T----------------SSGKSSVIN-----A---ML-WDKVLPSG---
IGHTTNCFL---------SVE--G--
TDGD---K------AYL--------------------------------M---------------------------------------TEGSD---------
----------EK------KSV-----KTVN---QLAHALHMDKD----------L---
-------KAGCLVHVFWPKA-------------K----CAL---L---
RDDLVLVDSPGT---DV---TTELD--------IWIDKFCLD------------
ADVFVLVANSEST---LM-NT--EKHFFHKVNERLSKPNIFILNNRWDASASE--------
PEYMEDVRRQH--M--------------------------------E---------------------------------RCLH---------------
-----------------FL--VEELKVV--S--P----SEAR---NRIFF--------
VSA-------KE--------------------VL----------------NS-R------K--------------------------HKA-QGM-----
PEGGGALAEGF------------
>XP_006162789.1
--------EVLSRRHMKVAFFGR----T----------------SSGKSSVIN-----A---ML-WDKVLPSG---
IGHTTNCFL---------SVE--G--
TDGD---K------AYL--------------------------------M---------------------------------------TEGSD---------
----------EK------KSV-----KTVN---QLAHALHMDKD----------L---
-------KAGCLVHVFWPKA-------------K----CAL---L---
RDDLVLVDSPGT---DV---TTELD--------SWIDKFCLD------------
ADVFVLVANSEST---LM-NT--EKHFFHKVNERLSKPNIFILNNRWDASASE--------
PEYMEDVRRQH--M--------------------------------E---------------------------------RCLH---------------
-----------------FL--VEELKVV--G--P----SEAR---NRIFF--------
VSA-------KE--------------------VL----------------SA-R------K--------------------------HRA-QGM-----
PEGGGALAEGF------------
>XP_005883071.1
--------EVLSRRHMKVAFFGR----T----------------SSGKSSVIN-----A---ML-WDKVLPSG---
IGHTTNCLL---------SVE--G--
TDGD---R------AYL--------------------------------M---------------------------------------TEGSD---------
----------EK------KSV-----KTVN---QLAHALHMDKD----------L---
-------KAGSLVHVFWPKA-------------K----CAL---L---
RDDLVLVDSPGT---DV---TTELD--------SWIDKFCLD------------
ADVFVLVANSEST---LM-NT--EKQFFHKVNERLSKPNIFILNNRWDASASE--------
PEYMEDVRRQH--T--------------------------------E---------------------------------RCLH-----------------
---------------FL--VEELKVV--D--P----SEAR---NRIFF--------VSA-
------KE--------------------VL----------------SA-R------M--------------------------HKA-QGM-----
PEGGGALAEGF------------
>NP_284941.2.2

--------EVLSRRHMKVAFFGR----T----------------SSGKSSVIN-----A---ML-WDKVLPSG---
IGHITNCFL---------SVE--G--
TDGD---K------AYL---------------------------------M--TEGSD---------
----------EK------KSV-----KTVN---QLAHALHMDKD---------L--
-------KAGCLVRVFWPKA------------K----CAL--L---
RDDLVLVDSPGT--DV---TTELD--------SWIDKFCLD------------
ADVFVLVANSEST---LM-NT--EKHFFHKVNERLSKPNIFILNNRWDASASE--------
PEYMEDVRRQH--M---------------------------------E----------------------------------RCLH---------------
-----------------FL--VEELKVV--N---A----LEAQ---NRIFF--------
VSA-------KE--------------------VL----------------SA-R------K--------------------------QKA-QGM-----
PESGVALAEGF------------
>XP_030846906.1
--------DVLERDHMKVAFFGR----T----------------SNGKSTVIN-----A---ML-RDKVLPSG---
IGHTTDCFL---------CVE--G--
CEGQ---E------GYM---------------------------------S--RQNSS--------
-----------EK------IST-----TSVS---QLANALAGERDH-------EDF---
--------QQRSILHIFWPKT------------Q----CHL--L---
KNDVVLLDSPGI--DV---EHDMD--------EWIDDHYMD-----------
-ADVFVLVSNAEST---LT-RT--ETSFFLKVSAKLSKPNIFILNNRWDASANE--------PENMEVVKRQH--
L---------------------------------E----------------------------------REIK--------------------------------FL--
VEELKVM--T---E----AQAK---DRIFF--------VSA-------KE----------
----------AL----------------NS-R------I--------------------------LQT-LST-----PNAN-PIVEGY------------
>XP_030847518.1
--------DVLERDHMKVAFFGR----T----------------SNGKSTVIN-----A---ML-RDKVLPSG---
IGHTTDCFL---------CVE--G--
CEGQ---E------GYM---------------------------------S--RQNSS--------
-----------EK------MSI-----TSVS---QLAHALAGERDH-------EEC--
---------QQSSILHIFWPKT------------Q----CHL--L---
MNDVVLLDSPGI--DV---EQDLD--------EWINTHCVD------------
ADVFVLVLNAEST---LM-RT--EKSFFHKVSEKLSKPNIFILNNRWDASANE--------PEFMEAVKRQH-
-L---------------------------------E----------------------------------RDVK--------------------------------FL--
VEELKVM--T---E----AQAK---DRVFF--------VSA-------KE---------
-----------AL----------------NS-R------I--------------------------PKT-LST-----PDAN-PVVEGY-----------
-
>NP_996357.1
--------EVLQRDHMKVAFFGR----T----------------SNGKSSVIN-----A---ML-REKILPSG---
IGHTTNCFC---------QVE--G--
SNGG---E------AYL---------------------------------M--TEGSE---------
----------EK------LNV-----VNIK---QLANALCQEK----------L--
-----CESSLVRIFWPRE------------R----CSL--L---
RDDVVFVDSPGV--DV---SANLD--------DWIDNHCLN-----------
-ADVFVLVLNAEST---MT-RA--EKQFFHTVSQKLSKPNIFILNNRWDASANE--------
PECQESVKSQH--T--------------------------------E----------------------------------RCID-----------------
-------------FL--TKELKVS--N---E----KEAA---ERVFF--------VSA-

------RE-------------------TL----------------QA-R------I-------------------------EEA-KGN-----
PPHMGAIAEGF------------
>XP_004365821.1
--------KLFERDHMKVVFVGQ----T----------------SNGKSTVVN-----A---ML-YNRILPSG---
IGHTTNCFV---------SVS--G---
SDAN---T------PYI--------------------------------I-------------------------------------DSLSS------------
-------EQ------QPI-----SNVL---QLANALHPEGS----------L--
----NQSGLIRVFWPTT-------------K----CRL--L---
GDDVDLIDSPGL--DL---SNDIN--------QWIDDYCMD-----------
ADVFVLVANAEAT---LK-VA--ERAFFFKVNEKLSKPNVFILNNRWDASDNEI-------
DDSPERVREQH--L-------------------------------E--------------------------------YASK-----------------
--------------FL--ADELKVV--S---R----SKIL---DRVFF--------VSA--
-----RE-------------------TL----------------LY-R------T---T---------------------ENW-TRF-----KESQAV-
----
>XP_014153836.1
---------MLRRDSMKVVFVGH----T----------------SNGKSTVIN-----S---ML-GQKVLPMG---
IGHTTSCFC---------SVT--G---
TDEE----------PYI--------------------------------ILG--------------
HEPKAKNSNSIRLNRVQRSDSPPSPPPE-------------------KR------MAI-----DNVK---
TVANALCPESD----------H---DAYQFVRVFWDKR---------
----K----CNL--L---GDGVLFVDTPGL-----------------------------
-----------DI---DENYD--------NWIDKFCMD------------ADVFVLVANGEST---IK-HT--
EMNFFTKVAEKLSRPNVFILFNRWDGSDME---------DDVTPVQEQH--K-----------------------------
--D-------------------------------RVRS------------------------------FF--KKELQQ---D-----------------
------------------------A----NIID---KRVFF--------VSG-------KE--------------------VL----------------
TH-R------T---K---------------------PD--KSVVKESNPSPMG------------------
