# Supplementary material for: Antiviral Mx proteins have an ancient origin and widespread distribution among eukaryotes
Source: Proc Natl Acad Sci U S A. 2025 Jan 24;122(4):e2416811122. doi: 10.1073/pnas.2416811122 (PMC11789081; doi:10.1073/pnas.2416811122)
Supplement: Supplementary file 9 — Dataset S08 (PDF) [file pnas.2416811122.sd08.pdf]

Dataset S8. Figure_3_FastTree

#NEXUS
begin taxa;
dimensions ntax=549;
taxlabels
GMI12809.1[&Organism="Triparma laevis f. longispina",Description="hypothetical protein TrLO_g525 [Triparma laevis f. longispina]","Genetic Code"="Standard",Taxonomy="Eukaryota; Sar; Stramenopiles; Ochrophyta; Bolidophyceae; Parmales; Triparmaceae; Triparma"]
GMI47362.1[&Organism="Triparma columacea",Description="hypothetical protein TrCOL_g11230 [Triparma columacea]","Genetic Code"="Standard",Taxonomy="Eukaryota; Sar; Stramenopiles; Ochrophyta; Bolidophyceae; Parmales; Triparmaceae; Triparma"]
OQR85161.1[&Organism="Achlya hypogyna",Description="dynamin-2 [Achlya hypogyna]","Genetic Code"="Standard",Taxonomy="Eukaryota; Sar; Stramenopiles; Oomycota; Saprolegniales; Saprolegniaceae; Achlya"]
RLO06844.1[&Organism="Aphanomyces astaci",Description="hypothetical protein DYB28_000925, partial [Aphanomyces astaci]","Genetic Code"="Standard",Taxonomy="Eukaryota; Sar; Stramenopiles; Oomycota; Saprolegniales; Saprolegniaceae; Aphanomyces"]
KAF0740912.1[&Organism="Aphanomyces euteiches",Description="hypothetical protein Ae201684_003801 [Aphanomyces euteiches]","Genetic Code"="Standard",Taxonomy="Eukaryota; Sar; Stramenopiles; Oomycota; Saprolegniales; Saprolegniaceae; Aphanomyces"]
CAH0521473.1[&Organism="Peronospora belbahrii",Description="unnamed protein product [Peronospora belbahrii]","Genetic Code"="Standard",Taxonomy="Eukaryota; Sar; Stramenopiles; Oomycota; Peronosporales; Peronosporaceae; Peronospora"]
KAG2764740.1[&Organism="Phytophthora cactorum",Description="Dynamin-1-like protein [Phytophthora cactorum]","Genetic Code"="Standard",Taxonomy="Eukaryota; Sar; Stramenopiles; Oomycota; Peronosporales; Peronosporaceae; Phytophthora"]
GMF38092.1[&Organism="Phytophthora lilii",Description="unnamed protein product [Phytophthora lilii]","Genetic Code"="Standard",Taxonomy="Eukaryota; Sar; Stramenopiles; Oomycota; Peronosporales; Peronosporaceae; Phytophthora"]
KAH7500220.1[&Organism="Phytophthora ramorum",Description="Dynamin-1-like protein [Phytophthora ramorum]","Genetic Code"="Standard",Taxonomy="Eukaryota; Sar; Stramenopiles; Oomycota; Peronosporales; Peronosporaceae; Phytophthora","Common Name"="sudden oak death agent"]
TMW56688.1[&Organism="Pythium oligandrum",Description="hypothetical protein Poli38472_006698 [Pythium oligandrum]","Genetic Code"="Standard",Taxonomy="Eukaryota; Sar; Stramenopiles; Oomycota; Pythiales; Pythiaceae; Pythium"]
CCI11042.1[&Organism="Albugo candida",Description="unnamed protein product [Albugo candida]","Genetic Code"="Standard",Taxonomy="Eukaryota; Sar; Stramenopiles; Oomycota; Albuginales; Albuginaceae; Albugo"]

CCA17876.1[&Organism="Albugo laibachii Nc14",Description="dynamin GTPase putative [Albugo laibachii Nc14]","Genetic Code"="Standard",Taxonomy="Eukaryota; Sar; Stramenopiles; Oomycota; Albuginales; Albuginaceae; Albugo"]

CEP02405.1[&Organism="Plasmodiophora brassicae",Description="hypothetical protein PBRA_008989 [Plasmodiophora brassicae]","Genetic Code"="Standard",Taxonomy="Eukaryota; Sar; Rhizaria; Endomyxa; Phytomyxea; Plasmodiophorida; Plasmodiophoridae; Plasmodiophora"]

XP_044553261.1[&Organism="Naegleria lovaniensis",Description="uncharacterized protein C9374_014669 [Naegleria lovaniensis]","Genetic Code"="Standard",Taxonomy="Eukaryota; Discoba; Heterolobosea; Tetramitia; Eutetramitia; Vahlkampfiidae; Naegleria"]

XP_044569353.1[&Organism="Naegleria fowleri",Description="uncharacterized protein FDP41_000539 [Naegleria fowleri]","Genetic Code"="Standard",Taxonomy="Eukaryota; Discoba; Heterolobosea; Tetramitia; Eutetramitia; Vahlkampfiidae; Naegleria"]

XP_044550536.1[&Organism="Naegleria lovaniensis",Description="uncharacterized protein C9374_002288 [Naegleria lovaniensis]","Genetic Code"="Standard",Taxonomy="Eukaryota; Discoba; Heterolobosea; Tetramitia; Eutetramitia; Vahlkampfiidae; Naegleria"]

XP_002681690.1[&Organism="Naegleria gruberi",Description="dynamin [Naegleria gruberi]","Genetic Code"="Standard",Taxonomy="Eukaryota; Discoba; Heterolobosea; Tetramitia; Eutetramitia; Vahlkampfiidae; Naegleria"]

KAH3761456.1[&Organism="Pelomyxa schiedti",Description="Dynamin GTPase [Pelomyxa schiedti]","Genetic Code"="Standard",Taxonomy="Eukaryota; Amoebozoa; Evosea; Archamoebae; Pelobiontida; Pelomyxidae; Pelomyxa"]

PRP82407.1[&Organism="Planoprotostelium fungivorum",Description="dynamin-2 [Planoprotostelium fungivorum]","Genetic Code"="Standard",Taxonomy="Eukaryota; Amoebozoa; Evosea; Variosea; Cavosteliida; Cavosteliaceae; Planoprotostelium"]

XP_004368323.1[&Organism="Acanthamoeba castellanii str. Neff",Description="dynamin domain containing protein [Acanthamoeba castellanii str. Neff]","Genetic Code"="Standard",Taxonomy="Eukaryota; Amoebozoa; Discosea; Longamoebia; Centramoebida; Acanthamoebidae; Acanthamoeba"]

XP_012754660.1[&Organism="Acytostelium subglobosum LB1",Description="hypothetical protein SAMD00019534_057150 [Acytostelium subglobosum LB1]","Genetic Code"="Standard",Taxonomy="Eukaryota; Amoebozoa; Evosea; Eumycetozoa; Dictyostelia; Acytosteliales; Acytosteliaceae; Acytostelium"]

KYR01170.1[&Organism="Tieghemostelium lacteum",Description="dynamin like protein [Tieghemostelium lacteum]","Genetic Code"="Standard",Taxonomy="Eukaryota; Amoebozoa; Evosea; Eumycetozoa; Dictyostelia; Dictyosteliales; Raperosteliaceae; Tieghemostelium"]

XP_003294436.1[&Organism="Dictyostelium purpureum",Description="uncharacterized protein DICPUDRAFT_51521 [Dictyostelium purpureum]","Genetic Code"="Standard",Taxonomy="Eukaryota; Amoebozoa; Evosea; Eumycetozoa; Dictyostelia; Dictyosteliales; Dictyosteliaceae; Dictyostelium"]

PRP81066.1[&Organism="Planoprotostelium fungivorum",Description="hypothetical protein PROFUN_11144 [Planoprotostelium fungivorum]","Genetic Code"="Standard",Taxonomy="Eukaryota; Amoebozoa; Evosea; Variosea; Cavosteliida; Cavosteliaceae; Planoprotostelium"]

XP_004348308.1[&Organism="Capsaspora owczarzaki ATCC 30864",Description="dynamin central region family protein [Capsaspora owczarzaki ATCC 30864]","Genetic Code"="Standard",Taxonomy="Eukaryota; Filasterea; Capsaspora"]

NP_012926.1[&Organism="Saccharomyces cerevisiae S288C",Description="dynamin-like GTPase VPS1 [Saccharomyces cerevisiae S288C]","Genetic Code"="Standard",Taxonomy="Eukaryota; Fungi; Dikarya; Ascomycota; Saccharomycotina; Saccharomycetes; Saccharomycetales; Saccharomycetaceae; Saccharomyces"]

OUM62108.1[&Organism="Piromyces sp. E2",Description="hypothetical protein PIROE2DRAFT_51763 [Piromyces sp. E2]","Genetic Code"="Standard",Taxonomy="Eukaryota; Fungi; Fungi incertae sedis; Chytridiomycota; Chytridiomycota incertae sedis; Neocallimastigomycetes; Neocallimastigales; Neocallimastigaceae; Piromyces; unclassified Piromyces"]

KXN66323.1[&Organism="Conidiobolus coronatus NRRL 28638",Description="vacuolar dynamin-like GTPase-like protein VpsA [Conidiobolus coronatus NRRL 28638]","Genetic Code"="Standard",Taxonomy="Eukaryota; Fungi; Fungi incertae sedis; Zoopagomycota; Entomophthoromycotina; Entomophthoromycetes; Entomophthorales; Ancylistaceae; Conidiobolus"]

XP_011389257.1[&Organism="Ustilago maydis 521",Description="putative dynamin-like GTPase VPS1 [Ustilago maydis 521]","Genetic Code"="Standard",Taxonomy="Eukaryota; Fungi; Dikarya; Basidiomycota; Ustilaginomycotina; Ustilaginomycetes; Ustilaginales; Ustilaginaceae; Ustilago"]

XP_006458578.1[&Organism="Agaricus bisporus var. bisporus H97",Description="hypothetical protein AGABI2DRAFT_190843 [Agaricus bisporus var. bisporus H97]","Genetic Code"="Standard",Taxonomy="Eukaryota; Fungi; Dikarya; Basidiomycota; Agaricomycotina; Agaricomycetes; Agaricomycetidae; Agaricales; Agaricaceae; Agaricus"]

KNE68830.1[&Organism="Allomyces macrogynus ATCC 38327",Description="hypothetical protein AMAG_13468 [Allomyces macrogynus ATCC 38327]","Genetic Code"="Standard",Taxonomy="Eukaryota; Fungi; Fungi incertae sedis; Blastocladiomycota; Blastocladiomycota incertae sedis; Blastocladiomycetes; Blastocladiales; Blastocladiaceae; Allomyces"]

XP_748106.1[&Organism="Aspergillus fumigatus Af293",Description="vacuolar dynamin-like GTPase VpsA, putative [Aspergillus fumigatus Af293]","Genetic Code"="Standard",Taxonomy="Eukaryota; Fungi; Dikarya; Ascomycota; Pezizomycotina; Eurotiomycetes; Eurotiomycetidae; Eurotiales; Aspergillaceae; Aspergillus; Aspergillus subgen. Fumigati"]

OAJ44422.1[&Organism="Batrachochytrium dendrobatidis JEL423",Description="hypothetical protein BDEG_27650 [Batrachochytrium dendrobatidis JEL423]","Genetic Code"="Standard",Taxonomy="Eukaryota; Fungi; Fungi incertae sedis;

Chytridiomycota; Chytridiomycota incertae sedis; Chytridiomycetes; Rhizophydiales; Rhizophydiales incertae sedis; Batrachochytrium"]

KAJ1432693.1[&Organism="Ochromonadaceae sp. CCMP2298",Description="dynamin-2 [Ochromonadaceae sp. CCMP2298]","Genetic Code"="Standard",Taxonomy="Eukaryota; Sar; Stramenopiles; Ochrophyta; Synurophyceae; Ochromonadales; Ochromonadaceae"]

XP_004347890.1[&Organism="Capsaspora owczarzaki ATCC 30864",Description="dynamin 1 [Capsaspora owczarzaki ATCC 30864]","Genetic Code"="Standard",Taxonomy="Eukaryota; Filasterea; Capsaspora"]

NP_001024332.1[&Organism="Caenorhabditis elegans",Description="Dynamin [Caenorhabditis elegans]","Genetic Code"="Standard",Taxonomy="Eukaryota; Metazoa; Ecdysozoa; Nematoda; Chromadorea; Rhabditida; Rhabditina; Rhabditomorpha; Rhabditoidea; Rhabditidae; Peloderinae; Caenorhabditis"]

KMZ10000.1[&Organism="Drosophila melanogaster",Description="dynamin [Drosophila melanogaster]","Genetic Code"="Standard",Taxonomy="Eukaryota; Metazoa; Ecdysozoa; Arthropoda; Hexapoda; Insecta; Pterygota; Neoptera; Holometabola; Diptera; Brachycera; Muscomorpha; Ephydroidea; Drosophilidae; Drosophila; Sophophora","Common Name"="fruit fly"]

XP_030853442.1[&Organism="Strongylocentrotus purpuratus",Description="dynamin-1 isoform X4 [Strongylocentrotus purpuratus]","Genetic Code"="Standard",Taxonomy="Eukaryota; Metazoa; Echinodermata; Eleutherozoa; Echinozoa; Echinoidea; Euechinoidea; Echinacea; Echinoida; Strongylocentrotidae; Strongylocentrotus","Common Name"="purple sea urchin"]

XP_030853442.1.2[&Organism="Strongylocentrotus purpuratus",Description="dynamin-1 isoform X1 [Strongylocentrotus purpuratus]","Genetic Code"="Standard",Taxonomy="Eukaryota; Metazoa; Echinodermata; Eleutherozoa; Echinozoa; Echinoidea; Euechinoidea; Echinacea; Echinoida; Strongylocentrotidae; Strongylocentrotus","Common Name"="purple sea urchin"]

XP_005165639.1[&Organism="Danio rerio",Description="dynamin-1 isoform X3 [Danio rerio]","Genetic Code"="Standard",Taxonomy="Eukaryota; Metazoa; Chordata; Craniata; Vertebrata; Euteleostomi; Actinopterygii; Neopterygii; Teleostei; Ostariophysi; Cypriniformes; Cyprinidae; Danio","Common Name"="zebrafish"]

XP_028570166.1[&Organism="Podarcis muralis",Description="dynamin-1 isoform X14 [Podarcis muralis]","Genetic Code"="Standard",Taxonomy="Eukaryota; Metazoa; Chordata; Craniata; Vertebrata; Euteleostomi; Lepidosauria; Squamata; Bifurcata; Unidentata; Episquamata; Laterata; Lacertibaenia; Lacertidae; Podarcis","Common Name"="Common wall lizard"]

EPQ17174.1[&Organism="Myotis brandtii",Description="PREDICTED: dynamin-1 [Myotis brandtii]","Genetic Code"="Standard",Taxonomy="Eukaryota; Metazoa; Chordata; Craniata; Vertebrata; Euteleostomi; Mammalia; Eutheria; Laurasiatheria; Chiroptera; Microchiroptera; Vespertilionidae; Myotis","Common Name"="Brandt's bat"]

BAB27759.1[&Organism="Mus musculus",Description="dynamin-1 isoform X5 [Mus musculus]","Genetic Code"="Standard",Taxonomy="Eukaryota; Metazoa; Chordata; Craniata; Vertebrata; Euteleostomi; Mammalia; Eutheria; Euarchontoglires; Glires;

Rodentia; Myomorpha; Muroidea; Muridae; Murinae; Mus; Mus","Common Name"="house mouse"]

ELW62001.1[&Organism="Tupaia chinensis",Description="dynamin-1 isoform X3 [Tupaia chinensis]","Genetic Code"="Standard",Taxonomy="Eukaryota; Metazoa; Chordata; Craniata; Vertebrata; Euteleostomi; Mammalia; Eutheria; Euarchontoglires; Scandentia; Tupaiidae; Tupaia","Common Name"="Chinese tree shrew"]

EAW87759.1[&Organism="Homo sapiens",Description="dynamin-1 isoform 2 [Homo sapiens]","Genetic Code"="Standard",Taxonomy="Eukaryota; Metazoa; Chordata; Craniata; Vertebrata; Euteleostomi; Mammalia; Eutheria; Euarchontoglires; Primates; Haplorrhini; Catarrhini; Hominidae; Homo","Common Name"="human"]

XP_025915522.1[&Organism="Apteryx rowi",Description="dynamin-1 isoform X1 [Apteryx rowi]","Genetic Code"="Standard",Taxonomy="Eukaryota; Metazoa; Chordata; Craniata; Vertebrata; Euteleostomi; Archelosauria; Archosauria; Dinosauria; Saurischia; Theropoda; Coelurosauria; Aves; Palaeognathae; Apterygiformes; Apterygidae; Apteryx","Common Name"="Okarito brown kiwi"]

XP_012378586.1[&Organism="Dasypus novemcinctus",Description="dynamin-1, partial [Dasypus novemcinctus]","Genetic Code"="Standard",Taxonomy="Eukaryota; Metazoa; Chordata; Craniata; Vertebrata; Euteleostomi; Mammalia; Eutheria; Xenarthra; Cingulata; Dasypodidae; Dasypus","Common Name"="nine-banded armadillo"]

KAE8583055.1[&Organism="Xenopus tropicalis",Description="dynamin-1 isoform X18 [Xenopus tropicalis]","Genetic Code"="Standard",Taxonomy="Eukaryota; Metazoa; Chordata; Craniata; Vertebrata; Euteleostomi; Amphibia; Batrachia; Anura; Pipoidea; Pipidae; Xenopodinae; Xenopus; Silurana","Common Name"="tropical clawed frog"]

XP_032814666.1[&Organism="Petromyzon marinus",Description="dynamin-1-like isoform X25 [Petromyzon marinus]","Genetic Code"="Standard",Taxonomy="Eukaryota; Metazoa; Chordata; Craniata; Vertebrata; Cyclostomata; Hyperoartia; Petromyzontiformes; Petromyzontidae; Petromyzon","Common Name"="sea lamprey"]

XP_025944940.1[&Organism="Apteryx rowi",Description="dynamin-3 isoform X1 [Apteryx rowi]","Genetic Code"="Standard",Taxonomy="Eukaryota; Metazoa; Chordata; Craniata; Vertebrata; Euteleostomi; Archelosauria; Archosauria; Dinosauria; Saurischia; Theropoda; Coelurosauria; Aves; Palaeognathae; Apterygiformes; Apterygidae; Apteryx","Common Name"="Okarito brown kiwi"]

EPQ08653.1[&Organism="Myotis brandtii",Description="PREDICTED: dynamin-3 isoform X1 [Myotis brandtii]","Genetic Code"="Standard",Taxonomy="Eukaryota; Metazoa; Chordata; Craniata; Vertebrata; Euteleostomi; Mammalia; Eutheria; Laurasiatheria; Chiroptera; Microchiroptera; Vespertilionidae; Myotis","Common Name"="Brandt's bat"]

XP_006496668.1[&Organism="Mus musculus",Description="dynamin-3 isoform X6 [Mus musculus]","Genetic Code"="Standard",Taxonomy="Eukaryota; Metazoa; Chordata; Craniata; Vertebrata; Euteleostomi; Mammalia; Eutheria; Euarchontoglires; Glires; Rodentia; Myomorpha; Muroidea; Muridae; Murinae; Mus; Mus","Common Name"="house mouse"]

XP_016856477.1[&Organism="Homo sapiens",Description="dynamin-3 isoform d [Homo sapiens]","Genetic Code"="Standard",Taxonomy="Eukaryota; Metazoa; Chordata;

Craniata; Vertebrata; Euteleostomi; Mammalia; Eutheria; Euarchontoglires; Primates; Haplorrhini; Catarrhini; Hominidae; Homo","Common Name"="human"]

XP_027623811.1[&Organism="Tupaia chinensis",Description="dynamin-3 isoform X3 [Tupaia chinensis]","Genetic Code"="Standard",Taxonomy="Eukaryota; Metazoa; Chordata; Craniata; Vertebrata; Euteleostomi; Mammalia; Eutheria; Euarchontoglires; Scandentia; Tupaiidae; Tupaia","Common Name"="Chinese tree shrew"]

XP_012379251.1[&Organism="Dasypus novemcinctus",Description="dynamin-3 [Dasypus novemcinctus]","Genetic Code"="Standard",Taxonomy="Eukaryota; Metazoa; Chordata; Craniata; Vertebrata; Euteleostomi; Mammalia; Eutheria; Xenarthra; Cingulata; Dasypodidae; Dasypus","Common Name"="nine-banded armadillo"]

XP_031753735.1[&Organism="Xenopus tropicalis",Description="dynamin-2 isoform X2 [Xenopus tropicalis]","Genetic Code"="Standard",Taxonomy="Eukaryota; Metazoa; Chordata; Craniata; Vertebrata; Euteleostomi; Amphibia; Batrachia; Anura; Pipoidea; Pipidae; Xenopodinae; Xenopus; Silurana","Common Name"="tropical clawed frog"]

XP_028568434.1[&Organism="Podarcis muralis",Description="dynamin-2 isoform X7 [Podarcis muralis]","Genetic Code"="Standard",Taxonomy="Eukaryota; Metazoa; Chordata; Craniata; Vertebrata; Euteleostomi; Lepidosauria; Squamata; Bifurcata; Unidentata; Episquamata; Laterata; Lacertibaenia; Lacertidae; Podarcis","Common Name"="Common wall lizard"]

XP_025920181.1[&Organism="Apteryx rowi",Description="dynamin-2 isoform X1 [Apteryx rowi]","Genetic Code"="Standard",Taxonomy="Eukaryota; Metazoa; Chordata; Craniata; Vertebrata; Euteleostomi; Archelosauria; Archosauria; Dinosauria; Saurischia; Theropoda; Coelurosauria; Aves; Palaeognathae; Apterygiformes; Apterygidae; Apteryx","Common Name"="Okarito brown kiwi"]

XP_006510037.1[&Organism="Mus musculus",Description="dynamin-2 isoform X18 [Mus musculus]","Genetic Code"="Standard",Taxonomy="Eukaryota; Metazoa; Chordata; Craniata; Vertebrata; Euteleostomi; Mammalia; Eutheria; Euarchontoglires; Glires; Rodentia; Myomorpha; Muroidea; Muridae; Murinae; Mus; Mus","Common Name"="house mouse"]

NP_001005360.1[&Organism="Homo sapiens",Description="dynamin-2 isoform 4 [Homo sapiens]","Genetic Code"="Standard",Taxonomy="Eukaryota; Metazoa; Chordata; Craniata; Vertebrata; Euteleostomi; Mammalia; Eutheria; Euarchontoglires; Primates; Haplorrhini; Catarrhini; Hominidae; Homo","Common Name"="human"]

XP_014389433.1[&Organism="Myotis brandtii",Description="PREDICTED: dynamin-2 isoform X6 [Myotis brandtii]","Genetic Code"="Standard",Taxonomy="Eukaryota; Metazoa; Chordata; Craniata; Vertebrata; Euteleostomi; Mammalia; Eutheria; Laurasiatheria; Chiroptera; Microchiroptera; Vespertilionidae; Myotis","Common Name"="Brandt's bat"]

XP_006161648.2.2[&Organism="Tupaia chinensis",Description="dynamin-2 [Tupaia chinensis]","Genetic Code"="Standard",Taxonomy="Eukaryota; Metazoa; Chordata; Craniata; Vertebrata; Euteleostomi; Mammalia; Eutheria; Euarchontoglires; Scandentia; Tupaiidae; Tupaia","Common Name"="Chinese tree shrew"]

XP_012381548.1[&Organism="Dasypus novemcinctus",Description="dynamin-2 [Dasypus novemcinctus]","Genetic Code"="Standard",Taxonomy="Eukaryota; Metazoa;

Chordata; Craniata; Vertebrata; Euteleostomi; Mammalia; Eutheria; Xenarthra; Cingulata; Dasypodidae; Dasypus","Common Name"="nine-banded armadillo"]

NP_001025299.1[&Organism="Danio rerio",Description="dynamin-3 [Danio rerio]","Genetic Code"="Standard",Taxonomy="Eukaryota; Metazoa; Chordata; Craniata; Vertebrata; Euteleostomi; Actinopterygii; Neopterygii; Teleostei; Ostariophysi; Cypriniformes; Cyprinidae; Danio","Common Name"="zebrafish"]

XP_021326548.1[&Organism="Danio rerio",Description="dynamin-2 isoform X5 [Danio rerio]","Genetic Code"="Standard",Taxonomy="Eukaryota; Metazoa; Chordata; Craniata; Vertebrata; Euteleostomi; Actinopterygii; Neopterygii; Teleostei; Ostariophysi; Cypriniformes; Cyprinidae; Danio","Common Name"="zebrafish"]

XP_035683496.1[&Organism="Branchiostoma floridae",Description="hypothetical protein BRAFLDRAFT_121263, partial [Branchiostoma floridae]","Genetic Code"="Standard",Taxonomy="Eukaryota; Metazoa; Chordata; Cephalochordata; Branchiostomidae; Branchiostoma","Common Name"="Florida lancelet"]

XP_026693152.1[&Organism="Ciona intestinalis",Description="dynamin-1 isoform X15 [Ciona intestinalis]","Genetic Code"="Standard",Taxonomy="Eukaryota; Metazoa; Chordata; Tunicata; Ascidiacea; Enterogona; Phlebobranchia; Cionidae; Ciona","Common Name"="vase tunicate"]

PAA65118.1[&Organism="Macrostomum lignano",Description="hypothetical protein BOX15_Mlig013747g2 [Macrostomum lignano]","Genetic Code"="Standard",Taxonomy="Eukaryota; Metazoa; Platyhelminthes; Rhabditophora; Macrostomorpha; Macrostomida; Macrostomidae; Macrostomum"]

PAA78248.1[&Organism="Macrostomum lignano",Description="hypothetical protein BOX15_Mlig010364g2 [Macrostomum lignano]","Genetic Code"="Standard",Taxonomy="Eukaryota; Metazoa; Platyhelminthes; Rhabditophora; Macrostomorpha; Macrostomida; Macrostomidae; Macrostomum"]

PAA59145.1[&Organism="Macrostomum lignano",Description="hypothetical protein BOX15_Mlig005677g1 [Macrostomum lignano]","Genetic Code"="Standard",Taxonomy="Eukaryota; Metazoa; Platyhelminthes; Rhabditophora; Macrostomorpha; Macrostomida; Macrostomidae; Macrostomum"]

PAA64382.1[&Organism="Macrostomum lignano",Description="hypothetical protein BOX15_Mlig016602g2 [Macrostomum lignano]","Genetic Code"="Standard",Taxonomy="Eukaryota; Metazoa; Platyhelminthes; Rhabditophora; Macrostomorpha; Macrostomida; Macrostomidae; Macrostomum"]

XP_001749319.1[&Organism="Monosiga brevicollis MX1",Description="uncharacterized protein MONBRDRAFT_28892 [Monosiga brevicollis MX1]","Genetic Code"="Standard",Taxonomy="Eukaryota; Choanoflagellata; Craspedida; Salpingoecidae; Monosiga"]

XP_031757197.1[&Organism="Xenopus tropicalis",Description="dynamin-3 [Xenopus tropicalis]","Genetic Code"="Standard",Taxonomy="Eukaryota; Metazoa; Chordata; Craniata; Vertebrata; Euteleostomi; Amphibia; Batrachia; Anura; Pipoidea; Pipidae; Xenopodinae; Xenopus; Silurana","Common Name"="tropical clawed frog"]

XP_014148725.1[&Organism="Sphaeroforma arctica JP610",Description="hypothetical protein SARC_12638, partial [Sphaeroforma arctica

JP610]","Genetic Code"="Standard",Taxonomy="Eukaryota; Ichthyosporea; Ichthyophonida; Sphaeroforma"]

XP_014153758.1[&Organism="Sphaeroforma arctica JP610",Description="dynamin-3 [Sphaeroforma arctica JP610]","Genetic Code"="Standard",Taxonomy="Eukaryota; Ichthyosporea; Ichthyophonida; Sphaeroforma"]

XP_006812840.1[&Organism="Saccoglossus kowalevskii",Description="PREDICTED: dynamin-1-like, partial [Saccoglossus kowalevskii]","Genetic Code"="Standard",Taxonomy="Eukaryota; Metazoa; Hemichordata; Enteropneusta; Harrimaniidae; Saccoglossus"]

NP_013100.1[&Organism="Saccharomyces cerevisiae S288C",Description="dynamin-related GTPase DNM1 [Saccharomyces cerevisiae S288C]","Genetic Code"="Standard",Taxonomy="Eukaryota; Fungi; Dikarya; Ascomycota; Saccharomycotina; Saccharomycetes; Saccharomycetales; Saccharomycetaceae; Saccharomyces"]

XP_011392073.1[&Organism="Ustilago maydis 521",Description="dynamin-related GTPase DNM1 [Ustilago maydis 521]","Genetic Code"="Standard",Taxonomy="Eukaryota; Fungi; Dikarya; Basidiomycota; Ustilaginomycotina; Ustilaginomycetes; Ustilaginales; Ustilaginaceae; Ustilago"]

XP_006461708.1[&Organism="Agaricus bisporus var. bisporus H97",Description="hypothetical protein AGABI2DRAFT_185821 [Agaricus bisporus var. bisporus H97]","Genetic Code"="Standard",Taxonomy="Eukaryota; Fungi; Dikarya; Basidiomycota; Agaricomycotina; Agaricomycetes; Agaricomycetidae; Agaricales; Agaricaceae; Agaricus"]

XP_746923.1[&Organism="Aspergillus fumigatus Af293",Description="dynamin-like GTPase Dnm1, putative [Aspergillus fumigatus Af293]","Genetic Code"="Standard",Taxonomy="Eukaryota; Fungi; Dikarya; Ascomycota; Pezizomycotina; Eurotiomycetes; Eurotiomycetidae; Eurotiales; Aspergillaceae; Aspergillus; Aspergillus subgen. Fumigati"]

KXN67416.1[&Organism="Conidiobolus coronatus NRRL 28638",Description="hypothetical protein CONCODRAFT_19627 [Conidiobolus coronatus NRRL 28638]","Genetic Code"="Standard",Taxonomy="Eukaryota; Fungi; Fungi incertae sedis; Zoopagomycota; Entomophthoromycotina; Entomophthoromycetes; Entomophthorales; Ancylistaceae; Conidiobolus"]

KNE61418.1[&Organism="Allomyces macrogynus ATCC 38327",Description="hypothetical protein AMAG_06247 [Allomyces macrogynus ATCC 38327]","Genetic Code"="Standard",Taxonomy="Eukaryota; Fungi; Fungi incertae sedis; Blastocladiomycota; Blastocladiomycota incertae sedis; Blastocladiomycetes; Blastocladiales; Blastocladiaceae; Allomyces"]

KNE67543.1[&Organism="Allomyces macrogynus ATCC 38327",Description="hypothetical protein AMAG_11997 [Allomyces macrogynus ATCC 38327]","Genetic Code"="Standard",Taxonomy="Eukaryota; Fungi; Fungi incertae sedis; Blastocladiomycota; Blastocladiomycota incertae sedis; Blastocladiomycetes; Blastocladiales; Blastocladiaceae; Allomyces"]

GAX23670.1[&Organism="Fistulifera solaris",Description="dynamin GTPase [Fistulifera solaris]","Genetic Code"="Standard",Taxonomy="Eukaryota; Sar; Stramenopiles; Ochrophyta; Bacillariophyta; Bacillariophyceae; Bacillariophycidae; Naviculales; Naviculaceae; Fistulifera"]

CAB9512103.1[&Organism="Seminavis robusta",Description="Dynamin-1-like protein [Seminavis robusta]","Genetic Code"="Standard",Taxonomy="Eukaryota; Sar; Stramenopiles; Ochrophyta; Bacillariophyta; Bacillariophyceae; Bacillariophycidae; Naviculales; Naviculaceae; Seminavis"]

XP_002296064.1[&Organism="Thalassiosira pseudonana CCMP1335",Description="dynamin GTPase, switch/motor protein [Thalassiosira pseudonana CCMP1335]","Genetic Code"="Standard",Taxonomy="Eukaryota; Sar; Stramenopiles; Ochrophyta; Bacillariophyta; Coscinodiscophyceae; Thalassiosirophycidae; Thalassiosirales; Thalassiosiraceae; Thalassiosira"]

XP_004184473.1[&Organism="Entamoeba invadens IP1",Description="dynamin, putative [Entamoeba invadens IP1]","Genetic Code"="Standard",Taxonomy="Eukaryota; Amoebozoa; Evosea; Archamoebae; Mastigamoebida; Entamoebidae; Entamoeba"]

EMS16943.1[&Organism="Entamoeba histolytica HM-3:IMSS",Description="dynamin-1 family protein [Entamoeba histolytica HM-3:IMSS]","Genetic Code"="Standard",Taxonomy="Eukaryota; Amoebozoa; Evosea; Archamoebae; Mastigamoebida; Entamoebidae; Entamoeba"]

NP_741403.2[&Organism="Caenorhabditis elegans",Description="Dynamin GTPase [Caenorhabditis elegans]","Genetic Code"="Standard",Taxonomy="Eukaryota; Metazoa; Ecdysozoa; Nematoda; Chromadorea; Rhabditida; Rhabditina; Rhabditomorpha; Rhabditoidea; Rhabditidae; Peloderinae; Caenorhabditis"]

XP_002129967.2[&Organism="Ciona intestinalis",Description="dynamin-1-like protein [Ciona intestinalis]","Genetic Code"="Standard",Taxonomy="Eukaryota; Metazoa; Chordata; Tunicata; Ascidiacea; Phlebobranchia; Cionidae; Ciona","Common Name"="vase tunicate"]

NP_001259946.1[&Organism="Drosophila melanogaster",Description="dynamin related protein 1, isoform B [Drosophila melanogaster]","Genetic Code"="Standard",Taxonomy="Eukaryota; Metazoa; Ecdysozoa; Arthropoda; Hexapoda; Insecta; Pterygota; Neoptera; Endopterygota; Diptera; Brachycera; Muscomorpha; Ephydroidea; Drosophilidae; Drosophila; Sophophora","Common Name"="fruit fly"]

XP_035676386.1[&Organism="Branchiostoma floridae",Description="dynamin-1-like protein isoform X5 [Branchiostoma floridae]","Genetic Code"="Standard",Taxonomy="Eukaryota; Metazoa; Chordata; Cephalochordata; Leptocardii; Amphioxiformes; Branchiostomatidae; Branchiostoma","Common Name"="Florida lancelet"]

XP_006821224.1[&Organism="Saccoglossus kowalevskii",Description="PREDICTED: dynamin-1-like protein-like [Saccoglossus kowalevskii]","Genetic Code"="Standard",Taxonomy="Eukaryota; Metazoa; Hemichordata; Enteropneusta; Harrimaniidae; Saccoglossus"]

XP_030827871.1[&Organism="Strongylocentrotus purpuratus",Description="dynamin-1-like protein isoform X2 [Strongylocentrotus

purpuratus]","Genetic Code"="Standard",Taxonomy="Eukaryota; Metazoa; Echinodermata; Eleutherozoa; Echinozoa; Echinoidea; Euechinoidea; Echinacea; Camarodonta; Echinidea; Strongylocentrotidae; Strongylocentrotus","Common Name"="purple sea urchin"]

XP_032819300.1[&Organism="Petromyzon marinus",Description="dynamin-1-like protein isoform X2 [Petromyzon marinus]","Genetic Code"="Standard",Taxonomy="Eukaryota; Metazoa; Chordata; Craniata; Vertebrata; Cyclostomata; Hyperoartia; Petromyzontiformes; Petromyzontidae; Petromyzon","Common Name"="sea lamprey"]

NP_957216.1[&Organism="Danio rerio",Description="dynamin-1-like protein [Danio rerio]","Genetic Code"="Standard",Taxonomy="Eukaryota; Metazoa; Chordata; Craniata; Vertebrata; Euteleostomi; Actinopterygii; Neopterygii; Teleostei; Ostariophysi; Cypriniformes; Danionidae; Danioninae; Danio","Common Name"="zebrafish"]

XP_025940269.1[&Organism="Apteryx rowi",Description="dynamin-1-like protein isoform X4 [Apteryx rowi]","Genetic Code"="Standard",Taxonomy="Eukaryota; Metazoa; Chordata; Craniata; Vertebrata; Euteleostomi; Archelosauria; Archosauria; Dinosauria; Saurischia; Theropoda; Coelurosauria; Aves; Palaeognathae; Apterygiformes; Apterygidae; Apteryx","Common Name"="Okarito brown kiwi"]

XP_006168142.1[&Organism="Tupaia chinensis",Description="dynamin-1-like protein isoform X1 [Tupaia chinensis]","Genetic Code"="Standard",Taxonomy="Eukaryota; Metazoa; Chordata; Craniata; Vertebrata; Euteleostomi; Mammalia; Eutheria; Euarchontoglires; Scandentia; Tupaiidae; Tupaia","Common Name"="Chinese tree shrew"]

NP_001317309.1[&Organism="Homo sapiens",Description="dynamin-1-like protein isoform 8 [Homo sapiens]","Genetic Code"="Standard",Taxonomy="Eukaryota; Metazoa; Chordata; Craniata; Vertebrata; Euteleostomi; Mammalia; Eutheria; Euarchontoglires; Primates; Haplorrhini; Catarrhini; Hominidae; Homo","Common Name"="human"]

NP_001392186.1[&Organism="Mus musculus",Description="dynamin-1-like protein isoform m [Mus musculus]","Genetic Code"="Standard",Taxonomy="Eukaryota; Metazoa; Chordata; Craniata; Vertebrata; Euteleostomi; Mammalia; Eutheria; Euarchontoglires; Glires; Rodentia; Myomorpha; Muroidea; Muridae; Murinae; Mus; Mus","Common Name"="house mouse"]

XP_014394711.1[&Organism="Myotis brandtii",Description="PREDICTED: dynamin-1-like protein isoform X6 [Myotis brandtii]","Genetic Code"="Standard",Taxonomy="Eukaryota; Metazoa; Chordata; Craniata; Vertebrata; Euteleostomi; Mammalia; Eutheria; Laurasiatheria; Chiroptera; Microchiroptera; Vespertilionidae; Myotis","Common Name"="Brandt's bat"]

XP_028602039.1[&Organism="Podarcis muralis",Description="dynamin-1-like protein isoform X3 [Podarcis muralis]","Genetic Code"="Standard",Taxonomy="Eukaryota; Metazoa; Chordata; Craniata; Vertebrata; Euteleostomi; Lepidosauria; Squamata; Bifurcata; Unidentata; Episquamata; Laterata; Lacertibaenia; Lacertidae; Podarcis","Common Name"="Common wall lizard"]

XP_012382650.2[&Organism="Dasypus novemcinctus",Description="dynamin-1-like protein, partial [Dasypus novemcinctus]","Genetic Code"="Standard",Taxonomy="Eukaryota; Metazoa; Chordata; Craniata; Vertebrata;

Euteleostomi; Mammalia; Eutheria; Xenarthra; Cingulata; Dasypodidae; Dasypus","Common Name"="nine-banded armadillo"]

XP_031753959.1[&Organism="Xenopus tropicalis",Description="dynamin-1-like protein [Xenopus tropicalis]","Genetic Code"="Standard",Taxonomy="Eukaryota; Metazoa; Chordata; Craniata; Vertebrata; Euteleostomi; Amphibia; Batrachia; Anura; Pipoidea; Pipidae; Xenopodinae; Xenopus; Silurana","Common Name"="tropical clawed frog"]

PAA85687.1[&Organism="Macrostomum lignano",Description="hypothetical protein BOX15_Mlig022202g1 [Macrostomum lignano]","Genetic Code"="Standard",Taxonomy="Eukaryota; Metazoa; Spiralia; Lophotrochozoa; Platyhelminthes; Rhabditophora; Macrostomorpha; Macrostomida; Macrostomidae; Macrostomum"]

XP_042914770.1[&Organism="Chlamydomonas reinhardtii",Description="uncharacterized protein CHLRE_17g724150v5 [Chlamydomonas reinhardtii]","Genetic Code"="Standard",Taxonomy="Eukaryota; Viridiplantae; Chlorophyta; core chlorophytes; Chlorophyceae; CS clade; Chlamydomonadales; Chlamydomonadaceae; Chlamydomonas"]

PWZ09977.1[&Organism="Zea mays",Description="Dynamin-related protein 3A [Zea mays]","Genetic Code"="Standard",Taxonomy="Eukaryota; Viridiplantae; Streptophyta; Embryophyta; Tracheophyta; Spermatophyta; Magnoliopsida; Liliopsida; Poales; Poaceae; PACMAD clade; Panicoideae; Andropogonodae; Andropogoneae; Tripsacinae; Zea"]

KAH9304002.1[&Organism="Taxus chinensis",Description="hypothetical protein KI387_008406 [Taxus chinensis]","Genetic Code"="Standard",Taxonomy="Eukaryota; Viridiplantae; Streptophyta; Embryophyta; Tracheophyta; Spermatophyta; Pinopsida; Pinidae; Conifers II; Cupressales; Taxaceae; Taxus"]

EFJ37641.1[&Organism="Selaginella moellendorffii",Description="hypothetical protein SELMODRAFT_437242 [Selaginella moellendorffii]","Genetic Code"="Standard",Taxonomy="Eukaryota; Viridiplantae; Streptophyta; Embryophyta; Tracheophyta; Lycopodiopsida; Selaginellales; Selaginellaceae; Selaginella"]

EFJ15047.1[&Organism="Selaginella moellendorffii",Description="hypothetical protein SELMODRAFT_119205 [Selaginella moellendorffii]","Genetic Code"="Standard",Taxonomy="Eukaryota; Viridiplantae; Streptophyta; Embryophyta; Tracheophyta; Lycopodiopsida; Selaginellales; Selaginellaceae; Selaginella"]

KAI5070758.1[&Organism="Adiantum capillus-veneris",Description="hypothetical protein GOP47_0015101 [Adiantum capillus-veneris]","Genetic Code"="Standard",Taxonomy="Eukaryota; Viridiplantae; Streptophyta; Embryophyta; Tracheophyta; Polypodiopsida; Polypodiidae; Polypodiales; Pteridineae; Pteridaceae; Vittarioideae; Adiantum"]

PTQ35749.1[&Organism="Marchantia polymorpha",Description="hypothetical protein MARPO_0069s0084 [Marchantia polymorpha]","Genetic Code"="Standard",Taxonomy="Eukaryota; Viridiplantae; Streptophyta; Embryophyta; Marchantiophyta; Marchantiopsida; Marchantiidae; Marchantiales; Marchantiaceae; Marchantia","Common Name"="liverwort"]

KAG0555682.1[&Organism="Ceratodon purpureus",Description="hypothetical protein KC19_12G188200 [Ceratodon purpureus]","Genetic

Code"="Standard",Taxonomy="Eukaryota; Viridiplantae; Streptophyta; Embryophyta; Bryophyta; Bryophytina; Bryopsida; Dicranidae; Pseudoditrichales; Ditrichaceae; Ceratodon"]

XP_024362051.1[&Organism="Physcomitrium patens",Description="dynamin-related protein 3A-like isoform X1 [Physcomitrium patens]","Genetic Code"="Standard",Taxonomy="Eukaryota; Viridiplantae; Streptophyta; Embryophyta; Bryophyta; Bryophytina; Bryopsida; Funariidae; Funariales; Funariaceae; Physcomitrium"]

KAG0554580.1[&Organism="Ceratodon purpureus",Description="hypothetical protein KC19_12G102200 [Ceratodon purpureus]","Genetic Code"="Standard",Taxonomy="Eukaryota; Viridiplantae; Streptophyta; Embryophyta; Bryophyta; Bryophytina; Bryopsida; Dicranidae; Pseudoditrichales; Ditrichaceae; Ceratodon"]

KAI5070335.1[&Organism="Adiantum capillus-veneris",Description="hypothetical protein GOP47_0014678 [Adiantum capillus-veneris]","Genetic Code"="Standard",Taxonomy="Eukaryota; Viridiplantae; Streptophyta; Embryophyta; Tracheophyta; Polypodiopsida; Polypodiidae; Polypodiales; Pteridineae; Pteridaceae; Vittarioideae; Adiantum"]

ONM18162.1[&Organism="Zea mays",Description="Dynamin-related protein 3A [Zea mays]","Genetic Code"="Standard",Taxonomy="Eukaryota; Viridiplantae; Streptophyta; Embryophyta; Tracheophyta; Spermatophyta; Magnoliopsida; Liliopsida; Poales; Poaceae; PACMAD clade; Panicoideae; Andropogonodae; Andropogoneae; Tripsacinae; Zea"]

XP_052310486.1[&Organism="Populus trichocarpa",Description="dynamin-related protein 3A [Populus trichocarpa]","Genetic Code"="Standard",Taxonomy="Eukaryota; Viridiplantae; Streptophyta; Embryophyta; Tracheophyta; Spermatophyta; Magnoliopsida; eudicotyledons; Gunneridae; Pentapetalae; rosids; fabids; Malpighiales; Salicaceae; Saliceae; Populus","Common Name"="Populus balsamifera subsp. trichocarpa"]

AAC61784.1[&Organism="Arabidopsis thaliana",Description="similar to dynamin-like protein encoded by GenBank Accession Number X99669 [Arabidopsis thaliana]","Genetic Code"="Standard",Taxonomy="Eukaryota; Viridiplantae; Streptophyta; Embryophyta; Tracheophyta; Spermatophyta; Magnoliopsida; eudicotyledons; Gunneridae; Pentapetalae; rosids; malvids; Brassicales; Brassicaceae; Camelineae; Arabidopsis","Common Name"="thale cress"]

KAH9327796.1[&Organism="Taxus chinensis",Description="hypothetical protein KI387_007974, partial [Taxus chinensis]","Genetic Code"="Standard",Taxonomy="Eukaryota; Viridiplantae; Streptophyta; Embryophyta; Tracheophyta; Spermatophyta; Pinopsida; Pinidae; Conifers II; Cupressales; Taxaceae; Taxus"]

XP_014148015.1[&Organism="Sphaeroforma arctica JP610",Description="hypothetical protein SARC_13330, partial [Sphaeroforma arctica JP610]","Genetic Code"="Standard",Taxonomy="Eukaryota; Ichthyosporea; Ichthyophonida; Sphaeroforma"]

GMH85941.1[&Organism="Triparma verrucosa",Description="hypothetical protein TrVE_jg2671 [Triparma verrucosa]","Genetic Code"="Standard",Taxonomy="Eukaryota; Sar; Stramenopiles; Ochrophyta; Bolidophyceae; Parmales; Triparmaceae; Triparma"]

GMI59178.1[&Organism="Parmales sp. scaly parma",Description="hypothetical protein ScalyP_jg8911 [Parmales sp. scaly parma]","Genetic Code"="Standard",Taxonomy="Eukaryota; Sar; Stramenopiles; Ochrophyta; Bolidophyceae; Parmales"]

GMI25649.1[&Organism="Tetraparma gracilis",Description="hypothetical protein TeGR_g6469 [Tetraparma gracilis]","Genetic Code"="Standard",Taxonomy="Eukaryota; Sar; Stramenopiles; Ochrophyta; Bolidophyceae; Parmales; Triparmaceae; Tetraparma"]

GMI62840.1[&Organism="Parmales sp. scaly parma",Description="hypothetical protein ScalyP_jg9771 [Parmales sp. scaly parma]","Genetic Code"="Standard",Taxonomy="Eukaryota; Sar; Stramenopiles; Ochrophyta; Bolidophyceae; Parmales"]

GMH55978.1[&Organism="Triparma strigata",Description="hypothetical protein TrST_g5167 [Triparma strigata]","Genetic Code"="Standard",Taxonomy="Eukaryota; Sar; Stramenopiles; Ochrophyta; Bolidophyceae; Parmales; Triparmaceae; Triparma"]

PRP80825.1[&Organism="Planoprotostelium fungivorum",Description="dynamin-2 [Planoprotostelium fungivorum]","Genetic Code"="Standard",Taxonomy="Eukaryota; Amoebozoa; Evosea; Variosea; Cavosteliida; Cavosteliaceae; Planoprotostelium"]

XP_004355605.1[&Organism="Cavenderia fasciculata",Description="dynamin B [Cavenderia fasciculata]","Genetic Code"="Standard",Taxonomy="Eukaryota; Amoebozoa; Evosea; Eumycetozoa; Dictyostelia; Acytosteliales; Cavenderiaceae; Cavenderia"]

XP_020436927.1[&Organism="Heterostelium album PN500",Description="dynamin B [Heterostelium album PN500]","Genetic Code"="Standard",Taxonomy="Eukaryota; Amoebozoa; Evosea; Eumycetozoa; Dictyostelia; Acytosteliales; Acytosteliaceae; Heterostelium"]

AER35077.1[&Organism="Tieghemostelium lacteum",Description="dynamin B [Tieghemostelium lacteum]","Genetic Code"="Standard",Taxonomy="Eukaryota; Amoebozoa; Evosea; Eumycetozoa; Dictyostelia; Dictyosteliales; Raperosteliaceae; Tieghemostelium"]

KAF2075389.1[&Organism="Polysphondylium violaceum",Description="hypothetical protein CYY_003313 [Polysphondylium violaceum]","Genetic Code"="Standard",Taxonomy="Eukaryota; Amoebozoa; Evosea; Eumycetozoa; Dictyostelia; Dictyosteliales; Dictyosteliaceae; Polysphondylium"]

XP_003292385.1[&Organism="Dictyostelium purpureum",Description="uncharacterized protein DICPUDRAFT_57918 [Dictyostelium purpureum]","Genetic Code"="Standard",Taxonomy="Eukaryota; Amoebozoa; Evosea; Eumycetozoa; Dictyostelia; Dictyosteliales; Dictyosteliaceae; Dictyostelium"]

KOO24608.1[&Organism="Chrysochromulina tobinii",Description="dynamin like protein [Chrysochromulina tobinii]","Genetic Code"="Standard",Taxonomy="Eukaryota; Haptista; Haptophyta; Prymnesiophyceae; Prymnesiales; Chrysochromulinaceae; Chrysochromulina"]

CCW59714.1[&Organism="Phytomonas sp. EM1",Description="unnamed protein product [Phytomonas sp. EM1]","Genetic Code"="Standard",Taxonomy="Eukaryota; Discoba; Euglenozoa; Kinetoplastea; Metakinetoplastina; Trypanosomatida; Trypanosomatidae; Phytomonas"]

KAG5490335.1[&Organism="Porcisia hertigi",Description="hypothetical protein JKF63_00455 [Porcisia hertigi]","Genetic Code"="Standard",Taxonomy="Eukaryota; Discoba; Euglenozoa; Kinetoplastea; Metakinetoplastina; Trypanosomatida; Trypanosomatidae; Leishmaniinae; Porcisia"]

XP_003872337.1[&Organism="Leishmania mexicana MHOM/GT/2001/U1103",Description="putative GTP-binding protein [Leishmania mexicana MHOM/GT/2001/U1103]","Genetic Code"="Standard",Taxonomy="Eukaryota; Discoba; Euglenozoa; Kinetoplastea; Metakinetoplastina; Trypanosomatida; Trypanosomatidae; Leishmaniinae; Leishmania"]

KAI5685071.1[&Organism="Leishmania braziliensis",Description="Dynamin family [Leishmania braziliensis]","Genetic Code"="Standard",Taxonomy="Eukaryota; Discoba; Euglenozoa; Kinetoplastea; Metakinetoplastina; Trypanosomatida; Trypanosomatidae; Leishmaniinae; Leishmania; Leishmania braziliensis species complex"]

XP_028887534.1[&Organism="Trypanosoma theileri",Description="putative dynamin, putative,vacuolar sortin protein 1 [Trypanosoma theileri]","Genetic Code"="Standard",Taxonomy="Eukaryota; Discoba; Euglenozoa; Kinetoplastea; Metakinetoplastina; Trypanosomatida; Trypanosomatidae; Trypanosoma"]

EKF32958.1[&Organism="Trypanosoma cruzi marinkellei",Description="dynamin, putative,vacuolar sortin protein 1, putative [Trypanosoma cruzi marinkellei]","Genetic Code"="Standard",Taxonomy="Eukaryota; Discoba; Euglenozoa; Kinetoplastea; Metakinetoplastina; Trypanosomatida; Trypanosomatidae; Trypanosoma; Schizotrypanum"]

ESL10883.1[&Organism="Trypanosoma rangeli SC58",Description="dynamin [Trypanosoma rangeli SC58]","Genetic Code"="Standard",Taxonomy="Eukaryota; Discoba; Euglenozoa; Kinetoplastea; Metakinetoplastina; Trypanosomatida; Trypanosomatidae; Trypanosoma; Herpetosoma"]

RHW73545.1[&Organism="Trypanosoma brucei equiperdum",Description="dynamin-1-like protein [Trypanosoma brucei equiperdum]","Genetic Code"="Standard",Taxonomy="Eukaryota; Discoba; Euglenozoa; Kinetoplastea; Metakinetoplastina; Trypanosomatida; Trypanosomatidae; Trypanosoma"]

CCC89860.1[&Organism="Trypanosoma congolense IL3000",Description="unnamed protein product, partial [Trypanosoma congolense IL3000]","Genetic Code"="Standard",Taxonomy="Eukaryota; Discoba; Euglenozoa; Kinetoplastea; Metakinetoplastina; Trypanosomatida; Trypanosomatidae; Trypanosoma; Nannomonas"]

KAH8605762.1[&Organism="Trypanosoma vivax",Description="50S ribosome binding GTPase putative Dynamin family Dynamin central region [Trypanosoma vivax]","Genetic Code"="Standard",Taxonomy="Eukaryota; Discoba; Euglenozoa; Kinetoplastea; Metakinetoplastina; Trypanosomatida; Trypanosomatidae; Trypanosoma; Duttonella"]

KAJ9467322.1[&Organism="Diplonema papillatum",Description="hypothetical protein DIPPA_70197 [Diplonema papillatum]","Genetic Code"="Standard",Taxonomy="Eukaryota; Discoba; Euglenozoa; Diplonemea; Diplonemidae; Diplonema"]

KAJ9466576.1[&Organism="Diplonema papillatum",Description="Vacuolar protein sorting-associated protein 1 [Diplonema papillatum]","Genetic Code"="Standard",Taxonomy="Eukaryota; Discoba; Euglenozoa; Diplonemea; Diplonemidae; Diplonema"]

XP_018636213.1[&Organism="Toxoplasma gondii ME49",Description="dynamin-related protein DRPA [Toxoplasma gondii ME49]","Genetic Code"="Standard",Taxonomy="Eukaryota; Sar; Alveolata; Apicomplexa; Conoidasida; Coccidia; Eucoccidiorida; Eimeriorina; Sarcocystidae; Toxoplasma"]

XP_001750431.1[&Organism="Monosiga brevicollis MX1",Description="uncharacterized protein MONBRDRAFT_34545 [Monosiga brevicollis MX1]","Genetic Code"="Standard",Taxonomy="Eukaryota; Choanoflagellata; Craspedida; Salpingoecidae; Monosiga"]

XP_042924642.1[&Organism="Chlamydomonas reinhardtii",Description="uncharacterized protein CHLRE_05g245950v5 [Chlamydomonas reinhardtii]","Genetic Code"="Standard",Taxonomy="Eukaryota; Viridiplantae; Chlorophyta; core chlorophytes; Chlorophyceae; CS clade; Chlamydomonadales; Chlamydomonadaceae; Chlamydomonas"]

PTQ29980.1[&Organism="Marchantia polymorpha",Description="hypothetical protein MARPO_0132s0051 [Marchantia polymorpha]","Genetic Code"="Standard",Taxonomy="Eukaryota; Viridiplantae; Streptophyta; Embryophyta; Marchantiophyta; Marchantiopsida; Marchantiidae; Marchantiales; Marchantiaceae; Marchantia","Common Name"="liverwort"]

XP_002302631.1[&Organism="Populus trichocarpa",Description="phragmoplastin DRP1B isoform X1 [Populus trichocarpa]","Genetic Code"="Standard",Taxonomy="Eukaryota; Viridiplantae; Streptophyta; Embryophyta; Tracheophyta; Spermatophyta; Magnoliopsida; eudicotyledons; Gunneridae; Pentapetalae; rosids; fabids; Malpighiales; Salicaceae; Saliceae; Populus","Common Name"="Populus balsamifera subsp. trichocarpa"]

XP_002299468.1[&Organism="Populus trichocarpa",Description="dynamin-related protein 5A isoform X2 [Populus trichocarpa]","Genetic Code"="Standard",Taxonomy="Eukaryota; Viridiplantae; Streptophyta; Embryophyta; Tracheophyta; Spermatophyta; Magnoliopsida; eudicotyledons; Gunneridae; Pentapetalae; rosids; fabids; Malpighiales; Salicaceae; Saliceae; Populus","Common Name"="Populus balsamifera subsp. trichocarpa"]

NP_001190448.1[&Organism="Arabidopsis thaliana",Description="dynamin-like protein [Arabidopsis thaliana]","Genetic Code"="Standard",Taxonomy="Eukaryota; Viridiplantae; Streptophyta; Embryophyta; Tracheophyta; Spermatophyta; Magnoliopsida; eudicotyledons; Gunneridae; Pentapetalae; rosids; malvids; Brassicales; Brassicaceae; Camelineae; Arabidopsis","Common Name"="thale cress"]

AQK88296.1[&Description="Dynamin-related protein 1A [Zea mays]"]

PTQ45603.1[&Organism="Marchantia polymorpha",Description="hypothetical protein MARPO_0014s0125 [Marchantia polymorpha]","Genetic Code"="Standard",Taxonomy="Eukaryota; Viridiplantae; Streptophyta; Embryophyta;

Marchantiophyta; Marchantiopsida; Marchantiidae; Marchantiales; Marchantiaceae; Marchantia","Common Name"="liverwort"]

KAI5072318.1[&Organism="Adiantum capillus-veneris",Description="hypothetical protein GOP47_0012424 [Adiantum capillus-veneris]","Genetic Code"="Standard",Taxonomy="Eukaryota; Viridiplantae; Streptophyta; Embryophyta; Tracheophyta; Polypodiopsida; Polypodiidae; Polypodiales; Pteridineae; Pteridaceae; Vittarioideae; Adiantum"]

KAI5058380.1[&Organism="Adiantum capillus-veneris",Description="hypothetical protein GOP47_0026550 [Adiantum capillus-veneris]","Genetic Code"="Standard",Taxonomy="Eukaryota; Viridiplantae; Streptophyta; Embryophyta; Tracheophyta; Polypodiopsida; Polypodiidae; Polypodiales; Pteridineae; Pteridaceae; Vittarioideae; Adiantum"]

XP_002987566.1[&Organism="Selaginella moellendorffii",Description="dynamin-related protein 1E [Selaginella moellendorffii]","Genetic Code"="Standard",Taxonomy="Eukaryota; Viridiplantae; Streptophyta; Embryophyta; Tracheophyta; Lycopodiopsida; Selaginellales; Selaginellaceae; Selaginella"]

KAI5602084.1[&Organism="Populus trichocarpa",Description="hypothetical protein BDE02_01G133700 [Populus trichocarpa]","Genetic Code"="Standard",Taxonomy="Eukaryota; Viridiplantae; Streptophyta; Embryophyta; Tracheophyta; Spermatophyta; Magnoliopsida; eudicotyledons; Gunneridae; Pentapetalae; rosids; fabids; Malpighiales; Salicaceae; Saliceae; Populus","Common Name"="Populus balsamifera subsp. trichocarpa"]

XP_006375094.1[&Organism="Populus trichocarpa",Description="phragmoplastin DRP1E [Populus trichocarpa]","Genetic Code"="Standard",Taxonomy="Eukaryota; Viridiplantae; Streptophyta; Embryophyta; Tracheophyta; Spermatophyta; Magnoliopsida; eudicotyledons; Gunneridae; Pentapetalae; rosids; fabids; Malpighiales; Salicaceae; Saliceae; Populus","Common Name"="Populus balsamifera subsp. trichocarpa"]

XP_002315854.1[&Organism="Populus trichocarpa",Description="phragmoplastin DRP1C [Populus trichocarpa]","Genetic Code"="Standard",Taxonomy="Eukaryota; Viridiplantae; Streptophyta; Embryophyta; Tracheophyta; Spermatophyta; Magnoliopsida; eudicotyledons; Gunneridae; Pentapetalae; rosids; fabids; Malpighiales; Salicaceae; Saliceae; Populus","Common Name"="Populus balsamifera subsp. trichocarpa"]

NP_001147100.1[&Organism="Zea mays",Description="dynamin-related protein 1C [Zea mays]","Genetic Code"="Standard",Taxonomy="Eukaryota; Viridiplantae; Streptophyta; Embryophyta; Tracheophyta; Spermatophyta; Magnoliopsida; Liliopsida; Poales; Poaceae; PACMAD clade; Panicoideae; Andropogonodae; Andropogoneae; Tripsacinae; Zea"]

AAF22292.1[&Organism="Arabidopsis thaliana",Description="dynamin-like protein 4 [Arabidopsis thaliana]","Genetic Code"="Standard",Taxonomy="Eukaryota; Viridiplantae; Streptophyta; Embryophyta; Tracheophyta; Spermatophyta; Magnoliopsida; eudicotyledons; Gunneridae; Pentapetalae; rosids; malvids; Brassicales; Brassicaceae; Camelineae; Arabidopsis","Common Name"="thale cress"]

KAH9306600.1[&Organism="Taxus chinensis",Description="hypothetical protein KI387_011004, partial [Taxus chinensis]","Genetic Code"="Standard",Taxonomy="Eukaryota; Viridiplantae; Streptophyta; Embryophyta;

Tracheophyta; Spermatophyta; Pinopsida; Pinidae; Conifers II; Cupressales; Taxaceae; Taxus"]

KAG0556007.1[&Organism="Ceratodon purpureus",Description="hypothetical protein KC19_11G019300 [Ceratodon purpureus]","Genetic Code"="Standard",Taxonomy="Eukaryota; Viridiplantae; Streptophyta; Embryophyta; Bryophyta; Bryophytina; Bryopsida; Dicranidae; Pseudoditrichales; Ditrichaceae; Ceratodon"]

PWZ36850.1[&Organism="Zea mays",Description="Dynamin-related protein 1E [Zea mays]","Genetic Code"="Standard",Taxonomy="Eukaryota; Viridiplantae; Streptophyta; Embryophyta; Tracheophyta; Spermatophyta; Magnoliopsida; Liliopsida; Poales; Poaceae; PACMAD clade; Panicoideae; Andropogonodae; Andropogoneae; Tripsacinae; Zea"]

AAF79238.1[&Organism="Arabidopsis thaliana",Description="F10B6.23 [Arabidopsis thaliana]","Genetic Code"="Standard",Taxonomy="Eukaryota; Viridiplantae; Streptophyta; Embryophyta; Tracheophyta; Spermatophyta; Magnoliopsida; eudicotyledons; Gunneridae; Pentapetalae; rosids; malvids; Brassicales; Brassicaceae; Camelineae; Arabidopsis","Common Name"="thale cress"]

NP_850420.1[&Organism="Arabidopsis thaliana",Description="DYNAMIN-like 1D [Arabidopsis thaliana]","Genetic Code"="Standard",Taxonomy="Eukaryota; Viridiplantae; Streptophyta; Embryophyta; Tracheophyta; Spermatophyta; Magnoliopsida; eudicotyledons; Gunneridae; Pentapetalae; rosids; malvids; Brassicales; Brassicaceae; Camelineae; Arabidopsis","Common Name"="thale cress"]

EFJ15761.1[&Organism="Selaginella moellendorffii",Description="hypothetical protein SELMODRAFT_451592 [Selaginella moellendorffii]","Genetic Code"="Standard",Taxonomy="Eukaryota; Viridiplantae; Streptophyta; Embryophyta; Tracheophyta; Lycopodiopsida; Selaginellales; Selaginellaceae; Selaginella"]

EFJ23099.1[&Organism="Selaginella moellendorffii",Description="hypothetical protein SELMODRAFT_232702 [Selaginella moellendorffii]","Genetic Code"="Standard",Taxonomy="Eukaryota; Viridiplantae; Streptophyta; Embryophyta; Tracheophyta; Lycopodiopsida; Selaginellales; Selaginellaceae; Selaginella"]

XP_008860500.1[&Organism="Entamoeba nuttalli P19",Description="dynamin family protein [Entamoeba nuttalli P19]","Genetic Code"="Standard",Taxonomy="Eukaryota; Amoebozoa; Evosea; Archamoebae; Mastigamoebida; Entamoebidae; Entamoeba"]

XP_004185630.1[&Organism="Entamoeba invadens IP1",Description="dynamin, putative [Entamoeba invadens IP1]","Genetic Code"="Standard",Taxonomy="Eukaryota; Amoebozoa; Evosea; Archamoebae; Mastigamoebida; Entamoebidae; Entamoeba"]

KAF0852279.1[&Organism="Andalucia godoyi",Description="mitochondrial dynamin family protein [Andalucia godoyi]","Genetic Code"="Standard",Taxonomy="Eukaryota; Discoba; Jakobida; Andalucina; Andaluciidae; Andalucia"]

ETO36135.1[&Organism="Reticulomyxa filosa",Description="hypothetical protein RFI_00927 [Reticulomyxa filosa]","Genetic Code"="Standard",Taxonomy="Eukaryota; Sar; Rhizaria; Retaria; Foraminifera; Monothalamids; Reticulomyxidae; Reticulomyxa"]

KNH06820.1[&Organism="Perkinsela sp. CCAP 1560/4",Description="hypothetical protein XU18_2422 [Perkinsela sp. CCAP 1560/4]","Genetic

Code"="Standard",Taxonomy="Eukaryota; Discoba; Euglenozoa; Kinetoplastea; Prokinetoplastina; Ichthyobodonidae; Perkinsela"]

CAD2212698.1[&Organism="Angomonas deanei",Description="Dynamin family/Dynamin central region/Dynamin GTPase effector domain containing protein, putative [Angomonas deanei]","Genetic Code"="Standard",Taxonomy="Eukaryota; Discoba; Euglenozoa; Kinetoplastea; Metakinetoplastina; Trypanosomatida; Trypanosomatidae; Strigomonadinae; Angomonas"]

ABB13595.1[&Organism="Tetrahymena thermophila",Description="Drp7p [Tetrahymena thermophila]","Genetic Code"="Ciliate",Taxonomy="Eukaryota; Sar; Alveolata; Ciliophora; Intramacronucleata; Oligohymenophorea; Hymenostomatida; Tetrahymenina; Tetrahymenidae; Tetrahymena"]

XP_001009829.2[&Organism="Tetrahymena thermophila SB210",Description="dynamin central region family protein [Tetrahymena thermophila SB210]","Genetic Code"="Ciliate",Taxonomy="Eukaryota; Sar; Alveolata; Ciliophora; Intramacronucleata; Oligohymenophorea; Hymenostomatida; Tetrahymenina; Tetrahymenidae; Tetrahymena"]

XP_002371703.1[&Organism="Toxoplasma gondii ME49",Description="dynamin-related protein DRPB [Toxoplasma gondii ME49]","Genetic Code"="Standard",Taxonomy="Eukaryota; Sar; Alveolata; Apicomplexa; Conoidasida; Coccidia; Eucoccidiorida; Eimeriorina; Sarcocystidae; Toxoplasma"]

'XP_019914840.1'[&Organism="Plasmodium coatneyi",Description="Dynamin protein [Plasmodium coatneyi]","Genetic Code"="Standard",Taxonomy="Eukaryota; Sar; Alveolata; Apicomplexa; Aconoidasida; Haemosporida; Plasmodiidae; Plasmodium"]

'XP_012763851.2_1'[&Organism="Plasmodium reichenowi",Description="dynamin-like protein [Plasmodium reichenowi]","Genetic Code"="Standard",Taxonomy="Eukaryota; Sar; Alveolata; Apicomplexa; Aconoidasida; Haemosporida; Plasmodiidae; Plasmodium; Plasmodium (Laverania)"]

'EUR69800.1_1'[&Organism="Plasmodium falciparum 7G8",Description="hypothetical protein PFBG_03636 [Plasmodium falciparum 7G8]","Genetic Code"="Standard",Taxonomy="Eukaryota; Sar; Alveolata; Apicomplexa; Aconoidasida; Haemosporida; Plasmodiidae; Plasmodium; Plasmodium (Laverania)"]

XP_028867889.1[&Organism="Babesia ovata",Description="dynamin [Babesia ovata]","Genetic Code"="Standard",Taxonomy="Eukaryota; Sar; Alveolata; Apicomplexa; Aconoidasida; Piroplasmida; Babesiidae; Babesia"]

'CAE8701890.1'[&Organism="Polarella glacialis",Description="unnamed protein product, partial [Polarella glacialis]","Genetic Code"="Standard",Taxonomy="Eukaryota; Sar; Alveolata; Dinophyceae; Suessiales; Suessiaceae; Polarella"]

XP_001016567.2[&Organism="Tetrahymena thermophila SB210",Description="dynamin central region family protein [Tetrahymena thermophila SB210]","Genetic Code"="Ciliate",Taxonomy="Eukaryota; Sar; Alveolata; Ciliophora; Intramacronucleata; Oligohymenophorea; Hymenostomatida; Tetrahymenina; Tetrahymenidae; Tetrahymena"]

XP_001032891.1[&Organism="Tetrahymena thermophila SB210",Description="dynamin central region family protein [Tetrahymena thermophila

SB210]","Genetic Code"="Ciliate",Taxonomy="Eukaryota; Sar; Alveolata; Ciliophora; Intramacronucleata; Oligohymenophorea; Hymenostomatida; Tetrahymenina; Tetrahymenidae; Tetrahymena"]

'CAD8140652.1_1'[&Organism="Paramecium pentaurelia",Description="unnamed protein product [Paramecium pentaurelia]","Genetic Code"="Ciliate",Taxonomy="Eukaryota; Sar; Alveolata; Ciliophora; Intramacronucleata; Oligohymenophorea; Peniculida; Parameciidae; Paramecium"]

XP_001029982.1[&Organism="Tetrahymena thermophila SB210",Description="dynamin central region family protein [Tetrahymena thermophila SB210]","Genetic Code"="Ciliate",Taxonomy="Eukaryota; Sar; Alveolata; Ciliophora; Intramacronucleata; Oligohymenophorea; Hymenostomatida; Tetrahymenina; Tetrahymenidae; Tetrahymena"]

XP_001029985.2[&Organism="Tetrahymena thermophila SB210",Description="dynamin central region family protein [Tetrahymena thermophila SB210]","Genetic Code"="Ciliate",Taxonomy="Eukaryota; Sar; Alveolata; Ciliophora; Intramacronucleata; Oligohymenophorea; Hymenostomatida; Tetrahymenina; Tetrahymenidae; Tetrahymena"]

XP_005788601.1[&Organism="Emiliania huxleyi CCMP1516",Description="hypothetical protein EMIHUDRAFT_43878, partial [Emiliania huxleyi CCMP1516]","Genetic Code"="Standard",Taxonomy="Eukaryota; Haptista; Haptophyta; Prymnesiophyceae; Isochrysidales; Noelaerhabdaceae; Emiliania"]

EFJ35472.1[&Organism="Selaginella moellendorffii",Description="hypothetical protein SELMODRAFT_404911 [Selaginella moellendorffii]","Genetic Code"="Standard",Taxonomy="Eukaryota; Viridiplantae; Streptophyta; Embryophyta; Tracheophyta; Lycopodiopsida; Selaginellales; Selaginellaceae; Selaginella"]

OUM67143.1[&Organism="Piromyces sp. E2",Description="hypothetical protein PIROE2DRAFT_40291 [Piromyces sp. E2]","Genetic Code"="Standard",Taxonomy="Eukaryota; Fungi; Fungi incertae sedis; Chytridiomycota; Chytridiomycota incertae sedis; Neocallimastigomycetes; Neocallimastigales; Neocallimastigaceae; Piromyces; unclassified Piromyces"]

KNE54706.1[&Organism="Allomyces macrogynus ATCC 38327",Description="hypothetical protein AMAG_00665 [Allomyces macrogynus ATCC 38327]","Genetic Code"="Standard",Taxonomy="Eukaryota; Fungi; Fungi incertae sedis; Blastocladiomycota; Blastocladiomycota incertae sedis; Blastocladiomycetes; Blastocladiales; Blastocladiaceae; Allomyces"]

OAJ38404.1[&Organism="Batrachochytrium dendrobatidis JEL423",Description="hypothetical protein BDEG_22342 [Batrachochytrium dendrobatidis JEL423]","Genetic Code"="Standard",Taxonomy="Eukaryota; Fungi; Fungi incertae sedis; Chytridiomycota; Chytridiomycota incertae sedis; Chytridiomycetes; Rhizophydiales; Rhizophydiales incertae sedis; Batrachochytrium"]

XP_006459124.1[&Organism="Agaricus bisporus var. bisporus H97",Description="hypothetical protein AGABI2DRAFT_201103 [Agaricus bisporus var. bisporus H97]","Genetic Code"="Standard",Taxonomy="Eukaryota; Fungi; Dikarya;

Basidiomycota; Agaricomycotina; Agaricomycetes; Agaricomycetidae; Agaricales; Agaricaceae; Agaricus"]

XP_011389557.1[&Organism="Ustilago maydis 521",Description="putative dynamin-related GTPase [Ustilago maydis 521]","Genetic Code"="Standard",Taxonomy="Eukaryota; Fungi; Dikarya; Basidiomycota; Ustilaginomycotina; Ustilaginomycetes; Ustilaginales; Ustilaginaceae; Ustilago"]

XP_752563.1[&Organism="Aspergillus fumigatus Af293",Description="mitochondrial dynamin GTPase (Msp1), putative [Aspergillus fumigatus Af293]","Genetic Code"="Standard",Taxonomy="Eukaryota; Fungi; Dikarya; Ascomycota; Pezizomycotina; Eurotiomycetes; Eurotiomycetidae; Eurotiales; Aspergillaceae; Aspergillus; Aspergillus subgen. Fumigati"]

KXN72852.1[&Organism="Conidiobolus coronatus NRRL 28638",Description="hypothetical protein CONCODRAFT_77587 [Conidiobolus coronatus NRRL 28638]","Genetic Code"="Standard",Taxonomy="Eukaryota; Fungi; Fungi incertae sedis; Zoopagomycota; Entomophthoromycotina; Entomophthoromycetes; Entomophthorales; Ancylistaceae; Conidiobolus"]

NP_014854.2[&Organism="Saccharomyces cerevisiae S288C",Description="dynamin-related GTPase MGM1 [Saccharomyces cerevisiae S288C]","Genetic Code"="Standard",Taxonomy="Eukaryota; Fungi; Dikarya; Ascomycota; Saccharomycotina; Saccharomycetes; Saccharomycetales; Saccharomycetaceae; Saccharomyces"]

PSC76263.1[&Organism="Micractinium conductrix",Description="dynamin-related 4C-like [Micractinium conductrix]","Genetic Code"="Standard",Taxonomy="Eukaryota; Viridiplantae; Chlorophyta; core chlorophytes; Trebouxiophyceae; Chlorellales; Chlorellaceae; Chlorella clade; Micractinium"]

PRW56740.1[&Organism="Chlorella sorokiniana",Description="chromatin-remodeling complex ATPase chain [Chlorella sorokiniana]","Genetic Code"="Standard",Taxonomy="Eukaryota; Viridiplantae; Chlorophyta; core chlorophytes; Trebouxiophyceae; Chlorellales; Chlorellaceae; Chlorella clade; Chlorella"]

XP_005849062.1[&Organism="Chlorella variabilis",Description="hypothetical protein CHLNCDRAFT_21811, partial [Chlorella variabilis]","Genetic Code"="Standard",Taxonomy="Eukaryota; Viridiplantae; Chlorophyta; core chlorophytes; Trebouxiophyceae; Chlorellales; Chlorellaceae; Chlorella clade; Chlorella"]

GHP04420.1[&Organism="Pycnococcus provasolii",Description="hypothetical protein PPROV_000317400 [Pycnococcus provasolii]","Genetic Code"="Standard",Taxonomy="Eukaryota; Viridiplantae; Chlorophyta; Pycnococcaceae; Pycnococcus"]

OSX73843.1[&Organism="Porphyra umbilicalis",Description="hypothetical protein BU14_0324s0007 [Porphyra umbilicalis]","Genetic Code"="Standard",Taxonomy="Eukaryota; Rhodophyta; Bangiophyceae; Bangiales; Bangiaceae; Porphyra","Common Name"="laver"]

OSX70108.1[&Organism="Porphyra umbilicalis",Description="hypothetical protein BU14_0900s0001 [Porphyra umbilicalis]","Genetic

Code"="Standard",Taxonomy="Eukaryota; Rhodophyta; Bangiophyceae; Bangiales; Bangiaceae; Porphyra","Common Name"="laver"]

KAJ1442373.1[&Organism="Ochromonadaceae sp. CCMP2298",Description="Mx2, Mx-like dynamin-related GTPase [Ochromonadaceae sp. CCMP2298]","Genetic Code"="Standard",Taxonomy="Eukaryota; Sar; Stramenopiles; Ochrophyta; Synurophyceae; Ochromonadales; Ochromonadaceae"]

CAH0370685.1[&Organism="Pelagomonas calceolata",Description="unnamed protein product [Pelagomonas calceolata]","Genetic Code"="Standard",Taxonomy="Eukaryota; Sar; Stramenopiles; Ochrophyta; Pelagophyceae; Pelagomonadales; Pelagomonas"]

KAH8053135.1[&Organism="Aureococcus anophagefferens",Description="hypothetical protein JL722_9801 [Aureococcus anophagefferens]","Genetic Code"="Standard",Taxonomy="Eukaryota; Sar; Stramenopiles; Ochrophyta; Pelagophyceae; Pelagomonadales; Aureococcus"]

KAJ8603921.1[&Organism="Chrysophaeum taylorii",Description="hypothetical protein CTAYLR_009714 [Chrysophaeum taylorii]","Genetic Code"="Standard",Taxonomy="Eukaryota; Sar; Stramenopiles; Ochrophyta; Pelagophyceae; Pelagomonadales; Chrysophaeum"]

KAJ1460259.1[&Organism="Pelagophyceae sp. CCMP2097",Description="P-loop containing nucleoside triphosphate hydrolase protein [Pelagophyceae sp. CCMP2097]","Genetic Code"="Standard",Taxonomy="Eukaryota; Sar; Stramenopiles; Ochrophyta; Pelagophyceae"]

KAG5178451.1[&Organism="Tribonema minus",Description="Mgm1-like protein [Tribonema minus]","Genetic Code"="Standard",Taxonomy="Eukaryota; Sar; Stramenopiles; Ochrophyta; PX clade; Xanthophyceae; Tribonematales; Tribonemataceae; Tribonema"]

CBN76986.1[&Organism="Ectocarpus siliculosus",Description="Mgm1 homolog, dynamin-related GTPase [Ectocarpus siliculosus]","Genetic Code"="Standard",Taxonomy="Eukaryota; Sar; Stramenopiles; Ochrophyta; PX clade; Phaeophyceae; Ectocarpales; Ectocarpaceae; Ectocarpus"]

KAG5185531.1[&Organism="Tribonema minus",Description="P-loop containing nucleoside triphosphate hydrolase protein [Tribonema minus]","Genetic Code"="Standard",Taxonomy="Eukaryota; Sar; Stramenopiles; Ochrophyta; PX clade; Xanthophyceae; Tribonematales; Tribonemataceae; Tribonema"]

XP_009038401.1[&Organism="Aureococcus anophagefferens",Description="hypothetical protein AURANDRAFT_28354, partial [Aureococcus anophagefferens]","Genetic Code"="Standard",Taxonomy="Eukaryota; Sar; Stramenopiles; Ochrophyta; Pelagophyceae; Pelagomonadales; Aureococcus"]

KAJ1460532.1[&Organism="Pelagophyceae sp. CCMP2097",Description="Mgm1-like protein [Pelagophyceae sp. CCMP2097]","Genetic Code"="Standard",Taxonomy="Eukaryota; Sar; Stramenopiles; Ochrophyta; Pelagophyceae"]

CBN78192.1[&Organism="Ectocarpus siliculosus",Description="Mx2, Mx-like dynamin-related GTPase [Ectocarpus siliculosus]","Genetic

Code"="Standard",Taxonomy="Eukaryota; Sar; Stramenopiles; Ochrophyta; PX clade; Phaeophyceae; Ectocarpales; Ectocarpaceae; Ectocarpus"]

KAG5184845.1[&Organism="Tribonema minus",Description="Mx2, Mx-like dynamin-related GTPase [Tribonema minus]","Genetic Code"="Standard",Taxonomy="Eukaryota; Sar; Stramenopiles; Ochrophyta; PX clade; Xanthophyceae; Tribonematales; Tribonemataceae; Tribonema"]

XP_009039543.1[&Organism="Aureococcus anophagefferens",Description="hypothetical protein AURANDRAFT_38339 [Aureococcus anophagefferens]","Genetic Code"="Standard",Taxonomy="Eukaryota; Sar; Stramenopiles; Ochrophyta; Pelagophyceae; Pelagomonadales; Aureococcus"]

KAJ8614481.1[&Organism="Chrysophaeum taylorii",Description="hypothetical protein CTAYLR_000866 [Chrysophaeum taylorii]","Genetic Code"="Standard",Taxonomy="Eukaryota; Sar; Stramenopiles; Ochrophyta; Pelagophyceae; Pelagomonadales; Chrysophaeum"]

XP_009039855.1[&Organism="Aureococcus anophagefferens",Description="hypothetical protein AURANDRAFT_38499 [Aureococcus anophagefferens]","Genetic Code"="Standard",Taxonomy="Eukaryota; Sar; Stramenopiles; Ochrophyta; Pelagophyceae; Pelagomonadales; Aureococcus"]

KAJ1428896.1[&Organism="Ochromonadaceae sp. CCMP2298",Description="P-loop containing nucleoside triphosphate hydrolase protein [Ochromonadaceae sp. CCMP2298]","Genetic Code"="Standard",Taxonomy="Eukaryota; Sar; Stramenopiles; Ochrophyta; Synurophyceae; Ochromonadales; Ochromonadaceae"]

KAG5183739.1[&Organism="Tribonema minus",Description="P-loop containing nucleoside triphosphate hydrolase protein [Tribonema minus]","Genetic Code"="Standard",Taxonomy="Eukaryota; Sar; Stramenopiles; Ochrophyta; PX clade; Xanthophyceae; Tribonematales; Tribonemataceae; Tribonema"]

KAK3283006.1[&Organism="Cymbomonas tetramitiformis",Description="hypothetical protein CYMTET_9279 [Cymbomonas tetramitiformis]","Genetic Code"="Standard",Taxonomy="Eukaryota; Viridiplantae; Chlorophyta; Pyramimonadophyceae; Pyramimonadales; Pyramimonadaceae; Cymbomonas"]

KAJ1474099.1[&Organism="Cryptophyta sp. CCMP2293",Description="P-loop containing nucleoside triphosphate hydrolase protein, partial [Cryptophyta sp. CCMP2293]","Genetic Code"="Standard",Taxonomy="Eukaryota; Cryptophyceae"]

KAJ1495567.1[&Organism="Cryptophyta sp. CCMP2293",Description="P-loop containing nucleoside triphosphate hydrolase protein [Cryptophyta sp. CCMP2293]","Genetic Code"="Standard",Taxonomy="Eukaryota; Cryptophyceae"]

KAJ1474882.1[&Organism="Cryptophyta sp. CCMP2293",Description="P-loop containing nucleoside triphosphate hydrolase protein [Cryptophyta sp. CCMP2293]","Genetic Code"="Standard",Taxonomy="Eukaryota; Cryptophyceae"]

KAJ1487321.1[&Organism="Cryptophyta sp. CCMP2293",Description="P-loop containing nucleoside triphosphate hydrolase protein, partial [Cryptophyta sp. CCMP2293]","Genetic Code"="Standard",Taxonomy="Eukaryota; Cryptophyceae"]

XP_005711749.1[&Organism="Chondrus crispus",Description="unnamed protein product [Chondrus crispus]","Genetic Code"="Standard",Taxonomy="Eukaryota; Rhodophyta; Florideophyceae; Rhodymeniophycidae; Gigartinales; Gigartinaceae; Chondrus","Common Name"="carragheen"]

KAI0564035.1[&Organism="Gracilaria domingensis",Description="Dynamin [Gracilaria domingensis]","Genetic Code"="Standard",Taxonomy="Eukaryota; Rhodophyta; Florideophyceae; Rhodymeniophycidae; Gracilariales; Gracilariaceae; Gracilaria"]

PXF49978.1[&Organism="Gracilariopsis chorda",Description="Interferon-induced GTP-binding protein Mx [Gracilariopsis chorda]","Genetic Code"="Standard",Taxonomy="Eukaryota; Rhodophyta; Florideophyceae; Rhodymeniophycidae; Gracilariales; Gracilariaceae; Gracilariopsis"]

XP_006815062.1[&Organism="Saccoglossus kowalevskii",Description="PREDICTED: interferon-induced GTP-binding protein Mx-like [Saccoglossus kowalevskii]","Genetic Code"="Standard",Taxonomy="Eukaryota; Metazoa; Hemichordata; Enteropneusta; Harrimaniidae; Saccoglossus"]

XP_035690836.1[&Organism="Branchiostoma floridae",Description="interferon-induced GTP-binding protein Mx3-like [Branchiostoma floridae]","Genetic Code"="Standard",Taxonomy="Eukaryota; Metazoa; Chordata; Cephalochordata; Leptocardii; Amphioxiformes; Branchiostomidae; Branchiostoma","Common Name"="Florida lancelet"]

XP_019617847.1[&Organism="Branchiostoma belcheri",Description="PREDICTED: interferon-induced GTP-binding protein Mx1-like [Branchiostoma belcheri]","Genetic Code"="Standard",Taxonomy="Eukaryota; Metazoa; Chordata; Cephalochordata; Branchiostomidae; Branchiostoma","Common Name"="Belcher's lancelet"]

XP_002608668.1[&Organism="Branchiostoma floridae",Description="hypothetical protein BRAFLDRAFT_58103 [Branchiostoma floridae]","Genetic Code"="Standard",Taxonomy="Eukaryota; Metazoa; Chordata; Cephalochordata; Branchiostomidae; Branchiostoma","Common Name"="Florida lancelet"]

XP_003973512.2.2[&Organism="Takifugu rubripes",Description="interferon-induced GTP-binding protein Mx [Takifugu rubripes]","Genetic Code"="Standard",Taxonomy="Eukaryota; Metazoa; Chordata; Craniata; Vertebrata; Euteleostomi; Actinopterygii; Neopterygii; Teleostei; Neoteleostei; Acanthomorphata; Eupercaria; Tetraodontiformes; Tetradontoidea; Tetraodontidae; Takifugu","Common Name"="torafugu"]

NP_891987.2.2[&Organism="Danio rerio",Description="interferon-induced GTP-binding protein MxA [Danio rerio]","Genetic Code"="Standard",Taxonomy="Eukaryota; Metazoa; Chordata; Craniata; Vertebrata; Euteleostomi; Actinopterygii; Neopterygii; Teleostei; Ostariophysi; Cypriniformes; Cyprinidae; Danio","Common Name"="zebrafish"]

XP_009304072.1[&Organism="Danio rerio",Description="interferon-induced GTP-binding protein MxB isoform X1 [Danio rerio]","Genetic Code"="Standard",Taxonomy="Eukaryota; Metazoa; Chordata; Craniata; Vertebrata; Euteleostomi; Actinopterygii; Neopterygii; Teleostei; Ostariophysi; Cypriniformes; Cyprinidae; Danio","Common Name"="zebrafish"]

AGU16245.1[&db_xref="taxon:27779",Organism="Protopterus dolloi",Description="Mx1 protein, partial [Protopterus dolloi]","Genetic Code"="Standard",Modified=Mon Mar 27 11:17:17 PDT 2023,Taxonomy="Eukaryota; Metazoa; Chordata; Craniata; Vertebrata; Euteleostomi; Dipnoi; Lepidosireniformes; Protopteridae; Protopterus",Accession="AGU16245.1","Common Name"="slender lungfish",Topology="linear","Molecule Type"="AA"]

XP_007904885.1[&Organism="Callorhinchus milii",Description="PREDICTED: interferon-induced GTP-binding protein Mx-like isoform X2 [Callorhinchus milii]","Genetic Code"="Standard",Taxonomy="Eukaryota; Metazoa; Chordata; Craniata; Vertebrata; Chondrichthyes; Holocephali; Chimaeriformes; Callorhinchidae; Callorhinchus","Common Name"="elephant shark"]

XP_032888405.1[&Organism="Amblyraja radiata",Description="interferon-induced GTP-binding protein Mx3-like isoform X1 [Amblyraja radiata]","Genetic Code"="Standard",Taxonomy="Eukaryota; Metazoa; Chordata; Craniata; Vertebrata; Chondrichthyes; Elasmobranchii; Batoidea; Rajiformes; Rajidae; Amblyraja","Common Name"="thorny skate"]

XP_009815891.1[&Organism="Gavia stellata",Description="PREDICTED: interferon-induced GTP-binding protein Mx-like [Gavia stellata]","Genetic Code"="Standard",Taxonomy="Eukaryota; Metazoa; Chordata; Craniata; Vertebrata; Euteleostomi; Archelosauria; Archosauria; Dinosauria; Saurischia; Theropoda; Coelurosauria; Aves; Neognathae; Gaviiformes; Gaviidae; Gavia","Common Name"="red-throated loon"]

XP_025933558.1[&Organism="Apteryx rowi",Description="interferon-induced GTP-binding protein Mx-like isoform X1 [Apteryx rowi]","Genetic Code"="Standard",Taxonomy="Eukaryota; Metazoa; Chordata; Craniata; Vertebrata; Euteleostomi; Archelosauria; Archosauria; Dinosauria; Saurischia; Theropoda; Coelurosauria; Aves; Palaeognathae; Apterygiformes; Apterygidae; Apteryx","Common Name"="Okarito brown kiwi"]

XP_015269256.1[&Organism="Gekko japonicus",Description="PREDICTED: interferon-induced GTP-binding protein Mx1 [Gekko japonicus]","Genetic Code"="Standard",Taxonomy="Eukaryota; Metazoa; Chordata; Craniata; Vertebrata; Euteleostomi; Lepidosauria; Squamata; Bifurcata; Gekkota; Gekkonidae; Gekkoninae; Gekko"]

XP_006156438.1[&Organism="Tupaia chinensis",Description="interferon-induced GTP-binding protein Mx2 [Tupaia chinensis]","Genetic Code"="Standard",Taxonomy="Eukaryota; Metazoa; Chordata; Craniata; Vertebrata; Euteleostomi; Mammalia; Eutheria; Euarchontoglires; Scandentia; Tupaiidae; Tupaia","Common Name"="Chinese tree shrew"]

NP_001003133.1[&Organism="Canis lupus familiaris",Description="interferon-induced GTP-binding protein Mx2 [Canis lupus familiaris]","Genetic Code"="Standard",Taxonomy="Eukaryota; Metazoa; Chordata; Craniata; Vertebrata; Euteleostomi; Mammalia; Eutheria; Laurasiatheria; Carnivora; Caniformia; Canidae; Canis","Common Name"="dog"]

XP_002830747.1[&Organism="Pongo abelii",Description="interferon-induced GTP-binding protein Mx2 [Pongo abelii]","Genetic Code"="Standard",Taxonomy="Eukaryota; Metazoa; Chordata; Craniata; Vertebrata; Euteleostomi; Mammalia; Eutheria; Euarchontoglires; Primates; Haplorrhini; Catarrhini; Hominidae; Pongo","Common Name"="Sumatran orangutan"]

NP_002454.1[&Organism="Homo sapiens",Description="interferon-induced GTP-binding protein Mx2 [Homo sapiens]","Genetic Code"="Standard",Taxonomy="Eukaryota; Metazoa; Chordata; Craniata; Vertebrata; Euteleostomi; Mammalia; Eutheria; Euarchontoglires; Primates; Haplorrhini; Catarrhini; Hominidae; Homo","Common Name"="human"]

XP_008569440.1[&Organism="Galeopterus variegatus",Description="PREDICTED: interferon-induced GTP-binding protein Mx2 [Galeopterus variegatus]","Genetic Code"="Standard",Taxonomy="Eukaryota; Metazoa; Chordata; Craniata; Vertebrata; Euteleostomi; Mammalia; Eutheria; Euarchontoglires; Dermoptera; Cynocephalidae; Galeopterus","Common Name"="Sunda flying lemur"]

XP_017508123.1[&Organism="Manis javanica",Description="PREDICTED: interferon-induced GTP-binding protein Mx2-like, partial [Manis javanica]","Genetic Code"="Standard",Taxonomy="Eukaryota; Metazoa; Chordata; Craniata; Vertebrata; Euteleostomi; Mammalia; Eutheria; Laurasiatheria; Pholidota; Manidae; Manis","Common Name"="Malayan pangolin"]

XP_005885748.1[&Organism="Myotis brandtii",Description="PREDICTED: interferon-induced GTP-binding protein Mx2 [Myotis brandtii]","Genetic Code"="Standard",Taxonomy="Eukaryota; Metazoa; Chordata; Craniata; Vertebrata; Euteleostomi; Mammalia; Eutheria; Laurasiatheria; Chiroptera; Microchiroptera; Vespertilionidae; Myotis","Common Name"="Brandt's bat"]

NP_776366.1[&Organism="Bos taurus",Description="interferon-induced GTP-binding protein Mx2 [Bos taurus]","Genetic Code"="Standard",Taxonomy="Eukaryota; Metazoa; Chordata; Craniata; Vertebrata; Euteleostomi; Mammalia; Eutheria; Laurasiatheria; Artiodactyla; Ruminantia; Pecora; Bovidae; Bovinae; Bos","Common Name"="cattle"]

XP_032211320.1[&Organism="Mustela erminea",Description="interferon-induced GTP-binding protein Mx2 isoform X1 [Mustela erminea]","Genetic Code"="Standard",Taxonomy="Eukaryota; Metazoa; Chordata; Craniata; Vertebrata; Euteleostomi; Mammalia; Eutheria; Laurasiatheria; Carnivora; Caniformia; Mustelidae; Mustelinae; Mustela","Common Name"="ermine"]

XP_004675614.2.2[&Organism="Condylura cristata",Description="PREDICTED: interferon-induced GTP-binding protein Mx1 [Condylura cristata]","Genetic Code"="Standard",Taxonomy="Eukaryota; Metazoa; Chordata; Craniata; Vertebrata; Euteleostomi; Mammalia; Eutheria; Laurasiatheria; Insectivora; Talpidae; Condylura","Common Name"="star-nosed mole"]

XP_004466363.1[&Organism="Dasypus novemcinctus",Description="interferon-induced GTP-binding protein Mx1 [Dasypus novemcinctus]","Genetic Code"="Standard",Taxonomy="Eukaryota; Metazoa; Chordata; Craniata; Vertebrata;

Euteleostomi; Mammalia; Eutheria; Xenarthra; Cingulata; Dasypodidae; Dasypus","Common Name"="nine-banded armadillo"]

NP_002453.2.2[&Organism="Homo sapiens",Description="interferon-induced GTP-binding protein Mx1 isoform a [Homo sapiens]","Genetic Code"="Standard",Taxonomy="Eukaryota; Metazoa; Chordata; Craniata; Vertebrata; Euteleostomi; Mammalia; Eutheria; Euarchontoglires; Primates; Haplorrhini; Catarrhini; Hominidae; Homo","Common Name"="human"]

NP_001127618.1[&Organism="Pongo abelii",Description="interferon-induced GTP-binding protein Mx1 [Pongo abelii]","Genetic Code"="Standard",Taxonomy="Eukaryota; Metazoa; Chordata; Craniata; Vertebrata; Euteleostomi; Mammalia; Eutheria; Euarchontoglires; Primates; Haplorrhini; Catarrhini; Hominidae; Pongo","Common Name"="Sumatran orangutan"]

XP_006156437.1[&Organism="Tupaia chinensis",Description="interferon-induced GTP-binding protein Mx1 [Tupaia chinensis]","Genetic Code"="Standard",Taxonomy="Eukaryota; Metazoa; Chordata; Craniata; Vertebrata; Euteleostomi; Mammalia; Eutheria; Euarchontoglires; Scandentia; Tupaiidae; Tupaia","Common Name"="Chinese tree shrew"]

XP_017508130.1[&Organism="Manis javanica",Description="PREDICTED: interferon-induced GTP-binding protein Mx1 [Manis javanica]","Genetic Code"="Standard",Taxonomy="Eukaryota; Metazoa; Chordata; Craniata; Vertebrata; Euteleostomi; Mammalia; Eutheria; Laurasiatheria; Pholidota; Manidae; Manis","Common Name"="Malayan pangolin"]

XP_032211398.1[&Organism="Mustela erminea",Description="interferon-induced GTP-binding protein Mx1 isoform X1 [Mustela erminea]","Genetic Code"="Standard",Taxonomy="Eukaryota; Metazoa; Chordata; Craniata; Vertebrata; Euteleostomi; Mammalia; Eutheria; Laurasiatheria; Carnivora; Caniformia; Mustelidae; Mustelinae; Mustela","Common Name"="ermine"]

NP_001003134.1[&Organism="Canis lupus familiaris",Description="interferon-induced GTP-binding protein Mx1 [Canis lupus familiaris]","Genetic Code"="Standard",Taxonomy="Eukaryota; Metazoa; Chordata; Craniata; Vertebrata; Euteleostomi; Mammalia; Eutheria; Laurasiatheria; Carnivora; Caniformia; Canidae; Canis","Common Name"="dog"]

XP_008569442.1[&Organism="Galeopterus variegatus",Description="PREDICTED: interferon-induced GTP-binding protein Mx1 [Galeopterus variegatus]","Genetic Code"="Standard",Taxonomy="Eukaryota; Metazoa; Chordata; Craniata; Vertebrata; Euteleostomi; Mammalia; Eutheria; Euarchontoglires; Dermoptera; Cynocephalidae; Galeopterus","Common Name"="Sunda flying lemur"]

XP_014388412.1[&Organism="Myotis brandtii",Description="PREDICTED: interferon-induced GTP-binding protein Mx1 isoform X1 [Myotis brandtii]","Genetic Code"="Standard",Taxonomy="Eukaryota; Metazoa; Chordata; Craniata; Vertebrata; Euteleostomi; Mammalia; Eutheria; Laurasiatheria; Chiroptera; Microchiroptera; Vespertilionidae; Myotis","Common Name"="Brandt's bat"]

XP_005202045.1[&Organism="Bos taurus",Description="interferon-induced GTP-binding protein Mx1 isoform X1 [Bos taurus]","Genetic

Code"="Standard",Taxonomy="Eukaryota; Metazoa; Chordata; Craniata; Vertebrata; Euteleostomi; Mammalia; Eutheria; Laurasiatheria; Cetartiodactyla; Ruminantia; Pecora; Bovidae; Bovinae; Bos","Common Name"="cattle"]

NP_034976.1[&Organism="Mus musculus",Description="interferon-induced GTP-binding protein Mx1 [Mus musculus]","Genetic Code"="Standard",Taxonomy="Eukaryota; Metazoa; Chordata; Craniata; Vertebrata; Euteleostomi; Mammalia; Eutheria; Euarchontoglires; Glires; Rodentia; Myomorpha; Muroidea; Muridae; Murinae; Mus; Mus","Common Name"="house mouse"]

NP_038634.1[&Organism="Mus musculus",Description="interferon-induced GTP-binding protein Mx2 [Mus musculus]","Genetic Code"="Standard",Taxonomy="Eukaryota; Metazoa; Chordata; Craniata; Vertebrata; Euteleostomi; Mammalia; Eutheria; Euarchontoglires; Glires; Rodentia; Myomorpha; Muroidea; Muridae; Murinae; Mus; Mus","Common Name"="house mouse"]

XP_028583072.1[&Organism="Podarcis muralis",Description="interferon-induced GTP-binding protein Mx2-like [Podarcis muralis]","Genetic Code"="Standard",Taxonomy="Eukaryota; Metazoa; Chordata; Craniata; Vertebrata; Euteleostomi; Lepidosauria; Squamata; Bifurcata; Unidentata; Episquamata; Laterata; Lacertibaenia; Lacertidae; Podarcis","Common Name"="Common wall lizard"]

XP_028583068.1[&Organism="Podarcis muralis",Description="interferon-induced GTP-binding protein Mx1-like isoform X1 [Podarcis muralis]","Genetic Code"="Standard",Taxonomy="Eukaryota; Metazoa; Chordata; Craniata; Vertebrata; Euteleostomi; Lepidosauria; Squamata; Bifurcata; Unidentata; Episquamata; Laterata; Lacertibaenia; Lacertidae; Podarcis","Common Name"="Common wall lizard"]

XP_031752404.1[&Organism="Xenopus tropicalis",Description="interferon-induced GTP-binding protein Mx2 [Xenopus tropicalis]","Genetic Code"="Standard",Taxonomy="Eukaryota; Metazoa; Chordata; Craniata; Vertebrata; Euteleostomi; Amphibia; Batrachia; Anura; Pipoidea; Pipidae; Xenopodinae; Xenopus; Silurana","Common Name"="tropical clawed frog"]

XP_005167721.2.2[&Organism="Danio rerio",Description="interferon-induced GTP-binding protein MxE isoform X1 [Danio rerio]","Genetic Code"="Standard",Taxonomy="Eukaryota; Metazoa; Chordata; Craniata; Vertebrata; Euteleostomi; Actinopterygii; Neopterygii; Teleostei; Ostariophysi; Cypriniformes; Cyprinidae; Danio","Common Name"="zebrafish"]

NP_001007285.1[&Organism="Danio rerio",Description="interferon-induced GTP-binding protein MxC [Danio rerio]","Genetic Code"="Standard",Taxonomy="Eukaryota; Metazoa; Chordata; Craniata; Vertebrata; Euteleostomi; Actinopterygii; Neopterygii; Teleostei; Ostariophysi; Cypriniformes; Cyprinidae; Danio","Common Name"="zebrafish"]

XP_012586448.1[&Organism="Condylura cristata",Description="PREDICTED: interferon-induced GTP-binding protein Mx2 [Condylura cristata]","Genetic Code"="Standard",Taxonomy="Eukaryota; Metazoa; Chordata; Craniata; Vertebrata; Euteleostomi; Mammalia; Eutheria; Laurasiatheria; Insectivora; Talpidae; Condylura","Common Name"="star-nosed mole"]

KAI0213370.1[&Organism="Lamellibrachia satsuma",Description="Interferon-induced GTP-binding protein Mx2 [Lamellibrachia satsuma]","Genetic

Code"="Standard",Taxonomy="Eukaryota; Metazoa; Spiralia; Lophotrochozoa; Annelida; Polychaeta; Sedentaria; Canalipalpata; Sabellida; Siboglinidae; Lamellibrachia"]

KAI0208044.1[&Organism="Lamellibrachia satsuma",Description="Interferon-induced GTP-binding protein Mx1 [Lamellibrachia satsuma]","Genetic Code"="Standard",Taxonomy="Eukaryota; Metazoa; Spiralia; Lophotrochozoa; Annelida; Polychaeta; Sedentaria; Canalipalpata; Sabellida; Siboglinidae; Lamellibrachia"]

KAI0218869.1[&Organism="Lamellibrachia satsuma",Description="hypothetical protein LSAT2_029455 [Lamellibrachia satsuma]","Genetic Code"="Standard",Taxonomy="Eukaryota; Metazoa; Spiralia; Lophotrochozoa; Annelida; Polychaeta; Sedentaria; Canalipalpata; Sabellida; Siboglinidae; Lamellibrachia"]

XP_032804093.1[&Organism="Petromyzon marinus",Description="interferon-induced GTP-binding protein Mx1-like isoform X2 [Petromyzon marinus]","Genetic Code"="Standard",Taxonomy="Eukaryota; Metazoa; Chordata; Craniata; Vertebrata; Cyclostomata; Hyperoartia; Petromyzontiformes; Petromyzontidae; Petromyzon","Common Name"="sea lamprey"]

XP_046565196.1[&Organism="Haliotis rubra",Description="interferon-induced GTP-binding protein Mx-like [Haliotis rubra]","Genetic Code"="Standard",Taxonomy="Eukaryota; Metazoa; Spiralia; Lophotrochozoa; Mollusca; Gastropoda; Vetigastropoda; Lepetellida; Haliotoidea; Haliotidae; Haliotis","Common Name"="blacklip abalone"]

XP_046563124.1[&Organism="Haliotis rubra",Description="interferon-induced GTP-binding protein Mx-like [Haliotis rubra]","Genetic Code"="Standard",Taxonomy="Eukaryota; Metazoa; Spiralia; Lophotrochozoa; Mollusca; Gastropoda; Vetigastropoda; Lepetellida; Haliotoidea; Haliotidae; Haliotis","Common Name"="blacklip abalone"]

XP_046562919.1[&Organism="Haliotis rubra",Description="interferon-induced GTP-binding protein Mx-like [Haliotis rubra]","Genetic Code"="Standard",Taxonomy="Eukaryota; Metazoa; Spiralia; Lophotrochozoa; Mollusca; Gastropoda; Vetigastropoda; Lepetellida; Haliotoidea; Haliotidae; Haliotis","Common Name"="blacklip abalone"]

XP_046563126.1[&Organism="Haliotis rubra",Description="interferon-induced GTP-binding protein Mx-like [Haliotis rubra]","Genetic Code"="Standard",Taxonomy="Eukaryota; Metazoa; Spiralia; Lophotrochozoa; Mollusca; Gastropoda; Vetigastropoda; Lepetellida; Haliotoidea; Haliotidae; Haliotis","Common Name"="blacklip abalone"]

XP_046565195.1[&Organism="Haliotis rubra",Description="interferon-induced GTP-binding protein Mx-like [Haliotis rubra]","Genetic Code"="Standard",Taxonomy="Eukaryota; Metazoa; Spiralia; Lophotrochozoa; Mollusca; Gastropoda; Vetigastropoda; Lepetellida; Haliotoidea; Haliotidae; Haliotis","Common Name"="blacklip abalone"]

XP_046563125.1[&Organism="Haliotis rubra",Description="interferon-induced GTP-binding protein Mx-like [Haliotis rubra]","Genetic Code"="Standard",Taxonomy="Eukaryota; Metazoa; Spiralia; Lophotrochozoa; Mollusca; Gastropoda; Vetigastropoda; Lepetellida; Haliotoidea; Haliotidae; Haliotis","Common Name"="blacklip abalone"]

XP_046352527.2[&Organism="Haliotis rufescens",Description="interferon-induced GTP-binding protein Mx1-like [Haliotis rufescens]","Genetic Code"="Standard",Taxonomy="Eukaryota; Metazoa; Spiralia; Lophotrochozoa; Mollusca; Gastropoda; Vetigastropoda; Lepetellida; Haliotoidea; Haliotidae; Haliotis","Common Name"="red abalone"]

XP_048248476.1[&Organism="Haliotis rufescens",Description="interferon-induced GTP-binding protein Mx-like [Haliotis rufescens]","Genetic Code"="Standard",Taxonomy="Eukaryota; Metazoa; Spiralia; Lophotrochozoa; Mollusca; Gastropoda; Vetigastropoda; Lepetellida; Haliotoidea; Haliotidae; Haliotis","Common Name"="red abalone"]

XP_048258111.1[&Organism="Haliotis rufescens",Description="interferon-induced GTP-binding protein Mx-like [Haliotis rufescens]","Genetic Code"="Standard",Taxonomy="Eukaryota; Metazoa; Spiralia; Lophotrochozoa; Mollusca; Gastropoda; Vetigastropoda; Lepetellida; Haliotoidea; Haliotidae; Haliotis","Common Name"="red abalone"]

XP_046352531.2[&Organism="Haliotis rufescens",Description="interferon-induced GTP-binding protein Mx-like [Haliotis rufescens]","Genetic Code"="Standard",Taxonomy="Eukaryota; Metazoa; Spiralia; Lophotrochozoa; Mollusca; Gastropoda; Vetigastropoda; Lepetellida; Haliotoidea; Haliotidae; Haliotis","Common Name"="red abalone"]

XP_048248472.1[&Organism="Haliotis rufescens",Description="LOW QUALITY PROTEIN: interferon-induced GTP-binding protein Mx-like [Haliotis rufescens]","Genetic Code"="Standard",Taxonomy="Eukaryota; Metazoa; Spiralia; Lophotrochozoa; Mollusca; Gastropoda; Vetigastropoda; Lepetellida; Haliotoidea; Haliotidae; Haliotis","Common Name"="red abalone"]

XP_048248474.1[&Organism="Haliotis rufescens",Description="interferon-induced GTP-binding protein Mx-like isoform X2 [Haliotis rufescens]","Genetic Code"="Standard",Taxonomy="Eukaryota; Metazoa; Spiralia; Lophotrochozoa; Mollusca; Gastropoda; Vetigastropoda; Lepetellida; Haliotoidea; Haliotidae; Haliotis","Common Name"="red abalone"]

XP_048248473.1[&Organism="Haliotis rufescens",Description="interferon-induced GTP-binding protein Mx-like isoform X1 [Haliotis rufescens]","Genetic Code"="Standard",Taxonomy="Eukaryota; Metazoa; Spiralia; Lophotrochozoa; Mollusca; Gastropoda; Vetigastropoda; Lepetellida; Haliotoidea; Haliotidae; Haliotis","Common Name"="red abalone"]

ABI53802.1[&Organism="Haliotis discus discus",Description="Mx [Haliotis discus discus]","Genetic Code"="Standard",Taxonomy="Eukaryota; Metazoa; Lophotrochozoa; Mollusca; Gastropoda; Vetigastropoda; Haliotoidea; Haliotidae; Haliotis","Common Name"="disc abalone"]

CAH1802128.1[&Organism="Owenia fusiformis",Description="unnamed protein product [Owenia fusiformis]","Genetic Code"="Standard",Taxonomy="Eukaryota; Metazoa; Spiralia; Lophotrochozoa; Annelida; Polychaeta; Sedentaria; Canalipalpata; Sabellida; Oweniida; Oweniidae; Owenia"]

PAA74204.1[&Organism="Macrostomum lignano",Description="hypothetical protein BOX15_Mlig022940g2 [Macrostomum lignano]","Genetic Code"="Standard",Taxonomy="Eukaryota; Metazoa; Platyhelminthes; Rhabditophora; Macrostomorpha; Macrostomida; Macrostomidae; Macrostomum"]

PAA76532.1[&Organism="Macrostomum lignano",Description="hypothetical protein BOX15_Mlig002592g2 [Macrostomum lignano]","Genetic

Code"="Standard",Taxonomy="Eukaryota; Metazoa; Platyhelminthes; Rhabditophora; Macrostomorpha; Macrostomida; Macrostomidae; Macrostomum"]
PAA92268.1[&Organism="Macrostomum lignano",Description="hypothetical protein BOX15_Mlig009769g1 [Macrostomum lignano]","Genetic Code"="Standard",Taxonomy="Eukaryota; Metazoa; Platyhelminthes; Rhabditophora; Macrostomorpha; Macrostomida; Macrostomidae; Macrostomum"]
PAA69582.1[&Organism="Macrostomum lignano",Description="hypothetical protein BOX15_Mlig021727g2 [Macrostomum lignano]","Genetic Code"="Standard",Taxonomy="Eukaryota; Metazoa; Platyhelminthes; Rhabditophora; Macrostomorpha; Macrostomida; Macrostomidae; Macrostomum"]
PAA83069.1[&Organism="Macrostomum lignano",Description="hypothetical protein BOX15_Mlig009247g1 [Macrostomum lignano]","Genetic Code"="Standard",Taxonomy="Eukaryota; Metazoa; Platyhelminthes; Rhabditophora; Macrostomorpha; Macrostomida; Macrostomidae; Macrostomum"]
PAA94353.1[&Organism="Macrostomum lignano",Description="hypothetical protein BOX15_Mlig014920g1 [Macrostomum lignano]","Genetic Code"="Standard",Taxonomy="Eukaryota; Metazoa; Platyhelminthes; Rhabditophora; Macrostomorpha; Macrostomida; Macrostomidae; Macrostomum"]
KAI9324922.1[&"% Charged Amino Acids"=24.91%,"% Acidic Amino Acids"=13.17%,"% Hydrophobic Amino Acids"=46.26%,Description="KAI9324922.1",Modified=Fri Jun 28 11:50:37 PDT 2024,"% GC-rich Amino Acids"=21.71%,"# Nucleotide Sequences With Quality"=0,"Extinction Coefficient"=13075.0,"Molecular Weight (kDa)"=30.80993478000001,Topology="linear","Alignment method"="MAFFT Alignment",Created=Fri Jun 28 11:46:17 PDT 2024,"Charge at pH 7"=-7.798989763504893,"Isoelectric Point"=4.897228240966797,"% Basic Amino Acids"=11.74%,"% AT-rich Amino Acids"=21.71%,"% Polar Uncharged Amino Acids"=29.18%,"Free end gaps"=true,"Molecule Type"="AA"]
'KAJ3066410.1'[&"% Charged Amino Acids"=27.50%,"% Acidic Amino Acids"=15.00%,"% Hydrophobic Amino Acids"=47.14%,Description="JEL0797 KAJ3066410.1",Modified=Fri Jun 28 11:50:27 PDT 2024,"% GC-rich Amino Acids"=22.14%,"# Nucleotide Sequences With Quality"=0,"Extinction Coefficient"=13075.0,"Molecular Weight (kDa)"=30.847098080000013,Topology="linear","Alignment method"="MAFFT Alignment",Created=Fri Jun 28 11:46:17 PDT 2024,"Charge at pH 7"=-10.798238550692695,"Isoelectric Point"=4.645633697509766,"% Basic Amino Acids"=12.50%,"% AT-rich Amino Acids"=21.07%,"% Polar Uncharged Amino Acids"=25.71%,"Free end gaps"=true,"Molecule Type"="AA"]
KAI8836453.1[&"% Charged Amino Acids"=29.14%,"% Acidic Amino Acids"=13.67%,"% Hydrophobic Amino Acids"=46.76%,Description="JEL632 KAI8836453.1",Modified=Fri Jun 28 11:50:45 PDT 2024,"% GC-rich Amino Acids"=22.30%,"# Nucleotide Sequences With Quality"=0,"Extinction Coefficient"=13200.0,"Molecular Weight (kDa)"=30.927449480000007,Topology="linear","Alignment method"="MAFFT Alignment",Created=Fri Jun 28 11:46:17 PDT 2024,"Charge at pH 7"=-3.4437602930013576,"Isoelectric Point"=6.172344207763672,"% Basic Amino

Acids"=15.47%,"% AT-rich Amino Acids"=21.94%,"% Polar Uncharged Amino Acids"=24.46%,"Free end gaps"=true,"Molecule Type"="AA"]

KAJ3350919.1[&"% Charged Amino Acids"=24.29%,"% Acidic Amino Acids"=12.14%,"% Hydrophobic Amino Acids"=49.64%,Description="KAJ3350919.1",Modified=Fri Jun 28 11:50:59 PDT 2024,"% GC-rich Amino Acids"=20.71%,"# Nucleotide Sequences With Quality"=0,"Extinction Coefficient"=13200.0,"Molecular Weight (kDa)"=30.724561180000016,Topology="linear","Alignment method"="MAFFT Alignment",Created=Fri Jun 28 11:46:17 PDT 2024,"Charge at pH 7"=-4.908779490673721,"Isoelectric Point"=5.475826263427734,"% Basic Amino Acids"=12.14%,"% AT-rich Amino Acids"=24.64%,"% Polar Uncharged Amino Acids"=26.43%,"Free end gaps"=true,"Molecule Type"="AA"]

TDH66190.1[&Organism="Bremia lactucae",Description="hypothetical protein CCR75_009300 [Bremia lactucae]","Genetic Code"="Standard",Taxonomy="Eukaryota; Sar; Stramenopiles; Oomycota; Peronosporales; Peronosporaceae; Bremia","Common Name"="lettuce downy mildew"]

XP_024574100.1[&Organism="Plasmopara halstedii",Description="interferon-induced gtp-binding protein mx [Plasmopara halstedii]","Genetic Code"="Standard",Taxonomy="Eukaryota; Sar; Stramenopiles; Oomycota; Peronosporales; Peronosporaceae; Plasmopara"]

KAG1692046.1[&Organism="Phytophthora capsici",Description="hypothetical protein DVH05_025855 [Phytophthora capsici]","Genetic Code"="Standard",Taxonomy="Eukaryota; Sar; Stramenopiles; Oomycota; Peronosporales; Peronosporaceae; Phytophthora"]

KAG3062152.1[&Organism="Phytophthora cactorum",Description="hypothetical protein PC121_g12701 [Phytophthora cactorum]","Genetic Code"="Standard",Taxonomy="Eukaryota; Sar; Stramenopiles; Oomycota; Peronosporales; Peronosporaceae; Phytophthora"]

XP_009533587.1[&Organism="Phytophthora sojae",Description="hypothetical protein PHYSODRAFT_520498 [Phytophthora sojae]","Genetic Code"="Standard",Taxonomy="Eukaryota; Sar; Stramenopiles; Oomycota; Peronosporales; Peronosporaceae; Phytophthora"]

KAI9918701.1[&Organism="Peronosclerospora sorghi",Description="hypothetical protein PsorP6_011823 [Peronosclerospora sorghi]","Genetic Code"="Standard",Taxonomy="Eukaryota; Sar; Stramenopiles; Oomycota; Peronosporales; Peronosporaceae; Peronosclerospora"]

RMX63821.1[&Organism="Peronospora effusa",Description="hypothetical protein DD238_005926 [Peronospora effusa]","Genetic Code"="Standard",Taxonomy="Eukaryota; Sar; Stramenopiles; Oomycota; Peronosporales; Peronosporaceae; Peronospora"]

GMH36208.1[&Organism="Bryopsis sp. KO-2023",Description="hypothetical protein BSKO_04076 [Bryopsis sp. KO-2023]","Genetic Code"="Standard",Taxonomy="Eukaryota; Viridiplantae; Chlorophyta; Ulvophyceae; TCBD clade; Bryopsidales; Bryopsidineae; Bryopsidaceae; Bryopsis"]

GMH43921.1[&Organism="Bryopsis sp. KO-2023",Description="hypothetical protein BSKO_11855 [Bryopsis sp. KO-2023]","Genetic Code"="Standard",Taxonomy="Eukaryota; Viridiplantae; Chlorophyta; Ulvophyceae; TCBD clade; Bryopsidales; Bryopsidineae; Bryopsidaceae; Bryopsis"]

CAG9460856.1[&Organism="Pedinophyceae sp. YPF-701",Description="unnamed protein product [Pedinophyceae sp. YPF-701]","Genetic Code"="Standard",Taxonomy="Eukaryota; Viridiplantae; Chlorophyta; Pedinophyceae"]

GJP35534.1[&Organism="Closterium sp. NIES-68",Description="hypothetical protein CLOM_g20043 [Closterium sp. NIES-68]","Genetic Code"="Standard",Taxonomy="Eukaryota; Viridiplantae; Streptophyta; Zygnemophyceae; Zygnematophycidae; Desmidiales; Closteriaceae; Closterium; Closterium peracerosum-strigosum-littorale complex"]

CAI5480041.1[&Organism="Closterium sp. Yama58-4",Description="unnamed protein product [Closterium sp. Yama58-4]","Genetic Code"="Standard",Taxonomy="Eukaryota; Viridiplantae; Streptophyta; Zygnemophyceae; Zygnematophycidae; Desmidiales; Closteriaceae; Closterium; Closterium peracerosum-strigosum-littorale complex"]

KAJ7294545.1[&Organism="Diphasiastrum complanatum",Description="hypothetical protein O6H91_Y251600 [Diphasiastrum complanatum]","Genetic Code"="Standard",Taxonomy="Eukaryota; Viridiplantae; Streptophyta; Embryophyta; Tracheophyta; Lycopodiopsida; Lycopodiales; Lycopodiaceae; Lycopodioideae; Diphasiastrum"]

XP_024380180.1[&Organism="Physcomitrium patens",Description="dynamin-related protein 4C-like [Physcomitrium patens]","Genetic Code"="Standard",Taxonomy="Eukaryota; Viridiplantae; Streptophyta; Embryophyta; Bryophyta; Bryophytina; Bryopsida; Funariidae; Funariales; Funariaceae; Physcomitrium"]

XP_024367947.1[&Organism="Physcomitrium patens",Description="dynamin-related protein 4C-like, partial [Physcomitrium patens]","Genetic Code"="Standard",Taxonomy="Eukaryota; Viridiplantae; Streptophyta; Embryophyta; Bryophyta; Bryophytina; Bryopsida; Funariidae; Funariales; Funariaceae; Physcomitrium"]

KAG0619429.1[&Organism="Ceratodon purpureus",Description="hypothetical protein M758_4G139100 [Ceratodon purpureus]","Genetic Code"="Standard",Taxonomy="Eukaryota; Viridiplantae; Streptophyta; Embryophyta; Bryophyta; Bryophytina; Bryopsida; Dicranidae; Pseudoditrichales; Ditrichaceae; Ceratodon"]

KAG0561847.1[&Organism="Ceratodon purpureus",Description="hypothetical protein KC19_9G097200 [Ceratodon purpureus]","Genetic Code"="Standard",Taxonomy="Eukaryota; Viridiplantae; Streptophyta; Embryophyta; Bryophyta; Bryophytina; Bryopsida; Dicranidae; Pseudoditrichales; Ditrichaceae; Ceratodon"]

KAH9290598.1[&Organism="Taxus chinensis",Description="hypothetical protein KI387_034715 [Taxus chinensis]","Genetic Code"="Standard",Taxonomy="Eukaryota; Viridiplantae; Streptophyta; Embryophyta; Tracheophyta; Spermatophyta; Pinopsida; Pinidae; Conifers II; Cupressales; Taxaceae; Taxus"]

KAH9320939.1[&Organism="Taxus chinensis",Description="hypothetical protein KI387_015578 [Taxus chinensis]","Genetic Code"="Standard",Taxonomy="Eukaryota; Viridiplantae; Streptophyta; Embryophyta; Tracheophyta; Spermatophyta; Pinopsida; Pinidae; Conifers II; Cupressales; Taxaceae; Taxus"]

KAF8079489.1[&Organism="Sinapis alba",Description="hypothetical protein N665_1024s0016 [Sinapis alba]","Genetic Code"="Standard",Taxonomy="Eukaryota; Viridiplantae; Streptophyta; Embryophyta; Tracheophyta; Spermatophyta; Magnoliopsida; eudicotyledons; Gunneridae; Pentapetalae; rosids; malvids; Brassicales; Brassicaceae; Brassiceae; Sinapis","Common Name"="white mustard"]

OAP19580.1[&Description="DRP4A [Arabidopsis thaliana]"]

OAP13353.1[&Description="hypothetical protein AXX17_AT1G53610 [Arabidopsis thaliana]"]

OAP13972.1[&Description="hypothetical protein AXX17_AT1G53540 [Arabidopsis thaliana]"]

KAF5727250.1[&Organism="Tripterygium wilfordii",Description="hypothetical protein HS088_TW22G00939 [Tripterygium wilfordii]","Genetic Code"="Standard",Taxonomy="Eukaryota; Viridiplantae; Streptophyta; Embryophyta; Tracheophyta; Spermatophyta; Magnoliopsida; eudicotyledons; Gunneridae; Pentapetalae; rosids; fabids; Celastrales; Celastraceae; Tripterygium"]

XP_002303204.3[&Organism="Populus trichocarpa",Description="dynamin-related protein 4C isoform X2 [Populus trichocarpa]","Genetic Code"="Standard",Taxonomy="Eukaryota; Viridiplantae; Streptophyta; Embryophyta; Tracheophyta; Spermatophyta; Magnoliopsida; eudicotyledons; Gunneridae; Pentapetalae; rosids; fabids; Malpighiales; Salicaceae; Saliceae; Populus","Common Name"="Populus balsamifera subsp. trichocarpa"]

KAF8391993.1[&Organism="Tetracentron sinense",Description="hypothetical protein HHK36_022333 [Tetracentron sinense]","Genetic Code"="Standard",Taxonomy="Eukaryota; Viridiplantae; Streptophyta; Embryophyta; Tracheophyta; Spermatophyta; Magnoliopsida; Trochodendrales; Trochodendraceae; Tetracentron"]

XP_058079501.1[&Organism="Magnolia sinica",Description="dynamin-related protein 4C-like [Magnolia sinica]","Genetic Code"="Standard",Taxonomy="Eukaryota; Viridiplantae; Streptophyta; Embryophyta; Tracheophyta; Spermatophyta; Magnoliopsida; Magnoliidae; Magnoliales; Magnoliaceae; Magnolia"]

KAK1401877.1[&Organism="Heracleum sosnowskyi",Description="Dynamin-related protein 4C [Heracleum sosnowskyi]","Genetic Code"="Standard",Taxonomy="Eukaryota; Viridiplantae; Streptophyta; Embryophyta; Tracheophyta; Spermatophyta; Magnoliopsida; eudicotyledons; Gunneridae; Pentapetalae; asterids; campanulids; Apiales; Apiaceae; Apioideae; apioid superclade; Tordylieae; Tordyliinae; Heracleum"]

XP_038984915.1[&Organism="Phoenix dactylifera",Description="dynamin-related protein 4C-like [Phoenix dactylifera]","Genetic Code"="Standard",Taxonomy="Eukaryota; Viridiplantae; Streptophyta; Embryophyta; Tracheophyta; Spermatophyta; Magnoliopsida; Liliopsida; Arecaceae; Coryphoideae; Phoeniceae; Phoenix","Common Name"="date palm"]

XP_002297993.1[&Description="dynamin-related protein 4C [Populus trichocarpa]"]

XP_024439231.1[&Description="dynamin-related protein 4C [Populus trichocarpa]"]

KAH0683503.1[&Organism="Solanum tuberosum",Description="hypothetical protein KY289_021255 [Solanum tuberosum]","Genetic Code"="Standard",Taxonomy="Eukaryota; Viridiplantae; Streptophyta; Embryophyta; Tracheophyta; Spermatophyta; Magnoliopsida; eudicotyledons; Gunneridae; Pentapetalae; asterids; lamiids; Solanales; Solanaceae; Solanoideae; Solaneae; Solanum","Common Name"="potato"]

PWZ56863.1[&Organism="Zea mays",Description="Dynamin-related protein 4C [Zea mays]","Genetic Code"="Standard",Taxonomy="Eukaryota; Viridiplantae; Streptophyta; Embryophyta; Tracheophyta; Spermatophyta; Magnoliopsida; Liliopsida; Poales; Poaceae; PACMAD clade; Panicoideae; Andropogonodae; Andropogoneae; Tripsacinae; Zea"]

PWZ56864.1[&Organism="Zea mays",Description="Dynamin-related protein 4C [Zea mays]","Genetic Code"="Standard",Taxonomy="Eukaryota; Viridiplantae; Streptophyta; Embryophyta; Tracheophyta; Spermatophyta; Magnoliopsida; Liliopsida; Poales; Poaceae; PACMAD clade; Panicoideae; Andropogonodae; Andropogoneae; Tripsacinae; Zea"]

KAH9291961.1[&Organism="Taxus chinensis",Description="hypothetical protein KI387_042849 [Taxus chinensis]","Genetic Code"="Standard",Taxonomy="Eukaryota; Viridiplantae; Streptophyta; Embryophyta; Tracheophyta; Spermatophyta; Pinopsida; Pinidae; Conifers II; Cupressales; Taxaceae; Taxus"]

KAH9325151.1[&Description="hypothetical protein KI387_005329, partial [Taxus chinensis]"]

KAH9300179.1[&Description="hypothetical protein KI387_011762, partial [Taxus chinensis]"]

KAH9314974.1[&Description="hypothetical protein KI387_023601, partial [Taxus chinensis]"]

EFJ22917.1[&Description="hypothetical protein SELMODRAFT_104286 [Selaginella moellendorffii]"]

XP_005823288.1[&Organism="Guillardia theta CCMP2712",Description="hypothetical protein GUITHDRAFT_79026, partial [Guillardia theta CCMP2712]","Genetic Code"="Standard",Taxonomy="Eukaryota; Cryptophyceae; Pyrenomonadales; Geminigeraceae; Guillardia"]

KAJ1441760.1[&Organism="Ochromonadaceae sp. CCMP2298",Description="P-loop containing nucleoside triphosphate hydrolase protein [Ochromonadaceae sp. CCMP2298]","Genetic Code"="Standard",Taxonomy="Eukaryota; Sar; Stramenopiles; Ochrophyta; Synurophyceae; Ochromonadales; Ochromonadaceae"]

KAG8471152.1[&Organism="Diacronema lutheri",Description="hypothetical protein KFE25_009573 [Diacronema lutheri]","Genetic Code"="Standard",Taxonomy="Eukaryota; Haptista; Haptophyta; Pavlovales; Pavlovaceae; Diacronema"]

KOO34643.1[&Organism="Chrysochromulina tobinii",Description="dynamin-like protein [Chrysochromulina tobinii]","Genetic Code"="Standard",Taxonomy="Eukaryota;

Haptista; Haptophyta; Prymnesiophyceae; Prymnesiales; Chrysochromulinaceae; Chrysochromulina"]

XP_005775544.1[&Organism="Emiliania huxleyi CCMP1516",Description="hypothetical protein EMIHUDRAFT_461229, partial [Emiliania huxleyi CCMP1516]","Genetic Code"="Standard",Taxonomy="Eukaryota; Haptista; Haptophyta; Prymnesiophyceae; Isochrysidales; Noelaerhabdaceae; Emiliania"]

ATZ81043.1[&Organism="Bodo saltans virus",Description="dynamin-like protein [Bodo saltans virus]","Genetic Code"="Standard",Taxonomy="Viruses; Varidnaviria; Bamfordvirae; Nucleocytoviricota; Megaviricetes; Imitervirales; Mimiviridae; Klosneuvirinae; Klosneuvirus"]

ARF10282.1[&Organism="Hokovirus HKV1",Description="dynamin family protein [Hokovirus HKV1]","Genetic Code"="Standard",Taxonomy="Viruses; Varidnaviria; Bamfordvirae; Nucleocytoviricota; Megaviricetes; Imitervirales; Mimiviridae; Klosneuvirinae; Hokovirus"]

QKF94243.1[&Organism="Fadolivirus 1",Description="dynamin GTPase [Fadolivirus 1]","Genetic Code"="Standard",Taxonomy="Viruses; unclassified viruses; unclassified DNA viruses"]

ARF11508.1[&Organism="Klosneuvirus KNV1",Description="dynamin family protein [Klosneuvirus KNV1]","Genetic Code"="Standard",Taxonomy="Viruses; Varidnaviria; Bamfordvirae; Nucleocytoviricota; Megaviricetes; Imitervirales; Mimiviridae; Klosneuvirinae; Klosneuvirus"]

ARF09562.1[&Organism="Indivirus ILV1",Description="dynamin family protein [Indivirus ILV1]","Genetic Code"="Standard",Taxonomy="Viruses; Varidnaviria; Bamfordvirae; Nucleocytoviricota; Megaviricetes; Imitervirales; Mimiviridae; Klosneuvirinae; Indivirus"]

AYV76902.1[&Organism="Barrevirus sp.",Description="MAG: dynamin family protein [Barrevirus sp.]","Genetic Code"="Standard",Taxonomy="Viruses; Varidnaviria; Bamfordvirae; Nucleocytoviricota; Megaviricetes; Imitervirales; Mimiviridae","Common Name"="soil metagenome"]

ARF08414.1[&Organism="Catovirus CTV1",Description="dynamin family protein [Catovirus CTV1]","Genetic Code"="Standard",Taxonomy="Viruses; Varidnaviria; Bamfordvirae; Nucleocytoviricota; Megaviricetes; Imitervirales; Mimiviridae; Klosneuvirinae; Catovirus"]

AYV78912.1[&Organism="Edafosvirus sp.",Description="MAG: dynamin family protein [Edafosvirus sp.]","Genetic Code"="Standard",Taxonomy="Viruses; Varidnaviria; Bamfordvirae; Nucleocytoviricota; Megaviricetes; Imitervirales; Mimiviridae","Common Name"="soil metagenome"]

VBB18790.1[&Organism="Yasminevirus sp. GU-2018",Description="dynamin family protein [Yasminevirus sp. GU-2018]","Genetic Code"="Standard",Taxonomy="Viruses"]

ABI33144.1[&Organism="Bigelowiella natans",Description="dynamin-like protein 1 [Bigelowiella natans]","Genetic Code"="Standard",Taxonomy="Eukaryota; Sar; Rhizaria; Cercozoa; Chlorarachniophyceae; Bigelowiella"]

KAI8587516.1[&"% Charged Amino Acids"=22.91%,"% Acidic Amino Acids"=11.27%,"% Hydrophobic Amino

Acids"=52.00%,Description="KAI8587516.1",Modified=Fri Jun 28 11:50:12 PDT 2024,"% GC-rich Amino Acids"=23.27%,"# Nucleotide Sequences With Quality"=0,"Extinction Coefficient"=24075.0,"Molecular Weight (kDa)"=30.21366338000001,Topology="linear","Alignment method"="MAFFT Alignment",Created=Fri Jun 28 11:46:17 PDT 2024,"Charge at pH 7"=-3.7092413986402963,"Isoelectric Point"=5.703121185302734,"% Basic Amino Acids"=11.64%,"% AT-rich Amino Acids"=21.82%,"% Polar Uncharged Amino Acids"=26.18%,"Free end gaps"=true,"Molecule Type"="AA"]

OLL24579.1[&"% Charged Amino Acids"=26.39%,"% Acidic Amino Acids"=12.15%,"% Hydrophobic Amino Acids"=44.79%,Description="OLL24579.1",Modified=Fri Jun 28 11:46:17 PDT 2024,"% GC-rich Amino Acids"=20.49%,"# Nucleotide Sequences With Quality"=0,"Extinction Coefficient"=14565.0,"Molecular Weight (kDa)"=31.81140938000001,Topology="linear","Alignment method"="MAFFT Alignment",Created=Fri Jun 28 11:46:17 PDT 2024,"Charge at pH 7"=3.0255167076407066,"Isoelectric Point"=8.445613861083984,"% Basic Amino Acids"=14.24%,"% AT-rich Amino Acids"=25.69%,"% Polar Uncharged Amino Acids"=29.17%,"Free end gaps"=true,"Molecule Type"="AA"]

KAI9096888.1[&"% Charged Amino Acids"=30.14%,"% Acidic Amino Acids"=15.07%,"% Hydrophobic Amino Acids"=44.18%,Description="KAI9096888.1",Modified=Fri Jun 28 11:51:56 PDT 2024,"% GC-rich Amino Acids"=17.47%,"# Nucleotide Sequences With Quality"=0,"Extinction Coefficient"=20065.0,"Molecular Weight (kDa)"=33.101868080000024,Topology="linear","Alignment method"="MAFFT Alignment",Created=Fri Jun 28 11:46:17 PDT 2024,"Charge at pH 7"=-4.760784008823309,"Isoelectric Point"=5.568431854248047,"% Basic Amino Acids"=15.07%,"% AT-rich Amino Acids"=25.68%,"% Polar Uncharged Amino Acids"=26.37%,"Free end gaps"=true,"Molecule Type"="AA"]

RSH87279.1[&"% Charged Amino Acids"=29.37%,"% Acidic Amino Acids"=15.03%,"% Hydrophobic Amino Acids"=47.20%,Description="RSH87279.1",Modified=Fri Jun 28 11:48:55 PDT 2024,"% GC-rich Amino Acids"=24.13%,"# Nucleotide Sequences With Quality"=0,"Extinction Coefficient"=7115.0,"Molecular Weight (kDa)"=31.83822928,Topology="linear","Alignment method"="MAFFT Alignment",Created=Fri Jun 28 11:46:17 PDT 2024,"Charge at pH 7"=-5.858968677562601,"Isoelectric Point"=5.208797454833984,"% Basic Amino Acids"=14.34%,"% AT-rich Amino Acids"=20.63%,"% Polar Uncharged Amino Acids"=23.78%,"Free end gaps"=true,"Molecule Type"="AA"]

XP_041144356.1[&"% Charged Amino Acids"=26.92%,"% Acidic Amino Acids"=12.24%,"% Hydrophobic Amino Acids"=48.25%,Description="XM_041290714.1",Modified=Fri Jun 28 11:46:17 PDT 2024,"% GC-rich Amino Acids"=22.03%,"# Nucleotide Sequences With Quality"=0,"Extinction Coefficient"=17085.0,"Molecular Weight (kDa)"=31.70809888000001,Topology="linear","Alignment method"="MAFFT Alignment",Created=Fri Jun 28 11:46:17 PDT 2024,"Charge at pH 7"=-

3.1075449530009323,"Isoelectric Point"=6.306392669677734,"% Basic Amino Acids"=14.69%,"% AT-rich Amino Acids"=19.58%,"% Polar Uncharged Amino Acids"=25.52%,"Free end gaps"=true,"Molecule Type"="AA"]
KAJ5704467.1[&"% Charged Amino Acids"=24.13%,"% Acidic Amino Acids"=11.89%,"% Hydrophobic Amino Acids"=49.30%,Description="KAJ5704467.1",Modified=Fri Jun 28 11:46:17 PDT 2024,"% GC-rich Amino Acids"=23.78%,"# Nucleotide Sequences With Quality"=0,"Extinction Coefficient"=19605.0,"Molecular Weight (kDa)"=31.15122537999999,Topology="linear","Alignment method"="MAFFT Alignment",Created=Fri Jun 28 11:46:17 PDT 2024,"Charge at pH 7"=-5.574711435859037,"Isoelectric Point"=5.575748443603516,"% Basic Amino Acids"=12.24%,"% AT-rich Amino Acids"=15.73%,"% Polar Uncharged Amino Acids"=27.62%,"Free end gaps"=true,"Molecule Type"="AA"]
KAI9774215.1[&"% Charged Amino Acids"=25.87%,"% Acidic Amino Acids"=12.24%,"% Hydrophobic Amino Acids"=47.90%,Description="KAI9774215.1",Modified=Fri Jun 28 11:46:17 PDT 2024,"% GC-rich Amino Acids"=21.68%,"# Nucleotide Sequences With Quality"=0,"Extinction Coefficient"=21095.0,"Molecular Weight (kDa)"=31.32162668,Topology="linear","Alignment method"="MAFFT Alignment",Created=Fri Jun 28 11:46:17 PDT 2024,"Charge at pH 7"=-4.3741271905474495,"Isoelectric Point"=6.005184173583984,"% Basic Amino Acids"=13.64%,"% AT-rich Amino Acids"=18.53%,"% Polar Uncharged Amino Acids"=27.27%,"Free end gaps"=true,"Molecule Type"="AA"]
MCJ1392161.1[&"% Charged Amino Acids"=26.22%,"% Acidic Amino Acids"=13.29%,"% Hydrophobic Amino Acids"=47.90%,Description="MCJ1392161.1",Modified=Fri Jun 28 11:46:17 PDT 2024,"% GC-rich Amino Acids"=21.68%,"# Nucleotide Sequences With Quality"=0,"Extinction Coefficient"=22585.0,"Molecular Weight (kDa)"=31.422763779999993,Topology="linear","Alignment method"="MAFFT Alignment",Created=Fri Jun 28 11:46:17 PDT 2024,"Charge at pH 7"=-6.599380872602497,"Isoelectric Point"=5.295635223388672,"% Basic Amino Acids"=12.94%,"% AT-rich Amino Acids"=17.83%,"% Polar Uncharged Amino Acids"=26.92%,"Free end gaps"=true,"Molecule Type"="AA"]
XP_002543522.1[&"% Charged Amino Acids"=26.92%,"% Acidic Amino Acids"=12.59%,"% Hydrophobic Amino Acids"=48.95%,Description="1704 XP_002543522.1",Modified=Fri Jun 28 11:46:17 PDT 2024,"% GC-rich Amino Acids"=23.78%,"# Nucleotide Sequences With Quality"=0,"Extinction Coefficient"=14105.0,"Molecular Weight (kDa)"=31.483921279999993,Topology="linear","Alignment method"="MAFFT Alignment",Created=Fri Jun 28 11:46:17 PDT 2024,"Charge at pH 7"=-2.40438839054951,"Isoelectric Point"=6.292827606201172,"% Basic Amino Acids"=14.34%,"% AT-rich Amino Acids"=19.58%,"% Polar Uncharged Amino Acids"=24.83%,"Free end gaps"=true,"Molecule Type"="AA"]
XP_746402.1[&Organism="Aspergillus fumigatus Af293",Description="dynamin GTPase, putative [Aspergillus fumigatus Af293]","Genetic

Code"="Standard",Taxonomy="Eukaryota; Fungi; Dikarya; Ascomycota; Pezizomycotina; Eurotiomycetes; Eurotiomycetidae; Eurotiales; Aspergillaceae; Aspergillus; Aspergillus subgen. Fumigati"]

XP_751069.1[&Organism="Aspergillus fumigatus Af293",Description="dynamin GTPase, putative [Aspergillus fumigatus Af293]","Genetic Code"="Standard",Taxonomy="Eukaryota; Fungi; Dikarya; Ascomycota; Pezizomycotina; Eurotiomycetes; Eurotiomycetidae; Eurotiales; Aspergillaceae; Aspergillus; Aspergillus subgen. Fumigati"]

XP_026607910.1[&"% Charged Amino Acids"=25.93%,"% Acidic Amino Acids"=11.11%,"% Hydrophobic Amino Acids"=48.82%,Description="XM_026742065.1",Modified=Fri Jun 28 11:46:17 PDT 2024,"% GC-rich Amino Acids"=24.58%,"# Nucleotide Sequences With Quality"=0,"Extinction Coefficient"=11710.0,"Molecular Weight (kDa)"=32.72361798000001,Topology="linear","Alignment method"="MAFFT Alignment",Created=Fri Jun 28 11:46:17 PDT 2024,"Charge at pH 7"=2.553564522492767,"Isoelectric Point"=7.953372955322266,"% Basic Amino Acids"=14.81%,"% AT-rich Amino Acids"=22.56%,"% Polar Uncharged Amino Acids"=25.59%,"Free end gaps"=true,"Molecule Type"="AA"]

XP_748757.2[&Organism="Aspergillus fumigatus Af293",Description="dynamin GTPase, putative [Aspergillus fumigatus Af293]","Genetic Code"="Standard",Taxonomy="Eukaryota; Fungi; Dikarya; Ascomycota; Pezizomycotina; Eurotiomycetes; Eurotiomycetidae; Eurotiales; Aspergillaceae; Aspergillus; Aspergillus subgen. Fumigati"]

XP_040633937.1[&"% Charged Amino Acids"=27.74%,"% Acidic Amino Acids"=14.04%,"% Hydrophobic Amino Acids"=51.71%,Description="XM_040785195.1",Modified=Fri Jun 28 11:46:17 PDT 2024,"% GC-rich Amino Acids"=27.40%,"# Nucleotide Sequences With Quality"=0,"Extinction Coefficient"=7115.0,"Molecular Weight (kDa)"=31.85812448,Topology="linear","Alignment method"="MAFFT Alignment",Created=Fri Jun 28 11:46:17 PDT 2024,"Charge at pH 7"=-11.101735485136608,"Isoelectric Point"=5.194911956787109,"% Basic Amino Acids"=13.70%,"% AT-rich Amino Acids"=17.12%,"% Polar Uncharged Amino Acids"=20.89%,"Free end gaps"=true,"Molecule Type"="AA"]

XP_754266.1[&Organism="Aspergillus fumigatus Af293",Description="dynamin GTPase, putative [Aspergillus fumigatus Af293]","Genetic Code"="Standard",Taxonomy="Eukaryota; Fungi; Dikarya; Ascomycota; Pezizomycotina; Eurotiomycetes; Eurotiomycetidae; Eurotiales; Aspergillaceae; Aspergillus; Aspergillus subgen. Fumigati"]

XP_043140374.1[&"% Charged Amino Acids"=27.74%,"% Acidic Amino Acids"=14.38%,"% Hydrophobic Amino Acids"=51.03%,Description="XM_043283056.1",Modified=Fri Jun 28 11:46:17 PDT 2024,"% GC-rich Amino Acids"=26.71%,"# Nucleotide Sequences With Quality"=0,"Extinction Coefficient"=7115.0,"Molecular Weight (kDa)"=32.242680480000004,Topology="linear","Alignment method"="MAFFT Alignment",Created=Fri Jun 28 11:46:17 PDT 2024,"Charge at pH 7"=-

8.599323914913342,"Isoelectric Point"=4.979366302490234,"% Basic Amino Acids"=13.36%,"% AT-rich Amino Acids"=20.21%,"% Polar Uncharged Amino Acids"=21.58%,"Free end gaps"=true,"Molecule Type"="AA"]
KXS17655.1[&"% Charged Amino Acids"=25.81%,"% Acidic Amino Acids"=13.26%,"% Hydrophobic Amino Acids"=53.05%,Description="KXS17655.1",Modified=Fri Jun 28 11:49:57 PDT 2024,"% GC-rich Amino Acids"=23.30%,"# Nucleotide Sequences With Quality"=0,"Extinction Coefficient"=14105.0,"Molecular Weight (kDa)"=30.090607580000007,Topology="linear","Alignment method"="MAFFT Alignment",Created=Fri Jun 28 11:46:17 PDT 2024,"Charge at pH 7"=-7.601807258076357,"Isoelectric Point"=5.110424041748047,"% Basic Amino Acids"=12.54%,"% AT-rich Amino Acids"=23.30%,"% Polar Uncharged Amino Acids"=21.86%,"Free end gaps"=true,"Molecule Type"="AA"]
XP_021869222.1[&"% Charged Amino Acids"=32.55%,"% Acidic Amino Acids"=15.77%,"% Hydrophobic Amino Acids"=44.30%,Description="XP_021869222.1",Modified=Fri Jun 28 11:49:08 PDT 2024,"% GC-rich Amino Acids"=21.48%,"# Nucleotide Sequences With Quality"=0,"Extinction Coefficient"=21095.0,"Molecular Weight (kDa)"=33.45680067999999,Topology="linear","Alignment method"="MAFFT Alignment",Created=Fri Jun 28 11:46:17 PDT 2024,"Charge at pH 7"=-6.272273006910691,"Isoelectric Point"=5.793697357177734,"% Basic Amino Acids"=16.78%,"% AT-rich Amino Acids"=21.48%,"% Polar Uncharged Amino Acids"=24.16%,"Free end gaps"=true,"Molecule Type"="AA"]
TVY17522.1[&"% Charged Amino Acids"=29.25%,"% Acidic Amino Acids"=13.95%,"% Hydrophobic Amino Acids"=50.00%,Description="TVY17522.1",Modified=Fri Jun 28 11:46:17 PDT 2024,"% GC-rich Amino Acids"=22.79%,"# Nucleotide Sequences With Quality"=0,"Extinction Coefficient"=19605.0,"Molecular Weight (kDa)"=32.82586588,Topology="linear","Alignment method"="MAFFT Alignment",Created=Fri Jun 28 11:46:17 PDT 2024,"Charge at pH 7"=-3.4030920147103245,"Isoelectric Point"=6.098857879638672,"% Basic Amino Acids"=15.31%,"% AT-rich Amino Acids"=18.71%,"% Polar Uncharged Amino Acids"=21.77%,"Free end gaps"=true,"Molecule Type"="AA"]
KAF9951223.1[&"% Charged Amino Acids"=29.71%,"% Acidic Amino Acids"=15.22%,"% Hydrophobic Amino Acids"=45.65%,Description="KAF9951223.1",Modified=Fri Jun 28 11:51:59 PDT 2024,"% GC-rich Amino Acids"=21.01%,"# Nucleotide Sequences With Quality"=0,"Extinction Coefficient"=27055.0,"Molecular Weight (kDa)"=31.347671079999994,Topology="linear","Alignment method"="MAFFT Alignment",Created=Fri Jun 28 11:46:17 PDT 2024,"Charge at pH 7"=-6.773009592825655,"Isoelectric Point"=5.118595123291016,"% Basic Amino Acids"=14.49%,"% AT-rich Amino Acids"=23.55%,"% Polar Uncharged Amino Acids"=25.72%,"Free end gaps"=true,"Molecule Type"="AA"]
XP_047808890.1[&"% Charged Amino Acids"=27.17%,"% Acidic Amino Acids"=12.68%,"% Hydrophobic Amino

Acids"=47.10%,Description="XP_047808890.1",Modified=Fri Jun 28 11:49:32 PDT 2024,"% GC-rich Amino Acids"=23.91%,"# Nucleotide Sequences With Quality"=0,"Extinction Coefficient"=13200.0,"Molecular Weight (kDa)"=30.56279258,Topology="linear","Alignment method"="MAFFT Alignment",Created=Fri Jun 28 11:46:17 PDT 2024,"Charge at pH 7"=-1.6440202409101712,"Isoelectric Point"=6.441722869873047,"% Basic Amino Acids"=14.49%,"% AT-rich Amino Acids"=22.83%,"% Polar Uncharged Amino Acids"=26.09%,"Free end gaps"=true,"Molecule Type"="AA"]

CAD7955538.1[&Organism="Amoebophrya sp. A120",Description="unnamed protein product [Amoebophrya sp. A120]","Genetic Code"="Standard",Taxonomy="Eukaryota; Sar; Alveolata; Dinophyceae; Syndiniales; Amoebophryaceae; Amoebophrya"]

CAD7971727.1[&Organism="Amoebophrya sp. A25",Description="unnamed protein product [Amoebophrya sp. A25]","Genetic Code"="Standard",Taxonomy="Eukaryota; Sar; Alveolata; Dinophyceae; Syndiniales; Amoebophryaceae; Amoebophrya"]

CAE6914669.1[&Organism="Symbiodinium sp. CCMP2592",Description="DRP4C [Symbiodinium sp. CCMP2592]","Genetic Code"="Standard",Taxonomy="Eukaryota; Sar; Alveolata; Dinophyceae; Suessiales; Symbiodiniaceae; Symbiodinium"]

CAI4004018.1[&Organism="Cladocopium goreaui",Description="unnamed protein product [Cladocopium goreaui]","Genetic Code"="Standard",Taxonomy="Eukaryota; Sar; Alveolata; Dinophyceae; Suessiales; Symbiodiniaceae; Cladocopium"]

CAI3978736.1[&Organism="Cladocopium goreaui",Description="unnamed protein product [Cladocopium goreaui]","Genetic Code"="Standard",Taxonomy="Eukaryota; Sar; Alveolata; Dinophyceae; Suessiales; Symbiodiniaceae; Cladocopium"]

OLP81297.1[&Organism="Symbiodinium microadriaticum",Description="Dynamin-related protein 4C [Symbiodinium microadriaticum]","Genetic Code"="Standard",Taxonomy="Eukaryota; Sar; Alveolata; Dinophyceae; Suessiales; Symbiodiniaceae; Symbiodinium"]

CAE7315868.1[&Organism="Symbiodinium natans",Description="DRP4C [Symbiodinium natans]","Genetic Code"="Standard",Taxonomy="Eukaryota; Sar; Alveolata; Dinophyceae; Suessiales; Symbiodiniaceae; Symbiodinium"]

CAE8582582.1[&Organism="Polarella glacialis",Description="unnamed protein product [Polarella glacialis]","Genetic Code"="Standard",Taxonomy="Eukaryota; Sar; Alveolata; Dinophyceae; Suessiales; Suessiaceae; Polarella"]

KAI0562045.1[&Organism="Gracilaria domingensis",Description="Dynamin [Gracilaria domingensis]","Genetic Code"="Standard",Taxonomy="Eukaryota; Rhodophyta; Florideophyceae; Rhodymeniophycidae; Gracilariales; Gracilariaceae; Gracilaria"]

KAI0557988.1[&Organism="Gracilaria domingensis",Description="Dynamin [Gracilaria domingensis]","Genetic Code"="Standard",Taxonomy="Eukaryota; Rhodophyta; Florideophyceae; Rhodymeniophycidae; Gracilariales; Gracilariaceae; Gracilaria"]

XP_005716602.1[&Organism="Chondrus crispus",Description="unnamed protein product [Chondrus crispus]","Genetic Code"="Standard",Taxonomy="Eukaryota; Rhodophyta; Florideophyceae; Rhodymeniophycidae; Gigartinales; Gigartinaceae; Chondrus","Common Name"="carragheen"]

KAI0559778.1[&Organism="Gracilaria domingensis",Description="Dynamin [Gracilaria domingensis]","Genetic Code"="Standard",Taxonomy="Eukaryota; Rhodophyta; Florideophyceae; Rhodymeniophycidae; Gracilariales; Gracilariaceae; Gracilaria"]

WGU15254.1[&Organism="Variosea sp.",Description="dynamin superfamily protein [Variosea sp.]","Genetic Code"="Standard",Taxonomy="Eukaryota; Amoebozoa; Evosea; Variosea"]

XP_013756556.1[&Organism="Thecamonas trahens ATCC 50062",Description="uncharacterized protein AMSG_12025 [Thecamonas trahens ATCC 50062]","Genetic Code"="Standard",Taxonomy="Eukaryota; Apusozoa; Apusomonadida; Apusomonadidae; Thecamonas"]

CAH6419740.1[&Organism="uncultured virus",Description="Dynamin family GTPase [uncultured virus]","Genetic Code"="Standard",Taxonomy="Viruses; environmental samples"]

AYV81982.1[&Organism="Homavirus sp.",Description="MAG: dynamin family protein [Homavirus sp.]","Genetic Code"="Standard",Taxonomy="Viruses; Varidnaviria; Bamfordvirae; Nucleocytoviricota; Megaviricetes; Imitervirales; Mimiviridae","Common Name"="soil metagenome"]

CAH6421112.1[&Organism="uncultured virus",Description="Dynamin family GTPase [uncultured virus]","Genetic Code"="Standard",Taxonomy="Viruses; environmental samples"]

ARF12445.1[&Organism="Klosneuvirus KNV1",Description="dynamin family protein [Klosneuvirus KNV1]","Genetic Code"="Standard",Taxonomy="Viruses; Varidnaviria; Bamfordvirae; Nucleocytoviricota; Megaviricetes; Imitervirales; Mimiviridae; Klosneuvirinae; Klosneuvirus"]

QKF93607.1[&Organism="Fadolivirus 1",Description="dynamin superfamily protein [Fadolivirus 1]","Genetic Code"="Standard",Taxonomy="Viruses"]

QKU35298.1[&Organism="Tupanvirus soda lake",Description="dynamin family protein [Tupanvirus soda lake]","Genetic Code"="Standard",Taxonomy="Viruses; Varidnaviria; Bamfordvirae; Nucleocytoviricota; Megaviricetes; Imitervirales; Mimiviridae; Tupanvirus"]

AYV83919.1[&Organism="Hyperionvirus sp.",Description="MAG: dynamin family protein [Hyperionvirus sp.]","Genetic Code"="Standard",Taxonomy="Viruses; Varidnaviria; Bamfordvirae; Nucleocytoviricota; Megaviricetes; Imitervirales; Mimiviridae","Common Name"="soil metagenome"]

QFG74079.1[&Organism="Megaviridae environmental sample",Description="MAG: dynamin family protein [Megaviridae environmental sample]","Genetic Code"="Standard",Taxonomy="Viruses; Varidnaviria; Bamfordvirae; Nucleocytoviricota; Megaviricetes; Imitervirales; Mimiviridae; environmental samples","Common Name"="marine metagenome"]

XP_750654.1[&Organism="Aspergillus fumigatus Af293",Description="dynamin GTPase, putative [Aspergillus fumigatus Af293]","Genetic Code"="Standard",Taxonomy="Eukaryota; Fungi; Dikarya; Ascomycota; Pezizomycotina; Eurotiomycetes; Eurotiomycetidae; Eurotiales; Aspergillaceae; Aspergillus; Aspergillus subgen. Fumigati"]

XP_006461472.1[&Organism="Agaricus bisporus var. bisporus H97",Description="hypothetical protein AGABI2DRAFT_222252 [Agaricus bisporus var. bisporus H97]","Genetic Code"="Standard",Taxonomy="Eukaryota; Fungi; Dikarya; Basidiomycota; Agaricomycotina; Agaricomycetes; Agaricomycetidae; Agaricales; Agaricaceae; Agaricus"]

XP_006461433.1[&Organism="Agaricus bisporus var. bisporus H97",Description="hypothetical protein AGABI2DRAFT_185678 [Agaricus bisporus var. bisporus H97]","Genetic Code"="Standard",Taxonomy="Eukaryota; Fungi; Dikarya; Basidiomycota; Agaricomycotina; Agaricomycetes; Agaricomycetidae; Agaricales; Agaricaceae; Agaricus"]

KAI3646081.1[&Organism="Amoeboaphelidium protococcarum",Description="hypothetical protein MP228_009009 [Amoeboaphelidium protococcarum]","Genetic Code"="Ciliate",Taxonomy="Eukaryota; Aphelida; Aphelidea; Amoeboaphelidium"]

KAJ9515210.1[&Description="KAJ9515210.1"]

ETO25748.1[&Organism="Reticulomyxa filosa",Description="myxovirus resistance 1 [Reticulomyxa filosa]","Genetic Code"="Standard",Taxonomy="Eukaryota; Sar; Rhizaria; Retaria; Foraminifera; Monothalamids; Reticulomyxidae; Reticulomyxa"]

OAJ38670.1[&Organism="Batrachochytrium dendrobatidis JEL423",Description="hypothetical protein BDEG_22578 [Batrachochytrium dendrobatidis JEL423]","Genetic Code"="Standard",Taxonomy="Eukaryota; Fungi; Fungi incertae sedis; Chytridiomycota; Chytridiomycota incertae sedis; Chytridiomycetes; Rhizophydiales; Rhizophydiales incertae sedis; Batrachochytrium"]

GAX85982.1[&Description="GAX85982.1"]

KAG2488600.1[&Description="KAG2488600.1"]

XP_042924875.1[&Description="uncharacterized protein CHLRE_05g238290v5 [Chlamydomonas reinhardtii]"]

XP_042924848.1[&Description="uncharacterized protein CHLRE_05g237200v5 [Chlamydomonas reinhardtii]"]

XP_042923301.1[&Description="uncharacterized protein CHLRE_06g250650v5 [Chlamydomonas reinhardtii]"]

XP_006457072.1[&Organism="Agaricus bisporus var. bisporus H97",Description="hypothetical protein AGABI2DRAFT_139599 [Agaricus bisporus var. bisporus H97]","Genetic Code"="Standard",Taxonomy="Eukaryota; Fungi; Dikarya; Basidiomycota; Agaricomycotina; Agaricomycetes; Agaricomycetidae; Agaricales; Agaricaceae; Agaricus"]

GBG30247.1[&Organism="Hondaea fermentalgiana",Description="Interferon-induced GTP-binding protein Mx1 [Hondaea fermentalgiana]","Genetic Code"="Standard",Taxonomy="Eukaryota; Sar; Stramenopiles; Bigyra; Labyrinthulomycetes; Thraustochytrida; Thraustochytriaceae; Hondaea"]

CEM15039.1[&Organism="Vitrella brassicaformis CCMP3155",Description="unnamed protein product [Vitrella brassicaformis CCMP3155]","Genetic Code"="Standard",Taxonomy="Eukaryota; Sar; Alveolata; Colpodellida; Vitrellaceae; Vitrella"]

PAA87312.1[&Organism="Macrostomum lignano",Description="hypothetical protein BOX15_Mlig004017g1, partial [Macrostomum lignano]","Genetic Code"="Standard",Taxonomy="Eukaryota; Metazoa; Platyhelminthes; Rhabditophora; Macrostomorpha; Macrostomida; Macrostomidae; Macrostomum"]

PAA68234.1[&Organism="Macrostomum lignano",Description="hypothetical protein BOX15_Mlig021919g1, partial [Macrostomum lignano]","Genetic Code"="Standard",Taxonomy="Eukaryota; Metazoa; Platyhelminthes; Rhabditophora; Macrostomorpha; Macrostomida; Macrostomidae; Macrostomum"]

NP_495986.3.3[&Organism="Caenorhabditis elegans",Description="Dynamin-type G domain-containing protein [Caenorhabditis elegans]","Genetic Code"="Standard",Taxonomy="Eukaryota; Metazoa; Ecdysozoa; Nematoda; Chromadorea; Rhabditida; Rhabditina; Rhabditomorpha; Rhabditoidea; Rhabditidae; Peloderinae; Caenorhabditis"]

XP_002602331.1[&Organism="Branchiostoma floridae",Description="hypothetical protein BRAFLDRAFT_60684 [Branchiostoma floridae]","Genetic Code"="Standard",Taxonomy="Eukaryota; Metazoa; Chordata; Cephalochordata; Branchiostomidae; Branchiostoma","Common Name"="Florida lancelet"]

XP_019637857.1[&Organism="Branchiostoma belcheri",Description="PREDICTED: dynamin-like 120 kDa protein, mitochondrial isoform X3 [Branchiostoma belcheri]","Genetic Code"="Standard",Taxonomy="Eukaryota; Metazoa; Chordata; Cephalochordata; Branchiostomidae; Branchiostoma","Common Name"="Belcher's lancelet"]

XP_006813643.1[&Organism="Saccoglossus kowalevskii",Description="PREDICTED: dynamin-like 120 kDa protein, mitochondrial-like [Saccoglossus kowalevskii]","Genetic Code"="Standard",Taxonomy="Eukaryota; Metazoa; Hemichordata; Enteropneusta; Harrimaniidae; Saccoglossus"]

XP_030843280.1[&Organism="Strongylocentrotus purpuratus",Description="dynamin-like 120 kDa protein, mitochondrial [Strongylocentrotus purpuratus]","Genetic Code"="Standard",Taxonomy="Eukaryota; Metazoa; Echinodermata; Eleutherozoa; Echinozoa; Echinoidea; Euechinoidea; Echinacea; Echinoida; Strongylocentrotidae; Strongylocentrotus","Common Name"="purple sea urchin"]

XP_018667792.1[&Organism="Ciona intestinalis",Description="dynamin-like 120 kDa protein, mitochondrial [Ciona intestinalis]","Genetic Code"="Standard",Taxonomy="Eukaryota; Metazoa; Chordata; Tunicata; Ascidiacea; Enterogona; Phlebobranchia; Cionidae; Ciona","Common Name"="vase tunicate"]

XP_032818114.1[&Organism="Petromyzon marinus",Description="dynamin-like 120 kDa protein, mitochondrial isoform X2 [Petromyzon marinus]","Genetic Code"="Standard",Taxonomy="Eukaryota; Metazoa; Chordata; Craniata; Vertebrata; Cyclostomata; Hyperoartia; Petromyzontiformes; Petromyzontidae; Petromyzon","Common Name"="sea lamprey"]

XP_021332524.1[&Organism="Danio rerio",Description="dynamin-like 120 kDa protein, mitochondrial isoform X5 [Danio rerio]","Genetic Code"="Standard",Taxonomy="Eukaryota; Metazoa; Chordata; Craniata; Vertebrata;

Euteleostomi; Actinopterygii; Neopterygii; Teleostei; Ostariophysi; Cypriniformes; Cyprinidae; Danio","Common Name"="zebrafish"]

XP_028587646.1[&Organism="Podarcis muralis",Description="LOW QUALITY PROTEIN: dynamin-like 120 kDa protein, mitochondrial [Podarcis muralis]","Genetic Code"="Standard",Taxonomy="Eukaryota; Metazoa; Chordata; Craniata; Vertebrata; Euteleostomi; Lepidosauria; Squamata; Bifurcata; Unidentata; Episquamata; Laterata; Lacertibaenia; Lacertidae; Podarcis","Common Name"="Common wall lizard"]

XP_025913835.1[&Organism="Apteryx rowi",Description="dynamin-like 120 kDa protein, mitochondrial isoform X10 [Apteryx rowi]","Genetic Code"="Standard",Taxonomy="Eukaryota; Metazoa; Chordata; Craniata; Vertebrata; Euteleostomi; Archelosauria; Archosauria; Dinosauria; Saurischia; Theropoda; Coelurosauria; Aves; Palaeognathae; Apterygiformes; Apterygidae; Apteryx","Common Name"="Okarito brown kiwi"]

XP_023440724.1[&Organism="Dasypus novemcinctus",Description="dynamin-like 120 kDa protein, mitochondrial [Dasypus novemcinctus]","Genetic Code"="Standard",Taxonomy="Eukaryota; Metazoa; Chordata; Craniata; Vertebrata; Euteleostomi; Mammalia; Eutheria; Xenarthra; Cingulata; Dasypodidae; Dasypus","Common Name"="nine-banded armadillo"]

XP_005873264.1[&Organism="Myotis brandtii",Description="PREDICTED: dynamin-like 120 kDa protein, mitochondrial isoform X5 [Myotis brandtii]","Genetic Code"="Standard",Taxonomy="Eukaryota; Metazoa; Chordata; Craniata; Vertebrata; Euteleostomi; Mammalia; Eutheria; Laurasiatheria; Chiroptera; Microchiroptera; Vespertilionidae; Myotis","Common Name"="Brandt's bat"]

NP_598513.1[&Organism="Mus musculus",Description="dynamin-like 120 kDa protein, mitochondrial isoform 2 precursor [Mus musculus]","Genetic Code"="Standard",Taxonomy="Eukaryota; Metazoa; Chordata; Craniata; Vertebrata; Euteleostomi; Mammalia; Eutheria; Euarchontoglires; Glires; Rodentia; Myomorpha; Muroidea; Muridae; Murinae; Mus; Mus","Common Name"="house mouse"]

XP_006163024.2.2[&Organism="Tupaia chinensis",Description="LOW QUALITY PROTEIN: dynamin-like 120 kDa protein, mitochondrial [Tupaia chinensis]","Genetic Code"="Standard",Taxonomy="Eukaryota; Metazoa; Chordata; Craniata; Vertebrata; Euteleostomi; Mammalia; Eutheria; Euarchontoglires; Scandentia; Tupaiidae; Tupaia","Common Name"="Chinese tree shrew"]

NP_056375.2.2[&Organism="Homo sapiens",Description="dynamin-like 120 kDa protein, mitochondrial isoform 1 preproprotein [Homo sapiens]","Genetic Code"="Standard",Taxonomy="Eukaryota; Metazoa; Chordata; Craniata; Vertebrata; Euteleostomi; Mammalia; Eutheria; Euarchontoglires; Primates; Haplorrhini; Catarrhini; Hominidae; Homo","Common Name"="human"]

XP_031757388.1[&Organism="Xenopus tropicalis",Description="dynamin-like 120 kDa protein, mitochondrial isoform X3 [Xenopus tropicalis]","Genetic Code"="Standard",Taxonomy="Eukaryota; Metazoa; Chordata; Craniata; Vertebrata; Euteleostomi; Amphibia; Batrachia; Anura; Pipoidea; Pipidae; Xenopodinae; Xenopus; Silurana","Common Name"="tropical clawed frog"]

NP_610941.1[&Organism="Drosophila melanogaster",Description="optic atrophy 1, isoform B [Drosophila melanogaster]","Genetic Code"="Standard",Taxonomy="Eukaryota; Metazoa; Ecdysozoa; Arthropoda; Hexapoda; Insecta; Pterygota; Neoptera; Holometabola; Diptera; Brachycera; Muscomorpha; Ephydroidea; Drosophilidae; Drosophila; Sophophora","Common Name"="fruit fly"]

MEN2496893[&Organism="Marteilia pararefringens",Description="MAG: Dynamin-like 120 kDa protein, mitochondrial, variant 2 [Marteilia pararefringens]","Genetic Code"="Standard",Taxonomy="Eukaryota; Sar; Rhizaria; Endomyxa; Ascetosporea; Paramyxea; Paramyxida; Marteiliidae; Marteilia","Common Name"="invertebrate metagenome"]

KAJ1637655.1[&Organism="Pavlovales sp. CCMP2436",Description="P-loop containing nucleoside triphosphate hydrolase protein [Pavlovales sp. CCMP2436]","Genetic Code"="Standard",Taxonomy="Eukaryota; Haptista; Haptophyta; Pavlovales"]

AYV75702.1[&Organism="Terrestrivirus sp.",Description="MAG: dynamin family protein [Terrestrivirus sp.]","Genetic Code"="Standard",Taxonomy="Viruses; Varidnaviria; Bamfordvirae; Nucleocytoviricota; Megaviricetes; Imitervirales; Mimiviridae","Common Name"="soil metagenome"]

KAI5073815.1[&Organism="Adiantum capillus-veneris",Description="hypothetical protein GOP47_0011828 [Adiantum capillus-veneris]","Genetic Code"="Standard",Taxonomy="Eukaryota; Viridiplantae; Streptophyta; Embryophyta; Tracheophyta; Polypodiopsida; Polypodiidae; Polypodiales; Pteridineae; Pteridaceae; Vittarioideae; Adiantum"]

XP_008646219.1[&Organism="Zea mays",Description="dynamin-2A [Zea mays]","Genetic Code"="Standard",Taxonomy="Eukaryota; Viridiplantae; Streptophyta; Embryophyta; Tracheophyta; Spermatophyta; Magnoliopsida; Liliopsida; Poales; Poaceae; PACMAD clade; Panicoideae; Andropogonodae; Andropogoneae; Tripsacinae; Zea"]

ACG47836.1[&Organism="Zea mays",Description="dynamin-2A [Zea mays]","Genetic Code"="Standard",Taxonomy="Eukaryota; Viridiplantae; Streptophyta; Embryophyta; Tracheophyta; Spermatophyta; Magnoliopsida; Liliopsida; Poales; Poaceae; PACMAD clade; Panicoideae; Andropogonodae; Andropogoneae; Tripsacinae; Zea"]

XP_006385192.1[&Organism="Populus trichocarpa",Description="dynamin-2A [Populus trichocarpa]","Genetic Code"="Standard",Taxonomy="Eukaryota; Viridiplantae; Streptophyta; Embryophyta; Tracheophyta; Spermatophyta; Magnoliopsida; eudicotyledons; Gunneridae; Pentapetalae; rosids; fabids; Malpighiales; Salicaceae; Saliceae; Populus","Common Name"="Populus balsamifera subsp. trichocarpa"]

'KAG7649995.1'[&Organism="Arabidopsis thaliana",Description="DRP2B [Arabidopsis thaliana]","Genetic Code"="Standard",Taxonomy="Eukaryota; Viridiplantae; Streptophyta; Embryophyta; Tracheophyta; Spermatophyta; Magnoliopsida; eudicotyledons; Gunneridae; Pentapetalae; rosids; malvids; Brassicales; Brassicaceae; Camelineae; Arabidopsis","Common Name"="thale cress"]

'NP_172500.1'[&Organism="Arabidopsis thaliana",Description="unnamed protein product [Arabidopsis thaliana]","Genetic Code"="Standard",Taxonomy="Eukaryota; Viridiplantae; Streptophyta; Embryophyta; Tracheophyta; Spermatophyta; Magnoliopsida;

eudicotyledons; Gunneridae; Pentapetalae; rosids; malvids; Brassicales; Brassicaceae; Camelineae; Arabidopsis","Common Name"="thale cress"]

KAH9330549.1[&Organism="Taxus chinensis",Description="hypothetical protein KI387_002657 [Taxus chinensis]","Genetic Code"="Standard",Taxonomy="Eukaryota; Viridiplantae; Streptophyta; Embryophyta; Tracheophyta; Spermatophyta; Pinopsida; Pinidae; Conifers II; Cupressales; Taxaceae; Taxus"]

ONM04707.1[&Organism="Zea mays",Description="Dynamin-2A [Zea mays]","Genetic Code"="Standard",Taxonomy="Eukaryota; Viridiplantae; Streptophyta; Embryophyta; Tracheophyta; Spermatophyta; Magnoliopsida; Liliopsida; Poales; Poaceae; PACMAD clade; Panicoideae; Andropogonodae; Andropogoneae; Tripsacinae; Zea"]

EFJ33653.1[&Organism="Selaginella moellendorffii",Description="hypothetical protein SELMODRAFT_439053 [Selaginella moellendorffii]","Genetic Code"="Standard",Taxonomy="Eukaryota; Viridiplantae; Streptophyta; Embryophyta; Tracheophyta; Lycopodiopsida; Selaginellales; Selaginellaceae; Selaginella"]

EFJ28901.1[&Organism="Selaginella moellendorffii",Description="hypothetical protein SELMODRAFT_171046 [Selaginella moellendorffii]","Genetic Code"="Standard",Taxonomy="Eukaryota; Viridiplantae; Streptophyta; Embryophyta; Tracheophyta; Lycopodiopsida; Selaginellales; Selaginellaceae; Selaginella"]

OAE31801.1[&Organism="Marchantia polymorpha subsp. ruderalis",Description="hypothetical protein AXG93_1838s1110 [Marchantia polymorpha subsp. ruderalis]","Genetic Code"="Standard",Taxonomy="Eukaryota; Viridiplantae; Streptophyta; Embryophyta; Marchantiophyta; Marchantiopsida; Marchantiidae; Marchantiales; Marchantiaceae; Marchantia"]

KAG0632288.1[&Organism="Ceratodon purpureus",Description="hypothetical protein M758_1G317400 [Ceratodon purpureus]","Genetic Code"="Standard",Taxonomy="Eukaryota; Viridiplantae; Streptophyta; Embryophyta; Bryophyta; Bryophytina; Bryopsida; Dicranidae; Pseudoditrichales; Ditrichaceae; Ceratodon"]

'KAG0555995.1'[&Organism="Ceratodon purpureus",Description="hypothetical protein KC19_11G018700 [Ceratodon purpureus]","Genetic Code"="Standard",Taxonomy="Eukaryota; Viridiplantae; Streptophyta; Embryophyta; Bryophyta; Bryophytina; Bryopsida; Dicranidae; Pseudoditrichales; Ditrichaceae; Ceratodon"]

XP_024368367.1[&Organism="Physcomitrium patens",Description="dynamin-2A-like [Physcomitrium patens]","Genetic Code"="Standard",Taxonomy="Eukaryota; Viridiplantae; Streptophyta; Embryophyta; Bryophyta; Bryophytina; Bryopsida; Funariidae; Funariales; Funariaceae; Physcomitrium"]

XP_024391061.1[&Organism="Physcomitrium patens",Description="dynamin-2B-like [Physcomitrium patens]","Genetic Code"="Standard",Taxonomy="Eukaryota; Viridiplantae; Streptophyta; Embryophyta; Bryophyta; Bryophytina; Bryopsida; Funariidae; Funariales; Funariaceae; Physcomitrium"]

ARF10781.1[&Organism="Hokovirus HKV1",Description="dynamin-like protein [Hokovirus HKV1]","Genetic Code"="Standard",Taxonomy="Viruses; Varidnaviria;

Bamfordvirae; Nucleocytoviricota; Megaviricetes; Imitervirales; Mimiviridae; Klosneuvirinae; Hokovirus"]

ARF10780.1[&Organism="Hokovirus HKV1",Description="dynamin-like protein [Hokovirus HKV1]","Genetic Code"="Standard",Taxonomy="Viruses; Varidnaviria; Bamfordvirae; Nucleocytoviricota; Megaviricetes; Imitervirales; Mimiviridae; Klosneuvirinae; Hokovirus"]

KAH8061769.1[&Organism="Aureococcus anophagefferens",Description="hypothetical protein JL722_3723 [Aureococcus anophagefferens]","Genetic Code"="Standard",Taxonomy="Eukaryota; Sar; Stramenopiles; Ochrophyta; Pelagophyceae; Pelagomonadales; Aureococcus"]

OUS45493.1[&Organism="Ostreococcus tauri",Description="P-loop containing nucleoside triphosphate hydrolase protein [Ostreococcus tauri]","Genetic Code"="Standard",Taxonomy="Eukaryota; Viridiplantae; Chlorophyta; Mamiellophyceae; Mamiellales; Bathycoccaceae; Ostreococcus"]

XP_001419538.1[&Organism="Ostreococcus lucimarinus CCE9901",Description="predicted protein [Ostreococcus lucimarinus CCE9901]","Genetic Code"="Standard",Taxonomy="Eukaryota; Viridiplantae; Chlorophyta; Mamiellophyceae; Mamiellales; Bathycoccaceae; Ostreococcus"]

PRP82121.1[&Organism="Planoprotostelium fungivorum",Description="hypothetical protein PROFUN_10329 [Planoprotostelium fungivorum]","Genetic Code"="Standard",Taxonomy="Eukaryota; Amoebozoa; Evosea; Variosea; Cavosteliida; Cavosteliaceae; Planoprotostelium"]

TMW65229.1[&Organism="Pythium oligandrum",Description="hypothetical protein Poli38472_009396 [Pythium oligandrum]","Genetic Code"="Standard",Taxonomy="Eukaryota; Sar; Stramenopiles; Oomycota; Pythiales; Pythiaceae; Pythium"]

KAG1689960.1[&Organism="Phytophthora capsici",Description="hypothetical protein DVH05_001790 [Phytophthora capsici]","Genetic Code"="Standard",Taxonomy="Eukaryota; Sar; Stramenopiles; Oomycota; Peronosporales; Peronosporaceae; Phytophthora"]

KAE9027747.1[&Organism="Phytophthora rubi",Description="hypothetical protein PR001_g11898 [Phytophthora rubi]","Genetic Code"="Standard",Taxonomy="Eukaryota; Sar; Stramenopiles; Oomycota; Peronosporales; Peronosporaceae; Phytophthora"]

KAG7385474.1[&Organism="Phytophthora boehmeriae",Description="hypothetical protein PHYBOEH_009011 [Phytophthora boehmeriae]","Genetic Code"="Standard",Taxonomy="Eukaryota; Sar; Stramenopiles; Oomycota; Peronosporales; Peronosporaceae; Phytophthora"]

KAH7489187.1[&Organism="Phytophthora ramorum",Description="Interferon-induced GTP-binding protein Mx1 [Phytophthora ramorum]","Genetic Code"="Standard",Taxonomy="Eukaryota; Sar; Stramenopiles; Oomycota; Peronosporales; Peronosporaceae; Phytophthora","Common Name"="sudden oak death agent"]

POM76499.1[&Organism="Phytophthora palmivora var. palmivora",Description="Dynamin domain containing protein, partial [Phytophthora

palmivora var. palmivora]","Genetic Code"="Standard",Taxonomy="Eukaryota; Sar; Stramenopiles; Oomycota; Peronosporales; Peronosporaceae; Phytophthora"]

KAG7377001.1[&Organism="Phytophthora pseudosyringae",Description="hypothetical protein PHYPSEUDO_012338 [Phytophthora pseudosyringae]","Genetic Code"="Standard",Taxonomy="Eukaryota; Sar; Stramenopiles; Oomycota; Peronosporales; Peronosporaceae; Phytophthora"]

XP_008910862.1[&Organism="Phytophthora parasitica INRA-310",Description="hypothetical protein PPTG_15242 [Phytophthora parasitica INRA-310]","Genetic Code"="Standard",Taxonomy="Eukaryota; Sar; Stramenopiles; Oomycota; Peronosporales; Peronosporaceae; Phytophthora"]

KAF1774311.1[&Organism="Phytophthora cactorum",Description="Dynamin-type guanine nucleotide-binding (G) domain [Phytophthora cactorum]","Genetic Code"="Standard",Taxonomy="Eukaryota; Sar; Stramenopiles; Oomycota; Peronosporales; Peronosporaceae; Phytophthora"]

KAI9982172.1[&Organism="Phytophthora infestans",Description="hypothetical protein PInf_008063 [Phytophthora infestans]","Genetic Code"="Standard",Taxonomy="Eukaryota; Sar; Stramenopiles; Oomycota; Peronosporales; Peronosporaceae; Phytophthora","Common Name"="potato late blight agent"]

KAE8986371.1[&Organism="Phytophthora rubi",Description="hypothetical protein PR001_g22620 [Phytophthora rubi]","Genetic Code"="Standard",Taxonomy="Eukaryota; Sar; Stramenopiles; Oomycota; Peronosporales; Peronosporaceae; Phytophthora"]

KAG8459568.1[&Organism="Diacronema lutheri",Description="hypothetical protein KFE25_000924 [Diacronema lutheri]","Genetic Code"="Standard",Taxonomy="Eukaryota; Haptista; Haptophyta; Pavlovales; Pavlovaceae; Diacronema"]

XP_004338334.1[&Organism="Acanthamoeba castellanii str. Neff",Description="dynamin domain containing protein [Acanthamoeba castellanii str. Neff]","Genetic Code"="Standard",Taxonomy="Eukaryota; Amoebozoa; Discosea; Longamoebia; Centramoebida; Acanthamoebidae; Acanthamoeba"]

KOO23261.1[&Organism="Chrysochromulina tobinii",Description="hypothetical protein Ctob_000830 [Chrysochromulina tobinii]","Genetic Code"="Standard",Taxonomy="Eukaryota; Haptista; Haptophyta; Prymnesiophyceae; Prymnesiales; Chrysochromulinaceae; Chrysochromulina"]

'XP_005785253.1'[&Organism="Emiliania huxleyi CCMP1516",Description="hypothetical protein EMIHUDRAFT_631932, partial [Emiliania huxleyi CCMP1516]","Genetic Code"="Standard",Taxonomy="Eukaryota; Haptista; Haptophyta; Prymnesiophyceae; Isochrysidales; Noelaerhabdaceae; Emiliania"]

XP_005792501.1[&Organism="Emiliania huxleyi CCMP1516",Description="hypothetical protein EMIHUDRAFT_522907 [Emiliania huxleyi CCMP1516]","Genetic Code"="Standard",Taxonomy="Eukaryota; Haptista; Haptophyta; Prymnesiophyceae; Isochrysidales; Noelaerhabdaceae; Emiliania"]

XP_042918632.1[&Organism="Chlamydomonas reinhardtii",Description="uncharacterized protein CHLRE_12g529450v5 [Chlamydomonas reinhardtii]","Genetic Code"="Standard",Taxonomy="Eukaryota; Viridiplantae; Chlorophyta;

core chlorophytes; Chlorophyceae; CS clade; Chlamydomonadales; Chlamydomonadaceae; Chlamydomonas"]

PWZ11893.1[&Organism="Zea mays",Description="Dynamin-related protein 5A [Zea mays]","Genetic Code"="Standard",Taxonomy="Eukaryota; Viridiplantae; Streptophyta; Embryophyta; Tracheophyta; Spermatophyta; Magnoliopsida; Liliopsida; Poales; Poaceae; PACMAD clade; Panicoideae; Andropogonodae; Andropogoneae; Tripsacinae; Zea"]

NP_001130364.1[&Organism="Zea mays",Description="Dynamin-related protein 5A-like [Zea mays]","Genetic Code"="Standard",Taxonomy="Eukaryota; Viridiplantae; Streptophyta; Embryophyta; Tracheophyta; Spermatophyta; Magnoliopsida; Liliopsida; Poales; Poaceae; PACMAD clade; Panicoideae; Andropogonodae; Andropogoneae; Tripsacinae; Zea"]

AAF87857.1[&Organism="Arabidopsis thaliana",Description="Hypothetical protein [Arabidopsis thaliana]","Genetic Code"="Standard",Taxonomy="Eukaryota; Viridiplantae; Streptophyta; Embryophyta; Tracheophyta; Spermatophyta; Magnoliopsida; eudicotyledons; Gunneridae; Pentapetalae; rosids; malvids; Brassicales; Brassicaceae; Camelineae; Arabidopsis","Common Name"="thale cress"]

XP_002317496.2[&Organism="Populus trichocarpa",Description="dynamin-related protein 5A [Populus trichocarpa]","Genetic Code"="Standard",Taxonomy="Eukaryota; Viridiplantae; Streptophyta; Embryophyta; Tracheophyta; Spermatophyta; Magnoliopsida; eudicotyledons; Gunneridae; Pentapetalae; rosids; fabids; Malpighiales; Salicaceae; Saliceae; Populus","Common Name"="Populus balsamifera subsp. trichocarpa"]

KAI5058044.1[&Organism="Adiantum capillus-veneris",Description="hypothetical protein GOP47_0026214 [Adiantum capillus-veneris]","Genetic Code"="Standard",Taxonomy="Eukaryota; Viridiplantae; Streptophyta; Embryophyta; Tracheophyta; Polypodiopsida; Polypodiidae; Polypodiales; Pteridineae; Pteridaceae; Vittarioideae; Adiantum"]

EFJ18064.1[&Organism="Selaginella moellendorffii",Description="hypothetical protein SELMODRAFT_113285 [Selaginella moellendorffii]","Genetic Code"="Standard",Taxonomy="Eukaryota; Viridiplantae; Streptophyta; Embryophyta; Tracheophyta; Lycopodiopsida; Selaginellales; Selaginellaceae; Selaginella"]

PTQ33908.1[&Organism="Marchantia polymorpha",Description="hypothetical protein MARPO_0084s0004 [Marchantia polymorpha]","Genetic Code"="Standard",Taxonomy="Eukaryota; Viridiplantae; Streptophyta; Embryophyta; Marchantiophyta; Marchantiopsida; Marchantiidae; Marchantiales; Marchantiaceae; Marchantia","Common Name"="liverwort"]

KAG0628798.1[&Organism="Ceratodon purpureus",Description="hypothetical protein M758_1G053700 [Ceratodon purpureus]","Genetic Code"="Standard",Taxonomy="Eukaryota; Viridiplantae; Streptophyta; Embryophyta; Bryophyta; Bryophytina; Bryopsida; Dicranidae; Pseudoditrichales; Ditrichaceae; Ceratodon"]

KAH9322298.1[&Organism="Taxus chinensis",Description="hypothetical protein KI387_016937, partial [Taxus chinensis]","Genetic Code"="Standard",Taxonomy="Eukaryota; Viridiplantae; Streptophyta; Embryophyta;

Tracheophyta; Spermatophyta; Pinopsida; Pinidae; Conifers II; Cupressales; Taxaceae; Taxus"]

XP_002683545.1[&Organism="Naegleria gruberi",Description="dynamin family GTPase [Naegleria gruberi]","Genetic Code"="Standard",Taxonomy="Eukaryota; Discoba; Heterolobosea; Tetramitia; Eutetramitia; Vahlkampfiidae; Naegleria"]

XP_044544418.1[&Organism="Naegleria lovaniensis",Description="uncharacterized protein C9374_009867 [Naegleria lovaniensis]","Genetic Code"="Standard",Taxonomy="Eukaryota; Discoba; Heterolobosea; Tetramitia; Eutetramitia; Vahlkampfiidae; Naegleria"]

XP_044559198.1[&Organism="Naegleria fowleri",Description="uncharacterized protein FDP41_006517 [Naegleria fowleri]","Genetic Code"="Standard",Taxonomy="Eukaryota; Discoba; Heterolobosea; Tetramitia; Eutetramitia; Vahlkampfiidae; Naegleria"]

XP_004182822.1[&Organism="Entamoeba invadens IP1",Description="hypothetical protein EIN_376410 [Entamoeba invadens IP1]","Genetic Code"="Standard",Taxonomy="Eukaryota; Amoebozoa; Evosea; Archamoebae; Mastigamoebida; Entamoebidae; Entamoeba"]

XP_008857507.1[&Organism="Entamoeba nuttalli P19",Description="dynamin family protein [Entamoeba nuttalli P19]","Genetic Code"="Standard",Taxonomy="Eukaryota; Amoebozoa; Evosea; Archamoebae; Mastigamoebida; Entamoebidae; Entamoeba"]

XP_004340186.1[&Organism="Acanthamoeba castellanii str. Neff",Description="dynamin domain containing protein [Acanthamoeba castellanii str. Neff]","Genetic Code"="Standard",Taxonomy="Eukaryota; Amoebozoa; Discosea; Longamoebia; Centramoebida; Acanthamoebidae; Acanthamoeba"]

KAH3742895.1[&Organism="Pelomyxa schiedti",Description="dynamin family GTPase [Pelomyxa schiedti]","Genetic Code"="Standard",Taxonomy="Eukaryota; Amoebozoa; Evosea; Archamoebae; Pelobiontida; Pelomyxidae; Pelomyxa"]

KYQ94066.1[&Organism="Tieghemostelium lacteum",Description="dynamin like protein [Tieghemostelium lacteum]","Genetic Code"="Standard",Taxonomy="Eukaryota; Amoebozoa; Evosea; Eumycetozoa; Dictyostelia; Dictyosteliales; Raperosteliaceae; Tieghemostelium"]

XP_004366192.1[&Organism="Cavenderia fasciculata",Description="dynamin like protein [Cavenderia fasciculata]","Genetic Code"="Standard",Taxonomy="Eukaryota; Amoebozoa; Evosea; Eumycetozoa; Dictyostelia; Acytosteliales; Cavenderiaceae; Cavenderia"]

XP_645576.2[&Organism="Dictyostelium discoideum AX4",Description="dynamin like protein [Dictyostelium discoideum AX4]","Genetic Code"="Standard",Taxonomy="Eukaryota; Amoebozoa; Evosea; Eumycetozoa; Dictyostelia; Dictyosteliales; Dictyosteliaceae; Dictyostelium"]

XP_003288319.1[&Organism="Dictyostelium purpureum",Description="hypothetical protein DICPUDRAFT_33855 [Dictyostelium purpureum]","Genetic Code"="Standard",Taxonomy="Eukaryota; Amoebozoa; Evosea; Eumycetozoa; Dictyostelia; Dictyosteliales; Dictyosteliaceae; Dictyostelium"]

KAF2073270.1[&Organism="Polysphondylium violaceum",Description="hypothetical protein CYY_005432 [Polysphondylium violaceum]","Genetic Code"="Standard",Taxonomy="Eukaryota; Amoebozoa; Evosea; Eumycetozoa; Dictyostelia; Dictyosteliales; Dictyosteliaceae; Polysphondylium"]

XP_020428321.1[&Organism="Heterostelium album PN500",Description="dynamin like protein [Heterostelium album PN500]","Genetic Code"="Standard",Taxonomy="Eukaryota; Amoebozoa; Evosea; Eumycetozoa; Dictyostelia; Acytosteliales; Acytosteliaceae; Heterostelium"]

XP_012754836.1[&Organism="Acytostelium subglobosum LB1",Description="hypothetical protein SAMD00019534_049110, partial [Acytostelium subglobosum LB1]","Genetic Code"="Standard",Taxonomy="Eukaryota; Amoebozoa; Evosea; Eumycetozoa; Dictyostelia; Acytosteliales; Acytosteliaceae; Acytostelium"]

PRP82286.1[&Organism="Planoprotostelium fungivorum",Description="hypothetical protein PROFUN_06298 [Planoprotostelium fungivorum]","Genetic Code"="Standard",Taxonomy="Eukaryota; Amoebozoa; Evosea; Variosea; Cavosteliida; Cavosteliaceae; Planoprotostelium"]

QYA18543.1[&Organism="Clandestinovirus",Description="dynamin 1-like protein [Clandestinovirus]","Genetic Code"="Standard",Taxonomy="Viruses; unclassified viruses"]

XP_005775651.1[&Organism="Emiliania huxleyi CCMP1516",Description="ARC5B, dynamin-related protein [Emiliania huxleyi CCMP1516]","Genetic Code"="Standard",Taxonomy="Eukaryota; Haptista; Haptophyta; Prymnesiophyceae; Isochrysidales; Noelaerhabdaceae; Emiliania"]

XP_005767412.1[&Organism="Emiliania huxleyi CCMP1516",Description="ARC5A, dynamin-related protein [Emiliania huxleyi CCMP1516]","Genetic Code"="Standard",Taxonomy="Eukaryota; Haptista; Haptophyta; Prymnesiophyceae; Isochrysidales; Noelaerhabdaceae; Emiliania"]

KOO34265.1[&Organism="Chrysochromulina tobinii",Description="protein arc5 [Chrysochromulina tobinii]","Genetic Code"="Standard",Taxonomy="Eukaryota; Haptista; Haptophyta; Prymnesiophyceae; Prymnesiales; Chrysochromulinaceae; Chrysochromulina"]

EWM28268.1[&Organism="Nannochloropsis gaditana",Description="Dynamin [Nannochloropsis gaditana]","Genetic Code"="Standard",Taxonomy="Eukaryota; Sar; Stramenopiles; Ochrophyta; Eustigmatophyceae; Eustigmatales; Monodopsidaceae; Nannochloropsis"]

KAG5184668.1[&Organism="Tribonema minus",Description="hypothetical protein JKP88DRAFT_348446 [Tribonema minus]","Genetic Code"="Standard",Taxonomy="Eukaryota; Sar; Stramenopiles; Ochrophyta; PX clade; Xanthophyceae; Tribonematales; Tribonemataceae; Tribonema"]

CBN78455.1[&Organism="Ectocarpus siliculosus",Description="ARC5, dynamin-related protein involved in plastid division [Ectocarpus siliculosus]","Genetic Code"="Standard",Taxonomy="Eukaryota; Sar; Stramenopiles; Ochrophyta; PX clade; Phaeophyceae; Ectocarpales; Ectocarpaceae; Ectocarpus"]

GMI61978.1[&Organism="Parmales sp. scaly parma",Description="hypothetical protein ScalyP_jg8233 [Parmales sp. scaly parma]","Genetic

Code"="Standard",Taxonomy="Eukaryota; Sar; Stramenopiles; Ochrophyta; Bolidophyceae; Parmales"]

GMH67967.1[&Organism="Triparma retinervis",Description="hypothetical protein TrRE_jg10005 [Triparma retinervis]","Genetic Code"="Standard",Taxonomy="Eukaryota; Sar; Stramenopiles; Ochrophyta; Bolidophyceae; Parmales; Triparmaceae; Triparma"]

GMH92561.1[&Organism="Triparma strigata",Description="hypothetical protein TrST_g7259 [Triparma strigata]","Genetic Code"="Standard",Taxonomy="Eukaryota; Sar; Stramenopiles; Ochrophyta; Bolidophyceae; Parmales; Triparmaceae; Triparma"]

GMI07688.1[&Organism="Triparma laevis f. longispina",Description="hypothetical protein TrLO_g13234 [Triparma laevis f. longispina]","Genetic Code"="Standard",Taxonomy="Eukaryota; Sar; Stramenopiles; Ochrophyta; Bolidophyceae; Parmales; Triparmaceae; Triparma"]

EJK67908.1[&Organism="Thalassiosira oceanica",Description="hypothetical protein THAOC_10987 [Thalassiosira oceanica]","Genetic Code"="Standard",Taxonomy="Eukaryota; Sar; Stramenopiles; Ochrophyta; Bacillariophyta; Coscinodiscophyceae; Thalassiosirophycidae; Thalassiosirales; Thalassiosiraceae; Thalassiosira"]

KAI2494507.1[&Organism="Fragilaria crotonensis",Description="hypothetical protein MHU86_20003 [Fragilaria crotonensis]","Genetic Code"="Standard",Taxonomy="Eukaryota; Sar; Stramenopiles; Ochrophyta; Bacillariophyta; Fragilariophyceae; Fragilariophycidae; Fragilariales; Fragilariaceae; Fragilaria"]

CAB9516894.1[&Organism="Seminavis robusta",Description="Dynamin-like protein ARC5 [Seminavis robusta]","Genetic Code"="Standard",Taxonomy="Eukaryota; Sar; Stramenopiles; Ochrophyta; Bacillariophyta; Bacillariophyceae; Bacillariophycidae; Naviculales; Naviculaceae; Seminavis"]

GKY99394.1[&Organism="Mayamaea pseudoterrestris",Description="hypothetical protein MPSEU_000894100 [Mayamaea pseudoterrestris]","Genetic Code"="Standard",Taxonomy="Eukaryota; Sar; Stramenopiles; Ochrophyta; Bacillariophyta; Bacillariophyceae; Bacillariophycidae; Naviculales; Naviculaceae; Mayamaea"]

XP_042920073.1[&Organism="Chlamydomonas reinhardtii",Description="uncharacterized protein CHLRE_10g433050v5 [Chlamydomonas reinhardtii]","Genetic Code"="Standard",Taxonomy="Eukaryota; Viridiplantae; Chlorophyta; core chlorophytes; Chlorophyceae; CS clade; Chlamydomonadales; Chlamydomonadaceae; Chlamydomonas"]

PWZ44616.1[&Organism="Zea mays",Description="Dynamin-like protein ARC5 [Zea mays]","Genetic Code"="Standard",Taxonomy="Eukaryota; Viridiplantae; Streptophyta; Embryophyta; Tracheophyta; Spermatophyta; Magnoliopsida; Liliopsida; Poales; Poaceae; PACMAD clade; Panicoideae; Andropogonodae; Andropogoneae; Tripsacinae; Zea"]

NP_001189935.1[&Organism="Arabidopsis thaliana",Description="P-loop containing nucleoside triphosphate hydrolases superfamily protein [Arabidopsis thaliana]","Genetic Code"="Standard",Taxonomy="Eukaryota; Viridiplantae; Streptophyta; Embryophyta; Tracheophyta; Spermatophyta; Magnoliopsida; eudicotyledons;

Gunneridae; Pentapetalae; rosids; malvids; Brassicales; Brassicaceae; Camelineae; Arabidopsis","Common Name"="thale cress"]

XP_002309632.3[&Organism="Populus trichocarpa",Description="dynamin-like protein ARC5 [Populus trichocarpa]","Genetic Code"="Standard",Taxonomy="Eukaryota; Viridiplantae; Streptophyta; Embryophyta; Tracheophyta; Spermatophyta; Magnoliopsida; eudicotyledons; Gunneridae; Pentapetalae; rosids; fabids; Malpighiales; Salicaceae; Saliceae; Populus","Common Name"="Populus balsamifera subsp. trichocarpa"]

EFJ19523.1[&Organism="Selaginella moellendorffii",Description="hypothetical protein SELMODRAFT_110974 [Selaginella moellendorffii]","Genetic Code"="Standard",Taxonomy="Eukaryota; Viridiplantae; Streptophyta; Embryophyta; Tracheophyta; Lycopodiopsida; Selaginellales; Selaginellaceae; Selaginella"]

KAH9308354.1[&Organism="Taxus chinensis",Description="hypothetical protein KI387_036265, partial [Taxus chinensis]","Genetic Code"="Standard",Taxonomy="Eukaryota; Viridiplantae; Streptophyta; Embryophyta; Tracheophyta; Spermatophyta; Pinopsida; Pinidae; Conifers II; Cupressales; Taxaceae; Taxus"]

KAI5064281.1[&Organism="Adiantum capillus-veneris",Description="hypothetical protein GOP47_0020951 [Adiantum capillus-veneris]","Genetic Code"="Standard",Taxonomy="Eukaryota; Viridiplantae; Streptophyta; Embryophyta; Tracheophyta; Polypodiopsida; Polypodiidae; Polypodiales; Pteridineae; Pteridaceae; Vittarioideae; Adiantum"]

PTQ34556.1[&Organism="Marchantia polymorpha",Description="hypothetical protein MARPO_0079s0059 [Marchantia polymorpha]","Genetic Code"="Standard",Taxonomy="Eukaryota; Viridiplantae; Streptophyta; Embryophyta; Marchantiophyta; Marchantiopsida; Marchantiidae; Marchantiales; Marchantiaceae; Marchantia","Common Name"="liverwort"]

KAG0561482.1[&Organism="Ceratodon purpureus",Description="hypothetical protein KC19_9G067700 [Ceratodon purpureus]","Genetic Code"="Standard",Taxonomy="Eukaryota; Viridiplantae; Streptophyta; Embryophyta; Bryophyta; Bryophytina; Bryopsida; Dicranidae; Pseudoditrichales; Ditrichaceae; Ceratodon"]

KAG0605142.1[&Organism="Ceratodon purpureus",Description="hypothetical protein M758_9G034900 [Ceratodon purpureus]","Genetic Code"="Standard",Taxonomy="Eukaryota; Viridiplantae; Streptophyta; Embryophyta; Bryophyta; Bryophytina; Bryopsida; Dicranidae; Pseudoditrichales; Ditrichaceae; Ceratodon"]

XP_009032466.1.2[&Organism="Aureococcus anophagefferens",Description="hypothetical protein AURANDRAFT_60924 [Aureococcus anophagefferens]","Genetic Code"="Standard",Taxonomy="Eukaryota; Sar; Stramenopiles; Ochrophyta; Pelagophyceae; Pelagomonadales; Aureococcus"]

XP_009032466.1[&Organism="Aureococcus anophagefferens",Description="hypothetical protein AURANDRAFT_60924 [Aureococcus anophagefferens]","Genetic Code"="Standard",Taxonomy="Eukaryota; Sar; Stramenopiles; Ochrophyta; Pelagophyceae; Pelagomonadales; Aureococcus"]

XP_004336224.1[&Organism="Acanthamoeba castellanii str. Neff",Description="dynamin family protein [Acanthamoeba castellanii str. Neff]","Genetic Code"="Standard",Taxonomy="Eukaryota; Amoebozoa; Discosea; Longamoebia; Centramoebida; Acanthamoebidae; Acanthamoeba"]

XP_002649212.1[&Organism="Dictyostelium discoideum AX4",Description="dynamin like protein [Dictyostelium discoideum AX4]","Genetic Code"="Standard",Taxonomy="Eukaryota; Amoebozoa; Evosea; Eumycetozoa; Dictyostelia; Dictyosteliales; Dictyosteliaceae; Dictyostelium"]

XP_003288465.1[&Organism="Dictyostelium purpureum",Description="uncharacterized protein DICPUDRAFT_34090 [Dictyostelium purpureum]","Genetic Code"="Standard",Taxonomy="Eukaryota; Amoebozoa; Evosea; Eumycetozoa; Dictyostelia; Dictyosteliales; Dictyosteliaceae; Dictyostelium"]

KAF2077035.1[&Organism="Polysphondylium violaceum",Description="hypothetical protein CYY_001667 [Polysphondylium violaceum]","Genetic Code"="Standard",Taxonomy="Eukaryota; Amoebozoa; Evosea; Eumycetozoa; Dictyostelia; Dictyosteliales; Dictyosteliaceae; Polysphondylium"]

XP_020436215.1[&Organism="Heterostelium album PN500",Description="dynamin like protein [Heterostelium album PN500]","Genetic Code"="Standard",Taxonomy="Eukaryota; Amoebozoa; Evosea; Eumycetozoa; Dictyostelia; Acytosteliales; Acytosteliaceae; Heterostelium"]

XP_012753198.1[&Organism="Acytostelium subglobosum LB1",Description="hypothetical protein SAMD00019534_073370 [Acytostelium subglobosum LB1]","Genetic Code"="Standard",Taxonomy="Eukaryota; Amoebozoa; Evosea; Eumycetozoa; Dictyostelia; Acytosteliales; Acytosteliaceae; Acytostelium"]

XP_004360608.1[&Organism="Cavenderia fasciculata",Description="dynamin like protein [Cavenderia fasciculata]","Genetic Code"="Standard",Taxonomy="Eukaryota; Amoebozoa; Evosea; Eumycetozoa; Dictyostelia; Acytosteliales; Cavenderiaceae; Cavenderia"]

KYQ90260.1[&Organism="Tieghemostelium lacteum",Description="dynamin like protein [Tieghemostelium lacteum]","Genetic Code"="Standard",Taxonomy="Eukaryota; Amoebozoa; Evosea; Eumycetozoa; Dictyostelia; Dictyosteliales; Raperosteliaceae; Tieghemostelium"]

KAH3767868.1[&Organism="Pelomyxa schiedti",Description="dynamin family protein [Pelomyxa schiedti]","Genetic Code"="Standard",Taxonomy="Eukaryota; Amoebozoa; Evosea; Archamoebae; Pelobiontida; Pelomyxidae; Pelomyxa"]

ATZ80405.1[&Organism="Bodo saltans virus",Description="putative dynamin-1-like protein [Bodo saltans virus]","Genetic Code"="Standard",Taxonomy="Viruses; Varidnaviria; Bamfordvirae; Nucleocytoviricota; Megaviricetes; Imitervirales; Mimiviridae; Klosneuvirinae; Klosneuvirus"]

KAF5834941.1[&Organism="Dunaliella salina",Description="P-loop containing nucleoside triphosphate hydrolase protein [Dunaliella salina]","Genetic Code"="Standard",Taxonomy="Eukaryota; Viridiplantae; Chlorophyta; core chlorophytes; Chlorophyceae; CS clade; Chlamydomonadales; Dunaliellaceae; Dunaliella"]

QFG74057.1[&Organism="Megaviridae environmental sample",Description="MAG: dynamin family protein [Megaviridae environmental sample]","Genetic Code"="Standard",Taxonomy="Viruses; Varidnaviria; Bamfordvirae; Nucleocytoviricota; Megaviricetes; Imitervirales; Mimiviridae; environmental samples","Common Name"="marine metagenome"]

CEM26963.1[&Organism="Vitrella brassicaformis CCMP3155",Description="unnamed protein product [Vitrella brassicaformis CCMP3155]","Genetic Code"="Standard",Taxonomy="Eukaryota; Sar; Alveolata; Colpodellida; Vitrellaceae; Vitrella"]

OII76931.1[&Organism="Cryptosporidium andersoni",Description="hypothetical protein cand_022740 [Cryptosporidium andersoni]","Genetic Code"="Standard",Taxonomy="Eukaryota; Sar; Alveolata; Apicomplexa; Conoidasida; Coccidia; Eucoccidiorida; Eimeriorina; Cryptosporidiidae; Cryptosporidium"]

KAH8582109.1[&Organism="Cryptosporidium sp. chipmunk genotype I",Description="dynamin like TRAFAC class GTpase domain [Cryptosporidium sp. chipmunk genotype I]","Genetic Code"="Standard",Taxonomy="Eukaryota; Sar; Alveolata; Apicomplexa; Conoidasida; Coccidia; Eucoccidiorida; Eimeriorina; Cryptosporidiidae; Cryptosporidium"]

POM84969.1[&Organism="Cryptosporidium meleagridis",Description="Dynamin family protein [Cryptosporidium meleagridis]","Genetic Code"="Standard",Taxonomy="Eukaryota; Sar; Alveolata; Apicomplexa; Conoidasida; Coccidia; Eucoccidiorida; Eimeriorina; Cryptosporidiidae; Cryptosporidium"]

XP_667128.1[&Organism="Cryptosporidium hominis TU502",Description="hypothetical protein [Cryptosporidium hominis TU502]","Genetic Code"="Standard",Taxonomy="Eukaryota; Sar; Alveolata; Apicomplexa; Conoidasida; Coccidia; Eucoccidiorida; Eimeriorina; Cryptosporidiidae; Cryptosporidium"]

XP_001617280.1[&Organism="Plasmodium vivax",Description="hypothetical protein, conserved [Plasmodium vivax]","Genetic Code"="Standard",Taxonomy="Eukaryota; Sar; Alveolata; Apicomplexa; Aconoidasida; Haemosporida; Plasmodiidae; Plasmodium; Plasmodium (Plasmodium)","Common Name"="malaria parasite P. vivax"]

XP_028539355.1[&Organism="Plasmodium sp. gorilla clade G2",Description="conserved Plasmodium protein, unknown function [Plasmodium sp. gorilla clade G2]","Genetic Code"="Standard",Taxonomy="Eukaryota; Sar; Alveolata; Apicomplexa; Aconoidasida; Haemosporida; Plasmodiidae; Plasmodium; Plasmodium (Laverania)"]

PHJ24853.1[&Organism="Cystoisospora suis",Description="arginyl-trna synthetase [Cystoisospora suis]","Genetic Code"="Standard",Taxonomy="Eukaryota; Sar; Alveolata; Apicomplexa; Conoidasida; Coccidia; Eucoccidiorida; Eimeriorina; Sarcocystidae; Cystoisospora"]

KFG43683.1[&Organism="Toxoplasma gondii GAB2-2007-GAL-DOM2",Description="arginyl-tRNA synthetase, partial [Toxoplasma gondii GAB2-2007-GAL-DOM2]","Genetic Code"="Standard",Taxonomy="Eukaryota; Sar; Alveolata; Apicomplexa; Conoidasida; Coccidia; Eucoccidiorida; Eimeriorina; Sarcocystidae; Toxoplasma"]

UKJ88078.2[&Organism="Theileria orientalis",Description="hypothetical protein MACJ_000521 [Theileria orientalis]","Genetic Code"="Standard",Taxonomy="Eukaryota; Sar; Alveolata; Apicomplexa; Aconoidasida; Piroplasmida; Theileriidae; Theileria"]
XP_004833148.1[&Organism="Theileria equi strain WA",Description="conserved hypothetical protein [Theileria equi strain WA]","Genetic Code"="Standard",Taxonomy="Eukaryota; Sar; Alveolata; Apicomplexa; Aconoidasida; Piroplasmida; Theileriidae; Theileria"]
GFE54186.1[&Organism="Babesia ovis",Description="arginyl-tRNA synthetase, putative [Babesia ovis]","Genetic Code"="Standard",Taxonomy="Eukaryota; Sar; Alveolata; Apicomplexa; Aconoidasida; Piroplasmida; Babesiidae; Babesia"]
XP_012766661.1[&Organism="Babesia bigemina",Description="Dynamin-1-like protein [Babesia bigemina]","Genetic Code"="Standard",Taxonomy="Eukaryota; Sar; Alveolata; Apicomplexa; Aconoidasida; Piroplasmida; Babesiidae; Babesia"]
GIX62800.1[&Organism="Babesia caballi",Description="arginyl-tRNA synthetase [Babesia caballi]","Genetic Code"="Standard",Taxonomy="Eukaryota; Sar; Alveolata; Apicomplexa; Aconoidasida; Piroplasmida; Babesiidae; Babesia"]
KAH0479249.1[&Organism="Porospora cf. gigantea B",Description="MAG: hypothetical protein KVP17_000372 [Porospora cf. gigantea B]","Genetic Code"="Standard",Taxonomy="Eukaryota; Sar; Alveolata; Apicomplexa; Conoidasida; Gregarinasina; Eugregarinorida; Porosporidae; Porospora","Common Name"="symbiont metagenome"]
;
end;

begin characters;
dimensions nchar=1103;
format datatype=protein missing=? gap=-;
matrix
GMI12809.1 ------------IDLPQ--------IVTI--GS----Q-
---------------SSGKSSVLE-----N--VV-G-K-GFLPRG--------------TG-IVTRRPL---------------VLQ-----
---------L-Y-NTS------------------------------NHS----------------IPT--SP-
NAISSSLRSSPTDTNRAATNPAADSDSSD-----ENNNENSNDVNTPP-
PKPASSRKSTEIASPPVLPGDE--------------W---------------------------GEF-----------------------------
---LHLPGEKFYSFD-----------EIRDEI------VRET-DR---S-TG-----
-------------------RN-------------------K-GISNK-SINLKIYSPR---
---------------VLNLTLVDLPGITK--VS-------------------V-------GD--------------Q--------PA--D--
IEDQIR--------EMCL-HYI---SN------------PNAIILAVTAGNT--D-LANSD-ALKMARSVD------
PEGNRTIGVLTKLDLMD-------PGT------D-------A----------------------------SDM--LN---NR---------
I-IP-----------------------L---RRGYVGVINR--G-QRD--ISQ----
-------R--------KS--------------------IK----------EGLRK---EMEFFKS--------HP-AY---------------------
R-S------L-----------QHR-CGTTTLAKMLNSILMH-----------
GMI47362.1 ------------IDLPQ--------IVTI--GS----Q-
---------------SSGKSSVLE-----N--VV-G-K-GFLPRG--------------TG-IVTRRPL---------------VLQ-----
---------L-Y-NTS------------------------------NHS----------------VPA--SP-

GSISSSLRSPGLDDVEEETNPA--EEEAD-----ENSNLN---
MHTPPESKPKRKSKVAADASPVVLPGDE-------------W-------------------------GEF----------------
---LHLPGEKFYSFD-----------EIRDEI------VRET-DR--
-S-TG-----------------------RN-----------------K-GISNK-SINLKIYSPR--------------------------------
--------------------------VLNLTLVDLPGITK--VS------------------V-------GD-------------Q--------PV-
-D--IEDQIR--------EMCL-HYI---SN-----------PNAIILAVTAGNT--D-LANSD-ALKMARSVD------
PEGNRTIGVLTKLDLMD-------PGT------D-------A--------------------------SDM--LN---NR---------
V-IP----------------------L---RRGYVGVINR--G-QRD--ISQ---
--------K--------RS-------------------IR----------EGLKK---EMEFFKN--------HP-AY--------------------
R-S------L-----------SHR-CGTSTLSKMLNSILMH-----------
OQR85161.1 ------------INLPQ--------IVVV--GS----
Q----------------SSGKSSVLE-----N--IV-G-K-DFLPRG-------------SG-IVTRRPL---------------VLQ---
-----------L-Y-NI---PAGD-----
DKEKE-----------------------------E-------------W-------------------------GEF----------------------
--NHLPNQKIYDFA-----------KLREEI------EKET-DR---M-
TG-----------------------KN------------------K-GISNK-PITLKIFSPY-------------------------------------
---------------------VLNLTLVDLPGITK--VP-----------------V-------GD-------------Q--------PA--N--
IEEQIR--------DMCT-EFI---SN------------PNSIILAVTAANT--D-LANSD-SLKMARAID------
PDGVRTIGVLTKLDLMD-------NGT------D-------A--------------------------MEM--LQ---GR---------
V-IP----------------------L---KKGYVGVVNR--S-QAD--INN--
---------N--------VG-------------------IR----------DSVAK---ETQFFKN--------HP-SY-------------------
-R-A------I-----------ASR-MGTQFLSKSLNTILMH-----------
RLO06844.1 ------------INLPQ--------IVVV--GS----
Q----------------SSGKSSVLE-----N--IV-G-K-DFLPRG-------------SG-IVTRRPL---------------VLQ---
-----------L-Y-NT---DGGV----
-SKD-E-----------------------------E-------------W-------------------------GEF----------------------
--NHLPNQKFNDFK-----------KIREEI------EKET-DR---M-
TG-----------------------KN------------------K-GISNK-PITLKIFSPF-------------------------------------
---------------------VLNLTLVDLPGITK--VP-----------------V-------GD-------------Q--------PA--N--
IEEQIR--------DMCL-EFI---GN------------PNSIILAVTAANT--D-LANSD-SLKMARAID------
PEGLRTIGVLTKLDLMD-------NGT------D-------A--------------------------MEM--LQ---GR---------
I-IP----------------------L---KKGYVGIVNR--S-QAD--INN----
-------G--------VG-------------------IR----------DSVSK---ETTFFKA--------HP-AY--------------------
R-G------I-----------ASR-MGTAFLSKSLNTILMH-----------
KAF0740912.1 ------------INLPQ--------IVVV--
GS----Q----------------SSGKSSVLE-----N--IV-G-K-DFLPRG-------------SG-IVTRRPL---------------
VLQ-------------L-Y-NT---
DGGV-----TKD-E-----------------------------E-------------W-------------------------GEF------------
--NHLPNQKFHDFK-----------KIREEI------EKET-
DR---M-TG-----------------------KN------------------K-GISNK-PITLKVFSPF--------------------------
--------------------------------VLNLTLVDLPGITK--VP-----------------V-------GD-------------Q------
--PA--N--IEEQIR--------DMCL-EFI---GN------------PNSIILAVTAANT--D-LANSD-SLKMARAID----
--PEGQRTIGVLTKLDLMD-------DGT------D-------A--------------------------MEM--LQ---GR--------
-V-IP----------------------L---KKGYVGIVNR--S-QAD--INN--

---------G--------VG--------------------IR----------DSVAK---EQTFFKS--------HP-AY--------------------
-R-G------I-----------ASR-MGTQYLSKSLNTILMH-----------
CAH0521473.1 ------------INLPQ--------IVVI--
GS----Q----------------SSGKSSVLE-----N--IV-G-K-DFLPRG--------------SG-IVTRRPL---------------
ILQ--------------L-Y-NSS-----------------------------TNG---------------V----------------------------
VENHEE-----TLRSE------------------------------E-------------W--------------------------GEF---------
---LHLPGQKFTDFN-----------EIRQEI------
EKET-DR---L-TG-----------------------KN------------------K-GISNK-SINLKVFSPY--------------------
-------------------------------------VLNLTLVDLPGITK--VP------------------V-------GD--------------
Q--------PV--N--IEEQIR--------DMCI-EFI---SN------------PNSIILAVTSANT--D-LANSD-
ALKMAREID------PDGQRTIGVLTKLDLMD-------AGT------D-------A---------------------------
MDM--LQ---GR---------V-IP-----------------------L---KRGYVGVVNR-----------------------------------
----------S-QAD--INA-----------Q--------LS--------------------IR---------DSLRK---EQTFFKT--------
HP-AY---------------------R-A------I-----------ASR-MGTQYLSKTLNTILMH-----------
KAG2764740.1 ------------INLPQ--------IVVI--
GS----Q----------------SSGKSSVLE-----N--IV-G-K-DFLPRG--------------SG-IVTRRPL---------------
VLQ--------------L-Y-NSS-----------------------------ATV---------------P------------------------------
GEGE-----DETVE------------------------------E-------------W--------------------------GEF------------
--LHLPGQKFSDFN-----------EIRREI------EKET-
DR---I-TG-----------------------KN-------------------K-GISNK-SINLKVFSPY---------------------------
------------------------------VLNLTLVDLPGITK--VP------------------V-------GD-------------Q-------
-PV--N--IEEQIR--------DMCT-EFI---TN------------PNSIILAVTSANT--D-LANSD-ALKMAREID------
PEGQRTIGVLTKLDLMD-------DGT------D-------A---------------------------MDM--LQ---GR---------
V-IP-----------------------L---KKGYVGVVNR---S-QAD--INS---
--------K--------LS--------------------IR---------DSLVK---EQNFFKT--------HP-AY--------------------
R-A------I-----------ASR-MGTQYLSKTLNTILMH-----------
GMF38092.1 ------------INLPQ--------IVVI--GS----Q-
---------------SSGKSSVLE-----N--IV-G-K-DFLPRG--------------SG-IVTRRPL---------------VLQ------
--------L-Y-NSS------------------------------ATV----------------P-----------------------------VDDADG--
----AEAE------------------------------E-------------W--------------------------GEF--------------------
--LHMPGQKFTDFD-----------EIRREI------EKET-DR---I-
TG-----------------------KN-------------------K-GISNK-SINLKVFSPH-----------------------------------
-----------------------VLNLTLVDLPGITK--VP------------------V-------GD-------------Q--------PV--
N--IEEQIR--------DMCT-EFI---SN------------PNSIILAVTSANT--D-LANSD-ALKMAREID------
PDGQRTIGVLTKLDLMD-------DGT------D-------A---------------------------MDM--LQ---GR---------
V-IP-----------------------L---KRGYVGVVNR---S-QAD--INA---
--------Q--------LS--------------------IR---------DSLSK---EQNFFKT--------HP-AY--------------------
R-A------I-----------ASR-MGTQYLSKTLNTILMH-----------
KAH7500220.1 ------------INLPQ--------IVVI--
GS----Q----------------SSGKSSVLE-----N--IV-G-K-DFLPRG--------------SG-IVTRRPL---------------
VLQ--------------L-Y-NSS-----------------------------TSV---------------PA----------------------------
AEDAGG-----DASAE------------------------------E-------------W--------------------------GEF--------
---LHVPGQKFTDFD-----------EIRREI------
EKET-DR---I-TG-----------------------KN------------------K-GISNK-SINLKVFSPY--------------------

-------------------------------------VLNLTLVDLPGITK--VP------------------V-------GD-------------
Q--------PV--N--IEEQIR--------DMCT-EFI---SN-----------PNSIILAVTSANT--D-LANSD-
SLKMAREID------PEGQRTIGVLTKLDLMD-------DGT------D-------A---------------------------MDM-
-LQ---GR---------V-IP----------------------L---KRGYVGVVNR---
---S-QAD--INA----------K--------LS-------------------IR----------ESLSK---EQNFFKT--------HP-AY-
--------------------R-A------I-----------ASR-MGTQYLSKTLNTILMH-----------
TMW56688.1 ------------INLPQ--------IVVI--GS---Q-
---------------SSGKSSVLE-----N--IV-G-R-DFLPRG-------------SG-IVTRRPL--------------VLQ-----
---------L-Y-NIS-----------------------------NT---DLAK-----
ESDVE------------------------------E-------------W--------------------------GEF-----------------------
--LHLPGQKISDFS-----------ELRKEI------ERET-ER---M-
TG------------------------KN------------------K-GISNK-SINLKIFSPH---------------------------------
----------------------VLNLTLVDLPGVTK--VP------------------V-------GD-------------Q--------PV--
N--IEEQIR--------DMCM-EFI---AN-----------PNSIILAVTSANT--D-LANSD-ALKMAREID------
PEGVRTIGVLTKLDLMD-------DGT------D-------A---------------------------LDM--LQ---GR---------I-
IP----------------------L---KRGFVGVVNR--S-QAD--INN-----
------S--------VS-------------------IR----------DALSK---ETSYFKN--------HP-SY--------------------R-
A------M-----------ASR-MGTQYLSKTLNTILMH-----------
CCI11042.1 ------------VNLPQ--------IVVI---GS----
Q----------------SSGKSSVLE-----N--IV-G-R-DFLPRG-------------SG-IVTRRPL---------------ILQ---
-----------L-Y-NSQ-----------------------------SN---LDETQ-
----LIGGES---------------------------NVE-------------W--------------------------GEF-----------------
--LHIPGKKFKDFS-----------EIRHEI------ERET-DR---
L-TG-----------------------KN------------------K-GISNK-TINLKIYSPH---------------------------------
------------------------VLNLTLVDLPGVTK--VP------------------V-------GD-------------Q--------PI--
D--IEEQIR--------DMCV-EFI---SN-----------PNSIILAVTSANT--D-LANSD-ALKLAREID------
PAGDRTIGVLTKIDLMD-------EGT------D-------A---------------------------LEM--LQ---GR---------I-
IS----------------------L---RRGFVGVVNR--S-QAD--INN-----
------N--------VS-------------------IR----------DSLQK---EQKFFQQ--------HS-AY--------------------
R-T------V-----------SST-MGTQYLSKTLNTILMH-----------
CCA17876.1 ------------VNLPQ--------IVVI---GS----
Q----------------SSGKSSVLE-----N--IV-G-R-DFLPRG-------------SG-IVTRRPL---------------ILQ---
-----------L-Y-NSQ-----------------------------SNS---
TLDESQ-----PLRGEN---------------------------DVE-------------W--------------------------GEF------
--LHIPGKKFTDFS-----------EIRREI------
ERET-DR---L-TG-----------------------KN------------------K-GISNK-TINLKIFSPY--------------------
-------------------------------------VLNLTLVDLPGVTK--VP------------------V-------GD-------------Q-
-------PV--N--IEEQIR--------DMCV-EFI---SN-----------PNSIILAVTSANT--D-LANSD-
ALKLAREID------PAGDRTIGVLTKIDLMD-------EGT------D-------A---------------------------LEM--
LQ---GR---------V-IS----------------------L---RRGFVGVVNR--
--S-QAD--INN-----------N--------VS-------------------IR----------DSLQK---EHLFFQN--------HT-
AY--------------------R-T------V-----------SSK-MGTQYLSKTLNTILMH-----------
CEP02405.1 ------------IDLPQ--------IVVI--GS---Q-
---------------SSGKSSVLE-----N--IV-G-R-DFLPRG-------------SG-IVTRRPL--------------ILQ-----

--------L-V-NTS------------------------------LDL---KLSGKD----
-DESGQ-------------------------------E-------------W---------------------------GEF----------------------
--LHKRDKKFTSFE-----------DIRAEI------VAET-DR---L-
TG------------------------TN-------------------K-GISDK-AINLKVYSPR-------------------------------------
-----------------------FLNLTLVDLPGITK--VP---------------V-------GD-------------Q--------PS--D-
-IENQIK--------GMIM-RFV---SN------------PRAIILAVTAANT--D-LANSD-ALKLAREVD------
PECNRTIGVITKIDLMD-------RGT------N-------A----------------------------LDV--LL---GK--------V-
IP-----------------------L---KLGFIGVINR--S-QED--INN-------
----N--------TS--------------------IN----------AALTA---EMQFFQK--------HP-AY---------------------R-
A------I-----------ASR-LGTSYLTKTLNSILIN-----------

XP_044553261.1 ----------VQIDLPQ--------IAVV---
-GS----Q----------------SSGKSSVLE-----T--IV-G-R-DFLPRG--------------SG-IVTRRPL---------------
VLQ--------------L-N-K---TPS------KQV-----------------------
---------DSTGPED-----------------------------E-------------W---------------------------GEF-------------
---LHVPNKKFYDFT-----------EIRNEI------IRET-
DR---V-TG------------------------SS-------------------K-GISDL-PINLKIYSPN----------------------------
--------------------------------VLNLTLVDLPGITK--VP---------------V-------GD-------------Q-------
-PK--D--IEAQIR--------NMIM-KFI---SK------------PNCLILAVTAANT--D-IANSD-ALKLAKEVD------
KNGSRTIGVLTKIDIMD-------QGT------D-------C----------------------------MDV--LR---GD---------I-
LP-----------------------L---RLGYIGVVCR--S-QND--INL------
-----N--------KS--------------------IR----------DALKD---EERFFAQ--------HP-TY---------------------K-
S------I-----------SDR-MGTKHLAKTLNRILLN-----------

XP_044569353.1 ----------VQIDLPQ--------IAVV---
-GS----Q----------------SSGKSSVLE-----T--IV-G-R-DFLPRG--------------SG-IVTRRPL---------------
VLQ--------------L-N-K---TPS------KQQ--------------------
-QTGD-----DSTGPEE-----------------------------E-------------W---------------------------GEF---------
---LHVPNKKFYDFT-----------EIRNEI------
IRET-DR---V-TG------------------------SS-------------------K-GISDL-PINLKIYSPN----------------------
--------------------------------------VLNLTLVDLPGITK--VP-------------------V-------GD-------------Q-
-------PK--D--IEAQIR--------NMIM-KFI---SK------------PNCLILAVTAANT--D-IANSD-
ALKLAKEVD------KNGSRTIGVLTKIDIMD-------QGT------D-------C----------------------------MDV--
LR---GE---------L-LP---------------------L---RLGYIGVVCR--
-S-QND--INL-----------N--------KS--------------------IR----------DALRD---EERFFAT--------HP-IY---
------------------K-S------I-----------SDR-MGTKYLAKTLNRILLN-----------

XP_044550536.1 -----------TIDLPQ--------IAVV--
GS----Q----------------SSGKSSVLE-----N--VV-G-R-DFLPRG--------------AG-IVTRRPL--------------
-VLQ--------------L-I-N---IPS------KYK--------------------
EEVS-----EQKKQEE-----------------------------E-------------Y---------------------------GEF------------
--LHLPNKKFYDFA-----------EIRQEI------IRET-
DR---V-TG------------------------SS-------------------K-GISPV-PINLKIYSPY-----------------------------
-------------------------------VLNLTLIDLPGITK--VP-------------------V-------GD-------------Q--------
PK--D--IELQIR--------NMIL-QYI---TK------------PNCLILAVTAANT--D-LANSD-ALKLAKQVD------
KSGLRTLGVLTKVDIMD-------KGV------D-------C----------------------------LDI--LR---GE--------V-
LP-----------------------L---KLGYIAVVNR--S-QND--INN-----

------N--------KS--------------------IR----------EALKD---EEAFFRN--------HP-SY--------------------
R-S------V-----------ADT-LGTKHLAKTLNKILLE-----------
XP_002681690.1 -----------SIDLPQ--------IAVV---
GS----Q----------------SSGKSSVLE-----N--VV-G-R-DFLPRG--------------SG-IVTRRPL--------------
-ILQ--------------L-I-T--IAS------KYKA------------------
VEEVT-----EQQKQQE----------------------------E-------------Y--------------------------GEF----------
---LHLPNKKFYNFS-----------EIREEI------
VRET-DR---I-TG-----------------------SN-------------------K-NISSA-PINLKIYSPY---------------------
-------------------------------------VLNLTLIDLPGITK--VP------------------V-------GD--------------Q--
------PK--D--IEQQIR--------KMIL-QFI---SK------------PTCIILAVTAANT--D-LANSD-ALKLAKEVD--
----RTGDRTLGVLTKVDIMD-------KGV------D-------C---------------------------MDI--IR---GE--------
-V-LP----------------------L---KMGYIGVINR---S-QND--INT--
---------N--------KS--------------------IR----------DALKD---EDAFFRN--------HP-SY------------------
--R-S------Y-----------ANN-MGTKYLAKTLNKILLN-----------
KAH3761456.1 ------------IDLPQ--------IVVV---
GS----Q----------------SSGKSSVLE-----H--IV-G-R-DFLPRG--------------SG-IVTRRPL--------------
ILQ--------------L--
STN-----KLD--SK-----------------------------E-------------W--------------------------GEF-------------
---LHKPNVRFADFS-----------EIREEI------KRET-
DR---A-AG------------------------SN-------------------K-GVNNT-PINLKISSPH-------------------------
---------------------------------VLNLTLVDLPGMTR--VP------------------V-------GD-------------Q---
----PS--N--IEDIIR--------KMIK-GYI---AK------------PNSIILALTAANT--D-LSNSD-ALQLASEVD-----
-PEGKRTLGVLTKLDIMD-------RGT------S-------A---------------------------IDV--LY---NR---------A-
IP----------------------L---ALGYVGVVNR---S-QED--IMR-----
------D--------KP--------------------IS----------EALKT---EALFFEN--------HP-NY--------------------S-
A------I-----------ASR-CGTPFLAQRLNEILLN-----------
PRP82407.1 ------------IDLPQ--------IVVI---GS----Q-
---------------SSGKSSVLE-----N--IV-G-K-DFLPRG--------------SG-IVTRRPL--------------VLQ------
--------L-F-N--VP--------SK---------------------NGAD-----
AAD-APQ-----------------------------D-------------W--------------------------GEF---------------------
---LHKPKERFYDFA-----------EIRNEI------ERET-DR---L-
TG------------------------KN-------------------K-GISHM-PINLKVYSTR------------------------------------
-----------------------VLNLTLVDLPGITK--VP------------------I-------GD-------------Q--------PK--D-
-IEAQIR--------NMIF-HYI---EK------------QSTIILAVTAANT--D-LTNSD-ALQLAREVD------
PEGKRTIGVITKLDLMD-------KGT------D-------A---------------------------LDM--LM---GR---------V-
VP----------------------L---RLGYIGVVNR---S-QAD--INS-----
------R--------RS--------------------IT----------SALEG---EREFFST--------HP-AY--------------------R-
N------V-----------ASR-CGTSFLAGNLSKILMN-----------
XP_004368323.1 ------------IDLPQ--------IVVV---
GS----Q----------------SSGKSSVLE-----N--IV-G-R-DFLPRG--------------QG-IVTRRPL-------------
-VLQ-------------L-I-N--LPT--DP-KKKK------------------
--NVDD-----DDDSALK-----------------------------E-------------W--------------------------GEF-------
---LHKPNEIFYDFN-----------QIREEI------
VRET-DR---M-SG------------------------KN-------------------K-GISHM-PINLKIYSPH------------------

--VLNLTLVDLPGITK--VP------------------V-------GD-------------
Q--------PA--D--IEIQIR--------TMVL-QYI---ER------------PNAVILAVTAANT--D-LSNSD-
ALQIAAVAD------PDGQRTIGVLTKIDLMD-------AGT------D-------A---------------------------MDM-
-LL---NR---------V-IP----------------------L---RLGYIGVINR---
S-QQD--IIK-----------K--------KP-------------------IR---------AALKA---EAEYFTT--------HP-LY------
---------------R-S------V-----------ASR-CGTPFLSKTLNKILMN-----------
XP_012754660.1 ------------LDLPQ--------IVVV--
GS----Q----------------SSGKSSVLE-----N--IV-G-R-DFLPRG--------------SG-IVTRRPL---------------
ILQ--------------L-T-H---LP-------------------------------
ITD-----EVDVSTQ-----------------------------E-------------W--------------------------GEF-------------
--LHRPNDMFYDFA-----------EIREEI------IKDT-
DR---L-TG------------------------KN-------------------K-GVSAQ-PINLKIYSPH--------------------------
--------------------------------VVNLTLVDLPGITK--VP------------------V-------GD-------------Q------
--PT--D--IEMQIR--------RMIM-AYI---KR------------PNAIILAVTPANT--D-LANSD-ALQLAREVD-----
-PDGKRTIGVITKLDLMD-------KGT------D-------A---------------------------MDV--LT---GR---------V-
VP----------------------L---ALGFVGVINR---S-QED--IIS-------
----K--------KS--------------------IR---------DALKS---EVQYFKN--------HP-IY---------------------K-T-
-----I-----------ANR-SGTAYLSKTLNKLLMF-----------
KYR01170.1 ------------LDLPQ--------IVVV---GS----
Q----------------SSGKSSVLE-----N--IV-G-K-DFLPRG--------------SG-IVTRRPL---------------ILQ---
-----------L-T-H---LP-------------------------------LAD-----
DGSGPTS-----------------------------E-------------W--------------------------GEF---------------------
--LHKPNDMYYDFA-----------EIREEI------IRDT-DR---M-
TG------------------------KN-------------------K-GISAQ-PINLKIYSPH---
----------------------VVNLTLVDLPGITK--VP------------------V-------GD-------------Q--------PT--D-
-IEQQIR--------RMVM-AYI---KK------------PNAIILAVTPANT--D-LANSD-ALQLAKEVD------
PEGKRTIGVITKLDLMD-------KGT------D-------A---------------------------MEV--LT---GK---------V-
IP----------------------L---SLGFIGVINR---S-QED--IIS---------
--K--------KS--------------------IR----------ESLKS---ELLYFKN--------HP-IY---------------------K-S----
--I-----------ANR-SGTAYLSKTLNKLLMF-----------
XP_003294436.1 ------------LDLPQ--------IVVV--
GS----Q----------------SSGKSSVLE-----N--IV-G-R-DFLPRG--------------SG-IVTRRPL---------------
ILQ--------------L-T-H---LP-------------------------------
MAD-----DGS-PTS----------------------------E-------------W--------------------------GEF------------
--LHRPNDMFYDFS-----------EIREEI------IRDT-
DR---L-TG------------------------KN-------------------K-GISAQ-PINLKIYSPH--------------------------
--------------------------------VVNLTLVDLPGITK--VP------------------V-------GD-------------Q------
--PS--D--IEQQIR--------RMIM-AYI---KK------------QNAIIVAVTPANT--D-LANSD-ALQLAKEVD----
--PEGKRTIGVITKLDLMD-------KGT------D-------A---------------------------MDV--LT---GR---------
V-IP----------------------L---TLGFIGVINR---S-QED--IIA-------
----K--------KS--------------------IR----------ESLKS---EVLFFKN--------HP-IY---------------------K-T--
----I-----------ANR-SGTAYLSKSLNKLLMF-----------
PRP81066.1 ------------LDLPQ--------IVVV---GS----
Q----------------SSGKSSVLE-----A--IV-G-R-DFLPRG--------------SG-IVTRRPL---------------VLQ---

-----------L-T-H---LP----P---------------------------SIN-----
DAE-SDV-----------------------------E--------------W---------------------------GEF-----------------------
---NHRPNEMFHDFD----------KIREEI------ERET-ER---T-
TG------------------------KN-------------------K-GISDI-PISLKIYSSH--------------------------------------
----------------------VLNLTLVDLPGITR--VP-------------------I-------GD--------------Q--------PP--D--
IERQIR--------SMVK-HYI---DK------------PNAIILAITAANT--D-LTNSD-ALQMARESD------
PDGIRTVGVITKIDIMD-------KGT------N-------A----------------------------LDM--LS---GR---------V-
VP-----------------------L---KLGFIGVVNR--S-QQD--IIK------
-----K--------KS--------------------IR----------DALVD---EQNFFHQ--------HS-LY---------------------K-
N------I-----------SGR-LGTAYLSRTLNKTLIQ-----------
XP_004348308.1 ------------IQLPQ--------IAVV--
GS----Q---------------SSGKSSVLE-----N--IV-G-K-DFLPRG--------------HG-IVTRRPL--------------
-ILQ--------------L-V-HRK------------------------------PGSPR------------PALPD--DP-SSSGGH-------
----------TDDGID-----GED--VE-----------------------------E--------------W---------------------------
GEF--LHAPGKRFISFA-----------
EIRKEI------EAET-DR---V-TG------------------------SN-------------------K-GISSK-PINLRIYSPN-------
---VLNLTLVDLPGITK--VP-------------------V-------GD---
-----------Q--------PE--D--IEKQIR--------TLVR-SYI---SN------------PNCIILAVTPANV--D-LANSD-
ALKLAKTID------PEGNRTIGVCTKIDLMD-------AGT------D-------A----------------------------MDI--
LS---GR---------V-VP-----------------------V---KLGFIGVVNR---
-S-QAD--INT-----------A--------KP--------------------IA----------DSLKS---EEQFFKS--------HP-AY---
------------------Q-A------I-----------AHR-CGTAYLSKALNKLLMHHIRDC-L----
NP_012926.1 ------------IDLPQ--------ITVV--GS----
Q----------------SSGKSSVLE-----N--IV-G-R-DFLPRG--------------TG-IVTRRPL---------------VLQ---
-----------L-I-NRR-------------------------------PKKSEHAKVNQTANELIDLNIN--DD-DKKKDE---------
--------SGKHQN-----EGQSEDN---------------------------KEE--------------W---------------------------
GEF--LHLPGKKFYNFD-----------
EIRKEI------VKET-DK---V-TG------------------------AN-------------------S-GISSV-PINLRIYSPH-------
---VLTLTLVDLPGLTK--VP-------------------V-------GD---
-----------Q--------PP--D--IERQIK--------DMLL-KYI---SK------------PNAIILSVNAANT--D-LANSD-
GLKLAREVD------PEGTRTIGVLTKVDLMD-------QGT------D-------V----------------------------IDI--
LA---GR---------V-IP-----------------------L---RYGYIPVINR---
G-QKD--IEH-----------K--------KT--------------------IR----------EALEN---ERKFFEN--------HP-SY----
-----------------S-S------K-----------AHY-CGTPYLAKKLNSILLHHIRQT-LP---
OUM62108.1 ------------IDLPQ--------IVVI--GS----Q-
---------------SSGKSSVLE-----N--IV-G-R-DFLPRG--------------TG-IVTRRPL---------------ILQ-------
-------L-I-NKR------------------------------DGLTT-------------------------NS-----------------------A-----
PANSEQN---------------------------MNE--------------W---------------------------GEF-------------------
---LHLPGKKFYDFT-----------EIRKEI------EKET-EL---K-
VG------------------------KN-------------------A-GISDQ-PINLRIFSPK------------------------------------
-----------------------VLTLTLVDLPGLTK--VP-------------------V-------GD--------------Q--------PK--
D--IERLIR--------DMIL-KYI---TK------------PNAIILAVTAANT--D-LANSD-GLKLAREVD------
PEGVRTIGVLTKVDLMD-------AGT------D-------V----------------------------IDI--LA---GR---------V-
IP-----------------------L---RLGYVPVVNR--G-QKD--IET------

-----N--------KS--------------------IS----------KALEA---EKQFFEH--------HS-SY--------------------K-
S------K-----------AQY-CGTPFLARKLNMILMHHIRNT-LP---
KXN66323.1 ------------VDLPQ--------IVVI---GS----
Q----------------SSGKSSVLE-----N--IV-G-R-DFLPRG--------------TG-IVTRRPL---------------VLQ---
-----------L-F-NVP-----------------------------QTQ---A---
--DSAKENE---------------------------PAE-------------Y--------------------------GEF-------------------
---LHLPGTKFTDFD-----------KIRKEI------ESET-ES---K-
TG-------------------------KN-------------------A-GISPL-PINLRIFSPK--------------------------------------
----------------------VLTLTLVDLPGLTK--VP------------------V-------GD--------------Q--------PK--D-
-IEKQIR--------DMIM-KYI---NK------------PNAIILAVTAANT--D-LANSD-GLKLAREVD------
PEGSRTIGVLTKVDLMD-------HGT------D-------V---------------------------VDI--LA---GR---------V-
IP-----------------------L---RLGYVPVINR--S-QRD--IES-------
----K--------KN--------------------IS----------AALDH---ERQFFEK--------HP-SY--------------------T-
S------K-----------VQY-CGTPFLARKLSMILMHHIRNT-LP---
XP_011389257.1 ------------IDLPQ--------ITVL---
GS----Q----------------SSGKSSVLE-----N--IV-G-R-DFLPRG--------------TG-IVTRRPL---------------
VLQ--------------L-I-NRP-----------------------------ATSKA-------------------------NDE----------------
-A-APAV-----SSKGANN---------------------------PDE-------------W---------------------------GEF-----
---LHLPGEKFFDFD-----------KIREEI------
VRDT-EL---K-TG-----------------------RN-------------------A-GISPQ-PINLRIYSPN--------------------
---------------------------------------VLTLTLVDLPGLTK--VP------------------V-------GD--------------
Q--------PR--D--IERQIR--------DTVL-KFI---SK------------PNAVILAVTAANT--D-LANSD-
GLKLAREVD------PEGTRTVGVLTKVDLMD-------AGT------D-------V---------------------------VDI--
LA---GR---------V-IP-----------------------L---RLGYVPVVNR--
-G-QRD--IDQ-----------K--------KL--------------------VS----------AALTA---EKEFFEN--------HP-SY--
-------------------R-S------K-----------AQY-CGTPFLARKLNTILMHHIRNT-LP---
XP_006458578.1 ------------IDLPQ--------ICVL---
GS----Q----------------SSGKSSVLE-----N--IV-G-R-DFLPRG--------------TG-IVTRRPL---------------
VLQ--------------L-I-NRP-----------------------------AGTPG-------------------------GPQ---------------
--INGTDK-----SSDKHAN---------------------------ADE-------------W---------------------------GEF---
---LHLPGEKFFDFN-----------KIRAEI-----
-VRDT-EV---K-TG-----------------------KN-------------------A-GISPL-PINLRVFSPN-------------------
---------------------------------------VLTLTLVDLPGLTK--VP-------------------V------GD--------------
Q--------PR--D--IEKQIR--------DMLL-KYI---SK------------SACIILAVTAGNT--D-LANSD-
GLKMAREVD------PEGLRTIGVLTKIDLMD-------KGT------D-------V---------------------------VDI--
LA---GR---------I-IP-----------------------L---RLGYVPVVNR--
G-QRD--IES-----------S--------KP--------------------IS----------AALEY---ERSFFEN--------HA-SY----
-----------------K-S------K-----------AQF-CGTPFLARKLNMILMHHIKAT-IP---
KNE68830.1 ------------IDLPQ--------ITVI---GS----Q-
---------------SSGKSSVLE-----N--IV-G-R-DFLPRG--------------TG-IVTRRPL---------------VLQ------
--------L-I-NRP-----------------------------ATPAAPGS-------------------PATDGE-----------------
PAADGT-----SGDNQDN---------------------------KNE-------------W---------------------------GEF----
---LHAPNKRFYNFD-----------EIRKEI------
IRDT-DE---K-AG-----------------------AN------------------T-GISHA-PINLRIYSPN--------------------

-------------------------------------VLTLTLVDLPGLTK--VP-----------------V-------GD-------------Q-
-------PK--D--IEIQIR--------EMLL-KYI---TK-----------PNAIILAVTAANT--D-LANSD-GLKLAREVD--
----PEGSRTIGVLTKVDLMD-------PGT------D-------V--------------------------VDI--LA---GR---------
V-IP---------------------L---RLGYVPVVNR---G-QKD--IDK---
--------R--------KS------------------IA----------LALEH---ERSYFEE--------HP-SY-------------------
R-S------K-----------AQY-CGTPFLARKLNMILLHHIKNT-LP---
XP_748106.1 ------------IDLPQ--------IVVV---GS----
Q---------------SSGKSSVLE-----N--IV-G-R-DFLPRG-------------SG-IVTRRPL--------------ILQ---
-----------L-I-NRP----------------------------SRNSV----------------------TNGV----------------
KEEKLE-----TTDSEAN--------------------------VDE-------------Y--------------------------GEF--------
--LHIPGQKFYDFN-----------KIREEI------
VRET-EQ---K-VG-----------------------RN------------------A-GISPA-PINLRIYSPN-------------------
--------------------------------------VLTLTLVDLPGLTK--VP------------------V-------GD-------------
Q--------PK--D--IERQIR--------DMVL-KYI---SK-----------PNAIILAVTSANQ--D-LANSD-
GLKLAREVD------PEGQRTIGVLTKVDLMD-------EGT------D-------V--------------------------VDI--
LA---GR---------I-IP---------------------L---RLGYVPVVNR---
G-QRD--IEN-----------K--------RP------------------IS---------YALEH---EKNFFES--------HK-AY----
-----------------R-N------K-----------ASY-CGTPYLARKLNLILMMHIKQT-LP---
OAJ44422.1 ------------IDLPQ--------IAVV--GS----
Q---------------SSGKSSVLE-----N--IV-G-K-DFLPRG-------------SG-IVTRRPL--------------VLQ---
-----------L-I-NRP----------------------------NSKGEIGGIAAGT----DKPPS--SP-STPGAK------------
----SATTSN-----AAVTVEE--------------------------GDE-------------W--------------------------GEF--
---LHIPGKRFTDFN-----------EIRDEI----
--DRET-EK---T-TG-----------------------KN------------------A-GVSSN-PINLRIYSPN-----------------
--VLTLTLVDLPGLTK--VP------------------V-------GD-------------
Q--------PK--D--IEKLIK--------DMIL-KYI---SK-----------SNAIILAVTSANT--D-LANSD-
GLKLAREVD------PEGVRTIGVLTKIDLMD-------QGT------D-------V--------------------------IDI--
LA---GR---------V-IP---------------------L---RLGYVPVVNR---
-G-QRD--IEN-----------K--------KK------------------IS---------LALEA---EKNYFEN--------HA-SY--
-------------------R-S------K-----------AQY-CA------------------------
KAJ1432693.1 ------------LDLPQ--------ICVV--
GS----Q---------------SAGKSSVLE-----N--IV-G-R-DFLPRG-------------TG-ICTRRPL--------------
VLQ-------------L-Y-CTA-----------------------------AAEE--
ADVDVD-----ELAAEEN--CNGS--------------------AKE-------------W-------------------------GEF-
---LHLPGQRFHDFS-----------EIRSEI--
----ERET-DR---V-SG-----------------------RN------------------K-GISNK-SINLKIFSPY-----------------
---VLNLTLVDLPGITK--VP------------------T-------GD------------
-Q--------PE--D--VEQQIL--------SMCR-EFI---SN-----------PNAIILAVSAANQ--D-LVNSE-
GLKLARSVD------PEGLRTIGVLTKVDIMD-------HGT------D-------C--------------------------CDV--
LN---NQ---------V-IP---------------------L---RRGYIAVINR---
S-QKD--IID-----------A--------LP------------------IR---------KALLK---EQKYFQS--------HP-KY-----
----------------R-S------Q-----------LAK-CGTGNMARTLNQLLMN-----------
XP_004347890.1 ------------LDLPQ--------IAVV--
GS----Q---------------SAGKSSVLE-----N--FV-G-K-DFLPRG-------------SG-IVTRRPL-------------

-VLQ--------------L-V-NSK-----------------------------GP---
--E--------------Y--------------------------GEF--------------------
---LHNKSKKFTDFD-----------EVRKEI------EAET-DR---
I-TG------------------------TN-------------------K-GISPV-PINLKVYSPN----------------------------------
------------------------VLNLTLVDLPGITK--VP-------------------I-------GD--------------Q--------PT--
N--IESLIR--------EMIM-QFI---GR------------PNCLILAVSPANS--D-LANSD-ALKLAREVD------
QQGIRTIGVITKLDLMD-------EGT------D-------A---------------------------REV--LE---NK---------L-
IP----------------------L---RRGFIGVVNR---S-QKD--IDG------
-----R--------KD--------------------IK----------AAMSA---ELRFFST--------HP-AY--------------------R-
D------L-----------ANK-NGTMYLQRVLNQQLTNHIRDT-L----
NP_001024332.1 ------------FELPQ--------IAVV---
GG----Q----------------SAGKSSVLE-----N--FV-G-K-DFLPRG--------------SG-IVTRRPL-------------
-ILQ--------------L-I-QDR-----------------------------N---
---E--------------Y--------------------------AEF--------------------
--LHKKGHRFVDFD-----------AVRKEI------EDET-DR---
V-TG------------------------QN-------------------K-GISPH-PINLRVFSPN---------------------------------
--------------------------VLNLTLIDLPGLTK--VP-------------------V-------GD--------------Q--------PA-
-D--IEQQIR--------DMIL-TFI---NR------------ETCLILAVTPANS--D-LATSD-ALKLAKEVD------
PQGLRTIGVLTKLDLMD-------EGT------D-------A---------------------------REI--LE---NK---------L-
FT----------------------L---RRGYVGVVNR---G-QKD--IVG----
-------R--------KD--------------------IR----------AALDA---ERKFFIS--------HP-SY--------------------
R-H------M-----------ADR-LGTSYLQHTLNQQLTNHIRDT-L----
KMZ10000.1 ------------LDLPQ--------IAVV---GG----
Q----------------SAGKSSVLE-----N--FV-G-K-DFLPRG--------------SG-IVTRRPL---------------ILQ---
-----------L-I-NGV-----------------------------T---
---------------------------------E--------------Y--------------------------GEF------------------------------
--LHIKGKKFSSFD-----------EIRKEI------EDET-DR---V-TG--------
----------------SN-------------------K-GISNI-PINLRVYSPH---
------------VLNLTLIDLPGLTK--VA-------------------I-------GD--------------Q--------PV--D--IEQQIK--
------QMIF-QFI---RK------------ETCLILAVTPANT--D-LANSD-ALKLAKEVD------
PQGVRTIGVITKLDLMD-------EGT------D-------A---------------------------RDI--LE---NK---------L-
LP----------------------L---RRGYIGVVNR---S-QKD--IEG-----
------R--------KD--------------------IH----------QALAA---ERKFFLS--------HP-SY--------------------
R-H------M-----------ADR-LGTPYLQRVLNQQLTNHIRDT-L----
XP_030853442.1 ------------LDLPQ--------IAVV---
GG----Q----------------SAGKSSVLE-----N--FV-G-R-DFLPRG--------------SG-IVTRRPL-------------
--VLQ--------------L-N-NSK-----------------------------T--
--E--------------Y--------------------------GEF--------------------
---LHCKGKKFTDFD-----------EIRKEI------EAET-DR---
V-TG------------------------SN-------------------K-GISNV-PINLRVYSPN---------------------------------
--------------------------VLNLTLVDLPGMTK--IA-------------------V-------GD--------------Q--------
PV--D--IEIQIR--------SMVM-EFV---TN------------ESTLILAVSPANQ--D-LANSD-ALKVAKEVD------
PKGVRTIGVITKLDLMD-------DGT------D-------A---------------------------KDI--LE---NK---------L-
LP----------------------L---RRGYVGVVNR---S-QRD--IEG----

-------K--------KD-------------------IK----------AALAA---ERKFFLS--------HP-SY--------------------
R-H------I-----------ADK-MGTPWLQKILNQQLTNHIRDS-L----
XP_030853442.1.2 ------------LDLPQ--------IAVV---
GG----Q----------------SAGKSSVLE-----N--FV-G-R-DFLPRG--------------SG-IVTRRPL-------------
--VLQ-------------L-N-NSK----------------------------T---
--E-------------Y-------------------------GEF--------------------
--LHCKGKKFTDFD-----------EIRKEI------EAET-DR---
V-TG------------------------SN-------------------K-GISNV-PINLRVYSPN--------------------------------
-------------------------VLNLTLVDLPGMTK--IA------------------V-------GD-------------Q--------
PV--D--IEIQIR--------SMVM-EFV---TN------------ESTLILAVSPANQ--D-LANSD-ALKVAKEVD------
PKGVRTIGVITKLDLMD-------DGT------D-------A---------------------------KDI--LE---NK---------L-
LP-----------------------L---RRGYVGVVNR--S-QRD--IEG----
-------K--------KD-------------------IK----------AALAA---ERKFFLS--------HP-SY--------------------
R-H------I-----------ADK-MGTPWLQKILNQQLTNHIRDS-L----
XP_005165639.1 ------------LDLPQ--------IAVV---
GG----Q----------------SAGKSSVLE-----N--FV-G-K-DFLPRG--------------SG-IVTRRPL--------------
-VLQ-------------L-I-NCP-----------------------------T---
---E-------------Y--------------------------AEF---------------------
---LHCKGKKFTDFD-----------EVRQEI------EAET-DR---I-
TG-----------------------QN-------------------K-GISPV-PINLRVYSPN-----------------------------------
-----------------------VLNLTLVDLPGMTK--VP------------------V-------GD-------------Q--------PA--
D--IEAQIR--------DMLM-QFV---TK------------ENCLLLAVSPANS--D-LANSD-ALKIAKEVD------
PQGMRTIGVITKLDLMD-------EGT------D-------A---------------------------REI--LE---NK---------L-
LP-----------------------L---RRGYIGVVNR--S-QKD--IDG-----
------K--------KD-------------------IT----------AAMSA---ERKFFLT--------HP-SY---------------------R-
H------L-----------ADR-MGTPYLQKALNQQLTNHIRDT-L----
XP_028570166.1 ------------LDLPQ--------IAVV---
GG----Q----------------SAGKSSVLE-----N--FV-G-R-DFLPRG--------------SG-IVTRRPL-------------
--VLQ-------------L-V-NSP-----------------------------T--
--E-------------Y--------------------------GEF--------------------
--LHCKGKKFTDFD-----------EIRQEI------EAET-DR---I-
TG-----------------------SN-------------------K-GISPV-PINLRVYSPH-----------------------------------
-----------------------VLSLTLVDLPGMTK--VP------------------V-------GD-------------Q--------PA--
D--IEFQIR--------EMLM-QFV---TK------------ENCLILAVSPANS--D-LANSD-ALKIAKEVD------
PQGQRTIGVITKLDLMD-------EGT------D-------A---------------------------RDV--LE---NK---------L-
LP-----------------------L---RRGYIGVVNR--S-QKD--IDG-----
------K--------KD-------------------IQ----------AALAA---ERKFFLT--------HP-AY---------------------R-
H------M-----------ADR-MGTPYLQKVLNQQLTNHIRDT-L----
EPQ17174.1 ------------LDLPQ--------IAVV---GG----
Q----------------SAGKSSVLE-----N--FV-G-R-DFLPRG--------------SG-IVTRRPL---------------VLQ--
------------L-V-NAS-----------------------------T---
---------------------------------E-------------Y-------------------------AEF-----------------------------
--LHCKGKKFTDFE-----------EVRLEI------EAET-DR---V-TG------
------------------TN-------------------K-GISPV-PINLRVYSPH---

--------------VLNLTLVDLPGMTK--VP-------------------V-------GD--------------Q--------PA--D--
IEFQIR--------DMLM-QFV---TK------------ENCLILAVSPANS--D-LANSD-ALKIAKEVD------
PQGQRTIGVITKLDLMD-------EGT------D-------A----------------------------RDV--LE---NK---------L-
LP-----------------------L---RRGYIGVVNR--S-QKD--IDG-----
------K--------KD--------------------IT----------AALAA---ERKFFLS--------HP-SY---------------------R-
H------L-----------ADR-MGTPYLQKVLNQQLTNHIRDT-L----
BAB27759.1 ------------LDLPQ--------IAVV---GG----
Q----------------SAGKSSVLE-----N--FV-G-R-DFLPRG--------------SG-IVTRRPL---------------VLQ--
------------L-V-NST------------------------------T---
-----------------------------------E--------------Y---------------------------AEF------------------------------
--LHCKGKKFTDFE-----------EVRLEI------EAET-DR---V-TG------
------------------TN-------------------K-GISPV-PINLRVYSPH--
--------------VLNLTLVDLPGMTK--VP-------------------V-------GD--------------Q--------PP--D--
IEFQIR--------DMLM-QFV---TK------------ENCLILAVSPANS--D-LANSD-ALKIAKEVD------
PQGQRTIGVITKLDLMD-------EGT------D-------A----------------------------RDV--LE---NK---------L-
LP-----------------------L---RRGYIGVVNR--S-QKD--IDG-----
------K--------KD--------------------IT----------AALAA---ERKFFLS--------HP-SY---------------------R-
H------L-----------ADR-MGTPYLQKVLNQQLTNHIRDT-L----
ELW62001.1 ------------LDLPQ--------IAVV---GG----
Q----------------SAGKSSVLE-----N--FV-G-R-DFLPRG--------------SG-IVTRRPL---------------VLQ--
------------L-V-NAT------------------------------T---
-----------------------------------E--------------Y---------------------------AEF------------------------------
--LHCKGKKFTDFE-----------EVRLEI------EAET-DR---V-TG------
------------------TN-------------------K-GISPV-PINLRVYSPH--
--------------VLNLTLVDLPGMTK--VP-------------------V-------GD--------------Q--------PP--D--
IEFQIR--------DMLM-QFV---TK------------ENCLILAVSPANS--D-LANSD-ALKIAKEVD------
PQGQRTIGVITKLDLMD-------EGT------D-------A----------------------------RDV--LE---NK---------L-
LP-----------------------L---RRGYIGVVNR--S-QKD--IDG-----
------K--------KD--------------------IT----------AALAA---ERKFFLS--------HP-SY---------------------R-
H------L-----------ADR-MGTPYLQKVLNQQLTNHIRDT-L----
EAW87759.1 ------------LDLPQ--------IAVV---GG----
Q----------------SAGKSSVLE-----N--FV-G-R-DFLPRG--------------SG-IVTRRPL---------------VLQ--
------------L-V-NAT------------------------------T---
-----------------------------------E--------------Y---------------------------AEF------------------------------
--LHCKGKKFTDFE-----------EVRLEI------EAET-DR---V-TG------
------------------TN-------------------K-GISPV-PINLRVYSPH--
--------------VLNLTLVDLPGMTK--VP-------------------V-------GD--------------Q--------PP--D--
IEFQIR--------DMLM-QFV---TK------------ENCLILAVSPANS--D-LANSD-ALKVAKEVD------
PQGQRTIGVITKLDLMD-------EGT------D-------A----------------------------RDV--LE---NK---------L-
LP-----------------------L---RRGYIGVVNR--S-QKD--IDG-----
------K--------KD--------------------IT----------AALAA---ERKFFLS--------HP-SY---------------------R-
H------L-----------ADR-MGTPYLQKVLNQQLTNHIRDT-L----
XP_025915522.1 --

--MTK--VP-----------------V-
------GD-------------Q--------PP--D--IEFQIR--------DMLM-QFV---TK-----------
ENCLILAVSPANS--D-LANSD-ALKIAKEVD------PQGQRTIGVITKLDLMD-------EGT------D-------
A--------------------------RDV--LE---NK--------L-LP---------------------L---RRGYIGVVNR--------
-------------------------------------S-QKD--IDG----------K--------KD------------------IQ---------
AALAA---ERKFFLS--------HP-AY------------------R-H------M----------ADR-
MGTPYLQKVLNQQLTNHIRDT-L----
XP_012378586.1 --------------------------A--
GGPRRRQ---------------SGRAAAAAEPEPERN--FV-G-R-DFLPRG-------------SG-IVTRRPL------
---------VLQ-------------L-V-NAT---------------------------T---------------------------------------
--E-------------Y------------------------AEF-------------
---LHCKGKKFTDFE----------EVRLEI------EAET-
DR---V-TG----------------------TN-----------------K-GISPV-PINLRVYSPH--------------------------
---------------------------------VLNLTLVDLPGMTK--VP----------------V-------GD------------Q-----
---PP--D--IEFQIR--------DMLM-QFV---TK-----------ENCLILAVSPANS--D-LANSD-ALKVAKEVD-
-----PQGQRTIGVITKLDLMD-------EGT------D-------A--------------------------RDV--LE---NK-------
--L-LP---------------------L---RRGYIGVVNR---S-QKD--
IDG----------K--------KD------------------IT---------AALAA---ERKFFLS--------HP-SY-------------
-------R-H------L----------ADR-MGTPYLQKVLNQQLTNHIRDT-L----
KAE8583055.1 ------------LDLPQ--------IAVV--
GG----Q---------------SAGKSSVLE-----N--FV-G-K-DFLPRG-------------SG-IVTRRPL-------------
-VLQ-------------L-V-NSS---------------------------T---
---E-------------Y------------------------GEF--------------------
---LHCKGKKFTDFD----------EIRLEI------EAET-DR---A-
TG----------------------TN-----------------K-GISPV-PINLRVYSPN------------------------------------
----------------------VLNLTLVDLPGMTK--VP----------------V-------GD------------Q--------PV--
D--IEFQIR--------DMLM-QFV---TK-----------ENCLVLAVSPANS--D-LANSD-ALKIAKEVD------
PKGLRTIGVITKLDLMD-------EGT------D-------A--------------------------RDV--LE---NK--------L-
LP---------------------L---RRGYIGVVNR---S-QKD--IDG-----
------K--------KD------------------IQ---------AALAA---ERKFFLS--------HP-SY-------------------R-
H------L----------ADR-MGTPYLQKALNQQLTNHIRDT-L----
XP_032814666.1 ------------LDLPQ--------IAVV--
GG----Q---------------SAGKSSVLE-----N--FV-G-R-DFLPRG-------------SG-IVTRRPL------------
--ILQ-------------L-M-FCK---------------------------A--
--E-------------Y------------------------AEF-------------------
--LHCKGKKFTDFE----------EVRAEI------EAET-DR---
L-TG----------------------SN-----------------K-GISPI-PINLRVYSPH-----------------------------------
------------------------VLNLTLIDLPGMTK--VP----------------V-------GD------------Q--------PV--
D--IEYQIR--------EMLM-QFV---TK-----------ENCLILAVSPANT--D-LANSD-ALKIAKEVD------
PQGLRTIGVITKLDLMD-------DGT------D-------A--------------------------RDI--LE---NK--------L-
LP---------------------L---RRGYIGVVNR---S-QKD--IDG-----

------R--------KD--------------------IN----------AAMAA---ERKFFLS--------HP-SY--------------------
R-H------M-----------ADR-MGTPYLQKTLNQQLTNHIRDT-L----
XP_025944940.1 --
---------------------------------MF-F-R-DFLPRG-------------SG-IVTRRPL--------------VLQ----------
----L-V-TAK-----------------------------T---
-------------------------E-------------Y-------------------------AEF--------------------------------------
--------------------------------LHCKGRKFTDFD-----------EVRQEI------EVET-DR---I-TG-------------
----------VN-------------------K-GISSI-PINLRIYSPH--
-----VLSLTLIDLPGITK--VP------------------V-------GD-------------Q--------PP--D--IEQQIR--------
DMIM-QFI---SR------------ENCLILAVTPANT--D-LANSD-ALKLAKEVD------
PQGLRTIGVITKLDLMD-------EGT------D-------A---------------------------REI--LE---NK---------L-
LP----------------------L---RRGYIGVVNR---S-QKD--IDG-----
------K--------KD--------------------IK----------AALLA---ERKFFLS--------HP-AY--------------------R-
H------M-----------ADR-MGTPYLQKVLNQQLTNHIRDT-L----
EPQ08653.1 ------------LELPQ--------IAVV--GG----
Q----------------SAGKSSVLE-----N--FV-G-R-DFLPRG-------------SG-IVTRRPL--------------VLQ--
------------L-V-TSK-----------------------------T---
---------------------------------E-------------Y-------------------------AEF--------------------------------
--LHCKGKKFTDFD-----------EVRHEI------EAET-DR---V-TG------
------------------MN-------------------K-GISSI-PINLRVYSPH--
-------------VLNLTLIDLPGITK--VP------------------V-------GD-------------Q--------PV--D--
IEHQIR--------EMIM-QFI---TR------------ENCLILAVTPANT--D-LANSD-ALKLAKDVD------
PQGLRTIGVITKLDLMD-------EGT------D-------A---------------------------RDI--LE---NK---------L-
LP----------------------L---RRGYVGVVNR---S-QKD--IDG----
-------K--------KD--------------------IK----------AAMLA---ERKFFLS--------HP-AY--------------------
R-H------I-----------ADR-MGTPHLQKVLNQQLTNHIRDT-L----
XP_006496668.1 ------------LELPQ--------IAVV--
GG----Q----------------SAGKSSVLE-----N--FV-G-R-DFLPRG-------------SG-IVTRRPL-------------
--VLQ-------------L-V-TSK-----------------------------A---
---E-------------Y-------------------------AEF--------------------
---LHCKGKKFTDFD-----------EVRHEI------EAET-DR---
V-TG-----------------------MN-------------------K-GISSI-PINLRVYSPH---------------------------------
-------------------------VLNLTLIDLPGITK--VP------------------V-------GD-------------Q--------PP--
D--IEYQIR--------DMIM-QFI---TR------------ENCLILAVTPANT--D-LANSD-ALKLAKEVD------
PQGLRTIGVITKLDLMD-------EGT------D-------A---------------------------RDV--LE---NK---------L-
LP----------------------L---RRGYVGVVNR---S-QKD--IDG----
-------K--------KD--------------------IK----------AAMLA---ERKFFLS--------HP-AY--------------------
R-H------I-----------ADR-MGTPHLQKVLNQQLTNHIRDT-L----
XP_016856477.1 ------------LELPQ--------IAVV--
GG----Q----------------SAGKSSVLE-----N--FV-G-R-DFLPRG-------------SG-IVTRRPL-------------
--VLQ-------------L-V-TSK-----------------------------A---
---E-------------Y-------------------------AEF--------------------
---LHCKGKKFTDFD-----------EVRLEI------EAET-DR---V-
TG-----------------------MN-------------------K-GISSI-PINLRVYSPH-----------------------------------

------------------------VLNLTLIDLPGITK--VP-------------------V-------GD-------------Q--------PP--D-
-IEYQIR--------EMIM-QFI---TR------------ENCLILAVTPANT--D-LANSD-ALKLAKEVD------
PQGLRTIGVITKLDLMD-------EGT------D-------A----------------------------RDV--LE---NK---------L-
LP----------------------L---RRGYVGVVNR--S-QKD--IDG----
-------K--------KD-------------------IK---------AAMLA---ERKFFLS--------HP-AY--------------------
R-H------I-----------ADR-MGTPHLQKVLNQQLTNHIRDT-L----
XP_027623811.1 ------------LELPQ--------IAVV--
GG----Q----------------SAGKSSVLE-----N--FV-G-R-DFLPRG-------------SG-IVTRRPL------------
--VLQ-------------L-V-TSK------------------------------A--
--E-------------Y---------------------------GEF----------------------
---LHCKGKKFTDFD-----------EIRHEI------EAET-DR---V-
TG-----------------------VN------------------K-GISSI-PINLRVYSPH-------------------------------------
-----------------------VLNLTLIDLPGITK--VP------------------V-------GD-------------Q--------PP--D--
IEYQIR--------EMIM-QFI---TR------------ENCLILAVTPANT--D-LANSD-ALKLAKEVD------
PQGLRTIGVITKLDLMD-------EGT------D-------A----------------------------RDV--LE---NK---------L-
LP----------------------L---RRGYVGVVNR--S-QKD--IDG----
-------K--------KD-------------------IK---------AAMLA---ERKFFLS--------HP-AY--------------------
R-H------I-----------ADR-MGTPHLQKVLNQQLTNHIRDT-L----
XP_012379251.1 ---
-----------------------------------V-G-R-DFLPRG-------------SG-IVTRRPL---------------VLQ----------
---L-V-TSK------------------------------A--
--------------------------E-------------Y---------------------------AEF---------------------------------------
---------------------------------LHCKGKKFTDFD-----------EVRHEI------EAET-DR---V-TG-------------
----------MN------------------K-GISSI-PINLRVYSPH--
------VLNLTLIDLPGITK--VP------------------V-------GD-------------Q--------PP--D--IEYQIR--------
EMIM-QFI---TR------------ENCLILAVTPANT--D-LANSD-ALKLAKEVD------
PQGLRTIGVITKLDLMD-------EGT------D-------A----------------------------RDV--LE---NK---------L-
LP----------------------L---RRGYVGVVNR--S-QKD--IDG----
-------K--------KD-------------------IK---------AAMLA---ERKFFLS--------HP-AY--------------------
R-H------I-----------ADR-MGTPHLQKVLNQQLTNHIRDT-L----
XP_031753735.1 ------------LDLPQ--------IAVV--
GG----Q----------------SAGKSSVLE-----N--FV-G-R-DFLPRG-------------SG-IVTRRPL------------
--ILQ-------------L-I-FSK------------------------------T---
--E-------------Y---------------------------AEF----------------------
---LHCKSKKFTDFD-----------EVRQEI------EAET-DR---V-
TG-----------------------TN------------------K-GISPV-PINLRVYSPN-------------------------------------
-----------------------VLNLTLIDLPGITK--VP------------------V-------GD-------------Q--------PH--D-
-IEYQIK--------DMIL-QFI---SR------------DSCLILAVTPGNT--D-LANSD-ALKMAKEVD------
PQGLRTIGVITKLDLMD-------EGT------D-------A---------------------------KDI--LE---NK---------L-
LP----------------------L---RRGYIGVVNR--S-QKD--IDG-----
------K--------KD-------------------IK---------AALGA---ERKFFLS--------HP-GY-------------------R-
H------I-----------AER-MGTPHLQKTLNQQLTNHIRET-L----
XP_028568434.1 ------------LDLPQ--------IAVV--
GG----Q----------------SAGKSSVLE-----N--FV-G-R-DFLPRG-------------SG-IVTRRPL------------

--ILQ--------------L-I-FSK-----------------------------T---
---E-------------Y--------------------------AEF----------------------
---LHCKSKKFTDFD-----------EVRQEI------EAET-DR---V-
TG-----------------------TN-------------------K-GISPV-PINLRVYSPH-------------------------------------
----------------------VLNLTLIDLPGITK--VP------------------V-------GD-------------Q--------PQ--D-
-IEYQIK--------DMIL-QFI---SR------------ESSLILAVTPANM--D-LANSD-ALKMAKEVD------
PQGLRTIGVITKLDLMD-------EGT------D-------A---------------------------RDV--LE---NK---------L-
LP----------------------L---RRGYIGVVNR--S-QKD--IDG-----
------K--------KD-------------------IR---------AALAA---ERKFFLS--------HP-AY--------------------R-
H------M-----------ADR-MGTPHLQKLLNQQLTNHIRET-L----
XP_025920181.1 ------------LDLPQ--------IAVV---
GG----Q----------------SAGKSSVLE-----N--FV-G-R-DFLPRG--------------SG-IVTRRPL-------------
--ILQ--------------L-I-FSK-----------------------------T---
---E-------------Y--------------------------AEF----------------------
---LHCKSKKFTDFD-----------EVRQEI------EAET-DR---V-
TG-----------------------TN-------------------K-GISPV-PINLRVYSPH-------------------------------------
----------------------VLNLTLIDLPGITK--VP------------------V-------GD-------------Q--------PQ--D-
-IEYQIK--------DMIM-QFI---SR------------ESSLILAVTPANM--D-LANSD-ALKMAKEVD------
PQGLRTIGVITKLDLMD-------EGT------D-------A---------------------------RDV--LE---NK---------L-
LP----------------------L---RRGYIGVVNR--S-QKD--IDG-----
------K--------KD-------------------IR---------AALAA---ERKFFLS--------HP-AY--------------------R-
H------M-----------ADR-MGTPHLQKVLNQQLTNHIRET-L----
XP_006510037.1 ------------LDLPQ--------IAVV---
GG----Q----------------SAGKSSVLE-----N--FV-G-R-DFLPRG--------------SG-IVTRRPL-------------
--ILQ--------------L-I-FSK-----------------------------T---
---E-------------Y--------------------------AEF----------------------
---LHCKSKKFTDFD-----------EVRQEI------EAET-DR---V-
TG-----------------------TN-------------------K-GISPV-PINLRVYSPH-------------------------------------
----------------------VLNLTLIDLPGITK--VP------------------V-------GD-------------Q--------PP--D--
IEYQIK--------DMIL-QFI---SR------------ESSLILAVTPANM--D-LANSD-ALKLAKEVD------
PQGLRTIGVITKLDLMD-------EGT------D-------A---------------------------RDV--LE---NK---------L-
LP----------------------L---RRGYIGVVNR--S-QKD--IEG-----
------K--------KD-------------------IR---------AALAA---ERKFFLS--------HP-AY--------------------R-
H------M-----------ADR-MGTPHLQKTLNQQLTNHIRES-L----
NP_001005360.1 ------------LDLPQ--------IAVV---
GG----Q----------------SAGKSSVLE-----N--FV-G-R-DFLPRG--------------SG-IVTRRPL-------------
--ILQ--------------L-I-FSK-----------------------------T---
---E-------------H--------------------------AEF---------------------
--LHCKSKKFTDFD-----------EVRQEI------EAET-DR---V-
TG-----------------------TN-------------------K-GISPV-PINLRVYSPH-------------------------------------
----------------------VLNLTLIDLPGITK--VP------------------V-------GD-------------Q--------PP--D--
IEYQIK--------DMIL-QFI---SR------------ESSLILAVTPANM--D-LANSD-ALKLAKEVD------
PQGLRTIGVITKLDLMD-------EGT------D-------A---------------------------RDV--LE---NK---------L-
LP----------------------L---RRGYIGVVNR--S-QKD--IEG-----

------K--------KD-------------------IR---------AALAA---ERKFFLS--------HP-AY--------------------R-
H------M-----------ADR-MGTPHLQKTLNQQLTNHIRES-L----

XP_014389433.1 ---
-----------------AGKGQI-----------T-A-W-DFLPRG-------------SG-IVTRRPL--------------ILQ-------
-------L-I-FSK-----------------------------T--
---------------------------E-------------Y-------------------------AEF-----------------------------------
----------------------------------LHCKSKKFTDFD-----------EVRQEI------EAET-DR---V-TG-----------
-------------TN-------------------K-GISPV-PINLRVYSPH--
---------VLNLTLIDLPGITK--VP-------------------V-------GD-------------Q--------PP--D--IEYQIK-----
---DMIL-QFI---SR------------ESSLILAVTPANM--D-LANSD-ALKMAKEVD------
PQGLRTIGVITKLDLMD-------EGT------D-------A---------------------------RDV--LE---NK---------L-
LP----------------------L---RRGYIGVVNR--S-QKD--IEG-----
------K--------KD-------------------IR---------AALAA---ERKFFLS--------HP-AY--------------------R-
H------M-----------ADR-MGTPHLQKTLNQQLTNHIRES-L----

XP_006161648.2.2 ---------------PH--------CTSV---
SR----QAYS------------SPQGPRKVIS-----VLFLP-H-R-DFLPRG-------------SG-IVTRRPL-----------
----ILQ--------------L-I-FSK-----------------------------T--
--E-------------Y-------------------------AEF--------------------
---LHCKSKKFTDFD-----------EVRQEI------EAET-DR---
V-TG-----------------------TN-------------------K-GISPV-PINLRVYSPH----------------------------------
-------------------------VLNLTLIDLPGITK--VP-------------------V-------GD-------------Q--------PP--
D--IEYQIK--------DMIL-QFI---SR------------ESSLILAVTPANM--D-LANSD-ALKLAKEVD------
PQGLRTIGVITKLDLMD-------EGT------D-------A---------------------------RDV--LE---NK---------L-
LP----------------------L---RRGYIGVVNR--S-QKD--IEG-----
------K--------KD-------------------IR---------AALAA---ERKFFLS--------HP-AY--------------------R-
H------M-----------ADR-MGTPHLQKTLNQQLTNHIRES-L----

XP_012381548.1 ---

---MIL-QFI---GR------------ESSLILAVTPANM--D-LANSD-
ALKLAKEVD------PQGLRTIGVITKLDLMD-------EGT------D-------A---------------------------RDV--
LE---NK---------L-LP----------------------L---RRGYIGVVNR---
-S-QKD--IEG-----------R--------KD-------------------IR---------SALAA---ERKFFFS--------HS-AY---
-----------------R-H------M-----------ADR-MGTLHLQKTLNQQLTNHIRES-L----

NP_001025299.1 ------------LDLPQ--------IAVV--
GG----Q----------------SAGKSSVLE-----N--FV-G-R-DFLPRG-------------SG-IVTRRPL-------------
--ILQ--------------L-V-NNK------------------------------A--
--E-------------Y-------------------------AEF-------------------
---LHCKGRKFVDFD-----------EVRQEI------EAET-DR---
I-TG-----------------------SN------------------K-GISPI-PINLRVYSPN----------------------------------
------------------------VLNLTLIDLPGMTK--VA-------------------V-------GD-------------Q--------PP--

D--IEHQIR--------DMIM-QFI---TR------------ESCLILAVTPANM--D-LANSD-ALKVAKEVD------
PQGLRTIGVITKLDLMD-------EGT------D-------A----------------------------RDI--LE---NK---------L-
LP-----------------------L---RRGYIGVVNR--S-QKD--IDG-----
------R--------KD--------------------IR----------AALAA---ERKFFLS--------HP-SY---------------------R-
H------M-----------AER-MGTPHLQKALNQQLTNHIRDT-L----
XP_021326548.1 ------------LDLPQ--------IAVV---
GG----Q----------------SAGKSSVLE-----N--FV-G-R-DFLPRG--------------SG-IVTRRPL-------------
--ILQ--------------L-V-NNK------------------------------A---
---E--------------Y--------------------------AEF--------------------
--LHCKGRKFVDFD-----------EVRQEI------EAET-DR---
I-TG------------------------SN-------------------K-GISPI-PINLRVYSPN-----------------------------------
-------------------------VLNLTLIDLPGMTK--VA-------------------V-------GD--------------Q--------PP--
D--IEHQIR--------DMIM-QFI---TR------------ESCLILAVTPANM--D-LANSD-ALKVAKEVD------
PQGLRTIGVITKLDLMD-------EGT------D-------A----------------------------RDI--LE---NK---------L-
LP-----------------------L---RRGYIGVVNR--S-QKD--IDG-----
------R--------KD--------------------IR----------AALAA---ERKFFLS--------HP-SY---------------------R-
H------M-----------AER-MGTPHLQKALNQQLTNHIRDT-L----
XP_035683496.1 ------------LDLPQ--------IAVV---
GG----Q----------------SAGKSSVLE-----N--FV-G-R-DFLPRG--------------SG-IVTRRPL-------------
--VLQ--------------L-IHNPK------------------------------A---
---E--------------Y--------------------------GEF--------------------
--LHAKGKMFSDFH-----------EIRAEI------EAET-DR---
M-TG------------------------SN-------------------K-GISPV-PINLRVYSPH--------------------------------
--------------------------VLNLTLIDLPGMTK--VP-------------------V-------GD--------------Q--------
PP--D--IEQQIR--------DMLL-QFI---TK------------DNCLILAVSPANQ--D-LANSD-ALKIAKEVD-----
-PQGMRTIGVITKLDLMD-------EGT------D-------A----------------------------RNI--LE---NR---------T-
YP-----------------------L---RRGYIGVVNR--S-QAD--IDG-----
------R--------KD--------------------IK----------AALAA---ERKFFLS--------HP-AY---------------------R-
H------L-----------ADR-MGTPYLQKTLNQQLTNHIRDT-L----
XP_026693152.1 ------------IDLPQ--------IAVV---
GG----Q----------------SAGKSSVLE-----N--FV-G-K-DFLPRG--------------SG-IVTRRPL--------------
-VLQ--------------L-ITAKN------------------------------G--
--E--------------W---------------------------GEF--------------------
--LHCKGKKFTDFN-----------EIRKEI------EEET-DR---
M-TG------------------------SN-------------------K-GISAI-PINLRVHSPH---------------------------------
--------------------------VLNLTLVDLPGMTK--VP-------------------V-------GD--------------Q--------
PA--D--IEQQIR--------DMIM-QFV---VK------------DNCLILAVSPANS--D-LANSD-ALKIAKEFD-----
-PQGIRTIGVITKLDLMD-------EGT------D-------A----------------------------KHI--LE---NK---------H-
LP-----------------------L---RRGYVGVVNR--S-QKD--IDG----
-------N--------KD--------------------IK---------AALSA---ERRFFLS--------HP-AY---------------------
R-H------M-----------ADK-LGTPYLQKILNQQLTNHIKET-L----
PAA65118.1 ------------FDLPQ--------IAVV--GS----
Q----------------SAGKSSVLE-----N--FV-G-K-DFLPRG--------------SG-IVTRRPL---------------ILQ---
-----------L-L-YNP------------------------------SA--

----------------------------------E-------------Y--------------------------AEF-----------------------------
---GHQRGRKYTNFE-----------EVRQEI------EAET-DR---L-TG----
--------------------RN-------------------K-GISNV-PIMLRVFSPH--
----------------VLNLTLVDLPGMTK--VA------------------V-------GD-------------Q--------PP--D--
IEVQIR--------NMLL-EFI---TK-----------ENCLILAVSPANS--D-LANSD-ALKLAKEVD------
PAGTRTIGVITKLDLMD-------QGT------D-------A---------------------------REV--LE---NK---------L-
LP----------------------L---RRGYIGVVNR--S-QKD--IEG-----
------R--------KD--------------------IK---------AAMAA---ERKFFLS--------HP-SY--------------------
R-H------M-----------AER-MGTPYLQRCLNQQLTNHIRET-L----
PAA78248.1 ------------FDLPQ--------IAVV---GS----
Q----------------SAGKSSVLE-----N--FV-G-K-DFLPRG--------------SG-IVTRRPL---------------VLQ--
------------L-L-THP-----------------------------S--
----------------------------------E-------------F--------------------------AEF-----------------------------
---GHLRGKKFTNFD-----------EVRQEI------ENET-DR---L-TG----
--------------------KN-------------------K-GISNV-PITLRVFSPH--
----------------VLNLTLVDLPGMTK--VA------------------V-------GD-------------Q--------PP--D--
IEQQIR--------AMLF-EFI---SK-----------ENCLILAVSPANS--D-LANSD-ALKIAKEVD------
PNGTRTIGVITKLDLMD-------QGT------D-------A---------------------------REV--LE---NK---------L-
LP----------------------L---RRGYIGVVNR--S-QKD--IEG-----
------K--------KD--------------------IA---------AAMAA---ERKFFLS--------HP-SY--------------------
R-H------M-----------AER-MGTPYLQRCLNQQLTNHIRET-L----
PAA59145.1 ------------FDLPQ--------IAVV---GS----
Q----------------SAGKSSVLE-----N--FV-G-K-DFLPRG--------------SG-IVTRRPL---------------VLQ--
------------L-I-NFH-----------------------------T---
----------------------------------E-------------Y--------------------------AEF-----------------------------
---GHIRGKRFTNFD-----------EVRQEI------ENET-DR---V-TG-----
-------------------KN-------------------K-GISNV-PIMLRVYSPQ---
----------------VLNLTLVDLPGLTK--VA------------------V-------GD-------------Q--------PQ--D--
IELLIR--------AMIL-EFV---SK-----------DNCLILAVTPANS--D-LANSD-ALKIAKEVD------
PSGTRTIGVITKLDLMD-------QGT------D-------A---------------------------RDV--LE---NR---------L-
LP----------------------L---RRGYIGVVNR--S-QKD--IEG-----
------K--------KD--------------------IV---------AAMAA---ERKFFLS--------HP-AY--------------------
R-H------M-----------AER-MGTSYLQRCLNQQLTNHIRET-L----
PAA64382.1 ------------IDLPQ--------IAVV---GS----
Q----------------SAGKSSVLE-----N--FV-G-R-DFLPRG--------------SG-IVTRRPL---------------ILQ--
------------L-M-NYQ-----------------------------T---
------------------------------------E-------------Y--------------------------AEF---------------------------
---GHIRGKKFVNFD-----------EVRREI------EVET-DR---L-TG---
--------------------QN-------------------K-GISNV-PITLRVYSPQ---
------------------VLNLTLVDLPGMTK--VA------------------V-------GD-------------Q--------PP--D--
IEQQVR--------AMIW-EFI---SK-----------DNCLILAVSPANS--D-LANSD-ALKLAKEAD------
PSGSRTIGVLTKLDLMD-------AGT------D-------A---------------------------RDV--LE---NR---------F-
LP----------------------L---RRGYVGVVNR--S-QKD--IDG----

-------R--------KD-------------------IS----------SAMAA---ERKFFLG--------HP-AY--------------------
R-H------M-----------AER-MGTAHLQRCLNQQLVGHIRDT-L----
XP_001749319.1 ------------LDLPQ--------IAVV--
GG----Q----------------SAGKSSVLE-----N--FV-G-K-DFLPRG--------------SG-IVTRRPL-------------
-VLQ-------------L-N-YHP-----------------------------SA---
---E-------------W-------------------------GEF------------------
---LHARGKKFTDFN-----------EIRQEI------EAET-DR---
M-TG------------------------SN-------------------K-GISNI-PINLRVYSPH--------------------------------
---------------------------VLNLTLVDLPGLTK--VA-------------------V-------GD-------------Q--------PA-
-D--IENQIR--------GMLM-EFI---TK------------DNCIILAVTPANQ--D-LANSD-ALKLAKEVD------
PEGVRTIGVITKLDLMD-------SGT------D-------A---------------------------RAI--LT---NE---------F-
LP-----------------------L---RRGYIGVVNR---S-QKD--IDG-----
------R--------KD--------------------IR----------AALDA---ERKFFLM--------HP-SY--------------------
K-D------I-----------ASK-NGTPYLQKALNQQLTNHIREC-L----
XP_031757197.1 ------------VDLPQ--------IAVV--
GG----Q----------------SAGKSSVLE-----N--LV-G-R--WIH---
-----------V-L--SS-------------------------------T---
---------------------------------E-------------Y-------------------------AEF-------------------------------
---LHCKGTKYTDFS-----------EVRQEI------EEET-ER---A-TG--------
----------------LN-------------------K-GISAI-PISLRIYSPH--
----------VLNLSLIDLPGVTK--VP-------------------V-------GD-------------Q--------PA--D--IETQIR---
-----DMIM-NFI---SR------------ENCLILAVTPANT--D-LANSD-ALKLAKEVD------
PQGLRTIGVITKLDLMD-------EGT------N-------A---------------------------QEI--LE---NK---------L-
LP-----------------------L---RRGYVGVVNR--S-QKD--IDG----
-------K--------KN-------------------IN----------AALQA---EQMFFLT--------HP-AY--------------------
R-H------M-----------ADR-MGTSHLQKMLNQQLTNHIRET-L----
XP_014148725.1 ---
---T--------

---------E-------------Y-------------------------GEF---
----------------LHKPGRLFDNFD-----------EIRNEI------EADT-AR---I-TG------------------------AN----
---------------K-GISHL-PINLKVYSPH---
VLDLTLVDLPGLTK--VA-------------------V-------GD-------------Q--------PA--D--IEMQIK--------
NMIM-EFI---TK------------PNCLILAVTPANS--D-LANSD-ALKLAKEVD------
PQGLRTIGVITKLDLMD-------AGT------D-------A---------------------------RDV--LE---NK---------L-
LP-----------------------L---RRGYVGVVNR--S-QKD--IAG----
-------N--------KD-------------------IR----------AAQAA---EKKFFKT--------HP-AY--------------------
R-H------L-----------ADK-MGTPKLQQVLNQQLTDHIRQT-L----
XP_014153758.1 ---
-------------------------------N---LY-------------LYI------

-----------E-------------W-------------------------GEF---
-------------------LHQPGRKYTDFE-----------EIMKEI------EAET-DR---M-TG-----------------------
SN-------------------K-GISNI-PINLKVMSPH---

VLDLTLVDLPGLTK--VA------------------V-------GD-------------Q--------PA--D--IEQQIL--------
GMIM-EFI---TR------------PNCLILAVSPANA--D-LANSD-ALKLAKEVD------
PQGLRTIGVITKLDLMD-------QGT------D-------A----------------------------REI--LE---NK---------L-
LP----------------------L---RRGYIGVVNR---S-QKD--ITG------
-----K--------KD-------------------IR----------AAQEA---ERRFFST--------HP-AY--------------------R-
H------L-----------AQN-MGTPKLQKVLNQQLTNHIRDS-L----

XP_006812840.1 ------------ITKPS--------CLIL--
AV---------------------TPGNSDLANS----DALKV-A-K-EVDPQGL-----------RTIG-VITKLDLL-
DDGTDAREILENKLLP-------------L---RRA----------------------------S-------------------------------
--E-------------W--------------------------
GEF---LHCKGKKFTNFD-----------
EIRMEI------EAET-DR---L-TG-----------------------KN-------------------K-GISPI-PINLRVYSPH------
---VLNLTLIDLPGMTK--VP------------------V-------GD-
-------------Q--------PA--D--IEQQIR--------SMLM-EFI---TK------------PSCLILAVTPGNS--D-
LANSD-ALKVAKEVD------PQGLRTIGVITKLDLLD-------DGT------D-------A---------------------------
-REI--LE---NK---------L-LP----------------------L---RRGYIGVVNR-------------------------------------
--------G-QKD--IEG-----------R--------KD-------------------IK----------SALAS---ERKFFLS--------
HP-SY--------------------R-H------M-----------ADR-LGTPYLQKALNQQLTNHIRDT-L----

NP_013100.1 ------------LDLPI--------LAVV---GS----
Q----------------SSGKSSILE-----T--LV-G-R-DFLPRG-------------TG-IVTRRPL---------------VLQ----
----------L-N-NIS------------------------------PNSPLIEEDDN------SVNPH--DEVTKI-SGFEA---------
-----GTKPLE-----YRGKERN-------------------------HADE-------------W--------------------------
GEF---LHIPGKRFYDFD-----------
DIKREI------ENET-AR---I-AG------------------------KD-------------------K-GISKI-PINLKVFSPH------
---VLNLTLVDLPGITK--VP------------------I-------GE---
-----------Q--------PP--D--IEKQIK--------NLIL-DYI---AT------------PNCLILAVSPANV--D-LVNSE-
SLKLAREVD------PQGKRTIGVITKLDLMD-------SGT------N-------A----------------------------LDI--
LS---GK---------M-YP-----------------------L---KLGFVGVVNR--
--S-QQD--IQL-----------N--------KT-------------------VE----------ESLDK---EEDYFRK--------HP-VY-
--------------------R-T------I-----------STK-CGTRYLAKLLNQTLLSHIRDK-LP---

XP_011392073.1 ------------VDLPQ--------IVVV--
GS----Q----------------SAGKSSVLE-----T--IV-G-R-DFLPRG-------------SG-IVTRRPL---------------
VLQ--------------L-I-HTP------------------------------STKEQPRQPKQ------SSRPY--
DLSDGLASDMQR--------------GGSHAS-----SADTRSP-------------------------TYEE-------------Y--
-------------------------GEF---LHL-
DKRFTDFN-----------EIRREI------ENET-FR---V-AG-----------------------QN-------------------K-
GVSKL-PIHLKIYSPN---VLNLTLVDLPGLTK--IP---
----------------V-------GD-------------Q--------PS--D--IERQIR--------NLVT-DYV---SK------------
PNCIILAVSPANV--D-LANSD-SLKLARTVD------PQGRRTIGVLTKLDLMD-------QGT------H-------
A----------------------------LDI--LT---GR---------V-YP-----------------------L---KLGFIGVVNR----------
-----------------------------------S-QQD--ING-----------N--------VS-------------------ML----------
AARRA---EEDFFRS--------HA-AY---------------------K-N------I-----------AHR-
CGTKYLAKTLNQVLMSHIRDK-LP---

XP_006461708.1 ------------LDMPQ--------IVVV---
-GS----Q----------------SAGKSSVLE-----T--IV-G-K-DFLPRG--------------SG-IVTRRPL--------------
VLQ--------------L-I-HTP-----------------------------V----PSEPSP------NALP---------------------------
---YTE-------------W--------------------------GQF-------------
--LHI-DKRFTDFN-----------DIRKEI------EQET-
FR---V-AG------------------------QN-------------------K-GISRL-PISLRVYSPN---------------------------
--------------------------------VLDLTLVDLPGLTK--IP-------------------V-------GD-------------Q-------
-PS--D--IEKQIR--------NLVV-DYI---SK------------PNSVILAVSAANV--D-LANSE-SLKLARSVD------
PQGRRTIGVLTKLDLMD-------AGT------N-------A--------------------------LDI--LT---GR---------V-
YP-----------------------L---KLGFIGIVNR---S-QQD--INV------
-----E--------KS--------------------LT----------DAVES---EAEFFRN--------HA-VY---------------------R-
N------I-----------AHK-NGTRYLAKTLNQVLMNHIRDK-LP---
XP_746923.1 ------------LDLPQ--------IVVV---GS----
Q----------------SSGKSSVLE-----N--IV-G-R-DFLPRG--------------SG-IVTRRPL---------------ILQ---
-----------L-I-NIP------------------------------SEH--------------NDRPG--DN------DVLA-------------
PHTAAS-----VAG--------------------------------QHE--------------W--------------------------AEF--------
---HHLPGRKFEDFA-----------LVKQEI------
EAET-AR---I-AG------------------------NN-------------------K-GINRQ-PINLKIFSPH--------------------
--VLNLTMVDLPGLTK--VP-------------------I-------GD-------------
Q--------PS--D--IEKQTR--------TLIL-EYI---AK------------PNSIILAVSPANV--D-LVNSE-
ALKLARQVD------PMGRRTIGVLTKLDLMD-------HGT------N-------A----------------------------MDI-
-LS---GR---------V-YP------------------------L---KLGFIGVVNR--
--S-QQD--IQS-----------G--------KS--------------------LS----------EALQA---EAEFFRH--------HP-
AY---------------------R-N------M-----------ANR-CGTQFLAKTLNTTLMAHIRDR-LP---
KXN67416.1 ------------LDLPQ--------IVVV---GS----
Q----------------SSGKSSVLE-----N--LV-G-R-DFLPRG--------------NG-IVTRRPL---------------VLQ-
-------------L-V-NLR------------------------------ED-------------------A--DL------DLT-------------------
---E--------------R--------------------------AQF---------------------
--LHNPTKFYTDFS-----------EVRQEI------EQET-NR---L-
AG------------------------EN-------------------K-GISRN-PIHLKIFSTQ-------------------------------------
----------------------VLNLTVVDLPGLTK--IP-------------------I-------GD-------------Q--------PT--D--
IEKQTK--------SLIL-DYI---SK-------------PNSIILAVSPANV--D-LVNSE-SLKLAKEVD------
PEGKRTIGIITKIDLMD-------AGT------N-------A----------------------------LDI--LT---GR---------V-LN-
----------------------L---KLGFIGVINR---S-QQD--TVA----------
-K--------KP--------------------IR----------ESLEA---ELEFFRT--------HP-AY---------------------R-N----
--I-----------SQR-CGTGHLSKTLNQVLVNHIRDR-LP---
KNE61418.1 ------------LDLPQ--------IAVV---GS----
Q----------------SSGKSSVLE-----A--IV-G-K-DFLPKG--------------NG-IVTRRPL---------------VLQ---
-----------L-R-NVP---------------------------------PHAVPE--
---GEE--------------AVD-------------------------VAEF---------------------
--SHCPDRVFTDFA-----------DVRREI------EAET-AR---I-
AG------------------------DN-------------------K-GIATD-PIRLCVRSPN------------------------------------
-------------------------VVDLTLVDLPGMTK--IP-------------------V-------GD-------------Q--------PS--
D--IELQIR--------ELIM-GFI---TK------------PNCLILAVSPANV--D-LANSD-SLKLAREVD------

PTGDRTLGLLTKVDLME-------PGT------H-------A---------------------------LDI--LA---GR---------V-
YP----------------------L---RLGFVGVVNR--S-QRD--IDQ----
-------G--------KS--------------------LE----------FARKR---EQQFFAT--------HP-VY---------------------
A-P------V-----------ASR-CGTTVLARTLNQVLLAHIRDR-LP---
KNE67543.1 ------------LDLPQ--------IAVV--GS----
Q----------------SSGKSSVLE-----A--IV-G-K-DFLPKG--------------AG-IVTRRPL----------------ILQ----
----------L-N-YAP------------------------------VDPETPDE---
--PEE--------------W---------------------------AEF-----------------------
---QHLPGQQFADFG-----------EVKREI------ERET-AR---I-
AG-------------------------DN-------------------K-GISDE-PITLRVHSPS--------------------------------------
-----------------------VVDLTLVDLPGLTK--IP------------------V-------GD--------------Q--------PS--
D--IELQIR--------DLIM-KFI---LQ------------PNCIILAVSPANV--D-LANSD-SLKLAREVD------
PQGLRTLGLLTKVDLME-------TGS------H-------A---------------------------LDI--LG---GR---------V-
YP----------------------L---RLGFVAVVNR--S-QRD--IEA-----
------R--------RT--------------------LE---------WSRKR---EQQFF---
----------------SGR-CGTAALARTLNSVLLDHIRAQ-LP---
GAX23670.1 -----------HLDLPQ--------IVVV--GG----
Q----------------SSGKSSVLE-----N--IV-G-R-SFLPRG--------------TG-IVTRRPL----------------VLQ---
-----------L-F-NTR------------------------------QAPADV-----------DEMP-----------------------------
NKNRII-----PPGGSAE-----------------------------E--------------W---------------------------GEF--------
---LHLPGKRMYDFA-----------DIRREI------
ADET-NR---I-TG-------------------------NN-------------------K-GVDST-PINLKIFSPR--------------------
--VVSLTLVDLPGIAK--VP-------------------V-------GD--------------
Q--------PE--D--IERQIN--------SMCM-SFI---SN------------PNAIILAVTSANT--D-LANSD-
ALKLAQAVD------PEGLRTIGVLTKVDLMD-------EGT------D-------C---------------------------TEI--
LL---NQ---------V-IP----------------------L---RRGYIAVVNR--
G-QKD--VVS-----------D--------VS--------------------IR----------DGLKK---EENFFRK--------HP-IYS-
R------------------DR-S------I-----------LSK-CGTGRLSKNLNSILMH-----------
CAB9512103.1 -----------AIDLPQ--------IVVV--
GG----Q----------------SSGKSSVLE-----A--IV-G-R-SFLPRG---------------SG-IVTRRPL---------------
VLQ--------------L-F-NTS------------------------------H---
--------DGQSET-----------------------------E--------------F---------------------------GEF------------------
---LHQPGKRYHDFV-----------SIRSEI------VRET-ER--
-L-TG------------------------PN-------------------K-GIDHA-PIHLKIYSPE-----------------------------------
--------------------------VLSLTLVDLPGIAK--VP-------------------V-------GD--------------Q--------PE-
-N--IEEQIR--------EMCM-EYI---SN------------PNAIILAVTSANQ--D-IANSD-ALKLAQAVD------
PHGTRTVGVLTKLDLMD-------DGT------D-------A---------------------------SDI--LM---NR---------
V-IP----------------------L---RRGYVAVVNR--G-QRD--VNN-
----------D--------LS--------------------IQ----------DGLKK---EESFFRA--------HS-VYG-R--------------
---DR-Q------L-----------LAK-CGTKRLSQHLNTMLMH-----------
XP_002296064.1 -----------NLDLPQ--------IVVI--
GG----Q----------------SSGKSSVLE-----S--VV-G-R-SFLPRG--------------TG-IVTRRPL---------------
VLQ--------------L-F-NTS-----------------------------G----------------KRHP----------------------------
KNDATS-----SSTEPVE-----------------------------E--------------W---------------------------GEF--------

---LHQPGKRYTDFS-----------QIRSEI------
SRDT-NR---LCSG------------------------PN------------------SK-GVSST-PIHLKIYSPR------------------
---VLSLTMVDLPGLTK--VA-------------------V-------KD------------
-Q--------PE--D--IEEQIY--------QINV-QYG---SN------------PNAILLAVTGANT--D-LASSD-
ALKLARELD------PRGERTIGVLTKLDLMD-------PGT------D-------A----------------------------GEI--
LH---NK---------V-IP-----------------------L---RRGYVAVVNR---
-G-QRD--IDA-----------D--------LS--------------------IQ----------VGLRN---EERYFRT--------HP-
VYS-R------------------DR-S------L-----------VGK-CGTMNLARNLNGILIH-----------

XP_004184473.1 ------------IDLPQ--------IVVV--
GA----Q----------------SAGKSSVLE-----S--IV-G-R-DFLPRG--------------CG-MVTKRPL-------------
--ILQ--------------L-V-NLP------------------------------PTE---
---TTE--------------W---------------------------GEF------------------
---AHKAGEVFKDFE-----------DIKKEI------ENET-IR---
L-TG------------------------KS-------------------K-TISTV-AIRLKIYSPY---------------------------------------
-----------------------VVDLTLVDLPGLTK--IS-------------------V-------EG--------------Q--------EK--D--
ISQQLK--------QMVL-KFI---ES------------PNAIILAVTSANV--D-LATSD-ALSIAREVD------
PEGDRTIGVLTKMDLMD-------KGT------D-------A----------------------------MDV--LY---GR---------
V-YP-----------------------L---KLGYIGVLNR---S-QED--IEK----
-------K--------VP---------------------IR----------QALKS---EKEWFTN--------HP-IY----------------------
G-K------I-----------ADR-LGVSYLSKTLNQMLMQ-----------

EMS16943.1 ------------IDLPQ--------IVVV--GS----
Q----------------SAGKSSVLE-----S--IV-G-R-DFLPRG--------------SG-MVTKRPL---------------ILQ--
------------L-V-NLP------------------------------STE---
-------------------------------------TKE--------------W----------------------------GEF---------------------------
--AHKPGIVYRDFE-----------EIKKEI------ENET-IR---L-TG----
--------------------TK-------------------K-TISPV-AIRLKIYSPY--
-------------VVDLTLVDLPGLTK--IS-------------------V-------GS--------------Q--------EK--D--
ISNQLK--------QMVL-KFI---ER------------PNAIILAVTSANV--D-LATSD-ALSIAREVD------
PDGDRTIGVLTKMDIMD-------KGT------D-------A----------------------------MDV--LY---GR---------
V-YP-----------------------L---KLGYIGVLNR---S-QHD--IDT----
-------N--------VP---------------------IK----------TALTK---EKEWFSN--------HP-IY----------------------S-
K------I-----------ADR-LGIPYLTKTLNEILMQ-----------

NP_741403.2 ------------IQLPQ--------IVVV--GS----
Q----------------SAGKSSVLE-----N--LV-G-R-DFLPRG--------------TG-IVTRRPL---------------ILQ---
-----------L-N-HVA------------------------------LDDESKRRRSN------G------------------------------------
-------------TLL-----------------------------TDD--------------W----------------------------AMF----------------
--EHTGSKVFTDFD-----------AVRKEI------EDET-
DR---V-TG-------------------------VN-------------------K-GISLL-PISLKIYSHR------------------------------
--------------------------------VVSLSLVDLPGITK--IP-------------------V-------GD--------------Q--------
PV--N--IEEQIR--------EMIL-LYI---SN------------PSSIILAVTPANQ--D-FATSE-PIKLAREVD------
AGGQRTLAVLTKLDLMD-------QGT------D-------A----------------------------MDV--LM---GK---------
V-IP-----------------------V---KLGIIGVVNR---S-QQN--ILD----
-------N--------KL---------------------IV----------DAVKD---EQSFMQK--------K---Y---------------------
P-T------L-----------ASR-NGTPYLAKRLNMLLMHHIRNC-L----

XP_002129967.2 ------------LQLPQ--------IVVV--
GV----Q----------------SSGKSSVLE-----N--LV-G-R-DFLPRG-------------TG-IVTRCPL--------------
VLQ-------------M-I-HTT------------------------------NEDTAQCSNEG------SSGNN--DS-------------
---------DSSGESF-----K-ETNEE--------------------------VKE-------------W---------------------------
VKF---QHTKGKIFRSFK----------
QVKKEI------ELET-QR---L-SG-----------------------NN------------------K-GISSE-AIRLKIFSPK-----
--VLNLTLVDLPGLMK--IP-----------------V-------
GD-------------Q--------PD--D--IEEQAR--------NLIL-RYI---SN-----------PNSIILAVTPANV--D-
FATSE-ALQMARIVD------PDGCRTLAVVTKLDLMD-------AGT------D-------A-------------------------
---IDV--LC---GR---------I-VP----------------------V---KLGIIGIVNR-------------------------------------
--------S-QLD--INK----------G--------KS-------------------VQ----------DAIKD---EQAFLQK--------
K---Y----------------------P-S------F-----------ANR-SGSRYLSITLNRLLMHHIRDC-L----
NP_001259946.1 ------------IQLPQ--------IVVL---
GS----Q----------------SSGKSSVIE-----S--VV-G-R-SFLPRG-------------TG-IVTRRPL--------------
VLQ-------------L-I-YSP------------------------------LDDRENRSAEN------------------------------------
-----GTS------------N---------------------------AEE---------------W---------------------------GRF---------
--LHTK-KCFTDFD----------EIRKEI------
ENET-ER---A-AG------------------------SN------------------K-GICPE-PINLKIFSTH--------------------
---------------------------------------VVNLTLVDLPGITK--VP------------------V-------GD-------------
Q--------PE--D--IEAQIK--------ELVL-KYI---EN------------PNSIILAVTAANT--D-MATSE-
ALKLAKDVD------PDGRRTLAVVTKLDLMD-------AGT------D-------A----------------------------IDI--
LC---GR---------V-IP----------------------V---KLGIIGVMNR---
S-QKD--IMD-----------Q--------KH--------------------ID----------DQMKD---EAAFLQR--------K---Y--
-------------------P-T------L-----------ATR-NGTPYLAKTLNRLLMHHIRDC-L----
XP_035676386.1 ------------IQLPQ--------IVVI---
GT----Q----------------SSGKSSVLE-----S--LV-G-R-DFLPRG-------------TG-IVTRRPL--------------
VLQ-------------L-V-HVN------------------------------SEEKKRPSEDE------D---------------------------
------GGHKQ-----DIKEHAH--------------------------VEE-------------W---------------------------
GKF--LHTKNKIYTDFD----------
EIRQEI------ENET-DR---V-TG-----------------------TN------------------K-GIIDD-AIHLKIYSPK-------
--VLNLTLVDLPGITK--VP------------------V-------GD---
-----------Q--------PP--D--IEVQIR--------EMCL-KYI---AN------------PNSIILAVTSANT--D-MATSE-
ALKFAKEVD------PDGRRTLAVITKLDLMD-------AGT------D-------A----------------------------HDV--
LM---GR---------V-IP----------------------V---KLGIIGVVNR--
S-QMD--INK-----------R--------KP--------------------IE----------EAIKD---EAAFMQR--------K---Y----
-----------------P-S------L-----------ASR-NGTSHLARTLNRLLMHHIRDC-L----
XP_006821224.1 --

---MATSE-
AIKLSREVD------EDGRRTLAVITKLDLMD-------AGT------D-------A----------------------------VEI--

IC---GR---------V-IP----------------------V---KLGIIGVINR--
S-QMD--INN-----------K--------KP--------------------IQ----------ESVKD---EAAFLQR--------K---Y----
------------------P-A------L-----------ASR-NGTPYLAKTLNRLLMHHIRDC-L----
XP_030827871.1 ------------IQLPQ--------IVVV--
GN----Q----------------SSGKSSVLE-----G--LV-G-K-DFLPRG--------------NG-IVTRRPL--------------
-VLQ--------------M-V-HVD------------------------------PEDKR---
---GASG-----EGEEEIT---------------------------ADE--------------W---------------------------GKF-------
--LHTKNKVYTDFE-----------EIREEI------
QNET-DR---M-AG-----------------------TN--------------------K-GIVHD-AIHLRIYSPK------------------
--VLNLTLVDLPGLTK--VP--------------------V-------GD-------------
-Q--------PE--D--IESQIR--------EMLV-KYI---GN------------PNSIILAVTSANT--D-MATSE-
SLKLAKEID------PDGRRTLAVITKLDLMD-------AGT------D-------A----------------------------VDV--
LC---GR---------V-IP----------------------V---KLGIIGVVNR--
S-QMD--INN-----------K--------KV--------------------ID----------DAVKD---ESAFLQR--------K---Y----
------------------P-A------L-----------ASR-NGTAYLARTLNRLLMHHIRDC-L----
XP_032819300.1 ------------IQLPQ--------IVVV--
GA----Q----------------SSGKSSVLE-----S--LV-G-R-DFLPRG--------------TG-IVTRRPL---------------
VLQ--------------L-V-HVI------------------------------PDERIRPGGEE------N------------------------------
------------------GVE---------------------------AEE--------------W----------------------------GKF----------
---LHTKNKVYSDFN-----------EIRQEI------
ENET-ER---I-TG-------------------------TN--------------------K-GISSE-AIHLKIFSPH----------------------
--------------------------------------VLNLTLVDLPGITK--VP--------------------V-------GD--------------Q-
-------PV--D--IEQQIR--------ELII-KFI---GN------------PNSIILAVTAANT--D-LATSE-ALKIAREVD----
--TDGRRTLAVITKLDLMD-------AGT------D-------A----------------------------MDI--LT---GR---------
V-IP----------------------V---KLGIIGVVNR--S-QLD--INT-----
------K--------KT--------------------IL----------DAMQD---EQSFMQK--------K---Y---------------------P-
S------L-----------ANR-NGTKFLGKTLNRLLMHHIRDC-L----
NP_957216.1 ------------IQLPQ--------IAVV--GT----
Q----------------SSGKSSVLE-----S--LV-G-R-DLLPRG--------------TG-IVTRRPL----------------ILQ----
----------L-V-HVD------------------------------PEDRRKTS-EE------N--------------------------------------
----------GVD---------------------------GEE--------------W----------------------------GKF-----------------
---LHTKNKIYTDFD-----------EIRQEI------ENET-ER---
V-SG-------------------------NN--------------------K-GISDE-PIHLKIFSPH---------------------------------
--------------------------VVNLTLVDLPGITK--VP--------------------V-------GD--------------Q--------PK-
-D--IELQIR--------ELIL-KYI---SN------------PNSIILAVTAANT--D-MATSE-ALKVAREVD------
PDGRRTLAVVTKLDLMD-------AGT------D-------A----------------------------MDV--LM---GR---------
V-IP----------------------V---KLGLIGVVNR--S-QLD--INN----
-------K--------KS--------------------VA----------DSIRD---EHGFLQK--------K---Y---------------------P-
S------L-----------ANR-NGTKYLARTLNRLLMHHIRDC-L----
XP_025940269.1 ------------IQLPQ--------IVVV--
GT----Q----------------SSGKSSVLE-----S--LV-G-R-DLLPRG--------------TG-VVTRRPL---------------
ILQ--------------L-V-HVS------------------------------PEDGRKTAGDE------N-----------------------------
-------------------EID---------------------------AEE--------------W---------------------------GKF----------
---LHTKNKVYTDFD-----------EIRQEI------

ENET-ER---I-SG------------------------NN-------------------K-GISPE-PIHLKIFSSN---------------------
---------------------------------------VVNLTLVDLPGMTK--VP-------------------V-------GD--------------
Q--------PK--D--IELQIR--------ELIL-QFI---SN------------PNSIILAVTAANT--D-MATSE-
ALKIAREVD------PDGRRTLAVITKLDLMD-------AGT------D-------A----------------------------MDV--
LM---GR---------V-IP-----------------------V---KLGIIGVVNR--
S-QLD--INN-----------K--------KS--------------------VA----------DSIRD---EYGFLQK--------K---Y-----
----------------P-S------L-----------ANR-NGTKYLARTLNRLLMHHIRDC-L----
XP_006168142.1 ------------IQLPQ--------IVVV--
GT----Q----------------SSGKSSVLE-----S--LV-G-R-DLLPRG--------------TG-IVTRRPL---------------
ILQ--------------L-V-HVS------------------------------PEDKRKTTGEE------N------DP-------------------
----ATWKNSR-----HLSKGVE---------------------------AEE--------------W---------------------------
GKF--LHTKNKLYTDFD-----------
EIRQEI------ENET-ER---I-SG------------------------NN-------------------K-GVSPE-PIHLKIFSPN-----
---VVNLTLVDLPGMTK--VP-------------------V-------
GD--------------Q--------PK--D--IELQIR--------ELIL-RFI---SN------------PNSIILAVTAANT--D-
MATSE-ALKISREVD------PDGRRTLAVITKLDLMD-------AGT------D-------A---------------------------
-MDV--LM---GR---------V-IP-----------------------V---KLGIIGVVNR-------------------------------------
---------S-QLD--INN-----------K--------KS--------------------VT----------DSIRD---EYAFLQK--------K-
--Y---------------------P-S------L-----------ANR-NGTKYLARTLNRLLMHHIRDC-L----
NP_001317309.1 ------------IQLPQ--------IVVV--
GT----Q----------------SSGKSSVLE-----S--LV-G-R-DLLPRG--------------TG-IVTRRPL---------------
ILQ--------------L-V-HVS------------------------------QEDKRKTTGEE------N------DP-------------------
----ATWKNSR-----HLSKGVE---------------------------AEE--------------W---------------------------
GKF--LHTKNKLYTDFD-----------
EIRQEI------ENET-ER---I-SG------------------------NN-------------------K-GVSPE-PIHLKIFSPN-----
---VVNLTLVDLPGMTK--VP-------------------V-------
GD--------------Q--------PK--D--IELQIR--------ELIL-RFI---SN------------PNSIILAVTAANT--D-
MATSE-ALKISREVD------PDGRRTLAVITKLDLMD-------AGT------D-------A---------------------------
-MDV--LM---GR---------V-IP-----------------------V---KLGIIGVVNR-------------------------------------
---------S-QLD--INN-----------K--------KS--------------------VT----------DSIRD---EYAFLQK--------K-
--Y---------------------P-S------L-----------ANR-NGTKYLARTLNRLLMHHIRDC-L----
NP_001392186.1 ------------IQLPQ--------IVVV--
GT----Q----------------SSGKSSVLE-----S--LV-G-R-DLLPRG--------------TG-VVTRRPL---------------
ILQ--------------L-V-HVS------------------------------PEDKRKTTGEE------NGKFQSWNP-------------
----------ATWKNSR-----HLSKGVE---------------------------AEE--------------W--------------------------
-GKF--LHTKNKLYTDFD-----------
EIRQEI------ENET-ER---I-SG------------------------NN-------------------K-GVSPE-PIHLKVFSPN----
--VVNLTLVDLPGMTK--VP-------------------V-------
GD--------------Q--------PK--D--IELQIR--------ELIL-RFI---SN------------PNSIILAVTAANT--D-
MATSE-ALKISREVD------PDGRRTLAVITKLDLMD-------AGT------D-------A---------------------------
-MDV--LM---GR---------V-IP-----------------------V---KLGIIGVVNR-------------------------------------
---------S-QLD--INN-----------K--------KS--------------------VT----------DSIRD---EYAFLQK--------K-
--Y---------------------P-S------L-----------ANR-NGTKYLARTLNRLLMHHIRDC-L----

XP_014394711.1 ------------LLCPV--------CVQI--
SS----L----------------SSGKSSVLE-----S--LV-G-R-DLLPRG-------------TG-IVTRRPL--------------
ILQ-------------L-V-HVS-----------------------------PEDQRKTSGEE------N------DP-----------------
-----ATWKNSR-----HLSKGVE--------------------------AEE-------------W--------------------------
GKF---LHTKNKLYTDFD-----------
EIRQEI------ENET-ER---I-SG-----------------------NN------------------K-GVSPE-PIHLKIFSPN-----
---VVNLTLVDLPGMTK--VP------------------V-------
GD-------------Q--------PK--D--IELQIR--------ELIL-RFI---SN------------PNSIILAVTAANT--D-
MATSE-ALKISREVD------PDGRRTLAVITKLDLMD-------AGT------D-------A-------------------------
-MDV--LM---GR---------V-IP----------------------V---KLGIIGVVNR-------------------------------------
---------S-QLD--INN-----------K--------KS-------------------VT----------DSIRD---EYAFLQK--------K-
--Y--------------------P-S------L-----------ANR-NGTKYLARTLNRLLMHHIRDC-L----
XP_028602039.1 ------------IQLPQ--------IVVV---
GT----Q----------------SSGKSSVLE-----S--LV-G-R-DLLPRG-------------TG-IVTRRPL--------------
ILQ-------------L-V-NVS-----------------------------AEDLRKKTGDE------N------DP-----------------
----ATWKHAR-----HLTKGVD--------------------------TEE-------------W--------------------------
GKF---LHTKNKLYSDFD-----------
EIRQEI------ESET-ER---I-SG-----------------------NN------------------K-GISPE-PIHLKVFSPN-----
---VVNLTLVDLPGMTK--VP------------------V-------
GD-------------Q--------PK--D--IELQIR--------ELIL-RFI---SN------------PNSIILAVTAANT--D-
MATSE-ALKIAREVD------PDGRRTLAVITKLDLMD-------AGT------D-------A-------------------------
-MDV--LM---GR---------V-IP----------------------V---KLGIIGVVNR-------------------------------------
---------S-QLD--INN-----------K--------KS-------------------VA----------DSIRD---EYGFLQK--------
K---Y--------------------P-S------L-----------ANR-NGTKYLARTLNRLLMHHIRDC-L----
XP_012382650.2 --
--G-------------FK-LVISTKL---------------ILC-------------C-
F-SLT-----------------------------T----------------------DP----------------------ATWKNSR-----
HLSKGVE--------------------------AEE-------------W-------------------------GKF-------------------
---LHTKNKLYTDFD-----------EIRQEI------ENET-ER---I-
SG------------------------NN------------------K-GVSPE-PIHLKIFSPN-----------------------------------
------------------------VVNLTLVDLPGMTK--VP------------------V-------GD-------------Q--------PK-
-D--IELQIR--------ELIL-RFI---SN------------PNSIILAVTAANT--D-MATSE-ALKISREVD------
PDGRRTLAVITKLDLMD-------AGT------D-------A---------------------------MDV--LM---GR---------
V-IP----------------------V---KLGIIGIVNR--S-QLD--INN-----
------K--------KS-------------------VT----------DSIRD---EYAFLQK--------K---Y--------------------P-S-
-----L-----------ANR-NGTKYLARTLNRLLMHHIRDC-L----
XP_031753959.1 ------------IGFGP--------VLRV---
PL----Q----------------PAKCWAAKE-----S--MVLS-A-SLLP-V--------------PH-LYERLLL--------------
SVV-------------F-F-SLI-----------------------------P----------------------DP---------------------
NAWKIPK-----HFSKGVE---------------------------TEE-------------W---------------------------GKF----
---LHTKNKIYTDFD-----------EIRQEI------
ENET-ER---I-SG-----------------------NN------------------K-GISSE-PIHLKIFSPN-------------------
-------------------------------------VVNLTLVDLPGMTK--VP------------------V-------GD-------------
Q--------PK--D--IEIQIR--------ELIL-RYI---SN------------PNSIILAVTAANT--D-MATSE-ALKIARESD-

-----PDGRRTLAVITKLDLMD-------AGT------D-------A---------------------------MDV--LL---GR------
---V-IP----------------------V---KLGIIGVVNR---S-QLD--INN--
---------K--------KS-------------------VA----------DSIRD---EYGFLQK--------K---Y--------------------
P-S------L-----------ANR-NGTKYLARTLNRLLMHHIRDC-L----
PAA85687.1 ------------IQLPQ--------IVVI---GT----Q-
---------------SSGKSSVLE-----S--LV-G-R-DFLPRG-------------TG-IVTRRPL---------------VLQ------
--------L-V-HLE-----------------------------ADEKDE-AGDR------P-------------------------------------
--------AAE--------------------------EEE-------------W-------------------------GKF-------------------
--LHTKGKIYTDFN-----------EIRDEI------ARET-DR---I-
AG------------------------SG-------------------K-CVSID-PINLKIYSPH-----------------------------------
----------------------VVSLTLVDLPGITK--VP------------------V-------AD-------------Q--------PE--D-
-IEVQIR--------ALCI-EYI---KN------------PNSIILAVTPANT--D-MATSE-SLKLAKEVD------
PQGKRTLAVITKLDLMD-------AGT------D-------A---------------------------HDL--LL---GR---------V-
IP----------------------V---KLGIIGVVNR---S-QAD--IKN-------
----Q--------KQ-------------------VK----------EAVRD---ESSFLQR--------R---Y--------------------P-
S------L-----------ASR-NGTPYLARTLNRLLMHHIRDC-L----
XP_042914770.1 ------------LDLPQ--------VAVV--
GS----Q----------------SSGKSSVLE-----A--LV-G-R-DFLPRG-------------ND-IVTRRPL--------------
-LLQ-------------L-V-KTT------------------------------PGPT---
--------------GR--------------------------PSE-------------W--------------------------GEF---------------
--LHAPGKMFYDFD-----------RIRDEI------HQET-
ER---L-VG-----------------------YN------------------K-NVSDK-PIRLKIFSPR---------------------------
------------------------------VLTMTLVDLPGLTR--VP------------------V-------GD-------------Q------
--PG--D--IEQRIR--------EMAL-EYI---RR------------PNCIILAVSPANV--D-LATSD-ALQLSQVAD----
--PEGVRTIGVLTKLDIMD-------RGT------D-------A---------------------------AHI--LR---NA---------
H-IP----------------------L---RLGYIGVVLR---A-QAD--IAA----
-------K--------LP-------------------MS----------ECRKR---EESFFAS--------RA-EY--------------------
R-D------V-----------AAH-CGVPTLARRLNVILVEHIR--------
PWZ09977.1 ------------IDLPQ--------VAVV---GS----
Q----------------SSGKSSVLE-----A--LV-G-R-DFLPRG-------------SD-ICTRRPL---------------VLQ--
------------L-V-HQP------------------------------RRPAD---
---------AE--------------------------ADE-------------W-------------------------GEF--------------------
---LHLSGRRFYDFR-----------EIRREI------QAET-DR---E-
AG------------------------GN------------------K-GVSDR-QIRLKIFSPN---------------------------------
------------------------VLNITLVDLPGITK--VP------------------V-------GD-------------Q--------PT--
D--IEARIR--------TMIM-SYI---KH------------KTCIILAVSPANA--D-LANSD-ALQMARVAD------
PDGSRTIGVITKLDIMD-------RGT------D-------A---------------------------RNF--LL---GN---------V-
IP----------------------L---KLGYVGVVNR---S-QQD--INS-----
------D--------LS-------------------IK----------DALAR---EEKFFRT--------QP-AY--------------------H-
G------L-----------AQY-CGIPQLAKKLNQILVQHIK--------
KAH9304002.1 --

--MV------QAET-ER---E-VG------------------------VN----------------
---K-GVSDK-QIRLKIYSPN---VLNITLVDLPGLTK-
-VP------------------V-------GD--------------Q--------PT--D--IEARIR--------TMIM-SYI---KH----------
--ETCIILAVSPANA--D-LANSD-ALQMARVAD------ADGHRTIGVITKLDIMD-------RGT------D-------
A----------------------------TNF--LL---GN---------V-IP----------------------L---RLGYVGVVNR--------
--------------------------------------S-QAD--ING-----------N--------KS--------------------IQ----------
DALAY---EERFFRS--------RP-VY--------------------H-R------L-----------ADR-
CGIPQLAKKLNYILVQHIR--------
EFJ37641.1 ------------IDLPQ--------VAVV--GS----
Q-----------------SSGKSSVLE-----A--LV-G-R-DFLPRG--------------CD-ICTRRPL---------------VLQ--
------------L-V-QTT------------------------------RRGD---
--------E---------------------------VVE--------------W---------------------------GEF------------------------
--LHLPNRRFTDFT-----------AIRKEI------QAET-DR---E-
LG------------------------SN-------------------K-GISDK-QIRLKIFSPK-------------------------------------
-----------------------VLNITLVDLPGITK--VP-------------------V-------GD--------------Q--------PT--D--
IEARIR--------TMIL-SYI---KH------------ETCIILAVSPANA--D-LANSD-ALQMARIAD------
PDGSRTIGVITKLDIMD-------RGT------D-------A----------------------------RNF--LL---GS---------V-
IP-----------------------L---RLGYIGVVNR--S-QED--ITS-------
----N--------RS--------------------IQ---------DALMY---EEQFFRS--------RP-VY---------------------H-
S------L-----------SDR-CGIPQLAKKLNQILVQHIR--------
EFJ15047.1 --------------LPQ--------VAVV--GS----
Q-----------------SSGKSSVLE-----A--LV-G-R-DFLPRG--------------CD-ICTRRPL---------------VLQ--
------------L-V-QTT------------------------------RRGD---
--------E---------------------------VVE--------------W---------------------------GEF------------------------
--LHLPNRRFTDFS-----------AIRKEI------QAET-DR---E-
LG------------------------SN-------------------K-GISDK-QIRLKIFSPK-------------------------------------
-----------------------VLNITLVDLPGITK--VP-------------------V-------GD--------------Q--------PT--D--
IEARIR--------TMIL-SYI---KH------------ETCIILAVSPANA--D-LANSD-ALQMARIAD------
PDGSRTIGVITKLDIMD-------RGT------D-------A----------------------------RNF--LL---GS---------V-
IP-----------------------L---RLGYIGVVNR--S-QED--ITS-------
----N--------RS--------------------IQ---------DALMY---EEQFFRS--------RP-VY---------------------H-
S------L-----------SDR-CGIPQLAKKLNQILVQHIRTI-LP---
KAI5070758.1------------VNLPQ--------VAVV---GS----
Q-----------------SSGKSSVLE-----G--LV-G-R-DFLPRG--------------SD-ICTRRPL---------------VLQ--
------------L-I-QTT------------------------------RRPDE--
------KSE---------------------------VVE--------------W---------------------------GEF----------------------
---LHAPGKRFTDFS-----------LIRKEI------EAET-ER---E-
LG------------------------SN-------------------K-GISDK-QIRLKIFSPN-------------------------------------
-----------------------VLTITLVDLPGITK--VP------------------V-------GD--------------Q--------PS--D--
IESRVR--------TMIL-SYI---KH------------ETCIILAVSPANA--D-LANSD-ALQMARLAD------
PDGSRTIGVITKLDIMD-------RGT------D-------A----------------------------RNL--LL---GN---------V-
IP-----------------------L---RLGYIGVVNR--S-QED--ILG-------
----N--------KS--------------------VV---------DALLY---EENFFRS--------RP-VY---------------------H-
N------L-----------ADR-CGIAQLAKKLNTILVQHIK--------

PTQ35749.1 ------------IDLPQ--------VAVV---GS----
Q----------------SSGKSSVLE-----A--MV-G-R-DFLPRG--------------AD-ICTRRPL---------------VLQ-
-------------L-V-QTV------------------------------RRPED---
--------RSE---------------------------LVE--------------W---------------------------GEF---------------------
--LHIPGRRFTDFT-----------AIRKEI------QAET-DR---E-
LG------------------------TN-------------------K-GISEK-QIRLKIFSPN--------------------------------------
-----------------------VLNITLVDLPGITK--VP-------------------V-------GD--------------Q--------PS--D--
IEARVR--------TMIL-NYI---KH------------ETCIILAVSPANA--D-LANSD-ALQMARIAD------
PDGSRTIGVITKLDIMD-------RGT------D-------A----------------------------RNF--LL---GN---------V-
VP-----------------------L---RLGYIGVVNR---S-QED--IQA-----
------N--------KT--------------------IR----------EALGY---EENFFRS--------RP-VY---------------------H-
S------L-----------SER-CGIPQLAKKLNSILVQHIR--------
KAG0555682.1 ------------IDLPQ--------VAVV---
GS----Q----------------SSGKSSVLE-----A--LV-G-R-DFLPRG--------------SD-ICTRRPL--------------
-VLQ--------------L-V-QTS------------------------------RRPED--
---------------RTE---------------------------LVE--------------W---------------------------GEF--------------
---LHIPGRRFTDFA-----------AIRKEI------QAET-
DR---E-LG------------------------TN-------------------K-GISEK-QIRLKIFSPN-----------------------------
--------------------------------VLNITLVDLPGITK--VP-------------------V-------GD--------------Q--------
PN--D--IEARVR--------TMIL-SYI---KH------------ETCIILAVSPANA--D-LANSD-ALQMARIAD------
PDGSRTIGVITKLDIMD-------RGT------D-------A----------------------------RNF--LL---GN---------V-
IP-----------------------L---RLGYIGVVNR---S-QED--IIA--------
---N--------KS--------------------IR----------DALVY---EEGFFRS--------KP-VY---------------------H-N-
-----L-----------ADR-CGVPQLAIRLNTILVQHIK--------
XP_024362051.1 ------------IDLPQ--------VAVV---
GS----Q----------------SSGKSSVLE-----A--LV-G-K-DFLPRS--------------SD-ICTRRPL---------------
VLQ--------------L-V-QTL------------------------------RRSDE---
--------------KSE---------------------------LVE--------------W---------------------------GEF---------------
--LHIPGRQFTNFS-----------AIRKEI------QLET-ER-
--D-MG------------------------TN-------------------K-GISEK-QIRLKIFSPD-------------------------------
-----------------------------VLNITLVDLPGITK--VP-------------------V-------GD--------------Q--------
PT--D--IEARVR--------TMIL-SYI---KH------------KTCIILAVSPANA--D-LANSD-ALQMARIAD------
PDGSRTIGVISKLDIMD-------RGT------D-------A----------------------------RSL--LL---GN---------V-
IP-----------------------L---RLGYVGVVNR---S-QED--ISR------
-----N--------RS--------------------IR----------DALTN---EENFFRS--------RP-VY---------------------H-
N------L-----------SDR-CGVPQLAKKLNTILVQHIK--------
KAG0554580.1 ------------IDLPQ--------VAVV---
GS----Q----------------SSGKSSVLE-----A--LV-G-R-DFLPRG--------------SD-ICTRRPL--------------
-VLQ--------------L-V-QTL------------------------------RRPDE--
---------------KSE---------------------------PVE--------------W---------------------------GEF-------------
--LHIPGRRFTDFS-----------AIRKEI------QLET-
ER---E-LG------------------------TN-------------------K-GISEK-QIRLKIFSPN----------------------------
--------------------------------VLNITLVDLPGITK--VP-------------------V-------GD--------------Q--------
PT--D--IEARVR--------TMIL-SYI---KH------------ETCIILAVSPANA--D-LANSD-ALQMARIAD------

PDGSRTIGVISKLDIMD-------RGT------D-------A----------------------------RSF--LL---GN---------I-
IP----------------------L---RLGYVGVVNR--S-QED--ISA------
-----N--------KS--------------------IR----------DALTY---EENFFRS--------RP-VY---------------------H-
N------L-----------SDR-CGVPQLAKKLNTILVQHIK--------
KAI5070335.1------------IDLPQ--------VAVI--GS----
Q----------------SSGKSSVLE-----A--LV-G-R-DFLPRG--------------SD-ICTRRPL---------------VLQ--
------------L-V-QLV------------------------------RRPDD---
-------KSD---------------------------VME-------------W---------------------------GEF--------------------
--LHTPGKRYTDFS-----------LIRKEI------QAET-ER---E-
LG------------------------SN-------------------R-GISDK-QIRLKIFSPS----------------------------------
----------------------VLNITLVDLPGITK--VP------------------V-------GD-------------Q--------PS--D--
IEARVR--------TMIL-SYI---KH------------ETCIILAVSPANA--D-LANSD-ALQMARMAD------
PDGSRTIGVITKLDIMD-------RGT------D-------A----------------------------RNF--LL---GS---------A-
IP----------------------L---RLGYIGVVNR--S-QAD--ILG-------
----N--------KS--------------------IR----------EALAY---EDNFFRS--------LP-VY---------------------H-
S------L-----------ADR-CGIPQLAKKLNNILVQHIK--------
ONM18162.1 ------------LELPQ--------VAAI--GG----
Q----------------SSGKSSVLE-----A--LV-G-R-DFLPRG--------------PD-ICTRRPL---------------VLQ--
------------L-V-RH---
----AA---------------------------PEE-------------W---------------------------GEF--------------------------
--LHVPGRQFHDFE-----------QIKREI------QLET-DK---E-
AG------------------------DN-------------------K-GVSEK-QIRLKIFSPN-----------------------------------
------------------------VLDITLVDLPGITR--VP------------------V-------GD-------------Q--------PS--
D--IESRIR--------AMIM-QYI---KH------------PSCIILAVSPANA--D-LANSD-ALQLARLAD------
PDGSRTIGVITKLDIMD-------RGT------D-------A----------------------------RNF--LL---GN---------V-
IP----------------------L---KFGYVGVVNR--S-QED--INF------
-----N--------RS--------------------VK----------DALAF---EEKYFLT--------LP-AY---------------------H-
G------L-----------AHC-CGVPQLAKKLNMILLKHVT--------
XP_052310486.1 ------------IELPQ--------VAVV--
GS---Q------------------SSGKSSVLE-----A--LV-G-R-DFLPRG--------------NE-ICTRRPL--------------
-VLQ--------------L-L-QTK------------------------------RKGDG--
-----------------SG---------------------------EDE--------------W---------------------------GEF------------
--LHLPGKRFYDFS-----------EIRSEI------QAET-
AK---E-AG------------------------GN-------------------K-GVSDK-QIRLKIFSPN--------------------------
----------------------------------VLDITLVDLPGITK--VP--------------------V-------GD--------------Q------
--PS--D--IEARIR--------TMIM-SYI---KK------------PSCLILAVTAANS--D-LANSD-ALQIAGNAD-----
-PDGYRTIGIITKLDIMD-------RGT------D-------A----------------------------RNL--LL---GK---------V-
IP----------------------L---RLGYVGVVNR--S-QED--IIL-------
----N--------RS--------------------IK----------DALAA---EEKFFRS--------RP-VY---------------------N-
G------L-----------ADR-CGVPQLAKKLNQILVQHIK--------
AAC61784.1 ------------IALPQ--------VVVV--GS----
Q----------------SSGKSSVLE-----A--LV-G-R-DFLPRG--------------ND-ICTRRPL---------------VLQ--
------------L-L-QTK------------------------------SRANG---
--------GS---------------------------DDE-------------W---------------------------GEF--------------------

--RHLPETRFYDFS-----------EIRREI------EAET-NR---L-
VG-----------------------EN-------------------K-GVADT-QIRLKISSPN-----------------------------------
----------------------VLNITLVDLPGITK--VP------------------V-------GD-------------Q--------PS--D-
-IEARIR--------TMIL-SYI---KQ------------DTCLILAVTPANT--D-LANSD-ALQIASIVD------
PDGHRTIGVITKLDIMD-------KGT------D-------A--------------------------RKL--LL---GN---------V-
VP----------------------L---RLGYVGVNR--C-QED--ILL-----
------N--------RT-------------------VK---------EALLA---EEKFFRS--------HP-VY--------------------H-
G------L-----------ADR-LGVPQLAKKLNQILVQHIK--------
KAH9327796.1 --
---------------------MYP-----W--LV-V-K-EF--------------------------PD---------------AIA--------------
L--

--QVET-ER---E-AG-----------------------GN------
------------K-GISAK-QIRLKIFSPF---
VLNINLVDLPGIMK--VP------------------V-------GD-------------Q--------PI--D--IEARIR--------
TMIM-SYI---KH------------PSCIILAVSPANS--D-LANSD-ALQIARVAD------
TDGSRTIGVITKLDIMD-------RGT------D-------A--------------------------RNF--LL---GN---------V-
IP----------------------L---QLGYIGVVNR--S-QED--IIA-------
----N--------QS--------------------IR---------DALAY---EENFFRG--------HP-VY--------------------C-
S------L-----------ADQ-CGIPQLARKLNQILVKHIR--------
XP_014148015.1 --
----------------------LQ--------LV---H-HPVQRGG--------------------PA--------------------------------
--A--
-------------------E-------------W------------------------GEF---
----------------------------LHQPGKIYTDFS-----------KIRDEI------ANET-DR---L-TG-------------------
-----TN-------------------K-GISHT-PINLKLYSPN---
-MLDLTLVDLPGITK--IA------------------V-------GD-------------Q--------PE--D--IEVQIH--------
QLIE-SYI---NN------------PNCIILAVTAANT--D-IANSD-ALKMAKKAD------
PKGLRTIGVATKLDLMD-------AGT------D-------A--------------------------LDI--LT---GK---------V-
VA----------------------S---KLGFIGVVNR--S-QAD--INQ-----
------K--------VS--------------------IE---------TAREA---EQEYFRT--------HP-AY--------------------K-
S------L-----------YKQ-SGTEYLTRRLNQLLMTHIRRC-L----
GMH85941.1 ------------IDLPQ--------IAVL--GG----
Q----------------SSGKSSVLE-----N--VV-G-K-SFLPRG--------------SG-IVTRRPL---------------ILQ---
-----------L-F-HEP-----------------------------SG--
-----------------------------------E-------------W-------------------------GEF---------------------------
---LHKPGEKFYDFD-----------MICEEI------EADT-AR---I-CG---
---------------------KN-------------------K-GLSTK-PINLRVYSPD--
------------------VLNLTLIDLPGATK--VA------------------V-------GD-------------Q--------PS--D--
IGKQIE--------SMIK-FYV---SK------------PNCLILAVTAANT--D-LANSD-AIAIAKEVD------
PKGERTLGVMTKLDLMD-------RGT------D-------A--------------------------RGI--FT---GESQ------
DV-PL----------------------L---KMGYIGVVNR--S-QAD--
INE-----------R--------KT-------------------IQ---------GARDA---ENAFFEG--------HP-GY------------
---------A-D------I-----------ADR-LGTAYLVKKCSNMLLK-----------

GMI59178.1 ------------IDLPQ--------IAVL--GG----
Q----------------SSGKSSVLE-----N--IV-G-K-SFLPRG-------------SG-IVTRRPL--------------VLQ---
-----------L-Y-HET-----------------------------SG---
----------------------------------E-------------W---------------------------GEF----------------------------
---LHAPGKKFFDFD-----------EICQEI------ENDT-AR---I-CG----
--------------------AN-------------------K-GLSTK-PINLRVYSPD---
-----------------VLNLTLIDLPGATK--VA------------------V-------GD-------------Q--------PS--D--
IGKQIE--------SMIK-FYV---SK------------PNCLILAVTAANT--D-LANSD-AIFIAKEVD------
PKGERTLGVMTKLDLMD-------RGT------D-------A--------------------------RGI--FT---GESQ------
DV-PH----------------------L---ALGYVGVVNR--A-QAD--
INE-----------K--------KT-------------------IQ---------AARNS---ENQFFEQ--------HP-GY------------
---------G-D------I-----------ADK-LGTQYLVKKCSTMLLK-----------
GMI25649.1 ------------IDLPQ--------IAVV--GG----
Q----------------SSGKSSVLE-----N--IV-G-K-SFLPRG-------------SG-IVTRRPL--------------VLQ---
-----------L-Y-NSP-----------------------------E---
----------------------------------E-------------Y--------------------------GEF----------------------------
--LHQPNKKYYDFN-----------EICGEI------EADT-DR---V-CG----
--------------------AN-------------------K-NLKNQ-PINLRIYSPD--
-----------------VLNLTLVDLPGATK--VA-------------------V-------GD-------------Q--------PK--D--
IGRQIK--------NMIM-SYA---SK------------PNCLILAVSAANV--D-LANSD-AIEIAKEVD------
PQGKRTLGVVTKLDLMD-------RGT------D-------A---------------------------RGI--FT---GEDS------
DL-PK----------------------L---ALGFVGVVNR--S-QAD--
INE-----------R--------KG--------------------IG---------QARDA---ENQYFSM--------HP-AY-----------
----------S-D------I-----------QER-LGTQYLVKKCSQMLLK-----------
GMI62840.1 ------------IDLPQ--------IAVV--GG----
Q----------------SSGKSSVLE-----N--IV-G-K-TFLPRG-------------SG-IVTRRPL--------------VLQ---
-----------L-Y-NSI-----------------------------E--
---------------------------------E-------------Y--------------------------GEF-------------------------------
---------------------------------------LHIPNKKFYDFN-----------EVCSEI------EADT-DR---V-CG------
------------------SN-------------------K-NLKNQ-PINLRIYSPD---
---------------VLNLTLIDLPGATK--VA-------------------V-------GD--------------Q--------PK--D--
IGRQIK--------NMIM-SYA---SK------------PNCMILAVTAANT--D-LANSD-AIEIAKEVD------
PEGKRTLGVLTKLDLMD-------RGT------D-------A---------------------------RGI--FT---GEDR------
DL-PF----------------------L---ALGYVGVVNR--S-QAD--
INE-----------R--------KG--------------------IA---------QARAA---EMQYFSM--------HP-GY-----------
----------S-D------I-----------QDK-LGTGYLVKKCSQMLLK-----------
GMH55978.1 ------------IDLPQ--------IAVV--GG----
Q----------------SSGKSSVLE-----N--IV-G-K-SFLPRG-------------SG-IVTRRPL--------------VLQ---
-----------L-Y-NSQ-----------------------------E---
----------------------------------E-------------Y--------------------------GEF----------------------------
--LHHPNKKWYDFE-----------EICQEI------ERDT-DR---V-CG---
---------------------SN-------------------K-NLKNQ-PINLRVYSPD---
-------------------VLNLTLIDLPGATK--VA------------------V-------GD-------------Q--------PK--D--
IGKQIR--------NMIM-SYA---SK------------PNCLILAVTAANT--D-LANSD-AIEIAKEVD------

PQGKRTLGVLTKLDLMD-------RGT------D-------A---------------------------RGI--FT---GEDT------
NL-PN----------------------L---ALGYVGVVNR---S-QAD--
INE-----------R--------KG--------------------IA----------SAREG---EMQYFSM--------HP-SY------------
---------S-D------I-----------QDK-LGTAYLVKKCSQMLLK-----------
PRP80825.1 ------------IDLPQ--------IVVI--GG----
Q----------------SSGKSSVLE-----N--LV-G-R-DFLPRG--------------NE-LVTRRPL---------------ILQ--
------------L---NRI-----------------------------ADSSESTI-------FYSIAPN--EN--------------------------
--TDE-------------W--------------------------GEF------------------
--LHKPGEKF-TFD-----------GIREEI------HRET-ER---
T-TG------------------------KN-------------------K-GISTE-PILLRIYSPN------------------------------------
-----------------------VLPLTLVDTPGMTR--VP------------------VDARFTPGGD-------------Q--------
PP--D--IEQRLR--------DMIM-QFI---SK------------PNSIILAVQSATQ--D-LATSD-ALKLAREVD------
PEGHRTIGVLTKIDIMD-------RGT------N-------A---------------------------MDT--LM---GK---------S-
IP----------------------L---RLGFVGTISR--S-QHD--INV-------
----G--------KS-------------------IR----------SSLDD---EQKFFRE--------HS-VY--------------------N-
S------L-----------QDL-CGTANLAAKCNRILAG-----------
XP_004355605.1 -----------EIKLPQ--------IVVV--
GS----Q----------------SSGKSSVLE-----N--LV-G-R-DFLPRG--------------SG-LVTRRPL--------------
-VLQ-------------L---NRI-----------------------------EPGHA--
---E-------------W--------------------------GEF------------------
--GHTGDSKF-NFD-----------EIKKEI------EIET-NR---
V-AG------------------------GN-------------------K-SISSE-PIILKIYSPN-------------------------------------
-----------------------VIPLTLVDTPGITR--IP------------------I-------GD-------------Q--------PT--N--
IEEKIR--------DMVV-DYI---SN------------PNSIILAISAANQ--D-IVTSD-ALKLAKEVD------
PTGKRTIGVLTKLDLMD-------KGV------D-------A---------------------------MDI--LI---GS---------V-
VP----------------------L---KLGFVGIVNR--S-QQD--INM----
-------K--------KQ-------------------IG----------QAIQD---ESAWFQS--------HP-IY---------------------
N-R------I-----------ANQ-SGSLFLGQRCNKILTK-----------
XP_020436927.1 -----------EIKLPQ--------IVVI--
GS----Q----------------SSGKSSVLE-----N--LV-G-R-DFLPRG--------------SG-LVTRRPL--------------
-VLQ-------------L---IRI-----------------------------E-DNA---
---E-------------W--------------------------GEF--------------------
--AHTGDVRF-NFA-----------GIRDEI------EAET-NR---
V-AG-----------------------AN-------------------K-EISSD-PIILKIFSPY------------------------------------
-----------------------VIPLTLVDLPGITR--IP------------------I-------GN-------------Q--------PT--N--
IEERIR--------DMVL-DYI---SN------------PNSIILAISAANQ--D-IVTSD-ALKLAKEVD------
PEGRRTIGVLTKLDLMD-------RGT------D-------A---------------------------MDI--LL---GH---------T-
VP----------------------L---KLGFVGIINR--S-QHD--IQT------
-----K--------KA-------------------IS----------TMLKD---EERWFQN--------HP-VY--------------------
S-R------I-----------ANQ-TGSIFLAQKCNKILTK-----------
AER35077.1 -----------EITLPQ--------IIVI--GS----Q--
--------------SSGKSSVLE-----N--LV-G-R-DFLPRG--------------SG-LVTRRPL---------------ILQ------
--------L---NKH-----------------------------D-SLE---
---------------------------------E-------------Y--------------------------GEF-----------------------------

--AHTGNKKF-DFD-----------GIKQEI------ERET-ER---L-AG------
------------------AN-------------------K-DISSE-PILLRIYSPN---
-------------VIPLTLVDTPGIAR--VP-------------------I-------GD-------------Q--------PS--N--IEEKLK--
------SMIM-EYI---SN------------PNSIILAITSANQ--D-IVTSD-GIKLAKEVD------
PEGKRTIGVLTKLDLMD-------KGT------D-------A---------------------------IDV--LL---GD---------Q-
IP----------------------L---KYGFVGIINR---S-QQD--INN------
-----R--------KP-------------------IS---------QMLKD---EQIWFDQ--------HP-AY--------------------
S-R------I-----------NNQ-LGTKYLAQKCNKILTK-----------
KAF2075389.1 -----------EITLPQ--------IVVI--
GS----Q----------------SSGKSSVLE-----N--LV-G-R-DFLPRG-------------SG-LVTRRPL--------------
-ILQ-------------L---NKS-----------------------------E-GQE---
---E-------------W-------------------------GEF------------------
---GHTGNIKF-TFE-----------GIKQEI------EAET-SR---
V-AG------------------------PN-------------------K-DISPE-PIVLKIYSPN---------------------------------------
-------------------------VVPLTLVDTPGITR--VP------------------I-------GD-------------Q--------AL--
N--IEEKIR--------TMIT-EYI---QN------------PNCIILAVTSANQ--D-IVTSD-AIQMARNID------
PLGQRTIGVLTKIDLMD-------KGT------D-------A---------------------------LDI--LL---GN---------T-
IP----------------------L---KLGFVGVVNR---S-QSD--INL------
-----N--------KP-------------------IH---------TMLKD---EMKFFES--------HP-VY--------------------
N-R------I-----------LHQ-AGTKYLAQKCNKILTK-----------
XP_003292385.1 -----------EITLPQ--------IIVV--
GS----Q----------------SSGKSSVLE-----S--LV-G-R-DFLPRG-------------SG-LVTRRPL--------------
-VLQ-------------L---YQN-----------------------------EDSNE--
---E-------------W-------------------------GEF------------------
---GHTGDRKF-SYF-----------EIKEEI------EKET-ER---
I-AG------------------------AK-------------------K-DISPE-PIILKIHSPN---------------------------------------
-----------------------VIPLTLVDLPGLTR--VA------------------V-------DD-------------Q--------PI--D-
-IEEKVR--------SMIL-SYI---NN------------PNSIILAITPANQ--D-IVTSD-ALKLAQQVD------
PLGKRTVGVLTKLDLMD-------KGT------D-------A---------------------------LDI--LL---GN---------E-
IP----------------------L---SMGFVGVVNR---S-QQD--INY----
-------G--------KP-------------------IS---------DSLKD---EVKWFQN--------HP-VY-------------------
-S-R------V-----------FNQ-SGSKYLAQKCNKILTK-----------
KOO24608.1 -----------APKLPQ--------IVVI--GS----
Q----------------SSGKSSVLE-----S--FV-G-R-DFLPRG-------------TG-IVTRRPL---------------VLQ--
------------L-V-RTA------------------------------ADGDT--
----ASATDEG-------------------------ATVE-------------W-------------------------GEF------------------
---LHAPGRRFTSFE-----------AIRAEI------EAET-ER---
K-LG------------------------KS-------------------K-SVSAD-PIRLAIYSPH---------------------------------------
-------------------------VVDLSLVDLPGMTK--VP------------------I-------AD-------------Q--------PA-
-N--IEEQLR--------AMAL-TYI---EP------------EESLILAVSAANA--D-LATSD-AIQLARRVD------
PEGLRTIGVLTKLDLMD-------AGT------D-------A---------------------------LAV--LQ---GR---------V-
IP----------------------L---KRGFVGVVNR---S-QQD--LFD----
-------G--------KS-------------------PQ---------AAREH---EARFFAN--------HP-QY-------------------
-Q-S------I-----------AAR-MGSRYLAHRLNELLLS-----------

CCW59714.1 -----------KLNLPQ--------IAVV--GS----
Q----------------SCGKSSVLE-----S--IV-G-K-DFLPRG--------------SG-IVTRCPL---------------VLQ--
------------L-I-QLP------------------------------KNS---
------------------------------------QEE--------------W--------------------------GEF------------------------
--LHLPNKKFFVFS-----------DIRNEI------TRRT-NE---V-AG-
-----------------------PS--------------------AITDK-PINLKVYSAH--
------------------VLNLTMVDLPGLVM--NA------------------V-------GD--------------Q---------PK--D--
IDRQIK--------EMVT-RYV---AP------------KNTIILAISPANT--D-LATSQ-SLRLAHQLD------
PEGNRTVGVLTKLDLMD-------RGT------D-------C---------------------------YDV--LT---NK---------V-
LP-----------------------L---RHGFVGVICR--S-QQD--INT-----
------E--------KG--------------------ME----------EARAS---EQEFFLN--------SS-VY---------------------
A-P------I-----------ANE-QGTVYLSKKLNGLLLD-----------
KAG5490335.1 -----------KLNLPQ--------IAVV--
GS----Q----------------SCGKSSVLE-----S--IV-G-K-DFLPRG--------------SG-IVTRCPL---------------
VLQ--------------L-V-QLP------------------------------KSN--
--NDE--------------W--------------------------GEF-----------------
--LHLPTKKFFDFS-----------EIREEI------TRRT-VE---
L-AG------------------------PS--------------------AITDK-PINLKVYSNM------------------------------------
---------------------------VLNLTLVDLPGLVM--NA-------------------V-------GD--------------Q--------
PK--D--IDRQIK--------EMVT-RYV---SP------------KNTIILAISPANT--D-LATSQ-SLRLAKQLD------
PDGKRTVGVLTKIDLMD-------KGT------D-------C---------------------------FDI--LN---NR---------V-
LN-----------------------L---HHGFIGVVCR--S-QQD--IND---
---------R--------KS--------------------ME----------AARQA---EHDFFAN--------SP-IY--------------------
-S-S------I-----------ADE-AGTVYLTKKLNMVLLD-----------
XP_003872337.1 -----------KLNLPQ--------IAVV--
GS----Q----------------SCGKSSVLE-----S--IV-G-K-DFLPRG--------------SG-IVTRCPL---------------
VLQ--------------L-V-QLP------------------------------KSN--
--NEE--------------W--------------------------GEF-----------------
--LHIPQKKFYDFN-----------EIQNEI------TRRT-IE---
M-AG------------------------PS--------------------AITDK-PISLKVYSKT------------------------------------
-------------------------VLNLTLVDLPGLVM--NA-------------------V-------GD--------------Q--------PK-
-D--IDRQIK--------DMVT-RYV---SP------------KNTIILAISPANT--D-LATSQ-SLRLAKQLD------
PDGLRTVGVLTKIDLMD-------KGT------D-------C---------------------------LDI--LQ---NR---------V-
LQ-----------------------L---RHGFIGVVCR--S-QQD--IND----
--------R--------KS--------------------ME----------GARRS---EYEFFAN--------SP-IY---------------------
S-P------I-----------AEE-AGTTYLSKKLNFLLLE-----------
KAI5685071.1 -----------KLNLPQ--------IAVV--GS----
Q----------------SCGKSSVLE-----S--IV-G-K-DFLPRG--------------SG-IVTRCPL---------------VLQ--
------------L-V-QLP------------------------------KSN---
------------------------------------SEE--------------W--------------------------GEF------------------------
--LHIPNKKFFDFN-----------AIQEEI------TRRT-IE---V-AG---
---------------------PH--------------------AITDK-PINLKVYSNM--
------------------VLNLTLVDLPGLVM--NA------------------V-------GD--------------Q---------PK--D--
IDRQIK--------DMVT-RYV---SP------------KNTIILAISPANT--D-LATSQ-SLRLAKQLD------

PEGTRTVGVLTKIDLMD-------KGT------D-------C--------------------------FDV--LQ---NK---------V-
LH----------------------L---RHGFVGVVCR---S-QQD--IND---
---------R--------KS--------------------ME----------AARQS---EYDFFAN--------SP-TY--------------------
-S-P------I-----------ADE-AGTVYLSKKLNNLLLE-----------
XP_028887534.1 -----------KLNLPQ--------IAVV---
GS----Q----------------SSGKSSVLE-----A--IV-G-R-DFLPRG--------------SG-IVTRCPL---------------
VLQ--------------L-V-QLP-----------------------------ASA---
---KEE--------------W--------------------------GEF-----------------
---LHKPGKKYYNFA-----------EINEEI------QNRT-VE--
-I-AG------------------------KS---------------------AITDR-PINLKVFSPH-----------------------------------
-------------------------VLNLTLVDLPGLVM--NA------------------V-------GD-------------Q--------
PK--D--IDRQIK--------DMVT-RYV---SP------------ANTIILAISPANT--D-LATSA-SLRLARQLD------
PEGLRTVGVLTKLDLMD-------RGT------D-------A---------------------------FDV--LT---GK---------V-
IG----------------------L---RHGFVGVVNR---S-QQD--IND---
---------S--------KG--------------------ME----------AARED---ERAFFHN--------HP-VY--------------------
--S-A------I-----------ADT-QGTEYLAKKLNHLLLE-----------
EKF32958.1 -----------KLNLPQ--------IAVV---GS----
Q----------------SSGKSSVLE-----A--IV-G-K-DFLPRG--------------SG-IVTRCPL---------------VLQ---
-----------L-V-QLP-----------------------------KTS---
---------------------------------EEE--------------W--------------------------GEF--------------------------
--LHKPNKKYFNFS-----------DINEEI------KHRT-VE---I-AG--
----------------------NS---------------------AISER-PINLKVYSKN---
------------------VLNLTLVDLPGLVM--NA------------------V-------GD-------------Q--------PK--D--
IDRQIK--------EMVT-RYV---AP------------VNTIILAISPANT--D-LATSS-SLRLAKQLD------
PEGIRTVGVLTKLDLMD-------RGT------D-------A---------------------------LDA--LT---GK---------L-
VS----------------------L---RHGFVGVVNR---S-QQD--IND---
---------S--------KG--------------------MV----------AARED---ERAFFHN--------HP-SY--------------------
--S-A------I-----------ADR-QGTEYLAKKLNQLLLQ-----------
ESL10883.1 -----------KLNLPQ--------IAVV---GS----
Q----------------SSGKSSVLE-----A--IV-G-K-DFLPRG--------------SG-IVTRCPL---------------VLQ---
-----------L-V-QLP-----------------------------STT---
---------------------------------DEE--------------W--------------------------GEF--------------------------
--LHKPHKKFFDFA-----------EINDEI------KNRT-VE---I-AG---
---------------------KS--------------------AISDR-PINLKVFSRH---
-----------------VLNLTLVDLPGLVM--NP------------------V-------GD-------------Q--------PK--D--
IDRQIK--------EMVT-RYV---AP------------VNTIILAISPANT--D-LATSA-SLRLAKQLD------
PEGLRTVGVLTKLDLMD-------RGT------D-------A---------------------------LDA--LT---GK---------L-
VG----------------------L---RHGFVGVVNR---S-QQD--IHD--
---------M--------KG--------------------ME----------SARAD---ERMFFCN--------HP-VY-----------------
----S-A------I-----------ADR-QGTEYLAKKLNQLLLQ-----------
RHW73545.1 -----------KLNLPQ--------IAVV---GS----
Q----------------SAGKSSVLE-----A--IV-G-K-DFLPRG--------------SG-IVTRCPL---------------VLQ---
-----------L-V-QLP-----------------------------RSN---
---------------------------------KDE--------------W--------------------------GEF------------------------

--LHRPNKKFFDFS-----------EINEEI------QNRT-TE---V-AG-
----------------------HS--------------------AITDK-PINLKIYSSH--
-----------------VLNLTLVDLPGLVM--NA-------------------V-------GD-------------Q--------PK--D--
IDRQIK--------SMVT-RYI---SP------------SNTIILAISPANA--D-LATSS-SLQIAKQLD------
PEGLRTLGVLTKLDLMD-------RGT------N-------A---------------------------YDI--LT---GK---------V-
LP-----------------------L---RHGFVGVVNR---S-QHD--INT----
-------S--------KG-------------------MQ----------AARDD---EKEFFRN--------HP-AY------------------
--A-S------I-----------ADT-QGTEYLTQKLNGLLLE-----------
CCC89860.1 -----------KLNLPQ--------IAVV--GS----
Q----------------SAGKSSVLE-----A--IV-G-K-DFLPRG--------------SG-IVTRCPL---------------VLQ---
-----------L-V-QLP------------------------------KSN---
---------------------------------TEE-------------W--------------------------GEF--------------------------
---LHMPGSKFYDFT-----------QINEEI------QNRT-ID---V-AG--
---------------------QT--------------------SITER-PINLKIYSSN--
----------------VLNLTLVDLPGLVM--NA-------------------V-------GD-------------Q--------PK--D--
IDRQIK--------NMVT-RYV---SP------------SNTIILAISPANA--D-LATSS-SLQIAKQLD------
PEGLRTVGVLTKLDLMD-------RGT------D-------A---------------------------YDI--LT---GK---------V-
VP-----------------------L---RHGFVGGVNR---S-QHD--INT---
--------S--------KG-------------------MR----------EARDD---EKEFFRS--------HP-AY------------------
--S-Q------I-----------ADT-QGTEYLTRKLNGLLLE-----------
KAH8605762.1 -----------SLPLPQ--------IAVV--
GS----Q----------------SAGKSSVLE-----A--IV-G-K-DFLPRG--------------SG-IVTRCPL---------------
VLQ--------------L-V-QLP------------------------------QTN---
--TEE-------------W--------------------------GEF------------------
---LHLPGKKFFYFP-----------DIDQEI------RSRT-RE---
I-AG------------------------EF--------------------SITDR-AINLKIYSAN-------------------------------------
------------------------ILNLTLVDLPGLVS--NA-------------------V-------GD-------------Q--------PA--
D--IDRQIK--------EMVT-RYI---SP------------PNTIILAVSPANA--D-LATSY-SLQLAKKVD------
PEGVRTVGVLTKLDLMD-------RGT------D-------A---------------------------SDI--LM---GK---------V-
MH-----------------------L---SHGFVGVVNR---S-QHD--INT---
--------S--------KS-------------------MQ----------SARAD---ERAFFQN--------HP-AY------------------
--S-A------I-----------ADT-QGTEYLAQKLNYILLE-----------
KAJ9467322.1 -----------EFDLPQ--------IVVV--
GA----Q----------------SSGKSSVLE-----N--VV-G-K-DFLPRG--------------SG-IVTRRPL--------------
-ILH--------------M-S-HLS------------------------------NEEHQ--
------------RKVNAGE---------------------------PTE-------------T--------------------------ASF-----------
--AHQPGKIYTDFD-----------AVKAEI------
EAET-AR---K-CG------------------------DS--------------------K-GVDSE-PIRLSVTSNY-------------------
---VVDLTLIDLPGLTK--VA-------------------V-------EG--------------
Q--------KE--T--VAADIE--------SMVI-EWS---KP------------RNTIILAVTAANT--D-IANSD-
ALQLAKRVD------PSGDRTIGVLTKIDLMD-------QGT------D-------C---------------------------LDI--
LK---GN---------V-VK-----------------------L---KKGFYGLVNR--
-S-QRD--IDS-----------K--------KD--------------------IK----------AALAA---ERQFFET--------HP-AY---
------------------K-A------N-----------AAR-MGTPFLTRVLSQELMF-----------

KAJ9466576.1 -----------ELDLPQ--------IAVV--
GV----Q----------------SSGKSSVLE-----N--IV-G-K-DFLPRG--------------TG-IVTRRPL---------------
VLQ--------------M-V-HMS------------------------------PEEKA--
-----------QREEKGE---------------------------ATE-------------V---------------------------ASF-----------
---LHEPGKEYTDFQ-----------EVKRQI------
EKET-ER---L-CG------------------------AR-------------------K-GISSE-PIRLRVLSHR--------------------
-------------------------------------VLDLTLVDLPGLTQ--IA------------------V-------EG--------------
Q--------PA--N--IGKEIK--------AMIL-EWI---EP------------KNTIILAVSAANV--D-IANSD-
ALKLAKQVD------PAGERTVGVLTKLDLMD-------QGT------D-------C---------------------------VDV-
-LS---GS---------V-VA----------------------L---KKGFIGLVNR---
-S-QRD--IDS-----------Q--------KD-------------------IN----------AALKA---EQDFFRT--------HP-SY-
--------------------S-K------I-----------SHK-LGTAYLTKMLSHHLLL-----------
XP_018636213.1 ------------LDLPQ--------IAVV--
GA----Q----------------SVGKSSVLE-----A--LV-G-R-SFLPRG--------------TG-IVTRRPL---------------
ILQ--------------L-R-NAS------------------------------D--
--------------------------------------LQEE-------------F--------------------------GEF-------------------
--LHCPSRKFTDFE-----------EIRREI------ERET-ER---
V-GG------------------------Q-------------------K-NISPS-PIVLKVSSPH---------------------------------
-------------------------VIDLTLVDLPGITK--VP------------------V-------GD--------------Q--------PS--
D--IEAQIR--------RIVF-QFI---SE------------PSTIILAVTAANT--D-IANSD-SLKIAREVD------
PEGLRTVGVVTKVDTLE-------EGA------D-------C---------------------------SEV--LR---NR---------V-
IP----------------------L---KRGYVGVVCR---G-QRQ---AA-----
------E--------MS--------------------IR----------DGLKE---EESFFRS--------HP-AY--------------------
R-A------I-----------ASK-QGIPFLAKMLNQILMKHIREA-LPEL-
XP_001750431.1 ------------IQLPQ--------IVVV--
GA----QASSPADHPALRMSHEQSSGKSSILE-----N--VV-G-K-DFLPRG--------------TG-IVTRVPL---
------------VLQ--------------L-V-QTA------------------------------DD--------------------------------------
--E--------------W--------------------------ATF--------
---QHAGGKVFRDFE-----------QVRQEI------
VDQT-ER---I-TG------------------------PG-------------------K-AVSNE-PIHLRVHSPN-------------------
--------------------------------------VVNLTLVDLPGLTK--VA------------------V-------AD--------------
Q--------PQ--D--IGPQIR--------RLVR-HYI---DN------------PNSLILAVSPANA--D-IANSD-
SLQIAKEVD------PQGDRTLAIVTKLDLMD-------RGT------D-------A---------------------------KAL--
LS---GE---------V-LP----------------------V---KLGIIGIVNR---
S-QND--INC-----------K--------TS-------------------IQ----------DSLDN---EKRFFRT--------H---Y----
-----------------P-E------M-----------ADR-CGCAFLADTLHHLLLQHIRAC-L----
XP_042924642.1 --------------LPT--------IVVV--
GG----Q----------------SSGKSSVLE-----A--VV-G-R-DFLPRG--------------TG-IVTRRPL--------------
-VLQ--------------L---VKT------------------------------DDP--
--NAVD-------------Y---------------------------GEF-----------------
--AHAPGRKFTNFD-----------DITTEI------EDET-
TRHLQR-QG------------------------GT-------------------K-VVSPD-PIYLTVYSVN------------------------
----------------------------------VPNLTLVDMPGLTK--VP------------------I-------DG--------------Q--
------PA--S--IVQELD--------DMAR-QYV---KS------------DNAIILAVTPANA--D-LATSD-

ALRMARDVD------PSGDRTIGVLTKVDIMD-------RGT------D-------C----------------------------RDV-
-LL---GK---------T-LK-----------------------L---KHGWVAVVNR---
---G-QAD--LNS-----------K--------VT--------------------MK----------DARAR---EQEFFKG--------KP-
EY---------------------Q-D------L-----------QN--TGTTFLAEKLSNHLINEIMKS-LP---
PTQ29980.1 --------------LPS--------VVVV--GG----
Q----------------SSGKSSVLE-----S--VV-G-K-DFLPRG--------------SG-IVTRRPL---------------VLQ--
------------L---QKT------------------------------ED---
---------------------------------GTRE--------------W---------------------------AEF--------------------------
--LHAPRKRFEDFA-----------LVRKEI------SDET-DR---V-GG-
-----------------------R--------------------K-GISKI-PIHLTVYSPN---
-----------------VVNLTLVDLPGLTK--VA-------------------V-------EG--------------Q--------SD--S--
IVADIE--------DMVR-SYV---EK------------PNSIILAVSPANQ--D-IATSD-AIKIAREVD------
PNGERTFGVATKLDLMD-------KGT------N-------A----------------------------LDV--LE---GR---------T-
YR-----------------------L---QLGWVGVVNR--S-QQD--INK---
--------N--------TD--------------------ML----------AARRR---EREYFQT--------SQ-DY-------------------
--G-H------L-----------ASR-MGSEYLGKLLSKHLEQVIKAR-IP---
XP_002302631.1 --------------LPS--------IAVV--
GG----Q----------------SSGKSSVLE-----S--IV-G-K-DFLPRG--------------AG-IVTRRPL---------------
VLQ--------------L---HKI------------------------------DE--
--G-KE--------------Y---------------------------AEF--------------------
--MHLPRKKFTDFA-----------AVRKEI------ADET-DR---
E-TG------------------------RS-------------------K-QISSV-PIHLSIFSPN-----------------------------------
-------------------------VVNLTLVDLPGLTK--VA-------------------I-------DG--------------Q--------PE--
S--IVHDIE--------NMVR-SYI---EK------------PNCIILAISPANQ--D-LATSD-AIKISREVD------
PRGERTFGVLTKIDLMD-------KGT------D-------A----------------------------VDI--LE---GK---------S-
YK-----------------------L---QFPWIGVVNR--S-QAD--INK----
-------S--------VD--------------------MI----------AARRR---EREYFQS--------SP-EY---------------------
G-H------L-----------ASR-MGSEHLGKMLSKHLEQVIKSR-IP---
XP_002299468.1 --------------LPS--------IAVV--
GG----Q----------------SSGKSSVLE-----S--VV-G-K-DFLPRG--------------SG-IVTRRPL--------------
-VLQ--------------L---HKI------------------------------DE---
---GSRE--------------Y---------------------------AEF-------------------
---LHLPRKRFTDFA-----------AVRREI------QDET-DR---
E-TG------------------------RS-------------------K-QISSV-PIHLSIYSPN-----------------------------------
-------------------------VVNLTLVDLPGLTK--VA-------------------V-------EG--------------Q--------PD--
S--IVQDIE--------NMVR-AYI---EK------------PNCIILAISPANQ--D-LATSD-AIKISREVD------
PTGERTLGVLTKIDLMD-------KGT------D-------A----------------------------VDM--LE---GK---------S-
YR-----------------------L---KFPWVGVVNR--S-QAD--INK----
-------N--------VD--------------------MI----------AARRR---EREYFSS--------TP-EY---------------------
K-H------L-----------AHR-MGSEHLAKMLSKHLEVVIKSK-IP---
NP_001190448.1 --------------LPA--------IAVV--
GG----Q----------------SSGKSSVLE-----S--IV-G-K-DFLPRG--------------SG-IVTRRPL---------------
VLQ--------------L---QKI------------------------------DD--
--GTRE--------------Y---------------------------AEF--------------------

---LHLPRKKFTDFA-----------AVRKEI------QDET-DR---
E-TG------------------------RS------------------K-AISSV-PIHLSIYSPN-----------------------------------
-------------------------VVNLTLIDLPGLTK--VA-------------------V-------DG--------------Q--------SD--
S--IVKDIE--------NMVR-SYI---EK------------PNCIILAISPANQ--D-LATSD-AIKISREVD------
PSGDRTFGVLTKIDLMD-------KGT------D-------A----------------------------VEI--LE---GR---------S-
FK----------------------L---KYPWVGVVNR---S-QAD--INK----
-------N--------VD--------------------MI----------AARKR---EREYFSN--------TT-EY--------------------
R-H------L-----------ANK-MGSEHLAKMLSKHLERVIKSR-IP---
AQK88296.1 --------------ISR--------VAGG---TA----R-
---------------SSGKSSVLE-----S--VV-G-K-DFLPRG--------------SG-IVTRRPL---------------VLQ-----
---------L---HRI------------------------------D---
-----------------------------GDRE--------------Y---------------------------AEF------------------------------
--MHLPRKRFTDFA-----------AVRKEI------ADET-DR---E-TG-----
-------------------RS------------------K-QISTV-PIHLSIFSPHG-----------------KMQTLEG--------------
------YIVTVALSHLNCPLPIVNLTLIDLPGLTK--VA-------------------V-------DG--------------Q--------
PE--S--IVHDIE--------NMVR-SYI---EK------------PNCIILAVSPANQ--D-LATSD-AIKISREVD------
PKGERTFGVLTKIDLMD-------KGT------D-------A----------------------------VDI--LE---GR---------S-
YR------------------------L---QTPWVGVVNR---S-QQD--INK---
--------N--------VD--------------------MI----------AARRR---EREYFAS--------TP-EY---------------------
K-H------M-----------ASR-MGSEYLGKMLSKHLEQVIKSR-IP---
PTQ45603.1 --------------LPS--------VVVV---GG----
Q----------------SSGKSSVLE-----S--IV-G-R-DFLPRG--------------SG-IVTRRPL---------------VLQ---
-----------L---HKT-------------------------------D--
-------------------------------DGSD--------------Y---------------------------AEF----------------------------
---LHHPRRRFADFA-----------AVRKEI------ADET-DR---V-TG---
---------------------RS------------------K-MISPV-PIHLSIYSPN--
----------------VVNLTLIDLPGLTK--VA-------------------V-------EG--------------Q--------PD--S--
IVHDIE--------NMVR-SYV---EK------------PNSIILAISPANQ--D-IATSD-AIKLAREVD------
PAGERTWGVLTKLDLMD-------RGT------N-------A----------------------------LDV--LE---GR---------
S-YR------------------------L---QLPWIGVVNR---S-QAD--INK--
---------N--------VD--------------------MI----------AARRR---EREYFQS--------SQ-DY------------------
---G-H------L-----------AGK-MGSEYLAKMLSKHLEAVIKSR-IP---
KAI5072318.1--------------LPS--------VAVV---GG----
Q----------------SSGKSSVLE-----S--IV-G-R-DFLPRG--------------SG-IVTRRPL---------------VLQ---
-----------L---YRT-------------------------------D--
-------------------------------KGPE--------------Y---------------------------AEF----------------------------
--LHAPKKRFTDFA----------AVRKEI------SDET-DR---I-TG-----
-------------------RS------------------K-QISPV-PIHLSIYSPN---
-------------VVNLTLIDLPGLTK--VA------------------V-------EG--------------Q--------PD--S--
IVADIE--------NMVR-SYV---EK------------PNSLILAISPANQ--D-IATSD-AIKLAREVD------
PSGERTFGVLTKLDLMD-------KGT------N-------A----------------------------LDT--LE---GR---------A-
YR-----------------------L---QHPWVGVVNR---S-QAD--INK---
--------S--------VD--------------------MM----------AARRR---EREYFAT--------SP-DY-------------------
-K-H------L-----------ASR-MGSEYLGQMLSKHLESVIKSR-IP---

KAI5058380.1--------------LPS--------VAVV--GG----
Q----------------SSGKSSVLE-----S--IV-G-R-DFLPRG--------------SG-IVTRRPL---------------VLQ---
-----------L---HKL-------------------------------D--
--------------------------------EGSE--------------Y---------------------------AEF----------------------------
--MHLPKRRFTDFA-----------AVRKEI------QDET-DR---V-TG--
----------------------RS-------------------K-QISPV-PIHLSIYSPH---
-----------------VVNLTLIDLPGLTK--VA-------------------V-------EG--------------Q--------PE--S--
IVADIE--------NMVR-LYV---DK-----------PNTIILAISPANQ--D-IATSD-AIKLAREVD------
PTGERTWGVLTKLDLMD-------KGT------N-------A----------------------------IDV--LE---GR---------A-
YH-----------------------L---KNPWIGVVNR--S-QAD--INK----
-------N--------VD--------------------MM----------AARRR---EREYFAT--------SS-DY--------------------
-S-H------L-----------TSR-MGSEYLGKMLSKHLEAVIKAR-IP---
XP_002987566.1 --------------LPS--------VVVV---
GG----Q----------------SSGKSSVLE-----S--IV-G-R-DFLPRG--------------SG-IVTRRPL---------------
VLQ--------------L---HKT-------------------------------E---
---------------------------------------GGAE--------------Y---------------------------AEF--------------------
--LHIPKKRFTDFS----------LVRKEI------QDET-DR---V-
TG------------------------RS-------------------K-QISPI-PIQLSIYSPN--------------------------------------
----------------------VVNLTLIDLPGLTK--IA-------------------I-------EG--------------Q--------PD--S--
IVADIE--------NMVR-SYV---EK-----------QNSVILAISPANQ--D-IATSD-AMKLAREVD------
PTGERTFGVLTKLDLMD-------KGT------N-------A----------------------------LDV--LE---GR----------S-
YK-----------------------L---QHPWVGVVNR--S-QAD--INR---
--------S--------VD--------------------MV----------AARRR---EREYFSS--------SA-DY--------------------
-G-H------L-----------TSR-MGSEYLAKILSKHLEAFIKAR-IP---
KAI5602084.1--------------LPS--------VAVV--GG----
Q----------------SSGKSSVLE-----S--IV-G-R-DFLPRG--------------SG-IVTRRPL---------------VLQ---
-----------L---HKT------------------------------EP---
--------------------------------GITE--------------Y---------------------------AEF-----------------------------
---LHKQRERFTDFA-----------MVRKEI------QDET-DK---I-TG----
--------------------KS-------------------K-QISPV-PIHLSIYSPN---
---------------VVNLTLIDLPGLTK--VA-------------------V-------EG--------------Q--------PE--S--
IVKDIE--------NMVR-LYV---EK----------PNCIILAITPANQ--D-IATSD-AIKLAREVD------
PAGERTFGVLTKLDLMD-------KGT------N-------A----------------------------QDV--LE---GR---------
A-YP------------------------L---QHPWVGIVNR--S-QAD--INK-
----------N--------VD--------------------MI----------AARRR---EREFFST--------SP-DY-------------------
--G-H------L-----------AGR-MGSEYLAKLLSKHLESVIKTR-IP---
XP_006375094.1 --------------LPS--------VAVV---
GG----Q----------------SSGKSSVLE-----S--IV-G-R-DFLPRG--------------SG-IVTRRPL---------------
VLQ--------------L---HKT------------------------------ED--
--GSQE--------------Y---------------------------AEF-------------------
---LHLPKRRFSDFA-----------VVRKEI------QDET-DR---
I-TG------------------------KT-------------------K-QISPV-PIHLSIYSPN------------------------------------
------------------------VVNLTLIDLPGLTK--VA-------------------V-------EG--------------Q--------PE--S-
-IVQDIE--------TMVR-TYV---EK------------PNCIILAISPANQ--D-IATSD-AIKLAREVD------

PSGERTFGVLTKLDLMD-------KGT------N-------A---------------------------LDV--IE---GR---------S-
YR----------------------L---QHPWVGIVNR---S-QAD--INK----
-------N--------VD-------------------MI---------VARRK---EREYFAT--------SP-DY--------------------
G-H------L-----------ANK-MGSEYLAKLLSKHLESAIRAR-IP---
XP_002315854.1 --------------LPS--------VAVV--
GG----Q----------------SSGKSSVLE-----S--VV-G-R-DFLPRG--------------SG-IVTRRPL--------------
-VLQ-------------L---HKI-----------------------------D--
--------------------------------------GGSD-------------Y-------------------------AEF-------------------
--LHAPRKKFTDFA-----------SVRKEI------ADET-DR---
I-TG------------------------KS-------------------K-QISNV-PIHLSIYSPN------------------------------------
-----------------------VVNLTLIDLPGLTK--VA------------------V-------EG--------------Q--------PE--S-
-IVEDIE--------NMVR-SYV---EK------------PNSIILAISPANQ--D-IATSD-AIKLAREVD------
PSGERTFGVLTKLDLMD-------KGT------N-------A---------------------------LDV--IE---GR---------S-
YR----------------------L---QHPWVGIVNR---S-QAD--INK----
-------N--------VD-------------------MI---------AARRK---EREYFET--------SP-EY--------------------
G-H------L-----------SSK-MGAEYLAKLLSKHLETVIRQR-IP---
NP_001147100.1 --------------LPS--------VAVV--
GG----Q----------------SSGKSSVLE-----S--IV-G-R-DFLPRG--------------SG-IVTRRPL---------------
VLQ-------------L---HKT------------------------------D--
--------------------------------------GGHE-------------Y-------------------------AEF-------------------
--LHAPRKRFTDFA-----------AVRKEI------ADET-DR---
I-TG------------------------KT-------------------K-AISNV-PIHLSIYSPH-----------------------------------
-----------------------VVNLTLIDLPGLTK--VA------------------V-------EG--------------Q--------PE--S-
-IVQDIE--------NMVR-AYV---DK------------PNCIILAISPANQ--D-IATSD-AIKLARDVD------
PSGDRTFGVLTKLDLMD-------KGT------N-------A---------------------------VDV--LE---GR---------
Q-YR----------------------L---QHPWVGIVNR---S-QAD--INK-
----------N--------VD-------------------ML---------SARRK---EKEYFES--------SP-EY------------------
---G-H------L-----------AHK-MGAEYLAKLLSQHLEAVIRAK-IP---
AAF22292.1 --------------LPT--------VAVV--GG----Q-
---------------SSGKSSVLE-----S--IV-G-R-DFLPRG--------------SG-IVTRRPL---------------VLQ------
--------L---HKT-----------------------------DD--
----------------------------GTEE--------------Y-------------------------AEF------------------------------
---LHLPKKQFTDFA-----------LVRREI------QDET-DR---I-TG-------
-----------------KN------------------K-QISPV-PIHLSIYSPN---
------------VVNLTLIDLPGLTK--VA------------------V-------EG--------------Q--------PE--T--IAEDIE---
-----SMVR-TYV---DK------------PNCIILAISPANQ--D-IATSD-AIKLAKDVD------
PTGERTFGVLTKLDLMD-------KGT------N-------A---------------------------LEV--LE---GR---------S-
YR----------------------L---QHPWVGISEP---FN-KQD--INK----
-------N--------VD-------------------MM---------LARRK---EREYFDT--------SP-DY-------------------
-G-H------L-----------ASK-MGSEYLAKLLSKHLESVIRTR-IP---
KAH9306600.1 --------------LPS--------VAVV--
GG----Q----------------SSGKSSVLE-----S--IV-G-R-DFLPRG--------------SG-IVTRRPL---------------
VLQ-------------L---HKT-----------------------------DE--
--------------------------------------GTPE-------------Y-------------------------GEF-------------------

---LHRPNKRITDFA-----------KVRSEI------QEET-DR---I-
TG-----------------------RT-------------------K-MISPV-PIHLSIYSPN---
----------------------VVNLTLIDLPGLTK--VA-------------------V-------EG--------------Q--------PE--S--
IVGDIE--------NMVR-SYV---EKLLKLVVTADFLLPNSIILAISPANQ--D-IATSD-AIKLAREVD------
PTGERTFGVLTKLDLMD-------KGT------N-------A---------------------------LDV--LD---GR---------S-
YR----------------------L---QHPWVGVVNR--S-QAD--INK---
--------S--------VD--------------------MI----------AARRR---EREYFST--------ST-DY--------------------
G-H------L-----------SSR-MGSEYLAKLLSKHLENVIKAR-IP---
KAG0556007.1 --------------LPS--------VAVV---
GG----Q----------------SSGKSSVLE-----S--IV-G-R-DFLPRG--------------SG-IVTRRPL---------------
VLQ--------------L---HKT------------------------------E--
--------------------------------------DKYE-------------Y--------------------------AEF--------------------
---LHMPKRRFTDFA-----------AVRKEI------SDET-DR---I-
TG-----------------------RS-------------------K-QISPV-PIHLSVYSPN------------------------------------
------------------------VVNLTLIDLPGLTK--IA-------------------V-------EG--------------Q--------SD--S-
-IVGDIE--------NMVR-SYI---EK------------PNCIILAVSPANQ--D-IATSD-AIKIAREVD------
PNGERTFGVLTKLDLMD-------KGT------N-------A---------------------------IDV--LE---GR---------S-
YK----------------------L---IQPWIGVVNR--S-QQD--INK-----
------N--------VD--------------------MI----------AARRR---EREYFQT--------SP-DY---------------------
S-H------L-----------QSK-MGSEYLGRVLSKHLEAVIRSR-IP---
PWZ36850.1 --------------LPT--------IAVV---GG----Q-
---------------SSGKSSVLE-----S--IV-G-T-DFLPRG--------------SG-IVTRRPL---------------VLQ-------
-------L---QQT-----------------------------ED--
---------------------------GSQE-------------Y--------------------------AEF-----------------------------
--LHMPKRRFSDFA-----------LVRQEI------ADET-DR---L-TG----
---------------------KT-------------------K-QISPV-PIHLSIYSPK---
----------------VVNLTMIDLPGLTK--VA-------------------V-------EG--------------Q--------SE--S--
IVQDIE--------NMVR-SYV---DK------------PNCIILAISPANQ--D-IATSD-AIKLSKEVD------
PTGERTFGVLTKLDLMD-------KGT------N-------A---------------------------LDV--LE---GR---------A-
YR----------------------L---QNPWVGIVNR--S-QAD--INR---
--------K--------VD--------------------MI----------SAREK---EREYFET--------SP-DY--------------------
A-H------L-----------SSR-MGSGYLAKLLSQHLESVIKVR-IP---
AAF79238.1 --------------LPT--------VAVV---GG----Q-
---------------SSGKSSVLE-----S--VV-G-R-DFLPRG--------------SG-IVTRRPL---------------VLQ-----
---------L---HKT-----------------------------ED---
----------------------------GTTE-------------Y--------------------------AEF-----------------------------
--LHAPKKRFADFA-----------AVRKEI------EDET-DR---I-TG------
------------------KS-------------------K-QISNI-
PIQLSIYSPNGLCLRPHFLLCIPIVVSSETNTFDTSCNEITSSTIRFEDSNFANLFHVTL-
ISHSTLFSTVVNLTLIDLPGLTK--VAVVTDMNLVLKLVTDMNIIRV-------DG--------------Q--------PE--
S--IVQDIE--------NMVR-SYV---EK------------PNCIILAISPANQ--D-IATSD-AIKLAREVD------
PTGERTFGVATKLDIMD-------KGT------D-------C---------------------------LDV--LE---GR---------S-
YR----------------------L---QHPWVGIVNR--S-QAD--INK----

-------R--------VD-------------------MI----------AARRK---EQEYFET--------SP-EY--------------------
G-H------L-----------ASR-MGSEYLAKLLSQHLETVIRQK-IP---
NP_850420.1 --------------LPS--------VAVV--GG----
Q----------------SSGKSSVLE-----S--IV-G-R-DFLPRG--------------SG-IVTRRPL---------------VLQ---
-----------L---HKT-----------------------------EN---
--------------------------------GTED--------------N--------------------------AEF---------------------------
--LHLTNKKFTNFS-----------LVRKEI------EDET-DR---I-TG----
--------------------KN-------------------K-QISSI-PIHLSIFSPN---
---------------VVNLTLIDLPGLTK--VA------------------V-------EG-------------Q--------PE--T--
IVEDIE--------SMVR-SYV---EK------------PNCLILAISPANQ--D-IATSD-AMKLAKEVD------
PIGDRTFGVLTKLDLMD-------KGT------N-------A---------------------------LDV--IN---GR---------S-
YK----------------------L---KYPWVGIVNR--S-QAD--INK-----
------N--------VD-------------------MM----------VARRK---EREYFET--------SP-DY--------------------
G-H------L-----------ATR-MGSEYLAKLLSKLLESVIRSR-IP---
EFJ15761.1 --------------LPS--------VVVV--GG----
Q----------------SSGKSSVLE-----S--IV-G-R-DFLPRG--------------SG-IVTRRPL---------------VLQ---
-----------L---HRT------------------------------E--
-------------------------------DGPD--------------Y--------------------------AEF----------------------------
--LHLPKKKFTDFA-----------LVRKEI------QDET-DR---I-TG----
--------------------RS-------------------K-QISPV-PIHLSIYSRN--
---------------VVNLTMIDLPGLTK--IA-------------------V-------DG--------------Q--------PE--S--
IVGDIE--------NMVR-SYV---EK------------ENTIILAISPANQ--D-IATSD-AMKLARENI------
ALGDRTFGVLTKLDLMD-------KGT------N-------A---------------------------IDV--LE---GH---------S-
YR----------------------L---QRPWIGVVNR---S-QAD--INK----
-------S--------VD-------------------MI----------VARRR---EREYFSS--------SP-DY--------------------
R-H------L-----------ASR-MGSEYLGRVLSKHLEAVIKAR-IP---
EFJ23099.1 --------------LPS--------VAVV--GG----
Q----------------SSGKSSVLE-----S--VV-G-R-DFLPRG--------------SG-IVTRRPL---------------VLQ--
------------L---HKT------------------------------E---
--------------------------------GGQE--------------Y--------------------------AEF---------------------------
--LHNPKTKFSDFS-----------LVRKEI------EDET-DR---M-TG--
----------------------HT-------------------K-QISPV-PIHLSIYSPNGTCLS-H-----PV----------------------
---KFSMPWFVYS----------VVNLTLIDLPGLTK--IA-------VGKCISF-----S-------KM--------------M-----
---CL--L--ILADIE--------NMVR-SYV---EK------------QNSIILAISPANQ--D-IATSD-AMKLAKEVD----
--PTGERTFGVLTKLDLMD-------KGT------N-------A---------------------------LEV--LE---GR---------
A-YR----------------------L---QFQWVGVVNR--S-QAD--
INK-----------S--------VD-------------------MI----------AARKK---EREFFAS--------SP-DY--------------
-------G-H------L-----------ANR-MGSEYLAKMLSKHLETVIKTR-LP---
XP_008860500.1 -----------TLPLPQ--------IVVV--
GS----Q----------------SSGKSSVLE-----H--VV-G-K-DFLPRG--------------SG-IVTRRPL---------------
-IVQ--------------C-V-RSN-----------------------------V---
---AED-------------Y--------------------------GQF------------------
---EHTGDRKFTDFG-----------EIRNEI------TRET-ER---
TCPG-----------------------RN---------------------VSSV-PIRLRIYSSS-----------------------------------

------------------------VVDLTLVDLPGLVK--VN-----------------I-------NG-------------Q--------TA--
E--MVKNLR--------DMVY-EYA---SP-----------SNALILAVTAGNI--D-IANSD-ALQVAKDVD------
PEGERTIGVLTKLDLED-------KGT------N-------S--------------------------MDV--LM---GR---------V-
YP---------------------L---KLGYIGVVNR---S-QQD--INN-----
------G--------VD------------------VK---------TSLRH---EKEFFEN--------HP-VY-------------------
C-S------I-----------AER-MGTEYMVNRLNVLLLQ-----------
XP_004185630.1 -----------TLPLPQ--------IVVV--
GS----Q---------------SSGKSSVLE-----H--VV-G-K-DFLPRG-------------SG-IVTRRPL--------------
-IVQ-------------C-V-RTD----------------------------V---
--PKE-------------Y-------------------------GLF-------------------
---EHQGDKQYFDFN-----------AIRDEI------TAET-QR---
TCPG----------------------RN--------------------VSPT-PIRLRIVSPN----------------------------------
------------------------VVDLTLVDLPGLVK--VT-----------------V-------VG-------------Q--------SN--
E--IVKNLR--------DMVY-QYA---AP------------ENALILAVTAGNV--D-IANSD-ALNVAKEVD------
PDGERTIGVLTKLDLED-------KGT------N-------S--------------------------MDV--LM---GR---------V-
YP---------------------L---KLGYIGVVNR---S-QQD--INN-----
------G--------MD------------------VQ---------TSLKN---EKKFFED--------HP-VY-------------------
C-S------I-----------ADR-MGTEYMVNRLNLLLLQ-----------
KAF0852279.1 -----------SIDLPQ--------IAVV--
GS----Q---------------SSGKSSVLE-----S--LV-G-K-GFLPRG-------------SG-IVTRRPT--------------
VLQ-------------L-I-HEP-----------------------------PGTAAAT--
--APVE-------------Y--------------------------AEF----------------
--VHLPNQRFTDWD-----------AVRIEI------ERET-AR-
--V-AG----------------------SG------------------K-GVSPS-PITLRIHSPY------------------------------
---------------------------VLNLTLVDLPGLIK--IP-----------------V-------GD-------------Q--------
PQ--N--IESIVR--------DLVL-KFI---AR-----------PSTIILAVTPANM--D-LANSD-AIQIARQVD------
PEGTRTLGVLTKLDLMD-------KGT------D-------A--------------------------ADI--LR---NN---------L-
LP---------------------L---RLGYVGVVCR---S-QAD--LDA----
-------K--------TP-------------------LA----------VSRAR---ENSFFAN--------HP-AYQ-
DLYLQQQQLQTASSSQFVDS-A------A-----------AGW-LGTEVLGKRLQTLLMG-----------
ETO36135.1 ------------RKLPG--------ISNI--QK----
K----------KLY---SVGKSSVLE-----N--IV-G-R-DFLPRG-------------TD-IVTRRPL---------------ILQ---
-----------L-I-NTS-----------------------------KPRPSTSSSDDEKS---EQLIT-----SSQSSSLQSPS------
----QQSSSTQS-----LLSESSE------------------------ANLE-------------W-------------------------
GEF--LHIKNKRFYDFN-----------
DIRKEI------EKET-DR-----VA----------------------PS------------------K-AVSEE-PISLKIYSPK--------
--VLNLTLVDLPGLTK--VA------------------V-------GN---
-----------Q--------PE--N--IEQIIG--------DLVY-SFI---KR------------PKCLILAVSAATS--D-LATSD-
GLQMALRID------PKGVRTLGVITKLDLMD-------QGT------D-------A--------------------------MKV-
-LN---GE---------V-IP----------------------L---QLGYVGVINR---
--S-QQN--IND-----------Q--------LH-------------------IT----------DALKN---EERFFKN--------HP-
AY--------------------R-N------I----------AHL-CGIPYLAKRLNQILIN-----------
KNH06820.1 ---------DFSQQLPQ--------IAVV--GS--
--Q---------------SSGKSSVLE-----S--IV-G-K-AFLPRG-------------SG-IVTRRPL--------------ILQ--

------------L-V-PMP------------------------------TDVSS--
-----------------------------------TVAE--------------Q-P------------------------FGEF----------------------
---LHEPGKKYYDFD-----------RIREVI------SEET-DS---I-
AG------------------------VN-------------------K-NISPE-PIRLRIHSPS--------------------------------------
-----------------------VAPLTLVDLPGLVR--NP-------------------I-------GD--------------Q--------PK--
D--IDRQVS--------NLVR-KYI---VH------------ENTLILAVSAANA--D-IATSD-GVQLAQQVD------
PHGRRTIGVLTKLDLME-------EGT------H-------V---------------------------VDI--IE---QR---------V-
IS-----------------------L---QRGFIPVVNM---S-QSD--LDQG--
--------S--------KT--------------------MA----------DQRRA---ELQFFAQ--------HP-RY--------------------
--A-P------M-----------RDR-CGMAFLQAKLSAVLLE-----------
CAD2212698.1 ---------SLDLTLPQ--------IAVV---
-GS----Q----------------SSGKSSVLE-----H--IV-G-E-EFLPRG--------------PT-MVTRCPI--------------
-VLQ--------------L-H-QLP------------------------------KND---
---KRK--------------W---------------------------GEF--------------
--LHLPNKRFTDFN-----------LIREEI------LRYT-RE-
--L-IG------------------------D--------------------R-TVTSQ-SITLKISSAA-----------------------------------
--------------------------VANLTLVDLPGLVT--TP-------------------I-------RG--------------Q--------PE--
T--IVTDIE--------DMVR-RYV---AD------------KNTVILAITPANQ--D-VATSA-ALSVSRMVD------
PHGERTMGVLTKLDLMD-------RGT------T-------A----------------------------HRT--LM---GD---------
E-YE-----------------------L---KFGFIGVVNR--S-QES--INS---
--------G--------QT---------------------MS----------DARAA---EEEFINE--------Y---Y---------------------
P-E------L-----------SGR-MGTKYLTAVLNSVLVS-----------
ABB13595.1 ------------LRLPQ--------IVVI---GS----
Q----------------STGKSSLLE-----S--IV-G-Q-EILPRG--------------KG-IVTRRPI---------------EIQ-----
---------L-K-NQQ-------------------------------N---
--------------------------------AEQD-------------Y---------------------------VEF-------------------------------
--SERRGEKITDMD-----------QVRKMI------DEDT-EK---I-AG---
---------------------KN-------------------K-AISNV-PLRLKFYSKN---
------------------VVDLILVDLPGMTK--NP-------------------V-------GD--------------Q--------PQ--D--
IEQQIL--------NLIE-PYI---KN------------PNSIIMAVSKGSD--D-LANSE-SLKLSRKID------
PQGNRTIGVITQLDLID-------EGA------D-------V---------------------------LND--LQ---NK---------T-
YP------------------------L---KLGYVGVIMR---G-QKD--IKI------
--------------KS-------------------IK----------EQIAD---EKAYFEN--------HS-IY---------------------K-R-
-----V-----------SNK-MGIPYLIKLLNLSLMNHIKKT-LPNI-
XP_001009829.2 ------------LRLPQ--------IVVI---
GS----Q----------------SSGKSSVLE-----S--II-G-K-DFLPRG--------------KG-IVTRRPI---------------
EIQ--------------L-T-NIS-------------------------------S---
--GEE-------------Y---------------------------AEI------------------------
---LDRKGEKVTDME----------VLTKII------EDET-EK---V-
AG-------------------------KQ-------------------K-GVSGV-PLKIRFYSKN------------------------------------
---------------------------VVDLLLVDLPGITK--NP-------------------V-------GD--------------Q--------PA--
D--IEQKLL--------EIVN-PYI---AN------------PNSIILAISKGTD--D-LANSE-SLKLAREFD------
INGQRTIGVITQIDLQD------FESE------N-------A---------------------------LND--IT---NK---------T-
YP------------------------L---RLGYVGVVMR---G-QNQ--LKT----

----------------KT-------------------IQ----------EQIVD---EAAFFEN--------HS-VY--------------------R-
K------V-----------ADK-MGIPYLIKTLNLIFMNHIKKC-LPKI-
XP_002371703.1 ------------IQLPR--------ICVV---
GT----Q----------------SAGKSSVLE-----A--IV-G-L-DFLPRG--------------DG-VVTRRPL--------------
ELR--------------L-V-HL-------------------------------SEAEHDL---
---NEA------------Y--------------------------AVF---------------
---ENDKERKIRDFE-----------QVRQEI------DRLT-
DQ---V-AG------------------------KN-------------------K-GIIDS-PIVLTIYATQ----------------------------
------------------------------CPDLSLIDLPGITR--VP-------------------L-----KGSD--------------Q-------
-CE--D--IEMLTR--------QMAL-RYA---SD------------PRTIILAVIPANV--D-MSTSD-ALQMSRRVD---
---PRGVRTIGVITKIDLMD-------RGT------D-------A---------------------------AKM--LM---GE---------
E-IP----------------------L---RLGYTGVRNR---S-QAD--IRE---
--------G--------KS-------------------VR----------ECLEE---EKTFFAT--------HP-TY--------------------
R-L------LP----------PHL-VGVHSLVDKLTKVLFRHIKNF-LPEI-
'XP_019914840.1' ------------INLPR--------ICVV---
GT----Q----------------SSGKSSVLE-----S--IV-G-L-DFLPRG--------------EG-IVTRRPI---------------
EFR--------------L-I-HI-------------------------------KEDSEI--
--KH-------------W--------------------------AIF--------------------
--EDDKSKKFTDFN-----------QVREHI------NNLT-DE---
L-AG------------------------KN-------------------K-GIIDE-PIVLNIYSTS-----------------------------------
-----------------------CPDLSLIDLPGITR--VP-------------------L-----KNSD--------------Q--------TD--
D--IERLTR--------EMAF-RYV---KD------------PRTIILAVLPANA--D-MSTSD-ALQIARKVD------
PKGLRTIGVITKIDLMD-------KGA------D-------A---------------------------SKM--LM---ND---------E-
IT----------------------L---RLGYTGVVNR---S-TAD--IKS--------
---G--------KS-------------------IA----------QSLKD---ELKYFQN--------HP-VY--------------------K-
K------LP----------PTL-YGTTSLTDKLTKVLLRHIKNF-LPDI-
'XP_012763851.2_1' ------------INLPR--------ICVV---
GT----Q----------------SSGKSSVLE-----S--IV-G-M-DFLPRG--------------EG-IVTRRPI---------------
EFR--------------L-I-HI-------------------------------KEDSEI--
--KY-------------W--------------------------AVF--------------------
--ENEKNKKYTDFN-----------EVREQI------NRLT-DE---I-
AG------------------------KN-------------------K-GIIDE-PIVLNIYSIK--------------------------------------
--------------------CPDLSLIDLPGITR--VP-------------------L-----KNSD--------------Q--------TD--D-
-IERLTR--------DMAL-RYV---KD------------PRTIILAVLPANA--D-MSTSD-ALQIARKVD------
PKGLRTIGVITKIDLMD-------KGA------D-------A---------------------------SKM--LL---ND---------E-
IN----------------------L---RLGYTGVVNR---S-TAD--IKK-------
----G--------KT-------------------IS----------QALKD---ELEFFQK--------HP-VY--------------------K-
K------LP----------PSL-YGTNSLTDKLTKVLLRHIKNF-LPDI-
'EUR69800.1_1' ------------INLPR--------ICVV---
GT----Q----------------SSGKSSVLE-----S--IV-G-M-DFLPRG--------------EG-IVTRRPI---------------
EFR--------------L-I-HI-------------------------------KEDSEI--
--KY-------------W--------------------------AVF--------------------
--ENEKNKKYTDFN-----------EVREQI------NRLT-DE---I-
AG------------------------KN-------------------K-GIIDE-PIVLNIYSIK--------------------------------------

--------------------CPDLSLIDLPGITR--VP------------------L-----KNSD-------------Q--------TD--D-
-IERLTR--------DMAL-RYV---KD------------PRTIILAVLPANA--D-MSTSD-ALQIARKVD------
PKGLRTIGVITKIDLMD-------KGA------D-------A---------------------------SKM--LL---ND---------E-
IN----------------------L---RLGYTGVVNR---S-TAD--IKK-------
----G--------KT-------------------IS----------QALKD---ELEFFQK--------HP-VY--------------------K-
K------LP----------PAL-YGTNSLTDKLTKVLLRHIKNF-LPDI-
XP_028867889.1 ------------INLPR--------ICVA--
GT----Q----------------SSGKSSVLE-----S--IV-G-I-DFLPRG--------------DG-IVTRRPV---------------
EFR-------------L-S-RLT-----------------------------GKDGDADL---
---RP------------Y--------------------------IIF-----------------
---EGNSE-KFYDFE-----------QARQHI------QELT-NE-
--K-AG-----------------------VN------------------K-GIIDD-PIVLSVFSPD--------------------------------
---------------------------CPDLSLIDLPGVTR--VP------------------L-----KNSD-------------Q--------
TD--D--IEALTK--------DMIM-RYA---RD------------PRTIILAVVAANV--D-MSTSD-ALQLARRAD-----
-PLGVRTLGVITKIDLMD-------RGA------D-------A---------------------------VAM--LQ---ND---------
E-VP----------------------L---RLGYTGVKNR---S-QKD--IAE---
--------G--------VT-------------------IK----------KALEL---ERQYFSE--------HS-VY--------------------
K-H------IK----------PSL-WGIPSLVEKLTKVLYRHISTV-LPDL-
'CAE8701890.1' ------------ISLPR--------IAAI--
GT----Q----------------SSGKSSLIE-----S--IV-G-M-DFLPRG--------------GG-VVTRRPL---------------
ELR-------------L-V-HLN-----------------------------TQEYPGE--
---QA------------W--------------------------AVF----------------
---DKISDKKFTDFD-----------LVRQEI------ERQT-DL-
--V-AG-----------------------AN------------------K-GIVND-PIILTVYATG--------------------------------
--------------------------APDLTLIDLPGITR--VP------------------V-----KGSD-------------Q--------TE-
-D--VEKLTR--------DMTM-HYV---ND------------PRTIILAVLPANQ--D-MSVSD-SLHVARMVD------
PQGHRSIGVITKIDIMD-------QGT------D-------A---------------------------ARM--LR---GE---------
D-VP----------------------L---KLGYVGVKMR---S-QQD--
IMD-----------K--------KP--------------------VQ----------DSLKE---EKAWFES--------HR-VY------------
---------G-K------LP----------PGM-VGTPVLIDKLTQILFKHIRRF-LPEI-
XP_001016567.2 ------------IDLPR--------IAVL--
GS----Q----------------SAGKSSLLE-----Q--IV-G-L-DFLPRG--------------EG-TVTRRPL---------------
EMR-------------L-F-YVP------------------------------KEKLS--
---MP------------Y-------------------------GVF-----------------
---EEIPGQKFTDFQ----------MVKQNI------DKLT-NN-
--V-AG-----------------------AN------------------K-GIVDK-PIVLTIYSST----------------------------------
-------------------------CPDLTIVDLPGITK--IP------------------I-----RGTD-------------Q--------TQ--
D--IEKITK--------EMAA-RYC---KD------------PKTIILCVIPANA--D-ITTSD-GLMMARQLD------
PQGSRTIGCITKIDIMD-------KGT------D-------A---------------------------RRL--LT---GE---------D-
VG-----------------------L---KLGYVGIKNR---S-QAD--INE-----
------K--------KT-------------------VL----------QSLDD---ERKFFST--------SP-IY--------------------S-
S------LP----------SSL-LGTRSLTNKLTDVLYTHIRTC-LPQI-
XP_001032891.1 ------------IRLPR--------IAVI--
GS----Q----------------SSGKSSLLE-----S--IV-G-I-DFLPRG--------------SG-VVTRRPL---------------

ELR--------------L-V-HVP------------------------------PNERQI--
---KP------------Y-------------------------AIF------------------
---DVDKSKKWENFD----------QVRQQI------DFLT-DQ-
--V-AG-----------------------KR------------------K-KIIND-PIVLTIYSND-------------------------------
-------------------------VIDLTIIDLPGITR--IP------------------L-----KDSD-------------Q--------QE--
D--IEKVTK--------DMAY-SYI---KD------------ERTIILCVVPGNQ--D-ISNSD-GLQLAREVD------
REGNRTIGVVTKLDIMD-------ADT------D-------A--------------------------RKM--IM---GL---------E-
IP----------------------L---KLGYVGVKGR---S-QKD--IND-----
------N--------KR-------------------VS----------KALDE---ERLFFAQ--------HK-VY--------------------
S-T------MD----------PKF-LGTKALTNKLSSVLFYHIRNI-LPAI-
'CAD8140652.1_1'   ------------IKLPR--------IVVL---
GI----Q----------------SAGKSSLLE-----H--IV-G-I-DFLPRG-------------SG-VVTRRPL--------------
ELR--------------L-S-YSP------------------------------QSVCA--
---QP------------T-------------------------AEF----------V-------
---EEIKGKKFTNFD----------EVRKNI------EELT-DK---
V-CG-----------------------SS------------------K-NIIDK-PIILAVTGPN--------------------------------
------------------------CPDLTLVDLPGITR--IP------------------I-------MD-------------Q--------PK--
D--IEQITT--------NMAK-RYC---ED-----------PSAIILCVVAANA--D-MTTSD-ALLLAKKLD------
PDGIRTVGVLTKIDIMD-------QGT------N-------A--------------------------IKM--LK---GE---------E-
VP----------------------L---KYGYVGVKLR---S-QQE--IKD-----
------N--------VP-------------------IV----------QAVQR---EKNFFAN--------HP-VY--------------------
S-S------IP----------GDI-FGTQVLTGKLTRILYRRIRSF-LPTL-
XP_001029982.1   ------------IELPK--------IVVI---
GV----Q----------------SSGKSSLLE-----Q--IV-Q-I-DFLPRG-------------TG-VVTRCPL--------------
EIR--------------L-I-EVS-----------------------------NYGKNF--
---KP------------F-------------------------AYF------------------
---FEERNITYDDFE----------EVKQRI------EIIT-KE---L-
AG-----------------------EG------------------K-KIVDE-TITLTIFQTN--------------------------------
----------------------CPTLTLIDLPGMT----------------------L-----NSIE-------------D--------QE--D--
VEIVTQ--------EMTL-KYI---QE-----------ETTIILCVIPINS--D-LENSL-ALKLSRNVD------
KVGSRTIGVLTMIDIMN-------PGT------N-------C--------------------------EKV--LR---NQ---------Q-
IP----------------------L---KHRYYGMKPR---N-QKD--INN----
-------N--------VT-------------------VE----------LAIQN---EYEYFSN--------HE-IY--------------------K-
E------F-----------ADY-TGTAALTHKLSTLLDSHIRHF-LPDI-
XP_001029985.2   ------------IQLPK--------IVVI---
GV----Q----------------SSGKSSLLE-----Q--IV-Q-L-DFLPRG-------------TG-VVTRCPL--------------
EIR--------------L-I-EVT------------------------------NQDSDF--
---KP------------Y-------------------------AYF------------------
---FEERQKIFHDFE----------LVKKEI------QKIT-ND---F-
AG-----------------------PG------------------K-KIVDK-VITLTIYQAQ--------------------------------
----------------------CPTLTLIDLPGMT----------------------L-----NSVG-------------D--------QK--D--
IERVTQ--------DMTK-KYI---IE------------KTTIILCVIPINQ--D-LENSL-ALNLARSVD------
ENGERTIGVLTMIDIMN-------PGT------N-------C--------------------------ENV--LK---NR---------Q-
IP----------------------L---LHRYYGMKPR---S-QKD--INE-----

------N--------VS-------------------VQ----------EAIQR---EQLYFTS--------HQ-IY--------------------S-
M------Y-----------PEY-TGTNALTNKLSTLLDSHIRTF-LPSI-
XP_005788601.1 ------------LDLPM--------FVVV--
-GA----Q----------------SSGKSSVLD-----N--VI-G-R-SFLPRG--------------SG-TVTRRPL-------------
--VLM--------------M-Q-HHA-----------------------------SE---
---E-------------Y--------------------------GEF-----------------
--NHLPGQRLTTDA-----------QICSAI------EKAT-
IDE----CG-----------------------E--------------------K-GFSSR-PIYLHYYSPT----------------------------
-------------------------------VPDLTLVDLPGLIK--AH------------------A-------AD-------------M------
--DP--S--APHIIK--------TMVL-EFA---QA------------PAAILLAVTPAHS--D-LVTSD-AIQLAREVD-----
-PDGQRTLGVLTKLDLMD-------EGT------D-------C----------------------------STA--LL---GEDP------
RA-PK-----------------------L---RLGYVGVVNR--G-QKD--
INN-----------G--------VD---------------------LT----------AARAR---ERTFFKE--------HG-VYG-R--------
----------P-A------MS----------GAR-LGTEALVATSSALLVE-----------
EFJ35472.1 ------------VDLPQ--------LVHV---QR----
K-----------------------MD-----------D-R-EF---S--

---------------E--------------W----------------------------GEF---
-----------------------LHLPGRRFTDFS-----------HIRKEI------QAET-ERE----LG------------------------
DK-------------------K-GVSDK-QIRLKIFSPN---
VLNITLVDLPGLTR--VP-------------------V-------GD--------------Q--------PS--D--IESRIR--------
AMIL-SYI---KH------------ATCIILAVSPANT--D-LSNSD-SLQMAKLVD------
PDGSRTIGVVTKLDIMD-------RGT------D-------A----------------------------RSI--LL---GT---------V-
IP-----------------------L---RLGYVGVVNR--S-QEV--
FRSPFFCLKILMKHG--------CR--------------------IF----------MPTNR---SRMLLLR--------KS-IFS-
EVERCTKAFWIDV------A-S------L-----------NSR-KSLIKYELEPVKILVQHIK--------
OUM67143.1 ------------LQLPS--------IVVI--GS----
Q----------------SSGKSSVLE-----A--IV-G-H-EFLPK-----------------------PI---------------ELT----------
----L-V-HTP-------------------------------NAEE---
-------------------------------E--------------Y---------------------------GEF-------------------------------------
---PEIGMKNITDFG-----------HIQKTL------SELN-MS-----VS----------
--------------ES-------------------E-CISTD-PIELKIFSPN---
---------VPDLTLIDLPGYIQ--VI--------------------N-------RK-------------Q--------PP--V--LKRKIV------
--ELCD-RYI---VE------------PN-IILAISSADV--D-LANSE-ALTHSRKVD------
PYGQRTIGVITKMDLVE--------PE------K-------G----------------------------VDL--LI---NN---------D-
YP-----------------------L---ELGYVGMVCK--PPG-KSG--FSR---
--------Q--------LS--------------------LT----------QK------SDEYFRK--------NP-IFH-QP-----------------
---------------------DVQ-VGLSALKKRLTTILEENMGQN-LY---
KNE54706.1 ------------LVLPS--------IVVI--GS----Q-
----------------SSGKSSVLE-----A--IV-G-R-EFLPKG--------------TN-MVTRRPL---------------ELT------
--------L-I-HTP-----------------------------DSTE--
----------------------------------E-------------Y---------------------------GEF------------------------------
--PQLGFGKVTNFQ-----------QIQRTL------YDLN-MA-----VQ-----
------------------D--------------------E-CISDK-PIELRIYSPN--

--------------VPDLTLVDLPGYIQ--IH-------------------S-------KN-------------Q--------PR--D--
LKEKIA--------DLCE-KYI---QE------------PN-VILAVCAADV--D-LANSE-ALRASRKAD------
PLGLRTIGVLTKLDLVS--------PE------M-------G---------------------------EQL--IA---HN---------D-
YP----------------------L---HLGYVGVVCG---GKN--PQA----
-------A--------LV-------------------PF----------QK------R---
-----------DLT-VGVPYLRQLLMRTLEERMGSR-LS---
OAJ38404.1 ------------LNLPS--------IVVV---GS----
Q----------------SSGKSSVLE-----A--VV-G-H-EFLPKG--------------AN-MVTRRPI----------------ELT--
------------L-I-HTP-----------------------------DSKE--
------------------------------------E-------------Y--------------------------SEF----------------------------
--PQLGLGKIKDFS-----------QVRRTL------TDLN-LA-----VS----
--------------------DA-------------------E-CVSEI-PIELRVYSPN--
----------------IPDLTLVDLPGYIQ--IH-------------------T-------KD-------------Q--------PP--I--
LKEKIA--------ALCQ-KYI---QE------------PN-IILAVCAADV--D-LANSE-ALRASRKID------
PLGLRTIGVITKMDLVE--------PQ------A-------A---------------------------VNI--LE---NK---------S-
YP----------------------L---ALGYIGVVNK---TSS--RSF-------
----S--------QA-------------------LT----------RQ------E-SYFRS--------HP-EFN-------------------------
----------------NAM-VGTATLRRRLVEVLEEHMGRS-LH---
XP_006459124.1 ------------LKLPS--------IVVI---
GS----Q----------------SSGKSSVLE-----A--IV-G-H-EFLPKG--------------NN-MVTRRPI--------------
-ELT--------------L-I-HTPTK-----------------------DGQLPT---
---E-------------Y---------------------------GEF-----------------
--PGLGMGKITNFA-----------DIQRTL------TDLN-LA--
---VP------------------------AS-------------------D-CVNND-PIDLRIYSPN-------------------------------
---------------------------VPDLTLIDLPGYVQ--IS-------------------S-------LD-------------Q--------
PE--S--LKEKIA--------SLCE-KYI---RE------------PN-IILAVCAADV--D-LANSP-ALRASRKVD------
PLGLRTIGVITKMDLVP--------PE------R-------G---------------------------AQI--LA---GN---------R-
YP----------------------L---HLGYVGVVAK--KS-PVE--PSS-----
------A--------LV--------------------TQ----------RA------EDSYFHQ--------NR-DFF------------------
GNS-S------------------QLL-VGTGTLRRRLMEVLESSMASS-LH---
XP_011389557.1 ------------LTLPS--------IVVI---
GS----Q----------------SSGKSSVLE-----A--IV-G-H-EFLPKG--------------NN-MVTRRPI--------------
-ELT--------------L-I-HTTTQ-----------------------PGR-PTKDT---
--VVE-------------Y--------------------------AEF----------------
--PGLGLGRITDFS-----------HVQKTL------YDLN-
MA-----VP-----------------------AS-------------------E-CVSDE-PIELRIHSPH--------------------------
--------------------------------VPDLTLIDLPGYVQ--IA-------------------S-------MD-------------Q------
--PD--E--LREKIQ--------KLCH-KYI---QE------------PN-IILAVCAADV--D-LANSP-ALRASRQVD----
--PLGLRTIGVVTKMDLVP--------PE------V-------G---------------------------AGI--LS---NN---------K-
YP----------------------L---ALGYVGVVCKNNLGVFQS-----------------------------------SKGHDRA-TGE-
-GRM-----------S--------SL-------------------VM----------KQ------ESDYFST--------NR-EHFCAPT---
----------RGRN-A------GV----------QPM-TGTDTLRRRLMSVLEESMGSS-LH---
XP_752563.1 ------------LTLPS--------IVVI---GS----Q-
--------------SSGKSSVLE-----A--IV-G-H-EFLPKG--------------TN-MVTRRPI---------------ELT-------

-------L-V-NTP-----------------------------NAQS--
--------------------------------E-------------Y--------------------------GEF--------------------------------
---------------------------------------PALGLGKITDFS-----------QIQRTL------TDLN-LA-----VP---------
---------------ER-------------------D-CVSDD-PIKLTIYSPN---
-----------VPDLSLIDLPGYIQ--VA-------------------G-------KD-------------Q--------PP--E--LKQKIA-
-------DLCD-KYI---QP------------PN-VILAISAADV--D-LANST-ALRASRRVD------
PRGERTIGVITKMDLVD--------PE------R-------G---------------------------FSI--LS---DQ---------K-
YP----------------------L---RLGYVGVVSR----VPQT-------------------------------TAL--FS-RGS--GNI-
----------T--------SA-------------------IL----------KN------ENAYFSA--------HP-SEF------------------
GPQ-S------------------GVS-VGVSTLRGKLMHVLEQTMAAS-LA---
KXN72852.1 ------------LNLPS--------IVVV---GS----
Q----------------SSGKSSVLE-----A--IV-G-K-EFLPKG--------------DN-MVTRRPI---------------ELT---
-----------L-I-HSP------------------------------NLTQ---
-----------------------------------E-------------F--------------------------GVF-----------------------------
---PQLGPSKITDFN-----------LITSQL------TELN-LQ-----VS------
------------------DK-------------------E-CISEE-PVQLHIYSPS---
--------------VPDLTLIDLPGYIQ--IH-------------------T-------KD-------------Q--------PS--D--
LKPKIR--------ALCR-KYL---AP------------PN-LILAVCPADV--D-LANSE-ALLESRRAD------
PSGQRTLGVVTKLDLVD--------PA------L-------G---------------------------ASI--LL---NH---------D-
YP----------------------L---NLGYVGVICR---KNK-PSA--VSF----
-------S--------RD-------------------PL----------Q---------------------HP-NYQ-SP---------------------
----------------DIR-VGIQTLQSTLIRVLEQRMLKS-LH---
NP_014854.2 ------------LTLPS--------IVVI---GS----Q-
---------------SSGKSSVLE-----S--IV-G-R-EFLPKG--------------SN-MVTRRPI---------------ELT-------
-------L-V-NTP-----------------------------NSNN---
---------------------------------V-------------T--------------------------ADF------------------------------
---------------------------------------PSMRLYNIKDFK-----------EVKRML------MELN-MA-----VP-----
-------------------TS-------------------E-AVSEE-PIQLTIKSSR---
-------------VPDLSLVDLPGYIQ--VE-------------------A-------AD-------------Q--------PI--E--
LKTKIR--------DLCE-KYL---TA------------PN-IILAISAADV--D-LANSS-ALKASKAAD------
PKGLRTIGVITKLDLVD--------PE------K-------A---------------------------RSI--LN---NK---------K-YP-
----------------------L---SMGYVGVITK----TPSSINR-----KHLG------LFGEAPSSSLSGIFSKG-QHG-
QSS--GEE-----------N--------TN-------------------GL----------KQIVSHQFEKAYFKE--------NK-KYF-
-----------------------------T----------NCQ-VSTKKLREKLIKILEISMSNA-LE---
PSC76263.1 RLLGGIESG---IDLPK--------IAVV---
GD----Q----------------SSGKSSVIE-----S--IF-G-I-SL-PRG--------------SG-IVTRTPI---------------
QVE--------------H---RYT-----------------------------EG---
---Q-------AY-------------------------AEL----------S--------
-------YKT-S-P--------------DD----------------------EEWTKRVVEDLD-----------GIEQAV------EEAT-
DA---V-TG-----------------------TT-------------------K-G--
---------------------------LLDLPGIMR--IA------------------V-------DD-------------Q--------PK--N--
IEEIVE--------NQIR-RHI---EG------------DNVVILCVLHGTS--D-PSTAS-AIKLAQEYD------
EDGDQTMGVVTKPDRCE-------KAQ------V-----EDL---------------------------IGS--VL---GAGS-----
--S-IK----------------------L---KLGFIPVRNR---T-TSE--LTD----

-------G--------TS-------------------LD----------QVREN---EAAFFKC--------HP-LL--------------------
S-Q------L--------A--KDR-RGIPALVDQLAHVQMQRVHKA-LPELK
PRW56740.1 ---SGMPSAKRSKDMYR--------VVVV--
GD----Q----------------SSGKSSLIE-----S--MF-S-F-EL-PRG-------------QD-IVTRTPI--------------
QVK-------------H---RFE----------------------------KG---
---P-------PR-------------------------AEI---------S--------
------FR--K-P-------------GE----------------------SDLTVVKLDSLD-----------EIEEAV------IQAT-DI--
-V-AG----------------------EG------------------K-DIVDS-TIFLRVFSDA------------------------------
--------------------------LPDLTLTDLPGITR--NA------------------T-------QG-------------Q--------PE-
-N--IEEIVT--------SVIE-NHI---KG------------EMVVILCVVPANV--D-FSTAG-GIKLARRHD------
ADGVRTMGVVTKIDLTE-------SGQ------------EM---------------------------LDR--LS---GAGK-------
N-LK----------------------L---KLGFIPVRNR--T-AQE--LKD---
--------G--------TP-------------------LE----------KVCKA---EGDFFSS--------HP-QL-------------------
-S-K------V--------N--PDR-RGISALVTELARLQMQRVTKS-LPGVK
XP_005849062.1 ------------LQIPK--------IAVV---
GD----Q----------------SSGKSSLIE-----S--IF-G-I-SL-PRG-------------ED-IVTRTPI--------------
QVE-------------H---RYS----------------------------DG--
---E-------AY-------------------------AEL---------S--------
------FRS-S-P-------------DS----------------------EELIKKRIADLS-----------TIDDEV------REAT-RR--
-V-AG----------------------SG------------------K-GVVDS-PIYLRVYTNK-----------------------------
---------------------------LPDLTVLDLPGITR--NA------------------V-------EG--------------Q--------
PE--D--IEEIIN--------TMIE-SHI---AG------------ETTVVLAIVQANV--D-FSTAA-AIKLAKKFD----------
----
---------PNRDR--T-M--

GHP04420.1 ---------QDAIAIPQ--------IAVM--GD---
-Q----------------SSGKSSVLE-----A--IS-G-V-PF-PRG-------------KG-LVTKCAT--------------QVI---
-----------M---RTA------------------------------PK---
-------------------------------GSP-------------WS-------------------------AVT---------S--------------
VRW-A-DGTT--------EHDQQ----------------------PEEAG-VIASPE-----------EVAGVI------ERLT-QV-
--L-LK----------------------KS--------------GH-QK-SFSEH-SIIIKLSSPE-----------------------------
----------------------------HPNLSMVDLPGLVR--TV------------------T-------ED-------------Q--------
DD--R--DIETVS--------ELIT-RFM---KQ------------ERTIILGVIPVNA--D-IATSE-VLQRAKHYD------
PSGMRTLAVLTEPDLVD-------PGS------E-----NEM---------------------------IEV--LM---NR---------
R-VN----------------------L---MLGFCMVKLR---G-QKE--
LDE-----------CEGKDVNSTEL-------------------TR----------RAREA---EEKFFRE--------NE-AL-------
--------------A-R------LG-------DEVDGQ-LGIPNLITRLSDTLSNRIREQ-FPTIK
OSX73843.1 ------------LPIPQ--------LSVM---GD----
Q----------------SSGKSSVLE-----F--LS-G-V-PF-PRG--------------TG-LVTRCAT--------------QLT----
----------M---KRV------------------------------Q--
-------------------------------GKS-------------FC-------------------------GKV---------E--------------
VLW-G-E--------------PH----------------------QPAASGPIYSKV-----------EVAAKI------EQLT-AV---L-
IG----------------------D-------------------G-EFSE-ATIVVKLEAAD-----------------------------------

-----------------------LPDLTIIDLPGLVR--TT-------------------T-------AG--------------Q--------SK--S--
IIAQVD--------GLVQ-RYL---ES------------ERTIVLCVVPANV--D-IATSD-ILERAHKVD------
PMGDRTVGVLTKLDLVG-------RGS------E-----DEV---------------------------VQV--LE---NV---------
R-KP----------------------L---QLGYVGIKCR--S-QAG--LKD--
---------N--------QS--------------------LE----------DARTD---ETTFFAT--------HP-VF--------------------
-K-R------L--------P--ADL-VGVETLTTKLTRVLVSRIREA-VPAM-
OSX70108.1 ------------MPIPQ--------LSVM---GD----
Q----------------SSGKSSVLE-----F--LS-G-V-PF-PRG--------------TG-LVTRCAT---------------QLS---
-----------M---KRV-----------------------------L---
---------------------------------GQS--------------FR--------------------------GSA----------K---------------
VRW-G-E--------------AH-----------------------QPDASGPIRSKA-----------EVADKI------EQLT-AA---
L-VG-------------------------D--------------------G-EFSDGGTIVVELEAAD--------------------------------
----------------------------LPDLTIIDLPGLVR--TT-------------------T-------AG--------------Q--------SK--
S--IIAQVD--------GLVQ-RYL---ES------------ERTIVLCVVPANV--D-IATSD-ILERAHKVD------
PMGDRTVGVLTKLDLVG-------RGS------E-----DEV---------------------------VQV--LE---NV---------
R-KP----------------------L---QLGYVGIKCR--S-QAG--LKD--
---------N--------QS--------------------LE----------DARHE---ESVFFRT--------HP-VF--------------------
--R-Q------L--------S--PDV-HGVETLSKKLTRVLVSRIRAA-TPTM-
KAJ1442373.1 --------------MPQ--------ICVM--
GD----Q----------------SSGKSSVLE-----A--LS-G-I-PF-PRG--------------AG-LVTRCPL---------------
RMV--------------M---RRA-----------------------------RE--
--GET--------------WS--------------------------AEV----------S-----
----------TTV-T-P--ESVQKAKDVV-----------HLSQLM------NRAM-
HT---L-CA-----------------------DD-------------------V-SFSTE-SVVINLVSPD---------------------------
---------------------------------ACDLTVVDLPGIIR--TV-------------------T------VG--------------Q-------
-DV--Q--AIEQVN--------RLIK-SCL---AD------------ERTVILAVIPANQ--D-IATVD-ILERAEGVD------
PTGERTIGVLTKTDLIG-------QGG------E-----DEI---------------------------IEV--VN---NR---------R-
KP----------------------L---ALGYTMVKNR--S-QKD--IKD-----
------N--------IS--------------------SA----------KAREN---EENFFST--------HP-VF---------------------
R-N------C--------N--ETL-YGADQLSKKLTDVLVA-----------
CAH0370685.1 -----------DLPLPQ--------IAVM--
-GD----Q----------------SAGKSSVLE-----A--IS-G-V-PF-PRG--------------TG-LVTRCAT---------------
QLI--------------M---SRA-----------------------------PP--
--GAA--------------WT-------------------------ASA----------C-------
--------VE-------------------PN----------------------DGAAPIKLEKAE-----------DVADVI------EKLT-
AK---L-CE-----------------------RE------------------GG-AFSTTAAVVIKLRSPD-------------------------
----------------------------------VPDLTLLDLPGIVR--TA-------------------V-------AG--------------Q-----
---SQ--S--VIGDVN--------SLIE-TYL---KQ------------ERTVVLAVIPANQ--D-VATID-ILQKAKGAD----
--PEGARTIAVLTKPDLID-------QGA------E-----KEV---------------------------LET--LL---NK---------
R-AP----------------------L---KLGYAMVRCR--N-QRE--NEA-
----------Q--------IS--------------------MT----------TARER---EKAFFDA--------HA-FW------------------
---G-S------DALHVPREQR--YTL-LGVDALTGRLSSLLVT-----------
KAH8053135.1 ---RARETPPVCRPLPQ--------IAVM-------------------------------------
-------GD----Q----------------SAGKSSVLE-----M--IS-G-V-PF-PRG--------------SG-LVTRCAT--------

-------QLI-------------M---KRT----------------------------AP---
---GSA-------------WA-------------------------ATA----------
S--------------VSW-S-R-------------PQ----------------------PAESG-PVADPA-----------ALSVAI-----
-ERLT-EA---L-CA----------------------VT-----------------TN-GFSTE-TIVINLSAPD-------------------
-------------------------------------VPDLTLLDLPGIVR--TA------------------V-------AG-------------
Q--------NA--D--VVGDVN--------GLIT-SYL---EQ-----------ERTIVLAVMPANQ--D-IATID-
ILERAAVAD------PGGMRTLAVLTKPDLVD-------DGA------E-----DEV---------------------------KEC-
-LM---NR---------R-KP----------------------L---RLGFAMVKCR---
----T-QRE--LDG-----------R--------IS-------------------LS----------KALVV---EREFFEA--------HP-
FW--------------------S-A------AV---HADER--KKL-MGVQALTGRLSSLLVERIKVA-LPEI-

KAJ8603921.1 -----------DLPLPQ--------IAVM--
-GD----Q----------------SSGKSSVLE-----M--IS-G-V-PF-PRG-------------SG-LVTRCAT-------------
-QLI-------------M---KRT----------------------------PP---
--GSE-------------WT-------------------------ARA----------S------
---------VSW-Q-G-------------PQ----------------------PKAAG-NVKDQA-----------SLCAAI------
EQLT-QV---L-CS----------------------ST-----------------MN-GFSRD-SIVIQLSSPD-----------------
---VPDLTLLDLPGIVR--TA------------------V-------SG-------------
Q--------SA--S--VVEDVN--------GLIE-AYL---SQ-----------ERTIVLAVMPANQ--D-IATID-
ILERAARAD------PAGERTLAVLTKPDLVD-------AGA------E-----EEV---------------------------KAV--
LA---NK---------R-KP----------------------L---RLGFAMVKCR--
--S-QRE--LDA-----------K--------ST-------------------LA----------DALRS---EADFFRR--------HP-
HW--------------------S-N------E--------D--PKK-LGVRALTMRLSTLLVD-----------

KAJ1460259.1 ------------LPLPQ--------IAVM--
GD----Q----------------SAGKSSVLE-----L--IS-G-V-PF-PRG-------------SG-LVTRCAT--------------
QLV-------------M---KRT----------------------------AP---
--GTV-------------WS-------------------------ATA----------S-------
-------VSW-P-G-------------PQ----------------------PPAAG-IVKDRE-----------SLGRAI------EQLT-
QV---L-CS----------------------GS-----------------SN-SFSTH-SIVIELRSPD-------------------------
---------------------------------VPDLTLLDLPGIVR--TN------------------V-------AG-------------Q-----
---DA--S--VVKDVN--------TLID-SYL---RQ-----------ERTIVLAVMPANQ--D-VATID-ILERAAAAD---
---PAGQRTLAVLTKPDLVD-------EGA------E-----DEV---------------------------ASV--LS---NR-------
--R-KP----------------------L---RLGYAMVRCR--T-QRQ--
LDS-----------N--------IS-------------------LT---------DALKA---EAAFFES--------HA-FW------------
--------G-K------AQ---GQEWQ--PQQ-LGVVALTRRLSSLLVDRIKVA-LPSI-

KAG5178451.1 ------------TPLPQ--------IAVM--
GD----Q----------------SSGKSSVLE-----A--LS-G-I-SF-PRG-------------SG-LVTRCPV--------------
QLV-------------M---KRA----------------------------RA---
--GEA-------------WR-------------------------ASA----------R-----
----------VAW-S-NSA-----------RAQ----------------------PPAAG-AVASPE-----------ALADVI------
AQLM-AA---A-CD-----------------------AT------------------AN-GFSTD-SLVVEVRAPE----------------
--CPDLTLVDLPGIVR--TA------------------V-------AG-----------
---Q--------SD--A--VIPEVS--------ALID-AYL---RQ-----------ERTIILAIVPANQ--D-VATVD-
ILERAARVD------PSGERTIGVLTKPDLIG-------PGN------E-----DEV---------------------------IAV--
LS---NR---------R-KP----------------------L---RLGYVAVRCR---

--T-QRE--LAA-----------G--------VT-------------------LR----------AAHAV---ERAFFAG--------HP-AF-
--------------------A-A------L--------D--PAL-LGIESLTRRCVGVLTTRIRAA-LPFM-
CBN76986.1 ------------LSLPQ--------IAVM---GD----
Q----------------SCGKSSVLE-----A--LS-G-V-QF-PRG--------------SG-LVTRCPV---------------QLI--
------------M---KRT-----------------------------KP--
-----------------------------------GDG--------------WH-------------------------GKT----------S-------------
--VEW-K-RG-------------PQ----------------------PPAAG-HVASPE-----------ALVGVI------EELM-NA-
--V-CE------------------------GQ------------------KN-GFSSD-FIAIEIKSPD-------------------------------
----------------------------CPDLTLIDLPGIVR--TA-------------------V-------SG--------------Q--------
SQ--G--VITEVN--------GLIE-NYL---RS------------ERTIILAIVPANQ--D-VATVD-ILERAKKVD------
PSGKRTIGVLTKPDLIN-------PGG------E-----AEV---------------------------LEV--LS---NN---------R-
KP-----------------------L---KLGYVMVKNR--N-QLQ--LRE----
-------G--------VS--------------------LK----------EAHDA---EMDFFKS--------HA-VF--------------------
-K-T------V--------D--STC-LGVESLTKRLVSLLTVRIKDA-LPNM-
KAG5185531.1 ------------IALPQ--------IAVM--
GD----Q----------------SSGKSSVLE-----A--IS-G-V-PF-PRG--------------GG-LVTRCAT---------------
QLI--------------M---KSA-----------------------------PE--
--GAE--------------WA-------------------------ARA----------S------
---------VTG-P-R--------------KN-----------------------LKGCGPVQGGPG-----------ALTRVI------
ADLQ-NA---L-TS------------------------GN------------------AS-AFSSE-SIVIEVRAPG------------------
---LPDLTLIDLPGIVR--TA-------------------T-------SG--------------
Q--------SA--G--VMAEVN--------TMVE-RYL---IQ------------ERTIILAVVPANQ--D-VATVD-
ILERAKRVD------PEGDRTIGVLTKPDLIG-------PGN------E-----DEV---------------------------VAV--
LR---NI---------R-KP-----------------------L---KLGYVMLKCR---
-S-QAD--INA-----------G--------MD--------------------NK----------AALAA---EHKFFRE--------HP-
AF---------------------S-G------L--------P--GTI-FGVQCLTGRLTDLLVKRIGAA-LPAI-
XP_009038401.1 ------------LPLPQ--------IAVM---
GD----Q----------------SSGKSSVLE-----A--LS-G-V-PF-PRG--------------TG-LVTRCPC---------------
-QLT--------------M---KRT------------------------------AD--
---GAP--------------RG-------------------------GRV----------P------
----------RR-P-G--NERPSRAPS-----------ELTAAI------ERVT-SA-
--L-TK------------------------GS------------------AS-GFSTD-SIVVTVNAPS-------------------------------
----------------------------VPDLTIIDLPGIVR--TA-------------------T-------QG--------------Q--------
DP--R--VIADVN--------SMVE-FYL---KQ------------ERTIVLAIVPSNQ--D-VATVD-ILERALTVD------
PTGERTIGVLTKPDLIG-------EGA------E-----DEV---------------------------VAV--LK---NE---------R-
KP-----------------------L---KLGYIMVKNR--S-AMQ--LKQ----
-------GA------INS-------------------PA----------ANERA---EREFFVQ--------H-------------------------
----
KAJ1460532.1 ------------LALPQ--------IAVM---
GD----Q----------------SSGKSSVLE-----A--LS-G-V-PF-PRG--------------TG-LVTRCPC---------------
-QLT--------------M---KHT------------------------------AP--
---GTP-------------WR-------------------------GEI----------G------
---------VMR-S-G-------------VF----------------------SKIGELAS--PA-----------ELTAAI------ERVT-
TS---L-TE------------------------KS------------------RS-GFSRD-SIVVSVSAPS--------------------------

--------------------------------VPDLTIIDLPGIVR--TV------------------T-------AG-------------Q--------
DS--N--VITEVN--------DMVE-SYL---MQ------------DRTIVLAIVPANQ--D-VATVD-ILERALRVD-----
-PTGERTIGVLTKPDLVG-------EGA------E-----DEV---------------------------LAV--LR---NE---------
R-KP----------------------L---KLGYVMVKNR---S-AKE--LKS--
---------GE------KTG-------------------LR----------EQAEA---ERRFFRT--------HA-AY-----------------
---S-A------V--------D--ASL-LGISNLTGKLTKLLTRRIEAV-LPEL-
CBN78192.1 ------------ISVPQ--------ICVM---GD----
Q----------------SSGKSSVLE-----A--LA-G-V-PF-PRG-------------SG-LVTRCPI---------------RLS---
-----------M---KRS-----------------------------AT---
--------------------------------GSR-------------WS-------------------------AVA---------H-------------
-ASN-E-P--SKSHSASTPG-----------QLTKLL------ERLT-DG---L-
TS-----------------------TS------------------S-NFSTE-TINVRLSSPD------------------------------------
----------------------VPDLTVVDLPGIIR--TS------------------T-------AG-------------Q--------DP--A--
VIAQVN--------NLIE-SFL---EQ------------ARTIILCVIPANQ--D-IATVD-ILERAQKVD------
PRGERTIGVLTKPDLIG-------PGN------E-----DEV---------------------------LAV--LH---NV---------R-
KP----------------------L---QLGYVMVKNR---S-QAQ--LKA---
--------G--------LT-------------------GT----------EAREA---EEAFFRQ--------HS-HF-------------------
-K-G------C--------D--PKL-FGVANLTTRLTQLLVTRIQHE-LVPM-
KAG5184845.1 ------------ISVPQ--------ICVM---
GD----Q----------------SSGKSSVLE-----S--LS-G-I-PF-PRG-------------SG-LVTRCPI--------------
RLS-------------M---KRT-----------------------------AA---
--GSA-------------WI-------------------------AHA---------H-------
--------TSS-S-P--GDVKRAETPA-----------HLTALM------EALT-DT-
--L-T---S-NFSTE-SINVKVTAPG---------------------------------
-------------------------VPDLTVVDLPGIIR--TS------------------T-------AG-------------Q--------DP--
R--MIQQVN--------ALID-SFL---MQ------------ERTIILCVIPANQ--D-IATVD-ILERALKVD------
PSGERTIGVLTKPDLIG-------PGN------E-----DEV---------------------------MAV--LL---NV---------
R-KP----------------------L---QLGYTMLRNR---T-QKE--LAA--
---------G--------AS-------------------AA----------EAKRL---EQEFFKA--------HP-HF------------------
--K-S------C--------D--PKL-FGIDNLTSRLTQLLVTRIQAE-LVPM-
XP_009039543.1 ------------LSVPQ--------ICVM--
-GD----Q----------------SCGKSSVLE-----A--LS-G-V-PF-PRG-------------SG-LVTRCPV------------
--RLI-------------M---RRS--
--DGD-------------WS-------------------------ATA---------S------
---------TTL-S--SHTVKANSPA-----------ELTAII------SRLT-DT---
L-TK-----------------------NS------------------H-GFSTE-SIVVRLGSSE---------------------------------
-------------------------SPDLTVVDLPGIVR--TA------------------T-------SG-------------Q--------DP--
K--VIYEVN--------ELID-TYL---RQ------------ERTIILAVIPSNQ--D-IATID-ILERAQTVD------
PGGERTIGVLTKPDLIG-------PGN------E-----EEV---------------------------MAV--LN---NL---------
R-KP----------------------L---RLGYIMVKNR---S-QAQ--VKQ--
---------G--------IS-------------------HK----------SALDN---EELFFQN--------HK-AF------------------
--S-C------L--------D--KRF-WGIKNLTSALTNLLVVRIQEQ-LAPM-
KAJ8614481.1 -----------TLAVPQ--------ICVM--
-GD----Q----------------SSGKSSVLE-----A--LS-G-V-PF-PRG-------------SG-LVTRCPC------------

--RMV-------------M---RRA----------------------------AP--
---DAP-------------WA------------------------AVA---------S---
-----------TTT-S-G-------------------------------------SSSITAESPE----------ELTSII------SRLT-ES-
--L-TR-----------------------NT------------------H-GFSTE-SIIVRLTSPL--------------------------------
-------------------------SPDLTVVDLPGIVR--TA-----------------T------VG-------------Q--------NV--
A--VIEEVN--------SLID-DYL---KQ------------ERTIILAVIPANQ--D-IATVD-ILERAQHVD------
PDGERTLGVLTKPDLIG-------PGN------E-----EEV--------------------------VAV--LS---NV--------
R-KP---------------------L---KLGYVMVKNR---S-QAQ--IKQ--
---------G--------TS------------------HE---------AARAE---ELRFFDS--------HP-VF-----------------
--S-R------L--------D--KRL-LGISNLTTSLTKLLVR-----------
XP_009039855.1 ------------LSVPQ--------ICVM--
-GD----Q----------------SSGKSSVLE-----A--LS-G-V-PF-PRG-------------SG-LVTRCPV------------
--RLV-------------M---RRA----------------------------AA--
---GMQ-------------WS------------------------ALV---------S---
-----------TTS-S--AQTVAAETPT----------ELAELI------SRLT-
ET---L-TR-----------------------NT------------------Q-GFSSE-AIIVRLSSPE--------------------------
------------------------------SPELTVVDLPGIVR--TA-----------------T------VG-------------Q--------
GL--Q--VIAEVD--------KLID-EYL---RQ------------DRTIILAVIPANQ--D-IATVD-ILERARLVD------
PTGERTLGVLTKPDLIG-------PGN------E-----EEV--------------------------AAV--LK---NI---------R-
KP---------------------L---KLGYVMVKNR---S-QAQ--IRN----
-------G--------TS------------------PE---------AAKED---EEAFFVS--------HP-IF--------------------
S-C------L--------D--QRL-LGIKNLTYALTKILVSRIKEE-LAPM-
KAJ1428896.1 ------------LPIPQ--------IVVF--
GD----Q----------------SSGKSSLLE-----S--IS-G-I-PF-PKG-------------TG-LVTRCPT--------------
RIS-------------M---SNC----------------------------GP--
---TEP-------------WT------------------------AHV---------K-------
--------LSP-N-IP-------------ES---------------------EAFNKRPVKDVE----------DLSKRL------AEAA-
AI---I-TP-----------------------HS-----------------NS-EFSSE-VIMVHVQSPN--------------------------
-------------------------------SPNLSLIDLPGIIR--TT-----------------T-------AG--------------Q--------
DR--S--VIASVN--------ELVE-SYL---RQ------------PETVILAVIPSNQ--D-IATID-ILERANEYD------
PAGLRTIGVLTKPDLID-------RGA------E-----DEV--------------------------LKI--VQ---NV---------R-
KP---------------------L---KLGYVMCKNR---S-QAE--LKS----
-------N--------VS------------------LA---------QSQKN---EDSYFLS--------HE-VW------------------
--Q-K------I--------V--PSS-RGIKPLCERLTSIIVSRALDR-GPFL-
KAG5183739.1 ------------VAIPQ--------IAVV--
GD----Q----------------SSGKSSVLE-----A--IS-G-I-PF-PRG-------------SG-LVTRCAT--------------
ELR-------------M---QSA--
---ER-------------FL-------------------------ARA---------Y--------
------TTA-A-R--DAVATLTTVA----------AVECAI------AQLT-EE---
L-CS-----------------------E------------------Q-GFSAE-SIVIQLAAPN---------------------------------
------------------------IPNLTVIDLPGIVR--TT-----------------T-------AG-------------Q--------SR--
S--VMQQVD--------GLLR-SYM---KQ------------PRTIMLVLVPSNQ--D-VATVG-GLELAAEHD------
PEGVRTMGVLTKPDLIN-------PGA------E-----REA--------------------------VAV--LL---NE--------T-
KP---------------------L---KLGYVMVKNR---S-HRD--IAN----

-------D--------MS-------------------LA---------NAHAA---EVAYFDE--------HC-LF-------------------
-G-A------L--------D--RAL-FTVANLKQRLSALLVARIRAE-LPRM-
KAK3283006.1 ----------QDIPIPQ--------IAVM--
-GD----Q----------------SSGKSSVLE-----A--LS-G-V-HF-PRG--------------TG-LVTKCAV--------------
-EVR--------------M---KRL-----------------------------QP--
--NED--------------WN-------------------------ASV----------S----
-----------LSW-D-R--------------PQ-----------------------PSEAG-VATTPN-----------EVGEKI------
SKLT-EV---L-LR------------------------AR---------------GN-RA-TFEPEHRIQVELKSPD------------------
---VSDLTIIDLPGIVR--TN-------------------V-------AG--------------
Q--------CK--K--VIAEVD--------ALLD-KYL---RQ------------ERTIILAVIPSTV--D-IATVD-
VIERAEKVD------PHGLRTIGVLTKADQIS-------SDD------E-----AER----------------------------VAV--
LR---GV---------R-KP-----------------------L---KLGYFMVKNR---
--T-QTE--LEA-----------G--------VT--------------------LA---------EARLA---EARYFSA--------HK-TF--
-------------------G-K------L--------H--PGL-FGSQNLAERLSDVLATRIRDD-LPLLV
KAJ1474099.1 ----------LEGGIPQ--------IAVM--
-GD----Q----------------SSGKSSVLE-----A--LT-G-V-EF-PRG--------------PG-LVTKCAT--------------
-EVR--------------M---RCC-----------------------------KN--
--GEP--------------AS--------------------------FKV----------S-----
----------LSW-S-K--------------AQ-----------------------PAEAG--FCSSE-----------EIGARI------ASLT-
ER---L-LS------------------------DRSE------------HGK-KA-SFEKEHAIVVEMVSPD--------------------
-------------------------------------VPDLTIIDLPGIVR--TA-------------------V-------AG--------------Q--
------NA--S--VVEDVR--------SLLD-RYL---KL------------NRTIILAVVPCNV--D-IATVD-IIELANNAD-
-----PLGQRTIGVLTKPDCIA-------QGE------E-----KDV----------------------------IET--LL---NK--------
-T-KP-----------------------F---KLGYIMMKNR--S-EQD--IKA--
---------G--------VS--------------------LR----------SPLEL---E---
----
KAJ1495567.1 ----------LEGGIPQ--------IAVM--
-GD----Q----------------SSGKSSVLE-----A--LS-G-V-QF-PRG--------------PG-LVTKCAT-------------
--EVR--------------M---RCC-----------------------------KD--
---GQP--------------AT--------------------------FKV----------S----
-----------LSW-S-K--------------PQ-----------------------PAEAG--FCSRE-----------EIGDKI------TSLT-
ER---L-LA------------------------DRTT------------RGE-SA-SFEKEHAIVVEMQALD--------------------
-------------------------------------VPDLTIIDLPGIVR--TA-------------------V-------EG--------------Q--
------NA--S--VIEDVR--------NLLN-RYL---QQ------------DRTVILAVVPCNV--D-IATVD-
IIEMARKAD------PQGQRTIGVLTKPDLID-------EGA------E-----KDV----------------------------VET--
LK---NA---------K-KP-----------------------L---ELGYIMVKNR---
-S-PKQ--IED-----------G--------VS--------------------LS---------DARRK---EREYFDS--------HP-VF--
-------------------G-K------E--------D--AAL-FGVDRLRMRLTEILVERIKFS-LPEL-
KAJ1474882.1 ----------LVGGIPQ--------IAVM--
-GD----Q----------------SSGKSSVLE-----A--LS-G-V-QF-PRG--------------PG-LVTKCAT-------------
--EVR--------------M---RSC-----------------------------KH--
---GQA--------------AS--------------------------FKV----------S---
------------LSW-S-K--------------AQ-----------------------PEEAG--PVLRE-----------EIGERI------
ARLT-TR---L-LA------------------------ER---------------GE-KA-SFEKEHAIVVDMRAPD----------------

---VPDLTIIDLPGIVR--TA------------------V-------DG-------------
-Q--------ES--S--VKDDVW--------SLLD-RYL---KQ-----------ERTIILAVVPSNV--D-IATVD-
IIDKARQAD------PQGQRTIGVLTKPDMIA-------EGE------E-----LDV----------------------------VDT--
LR---NR---------K-AP----------------------L---ELGYIMVKNR--
-S-PKE--IEA-----------G--------VS--------------------LS----------EARRT---EAAYFDA--------HP-VF---
------------------G-R------E--------DLPRAL-FGVERLRERLTDILVERIK--------
KAJ1487321.1 ----------LQGGIPQ--------IAVM---
--GD----Q----------------SSGKSSVLE-----A--LS-G-V-QF-PRG--------------TG-LVTKCAT-------------
--EVR--------------M---RSC------------------------------KN---
--SEL--------------SS-------------------------YRV----------S-----
----------LSW-S-K--------------PQ-----------------------PEEAG-TVVTRE-----------DIEGKI------ALLT-
EL---L-LA------------------------DR---------------GG-AS-ALNQ----VVEMVAPD------------------------
-----------------------------------IPDLTIIDLPGMVR--TA------------------V-------DG--------------Q----
----DE--S--VIQDVK--------SLLD-RYL---KQ-----------ERTVILAVVPCNV--D-IATVG-IINDARKAD---
---PQGQRTIGVLTKPDLIA-------RGE------E-----ESV---------------------------VET--LR---NV---------
K-AP----------------------L---ELGYIMVKNR---G-QDE--ING--
---------K--------VA--------------------L--
----------------------RFPTVLL------------
XP_005711749.1 -----------IAIPQ--------VAVI---
GD----Q----------------SSGKSSVLE-----A--IC-S-V-PL-PRG--------------AG-LTTRCAI---------------
ELR--------------L---SDV-----------------------------HP---
--DDLHLPPDTPRPERDMFWV--------------------------GSI--------
--F---------------TSV-D-S---GQVPIEKKD---------DLEQAI------
AAKA-KS---L-TN------------------------SR----------------H-AG-GFSNE-RVIVQIAASG-----------------
--SPNLTIIDLPGIIR--TK-----------------T-------FG--------------
Q--------NS--N--AIREVT--------NLIR-GYI---SQ------------ERTVMLVVVPATQ--D-VATIE-
ALEWAARSD------PSGERTIGVITKPDLID-------DGA------E-----EEV---------------------------GVV--
LS---NK---------R-KP----------------------L---KLGYVMVKNR---
--S-QRE--VEE-----------H--------VS--------------------VR----------EARKS---EAEFFAQ--------HR-
VF---------------------S-T------M--------S--KDL-FGVEKLVRKLTDVLVSRVYNA-LPGM-
KAI0564035.1------------IAIPQ--------VAVI---GD----Q-
---------------SSGKSSVLE-----A--IC-S-V-PL-PRG--------------AG-LTTRCAI---------------ELR--------
------L---SST-----------------------------HP--
----------------------------EDLALPPDSEPPEREEFWV--------------------------ASI----------S-----------
----TSL-N-P--KQVPLEGKA----------ELEDAI------AARA-AS---
L-VD------------------------TR----------------Q-HG-GFSKE-RVIVHIAATG-------------------------------
----------------------------SPNLTIIDLPGIIR--TK-----------------T-------FGDFMPFIPNPFGRDSQ---
-----DR--N--AIRQVS--------ELIQ-GYA---AQ------------ERTIMLVVVPATQ--D-VATIE-
ALEWAARYD------HAGRRTIGVITKPDLVD-------KGA------E-----KEV----------------------------ASV-
-LM---NR---------R-KP----------------------L---KLGYVMVKNR---
----S-QIE--VEE-----------Q--------VS-------------------VQ----------QARKN---EYDFFAT--------HH-
VF---------------------S-K------M--------S--RSY-FGVENLVKKVTRVLVARVHDE-VPKM-
PXF49978.1 ------------IAIPQ--------VAVI---GD----Q-
---------------SSGKSSVLE-----A--IC-S-V-PL-PRG--------------AG-LTTRCAI---------------ELR--------

------L---TNT----------------------------HP--
----------------------------EDAALPSDTEPPKREPFWL-------------------------ASI----------S------------
---TSL-N-P---KQVPLSGKA-----------ELEDAI------AAHA-AS---L-
TD---------------------------PR---------------Q-NS-GFSKE-RVIVHIAATG----------------------------------
--------------------------SPNLTIIDLPGIIR--TK-----------------T-------FGDFFPFIP-PFG-DSQ--------
DR--N--AIRQVS--------QLIQ-GYA---AQ------------ERTILLVVIPATQ--D-VATIE-ALEWAARYD------
PSGRRTIGVITKPDLID-------KGA------E-----REV---------------------------ASV--LN---NR---------R-
KP----------------------L---KLGYIMVKNR--S-QKE--VEE-----
------H--------VS--------------------VQ----------QAREN---EYEFFSN--------HP-VF--------------------
S-K------M--------S--RSY-FGVDNLVKKVTGVLVSRLHDE-LPKM-

XP_006815062.1 ------------VDLPA--------VVVI---
GD----Q----------------SVGKSSVLE-----A--IS-G-V-QL-PRG-------------NE-IVTRCPI---------------
ELR-------------L---KTL------------------------------DN--
--DE-------------WC-------------------------GKI----------L--------
-------YIN-Y-S--------------KE-------------------------QVNKYIDSPD-----------ELGAAI------RTAQ-
QD---I-TN------------------------SQ-------------------K-GISKT-SITVEIQSAH---------------------------
-------------------------------VPNLTLIDLPGIAR--VP-------------------Q-------EG-------------Q-------
-SR--N--IADETK--------DLIK-KYI---SK------------DDAIVLCVIPCNV--D-IATTE-AIKMAQEVD------
PTGSRTLGVLTKPDLVD-------KGS------E-----NVV---------------------------VRI--AE---NK---------V-
IN----------------------L---KKGYTIMKCR---S-QRN--LED-----
------A--------MS--------------------LE----------EAMDE---EERFFRE--------HK-HY--------------------
S-V------L-----------SGQ-AGSRLLAHRLTTELVEQILKS-V----

XP_035690836.1 ------------VSLPS--------VVVI---
GD----Q----------------SAGKSSTLE-----A--IS-G-V-QL-PRG-------------SG-IVTRCPL---------------
ELR-------------L---KKS------------------------------QKK--
--DAP-------------WK-------------------------GCI----------R------
---------YV----K--------------NK-----------------------KDVRFDVDEPG-----------NVGDAV------
KKAQ-ND---L-AG-----------------------TT-------------------N-GISDS-LITLDVESPD-------------------
---IPDLTLIDLPGIAR--IA-------------------A-------EG-------------
Q--------PT--D--IGQQIK--------DLIS-KYI---QK------------KDTIILAVVPCNV--D-IATTE-
ALQMAQEVD------ADGSRTLGVLTKPDLID-------PGT------E-----RGV---------------------------LQI-
-LN---NE---------K-YK----------------------L---RKGYTIIKCR---
G-QMD--IEK-----------G--------MS-------------------LE----------EAMDK---EQSYFKS--------HE-
HF---------------------K-S------VY-------K--EKK-AGVRTLAGRLSTELVGQIKNS-I----

XP_019617847.1 ------------VTLPS--------VVVI---
GD----Q----------------SAGKSSCLE-----A--IS-G-V-QL-PRG-------------SG-IVTRCPL---------------
ELR-------------L---KKS------------------------------PDP--
--ESG-------------WR-------------------------GYI----------H------
---------FE-------------------DK-----------------------GETRWELDSPE-----------DVGEAV------KKAQ-
NQ---L-AG------------------------ES-------------------L-CISPR-LITLDVESPD---------------------------
-------------------------------IPDLTLIDLPGIAR--VP-------------------V-------GG-------------Q-------
-PD--D--IGDQTK--------ALIR-EYI---QM------------DETIILAVVPCNV--D-IATTE-ALKMAKEVD------
PDGSRTLGVLTKPDLID-------RGT------E-----NMT---------------------------VDI--VN---NR---------
K-YA----------------------L---KKGYTIIKCR---G-QVD--IEN----

-------K--------VS-------------------LS---------DAMDK---EEMFFQK--------HE-HF-------------------
-K-I------LY-------E--EKK-TGTKTLAGKLTTELVEQIKKS-I----
XP_002608668.1 ------------VTLPS--------VVVI--
GD----Q----------------SAGKSSCLE-----A--MS-G-V-QL-PRG--------------SG-IVTRCPL-------------
--ELR--------------L---KKS-----------------------------QDP---
---ESP-------------WK------------------------GYI---------H-----
----------YHF-E-G--------------DR-----------------------DETGWKLTDPS-----------DVGEAV------
RKAQ-NN---L-AG------------------------DS-------------------H-GISPR-LITLDVESPD------------------
---IPDLTLIDLPGIAR--IA-------------------V-------DG-------------
Q--------PP--D--IGDQIK--------DLIK-EYI---QK------------DETIILAVVPCNV--D-IATTE-
ALQMAKDVD------PTGSRTLGVLTKPDLID-------RGT------E-----NTI---------------------------VDI--
VN---NQ---------K-YP----------------------L---KKGYTIIRCR---
G-QED--INE-----------N--------VT--------------------LS---------EAMEK---EERFFKT--------HE-HF--
-------------------K-L------PY-------H--EKK-TGTRTLAGKLTTELVEQIK--------
XP_003973512.2.2 ------------LALPA--------IAVI--
GD----Q----------------SSGKSSVLE-----A--LS-G-V-AL-PRG--------------SG-IVTRCPL---------------
ELK--------------M---KRR-----------------------------KV--
--GEP-------------WY------------------------GNI---------S-------
--------YL--DQEEVIEDPA-----------DVEKKI------QEAQ-NE--
-M-AG------------------------VG-------------------V-GISDD-LISLEIASPE--------------------------------
--------------------------VPDLTLIDLPGIAR--VA-------------------V-------KG-------------Q--------PE--
N--IGEQIK--------RLIR-KFI---TK------------QETISLVVVPCNV--D-IATTE-ALKMAQEVD------
PDGERTLGILTKPDLVD-------KGT------E-----ETV---------------------------VDI--IH---NE---------V-
IH----------------------L---KKGYMIVRCR---G-QKE--IID------
-----K--------VS--------------------LA---------EATET---ETAFFRD--------HA-HF---------------------Q-
T------LY-------D--DGQ-ATILKLAEKLTLELVNHIEKS-L----
NP_891987.2.2 ------------LALPA--------IAVI--
GD----Q----------------SSGKSSVLE-----A--LS-G-V-PL-PRG--------------SG-IVTRCPL---------------
ELK--------------M---IRT-----------------------------KD--
---------------------------------------QDK-------------WH------------------------GRI---------S-------
--------YK--TYEEDFDDPA-----------EVEKKI------RQAQ-DE--
-M-AG------------------------AG-------------------V-GISEE-LISLQITSAN--------------------------------
--------------------------VPDLTLIDLPGIAR--VA-------------------V-------KG-------------Q--------PE--
N--IGDQIK--------RLIR-KFV---TR------------QETINLVVVPCNV--D-IATTE-ALQMAQAED------
PDGERTLGILTKPDLVD-------KGT------E-----GTV---------------------------VDI--VH---NE---------V-
IH----------------------L---TKGYMIVRCR---G-QKE--IMD-----
------Q--------VT--------------------LN---------EATET---ESAFFKD--------HP-HF--------------------
S-K------LY-------E--EGF-ATIPKLAEKLTIELVHHIQKS-L----
XP_009304072.1 ------------LALPA--------IAVI--
GD----Q----------------SSGKSSVLE-----A--LS-G-V-PL-PRG--------------SG-IVTRCPL---------------
ELK--------------M---IRS-----------------------------KE--
---------------------------------------DEK-------------WH------------------------GRI---------S-------
-------YQ--NHEEDFDDPA-----------EVEKKI------REAQ-DE-
--M-AG------------------------AG-------------------V-GISEE-LISLQITSAN-------------------------------

---------------------------VPDLTLIDLPGIAR--VA------------------V-------KG-------------Q--------PE-
-N--IGDQIK--------RLIR-MFI---TK-----------QETINLVVVPCNV--D-IATTE-ALQMAQAED------
PEGERTLGILTKPDLVD-------KGT------E-----GTV---------------------------VDI--VH---NE--------V-
IH----------------------L---TKGYMIVRCR--G-QKE--IMD-----
------Q--------VT--------------------LN----------EATET---ESAFFKD--------HP-HF--------------------
R-K------LY-------E--EGF-ATIPKLAEKLTIELVHHIQRS-L----
AGU16245.1 --------------LPA--------IAVI---GD----Q--
-------------SSGKSSVLE-----A--LS-G-V-GL-PRG-------------SG-IVTRCPL---------------ELK-------
-------L---KKA-----------------------------KK--
----------------------------ETE-------------WK-------------------------ATI----------R--------------YE---
--DEYKELTSPS-----------EVEKEI------RTAQ-NA---M-AG-------
----------------SG------------------K-GISDK-LISLEIESDN--
-----------VPDLTLIDLPGIAR--VA------------------V-------QG-------------Q--------PY--D--IGEQIK-
-------KLIR-KFI---EK-----------EETINLVVVPCNV--D-IATTE-ALKMAQDVD------
QSGERTLGILTKPDLVD-------KGA------E-----QNI---------------------------VDV--VN---NM--------
V-IP----------------------L---KKGYMIVKCR---G-QQD--INE---
--------N--------LT--------------------LA----------EAT--

XP_007904885.1 ------------LSLPA--------IAVI---
GD----Q---------------SSGKSSVLE-----A--LS-G-V-SL-PRG-------------TG-IVTRCPL--------------
ELK-------------L---KKA-----------------------------KK---
---------------------------------------ANV-------------WK-------------------------GAI----------S--------
-------FR--EYSKEITNAS-----------EVEQEI------RKAQ-NS---
M-AG-----------------------KE--------------------GISHD-LISLKIESSN---------------------------------
-------------------------VPDLTLIDLPGIAR--VA------------------V-------GN-------------Q--------PL--
D--IGDQIK--------KMIR-SFI---NK----------QETINLVVVPCNV--D-IATTE-ALKMAQEVD------
PSGERTVGILTKPDLVD-------KGT------E-----STI---------------------------VDI--VQ---NL--------V-
VE----------------------L---KKGYMIVKCR--G-QKE--IND-----
------K--------LT-------------------LQ----------DAIAR---ENRYFEE--------HE-QF--------------------
R-T------LL-------D--EKK-ASIPHLAERLTNELVYHISKC-L----
XP_032888405.1 ------------LGLPA--------IAVI---
GD----Q---------------SSGKSSVLE-----A--LS-G-V-AF-PRG-------------SG-IVTRCPL--------------
ELK-------------L---KNV-----------------------------KK--
--ANV-------------WK-------------------------GKI----------S-------
--------YK--DYSNKLSSAA-----------EVEQAI------LKAQ-DS--
-I-AG-----------------------KG------------------V-GISHE-LISLEIESTN----------------------------------
------------------------VPDLTLIDLPGIAR--VA------------------V-------GN-------------Q--------PQ--
D--IGDQIK--------RLIR-LFI---QK----------QETVNLVVVPCNV--D-IATTE-ALKMAQEVD------
PTGDRTLGILTKPDLVD-------KGT------E-----KNV---------------------------VDI--VK---NL--------T-
VE----------------------L---EKGYMIVKCR--G-QND--INE----
-------N--------IS-------------------LV---------DAIAK---EKEFFED--------HE-QF--------------------
R-P------LL-------E--DGK-AGIPNLAVRLTKELVNHINKS-L----
XP_009815891.1 ------------LALPA--------IAVI---
GD----Q---------------SSGKSSVLE-----A--LS-G-I-AL-PRG-------------NG-IVTRCPL--------------

ELK-------------L---KRI-----------------------------PA--
---------------------------------------TQA-------------WK-------------------------GKI----------C--------
-------YR--NISSELQNAS-----------EVEKAI------REAQ-DI---
V-AG-----------------------TR-------------------G-AISGE-LISLEIWSPD----------------------------------
-------------------------VPDLTLIDLPGIAR--VA------------------V-------GN-------------Q--------PK--
D--IGEQIK--------MLLK-KII---GC------------KETLNLVVVPCNV--D-IATTE-ALKMAQEVD------
PSGERTLGILTKPDLVD-------RGT------E-----ESI---------------------------INI--IR---NL---------V-IP-
----------------------L---KKGYMIVKCR--G-QQD--IHN--------
---K--------LA-------------------LA----------AAIQQ---ERKFFET--------HE-HF---------------------S-I--
----LL-------E--EGK-ATVPHLAEKLTNELVRHIIKT-L----
XP_025933558.1 ------------LALPS--------IAVI---
GD----Q----------------SSGKSSVLE-----A--LS-G-I-AL-PRG--------------NG-IVTRCPL--------------
ELK-------------L---KKT-----------------------------PA--
---------------------------------------TQK-------------WK-------------------------GKI----------S---------
------YH--NTSEELKNPS-----------EVEKAI------RGAQ-DV---
V-AG-----------------------TK-------------------G-AISRE-LISLEVWSPT----------------------------------
-------------------------VPDLTLIDLPGIAR--VA------------------V-------GD-------------Q--------PE--
D--IGEQIK--------KLLK-NII---GN------------KETLNLVVVPCNV--D-IATTE-ALKMAQEVD------
PKGERTLGILTKPDLVD-------KGT------E-----ESI---------------------------VNI--IR---NL---------T-
VP----------------------L---KKGYMIVKCR--G-QQD--IHN----
-------N--------LT-------------------LA----------SAIQQ---EKEFFET--------HQ-HF---------------------
S-I------LL-------N--EGK-ATVPLLAEKLTXELVGHIIKT-L----
XP_015269256.1 ------------LALPA--------IAVI---
GD----Q----------------SSGKSSVLE-----A--LS-G-V-AL-PRG--------------NG-IVTRCPL--------------
-ALK-------------L---KKT-----------------------------RQ--
---GCG-------------WK-------------------------GKI----------S------
---------YR--DINEELNHPS-----------EVEKEI------RKAQ-IS--
-I-AG-----------------------EG-------------------V-GISHE-LITLEIRSSE---------------------------------
------------------------VPDLTLIDLPGIAR--VA------------------V-------GN-------------Q--------PQ--
D--IGHQIK--------RLIK-KII---AK------------DETINLVVVPCNV--D-IATTE-ALKMAQEVD------
PDGERTLGILTKPDLVD-------KGT------E-----EAV---------------------------VDI--VR---NL---------I-
IH----------------------L---KKGYMIVKCR--G-QQD--IQS-----
------N--------LD-------------------LA----------SAIQK---EKAFFED--------NR-HF---------------------
R-I------LL-------A--EKR-ATIPLLAEKLTSELVEHINKS-L----
XP_006156438.1 ------------LALPA--------IAVI---
GD----Q----------------SSGKSSVLE-----A--LS-G-V-AL-PRG--------------SG-IVTRCPL--------------
VLK-------------L---MKQ-----------------------------SQ--
---EPV-------------WR-------------------------GKI----------R-------
--------YR--NTEKKLGDPT-----------QVEAEI------CKAQ-NI--
-I-AG-----------------------SG-------------------V-GISHE-LITLEITSPE---------------------------------
------------------------VPDLTLIDLPGITR--VA------------------L-------GN-------------Q--------PQ--
D--ISLQIK--------ALIK-KYI---KR------------QQTINLVVVPCNV--D-IATTE-ALSMAQEVD------
PEGDRTLGILTKPDLVD-------KGS------E-----KSV---------------------------MNV--LQ---NL--------
T-FP----------------------L---KKGYMIVKCR--G-QQE--IMN--

---------N--------LS--------------------LA----------EATRK---ELMFFQS--------HP-HF-------------------
--R-V------FL-------E--EKK-ATVPHLAERLTAELIAHIRKS-L----
NP_001003133.1 ------------LALPA--------IAVI--
GD----Q----------------SSGKSSVLE-----A--LS-G-V-AL-PRG--------------SG-IVTRCPL---------------
VLK--------------L---KRD------------------------------PH--
--KA--------------WR--------------------------GRI----------S--------
-------YR--KTELQFQDPS-----------QVEKEI------RQAQ-NI---
I-AG------------------------QG-------------------L-GISHE-LISLEITSPE-----------------------------------
-------------------------VPDLTLIDLPGITR--VA-------------------V-------GN--------------Q--------PQ--
D--IGVQIK--------ALIK-NYI---QK------------QETINLVVVPCNV--D-IATTE-ALSMAQEVD------
PNGDRTIGVLTKPDLVD-------RGT------E-----KTV----------------------------VNV--AQ---NL---------T-
YH-----------------------L---QKGYMIVRCR--G-QEE--ITN----
-------Q--------LS--------------------LA----------EATEK---ERMFFQT--------HP-YF---------------------
R-A------LL-------E--EGK-ATVPCLAERLTKELILHINKS-L----
XP_002830747.1 ------------LALPA--------IAVI--
GD----Q----------------SSGKSSVLE-----A--LS-G-V-AL-PRG--------------SG-IVTRCPL---------------
VLK--------------L---KKQ------------------------------PC--
--EA--------------WA--------------------------GRI----------S--------
-------YR--NTELELQDPG-----------QVEKEI------HKAQ-NI--
-M-AG------------------------NG-------------------R-GISHE-LISLEITSPE---------------------------------
---------------------------VPDLTIIDLPGITR--VA-------------------V-------DN--------------Q--------PR--
D--IGLQIK--------ALIK-KYI---QR------------QQTINLVVVPCNV--D-IATTE-ALSMAHEVD------
PEGDRTIGILTKPDLMD-------KGT------E-----KSV----------------------------MNV--VR---NL---------T-
YP-----------------------L---KKGYMIVRCR--G-QQE--LTN----
-------R--------LS--------------------LA----------EATKK---EITFFQT--------HP-YF---------------------R-
V------LL-------E--EGS-ATVPRLAERLTSELIMHIQKS-L----
NP_002454.1 ------------LALPA--------IAVI--GD---
Q----------------SSGKSSVLE-----A--LS-G-V-AL-PRG--------------SG-IVTRCPL---------------VLK---
-----------L---KKQ------------------------------PC---
-----------------------------------EA--------------WA--------------------------GRI----------S---------------
YR--NTELELQDPG-----------QVEKEI------HKAQ-NV---M-
AG------------------------NG-------------------R-GISHE-LISLEITSPE-------------------------------------
-----------------------VPDLTIIDLPGITR--VA-------------------V-------DN--------------Q--------PR--D--
IGLQIK--------ALIK-KYI---QR------------QQTINLVVVPCNV--D-IATTE-ALSMAHEVD------
PEGDRTIGILTKPDLMD-------RGT------E-----KSV----------------------------MNV--VR---NL---------T-
YP-----------------------L---KKGYMIVKCR--G-QQE--ITN-----
------R--------LS--------------------LA----------EATKK---EITFFQT--------HP-YF---------------------R-
V------LL-------E--EGS-ATVPRLAERLTTELIMHIQKS-L----
XP_008569440.1 ------------LALPA--------IAVI--
GD----Q----------------SSGKSSVLE-----A--LS-G-V-AL-PRG--------------SG-IVTRCPL---------------
VLK--------------L---KKH------------------------------LQ--
---EDG--------------WK--------------------------GKI----------S-------
--------YR--HTELLLQDPS-----------QVEKEI------HKAQ-NT-
--I-AG------------------------NG-------------------V-GISHE-LISLEITSPE----------------------------------

--------------------------VPDLTLIDLPGITR--VA-------------------V-------GN-------------Q--------PQ--
D--IGQQVK--------ALIK-KYI---QR------------QQTINLVVVPCNV--D-IATTE-ALSMAQEVD------
PDGDRTIGILTKPDLVD-------KGT------E-----KGV---------------------------MNV--AR---NL---------T-
YH-----------------------L---KKGYMIVKCR--G-QQD--ITN----
-------K--------LS--------------------LA----------EATKK---EMAFFQT--------HP-YF--------------------
R-V------LL-------E--EGK-ATVPCVAEKLTAELIVHINKS-L----
XP_017508123.1 ------------LALPA--------IAVI--
GD----Q----------------SSGKSSVLE-----A--LS-G-V-AL-PRG--------------SG-IVTRCPL---------------
VLK-------------L---KKQ-----------------------------LH---
--EPA-------------WT-------------------------GRL----------S-------
--------YQ--TTELQLHNPS-----------QVEKEI------QKAQ-NA-
--I-AG-------------------------DG-------------------V-GISHE-LINLEITSPD---------------------------------
--------------------------VPDLTLIDLPGIAR--VA-------------------V-------GN-------------Q--------PQ-
-D--IGLQIK--------ALIK-KYI---QR------------QQTINLVVVPCNV--D-IATTE-ALSMAQEVD------
PDGDRTIGILTKPDLVD-------KGT------E-----RVI---------------------------VNV--VQ---NL---------T-
YH-----------------------L---KKGYMIVKCR--G-QQE--VTN----
-------K--------LS--------------------LA----------EATSK---EMTFFQT--------HP-YF--------------------
R-I------LL-------E--EGK-ATVPRLAEKLTTELISHINKS-L----
XP_005885748.1 ------------LALPA--------IAVI--
GD----Q----------------SSGKSSVLE-----A--LS-G-V-AL-PRG--------------SG-IVTRCPL---------------
VLK-------------L---KKQ-----------------------------LAG---
---ESL-------------WT-------------------------GKI----------S-------
--------YR--STELQLQDPS-----------QVEREI------YKAQ-NT--
-I-AG-------------------------NG-------------------V-GISHE-LINLEITSPE----------------------------------
-------------------------VPDLTLIDLPGIAR--VA-------------------V-------GN-------------Q--------PQ--
D--IGLQIK--------ALIK-KYI---QR------------QQTINLVVVPCNV--D-IATTE-ALSMAHEVD------
PDGDRTIGILTKPDLVD-------KGA------E-----KNV---------------------------VNV--AQ---NL---------
T-YR-----------------------L---KKGYMIVKCR--G-QQE--ITD---
--------K--------LS--------------------LA----------EATKK---EMMFFQT--------HP-YF--------------------
R-V------LL-------E--EGK-ATVPRLAERLTTELIWHINKS-L----
NP_776366.1 ------------LALPA--------IAVI--GD----
Q----------------SSGKSSVLE-----A--LS-G-V-AL-PRG--------------SG-IITRCPL---------------VLK----
----------L---TKR--
-----------------------------ECE-------------WT-------------------------GKI----------T---------------YR-
---NITQQLQNPS-----------EVEWEI------RRAQ-NI---I-AG-----
-------------------NG-------------------L-GISHE-LINLEITSPE---
--------------VPDLTLIDLPGITR--VA-------------------V-------EN-------------Q--------PQ--D--
IGLQIK--------ALIK-KYI---QR------------QETINLVVVPCNV--D-IATTE-ALSMAQEVD------
PDGDRTIGILTKPDLVD-------KGT------E-----KGV---------------------------LKV--MQ---NL---------T-
YH-----------------------L---KKGYMIVKCR--G-QQD--ITN----
-------K--------LS--------------------LA----------EATRK---ETMFFET--------HP-YF--------------------
R-I------LL-------D--EGK-ATVPLLAERLTTELIWHINKS-L----
XP_032211320.1 ------------LALPA--------IAVI--
GD----Q----------------SSGKSSVLE-----A--LS-G-V-AL-PRG--------------SG-IVTRCPL---------------

VLK-------------L---KRQ----------------------------PQ--
---ESA-------------WK------------------------GRV---------I-------
--------YG--TREVRLQDPS-----------QVEKEI------LKAQ-NT--
-L-AG------------------------DG------------------V-SISHE-LISVDIISPE---------------------------------
-------------------------VPDLTLIDLPGITR--VP------------------V-------GN-------------Q--------PQ--
D--IGLQIK--------ALIK-KYI---QR------------QETINLVVVPCNV--D-IATTE-ALSMAQEVD------
PRGDRTIGILTKPDLVD-------KGA------E-----PIV--------------------------MKV--AQ---NL---------T-
YH----------------------L---QKGYMMVRCR--G-QEE--ITN---
--------R--------LS-------------------LA---------EATRK---ETMFFQK--------HP-HF--------------------
R-A------LL-------Q--EGK-ATVPCLAERLTNELILHINKS-L----

XP_004675614.2.2 ------------LALPA--------IAVI--
GD----Q----------------SSGKSSVLE-----A--LS-G-V-AL-PRG-------------SG-IVTRCPL--------------
VLK-------------L---KKL----------------------------MN---
--EDS-------------WK------------------------GKI----------N-------
--------YQ--GVEVTIAKAS-----------DVEQEV------NKAQ-AV-
--I-AG------------------------DG------------------L-GISHE-LITLEVSSPE---------------------------------
--------------------------VPDLTLIDLPGITR--VA------------------V-------GN-------------Q--------PQ-
-D--IGEQIK--------ALIR-KYI---QR------------QQTINLVVVPCNV--D-IATTE-ALSMAREVD------
PDGDRTLGILTKPDLVD-------RGT------E-----DRV--------------------------VDV--IR---NF---------I-
CP----------------------L---KKGYMIVKCR--G-QKD--IQD----
-------R--------LS-------------------LA---------QALQK---EQAFFEE--------HP-HF--------------------
R-Q------LL-------E--EGR-ASIPKLADRLTSELIRHISKS-L----

XP_004466363.1 ------------LSLPA--------IAVI--
GD----Q----------------SSGKSSVLE-----A--LS-G-V-AL-PRG-------------SG-IVTRCPL--------------
VLK-------------L---KKL----------------------------TN---
---------------------------------------EEK-------------WR------------------------GKV---------T--------
------YE--DYEIDISDAS-----------EVEEEI------NKAQ-NV---I-
AG------------------------EG------------------L-GISQK-LINLEVCSPD---------------------------------
------------------------VPDLTLIDLPGITR--VA------------------V-------GN-------------Q--------PA--
D--IGWQIK--------CLIK-KYI---TR------------QETINLVVVPSNV--D-IATTE-ALSMAQEVD------
PNGDRTIGILTKPDLVD-------RGT------E-----DKV--------------------------VDV--VR---NL---------V-
CH----------------------L---KKGYMIVRCR--G-QQD--IQD---
--------R--------LS-------------------LA---------TALQK---ERAFFEN--------HE-NF--------------------
R-V------LL-------E--EGK-ATVPHLAERLTTELITHISKT-L----

NP_002453.2.2 ------------LALPA--------IAVI--
GD----Q----------------SSGKSSVLE-----A--LS-G-V-AL-PRG-------------SG-IVTRCPL--------------
VLK-------------L---KKL----------------------------VN---
--EDK-------------WR------------------------GKV----------S-------
--------YQ--DYEIEISDAS-----------EVEKEI------NKAQ-NA---
I-AG------------------------EG------------------M-GISHE-LITLEISSRD---------------------------------
------------------------VPDLTLIDLPGITR--VA------------------V-------GN-------------Q--------PA--
D--IGYKIK--------TLIK-KYI---QR------------QETISLVVVPSNV--D-IATTE-ALSMAQEVD------
PEGDRTIGILTKPDLVD-------KGT------E-----DKV--------------------------VDV--VR---NL---------V-
FH----------------------L---KKGYMIVKCR--G-QQE--IQD----

-------Q--------LS--------------------LS----------EALQR---EKIFFEN--------HP-YF---------------------
R-D------LL-------E--EGK-ATVPCLAEKLTSELITHICKS-L----
NP_001127618.1 ------------LALPA--------IAVI--
GD----Q----------------SSGKSSVLE-----A--LS-G-V-AL-PRG--------------SG-IVTRCPL---------------
VLK-------------L---KKL-----------------------------VN---
--EDK-------------WR--------------------------GKV----------S-------
--------YQ---DYEIEISDAS----------EVEKEI------NKAQ-NT---
I-AG------------------------EG-------------------M-GISHE-LITLEISSRD----------------------------------
-------------------------VPDLTLIDLPGITR--VA-------------------V-------GN--------------Q--------PA--
D--IGYKIK--------TLIK-KYI---QR------------QETISLVVVPSNV--D-IATTE-ALSMAQEVD------
PEGDRTIGILTKPDLVD-------KGT------E-----DKV---------------------------VDV--VR---NL--------V-
FH----------------------L---KKGYMIVKCR---G-QQE--IQD----
-------Q--------LS--------------------LS----------EALQR---EKIFFED--------HP-YF---------------------
R-D------LL-------E--EGK-ATVPCLAEKLTSELITHICKS-L----
XP_006156437.1 ------------LALPA--------IAVI--
GD----Q----------------SSGKSSVLE-----A--LS-G-V-AL-PRG--------------SG-IVTRCPL---------------
VLK-------------L---KKL-----------------------------IN--
---------------------------------------EDK-------------WR--------------------------GKV----------S--------
-------YQ---DIEVEITDPS----------KVEPEI------NKAQ-NV---
I-AG------------------------EG-------------------M-GISHE-LISLEVSSPH---------------------------------
--------------------------VPDLTLIDLPGITR--VA-------------------V-------GN--------------Q--------PA--
D--IGRQIK--------TLIK-KYI---HK------------QETINLVVVPSNV--D-IATTE-ALSMAQEVD------
PDGDRTIGILTKPDLVD-------KGT------E-----EKV---------------------------VDV--VR---NL--------V-
CH----------------------L---KKGYMIVKCR---G-QQD--IQD---
--------R--------LS--------------------LA----------EALQR---EKVFFEE--------HP-YF---------------------
S-F------LL-------E--EGK-ATIPCLAERLTTELIMHISKS-L----
XP_017508130.1 ------------LALPA--------IAVI--
GD----Q----------------SSGKSSVLE-----A--LS-G-V-AL-PRG--------------SG-IVTRCPL---------------
VLK-------------L---KKL-----------------------------TN--
---------------------------------------EET-------------WR--------------------------GKV----------S--------
-------YQ---DFEAELSDPS-----------EVEREI------NRAQ-NS--
-I-AG------------------------EG-------------------T-GISHE-LISLEISSPH----------------------------------
-------------------------VPDLTLIDLPGITR--VA-------------------V-------GN--------------Q--------PA--
D--IGRQIK--------ALIR-KYI---YK------------QETINLVVVPSNV--D-IATTE-ALSMAQEVD------
PDGDRTIGILTKPDLVD-------RGT------E-----DKV---------------------------VDV--VR---NL--------V-
CH----------------------L---KKGYMIVKCR---G-QQD--IQD---
--------Q--------LS--------------------LA----------EALKK---ERAFFED--------NP-YF---------------------
R-D------LL-------E--EGR-ATVPCLADKLTVELITHICKS-L----
XP_032211398.1 ------------LALPA--------IAVI--
GD----Q----------------SSGKSSVLE-----A--LS-G-V-AL-PRG--------------SG-IVTRCPL---------------
VLK-------------L---KKV-----------------------------TN--
---------------------------------------QDE-------------WR--------------------------GKV----------S-------
--------YQ---DFEKEISDPS-----------EVEAEI------NKAQ-NA--
-V-AG------------------------EG-------------------Q-GISHE-LISLEVSSSH-------------------------------

---------------------------VPDLTLIDLPGITR--VA------------------V-------GN--------------Q--------
PA--D--IGRQTK--------QLIR-KYI---LR------------QETINLVVVPCNV--D-IATTE-ALSMAQEVD------
PDGDRTIGILTKPDLVD-------RGT------E-----SKV--------------------------VDV--AQ---NL--------V-
CH----------------------L---KKGYMIVKCR---G-QQD--IQD---
--------Q--------VT-------------------LA----------EALQK---ERDFFED--------HP-HF-------------------
-R-V------LL-------E--EGR-ATVPCLADKLTSELIMHICKT-L----
NP_001003134.1 ------------LALPA--------IAVI--
GD----Q----------------SSGKSSVLE-----A--LS-G-V-AL-PRG-------------SG-IVTRCPL--------------
VLK-------------L---KKL-----------------------------IN---
---------------------------------------EDE-------------WR-------------------------GKV----------S-------
--------YQ---DTEMEISDPS-----------EVEVEI------NKAQ-DA-
--I-AG-----------------------EG------------------Q-GISHE-LISLEVSSPH-----------------------------
---------------------------VPDLTLIDLPGITR--VA------------------V-------GN--------------Q--------
PA--D--IGRQTK--------QLIR-KYI---LK------------QETINLVVVPCNV--D-IATTE-ALSMAQEVD------
PDGDRTIGILTKPDLVD-------RGT------E-----GKV--------------------------VDV--AQ---NL--------V-
CH----------------------L---KKGYMIVKCR---G-QQD--IQD---
--------Q--------VS-------------------LA----------EALQK---EKDFFED--------HP-HF-------------------
-R-V------LL-------E--EGR-ATVPNLAEKLTSELITHICKT-L----
XP_008569442.1 ------------LALPA--------IAVI--
GD----Q----------------SSGKSSVLE-----A--LS-G-V-AL-PRG-------------SG-IVTRCPL--------------
VLK-------------L---KKL-----------------------------VH---
--GEE-------------WK-------------------------GKV----------S-------
--------YR--DLEIKISDAL-----------EVEEEV------RKAQ-TI---I-
AG-----------------------EG-------------------M-GISHE-LINLEISSPH-----------------------------------
-----------------------VPDLTLIDLPGIAR--VA------------------M-------GN--------------Q--------PA--
D--IGYQVK--------XLIR-KYI---QR------------QETINLVVVPSNV--D-IATTE-ALSMAQEVD------
PEGDRTIGILTKPDLVD-------KGT------E-----DKV--------------------------VDV--VR---NL---------V-
YH----------------------L---KKGYMIVKCR---G-QQD--IQD----
-------Q--------LS-------------------LA----------TALQR---EKDFFED--------HP-QF--------------------
R-D------LL-------E--EGR-ATIPCLAERLTTELITHICKS-L----
XP_014388412.1 ------------LALPA--------IAVI--
GD----Q----------------SSGKSSVLE-----A--LS-G-V-SL-PRG-------------SG-IVTRCPL--------------
VLK-------------L---RKL-----------------------------RH---
--DDE-------------WK-------------------------GKV----------T-------
--------YR--DLEIDLSAAS-----------EVEQEI------RKAQ-NV--
-I-AG-----------------------EG------------------V-GISQE-LINLEVSSPH--------------------------------
--------------------------VPDLTLIDLPGITR--VA------------------V-------GN--------------Q--------PA-
-D--IGRQIT--------ALIK-KYI---LR------------QQTIMLVVVPSNV--D-IATTE-ALSMAHEVD------
PDGDRTIGILTKPDLVD-------RGT------E-----DKV--------------------------VDV--VR---NL---------V-
YH----------------------L---KKGYMIVKCR---G-QQD--IQY----
-------Q--------MS-------------------LS----------KALQR---ERAFFED--------HP-YF--------------------
R-D------LL-------E--EGK-ATIPCLAERLTNELIAHISKS-L----
XP_005202045.1 ------------LALPA--------IAVI--
GD----Q----------------SSGKSSVLE-----A--LS-G-V-AL-PRG-------------SG-IVTRCPL--------------

VLR-------------L---KKL----------------------------GN---
---------------------------------------EDE-------------WK-------------------------GKV----------S-------
--------FL--DKEIEIPDAS-----------QVEKEI------SEAQ-IA---I-
AG-----------------------EG------------------T-GISHE-LISLEVSSPH-------------------------------------
----------------------VPDLTLIDLPGITR--VA------------------V-------GN-------------Q--------PP--D-
-IEYQIK--------SLIR-KYI---LR------------QETINLVVVPANV--D-IATTE-ALRMAQEVD------
PQGDRTIGILTKPDLVD-------KGT------E-----DKV---------------------------VDV--VR---NL---------V-
FH----------------------L---KKGYMIVKCR---G-QQD--IKH----
-------R--------MS-------------------LD----------KALQR---ERIFFED--------HA-HF--------------------
R-D------LL-------E--EGK-ATIPCLAERLTSELIMHICKT-L----

NP_034976.1 ------------LALPA--------IAVI---GD----
Q----------------SSGKSSVLE-----A--LS-G-V-AL-PRG--------------SG-IVTRCPL---------------VLK---
-----------L---RKL-----------------------------KE---
---------------------------------GEE-------------WR-------------------------GKV----------S-------------
-YD---DIEVELSDPS-----------EVEEAI------NKGQ-NF---I-
AG-----------------------VG------------------L-GISDK-LISLDVSSPN------------------------------------
-----------------------VPDLTLIDLPGITR--VA------------------V-------GN-------------Q--------PA--
D--IGRQIK--------RLIK-TYI---QK------------QETINLVVVPSNV--D-IATTE-ALSMAQEVD------
PEGDRTIGVLTKPDLVD-------RGA------E-----GKV---------------------------LDV--MR---NL---------
V-YP----------------------L---KKGYMIVKCR---G-QQD--IQE--
---------Q--------LS--------------------LT----------EAFQK---EQVFFKD--------HS-YF-------------------
-S-I------LL-------E--DGK-ATVPCLAERLTEELTSHICKS-L----

NP_038634.1 ------------LALPA--------IAVI---GD----
Q----------------SSGKSSVLE-----A--LS-G-V-AL-PRG--------------SG-IVTRCPL---------------VLK---
-----------L---RKL-----------------------------NE--
---------------------------------GEE-------------WR-------------------------GKV----------S-------------
-YD---DIEVELSDPS-----------EVEEAI------NKGQ-NF---I-
AG-----------------------VG------------------L-GISDK-LISLDVSSPN------------------------------------
-----------------------VPDLTLIDLPGITR--VA------------------V-------GN-------------Q--------PA--
D--IGRQIK--------RLIK-TYI---QK------------QETINLVVVPSNV--D-IATTE-ALSMAQEVD------
PEGDRTIGILTKPDLVD-------RGT------E-----DKV---------------------------VDV--VR---NL---------V-
YH----------------------L---KKGYMIVKCR---G-QQD--IQE----
-------Q--------LS--------------------LT----------EALQN---EQIFFKE--------HP-HF--------------------
R-V------LL-------E--DGK-ATVPCLAERLTAELILHICKS-L----

XP_028583072.1 ------------LALPA--------IAVI---
GD----Q----------------SSGKSSVLE-----A--LS-G-V-AL-PRG--------------SG-IVTRCPL---------------
ELR-------------L---KKL----------------------------LP---
---------------------------------------GEK-------------WN-------------------------GKI----------S--------
-------YL--GKYMELANPS-----------MVEIEI------RKAQ-NI---
I-AG-----------------------DG------------------V-AISDK-LITLEIRSPE-------------------------------------
-----------------------VPDLTLIDLPGIAR--VA------------------V-------GN-------------Q--------PV--
N--IGDQIK--------KLIK-TFI---DK------------QETINLVVVPSNV--D-IATTE-ALKMAQEVD------
PNGERTLGIVTKPDLMD-------RGT------E-----GTV---------------------------VNI--VR---NQ---------
V-IP----------------------L---KKGYMIVKCR---G-QQD--IQS---

--------N--------MT-------------------LA----------SALKE---ERAFFEK--------HK-CF-------------------
-S-I------LL-------Q--EKK-ATVPLLAEKLTSELVEHISKS-L----
XP_028583068.1 ------------LALPA--------IAVI--
GD----Q----------------SSGKSSVLE-----A--LS-G-V-AL-PRG--------------SG-IVTRCPL---------------
ELK--------------L---KKT------------------------------HN--
--TKE-------------WK-------------------------GKI----------S--------
-------YL--NTVEEMNSSR-----------QVEEQI------IRAQ-NA--
-M-AG-----------------------SG-------------------S-GISSE-LISLEISSSD---------------------------------
--------------------------VPDLTLIDLPGIAR--VA-------------------V-------GD--------------Q--------PK-
-D--IGQQII--------KLIK-KYI---NK------------QETINLVVVPSNV--D-IATTE-ALKMAQEVD------
PTGERTLGILTKPDLVD-------KGT------E-----AEV--------------------------VDI--IR---NQ---------R-
VP-----------------------L---RKGYMIVKCR---G-QSD--IND----
-------K--------VT-------------------LG----------DAIEK---EREFFEE--------HD-FF--------------------
R-S------LL-------E--EGR-ATIPLLAERLTQELIEHISKT-L----
XP_031752404.1 ------------LALPA--------IAVI--
GD----Q----------------SSGKSSVLE-----A--LS-G-V-TL-PRG--------------SG-IVTRCPL---------------
ELK--------------L---KKA------------------------------MK--
--KTT-------------WS-------------------------GKI----------S--------
-------YR--DHEIKIASAA-----------DVEEEV------KRAQ-NL---
M-AG-----------------------SG-------------------K-GISDE-LISLEVISPD----------------------------------
--------------------------VPDLTLIDLPGITR--VA-------------------L-------PD--------------Q--------PK--
D--IEQQIK--------KMIR-KYI---QK------------QETINLVVVPSNV--D-IATTE-ALEMAREVD------
PNGERTLGILTKPDLVD-------RGA------E-----TDV--------------------------ISV--VR---NL---------V-
YS-----------------------L---NKGYMIVKCR---G-QQE--IQE----
-------N--------LS-------------------LK----------DALVN---EQNFFKE--------HE-HF--------------------
S-V------LL-------E--EGY-ATIACLAGKLTNELVAHIVRN-L----
XP_005167721.2.2 ------------LNLPA--------IAVI--
GD----Q----------------SSGKSSVLE-----A--LS-G-V-AL-PRG--------------TG-IVTRCPL---------------
VLK--------------L---KKI------------------------------TK--
--------------------------------------DKS-------------WH-------------------------GLL----------T---------
------YN--DKIRELKDPA-----------KIEKAV------LNAQ-TA---L-
AG------------------------IG-------------------E-GISHE-MITLEIQSCD-----------------------------------
-----------------------VPDLTLIDLPGIAR--VA-------------------T-------GN--------------Q--------PE--
D--IEKQIK--------SLIE-KFI---KR------------QETISLVVVPANI--D-IATTE-ALKMASTVD------
PTGQRTLGILTKPDLVD-------RGM------E-----DTV--------------------------VRT--VN---NE---------
V-IP-----------------------L---KKGYMIVKCR---G-QQD--IND--
---------K--------LG-------------------LV----------EALEK---ERRFFDE--------NV-HF-------------------
-R-S------LL-------E--DRK-ATIPLLAERLTKELVEHIAKN-L----
NP_001007285.1 ------------LNLPA--------IAVI--
GD----Q----------------SSGKSSVLE-----A--LS-G-V-AL-PRG--------------IG-IVTRCPL---------------
ILK--------------L---KKI------------------------------TR--
-------------------------------------DKN-------------WS-------------------------GLL----------T---------
-----YK--DQTEILKEPT-----------GIENAV------LKAQ-IA---L-
AG-----------------------TG-------------------E-GISHE-MITLEIQSCD-----------------------------------

------------------------VPDLTLIDLPGIAR--VA-------------------T-------GN--------------Q--------PE--
D--IEKQIK--------DLIE-KFI---KR------------QETISLVVVPANI--D-IATTE-ALKMASTVD------
PTGQRTLCILTKPDLVD-------RGM------E-----DTV----------------------------VRT--VN---NE---------
V-IR-----------------------L---EKGYMIVKCR--G-QQD--IND--
---------K--------LN--------------------LV----------EALEK---ERRFFDE--------HP-QF--------------------
-S-S------LL-------E--DGK-ATIPLLGQRLTEELVEHIAKN-V----
XP_012586448.1 ------------LALPA--------IAVI---
GD----Q----------------SSGKSSVLE-----A--LS-G-V-AL-PRG--------------SG-IVTRCPL---------------
VLK--------------L---KKQ------------------------------LQ--
---GAP--------------WT--------------------------GTI----------S--------
-------YR--GVTLGLQDPS-----------AVEREI------HVAQ-NV--
-I-AG------------------------HG-------------------V-GISHE-LITLEVSSPE---------------------------------
--------------------------VPDLTLIDLPGIAR--VA-------------------V-------GN--------------Q--------PQ--
D--IGAQVSLSGAGRWGALVS-GLP---RS------------ERCTGQ------------ETAE-ALGGGRAAX------
XDGDRTLGILTKPDLVD-------KGA------E-----KAV----------------------------VNV--AQ---NL---------
T-YR-----------------------L---KKGYMVVKCR--G-QQD--IMD-
----------R--------LS--------------------LA----------QATEK---EVAFFQT--------HP-HF-------------------
--R-A------LL-------E--EGK-ATVPRLAEKLTSELILHINKS-L----
KAI0213370.1------------ISLPE--------VAVI--GD---Q-
---------------SAGKSSVLE-----A--IS-G-V-QL-PRG--------------SG-IVTRCPL---------------ALQ------
--------L---KSH------------------------------DT---
------------------------------PGY--------------WN--------------------------GVI----------K---------------
YE--T-Y--------------NH-------------------------PVEKTIEGPT-----------EVGAEV------REAQ-DV---I-
AG------------------------KN-------------------V-GISDT-LISLQITSHG-------------------------------------
-----------------------VPDLTLIDLPGITR--VA-------------------V-------EG--------------Q--------PK--D--
IGDQIK--------RLIG-HYI---KK------------EETIILAVVPANV--D-IATTE-ALKMAKEVD------
PSGGRTLGVVTKPDLID-------IGT------E-----KGL----------------------------IDI--IN---NE---------T-
YP-----------------------L---EKGYSCVRCR--G-QKA--INE-----
------G--------QT--------------------LA----------EAIQQ---DTDFFSS--------AP-HF---------------------
S-D------V--------D--ESI-LGVKNLAMKLTYELVKQIKRA-L----
KAI0208044.1------------ISLPE--------VAVI--GD---Q-
---------------SAGKSSVLE-----A--IS-G-V-QL-PRG--------------SG-IVTRCPL---------------ALQ------
--------L---KSH------------------------------DT---
------------------------------PGY--------------WN--------------------------SVI----------K---------------YK-
-Y-G--------------DEF-------------------DFEDIVVEKTIEGPT-----------KVDAGV------REAQ-DA---I-
AG------------------------KN-------------------V-GISDT-LISLQITAYG--------------------------------------
----------------------VPDLTLIDLPGITR--VA-------------------V-------QG--------------Q--------PP--D--
IGDQIK--------RLIG-NYI---KK------------EETIILAVVPANV--D-IATTE-ALKMAKEVD------
PSGGRTLGVVTKPDLID-------IGT------E-----KGL----------------------------IDI--IN---NE---------T-
YP-----------------------L---EKGYSCVRCR--G-QKA--INE-----
------G--------QT--------------------LA----------DAVQE---DTDFFSS--------AP-HF---------------------
S-A------V--------D--ESI-LGVKNLAMKLTFELVKQIKRA-L----
KAI0218869.1------------IALPE--------VAVI--GD----
Q----------------SAGKSSVLE-----A--IS-G-V-QL-PRG--------------SG-IVTRCPL---------------ALQ---

-----------L---KSD-----------------------------KT--
--------------------------------PGY-------------WN-------------------------GVI----------K--------------
YE--I-N--------------ER------------------------LVEKTIVGPA-----------EVDAEV------RNAQ-DV---I-
AG------------------------KN------------------V-GISSK-LISLQITSYG-----------------------------------
---------------------IPDLTLIDLPGITR--VA-----------------V-------EG-------------Q--------PQ--N--
IGEQIK--------RLIE-KYI---KK------------EETIILAVVPANV--D-IATTE-ALKMAKEVD------
PSGSRTLGVVTKPDLID-------AGT------E-----KGL--------------------------ISI--IN---NE---------T-
YP----------------------L---KKGYSCVRCR---G-QKA--IDE-----
------G--------QT------------------LA----------QAIQQ---DTDFFSI--------AS-HF--------------------
S-D------V--------D--QST-LGVKNLAMKLTYELVRQIKRA-L----

XP_032804093.1 -----------VGLPA--------VAVI--
GD----Q----------------SSGKSSVLE-----A--LS-G-V-QL-PRG-------------SG-IVTRCPL--------------
-ALK-------------L---KRA--
--------------------------------------PGP-------------WH-------------------------GRI----------K--------
-------YR--V-Q--------------GR------------------------TVNTKLDTPE-----------SVGDAV------LQAQ-
SE---L-TG-----------------------DD------------------L-GVSKS-LIELEVTSDS--------------------------
--------------------------------VPDLTLIDLPGIAR--VA-----------------L-------AG-------------Q-------
-AV--D--IETQIK--------DLIR-DHI---GR------------QETINLVVIPCNV--D-IATTE-ALKMAQAVD------
PTGVRTLGVLTKPDLMD-------EGT------E-----RNA--------------------------LRI--LQ---NQ---------
V-FP----------------------L---SKGYVLVKCR---S-QRD--VEA--
---------H--------QT-------------------LA---------EASRV---EAAFFKK--------HP-VF--------------------
-C-H------VH-------N--GGKLTTTTVLAAKLTEELVDNIKRT-L----

XP_046565196.1 ------------INLPA--------VAVI---
GD----Q----------------SAGKSSVLE-----A--IS-G-V-QL-PRG-------------TG-IVTRCPL---------------
EMR-------------M---KHS-----------------------------ED---
---EDK-------------WE-------------------------GKI----------M-----
----------YKD-K-H-------------DE------------------------LHKEDIQDRE-----------SVGDLV------
RKAQ-DE---M-TD-----------------------CE------------------K-GISDD-LITLEVTSSD------------------
--VPDLTLIDLPGIAR--NA-------------------V-------KG--------------
Q--------PV--D--IEKRIK--------DMIR-KYI---RR-----------QETIILAVLQCNV--D-IATCE-
ALKMAKEFD------EEGRRTLGVLTKPDLLD-------KGA------E-----NGV---------------------------
MRI--LN---NM---------E-FS---------------------L---SKGYIIVKCR-------------------------------------
-------G-QEA--ISK-----------G--------QS------------------LT---------EALGD---EDNFFKD--------
HS-HF---------------------R-S------L--------K--VSQ-WGILTLSSRLSLELQKHIK--------

XP_046563124.1 ------------INLPA--------VAVI---
GD----Q----------------SAGKSSVLE-----A--IS-G-V-QL-PRG-------------TG-IVTRCPL---------------
EMR-------------L---KHS-----------------------------ED---
---EDK-------------WE-------------------------GKI----------L-------
--------YKD-K-H-------------DM-------------------------RQEEVILNRE-----------SVGDLV------RKAQ-
KE---M-TD-----------------------SA-------------------K-GISDE-LITLEVTSSD---------------------------
------------------------------VPDLTVIDLPGIAR--NA-----------------V-------EG-------------Q-------
-PF--D--IEARIK--------NMIR-RYI---GR------------QETIILAVLQCNV--D-IATCE-ALKMAKEFD------
DEGGRTLGVLTKPDLLD-------KGA------E-----TGV---------------------------MRI--LN---NM---------
E-FT----------------------L---SKGYIIVKCR---G-QEA--ISE-----

------G--------QS-------------------LK----------HALDV---EEDFFKS--------HR-HF--------------------
S-S------L--------R--PSQ-WGIPNLSARLSRELKKHIKKL-L----
XP_046562919.1 ------------INLPA--------VAVI--
GD----Q----------------SAGKSSVLE-----A--IS-G-V-QL-PRG-------------TG-IVTRCPL---------------
EMR-------------M---KHS-----------------------------ED---
---EDK-------------WE-------------------------GKI----------M-----
----------YKD-K-H-------------DM------------------------RQEEVILNRE-----------SVGDLV------
RKAQ-KE---M-TD-----------------------GA------------------K-GISDE-LITLEVTSSD------------------
--VPDLTVIDLPGITR--NA------------------V-------EG-------------
Q--------PF--D--IEARIK--------NMIR-KYI---KR------------QETIILAVLQCNV--D-IATCE-
ALKMAKEFD------GEGGRTLGVLTKPDLMD-------KGA------E-----TGV---------------------------IRI-
-LN---NM---------E-FT----------------------L---SKGYIIVKCR--
-G-QEA--ISE-----------G--------QS-------------------LK----------QALDI---EEDFFKS--------HR-HF-
--------------------S-S------L--------R--PSQ-WGIPDLSSRLSRELKRHIKKL-L----
XP_046563126.1 ------------INLPA--------VAVI--
GD----Q----------------SAGKSSVLE-----A--IS-G-V-QL-PRG-------------TG-IVTRCPL---------------
EMR-------------M---KHS-----------------------------ED---
---EDK-------------WE-------------------------GKI----------M-----
----------YKD-K-H-------------DM------------------------PHEEVILNRE-----------SVGDLV------
RKAQ-TE---M-TD-----------------------GA------------------T-GISDE-LITLEVTSSD-------------------
---------------------------------------VPDLTVIDLPGIAR--NA------------------V-------EG-------------
Q--------PF--D--IEARIK--------NMIR-QYI---ER------------QETIILAVLQCNV--D-IATCE-
ALKMAKEFD------DEGGRTLGVLTKPDLLD-------KGA------E-----TSV---------------------------IRI--
LN---NM---------E-FT----------------------L---SKGYVIVKCR---
-G-QEA--ISE-----------G--------QS-------------------LK----------HALDV---EEDFFRS--------HR-
HF--------------------S-A------L--------R--PSQ-WGIPNLSERLSRELKKHIKKL-L----
XP_046565195.1 ------------INLPA--------VAVI--
GD----Q----------------SAGKSSVLE-----A--IS-G-V-QL-PRG-------------TG-IVTRCPL---------------
EMR-------------M---KHS-----------------------------ED---
---EDK-------------WE-------------------------GKI----------M-----
----------YKD-K-H-------------DM------------------------PHEEVILNRE-----------SVGDLV------
RKAQ-KE---M-TD-----------------------GA------------------T-GISDE-LITLEVMSSD------------------
--VPDLTVIDLPGIAR--NA------------------V-------EG-------------
Q--------PF--D--IEARIK--------NMIR-QYI---QR------------QETIILAVLQCNV--D-IATCE-
ALKMAKEFD------DEGGRTLGVLTKPDLLD-------KGA------E-----TGV---------------------------IRI--
LN---NM---------E-FT----------------------L---SKGYVIVKCR---
-G-QEA--ISE-----------G--------QS-------------------LK----------HALDV---EEDFFRS--------HR-
HF--------------------S-A------M--------R--PSQ-WGIPNLSERLSRELKKHIKKL-L----
XP_046563125.1 ------------INLPA--------VAVI--
GD----Q----------------SAGKSSVLE-----A--IS-G-V-QL-PRG-------------TG-IVTRCPL---------------
EMR-------------M---KHS-----------------------------ED---
---EDK-------------WE-------------------------GKI----------M-----
----------YTD-K-H-------------DE------------------------PHQEVILNRE-----------SVGDLV------
RKAQ-KE---M-TD-----------------------SA------------------K-GISDE-LITLEVTSSD------------------

---------------------------------------VPDLTVIDLPGIAR--NA------------------V-------EG-------------
Q--------PV--D--IEARIK--------NMIR-QYI---ER-----------QETIILAVLQCNV--D-IATCE-
ALKMAKEFD------DEGGRTLGVLTKPDLLD-------RGA------E-----TGV---------------------------
MRI--LN---NM---------E-FT----------------------L---SKGYIIVKCR--------------------------------------
-------G-QEA--ISE-----------G--------QS--------------------LK----------HALDV---EEDFFKS-------
HR-HF---------------------S-S------L--------G--PSQ-WGIPNLSRRLSRELKKHIKKL-L----
XP_046352527.2 ------------INLPA--------VAVI---
GD----Q----------------SAGKSSVLE-----A--IS-G-V-QL-PRG-------------TG-IVTRCPL--------------
EMR--------------M---KHS------------------------------ED---
--EDM--------------WE--------------------------GKI-----------M----
----------YKD-M-H--------------GE-------------------------AHEEIILNRE-----------SVGELV------
RKAQ-KE---M-TD------------------------SA------------------K-GISDE-LITLEVTSSD--------------------
---------------------------------------VPDLTVIDLPGIAR--NA------------------V-------EG-------------
Q--------PV--D--IEARIK--------QMIR-KYI---GR-----------QETIILAVLQCNV--D-IATCE-
ALKMAKEFD------DEGGRTLGVLTKPDLLD-------KGA------E-----SGV---------------------------VRI-
-LN---NM---------E-FT----------------------L---SKGYIIVKCR--
-G-QEA--ISD-----------G--------QS--------------------LK----------QALEV---EEDFFKS--------HR-
HF---------------------S-S------L--------R--PSQ-WGIPNLSTRLSRELKKHIKKL-L----
XP_048248476.1 ------------INLPA--------VAVI---
GD----Q----------------SAGKSSVLE-----A--IS-G-V-QL-PRG-------------TG-IVTRCPL--------------
EMR--------------M---KHS------------------------------ED---
--GDM--------------WE--------------------------GKI-----------M----
----------YKD-M-H--------------DM-------------------------AHEEIILNRE-----------SVGELV------
RKAQ-IE---M-TD------------------------SA------------------K-GISDE-LITLEVTSSD---------------------
--------------------------------------VPDLTVIDLPGIAR--NA------------------V-------EG-------------
Q--------PF--D--IEARIK--------NMIR-RYI---GR-----------QETIILAVLQCNV--D-IATCE-
ALKMAKEFD------TEGGRTLGVLTKPDLLD-------KGA------E-----SGV---------------------------VRI--
LN---NK---------E-FT----------------------L---SKGYIIVKCR---
G-QEA--ISD-----------G--------QS--------------------LK----------QALEV---EEDFFKS--------HR-HF-
--------------------S-S------L--------R--PSQ-WGIPNLSMRLSRELKKHIKKL-L----
XP_048258111.1 ------------INLPA--------VAVI---
GD----Q----------------SAGKSSVLE-----A--IS-G-V-QL-PRG-------------TG-IVTRCPL--------------
EMR--------------M---KHS------------------------------ED---
--EDM--------------WE--------------------------GKI-----------M----
----------YKD-M-H--------------DM-------------------------AHEEIILNRE-----------SVGELV------
RKAQ-KE---M-TD------------------------SA------------------K-GISDE-LITLEVTSSD--------------------
---------------------------------------VPDLTVIDLPGIAR--NA------------------V-------EG-------------
Q--------PF--D--IEARIK--------NMIR-RYI---GR-----------QETIILAVLQCNV--D-IATCE-
ALKMAKEFD------AEGGRTLGVLTKPDLLD-------KGA------E-----AGV---------------------------VRI-
-LN---NM---------E-FT----------------------L---SKGYIIVKCR--
-G-QEA--ISY-----------G--------QS--------------------LK----------QALEV---EEDFFKS--------HR-HF-
--------------------S-S------L--------R--PSQ-WGIPNLSARLSRELKKHIKKL-L----
XP_046352531.2 ------------INLPS--------VAVI---
GD----Q----------------SAGKSSVLE-----A--IS-G-V-QL-PRG-------------TG-IVTRCPL--------------

EMR--------------M---KHS------------------------------ED--
---EDM--------------WE--------------------------GKI----------M----
-----------YKD-M-Y--------------DM-------------------------AHEEIILNRE-----------SVGELV------
RKAQ-KE---M-TD------------------------SA-------------------K-GISDE-LITLEVTSSD--------------------
--VPDLTVIDLPGIAR--NA-------------------V-------EG--------------
Q--------PF--D--IEARIK--------NMIR-KYI---GR-----------QETIILAVLQCNV--D-IATCE-
ALKMAKEFD------AEGGRTLGVLTKPDLLD-------KGA------E-----SGV-----------------------------VRI-
-LN---NM---------E-FT-----------------------L---SKGYIIVKCR---
-G-QEA--ISD----------G--------QT--------------------LK----------QALEV---EEDFFKS--------HR-HF-
--------------------S-S------L--------R--PSQ-WGIPNLSGRLSRELKIHIKKL-L----
XP_048248472.1 ------------INLPS--------VAVI--
GD----Q----------------SAGKSSVLE-----A--IS-G-V-QL-PRG--------------TG-IVTRCPL---------------
EMR--------------M---KHS------------------------------ED--
---EDM--------------WA--------------------------GKI----------M----
-----------YKD-M-Y--------------DM-------------------------THEEIILNRE-----------SVGELV------
RKAQ-KE---M-TD------------------------SA-------------------K-GISDE-LITLEVTSSD--------------------
--VPDLTVIDLPGIAR--NA-------------------V-------EG--------------
Q--------PL--D--IEARIK--------NMIR-RYI---RR-----------QETIILAVLQCNV--D-IATCE-
ALKMAKEFD------AEGGRTLGVLTKPDLLD-------KGA------E-----TGV-----------------------------VRI--
LN---NM---------E-FT-----------------------L---SKGYIIVKCR---
G-QEA--ISD----------G--------QS--------------------LK----------QALEV---EEEFFKS--------HR-HF--
-------------------S-S------L--------R--PSQ-XGIPNLSGRLSRELKIHIKKR-L----
XP_048248474.1 ------------INLPS--------VAVI--
GD----Q----------------SAGKSSVLE-----A--IS-G-V-QL-PRG--------------TG-IVTRCPL---------------
EMR--------------M---KHS------------------------------ED--
---EDM--------------WA--------------------------GKI----------M----
-----------YKD-M-Y--------------DM-------------------------THEEIILNRE-----------SVGELV------
RKAQ-KE---M-TD------------------------SA-------------------K-GISDE-LITLEVTSSD--------------------
--VPDLTVIDLPGIAR--NA-------------------V-------EG--------------
Q--------PL--D--IEARIK--------NMIR-RYI---RR-----------QETIILAVLQCNV--D-IATCE-
ALKMAKEFD------AEGGRTLGVLTKPDLLD-------KGA------E-----TGV-----------------------------VRI--
LN---NM---------E-FT-----------------------L---SKGYIIVKCR---
G-QEA--ISD----------G--------QS--------------------LK----------QALEV---EEEFFKS--------HR-HF--
-------------------S-S------L--------R--PSQ-WGIPNLSGRLSRELKIHIKKR-L----
XP_048248473.1 ------------INLPS--------VAVI--
GD----Q----------------SAGKSSVLE-----A--IS-G-V-QL-PRG--------------TG-IVTRCPL---------------
EMR--------------M---KHS------------------------------ED--
---EDM--------------WA--------------------------GKI----------M----
-----------YKD-M-Y--------------DM-------------------------THEEIILNRE-----------SVGELV------
RKAQ-KE---M-TD------------------------SA-------------------K-GISDE-LITLEVTSSD--------------------
--VPDLTVIDLPGIAR--NA-------------------V-------EG--------------
Q--------PL--D--IEARIK--------NMIR-RYI---RR-----------QETIILAVLQCNV--D-IATCE-
ALKMAKEFD------AEGGRTLGVLTKPDLLD-------KGA------E-----TGV-----------------------------VRI--
LN---NM---------E-FT-----------------------L---SKGYIIVKCR---

G-QEA--ISD-----------G--------QS-------------------LK---------QALEV---EEEFFKS--------HR-HF--
-------------------S-S------L--------R--PSQ-WGIPNLSGRLSRELKIHIKKR-L----
ABI53802.1 ------------INLPA--------VAVI--GD----
Q----------------SAGKSSVLE-----A--IS-G-V-QL-PRG--------------TG-IVTRCPL---------------EMR---
-----------M---KHS------------------------------EA--
----------------------------------EDM--------------WE--------------------------GKI----------M-------------
--YKD-M-Y--------------DV-------------------------AHEEIILNRE-----------SVEELV------RKAQ-KE---
M-TD------------------------SA-------------------K-GISDE-LITLEVTSSD----------------------------------
-------------------------VPDLTVIDLPGIAR--NA------------------V-------EG--------------Q--------PF--
D--IEARIK--------NMIR-RYI---GR------------QETIILAVLQCNV--D-IATCE-ALKMAKEFD------
AEGGRTLGVLTKPDLLD-------KGA------E-----TGV---------------------------VRI--LN---NM---------
E-FT-----------------------L---SKGYIIATCR--G-QEA--ISD-----
------G--------QS-------------------LT----------QALEV---EEDFFKS--------HR-YF--------------------
S-S------L--------R--PSQ-WGIPNLSGRLSRELKKHIKKL-L----
CAH1802128.1 ------------VALPA--------VVVI--
GD----Q----------------SVGKSSVLE-----A--MS-G-V-QL-PRG--------------TG-IVTRCPL--------------
-ELR--------------M---KQC------------------------------D--
--PGN--------------FH--------------------------AKI----------S------
---------YDI-Q-G--------------GH------------------------QPLEKTITDPS-----------NIDFEI------RQAQ-
RA---L-VG-------------------------DS-------------------G-GVSDR-LIRLEVQADY--------------------------
----------------------------------VPDLTLIDLPGIVR--YS-------------------E-------GS--------------D------
------T--IVEETK--------NLIK-TYV---SR------------PETIILVVIPCNV--D-IDTVE-ACNLAKQVD------
PNGDRTIGVLTRPDLID-------HGV------GP---IKEV---------------------------LDI--LE---NK---------K-
MK-----------------------L---KKGFYVVKCR---S-QKR--IEE-----
------G--------QS-------------------LE----------QALAE---EVQFFRS--------DE-RF--------------------
R-V------I--------N--PSQ-CGVKQLSSKLTNELFLHIKNC-V----
PAA74204.1 ------------VRLPA--------IAVV--GD----
Q----------------SVGKSSVLE-----S--IS-G-I-DL-PRG--------------LG-IVTRCPL---------------MLS----
----------M---R---------------------------------NRE---
---------------------------------EAG--------------WS--------------------------ARI----------K---------------
YKT-K-T--------------GE-------------------------GREKKLTGAS-----------EVGQAI------RDAQ-NE---M-
TN------------------------SS-------------------G-EVSEQ-LIELWVESPE-----------------------------------
-------------------------SPDLTLIDLPGIAR--YS-------------------I-------DG--------------G--------G---
A--IAGLTK--------SLIL-SYI---EK------------EEILILVVIPCHV--D-IETVE-ALSLAKEVD------
PESKRTIGVLTCPDLVN-------PGS------E-----SEV---------------------------LAL--MQ---NR---------
K-IP-----------------------L---KKGYVSVRCR--T-PQQ--LKD---
---------N--------MS-------------------LQ----------QAARE---EEVFFRT--------HP-HF-------------------
--R-A------L--------D--KFE-YGTKTLAVKLSSELYEAIKHN-I----
PAA76532.1 ------------ISLPA--------IAVV--GD----
Q----------------SVGKSSVLE-----A--IS-G-V-EF-PRG--------------LG-IVTRCPL---------------MLS----
----------M---R-------------------------------GRE---
---------------------------------DSG--------------WT--------------------------ARI----------R---------------
YET-K-S--------------GQ-------------------------ARDKPLSTPA-----------EIGQAI------RDAQ-EE---M-
TS------------------------SS-------------------G-EISEK-LIELHIEGAD--------------------------------------

---------------------TPDLTLIDLPGIAR--FS------------------I-------AN-------------A--------G---D--
IATVSK--------SLIM-SYI---LK------------PEVLILVVIPCNV--D-VETVE-AISLAREVD------
PECKRTLGVLTCPDLVN-------PGS------E-----TEV--------------------------LAM--MR---NE---------
R-LK----------------------L---RKGFVTVRCR--T-PQQ--LKD--
---------N--------MG-------------------LR----------EACKA---EEEFFKL--------HP-QF-----------------
---C-A------L--------G--DYQ-RGCKTLANKLSVELYQAVKER-I----
PAA92268.1 ------------LKLPM--------VAVV---GD----
Q----------------SVGKSSVLE-----A--IS-G-V-EF-PRG-------------TG-MVTRCAL---------------QLS--
------------M---QWN----------------------------ADP--
-----------------------------------EAP-------------WH-------------------------GRI---------S-----------
----YKD-V-N-------------GH------------------------KVDKELNSPG-----------EVDGAV------REAQ-
QR---M-TH-----------------------GD------------------N-EISSE-QIDLAIKGPD-------------------------
---------------------------------VPDLTLIDLPGIAR--YS------------------A-------TG-------------G------
--S---G--IAQITK--------SLIA-KYV---SQ------------PQVLILVVVPCHQ--D-IETVE-ALSLAKEAD------
PQGERTIGVLTCPDMVN-------KGA------E-----QET--------------------------LKI--AN---NE--------
K-IP----------------------L---KKGYVMVKCR---S-PEE--LNN--
---------G--------VT-------------------LS----------ESVAN---EAAFFKT--------HR-HF-------------------
-S-L------L--------P--EQS-VGIRTLADKLTEELFESVKRN-I----
PAA69582.1 ------------LRLPT--------VAVV---GD----
Q----------------SVGKSSVLE-----A--IS-G-V-DL-PRG-------------TG-IVTRCPL---------------QLS----
----------M---R-------------------------------SKP--
--------------------------------TGD-------------WT-------------------------GRI---------S--------------
YQN-R-K-------------GE------------------------HVEREISKKC-----------EVDEMV------RKVQ-NE---
I-TG----------------------DS------------------N-GVSTE-QIDLTIESAD---------------------------------
-------------------------VSDLTLVDLPGIAR--YS------------------E-------KN----------------------P---
K--INEVTK--------QLIL-SYI---SQ------------DQVIILVVVPCSV--D-IETVE-AIALAKQVD------
PGGTRTIGVLTCPDLTN-------PGS------E-----EDI--------------------------KAI--VN---NQG--------
R-VR----------------------L---HKGFVMVKCR--S-PKE--LRN-
----------N--------IS-------------------LS----------EVAKI---EEDYFKN--------DP-HF-------------------
-S-Q------L--------P--KDI-VGTKTLAEKLTNELFKAVAAG-I----
PAA83069.1 ------------LKLPS--------IVVI---GD----
Q----------------SSGKSSVLE-----T--IS-G-V-SF-PRG-------------NG-VVTLCPL---------------QLS---
-----------M---RSS----------------------------DK---
-----------------------------------K-------------WR-------------------------GTV---------R--------------
YFD-A-Q-------------GK------------------------EVHWDIDSPD-----------DVENAI------QNAQ-MR--
-I-TG----------------------HK------------------K-AISKN-IIEMTLEAPD----------------------------------
-------------------------LPNLTLVDLPGIAR--YS------------------H-------SD-------------G--------GS-
VN--LYKLTT--------DIIK-EYI---QR------------EETIILTVIPLSA--D-TATME-ALQLAKDVD------
PYGLRTIGVLTFPDLVN-------KGA------E-----EEK--------------------------LQI--AR---NI---------T-
FP----------------------L---SKGYITVKCR---N-QED--IKS------
-----R--------KS-------------------LR----------EAKVD---EMRFFSN--------DP-FF--------------------
S-Q------L--------D--PSQ-RGTDTLAKRLSTELLTLIKKF-I----
PAA94353.1 ------------LRLPS--------IVVI---GD----
Q----------------STGKSSVLE-----S--IS-G-V-RF-PRG-------------NG-VVTLCPL---------------QLS---

-----------M---RTS-----------------------------DD--
----------------------------------GK-------------WR-------------------------GNI---------R-------------
-YYD-T-Y-------------GK------------------------LMKWDIDGPE----------DVEDAI------QEAQ-MR---
I-TG-----------------------NQ------------------R-NVSKS-IIEMTLESPE---------------------------------
-------------------------LPNLTLIDLPGIAR--YN------------------H-------NS-------------A--------
ESGAS--LHQLTT--------DIIK-EYI---RR-----------EETIILVVIPLTS--D-TATME-ALQLAKDAD------
PYGMRTIGVLTFPDLVN-------KGA------Q-----EEK--------------------------LMI--AR---NI--------T-
YP----------------------L---SKGYVTKCR--N-QED--IKN-----
------R--------KS-------------------LK---------DAKAD---EALFFNT--------DP-FF--------------------
K-Q------L--------D--SMY-RGSDTLARRLSEELLYLVKKF-I----
KAI9324922.1---------DRYVELPQ--------IATM---GD--
--T---------------SSGKSSVLS-----A--IS-G-I-TF-PSS-------------SE-LTTRCPT--------------QLI-----
---------L---SQS-----------------------------ET---
---FS-------------------------GSV---------R--------------
LQR-F-KP------------QQN-------------------EEPPETKTLTNAN----------EIEHEI------ERLT-RQ---
L-VQ-----------------------EN------------------Q-SISDD-SIIINVSGPN---------------------------------
-------------------------YPNLTLTDLPGLIR--TV------------------E-------DS-------------E--------DP--
A--IIGRVR--------ALVD-RYL---VQ-----------SRTVILAVVPANV--D-VHNTE-ILQAAEAAD------
PQGVRTISIITKPDLID-------SGA------E-----SQV--------------------------VEL--LL---NR---------K-
KM----------------------L---KLGYHAVKCR--G-QKD--LNN---
--------G--------VS--------------------IA---------DGIAK---EAEFFET--------HA-VW--------------------
R-K------V--------D--SSY-FGISKLTEKLVKILETVIGGS-L----
'KAJ3066410.1' ---------DKYVELPQ--------IAVM---
--GD----T---------------SSGKSSVLS-----A--IS-G-I-TF-PSS-------------SE-LTTRCPT--------------
QLI-------------L---SEA-----------------------------EE--
---FS-------------------------GTV---------R----------
----LMR-F-KP------------QEG--------------------EILESTTLSSPA----------DITGEI------ERLT-KQ---
I-VS-----------------------EQ------------------Q-LISDD-AIIIEVRGPG---------------------------------
------------------------YPNLTLTDLPGLIR--TV------------------E-------DH-------------E--------DK--
D--IIRRVR--------GLVD-RYL---VQ-----------NRTVILAVVPANV--D-VHNTE-ILQAAQDAD------
PEGIRTISIITKPDRID-------PGA------E-----SQV--------------------------VDL--LM---NR---------K-
KK----------------------L---KLGYHAVRCR--G-QQD--LDD---
--------G--------VT-------------------IA---------DGIVN---ETKFFSE--------HK-AW--------------------
S-D------V--------D--PSY-VGINRLTEKLVKILQSIIASS-L----
KAI8836453.1---------DKYIELPQ--------IAVM---GD---
-T---------------SSGKSSVLS-----A--IS-G-I-EF-PSS-------------DT-LTTRCPT--------------QIV------
--------L---SEA-----------------------------DK--
---FS-------------------------GTV---------C--------------LVR-
F-GS------------GIS----------------------SHLTHLKNRN----------EITTEI------ARLT-QV---I-RD-----
-------------------EG------------------Q-TISDD-AIVIEVRGPE---
---------------YPNLTLTDLPGIIR--TV------------------Q-------DN-------------E--------DP--A--
MIPRVR--------QLVD-RYL---VQ-----------KRTVILAVVPANV--D-FHNSE-ILQAAEKVD------
PKGERTIAIITKPDAID-------PGA------E-----QSV--------------------------LDL--LM---NK---------K-
KA----------------------L---RLGYHAVRCR--G-KQH--HDD--

---------K--------MT-------------------IP----------EGLEM---EHDFFHN--------HK-VW------------------
---K-S------V--------A--PSY-VGCERLTEKLVKVLRNIITDS-L----
KAJ3350919.1 ---------DQYVELPQ--------IAVM--
---GD----T----------------SSGKSSVLS-----A--IS-G-I-EF-PSH--------------AE-LTTRCPT---------------
QLV--------------L---STA-----------------------------EA---
---FT-------------------------CTV----------C-----------
----IIR-V-KN-------------SKA---------------------SSPPSLKLKEPG------------EIRGAI------TELT-KI---I-
ID------------------------DG-------------------P-AISED-MISIEVSGPK-------------------------------------
-----------------------YPNLTLIDLPGIVR--TV-------------------A-------DN--------------E--------DP--S--
MILNIR--------DLVT-SYL---QK------------KRTVILAVIPANV--D-MHNVE-ILQLAESVD------
PSGDRTIAIITKPDAVD-------CGA------E-----KQI---------------------------VDL--LL---NR---------K-
KF------------------------L---KLGYHAMRCR--G-QQD--LNE--
---------K--------MS--------------------IH----------EGVIK---ESKFFYN--------HP-VW-------------------
--R-N------I--------S--PEL-LGVESLVPKLVNTLQNVINQS-L----
TDH66190.1 ------------IELPT--------IAVM---GD----R-
---------------SSGKSSLLS-----A--LS-G-I-SF-PSN--------------DQ-LTTRCPT---------------QLI-------
-------L---TRG------------------------------DV---
---FH--------------------------GTV----------R---------------LVR-
F-QT-------------GSDREQIEATEDLK-----HLKDVEQIEATEDLKHLK-----------DVPNAI------SKLT-
KK---I-IN------------------------DG-------------------L-IISND-QIVIEVCGPE-----------------------------
-------------------------------IPELTLVDLPGLVR--TA-------------------K-------KL--------------K--------
DK--S--MIPRVQ--------KMVD-RYM---KK------------EQIVVIAVVPANK--D-MHNSE-ILKTVQAAD---
---PDGTRTIAVVTKLDLVN-------AGA------E-----KTV---------------------------LEF--LL---NK---------
N-KQ------------------------M---HLGYHAVKCR---N-QRD--
LTT-----------G--------MS--------------------IE----------IGLAN---ERTFFSQ--------HE-YW-------------
--------S-Q------L--------P--SHL-WGIPTLTKRLISILQDNTRKT-FQNA-
XP_024574100.1 ------------IELPQ--------IAVM---
GD----T----------------SSGKSSLLS-----A--LS-G-V-AF-PSN--------------NQ-LTTRCPT---------------
QLV--------------L---TRA------------------------------DT---
---FQ--------------------------GTV----------K-----------
----LVR-F-ES-----------------------------------SDNDENNEAEDLKRME-----------DVPDAI------TKLT-
QK---L-VD------------------------EG-------------------Q-YISDD-QIVIEMCGPE--------------------------
---------------------------------LPDLTLTDLPGLVR--TV-------------------G-------DH--------------E-----
---DQ--S--IISRVR--------EMVN-RYM---KQ------------ERTVIIAVVPANV--D-MHNTE-ILQAAQEAD-
-----PTGTRTIAVVTKMDLVD-------GGA------E-----SAV---------------------------HDL--LL---NK------
---K-KK-----------------------M---RLGYHAVKCR--N-QRE--
LTE-----------G--------AS--------------------IE----------KGLEN---EISFFSR--------HE-YW-------------
-------R-R------L--------P--SHL-WGIATLTDRLVLILQDNIRRS-LPKV-
KAG1692046.1 ------------IELPQ--------IAVM---
GD----T----------------SSGKSSLLS-----A--LS-G-V-SF-PSN--------------DQ-LTTRCPT---------------
QLV--------------L---TRA------------------------------DT---
---FR-------------------------GTV----------R-----------
----LVR-F-QS-----------------------------------NGDSNDGEEKEDLNRLE-----------DVPDAI------TKLT-
NK---L-IA------------------------EG-------------------Q-YISDD-QIVIEMCGPE--------------------------

---------------------------------LPNLTLTDLPGLVR--TV------------------G-------DH-------------E------
--DQ--S--IIPRVR--------QMVD-RYM---QQ-----------ERTVIIAVVPANV--D-MHNTE-
ILQAAQQAD------PNGTRTIAVVTKVDLVD-------PGA------E-----LAV---------------------------HEL--
LL---NR---------K-KK----------------------M---HLGYHAVKCR--
---N-QRE--LTK----------G--------TS-------------------IE---------KGLVN---ETMFFSQ--------HE-
YW--------------------R-R------L--------P--SHL-WGVPRLTDRLVSILQDNIRRS-LPKV-
KAG3062152.1 ------------IELPQ--------IAVM--
GD----T---------------SSGKSSLLS-----A--LS-G-V-SF-PSN-------------DQ-LTTRCPT--------------
QLI-------------L---TRA----------------------------DT---
--FR------------------------GTV---------R-----------
----LVR-F-QS--------------------------------NGEHDEGEEKQDLNRLE-----------DVPDAI------TKLT-
QK---L-VD-----------------------EG------------------Q-HISDD-QIVIEMCGPE-----------------------
----------------------------------LPNLTLTDLPGLVR--TI------------------G-------DH-------------E-----
---DQ--S--IIPRVR--------QMVD-RYM---KQ-----------ERTVIIAVVPANV--D-MHNTE-
ILQAAQEAD------PNGTRTIAVVTKVDLVD-------AGA------E-----LAV---------------------------HEL--
LL---NK---------K-KK----------------------M---HLGYHAVKCR--
--S-QRE--LTK-----------G--------TN-------------------IE---------KGLAN---EMTFFGQ--------HE-
YW--------------------C-R------L--------P--THL-WGVSRLTERLVSILQDNIRRS-LPKV-
XP_009533587.1 ------------VELPQ--------IAVM--
GD----T---------------SSGKSSLLS-----A--LS-G-V-SF-PSS-------------DQ-LTTRCPT--------------
QLV-------------L---TRA-----------------------------DA---
---FR------------------------GTV---------R----------
-----LVR-F-QS-------------G-----------------NNSDNDDGEEKADLQRLE-----------DVPDAI------
TKLT-QK---L-VD-----------------------EG------------------Q-YISDD-QIVIEMCGPD------------------
---LPNLTLTDLPGLVR--TV------------------G-------DH-------------
-E--------DQ--S--IIPRVR--------QMVD-RYM---QQ-----------ERTVIIAVVPANV--D-MHNME-
ILQAAQEAD------PNGTRTIAVVTKVDLVD-------AGA------E-----LAV---------------------------HEL--
LL---NK---------K-KR----------------------M---HLGYHAVKCR--
---S-QRE--LTK-----------G--------TS-------------------IE---------KGVAN---ELAFFGQ--------HE-
YW--------------------C-K------L--------P--THL-WGVPRLSERLVSILQDNIRRS-LPKV-
KAI9918701.1------------IELPQ--------IAVM--GD----
T---------------SSGKSSLLS-----A--LS-G-V-SF-PSS-------------DQ-LTTRCPT---------------QLI-----
---------L---SRS----------------------------DS---
--YH-----------------------AHV----------R--------------
LVR-F-QS------------AG----------------MTDENDDGEERAILNRLE-----------DVPDAI------SKLT-
QK---L-ID----------------------EG------------------Q-YISDD-RIVIEISSPD---------------------------
------------------------------LPDLTLTDLPGLVR--TV------------------G-------DH-------------E--------
DH--S--IIPRVR--------QMVD-RYM---KQ-----------ERTVIIAVVPANV--D-MHNTE-ILQAAQEAD---
---PNGTRTIAVVTKMDLVD-------AGA------E-----LAV--------------------------HEL--LL---NR--------
-K-KK----------------------M---HLGYHAVKCR---N-QRE--
LTK-----------G--------TS-------------------IE---------KGLAN---EMTFFGQ--------HE-YW------------
---------C-R------L--------P--KHL-WGVPRLSERLVSILQNNIRQS-LPKV-
RMX63821.1 ------------IELPQ--------IAVM--GD----
T---------------SSGKSSLLS-----A--LS-G-I-SF-PSS-------------DQ-LTTRCPT--------------RVL-----

---------L---TRA-------------------------------DT--
--FR--------------------------GSV----------R---------------LIR-
F-QT--------------SA-----------N-----DGGENKKREEIESLNRME-----------DVPDAI------TKLT-KK---L-
VS------------------------EG-------------------Q-YISDD-EIVIEMSGPN-------------------------------------
------------------------LPNLTLTDLPGLVR--TV------------------G-------DN-------------E--------DQ--
S--IISRIR--------QIVN-RYM---QQ------------ERTIILAVVPANV--D-MHNTE-ILQAAQEAD------
PSGTRTIAVITKLDLVD-------TGA------E-----AGV----------------------------HEL--LL---NK---------K-
KC-----------------------M---KLGYHAVKCR--N-QLE--LTN----
-------G--------TT--------------------IE----------KSVVN---EMSFFSE--------HP-YW---------------------
R-R------L--------P--NHL-WGVSRLVERLVSILQDNVRRS-LPKV-

GMH36208.1 -------------MLPA--------IVVV---GD----
Q-----------------SSGKSSLLE-----I--LS-G-V-TL-PRG--------------EG-ICTRVPL---------------ELQ-----
---------L---RNG------------------------------TEV---
---S---------------------------AQI----------E---------------YQT-
D-L--------------------------------------DAPRVSKHIM-VE-----------EVKNEI------LLAT-KR---I-AG-----
-------------------ME-------------------L-NIKDL-PIVLRMTGPT---
---------------YQDLTLIDLPGIAR--MP-------------------L-------RG--------------Q--------PD--N--
IEELTM--------EMIQ-KYI---NG------------DSKVILCAVPANN--E-FVTSA-ALKLASNVD------
PLGLRTLGVVTKADQFS-------RGM------R-----RRL----------------------------EGL--DD---T----------
D-VK-----------------------L---KLGFVAVRCR--T-QKE--LEE---
--------G--------IS-------------------LQ----------DVRMR---EELLFET--------DP-EL---------------------
R-D------V--------Q--PHC-RGISTLVDKLVDIQKERLIEQ-LPRIV

GMH43921.1 -----------SQMLTA--------IVVV---GD----
Q-----------------SSGKSSTLE-----R--IA-G-I-DL-PRG--------------QG-ICTRVPL---------------EMQ---
-----------M---RKG-------------------------------SKF--
--S---------------------------ATL----------E---------------
YQQ-E-K---------------------------------------GGSKQSVEIKDAS----------KISDAI------QAAT-RD---I-
VG-------------------------NS-------------------K-NVEDL-PLVLRISSPI--------------------------------------
-----------------------YQDLTLIDLPGIAR--AP-------------------L-------PG--------------Q--------RS--D-
-IEEQTL--------EMMR-RYI---TG------------EAKVILCALPATN--D-FVTSA-ALKLALQLD------
PDGERTLGAVTKIDQAR-------KGI------A-----KKL----------------------------EGT--DA---S----------E-
IT----------------------L---HLGFAGVRCR--T-ENE--TDA------
-----G--------IT--------------------LE----------QVRQA---EEELFRT--------HD-EL---------------------K-
H------V--------D--DSC-KGVSALLQKLVAVQRGRLISH-LPKVL

CAG9460856.1 ------------DKLPT--------IVVT---
GD----Q-----------------SAGKSSVLE-----S--LS-G-I-AF-PVG--------------DG-IVTRLPC---------------
QVA--------------L---REG-------------------------------PAF---
--RA--------------VC--------------------------TPP----------E--------
-------G--HGEA-VTLTDPK----------AVTKWI------EDTT-AA---
V-AG-------------------------DK-------------------K-GVLDK-PLSIKVEREG----------------------------------
---------------------------SADLTLVDLPGITR--VA-------------------V-------DG--------------Q--------
AD--D--IEEQVK--------RMIQ-RYI---SR------------EAAVVLCVLPANV--D-FSTAE-CIKMARAVD-----
-PGGERTLGVVTKVDRAE-------RGI------V-----TRL----------------------------NAF--GT---T---------
G-WA-----------------------L---RLGYVAVKNL--S-QDE--RAK-

---------HG--------VS-------------------TT----------KVLEL---EDAFFDDGVG-RPAHLA-EL-------------
--------A-D------L--------D--ADM-RGLRTLVQKLVQVQGERIEAF-MPSLV
GJP35534.1 ----------EGIELPT--------IVVV--GD----
Q----------------SSGKSSVLE-----N--LS-G-I-SL-PRG--------------KG-IVTRVPL---------------ILR-----
---------L---QSC-----------------------------VK--
-------------------------------GK-------------DM-------------------------ITI---------E---------------Y---
T-P-----------------------------------VTGKVSKVLSDEE-----------MIEEEI------SEAT-IA---L-AG------
------------------SR-------------------K-GVMNC-PITLQVQRPD---
-----------------LPDLTLVDLPGITR--VP------------------I-------ED--------------Q--------PK--D--
IYNQVK--------NMIM-HYI---TP------------KESVILNVLAAEV--D-FSTCE-SIVMSQEVD------
SDGDRTLAVVTKVDRAP-------DGL------Y-----EKI----------------------------QGN------------------S-
VR-----------------------I---GLGYVCVRNK---TDADA--SHD----
---DARRA---EAAFFNS--------HP-EL---------------------S-
Q------I--------E--SHC-LGIPALAQRLTEIQAKRVADS-IPRIR
CAI5480041.1 ----------EGIELPT--------IVVV--
GD----Q----------------SSGKSSVLE-----N--LS-G-I-SL-PRG--------------NG-IVTRVPL---------------
ILR--------------L---QSC-----------------------------TS--
--KD-------------GE-------------------------ITI---------E----------
----YN--N-P------------------------------------SSGKIFKILPDEE-----------SIQEEI------SKAT-VT---L-
AG------------------------SR-------------------K-GVMDR-PITLQVKRSG----------------------------------
--------------------------LPDLTLVDLPGITR--VP------------------V-------DD--------------Q--------PK--
D--IYNQVK--------KMIM-QYI---TP------------EESVILNVLAAEV--D-FSTCE-SIVMSQEVD------
QDGDRTLAVVTKVDRAP-------DGL------Y-----EKI----------------------------QGN------------------S-
VR-----------------------I---GLGYVCVRNK---TDADA--SHA----
---AARLA---ETDFFDR--------HP-EL---------------------S-
R------I--------E--TDS-RGIPALAQRLSEIQAKRVAES-IPRIR
KAJ7294545.1 -----------GIQLPT--------IVVV--
GD----Q----------------SHGKSSVLE-----S--LA-E-I-TL-PRR--------------QG-IATRVPL--------------
ILR--------------L---QSC------------------------------KVA--
--SE--------------QS-------------------------ITI---------E----------
-----Y---LNVKD--EIKSEE-----------LIEAAI------DEAT-NV---L-
AG------------------------PR-------------------K-DVRDT-PISLHVRKLG-----------------------------------
-------------------------APDLTMVDLPGITR--VP------------------V-------HG--------------Q--------PE-
-N--IYEQIA--------AMIQ-KYI---NP------------PESIILNVISATV--D-FPTCE-SIRMSQLAD------
KEGKRTLAVVTKVDKAP-------EGL------Y-----EKV----------------------------TSD------------------A-
VN-----------------------I---GLGYICVRNR---TEKEN--SNE-----
---QARFV---EKHLFDT--------HP-SL---------------------C-
K------L--------D--KSM-VGIPMLAYRLTCIQAQMIQGC-LPGL-
XP_024380180.1 ----------ENVNIPT--------IVVV--
GD----Q----------------SSGKSSVLE-----S--LA-G-I-TL-PRG--------------QG-IATRVPL---------------
ILR--------------L---QSC-----------------------------LSE---
---QD-------------SK-------------------------ILM---------E-------
-------Y---GSVKE-MRINSED-----------DIEAAI------NAAT-DD--
-L-AG-----------------------SN-------------------K-NIRDT-PILLHIRKPD--------------------------------

--------------------------APDLTMVDLPGITR--VP------------------V-------HG-------------Q--------
PE--N--IYEQVR--------DMIM-HYI---KP-----------EESIILNVLPAEV--D-FSTCE-SIRLSQTVD------
KKGVRTLAVVTKVDKAP-------EGL------F-----EKV--------------------------TSD-----------------A-
VS----------------------I---GLGYVCVRNR--TPADD--SIA-----
--VARCR---ELELFND--------HP-DL--------------------R-
N------I--------D--RSM-VGIPTLGRRLVKIQSDMVRGC-LPRIR
XP_024367947.1 ----------ENVNIPT--------IVVV--
GD----Q---------------SSGKSSVLE-----S--LA-G-I-TL-PRG-------------QG-IATRVPL--------------
ILR-------------L---QSC----------------------------LSE---
--QD-------------SK------------------------ILM---------E--------
-------Y--ENVKE-MRINSED-----------DIEAAI------NAAT-DD--
-L-AG-----------------------SN------------------K-NIRNT-PISLHIRKPD--------------------------------
--------------------------APDLTMVDLPGITR--VP------------------V-------HG-------------Q--------
PE--N--IYEQVR--------DMIM-HYI---KP-----------EESIILNVLPAEV--D-FSTCE-SIRLSQTVD------
KKGVRTLAVVTKVDKAP-------EGL------F-----EKV--------------------------TSD-----------------A-
VS----------------------I---GLGYVCVRNR--TPADD--SIA-----
--VARCR---ELELFND--------HP-DL--------------------R-
N------I--------D--RSM-VGIPTLARRLVKIQSDMVRGC-LPRIR
KAG0619429.1 -----------NVQLPT--------IVVV---
GD----Q---------------SSGKSSVLE-----S--LA-G-I-TL-PRG-------------QG-IATRVPL--------------
ILR-------------L---QSC----------------------------DST--
--EE-------------SL------------------------IRM---------D---------
------Y--GNVKD-REIDGEE-----------QIEAAI------NEAT-NV---
L-AG-----------------------GN------------------K-DVKDT-PISLHIRKPH--------------------------------
--------------------------APDLTMVDLPGITR--VP------------------V-------HG-------------Q--------
PQ--N--IYEQIQ--------AMIM-KHI---SP-----------EESIILNVLSAQV--D-FPTCE-SIRMSQQVD------
KDGKRTLAVVTKVDKAP-------EGL------L-----EKV--------------------------TTD-----------------A-
VN----------------------I---GLGYVCVRNR--TDDDD--TIS----
--VARIR---EQRLFES--------HP-AL--------------------K-
D------L--------D--RSM-VGIPALARKLTKIQSDMVKGC-LPRI-
KAG0561847.1 -----------NIQLPT--------IVVV--
GD----Q---------------SSGKSSVLE-----S--LA-G-I-TL-PRG-------------QG-IATRVPL--------------
VLR-------------L---QSC------------------------------QL---
--EE-------------SI------------------------IKM---------D--------
------Y--GNVKD-QEISGEE-----------QIEAAI------NAAT-NA---
L-AG-----------------------SG------------------K-GVKDS-PIQLLIRKPN--------------------------------
--------------------------SPDLTMVDLPGITR--VP------------------V-------HG-------------Q--------
PK--N--IYEQIR--------GMIM-RYI---TP-----------EESIILNVLSAQV--D-FPTCE-SIRMSQQVD------
KEGNRTLAVVTKVDKAP-------EGL------L-----EKV--------------------------TTD-----------------A-
VN----------------------I---GLGYICVRNR--IDVDD--SIA------
--IARQR---ERELFES--------HP-AL--------------------K-E--
----L--------D--GSM-VGIPALARKLTKIQSDMVKEC-LPRI-
KAH9290598.1 -----------GIHLPT--------IVVV--
GD----Q---------------SSGKSSVLE-----S--LS-G-I-DL-PRG-------------QG-ICTRVPL--------------

IMR-------------L---QNS----------------------------TE---
--EY-------------SV-------------------------ISV---------E---------
-----Y--KDRK--LSINEH-----------QIVDTI------NLVT-EE---I-
AG-----------------------RN------------------K-GISDD-PITLHVRKKN-----------------------------------
------------------------VPDLTLVDLPGITR--VP------------------V-------YG-------------Q--------PK--
D--IYEQVY--------KIIM-KYI---SP------------RDSIILNVLSATV--D-FPTCE-SIRMSQKVD------
EDGERTLAVVTKVDKAP-------EGL------R-----EKV---------------------------AED-----------------A-
MN----------------------I---GLGYVCVRNR--V-EGE--SIV-----
---KARKK---ENELFKT--------HP-LL--------------------S-G-
-----I--------D--KSI-VGIPILAHKLMKIQAAGITNS-LPRI-
KAH9320939.1 -----------GIQLPS--------IVVV--
GD----Q----------------SSGKSSVLE-----S--LA-G-I-KL-PRG-------------QG-ICTRVPL--------------
VMR-------------L---QSC-----------------------------AEE---
---SE-------------EE-------------------------ISI---------E--------
------F---NGVE--KFIQES----------DITSSI------DTAT-QE---I-
AG-----------------------NG------------------K-GISHT-PITLHVTKVG------------------------------------
-----------------------APDLTMVDLPGITR--VP------------------V-------GG-------------Q--------PG--
D--IFEQIC--------EIIK-EYI---TP------------KESIILNVLAANV--D-FPTCE-SIRMSQKVD------
ELGERTLAVVTKSDRAP-------DGL------K-----EKV---------------------------TTD-----------------A-
VN----------------------I---GLGYVCVRNG--I-GDE--SNA----
--EAREK---EKNLFDF--------HP-LL--------------------K-
D------L--------D--KSM-VGIPTLAKKLMQIQATTISAT-LPQI-
KAF8079489.1 ----------EGIQLPT--------IVVV--
GD----Q----------------SSGKSSVLE-----S--LA-G-I-SL-PRG-------------QG-ICTRVPL--------------
VMR-------------L---QGS-----------------------------AS---
---SE-------------PE-------------------------IWL---------E-------
--------Y---SDNV--VPTDEE-----------HIAEAI------SAAT-DV---
I-AG-----------------------SH------------------K-GVSDA-PLTLHVKKAG-------------------------------
----------------------------APDLTMVDLPGITR--VP------------------V-------KG-------------Q--------
PE--N--IYEQIS--------GMIM-KYI---KP------------QESIILNVLSATV--D-FTTCE-SIRMSRQVD------
KTGERTLAVVTKADMAP-------EGL------L-----QKV---------------------------TSD-----------------D-
VS----------------------I---GLGYVCVRNR--V-GEE--TYE------
--EARKQ---EELLFET--------HP-TL--------------------S-M--
----I--------D--ENI-VGVPVLAQKLIQIQTTMIARC-LPKIV
OAP19580.1 ------------IQLPT--------IVVV--GD----
Q----------------SSGKSSVLE-----S--LA-G-I-NL-PRG-------------QG-ICTRVPL--------------VMR--
------------L---QRS-----------------------------SS--
-----------------------------------PE-------------PE-------------------------IWL---------E---------------
Y---SDKV--VPTDEE-----------HVAEAI------CAAT-DV---I-AG---
--------------------K---

------------------------FSLSP-S---
---QCSVKCV--------------------------------------

--LLQK------------------
OAP13353.1 ------------IQLPT--------IVVV---GD----
Q----------------SSGKSSVLE-----S--LA-G-I-SL-PRG--------------QG-ICTRVPL---------------VMR--
-------------L---QRR------------------------------RS---
---PE-------------PE---------------------------IWL----------E--------------
Y--GDKI--VPTDEE-----------HIAQTI------CAAT-DV---I-AG----
-----------------------M--

-----------------------------F---

OAP13972.1 ------------IQLPT--------IVVV---GD----
Q----------------SSGKSSVLD-----S--LA-G-I-SL-PRG--------------QG-ICTRVPL---------------VMR--
-------------L---QRS------------------------------SS---
--PV-------------PE---------------------------IWL----------E--------------
Y--SDKI--VPTDEE-----------HIAEAI------CAAT-DV---I-AG----
-----------------------K---

----------------------------FTL--
--YLGIKCV---

KAF5727250.1 ----------EGIQLPT--------IVVV---
GD----Q----------------SSGKSSVLE-----S--LA-G-I-SL-PRG--------------QG-ICTRVPL---------------
VMR--------------L---QHH------------------------------HI--
---PE-------------PE---------------------------LYL----------E--------
-------F---NGKT--VQTTES-----------RISEAI------NLAT-DE---I-
AG-------------------------NG--------------------K-GVSNT-PLTLVVKKHG------------------------------------
------------------------------VPDLTMVDLPGITR--VP-------------------V-------HD--------------Q--------PE-
-N--IYEQIA--------GIIM-EYI---KP------------DESIILNVLSATV--D-FSTCE-SIRMSQQVD------
KTGERTLAVVTKVDKAP-------EGL------L-----EKV-----------------------------TAD-------------------D-
VN------------------------V---GLGYVCVRNR--I-GDE--TYE-----
--EARRK---EAALFES--------HP-LL---------------------C-L-
-----I--------D--KSI-VGVPVLAQKLVQIQAAIIMKC-LPEIV
XP_002303204.3 -----------GIQLPT--------IVVV---
GD----Q----------------SSGKSSVLE-----S--LA-G-I-SL-PRG--------------QG-ICTRVPL---------------
IMR--------------L---QHH------------------------------TA--
---PE-------------PE---------------------------LSL----------E--------
------F---NGKT--VPTSEA-----------KIANAI------SLAT-DE---I-
AG------------------------NA-------------------K-GISNT-PLTLVVKKNG-----------------------------------
----------------------------VPDLTMVDLPGITR--VP------------------V-------HG--------------Q--------PE--
N--IYEQIA--------DIIM-EYI---RP------------EESIILNVLSATV--D-FTTCE-SIRMSQKVD------

KNGERTLAVVTKADRAP-------EGL------L-----EKV---------------------------TAD------------------D-
VN----------------------I---GLGYVCVRNR--I-GDE--SYK-----
--EARKE---EADLFEN--------HP-LL---------------------S-K-
-----I--------D--KSM-VGIPVLAQKLVQIQATIIARC-LPEI-
KAF8391993.1 ----------EGIELPT--------IVVV---
GD----Q----------------SSGKSSVLE-----S--LA-G-I-SL-PRG--------------QG-ICTRVPL---------------
IMR--------------L---QHH-----------------------------ST--
--PS--------------PE-------------------------LHL----------E---------
------Y--HNKI--IPTNET-----------HVAEAI------NMAT-NE---I-
AG-------------------------NG-------------------K-GISNT-PLTLVVKKKG-------------------------------------
----------------------VPDLTMVDLPGITR--VP-------------------V-------HG--------------Q--------PE--
D--IYEQIS--------SIIM-EYI---KP------------KESTILNVLSATV--D-FPTCE-SIRMSQRVD------
KTGERTLAVVTKCDKAP-------EGL------L-----EKV---------------------------TAD------------------D-
VN----------------------I---GLGYVCVRNR--I-GEE--LYE------
---EARME---EATLFES--------HQ-LL---------------------S-K--
----I--------D--KSI-VGVPILAQKLVQIQASIIAKC-LPDIV
XP_058079501.1 ----------EGIELPT--------IVVV---
GD----Q----------------SSGKSSVIE-----S--LA-G-I-SL-PRG--------------QG-ICTRVPL---------------
IMC--------------L---QNV------------------------------PT--
--DK--------------PQ-------------------------MHL----------E-------
--------Y--QGKI--VLTSEN-----------QISDSI------SMAT-DE---
I-AG-------------------------NG-------------------K-GISNI-PLTLVVKKKG----------------------------------
-------------------------VPDLTIVDLPGITR--VP-------------------V-------HG--------------Q--------PE--
D--IYEQIS--------NIIM-DYI---KP------------KESIILNVLSATV--D-FPTCE-SIRMSQHVD------
KTGERTLAVVTKADKAP-------EGL------L-----EKV---------------------------TAD------------------D-
VN----------------------I---GLGYVCVRNR--I-GDE--TYE-----
---EARIE---EATLFKS--------HP-LL---------------------S-K---
---I--------D--KSI-VGIPVLAQKLFQIQANSLSQC-LPDIV
KAK1401877.1 ----------EGIQLPT--------IVVV---
GD----Q----------------SSGKSSVLE-----S--LA-G-I-SL-PRG--------------QG-ICTRVPL---------------
IMR--------------L---HHH-----------------------------SK---
---PE--------------SE-------------------------LHL----------E--------
-------Y--CGKV--VSTDEL-----------KIAESI------NMAT-AE---I-
AG-----------------------DG------------------K-GISHT-PLTLVVKKNG----------------------------------
------------------------VPDLTMVDLPGITR--VP-------------------V-------HG--------------Q--------PE--
N--IYEQIS--------GIIM-EYI---KP------------EESIILNVLSATV--D-FPTCE-SIRMSQSVD------
KTGDRTLAVVTKSDKSP-------EGL------R-----DKV--------------------------MAD------------------D-
VN----------------------I---GLGYVCVRNR--I-GDE--SYE-----
--EARMA---ESMLFES--------HP-LL---------------------S-
K------I--------D--KSM-VSVPVLARKLVQIQARIIAKC-LPEIV
XP_038984915.1 ----------EGIELPT--------IVVV---
GD----Q----------------SSGKSSVLE-----S--LA-G-I-SL-PRG--------------QG-ICTRVPL---------------
IMR--------------L---QDD-----------------------------PSL---
---SQ--------------PQ-------------------------LQL----------E-------

--------Y--KDKA--IHTSED-----------GIADAI------NSAT-DD---
I-AG------------------------SG-------------------K-GISNA-PLTLVVRKRG---------------------------------
---------------------------VPDLTMVDLPGITR--VP-------------------V-------HG--------------Q--------
PD--N--IYEQIS--------NIIM-EYI---AP------------KASIILNVLSATV--D-FPTCE-SIRMSQSVD------
RTGERTLAVVTKADKAP-------EGL------L-----EKV---------------------------TAD------------------D-
VN-----------------------I---GLGYVCVRNR--I-GDE--SYE-----
---EARAE---ERNLFKR--------HP-LL---------------------S-R-
-----I--------D--KSI-VGIPVLAQRLMQIQAASIAKS-LPDIV
XP_002297993.1 ------------IQLPT--------IVVV---
GD----Q----------------SSGKSSVLE-----S--LA-G-I-SL-PRG--------------QG-ICTRVPL---------------
IMR--------------L---QHH------------------------------TS--
--LI--------------PE--------------------------MFL----------E----------
-----F---NGKT--TQTDEA-----------NVADDI------NIAT-EE---I-
AG------------------------SG-------------------K-GISDA-PLTLVIKKNG------------------------------------
------------------------VPDLTMVDLPGITR--VP-------------------V-------HG--------------Q--------PD--
N--IYEQIA--------GIVM-QYI---QP------------EESIILNVLPASV--D-FTTCE-SIRMSRQVD------
KTGERTLAVVTKADKAP-------EGL------L-----EKV---------------------------TAD------------------D-
VN-----------------------I---GLGYVCVRNR--I-GDE--SYD-----
---NARME---EANLFAT--------HP-LL---------------------S-
R------I--------D--KSI-VGIPVLAKKLMQVQATIMAKC-WP---
XP_024439231.1 ------------IQLPT--------IVVV---
GD----Q----------------SSGKSSVLE-----S--LA-C-I-NL-PRG--------------DG-ICTRVPL---------------
IVR--------------L---KHH------------------------------PS--
--LV--------------PE--------------------------IFL----------Q----------
-----F---NGKT--VPTDEA-----------HVADAI------NLVT-DE---I-
AG------------------------NG-------------------K-GISNT-ELTLVVKKNG-----------------------------------
-------------------------VPDLTLVDLPGITR--VP-------------------V-------HG--------------Q--------PE--
N--IYEQIA--------YIIM-KYI---SP------------DESVILNVLSASV--D-FSTCE-SIRMSQKVD------
KNGQRTLAVVTKVDKSP-------EGL------L-----EKV---------------------------TRN------------------D-
VN-----------------------I---GLGYVCVRNR--I-GNE--SYE-----
---DARKE---EAALFAT--------HQ-LL---------------------S-K-
-----I--------D--KST-VGIQVLAQKLVQIQANIIAKC-LP---
KAH0683503.1 ----------EGIQLPT--------IVVV---
GD----Q----------------SSGKSSVLE-----S--LA-G-I-SL-PRG--------------QG-ICTRVPL---------------
VMR--------------L---KND------------------------------PNI---
---TA--------------PN--------------------------LQL----------E-------
--------Y--NNKS--LPVDEI-----------GIADAI------ILAT-DE---I-
AG------------------------HG-------------------K-GISNN-PLTLVVKKNG----------------------------------
--------------------------VPDLTMVDLPGITR--VA-------------------V-------QG--------------Q--------
PE--D--IYEQVY--------DIIM-KYI---VP------------EESIILNVLSATV--D-FPTCE-SIRMSQKVD------
KTGERTLAVVTKADKAP-------EGL------L-----EKV---------------------------TAD------------------E-
VN-----------------------I---GLGYVCVRNR--I-GNE--SYE-----
---EARSD---EQRLFST--------HP-LL---------------------S-K-
-----I--------D--KSM-VSVPILAQKLVRIQASIISKC-LPEIV

PWZ56863.1 -----------GIQLPT--------IVVV--GD----
Q---------------SSGKSSVLE-----S--LA-G-I-SL-PRG-------------QG-ICTRVPL--------------VMR--
------------L---QDD------------------------------PSA---
---DS-------------PK--------------------------LQL---------E------------
--Y--SNGRV--VTTTEA----------DVADAI------NAAT-AE---I-
AG----------------------SG-------------------K-GISDA-PITLVVRKRG------------------------------------
------------------------VPDLTLVDLPGITR--VP------------------V-------HG-------------Q--------PE--
D--IYDQVA--------KIIK-EYI---AP------------KESIILNVLSATV--D-FPTCE-SIRMSQQVD------
RSGERTLAVVTKVDKNP-------EGL------L-----EKV---------------------------TMD------------------D-
VN----------------------I---GLGYVCVRNR---I-GDE--TYD-----
---QARVE---EERLFKY--------HP-LL--------------------S-K-
-----I--------D--KDM-VGIPVLANRLMQIQSTIIAKC-LPDI-

PWZ56864.1 -----------GIQLPT--------IVVV--GD----
Q---------------SSGKSSVLE-----S--LA-G-I-SL-PRG-------------QG-ICTRVPL--------------VMR--
------------L---QGD------------------------------PST---
--DS-------------PK--------------------------LQL---------E-------------
-Y--SNGRV--VTTTEA----------KVADAI------NAAT-AE---I-AG-
----------------------SG-------------------K-GISDA-PITLVVRKSG--
-------------------VPDLTLVDLPGITR--VP------------------V-------QG-------------Q--------PE--D--
IYDQIA--------NIIK-EYI---TP------------KESIILNVLSATV--D-FPTCE-SIRMSQQVD------
RTGERTLAVVTKVDKAP-------EGL------L-----EKV---------------------------TMD------------------D-
VH----------------------I---GLGYVCVRNR---V-GEE--TYD-----
---QARVA---EAQLFKN--------HP-LL--------------------S-
Q------I--------D--KSM-VGIPVLAQRLMQIQASIIAKC-LPDI-

KAH9291961.1 -----------GIQLPY--------IVVV--
GD----Q----------------SSGKSSVLE-----C--LT-G-I-SL-PRG-------------VG-ICTRVPL---------------
IMR-------------L---QNS-----------------------------SE--
---QD-------------SE--------------------------IVV----------E---------
------Y--NDTV--EHIIES-----------QITERI------DSIT-KE---I-
AG------------------------TN-------------------K-GISHV-PIRLNVKKMN------------------------------------
------------------------APDLTLVDLPGIAR--VS-------------------L-------NG-------------N--------PD--
D---HELIS--------KIVM-EYI---SP-----------ADSIILNVLSATV--N-FRTCE-SIRMSQRVD------
VHGERTLGVVTKVDIAP-------EGL------L-----EKV---------------------------ALD------------------D-
VN----------------------T---GLGYVCVRNR--V-GDE--CNE---
---EAREA---EAELFRS--------HT-QL--------------------N-
K------F--------D--EAM-VGIPMLARRLMQIQTKRISKC-FPDI-

KAH9325151.1 ------------IQLLS--------IVVV---
GD----Q----------------SSGKSSMLR-----S--LA-G-I-NL-PKG--------------QW-ICTRVPL-------------
-IMR-------------L---QIS-----------------------------KS--
---QE-------------TE--------------------------ITI---------E-----------
----Y--SGVK--NNIFEY-----------KIIEAL------NAAT-DE---I-
AG-----------------------VG-------------------K-GISDT-PITLNVSKSN-------------------------------------
------------------------IPNITMVDFPCITK--MP------------------V-------HD-------------Q--------PQ--
D--IYDQIS--------QVIK-QYI---TP------------KESIILNVLFTSV--D-FPTCE-SIRMSQLVD------

VKGEKTLAVVTKVDKAV-------EGL------F-----EKV---------------------------TVD--V----V---------S-
VN----------------------I---GLGYVCVRNK---I-GNE--SNA-----

KAH9300179.1 -------------NCLV--------LLL--
---W-ETSRLE----------------------------------

--PG--------------
-----R-SINDT-PITLNVGKSN--
APDLTMVDFPSITR--VP-------------------V-------HG--------------Q--------PQ--D--IYDQIS--------
QVIK-QYI---TP------------KESIIMNVLSASV--D-FPTCE-SIRMSQLVD------
EKGERTLAVFTKVNKAA-------EVL------F-----EKV---------------------------TVD------------------A-
VN----------------------I---GLGYVCVRNR---I-GNE--SNA----

KAH9314974.1 ---

--AL------NAAT-NE---F-AG------------------------AG----------------
---K-SISDT-PITLNVSKSN--
APNLTMVDLPGITW--VP-------------------V-------HG--------------Q--------PQ--D--IYDQIS--------
QVIK-QYI---TP------------KESIIMNVLSASV--D-FPTCE-SIRMSQLVD------
EKGERTLAVVTKVDNAA-------EGL------F-----EKV---------------------------TVD------------------V-
VN----------------------I---GLGYTCVYSF---A-KRS--RNLA----
-VQACDY--------LD--------------------RI----------ICRVI---DPQLQASS------RRA-FQ------------------
---A-L------I--------D--RKR-DKCIQYVEDAMEMQKSIVYTE-NP---
EFJ22917.1 ------------IKLPT--------IVVV--GD----Q-
---------------SSGKSSVLE-----S--LA-Q-V-DL-PRG--------------QG-VVTRVPL-----------------VLR-----
---------L---QNT------------------------------SVT--
-------------------------------DQS--------------HQ--------------------------VVI----------Q---------------
Y---GGKK--RVIEEA-----------EISAAV------VEAT-IE---L-AG----
--------------------D--------------------K-HIVNK-PISLHITKPG---
---------------APDLTMIDLPGITR--VP-------------------V-------HG--------------Q--------PE--D--
IEEQIK--------KIIQ-EYI---SP------------KETIILNVICSTV--D-FPTCE-SILMSRQVD------
REGERTMAVVTKVDMSP-------KDL------K-----EKV---------------------------MAD-----------------V-
VG----------------------I---GLGYICVRNR---I-GDE--THE------
--EGRDR---EAELFRT--------DP-HL---------------------R-D-
-----L--------P--ESM-LGIRQLAKRLTEFQADSLRKN-LP---
XP_005823288.1 ------------LDLPQ--------IAAL--
GD----T----------------SSGKSSVLS-----A--MS-S-V-VF-PSR--------------SD-ITTRCPT---------------
RLC--------------M---DTG------------------------------EQF---
---QCK----------MQVLWH--------------------------SKP----------

A---------------SGS-N---PEVKKSVTQAS-----------DVTAAI------
EELQ-KS---V-LD------------------------HE---------------KT-ES-GVSKS-IIEICLTGPN-------------------
---------------------------------------YPNLTLIDLPGIVR--ST-------------------G-------DG--------------
E--------SL--E--MIRDIQ--------SVME-QYL---NN------------KRCILLAVHPANI--D-RHNNE-
VFRLTKEVD------PDSARTIPVITKIDLVE-------KGT------E-----GNV---------------------------MRL--
IQ---GK---------E-AK----------------------F---SLGFSVVKCR--
-T-QTE--LDQ-----------G--------VT-------------------LE----------KSLEQ---EMIFFRD--------TS-PW--
-------------------N-Q--------M------KQ-DNM-FGISNLTGKLSQIYVEMIQST-MPAV-
KAJ1441760.1 ---------------------------M--
GD----T----------------SSGKSSLLS-----A--LS-G-I-LL-PAN--------------DQ-ITTRCPT---------------
RLR--------------M---ENA------------------------------EQR---
--RAS----------ISVRWQ-------------------------FTS----------G---
------------YKS-E-AAYK--------L--TH-----------------------LSEEKDGASFYS-----------NITSEI------
EKAQ-KA---I-ID------------------------TS---------------KL-E---VTRD-IIEVEYCGPD---------------------
-------------------------------------CYNLTLTDLPGIVR--VA-------------------G-------KG--------------
E--------SA--S--IIVDIQ--------LLIK-EYL---EN------------ERCVVLAVVPANV--D-FHNSG-
IMADAEKYD------PTTRRTIPVITKPDLID-------KGA------E-----GGV---------------------------LKL--
LL---GEM--------T-CT----------------------F---QMGFHMVKCR---
----G-QAQ--LNE-----------G--------VT-------------------LE----------QGVEK---EAQFFKN--------ED-
PW--------------------R-K------ESE------KR-ADL-FGVPALRRKLEALQMRMIQES-IPSI-
KAG8471152.1 --------DFRMPELPQ--------MVVV-------------------------------------
-----GT----Q----------------SSGKSSLLN-----A--IM-A-C-DILPLG--------------ES-MVTRCPL----------
-----HLQ--------------L-V-HAP-----------------------------SE-SAG---------------------------------------
---VMRAE--------------F--------------------------GTY----------
A---------------GGS---------------WQAQRT--------------------IILTAPTPSASDIT-----------QIRREI------
EAQT-NA---L-AG-----------------------PG-------------------K-CIASQ-PIHLRIHSAY-------------------
---------------------------------------VPDLSLVDLPGLTQ--MAL------------------TT------QG-----------
---Q--------PR--D--IKEQIR--------ALVL-EHI---TK------------PRAVILAVLPART--D-LEADA-
ALELVKEVD------PRGERTIGVLTKVDLMN-------QGS------D-------I---------------------------ASF--
LVD-DGRTP------SD-LR-----------------------M---EYGYYAVRNR---
------S-TAEVVKDG--------------------MS-------------------VD----------GGFER---EATFFAM--------
HP-IY--------------------G-A------LR-------PAARAR-LGVPALARALSKLLVN-----------
KOO34643.1 --------RLKQPEVPR--------LVVV--GT---
-Q----------------SSGKSSLLN-----G--IM-G-A-DILPLG--------------EE-MVTRAPL---------------
ALQ--------------L-V-HHA-----------------------------EP-SE---
--MRAE--------------F--------------------------GTF----------V-----
----------NGG---------------WCVEET--------------------VELACPDPKAAQLE-----------RIRKAI------
EAQT-AA---R-AG------------------------SQ-------------------K-GVTNE-AIFLRLYSPH-------------------
---------------------------------------VPNLSLVDLPGLTM--TAL------------------TA------QG------------
--Q--------PK--D--IKQQIR--------QMIA-SYI---QP------------ARTIILMVCPARA--D-LEADP-
AVELAREYD------PQGTRTVGVLTKVDLMN-------KGT------D-------V---------------------------SKY--
L---TNALP------SD-LQ----------------------L---SLGYFAVKMR---
---G-PA---EKG--------------------LT-------------------VR----------EGYGS---EAEYFKA--------HA-TY--
-------------------G-R------AF-------APFAER-LGVPPLSKFLARVLLG-----------

XP_005775544.1 --------RLREPELPR--------
LVVVGARRSRSGIRSKNSRSPHRRAPSQPFREKKQRSRSSTVVHLPSSGT----Q---------------
SSGKSSLLN-----G--FL-A-A-DILPLG-------------EQ-MVTRAPL--------------NLQ-------------L-V-
HSP-----------------------------DP-AE--
------------------MRAE-------------F-------------------------GDY---------S--------------CGA--------
-------WQCAAA-------------------VPLAFPDPTPPQLQ----------QIRASI------EAQT-EA---R-AG-----
-------------------AQ------------------K-GVSAA-PIFVRYYSPN--
----------------VPNLSLVDLPGLTM--TAL-----------------TD------HG-------------QHRDIPYPTPR--
D--IKRQIR--------DMIA-AYI---RP-----------ERTIILMVCGARA--D-LEA-P-C-----QRS------
SGGARTVGVLTKVDLMN-------AGT------D-------VARDLEGSARYPEGSARYLEGSARYLEGSARY--
L---EGSVP------SD-LR---------------------L---ALGYFAAK--
--G-P-----QG-------------------LT------------------VL---------DGFRV---EREYFGK--------HA-AY---
------------------S-A------LG-------AAGRVR-LGVPLLSRFLSRVLLQ-----------
ATZ81043.1 ------------LKFPK--------IVVV--GG----
Q----------------SSGKSTVLN-----N--LI-T-M-NILPTG-------------SE-MVTRTPL--------------CME--
------------M-N-NSD-----------------------------IP---
----------------------------------HIE-------------F-------------------------GQY---------N-------------
-IAN-E-SDGN-------VWKCGRN-------------------IKVTYPEPTQEEIN-----------AIRIEI------ERQT-
IL---L-AG-----------------------KD------------------K-NISHN-PITIKIFLPN----------------------------
-----------------------------VPNLTLVDLPGITQ--VAC-----------------KD------KG-------------Q-------
-PD--D--IKEQIE--------KLIG-SYI---KS-----------EETIILSIIPART--D-VEADV-GVGLVKKYD------
PDFSRSIGVLTKVDLMN-------VDT------D-------V--------------------------SSY--VC---GNIS------
KN-LR----------------------M---NYGYYLVRNR--T-NKE--
MNE-------------------IT------------------MR---------EGFLK---EQAFFEE--------HQ-VY------------
--------G-R------MS-------DVDKRK-MGTNNLRDKLVNVLSNKIKEL-MPII-
ARF10282.1 ------------LSLPR--------LVVV--GT----
Q----------------SSGKSSVLN-----S--II-T-M-DILPTG-------------KL-MTTRTPL--------------ELN-----
---------L-I-KIP-----------------------------KN-NA--
-------------------------------SYVE-------------F-------------------------GNY---------D--------------
NEK--------------WFQEKK-------------------ITITVPIPTPNEIT----------TIRNHI------QDKT-NE---I-
CG-----------------------NN------------------M-NISNV-PIILKIYSPN-----------------------------------
-----------------------VPDLSLIDLPGLTM--VAC-----------------ED------KG-------------Q--------PV-
-D--IKERIE--------NLVS-SYI---IQ-----------PKTIIIAVMQARA--D-LETDL-GLALVKKYD------
KSGSRTIGVLTKPDLMN-------QDS------H-------V-------------------------GDY--LL---NKIS------
KN-LM----------------------L---NYGYYLLKNR--N-DIQ----E-
-------------------ID------------------IL---------TGFKL---ETEYFNA--------HN-EY-------------------
K-K--------------SLYKPK-LGINNLTNNLTKILVQSLNEA-IPVS-
QKF94243.1 ------------LALPR--------LCVV--GT----
Q----------------SSGKSSVLN-----A--IM-S-M-DLLPTG-------------KN-MVTRTPI--------------DLR-
-------------L-H-QLK------------------------------GS-TD---
------------------------------------GWVE-------------F-------------------------GDY---------T---------
-----GEG--------------WITEKK-------------------ISIKMPIPLDSEIT----------EIREYI------MKKT-ND-
--V-AG-----------------------EG------------------M-NISPK-PIIINIYSPN-------------------------------
--------------------------VPNLSLVDLPGLTM--VAC-----------------TD------KG-------------Q--------

PE--D--IRERIE--------NLVV-SYI---KE------------KKTIVIAVMQARS--D-LETDI-GLALIKKYD------
ISGQRIVGVLTKPDLMN-------HET------N-------I---------------------------GEY--LT---NNIS------KN-
LM----------------------L---TYGYYVVKNR--N-GQE--MKD---
-----------------VN-------------------IL----------KGFEL---EKEYFNN--------HY-EY---------------------
K-K---------------PLYKDR-IGSHNLTASLSKILIASITEV-LPSV-
ARF11508.1 ------------LTLPR--------IAVV---GT----Q-
---------------SAGKSSVLN-----G--IM-G-L-DLLPAG--------------RY-MTTRTPL---------------DIR-----
---------L-H-QLK------------------------------GDYKE---
-----------------------------------GYVE--------------F----------------------------GNY----------T--------------
-EEG---------------WETEKK--------------------IGIKIPIPLDSEIT-----------EIREFI------SKKT-NI---I-
AG------------------------DG-------------------M-NISSQ-PIIINIYSPN-------------------------------------
-----------------------VPNLSLIDLPGLTM--VAC------------------VD------KG--------------Q--------PD--
D--IKERIE--------DLVV-SYI---KQ------------PKTIILAVMQSRS--D-LETDI-GLALTKKYD------
VNGQRIIGVLTKPDLLN-------PDT------N-------I---------------------------GEY--LI---NNIS------KN-
LM----------------------L---TYGYYVVKNR--N-GQE--MKD---
-----------------CN-------------------II----------KGFEL---EKEYFNN--------HN-EY---------------------
K-K---------------SIYKDR-IGTNNLTNNLSKILITSITEM-LPNV-
ARF09562.1 ------------LSLPK--------LVVI---GC----
Q----------------SAGKSSVLN-----S--II-S-M-DILPTG--------------KN-MVTRTPL---------------ELQ---
-----------L-H-QLT------------------------------QDSKD---
-----------------------------------GWVE--------------F----------------------------GQY----------N----------
-----QNG---------------WTSEKK--------------------IPITTPIPTETEIK-----------NVRDYI------SAKT-IE--
-L-AG------------------------DG-------------------M-NISHI-PIMMNIYSPN--------------------------------
-----------------------------VPNLSLVDLPGLTV--IPC------------------VD------KG--------------Q--------
PV--D--IKEKIE--------ELVA-SYI---KQ------------ERTIVLAVMQSRN--D-LETDI-GLGLIKKYD------
NGNQRIIGVLTKPDLMN-------QET------H-------I---------------------------GEY--LI---NNIS------
KN-LM----------------------L---TYGYYVVKNR--A-GIQ---GS-
-------------------MD-------------------VF----------KGYEL---EKEYFNS--------HF-EY--------------------
--K-K---------------IIYKDR-IGTDNLTKNLSKILISSITEL-LPSV-
AYV76902.1 ------------LTLPQ--------IVVC---GT----
Q----------------SSGKSSVLN-----S--II-S-M-DILPTG--------------KT-MVTRTPL---------------SLR----
----------L-H-QLS------------------------------PDTKE---
-----------------------------------GLVE--------------F----------------------------GNY----------E--------------
-TGT---------------FISEKK--------------------ISITVPIPTENEIK-----------TIRDFI------MAKT-DE---I-
CG------------------------VG-------------------M-NISTK-PITINIYSPN-------------------------------------
-----------------------VPNLSLVDLPGLTV--VAC------------------QD------KG--------------Q--------PV--
D--IKDRIE--------ALVS-SYL---KQ------------ERTIVLLVMQAKC--D-LETDL-ALGLIKKHD------
SGNQKIIGVLTKPDLMN-------MDT------H-------I---------------------------GDY--LC---NNIS------
KN-LK----------------------L---AFGYYVVKNR--S-NL-----S--
-------------------ID--------------------IF----------KGLEL---EKEYFIN--------HN-EY--------------------
K-K---------------SIYKDK-LGTVNLTQNLNKILVSEITDL-LPSV-
ARF08414.1 ------------ITLPK--------IVVV---GT----Q--
--------------SSGKSTVLN-----A--IM-A-L-DILPTG--------------KN-MVTRTPL---------------DIR-------
-------L-H-KNK------------------------------IN--E---

-----------------------------ARIE-------------L-------------------------GFY---------G--------------
DSG---------------WISEKN-------------------IKLSLPNASKDEID-----------QIRQYI------SQKT-IE---
L-AG-----------------------NE------------------M-NISKN-PIYIQIFSPN---------------------------------
-------------------------VPDLSLVDLPGLIM--VAC-----------------TD------KG-------------Q--------
PK--N--MPEQIE--------KLVE-SYI---KD-----------NNTITLAIMQSRS--D-LETDL-GLALIKKYK------
CS--KTVGVLTKPDLMN-------NDS------H-------I---------------------------GCY--LN--GNNIS------
KD-LM----------------------L---DYGYFVVKNK---S-SMD--
NDE------------------YD------------------IQ---------KIIDM---EKKYFAN--------HN-EY------------
--------N-K--------------PIYQNK-IGYNSLIKELTKVLISAIRDT-MPKA-

AYV78912.1 ------------IMMPQ--------LAVV---GS----
Q----------------SSGKSSLLN-----N--IM-A-MPDLLPTG-------------RD-MVTRSPI--------------
RLC--------------L-H-KTK-----------------------------TT--D--
---GYIE-------------F-------------------------GTY---------N-------
-------DAG---------------WAVEKK-------------------ITLTMPNPTTAEIQ-----------EVRENI------KAKT-
IE---I-AG----------------------AQ------------------M-NISHT-PIVLQIYTPN---------------------------
------------------------------VPDLILIDLPGLVM--TSC-----------------VD------KG-------------Q------
--PS--D--IDEQIQ--------NLAL-SYV---KN-----------PKTIVLLVMQASN--D-LQTDI-GLAFLKKHD---
---VN-RNIVGIFTKPDLMN-------SDS------H-------V---------------------------GDY--LL---GKTS------
SN-LM----------------------L---QHGYFVVKNK---S-SSD---
TS--------------------SD------------------IL----------KSTDI---EKKYFQN--------HF-EY----------------
----K-K--------------SIYQNR-VGYNSLIAELTKILVAAINNN-IPSV-

VBB18790.1 -----------FLDPPT--------LCVV---GS----
Q----------------SSGKSITLN-----G--LT-G-I-DILPNG-------------KS-IVTRTPI---------------HLR------
--------L-I-HVK-----------------------------ES-KN---
------------------------------IVVE-------------F-------------------------FDK---------D--------------
DSQ------K-------LISTFTV-------------------DALTTP---ADQLT-----------PIREEI------VKLT-EM---Y-
AG----------------------RS------------------K-NVVDT-PINVRIKSPS----------------------------------
-----------------------VPNLSVIDLPGLTH--IAL-----------------TD------QG-------------Q--------PE--
N--IKESIE--------NMII-KYI---KN-----------PRTIILSIIPATV--D-VESDA-GLGLIKKHD------
PDFKRTIGVLTKVDML--------KDS------N-------V---------------------------ENY--LC---GKIS------
RN-LQ----------------------L---GYGYYAVRNR---S-SDE--
VKV--------------------MS------------------VK---------DGHAL---EAKFFAE-------TE-PY------------
--------K-S--------------SEMRQR-TGSINLGIKLSEVLLAHLRSC-LPAV-

ABI33144.1 -----------GIALPQ--------IAVV---GD----
Q----------------SSGKSSVLE-----N--IT-G-I-PF-PKG-------------TG-TVTKCAT--------------RIT------
--------I---RRA----------------------------LKR---
-----------------------------TTP-------------FS------------------------ARV---------S--------------FVI-
N-GK-------------NSP-------------------FKNEGPKMNHTSVG-----------KLQKTL------ERLN-EL---
IHQN-----------------------ES------------------K-TDIDD-IIEVDIEERH----------------------DSD------
----------------------------AVDLSIVDLPGLIA--TT-------------------T-------EG-------------Q--------SK-
-D--LVGSIE--------KMVS-RYA---ED-----------KRTVILAIMEAHR--D-ISNSR-ALAMAEKAD------
EKGERTVGVLTKMDLVE-------EAT------E-----DDV---------------------------LNV--LN---NS--------
R-KP----------------------L---YHGYFAVVNR---A-QKT--LNE--

---------G--------KS-------------------LK---------DARRD---EKEWFER--------NK-HY-----------------
---M-S------M-----------DGR-CGIDNLKRGISKRLRV-----------
KAI8587516.1---------GQDVSLPQ--------LVVA--GD-
---Q---------------SSGKSSLLE-----S--LS-G-I-SF-PKA-------------AE-LCTTFPT--------------QIV--
------------M---RTK-----------------------------KTWE-ARV---
----------Y-----------------------TVPE---
-------I------------------------------------ANFPATNCASKV-----------AVQNII------QFIK-RD---VLKA-
----------------------TAG------------------VSET-VLVIELGSPE--
----------------LPNLTIIDLPGYVH--TL-----------------V-------KG-------------Q--------SE-NF---
KQKID--------NMVD-KFI---QD-----------RRSIILAVIPANK--D-FATNV-VLQRAQEWD------
PEGDRTIGVVTKPDLVD-------QGT------E-----AAV--------------------------IRM--IQ---GH---------
Y-KE----------------------L---KLGYYMVHNR--S-HMD--
LQN-----------G--------VD--------------------LA---------AAVAK---EAEFFS---------QP-AW----------
----------D-A------L--------N--ARQ-LGTQQLQTAVVEVLAAHVVKE-F----
OLL24579.1 ---------NSVISLPQ--------IVVC--GD---
-Q---------------SSGKSSVLE-----A--LT-Q-I-PF-PRS-------------DG-LCTKFAT--------------QVI----
----------L---RRA-----------------------------SKTS-VRV--
-------Q------------------------IIPDC---KRP---------E-------------
-AEQ-R-AL-------------------------------------QSVD-IKLKKLE-----------DMTILI------EEAS-KH---
MGVQ-----------------------SSSS-T--------S--------TFSSD-ILSIEVSGPK-----------------------------
---------------------------QPHLTVVDLPGYIR--TT-----------------S-------GN-------------Q--------
TK-KD---ITLIY--------DLVK-DYI---SD------------KRSIILAVIPANV--D-VANAE-ILEKASEAD------
PNKTRTLGVITKPDLVD-------NGA------E-----NQV--------------------------LDL--AA---NV---------
T-KP----------------------L---KLGYFIVRNR--N-YNE--LKS----
-------A--------SD------------------SK---------ARNKS---EAAFFA---------QS-PW--------------------
S-E------I--------N--KTR-IGIDRLRLYLSDLLQEHIFKE-L----
KAI9096888.1---------SEYVSLPQ--------LVVC--GD--
--Q---------------SAGKSSVLE-----A--IT-E-V-PF-PQN-------------QG-TCTRFAT--------------QIV--
------------L---RRS-----------------------------VVTE-AIV--
--------T-----------------------IIPES--RRT---------D--------------
NEV-A-KF------------------------------------AQFK-KKIEDLK-----------DLPSII------TEAE-AL---ILFS-
----------------------NNVR-T--------K---------FSKD-VLNVEISGPK--------------------------------------
---------------------QPHLTVVDLPGIIHTSSS------------------T------TE-------------D--------EE-
GDEFEVEVVK--------ELVK-GYM---KE------------DRTIILAIVAGNY--D-YNNQI-ILQMAKELD------
QDRTRTLGIVTKPDLQE-------VGSD----YE-----KTL--------------------------VKM--VK---NE---------
V-KH----------------------L---SLGWHVLKNR--G-FKE----R--
---------E--------CS------------------IE---------QRNIA---EEKFFN---------QG-VW------------------
--T-S------L--------P--RKD-VGVESLRIKLSNLLYQHIKRE-L----
RSH87279.1 ---------DSVLSLPQ--------IVVC--GD---
-Q---------------SSGKSSLLE-----A--LT-E-I-PF-PRN-------------DN-LCTRFAT--------------EIS-----
---------L---RRE-----------------------------SESS-LTI---
-----R------------------------IIPAH---SRP---------Q--------------
EEQ-K-RL-------------------------------------ERFS-EVITDFE-----------DLPAVM------DKAM-QE---
LGIS-----------------------EGTG-A-----------------FAKD-TLSVEIQGPD------------------------------

----------------------------RPQLTLVDIPGLIQ--TS-------------------T-------RG--------------V--------SE-
AD---VALVA--------EITD-SYI---KQ------------PRTICLAVVSATN--D-AANQP-ILSRVRKFD------
PHGKRTLGVITKPDRLP-------SGSA----SE-----SKF----------------------------LEL--AR---NE---------
D-VF-----------------------F---KLGWHVIKNR--K-FEE----T----
-------A--------FS--------------------ID----------ERNLV---ERTFFA---------TS-NF---------------------K-
A------L--------P--RED-VGIDALRVKLSTLLFDHVKKE-L----
XP_041144356.1 ---------SHYISLPQ--------IIVC--
GD----Q----------------SSGKSSVLE-----A--IS-G-V-SF-PVK--------------SN-LCTRFPT---------------
ELV--------------L---RKS------------------------------SHIG-VKV---
---------------S-------------------------IVPHR--SRS----------H-----
----------VEQ-D-AL---------------------------------------SRFH-EELESFE-----------GLPTLI------ENAK-
AA---MGIF------------------------THGK-A------------------FSND-LLRVEVSGPD-----------------------
-------------------------------------RPHLTIVDLPGLIH--SE-------------------T-------KL--------------Q----
----SA-AD---VALVQ--------DVVQ-SYM---KE------------PRSIILAVVSAKN--D-FANQI-
VLRLAREAD------SFGHRTLGVITKPDTLV-------EGSE----SE-----YQF----------------------------VSL-
-AK---NQ---------E-VT-----------------------F---RLGWHVLKNM---
-----D-TEK----G-----------N--------YN-------------------LS---------VRGQE---EAEFFS---------RG-
IW---------------------E-D------L--------P--RSH-VGIDTLRQRLSKLLLGQIATE-L----
KAJ5704467.1 ---------SHYVSLPQ--------IIVC---
-GD----Q----------------SSGKSSVLE-----A--IS-G-V-SF-PVK--------------SS-LCTRFPT---------------
ELV--------------L---RKN------------------------------SQVG-VRV--
-----------------S-------------------------IVPHQ--SRS----------D---
------------AEQ-H-SL--GSFC-EQLDGFD-----------GLANLI------
ENAK-AA---MGIS------------------------THGK-A------------------FSND-LLRVEVSGPD----------------
---RPHLTIVDLPGLIH--SE-------------------T-------RQ------------
--Q--------SA-AD---VQLVQ--------DVVQ-SYM---RE------------PRSVILAVVSAKN--D-FANQI-
VLRLARDAD------PSGNRTLGVISKPDTLV-------PGSE----SE-----ASF---------------------------VSL-
-AK---NQ---------D-VE-----------------------F---RLGWHVLMNM--
------D-SEK----G-----------Q--------WS--------------------LS----------DRDIQ---ERKFFS---------GG-
IW---------------------E-D------L--------P--RSL-VGVDSLRTRMSSLLLGQIAGE-L----
KAI9774215.1---------SHYVSLPQ--------IIVC---GD---
-Q----------------SSGKSSVLE-----A--IS-G-V-SF-PIK--------------SN-LCTRFPT---------------ELV-----
---------L---RKT------------------------------SQIG-VSV--
-----S-------------------------IVPHQ--SRS----------E---------------
SEQ-H-TL--SSFH-EELDGFE-----------GLPALI------ENAK-AV---
MGIS------------------------THGK-A------------------FSKD-LLRVEVSGPD------------------------------
------------------------------RPHLTIVDLPGLIH--SE-------------------T-------KH--------------Q--------
SA-SD---VELVQ--------DVVQ-AYM---KE------------PRSIILAVVSAKN--D-AANQI-VLKLARAAD----
--KKGNRTLGVITKPDVLI-------PGSE----SE-----ASY---------------------------ICL--AR---NQ---------
N-VE-----------------------F---RLGWHVLRNT--D-SEM----G-
----------D--------WS-------------------LA----------DRDAQ---EKEFFS---------QG-IW------------------
----E-Q------M--------S--QSL-LGVDKLRSRLSKVLLGQIATE-L----
MCJ1392161.1 ---------SHYVSLPQ--------IIVC---
-GD----Q----------------SSGKSSVLE-----A--IS-G-V-SF-PVK--------------SN-LSTRFPT---------------

ELV--------------L---RKT-----------------------------SQIS-VSV--
---------------S------------------------IVPHQ--ARS----------E-----
----------SER-L-IL-------------------------------------SSFH-EELEGFE-----------GLPSLI------ENAK-
SA---MGIS------------------------TLGK-A------------------FSKD-LLRVEISGPD-------------------------
----------------------------------RPHLTIVDLPGLIH--SE------------------T-------KQ--------------Q------
--SA-SD---VELVQ--------DVVQ-SYM---KE------------PRCIILAVVSAKN--D-YANQI-VLKLARAAD--
----KKGTRTLGVITKPDTLI-------AGSE----SE-----AMY---------------------------VSL--AR---NQ-------
--D-VE-----------------------F---RLGWHALKNM--D-SET----
G-----------E--------WS--------------------LA----------ERDVE---EQEFFS---------QG-IW--------------
-------R-D------M--------S--RSL-LGVDDLRSRLSKVLLGQIAAE-L----

XP_002543522.1 ---------SHYISLPQ--------IIVC---
GD----Q----------------SSGKSSVLE-----A--IS-G-V-AF-PVK--------------SN-LCTRFPT---------------
ELV--------------L---RKS-----------------------------PEVH-VSV---
----------------S------------------------IVPHR--SRS----------E-----
----------SEQ-H-SL-------------------------------------ESFR-EDLDSFE-----------GLPELI------ENVK-
TA---LGIS------------------------THGR-A------------------FSND-ILRIEISGPD--------------------------
---------------------------------RPHLTIVDLPGLIH--SE------------------T-------KQ--------------Q-------
-SA-SD---VDLVQ--------DVVQ-AYM---RE------------PRSIILAVISAKN--D-IANQI-VLKLARGAD----
--RFGRRTLGVITKPDTLI-------PGSA----TE-----SIF---------------------------VSL--AK---NQ---------
E-VD-----------------------F---RLGWHVLKNM--D-SEK----G-
----------I--------ST--------------------LA----------DRDVE---ERQFFS---------QG-VW------------------
---K-D------I--------A--PST-MGIARLRGRLSKVLLGQIATE-L----

XP_746402.1 ------------ISLPQ--------IIVC---GD----
Q----------------SSGKSSVLE-----A--IS-G-V-SF-PIR--------------SS-LCTRFPT---------------ELV------
--------L---RKS-----------------------------SQVG-VCV---
------S------------------------IVPHR--SRS----------E--------------
SER-E-AL-------------------------------------AQFH-EELDSFE-----------GLPQLI------ENAK-SA---
MGIY------------------------TNAK-S------------------FSND-LLRVEVSGPD------------------------------
-----------------------------RPHLTIVDLPGLIH--SE-------------------T-------KQ--------------Q--------
SA-AD---VELVH--------DVVK-SYM---EE------------PRSIILAVVSAKN--D-VPNQI-VLKLARAAD-----
-PHGTRTLGVITKPDTLV-------RGSD----SE-----AQF---------------------------VSL--AK---NQ---------
E-VE-----------------------F---RLGWHALKNM--D-TDK----G--
---------A--------WT-------------------LA----------ERDKE---EHAFFA---------SG-VW------------------
---E-A------L--------P--RSH-VGIDQLRKRLSKLLLAQIATE-LPSL-

XP_751069.1 ------------VELPQ--------LIVC---GN----
Q----------------SSGKSSVLE-----A--IS-R-V-RF-PAK--------------SN-VCTRFAT---------------EVI-----
---------L---RRN-----------------------------AAFSKIKV---
------S-------------------------IEPGP--SRTD---------E--------------
-DER-R-RL--------------------------------------RSFTYEDFSNGD----------DLPPLI------EKAK-VH---
MGIT-------------------------ESVN-T--------G---------FSDD-VLKVEISGPD------------------------------
-----------------------------KPELTLVDLPGLYY--ST------------------S-------QE--------------Q--------
DL-QG---ILIVR--------KLTE-RYM---SN------------PRSIILAVISAKT--D-YHLQE-VLNIAEQFD------
PKRERTLGIITQPDILE-------ANSE----EE-----DTY---------------------------LHF--VK---NE---------K-
IP----------------------L---ELGWHVLRNR--S-FET----R-------

----D--------IS--------------------DD----------ARDEM---EKAFFN---------QG-RW--------------------
A-S------L--------S--REC-VGIESLRRRLSGVLLRLIRRN-LPGL-
XP_026607910.1 ---------GEVVALPQ--------LAVC---
---GD----Q----------------SAGKSSVLE-----A--IT-G-I-PF-PQQ-------------DG-LCTRFPT-------------
--EIT-------------L---RHS-----------------------------EATQSITIF--
---------------AS-------------------------IRPHS---VRS---------R----
-----------KEK-D-YL---ASYQ-KTLGAIS-----------ELPSII------ADAS-
KL---MGIR--------------------------GYGG-Q--------KNGP-----AFAAD-VLRIEITGPI---------------------
-----------------------------------GLQLSVVDLPGLIS--VV------------------S-------EE-------------
Q--------NE-ND---VVMIH--------DMVT-SYL---QS------------SRTIILAVVQASN--D-FANQC-
IIRMARKHD------PEGQRTVGIITKPDLIN-------QGT------E-----SKI----------------------------ARI--
AK---NL---------DTIK----------------------L---KLGFFLLKNP---
S-PME--RKD-----------C--------HS---------------------MT----------ARSAL---EDRFFS---------RP-SW-
--------------------A-IH----HL--------D--KKR-IGSESLRTFLQKLLDSHIEHE-L----
XP_748757.2 ------------ISLPQ--------LVVS--GD----
Q----------------SSGKSSVLT-----A--VT-G-F-SF-PRR-------------EG-TCTRFAT---------------EII------
--------L---RHS------------------------------KETETIIT--
--AS-------------------------IIPSL--------------------------------------SRH---------D---------------
GSE-E-AL--KRFK-KVLKSTE-----------ELPSVI------HEAS-VA---
MGIR-------------------------GYSD-S--------DDSP-----AFTAD-VLRIEVVGDT---------------------------
-------------------------------GLCLTIVDLPGLIS--VS-------------------D-------YD-------------E--------
GE-AD---VQLVN--------TLID-SYL---AN------------TRSIILAVVQASN--D-IQNQN-IIQRARRFD------
KLGERTVGIITKPDLVN-------KGT------E-----SHI----------------------------VRL--AN---NL---------
DIVR-----------------------L---KLGFFLMKNP---S-PEQ--LKN--
---------N--------IS-------------------MF----------EWKQK---ELEFFN---------SP-PW------------------
--K-DL----ML--------D--HNR-VGAECLRSFLEKILEEHIERE-LP---
XP_040633937.1 ---------GDHVALPQ--------LVVC---------------------------------------
----GD----Q----------------SAGKSSVLE-----G--IS-G-I-PF-PRQ-------------DG-LCTRFAT-----------
----EII-------------L---RH-------------------------------EPGEQRAT--------------------------------------
------------------AM-------------------------IIPHV--SRT----------D-
--------------EEK-S-SL--GAFH-RNISDFT-----------ELPIIV------
EEAR-SI---MDIH-------------------------GHGI-G--------SNAS-----AFSAD-VLRLELVGST-------------
--GLHLTMVDLPGLIS--VS------------------------------------
----E--------NE-HD---VQLVR--------DLVD-SYL---EN------------SRTIIMAVVPASS--D-VDTQG-
ILQRARHFD------KTGLRTVGVITKPDLIN-------AGT------E-----PRV----------------------------ARL--
AR---NL---------DGAK----------------------L---NLGFFLLKNP--
---S-PAE--LEA-----------G--------TT--------------------LP----------ERRKV---ELEFFS---------SG-AW-
--------------------K-GQ----GL--------D--PSR-IGIDNLRSFLQDLLDHHIERE-L----
XP_754266.1 ------------IALPQ--------LVVC--GD----
Q----------------SAGKSSVLE-----G--IT-G-I-PF-PRQ-------------DG-VCTRFAT---------------EII------
--------L---RH-------------------------------EPNHRRNT---
------AT------------------------ILPHI---SRT---------E---------------
EEK-A-KL-------------------------------------SAFR-REVSDLA-----------QLPGII------EEAA-RL---
MGVQ-----------------------GMND-L--------ADAP-----TFAAD-VLRLEIVGDT-------------------------

---------------------------------GLHLTLVDLPGLIS--VS---E--------
ND-DD---VQLVG--------DLVN-SYL---EN------------SRSIILAVVPASS--D-VDTQS-IIQRARRFD-----
-KDGFRTVGIITKPDLIN-------DGT------E-----GRI---------------------------AKL--AN---NA---------
DRTK-----------------------L---RLGFFLVKNP--R-PID--LEK---
--------G--------MT-------------------TA----------ERRKV---EAEFFA---------HP-PW--------------------
-N-KL----GL--------D--PSR-VGIDNLRIFMQDLLDRHIERE-LPKV-
XP_043140374.1 ---------GDHIALPQ--------LVVC---
---GD----Q----------------SAGKSSVLE-----G--IS-G-I-PF-PRQ--------------DG-VCTRFAT------------
---EII--------------L---RH-------------------------------EPNDQRNT---------------------------------------
------------------AT-------------------------IIPHM---SRA----------E--
-------------DEK-V-RL--SAFH-RDINDLV-----------NLPGIV------
DEAA-RL---MGVQ------------------------GAND-S--------TDAP-----TFAAD-VLRLEVVGDT------------
--GLHLTLVDLPGLIS--VS---
-----E--------NE-ED---VQLVG--------NLVN-SYL---EN------------SRTIILAVVPASS--D-VDTQS-
IIQRARRFD------KDGLRTVGIITKPDLIN-------DGT------E-----SRV----------------------------AKL--
AN---NA---------DRTK-----------------------L---KLGFFLLKNP--
--R-PID--LEK-----------G--------MT-------------------MV----------ERRKM---EAQFFA---------NP-
PW---------------------N-NL----GL--------N--PSR-VGIDNLRVFMQDLLDRHIERE-L----
KXS17655.1 ---------NHLISLPQ--------IAVV---GD---
-Q----------------SAGKSSLLE-----A--IS-G-I-SF-PKD--------------KE-MCTTFAT---------------QIV----
----------M---AKG-------------------------------ASFA-AKV--
--------T-------------------------IDPDP---SNI-------------------------
--SVGLPVPKSPL-----------DVAAVI------EEAK-NL---
MSEG------------------------NSNL-I------------------IADK-ILTIELTGPN------------------------------------
--------------------------YPRLTLVDLPGYVQ--SV-------------------I-------KG--------------Q--------SE-
TI---IEDIA--------DIVD-RHL---KD------------ERTITLAVIPANK--D-LATNV-VVGKVDKLG------
SNGARTLGVITKVDVID-------AGE------E-----EAV----------------------------LEI--LH---GR---------R-
CD-----------------------F---GLGFHAVRNR--N-WAE--VNG--
---------S--------LS--------------------TE----------ELLVK---EAQFFA---------RA-PW---------------------
S-Q------L--------D--KSM-KGIVSLRSKLVEILHNHVEKE-L----
XP_021869222.1 ---------GAEVELPQ--------LVVV---
---GN----Q----------------SSGKSSVLE-----A--LT-G-I-PF-PRD--------------DG-LCTRFAT-------------
--RIT--------------F---RRA-------------------------------LETR-YQA---
-----------------K-------------------------IVPDK---LSS----------K----
-----------EHQ-D-KC-------------------------------------QQWGQ-ELESFDL---------FQIADLM------
KKVR-TV---MGVS-----------------------DKTS-D--------STYPA-GS-AFSND-VLSLEITGPG------------
--EEHFSIVDVPGTFKV-EA-------------------E-------GV------
--------T--------TK-ED---IKLVD--------DMVK-RYM---TN------------SRSIMLTVVNCND--D-ISSHD-
IIQKARDID------PHGERTLGILTKPDLAD-------EGA------E-----QKI----------------------------IDI--
LD---GK---------Q-HR------------------------L---FHGWHILRNR--
----G-QKD--LRD-----------A--------TS--------------------LS----------DRHAT---ERKFFTD--------KD-
PW---------------------N-K------L--------D--KSL-VGIDALNHRLHAVLATQLNKE-F----
TVY17522.1 ---------GDYIDLPQ--------VVVV---GD--
--Q----------------SSGKSSVLE-----G--LT-N-L-PF-PRD--------------SG-LCTKFVT---------------KIT--

------------F---RRS-----------------------------PMTR-IAI--
--------T------------------------IIPAK--NSS---------T--------------
EHV-E-HV-------------------------------------RGWAKADLRSLDE---------KTFADIM------KEVH-TV---
MGLS-----------------------EQVD-G--------MTPP-----TFSDD-VLSLEVCGPK-------------------------
---------------------------------QEHLSIIDVPGIFKK-RT------------------Q-------GL-------------T------
--SK-AD---IQMVK--------SMVL-GYM---KN------------PRSVMLTVIPANV--D-IVTQE-ILEMAEEVD--
----PDGQRTLGVLTKPDLVD-------KGA------E-----KTI--------------------------VDL--IE---GR--------
-R-HR----------------------L---ALGWLLVRNP---G-QQE--
LTD--------------------P-------------------MT----------DRHAL---EKSFFSH--------EQ-PW------------
---------N-S------L--------D--KEK-VGIPALQVRVRVILAEHIRRD-F----

KAF9951223.1 ---------NHVLSVPQ--------IAIV---
--GD----Q----------------SSGKSSVLE-----A--FT-Q-L-SF-PRD-------------KG-MCTRFAT------------
---QVN--------------L---CRD-----------------------------LTLDKDTL-------------------------------------
--S--ARI----------D---
------------DED--SFN-ERWKTFQV---------DQFYAVI------
KEAV-SL---LCGT-----------------------S---------------------DISDK-VLELTLSGPH--------------------
-------------------------------------QSPLTVVDLPGFIN--TT------------------L-------DG-------------
Q--------DK-NI---PHTIR--------DINE-RYM---KD------------PRTIILAVIPANV--D-LNNSY-
VLARAEEHD------PKNERTVPIVTKPDMID-------KGT------L-----SEL---------------------------IDM--
VL---NN---------R-KK----------------------M---PLGYLVMRNT---
---G-YAD----R-----------D--------LS--------------------WE----------EAQRA---EEDYFAQ--------DK-
AW---------------------E-A------V--------P--RSN-RGRVMVKKFLGDLLYFHIKKE-L----

XP_047808890.1 ---------QDMVSLPQ--------IAVV---
---GD----Q----------------SSGKSTLLE-----Y--IS-G-V-TF-PKD-------------AG-MCTCFAT-------------
--EVS--------------M---RPS-----------------------------TQFS-ARVF--------------------------------------
-------------------IN-------------------------HQPD---SRI------------
--KQPRSPE-----------DVAGVI------QQAK-
KL---FVEA------------------------SGQK-A------------------IYDD-ILTVELNGPG-------------------------
---------------------------------LPILTLVDLPGYIH--TH------------------A-------TG-------------Q------
--PE-SI---VKDIE--------QLVE-RYL---NS------------PRTVIMAVIPVNR--D-FETNV-AIKHIRRFD------
PSGKRTLCVLTKPDQVD-------AGT------E-----RNV--------------------------LDV--LA---GK---------
K-MH-----------------------L---DRGYHIIKNK---N-FEE--CQA--
---------G--------DN-------------------RE---------ATSKK---EGHFFA---------RS-PW-----------------
---S-S------I--------S--PTE-KGIASLVDRLSDTLNAQVEKE-F----

CAD7955538.1 ------------VEVPG--------VIVV--
GN----Q----------------SAGKSSVLE-----A--IS-G-I-NF-PRG--------------EN-TCTRCAS--------------
IVR-------------L---ECH-----------------------------PAEA--
---------------------------------------QDDRP--------------Y--------------------------ALL----------S------
---------CND-A-EQ--------------------------------------KQAQKIYDFA-----------KIGQQI------DALT-
KK---LGEGR----------------------DSG-------------------T-ILGDD-VIYITVRNKS--------------------------
---------------------------------GPTMTLIDLPGLTF--VH-------------------K-------TQ-------------K-----
-------N--IHDVTV--------ELIR-KYI---KN------------EQAVILAVIPATE--D-FGNCE-ALNLASEVD------
PEGERTLGVATKCDMIS-------EDS------D-------L--------------------------VQK--LT--MSRSS-------
D-IK----------------------L---KLGFVGVRCR---G-PAE--VKD--

---------N--------LP-------------------FD---------QMAVR---ERELFTT--------HR-AFTKNIT-----------
GTTSK-K------F--------P--RHC-WGMDTLVAKICQIQEQTVEKW-LPRV-
CAD7971727.1 ------------VEVPG--------VIVV--
GN----Q---------------SAGKSSVLE-----A--IS-G-I-NF-PRG-------------EN-TCTRCAS--------------
IVR-------------L---ESG----------------------------NLEA---
--NERA-------------Y-------------------------ALL---------S------
--------VND-P-EQ------------------------------------KQCDKIRDFS-----------AIGVQI------EKLT-
KK---LGEGQ----------------------DGG------------------T-ILADD-VIYITVRNRT------------------------
---------------------------------GPTMTLIDLPGLTF--VH------------------K-------TQ-------------K-----
-------N--IHDVTV--------ELIR-RYI---KN------------EQAVILAVIPATE--D-FGNCE-ALNLAADVD-----
-PDGERTLGVATKCDMIG-------NDS------D-------L--------------------------CAK--LR--MERTS-----
--D-IK----------------------L---KLGFVGVRCR---G-PGE--
VRD-----------G--------IS--------------------FQ---------EMAKR---ERELFNS--------HP-AL----------
--------TGK-G------L--------K--KEH-WGMDTLVEKICNIQEQTVERW-LPKV-
CAE6914669.1 ------------IPIPG--------VVVV---
GE----Q---------------SAGKSSLLE-----S--IS-G-I-QF-PRA-------------QN-TCTRMPC--------------
VVS-------------L---LTD----------------------------RS--
--------------------------------------VKQP-------------Y-------------------------AIV---------S--------
------M-D-P-AF-------------------------------------SEAEKC-TL--------------------------------------
--QQ-AIYVRVTRAE---
-------------GPQMSLIDLPGITH--NS-------------------T-------KM-------------A-----------N--
IHQVTR--------ELVE-QYI---AP-----------TEMVILCVIPAAN--D-FGNAE-VLKLASKFD------
PSGERTLGVVTKCDDDAK-----REAS------D-------L--------------------------VDK--VM--MKRDL----
---D-VK---------------------L---DLGFHCVVSG--S-PKD--
IKE-----------A--------TS--------------------RE---------DL---
------------------------RRLMEKIAKLQEARVDAH-LPNI-
CAI4004018.1 ------------IPIPG--------VVVV---
GE----Q---------------SAGKSSLLE-----N--IS-G-I-QF-PRA-------------QN-TCTRMPC-------------
-VLT-------------L---LTD----------------------------PA---
-------------------------------------VEES-------------Y-------------------------AMV---------S--------
------M-D-P-NF-------------------------------------ENVKRC-SIP----------EVEEQI------KALT-EK---
HAT-----------------------GD------------------Q-FISSQ-AMYISVVRRE------------------------------
--------------------------GPQLSLIDLPGITH--NS------------------S-------KM-------------T----------
-K--IHEVTV--------KLVE-NYI---KP-----------EEMVILCVIPASN--D-FGNAE-VVELAKKYD------
PDGERTLGVVTKCDDVAK-----TESS------D-------I--------------------------VEK--VL--MERTD-------
D-VR---------------------L---ELGFHCVVNR---S-QKD--IDE-
----------G--------MS--------------------RE---------DLLEK---GRKVFSE--------SD-RM----------------
----K-R------L--------P--KEN-WGTLALTEKIAKLQEHRVDAH-LPKI-
CAI3978736.1 ------------IPLPG--------VVVV---
GE----Q---------------SAGKSSLLE-----N--IS-G-I-QF-PRA-------------QN-TCTRMPC-------------
-VLS-------------L---LTD----------------------------PT---
--------------------------------------VKES-------------H-------------------------AMV---------S------
--------I-D-P-GF-------------------------------------ANATTC-TVP----------EVEKQI------KQLT-EE--
-YATG----------------------GD------------------Q-FISSQ-AMYIRVVRKE-----------------------------

----------------------------GPQLSLIDLPGVTH--NS------------------D-------EM-------------K--------
----D--IHEVTV--------NLVE-EYI---RP-----------KEMVILCVIPAMS--D-FGNAE-VVKLARKYD------
PDGIRTLGVVTKCDDAAN-----AEAS------D-------I--------------------------VKK--VL--MSRSS-------
D-VR----------------------L---ELGFHCVVNR--S-QKN--IDE-
----------E--------MS------------------RE---------DLWAK---ERKIFTK--------NE-RM-----------------
---K-R------L--------P--EKN-WGTLRLMEKVAKIQEARVDEC-LPKI-

OLP81297.1 -----------PDPPPG--------VVVV---GE---
-Q----------------SAGKSSLLE-----N--IS-G-I-QF-PRA-------------QN-TCTRMPC--------------VLT--
------------M---LTD----------------------------PT--
---------------------------------VNES-------------F-------------------------ALV---------S--------------
M-D-S-SF-------------------------------------TDAKRC-AVA-----------DVEGQI------KELT-DQ---
HTT------------------------GA-------------------A-FISTE-ALYIRVVRRD-----------------------------------
------------------------GLQLSLIDLPGVTH--NA------------------E-------KM-------------S-----------
N--IHEVTV--------SLVK-EYI---EP-----------EEMVILCVIPAMS--D-FGNAE-VVKLAREYD------
PEGIRTLGVVTKCDDAAN-----AEAS------D-------I--------------------------VEK--VT--MSRDS-------
D-VR----------------------L---AFGFHCVVNR--S-QKN--IDE-
----------G--------MS------------------RE---------DLWQK---EEKTFTA--------SD-R------------------
-----K------L--------P--ANN-WGTLRLMEKVAKIQEARVDEC-LPKI-

CAE7315868.1 ------------IPLPG--------VVVV--
GE----Q----------------SAGKSSLLE-----S--IS-G-I-QF-PRS-------------QN-TCTRMPC--------------
VLT-------------M---LTD----------------------------PA--
-------------------------------------TDDP-------------Y-------------------------ALV---------S---------
-----M-D-P-SF--------------------------------------EDAGPC-PVM-----------QVEGKI------KQLT-EK--
-HAT------------------------GD------------------D-FISKQ-ALFVRVVRKE------------------------------
---------------------------GPQLSLIDLPGVTH--NA------------------E-------CM-------------E---------
---N--IHEVTV--------ALVE-EYI---KP-----------DEMVILCVIPAMS--D-FGNAE-VVKLAKKYD------
PDGIRTLGVVTKCDDAAH-----AEAS------D-------V--------------------------VEK--VL--MRRET------
-D-MR----------------------L---QLGFHCVVNR--S-QKN--
IDE-----------G--------MS------------------RV---------DLWNK---EKKIFS---------SE-RL-------------
--------R-S------L--------P--EDY-WGTRRLMEKIAKIQEDRVDAC-LPKI-

CAE8582582.1 ------------IALPG--------VVVV--
GE----Q----------------SAGKSSLLE-----N--IS-G-I-QF-PRA-------------QN-TCTRMPC--------------
-ILT-------------M---LTD----------------------------PS---
--------------------------------------ILEP-------------Y------------------------AKV--------------------
---RQC-AVG----------EVEEKI------RALT-AQ---HAT-
-----------------------GD------------------T-FISRK-ALYVRVVRKD--
-------------------GPQLSLIDLPGVTH--NA------------------D-------KM-------------A-----------D--
IHEVTV--------SLVE-EYV---RH-----------EEVVILCVIPAMS--D-FGNAE-VIKLARKYD------
PEGKRTLGVVTKCDDAAR-----AEAS------D-------V--------------------------VDK--TL--MRRDS-----
--D-VQ----------------------L---KLGFHCVVNR--S-QKN--
IDD-----------N--------MA-------------------RK---------DLWVK---EQQLFET--------SE-RL-----------
----------R-G------L--------P--SDH-WGTLRLMEKIARIQASRVDEC-LPKI-

KAI0562045.1------------IECPG--------IVVV---GA----
Q---------------SAGKSSVLQ-----R--LT-G-I-SF-PRA-------------EN-TCTRLPA--------------IVS-----

---------L---QTD------------------------------PS---
------------------------------INGS--------------R---------------------------IYV----------S---------------R-
D-A-AF--RNPLECDSMA-----------VLENRI------MAMT-MD---
LEN-------------------------SN-------------------F-SVKNE-PVYIRYIRKE----------------------------------
--------------------------GPVFSLIDLPGITY--MD-------------------P-------KN--------------Q---------N-
FD--IHEETV--------SMVR-QYV---SN------------PNTIILVVIPAVE--D-FSNAE-ALKIAMECD------
KEGNRTIGVVTKCDMVS-------ESS------Y-------F----------------------------LRK--MR--MTGPN------
-D-IK----------------------L---KLGFVAVRNR--G-PGE----E---
--------N--------IN--------------------ID----------EA------ERNMFSN--------HS-LL--------------------
K-Q------L--------D--PTQ-KGYVALTKKIVELQSELVDAF-IPKT-
KAI0557988.1------------IQCPG--------VLVV--GA----
Q----------------SAGKSSVLE-----R--LT-S-I-RF-PRA--------------QN-TCTRVPT---------------IVQ-----
---------L---HTN------------------------------PL---
------------------------------VKVA--------------T---------------------------ALV----------S---------------K-
N-A-DF--SDAKTCEDMK-----------SVEDAI------LAFS-SS---IME--
-----------------------NT-------------------A-PISDS-PIHIRYTRKK--
------------------GPVMTLIDLPGITH--VD-------------------V-------DG--------------R--------DD-FD--
IHDVTS--------SMVH-KYV---KN------------DNMVVLVVIPAND--D-FGNAE-ALRIAQLYD------
KEGKRTIGVVSKCDLVP-------QNS------D-------I----------------------------LHK--IR--MSRQG-------
D-VK----------------------L---ALGFIAVRNK--D-IGE----D-----
------G--------KD--------------------IE----------KI------ESKLFST--------HE-LL---------------------R-H-
-----L--------A--EEQ-RGYAALTRKIVDLQSQRVDEF-IPEA-
XP_005716602.1 ------------IPCPG--------IVVV---
GA----Q----------------SSGKSSVLE-----R--LT-D-I-AF-PRD--------------TN-TCTRVPI---------------
IVQ-------------L---QRD------------------------------AS--
--VTLP--------------T---------------------------ATI----------C----------
-----G-D-E-HF---------------------------------------ETDVINCSTKQ-----------DITNAI------HDLTEKA--
-LVS-------------------------TR-------------------S-RVVDK-PIHIRYVRSK----------------------------------
--------------------------GPVMTLIDLPGITH--DD-------------------G-------EG--------------R------------
D--IHGITA--------DIVK-KYL---KY------------ENMIALVVIPAND--D-FGNSE-ALKIAKTFD------
TTGARTLGVISKCDLVP------EQHS------D-------I----------------------------VEK--IQ--MTPSN-------
A-IK----------------------L---GLGYIAIRNK--G-PGD----D-----
------L--------AN--------------------ID----------NI------ETELFKT--------HP-LL---------------------K-N-
-----L--------A--PHE-RGCGALRRKIISLQENSIKHA-IPRI-
KAI0559778.1------------IACPGLEAIERKYLVTM--
GG----V----------------GCGKSSVLE-----D--LA-G-I-RF-PRA--------------EN-TCTRTPT---------------
VVH--------------L---QVD------------------------------PD--
---INKN--------------T---------------------------IAI---------S--------
-------R-Q-E-DF--RNPKSCEDME-----------ELEKEL------RKIA-
LD---VKQ-------------------------SG-------------------I-PVKDE-PVYTRYVRRT---------------------------
---------------------------------GPTMTLIDLPGISY--FD-------------------P-------NG----------------------
----FD--VHEATT-------SMIR-KYA---AH----------ENMILLIVFRATE--E-FGR-K-AFALAKKYD------
EDSIRTIGVATRCDTVE------TDSA------T-------F----------------------------LPQ--MR--MEDPN-------
P--R-----------------------L---LHGFTAIRNR--L-RGE----E-----

------S--------LR-------------------IE----------DF------E-QVLST--------HA-LL--------------------Q-
Q------L--------D--CTT-TGLNALAKKVVHVQSNVVDEF-ISNT-
WGU15254.1 ---------GTSLNYPR--------IVVV--GN---
-Q----------------SAGKSSVLE-----A--IV-G-E-SFLPKG--------------TN-MVTRRPL---------------EIT---
-----------L-V-RDE--
-------------------------------SAK--------------Y--------------------------FLF------------------------------
---EDGHKLHDID-----------AVRDRL------NEEN-----------------
-----------------KD-------------------F-EFSDS-PIRLEVHSPD---
-------------VQNVTLVDLPGYIHA-VT--------------------------KD--------------Q--------SK--D--
LPKKIQ--------KLCK-NYI---DK------------KDNLILVIVSAAE--D-TAMSV-GLKEVYKLE------
DWESRTLGVLTKMDL---------RNT------------KDI---------------------------VKT--LK---NN---------E-
YP-----------------------L---GMGYIGVRCR---T-DKE--IDD-----
------K--------IN-------------------FN----------ELIKK---EDEFIKS----------------------------------R-K---
---LH-------Q--SEVKVGIPVLREELSKELLS-----------
XP_013756556.1 ---------KAKLELPK--------IVVV--
-GN----Q----------------SSGKSSVLE-----A--VA-G-F-DFLPKS--------------TS-MCTTRPL--------------
--ELN--------------L-L-RSD--
---SGT--------------W--------------------------AEF-----------------
---DDGRKIFDFE-----------QVEAIL------REKN-------
-------------------------QD-------------------E-EFSEL-PVTMSIYAPH------------------------------------
-----------------------VYNVSLIDLPGYIHA-VR--------------------------AG--------------Q--------SP--
E--LPERIA--------ALCQ-KYI---SD------------PNNIIVAVASAAD--D-VAMSM-GIKNAQAVD------
ANMDRSLGVLTKMDLI--------KSR------------KHI---------------------------HSI--LR---NE---------D-
YP-----------------------L---GLGYVGVRCR---S-QKE--LED----
-------G--------KS-------------------FD----------DVLRI---EEAFIRR----------------------------------A-
K------LR-------DV-GDLRLGIPTLRRVLSEEQLNKVAAD-FPRIL
CAH6419740.1 -----------IKNWPQ--------IVIL---
GQ----Q----------------NEGKSSLIE-----G--VT-Q-V-EILPKC--------------DG-LCTRKPI---------------
HMT--------------L-I-NDP--
---DTK--
--FIIGDR-MYTET-----------QASEEL------NRLN-------------
---------------------LN-------------------P-KVD---HINCTILSPI--
---------------VYNCTIIDTVGLIH--VS--------------------------EQ--------------D--------NT--L--
DPKKIK--------QDTI-RYL---KD------------KNNIFVLVSSAPS--D-LANSQ-MLQLIKKYN------RVDD-
TLGVMTKIDLIE------NQNQ------------NSI---------------------------NDI--LC---GK---------N-YK-----
------------------L---GFGWIPTKLR---S-DRD--IQD-----------
G--------IT-------------------IE----------QSIIK---EHEYCQG--------RN-FS--------------------S-F-----
-------------S---YGVSEVRRTISQIQLERIKAN-IPSI-
AYV81982.1 -----------LKGLPK--------PVAI---GP----
Q----------------SAGKSSVVE-----A--II-G-F-DILPKK--------------MG-MCTMKPI---------------NIT----
----------T-I-RDD---
------------------------------MVK---
-------------------------------------FKVGDKELSTIE-----------SAKHEV------NRLN-------------------
-------------SN-------------------S-NVT---IINLTIYSPD--

-------VTNISMVDLPGLFV--VT--------------------------EG-------------V--------SD--N--LPKLVK------
--EMTI-EYI---QN------------RNNIPLVITAAPS--D-PATNM-ALQLVSKYK------RREE-
SIGVITKMDLTV------KQHT-----------EII---------------------------EQM--LK---GN---------K-FA------
-----------------L---GNGWVSVLLK--N-NND--DDN-----------
G--------IT-------------------VQ----------QKILE---EEEFFKD--------KP-NF--------------------S-P-----
-------------S----GVKMLRKKIGDLQLSKIQHN-IPQL-

CAH6421112.1 -----------LKSFPG--------CAVF--
-GP----Q----------------SVGKTSVLE-----A--IC-DIQ-DFLPKG--------------EG-MVTKKPI--------------
-HVT--------------M-I-KSQ--
---NIL---
---YKIGDKEIKNES-----------DAREEI------NHLN-------------
---------------------YN-------------------D-SIK---KIDVRIHSPN---
----------------VCNSSLIDLPGLFV--VS--------------------------DK--------------N--------DP--D--
LPNKVK--------KMNM-EYI---SN------------EKIIPIVITSAAM--D-PATNQ-ALKMLGRFQ------REKD-
SLGIITKIDLME------KQNM-----------IET---------------------------EQM--LS---GK---------K-YP------
-----------------L---GHGYVTVILR---N-KED--IDK-----------K---
-----IS-------------------MK----------DKILM---EKEYFSS--------RP-NL--------------------H-P---------
---------S----GVPEMRQIISNIQFEKLKDQ-IPFL-

ARF12445.1 -----------LKHAPN--------FVVI---GQ----
Q----------------SSGKTSVNE-----A--LT-N-T-NIFPKA--------------MK-MATMKPM---------------KIT---
-----------T-I-RSE---
-------------------------------ETK---
--------------------------------------YKIGDKEFRNEN-----------EAADEV------NRLN---------------------
-------------NN-------------------P-SVQ---QVVVTVWSPK---
-----------VYNSSYTDTPGLFS--VT--------------------------NK--------------S--------NT--D--MPKKIK--
------ELIF-QQL---QD------------PNVIPVVIHSGPS--D-PATDQ-ALKFVEKFN------RQDD-
ALGVITKVDLLE------KQNT-----------DFI---------------------------AKL--LN---GE---------E-YA------
-----------------L---GHGWVGVSLR---S-DKD--IEA-----------
G--------MS--------------------IE----------DKIMI---EKNLMAK--------MK--L---------------------K-P----
--------------S----GVETLRKMIADIHAHKIKDQ-LPNI-

QKF93607.1 -----------LKSFPS--------VVVV---GP----
Q----------------SSGKSSVIE-----A--IC-G-E-TILPKA--------------MK-MATMKPM---------------HLT---
-----------T-I-RSP---
------------------------------EKK---
--------------------------------------FKVGDRELKTER-----------EAADEI------DRVN---------------------
-------------NN-------------------T-HIQ---KVNDTIWSPD---
----------VYNAILIDLPGLFV--VA--------------------------GK--------------N--------EA--D--LPKKVK---
-----DMSI-QHL---QD------------VSNIPLVVHAAPS--D-PATNH-AIKLVGKYG------REGD-
TLGIITKVDMLE------RQKT-----------SFI---------------------------EDM--LR---GD---------T-CP------
-----------------M---GHGYCTVVLR---N-DKD--VEA-----------
G--------MT-------------------VN----------DKIKI---EKEFFTR--------VP--L--------------------K-P------
------------S----GVPQMRKMISNIQFSRVKEQ-IPNL-

QKU35298.1 -----------LKNFPT--------MVFF---GP----
Q----------------SSGKSSAIQ-----A--IT-G-D-YILPTD--------------MK-IATRKPT---------------HIT------

--------T-L-RSD--
-----------------------------NVK---
--YKVGDREFFTAK-----------ETSNEI------DRLN-----------------------
----------RN-------------------D-HVE---KIDVVVKSPH--
-------VHNSVFIDLPGLFS--IS---------------------------DN--------------D--------TD--D-HFRKKVK----
----QMST-AYT---SN------------RNFIPMIVHAAPS--D-PATNA-AIKLVSKID------RRND-
AFGILTKFDMVK------NQKT------------AYL----------------------------ERM--LK---GV---------D-YK----
-------------------L---GHGYCAVVLP---N-DLD--IER-----------
G--------VS--------------------VE----------EKIKE---EEEFFKK--------YP-NV---------------------K-P-----
-------------S----GVPILRKMISDIQATKIMEH-IPAI-
AYV83919.1 -----------IKSFPI--------MTFF--AP----
Q----------------SAGKTSSLE-----A--LC-R-K-SLFPKN--------------SG-MSTMKPI---------------YVV--
------------M-L-PSP---
-----------------------------------TEK---
---IVVNGKEL-NEK-----------MATDEI------QRLN-------------------
---------------SN-------------------V-NID---MINVVIYGPD--
-----------EITSNYGDLPGLIA--LS--------------------------SS--------------H--------PE-----LPEKIK----
----KLCY-SHM---EN------------PNTIPIIVHDASG--D-PEINK-ALQIVMKLR------RSAD-ACGIITKID-
KQ------KSHN------------SSI----------------------------KSM--LE---GK---------T-YP---------------------
--L---GYGYVPVTLR--N-MEE--VDA-----------G--------MT----
----------------VE----------EKEEQ---EKQFFER--------NP-AL----------------------K-P------------------
VNGPYGVAALRSKLADIQVDKIKQN-VPEI-
QFG74079.1 ------------IKIPT--------CVVV---GT----Q-
---------------SSGKSSLIN-----M--LI-G-M-PILPMG--------------KS-MVTRVPL---------------NLQ----
----------L-I-EGG------------------------------SR--
---------------------------------KIE--------------F---------------------------GKY-------------------------------
-----------------EGG----------------------TWIVIASTKTPS-----------EVSQII------ERET-IN---I-AG-------
-----------------DS-------------------K-NIGHK-EICIRVYGAE--
--------------TSLNFIDLPGLTM--VAC-------------------TD------LG--------------Q--------PP--D--
IKQQIK---------NLIE-KYT---SV------------ESNIIIGVFPARQ--D-LEADY-GLDFIKKYD------
SNFNRSIGVLTKVDLMQGDLIPYLQGG------S---HDA--LNT-TNPSI---
---SDNLK------------------------L---KWGYYAVNTR---DGD---
---TYFDN--I-EWAKTP-SL---------------------
---------------------TNR-IGVKNLSKQLYSILLQSIRES-LPKV-
XP_750654.1 ------------IALPK--------ICVI---GD----Q-
---------------STGKSSLIE-----G--MS-Q-I-KV-PRS--------------AG-TCTRCPM---------------EIN------
--------L---SEGE-----------------------------PG--
-------------------------------QD--------------WN----------------------------CRIFLS------RK---------------
YIF-D-GSRKVTKLPKKSQPLGPW-----------------IEQDQEDEHFTDVRDKD-----------GVQAAI------
KWAQ-LA--ILNPGRPSTD--------YQP--------GHNGD----------TDESYCQV-KFSPN-VVRLDISAPN--
---FPNLSFYDLPGVISQ-AE---------------------------
----------------H--------DH--ERYLVSLVE--------NLVR-EYI---SQ------------ENCIVLLALPMTD--D-
ATNSS-AAKIMRDVP------GAKERTLGVLTKPDRIQ-------TGE------S-----YDQW---------------------
-----VEI--LE---GD---------K-FA----------------------L---GHGYYIVRNN-----------------------------------

-------------P-NPA--IEH-----------S---RAREE---EAVFFAK-------
SP---------------------WATDLS-----AY-----------QNR-FGTRNLQSALSSLLLEQIQGC-LP---
XP_006461472.1 ------------IDLPQ--------IAVV---
GN----Q----------------SAGKSSLIE-----S--IS-G-I-TL-PRA--------------SG-TCTRCPT--------------
ECR-------------L---SYS------------------------------S---
--QP-------------WK-------------------------CVVSL--------R-------
--------ITT-D-RSGQ---------PLG---------------------QSRNETFGSTIYDKK-----------EVDDRI------
RRAQ-LA--ILNPDKPAKS-------FLN--------DDEPS----------LMEGN-FL-TFSKN-CVSLAISGPD----
---VADLSFVDLPGLIAS-VG-----------------------------
--------------R--------GG--NAGDIKLVE--------GLVT-TYI---HK------------TNCIILLTVACET--D-
FENQG-AHQLAKQYD------PEGKRTIGVLTKPDRIP-------AGE------E------QNW--------------------
------LKF--IR---NE---------K-EP-----------------------L---QNNWFCVKQP-----------------------------
----------------A-SSD--LKN-----------N--------WT--------------------WQ----------QARQK---
EDEFFTA--------TS-PW------------------NELE-A-----MY-----------VRY-
LRTKNLVERLSQVLSDLIAKT-LP---
XP_006461433.1 ------------IDLPQ--------IAVI--
GS----Q----------------SAGKSSLIE-----S--IS-G-I-TL-PRA--------------AG-TCTRCPT---------------
ECR-------------L---SYS------------------------------P--
--LP-------------WK-------------------------CTVHL--------R-------
--------FTT-D-GNGT---------PLG---------------------QSRNEIFGPTIYEKS-----------EVEERI------
RRAQ-RA--ILNPGKPTKL-------FLE--------DDDEM----------SGEAE--L-SFSNN-CVSLQISGPD----
---VADLSFCDLPGLIAS-VG-----------------------------
--------------R--------GG--NTNDIKLVE--------SLVT-SYI---KK------------PSCIILLTVACET--D-
FENQG-AHQISKAYD------PEGKRTIGVLTKPDRIP-------LGE------E------LNW-----------------------
---LKF--LK---NE---------R-EP-----------------------L---ENNWYCVKQP--------------------------------
-------------S-SND--LKN-----------N--------WT--------------------WQ----------EAREK---EQQFFAA--
------TA-PW------------------CELE-G-----MY-----------QKF-LRTTNLVERLSGVLSDLIAKR-LP---
KAI3646081.1------------LNLPG--------IVVC---GN----
Q----------------SSGKSSLIE-----A--IS-R-V-PL-PRA--------------AG-TCTRCPF---------------ECR----
----------L---SES------------------------------K---
------------------------------NP-------------WS-------------------------CKVML--------R--------------
YEV-D--AKGQAL------T--L-KA--
VLNPGLEAQQ-------FLT--------DTNWE----------KIE-D-QQ-KFTEN-VVCLTIEGEG-----------------
--VGYLTLTDLPGIIQS-TN---
D--------AA--DEHFVELIK--------NLVE-KYV---SC------------PTNIIVEVITCKD--D-MENQI-
VHTLARKAD------PSGLRTVGVLTKPDMIE-------EGC------T------DDW--------------------------
LNV--LR---NN---------T-CP-----------------------L---KHGYYMVKNP-----------------------------------
----------A-TKD--LQA-----------N--------IT--------------------FS----------QARQK---EVAFFSQ--------
KP---------------------WSTE-R-----AL-----------SKR-FGIYNLKEFLSNLLSNLLKQN-LPQL-
KAJ9515210.1 ---LADFGAAHEISYPT--------IVVC---
-----GD----Q----------------SAGKSSIIQ-----R--IS-G-I-DL-PRS--------------SG-TCTRCPM-----------
----EVRM------------TL---SEG------------------------------G---
---VP-------------WS-------------------------CKIKI--------R--
-------------REW-DDGKRK---------TLS--------------------KVSWEDFGAPLLDKE-----------AVGPAV-

-----SRAQ-KA--VLNPGKGYAS-------FVD--------PTSPI----------LADAD-EL-GFSRN-
VVVLEIQGAD--IS-LSLIDLPGIINS-TE------------
--------------------------------K--------KE--DQYLVNMIK--------DMVK-QYI---EA------------
SQTIIVLAVHALS--D-IQNQV-VYQMAREAD------PHQQRTLGVITKVDVIP-------PGS------H------
SMW----------------------------IRM--MR---GE---------L-FP------------------------L---DLGYYMVVNP---
--N-QVD--LDQ-----------G--------TS--------------------
HEVGHTGRMWKDDAVDK---EMRFFET--------DA-NLG----------------VLAQS-V-----VW-----------
SSH-LGLSNLTAALSKQLVDRTMAE-LPHMR

ETO25748.1 -----------TVDLPT--------IVLC---GQ----
Q----------------SSGKSSVSE-----A--IS-G-V-AL-PRS--------------SG-TCTRCPT---------------EVR---
-----------L---IHTQD---
-----------------------------------TK--------------WK---------------------------CTIKV--------R---------------
WEWND-RKKT---------HLHQ---------------------VREEEIA-TVTERE-----------AVAGIV------EKAQ-
KV--LL--EKA-EV-KFSRN-VICVEVQTSD----------------------------
----------------------------------CVDLTVVDLPGLIQA-VE--N-------
-EE--DFQYIQLTQ--FHCNN--D-MENQA-VNSLIRQHD------
PKGLRTLGILTKVDTIE-------TDS------E------QIW----------------------------INV--LK---GK---------S-
YP-----------------------L---KLGYVAVRNI---T-QHE--LKH------
-----G--------KS--------------------FK----------ETRAI---EAQFFAS--------HP-TWS------------------
KLS-S-----SL-----------QSR-LGCSN--------LVDILSCK-LSS--

OAJ38670.1 ------------LSLPT--------LVVC---GN----
Q----------------SVGKSSLVE-----A--IC-G-I-TL-PKA--------------AG-TCTRCVT---------------EVR-----
---------L---SEYSDV--------GPVDSVRKKSVAGDRNSTEST-------ITQGV-YIDALDT--------------------
-------VSSGSVPSAF--HIDGHEK--------------------------ESST--------------WS---------------------------
CTITL--------R---------------FEY-D-EAGI---------PLRS---------------------IREVLFGPPLVEKS---------
--LVALAV------RRAQ-KA--LLNPTLDPSV-------FLTHIFTDND--QSNS----------DTKSN-QL-KFTKN-
IVCLDIQGAG---INLALVDLPGIIRN-VE-----------
---------------------------------H--------PD--DAMFIPMIE--------DLVK-SYI---QK------------
ERTIIVATITCKD--E-MENQA-IVHLAREVD------PTGIRTIGVLTKPDTIE-------SGT------A------ARW--
-------------------------ADI--LM---GN---------L-YP-----------------------L---KLGYFMVRCL------------
-----------------------------------S-KAE--LAA-----------G--------NT--------------------LQ----------
DAQKL---ENAFFAQ--------SQ-PWS------------------TLR-R-----K------------SAR-
FGAPALRFELSRLLINLVDMS-LP---

GAX85982.1 ----RQLGVSNDIKLPT--------LVTA---
GN----Q----------------SSGKSSVVE-----A--IA-G-I-PL-PRS--------------SG-TCTRCPT---------------
EVR--------------M---RS--

--VHQ-VV--RLTLTDVDQK----
-----------KP--KEPE-HSRSFLEL--PTEY-QL-EFTRN-SVVLEIEGAD--------------------------------------
------------------------ADLTIIDLPGIIQ---S--H--------HK--
GPHYVEMIK--------SMVL-NSI---ES------------DHVIIVMVITAMD--D-VENQA-INLEARNVD------
PEGQRTIGVITKPDNIP-------KGE------H-------------------------------------DKWVALA---SN---------
R-RP--------------------GQEL---SLGYYVVRNP---G-QNE--

LDE-----------S--------IR-------------------FE---------DARVK---ESEYFET--------SP-YW-------------
-----PSN-G-----EL-----------QGR-LGTTFLRNALSESLVQGIKKG-LPGMQ
KAG2488600.1 ---LRALGVGSALQLPA--------LVIA--------------------------------------
-----GD----Q----------------SSGKSSVVE-----A--IA-G-V-SL-PRS-------------DG-TCTRCPT---------
-----EVR-------------L---RT-------------------------HAGPDA---
-----NGDSP-----MPDGD-----------------------------VP-------------WT-------------------------
CRIKL--------H---------------REY-D-SDGH---------PLTE-------------------LPPEELFA-TLTNKA------
-----HIAAFV------TAAQ-AV--LLNPRAADAA-PGGARAFVPDVSGDRP--RDPQ-P---LRALGHPTTY-
EL-SFTAN-KVVLEVDGAD--ADLTIIDLPGIIH---
D---H--------PK--GKQYVDMVE--------RMTK-AQL---RP---------
---EHHIIAMALPAGL--D-PETQA-IRLWVREVD------PSGSRSIGIITKPDTIA-------DDA------H------
ITY--------------------------GKLVKLV---GG---------S-TM-AGGAAGAAASAGPAAGHDESHQL---
TLGYYVVRNP---G-QEQ--LED-----------C--------IG-----------
---------FA----------EARAA---EQRYFAT--------NT-HWV---------------QAVAAL-P-----SL-----------
KQR-LGANHLRSGLSALLVERIETQ-LPHMR
XP_042924875.1 ---MRTQAANADL---------------A--
---SD---ME----------------SSGGG-----------------------E-------------DG-SGME--------------------
---EDG--EGAAN-
----GADGKRR----------------------------PA-------------WR-------------------------CRIKL--------C----
-----------RDY-D-SEDK---------PLAE--------------------KPPEQPFC-VVRDKA-----------HIAACV-----
-SAAQ-AV--LLNPRAVEAA-AGGPQAFVPELSS--A--HSPS----------HP-----------N-KVVLEIDGAE--
--ADLTIIDLPGIIH---D------------------------------
------------H--------PK--GRHLVEVVE--------RMTK-TNL---AP-----------AHHIIAMALPAGL--D-
PETQA-IRLWAREVD------PDGHRSIGIITKPDMIA-------EEA------H------IVC------------------------
--NKLVKLV---G---------------ARGELGPGGTRSQPDGH-----L---RLGYYVVKNP----------------------
----------------------S-QEQ--LVE-----------G--------IT-------------------FE----------KAREI---
EARYFAN--------HV-HWR---------------PAMATS-P-----GL-----------VQR-
LGANALRSGLSLLLVERIEEQ-MP---
XP_042924848.1 ------------LQVPT--------LVIA--
GD----Q----------------SSGKSSVVE-----A--SA-G-V-PL-PRS-------------DG-TCTRCPT--------------
-EVR--------------M---RTTQQLPAATGVCGGSALSAAAASSPPGPHSGEDGEDDEDDEDEEDE-
EDDAVVN---------------------------NAAGLPPGTTRGGGGGSSA---------------------------SA---------
-----WQ-------------------------CRIKL--------C---------------REF-D-SDGV---------RLAT--------------
------KPPEQPFC-VVRDKA-----------HIATCV------LAAQ-AV--LLNPRAVEDT-
PGGAGAFVPLLSSAQP-GRQPA-AAAKLLALRDASHY-EL-PFTPN-KVVLEIDGAE-------------------
--------------------------------------ADLTIIDLPGIIH---S---H-----
----E--DPRLIELVK--------DMVK-ANL---AP-----------EHHIIAMALPAGQ--D-AETQA-IRLMTREVD-
-----PDGRRSIGIITKPDRVP-------EHE------A------GET--------------------------LKLIRLV---GA-------
--C-GAPPAGAGAAGGS--ARVAH---PQH---PLGHYVVKNP--
-S-QDG--LAM-----------N--------IT-------------------FE----------QARAD---EAAYFAG--------HK-
HWA---------------AALRRQ-P-----EL-----------QRR-MGAAALRRGLSGLLVELVIAQ-LP---
XP_042923301.1 ------------LKVPA--------LVIA--
GD----Q----------------SSGKSSVVE-----A--IA-G-V-PL-PRS-------------DG-TCTRCPT--------------
EVR-------------M---RT--

HGAP------GEGGS-----------------------------AV-------------WQ-------------------------CRIKV-----
---V---------------RNF-D-STGK---------PLAPG-------------------EAHEKLFC-TVTDKA-----------
HITACI------SAAQ-AV--LLNPTVVGDAVADGAERFVPLLSAAEPGGRAPE-ASSAMRGLGDAAGY-
EL-QFTAN-KVVLEIVGAE---ADLTIIDLPGIIH---
S--H--------PK--DPSLIDVVK--------SLVK-CYL---AP-----------
AHHIIVMTLPAGM--D-AETQA-ILQFAREAD------PEGRRSIGIITKPDKIG-------TDE------R------
TEW--------------------------GKLCNLV---AG---------A-RA-PTGVPAAGGSRAAAAPN---PHL---
QLGYYVVKNP---G-QEQ--LAA-----------G--------IS-----------
---------FE----------QARAA---EERYFAD--------HP-LWA---------------SAMKAN-S-----LL-----------
SQR-LGTNALRDGLSALLVDKIGEH-MP---
XP_006457072.1 ------------FDLPK--------IVVI---
GN----Q----------------SAGKSSLIE-----A--VT-G-I-NV-PRD-------------SG-
TCTRCPMECSMSSDTRSWSCTISLRSGTSTSIPPTSPRVL---RSTRGT----------------------
SIASASSTASAGV---------TPTPA-------------------------RASTGVRNITTQ-SFGPTITD------------------
------KSQVE-------------LWLR----R-----------------AQGAI-L---------------------------S--------------
-------------------------TDADKSQWLNKSA---------EEIRQAI------QNKT-----GM----------------------
------------------------R-DFTED-TIVVDIQDPT---
ATDLSFVDLPGLISN-----------------------------AD----------------------PG-----SIDLIK--------NLVR-
QHV---AG------------ENTLILVTIPASD--D-IQNHG-AVVLAKEAD------GNGDRTIVVLTKPDSLG-----
--PGD------TG---LQETW-------------------------RQT--FK---NP---------N-VP------------ENQNY---
--L---RHGYYCVQLP---NDQQR----------------Q---------------
--------------------------QGLTAHT-LPNYLGV--------TW-PW--------------------S-E------FAG---------
QGR-FGVTNLVKNVSALLVQMIEAN-LP---
GBG30247.1 ------------FHAPG--------VAVI---GN----
Q----------------SSGKSSVLS-----A--LT-R-V-DL-PRG-------------TG-TVTRCPI---------------VVH----
----------C---ERS-----------------------------QECKA--
-------------------------------LVSRD---------------------------------------ATF-------------------------
---SEATEVRDMA-----------YLAEAI------ARKQ----------------
-----------------PSS------------------G-AIENK-TVHVKVMRPD---
-----------------VHKLVLIDLPGIKQ----------------------F-------GG------------SK--------GA--D---
EHELTV-------GLVK-EYI---SN------------PRMVILVVLPAES--D-LETCE-ALRLAREHD------
PQGKRTLGVFTKVDCLP-------DSDP----IQ-----KRSF--------------------------CER--LT---KK---------
G-AP--------------------QALL---AHGYFAVRTR--T-ETE--AQE-
----------G--------DS-------------------HT---------DVLDR---ERALFTK--------DK-YF------------------
---R-E------L--------S--PDL-WGIQTLGEKVSIIQVKMVRQF-INEI-
CEM15039.1 ------------IEVPG--------VVVC---GD----
Q----------------SAGKSSVLE-----R--IM-G-V-PF-PRA-------------QG-TCTKTPT---------------ILMG-
----------------ETD-----------------------------ASAD---
-----------------------------------GVSV------------W-------------------------VSLK----------------------
------E---DITDAEELNDIK-----------DIGNKI------TELT-DD---L-VR-
----------------------QEG------------------S-AIVNR-PIYVRIRGKN---------------------------------------
--------------------VPTLTVTDLPGLLY--NK------------------K-------GD-------------E------------N--
IHAEVV--------AMVN-EYI---EK------------EKTVILCVVPAGG--DFLSTSE-AIKLAKDVD------
PERHRTIVVVTKTDKIE-------PGE------EG----RHF---------------------------LKS--IK--QGQ---------

DEIG---G-------------------K---KLKCIALRNR---KPD---------
-------E--------IV-------------------LDI---------DAEQE---EAAFFER--------HP-DLC-------------------
K--------LL----------SEE-WGITTLVKRITDIQVVYVDEF-MKDI-
PAA87312.1 ------------DHLPR--------VVVV--GD----
Q----------------SSGKTSVLE-----M--IA-K-A-RIFPRG-------------AGE-MMTRAPV---------------QVT-
-------------L---AEG---
---P-------YHV------------------------ARF----------K---------------
DNP-S---REYDLTQESELA-----------ALRDTI------ERRM-RS--
VVQSG-----------------------G---------------------TVSAE-TISLSVQGPG------------------------------
----------------------------LPRMVLVDLPGIIS--TE-------------------T-------RG--------------M--------
AS--Q--TREAIR--------QLAS-QHM---RN------------PNSIILCVADACV--D-PERSN-AFDLVARHD---
---PSGRRTIFVLTKLDLAE-------RDR-----ISP----DRI---------------------------GRL--LA---GR--------
-L-LP----------------------LK---ALGYFAVVTGS---G-GAD-------
------------------ES--------------------IP----------AIQRY---EEQFFRN--------SQ-FF----------------------
K-EG----VL--------S--VSQ-MTAANMAQAVSRRFWALVQES-V----
PAA68234.1 ------------DHLPR--------VVVI--GD----
Q----------------SSGKTSVLE-----A--VA-R-A-RLFPRG-------------AGE-MMTRAPV---------------
QVT--------------L---ADG---
--P-------YHV-------------------------ARF----------K----------
-----DDP-D---REFDLTKESELA-----------ALRDAI------ERRM-RA-
-AVRSS-------------------------GP-----------------DA-AVSTE-AIPLSVQGPG----------------------------
--------------------------------LPRMVLVDLPGIIS--TE-------------------T-------AG--------------M--------
AA--Q--TRESIR--------QLAR-QYM---RN------------PNAIILCVADACV--D-PERSN-AFDLVAKHD---
---PAGRRTIFVLTKMDLAE-------RDK-----VSP----DRV---------------------------AKL--LA---GR-------
--L-LP----------------------LK---ALGYFAVVTGS---G-SQD------
-------------------ES-------------------VE---------AIERH---EAEYFAS--------SR-LF--------------------
K-DG----RL--------S--PNQ-VTAANMARAVSRRFWALVRES-V----
NP_495986.3.3 ------------DNLPR--------VVVV--
-GD----Q----------------SAGKTSVLE-----M--VA-Q-A-RIFPRG-------------SGE-MMTRAPV-----------
----KVT--------------L---SEG---
--P-------YHV-------------------------AQF----------R------
---------DS--S---REFDLTKETDLQ-----------QLRNET------EVRM-
RN--SVRDG-----------------------K---------------------TVSNE-VISLTVKGPN--------------------------
---------------------------------LPRMVLVDLPGVIS--TV------------------T-------AD--------------M-----
---AR--E--TKDDII--------RMSK-AHM---EN------------PNAIILCIQDGSV--D-AERSN-VTDLVSSID---
---PSGKRTILVLTKVDMAE-------KNL-----ANP----DRI---------------------------KKI--LE---GK--------
-L-FP----------------------MK---ALGYFGVVTGR---G-NSS------
-------------------DS--------------------ID----------EIRKY---EENFFST--------SQ-LL---------------------
R-DG----VL--------K--PSQ-MTTRNMSLAVSDCFWRMVRDS-I----
XP_002602331.1 ------------DELPR--------VVVV--
GD----Q----------------SAGKTSVLE-----M--VA-Q-A-RIFPRG-------------AGE-MMTRAPV------------
---KVT--------------L---SEG--
--P-------HHI------------------------AMF----------K-------
--------DS--D---REFDLTKESELE-----------ALRREV------EIRM-

KA--SVRPG------------------------Q----------------------TVSME-TIAMSVKGPG-------------------------
-----------------------------------LQRMVLVDLPGIIS--TE-------------------T-------QG--------------M----
----AS--A--TKESIK--------MMCE-HYM---SN------------PNAIILCIQDGSV--D-AERSN-
VTDLVSQMD------PQGKRTIFVLTKVDLAE-------KNI-----TNP----RRI----------------------------KQI--
LE---GK---------L-FP----------------------MK---ALGYFAVVTGR--
---G-NKD-------------------------DS--------------------ID----------TIRGY---EEEFFRN--------SQ-LF---
------------------R-SG----VL--------K--ASQ-MTTQNLSFAVSDCFWKMVKAS-V----
XP_019637857.1 ------------DELPR--------VVVV--
GD----Q----------------SAGKTSVLE-----M--VA-Q-A-RIFPRG-------------AGE-MMTRAPV------------
---KVT--------------L---SEG--
--P-------HHI-------------------------AMF----------K-------
--------DS--D--REFDLTKESELE-----------ALRREV------EIRM-
KA--SVRPG------------------------Q----------------------TVSME-TISMSVKGPG-------------------------
-----------------------------------LQRMVLVDLPGIIS--TE-------------------T-------QG--------------M----
----AS--A--TKESIK--------MMCE-HYM---SN------------PNAIILCIQDGSV--D-AERSN-
VTDLVSQMD------PQGKRTIFVLTKVDLAE-------KNI-----TNP----HRI----------------------------KQI--
LE---GK---------L-FP----------------------MK---ALGYFAVVMGR---
----G-NKD-------------------------DS--------------------ID----------TIRGY---EEEFFRT--------SQ-LF---
------------------R-SG----VL--------K--ASQ-MTTQNLSFAVSDCFWKMVKAS-V----
XP_006813643.1 ------------DHLPR--------VVVV---
-GD----Q----------------SSGKTSVLE-----M--IA-Q-A-RIFPRG-------------SGE-MMTRTPV------------
---KVT--------------L---SEG--
--P-------YHV-------------------------AQF----------K-------
--------DS--N--KEYDLSKESELQ-----------SLRQEI------ELRM-
KN--RVKKG------------------------Q----------------------TVSND-TISLSVRGPG--------------------------
----------------------------------IQRMVLVDLPGMIS--TV-------------------T-------TG--------------M-----
---AA--D--TREAIH--------NMSK-SYM---KN------------PNAIILCIQDGSV--D-AERSI-VTDLATTMD--
----PEGKRTIFVLTKVDLAE-------KNS-----ANP----SRI----------------------------KQI--LD---GK-------
--L-FP----------------------MK---ALGYFAVVTGR--G-NTN-----
--------------------ES--------------------IE----------QIKNY---EETFFRS--------SK-LF---------------------
K-TG----TL--------K--PSQ-MTTQNLSFAVSDCFWKMVRES-V----
XP_030843280.1 ------------DHLPR--------VVVV---
-GD----Q----------------SAGKTSVLE-----M--IA-Q-A-RIFPRG-------------AGQ-MMTRAPV-----------
----KVT--------------L---SEG---
---P-------NHI-------------------------AQF----------K-------
--------DS--G--KEFDLTKESELK-----------ALRQEI------EARM-
KG--SVKEG------------------------Q----------------------TISPE-VISLSVRGPG---------------------------
---------------------------------IQRMVLVDLPGMIS--TV-------------------T-------TG--------------M------
--AA--D--TKTSIQ--------KMIN-GYM---GN------------PNAIILCIQDGAI--D-AERSI-VTDLVNEID-----
-PTGKRTIFVLTKVDLAE-------KNH-----LNP----NRI----------------------------RQI--LD---GR---------
L-FP----------------------MK---ALGYFAVVTGK---G-NTS--------
-----------------DS--------------------ID----------SIKQY---EEQFFRH--------SA-LF---------------------
K-SG----VF--------K--PSQ-LNTQNLSFAVSDCFWKMVRES-V----

XP_018667792.1 ------------DHLPR--------VVVV---
-GD----Q----------------SAGKTSVLE-----M--IA-Q-A-RIFPRG-------------SGE-MMTRAPV-----------
----KVT-------------L---SEG---
--P-------NHV-------------------------AQF----------R------
---------DS--S---------------------------------------REFDLSKEEELK-----------SLRHEI------ELRM-
KS--SCSDG------------------------K---------------------TVSND-TISLTVKGPG---------------------------
---------------------------------LQRMVLVDLPGMIS--TV------------------T-------SG--------------M-----
---AP--D--TKDAIC--------NMSK-HYM---EN------------PNAIILCIQDGSV--D-AERSI-VTDLVSQMD-
-----PSGRRTIFVLTKVDLAE-------KNI-----TNP----SRI---------------------------QEI--LD---GK-------
--L-FP----------------------MK---ALGYFAVVTGQ---G-SAN-----
--------------------SS--------------------IT----------DIKEY---EEEFFSN--------SK-VF---------------------
K-SG----LL--------K--ASQ-LTTANLSYAVSNCFWKMVRES-V----
XP_032818114.1 ------------DQLPR--------VVVV---
-GD----Q----------------SSGKTSVLE-----M--IA-Q-A-RIFPRG-------------SGE-MMTRSPV-----------
----KVT-------------L---SEG---
--P-------HHV-------------------------AIF----------K-------
--------DS--S---------------------------------------REFDLTKEDDLA-----------ALRKEI------EIRM-KK-
-SVKEG-----------------------H---------------------TVSAE-TISLSVKGPG-------------------------------
------------------------------LQRMVLVDLPGVIS--TM-------------------T-------SG--------------M--------
AP--D--TKDAIF--------AMSK-GYM---QN------------PNAIILCIQDGSV--D-AERSI-VTDLVSNMD----
--PQGKRTIFVLTKVDLAE-------KNL-----ASP----NRI---------------------------QQI--LD---GK--------
-L-FP---------------------MK---ALGYFAVVTGK--G-NRD------
-------------------ES--------------------IE----------SIKDY---EEEFFQK--------SK-LC---------------------
R-SG----ML--------K--AHQ-VTTKNLSLAVSDCFWKMVRES-V----
XP_021332524.1 ------------DHLPR--------VVVV---
-GD----Q----------------SAGKTSVLE-----M--IA-Q-A-RIFPRG-------------SGE-MMTRSPV-----------
----KVT-------------L---SEG---
--P-------HHV-------------------------AMF----------K------
---------DS--S---------------------------------------REFDLGKEEDLA-----------ALRHEI------ELRM-
RK--SVKEG------------------------Q---------------------TVSPE-TISLSVKGPG---------------------------
---------------------------------IQRMVLVDLPGVIS--TV------------------T-------TG--------------M-------
-AA--D--TKETIF--------SISK-AYM---QN------------PNAIILCIQDGSV--D-AERSI-VTDLVSQMD------
PQGKRTIFVLTKVDLAE-------KNL-----ASP----SRI---------------------------QQI--VE---GK---------L-
FP---------------------MK---ALGYFAVVTGK--G-SPN----------
---------------ES-------------------ID----------SIKDY---EEDFFQN--------SR-LL---------------------K-
DG----ML--------K--AHQ-VTTKNLSLAVSDCFWKMVRES-V----
XP_028587646.1 ------------DHLPR--------VVVV---
-GD----Q----------------SAGKTSVLE-----M--IA-Q-A-RIFPRG-------------SGE-MMTRSPV-----------
----KVT-------------L---SEG---
--P-------HHV-------------------------AMF----------K------
---------DS--S---------------------------------------REFDLTKEEDLA-----------ALRNEI------EIRM-
RK--SVSDG------------------------C---------------------TVSTE-TISLSVKGPG---------------------------
---------------------------------LQRMVLVDLPGVIS--TV------------------T-------SG--------------M-----
---AP--D--TKETIF--------SISK-AYM---QN------------PNAIILCIQDGSV--D-AERSI-VTDMVSQMD---

---PQGKRTIFVLTKVDLAE-------KNV-----ASP----SRI---------------------------QQI--IE---GK---------
L-XP---------------------MK---ALGYFAVVTGK---G-NSC-------
------------------ES--------------------IE----------SIKEY---EEEFFQN--------SK-LL---------------------
K-NC----ML--------K--AHQ-VTTRNLSLAVSDCFWKMVRES-V----
XP_025913835.1 ------------DHLPR--------VVVV--
-GD----Q----------------SAGKTSVLE-----M--IA-Q-A-RIFPRG-------------SGE-MMTRSPV-----------
----KVT-------------L---SEG--
--P-------HHV------------------------ALF----------K------
---------DS--S---------------------------------------REFDLTKEEDLA-----------ALRNEI------EIRM-
RN--SVKEG------------------------C---------------------TVSTE-TISLSVKGPG---------------------------
--------------------------------LQRMVLVDLPGVIS--TV------------------T-------SG--------------M-----
---AP--D--TKETIF--------SISK-AYM---QN------------PNAIILCIQDGSV--D-AERSI-VTDLVSQMD----
--PQGKRTIFVLTKVDLAE-------KNV-----ASP----SRI---------------------------QQI--IE---GK---------
L-FP---------------------MK---ALGYFAVVTGK--G-NSS--------
-----------------ES-------------------ID----------SIKEY---EEEFFQN--------SK-LL---------------------K-
TC----ML--------K--AHQ-VTTKNLSLAVSDCFWKMVRES-V----
XP_023440724.1 ------------DHLPR--------VVVV--
-GD----Q----------------SAGKTSVLE-----M--IA-Q-A-RIFPRG-------------SGE-MMTRSPV-----------
----KVT-------------L---SEG--
--P-------HHV------------------------ALF----------K------
---------DS--S---------------------------------------REFDLTKEEDLA-----------ALRREI------EIRM-
RK--SVKEG------------------------C---------------------TVSPE-TISLNVKGPG---------------------------
--------------------------------LQRMVLVDLPGVIN--TV------------------T-------SG--------------M-----
---AP--D--TKETIF--------SISK-AYM---QN------------PNAIILCIQDGSV--D-AERSI-VTDLVSQMD----
--PHGRRTIFVLTKVDLAE-------KNV-----TSP----SRI---------------------------QQI--IE---GK---------
L-FP---------------------MK---ALGYFAVVTGK--G-NSS--------
-----------------ES-------------------IE----------AIREY---EEEFFQN--------SK-LL---------------------K-
TS----ML--------K--AHQ-VTTRNLSLAVSDCFWKMVRES-V----
XP_005873264.1 ------------DHLPR--------VVVV--
-GD----Q----------------SAGKTSVLE-----M--IA-Q-A-RIFPRG-------------SGE-MMTRSPV-----------
----KVT-------------L---SEG--
--P-------HHV------------------------ALF----------K------
---------DS--S---------------------------------------REFDLTKEEDLA-----------ALRHEI------ELRM-
RK--NVKEG------------------------C---------------------TVSPE-TISLNVKGPG-------------------------
---------------------------------LQRMVLVDLPGVIN--TV------------------T-------SG--------------M----
----AP--D--TKETIF--------SMSK-AYM---QN------------PNAIILCIQDGSV--D-AERSI-VTDLVSQMD-
-----PHGRRTIFVLTKVDLAE-------KNV-----ASP----SRI---------------------------QQI--IE---GK-------
--L-FP---------------------MK---ALGYFAVVTGK--G-NSS------
-------------------ES-------------------IE----------AIREY---EEEFFQN--------SK-LL--------------------
K-AS----ML--------K--AHQ-VTTRNLSLAVSDCFWKMVRES-V----
NP_598513.1 ------------DHLPR--------VVVV--GD----
Q----------------SAGKTSVLE-----M--IA-Q-A-RIFPRG-------------SGE-MMTRSPV---------------KVT-
-------------L---SEG--
---P-------HHV------------------------ALF----------K---------------

DS--S---------------------------------------REFDLTKEEDLA-----------ALRHEI------ELRM-RK--
NVKEG-----------------------C---------------------TVSPE-TISLNVKGPG-------------------------------
---------------------------LQRMVLVDLPGVIN--TV------------------T-------SG--------------M--------
AP--D--TKETIF--------SISK-AYM---QN------------PNAIILCIQDGSV--D-AERSI-VTDLVSQMD------
PHGRRTIFVLTKVDLAE-------KNV-----ASP----SRI--------------------------QQI--IE---GK---------L-
FP---------------------MK---ALGYFAVVTGK---G-NSS----------
---------------ES--------------------IE----------AIREY---EEEFFQN--------SK-LL--------------------K-
TS----ML--------K--AHQ-VTTRNLSLAVSDCFWKMVRES-V----
XP_006163024.2.2 ------------DHLPR--------VVVV---
-GD----Q----------------SAGKTSVLE-----M--IA-Q-A-RIFPRG------------SGE-MMTRSPV----------
----KVT-------------L---SEG---
--P-------HHV------------------------ALF-----------K------
---------DS--S---------------------------------------REFDLTKEEDLA-----------ALRHEI------ELRM-
RK--NVKEG-----------------------C---------------------TVSPE-TISLNVKGPG-------------------------
---------------------------------LQRMVLVDLPGVIN--TV------------------T-------SG--------------M----
----AP--D--TKETIF--------SISK-AYM---QN------------PNAIILCIQDGSV--D-AERSI-VTDLVSQMD---
---PHGRRTIFVLTKVDLAE-------KNV-----ASP----SRI--------------------------QQI--IE---GK---------
L-FP---------------------MK---ALGYFAVVTGK---G-NSS--------
-----------------ES--------------------IE----------AIREY---EEEFFQN--------SK-LL--------------------K-
TS----ML--------K--AHQ-VTTRNLSLAVSDCFWKMVRES-V----
NP_056375.2.2 ------------DHLPR--------VVVV---
-GD----Q----------------SAGKTSVLE-----M--IA-Q-A-RIFPRG------------SGE-MMTRSPV----------
----KVT-------------L---SEG---
--P-------HHV------------------------ALF-----------K------
---------DS--S---------------------------------------REFDLTKEEDLA-----------ALRHEI------ELRM-
RK--NVKEG-----------------------C---------------------TVSPE-TISLNVKGPG-------------------------
---------------------------------LQRMVLVDLPGVIN--TV------------------T-------SG--------------M----
----AP--D--TKETIF--------SISK-AYM---QN------------PNAIILCIQDGSV--D-AERSI-VTDLVSQMD---
---PHGRRTIFVLTKVDLAE-------KNV-----ASP----SRI--------------------------QQI--IE---GK---------
L-FP---------------------MK---ALGYFAVVTGK---G-NSS--------
-----------------ES--------------------IE----------AIREY---EEEFFQN--------SK-LL--------------------K-
TS----ML--------K--AHQ-VTTRNLSLAVSDCFWKMVRES-V----
XP_031757388.1 ------------DHLPR--------VVVV---
-GD----Q----------------SAGKTSVLE-----M--IA-Q-A-RIFPRG------------SGE-MMTRSPV----------
----KVT-------------L---SEG---
--P-------HHV------------------------AMF-----------K------
---------DS--S---------------------------------------REFDLSKETDLA-----------ALRNEI------EVRM-
RK--SVKNG-----------------------Q---------------------TVSPE-TISLSVKGPG-------------------------
--------------------------------IQRMVLVDLPGVIN--TV------------------T-------SG--------------M------
--AP--D--TKDTIF--------NISK-AYM---LN------------PNAIILCIQDGSV--D-AERSI-VTDLVSQMD-----
-PQGRRTIFVLTKVDLAE-------KNV-----ASP----NRI---------------------------QQI--IE---GK---------
L-FP---------------------MK---ALGYFAVVTGK---G-NSN-------
-----------------ES--------------------ID----------SIKDY---EEEFFQG--------SS-LL--------------------
K-KG----ML--------K--AHQ-VTTKNLSLAVSDCFWKMVRES-I----

NP_610941.1 ------------DHLPR--------VVVV---GD----
Q----------------SSGKTSVLE-----S--IA-K-A-RIFPRG-------------SGE-MMTRAPV---------------KVT--
------------L---AEG--
---P-------YHV-------------------------AQF----------R---------------
DS--D--REYDLTKESDLQ-----------DLRRDV------EFRM-KA--
SVRGG-------------------------K----------------------TVSNE-VIAMTVKGPG--------------------------------
-----------------------------LQRMVLVDLPGIIS--TM-------------------T------VD-------------M--------
AS--D--TKDSIH--------QMTK-HYM---SN------------PNAIILCIQDGSV--D-AERSN-VTDLVMQCD--
----PLGRRTIFVLTKVDLAE-------E-L-----ADP----DRI----------------------------RKI--LS---GK---------
L-FP----------------------MK---ALGYYAVVTGR--G-RKD--------
-----------------DS--------------------ID----------AIRQY---EEDFFKN--------SK-LFH--------------------
R-RG----VI--------M--PHQ-VTSRNLSLAVSDRFWKMVRET-I----
MEN2496893 ---------NLVDHLPR--------IIVV---GD----
Q----------------SSGKTSVLE-----M--LI-N-A-RIFPRG-------------SGK-MMTKSPI---------------QVT--
------------L---SNG--
---P-------RHI-------------------------ACF----------K---------------
GGR-S--HEYSLDSQADLL-----------ALRLEI------ERRM-LA--
QIEPH-------------------------Q----------------------TVSDR-PIYLDISGPG--------------------------------
-----------------------------LQQMILIDLPGLIN--TV-------------------T------TE-------------L--------AE--
T--TKDDIE--------TMVK-KQI---EN------------PNAVILCIQDGSI--D-AERSS-VSKLVAQAD------
PKGNRTIFVLNKSDLAE-------RNL------SK----QTV----------------------------EKI--LS---GK---------L-
FP----------------------MR---ASAYFAIVSGT--D-DRN-----------
--------------SS--------------------IE----------EIKKH---EEKFFRN--------SK-FF------------------------K-T--
----I--------Q--DKN-KTSKNFSEQVSECFWS-----------
KAJ1637655.1 ------------IKLPQ--------IAVV--
GN----Q----------------SVGKSTVMS-----A--LS-G-I-P-FPVD-------------A-K-LTTRCAT---------------
QVT--------------M---RKA---
--E------TLQ---------------------------VSI-----------G-----------
----LSD-Q--AEAESIILDSLE-----------QVVSAI------EAKT-NE--
LVPEH-------------------------G---------------------A-IETER-FVKIEVSGPN--------------------------------
-----------------------------CPNLTVIDLPGLIQ--TV-------------------D------DG---------------------------
-------QIK--------GLVT-RTM---KS------------ERTINLLIMRADV--D-PAGNE-AFQLAREHD------
PLG---
-----------------VTNA--G-RSH--------------------------------------
----

AYV75702.1 -----------LENIPS--------IVMI--GS----
Q----------------SSGKSTLIN-----R--IA-G-F-NVSPTG--------------SG-LVTTAPL---------------NIL----
----------M---KTS-------------------------------ENRR--
----------------------------------VVIE---TKY------------------------------
--FDNFKQTAEEVE---------MVQQYI------RTIS-KQ---F-AD----
---------------------EQ------------------HS-PISSK-SINIVIESPD---
----------------VCNISMIDLPGLIA--VP---------------------------KQ--------------Q--------NQ-TE--
LIDKIN--------NIAV-QFI---SR------------PNSIVAVMIQAGS--D-LETNI-ALALLKKYN------

YQLSNCVGVLTKVDLTD-----ISDGI---CET--LN---GN--------T-
VP--------------------DAFKM---EYGYFAIQCK---TLD-KINN---

-------HNR-FGVDNLLQFLAQILMKSLKIH-HNKF-
KAI5073815.1---------------------------VVAL--GNV------
--------------GAGKSAVLN-----S--LT-G-H-PVLPTG--------------EN-GATRVPI---------------VID-------
-------M---ERD------------------------------EG--
------------------------------LNSK---QLV-------------------------------
---LQIE-SKTQQVSA---------SSIRRSL------QEKL-TS---A-----------
----------------SE---------------------R-GRLDE--IYLKLRSST--
-----------APPLKLVDLPGLDQ--RG-------------------S-----------------------D-----------E-------------
--STVS-AYT---DQ-----------GDAILLVVVPAFQTSE-ISNSR-ALRLAHDLD------
PDGSRTVGVISKVDQAASD----SRNL-----------AAV-----------------------------QAL--LV---GQ--------
-G--P--------------------SITL---DVPWVALIGQS---ASIA--TAHSS-
STGG-----------D--------NS---------------------LE---------TAWRA---EMENLKS---------------------------
------------------VLGSA-----P--QSK-LGRIALVDTLSKQIRKRLKLR-LP---
XP_008646219.1 ---------------------------AVAL--
GNV---------------------GAGKSAVLN-----S--LI-G-H-PVLPTG--------------EN-GATRAPI---------------
VVD--------------L---ARD------------------------------PG--
---LSSK---SIV---------------------
--LQID-SKSQQVSA---------SALRHSL------QDRL-SK---
GASS-------------------------GSG---------------------R-SRSDE--IYLKLRTST-------------------------------------
----------------------------APPLKLIDLPGIDQ--RV-------------------M-----------------------D------------
D----------------STIS-EYA---GH------------NDALLIVVIPAMQAAD-VASSR-ALRLAKDID------
PDGTRTIGVLSKIDQAAAD----AKTV-----------SCV-----------------------------QSI--LS---NK---------
G-AP--------------------RAAA---DIEWVALIGQS---VSIA--SAQSG-
SVGS-----------D--------NS---------------------LE---------TAWRA---EAETLKS----------------------------
----------------ILTGA-----P--QSK-LGRIALVDTIAKQIRKRMKVR-LP---
ACG47836.1 ---------------------------AVAL--GNV------
--------------GAGKSAVLN-----S--LI-G-H-PVLPTG--------------EN-GATRAPI---------------VVD------
--------L---QRE------------------------------PG--
--------------------------------LSSK---SIV-------------------------------
---LQID-SKSQQVSA---------SALRHSL------QDRL-SR---GASG---
--------------------GSG-------------------R-GRVDE--IYLKLRTST---
------------------APSLKLIDLPGIDQ--RA-------------------V-----------------------D-----------D--------
--------SMIN-EYA---GH------------NDAILLIVIPAMQAAD-VASSR-ALRLAKDID------
ADGTRTVGVISKVDQANGD----AKTI------------ACV----------------------------QAL--LS---NK---------
G--P--------------------KNLP---DIEWVALIGQS---VAIA--SAQ---
SVGS-----------E--------NS---------------------LE---------TAWRA---EAESLKN----------------------------
----------------ILTGS-----P--QNK-LGRIALVDTIAKQIRKRMKVR-VP---
XP_006385192.1 ---------------------------VVAL--
GNV---------------------GAGKSAVLN-----S--LI-G-H-PVLPTG--------------EN-GATRAPI---------------
SID--------------L---SRD------------------------------SS--
--VSSK---SII---------------------

---LQID-SKNQQVSA---------SALRHSL------QERL-SK---
VS---------------------------SG--------------------R-SR-DE--IYLKLRTST--------------------------------------
-------------------------APPLKLIDLPGVDQ--RI------------------V----------------------D------------D--
--------------SMIS-EYV---QH------------NDAILLVVIPAIQAPE-ISSSR-ALRIAKEYD------
AESTRTVGIISKIDQAATE----SKAI------------AAV----------------------------QAL--LL---NQ---------G-
-P---------------------PKTS---DIPWVALIGQS---VSIA---------------------------------------SVQSG-
SASS-----------E--------SS--------------------LE----------TAWRA---ESESLKS---------------------------
---------------ILTGA-----P--QSK-LGRVALVDVLAGQIRSRMKLR-LP---
'KAG7649995.1' -------------------------VVAL---
GNV---------------------GAGKSAVLN-----S--LI-G-H-PVLPTG--------------EN-GATRAPI---------------
IID--------------L---SRE-------------------------------ES---
---------------------------------------LSSK--AII------------------------
---LQID-NKNQQVSA---------SALRHSL------QDRL-SK---
GA---------------------------SG--------------------R-GR-DE--IYLKLRTST------------------------------------
--------------------------APPLKLIDLPGLDQ--RI-------------------V-----------------------D------------E--
--------------SMIG-EHA---QH------------NDAILLVVVPASQASE-ISSSR-ALKIAKEYD------
PDSTRTVGIISKIDQAAEN----PKSL------------AAV-----------------------------QAL--LS---NQ---------
G--P--------------------PKTT---DIPWVALIGQS---VSIA---------------------------------------SAQSG---
GS------------E--------NS--------------------LE----------TAWRA---ESESLKS------------------------------
------------ILTGA-----P--QSK-LGRIALVDTLASQIRSRMKLR-LP---
'NP_172500.1' -------------------------VVAL---
GNV---------------------GAGKSAVLN-----S--LI-G-H-PVLPTG--------------EN-GATRAPI---------------
IIE--------------L---SRE-------------------------------SS---
---------------------------------------LSSK--AII------------------------
---LQID-NKSQQVSA---------SALRHSL------QDRL-SK---
GA---------------------------SG--------------------K-NR-DE--INLKLRTST------------------------------------
--------------------------APPLKLVDLPGLDQ--RI-------------------V-----------------------D------------E-
---------------SMIA-EYA---QH------------NDAILLVIVPASQASE-ISSSR-ALKIAKEYD------
PESTRTIGIIGKIDQAAEN----SKAL------------AAV----------------------------QAL--LS---NQ---------
G--P--------------------PKTT---DIPWVAVIGQS---VSIA---------------------------------------SAQSG---
SG------------E--------NS--------------------LE----------TAWRA---ESESLKS------------------------------
------------ILTGA-----P--QSK-LGRIALVDTLASQIRSRMKLR-LP---
KAH9330549.1 -------------------------VVAL---
GNV---------------------GAGKSAVLN-----S--LI-G-H-PLLPTG--------------EN-GATRAPI---------------
IID--------------L---QRD-------------------------------SS--
--VSSR---AIF-----------------------
---LQIE-NKTQQVSA---------SALRHSL------QDKL-SK---
GL---GI--PPMKLRTST---------------------------------------
---------------------SPPLKLIDLPGLDQ--RV-------------------A-----------------------D------------D-----
-----------SMIS-NVA---EH------------NDAILLVVVPASQAPE-ISSSR-ALKLALEFD------PD------------
----
----------AIA--SAQSG-SVGG-----------E--------SS--------------------
LE----------TAWRA---ESESLKA---ILTGA-----P--QTK-
LGRIALVETLARQIRKRIKIR-LP---

ONM04707.1 -------------------------VVAI---GNV------
-------------GAGKSAVLN-----G--LI-G-H-PVLPTG--------------EN-GATRAPI---------------CVE-------
-------L---QRD------------------------------AS--
-----------------------------LSSK---AIV---------------------------------
---------------------------------------LQID-SKSQQVSA---------SSIRHSL------QDRL-SK---V----------
----------------SG-------------------K-GRPDE--ITVKLCTST---
------------APPLKLIDIPGVDQ--RS-------------------T-----------------------D------------E---------------
-S-IS-NYA---AR-----------NDAILLVIIPALQAPD-VASSR-ALRIARELD------
SEGTRTIGVLSKIDQASGE----QKAL------------GAV----------------------------QAL--LV---NQ---------
G--P--------------------RTAA---DIQWVATIGHS---VPTA--------------------------------------SAQS--
EAGS-----------E--------TP--------------------PE----------AYWQA---EVKTLVS---------------------------
---------------TLGGA-----P--ESK-LGRVALVDSLSKQIKARIKAR-LP---
EFJ33653.1 -------------------------VVTI---GNT--------
------------GAGKSAVLN-----S--II-G-Y-TVMPTG--------------EN-GATRAPI---------------VVE---------
-----L---ERD------------------------------QS--
---------------------------EGK---GLA----------------------------------
--------------------------------------VMTE-GRARPSSA---------NEIRLSL------QSRI-SR---I-AS---------
---------------SR-------------------S-GRPEE--IRLRLRSSA--
-----------APPLTLIDLPGLA----S--------------------L------------------------D------------D----------------
QFVN-EYG---SH------------NDAVLLVVVPATSVRD-ITGSQ-ALKMARELD------
PEFSRTVGVISKVDQSASD----PKSL------------AAV----------------------------QAV--LS---GQ---------
G--P--------------------SASA---DITWVALIGQS---VSIA--------------------------------------AAHAG-
SVGT-----------D--------DS---------------------LE----------TAWKA---ETETLRS---------------------------
---------------ILTAA-----P--STR-LGRAALVDVISKQIRKRIRQR-LP---
EFJ28901.1 -------------------------VVAL---GNT-------
-------------GAGKSAVLN-----S--LI-G-Y-AVLPTG--------------EN-GATRAPV---------------TID-------
------L---EAD-------------------------------DS--
---------------------------GNKR---GLT---------------------------------
---------------------------------------VQME-GKSMQVSA---------TDIRHSL------QNKF-GR---M--------
-------------------ST-------------------G-AVKEN--IHLKLKCSS--
---------------APPLKLVDLPGLES--RS-------------------V-----------------------S------------D------------
-----SLVR-EYI---DS-------------NDALLLVVIPATSVRD-ITGSQ-ALKIAQDID------
HEGSRTVGVISKIDQAASD----PKSL------------AAV----------------------------QAV--LS---GQ---------
G--P--------------------SITS---KFTWIALIGQS---VSIA---------------------------------------GAHS-----K-
----------D--------DS---------------------LE----------TAWKA---EMESLKS----------------------------------
---------ILGGA-----S--SSR-LGRSSLVEAIAKQIRQRMQQR-LP---
OAE31801.1 -------------------------VVAV---GHI-------
-------------SAGKSAVLN-----S--LV-G-Y-PVLPTG--------------EN-GATRVPI---------------IIE----------
----M---KRD------------------------------ES---
--------------------------GNRK--GLA----------------------------------
---------------------------------------IQVEGGRIQSVSA---------SDLRHNL------QGRL-QK---WTPN---
---------------------A---------------------K-GRPDE--IKLRLVSSA---
----------------APPLKLIDLPGMEVR-AP-------------------I------------------------E------------D----------
------SIVR-DYA---EH------------NDAVLLLVIPATQAAN-ILGAR-ALKLVQDLD------

GEGTRTVGVISKVDQAAAD----PRSL-----------AAV--------------------------QAL--LS---GQ--------
-G--P-------------------SSTA---EFPWVALIGQS---VSIA-----------------------------------AAHAG-
G-GA----------E--------DS-------------------LE---------TAWRA---EAESLKQ-------------------------
----------------ILPQA-----S--PSK-LGRVALVETLSSQIRKRLKNR-LP---
KAG0632288.1 ------------------------VVTL--
GSV-------------------GAGKSAVLN-----S--LM-G-Y-PVLPTG-------------EN-GATRAPI--------------
-IIQ-------------M---QRG---------------------------SS---
---STNR-------------------------------------GLY------------------
--VVLD-GRTSNVTA---------SDVRHSL------QGRL-
KS---WTPN----------------------AR------------------S-GRTEG--IQLTLQSSA-------------------------
----------------------------------APPLKLFDLPGLDTR-AT-----------------S---------------------D-----
-------D----------------SLVQ-EFA---EH-----------SDAILLVVVPAASVRE-VGTSK-ALKLAQELD------
SDATRTVGVISKVDQAASD----RRSL-----------DAV---------------------------AAL--LS---GN---------
G--P-------------------AITQ---EIPWVAMIGQS---VSIA------------------------------------AAHGS----
------------E--------DS-------------------LD---------TAWKA---EAESLKS-------------------------------
----------LLTQA-----A--PTK-LGRVALVEAIAKQIRKRLKQR-IP---
'KAG0555995.1' ------------------------VVAL--
GSV-------------------SAGKSAVLN-----S--LI-G-H-PVLPTG-------------EN-GATRAPI---------------
ILD-------------M---ERD----------------------------KS---
-------------------------------------SSSR------------------------------------GLA-------------------
---VVLE-GRTQNVSA---------SDVRHSL------QGRL-KN-
----AS----------------------SS------------------K-GRTEG--IRLTLRSAS-------------------------------
--------------------------TPPLKLIDLPGVS---GS------------------I---------------------D------------D-
---------------SPAH-DLA---AN-----------NDTILLIVIPATSCRD-VAASK-ALKLAQELD------
SDGTRTVGVISKVDQAASD----PRSL-----------AAV--------------------------NAL--IS---GQ---------
G--P-------------------PSTA---DIPWVALIGQS---VSIA------------------------------------AAHSS-G--
------------E--------DS-------------------LD---------TAWKA---EMESLKS-----------------------------
-----------ILNGA-----P--SAK-LGRIALVETLSHQIRTRLKQR-LP---
XP_024368367.1 ------------------------AIAL---
GSV-------------------SAGKSAVLN-----T--II-G-H-PVLPTG-------------EN-GATRAPI--------------
IID-------------M---ERD----------------------------KS---
-------------------------------------GRPG---GLA-------------------
--VVLE-GRTQNVSA---------SDVRHSL------QGRL-
KS-----VS----------------------SS------------------K-SRGDG--IRLTLRSNS----------------------------
-------------------------------GPPLKLIDLPGIDSR-GS-----------------L---------------------D-------
-----D----------------SPAH-DLA---AN-----------NDTILLVVIAATSCRD-VAVNR-ALKLAQELD------
SDGSRTVGVISKVDQAASD----PRSL-----------AAV--------------------------NAL--IS---GQ---------
G--P-------------------SNTQ---DIPWVALIGQS---VSIA------------------------------------AAHSS-P-
-------------E--------DS-------------------LD---------TAWKA---EMESLKT-----------------------------
-----------ILKGA-----P--SAK-LGRIALLETLASKIQSRLKQR-IP---
XP_024391061.1 ------------------------AIAL---
GNV-------------------SAGKSAVLN-----S--LI-G-H-PVLPTG-------------EN-GATRAPI---------------
IID-------------M---ERD----------------------------KS---
-------------------------------------GRPG---GLA------------------

---VVLE-GRTQNVSA---------SDIRHSL------QGRL-KS-
----VS-----------------------SS------------------K-GRGDG--IRLTLRSNS------------------------------
--------------------------GPPLKLIDLPGIDSR-GS------------------I---------------------D------------
D---------------SPAH-DLA---AN------------NDTILLVVIAATSCRD-VAVNR-ALKLAQELD------
SDGSRTIGVISKVDQAASD----PRSL-----------AAV--------------------------NAL--IS---GQ---------
G--P------------------SNTQ---DMPWVALIGQS---VSIA-----------------------------------AAHSS-
S--------------E--------DP-------------------LD----------TAWKA---EMESLNS--------------------------
--------------ILKGA-----P--PAK-LGRIALLETLASKIQSRLKQR-IP---
ARF10781.1 ------------FILPR--------IITV--GN----E--
-------------STGKSSLFE-----K--IL-K-C-PIFPKN-------------NI-ICTKSPI--------------RLI-----------
--L---KDG----------------------------PNKY-V---
---IKH---------------------------KN-N-----
-------------------------------IIELNNK-------Q-----------EILDNI------II-I-NN--SI-SP-----------------
-----KNSD------------------IISDD-EIVIEFSEPN---
-LLPLELIDLPGLRA--F--PP--D--LAAKTT--------SLCK-
KYL----T------------DKDIILCVVPATVT-R-LTSCQ-PLALINELN-------LCKNTILALTMADRLI-------
MTEYS--QSQKVNIDNLL--------------------------LNR--IL---GTSD-------EMES---------------------
-LK--LKGCVAIINR---T-HND--IID------------------------------
----------LE----------ESDNM---EKKYFDKL----LNDLPISYI-------------------K-E-----SI-----------
NDN-LSVKNLLIMINNLVDDCIQNN-WKQMI
ARF10780.1 ------------FKLPR--------IIAI---GN----E--
--------------STGKSSLFE-----K--IL-K-C-PIFPRN-------------SI-ICTKAPI--------------RLV-----------
---L---NSG----------------------------QQKY-V---
---IRNTM----------P--------------GSE-
K------------------------------------EIVLYDK-------Q-----------EIYKNM------ME-I-NN--SI-SP--------
---------------KHSD--------------------VITEH-EIIIEFTEPN--
-----------LPSLEFIDLPGLRA--Y--PP--D--LATITT-------
-NLCK-KYL----S------------IQDIILCVIPATVT-R-LTSCQ-PLALINELN-------LCQNTILALTMADRLV--
-----MSEYA--QEE--NINELL--------------------------LNR--IL---GTSD-------EMTY-------------------
---LK--LKGCVAVINR---T-HND--TMN-------------------------
---------------LE----------ESDDM---EIQCFEKL----LNNLPTEYL-------------------K-DK---NTI--------
---EEN-ITVKNLLTIINNFYVHHIQNN-WKPII
KAH8061769.1 ---------LGHVALPS--------IIVV--------------------------------------
-GS----E---------------SAGKSSTLE-----R--IA-G-L-TLFPRD-------------AT-ICTRMPI--------------
QLR-------------L---IQD----------------------------DAAA-G--
---SC-------------------VTVRL----------A------
---------GRE-D------------------------------------AVVSEADAART-------------------V------AAFM-NE-
-AV-AA----------------------RHG--GT------------VR-GVVND-VLTIEVRKPD-------------------------
-------------------------------VPTLDLIDLPGIVA--AS------------------V-------EG--------------E-------
-PA--D--MMEQTR--------KITE-HYM---CR------------ADTVCVCVVPANAT-R-VRDSQ-
AMQLVQRHG-------KEPLTIGVLAKADLAY-------DPRFKQRKKK--SPFWEL-------------------------
-KDR--LA---GRAD-------DMVA----------------------L---PQGWVGVKNR----------------------------
---------------D-TMV--VEE-------------------EGSS-----------------LH----------DSAEE---

EQRWFSE-------D-A-KIQ--DG-VGINQLLVKIDALFSSHIRDN-
WVPRA
OUS45493.1 ----------LRYSVPR--------IVCV--GE----
E-----------------SSGKSSTLE-----R--VA-M-M-KVFPSD---------------EK-LCTRVPI----------------ELR---
------------L---RYR--------------------------------DPES-L---
-------------------------------------EER---------------FRESGY---------------------VVMRM----------A---------
------PGL-R--STVPEDESPPMSP-D----------HVPNQI------
RQWM-ED--LV-RA------------------------AN---GT-------------LT-GVTDD-KIIVELYSSR----------------
--RVNLDLIDLPGIVA--GS-------------------M-------PG----------
----E--------PA--D--MMERTR--------QLSS-SFMDDAAN------------PHTFVIAVVSAMDA-R-IRNSQ-
AMELVQRHN-------KVQFTIGALTKADLSA-------DTR-----RE--NPYSKL----------------------------
LER--LN---GVAE-------DAPE-----------------------L---ALGYVALKNR-----------------------------------
------------D-TVI--DTD---------------------IS---------------------LT-----------QVNAE---EKEWFQR-------
HLP-EHV--KS-CGIDSLIDKLVDKVEEYTRGP-WIEAE
XP_001419538.1 ----------LRYSVPR--------IVCI--
GE----E-----------------SSGKSSTLE-----R--VA-M-M-SVFPSD---------------ER-LCTRVPI----------------
ELR---------------L---RHR--------------------------------DRES-LA--
--KEEER---------------FRD-GY---------------------VVMKM-----------
A----------------PGE-D--SKLNADVSSQMHP-D-----------EVPAQV---
---RTWM-EE--LV-AA-------------------------AN---GK-------------VT-GVTDD-RIIIELYSPL---------------
---CVNLDLIDLPGIVA--GS--------------------I-------PG----------
----E--------PT--D--MMDRTR--------NLSA-SFLNDKAH------------PHTFVIAVASAREA-R-IRNSQ-
AMELVQRYN-------KVQFTIGALTMADLSA-------DSR-----RE--SPYTRL----------------------------
IER--LN---AQAD-------DTPE-----------------------L---GLGYVALKNR-----------------------------------
------------D-TVT--ATR---------------------ENQSR----------------EELE-----------KANQD---EKEWFAE---
----HLS-GHT--ER-CGIESLVDRLVQKVDEYTRGP-WIVAE
PRP82121.1 ---------KLKLDFPQ--------LVVV--GE---
-E-----------------SCGKSSTLE-----R--IC-M-M-PLFPRD-----------RTEA-ICTRMPI----------------KLR-
------------L---KHL--------------------------------SVPE-MKTFCA---
---QNGIG--------------YQD-GT---------------------AAVRL----------C--
-------------YED-A--------------------------------------HKPANKQWSPFYTIA-----------EIEEKV------
KQFM-DE--AV-RQ------------------------KH---GQ-------------VK-GIIED-VMVVEITSFE---------------
---VPNLTLVDLPGIIG--GS-------------------V-------AG----------
----E--------PI--D--MPQQTR--------ALAE-KYL---NN------------QHTLAIAVVPAYQ--R-IRNAQ-
VMDLIERSG-------KKDQTIGVLTMCDKAD-------QSNLR---PR--DPFFEL----------------------------
KER--LS---GRAK-------DVIP-----------------------L---KNNYIAVMNR-----------------------------------
------------D-NTL--HAG---------------------MD---------------------LT----------TAATR---ETEWFER-------
NLP-GYT---------------------A-Q-------------------GKA-TG-DQLVLKLTDMLCQ------------
TMW65229.1 -------------LDLPQ--------IVVV--GQ----
E-----------------SSGKSSVLE-----S--LA-M-M-PLFPRD---------------ES-ICTRMPI----------------HLK--
-------------M---RHR--------------------------------QTRTGVSG--
--SGSGS--------------DSP-TS----------EVETPQ-----IKMRL---------
V----------------FSD-G--RAPVESDDGFQT--E----------EVAQQM----
--RTWM-DE--IV-RG-------------------------TNSDGSA-------------VT-GVVDH-VLEIEVTSSQ-----------

---VPTLDLIDLPGIVA--GR------------------L-------VD------
--------E--------PE--D--MMHQTR--------ALVE-KYL---KM------------PHTLVLAVVPAFE--R-VRNSQ-
AFQLVQQYG-------LEDRTIGVLTMVDRAI-------DVS----NPK--GPLVAV---------------------------
MDR--LD---EVSS-------DVVK----------------------L---KHGYVAVKNR----------------------------------
------------D-TRT--DQQ--------------------PP-------------------LE----------EFKRD---EVAWLEE------
-NLA-GYV--------------------E-K-------------------KL-ASSGILASKLEMLLAEHVRTT-WVSQT
KAG1689960.1 -----------ELALPQ--------IVVI--
GQ----E----------------SSGKSSVLE-----S--LA-M-M-PLFPRD--------------RD-LCTRMPI-------------
--HLK--------------M---RHV-----------------------------SQAE-AD---------------------------------------
---ESAE--------------LMP-H--RPP------LDTHQ-----IKMKL---
-------V---------------YAD-G--------------------------------------REPVESEKNFTA--E-----------
EAAELM------SQWM-EQ--IV-KE------------------------EDED-KK-------------LQ-GVTEH-
VLEIEVRSSK---VPNLNLIDLPGIVA--GR----------
---------L-------VD--------------E--------PD--D--MMERTR--------ALVE-KYL---QL------------
PHTLVLAVIPAYE--R-VRNSQ-AFQLVQKFN-------LMDKTIGVLTMVDRAM-------DAT----NPE--
GPLGEV----------------------------MNR--LD---GTSR-------DIVY-----------------------L---
KEGYVAVMNR---D-TRL--SPE--------------------LA------------
--------LD----------KFKAE---EMAWLEE-------NLP-GYI--------------------G-R-------------------GL-
ASSSVLATKLEKMLAD-----------
KAE9027747.1 -----------VLALPQ--------IVVI---
GQ----M----------------SSGKSSVLE-----S--LA-M-M-PLFPRD--------------RD-ICTRMPI-------------
--HLK--------------M---RHV------------------------------PKSE-VD--
--EDAD--------------LMP-H--R-T------VDKHQ-----IKMKL----
------I---------------YSD-G---------------------------------------RKPVVSNKDFTP--E-----------
EAAERM------SEWM-QQ--IV-KE------------------------ESQE-NK-------------LQ-GVVDH-
ILEIEVRSSK--VPNLNLIDLPGTVA--RR-----------
--------L-------ID--------------E--------PD--D--MMQRTR--------TLVE-KYL---QM------------
PHTLVLAVVPAFE--R-VRNSQ-AFQLVQQYN-------LMDSTIGVLTMVDRAT-------DAS----DPN--
GPLAEV----------------------------ISR--LE---GTSR-------DIVY-----------------------L---
KEGYVAVKNR---D-TRL--SPE--------------------CL------------
-------LD----------EFKAE---ENAWMEE-------NMP-GFT--------------------E-R-------------------RL-
ASSSVLAPKLEKMLAD-----------
KAG7385474.1 -----------ELALPQ--------IVVV--
GQ----E----------------SSGKSSVLE-----S--LA-M-M-PLFPRK--------------ED-VCTRLPI--------------
-HLK--------------M---RHV------------------------------PKRDVVD---
--GDQE--------------LMP-H--RRTPHSV-DVAKHQ-----
IKMRL----------V---------------YAD-D-------------------------------------REPVVSEKELTA--E----------
QAAERM------SEWM-EM--IV-KE------------------------ENED-KK-------------LK-GVVDH-
VLEIEVRSPH---VPNLNLIDLPGIVA--GR----------
---------L-------ID--------------E--------PD--D--MMQRTR--------ALVE-KYL---QM------------
PHTLVLAVVPAFE--R-VRNSQ-AFQLVQQYD-------LMDSTIGVLTMADRSL-------DTS----NPE--
GPLAEV----------------------------MSR--LD---GTSR-------DIVY-----------------------L---
KEGYVAVKNR---D-TRV--SPE-------------------WS------------

--------LD----------AFKTE---ENAWLEE-------NLP-GCI--------------------E-R-------------------KL-
ASSSVLATKLEKMLAD-----------
KAH7489187.1 -----------EFALPQ--------IVVV--
GQ----E----------------SSGKSSVLE-----A--LA-M-L-PLFPRD--------------SV-MCTKLPI---------------
LLK--------------L------------------------------------RHGN-VDV--
---EGDPD--------------LMP-HC--GKPQ-----AKQQ-----IKMKL----
------A---------------YSD-G--------------------------------------RKPVESDRNFTP--D-----------
EAAQLM------RKWM-EE--IV-AQ------------------------EGEE-------------------GVVDH-
VLEIEVQSPH--VPNLNVVDLPGIVA--GR--------
-----------R-------VG--------------E--------PV--D--MMQRTR--------ALVE-KYL---KM------------
PNTLVLAVVPAFE--R-VRNSQ-AFQLVQQYN-------LMDSTIGVLTMMDRAV-------DAT----NPD--
APLSEV----------------------------MSR--LE---GTSQ-------DVVD-----------------------L---
KHGYVAVKNR---D-TRL--APA--------------------LS-------------
-------LD----------EAKAE---ENAWLEE-------KMP-GYI--------------------D-R-------------------GL-
ASSSVLAKKLEEILAD-----------
POM76499.1 ------------LTLPQ--------IVVI--GQ----E-
---------------SSGKSSVLE-----S--LA-M-L-PLFPRD--------------SD-ICTRLPI---------------LLK-------
-------M---RHM------------------------------SHGN-VEV--
---------------------------------EGDPE--------------LMP-HCPRGDIVMT----EQQ-----IKMRL----------
I---------------YSD-S--------------------------------------REPVESERNFTP--E-----------EAAQLM------
RKWM-DQ--IV-KD------------------------EHDD-EQ-------------LK-GVVDH-VLEIEVRSPN-----------
---MPNLNLIDLPGIVA--GK-------------------L-------ID------
--------E--------PD--D--MMQRTR--------ALVE-KYL---QQ------------SDTLVLAVVPAFE--R-VRNSQ-
AFQLVQQYN-------LTEKTIGVLTMVDRAI-------DET---NPE--GPLAEV----------------------------
MGR--LD---GMAQ-------DLVY-----------------------L---KQGYVAVKNR--------------------------------
--------------D-TRV--PPE--------------------RS--------------------LE----------KSKDE---EIAWLEE------
-NLS-GYV--------------------D-R-------------------GL-ASSTALVMKIEKMLVDHICTT-WVPQM
KAG7377001.1 -----------ELTLPQ--------IVVI--
GE----E----------------GSGKSSVLE-----S--VA-M-L-PLFPRH--------------VD-ICTRLPI---------------
LLK--------------L---RRV------------------------------SHGN-VEV--
---KGDPE--------------LMP-HC-RGNSQPVNPPDKQQ---Q-
IKMRL----------L---------------YSD-G--------------------------------------REPIESERNFTP--Q----------
-EAAQLM------SQWM-EQ--IV-TE------------------------QHDD-NK-------------LK-GVVDH-
VLEIQVRSPH--VPNLNLLDLPGIVA--GK---------
----------L-------ID--------------E--------PE--D--MMQRTR--------ALVE-KYL---KM------------
PDTLVLAVVPAFE--R-VRNSQ-AFQLVQQFK-------LTDKTIGVLTMVDRAL-------DDA---DPE--
GPLAQV----------------------------MSR--LN---GTSS-------DIVY-----------------------L---
KEGYVAVRNR---D-TRL--VPE--------------------VT-------------
-------LE----------EFKDE---EDAWMEE-------NLP-RYI--------------------D-R-------------------RL-
ASSSVLVKKIEEMLAD-----------
XP_008910862.1 ------------LKLPQ--------IVVI--
GE----E----------------SSGKSSVLE-----N--VA-M-L-PLFPRD--------------SD-ICTRMPI---------------
LLK--------------M------------------------------------SHGN-IE---
---GDPE--------------LMP-HC-KGNPHPA---DQQQ-----IKMRL---

-------I--------------YSD-A-------------------------------------RAPIESERLFTL--Q----------EAAQLM-
-----SKWM-KQ--IV-QE-----------------------EHDA-NK------------LA-GVVDH-VLEIEVRAPN-------
---LPNLKVVDLPGIVA--GK------------------L-------ID--
------------E--------PE--D--MMQRTR--------ALVE-KYL---KM------------PDTLVLAVVPAFE--R-
ARNSQ-AFQLVQQYK-------LADKTIGVLTMVDRAL-------DET----NPE--GPLTQV--------------------
-------KSR--LD---GTSS-------DIVY----------------------L---KEGYVAVRNR----------------------------
-----------------N-SRL--VPE-------------------VS-------------------LE---------EFKEE---EDAWLEG--
-----NLP-GYI--------------------D-R-------------------RL-ASSTVLVDKLEKMLATHIRQY-WVPAT
KAF1774311.1 -----------ELKLPQ--------IVVI--
GE----E----------------SSGKSSVLE-----S--VA-M-L-PLFPRE-------------SD-ICTRMPI--------------
LLK-------------M-----------------------------------SHGS-FEV--
--QGDPE-------------LMP-HC-KGNPQPT---
DQQTPTYQLIKMRL----------I--------------YSD-D-------------------------------------
RAPIESERLFTL--Q-----------QAAELM------RKWM-EQ--IV-RE----------------------EHDA-NK-----
--------LA-GVVDH-VLEIEVRAPN--
LPNLKLVDLPGIVA--GK------------------L-------ID-------------E--------PE--D--MMQRTR--------
ALVE-KYL---KL------------PDTLVLAVVPAFE--R-VRNSQ-AFQLVQQYK-------
LADKTIGVLTMVDRAQ-------DET----NPE--GPLTQV---------------------------KNR--LD---GTSS-----
--DIVY----------------------L---KEGYVAVRNR---N-TRL--VPE-
------------------VS--------------------LE---------EFKEE---EDAWLEE-------NLP-GYI------------------
--N-R------------------HL-ASSTVLVENLEKMLAD-----------
KAI9982172.1------------LKLPQ--------IVVI---GE----E-
---------------SSGKSSVLE-----S--VA-M-L-PLFPRE-------------VD-ICTRMPI---------------LLQ------
--------M-------------------------------------SHGN-IE--
------------------------------GDPE-------------LMP-HC-KGNPPHA---DELQ-----IKMRL----------V---
------------YSD-K-------------------------------------RAPIEFDKLCTL--Q----------DVAQRM------
SQLM-KQ--VA-QE----------------------EHDA-NK------------LA-GVVDH-VLEIEVRAPN------------
---LPNLKLVDLPGIVA--RK-----------------L-------ID--------
------E--------PG--D--MMQRTR--------TLVE-KYL---KM------------PDTLVLAVTPAFV--R-VRNSQ-
AFQLVQQYK-------LTDKTIGVLTMVDRAR-------DET----NPE--GPLTQV---------------------------
QNR--LD---GTSS-------DIVY----------------------L---KEGYVAVRCR---------------------------------
-----------N-TRV--VPA-------------------VS------------------LE---------EFKEE---EDLGLR-----------
---KTLLVGKLEKLLAHHVRQS-WVPPA
KAE8986371.1 ------------LLLPQ--------IVVV--
GQ----E----------------SSGKSTVLE-----S--VA-M-L-PLFPRD-------------SA-MCTRLPI--------------
LLK-------------L---RHT-----------------------------SQIH-VEV--
--EGDLE-------------LMP-HC--GAPQ-----STEQ-----IQMRL-----
-----L---------------YAD-G--------------------------------------RAPIGSSARVTR--D-----------
QAAQLM------RQWM-DQ--IV-AE----------------------QHEH-EQ-------------LR-GVVEH-
VLEVRVQSPH---VPNLTLVDLPGIVA--GR--------
-----------L-------ID-------------E--------PN--D--MMQRTR--------AIAE-KFL---QM------------
PHTLVLAVAPASE--R-VRNSQ-AFQLVQQLG-------LTDKTIGVLTMTDRAV-------DAT----NPD--
GPLADV---------------------------TSR--LD---GTSS-------DIVF----------------------L---
KQGYVAVKSR--D-SRA--KTQ-------------------LS-----------

--------LE----------EAKAE---ENAWLEE-------NLP-GYI-------------------G-R------------------GL-
ASSSALVAKIEQMLAEHVRTS-WVPQA
KAG8459568.1 -------DILKGLSPPR--------IIVI---
GA----E----------------STGKSTVLE-----R--LA-M-M-PLFPRS-------------AR-LCTRVPI---------------
HLR--------------L---RRC------------------------------APGE---
--SHARL----------T--------
-------AFD-S--GCEPADAGPIIAI-E-----------QGWERV------QEQM-
SA--LL-AE-------------------------CE---HR-------------VR-A--DK-TIMLTVYHSR-------------------------
---VPSIELVDLPGI------------------------------------QQ-------------R--------
TA--D--ARARTE--------KIVH-EYVRN-SR------------PSDMFLLVHRWGP--T-CRTAE-
AGGLSCLDGC----EAAQRRTVGVLTYCDRVD-------KPR-----DC--AHLRAV---------------------------
-LAR--EH---SEAQI---DELGIAP----------------------LA--PHGWVAVANV-----------------------------
----------------D-PLD--DAD--------------------GDGGGGGGDAIAGGGCVGLARLLR----------
TARAE---RAHFDSN-------DVL-REL--------------------A-R------------------EGS-
ATCDALVDRLECMYVK-----------
XP_004338334.1 ---------VAELSFPQ--------IIVV---
GQ----E----------------SSGKSSVLE-----R--IA-M-L-KFFPRS-------------DK-MCTRMPI--------------
-KLQ--------------L---KHM------------------------------SPTD-MTRFCE-------------------------------------
--DHEEA-------------YRD-GV-------------------AFVRARM----
------E---------------YAD-R---ST---SSWSVFLPVE-----------AVEKLV--
----RDTM-EQ--AV-QA------------------------RN---AT------------LA-GT-------------------------------------
---VPNLTLIDLPGTCE--VA--------------------R-------AG--------------
E--------PE--N--IKESTE--------SLVE-KYL---KQ------------DHTLVLLVMSACD--R-MHNYR-
PLTVVKKHH-------KLGNTIGVLTKSDLAE-------NSR----YEH--DKFYEF---------------------------
KRQ--LN---GQSE-------DYIP----------------------L---ANGYVAVRNR-------------------------------------
------------D-TQG--TRG--------------------YS--------------------LE----------QAAIE---EVEWFNR------
-NLP-GYV--------------------D-K-------------------KL-ASSLVLVEKLVEMMCT-----------
KOO23261.1 ------AATLEGWKTPV--------VIVF---GQ-
---E----------------TSGKSSLLE-----R--LA---MMPLLPRG-------------ED-TCTRLPI---------------LLK-
-------------L---RHT------------------------------KE---
--AQLPVL-------V---------------
VRD-S-AT--GKEEVRRTVSLAG------G-QVDVRHEM------ERVL-----
---AA------------------------EQGG----------------LT-SVSMTRTIEVHVHSPA-----------------------------
-----------------------------VPSIDLVDLPGLRL--SA--------------------------GS-------------D--------
AA--D--MPEKAK--------ALVK-AQV---EQ-HK---------GSAIFLVSCTAST--P-PSQSL-GLQLVEELG---
----LQRDTIGVVTMCDNIG------KKDM-----------TKL---------------------------PDR--LRQTSGD----
-----A-FQ-----------------------LA--PHGFIGTMTD---P------
IKD----------EG--------LSN-------------------VQ-----RL---EMMATA--ERKWFAS--------QP-LL--------
-------------S-P-----LL--------S--DGL-LTCDALVDKLSSVYAR-----------
'XP_005785253.1' ------------LLLPQ--------IVVV---
GS----E----------------SAGKSSLME-----R--IA---MQAFFPRA--------------EG-FCTRMAI--------------
-RLK-------------M-M-HRP-----H-------------------------ESRVMIRC--
--LRTSN--GQV----------
-------------------A-MF---NGMPAEQRFEVDG------VQHTVIQRII----

--ERFI-AQ---V-HG------------------------QDDG-----------------R-SVLTDIEIEIEFRACN---------------
---VPTLTLVDLPGIVS-----------------------M----------------------
----------PA--D--VRQQTL--------ELTR-RYL---RD-----------PNTLVLCVINGNEA-A-LRGSR-
ALEEISEHAV---ARPAFSKTIVVTTRTDLWV-----QSNGL------------VSL----------------------------
ARR--LADPKNE-----------VG-----------------------CE--PVALIPVINRE-----------------------------------
-----------A-PKENQVDG--------------------YS--------------------LP----------DLVKK---ERAKFEE----
WQAKEP---------------------------------GL-----------RGKPLGILSVLDALNDLFETHMSEV-WVPQA
XP_005792501.1 ----------DLLLPQ--------IVVV---
GS----E-----------------SAGKSSLME-----R--IA---MQAFFPRA--------------EG-FCTRMAI--------------
-RLK--------------M-M-HRP-----H------------------------ESRVMIRC--------------------------------------
---LRTSN---GQV----------
--------------------A-MF---------------------------------------NGMPAEQRFEVDG------VQHTVIQGII----
--ERFI-VQ---I-HG------------------------QDDG-----------------R-SVLTDIEIEIEFRACN----------------
---VPTLTLVDLPGIVS-----------------------M------------------------
---------PA--D--VRQQTL--------ELTR-RYL---RD------------PNTLVLCVINGNEA-A-LRGSR-
ALEEISEHAV---VRPAFSKTIVVTTRTDLWV-----HSNGL------------ATL-----------------------------ARR-
-LADPKKE-----------VG-----------------------CE--PVALIPVINRF--
----G-------DG--------------------IT--------------------LP----------DLVNA---ERAKFEE----WQATEP---
---------------------G-A-----GL-----------RGKPLGILSVLNALNDLFET-----------
XP_042918632.1 ------------LAIPE--------IVAI---
GG----Q-----------------SDGKSSLLE-----A--FL-G-F-RFNVRE--------------VE-MGTRRPL-------------
--IVQ--------------M-V-HDP--
--TAQEPR--CRL----------Q-----
-----------EE---DSDEYGPPIV-------PETAVADAI------QRRT-
EE---H-LR--------------------------KM------------------GGIAVSSK-PIVMRAEYAY-------------------------
------------------------------------CPNLTIIDTPGFIL--KA-------------------K--------TG-----------------E---
--LD--N--TPDEIM--------SMVK-AQA---SP------------PHRMILFLQQSSV--E-WASSL-
WLRVVQEVD------PYFQRTVIVASKFDNRL------KEFA------ER----WEV-----------------------------
DKY--LS---ATGY------LPPNV---------------------------RPFFVALPK---------------------------------------
----DRVIQS-SAE--WRR-----------S-------------------------------MT----------EVDTA---IYKHMRDGI---
---KG-GFD---------------------E-E-----RF-----------ASR-IGFSNLKKFLEEELSRRYREA-AP---
PWZ11893.1 ------------LPIPE--------IVAI---GG----Q-
---------------SDGKSSLLE-----A--LL-G-F-RFNVRE--------------VE-MGTRRPL---------------VLQ----
----------M-V-HDP---
-------------------------------TALEPR--CRF----------Q-----------------
EE---DSEEYGSPMV-------LATAIADLI------KQRT-ES---H-LR-
------------------------KI-------------------QA-AVSSK-PIVMRAEYAH---
--------------------CPNLTIIDTPGFVL--KA------------------K--------RG-----------------E-----PG--S--
TPDEIR---LQQSSV--E-WCSSI-WLDTLKEID------
PTFRRTIIVISKFDNRL------KEFT------ER----WEV----------------------------DTF--LS---ASGY------
LGDNI-------------------------------HPFFVALPK---DHGTIS-NEE--
FRR-----------Q-------------------------------IC----------QVDID---VLRHLRENV------KG-GFN----------
----------E-E-----KY-----------VSC-IGFSCLKKYLESELQKRYKEA-AP---

NP_001130364.1 ------------LPIPE--------IVVI--
GG----Q----------------SDGKSSLLE-----A--LL-G-F-RFNVRE-------------VE-MGTRRPL-------------
--VLQ-------------M-V-HDP---
---------------------------------------TALEPR---------------------------------------CRF---------Q-----
-----------EE--DSEEYGSPMV-------VATAIADLI------KQRT-
ES---H-LR-----------------------KI-----------------QA-AVSSK-PIVMRAEYAH------------------------
-----------------------------CPNLTIIDTPGFVL--KA-----------------K--------RG---------------E---
--PE--S--TPDEIR--------SMVK-SLA---TP-----------PHRLVLFLQQSSV--E-WCSSI-WLDTLKEID----
--PTFRRTMIVISKFDNRL------KEFT------ER----WEV-------------------------DAF--LS---ASGY-----
-LGDNI-------------------------HPFFVALPK--------------------------------------DRGTIS-NEE--
FRR-----------Q---------------------------IC---------HVDID---VLRHLRDNV------KG-GFN---------
-----------E-E-----KY----------GSH-IGFSCLRKYLESELQKRYKEA-AP---
AAF87857.1 ------------LPIPE--------IVAI--GG----Q-
---------------SDGKSSLLE-----A--LL-G-F-RFNVRE-------------VE-MGTRRPL---------------ILQ-----
---------M-V-HDL--
---------------------------SALEPR---------------------------------------CRF----------
QISRIFFVELAILITDLDE---DSEEYGSPIV-------SATAVADVI---
---RSRT-EA---L-LK-----------------------KT-----------------KT-AVSPK-PIVMRAEYAH-----------------
--CPNLTIIDTPGFVL--KA-----------------K--------KG------------
----E-----PE--T--TPDEIL--------SMVK-SLA---SP-----------PHRILLFLQQSSV--E-WCSSL-
WLDAVREID------SSFRRTIVVVSKFDNRL------KEFS------DR----GEV-------------------------
DRY--LS---ASGY------LGENT-------------------------RPYFVALPK------------------------------------
----DRSTIS-NDE--FRR-----------Q---------------------------IS---------QVDTE---VIRHLREGV-----
-KG-GFD-------------------E-E-----KF-----------RSC-IGFGSLRDFLESELQKRYKEA-AP---
XP_002317496.2 ------------LPIPE--------IVAL--
GG----Q----------------SDGKSSLLE-----A--LL-G-F-RFNVRE-------------VE-MGTRRPL-------------
--ILQ-------------M-V-HDP---
---------------------------------------SALEPR---------------------------------------CRF----------Q-----
-----------EE--DSEEYGSSVV-------SSTTIADII------KSRT-EV-
--L-LK-----------------------RT-----------------KT-AVSSK-PIVMRAEYAH------------------------------
-------------------------CPNLTIIDTPGFVL--KA-----------------R--------KG----------------E-----
PE--N--TPDEIL--------SMVK-SLA---SP-----------PHRILLFLQQSSV--E-WCSSL-WLDAIKDID-----
-PNFRRTVIVVSKFDNRL------KEFS------DR----WEV-------------------------DRY--LS---ASGY-----
-LGENT-------------------------RPFFVALPK--------------------------------------DKNTIT-NDE--
FRR-----------Q---------------------------IS---------QVDSE---ILHHLRDGV------KG-GFD----------
----------E-E-----KF-----------RPY-IGFSTLRDYLESELQKRYKEA-AP---
KAI5058044.1------------LPIPE--------IVAL--GG----
Q----------------SDGKSSLLE-----A--LL-G-F-RFNIRE-------------VE-MGTRRPL---------------MIQ--
------------M-V-HDA--
-------------------------------SALEPL--CRL---------Q--------------
--DE--DSDDYGPVIA-------PASAVAEAI------KLRT-EE---H-
LK-----------------------KE-----------------RT-AVSSK-PIVMRVEYAY-----------------------------------
----------------------CPNLTIIDTPGFVL--KA-----------------K--------KG---------------E-----PE--N--
TPEDIM--------SMVR-TLA---AP-----------QHRLLLFLQQSSV--E-WCSSL-WLDSIRTVD------

PSLRRTIIVISKFDNRL------KEFG------ER----WEV--------------------------DRY--LS---AGGY------
LGDTA--------------------------HPFFVALPK--DRTMTS-NEE--
FRR-----------Q-----------------------------IG---------AVDSD---VHRYLRNNI------KG-GFD---------
-----------E-E-----KF-----------GDF-IGFLNLKQYLELELQRRYRDA-AP---
EFJ18064.1 ------------LSIPE--------IVAV--GG----
Q----------------SDGKSSLLE-----A--LL-G-F-RFNVRE-------------VE-MGTRRPL---------------VLQ-
-------------M-I-HEP---
------------------------------AAVDPR---------------------------------------CRL---------Q-------------
---HE---DDEEYGPVIV-------PHYAVAEAI------KLRT-EE---H-
LK-----------------------KI------------------RA-AVSSK-PIVMRVEYAY-------------------------------------
-----------------------CPNLTIIDTPGFIL--KA------------------K--------KG----------------E-----PE--S--
TPDDIL--------QMVR-ALA---LP------------PNRLLLFLQQSSV--E-WCSSL-WLDTVRSID------
PGFHRTVVVVSKFDNRL------GEFA------EK----WEV--------------------------DRY--LS---AGGY----
--LGDHV--------------------------RPFFVALPK---DRGSVT-NEE--
FRS-----------Q-----------------------------IA---------SVDAE---VLKHLRERI------SG-GFS-----------
---------E-D-----KY-----------SGS-IGFGNLRNYLEAELQRRYREA-AP---
PTQ33908.1 ------------LPIPE--------IVAV--GG----
Q----------------SDGKSSLLE-----A--LL-G-F-RFNVRE-------------VE-MGTRRPL--------------
MLQ-------------M-I-HDP--
--------------------------------------GALEPR---------------------------------------CRL---------Q-----
-----------DE---DSDEYGPAIV-------PASAVAEAI------RSRT-
EL---F-LK-----------------------RT------------------GT-AVASK-PIVMRAEYAF---------------------------
-------------------------------CSNLTIIDTPGFIL--KA------------------K--------KG----------------E-----
SE--S--TPDDIV--------AMVR-ELA---AP------------PNRILVFLQQSSV--E-WCSSL-WLDTVRAID----
--PALRRTIVVVSKFDNRL------KEFA------EK----WEV--------------------------DRY--LS---AGGY----
--LGDST--------------------------RPFFVALPK---ERSSVS-NEE--
FRR-----------S-----------------------------IA---------RVDNE---VVRHLRENV------SG-GFD---------
-----------E-D-----QF-----------GDR-IGFSNLRRFLEAELQRRYRQS-AP---
KAG0628798.1 ------------LPIPE--------IVAV--
GG----Q----------------SDGKSSLLE-----A--LL-G-F-RFNVKE-------------VE-MGTRRPL-------------
--MLQ-------------M-I-HDP--
---EALEPR---------------------------------------CRL----------Q----
------------DE---DADDYGPVIT-------PVSSVADHI------RIRT-
EG---F-LK-----------------------KL------------------GT-AVSAK-PIVMRAEYAY--------------------------
--------------------------------CPNLTIIDTPGFIL--KA------------------K--------KG----------------E-----
PD--S--TPDEIE--------AMVR-ELA---AP-----------QHRLLLFLQQSSV--E-WCSSL-WLDVVKSID---
---PSLQRTMVVVSKFDNRL------KEFT------ER----WEV--------------------------DRY--LS---TGGY--
----LGENA--------------------------RPFFVALPK---DRGTTT-NDD-
-YRH-----------Q-----------------------------IS---------VVDID---ILKQLRENV------AG-GFD---------
----------E-E-----RF-----------GNY-VGFGKLRQFLEAELQRRYRDA-AP---
KAH9322298.1 ---

--DE--------------------------

------------------DSDDYGAPVA-------LASAVADAI------KSRT-EE---H-LK-----------------------KI-----
-------------RA-AVSAK-PIIMRAEYAH---
CPNLTIIDTPGFVL--KA-----------------K--------KG-----------------E-----PD--S--TPEDIL--------
SMVK-ALA---AP------------PNRLLLFLQQSSV--E-WCSSL-WLDTIRAVD------
PTLRRTIVVVSKFDNRL------KEFG------ER----WEV---------------------------DRY--LS---ASGY------
LGENT--------------------------RPFFVALPK---DRGAIT-NEE--
FRT-----------Q------------------------------IA----------QVDAE---VNCYLKESV------IG-GYD-----------
---------E-E-----KF-----------GAY-VGFGNLRGYLENELQRRYRDA-AP---

XP_002683545.1 ------------FEAPE--------LVVV---
GM----Q----------------SDGKSSFVE-----A--LL-G-F-QFNTVD--------------TQ-IGTRRPL-------------
--ILQ-------------M-V-NDP--
--SSEKPL--CHF----------F-----
-----------KE--ASPSDIEEEAT-------PVPLLEKEI------RRRT-
DE---V-CG------------------------K-------------------G-GVSSR-PIVLRVKYKY--------------------------
--------------------------------CANLTIYDTPGF------------------------R--------KG-----------------D-----
TD--P--LGERIH--------KTVM-GLI---KP------------QNRIIVALEQSTV--E-WCNTQ-VRPIIKKAD------
PNFERTIFVITKFNNRN------NQFR------DG----KEA---------------------------NDY--IS---TDGN------
IQDLS--------------------------KVFYISLPSGH--------------------------------------GTRNI-A-EEE--
FKN-----------E------------------------------IV----------NTYLK---DFKKLTKV----------GFD-------------
------E-Q-----KY-----------KSQ-LGFYNLKRYLEKLLNEKYVENISPV--

XP_044544418.1 ---------KMKFEAPE--------LVVV--
--GM----Q----------------SDGKSSFVE-----A--LL-G-F-QFNTVD--------------TQ-IGTRRPL-----------
----ILQ-------------M-T-NDP---
---TAEKPI--CNF----------F-----
-----------KE--ATPSEIEEEPT-------PVPELEKEI------RKRT-
ED---L-CG------------------------K-------------------S-NVNSR-PIVLRVRYKY--------------------------
--------------------------------CANLTIFDTPGF------------------------R--------KG-----------------E-----
QD--P--LAERIH--------KIVM-NTI---KP------------QNRIIIALEQSTV--E-WCNTQ-VRPLIKQVD------
PNYERTIFVVTKFNNRN------NQFR------DA----KEA---------------------------NDY--LA---TDGH----
--IPDLS--------------------------KVFYISLPSGH--------------------------------------GTRNL-A-EEE--
FKN-----------E------------------------------II----------NTYLK---DYKKLAKV----------GFD---------------
-----E-Q-----KF-----------KPQ-IGFFNLKRHLERMLNE-----------

XP_044559198.1 ---------KMKFEAPE--------LVVV--
--GM----Q----------------SDGKSSFVE-----A--LL-G-F-QFNTVD--------------TQ-IGTRRPL-----------
----ILQ-------------M-T-NDP---
---SAEKPI--CNF----------F-----
-----------KE--ATPSEIEEEPT-------PVPDLEKEI------RKRT-
ED---L-CG------------------------K-------------------S-NVNSR-PIVLRVRYKY--------------------------
--------------------------------CANLTIFDTPGF------------------------R--------KG-----------------E-----
QD--P--LAERIH--------KIVM-NTI---KP------------QNRIIIALEQSTV--E-WCNTQ-VRPLIKQVD------
PNYERTIFVVTKFNNRN------NQFR------DA----KEA---------------------------NDY--LA---TDGH----
--IPDLS--------------------------KVFYISLPSGH--------------------------------------GTRNL-A-EEE--
FKN-----------E------------------------------II----------NTYLK---DYKKLAKV----------GFD---------------
-----E-Q-----KY-----------KPQ-IGFFNLKRHLERMLNE-----------

XP_004182822.1 ------------IETPE--------IVVV--
GM----Q----------------SDGKSSFIE-----A--LV-G-F-QFNVVE--------------ST-IGTRRPL---------------
ILQ--------------M-F-NNP--
--------------------------------------DKRTPR---CCF----------A------
----------DE--NGVFEEREI-------PVEYLSREI------ANRT-
CD---V-AG------------------------R--------------------N-NVSNK-PLILRVEFAG--------------------------
-----------------------------------CSNLNIIDTPGF------------------------R--------IG-----------------G-----
EE--G--LRDDIS--------KMVK-ELI---TP------------PNRIIVCLEQSTT--E-WANSV-SRPIVREVD------
PEFKRTVLINTKFDNRV------KELT------DK----NSV---------------------------DVY--LS---GDKL------
IIGDK---------------------------KPFFISLPC---KRNI-S-MEQ--YPD-
----------Y------------------------------IT----------DSYIS---DYRQLLEI----------GFE--------------------
E-Q-----KF-----------VEQ-IGFPRAKMFLENLLQNKYKESLAPT--
XP_008857507.1 ------------IETPE--------IVVV--
GM----Q----------------SDGKSSFIE-----A--LV-G-F-QFNVVE--------------ST-IGTRRPL---------------
YLQ--------------M-F-NNP---
---------------------------------------KQRTPK---CCF----------A------
----------NE--NGVFEEREI-------AVEYLSKEI------STRT-CD-
--V-AG------------------------R--------------------T-SVSNK-PLILRIEFSG-----------------------------------
--------------------------CSNLTIIDTPGF------------------------R--------LG------------------G-----DE--T-
-LKEDID--------QMVK-ELI---TP------------SNRIIVCLEQSTT--E-WANSV-SRPLVKEVD------
PNFNRTILINTKFDNRV------KELT------DA----QTV---------------------------ANY--LN---GDEL------
IIGDK---------------------------KPFFISLPC---KRNI-P-MEQ--FPD-
----------Y------------------------------II----------DSYIS---DYRQLLEI----------GFD--------------------E-
T-----KY-----------HEQ-LGFPRAKIFLENLLQKKYKEALVPT--
XP_004340186.1 ------------FETPE--------LVVV--
GM----Q----------------SDGKSSFIE-----A--LL-G-F-QFNIVE--------------TN-IGTRRPL---------------
ILQ--------------M-I-NNP--
-------------------------------------ERDIPN--CRF----------R--------
--------RE-N-AYNE-------------------------------GEAGQQAVEADTWEPRDT-------PIEDLVHEI------
VRRT-NE---K-AG------------------------R-------------------GE-HVSAA-PIILRVEYAH-------------------
---CANLTIYDTPGF------------------------R--------LG-----------------
-G-----DE--K--LRADIQ--------RMVE-RLM---QP------------ANRIIVCLEQSTV--E-WANTS-
SRPIVRRFD------PTFSRTVLVNTKFDNRV------KELR------TP----ESA---------------------------AKY--
LM---GENL------PEG-K--------------------------KPFFISLPV--
RRNL-D-SER--FRD-----------G------------------------------IK----------ECYLD---DFRRLLEI----------
KFQ--------------------E-Q-----DF-----------AEQ-VGFHRVKAYLERMLTEKYYASVPPT--
KAH3742895.1 ------------FSTPE--------LVVV--
GM----Q----------------SDGKSSFIE-----A--LL-G-F-QFNIVD--------------SN-IGTRRPL---------------
ILQ--------------M-I-NNP--
-------------------------------------HREQPS--CRF----------R-------
---------KE---------------------------------------VLRSADEDPFEATET-------PVESLSQEI------IRRT-
NE---K-AG------------------------R-------------------GD-SVSPS-PIILRVEYCN--------------------------
-----------------------------------CSNLNIYDTPGF------------------------R--------LG-----------------G---
--DD--K--LRSEIR--------EMVQ-RII---EP------------KHRIIICLEQSTV--E-AVNTV-SRPLVQEVD------

PTFSRTVLINTKFDNRV------KEFS------SQ----ASA----------------------------DKY--LA---GEYL------
TVK-K---------------------------KPFFISLPV---RRNL-D-PRS--YRE-
----------A-------------------------------MR----------ECYLE---DYRTLLQV----------GFD------------------
--E-K-----RF-----------TPQ-IGICKAKNHLEHLLSDLYQQSLAPT--
KYQ94066.1 ------------FDTPE--------LVVV--GM----
Q-----------------SDGKSSFVE-----S--LL-G-F-QFNIVE--------------SN-IGTRRPL---------------IIQ----
----------M-I-NNP--
-------------------------------DKVEPS--CRF----------K----------------
KE-H-YFPL---VPSSSHSNSNNNNNSEYGSSQNSSIQ---NTLYSGTDQDKWEEYET-------
PVDELTEEI------VRRT-NE---R-AG------------------------RS------------------GD-RVSSV-
PIFLRVEFAN---CSNCSIFDLPGL------------------
------R--------KG-----------------G-----DD--R--LKHEIL--------DMVK-KLI---EP-----------
KNRIIICLEQSTV--E-HVNST-SRPFVKKID------PDFSRTILINTKFDNRV------KELR------TR----ESA--
---------------------------HKY--LE---GEGI------IQQ-K---------------------------KPFFISLPL--------------
-----------------------------KRNL-D-PHR--FKD-----------A------------------------------IK----------
ECFLD---DLRKLHEV----------NFD--------------------E-N-----RF-----------QGQ-
VGIYKVKNYIETLLQEKYQQNLLPS--
XP_004366192.1 ------------FETPE--------LVVV--
GM----Q----------------SDGKSSFIE-----S--LL-G-F-QFNIVE--------------SN-IGTRRPL---------------
IIQ--------------M-I-NNQ---
---------------------------------------SKQEPS---CRF----------K--------
--------KE-C-HLI----------------------------------DGTSIGSLEDKWEEHET-------PAEELTEEI------
VRRT-ND---L-TG------------------------AR------------------GD-RVSSF-PIFLRVEFAH------------------
---CANLNIYDTPGF------------------------R--------KG--------------
---G-----DE--R--LKYEIL--------DMVK-KLI---EP-----------KHRIIVCLEQSNV--E-WANTI-
SRPLIKKID------PDFTRTILINTKFDNRV------KELR------NR----ESA----------------------------HKY--
LE---GEGI------VTG-K---------------------------KIFFISLPL---
KRNL-D-PYK--FKE-----------A-------------------------------CK----------ECYLE---DYRKLLEI----------
GFD--------------------E-N-----RF-----------GAQ-IGIYKVKEYVERHLHERYQQNLVPS--
XP_645576.2 ------------FDTPE--------LVVV--GM----
Q-----------------SDGKSSFIE-----S--LL-G-F-QFNIVE--------------TN-IGTRRPL---------------IIQ-----
---------M-I-NNP--
-----------------------------SKQQPS--CRF----------K----------------
KE-D-YSNSY-GGSSSSTSTTSGNSNHNTDKQQNVSSSQGGGGGSNNLNEDKWEEYET-------
PVNELTEEI------IRRT-NE---R-TG------------------------RA------------------GD-RVSSI-
PIFLRVEFAH---CSNLNIYDTPGF------------------
------R--------KG-----------------G-----DE--R--LKYEIS--------EMVK-KLI---EP----------
KNRIIVCLEQSNV--E-WANTI-SRPLVKKID------PDFSRTILVNTKFDNRV------KELR------NR----
ESA----------------------------HKY--LE---GEGI------IAQ-K---------------------------KPFFISLPL--------
-----------------------------------KRNL-E-THR--FKD----------A------------------------------MK----------
ETFLD---DYRKLLEI----------GFD--------------------E-N-----RF-----------GGQ-
IGIYKVRQYVENLLHEKYQQNLLPS--
XP_003288319.1 ------------FDTPE--------LVVV--
GM----Q----------------SDGKSSFIE-----S--LL-G-F-QFNIVE--------------TN-IGTRRPL---------------

IIQ-------------M-I-NNP--
-----------------------------------LKQQPS---------------------------------------CRF----------K--------
--------KE-D-YSS----GESSRQPVSSSQGSTTNGKELSASS-------ANNSLDDKWEEYET-------
PVNELTEEI------VRRT-NE---R-TG-----------------------RS-----------------GD-RVSAV-
PIFLRVEYAH---CSNLNIYDTPGF-----------------
------R--------KG----------------G-----DE--R--LKHEIS--------EMVK-KLI---EP------------
KNRIIVCLEQSNV--E-WANTI-SRPLVKKID------PDFSRTILVNTKFDNRV------KELR------NR----
ESA---------------------------HKY--LE---GEGI------VSQ-K--------------------------KPFFISLPL-------
---------------------------------KRDL-E-NNK--FKE----------S----------------------------MK---------
-DCFLD---DYKKLLEV----------GFD------------------E-N-----RF-----------GGQ-
IGIYRVKSFIENLLHEKYQQNLLPS--
KAF2073270.1 ------------FDTPE--------LVVV--
GM----Q----------------SDGKSSFIE-----S--LL-G-F-QFNIVE-------------TN-IGTRRPL---------------
IIQ-------------M-I-NNP--
-----------------------------------QKLEPS---------------------------------------CRF----------K---------
-------KE----------IDSYDRPSSQQQ-------------------QTNNLDDRWEEHET-------PVNELTEEI------
VKRT-NE---K-TG-----------------------SS-----------------GE-RVSAE-PIFLRVEFAG------------------
---------------------------------------CSNLNIYDTPGF----------------------R--------KG---------------
--G-----DE--R--LKHEIG--------EMVK-KLI---QP------------KNRIIVCLEQSNV--E-WANTI-
SRPLVKKID------QDFSRTILISTKFDNRV------KELR------NR----ESA---------------------------HKY--
LE---GEGI------VAK-K--------------------------KPFFISLPL--
KRNL-E-PHK--FKE-----------A-----------------------------MK----------DCFLE---DYRKLLEV----------
NID--------------------E-A-----RF-----------SSQ-IGIFKVRSYIENLLHEKYQQNLLPS--
XP_020428321.1 ------------FETPE--------LVVV--
GM----Q----------------SDGKSSFIE-----S--LL-G-F-QFNIVE-------------TN-IGTRRPL---------------
IIQ-------------M-M-NNP--
-------------------------------------KKLHPS---------------------------------------CRF----------K-------
---------KE---AAN-----------------------------------GEYITEGDKWEDHET-------PVEELTEEI------
VKRT-ND---L-TG-----------------------SR-----------------GD-KVSAQ-PIFLRVEFAQ-----------------
---CSNLNIYDTPGF-----------------------R--------KG--------------
---G-----DE--K--LKLEIG--------DMVK-KLI---EP------------KHRIIVCLEQSNV--E-WANTI-
SRPLVKKID------TDFSRTILINTKFDNRV------KELR------TR----ESA---------------------------HKY--
LE---GEGI------IAN-K--------------------------KPFFISLPL--
KRNL-E-PHR--FKD-----------A-----------------------------MK----------ESYLE---DYRKLLEV----------
NFD--------------------E-N-----RF-----------GHQ-IGIYNVKEHIESLLHEKYQQNLLPS--
XP_012754836.1 ---------NISFETPE--------LVVV--
-GM----Q----------------SDGKSSFIE-----S--LL-G-F-QFNIVE-------------TN-IGTRRPL--------------
-IIQ-------------M-I-NNP---
-------------------------------------IKHQPS---------------------------------------CRF----------K--------
--------KE---FS------------------------------------GEASALENKWEEHET-------PVEELTEEI------IKRT-
NN---L-TG-----------------------TR-----------------GD-KVSAQ-PIFLRVEFAH------------------------
---------------------------------CANLNIYDTPGF-----------------------R--------KG----------------G--
---DE--K--LKTEIG--------DMVK-KLI---EP------------KHRIIVCLEQSNV--E-WANTI-SRPLVKKID----
--QEFGRTILVNTKFDNRV------KELR------SR----ESA---------------------------HKY--LE---GEGI-----

-VAS-K--------------------------KPFFISLPL--KRNL-E-PHR--
FKE-----------A-----------------------------MK----------DSYLE---DYRKLLEV----------NFD------------
-------E-N-----RF-----------GGQ-IGIYKVKDYIEGLLHE-----------
PRP82286.1 ---------DIPVEYPE--------IVVI--GM----
Q----------------SDGKSSFVE-----G--LL-G-F-QFNIVESSEPQLYSLSISSITD-IGTRRPL---------------
ILQ--------------M-I-NNP---
------------------------------------DRDQPR--------------------------------------CRF----------R-------
---------KE--NPSVHEEPFEDSDT-------PVHRLVDEI------CRRT-
NE---K-AG-----------------------SA------------------GD-RVSDV-PIILRVEFAN-------------------------
---------------------------------CANLTIYDTPGF-----------------------R--------LG-----------------G----
-DE--R--LKNEIK--------DMVH-KLI---LP------------KHRIIVCLEQSTV--E-WANSV-SRPMARQID----
--PDFSRTILVNTKFDNRV------KELR------NK----ESA---------------------------NKY--LG---GENL----
--PQG-V---------------------------QPFFISMPL--RRDL-E-ASR--
FRD-----------G-----------------------------IR----------DASLD---DYSKLMEV----------NFD-----------
--------E-G-----RF-----------LPH-LGLFRVKQHLEKALNE-----------
QYA18543.1 ------------FKAPE--------IVVV--GM----
Q----------------SDGKSTFIE-----G--LL-G-F-QFNIVE--------------TN-IGTRRPL---------------IIQ-----
---------M-V-NDP---
----------------------------ECDVPQ--CRF----------K----------------
RE------------------------------------DENGDPKSEDDDLFEHDTV-------PVQRLSAEL------MRRT-
DK---K-TG------------------------KN------------------KH-CVSDS-PIVLRVRFAK-------------------------
----------------------------------CANLTIYDTPGF-----------------------R--------LG-----------------G---
--LE--S--LRQDIE--------NMVL-KLI---QP------------EHRIIVCLEQSTI--E-WANTN-SRPFVQRVD---
---PNLERTVVVCTKFDNRV------KEMR------DA----DST---------------------------NAY--LD---
GEGM------PAG-V---------------------------RPFFVSMPV--
SRNLGN-PNK--FAL-----------V-----------------------------TQ----------DCYLN---DYRQLLEV----------
-KFD--------------------E-K-----RF-----------ADR-LGFHRVKKYIENLLKKKYMDSLKPT--
XP_005775651.1 ----------GGANLPT--------VVLV---
--GH----Q----------------NAGKSSLLE-----A--LL-G-I-KLTHVG--------------NE-GLTRRPL------------
---QIT--------------A-Q-RDD---
---SAAEPA---LFL----------T-----
--RDGG--GSGGG--EEQVR--------PEQLRSLI------
EREN-AR---L-EA-----------------------AG--------------------EYEAE-PLRVRLSWRR------------------
--APTLVLIDTPGLIG--PA------------------D---------G--------------
--LE-----LG--E--AAAAVE--------GLVL-QHL---TP------------PKRLILCLEDTS---D-WAISR-
TMAVVSRAD------TDLRRTLLVGTKLDAKM------AQFS------LP----EDL---------------------------
RGL--VD---PPAL------AAERP--------------------HLLG--GPIFTSVPPLR---SDSRH------------------
AGRKA----------AAGGGG-AGG--YSD-----------L------------------------------IE----------EHEAS---
LRRLLGSKLQ----------S--------------------S-E------F-----------DAR-IGVSALWRLMAPHLTA----------
--
XP_005767412.1 ---------QLPIKTPQ--------IVVV---
-GQ----Q----------------TDGKSALIE-----A--LM-G-F-QFNHVG--------------GG-TKTRRPI------------
---ALQ--------------M-Q-YRA---
---ECEEPR--CFL----------F---

--EGGR--ERQVG--------LQELKAYI------EAEN-
SR---L-EA------------------------EG--------------------AFEAE-ELIVRMDYKY--------------------------------
--------------------------------CPNLSIIDTPGLLD--FD------------------SAA------QS-TAPSA-----------
------A--T--NARAVR--------ELVV-AQM---SA------------AERVILCVEETR---C-WQSSA-
VRQLVGEVD------PTFGRTVVVSTKLDTKL------AQLS------GT----SDL---------------------------
LHF--LA---ARPL------RESHP---------------------RLLG--GPFFTSVPSGR---VGSSP-------------------
-------------LHGFAT-DGE--YQD-----------A----------------------------LA----------RTEHA---
NLEYLREVAA---PAAD-AAT--------------------Q-P-----GA-----------RSA-VGISRLRRFLEDLLRE-----
------

KOO34265.1 ---------KLPVDSPT--------VVVV--GR---
-Q----------------TDGKSALVE-----A--LM-G-F-QFNHVG--------------GG-TKTRRPI---------------
ALQ--------------M-Q-YHP---
---AREQPV---CYL----------H-----
---TADG--EKQLS--------LADLQAHI------EAEN-
VR---L-ER------------------------AG--------------------AFATD-EIVVRIEYRY--------------------------------
------------------------------CPNLSIVDTPGLLS--TA------------------DDY------GG-GFLQA-----------
------A--T--TCSDVE--------QLVM-SKI---AS------------PEAIILCVEETN---N-WDVAA-ARAVVARVD-
-----ADLLRTVVVSTKLDTKF------AQFG------SP----SEL---------------------------AQF--LD---AAPL-
-----HQRHA----------------------ELLG--GPFFTSVPAGR---VG--
--AGG-------------------
G-A-----GA-----------RD----------------------------

EWM28268.1 ------------FDATA--------ILIV--GQ----Q-
---------------TGGKSALIE-----A--LM-G-F-QFNQVG--------------GG-TKTRRPI---------------ALR-----
---------M-Q-YNP---
------------------------------RCSQPH--CFL----------T---------------
--LDNGK--EEARS--------LKEIQQYI------EGEN-KR---L-EN-
-----------------------DE------------------LR-CFDPR-EIMVRVEYRF---------------------------------------
---------------------CPNMIVIDTPGLLS--AP------------------A---------KLRHMNE--------QQRQ-----
LH--Q--AAKEAE--------HLVL-EKM---KC------------QDYLILCVEDTT---D-WKHST-TRNIVMQAD---
---PDLRRTVLVTTKFDTKL------PQFG------TS----EDL---------------------------EDF--LR---APLI-----
-QKLYP----------------------QMLG--GPFYTSVPCGR---VGMGK--------------------------------DSAFIS-
NEV--FVE-----------A-----------------------------VK----------KVEKY---DHARIEARL------GS-VGA----
-----------------A-P------L-----------LQR-VGVSRLRRFLEKRVEECYRRN-VAKI-

KAG5184668.1 ---------HKPFDSPA--------VLVV--
--GH----Q----------------TSGKSALIE-----A--LM-G-F-QFNQVG--------------GG-TKTRRPI------------
---ALR--------------M-Q-YNP---
--DCDQPR--CYL----------T---
---LENGR--EEPRS--------LAEIQAYI------EAEN-
KR---L-ER------------------------DP------------------IR-SFDAR-EIMIRMEYRF---------------------------
---------------------------------------CPNMILIDTPGLIQ--AP------------------R---------G-KHLNA--------
QQQA-----LL--Q--AAKECE--------QLVI-QKM---RC------------QDFIILCVEDTT---D-WKHAT-
TRNVVMQVD------PSLERTVLVCTKLDTKL------PQFS------SG----EDL---------------------------
EDF--ID---APVM------RRMYS--------------------GLLG--GPFFTSVPSGR---VGRGR-----------------
---------------ESAYFS-NEA--FVA-----------G-----------------------------VR----------RAERD---

DSMHVASKL------GP-TAG--------------------T-Q------C-----------LPH-VGVSRLRRFLERRVEDC---
----
CBN78455.1 ---------HKPFDSPA--------VLVV---GH--
--Q----------------TSGKSALIE-----A--LM-G-F-QFNQVG--------------GG-TKTRRPI---------------
ALR--------------M-Q-YNP--
--DCDQPR---CYL----------A------
---LEDGK--EEPRS--------LQEIQAYI------ESEN-RR-
--L-ER------------------------DP------------------VR-SFDSR-EINIRMEYRF-------------------------------
------------------------------CPNMILIDTPGLIH--AP------------------S---------G-SNLNA--------EQRA-
----VA--A--AAKEAE--------NLVI-QKM---RC------------QDYVILCVEDTT---D-WKHAT-TRNIVTQVD-
-----HDLSRTVLVTTKLDTKL------PQFG------GG----DDL-----------------------------QDF--LR---APSI-
-----QRMYR---------------------CMLG--GPFFTTVPSGR---VGRAR--------------------------------
DSAYFS-NEA--FVH-----------G------------------------------VR----------RSERD---DQMLVAAKL------
GP-QAA---------------------P-S------C-----------LPR-VGVSRLRRFLERRVEE-----------
GMI61978.1 ---------KKPFDSPA--------VLVV---GH---
-Q----------------SSGKSALIE-----A--II-G-F-QFNQVG--------------GG-TKTRRPV---------------ALK--
------------M-Q-YNP---
-----------------------------------KFDTPV---CYL----------Q------------
---GDDGK--ERPAS--------LQEIQRYI------ESEN-NR---L-
EM------------------------DK------------------VR-CFDSR-EINIRMEYKE-----------------------------------
----------------------------CPNMILIDTPGLIS--AP------------------KTP------KG-SLANA--------QSRS---
--LQ--V--AAREAE--------KLVI-DKM---NC------------QDYIILCVEDTS---D-WKHGA-TREIVQKAD---
---PELSRTVIVNTKLDTKV------PQFG------DP----ADM-----------------------------EEF--LK---APII-----
-SRLAP---------------------HKLG--GPFFTSVPSGR---V-----------------------GQSE---------SHAFRS-
DKE--FVK-----------E------------------------------VK----------SKENS---DRAYCQKTL----KADP-RLE--
-------------------R-A------L-----------LPR-IGVLRLKGFLERRVHE-----------
GMH67967.1 ---------QKPFDAPA--------VLVV---GH--
--Q----------------SSGKSALIE-----A--LM-G-F-QFNQVG--------------GG-TKTRRPV---------------
ALR--------------M-Q-YNP--
--KCKEPK---CFL----------Q-------
---GDDGV--ERPTS--------LTEIQEYI------QAEN-LR--
-L-EK------------------------DP------------------VR-CFDSR-EINIRMEYKY-------------------------------
-----------------------------CPNLILIDTPGLIA--AP------------------RTP------KG-STSNM--------QTKA--
---LL--A--SAKEAE--------KLVI-NKM---RC------------QDYIILCVEDTA---D-WKHGQ-TREIVQKAD--
----PDLSRTVIVNTKLDTKV------PQFG------DP----ADV-----------------------------EEF--LK---APIV----
--SKLAP---------------------HKLG--GPFFTSVPSGR---V-----------------------GNGD---------
SFLFRD-DDE--FVG-----------A------------------------------CA----------DNEET---DREVVRSRL----
VSGP-AIT---------------------K-A------L-----------LPR-VGISRLRGFLERRVDE-----------
GMH92561.1 ---------QKPFDAPA--------VLVV---GH--
--Q----------------SSGKSALIE-----A--LM-G-F-QFNQVG--------------GG-TKTRRPV---------------
ALR--------------M-Q-YNP--
--KCESPQ---CFL----------Q------
---GDDGV--ERAKT--------LGEIQEYI------ESEN-SR-
--L-EN------------------------DP------------------VR-CFDSR-EINIRMEYKF-------------------------------

------------------------------CPNLILIDTPGLIA--AP------------------KTP------KG-SSANM--------
QSRA-----LQ--A--SAKEAE--------KLVI-SKM---RC------------KDYIILCVEDTA---D-WKHGQ-
TREIVQKAD------PDLSRTVIVNTKLDTKI------PQFG------DP----EDV----------------------------EEF--
LK---APIV------SKLAP---------------------HKLG--GPFFTSVPSGR---V-----------------------GSGD----
-----SYLFRT-DDE--FVS-----------S------------------------------CA----------DNEEK---DREVVKSRL---
-KAGP-LVT--------------------K-A------L-----------LPR-VGISRLRGFLERRVDE-----------
GMI07688.1 ---------QKPFDAPA--------VLVV--GH--
--Q----------------SSGKSALIE-----A--LM-G-F-QFNQVG--------------GG-TKTRRPV---------------
ALR--------------M-Q-YNP---
---------------------------------------KCDSPS---CFL----------Q------
---GDDGV--ECPKS--------LGEIQEYI------ESEN-
SR---L-EH------------------------DP------------------VR-CFDSR-EINIRMEYKF--------------------------
----------------------------------CPNLILIDTPGLIA--AP------------------KTP------KG-SQANM--------
QSRA-----LQ--A--SAKEAE--------KLVI-AKM---RC------------KDYIILCVEDTA---D-WKHGQ-
TREIVQKAD------PDLSRTVIVNTKLDTKI------PQFG------DP----EDV----------------------------EEF--
LK---APLV------SKLAP---------------------HKLG--GPFFTSVPSGR---V-----------------------GSGD---
-------SYLFRN-DEE--FVS-----------S------------------------------CA----------NNEDA---
DREVVKKRL----TAGP-HVT--------------------K-A------L-----------LPR-VGISRLRGFLERRVDE-------
----
EJK67908.1 ------------FDAPA--------VVVV--GH----
Q----------------SSGKSALIE-----A--LM-G-F-QFNQVG--------------GG-TKTRRPV---------------
ALR--------------M-Q-YNP---
---------------------------------------RCSSPR---CFL----------Q------
---GDDGV--ERPMS--------LVEIQEHI------EAEN-
RR---L-EK------------------------DP------------------VR-SFDSR-EINVRMEYRH-------------------------
-----------------------------------CPNMILIDTPGLIS--AP------------------RLR------RE-SGGNA--------
QQRA-----LL--H--AAKEAE--------RLVV-GKM---RC------------PDYIILCVEDTM---D-WKHGT-
TREVVQKAD------PDLSRTVIVNTKLDTKL------PQFG------TP----KDV----------------------------
ADF--VS---ATIV------DRMSP---------------------HKLG--GPFYTSVPSGR---VRHHSAP------PQG---
----RNSDD---------DYLFDD-DEE--FVA-----------A------------------------------CA----------EKEDA---
DRELVFGRIR---RASE-ADL--------------------K-R-----T-----------MPR-
VGISRLRGFLERRVDECYRRN-VAKI-
KAI2494507.1---------NKGFDAPA--------VVVV--GH--
--Q----------------SSGKSALIE-----A--LM-G-F-QFNQVG--------------GG-TKTRRPI---------------
ALR--------------M-Q-YNP---
---------------------------------------KYSTPR---CYL----------V--------
---GEDGI--ERELS--------LHEIQDYI------EREN-QR---
L-EK------------------------DP------------------LR-SFESR-EINIRMEYRH---------------------------------
---------------------------CPNLILIDTPGLIL--AP------------------RVP------KG-RAGSS-----
AVSQQRA-----LQ--A--SAKEAE--------RLVI-DKL---SC------------PDYIILCVEDTA---D-WKHGS-
TREVVQHAD------PDLSRTVIVNTKFDTKV------PQFG------TP----SDL----------------------------
EDF--IS---AGIL------DRLSP---------------------TKLG--GPYFTSVPSGR---VGHSS-------------------
SNPD----------AMYDS-DLD--FVN-----------A------------------------------CS----------EAEMT---

DRAVVNQKMRRMGKQGE-IAY--------------------A-T------L-----------QKR-VGLSKLRAFLEQKVDE----------
CAB9516894.1 ------------FDAPA--------VVVV--GH----Q----------------SSGKSALIE-----A--LM-G-F-QFNQVG--------------GG-TKTRRPV---------------ALR--------------M-Q-YNP--KCSTPK---------------------------------------WYL----------V--GDDGV--ERPMS--------LKDIQDLI------EREN-KR---L-ER------------------------DP------------------MR-SFDPR-EINIRMEYKH---CPNMILIDTPGLIS--AP------------------RTP------KG-RTSSST----AMAQQRA-----LQ--A--SAKEAE--------RLVI-EKM---KC------------EDYIILCVEDTS---D-WKHGA-TREVVQKAD------PDLSRTVIVNTKFDTKL------PQFG------TP----SDV---------------------------EEF--LR---AQIL------DRISP---------------------HRLG--GPFYTSVPSGR---VGRVDGG------SL--------LDQSN----------YMYNS-DSA--FVA-----------T------------------------------CR----------EAENT---DRRSVVQRLSRMKYSNE-VTN--------------------A-A------I-----------TSK-IGLAKLRTFLEQRVDECYRRN-VNKI-
GKY99394.1 ---------DKPFDAPA--------VVVV--GH----Q----------------SSGKSALIE-----A--LM-G-F-QFNQVG--------------GG-TKTRRPI---------------ALR--------------M-Q-YNP--SCRQPR--WFL----------L---GEDGV--ERPMQ--------LSEIQDYI------EKEN-RR---L-ER------------------------DP------------------MR-SFDPK-EINIRMEYKH---CPNMILIDTPGLIA--AP------------------RIA------KG-RSSAGA----AIAQQRA-----LQ--L--AAKEAE--------RMVV-EKM---RC------------EDYIILCVEDNN---D-WKHGA-TREIVAKAD------PDLSRTVIVNTKFDTKI------PQFS------SP----SDV---------------------------QDF--LK---ASIL------DRICP---------------------QKLG--GPFFTSVPSGR---VGRTG------------------DQDDE---DDDGYTKVYNN-DDD--FVA-----------G------------------------------CA----------DSENA---DRAVVMQRLKKIGLVGD-AAT--------------------G-S-----VV-----------ASR-IGLTKLRTFLEQRVDE----------
XP_042920073.1 ------------FDAPA--------ILVV--GH----Q----------------TDGKSALVE-----G--LM-G-F-QFNSVG--------------GG-TKTRRPI---------------AIN--------------M-K-YNG---ACSTPA--CFL----------K--LEDGVS-EQEMS--------LAELQAYI------DADN-AA---L-ER------------------------E-------------------Q-RFAAK-EIVVRMEYKH---CPNLTIIDTPGLIS--PA-----------------P---------G-------------KKNCA-----LQ--N--CAAQVE--------EIVR-AKA---QV------------PEYVILCLEDCS---D-WSNAT-TRRLVMQVD------PNLVRTVLVSTKFDTRI------PQFA------RA----ADC---------------------------EMF--LR---PSA--------LDSM---------------------GMLG-DGPFFTSVPSGR---VGSGA-------------------------------DCVFPS-HDV--FRE-----------R------------------------------LA----------DREAT---DVAELESKL------AR-KLS---------------------R-G------E-----------RDH-IGVGALRRYLEQLLQKRYLDA-VP---
PWZ44616.1 ------------AAAPA--------VVVV--GH----Q----------------TDGKSALVE-----A--LM-G-F-QFNHVG--------------GG-TKTRRPV---------------ALH--------------L-R-FNP---

--RCDEPQ--CRL----------L------
---SATGDAEEHDEAGVAARPMPLADIQAYI------
EAEN-LR---L-EN------------------------DP------------------CQ--FSEK-EIIIKVEYKH--------------------
--SPNLTIIDTPGLIL--PA------------------P---------G-------------
RKNRV-----LQ--S--QASAVE--------SLVR-AKI---QH------------KETIILCLEDCS---D-WSNAT-
TRRVVMQVD------PDLARTVLVSTKLDTKI------PQFA------RP----SDV----------------------------
EVF--LH---PPNC------VLDV----------------------SLLG-DSPFFTSVPSGR---VGSCH------------------
--------------EAVFRS-NGE--FKK-----------A------------------------------IL----------SRELE---
DIASIEDKL------GR-SLT--------------------T-M------E-----------KDR-IGVGNLRLYLEELLQKRYVKS-
VP---

NP_001189935.1 ------------FEAPA--------VLVV--
GQ----Q----------------TDGKSALVE-----A--LM-G-F-QFNHVG--------------GG-TKTRRPI-------------
---TLH--------------M-K-YDP---
---QCQFPL--CHL----------G--
--SDDDPSVSLPKS--------LSQIQAYI------
EAEN-MR---L-EQ------------------------EP------------------CS-PFSAK-EIIVKVQYKY------------------
--CPNLTIIDTPGLIA--PA------------------P---------G-------------
LKNRA-----LQ--V--QARAVE--------ALVR-AKM---QH------------KEFIILCLEDSS---D-WSIAT-
TRRIVMQVD------PELSRTIVVSTKLDTKI------PQFS------CS----SDV----------------------------EVF--
LS---PPAS------ALDS----------------------SLLG-DSPFFTSVPSGR---VGYGQ--------------------------
------DSVYKS-NDE--FKQ-----------A------------------------------VS----------LREME---DIASLEKKL--
----GR-LLT--------------------K-Q------E-----------KSR-IGISKLRLFLEELLWKRYKES-VP---

XP_002309632.3 ------------FDAPA--------VLVV---
GH----Q----------------TDGKSALVE-----G--LM-G-F-QFNHVG--------------GG-TKTRRPI-------------
---TLH--------------M-K-YDP---
---ECEVPT--CHL----------V---
--SDDDPSFAQEKS--------LHEIQAYI------EYEN-
MR---L-EK------------------------ES------------------FQ--FSAK-EIIIRVEYKH----------------------------
--------------------------------CPNLTIIDTPGLIA--PA------------------P---------G-------------RKNQA---
--LQ--S--QAHAVE--------SLVR-AKM---QH------------KEFIILCLEDCS---D-WSNAT-TRRVVMQID-
-----PELSRTIVVSTKLDTRI------PQFA------RA----SDV----------------------------EVF--LS---PPAH----
--TLDG----------------------FILG-DSPFFTSVPSGR---VGSGH--------------------------------DSVYSS-
NDE--FKQ-----------A------------------------------IS----------LREVE---DIASLEEKL------CR-PLS-----
---------------M-Q------E-----------RNR-IGVSKLRSFLEELLQKRYMDS-VP---

EFJ19523.1 ------------FEAPA--------ILLV---GH----
Q----------------TDGKSALIE-----A--LM-G-F-QFNHVG--------------GG-TKTRRPI----------------TLH--
------------M-K-YNA--
--------------------------------ACSEPL--CYL----------M--------------
--TEDGLPREEERS--------LDDIQAHI------ESEN-LR---L-
ER------------------------DT------------------HQ--FWAK-EIIIKIEYKY--------------------------------------
----------------------CPNLTIIDTPGLIA--PP------------------P--------QS------------NATTA-----LQ--A-
-QAKAVE--------ALVR-SKM---QH------------KEFIILCLEDCS---D-WSNAT-TRRVVMQVD------
PELSRTIVVSTKLDTRI------PQFA------RA----ADV----------------------------ELF--LR---PPPR------
LLDG----------------------DILG-GTPFFTSVPSGR---VGSGR--------------------------------DAVYKS-

NEQ--FRE-----------A----------------------------SP---------ARELE---DVSSLEEKL------GR-PLL----
----------------R-E------E-----------RNH-VGVSRLRWFLEQILQRKYMES-VP---
KAH9308354.1 --

-------------------------------A--QARAVE--------MLVR-TKM---QH-----------KEYIILCLEDCS---D-
WSNAT-TRRVVMQVD------PELSRTVMVATKLDTKI------PQFG------RS----SDV---------------------
------ELF--LR---PPTR------LLDG---------------------SILG-ETPFFTSVPSGR---VGTNR--------------
-----------------DAVYRS-NDQ--FKE-----------A----------------------------VA---------SREAQ---
DVAALEDKL------GR-SLL--------------------K-E------E-----------RAR-
VGVSRLGCFLEELLQRRYIDS-VP---
KAI5064281.1------------FDAPA--------ILVV---GH----
Q----------------TDGKSALVE-----A--LM-G-F-QFNHVG-------------GG-TKTRRPI---------------TLH-
-------------M-K-YNA--
----------------------------------GCSEPV--------------------------------------CYL----------M------------
--TDDKPPIEEERS--------LEELQAFI------EAEN-MR---L-
EQ-------------------------EA------------------CQ--FWAK-EIVVKIEYKY-----------------------------------
-------------------------CPNLTIIDTPGLIA--AA-----------------P---------G-------------RKNHL-----LQ--
A--QARAVE--------ALVR-TKM---QQ-----------KEFIILCLEDCS---D-WSNAT-TRRVVMQMD------
PELSRTVVVSTKLDTKI------PQFA------RA----ADV---------------------------ELF--LR---PPPR------
LLDG---------------------NILG-ETPFFTSVPSGR---VGTSR-------------------------------DSVFRS-
NEQ--FRE-----------A----------------------------VA---------AREAQ---DLSALEEKM------DR-HLL--
------------------P-D------E-----------RAR-VGVSRLRWFLEQLLQQRYMES-VP---
PTQ34556.1 ------------FDAPA--------ILVV---GH----
Q----------------TDGKSALVE-----A--LM-G-F-QFNHVG-------------GG-TKTRRPI---------------TLH-
-------------M-T-YNA--
----------------------------------ECTEPR--------------------------------------CFL----------L-------------
--SEDAPPKEEEKS--------LDDLQAYI------ESEN-MR---L-
EL-------------------------EP------------------SQ--FWAK-EIVVKIEYKF-----------------------------------
------------------------CPNLTIIDTPGLIS--AA-----------------P---------G-------------RKNIS-----LQ--S-
-QARAVE--------ALVR-SKM---QQ-----------KEFIILCLEDCS---D-WTNAT-TRRFVMQMD------
PELTRTVIVSTKLDTRI------PQFA------RP----ADV---------------------------ELF--LR---PPSR------
LLDG---------------------NILG-DTPFFTSVPSGR---VGSGR-------------------------------DSVYRT-
NES--FRE-----------A----------------------------VA---------MREAL---DVAMLEEKM------DR-PLL--
------------------N-E------E-----------RNR-VGISRLRCFLEQLLQRRYMDS-VP---
KAG0561482.1 ------------FDAPA--------IVVV---
GH----Q----------------TDGKSALVE-----A--LM-G-F-QFNHVG-------------GG-TKTRRPI-------------
---TLH-------------M-K-YNA---
---MCAEPR--------------------------------------CYL----------I----
---TEDRPPREEEKS--------LEELQAYI------EAEN-
LR---L-ER-----------------------EV-----------------CQ--FWEK-EIILKIEYKF-----------------------------

------------------------------CPNLTIIDTPGLIS--AA-----------------P---------G------------RKNQS----
-LQ--S--QARSVE--------VLVR-TKM---QH-----------KEFIILCLEDSS---D-WSNAT-TRSIVMQID-----
-PDLSRTVVVSTKLDTRI------PQFA------CR----ADV--------------------------ELF--LR---PSQR------
LLEG--------------------NILS-GSPFFTSVPSGR---VGVTR-----------------------------DSVHRS-
NDH--FRE-----------A----------------------------IA---------LREAQ---DIALLEEKL------DR-QLT-----
---------------K-E------E-----------LAH-VGVSRLRLFLEQLLQRQYMDS-VP---
KAG0605142.1 ------------FDAPA--------ILVV--
GH----Q----------------TDGKSALVE-----A--LM-G-F-QFNHVG-------------GG-TKTRRPI------------
---TLH-------------M-K-YNA---
---NCAQPR---------------------------------------CFL----------I----
--SEDQPHREKEQS--------LEEIQAYI------EAEN-
KR---L-ER-----------------------EA-----------------CQ--FSAK-EIILKIEYKF----------------------------
------------------------------CPNLTIIDTPGLIS--AA-----------------P---------G------------RKNQS----
-LQ--S--QGGAVE--------ALVQ-LKM---QQ-----------KEFIILCLEDSS---D-WSNAT-TRRVVMQAD-
-----PELRRTVLVSTKLDTRI------PQFA------RP----DDV--------------------------ELF--LK---PPSC---
---LLDG--------------------IILG-GSPFFTSVPSGR---VGSSK------------------------------DSVFRS-
NTD--FQE-----------A----------------------------VA---------ARQIQ---DLALLEEKL------NR-PLT----
----------------R-D------E-----------CSR-IGISRLRWFLEQLLQCRYMDS-VP---
XP_009032466.1.2 ------------FDVPS--------VVVI--
GA----Q----------------SSGKSALVE-----A--LM-G-F-QFNEVG-------------GG-TRTRRPI-------------
--ALQ-------------M-H-YNA--
---ACDEPA---------------------------------------CYI----------M----
------------DE-----------------------------------RFSGGEPVDGGAPFERRAT--------LAEARRFI------
EEEN-RR---L-ER-----------------------DQ-----------------HR-SFEAR-EIVMRVEYRH----------------
---CPNLVLVDTPGLVG--GG-----------------GDV------
FGDDFGEES---HESPHARG-----MK--R--QAREAY--------ELAL-GKA---RA-----------
RNAVLLCVDDGN---D-WKLGSIARRLCADAD------PTLSRTVVVSTKLDTKL------VQFG------SG----
RDV---------------------------ASF--LR---AKVL------HDLHP--------------------RLLA--
GPFFTSVPCGR---VAGAISPGGDAWDPQGGAPENQPWDLDDEGFYEDD--GVAFRG-DAE--FRA-
----------A----------------------------TA----------RASRA---DRSLVKSKV------GF-EFF-----------------
--D-K------A-----------APQ-LGVGALRQFLERHVELQYRSN-VARV-
XP_009032466.1 ------------FDVPS--------VVVI--
GA----Q----------------SSGKSALVE-----A--LM-G-F-QFNEVG-------------GG-TRTRRPI-------------
--ALQ-------------M-H-YNA--
---ACDEPA---------------------------------------CYI----------M----
------------DE-----------------------------------RFSGGEPVDGGAPFERRAT--------LAEARRFI------
EEEN-RR---L-ER-----------------------DQ-----------------HR-SFEAR-EIVMRVEYRH----------------
---CPNLVLVDTPGLVG--GG-----------------GDV------
FGDDFGEES---HESPHARG-----MK--R--QAREAY--------ELAL-GKA---RA-----------
RNAVLLCVDDGN---D-WKLGSIARRLCADAD------PTLSRTVVVSTKLDTKL------VQFG------SG----
RDV---------------------------ASF--LR---AKVL------HDLHP--------------------RLLA--
GPFFTSVPCGR---VAGAISPGGDAWDPQGGAPENQPWDLDDEGFYEDD--GVAFRG-DAE--FRA-
----------A----------------------------TA----------RASRA---DRSLVKSKV------GF-EFF-----------------
--D-K------A-----------APQ-LGVGALRQFLERHVELQYRSN-VA---

XP_004336224.1 ------------VAAPE--------IVVI--
GH----Q---------------GHGKSSIIE-----G--IL-G-H-HVTFTG--------------Y-GATKRPL--------------
FLN-------------L-I-NNP---
-------------------------------------KCDRPR---------------------------------------VTL----------K--------
--------RD---PLLKGPEYDHDVV------VALSDLPEEL------QKRN-
KL--N-KLSEE-PVFLQYEYRY---------------------------
---------------------------CSNLTLIDTPGLLK--EE-----------------D--------------------A-------------
-D--ATGEVQ--------AMIN-NLV---KH-----------PDRFILCVEEAK---D-WDKLD-MMDFIKKFD------
PEFSRTTFVYTKLHFHL------QRFT------SA----R------------------------------Y--LQ---GT-I------
PDA---------------------------HCFFTTMLPSR---V-----------------------------------RARYAD-PEK--
FQE-----------K-----------------------------IY----------QCTRR---DIKALEQL----------QYD-------------
-------R-R------H----------ESN-IGAVQLRQYLLNLAWKRYQDD-IPQI-
XP_002649212.1 ---------NTAVSHPE--------IVFV--
-GP----R---------------SSGKSSLIE-----A--FI-G-R-ALNIVGGGNIVGVGGSN---AN-GCSKRVL-----
----------YLQ-------------F-T-NNI--
---DFEVPK---------------------------------------VTI----------K-
---------------KD--NTIK--EFDHDII------VSIEQLNENL------
AKRN-QL---TN-DYIEE-PIYVSIESRT-----------------------
----------------------------------TLNLTLIDSPGLLF--DQ------------------S--------------------Q-------
--------A--ESNKIE--------SIVS-SLL---RP-----------SHRLIIAVESCSQ--D-WKSMS-MGQYLKKID---
---PELSRSTFVFTKFHHTV------RGFS------ST----RDI---------------------------NKY--LS---GT-V------
PDI---------------------------KGFFVTLPNHQ---V-----------------------------------RASYSE-ANR--
FQE-----------K-----------------------------IY----------QAHKR---DMHALEQL----------QYD-----------
---------K-R------Y----------ERT-IGVAPLRRYILNIVWK-----------
XP_003288465.1 ---------NTSVLHPE--------IVFV--
-GP----R---------------SSGKSSLIE-----A--FI-G-R-ALNIVGASSTL---------AN-GCSKRVL------------
---YLQ-------------F-N-NNN--
--DCDIPK---------------------------------------VTI----------K-----
-----------KD--NNLK--EFDHDII------IPLEQLNDSL------AKRN-
--SS-DFSEE-PIYVTIESRN--------------------------------
---------------------------TLNMTLIDSPGLLF--TP------------------T--------------------E-----------T--
A--ESTKIE--------SIVS-SLL---RP-----------THRLIIAVESCNQ--D-WKTMS-MNQYLKKID------
PELSRSTFVFTKFFNTV------RGFS------NT----RDI---------------------------NKY--LS---GT-V------
PDI---------------------------KPFFVTLPNYQ---V-----------------------------------RASFSE-PNR--
FQE-----------K-----------------------------IY----------QAHKR---DMHALEQL----------QYD-----------
---------K-R------Y----------ERS-IGVQPLRKYILNIVWK-----------
KAF2077035.1 ---------NTSVSHPE--------IVFV---
--GP----R---------------GAGKSSLIE-----A--FI-G-R-ALNIVGASTNL---------TS-GTSKRPV-----------
----YLQ-------------F-V-NNV---
--ECETPK--VTM----------K---
-------------KD--NILK--EFDHDLV------IPFEQLNDNL------
AKRN--S-IYSEE-PIYVSIEVKT--------------------------
--------------------------------ALNLTIIDTPGLVA--NE------------------S--------------------A-----------
---A--DQQKID--------QIVN-GIL---RP-----------THRLIIAVEACG---D-WSNMT-MLSFVKKVD------

PELSRSTFVFTKFFNMI------QDFN------NT----RGV---------------------------NRF--LS---GT-M------
SEI-----------------------------KSFFVTLPNHK---I-----------------------------------RARFSE-PAR--
YQE-----------K-----------------------------IN----------QAYKR---DMQSLEQF----------QYD-----------
---------K-R------Y-----------ERN-IGVHPFRRYILNIVWK-----------
XP_020436215.1 ---------NTSIPQPE--------IVFV---
-GP----R----------------GSGKSSLIE-----S--FI-G-R-PLNIVGA--------------M-GCSKRAL--------------
-HLQ--------------F-V-NNT--
--ECETPK---ITI----------K---------
-------RD--GFIK--EFDHDVV------IPIEQLNDTI------TRRN------
--T-AITEE-PIYIMIEAKN---
--------------------TLNLTLIDTPGLVA---E------------------G--------------------T---------------A--
DQAKID--------AIVN-SIL---RP------------THRLIVAVEACG---D-WSNMT-MLSFVKRVD------
PELSRSTFVFTKFFNII------QDFN------ST----RSV---------------------------NRF--LA---GT-M------
SEI-----------------------------KSFFVTIPNHK---I-----------------------------------RARFSE-PAN--
FQE-----------K-----------------------------LT----------QAYKR---DMNALEQL----------QYD-----------
---------K-R------Y-----------ERN-IGVHPFRRYILNITWK-----------
XP_012753198.1 ---------NTSIAQPE--------IVFV---
-GP----K----------------SSGKSSLVE-----A--FI-G-R-PLNIVGA--------------L-GCSKRSI---------------
HFQ--------------F-V-NNA---
--ECEVAK---VTV----------K-------
---------RD--ALIK--ELDHDVV------ITVEQLNDTL------NRRN---
--T-VISDE-PIYVLIESRS---
----------------------TLNLTLIDTPGLVA---D------------------G--------------------A---------------N--
DHAKID--------AIVS-AIL---RP------------NHRLIVAVEPCG---D-WANMT-MLPFVKRVD------
PELSRSTFVFTKFFNVI------QDFN------ST----RSV---------------------------NRF--LA---GA-V------
SDI-----------------------------KSFFVTIPNHK---I-----------------------------------RARFAD-PAS--
FQE-----------K-----------------------------LA----------QAYKR---DMNALEQF----------QYD-----------
---------K-R------Y-----------ERN-IGVTPFRRYILNIIWK-----------
XP_004360608.1 ------------ISQPE--------IVFV---
GP----R----------------SSGKSSLIE-----S--FI-G-R-PLNIVAGQSG-----------G-ACSKRAV-------------
--HLQ--------------F-M-NNP---
--EVEGTK---ITI----------K-------
---------RD--SFIK--EFDHDIV------VPLEQLNDNL------ARRS---
--N-QFHEE-PIYIIFESKS-------------------------------------
------------------------TLNLTLIDTPGLLF---D------------------G--------------------Q---------------P--
EQSKIE--------AIVQ-QAV---RP------------SHRLIVAVEASG---E-WAEMT-MLPFVKKVD------
PELSRSTFVFTKFFNMI------QDFN------ST----RGV---------------------------NRF--LA---GT-I------
NEI-----------------------------KTFFVTLPNHK---I-----------------------------------RARYSE-PAA--
FQE-----------K-----------------------------LT----------QAFRR---DMHSLEQF----------QYD-----------
---------K-R------Y-----------ERT-IGVHPFRRYMLNIVWKSYQDA-IPRI-
KYQ90260.1 ------------ISHPE--------IVFV---GP----R-
---------------TSGKSSLIE-----A--FI-G-R-SLNIVGQ--------------N-GCSKRPI---------------YFQ-------
-------F-V-NNI---
-------------------------ECEVPK---ITV----------K----------------RD--

---------------------------------------SNIK--DFDHDIV------IPLEQLNDNI------FRRN-----------------
-----------------------------------Q-QLSDD-PIYITIETKS---
----------SLNLTIIDTPGLLI--DS------------------N---------------------S---------------Q--EQQKID------
--NIVQ-SIL---KP-----------NNRLIIAVENCG---D-WSQMS-MLTFIKKVD------
PELSRSTFVFTKFFNQI------QDFT------ST----RSV--------------------------NRF--LA---GT-M------
SEI-----------------------------KSFFVTIPNHK---I----------------------------------RARFSE-PAR--
FQE-----------K-----------------------------LQ---------QAYKR---DMYHLEQI----------QYD-----------
---------K-R------Y-----------ERS-IGVNPFRKYILNIVWKSYQDG-IPRI-
KAH3767868.1 ------------VQVPE--------IVLV--
GK----K----------------GVGKTSLLE-----A--IL-G-H-KFGDTGT-----------------STMRPI---------------
EVK-------------M-V-NNQ--
--------------------------------------QCATPK--ITF----------M-------
---------HD---LALP--QFMEDTE------IPLNKVPETI------RDRN---
--KKSSV-PIRILYEFKY--------------------------------------
----------------------CWGMTIFDTPGLLP--KS-----------------D--------------------P-----------A--E-
-AIEEVE--------SIVL-ELM---TP------------PTRMLLCVEEAT---D-WERVQ-IVDFVSKVD------
PKRSRSVFVFNKFAGLL------KNFG------SF----REL---------------------------QLF--LS---ANPL------
MDT----------------------------PVFFTSLPAGA---I-----------------------------------EAHL--------YKR--
---------R-----------------------------LA---------QLLKG---DKDALELL----------QYD------------------
-R-R------Y-----------EKS-IGVVALQKYVLDWTLNKYQDL-IPEI-
ATZ80405.1 ------------INFPR--------IVLV---GT----Q-
--------------TSGKSSFIN-----N--LI-N-M-ELMPTG--------------DN-MVTRSPV---------------HIK-----
---------V-I-NNT--
-------------------------TNSTDR---------------------------------------VSI---------FT--------------
MTN--GNKNLVYCADLNST---------NTFNTLIFQKKMREAT-
DM---I-AH-----------------------KN--------------------CISEE-PIIVEILTKD---------------------
TNNNMS--------------------------------AKNLIIVDLPGLVT--IP-----------------KTD------IG------------
--Q--------PA--S--IVEDLK--------NLVM-KEI---SH------------PNVYVLVAIQAK-T-D-LETDV-
GLAVVKEIQ----RTNKSLKAIGLLTKLDLLD-------KRSLK--QFD-----NNI---------------------------
FNPAVLS--KS------------TA-----------------------L----DGGFFVINN-------------------------------------
----------HND----------------N---DEYYL---NKNMFDK--------
SLRFIQ-------------G------N-RFGSHNLMI-------QL-KK------NLITGIRAILPDF----------
KAF5834941.1 ------------FQMPR--------IVVV---
GG----E----------------STGKSSLLE-----N--IT-K-C-AVFPRD--------------KD-ICTRMPI---------------
RLQ-------------L-T-NAT--
-----------------------------------DASDTA---------------------------------------VEV-------------------
-------QF---GSQPATRLQD--SS---------QVLGAV------EGAM-
DK---L-P------------------------KD--------------------SICET-EFVVRI--RE---------------------T----G--
-----------------------------IPTFEFIDLPGIRA--YP-------------------LD------MA-------------Q--------
KT----------E--------SLVR-KYL---KV------------PNTLVIAVVRATDT-R-ITNDR-GYALVQEMG----
LESKTVMA---LTR---TD-------RVSAA--EFQ-----EMV--------------------------LDRIMMS--SSEFP---
------KP----------------------L----FACIAIVNR--TSD---------
-------DS-------VT-------------------LA---------EHQAM---EAEWFEK--------HVGVWY-------------S-
-----E-R--PWEQLV-------EM-ASR-MTLFNLIKRVDVLYSQYISAVWKPRA-

QFG74057.1 --------FISTSKLPT--------IVMI--GG----
E----------------STGKSATIE-----N--IT---MLSIFPTD--------------KS-VCTRCPV--------R------IVM-----
-VPS-----M-V-SHT--
-------------------------------EIEIT--------------WR---------------------------GEK----------S--------------
HVNE-----------------------------------Y-------------------------------DAREYI-----------SR---I----------------
--F------------TD------------------IE-SYSKD-ELLIEIYGNY--
---------SCRFEFVDLPGIVN--YP--Q------------S--AHEFTT------
--ALAD-DYI---KN------------SDNFIMCVVNATIP-R-LTSYY-AINRIINFN-------
ANMRTVLVLTKYDKLV--------------LVDF-----------------------------------DEY--LF---NR--------LD-
MS-------------------SDEFISHKYIGCCGVHNRD--S-ART------
-------------------ST--------------------LI----------NHVQN---EQALINYF-------HENNY------------------
---H-N------------------VEK-LGVEYLMNFVLHHYDNHIKQHWIPKV-
CEM26963.1 ----LLRLVGLGFMIPR--------IVTI---GQ-
---Q----------------SDGKTTFLE-----A--ML-R-L----CFG-----------YTRNGAAATKGPV---------------
RID--------------V---KND------------------------------ESS--

---PTPRCQLNQEHIRLA-----------AIQGRM------RELM-EH---
R--SLSKE-ETHLEISYSG-------------------------------------
-----------------------APNLMCVDLPGIVQ----------------------ET-------GD--------------T--------TR--E-
-DVELTM--------DVVR-HYV---RH-----------SPNDLYLVFKRADV--D-PGTWP--IHDFIQS-----
LGLHREQTIVACTRAENFL-----RENRV------IY-KT--EL----------------------------LKL--IK---QKRV--
HDCSGEPIP--------------------MYFV---ELHNLSDQEK--A-L-
P--FAE-----------RT-----AVMEGQ------------------LA----------ARRLD---ILDRLNKLEN--GAATD-E--
-------------------DT-T-----EL-----------YRY-FDVESLEAHLNATFRRLLLRQ-LHAL-
OII76931.1   ----FLKLANLSDQIPR--------IVVI--GQ-
---Q----------------SMGKTTLLD-----S--LI-G-Y----PLG-----------YSSSD-IGTCCPI---------------VFH-
-------------IYAKSPR------------------------------SALNSPI---
-------------------------------------SCHTDS-------------TSS-------------------------SDI---------DK-------
------LALNV--ETIDCIIDDEIIEFD-----------TLPEII------AKKT-LE---
K---------------------------NG---------------------VVDSN-EINIKIKSEK------------------------------------
------------------------SVNMILVDLPGL---------------------------------KE--------------N--------TR--E--
GAALTQ--------QIVR-EYV---NK-----------HPNDIYILVKRSLD--D-PANWSWKQKEFILND----
LGLGCDQTITVGTKGLEYA-----TEEIK------EV-STADEL----------------------------IER--IE---
KRKIGYNNDKWNSLP--------------------LYIL---EFFSLSKEER---
---S-LKN--MKL-----------RR-----EAMYRN------------------IN----------DGNIK---LQAILGEFC---
KDTDP-L---------------------TR-Q-----AV-----------FNY-FSYDLFQREVNLKFVKIFLRQ-LDLL-
KAH8582109.1   ----LLKLKGLEREIPR--------IVIL--
--GQ----Q----------------TIGKTTVID-----Y--LI-N-H----PLG-----------YSTNN-TGTCCPI---------------
VFH--------------I---SPS------------------------------NEGSSES--
--FLPLEL-------------TSK-------------------------TLV---------GS----
---------GILHE--EITECLIGGEKVSFE-----------TLPKKN------
SERM-KK---M---------------------------RM---------------------QISPE-ELRIDIKSRG---------------------
---------------------------------------AMEMIIVDLPGL---------------------------------KE--------------D---
-----TN--Q--GSKMTQ--------KIVQ-EY---------------------------RSID--D-PTNWSWRQKLFLFEE----

LRLRKEQTIVVGTRALEFL-----QQEVE------EI-SNGDEL---------------------------LER--IK---
KRELSSTYGFGAPLP-------------------LYML---ELFSLSKEER---
---A-IQK--MSC-----------RK-----NAMDRR-----------------IT---------AGEKN---IKEIINQICD--
QDHEK-E--------------------VK-E-----KL-----------LSY-FSRDLFEMDLSSKFGQILLKE-LNSL-
POM84969.1 ----LLRLNFSGGEIPR--------IVVL--
GQ----Q---------------SMGKTTVID-----Y--LI-G-H----PLA-----------YSTND-NGTCCPI--------------
VFH-------------I---SPS---------------------------EEKDRES---
---YLTFKL-------------ESD-----------------------CLL---------GS--
-----------GILNE-K-----------------------------------EITECLIGGEKVTFE-----------ALPEKI------
LEKM-KE---I--------------------------KM--------------------QTSSQ-ELRIDIRSRG--------------------
-------------------------------------AIEMIIVDLPGL--------------------------------KE-------------D----
----T---D--GSKITQ--------KILYDEYV---RN-----------HPNDIYILVQRLND--N-
PANWSWKQKPFLLEE----LGLGKEQAIVVGTRALEYL-----QKEVK------EI-SNGKQL-----------------
----------LER--II---KRGPSSGDESGTPIP-------------------FFML---ELFGLSMEER-------------------
-------------------------A-IKK--MSS-----------RK-----SAMERR-----------------IS---------AGEKA---
IKEIITQLCN--QDHET-K--------------------IK-E-----RI-----------LSY-FSRDLFEKELRSKFGQILFKQ-
LNSL-
XP_667128.1 ----LLKLNGLGGEIPR--------IVIL--GQ-
---Q---------------SMGKTTVID-----Y--LI-G-H----PLG-----------YSTND-IGTCCPI--------------VFH-
-------------I---SPS-----------------------------EEKDRES--
----------------------------------CLSSKL-------------VSD-----------------------CLL---------GS-------
------GILNE-E-------------------------------------EITECLIGGEKVTFE-----------TLPEKI------LERM-
KE---I--------------------------KM--------------------QISSQ-ELRIDIRSKG-----------------------------
-----------------------------AIEMIIVDLPGL--------------------------------KE-------------D--------TK--
E--GSKITQ--------KIVH-EYV---KN-----------HPNDIYILVKRSID--D-PANWSWRQKSFLLEE----
LGLGKEQAIVVGTRALEYL-----QEEVK------EI-SNGKQL---------------------------LER--IK---
KRELSSGDESGAPLP-------------------LFML---ELFSLSREER---
----A-IKK--MSS-----------RK-----NAMERR-----------------IS---------AGEKA---IKEIITQLCD--
QDHET-E--------------------VK-E-----RL-----------LSY-FSRDLFEMELRSKFGRILLKQ-LNSL-
XP_001617280.1 ----IFKICNIHNELPR--------LVVF---------------------------------------
----GQ----Q---------------SMGKTTLLD-----F--IM-G-G----PMG----------YTSSD-TGTKQPI---------
------VII-------------L---KPS-----------------------------DTN---
----
---KIECYLNKKKVSID-----------DLHEKM------KAIM-VN---
L--------------------------SE-------------------SIIPK-ELEVEISIPG--------------------------------------
---------------------GIYATFVDLPGI--------------------------------KD-------------D--------SK--S--
GSELTR--------KIVR-NYV---QN-----------FPNDIYILVKKASD--D-
PANWPYHLREFFMKPKPMGLGLQNKQCIVVGTRALEFL-----NNELS------TI-KTLTEL----------------
------------HDR--VK---KRGI--TDHNDNILS-------------------LYLL---ELFSIPIEQK--------------------
-------------------------E-KND--FLT-----------NR-----ISMYSK-----------------IL---------NGRKN---
VLDLLLNKFE--NDCND-S--------------------IK-K-----EL----------LDC-
FDVEKFKQEVNSKFMNILIQQ-LRKV-
XP_028539355.1 ----IFKICNIHNELPR--------LVVF---------------------------------------
----GQ----Q---------------SMGKTTLLD-----F--IM-G-G----PMG----------YTSTD-TGTKQPI---------

-----VII-------------L---KPS----------------------------DTN---
----
--KIECYLNKKKVNID----------DLHEKM------KAIM-LN---
L-------------------------NE-------------------SIIHK-ELEVELSIPG-----------------------------------
----------------------GIYATFVDLPGI-------------------------------KD------------D--------SK--A--
GSELTR--------KIVR-NYV---QN----------FPNDIYILVKKASD--D-
PANWPYNLREFILKPRPLGLGLQPKQCIVVGTRALEFL-----NNELT------NI-KTLSEL----------------
----------YER--VK---KRSI--TDNNDNVLP------------------LYLL---ELFSIPIEQK---------------------
----------------------E-KND--FLT----------NR-----ISMYSK-----------------IL---------NGRKN---
VLDLLLNKFE--NDCND-S--------------------IK-K-----EL----------IDC-
FDVEKFKQEVNSKFMNILIQQ-LRKV-
PHJ24853.1 ----LLKLAGLEAEIPR--------LVVF---GQ-
---Q---------------SMGKTTLLD-----Y--IM-G-G----PIG----------YSSTD-TGTRQPV--------------VIV-
-------------L---RPT----------------------------DS--
---D-----------------------
--------------------------------------TVQCWLAGEEVSVQ----------ELQARM------KNIM-SS---Q----
----------------------GE-------------------QISSR-ELEVELAVPR--------------------------------------
-----------------GVHAVFVDLPGI-------------------------------KD------------D--------SK--A--
GALQTR--------SVVR-TYV---QN----------NPNDLYILVKKASD--D-
PANWPWSLREFILSAPPTGLGLTPRQTVVVGTRAREFL-----TNERS------DI-RTQAQL---------------
------------LER--VF---KRAV--KDSTGAPLP-------------------LFLL---ELFSLSIEEK--------------------
------------------------D-QND--FAA----------KR-----AAMNRQ-----------------ME---------DGGRT-
--VRELLNS-FE--AGANS-Q--------------------QT-K-----TL----------MEF-
FSPVRFKKELNSKFQGLLSDQ-LGLL-
KFG43683.1 ----LLKLAGLEAEIPR--------LVVF---GQ-
---Q---------------SMGKTTLLD-----Y--IM-G-G----PIG----------YSSTD-TGTRQPV--------------VIL-
-------------L---RPS----------------------------ESA---
---D-----------------------
---------------------------------------SVQCWLGGEEIEVK----------ELQARM------KEIM-SS---Q-----
----------------------GE-------------------RISSQ-ELEVELAVPN--
-----------------GVHAVFVDLPGV-------------------------------KD------------D--------SK--A--
GATQTR--------SVVR-TYV---QN----------NPNDLYILVKKASD--D-
PANWPWSLREFILSAPPKGLGLTPRQTVVVGTRAKEFL-----VNEKN------DI-RTQSQL--------------
-------------LER--VL---KRAV--KDSSGAPLP-------------------LFLL---ELFSLSIEEK------------------
------------------------D-ALD--FAA----------KR-----AAMNRQ-----------------MD---------EGGRT-
--VRQLLETAFE--PGPNP-Q--------------------LS-R-----KL----------TEF-
FSPSRFKKELNHKFQSLLSEQ-MGIL-
UKJ88078.2 ----LMKLGGLEAEVPR--------LVVF---
GQ----Q---------------SMGKTTLLD-----F--IM-G-G----PIG----------YSSTD-TGTKQPV-------------
-SII-------------M---KPL-----------------------------TTLNTLKE--
---SLKENL------------KEG-----------------------
SKANLKDKYKGNEE------------ILLNE-T-----------------------------------NAIVCKFNGRFMTIH----
-------EVQDAM------RVHM-QS---L------------------------GQ------------------TILSD-
ELEVEVYVPN---ALYAIFVDLPGI------------------

--------------KD-------------D--------SK--V--GAELTR--------SVVR-NYV---SN-----------
NPNDLYILVKKASD--D-PSNWPWSLKEFITSSAPAGLGLTPQQTMVVGTRAKEFL-----LNEKT------
DI-KTYEEL---------------------------VER--VY---KRGV--VDSKGQMLP--------------------LHLL---
ELFSLSIQAK---E-SGD--FLS-----------NR-----DDMKAQ------
------------IS----------SSQKQ---IYDLLINSF-----NST-NLG------------D-EDR-LK-D-----EL-----------
FNI-FSIDSFLKTLNHKFQSLMTNQ-LNNL-

XP_004833148.1 ----LMKLGGLEAEVPR--------LVVF-------------------------------------
------GQ----Q----------------SMGKTTLLD-----F--IM-G-G----PIG-----------YSSTD-TGTKQPV--------
-------SII-------------M---KPS----------------------------SSLQN---
---YEGSGP--
-----------PPVTD-T-------------------------------------SIICKFGGRLMNIH-----------EVQDAM------
RNHM-QS---L--------------------------GS--------------------SILSD-ELEVEVYVPN-------------------
--ALYAIFVDLPGI--------------------------------KD--------------D--
------SK--A--GAELTR--------SVVR-NYV---SN-----------NPNDLYILVKKASD--D-
PSNWPWSLKEFITASAPAGLGLTPQQTMVVGTRAKEFL-----INEKT------DI-KTQEQL----------------
------------MER--VY---KRGV--VDSKGQMLP--------------------LHLL---ELFSLSIQAK-----------------
---------------------------E-SCD--FLS-----------NR-----DDMKAQ------------------IS----------
ASQRE---VYDLITNGFE--LSSSS-NLN-------------D-EGKTVR-E-----EL-----------MQI-
FSIDSFLKTLNNKFQHLMNNQ-LTNL-

GFE54186.1 ----IMKLGGLDAEVPR--------LVVF--
GQ----Q----------------SMGKTTVLD-----F--IM-G-G----PIG-----------YSSTD-TGTKQPV--------------
-VII--------------M---RPL-----------------------------EVIQEVAA---
---SLNVQL---SP-----
--------DAVNG-S--------------------------------------TIWCLFDGKLMDIR-----------SVQDAM------
RVHM-LN---I--------------------------GD--------------------TIIAD-ELEVEVFVPN---------------------
--------------------------------------GLNAIFVDLPGI--------------------------------KD-------------D---
-----SK--M--GAEFTR--------NVVR-NYV---KN-----------NPNDLYILVKKSSD--D-
PANWPWSLREFITTAPPTGLGLSPHQTVVVGTRARDFL-----INEKT------DI-RTAEQL-----------------
-----------LER--VL---KRSV--IDSRGNMLP--------------------IHLL---ELFSLSIQAK--------------------
------------------------E-SGD--FTA-----------NK-----AEMKRQ------------------IA---------TGQQE---
VEDMIRHSFE--L-KSG-SVD-------------M-DGLTVQ-E-----RL-----------IQM-
FSVNGFLTTLDSKYQTLVANT-FRNL-

XP_012766661.1 ----IMKLGGLDAEVPR--------LVVF------------------------------------
------GQ----Q----------------SMGKTTVLD-----F--IM-G-G----PIG-----------YSSTD-TGTKQPV--------
-------VII-------------M---RPL-----------------------------EVIMEVAS-------------------------------------
---ALKLQI---
DP-------------ASLKG-G---------------------------------------TIWCLFNGKLMDIR-----------SVQDAM-
-----RLHM-QS---M--------------------------GE--------------------RIVAD-ELEVEVFVPN--------------
---GVNAIFVDLPGI--------------------------------KD------------
--D--------SK--T--GAEFTR--------NVVR-NYV---KS-----------NPNDLYLLVKKSSD--D-
PANWPWSLKEFITTAPPTGLGLSPHQTIVVGTRAREFL-----INEKT------DI-RTVEQL------------------
----------MDR--VL---KRTI--TDSKGNILP-------------------LHLL---ELFSLSIEAK-----------------------
----------------------E-SGD--FLA-----------NK-----REMKRQ------------------IA----------KGQEE---

VENMIRTSFE--V-TND-TVG-------------R-DGMTVV-D-----RL-----------LQM-FSINGFLTTLDTRYQALVATT-FRNL-
GIX62800.1 ----IMKLGGLDAEVPR--------LVVF---GQ----Q----------------SMGKTTVLD-----F--IM-G-G----PIG-----------YSSTD-TGTKQPV---------------VII--------------M---RPL-----------------------------EVILEVAD---ALKVQI---DP-------------TNLKN-G--------------------------------------TIWCLFNGQLMDIR-----------SVQDAM------RAHM-QG---I---------------------------GD---------------------RILAE-ELEVEVFVPS---GVNAIFVDLPGI---------------------------------KD--------------D--------SK--S--GAEFTR--------NVVR-NYV---QN-----------NPNDLYILVKKSSD--D-PANWPWSLKEFITTAPPTGLGLSPHQTVVVGTRAREFL-----LNEKT------DI-RTAEKL----------------------------MER--VL---KRSI--TDSKGTMLP--------------------LHLL---ELFSLSIQAK---E-SGD--FLA-----------NK-----AEMKRQ------------------IA----------AGQQD---VEEIIRSSFE--V-TNG-SVN-------------R-DGMTVV-E-----RL-----------VQM-FSIHGFLTTLDAKYQSLVATT-FRNL-
KAH0479249.1 ----LLKLGGLEAEIPR--------LVVF---GQ----Q----------------SMGKTTLLD-----F--IM-G-G----PIG-----------YSSTT-TGTKMPV---------------VIM--------------L---RPP-----------------------------QGNEDRA--RP--SAMCK--GQHMSID-----------SLQHFM------SDVM-IK---Q---------------------------GD---------------------KITAE-ELDLEIVVPD--GVHAVFVDLPGI---------------------------------KD--------------D--------SK--D--GAEVTR--------NVVR-TYV---SN-----------NPNDLYILVKKASD--D-PANWPWSLREFIISPAPVGLGLSPKQTLVVGTRARDFL-----TAEKN------DI-KTQDEL----------------------------VLR--VR---NRAV--KDQYGTRLP--------------------LHLL---ELFSLSMSTK---E-SKN--FQL-----------KK-----AEMLNQ------------------IR----------LAQRS---CLSALTKDFE--YSRSQ-E---------------------VR-R-----DL-----------RQF-FDIDHFKTSLNVKFQSLLSEQ-ITLL-

;
end;
begin trees;
tree tree_1 = [&R] [&branchAttributeNames={"FastTree support value"}]((XP_004347890.1[&Organism="Capsaspora owczarzaki ATCC 30864","Genetic Code"="Standard",Taxonomy="Eukaryota; Filasterea; Capsaspora"]:0.13488999999999995,((NP_001024332.1[&Organism="Caenorhabditis elegans","Genetic Code"="Standard",Taxonomy="Eukaryota; Metazoa; Ecdysozoa; Nematoda; Chromadorea; Rhabditida; Rhabditina; Rhabditomorpha; Rhabditoidea; Rhabditidae; Peloderinae; Caenorhabditis"]:0.14437999999999995,XP_031757197.1[&Organism="Xenopus tropicalis","Genetic Code"="Standard",Taxonomy="Eukaryota; Metazoa; Chordata; Craniata; Vertebrata; Euteleostomi; Amphibia; Batrachia; Anura; Pipoidea; Pipidae; Xenopodinae; Xenopus; Silurana","Common Name"="tropical clawed frog"]:0.1572)[&"FastTree support

value"=0.931]:0.04847000000000001,((((XP_030853442.1[&Organism="Strongylocentrotu
s purpuratus","Genetic Code"="Standard",Taxonomy="Eukaryota; Metazoa; Echinodermata;
Eleutherozoa; Echinozoa; Echinoidea; Euechinoidea; Echinacea; Echinoida;
Strongylocentrotidae; Strongylocentrotus","Common Name"="purple sea
urchin"]:0.0,XP_030853442.1.2[&Organism="Strongylocentrotus purpuratus","Genetic
Code"="Standard",Taxonomy="Eukaryota; Metazoa; Echinodermata; Eleutherozoa;
Echinozoa; Echinoidea; Euechinoidea; Echinacea; Echinoida; Strongylocentrotidae;
Strongylocentrotus","Common Name"="purple sea
urchin"]:0.0):0.10166999999999993,((XP_001749319.1[&Organism="Monosiga brevicollis
MX1","Genetic Code"="Standard",Taxonomy="Eukaryota; Choanoflagellata; Craspedida;
Salpingoecidae;
Monosiga"]:0.12369999999999992,(XP_006812840.1[&Organism="Saccoglossus
kowalevskii","Genetic Code"="Standard",Taxonomy="Eukaryota; Metazoa; Hemichordata;
Enteropneusta; Harrimaniidae;
Saccoglossus"]:0.23876999999999997,(XP_014148725.1[&Organism="Sphaeroforma
arctica JP610","Genetic Code"="Standard",Taxonomy="Eukaryota; Ichthyosporea;
Ichthyophonida;
Sphaeroforma"]:0.11654000000000009,XP_014153758.1[&Organism="Sphaeroforma
arctica JP610","Genetic Code"="Standard",Taxonomy="Eukaryota; Ichthyosporea;
Ichthyophonida; Sphaeroforma"]:0.07362000000000002)[&"FastTree support
value"=0.983]:0.07342999999999966)[&"FastTree support
value"=0.009]:0.01957000000000031)[&"FastTree support
value"=0.403]:0.007899999999999796,(((PAA59145.1[&Organism="Macrostomum
lignano","Genetic Code"="Standard",Taxonomy="Eukaryota; Metazoa; Platyhelminthes;
Rhabditophora; Macrostomorpha; Macrostomida; Macrostomidae;
Macrostomum"]:0.04702999999999905,PAA64382.1[&Organism="Macrostomum
lignano","Genetic Code"="Standard",Taxonomy="Eukaryota; Metazoa; Platyhelminthes;
Rhabditophora; Macrostomorpha; Macrostomida; Macrostomidae;
Macrostomum"]:0.11000999999999994)[&"FastTree support
value"=0.935]:0.04535,PAA78248.1[&Organism="Macrostomum lignano","Genetic
Code"="Standard",Taxonomy="Eukaryota; Metazoa; Platyhelminthes; Rhabditophora;
Macrostomorpha; Macrostomida; Macrostomidae; Macrostomum"]:0.01654)[&"FastTree
support value"=0.246]:0.026699999999999946,PAA65118.1[&Organism="Macrostomum
lignano","Genetic Code"="Standard",Taxonomy="Eukaryota; Metazoa; Platyhelminthes;
Rhabditophora; Macrostomorpha; Macrostomida; Macrostomidae;
Macrostomum"]:0.03900000000000146)[&"FastTree support
value"=1.0]:0.10143999999999975)[&"FastTree support
value"=0.891]:0.028570000000000206)[&"FastTree support
value"=0.753]:0.003820000000001566,KMZ10000.1[&Organism="Drosophila
melanogaster","Genetic Code"="Standard",Taxonomy="Eukaryota; Metazoa; Ecdysozoa;
Arthropoda; Hexapoda; Insecta; Pterygota; Neoptera; Holometabola; Diptera; Brachycera;
Muscomorpha; Ephydroidea; Drosophilidae; Drosophila; Sophophora","Common
Name"="fruit fly"]:0.09075000000000033)[&"FastTree support
value"=0.887]:0.01902999999999988,(XP_026693152.1[&Organism="Ciona

intestinalis","Genetic Code"="Standard",Taxonomy="Eukaryota; Metazoa; Chordata; Tunicata; Ascidiacea; Enterogona; Phlebobranchia; Cionidae; Ciona","Common Name"="vase tunicate"]:0.11643000000000026,((XP_005165639.1[&Organism="Danio rerio","Genetic Code"="Standard",Taxonomy="Eukaryota; Metazoa; Chordata; Craniata; Vertebrata; Euteleostomi; Actinopterygii; Neopterygii; Teleostei; Ostariophysi; Cypriniformes; Cyprinidae; Danio","Common Name"="zebrafish"]:0.05400000000000027,(XP_028570166.1[&Organism="Podarcis muralis","Genetic Code"="Standard",Taxonomy="Eukaryota; Metazoa; Chordata; Craniata; Vertebrata; Euteleostomi; Lepidosauria; Squamata; Bifurcata; Unidentata; Episquamata; Laterata; Lacertibaenia; Lacertidae; Podarcis","Common Name"="Common wall lizard"]:0.014839999999999964,(KAE8583055.1[&Organism="Xenopus tropicalis","Genetic Code"="Standard",Taxonomy="Eukaryota; Metazoa; Chordata; Craniata; Vertebrata; Euteleostomi; Amphibia; Batrachia; Anura; Pipoidea; Pipidae; Xenopodinae; Xenopus; Silurana","Common Name"="tropical clawed frog"]:0.02915000000000001,(EPQ17174.1[&Organism="Myotis brandtii","Genetic Code"="Standard",Taxonomy="Eukaryota; Metazoa; Chordata; Craniata; Vertebrata; Euteleostomi; Mammalia; Eutheria; Laurasiatheria; Chiroptera; Microchiroptera; Vespertilionidae; Myotis","Common Name"="Brandt's bat"]:0.000550,((XP_025915522.1[&Organism="Apteryx rowi","Genetic Code"="Standard",Taxonomy="Eukaryota; Metazoa; Chordata; Craniata; Vertebrata; Euteleostomi; Archelosauria; Archosauria; Dinosauria; Saurischia; Theropoda; Coelurosauria; Aves; Palaeognathae; Apterygiformes; Apterygidae; Apteryx","Common Name"="Okarito brown kiwi"]:0.017609999999999946,BAB27759.1[&Organism="Mus musculus","Genetic Code"="Standard",Taxonomy="Eukaryota; Metazoa; Chordata; Craniata; Vertebrata; Euteleostomi; Mammalia; Eutheria; Euarchontoglires; Glires; Rodentia; Myomorpha; Muroidea; Muridae; Murinae; Mus; Mus","Common Name"="house mouse"]:0.002589999999999648)[&"FastTree support value"=0.936]:0.0010400000000000152,(ELW62001.1[&Organism="Tupaia chinensis","Genetic Code"="Standard",Taxonomy="Eukaryota; Metazoa; Chordata; Craniata; Vertebrata; Euteleostomi; Mammalia; Eutheria; Euarchontoglires; Scandentia; Tupaiidae; Tupaia","Common Name"="Chinese tree shrew"]:0.000550,(EAW87759.1[&Organism="Homo sapiens","Genetic Code"="Standard",Taxonomy="Eukaryota; Metazoa; Chordata; Craniata; Vertebrata; Euteleostomi; Mammalia; Eutheria; Euarchontoglires; Primates; Haplorrhini; Catarrhini; Hominidae; Homo","Common Name"="human"]:0.000540,XP_012378586.1[&Organism="Dasypus novemcinctus","Genetic Code"="Standard",Taxonomy="Eukaryota; Metazoa; Chordata; Craniata; Vertebrata; Euteleostomi; Mammalia; Eutheria; Xenarthra; Cingulata; Dasypodidae; Dasypus","Common Name"="nine-banded armadillo"]:0.029630000000000045)[&"FastTree support value"=0.779]:0.003609999999999669)[&"FastTree support value"=0.0]:0.000540)[&"FastTree support value"=0.618]:0.007230000000000292)[&"FastTree support value"=0.942]:0.019269999999999676)[&"FastTree support

value"=0.697]:0.01855999999999991)[&"FastTree support value"=0.602]:0.017900000000000027)[&"FastTree support value"=0.512]:0.018899999999999917,((XP_032814666.1[&Organism="Petromyzon marinus","Genetic Code"="Standard",Taxonomy="Eukaryota; Metazoa; Chordata; Craniata; Vertebrata; Cyclostomata; Hyperoartia; Petromyzontiformes; Petromyzontidae; Petromyzon","Common Name"="sea lamprey"]:0.05137999999999998,((NP_001025299.1[&Organism="Danio rerio","Genetic Code"="Standard",Taxonomy="Eukaryota; Metazoa; Chordata; Craniata; Vertebrata; Euteleostomi; Actinopterygii; Neopterygii; Teleostei; Ostariophysi; Cypriniformes; Cyprinidae; Danio","Common Name"="zebrafish"]:0.0,XP_021326548.1[&Organism="Danio rerio","Genetic Code"="Standard",Taxonomy="Eukaryota; Metazoa; Chordata; Craniata; Vertebrata; Euteleostomi; Actinopterygii; Neopterygii; Teleostei; Ostariophysi; Cypriniformes; Cyprinidae; Danio","Common Name"="zebrafish"]:0.0):0.04506999999999994,((XP_025944940.1[&Organism="Apteryx rowi","Genetic Code"="Standard",Taxonomy="Eukaryota; Metazoa; Chordata; Craniata; Vertebrata; Euteleostomi; Archelosauria; Archosauria; Dinosauria; Saurischia; Theropoda; Coelurosauria; Aves; Palaeognathae; Apterygiformes; Apterygidae; Apteryx","Common Name"="Okarito brown kiwi"]:0.0524,(EPQ08653.1[&Organism="Myotis brandtii","Genetic Code"="Standard",Taxonomy="Eukaryota; Metazoa; Chordata; Craniata; Vertebrata; Euteleostomi; Mammalia; Eutheria; Laurasiatheria; Chiroptera; Microchiroptera; Vespertilionidae; Myotis","Common Name"="Brandt's bat"]:0.008020000000000138,(XP_006496668.1[&Organism="Mus musculus","Genetic Code"="Standard",Taxonomy="Eukaryota; Metazoa; Chordata; Craniata; Vertebrata; Euteleostomi; Mammalia; Eutheria; Euarchontoglires; Glires; Rodentia; Myomorpha; Muroidea; Muridae; Murinae; Mus; Mus","Common Name"="house mouse"]:0.003390000000000004,(XP_027623811.1[&Organism="Tupaia chinensis","Genetic Code"="Standard",Taxonomy="Eukaryota; Metazoa; Chordata; Craniata; Vertebrata; Euteleostomi; Mammalia; Eutheria; Euarchontoglires; Scandentia; Tupaiidae; Tupaia","Common Name"="Chinese tree shrew"]:0.01092000000000004,(XP_016856477.1[&Organism="Homo sapiens","Genetic Code"="Standard",Taxonomy="Eukaryota; Metazoa; Chordata; Craniata; Vertebrata; Euteleostomi; Mammalia; Eutheria; Euarchontoglires; Primates; Haplorrhini; Catarrhini; Hominidae; Homo","Common Name"="human"]:0.0036399999999998656,XP_012379251.1[&Organism="Dasypus novemcinctus","Genetic Code"="Standard",Taxonomy="Eukaryota; Metazoa; Chordata; Craniata; Vertebrata; Euteleostomi; Mammalia; Eutheria; Xenarthra; Cingulata; Dasypodidae; Dasypus","Common Name"="nine-banded armadillo"]:0.000530)[&"FastTree support value"=0.0]:0.000550)[&"FastTree support value"=1.0]:0.000550)[&"FastTree support value"=0.449]:0.010489999999999888)[&"FastTree support value"=0.934]:0.022710000000000008)[&"FastTree support value"=0.936]:0.028579999999999828,(XP_031753735.1[&Organism="Xenopus tropicalis","Genetic Code"="Standard",Taxonomy="Eukaryota; Metazoa; Chordata; Craniata; Vertebrata; Euteleostomi; Amphibia; Batrachia; Anura; Pipoidea; Pipidae; Xenopodinae; Xenopus; Silurana","Common Name"="tropical clawed

frog"]:0.03391999999999973,(((XP_014389433.1[&Organism="Myotis brandtii","Genetic Code"="Standard",Taxonomy="Eukaryota; Metazoa; Chordata; Craniata; Vertebrata; Euteleostomi; Mammalia; Eutheria; Laurasiatheria; Chiroptera; Microchiroptera; Vespertilionidae; Myotis","Common Name"="Brandt's bat"]:0.01122000000000023,(XP_006161648.2.2[&Organism="Tupaia chinensis","Genetic Code"="Standard",Taxonomy="Eukaryota; Metazoa; Chordata; Craniata; Vertebrata; Euteleostomi; Mammalia; Eutheria; Euarchontoglires; Scandentia; Tupaiidae; Tupaia","Common Name"="Chinese tree shrew"]:0.00848000000000043,XP_012381548.1[&Organism="Dasypus novemcinctus","Genetic Code"="Standard",Taxonomy="Eukaryota; Metazoa; Chordata; Craniata; Vertebrata; Euteleostomi; Mammalia; Eutheria; Xenarthra; Cingulata; Dasypodidae; Dasypus","Common Name"="nine-banded armadillo"]:0.03946000000000005)[&"FastTree support value"=0.867]:0.04524000000000017)[&"FastTree support value"=0.269]:0.02656999999999954,(XP_006510037.1[&Organism="Mus musculus","Genetic Code"="Standard",Taxonomy="Eukaryota; Metazoa; Chordata; Craniata; Vertebrata; Euteleostomi; Mammalia; Eutheria; Euarchontoglires; Glires; Rodentia; Myomorpha; Muroidea; Muridae; Murinae; Mus; Mus","Common Name"="house mouse"]:0.000550,NP_001005360.1[&Organism="Homo sapiens","Genetic Code"="Standard",Taxonomy="Eukaryota; Metazoa; Chordata; Craniata; Vertebrata; Euteleostomi; Mammalia; Eutheria; Euarchontoglires; Primates; Haplorrhini; Catarrhini; Hominidae; Homo","Common Name"="human"]:0.003589999999999982)[&"FastTree support value"=0.783]:0.0030399999999994876)[&"FastTree support value"=0.887]:0.00816000000000167,(XP_028568434.1[&Organism="Podarcis muralis","Genetic Code"="Standard",Taxonomy="Eukaryota; Metazoa; Chordata; Craniata; Vertebrata; Euteleostomi; Lepidosauria; Squamata; Bifurcata; Unidentata; Episquamata; Laterata; Lacertibaenia; Lacertidae; Podarcis","Common Name"="Common wall lizard"]:0.0027100000000004343,XP_025920181.1[&Organism="Apteryx rowi","Genetic Code"="Standard",Taxonomy="Eukaryota; Metazoa; Chordata; Craniata; Vertebrata; Euteleostomi; Archelosauria; Archosauria; Dinosauria; Saurischia; Theropoda; Coelurosauria; Aves; Palaeognathae; Apterygiformes; Apterygidae; Apteryx","Common Name"="Okarito brown kiwi"]:0.004700000000001485)[&"FastTree support value"=0.842]:0.006759999999999877)[&"FastTree support value"=0.912]:0.01454999999999984)[&"FastTree support value"=0.979]:0.03986000000000001)[&"FastTree support value"=0.703]:0.02090000000000014)[&"FastTree support value"=0.88]:0.01961999999999975)[&"FastTree support value"=0.834]:0.02157000000000533,XP_035683496.1[&Organism="Branchiostoma floridae","Genetic Code"="Standard",Taxonomy="Eukaryota; Metazoa; Chordata; Cephalochordata; Branchiostomidae; Branchiostoma","Common Name"="Florida lancelet"]:0.07413000000000025)[&"FastTree support value"=0.669]:0.017919999999999714)[&"FastTree support value"=0.694]:0.01371000000000111)[&"FastTree support value"=0.805]:0.007489999999999775)[&"FastTree support

value"=0.284]:0.000540)[&"FastTree support
value"=0.888]:0.04687999999999981)[&"FastTree support
value"=1.0]:0.10415000000000019,((((GMI12809.1[&Organism="Triparma laevis f.
longispina","Genetic Code"="Standard",Taxonomy="Eukaryota; Sar; Stramenopiles;
Ochrophyta; Bolidophyceae; Parmales; Triparmaceae;
Triparma"]:0.06823999999999986,GMI47362.1[&Organism="Triparma
columacea","Genetic Code"="Standard",Taxonomy="Eukaryota; Sar; Stramenopiles;
Ochrophyta; Bolidophyceae; Parmales; Triparmaceae;
Triparma"]:0.05138999999999996)[&"FastTree support
value"=1.0]:0.19388000000000005,(((CAH0521473.1[&Organism="Peronospora
belbahrii","Genetic Code"="Standard",Taxonomy="Eukaryota; Sar; Stramenopiles;
Oomycota; Peronosporales; Peronosporaceae;
Peronospora"]:0.05980000000000075,(KAG2764740.1[&Organism="Phytophthora
cactorum","Genetic Code"="Standard",Taxonomy="Eukaryota; Sar; Stramenopiles;
Oomycota; Peronosporales; Peronosporaceae;
Phytophthora"]:0.03268000000000004,(GMF38092.1[&Organism="Phytophthora
lilii","Genetic Code"="Standard",Taxonomy="Eukaryota; Sar; Stramenopiles; Oomycota;
Peronosporales; Peronosporaceae;
Phytophthora"]:0.02178000000000133,KAH7500220.1[&Organism="Phytophthora
ramorum","Genetic Code"="Standard",Taxonomy="Eukaryota; Sar; Stramenopiles;
Oomycota; Peronosporales; Peronosporaceae; Phytophthora","Common Name"="sudden
oak death agent"]:0.01780000000000026)[&"FastTree support
value"=0.715]:0.01101999999999808)[&"FastTree support
value"=0.571]:0.01404999999999674)[&"FastTree support
value"=0.955]:0.04192000000000018,(TMW56688.1[&Organism="Pythium
oligandrum","Genetic Code"="Standard",Taxonomy="Eukaryota; Sar; Stramenopiles;
Oomycota; Pythiales; Pythiaceae;
Pythium"]:0.07204999999999995,(CCI11042.1[&Organism="Albugo candida","Genetic
Code"="Standard",Taxonomy="Eukaryota; Sar; Stramenopiles; Oomycota; Albuginales;
Albuginaceae; Albugo"]:0.04805999999999999,CCA17876.1[&Organism="Albugo laibachii
Nc14","Genetic Code"="Standard",Taxonomy="Eukaryota; Sar; Stramenopiles; Oomycota;
Albuginales; Albuginaceae; Albugo"]:0.01034000000000238)[&"FastTree support
value"=1.0]:0.12685000000000013)[&"FastTree support
value"=0.843]:0.02700999999999756)[&"FastTree support
value"=0.522]:0.02092000000000027,(OQR85161.1[&Organism="Achlya
hypogyna","Genetic Code"="Standard",Taxonomy="Eukaryota; Sar; Stramenopiles;
Oomycota; Saprolegniales; Saprolegniaceae;
Achlya"]:0.04820000000000465,(RLO06844.1[&Organism="Aphanomyces
astaci","Genetic Code"="Standard",Taxonomy="Eukaryota; Sar; Stramenopiles; Oomycota;
Saprolegniales; Saprolegniaceae;
Aphanomyces"]:0.02762999999999982,KAF0740912.1[&Organism="Aphanomyces
euteiches","Genetic Code"="Standard",Taxonomy="Eukaryota; Sar; Stramenopiles;
Oomycota; Saprolegniales; Saprolegniaceae;
Aphanomyces"]:0.01073999999999975)[&"FastTree support

value"=0.852]:0.04275000000000029)[&"FastTree support value"=0.994]:0.07477)[&"FastTree support value"=0.991]:0.10173999999999995)[&"FastTree support value"=0.17]:0.04410000000000025,((KAJ1432693.1[&Organism="Ochromonadaceae sp. CCMP2298","Genetic Code"="Standard",Taxonomy="Eukaryota; Sar; Stramenopiles; Ochrophyta; Synurophyceae; Ochromonadales; Ochromonadaceae"]:0.2671699999999997,(GAX23670.1[&Organism="Fistulifera solaris","Genetic Code"="Standard",Taxonomy="Eukaryota; Sar; Stramenopiles; Ochrophyta; Bacillariophyta; Bacillariophyceae; Bacillariophycidae; Naviculales; Naviculaceae; Fistulifera"]:0.11753999999999998,(XP_002296064.1[&Organism="Thalassiosira pseudonana CCMP1335","Genetic Code"="Standard",Taxonomy="Eukaryota; Sar; Stramenopiles; Ochrophyta; Bacillariophyta; Coscinodiscophyceae; Thalassiosirophycidae; Thalassiosirales; Thalassiosiraceae; Thalassiosira"]:0.2442000000000002,CAB9512103.1[&Organism="Seminavis robusta","Genetic Code"="Standard",Taxonomy="Eukaryota; Sar; Stramenopiles; Ochrophyta; Bacillariophyta; Bacillariophyceae; Bacillariophycidae; Naviculales; Naviculaceae; Seminavis"]:0.14927)[&"FastTree support value"=0.738]:0.08892999999999995)[&"FastTree support value"=0.984]:0.13120999999999983)[&"FastTree support value"=0.02]:0.03475000000000028,XP_018636213.1[&Organism="Toxoplasma gondii ME49","Genetic Code"="Standard",Taxonomy="Eukaryota; Sar; Alveolata; Apicomplexa; Conoidasida; Coccidia; Eucoccidiorida; Eimeriorina; Sarcocystidae; Toxoplasma"]:0.3492600000000001)[&"FastTree support value"=0.849]:0.04744999999999999)[&"FastTree support value"=0.95]:0.05487000000000196,((((XP_044553261.1[&Organism="Naegleria lovaniensis","Genetic Code"="Standard",Taxonomy="Eukaryota; Discoba; Heterolobosea; Tetramitia; Eutetramitia; Vahlkampfiidae; Naegleria"]:0.01753999999999989,XP_044569353.1[&Organism="Naegleria fowleri","Genetic Code"="Standard",Taxonomy="Eukaryota; Discoba; Heterolobosea; Tetramitia; Eutetramitia; Vahlkampfiidae; Naegleria"]:0.009489999999999998)[&"FastTree support value"=0.999]:0.13012999999999986,(XP_044550536.1[&Organism="Naegleria lovaniensis","Genetic Code"="Standard",Taxonomy="Eukaryota; Discoba; Heterolobosea; Tetramitia; Eutetramitia; Vahlkampfiidae; Naegleria"]:0.06181000000000365,XP_002681690.1[&Organism="Naegleria gruberi","Genetic Code"="Standard",Taxonomy="Eukaryota; Discoba; Heterolobosea; Tetramitia; Eutetramitia; Vahlkampfiidae; Naegleria"]:0.08654000000000028)[&"FastTree support value"=0.975]:0.07895999999999948)[&"FastTree support value"=0.995]:0.11298999999999992,(NP_012926.1[&Organism="Saccharomyces cerevisiae S288C","Genetic Code"="Standard",Taxonomy="Eukaryota; Fungi; Dikarya; Ascomycota; Saccharomycotina; Saccharomycetes; Saccharomycetales; Saccharomycetaceae; Saccharomyces"]:0.13675000000000015,(KNE68830.1[&Organism="Allomyces macrogynus ATCC 38327","Genetic Code"="Standard",Taxonomy="Eukaryota; Fungi; Fungi

incertae sedis; Blastocladiomycota; Blastocladiomycota incertae sedis; Blastocladiomycetes; Blastocladiales; Blastocladiaceae; Allomyces"]:0.12562000000000006,((KXN66323.1[&Organism="Conidiobolus coronatus NRRL 28638","Genetic Code"="Standard",Taxonomy="Eukaryota; Fungi; Fungi incertae sedis; Zoopagomycota; Entomophthoromycotina; Entomophthoromycetes; Entomophthorales; Ancylistaceae; Conidiobolus"]:0.12718000000000007,(OUM62108.1[&Organism="Piromyces sp. E2","Genetic Code"="Standard",Taxonomy="Eukaryota; Fungi; Fungi incertae sedis; Chytridiomycota; Chytridiomycota incertae sedis; Neocallimastigomycetes; Neocallimastigales; Neocallimastigaceae; Piromyces; unclassified Piromyces"]:0.09913999999999978,OAJ44422.1[&Organism="Batrachochytrium dendrobatidis JEL423","Genetic Code"="Standard",Taxonomy="Eukaryota; Fungi; Fungi incertae sedis; Chytridiomycota; Chytridiomycota incertae sedis; Chytridiomycetes; Rhizophydiales; Rhizophydiales incertae sedis; Batrachochytrium"]:0.17342999999999975)[&"FastTree support value"=0.501]:0.03461000000000025)[&"FastTree support value"=0.061]:0.010960000000000303,(XP_748106.1[&Organism="Aspergillus fumigatus Af293","Genetic Code"="Standard",Taxonomy="Eukaryota; Fungi; Dikarya; Ascomycota; Pezizomycotina; Eurotiomycetes; Eurotiomycetidae; Eurotiales; Aspergillaceae; Aspergillus; Aspergillus subgen. Fumigati"]:0.14656000000000002,(XP_011389257.1[&Organism="Ustilago maydis 521","Genetic Code"="Standard",Taxonomy="Eukaryota; Fungi; Dikarya; Basidiomycota; Ustilaginomycotina; Ustilaginomycetes; Ustilaginales; Ustilaginaceae; Ustilago"]:0.0858800000000004,XP_006458578.1[&Organism="Agaricus bisporus var. bisporus H97","Genetic Code"="Standard",Taxonomy="Eukaryota; Fungi; Dikarya; Basidiomycota; Agaricomycotina; Agaricomycetes; Agaricomycetidae; Agaricales; Agaricaceae; Agaricus"]:0.15537000000000045)[&"FastTree support value"=0.628]:0.02110999999999974)[&"FastTree support value"=0.222]:0.019500000000000295)[&"FastTree support value"=0.823]:0.02788999999999975)[&"FastTree support value"=0.823]:0.07679999999999998)[&"FastTree support value"=1.0]:0.15512999999999977)[&"FastTree support value"=0.823]:0.03380999999999896,((((NP_013100.1[&Organism="Saccharomyces cerevisiae S288C","Genetic Code"="Standard",Taxonomy="Eukaryota; Fungi; Dikarya; Ascomycota; Saccharomycotina; Saccharomycetes; Saccharomycetales; Saccharomycetaceae; Saccharomyces"]:0.30362,(XP_746923.1[&Organism="Aspergillus fumigatus Af293","Genetic Code"="Standard",Taxonomy="Eukaryota; Fungi; Dikarya; Ascomycota; Pezizomycotina; Eurotiomycetes; Eurotiomycetidae; Eurotiales; Aspergillaceae; Aspergillus; Aspergillus subgen. Fumigati"]:0.1753899999999997,KXN67416.1[&Organism="Conidiobolus coronatus NRRL 28638","Genetic Code"="Standard",Taxonomy="Eukaryota; Fungi; Fungi incertae sedis; Zoopagomycota; Entomophthoromycotina; Entomophthoromycetes; Entomophthorales; Ancylistaceae; Conidiobolus"]:0.22438000000000002)[&"FastTree support value"=0.626]:0.03764000000000012)[&"FastTree support

value"=0.825]:0.04756000000000027,((KNE61418.1[&Organism="Allomyces macrogynus
ATCC 38327","Genetic Code"="Standard",Taxonomy="Eukaryota; Fungi; Fungi incertae
sedis; Blastocladiomycota; Blastocladiomycota incertae sedis; Blastocladiomycetes;
Blastocladiales; Blastocladiaceae;
Allomyces"]:0.1457200000000003,KNE67543.1[&Organism="Allomyces macrogynus ATCC
38327","Genetic Code"="Standard",Taxonomy="Eukaryota; Fungi; Fungi incertae sedis;
Blastocladiomycota; Blastocladiomycota incertae sedis; Blastocladiomycetes;
Blastocladiales; Blastocladiaceae; Allomyces"]:0.1413500000000001)[&"FastTree support
value"=1.0]:0.24378999999999973,(XP_011392073.1[&Organism="Ustilago maydis
521","Genetic Code"="Standard",Taxonomy="Eukaryota; Fungi; Dikarya; Basidiomycota;
Ustilaginomycotina; Ustilaginomycetes; Ustilaginales; Ustilaginaceae;
Ustilago"]:0.1201800000000004,XP_006461708.1[&Organism="Agaricus bisporus var.
bisporus H97","Genetic Code"="Standard",Taxonomy="Eukaryota; Fungi; Dikarya;
Basidiomycota; Agaricomycotina; Agaricomycetes; Agaricomycetidae; Agaricales;
Agaricaceae; Agaricus"]:0.11559000000000008)[&"FastTree support
value"=0.941]:0.08434999999999997)[&"FastTree support
value"=0.633]:0.03047000000000022)[&"FastTree support
value"=0.996]:0.11780999999999953,(((XP_001750431.1[&Organism="Monosiga
brevicollis MX1","Genetic Code"="Standard",Taxonomy="Eukaryota; Choanoflagellata;
Craspedida; Salpingoecidae;
Monosiga"]:0.40732999999999997,((((XP_032819300.1[&Organism="Petromyzon
marinus","Genetic Code"="Standard",Taxonomy="Eukaryota; Metazoa; Chordata; Craniata;
Vertebrata; Cyclostomata; Hyperoartia; Petromyzontiformes; Petromyzontidae;
Petromyzon","Common Name"="sea
lamprey"]:0.10130000000000017,(((((XP_006168142.1[&Organism="Tupaia
chinensis","Genetic Code"="Standard",Taxonomy="Eukaryota; Metazoa; Chordata;
Craniata; Vertebrata; Euteleostomi; Mammalia; Eutheria; Euarchontoglires; Scandentia;
Tupaiidae; Tupaia","Common Name"="Chinese tree
shrew"]:0.000550,(NP_001392186.1[&Organism="Mus musculus","Genetic
Code"="Standard",Taxonomy="Eukaryota; Metazoa; Chordata; Craniata; Vertebrata;
Euteleostomi; Mammalia; Eutheria; Euarchontoglires; Glires; Rodentia; Myomorpha;
Muroidea; Muridae; Murinae; Mus; Mus","Common Name"="house
mouse"]:0.008550000000000058,NP_001317309.1[&Organism="Homo sapiens","Genetic
Code"="Standard",Taxonomy="Eukaryota; Metazoa; Chordata; Craniata; Vertebrata;
Euteleostomi; Mammalia; Eutheria; Euarchontoglires; Primates; Haplorrhini; Catarrhini;
Hominidae; Homo","Common Name"="human"]:0.0027599999999998737)[&"FastTree
support value"=0.828]:0.000550)[&"FastTree support
value"=0.916]:0.000530,(XP_014394711.1[&Organism="Myotis brandtii","Genetic
Code"="Standard",Taxonomy="Eukaryota; Metazoa; Chordata; Craniata; Vertebrata;
Euteleostomi; Mammalia; Eutheria; Laurasiatheria; Chiroptera; Microchiroptera;
Vespertilionidae; Myotis","Common Name"="Brandt's
bat"]:0.026120000000000143,(XP_012382650.2[&Organism="Dasypus
novemcinctus","Genetic Code"="Standard",Taxonomy="Eukaryota; Metazoa; Chordata;
Craniata; Vertebrata; Euteleostomi; Mammalia; Eutheria; Xenarthra; Cingulata;

Dasypodidae; Dasypus","Common Name"="nine-banded
armadillo"]:0.023490000000000233,XP_031753959.1[&Organism="Xenopus
tropicalis","Genetic Code"="Standard",Taxonomy="Eukaryota; Metazoa; Chordata;
Craniata; Vertebrata; Euteleostomi; Amphibia; Batrachia; Anura; Pipoidea; Pipidae;
Xenopodinae; Xenopus; Silurana","Common Name"="tropical clawed
frog"]:0.1543300000000003)[&"FastTree support
value"=0.959]:0.044099999999999806)[&"FastTree support
value"=0.796]:0.008869999999999933)[&"FastTree support
value"=0.971]:0.021649999999999725,XP_028602039.1[&Organism="Podarcis
muralis","Genetic Code"="Standard",Taxonomy="Eukaryota; Metazoa; Chordata; Craniata;
Vertebrata; Euteleostomi; Lepidosauria; Squamata; Bifurcata; Unidentata; Episquamata;
Laterata; Lacertibaenia; Lacertidae; Podarcis","Common Name"="Common wall
lizard"]:0.030380000000000074)[&"FastTree support
value"=0.046]:0.003770000000000273,XP_025940269.1[&Organism="Apteryx
rowi","Genetic Code"="Standard",Taxonomy="Eukaryota; Metazoa; Chordata; Craniata;
Vertebrata; Euteleostomi; Archelosauria; Archosauria; Dinosauria; Saurischia; Theropoda;
Coelurosauria; Aves; Palaeognathae; Apterygiformes; Apterygidae; Apteryx","Common
Name"="Okarito brown kiwi"]:0.024550000000000072)[&"FastTree support
value"=0.95]:0.031179999999999986,NP_957216.1[&Organism="Danio rerio","Genetic
Code"="Standard",Taxonomy="Eukaryota; Metazoa; Chordata; Craniata; Vertebrata;
Euteleostomi; Actinopterygii; Neopterygii; Teleostei; Ostariophysi; Cypriniformes;
Danionidae; Danioninae; Danio","Common
Name"="zebrafish"]:0.024459999999999926)[&"FastTree support
value"=0.96]:0.05783000000000005)[&"FastTree support
value"=0.853]:0.0339400000000003,((XP_035676386.1[&Organism="Branchiostoma
floridae","Genetic Code"="Standard",Taxonomy="Eukaryota; Metazoa; Chordata;
Cephalochordata; Leptocardii; Amphioxiformes; Branchiostomatidae;
Branchiostoma","Common Name"="Florida
lancelet"]:0.07545000000000002,(XP_006821224.1[&Organism="Saccoglossus
kowalevskii","Genetic Code"="Standard",Taxonomy="Eukaryota; Metazoa; Hemichordata;
Enteropneusta; Harrimaniidae;
Saccoglossus"]:0.08854999999999968,XP_030827871.1[&Organism="Strongylocentrotus
purpuratus","Genetic Code"="Standard",Taxonomy="Eukaryota; Metazoa; Echinodermata;
Eleutherozoa; Echinozoa; Echinoidea; Euechinoidea; Echinacea; Camarodonta; Echinidea;
Strongylocentrotidae; Strongylocentrotus","Common Name"="purple sea
urchin"]:0.08443000000000023)[&"FastTree support
value"=0.809]:0.04530000000000012)[&"FastTree support
value"=0.613]:0.03824000000000005,PAA85687.1[&Organism="Macrostomum
lignano","Genetic Code"="Standard",Taxonomy="Eukaryota; Metazoa; Spiralia;
Lophotrochozoa; Platyhelminthes; Rhabditophora; Macrostomorpha; Macrostomida;
Macrostomidae; Macrostomum"]:0.18686000000000025)[&"FastTree support
value"=0.736]:0.04933999999999994)[&"FastTree support
value"=0.791]:0.04950999999999972,NP_001259946.1[&Organism="Drosophila
melanogaster","Genetic Code"="Standard",Taxonomy="Eukaryota; Metazoa; Ecdysozoa;

Arthropoda; Hexapoda; Insecta; Pterygota; Neoptera; Endopterygota; Diptera; Brachycera; Muscomorpha; Ephydroidea; Drosophilidae; Drosophila; Sophophora","Common Name"="fruit fly"]:0.16145000000000032)[&"FastTree support value"=0.707]:0.014289999999999914,(NP_741403.2[&Organism="Caenorhabditis elegans","Genetic Code"="Standard",Taxonomy="Eukaryota; Metazoa; Ecdysozoa; Nematoda; Chromadorea; Rhabditida; Rhabditina; Rhabditomorpha; Rhabditoidea; Rhabditidae; Peloderinae; Caenorhabditis"]:0.2637100000000001,XP_002129967.2[&Organism="Ciona intestinalis","Genetic Code"="Standard",Taxonomy="Eukaryota; Metazoa; Chordata; Tunicata; Ascidiacea; Phlebobranchia; Cionidae; Ciona","Common Name"="vase tunicate"]:0.27249999999999996)[&"FastTree support value"=0.802]:0.02458999999999989)[&"FastTree support value"=0.952]:0.09132000000000007)[&"FastTree support value"=0.947]:0.06794000000000011,XP_014148015.1[&Organism="Sphaeroforma arctica JP610","Genetic Code"="Standard",Taxonomy="Eukaryota; Ichthyosporea; Ichthyophonida; Sphaeroforma"]:0.35202)[&"FastTree support value"=0.429]:0.016270000000000007,XP_004348308.1[&Organism="Capsaspora owczarzaki ATCC 30864","Genetic Code"="Standard",Taxonomy="Eukaryota; Filasterea; Capsaspora"]:0.20153)[&"FastTree support value"=0.962]:0.07087999999999983)[&"FastTree support value"=0.95]:0.04885000000000028,((CEP02405.1[&Organism="Plasmodiophora brassicae","Genetic Code"="Standard",Taxonomy="Eukaryota; Sar; Rhizaria; Endomyxa; Phytomyxea; Plasmodiophorida; Plasmodiophoridae; Plasmodiophora"]:0.26495000000000024,(PRP82407.1[&Organism="Planoprotostelium fungivorum","Genetic Code"="Standard",Taxonomy="Eukaryota; Amoebozoa; Evosea; Variosea; Cavosteliida; Cavosteliaceae; Planoprotostelium"]:0.18337000000000003,(XP_004368323.1[&Organism="Acanthamoeba castellanii str. Neff","Genetic Code"="Standard",Taxonomy="Eukaryota; Amoebozoa; Discosea; Longamoebia; Centramoebida; Acanthamoebidae; Acanthamoeba"]:0.16565999999999992,(PRP81066.1[&Organism="Planoprotostelium fungivorum","Genetic Code"="Standard",Taxonomy="Eukaryota; Amoebozoa; Evosea; Variosea; Cavosteliida; Cavosteliaceae; Planoprotostelium"]:0.22750000000000004,(XP_012754660.1[&Organism="Acytostelium subglobosum LB1","Genetic Code"="Standard",Taxonomy="Eukaryota; Amoebozoa; Evosea; Eumycetozoa; Dictyostelia; Acytosteliales; Acytosteliaceae; Acytostelium"]:0.038899999999999935,(KYR01170.1[&Organism="Tieghemostelium lacteum","Genetic Code"="Standard",Taxonomy="Eukaryota; Amoebozoa; Evosea; Eumycetozoa; Dictyostelia; Dictyosteliales; Raperosteliaceae; Tieghemostelium"]:0.0354000000000001,XP_003294436.1[&Organism="Dictyostelium purpureum","Genetic Code"="Standard",Taxonomy="Eukaryota; Amoebozoa; Evosea; Eumycetozoa; Dictyostelia; Dictyosteliales; Dictyosteliaceae; Dictyostelium"]:0.02662000000000031)[&"FastTree support value"=0.926]:0.032489999999999686)[&"FastTree support value"=0.995]:0.11380999999999997)[&"FastTree support

value"=0.727]:0.036519999999999886)[&"FastTree support
value"=0.401]:0.026629999999999993)[&"FastTree support
value"=0.866]:0.0388200000000003)[&"FastTree support
value"=0.856]:0.028089999999999726,((((PRP80825.1[&Organism="Planoprotostelium
fungivorum","Genetic Code"="Standard",Taxonomy="Eukaryota; Amoebozoa; Evosea;
Variosea; Cavosteliida; Cavosteliaceae;
Planoprotostelium"]:0.2383500000000005,((XP_004355605.1[&Organism="Cavenderia
fasciculata","Genetic Code"="Standard",Taxonomy="Eukaryota; Amoebozoa; Evosea;
Eumycetozoa; Dictyostelia; Acytosteliales; Cavenderiaceae;
Cavenderia"]:0.11452000000000018,XP_020436927.1[&Organism="Heterostelium album
PN500","Genetic Code"="Standard",Taxonomy="Eukaryota; Amoebozoa; Evosea;
Eumycetozoa; Dictyostelia; Acytosteliales; Acytosteliaceae;
Heterostelium"]:0.1034900000000003)[&"FastTree support
value"=0.975]:0.08106999999999998,(AER35077.1[&Organism="Tieghemostelium
lacteum","Genetic Code"="Standard",Taxonomy="Eukaryota; Amoebozoa; Evosea;
Eumycetozoa; Dictyostelia; Dictyosteliales; Raperosteliaceae;
Tieghemostelium"]:0.12647999999999993,(KAF2075389.1[&Organism="Polysphondylium
violaceum","Genetic Code"="Standard",Taxonomy="Eukaryota; Amoebozoa; Evosea;
Eumycetozoa; Dictyostelia; Dictyosteliales; Dictyosteliaceae;
Polysphondylium"]:0.16382999999999992,XP_003292385.1[&Organism="Dictyostelium
purpureum","Genetic Code"="Standard",Taxonomy="Eukaryota; Amoebozoa; Evosea;
Eumycetozoa; Dictyostelia; Dictyosteliales; Dictyosteliaceae;
Dictyostelium"]:0.17550000000000043)[&"FastTree support
value"=0.385]:0.033360000000000056)[&"FastTree support
value"=0.775]:0.021329999999999985)[&"FastTree support
value"=0.998]:0.16298000000000012)[&"FastTree support
value"=0.989]:0.13333999999999957,((KOO24608.1[&Organism="Chrysochromulina
tobinii","Genetic Code"="Standard",Taxonomy="Eukaryota; Haptista; Haptophyta;
Prymnesiophyceae; Prymnesiales; Chrysochromulinaceae;
Chrysochromulina"]:0.3957900000000003,((XP_004184473.1[&Organism="Entamoeba
invadens IP1","Genetic Code"="Standard",Taxonomy="Eukaryota; Amoebozoa; Evosea;
Archamoebae; Mastigamoebida; Entamoebidae;
Entamoeba"]:0.06332000000000004,EMS16943.1[&Organism="Entamoeba histolytica
HM-3:IMSS","Genetic Code"="Standard",Taxonomy="Eukaryota; Amoebozoa; Evosea;
Archamoebae; Mastigamoebida; Entamoebidae;
Entamoeba"]:0.07438999999999973)[&"FastTree support
value"=1.0]:0.27093999999999996,((XP_008860500.1[&Organism="Entamoeba nuttalli
P19","Genetic Code"="Standard",Taxonomy="Eukaryota; Amoebozoa; Evosea;
Archamoebae; Mastigamoebida; Entamoebidae;
Entamoeba"]:0.06644000000000005,XP_004185630.1[&Organism="Entamoeba invadens
IP1","Genetic Code"="Standard",Taxonomy="Eukaryota; Amoebozoa; Evosea;
Archamoebae; Mastigamoebida; Entamoebidae;
Entamoeba"]:0.10976000000000008)[&"FastTree support
value"=1.0]:0.29849000000000014,((KAJ9467322.1[&Organism="Diplonema

papillatum","Genetic Code"="Standard",Taxonomy="Eukaryota; Discoba; Euglenozoa; Diplonemea; Diplonemidae;
Diplonema"]:0.2753700000000001,KAJ9466576.1[&Organism="Diplonema papillatum","Genetic Code"="Standard",Taxonomy="Eukaryota; Discoba; Euglenozoa; Diplonemea; Diplonemidae; Diplonema"]:0.17383000000000015)[&"FastTree support value"=0.999]:0.25708999999999998,(XP_005788601.1[&Organism="Emiliania huxleyi CCMP1516","Genetic Code"="Standard",Taxonomy="Eukaryota; Haptista; Haptophyta; Prymnesiophyceae; Isochrysidales; Noelaerhabdaceae;
Emiliania"]:0.6382100000000004,(XP_042924642.1[&Organism="Chlamydomonas reinhardtii","Genetic Code"="Standard",Taxonomy="Eukaryota; Viridiplantae; Chlorophyta; core chlorophytes; Chlorophyceae; CS clade; Chlamydomonadales; Chlamydomonadaceae;
Chlamydomonas"]:0.38491999999999997,(PTQ29980.1[&Organism="Marchantia polymorpha","Genetic Code"="Standard",Taxonomy="Eukaryota; Viridiplantae; Streptophyta; Embryophyta; Marchantiophyta; Marchantiopsida; Marchantiidae; Marchantiales; Marchantiaceae; Marchantia","Common
Name"="liverwort"]:0.07580999999999971,((((XP_002299468.1[&Organism="Populus trichocarpa","Genetic Code"="Standard",Taxonomy="Eukaryota; Viridiplantae; Streptophyta; Embryophyta; Tracheophyta; Spermatophyta; Magnoliopsida; eudicotyledons; Gunneridae; Pentapetalae; rosids; fabids; Malpighiales; Salicaceae; Saliceae; Populus","Common Name"="Populus balsamifera subsp.
trichocarpa"]:0.03943999999999992,NP_001190448.1[&Organism="Arabidopsis thaliana","Genetic Code"="Standard",Taxonomy="Eukaryota; Viridiplantae; Streptophyta; Embryophyta; Tracheophyta; Spermatophyta; Magnoliopsida; eudicotyledons; Gunneridae; Pentapetalae; rosids; malvids; Brassicales; Brassicaceae; Camelineae; Arabidopsis","Common Name"="thale cress"]:0.07143999999999995)[&"FastTree support value"=0.917]:0.025650000000000173,AQK88296.1:0.09719999999999995)[&"FastTree support value"=0.192]:0.012980000000000214,XP_002302631.1[&Organism="Populus trichocarpa","Genetic Code"="Standard",Taxonomy="Eukaryota; Viridiplantae; Streptophyta; Embryophyta; Tracheophyta; Spermatophyta; Magnoliopsida; eudicotyledons; Gunneridae; Pentapetalae; rosids; fabids; Malpighiales; Salicaceae; Saliceae; Populus","Common Name"="Populus balsamifera subsp.
trichocarpa"]:0.03725000000000023)[&"FastTree support
value"=0.999]:0.08665999999999974,((KAG0556007.1[&Organism="Ceratodon purpureus","Genetic Code"="Standard",Taxonomy="Eukaryota; Viridiplantae; Streptophyta; Embryophyta; Bryophyta; Bryophytina; Bryopsida; Dicranidae; Pseudoditrichales; Ditrichaceae; Ceratodon"]:0.09479000000000015,(((((KAI5602084.1[&Organism="Populus trichocarpa","Genetic Code"="Standard",Taxonomy="Eukaryota; Viridiplantae; Streptophyta; Embryophyta; Tracheophyta; Spermatophyta; Magnoliopsida; eudicotyledons; Gunneridae; Pentapetalae; rosids; fabids; Malpighiales; Salicaceae; Saliceae; Populus","Common Name"="Populus balsamifera subsp.
trichocarpa"]:0.0708000000000002,((PWZ36850.1[&Organism="Zea mays","Genetic Code"="Standard",Taxonomy="Eukaryota; Viridiplantae; Streptophyta; Embryophyta; Tracheophyta; Spermatophyta; Magnoliopsida; Liliopsida; Poales; Poaceae; PACMAD

clade; Panicoideae; Andropogonodae; Andropogoneae; Tripsacinae;
Zea"]:0.10711000000000004,(AAF79238.1[&Organism="Arabidopsis thaliana","Genetic
Code"="Standard",Taxonomy="Eukaryota; Viridiplantae; Streptophyta; Embryophyta;
Tracheophyta; Spermatophyta; Magnoliopsida; eudicotyledons; Gunneridae;
Pentapetalae; rosids; malvids; Brassicales; Brassicaceae; Camelineae;
Arabidopsis","Common Name"="thale
cress"]:0.07098000000000004,(XP_002315854.1[&Organism="Populus
trichocarpa","Genetic Code"="Standard",Taxonomy="Eukaryota; Viridiplantae;
Streptophyta; Embryophyta; Tracheophyta; Spermatophyta; Magnoliopsida;
eudicotyledons; Gunneridae; Pentapetalae; rosids; fabids; Malpighiales; Salicaceae;
Saliceae; Populus","Common Name"="Populus balsamifera subsp.
trichocarpa"]:0.03885000000000005,NP_001147100.1[&Organism="Zea mays","Genetic
Code"="Standard",Taxonomy="Eukaryota; Viridiplantae; Streptophyta; Embryophyta;
Tracheophyta; Spermatophyta; Magnoliopsida; Liliopsida; Poales; Poaceae; PACMAD
clade; Panicoideae; Andropogonodae; Andropogoneae; Tripsacinae;
Zea"]:0.06677)[&"FastTree support value"=0.103]:0.01711000000000018)[&"FastTree
support value"=0.923]:0.03467999999999982)[&"FastTree support
value"=0.311]:0.008589999999999876,(XP_006375094.1[&Organism="Populus
trichocarpa","Genetic Code"="Standard",Taxonomy="Eukaryota; Viridiplantae;
Streptophyta; Embryophyta; Tracheophyta; Spermatophyta; Magnoliopsida;
eudicotyledons; Gunneridae; Pentapetalae; rosids; fabids; Malpighiales; Salicaceae;
Saliceae; Populus","Common Name"="Populus balsamifera subsp.
trichocarpa"]:0.04144000000000014,(NP_850420.1[&Organism="Arabidopsis
thaliana","Genetic Code"="Standard",Taxonomy="Eukaryota; Viridiplantae; Streptophyta;
Embryophyta; Tracheophyta; Spermatophyta; Magnoliopsida; eudicotyledons;
Gunneridae; Pentapetalae; rosids; malvids; Brassicales; Brassicaceae; Camelineae;
Arabidopsis","Common Name"="thale
cress"]:0.0908500000000001,AAF22292.1[&Organism="Arabidopsis thaliana","Genetic
Code"="Standard",Taxonomy="Eukaryota; Viridiplantae; Streptophyta; Embryophyta;
Tracheophyta; Spermatophyta; Magnoliopsida; eudicotyledons; Gunneridae;
Pentapetalae; rosids; malvids; Brassicales; Brassicaceae; Camelineae;
Arabidopsis","Common Name"="thale cress"]:0.06793000000000005)[&"FastTree support
value"=0.871]:0.026009999999999867)[&"FastTree support
value"=0.662]:0.008669999999999956)[&"FastTree support
value"=0.858]:0.01880000000000015)[&"FastTree support
value"=0.922]:0.02436000000000016,KAH9306600.1[&Organism="Taxus
chinensis","Genetic Code"="Standard",Taxonomy="Eukaryota; Viridiplantae; Streptophyta;
Embryophyta; Tracheophyta; Spermatophyta; Pinopsida; Pinidae; Conifers II; Cupressales;
Taxaceae; Taxus"]:0.05803000000000026)[&"FastTree support
value"=0.736]:0.015149999999999775,(EFJ23099.1[&Organism="Selaginella
moellendorffii","Genetic Code"="Standard",Taxonomy="Eukaryota; Viridiplantae;
Streptophyta; Embryophyta; Tracheophyta; Lycopodiopsida; Selaginellales;
Selaginellaceae;
Selaginella"]:0.1517999999999997,(XP_002987566.1[&Organism="Selaginella

moellendorffii","Genetic Code"="Standard",Taxonomy="Eukaryota; Viridiplantae; Streptophyta; Embryophyta; Tracheophyta; Lycopodiopsida; Selaginellales; Selaginellaceae; Selaginella"]:0.05451999999999968,EFJ15761.1[&Organism="Selaginella moellendorffii","Genetic Code"="Standard",Taxonomy="Eukaryota; Viridiplantae; Streptophyta; Embryophyta; Tracheophyta; Lycopodiopsida; Selaginellales; Selaginellaceae; Selaginella"]:0.09214999999999973)[&"FastTree support value"=0.739]:0.004480000000000395)[&"FastTree support value"=0.949]:0.02537000000000115)[&"FastTree support value"=0.875]:0.018540000000000223,KAI5058380.1[&Organism="Adiantum capillus-veneris","Genetic Code"="Standard",Taxonomy="Eukaryota; Viridiplantae; Streptophyta; Embryophyta; Tracheophyta; Polypodiopsida; Polypodiidae; Polypodiales; Pteridineae; Pteridaceae; Vittarioideae; Adiantum"]:0.0754999999999999)[&"FastTree support value"=0.196]:0.012279999999999625,KAI5072318.1[&Organism="Adiantum capillus-veneris","Genetic Code"="Standard",Taxonomy="Eukaryota; Viridiplantae; Streptophyta; Embryophyta; Tracheophyta; Polypodiopsida; Polypodiidae; Polypodiales; Pteridineae; Pteridaceae; Vittarioideae; Adiantum"]:0.04767999999999972)[&"FastTree support value"=0.745]:0.011900000000000244)[&"FastTree support value"=0.228]:0.02073999999999998,PTQ45603.1[&Organism="Marchantia polymorpha","Genetic Code"="Standard",Taxonomy="Eukaryota; Viridiplantae; Streptophyta; Embryophyta; Marchantiophyta; Marchantiopsida; Marchantiidae; Marchantiales; Marchantiaceae; Marchantia","Common Name"="liverwort"]:0.05561000000000016)[&"FastTree support value"=0.563]:0.02381000000000011)[&"FastTree support value"=0.887]:0.04257)[&"FastTree support value"=0.999]:0.2385799999999998)[&"FastTree support value"=0.824]:0.09634000000000054)[&"FastTree support value"=0.716]:0.0707399999999998)[&"FastTree support value"=0.513]:0.055760000000000254)[&"FastTree support value"=0.596]:0.045869999999999855)[&"FastTree support value"=0.898]:0.061670000000000336)[&"FastTree support value"=0.794]:0.027639999999999887,((ABB13595.1[&Organism="Tetrahymena thermophila","Genetic Code"="Ciliate",Taxonomy="Eukaryota; Sar; Alveolata; Ciliophora; Intramacronucleata; Oligohymenophorea; Hymenostomatida; Tetrahymenina; Tetrahymenidae; Tetrahymena"]:0.16249999999999964,XP_001009829.2[&Organism="Tetrahymena thermophila SB210","Genetic Code"="Ciliate",Taxonomy="Eukaryota; Sar; Alveolata; Ciliophora; Intramacronucleata; Oligohymenophorea; Hymenostomatida; Tetrahymenina; Tetrahymenidae; Tetrahymena"]:0.22879000000000005)[&"FastTree support value"=1.0]:0.3732800000000003,(((XP_002371703.1[&Organism="Toxoplasma gondii ME49","Genetic Code"="Standard",Taxonomy="Eukaryota; Sar; Alveolata; Apicomplexa; Conoidasida; Coccidia; Eucoccidiorida; Eimeriorina; Sarcocystidae; Toxoplasma"]:0.17969999999999997,(XP_028867889.1[&Organism="Babesia ovata","Genetic Code"="Standard",Taxonomy="Eukaryota; Sar; Alveolata; Apicomplexa; Aconoidasida; Piroplasmida; Babesiidae;

Babesia"]:0.32657999999999987,('XP_019914840.1'[&Organism="Plasmodium coatneyi","Genetic Code"="Standard",Taxonomy="Eukaryota; Sar; Alveolata; Apicomplexa; Aconoidasida; Haemosporida; Plasmodiidae; Plasmodium"]:0.04422000000000015,('XP_012763851.2_1'[&Organism="Plasmodium reichenowi","Genetic Code"="Standard",Taxonomy="Eukaryota; Sar; Alveolata; Apicomplexa; Aconoidasida; Haemosporida; Plasmodiidae; Plasmodium; Plasmodium (Laverania)"]:0.000530,'EUR69800.1_1'[&Organism="Plasmodium falciparum 7G8","Genetic Code"="Standard",Taxonomy="Eukaryota; Sar; Alveolata; Apicomplexa; Aconoidasida; Haemosporida; Plasmodiidae; Plasmodium; Plasmodium (Laverania)"]:0.003089999999999815)[&"FastTree support value"=0.965]:0.05113000000000012)[&"FastTree support value"=0.995]:0.14936999999999978)[&"FastTree support value"=0.896]:0.06799)[&"FastTree support value"=0.924]:0.0631999999999997,('CAE8701890.1'[&Organism="Polarella glacialis","Genetic Code"="Standard",Taxonomy="Eukaryota; Sar; Alveolata; Dinophyceae; Suessiales; Suessiaceae; Polarella"]:0.31191999999999975,((XP_001032891.1[&Organism="Tetrahymena thermophila SB210","Genetic Code"="Ciliate",Taxonomy="Eukaryota; Sar; Alveolata; Ciliophora; Intramacronucleata; Oligohymenophorea; Hymenostomatida; Tetrahymenina; Tetrahymenidae; Tetrahymena"]:0.2164900000000003,(XP_001029982.1[&Organism="Tetrahymena thermophila SB210","Genetic Code"="Ciliate",Taxonomy="Eukaryota; Sar; Alveolata; Ciliophora; Intramacronucleata; Oligohymenophorea; Hymenostomatida; Tetrahymenina; Tetrahymenidae; Tetrahymena"]:0.15904000000000007,XP_001029985.2[&Organism="Tetrahymena thermophila SB210","Genetic Code"="Ciliate",Taxonomy="Eukaryota; Sar; Alveolata; Ciliophora; Intramacronucleata; Oligohymenophorea; Hymenostomatida; Tetrahymenina; Tetrahymenidae; Tetrahymena"]:0.16249000000000002)[&"FastTree support value"=1.0]:0.52515)[&"FastTree support value"=0.946]:0.12084999999999999,(XP_001016567.2[&Organism="Tetrahymena thermophila SB210","Genetic Code"="Ciliate",Taxonomy="Eukaryota; Sar; Alveolata; Ciliophora; Intramacronucleata; Oligohymenophorea; Hymenostomatida; Tetrahymenina; Tetrahymenidae; Tetrahymena"]:0.27174999999999994,'CAD8140652.1_1'[&Organism="Paramecium pentaurelia","Genetic Code"="Ciliate",Taxonomy="Eukaryota; Sar; Alveolata; Ciliophora; Intramacronucleata; Oligohymenophorea; Peniculida; Parameciidae; Paramecium"]:0.3664200000000002)[&"FastTree support value"=0.979]:0.11900000000000022)[&"FastTree support value"=0.819]:0.05239999999999956)[&"FastTree support value"=0.856]:0.03838000000000008)[&"FastTree support value"=1.0]:0.2581800000000003,((((KAG8471152.1[&Organism="Diacronema lutheri","Genetic Code"="Standard",Taxonomy="Eukaryota; Haptista; Haptophyta; Pavlovales; Pavlovaceae; Diacronema"]:0.32535999999999987,(KOO34643.1[&Organism="Chrysochromulina

tobinii","Genetic Code"="Standard",Taxonomy="Eukaryota; Haptista; Haptophyta; Prymnesiophyceae; Prymnesiales; Chrysochromulinaceae; Chrysochromulina"]:0.21397999999999984,XP_005775544.1[&Organism="Emiliania huxleyi CCMP1516","Genetic Code"="Standard",Taxonomy="Eukaryota; Haptista; Haptophyta; Prymnesiophyceae; Isochrysidales; Noelaerhabdaceae; Emiliania"]:0.22355999999999998)[&"FastTree support value"=0.996]:0.30123999999999995)[&"FastTree support value"=0.982]:0.28155999999999998,((((ARF08414.1[&Organism="Catovirus CTV1","Genetic Code"="Standard",Taxonomy="Viruses; Varidnaviria; Bamfordvirae; Nucleocytoviricota; Megaviricetes; Imitervirales; Mimiviridae; Klosneuvirinae; Catovirus"]:0.28374999999999995,AYV78912.1[&Organism="Edafosvirus sp.","Genetic Code"="Standard",Taxonomy="Viruses; Varidnaviria; Bamfordvirae; Nucleocytoviricota; Megaviricetes; Imitervirales; Mimiviridae","Common Name"="soil metagenome"]:0.34341)[&"FastTree support value"=0.986]:0.16532999999999998,((QKF94243.1[&Organism="Fadolivirus 1","Genetic Code"="Standard",Taxonomy="Viruses; unclassified viruses; unclassified DNA viruses"]:0.1109,ARF11508.1[&Organism="Klosneuvirus KNV1","Genetic Code"="Standard",Taxonomy="Viruses; Varidnaviria; Bamfordvirae; Nucleocytoviricota; Megaviricetes; Imitervirales; Mimiviridae; Klosneuvirinae; Klosneuvirus"]:0.10428000000000015)[&"FastTree support value"=0.95]:0.06780000000000008,(ARF09562.1[&Organism="Indivirus ILV1","Genetic Code"="Standard",Taxonomy="Viruses; Varidnaviria; Bamfordvirae; Nucleocytoviricota; Megaviricetes; Imitervirales; Mimiviridae; Klosneuvirinae; Indivirus"]:0.1423700000000001,AYV76902.1[&Organism="Barrevirus sp.","Genetic Code"="Standard",Taxonomy="Viruses; Varidnaviria; Bamfordvirae; Nucleocytoviricota; Megaviricetes; Imitervirales; Mimiviridae","Common Name"="soil metagenome"]:0.19146000000000019)[&"FastTree support value"=0.964]:0.07935999999999988)[&"FastTree support value"=0.043]:0.05688000000000004)[&"FastTree support value"=0.512]:0.06221999999999994,ARF10282.1[&Organism="Hokovirus HKV1","Genetic Code"="Standard",Taxonomy="Viruses; Varidnaviria; Bamfordvirae; Nucleocytoviricota; Megaviricetes; Imitervirales; Mimiviridae; Klosneuvirinae; Hokovirus"]:0.22999999999999998)[&"FastTree support value"=1.0]:0.3020200000000002,(QFG74079.1[&Organism="Megaviridae environmental sample","Genetic Code"="Standard",Taxonomy="Viruses; Varidnaviria; Bamfordvirae; Nucleocytoviricota; Megaviricetes; Imitervirales; Mimiviridae; environmental samples","Common Name"="marine metagenome"]:0.8462100000000001,(ATZ81043.1[&Organism="Bodo saltans virus","Genetic Code"="Standard",Taxonomy="Viruses; Varidnaviria; Bamfordvirae; Nucleocytoviricota; Megaviricetes; Imitervirales; Mimiviridae; Klosneuvirinae; Klosneuvirus"]:0.4755099999999999,VBB18790.1[&Organism="Yasminevirus sp. GU-2018","Genetic Code"="Standard",Taxonomy="Viruses"]:0.6141899999999998)[&"FastTree support value"=0.664]:0.06628999999999996)[&"FastTree support value"=0.162]:0.02918000000000206)[&"FastTree support

value"=0.846]:0.08300000000000018)[&"FastTree support value"=0.869]:0.15155999999999992,(AYV75702.1[&Organism="Terrestrivirus sp.","Genetic Code"="Standard",Taxonomy="Viruses; Varidnaviria; Bamfordvirae; Nucleocytoviricota; Megaviricetes; Imitervirales; Mimiviridae","Common Name"="soil metagenome"]:1.14283,ATZ80405.1[&Organism="Bodo saltans virus","Genetic Code"="Standard",Taxonomy="Viruses; Varidnaviria; Bamfordvirae; Nucleocytoviricota; Megaviricetes; Imitervirales; Mimiviridae; Klosneuvirinae; Klosneuvirus"]:1.20649)[&"FastTree support value"=0.853]:0.19586000000000015)[&"FastTree support value"=0.997]:0.33088000000000006,(((((GBG30247.1[&Organism="Hondaea fermentalgiana","Genetic Code"="Standard",Taxonomy="Eukaryota; Sar; Stramenopiles; Bigyra; Labyrinthulomycetes; Thraustochytrida; Thraustochytriaceae; Hondaea"]:1.10732,(CEM15039.1[&Organism="Vitrella brassicaformis CCMP3155","Genetic Code"="Standard",Taxonomy="Eukaryota; Sar; Alveolata; Colpodellida; Vitrellaceae; Vitrella"]:0.7524000000000002,((CAD7955538.1[&Organism="Amoebophrya sp. A120","Genetic Code"="Standard",Taxonomy="Eukaryota; Sar; Alveolata; Dinophyceae; Syndiniales; Amoebophryaceae; Amoebophrya"]:0.11782000000000004,CAD7971727.1[&Organism="Amoebophrya sp. A25","Genetic Code"="Standard",Taxonomy="Eukaryota; Sar; Alveolata; Dinophyceae; Syndiniales; Amoebophryaceae; Amoebophrya"]:0.11936999999999998)[&"FastTree support value"=0.999]:0.41923999999999984,(((KAI0562045.1[&Organism="Gracilaria domingensis","Genetic Code"="Standard",Taxonomy="Eukaryota; Rhodophyta; Florideophyceae; Rhodymeniophycidae; Gracilariales; Gracilariaceae; Gracilaria"]:0.21760000000000002,KAI0559778.1[&Organism="Gracilaria domingensis","Genetic Code"="Standard",Taxonomy="Eukaryota; Rhodophyta; Florideophyceae; Rhodymeniophycidae; Gracilariales; Gracilariaceae; Gracilaria"]:0.63361)[&"FastTree support value"=0.969]:0.17691999999999997,(KAI0557988.1[&Organism="Gracilaria domingensis","Genetic Code"="Standard",Taxonomy="Eukaryota; Rhodophyta; Florideophyceae; Rhodymeniophycidae; Gracilariales; Gracilariaceae; Gracilaria"]:0.23843000000000014,XP_005716602.1[&Organism="Chondrus crispus","Genetic Code"="Standard",Taxonomy="Eukaryota; Rhodophyta; Florideophyceae; Rhodymeniophycidae; Gigartinales; Gigartinaceae; Chondrus","Common Name"="carragheen"]:0.52128)[&"FastTree support value"=0.925]:0.1282700000000001)[&"FastTree support value"=0.992]:0.25992000000000015,(CAE6914669.1[&Organism="Symbiodinium sp. CCMP2592","Genetic Code"="Standard",Taxonomy="Eukaryota; Sar; Alveolata; Dinophyceae; Suessiales; Symbiodiniaceae; Symbiodinium"]:0.19754000000000005,(CAI4004018.1[&Organism="Cladocopium goreaui","Genetic Code"="Standard",Taxonomy="Eukaryota; Sar; Alveolata; Dinophyceae; Suessiales; Symbiodiniaceae; Cladocopium"]:0.0983900000000002,(((CAE7315868.1[&Organism="Symbiodinium natans","Genetic Code"="Standard",Taxonomy="Eukaryota; Sar; Alveolata; Dinophyceae;

Suessiales; Symbiodiniaceae;
Symbiodinium"]:0.12995,CAE8582582.1[&Organism="Polarella glacialis","Genetic Code"="Standard",Taxonomy="Eukaryota; Sar; Alveolata; Dinophyceae; Suessiales; Suessiaceae; Polarella"]:0.18150000000000022)[&"FastTree support value"=0.937]:0.0488900000000001,OLP81297.1[&Organism="Symbiodinium microadriaticum","Genetic Code"="Standard",Taxonomy="Eukaryota; Sar; Alveolata; Dinophyceae; Suessiales; Symbiodiniaceae;
Symbiodinium"]:0.12075000000000014)[&"FastTree support value"=0.936]:0.05211999999999944,CAI3978736.1[&Organism="Cladocopium goreaui","Genetic Code"="Standard",Taxonomy="Eukaryota; Sar; Alveolata; Dinophyceae; Suessiales; Symbiodiniaceae; Cladocopium"]:0.08116000000000012)[&"FastTree support value"=0.979]:0.07867999999999986)[&"FastTree support value"=0.885]:0.08106999999999998)[&"FastTree support value"=1.0]:0.45042999999999999)[&"FastTree support value"=0.918]:0.12417999999999996)[&"FastTree support value"=0.984]:0.21456000000000008)[&"FastTree support value"=0.845]:0.12354999999999983)[&"FastTree support value"=0.936]:0.21606000000000014,((PSC76263.1[&Organism="Micractinium conductrix","Genetic Code"="Standard",Taxonomy="Eukaryota; Viridiplantae; Chlorophyta; core chlorophytes; Trebouxiophyceae; Chlorellales; Chlorellaceae; Chlorella clade; Micractinium"]:0.2732000000000001,(PRW56740.1[&Organism="Chlorella sorokiniana","Genetic Code"="Standard",Taxonomy="Eukaryota; Viridiplantae; Chlorophyta; core chlorophytes; Trebouxiophyceae; Chlorellales; Chlorellaceae; Chlorella clade; Chlorella"]:0.38899000000000017,XP_005849062.1[&Organism="Chlorella variabilis","Genetic Code"="Standard",Taxonomy="Eukaryota; Viridiplantae; Chlorophyta; core chlorophytes; Trebouxiophyceae; Chlorellales; Chlorellaceae; Chlorella clade; Chlorella"]:0.28844000000000003)[&"FastTree support value"=0.656]:0.07525000000000004)[&"FastTree support value"=0.997]:0.33306000000000013,((GMH36208.1[&Organism="Bryopsis sp. KO-2023","Genetic Code"="Standard",Taxonomy="Eukaryota; Viridiplantae; Chlorophyta; Ulvophyceae; TCBD clade; Bryopsidales; Bryopsidineae; Bryopsidaceae;
Bryopsis"]:0.30789,GMH43921.1[&Organism="Bryopsis sp. KO-2023","Genetic Code"="Standard",Taxonomy="Eukaryota; Viridiplantae; Chlorophyta; Ulvophyceae; TCBD clade; Bryopsidales; Bryopsidineae; Bryopsidaceae;
Bryopsis"]:0.4081999999999999)[&"FastTree support value"=1.0]:0.38656999999999986,(CAG9460856.1[&Organism="Pedinophyceae sp. YPF-701","Genetic Code"="Standard",Taxonomy="Eukaryota; Viridiplantae; Chlorophyta; Pedinophyceae"]:0.65998,((GJP35534.1[&Organism="Closterium sp. NIES-68","Genetic Code"="Standard",Taxonomy="Eukaryota; Viridiplantae; Streptophyta; Zygnemophyceae; Zygnematophycidae; Desmidiales; Closteriaceae; Closterium; Closterium peracerosum-strigosum-littorale
complex"]:0.10535000000000005,CAI5480041.1[&Organism="Closterium sp. Yama58-4","Genetic Code"="Standard",Taxonomy="Eukaryota; Viridiplantae; Streptophyta; Zygnemophyceae; Zygnematophycidae; Desmidiales; Closteriaceae; Closterium;

Closterium peracerosum-strigosum-littorale complex"]:0.07355)[&"FastTree support value"=0.988]:0.18855999999999984,((KAJ7294545.1[&Organism="Diphasiastrum complanatum","Genetic Code"="Standard",Taxonomy="Eukaryota; Viridiplantae; Streptophyta; Embryophyta; Tracheophyta; Lycopodiopsida; Lycopodiales; Lycopodiaceae; Lycopodioideae;
Diphasiastrum"]:0.20962000000000014,((XP_024380180.1[&Organism="Physcomitrium patens","Genetic Code"="Standard",Taxonomy="Eukaryota; Viridiplantae; Streptophyta; Embryophyta; Bryophyta; Bryophytina; Bryopsida; Funariidae; Funariales; Funariaceae; Physcomitrium"]:0.010460000000000136,XP_024367947.1[&Organism="Physcomitrium patens","Genetic Code"="Standard",Taxonomy="Eukaryota; Viridiplantae; Streptophyta; Embryophyta; Bryophyta; Bryophytina; Bryopsida; Funariidae; Funariales; Funariaceae; Physcomitrium"]:0.006079999999999863)[&"FastTree support value"=1.0]:0.17345999999999995,(KAG0619429.1[&Organism="Ceratodon purpureus","Genetic Code"="Standard",Taxonomy="Eukaryota; Viridiplantae; Streptophyta; Embryophyta; Bryophyta; Bryophytina; Bryopsida; Dicranidae; Pseudoditrichales; Ditrichaceae;
Ceratodon"]:0.05869999999999974,KAG0561847.1[&Organism="Ceratodon purpureus","Genetic Code"="Standard",Taxonomy="Eukaryota; Viridiplantae; Streptophyta; Embryophyta; Bryophyta; Bryophytina; Bryopsida; Dicranidae; Pseudoditrichales; Ditrichaceae; Ceratodon"]:0.09913999999999978)[&"FastTree support value"=0.883]:0.04718)[&"FastTree support value"=0.926]:0.07041999999999993)[&"FastTree support value"=0.96]:0.08659000000000017,(EFJ22917.1:0.40147999999999984,(((KAF8079489.1[&Organism="Sinapis alba","Genetic Code"="Standard",Taxonomy="Eukaryota; Viridiplantae; Streptophyta; Embryophyta; Tracheophyta; Spermatophyta; Magnoliopsida; eudicotyledons; Gunneridae; Pentapetalae; rosids; malvids; Brassicales; Brassicaceae; Brassiceae; Sinapis","Common Name"="white mustard"]:0.02635999999999994,(OAP19580.1:0.08923000000000014,(OAP13353.1:0.07311999999999985,OAP13972.1:0.03117999999999986)[&"FastTree support value"=0.229]:0.001040000000000152)[&"FastTree support value"=0.967]:0.11460999999999988)[&"FastTree support value"=0.987]:0.15702000000000016,(((KAF8391993.1[&Organism="Tetracentron sinense","Genetic Code"="Standard",Taxonomy="Eukaryota; Viridiplantae; Streptophyta; Embryophyta; Tracheophyta; Spermatophyta; Magnoliopsida; Trochodendrales; Trochodendraceae;
Tetracentron"]:0.0632600000000001,XP_058079501.1[&Organism="Magnolia sinica","Genetic Code"="Standard",Taxonomy="Eukaryota; Viridiplantae; Streptophyta; Embryophyta; Tracheophyta; Spermatophyta; Magnoliopsida; Magnoliidae; Magnoliales; Magnoliaceae; Magnolia"]:0.10823999999999989)[&"FastTree support value"=0.55]:0.02461000000000002,(((KAF5727250.1[&Organism="Tripterygium wilfordii","Genetic Code"="Standard",Taxonomy="Eukaryota; Viridiplantae; Streptophyta; Embryophyta; Tracheophyta; Spermatophyta; Magnoliopsida; eudicotyledons; Gunneridae; Pentapetalae; rosids; fabids; Celastrales; Celastraceae; Tripterygium"]:0.09540999999999977,XP_002303204.3[&Organism="Populus

trichocarpa","Genetic Code"="Standard",Taxonomy="Eukaryota; Viridiplantae; Streptophyta; Embryophyta; Tracheophyta; Spermatophyta; Magnoliopsida; eudicotyledons; Gunneridae; Pentapetalae; rosids; fabids; Malpighiales; Salicaceae; Saliceae; Populus","Common Name"="Populus balsamifera subsp. trichocarpa"]:0.06084999999999985)[&"FastTree support value"=0.142]:0.0011199999999997878,(XP_002297993.1:0.1127600000000002,XP_024439231.1:0.11716000000000015)[&"FastTree support value"=0.918]:0.03705999999999987)[&"FastTree support value"=0.924]:0.027390000000000025,(KAK1401877.1[&Organism="Heracleum sosnowskyi","Genetic Code"="Standard",Taxonomy="Eukaryota; Viridiplantae; Streptophyta; Embryophyta; Tracheophyta; Spermatophyta; Magnoliopsida; eudicotyledons; Gunneridae; Pentapetalae; asterids; campanulids; Apiales; Apiaceae; Apioideae; apioid superclade; Tordylieae; Tordyliinae; Heracleum"]:0.09753999999999996,KAH0683503.1[&Organism="Solanum tuberosum","Genetic Code"="Standard",Taxonomy="Eukaryota; Viridiplantae; Streptophyta; Embryophyta; Tracheophyta; Spermatophyta; Magnoliopsida; eudicotyledons; Gunneridae; Pentapetalae; asterids; lamiids; Solanales; Solanaceae; Solanoideae; Solaneae; Solanum","Common Name"="potato"]:0.16480000000000006)[&"FastTree support value"=0.585]:0.012010000000000076)[&"FastTree support value"=0.837]:0.019620000000000193)[&"FastTree support value"=0.316]:0.026009999999999867,(XP_038984915.1[&Organism="Phoenix dactylifera","Genetic Code"="Standard",Taxonomy="Eukaryota; Viridiplantae; Streptophyta; Embryophyta; Tracheophyta; Spermatophyta; Magnoliopsida; Liliopsida; Arecaceae; Coryphoideae; Phoeniceae; Phoenix","Common Name"="date palm"]:0.09492000000000012,(PWZ56863.1[&Organism="Zea mays","Genetic Code"="Standard",Taxonomy="Eukaryota; Viridiplantae; Streptophyta; Embryophyta; Tracheophyta; Spermatophyta; Magnoliopsida; Liliopsida; Poales; Poaceae; PACMAD clade; Panicoideae; Andropogonodae; Andropogoneae; Tripsacinae; Zea"]:0.04206999999999983,PWZ56864.1[&Organism="Zea mays","Genetic Code"="Standard",Taxonomy="Eukaryota; Viridiplantae; Streptophyta; Embryophyta; Tracheophyta; Spermatophyta; Magnoliopsida; Liliopsida; Poales; Poaceae; PACMAD clade; Panicoideae; Andropogonodae; Andropogoneae; Tripsacinae; Zea"]:0.03878000000000004)[&"FastTree support value"=0.999]:0.08811999999999998)[&"FastTree support value"=0.94]:0.05061999999999989)[&"FastTree support value"=0.845]:0.040309999999999846)[&"FastTree support value"=0.99]:0.09423000000000004,((KAH9320939.1[&Organism="Taxus chinensis","Genetic Code"="Standard",Taxonomy="Eukaryota; Viridiplantae; Streptophyta; Embryophyta; Tracheophyta; Spermatophyta; Pinopsida; Pinidae; Conifers II; Cupressales; Taxaceae; Taxus"]:0.2209699999999999,(KAH9325151.1:0.10380000000000011,(KAH9300179.1:0.1217000000000014,KAH9314974.1:0.2149000000000001)[&"FastTree support value"=0.767]:0.08804999999999996)[&"FastTree support value"=0.998]:0.25303999999999993)[&"FastTree support

value"=0.794]:0.03893000000000013,(KAH9290598.1[&Organism="Taxus chinensis","Genetic Code"="Standard",Taxonomy="Eukaryota; Viridiplantae; Streptophyta; Embryophyta; Tracheophyta; Spermatophyta; Pinopsida; Pinidae; Conifers II; Cupressales; Taxaceae; Taxus"]:0.20793999999999998,KAH9291961.1[&Organism="Taxus chinensis","Genetic Code"="Standard",Taxonomy="Eukaryota; Viridiplantae; Streptophyta; Embryophyta; Tracheophyta; Spermatophyta; Pinopsida; Pinidae; Conifers II; Cupressales; Taxaceae; Taxus"]:0.27728999999999998)[&"FastTree support value"=0.873]:0.06160999999999994)[&"FastTree support value"=0.684]:0.0182500000000001)[&"FastTree support value"=0.95]:0.07837000000000005)[&"FastTree support value"=0.923]:0.06455000000000011)[&"FastTree support value"=0.988]:0.17684999999999995)[&"FastTree support value"=1.0]:0.3440500000000002)[&"FastTree support value"=0.582]:0.04813000000000006)[&"FastTree support value"=0.903]:0.14145999999999992)[&"FastTree support value"=0.944]:0.15702000000000016)[&"FastTree support value"=0.974]:0.18603999999999976,((((XP_750654.1[&Organism="Aspergillus fumigatus Af293","Genetic Code"="Standard",Taxonomy="Eukaryota; Fungi; Dikarya; Ascomycota; Pezizomycotina; Eurotiomycetes; Eurotiomycetidae; Eurotiales; Aspergillaceae; Aspergillus; Aspergillus subgen. Fumigati"]:0.7652900000000002,((XP_006461472.1[&Organism="Agaricus bisporus var. bisporus H97","Genetic Code"="Standard",Taxonomy="Eukaryota; Fungi; Dikarya; Basidiomycota; Agaricomycotina; Agaricomycetes; Agaricomycetidae; Agaricales; Agaricaceae; Agaricus"]:0.12257000000000007,XP_006461433.1[&Organism="Agaricus bisporus var. bisporus H97","Genetic Code"="Standard",Taxonomy="Eukaryota; Fungi; Dikarya; Basidiomycota; Agaricomycotina; Agaricomycetes; Agaricomycetidae; Agaricales; Agaricaceae; Agaricus"]:0.12647999999999993)[&"FastTree support value"=1.0]:0.5085500000000001,XP_006457072.1[&Organism="Agaricus bisporus var. bisporus H97","Genetic Code"="Standard",Taxonomy="Eukaryota; Fungi; Dikarya; Basidiomycota; Agaricomycotina; Agaricomycetes; Agaricomycetidae; Agaricales; Agaricaceae; Agaricus"]:0.9730699999999999)[&"FastTree support value"=0.003]:0.0661900000000002)[&"FastTree support value"=0.897]:0.1328800000000001,((KAI3646081.1[&Organism="Amoeboaphelidium protococcarum","Genetic Code"="Ciliate",Taxonomy="Eukaryota; Aphelida; Aphelidea; Amoeboaphelidium"]:0.5251999999999999,OAJ38670.1[&Organism="Batrachochytrium dendrobatidis JEL423","Genetic Code"="Standard",Taxonomy="Eukaryota; Fungi; Fungi incertae sedis; Chytridiomycota; Chytridiomycota incertae sedis; Chytridiomycetes; Rhizophydiales; Rhizophydiales incertae sedis; Batrachochytrium"]:0.5162999999999998)[&"FastTree support value"=0.346]:0.11051000000000011,(ETO25748.1[&Organism="Reticulomyxa filosa","Genetic Code"="Standard",Taxonomy="Eukaryota; Sar; Rhizaria; Retaria; Foraminifera; Monothalamids; Reticulomyxidae; Reticulomyxa"]:0.6195399999999998,((GAX85982.1:0.3405300000000002,(((XP_042924848.1:0.23565000000000014,XP_042923301.1:0.23902)[&"FastTree support

value"=0.993]:0.11841999999999997,XP_042924875.1:0.18788999999999999)[&"FastTree support value"=0.044]:0.09004000000000012,KAG2488600.1:0.18018)[&"FastTree support value"=0.999]:0.32224999999999999)[&"FastTree support value"=0.999]:0.33030999999999999,KAJ9515210.1:0.5135800000000001)[&"FastTree support value"=0.814]:0.14381000000000022)[&"FastTree support value"=0.194]:0.05862999999999996)[&"FastTree support value"=0.736]:0.11413999999999991)[&"FastTree support value"=1.0]:0.45754999999999999,(((((XP_032804093.1[&Organism="Petromyzon marinus","Genetic Code"="Standard",Taxonomy="Eukaryota; Metazoa; Chordata; Craniata; Vertebrata; Cyclostomata; Hyperoartia; Petromyzontiformes; Petromyzontidae; Petromyzon","Common Name"="sea lamprey"]:0.4513600000000002,((XP_003973512.2.2[&Organism="Takifugu rubripes","Genetic Code"="Standard",Taxonomy="Eukaryota; Metazoa; Chordata; Craniata; Vertebrata; Euteleostomi; Actinopterygii; Neopterygii; Teleostei; Neoteleostei; Acanthomorphata; Eupercaria; Tetraodontiformes; Tetradontoidea; Tetraodontidae; Takifugu","Common Name"="torafugu"]:0.09996999999999989,(NP_891987.2.2[&Organism="Danio rerio","Genetic Code"="Standard",Taxonomy="Eukaryota; Metazoa; Chordata; Craniata; Vertebrata; Euteleostomi; Actinopterygii; Neopterygii; Teleostei; Ostariophysi; Cypriniformes; Cyprinidae; Danio","Common Name"="zebrafish"]:0.03591999999999995,XP_009304072.1[&Organism="Danio rerio","Genetic Code"="Standard",Taxonomy="Eukaryota; Metazoa; Chordata; Craniata; Vertebrata; Euteleostomi; Actinopterygii; Neopterygii; Teleostei; Ostariophysi; Cypriniformes; Cyprinidae; Danio","Common Name"="zebrafish"]:0.012599999999999945)[&"FastTree support value"=0.992]:0.09855999999999998)[&"FastTree support value"=0.983]:0.09868999999999994,(AGU16245.1[&db_xref="taxon:27779",Organism="Protopterus dolloi","Genetic Code"="Standard",Modified=Mon Mar 27 11:17:17 PDT 2023,Taxonomy="Eukaryota; Metazoa; Chordata; Craniata; Vertebrata; Euteleostomi; Dipnoi; Lepidosireniformes; Protopteridae; Protopterus",Accession="AGU16245.1","Common Name"="slender lungfish",Topology="linear","Molecule Type"="AA"]:0.1545200000000002,(((XP_028583068.1[&Organism="Podarcis muralis","Genetic Code"="Standard",Taxonomy="Eukaryota; Metazoa; Chordata; Craniata; Vertebrata; Euteleostomi; Lepidosauria; Squamata; Bifurcata; Unidentata; Episquamata; Laterata; Lacertibaenia; Lacertidae; Podarcis","Common Name"="Common wall lizard"]:0.16963000000000017,(XP_005167721.2.2[&Organism="Danio rerio","Genetic Code"="Standard",Taxonomy="Eukaryota; Metazoa; Chordata; Craniata; Vertebrata; Euteleostomi; Actinopterygii; Neopterygii; Teleostei; Ostariophysi; Cypriniformes; Cyprinidae; Danio","Common Name"="zebrafish"]:0.034720000000000084,NP_001007285.1[&Organism="Danio rerio","Genetic Code"="Standard",Taxonomy="Eukaryota; Metazoa; Chordata; Craniata; Vertebrata; Euteleostomi; Actinopterygii; Neopterygii; Teleostei; Ostariophysi; Cypriniformes; Cyprinidae; Danio","Common

Name"="zebrafish"]:0.08470999999999984)[&"FastTree support
value"=1.0]:0.26496999999999993)[&"FastTree support
value"=0.797]:0.030609999999999804,((XP_031752404.1[&Organism="Xenopus
tropicalis","Genetic Code"="Standard",Taxonomy="Eukaryota; Metazoa; Chordata;
Craniata; Vertebrata; Euteleostomi; Amphibia; Batrachia; Anura; Pipoidea; Pipidae;
Xenopodinae; Xenopus; Silurana","Common Name"="tropical clawed
frog"]:0.2679300000000002,((((NP_001003133.1[&Organism="Canis lupus
familiaris","Genetic Code"="Standard",Taxonomy="Eukaryota; Metazoa; Chordata;
Craniata; Vertebrata; Euteleostomi; Mammalia; Eutheria; Laurasiatheria; Carnivora;
Caniformia; Canidae; Canis","Common
Name"="dog"]:0.06935000000000002,XP_032211320.1[&Organism="Mustela
erminea","Genetic Code"="Standard",Taxonomy="Eukaryota; Metazoa; Chordata; Craniata;
Vertebrata; Euteleostomi; Mammalia; Eutheria; Laurasiatheria; Carnivora; Caniformia;
Mustelidae; Mustelinae; Mustela","Common
Name"="ermine"]:0.10658000000000012)[&"FastTree support
value"=0.932]:0.033360000000000056,(((XP_008569440.1[&Organism="Galeopterus
variegatus","Genetic Code"="Standard",Taxonomy="Eukaryota; Metazoa; Chordata;
Craniata; Vertebrata; Euteleostomi; Mammalia; Eutheria; Euarchontoglires; Dermoptera;
Cynocephalidae; Galeopterus","Common Name"="Sunda flying
lemur"]:0.05720999999999998,((XP_012586448.1[&Organism="Condylura
cristata","Genetic Code"="Standard",Taxonomy="Eukaryota; Metazoa; Chordata; Craniata;
Vertebrata; Euteleostomi; Mammalia; Eutheria; Laurasiatheria; Insectivora; Talpidae;
Condylura","Common Name"="star-nosed
mole"]:0.22960000000000003,XP_005885748.1[&Organism="Myotis brandtii","Genetic
Code"="Standard",Taxonomy="Eukaryota; Metazoa; Chordata; Craniata; Vertebrata;
Euteleostomi; Mammalia; Eutheria; Laurasiatheria; Chiroptera; Microchiroptera;
Vespertilionidae; Myotis","Common Name"="Brandt's
bat"]:0.0205700000000002)[&"FastTree support
value"=0.874]:0.01933000000000007,XP_017508123.1[&Organism="Manis
javanica","Genetic Code"="Standard",Taxonomy="Eukaryota; Metazoa; Chordata; Craniata;
Vertebrata; Euteleostomi; Mammalia; Eutheria; Laurasiatheria; Pholidota; Manidae;
Manis","Common Name"="Malayan pangolin"]:0.07385999999999981)[&"FastTree support
value"=0.858]:0.016270000000000007)[&"FastTree support
value"=0.065]:0.009030000000000094,(XP_002830747.1[&Organism="Pongo
abelii","Genetic Code"="Standard",Taxonomy="Eukaryota; Metazoa; Chordata; Craniata;
Vertebrata; Euteleostomi; Mammalia; Eutheria; Euarchontoglires; Primates; Haplorrhini;
Catarrhini; Hominidae; Pongo","Common Name"="Sumatran
orangutan"]:0.010689999999999866,NP_002454.1[&Organism="Homo sapiens","Genetic
Code"="Standard",Taxonomy="Eukaryota; Metazoa; Chordata; Craniata; Vertebrata;
Euteleostomi; Mammalia; Eutheria; Euarchontoglires; Primates; Haplorrhini; Catarrhini;
Hominidae; Homo","Common Name"="human"]:0.006190000000000014)[&"FastTree
support value"=1.0]:0.08632999999999998)[&"FastTree support
value"=0.83]:0.010720000000000063,NP_776366.1[&Organism="Bos taurus","Genetic
Code"="Standard",Taxonomy="Eukaryota; Metazoa; Chordata; Craniata; Vertebrata;

Euteleostomi; Mammalia; Eutheria; Laurasiatheria; Artiodactyla; Ruminantia; Pecora; Bovidae; Bovinae; Bos","Common Name"="cattle"]:0.10275999999999996)[&"FastTree support value"=0.733]:0.004570000000000185)[&"FastTree support value"=0.696]:0.02434000000000003,XP_006156438.1[&Organism="Tupaia chinensis","Genetic Code"="Standard",Taxonomy="Eukaryota; Metazoa; Chordata; Craniata; Vertebrata; Euteleostomi; Mammalia; Eutheria; Euarchontoglires; Scandentia; Tupaiidae; Tupaia","Common Name"="Chinese tree shrew"]:0.11172000000000004)[&"FastTree support value"=0.993]:0.10925999999999991,(XP_004675614.2.2[&Organism="Condylura cristata","Genetic Code"="Standard",Taxonomy="Eukaryota; Metazoa; Chordata; Craniata; Vertebrata; Euteleostomi; Mammalia; Eutheria; Laurasiatheria; Insectivora; Talpidae; Condylura","Common Name"="star-nosed mole"]:0.12835999999999998,((((NP_002453.2.2[&Organism="Homo sapiens","Genetic Code"="Standard",Taxonomy="Eukaryota; Metazoa; Chordata; Craniata; Vertebrata; Euteleostomi; Mammalia; Eutheria; Euarchontoglires; Primates; Haplorrhini; Catarrhini; Hominidae; Homo","Common Name"="human"]:0.005079999999999973,NP_001127618.1[&Organism="Pongo abelii","Genetic Code"="Standard",Taxonomy="Eukaryota; Metazoa; Chordata; Craniata; Vertebrata; Euteleostomi; Mammalia; Eutheria; Euarchontoglires; Primates; Haplorrhini; Catarrhini; Hominidae; Pongo","Common Name"="Sumatran orangutan"]:0.0015999999999998238)[&"FastTree support value"=0.986]:0.05319999999999914,((XP_008569442.1[&Organism="Galeopterus variegatus","Genetic Code"="Standard",Taxonomy="Eukaryota; Metazoa; Chordata; Craniata; Vertebrata; Euteleostomi; Mammalia; Eutheria; Euarchontoglires; Dermoptera; Cynocephalidae; Galeopterus","Common Name"="Sunda flying lemur"]:0.06852999999999998,XP_014388412.1[&Organism="Myotis brandtii","Genetic Code"="Standard",Taxonomy="Eukaryota; Metazoa; Chordata; Craniata; Vertebrata; Euteleostomi; Mammalia; Eutheria; Laurasiatheria; Chiroptera; Microchiroptera; Vespertilionidae; Myotis","Common Name"="Brandt's bat"]:0.09446000000000021)[&"FastTree support value"=0.27]:0.020610000000000017,XP_005202045.1[&Organism="Bos taurus","Genetic Code"="Standard",Taxonomy="Eukaryota; Metazoa; Chordata; Craniata; Vertebrata; Euteleostomi; Mammalia; Eutheria; Laurasiatheria; Cetartiodactyla; Ruminantia; Pecora; Bovidae; Bovinae; Bos","Common Name"="cattle"]:0.1103200000000002)[&"FastTree support value"=0.143]:0.018069999999999981)[&"FastTree support value"=0.884]:0.02895000000000003,((XP_006156437.1[&Organism="Tupaia chinensis","Genetic Code"="Standard",Taxonomy="Eukaryota; Metazoa; Chordata; Craniata; Vertebrata; Euteleostomi; Mammalia; Eutheria; Euarchontoglires; Scandentia; Tupaiidae; Tupaia","Common Name"="Chinese tree shrew"]:0.061819999999999986,(NP_034976.1[&Organism="Mus musculus","Genetic Code"="Standard",Taxonomy="Eukaryota; Metazoa; Chordata; Craniata; Vertebrata; Euteleostomi; Mammalia; Eutheria; Euarchontoglires; Glires; Rodentia; Myomorpha; Muroidea; Muridae; Murinae; Mus; Mus","Common Name"="house mouse"]:0.05559000000000003,NP_038634.1[&Organism="Mus musculus","Genetic

Code"="Standard",Taxonomy="Eukaryota; Metazoa; Chordata; Craniata; Vertebrata;
Euteleostomi; Mammalia; Eutheria; Euarchontoglires; Glires; Rodentia; Myomorpha;
Muroidea; Muridae; Murinae; Mus; Mus","Common Name"="house
mouse"]:0.00803000000000204)[&"FastTree support
value"=1.0]:0.11167999999999978)[&"FastTree support
value"=0.641]:0.007249999999999979,(XP_017508130.1[&Organism="Manis
javanica","Genetic Code"="Standard",Taxonomy="Eukaryota; Metazoa; Chordata; Craniata;
Vertebrata; Euteleostomi; Mammalia; Eutheria; Laurasiatheria; Pholidota; Manidae;
Manis","Common Name"="Malayan
pangolin"]:0.05636999999999981,(XP_032211398.1[&Organism="Mustela
erminea","Genetic Code"="Standard",Taxonomy="Eukaryota; Metazoa; Chordata; Craniata;
Vertebrata; Euteleostomi; Mammalia; Eutheria; Laurasiatheria; Carnivora; Caniformia;
Mustelidae; Mustelinae; Mustela","Common
Name"="ermine"]:0.03530999999999995,NP_001003134.1[&Organism="Canis lupus
familiaris","Genetic Code"="Standard",Taxonomy="Eukaryota; Metazoa; Chordata;
Craniata; Vertebrata; Euteleostomi; Mammalia; Eutheria; Laurasiatheria; Carnivora;
Caniformia; Canidae; Canis","Common
Name"="dog"]:0.020239999999999814)[&"FastTree support
value"=0.986]:0.04936000000000007)[&"FastTree support
value"=0.904]:0.021469999999999878)[&"FastTree support
value"=0.291]:0.010240000000000027)[&"FastTree support
value"=0.437]:0.013030000000000097,XP_004466363.1[&Organism="Dasypus
novemcinctus","Genetic Code"="Standard",Taxonomy="Eukaryota; Metazoa; Chordata;
Craniata; Vertebrata; Euteleostomi; Mammalia; Eutheria; Xenarthra; Cingulata;
Dasypodidae; Dasypus","Common Name"="nine-banded
armadillo"]:0.08924000000000021)[&"FastTree support
value"=0.94]:0.03920000000000124)[&"FastTree support
value"=0.927]:0.06303999999999998)[&"FastTree support
value"=0.737]:0.04909000000000008)[&"FastTree support
value"=0.959]:0.06705000000000005,((XP_009815891.1[&Organism="Gavia
stellata","Genetic Code"="Standard",Taxonomy="Eukaryota; Metazoa; Chordata; Craniata;
Vertebrata; Euteleostomi; Archelosauria; Archosauria; Dinosauria; Saurischia; Theropoda;
Coelurosauria; Aves; Neognathae; Gaviiformes; Gaviidae; Gavia","Common Name"="red-
throated loon"]:0.07502999999999993,XP_025933558.1[&Organism="Apteryx
rowi","Genetic Code"="Standard",Taxonomy="Eukaryota; Metazoa; Chordata; Craniata;
Vertebrata; Euteleostomi; Archelosauria; Archosauria; Dinosauria; Saurischia; Theropoda;
Coelurosauria; Aves; Palaeognathae; Apterygiformes; Apterygidae; Apteryx","Common
Name"="Okarito brown kiwi"]:0.05842999999999998)[&"FastTree support
value"=0.999]:0.1494200000000001,(XP_015269256.1[&Organism="Gekko
japonicus","Genetic Code"="Standard",Taxonomy="Eukaryota; Metazoa; Chordata;
Craniata; Vertebrata; Euteleostomi; Lepidosauria; Squamata; Bifurcata; Gekkota;
Gekkonidae; Gekkoninae;
Gekko"]:0.10238000000000014,XP_028583072.1[&Organism="Podarcis muralis","Genetic
Code"="Standard",Taxonomy="Eukaryota; Metazoa; Chordata; Craniata; Vertebrata;

Euteleostomi; Lepidosauria; Squamata; Bifurcata; Unidentata; Episquamata; Laterata; Lacertibaenia; Lacertidae; Podarcis","Common Name"="Common wall lizard"]:0.1725500000000002)[&"FastTree support value"=0.13]:0.028589999999999893)[&"FastTree support value"=0.841]:0.02876000000000012)[&"FastTree support value"=0.872]:0.03354999999999997)[&"FastTree support value"=0.504]:0.027229999999999865,(XP_007904885.1[&Organism="Callorhinchus milii","Genetic Code"="Standard",Taxonomy="Eukaryota; Metazoa; Chordata; Craniata; Vertebrata; Chondrichthyes; Holocephali; Chimaeriformes; Callorhinchidae; Callorhinchus","Common Name"="elephant shark"]:0.12238000000000016,XP_032888405.1[&Organism="Amblyraja radiata","Genetic Code"="Standard",Taxonomy="Eukaryota; Metazoa; Chordata; Craniata; Vertebrata; Chondrichthyes; Elasmobranchii; Batoidea; Rajiformes; Rajidae; Amblyraja","Common Name"="thorny skate"]:0.13580000000000014)[&"FastTree support value"=0.979]:0.07927000000000017)[&"FastTree support value"=0.593]:0.03555000000000019)[&"FastTree support value"=0.954]:0.09227000000000007)[&"FastTree support value"=0.994]:0.13430000000000009)[&"FastTree support value"=0.936]:0.09038000000000013,(KAI0213370.1[&Organism="Lamellibrachia satsuma","Genetic Code"="Standard",Taxonomy="Eukaryota; Metazoa; Spiralia; Lophotrochozoa; Annelida; Polychaeta; Sedentaria; Canalipalpata; Sabellida; Siboglinidae; Lamellibrachia"]:0.007769999999999833,(KAI0218869.1[&Organism="Lamellibrachia satsuma","Genetic Code"="Standard",Taxonomy="Eukaryota; Metazoa; Spiralia; Lophotrochozoa; Annelida; Polychaeta; Sedentaria; Canalipalpata; Sabellida; Siboglinidae; Lamellibrachia"]:0.10307999999999984,KAI0208044.1[&Organism="Lamellibrachia satsuma","Genetic Code"="Standard",Taxonomy="Eukaryota; Metazoa; Spiralia; Lophotrochozoa; Annelida; Polychaeta; Sedentaria; Canalipalpata; Sabellida; Siboglinidae; Lamellibrachia"]:0.060169999999999835)[&"FastTree support value"=0.41]:0.013020000000000032)[&"FastTree support value"=1.0]:0.3864000000000001)[&"FastTree support value"=0.77]:0.026040000000000063,(XP_046565196.1[&Organism="Haliotis rubra","Genetic Code"="Standard",Taxonomy="Eukaryota; Metazoa; Spiralia; Lophotrochozoa; Mollusca; Gastropoda; Vetigastropoda; Lepetellida; Haliotoidea; Haliotidae; Haliotis","Common Name"="blacklip abalone"]:0.053389999999999826,(((XP_046563124.1[&Organism="Haliotis rubra","Genetic Code"="Standard",Taxonomy="Eukaryota; Metazoa; Spiralia; Lophotrochozoa; Mollusca; Gastropoda; Vetigastropoda; Lepetellida; Haliotoidea; Haliotidae; Haliotis","Common Name"="blacklip abalone"]:0.008399999999999963,XP_046562919.1[&Organism="Haliotis rubra","Genetic Code"="Standard",Taxonomy="Eukaryota; Metazoa; Spiralia; Lophotrochozoa; Mollusca; Gastropoda; Vetigastropoda; Lepetellida; Haliotoidea; Haliotidae; Haliotis","Common Name"="blacklip abalone"]:0.03783000000000003)[&"FastTree support value"=0.253]:0.005900000000000016,((XP_046563126.1[&Organism="Haliotis rubra","Genetic Code"="Standard",Taxonomy="Eukaryota; Metazoa; Spiralia;

Lophotrochozoa; Mollusca; Gastropoda; Vetigastropoda; Lepetellida; Haliotoidea;
Haliotidae; Haliotis","Common Name"="blacklip
abalone"]:0.006479999999999819,XP_046565195.1[&Organism="Haliotis rubra","Genetic
Code"="Standard",Taxonomy="Eukaryota; Metazoa; Spiralia; Lophotrochozoa; Mollusca;
Gastropoda; Vetigastropoda; Lepetellida; Haliotoidea; Haliotidae; Haliotis","Common
Name"="blacklip abalone"]:0.009669999999999845)[&"FastTree support
value"=0.973]:0.022479999999999833,XP_046563125.1[&Organism="Haliotis
rubra","Genetic Code"="Standard",Taxonomy="Eukaryota; Metazoa; Spiralia;
Lophotrochozoa; Mollusca; Gastropoda; Vetigastropoda; Lepetellida; Haliotoidea;
Haliotidae; Haliotis","Common Name"="blacklip
abalone"]:0.023070000000000146)[&"FastTree support
value"=0.866]:0.008700000000000152)[&"FastTree support
value"=0.698]:0.0014099999999999113,(XP_046352527.2[&Organism="Haliotis
rufescens","Genetic Code"="Standard",Taxonomy="Eukaryota; Metazoa; Spiralia;
Lophotrochozoa; Mollusca; Gastropoda; Vetigastropoda; Lepetellida; Haliotoidea;
Haliotidae; Haliotis","Common Name"="red
abalone"]:0.019159999999999844,((XP_048258111.1[&Organism="Haliotis
rufescens","Genetic Code"="Standard",Taxonomy="Eukaryota; Metazoa; Spiralia;
Lophotrochozoa; Mollusca; Gastropoda; Vetigastropoda; Lepetellida; Haliotoidea;
Haliotidae; Haliotis","Common Name"="red
abalone"]:0.00641999999999987,((XP_046352531.2[&Organism="Haliotis
rufescens","Genetic Code"="Standard",Taxonomy="Eukaryota; Metazoa; Spiralia;
Lophotrochozoa; Mollusca; Gastropoda; Vetigastropoda; Lepetellida; Haliotoidea;
Haliotidae; Haliotis","Common Name"="red
abalone"]:0.00719000000000003,(XP_048248472.1[&Organism="Haliotis
rufescens","Genetic Code"="Standard",Taxonomy="Eukaryota; Metazoa; Spiralia;
Lophotrochozoa; Mollusca; Gastropoda; Vetigastropoda; Lepetellida; Haliotoidea;
Haliotidae; Haliotis","Common Name"="red
abalone"]:0.000550,(XP_048248474.1[&Organism="Haliotis rufescens","Genetic
Code"="Standard",Taxonomy="Eukaryota; Metazoa; Spiralia; Lophotrochozoa; Mollusca;
Gastropoda; Vetigastropoda; Lepetellida; Haliotoidea; Haliotidae; Haliotis","Common
Name"="red abalone"]:0.0,XP_048248473.1[&Organism="Haliotis rufescens","Genetic
Code"="Standard",Taxonomy="Eukaryota; Metazoa; Spiralia; Lophotrochozoa; Mollusca;
Gastropoda; Vetigastropoda; Lepetellida; Haliotoidea; Haliotidae; Haliotis","Common
Name"="red abalone"]:0.0):0.000550)[&"FastTree support
value"=0.984]:0.021929999999999783)[&"FastTree support
value"=0.872]:0.006639999999999979,ABI53802.1[&Organism="Haliotis discus
discus","Genetic Code"="Standard",Taxonomy="Eukaryota; Metazoa; Lophotrochozoa;
Mollusca; Gastropoda; Vetigastropoda; Haliotoidea; Haliotidae; Haliotis","Common
Name"="disc abalone"]:0.025560000000000027)[&"FastTree support
value"=0.867]:0.006609999999999783)[&"FastTree support
value"=0.655]:0.000540,XP_048248476.1[&Organism="Haliotis rufescens","Genetic
Code"="Standard",Taxonomy="Eukaryota; Metazoa; Spiralia; Lophotrochozoa; Mollusca;
Gastropoda; Vetigastropoda; Lepetellida; Haliotoidea; Haliotidae; Haliotis","Common

Name"="red abalone"]:0.01689000000000007)[&"FastTree support value"=0.691]:0.004239999999999997995)[&"FastTree support value"=0.614]:0.030269999999999797)[&"FastTree support value"=0.983]:0.09678999999999993)[&"FastTree support value"=1.0]:0.35706000000000016)[&"FastTree support value"=0.81]:0.03685999999999989,(XP_035690836.1[&Organism="Branchiostoma floridae","Genetic Code"="Standard",Taxonomy="Eukaryota; Metazoa; Chordata; Cephalochordata; Leptocardii; Amphioxiformes; Branchiostomidae; Branchiostoma","Common Name"="Florida lancelet"]:0.19198000000000004,(XP_019617847.1[&Organism="Branchiostoma belcheri","Genetic Code"="Standard",Taxonomy="Eukaryota; Metazoa; Chordata; Cephalochordata; Branchiostomidae; Branchiostoma","Common Name"="Belcher's lancelet"]:0.12152999999999992,XP_002608668.1[&Organism="Branchiostoma floridae","Genetic Code"="Standard",Taxonomy="Eukaryota; Metazoa; Chordata; Cephalochordata; Branchiostomidae; Branchiostoma","Common Name"="Florida lancelet"]:0.08363999999999994)[&"FastTree support value"=0.98]:0.11534999999999984)[&"FastTree support value"=0.997]:0.1790400000000001)[&"FastTree support value"=0.845]:0.06083999999999978,((CAH1802128.1[&Organism="Owenia fusiformis","Genetic Code"="Standard",Taxonomy="Eukaryota; Metazoa; Spiralia; Lophotrochozoa; Annelida; Polychaeta; Sedentaria; Canalipalpata; Sabellida; Oweniida; Oweniidae; Owenia"]:0.5095000000000001,((PAA83069.1[&Organism="Macrostomum lignano","Genetic Code"="Standard",Taxonomy="Eukaryota; Metazoa; Platyhelminthes; Rhabditophora; Macrostomorpha; Macrostomida; Macrostomidae; Macrostomum"]:0.09798000000000018,PAA94353.1[&Organism="Macrostomum lignano","Genetic Code"="Standard",Taxonomy="Eukaryota; Metazoa; Platyhelminthes; Rhabditophora; Macrostomorpha; Macrostomida; Macrostomidae; Macrostomum"]:0.16916999999999982)[&"FastTree support value"=1.0]:0.5521400000000001,((PAA74204.1[&Organism="Macrostomum lignano","Genetic Code"="Standard",Taxonomy="Eukaryota; Metazoa; Platyhelminthes; Rhabditophora; Macrostomorpha; Macrostomida; Macrostomidae; Macrostomum"]:0.12783999999999995,PAA76532.1[&Organism="Macrostomum lignano","Genetic Code"="Standard",Taxonomy="Eukaryota; Metazoa; Platyhelminthes; Rhabditophora; Macrostomorpha; Macrostomida; Macrostomidae; Macrostomum"]:0.26019000000000014)[&"FastTree support value"=0.998]:0.25215999999999994,(PAA92268.1[&Organism="Macrostomum lignano","Genetic Code"="Standard",Taxonomy="Eukaryota; Metazoa; Platyhelminthes; Rhabditophora; Macrostomorpha; Macrostomida; Macrostomidae; Macrostomum"]:0.30767999999999995,PAA69582.1[&Organism="Macrostomum lignano","Genetic Code"="Standard",Taxonomy="Eukaryota; Metazoa; Platyhelminthes; Rhabditophora; Macrostomorpha; Macrostomida; Macrostomidae; Macrostomum"]:0.3210099999999998)[&"FastTree support value"=0.909]:0.11683000000000021)[&"FastTree support value"=0.946]:0.12062999999999979)[&"FastTree support

value"=0.951]:0.11621000000000015)[&"FastTree support value"=0.921]:0.08004999999999995,XP_006815062.1[&Organism="Saccoglossus kowalevskii","Genetic Code"="Standard",Taxonomy="Eukaryota; Metazoa; Hemichordata; Enteropneusta; Harrimaniidae; Saccoglossus"]:0.42920999999999987)[&"FastTree support value"=0.637]:0.05820999999999987)[&"FastTree support value"=0.986]:0.18977999999999984)[&"FastTree support value"=0.845]:0.07169999999999987,(ABI33144.1[&Organism="Bigelowiella natans","Genetic Code"="Standard",Taxonomy="Eukaryota; Sar; Rhizaria; Cercozoa; Chlorarachniophyceae; Bigelowiella"]:0.9564599999999999,((GHP04420.1[&Organism="Pycnococcus provasolii","Genetic Code"="Standard",Taxonomy="Eukaryota; Viridiplantae; Chlorophyta; Pycnococcaceae; Pycnococcus"]:0.52183,((OSX73843.1[&Organism="Porphyra umbilicalis","Genetic Code"="Standard",Taxonomy="Eukaryota; Rhodophyta; Bangiophyceae; Bangiales; Bangiaceae; Porphyra","Common Name"="laver"]:0.06469000000000014,OSX70108.1[&Organism="Porphyra umbilicalis","Genetic Code"="Standard",Taxonomy="Eukaryota; Rhodophyta; Bangiophyceae; Bangiales; Bangiaceae; Porphyra","Common Name"="laver"]:0.06605000000000016)[&"FastTree support value"=1.0]:0.4036200000000001,((KAK3283006.1[&Organism="Cymbomonas tetramitiformis","Genetic Code"="Standard",Taxonomy="Eukaryota; Viridiplantae; Chlorophyta; Pyramimonadophyceae; Pyramimonadales; Pyramimonadaceae; Cymbomonas"]:0.31252999999999975,(KAJ1487321.1[&Organism="Cryptophyta sp. CCMP2293","Genetic Code"="Standard",Taxonomy="Eukaryota; Cryptophyceae"]:0.21437000000000017,(KAJ1474882.1[&Organism="Cryptophyta sp. CCMP2293","Genetic Code"="Standard",Taxonomy="Eukaryota; Cryptophyceae"]:0.10035999999999978,(KAJ1495567.1[&Organism="Cryptophyta sp. CCMP2293","Genetic Code"="Standard",Taxonomy="Eukaryota; Cryptophyceae"]:0.08374000000000015,KAJ1474099.1[&Organism="Cryptophyta sp. CCMP2293","Genetic Code"="Standard",Taxonomy="Eukaryota; Cryptophyceae"]:0.18086000000000002)[&"FastTree support value"=0.789]:0.04186999999999985)[&"FastTree support value"=0.108]:0.04396999999999984)[&"FastTree support value"=0.997]:0.22467000000000015)[&"FastTree support value"=0.996]:0.18757000000000001,(((KAG5185531.1[&Organism="Tribonema minus","Genetic Code"="Standard",Taxonomy="Eukaryota; Sar; Stramenopiles; Ochrophyta; PX clade; Xanthophyceae; Tribonematales; Tribonemataceae; Tribonema"]:0.34384999999999977,((KAG5178451.1[&Organism="Tribonema minus","Genetic Code"="Standard",Taxonomy="Eukaryota; Sar; Stramenopiles; Ochrophyta; PX clade; Xanthophyceae; Tribonematales; Tribonemataceae; Tribonema"]:0.20156000000000018,CBN76986.1[&Organism="Ectocarpus siliculosus","Genetic Code"="Standard",Taxonomy="Eukaryota; Sar; Stramenopiles; Ochrophyta; PX clade; Phaeophyceae; Ectocarpales; Ectocarpaceae; Ectocarpus"]:0.23192000000000013)[&"FastTree support value"=0.968]:0.08273999999999981,(CAH0370685.1[&Organism="Pelagomonas

calceolata","Genetic Code"="Standard",Taxonomy="Eukaryota; Sar; Stramenopiles; Ochrophyta; Pelagophyceae; Pelagomonadales; Pelagomonas"]:0.3149799999999998,(KAJ1460259.1[&Organism="Pelagophyceae sp. CCMP2097","Genetic Code"="Standard",Taxonomy="Eukaryota; Sar; Stramenopiles; Ochrophyta; Pelagophyceae"]:0.1415000000000018,(KAJ8603921.1[&Organism="Chrysophaeum taylorii","Genetic Code"="Standard",Taxonomy="Eukaryota; Sar; Stramenopiles; Ochrophyta; Pelagophyceae; Pelagomonadales; Chrysophaeum"]:0.1453500000000001,KAH8053135.1[&Organism="Aureococcus anophagefferens","Genetic Code"="Standard",Taxonomy="Eukaryota; Sar; Stramenopiles; Ochrophyta; Pelagophyceae; Pelagomonadales; Aureococcus"]:0.19869000000000003)[&"FastTree support value"=0.106]:0.03929999999999989)[&"FastTree support value"=0.974]:0.1020500000000002)[&"FastTree support value"=0.992]:0.13723000000000019)[&"FastTree support value"=0.738]:0.04364999999999997)[&"FastTree support value"=0.264]:0.05927999999999978,((XP_009038401.1[&Organism="Aureococcus anophagefferens","Genetic Code"="Standard",Taxonomy="Eukaryota; Sar; Stramenopiles; Ochrophyta; Pelagophyceae; Pelagomonadales; Aureococcus"]:0.15290999999999988,KAJ1460532.1[&Organism="Pelagophyceae sp. CCMP2097","Genetic Code"="Standard",Taxonomy="Eukaryota; Sar; Stramenopiles; Ochrophyta; Pelagophyceae"]:0.15932000000000013)[&"FastTree support value"=0.995]:0.19921999999999995,((XP_009039543.1[&Organism="Aureococcus anophagefferens","Genetic Code"="Standard",Taxonomy="Eukaryota; Sar; Stramenopiles; Ochrophyta; Pelagophyceae; Pelagomonadales; Aureococcus"]:0.1652300000000002,(KAJ8614481.1[&Organism="Chrysophaeum taylorii","Genetic Code"="Standard",Taxonomy="Eukaryota; Sar; Stramenopiles; Ochrophyta; Pelagophyceae; Pelagomonadales; Chrysophaeum"]:0.12109000000000014,XP_009039855.1[&Organism="Aureococcus anophagefferens","Genetic Code"="Standard",Taxonomy="Eukaryota; Sar; Stramenopiles; Ochrophyta; Pelagophyceae; Pelagomonadales; Aureococcus"]:0.14856999999999987)[&"FastTree support value"=0.831]:0.04088000000000003)[&"FastTree support value"=0.987]:0.12487000000000004,(CBN78192.1[&Organism="Ectocarpus siliculosus","Genetic Code"="Standard",Taxonomy="Eukaryota; Sar; Stramenopiles; Ochrophyta; PX clade; Phaeophyceae; Ectocarpales; Ectocarpaceae; Ectocarpus"]:0.12077999999999989,KAG5184845.1[&Organism="Tribonema minus","Genetic Code"="Standard",Taxonomy="Eukaryota; Sar; Stramenopiles; Ochrophyta; PX clade; Xanthophyceae; Tribonematales; Tribonemataceae; Tribonema"]:0.16468000000000016)[&"FastTree support value"=0.988]:0.13194000000000017)[&"FastTree support value"=0.987]:0.12331000000000003)[&"FastTree support value"=0.721]:0.0884999999999998)[&"FastTree support value"=0.716]:0.08598000000000017,((KAJ1442373.1[&Organism="Ochromonadaceae sp.

CCMP2298","Genetic Code"="Standard",Taxonomy="Eukaryota; Sar; Stramenopiles; Ochrophyta; Synurophyceae; Ochromonadales; Ochromonadaceae"]:0.3897599999999999,KAJ1428896.1[&Organism="Ochromonadaceae sp. CCMP2298","Genetic Code"="Standard",Taxonomy="Eukaryota; Sar; Stramenopiles; Ochrophyta; Synurophyceae; Ochromonadales; Ochromonadaceae"]:0.5843599999999998)[&"FastTree support value"=0.449]:0.11460999999999988,(KAG5183739.1[&Organism="Tribonema minus","Genetic Code"="Standard",Taxonomy="Eukaryota; Sar; Stramenopiles; Ochrophyta; PX clade; Xanthophyceae; Tribonematales; Tribonemataceae; Tribonema"]:0.41462999999999983,(XP_005711749.1[&Organism="Chondrus crispus","Genetic Code"="Standard",Taxonomy="Eukaryota; Rhodophyta; Florideophyceae; Rhodymeniophycidae; Gigartinales; Gigartinaceae; Chondrus","Common Name"="carragheen"]:0.1501100000000002,(KAI0564035.1[&Organism="Gracilaria domingensis","Genetic Code"="Standard",Taxonomy="Eukaryota; Rhodophyta; Florideophyceae; Rhodymeniophycidae; Gracilariales; Gracilariaceae; Gracilaria"]:0.05076999999999998,PXF49978.1[&Organism="Gracilariopsis chorda","Genetic Code"="Standard",Taxonomy="Eukaryota; Rhodophyta; Florideophyceae; Rhodymeniophycidae; Gracilariales; Gracilariaceae; Gracilariopsis"]:0.06759000000000004)[&"FastTree support value"=0.94]:0.11348000000000003)[&"FastTree support value"=1.0]:0.40302000000000016)[&"FastTree support value"=0.94]:0.1718799999999998)[&"FastTree support value"=0.337]:0.05091000000000001)[&"FastTree support value"=0.8]:0.03827999999999987)[&"FastTree support value"=0.604]:0.031140000000000168)[&"FastTree support value"=0.915]:0.1225299999999998)[&"FastTree support value"=0.975]:0.22162000000000015,(((((TDH66190.1[&Organism="Bremia lactucae","Genetic Code"="Standard",Taxonomy="Eukaryota; Sar; Stramenopiles; Oomycota; Peronosporales; Peronosporaceae; Bremia","Common Name"="lettuce downy mildew"]:0.2889900000000001,(XP_024574100.1[&Organism="Plasmopara halstedii","Genetic Code"="Standard",Taxonomy="Eukaryota; Sar; Stramenopiles; Oomycota; Peronosporales; Peronosporaceae; Plasmopara"]:0.08903000000000016,(RMX63821.1[&Organism="Peronospora effusa","Genetic Code"="Standard",Taxonomy="Eukaryota; Sar; Stramenopiles; Oomycota; Peronosporales; Peronosporaceae; Peronospora"]:0.18272000000000022,(KAG1692046.1[&Organism="Phytophthora capsici","Genetic Code"="Standard",Taxonomy="Eukaryota; Sar; Stramenopiles; Oomycota; Peronosporales; Peronosporaceae; Phytophthora"]:0.04228999999999994,(KAG3062152.1[&Organism="Phytophthora cactorum","Genetic Code"="Standard",Taxonomy="Eukaryota; Sar; Stramenopiles; Oomycota; Peronosporales; Peronosporaceae; Phytophthora"]:0.027470000000000105,(KAI9918701.1[&Organism="Peronosclerospora sorghi","Genetic Code"="Standard",Taxonomy="Eukaryota; Sar; Stramenopiles; Oomycota; Peronosporales; Peronosporaceae;

Peronosclerospora"]:0.09710999999999981,XP_009533587.1[&Organism="Phytophthora sojae","Genetic Code"="Standard",Taxonomy="Eukaryota; Sar; Stramenopiles; Oomycota; Peronosporales; Peronosporaceae; Phytophthora"]:0.03860999999999981)[&"FastTree support value"=0.687]:0.01506000000000073)[&"FastTree support value"=0.892]:0.01603000000000021)[&"FastTree support value"=0.343]:0.01567000000000073)[&"FastTree support value"=0.093]:0.02010999999999985)[&"FastTree support value"=0.299]:0.02964000000000011)[&"FastTree support value"=0.995]:0.24834999999999985,((KAI9324922.1[&"% Charged Amino Acids"=24.91%,"% Acidic Amino Acids"=13.17%,"% Hydrophobic Amino Acids"=46.26%,Modified=Fri Jun 28 11:50:37 PDT 2024,"% GC-rich Amino Acids"=21.71%,"Molecular Weight (kDa)"=30.80993478000001,"Extinction Coefficient"=13075.0,"# Nucleotide Sequences With Quality"=0,Topology="linear","Alignment method"="MAFFT Alignment",Created=Fri Jun 28 11:46:17 PDT 2024,"Charge at pH 7"=-7.798989763504893,"Isoelectric Point"=4.897228240966797,"% Basic Amino Acids"=11.74%,"% AT-rich Amino Acids"=21.71%,"% Polar Uncharged Amino Acids"=29.18%,"Free end gaps"=true,"Molecule Type"="AA"]:0.18043999999999993,'KAJ3066410.1'[&"% Charged Amino Acids"=27.50%,"% Acidic Amino Acids"=15.00%,"% Hydrophobic Amino Acids"=47.14%,Modified=Fri Jun 28 11:50:27 PDT 2024,"% GC-rich Amino Acids"=22.14%,"Molecular Weight (kDa)"=30.847098080000013,"Extinction Coefficient"=13075.0,"# Nucleotide Sequences With Quality"=0,Topology="linear","Alignment method"="MAFFT Alignment",Created=Fri Jun 28 11:46:17 PDT 2024,"Charge at pH 7"=-10.798238550692695,"Isoelectric Point"=4.645633697509766,"% Basic Amino Acids"=12.50%,"% AT-rich Amino Acids"=21.07%,"% Polar Uncharged Amino Acids"=25.71%,"Free end gaps"=true,"Molecule Type"="AA"]:0.10937999999999981)[&"FastTree support value"=0.573]:0.10460000000000003,(KAI8836453.1[&"% Charged Amino Acids"=29.14%,"% Acidic Amino Acids"=13.67%,"% Hydrophobic Amino Acids"=46.76%,Modified=Fri Jun 28 11:50:45 PDT 2024,"% GC-rich Amino Acids"=22.30%,"Molecular Weight (kDa)"=30.927449480000007,"Extinction Coefficient"=13200.0,"# Nucleotide Sequences With Quality"=0,Topology="linear","Alignment method"="MAFFT Alignment",Created=Fri Jun 28 11:46:17 PDT 2024,"Charge at pH 7"=-3.4437602930013576,"Isoelectric Point"=6.172344207763672,"% Basic Amino Acids"=15.47%,"% AT-rich Amino Acids"=21.94%,"% Polar Uncharged Amino Acids"=24.46%,"Free end gaps"=true,"Molecule Type"="AA"]:0.17520999999999987,KAJ3350919.1[&"% Charged Amino Acids"=24.29%,"% Acidic Amino Acids"=12.14%,"% Hydrophobic Amino Acids"=49.64%,Modified=Fri Jun 28 11:50:59 PDT 2024,"% GC-rich Amino Acids"=20.71%,"Molecular Weight (kDa)"=30.724561180000016,"Extinction Coefficient"=13200.0,"# Nucleotide Sequences With Quality"=0,Topology="linear","Alignment method"="MAFFT Alignment",Created=Fri Jun 28 11:46:17 PDT 2024,"Charge at pH 7"=-4.908779490673721,"Isoelectric Point"=5.475826263427734,"% Basic Amino Acids"=12.14%,"% AT-rich Amino Acids"=24.64%,"% Polar Uncharged Amino Acids"=26.43%,"Free end gaps"=true,"Molecule Type"="AA"]:0.3765100000000001)[&"FastTree support value"=0.607]:0.10568)[&"FastTree

support value"=0.956]:0.17776000000000014)[&"FastTree support value"=0.984]:0.24634,(XP_005823288.1[&Organism="Guillardia theta CCMP2712","Genetic Code"="Standard",Taxonomy="Eukaryota; Cryptophyceae; Pyrenomonadales; Geminigeraceae; Guillardia"]:0.50074,KAJ1441760.1[&Organism="Ochromonadaceae sp. CCMP2298","Genetic Code"="Standard",Taxonomy="Eukaryota; Sar; Stramenopiles; Ochrophyta; Synurophyceae; Ochromonadales; Ochromonadaceae"]:0.5162)[&"FastTree support value"=0.996]:0.39579999999999993)[&"FastTree support value"=0.951]:0.20136999999999983,KAJ1637655.1[&Organism="Pavlovales sp. CCMP2436","Genetic Code"="Standard",Taxonomy="Eukaryota; Haptista; Haptophyta; Pavlovales"]:0.9694400000000001)[&"FastTree support value"=0.841]:0.09257999999999988,((KAI8587516.1[&"% Charged Amino Acids"=22.91%,"% Acidic Amino Acids"=11.27%,"% Hydrophobic Amino Acids"=52.00%,Modified=Fri Jun 28 11:50:12 PDT 2024,"% GC-rich Amino Acids"=23.27%,"Molecular Weight (kDa)"=30.21366338000001,"Extinction Coefficient"=24075.0,"# Nucleotide Sequences With Quality"=0,Topology="linear","Alignment method"="MAFFT Alignment",Created=Fri Jun 28 11:46:17 PDT 2024,"Charge at pH 7"=-3.7092413986402963,"Isoelectric Point"=5.703121185302734,"% Basic Amino Acids"=11.64%,"% AT-rich Amino Acids"=21.82%,"% Polar Uncharged Amino Acids"=26.18%,"Free end gaps"=true,"Molecule Type"="AA"]:0.67137,(KXS17655.1[&"% Charged Amino Acids"=25.81%,"% Acidic Amino Acids"=13.26%,"% Hydrophobic Amino Acids"=53.05%,Modified=Fri Jun 28 11:49:57 PDT 2024,"% GC-rich Amino Acids"=23.30%,"Molecular Weight (kDa)"=30.090607580000007,"Extinction Coefficient"=14105.0,"# Nucleotide Sequences With Quality"=0,Topology="linear","Alignment method"="MAFFT Alignment",Created=Fri Jun 28 11:46:17 PDT 2024,"Charge at pH 7"=-7.601807258076357,"Isoelectric Point"=5.110424041748047,"% Basic Amino Acids"=12.54%,"% AT-rich Amino Acids"=23.30%,"% Polar Uncharged Amino Acids"=21.86%,"Free end gaps"=true,"Molecule Type"="AA"]:0.40758999999999999,XP_047808890.1[&"% Charged Amino Acids"=27.17%,"% Acidic Amino Acids"=12.68%,"% Hydrophobic Amino Acids"=47.10%,Modified=Fri Jun 28 11:49:32 PDT 2024,"% GC-rich Amino Acids"=23.91%,"Molecular Weight (kDa)"=30.56279258,"Extinction Coefficient"=13200.0,"# Nucleotide Sequences With Quality"=0,Topology="linear","Alignment method"="MAFFT Alignment",Created=Fri Jun 28 11:46:17 PDT 2024,"Charge at pH 7"=-1.6440202409101712,"Isoelectric Point"=6.441722869873047,"% Basic Amino Acids"=14.49%,"% AT-rich Amino Acids"=22.83%,"% Polar Uncharged Amino Acids"=26.09%,"Free end gaps"=true,"Molecule Type"="AA"]:0.52587999999999999)[&"FastTree support value"=0.886]:0.12447000000000008)[&"FastTree support value"=0.98]:0.20122000000000018,(KAF9951223.1[&"% Charged Amino Acids"=29.71%,"% Acidic Amino Acids"=15.22%,"% Hydrophobic Amino Acids"=45.65%,Modified=Fri Jun 28 11:51:59 PDT 2024,"% GC-rich Amino Acids"=21.01%,"Molecular Weight (kDa)"=31.347671079999994,"Extinction Coefficient"=27055.0,"# Nucleotide Sequences With

Quality"=0,Topology="linear","Alignment method"="MAFFT Alignment",Created=Fri Jun 28 11:46:17 PDT 2024,"Charge at pH 7"=-6.773009592825655,"Isoelectric Point"=5.118595123291016,"% Basic Amino Acids"=14.49%,"% AT-rich Amino Acids"=23.55%,"% Polar Uncharged Amino Acids"=25.72%,"Free end gaps"=true,"Molecule Type"="AA"]:0.82389,(OLL24579.1[&"% Charged Amino Acids"=26.39%,"% Acidic Amino Acids"=12.15%,"% Hydrophobic Amino Acids"=44.79%,Modified=Fri Jun 28 11:46:17 PDT 2024,"% GC-rich Amino Acids"=20.49%,"Molecular Weight (kDa)"=31.81140938000001,"Extinction Coefficient"=14565.0,"# Nucleotide Sequences With Quality"=0,Topology="linear","Alignment method"="MAFFT Alignment",Created=Fri Jun 28 11:46:17 PDT 2024,"Charge at pH 7"=3.0255167076407066,"Isoelectric Point"=8.445613861083984,"% Basic Amino Acids"=14.24%,"% AT-rich Amino Acids"=25.69%,"% Polar Uncharged Amino Acids"=29.17%,"Free end gaps"=true,"Molecule Type"="AA"]:0.46069000000000004,((XP_021869222.1[&"% Charged Amino Acids"=32.55%,"% Acidic Amino Acids"=15.77%,"% Hydrophobic Amino Acids"=44.30%,Modified=Fri Jun 28 11:49:08 PDT 2024,"% GC-rich Amino Acids"=21.48%,"Molecular Weight (kDa)"=33.45680067999999,"Extinction Coefficient"=21095.0,"# Nucleotide Sequences With Quality"=0,Topology="linear","Alignment method"="MAFFT Alignment",Created=Fri Jun 28 11:46:17 PDT 2024,"Charge at pH 7"=-6.272273006910691,"Isoelectric Point"=5.793697357177734,"% Basic Amino Acids"=16.78%,"% AT-rich Amino Acids"=21.48%,"% Polar Uncharged Amino Acids"=24.16%,"Free end gaps"=true,"Molecule Type"="AA"]:0.40093000000000023,TVY17522.1[&"% Charged Amino Acids"=29.25%,"% Acidic Amino Acids"=13.95%,"% Hydrophobic Amino Acids"=50.00%,Modified=Fri Jun 28 11:46:17 PDT 2024,"% GC-rich Amino Acids"=22.79%,"Molecular Weight (kDa)"=32.82586588,"Extinction Coefficient"=19605.0,"# Nucleotide Sequences With Quality"=0,Topology="linear","Alignment method"="MAFFT Alignment",Created=Fri Jun 28 11:46:17 PDT 2024,"Charge at pH 7"=-3.4030920147103245,"Isoelectric Point"=6.098857879638672,"% Basic Amino Acids"=15.31%,"% AT-rich Amino Acids"=18.71%,"% Polar Uncharged Amino Acids"=21.77%,"Free end gaps"=true,"Molecule Type"="AA"]:0.33081000000000005)[&"FastTree support value"=1.0]:0.4350999999999998,((XP_026607910.1[&"% Charged Amino Acids"=25.93%,"% Acidic Amino Acids"=11.11%,"% Hydrophobic Amino Acids"=48.82%,Modified=Fri Jun 28 11:46:17 PDT 2024,"% GC-rich Amino Acids"=24.58%,"Molecular Weight (kDa)"=32.72361798000001,"Extinction Coefficient"=11710.0,"# Nucleotide Sequences With Quality"=0,Topology="linear","Alignment method"="MAFFT Alignment",Created=Fri Jun 28 11:46:17 PDT 2024,"Charge at pH 7"=2.553564522492767,"Isoelectric Point"=7.953372955322266,"% Basic Amino Acids"=14.81%,"% AT-rich Amino Acids"=22.56%,"% Polar Uncharged Amino Acids"=25.59%,"Free end gaps"=true,"Molecule Type"="AA"]:0.3266300000000002,(XP_748757.2[&Organism="Aspergillus fumigatus Af293","Genetic Code"="Standard",Taxonomy="Eukaryota; Fungi; Dikarya; Ascomycota; Pezizomycotina; Eurotiomycetes; Eurotiomycetidae; Eurotiales; Aspergillaceae; Aspergillus; Aspergillus subgen. Fumigati"]:0.34559000000000006,(XP_040633937.1[&"% Charged Amino Acids"=27.74%,"% Acidic Amino Acids"=14.04%,"% Hydrophobic Amino

Acids"=51.71%,Modified=Fri Jun 28 11:46:17 PDT 2024,"% GC-rich Amino Acids"=27.40%,"Molecular Weight (kDa)"=31.85812448,"Extinction Coefficient"=7115.0,"# Nucleotide Sequences With Quality"=0,Topology="linear","Alignment method"="MAFFT Alignment",Created=Fri Jun 28 11:46:17 PDT 2024,"Charge at pH 7"=-11.101735485136608,"Isoelectric Point"=5.194911956787109,"% Basic Amino Acids"=13.70%,"% AT-rich Amino Acids"=17.12%,"% Polar Uncharged Amino Acids"=20.89%,"Free end gaps"=true,"Molecule Type"="AA"]:0.17605000000000004,(XP_754266.1[&Organism="Aspergillus fumigatus Af293","Genetic Code"="Standard",Taxonomy="Eukaryota; Fungi; Dikarya; Ascomycota; Pezizomycotina; Eurotiomycetes; Eurotiomycetidae; Eurotiales; Aspergillaceae; Aspergillus; Aspergillus subgen. Fumigati"]:0.07299999999999995,XP_043140374.1[&"% Charged Amino Acids"=27.74%,"% Acidic Amino Acids"=14.38%,"% Hydrophobic Amino Acids"=51.03%,Modified=Fri Jun 28 11:46:17 PDT 2024,"% GC-rich Amino Acids"=26.71%,"Molecular Weight (kDa)"=32.242680480000004,"Extinction Coefficient"=7115.0,"# Nucleotide Sequences With Quality"=0,Topology="linear","Alignment method"="MAFFT Alignment",Created=Fri Jun 28 11:46:17 PDT 2024,"Charge at pH 7"=-8.599323914913342,"Isoelectric Point"=4.979366302490234,"% Basic Amino Acids"=13.36%,"% AT-rich Amino Acids"=20.21%,"% Polar Uncharged Amino Acids"=21.58%,"Free end gaps"=true,"Molecule Type"="AA"]:0.045700000000000074)[&"FastTree support value"=0.976]:0.10695999999999994)[&"FastTree support value"=0.994]:0.1629900000000002)[&"FastTree support value"=0.838]:0.05196999999999985)[&"FastTree support value"=1.0]:0.3333400000000002,(((XP_751069.1[&Organism="Aspergillus fumigatus Af293","Genetic Code"="Standard",Taxonomy="Eukaryota; Fungi; Dikarya; Ascomycota; Pezizomycotina; Eurotiomycetes; Eurotiomycetidae; Eurotiales; Aspergillaceae; Aspergillus; Aspergillus subgen. Fumigati"]:0.54861,((XP_041144356.1[&"% Charged Amino Acids"=26.92%,"% Acidic Amino Acids"=12.24%,"% Hydrophobic Amino Acids"=48.25%,Modified=Fri Jun 28 11:46:17 PDT 2024,"% GC-rich Amino Acids"=22.03%,"Molecular Weight (kDa)"=31.70809888000001,"Extinction Coefficient"=17085.0,"# Nucleotide Sequences With Quality"=0,Topology="linear","Alignment method"="MAFFT Alignment",Created=Fri Jun 28 11:46:17 PDT 2024,"Charge at pH 7"=-3.1075449530009323,"Isoelectric Point"=6.306392669677734,"% Basic Amino Acids"=14.69%,"% AT-rich Amino Acids"=19.58%,"% Polar Uncharged Amino Acids"=25.52%,"Free end gaps"=true,"Molecule Type"="AA"]:0.08030999999999988,XP_746402.1[&Organism="Aspergillus fumigatus Af293","Genetic Code"="Standard",Taxonomy="Eukaryota; Fungi; Dikarya; Ascomycota; Pezizomycotina; Eurotiomycetes; Eurotiomycetidae; Eurotiales; Aspergillaceae; Aspergillus; Aspergillus subgen. Fumigati"]:0.12617000000000012)[&"FastTree support value"=0.858]:0.034089999999999954,(KAJ5704467.1[&"% Charged Amino Acids"=24.13%,"% Acidic Amino Acids"=11.89%,"% Hydrophobic Amino Acids"=49.30%,Modified=Fri Jun 28 11:46:17 PDT 2024,"% GC-rich Amino Acids"=23.78%,"Molecular Weight (kDa)"=31.15122537999999,"Extinction Coefficient"=19605.0,"# Nucleotide Sequences With

Quality"=0,Topology="linear","Alignment method"="MAFFT Alignment",Created=Fri Jun 28 11:46:17 PDT 2024,"Charge at pH 7"=-5.574711435859037,"Isoelectric Point"=5.575748443603516,"% Basic Amino Acids"=12.24%,"% AT-rich Amino Acids"=15.73%,"% Polar Uncharged Amino Acids"=27.62%,"Free end gaps"=true,"Molecule Type"="AA"]:0.0963099999999999,(XP_002543522.1[&"% Charged Amino Acids"=26.92%,"% Acidic Amino Acids"=12.59%,"% Hydrophobic Amino Acids"=48.95%,Modified=Fri Jun 28 11:46:17 PDT 2024,"% GC-rich Amino Acids"=23.78%,"Molecular Weight (kDa)"=31.483921279999993,"Extinction Coefficient"=14105.0,"# Nucleotide Sequences With Quality"=0,Topology="linear","Alignment method"="MAFFT Alignment",Created=Fri Jun 28 11:46:17 PDT 2024,"Charge at pH 7"=-2.40438839054951,"Isoelectric Point"=6.292827606201172,"% Basic Amino Acids"=14.34%,"% AT-rich Amino Acids"=19.58%,"% Polar Uncharged Amino Acids"=24.83%,"Free end gaps"=true,"Molecule Type"="AA"]:0.15759999999999996,(KAI9774215.1[&"% Charged Amino Acids"=25.87%,"% Acidic Amino Acids"=12.24%,"% Hydrophobic Amino Acids"=47.90%,Modified=Fri Jun 28 11:46:17 PDT 2024,"% GC-rich Amino Acids"=21.68%,"Molecular Weight (kDa)"=31.32162668,"Extinction Coefficient"=21095.0,"# Nucleotide Sequences With Quality"=0,Topology="linear","Alignment method"="MAFFT Alignment",Created=Fri Jun 28 11:46:17 PDT 2024,"Charge at pH 7"=-4.3741271905474495,"Isoelectric Point"=6.005184173583984,"% Basic Amino Acids"=13.64%,"% AT-rich Amino Acids"=18.53%,"% Polar Uncharged Amino Acids"=27.27%,"Free end gaps"=true,"Molecule Type"="AA"]:0.056560000000000166,MCJ1392161.1[&"% Charged Amino Acids"=26.22%,"% Acidic Amino Acids"=13.29%,"% Hydrophobic Amino Acids"=47.90%,Modified=Fri Jun 28 11:46:17 PDT 2024,"% GC-rich Amino Acids"=21.68%,"Molecular Weight (kDa)"=31.422763779999993,"Extinction Coefficient"=22585.0,"# Nucleotide Sequences With Quality"=0,Topology="linear","Alignment method"="MAFFT Alignment",Created=Fri Jun 28 11:46:17 PDT 2024,"Charge at pH 7"=-6.599380872602497,"Isoelectric Point"=5.295635223388672,"% Basic Amino Acids"=12.94%,"% AT-rich Amino Acids"=17.83%,"% Polar Uncharged Amino Acids"=26.92%,"Free end gaps"=true,"Molecule Type"="AA"]:0.06973999999999991)[&"FastTree support value"=0.93]:0.039670000000000094)[&"FastTree support value"=0.308]:0.03283000000000014)[&"FastTree support value"=0.719]:0.012950000000000017)[&"FastTree support value"=0.996]:0.29590000000000005)[&"FastTree support value"=0.834]:0.11409000000000002,RSH87279.1[&"% Charged Amino Acids"=29.37%,"% Acidic Amino Acids"=15.03%,"% Hydrophobic Amino Acids"=47.20%,Modified=Fri Jun 28 11:48:55 PDT 2024,"% GC-rich Amino Acids"=24.13%,"Molecular Weight (kDa)"=31.83822928,"Extinction Coefficient"=7115.0,"# Nucleotide Sequences With Quality"=0,Topology="linear","Alignment method"="MAFFT Alignment",Created=Fri Jun 28 11:46:17 PDT 2024,"Charge at pH 7"=-5.858968677562601,"Isoelectric Point"=5.208797454833984,"% Basic Amino Acids"=14.34%,"% AT-rich Amino Acids"=20.63%,"% Polar Uncharged Amino Acids"=23.78%,"Free end gaps"=true,"Molecule Type"="AA"]:0.4797600000000002)[&"FastTree support

value"=0.376]:0.06965000000000021,KAI9096888.1[&"% Charged Amino Acids"=30.14%,"% Acidic Amino Acids"=15.07%,"% Hydrophobic Amino Acids"=44.18%,Modified=Fri Jun 28 11:51:56 PDT 2024,"% GC-rich Amino Acids"=17.47%,"Molecular Weight (kDa)"=33.101868080000024,"Extinction Coefficient"=20065.0,"# Nucleotide Sequences With Quality"=0,Topology="linear","Alignment method"="MAFFT Alignment",Created=Fri Jun 28 11:46:17 PDT 2024,"Charge at pH 7"=-4.760784008823309,"Isoelectric Point"=5.568431854248047,"% Basic Amino Acids"=15.07%,"% AT-rich Amino Acids"=25.68%,"% Polar Uncharged Amino Acids"=26.37%,"Free end gaps"=true,"Molecule Type"="AA"]:0.4826999999999999)[&"FastTree support value"=0.76]:0.12352000000000007)[&"FastTree support value"=0.9]:0.11972000000000005)[&"FastTree support value"=0.061]:0.055689999999999795)[&"FastTree support value"=0.902]:0.13669999999999982)[&"FastTree support value"=0.408]:0.0871599999999999)[&"FastTree support value"=0.991]:0.24147000000000007)[&"FastTree support value"=0.951]:0.12263000000000002)[&"FastTree support value"=0.27]:0.023219999999999796)[&"FastTree support value"=0.97]:0.17498000000000014)[&"FastTree support value"=0.975]:0.12927)[&"FastTree support value"=0.647]:0.07604999999999995,((((((TMW65229.1[&Organism="Pythium oligandrum","Genetic Code"="Standard",Taxonomy="Eukaryota; Sar; Stramenopiles; Oomycota; Pythiales; Pythiaceae; Pythium"]:0.20099,((KAG1689960.1[&Organism="Phytophthora capsici","Genetic Code"="Standard",Taxonomy="Eukaryota; Sar; Stramenopiles; Oomycota; Peronosporales; Peronosporaceae; Phytophthora"]:0.10802000000000023,(KAE9027747.1[&Organism="Phytophthora rubi","Genetic Code"="Standard",Taxonomy="Eukaryota; Sar; Stramenopiles; Oomycota; Peronosporales; Peronosporaceae; Phytophthora"]:0.10612000000000021,KAG7385474.1[&Organism="Phytophthora boehmeriae","Genetic Code"="Standard",Taxonomy="Eukaryota; Sar; Stramenopiles; Oomycota; Peronosporales; Peronosporaceae; Phytophthora"]:0.08560999999999996)[&"FastTree support value"=0.426]:0.026450000000000085)[&"FastTree support value"=0.24]:0.03445000000000009,((POM76499.1[&Organism="Phytophthora palmivora var. palmivora","Genetic Code"="Standard",Taxonomy="Eukaryota; Sar; Stramenopiles; Oomycota; Peronosporales; Peronosporaceae; Phytophthora"]:0.11807999999999996,(KAG7377001.1[&Organism="Phytophthora pseudosyringae","Genetic Code"="Standard",Taxonomy="Eukaryota; Sar; Stramenopiles; Oomycota; Peronosporales; Peronosporaceae; Phytophthora"]:0.06985999999999981,(XP_008910862.1[&Organism="Phytophthora parasitica INRA-310","Genetic Code"="Standard",Taxonomy="Eukaryota; Sar; Stramenopiles; Oomycota; Peronosporales; Peronosporaceae; Phytophthora"]:0.03135999999999983,(KAF1774311.1[&Organism="Phytophthora

cactorum","Genetic Code"="Standard",Taxonomy="Eukaryota; Sar; Stramenopiles; Oomycota; Peronosporales; Peronosporaceae; Phytophthora"]:0.04163000000000056,KAI9982172.1[&Organism="Phytophthora infestans","Genetic Code"="Standard",Taxonomy="Eukaryota; Sar; Stramenopiles; Oomycota; Peronosporales; Peronosporaceae; Phytophthora","Common Name"="potato late blight agent"]:0.14156000000000013)[&"FastTree support value"=0.391]:0.008869999999999933)[&"FastTree support value"=1.0]:0.07982999999999985)[&"FastTree support value"=0.986]:0.06231000000000009)[&"FastTree support value"=0.707]:0.031210000000000182,(KAH7489187.1[&Organism="Phytophthora ramorum","Genetic Code"="Standard",Taxonomy="Eukaryota; Sar; Stramenopiles; Oomycota; Peronosporales; Peronosporaceae; Phytophthora","Common Name"="sudden oak death agent"]:0.12908000000000008,KAE8986371.1[&Organism="Phytophthora rubi","Genetic Code"="Standard",Taxonomy="Eukaryota; Sar; Stramenopiles; Oomycota; Peronosporales; Peronosporaceae; Phytophthora"]:0.20874000000000015)[&"FastTree support value"=0.808]:0.04103000000000012)[&"FastTree support value"=0.922]:0.05310000000000015)[&"FastTree support value"=0.983]:0.15644999999999998)[&"FastTree support value"=1.0]:0.4383300000000001,(OUS45493.1[&Organism="Ostreococcus tauri","Genetic Code"="Standard",Taxonomy="Eukaryota; Viridiplantae; Chlorophyta; Mamiellophyceae; Mamiellales; Bathycoccaceae; Ostreococcus"]:0.13877000000000006,XP_001419538.1[&Organism="Ostreococcus lucimarinus CCE9901","Genetic Code"="Standard",Taxonomy="Eukaryota; Viridiplantae; Chlorophyta; Mamiellophyceae; Mamiellales; Bathycoccaceae; Ostreococcus"]:0.16000000000000014)[&"FastTree support value"=1.0]:0.5644299999999998)[&"FastTree support value"=0.517]:0.02259000000000011,(PRP82121.1[&Organism="Planoprotostelium fungivorum","Genetic Code"="Standard",Taxonomy="Eukaryota; Amoebozoa; Evosea; Variosea; Cavosteliida; Cavosteliaceae; Planoprotostelium"]:0.4748000000000001,XP_004338334.1[&Organism="Acanthamoeba castellanii str. Neff","Genetic Code"="Standard",Taxonomy="Eukaryota; Amoebozoa; Discosea; Longamoebia; Centramoebida; Acanthamoebidae; Acanthamoeba"]:0.6297000000000001)[&"FastTree support value"=0.879]:0.14516999999999998)[&"FastTree support value"=0.916]:0.17316000000000002,KAH8061769.1[&Organism="Aureococcus anophagefferens","Genetic Code"="Standard",Taxonomy="Eukaryota; Sar; Stramenopiles; Ochrophyta; Pelagophyceae; Pelagomonadales; Aureococcus"]:0.5608)[&"FastTree support value"=0.901]:0.1637900000000001,('XP_005785253.1'[&Organism="Emiliania huxleyi CCMP1516","Genetic Code"="Standard",Taxonomy="Eukaryota; Haptista; Haptophyta; Prymnesiophyceae; Isochrysidales; Noelaerhabdaceae; Emiliania"]:0.018990000000000062,XP_005792501.1[&Organism="Emiliania huxleyi CCMP1516","Genetic Code"="Standard",Taxonomy="Eukaryota; Haptista; Haptophyta; Prymnesiophyceae; Isochrysidales; Noelaerhabdaceae; Emiliania"]:0.0312800000000002)[&"FastTree support value"=1.0]:1.15504)[&"FastTree

support value"=0.829]:0.16366000000000014,((KAG8459568.1[&Organism="Diacronema lutheri","Genetic Code"="Standard",Taxonomy="Eukaryota; Haptista; Haptophyta; Pavlovales; Pavlovaceae; Diacronema"]:1.32491,KOO23261.1[&Organism="Chrysochromulina tobinii","Genetic Code"="Standard",Taxonomy="Eukaryota; Haptista; Haptophyta; Prymnesiophyceae; Prymnesiales; Chrysochromulinaceae; Chrysochromulina"]:0.95573)[&"FastTree support value"=0.907]:0.2870400000000002,(KAF5834941.1[&Organism="Dunaliella salina","Genetic Code"="Standard",Taxonomy="Eukaryota; Viridiplantae; Chlorophyta; core chlorophytes; Chlorophyceae; CS clade; Chlamydomonadales; Dunaliellaceae; Dunaliella"]:0.9129800000000001,(QFG74057.1[&Organism="Megaviridae environmental sample","Genetic Code"="Standard",Taxonomy="Viruses; Varidnaviria; Bamfordvirae; Nucleocytoviricota; Megaviricetes; Imitervirales; Mimiviridae; environmental samples","Common Name"="marine metagenome"]:1.4666000000000001,(ARF10781.1[&Organism="Hokovirus HKV1","Genetic Code"="Standard",Taxonomy="Viruses; Varidnaviria; Bamfordvirae; Nucleocytoviricota; Megaviricetes; Imitervirales; Mimiviridae; Klosneuvirinae; Hokovirus"]:0.21300000000000008,ARF10780.1[&Organism="Hokovirus HKV1","Genetic Code"="Standard",Taxonomy="Viruses; Varidnaviria; Bamfordvirae; Nucleocytoviricota; Megaviricetes; Imitervirales; Mimiviridae; Klosneuvirinae; Hokovirus"]:0.13137)[&"FastTree support value"=0.996]:0.7075900000000002)[&"FastTree support value"=0.538]:0.17702999999999998)[&"FastTree support value"=0.966]:0.32907999999999998)[&"FastTree support value"=0.877]:0.09935999999999989)[&"FastTree support value"=0.998]:0.3645900000000002,((((((OAE31801.1[&Organism="Marchantia polymorpha subsp. ruderalis","Genetic Code"="Standard",Taxonomy="Eukaryota; Viridiplantae; Streptophyta; Embryophyta; Marchantiophyta; Marchantiopsida; Marchantiidae; Marchantiales; Marchantiaceae; Marchantia"]:0.21699000000000002,(KAG0632288.1[&Organism="Ceratodon purpureus","Genetic Code"="Standard",Taxonomy="Eukaryota; Viridiplantae; Streptophyta; Embryophyta; Bryophyta; Bryophytina; Bryopsida; Dicranidae; Pseudoditrichales; Ditrichaceae; Ceratodon"]:0.20935999999999977,('KAG0555995.1'[&Organism="Ceratodon purpureus","Genetic Code"="Standard",Taxonomy="Eukaryota; Viridiplantae; Streptophyta; Embryophyta; Bryophyta; Bryophytina; Bryopsida; Dicranidae; Pseudoditrichales; Ditrichaceae; Ceratodon"]:0.03503999999999996,(XP_024368367.1[&Organism="Physcomitrium patens","Genetic Code"="Standard",Taxonomy="Eukaryota; Viridiplantae; Streptophyta; Embryophyta; Bryophyta; Bryophytina; Bryopsida; Funariidae; Funariales; Funariaceae; Physcomitrium"]:0.02084000000000019,XP_024391061.1[&Organism="Physcomitrium patens","Genetic Code"="Standard",Taxonomy="Eukaryota; Viridiplantae; Streptophyta; Embryophyta; Bryophyta; Bryophytina; Bryopsida; Funariidae; Funariales; Funariaceae; Physcomitrium"]:0.02240999999999982)[&"FastTree support value"=0.999]:0.08942000000000005)[&"FastTree support value"=0.982]:0.08761999999999981)[&"FastTree support

value"=0.953]:0.07377000000000011)[&"FastTree support
value"=0.137]:0.04112999999999989,(KAI5073815.1[&Organism="Adiantum capillus-
veneris","Genetic Code"="Standard",Taxonomy="Eukaryota; Viridiplantae; Streptophyta;
Embryophyta; Tracheophyta; Polypodiopsida; Polypodiidae; Polypodiales; Pteridineae;
Pteridaceae; Vittarioideae;
Adiantum"]:0.18188000000000004,((KAH9330549.1[&Organism="Taxus
chinensis","Genetic Code"="Standard",Taxonomy="Eukaryota; Viridiplantae; Streptophyta;
Embryophyta; Tracheophyta; Spermatophyta; Pinopsida; Pinidae; Conifers II; Cupressales;
Taxaceae; Taxus"]:0.12277000000000005,(XP_006385192.1[&Organism="Populus
trichocarpa","Genetic Code"="Standard",Taxonomy="Eukaryota; Viridiplantae;
Streptophyta; Embryophyta; Tracheophyta; Spermatophyta; Magnoliopsida;
eudicotyledons; Gunneridae; Pentapetalae; rosids; fabids; Malpighiales; Salicaceae;
Saliceae; Populus","Common Name"="Populus balsamifera subsp.
trichocarpa"]:0.08637000000000006,('KAG7649995.1'[&Organism="Arabidopsis
thaliana","Genetic Code"="Standard",Taxonomy="Eukaryota; Viridiplantae; Streptophyta;
Embryophyta; Tracheophyta; Spermatophyta; Magnoliopsida; eudicotyledons;
Gunneridae; Pentapetalae; rosids; malvids; Brassicales; Brassicaceae; Camelineae;
Arabidopsis","Common Name"="thale
cress"]:0.016129999999999978,'NP_172500.1'[&Organism="Arabidopsis
thaliana","Genetic Code"="Standard",Taxonomy="Eukaryota; Viridiplantae; Streptophyta;
Embryophyta; Tracheophyta; Spermatophyta; Magnoliopsida; eudicotyledons;
Gunneridae; Pentapetalae; rosids; malvids; Brassicales; Brassicaceae; Camelineae;
Arabidopsis","Common Name"="thale cress"]:0.04508000000000001)[&"FastTree support
value"=0.264]:0.02049000000000012)[&"FastTree support
value"=0.906]:0.04266000000000014)[&"FastTree support
value"=0.793]:0.027029999999999887,((XP_008646219.1[&Organism="Zea
mays","Genetic Code"="Standard",Taxonomy="Eukaryota; Viridiplantae; Streptophyta;
Embryophyta; Tracheophyta; Spermatophyta; Magnoliopsida; Liliopsida; Poales; Poaceae;
PACMAD clade; Panicoideae; Andropogonodae; Andropogoneae; Tripsacinae;
Zea"]:0.05087000000000019,ACG47836.1[&Organism="Zea mays","Genetic
Code"="Standard",Taxonomy="Eukaryota; Viridiplantae; Streptophyta; Embryophyta;
Tracheophyta; Spermatophyta; Magnoliopsida; Liliopsida; Poales; Poaceae; PACMAD
clade; Panicoideae; Andropogonodae; Andropogoneae; Tripsacinae;
Zea"]:0.07233)[&"FastTree support
value"=0.969]:0.05390000000000006,ONM04707.1[&Organism="Zea mays","Genetic
Code"="Standard",Taxonomy="Eukaryota; Viridiplantae; Streptophyta; Embryophyta;
Tracheophyta; Spermatophyta; Magnoliopsida; Liliopsida; Poales; Poaceae; PACMAD
clade; Panicoideae; Andropogonodae; Andropogoneae; Tripsacinae;
Zea"]:0.24454999999999982)[&"FastTree support
value"=0.781]:0.049269999999999925)[&"FastTree support
value"=0.976]:0.08214999999999995)[&"FastTree support
value"=0.996]:0.1267999999999998)[&"FastTree support
value"=0.963]:0.07722000000000007,EFJ28901.1[&Organism="Selaginella
moellendorffii","Genetic Code"="Standard",Taxonomy="Eukaryota; Viridiplantae;

Streptophyta; Embryophyta; Tracheophyta; Lycopodiopsida; Selaginellales;
Selaginellaceae; Selaginella"]:0.21460999999999997)[&"FastTree support
value"=0.266]:0.046619999999999884,EFJ33653.1[&Organism="Selaginella
moellendorffii","Genetic Code"="Standard",Taxonomy="Eukaryota; Viridiplantae;
Streptophyta; Embryophyta; Tracheophyta; Lycopodiopsida; Selaginellales;
Selaginellaceae; Selaginella"]:0.15928999999999993)[&"FastTree support
value"=1.0]:1.3084500000000001,((CEM26963.1[&Organism="Vitrella brassicaformis
CCMP3155","Genetic Code"="Standard",Taxonomy="Eukaryota; Sar; Alveolata;
Colpodellida; Vitrellaceae;
Vitrella"]:0.7260499999999999,(((XP_001617280.1[&Organism="Plasmodium
vivax","Genetic Code"="Standard",Taxonomy="Eukaryota; Sar; Alveolata; Apicomplexa;
Aconoidasida; Haemosporida; Plasmodiidae; Plasmodium; Plasmodium
(Plasmodium)","Common Name"="malaria parasite P.
vivax"]:0.06646999999999981,XP_028539355.1[&Organism="Plasmodium sp. gorilla clade
G2","Genetic Code"="Standard",Taxonomy="Eukaryota; Sar; Alveolata; Apicomplexa;
Aconoidasida; Haemosporida; Plasmodiidae; Plasmodium; Plasmodium
(Laverania)"]:0.0032500000000004192)[&"FastTree support
value"=1.0]:0.4323199999999998,((PHJ24853.1[&Organism="Cystoisospora suis","Genetic
Code"="Standard",Taxonomy="Eukaryota; Sar; Alveolata; Apicomplexa; Conoidasida;
Coccidia; Eucoccidiorida; Eimeriorina; Sarcocystidae;
Cystoisospora"]:0.05916000000000032,KFG43683.1[&Organism="Toxoplasma gondii
GAB2-2007-GAL-DOM2","Genetic Code"="Standard",Taxonomy="Eukaryota; Sar; Alveolata;
Apicomplexa; Conoidasida; Coccidia; Eucoccidiorida; Eimeriorina; Sarcocystidae;
Toxoplasma"]:0.09527999999999981)[&"FastTree support
value"=0.998]:0.17865999999999982,(KAH0479249.1[&Organism="Porospora cf. gigantea
B","Genetic Code"="Standard",Taxonomy="Eukaryota; Sar; Alveolata; Apicomplexa;
Conoidasida; Gregarinasina; Eugregarinorida; Porosporidae; Porospora","Common
Name"="symbiont
metagenome"]:0.3367800000000001,((GFE54186.1[&Organism="Babesia ovis","Genetic
Code"="Standard",Taxonomy="Eukaryota; Sar; Alveolata; Apicomplexa; Aconoidasida;
Piroplasmida; Babesiidae;
Babesia"]:0.06883999999999979,(XP_012766661.1[&Organism="Babesia
bigemina","Genetic Code"="Standard",Taxonomy="Eukaryota; Sar; Alveolata; Apicomplexa;
Aconoidasida; Piroplasmida; Babesiidae;
Babesia"]:0.0743999999999998,GIX62800.1[&Organism="Babesia caballi","Genetic
Code"="Standard",Taxonomy="Eukaryota; Sar; Alveolata; Apicomplexa; Aconoidasida;
Piroplasmida; Babesiidae; Babesia"]:0.05032000000000014)[&"FastTree support
value"=0.907]:0.04148999999999958)[&"FastTree support
value"=0.998]:0.18449000000000026,(UKJ88078.2[&Organism="Theileria
orientalis","Genetic Code"="Standard",Taxonomy="Eukaryota; Sar; Alveolata; Apicomplexa;
Aconoidasida; Piroplasmida; Theileriidae;
Theileria"]:0.1251300000000004,XP_004833148.1[&Organism="Theileria equi strain
WA","Genetic Code"="Standard",Taxonomy="Eukaryota; Sar; Alveolata; Apicomplexa;
Aconoidasida; Piroplasmida; Theileriidae; Theileria"]:0.0329600000000001)[&"FastTree

support value"=0.976]:0.12664999999999997)[&"FastTree support value"=0.997]:0.19062999999999963)[&"FastTree support value"=0.795]:0.08690999999999996)[&"FastTree support value"=0.829]:0.07521000000000022)[&"FastTree support value"=0.987]:0.2902899999999997,(OII76931.1[&Organism="Cryptosporidium andersoni","Genetic Code"="Standard",Taxonomy="Eukaryota; Sar; Alveolata; Apicomplexa; Conoidasida; Coccidia; Eucoccidiorida; Eimeriorina; Cryptosporidiidae; Cryptosporidium"]:0.49260000000000037,(KAH8582109.1[&Organism="Cryptosporidium sp. chipmunk genotype I","Genetic Code"="Standard",Taxonomy="Eukaryota; Sar; Alveolata; Apicomplexa; Conoidasida; Coccidia; Eucoccidiorida; Eimeriorina; Cryptosporidiidae; Cryptosporidium"]:0.16917999999999989,(POM84969.1[&Organism="Cryptosporidium meleagridis","Genetic Code"="Standard",Taxonomy="Eukaryota; Sar; Alveolata; Apicomplexa; Conoidasida; Coccidia; Eucoccidiorida; Eimeriorina; Cryptosporidiidae; Cryptosporidium"]:0.12087000000000003,XP_667128.1[&Organism="Cryptosporidium hominis TU502","Genetic Code"="Standard",Taxonomy="Eukaryota; Sar; Alveolata; Apicomplexa; Conoidasida; Coccidia; Eucoccidiorida; Eimeriorina; Cryptosporidiidae; Cryptosporidium"]:0.000540)[&"FastTree support value"=0.916]:0.05318999999999985)[&"FastTree support value"=0.99]:0.27501)[&"FastTree support value"=0.994]:0.3790699999999996)[&"FastTree support value"=0.92]:0.28878999999999966)[&"FastTree support value"=1.0]:1.3760599999999998,(((XP_042918632.1[&Organism="Chlamydomonas reinhardtii","Genetic Code"="Standard",Taxonomy="Eukaryota; Viridiplantae; Chlorophyta; core chlorophytes; Chlorophyceae; CS clade; Chlamydomonadales; Chlamydomonadaceae; Chlamydomonas"]:0.14283,(((PWZ11893.1[&Organism="Zea mays","Genetic Code"="Standard",Taxonomy="Eukaryota; Viridiplantae; Streptophyta; Embryophyta; Tracheophyta; Spermatophyta; Magnoliopsida; Liliopsida; Poales; Poaceae; PACMAD clade; Panicoideae; Andropogonodae; Andropogoneae; Tripsacinae; Zea"]:0.017739999999999867,NP_001130364.1[&Organism="Zea mays","Genetic Code"="Standard",Taxonomy="Eukaryota; Viridiplantae; Streptophyta; Embryophyta; Tracheophyta; Spermatophyta; Magnoliopsida; Liliopsida; Poales; Poaceae; PACMAD clade; Panicoideae; Andropogonodae; Andropogoneae; Tripsacinae; Zea"]:0.020019999999999705)[&"FastTree support value"=1.0]:0.1075999999999997,(AAF87857.1[&Organism="Arabidopsis thaliana","Genetic Code"="Standard",Taxonomy="Eukaryota; Viridiplantae; Streptophyta; Embryophyta; Tracheophyta; Spermatophyta; Magnoliopsida; eudicotyledons; Gunneridae; Pentapetalae; rosids; malvids; Brassicales; Brassicaceae; Camelineae; Arabidopsis","Common Name"="thale cress"]:0.04190000000000005,XP_002317496.2[&Organism="Populus trichocarpa","Genetic Code"="Standard",Taxonomy="Eukaryota; Viridiplantae; Streptophyta; Embryophyta; Tracheophyta; Spermatophyta; Magnoliopsida; eudicotyledons; Gunneridae; Pentapetalae; rosids; fabids; Malpighiales; Salicaceae; Saliceae; Populus","Common Name"="Populus balsamifera subsp.

trichocarpa"]:0.08134999999999959)[&"FastTree support
value"=0.947]:0.04961000000000376)[&"FastTree support
value"=0.877]:0.04376000000000024,((KAI5058044.1[&Organism="Adiantum capillus-
veneris","Genetic Code"="Standard",Taxonomy="Eukaryota; Viridiplantae; Streptophyta;
Embryophyta; Tracheophyta; Polypodiopsida; Polypodiidae; Polypodiales; Pteridineae;
Pteridaceae; Vittarioideae;
Adiantum"]:0.1295200000000003,KAH9322298.1[&Organism="Taxus chinensis","Genetic
Code"="Standard",Taxonomy="Eukaryota; Viridiplantae; Streptophyta; Embryophyta;
Tracheophyta; Spermatophyta; Pinopsida; Pinidae; Conifers II; Cupressales; Taxaceae;
Taxus"]:0.11385000000000023)[&"FastTree support
value"=0.099]:0.03629999999999978,((PTQ33908.1[&Organism="Marchantia
polymorpha","Genetic Code"="Standard",Taxonomy="Eukaryota; Viridiplantae;
Streptophyta; Embryophyta; Marchantiophyta; Marchantiopsida; Marchantiidae;
Marchantiales; Marchantiaceae; Marchantia","Common
Name"="liverwort"]:0.10622000000000043,KAG0628798.1[&Organism="Ceratodon
purpureus","Genetic Code"="Standard",Taxonomy="Eukaryota; Viridiplantae; Streptophyta;
Embryophyta; Bryophyta; Bryophytina; Bryopsida; Dicranidae; Pseudoditrichales;
Ditrichaceae; Ceratodon"]:0.16798000000000002)[&"FastTree support
value"=0.718]:0.02587000000000028,EFJ18064.1[&Organism="Selaginella
moellendorffii","Genetic Code"="Standard",Taxonomy="Eukaryota; Viridiplantae;
Streptophyta; Embryophyta; Tracheophyta; Lycopodiopsida; Selaginellales;
Selaginellaceae; Selaginella"]:0.15322999999999976)[&"FastTree support
value"=0.463]:0.028999999999999915)[&"FastTree support
value"=0.919]:0.05228000000000104)[&"FastTree support
value"=0.935]:0.1004299999999998)[&"FastTree support
value"=0.999]:0.5425299999999997,((XP_002683545.1[&Organism="Naegleria
gruberi","Genetic Code"="Standard",Taxonomy="Eukaryota; Discoba; Heterolobosea;
Tetramitia; Eutetramitia; Vahlkampfiidae;
Naegleria"]:0.06547000000000036,(XP_044544418.1[&Organism="Naegleria
lovaniensis","Genetic Code"="Standard",Taxonomy="Eukaryota; Discoba; Heterolobosea;
Tetramitia; Eutetramitia; Vahlkampfiidae;
Naegleria"]:0.009619999999999962,XP_044559198.1[&Organism="Naegleria
fowleri","Genetic Code"="Standard",Taxonomy="Eukaryota; Discoba; Heterolobosea;
Tetramitia; Eutetramitia; Vahlkampfiidae; Naegleria"]:0.000540)[&"FastTree support
value"=0.958]:0.10217999999999972)[&"FastTree support
value"=1.0]:0.4494799999999999,((XP_004182822.1[&Organism="Entamoeba invadens
IP1","Genetic Code"="Standard",Taxonomy="Eukaryota; Amoebozoa; Evosea;
Archamoebae; Mastigamoebida; Entamoebidae;
Entamoeba"]:0.05579999999999963,XP_008857507.1[&Organism="Entamoeba nuttalli
P19","Genetic Code"="Standard",Taxonomy="Eukaryota; Amoebozoa; Evosea;
Archamoebae; Mastigamoebida; Entamoebidae;
Entamoeba"]:0.12622999999999962)[&"FastTree support
value"=1.0]:0.2764899999999999,(QYA18543.1[&Organism="Clandestinovirus","Genetic
Code"="Standard",Taxonomy="Viruses; unclassified

viruses"]:0.39929999999999977,(((KAH3742895.1[&Organism="Pelomyxa
schiedti","Genetic Code"="Standard",Taxonomy="Eukaryota; Amoebozoa; Evosea;
Archamoebae; Pelobiontida; Pelomyxidae;
Pelomyxa"]:0.2209500000000002,(KYQ94066.1[&Organism="Tieghemostelium
lacteum","Genetic Code"="Standard",Taxonomy="Eukaryota; Amoebozoa; Evosea;
Eumycetozoa; Dictyostelia; Dictyosteliales; Raperosteliaceae;
Tieghemostelium"]:0.15817000000000014,(XP_004366192.1[&Organism="Cavenderia
fasciculata","Genetic Code"="Standard",Taxonomy="Eukaryota; Amoebozoa; Evosea;
Eumycetozoa; Dictyostelia; Acytosteliales; Cavenderiaceae;
Cavenderia"]:0.13541999999999987,(XP_645576.2[&Organism="Dictyostelium
discoideum AX4","Genetic Code"="Standard",Taxonomy="Eukaryota; Amoebozoa; Evosea;
Eumycetozoa; Dictyostelia; Dictyosteliales; Dictyosteliaceae;
Dictyostelium"]:0.06266000000000016,((KAF2073270.1[&Organism="Polysphondylium
violaceum","Genetic Code"="Standard",Taxonomy="Eukaryota; Amoebozoa; Evosea;
Eumycetozoa; Dictyostelia; Dictyosteliales; Dictyosteliaceae;
Polysphondylium"]:0.07704000000000022,(XP_020428321.1[&Organism="Heterostelium
album PN500","Genetic Code"="Standard",Taxonomy="Eukaryota; Amoebozoa; Evosea;
Eumycetozoa; Dictyostelia; Acytosteliales; Acytosteliaceae;
Heterostelium"]:0.07761999999999958,XP_012754836.1[&Organism="Acytostelium
subglobosum LB1","Genetic Code"="Standard",Taxonomy="Eukaryota; Amoebozoa;
Evosea; Eumycetozoa; Dictyostelia; Acytosteliales; Acytosteliaceae;
Acytostelium"]:0.04940999999999954)[&"FastTree support
value"=0.987]:0.06695000000000029)[&"FastTree support
value"=0.957]:0.04847999999999634,XP_003288319.1[&Organism="Dictyostelium
purpureum","Genetic Code"="Standard",Taxonomy="Eukaryota; Amoebozoa; Evosea;
Eumycetozoa; Dictyostelia; Dictyosteliales; Dictyosteliaceae;
Dictyostelium"]:0.06130000000000013)[&"FastTree support
value"=0.241]:0.03575000000000017)[&"FastTree support
value"=0.468]:0.03338000000000019)[&"FastTree support
value"=0.087]:0.02908000000000044)[&"FastTree support
value"=0.996]:0.16906999999999996)[&"FastTree support
value"=0.73]:0.07209999999999983,PRP82286.1[&Organism="Planoprotostelium
fungivorum","Genetic Code"="Standard",Taxonomy="Eukaryota; Amoebozoa; Evosea;
Variosea; Cavosteliida; Cavosteliaceae;
Planoprotostelium"]:0.25041000000000047)[&"FastTree support
value"=0.355]:0.05834999999999999,XP_004340186.1[&Organism="Acanthamoeba
castellanii str. Neff","Genetic Code"="Standard",Taxonomy="Eukaryota; Amoebozoa;
Discosea; Longamoebia; Centramoebida; Acanthamoebidae;
Acanthamoeba"]:0.23125000000000018)[&"FastTree support
value"=0.236]:0.05364000000000013)[&"FastTree support
value"=0.298]:0.10349999999999993)[&"FastTree support
value"=0.776]:0.1766000000000001)[&"FastTree support
value"=0.962]:0.19451000000000018)[&"FastTree support
value"=0.92]:0.2195800000000001,((((XP_002649212.1[&Organism="Dictyostelium

discoideum AX4","Genetic Code"="Standard",Taxonomy="Eukaryota; Amoebozoa; Evosea; Eumycetozoa; Dictyostelia; Dictyosteliales; Dictyosteliaceae; Dictyostelium"]:0.10060000000000002,XP_003288465.1[&Organism="Dictyostelium purpureum","Genetic Code"="Standard",Taxonomy="Eukaryota; Amoebozoa; Evosea; Eumycetozoa; Dictyostelia; Dictyosteliales; Dictyosteliaceae; Dictyostelium"]:0.0636000000000001)[&"FastTree support value"=0.967]:0.12772000000000006,(KAF2077035.1[&Organism="Polysphondylium violaceum","Genetic Code"="Standard",Taxonomy="Eukaryota; Amoebozoa; Evosea; Eumycetozoa; Dictyostelia; Dictyosteliales; Dictyosteliaceae; Polysphondylium"]:0.10334000000000021,(KYQ90260.1[&Organism="Tieghemostelium lacteum","Genetic Code"="Standard",Taxonomy="Eukaryota; Amoebozoa; Evosea; Eumycetozoa; Dictyostelia; Dictyosteliales; Raperosteliaceae; Tieghemostelium"]:0.16019999999999968,(XP_004360608.1[&Organism="Cavenderia fasciculata","Genetic Code"="Standard",Taxonomy="Eukaryota; Amoebozoa; Evosea; Eumycetozoa; Dictyostelia; Acytosteliales; Cavenderiaceae; Cavenderia"]:0.16518999999999995,(XP_020436215.1[&Organism="Heterostelium album PN500","Genetic Code"="Standard",Taxonomy="Eukaryota; Amoebozoa; Evosea; Eumycetozoa; Dictyostelia; Acytosteliales; Acytosteliaceae; Heterostelium"]:0.048659999999999926,XP_012753198.1[&Organism="Acytostelium subglobosum LB1","Genetic Code"="Standard",Taxonomy="Eukaryota; Amoebozoa; Evosea; Eumycetozoa; Dictyostelia; Acytosteliales; Acytosteliaceae; Acytostelium"]:0.14177000000000017)[&"FastTree support value"=0.97]:0.06027999999999967)[&"FastTree support value"=0.953]:0.05011999999999972)[&"FastTree support value"=0.692]:0.03509999999999991)[&"FastTree support value"=0.953]:0.0922799999999997)[&"FastTree support value"=1.0]:0.4308899999999998,(XP_004336224.1[&Organism="Acanthamoeba castellanii str. Neff","Genetic Code"="Standard",Taxonomy="Eukaryota; Amoebozoa; Discosea; Longamoebia; Centramoebida; Acanthamoebidae; Acanthamoeba"]:0.3453000000000004,KAH3767868.1[&Organism="Pelomyxa schiedti","Genetic Code"="Standard",Taxonomy="Eukaryota; Amoebozoa; Evosea; Archamoebae; Pelobiontida; Pelomyxidae; Pelomyxa"]:0.92109)[&"FastTree support value"=0.161]:0.08522000000000007)[&"FastTree support value"=1.0]:0.54101,(XP_005775651.1[&Organism="Emiliania huxleyi CCMP1516","Genetic Code"="Standard",Taxonomy="Eukaryota; Haptista; Haptophyta; Prymnesiophyceae; Isochrysidales; Noelaerhabdaceae; Emiliania"]:1.0780600000000002,((XP_005767412.1[&Organism="Emiliania huxleyi CCMP1516","Genetic Code"="Standard",Taxonomy="Eukaryota; Haptista; Haptophyta; Prymnesiophyceae; Isochrysidales; Noelaerhabdaceae; Emiliania"]:0.3970600000000002,KOO34265.1[&Organism="Chrysochromulina tobinii","Genetic Code"="Standard",Taxonomy="Eukaryota; Haptista; Haptophyta; Prymnesiophyceae; Prymnesiales; Chrysochromulinaceae; Chrysochromulina"]:0.36938999999999966)[&"FastTree support value"=0.903]:0.12360000000000015,((XP_042920073.1[&Organism="Chlamydomonas

reinhardtii","Genetic Code"="Standard",Taxonomy="Eukaryota; Viridiplantae; Chlorophyta; core chlorophytes; Chlorophyceae; CS clade; Chlamydomonadales; Chlamydomonadaceae; Chlamydomonas"]:0.2731500000000002,((EFJ19523.1[&Organism="Selaginella moellendorffii","Genetic Code"="Standard",Taxonomy="Eukaryota; Viridiplantae; Streptophyta; Embryophyta; Tracheophyta; Lycopodiopsida; Selaginellales; Selaginellaceae; Selaginella"]:0.13504000000000005,(KAH9308354.1[&Organism="Taxus chinensis","Genetic Code"="Standard",Taxonomy="Eukaryota; Viridiplantae; Streptophyta; Embryophyta; Tracheophyta; Spermatophyta; Pinopsida; Pinidae; Conifers II; Cupressales; Taxaceae; Taxus"]:0.11695000000000011,((PTQ34556.1[&Organism="Marchantia polymorpha","Genetic Code"="Standard",Taxonomy="Eukaryota; Viridiplantae; Streptophyta; Embryophyta; Marchantiophyta; Marchantiopsida; Marchantiidae; Marchantiales; Marchantiaceae; Marchantia","Common Name"="liverwort"]:0.10381000000000018,(KAG0561482.1[&Organism="Ceratodon purpureus","Genetic Code"="Standard",Taxonomy="Eukaryota; Viridiplantae; Streptophyta; Embryophyta; Bryophyta; Bryophytina; Bryopsida; Dicranidae; Pseudoditrichales; Ditrichaceae; Ceratodon"]:0.10543000000000013,KAG0605142.1[&Organism="Ceratodon purpureus","Genetic Code"="Standard",Taxonomy="Eukaryota; Viridiplantae; Streptophyta; Embryophyta; Bryophyta; Bryophytina; Bryopsida; Dicranidae; Pseudoditrichales; Ditrichaceae; Ceratodon"]:0.13504999999999967)[&"FastTree support value"=0.91]:0.030579999999999607)[&"FastTree support value"=0.635]:0.03324999999999978,KAI5064281.1[&Organism="Adiantum capillus-veneris","Genetic Code"="Standard",Taxonomy="Eukaryota; Viridiplantae; Streptophyta; Embryophyta; Tracheophyta; Polypodiopsida; Polypodiidae; Polypodiales; Pteridineae; Pteridaceae; Vittarioideae; Adiantum"]:0.07157999999999998)[&"FastTree support value"=0.136]:0.020459999999999923)[&"FastTree support value"=0.281]:0.02245000000000008)[&"FastTree support value"=0.948]:0.08145000000000024,((NP_001189935.1[&Organism="Arabidopsis thaliana","Genetic Code"="Standard",Taxonomy="Eukaryota; Viridiplantae; Streptophyta; Embryophyta; Tracheophyta; Spermatophyta; Magnoliopsida; eudicotyledons; Gunneridae; Pentapetalae; rosids; malvids; Brassicales; Brassicaceae; Camelineae; Arabidopsis","Common Name"="thale cress"]:0.14240999999999993,XP_002309632.3[&Organism="Populus trichocarpa","Genetic Code"="Standard",Taxonomy="Eukaryota; Viridiplantae; Streptophyta; Embryophyta; Tracheophyta; Spermatophyta; Magnoliopsida; eudicotyledons; Gunneridae; Pentapetalae; rosids; fabids; Malpighiales; Salicaceae; Saliceae; Populus","Common Name"="Populus balsamifera subsp. trichocarpa"]:0.07190999999999992)[&"FastTree support value"=0.857]:0.03291000000000022,PWZ44616.1[&Organism="Zea mays","Genetic Code"="Standard",Taxonomy="Eukaryota; Viridiplantae; Streptophyta; Embryophyta; Tracheophyta; Spermatophyta; Magnoliopsida; Liliopsida; Poales; Poaceae; PACMAD clade; Panicoideae; Andropogonodae; Andropogoneae; Tripsacinae; Zea"]:0.25251999999999963)[&"FastTree support value"=0.9]:0.06616)[&"FastTree support value"=0.933]:0.07946000000000009)[&"FastTree support

value"=0.997]:0.21130999999999966,((XP_009032466.1.2[&Organism="Aureococcus anophagefferens","Genetic Code"="Standard",Taxonomy="Eukaryota; Sar; Stramenopiles; Ochrophyta; Pelagophyceae; Pelagomonadales; Aureococcus"]:0.000550,XP_009032466.1[&Organism="Aureococcus anophagefferens","Genetic Code"="Standard",Taxonomy="Eukaryota; Sar; Stramenopiles; Ochrophyta; Pelagophyceae; Pelagomonadales; Aureococcus"]:0.000550)[&"FastTree support value"=1.0]:0.6176599999999999,((EWM28268.1[&Organism="Nannochloropsis gaditana","Genetic Code"="Standard",Taxonomy="Eukaryota; Sar; Stramenopiles; Ochrophyta; Eustigmatophyceae; Eustigmatales; Monodopsidaceae; Nannochloropsis"]:0.24943999999999988,(KAG5184668.1[&Organism="Tribonema minus","Genetic Code"="Standard",Taxonomy="Eukaryota; Sar; Stramenopiles; Ochrophyta; PX clade; Xanthophyceae; Tribonematales; Tribonemataceae; Tribonema"]:0.11151999999999962,CBN78455.1[&Organism="Ectocarpus siliculosus","Genetic Code"="Standard",Taxonomy="Eukaryota; Sar; Stramenopiles; Ochrophyta; PX clade; Phaeophyceae; Ectocarpales; Ectocarpaceae; Ectocarpus"]:0.09529999999999994)[&"FastTree support value"=0.717]:0.05337999999999976)[&"FastTree support value"=0.962]:0.10038999999999998,((GMI61978.1[&Organism="Parmales sp. scaly parma","Genetic Code"="Standard",Taxonomy="Eukaryota; Sar; Stramenopiles; Ochrophyta; Bolidophyceae; Parmales"]:0.21358999999999995,(GMH67967.1[&Organism="Triparma retinervis","Genetic Code"="Standard",Taxonomy="Eukaryota; Sar; Stramenopiles; Ochrophyta; Bolidophyceae; Parmales; Triparmaceae; Triparma"]:0.06315999999999988,(GMH92561.1[&Organism="Triparma strigata","Genetic Code"="Standard",Taxonomy="Eukaryota; Sar; Stramenopiles; Ochrophyta; Bolidophyceae; Parmales; Triparmaceae; Triparma"]:0.0176400000000001,GMI07688.1[&Organism="Triparma laevis f. longispina","Genetic Code"="Standard",Taxonomy="Eukaryota; Sar; Stramenopiles; Ochrophyta; Bolidophyceae; Parmales; Triparmaceae; Triparma"]:0.03950999999999934)[&"FastTree support value"=0.935]:0.04161000000000037)[&"FastTree support value"=0.93]:0.05100999999999978)[&"FastTree support value"=0.634]:0.07538999999999962,(EJK67908.1[&Organism="Thalassiosira oceanica","Genetic Code"="Standard",Taxonomy="Eukaryota; Sar; Stramenopiles; Ochrophyta; Bacillariophyta; Coscinodiscophyceae; Thalassiosirophycidae; Thalassiosirales; Thalassiosiraceae; Thalassiosira"]:0.15036000000000005,(KAI2494507.1[&Organism="Fragilaria crotonensis","Genetic Code"="Standard",Taxonomy="Eukaryota; Sar; Stramenopiles; Ochrophyta; Bacillariophyta; Fragilariophyceae; Fragilariophycidae; Fragilariales; Fragilariaceae; Fragilaria"]:0.1797500000000003,(CAB9516894.1[&Organism="Seminavis robusta","Genetic Code"="Standard",Taxonomy="Eukaryota; Sar; Stramenopiles; Ochrophyta; Bacillariophyta; Bacillariophyceae; Bacillariophycidae; Naviculales; Naviculaceae; Seminavis"]:0.11756999999999973,GKY99394.1[&Organism="Mayamaea pseudoterrestris","Genetic Code"="Standard",Taxonomy="Eukaryota; Sar; Stramenopiles;

Ochrophyta; Bacillariophyta; Bacillariophyceae; Bacillariophycidae; Naviculales; Naviculaceae; Mayamaea"]:0.17175000000000003)[&"FastTree support value"=0.81]:0.05264000000000024)[&"FastTree support value"=0.991]:0.10407000000000001)[&"FastTree support value"=0.137]:0.03343999999999969)[&"FastTree support value"=0.995]:0.15721999999999997)[&"FastTree support value"=0.812]:0.11462000000000039)[&"FastTree support value"=0.986]:0.19018000000000024)[&"FastTree support value"=0.861]:0.08960999999999997)[&"FastTree support value"=0.855]:0.17083000000000004)[&"FastTree support value"=0.988]:0.43073000000000006)[&"FastTree support value"=0.77]:0.15465000000000018)[&"FastTree support value"=0.995]:0.7263000000000002)[&"FastTree support value"=0.957]:0.3974700000000002)[&"FastTree support value"=0.421]:0.11840999999999999,((PAA87312.1[&Organism="Macrostomum lignano","Genetic Code"="Standard",Taxonomy="Eukaryota; Metazoa; Platyhelminthes; Rhabditophora; Macrostomorpha; Macrostomida; Macrostomidae; Macrostomum"]:0.09492000000000012,PAA68234.1[&Organism="Macrostomum lignano","Genetic Code"="Standard",Taxonomy="Eukaryota; Metazoa; Platyhelminthes; Rhabditophora; Macrostomorpha; Macrostomida; Macrostomidae; Macrostomum"]:0.11172000000000004)[&"FastTree support value"=0.865]:0.14550000000000018,(MEN2496893[&Organism="Marteilia pararefringens","Genetic Code"="Standard",Taxonomy="Eukaryota; Sar; Rhizaria; Endomyxa; Ascetosporea; Paramyxea; Paramyxida; Marteiliidae; Marteilia","Common Name"="invertebrate metagenome"]:0.5962100000000001,(((XP_002602331.1[&Organism="Branchiostoma floridae","Genetic Code"="Standard",Taxonomy="Eukaryota; Metazoa; Chordata; Cephalochordata; Branchiostomidae; Branchiostoma","Common Name"="Florida lancelet"]:0.005679999999999907,XP_019637857.1[&Organism="Branchiostoma belcheri","Genetic Code"="Standard",Taxonomy="Eukaryota; Metazoa; Chordata; Cephalochordata; Branchiostomidae; Branchiostoma","Common Name"="Belcher's lancelet"]:0.007629999999999804)[&"FastTree support value"=0.999]:0.1109300000000002,((XP_018667792.1[&Organism="Ciona intestinalis","Genetic Code"="Standard",Taxonomy="Eukaryota; Metazoa; Chordata; Tunicata; Ascidiacea; Enterogona; Phlebobranchia; Cionidae; Ciona","Common Name"="vase tunicate"]:0.12145999999999999,(XP_032818114.1[&Organism="Petromyzon marinus","Genetic Code"="Standard",Taxonomy="Eukaryota; Metazoa; Chordata; Craniata; Vertebrata; Cyclostomata; Hyperoartia; Petromyzontiformes; Petromyzontidae; Petromyzon","Common Name"="sea lamprey"]:0.061189999999999856,((XP_028587646.1[&Organism="Podarcis muralis","Genetic Code"="Standard",Taxonomy="Eukaryota; Metazoa; Chordata; Craniata; Vertebrata; Euteleostomi; Lepidosauria; Squamata; Bifurcata; Unidentata; Episquamata; Laterata; Lacertibaenia; Lacertidae; Podarcis","Common Name"="Common wall lizard"]:0.019680000000000142,((XP_023440724.1[&Organism="Dasypus

novemcinctus","Genetic Code"="Standard",Taxonomy="Eukaryota; Metazoa; Chordata; Craniata; Vertebrata; Euteleostomi; Mammalia; Eutheria; Xenarthra; Cingulata; Dasypodidae; Dasypus","Common Name"="nine-banded armadillo"]:0.006149999999999878,(XP_005873264.1[&Organism="Myotis brandtii","Genetic Code"="Standard",Taxonomy="Eukaryota; Metazoa; Chordata; Craniata; Vertebrata; Euteleostomi; Mammalia; Eutheria; Laurasiatheria; Chiroptera; Microchiroptera; Vespertilionidae; Myotis","Common Name"="Brandt's bat"]:0.006660000000000011,(NP_598513.1[&Organism="Mus musculus","Genetic Code"="Standard",Taxonomy="Eukaryota; Metazoa; Chordata; Craniata; Vertebrata; Euteleostomi; Mammalia; Eutheria; Euarchontoglires; Glires; Rodentia; Myomorpha; Muroidea; Muridae; Murinae; Mus; Mus","Common Name"="house mouse"]:0.0,XP_006163024.2.2[&Organism="Tupaia chinensis","Genetic Code"="Standard",Taxonomy="Eukaryota; Metazoa; Chordata; Craniata; Vertebrata; Euteleostomi; Mammalia; Eutheria; Euarchontoglires; Scandentia; Tupaiidae; Tupaia","Common Name"="Chinese tree shrew"]:0.0,NP_056375.2.2[&Organism="Homo sapiens","Genetic Code"="Standard",Taxonomy="Eukaryota; Metazoa; Chordata; Craniata; Vertebrata; Euteleostomi; Mammalia; Eutheria; Euarchontoglires; Primates; Haplorrhini; Catarrhini; Hominidae; Homo","Common Name"="human"]:0.0):0.000550)[&"FastTree support value"=0.875]:0.007260000000000044)[&"FastTree support value"=0.999]:0.036150000000000126,XP_025913835.1[&Organism="Apteryx rowi","Genetic Code"="Standard",Taxonomy="Eukaryota; Metazoa; Chordata; Craniata; Vertebrata; Euteleostomi; Archelosauria; Archosauria; Dinosauria; Saurischia; Theropoda; Coelurosauria; Aves; Palaeognathae; Apterygiformes; Apterygidae; Apteryx","Common Name"="Okarito brown kiwi"]:0.004519999999998575)[&"FastTree support value"=0.273]:0.006450000000000067)[&"FastTree support value"=0.907]:0.016669999999999963,(XP_021332524.1[&Organism="Danio rerio","Genetic Code"="Standard",Taxonomy="Eukaryota; Metazoa; Chordata; Craniata; Vertebrata; Euteleostomi; Actinopterygii; Neopterygii; Teleostei; Ostariophysi; Cypriniformes; Cyprinidae; Danio","Common Name"="zebrafish"]:0.040389999999999926,XP_031757388.1[&Organism="Xenopus tropicalis","Genetic Code"="Standard",Taxonomy="Eukaryota; Metazoa; Chordata; Craniata; Vertebrata; Euteleostomi; Amphibia; Batrachia; Anura; Pipoidea; Pipidae; Xenopodinae; Xenopus; Silurana","Common Name"="tropical clawed frog"]:0.0455899999999998)[&"FastTree support value"=0.589]:0.01208000000000009)[&"FastTree support value"=0.916]:0.030990000000000073)[&"FastTree support value"=1.0]:0.08169000000000004)[&"FastTree support value"=0.854]:0.03230999999999984,(XP_006813643.1[&Organism="Saccoglossus kowalevskii","Genetic Code"="Standard",Taxonomy="Eukaryota; Metazoa; Hemichordata; Enteropneusta; Harrimaniidae; Saccoglossus"]:0.10110999999999981,XP_030843280.1[&Organism="Strongylocentrotus purpuratus","Genetic Code"="Standard",Taxonomy="Eukaryota; Metazoa; Echinodermata; Eleutherozoa; Echinozoa; Echinoidea; Euechinoidea; Echinacea; Echinoida; Strongylocentrotidae; Strongylocentrotus","Common Name"="purple sea

urchin"]:0.15044000000000013)[&"FastTree support
value"=0.5]:0.022139999999999826)[&"FastTree support
value"=0.928]:0.05317999999999978)[&"FastTree support
value"=0.182]:0.0259999999999998,(NP_495986.3.3[&Organism="Caenorhabditis
elegans","Genetic Code"="Standard",Taxonomy="Eukaryota; Metazoa; Ecdysozoa;
Nematoda; Chromadorea; Rhabditida; Rhabditina; Rhabditomorpha; Rhabditoidea;
Rhabditidae; Peloderinae;
Caenorhabditis"]:0.16041000000000016,NP_610941.1[&Organism="Drosophila
melanogaster","Genetic Code"="Standard",Taxonomy="Eukaryota; Metazoa; Ecdysozoa;
Arthropoda; Hexapoda; Insecta; Pterygota; Neoptera; Holometabola; Diptera; Brachycera;
Muscomorpha; Ephydroidea; Drosophilidae; Drosophila; Sophophora","Common
Name"="fruit fly"]:0.15097000000000005)[&"FastTree support
value"=0.407]:0.038590000000000124)[&"FastTree support
value"=0.665]:0.0531299999999999)[&"FastTree support
value"=0.922]:0.13635999999999981)[&"FastTree support
value"=1.0]:1.0778600000000003)[&"FastTree support
value"=0.706]:0.020010000000000083)[&"FastTree support
value"=0.913]:0.1148699999999998)[&"FastTree support
value"=0.916]:0.11427000000000032,((((KNE54706.1[&Organism="Allomyces macrogynus
ATCC 38327","Genetic Code"="Standard",Taxonomy="Eukaryota; Fungi; Fungi incertae
sedis; Blastocladiomycota; Blastocladiomycota incertae sedis; Blastocladiomycetes;
Blastocladiales; Blastocladiaceae;
Allomyces"]:0.23439999999999994,KXN72852.1[&Organism="Conidiobolus coronatus
NRRL 28638","Genetic Code"="Standard",Taxonomy="Eukaryota; Fungi; Fungi incertae
sedis; Zoopagomycota; Entomophthoromycotina; Entomophthoromycetes;
Entomophthorales; Ancylistaceae; Conidiobolus"]:0.38027999999999995)[&"FastTree
support
value"=0.575]:0.04198000000000013,(OAJ38404.1[&Organism="Batrachochytrium
dendrobatidis JEL423","Genetic Code"="Standard",Taxonomy="Eukaryota; Fungi; Fungi
incertae sedis; Chytridiomycota; Chytridiomycota incertae sedis; Chytridiomycetes;
Rhizophydiales; Rhizophydiales incertae sedis;
Batrachochytrium"]:0.1818599999999999,((NP_014854.2[&Organism="Saccharomyces
cerevisiae S288C","Genetic Code"="Standard",Taxonomy="Eukaryota; Fungi; Dikarya;
Ascomycota; Saccharomycotina; Saccharomycetes; Saccharomycetales;
Saccharomycetaceae;
Saccharomyces"]:0.4665499999999998,XP_752563.1[&Organism="Aspergillus fumigatus
Af293","Genetic Code"="Standard",Taxonomy="Eukaryota; Fungi; Dikarya; Ascomycota;
Pezizomycotina; Eurotiomycetes; Eurotiomycetidae; Eurotiales; Aspergillaceae;
Aspergillus; Aspergillus subgen. Fumigati"]:0.17031000000000018)[&"FastTree support
value"=0.96]:0.11599000000000004,(XP_006459124.1[&Organism="Agaricus bisporus var.
bisporus H97","Genetic Code"="Standard",Taxonomy="Eukaryota; Fungi; Dikarya;
Basidiomycota; Agaricomycotina; Agaricomycetes; Agaricomycetidae; Agaricales;
Agaricaceae; Agaricus"]:0.17734000000000005,XP_011389557.1[&Organism="Ustilago
maydis 521","Genetic Code"="Standard",Taxonomy="Eukaryota; Fungi; Dikarya;

Basidiomycota; Ustilaginomycotina; Ustilaginomycetes; Ustilaginales; Ustilaginaceae; Ustilago"]:0.16968000000000005)[&"FastTree support value"=0.964]:0.09134999999999982)[&"FastTree support value"=0.942]:0.07140000000000013)[&"FastTree support value"=0.851]:0.040509999999999824)[&"FastTree support value"=0.915]:0.12511999999999999,OUM67143.1[&Organism="Piromyces sp. E2","Genetic Code"="Standard",Taxonomy="Eukaryota; Fungi; Fungi incertae sedis; Chytridiomycota; Chytridiomycota incertae sedis; Neocallimastigomycetes; Neocallimastigales; Neocallimastigaceae; Piromyces; unclassified Piromyces"]:0.25739)[&"FastTree support value"=1.0]:0.54941,((CAH6419740.1[&Organism="uncultured virus","Genetic Code"="Standard",Taxonomy="Viruses; environmental samples"]:0.6571500000000001,((((ARF12445.1[&Organism="Klosneuvirus KNV1","Genetic Code"="Standard",Taxonomy="Viruses; Varidnaviria; Bamfordvirae; Nucleocytoviricota; Megaviricetes; Imitervirales; Mimiviridae; Klosneuvirinae; Klosneuvirus"]:0.41111999999999993,(QKU35298.1[&Organism="Tupanvirus soda lake","Genetic Code"="Standard",Taxonomy="Viruses; Varidnaviria; Bamfordvirae; Nucleocytoviricota; Megaviricetes; Imitervirales; Mimiviridae; Tupanvirus"]:0.5004300000000002,QKF93607.1[&Organism="Fadolivirus 1","Genetic Code"="Standard",Taxonomy="Viruses"]:0.23480999999999996)[&"FastTree support value"=0.647]:0.06660000000000021)[&"FastTree support value"=0.66]:0.08677000000000001,AYV83919.1[&Organism="Hyperionvirus sp.","Genetic Code"="Standard",Taxonomy="Viruses; Varidnaviria; Bamfordvirae; Nucleocytoviricota; Megaviricetes; Imitervirales; Mimiviridae","Common Name"="soil metagenome"]:0.8416700000000001)[&"FastTree support value"=0.163]:0.05506000000000011,CAH6421112.1[&Organism="uncultured virus","Genetic Code"="Standard",Taxonomy="Viruses; environmental samples"]:0.4425500000000002)[&"FastTree support value"=0.96]:0.14765000000000006,AYV81982.1[&Organism="Homavirus sp.","Genetic Code"="Standard",Taxonomy="Viruses; Varidnaviria; Bamfordvirae; Nucleocytoviricota; Megaviricetes; Imitervirales; Mimiviridae","Common Name"="soil metagenome"]:0.41879)[&"FastTree support value"=0.945]:0.1894)[&"FastTree support value"=0.999]:0.5444800000000001,(WGU15254.1[&Organism="Variosea sp.","Genetic Code"="Standard",Taxonomy="Eukaryota; Amoebozoa; Evosea; Variosea"]:0.4566699999999999,XP_013756556.1[&Organism="Thecamonas trahens ATCC 50062","Genetic Code"="Standard",Taxonomy="Eukaryota; Apusozoa; Apusomonadida; Apusomonadidae; Thecamonas"]:0.48187999999999986)[&"FastTree support value"=0.908]:0.22258999999999984)[&"FastTree support value"=0.358]:0.16012000000000004)[&"FastTree support value"=0.989]:0.24734000000000034)[&"FastTree support value"=0.276]:0.08333999999999975)[&"FastTree support value"=0.887]:0.08514)[&"FastTree support value"=0.989]:0.1641300000000001)[&"FastTree support value"=0.902]:0.06953999999999994)[&"FastTree support

value"=0.901]:0.06048999999999971)[&"FastTree support
value"=0.326]:0.029180000000000206,(ETO36135.1[&Organism="Reticulomyxa filosa","Genetic Code"="Standard",Taxonomy="Eukaryota; Sar; Rhizaria; Retaria; Foraminifera; Monothalamids; Reticulomyxidae;
Reticulomyxa"]:0.40329999999999977,(XP_042914770.1[&Organism="Chlamydomonas reinhardtii","Genetic Code"="Standard",Taxonomy="Eukaryota; Viridiplantae; Chlorophyta; core chlorophytes; Chlorophyceae; CS clade; Chlamydomonadales; Chlamydomonadaceae;
Chlamydomonas"]:0.35358,((((XP_052310486.1[&Organism="Populus trichocarpa","Genetic Code"="Standard",Taxonomy="Eukaryota; Viridiplantae; Streptophyta; Embryophyta; Tracheophyta; Spermatophyta; Magnoliopsida; eudicotyledons; Gunneridae; Pentapetalae; rosids; fabids; Malpighiales; Salicaceae; Saliceae; Populus","Common Name"="Populus balsamifera subsp. trichocarpa"]:0.05848999999999993,AAC61784.1[&Organism="Arabidopsis thaliana","Genetic Code"="Standard",Taxonomy="Eukaryota; Viridiplantae; Streptophyta; Embryophyta; Tracheophyta; Spermatophyta; Magnoliopsida; eudicotyledons; Gunneridae; Pentapetalae; rosids; malvids; Brassicales; Brassicaceae; Camelineae; Arabidopsis","Common Name"="thale cress"]:0.13491999999999997)[&"FastTree support
value"=0.981]:0.06661000000000028,KAH9327796.1[&Organism="Taxus chinensis","Genetic Code"="Standard",Taxonomy="Eukaryota; Viridiplantae; Streptophyta; Embryophyta; Tracheophyta; Spermatophyta; Pinopsida; Pinidae; Conifers II; Cupressales; Taxaceae; Taxus"]:0.17598000000000003)[&"FastTree support
value"=0.728]:0.01662999999999997,(PWZ09977.1[&Organism="Zea mays","Genetic Code"="Standard",Taxonomy="Eukaryota; Viridiplantae; Streptophyta; Embryophyta; Tracheophyta; Spermatophyta; Magnoliopsida; Liliopsida; Poales; Poaceae; PACMAD clade; Panicoideae; Andropogonodae; Andropogoneae; Tripsacinae;
Zea"]:0.041749999999999954,ONM18162.1[&Organism="Zea mays","Genetic Code"="Standard",Taxonomy="Eukaryota; Viridiplantae; Streptophyta; Embryophyta; Tracheophyta; Spermatophyta; Magnoliopsida; Liliopsida; Poales; Poaceae; PACMAD clade; Panicoideae; Andropogonodae; Andropogoneae; Tripsacinae;
Zea"]:0.18482999999999983)[&"FastTree support
value"=0.927]:0.034969999999999946)[&"FastTree support
value"=0.493]:0.007000000000000117,((EFJ35472.1[&Organism="Selaginella moellendorffii","Genetic Code"="Standard",Taxonomy="Eukaryota; Viridiplantae; Streptophyta; Embryophyta; Tracheophyta; Lycopodiopsida; Selaginellales; Selaginellaceae;
Selaginella"]:0.42010999999999976,((EFJ37641.1[&Organism="Selaginella moellendorffii","Genetic Code"="Standard",Taxonomy="Eukaryota; Viridiplantae; Streptophyta; Embryophyta; Tracheophyta; Lycopodiopsida; Selaginellales; Selaginellaceae;
Selaginella"]:0.0032900000000000237,EFJ15047.1[&Organism="Selaginella moellendorffii","Genetic Code"="Standard",Taxonomy="Eukaryota; Viridiplantae; Streptophyta; Embryophyta; Tracheophyta; Lycopodiopsida; Selaginellales; Selaginellaceae; Selaginella"]:0.000550)[&"FastTree support

value"=0.967]:0.03160999999999694,((KAI5070758.1[&Organism="Adiantum capillus-veneris","Genetic Code"="Standard",Taxonomy="Eukaryota; Viridiplantae; Streptophyta; Embryophyta; Tracheophyta; Polypodiopsida; Polypodiidae; Polypodiales; Pteridineae; Pteridaceae; Vittarioideae; Adiantum"]:0.05523999999999956,KAI5070335.1[&Organism="Adiantum capillus-veneris","Genetic Code"="Standard",Taxonomy="Eukaryota; Viridiplantae; Streptophyta; Embryophyta; Tracheophyta; Polypodiopsida; Polypodiidae; Polypodiales; Pteridineae; Pteridaceae; Vittarioideae; Adiantum"]:0.05630999999999986)[&"FastTree support value"=0.914]:0.02398999999999956,(PTQ35749.1[&Organism="Marchantia polymorpha","Genetic Code"="Standard",Taxonomy="Eukaryota; Viridiplantae; Streptophyta; Embryophyta; Marchantiophyta; Marchantiopsida; Marchantiidae; Marchantiales; Marchantiaceae; Marchantia","Common Name"="liverwort"]:0.05099000000000535,((XP_024362051.1[&Organism="Physcomitrium patens","Genetic Code"="Standard",Taxonomy="Eukaryota; Viridiplantae; Streptophyta; Embryophyta; Bryophyta; Bryophytina; Bryopsida; Funariidae; Funariales; Funariaceae; Physcomitrium"]:0.0438799999999997,KAG0554580.1[&Organism="Ceratodon purpureus","Genetic Code"="Standard",Taxonomy="Eukaryota; Viridiplantae; Streptophyta; Embryophyta; Bryophyta; Bryophytina; Bryopsida; Dicranidae; Pseudoditrichales; Ditrichaceae; Ceratodon"]:0.00577000000000053)[&"FastTree support value"=0.975]:0.0329600000000001,KAG0555682.1[&Organism="Ceratodon purpureus","Genetic Code"="Standard",Taxonomy="Eukaryota; Viridiplantae; Streptophyta; Embryophyta; Bryophyta; Bryophytina; Bryopsida; Dicranidae; Pseudoditrichales; Ditrichaceae; Ceratodon"]:0.04008999999999974)[&"FastTree support value"=0.279]:0.00731000000000372)[&"FastTree support value"=0.849]:0.01488999999999848)[&"FastTree support value"=0.929]:0.02621999999999991)[&"FastTree support value"=0.743]:0.01161999999999742)[&"FastTree support value"=0.847]:0.02779000000000425,KAH9304002.1[&Organism="Taxus chinensis","Genetic Code"="Standard",Taxonomy="Eukaryota; Viridiplantae; Streptophyta; Embryophyta; Tracheophyta; Spermatophyta; Pinopsida; Pinidae; Conifers II; Cupressales; Taxaceae; Taxus"]:0.08108000000000004)[&"FastTree support value"=0.756]:0.01654)[&"FastTree support value"=0.985]:0.14515999999999973)[&"FastTree support value"=0.953]:0.10203000000000007)[&"FastTree support value"=0.944]:0.09349000000000007)[&"FastTree support value"=0.847]:0.0351600000000003,(KAH3761456.1[&Organism="Pelomyxa schiedti","Genetic Code"="Standard",Taxonomy="Eukaryota; Amoebozoa; Evosea; Archamoebae; Pelobiontida; Pelomyxidae; Pelomyxa"]:0.26632999999999996,(((GMI25649.1[&Organism="Tetraparma gracilis","Genetic Code"="Standard",Taxonomy="Eukaryota; Sar; Stramenopiles; Ochrophyta; Bolidophyceae; Parmales; Triparmaceae; Tetraparma"]:0.04735999999999985,(GMI62840.1[&Organism="Parmales sp. scaly parma","Genetic Code"="Standard",Taxonomy="Eukaryota; Sar; Stramenopiles; Ochrophyta; Bolidophyceae;

Parmales"]:0.04389999999999983,GMH55978.1[&Organism="Triparma strigata","Genetic Code"="Standard",Taxonomy="Eukaryota; Sar; Stramenopiles; Ochrophyta; Bolidophyceae; Parmales; Triparmaceae; Triparma"]:0.05186000000000002)[&"FastTree support value"=0.305]:0.014759999999999884)[&"FastTree support value"=0.975]:0.0786800000000003,(GMH85941.1[&Organism="Triparma verrucosa","Genetic Code"="Standard",Taxonomy="Eukaryota; Sar; Stramenopiles; Ochrophyta; Bolidophyceae; Parmales; Triparmaceae; Triparma"]:0.0642100000000001,GMI59178.1[&Organism="Parmales sp. scaly parma","Genetic Code"="Standard",Taxonomy="Eukaryota; Sar; Stramenopiles; Ochrophyta; Bolidophyceae; Parmales"]:0.048859999999999904)[&"FastTree support value"=0.984]:0.1083400000000001)[&"FastTree support value"=1.0]:0.22693999999999992,(KAF0852279.1[&Organism="Andalucia godoyi","Genetic Code"="Standard",Taxonomy="Eukaryota; Discoba; Jakobida; Andalucina; Andaluciidae; Andalucia"]:0.43591000000000024,(KNH06820.1[&Organism="Perkinsela sp. CCAP 1560/4","Genetic Code"="Standard",Taxonomy="Eukaryota; Discoba; Euglenozoa; Kinetoplastea; Prokinetoplastina; Ichthyobodonidae; Perkinsela"]:0.5139100000000001,(CAD2212698.1[&Organism="Angomonas deanei","Genetic Code"="Standard",Taxonomy="Eukaryota; Discoba; Euglenozoa; Kinetoplastea; Metakinetoplastina; Trypanosomatida; Trypanosomatidae; Strigomonadinae; Angomonas"]:0.6289099999999999,((CCW59714.1[&Organism="Phytomonas sp. EM1","Genetic Code"="Standard",Taxonomy="Eukaryota; Discoba; Euglenozoa; Kinetoplastea; Metakinetoplastina; Trypanosomatida; Trypanosomatidae; Phytomonas"]:0.0986400000000005,(KAG5490335.1[&Organism="Porcisia hertigi","Genetic Code"="Standard",Taxonomy="Eukaryota; Discoba; Euglenozoa; Kinetoplastea; Metakinetoplastina; Trypanosomatida; Trypanosomatidae; Leishmaniinae; Porcisia"]:0.05316000000000096,(XP_003872337.1[&Organism="Leishmania mexicana MHOM/GT/2001/U1103","Genetic Code"="Standard",Taxonomy="Eukaryota; Discoba; Euglenozoa; Kinetoplastea; Metakinetoplastina; Trypanosomatida; Trypanosomatidae; Leishmaniinae; Leishmania"]:0.06839999999999957,KAI5685071.1[&Organism="Leishmania braziliensis","Genetic Code"="Standard",Taxonomy="Eukaryota; Discoba; Euglenozoa; Kinetoplastea; Metakinetoplastina; Trypanosomatida; Trypanosomatidae; Leishmaniinae; Leishmania; Leishmania braziliensis species complex"]:0.025599999999999845)[&"FastTree support value"=0.573]:0.024570000000000203)[&"FastTree support value"=0.972]:0.05889000000000033)[&"FastTree support value"=0.968]:0.06443999999999983,((XP_028887534.1[&Organism="Trypanosoma theileri","Genetic Code"="Standard",Taxonomy="Eukaryota; Discoba; Euglenozoa; Kinetoplastea; Metakinetoplastina; Trypanosomatida; Trypanosomatidae; Trypanosoma"]:0.030610000000000248,(EKF32958.1[&Organism="Trypanosoma cruzi marinkellei","Genetic Code"="Standard",Taxonomy="Eukaryota; Discoba; Euglenozoa; Kinetoplastea; Metakinetoplastina; Trypanosomatida; Trypanosomatidae; Trypanosoma; Schizotrypanum"]:0.04917000000000016,ESL10883.1[&Organism="Trypanosoma rangeli

SC58","Genetic Code"="Standard",Taxonomy="Eukaryota; Discoba; Euglenozoa;
Kinetoplastea; Metakinetoplastina; Trypanosomatida; Trypanosomatidae; Trypanosoma;
Herpetosoma"]:0.05418000000000012)[&"FastTree support
value"=0.972]:0.05393999999999988)[&"FastTree support
value"=0.945]:0.0644000000000001,((KAH8605762.1[&Organism="Trypanosoma
vivax","Genetic Code"="Standard",Taxonomy="Eukaryota; Discoba; Euglenozoa;
Kinetoplastea; Metakinetoplastina; Trypanosomatida; Trypanosomatidae; Trypanosoma;
Duttonella"]:0.1851900000000004,CCC89860.1[&Organism="Trypanosoma congolense
IL3000","Genetic Code"="Standard",Taxonomy="Eukaryota; Discoba; Euglenozoa;
Kinetoplastea; Metakinetoplastina; Trypanosomatida; Trypanosomatidae; Trypanosoma;
Nannomonas"]:0.05808999999999975)[&"FastTree support
value"=0.22]:0.01908999999999983,RHW73545.1[&Organism="Trypanosoma brucei
equiperdum","Genetic Code"="Standard",Taxonomy="Eukaryota; Discoba; Euglenozoa;
Kinetoplastea; Metakinetoplastina; Trypanosomatida; Trypanosomatidae;
Trypanosoma"]:0.03862000000000032)[&"FastTree support
value"=0.944]:0.05896999999999997)[&"FastTree support
value"=0.902]:0.05071000000000003)[&"FastTree support
value"=0.893]:0.0770900000000001)[&"FastTree support
value"=0.966]:0.1377900000000003)[&"FastTree support
value"=0.887]:0.07904)[&"FastTree support
value"=0.718]:0.05466999999999774)[&"FastTree support
value"=0.924]:0.0632299999999999)[&"FastTree support
value"=0.81]:0.02836999999999784)[&"FastTree support
value"=0.303]:0.01670000000000016)[&"FastTree support
value"=0.825]:0.01591999999999934)[&"FastTree support
value"=0.795]:0.02147000000000322)[&"FastTree support
value"=0.541]:0.02194999999999947):0.10415000000000019);
end;
