# Supplementary material for: Antiviral Mx proteins have an ancient origin and widespread distribution among eukaryotes
Source: Proc Natl Acad Sci U S A. 2025 Jan 24;122(4):e2416811122. doi: 10.1073/pnas.2416811122 (PMC11789081; doi:10.1073/pnas.2416811122)
Supplement: Supplementary file 10 — Dataset S09 (PDF) [file pnas.2416811122.sd09.pdf]

Dataset S9. Figure_3_MAFFT

>GMI12809.1
------------IDLPQ--------IVTI---GS----Q---------------
SSGKSSVLE-----N--VV-G-K-GFLPRG-------------TG-IVTRRPL--------------VLQ-------------L-Y-
NTS-----------------------------NHS---------------IPT--SP-
NAISSSLRSSPTDTNRAATNPAADSDSSD-----ENNNENSNDVNTPP-
PKPASSRKSTEIASPPVLPGDE-------------W--------------------------GEF----------------------------
---LHLPGEKFYSFD-----------EIRDEI------VRET-DR---S-TG-----
-------------------RN-------------------K-GISNK-SINLKIYSPR--
--------------VLNLTLVDLPGITK--VS------------------V-------GD-------------Q--------PA--D--
IEDQIR--------EMCL-HYI---SN------------PNAIILAVTAGNT--D-LANSD-ALKMARSVD------
PEGNRTIGVLTKLDLMD-------PGT------D-------A---------------------------SDM--LN---NR---------
I-IP----------------------L---RRGYVGVINR--G-QRD--ISQ----
-------R--------KS-------------------IK---------EGLRK---EMEFFKS--------HP-AY--------------------
R-S------L-----------QHR-CGTTTLAKMLNSILMH-----------
>GMI47362.1
------------IDLPQ--------IVTI---GS----Q---------------
SSGKSSVLE-----N--VV-G-K-GFLPRG-------------TG-IVTRRPL--------------VLQ-------------L-Y-
NTS-----------------------------NHS---------------VPA--SP-GSISSSLRSPGLDDVEEETNPA--
EEEAD-----ENSNLN---MHTPPESKPKRKSKVAADASPVVLPGDE-------------W--------------------
------GEF---LHLPGEKFYSFD----------
-EIRDEI------VRET-DR---S-TG-----------------------RN-------------------K-GISNK-SINLKIYSPR-----
---VLNLTLVDLPGITK--VS------------------V-------GD-
-------------Q--------PV--D--IEDQIR--------EMCL-HYI---SN------------PNAIILAVTAGNT--D-
LANSD-ALKMARSVD------PEGNRTIGVLTKLDLMD-------PGT------D-------A------------------------
---SDM--LN---NR---------V-IP----------------------L---RRGYVGVINR---------------------------------
------------G-QRD--ISQ-----------K--------RS-------------------IR---------EGLKK---EMEFFKN------
--HP-AY---------------------R-S------L-----------SHR-CGTSTLSKMLNSILMH-----------
>OQR85161.1
------------INLPQ--------IVVV---GS----Q---------------
SSGKSSVLE-----N--IV-G-K-DFLPRG-------------SG-IVTRRPL--------------VLQ-------------L-Y-
NI---PAGD-----DKEKE-------
-----------------------E-------------W--------------------------GEF--------------------------------------
--------------------------------NHLPNQKIYDFA----------KLREEI------EKET-DR---M-TG--------------
---------KN-------------------K-GISNK-PITLKIFSPY---
---VLNLTLVDLPGITK--VP------------------V-------GD-------------Q--------PA--N--IEEQIR--------
DMCT-EFI---SN------------PNSIILAVTAANT--D-LANSD-SLKMARAID------
PDGVRTIGVLTKLDLMD-------NGT------D-------A--------------------------MEM--LQ---GR---------
V-IP----------------------L---KKGYVGVVNR---S-QAD--INN--
---------N--------VG-------------------IR---------DSVAK---ETQFFKN--------HP-SY-------------------
-R-A------I-----------ASR-MGTQFLSKSLNTILMH-----------
>RLO06844.1

------------INLPQ--------IVVV---GS----Q---------------
SSGKSSVLE-----N--IV-G-K-DFLPRG--------------SG-IVTRRPL---------------VLQ--------------L-Y-
NT--DGGV-----SKD-E-------
------------------------E-------------W---------------------------GEF--
--------------------------------NHLPNQKFNDFK-----------KIREEI------EKET-DR---M-TG--------------
----------KN-------------------K-GISNK-PITLKIFSPF---
-----VLNLTLVDLPGITK--VP------------------V-------GD-------------Q--------PA--N--IEEQIR--------
DMCL-EFI---GN------------PNSIILAVTAANT--D-LANSD-SLKMARAID------
PEGLRTIGVLTKLDLMD-------NGT------D-------A---------------------------MEM--LQ---GR---------
I-IP----------------------L---KKGYVGIVNR--S-QAD--INN----
-------G--------VG-------------------IR----------DSVSK---ETTFFKA--------HP-AY--------------------
R-G------I-----------ASR-MGTAFLSKSLNTILMH-----------
>KAF0740912.1
------------INLPQ--------IVVV---GS----Q---------------
SSGKSSVLE-----N--IV-G-K-DFLPRG--------------SG-IVTRRPL---------------VLQ--------------L-Y-
NT--DGGV-----TKD-E-------
------------------------E-------------W---------------------------GEF--
--------------------------------NHLPNQKFHDFK-----------KIREEI------EKET-DR---M-TG--------------
----------KN-------------------K-GISNK-PITLKVFSPF--
------VLNLTLVDLPGITK--VP------------------V-------GD-------------Q--------PA--N--IEEQIR-------
-DMCL-EFI---GN------------PNSIILAVTAANT--D-LANSD-SLKMARAID------
PEGQRTIGVLTKLDLMD-------DGT------D-------A---------------------------MEM--LQ---GR---------
V-IP----------------------L---KKGYVGIVNR---S-QAD--INN---
--------G--------VG-------------------IR----------DSVAK---EQTFFKS--------HP-AY--------------------
R-G------I-----------ASR-MGTQYLSKSLNTILMH-----------
>CAH0521473.1
------------INLPQ--------IVVI---GS----Q---------------
SSGKSSVLE-----N--IV-G-K-DFLPRG--------------SG-IVTRRPL---------------ILQ--------------L-Y-
NSS------------------------------TNG----------------V------------------------------VENHEE-----TLRSE--
-----------------------------E-------------W---------------------------GEF----------------------------------
-----------------------------------LHLPGQKFTDFN-----------EIRQEI------EKET-DR---L-TG----------
--------------KN-------------------K-GISNK-SINLKVFSPY--
----------VLNLTLVDLPGITK--VP------------------V-------GD-------------Q--------PV--N--IEEQIR---
-----DMCI-EFI---SN------------PNSIILAVTSANT--D-LANSD-ALKMAREID------
PDGQRTIGVLTKLDLMD-------AGT------D-------A---------------------------MDM--LQ---GR---------
V-IP----------------------L---KRGYVGVVNR--S-QAD--INA---
--------Q--------LS-------------------IR----------DSLRK---EQTFFKT--------HP-AY--------------------
R-A------I-----------ASR-MGTQYLSKTLNTILMH-----------
>KAG2764740.1
------------INLPQ--------IVVI---GS----Q---------------
SSGKSSVLE-----N--IV-G-K-DFLPRG--------------SG-IVTRRPL---------------VLQ--------------L-Y-
NSS------------------------------ATV----------------P-------------------------------GEGE-----DETVE----
---------------------------E-------------W---------------------------GEF---------------------------------------
---------------------------------LHLPGQKFSDFN-----------EIRREI------EKET-DR---I-TG-------------

-----------KN-------------------K-GISNK-SINLKVFSPY--
-------VLNLTLVDLPGITK--VP-------------------V-------GD--------------Q--------PV--N--IEEQIR------
--DMCT-EFI---TN------------PNSIILAVTSANT--D-LANSD-ALKMAREID------
PEGQRTIGVLTKLDLMD-------DGT------D-------A----------------------------MDM--LQ---GR---------
V-IP-----------------------L---KKGYVGVVNR---S-QAD--INS---
--------K--------LS--------------------IR----------DSLVK---EQNFFKT--------HP-AY---------------------
R-A------I-----------ASR-MGTQYLSKTLNTILMH-----------
>GMF38092.1
------------INLPQ--------IVVI---GS----Q----------------
SSGKSSVLE-----N--IV-G-K-DFLPRG--------------SG-IVTRRPL---------------VLQ--------------L-Y-
NSS------------------------------ATV----------------P------------------------------VDDADG------AEAE--
-----------------------------E--------------W---------------------------GEF--------------------------------------
--LHMPGQKFTDFD-----------EIRREI------EKET-DR---I-TG-----------
-------------KN-------------------K-GISNK-SINLKVFSPH---
----------VLNLTLVDLPGITK--VP-------------------V-------GD--------------Q--------PV--N--IEEQIR---
-----DMCT-EFI---SN------------PNSIILAVTSANT--D-LANSD-ALKMAREID------
PDGQRTIGVLTKLDLMD-------DGT------D-------A----------------------------MDM--LQ---GR---------
V-IP-----------------------L---KRGYVGVVNR---S-QAD--INA---
--------Q--------LS--------------------IR----------DSLSK---EQNFFKT--------HP-AY---------------------
R-A------I-----------ASR-MGTQYLSKTLNTILMH-----------
>KAH7500220.1
------------INLPQ--------IVVI---GS----Q----------------
SSGKSSVLE-----N--IV-G-K-DFLPRG--------------SG-IVTRRPL---------------VLQ--------------L-Y-
NSS------------------------------TSV----------------PA-----------------------------AEDAGG-----DASAE-
------------------------------E--------------W---------------------------GEF------------------------------------
---LHVPGQKFTDFD-----------EIRREI------EKET-DR---I-TG----------
--------------KN-------------------K-GISNK-SINLKVFSPY---
----------VLNLTLVDLPGITK--VP-------------------V-------GD--------------Q--------PV--N--IEEQIR---
-----DMCT-EFI---SN------------PNSIILAVTSANT--D-LANSD-SLKMAREID------
PEGQRTIGVLTKLDLMD-------DGT------D-------A----------------------------MDM--LQ---GR---------
V-IP-----------------------L---KRGYVGVVNR---S-QAD--INA---
--------K--------LS--------------------IR----------ESLSK---EQNFFKT--------HP-AY---------------------
R-A------I-----------ASR-MGTQYLSKTLNTILMH-----------
>TMW56688.1
------------INLPQ--------IVVI---GS----Q----------------
SSGKSSVLE-----N--IV-G-R-DFLPRG--------------SG-IVTRRPL---------------VLQ--------------L-Y-
NIS------------------------------NT--DLAK-----ESDVE------
-------------------------E--------------W---------------------------GEF--
---------------------------------LHLPGQKISDFS-----------ELRKEI------ERET-ER---M-TG---------------
---------KN-------------------K-GISNK-SINLKIFSPH---
-----VLNLTLVDLPGVTK--VP-------------------V-------GD--------------Q--------PV--N--IEEQIR-------
-DMCM-EFI---AN------------PNSIILAVTSANT--D-LANSD-ALKMAREID------
PEGVRTIGVLTKLDLMD-------DGT------D-------A---------------------------LDM--LQ---GR---------I-
IP-----------------------L---KRGFVGVVNR---S-QAD--INN-----

------S--------VS-------------------IR----------DALSK---ETSYFKN--------HP-SY--------------------R-
A------M-----------ASR-MGTQYLSKTLNTILMH-----------
>CCI11042.1
------------VNLPQ--------IVVI--GS----Q---------------
SSGKSSVLE-----N--IV-G-R-DFLPRG-------------SG-IVTRRPL---------------ILQ-------------L-Y-
NSQ-----------------------------SN---LDETQ-----LIGGES--
-------------------------NVE-------------W-------------------------GEF--------------------------------
-------------------------------------LHIPGKKFKDFS-----------EIRHEI------ERET-DR---L-TG-----------
-------------KN------------------K-GISNK-TINLKIYSPH---
--------VLNLTLVDLPGVTK--VP------------------V-------GD-------------Q--------PI--D--IEEQIR-----
---DMCV-EFI---SN------------PNSIILAVTSANT--D-LANSD-ALKLAREID------
PAGDRTIGVLTKIDLMD-------EGT------D-------A--------------------------LEM--LQ---GR---------I-
IS----------------------L---RRGFVGVVNR--S-QAD--INN-----
------N--------VS-------------------IR----------DSLQK---EQKFFQQ--------HS-AY--------------------
R-T------V-----------SST-MGTQYLSKTLNTILMH-----------
>CCA17876.1
------------VNLPQ--------IVVI--GS----Q---------------
SSGKSSVLE-----N--IV-G-R-DFLPRG-------------SG-IVTRRPL---------------ILQ-------------L-Y-
NSQ-----------------------------SNS--TLDESQ-----
PLRGEN---------------------------DVE-------------W-------------------------GEF--------------------
--LHIPGKKFTDFS----------EIRREI------ERET-DR---L-
TG------------------------KN-------------------K-GISNK-TINLKIFSPY--------------------------------------
----------------------VLNLTLVDLPGVTK--VP------------------V-------GD-------------Q--------PV--N-
-IEEQIR--------DMCV-EFI---SN------------PNSIILAVTSANT--D-LANSD-ALKLAREID------
PAGDRTIGVLTKIDLMD-------EGT------D-------A--------------------------LEM--LQ---GR---------V-
IS----------------------L---RRGFVGVVNR--S-QAD--INN-----
------N--------VS-------------------IR----------DSLQK---EHLFFQN--------HT-AY--------------------
R-T------V-----------SSK-MGTQYLSKTLNTILMH-----------
>CEP02405.1
------------IDLPQ--------IVVI--GS----Q---------------
SSGKSSVLE-----N--IV-G-R-DFLPRG-------------SG-IVTRRPL---------------ILQ-------------L-V-
NTS------------------------------LDL--KLSGKD-----DESGQ--
-----------------------------E-------------W-------------------------GEF-------------------------------
-------------------------------------LHKRDKKFTSFE-----------DIRAEI------VAET-DR---L-TG------------
------------TN------------------K-GISDK-AINLKVYSPR---
--------FLNLTLVDLPGITK--VP------------------V-------GD-------------Q--------PS--D--IENQIK-----
---GMIM-RFV---SN------------PRAIILAVTAANT--D-LANSD-ALKLAREVD------
PECNRTIGVITKIDLMD-------RGT------N-------A--------------------------LDV--LL---GK---------V-
IP----------------------L---KLGFIGVINR--S-QED--INN-------
----N--------TS--------------------IN----------AALTA---EMQFFQK--------HP-AY--------------------R-
A------I-----------ASR-LGTSYLTKTLNSILIN-----------
>XP_044553261.1
----------VQIDLPQ--------IAVV--GS----Q---------------
SSGKSSVLE-----T--IV-G-R-DFLPRG-------------SG-IVTRRPL---------------VLQ-------------L-N-

K--TPS------KQV-----------------------------DSTGPED---
-------------------------E-------------W-------------------------GEF-------------------------------------
-----------------------------------LHVPNKKFYDFT-----------EIRNEI------IRET-DR---V-TG--------------
---------SS------------------K-GISDL-PINLKIYSPN---
----VLNLTLVDLPGITK--VP------------------V-------GD-------------Q--------PK--D--IEAQIR--------
NMIM-KFI---SK------------PNCLILAVTAANT--D-IANSD-ALKLAKEVD------
KNGSRTIGVLTKIDIMD-------QGT------D-------C--------------------------MDV--LR---GD---------I-
LP-----------------------L---RLGYIGVVCR--S-QND--INL------
-----N--------KS-------------------IR----------DALKD---EERFFAQ--------HP-TY--------------------K-
S------I-----------SDR-MGTKHLAKTLNRILLN-----------
>XP_044569353.1
----------VQIDLPQ--------IAVV---GS----Q---------------
SSGKSSVLE-----T--IV-G-R-DFLPRG--------------SG-IVTRRPL---------------VLQ--------------L-N-
K--TPS------KQQ---------------------QTGD-----
DSTGPEE----------------------------E-------------W-------------------------GEF---------------------
--LHVPNKKFYDFT-----------EIRNEI------IRET-DR---V-
TG------------------------SS------------------K-GISDL-PINLKIYSPN-----------------------------------
----------------------VLNLTLVDLPGITK--VP------------------V-------GD-------------Q--------PK--D-
-IEAQIR--------NMIM-KFI---SK------------PNCLILAVTAANT--D-IANSD-ALKLAKEVD------
KNGSRTIGVLTKIDIMD-------QGT------D-------C--------------------------MDV--LR---GE---------L-
LP-----------------------L---RLGYIGVVCR--S-QND--INL------
-----N--------KS-------------------IR----------DALRD---EERFFAT--------HP-IY--------------------K-
S------I-----------SDR-MGTKYLAKTLNRILLN-----------
>XP_044550536.1
-----------TIDLPQ--------IAVV---GS----Q---------------
SSGKSSVLE-----N--VV-G-R-DFLPRG--------------AG-IVTRRPL---------------VLQ--------------L-I-
N--IPS------KYK---------------------EEVS-----EQKKQEE-
---------------------------E-------------Y-------------------------GEF-------------------------------------
-----------------------------------LHLPNKKFYDFA-----------EIRQEI------IRET-DR---V-TG-------------
----------SS------------------K-GISPV-PINLKIYSPY---
----VLNLTLIDLPGITK--VP------------------V-------GD-------------Q--------PK--D--IELQIR--------
NMIL-QYI---TK------------PNCLILAVTAANT--D-LANSD-ALKLAKQVD------
KSGLRTLGVLTKVDIMD-------KGV------D-------C--------------------------LDI--LR---GE---------V-
LP-----------------------L---KLGYIAVVNR--S-QND--INN-----
------N--------KS-------------------IR----------EALKD---EEAFFRN--------HP-SY--------------------
R-S------V-----------ADT-LGTKHLAKTLNKILLE-----------
>XP_002681690.1
-----------SIDLPQ--------IAVV---GS----Q---------------
SSGKSSVLE-----N--VV-G-R-DFLPRG--------------SG-IVTRRPL---------------ILQ--------------L-I-T-
--IAS------KYKA-------------------VEEVT-----EQQKQQE--
--------------------------E-------------Y-------------------------GEF-------------------------------------
-----------------------------------LHLPNKKFYNFS-----------EIREEI------VRET-DR---I-TG--------------
---------SN------------------K-NISSA-PINLKIYSPY---
----VLNLTLIDLPGITK--VP------------------V-------GD-------------Q--------PK--D--IEQQIR--------

KMIL-QFI---SK------------PTCIILAVTAANT--D-LANSD-ALKLAKEVD------
RTGDRTLGVLTKVDIMD-------KGV------D-------C----------------------------MDI--IR---GE--------V-
LP-----------------------L---KMGYIGVINR--S-QND--INT------
-----N--------KS--------------------IR----------DALKD---EDAFFRN--------HP-SY---------------------R-
S------Y-----------ANN-MGTKYLAKTLNKILLN-----------
>KAH3761456.1
------------IDLPQ--------IVVV--GS---Q----------------
SSGKSSVLE-----H--IV-G-R-DFLPRG--------------SG-IVTRRPL---------------ILQ--------------L------
--STN-----KLD--SK-----------
------------------E--------------W---------------------------GEF---
--------------------------LHKPNVRFADFS-----------EIREEI------KRET-DR---A-AG---------------------
---SN-------------------K-GVNNT-PINLKISSPH---
VLNLTLVDLPGMTR--VP-------------------V-------GD--------------Q--------PS--N--IEDIIR--------
KMIK-GYI---AK------------PNSIILALTAANT--D-LSNSD-ALQLASEVD------
PEGKRTLGVLTKLDIMD-------RGT------S-------A----------------------------IDV--LY---NR---------A-
IP-----------------------L---ALGYVGVVNR--S-QED--IMR-----
------D--------KP--------------------IS----------EALKT---EALFFEN--------HP-NY---------------------S-
A------I-----------ASR-CGTPFLAQRLNEILLN-----------
>PRP82407.1
------------IDLPQ--------IVVI--GS---Q----------------
SSGKSSVLE-----N--IV-G-K-DFLPRG--------------SG-IVTRRPL---------------VLQ--------------L-F-
N---VP--------SK---------------------NGAD-----AAD-APQ-
----------------------------D--------------W---------------------------GEF----------------------------------
-------------------------------------LHKPKERFYDFA-----------EIRNEI------ERET-DR---L-TG-----------
-------------KN-------------------K-GISHM-PINLKVYSTR---
----------VLNLTLVDLPGITK--VP-------------------I-------GD--------------Q--------PK--D--IEAQIR----
----NMIF-HYI---EK------------QSTIILAVTAANT--D-LTNSD-ALQLAREVD------
PEGKRTIGVITKLDLMD-------KGT------D-------A----------------------------LDM--LM---GR---------V-
VP-----------------------L---RLGYIGVVNR--S-QAD--INS-----
------R--------RS--------------------IT----------SALEG---EREFFST--------HP-AY---------------------R-
N------V-----------ASR-CGTSFLAGNLSKILMN-----------
>XP_004368323.1
------------IDLPQ--------IVVV--GS---Q----------------
SSGKSSVLE-----N--IV-G-R-DFLPRG--------------QG-IVTRRPL---------------VLQ--------------L-I-
N---LPT--DP-KKKK---------------------NVDD-----
DDDSALK-----------------------------E--------------W---------------------------GEF---------------------
--LHKPNEIFYDFN-----------QIREEI------VRET-DR---M-
SG------------------------KN-------------------K-GISHM-PINLKIYSPH-----------------------------------
------------------------VLNLTLVDLPGITK--VP-------------------V-------GD--------------Q--------PA--
D--IEIQIR--------TMVL-QYI---ER------------PNAVILAVTAANT--D-LSNSD-ALQIAAVAD------
PDGQRTIGVLTKIDLMD-------AGT------D-------A----------------------------MDM--LL---NR---------
V-IP-----------------------L---RLGYIGVINR--S-QQD--IIK------
-----K--------KP--------------------IR----------AALKA---EAEYFTT--------HP-LY---------------------R-S-
-----V-----------ASR-CGTPFLSKTLNKILMN-----------

>XP_012754660.1
------------LDLPQ--------IVVV---GS----Q----------------
SSGKSSVLE-----N--IV-G-R-DFLPRG---------------SG-IVTRRPL---------------ILQ--------------L-T-H-
--LP---------------------------------ITD-----EVDVSTQ--------
---------------------E--------------W----------------------------GEF---
-----------------------------LHRPNDMFYDFA------------EIREEI------IKDT-DR---L-TG-------------------
-----KN-------------------K-GVSAQ-PINLKIYSPH--
--VVNLTLVDLPGITK--VP-------------------V-------GD--------------Q--------PT--D--IEMQIR--------
RMIM-AYI---KR------------PNAIILAVTPANT--D-LANSD-ALQLAREVD------
PDGKRTIGVITKLDLMD-------KGT------D-------A----------------------------MDV--LT---GR---------V-
VP-----------------------L---ALGFVGVINR---S-QED--IIS-------
----K--------KS--------------------IR----------DALKS---EVQYFKN--------HP-IY---------------------K-T-
-----I-----------ANR-SGTAYLSKTLNKLLMF-----------
>KYR01170.1
------------LDLPQ--------IVVV---GS----Q----------------
SSGKSSVLE-----N--IV-G-K-DFLPRG---------------SG-IVTRRPL---------------ILQ--------------L-T-H-
--LP---------------------------------LAD-----DGSGPTS------
-----------------------E--------------W----------------------------GEF---
-------------------------------LHKPNDMYYDFA------------EIREEI------IRDT-DR---M-TG----------------
--------KN-------------------K-GISAQ-PINLKIYSPH--
----VVNLTLVDLPGITK--VP-------------------V-------GD--------------Q--------PT--D--IEQQIR--------
RMVM-AYI---KK------------PNAIILAVTPANT--D-LANSD-ALQLAKEVD------
PEGKRTIGVITKLDLMD-------KGT------D-------A----------------------------MEV--LT---GK--------V-
IP-----------------------L---SLGFIGVINR---S-QED--IIS---------
--K--------KS--------------------IR----------ESLKS---ELLYFKN--------HP-IY---------------------K-S----
--I-----------ANR-SGTAYLSKTLNKLLMF-----------
>XP_003294436.1
------------LDLPQ--------IVVV---GS----Q----------------
SSGKSSVLE-----N--IV-G-R-DFLPRG---------------SG-IVTRRPL---------------ILQ--------------L-T-H-
--LP---------------------------------MAD-----DGS-PTS-------
----------------------E--------------W----------------------------GEF--
------------------------------LHRPNDMFYDFS------------EIREEI------IRDT-DR---L-TG------------------
------KN-------------------K-GISAQ-PINLKIYSPH--
--VVNLTLVDLPGITK--VP-------------------V-------GD--------------Q--------PS--D--IEQQIR--------
RMIM-AYI---KK------------QNAIIVAVTPANT--D-LANSD-ALQLAKEVD------
PEGKRTIGVITKLDLMD-------KGT------D-------A----------------------------MDV--LT---GR---------V-
IP-----------------------L---TLGFIGVINR---S-QED--IIA---------
--K--------KS--------------------IR----------ESLKS---EVLFFKN--------HP-IY---------------------K-T----
--I-----------ANR-SGTAYLSKSLNKLLMF-----------
>PRP81066.1
------------LDLPQ--------IVVV---GS----Q----------------
SSGKSSVLE-----A--IV-G-R-DFLPRG---------------SG-IVTRRPL---------------VLQ--------------L-T-
H---LP----P---------------------------SIN-----DAE-SDV----
-------------------------E--------------W----------------------------GEF---------------------------------------

```
-------------------------------NHRPNEMFHDFD-----------KIREEI------ERET-ER---T-TG--------------
----------KN-------------------K-GISDI-PISLKIYSSH------------------------------------------------------
----VLNLTLVDLPGITR--VP-------------------I-------GD-------------Q--------PP--D--IERQIR--------
SMVK-HYI---DK-----------PNAIILAITAANT--D-LTNSD-ALQMARESD------
PDGIRTVGVITKIDIMD-------KGT------N-------A--------------------------LDM--LS---GR---------V-
VP----------------------L---KLGFIGVVNR-------------------------------------------S-QQD--IIK------
-----K--------KS-------------------IR----------DALVD---EQNFFHQ--------HS-LY--------------------K-
N------I-----------SGR-LGTAYLSRTLNKTLIQ-----------
>XP_004348308.1
------------IQLPQ--------IAVV------------------------------------------GS----Q----------------
SSGKSSVLE-----N--IV-G-K-DFLPRG-------------HG-IVTRRPL---------------ILQ-------------L-V-
HRK-----------------------------PGSPR------------PALPD--DP-SSSGGH-----------------TDDGID----
-GED--VE----------------------------E-------------W--------------------------GEF--------------------
----------------------------------------------LHAPGKRFISFA-----------EIRKEI------EAET-DR---V-
TG-----------------------SN-------------------K-GISSK-PINLRIYSPN-------------------------------------
----------------------VLNLTLVDLPGITK--VP------------------V-------GD-------------Q--------PE--D-
-IEKQIR--------TLVR-SYI---SN------------PNCIILAVTPANV--D-LANSD-ALKLAKTID------
PEGNRTIGVCTKIDLMD-------AGT------D-------A---------------------------MDI--LS---GR---------V-
VP----------------------V---KLGFIGVVNR--------------------------------------------S-QAD--INT------
-----A--------KP-------------------IA----------DSLKS---EEQFFKS--------HP-AY--------------------Q-
A------I-----------AHR-CGTAYLSKALNKLLMHHIRDC-L----
>NP_012926.1
------------IDLPQ--------ITVV------------------------------------------GS----Q----------------
SSGKSSVLE-----N--IV-G-R-DFLPRG-------------TG-IVTRRPL---------------VLQ-------------L-I-
NRR-----------------------------PKKSEHAKVNQTANELIDLNIN--DD-DKKKDE-----------------
SGKHQN-----EGQSEDN--------------------------KEE-------------W--------------------------GEF---
---------------------------------------------------------------LHLPGKKFYNFD-----------EIRKEI-----
-VKET-DK---V-TG-----------------------AN------------------S-GISSV-PINLRIYSPH--------------------
---------------------------------------VLTLTLVDLPGLTK--VP------------------V-------GD--------------
Q--------PP--D--IERQIK--------DMLL-KYI---SK------------PNAIILSVNAANT--D-LANSD-
GLKLAREVD------PEGTRTIGVLTKVDLMD-------QGT------D-------V---------------------------IDI--
LA---GR---------V-IP----------------------L---RYGYIPVINR--------------------------------------------
G-QKD--IEH-----------K--------KT-------------------IR----------EALEN---ERKFFEN--------HP-SY----
-----------------S-S------K-----------AHY-CGTPYLAKKLNSILLHHIRQT-LP---
>OUM62108.1
------------IDLPQ--------IVVI------------------------------------------GS----Q----------------
SSGKSSVLE-----N--IV-G-R-DFLPRG-------------TG-IVTRRPL---------------ILQ-------------L-I-
NKR-----------------------------DGLTT-------------------------NS-----------------------A-----
PANSEQN--------------------------MNE-------------W--------------------------GEF------------------
---------------------------------------------------LHLPGKKFYDFT-----------EIRKEI------EKET-EL---K-
VG-----------------------KN------------------A-GISDQ-PINLRIFSPK-----------------------------------
-----------------------VLTLTLVDLPGLTK--VP------------------V-------GD-------------Q--------PK--
D--IERLIR--------DMIL-KYI---TK------------PNAIILAVTAANT--D-LANSD-GLKLAREVD------
PEGVRTIGVLTKVDLMD-------AGT------D-------V--------------------------IDI--LA---GR---------V-
```

IP------------------------L---RLGYVPVVNR---G-QKD--IET------
-----N--------KS--------------------IS----------KALEA---EKQFFEH--------HS-SY---------------------K-
S------K-----------AQY-CGTPFLARKLNMILMHHIRNT-LP---
>KXN66323.1
------------VDLPQ--------IVVI---GS----Q----------------
SSGKSSVLE-----N--IV-G-R-DFLPRG--------------TG-IVTRRPL---------------VLQ--------------L-F-
NVP------------------------------QTQ---A-----DSAKENE--
--------------------------PAE--------------Y----------------------------GEF--------------------------------------
------------------------------------LHLPGTKFTDFD-----------KIRKEI------ESET-ES---K-TG--------------
----------KN-------------------A-GISPL-PINLRIFSPK---
-----VLTLTLVDLPGLTK--VP-------------------V-------GD--------------Q--------PK--D--IEKQIR--------
DMIM-KYI---NK------------PNAIILAVTAANT--D-LANSD-GLKLAREVD------
PEGSRTIGVLTKVDLMD-------HGT------D-------V----------------------------VDI--LA---GR--------V-
IP------------------------L---RLGYVPVINR---S-QRD--IES-------
----K--------KN--------------------IS----------AALDH---ERQFFEK--------HP-SY---------------------T-
S------K-----------VQY-CGTPFLARKLSMILMHHIRNT-LP---
>XP_011389257.1
------------IDLPQ--------ITVL---GS----Q----------------
SSGKSSVLE-----N--IV-G-R-DFLPRG--------------TG-IVTRRPL---------------VLQ--------------L-I-
NRP------------------------------ATSKA-------------------------NDE-----------------A-APAV-----
SSKGANN----------------------------PDE--------------W----------------------------GEF--------------------
---LHLPGEKFFDFD-----------KIREEI------VRDT-EL---K-
TG-------------------------RN-------------------A-GISPQ-PINLRIYSPN-------------------------------------
------------------------VLTLTLVDLPGLTK--VP-------------------V-------GD--------------Q--------PR--
D--IERQIR--------DTVL-KFI---SK------------PNAVILAVTAANT--D-LANSD-GLKLAREVD------
PEGTRTVGVLTKVDLMD-------AGT------D-------V----------------------------VDI--LA---GR--------V-
IP------------------------L---RLGYVPVVNR---G-QRD--IDQ-----
------K--------KL--------------------VS----------AALTA---EKEFFEN--------HP-SY---------------------R-
S------K-----------AQY-CGTPFLARKLNTILMHHIRNT-LP---
>XP_006458578.1
------------IDLPQ--------ICVL---GS----Q----------------
SSGKSSVLE-----N--IV-G-R-DFLPRG--------------TG-IVTRRPL---------------VLQ--------------L-I-
NRP------------------------------AGTPG-------------------------GPQ-----------------INGTDK-----
SSDKHAN----------------------------ADE--------------W---------------------------GEF--------------------
---LHLPGEKFFDFN-----------KIRAEI------VRDT-EV---K-
TG------------------------KN-------------------A-GISPL-PINLRVFSPN-------------------------------------
------------------------VLTLTLVDLPGLTK--VP-------------------V-------GD--------------Q--------PR--
D--IEKQIR--------DMLL-KYI---SK------------SACIILAVTAGNT--D-LANSD-GLKMAREVD------
PEGLRTIGVLTKIDLMD-------KGT------D-------V----------------------------VDI--LA---GR---------I-IP-
----------------------L---RLGYVPVVNR---G-QRD--IES---------
--S--------KP--------------------IS----------AALEY---ERSFFEN--------HA-SY---------------------K-S---
---K-----------AQF-CGTPFLARKLNMILMHHIKAT-IP---
>KNE68830.1

------------IDLPQ--------ITVI--GS----Q----------------
SSGKSSVLE-----N--IV-G-R-DFLPRG--------------TG-IVTRRPL---------------VLQ--------------L-I-
NRP------------------------------ATPAAPGS------------------PATDGE-----------------PAADGT-----
SGDNQDN---------------------------KNE--------------W---------------------------GEF-------------------
---LHAPNKRFYNFD-----------EIRKEI------IRDT-DE---
K-AG------------------------AN-------------------T-GISHA-PINLRIYSPN----------------------------------
--------------------------VLTLTLVDLPGLTK--VP-------------------V-------GD--------------Q--------PK--
D--IEIQIR--------EMLL-KYI---TK------------PNAIILAVTAANT--D-LANSD-GLKLAREVD------
PEGSRTIGVLTKVDLMD-------PGT------D-------V----------------------------VDI--LA---GR--------V-
IP-----------------------L---RLGYVPVVNR--G-QKD--IDK-----
------R--------KS--------------------IA----------LALEH---ERSYFEE--------HP-SY---------------------R-
S------K-----------AQY-CGTPFLARKLNMILLHHIKNT-LP---
>XP_748106.1
------------IDLPQ--------IVVV--GS----Q----------------
SSGKSSVLE-----N--IV-G-R-DFLPRG--------------SG-IVTRRPL---------------ILQ--------------L-I-
NRP------------------------------SRNSV------------------------TNGV------------------KEEKLE-----
TTDSEAN---------------------------VDE--------------Y---------------------------GEF----------------------
--LHIPGQKFYDFN-----------KIREEI------VRET-EQ---K-
VG------------------------RN-------------------A-GISPA-PINLRIYSPN------------------------------------
------------------------VLTLTLVDLPGLTK--VP-------------------V-------GD--------------Q--------PK--
D--IERQIR--------DMVL-KYI---SK------------PNAIILAVTSANQ--D-LANSD-GLKLAREVD------
PEGQRTIGVLTKVDLMD-------EGT------D-------V----------------------------VDI--LA---GR---------I-
IP-----------------------L---RLGYVPVVNR--G-QRD--IEN-----
------K--------RP--------------------IS----------YALEH---EKNFFES--------HK-AY---------------------R-
N------K-----------ASY-CGTPYLARKLNLILMMHIKQT-LP---
>OAJ44422.1
------------IDLPQ--------IAVV--GS----Q----------------
SSGKSSVLE-----N--IV-G-K-DFLPRG--------------SG-IVTRRPL---------------VLQ--------------L-I-
NRP------------------------------NSKGEIGGIAAGT----DKPPS--SP-STPGAK-----------------SATTSN-
----AAVTVEE---------------------------GDE--------------W---------------------------GEF-----------------
---LHIPGKRFTDFN-----------EIRDEI------DRET-EK---
T-TG------------------------KN-------------------A-GVSSN-PINLRIYSPN---------------------------------
---------------------------VLTLTLVDLPGLTK--VP-------------------V-------GD--------------Q--------PK-
-D--IEKLIK--------DMIL-KYI---SK------------SNAIILAVTSANT--D-LANSD-GLKLAREVD------
PEGVRTIGVLTKIDLMD-------QGT------D-------V----------------------------IDI--LA---GR---------V-
IP-----------------------L---RLGYVPVVNR--G-QRD--IEN-----
------K--------KK--------------------IS----------LALEA---EKNYFEN--------HA-SY---------------------R-
S------K-----------AQY-CA-------------------------
>KAJ1432693.1
------------LDLPQ--------ICVV--GS----Q----------------
SAGKSSVLE-----N--IV-G-R-DFLPRG--------------TG-ICTRRPL---------------VLQ--------------L-Y-
CTA------------------------------AAEE--ADVDVD-----
ELAAEEN--CNGS-----------------AKE--------------W---------------------------GEF-----------------
---LHLPGQRFHDFS-----------EIRSEI------ERET-DR-

--V-SG------------------------RN-------------------K-GISNK-SINLKIFSPY---------------------------------
---------------------------VLNLTLVDLPGITK--VP-------------------T-------GD--------------Q--------PE-
-D--VEQQIL--------SMCR-EFI---SN----------PNAIILAVSAANQ--D-LVNSE-GLKLARSVD------
PEGLRTIGVLTKVDIMD-------HGT------D-------C-----------------------------CDV--LN---NQ---------
V-IP------------------------L---RRGYIAVINR--S-QKD--IID------
-----A--------LP--------------------IR----------KALLK---EQKYFQS--------HP-KY---------------------R-
S------Q-----------LAK-CGTGNMARTLNQLLMN-----------
>XP_004347890.1
------------LDLPQ--------IAVV---GS----Q----------------
SAGKSSVLE-----N--FV-G-K-DFLPRG--------------SG-IVTRRPL---------------VLQ--------------L-V-
NSK------------------------------GP---
--------------------E--------------Y---------------------------GEF--
---------------------------LHNKSKKFTDFD-----------EVRKEI------EAET-DR---I-TG---------------------
---TN--------------------K-GISPV-PINLKVYSPN---
VLNLTLVDLPGITK--VP-------------------I-------GD--------------Q--------PT--N--IESLIR--------
EMIM-QFI---GR----------PNCLILAVSPANS--D-LANSD-ALKLAREVD------
QQGIRTIGVITKLDLMD-------EGT------D-------A-----------------------------REV--LE---NK---------L-
IP-----------------------L---RRGFIGVVNR---S-QKD--IDG------
-----R--------KD--------------------IK----------AAMSA---ELRFFST--------HP-AY---------------------R-
D------L-----------ANK-NGTMYLQRVLNQQLTNHIRDT-L----
>NP_001024332.1
------------FELPQ--------IAVV---GG----Q----------------
SAGKSSVLE-----N--FV-G-K-DFLPRG--------------SG-IVTRRPL---------------ILQ--------------L-I-
QDR------------------------------N--
--------------------E--------------Y---------------------------AEF--
---------------------------LHKKGHRFVDFD-----------AVRKEI------EDET-DR---V-TG--------------------
-----QN---------------------K-GISPH-PINLRVFSPN---
--VLNLTLIDLPGLTK--VP-------------------V-------GD--------------Q--------PA--D--IEQQIR--------
DMIL-TFI---NR------------ETCLILAVTPANS--D-LATSD-ALKLAKEVD------
PQGLRTIGVLTKLDLMD-------EGT------D-------A----------------------------REI--LE---NK---------L-
FT-----------------------L---RRGYVGVVNR--G-QKD--IVG----
-------R--------KD--------------------IR----------AALDA---ERKFFIS--------HP-SY---------------------
R-H------M-----------ADR-LGTSYLQHTLNQQLTNHIRDT-L----
>KMZ10000.1
------------LDLPQ--------IAVV---GG----Q----------------
SAGKSSVLE-----N--FV-G-K-DFLPRG--------------SG-IVTRRPL---------------ILQ--------------L-I-
NGV------------------------------T--
-------------------E--------------Y---------------------------GEF---
--------------------------LHIKGKKFSSFD-----------EIRKEI------EDET-DR---V-TG-----------------------
-SN-------------------K-GISNI-PINLRVYSPH---
VLNLTLIDLPGLTK--VA-------------------I-------GD--------------Q--------PV--D--IEQQIK--------
QMIF-QFI---RK------------ETCLILAVTPANT--D-LANSD-ALKLAKEVD------
PQGVRTIGVITKLDLMD-------EGT------D-------A----------------------------RDI--LE---NK---------L-
LP-----------------------L---RRGYIGVVNR---S-QKD--IEG-----

------R--------KD-------------------IH----------QALAA---ERKFFLS--------HP-SY--------------------
R-H------M-----------ADR-LGTPYLQRVLNQQLTNHIRDT-L----
>XP_030853442.1
------------LDLPQ--------IAVV--GG----Q---------------
SAGKSSVLE-----N--FV-G-R-DFLPRG--------------SG-IVTRRPL---------------VLQ-------------L-N-
NSK-----------------------------T---
------------------E-------------Y--------------------------GEF--
-------------------------LHCKGKKFTDFD-----------EIRKEI------EAET-DR---V-TG-----------------------
-SN-------------------K-GISNV-PINLRVYSPN---
VLNLTLVDLPGMTK--IA-------------------V-------GD--------------Q--------PV--D--IEIQIR--------
SMVM-EFV---TN------------ESTLILAVSPANQ--D-LANSD-ALKVAKEVD------
PKGVRTIGVITKLDLMD-------DGT------D-------A----------------------------KDI--LE---NK---------L-
LP-----------------------L---RRGYVGVVNR---S-QRD--IEG----
-------K--------KD-------------------IK---------AALAA---ERKFFLS--------HP-SY-------------------
R-H------I-----------ADK-MGTPWLQKILNQQLTNHIRDS-L----
>XP_030853442.1.2
------------LDLPQ--------IAVV--GG----Q---------------
SAGKSSVLE-----N--FV-G-R-DFLPRG--------------SG-IVTRRPL---------------VLQ-------------L-N-
NSK-----------------------------T---
------------------E-------------Y--------------------------GEF--
-------------------------LHCKGKKFTDFD-----------EIRKEI------EAET-DR---V-TG-----------------------
-SN-------------------K-GISNV-PINLRVYSPN---
VLNLTLVDLPGMTK--IA-------------------V-------GD--------------Q--------PV--D--IEIQIR--------
SMVM-EFV---TN------------ESTLILAVSPANQ--D-LANSD-ALKVAKEVD------
PKGVRTIGVITKLDLMD-------DGT------D-------A----------------------------KDI--LE---NK---------L-
LP-----------------------L---RRGYVGVVNR---S-QRD--IEG----
-------K--------KD-------------------IK---------AALAA---ERKFFLS--------HP-SY-------------------
R-H------I-----------ADK-MGTPWLQKILNQQLTNHIRDS-L----
>XP_005165639.1
------------LDLPQ--------IAVV--GG----Q---------------
SAGKSSVLE-----N--FV-G-K-DFLPRG--------------SG-IVTRRPL---------------VLQ-------------L-I-
NCP-----------------------------T--
-------------------E-------------Y--------------------------AEF---
--------------------------LHCKGKKFTDFD-----------EVRQEI------EAET-DR---I-TG---------------------
---QN-------------------K-GISPV-PINLRVYSPN---
VLNLTLVDLPGMTK--VP-------------------V-------GD--------------Q--------PA--D--IEAQIR--------
DMLM-QFV---TK------------ENCLLLAVSPANS--D-LANSD-ALKIAKEVD------
PQGMRTIGVITKLDLMD-------EGT------D-------A----------------------------REI--LE---NK---------L-
LP-----------------------L---RRGYIGVVNR---S-QKD--IDG-----
------K--------KD--------------------IT---------AAMSA---ERKFFLT--------HP-SY---------------------R-
H------L-----------ADR-MGTPYLQKALNQQLTNHIRDT-L----
>XP_028570166.1
------------LDLPQ--------IAVV--GG----Q---------------
SAGKSSVLE-----N--FV-G-R-DFLPRG--------------SG-IVTRRPL---------------VLQ-------------L-V-

NSP----------------------------T---
-----------------E-------------Y--------------------------GEF--
------------------------LHCKGKKFTDFD-----------EIRQEI------EAET-DR---I-TG----------------------
-SN------------------K-GISPV-PINLRVYSPH--
VLSLTLVDLPGMTK--VP-------------------V-------GD--------------Q--------PA--D--IEFQIR--------
EMLM-QFV---TK------------ENCLILAVSPANS--D-LANSD-ALKIAKEVD------
PQGQRTIGVITKLDLMD-------EGT------D-------A--------------------------RDV--LE---NK---------L-
LP----------------------L---RRGYIGVVNR---S-QKD--IDG-----
------K--------KD-------------------IQ----------AALAA---ERKFFLT--------HP-AY--------------------R-
H------M-----------ADR-MGTPYLQKVLNQQLTNHIRDT-L----
>EPQ17174.1
------------LDLPQ--------IAVV---GG----Q---------------
SAGKSSVLE-----N--FV-G-R-DFLPRG--------------SG-IVTRRPL---------------VLQ-------------L-V-
NAS----------------------------T---
-----------------E-------------Y--------------------------AEF--
------------------------LHCKGKKFTDFE-----------EVRLEI------EAET-DR---V-TG----------------------
-TN------------------K-GISPV-PINLRVYSPH--
VLNLTLVDLPGMTK--VP-------------------V-------GD--------------Q--------PA--D--IEFQIR--------
DMLM-QFV---TK------------ENCLILAVSPANS--D-LANSD-ALKIAKEVD------
PQGQRTIGVITKLDLMD-------EGT------D-------A--------------------------RDV--LE---NK---------L-
LP----------------------L---RRGYIGVVNR---S-QKD--IDG-----
------K--------KD-------------------IT----------AALAA---ERKFFLS--------HP-SY--------------------R-
H------L-----------ADR-MGTPYLQKVLNQQLTNHIRDT-L----
>BAB27759.1
------------LDLPQ--------IAVV---GG----Q---------------
SAGKSSVLE-----N--FV-G-R-DFLPRG--------------SG-IVTRRPL---------------VLQ-------------L-V-
NST----------------------------T---
-----------------E-------------Y--------------------------AEF--
------------------------LHCKGKKFTDFE-----------EVRLEI------EAET-DR---V-TG----------------------
-TN------------------K-GISPV-PINLRVYSPH--
VLNLTLVDLPGMTK--VP-------------------V-------GD--------------Q--------PP--D--IEFQIR--------
DMLM-QFV---TK------------ENCLILAVSPANS--D-LANSD-ALKIAKEVD------
PQGQRTIGVITKLDLMD-------EGT------D-------A--------------------------RDV--LE---NK---------L-
LP----------------------L---RRGYIGVVNR---S-QKD--IDG-----
------K--------KD-------------------IT----------AALAA---ERKFFLS--------HP-SY--------------------R-
H------L-----------ADR-MGTPYLQKVLNQQLTNHIRDT-L----
>ELW62001.1
------------LDLPQ--------IAVV---GG----Q---------------
SAGKSSVLE-----N--FV-G-R-DFLPRG--------------SG-IVTRRPL---------------VLQ-------------L-V-
NAT----------------------------T---
-----------------E-------------Y--------------------------AEF--
------------------------LHCKGKKFTDFE-----------EVRLEI------EAET-DR---V-TG----------------------
-TN------------------K-GISPV-PINLRVYSPH--
VLNLTLVDLPGMTK--VP-------------------V-------GD--------------Q--------PP--D--IEFQIR--------

DMLM-QFV---TK------------ENCLILAVSPANS--D-LANSD-ALKIAKEVD------
PQGQRTIGVITKLDLMD-------EGT------D-------A---------------------------RDV--LE---NK---------L-
LP----------------------L---RRGYIGVVNR--S-QKD--IDG-----
------K--------KD--------------------IT----------AALAA---ERKFFLS--------HP-SY---------------------R-
H------L-----------ADR-MGTPYLQKVLNQQLTNHIRDT-L----
>EAW87759.1
------------LDLPQ--------IAVV---GG----Q----------------
SAGKSSVLE-----N--FV-G-R-DFLPRG--------------SG-IVTRRPL---------------VLQ--------------L-V-
NAT-----------------------------T---
------------------E--------------Y---------------------------AEF--
------------------------LHCKGKKFTDFE-----------EVRLEI------EAET-DR---V-TG----------------------
-TN-------------------K-GISPV-PINLRVYSPH---
VLNLTLVDLPGMTK--VP-------------------V-------GD--------------Q--------PP--D--IEFQIR--------
DMLM-QFV---TK------------ENCLILAVSPANS--D-LANSD-ALKVAKEVD------
PQGQRTIGVITKLDLMD-------EGT------D-------A---------------------------RDV--LE---NK---------L-
LP----------------------L---RRGYIGVVNR--S-QKD--IDG-----
------K--------KD--------------------IT----------AALAA---ERKFFLS--------HP-SY---------------------R-
H------L-----------ADR-MGTPYLQKVLNQQLTNHIRDT-L----
>XP_025915522.1

---MTK--VP-------------------V-------GD--------------Q--------
PP--D--IEFQIR--------DMLM-QFV---TK------------ENCLILAVSPANS--D-LANSD-ALKIAKEVD-----
-PQGQRTIGVITKLDLMD-------EGT------D-------A---------------------------RDV--LE---NK---------
L-LP----------------------L---RRGYIGVVNR--S-QKD--IDG--
---------K--------KD--------------------IQ----------AALAA---ERKFFLS--------HP-AY--------------------
-R-H------M-----------ADR-MGTPYLQKVLNQQLTNHIRDT-L----
>XP_012378586.1
----------------------------A---GGPRRRQ----------------
SGRAAAAAEPEPERN--FV-G-R-DFLPRG--------------SG-IVTRRPL---------------VLQ--------------
L-V-NAT-----------------------------T---
------------------------E--------------Y---------------------------AEF--
------------------------------LHCKGKKFTDFE-----------EVRLEI------EAET-DR---V-TG------------------
------TN-------------------K-GISPV-PINLRVYSPH---
--VLNLTLVDLPGMTK--VP-------------------V-------GD--------------Q--------PP--D--IEFQIR--------
DMLM-QFV---TK------------ENCLILAVSPANS--D-LANSD-ALKVAKEVD------
PQGQRTIGVITKLDLMD-------EGT------D-------A---------------------------RDV--LE---NK---------L-
LP----------------------L---RRGYIGVVNR--S-QKD--IDG-----
------K--------KD--------------------IT----------AALAA---ERKFFLS--------HP-SY---------------------R-
H------L-----------ADR-MGTPYLQKVLNQQLTNHIRDT-L----
>KAE8583055.1

```
------------LDLPQ--------IAVV-------------------------------------------GG----Q---------------
SAGKSSVLE-----N--FV-G-K-DFLPRG--------------SG-IVTRRPL---------------VLQ--------------L-V-
NSS-----------------------------T------------------------------------------------------------------------------
------------------E-------------Y---------------------------GEF-----------------------------------------------
-------------------------LHCKGKKFTDFD-----------EIRLEI------EAET-DR---A-TG-----------------------
-TN-------------------K-GISPV-PINLRVYSPN------------------------------------------------------------
VLNLTLVDLPGMTK--VP------------------V-------GD--------------Q--------PV--D--IEFQIR--------
DMLM-QFV---TK------------ENCLVLAVSPANS--D-LANSD-ALKIAKEVD------
PKGLRTIGVITKLDLMD-------EGT------D-------A----------------------------RDV--LE---NK---------L-
LP----------------------L---RRGYIGVVNR----------------------------------------------S-QKD--IDG-----
------K--------KD--------------------IQ----------AALAA---ERKFFLS--------HP-SY---------------------R-
H------L-----------ADR-MGTPYLQKALNQQLTNHIRDT-L----
>XP_032814666.1
------------LDLPQ--------IAVV-------------------------------------------GG----Q---------------
SAGKSSVLE-----N--FV-G-R-DFLPRG--------------SG-IVTRRPL---------------ILQ--------------L-M-
FCK-----------------------------A-----------------------------------------------------------------------------
-------------------E--------------Y---------------------------AEF----------------------------------------------
--------------------------LHCKGKKFTDFE-----------EVRAEI------EAET-DR---L-TG---------------------
---SN-------------------K-GISPI-PINLRVYSPH------------------------------------------------------------
VLNLTLIDLPGMTK--VP-------------------V-------GD--------------Q--------PV--D--IEYQIR--------
EMLM-QFV---TK------------ENCLILAVSPANT--D-LANSD-ALKIAKEVD------
PQGLRTIGVITKLDLMD-------DGT------D-------A----------------------------RDI--LE---NK---------L-
LP----------------------L---RRGYIGVVNR----------------------------------------------S-QKD--IDG-----
------R--------KD--------------------IN----------AAMAA---ERKFFLS--------HP-SY---------------------
R-H------M-----------ADR-MGTPYLQKTLNQQLTNHIRDT-L----
>XP_025944940.1
----------------------------------------------------------------------------------------------------------------
MF-F-R-DFLPRG--------------SG-IVTRRPL---------------VLQ--------------L-V-TAK--------------------
----------T-----------------------------------------------------------------------------------------------E-----
---------Y---------------------------AEF------------------------------------------------------------------------
LHCKGRKFTDFD-----------EVRQEI------EVET-DR---I-TG------------------------VN-------------------
K-GISSI-PINLRIYSPH------------------------------------------------------------VLSLTLIDLPGITK--VP--
----------------V-------GD--------------Q--------PP--D--IEQQIR--------DMIM-QFI---SR-------------------
ENCLILAVTPANT--D-LANSD-ALKLAKEVD------PQGLRTIGVITKLDLMD-------EGT------D-------
A----------------------------REI--LE---NK---------L-LP-----------------------L---RRGYIGVVNR---------
-------------------------------------S-QKD--IDG-----------K--------KD--------------------IK----------
AALLA---ERKFFLS--------HP-AY---------------------R-H------M-----------ADR-
MGTPYLQKVLNQQLTNHIRDT-L----
>EPQ08653.1
------------LELPQ--------IAVV-------------------------------------------GG----Q---------------
SAGKSSVLE-----N--FV-G-R-DFLPRG--------------SG-IVTRRPL---------------VLQ--------------L-V-
TSK-----------------------------T-----------------------------------------------------------------------------
------------------E-------------Y---------------------------AEF-----------------------------------------------
-------------------------LHCKGKKFTDFD-----------EVRHEI------EAET-DR---V-TG----------------------
```

--MN-------------------K-GISSI-PINLRVYSPH--
VLNLTLIDLPGITK--VP------------------V-------GD--------------Q--------PV--D--IEHQIR--------
EMIM-QFI---TR------------ENCLILAVTPANT--D-LANSD-ALKLAKDVD------
PQGLRTIGVITKLDLMD-------EGT------D-------A---------------------------RDI--LE---NK---------L-
LP----------------------L---RRGYVGVVNR---S-QKD--IDG----
-------K--------KD--------------------IK---------AAMLA---ERKFFLS--------HP-AY--------------------
R-H------I-----------ADR-MGTPHLQKVLNQQLTNHIRDT-L----
>XP_006496668.1
------------LELPQ--------IAVV---GG---Q---------------
SAGKSSVLE-----N--FV-G-R-DFLPRG--------------SG-IVTRRPL--------------VLQ--------------L-V-
TSK-----------------------------A--
------------------E--------------Y--------------------------AEF--
------------------------LHCKGKKFTDFD-----------EVRHEI------EAET-DR---V-TG---------------------
--MN-------------------K-GISSI-PINLRVYSPH--
VLNLTLIDLPGITK--VP------------------V-------GD--------------Q--------PP--D--IEYQIR--------
DMIM-QFI---TR------------ENCLILAVTPANT--D-LANSD-ALKLAKEVD------
PQGLRTIGVITKLDLMD-------EGT------D-------A---------------------------RDV--LE---NK---------L-
LP----------------------L---RRGYVGVVNR---S-QKD--IDG----
-------K--------KD--------------------IK---------AAMLA---ERKFFLS--------HP-AY--------------------
R-H------I-----------ADR-MGTPHLQKVLNQQLTNHIRDT-L----
>XP_016856477.1
------------LELPQ--------IAVV---GG---Q---------------
SAGKSSVLE-----N--FV-G-R-DFLPRG--------------SG-IVTRRPL--------------VLQ--------------L-V-
TSK-----------------------------A--
------------------E--------------Y--------------------------AEF--
------------------------LHCKGKKFTDFD-----------EVRLEI------EAET-DR---V-TG---------------------
--MN-------------------K-GISSI-PINLRVYSPH--
VLNLTLIDLPGITK--VP------------------V-------GD--------------Q--------PP--D--IEYQIR--------
EMIM-QFI---TR------------ENCLILAVTPANT--D-LANSD-ALKLAKEVD------
PQGLRTIGVITKLDLMD-------EGT------D-------A---------------------------RDV--LE---NK---------L-
LP----------------------L---RRGYVGVVNR---S-QKD--IDG----
-------K--------KD--------------------IK---------AAMLA---ERKFFLS--------HP-AY--------------------
R-H------I-----------ADR-MGTPHLQKVLNQQLTNHIRDT-L----
>XP_027623811.1
------------LELPQ--------IAVV---GG---Q---------------
SAGKSSVLE-----N--FV-G-R-DFLPRG--------------SG-IVTRRPL--------------VLQ--------------L-V-
TSK-----------------------------A--
------------------E--------------Y--------------------------GEF--
------------------------LHCKGKKFTDFD-----------EIRHEI------EAET-DR---V-TG---------------------
-VN-------------------K-GISSI-PINLRVYSPH--
VLNLTLIDLPGITK--VP------------------V-------GD--------------Q--------PP--D--IEYQIR--------
EMIM-QFI---TR------------ENCLILAVTPANT--D-LANSD-ALKLAKEVD------
PQGLRTIGVITKLDLMD-------EGT------D-------A---------------------------RDV--LE---NK---------L-
LP----------------------L---RRGYVGVVNR---S-QKD--IDG----

-------K--------KD-------------------IK----------AAMLA---ERKFFLS--------HP-AY--------------------
R-H------I-----------ADR-MGTPHLQKVLNQQLTNHIRDT-L----
>XP_012379251.1

V-G-R-DFLPRG--------------SG-IVTRRPL---------------VLQ--------------L-V-TSK---------------------
--------A--E-------
-------Y---------------------------AEF---
LHCKGKKFTDFD-----------EVRHEI------EAET-DR---V-TG-----------------------MN-----------------
-K-GISSI-PINLRVYSPH---VLNLTLIDLPGITK--
VP-------------------V-------GD-------------Q--------PP--D--IEYQIR--------EMIM-QFI---TR-----------
-ENCLILAVTPANT--D-LANSD-ALKLAKEVD------PQGLRTIGVITKLDLMD-------EGT------D-------
A---------------------------RDV--LE---NK---------L-LP----------------------L---RRGYVGVVNR-------
--------------------------------------S-QKD--IDG-----------K--------KD-------------------IK----------
AAMLA---ERKFFLS--------HP-AY---------------------R-H------I-----------ADR-
MGTPHLQKVLNQQLTNHIRDT-L----
>XP_031753735.1
------------LDLPQ--------IAVV--GG----Q----------------
SAGKSSVLE-----N--FV-G-R-DFLPRG--------------SG-IVTRRPL---------------ILQ--------------L-I-
FSK------------------------------T---
------------------E-------------Y---------------------------AEF---------------------------------------
------------------------LHCKSKKFTDFD-----------EVRQEI------EAET-DR---V-TG---------------------
--TN-------------------K-GISPV-PINLRVYSPN---
VLNLTLIDLPGITK--VP-------------------V-------GD-------------Q--------PH--D--IEYQIK--------
DMIL-QFI---SR------------DSCLILAVTPGNT--D-LANSD-ALKMAKEVD------
PQGLRTIGVITKLDLMD-------EGT------D-------A---------------------------KDI--LE---NK---------L-
LP----------------------L---RRGYIGVVNR--S-QKD--IDG-----
------K--------KD-------------------IK----------AALGA---ERKFFLS--------HP-GY---------------------R-
H------I-----------AER-MGTPHLQKTLNQQLTNHIRET-L----
>XP_028568434.1
------------LDLPQ--------IAVV--GG----Q----------------
SAGKSSVLE-----N--FV-G-R-DFLPRG--------------SG-IVTRRPL---------------ILQ--------------L-I-
FSK------------------------------T---
------------------E-------------Y---------------------------AEF---------------------------------------
------------------------LHCKSKKFTDFD-----------EVRQEI------EAET-DR---V-TG---------------------
--TN-------------------K-GISPV-PINLRVYSPH---
VLNLTLIDLPGITK--VP-------------------V-------GD-------------Q--------PQ--D--IEYQIK--------
DMIL-QFI---SR------------ESSLILAVTPANM--D-LANSD-ALKMAKEVD------
PQGLRTIGVITKLDLMD-------EGT------D-------A---------------------------RDV--LE---NK---------L-
LP----------------------L---RRGYIGVVNR--S-QKD--IDG-----
------K--------KD-------------------IR----------AALAA---ERKFFLS--------HP-AY---------------------R-
H------M-----------ADR-MGTPHLQKLLNQQLTNHIRET-L----
>XP_025920181.1
------------LDLPQ--------IAVV--GG----Q----------------
SAGKSSVLE-----N--FV-G-R-DFLPRG--------------SG-IVTRRPL---------------ILQ--------------L-I-

FSK-----------------------------T---
------------------E-------------Y--------------------------AEF--
-------------------------LHCKSKKFTDFD-----------EVRQEI------EAET-DR---V-TG---------------------
--TN-------------------K-GISPV-PINLRVYSPH---
VLNLTLIDLPGITK--VP-------------------V-------GD-------------Q--------PQ--D--IEYQIK--------
DMIM-QFI---SR------------ESSLILAVTPANM--D-LANSD-ALKMAKEVD------
PQGLRTIGVITKLDLMD-------EGT------D-------A---------------------------RDV--LE---NK---------L-
LP-----------------------L---RRGYIGVVNR--S-QKD--IDG-----
------K--------KD-------------------IR---------AALAA---ERKFFLS--------HP-AY--------------------R-
H------M-----------ADR-MGTPHLQKVLNQQLTNHIRET-L----
>XP_006510037.1
------------LDLPQ--------IAVV---GG----Q----------------
SAGKSSVLE-----N--FV-G-R-DFLPRG--------------SG-IVTRRPL---------------ILQ--------------L-I-
FSK-----------------------------T---
------------------E-------------Y--------------------------AEF--
-------------------------LHCKSKKFTDFD-----------EVRQEI------EAET-DR---V-TG---------------------
--TN-------------------K-GISPV-PINLRVYSPH---
VLNLTLIDLPGITK--VP-------------------V-------GD-------------Q--------PP--D--IEYQIK--------
DMIL-QFI---SR------------ESSLILAVTPANM--D-LANSD-ALKLAKEVD------
PQGLRTIGVITKLDLMD-------EGT------D-------A---------------------------RDV--LE---NK---------L-
LP-----------------------L---RRGYIGVVNR--S-QKD--IEG-----
------K--------KD-------------------IR---------AALAA---ERKFFLS--------HP-AY--------------------R-
H------M-----------ADR-MGTPHLQKTLNQQLTNHIRES-L----
>NP_001005360.1
------------LDLPQ--------IAVV---GG----Q----------------
SAGKSSVLE-----N--FV-G-R-DFLPRG--------------SG-IVTRRPL---------------ILQ--------------L-I-
FSK-----------------------------T---
------------------E-------------H--------------------------AEF--
--------------------------LHCKSKKFTDFD-----------EVRQEI------EAET-DR---V-TG--------------------
---TN-------------------K-GISPV-PINLRVYSPH---
VLNLTLIDLPGITK--VP-------------------V-------GD-------------Q--------PP--D--IEYQIK--------
DMIL-QFI---SR------------ESSLILAVTPANM--D-LANSD-ALKLAKEVD------
PQGLRTIGVITKLDLMD-------EGT------D-------A---------------------------RDV--LE---NK---------L-
LP-----------------------L---RRGYIGVVNR--S-QKD--IEG-----
------K--------KD-------------------IR---------AALAA---ERKFFLS--------HP-AY--------------------R-
H------M-----------ADR-MGTPHLQKTLNQQLTNHIRES-L----
>XP_014389433.1
---AGKGQI-------
----T-A-W-DFLPRG--------------SG-IVTRRPL---------------ILQ--------------L-I-FSK-------------------
-----------T---E----
----------Y--------------------------AEF---
-LHCKSKKFTDFD-----------EVRQEI------EAET-DR---V-TG-----------------------TN------------------
K-GISPV-PINLRVYSPH---VLNLTLIDLPGITK--
VP-------------------V-------GD-------------Q--------PP--D--IEYQIK--------DMIL-QFI---SR-----------

-ESSLILAVTPANM--D-LANSD-ALKMAKEVD------PQGLRTIGVITKLDLMD-------EGT------D-------
A---------------------------RDV--LE---NK---------L-LP----------------------L---RRGYIGVVNR--------
-------------------------------------S-QKD--IEG-----------K--------KD--------------------IR----------
AALAA---ERKFFLS--------HP-AY--------------------R-H------M-----------ADR-
MGTPHLQKTLNQQLTNHIRES-L----
>XP_006161648.2.2
--------------PH--------CTSV--SR----QAYS------------
SPQGPRKVIS-----VLFLP-H-R-DFLPRG--------------SG-IVTRRPL---------------ILQ--------------L-I-
FSK-----------------------------T---
-----------------E-------------Y--------------------------AEF---
------------------------LHCKSKKFTDFD-----------EVRQEI------EAET-DR---V-TG----------------------
--TN-------------------K-GISPV-PINLRVYSPH--
VLNLTLIDLPGITK--VP-------------------V-------GD--------------Q--------PP--D--IEYQIK--------
DMIL-QFI---SR------------ESSLILAVTPANM--D-LANSD-ALKLAKEVD------
PQGLRTIGVITKLDLMD-------EGT------D-------A---------------------------RDV--LE---NK---------L-
LP----------------------L---RRGYIGVVNR--S-QKD--IEG-----
------K--------KD--------------------IR----------AALAA---ERKFFLS--------HP-AY--------------------R-
H------M-----------ADR-MGTPHLQKTLNQQLTNHIRES-L----
>XP_012381548.1

-------------MIL-QFI---GR------------ESSLILAVTPANM--D-LANSD-ALKLAKEVD------
PQGLRTIGVITKLDLMD-------EGT------D-------A---------------------------RDV--LE---NK---------L-
LP----------------------L---RRGYIGVVNR--S-QKD--IEG-----
------R--------KD--------------------IR----------SALAA---ERKFFFS--------HS-AY--------------------R-
H------M-----------ADR-MGTLHLQKTLNQQLTNHIRES-L----
>NP_001025299.1
------------LDLPQ--------IAVV--GG----Q----------------
SAGKSSVLE-----N--FV-G-R-DFLPRG--------------SG-IVTRRPL---------------ILQ--------------L-V-
NNK-----------------------------A--
------------------E-------------Y-------------------------AEF--
-------------------------LHCKGRKFVDFD-----------EVRQEI------EAET-DR---I-TG--------------------
---SN-------------------K-GISPI-PINLRVYSPN---
VLNLTLIDLPGMTK--VA-------------------V-------GD--------------Q--------PP--D--IEHQIR--------
DMIM-QFI---TR------------ESCLILAVTPANM--D-LANSD-ALKVAKEVD------
PQGLRTIGVITKLDLMD-------EGT------D-------A----------------------------RDI--LE---NK---------L-
LP----------------------L---RRGYIGVVNR--S-QKD--IDG-----
------R--------KD--------------------IR---------AALAA---ERKFFLS--------HP-SY--------------------R-
H------M-----------AER-MGTPHLQKALNQQLTNHIRDT-L----
>XP_021326548.1

------------LDLPQ--------IAVV--GG----Q----------------
SAGKSSVLE-----N--FV-G-R-DFLPRG--------------SG-IVTRRPL---------------ILQ--------------L-V-
NNK------------------------------A---
-------------------E--------------Y---------------------------AEF--
--------------------------LHCKGRKFVDFD-----------EVRQEI------EAET-DR---I-TG---------------------
---SN-------------------K-GISPI-PINLRVYSPN--
VLNLTLIDLPGMTK--VA-------------------V-------GD--------------Q--------PP--D--IEHQIR--------
DMIM-QFI---TR-------------ESCLILAVTPANM--D-LANSD-ALKVAKEVD------
PQGLRTIGVITKLDLMD-------EGT------D-------A----------------------------RDI--LE---NK---------L-
LP-----------------------L---RRGYIGVVNR--S-QKD--IDG-----
------R--------KD--------------------IR----------AALAA---ERKFFLS--------HP-SY---------------------R-
H------M-----------AER-MGTPHLQKALNQQLTNHIRDT-L----
>XP_035683496.1
------------LDLPQ--------IAVV--GG----Q----------------
SAGKSSVLE-----N--FV-G-R-DFLPRG--------------SG-IVTRRPL---------------VLQ--------------L-
IHNPK-----------------------------A--
----------------------E--------------Y---------------------------GEF---
-----------------------------LHAKGKMFSDFH-----------EIRAEI------EAET-DR---M-TG-----------------
-------SN-------------------K-GISPV-PINLRVYSPH---
---VLNLTLIDLPGMTK--VP------------------V-------GD--------------Q--------PP--D--IEQQIR--------
DMLL-QFI---TK-------------DNCLILAVSPANQ--D-LANSD-ALKIAKEVD------
PQGMRTIGVITKLDLMD-------EGT------D-------A----------------------------RNI--LE---NR---------T-
YP-----------------------L---RRGYIGVVNR--S-QAD--IDG-----
------R--------KD--------------------IK----------AALAA---ERKFFLS--------HP-AY---------------------R-
H------L-----------ADR-MGTPYLQKTLNQQLTNHIRDT-L----
>XP_026693152.1
------------IDLPQ--------IAVV--GG----Q----------------
SAGKSSVLE-----N--FV-G-K-DFLPRG--------------SG-IVTRRPL---------------VLQ--------------L-
ITAKN------------------------------G--
----------------------E--------------W----------------------------GEF--
------------------------------LHCKGKKFTDFN-----------EIRKEI------EEET-DR---M-TG-----------------
-------SN-------------------K-GISAI-PINLRVHSPH---
---VLNLTLVDLPGMTK--VP-------------------V-------GD--------------Q--------PA--D--IEQQIR--------
DMIM-QFV---VK------------DNCLILAVSPANS--D-LANSD-ALKIAKEFD------
PQGIRTIGVITKLDLMD-------EGT------D-------A----------------------------KHI--LE---NK---------H-
LP-----------------------L---RRGYVGVVNR--S-QKD--IDG----
-------N--------KD--------------------IK----------AALSA---ERRFFLS--------HP-AY--------------------
R-H------M-----------ADK-LGTPYLQKILNQQLTNHIKET-L----
>PAA65118.1
------------FDLPQ--------IAVV--GS----Q----------------
SAGKSSVLE-----N--FV-G-K-DFLPRG--------------SG-IVTRRPL---------------ILQ--------------L-L-
YNP------------------------------SA---
--------------------E--------------Y---------------------------AEF---
---------------------------GHQRGRKYTNFE-----------EVRQEI------EAET-DR---L-TG-------------------

-----RN------------------K-GISNV-PIMLRVFSPH---
--VLNLTLVDLPGMTK--VA-----------------V-------GD-------------Q--------PP--D--IEVQIR--------
NMLL-EFI---TK------------ENCLILAVSPANS--D-LANSD-ALKLAKEVD------
PAGTRTIGVITKLDLMD-------QGT------D-------A---------------------------REV--LE---NK---------L-
LP----------------------L---RRGYIGVVNR--S-QKD--IEG-----
------R--------KD-------------------IK---------AAMAA---ERKFFLS--------HP-SY--------------------
R-H------M-----------AER-MGTPYLQRCLNQQLTNHIRET-L----
>PAA78248.1
------------FDLPQ--------IAVV---GS----Q---------------
SAGKSSVLE-----N--FV-G-K-DFLPRG-------------SG-IVTRRPL--------------VLQ-------------L-L-
THP-----------------------------S---
------------------E-------------F-------------------------AEF--
-------------------------GHLRGKKFTNFD-----------EVRQEI------ENET-DR---L-TG-------------------
----KN------------------K-GISNV-PITLRVFSPH---
VLNLTLVDLPGMTK--VA-----------------V-------GD-------------Q--------PP--D--IEQQIR--------
AMLF-EFI---SK------------ENCLILAVSPANS--D-LANSD-ALKIAKEVD------
PNGTRTIGVITKLDLMD-------QGT------D-------A---------------------------REV--LE---NK---------L-
LP----------------------L---RRGYIGVVNR--S-QKD--IEG-----
------K--------KD-------------------IA---------AAMAA---ERKFFLS--------HP-SY--------------------
R-H------M-----------AER-MGTPYLQRCLNQQLTNHIRET-L----
>PAA59145.1
------------FDLPQ--------IAVV---GS----Q---------------
SAGKSSVLE-----N--FV-G-K-DFLPRG-------------SG-IVTRRPL--------------VLQ-------------L-I-
NFH-----------------------------T---
------------------E-------------Y-------------------------AEF--
-------------------------GHIRGKRFTNFD-----------EVRQEI------ENET-DR---V-TG--------------------
---KN------------------K-GISNV-PIMLRVYSPQ---
VLNLTLVDLPGLTK--VA-----------------V-------GD-------------Q--------PQ--D--IELLIR--------
AMIL-EFV---SK------------DNCLILAVTPANS--D-LANSD-ALKIAKEVD------
PSGTRTIGVITKLDLMD-------QGT------D-------A---------------------------RDV--LE---NR---------L-
LP----------------------L---RRGYIGVVNR--S-QKD--IEG-----
------K--------KD-------------------IV---------AAMAA---ERKFFLS--------HP-AY--------------------
R-H------M-----------AER-MGTSYLQRCLNQQLTNHIRET-L----
>PAA64382.1
------------IDLPQ--------IAVV---GS----Q---------------
SAGKSSVLE-----N--FV-G-R-DFLPRG-------------SG-IVTRRPL--------------ILQ-------------L-M-
NYQ-----------------------------T---
------------------E-------------Y-------------------------AEF--
-------------------------GHIRGKKFVNFD-----------EVRREI------EVET-DR---L-TG--------------------
---QN------------------K-GISNV-PITLRVYSPQ---
VLNLTLVDLPGMTK--VA-----------------V-------GD-------------Q--------PP--D--IEQQVR--------
AMIW-EFI---SK------------DNCLILAVSPANS--D-LANSD-ALKLAKEAD------
PSGSRTIGVLTKLDLMD-------AGT------D-------A---------------------------RDV--LE---NR---------F-
LP----------------------L---RRGYVGVVNR--S-QKD--IDG----

-------R--------KD-------------------IS----------SAMAA---ERKFFLG--------HP-AY--------------------
R-H------M-----------AER-MGTAHLQRCLNQQLVGHIRDT-L----
>XP_001749319.1
-----------LDLPQ--------IAVV---GG----Q----------------
SAGKSSVLE-----N--FV-G-K-DFLPRG--------------SG-IVTRRPL---------------VLQ--------------L-N-
YHP----------------------------SA---
-------------------E-------------W-------------------------GEF---
----------------------------LHARGKKFTDFN-----------EIRQEI------EAET-DR---M-TG------------------
-----SN-------------------K-GISNI-PINLRVYSPH---
-VLNLTLVDLPGLTK--VA-------------------V-------GD-------------Q--------PA--D--IENQIR--------
GMLM-EFI---TK------------DNCIILAVTPANQ--D-LANSD-ALKLAKEVD------
PEGVRTIGVITKLDLMD-------SGT------D-------A----------------------------RAI--LT---NE---------F-
LP----------------------L---RRGYIGVVNR---S-QKD--IDG-----
------R--------KD-------------------IR----------AALDA---ERKFFLM--------HP-SY--------------------
K-D------I-----------ASK-NGTPYLQKALNQQLTNHIREC-L----
>XP_031757197.1
-----------VDLPQ--------IAVV---GG----Q----------------
SAGKSSVLE-----N--LV-G-R---WIH-------------V-L--SS------
-----------------------T---
-------E-------------Y-------------------------AEF---
-------------LHCKGTKYTDFS-----------EVRQEI------EEET-ER---A-TG------------------------LN------
-------------K-GISAI-PISLRIYSPH---
VLNLSLIDLPGVTK--VP-------------------V-------GD-------------Q--------PA--D--IETQIR--------
DMIM-NFI---SR------------ENCLILAVTPANT--D-LANSD-ALKLAKEVD------
PQGLRTIGVITKLDLMD-------EGT------N-------A----------------------------QEI--LE---NK---------L-
LP----------------------L---RRGYVGVVNR---S-QKD--IDG----
-------K--------KN-------------------IN----------AALQA---EQMFFLT--------HP-AY--------------------
R-H------M-----------ADR-MGTSHLQKMLNQQLTNHIRET-L----
>XP_014148725.1

---T-------------------------------------
--E-------------Y---------
------------------GEF--
LHKPGRLFDNFD-----------EIRNEI------EADT-AR---I-TG------------------------AN------------------
K-GISHL-PINLKVYSPH--VLDLTLVDLPGLTK--
VA-------------------V-------GD-------------Q--------PA--D--IEMQIK--------NMIM-EFI---TK----------
--PNCLILAVTPANS--D-LANSD-ALKLAKEVD------PQGLRTIGVITKLDLMD-------AGT------D-------
A----------------------------RDV--LE---NK---------L-LP----------------------L---RRGYVGVVNR-------
-------------------------------------S-QKD--IAG-----------N--------KD-------------------IR----------
AAQAA---EKKFFKT--------HP-AY--------------------R-H------L-----------ADK-
MGTPKLQQVLNQQLTDHIRQT-L----
>XP_014153758.1
--N--
--LY-------------LYI---------------------------------------

--E-------------W------
---------------------GEF--
LHQPGRKYTDFE-----------EIMKEI------EAET-DR---M-TG-----------------------SN------------------
K-GISNI-PINLKVMSPH---VLDLTLVDLPGLTK--
VA------------------V-------GD-------------Q--------PA--D--IEQQIL--------GMIM-EFI---TR----------
--PNCLILAVSPANA--D-LANSD-ALKLAKEVD------PQGLRTIGVITKLDLMD-------QGT------D------
-A--------------------------REI--LE---NK---------L-LP----------------------L---RRGYIGVVNR--------
------------------------------------S-QKD--ITG-----------K--------KD-------------------IR----------
AAQEA---ERRFFST--------HP-AY--------------------R-H------L-----------AQN-
MGTPKLQKVLNQQLTNHIRDS-L----
>XP_006812840.1
------------ITKPS--------CLIL--AV--------------------
TPGNSDLANS----DALKV-A-K-EVDPQGL-----------RTIG-VITKLDLL-DDGTDAREILENKLLP-------
-------L---RRA----------------------------S--
------------------------------E-------------W------------------------GEF-----------------------------
--------------------------------------LHCKGKKFTNFD-----------EIRMEI------EAET-DR---L-TG---------
---------------KN------------------K-GISPI-PINLRVYSPH--
-----------VLNLTLIDLPGMTK--VP------------------V-------GD-------------Q--------PA--D--IEQQIR-
-------SMLM-EFI---TK------------PSCLILAVTPGNS--D-LANSD-ALKVAKEVD------
PQGLRTIGVITKLDLLD-------DGT------D-------A--------------------------REI--LE---NK---------L-
LP----------------------L---RRGYIGVVNR--G-QKD--IEG-----
------R--------KD-------------------IK---------SALAS---ERKFFLS--------HP-SY--------------------R-
H------M-----------ADR-LGTPYLQKALNQQLTNHIRDT-L----
>NP_013100.1
------------LDLPI--------LAVV--GS----Q----------------
SSGKSSILE-----T--LV-G-R-DFLPRG-------------TG-IVTRRPL--------------VLQ-------------L-N-
NIS-----------------------------PNSPLIEEDDN------SVNPH--DEVTKI-SGFEA-------------GTKPLE-
----YRGKERN-------------------------HADE-------------W-------------------------GEF---------------
--LHIPGKRFYDFD-----------DIKREI------ENET-
AR---I-AG-----------------------KD------------------K-GISKI-PINLKVFSPH-----------------------------
-------------------------------VLNLTLVDLPGITK--VP------------------I-------GE-------------Q--------
PP--D--IEKQIK--------NLIL-DYI---AT------------PNCLILAVSPANV--D-LVNSE-SLKLAREVD------
PQGKRTIGVITKLDLMD-------SGT------N-------A--------------------------LDI--LS---GK---------M-
YP----------------------L---KLGFVGVVNR---S-QQD--IQL----
-------N--------KT------------------VE---------ESLDK---EEDYFRK--------HP-VY--------------------
R-T------I-----------STK-CGTRYLAKLLNQTLLSHIRDK-LP---
>XP_011392073.1
------------VDLPQ--------IVVV--GS----Q----------------
SAGKSSVLE-----T--IV-G-R-DFLPRG-------------SG-IVTRRPL--------------VLQ-------------L-I-
HTP------------------------------STKEQPRQPKQ------SSRPY--DLSDGLASDMQR-------------
GGSHAS-----SADTRSP-------------------------TYEE-------------Y-------------------------GEF------
---LHL-DKRFTDFN-----------EIRREI------
ENET-FR---V-AG-----------------------QN------------------K-GVSKL-PIHLKIYSPN-------------------
--------------------------------------VLNLTLVDLPGLTK--IP------------------V-------GD-------------

Q--------PS--D--IERQIR--------NLVT-DYV---SK------------PNCIILAVSPANV--D-LANSD-
SLKLARTVD------PQGRRTIGVLTKLDLMD-------QGT------H-------A---------------------------LDI--
LT---GR---------V-YP----------------------L---KLGFIGVVNR--
S-QQD--ING-----------N--------VS-------------------ML----------AARRA---EEDFFRS--------HA-AY-
--------------------K-N------I-----------AHR-CGTKYLAKTLNQVLMSHIRDK-LP---
>XP_006461708.1
------------LDMPQ--------IVVV---GS----Q---------------
SAGKSSVLE-----T--IV-G-K-DFLPRG--------------SG-IVTRRPL---------------VLQ-------------L-I-
HTP------------------------------V----PSEPSP------NALP---
----------------------YTE-------------W-------------------------GQF-------------------------------------
-----------------------------------LHI-DKRFTDFN-----------DIRKEI------EQET-FR---V-AG-------------
----------QN------------------K-GISRL-PISLRVYSPN--
-------VLDLTLVDLPGLTK--IP------------------V-------GD-------------Q--------PS--D--IEKQIR-------
-NLVV-DYI---SK------------PNSVILAVSAANV--D-LANSE-SLKLARSVD------
PQGRRTIGVLTKLDLMD-------AGT------N-------A---------------------------LDI--LT---GR---------V-
YP----------------------L---KLGFIGIVNR--S-QQD--INV------
-----E--------KS--------------------LT---------DAVES---EAEFFRN--------HA-VY--------------------R-
N------I-----------AHK-NGTRYLAKTLNQVLMNHIRDK-LP---
>XP_746923.1
------------LDLPQ--------IVVV---GS----Q---------------
SSGKSSVLE-----N--IV-G-R-DFLPRG--------------SG-IVTRRPL---------------ILQ-------------L-I-
NIP------------------------------SEH--------------NDRPG--DN------DVLA--------------PHTAAS-----
VAG-------------------------------QHE-------------W-------------------------AEF----------------------
--HHLPGRKFEDFA-----------LVKQEI------EAET-AR---I-
AG------------------------NN------------------K-GINRQ-PINLKIFSPH----------------------------------
-----------------------VLNLTMVDLPGLTK--VP-------------------I-------GD-------------Q--------PS--
D--IEKQTR--------TLIL-EYI---AK------------PNSIILAVSPANV--D-LVNSE-ALKLARQVD------
PMGRRTIGVLTKLDLMD-------HGT------N-------A---------------------------MDI--LS---GR---------
V-YP----------------------L---KLGFIGVVNR--S-QQD--IQS---
--------G--------KS--------------------LS----------EALQA---EAEFFRH--------HP-AY-------------------
-R-N------M-----------ANR-CGTQFLAKTLNTTLMAHIRDR-LP---
>KXN67416.1
------------LDLPQ--------IVVV---GS----Q---------------
SSGKSSVLE-----N--LV-G-R-DFLPRG--------------NG-IVTRRPL---------------VLQ-------------L-V-
NLR------------------------------ED-------------------A--DL------DLT--------------------------------------
------------------------E-------------R--------------------------AQF---
-------------------------------LHNPTKFYTDFS-----------EVRQEI------EQET-NR---L-AG---------------
---------EN-------------------K-GISRN-PIHLKIFSTQ---
-----VLNLTVVDLPGLTK--IP------------------I-------GD-------------Q--------PT--D--IEKQTK--------
SLIL-DYI---SK------------PNSIILAVSPANV--D-LVNSE-SLKLAKEVD------PEGKRTIGIITKIDLMD---
----AGT------N-------A---------------------------LDI--LT---GR---------V-LN----------------------L---
KLGFIGVINR--S-QQD--TVA-----------K--------KP------------
-------IR----------ESLEA---ELEFFRT--------HP-AY--------------------R-N------I-----------SQR-
CGTGHLSKTLNQVLVNHIRDR-LP---

>KNE61418.1
------------LDLPQ--------IAVV--GS----Q----------------
SSGKSSVLE-----A--IV-G-K-DFLPKG--------------NG-IVTRRPL---------------VLQ--------------L-R-
NVP--------------------------------PHAVPE--
---------------------GEE--------------AVD------------------------VAEF--------------------------------------
----------------------------------SHCPDRVFTDFA-----------DVRREI------EAET-AR---I-AG-------------
-----------DN-------------------K-GIATD-PIRLCVRSPN--
--------VVDLTLVDLPGMTK--IP------------------V-------GD--------------Q--------PS--D--IELQIR-----
---ELIM-GFI---TK------------PNCLILAVSPANV--D-LANSD-SLKLAREVD------
PTGDRTLGLLTKVDLME-------PGT------H-------A----------------------------LDI--LA---GR---------V-
YP-----------------------L---RLGFVGVVNR---S-QRD--IDQ----
-------G--------KS--------------------LE----------FARKR---EQQFFAT--------HP-VY--------------------
A-P------V-----------ASR-CGTTVLARTLNQVLLAHIRDR-LP---
>KNE67543.1
------------LDLPQ--------IAVV--GS----Q----------------
SSGKSSVLE-----A--IV-G-K-DFLPKG--------------AG-IVTRRPL---------------ILQ--------------L-N-
YAP------------------------------VDPETPDE--
---------------------PEE--------------W----------------------------AEF--
--------------------------------QHLPGQQFADFG-----------EVKREI------ERET-AR---I-AG--------------
----------DN------------------K-GISDE-PITLRVHSPS---
-------VVDLTLVDLPGLTK--IP------------------V-------GD--------------Q--------PS--D--IELQIR-------
-DLIM-KFI---LQ------------PNCIILAVSPANV--D-LANSD-SLKLAREVD------
PQGLRTLGLLTKVDLME-------TGS------H-------A----------------------------LDI--LG---GR---------V-
YP-----------------------L---RLGFVAVVNR--S-QRD--IEA-----
------R--------RT-------------------LE----------WSRKR---EQQFF--
----------------SGR-CGTAALARTLNSVLLDHIRAQ-LP---
>GAX23670.1
-----------HLDLPQ--------IVVV--GG----Q----------------
SSGKSSVLE-----N--IV-G-R-SFLPRG--------------TG-IVTRRPL---------------VLQ--------------L-F-
NTR------------------------------QAPADV-----------DEMP-----------------------------NKNRII-----
PPGGSAE-----------------------------E--------------W---------------------------GEF----------------------
--LHLPGKRMYDFA-----------DIRREI------ADET-NR---I-
TG------------------------NN-------------------K-GVDST-PINLKIFSPR------------------------------------
------------------------VVSLTLVDLPGIAK--VP------------------V-------GD--------------Q--------PE--
D--IERQIN--------SMCM-SFI---SN-----------PNAIILAVTSANT--D-LANSD-ALKLAQAVD------
PEGLRTIGVLTKVDLMD-------EGT------D-------C----------------------------TEI--LL---NQ---------V-
IP-----------------------L---RRGYIAVVNR--G-QKD--VVS------
-----D--------VS--------------------IR----------DGLKK---EENFFRK--------HP-IYS-R-----------------
DR-S------I-----------LSK-CGTGRLSKNLNSILMH-----------
>CAB9512103.1
-----------AIDLPQ--------IVVV--GG----Q----------------
SSGKSSVLE-----A--IV-G-R-SFLPRG--------------SG-IVTRRPL---------------VLQ--------------L-F-
NTS------------------------------H--DGQSET-----
------------------------E--------------F---------------------------GEF---

-------------------------------LHQPGKRYHDFV-----------SIRSEI------VRET-ER---L-TG-----------------
-------PN-------------------K-GIDHA-PIHLKIYSPE---
---VLSLTLVDLPGIAK--VP------------------V-------GD-------------Q--------PE--N--IEEQIR--------
EMCM-EYI---SN------------PNAIILAVTSANQ--D-IANSD-ALKLAQAVD------
PHGTRTVGVLTKLDLMD-------DGT------D-------A---------------------------SDI--LM---NR---------
V-IP----------------------L---RRGYVAVVNR---G-QRD--VNN-
----------D--------LS--------------------IQ---------DGLKK---EESFFRA--------HS-VYG-R--------------
---DR-Q------L-----------LAK-CGTKRLSQHLNTMLMH-----------
>XP_002296064.1
-----------NLDLPQ--------IVVI---GG----Q---------------
SSGKSSVLE-----S--VV-G-R-SFLPRG-------------TG-IVTRRPL--------------VLQ-------------L-F-
NTS------------------------------G----------------KRHP-----------------------------KNDATS-----
SSTEPVE-----------------------------E-------------W--------------------------GEF-----------------------
--LHQPGKRYTDFS-----------QIRSEI------SRDT-NR---
LCSG------------------------PN------------------SK-GVSST-PIHLKIYSPR---------------------------------
--------------------------VLSLTMVDLPGLTK--VA------------------V-------KD-------------Q--------
PE--D--IEEQIY--------QINV-QYG---SN------------PNAILLAVTGANT--D-LASSD-ALKLARELD------
PRGERTIGVLTKLDLMD-------PGT------D-------A---------------------------GEI--LH---NK---------V-
IP----------------------L---RRGYVAVVNR---G-QRD--IDA-----
------D--------LS--------------------IQ---------VGLRN---EERYFRT--------HP-VYS-R----------------
DR-S------L-----------VGK-CGTMNLARNLNGILIH-----------
>XP_004184473.1
------------IDLPQ--------IVVV---GA----Q---------------
SAGKSSVLE-----S--IV-G-R-DFLPRG--------------CG-MVTKRPL---------------ILQ--------------L-V-
NLP------------------------------PTE--
------------------TTE-------------W--------------------------GEF--
-----------------------------AHKAGEVFKDFE-----------DIKKEI------ENET-IR---L-TG--------------------
----KS----------------K-TISTV-AIRLKIYSPY---
VVDLTLVDLPGLTK--IS------------------V-------EG-------------Q--------EK--D--ISQQLK--------
QMVL-KFI---ES------------PNAIILAVTSANV--D-LATSD-ALSIAREVD------
PEGDRTIGVLTKMDLMD-------KGT------D-------A---------------------------MDV--LY---GR---------
V-YP----------------------L---KLGYIGVLNR---S-QED--IEK----
-------K--------VP--------------------IR---------QALKS---EKEWFTN--------HP-IY--------------------
G-K------I-----------ADR-LGVSYLSKTLNQMLMQ-----------
>EMS16943.1
------------IDLPQ--------IVVV---GS----Q---------------
SAGKSSVLE-----S--IV-G-R-DFLPRG--------------SG-MVTKRPL---------------ILQ--------------L-V-
NLP------------------------------STE--
------------------TKE-------------W--------------------------GEF--
-----------------------------AHKPGIVYRDFE-----------EIKKEI------ENET-IR---L-TG---------------------
---TK-----------------K-TISPV-AIRLKIYSPY---
VVDLTLVDLPGLTK--IS------------------V-------GS-------------Q--------EK--D--ISNQLK--------
QMVL-KFI---ER------------PNAIILAVTSANV--D-LATSD-ALSIAREVD------
PDGDRTIGVLTKMDIMD-------KGT------D-------A---------------------------MDV--LY---GR---------

V-YP-----------------------L---KLGYIGVLNR--S-QHD--IDT----
-------N--------VP-------------------IK----------TALTK---EKEWFSN--------HP-IY--------------------S-
K------I-----------ADR-LGIPYLTKTLNEILMQ-----------
>NP_741403.2
------------IQLPQ--------IVVV--GS----Q---------------
SAGKSSVLE-----N--LV-G-R-DFLPRG-------------TG-IVTRRPL---------------ILQ-------------L-N-
HVA------------------------------LDDESKRRRSN------G--
TLL---------------------------TDD-------------W--------------------------AMF---------------------------
--EHTGSKVFTDFD-----------AVRKEI------EDET-DR---V-TG---
---------------------VN-------------------K-GISLL-PISLKIYSHR--
----------------VVSLSLVDLPGITK--IP------------------V-------GD-------------Q--------PV--N--
IEEQIR--------EMIL-LYI---SN------------PSSIILAVTPANQ--D-FATSE-PIKLAREVD------
AGGQRTLAVLTKLDLMD-------QGT------D-------A---------------------------MDV--LM---GK---------
V-IP----------------------V---KLGIIGVVNR--S-QQN--ILD----
-------N--------KL-------------------IV---------DAVKD---EQSFMQK--------K---Y--------------------
P-T------L-----------ASR-NGTPYLAKRLNMLLMHHIRNC-L----
>XP_002129967.2
------------LQLPQ--------IVVV---GV----Q---------------
SSGKSSVLE-----N--LV-G-R-DFLPRG-------------TG-IVTRCPL---------------VLQ-------------M-I-
HTT------------------------------NEDTAQCSNEG------SSGNN--DS----------------------DSSGESF---
--K-ETNEE--------------------------VKE-------------W--------------------------VKF--------------------
---QHTKGKIFRSFK-----------QVKKEI------ELET-QR---L-
SG------------------------NN-------------------K-GISSE-AIRLKIFSPK------------------------------------
----------------------VLNLTLVDLPGLMK--IP------------------V-------GD-------------Q--------PD--
D--IEEQAR--------NLIL-RYI---SN------------PNSIILAVTPANV--D-FATSE-ALQMARIVD------
PDGCRTLAVVTKLDLMD-------AGT------D-------A---------------------------IDV--LC---GR---------I-
VP----------------------V---KLGIIGIVNR--S-QLD--INK--------
---G--------KS-------------------VQ---------DAIKD---EQAFLQK--------K---Y--------------------P-S--
----F-----------ANR-SGSRYLSITLNRLLMHHIRDC-L----
>NP_001259946.1
------------IQLPQ--------IVVL---GS----Q---------------
SSGKSSVIE-----S--VV-G-R-SFLPRG-------------TG-IVTRRPL---------------VLQ-------------L-I-
YSP------------------------------LDDRENRSAEN--------------------------------------GTS------------
N--------------------------AEE-------------W--------------------------GRF------------------------------
--LHTK-KCFTDFD-----------EIRKEI------ENET-ER---A-AG--------
----------------SN-------------------K-GICPE-PINLKIFSTH--
------------VVNLTLVDLPGITK--VP------------------V-------GD-------------Q--------PE--D--IEAQIK-
-------ELVL-KYI---EN------------PNSIILAVTAANT--D-MATSE-ALKLAKDVD------
PDGRRTLAVVTKLDLMD-------AGT------D-------A---------------------------IDI--LC---GR---------V-
IP----------------------V---KLGIIGVMNR---S-QKD--IMD------
-----Q--------KH-------------------ID---------DQMKD---EAAFLQR--------K---Y--------------------P-
T------L-----------ATR-NGTPYLAKTLNRLLMHHIRDC-L----
>XP_035676386.1

------------IQLPQ--------IVVI---GT----Q---------------
SSGKSSVLE-----S--LV-G-R-DFLPRG-------------TG-IVTRRPL--------------VLQ-------------L-V-
HVN-----------------------------SEEKKRPSEDE------D--------------------------------GGHKQ-----
DIKEHAH--------------------------VEE-------------W--------------------------GKF--------------------
--LHTKNKIYTDFD----------EIRQEI------ENET-DR---V-
TG-----------------------TN------------------K-GIIDD-AIHLKIYSPK-------------------------------------
---------------------VLNLTLVDLPGITK--VP------------------V-------GD-------------Q--------PP--D--
IEVQIR--------EMCL-KYI---AN------------PNSIILAVTSANT--D-MATSE-ALKFAKEVD------
PDGRRTLAVITKLDLMD-------AGT------D-------A---------------------------HDV--LM---GR---------
V-IP----------------------V---KLGIIGVVNR---S-QMD--INK----
-------R--------KP-------------------IE----------EAIKD---EAAFMQR--------K---Y--------------------P-
S------L-----------ASR-NGTSHLARTLNRLLMHHIRDC-L----
>XP_006821224.1

--MATSE-AIKLSREVD------
EDGRRTLAVITKLDLMD-------AGT------D-------A---------------------------VEI--IC---GR---------V-
IP----------------------V---KLGIIGVINR---S-QMD--INN-------
----K--------KP-------------------IQ----------ESVKD---EAAFLQR--------K---Y--------------------P-A--
----L-----------ASR-NGTPYLAKTLNRLLMHHIRDC-L----
>XP_030827871.1
------------IQLPQ--------IVVV---GN----Q---------------
SSGKSSVLE-----G--LV-G-K-DFLPRG-------------NG-IVTRRPL--------------VLQ-------------M-V-
HVD-----------------------------PEDKR--GASG-----
EGEEEIT--------------------------ADE-------------W--------------------------GKF---------------------
---LHTKNKVYTDFE----------EIREEI------QNET-DR---M-
AG-----------------------TN------------------K-GIVHD-AIHLRIYSPK------------------------------------
----------------------VLNLTLVDLPGLTK--VP------------------V-------GD-------------Q--------PE--D-
-IESQIR--------EMLV-KYI---GN------------PNSIILAVTSANT--D-MATSE-SLKLAKEID------
PDGRRTLAVITKLDLMD-------AGT------D-------A---------------------------VDV--LC---GR---------V-
IP----------------------V---KLGIIGVVNR---S-QMD--INN------
-----K--------KV-------------------ID----------DAVKD---ESAFLQR--------K---Y--------------------P-
A------L-----------ASR-NGTAYLARTLNRLLMHHIRDC-L----
>XP_032819300.1
------------IQLPQ--------IVVV---GA----Q---------------
SSGKSSVLE-----S--LV-G-R-DFLPRG-------------TG-IVTRRPL--------------VLQ-------------L-V-
HVI-----------------------------PDERIRPGGEE------N--
GVE--------------------------AEE-------------W--------------------------GKF---------------------------
---LHTKNKVYSDFN----------EIRQEI------ENET-ER---I-TG----
------------------TN------------------K-GISSE-AIHLKIFSPH--

---------------VLNLTLVDLPGITK--VP------------------V-------GD-------------Q--------PV--D--
IEQQIR--------ELII-KFI---GN------------PNSIILAVTAANT--D-LATSE-ALKIAREVD------
TDGRRTLAVITKLDLMD-------AGT------D-------A---------------------------MDI--LT---GR---------V-
IP----------------------V---KLGIIGVVNR--S-QLD--INT--------
---K--------KT-------------------IL----------DAMQD---EQSFMQK--------K---Y--------------------P-S-
-----L-----------ANR-NGTKFLGKTLNRLLMHHIRDC-L----
>NP_957216.1
------------IQLPQ--------IAVV---GT----Q---------------
SSGKSSVLE-----S--LV-G-R-DLLPRG-------------TG-IVTRRPL--------------ILQ-------------L-V-
HVD------------------------------PEDRRKTS-EE------N---
GVD--------------------------GEE-------------W--------------------------GKF--------------------------
--LHTKNKIYTDFD-----------EIRQEI------ENET-ER---V-SG---
---------------------NN------------------K-GISDE-PIHLKIFSPH---
-----------------VVNLTLVDLPGITK--VP------------------V-------GD-------------Q--------PK--D--
IELQIR--------ELIL-KYI---SN------------PNSIILAVTAANT--D-MATSE-ALKVAREVD------
PDGRRTLAVVTKLDLMD-------AGT------D-------A---------------------------MDV--LM---GR---------
V-IP----------------------V---KLGLIGVVNR--S-QLD--INN----
-------K--------KS-------------------VA----------DSIRD---EHGFLQK--------K---Y--------------------P-
S------L-----------ANR-NGTKYLARTLNRLLMHHIRDC-L----
>XP_025940269.1
------------IQLPQ--------IVVV---GT----Q---------------
SSGKSSVLE-----S--LV-G-R-DLLPRG-------------TG-VVTRRPL--------------ILQ-------------L-V-
HVS------------------------------PEDGRKTAGDE------N---
EID--------------------------AEE-------------W--------------------------GKF----------------------------
---LHTKNKVYTDFD-----------EIRQEI------ENET-ER---I-SG-----
-------------------NN------------------K-GISPE-PIHLKIFSSN--
---------------VVNLTLVDLPGMTK--VP------------------V-------GD-------------Q--------PK--D--
IELQIR--------ELIL-QFI---SN------------PNSIILAVTAANT--D-MATSE-ALKIAREVD------
PDGRRTLAVITKLDLMD-------AGT------D-------A---------------------------MDV--LM---GR---------
V-IP----------------------V---KLGIIGVVNR--S-QLD--INN----
-------K--------KS-------------------VA----------DSIRD---EYGFLQK--------K---Y--------------------P-
S------L-----------ANR-NGTKYLARTLNRLLMHHIRDC-L----
>XP_006168142.1
------------IQLPQ--------IVVV---GT----Q---------------
SSGKSSVLE-----S--LV-G-R-DLLPRG-------------TG-IVTRRPL--------------ILQ-------------L-V-
HVS------------------------------PEDKRKTTGEE------N------DP----------------------ATWKNSR-----
HLSKGVE--------------------------AEE-------------W--------------------------GKF--------------------
--LHTKNKLYTDFD-----------EIRQEI------ENET-ER---I-
SG------------------------NN------------------K-GVSPE-PIHLKIFSPN-----------------------------------
------------------------VVNLTLVDLPGMTK--VP------------------V-------GD-------------Q--------PK-
-D--IELQIR--------ELIL-RFI---SN------------PNSIILAVTAANT--D-MATSE-ALKISREVD------
PDGRRTLAVITKLDLMD-------AGT------D-------A---------------------------MDV--LM---GR--------
V-IP----------------------V---KLGIIGVVNR--S-QLD--INN----

-------K--------KS-------------------VT----------DSIRD---EYAFLQK--------K---Y---------------------P-
S------L-----------ANR-NGTKYLARTLNRLLMHHIRDC-L----
>NP_001317309.1
------------IQLPQ--------IVVV---GT----Q---------------
SSGKSSVLE-----S--LV-G-R-DLLPRG--------------TG-IVTRRPL---------------ILQ--------------L-V-
HVS------------------------------QEDKRKTTGEE------N------DP-----------------------ATWKNSR-----
HLSKGVE----------------------------AEE--------------W---------------------------GKF---------------------
---LHTKNKLYTDFD-----------EIRQEI------ENET-ER---I-
SG------------------------NN-------------------K-GVSPE-PIHLKIFSPN-------------------------------------
-------------------------VVNLTLVDLPGMTK--VP-------------------V-------GD--------------Q--------PK-
-D--IELQIR--------ELIL-RFI---SN------------PNSIILAVTAANT--D-MATSE-ALKISREVD------
PDGRRTLAVITKLDLMD-------AGT------D-------A----------------------------MDV--LM---GR---------
V-IP-------------------------V---KLGIIGVVNR---S-QLD--INN----
-------K--------KS-------------------VT----------DSIRD---EYAFLQK--------K---Y---------------------P-
S------L-----------ANR-NGTKYLARTLNRLLMHHIRDC-L----
>NP_001392186.1
------------IQLPQ--------IVVV---GT----Q---------------
SSGKSSVLE-----S--LV-G-R-DLLPRG--------------TG-VVTRRPL---------------ILQ--------------L-V-
HVS------------------------------PEDKRKTTGEE------NGKFQSWNP-----------------------ATWKNSR-
----HLSKGVE----------------------------AEE--------------W---------------------------GKF-----------------
---LHTKNKLYTDFD-----------EIRQEI------ENET-ER--
-I-SG------------------------NN-------------------K-GVSPE-PIHLKVFSPN----------------------------------
----------------------------VVNLTLVDLPGMTK--VP-------------------V-------GD--------------Q--------
PK--D--IELQIR--------ELIL-RFI---SN------------PNSIILAVTAANT--D-MATSE-ALKISREVD------
PDGRRTLAVITKLDLMD-------AGT------D-------A----------------------------MDV--LM---GR---------
V-IP-------------------------V---KLGIIGVVNR---S-QLD--INN----
-------K--------KS-------------------VT----------DSIRD---EYAFLQK--------K---Y---------------------P-
S------L-----------ANR-NGTKYLARTLNRLLMHHIRDC-L----
>XP_014394711.1
------------LLCPV--------CVQI---SS----L---------------
SSGKSSVLE-----S--LV-G-R-DLLPRG--------------TG-IVTRRPL---------------ILQ--------------L-V-
HVS------------------------------PEDQRKTSGEE------N------DP-----------------------ATWKNSR-----
HLSKGVE----------------------------AEE--------------W---------------------------GKF---------------------
---LHTKNKLYTDFD-----------EIRQEI------ENET-ER---I-
SG------------------------NN-------------------K-GVSPE-PIHLKIFSPN-------------------------------------
-------------------------VVNLTLVDLPGMTK--VP-------------------V-------GD--------------Q--------PK-
-D--IELQIR--------ELIL-RFI---SN------------PNSIILAVTAANT--D-MATSE-ALKISREVD------
PDGRRTLAVITKLDLMD-------AGT------D-------A----------------------------MDV--LM---GR---------
V-IP-------------------------V---KLGIIGVVNR---S-QLD--INN----
-------K--------KS-------------------VT----------DSIRD---EYAFLQK--------K---Y---------------------P-
S------L-----------ANR-NGTKYLARTLNRLLMHHIRDC-L----
>XP_028602039.1
------------IQLPQ--------IVVV---GT----Q---------------
SSGKSSVLE-----S--LV-G-R-DLLPRG--------------TG-IVTRRPL---------------ILQ--------------L-V-

NVS--------------------------------AEDLRKKTGDE------N------DP-----------------------ATWKHAR-----
HLTKGVD---------------------------TEE--------------W---------------------------GKF-------------------------
---LHTKNKLYSDFD-----------EIRQEI------ESET-ER---I-
SG------------------------NN-------------------K-GISPE-PIHLKVFSPN-----------------------------------
-------------------------VVNLTLVDLPGMTK--VP-------------------V-------GD-------------Q--------PK-
-D--IELQIR--------ELIL-RFI---SN------------PNSIILAVTAANT--D-MATSE-ALKIAREVD------
PDGRRTLAVITKLDLMD-------AGT------D-------A----------------------------MDV--LM---GR---------
V-IP-----------------------V---KLGIIGVVNR---S-QLD--INN----
-------K--------KS--------------------VA----------DSIRD---EYGFLQK--------K---Y--------------------P-
S------L-----------ANR-NGTKYLARTLNRLLMHHIRDC-L----
>XP_012382650.2

-----------G--------------FK-LVISTKL---------------ILC--------------C-F-SLT------------------------------
T-----------------------DP-----------------------ATWKNSR-----HLSKGVE---------------------------AEE-
-------------W---------------------------GKF---
------LHTKNKLYTDFD-----------EIRQEI------ENET-ER---I-SG------------------------NN---------------
----K-GVSPE-PIHLKIFSPN--
VVNLTLVDLPGMTK--VP-------------------V-------GD-------------Q--------PK--D--IELQIR--------
ELIL-RFI---SN------------PNSIILAVTAANT--D-MATSE-ALKISREVD------PDGRRTLAVITKLDLMD-
------AGT------D-------A----------------------------MDV--LM---GR---------V-IP-----------------------V--
-KLGIIGIVNR---S-QLD--INN-----------K--------KS-------------
-------VT----------DSIRD---EYAFLQK--------K---Y--------------------P-S------L-----------ANR-
NGTKYLARTLNRLLMHHIRDC-L----
>XP_031753959.1
------------IGFGP--------VLRV--PL----Q---------------
PAKCWAAKE-----S--MVLS-A-SLLP-V-------------PH-LYERLLL-------------SVV-------------F-F-
SLI-----------------------------P-----------------------DP-----------------------NAWKIPK-----
HFSKGVE---------------------------TEE-------------W---------------------------GKF-------------------------
---LHTKNKIYTDFD-----------EIRQEI------ENET-ER---I-
SG------------------------NN-------------------K-GISSE-PIHLKIFSPN------------------------------------
------------------------VVNLTLVDLPGMTK--VP-------------------V-------GD-------------Q--------PK--
D--IEIQIR--------ELIL-RYI---SN------------PNSIILAVTAANT--D-MATSE-ALKIARESD------
PDGRRTLAVITKLDLMD-------AGT------D-------A----------------------------MDV--LL---GR---------V-
IP-----------------------V---KLGIIGVVNR---S-QLD--INN-------
----K--------KS--------------------VA----------DSIRD---EYGFLQK--------K---Y--------------------P-S--
----L-----------ANR-NGTKYLARTLNRLLMHHIRDC-L----
>PAA85687.1
------------IQLPQ--------IVVI--GT----Q---------------
SSGKSSVLE-----S--LV-G-R-DFLPRG-------------TG-IVTRRPL--------------VLQ-------------L-V-
HLE------------------------------ADEKDE-AGDR------P---
AAE---------------------------EEE-------------W---------------------------GKF-----------------------------
--LHTKGKIYTDFN-----------EIRDEI------ARET-DR---I-AG-----
-------------------SG-------------------K-CVSID-PINLKIYSPH--
---------------VVSLTLVDLPGITK--VP-------------------V-------AD-------------Q--------PE--D--

IEVQIR--------ALCI-EYI---KN------------PNSIILAVTPANT--D-MATSE-SLKLAKEVD------
PQGKRTLAVITKLDLMD-------AGT------D-------A----------------------------HDL--LL---GR---------V-
IP----------------------V---KLGIIGVVNR--S-QAD--IKN-------
----Q--------KQ--------------------VK----------EAVRD---ESSFLQR--------R---Y---------------------P-
S------L-----------ASR-NGTPYLARTLNRLLMHHIRDC-L----
>XP_042914770.1
------------LDLPQ--------VAVV--GS----Q----------------
SSGKSSVLE-----A--LV-G-R-DFLPRG--------------ND-IVTRRPL---------------LLQ--------------L-V-
KTT------------------------------PGPT--GR-------
--------------------PSE--------------W---------------------------GEF--
--------------------------------LHAPGKMFYDFD-----------RIRDEI------HQET-ER---L-VG--------------
----------YN-------------------K-NVSDK-PIRLKIFSPR--
------VLTMTLVDLPGLTR--VP-------------------V-------GD--------------Q--------PG--D--IEQRIR------
--EMAL-EYI---RR------------PNCIILAVSPANV--D-LATSD-ALQLSQVAD------
PEGVRTIGVLTKLDIMD-------RGT------D-------A----------------------------AHI--LR---NA---------H-
IP----------------------L---RLGYIGVVLR--A-QAD--IAA-------
----K--------LP--------------------MS----------ECRKR---EESFFAS--------RA-EY---------------------R-
D------V-----------AAH-CGVPTLARRLNVILVEHIR--------
>PWZ09977.1
------------IDLPQ--------VAVV--GS----Q----------------
SSGKSSVLE-----A--LV-G-R-DFLPRG--------------SD-ICTRRPL---------------VLQ--------------L-V-
HQP------------------------------RRPAD---AE-----
----------------------ADE--------------W---------------------------GEF--------------------------------------
----------------------------------LHLSGRRFYDFR-----------EIRREI------QAET-DR---E-AG-------------
-----------GN-------------------K-GVSDR-QIRLKIFSPN--
--------VLNITLVDLPGITK--VP-------------------V-------GD--------------Q--------PT--D--IEARIR-------
-TMIM-SYI---KH------------KTCIILAVSPANA--D-LANSD-ALQMARVAD------
PDGSRTIGVITKLDIMD-------RGT------D-------A----------------------------RNF--LL---GN---------V-
IP----------------------L---KLGYVGVVNR--S-QQD--INS-----
------D--------LS--------------------IK----------DALAR---EEKFFRT--------QP-AY---------------------H-
G------L-----------AQY-CGIPQLAKKLNQILVQHIK--------
>KAH9304002.1

-----MV------QAET-ER---E-VG------------------------VN-------------------K-GVSDK-QIRLKIYSPN----
--VLNITLVDLPGLTK--VP-------------------V-------
GD--------------Q--------PT--D--IEARIR--------TMIM-SYI---KH------------ETCIILAVSPANA--D-
LANSD-ALQMARVAD------ADGHRTIGVITKLDIMD-------RGT------D-------A-------------------------
---TNF--LL---GN---------V-IP----------------------L---RLGYVGVVNR-----------------------------------
-----------S-QAD--ING-----------N--------KS--------------------IQ----------DALAY---EERFFRS-------
-RP-VY---------------------H-R------L-----------ADR-CGIPQLAKKLNYILVQHIR--------
>EFJ37641.1

------------IDLPQ--------VAVV--GS----Q----------------
SSGKSSVLE-----A--LV-G-R-DFLPRG--------------CD-ICTRRPL---------------VLQ--------------L-V-
QTT------------------------------RRGD--E-------
--------------------VVE--------------W---------------------------GEF--
--------------------------------LHLPNRRFTDFT-----------AIRKEI------QAET-DR---E-LG----------------
--------SN-------------------K-GISDK-QIRLKIFSPK---
---VLNITLVDLPGITK--VP-------------------V-------GD--------------Q--------PT--D--IEARIR--------
TMIL-SYI---KH------------ETCIILAVSPANA--D-LANSD-ALQMARIAD------
PDGSRTIGVITKLDIMD-------RGT------D-------A----------------------------RNF--LL---GS--------V-
IP-----------------------L---RLGYIGVVNR--S-QED--ITS-------
----N--------RS--------------------IQ----------DALMY---EEQFFRS--------RP-VY---------------------H-
S------L-----------SDR-CGIPQLAKKLNQILVQHIR--------
>EFJ15047.1
--------------LPQ--------VAVV--GS----Q----------------
SSGKSSVLE-----A--LV-G-R-DFLPRG--------------CD-ICTRRPL---------------VLQ--------------L-V-
QTT------------------------------RRGD--E-------
--------------------VVE--------------W---------------------------GEF--
--------------------------------LHLPNRRFTDFS-----------AIRKEI------QAET-DR---E-LG----------------
--------SN-------------------K-GISDK-QIRLKIFSPK---
---VLNITLVDLPGITK--VP-------------------V-------GD--------------Q--------PT--D--IEARIR--------
TMIL-SYI---KH------------ETCIILAVSPANA--D-LANSD-ALQMARIAD------
PDGSRTIGVITKLDIMD-------RGT------D-------A----------------------------RNF--LL---GS--------V-
IP-----------------------L---RLGYIGVVNR--S-QED--ITS-------
----N--------RS--------------------IQ----------DALMY---EEQFFRS--------RP-VY---------------------H-
S------L-----------SDR-CGIPQLAKKLNQILVQHIRTI-LP---
>KAI5070758.1
------------VNLPQ--------VAVV--GS----Q----------------
SSGKSSVLE-----G--LV-G-R-DFLPRG--------------SD-ICTRRPL---------------VLQ--------------L-I-
QTT------------------------------RRPDE--KSE------
---------------------VVE--------------W---------------------------GEF---------------------------------------
---------------------------------LHAPGKRFTDFS-----------LIRKEI------EAET-ER---E-LG----------------
--------SN-------------------K-GISDK-QIRLKIFSPN---
----VLTITLVDLPGITK--VP-------------------V-------GD--------------Q--------PS--D--IESRVR--------
TMIL-SYI---KH------------ETCIILAVSPANA--D-LANSD-ALQMARLAD------
PDGSRTIGVITKLDIMD-------RGT------D-------A----------------------------RNL--LL---GN--------V-
IP-----------------------L---RLGYIGVVNR--S-QED--ILG-------
----N--------KS--------------------VV----------DALLY---EENFFRS--------RP-VY---------------------H-
N------L-----------ADR-CGIAQLAKKLNTILVQHIK--------
>PTQ35749.1
------------IDLPQ--------VAVV--GS----Q----------------
SSGKSSVLE-----A--MV-G-R-DFLPRG--------------AD-ICTRRPL---------------VLQ--------------L-V-
QTV------------------------------RRPED--RSE-----
----------------------LVE--------------W---------------------------GEF---------------------------------------
---------------------------------LHIPGRRFTDFT-----------AIRKEI------QAET-DR---E-LG----------------

--------TN-------------------K-GISEK-QIRLKIFSPN--
---VLNITLVDLPGITK--VP-------------------V-------GD--------------Q--------PS--D--IEARVR--------
TMIL-NYI---KH------------ETCIILAVSPANA--D-LANSD-ALQMARIAD------
PDGSRTIGVITKLDIMD-------RGT------D-------A----------------------------RNF--LL---GN---------V-
VP-----------------------L---RLGYIGVVNR---S-QED--IQA-----
------N--------KT--------------------IR----------EALGY---EENFFRS--------RP-VY---------------------H-
S------L-----------SER-CGIPQLAKKLNSILVQHIR--------
>KAG0555682.1
------------IDLPQ--------VAVV---GS---Q----------------
SSGKSSVLE-----A--LV-G-R-DFLPRG--------------SD-ICTRRPL---------------VLQ--------------L-V-
QTS------------------------------RRPED---RTE-----
----------------------LVE--------------W---------------------------GEF---------------------------------------
---------------------------------LHIPGRRFTDFA-----------AIRKEI------QAET-DR---E-LG---------------
---------TN-------------------K-GISEK-QIRLKIFSPN---
----VLNITLVDLPGITK--VP-------------------V-------GD--------------Q--------PN--D--IEARVR--------
TMIL-SYI---KH------------ETCIILAVSPANA--D-LANSD-ALQMARIAD------
PDGSRTIGVITKLDIMD-------RGT------D-------A----------------------------RNF--LL---GN---------V-
IP-----------------------L---RLGYIGVVNR---S-QED--IIA--------
---N--------KS--------------------IR----------DALVY---EEGFFRS--------KP-VY---------------------H-N-
-----L-----------ADR-CGVPQLAIRLNTILVQHIK--------
>XP_024362051.1
------------IDLPQ--------VAVV---GS---Q----------------
SSGKSSVLE-----A--LV-G-K-DFLPRS--------------SD-ICTRRPL---------------VLQ--------------L-V-
QTL------------------------------RRSDE---KSE-----
----------------------LVE--------------W---------------------------GEF---------------------------------------
---------------------------------LHIPGRQFTNFS-----------AIRKEI------QLET-ER---D-MG--------------
----------TN-------------------K-GISEK-QIRLKIFSPD--
-----VLNITLVDLPGITK--VP-------------------V-------GD--------------Q--------PT--D--IEARVR--------
TMIL-SYI---KH------------KTCIILAVSPANA--D-LANSD-ALQMARIAD------
PDGSRTIGVISKLDIMD-------RGT------D-------A----------------------------RSL--LL---GN---------V-
IP-----------------------L---RLGYVGVVNR---S-QED--ISR------
-----N--------RS--------------------IR----------DALTN---EENFFRS--------RP-VY---------------------H-
N------L-----------SDR-CGVPQLAKKLNTILVQHIK--------
>KAG0554580.1
------------IDLPQ--------VAVV---GS---Q----------------
SSGKSSVLE-----A--LV-G-R-DFLPRG--------------SD-ICTRRPL---------------VLQ--------------L-V-
QTL------------------------------RRPDE---KSE-----
----------------------PVE--------------W---------------------------GEF--------------------------------------
----------------------------------LHIPGRRFTDFS-----------AIRKEI------QLET-ER---E-LG---------------
---------TN-------------------K-GISEK-QIRLKIFSPN---
----VLNITLVDLPGITK--VP-------------------V-------GD--------------Q--------PT--D--IEARVR--------
TMIL-SYI---KH------------ETCIILAVSPANA--D-LANSD-ALQMARIAD------
PDGSRTIGVISKLDIMD-------RGT------D-------A----------------------------RSF--LL---GN---------I-
IP-----------------------L---RLGYVGVVNR---S-QED--ISA------

-----N--------KS-------------------IR----------DALTY---EENFFRS--------RP-VY--------------------H-
N------L-----------SDR-CGVPQLAKKLNTILVQHIK--------
>KAI5070335.1
------------IDLPQ--------VAVI---GS----Q---------------
SSGKSSVLE-----A--LV-G-R-DFLPRG-------------SD-ICTRRPL--------------VLQ-------------L-V-
QLV-----------------------------RRPDD---KSD----
----------------------VME-------------W------------------------GEF-------------------------------------
-----------------------------------LHTPGKRYTDFS-----------LIRKEI------QAET-ER---E-LG------------
-----------SN------------------R-GISDK-QIRLKIFSPS--
-------VLNITLVDLPGITK--VP------------------V-------GD-------------Q--------PS--D--IEARVR------
--TMIL-SYI---KH------------ETCIILAVSPANA--D-LANSD-ALQMARMAD------
PDGSRTIGVITKLDIMD-------RGT------D-------A---------------------------RNF--LL---GS---------A-
IP----------------------L---RLGYIGVVNR---S-QAD--ILG-------
----N--------KS-------------------IR----------EALAY---EDNFFRS--------LP-VY--------------------H-
S------L-----------ADR-CGIPQLAKKLNNILVQHIK--------
>ONM18162.1
------------LELPQ--------VAAI---GG----Q---------------
SSGKSSVLE-----A--LV-G-R-DFLPRG-------------PD-ICTRRPL--------------VLQ-------------L-V-
RH---AA---------
-----------------PEE-------------W-------------------------GEF--
----------------------------LHVPGRQFHDFE-----------QIKREI------QLET-DK---E-AG-----------------
-------DN-------------------K-GVSEK-QIRLKIFSPN--
----VLDITLVDLPGITR--VP------------------V-------GD-------------Q--------PS--D--IESRIR--------
AMIM-QYI---KH------------PSCIILAVSPANA--D-LANSD-ALQLARLAD------
PDGSRTIGVITKLDIMD-------RGT------D-------A---------------------------RNF--LL---GN---------V-
IP----------------------L---KFGYVGVVNR---S-QED--INF------
-----N--------RS-------------------VK---------DALAF---EEKYFLT--------LP-AY--------------------H-
G------L-----------AHC-CGVPQLAKKLNMILLKHVT--------
>XP_052310486.1
------------IELPQ--------VAVV---GS----Q---------------
SSGKSSVLE-----A--LV-G-R-DFLPRG-------------NE-ICTRRPL--------------VLQ-------------L-L-
QTK-----------------------------RKGDG---SG-----
---------------------EDE-------------W-------------------------GEF-------------------------------------
---------------------------------LHLPGKRFYDFS-----------EIRSEI------QAET-AK---E-AG-------------
----------GN------------------K-GVSDK-QIRLKIFSPN--
-------VLDITLVDLPGITK--VP------------------V-------GD-------------Q--------PS--D--IEARIR-------
-TMIM-SYI---KK------------PSCLILAVTAANS--D-LANSD-ALQIAGNAD------
PDGYRTIGIITKLDIMD-------RGT------D-------A---------------------------RNL--LL---GK---------V-
IP----------------------L---RLGYVGVVNR---S-QED--IIL-------
----N--------RS--------------------IK----------DALAA---EEKFFRS--------RP-VY--------------------N-
G------L-----------ADR-CGVPQLAKKLNQILVQHIK--------
>AAC61784.1
------------IALPQ--------VVVV---GS----Q---------------
SSGKSSVLE-----A--LV-G-R-DFLPRG-------------ND-ICTRRPL--------------VLQ-------------L-L-

QTK------------------------------SRANG---GS-----
----------------------DDE--------------W---------------------------GEF--------------------------------------
----------------------------------RHLPETRFYDFS-----------EIRREI------EAET-NR---L-VG--------------
----------EN-------------------K-GVADT-QIRLKISSPN---
------VLNITLVDLPGITK--VP-------------------V-------GD--------------Q--------PS--D--IEARIR--------
TMIL-SYI---KQ------------DTCLILAVTPANT--D-LANSD-ALQIASIVD------PDGHRTIGVITKLDIMD-
------KGT------D-------A----------------------------RKL--LL---GN---------V-VP-----------------------L--
-RLGYVGVVNR--C-QED--ILL-----------N--------RT-----------
---------VK----------EALLA---EEKFFRS--------HP-VY---------------------H-G------L-----------ADR-
LGVPQLAKKLNQILVQHIK--------
>KAH9327796.1
---MYP-----
W--LV-V-K-EF--------------------------PD---------------AIA--------------L---------------------------------
----

-------------------------QVET-ER---E-AG------------------------GN-------------------K-GISAK-
QIRLKIFSPF--VLNINLVDLPGIMK--VP-----------
--------V-------GD--------------Q--------PI--D--IEARIR--------TMIM-SYI---KH------------
PSCIILAVSPANS--D-LANSD-ALQIARVAD------TDGSRTIGVITKLDIMD-------RGT------D-------A--
--------------------------RNF--LL---GN---------V-IP-----------------------L---QLGYIGVVNR------------
----------------------------------S-QED--IIA-----------N--------QS--------------------IR----------DALAY-
--EENFFRG--------HP-VY---------------------C-S------L-----------ADQ-CGIPQLARKLNQILVKHIR--
------
>XP_014148015.1
---LQ--------
LV---H-HPVQRGG---------------------PA----------------------------------A--------------------------------
--E-------------
-W---------------------------GEF--
LHQPGKIYTDFS-----------KIRDEI------ANET-DR---L-TG------------------------TN-------------------
K-GISHT-PINLKLYSPN--MLDLTLVDLPGITK--
IA-------------------V-------GD--------------Q--------PE--D--IEVQIH--------QLIE-SYI---NN-----------
-PNCIILAVTAANT--D-IANSD-ALKMAKKAD------PKGLRTIGVATKLDLMD-------AGT------D-------
A----------------------------LDI--LT---GK---------V-VA-----------------------S---KLGFIGVVNR----------
------------------------------------S-QAD--INQ-----------K--------VS--------------------IE----------
TAREA---EQEYFRT--------HP-AY---------------------K-S------L-----------YKQ-
SGTEYLTRRLNQLLMTHIRRC-L----
>GMH85941.1
------------IDLPQ--------IAVL--GG----Q----------------
SSGKSSVLE-----N--VV-G-K-SFLPRG--------------SG-IVTRRPL---------------ILQ--------------L-F-
HEP------------------------------SG---
--------------------E--------------W---------------------------GEF--
----------------------------LHKPGEKFYDFD-----------MICEEI------EADT-AR---I-CG-------------------
-----KN-------------------K-GLSTK-PINLRVYSPD--
--VLNLTLIDLPGATK--VA-------------------V-------GD--------------Q--------PS--D--IGKQIE--------

SMIK-FYV---SK------------PNCLILAVTAANT--D-LANSD-AIAIAKEVD------
PKGERTLGVMTKLDLMD-------RGT------D-------A----------------------------RGI--FT---GESQ------
DV-PL-----------------------L---KMGYIGVVNR--S-QAD--
INE-----------R--------KT--------------------IQ----------GARDA---ENAFFEG--------HP-GY------------
---------A-D------I-----------ADR-LGTAYLVKKCSNMLLK-----------
>GMI59178.1
------------IDLPQ--------IAVL--GG----Q----------------
SSGKSSVLE-----N--IV-G-K-SFLPRG--------------SG-IVTRRPL---------------VLQ--------------L-Y-
HET------------------------------SG---
--------------------E--------------W---------------------------GEF--
----------------------------LHAPGKKFFDFD-----------EICQEI------ENDT-AR---I-CG-------------------
-----AN-------------------K-GLSTK-PINLRVYSPD--
--VLNLTLIDLPGATK--VA-------------------V-------GD--------------Q--------PS--D--IGKQIE--------
SMIK-FYV---SK------------PNCLILAVTAANT--D-LANSD-AIFIAKEVD------
PKGERTLGVMTKLDLMD-------RGT------D-------A----------------------------RGI--FT---GESQ------
DV-PH-----------------------L---ALGYVGVVNR--A-QAD--
INE-----------K--------KT--------------------IQ----------AARNS---ENQFFEQ--------HP-GY------------
---------G-D------I-----------ADK-LGTQYLVKKCSTMLLK-----------
>GMI25649.1
------------IDLPQ--------IAVV--GG----Q----------------
SSGKSSVLE-----N--IV-G-K-SFLPRG--------------SG-IVTRRPL---------------VLQ--------------L-Y-
NSP------------------------------E---
-------------------E--------------Y---------------------------GEF--
--------------------------LHQPNKKYYDFN-----------EICGEI------EADT-DR---V-CG--------------------
----AN-------------------K-NLKNQ-PINLRIYSPD---
-VLNLTLVDLPGATK--VA-------------------V-------GD--------------Q--------PK--D--IGRQIK--------
NMIM-SYA---SK------------PNCLILAVSAANV--D-LANSD-AIEIAKEVD------
PQGKRTLGVVTKLDLMD-------RGT------D-------A----------------------------RGI--FT---GEDS------
DL-PK-----------------------L---ALGFVGVVNR--S-QAD--
INE-----------R--------KG--------------------IG----------QARDA---ENQYFSM--------HP-AY-----------
----------S-D------I-----------QER-LGTQYLVKKCSQMLLK-----------
>GMI62840.1
------------IDLPQ--------IAVV--GG----Q----------------
SSGKSSVLE-----N--IV-G-K-TFLPRG--------------SG-IVTRRPL---------------VLQ--------------L-Y-
NSI------------------------------E---
------------------E--------------Y---------------------------GEF---
-------------------------LHIPNKKFYDFN-----------EVCSEI------EADT-DR---V-CG----------------------
--SN-------------------K-NLKNQ-PINLRIYSPD--
VLNLTLIDLPGATK--VA-------------------V-------GD--------------Q--------PK--D--IGRQIK--------
NMIM-SYA---SK------------PNCMILAVTAANT--D-LANSD-AIEIAKEVD------
PEGKRTLGVLTKLDLMD-------RGT------D-------A----------------------------RGI--FT---GEDR------
DL-PF-----------------------L---ALGYVGVVNR--S-QAD--
INE-----------R--------KG--------------------IA----------QARAA---EMQYFSM--------HP-GY-----------
----------S-D------I-----------QDK-LGTGYLVKKCSQMLLK-----------

>GMH55978.1
------------IDLPQ--------IAVV---GG----Q---------------
SSGKSSVLE-----N--IV-G-K-SFLPRG-------------SG-IVTRRPL--------------VLQ-------------L-Y-
NSQ----------------------------E--
------------------E-------------Y-------------------------GEF---
------------------------LHHPNKKWYDFE----------EICQEI------ERDT-DR---V-CG------------------
-----SN-------------------K-NLKNQ-PINLRVYSPD---
---VLNLTLIDLPGATK--VA-----------------V------GD-------------Q--------PK--D--IGKQIR-------
NMIM-SYA---SK------------PNCLILAVTAANT--D-LANSD-AIEIAKEVD------
PQGKRTLGVLTKLDLMD-------RGT------D-------A---------------------------RGI--FT---GEDT------
NL-PN----------------------L---ALGYVGVVNR---S-QAD--
INE-----------R--------KG--------------------IA---------SAREG---EMQYFSM--------HP-SY-----------
---------S-D------I-----------QDK-LGTAYLVKKCSQMLLK-----------
>PRP80825.1
------------IDLPQ--------IVVI---GG----Q---------------
SSGKSSVLE-----N--LV-G-R-DFLPRG-------------NE-LVTRRPL--------------ILQ-------------L---
NRI----------------------------ADSSESTI-------FYSIAPN--EN--
------------------------TDE-------------W-------------------------GEF--------------------------------
-----------------------------------LHKPGEKF-TFD----------GIREEI------HRET-ER---T-TG-----------
------------KN------------------K-GISTE-PILLRIYSPN---
--------VLPLTLVDTPGMTR--VP-----------------VDARFTPGGD-----------Q--------PP--D--
IEQRLR--------DMIM-QFI---SK------------PNSIILAVQSATQ--D-LATSD-ALKLAREVD------
PEGHRTIGVLTKIDIMD-------RGT------N-------A--------------------------MDT--LM---GK---------S-
IP----------------------L---RLGFVGTISR---S-QHD--INV-------
----G--------KS-------------------IR----------SSLDD---EQKFFRE--------HS-VY--------------------N-
S------L-----------QDL-CGTANLAAKCNRILAG-----------
>XP_004355605.1
-----------EIKLPQ--------IVVV---GS----Q---------------
SSGKSSVLE-----N--LV-G-R-DFLPRG-------------SG-LVTRRPL--------------VLQ-------------L---
NRI----------------------------EPGHA---
---------------------E-------------W-------------------------GEF---------------------------------------
----------------------------GHTGDSKF-NFD----------EIKKEI------EIET-NR---V-AG------------------
-----GN------------------K-SISSE-PIILKIYSPN--
VIPLTLVDTPGITR--IP------------------I-------GD-------------Q--------PT--N--IEEKIR--------DMVV-
DYI---SN------------PNSIILAISAANQ--D-IVTSD-ALKLAKEVD------PTGKRTIGVLTKLDLMD-------
KGV------D-------A--------------------------MDI--LI---GS--------V-VP----------------------L---
KLGFVGIVNR---S-QQD--INM------------K--------KQ----------
---------IG----------QAIQD---ESAWFQS--------HP-IY--------------------N-R------I-----------ANQ-
SGSLFLGQRCNKILTK-----------
>XP_020436927.1
-----------EIKLPQ--------IVVI---GS----Q---------------
SSGKSSVLE-----N--LV-G-R-DFLPRG-------------SG-LVTRRPL--------------VLQ-------------L---
IRI----------------------------E-DNA---
-------------------E-------------W-------------------------GEF---

----------------------------AHTGDVRF-NFA-----------GIRDEI------EAET-NR---V-AG-------------------
-----AN-------------------K-EISSD-PIILKIFSPY--
VIPLTLVDLPGITR--IP-------------------I-------GN--------------Q--------PT--N--IEERIR--------DMVL-
DYI---SN------------PNSIILAISAANQ--D-IVTSD-ALKLAKEVD------PEGRRTIGVLTKLDLMD-------
RGT------D-------A---------------------------MDI--LL---GH---------T-VP----------------------L---
KLGFVGIINR---S-QHD--IQT-----------K--------KA-------------
-------IS----------TMLKD---EERWFQN--------HP-VY--------------------S-R------I-----------ANQ-
TGSIFLAQKCNKILTK-----------
>AER35077.1
-----------EITLPQ--------IIVI---GS----Q----------------
SSGKSSVLE-----N--LV-G-R-DFLPRG--------------SG-LVTRRPL---------------ILQ--------------L---
NKH------------------------------D-SLE--
----------------------E-------------Y---------------------------GEF---
-----------------------------AHTGNKKF-DFD-----------GIKQEI------ERET-ER---L-AG------------------
------AN-------------------K-DISSE-PILLRIYSPN--
-VIPLTLVDTPGIAR--VP-------------------I-------GD--------------Q--------PS--N--IEEKLK--------
SMIM-EYI---SN------------PNSIILAITSANQ--D-IVTSD-GIKLAKEVD------PEGKRTIGVLTKLDLMD-
------KGT------D-------A---------------------------IDV--LL---GD---------Q-IP----------------------L---
KYGFVGIINR---S-QQD--INN-----------R--------KP------------
--------IS----------QMLKD---EQIWFDQ--------HP-AY--------------------S-R------I-----------NNQ-
LGTKYLAQKCNKILTK-----------
>KAF2075389.1
-----------EITLPQ--------IVVI---GS----Q----------------
SSGKSSVLE-----N--LV-G-R-DFLPRG--------------SG-LVTRRPL---------------ILQ--------------L---
NKS------------------------------E-GQE--
----------------------E-------------W--------------------------GEF---
------------------------------GHTGNIKF-TFE-----------GIKQEI------EAET-SR---V-AG-------------------
-----PN-------------------K-DISPE-PIVLKIYSPN---
VVPLTLVDTPGITR--VP-------------------I-------GD--------------Q--------AL--N--IEEKIR--------TMIT-
EYI---QN------------PNCIILAVTSANQ--D-IVTSD-AIQMARNID------PLGQRTIGVLTKIDLMD-------
KGT------D-------A---------------------------LDI--LL---GN---------T-IP----------------------L---
KLGFVGVVNR---S-QSD--INL-----------N--------KP-----------
---------IH----------TMLKD---EMKFFES--------HP-VY---------------------N-R------I-----------LHQ-
AGTKYLAQKCNKILTK-----------
>XP_003292385.1
-----------EITLPQ--------IIVV---GS----Q----------------
SSGKSSVLE-----S--LV-G-R-DFLPRG--------------SG-LVTRRPL---------------VLQ--------------L---
YQN------------------------------EDSNE--
----------------------E-------------W--------------------------GEF--
--------------------------------GHTGDRKF-SYF-----------EIKEEI------EKET-ER---I-AG-------------------
-----AK-------------------K-DISPE-PIILKIHSPN--
VIPLTLVDLPGLTR--VA-------------------V-------DD--------------Q--------PI--D--IEEKVR--------
SMIL-SYI---NN------------PNSIILAITPANQ--D-IVTSD-ALKLAQQVD------
PLGKRTVGVLTKLDLMD-------KGT------D-------A---------------------------LDI--LL---GN---------E-

IP----------------------L---SMGFVGVVNR---S-QQD--INY----
-------G--------KP-------------------IS----------DSLKD---EVKWFQN--------HP-VY-------------------
-S-R------V-----------FNQ-SGSKYLAQKCNKILTK-----------
>KOO24608.1
-----------APKLPQ--------IVVI---GS----Q---------------
SSGKSSVLE-----S--FV-G-R-DFLPRG--------------TG-IVTRRPL---------------VLQ--------------L-V-
RTA-----------------------------ADGDT---ASATDEG-
------------------------ATVE--------------W--------------------------GEF-----------------------------------
-------------------------------------LHAPGRRFTSFE-----------AIRAEI------EAET-ER---K-LG-----------
------------KS-------------------K-SVSAD-PIRLAIYSPH--
--------VVDLSLVDLPGMTK--VP-------------------I-------AD--------------Q--------PA--N--IEEQLR---
-----AMAL-TYI---EP------------EESLILAVSAANA--D-LATSD-AIQLARRVD------
PEGLRTIGVLTKLDLMD-------AGT------D-------A---------------------------LAV--LQ---GR---------V-
IP----------------------L---KRGFVGVVNR---S-QQD--LFD----
-------G--------KS-------------------PQ----------AAREH---EARFFAN--------HP-QY-------------------
-Q-S------I-----------AAR-MGSRYLAHRLNELLLS-----------
>CCW59714.1
-----------KLNLPQ--------IAVV---GS----Q---------------
SCGKSSVLE-----S--IV-G-K-DFLPRG--------------SG-IVTRCPL---------------VLQ--------------L-I-
QLP-----------------------------KNS--
-------------------QEE--------------W--------------------------GEF---
------------------------------LHLPNKKFFVFS-----------DIRNEI------TRRT-NE---V-AG-----------------
-------PS---------------------AITDK-PINLKVYSAH--
--VLNLTMVDLPGLVM--NA-------------------V-------GD--------------Q--------PK--D--IDRQIK--------
EMVT-RYV---AP------------KNTIILAISPANT--D-LATSQ-SLRLAHQLD------
PEGNRTVGVLTKLDLMD-------RGT------D-------C---------------------------YDV--LT---NK---------V-
LP----------------------L---RHGFVGVICR---S-QQD--INT-----
------E--------KG-------------------ME----------EARAS---EQEFFLN--------SS-VY--------------------
A-P------I-----------ANE-QGTVYLSKKLNGLLLD-----------
>KAG5490335.1
-----------KLNLPQ--------IAVV---GS----Q---------------
SCGKSSVLE-----S--IV-G-K-DFLPRG--------------SG-IVTRCPL---------------VLQ--------------L-V-
QLP-----------------------------KSN--
------------------NDE--------------W--------------------------GEF---
-----------------------------LHLPTKKFFDFS-----------EIREEI------TRRT-VE---L-AG--------------------
----PS---------------------AITDK-PINLKVYSNM---
VLNLTLVDLPGLVM--NA-------------------V-------GD--------------Q--------PK--D--IDRQIK--------
EMVT-RYV---SP------------KNTIILAISPANT--D-LATSQ-SLRLAKQLD------
PDGKRTVGVLTKIDLMD-------KGT------D-------C---------------------------FDI--LN---NR---------V-
LN----------------------L---HHGFIGVVCR---S-QQD--IND---
---------R--------KS-------------------ME----------AARQA---EHDFFAN--------SP-IY-------------------
-S-S------I-----------ADE-AGTVYLTKKLNMVLLD-----------
>XP_003872337.1

-----------KLNLPQ--------IAVV--GS----Q----------------
SCGKSSVLE-----S--IV-G-K-DFLPRG--------------SG-IVTRCPL---------------VLQ--------------L-V-
QLP------------------------------KSN---
------------------NEE--------------W---------------------------GEF--
------------------------------LHIPQKKFYDFN-----------EIQNEI------TRRT-IE---M-AG------------------
------PS---------------------AITDK-PISLKVYSKT--
VLNLTLVDLPGLVM--NA-------------------V-------GD--------------Q--------PK--D--IDRQIK--------
DMVT-RYV---SP------------KNTIILAISPANT--D-LATSQ-SLRLAKQLD------
PDGLRTVGVLTKIDLMD-------KGT------D-------C----------------------------LDI--LQ---NR---------V-
LQ-----------------------L---RHGFIGVVCR--S-QQD--IND----
-------R--------KS--------------------ME----------GARRS---EYEFFAN--------SP-IY---------------------
S-P------I-----------AEE-AGTTYLSKKLNFLLLE-----------
>KAI5685071.1
-----------KLNLPQ--------IAVV--GS----Q----------------
SCGKSSVLE-----S--IV-G-K-DFLPRG--------------SG-IVTRCPL---------------VLQ--------------L-V-
QLP------------------------------KSN---
------------------SEE--------------W---------------------------GEF--
------------------------------LHIPNKKFFDFN-----------AIQEEI------TRRT-IE---V-AG--------------------
----PH---------------------AITDK-PINLKVYSNM---
VLNLTLVDLPGLVM--NA-------------------V-------GD--------------Q--------PK--D--IDRQIK--------
DMVT-RYV---SP------------KNTIILAISPANT--D-LATSQ-SLRLAKQLD------
PEGTRTVGVLTKIDLMD-------KGT------D-------C----------------------------FDV--LQ---NK---------V-
LH-----------------------L---RHGFVGVVCR--S-QQD--IND---
--------R--------KS--------------------ME----------AARQS---EYDFFAN--------SP-TY--------------------
-S-P------I-----------ADE-AGTVYLSKKLNNLLLE-----------
>XP_028887534.1
-----------KLNLPQ--------IAVV--GS----Q----------------
SSGKSSVLE-----A--IV-G-R-DFLPRG--------------SG-IVTRCPL---------------VLQ--------------L-V-
QLP------------------------------ASA---
------------------KEE--------------W---------------------------GEF--
------------------------------LHKPGKKYYNFA-----------EINEEI------QNRT-VE---I-AG------------------
------KS---------------------AITDR-PINLKVFSPH---
-VLNLTLVDLPGLVM--NA-------------------V-------GD--------------Q--------PK--D--IDRQIK--------
DMVT-RYV---SP------------ANTIILAISPANT--D-LATSA-SLRLARQLD------
PEGLRTVGVLTKLDLMD-------RGT------D-------A----------------------------FDV--LT---GK---------V-
IG-----------------------L---RHGFVGVVNR--S-QQD--IND---
--------S--------KG--------------------ME----------AARED---ERAFFHN--------HP-VY-------------------
--S-A------I-----------ADT-QGTEYLAKKLNHLLLE-----------
>EKF32958.1
-----------KLNLPQ--------IAVV--GS----Q----------------
SSGKSSVLE-----A--IV-G-K-DFLPRG--------------SG-IVTRCPL---------------VLQ--------------L-V-
QLP------------------------------KTS---
------------------EEE--------------W---------------------------GEF--
------------------------------LHKPNKKYFNFS-----------DINEEI------KHRT-VE---I-AG------------------

------NS---------------------AISER-PINLKVYSKN---
--VLNLTLVDLPGLVM--NA------------------V-------GD--------------Q--------PK--D--IDRQIK--------
EMVT-RYV---AP------------VNTIILAISPANT--D-LATSS-SLRLAKQLD------PEGIRTVGVLTKLDLMD-
------RGT------D-------A---------------------------LDA--LT---GK---------L-VS----------------------L---
RHGFVGVVNR---S-QQD--IND----------S--------KG---------
-----------MV----------AARED---ERAFFHN--------HP-SY--------------------S-A------I----------
ADR-QGTEYLAKKLNQLLLQ-----------
>ESL10883.1
-----------KLNLPQ--------IAVV---GS---Q---------------
SSGKSSVLE-----A--IV-G-K-DFLPRG--------------SG-IVTRCPL--------------VLQ-------------L-V-
QLP-----------------------------STT---
-----------------DEE--------------W--------------------------GEF--
----------------------------LHKPHKKFFDFA-----------EINDEI------KNRT-VE---I-AG------------------
-----KS--------------------AISDR-PINLKVFSRH---
-VLNLTLVDLPGLVM--NP------------------V-------GD--------------Q--------PK--D--IDRQIK--------
EMVT-RYV---AP------------VNTIILAISPANT--D-LATSA-SLRLAKQLD------
PEGLRTVGVLTKLDLMD-------RGT------D-------A---------------------------LDA--LT---GK---------L-
VG----------------------L---RHGFVGVVNR---S-QQD--IHD--
---------M--------KG-------------------ME----------SARAD---ERMFFCN--------HP-VY----------------
----S-A------I-----------ADR-QGTEYLAKKLNQLLLQ-----------
>RHW73545.1
-----------KLNLPQ--------IAVV---GS---Q---------------
SAGKSSVLE-----A--IV-G-K-DFLPRG--------------SG-IVTRCPL--------------VLQ-------------L-V-
QLP-----------------------------RSN---
-------------------KDE--------------W--------------------------GEF--
------------------------------LHRPNKKFFDFS-----------EINEEI------QNRT-TE---V-AG----------------
-------HS--------------------AITDK-PINLKIYSSH---
-VLNLTLVDLPGLVM--NA------------------V-------GD--------------Q--------PK--D--IDRQIK--------
SMVT-RYI---SP------------SNTIILAISPANA--D-LATSS-SLQIAKQLD------PEGLRTLGVLTKLDLMD-
------RGT------N-------A---------------------------YDI--LT---GK--------V-LP----------------------L---
RHGFVGVVNR---S-QHD--INT----------S--------KG----------
----------MQ----------AARDD---EKEFFRN--------HP-AY--------------------A-S------I-----------ADT-
QGTEYLTQKLNGLLLE-----------
>CCC89860.1
-----------KLNLPQ--------IAVV---GS---Q---------------
SAGKSSVLE-----A--IV-G-K-DFLPRG--------------SG-IVTRCPL--------------VLQ-------------L-V-
QLP-----------------------------KSN--
-----------------TEE--------------W--------------------------GEF--
----------------------------LHMPGSKFYDFT-----------QINEEI------QNRT-ID---V-AG------------------
------QT--------------------SITER-PINLKIYSSN--
VLNLTLVDLPGLVM--NA------------------V-------GD--------------Q--------PK--D--IDRQIK--------
NMVT-RYV---SP------------SNTIILAISPANA--D-LATSS-SLQIAKQLD------
PEGLRTVGVLTKLDLMD-------RGT------D-------A---------------------------YDI--LT---GK--------V-
VP----------------------L---RHGFVGGVNR---S-QHD--INT---

--------S--------KG--------------------MR----------EARDD---EKEFFRS--------HP-AY------------------
--S-Q------I-----------ADT-QGTEYLTRKLNGLLLE-----------
>KAH8605762.1
-----------SLPLPQ--------IAVV--GS----Q----------------
SAGKSSVLE-----A--IV-G-K-DFLPRG-------------SG-IVTRCPL--------------VLQ-------------L-V-
QLP------------------------------QTN---
-------------------TEE-------------W---------------------------GEF---
------------------------------LHLPGKKFFYFP-----------DIDQEI------RSRT-RE---I-AG------------------
------EF--------------------SITDR-AINLKIYSAN---
ILNLTLVDLPGLVS--NA------------------V-------GD-------------Q--------PA--D--IDRQIK--------
EMVT-RYI---SP------------PNTIILAVSPANA--D-LATSY-SLQLAKKVD------
PEGVRTVGVLTKLDLMD-------RGT------D-------A----------------------------SDI--LM---GK---------V-
MH-----------------------L---SHGFVGVVNR--S-QHD--INT---
--------S--------KS--------------------MQ----------SARAD---ERAFFQN--------HP-AY------------------
--S-A------I-----------ADT-QGTEYLAQKLNYILLE-----------
>KAJ9467322.1
-----------EFDLPQ--------IVVV--GA----Q----------------
SSGKSSVLE-----N--VV-G-K-DFLPRG-------------SG-IVTRRPL--------------ILH-------------M-S-
HLS------------------------------NEEHQ--RKVNAGE-
--------------------------PTE-------------T--------------------------ASF-------------------------------------
-------------------------------------AHQPGKIYTDFD-----------AVKAEI------EAET-AR---K-CG-----------
-------------DS-------------------K-GVDSE-PIRLSVTSNY---
----------VVDLTLIDLPGLTK--VA------------------V-------EG-------------Q--------KE--T--VAADIE----
----SMVI-EWS---KP------------RNTIILAVTAANT--D-IANSD-ALQLAKRVD------
PSGDRTIGVLTKIDLMD-------QGT------D-------C----------------------------LDI--LK---GN---------V-
VK-----------------------L---KKGFYGLVNR--S-QRD--IDS-----
------K--------KD--------------------IK----------AALAA---ERQFFET--------HP-AY-------------------K-
A------N-----------AAR-MGTPFLTRVLSQELMF-----------
>KAJ9466576.1
-----------ELDLPQ--------IAVV--GV----Q----------------
SSGKSSVLE-----N--IV-G-K-DFLPRG-------------TG-IVTRRPL--------------VLQ-------------M-V-
HMS------------------------------PEEKA---QREEKGE-
--------------------------ATE-------------V--------------------------ASF-------------------------------------
-------------------------------------LHEPGKEYTDFQ-----------EVKRQI------EKET-ER---L-CG----------
--------------AR-------------------K-GISSE-PIRLRVLSHR---
----------VLDLTLVDLPGLTQ--IA------------------V-------EG-------------Q--------PA--N--IGKEIK----
----AMIL-EWI---EP------------KNTIILAVSAANV--D-IANSD-ALKLAKQVD------
PAGERTVGVLTKLDLMD-------QGT------D-------C----------------------------VDV--LS---GS---------
V-VA-----------------------L---KKGFIGLVNR--S-QRD--IDS---
--------Q--------KD--------------------IN----------AALKA---EQDFFRT--------HP-SY------------------
-S-K------I-----------SHK-LGTAYLTKMLSHHLLL-----------
>XP_018636213.1
------------LDLPQ--------IAVV--GA----Q----------------
SVGKSSVLE-----A--LV-G-R-SFLPRG-------------TG-IVTRRPL--------------ILQ-------------L-R-

NAS-------------------------------D---
----------------LQEE--------------F----------------------------GEF--
------------------------------LHCPSRKFTDFE----------EIRREI------ERET-ER---V-GG--------------------
-----Q--------------------K-NISPS-PIVLKVSSPH--
-VIDLTLVDLPGITK--VP--------------------V-------GD--------------Q--------PS--D--IEAQIR--------
RIVF-QFI---SE------------PSTIILAVTAANT--D-IANSD-SLKIAREVD------PEGLRTVGVVTKVDTLE--
-----EGA------D-------C----------------------------SEV--LR---NR-------V-IP------------------------L---
KRGYVGVVCR---G-QRQ---AA-----------E--------MS----------
----------IR----------DGLKE---EESFFRS--------HP-AY---------------------R-A------I-----------ASK-
QGIPFLAKMLNQILMKHIREA-LPEL-
>XP_001750431.1
------------IQLPQ--------IVVV---GA----
QASSPADHPALRMSHEQSSGKSSILE-----N--VV-G-K-DFLPRG--------------TG-IVTRVPL-----------
----VLQ--------------L-V-QTA-------------------------------DD--
---E--------------W----------------------------ATF----------------
---QHAGGKVFRDFE-----------QVRQEI------VDQT-
ER---I-TG------------------------PG-------------------K-AVSNE-PIHLRVHSPN----------------------------
----------------------------------VVNLTLVDLPGLTK--VA-------------------V-------AD--------------Q------
--PQ--D--IGPQIR--------RLVR-HYI---DN----------PNSLILAVSPANA--D-IANSD-SLQIAKEVD----
--PQGDRTLAIVTKLDLMD-------RGT------D-------A----------------------------KAL--LS---GE---------
V-LP------------------------V---KLGIIGIVNR---S-QND--INC----
-------K--------TS--------------------IQ----------DSLDN---EKRFFRT--------H---Y---------------------P-
E------M-----------ADR-CGCAFLADTLHHLLLQHIRAC-L----
>XP_042924642.1
--------------LPT--------IVVV---GG----Q----------------
SSGKSSVLE-----A--VV-G-R-DFLPRG--------------TG-IVTRRPL---------------VLQ--------------L---
VKT------------------------------DDP--
-----------------NAVD--------------Y----------------------------GEF---
-------------------------------AHAPGRKFTNFD-----------DITTEI------EDET-TRHLQR-QG---------------
---------GT-------------------K-VVSPD-PIYLTVYSVN--
-----VPNLTLVDMPGLTK--VP-------------------I-------DG--------------Q--------PA--S--IVQELD-------
-DMAR-QYV---KS------------DNAIILAVTPANA--D-LATSD-ALRMARDVD------
PSGDRTIGVLTKVDIMD-------RGT------D-------C----------------------------RDV--LL---GK---------T-
LK------------------------L---KHGWVAVVNR---G-QAD--LNS--
---------K--------VT--------------------MK----------DARAR---EQEFFKG--------KP-EY-------------------
--Q-D------L-----------QN--TGTTFLAEKLSNHLINEIMKS-LP---
>PTQ29980.1
--------------LPS--------VVVV---GG----Q----------------
SSGKSSVLE-----S--VV-G-K-DFLPRG--------------SG-IVTRRPL---------------VLQ--------------L---
QKT------------------------------ED---
-----------------GTRE--------------W----------------------------AEF---
-------------------------------LHAPRKRFEDFA-----------LVRKEI------SDET-DR---V-GG-----------------
-------R--------------------K-GISKI-PIHLTVYSPN--
-VVNLTLVDLPGLTK--VA-------------------V-------EG--------------Q--------SD--S--IVADIE--------

DMVR-SYV---EK------------PNSIILAVSPANQ--D-IATSD-AIKIAREVD------
PNGERTFGVATKLDLMD-------KGT------N-------A---------------------------LDV--LE---GR--------T-
YR----------------------L---QLGWVGVVNR--S-QQD--INK---
--------N--------TD-------------------ML----------AARRR---EREYFQT--------SQ-DY------------------
--G-H------L-----------ASR-MGSEYLGKLLSKHLEQVIKAR-IP---
>XP_002302631.1
--------------LPS--------IAVV---GG----Q---------------
SSGKSSVLE-----S--IV-G-K-DFLPRG--------------AG-IVTRRPL---------------VLQ-------------L---
HKI------------------------------DE--
----------------G-KE-------------Y--------------------------AEF--
----------------------------MHLPRKKFTDFA-----------AVRKEI------ADET-DR---E-TG-------------------
-----RS-------------------K-QISSV-PIHLSIFSPN--
VVNLTLVDLPGLTK--VA-------------------I-------DG--------------Q--------PE--S--IVHDIE--------
NMVR-SYI---EK------------PNCIILAISPANQ--D-LATSD-AIKISREVD------
PRGERTFGVLTKIDLMD-------KGT------D-------A---------------------------VDI--LE---GK---------S-
YK----------------------L---QFPWIGVVNR--S-QAD--INK----
-------S--------VD-------------------MI----------AARRR---EREYFQS--------SP-EY--------------------
G-H------L-----------ASR-MGSEHLGKMLSKHLEQVIKSR-IP---
>XP_002299468.1
--------------LPS--------IAVV---GG----Q---------------
SSGKSSVLE-----S--VV-G-K-DFLPRG--------------SG-IVTRRPL---------------VLQ-------------L---
HKI------------------------------DE--
----------------GSRE-------------Y--------------------------AEF--
----------------------------LHLPRKRFTDFA-----------AVRREI------QDET-DR---E-TG-------------------
-----RS-------------------K-QISSV-PIHLSIYSPN--
VVNLTLVDLPGLTK--VA-------------------V-------EG--------------Q--------PD--S--IVQDIE--------
NMVR-AYI---EK------------PNCIILAISPANQ--D-LATSD-AIKISREVD------PTGERTLGVLTKIDLMD-
------KGT------D-------A---------------------------VDM--LE---GK---------S-YR----------------------L-
--KFPWVGVVNR--S-QAD--INK-----------N--------VD--------
------------MI----------AARRR---EREYFSS--------TP-EY---------------------K-H------L-----------AHR-
MGSEHLAKMLSKHLEVVIKSK-IP---
>NP_001190448.1
--------------LPA--------IAVV---GG----Q---------------
SSGKSSVLE-----S--IV-G-K-DFLPRG--------------SG-IVTRRPL---------------VLQ-------------L---
QKI------------------------------DD--
-----------------GTRE-------------Y--------------------------AEF---
-----------------------------LHLPRKKFTDFA-----------AVRKEI------QDET-DR---E-TG------------------
------RS-------------------K-AISSV-PIHLSIYSPN---
-VVNLTLIDLPGLTK--VA-------------------V-------DG--------------Q--------SD--S--IVKDIE--------
NMVR-SYI---EK------------PNCIILAISPANQ--D-LATSD-AIKISREVD------
PSGDRTFGVLTKIDLMD-------KGT------D-------A---------------------------VEI--LE---GR---------S-
FK----------------------L---KYPWVGVVNR--S-QAD--INK----
-------N--------VD-------------------MI----------AARKR---EREYFSN--------TT-EY--------------------
R-H------L-----------ANK-MGSEHLAKMLSKHLERVIKSR-IP---

>AQK88296.1
--------------ISR--------VAGG--TA----R----------------
SSGKSSVLE-----S--VV-G-K-DFLPRG--------------SG-IVTRRPL---------------VLQ--------------L---
HRI------------------------------D---
----------------GDRE--------------Y---------------------------AEF---
-----------------------------MHLPRKRFTDFA-----------AVRKEI------ADET-DR---E-TG------------------
------RS-------------------K-QISTV-PIHLSIFSPHG-----------------KMQTLEG--------------------
YIVTVALSHLNCPLPIVNLTLIDLPGLTK--VA------------------V-------DG--------------Q--------PE--S-
-IVHDIE--------NMVR-SYI---EK------------PNCIILAVSPANQ--D-LATSD-AIKISREVD------
PKGERTFGVLTKIDLMD-------KGT------D-------A----------------------------VDI--LE---GR---------S-
YR-----------------------L---QTPWVGVVNR--S-QQD--INK---
--------N--------VD--------------------MI----------AARRR---EREYFAS--------TP-EY--------------------
K-H------M-----------ASR-MGSEYLGKMLSKHLEQVIKSR-IP---
>PTQ45603.1
--------------LPS--------VVVV--GG----Q----------------
SSGKSSVLE-----S--IV-G-R-DFLPRG--------------SG-IVTRRPL---------------VLQ--------------L---
HKT------------------------------D---
----------------DGSD--------------Y---------------------------AEF---
-----------------------------LHHPRRRFADFA-----------AVRKEI------ADET-DR---V-TG------------------
------RS-------------------K-MISPV-PIHLSIYSPN---
-VVNLTLIDLPGLTK--VA------------------V-------EG--------------Q--------PD--S--IVHDIE--------
NMVR-SYV---EK------------PNSIILAISPANQ--D-IATSD-AIKLAREVD------
PAGERTWGVLTKLDLMD-------RGT------N-------A----------------------------LDV--LE---GR---------
S-YR-----------------------L---QLPWIGVVNR--S-QAD--INK--
---------N--------VD--------------------MI----------AARRR---EREYFQS--------SQ-DY-----------------
---G-H------L-----------AGK-MGSEYLAKMLSKHLEAVIKSR-IP---
>KAI5072318.1
--------------LPS--------VAVV--GG----Q----------------
SSGKSSVLE-----S--IV-G-R-DFLPRG--------------SG-IVTRRPL---------------VLQ--------------L---
YRT------------------------------D--
---------------KGPE--------------Y---------------------------AEF---
---------------------------LHAPKKRFTDFA-----------AVRKEI------SDET-DR---I-TG---------------------
---RS-------------------K-QISPV-PIHLSIYSPN--
VVNLTLIDLPGLTK--VA------------------V-------EG--------------Q--------PD--S--IVADIE--------
NMVR-SYV---EK------------PNSLILAISPANQ--D-IATSD-AIKLAREVD------
PSGERTFGVLTKLDLMD-------KGT------N-------A----------------------------LDT--LE---GR---------A-
YR-----------------------L---QHPWVGVVNR--S-QAD--INK---
--------S--------VD--------------------MM----------AARRR---EREYFAT--------SP-DY-------------------
-K-H------L-----------ASR-MGSEYLGQMLSKHLESVIKSR-IP---
>KAI5058380.1
--------------LPS--------VAVV--GG----Q----------------
SSGKSSVLE-----S--IV-G-R-DFLPRG--------------SG-IVTRRPL---------------VLQ--------------L---
HKL------------------------------D---
----------------EGSE--------------Y---------------------------AEF---

---------------------------MHLPKRRFTDFA-----------AVRKEI------QDET-DR---V-TG-----------------
------RS------------------K-QISPV-PIHLSIYSPH---
-VVNLTLIDLPGLTK--VA------------------V-------EG-------------Q--------PE--S--IVADIE--------
NMVR-LYV---DK------------PNTIILAISPANQ--D-IATSD-AIKLAREVD------
PTGERTWGVLTKLDLMD-------KGT------N-------A---------------------------IDV--LE---GR---------A-
YH----------------------L---KNPWIGVVNR--S-QAD--INK----
-------N--------VD--------------------MM----------AARRR---EREYFAT--------SS-DY-------------------
-S-H------L-----------TSR-MGSEYLGKMLSKHLEAVIKAR-IP---
>XP_002987566.1
--------------LPS--------VVVV---GG----Q----------------
SSGKSSVLE-----S--IV-G-R-DFLPRG--------------SG-IVTRRPL---------------VLQ-------------L---
HKT------------------------------E--
--------------GGAE-------------Y--------------------------AEF---
---------------------------LHIPKKRFTDFS-----------LVRKEI------QDET-DR---V-TG-------------------
----RS------------------K-QISPI-PIQLSIYSPN---
VVNLTLIDLPGLTK--IA------------------I-------EG-------------Q--------PD--S--IVADIE--------
NMVR-SYV---EK------------QNSVILAISPANQ--D-IATSD-AMKLAREVD------
PTGERTFGVLTKLDLMD-------KGT------N-------A---------------------------LDV--LE---GR---------S-
YK----------------------L---QHPWVGVVNR--S-QAD--INR---
--------S--------VD--------------------MV----------AARRR---EREYFSS--------SA-DY-------------------
-G-H------L-----------TSR-MGSEYLAKILSKHLEAFIKAR-IP---
>KAI5602084.1
--------------LPS--------VAVV---GG----Q----------------
SSGKSSVLE-----S--IV-G-R-DFLPRG--------------SG-IVTRRPL---------------VLQ-------------L---
HKT-----------------------------EP---
---------------GITE-------------Y--------------------------AEF---
--------------------------LHKQRERFTDFA-----------MVRKEI------QDET-DK---I-TG-------------------
----KS------------------K-QISPV-PIHLSIYSPN--
VVNLTLIDLPGLTK--VA------------------V-------EG-------------Q--------PE--S--IVKDIE--------
NMVR-LYV---EK------------PNCIILAITPANQ--D-IATSD-AIKLAREVD------
PAGERTFGVLTKLDLMD-------KGT------N-------A---------------------------QDV--LE---GR---------
A-YP----------------------L---QHPWVGIVNR--S-QAD--INK-
----------N--------VD--------------------MI----------AARRR---EREFFST--------SP-DY------------------
--G-H------L-----------AGR-MGSEYLAKLLSKHLESVIKTR-IP---
>XP_006375094.1
--------------LPS--------VAVV---GG----Q----------------
SSGKSSVLE-----S--IV-G-R-DFLPRG--------------SG-IVTRRPL---------------VLQ-------------L---
HKT-----------------------------ED--
----------------GSQE-------------Y--------------------------AEF---
-----------------------------LHLPKRRFSDFA----------VVRKEI------QDET-DR---I-TG-----------------
------KT------------------K-QISPV-PIHLSIYSPN--
-VVNLTLIDLPGLTK--VA------------------V-------EG-------------Q--------PE--S--IVQDIE--------
TMVR-TYV---EK------------PNCIILAISPANQ--D-IATSD-AIKLAREVD------
PSGERTFGVLTKLDLMD-------KGT------N-------A---------------------------LDV--IE---GR---------S-

YR----------------------L---QHPWVGIVNR--S-QAD--INK----
-------N--------VD-------------------MI----------VARRK---EREYFAT--------SP-DY--------------------
G-H------L-----------ANK-MGSEYLAKLLSKHLESAIRAR-IP---
>XP_002315854.1
--------------LPS--------VAVV--GG----Q----------------
SSGKSSVLE-----S--VV-G-R-DFLPRG--------------SG-IVTRRPL---------------VLQ--------------L---
HKI------------------------------D--
---------------GGSD--------------Y--------------------------AEF---
----------------------------LHAPRKKFTDFA-----------SVRKEI------ADET-DR---I-TG--------------------
----KS-------------------K-QISNV-PIHLSIYSPN---
VVNLTLIDLPGLTK--VA-------------------V-------EG--------------Q--------PE--S--IVEDIE--------
NMVR-SYV---EK------------PNSIILAISPANQ--D-IATSD-AIKLAREVD------
PSGERTFGVLTKLDLMD-------KGT------N-------A----------------------------LDV--IE---GR---------S-
YR----------------------L---QHPWVGIVNR--S-QAD--INK----
-------N--------VD-------------------MI----------AARRK---EREYFET--------SP-EY--------------------
G-H------L-----------SSK-MGAEYLAKLLSKHLETVIRQR-IP---
>NP_001147100.1
--------------LPS--------VAVV--GG----Q----------------
SSGKSSVLE-----S--IV-G-R-DFLPRG--------------SG-IVTRRPL---------------VLQ--------------L---
HKT------------------------------D--
----------------GGHE--------------Y--------------------------AEF---
-----------------------------LHAPRKRFTDFA-----------AVRKEI------ADET-DR---I-TG-------------------
-----KT-------------------K-AISNV-PIHLSIYSPH---
VVNLTLIDLPGLTK--VA-------------------V-------EG--------------Q--------PE--S--IVQDIE--------
NMVR-AYV---DK------------PNCIILAISPANQ--D-IATSD-AIKLARDVD------
PSGDRTFGVLTKLDLMD-------KGT------N-------A---------------------------VDV--LE---GR---------
Q-YR----------------------L---QHPWVGIVNR---S-QAD--INK-
----------N--------VD-------------------ML----------SARRK---EKEYFES--------SP-EY------------------
---G-H------L-----------AHK-MGAEYLAKLLSQHLEAVIRAK-IP---
>AAF22292.1
--------------LPT--------VAVV--GG----Q----------------
SSGKSSVLE-----S--IV-G-R-DFLPRG--------------SG-IVTRRPL---------------VLQ--------------L---
HKT-----------------------------DD---
-----------------GTEE--------------Y-------------------------AEF---
-----------------------------LHLPKKQFTDFA----------LVRREI------QDET-DR---I-TG-------------------
-----KN-------------------K-QISPV-PIHLSIYSPN---
VVNLTLIDLPGLTK--VA-------------------V-------EG-------------Q--------PE--T--IAEDIE--------
SMVR-TYV---DK------------PNCIILAISPANQ--D-IATSD-AIKLAKDVD------
PTGERTFGVLTKLDLMD-------KGT------N-------A---------------------------LEV--LE---GR---------S-
YR----------------------L---QHPWVGISEP---FN-KQD--INK----
-------N--------VD-------------------MM----------LARRK---EREYFDT--------SP-DY-------------------
-G-H------L-----------ASK-MGSEYLAKLLSKHLESVIRTR-IP---
>KAH9306600.1

--------------LPS--------VAVV---GG----Q----------------
SSGKSSVLE-----S--IV-G-R-DFLPRG--------------SG-IVTRRPL---------------VLQ-------------L---
HKT------------------------------DE---
-----------------GTPE-------------Y-------------------------GEF--
----------------------------LHRPNKRITDFA-----------KVRSEI------QEET-DR---I-TG------------------
-----RT------------------K-MISPV-PIHLSIYSPN---
VVNLTLIDLPGLTK--VA------------------V-------EG-------------Q--------PE--S--IVGDIE--------
NMVR-SYV---EKLLKLVVTADFLLPNSIILAISPANQ--D-IATSD-AIKLAREVD------
PTGERTFGVLTKLDLMD-------KGT------N-------A--------------------------LDV--LD---GR---------S-
YR----------------------L---QHPWVGVVNR---S-QAD--INK---
--------S--------VD-------------------MI----------AARRR---EREYFST--------ST-DY--------------------
G-H------L-----------SSR-MGSEYLAKLLSKHLENVIKAR-IP---
>KAG0556007.1
--------------LPS--------VAVV---GG----Q----------------
SSGKSSVLE-----S--IV-G-R-DFLPRG--------------SG-IVTRRPL---------------VLQ-------------L---
HKT-------------------------------E--
---------------DKYE-------------Y-------------------------AEF---
--------------------------LHMPKRRFTDFA-----------AVRKEI------SDET-DR---I-TG--------------------
---RS------------------K-QISPV-PIHLSVYSPN---
VVNLTLIDLPGLTK--IA------------------V-------EG-------------Q--------SD--S--IVGDIE--------
NMVR-SYI---EK------------PNCIILAVSPANQ--D-IATSD-AIKIAREVD------
PNGERTFGVLTKLDLMD-------KGT------N-------A---------------------------IDV--LE---GR---------S-
YK----------------------L---IQPWIGVVNR---S-QQD--INK-----
------N--------VD-------------------MI----------AARRR---EREYFQT--------SP-DY--------------------
S-H------L-----------QSK-MGSEYLGRVLSKHLEAVIRSR-IP---
>PWZ36850.1
--------------LPT--------IAVV---GG----Q----------------
SSGKSSVLE-----S--IV-G-T-DFLPRG--------------SG-IVTRRPL---------------VLQ-------------L---
QQT------------------------------ED---
-----------------GSQE-------------Y-------------------------AEF--
-----------------------------LHMPKRRFSDFA-----------LVRQEI------ADET-DR---L-TG----------------
--------KT-------------------K-QISPV-PIHLSIYSPK---
--VVNLTMIDLPGLTK--VA------------------V-------EG-------------Q--------SE--S--IVQDIE--------
NMVR-SYV---DK------------PNCIILAISPANQ--D-IATSD-AIKLSKEVD------
PTGERTFGVLTKLDLMD-------KGT------N-------A--------------------------LDV--LE---GR---------A-
YR----------------------L---QNPWVGIVNR---S-QAD--INR---
--------K--------VD-------------------MI----------SAREK---EREYFET--------SP-DY--------------------
A-H------L-----------SSR-MGSGYLAKLLSQHLESVIKVR-IP---
>AAF79238.1
--------------LPT--------VAVV---GG----Q----------------
SSGKSSVLE-----S--VV-G-R-DFLPRG--------------SG-IVTRRPL---------------VLQ-------------L---
HKT------------------------------ED---
-----------------GTTE-------------Y-------------------------AEF--
----------------------------LHAPKKRFADFA-----------AVRKEI------EDET-DR---I-TG------------------

-----KS-------------------K-QISNI-
PIQLSIYSPNGLCLRPHFLLCIPIVVSSETNTFDTSCNEITSSTIRFEDSNFANLFHVTL-
ISHSTLFSTVVNLTLIDLPGLTK--VAVVTDMNLVLKLVTDMNIIRV-------DG--------------Q--------PE--
S--IVQDIE--------NMVR-SYV---EK------------PNCIILAISPANQ--D-IATSD-AIKLAREVD------
PTGERTFGVATKLDIMD-------KGT------D-------C----------------------------LDV--LE---GR---------S-
YR-----------------------L---QHPWVGIVNR---S-QAD--INK----
-------R--------VD--------------------MI----------AARRK---EQEYFET--------SP-EY---------------------
G-H------L-----------ASR-MGSEYLAKLLSQHLETVIRQK-IP---
>NP_850420.1
--------------LPS--------VAVV---GG----Q----------------
SSGKSSVLE-----S--IV-G-R-DFLPRG--------------SG-IVTRRPL---------------VLQ--------------L---
HKT-------------------------------EN--
-----------------GTED--------------N----------------------------AEF---
------------------------------LHLTNKKFTNFS-----------LVRKEI------EDET-DR---I-TG-------------------
-----KN-------------------K-QISSI-PIHLSIFSPN---
VVNLTLIDLPGLTK--VA-------------------V-------EG--------------Q--------PE--T--IVEDIE--------
SMVR-SYV---EK------------PNCLILAISPANQ--D-IATSD-AMKLAKEVD------
PIGDRTFGVLTKLDLMD-------KGT------N-------A----------------------------LDV--IN---GR---------S-
YK-----------------------L---KYPWVGIVNR---S-QAD--INK-----
------N--------VD--------------------MM----------VARRK---EREYFET--------SP-DY---------------------
G-H------L-----------ATR-MGSEYLAKLLSKLLESVIRSR-IP---
>EFJ15761.1
--------------LPS--------VVVV---GG----Q----------------
SSGKSSVLE-----S--IV-G-R-DFLPRG--------------SG-IVTRRPL---------------VLQ--------------L---
HRT--------------------------------E---
----------------DGPD--------------Y---------------------------AEF--
-----------------------------LHLPKKKFTDFA-----------LVRKEI------QDET-DR---I-TG-------------------
-----RS-------------------K-QISPV-PIHLSIYSRN---
VVNLTMIDLPGLTK--IA-------------------V-------DG--------------Q--------PE--S--IVGDIE--------
NMVR-SYV---EK------------ENTIILAISPANQ--D-IATSD-AMKLARENI------
ALGDRTFGVLTKLDLMD-------KGT------N-------A----------------------------IDV--LE---GH---------S-
YR-----------------------L---QRPWIGVVNR---S-QAD--INK----
-------S--------VD--------------------MI----------VARRR---EREYFSS--------SP-DY---------------------
R-H------L-----------ASR-MGSEYLGRVLSKHLEAVIKAR-IP---
>EFJ23099.1
--------------LPS--------VAVV---GG----Q----------------
SSGKSSVLE-----S--VV-G-R-DFLPRG--------------SG-IVTRRPL---------------VLQ--------------L---
HKT--------------------------------E--
---------------GGQE--------------Y---------------------------AEF---
----------------------------LHNPKTKFSDFS-----------LVRKEI------EDET-DR---M-TG-------------------
-----HT-------------------K-QISPV-PIHLSIYSPNGTCLS-H-----PV--------------------------
KFSMPWFVYS----------VVNLTLIDLPGLTK--IA-------VGKCISF-----S-------KM--------------M--------
CL--L--ILADIE--------NMVR-SYV---EK------------QNSIILAISPANQ--D-IATSD-AMKLAKEVD------
PTGERTFGVLTKLDLMD-------KGT------N-------A----------------------------LEV--LE---GR---------A-

YR----------------------L---QFQWVGVVNR--S-QAD--INK---
--------S--------VD-------------------MI----------AARKK---EREFFAS--------SP-DY--------------------
G-H------L-----------ANR-MGSEYLAKMLSKHLETVIKTR-LP---
>XP_008860500.1
-----------TLPLPQ--------IVVV--GS----Q----------------
SSGKSSVLE-----H--VV-G-K-DFLPRG--------------SG-IVTRRPL---------------IVQ--------------C-V-
RSN-----------------------------V--
-----------------AED--------------Y---------------------------GQF---
----------------------------EHTGDRKFTDFG-----------EIRNEI------TRET-ER---TCPG-------------------
-----RN----------------------VSSV-PIRLRIYSSS---
VVDLTLVDLPGLVK--VN-------------------I-------NG--------------Q--------TA--E--MVKNLR--------
DMVY-EYA---SP------------SNALILAVTAGNI--D-IANSD-ALQVAKDVD------
PEGERTIGVLTKLDLED-------KGT------N-------S----------------------------MDV--LM---GR---------V-
YP----------------------L---KLGYIGVVNR---S-QQD--INN-----
------G--------VD--------------------VK---------TSLRH---EKEFFEN--------HP-VY--------------------
C-S------I-----------AER-MGTEYMVNRLNVLLLQ-----------
>XP_004185630.1
-----------TLPLPQ--------IVVV--GS----Q----------------
SSGKSSVLE-----H--VV-G-K-DFLPRG--------------SG-IVTRRPL---------------IVQ--------------C-V-
RTD-----------------------------V---
----------------PKE--------------Y---------------------------GLF--
--------------------------EHQGDKQYFDFN-----------AIRDEI------TAET-QR---TCPG-------------------
-----RN----------------------VSPT-PIRLRIVSPN--
VVDLTLVDLPGLVK--VT-------------------V------VG--------------Q--------SN--E--IVKNLR--------
DMVY-QYA---AP------------ENALILAVTAGNV--D-IANSD-ALNVAKEVD------
PDGERTIGVLTKLDLED-------KGT------N-------S----------------------------MDV--LM---GR---------V-
YP----------------------L---KLGYIGVVNR---S-QQD--INN-----
------G--------MD-------------------VQ---------TSLKN---EKKFFED--------HP-VY--------------------
C-S------I-----------ADR-MGTEYMVNRLNLLLLQ-----------
>KAF0852279.1
-----------SIDLPQ--------IAVV--GS----Q----------------
SSGKSSVLE-----S--LV-G-K-GFLPRG--------------SG-IVTRRPT---------------VLQ--------------L-I-
HEP------------------------------PGTAAAT---
-------------------APVE--------------Y--------------------------AEF---
------------------------------VHLPNQRFTDWD-----------AVRIEI------ERET-AR---V-AG---------------
---------SG-------------------K-GVSPS-PITLRIHSPY--
-----VLNLTLVDLPGLIK--IP------------------V-------GD-------------Q--------PQ--N--IESIVR--------
DLVL-KFI---AR------------PSTIILAVTPANM--D-LANSD-AIQIARQVD------
PEGTRTLGVLTKLDLMD-------KGT------D-------A----------------------------ADI--LR---NN---------L-
LP----------------------L---RLGYVGVVCR---S-QAD--LDA----
-------K--------TP-------------------LA----------VSRAR---ENSFFAN--------HP-AYQ-
DLYLQQQQLQTASSSQFVDS-A------A-----------AGW-LGTEVLGKRLQTLLMG-----------
>ETO36135.1

------------RKLPG--------ISNI--QK----K----------KLY---
SVGKSSVLE-----N--IV-G-R-DFLPRG--------------TD-IVTRRPL---------------ILQ--------------L-I-
NTS------------------------------KPRPSTSSSDDEKS---EQLIT-----SSQSSSLQSPS----------
QQSSSTQS-----LLSESSE--------------------------ANLE--------------W---------------------------GEF--
--LHIKNKRFYDFN-----------DIRKEI----
--EKET-DR-----VA------------------------PS-------------------K-AVSEE-PISLKIYSPK--------------------
--VLNLTLVDLPGLTK--VA-------------------V-------GN--------------
Q--------PE--N--IEQIIG-------DLVY-SFI---KR----------PKCLILAVSAATS--D-LATSD-
GLQMALRID------PKGVRTLGVITKLDLMD-------QGT------D-------A----------------------------MKV-
-LN---GE--------V-IP-----------------------L---QLGYVGVINR--
--S-QQN--IND-----------Q--------LH--------------------IT----------DALKN---EERFFKN-------HP-
AY---------------------R-N------I-----------AHL-CGIPYLAKRLNQILIN-----------
>KNH06820.1
---------DFSQQLPQ--------IAVV--GS----Q---------------
SSGKSSVLE-----S--IV-G-K-AFLPRG--------------SG-IVTRRPL---------------ILQ--------------L-V-
PMP------------------------------TDVSS--
-------------------TVAE--------------Q-P-------------------------FGEF---------------------------------------
---------------------------------LHEPGKKYYDFD-----------RIREVI------SEET-DS---I-AG---------------
---------VN-------------------K-NISPE-PIRLRIHSPS--
----VAPLTLVDLPGLVR--NP-------------------I-------GD--------------Q--------PK--D--IDRQVS-------
-NLVR-KYI---VH---------------ENTLILAVSAANA--D-IATSD-GVQLAQQVD------
PHGRRTIGVLTKLDLME-------EGT------H-------V----------------------------VDI--IE---QR---------V-
IS-----------------------L---QRGFIPVVNM--S-QSD--LDQG--
--------S--------KT--------------------MA----------DQRRA---ELQFFAQ--------HP-RY-------------------
--A-P------M-----------RDR-CGMAFLQAKLSAVLLE-----------
>CAD2212698.1
---------SLDLTLPQ--------IAVV--GS----Q---------------
SSGKSSVLE-----H--IV-G-E-EFLPRG--------------PT-MVTRCPI---------------VLQ--------------L-H-
QLP------------------------------KND---
-------------------KRK--------------W---------------------------GEF---
-------------------------------LHLPNKRFTDFN-----------LIREEI------LRYT-RE---L-IG------------------
------D--------------------R-TVTSQ-SITLKISSAA--
VANLTLVDLPGLVT--TP-------------------I-------RG--------------Q--------PE--T--IVTDIE--------
DMVR-RYV---AD---------------KNTVILAITPANQ--D-VATSA-ALSVSRMVD------
PHGERTMGVLTKLDLMD-------RGT------T-------A----------------------------HRT--LM---GD---------
E-YE-----------------------L---KFGFIGVVNR--S-QES--INS---
--------G--------QT--------------------MS----------DARAA---EEEFINE--------Y---Y--------------------
P-E------L-----------SGR-MGTKYLTAVLNSVLVS-----------
>ABB13595.1
------------LRLPQ--------IVVI--GS----Q---------------
STGKSSLLE-----S--IV-G-Q-EILPRG--------------KG-IVTRRPI---------------EIQ--------------L-K-
NQQ------------------------------N--
------------------AEQD-------------Y---------------------------VEF---
-----------------------------SERRGEKITDMD-----------QVRKMI------DEDT-EK---I-AG-----------------

-------KN-------------------K-AISNV-PLRLKFYSKN---
----VVDLILVDLPGMTK--NP-------------------V-------GD-------------Q--------PQ--D--IEQQIL-------
-NLIE-PYI---KN------------PNSIIMAVSKGSD--D-LANSE-SLKLSRKID------
PQGNRTIGVITQLDLID-------EGA------D-------V---------------------------LND--LQ---NK--------T-
YP----------------------L---KLGYVGVIMR---G-QKD--IKI------
--------------KS--------------------IK----------EQIAD---EKAYFEN--------HS-IY---------------------K-R-
-----V-----------SNK-MGIPYLIKLLNLSLMNHIKKT-LPNI-
>XP_001009829.2
------------LRLPQ--------IVVI---GS----Q---------------
SSGKSSVLE-----S--II-G-K-DFLPRG--------------KG-IVTRRPI---------------EIQ-------------L-T-
NIS-----------------------------S--
----------------GEE-------------Y-------------------------AEI---
-------------------------LDRKGEKVTDME-----------VLTKII------EDET-EK---V-AG---------------------
--KQ-------------------K-GVSGV-PLKIRFYSKN---
VVDLLLVDLPGITK--NP-------------------V-------GD-------------Q--------PA--D--IEQKLL--------
EIVN-PYI---AN------------PNSIILAISKGTD--D-LANSE-SLKLAREFD------INGQRTIGVITQIDLQD--
----FESE------N-------A---------------------------LND--IT---NK--------T-YP----------------------L---
RLGYVGVVMR---G-QNQ--LKT--------------------KT-----------
---------IQ----------EQIVD---EAAFFEN--------HS-VY---------------------R-K------V-----------ADK-
MGIPYLIKTLNLIFMNHIKKC-LPKI-
>XP_002371703.1
------------IQLPR--------ICVV---GT----Q---------------
SAGKSSVLE-----A--IV-G-L-DFLPRG--------------DG-VVTRRPL---------------ELR-------------L-V-
HL-------------------------------SEAEHDL--
----------------------NEA-------------Y-------------------------AVF---------------------------------------
-------------------------------ENDKERKIRDFE-----------QVRQEI------DRLT-DQ---V-AG--------------
----------KN-------------------K-GIIDS-PIVLTIYATQ---
----CPDLSLIDLPGITR--VP-------------------L-----KGSD-------------Q--------CE--D--IEMLTR-------
-QMAL-RYA---SD------------PRTIILAVIPANV--D-MSTSD-ALQMSRRVD------
PRGVRTIGVITKIDLMD-------RGT------D-------A---------------------------AKM--LM---GE---------E-
IP----------------------L---RLGYTGVRNR---S-QAD--IRE------
-----G--------KS--------------------VR----------ECLEE---EKTFFAT--------HP-TY---------------------R-
L------LP----------PHL-VGVHSLVDKLTKVLFRHIKNF-LPEI-
>XP_019914840.1
------------INLPR--------ICVV---GT----Q---------------
SSGKSSVLE-----S--IV-G-L-DFLPRG--------------EG-IVTRRPI---------------EFR-------------L-I-HI-
-------------------------------KEDSEI---
----------------KH-------------W--------------------------AIF---
------------------------EDDKSKKFTDFN-----------QVREHI------NNLT-DE---L-AG--------------------
---KN-------------------K-GIIDE-PIVLNIYSTS---
CPDLSLIDLPGITR--VP-------------------L-----KNSD-------------Q--------TD--D--IERLTR--------
EMAF-RYV---KD------------PRTIILAVLPANA--D-MSTSD-ALQIARKVD------
PKGLRTIGVITKIDLMD-------KGA------D-------A---------------------------SKM--LM---ND---------E-
IT----------------------L---RLGYTGVVNR---S-TAD--IKS--------

---G--------KS--------------------IA----------QSLKD---ELKYFQN--------HP-VY--------------------K-
K------LP----------PTL-YGTTSLTDKLTKVLLRHIKNF-LPDI-
>XP_012763851.2_1
------------INLPR--------ICVV--GT----Q---------------
SSGKSSVLE-----S--IV-G-M-DFLPRG--------------EG-IVTRRPI---------------EFR--------------L-I-
HI---------------------------------KEDSEI---
--------------------KY-------------W---------------------------AVF---
----------------------------ENEKNKKYTDFN-----------EVREQI------NRLT-DE---I-AG--------------------
-----KN-------------------K-GIIDE-PIVLNIYSIK--
CPDLSLIDLPGITR--VP-------------------L-----KNSD--------------Q--------TD--D--IERLTR--------
DMAL-RYV---KD------------PRTIILAVLPANA--D-MSTSD-ALQIARKVD------
PKGLRTIGVITKIDLMD-------KGA------D-------A----------------------------SKM--LL---ND---------E-
IN-----------------------L---RLGYTGVVNR--S-TAD--IKK-------
----G--------KT--------------------IS----------QALKD---ELEFFQK--------HP-VY--------------------K-
K------LP----------PSL-YGTNSLTDKLTKVLLRHIKNF-LPDI-
>EUR69800.1_1
------------INLPR--------ICVV--GT----Q---------------
SSGKSSVLE-----S--IV-G-M-DFLPRG--------------EG-IVTRRPI---------------EFR--------------L-I-
HI---------------------------------KEDSEI---
--------------------KY-------------W---------------------------AVF---
----------------------------ENEKNKKYTDFN-----------EVREQI------NRLT-DE---I-AG--------------------
-----KN-------------------K-GIIDE-PIVLNIYSIK--
CPDLSLIDLPGITR--VP-------------------L-----KNSD--------------Q--------TD--D--IERLTR--------
DMAL-RYV---KD------------PRTIILAVLPANA--D-MSTSD-ALQIARKVD------
PKGLRTIGVITKIDLMD-------KGA------D-------A----------------------------SKM--LL---ND---------E-
IN-----------------------L---RLGYTGVVNR--S-TAD--IKK-------
----G--------KT--------------------IS----------QALKD---ELEFFQK--------HP-VY--------------------K-
K------LP----------PAL-YGTNSLTDKLTKVLLRHIKNF-LPDI-
>XP_028867889.1
------------INLPR--------ICVA--GT----Q---------------
SSGKSSVLE-----S--IV-G-I-DFLPRG--------------DG-IVTRRPV---------------EFR--------------L-S-
RLT--------------------------------GKDGDADL---
------------------------RP-------------Y---------------------------IIF---
------------------------------EGNSE-KFYDFE-----------QARQHI------QELT-NE---K-AG----------------
--------VN-------------------K-GIIDD-PIVLSVFSPD---
----CPDLSLIDLPGVTR--VP-------------------L-----KNSD--------------Q--------TD--D--IEALTK-------
-DMIM-RYA---RD------------PRTIILAVVAANV--D-MSTSD-ALQLARRAD------
PLGVRTLGVITKIDLMD-------RGA------D-------A----------------------------VAM--LQ---ND---------
E-VP-----------------------L---RLGYTGVKNR--S-QKD--IAE---
--------G--------VT---------------------IK----------KALEL---ERQYFSE--------HS-VY---------------------
K-H------IK----------PSL-WGIPSLVEKLTKVLYRHISTV-LPDL-
>CAE8701890.1
------------ISLPR--------IAAI--GT----Q---------------
SSGKSSLIE-----S--IV-G-M-DFLPRG--------------GG-VVTRRPL---------------ELR--------------L-V-

HLN------------------------------TQEYPGE--
-----------------------QA-------------W---------------------------AVF---
--------------------------------DKISDKKFTDFD-----------LVRQEI------ERQT-DL---V-AG---------------
---------AN-------------------K-GIVND-PIILTVYATG---
----APDLTLIDLPGITR--VP------------------V-----KGSD--------------Q--------TE--D--VEKLTR----------
DMTM-HYV---ND------------PRTIILAVLPANQ--D-MSVSD-SLHVARMVD------
PQGHRSIGVITKIDIMD-------QGT------D-------A----------------------------ARM--LR---GE---------
D-VP-----------------------L---KLGYVGVKMR---S-QQD--
IMD-----------K--------KP--------------------VQ----------DSLKE---EKAWFES--------HR-VY------------
---------G-K------LP----------PGM-VGTPVLIDKLTQILFKHIRRF-LPEI-
>XP_001016567.2
------------IDLPR--------IAVL---GS----Q----------------
SAGKSSLLE-----Q--IV-G-L-DFLPRG--------------EG-TVTRRPL---------------EMR--------------L-F-
YVP--------------------------------KEKLS---
---------------------MP-------------Y---------------------------GVF--
-----------------------------EEIPGQKFTDFQ-----------MVKQNI------DKLT-NN---V-AG-----------------
-------AN-------------------K-GIVDK-PIVLTIYSST--
-CPDLTIVDLPGITK--IP-------------------I-----RGTD--------------Q--------TQ--D--IEKITK--------
EMAA-RYC---KD------------PKTIILCVIPANA--D-ITTSD-GLMMARQLD------
PQGSRTIGCITKIDIMD-------KGT------D-------A----------------------------RRL--LT---GE---------D-
VG-----------------------L---KLGYVGIKNR---S-QAD--INE-----
------K--------KT--------------------VL----------QSLDD---ERKFFST--------SP-IY--------------------S-
S------LP----------SSL-LGTRSLTNKLTDVLYTHIRTC-LPQI-
>XP_001032891.1
------------IRLPR--------IAVI---GS----Q----------------
SSGKSSLLE-----S--IV-G-I-DFLPRG--------------SG-VVTRRPL---------------ELR--------------L-V-
HVP------------------------------PNERQI--
----------------------KP-------------Y---------------------------AIF--
----------------------------DVDKSKKWENFD-----------QVRQQI------DFLT-DQ---V-AG----------------
--------KR-------------------K-KIIND-PIVLTIYSND---
--VIDLTIIDLPGITR--IP-------------------L-----KDSD--------------Q--------QE--D--IEKVTK--------
DMAY-SYI---KD------------ERTIILCVVPGNQ--D-ISNSD-GLQLAREVD------
REGNRTIGVVTKLDIMD-------ADT------D-------A----------------------------RKM--IM---GL---------E-
IP-----------------------L---KLGYVGVKGR--S-QKD--IND-----
------N--------KR--------------------VS----------KALDE---ERLFFAQ--------HK-VY---------------------
S-T------MD----------PKF-LGTKALTNKLSSVLFYHIRNI-LPAI-
>CAD8140652.1_1
------------IKLPR--------IVVL---GI----Q----------------
SAGKSSLLE-----H--IV-G-I-DFLPRG--------------SG-VVTRRPL---------------ELR--------------L-S-
YSP-------------------------------QSVCA--
----------------------QP-------------T---------------------------AEF---------V-------------------------------
------------------------------EEIKGKKFTNFD----------EVRKNI------EELT-DK---V-CG------------------
------SS-------------------K-NIIDK-PIILAVTGPN---
CPDLTLVDLPGITR--IP-------------------I-------MD--------------Q--------PK--D--IEQITT--------

NMAK-RYC---ED------------PSAIILCVVAANA--D-MTTSD-ALLLAKKLD------
PDGIRTVGVLTKIDIMD-------QGT------N-------A----------------------------IKM--LK---GE---------E-
VP-----------------------L---KYGYVGVKLR--S-QQE--IKD-----
------N--------VP--------------------IV----------QAVQR---EKNFFAN--------HP-VY---------------------
S-S------IP----------GDI-FGTQVLTGKLTRILYRRIRSF-LPTL-
>XP_001029982.1
------------IELPK--------IVVI--GV---Q----------------
SSGKSSLLE-----Q--IV-Q-I-DFLPRG--------------TG-VVTRCPL---------------EIR--------------L-I-
EVS------------------------------NYGKNF--
-----------------------KP-------------F---------------------------AYF--
------------------------------FEERNITYDDFE-----------EVKQRI------EIIT-KE---L-AG--------------------
----EG-------------------K-KIVDE-TITLTIFQTN--
CPTLTLIDLPGMT------------------------L-----NSIE--------------D--------QE--D--VEIVTQ--------
EMTL-KYI---QE------------ETTIILCVIPINS--D-LENSL-ALKLSRNVD------KVGSRTIGVLTMIDIMN--
-----PGT------N-------C----------------------------EKV--LR---NQ---------Q-IP-----------------------L---
KHRYYGMKPR--N-QKD--INN-----------N--------VT----------
----------VE----------LAIQN---EYEYFSN--------HE-IY---------------------K-E------F-----------ADY-
TGTAALTHKLSTLLDSHIRHF-LPDI-
>XP_001029985.2
------------IQLPK--------IVVI--GV---Q----------------
SSGKSSLLE-----Q--IV-Q-L-DFLPRG--------------TG-VVTRCPL---------------EIR--------------L-I-
EVT------------------------------NQDSDF--
-----------------------KP-------------Y---------------------------AYF--
------------------------------FEERQKIFHDFE-----------LVKKEI------QKIT-ND---F-AG------------------
------PG-------------------K-KIVDK-VITLTIYQAQ---
-CPTLTLIDLPGMT------------------------L-----NSVG--------------D--------QK--D--IERVTQ--------
DMTK-KYI---IE------------KTTIILCVIPINQ--D-LENSL-ALNLARSVD------ENGERTIGVLTMIDIMN--
-----PGT------N-------C----------------------------ENV--LK---NR---------Q-IP-----------------------L---
LHRYYGMKPR--S-QKD--INE-----------N--------VS-----------
---------VQ----------EAIQR---EQLYFTS--------HQ-IY---------------------S-M------Y-----------PEY-
TGTNALTNKLSTLLDSHIRTF-LPSI-
>XP_005788601.1
------------LDLPM--------FVVV--GA---Q----------------
SSGKSSVLD-----N--VI-G-R-SFLPRG--------------SG-TVTRRPL---------------VLM--------------M-
Q-HHA------------------------------SE--
-----------------------E--------------Y---------------------------GEF--
------------------------------NHLPGQRLTTDA-----------QICSAI------EKAT-IDE----CG-----------------
-------E--------------------K-GFSSR-PIYLHYYSPT--
--VPDLTLVDLPGLIK--AH-------------------A-------AD--------------M--------DP--S--APHIIK--------
TMVL-EFA---QA------------PAAILLAVTPAHS--D-LVTSD-AIQLAREVD------
PDGQRTLGVLTKLDLMD-------EGT------D-------C----------------------------STA--LL---GEDP------
RA-PK-----------------------L---RLGYVGVVNR--G-QKD--
INN-----------G--------VD--------------------LT----------AARAR---ERTFFKE--------HG-VYG-R--------
----------P-A------MS----------GAR-LGTEALVATSSALLVE-----------

>EFJ35472.1
------------VDLPQ--------LVHV--QR----K----------------------
MD-----------D-R-EF---S--------------------------
---E-------
-------W---------------------------GEF---
LHLPGRRFTDFS-----------HIRKEI------QAET-ERE----LG-----------------------DK-------------------
K-GVSDK-QIRLKIFSPN--VLNITLVDLPGLTR--
VP-------------------V-------GD-------------Q--------PS--D--IESRIR--------AMIL-SYI---KH-----------
-ATCIILAVSPANT--D-LSNSD-SLQMAKLVD------PDGSRTIGVVTKLDIMD-------RGT------D-------
A---------------------------RSI--LL---GT---------V-IP----------------------L---RLGYVGVVNR----------
-----------------------------------S-QEV--FRSPFFCLKILMKHG--------CR-------------------IF---------
-MPTNR---SRMLLLR--------KS-IFS-EVERCTKAFWIDV------A-S------L-----------NSR-
KSLIKYELEPVKILVQHIK--------
>OUM67143.1
------------LQLPS--------IVVI--GS----Q----------------
SSGKSSVLE-----A--IV-G-H-EFLPK----------------------PI---------------ELT-------------L-V-HTP----
--------------------------NAEE--
------------E-------------Y--------------------------GEF---
-------------------PEIGMKNITDFG-----------HIQKTL------SELN-MS-----VS------------------------ES-
------------------E-CISTD-PIELKIFSPN---
VPDLTLIDLPGYIQ--VI-------------------N-------RK-------------Q--------PP--V--LKRKIV--------
ELCD-RYI---VE------------PN-IILAISSADV--D-LANSE-ALTHSRKVD------PYGQRTIGVITKMDLVE-
-------PE------K-------G---------------------------VDL--LI---NN---------D-YP----------------------L---
ELGYVGMVCK---PPG-KSG--FSR-----------Q--------LS---------
-----------LT----------QK------SDEYFRK--------NP-IFH-QP-------------------------------------DVQ-
VGLSALKKRLTTILEENMGQN-LY---
>KNE54706.1
------------LVLPS--------IVVI--GS----Q----------------
SSGKSSVLE-----A--IV-G-R-EFLPKG--------------TN-MVTRRPL---------------ELT-------------L-I-
HTP-----------------------------DSTE--
---------------------E-------------Y--------------------------GEF---
----------------------------PQLGFGKVTNFQ-----------QIQRTL------YDLN-MA-----VQ-----------------
-------D--------------------E-CISDK-PIELRIYSPN---
--VPDLTLVDLPGYIQ--IH------------------S-------KN-------------Q--------PR--D--LKEKIA--------
DLCE-KYI---QE------------PN-VILAVCAADV--D-LANSE-ALRASRKAD------
PLGLRTIGVLTKLDLVS--------PE------M-------G---------------------------EQL--IA---HN---------D-
YP----------------------L---HLGYVGVVCG---GKN--PQA----
-------A--------LV--------------------PF----------QK------R--
-----------DLT-VGVPYLRQLLMRTLEERMGSR-LS---
>OAJ38404.1
------------LNLPS--------IVVV--GS----Q----------------
SSGKSSVLE-----A--VV-G-H-EFLPKG--------------AN-MVTRRPI---------------ELT-------------L-I-
HTP-----------------------------DSKE--
---------------------E-------------Y--------------------------SEF---

---------------------------PQLGLGKIKDFS-----------QVRRTL------TDLN-LA-----VS-------------------
----DA-------------------E-CVSEI-PIELRVYSPN---
IPDLTLVDLPGYIQ--IH-------------------T-------KD--------------Q--------PP--I--LKEKIA--------
ALCQ-KYI---QE------------PN-IILAVCAADV--D-LANSE-ALRASRKID------PLGLRTIGVITKMDLVE-
-------PQ------A-------A---------------------------VNI--LE---NK---------S-YP----------------------L---
ALGYIGVVNK---TSS--RSF-----------S--------QA-------------
-------LT----------RQ------E-SYFRS--------HP-EFN--NAM-
VGTATLRRRLVEVLEEHMGRS-LH---
>XP_006459124.1
------------LKLPS--------IVVI---GS----Q----------------
SSGKSSVLE-----A--IV-G-H-EFLPKG--------------NN-MVTRRPI---------------ELT--------------L-I-
HTPTK------------------------DGQLPT---
------------------------E--------------Y---------------------------GEF---
-------------------------------PGLGMGKITNFA-----------DIQRTL------TDLN-LA-----VP-----------------
-------AS-------------------D-CVNND-PIDLRIYSPN---
-----VPDLTLIDLPGYVQ--IS-------------------S-------LD-------------Q--------PE--S--LKEKIA--------
SLCE-KYI---RE------------PN-IILAVCAADV--D-LANSP-ALRASRKVD------PLGLRTIGVITKMDLVP-
-------PE------R-------G---------------------------AQI--LA---GN---------R-YP----------------------L---
HLGYVGVVAK--KS-PVE--PSS-----------A--------LV------------
--------TQ----------RA------EDSYFHQ--------NR-DFF------------------GNS-S------------------QLL-
VGTGTLRRRLMEVLESSMASS-LH---
>XP_011389557.1
------------LTLPS--------IVVI---GS----Q----------------
SSGKSSVLE-----A--IV-G-H-EFLPKG--------------NN-MVTRRPI---------------ELT--------------L-I-
HTTTQ------------------------PGR-PTKDT--
----------------------VVE--------------Y--------------------------AEF---
--------------------------------PGLGLGRITDFS-----------HVQKTL------YDLN-MA-----VP--------------
----------AS-------------------E-CVSDE-PIELRIHSPH---
-------VPDLTLIDLPGYVQ--IA-------------------S-------MD-------------Q--------PD--E--LREKIQ-----
---KLCH-KYI---QE------------PN-IILAVCAADV--D-LANSP-ALRASRQVD------
PLGLRTIGVVTKMDLVP--------PE------V-------G---------------------------AGI--LS---NN---------K-
YP----------------------L---ALGYVGVVCKNNLGVFQS--------------------------------SKGHDRA-TGE-
-GRM-----------S--------SL--------------------VM----------KQ------ESDYFST--------NR-EHFCAPT---
----------RGRN-A------GV----------QPM-TGTDTLRRRLMSVLEESMGSS-LH---
>XP_752563.1
------------LTLPS--------IVVI---GS----Q----------------
SSGKSSVLE-----A--IV-G-H-EFLPKG--------------TN-MVTRRPI---------------ELT--------------L-V-
NTP-------------------------------NAQS--
---------------------E--------------Y---------------------------GEF---
-----------------------------PALGLGKITDFS-----------QIQRTL------TDLN-LA-----VP--------------------
----ER-------------------D-CVSDD-PIKLTIYSPN---
VPDLSLIDLPGYIQ--VA-------------------G-------KD-------------Q--------PP--E--LKQKIA--------
DLCD-KYI---QP------------PN-VILAISAADV--D-LANST-ALRASRRVD------
PRGERTIGVITKMDLVD--------PE------R-------G---------------------------FSI--LS---DQ---------K-

YP----------------------L---RLGYVGVVSR----VPQT------------------------------TAL--FS-RGS--GNI-
----------T--------SA-------------------IL----------KN------ENAYFSA--------HP-SEF-----------------
GPQ-S------------------GVS-VGVSTLRGKLMHVLEQTMAAS-LA---
>KXN72852.1
------------LNLPS--------IVVV---GS----Q---------------
SSGKSSVLE-----A--IV-G-K-EFLPKG--------------DN-MVTRRPI---------------ELT-------------L-I-
HSP-----------------------------NLTQ--
---------------------E-------------F-------------------------GVF--
---------------------------PQLGPSKITDFN-----------LITSQL------TELN-LQ-----VS---------------------
---DK-------------------E-CISEE-PVQLHIYSPS--
VPDLTLIDLPGYIQ--IH-------------------T-------KD-------------Q--------PS--D--LKPKIR--------
ALCR-KYL---AP------------PN-LILAVCPADV--D-LANSE-ALLESRRAD------
PSGQRTLGVVTKLDLVD--------PA------L-------G---------------------------ASI--LL---NH---------D-
YP----------------------L---NLGYVGVICR--KNK-PSA--VSF----
-------S--------RD-------------------PL----------Q---------------------HP-NYQ-SP---------------------
----------------DIR-VGIQTLQSTLIRVLEQRMLKS-LH---
>NP_014854.2
------------LTLPS--------IVVI--GS----Q---------------
SSGKSSVLE-----S--IV-G-R-EFLPKG--------------SN-MVTRRPI---------------ELT-------------L-V-
NTP-----------------------------NSNN--
----------------------V-------------T-------------------------ADF--
----------------------------PSMRLYNIKDFK-----------EVKRML------MELN-MA-----VP-----------------
-------TS-------------------E-AVSEE-PIQLTIKSSR---
-VPDLSLVDLPGYIQ--VE-------------------A-------AD-------------Q--------PI--E--LKTKIR--------
DLCE-KYL---TA------------PN-IILAISAADV--D-LANSS-ALKASKAAD------PKGLRTIGVITKLDLVD--
------PE------K-------A---------------------------RSI--LN---NK---------K-YP----------------------L---
SMGYVGVITK----TPSSINR-----KHLG------LFGEAPSSSLSGIFSKG-QHG-QSS--GEE-----------N--
------TN-------------------GL----------KQIVSHQFEKAYFKE--------NK-KYF-----------------------------
-T----------NCQ-VSTKKLREKLIKILEISMSNA-LE---
>PSC76263.1
RLLGGIESG---IDLPK--------IAVV--GD----Q---------------
SSGKSSVIE-----S--IF-G-I-SL-PRG--------------SG-IVTRTPI---------------QVE--------------H---RYT-
----------------------------EG--
---------------------Q-------AY-------------------------AEL----------S--------------YKT-S-P--------------
DD----------------------EEWTKRVVEDLD-----------GIEQAV------EEAT-DA---V-TG-------------------
-----TT-------------------K-G---
LLDLPGIMR--IA-------------------V-------DD-------------Q--------PK--N--IEEIVE--------NQIR-RHI-
--EG------------DNVVILCVLHGTS--D-PSTAS-AIKLAQEYD------EDGDQTMGVVTKPDRCE-------
KAQ------V-----EDL---------------------------IGS--VL---GAGS-------S-IK----------------------L---
KLGFIPVRNR---T-TSE--LTD-----------G--------TS---------------
------LD----------QVREN---EAAFFKC--------HP-LL--------------------S-Q------L--------A--KDR-
RGIPALVDQLAHVQMQRVHKA-LPELK
>PRW56740.1

---SGMPSAKRSKDMYR--------VVVV---GD----Q----------------
SSGKSSLIE-----S--MF-S-F-EL-PRG--------------QD-IVTRTPI---------------QVK--------------H---
RFE-------------------------------KG---
----------------------------P-------PR---------------------------AEI----------S---------------FR--K-P---------
-----GE------------------------SDLTVVKLDSLD-----------EIEEAV------IQAT-DI---V-AG------------------
------EG--------------------K-DIVDS-TIFLRVFSDA---
--LPDLTLTDLPGITR--NA-------------------T-------QG--------------Q--------PE--N--IEEIVT--------
SVIE-NHI---KG------------EMVVILCVVPANV--D-FSTAG-GIKLARRHD------
ADGVRTMGVVTKIDLTE-------SGQ-------------EM-----------------------------LDR--LS---GAGK-------
N-LK------------------------L---KLGFIPVRNR---T-AQE--LKD---
--------G--------TP--------------------LE----------KVCKA---EGDFFSS--------HP-QL--------------------
-S-K------V--------N--PDR-RGISALVTELARLQMQRVTKS-LPGVK
>XP_005849062.1
------------LQIPK--------IAVV---GD----Q---------------
SSGKSSLIE-----S--IF-G-I-SL-PRG--------------ED-IVTRTPI---------------QVE--------------H---RYS-
------------------------------DG---
---------------------E-------AY---------------------------AEL----------S---------------FRS-S-P--------------
DS------------------------EELIKKRIADLS-----------TIDDEV------REAT-RR---V-AG-----------------------
-SG-------------------K-GVVDS-PIYLRVYTNK---
LPDLTVLDLPGITR--NA-------------------V-------EG--------------Q--------PE--D--IEEIIN--------
TMIE-SHI---AG------------ETTVVLAIVQANV--D-FSTAA-AIKLAKKFD-----------------------------------
--PNRDR------
---T-M--

>GHP04420.1
---------QDAIAIPQ--------IAVM---GD----Q----------------
SSGKSSVLE-----A--IS-G-V-PF-PRG--------------KG-LVTKCAT---------------QVI--------------M---
RTA-------------------------------PK--
-----------------GSP--------------WS---------------------------AVT----------S---------------VRW-A-
DGTT--------EHDQQ------------------------PEEAG-VIASPE-----------EVAGVI------ERLT-QV---L-LK--
----------------------KS---------------GH-QK-SFSEH-SIIIKLSSPE--
-------------------HPNLSMVDLPGLVR--TV-------------------T-------ED--------------Q--------DD--R--
DIETVS--------ELIT-RFM---KQ------------ERTIILGVIPVNA--D-IATSE-VLQRAKHYD------
PSGMRTLAVLTEPDLVD-------PGS------E-----NEM-----------------------------IEV--LM---NR---------
R-VN------------------------L---MLGFCMVKLR---G-QKE--
LDE-----------CEGKDVNSTEL--------------------TR----------RAREA---EEKFFRE--------NE-AL-------
--------------A-R------LG-------DEVDGQ-LGIPNLITRLSDTLSNRIREQ-FPTIK
>OSX73843.1
------------LPIPQ--------LSVM---GD----Q----------------
SSGKSSVLE-----F--LS-G-V-PF-PRG--------------TG-LVTRCAT---------------QLT--------------M---
KRV-------------------------------Q---
-----------------GKS--------------FC---------------------------GKV----------E---------------VLW-G-E-----
---------PH------------------------QPAASGPIYSKV-----------EVAAKI------EQLT-AV---L-IG--------------
----------D--------------------G-EFSE-ATIVVKLEAAD---

------LPDLTIIDLPGLVR--TT-------------------T-------AG--------------Q--------SK--S--IIAQVD--------
GLVQ-RYL---ES-------------ERTIVLCVVPANV--D-IATSD-ILERAHKVD------
PMGDRTVGVLTKLDLVG-------RGS------E-----DEV---------------------------VQV--LE---NV---------
R-KP-----------------------L---QLGYVGIKCR--S-QAG--LKD--
---------N--------QS--------------------LE----------DARTD---ETTFFAT--------HP-VF--------------------
-K-R------L--------P--ADL-VGVETLTTKLTRVLVSRIREA-VPAM-
>OSX70108.1
------------MPIPQ--------LSVM--GD----Q----------------
SSGKSSVLE-----F--LS-G-V-PF-PRG--------------TG-LVTRCAT---------------QLS--------------M---
KRV------------------------------L--
----------------GQS--------------FR--------------------------GSA----------K---------------VRW-G-E-----
---------AH-----------------------QPDASGPIRSKA-----------EVADKI------EQLT-AA---L-VG-----------
-------------D---------------------G-EFSDGGTIVVELEAAD--
-----------LPDLTIIDLPGLVR--TT-------------------T-------AG--------------Q--------SK--S--IIAQVD----
----GLVQ-RYL---ES-------------ERTIVLCVVPANV--D-IATSD-ILERAHKVD------
PMGDRTVGVLTKLDLVG-------RGS------E-----DEV---------------------------VQV--LE---NV---------
R-KP-----------------------L---QLGYVGIKCR--S-QAG--LKD--
---------N--------QS--------------------LE----------DARHE---ESVFFRT--------HP-VF--------------------
--R-Q------L--------S--PDV-HGVETLSKKLTRVLVSRIRAA-TPTM-
>KAJ1442373.1
--------------MPQ--------ICVM---GD----Q----------------
SSGKSSVLE-----A--LS-G-I-PF-PRG--------------AG-LVTRCPL---------------RMV--------------M---
RRA------------------------------RE--
------------------GET--------------WS--------------------------AEV----------S---------------TTV-T-P------
----------------------------------ESVQKAKDVV-----------HLSQLM------NRAM-HT---L-CA------------
------------DD--------------------V-SFSTE-SVVINLVSPD---
---------ACDLTVVDLPGIIR--TV-------------------T-------VG--------------Q--------DV--Q--AIEQVN---
-----RLIK-SCL---AD-------------ERTVILAVIPANQ--D-IATVD-ILERAEGVD------
PTGERTIGVLTKTDLIG-------QGG------E-----DEI---------------------------IEV--VN---NR---------R-
KP-----------------------L---ALGYTMVKNR--S-QKD--IKD-----
------N--------IS--------------------SA----------KAREN---EENFFST--------HP-VF---------------------
R-N------C--------N--ETL-YGADQLSKKLTDVLVA-----------
>CAH0370685.1
-----------DLPLPQ--------IAVM--GD----Q----------------
SAGKSSVLE-----A--IS-G-V-PF-PRG--------------TG-LVTRCAT---------------QLI--------------M---
SRA------------------------------PP---
-----------------GAA--------------WT--------------------------ASA----------C---------------VE------------
-------PN-----------------------DGAAPIKLEKAE-----------DVADVI------EKLT-AK---L-CE--------------
----------RE------------------GG-AFSTTAAVVIKLRSPD---
--------VPDLTLLDLPGIVR--TA-------------------V-------AG--------------Q--------SQ--S--VIGDVN----
----SLIE-TYL---KQ-------------ERTVVLAVIPANQ--D-VATID-ILQKAKGAD------
PEGARTIAVLTKPDLID-------QGA------E-----KEV---------------------------LET--LL---NK---------R-
AP-----------------------L---KLGYAMVRCR--N-QRE--NEA---

--------Q--------IS--------------------MT----------TARER---EKAFFDA--------HA-FW-------------------
-G-S------DALHVPREQR--YTL-LGVDALTGRLSSLLVT-----------
>KAH8053135.1
---RARETPPVCRPLPQ--------IAVM---GD----Q----------------
SAGKSSVLE-----M--IS-G-V-PF-PRG--------------SG-LVTRCAT---------------QLI-------------M---
KRT-----------------------------AP--
-----------------GSA--------------WA-------------------------ATA----------S--------------VSW-S-R-----
---------PQ-----------------------PAESG-PVADPA-----------ALSVAI------ERLT-EA---L-CA-------------
-----------VT------------------TN-GFSTE-TIVINLSAPD---
------VPDLTLLDLPGIVR--TA-------------------V-------AG-------------Q--------NA--D--VVGDVN-----
---GLIT-SYL---EQ------------ERTIVLAVMPANQ--D-IATID-ILERAAVAD------
PGGMRTLAVLTKPDLVD-------DGA------E-----DEV---------------------------KEC--LM---NR--------
-R-KP----------------------L---RLGFAMVKCR---T-QRE--
LDG-----------R--------IS--------------------LS----------KALVV---EREFFEA--------HP-FW------------
--------S-A------AV---HADER--KKL-MGVQALTGRLSSLLVERIKVA-LPEI-
>KAJ8603921.1
-----------DLPLPQ--------IAVM--GD----Q----------------
SSGKSSVLE-----M--IS-G-V-PF-PRG--------------SG-LVTRCAT---------------QLI-------------M---
KRT-----------------------------PP---
-----------------GSE--------------WT-------------------------ARA----------S--------------VSW-Q-G----
----------PQ-----------------------PKAAG-NVKDQA-----------SLCAAI------EQLT-QV---L-CS----------
--------------ST------------------MN-GFSRD-SIVIQLSSPD---
------------VPDLTLLDLPGIVR--TA-------------------V-------SG-------------Q--------SA--S--
VVEDVN--------GLIE-AYL---SQ------------ERTIVLAVMPANQ--D-IATID-ILERAARAD------
PAGERTLAVLTKPDLVD-------AGA------E-----EEV---------------------------KAV--LA---NK---------R-
KP----------------------L---RLGFAMVKCR---S-QRE--LDA----
-------K--------ST--------------------LA----------DALRS---EADFFRR--------HP-HW-------------------
-S-N------E--------D--PKK-LGVRALTMRLSTLLVD-----------
>KAJ1460259.1
------------LPLPQ--------IAVM--GD----Q----------------
SAGKSSVLE-----L--IS-G-V-PF-PRG--------------SG-LVTRCAT---------------QLV-------------M---
KRT-----------------------------AP---
-----------------GTV--------------WS-------------------------ATA----------S--------------VSW-P-G-----
---------PQ-----------------------PPAAG-IVKDRE-----------SLGRAI------EQLT-QV---L-CS------------
------------GS------------------SN-SFSTH-SIVIELRSPD---
---------VPDLTLLDLPGIVR--TN-------------------V-------AG-------------Q--------DA--S--VVKDVN--
------TLID-SYL---RQ------------ERTIVLAVMPANQ--D-VATID-ILERAAAAD------
PAGQRTLAVLTKPDLVD-------EGA------E-----DEV---------------------------ASV--LS---NR---------
R-KP----------------------L---RLGYAMVRCR---T-QRQ--LDS-
----------N--------IS--------------------LT----------DALKA---EAAFFES--------HA-FW-------------------
--G-K------AQ---GQEWQ--PQQ-LGVVALTRRLSSLLVDRIKVA-LPSI-
>KAG5178451.1
-----------TPLPQ--------IAVM--GD----Q----------------
SSGKSSVLE-----A--LS-G-I-SF-PRG--------------SG-LVTRCPV---------------QLV-------------M---

KRA------------------------------RA--
------------------GEA--------------WR--------------------------ASA----------R---------------VAW-S-
NSA-----------RAQ-----------------------PPAAG-AVASPE-----------ALADVI------AQLM-AA---A-CD-
-----------------------AT------------------AN-GFSTD-SLVVEVRAPE---------------------------------------
---------------------CPDLTLVDLPGIVR--TA-------------------V-------AG--------------Q--------SD--A--
VIPEVS--------ALID-AYL---RQ------------ERTIILAIVPANQ--D-VATVD-ILERAARVD------
PSGERTIGVLTKPDLIG-------PGN------E-----DEV---------------------------IAV--LS---NR---------R-
KP-----------------------L---RLGYVAVRCR--T-QRE--LAA-----
------G--------VT--------------------LR----------AAHAV---ERAFFAG--------HP-AF-----------------------
A-A------L--------D--PAL-LGIESLTRRCVGVLTTRIRAA-LPFM-
>CBN76986.1
------------LSLPQ--------IAVM--GD----Q----------------
SCGKSSVLE-----A--LS-G-V-QF-PRG--------------SG-LVTRCPV---------------QLI--------------M---
KRT------------------------------KP--
-----------------GDG--------------WH--------------------------GKT----------S---------------VEW-K-RG-
------------PQ-----------------------PPAAG-HVASPE-----------ALVGVI------EELM-NA---V-CE--------
----------------GQ------------------KN-GFSSD-FIAIEIKSPD---
-------------CPDLTLIDLPGIVR--TA-------------------V-------SG--------------Q--------SQ--G--
VITEVN--------GLIE-NYL---RS------------ERTIILAIVPANQ--D-VATVD-ILERAKKVD------
PSGKRTIGVLTKPDLIN-------PGG------E-----AEV---------------------------LEV--LS---NN---------R-
KP-----------------------L---KLGYVMVKNR--N-QLQ--LRE----
-------G--------VS--------------------LK----------EAHDA---EMDFFKS--------HA-VF--------------------
-K-T------V--------D--STC-LGVESLTKRLVSLLTVRIKDA-LPNM-
>KAG5185531.1
------------IALPQ--------IAVM--GD----Q----------------
SSGKSSVLE-----A--IS-G-V-PF-PRG--------------GG-LVTRCAT---------------QLI--------------M---
KSA------------------------------PE--
-----------------GAE--------------WA--------------------------ARA----------S---------------VTG-P-R-----
---------KN-----------------------LKGCGPVQGGPG-----------ALTRVI------ADLQ-NA---L-TS----------
--------------GN------------------AS-AFSSE-SIVIEVRAPG---
-----------LPDLTLIDLPGIVR--TA-------------------T-------SG--------------Q--------SA--G--VMAEVN-
-------TMVE-RYL---IQ------------ERTIILAVVPANQ--D-VATVD-ILERAKRVD------
PEGDRTIGVLTKPDLIG-------PGN------E-----DEV---------------------------VAV--LR---NI---------R-
KP-----------------------L---KLGYVMLKCR--S-QAD--INA-----
------G--------MD--------------------NK----------AALAA---EHKFFRE--------HP-AF---------------------
S-G------L--------P--GTI-FGVQCLTGRLTDLLVKRIGAA-LPAI-
>XP_009038401.1
------------LPLPQ--------IAVM--GD----Q----------------
SSGKSSVLE-----A--LS-G-V-PF-PRG--------------TG-LVTRCPC---------------QLT--------------M---
KRT------------------------------AD--
-----------------GAP--------------RG--------------------------GRV----------P----------------RR-P-G------
------------------------------------NERPSRAPS-----------ELTAAI------ERVT-SA---L-TK------------------
------GS------------------AS-GFSTD-SIVVTVNAPS---
----VPDLTIIDLPGIVR--TA-------------------T-------QG--------------Q--------DP--R--VIADVN--------

SMVE-FYL---KQ------------ERTIVLAIVPSNQ--D-VATVD-ILERALTVD------PTGERTIGVLTKPDLIG-
------EGA------E-----DEV---------------------------VAV--LK---NE---------R-KP----------------------L-
--KLGYIMVKNR--S-AMQ--LKQ-----------GA------INS--------
------------PA----------ANERA---EREFFVQ--------H---

>KAJ1460532.1
------------LALPQ--------IAVM---GD----Q---------------
SSGKSSVLE-----A--LS-G-V-PF-PRG--------------TG-LVTRCPC---------------QLT--------------M---
KHT-----------------------------AP--
-----------------GTP--------------WR-------------------------GEI----------G---------------VMR-S-G-----
---------VF----------------------SKIGELAS--PA-----------ELTAAI------ERVT-TS---L-TE-----------------
-------KS------------------RS-GFSRD-SIVVSVSAPS---
-----VPDLTIIDLPGIVR--TV-------------------T-------AG--------------Q--------DS--N--VITEVN--------
DMVE-SYL---MQ------------DRTIVLAIVPANQ--D-VATVD-ILERALRVD------
PTGERTIGVLTKPDLVG-------EGA------E-----DEV---------------------------LAV--LR---NE---------R-
KP----------------------L---KLGYVMVKNR--S-AKE--LKS-----
------GE------KTG-------------------LR----------EQAEA---ERRFFRT--------HA-AY---------------------
S-A------V--------D--ASL-LGISNLTGKLTKLLTRRIEAV-LPEL-
>CBN78192.1
------------ISVPQ--------ICVM---GD----Q---------------
SSGKSSVLE-----A--LA-G-V-PF-PRG--------------SG-LVTRCPI---------------RLS--------------M---
KRS------------------------------AT--
-----------------GSR--------------WS-------------------------AVA----------H---------------ASN-E-P-----
-------------------------------------SKSHSASTPG-----------QLTKLL------ERLT-DG---L-TS---------------
---------TS-------------------S-NFSTE-TINVRLSSPD---
-----VPDLTVVDLPGIIR--TS-------------------T-------AG--------------Q--------DP--A--VIAQVN--------
NLIE-SFL---EQ------------ARTIILCVIPANQ--D-IATVD-ILERAQKVD------PRGERTIGVLTKPDLIG--
-----PGN------E-----DEV---------------------------LAV--LH---NV---------R-KP----------------------L-
--QLGYVMVKNR--S-QAQ--LKA-----------G--------LT---------
-----------GT----------EAREA---EEAFFRQ--------HS-HF---------------------K-G------C--------D--
PKL-FGVANLTTRLTQLLVTRIQHE-LVPM-
>KAG5184845.1
------------ISVPQ--------ICVM---GD----Q---------------
SSGKSSVLE-----S--LS-G-I-PF-PRG--------------SG-LVTRCPI---------------RLS--------------M---
KRT-----------------------------AA--
-----------------GSA--------------WI-------------------------AHA----------H---------------TSS-S-P------
-----------------------------------GDVKRAETPA-----------HLTALM------EALT-DT---L-T------------------
-----------------------------S-NFSTE-SINVKVTAPG---
--VPDLTVVDLPGIIR--TS-------------------T-------AG--------------Q--------DP--R--MIQQVN--------
ALID-SFL---MQ------------ERTIILCVIPANQ--D-IATVD-ILERALKVD------PSGERTIGVLTKPDLIG--
-----PGN------E-----DEV---------------------------MAV--LL---NV---------R-KP----------------------L-
--QLGYTMLRNR--T-QKE--LAA-----------G--------AS---------
-----------AA----------EAKRL---EQEFFKA--------HP-HF---------------------K-S------C--------D--
PKL-FGIDNLTSRLTQLLVTRIQAE-LVPM-

>XP_009039543.1
------------LSVPQ--------ICVM--GD----Q----------------
SCGKSSVLE-----A--LS-G-V-PF-PRG--------------SG-LVTRCPV---------------RLI--------------M---
RRS---
----------------DGD-------------WS--------------------------ATA----------S---------------TTL-S----------
---------------------------------SHTVKANSPA-----------ELTAII------SRLT-DT---L-TK---------------------
---NS-------------------H-GFSTE-SIVVRLGSSE--
SPDLTVVDLPGIVR--TA-------------------T-------SG---------------Q--------DP--K--VIYEVN--------
ELID-TYL---RQ------------ERTIILAVIPSNQ--D-IATID-ILERAQTVD------PGGERTIGVLTKPDLIG----
---PGN------E-----EEV----------------------------MAV--LN---NL---------R-KP-----------------------L---
RLGYIMVKNR--S-QAQ--VKQ-----------G--------IS------------
--------HK----------SALDN---EELFFQN--------HK-AF---------------------S-C------L--------D--KRF-
WGIKNLTSALTNLLVVRIQEQ-LAPM-
>KAJ8614481.1
-----------TLAVPQ--------ICVM--GD----Q----------------
SSGKSSVLE-----A--LS-G-V-PF-PRG--------------SG-LVTRCPC---------------RMV---------------M---
RRA------------------------------AP---
------------------DAP-------------WA--------------------------AVA----------S---------------TTT-S-G-----
------------------------------------SSSITAESPE----------ELTSII------SRLT-ES---L-TR-------------------
-----NT-------------------H-GFSTE-SIIVRLTSPL--
SPDLTVVDLPGIVR--TA-------------------T-------VG---------------Q--------NV--A--VIEEVN--------
SLID-DYL---KQ------------ERTIILAVIPANQ--D-IATVD-ILERAQHVD------PDGERTLGVLTKPDLIG-
------PGN------E-----EEV----------------------------VAV--LS---NV---------R-KP-----------------------L-
--KLGYVMVKNR--S-QAQ--IKQ-----------G--------TS---------
-----------HE----------AARAE---ELRFFDS--------HP-VF---------------------S-R------L--------D--
KRL-LGISNLTTSLTKLLVR-----------
>XP_009039855.1
------------LSVPQ--------ICVM--GD----Q----------------
SSGKSSVLE-----A--LS-G-V-PF-PRG--------------SG-LVTRCPV---------------RLV--------------M---
RRA------------------------------AA---
------------------GMQ--------------WS--------------------------ALV----------S---------------TTS-S-------
------------------------------------AQTVAAETPT-----------ELAELI------SRLT-ET---L-TR------------------
------NT-------------------Q-GFSSE-AIIVRLSSPE--
--SPELTVVDLPGIVR--TA-------------------T-------VG--------------Q--------GL--Q--VIAEVD--------
KLID-EYL---RQ------------DRTIILAVIPANQ--D-IATVD-ILERARLVD------PTGERTLGVLTKPDLIG---
----PGN------E-----EEV----------------------------AAV--LK---NI---------R-KP-----------------------L---
KLGYVMVKNR--S-QAQ--IRN-----------G--------TS-----------
---------PE----------AAKED---EEAFFVS--------HP-IF--------------------S-C------L--------D--QRL-
LGIKNLTYALTKILVSRIKEE-LAPM-
>KAJ1428896.1
------------LPIPQ--------IVVF--GD----Q----------------
SSGKSSLLE-----S--IS-G-I-PF-PKG--------------TG-LVTRCPT---------------RIS--------------M---
SNC------------------------------GP---
------------------TEP--------------WT--------------------------AHV----------K---------------LSP-N-IP-----

--------ES-----------------------EAFNKRPVKDVE-----------DLSKRL------AEAA-AI---I-TP--------------
----------HS------------------NS-EFSSE-VIMVHVQSPN---
---------SPNLSLIDLPGIIR--TT------------------T-------AG--------------Q--------DR--S--VIASVN-----
---ELVE-SYL---RQ------------PETVILAVIPSNQ--D-IATID-ILERANEYD------
PAGLRTIGVLTKPDLID-------RGA------E-----DEV---------------------------LKI--VQ---NV---------R-
KP----------------------L---KLGYVMCKNR---S-QAE--LKS----
-------N--------VS--------------------LA----------QSQKN---EDSYFLS--------HE-VW-------------------
--Q-K------I--------V--PSS-RGIKPLCERLTSIIVSRALDR-GPFL-
>KAG5183739.1
------------VAIPQ--------IAVV---GD----Q---------------
SSGKSSVLE-----A--IS-G-I-PF-PRG--------------SG-LVTRCAT---------------ELR--------------M---
QSA--
-----------------ER--------------FL--------------------------ARA----------Y--------------TTA-A-R---------
--------------------------------DAVATLTTVA-----------AVECAI------AQLT-EE---L-CS--------------------
----E--------------------Q-GFSAE-SIVIQLAAPN---
IPNLTVIDLPGIVR--TT------------------T-------AG--------------Q--------SR--S--VMQQVD--------
GLLR-SYM---KQ------------PRTIMLVLVPSNQ--D-VATVG-GLELAAEHD------
PEGVRTMGVLTKPDLIN-------PGA------E-----REA---------------------------VAV--LL---NE---------T-
KP----------------------L---KLGYVMVKNR---S-HRD--IAN----
-------D--------MS--------------------LA----------NAHAA---EVAYFDE--------HC-LF--------------------
-G-A------L--------D--RAL-FTVANLKQRLSALLVARIRAE-LPRM-
>KAK3283006.1
----------QDIPIPQ--------IAVM--GD----Q---------------
SSGKSSVLE-----A--LS-G-V-HF-PRG--------------TG-LVTKCAV---------------EVR--------------M---
KRL------------------------------QP--
------------------NED--------------WN--------------------------ASV----------S---------------LSW-D-R---
-----------PQ-----------------------PSEAG-VATTPN-----------EVGEKI------SKLT-EV---L-LR------------
------------AR---------------GN-RA-TFEPEHRIQVELKSPD--
------------VSDLTIIDLPGIVR--TN------------------V-------AG--------------Q--------CK--K--VIAEVD--
------ALLD-KYL---RQ------------ERTIILAVIPSTV--D-IATVD-VIERAEKVD------
PHGLRTIGVLTKADQIS-------SDD------E-----AER---------------------------VAV--LR---GV---------R-
KP----------------------L---KLGYFMVKNR---T-QTE--LEA------
-----G--------VT--------------------LA----------EARLA---EARYFSA--------HK-TF--------------------G-
K------L--------H--PGL-FGSQNLAERLSDVLATRIRDD-LPLLV
>KAJ1474099.1
----------LEGGIPQ--------IAVM--GD----Q---------------
SSGKSSVLE-----A--LT-G-V-EF-PRG--------------PG-LVTKCAT---------------EVR--------------M---
RCC------------------------------KN---
------------------GEP--------------AS--------------------------FKV----------S---------------LSW-S-K-----
---------AQ-----------------------PAEAG--FCSSE-----------EIGARI------ASLT-ER---L-LS--------------
----------DRSE------------HGK-KA-SFEKEHAIVVEMVSPD---
-------------VPDLTIIDLPGIVR--TA------------------V-------AG--------------Q--------NA--S--
VVEDVR--------SLLD-RYL---KL------------NRTIILAVVPCNV--D-IATVD-IIELANNAD------
PLGQRTIGVLTKPDCIA-------QGE------E-----KDV---------------------------IET--LL---NK---------T-

KP----------------------F---KLGYIMMKNR--S-EQD--IKA-----
------G--------VS-------------------LR----------SPLEL---E--

>KAJ1495567.1
----------LEGGIPQ--------IAVM---GD----Q---------------
SSGKSSVLE-----A--LS-G-V-QF-PRG--------------PG-LVTKCAT---------------EVR--------------M---
RCC-----------------------------KD---
------------------GQP--------------AT--------------------------FKV----------S---------------LSW-S-K-----
---------PQ-----------------------PAEAG--FCSRE-----------EIGDKI------TSLT-ER---L-LA--------------
----------DRTT------------RGE-SA-SFEKEHAIVVEMQALD---
-------------VPDLTIIDLPGIVR--TA-------------------V-------EG--------------Q--------NA--S--VIEDVR-
-------NLLN-RYL---QQ------------DRTVILAVVPCNV--D-IATVD-IIEMARKAD------
PQGQRTIGVLTKPDLID-------EGA------E-----KDV-----------------------------VET--LK---NA---------K-
KP----------------------L---ELGYIMVKNR--S-PKQ--IED------
-----G--------VS-------------------LS----------DARRK---EREYFDS--------HP-VF---------------------
G-K------E--------D--AAL-FGVDRLRMRLTEILVERIKFS-LPEL-
>KAJ1474882.1
----------LVGGIPQ--------IAVM---GD----Q---------------
SSGKSSVLE-----A--LS-G-V-QF-PRG--------------PG-LVTKCAT---------------EVR--------------M---
RSC-----------------------------KH---
------------------GQA--------------AS--------------------------FKV----------S---------------LSW-S-K----
----------AQ-----------------------PEEAG--PVLRE-----------EIGERI------ARLT-TR---L-LA--------------
----------ER---------------GE-KA-SFEKEHAIVVDMRAPD--
-----------VPDLTIIDLPGIVR--TA-------------------V-------DG--------------Q--------ES--S--VKDDVW-
-------SLLD-RYL---KQ-------------ERTIILAVVPSNV--D-IATVD-IIDKARQAD------
PQGQRTIGVLTKPDMIA-------EGE------E-----LDV-----------------------------VDT--LR---NR---------
K-AP----------------------L---ELGYIMVKNR--S-PKE--IEA----
-------G--------VS-------------------LS----------EARRT---EAAYFDA--------HP-VF---------------------
G-R------E--------DLPRAL-FGVERLRERLTDILVERIK--------
>KAJ1487321.1
----------LQGGIPQ--------IAVM---GD----Q---------------
SSGKSSVLE-----A--LS-G-V-QF-PRG--------------TG-LVTKCAT---------------EVR--------------M---
RSC-----------------------------KN---
------------------SEL--------------SS-------------------------YRV----------S---------------LSW-S-K------
--------PQ-----------------------PEEAG-TVVTRE-----------DIEGKI------ALLT-EL---L-LA----------------
--------DR---------------GG-AS-ALNQ----VVEMVAPD--
--------IPDLTIIDLPGMVR--TA-------------------V-------DG--------------Q--------DE--S--VIQDVK-----
---SLLD-RYL---KQ------------ERTVILAVVPCNV--D-IATVG-IINDARKAD------
PQGQRTIGVLTKPDLIA-------RGE------E-----ESV-----------------------------VET--LR---NV---------K-
AP----------------------L---ELGYIMVKNR--G-QDE--ING----
-------K--------VA-------------------L--
---------------------RFPTVLL-------------
>XP_005711749.1

------------IAIPQ--------VAVI--GD----Q----------------
SSGKSSVLE-----A--IC-S-V-PL-PRG--------------AG-LTTRCAI---------------ELR-------------L---
SDV------------------------------HP---
------------------DDLHLPPDTPRPERDMFWV--------------------------GSI----------F---------------
TSV-D-S--GQVPIEKKD-----------DLEQAI------AAKA-KS---L-
TN------------------------SR----------------H-AG-GFSNE-RVIVQIAASG---------------------------------
---------------------------SPNLTIIDLPGIIR--TK-------------------T-------FG--------------Q--------NS--
N--AIREVT--------NLIR-GYI---SQ------------ERTVMLVVVPATQ--D-VATIE-ALEWAARSD------
PSGERTIGVITKPDLID-------DGA------E-----EEV----------------------------GVV--LS---NK---------R-
KP-----------------------L---KLGYVMVKNR--S-QRE--VEE----
-------H--------VS--------------------VR----------EARKS---EAEFFAQ--------HR-VF-----------------------
S-T------M--------S--KDL-FGVEKLVRKLTDVLVSRVYNA-LPGM-
>KAI0564035.1
------------IAIPQ--------VAVI--GD----Q----------------
SSGKSSVLE-----A--IC-S-V-PL-PRG--------------AG-LTTRCAI---------------ELR-------------L---
SST------------------------------HP---
-----------------EDLALPPDSEPPEREEFWV--------------------------ASI----------S---------------TSL-
N-P--KQVPLEGKA-----------ELEDAI------AARA-AS---L-VD-----
-------------------TR----------------Q-HG-GFSKE-RVIVHIAATG---
-----------------SPNLTIIDLPGIIR--TK-------------------T-------FGDFMPFIPNPFGRDSQ--------DR--
N--AIRQVS--------ELIQ-GYA---AQ------------ERTIMLVVVPATQ--D-VATIE-ALEWAARYD------
HAGRRTIGVITKPDLVD-------KGA------E-----KEV----------------------------ASV--LM---NR---------
R-KP-----------------------L---KLGYVMVKNR--S-QIE--VEE---
--------Q--------VS--------------------VQ----------QARKN---EYDFFAT--------HH-VF--------------------
--S-K------M--------S--RSY-FGVENLVKKVTRVLVARVHDE-VPKM-
>PXF49978.1
------------IAIPQ--------VAVI--GD----Q----------------
SSGKSSVLE-----A--IC-S-V-PL-PRG--------------AG-LTTRCAI---------------ELR-------------L---
TNT------------------------------HP---
-----------------EDAALPSDTEPPKREPFWL--------------------------ASI----------S---------------TSL-
N-P--KQVPLSGKA-----------ELEDAI------AAHA-AS---L-TD-----
-------------------PR----------------Q-NS-GFSKE-RVIVHIAATG---
------------------SPNLTIIDLPGIIR--TK-------------------T-------FGDFFPFIP-PFG-DSQ--------DR--N-
-AIRQVS--------QLIQ-GYA---AQ------------ERTILLVVIPATQ--D-VATIE-ALEWAARYD------
PSGRRTIGVITKPDLID-------KGA------E-----REV----------------------------ASV--LN---NR---------R-
KP-----------------------L---KLGYIMVKNR--S-QKE--VEE-----
------H--------VS--------------------VQ----------QAREN---EYEFFSN--------HP-VF---------------------
S-K------M--------S--RSY-FGVDNLVKKVTGVLVSRLHDE-LPKM-
>XP_006815062.1
------------VDLPA--------VVVI--GD----Q----------------
SVGKSSVLE-----A--IS-G-V-QL-PRG--------------NE-IVTRCPI---------------ELR-------------L---
KTL------------------------------DN---
-------------------DE--------------WC--------------------------GKI----------L---------------YIN-Y-S-------
-------KE-------------------------QVNKYIDSPD-----------ELGAAI------RTAQ-QD---I-TN---------------

---------SQ-------------------K-GISKT-SITVEIQSAH---
----VPNLTLIDLPGIAR--VP-------------------Q-------EG-------------Q--------SR--N--IADETK--------
DLIK-KYI---SK------------DDAIVLCVIPCNV--D-IATTE-AIKMAQEVD------PTGSRTLGVLTKPDLVD-
------KGS------E-----NVV---------------------------VRI--AE---NK---------V-IN----------------------L--
-KKGYTIMKCR---S-QRN--LED-----------A--------MS----------
----------LE----------EAMDE---EERFFRE--------HK-HY---------------------S-V------L-----------SGQ-
AGSRLLAHRLTTELVEQILKS-V----
>XP_035690836.1
------------VSLPS--------VVVI---GD----Q----------------
SAGKSSTLE-----A--IS-G-V-QL-PRG--------------SG-IVTRCPL---------------ELR--------------L---
KKS------------------------------QKK---
------------------DAP--------------WK-------------------------GCI----------R---------------YV----K------
--------NK------------------------KDVRFDVDEPG-----------NVGDAV------KKAQ-ND---L-AG---------
---------------TT-------------------N-GISDS-LITLDVESPD--
-----------IPDLTLIDLPGIAR--IA-------------------A-------EG-------------Q--------PT--D--IGQQIK----
----DLIS-KYI---QK------------KDTIILAVVPCNV--D-IATTE-ALQMAQEVD------
ADGSRTLGVLTKPDLID-------PGT------E-----RGV---------------------------LQI--LN---NE---------K-
YK----------------------L---RKGYTIIKCR---G-QMD--IEK-------
----G--------MS--------------------LE----------EAMDK---EQSYFKS--------HE-HF---------------------
K-S------VY-------K--EKK-AGVRTLAGRLSTELVGQIKNS-I----
>XP_019617847.1
------------VTLPS--------VVVI---GD----Q----------------
SAGKSSCLE-----A--IS-G-V-QL-PRG--------------SG-IVTRCPL---------------ELR--------------L---
KKS------------------------------PDP---
------------------ESG--------------WR-------------------------GYI----------H---------------FE------------
-------DK------------------------GETRWELDSPE-----------DVGEAV------KKAQ-NQ---L-AG-----------
-------------ES-------------------L-CISPR-LITLDVESPD---
--------IPDLTLIDLPGIAR--VP-------------------V-------GG-------------Q--------PD--D--IGDQTK-----
---ALIR-EYI---QM------------DETIILAVVPCNV--D-IATTE-ALKMAKEVD------
PDGSRTLGVLTKPDLID-------RGT------E-----NMT---------------------------VDI--VN---NR---------
K-YA----------------------L---KKGYTIIKCR---G-QVD--IEN----
-------K--------VS--------------------LS----------DAMDK---EEMFFQK--------HE-HF--------------------
-K-I------LY-------E--EKK-TGTKTLAGKLTTELVEQIKKS-I----
>XP_002608668.1
------------VTLPS--------VVVI---GD----Q----------------
SAGKSSCLE-----A--MS-G-V-QL-PRG--------------SG-IVTRCPL---------------ELR--------------L---
KKS------------------------------QDP--
-------------------ESP--------------WK-------------------------GYI----------H---------------YHF-E-G----
----------DR------------------------DETGWKLTDPS-----------DVGEAV------RKAQ-NN---L-AG--------
----------------DS-------------------H-GISPR-LITLDVESPD--
------------IPDLTLIDLPGIAR--IA-------------------V-------DG-------------Q--------PP--D--IGDQIK--
------DLIK-EYI---QK------------DETIILAVVPCNV--D-IATTE-ALQMAKDVD------
PTGSRTLGVLTKPDLID-------RGT------E-----NTI---------------------------VDI--VN---NQ---------K-
YP----------------------L---KKGYTIIRCR---G-QED--INE-------

----N--------VT--------------------LS----------EAMEK---EERFFKT--------HE-HF---------------------K-
L------PY-------H--EKK-TGTRTLAGKLTTELVEQIK--------
>XP_003973512.2.2
------------LALPA--------IAVI--GD----Q----------------
SSGKSSVLE-----A--LS-G-V-AL-PRG--------------SG-IVTRCPL---------------ELK--------------M---
KRR------------------------------KV---
-----------------GEP--------------WY--------------------------GNI----------S---------------YL-------------
---------------------------------DQEEVIEDPA-----------DVEKKI------QEAQ-NE---M-AG---------------
---------VG-------------------V-GISDD-LISLEIASPE---
----VPDLTLIDLPGIAR--VA-------------------V-------KG--------------Q--------PE--N--IGEQIK--------
RLIR-KFI---TK------------QETISLVVVPCNV--D-IATTE-ALKMAQEVD------PDGERTLGILTKPDLVD-
------KGT------E-----ETV----------------------------VDI--IH---NE---------V-IH-----------------------L---
KKGYMIVRCR---G-QKE--IID-----------K--------VS-------------
-------LA----------EATET---ETAFFRD--------HA-HF---------------------Q-T------LY-------D--DGQ-
ATILKLAEKLTLELVNHIEKS-L----
>NP_891987.2.2
------------LALPA--------IAVI--GD----Q----------------
SSGKSSVLE-----A--LS-G-V-PL-PRG--------------SG-IVTRCPL---------------ELK--------------M---
IRT-------------------------------KD--
----------------QDK--------------WH--------------------------GRI----------S---------------YK-------------
---------------------------------TYEEDFDDPA-----------EVEKKI------RQAQ-DE---M-AG----------------
--------AG--------------------V-GISEE-LISLQITSAN--
---VPDLTLIDLPGIAR--VA-------------------V-------KG--------------Q--------PE--N--IGDQIK--------
RLIR-KFV---TR------------QETINLVVVPCNV--D-IATTE-ALQMAQAED------
PDGERTLGILTKPDLVD-------KGT------E-----GTV----------------------------VDI--VH---NE---------V-
IH-----------------------L---TKGYMIVRCR--G-QKE--IMD-----
------Q--------VT--------------------LN----------EATET---ESAFFKD--------HP-HF---------------------
S-K------LY-------E--EGF-ATIPKLAEKLTIELVHHIQKS-L----
>XP_009304072.1
------------LALPA--------IAVI--GD----Q----------------
SSGKSSVLE-----A--LS-G-V-PL-PRG--------------SG-IVTRCPL---------------ELK--------------M---
IRS-------------------------------KE--
----------------DEK--------------WH--------------------------GRI----------S---------------YQ-------------
---------------------------------NHEEDFDDPA-----------EVEKKI------REAQ-DE---M-AG---------------
---------AG-------------------V-GISEE-LISLQITSAN--
----VPDLTLIDLPGIAR--VA-------------------V-------KG--------------Q--------PE--N--IGDQIK--------
RLIR-MFI---TK------------QETINLVVVPCNV--D-IATTE-ALQMAQAED------
PEGERTLGILTKPDLVD-------KGT------E-----GTV----------------------------VDI--VH---NE---------V-
IH-----------------------L---TKGYMIVRCR--G-QKE--IMD-----
------Q--------VT--------------------LN----------EATET---ESAFFKD--------HP-HF---------------------
R-K------LY-------E--EGF-ATIPKLAEKLTIELVHHIQRS-L----
>AGU16245.1
--------------LPA--------IAVI--GD----Q----------------
SSGKSSVLE-----A--LS-G-V-GL-PRG--------------SG-IVTRCPL---------------ELK--------------L---

KKA-----------------------------------KK---
--------------------ETE----------------WK------------------------------ATI-----------R------------------YE-----------------
------------------------------------DEYKELTSPS------------EVEKEI-------RTAQ-NA---M-AG----------------------
-----SG---------------------K-GISDK-LISLEIESDN---
VPDLTLIDLPGIAR--VA----------------------V--------QG-----------------Q----------PY--D--IGEQIK--------
KLIR-KFI---EK--------------EETINLVVVPCNV--D-IATTE-ALKMAQDVD------
QSGERTLGILTKPDLVD-------KGA------E-----QNI------------------------------VDV--VN---NM---------
V-IP------------------------L---KKGYMIVKCR--G-QQD--INE---
--------N--------LT---------------------LA-----------EAT---

>XP_007904885.1
------------LSLPA--------IAVI--GD----Q-----------------
SSGKSSVLE-----A--LS-G-V-SL-PRG----------------TG-IVTRCPL------------------ELK----------------L---
KKA-----------------------------------KK---
--------------------ANV---------------WK-----------------------------GAI----------S------------------FR-----------------
--------------------------------------EYSKEITNAS------------EVEQEI-------RKAQ-NS---M-AG---------------------
-------KE-----------------------GISHD-LISLKIESSN---
--VPDLTLIDLPGIAR--VA----------------------V--------GN-----------------Q----------PL--D--IGDQIK--------
KMIR-SFI---NK--------------QETINLVVVPCNV--D-IATTE-ALKMAQEVD------
PSGERTVGILTKPDLVD-------KGT------E-----STI------------------------------VDI--VQ---NL---------V-
VE-------------------------L---KKGYMIVKCR---G-QKE--IND-----
------K--------LT---------------------LQ-----------DAIAR---ENRYFEE--------HE-QF------------------------
R-T------LL-------D--EKK-ASIPHLAERLTNELVYHISKC-L----
>XP_032888405.1
------------LGLPA--------IAVI--GD----Q-----------------
SSGKSSVLE-----A--LS-G-V-AF-PRG----------------SG-IVTRCPL------------------ELK----------------L---
KNV-----------------------------------KK---
--------------------ANV---------------WK-----------------------------GKI----------S------------------YK-----------------
--------------------------------------DYSNKLSSAA------------EVEQAI-------LKAQ-DS---I-AG---------------------
-------KG----------------------V-GISHE-LISLEIESTN---
--VPDLTLIDLPGIAR--VA----------------------V--------GN-----------------Q----------PQ--D--IGDQIK--------
RLIR-LFI---QK--------------QETVNLVVVPCNV--D-IATTE-ALKMAQEVD------
PTGDRTLGILTKPDLVD-------KGT------E-----KNV------------------------------VDI--VK---NL---------T-
VE-------------------------L---EKGYMIVKCR---G-QND--INE----
-------N--------IS---------------------LV-----------DAIAK---EKEFFED--------HE-QF------------------------
R-P------LL-------E--DGK-AGIPNLAVRLTKELVNHINKS-L----
>XP_009815891.1
------------LALPA--------IAVI--GD----Q-----------------
SSGKSSVLE-----A--LS-G-I-AL-PRG----------------NG-IVTRCPL------------------ELK----------------L---
KRI-----------------------------------PA---
-------------------TQA---------------WK-----------------------------GKI----------C------------------YR-----------------
-------------------------------------NISSELQNAS------------EVEKAI-------REAQ-DI---V-AG----------------------
-----TR---------------------G-AISGE-LISLEIWSPD---
-VPDLTLIDLPGIAR--VA----------------------V--------GN-----------------Q----------PK--D--IGEQIK--------

MLLK-KII---GC------------KETLNLVVVPCNV--D-IATTE-ALKMAQEVD------
PSGERTLGILTKPDLVD-------RGT------E-----ESI----------------------------INI--IR---NL---------V-IP-
----------------------L---KKGYMIVKCR--G-QQD--IHN--------
---K--------LA--------------------LA----------AAIQQ---ERKFFET--------HE-HF---------------------S-I--
----LL-------E--EGK-ATVPHLAEKLTNELVRHIIKT-L----
>XP_025933558.1
------------LALPS--------IAVI--GD---Q----------------
SSGKSSVLE-----A--LS-G-I-AL-PRG--------------NG-IVTRCPL---------------ELK--------------L---
KKT------------------------------PA---
-----------------TQK--------------WK--------------------------GKI----------S---------------YH-------------
---------------------------------NTSEELKNPS-----------EVEKAI------RGAQ-DV---V-AG-----------------
-------TK-------------------G-AISRE-LISLEVWSPT---
--VPDLTLIDLPGIAR--VA-------------------V-------GD--------------Q--------PE--D--IGEQIK--------
KLLK-NII---GN------------KETLNLVVVPCNV--D-IATTE-ALKMAQEVD------
PKGERTLGILTKPDLVD-------KGT------E-----ESI----------------------------VNI--IR---NL---------T-
VP-----------------------L---KKGYMIVKCR--G-QQD--IHN----
-------N--------LT--------------------LA----------SAIQQ---EKEFFET--------HQ-HF---------------------
S-I------LL-------N--EGK-ATVPLLAEKLTXELVGHIIKT-L----
>XP_015269256.1
------------LALPA--------IAVI--GD---Q----------------
SSGKSSVLE-----A--LS-G-V-AL-PRG--------------NG-IVTRCPL---------------ALK--------------L---
KKT------------------------------RQ---
-----------------GCG--------------WK--------------------------GKI----------S---------------YR------------
----------------------------------DINEELNHPS-----------EVEKEI------RKAQ-IS---I-AG------------------
------EG-------------------V-GISHE-LITLEIRSSE---
-VPDLTLIDLPGIAR--VA-------------------V-------GN--------------Q--------PQ--D--IGHQIK--------
RLIK-KII---AK------------DETINLVVVPCNV--D-IATTE-ALKMAQEVD------PDGERTLGILTKPDLVD-
------KGT------E-----EAV----------------------------VDI--VR---NL---------I-IH-----------------------L---
KKGYMIVKCR--G-QQD--IQS-----------N--------LD----------
----------LA----------SAIQK---EKAFFED--------NR-HF---------------------R-I------LL-------A--EKR-
ATIPLLAEKLTSELVEHINKS-L----
>XP_006156438.1
------------LALPA--------IAVI--GD---Q----------------
SSGKSSVLE-----A--LS-G-V-AL-PRG--------------SG-IVTRCPL---------------VLK--------------L---
MKQ------------------------------SQ---
-------------------EPV--------------WR--------------------------GKI----------R---------------YR-----------
-----------------------------------NTEKKLGDPT-----------QVEAEI------CKAQ-NI---I-AG----------------
--------SG-------------------V-GISHE-LITLEITSPE--
--VPDLTLIDLPGITR--VA-------------------L-------GN--------------Q--------PQ--D--ISLQIK--------
ALIK-KYI---KR------------QQTINLVVVPCNV--D-IATTE-ALSMAQEVD------
PEGDRTLGILTKPDLVD-------KGS------E-----KSV----------------------------MNV--LQ---NL---------
T-FP-----------------------L---KKGYMIVKCR--G-QQE--IMN--
---------N--------LS--------------------LA----------EATRK---ELMFFQS--------HP-HF-------------------
--R-V------FL-------E--EKK-ATVPHLAERLTAELIAHIRKS-L----

>NP_001003133.1
------------LALPA--------IAVI--GD----Q---------------
SSGKSSVLE-----A--LS-G-V-AL-PRG--------------SG-IVTRCPL---------------VLK-------------L---
KRD------------------------------PH--
-------------------KA--------------WR--------------------------GRI----------S---------------YR------------
----------------------------------KTELQFQDPS-----------QVEKEI------RQAQ-NI---I-AG-----------------
-------QG-------------------L-GISHE-LISLEITSPE---
--VPDLTLIDLPGITR--VA------------------V-------GN--------------Q--------PQ--D--IGVQIK--------
ALIK-NYI---QK------------QETINLVVVPCNV--D-IATTE-ALSMAQEVD------
PNGDRTIGVLTKPDLVD-------RGT------E-----KTV----------------------------VNV--AQ---NL---------T-
YH----------------------L---QKGYMIVRCR---G-QEE--ITN----
-------Q--------LS--------------------LA----------EATEK---ERMFFQT--------HP-YF--------------------
R-A------LL-------E--EGK-ATVPCLAERLTKELILHINKS-L----
>XP_002830747.1
------------LALPA--------IAVI--GD----Q---------------
SSGKSSVLE-----A--LS-G-V-AL-PRG--------------SG-IVTRCPL---------------VLK-------------L---
KKQ------------------------------PC--
-------------------EA--------------WA--------------------------GRI----------S---------------YR------------
----------------------------------NTELELQDPG-----------QVEKEI------HKAQ-NI---M-AG---------------
---------NG-------------------R-GISHE-LISLEITSPE---
----VPDLTIIDLPGITR--VA------------------V-------DN--------------Q--------PR--D--IGLQIK--------
ALIK-KYI---QR------------QQTINLVVVPCNV--D-IATTE-ALSMAHEVD------
PEGDRTIGILTKPDLMD-------KGT------E-----KSV----------------------------MNV--VR---NL---------T-
YP----------------------L---KKGYMIVRCR---G-QQE--LTN----
-------R--------LS--------------------LA----------EATKK---EITFFQT--------HP-YF---------------------R-
V------LL-------E--EGS-ATVPRLAERLTSELIMHIQKS-L----
>NP_002454.1
------------LALPA--------IAVI--GD----Q---------------
SSGKSSVLE-----A--LS-G-V-AL-PRG--------------SG-IVTRCPL---------------VLK-------------L---
KKQ------------------------------PC--
-------------------EA--------------WA--------------------------GRI----------S---------------YR------------
----------------------------------NTELELQDPG-----------QVEKEI------HKAQ-NV---M-AG---------------
---------NG-------------------R-GISHE-LISLEITSPE---
----VPDLTIIDLPGITR--VA------------------V-------DN--------------Q--------PR--D--IGLQIK--------
ALIK-KYI---QR------------QQTINLVVVPCNV--D-IATTE-ALSMAHEVD------
PEGDRTIGILTKPDLMD-------RGT------E-----KSV----------------------------MNV--VR---NL---------T-
YP----------------------L---KKGYMIVKCR---G-QQE--ITN-----
------R--------LS--------------------LA----------EATKK---EITFFQT--------HP-YF---------------------R-
V------LL-------E--EGS-ATVPRLAERLTTELIMHIQKS-L----
>XP_008569440.1
------------LALPA--------IAVI--GD----Q---------------
SSGKSSVLE-----A--LS-G-V-AL-PRG--------------SG-IVTRCPL---------------VLK-------------L---
KKH------------------------------LQ--
------------------EDG--------------WK--------------------------GKI----------S---------------YR-----------

-----------------------------------HTELLLQDPS-----------QVEKEI------HKAQ-NT---I-AG----------------
--------NG-------------------V-GISHE-LISLEITSPE--
---VPDLTLIDLPGITR--VA-------------------V-------GN-------------Q--------PQ--D--IGQQVK--------
ALIK-KYI---QR------------QQTINLVVVPCNV--D-IATTE-ALSMAQEVD------
PDGDRTIGILTKPDLVD-------KGT------E-----KGV----------------------------MNV--AR---NL---------T-
YH-----------------------L---KKGYMIVKCR---G-QQD--ITN----
-------K--------LS--------------------LA----------EATKK---EMAFFQT--------HP-YF--------------------
R-V------LL-------E--EGK-ATVPCVAEKLTAELIVHINKS-L----
>XP_017508123.1
------------LALPA--------IAVI---GD----Q-----------------
SSGKSSVLE-----A--LS-G-V-AL-PRG--------------SG-IVTRCPL---------------VLK--------------L---
KKQ-------------------------------LH--
------------------EPA--------------WT--------------------------GRL----------S----------------YQ------------
-----------------------------------TTELQLHNPS-----------QVEKEI------QKAQ-NA---I-AG----------------
--------DG-------------------V-GISHE-LINLEITSPD---
----VPDLTLIDLPGIAR--VA-------------------V-------GN--------------Q---------PQ--D--IGLQIK--------
ALIK-KYI---QR------------QQTINLVVVPCNV--D-IATTE-ALSMAQEVD------
PDGDRTIGILTKPDLVD-------KGT------E-----RVI----------------------------VNV--VQ---NL---------T-
YH-----------------------L---KKGYMIVKCR---G-QQE--VTN----
-------K--------LS--------------------LA----------EATSK---EMTFFQT--------HP-YF--------------------
R-I------LL-------E--EGK-ATVPRLAEKLTTELISHINKS-L----
>XP_005885748.1
------------LALPA--------IAVI---GD----Q-----------------
SSGKSSVLE-----A--LS-G-V-AL-PRG--------------SG-IVTRCPL---------------VLK--------------L---
KKQ-------------------------------LAG---
-------------------ESL--------------WT--------------------------GKI----------S----------------YR------------
-----------------------------------STELQLQDPS-----------QVEREI------YKAQ-NT---I-AG-----------------
-------NG-------------------V-GISHE-LINLEITSPE---
--VPDLTLIDLPGIAR--VA-------------------V-------GN--------------Q---------PQ--D--IGLQIK--------
ALIK-KYI---QR------------QQTINLVVVPCNV--D-IATTE-ALSMAHEVD------
PDGDRTIGILTKPDLVD-------KGA------E-----KNV----------------------------VNV--AQ---NL---------
T-YR-----------------------L---KKGYMIVKCR---G-QQE--ITD---
--------K--------LS--------------------LA----------EATKK---EMMFFQT--------HP-YF---------------------
R-V------LL-------E--EGK-ATVPRLAERLTTELIWHINKS-L----
>NP_776366.1
------------LALPA--------IAVI---GD----Q-----------------
SSGKSSVLE-----A--LS-G-V-AL-PRG--------------SG-IITRCPL---------------VLK--------------L---
TKR---
----------------ECE--------------WT--------------------------GKI----------T---------------YR---------------
--------------------------------NITQQLQNPS-----------EVEWEI------RRAQ-NI---I-AG-------------------
-----NG-------------------L-GISHE-LINLEITSPE--
VPDLTLIDLPGITR--VA-------------------V-------EN--------------Q---------PQ--D--IGLQIK--------
ALIK-KYI---QR------------QETINLVVVPCNV--D-IATTE-ALSMAQEVD------
PDGDRTIGILTKPDLVD-------KGT------E-----KGV----------------------------LKV--MQ---NL---------T-

YH-----------------------L---KKGYMIVKCR--G-QQD--ITN----
-------K--------LS--------------------LA----------EATRK---ETMFFET--------HP-YF---------------------
R-I------LL-------D--EGK-ATVPLLAERLTTELIWHINKS-L----
>XP_032211320.1
------------LALPA--------IAVI--GD----Q----------------
SSGKSSVLE-----A--LS-G-V-AL-PRG--------------SG-IVTRCPL---------------VLK--------------L---
KRQ------------------------------PQ--
------------------ESA--------------WK--------------------------GRV----------I---------------YG------------
----------------------------------TREVRLQDPS-----------QVEKEI------LKAQ-NT---L-AG----------------
--------DG-------------------V-SISHE-LISVDIISPE---
--VPDLTLIDLPGITR--VP-------------------V-------GN--------------Q--------PQ--D--IGLQIK--------
ALIK-KYI---QR------------QETINLVVVPCNV--D-IATTE-ALSMAQEVD------
PRGDRTIGILTKPDLVD-------KGA------E-----PIV----------------------------MKV--AQ---NL--------T-
YH-----------------------L---QKGYMMVRCR--G-QEE--ITN---
--------R--------LS--------------------LA----------EATRK---ETMFFQK--------HP-HF---------------------
R-A------LL-------Q--EGK-ATVPCLAERLTNELILHINKS-L----
>XP_004675614.2.2
------------LALPA--------IAVI--GD----Q----------------
SSGKSSVLE-----A--LS-G-V-AL-PRG--------------SG-IVTRCPL---------------VLK--------------L---
KKL------------------------------MN--
------------------EDS--------------WK--------------------------GKI----------N---------------YQ-----------
-----------------------------------GVEVTIAKAS-----------DVEQEV------NKAQ-AV---I-AG---------------
---------DG-------------------L-GISHE-LITLEVSSPE---
-----VPDLTLIDLPGITR--VA-------------------V-------GN--------------Q--------PQ--D--IGEQIK--------
ALIR-KYI---QR------------QQTINLVVVPCNV--D-IATTE-ALSMAREVD------
PDGDRTLGILTKPDLVD-------RGT------E-----DRV----------------------------VDV--IR---NF---------I-
CP-----------------------L---KKGYMIVKCR--G-QKD--IQD----
-------R--------LS--------------------LA----------QALQK---EQAFFEE--------HP-HF---------------------
R-Q------LL-------E--EGR-ASIPKLADRLTSELIRHISKS-L----
>XP_004466363.1
------------LSLPA--------IAVI--GD----Q----------------
SSGKSSVLE-----A--LS-G-V-AL-PRG--------------SG-IVTRCPL---------------VLK--------------L---
KKL------------------------------TN---
-----------------EEK--------------WR--------------------------GKV----------T---------------YE-------------
---------------------------------DYEIDISDAS-----------EVEEEI------NKAQ-NV---I-AG-------------------
-----EG-------------------L-GISQK-LINLEVCSPD---
--VPDLTLIDLPGITR--VA-------------------V-------GN--------------Q--------PA--D--IGWQIK--------
CLIK-KYI---TR------------QETINLVVVPSNV--D-IATTE-ALSMAQEVD------PNGDRTIGILTKPDLVD-
------RGT------E-----DKV----------------------------VDV--VR---NL---------V-CH-----------------------
L---KKGYMIVRCR--G-QQD--IQD-----------R--------LS------
--------------LA----------TALQK---ERAFFEN--------HE-NF---------------------R-V------LL-------E--
EGK-ATVPHLAERLTTELITHISKT-L----
>NP_002453.2.2

------------LALPA--------IAVI--GD----Q---------------
SSGKSSVLE-----A--LS-G-V-AL-PRG--------------SG-IVTRCPL---------------VLK--------------L---
KKL------------------------------VN---
-----------------EDK-------------WR--------------------------GKV----------S--------------YQ------------
---------------------------------DYEIEISDAS-----------EVEKEI------NKAQ-NA---I-AG-----------------
------EG-------------------M-GISHE-LITLEISSRD---
--VPDLTLIDLPGITR--VA------------------V-------GN--------------Q--------PA--D--IGYKIK--------
TLIK-KYI---QR------------QETISLVVVPSNV--D-IATTE-ALSMAQEVD------PEGDRTIGILTKPDLVD-
------KGT------E-----DKV---------------------------VDV--VR---NL---------V-FH-----------------------L-
--KKGYMIVKCR---G-QQE--IQD-----------Q--------LS---------
-----------LS----------EALQR---EKIFFEN--------HP-YF--------------------R-D------LL-------E--EGK-
ATVPCLAEKLTSELITHICKS-L----
>NP_001127618.1
------------LALPA--------IAVI--GD----Q---------------
SSGKSSVLE-----A--LS-G-V-AL-PRG--------------SG-IVTRCPL---------------VLK--------------L---
KKL------------------------------VN---
-----------------EDK-------------WR--------------------------GKV----------S--------------YQ------------
---------------------------------DYEIEISDAS-----------EVEKEI------NKAQ-NT---I-AG-----------------
------EG-------------------M-GISHE-LITLEISSRD---
--VPDLTLIDLPGITR--VA------------------V-------GN--------------Q--------PA--D--IGYKIK--------
TLIK-KYI---QR------------QETISLVVVPSNV--D-IATTE-ALSMAQEVD------PEGDRTIGILTKPDLVD-
------KGT------E-----DKV---------------------------VDV--VR---NL---------V-FH-----------------------L-
--KKGYMIVKCR---G-QQE--IQD-----------Q--------LS---------
-----------LS----------EALQR---EKIFFED--------HP-YF--------------------R-D------LL-------E--EGK-
ATVPCLAEKLTSELITHICKS-L----
>XP_006156437.1
------------LALPA--------IAVI--GD----Q---------------
SSGKSSVLE-----A--LS-G-V-AL-PRG--------------SG-IVTRCPL---------------VLK--------------L---
KKL------------------------------IN--
-----------------EDK-------------WR--------------------------GKV----------S--------------YQ------------
---------------------------------DIEVEITDPS-----------KVEPEI------NKAQ-NV---I-AG-----------------
------EG-------------------M-GISHE-LISLEVSSPH--
---VPDLTLIDLPGITR--VA------------------V-------GN--------------Q--------PA--D--IGRQIK--------
TLIK-KYI---HK------------QETINLVVVPSNV--D-IATTE-ALSMAQEVD------PDGDRTIGILTKPDLVD-
------KGT------E-----EKV---------------------------VDV--VR---NL---------V-CH-----------------------L-
--KKGYMIVKCR---G-QQD--IQD-----------R--------LS---------
-----------LA----------EALQR---EKVFFEE--------HP-YF--------------------S-F------LL-------E--EGK-
ATIPCLAERLTTELIMHISKS-L----
>XP_017508130.1
------------LALPA--------IAVI--GD----Q---------------
SSGKSSVLE-----A--LS-G-V-AL-PRG--------------SG-IVTRCPL---------------VLK--------------L---
KKL------------------------------TN---
-----------------EET-------------WR--------------------------GKV----------S--------------YQ-------------
--------------------------------DFEAELSDPS-----------EVEREI------NRAQ-NS---I-AG-----------------

-------EG-------------------T-GISHE-LISLEISSPH--
--VPDLTLIDLPGITR--VA-------------------V-------GN--------------Q--------PA--D--IGRQIK--------
ALIR-KYI---YK------------QETINLVVVPSNV--D-IATTE-ALSMAQEVD------PDGDRTIGILTKPDLVD-
------RGT------E-----DKV---------------------------VDV--VR---NL---------V-CH-----------------------
L---KKGYMIVKCR--G-QQD--IQD-----------Q--------LS------
--------------LA----------EALKK---ERAFFED--------NP-YF--------------------R-D------LL-------E--
EGR-ATVPCLADKLTVELITHICKS-L----
>XP_032211398.1
------------LALPA--------IAVI---GD----Q----------------
SSGKSSVLE-----A--LS-G-V-AL-PRG--------------SG-IVTRCPL---------------VLK--------------L---
KKV------------------------------TN--
-----------------QDE--------------WR--------------------------GKV----------S---------------YQ-----------
-----------------------------------DFEKEISDPS-----------EVEAEI------NKAQ-NA---V-AG---------------
---------EG-------------------Q-GISHE-LISLEVSSSH---
------VPDLTLIDLPGITR--VA-------------------V-------GN--------------Q--------PA--D--IGRQTK-------
-QLIR-KYI---LR------------QETINLVVVPCNV--D-IATTE-ALSMAQEVD------
PDGDRTIGILTKPDLVD-------RGT------E-----SKV---------------------------VDV--AQ---NL---------V-
CH-----------------------L---KKGYMIVKCR--G-QQD--IQD---
--------Q--------VT--------------------LA----------EALQK---ERDFFED--------HP-HF--------------------
-R-V------LL-------E--EGR-ATVPCLADKLTSELIMHICKT-L----
>NP_001003134.1
------------LALPA--------IAVI---GD----Q----------------
SSGKSSVLE-----A--LS-G-V-AL-PRG--------------SG-IVTRCPL---------------VLK--------------L---
KKL------------------------------IN--
-----------------EDE--------------WR--------------------------GKV----------S---------------YQ-----------
-----------------------------------DTEMEISDPS-----------EVEVEI------NKAQ-DA---I-AG-----------------
--------EG-------------------Q-GISHE-LISLEVSSPH---
-----VPDLTLIDLPGITR--VA-------------------V-------GN--------------Q--------PA--D--IGRQTK--------
QLIR-KYI---LK------------QETINLVVVPCNV--D-IATTE-ALSMAQEVD------
PDGDRTIGILTKPDLVD-------RGT------E-----GKV---------------------------VDV--AQ---NL---------V-
CH-----------------------L---KKGYMIVKCR--G-QQD--IQD---
--------Q--------VS--------------------LA----------EALQK---EKDFFED--------HP-HF--------------------
-R-V------LL-------E--EGR-ATVPNLAEKLTSELITHICKT-L----
>XP_008569442.1
------------LALPA--------IAVI---GD----Q----------------
SSGKSSVLE-----A--LS-G-V-AL-PRG--------------SG-IVTRCPL---------------VLK--------------L---
KKL------------------------------VH--
-----------------GEE--------------WK--------------------------GKV----------S---------------YR-----------
-----------------------------------DLEIKISDAL-----------EVEEEV------RKAQ-TI---I-AG--------------------
-----EG-------------------M-GISHE-LINLEISSPH--
-VPDLTLIDLPGIAR--VA-------------------M-------GN--------------Q--------PA--D--IGYQVK--------
XLIR-KYI---QR------------QETINLVVVPSNV--D-IATTE-ALSMAQEVD------PEGDRTIGILTKPDLVD-
------KGT------E-----DKV---------------------------VDV--VR---NL---------V-YH-----------------------L-
--KKGYMIVKCR--G-QQD--IQD-----------Q--------LS--------

------------LA----------TALQR---EKDFFED--------HP-QF--------------------R-D------LL-------E--
EGR-ATIPCLAERLTTELITHICKS-L----
>XP_014388412.1
------------LALPA--------IAVI--GD----Q---------------
SSGKSSVLE-----A--LS-G-V-SL-PRG-------------SG-IVTRCPL--------------VLK-------------L---
RKL------------------------------RH--
-----------------DDE-------------WK-------------------------GKV---------T--------------YR-----------
---------------------------------DLEIDLSAAS----------EVEQEI------RKAQ-NV---I-AG-----------------
-------EG------------------V-GISQE-LINLEVSSPH--
----VPDLTLIDLPGITR--VA------------------V-------GN-------------Q--------PA--D--IGRQIT--------
ALIK-KYI---LR------------QQTIMLVVVPSNV--D-IATTE-ALSMAHEVD------
PDGDRTIGILTKPDLVD-------RGT------E-----DKV--------------------------VDV--VR---NL---------V-
YH----------------------L---KKGYMIVKCR---G-QQD--IQY----
-------Q--------MS-------------------LS---------KALQR---ERAFFED--------HP-YF-------------------
R-D------LL-------E--EGK-ATIPCLAERLTNELIAHISKS-L----
>XP_005202045.1
------------LALPA--------IAVI--GD----Q---------------
SSGKSSVLE-----A--LS-G-V-AL-PRG-------------SG-IVTRCPL--------------VLR-------------L---
KKL------------------------------GN---
-----------------EDE-------------WK-------------------------GKV---------S--------------FL-----------
---------------------------------DKEIEIPDAS----------QVEKEI------SEAQ-IA---I-AG------------------
-----EG------------------T-GISHE-LISLEVSSPH--
-VPDLTLIDLPGITR--VA------------------V-------GN-------------Q--------PP--D--IEYQIK-------
SLIR-KYI---LR------------QETINLVVVPANV--D-IATTE-ALRMAQEVD------PQGDRTIGILTKPDLVD-
------KGT------E-----DKV--------------------------VDV--VR---NL---------V-FH----------------------L-
--KKGYMIVKCR---G-QQD--IKH-----------R--------MS--------
------------LD----------KALQR---ERIFFED--------HA-HF--------------------R-D------LL-------E--
EGK-ATIPCLAERLTSELIMHICKT-L----
>NP_034976.1
------------LALPA--------IAVI--GD----Q---------------
SSGKSSVLE-----A--LS-G-V-AL-PRG-------------SG-IVTRCPL--------------VLK-------------L---
RKL------------------------------KE---
----------------GEE-------------WR-------------------------GKV---------S--------------YD-----------
---------------------------------DIEVELSDPS----------EVEEAI------NKGQ-NF---I-AG-----------------
-------VG------------------L-GISDK-LISLDVSSPN---
---VPDLTLIDLPGITR--VA------------------V-------GN-------------Q--------PA--D--IGRQIK--------
RLIK-TYI---QK------------QETINLVVVPSNV--D-IATTE-ALSMAQEVD------
PEGDRTIGVLTKPDLVD-------RGA------E-----GKV--------------------------LDV--MR---NL---------
V-YP----------------------L---KKGYMIVKCR---G-QQD--IQE--
---------Q--------LS-------------------LT---------EAFQK---EQVFFKD--------HS-YF--------------------
-S-I------LL-------E--DGK-ATVPCLAERLTEELTSHICKS-L----
>NP_038634.1
------------LALPA--------IAVI--GD----Q---------------
SSGKSSVLE-----A--LS-G-V-AL-PRG-------------SG-IVTRCPL--------------VLK-------------L---

RKL------------------------------NE---
------------------GEE-------------WR-------------------------GKV---------S--------------YD----------
----------------------------------DIEVELSDPS----------EVEEAI------NKGQ-NF---I-AG---------------
--------VG-------------------L-GISDK-LISLDVSSPN---
----VPDLTLIDLPGITR--VA------------------V-------GN-------------Q--------PA--D--IGRQIK--------
RLIK-TYI---QK-----------QETINLVVVPSNV--D-IATTE-ALSMAQEVD------PEGDRTIGILTKPDLVD-
------RGT------E-----DKV--------------------------VDV--VR---NL---------V-YH---------------------L-
--KKGYMIVKCR---G-QQD--IQE----------Q--------LS---------
-----------LT----------EALQN---EQIFFKE--------HP-HF--------------------R-V------LL-------E--DGK-
ATVPCLAERLTAELILHICKS-L----
>XP_028583072.1
------------LALPA--------IAVI---GD----Q---------------
SSGKSSVLE-----A--LS-G-V-AL-PRG-------------SG-IVTRCPL---------------ELR-------------L---
KKL------------------------------LP--
-----------------GEK-------------WN-------------------------GKI---------S--------------YL-------------
---------------------------------GKYMELANPS----------MVEIEI------RKAQ-NI---I-AG-----------------
------DG------------------V-AISDK-LITLEIRSPE--
VPDLTLIDLPGIAR--VA------------------V-------GN-------------Q--------PV--N--IGDQIK--------
KLIK-TFI---DK-----------QETINLVVVPSNV--D-IATTE-ALKMAQEVD------
PNGERTLGIVTKPDLMD-------RGT------E-----GTV--------------------------VNI--VR---NQ---------
V-IP-----------------------L---KKGYMIVKCR--G-QQD--IQS---
---------N--------MT--------------------LA----------SALKE---ERAFFEK--------HK-CF-------------------
-S-I------LL-------Q--EKK-ATVPLLAEKLTSELVEHISKS-L----
>XP_028583068.1
------------LALPA--------IAVI---GD----Q---------------
SSGKSSVLE-----A--LS-G-V-AL-PRG-------------SG-IVTRCPL---------------ELK-------------L---
KKT------------------------------HN--
------------------TKE-------------WK-------------------------GKI---------S--------------YL-------------
---------------------------------NTVEEMNSSR----------QVEEQI------IRAQ-NA---M-AG---------------
---------SG-------------------S-GISSE-LISLEISSSD---
----VPDLTLIDLPGIAR--VA------------------V-------GD-------------Q--------PK--D--IGQQII--------
KLIK-KYI---NK-----------QETINLVVVPSNV--D-IATTE-ALKMAQEVD------PTGERTLGILTKPDLVD-
------KGT------E-----AEV--------------------------VDI--IR---NQ---------R-VP---------------------L--
-RKGYMIVKCR---G-QSD--IND----------K--------VT-----------
---------LG----------DAIEK---EREFFEE--------HD-FF--------------------R-S------LL-------E--EGR-
ATIPLLAERLTQELIEHISKT-L----
>XP_031752404.1
------------LALPA--------IAVI---GD----Q---------------
SSGKSSVLE-----A--LS-G-V-TL-PRG-------------SG-IVTRCPL---------------ELK-------------L---
KKA------------------------------MK--
------------------KTT-------------WS-------------------------GKI---------S--------------YR-------------
---------------------------------DHEIKIASAA----------DVEEEV------KRAQ-NL---M-AG----------------
--------SG-------------------K-GISDE-LISLEVISPD---
---VPDLTLIDLPGITR--VA------------------L-------PD-------------Q--------PK--D--IEQQIK--------

KMIR-KYI---QK------------QETINLVVVPSNV--D-IATTE-ALEMAREVD------
PNGERTLGILTKPDLVD-------RGA------E-----TDV--------------------------ISV--VR---NL--------V-
YS----------------------L---NKGYMIVKCR--G-QQE--IQE----
-------N--------LS-------------------LK---------DALVN---EQNFFKE--------HE-HF--------------------
S-V------LL-------E--EGY-ATIACLAGKLTNELVAHIVRN-L----
>XP_005167721.2.2
------------LNLPA--------IAVI---GD----Q---------------
SSGKSSVLE-----A--LS-G-V-AL-PRG-------------TG-IVTRCPL---------------VLK-------------L---
KKI-----------------------------TK--
----------------DKS-------------WH-------------------------GLL---------T--------------YN-------------
-------------------------------DKIRELKDPA----------KIEKAV------LNAQ-TA---L-AG-----------------
------IG------------------E-GISHE-MITLEIQSCD--
--VPDLTLIDLPGIAR--VA------------------T-------GN-------------Q--------PE--D--IEKQIK--------
SLIE-KFI---KR------------QETISLVVVPANI--D-IATTE-ALKMASTVD------PTGQRTLGILTKPDLVD---
----RGM------E-----DTV--------------------------VRT--VN---NE---------V-IP----------------------L---
KKGYMIVKCR--G-QQD--IND----------K--------LG----------
---------LV---------EALEK---ERRFFDE--------NV-HF--------------------R-S------LL-------E--DRK-
ATIPLLAERLTKELVEHIAKN-L----
>NP_001007285.1
------------LNLPA--------IAVI---GD----Q---------------
SSGKSSVLE-----A--LS-G-V-AL-PRG-------------IG-IVTRCPL---------------ILK-------------L---KKI-
-----------------------------TR--
-----------DKN-------------WS-------------------------GLL---------T--------------YK------------------
--------------------------DQTEILKEPT----------GIENAV------LKAQ-IA---L-AG-----------------------
TG------------------E-GISHE-MITLEIQSCD--
VPDLTLIDLPGIAR--VA------------------T-------GN-------------Q--------PE--D--IEKQIK--------
DLIE-KFI---KR------------QETISLVVVPANI--D-IATTE-ALKMASTVD------PTGQRTLCILTKPDLVD--
-----RGM------E-----DTV--------------------------VRT--VN---NE---------V-IR----------------------L--
-EKGYMIVKCR--G-QQD--IND----------K--------LN---------
----------LV---------EALEK---ERRFFDE--------HP-QF--------------------S-S------LL-------E--DGK-
ATIPLLGQRLTEELVEHIAKN-V----
>XP_012586448.1
------------LALPA--------IAVI---GD----Q---------------
SSGKSSVLE-----A--LS-G-V-AL-PRG-------------SG-IVTRCPL---------------VLK-------------L---
KKQ-----------------------------LQ--
------------------GAP-------------WT-------------------------GTI---------S--------------YR-------------
-------------------------------GVTLGLQDPS----------AVEREI------HVAQ-NV---I-AG-----------------
-------HG-------------------V-GISHE-LITLEVSSPE--
---VPDLTLIDLPGIAR--VA------------------V-------GN-------------Q--------PQ--D--
IGAQVSLSGAGRWGALVS-GLP---RS------------ERCTGQ------------ETAE-ALGGGRAAX------
XDGDRTLGILTKPDLVD-------KGA------E-----KAV--------------------------VNV--AQ---NL---------
T-YR----------------------L---KKGYMVVKCR--G-QQD--IMD-
----------R--------LS-------------------LA---------QATEK---EVAFFQT--------HP-HF------------------
--R-A------LL-------E--EGK-ATVPRLAEKLTSELILHINKS-L----

>KAI0213370.1
------------ISLPE--------VAVI--GD----Q---------------
SAGKSSVLE-----A--IS-G-V-QL-PRG-------------SG-IVTRCPL--------------ALQ-------------L---
KSH-----------------------------DT--
-----------------PGY-------------WN------------------------GVI---------K--------------YE--T-Y-------
------NH------------------------PVEKTIEGPT----------EVGAEV------REAQ-DV---I-AG---------------
--------KN------------------V-GISDT-LISLQITSHG--
---VPDLTLIDLPGITR--VA------------------V-------EG-------------Q--------PK--D--IGDQIK--------
RLIG-HYI---KK------------EETIILAVVPANV--D-IATTE-ALKMAKEVD------PSGGRTLGVVTKPDLID-
------IGT------E-----KGL---------------------------IDI--IN---NE---------T-YP----------------------L---
EKGYSCVRCR--G-QKA--INE----------G--------QT----------
---------LA---------EAIQQ---DTDFFSS--------AP-HF--------------------S-D------V--------D--ESI-
LGVKNLAMKLTYELVKQIKRA-L----
>KAI0208044.1
------------ISLPE--------VAVI--GD----Q---------------
SAGKSSVLE-----A--IS-G-V-QL-PRG-------------SG-IVTRCPL--------------ALQ-------------L---
KSH-----------------------------DT--
-----------------PGY-------------WN------------------------SVI---------K--------------YK--Y-G-------
-------DEF------------------DFEDIVVEKTIEGPT----------KVDAGV------REAQ-DA---I-AG-----------
-------------KN------------------V-GISDT-LISLQITAYG---
-------VPDLTLIDLPGITR--VA------------------V-------QG-------------Q--------PP--D--IGDQIK------
--RLIG-NYI---KK------------EETIILAVVPANV--D-IATTE-ALKMAKEVD------
PSGGRTLGVVTKPDLID-------IGT------E-----KGL---------------------------IDI--IN---NE---------T-
YP----------------------L---EKGYSCVRCR--G-QKA--INE-----
------G--------QT-------------------LA---------DAVQE---DTDFFSS--------AP-HF--------------------
S-A------V--------D--ESI-LGVKNLAMKLTFELVKQIKRA-L----
>KAI0218869.1
------------IALPE--------VAVI--GD----Q---------------
SAGKSSVLE-----A--IS-G-V-QL-PRG-------------SG-IVTRCPL--------------ALQ-------------L---
KSD------------------------------KT--
-----------------PGY-------------WN------------------------GVI---------K--------------YE--I-N-------
-------ER------------------------LVEKTIVGPA----------EVDAEV------RNAQ-DV---I-AG---------------
---------KN------------------V-GISSK-LISLQITSYG---
----IPDLTLIDLPGITR--VA------------------V-------EG-------------Q--------PQ--N--IGEQIK--------
RLIE-KYI---KK------------EETIILAVVPANV--D-IATTE-ALKMAKEVD------PSGSRTLGVVTKPDLID---
----AGT------E-----KGL---------------------------ISI--IN---NE---------T-YP----------------------L---
KKGYSCVRCR--G-QKA--IDE----------G--------QT----------
---------LA---------QAIQQ---DTDFFSI--------AS-HF--------------------S-D------V--------D--QST-
LGVKNLAMKLTYELVRQIKRA-L----
>XP_032804093.1
------------VGLPA--------VAVI--GD----Q---------------
SSGKSSVLE-----A--LS-G-V-QL-PRG-------------SG-IVTRCPL--------------ALK-------------L---
KRA---
----------------PGP-------------WH------------------------GRI---------K--------------YR--V-Q-------

-------GR-----------------------TVNTKLDTPE----------SVGDAV------LQAQ-SE---L-TG-------------
----------DD-----------------L-GVSKS-LIELEVTSDS--
------VPDLTLIDLPGIAR--VA-----------------L-------AG-------------Q--------AV--D--IETQIK--------
DLIR-DHI---GR-----------QETINLVVIPCNV--D-IATTE-ALKMAQAVD------
PTGVRTLGVLTKPDLMD-------EGT------E-----RNA------------------------LRI--LQ---NQ---------
V-FP----------------------L---SKGYVLVKCR---S-QRD--VEA--
---------H--------QT------------------LA---------EASRV---EAAFFKK--------HP-VF-----------------
-C-H------VH-------N--GGKLTTTTVLAAKLTEELVDNIKRT-L----
>XP_046565196.1
------------INLPA--------VAVI---GD----Q---------------
SAGKSSVLE-----A--IS-G-V-QL-PRG-------------TG-IVTRCPL---------------EMR-------------M---
KHS-----------------------------ED--
------------------EDK-------------WE------------------------GKI---------M--------------YKD-K-H----
----------DE------------------------LHKEDIQDRE----------SVGDLV------RKAQ-DE---M-TD---------
---------------CE------------------K-GISDD-LITLEVTSSD--
-----------VPDLTLIDLPGIAR--NA------------------V-------KG-------------Q--------PV--D--IEKRIK---
-----DMIR-KYI---RR-----------QETIILAVLQCNV--D-IATCE-ALKMAKEFD------
EEGRRTLGVLTKPDLLD-------KGA------E-----NGV------------------------MRI--LN---NM---------
E-FS----------------------L---SKGYIIVKCR---G-QEA--ISK-----
------G--------QS------------------LT---------EALGD---EDNFFKD--------HS-HF-------------------
R-S------L--------K--VSQ-WGILTLSSRLSLELQKHIK--------
>XP_046563124.1
------------INLPA--------VAVI---GD----Q---------------
SAGKSSVLE-----A--IS-G-V-QL-PRG-------------TG-IVTRCPL---------------EMR-------------L---
KHS-----------------------------ED--
------------------EDK-------------WE------------------------GKI---------L--------------YKD-K-H-----
---------DM------------------------RQEEVILNRE----------SVGDLV------RKAQ-KE---M-TD-----------
-------------SA------------------K-GISDE-LITLEVTSSD--
--------VPDLTVIDLPGIAR--NA------------------V-------EG-------------Q--------PF--D--IEARIK-----
---NMIR-RYI---GR-----------QETIILAVLQCNV--D-IATCE-ALKMAKEFD------
DEGGRTLGVLTKPDLLD-------KGA------E-----TGV------------------------MRI--LN---NM---------
E-FT----------------------L---SKGYIIVKCR---G-QEA--ISE-----
------G--------QS------------------LK---------HALDV---EEDFFKS--------HR-HF-------------------
S-S------L--------R--PSQ-WGIPNLSARLSRELKKHIKKL-L----
>XP_046562919.1
------------INLPA--------VAVI---GD----Q---------------
SAGKSSVLE-----A--IS-G-V-QL-PRG-------------TG-IVTRCPL---------------EMR-------------M---
KHS-----------------------------ED--
------------------EDK-------------WE------------------------GKI---------M--------------YKD-K-H----
----------DM------------------------RQEEVILNRE----------SVGDLV------RKAQ-KE---M-TD----------
--------------GA------------------K-GISDE-LITLEVTSSD--
----------VPDLTVIDLPGITR--NA------------------V-------EG-------------Q--------PF--D--IEARIK----
----NMIR-KYI---KR-----------QETIILAVLQCNV--D-IATCE-ALKMAKEFD------
GEGGRTLGVLTKPDLMD-------KGA------E-----TGV------------------------IRI--LN---NM---------

E-FT-----------------------L---SKGYIIVKCR--G-QEA--ISE-----
------G--------QS--------------------LK----------QALDI---EEDFFKS--------HR-HF---------------------
S-S------L--------R--PSQ-WGIPDLSSRLSRELKRHIKKL-L----
>XP_046563126.1
------------INLPA--------VAVI--GD----Q----------------
SAGKSSVLE-----A--IS-G-V-QL-PRG--------------TG-IVTRCPL---------------EMR--------------M---
KHS------------------------------ED--
------------------EDK--------------WE--------------------------GKI----------M---------------YKD-K-H----
----------DM-------------------------PHEEVILNRE-----------SVGDLV------RKAQ-TE---M-TD----------
--------------GA-------------------T-GISDE-LITLEVTSSD---
---------VPDLTVIDLPGIAR--NA-------------------V-------EG--------------Q--------PF--D--IEARIK----
----NMIR-QYI---ER------------QETIILAVLQCNV--D-IATCE-ALKMAKEFD------
DEGGRTLGVLTKPDLLD-------KGA------E-----TSV----------------------------IRI--LN---NM---------E-
FT-----------------------L---SKGYVIVKCR--G-QEA--ISE-------
----G--------QS--------------------LK----------HALDV---EEDFFRS--------HR-HF---------------------
S-A------L--------R--PSQ-WGIPNLSERLSRELKKHIKKL-L----
>XP_046565195.1
------------INLPA--------VAVI--GD----Q----------------
SAGKSSVLE-----A--IS-G-V-QL-PRG--------------TG-IVTRCPL---------------EMR--------------M---
KHS------------------------------ED--
------------------EDK--------------WE--------------------------GKI----------M---------------YKD-K-H----
----------DM-------------------------PHEEVILNRE-----------SVGDLV------RKAQ-KE---M-TD----------
--------------GA-------------------T-GISDE-LITLEVMSSD--
----------VPDLTVIDLPGIAR--NA-------------------V-------EG--------------Q--------PF--D--IEARIK---
-----NMIR-QYI---QR------------QETIILAVLQCNV--D-IATCE-ALKMAKEFD------
DEGGRTLGVLTKPDLLD-------KGA------E-----TGV----------------------------IRI--LN---NM---------
E-FT-----------------------L---SKGYVIVKCR--G-QEA--ISE----
-------G--------QS--------------------LK----------HALDV---EEDFFRS--------HR-HF--------------------
-S-A------M--------R--PSQ-WGIPNLSERLSRELKKHIKKL-L----
>XP_046563125.1
------------INLPA--------VAVI--GD----Q----------------
SAGKSSVLE-----A--IS-G-V-QL-PRG--------------TG-IVTRCPL---------------EMR--------------M---
KHS------------------------------ED--
------------------EDK--------------WE--------------------------GKI----------M---------------YTD-K-H----
----------DE-------------------------PHQEVILNRE-----------SVGDLV------RKAQ-KE---M-TD----------
--------------SA-------------------K-GISDE-LITLEVTSSD---
---------VPDLTVIDLPGIAR--NA-------------------V-------EG--------------Q--------PV--D--IEARIK----
----NMIR-QYI---ER------------QETIILAVLQCNV--D-IATCE-ALKMAKEFD------
DEGGRTLGVLTKPDLLD-------RGA------E-----TGV----------------------------MRI--LN---NM---------
E-FT-----------------------L---SKGYIIVKCR--G-QEA--ISE-----
------G--------QS--------------------LK----------HALDV---EEDFFKS--------HR-HF---------------------
S-S------L--------G--PSQ-WGIPNLSRRLSRELKKHIKKL-L----
>XP_046352527.2

------------INLPA--------VAVI--GD----Q----------------
SAGKSSVLE-----A--IS-G-V-QL-PRG-------------TG-IVTRCPL---------------EMR-------------M---
KHS------------------------------ED---
------------------EDM-------------WE--------------------------GKI----------M---------------YKD-M-H---
-----------GE-------------------------AHEEIILNRE-----------SVGELV------RKAQ-KE---M-TD-----------
-------------SA-------------------K-GISDE-LITLEVTSSD--
--------VPDLTVIDLPGIAR--NA------------------V-------EG--------------Q--------PV--D--IEARIK-----
---QMIR-KYI---GR------------QETIILAVLQCNV--D-IATCE-ALKMAKEFD------
DEGGRTLGVLTKPDLLD-------KGA------E-----SGV---------------------------VRI--LN---NM---------
E-FT----------------------L---SKGYIIVKCR--G-QEA--ISD-----
------G--------QS--------------------LK----------QALEV---EEDFFKS--------HR-HF---------------------
S-S------L--------R--PSQ-WGIPNLSTRLSRELKKHIKKL-L----
>XP_048248476.1
------------INLPA--------VAVI--GD----Q----------------
SAGKSSVLE-----A--IS-G-V-QL-PRG-------------TG-IVTRCPL---------------EMR-------------M---
KHS------------------------------ED---
------------------GDM-------------WE--------------------------GKI----------M---------------YKD-M-H--
------------DM-------------------------AHEEIILNRE-----------SVGELV------RKAQ-IE---M-TD----------
--------------SA-------------------K-GISDE-LITLEVTSSD---
---------VPDLTVIDLPGIAR--NA------------------V-------EG--------------Q--------PF--D--IEARIK----
----NMIR-RYI---GR------------QETIILAVLQCNV--D-IATCE-ALKMAKEFD------
TEGGRTLGVLTKPDLLD-------KGA------E-----SGV----------------------------VRI--LN---NK---------
E-FT----------------------L---SKGYIIVKCR--G-QEA--ISD-----
------G--------QS--------------------LK----------QALEV---EEDFFKS--------HR-HF---------------------
S-S------L--------R--PSQ-WGIPNLSMRLSRELKKHIKKL-L----
>XP_048258111.1
------------INLPA--------VAVI--GD----Q----------------
SAGKSSVLE-----A--IS-G-V-QL-PRG-------------TG-IVTRCPL---------------EMR-------------M---
KHS------------------------------ED---
------------------EDM-------------WE--------------------------GKI----------M---------------YKD-M-H---
-----------DM-------------------------AHEEIILNRE-----------SVGELV------RKAQ-KE---M-TD----------
--------------SA-------------------K-GISDE-LITLEVTSSD---
---------VPDLTVIDLPGIAR--NA------------------V-------EG--------------Q--------PF--D--IEARIK----
----NMIR-RYI---GR------------QETIILAVLQCNV--D-IATCE-ALKMAKEFD------
AEGGRTLGVLTKPDLLD-------KGA------E-----AGV---------------------------VRI--LN---NM---------
E-FT----------------------L---SKGYIIVKCR--G-QEA--ISY-----
------G--------QS--------------------LK----------QALEV---EEDFFKS--------HR-HF---------------------
S-S------L--------R--PSQ-WGIPNLSARLSRELKKHIKKL-L----
>XP_046352531.2
------------INLPS--------VAVI--GD----Q----------------
SAGKSSVLE-----A--IS-G-V-QL-PRG-------------TG-IVTRCPL---------------EMR-------------M---
KHS------------------------------ED---
------------------EDM-------------WE--------------------------GKI----------M---------------YKD-M-Y---
-----------DM-------------------------AHEEIILNRE-----------SVGELV------RKAQ-KE---M-TD----------

--------------SA-------------------K-GISDE-LITLEVTSSD--
---------VPDLTVIDLPGIAR--NA-------------------V-------EG--------------Q--------PF--D--IEARIK----
----NMIR-KYI---GR------------QETIILAVLQCNV--D-IATCE-ALKMAKEFD------
AEGGRTLGVLTKPDLLD-------KGA------E-----SGV---------------------------VRI--LN---NM---------
E-FT-----------------------L---SKGYIIVKCR---G-QEA--ISD-----
------G--------QT--------------------LK----------QALEV---EEDFFKS--------HR-HF---------------------
S-S------L--------R--PSQ-WGIPNLSGRLSRELKIHIKKL-L----
>XP_048248472.1
------------INLPS--------VAVI--GD----Q----------------
SAGKSSVLE-----A--IS-G-V-QL-PRG--------------TG-IVTRCPL---------------EMR--------------M---
KHS------------------------------ED--
------------------EDM--------------WA--------------------------GKI----------M---------------YKD-M-Y---
-----------DM-------------------------THEEIILNRE-----------SVGELV------RKAQ-KE---M-TD-----------
-------------SA-------------------K-GISDE-LITLEVTSSD---
--------VPDLTVIDLPGIAR--NA-------------------V-------EG--------------Q--------PL--D--IEARIK------
--NMIR-RYI---RR------------QETIILAVLQCNV--D-IATCE-ALKMAKEFD------
AEGGRTLGVLTKPDLLD-------KGA------E-----TGV---------------------------VRI--LN---NM---------
E-FT-----------------------L---SKGYIIVKCR---G-QEA--ISD-----
------G--------QS--------------------LK----------QALEV---EEEFFKS--------HR-HF---------------------
S-S------L--------R--PSQ-XGIPNLSGRLSRELKIHIKKR-L----
>XP_048248474.1
------------INLPS--------VAVI--GD----Q----------------
SAGKSSVLE-----A--IS-G-V-QL-PRG--------------TG-IVTRCPL---------------EMR--------------M---
KHS------------------------------ED--
------------------EDM--------------WA--------------------------GKI----------M---------------YKD-M-Y---
-----------DM-------------------------THEEIILNRE-----------SVGELV------RKAQ-KE---M-TD-----------
-------------SA-------------------K-GISDE-LITLEVTSSD---
--------VPDLTVIDLPGIAR--NA-------------------V-------EG--------------Q--------PL--D--IEARIK------
--NMIR-RYI---RR------------QETIILAVLQCNV--D-IATCE-ALKMAKEFD------
AEGGRTLGVLTKPDLLD-------KGA------E-----TGV---------------------------VRI--LN---NM---------
E-FT-----------------------L---SKGYIIVKCR---G-QEA--ISD-----
------G--------QS--------------------LK----------QALEV---EEEFFKS--------HR-HF---------------------
S-S------L--------R--PSQ-WGIPNLSGRLSRELKIHIKKR-L----
>XP_048248473.1
------------INLPS--------VAVI--GD----Q----------------
SAGKSSVLE-----A--IS-G-V-QL-PRG--------------TG-IVTRCPL---------------EMR--------------M---
KHS------------------------------ED--
------------------EDM--------------WA--------------------------GKI----------M---------------YKD-M-Y---
-----------DM-------------------------THEEIILNRE-----------SVGELV------RKAQ-KE---M-TD-----------
-------------SA-------------------K-GISDE-LITLEVTSSD---
--------VPDLTVIDLPGIAR--NA-------------------V-------EG--------------Q--------PL--D--IEARIK------
--NMIR-RYI---RR------------QETIILAVLQCNV--D-IATCE-ALKMAKEFD------
AEGGRTLGVLTKPDLLD-------KGA------E-----TGV---------------------------VRI--LN---NM---------
E-FT-----------------------L---SKGYIIVKCR---G-QEA--ISD-----

------G--------QS-------------------LK---------QALEV---EEEFFKS--------HR-HF--------------------
S-S------L--------R--PSQ-WGIPNLSGRLSRELKIHIKKR-L----
>ABI53802.1
------------INLPA--------VAVI---GD----Q---------------
SAGKSSVLE-----A--IS-G-V-QL-PRG-------------TG-IVTRCPL---------------EMR-------------M---
KHS-----------------------------EA---
------------------EDM-------------WE-------------------------GKI----------M--------------YKD-M-Y---
-----------DV------------------------AHEEIILNRE-----------SVEELV------RKAQ-KE---M-TD-----------
-------------SA-------------------K-GISDE-LITLEVTSSD---
--------VPDLTVIDLPGIAR--NA------------------V-------EG-------------Q--------PF--D--IEARIK-----
---NMIR-RYI---GR------------QETIILAVLQCNV--D-IATCE-ALKMAKEFD------
AEGGRTLGVLTKPDLLD-------KGA------E-----TGV--------------------------VRI--LN---NM---------
E-FT----------------------L---SKGYIIATCR---G-QEA--ISD-----
------G--------QS-------------------LT---------QALEV---EEDFFKS--------HR-YF--------------------
S-S------L--------R--PSQ-WGIPNLSGRLSRELKKHIKKL-L----
>CAH1802128.1
------------VALPA--------VVVI---GD----Q---------------
SVGKSSVLE-----A--MS-G-V-QL-PRG-------------TG-IVTRCPL---------------ELR-------------M---
KQC-----------------------------D---
------------------PGN-------------FH-------------------------AKI----------S--------------YDI-Q-G-----
---------GH-----------------------QPLEKTITDPS-----------NIDFEI------RQAQ-RA---L-VG------------
------------DS-------------------G-GVSDR-LIRLEVQADY---
----------VPDLTLIDLPGIVR--YS------------------E-------GS-------------D-----------T--IVEETK------
--NLIK-TYV---SR------------PETIILVVIPCNV--D-IDTVE-ACNLAKQVD------
PNGDRTIGVLTRPDLID-------HGV------GP---IKEV---------------------------LDI--LE---NK---------K-
MK----------------------L---KKGFYVVKCR---S-QKR--IEE-----
------G--------QS-------------------LE---------QALAE---EVQFFRS--------DE-RF--------------------
R-V------I--------N--PSQ-CGVKQLSSKLTNELFLHIKNC-V----
>PAA74204.1
------------VRLPA--------IAVV---GD----Q---------------
SVGKSSVLE-----S--IS-G-I-DL-PRG-------------LG-IVTRCPL---------------MLS-------------M---R-
--------------------------------NRE--
--------------EAG-------------WS-------------------------ARI----------K--------------YKT-K-T-----------
---GE-------------------------GREKKLTGAS-----------EVGQAI------RDAQ-NE---M-TN-----------------
-------SS-------------------G-EVSEQ-LIELWVESPE---
----SPDLTLIDLPGIAR--YS------------------I-------DG-------------G--------G---A--IAGLTK--------
SLIL-SYI---EK------------EEILILVVIPCHV--D-IETVE-ALSLAKEVD------PESKRTIGVLTCPDLVN----
---PGS------E-----SEV---------------------------LAL--MQ---NR---------K-IP----------------------L---
KKGYVSVRCR---T-PQQ--LKD-----------N--------MS----------
----------LQ----------QAARE---EEVFFRT--------HP-HF--------------------R-A------L--------D--KFE-
YGTKTLAVKLSSELYEAIKHN-I----
>PAA76532.1
------------ISLPA--------IAVV---GD----Q---------------
SVGKSSVLE-----A--IS-G-V-EF-PRG-------------LG-IVTRCPL---------------MLS-------------M---R-

-------------------------------GRE--
--------------DSG--------------WT---------------------------ARI-----------R---------------YET-K-S----------
----GQ--------------------------ARDKPLSTPA-----------EIGQAI------RDAQ-EE---M-TS-----------------
-------SS-------------------G-EISEK-LIELHIEGAD---
--TPDLTLIDLPGIAR--FS--------------------I-------AN--------------A--------G---D--IATVSK--------
SLIM-SYI---LK------------PEVLILVVIPCNV--D-VETVE-AISLAREVD------PECKRTLGVLTCPDLVN--
-----PGS------E-----TEV----------------------------LAM--MR---NE---------R-LK-----------------------L-
--RKGFVTVRCR---T-PQQ--LKD-----------N--------MG--------
------------LR----------EACKA---EEEFFKL--------HP-QF----------------------C-A------L--------G--
DYQ-RGCKTLANKLSVELYQAVKER-I----
>PAA92268.1
------------LKLPM--------VAVV---GD----Q---------------
SVGKSSVLE-----A--IS-G-V-EF-PRG-------------TG-MVTRCAL---------------QLS--------------M---
QWN-------------------------------ADP--
--------------------EAP--------------WH---------------------------GRI----------S---------------YKD-V-N---
-----------GH-------------------------KVDKELNSPG-----------EVDGAV------REAQ-QR---M-TH-------
-----------------GD-------------------N-EISSE-QIDLAIKGPD---
--------------VPDLTLIDLPGIAR--YS-------------------A-------TG--------------G--------S---G--IAQITK-
-------SLIA-KYV---SQ------------PQVLILVVVPCHQ--D-IETVE-ALSLAKEAD------
PQGERTIGVLTCPDMVN-------KGA------E-----QET----------------------------LKI--AN---NE---------
K-IP-----------------------L---KKGYVMVKCR---S-PEE--LNN--
---------G--------VT--------------------LS----------ESVAN---EAAFFKT--------HR-HF--------------------
-S-L------L--------P--EQS-VGIRTLADKLTEELFESVKRN-I----
>PAA69582.1
------------LRLPT--------VAVV---GD----Q---------------
SVGKSSVLE-----A--IS-G-V-DL-PRG-------------TG-IVTRCPL---------------QLS--------------M---R-
-------------------------------SKP--
--------------TGD--------------WT---------------------------GRI----------S---------------YQN-R-K---------
-----GE-------------------------HVEREISKKC-----------EVDEMV------RKVQ-NE---I-TG----------------
--------DS-------------------N-GVSTE-QIDLTIESAD---
----VSDLTLVDLPGIAR--YS-------------------E-------KN-----------------------P---K--INEVTK--------
QLIL-SYI---SQ---------DQVIILVVVPCSV--D-IETVE-AIALAKQVD------PGGTRTIGVLTCPDLTN--
-----PGS------E-----EDI----------------------------KAI--VN---NQG--------R-VR-----------------------L-
--HKGFVMVKCR---S-PKE--LRN----------N--------IS--------
-----------LS---------EVAKI---EEDYFKN--------DP-HF---------------------S-Q------L--------P--KDI-
VGTKTLAEKLTNELFKAVAAG-I----
>PAA83069.1
------------LKLPS--------IVVI---GD----Q---------------
SSGKSSVLE-----T--IS-G-V-SF-PRG-------------NG-VVTLCPL---------------QLS--------------M---
RSS-------------------------------DK---
--------------------K--------------WR---------------------------GTV----------R---------------YFD-A-Q-----
---------GK-------------------------EVHWDIDSPD----------DVENAI------QNAQ-MR---I-TG-------------
--------------HK-------------------K-AISKN-IIEMTLEAPD--
---------LPNLTLVDLPGIAR--YS-------------------H-------SD--------------G--------GS-VN--LYKLTT---

-----DIIK-EYI---QR------------EETIILTVIPLSA--D-TATME-ALQLAKDVD------
PYGLRTIGVLTFPDLVN-------KGA------E-----EEK----------------------------LQI--AR---NI---------T-
FP----------------------L---SKGYITVKCR---N-QED--IKS------
-----R--------KS-------------------LR----------EAKVD---EMRFFSN--------DP-FF---------------------
S-Q------L--------D--PSQ-RGTDTLAKRLSTELLTLIKKF-I----
>PAA94353.1
------------LRLPS--------IVVI---GD----Q---------------
STGKSSVLE-----S--IS-G-V-RF-PRG--------------NG-VVTLCPL---------------QLS--------------M---
RTS------------------------------DD---
-------------------GK-------------WR---------------------------GNI----------R---------------YYD-T-Y------
--------GK-------------------------LMKWDIDGPE-----------DVEDAI------QEAQ-MR---I-TG------------
------------NQ-------------------R-NVSKS-IIEMTLESPE---
---------LPNLTLIDLPGIAR--YN-------------------H-------NS--------------A--------ESGAS--LHQLTT--
------DIIK-EYI---RR------------EETIILVVIPLTS--D-TATME-ALQLAKDAD------
PYGMRTIGVLTFPDLVN-------KGA------Q-----EEK----------------------------LMI--AR---NI---------T-
YP----------------------L---SKGYVTVKCR---N-QED--IKN-----
------R--------KS-------------------LK---------DAKAD---EALFFNT--------DP-FF---------------------
K-Q------L--------D--SMY-RGSDTLARRLSEELLYLVKKF-I----
>KAI9324922.1
---------DRYVELPQ--------IATM---GD----T---------------
SSGKSSVLS-----A--IS-G-I-TF-PSS--------------SE-LTTRCPT---------------QLI--------------L---SQS-
-----------------------------ET---
----------------------------FS--------------------------GSV----------R---------------LQR-F-KP-------------
QQN---------------------EEPPETKTLTNAN-----------EIEHEI------ERLT-RQ---L-VQ-------------------
----EN-------------------Q-SISDD-SIIINVSGPN--
YPNLTLTDLPGLIR--TV-------------------E-------DS-------------E--------DP--A--IIGRVR--------
ALVD-RYL---VQ------------SRTVILAVVPANV--D-VHNTE-ILQAAEAAD------PQGVRTISIITKPDLID-
------SGA------E-----SQV----------------------------VEL--LL---NR---------K-KM-----------------------
L---KLGYHAVKCR---G-QKD--LNN-----------G--------VS-----
---------------IA----------DGIAK---EAEFFET--------HA-VW---------------------R-K------V--------D--
SSY-FGISKLTEKLVKILETVIGGS-L----
>KAJ3066410.1
---------DKYVELPQ--------IAVM---GD----T---------------
SSGKSSVLS-----A--IS-G-I-TF-PSS--------------SE-LTTRCPT---------------QLI--------------L---SEA-
-----------------------------EE---
----------------------------FS--------------------------GTV----------R---------------LMR-F-KP-------------
QEG----------------------EILESTTLSSPA-----------DITGEI------ERLT-KQ---I-VS------------------------
EQ-------------------Q-LISDD-AIIIEVRGPG---
YPNLTLTDLPGLIR--TV-------------------E-------DH-------------E--------DK--D--IIRRVR--------
GLVD-RYL---VQ------------NRTVILAVVPANV--D-VHNTE-ILQAAQDAD------
PEGIRTISIITKPDRID-------PGA------E-----SQV----------------------------VDL--LM---NR---------K-
KK----------------------L---KLGYHAVRCR---G-QQD--LDD---
--------G--------VT-------------------IA----------DGIVN---ETKFFSE--------HK-AW---------------------
S-D------V--------D--PSY-VGINRLTEKLVKILQSIIASS-L----

>KAI8836453.1
---------DKYIELPQ--------IAVM---GD----T---------------
SSGKSSVLS-----A--IS-G-I-EF-PSS--------------DT-LTTRCPT---------------QIV-------------L---SEA-
----------------------------DK---
----------------------------FS--------------------------GTV----------C---------------LVR-F-GS-------------
GIS-----------------------SHLTHLKNRN-----------EITTEI------ARLT-QV---I-RD------------------------
EG-------------------Q-TISDD-AIVIEVRGPE---
YPNLTLTDLPGIIR--TV-------------------Q-------DN-------------E--------DP--A--MIPRVR--------
QLVD-RYL---VQ------------KRTVILAVVPANV--D-FHNSE-ILQAAEKVD------PKGERTIAIITKPDAID-
------PGA------E-----QSV----------------------------LDL--LM---NK---------K-KA-----------------------
L---RLGYHAVRCR---G-KQH--HDD-----------K--------MT-----
---------------IP----------EGLEM---EHDFFHN--------HK-VW---------------------K-S------V--------A--
PSY-VGCERLTEKLVKVLRNIITDS-L----
>KAJ3350919.1
---------DQYVELPQ--------IAVM---GD----T---------------
SSGKSSVLS-----A--IS-G-I-EF-PSH--------------AE-LTTRCPT---------------QLV-------------L---
STA------------------------------EA---
---------------------------------FT--------------------------CTV----------C---------------IIR-V-KN---------
----SKA----------------------SSPPSLKLKEPG-----------EIRGAI------TELT-KI---I-ID--------------------
---DG-------------------P-AISED-MISIEVSGPK--
YPNLTLIDLPGIVR--TV-------------------A-------DN-------------E--------DP--S--MILNIR--------
DLVT-SYL---QK------------KRTVILAVIPANV--D-MHNVE-ILQLAESVD------
PSGDRTIAIITKPDAVD-------CGA------E-----KQI---------------------------VDL--LL---NR---------K-
KF----------------------L---KLGYHAMRCR---G-QQD--LNE--
---------K--------MS--------------------IH----------EGVIK---ESKFFYN--------HP-VW-------------------
--R-N------I--------S--PEL-LGVESLVPKLVNTLQNVINQS-L----
>TDH66190.1
------------IELPT--------IAVM---GD----R---------------
SSGKSSLLS-----A--LS-G-I-SF-PSN--------------DQ-LTTRCPT---------------QLI-------------L---
TRG------------------------------DV---
----------------------------------FH--------------------------GTV----------R---------------LVR-F-QT------
-------GSDREQIEATEDLK-----HLKDVEQIEATEDLKHLK-----------DVPNAI------SKLT-KK---I-IN----
--------------------DG-------------------L-IISND-QIVIEVCGPE--
----------------IPELTLVDLPGLVR--TA-------------------K-------KL-------------K--------DK--S--
MIPRVQ--------KMVD-RYM---KK------------EQIVVIAVVPANK--D-MHNSE-ILKTVQAAD------
PDGTRTIAVVTKLDLVN-------AGA------E-----KTV---------------------------LEF--LL---NK---------N-
KQ----------------------M---HLGYHAVKCR---N-QRD--LTT---
--------G--------MS--------------------IE----------IGLAN---ERTFFSQ--------HE-YW-------------------
-S-Q------L--------P--SHL-WGIPTLTKRLISILQDNTRKT-FQNA-
>XP_024574100.1
------------IELPQ--------IAVM---GD----T---------------
SSGKSSLLS-----A--LS-G-V-AF-PSN--------------NQ-LTTRCPT---------------QLV-------------L---
TRA------------------------------DT---
---------------------------------FQ--------------------------GTV----------K---------------LVR-F-ES-------

---------------------------SDNDENNEAEDLKRME-----------DVPDAI------TKLT-QK---L-VD------------
------------EG------------------Q-YISDD-QIVIEMCGPE---
----------LPDLTLTDLPGLVR--TV------------------G-------DH-------------E--------DQ--S--IISRVR---
-----EMVN-RYM---KQ-----------ERTVIIAVVPANV--D-MHNTE-ILQAAQEAD------
PTGTRTIAVVTKMDLVD-------GGA------E-----SAV---------------------------HDL--LL---NK---------
K-KK----------------------M---RLGYHAVKCR---N-QRE--LTE-
----------G--------AS-------------------IE---------KGLEN---EISFFSR--------HE-YW------------------
--R-R------L--------P--SHL-WGIATLTDRLVLILQDNIRRS-LPKV-
>KAG1692046.1
------------IELPQ--------IAVM---GD----T---------------
SSGKSSLLS-----A--LS-G-V-SF-PSN-------------DQ-LTTRCPT--------------QLV-------------L---
TRA-----------------------------DT--
----------------------------------FR--------------------------GTV---------R--------------LVR-F-QS-------
--------------------------NGDSNDGEEKEDLNRLE-----------DVPDAI------TKLT-NK---L-IA------------
------------EG------------------Q-YISDD-QIVIEMCGPE---
----------LPNLTLTDLPGLVR--TV------------------G-------DH-------------E--------DQ--S--IIPRVR--
------QMVD-RYM---QQ-----------ERTVIIAVVPANV--D-MHNTE-ILQAAQQAD------
PNGTRTIAVVTKVDLVD-------PGA------E-----LAV---------------------------HEL--LL---NR---------K-
KK----------------------M---HLGYHAVKCR---N-QRE--LTK----
-------G--------TS--------------------IE----------KGLVN---ETMFFSQ--------HE-YW--------------------
R-R------L--------P--SHL-WGVPRLTDRLVSILQDNIRRS-LPKV-
>KAG3062152.1
------------IELPQ--------IAVM---GD----T---------------
SSGKSSLLS-----A--LS-G-V-SF-PSN-------------DQ-LTTRCPT--------------QLI-------------L---
TRA-----------------------------DT--
----------------------------------FR--------------------------GTV---------R--------------LVR-F-QS-------
--------------------------NGEHDEGEEKQDLNRLE-----------DVPDAI------TKLT-QK---L-VD-----------
-------------EG------------------Q-HISDD-QIVIEMCGPE--
-----------LPNLTLTDLPGLVR--TI------------------G-------DH-------------E--------DQ--S--IIPRVR--
------QMVD-RYM---KQ-----------ERTVIIAVVPANV--D-MHNTE-ILQAAQEAD------
PNGTRTIAVVTKVDLVD-------AGA------E-----LAV---------------------------HEL--LL---NK---------K-
KK----------------------M---HLGYHAVKCR---S-QRE--LTK----
-------G--------TN-------------------IE---------KGLAN---EMTFFGQ--------HE-YW-------------------
-C-R------L--------P--THL-WGVSRLTERLVSILQDNIRRS-LPKV-
>XP_009533587.1
------------VELPQ--------IAVM---GD----T---------------
SSGKSSLLS-----A--LS-G-V-SF-PSS-------------DQ-LTTRCPT--------------QLV-------------L---
TRA-----------------------------DA---
----------------------------------FR--------------------------GTV---------R--------------LVR-F-QS------
-------G------------------NNSDNDDGEEKADLQRLE-----------DVPDAI------TKLT-QK---L-VD--------
----------------EG------------------Q-YISDD-QIVIEMCGPD---
--------------LPNLTLTDLPGLVR--TV------------------G-------DH-------------E--------DQ--S--
IIPRVR--------QMVD-RYM---QQ-----------ERTVIIAVVPANV--D-MHNME-ILQAAQEAD------
PNGTRTIAVVTKVDLVD-------AGA------E-----LAV---------------------------HEL--LL---NK---------K-

KR----------------------M---HLGYHAVKCR--S-QRE--LTK----
-------G--------TS-------------------IE----------KGVAN---ELAFFGQ--------HE-YW--------------------
C-K------L--------P--THL-WGVPRLSERLVSILQDNIRRS-LPKV-
>KAI9918701.1
------------IELPQ--------IAVM--GD----T---------------
SSGKSSLLS-----A--LS-G-V-SF-PSS-------------DQ-LTTRCPT--------------QLI-------------L---
SRS-----------------------------DS---
---------------------------------YH-------------------------AHV----------R---------------LVR-F-QS-----
--------AG----------------MTDENDDGEERAILNRLE----------DVPDAI------SKLT-QK---L-ID-------
---------------EG------------------Q-YISDD-RIVIEISSPD---
----------LPDLTLTDLPGLVR--TV------------------G-------DH--------------E--------DH--S--IIPRVR--
------QMVD-RYM---KQ-----------ERTVIIAVVPANV--D-MHNTE-ILQAAQEAD------
PNGTRTIAVVTKMDLVD-------AGA------E-----LAV--------------------------HEL--LL---NR---------
K-KK----------------------M---HLGYHAVKCR--N-QRE--LTK-
----------G--------TS-------------------IE----------KGLAN---EMTFFGQ--------HE-YW----------------
----C-R------L--------P--KHL-WGVPRLSERLVSILQNNIRQS-LPKV-
>RMX63821.1
------------IELPQ--------IAVM--GD----T---------------
SSGKSSLLS-----A--LS-G-I-SF-PSS-------------DQ-LTTRCPT--------------RVL-------------L---
TRA-----------------------------DT--
--------------------------------FR-------------------------GSV----------R---------------LIR-F-QT-------
-----SA-----------N-----DGGENKKREEIESLNRME----------DVPDAI------TKLT-KK---L-VS----------
------------EG------------------Q-YISDD-EIVIEMSGPN--
----------LPNLTLTDLPGLVR--TV------------------G-------DN-------------E--------DQ--S--IISRIR---
-----QIVN-RYM---QQ------------ERTIILAVVPANV--D-MHNTE-ILQAAQEAD------
PSGTRTIAVITKLDLVD-------TGA------E-----AGV--------------------------HEL--LL---NK---------K-
KC----------------------M---KLGYHAVKCR--N-QLE--LTN----
-------G--------TT-------------------IE----------KSVVN---EMSFFSE--------HP-YW--------------------
R-R------L--------P--NHL-WGVSRLVERLVSILQDNVRRS-LPKV-
>GMH36208.1
-------------MLPA--------IVVV--GD----Q---------------
SSGKSSLLE-----I--LS-G-V-TL-PRG-------------EG-ICTRVPL--------------ELQ-------------L---
RNG-----------------------------TEV--
----------------------------------S-------------------------AQI----------E--------------YQT-D-L--------
---------------------------DAPRVSKHIM-VE----------EVKNEI------LLAT-KR---I-AG-----------------
------ME------------------L-NIKDL-PIVLRMTGPT---
--YQDLTLIDLPGIAR--MP----------------L-------RG-------------Q--------PD--N--IEELTM--------
EMIQ-KYI---NG------------DSKVILCAVPANN--E-FVTSA-ALKLASNVD------
PLGLRTLGVVTKADQFS-------RGM------R-----RRL----------------------------EGL--DD---T---------
D-VK----------------------L---KLGFVAVRCR--T-QKE--LEE---
--------G--------IS-------------------LQ----------DVRMR---EELLFET--------DP-EL--------------------
R-D------V--------Q--PHC-RGISTLVDKLVDIQKERLIEQ-LPRIV
>GMH43921.1

-----------SQMLTA--------IVVV--GD----Q----------------
SSGKSSTLE-----R--IA-G-I-DL-PRG--------------QG-ICTRVPL---------------EMQ--------------M---
RKG------------------------------SKF--
------------------------------------S--------------------------ATL----------E---------------YQQ-E-K--------
------------------------------GGSKQSVEIKDAS-----------KISDAI------QAAT-RD---I-VG----------------
--------NS-------------------K-NVEDL-PLVLRISSPI--
----YQDLTLIDLPGIAR--AP-------------------L-------PG--------------Q--------RS--D--IEEQTL--------
EMMR-RYI---TG------------EAKVILCALPATN--D-FVTSA-ALKLALQLD------
PDGERTLGAVTKIDQAR-------KGI------A-----KKL----------------------------EGT--DA---S----------E-
IT-----------------------L---HLGFAGVRCR--T-ENE--TDA------
-----G--------IT--------------------LE----------QVRQA---EEELFRT--------HD-EL---------------------K-
H------V--------D--DSC-KGVSALLQKLVAVQRGRLISH-LPKVL
>CAG9460856.1
------------DKLPT--------IVVT--GD----Q----------------
SAGKSSVLE-----S--LS-G-I-AF-PVG--------------DG-IVTRLPC---------------QVA--------------L---
REG------------------------------PAF--
-------------------RA--------------VC--------------------------TPP----------E---------------G---------------
------------------------------HGEA-VTLTDPK-----------AVTKWI------EDTT-AA---V-AG------------------
------DK-------------------K-GVLDK-PLSIKVEREG---
---SADLTLVDLPGITR--VA-------------------V-------DG--------------Q--------AD--D--IEEQVK--------
RMIQ-RYI---SR------------EAAVVLCVLPANV--D-FSTAE-CIKMARAVD------
PGGERTLGVVTKVDRAE-------RGI------V-----TRL----------------------------NAF--GT---T----------G-
WA-----------------------L---RLGYVAVKNL--S-QDE--RAK----
------HG--------VS--------------------TT----------KVLEL---EDAFFDDGVG-RPAHLA-EL----------------
-----A-D------L--------D--ADM-RGLRTLVQKLVQVQGERIEAF-MPSLV
>GJP35534.1
----------EGIELPT--------IVVV--GD----Q----------------
SSGKSSVLE-----N--LS-G-I-SL-PRG--------------KG-IVTRVPL---------------ILR--------------L---
QSC------------------------------VK--
-------------------GK--------------DM--------------------------ITI----------E---------------Y---T-P---------
----------------------------VTGKVSKVLSDEE-----------MIEEEI------SEAT-IA---L-AG--------------------
----SR-------------------K-GVMNC-PITLQVQRPD---
---LPDLTLVDLPGITR--VP-------------------I-------ED--------------Q--------PK--D--IYNQVK--------
NMIM-HYI---TP------------KESVILNVLAAEV--D-FSTCE-SIVMSQEVD------
SDGDRTLAVVTKVDRAP-------DGL------Y-----EKI----------------------------QGN------------------S-
VR-----------------------I---GLGYVCVRNK---TDADA--SHD----
--DARRA---EAAFFNS--------HP-EL---------------------S-
Q------I--------E--SHC-LGIPALAQRLTEIQAKRVADS-IPRIR
>CAI5480041.1
----------EGIELPT--------IVVV--GD----Q----------------
SSGKSSVLE-----N--LS-G-I-SL-PRG--------------NG-IVTRVPL---------------ILR--------------L---
QSC------------------------------TS--
-------------------KD--------------GE--------------------------ITI----------E---------------YN--N-P--------
------------------------------SSGKIFKILPDEE-----------SIQEEI------SKAT-VT---L-AG-------------------

-----SR------------------K-GVMDR-PITLQVKRSG---
---LPDLTLVDLPGITR--VP------------------V-------DD-------------Q--------PK--D--IYNQVK--------
KMIM-QYI---TP------------EESVILNVLAAEV--D-FSTCE-SIVMSQEVD------
QDGDRTLAVVTKVDRAP-------DGL------Y-----EKI--------------------------QGN-----------------S-
VR----------------------I---GLGYVCVRNK---TDADA--SHA----
---AARLA---ETDFFDR--------HP-EL--------------------S-
R------I--------E--TDS-RGIPALAQRLSEIQAKRVAES-IPRIR
>KAJ7294545.1
-----------GIQLPT--------IVVV--GD----Q---------------
SHGKSSVLE-----S--LA-E-I-TL-PRR-------------QG-IATRVPL---------------ILR-------------L---
QSC----------------------------KVA---
--------------------SE-------------QS------------------------ITI---------E--------------Y---------------
----------------------------LNVKD--EIKSEE-----------LIEAAI------DEAT-NV---L-AG--------------------
---PR------------------K-DVRDT-PISLHVRKLG---
APDLTMVDLPGITR--VP------------------V-------HG-------------Q--------PE--N--IYEQIA--------
AMIQ-KYI---NP------------PESIILNVISATV--D-FPTCE-SIRMSQLAD------
KEGKRTLAVVTKVDKAP-------EGL------Y-----EKV--------------------------TSD-----------------A-
VN----------------------I---GLGYICVRNR---TEKEN--SNE-----
---QARFV---EKHLFDT--------HP-SL--------------------C-
K------L--------D--KSM-VGIPMLAYRLTCIQAQMIQGC-LPGL-
>XP_024380180.1
----------ENVNIPT--------IVVV--GD----Q---------------
SSGKSSVLE-----S--LA-G-I-TL-PRG-------------QG-IATRVPL---------------ILR-------------L---
QSC----------------------------LSE--
--------------------QD-------------SK------------------------ILM---------E--------------Y-------------
------------------------------GSVKE-MRINSED-----------DIEAAI------NAAT-DD---L-AG---------------
---------SN-------------------K-NIRDT-PILLHIRKPD---
----APDLTMVDLPGITR--VP------------------V-------HG-------------Q--------PE--N--IYEQVR-------
-DMIM-HYI---KP------------EESIILNVLPAEV--D-FSTCE-SIRLSQTVD------
KKGVRTLAVVTKVDKAP-------EGL------F-----EKV--------------------------TSD-----------------A-
VS----------------------I---GLGYVCVRNR---TPADD--SIA-----
---VARCR---ELELFND--------HP-DL--------------------R-
N------I--------D--RSM-VGIPTLGRRLVKIQSDMVRGC-LPRIR
>XP_024367947.1
----------ENVNIPT--------IVVV--GD----Q---------------
SSGKSSVLE-----S--LA-G-I-TL-PRG-------------QG-IATRVPL---------------ILR-------------L---
QSC----------------------------LSE--
--------------------QD-------------SK------------------------ILM---------E--------------Y-------------
------------------------------ENVKE-MRINSED-----------DIEAAI------NAAT-DD---L-AG---------------
---------SN-------------------K-NIRNT-PISLHIRKPD---
-----APDLTMVDLPGITR--VP------------------V-------HG-------------Q--------PE--N--IYEQVR------
--DMIM-HYI---KP------------EESIILNVLPAEV--D-FSTCE-SIRLSQTVD------
KKGVRTLAVVTKVDKAP-------EGL------F-----EKV--------------------------TSD-----------------A-
VS----------------------I---GLGYVCVRNR---TPADD--SIA-----

--VARCR---ELELFND--------HP-DL---------------------R-
N------I--------D--RSM-VGIPTLARRLVKIQSDMVRGC-LPRIR
>KAG0619429.1
-----------NVQLPT--------IVVV---GD----Q----------------
SSGKSSVLE-----S--LA-G-I-TL-PRG--------------QG-IATRVPL---------------ILR--------------L---
QSC--------------------------------DST--
--------------------EE--------------SL-------------------------IRM----------D---------------Y--------------
------------------------------GNVKD-REIDGEE-----------QIEAAI------NEAT-NV---L-AG----------------
--------GN-------------------K-DVKDT-PISLHIRKPH---
-----APDLTMVDLPGITR--VP-----------------V-------HG--------------Q--------PQ--N--IYEQIQ------
--AMIM-KHI---SP------------EESIILNVLSAQV--D-FPTCE-SIRMSQQVD------
KDGKRTLAVVTKVDKAP-------EGL------L-----EKV---------------------------TTD------------------A-
VN-----------------------I---GLGYVCVRNR---TDDDD--TIS----
---VARIR---EQRLFES--------HP-AL---------------------K-
D------L--------D--RSM-VGIPALARKLTKIQSDMVKGC-LPRI-
>KAG0561847.1
-----------NIQLPT--------IVVV---GD----Q----------------
SSGKSSVLE-----S--LA-G-I-TL-PRG--------------QG-IATRVPL---------------VLR-------------L---
QSC---------------------------------QL---
-------------------EE--------------SI--------------------------IKM----------D---------------Y---------------
----------------------------GNVKD-QEISGEE-----------QIEAAI------NAAT-NA---L-AG-----------------
-------SG-------------------K-GVKDS-PIQLLIRKPN--
----SPDLTMVDLPGITR--VP-----------------V-------HG--------------Q--------PK--N--IYEQIR--------
GMIM-RYI---TP------------EESIILNVLSAQV--D-FPTCE-SIRMSQQVD------
KEGNRTLAVVTKVDKAP-------EGL------L-----EKV---------------------------TTD------------------A-
VN-----------------------I---GLGYICVRNR---IDVDD--SIA------
--IARQR---ERELFES--------HP-AL---------------------K-E--
----L--------D--GSM-VGIPALARKLTKIQSDMVKEC-LPRI-
>KAH9290598.1
-----------GIHLPT--------IVVV---GD----Q----------------
SSGKSSVLE-----S--LS-G-I-DL-PRG--------------QG-ICTRVPL---------------IMR--------------L---
QNS--------------------------------TE--
-------------------EY--------------SV--------------------------ISV---------E---------------Y-----------------
----------------------------KDRK--LSINEH-----------QIVDTI------NLVT-EE---I-AG----------------------
--RN-------------------K-GISDD-PITLHVRKKN--
VPDLTLVDLPGITR--VP------------------V-------YG-------------Q--------PK--D--IYEQVY--------
KIIM-KYI---SP------------RDSIILNVLSATV--D-FPTCE-SIRMSQKVD------EDGERTLAVVTKVDKAP-
------EGL------R-----EKV---------------------------AED------------------A-MN-----------------------I---
GLGYVCVRNR---V-EGE--SIV-----------------------------------
------------------KARKK---ENELFKT--------HP-LL---------------------S-G------I--------D--KSI-
VGIPILAHKLMKIQAAGITNS-LPRI-
>KAH9320939.1
-----------GIQLPS--------IVVV---GD----Q----------------
SSGKSSVLE-----S--LA-G-I-KL-PRG--------------QG-ICTRVPL---------------VMR--------------L---

QSC-----------------------------AEE--
-------------------SE-------------EE--------------------------ISI----------E----------------F-----------------
----------------------------NGVE--KFIQES-----------DITSSI------DTAT-QE---I-AG-----------------------
--NG-------------------K-GISHT-PITLHVTKVG--
APDLTMVDLPGITR--VP-------------------V-------GG-------------Q--------PG--D--IFEQIC--------
EIIK-EYI---TP------------KESIILNVLAANV--D-FPTCE-SIRMSQKVD------ELGERTLAVVTKSDRAP--
-----DGL------K-----EKV----------------------------TTD-------------------A-VN------------------------I---
GLGYVCVRNG---I-GDE--SNA-------------------------------------
-------------------EAREK---EKNLFDF--------HP-LL----------------------K-D-----L--------D--KSM-
VGIPTLAKKLMQIQATTISAT-LPQI-
>KAF8079489.1
----------EGIQLPT--------IVVV--GD----Q----------------
SSGKSSVLE-----S--LA-G-I-SL-PRG--------------QG-ICTRVPL--------------VMR-------------L---
QGS-----------------------------AS---
-------------------SE-------------PE---------------------------IWL----------E--------------Y------------------
----------------------------SDNV--VPTDEE-----------HIAEAI------SAAT-DV---I-AG---------------------
---SH-------------------K-GVSDA-PLTLHVKKAG--
-APDLTMVDLPGITR--VP-------------------V-------KG--------------Q--------PE--N--IYEQIS--------
GMIM-KYI---KP------------QESIILNVLSATV--D-FTTCE-SIRMSRQVD------
KTGERTLAVVTKADMAP-------EGL------L-----QKV---------------------------TSD-----------------D-
VS----------------------I---GLGYVCVRNR--V-GEE--TYE------
--EARKQ---EELLFET--------HP-TL---------------------S-M--
----I--------D--ENI-VGVPVLAQKLIQIQTTMIARC-LPKIV
>OAP19580.1
------------IQLPT--------IVVV--GD----Q----------------
SSGKSSVLE-----S--LA-G-I-NL-PRG--------------QG-ICTRVPL--------------VMR-------------L---
QRS-----------------------------SS--
-------------------PE-------------PE---------------------------IWL----------E--------------Y------------------
----------------------------SDKV--VPTDEE-----------HVAEAI------CAAT-DV---I-AG--------------------
----K--

--------FSLSP-S---
---QCSVKCV--

------------------LLQK-----------------
>OAP13353.1
------------IQLPT--------IVVV--GD----Q----------------
SSGKSSVLE-----S--LA-G-I-SL-PRG--------------QG-ICTRVPL--------------VMR-------------L---
QRR-----------------------------RS---
-------------------PE-------------PE---------------------------IWL----------E--------------Y------------------
----------------------------GDKI--VPTDEE-----------HIAQTI------CAAT-DV---I-AG---------------------
---M--

--------F---

>OAP13972.1
------------IQLPT--------IVVV---GD----Q---------------
SSGKSSVLD-----S--LA-G-I-SL-PRG--------------QG-ICTRVPL---------------VMR--------------L---
QRS------------------------------SS---
-------------------PV--------------PE--------------------------IWL----------E---------------Y----------------
-----------------------------SDKI--VPTDEE----------HIAEAI------CAAT-DV---I-AG----------------------
--K---

------FTL---
------------------------------------YLGIKCV---

>KAF5727250.1
----------EGIQLPT--------IVVV---GD----Q---------------
SSGKSSVLE-----S--LA-G-I-SL-PRG--------------QG-ICTRVPL---------------VMR--------------L---
QHH------------------------------HI---
-------------------PE--------------PE--------------------------LYL----------E---------------F----------------
-----------------------------NGKT--VQTTES----------RISEAI------NLAT-DE---I-AG---------------------
---NG-------------------K-GVSNT-PLTLVVKKHG---
-VPDLTMVDLPGITR--VP------------------V-------HD--------------Q--------PE--N--IYEQIA--------
GIIM-EYI---KP------------DESIILNVLSATV--D-FSTCE-SIRMSQQVD------KTGERTLAVVTKVDKAP-
------EGL------L-----EKV---------------------------TAD------------------D-VN----------------------V---
GLGYVCVRNR--I-GDE--TYE------------------------------------
------------------EARRK---EAALFES--------HP-LL---------------------C-L------I--------D--KSI-
VGVPVLAQKLVQIQAAIIMKC-LPEIV
>XP_002303204.3
-----------GIQLPT--------IVVV---GD----Q---------------
SSGKSSVLE-----S--LA-G-I-SL-PRG--------------QG-ICTRVPL---------------IMR--------------L---
QHH------------------------------TA---
-------------------PE--------------PE--------------------------LSL----------E---------------F----------------
-----------------------------NGKT--VPTSEA----------KIANAI------SLAT-DE---I-AG----------------------
--NA-------------------K-GISNT-PLTLVVKKNG---
VPDLTMVDLPGITR--VP------------------V-------HG--------------Q--------PE--N--IYEQIA--------
DIIM-EYI---RP------------EESIILNVLSATV--D-FTTCE-SIRMSQKVD------KNGERTLAVVTKADRAP-
------EGL------L-----EKV---------------------------TAD------------------D-VN----------------------I---
GLGYVCVRNR--I-GDE--SYK------------------------------------
------------------EARKE---EADLFEN--------HP-LL---------------------S-K------I--------D--KSM-
VGIPVLAQKLVQIQATIIARC-LPEI-
>KAF8391993.1
----------EGIELPT--------IVVV---GD----Q---------------
SSGKSSVLE-----S--LA-G-I-SL-PRG--------------QG-ICTRVPL---------------IMR--------------L---

QHH------------------------------ST---
-------------------PS--------------PE--------------------------LHL----------E---------------Y---------------
------------------------------HNKI--IPTNET-----------HVAEAI------NMAT-NE---I-AG--------------------
----NG-------------------K-GISNT-PLTLVVKKKG---
-VPDLTMVDLPGITR--VP-------------------V-------HG---------------Q---------PE--D--IYEQIS-------
SIIM-EYI---KP------------KESTILNVLSATV--D-FPTCE-SIRMSQRVD------KTGERTLAVVTKCDKAP-
------EGL------L-----EKV----------------------------TAD------------------D-VN-----------------------I---
GLGYVCVRNR--I-GEE--LYE------------------------------------
------------------EARME---EATLFES--------HQ-LL---------------------S-K------I--------D--KSI-
VGVPILAQKLVQIQASIIAKC-LPDIV
>XP_058079501.1
----------EGIELPT--------IVVV--GD----Q-----------------
SSGKSSVIE-----S--LA-G-I-SL-PRG--------------QG-ICTRVPL---------------IMC--------------L---
QNV------------------------------PT---
-------------------DK--------------PQ--------------------------MHL----------E---------------Y--------------
-------------------------------QGKI--VLTSEN-----------QISDSI------SMAT-DE---I-AG-------------------
-----NG-------------------K-GISNI-PLTLVVKKKG---
-VPDLTIVDLPGITR--VP-------------------V-------HG--------------Q---------PE--D--IYEQIS--------
NIIM-DYI---KP------------KESIILNVLSATV--D-FPTCE-SIRMSQHVD------KTGERTLAVVTKADKAP-
------EGL------L-----EKV----------------------------TAD------------------D-VN-----------------------I---
GLGYVCVRNR--I-GDE--TYE------------------------------------
------------------EARIE---EATLFKS--------HP-LL---------------------S-K------I--------D--KSI-
VGIPVLAQKLFQIQANSLSQC-LPDIV
>KAK1401877.1
----------EGIQLPT--------IVVV--GD----Q-----------------
SSGKSSVLE-----S--LA-G-I-SL-PRG--------------QG-ICTRVPL---------------IMR--------------L---
HHH------------------------------SK---
-------------------PE--------------SE--------------------------LHL----------E---------------Y----------------
-----------------------------CGKV--VSTDEL-----------KIAESI------NMAT-AE---I-AG---------------------
---DG-------------------K-GISHT-PLTLVVKKNG--
VPDLTMVDLPGITR--VP-------------------V-------HG--------------Q--------PE--N--IYEQIS--------
GIIM-EYI---KP-------------EESIILNVLSATV--D-FPTCE-SIRMSQSVD------KTGDRTLAVVTKSDKSP-
------EGL------R-----DKV----------------------------MAD------------------D-VN-----------------------I--
-GLGYVCVRNR--I-GDE--SYE-----------------------------------
-------------------EARMA---ESMLFES--------HP-LL---------------------S-K------I--------D--KSM-
VSVPVLARKLVQIQARIIAKC-LPEIV
>XP_038984915.1
----------EGIELPT--------IVVV--GD----Q-----------------
SSGKSSVLE-----S--LA-G-I-SL-PRG--------------QG-ICTRVPL---------------IMR--------------L---
QDD------------------------------PSL---
--------------------SQ--------------PQ--------------------------LQL----------E---------------Y-------------
--------------------------------KDKA--IHTSED-----------GIADAI------NSAT-DD---I-AG-----------------
-------SG-------------------K-GISNA-PLTLVVRKRG--
----VPDLTMVDLPGITR--VP-------------------V-------HG--------------Q--------PD--N--IYEQIS--------

NIIM-EYI---AP------------KASIILNVLSATV--D-FPTCE-SIRMSQSVD------RTGERTLAVVTKADKAP-
------EGL------L-----EKV---------------------------TAD-----------------D-VN----------------------I---
GLGYVCVRNR---I-GDE--SYE-----------------------------------
-----------------EARAE---ERNLFKR--------HP-LL--------------------S-R------I--------D--KSI-
VGIPVLAQRLMQIQAASIAKS-LPDIV
>XP_002297993.1
------------IQLPT--------IVVV---GD----Q---------------
SSGKSSVLE-----S--LA-G-I-SL-PRG--------------QG-ICTRVPL---------------IMR-------------L---
QHH-----------------------------TS---
-------------------LI--------------PE-------------------------MFL----------E---------------F----------------
----------------------------NGKT--TQTDEA-----------NVADDI------NIAT-EE---I-AG--------------------
----SG-------------------K-GISDA-PLTLVIKKNG---
VPDLTMVDLPGITR--VP-------------------V-------HG--------------Q--------PD--N--IYEQIA--------
GIVM-QYI---QP------------EESIILNVLPASV--D-FTTCE-SIRMSRQVD------
KTGERTLAVVTKADKAP-------EGL------L-----EKV---------------------------TAD-----------------D-
VN----------------------I---GLGYVCVRNR---I-GDE--SYD-----
---NARME---EANLFAT--------HP-LL---------------------S-
R------I--------D--KSI-VGIPVLAKKLMQVQATIMAKC-WP---
>XP_024439231.1
------------IQLPT--------IVVV---GD----Q---------------
SSGKSSVLE-----S--LA-C-I-NL-PRG--------------DG-ICTRVPL---------------IVR-------------L---
KHH-----------------------------PS---
-------------------LV--------------PE-------------------------IFL----------Q---------------F----------------
----------------------------NGKT--VPTDEA-----------HVADAI------NLVT-DE---I-AG--------------------
----NG-------------------K-GISNT-ELTLVVKKNG---
-VPDLTLVDLPGITR--VP-------------------V-------HG--------------Q--------PE--N--IYEQIA--------
YIIM-KYI---SP------------DESVILNVLSASV--D-FSTCE-SIRMSQKVD------
KNGQRTLAVVTKVDKSP-------EGL------L-----EKV---------------------------TRN-----------------D-
VN----------------------I---GLGYVCVRNR---I-GNE--SYE-----
---DARKE---EAALFAT--------HQ-LL---------------------S-K-
-----I--------D--KST-VGIQVLAQKLVQIQANIIAKC-LP---
>KAH0683503.1
----------EGIQLPT--------IVVV---GD----Q---------------
SSGKSSVLE-----S--LA-G-I-SL-PRG--------------QG-ICTRVPL---------------VMR-------------L---
KND-----------------------------PNI---
-------------------TA--------------PN-------------------------LQL----------E---------------Y----------------
----------------------------NNKS--LPVDEI-----------GIADAI------ILAT-DE---I-AG---------------------
--HG-------------------K-GISNN-PLTLVVKKNG---
VPDLTMVDLPGITR--VA-------------------V-------QG--------------Q--------PE--D--IYEQVY--------
DIIM-KYI---VP------------EESIILNVLSATV--D-FPTCE-SIRMSQKVD------KTGERTLAVVTKADKAP-
------EGL------L-----EKV---------------------------TAD-----------------E-VN----------------------I---
GLGYVCVRNR---I-GNE--SYE-----------------------------------
-----------------EARSD---EQRLFST--------HP-LL--------------------S-K------I--------D--KSM-
VSVPILAQKLVRIQASIISKC-LPEIV

>PWZ56863.1
-----------GIQLPT--------IVVV--GD----Q----------------
SSGKSSVLE-----S--LA-G-I-SL-PRG--------------QG-ICTRVPL---------------VMR--------------L---
QDD------------------------------PSA---
--------------------DS--------------PK-------------------------LQL----------E---------------Y--------------
------------------------------SNGRV--VTTTEA-----------DVADAI------NAAT-AE---I-AG-----------------
-------SG-------------------K-GISDA-PITLVVRKRG---
---VPDLTLVDLPGITR--VP---------------------V-------HG--------------Q--------PE--D--IYDQVA--------
KIIK-EYI---AP------------KESIILNVLSATV--D-FPTCE-SIRMSQQVD------RSGERTLAVVTKVDKNP-
------EGL------L-----EKV---------------------------TMD------------------D-VN----------------------I---
GLGYVCVRNR--I-GDE--TYD------------------------------------
------------------QARVE---EERLFKY--------HP-LL---------------------S-K------I--------D--KDM-
VGIPVLANRLMQIQSTIIAKC-LPDI-
>PWZ56864.1
-----------GIQLPT--------IVVV--GD----Q----------------
SSGKSSVLE-----S--LA-G-I-SL-PRG--------------QG-ICTRVPL---------------VMR--------------L---
QGD------------------------------PST---
--------------------DS--------------PK-------------------------LQL----------E---------------Y--------------
------------------------------SNGRV--VTTTEA-----------KVADAI------NAAT-AE---I-AG------------------
------SG-------------------K-GISDA-PITLVVRKSG--
--VPDLTLVDLPGITR--VP---------------------V-------QG--------------Q--------PE--D--IYDQIA--------
NIIK-EYI---TP------------KESIILNVLSATV--D-FPTCE-SIRMSQQVD------RTGERTLAVVTKVDKAP--
-----EGL------L-----EKV---------------------------TMD------------------D-VH----------------------I---
GLGYVCVRNR---V-GEE--TYD-----------------------------------
-------------------QARVA---EAQLFKN--------HP-LL---------------------S-Q------I--------D--KSM-
VGIPVLAQRLMQIQASIIAKC-LPDI-
>KAH9291961.1
-----------GIQLPY--------IVVV--GD----Q----------------
SSGKSSVLE-----C--LT-G-I-SL-PRG--------------VG-ICTRVPL---------------IMR--------------L---
QNS------------------------------SE---
-------------------QD--------------SE--------------------------IVV----------E---------------Y----------------
-----------------------------NDTV--EHIIES-----------QITERI------DSIT-KE---I-AG------------------------
TN-------------------K-GISHV-PIRLNVKKMN--
APDLTLVDLPGIAR--VS-------------------L-------NG--------------N--------PD--D---HELIS--------
KIVM-EYI---SP------------ADSIILNVLSATV--N-FRTCE-SIRMSQRVD------
VHGERTLGVVTKVDIAP-------EGL------L-----EKV---------------------------ALD------------------D-
VN----------------------T---GLGYVCVRNR--V-GDE--CNE---
--EAREA---EAELFRS--------HT-QL---------------------N-
K------F--------D--EAM-VGIPMLARRLMQIQTKRISKC-FPDI-
>KAH9325151.1
------------IQLLS--------IVVV---GD----Q----------------
SSGKSSMLR-----S--LA-G-I-NL-PKG--------------QW-ICTRVPL---------------IMR--------------L---
QIS-------------------------------KS---
------------------QE--------------TE--------------------------ITI----------E---------------Y------------------

---------------------------SGVK--NNIFEY-----------KIIEAL------NAAT-DE---I-AG-------------------------
VG-------------------K-GISDT-PITLNVSKSN---
IPNITMVDFPCITK--MP-------------------V-------HD-------------Q--------PQ--D--IYDQIS--------
QVIK-QYI---TP------------KESIILNVLFTSV--D-FPTCE-SIRMSQLVD------VKGEKTLAVVTKVDKAV--
-----EGL------F-----EKV---------------------------TVD--V----V---------S-VN----------------------I---
GLGYVCVRNK--I-GNE--SNA-------------------------------------

>KAH9300179.1
-------------NCLV--------LLL--
------------------------------W-ETSRLE--

---PG-------------------R-SINDT-PITLNVGKSN---
--APDLTMVDFPSITR--VP-------------------V-------
HG--------------Q--------PQ--D--IYDQIS--------QVIK-QYI---TP------------KESIIMNVLSASV--D-
FPTCE-SIRMSQLVD------EKGERTLAVFTKVNKAA-------EVL------F-----EKV---------------------------
--TVD------------------A-VN-----------------------I---GLGYVCVRNR--
--------I-GNE--SNA--

>KAH9314974.1

-----AL------NAAT-NE---F-AG-----------------------AG------------------K-SISDT-PITLNVSKSN-----
--APNLTMVDLPGITW--VP------------------V-------
HG--------------Q--------PQ--D--IYDQIS--------QVIK-QYI---TP------------KESIIMNVLSASV--D-
FPTCE-SIRMSQLVD------EKGERTLAVVTKVDNAA-------EGL------F-----EKV-------------------------
---TVD------------------V-VN----------------------I---GLGYTCVYSF---
--------A-KRS--RNLA-----VQACDY--------LD--------------------RI----------ICRVI---DPQLQASS-----
-RRA-FQ---------------------A-L------I--------D--RKR-DKCIQYVEDAMEMQKSIVYTE-NP---
>EFJ22917.1
------------IKLPT--------IVVV---GD----Q---------------
SSGKSSVLE-----S--LA-Q-V-DL-PRG--------------QG-VVTRVPL---------------VLR--------------L---
QNT-------------------------------SVT--
------------------DQS--------------HQ-------------------------VVI----------Q--------------Y-------------
----------------------------------GGKK--RVIEEA-----------EISAAV------VEAT-IE---L-AG--------------------
-----D--------------------K-HIVNK-PISLHITKPG---
APDLTMIDLPGITR--VP--------------------V-------HG--------------Q--------PE--D--IEEQIK--------
KIIQ-EYI---SP------------KETIILNVICSTV--D-FPTCE-SILMSRQVD------REGERTMAVVTKVDMSP-
------KDL------K-----EKV-----------------------------MAD------------------V-VG-----------------------I---
GLGYICVRNR--I-GDE--THE--------------------------------------

-----------------EGRDR---EAELFRT--------DP-HL--------------------R-D------L--------P--ESM-
LGIRQLAKRLTEFQADSLRKN-LP---
>XP_005823288.1
------------LDLPQ--------IAAL--GD----T---------------
SSGKSSVLS-----A--MS-S-V-VF-PSR-------------SD-ITTRCPT--------------RLC-------------M---
DTG----------------------------EQF---
-------------------QCK----------MQVLWH------------------------SKP---------A--------------SGS-N-
---PEVKKSVTQAS-----------DVTAAI------EELQ-KS---V-LD--------
---------------HE--------------KT-ES-GVSKS-IIEICLTGPN---
------------YPNLTLIDLPGIVR--ST-------------------G-------DG-------------E--------SL--E--
MIRDIQ--------SVME-QYL---NN------------KRCILLAVHPANI--D-RHNNE-VFRLTKEVD------
PDSARTIPVITKIDLVE-------KGT------E-----GNV-------------------------------MRL--IQ---GK---------E-
AK----------------------F---SLGFSVVKCR---T-QTE--LDQ-----
------G--------VT--------------------LE----------KSLEQ---EMIFFRD-------TS-PW--------------------
N-Q--------M------KQ-DNM-FGISNLTGKLSQIYVEMIQST-MPAV-
>KAJ1441760.1
---------------------------M---GD----T---------------
SSGKSSLLS-----A--LS-G-I-LL-PAN-------------DQ-ITTRCPT--------------RLR-------------M---
ENA---------------------------EQR---
-------------------RAS----------ISVRWQ------------------------FTS---------G--------------YKS-E-
AAYK--------L--TH----------------------LSEEKDGASFYS-----------NITSEI------EKAQ-KA---I-ID-----
-------------------TS---------------KL-E---VTRD-IIEVEYCGPD--
---------------CYNLTLTDLPGIVR--VA-------------------G-------KG-------------E--------SA--S--
IIVDIQ--------LLIK-EYL---EN------------ERCVVLAVVPANV--D-FHNSG-IMADAEKYD------
PTTRRTIPVITKPDLID-------KGA------E-----GGV-------------------------------LKL--LL---GEM--------T-
CT----------------------F---QMGFHMVKCR---G-QAQ--LNE-
----------G--------VT--------------------LE----------QGVEK---EAQFFKN--------ED-PW-----------------
----R-K------ESE------KR-ADL-FGVPALRRKLEALQMRMIQES-IPSI-
>KAG8471152.1
--------DFRMPELPQ--------MVVV--GT----Q---------------
SSGKSSLLN-----A--IM-A-C-DILPLG-------------ES-MVTRCPL--------------HLQ-------------L-V-
HAP---------------------------SE-SAG---
-----------------VMRAE-------------F-------------------------GTY---------A--------------GGS-------
--------WQAQRT--------------------IILTAPTPSASDIT-----------QIRREI------EAQT-NA---L-AG--------
---------------PG------------------K-CIASQ-PIHLRIHSAY---
------------VPDLSLVDLPGLTQ--MAL------------------TT------QG-------------Q--------PR--D--
IKEQIR--------ALVL-EHI---TK------------PRAVILAVLPART--D-LEADA-ALELVKEVD------
PRGERTIGVLTKVDLMN-------QGS------D-------I-------------------------------ASF--LVD-DGRTP------
SD-LR----------------------M---EYGYYAVRNR--S-
TAEVVKDG--------------------MS---------------------VD----------GGFER---EATFFAM--------HP-IY----
-----------------G-A------LR-------PAARAR-LGVPALARALSKLLVN-----------
>KOO34643.1
--------RLKQPEVPR--------LVVV--GT----Q---------------
SSGKSSLLN-----G--IM-G-A-DILPLG-------------EE-MVTRAPL--------------ALQ-------------L-V-

HHA------------------------------EP-SE--
------------------MRAE-------------F-------------------------GTF---------V--------------NGG--------
-------WCVEET-------------------VELACPDPKAAQLE-----------RIRKAI------EAQT-AA---R-AG------
------------------SQ------------------K-GVTNE-AIFLRLYSPH---
---------------VPNLSLVDLPGLTM--TAL-----------------TA------QG-------------Q--------PK--D--
IKQQIR--------QMIA-SYI---QP-----------ARTIILMVCPARA--D-LEADP-AVELAREYD------
PQGTRTVGVLTKVDLMN-------KGT------D-------V-------------------------SKY--L---TNALP------
SD-LQ-----------------------L---SLGYFAVKMR---G-PA---
EKG-------------------LT------------------VR---------EGYGS---EAEYFKA--------HA-TY-------------
-------G-R------AF-------APFAER-LGVPPLSKFLARVLLG-----------
>XP_005775544.1
--------RLREPELPR--------
LVVVGARRSRSGIRSKNSRSPHRRAPSQPFREKKQRSRSSTVVHLPSSGT----Q---------------
SSGKSSLLN-----G--FL-A-A-DILPLG-------------EQ-MVTRAPL--------------NLQ-------------L-V-
HSP-----------------------------DP-AE--
------------------MRAE-------------F-------------------------GDY---------S--------------CGA--------
-------WQCAAA-------------------VPLAFPDPTPPQLQ-----------QIRASI------EAQT-EA---R-AG-----
-------------------AQ------------------K-GVSAA-PIFVRYYSPN---
----------------VPNLSLVDLPGLTM--TAL-----------------TD------HG-------------QHRDIPYPTPR--
D--IKRQIR--------DMIA-AYI---RP-----------ERTIILMVCGARA--D-LEA-P-C-----QRS------
SGGARTVGVLTKVDLMN-------AGT------D-------VARDLEGSARYPEGSARYLEGSARYLEGSARY--
L---EGSVP------SD-LR-----------------------L---ALGYFAAK---
--G-P-----QG-------------------LT------------------VL---------DGFRV---EREYFGK--------HA-AY---
------------------S-A------LG-------AAGRVR-LGVPLLSRFLSRVLLQ-----------
>ATZ81043.1
------------LKFPK--------IVVV--GG----Q---------------
SSGKSTVLN-----N--LI-T-M-NILPTG-------------SE-MVTRTPL--------------CME-------------M-N-
NSD-----------------------------IP--
-----------------HIE-------------F-------------------------GQY----------N--------------IAN-E-SDGN-
------VWKCGRN-------------------IKVTYPEPTQEEIN-----------AIRIEI------ERQT-IL---L-AG---------
---------------KD------------------K-NISHN-PITIKIFLPN---
----------VPNLTLVDLPGITQ--VAC-----------------KD------KG-------------Q--------PD--D--
IKEQIE--------KLIG-SYI---KS-----------EETIILSIIPART--D-VEADV-GVGLVKKYD------
PDFSRSIGVLTKVDLMN-------VDT------D-------V-------------------------SSY--VC---GNIS------
KN-LR-----------------------M---NYGYYLVRNR---T-NKE--
MNE-------------------IT------------------MR---------EGFLK---EQAFFEE--------HQ-VY-------------
--------G-R------MS-------DVDKRK-MGTNNLRDKLVNVLSNKIKEL-MPII-
>ARF10282.1
------------LSLPR--------LVVV--GT----Q---------------
SSGKSSVLN-----S--II-T-M-DILPTG-------------KL-MTTRTPL--------------ELN-------------L-I-
KIP-----------------------------KN-NA---
-----------------SYVE-------------F-------------------------GNY---------D--------------NEK---------
-----WFQEKK-------------------ITITVPIPTPNEIT-----------TIRNHI------QDKT-NE---I-CG------------
-----------NN------------------M-NISNV-PIILKIYSPN---

-------VPDLSLIDLPGLTM--VAC-----------------ED------KG-------------Q--------PV--D--IKERIE----
----NLVS-SYI---IQ------------PKTIIIAVMQARA--D-LETDL-GLALVKKYD------
KSGSRTIGVLTKPDLMN-------QDS------H-------V---------------------------GDY--LL---NKIS------
KN-LM----------------------L---NYGYYLLKNR---N-DIQ----E-
-------------------ID-------------------IL---------TGFKL---ETEYFNA--------HN-EY--------------------
K-K---------------SLYKPK-LGINNLTNNLTKILVQSLNEA-IPVS-
>QKF94243.1
------------LALPR--------LCVV--GT----Q---------------
SSGKSSVLN-----A--IM-S-M-DLLPTG-------------KN-MVTRTPI--------------DLR-------------L-H-
QLK-----------------------------GS-TD---
------------------GWVE-------------F--------------------------GDY---------T--------------GEG--------
-------WITEKK--------------------ISIKMPIPLDSEIT-----------EIREYI------MKKT-ND---V-AG-----------
-------------EG-------------------M-NISPK-PIIINIYSPN--
-------VPNLSLVDLPGLTM--VAC-----------------TD------KG-------------Q--------PE--D--IRERIE---
-----NLVV-SYI---KE------------KKTIVIAVMQARS--D-LETDI-GLALIKKYD------
ISGQRIVGVLTKPDLMN-------HET------N-------I---------------------------GEY--LT---NNIS------KN-
LM----------------------L---TYGYYVVKNR---N-GQE--MKD---
-----------------VN-------------------IL---------KGFEL---EKEYFNN--------HY-EY--------------------
K-K---------------PLYKDR-IGSHNLTASLSKILIASITEV-LPSV-
>ARF11508.1
------------LTLPR--------IAVV--GT----Q---------------
SAGKSSVLN-----G--IM-G-L-DLLPAG-------------RY-MTTRTPL---------------DIR-------------L-H-
QLK-----------------------------GDYKE---
-------------------GYVE-------------F--------------------------GNY---------T--------------EEG---------
------WETEKK--------------------IGIKIPIPLDSEIT-----------EIREFI------SKKT-NI---I-AG--------------
---------DG-------------------M-NISSQ-PIIINIYSPN---
----VPNLSLIDLPGLTM--VAC-----------------VD------KG-------------Q--------PD--D--IKERIE------
--DLVV-SYI---KQ------------PKTIILAVMQSRS--D-LETDI-GLALTKKYD------
VNGQRIIGVLTKPDLLN-------PDT------N-------I---------------------------GEY--LI---NNIS------KN-
LM----------------------L---TYGYYVVKNR---N-GQE--MKD---
-----------------CN-------------------II---------KGFEL---EKEYFNN--------HN-EY--------------------
K-K---------------SIYKDR-IGTNNLTNNLSKILITSITEM-LPNV-
>ARF09562.1
------------LSLPK--------LVVI--GC----Q---------------
SAGKSSVLN-----S--II-S-M-DILPTG-------------KN-MVTRTPL---------------ELQ-------------L-H-
QLT-----------------------------QDSKD---
-------------------GWVE-------------F--------------------------GQY---------N--------------QNG-----
----------WTSEKK--------------------IPITTPIPTETEIK-----------NVRDYI------SAKT-IE---L-AG---------
---------------DG-------------------M-NISHI-PIMMNIYSPN--
-------------VPNLSLVDLPGLTV--IPC------------------VD------KG-------------Q--------PV--D--
IKEKIE--------ELVA-SYI---KQ------------ERTIVLAVMQSRN--D-LETDI-GLGLIKKYD------
NGNQRIIGVLTKPDLMN-------QET------H-------I---------------------------GEY--LI---NNIS------
KN-LM----------------------L---TYGYYVVKNR---A-GIQ---GS-

-------------------MD-------------------VF----------KGYEL---EKEYFNS--------HF-EY-------------------
--K-K---------------IIYKDR-IGTDNLTKNLSKILISSITEL-LPSV-
>AYV76902.1
------------LTLPQ--------IVVC--GT----Q----------------
SSGKSSVLN-----S--II-S-M-DILPTG--------------KT-MVTRTPL---------------SLR--------------L-H-
QLS------------------------------PDTKE---
-------------------GLVE--------------F----------------------------GNY----------E---------------TGT---------
------FISEKK--------------------ISITVPIPTENEIK-----------TIRDFI------MAKT-DE---I-CG--------------
----------VG-------------------M-NISTK-PITINIYSPN---
-----VPNLSLVDLPGLTV--VAC------------------QD------KG--------------Q--------PV--D--IKDRIE-----
---ALVS-SYL---KQ------------ERTIVLLVMQAKC--D-LETDL-ALGLIKKHD------
SGNQKIIGVLTKPDLMN-------MDT------H-------I----------------------------GDY--LC---NNIS------
KN-LK------------------------L---AFGYYVVKNR--S-NL-----S--
------------------ID-------------------IF----------KGLEL---EKEYFIN--------HN-EY--------------------
K-K---------------SIYKDK-LGTVNLTQNLNKILVSEITDL-LPSV-
>ARF08414.1
------------ITLPK--------IVVV---GT----Q----------------
SSGKSTVLN-----A--IM-A-L-DILPTG--------------KN-MVTRTPL---------------DIR--------------L-H-
KNK------------------------------IN--E--
-----------------ARIE--------------L----------------------------GFY----------G---------------DSG-----------
----WISEKN--------------------IKLSLPNASKDEID-----------QIRQYI------SQKT-IE---L-AG------------
------------NE-------------------M-NISKN-PIYIQIFSPN---
--------VPDLSLVDLPGLIM--VAC------------------TD------KG--------------Q--------PK--N--MPEQIE-
-------KLVE-SYI---KD------------NNTITLAIMQSRS--D-LETDL-GLALIKKYK------CS--
KTVGVLTKPDLMN-------NDS------H-------I----------------------------GCY--LN--GNNIS------KD-
LM-------------------------L---DYGYFVVKNK--S-SMD--NDE---
-----------------YD-------------------IQ----------KIIDM---EKKYFAN--------HN-EY--------------------
N-K---------------PIYQNK-IGYNSLIKELTKVLISAIRDT-MPKA-
>AYV78912.1
------------IMMPQ--------LAVV--GS----Q----------------
SSGKSSLLN-----N--IM-A-MPDLLPTG--------------RD-MVTRSPI---------------RLC--------------L-
H-KTK------------------------------TT--D---
-------------------GYIE--------------F----------------------------GTY----------N---------------DAG---------
------WAVEKK--------------------ITLTMPNPTTAEIQ-----------EVRENI------KAKT-IE---I-AG-----------
-------------AQ-------------------M-NISHT-PIVLQIYTPN--
---------VPDLILIDLPGLVM--TSC------------------VD------KG--------------Q--------PS--D--IDEQIQ--
------NLAL-SYV---KN------------PKTIVLLVMQASN--D-LQTDI-GLAFLKKHD------VN-
RNIVGIFTKPDLMN-------SDS------H-------V----------------------------GDY--LL---GKTS------SN-
LM-------------------------L---QHGYFVVKNK--S-SSD---TS-----
---------------SD--------------------IL----------KSTDI---EKKYFQN--------HF-EY---------------------K-
K---------------SIYQNR-VGYNSLIAELTKILVAAINNN-IPSV-
>VBB18790.1
-----------FLDPPT--------LCVV---GS----Q----------------
SSGKSITLN-----G--LT-G-I-DILPNG--------------KS-IVTRTPI---------------HLR--------------L-I-

HVK------------------------------ES-KN---
------------------IVVE-------------F---------------------------FDK---------D--------------DSQ------K--
-----LISTFTV--------------------DALTTP---ADQLT----------PIREEI------VKLT-EM---Y-AG-------------
-----------RS-------------------K-NVVDT-PINVRIKSPS---
-------VPNLSVIDLPGLTH--IAL------------------TD------QG-------------Q--------PE--N--IKESIE-----
---NMII-KYI---KN------------PRTIILSIIPATV--D-VESDA-GLGLIKKHD------PDFKRTIGVLTKVDML--
------KDS------N------V---------------------------ENY--LC---GKIS------RN-LQ----------------------
L---GYGYYAVRNR--S-SDE--VKV-------------------MS-------
-------------VK----------DGHAL---EAKFFAE--------TE-PY--------------------K-S--------------
SEMRQR-TGSINLGIKLSEVLLAHLRSC-LPAV-
>ABI33144.1
-----------GIALPQ--------IAVV---GD----Q---------------
SSGKSSVLE-----N--IT-G-I-PF-PKG-------------TG-TVTKCAT--------------RIT-------------I---RRA-
-----------------------------LKR--
------------TTP-------------FS-------------------------ARV---------S--------------FVI-N-GK----------
--NSP--------------------FKNEGPKMNHTSVG-----------KLQKTL------ERLN-EL---IHQN-------------
-----------ES-------------------K-TDIDD-IIEVDIEERH----------------------DSD-------------------------
--------AVDLSIVDLPGLIA--TT-------------------T-------EG-------------Q--------SK--D--LVGSIE------
--KMVS-RYA---ED------------KRTVILAIMEAHR--D-ISNSR-ALAMAEKAD------
EKGERTVGVLTKMDLVE-------EAT------E-----DDV---------------------------LNV--LN---NS---------
R-KP-----------------------L---YHGYFAVVNR---A-QKT--LNE--
---------G--------KS--------------------LK---------DARRD---EKEWFER--------NK-HY-----------------
---M-S------M-----------DGR-CGIDNLKRGISKRLRV-----------
>KAI8587516.1
---------GQDVSLPQ--------LVVA---GD----Q----------------
SSGKSSLLE-----S--LS-G-I-SF-PKA-------------AE-LCTTFPT--------------QIV-------------M---
RTK------------------------------KTWE-ARV---Y------
-------------------TVPE--I---------
----------------------------ANFPATNCASKV-----------AVQNII------QFIK-RD---VLKA------------------
------TAG--------------------VSET-VLVIELGSPE---
LPNLTIIDLPGYVH--TL-------------------V-------KG-------------Q--------SE-NF---KQKID--------
NMVD-KFI---QD------------RRSIILAVIPANK--D-FATNV-VLQRAQEWD------
PEGDRTIGVVTKPDLVD-------QGT------E-----AAV---------------------------IRM--IQ---GH---------
Y-KE-----------------------L---KLGYYMVHNR--S-HMD--
LQN-----------G--------VD--------------------LA---------AAVAK---EAEFFS---------QP-AW----------
----------D-A------L--------N--ARQ-LGTQQLQTAVVEVLAAHVVKE-F----
>OLL24579.1
---------NSVISLPQ--------IVVC---GD----Q---------------
SSGKSSVLE-----A--LT-Q-I-PF-PRS-------------DG-LCTKFAT--------------QVI-------------L---
RRA-------------------------------SKTS-VRV---Q-----
--------------------IIPDC--KRP---------E--------------AEQ-R-AL-
-------------------------------------QSVD-IKLKKLE----------DMTILI------EEAS-KH---MGVQ----------
--------------SSSS-T--------S--------TFSSD-ILSIEVSGPK---
----------QPHLTVVDLPGYIR--TT-------------------S-------GN-------------Q--------TK-KD---ITLIY----

----DLVK-DYI---SD------------KRSIILAVIPANV--D-VANAE-ILEKASEAD------
PNKTRTLGVITKPDLVD-------NGA------E-----NQV---------------------------LDL--AA---NV---------
T-KP----------------------L---KLGYFIVRNR---N-YNE--LKS----
-------A--------SD-------------------SK----------ARNKS---EAAFFA---------QS-PW--------------------
S-E------I--------N--KTR-IGIDRLRLYLSDLLQEHIFKE-L----
>KAI9096888.1
---------SEYVSLPQ--------LVVC--GD----Q----------------
SAGKSSVLE-----A--IT-E-V-PF-PQN--------------QG-TCTRFAT---------------QIV--------------L---
RRS-----------------------------VVTE-AIV---T-------
------------------IIPES--RRT----------D---------------NEV-A-KF----
---------------------------------AQFK-KKIEDLK-----------DLPSII------TEAE-AL---ILFS-----------------
-------NNVR-T--------K---------FSKD-VLNVEISGPK--
-----QPHLTVVDLPGIIHTSSS-------------------T-------TE--------------D--------EE-GDEFEVEVVK----
----ELVK-GYM---KE------------DRTIILAIVAGNY--D-YNNQI-ILQMAKELD------
QDRTRTLGIVTKPDLQE-------VGSD----YE-----KTL---------------------------VKM--VK---NE---------
V-KH----------------------L---SLGWHVLKNR---G-FKE----R--
---------E--------CS-------------------IE----------QRNIA---EEKFFN---------QG-VW-------------------
--T-S------L--------P--RKD-VGVESLRIKLSNLLYQHIKRE-L----
>RSH87279.1
---------DSVLSLPQ--------IVVC--GD----Q----------------
SSGKSSLLE-----A--LT-E-I-PF-PRN--------------DN-LCTRFAT---------------EIS--------------L---
RRE-----------------------------SESS-LTI--R-------
------------------IIPAH--SRP----------Q---------------EEQ-K-RL---
----------------------------------ERFS-EVITDFE-----------DLPAVM------DKAM-QE---LGIS------------
------------EGTG-A------------------FAKD-TLSVEIQGPD---
----------RPQLTLVDIPGLIQ--TS------------------T-------RG--------------V--------SE-AD---VALVA----
----EITD-SYI---KQ------------PRTICLAVVSATN--D-AANQP-ILSRVRKFD------
PHGKRTLGVITKPDRLP-------SGSA----SE-----SKF---------------------------LEL--AR---NE---------
D-VF----------------------F---KLGWHVIKNR---K-FEE----T----
-------A--------FS--------------------ID----------ERNLV---ERTFFA---------TS-NF---------------------K-
A------L--------P--RED-VGIDALRVKLSTLLFDHVKKE-L----
>XP_041144356.1
---------SHYISLPQ--------IIVC--GD----Q----------------
SSGKSSVLE-----A--IS-G-V-SF-PVK--------------SN-LCTRFPT---------------ELV--------------L---
RKS-----------------------------SHIG-VKV--S------
-------------------IVPHR--SRS----------H---------------VEQ-D-
AL-------------------------------------SRFH-EELESFE-----------GLPTLI------ENAK-AA---MGIF-------
-----------------THGK-A------------------FSND-LLRVEVSGPD--
----------------RPHLTIVDLPGLIH--SE------------------T-------KL--------------Q--------SA-AD---
VALVQ--------DVVQ-SYM---KE------------PRSIILAVVSAKN--D-FANQI-VLRLAREAD------
SFGHRTLGVITKPDTLV-------EGSE----SE-----YQF---------------------------VSL--AK---NQ---------
E-VT----------------------F---RLGWHVLKNM---D-TEK----G--
---------N--------YN-------------------LS----------VRGQE---EAEFFS---------RG-IW-------------------
--E-D------L--------P--RSH-VGIDTLRQRLSKLLLGQIATE-L----

>KAJ5704467.1
---------SHYVSLPQ--------IIVC---GD----Q----------------
SSGKSSVLE-----A--IS-G-V-SF-PVK--------------SS-LCTRFPT---------------ELV--------------L---
RKN------------------------------SQVG-VRV--S----
---------------------IVPHQ---SRS----------D---------------AEQ-H-
SL---------------------------------------GSFC-EQLDGFD-----------GLANLI------ENAK-AA---MGIS----
--------------------THGK-A------------------FSND-LLRVEVSGPD--
-------------------RPHLTIVDLPGLIH--SE-------------------T-------RQ--------------Q--------SA-AD---
VQLVQ--------DVVQ-SYM---RE------------PRSVILAVVSAKN--D-FANQI-VLRLARDAD------
PSGNRTLGVISKPDTLV-------PGSE----SE-----ASF----------------------------VSL--AK---NQ---------
D-VE-----------------------F---RLGWHVLMNM---D-SEK----
G-----------Q--------WS--------------------LS----------DRDIQ---ERKFFS---------GG-IW--------------
-------E-D------L--------P--RSL-VGVDSLRTRMSSLLLGQIAGE-L----
>KAI9774215.1
---------SHYVSLPQ--------IIVC---GD----Q----------------
SSGKSSVLE-----A--IS-G-V-SF-PIK--------------SN-LCTRFPT---------------ELV--------------L---RKT-
-----------------------------SQIG-VSV--S------------
-------------IVPHQ---SRS----------E---------------SEQ-H-TL-------
--------------------------------SSFH-EELDGFE-----------GLPALI------ENAK-AV---MGIS----------------
--------THGK-A------------------FSKD-LLRVEVSGPD--
-------RPHLTIVDLPGLIH--SE-------------------T-------KH--------------Q--------SA-SD---VELVQ-----
---DVVQ-AYM---KE------------PRSIILAVVSAKN--D-AANQI-VLKLARAAD------
KKGNRTLGVITKPDVLI-------PGSE----SE-----ASY----------------------------ICL--AR---NQ---------
N-VE-----------------------F---RLGWHVLRNT---D-SEM----G-
----------D--------WS--------------------LA----------DRDAQ---EKEFFS---------QG-IW-----------------
----E-Q------M--------S--QSL-LGVDKLRSRLSKVLLGQIATE-L----
>MCJ1392161.1
---------SHYVSLPQ--------IIVC---GD----Q----------------
SSGKSSVLE-----A--IS-G-V-SF-PVK--------------SN-LSTRFPT---------------ELV--------------L---
RKT------------------------------SQIS-VSV--S-------
------------------IVPHQ---ARS----------E---------------SER-L-IL---
------------------------------------SSFH-EELEGFE-----------GLPSLI------ENAK-SA---MGIS------------
------------TLGK-A------------------FSKD-LLRVEISGPD--
---------RPHLTIVDLPGLIH--SE-------------------T-------KQ--------------Q--------SA-SD---VELVQ---
-----DVVQ-SYM---KE------------PRCIILAVVSAKN--D-YANQI-VLKLARAAD------
KKGTRTLGVITKPDTLI-------AGSE----SE-----AMY----------------------------VSL--AR---NQ---------
D-VE-----------------------F---RLGWHALKNM---D-SET----G-
----------E--------WS--------------------LA----------ERDVE---EQEFFS---------QG-IW-----------------
----R-D------M--------S--RSL-LGVDDLRSRLSKVLLGQIAAE-L----
>XP_002543522.1
---------SHYISLPQ--------IIVC---GD----Q----------------
SSGKSSVLE-----A--IS-G-V-AF-PVK--------------SN-LCTRFPT---------------ELV--------------L---
RKS------------------------------PEVH-VSV--S-----
--------------------IVPHR---SRS----------E---------------SEQ-H-

SL-------------------------------------ESFR-EDLDSFE-----------GLPELI------ENVK-TA---LGIS--------
----------------THGR-A------------------FSND-ILRIEISGPD--
-------------RPHLTIVDLPGLIH--SE-------------------T-------KQ--------------Q--------SA-SD---
VDLVQ--------DVVQ-AYM---RE------------PRSIILAVISAKN--D-IANQI-VLKLARGAD------
RFGRRTLGVITKPDTLI-------PGSA----TE-----SIF---------------------------VSL--AK---NQ---------E-
VD-----------------------F---RLGWHVLKNM---D-SEK----G----
-------I--------ST-------------------LA----------DRDVE---ERQFFS---------QG-VW--------------------
K-D------I--------A--PST-MGIARLRGRLSKVLLGQIATE-L----
>XP_746402.1
------------ISLPQ--------IIVC---GD----Q----------------
SSGKSSVLE-----A--IS-G-V-SF-PIR--------------SS-LCTRFPT---------------ELV-------------L---
RKS------------------------------SQVG-VCV--S-----
--------------------IVPHR---SRS----------E---------------SER-E-
AL-------------------------------------AQFH-EELDSFE-----------GLPQLI------ENAK-SA---MGIY-----
------------------TNAK-S------------------FSND-LLRVEVSGPD---
------------------RPHLTIVDLPGLIH--SE-------------------T-------KQ--------------Q--------SA-AD---
VELVH--------DVVK-SYM---EE------------PRSIILAVVSAKN--D-VPNQI-VLKLARAAD------
PHGTRTLGVITKPDTLV-------RGSD----SE-----AQF---------------------------VSL--AK---NQ---------
E-VE-----------------------F---RLGWHALKNM---D-TDK----G--
---------A--------WT-------------------LA----------ERDKE---EHAFFA---------SG-VW------------------
---E-A------L--------P--RSH-VGIDQLRKRLSKLLLAQIATE-LPSL-
>XP_751069.1
------------VELPQ--------LIVC---GN----Q----------------
SSGKSSVLE-----A--IS-R-V-RF-PAK--------------SN-VCTRFAT---------------EVI--------------L---
RRN------------------------------AAFSKIKV--S-----
--------------------IEPGP---SRTD---------E---------------DER-R-
RL-------------------------------------RSFTYEDFSNGD-----------DLPPLI------EKAK-VH---MGIT-----
------------------ESVN-T--------G---------FSDD-VLKVEISGPD---
------------------KPELTLVDLPGLYY--ST-------------------S-------QE--------------Q--------DL-QG---
ILIVR--------KLTE-RYM---SN------------PRSIILAVISAKT--D-YHLQE-VLNIAEQFD------
PKRERTLGIITQPDILE-------ANSE----EE-----DTY---------------------------LHF--VK---NE---------K-
IP-----------------------L---ELGWHVLRNR---S-FET----R-------
----D--------IS-------------------DD----------ARDEM---EKAFFN---------QG-RW--------------------
A-S------L--------S--REC-VGIESLRRRLSGVLLRLIRRN-LPGL-
>XP_026607910.1
---------GEVVALPQ--------LAVC---GD----Q----------------
SAGKSSVLE-----A--IT-G-I-PF-PQQ--------------DG-LCTRFPT---------------EIT--------------L---
RHS------------------------------EATQSITIF--AS-----
--------------------IRPHS---VRS----------R---------------KEK-D-
YL-------------------------------------ASYQ-KTLGAIS-----------ELPSII------ADAS-KL---MGIR--------
----------------GYGG-Q--------KNGP-----AFAAD-VLRIEITGPI--
-----------------GLQLSVVDLPGLIS--VV-------------------S-------EE--------------Q--------NE-ND---
VVMIH--------DMVT-SYL---QS------------SRTIILAVVQASN--D-FANQC-IIRMARKHD------
PEGQRTVGIITKPDLIN-------QGT------E-----SKI---------------------------ARI--AK---NL---------

DTIK----------------------L---KLGFFLLKNP--S-PME--RKD---
--------C--------HS-------------------MT---------ARSAL---EDRFFS---------RP-SW-------------------
-A-IH----HL--------D--KKR-IGSESLRTFLQKLLDSHIEHE-L----
>XP_748757.2
------------ISLPQ--------LVVS--GD----Q---------------
SSGKSSVLT-----A--VT-G-F-SF-PRR-------------EG-TCTRFAT--------------EII-------------L---
RHS-----------------------------KETETIIT--AS-------
------------------IIPSL---SRH----------D--------------GSE-E-AL---
----------------------------------KRFK-KVLKSTE-----------ELPSVI------HEAS-VA---MGIR-------------
-----------GYSD-S--------DDSP-----AFTAD-VLRIEVVGDT---
------------GLCLTIVDLPGLIS--VS------------------D-------YD-------------E--------GE-AD---
VQLVN--------TLID-SYL---AN-----------TRSIILAVVQASN--D-IQNQN-IIQRARRFD------
KLGERTVGIITKPDLVN-------KGT------E-----SHI---------------------------VRL--AN---NL---------
DIVR----------------------L---KLGFFLMKNP--S-PEQ--LKN--
---------N--------IS--------------------MF----------EWKQK---ELEFFN---------SP-PW------------------
--K-DL----ML--------D--HNR-VGAECLRSFLEKILEEHIERE-LP---
>XP_040633937.1
---------GDHVALPQ--------LVVC---GD----Q---------------
SAGKSSVLE-----G--IS-G-I-PF-PRQ-------------DG-LCTRFAT--------------EII-------------L---RH-
------------------------------EPGEQRAT---AM--------
-----------------IIPHV---SRT----------D--------------EEK-S-SL-----
--------------------------------GAFH-RNISDFT-----------ELPIIV------EEAR-SI---MDIH----------------
--------GHGI-G--------SNAS-----AFSAD-VLRLELVGST---
----------GLHLTMVDLPGLIS--VS---E--------NE-HD---VQLVR--
------DLVD-SYL---EN------------SRTIIMAVVPASS--D-VDTQG-ILQRARHFD------
KTGLRTVGVITKPDLIN-------AGT------E-----PRV---------------------------ARL--AR---NL---------
DGAK----------------------L---NLGFFLLKNP--S-PAE--LEA--
---------G--------TT-------------------LP----------ERRKV---ELEFFS---------SG-AW-------------------
-K-GQ----GL--------D--PSR-IGIDNLRSFLQDLLDHHIERE-L----
>XP_754266.1
------------IALPQ--------LVVC---GD----Q---------------
SAGKSSVLE-----G--IT-G-I-PF-PRQ-------------DG-VCTRFAT--------------EII-------------L---RH-
------------------------------EPNHRRNT---AT---------
----------------ILPHI---SRT----------E--------------EEK-A-KL------
-------------------------------SAFR-REVSDLA-----------QLPGII------EEAA-RL---MGVQ--------------
----------GMND-L--------ADAP-----TFAAD-VLRLEIVGDT---
------------GLHLTLVDLPGLIS--VS---E--------ND-DD---VQLVG-
-------DLVN-SYL---EN------------SRSIILAVVPASS--D-VDTQS-IIQRARRFD------
KDGFRTVGIITKPDLIN-------DGT------E-----GRI--------------------------AKL--AN---NA---------
DRTK----------------------L---RLGFFLVKNP---R-PID--LEK---
--------G--------MT-------------------TA----------ERRKV---EAEFFA---------HP-PW-------------------
-N-KL----GL--------D--PSR-VGIDNLRIFMQDLLDRHIERE-LPKV-
>XP_043140374.1

---------GDHIALPQ--------LVVC---GD----Q----------------
SAGKSSVLE-----G--IS-G-I-PF-PRQ--------------DG-VCTRFAT---------------EII--------------L---RH-
------------------------------EPNDQRNT---AT---------
----------------IIPHM---SRA----------E---------------DEK-V-RL-----
---------------------------------SAFH-RDINDLV-----------NLPGIV------DEAA-RL---MGVQ----------
------------GAND-S--------TDAP-----TFAAD-VLRLEVVGDT---
--------------GLHLTLVDLPGLIS--VS--E--------NE-ED---
VQLVG--------NLVN-SYL---EN----------SRTIILAVVPASS--D-VDTQS-IIQRARRFD------
KDGLRTVGIITKPDLIN-------DGT------E-----SRV-----------------------------AKL--AN---NA---------
DRTK------------------------L---KLGFFLLKNP---R-PID--LEK---
--------G---------MT--------------------MV----------ERRKM---EAQFFA---------NP-PW------------------
---N-NL----GL--------N--PSR-VGIDNLRVFMQDLLDRHIERE-L----
>KXS17655.1
---------NHLISLPQ--------IAVV--GD----Q----------------
SAGKSSLLE-----A--IS-G-I-SF-PKD--------------KE-MCTTFAT---------------QIV--------------M---
AKG-------------------------------ASFA-AKV---T------
-------------------IDPDP---SNI--
---------------------------------SVGLPVPKSPL-----------DVAAVI------EEAK-NL---MSEG---------------
---------NSNL-I-------------------IADK-ILTIELTGPN--
---YPRLTLVDLPGYVQ--SV-------------------I-------KG--------------Q---------SE-TI---IEDIA--------
DIVD-RHL---KD----------ERTITLAVIPANK--D-LATNV-VVGKVDKLG------
SNGARTLGVITKVDVID-------AGE------E-----EAV----------------------------LEI--LH---GR---------R-
CD-------------------------F---GLGFHAVRNR---N-WAE--VNG--
---------S--------LS--------------------TE----------ELLVK---EAQFFA---------RA-PW---------------------
S-Q------L--------D--KSM-KGIVSLRSKLVEILHNHVEKE-L----
>XP_021869222.1
---------GAEVELPQ--------LLVV---GN----Q-----------------
SSGKSSVLE-----A--LT-G-I-PF-PRD--------------DG-LCTRFAT---------------RIT--------------F---
RRA-------------------------------LETR-YQA--K------
-------------------IVPDK---LSS----------K---------------EHQ-D-
KC---------------------------------------QQWGQ-ELESFDL---------FQIADLM------KKVR-TV---MGVS-
-----------------------DKTS-D--------STYPA-GS-AFSND-VLSLEITGPG-----------------------------------
--------------------------EEHFSIVDVPGTFKV-EA-------------------E-------GV--------------T--------TK-
ED---IKLVD--------DMVK-RYM---TN----------SRSIMLTVVNCND--D-ISSHD-IIQKARDID------
PHGERTLGILTKPDLAD-------EGA------E-----QKI----------------------------IDI--LD---GK---------Q-
HR-----------------------L---FHGWHILRNR---G-QKD--LRD--
---------A--------TS--------------------LS----------DRHAT---ERKFFTD--------KD-PW-------------------
--N-K------L--------D--KSL-VGIDALNHRLHAVLATQLNKE-F----
>TVY17522.1
---------GDYIDLPQ--------VVVV---GD----Q-----------------
SSGKSSVLE-----G--LT-N-L-PF-PRD--------------SG-LCTKFVT---------------KIT--------------F---
RRS-------------------------------PMTR-IAI---T--------
------------------IIPAK---NSS----------T---------------EHV-E-HV----
-----------------------------------RGWAKADLRSLDE---------KTFADIM------KEVH-TV---MGLS---------

---------------EQVD-G--------MTPP-----TFSDD-VLSLEVCGPK---
------------------QEHLSIIDVPGIFKK-RT-------------------Q-------GL-------------T--------SK-AD---
IQMVK--------SMVL-GYM---KN------------PRSVMLTVIPANV--D-IVTQE-ILEMAEEVD------
PDGQRTLGVLTKPDLVD-------KGA------E-----KTI---------------------------VDL--IE---GR---------R-
HR----------------------L---ALGWLLVRNP---G-QQE--LTD---
------------------P--------------------MT----------DRHAL---EKSFFSH--------EQ-PW--------------------
-N-S------L--------D--KEK-VGIPALQVRVRVILAEHIRRD-F----
>KAF9951223.1
---------NHVLSVPQ--------IAIV---GD----Q----------------
SSGKSSVLE-----A--FT-Q-L-SF-PRD--------------KG-MCTRFAT---------------QVN--------------L---
CRD------------------------------LTLDKDTL--
-----------------------S--ARI----------D--------------DED----------
----------------------------------SFN-ERWKTFQV---------DQFYAVI------KEAV-SL---LCGT------------
-----------S----------------------DISDK-VLELTLSGPH--
------QSPLTVVDLPGFIN--TT-------------------L-------DG--------------Q--------DK-NI---PHTIR------
--DINE-RYM---KD------------PRTIILAVIPANV--D-LNNSY-VLARAEEHD------
PKNERTVPIVTKPDMID-------KGT------L-----SEL---------------------------IDM--VL---NN---------R-
KK----------------------M---PLGYLVMRNT--G-YAD----R-----
------D--------LS--------------------WE----------EAQRA---EEDYFAQ--------DK-AW--------------------
-E-A------V--------P--RSN-RGRVMVKKFLGDLLYFHIKKE-L----
>XP_047808890.1
---------QDMVSLPQ--------IAVV---GD----Q----------------
SSGKSTLLE-----Y--IS-G-V-TF-PKD--------------AG-MCTCFAT---------------EVS--------------M---
RPS------------------------------TQFS-ARVF---IN-----
--------------------HQPD--SRI---------------------------------------
-------------------------------------KQPRSPE-----------DVAGVI------QQAK-KL---FVEA----------------
--------SGQK-A------------------IYDD-ILTVELNGPG--
------LPILTLVDLPGYIH--TH-------------------A-------TG--------------Q--------PE-SI---VKDIE--------
QLVE-RYL---NS------------PRTVIMAVIPVNR--D-FETNV-AIKHIRRFD------
PSGKRTLCVLTKPDQVD-------AGT------E-----RNV---------------------------LDV--LA---GK---------
K-MH-----------------------L---DRGYHIIKNK---N-FEE--CQA--
---------G--------DN--------------------RE----------ATSKK---EGHFFA---------RS-PW------------------
---S-S------I--------S--PTE-KGIASLVDRLSDTLNAQVEKE-F----
>CAD7955538.1
------------VEVPG--------VIVV---GN----Q----------------
SAGKSSVLE-----A--IS-G-I-NF-PRG--------------EN-TCTRCAS---------------IVR--------------L---
ECH------------------------------PAEA---
-----------------QDDRP--------------Y--------------------------ALL----------S---------------CND-A-
EQ--KQAQKIYDFA-----------KIGQQI------DALT-KK---LGEGR-----
-----------------DSG-------------------T-ILGDD-VIYITVRNKS---
--------------GPTMTLIDLPGLTF--VH-------------------K-------TQ--------------K------------N--
IHDVTV--------ELIR-KYI---KN------------EQAVILAVIPATE--D-FGNCE-ALNLASEVD------
PEGERTLGVATKCDMIS-------EDS------D-------L---------------------------VQK--LT--MSRSS-------
D-IK-----------------------L---KLGFVGVRCR---G-PAE--VKD--

---------N--------LP-------------------FD----------QMAVR---ERELFTT--------HR-AFTKNIT------------
GTTSK-K------F--------P--RHC-WGMDTLVAKICQIQEQTVEKW-LPRV-
>CAD7971727.1
------------VEVPG--------VIVV--GN----Q----------------
SAGKSSVLE-----A--IS-G-I-NF-PRG--------------EN-TCTRCAS---------------IVR--------------L---
ESG------------------------------NLEA---
------------------NERA-------------Y-------------------------ALL----------S---------------VND-P-EQ--
-------------------------------------KQCDKIRDFS-----------AIGVQI------EKLT-KK---LGEGQ----------
------------DGG-------------------T-ILADD-VIYITVRNRT---
---------GPTMTLIDLPGLTF--VH-------------------K-------TQ--------------K------------N--IHDVTV-----
---ELIR-RYI---KN------------EQAVILAVIPATE--D-FGNCE-ALNLAADVD------
PDGERTLGVATKCDMIG-------NDS------D-------L---------------------------CAK--LR--MERTS------
-D-IK-----------------------L---KLGFVGVRCR---G-PGE--VRD-
----------G--------IS--------------------FQ----------EMAKR---ERELFNS--------HP-AL------------------
-TGK-G------L--------K--KEH-WGMDTLVEKICNIQEQTVERW-LPKV-
>CAE6914669.1
------------IPIPG--------VVVV--GE----Q----------------
SAGKSSLLE-----S--IS-G-I-QF-PRA--------------QN-TCTRMPC---------------VVS--------------L---
LTD------------------------------RS---
----------------VKQP--------------Y-------------------------AIV----------S---------------M-D-P-AF------
-----------------------------------SEAEKC-TL--
----------------------QQ-AIYVRVTRAE---
GPQMSLIDLPGITH--NS-------------------T-------KM--------------A------------N--IHQVTR--------
ELVE-QYI---AP------------TEMVILCVIPAAN--D-FGNAE-VLKLASKFD------
PSGERTLGVVTKCDDDAK-----REAS------D-------L---------------------------VDK--VM--MKRDL----
---D-VK-----------------------L---DLGFHCVVSG--S-PKD--
IKE-----------A--------TS--------------------RE----------DL---
-------------------------RRLMEKIAKLQEARVDAH-LPNI-
>CAI4004018.1
------------IPIPG--------VVVV--GE----Q----------------
SAGKSSLLE-----N--IS-G-I-QF-PRA--------------QN-TCTRMPC---------------VLT--------------L---
LTD------------------------------PA---
----------------VEES--------------Y-------------------------AMV----------S---------------M-D-P-NF----
-----------------------------------ENVKRC-SIP-----------EVEEQI------KALT-EK---HAT------------------
-------GD-------------------Q-FISSQ-AMYISVVRRE---
-----GPQLSLIDLPGITH--NS-------------------S-------KM--------------T------------K--IHEVTV--------
KLVE-NYI---KP------------EEMVILCVIPASN--D-FGNAE-VVELAKKYD------
PDGERTLGVVTKCDDVAK-----TESS------D-------I---------------------------VEK--VL--MERTD-------
D-VR-----------------------L---ELGFHCVVNR--S-QKD--IDE-
----------G--------MS--------------------RE----------DLLEK---GRKVFSE--------SD-RM------------------
----K-R------L--------P--KEN-WGTLALTEKIAKLQEHRVDAH-LPKI-
>CAI3978736.1
------------IPLPG--------VVVV--GE----Q----------------
SAGKSSLLE-----N--IS-G-I-QF-PRA--------------QN-TCTRMPC---------------VLS--------------L---

LTD------------------------------PT---
----------------VKES---------------H-------------------------------AMV-----------S-----------------I-D-P-GF-----
-----------------------------------ANATTC-TVP-----------EVEKQI------KQLT-EE---YATG-------------------
-------GD-------------------Q-FISSQ-AMYIRVVRKE--
-----GPQLSLIDLPGVTH--NS-------------------D-------EM---------------K------------D--IHEVTV-------
-NLVE-EYI---RP------------KEMVILCVIPAMS--D-FGNAE-VVKLARKYD------
PDGIRTLGVVTKCDDAAN-----AEAS------D-------I-------------------------------VKK--VL--MSRSS-------
D-VR--------------------------L---ELGFHCVVNR--S-QKN--IDE-
----------E--------MS----------------------RE-----------DLWAK---ERKIFTK--------NE-RM--------------------
---K-R------L--------P--EKN-WGTLRLMEKVAKIQEARVDEC-LPKI-
>OLP81297.1
-----------PDPPPG--------VVVV---GE----Q------------------
SAGKSSLLE-----N--IS-G-I-QF-PRA---------------QN-TCTRMPC----------------VLT---------------M---
LTD------------------------------PT---
----------------VNES---------------F------------------------------ALV-----------S-----------------M-D-S-SF-----
-----------------------------------TDAKRC-AVA-----------DVEGQI------KELT-DQ---HTT---------------------
--------GA---------------------A-FISTE-ALYIRVVRRD---
----GLQLSLIDLPGVTH--NA---------------------E-------KM---------------S-------------N--IHEVTV--------
SLVK-EYI---EP------------EEMVILCVIPAMS--D-FGNAE-VVKLAREYD------
PEGIRTLGVVTKCDDAAN-----AEAS------D-------I------------------------------VEK--VT--MSRDS-------
D-VR--------------------------L---AFGFHCVVNR---S-QKN--IDE-
----------G--------MS----------------------RE-----------DLWQK---EEKTFTA--------SD-R---------------------
-----K------L--------P--ANN-WGTLRLMEKVAKIQEARVDEC-LPKI-
>CAE7315868.1
------------IPLPG--------VVVV---GE----Q------------------
SAGKSSLLE-----S--IS-G-I-QF-PRS---------------QN-TCTRMPC----------------VLT---------------M---
LTD------------------------------PA---
----------------TDDP---------------Y------------------------------ALV-----------S-----------------M-D-P-SF------
--------------------------------------EDAGPC-PVM------------QVEGKI------KQLT-EK---HAT------------------
--------GD---------------------D-FISKQ-ALFVRVVRKE---
------GPQLSLIDLPGVTH--NA---------------------E-------CM---------------E-------------N--IHEVTV------
--ALVE-EYI---KP------------DEMVILCVIPAMS--D-FGNAE-VVKLAKKYD------
PDGIRTLGVVTKCDDAAH-----AEAS------D-------V-------------------------------VEK--VL--MRRET------
-D-MR--------------------------L---QLGFHCVVNR--S-QKN--
IDE------------G--------MS----------------------RV-----------DLWNK---EKKIFS---------SE-RL--------------
--------R-S------L--------P--EDY-WGTRRLMEKIAKIQEDRVDAC-LPKI-
>CAE8582582.1
------------IALPG--------VVVV---GE----Q------------------
SAGKSSLLE-----N--IS-G-I-QF-PRA---------------QN-TCTRMPC----------------ILT---------------M---
LTD------------------------------PS---
----------------ILEP---------------Y------------------------------AKV--
------------------------------------RQC-AVG-----------EVEEKI------RALT-AQ---HAT---------------------------
GD---------------------T-FISRK-ALYVRVVRKD---
GPQLSLIDLPGVTH--NA---------------------D-------KM---------------A-------------D--IHEVTV--------

SLVE-EYV---RH------------EEVVILCVIPAMS--D-FGNAE-VIKLARKYD------
PEGKRTLGVVTKCDDAAR-----AEAS------D-------V---------------------------VDK--TL--MRRDS-----
--D-VQ-----------------------L---KLGFHCVVNR---S-QKN--
IDD-----------N--------MA--------------------RK----------DLWVK---EQQLFET--------SE-RL-----------
----------R-G------L--------P--SDH-WGTLRLMEKIARIQASRVDEC-LPKI-
>KAI0562045.1
------------IECPG--------IVVV---GA----Q----------------
SAGKSSVLQ-----R--LT-G-I-SF-PRA--------------EN-TCTRLPA---------------IVS--------------L---
QTD------------------------------PS--
-----------------INGS--------------R---------------------------IYV----------S---------------R-D-A-AF-----
-----------------------------------RNPLECDSMA-----------VLENRI------MAMT-MD---LEN-------------
------------SN-------------------F-SVKNE-PVYIRYIRKE---
--------GPVFSLIDLPGITY--MD-------------------P-------KN--------------Q---------N-FD--IHEETV----
----SMVR-QYV---SN------------PNTIILVVIPAVE--D-FSNAE-ALKIAMECD------
KEGNRTIGVVTKCDMVS-------ESS------Y-------F----------------------------LRK--MR--MTGPN------
-D-IK-----------------------L---KLGFVAVRNR---G-PGE----E---
--------N--------IN--------------------ID----------EA------ERNMFSN--------HS-LL---------------------
K-Q------L--------D--PTQ-KGYVALTKKIVELQSELVDAF-IPKT-
>KAI0557988.1
------------IQCPG--------VLVV---GA----Q----------------
SAGKSSVLE-----R--LT-S-I-RF-PRA--------------QN-TCTRVPT---------------IVQ--------------L---
HTN------------------------------PL--
-----------------VKVA--------------T---------------------------ALV----------S---------------K-N-A-DF-----
-----------------------------------SDAKTCEDMK-----------SVEDAI------LAFS-SS---IME----------------
---------NT-------------------A-PISDS-PIHIRYTRKK---
----GPVMTLIDLPGITH--VD-------------------V-------DG--------------R--------DD-FD--IHDVTS------
--SMVH-KYV---KN------------DNMVVLVVIPAND--D-FGNAE-ALRIAQLYD------
KEGKRTIGVVSKCDLVP-------QNS------D-------I----------------------------LHK--IR--MSRQG-------
D-VK-----------------------L---ALGFIAVRNK---D-IGE----D-----
------G--------KD--------------------IE----------KI------ESKLFST--------HE-LL---------------------R-H-
-----L--------A--EEQ-RGYAALTRKIVDLQSQRVDEF-IPEA-
>XP_005716602.1
------------IPCPG--------IVVV---GA----Q----------------
SSGKSSVLE-----R--LT-D-I-AF-PRD--------------TN-TCTRVPI---------------IVQ--------------L---
QRD------------------------------AS--
-----------------VTLP--------------T---------------------------ATI----------C---------------G-D-E-HF------
---------------------------------ETDVINCSTKQ-----------DITNAI------HDLTEKA---LVS------------------
-------TR-------------------S-RVVDK-PIHIRYVRSK--
---GPVMTLIDLPGITH--DD-------------------G-------EG--------------R------------D--IHGITA--------
DIVK-KYL---KY------------ENMIALVVIPAND--D-FGNSE-ALKIAKTFD------TTGARTLGVISKCDLVP-
-----EQHS------D-------I----------------------------VEK--IQ--MTPSN-------A-IK-----------------------
L---GLGYIAIRNK---G-PGD----D-----------L--------AN---------
-----------ID----------NI------ETELFKT--------HP-LL---------------------K-N------L--------A--PHE-
RGCGALRRKIISLQENSIKHA-IPRI-

>KAI0559778.1
------------IACPGLEAIERKYLVTM---GG----V----------------
GCGKSSVLE-----D--LA-G-I-RF-PRA--------------EN-TCTRTPT---------------VVH--------------L---
QVD-------------------------------PD--
------------------INKN---------------T----------------------------IAI------------S----------------R-Q-E-DF------
------------------------------------RNPKSCEDME-----------ELEKEL------RKIA-LD---VKQ-----------------
---------SG--------------------I-PVKDE-PVYTRYVRRT--
-----GPTMTLIDLPGISY--FD--------------------P-------NG---------------------------FD--VHEATT--------
SMIR-KYA---AH------------ENMILLIVFRATE--E-FGR-K-AFALAKKYD------EDSIRTIGVATRCDTVE-
-----TDSA------T-------F------------------------------LPQ--MR--MEDPN-------P--R------------------------
L---LHGFTAIRNR--L-RGE----E-----------S--------LR----------
----------IE----------DF------E-QVLST--------HA-LL----------------------Q-Q-----L--------D--CTT-
TGLNALAKKVVHVQSNVVDEF-ISNT-
>WGU15254.1
---------GTSLNYPR--------IVVV--GN----Q----------------
SAGKSSVLE-----A--IV-G-E-SFLPKG--------------TN-MVTRRPL---------------EIT--------------L-V-
RDE--
----------------SAK---------------Y----------------------------FLF--
-----------------------------EDGHKLHDID-----------AVRDRL------NEEN-----------------------------------
-KD--------------------F-EFSDS-PIRLEVHSPD---
VQNVTLVDLPGYIHA-VT----------------------------KD--------------Q-------SK--D--LPKKIQ--------
KLCK-NYI---DK-----------KDNLILVIVSAAE--D-TAMSV-GLKEVYKLE------DWESRTLGVLTKMDL--
-------RNT------------KDI-----------------------------VKT--LK---NN---------E-YP-----------------------L--
-GMGYIGVRCR--T-DKE--IDD----------K--------IN-----------
---------FN----------ELIKK---EDEFIKS-----------------------------------R-K------LH-------Q--
SEVKVGIPVLREELSKELLS-----------
>XP_013756556.1
---------KAKLELPK--------IVVV--GN----Q----------------
SSGKSSVLE-----A--VA-G-F-DFLPKS--------------TS-MCTTRPL---------------ELN--------------L-L-
RSD--
----------------SGT--------------W---------------------------AEF---
------------------------------DDGRKIFDFE-----------QVEAIL------REKN-----------------------------------
QD--------------------E-EFSEL-PVTMSIYAPH---
VYNVSLIDLPGYIHA-VR----------------------------AG--------------Q-------SP--E--LPERIA--------
ALCQ-KYI---SD-----------PNNIIVAVASAAD--D-VAMSM-GIKNAQAVD------
ANMDRSLGVLTKMDLI--------KSR-----------KHI-----------------------------HSI--LR---NE---------D-
YP----------------------L---GLGYVGVRCR--S-QKE--LED----
-------G--------KS--------------------FD----------DVLRI---EEAFIRR-----------------------------------A-
K------LR-------DV-GDLRLGIPTLRRVLSEEQLNKVAAD-FPRIL
>CAH6419740.1
-----------IKNWPQ--------IVIL--GQ----Q-----------------
NEGKSSLIE-----G--VT-Q-V-EILPKC--------------DG-LCTRKPI---------------HMT--------------L-I-
NDP---
-----------------DTK--

-------------------------FIIGDR-MYTET-----------QASEEL------NRLN----------------------------------
LN-------------------P-KVD---HINCTILSPI--
VYNCTIIDTVGLIH--VS---------------------------EQ--------------D--------NT--L--DPKKIK--------
QDTI-RYL---KD------------KNNIFVLVSSAPS--D-LANSQ-MLQLIKKYN------RVDD-
TLGVMTKIDLIE------NQNQ------------NSI----------------------------NDI--LC---GK---------N-YK-----
------------------L---GFGWIPTKLR--S-DRD--IQD-----------
G--------IT--------------------IE----------QSIIK---EHEYCQG--------RN-FS---------------------S-F-----
-------------S---YGVSEVRRTISQIQLERIKAN-IPSI-
>AYV81982.1
-----------LKGLPK--------PVAI--GP----Q----------------
SAGKSSVVE-----A--II-G-F-DILPKK--------------MG-MCTMKPI---------------NIT-------------T-I-
RDD---
-----------------MVK---
--------------------------FKVGDKELSTIE-----------SAKHEV------NRLN----------------------------------
SN-------------------S-NVT---IINLTIYSPD--
VTNISMVDLPGLFV--VT---------------------------EG--------------V--------SD--N--LPKLVK--------
EMTI-EYI---QN------------RNNIPLVITAAPS--D-PATNM-ALQLVSKYK------RREE-SIGVITKMDLTV-
-----KQHT------------EII----------------------------EQM--LK---GN---------K-FA-----------------------L-
--GNGWVSVLLK--N-NND--DDN-----------G--------IT-------
-------------VQ----------QKILE---EEEFFKD--------KP-NF---------------------S-P------------------S----
GVKMLRKKIGDLQLSKIQHN-IPQL-
>CAH6421112.1
-----------LKSFPG--------CAVF--GP----Q----------------
SVGKTSVLE-----A--IC-DIQ-DFLPKG--------------EG-MVTKKPI---------------HVT-------------M-I-
KSQ---
----------------NIL--
-----------------------YKIGDKEIKNES-----------DAREEI------NHLN----------------------------------
YN-------------------D-SIK---KIDVRIHSPN--
VCNSSLIDLPGLFV--VS---------------------------DK--------------N--------DP--D--LPNKVK--------
KMNM-EYI---SN------------EKIIPIVITSAAM--D-PATNQ-ALKMLGRFQ------REKD-
SLGIITKIDLME------KQNM------------IET----------------------------EQM--LS---GK---------K-YP------
-----------------L---GHGYVTVILR--N-KED--IDK-----------K---
-----IS--------------------MK----------DKILM---EKEYFSS--------RP-NL---------------------H-P---------
---------S----GVPEMRQIISNIQFEKLKDQ-IPFL-
>ARF12445.1
-----------LKHAPN--------FVVI--GQ----Q----------------
SSGKTSVNE-----A--LT-N-T-NIFPKA--------------MK-MATMKPM---------------KIT-------------T-I-
RSE---
----------------ETK--
-----------------------YKIGDKEFRNEN-----------EAADEV------NRLN----------------------------------
NN-------------------P-SVQ---QVVVTVWSPK--
VYNSSYTDTPGLFS--VT---------------------------NK--------------S--------NT--D--MPKKIK--------
ELIF-QQL---QD------------PNVIPVVIHSGPS--D-PATDQ-ALKFVEKFN------RQDD-
ALGVITKVDLLE------KQNT------------DFI----------------------------AKL--LN---GE---------E-YA------

-----------------L---GHGWVGVSLR---S-DKD--IEA-----------
G--------MS--------------------IE----------DKIMI---EKNLMAK--------MK--L---------------------K-P----
--------------S----GVETLRKMIADIHAHKIKDQ-LPNI-
>QKF93607.1
-----------LKSFPS--------VVVV--GP----Q----------------
SSGKSSVIE-----A--IC-G-E-TILPKA--------------MK-MATMKPM---------------HLT--------------T-I-
RSP--
----------------EKK--
-----------------------FKVGDRELKTER-----------EAADEI------DRVN-----------------------------------
NN-------------------T-HIQ---KVNDTIWSPD--
VYNAILIDLPGLFV--VA---------------------------GK--------------N--------EA--D--LPKKVK--------
DMSI-QHL---QD------------VSNIPLVVHAAPS--D-PATNH-AIKLVGKYG------REGD-
TLGIITKVDMLE------RQKT------------SFI-----------------------------EDM--LR---GD---------T-CP------
-----------------M---GHGYCTVVLR---N-DKD--VEA-----------
G--------MT-------------------VN----------DKIKI---EKEFFTR--------VP--L---------------------K-P------
------------S----GVPQMRKMISNIQFSRVKEQ-IPNL-
>QKU35298.1
-----------LKNFPT--------MVFF--GP----Q----------------
SSGKSSAIQ-----A--IT-G-D-YILPTD--------------MK-IATRKPT---------------HIT--------------T-L-
RSD--
----------------NVK--
-----------------------YKVGDREFFTAK-----------ETSNEI------DRLN-----------------------------------
RN-------------------D-HVE---KIDVVVKSPH--
VHNSVFIDLPGLFS--IS---------------------------DN--------------D--------TD--D-HFRKKVK--------
QMST-AYT---SN------------RNFIPMIVHAAPS--D-PATNA-AIKLVSKID------RRND-
AFGILTKFDMVK------NQKT------------AYL-----------------------------ERM--LK---GV---------D-YK----
-------------------L---GHGYCAVVLP---N-DLD--IER-----------
G--------VS--------------------VE----------EKIKE---EEEFFKK--------YP-NV---------------------K-P-----
-------------S----GVPILRKMISDIQATKIMEH-IPAI-
>AYV83919.1
-----------IKSFPI--------MTFF--AP----Q----------------
SAGKTSSLE-----A--LC-R-K-SLFPKN--------------SG-MSTMKPI---------------YVV--------------M-L-
PSP--
----------------TEK--
-----------------------IVVNGKEL-NEK-----------MATDEI------QRLN-----------------------------------
SN-------------------V-NID---MINVVIYGPD--
EITSNYGDLPGLIA--LS---------------------------SS--------------H--------PE-----LPEKIK--------KLCY-
SHM---EN------------PNTIPIIVHDASG--D-PEINK-ALQIVMKLR------RSAD-ACGIITKID-KQ------
KSHN------------SSI----------------------------KSM--LE---GK---------T-YP----------------------L---
GYGYVPVTLR---N-MEE--VDA-----------G--------MT-----------
---------VE----------EKEEQ---EKQFFER--------NP-AL---------------------K-P------------------
VNGPYGVAALRSKLADIQVDKIKQN-VPEI-
>QFG74079.1

------------IKIPT--------CVVV--GT----Q----------------
SSGKSSLIN-----M--LI-G-M-PILPMG--------------KS-MVTRVPL---------------NLQ--------------L-I-
EGG------------------------------SR---
------------------KIE--------------F---------------------------GKY--
---EGG----------------------TWIVIASTKTPS-----------EVSQII------ERET-IN---I-AG---------------------
---DS--------------------K-NIGHK-EICIRVYGAE--
TSLNFIDLPGLTM--VAC------------------TD------LG--------------Q--------PP--D--IKQQIK--------
NLIE-KYT---SV------------ESNIIIGVFPARQ--D-LEADY-GLDFIKKYD------
SNFNRSIGVLTKVDLMQGDLIPYLQGG------S-----------------------------------HDA--LNT-TNPSI---
---SDNLK------------------------L---KWGYYAVNTR---DGD---
--TYFDN--I-EWAKTP-SL----------------------
---------------------TNR-IGVKNLSKQLYSILLQSIRES-LPKV-
>XP_750654.1
------------IALPK--------ICVI---GD----Q----------------
STGKSSLIE-----G--MS-Q-I-KV-PRS--------------AG-TCTRCPM---------------EIN--------------L---
SEGE-----------------------------PG---
--------------------QD--------------WN--------------------------CRIFLS------RK---------------YIF-D-
GSRKVTKLPKKSQPLGPW------------------IEQDQEDEHFTDVRDKD-----------GVQAAI------
KWAQ-LA--ILNPGRPSTD-------YQP--------GHNGD----------TDESYCQV-KFSPN-VVRLDISAPN--
---FPNLSFYDLPGVISQ-AE---------------------------
----------------H--------DH--ERYLVSLVE--------NLVR-EYI---SQ------------ENCIVLLALPMTD--D-
ATNSS-AAKIMRDVP------GAKERTLGVLTKPDRIQ-------TGE------S-----YDQW----------------------
-----VEI--LE---GD---------K-FA-----------------------L---GHGYYIVRNN---------------------------------
-------------P-NPA--IEH-----------S---RAREE---EAVFFAK--------
SP---------------------WATDLS-----AY-----------QNR-FGTRNLQSALSSLLLEQIQGC-LP---
>XP_006461472.1
------------IDLPQ--------IAVV---GN----Q----------------
SAGKSSLIE-----S--IS-G-I-TL-PRA--------------SG-TCTRCPT---------------ECR--------------L---
SYS-------------------------------S---
-----------------QP--------------WK--------------------------CVVSL--------R---------------ITT-D-RSGQ-
--------PLG---------------------QSRNETFGSTIYDKK-----------EVDDRI------RRAQ-LA--
ILNPDKPAKS-------FLN--------DDEPS----------LMEGN-FL-TFSKN-CVSLAISGPD------------------
---VADLSFVDLPGLIAS-VG--
R--------GG--NAGDIKLVE--------GLVT-TYI---HK------------TNCIILLTVACET--D-FENQG-
AHQLAKQYD------PEGKRTIGVLTKPDRIP-------AGE------E------QNW---------------------------
LKF--IR---NE---------K-EP-----------------------L---QNNWFCVKQP-----------------------------------
----------A-SSD--LKN-----------N--------WT--------------------WQ----------QARQK---EDEFFTA-----
---TS-PW------------------NELE-A-----MY-----------VRY-LRTKNLVERLSQVLSDLIAKT-LP---
>XP_006461433.1
------------IDLPQ--------IAVI---GS----Q----------------
SAGKSSLIE-----S--IS-G-I-TL-PRA--------------AG-TCTRCPT---------------ECR--------------L---
SYS-------------------------------P---
-----------------LP--------------WK--------------------------CTVHL--------R---------------FTT-D-GNGT-
--------PLG---------------------QSRNEIFGPTIYEKS-----------EVEERI------RRAQ-RA--

ILNPGKPTKL-------FLE--------DDDEM----------SGEAE--L-SFSNN-CVSLQISGPD-----------------
--VADLSFCDLPGLIAS-VG---
R--------GG--NTNDIKLVE--------SLVT-SYI---KK------------PSCIILLTVACET--D-FENQG-
AHQISKAYD------PEGKRTIGVLTKPDRIP-------LGE------E------LNW-------------------------LKF--
LK---NE---------R-EP----------------------L---ENNWYCVKQP---
---S-SND--LKN-----------N--------WT-------------------WQ----------EAREK---EQQFFAA--------TA-
PW-----------------CELE-G-----MY-----------QKF-LRTTNLVERLSGVLSDLIAKR-LP---
>KAI3646081.1
------------LNLPG--------IVVC--GN----Q---------------
SSGKSSLIE-----A--IS-R-V-PL-PRA--------------AG-TCTRCPF---------------ECR-------------L---
SES------------------------------K---
-----------------NP-------------WS-------------------------CKVML--------R--------------YEV-D--------
---AKGQAL------T--L-KA--VLNPGLEAQQ-------FLT-
-------DTNWE----------KIE-D-QQ-KFTEN-VVCLTIEGEG--
-----------VGYLTLTDLPGIIQS-TN---D--------AA--DEHFVELIK---
-----NLVE-KYV---SC------------PTNIIVEVITCKD--D-MENQI-VHTLARKAD------
PSGLRTVGVLTKPDMIE-------EGC------T------DDW-------------------------LNV--LR---NN---------
T-CP----------------------L---KHGYYMVKNP--A-TKD--LQA--
---------N--------IT--------------------FS----------QARQK---EVAFFSQ--------KP--------------------
WSTE-R-----AL-----------SKR-FGIYNLKEFLSNLLSNLLKQN-LPQL-
>KAJ9515210.1
---LADFGAAHEISYPT--------IVVC---GD----Q---------------
SAGKSSIIQ-----R--IS-G-I-DL-PRS--------------SG-TCTRCPM---------------EVRM------------TL---
SEG------------------------------G---
------------------VP-------------WS-------------------------CKIKI--------R--------------REW-
DDGKRK---------TLS---------------------KVSWEDFGAPLLDKE-----------AVGPAV------SRAQ-KA--
VLNPGKGYAS-------FVD--------PTSPI----------LADAD-EL-GFSRN-VVVLEIQGAD-----------------
--IS-LSLIDLPGIINS-TE--K--
------KE--DQYLVNMIK--------DMVK-QYI---EA------------SQTIIVLAVHALS--D-IQNQV-
VYQMAREAD------PHQQRTLGVITKVDVIP-------PGS------H------SMW--------------------------
IRM--MR---GE---------L-FP----------------------L---DLGYYMVVNP-----------------------------------
----------N-QVD--LDQ-----------G--------TS-------------------HEVGHTGRMWKDDAVDK---
EMRFFET--------DA-NLG----------------VLAQS-V-----VW-----------SSH-
LGLSNLTAALSKQLVDRTMAE-LPHMR
>ETO25748.1
-----------TVDLPT--------IVLC--GQ----Q---------------
SSGKSSVSE-----A--IS-G-V-AL-PRS--------------SG-TCTRCPT---------------EVR-------------L---
IHTQD---
------------------TK-------------WK-------------------------CTIKV--------R--------------WEWND-
RKKT---------HLHQ---------------------VREEEIA-TVTERE-----------AVAGIV------EKAQ-KV--LL------
-------------------------------------EKA-EV-KFSRN-VICVEVQTSD---
-------------------CVDLTVVDLPGLIQA-VE---N--------EE--
DFQYIQLTQ---------------------------------------FHCNN--D-MENQA-VNSLIRQHD------
PKGLRTLGILTKVDTIE-------TDS------E------QIW-------------------------INV--LK---GK---------S-

YP-----------------------L---KLGYVAVRNI--T-QHE--LKH------
-----G--------KS--------------------FK----------ETRAI---EAQFFAS--------HP-TWS------------------
KLS-S-----SL-----------QSR-LGCSN--------LVDILSCK-LSS--
>OAJ38670.1
------------LSLPT--------LVVC---GN----Q----------------
SVGKSSLVE-----A--IC-G-I-TL-PKA--------------AG-TCTRCVT---------------EVR--------------L---
SEYSDV--------GPVDSVRKKSVAGDRNSTEST-------ITQGV-YIDALDT---------------------------
VSSGSVPSAF--HIDGHEK--------------------------ESST--------------WS--------------------------
CTITL--------R---------------FEY-D-EAGI---------PLRS---------------------IREVLFGPPLVEKS---------
--LVALAV------RRAQ-KA--LLNPTLDPSV-------FLTHIFTDND--QSNS----------DTKSN-QL-KFTKN-
IVCLDIQGAG--INLALVDLPGIIRN-VE-----------
--------------------------------H--------PD--DAMFIPMIE--------DLVK-SYI---QK------------
ERTIIVATITCKD--E-MENQA-IVHLAREVD------PTGIRTIGVLTKPDTIE-------SGT------A------ARW--
-------------------------ADI--LM---GN---------L-YP-----------------------L---KLGYFMVRCL------------
----------------------------------S-KAE--LAA-----------G--------NT--------------------LQ----------
DAQKL---ENAFFAQ--------SQ-PWS------------------TLR-R-----K------------SAR-
FGAPALRFELSRLLINLVDMS-LP---
>GAX85982.1
----RQLGVSNDIKLPT--------LVTA---GN----Q----------------
SSGKSSVVE-----A--IA-G-I-PL-PRS--------------SG-TCTRCPT---------------EVR--------------M---
RS--

---VHQ-VV--RLTLTDVDQK---------------KP--KEPE-
HSRSFLEL--PTEY-QL-EFTRN-SVVLEIEGAD--
ADLTIIDLPGIIQ---S---H--------HK--GPHYVEMIK--------SMVL-
NSI---ES------------DHVIIVMVITAMD--D-VENQA-INLEARNVD------PEGQRTIGVITKPDNIP------
-KGE------H------------------------------------DKWVALA---SN---------R-RP--------------------GQEL--
-SLGYYVVRNP--G-QNE--LDE-----------S--------IR-----------
---------FE----------DARVK---ESEYFET--------SP-YW-------------------PSN-G-----EL-----------
QGR-LGTTFLRNALSESLVQGIKKG-LPGMQ
>KAG2488600.1
---LRALGVGSALQLPA--------LVIA---GD----Q----------------
SSGKSSVVE-----A--IA-G-V-SL-PRS--------------DG-TCTRCPT---------------EVR--------------L---
RT---------------------------HAGPDA--NGDSP-----
MPDGD------------------------------VP--------------WT--------------------------CRIKL--------H---------
------REY-D-SDGH---------PLTE--------------------LPPEELFA-TLTNKA-----------HIAAFV------
TAAQ-AV--LLNPRAADAA-PGGARAFVPDVSGDRP--RDPQ-P---LRALGHPTTY-EL-SFTAN-
KVVLEVDGAD--ADLTIIDLPGIIH---D------------
-------------------------------H--------PK--GKQYVDMVE--------RMTK-AQL---RP------------
EHHIIAMALPAGL--D-PETQA-IRLWVREVD------PSGSRSIGIITKPDTIA-------DDA------H------ITY-
--------------------------GKLVKLV---GG---------S-TM-AGGAAGAAASAGPAAGHDESHQL---
TLGYYVVRNP--G-QEQ--LED-----------C--------IG-----------
---------FA----------EARAA---EQRYFAT--------NT-HWV---------------QAVAAL-P-----SL-----------
KQR-LGANHLRSGLSALLVERIETQ-LPHMR

>XP_042924875.1
---MRTQAANADL---------------A--SD---ME----------------
SSGGG------------------------E--------------DG-SGME---
-------------------EDG---EGAAN-----GADGKRR-------------
---------------PA--------------WR--------------------------CRIKL--------C---------------RDY-D-SEDK---
------PLAE---------------------KPPEQPFC-VVRDKA-----------HIAACV------SAAQ-AV--
LLNPRAVEAA-AGGPQAFVPELSS--A--HSPS----------HP-----------N-KVVLEIDGAE----------------
---ADLTIIDLPGIIH---D--
H--------PK--GRHLVEVVE--------RMTK-TNL---AP------------AHHIIAMALPAGL--D-PETQA-
IRLWAREVD------PDGHRSIGIITKPDMIA-------EEA------H------IVC---------------------------
NKLVKLV---G---------------ARGELGPGGTRSQPDGH-----L---RLGYYVVKNP------------------------
---------------------S-QEQ--LVE-----------G--------IT--------------------FE----------KAREI---
EARYFAN--------HV-HWR---------------PAMATS-P-----GL-----------VQR-
LGANALRSGLSLLLVERIEEQ-MP---
>XP_042924848.1
------------LQVPT--------LVIA---GD----Q----------------
SSGKSSVVE-----A--SA-G-V-PL-PRS--------------DG-TCTRCPT---------------EVR--------------M---
RTTQQLPAATGVCGGSALSAAAASSPPGPHSGEDGEDDEDDEDEEDE-EDDAVVN-----------------
----------NAAGLPPGTTRGGGGGSSA----------------------------SA--------------WQ--------------------
------CRIKL--------C---------------REF-D-SDGV---------RLAT--------------------KPPEQPFC-
VVRDKA-----------HIATCV------LAAQ-AV--LLNPRAVEDT-PGGAGAFVPLLSSAQP-GRQPA-
AAAKLLALRDASHY-EL-PFTPN-KVVLEIDGAE---
--ADLTIIDLPGIIH---S--H---------E--DPRLIELVK--------DMVK-
ANL---AP------------EHHIIAMALPAGQ--D-AETQA-IRLMTREVD------PDGRRSIGIITKPDRVP------
-EHE------A------GET--------------------------LKLIRLV---GA---------C-GAPPAGAGAAGGS--
ARVAH---PQH---PLGHYVVKNP---S-QDG--LAM-----------
N--------IT--------------------FE----------QARAD---EAAYFAG--------HK-HWA---------------
AALRRQ-P-----EL-----------QRR-MGAAALRRGLSGLLVELVIAQ-LP---
>XP_042923301.1
------------LKVPA--------LVIA---GD----Q----------------
SSGKSSVVE-----A--IA-G-V-PL-PRS--------------DG-TCTRCPT---------------EVR--------------M---
RT--HGAP------GEGGS------
------------------------AV--------------WQ-------------------------CRIKV--------V---------------RNF-D-
STGK---------PLAPG-------------------EAHEKLFC-TVTDKA-----------HITACI------SAAQ-AV--
LLNPTVVGDAVADGAERFVPLLSAAEPGGRAPE-ASSAMRGLGDAAGY-EL-QFTAN-
KVVLEIVGAE---ADLTIIDLPGIIH---S--------------
-----------------------------H--------PK--DPSLIDVVK--------SLVK-CYL---AP------------
AHHIIVMTLPAGM--D-AETQA-ILQFAREAD------PEGRRSIGIITKPDKIG-------TDE------R------
TEW--------------------------GKLCNLV---AG---------A-RA-PTGVPAAGGSRAAAAPN---PHL---
QLGYYVVKNP--G-QEQ--LAA-----------G--------IS-----------
---------FE----------QARAA---EERYFAD--------HP-LWA---------------SAMKAN-S-----LL-----------
SQR-LGTNALRDGLSALLVDKIGEH-MP---
>XP_006457072.1

------------FDLPK--------IVVI--GN----Q-----------------
SAGKSSLIE-----A--VT-G-I-NV-PRD--------------SG-
TCTRCPMECSMSSDTRSWSCTISLRSGTSTSIPPTSPRVL---RSTRGT----------------------
SIASASSTASAGV---------TPTPA--------------------------RASTGVRNITTQ-SFGPTITD------------------
------KSQVE-------------LWLR----R------------------AQGAI-L---------------------------S---------------
---------------------------TDADKSQWLNKSA---------EEIRQAI------QNKT-----GM-----------------------
--------------------------R-DFTED-TIVVDIQDPT---
ATDLSFVDLPGLISN-------------------------------AD------------------------PG-----SIDLIK--------NLVR-
QHV---AG------------ENTLILVTIPASD--D-IQNHG-AVVLAKEAD------GNGDRTIVVLTKPDSLG-----
--PGD------TG---LQETW---------------------------RQT--FK---NP---------N-VP------------ENQNY---
--L---RHGYYCVQLP---NDQQR---------------Q---------------
----------------------------QGLTAHT-LPNYLGV--------TW-PW----------------------S-E------FAG---------
QGR-FGVTNLVKNVSALLVQMIEAN-LP---
>GBG30247.1
------------FHAPG--------VAVI--GN---Q-----------------
SSGKSSVLS-----A--LT-R-V-DL-PRG--------------TG-TVTRCPI---------------VVH--------------C---
ERS------------------------------QECKA--
------------------LVSRD---ATF---
---------------------------------SEATEVRDMA----------YLAEAI------ARKQ---------------------------------
--PSS--------------------G-AIENK-TVHVKVMRPD--
--VHKLVLIDLPGIKQ------------------------F-------GG-------------SK--------GA--D---EHELTV-------
GLVK-EYI---SN------------PRMVILVVLPAES--D-LETCE-ALRLAREHD------
PQGKRTLGVFTKVDCLP-------DSDP----IQ-----KRSF----------------------------CER--LT---KK---------
G-AP--------------------QALL---AHGYFAVRTR---T-ETE--AQE-
----------G--------DS--------------------HT----------DVLDR---ERALFTK--------DK-YF------------------
---R-E------L--------S--PDL-WGIQTLGEKVSIIQVKMVRQF-INEI-
>CEM15039.1
------------IEVPG--------VVVC--GD---Q-----------------
SAGKSSVLE-----R--IM-G-V-PF-PRA--------------QG-TCTKTPT---------------ILMG-----------------
ETD------------------------------ASAD--
-------------------GVSV-------------W---------------------------VSLK-----------------------------E---------
--------------------------------DITDAEELNDIK----------DIGNKI------TELT-DD---L-VR------------------
------QEG--------------------S-AIVNR-PIYVRIRGKN--
----VPTLTVTDLPGLLY--NK-------------------K-------GD--------------E------------N--IHAEVV--------
AMVN-EYI---EK------------EKTVILCVVPAGG--DFLSTSE-AIKLAKDVD------
PERHRTIVVVTKTDKIE-------PGE------EG----RHF-----------------------------LKS--IK--QGQ---------
DEIG---G--------------------K---KLKCIALRNR--KPD---------
-------E--------IV----------------------LDI---------DAEQE---EAAFFER--------HP-DLC---------------------
K--------LL----------SEE-WGITTLVKRITDIQVVYVDEF-MKDI-
>PAA87312.1
------------DHLPR--------VVVV---GD----Q-----------------
SSGKTSVLE-----M--IA-K-A-RIFPRG-------------AGE-MMTRAPV----------------QVT--------------L---
AEG---
---------------------------P-------YHV--------------------------ARF----------K---------------DNP-S----------

-------------------------------REYDLTQESELA-----------ALRDTI------ERRM-RS--VVQSG--------------
----------G----------------------TVSAE-TISLSVQGPG--
-----LPRMVLVDLPGIIS--TE-------------------T-------RG-------------M--------AS--Q--TREAIR-------
QLAS-QHM---RN------------PNSIILCVADACV--D-PERSN-AFDLVARHD------
PSGRRTIFVLTKLDLAE-------RDR-----ISP----DRI----------------------------GRL--LA---GR---------L-
LP----------------------LK---ALGYFAVVTGS--G-GAD-----------
--------------ES--------------------IP---------AIQRY---EEQFFRN--------SQ-FF---------------------K-
EG----VL--------S--VSQ-MTAANMAQAVSRRFWALVQES-V----
>PAA68234.1
------------DHLPR--------VVVI--GD----Q-----------------
SSGKTSVLE-----A--VA-R-A-RLFPRG-------------AGE-MMTRAPV-------------QVT----------------L---
ADG--
--------------------------P-------YHV-------------------------ARF----------K---------------DDP-D---------
--------------------------------REFDLTKESELA-----------ALRDAI------ERRM-RA--AVRSS--------------
----------GP------------------DA-AVSTE-AIPLSVQGPG--
--------LPRMVLVDLPGIIS--TE-------------------T-------AG--------------M--------AA--Q--TRESIR-----
---QLAR-QYM---RN------------PNAIILCVADACV--D-PERSN-AFDLVAKHD------
PAGRRTIFVLTKMDLAE-------RDK-----VSP----DRV----------------------------AKL--LA---GR---------
L-LP----------------------LK---ALGYFAVVTGS---G-SQD--------
-----------------ES--------------------VE----------AIERH---EAEYFAS--------SR-LF---------------------
K-DG----RL--------S--PNQ-VTAANMARAVSRRFWALVRES-V----
>NP_495986.3.3
------------DNLPR--------VVVV--GD----Q----------------
SAGKTSVLE-----M--VA-Q-A-RIFPRG-------------SGE-MMTRAPV---------------KVT--------------L---
SEG---
-------------------------P-------YHV-------------------------AQF----------R------------------DS--S-----------
------------------------------REFDLTKETDLQ-----------QLRNET------EVRM-RN--SVRDG-------------
-----------K----------------------TVSNE-VISLTVKGPN--
------LPRMVLVDLPGVIS--TV-------------------T-------AD--------------M--------AR--E--TKDDII-------
-RMSK-AHM---EN------------PNAIILCIQDGSV--D-AERSN-VTDLVSSID------
PSGKRTILVLTKVDMAE-------KNL-----ANP----DRI----------------------------KKI--LE---GK---------L-
FP----------------------MK---ALGYFGVVTGR---G-NSS----------
---------------DS--------------------ID----------EIRKY---EENFFST--------SQ-LL---------------------R-
DG----VL--------K--PSQ-MTTRNMSLAVSDCFWRMVRDS-I----
>XP_002602331.1
------------DELPR--------VVVV--GD----Q----------------
SAGKTSVLE-----M--VA-Q-A-RIFPRG-------------AGE-MMTRAPV---------------KVT--------------L---
SEG---
-------------------------P-------HHI-------------------------AMF----------K---------------DS--D-----------
------------------------------REFDLTKESELE-----------ALRREV------EIRM-KA--SVRPG----------------
--------Q----------------------TVSME-TIAMSVKGPG--
----LQRMVLVDLPGIIS--TE-------------------T-------QG--------------M--------AS--A--TKESIK--------
MMCE-HYM---SN------------PNAIILCIQDGSV--D-AERSN-VTDLVSQMD------
PQGKRTIFVLTKVDLAE-------KNI-----TNP----RRI----------------------------KQI--LE---GK---------L-

FP---------------------MK---ALGYFAVVTGR--G-NKD---------
---------------DS-------------------ID----------TIRGY---EEEFFRN--------SQ-LF--------------------R-
SG----VL--------K--ASQ-MTTQNLSFAVSDCFWKMVKAS-V----
>XP_019637857.1
------------DELPR--------VVVV---GD----Q---------------
SAGKTSVLE-----M--VA-Q-A-RIFPRG-------------AGE-MMTRAPV---------------KVT-------------L---
SEG--
------------------------P-------HHI------------------------AMF----------K--------------DS--D----------
-----------------------------REFDLTKESELE-----------ALRREV------EIRM-KA--SVRPG----------------
--------Q---------------------TVSME-TISMSVKGPG--
----LQRMVLVDLPGIIS--TE------------------T-------QG-------------M--------AS--A--TKESIK--------
MMCE-HYM---SN------------PNAIILCIQDGSV--D-AERSN-VTDLVSQMD------
PQGKRTIFVLTKVDLAE-------KNI-----TNP----HRI---------------------------KQI--LE---GK---------L-
FP---------------------MK---ALGYFAVVMGR--G-NKD---------
----------------DS-------------------ID----------TIRGY---EEEFFRT--------SQ-LF--------------------R-
SG----VL--------K--ASQ-MTTQNLSFAVSDCFWKMVKAS-V----
>XP_006813643.1
------------DHLPR--------VVVV---GD----Q---------------
SSGKTSVLE-----M--IA-Q-A-RIFPRG-------------SGE-MMTRTPV---------------KVT-------------L---
SEG--
------------------------P-------YHV------------------------AQF----------K--------------DS--N----------
-----------------------------KEYDLSKESELQ-----------SLRQEI------ELRM-KN--RVKKG---------------
---------Q---------------------TVSND-TISLSVRGPG---
-----IQRMVLVDLPGMIS--TV------------------T-------TG-------------M--------AA--D--TREAIH-------
-NMSK-SYM---KN------------PNAIILCIQDGSV--D-AERSI-VTDLATTMD------
PEGKRTIFVLTKVDLAE-------KNS-----ANP----SRI---------------------------KQI--LD---GK---------L-
FP---------------------MK---ALGYFAVVTGR--G-NTN---------
---------------ES-------------------IE----------QIKNY---EETFFRS--------SK-LF--------------------K-
TG----TL--------K--PSQ-MTTQNLSFAVSDCFWKMVRES-V----
>XP_030843280.1
------------DHLPR--------VVVV---GD----Q---------------
SAGKTSVLE-----M--IA-Q-A-RIFPRG-------------AGQ-MMTRAPV---------------KVT-------------L---
SEG--
------------------------P-------NHI------------------------AQF----------K--------------DS--G----------
-----------------------------KEFDLTKESELK-----------ALRQEI------EARM-KG--SVKEG----------------
--------Q---------------------TISPE-VISLSVRGPG--
---IQRMVLVDLPGMIS--TV------------------T-------TG-------------M--------AA--D--TKTSIQ--------
KMIN-GYM---GN------------PNAIILCIQDGAI--D-AERSI-VTDLVNEID------
PTGKRTIFVLTKVDLAE-------KNH-----LNP----NRI---------------------------RQI--LD---GR---------
L-FP---------------------MK---ALGYFAVVTGK--G-NTS--------
-----------------DS-------------------ID----------SIKQY---EEQFFRH--------SA-LF--------------------
K-SG----VF--------K--PSQ-LNTQNLSFAVSDCFWKMVRES-V----
>XP_018667792.1

------------DHLPR--------VVVV--GD----Q---------------
SAGKTSVLE-----M--IA-Q-A-RIFPRG------------SGE-MMTRAPV---------------KVT-------------L---
SEG---
------------------------P-------NHV------------------------AQF----------R--------------DS--S----------
------------------------------REFDLSKEEELK----------SLRHEI------ELRM-KS--SCSDG--------------
----------K---------------------TVSND-TISLTVKGPG---
-----LQRMVLVDLPGMIS--TV------------------T-------SG-------------M--------AP--D--TKDAIC------
--NMSK-HYM---EN------------PNAIILCIQDGSV--D-AERSI-VTDLVSQMD------
PSGRRTIFVLTKVDLAE-------KNI-----TNP----SRI---------------------------QEI--LD---GK---------L-
FP---------------------MK---ALGYFAVVTGQ---G-SAN----------
---------------SS-------------------IT---------DIKEY---EEEFFSN--------SK-VF--------------------K-
SG----LL--------K--ASQ-LTTANLSYAVSNCFWKMVRES-V----
>XP_032818114.1
------------DQLPR--------VVVV--GD----Q---------------
SSGKTSVLE-----M--IA-Q-A-RIFPRG------------SGE-MMTRSPV---------------KVT-------------L---
SEG---
------------------------P-------HHV------------------------AIF----------K--------------DS--S------------
----------------------------REFDLTKEDDLA-----------ALRKEI------EIRM-KK--SVKEG------------------
-------H----------------------TVSAE-TISLSVKGPG---
--LQRMVLVDLPGVIS--TM-------------------T-------SG-------------M--------AP--D--TKDAIF--------
AMSK-GYM---QN------------PNAIILCIQDGSV--D-AERSI-VTDLVSNMD------
PQGKRTIFVLTKVDLAE-------KNL-----ASP----NRI---------------------------QQI--LD---GK---------
L-FP---------------------MK---ALGYFAVVTGK---G-NRD-------
------------------ES-------------------IE---------SIKDY---EEEFFQK--------SK-LC--------------------
R-SG----ML--------K--AHQ-VTTKNLSLAVSDCFWKMVRES-V----
>XP_021332524.1
------------DHLPR--------VVVV--GD----Q---------------
SAGKTSVLE-----M--IA-Q-A-RIFPRG------------SGE-MMTRSPV---------------KVT-------------L---
SEG---
------------------------P-------HHV------------------------AMF----------K--------------DS--S----------
------------------------------REFDLGKEEDLA-----------ALRHEI------ELRM-RK--SVKEG--------------
----------Q---------------------TVSPE-TISLSVKGPG---
-----IQRMVLVDLPGVIS--TV-------------------T------TG-------------M--------AA--D--TKETIF--------
SISK-AYM---QN------------PNAIILCIQDGSV--D-AERSI-VTDLVSQMD------
PQGKRTIFVLTKVDLAE-------KNL-----ASP----SRI---------------------------QQI--VE---GK---------L-
FP---------------------MK---ALGYFAVVTGK---G-SPN----------
---------------ES-------------------ID---------SIKDY---EEDFFQN--------SR-LL--------------------K-
DG----ML--------K--AHQ-VTTKNLSLAVSDCFWKMVRES-V----
>XP_028587646.1
------------DHLPR--------VVVV--GD----Q---------------
SAGKTSVLE-----M--IA-Q-A-RIFPRG------------SGE-MMTRSPV---------------KVT-------------L---
SEG---
------------------------P-------HHV------------------------AMF----------K--------------DS--S----------
------------------------------REFDLTKEEDLA-----------ALRNEI------EIRM-RK--SVSDG--------------

---------C----------------------TVSTE-TISLSVKGPG---
---LQRMVLVDLPGVIS--TV-------------------T-------SG--------------M--------AP--D--TKETIF--------
SISK-AYM---QN------------PNAIILCIQDGSV--D-AERSI-VTDMVSQMD------
PQGKRTIFVLTKVDLAE-------KNV-----ASP----SRI----------------------------QQI--IE---GK---------L-
XP----------------------MK---ALGYFAVVTGK---G-NSC---------
---------------ES--------------------IE----------SIKEY---EEEFFQN--------SK-LL---------------------K-
NC----ML--------K--AHQ-VTTRNLSLAVSDCFWKMVRES-V----
>XP_025913835.1
------------DHLPR--------VVVV--GD----Q----------------
SAGKTSVLE-----M--IA-Q-A-RIFPRG-------------SGE-MMTRSPV---------------KVT--------------L---
SEG--
-------------------------P-------HHV-------------------------ALF----------K---------------DS--S-----------
------------------------------REFDLTKEEDLA-----------ALRNEI------EIRM-RN--SVKEG----------------
--------C----------------------TVSTE-TISLSVKGPG---
--LQRMVLVDLPGVIS--TV-------------------T-------SG--------------M--------AP--D--TKETIF--------
SISK-AYM---QN------------PNAIILCIQDGSV--D-AERSI-VTDLVSQMD------
PQGKRTIFVLTKVDLAE-------KNV-----ASP----SRI----------------------------QQI--IE---GK---------L-
FP----------------------MK---ALGYFAVVTGK---G-NSS---------
---------------ES--------------------ID----------SIKEY---EEEFFQN--------SK-LL---------------------K-
TC----ML--------K--AHQ-VTTKNLSLAVSDCFWKMVRES-V----
>XP_023440724.1
------------DHLPR--------VVVV--GD----Q----------------
SAGKTSVLE-----M--IA-Q-A-RIFPRG-------------SGE-MMTRSPV---------------KVT--------------L---
SEG--
-------------------------P-------HHV-------------------------ALF----------K---------------DS--S-----------
------------------------------REFDLTKEEDLA-----------ALRREI------EIRM-RK--SVKEG----------------
--------C----------------------TVSPE-TISLNVKGPG---
---LQRMVLVDLPGVIN--TV-------------------T-------SG--------------M--------AP--D--TKETIF--------
SISK-AYM---QN------------PNAIILCIQDGSV--D-AERSI-VTDLVSQMD------
PHGRRTIFVLTKVDLAE-------KNV-----TSP----SRI----------------------------QQI--IE---GK---------L-
FP----------------------MK---ALGYFAVVTGK---G-NSS---------
---------------ES--------------------IE----------AIREY---EEEFFQN--------SK-LL---------------------K-
TS----ML--------K--AHQ-VTTRNLSLAVSDCFWKMVRES-V----
>XP_005873264.1
------------DHLPR--------VVVV--GD----Q----------------
SAGKTSVLE-----M--IA-Q-A-RIFPRG-------------SGE-MMTRSPV---------------KVT--------------L---
SEG--
-------------------------P-------HHV-------------------------ALF----------K---------------DS--S-----------
------------------------------REFDLTKEEDLA-----------ALRHEI------ELRM-RK--NVKEG---------------
---------C----------------------TVSPE-TISLNVKGPG--
----LQRMVLVDLPGVIN--TV-------------------T-------SG--------------M--------AP--D--TKETIF--------
SMSK-AYM---QN------------PNAIILCIQDGSV--D-AERSI-VTDLVSQMD------
PHGRRTIFVLTKVDLAE-------KNV-----ASP----SRI----------------------------QQI--IE---GK---------L-
FP----------------------MK---ALGYFAVVTGK---G-NSS---------

---------------ES--------------------IE----------AIREY---EEEFFQN--------SK-LL---------------------K-
AS----ML--------K--AHQ-VTTRNLSLAVSDCFWKMVRES-V----
>NP_598513.1
------------DHLPR--------VVVV---GD----Q----------------
SAGKTSVLE-----M--IA-Q-A-RIFPRG-------------SGE-MMTRSPV---------------KVT--------------L---
SEG---
-------------------------P-------HHV-------------------------ALF----------K---------------DS--S-----------
------------------------------REFDLTKEEDLA-----------ALRHEI------ELRM-RK--NVKEG---------------
---------C----------------------TVSPE-TISLNVKGPG--
----LQRMVLVDLPGVIN--TV-------------------T-------SG--------------M--------AP--D--TKETIF--------
SISK-AYM---QN------------PNAIILCIQDGSV--D-AERSI-VTDLVSQMD------
PHGRRTIFVLTKVDLAE-------KNV-----ASP----SRI----------------------------QQI--IE---GK---------L-
FP----------------------MK---ALGYFAVVTGK---G-NSS----------
---------------ES--------------------IE----------AIREY---EEEFFQN--------SK-LL---------------------K-
TS----ML--------K--AHQ-VTTRNLSLAVSDCFWKMVRES-V----
>XP_006163024.2.2
------------DHLPR--------VVVV---GD----Q----------------
SAGKTSVLE-----M--IA-Q-A-RIFPRG-------------SGE-MMTRSPV---------------KVT--------------L---
SEG---
-------------------------P-------HHV-------------------------ALF----------K---------------DS--S-----------
------------------------------REFDLTKEEDLA-----------ALRHEI------ELRM-RK--NVKEG---------------
---------C----------------------TVSPE-TISLNVKGPG--
----LQRMVLVDLPGVIN--TV-------------------T-------SG--------------M--------AP--D--TKETIF--------
SISK-AYM---QN------------PNAIILCIQDGSV--D-AERSI-VTDLVSQMD------
PHGRRTIFVLTKVDLAE-------KNV-----ASP----SRI----------------------------QQI--IE---GK---------L-
FP----------------------MK---ALGYFAVVTGK---G-NSS----------
---------------ES--------------------IE----------AIREY---EEEFFQN--------SK-LL---------------------K-
TS----ML--------K--AHQ-VTTRNLSLAVSDCFWKMVRES-V----
>NP_056375.2.2
------------DHLPR--------VVVV---GD----Q----------------
SAGKTSVLE-----M--IA-Q-A-RIFPRG-------------SGE-MMTRSPV---------------KVT--------------L---
SEG---
-------------------------P-------HHV-------------------------ALF----------K---------------DS--S-----------
------------------------------REFDLTKEEDLA-----------ALRHEI------ELRM-RK--NVKEG---------------
---------C----------------------TVSPE-TISLNVKGPG--
----LQRMVLVDLPGVIN--TV-------------------T-------SG--------------M--------AP--D--TKETIF--------
SISK-AYM---QN------------PNAIILCIQDGSV--D-AERSI-VTDLVSQMD------
PHGRRTIFVLTKVDLAE-------KNV-----ASP----SRI----------------------------QQI--IE---GK---------L-
FP----------------------MK---ALGYFAVVTGK---G-NSS----------
---------------ES--------------------IE----------AIREY---EEEFFQN--------SK-LL---------------------K-
TS----ML--------K--AHQ-VTTRNLSLAVSDCFWKMVRES-V----
>XP_031757388.1
------------DHLPR--------VVVV---GD----Q----------------
SAGKTSVLE-----M--IA-Q-A-RIFPRG-------------SGE-MMTRSPV---------------KVT--------------L---

SEG---
-------------------------P-------HHV--------------------------AMF----------K---------------DS--S----------
-------------------------------REFDLSKETDLA-----------ALRNEI------EVRM-RK--SVKNG--------------
----------Q----------------------TVSPE-TISLSVKGPG--
-----IQRMVLVDLPGVIN--TV-------------------T-------SG--------------M--------AP--D--TKDTIF--------
NISK-AYM---LN------------PNAIILCIQDGSV--D-AERSI-VTDLVSQMD------
PQGRRTIFVLTKVDLAE-------KNV-----ASP----NRI----------------------------QQI--IE---GK---------L-
FP----------------------MK---ALGYFAVVTGK--G-NSN----------
---------------ES--------------------ID----------SIKDY---EEEFFQG--------SS-LL---------------------K-
KG----ML--------K--AHQ-VTTKNLSLAVSDCFWKMVRES-I----
>NP_610941.1
------------DHLPR--------VVVV--GD----Q----------------
SSGKTSVLE-----S--IA-K-A-RIFPRG-------------SGE-MMTRAPV---------------KVT--------------L---
AEG---
-------------------------P-------YHV-------------------------AQF----------R---------------DS--D-----------
------------------------------REYDLTKESDLQ----------DLRRDV------EFRM-KA--SVRGG-------------
-----------K----------------------TVSNE-VIAMTVKGPG--
-------LQRMVLVDLPGIIS--TM-------------------T-------VD--------------M---------AS--D--TKDSIH-----
---QMTK-HYM---SN----------PNAIILCIQDGSV--D-AERSN-VTDLVMQCD------
PLGRRTIFVLTKVDLAE-------E-L-----ADP----DRI-----------------------------RKI--LS---GK---------L-
FP----------------------MK---ALGYYAVVTGR--G-RKD----------
---------------DS--------------------ID----------AIRQY---EEDFFKN--------SK-LFH--------------------R-
RG----VI--------M--PHQ-VTSRNLSLAVSDRFWKMVRET-I----
>MEN2496893
---------NLVDHLPR--------IIVV--GD----Q----------------
SSGKTSVLE-----M--LI-N-A-RIFPRG-------------SGK-MMTKSPI---------------QVT---------------L---
SNG--
--------------------------P-------RHI-------------------------ACF----------K---------------GGR-S-------
--------------------------------HEYSLDSQADLL-----------ALRLEI------ERRM-LA--QIEPH-------------
-----------Q-----------------------TVSDR-PIYLDISGPG---
------LQQMILIDLPGLIN--TV-------------------T-------TE--------------L--------AE--T--TKDDIE--------
TMVK-KQI---EN------------PNAVILCIQDGSI--D-AERSS-VSKLVAQAD------
PKGNRTIFVLNKSDLAE-------RNL------SK----QTV----------------------------EKI--LS---GK---------L-
FP----------------------MR---ASAYFAIVSGT--D-DRN-----------
--------------SS---------------------IE----------EIKKH---EEKFFRN--------SK-FF---------------------K-T--
----I--------Q--DKN-KTSKNFSEQVSECFWS-----------
>KAJ1637655.1
------------IKLPQ--------IAVV--GN----Q----------------
SVGKSTVMS-----A--LS-G-I-P-FPVD-------------A-K-LTTRCAT-------------QVT---------------M---
RKA---
-------------------------E-------TLQ-------------------------VSI----------G----------------LSD-Q----------
------------------------------AEAESIILDSLE-----------QVVSAI------EAKT-NE--LVPEH------------------
------G--------------------A-IETER-FVKIEVSGPN--
-CPNLTVIDLPGLIQ--TV-------------------D-------DG----------------------------------QIK---------GLVT-

RTM---KS------------ERTINLLIMRADV--D-PAGNE-AFQLAREHD------PLG---------------------------
--VTNA--------
-------------------------------------G-RSH--

>AYV75702.1
-----------LENIPS--------IVMI--GS----Q----------------
SSGKSTLIN-----R--IA-G-F-NVSPTG--------------SG-LVTTAPL---------------NIL--------------M---
KTS------------------------------ENRR--
------------------VVIE--TKY---
----------------------------FDNFKQTAEEVE-----------MVQQYI------RTIS-KQ---F-AD-------------------
-----EQ------------------HS-PISSK-SINIVIESPD---
-VCNISMIDLPGLIA--VP---------------------------KQ--------------Q--------NQ-TE--LIDKIN--------
NIAV-QFI---SR------------PNSIVAVMIQAGS--D-LETNI-ALALLKKYN------
YQLSNCVGVLTKVDLTD-----ISDGI---CET--LN---GN---------T-
VP-------------------DAFKM---EYGYFAIQCK---TLD-KINN---

-------HNR-FGVDNLLQFLAQILMKSLKIH-HNKF-
>KAI5073815.1
-------------------------VVAL--GNV--------------------
GAGKSAVLN-----S--LT-G-H-PVLPTG--------------EN-GATRVPI---------------VID--------------M---
ERD------------------------------EG---
-----------------LNSK--QLV---
-----------------------------LQIE-SKTQQVSA---------SSIRRSL------QEKL-TS---A-----------------------
----SE-------------------R-GRLDE--IYLKLRSST---
APPLKLVDLPGLDQ--RG-------------------S-----------------------D------------E----------------STVS-
AYT---DQ------------GDAILLVVVPAFQTSE-ISNSR-ALRLAHDLD------
PDGSRTVGVISKVDQAASD----SRNL------------AAV----------------------------QAL--LV---GQ--------
-G--P--------------------SITL---DVPWVALIGQS---ASIA--------------------------------------TAHSS-
STGG-----------D--------NS--------------------LE----------TAWRA---EMENLKS-------------------------
-----------------VLGSA-----P--QSK-LGRIALVDTLSKQIRKRLKLR-LP---
>XP_008646219.1
-------------------------AVAL--GNV--------------------
GAGKSAVLN-----S--LI-G-H-PVLPTG--------------EN-GATRAPI---------------VVD--------------L---
ARD------------------------------PG---
-----------------LSSK--SIV---
---------------------------LQID-SKSQQVSA---------SALRHSL------QDRL-SK---GASS-----------------
------GSG-------------------R-SRSDE--IYLKLRTST---
---APPLKLIDLPGIDQ--RV-------------------M-----------------------D------------D----------------STIS-
EYA---GH------------NDALLIVVIPAMQAAD-VASSR-ALRLAKDID------PDGTRTIGVLSKIDQAAAD-
---AKTV------------SCV----------------------------QSI--LS---NK---------G-AP--------------------RAAA-
--DIEWVALIGQS---VSIA--------------------------------------SAQSG-SVGS-----------D--------NS-----
---------------LE----------TAWRA---EAETLKS--ILTGA-----P--
QSK-LGRIALVDTIAKQIRKRMKVR-LP---
>ACG47836.1

------------------------AVAL--GNV---------------------
GAGKSAVLN-----S--LI-G-H-PVLPTG--------------EN-GATRAPI---------------VVD--------------L---
QRE-----------------------------PG--
------------------LSSK---SIV---
--------------------------LQID-SKSQQVSA---------SALRHSL------QDRL-SR---GASG----------------
-------GSG-------------------R-GRVDE--IYLKLRTST--
-----APSLKLIDLPGIDQ--RA-------------------V----------------------D------------D----------------
SMIN-EYA---GH------------NDAILLIVIPAMQAAD-VASSR-ALRLAKDID------
ADGTRTVGVISKVDQANGD----AKTI------------ACV---------------------------QAL--LS---NK---------
G--P--------------------KNLP---DIEWVALIGQS---VAIA---------------------------------------SAQ---
SVGS-----------E--------NS--------------------LE----------TAWRA---EAESLKN--------------------------
----------------ILTGS-----P--QNK-LGRIALVDTIAKQIRKRMKVR-VP---
>XP_006385192.1
------------------------VVAL--GNV---------------------
GAGKSAVLN-----S--LI-G-H-PVLPTG--------------EN-GATRAPI---------------SID--------------L---
SRD-----------------------------SS--
------------------VSSK---SII---
--------------------------LQID-SKNQQVSA---------SALRHSL------QERL-SK---VS--------------------
-----SG-------------------R-SR-DE--IYLKLRTST--
APPLKLIDLPGVDQ--RI-------------------V----------------------D------------D----------------SMIS-
EYV---QH------------NDAILLVVIPAIQAPE-ISSSR-ALRIAKEYD------AESTRTVGIISKIDQAATE----
SKAI------------AAV---------------------------QAL--LL---NQ---------G--P--------------------PKTS---
DIPWVALIGQS---VSIA---------------------------------------SVQSG-SASS-----------E--------SS--------
------------LE----------TAWRA---ESESLKS--ILTGA-----P--QSK-
LGRVALVDVLAGQIRSRMKLR-LP---
>KAG7649995.1
------------------------VVAL--GNV---------------------
GAGKSAVLN-----S--LI-G-H-PVLPTG--------------EN-GATRAPI---------------IID--------------L---
SRE-----------------------------ES--
-----------------LSSK---AII---
-------------------------LQID-NKNQQVSA---------SALRHSL------QDRL-SK---GA--------------------
------SG-------------------R-GR-DE--IYLKLRTST--
-APPLKLIDLPGLDQ--RI-------------------V----------------------D------------E----------------SMIG-
EHA---QH------------NDAILLVVVPASQASE-ISSSR-ALKIAKEYD------PDSTRTVGIISKIDQAAEN---
-PKSL------------AAV---------------------------QAL--LS---NQ---------G--P--------------------PKTT---
DIPWVALIGQS---VSIA---------------------------------------SAQSG---GS-----------E--------NS--------
------------LE----------TAWRA---ESESLKS--ILTGA-----P--QSK-
LGRIALVDTLASQIRSRMKLR-LP---
>NP_172500.1
------------------------VVAL--GNV---------------------
GAGKSAVLN-----S--LI-G-H-PVLPTG--------------EN-GATRAPI---------------IIE--------------L---
SRE-----------------------------SS--
-----------------LSSK---AII---
-------------------------LQID-NKSQQVSA---------SALRHSL------QDRL-SK---GA--------------------

-----SG--------------------K-NR-DE--INLKLRTST---
APPLKLVDLPGLDQ--RI------------------V-----------------------D------------E----------------SMIA-
EYA---QH------------NDAILLVIVPASQASE-ISSSR-ALKIAKEYD------PESTRTIGIIGKIDQAAEN----
SKAL------------AAV---------------------------QAL--LS---NQ---------G--P--------------------PKTT---
DIPWVAVIGQS---VSIA-------------------------------------SAQSG---SG---------E--------NS---------
------------LE----------TAWRA---ESESLKS---ILTGA-----P--QSK-
LGRIALVDTLASQIRSRMKLR-LP---
>KAH9330549.1
------------------------VVAL---GNV-------------------
GAGKSAVLN-----S--LI-G-H-PLLPTG--------------EN-GATRAPI---------------IID--------------L---
QRD-----------------------------SS---
----------------VSSR---AIF---
---------------------------LQIE-NKTQQVSA---------SALRHSL------QDKL-SK---GL-------------------
-------------------------------GI--PPMKLRTST--
SPPLKLIDLPGLDQ--RV------------------A----------------------D------------D----------------SMIS-
NVA---EH------------NDAILLVVVPASQAPE-ISSSR-ALKLALEFD------PD-----------------------------
--AIA----
---------------------------------SAQSG-SVGG-----------E--------SS--------------------LE----------
TAWRA---ESESLKA---ILTGA-----P--QTK-
LGRIALVETLARQIRKRIKIR-LP---
>ONM04707.1
------------------------VVAI---GNV-------------------
GAGKSAVLN-----G--LI-G-H-PVLPTG--------------EN-GATRAPI---------------CVE--------------L---
QRD-----------------------------AS---
----------------LSSK---AIV---
--------------------------LQID-SKSQQVSA---------SSIRHSL------QDRL-SK---V----------------------
----SG-------------------K-GRPDE--ITVKLCTST--
APPLKLIDIPGVDQ--RS-------------------T----------------------D------------E----------------S-IS-
NYA---AR------------NDAILLVIIPALQAPD-VASSR-ALRIARELD------SEGTRTIGVLSKIDQASGE----
QKAL------------GAV---------------------------QAL--LV---NQ---------G--P--------------------RTAA---
DIQWVATIGHS---VPTA-------------------------------------SAQS--EAGS-----------E--------TP--------
------------PE----------AYWQA---EVKTLVS---TLGGA-----P--ESK-
LGRVALVDSLSKQIKARIKAR-LP---
>EFJ33653.1
------------------------VVTI--GNT-------------------
GAGKSAVLN-----S--II-G-Y-TVMPTG--------------EN-GATRAPI--------------VVE--------------L---
ERD-----------------------------QS---
----------------EGK--GLA---
---------------------------VMTE-GRARPSSA---------NEIRLSL------QSRI-SR---I-AS------------------
-----SR-------------------S-GRPEE--IRLRLRSSA---
-APPLTLIDLPGLA----S-------------------L-----------------------D------------D----------------QFVN-
EYG---SH------------NDAVLLVVVPATSVRD-ITGSQ-ALKMARELD------
PEFSRTVGVISKVDQSASD----PKSL-----------AAV---------------------------QAV--LS---GQ--------
G--P-------------------SASA---DITWVALIGQS---VSIA-------------------------------------AAHAG-

SVGT-----------D--------DS-------------------LE---------TAWKA---ETETLRS--------------------------
---------------ILTAA-----P--STR-LGRAALVDVISKQIRKRIRQR-LP---
>EFJ28901.1
------------------------VVAL--GNT-------------------
GAGKSAVLN-----S--LI-G-Y-AVLPTG--------------EN-GATRAPV---------------TID-------------L---
EAD-----------------------------DS--
-----------------GNKR---GLT---------------------------------------
-----------------------------VQME-GKSMQVSA---------TDIRHSL------QNKF-GR---M------------------
---------ST------------------G-AVKEN--IHLKLKCSS--
-----APPLKLVDLPGLES--RS------------------V---------------------S-----------D----------------
SLVR-EYI---DS------------NDALLLVVIPATSVRD-ITGSQ-ALKIAQDID------
HEGSRTVGVISKIDQAASD----PKSL------------AAV---------------------------QAV--LS---GQ---------
G--P--------------------SITS---KFTWIALIGQS---VSIA--------------------------------------GAHS-----K-
----------D--------DS-------------------LE---------TAWKA---EMESLKS--------------------------------
---------ILGGA-----S--SSR-LGRSSLVEAIAKQIRQRMQQR-LP---
>OAE31801.1
------------------------VVAV--GHI-------------------
SAGKSAVLN-----S--LV-G-Y-PVLPTG--------------EN-GATRVPI---------------IIE-------------M---
KRD-----------------------------ES--
-----------------GNRK---GLA---------------------------------------
------------------------------IQVEGGRIQSVSA---------SDLRHNL------QGRL-QK---WTPN------------
------------A--------------------K-GRPDE--IKLRLVSSA---
-------APPLKLIDLPGMEVR-AP------------------I---------------------E-----------D----------------
SIVR-DYA---EH------------NDAVLLLVIPATQAAN-ILGAR-ALKLVQDLD------
GEGTRTVGVISKVDQAAAD----PRSL------------AAV---------------------------QAL--LS---GQ--------
-G--P--------------------SSTA---EFPWVALIGQS---VSIA--------------------------------------AAHAG-
G-GA-----------E--------DS-------------------LE---------TAWRA---EAESLKQ-------------------------
----------------ILPQA-----S--PSK-LGRVALVETLSSQIRKRLKNR-LP---
>KAG0632288.1
------------------------VVTL--GSV-------------------
GAGKSAVLN-----S--LM-G-Y-PVLPTG--------------EN-GATRAPI---------------IIQ-------------M---
QRG-----------------------------SS--
-----------------STNR---GLY---------------------------------------
----------------------------VVLD-GRTSNVTA---------SDVRHSL------QGRL-KS---WTPN--------------
----------AR------------------S-GRTEG--IQLTLQSSA---
------APPLKLFDLPGLDTR-AT------------------S---------------------D-----------D----------------
SLVQ-EFA---EH------------SDAILLVVVPAASVRE-VGTSK-ALKLAQELD------
SDATRTVGVISKVDQAASD----RRSL------------DAV---------------------------AAL--LS---GN---------
G--P--------------------AITQ---EIPWVAMIGQS---VSIA--------------------------------------AAHGS----
------------E--------DS-------------------LD---------TAWKA---EAESLKS--------------------------------
----------LLTQA-----A--PTK-LGRVALVEAIAKQIRKRLKQR-IP---
>KAG0555995.1
------------------------VVAL--GSV-------------------
SAGKSAVLN-----S--LI-G-H-PVLPTG--------------EN-GATRAPI---------------ILD-------------M---

ERD------------------------------KS---
-----------------SSSR--GLA---
-----------------------------VVLE-GRTQNVSA---------SDVRHSL------QGRL-KN-----AS--------------
---------SS-------------------K-GRTEG--IRLTLRSAS---
----TPPLKLIDLPGVS---GS-------------------I-------------------------D-----------D---------------SPAH-
DLA---AN------------NDTILLIVIPATSCRD-VAASK-ALKLAQELD------SDGTRTVGVISKVDQAASD-
---PRSL-----------AAV----------------------------NAL--IS---GQ---------G--P-------------------PSTA--
-DIPWVALIGQS---VSIA------------------------------------AAHSS-G-------------E--------DS---------
-----------LD----------TAWKA---EMESLKS---ILNGA-----P--SAK-
LGRIALVETLSHQIRTRLKQR-LP---
>XP_024368367.1
-------------------------AIAL--GSV-------------------
SAGKSAVLN-----T--II-G-H-PVLPTG--------------EN-GATRAPI---------------IID-------------M---
ERD------------------------------KS---
-----------------GRPG--GLA--
------------------------------VVLE-GRTQNVSA---------SDVRHSL------QGRL-KS-----VS--------------
---------SS-------------------K-SRGDG--IRLTLRSNS--
-----GPPLKLIDLPGIDSR-GS-------------------L----------------------D-----------D---------------
SPAH-DLA---AN------------NDTILLVVIAATSCRD-VAVNR-ALKLAQELD------
SDGSRTVGVISKVDQAASD----PRSL-----------AAV----------------------------NAL--IS---GQ---------
G--P--------------------SNTQ---DIPWVALIGQS---VSIA--AAHSS-P-
-------------E--------DS--------------------LD---------TAWKA---EMESLKT------------------------------
-----------ILKGA-----P--SAK-LGRIALLETLASKIQSRLKQR-IP---
>XP_024391061.1
-------------------------AIAL--GNV--------------------
SAGKSAVLN-----S--LI-G-H-PVLPTG--------------EN-GATRAPI---------------IID-------------M---
ERD------------------------------KS---
-----------------GRPG--GLA---
------------------------------VVLE-GRTQNVSA---------SDIRHSL------QGRL-KS-----VS----------------
--------SS-------------------K-GRGDG--IRLTLRSNS--
-----GPPLKLIDLPGIDSR-GS-------------------I----------------------D-----------D---------------
SPAH-DLA---AN------------NDTILLVVIAATSCRD-VAVNR-ALKLAQELD------
SDGSRTIGVISKVDQAASD----PRSL-----------AAV----------------------------NAL--IS---GQ---------
G--P--------------------SNTQ---DMPWVALIGQS---VSIA--AAHSS-
S-------------E--------DP-------------------LD----------TAWKA---EMESLNS---------------------------
---------------ILKGA-----P--PAK-LGRIALLETLASKIQSRLKQR-IP---
>ARF10781.1
------------FILPR--------IITV--GN----E----------------
STGKSSLFE-----K--IL-K-C-PIFPKN--------------NI-ICTKSPI---------------RLI-------------L---KDG--
----------------------------PNKY-V--
---IKH----------------------------KN-N--------------------
-------------------IIELNNK-------Q-----------EILDNI------II-I-NN--SI-SP------------------------KNSD-
------------------IISDD-EIVIEFSEPN---
LLPLELIDLPGLRA--F---PP--D--LAAKTT--------SLCK-

KYL----T------------DKDIILCVVPATVT-R-LTSCQ-PLALINELN-------LCKNTILALTMADRLI-------
MTEYS--QSQKVNIDNLL---------------------------LNR--IL---GTSD-------EMES---------------------
-LK--LKGCVAIINR---T-HND--IID-------------------------------
----------LE----------ESDNM---EKKYFDKL----LNDLPISYI-------------------K-E-----SI-----------
NDN-LSVKNLLIMINNLVDDCIQNN-WKQMI
>ARF10780.1
------------FKLPR--------IIAI--GN----E---------------
STGKSSLFE-----K--IL-K-C-PIFPRN--------------SI-ICTKAPI---------------RLV-------------L---NSG-
----------------------------QQKY-V---
---IRNTM----------P--------------GSE-K-------------
-----------------------EIVLYDK-------Q-----------EIYKNM------ME-I-NN--SI-SP-----------------------
KHSD------------------VITEH-EIIIEFTEPN---
LPSLEFIDLPGLRA--Y--PP--D--LATITT--------NLCK-
KYL----S------------IQDIILCVIPATVT-R-LTSCQ-PLALINELN-------LCQNTILALTMADRLV-------
MSEYA--QEE--NINELL----------------------------LNR--IL---GTSD-------EMTY----------------------
LK--LKGCVAVINR---T-HND--TMN-----------------------------
------------LE----------ESDDM---EIQCFEKL----LNNLPTEYL--------------------K-DK---NTI-----------
EEN-ITVKNLLTIINNFYVHHIQNN-WKPII
>KAH8061769.1
---------LGHVALPS--------IIVV---GS----E---------------
SAGKSSTLE-----R--IA-G-L-TLFPRD--------------AT-ICTRMPI---------------QLR-------------L---
IQD------------------------------DAAA-G--
--SC-------------------VTVRL----------A---------------GRE-D------
---------------------------------AVVSEADAART-------------------V------AAFM-NE--AV-AA---------------
---------RHG--GT-------------VR-GVVND-VLTIEVRKPD---
----------VPTLDLIDLPGIVA--AS------------------V-------EG-------------E--------PA--D--MMEQTR-
-------KITE-HYM---CR------------ADTVCVCVVPANAT-R-VRDSQ-AMQLVQRHG-------
KEPLTIGVLAKADLAY-------DPRFKQRKKK--SPFWEL---------------------------KDR--LA---GRAD--
-----DMVA----------------------L---PQGWVGVKNR---D-
TMV--VEE--------------------EGSS------------------LH----------DSAEE---EQRWFSE-------D-A-KIQ-
---DG-VGINQLLVKIDALFSSHIRDN-WVPRA
>OUS45493.1
----------LRYSVPR--------IVCV---GE----E---------------
SSGKSSTLE-----R--VA-M-M-KVFPSD--------------EK-LCTRVPI---------------ELR-------------L---
RYR-----------------------------DPES-L--
-------------------EER--------------FRESGY--------------------VVMRM----------A----------------PGL-R-
--------------------------------------STVPEDESPPMSP-D-----------HVPNQI------RQWM-ED--LV-RA-
-----------------------AN---GT-------------LT-GVTDD-KIIVELYSSR---
---------------------RVNLDLIDLPGIVA--GS------------------M-------PG-------------E--------PA--D--
MMERTR--------QLSS-SFMDDAAN------------PHTFVIAVVSAMDA-R-IRNSQ-AMELVQRHN------
-KVQFTIGALTKADLSA-------DTR-----RE--NPYSKL--------------------------LER--LN---GVAE------
-DAPE----------------------L---ALGYVALKNR--D-TVI--DTD-
-------------------IS-------------------LT----------QVNAE---EKEWFQR-------HLP-EHV---------------
--------------------------KS-CGIDSLIDKLVDKVEEYTRGP-WIEAE

>XP_001419538.1
----------LRYSVPR--------IVCI--GE----E---------------
SSGKSSTLE-----R--VA-M-M-SVFPSD--------------ER-LCTRVPI---------------ELR-------------L---
RHR------------------------------DRES-LA---
-------------------KEEER--------------FRD-GY-------------------VVMKM----------A--------------PGE-
D--SKLNADVSSQMHP-D-----------EVPAQV------RTWM-EE--LV-
AA-----------------------AN---GK------------VT-GVTDD-RIIIELYSPL-------------------------------------
------------------------CVNLDLIDLPGIVA--GS------------------I-------PG-------------E--------PT--
D--MMDRTR--------NLSA-SFLNDKAH------------PHTFVIAVASAREA-R-IRNSQ-AMELVQRYN----
---KVQFTIGALTMADLSA-------DSR-----RE--SPYTRL----------------------------IER--LN---AQAD----
---DTPE----------------------L---GLGYVALKNR---D-TVT--
ATR--------------------ENQSR--------------EELE----------KANQD---EKEWFAE-------HLS-GHT-----
--------------------------------------ER-CGIESLVDRLVQKVDEYTRGP-WIVAE
>PRP82121.1
---------KLKLDFPQ--------LVVV---GE----E---------------
SCGKSSTLE-----R--IC-M-M-PLFPRD------------RTEA-ICTRMPI---------------KLR-------------L---
KHL------------------------------SVPE-MKTFCA---
---------------------QNGIG--------------YQD-GT--------------------AAVRL----------C--------------
YED-A---------------------------------------HKPANKQWSPFYTIA-----------EIEEKV------KQFM-DE--
AV-RQ------------------------KH---GQ------------VK-GIIED-VMVVEITSFE------------------------------
------------------------------VPNLTLVDLPGIIG--GS------------------V-------AG-------------E--------
PI--D--MPQQTR--------ALAE-KYL---NN------------QHTLAIAVVPAYQ--R-IRNAQ-VMDLIERSG----
---KKDQTIGVLTMCDKAD-------QSNLR---PR--DPFFEL---------------------------KER--LS---
GRAK-------DVIP----------------------L---KNNYIAVMNR---D-
NTL--HAG--------------------MD-------------------LT----------TAATR---ETEWFER-------NLP-GYT----
----------------A-Q------------------GKA-TG-DQLVLKLTDMLCQ-----------
>TMW65229.1
------------LDLPQ--------IVVV--GQ----E---------------
SSGKSSVLE-----S--LA-M-M-PLFPRD--------------ES-ICTRMPI---------------HLK-------------M---
RHR------------------------------QTRTGVSG--
---------------------SGSGS-------------DSP-TS---------EVETPQ-----IKMRL----------V--------------
FSD-G---------------------------------------RAPVESDDGFQT--E-----------EVAQQM------RTWM-DE--
IV-RG------------------------TNSDGSA------------VT-GVVDH-VLEIEVTSSQ--------------------------
--------------------------------VPTLDLIDLPGIVA--GR------------------L-------VD-------------E-------
-PE--D--MMHQTR--------ALVE-KYL---KM-----------PHTLVLAVVPAFE--R-VRNSQ-
AFQLVQQYG-------LEDRTIGVLTMVDRAI-------DVS----NPK--GPLVAV---------------------------
MDR--LD---EVSS-------DVVK----------------------L---KHGYVAVKNR--------------------------------
------------D-TRT--DQQ--------------------PP-------------------LE----------EFKRD---EVAWLEE------
-NLA-GYV--------------------E-K------------------KL-ASSGILASKLEMLLAEHVRTT-WVSQT
>KAG1689960.1
-----------ELALPQ--------IVVI--GQ----E---------------
SSGKSSVLE-----S--LA-M-M-PLFPRD--------------RD-LCTRMPI---------------HLK-------------M---
RHV------------------------------SQAE-AD---
--------------------ESAE--------------LMP-H--RPP------LDTHQ-----IKMKL----------V--------------

YAD-G--------------------------------------REPVESEKNFTA--E-----------EAAELM------SQWM-EQ--
IV-KE------------------------EDED-KK-------------LQ-GVTEH-VLEIEVRSSK------------------------------
-------------------------------VPNLNLIDLPGIVA--GR--------------------L-------VD----------------E--------
PD--D--MMERTR--------ALVE-KYL---QL------------PHTLVLAVIPAYE--R-VRNSQ-AFQLVQKFN---
----LMDKTIGVLTMVDRAM-------DAT----NPE--GPLGEV-------------------------------MNR--LD---
GTSR-------DIVY-----------------------L---KEGYVAVMNR---D-
TRL--SPE--------------------LA--------------------LD---------KFKAE---EMAWLEE-------NLP-GYI----
----------------G-R---------------------GL-ASSSVLATKLEKMLAD-----------
>KAE9027747.1
-----------VLALPQ--------IVVI---GQ---M----------------
SSGKSSVLE-----S--LA-M-M-PLFPRD--------------RD-ICTRMPI---------------HLK--------------M---
RHV-------------------------------PKSE-VD--
--------------------EDAD--------------LMP-H--R-T------VDKHQ-----IKMKL----------I---------------
YSD-G--------------------------------------RKPVVSNKDFTP--E-----------EAAERM------SEWM-QQ--
IV-KE------------------------ESQE-NK-------------LQ-GVVDH-ILEIEVRSSK-----------------------------
-------------------------------VPNLNLIDLPGTVA--RR-------------------L-------ID---------------E--------
PD--D--MMQRTR--------TLVE-KYL---QM------------PHTLVLAVVPAFE--R-VRNSQ-AFQLVQQYN-
------LMDSTIGVLTMVDRAT-------DAS----DPN--GPLAEV----------------------------ISR--LE---
GTSR-------DIVY-----------------------L---KEGYVAVKNR--D-
TRL--SPE--------------------CL--------------------LD----------EFKAE---ENAWMEE-------NMP-GFT--
------------------E-R--------------------RL-ASSSVLAPKLEKMLAD-----------
>KAG7385474.1
-----------ELALPQ--------IVVV--GQ----E----------------
SSGKSSVLE-----S--LA-M-M-PLFPRK--------------ED-VCTRLPI---------------HLK--------------M---
RHV-------------------------------PKRDVVD--
---------------------GDQE--------------LMP-H--RRTPHSV-DVAKHQ-----IKMRL----------V------------
---YAD-D-------------------------------------REPVVSEKELTA--E-----------QAAERM------SEWM-
EM--IV-KE------------------------ENED-KK-------------LK-GVVDH-VLEIEVRSPH----------------------
--------------------------------------VPNLNLIDLPGIVA--GR-------------------L-------ID--------------E--
------PD--D--MMQRTR--------ALVE-KYL---QM------------PHTLVLAVVPAFE--R-VRNSQ-
AFQLVQQYD-------LMDSTIGVLTMADRSL-------DTS----NPE--GPLAEV----------------------------
MSR--LD---GTSR-------DIVY-----------------------L---KEGYVAVKNR------------------------------------
-----------D-TRV--SPE--------------------WS--------------------LD----------AFKTE---ENAWLEE-------
NLP-GCI------------------E-R--------------------KL-ASSSVLATKLEKMLAD-----------
>KAH7489187.1
-----------EFALPQ--------IVVV--GQ----E----------------
SSGKSSVLE-----A--LA-M-L-PLFPRD--------------SV-MCTKLPI---------------LLK--------------L-----
-------------------------------RHGN-VDV---
----------------EGDPD--------------LMP-HC--GKPQ-----AKQQ-----IKMKL----------A--------------
YSD-G--------------------------------------RKPVESDRNFTP--D-----------EAAQLM------RKWM-EE--
IV-AQ------------------------EGEE------------------GVVDH-VLEIEVQSPH------------------------------
------------------------------VPNLNVVDLPGIVA--GR-------------------R------VG--------------E-------
-PV--D--MMQRTR--------ALVE-KYL---KM------------PNTLVLAVVPAFE--R-VRNSQ-AFQLVQQYN-
------LMDSTIGVLTMMDRAV-------DAT----NPD--APLSEV----------------------------MSR--LE---

GTSQ-------DVVD----------------------L---KHGYVAVKNR---
D-TRL--APA--------------------LS-------------------LD----------EAKAE---ENAWLEE-------KMP-GYI-
-------------------D-R-------------------GL-ASSSVLAKKLEEILAD-----------
>POM76499.1
------------LTLPQ--------IVVI---GQ----E----------------
SSGKSSVLE-----S--LA-M-L-PLFPRD--------------SD-ICTRLPI---------------LLK--------------M---
RHM-----------------------------SHGN-VEV--
---------------------EGDPE--------------LMP-HCPRGDIVMT----EQQ-----IKMRL----------I------------
---YSD-S-------------------------------------REPVESERNFTP--E-----------EAAQLM------RKWM-
DQ--IV-KD------------------------EHDD-EQ-------------LK-GVVDH-VLEIEVRSPN---------------------
---------------------------------------MPNLNLIDLPGIVA--GK-------------------L-------ID--------------
E--------PD--D--MMQRTR--------ALVE-KYL---QQ------------SDTLVLAVVPAFE--R-VRNSQ-
AFQLVQQYN-------LTEKTIGVLTMVDRAI-------DET----NPE--GPLAEV-----------------------------
MGR--LD---GMAQ-------DLVY-----------------------L---KQGYVAVKNR--------------------------------
--------------D-TRV--PPE---------------------RS--------------------LE----------KSKDE---EIAWLEE------
-NLS-GYV---------------------D-R-------------------GL-ASSTALVMKIEKMLVDHICTT-WVPQM
>KAG7377001.1
-----------ELTLPQ--------IVVI---GE----E----------------
GSGKSSVLE-----S--VA-M-L-PLFPRH--------------VD-ICTRLPI---------------LLK--------------L---
RRV-----------------------------SHGN-VEV--
--------------------KGDPE--------------LMP-HC-RGNSQPVNPPDKQQ---Q-IKMRL----------L-------
--------YSD-G---------------------------------------REPIESERNFTP--Q-----------EAAQLM------
SQWM-EQ--IV-TE------------------------QHDD-NK-------------LK-GVVDH-VLEIQVRSPH-----------
---VPNLNLLDLPGIVA--GK-------------------L-------ID------
--------E--------PE--D--MMQRTR--------ALVE-KYL---KM------------PDTLVLAVVPAFE--R-VRNSQ-
AFQLVQQFK-------LTDKTIGVLTMVDRAL-------DDA----DPE--GPLAQV----------------------------
MSR--LN---GTSS-------DIVY----------------------L---KEGYVAVRNR----------------------------------
-----------D-TRL--VPE-------------------VT-------------------LE----------EFKDE---EDAWMEE-------
NLP-RYI--------------------D-R-------------------RL-ASSSVLVKKIEEMLAD-----------
>XP_008910862.1
------------LKLPQ--------IVVI---GE----E----------------
SSGKSSVLE-----N--VA-M-L-PLFPRD--------------SD-ICTRMPI---------------LLK--------------M----
---------------------------------SHGN-IE--
----------------GDPE--------------LMP-HC-KGNPHPA---DQQQ-----IKMRL----------I---------------
YSD-A-------------------------------------RAPIESERLFTL--Q-----------EAAQLM------SKWM-KQ--
IV-QE------------------------EHDA-NK-------------LA-GVVDH-VLEIEVRAPN---------------------------
---------------------------------LPNLKVVDLPGIVA--GK-------------------L-------ID--------------E------
--PE--D--MMQRTR--------ALVE-KYL---KM------------PDTLVLAVVPAFE--R-ARNSQ-
AFQLVQQYK-------LADKTIGVLTMVDRAL-------DET----NPE--GPLTQV---------------------------
KSR--LD---GTSS-------DIVY-----------------------L---KEGYVAVRNR------------------------------------
----------N-SRL--VPE--------------------VS--------------------LE----------EFKEE---EDAWLEG-------
NLP-GYI--------------------D-R-------------------RL-ASSTVLVDKLEKMLATHIRQY-WVPAT
>KAF1774311.1

-----------ELKLPQ--------IVVI--GE----E----------------
SSGKSSVLE-----S--VA-M-L-PLFPRE--------------SD-ICTRMPI---------------LLK--------------M-----
--------------------------------SHGS-FEV---
---------------QGDPE--------------LMP-HC-KGNPQPT---DQQTPTYQLIKMRL----------I--------------
-YSD-D------------------------------------RAPIESERLFTL--Q-----------QAAELM------RKWM-EQ--
IV-RE-------------------------EHDA-NK-------------LA-GVVDH-VLEIEVRAPN---------------------------
----------------------------------LPNLKLVDLPGIVA--GK-------------------L-------ID--------------E-------
-PE--D--MMQRTR--------ALVE-KYL---KL------------PDTLVLAVVPAFE--R-VRNSQ-AFQLVQQYK--
-----LADKTIGVLTMVDRAQ-------DET----NPE--GPLTQV---------------------------KNR--LD---
GTSS-------DIVY-----------------------L---KEGYVAVRNR--N-
TRL--VPE--------------------VS--------------------LE----------EFKEE---EDAWLEE-------NLP-GYI-----
---------------N-R--------------------HL-ASSTVLVENLEKMLAD-----------
>KAI9982172.1
------------LKLPQ--------IVVI--GE----E----------------
SSGKSSVLE-----S--VA-M-L-PLFPRE--------------VD-ICTRMPI---------------LLQ--------------M-----
--------------------------------SHGN-IE--
---------------GDPE--------------LMP-HC-KGNPPHA---DELQ-----IKMRL----------V---------------
YSD-K-------------------------------------RAPIEFDKLCTL--Q-----------DVAQRM------SQLM-KQ--
VA-QE-------------------------EHDA-NK-------------LA-GVVDH-VLEIEVRAPN--------------------------
-----------------------------------LPNLKLVDLPGIVA--RK-------------------L-------ID--------------E------
--PG--D--MMQRTR--------TLVE-KYL---KM------------PDTLVLAVTPAFV--R-VRNSQ-AFQLVQQYK-
------LTDKTIGVLTMVDRAR-------DET----NPE--GPLTQV----------------------------QNR--LD---
GTSS-------DIVY-----------------------L---KEGYVAVRCR--N-
TRV--VPA--------------------VS--------------------LE----------EFKEE---EDLGLR-------------------------
--KTLLVGKLEKLLAHHVRQS-WVPPA
>KAE8986371.1
------------LLLPQ--------IVVV--GQ----E----------------
SSGKSTVLE-----S--VA-M-L-PLFPRD--------------SA-MCTRLPI---------------LLK--------------L---
RHT------------------------------SQIH-VEV--
------------------EGDLE--------------LMP-HC--GAPQ-----STEQ-----IQMRL----------L---------------
YAD-G-------------------------------------RAPIGSSARVTR--D-----------QAAQLM------RQWM-DQ-
-IV-AE-------------------------QHEH-EQ-------------LR-GVVEH-VLEVRVQSPH--------------------------
------------------------------------VPNLTLVDLPGIVA--GR-------------------L-------ID--------------E-----
---PN--D--MMQRTR--------AIAE-KFL---QM------------PHTLVLAVAPASE--R-VRNSQ-
AFQLVQQLG-------LTDKTIGVLTMTDRAV-------DAT----NPD--GPLADV---------------------------
TSR--LD---GTSS-------DIVF-----------------------L---KQGYVAVKSR--------------------------------------
----------D-SRA--KTQ--------------------LS--------------------LE----------EAKAE---ENAWLEE-------
NLP-GYI--------------------G-R--------------------GL-ASSSALVAKIEQMLAEHVRTS-WVPQA
>KAG8459568.1
-------DILKGLSPPR--------IIVI--GA----E----------------
STGKSTVLE-----R--LA-M-M-PLFPRS--------------AR-LCTRVPI---------------HLR--------------L---
RRC------------------------------APGE---
--SHARL----------T---------------AFD-S--------
------------------------------GCEPADAGPIIAI-E-----------QGWERV------QEQM-SA--LL-AE----------

--------------CE---HR------------VR-A--DK-TIMLTVYHSR---
------------VPSIELVDLPGI--------------------------------QQ-------------R--------TA--D--ARARTE----
----KIVH-EYVRN-SR------------PSDMFLLVHRWGP--T-CRTAE-AGGLSCLDGC----
EAAQRRTVGVLTYCDRVD-------KPR-----DC--AHLRAV---------------------------LAR--EH---
SEAQI---DELGIAP----------------------LA--PHGWVAVANV---
-D-PLD--DAD-------------------GDGGGGGGDAIAGGGCVGLARLLR----------TARAE---
RAHFDSN-------DVL-REL-------------------A-R------------------EGS-ATCDALVDRLECMYVK-------
----
>XP_004338334.1
---------VAELSFPQ--------IIVV--GQ----E----------------
SSGKSSVLE-----R--IA-M-L-KFFPRS-------------DK-MCTRMPI---------------KLQ-------------L---
KHM-----------------------------SPTD-MTRFCE---
----------------------DHEEA-------------YRD-GV------------------AFVRARM----------E---------------
YAD-R-------------------------------------ST---SSWSVFLPVE-----------AVEKLV------RDTM-EQ--AV-
QA-----------------------RN---AT------------LA-GT---
------------------VPNLTLIDLPGTCE--VA-------------------R-------AG-------------E--------PE--N--
IKESTE--------SLVE-KYL---KQ------------DHTLVLLVMSACD--R-MHNYR-PLTVVKKHH-------
KLGNTIGVLTKSDLAE-------NSR----YEH--DKFYEF---------------------------KRQ--LN---GQSE-----
--DYIP----------------------L---ANGYVAVRNR--D-TQG--
TRG--------------------YS-------------------LE----------QAAIE---EVEWFNR-------NLP-GYV----------
----------D-K-------------------KL-ASSLVLVEKLVEMMCT-----------
>KOO23261.1
------AATLEGWKTPV--------VIVF--GQ----E---------------
TSGKSSLLE-----R--LA---MMPLLPRG-------------ED-TCTRLPI---------------LLK-------------L---
RHT-----------------------------KE---
---AQLPVL-------V--------------VRD-S-AT-------
------------------------------GKEEVRRTVSLAG------G-QVDVRHEM------ERVL--------AA------------
------------EQGG----------------LT-SVSMTRTIEVHVHSPA--
------------VPSIDLVDLPGLRL--SA--------------------------GS-------------D--------AA--D--
MPEKAK--------ALVK-AQV---EQ-HK---------GSAIFLVSCTAST--P-PSQSL-GLQLVEELG-------
LQRDTIGVVTMCDNIG------KKDM-----------TKL---------------------------PDR--LRQTSGD--------
-A-FQ----------------------LA--PHGFIGTMTD--P------IKD---
-------EG--------LSN-------------------VQ-----RL---EMMATA--ERKWFAS--------QP-LL---------------
------S-P-----LL--------S--DGL-LTCDALVDKLSSVYAR-----------
>XP_005785253.1
------------LLLPQ--------IVVV--GS----E----------------
SAGKSSLME-----R--IA---MQAFFPRA-------------EG-FCTRMAI---------------RLK-------------M-
M-HRP-----H-----------------------ESRVMIRC---
-----------------------LRTSN--GQV-----------------------------A-
MF-------------------------------------NGMPAEQRFEVDG------VQHTVIQRII------ERFI-AQ---V-HG-
-----------------------QDDG-----------------R-SVLTDIEIEIEFRACN---------------------------------------
---------------------VPTLTLVDLPGIVS----------------------M------------------------------PA--D--
VRQQTL--------ELTR-RYL---RD------------PNTLVLCVINGNEA-A-LRGSR-ALEEISEHAV---
ARPAFSKTIVVTTRTDLWV-----QSNGL-----------VSL---------------------------ARR--LADPKNE-----

------VG----------------------CE--PVALIPVINRE--A-
PKENQVDG-------------------YS------------------LP----------DLVKK---ERAKFEE----WQAKEP-----
--------------------------GL-----------RGKPLGILSVLDALNDLFETHMSEV-WVPQA
>XP_005792501.1
-----------DLLLPQ--------IVVV--GS----E---------------
SAGKSSLME-----R--IA---MQAFFPRA-------------EG-FCTRMAI--------------RLK-------------M-
M-HRP-----H----------------------ESRVMIRC--
-----------------------LRTSN---------------------------------------GQV----------------------------A-
MF-----------------------------------NGMPAEQRFEVDG------VQHTVIQGII------ERFI-VQ---I-HG-
----------------------QDDG----------------R-SVLTDIEIEIEFRACN--------------------------------------
-------------------VPTLTLVDLPGIVS---------------------M------------------------------PA--D--
VRQQTL--------ELTR-RYL---RD------------PNTLVLCVINGNEA-A-LRGSR-ALEEISEHAV---
VRPAFSKTIVVTTRTDLWV-----HSNGL------------ATL---------------------------ARR--LADPKKE-----
------VG----------------------CE--PVALIPVINRF--G-------DG-
-------------------IT-------------------LP----------DLVNA---ERAKFEE----WQATEP---------------------
--G-A-----GL-----------RGKPLGILSVLNALNDLFET-----------
>XP_042918632.1
------------LAIPE--------IVAI--GG----Q---------------
SDGKSSLLE-----A--FL-G-F-RFNVRE-------------VE-MGTRRPL--------------IVQ-------------M-V-
HDP--
---------------TAQEPR---------------------------------------CRL----------Q---------------EE-----------
----------------------------------DSDEYGPPIV-------PETAVADAI------QRRT-EE---H-LR--------------
---------KM-----------------GGIAVSSK-PIVMRAEYAY---
-------CPNLTIIDTPGFIL--KA------------------K--------TG----------------E-----LD--N--TPDEIM-------
-SMVK-AQA---SP------------PHRMILFLQQSSV--E-WASSL-WLRVVQEVD------
PYFQRTVIVASKFDNRL------KEFA------ER----WEV---------------------------DKY--LS---ATGY------
LPPNV-------------------------RPFFVALPK---------------------------------------DRVIQS-SAE--
WRR-----------S----------------------------MT----------EVDTA---IYKHMRDGI------KG-GFD---------
-----------E-E-----RF-----------ASR-IGFSNLKKFLEEELSRRYREA-AP---
>PWZ11893.1
------------LPIPE--------IVAI--GG----Q---------------
SDGKSSLLE-----A--LL-G-F-RFNVRE-------------VE-MGTRRPL--------------VLQ-------------M-
V-HDP---
-----------------TALEPR-------------------------------------CRF----------Q---------------EE----------
-----------------------------------DSEEYGSPMV-------LATAIADLI------KQRT-ES---H-LR-------------
----------KI-----------------QA-AVSSK-PIVMRAEYAH--
-------CPNLTIIDTPGFVL--KA------------------K--------RG----------------E-----PG--S--TPDEIR------
---------------------------------LQQSSV--E-WCSSI-WLDTLKEID------PTFRRTIIVISKFDNRL------
KEFT------ER----WEV---------------------------DTF--LS---ASGY------LGDNI-------------------------
HPFFVALPK---DHGTIS-NEE--FRR-----------Q--------------------
---------IC----------QVDID---VLRHLRENV------KG-GFN-------------------E-E-----KY-----------
VSC-IGFSCLKKYLESELQKRYKEA-AP---
>NP_001130364.1

------------LPIPE--------IVVI--GG----Q---------------
SDGKSSLLE-----A--LL-G-F-RFNVRE--------------VE-MGTRRPL---------------VLQ--------------M-
V-HDP---
-----------------TALEPR--CRF----------Q----------------EE----------
-------------------------------------DSEEYGSPMV-------VATAIADLI------KQRT-ES---H-LR--------------
----------KI------------------QA-AVSSK-PIVMRAEYAH--
-------CPNLTIIDTPGFVL--KA------------------K--------RG------------------E-----PE--S--TPDEIR-------
-SMVK-SLA---TP------------PHRLVLFLQQSSV--E-WCSSI-WLDTLKEID------
PTFRRTMIVISKFDNRL------KEFT------ER----WEV----------------------------DAF--LS---ASGY------
LGDNI--------------------------HPFFVALPK---DRGTIS-NEE--
FRR-----------Q-------------------------------IC----------HVDID---VLRHLRDNV------KG-GFN---------
-----------E-E-----KY-----------GSH-IGFSCLRKYLESELQKRYKEA-AP---
>AAF87857.1
------------LPIPE--------IVAI---GG----Q---------------
SDGKSSLLE-----A--LL-G-F-RFNVRE--------------VE-MGTRRPL---------------ILQ--------------M-V-
HDL---
--------------SALEPR--CRF----------QISRIFFVELAILITDLDE-----
--DSEEYGSPIV-------SATAVADVI------RSRT-EA---L-LK-----------
-------------KT------------------KT-AVSPK-PIVMRAEYAH---
----------CPNLTIIDTPGFVL--KA------------------K--------KG------------------E-----PE--T--TPDEIL-----
---SMVK-SLA---SP------------PHRILLFLQQSSV--E-WCSSL-WLDAVREID------
SSFRRTIVVVSKFDNRL------KEFS------DR----GEV----------------------------DRY--LS---ASGY------
LGENT---------------------------RPYFVALPK---DRSTIS-NDE--
FRR-----------Q-------------------------------IS----------QVDTE---VIRHLREGV------KG-GFD-----------
---------E-E-----KF----------RSC-IGFGSLRDFLESELQKRYKEA-AP---
>XP_002317496.2
------------LPIPE--------IVAL---GG----Q----------------
SDGKSSLLE-----A--LL-G-F-RFNVRE--------------VE-MGTRRPL---------------ILQ--------------M-V-
HDP--
---------------SALEPR--CRF----------Q----------------EE-----------
------------------------------------DSEEYGSSVV-------SSTTIADII------KSRT-EV---L-LK------------------
------RT------------------KT-AVSSK-PIVMRAEYAH--
---CPNLTIIDTPGFVL--KA------------------R--------KG------------------E-----PE--N--TPDEIL--------
SMVK-SLA---SP------------PHRILLFLQQSSV--E-WCSSL-WLDAIKDID------
PNFRRTVIVVSKFDNRL------KEFS------DR----WEV----------------------------DRY--LS---ASGY------
LGENT---------------------------RPFFVALPK---DKNTIT-NDE--
FRR-----------Q-------------------------------IS----------QVDSE---ILHHLRDGV------KG-GFD----------
----------E-E-----KF-----------RPY-IGFSTLRDYLESELQKRYKEA-AP---
>KAI5058044.1
------------LPIPE--------IVAL---GG----Q----------------
SDGKSSLLE-----A--LL-G-F-RFNIRE--------------VE-MGTRRPL---------------MIQ--------------M-V-
HDA--
---------------SALEPL--CRL----------Q----------------DE-----------
------------------------------------DSDDYGPVIA-------PASAVAEAI------KLRT-EE---H-LK---------------

---------KE------------------RT-AVSSK-PIVMRVEYAY---
-----CPNLTIIDTPGFVL--KA------------------K--------KG------------------E-----PE--N--TPEDIM--------
SMVR-TLA---AP------------QHRLLLFLQQSSV--E-WCSSL-WLDSIRTVD------
PSLRRTIIVISKFDNRL------KEFG------ER----WEV----------------------------DRY--LS---AGGY------
LGDTA----------------------------HPFFVALPK--DRTMTS-NEE--
FRR-----------Q------------------------------IG----------AVDSD---VHRYLRNNI------KG-GFD---------
-----------E-E-----KF-----------GDF-IGFLNLKQYLELELQRRYRDA-AP---
>EFJ18064.1
------------LSIPE--------IVAV---GG----Q----------------
SDGKSSLLE-----A--LL-G-F-RFNVRE--------------VE-MGTRRPL----------------VLQ--------------M-I-
HEP--
--------------AAVDPR--CRL----------Q----------------HE-----------
-----------------------------------DDEEYGPVIV-------PHYAVAEAI------KLRT-EE---H-LK----------------
--------KI------------------RA-AVSSK-PIVMRVEYAY---
----CPNLTIIDTPGFIL--KA------------------K--------KG------------------E-----PE--S--TPDDIL--------
QMVR-ALA---LP------------PNRLLLFLQQSSV--E-WCSSL-WLDTVRSID------
PGFHRTVVVVSKFDNRL------GEFA------EK----WEV----------------------------DRY--LS---AGGY----
--LGDHV----------------------------RPFFVALPK--DRGSVT-NEE--
FRS-----------Q------------------------------IA----------SVDAE---VLKHLRERI------SG-GFS-----------
---------E-D-----KY-----------SGS-IGFGNLRNYLEAELQRRYREA-AP---
>PTQ33908.1
------------LPIPE--------IVAV---GG----Q----------------
SDGKSSLLE-----A--LL-G-F-RFNVRE--------------VE-MGTRRPL----------------MLQ--------------M-
I-HDP--
----------------GALEPR--CRL----------Q----------------DE----------
-------------------------------------DSDEYGPAIV-------PASAVAEAI------RSRT-EL---F-LK---------------
---------RT------------------GT-AVASK-PIVMRAEYAF--
------CSNLTIIDTPGFIL--KA------------------K--------KG------------------E-----SE--S--TPDDIV--------
AMVR-ELA---AP------------PNRILVFLQQSSV--E-WCSSL-WLDTVRAID------
PALRRTIVVVSKFDNRL------KEFA------EK----WEV----------------------------DRY--LS---AGGY------
LGDST----------------------------RPFFVALPK--ERSSVS-NEE--
FRR-----------S------------------------------IA----------RVDNE---VVRHLRENV------SG-GFD---------
-----------E-D-----QF-----------GDR-IGFSNLRRFLEAELQRRYRQS-AP---
>KAG0628798.1
------------LPIPE--------IVAV---GG----Q----------------
SDGKSSLLE-----A--LL-G-F-RFNVKE--------------VE-MGTRRPL----------------MLQ--------------M-
I-HDP--
----------------EALEPR--CRL----------Q----------------DE----------
-------------------------------------DADDYGPVIT-------PVSSVADHI------RIRT-EG---F-LK---------------
---------KL------------------GT-AVSAK-PIVMRAEYAY--
------CPNLTIIDTPGFIL--KA------------------K--------KG------------------E-----PD--S--TPDEIE--------
AMVR-ELA---AP------------QHRLLLFLQQSSV--E-WCSSL-WLDVVKSID------
PSLQRTMVVVSKFDNRL------KEFT------ER----WEV----------------------------DRY--LS---TGGY-----
-LGENA----------------------------RPFFVALPK--DRGTTT-NDD--

YRH-----------Q------------------------------IS----------VVDID---ILKQLRENV------AG-GFD-----------
---------E-E-----RF-----------GNY-VGFGKLRQFLEAELQRRYRDA-AP---
>KAH9322298.1

---DE---
DSDDYGAPVA-------LASAVADAI------KSRT-EE---H-LK-----------------------KI------------------RA-
AVSAK-PIIMRAEYAH---CPNLTIIDTPGFVL--KA--
----------------K--------KG-----------------E-----PD--S--TPEDIL--------SMVK-ALA---AP------------
PNRLLLFLQQSSV--E-WCSSL-WLDTIRAVD------PTLRRTIVVVSKFDNRL------KEFG------ER----
WEV-----------------------------DRY--LS---ASGY------LGENT-----------------------------RPFFVALPK----
---DRGAIT-NEE--FRT-----------Q-------------------------------IA--------
--QVDAE---VNCYLKESV------IG-GYD--------------------E-E-----KF-----------GAY-
VGFGNLRGYLENELQRRYRDA-AP---
>XP_002683545.1
------------FEAPE--------LVVV---GM----Q----------------
SDGKSSFVE-----A--LL-G-F-QFNTVD--------------TQ-IGTRRPL----------------ILQ--------------M-V-
NDP--
---------------SSEKPL--CHF----------F----------------KE------------
---------------------------------ASPSDIEEEAT-------PVPLLEKEI------RRRT-DE---V-CG----------------
--------K--------------------G-GVSSR-PIVLRVKYKY---
----CANLTIYDTPGF------------------------R--------KG-----------------D-----TD--P--LGERIH--------
KTVM-GLI---KP------------QNRIIVALEQSTV--E-WCNTQ-VRPIIKKAD------
PNFERTIFVITKFNNRN------NQFR------DG----KEA-----------------------------NDY--IS---TDGN------
IQDLS----------------------------KVFYISLPSGH---GTRNI-A-EEE--
FKN-----------E--------------------------------IV----------NTYLK---DFKKLTKV----------GFD--------------
------E-Q-----KY-----------KSQ-LGFYNLKRYLEKLLNEKYVENISPV--
>XP_044544418.1
---------KMKFEAPE--------LVVV---GM----Q----------------
SDGKSSFVE-----A--LL-G-F-QFNTVD--------------TQ-IGTRRPL----------------ILQ--------------M-T-
NDP--
---------------TAEKPI--CNF----------F----------------KE-------------
--------------------------------ATPSEIEEEPT-------PVPELEKEI------RKRT-ED---L-CG------------------
------K--------------------S-NVNSR-PIVLRVRYKY--
--CANLTIFDTPGF------------------------R--------KG-----------------E-----QD--P--LAERIH--------
KIVM-NTI---KP------------QNRIIIALEQSTV--E-WCNTQ-VRPLIKQVD------
PNYERTIFVVTKFNNRN------NQFR------DA----KEA-----------------------------NDY--LA---TDGH----
--IPDLS----------------------------KVFYISLPSGH---GTRNL-A-EEE--
FKN-----------E--------------------------------II----------NTYLK---DYKKLAKV----------GFD----------------
-----E-Q-----KF-----------KPQ-IGFFNLKRHLERMLNE-----------
>XP_044559198.1
---------KMKFEAPE--------LVVV---GM----Q----------------
SDGKSSFVE-----A--LL-G-F-QFNTVD--------------TQ-IGTRRPL----------------ILQ--------------M-T-

NDP---
---------------SAEKPI--CNF----------F----------------KE------------
--------------------------------ATPSEIEEEPT-------PVPDLEKEI------RKRT-ED---L-CG-----------------
-------K--------------------S-NVNSR-PIVLRVRYKY---
---CANLTIFDTPGF-----------------------R--------KG-----------------E-----QD--P--LAERIH--------
KIVM-NTI---KP------------QNRIIIALEQSTV--E-WCNTQ-VRPLIKQVD------
PNYERTIFVVTKFNNRN------NQFR------DA----KEA---------------------------NDY--LA---TDGH----
--IPDLS--------------------------KVFYISLPSGH--------------------------------------GTRNL-A-EEE--
FKN-----------E-----------------------------II----------NTYLK---DYKKLAKV----------GFD--------------
-----E-Q-----KY-----------KPQ-IGFFNLKRHLERMLNE-----------
>XP_004182822.1
------------IETPE--------IVVV---GM----Q---------------
SDGKSSFIE-----A--LV-G-F-QFNVVE--------------ST-IGTRRPL---------------ILQ-------------M-F-
NNP---
---------------DKRTPR--CCF----------A---------------DE-----------
-----------------------------------NGVFEEREI-------PVEYLSREI------ANRT-CD---V-AG---------------
---------R--------------------N-NVSNK-PLILRVEFAG--
------CSNLNIIDTPGF-----------------------R--------IG-----------------G-----EE--G--LRDDIS--------
KMVK-ELI---TP------------PNRIIVCLEQSTT--E-WANSV-SRPIVREVD------
PEFKRTVLINTKFDNRV------KELT------DK----NSV---------------------------DVY--LS---GDKL------
IIGDK--------------------------KPFFISLPC---KRNI-S-MEQ--YPD-
----------Y-----------------------------IT----------DSYIS---DYRQLLEI----------GFE-------------------
E-Q-----KF-----------VEQ-IGFPRAKMFLENLLQNKYKESLAPT--
>XP_008857507.1
------------IETPE--------IVVV---GM----Q---------------
SDGKSSFIE-----A--LV-G-F-QFNVVE--------------ST-IGTRRPL---------------YLQ-------------M-F-
NNP---
---------------KQRTPK--CCF----------A---------------NE-----------
-----------------------------------NGVFEEREI-------AVEYLSKEI------STRT-CD---V-AG----------------
--------R--------------------T-SVSNK-PLILRIEFSG--
--CSNLTIIDTPGF-----------------------R--------LG-----------------G-----DE--T--LKEDID--------
QMVK-ELI---TP------------SNRIIVCLEQSTT--E-WANSV-SRPLVKEVD------
PNFNRTILINTKFDNRV------KELT------DA----QTV---------------------------ANY--LN---GDEL------
IIGDK--------------------------KPFFISLPC---KRNI-P-MEQ--FPD-
----------Y-----------------------------II----------DSYIS---DYRQLLEI----------GFD--------------------E-
T-----KY-----------HEQ-LGFPRAKIFLENLLQKKYKEALVPT--
>XP_004340186.1
------------FETPE--------LVVV---GM----Q----------------
SDGKSSFIE-----A--LL-G-F-QFNIVE-------------TN-IGTRRPL---------------ILQ-------------M-I-
NNP---
---------------ERDIPN--CRF----------R---------------RE-N-AYNE-
-----------------------------GEAGQQAVEADTWEPRDT-------PIEDLVHEI------VRRT-NE---K-AG----
-------------------R-------------------GE-HVSAA-PIILRVEYAH---
---------------CANLTIYDTPGF-----------------------R--------LG-----------------G-----DE--K--

LRADIQ--------RMVE-RLM---QP------------ANRIIVCLEQSTV--E-WANTS-SRPIVRRFD------
PTFSRTVLVNTKFDNRV------KELR------TP----ESA---------------------------AKY--LM---GENL------
PEG-K--------------------------KPFFISLPV---RRNL-D-SER--
FRD-----------G-----------------------------IK----------ECYLD---DFRRLLEI----------KFQ-------------
------E-Q-----DF-----------AEQ-VGFHRVKAYLERMLTEKYYASVPPT--
>KAH3742895.1
------------FSTPE--------LVVV--GM----Q---------------
SDGKSSFIE-----A--LL-G-F-QFNIVD--------------SN-IGTRRPL---------------ILQ-------------M-I-
NNP---
---------------HREQPS--CRF----------R----------------KE-----------
-----------------------------VLRSADEDPFEATET-------PVESLSQEI------IRRT-NE---K-AG-------------
-----------R-------------------GD-SVSPS-PIILRVEYCN---
--------CSNLNIYDTPGF-----------------------R--------LG-----------------G-----DD--K--LRSEIR------
--EMVQ-RII---EP------------KHRIIICLEQSTV--E-AVNTV-SRPLVQEVD------
PTFSRTVLINTKFDNRV------KEFS------SQ----ASA---------------------------DKY--LA---GEYL------
TVK-K--------------------------KPFFISLPV---RRNL-D-PRS--YRE-
----------A-----------------------------MR----------ECYLE---DYRTLLQV----------GFD------------------
--E-K-----RF-----------TPQ-IGICKAKNHLEHLLSDLYQQSLAPT--
>KYQ94066.1
------------FDTPE--------LVVV--GM----Q---------------
SDGKSSFVE-----S--LL-G-F-QFNIVE--------------SN-IGTRRPL---------------IIQ-------------M-I-
NNP---
---------------DKVEPS--CRF----------K----------------KE-H-YFPL-
--VPSSSHSNSNNNNNSEYGSSQNSSIQ---NTLYSGTDQDKWEEYET-------PVDELTEEI------
VRRT-NE---R-AG-----------------------RS------------------GD-RVSSV-PIFLRVEFAN------------------
---CSNCSIFDLPGL-----------------------R--------KG--------------
---G-----DD--R--LKHEIL--------DMVK-KLI---EP------------KNRIIICLEQSTV--E-HVNST-
SRPFVKKID------PDFSRTILINTKFDNRV------KELR------TR----ESA---------------------------HKY--
LE---GEGI------IQQ-K--------------------------KPFFISLPL---
KRNL-D-PHR--FKD-----------A-----------------------------IK----------ECFLD---DLRKLHEV----------
NFD--------------------E-N-----RF-----------QGQ-VGIYKVKNYIETLLQEKYQQNLLPS--
>XP_004366192.1
------------FETPE--------LVVV--GM----Q---------------
SDGKSSFIE-----S--LL-G-F-QFNIVE--------------SN-IGTRRPL---------------IIQ-------------M-I-
NNQ---
---------------SKQEPS--CRF----------K----------------KE-C-HLI---
-----------------------------DGTSIGSLEDKWEEHET-------PAEELTEEI------VRRT-ND---L-TG--------
----------------AR------------------GD-RVSSF-PIFLRVEFAH---
--------------CANLNIYDTPGF-----------------------R--------KG-----------------G-----DE--R--
LKYEIL--------DMVK-KLI---EP------------KHRIIVCLEQSNV--E-WANTI-SRPLIKKID------
PDFTRTILINTKFDNRV------KELR------NR----ESA---------------------------HKY--LE---GEGI------
VTG-K--------------------------KIFFISLPL---KRNL-D-PYK--FKE--
---------A-----------------------------CK----------ECYLE---DYRKLLEI----------GFD-------------------
E-N-----RF-----------GAQ-IGIYKVKEYVERHLHERYQQNLVPS--

>XP_645576.2
------------FDTPE--------LVVV--GM----Q----------------
SDGKSSFIE-----S--LL-G-F-QFNIVE--------------TN-IGTRRPL---------------IIQ--------------M-I-
NNP--
---------------SKQQPS---CRF----------K----------------KE-D-
YSNSY-GGSSSSTSTTSGNSNHNTDKQQNVSSSQGGGGGSNNLNEDKWEEYET-------
PVNELTEEI------IRRT-NE---R-TG------------------------RA------------------GD-RVSSI-
PIFLRVEFAH--CSNLNIYDTPGF------------------
------R--------KG-----------------G-----DE--R--LKYEIS--------EMVK-KLI---EP------------
KNRIIVCLEQSNV--E-WANTI-SRPLVKKID------PDFSRTILVNTKFDNRV------KELR------NR----
ESA----------------------------HKY--LE---GEGI------IAQ-K---------------------------KPFFISLPL--------
----------------------------------KRNL-E-THR--FKD-----------A------------------------------MK----------
ETFLD---DYRKLLEI----------GFD--------------------E-N-----RF-----------GGQ-
IGIYKVRQYVENLLHEKYQQNLLPS--
>XP_003288319.1
------------FDTPE--------LVVV--GM----Q----------------
SDGKSSFIE-----S--LL-G-F-QFNIVE--------------TN-IGTRRPL---------------IIQ--------------M-I-
NNP--
---------------LKQQPS---CRF----------K----------------KE-D-YSS--
--GESSRQPVSSSQGSTTNGKELSASS-------ANNSLDDKWEEYET-------PVNELTEEI------VRRT-
NE---R-TG------------------------RS------------------GD-RVSAV-PIFLRVEYAH--------------------------
----------------------------------CSNLNIYDTPGF------------------------R--------KG-----------------G---
--DE--R--LKHEIS--------EMVK-KLI---EP------------KNRIIVCLEQSNV--E-WANTI-SRPLVKKID-----
-PDFSRTILVNTKFDNRV------KELR------NR----ESA----------------------------HKY--LE---GEGI------
VSQ-K---------------------------KPFFISLPL--KRDL-E-NNK--
FKE-----------S------------------------------MK----------DCFLD---DYKKLLEV----------GFD------------
--------E-N-----RF-----------GGQ-IGIYRVKSFIENLLHEKYQQNLLPS--
>KAF2073270.1
------------FDTPE--------LVVV--GM----Q----------------
SDGKSSFIE-----S--LL-G-F-QFNIVE--------------TN-IGTRRPL---------------IIQ--------------M-I-
NNP--
---------------QKLEPS---CRF----------K----------------KE----------
IDSYDRPSSQQQ--------------------QTNNLDDRWEEHET-------PVNELTEEI------VKRT-NE---K-
TG------------------------SS------------------GE-RVSAE-PIFLRVEFAG-----------------------------------
-------------------------CSNLNIYDTPGF------------------------R--------KG-----------------G-----DE--
R--LKHEIG--------EMVK-KLI---QP------------KNRIIVCLEQSNV--E-WANTI-SRPLVKKID------
QDFSRTILISTKFDNRV------KELR------NR----ESA----------------------------HKY--LE---GEGI------
VAK-K---------------------------KPFFISLPL--KRNL-E-PHK--FKE-
----------A------------------------------MK----------DCFLE---DYRKLLEV----------NID-------------------
-E-A-----RF-----------SSQ-IGIFKVRSYIENLLHEKYQQNLLPS--
>XP_020428321.1
------------FETPE--------LVVV--GM----Q----------------
SDGKSSFIE-----S--LL-G-F-QFNIVE--------------TN-IGTRRPL---------------IIQ--------------M-M-
NNP--

---------------KKLHPS--CRF----------K----------------KE---AAN---
--------------------------------GEYITEGDKWEDHET-------PVEELTEEI------VKRT-ND---L-TG----------
--------------SR------------------GD-KVSAQ-PIFLRVEFAQ---
------------CSNLNIYDTPGF------------------------R--------KG-----------------G-----DE--K--LKLEIG--
------DMVK-KLI---EP----------KHRIIVCLEQSNV--E-WANTI-SRPLVKKID------
TDFSRTILINTKFDNRV------KELR------TR----ESA----------------------------HKY--LE---GEGI------
IAN-K---------------------------KPFFISLPL--KRNL-E-PHR--FKD-
----------A------------------------------MK----------ESYLE---DYRKLLEV----------NFD-------------------
-E-N-----RF-----------GHQ-IGIYNVKEHIESLLHEKYQQNLLPS--
>XP_012754836.1
---------NISFETPE--------LVVV--GM----Q----------------
SDGKSSFIE-----S--LL-G-F-QFNIVE--------------TN-IGTRRPL--------------IIQ--------------M-I-
NNP--
---------------IKHQPS--CRF----------K----------------KE---FS------
------------------------------GEASALENKWEEHET-------PVEELTEEI------IKRT-NN---L-TG------------
------------TR------------------GD-KVSAQ-PIFLRVEFAH--
----------CANLNIYDTPGF------------------------R--------KG-----------------G-----DE--K--LKTEIG----
----DMVK-KLI---EP----------KHRIIVCLEQSNV--E-WANTI-SRPLVKKID------
QEFGRTILVNTKFDNRV------KELR------SR----ESA----------------------------HKY--LE---GEGI------
VAS-K---------------------------KPFFISLPL--KRNL-E-PHR--FKE-
----------A------------------------------MK----------DSYLE---DYRKLLEV----------NFD------------------
--E-N-----RF-----------GGQ-IGIYKVKDYIEGLLHE-----------
>PRP82286.1
---------DIPVEYPE--------IVVI--GM----Q----------------
SDGKSSFVE-----G--LL-G-F-QFNIVESSEPQLYSLSISSITD-IGTRRPL---------------ILQ--------------
M-I-NNP--
--------------------DRDQPR---CRF----------R----------------KE-----
-------------------------------------NPSVHEEPFEDSDT-------PVHRLVDEI------CRRT-NE---K-AG----
--------------------SA------------------GD-RVSDV-PIILRVEFAN--
-----------------CANLTIYDTPGF------------------------R--------LG-----------------G-----DE--R--
LKNEIK--------DMVH-KLI---LP----------KHRIIVCLEQSTV--E-WANSV-SRPMARQID------
PDFSRTILVNTKFDNRV------KELR------NK----ESA----------------------------NKY--LG---GENL------
PQG-V---------------------------QPFFISMPL--RRDL-E-ASR--
FRD-----------G------------------------------IR----------DASLD---DYSKLMEV----------NFD------------
--------E-G-----RF-----------LPH-LGLFRVKQHLEKALNE-----------
>QYA18543.1
------------FKAPE--------IVVV--GM----Q----------------
SDGKSTFIE-----G--LL-G-F-QFNIVE--------------TN-IGTRRPL---------------IIQ--------------M-V-
NDP--
---------------ECDVPQ--CRF----------K----------------RE-----------
--------------------------DENGDPKSEDDDLFEHDTV-------PVQRLSAEL------MRRT-DK---K-TG-----
-------------------KN------------------KH-CVSDS-PIVLRVRFAK--
-----------------CANLTIYDTPGF------------------------R--------LG-----------------G-----LE--S--
LRQDIE--------NMVL-KLI---QP----------EHRIIVCLEQSTI--E-WANTN-SRPFVQRVD------

PNLERTVVVCTKFDNRV------KEMR------DA----DST----------------------------NAY--LD---GEGM---
---PAG-V---------------------------RPFFVSMPV--SRNLGN-PNK-
-FAL-----------V------------------------------TQ----------DCYLN---DYRQLLEV----------KFD------------
--------E-K-----RF-----------ADR-LGFHRVKKYIENLLKKKYMDSLKPT--
>XP_005775651.1
----------GGANLPT--------VVLV--GH----Q----------------
NAGKSSLLE-----A--LL-G-I-KLTHVG--------------NE-GLTRRPL---------------QIT--------------A-Q-
RDD--
---------------SAAEPA--LFL----------T--------------------------------
------------------------RDGG--GSGGG--EEQVR--------PEQLRSLI------EREN-AR---L-EA------------
------------AG---------------------EYEAE-PLRVRLSWRR---
----------APTLVLIDTPGLIG--PA------------------D---------G----------------LE-----LG--E--AAAAVE---
-----GLVL-QHL---TP------------PKRLILCLEDTS---D-WAISR-TMAVVSRAD------
TDLRRTLLVGTKLDAKM------AQFS------LP----EDL----------------------------RGL--VD---PPAL------
AAERP---------------------HLLG--GPIFTSVPPLR---SDSRH------------------AGRKA---------
AAGGGG-AGG--YSD-----------L-------------------------------IE----------EHEAS---LRRLLGSKLQ-----
-----S--------------------S-E------F-----------DAR-IGVSALWRLMAPHLTA-----------
>XP_005767412.1
---------QLPIKTPQ--------IVVV---GQ---Q----------------
TDGKSALIE-----A--LM-G-F-QFNHVG--------------GG-TKTRRPI---------------ALQ--------------M-
Q-YRA--
-----------------ECEEPR--CFL----------F-----------------------------
----------------------------------EGGR--ERQVG--------LQELKAYI------EAEN-SR---L-EA---------------
---------EG---------------------AFEAE-ELIVRMDYKY--
-----CPNLSIIDTPGLLD--FD------------------SAA------QS-TAPSA------------------A--T--NARAVR----
----ELVV-AQM---SA------------AERVILCVEETR---C-WQSSA-VRQLVGEVD------
PTFGRTVVVSTKLDTKL------AQLS------GT----SDL----------------------------LHF--LA---ARPL------
RESHP---------------------RLLG--GPFFTSVPSGR---VGSSP--------------------------------LHGFAT-
DGE--YQD-----------A------------------------------LA----------RTEHA---NLEYLREVAA---PAAD-AAT-
-------------------Q-P-----GA-----------RSA-VGISRLRRFLEDLLRE-----------
>KOO34265.1
---------KLPVDSPT--------VVVV---GR---Q----------------
TDGKSALVE-----A--LM-G-F-QFNHVG--------------GG-TKTRRPI---------------ALQ--------------M-
Q-YHP--
-----------------AREQPV--CYL----------H----------------------------
-----------------------------------TADG--EKQLS--------LADLQAHI------EAEN-VR---L-ER--------------
----------AG---------------------AFATD-EIVVRIEYRY--
-----CPNLSIVDTPGLLS--TA------------------DDY------GG-GFLQA------------------A--T--TCSDVE---
-----QLVM-SKI---AS------------PEAIILCVEETN---N-WDVAA-ARAVVARVD------
ADLLRTVVVSTKLDTKF------AQFG------SP----SEL----------------------------AQF--LD---AAPL------
HQRHA---------------------ELLG--GPFFTSVPAGR---VG--
--AGG--------------------G-A--
---GA-----------RD-----------------------------
>EWM28268.1

------------FDATA--------ILIV--GQ----Q----------------
TGGKSALIE-----A--LM-G-F-QFNQVG--------------GG-TKTRRPI---------------ALR--------------M-
Q-YNP---
-----------------RCSQPH---CFL----------T----------------------------
---------------------------------LDNGK--EEARS--------LKEIQQYI------EGEN-KR---L-EN--------------
----------DE------------------LR-CFDPR-EIMVRVEYRF--
--------CPNMIVIDTPGLLS--AP------------------A---------KLRHMNE--------QQRQ-----LH--Q--
AAKEAE--------HLVL-EKM---KC------------QDYLILCVEDTT---D-WKHST-TRNIVMQAD------
PDLRRTVLVTTKFDTKL------PQFG------TS----EDL----------------------------EDF--LR---APLI------
QKLYP--------------------QMLG--GPFYTSVPCGR---VGMGK--------------------------------DSAFIS-
NEV--FVE-----------A------------------------------VK----------KVEKY---DHARIEARL------GS-VGA----
----------------A-P------L-----------LQR-VGVSRLRRFLEKRVEECYRRN-VAKI-
>KAG5184668.1
---------HKPFDSPA--------VLVV--GH----Q----------------
TSGKSALIE-----A--LM-G-F-QFNQVG--------------GG-TKTRRPI---------------ALR--------------M-
Q-YNP---
-----------------DCDQPR---CYL---------T----------------------------
----------------------------------LENGR--EEPRS--------LAEIQAYI------EAEN-KR---L-ER---------------
---------DP------------------IR-SFDAR-EIMIRMEYRF--
------CPNMILIDTPGLIQ--AP------------------R---------G-KHLNA--------QQQA-----LL--Q--
AAKECE--------QLVI-QKM---RC------------QDFIILCVEDTT---D-WKHAT-TRNVVMQVD------
PSLERTVLVCTKLDTKL------PQFS------SG----EDL----------------------------EDF--ID---APVM------
RRMYS--------------------GLLG--GPFFTSVPSGR---VGRGR--------------------------------ESAYFS-
NEA--FVA-----------G------------------------------VR----------RAERD---DSMHVASKL------GP-TAG--
------------------T-Q------C-----------LPH-VGVSRLRRFLERRVEDC----------
>CBN78455.1
---------HKPFDSPA--------VLVV--GH----Q----------------
TSGKSALIE-----A--LM-G-F-QFNQVG--------------GG-TKTRRPI---------------ALR--------------M-
Q-YNP---
-----------------DCDQPR---CYL----------A----------------------------
----------------------------------LEDGK--EEPRS--------LQEIQAYI------ESEN-RR---L-ER---------------
---------DP------------------VR-SFDSR-EINIRMEYRF--
------CPNMILIDTPGLIH--AP------------------S---------G-SNLNA--------EQRA-----VA--A--
AAKEAE--------NLVI-QKM---RC------------QDYVILCVEDTT---D-WKHAT-TRNIVTQVD------
HDLSRTVLVTTKLDTKL------PQFG------GG----DDL-----------------------------QDF--LR---APSI------
QRMYR---------------------CMLG--GPFFTTVPSGR---VGRAR--------------------------------DSAYFS-
NEA--FVH-----------G------------------------------VR----------RSERD---DQMLVAAKL------GP-QAA-
-------------------P-S------C-----------LPR-VGVSRLRRFLERRVEE-----------
>GMI61978.1
---------KKPFDSPA--------VLVV--GH----Q----------------
SSGKSALIE-----A--II-G-F-QFNQVG--------------GG-TKTRRPV---------------ALK--------------M-Q-
YNP--
--------------KFDTPV---CYL----------Q--------------------------------
------------------------------GDDGK--ERPAS--------LQEIQRYI------ESEN-NR---L-EM-----------------

-------DK------------------VR-CFDSR-EINIRMEYKE---
-----CPNMILIDTPGLIS--AP------------------KTP------KG-SLANA--------QSRS-----LQ--V--
AAREAE--------KLVI-DKM---NC------------QDYIILCVEDTS---D-WKHGA-TREIVQKAD------
PELSRTVIVNTKLDTKV------PQFG------DP----ADM----------------------------EEF--LK---APII------
SRLAP---------------------HKLG--GPFFTSVPSGR---V-----------------------GQSE---------SHAFRS-
DKE--FVK-----------E------------------------------VK----------SKENS---DRAYCQKTL----KADP-RLE--
------------------R-A------L-----------LPR-IGVLRLKGFLERRVHE-----------
>GMH67967.1
---------QKPFDAPA--------VLVV--GH----Q----------------
SSGKSALIE-----A--LM-G-F-QFNQVG--------------GG-TKTRRPV---------------ALR--------------M-
Q-YNP--
-----------------KCKEPK---CFL----------Q-----------------------------
---------------------------------GDDGV--ERPTS--------LTEIQEYI------QAEN-LR---L-EK----------------
--------DP------------------VR-CFDSR-EINIRMEYKY--
------CPNLILIDTPGLIA--AP------------------RTP------KG-STSNM--------QTKA-----LL--A--
SAKEAE--------KLVI-NKM---RC------------QDYIILCVEDTA---D-WKHGQ-TREIVQKAD------
PDLSRTVIVNTKLDTKV------PQFG------DP----ADV----------------------------EEF--LK---APIV------
SKLAP---------------------HKLG--GPFFTSVPSGR---V-----------------------GNGD---------SFLFRD-
DDE--FVG-----------A------------------------------CA----------DNEET---DREVVRSRL----VSGP-AIT--
------------------K-A------L-----------LPR-VGISRLRGFLERRVDE-----------
>GMH92561.1
---------QKPFDAPA--------VLVV--GH----Q----------------
SSGKSALIE-----A--LM-G-F-QFNQVG--------------GG-TKTRRPV---------------ALR--------------M-
Q-YNP--
-----------------KCESPQ---CFL----------Q----------------------------
----------------------------------GDDGV--ERAKT--------LGEIQEYI------ESEN-SR---L-EN--------------
----------DP------------------VR-CFDSR-EINIRMEYKF--
--------CPNLILIDTPGLIA--AP------------------KTP------KG-SSANM--------QSRA-----LQ--A--
SAKEAE--------KLVI-SKM---RC------------KDYIILCVEDTA---D-WKHGQ-TREIVQKAD------
PDLSRTVIVNTKLDTKI------PQFG------DP----EDV----------------------------EEF--LK---APIV------
SKLAP---------------------HKLG--GPFFTSVPSGR---V-----------------------GSGD---------SYLFRT-
DDE--FVS-----------S------------------------------CA----------DNEEK---DREVVKSRL----KAGP-LVT-
-------------------K-A------L-----------LPR-VGISRLRGFLERRVDE-----------
>GMI07688.1
---------QKPFDAPA--------VLVV--GH----Q----------------
SSGKSALIE-----A--LM-G-F-QFNQVG--------------GG-TKTRRPV---------------ALR--------------M-
Q-YNP--
-----------------KCDSPS---CFL----------Q----------------------------
----------------------------------GDDGV--ECPKS--------LGEIQEYI------ESEN-SR---L-EH-------------
-----------DP------------------VR-CFDSR-EINIRMEYKF---
---------CPNLILIDTPGLIA--AP------------------KTP------KG-SQANM--------QSRA-----LQ--A--
SAKEAE--------KLVI-AKM---RC------------KDYIILCVEDTA---D-WKHGQ-TREIVQKAD------
PDLSRTVIVNTKLDTKI------PQFG------DP----EDV----------------------------EEF--LK---APLV------
SKLAP---------------------HKLG--GPFFTSVPSGR---V-----------------------GSGD---------SYLFRN-

DEE--FVS-----------S-------------------------------CA----------NNEDA---DREVVKKRL----TAGP-HVT-
-------------------K-A------L-----------LPR-VGISRLRGFLERRVDE-----------
>EJK67908.1
------------FDAPA--------VVVV---GH----Q----------------
SSGKSALIE-----A--LM-G-F-QFNQVG--------------GG-TKTRRPV---------------ALR--------------M-
Q-YNP--
-----------------RCSSPR--CFL----------Q----------------------------
-----------------------------------GDDGV--ERPMS--------LVEIQEHI------EAEN-RR---L-EK-------------
-----------DP------------------VR-SFDSR-EINVRMEYRH---
----------CPNMILIDTPGLIS--AP------------------RLR------RE-SGGNA--------QQRA-----LL--H--
AAKEAE--------RLVV-GKM---RC------------PDYIILCVEDTM---D-WKHGT-TREVVQKAD------
PDLSRTVIVNTKLDTKL------PQFG------TP----KDV----------------------------ADF--VS---ATIV------
DRMSP---------------------HKLG--GPFYTSVPSGR---VRHHSAP------PQG-------RNSDD---------
DYLFDD-DEE--FVA-----------A------------------------------CA----------EKEDA---DRELVFGRIR---
RASE-ADL--------------------K-R------T-----------MPR-VGISRLRGFLERRVDECYRRN-VAKI-
>KAI2494507.1
---------NKGFDAPA--------VVVV---GH----Q----------------
SSGKSALIE-----A--LM-G-F-QFNQVG--------------GG-TKTRRPI---------------ALR--------------M-
Q-YNP--
-----------------KYSTPR--CYL----------V------------------------------
---------------------------------GEDGI--ERELS--------LHEIQDYI------EREN-QR---L-EK----------------
--------DP------------------LR-SFESR-EINIRMEYRH--
-----CPNLILIDTPGLIL--AP------------------RVP------KG-RAGSS-----AVSQQRA-----LQ--A--
SAKEAE--------RLVI-DKL---SC------------PDYIILCVEDTA---D-WKHGS-TREVVQHAD------
PDLSRTVIVNTKFDTKV------PQFG------TP----SDL----------------------------EDF--IS---AGIL------
DRLSP---------------------TKLG--GPYFTSVPSGR---VGHSS-----------------SNPD----------
AMYDS-DLD--FVN-----------A------------------------------CS----------EAEMT---
DRAVVNQKMRRMGKQGE-IAY--------------------A-T------L-----------QKR-VGLSKLRAFLEQKVDE-
----
>CAB9516894.1
------------FDAPA--------VVVV---GH----Q----------------
SSGKSALIE-----A--LM-G-F-QFNQVG--------------GG-TKTRRPV---------------ALR--------------M-
Q-YNP--
-----------------KCSTPK--WYL----------V-----------------------------
----------------------------------GDDGV--ERPMS--------LKDIQDLI------EREN-KR---L-ER-------------
-----------DP------------------MR-SFDPR-EINIRMEYKH---
----------CPNMILIDTPGLIS--AP------------------RTP------KG-RTSSST----AMAQQRA-----LQ--A--
SAKEAE--------RLVI-EKM---KC------------EDYIILCVEDTS---D-WKHGA-TREVVQKAD------
PDLSRTVIVNTKFDTKL------PQFG------TP----SDV----------------------------EEF--LR---AQIL------
DRISP---------------------HRLG--GPFYTSVPSGR---VGRVDGG------SL--------LDQSN----------
YMYNS-DSA--FVA-----------T------------------------------CR----------EAENT---
DRRSVVQRLSRMKYSNE-VTN--------------------A-A------I-----------TSK-
IGLAKLRTFLEQRVDECYRRN-VNKI-
>GKY99394.1

---------DKPFDAPA--------VVVV--GH----Q---------------
SSGKSALIE-----A--LM-G-F-QFNQVG--------------GG-TKTRRPI---------------ALR--------------M-
Q-YNP---
-----------------SCRQPR--WFL----------L----------------------------
----------------------------------GEDGV--ERPMQ--------LSEIQDYI------EKEN-RR---L-ER-------------
-----------DP------------------MR-SFDPK-EINIRMEYKH---
---------CPNMILIDTPGLIA--AP------------------RIA------KG-RSSAGA----AIAQQRA-----LQ--L--
AAKEAE--------RMVV-EKM---RC------------EDYIILCVEDNN---D-WKHGA-TREIVAKAD------
PDLSRTVIVNTKFDTKI------PQFS------SP----SDV---------------------------QDF--LK---ASIL------
DRICP---------------------QKLG--GPFFTSVPSGR---VGRTG-------------------DQDDE---
DDDGYTKVYNN-DDD--FVA-----------G------------------------------CA----------DSENA---
DRAVVMQRLKKIGLVGD-AAT--------------------G-S-----VV-----------ASR-IGLTKLRTFLEQRVDE---
----
>XP_042920073.1
------------FDAPA--------ILVV--GH----Q----------------
TDGKSALVE-----G--LM-G-F-QFNSVG--------------GG-TKTRRPI---------------AIN--------------M-
K-YNG---
-----------------ACSTPA--CFL----------K-----------------------------
---------------------------------LEDGVS-EQEMS--------LAELQAYI------DADN-AA---L-ER-------------
-----------E--------------------Q-RFAAK-EIVVRMEYKH--
--------CPNLTIIDTPGLIS--PA------------------P---------G-------------KKNCA-----LQ--N--CAAQVE-
-------EIVR-AKA---QV------------PEYVILCLEDCS---D-WSNAT-TRRLVMQVD------
PNLVRTVLVSTKFDTRI------PQFA------RA----ADC---------------------------EMF--LR---PSA--------
LDSM---------------------GMLG-DGPFFTSVPSGR---VGSGA-------------------------------DCVFPS-
HDV--FRE-----------R------------------------------LA----------DREAT---DVAELESKL------AR-KLS----
----------------R-G------E-----------RDH-IGVGALRRYLEQLLQKRYLDA-VP---
>PWZ44616.1
------------AAAPA--------VVVV--GH----Q----------------
TDGKSALVE-----A--LM-G-F-QFNHVG--------------GG-TKTRRPV---------------ALH--------------L-
R-FNP---
-----------------RCDEPQ--CRL----------L----------------------------
----------------------------------SATGDAEEHDEAGVAARPMPLADIQAYI------EAEN-LR---L-EN------
------------------DP------------------CQ--FSEK-EIIIKVEYKH---
-------------SPNLTIIDTPGLIL--PA------------------P---------G-------------RKNRV-----LQ--S--
QASAVE--------SLVR-AKI---QH------------KETIILCLEDCS---D-WSNAT-TRRVVMQVD------
PDLARTVLVSTKLDTKI------PQFA------RP----SDV---------------------------EVF--LH---PPNC------
VLDV---------------------SLLG-DSPFFTSVPSGR---VGSCH-------------------------------EAVFRS-
NGE--FKK-----------A------------------------------IL----------SRELE---DIASIEDKL------GR-SLT-------
-------------T-M------E-----------KDR-IGVGNLRLYLEELLQKRYVKS-VP---
>NP_001189935.1
------------FEAPA--------VLVV--GQ----Q----------------
TDGKSALVE-----A--LM-G-F-QFNHVG--------------GG-TKTRRPI---------------TLH--------------M-
K-YDP---
-----------------QCQFPL--CHL----------G---------------------------

-----------------------------------SDDDPSVSLPKS--------LSQIQAYI------EAEN-MR---L-EQ-----------
-------------EP------------------CS-PFSAK-EIIVKVQYKY---
---------CPNLTIIDTPGLIA--PA------------------P---------G-------------LKNRA-----LQ--V--QARAVE--
------ALVR-AKM---QH------------KEFIILCLEDSS---D-WSIAT-TRRIVMQVD------
PELSRTIVVSTKLDTKI------PQFS------CS----SDV----------------------------EVF--LS---PPAS------
ALDS----------------------SLLG-DSPFFTSVPSGR---VGYGQ--------------------------------DSVYKS-
NDE--FKQ-----------A------------------------------VS----------LREME---DIASLEKKL------GR-LLT----
----------------K-Q------E-----------KSR-IGISKLRLFLEELLWKRYKES-VP---
>XP_002309632.3
------------FDAPA--------VLVV--GH----Q----------------
TDGKSALVE-----G--LM-G-F-QFNHVG--------------GG-TKTRRPI---------------TLH--------------M-
K-YDP---
-----------------ECEVPT---CHL----------V-----------------------------
---------------------------------SDDDPSFAQEKS--------LHEIQAYI------EYEN-MR---L-EK-------------
-----------ES------------------FQ--FSAK-EIIIRVEYKH--
------CPNLTIIDTPGLIA--PA------------------P---------G-------------RKNQA-----LQ--S--QAHAVE----
----SLVR-AKM---QH------------KEFIILCLEDCS---D-WSNAT-TRRVVMQID------
PELSRTIVVSTKLDTRI------PQFA------RA----SDV----------------------------EVF--LS---PPAH------
TLDG----------------------FILG-DSPFFTSVPSGR---VGSGH--------------------------------DSVYSS-
NDE--FKQ-----------A------------------------------IS----------LREVE---DIASLEEKL------CR-PLS-----
---------------M-Q------E-----------RNR-IGVSKLRSFLEELLQKRYMDS-VP---
>EFJ19523.1
------------FEAPA--------ILLV--GH----Q----------------
TDGKSALIE-----A--LM-G-F-QFNHVG--------------GG-TKTRRPI---------------TLH--------------M-K-
YNA--
--------------ACSEPL---CYL----------M--------------------------------
------------------------------TEDGLPREEERS--------LDDIQAHI------ESEN-LR---L-ER-----------------
-------DT------------------HQ--FWAK-EIIIKIEYKY---
-CPNLTIIDTPGLIA--PP------------------P--------QS-------------NATTA-----LQ--A--QAKAVE--------
ALVR-SKM---QH------------KEFIILCLEDCS---D-WSNAT-TRRVVMQVD------
PELSRTIVVSTKLDTRI------PQFA------RA----ADV----------------------------ELF--LR---PPPR------
LLDG----------------------DILG-GTPFFTSVPSGR---VGSGR--------------------------------DAVYKS-
NEQ--FRE-----------A------------------------------SP----------ARELE---DVSSLEEKL------GR-PLL----
----------------R-E------E-----------RNH-VGVSRLRWFLEQILQRKYMES-VP---
>KAH9308354.1

---A--
QARAVE--------MLVR-TKM---QH------------KEYIILCLEDCS---D-WSNAT-TRRVVMQVD------
PELSRTVMVATKLDTKI------PQFG------RS----SDV----------------------------ELF--LR---PPTR------
LLDG----------------------SILG-ETPFFTSVPSGR---VGTNR--------------------------------DAVYRS-

NDQ--FKE-----------A------------------------------VA----------SREAQ---DVAALEDKL------GR-SLL--
------------------K-E------E-----------RAR-VGVSRLGCFLEELLQRRYIDS-VP---
>KAI5064281.1
------------FDAPA--------ILVV--GH----Q----------------
TDGKSALVE-----A--LM-G-F-QFNHVG--------------GG-TKTRRPI---------------TLH--------------M-
K-YNA---
-----------------GCSEPV---CYL----------M----------------------------
----------------------------------TDDKPPIEEERS--------LEELQAFI------EAEN-MR---L-EQ-------------
-----------EA------------------CQ--FWAK-EIVVKIEYKY---
-------CPNLTIIDTPGLIA--AA------------------P---------G-------------RKNHL-----LQ--A--QARAVE---
-----ALVR-TKM---QQ------------KEFIILCLEDCS---D-WSNAT-TRRVVMQMD------
PELSRTVVVSTKLDTKI------PQFA------RA----ADV----------------------------ELF--LR---PPPR------
LLDG----------------------NILG-ETPFFTSVPSGR---VGTSR--------------------------------DSVFRS-
NEQ--FRE-----------A------------------------------VA----------AREAQ---DLSALEEKM------DR-HLL--
------------------P-D------E-----------RAR-VGVSRLRWFLEQLLQQRYMES-VP---
>PTQ34556.1
------------FDAPA--------ILVV--GH----Q----------------
TDGKSALVE-----A--LM-G-F-QFNHVG--------------GG-TKTRRPI---------------TLH--------------M-
T-YNA--
----------------ECTEPR---CFL----------L------------------------------
--------------------------------SEDAPPKEEEKS--------LDDLQAYI------ESEN-MR---L-EL--------------
----------EP------------------SQ--FWAK-EIVVKIEYKF--
------CPNLTIIDTPGLIS--AA------------------P---------G-------------RKNIS-----LQ--S--QARAVE-----
---ALVR-SKM---QQ------------KEFIILCLEDCS---D-WTNAT-TRRFVMQMD------
PELTRTVIVSTKLDTRI------PQFA------RP----ADV----------------------------ELF--LR---PPSR------
LLDG----------------------NILG-DTPFFTSVPSGR---VGSGR--------------------------------DSVYRT-
NES--FRE-----------A------------------------------VA----------MREAL---DVAMLEEKM------DR-PLL--
------------------N-E------E-----------RNR-VGISRLRCFLEQLLQRRYMDS-VP---
>KAG0561482.1
------------FDAPA--------IVVV--GH----Q----------------
TDGKSALVE-----A--LM-G-F-QFNHVG--------------GG-TKTRRPI---------------TLH--------------M-
K-YNA---
-----------------MCAEPR---CYL----------I-----------------------------
---------------------------------TEDRPPREEEKS--------LEELQAYI------EAEN-LR---L-ER---------------
---------EV------------------CQ--FWEK-EIILKIEYKF--
----CPNLTIIDTPGLIS--AA------------------P---------G-------------RKNQS-----LQ--S--QARSVE------
--VLVR-TKM---QH------------KEFIILCLEDSS---D-WSNAT-TRSIVMQID------
PDLSRTVVVSTKLDTRI------PQFA------CR----ADV----------------------------ELF--LR---PSQR------
LLEG----------------------NILS-GSPFFTSVPSGR---VGVTR--------------------------------DSVHRS-
NDH--FRE-----------A------------------------------IA----------LREAQ---DIALLEEKL------DR-QLT-----
---------------K-E------E-----------LAH-VGVSRLRLFLEQLLQRQYMDS-VP---
>KAG0605142.1
------------FDAPA--------ILVV--GH----Q----------------
TDGKSALVE-----A--LM-G-F-QFNHVG--------------GG-TKTRRPI---------------TLH--------------M-

K-YNA--
-----------------NCAQPR--CFL----------I------------------------------
----------------------------------SEDQPHREKEQS--------LEEIQAYI------EAEN-KR---L-ER-------------
-----------EA------------------CQ--FSAK-EIILKIEYKF--
-----CPNLTIIDTPGLIS--AA------------------P---------G--------------RKNQS-----LQ--S--QGGAVE----
----ALVQ-LKM---QQ------------KEFIILCLEDSS---D-WSNAT-TRRVVMQAD------
PELRRTVLVSTKLDTRI------PQFA------RP----DDV----------------------------ELF--LK---PPSC------
LLDG----------------------IILG-GSPFFTSVPSGR---VGSSK--------------------------------DSVFRS-
NTD--FQE-----------A------------------------------VA----------ARQIQ---DLALLEEKL------NR-PLT----
----------------R-D------E-----------CSR-IGISRLRWFLEQLLQCRYMDS-VP---
>XP_009032466.1
------------FDVPS--------VVVI---GA----Q----------------
SSGKSALVE-----A--LM-G-F-QFNEVG--------------GG-TRTRRPI---------------ALQ--------------M-
H-YNA--
-----------------ACDEPA--CYI----------M----------------DE---------
-----------------------------RFSGGEPVDGGAPFERRAT--------LAEARRFI------EEEN-RR---L-ER------
------------------DQ------------------HR-SFEAR-EIVMRVEYRH--
-----------------CPNLVLVDTPGLVG--GG------------------GDV------FGDDFGEES---HESPHARG---
--MK--R--QAREAY--------ELAL-GKA---RA----------RNAVLLCVDDGN---D-
WKLGSIARRLCADAD------PTLSRTVVVSTKLDTKL------VQFG------SG----RDV----------------------
------ASF--LR---AKVL------HDLHP---------------------RLLA--GPFFTSVPCGR---
VAGAISPGGDAWDPQGGAPENQPWDLDDEGFYEDD--GVAFRG-DAE--FRA-----------A----------
--------------------TA----------RASRA---DRSLVKSKV------GF-EFF--------------------D-K------A-------
----APQ-LGVGALRQFLERHVELQYRSN-VARV-
>XP_009032466.1.2
------------FDVPS--------VVVI---GA----Q----------------
SSGKSALVE-----A--LM-G-F-QFNEVG--------------GG-TRTRRPI---------------ALQ--------------M-
H-YNA--
-----------------ACDEPA--CYI----------M----------------DE---------
-----------------------------RFSGGEPVDGGAPFERRAT--------LAEARRFI------EEEN-RR---L-ER------
------------------DQ------------------HR-SFEAR-EIVMRVEYRH--
-----------------CPNLVLVDTPGLVG--GG------------------GDV------FGDDFGEES---HESPHARG---
--MK--R--QAREAY--------ELAL-GKA---RA----------RNAVLLCVDDGN---D-
WKLGSIARRLCADAD------PTLSRTVVVSTKLDTKL------VQFG------SG----RDV----------------------
------ASF--LR---AKVL------HDLHP---------------------RLLA--GPFFTSVPCGR---
VAGAISPGGDAWDPQGGAPENQPWDLDDEGFYEDD--GVAFRG-DAE--FRA-----------A----------
--------------------TA----------RASRA---DRSLVKSKV------GF-EFF--------------------D-K------A-------
----APQ-LGVGALRQFLERHVELQYRSN-VA---
>XP_004336224.1
------------VAAPE--------IVVI---GH----Q----------------
GHGKSSIIE-----G--IL-G-H-HVTFTG---------------Y-GATKRPL------------FLN---------------L-I-
NNP--
---------------KCDRPR--VTL----------K----------------RD-----------
---------------------------------PLLKGPEYDHDVV------VALSDLPEEL------QKRN-KL-------------------

----------------------------------N-KLSEE-PVFLQYEYRY--
-------CSNLTLIDTPGLLK--EE------------------D---------------------A---------------D--ATGEVQ-------
-AMIN-NLV---KH------------PDRFILCVEEAK---D-WDKLD-MMDFIKKFD------
PEFSRTTFVYTKLHFHL------QRFT------SA----R--------------------------------Y--LQ---GT-I------
PDA-----------------------------HCFFTTMLPSR---V-----------------------------------RARYAD-PEK--
FQE-----------K-------------------------------IY----------QCTRR---DIKALEQL----------QYD-------------
-------R-R------H-----------ESN-IGAVQLRQYLLNLAWKRYQDD-IPQI-
>XP_002649212.1
---------NTAVSHPE--------IVFV---GP----R----------------
SSGKSSLIE-----A--FI-G-R-ALNIVGGGNIVGVGGSN---AN-GCSKRVL---------------YLQ------------
--F-T-NNI---
--------------------DFEVPK---VTI----------K----------------KD--------
------------------------------------NTIK--EFDHDII------VSIEQLNENL------AKRN-QL--------------------
-------------------------------TN-DYIEE-PIYVSIESRT--
----TLNLTLIDSPGLLF--DQ-------------------S--------------------Q---------------A--ESNKIE--------
SIVS-SLL---RP------------SHRLIIAVESCSQ--D-WKSMS-MGQYLKKID------
PELSRSTFVFTKFHHTV------RGFS------ST----RDI----------------------------NKY--LS---GT-V------
PDI------------------------------KGFFVTLPNHQ---V------------------------------------RASYSE-ANR--
FQE-----------K-------------------------------IY----------QAHKR---DMHALEQL----------QYD-----------
---------K-R------Y-----------ERT-IGVAPLRRYILNIVWK-----------
>XP_003288465.1
---------NTSVLHPE--------IVFV---GP----R----------------
SSGKSSLIE-----A--FI-G-R-ALNIVGASSTL---------AN-GCSKRVL---------------YLQ-------------F-
N-NNN--
------------------DCDIPK---VTI----------K----------------KD----------
----------------------------------NNLK--EFDHDII------IPLEQLNDSL------AKRN-------------------------
-----------------------------SS-DFSEE-PIYVTIESRN--
----TLNMTLIDSPGLLF--TP------------------T---------------------E------------T--A--ESTKIE--------
SIVS-SLL---RP------------THRLIIAVESCNQ--D-WKTMS-MNQYLKKID------
PELSRSTFVFTKFFNTV------RGFS------NT----RDI----------------------------NKY--LS---GT-V------
PDI------------------------------KPFFVTLPNYQ---V------------------------------------RASFSE-PNR--
FQE-----------K-------------------------------IY----------QAHKR---DMHALEQL----------QYD-----------
---------K-R------Y-----------ERS-IGVQPLRKYILNIVWK-----------
>KAF2077035.1
---------NTSVSHPE--------IVFV---GP----R----------------
GAGKSSLIE-----A--FI-G-R-ALNIVGASTNL---------TS-GTSKRPV---------------YLQ-------------F-V-
NNV---
---------------ECETPK---VTM----------K----------------KD------------
------------------------------NILK--EFDHDLV------IPFEQLNDNL------AKRN---------------------------
-----------------------------S-IYSEE-PIYVSIEVKT--
ALNLTIIDTPGLVA--NE------------------S--------------------A---------------A--DQQKID--------QIVN-
GIL---RP-----------THRLIIAVEACG---D-WSNMT-MLSFVKKVD------PELSRSTFVFTKFFNMI------
QDFN------NT----RGV---------------------------NRF--LS---GT-M------SEI-----------------------------
KSFFVTLPNHK---I------------------------------------RARFSE-PAR--YQE-----------K-------------------

----------IN----------QAYKR---DMQSLEQF----------QYD--------------------K-R------Y-----------ERN-
IGVHPFRRYILNIVWK-----------
>XP_020436215.1
---------NTSIPQPE--------IVFV--GP----R----------------
GSGKSSLIE-----S--FI-G-R-PLNIVGA--------------M-GCSKRAL---------------HLQ--------------F-V-
NNT---
--------------ECETPK---ITI----------K----------------RD---------------
----------------------------GFIK--EFDHDVV------IPIEQLNDTI------TRRN--------------------------------
-----------------------T-AITEE-PIYIMIEAKN--
TLNLTLIDTPGLVA---E--------------------G---------------------T---------------A--DQAKID--------AIVN-
SIL---RP------------THRLIVAVEACG---D-WSNMT-MLSFVKRVD------PELSRSTFVFTKFFNII------
QDFN------ST----RSV-------------------------NRF--LA---GT-M------SEI-----------------------------
KSFFVTIPNHK---I------------------------------------RARFSE-PAN--FQE-----------K--------------------
----------LT----------QAYKR---DMNALEQL----------QYD--------------------K-R------Y-----------ERN-
IGVHPFRRYILNITWK-----------
>XP_012753198.1
---------NTSIAQPE--------IVFV--GP----K----------------
SSGKSSLVE-----A--FI-G-R-PLNIVGA--------------L-GCSKRSI---------------HFQ--------------F-V-
NNA---
---------------ECEVAK---VTV----------K----------------RD------------
-------------------------------ALIK--ELDHDVV------ITVEQLNDTL------NRRN---------------------------
----------------------------T-VISDE-PIYVLIESRS--
TLNLTLIDTPGLVA---D--------------------G---------------------A---------------N--DHAKID--------AIVS-
AIL---RP------------NHRLIVAVEPCG---D-WANMT-MLPFVKRVD------PELSRSTFVFTKFFNVI------
QDFN------ST----RSV-------------------------NRF--LA---GA-V------SDI-----------------------------
KSFFVTIPNHK---I------------------------------------RARFAD-PAS--FQE-----------K--------------------
----------LA----------QAYKR---DMNALEQF----------QYD--------------------K-R------Y-----------
ERN-IGVTPFRRYILNIIWK-----------
>XP_004360608.1
------------ISQPE--------IVFV--GP----R----------------
SSGKSSLIE-----S--FI-G-R-PLNIVAGQSG-----------G-ACSKRAV---------------HLQ--------------F-
M-NNP---
------------------EVEGTK---ITI----------K----------------RD-----------
--------------------------------SFIK--EFDHDIV------VPLEQLNDNL------ARRS--------------------------
-----------------------------N-QFHEE-PIYIIFESKS---
--TLNLTLIDTPGLLF---D--------------------G---------------------Q---------------P--EQSKIE--------
AIVQ-QAV---RP------------SHRLIVAVEASG---E-WAEMT-MLPFVKKVD------
PELSRSTFVFTKFFNMI------QDFN------ST----RGV-------------------------NRF--LA---GT-I------
NEI-----------------------------KTFFVTLPNHK---I------------------------------------RARYSE-PAA--
FQE-----------K------------------------------LT----------QAFRR---DMHSLEQF----------QYD-----------
---------K-R------Y-----------ERT-IGVHPFRRYMLNIVWKSYQDA-IPRI-
>KYQ90260.1
------------ISHPE--------IVFV--GP----R----------------
TSGKSSLIE-----A--FI-G-R-SLNIVGQ--------------N-GCSKRPI---------------YFQ--------------F-V-

NNI--
-------------ECEVPK-------------------------------------ITV---------K---------------RD-------------
----------------------------SNIK--DFDHDIV------IPLEQLNDNI------FRRN-----------------------------
------------------------Q-QLSDD-PIYITIETKS---
SLNLTIIDTPGLLI--DS-----------------N------------------S--------------Q--EQQKID--------NIVQ-
SIL---KP-----------NNRLIIAVENCG---D-WSQMS-MLTFIKKVD------PELSRSTFVFTKFFNQI------
QDFT------ST----RSV---------------------------NRF--LA---GT-M------SEI---------------------------
KSFFVTIPNHK---I----------------------------------RARFSE-PAR--FQE----------K-------------------
---------LQ----------QAYKR---DMYHLEQI----------QYD------------------K-R------Y----------ERS-
IGVNPFRKYILNIVWKSYQDG-IPRI-
>KAH3767868.1
-----------VQVPE--------IVLV---GK----K---------------
GVGKTSLLE-----A--IL-G-H-KFGDTGT----------------STMRPI--------------EVK------------M-V-
NNQ---
--------------QCATPK-------------------------------------ITF---------M--------------HD-----------
-----------------------------LALP--QFMEDTE------IPLNKVPETI------RDRN--------------------------
----------------------------KKSSV-PIRILYEFKY---
CWGMTIFDTPGLLP--KS-----------------D------------------P-----------A--E--AIEEVE--------
SIVL-ELM---TP-----------PTRMLLCVEEAT---D-WERVQ-IVDFVSKVD------
PKRSRSVFVFNKFAGLL------KNFG------SF----REL--------------------------QLF--LS---ANPL------
MDT----------------------------PVFFTSLPAGA---I-----------------------------------EAHL--------YKR--
---------R-----------------------------LA----------QLLKG---DKDALELL----------QYD-----------------
-R-R------Y-----------EKS-IGVVALQKYVLDWTLNKYQDL-IPEI-
>ATZ80405.1
-----------INFPR--------IVLV---GT----Q---------------
TSGKSSFIN-----N--LI-N-M-ELMPTG-------------DN-MVTRSPV--------------HIK------------V-I-
NNT---
-------------TNSTDR------------------------------------VSI---------FT-------------MTN-----------
------------------------------GNKNLVYCADLNST---------NTFNTLIFQKKMREAT-DM---I-AH---------
--------------KN-------------------CISEE-PIIVEILTKD--------------------TNNNMS------------------
-------------AKNLIIVDLPGLVT--IP-----------------KTD------IG------------Q--------PA--S--
IVEDLK--------NLVM-KEI---SH-----------PNVYVLVAIQAK-T-D-LETDV-GLAVVKEIQ----
RTNKSLKAIGLLTKLDLLD-------KRSLK--QFD-----NNI--------------------------FNPAVLS--KS------
------TA--------------------L----DGGFFVINN---HND-------
---------N--DEYYL---NKNMFDK--------SLRFIQ------------G----
--N-RFGSHNLMI-------QL-KK------NLITGIRAILPDF----------
>KAF5834941.1
-----------FQMPR--------IVVV---GG----E---------------
STGKSSLLE-----N--IT-K-C-AVFPRD-------------KD-ICTRMPI--------------RLQ------------L-T-
NAT---
-------------DASDTA-------------------------------------VEV-------------------------QF------------
----------------------------GSQPATRLQD--SS---------QVLGAV------EGAM-DK---L-P---------------
---------KD-------------------SICET-EFVVRI--RE--------------------T----G---------------------------
----IPTFEFIDLPGIRA--YP-----------------LD------MA-------------Q--------KT---------E--------

SLVR-KYL---KV------------PNTLVIAVVRATDT-R-ITNDR-GYALVQEMG----LESKTVMA---LTR---TD-
------RVSAA--EFQ-----EMV---------------------------LDRIMMS--SSEFP---------KP------------------
----L----FACIAIVNR--TSD----------------DS-------VT--------
------------LA----------EHQAM---EAEWFEK--------HVGVWY------------S------E-R--PWEQLV------
-EM-ASR-MTLFNLIKRVDVLYSQYISAVWKPRA-
>QFG74057.1
--------FISTSKLPT--------IVMI---GG----E---------------
STGKSATIE-----N--IT---MLSIFPTD--------------KS-VCTRCPV--------R------IVM------VPS-----M-V-
SHT---
--------------EIEIT--------------WR-------------------------GEK----------S-------------HVNE-----------
----------------------Y-----------------------------DAREYI-----------SR---I--------------F-----------
TD------------------IE-SYSKD-ELLIEIYGNY--
SCRFEFVDLPGIVN--YP---Q-----------S--AHEFTT--------
ALAD-DYI---KN------------SDNFIMCVVNATIP-R-LTSYY-AINRIINFN-------ANMRTVLVLTKYDKLV-
-------------LVDF-----------------------------------DEY--LF---NR--------LD-MS------------------
SDEFISHKYIGCCGVHNRD--S-ART------------------------ST-
-------------------LI----------NHVQN---EQALINYF-------HENNY---------------------H-N----------------
---VEK-LGVEYLMNFVLHHYDNHIKQHWIPKV-
>CEM26963.1
----LLRLVGLGFMIPR--------IVTI--GQ----Q---------------
SDGKTTFLE-----A--ML-R-L----CFG-----------YTRNGAAATKGPV---------------RID-------------V---
KND------------------------------ESS---

----------------------PTPRCQLNQEHIRLA-----------AIQGRM------RELM-EH---R---------------------
----------------------------SLSKE-ETHLEISYSG---
APNLMCVDLPGIVQ----------------------ET-------GD-------------T--------TR--E--DVELTM--------
DVVR-HYV---RH-----------SPNDLYLVFKRADV--D-PGTWP--IHDFIQS-----
LGLHREQTIVACTRAENFL-----RENRV------IY-KT--EL---------------------------LKL--IK---QKRV--
HDCSGEPIP--------------------MYFV---ELHNLSDQEK---A-L-
P--FAE-----------RT-----AVMEGQ------------------LA----------ARRLD---ILDRLNKLEN--GAATD-E--
-------------------DT-T-----EL-----------YRY-FDVESLEAHLNATFRRLLLRQ-LHAL-
>OII76931.1
----FLKLANLSDQIPR--------IVVI--GQ----Q---------------
SMGKTTLLD-----S--LI-G-Y----PLG-----------YSSSD-IGTCCPI--------------VFH-------------
IYAKSPR------------------------------SALNSPI---
------------------------SCHTDS-------------TSS-------------------------SDI---------DK-------------
LALNV--ETIDCIIDDEIIEFD----------TLPEII------AKKT-LE---K----
-----------------------NG--------------------VVDSN-EINIKIKSEK--
------------------SVNMILVDLPGL-----------------------------------KE-------------N--------TR--E--
GAALTQ--------QIVR-EYV---NK-----------HPNDIYILVKRSLD--D-PANWSWKQKEFILND----
LGLGCDQTITVGTKGLEYA-----TEEIK------EV-STADEL----------------------------IER--IE---
KRKIGYNNDKWNSLP--------------------LYIL---EFFSLSKEER---
---S-LKN--MKL-----------RR-----EAMYRN------------------IN----------DGNIK---LQAILGEFC---
KDTDP-L---------------------TR-Q-----AV-----------FNY-FSYDLFQREVNLKFVKIFLRQ-LDLL-

>KAH8582109.1
----LLKLKGLEREIPR--------IVIL--GQ----Q----------------
TIGKTTVID-----Y--LI-N-H----PLG----------YSTNN-TGTCCPI---------------VFH--------------I---
SPS------------------------------NEGSSES---
-------------------FLPLEL-------------TSK-------------------------TLV---------GS--------------GILHE-----
-----------------------------------EITECLIGGEKVSFE-----------TLPKKN------SERM-KK---M------------
---------------RM---------------------QISPE-ELRIDIKSRG---
-----------AMEMIIVDLPGL---------------------------------KE--------------D--------TN--Q--GSKMTQ--
------KIVQ-EY---------------------------RSID--D-PTNWSWRQKLFLFEE----
LRLRKEQTIVVGTRALEFL-----QQEVE------EI-SNGDEL----------------------------LER--IK---
KRELSSTYGFGAPLP--------------------LYML---ELFSLSKEER---
---A-IQK--MSC----------RK-----NAMDRR---------------IT----------AGEKN---IKEIINQICD--
QDHEK-E---------------------VK-E-----KL-----------LSY-FSRDLFEMDLSSKFGQILLKE-LNSL-
>POM84969.1
----LLRLNFSGGEIPR--------IVVL--GQ----Q----------------
SMGKTTVID-----Y--LI-G-H----PLA----------YSTND-NGTCCPI--------------VFH--------------I---
SPS------------------------------EEKDRES---
-------------------YLTFKL-------------ESD-------------------------CLL---------GS--------------GILNE-K--
------------------------------------EITECLIGGEKVTFE-----------ALPEKI------LEKM-KE---I--------------
-------------KM---------------------QTSSQ-ELRIDIRSRG--
----------AIEMIIVDLPGL---------------------------------KE--------------D--------T---D--GSKITQ--------
KILYDEYV---RN-----------HPNDIYILVQRLND--N-PANWSWKQKPFLLEE----
LGLGKEQAIVVGTRALEYL-----QKEVK------EI-SNGKQL----------------------------LER--II---
KRGPSSGDESGTPIP--------------------FFML---ELFGLSMEER--
----A-IKK--MSS----------RK-----SAMERR-----------------IS----------AGEKA---IKEIITQLCN--
QDHET-K---------------------IK-E-----RI-----------LSY-FSRDLFEKELRSKFGQILFKQ-LNSL-
>XP_667128.1
----LLKLNGLGGEIPR--------IVIL--GQ----Q----------------
SMGKTTVID-----Y--LI-G-H----PLG----------YSTND-IGTCCPI---------------VFH--------------I---
SPS------------------------------EEKDRES---
-------------------CLSSKL-------------VSD-------------------------CLL---------GS--------------GILNE-
E--------------------------------------EITECLIGGEKVTFE-----------TLPEKI------LERM-KE---I----------
-----------------KM---------------------QISSQ-ELRIDIRSKG---
-------------AIEMIIVDLPGL---------------------------------KE--------------D--------TK--E--GSKITQ----
----KIVH-EYV---KN-----------HPNDIYILVKRSID--D-PANWSWRQKSFLLEE----
LGLGKEQAIVVGTRALEYL-----QEEVK------EI-SNGKQL----------------------------LER--IK---
KRELSSGDESGAPLP--------------------LFML---ELFSLSREER--
----A-IKK--MSS----------RK-----NAMERR------------------IS----------AGEKA---IKEIITQLCD--
QDHET-E---------------------VK-E-----RL-----------LSY-FSRDLFEMELRSKFGRILLKQ-LNSL-
>XP_001617280.1
----IFKICNIHNELPR--------LVVF--GQ----Q----------------
SMGKTTLLD-----F--IM-G-G----PMG----------YTSSD-TGTKQPI--------------VII--------------L---
KPS------------------------------DTN--

----------------------KIECYLNKKKVSID-----------DLHEKM------KAIM-VN---L--------------------------
-SE---------------------SIIPK-ELEVEISIPG---
GIYATFVDLPGI---------------------------------KD--------------D--------SK--S--GSELTR--------KIVR-
NYV---QN-----------FPNDIYILVKKASD--D-
PANWPYHLREFFMKPKPMGLGLQNKQCIVVGTRALEFL-----NNELS------TI-KTLTEL----------------
------------HDR--VK---KRGI--TDHNDNILS--------------------LYLL---ELFSIPIEQK--------------------
-------------------------E-KND--FLT-----------NR-----ISMYSK------------------IL----------NGRKN---
VLDLLLNKFE--NDCND-S--------------------IK-K-----EL----------LDC-
FDVEKFKQEVNSKFMNILIQQ-LRKV-
>XP_028539355.1
----IFKICNIHNELPR--------LVVF---GQ----Q----------------
SMGKTTLLD-----F--IM-G-G----PMG--------------YTSTD-TGTKQPI---------------VII--------------L---
KPS------------------------------DTN---

----------------------KIECYLNKKKVNID-----------DLHEKM------KAIM-LN---L--------------------------
-NE---------------------SIIHK-ELEVELSIPG---
GIYATFVDLPGI---------------------------------KD--------------D--------SK--A--GSELTR--------KIVR-
NYV---QN-----------FPNDIYILVKKASD--D-
PANWPYNLREFILKPRPLGLGLQPKQCIVVGTRALEFL-----NNELT------NI-KTLSEL------------------
----------YER--VK---KRSI--TDNNDNVLP--------------------LYLL---ELFSIPIEQK----------------------
-----------------------E-KND--FLT-----------NR-----ISMYSK------------------IL----------NGRKN---
VLDLLLNKFE--NDCND-S--------------------IK-K-----EL----------IDC-
FDVEKFKQEVNSKFMNILIQQ-LRKV-
>PHJ24853.1
----LLKLAGLEAEIPR--------LVVF---GQ----Q----------------
SMGKTTLLD-----Y--IM-G-G----PIG-----------YSSTD-TGTRQPV---------------VIV--------------L---
RPT------------------------------DS---
---D--
---------------------TVQCWLAGEEVSVQ-----------ELQARM------KNIM-SS---Q----------------------
-----GE--------------------QISSR-ELEVELAVPR--
-GVHAVFVDLPGI---------------------------------KD--------------D--------SK--A--GALQTR--------
SVVR-TYV---QN-----------NPNDLYILVKKASD--D-
PANWPWSLREFILSAPPTGLGLTPRQTVVVGTRAREFL-----TNERS------DI-RTQAQL----------------
------------LER--VF---KRAV--KDSTGAPLP--------------------LFLL---ELFSLSIEEK--------------------
-------------------------D-QND--FAA-----------KR-----AAMNRQ------------------ME----------DGGRT-
--VRELLNS-FE--AGANS-Q---------------------QT-K-----TL----------MEF-
FSPVRFKKELNSKFQGLLSDQ-LGLL-
>KFG43683.1
----LLKLAGLEAEIPR--------LVVF---GQ----Q----------------
SMGKTTLLD-----Y--IM-G-G----PIG-----------YSSTD-TGTRQPV---------------VIL--------------L---
RPS------------------------------ESA---
--D---
-----------------------SVQCWLGGEEIEVK-----------ELQARM------KEIM-SS---Q----------------------
----GE---------------------RISSQ-ELEVELAVPN---

GVHAVFVDLPGV---------------------------------KD--------------D--------SK--A--GATQTR--------
SVVR-TYV---QN-----------NPNDLYILVKKASD--D-
PANWPWSLREFILSAPPKGLGLTPRQTVVVGTRAKEFL-----VNEKN------DI-RTQSQL---------------
-------------LER--VL---KRAV--KDSSGAPLP--------------------LFLL---ELFSLSIEEK--------------------
--------------------------D-ALD--FAA-----------KR-----AAMNRQ------------------MD----------EGGRT-
--VRQLLETAFE--PGPNP-Q---------------------LS-R-----KL-----------TEF-
FSPSRFKKELNHKFQSLLSEQ-MGIL-
>UKJ88078.2
----LMKLGGLEAEVPR--------LVVF--GQ----Q----------------
SMGKTTLLD-----F--IM-G-G----PIG-----------YSSTD-TGTKQPV---------------SII--------------M---
KPL------------------------------TTLNTLKE---
------------------SLKENL-------------KEG-------------------------SKANLKDKYKGNEE-------------
ILLNE-T--------------------------------------NAIVCKFNGRFMTIH-----------EVQDAM------RVHM-
QS---L---------------------------GQ---------------------TILSD-ELEVEVYVPN-----------------------------
-------------------------------ALYAIFVDLPGI---------------------------------KD--------------D--------SK-
-V--GAELTR--------SVVR-NYV---SN-----------NPNDLYILVKKASD--D-
PSNWPWSLKEFITSSAPAGLGLTPQQTMVVGTRAKEFL-----LNEKT------DI-KTYEEL-----------------
-----------VER--VY---KRGV--VDSKGQMLP--------------------LHLL---ELFSLSIQAK--------------------
--------------------------E-SGD--FLS-----------NR-----DDMKAQ------------------IS----------SSQKQ--
-IYDLLINSF-----NST-NLG-------------D-EDR-LK-D-----EL-----------FNI-
FSIDSFLKTLNHKFQSLMTNQ-LNNL-
>XP_004833148.1
----LMKLGGLEAEVPR--------LVVF--GQ----Q----------------
SMGKTTLLD-----F--IM-G-G----PIG-----------YSSTD-TGTKQPV---------------SII--------------M---
KPS------------------------------SSLQN--
---YEGSGP-------------PPVTD-T-------
--------------------------------SIICKFGGRLMNIH-----------EVQDAM------RNHM-QS---L-------------
--------------GS---------------------SILSD-ELEVEVYVPN--
----------ALYAIFVDLPGI---------------------------------KD--------------D--------SK--A--GAELTR------
--SVVR-NYV---SN-----------NPNDLYILVKKASD--D-
PSNWPWSLKEFITASAPAGLGLTPQQTMVVGTRAKEFL-----INEKT------DI-KTQEQL----------------
------------MER--VY---KRGV--VDSKGQMLP--------------------LHLL---ELFSLSIQAK------------------
----------------------------E-SCD--FLS-----------NR-----DDMKAQ------------------IS----------
ASQRE---VYDLITNGFE--LSSSS-NLN-------------D-EGKTVR-E-----EL-----------MQI-
FSIDSFLKTLNNKFQHLMNNQ-LTNL-
>GFE54186.1
----IMKLGGLDAEVPR--------LVVF--GQ----Q----------------
SMGKTTVLD-----F--IM-G-G----PIG-----------YSSTD-TGTKQPV---------------VII--------------M---
RPL------------------------------EVIQEVAA--
-------------------SLNVQL---SP-------------DAVNG-S---
------------------------------------TIWCLFDGKLMDIR-----------SVQDAM------RVHM-LN---I----------
-----------------GD---------------------TIIAD-ELEVEVFVPN--
------------GLNAIFVDLPGI---------------------------------KD--------------D--------SK--M--GAEFTR--
------NVVR-NYV---KN-----------NPNDLYILVKKSSD--D-

PANWPWSLREFITTAPPTGLGLSPHQTVVVGTRARDFL-----INEKT------DI-RTAEQL-----------------
-----------LER--VL---KRSV--IDSRGNMLP--------------------IHLL---ELFSLSIQAK---------------------
-------------------------E-SGD--FTA-----------NK-----AEMKRQ------------------IA----------TGQQE---
VEDMIRHSFE--L-KSG-SVD-------------M-DGLTVQ-E-----RL-----------IQM-
FSVNGFLTTLDSKYQTLVANT-FRNL-
>XP_012766661.1
----IMKLGGLDAEVPR--------LVVF--GQ----Q----------------
SMGKTTVLD-----F--IM-G-G----PIG-----------YSSTD-TGTKQPV---------------VII--------------M---
RPL------------------------------EVIMEVAS--
-------------------ALKLQI--DP-------------ASLKG-G-----
----------------------------------TIWCLFNGKLMDIR-----------SVQDAM------RLHM-QS---M----------
-----------------GE---------------------RIVAD-ELEVEVFVPN---
-------------GVNAIFVDLPGI---------------------------------KD--------------D--------SK--T--GAEFTR--
------NVVR-NYV---KS-----------NPNDLYLLVKKSSD--D-
PANWPWSLKEFITTAPPTGLGLSPHQTIVVGTRAREFL-----INEKT------DI-RTVEQL------------------
----------MDR--VL---KRTI--TDSKGNILP--------------------LHLL---ELFSLSIEAK-----------------------
-----------------------E-SGD--FLA-----------NK-----REMKRQ------------------IA----------KGQEE---
VENMIRTSFE--V-TND-TVG-------------R-DGMTVV-D-----RL-----------LQM-
FSINGFLTTLDTRYQALVATT-FRNL-
>GIX62800.1
----IMKLGGLDAEVPR--------LVVF--GQ----Q----------------
SMGKTTVLD-----F--IM-G-G----PIG-----------YSSTD-TGTKQPV---------------VII--------------M---
RPL------------------------------EVILEVAD---
------------------ALKVQI--DP-------------TNLKN-G-----
----------------------------------TIWCLFNGQLMDIR-----------SVQDAM------RAHM-QG---I----------
-----------------GD---------------------RILAE-ELEVEVFVPS---
-------------GVNAIFVDLPGI---------------------------------KD--------------D--------SK--S--GAEFTR--
------NVVR-NYV---QN-----------NPNDLYILVKKSSD--D-
PANWPWSLKEFITTAPPTGLGLSPHQTVVVGTRAREFL-----LNEKT------DI-RTAEKL-----------------
-----------MER--VL---KRSI--TDSKGTMLP--------------------LHLL---ELFSLSIQAK---------------------
-------------------------E-SGD--FLA-----------NK-----AEMKRQ------------------IA----------AGQQD--
-VEEIIRSSFE--V-TNG-SVN-------------R-DGMTVV-E-----RL-----------VQM-
FSIHGFLTTLDAKYQSLVATT-FRNL-
>KAH0479249.1
----LLKLGGLEAEIPR--------LVVF--GQ----Q----------------
SMGKTTLLD-----F--IM-G-G----PIG-----------YSSTT-TGTKMPV---------------VIM--------------L---
RPP------------------------------QGNEDRA---
--RP--------------------------------
---------------------------SAMCK--GQHMSID-----------SLQHFM------SDVM-IK---Q------------------
---------GD---------------------KITAE-ELDLEIVVPD---
----GVHAVFVDLPGI---------------------------------KD--------------D--------SK--D--GAEVTR--------
NVVR-TYV---SN-----------NPNDLYILVKKASD--D-
PANWPWSLREFIISPAPVGLGLSPKQTLVVGTRARDFL-----TAEKN------DI-KTQDEL-----------------
-----------VLR--VR---NRAV--KDQYGTRLP--------------------LHLL---ELFSLSMSTK--------------------

--------------------------E-SKN--FQL-----------KK-----AEMLNQ------------------IR----------LAQRS---
CLSALTKDFE--YSRSQ-E---------------------VR-R-----DL-----------RQF-
FDIDHFKTSLNVKFQSLLSEQ-ITLL-
