# Supplementary material for: Antiviral Mx proteins have an ancient origin and widespread distribution among eukaryotes
Source: Proc Natl Acad Sci U S A. 2025 Jan 24;122(4):e2416811122. doi: 10.1073/pnas.2416811122 (PMC11789081; doi:10.1073/pnas.2416811122)
Supplement: Supplementary file 11 — Dataset S10 (PDF) [file pnas.2416811122.sd10.pdf]

Dataset S10. Suppl_Figure_1_IQTree

```
#NEXUS
begin taxa;
	dimensions ntax=180;
	taxlabels
	XP_006812840.1
	XP_012378586.1
	EAW87759.1
	ELW62001.1
	EPQ17174.1
	BAB27759.1
	KAE8583055.1
	XP_025915522.1
	XP_028570166.1
	XP_032814666.1
	XP_012379251.1
	XP_016856477.1
	XP_027623811.1
	XP_006496668.1
	EPQ08653.1
	XP_025944940.1
	XP_031757197.1
	XP_014389433.1
	NP_001005360.1
	XP_006510037.1
	XP_012381548.1
	XP_006161648.2.2
	XP_025920181.1
	XP_028568434.1
	XP_031753735.1
	XP_021326548.1
	NP_001025299.1
	NP_001024332.1
	XP_035683496.1
	XP_030853442.1.2
	XP_030853442.1
	KMZ10000.1
	XP_001749319.1
	NP_741403.2
	XP_002129967.2
	NP_957216.1
	NP_001317309.1
	XP_012382650.2
```

NP_001392186.1
XP_006168142.1
XP_014394711.1
XP_028602039.1
XP_025940269.1
XP_031753959.1
XP_032819300.1
XP_035676386.1
XP_006821224.1
XP_030827871.1
PAA85687.1
NP_001259946.1
XP_001750431.1
XP_004348308.1
XP_014148015.1
XP_004466363.1
NP_002453.2.2
NP_001127618.1
XP_008569442.1
XP_014388412.1
XP_005202045.1
XP_017508130.1
NP_001003134.1
XP_032211398.1
NP_038634.1
NP_034976.1
XP_006156437.1
XP_005885748.1
XP_012586448.1
XP_017508123.1
NP_776366.1
NP_002454.1
XP_002830747.1
XP_006156438.1
NP_001003133.1
XP_032211320.1
XP_008569440.1
XP_004675614.2.2
XP_031752404.1
XP_015269256.1
XP_028583072.1
XP_025933558.1
XP_009815891.1
AGU16245.1

XP_007904885.1
XP_032888405.1
XP_028583068.1
NP_001007285.1
XP_005167721.2.2
XP_003973512.2.2
NP_891987.2.2
XP_009304072.1
XP_032804093.1
XP_002608668.1
XP_019617847.1
XP_035690836.1
XP_006815062.1
CAH1802128.1
PAA92268.1
PAA69582.1
PAA74204.1
PAA76532.1
PAA83069.1
PAA94353.1
ABI53802.1
XP_046352531.2
XP_048248472.1
XP_048248473.1
XP_048248474.1
XP_048258111.1
XP_048248476.1
XP_046352527.2
XP_046562919.1
XP_046563124.1
XP_046563126.1
XP_046565195.1
XP_046563125.1
XP_046565196.1
KAI0208044.1
KAI0218869.1
KAI0213370.1
XP_006813643.1
XP_030843280.1
XP_023440724.1
XP_005873264.1
XP_006163024.2.2
NP_598513.1
NP_056375.2.2

XP_028587646.1
XP_025913835.1
XP_021332524.1
XP_031757388.1
XP_032818114.1
XP_018667792.1
NP_495986.3.3
NP_610941.1
XP_002602331.1
XP_019637857.1
PAA68234.1
PAA87312.1
XP_004479029.1
NP_284941.2.2
NP_001193437.1
XP_005883071.1
NP_077162.2.2
XP_006162789.1
XP_025917892.1
XP_028587453.1
NP_001016189.1
XP_017213868.2.2
XP_004482574.1
NP_001272849.1
XP_006145367.1
XP_014400986.1
NP_001177198.1
NP_001121132.1
XP_025929938.1
XP_028597443.1
XP_015268039.1
NP_001121726.1
XP_002591612.1
XP_019628129.1
NP_996357.1
XP_006819998.1
XP_030846906.1
XP_030847518.1
XP_002126852.1
NP_495161.1
PAA75551.1
PAA75258.1
XP_004365821.1
XP_014153836.1

XP_001745740.1
XP_014153758.1
XP_014148725.1
PAA65118.1
PAA78248.1
PAA59145.1
PAA64382.1
XP_005165639.1
XP_004347890.1
XP_026693152.1
;
end;

begin trees;
tree tree_1 = [&R]
[&branchAttributeNames={"Value"}](XP_006812840.1:0.3609014682,((((((((XP_012378586.
1:0.0982247712,EAW87759.1:0.000002)[&Value="83.4/100"]:0.0051543872,ELW62001.1:
0.000002)[&Value="0/94"]:0.000002,EPQ17174.1:0.0103094455)[&Value="41.8/56"]:0.005
1516067,BAB27759.1:0.000003)[&Value="95/94"]:0.0263825785,KAE8583055.1:0.043902
214)[&Value="89.1/82"]:0.0199896535,(XP_025915522.1:0.000002,XP_028570166.1:0.024
0294175)[&Value="77.2/75"]:0.0079363061)[&Value="95.5/80"]:0.0309127821,((XP_03281
4666.1:0.0739189186,(((((((XP_012379251.1:0.000002,XP_016856477.1:0.0051306558)[&
Value="0/95"]:0.000002,XP_027623811.1:0.015614047)[&Value="0/94"]:0.000003,XP_006
496668.1:0.005082988)[&Value="79/96"]:0.0151182006,EPQ08653.1:0.0105344563)[&Val
ue="95.2/100"]:0.0342003247,(XP_025944940.1:0.057162481,XP_031757197.1:0.200683
4911)[&Value="72.9/100"]:0.018743471)[&Value="94/100"]:0.0391454013,(((XP_01438943
3.1:0.0319805083,((NP_001005360.1:0.0051232599,XP_006510037.1:0.000002)[&Value="
0/48"]:0.000002,(XP_012381548.1:0.0629140041,XP_006161648.2.2:0.0752458979)[&Val
ue="0/85"]:0.0174439704)[&Value="68.4/89"]:0.0051382433)[&Value="88.3/98"]:0.010532
1617,(XP_025920181.1:0.0061542708,XP_028568434.1:0.0044622969)[&Value="81.4/95"]
:0.0097192035)[&Value="88.7/95"]:0.0197321758,XP_031753735.1:0.0491660953)[&Value
="97.1/100"]:0.0519017128)[&Value="79.4/97"]:0.0335348186,((XP_021326548.1:0.00000
2,NP_001025299.1:0.000002)[&Value="93/100"]:0.0416776276,NP_001024332.1:0.26295
58477)[&Value="62/45"]:0.0252286232)[&Value="86.2/45"]:0.0304752796)[&Value="76.3/4
2"]:0.0306608494,XP_035683496.1:0.1090173388)[&Value="90.9/42"]:0.0287318777)[&Va
lue="84.7/30"]:0.0134034256,((XP_030853442.1.2:0.000002,XP_030853442.1:0.000002)[&
Value="100/100"]:0.1480532012,((KMZ10000.1:0.140734925,((XP_001749319.1:0.119238
455,((((((NP_741403.2:0.4305071658,XP_002129967.2:0.4458938091)[&Value="77.6/78"]:
0.0430261931,(((NP_957216.1:0.0355056597,((((((NP_001317309.1:0.000003,XP_0123826
50.2:0.0730511558)[&Value="36.3/89"]:0.003939715,NP_001392186.1:0.0119904913)[&V
alue="0/68"]:0.000002,XP_006168142.1:0.000002)[&Value="0/84"]:0.000002,XP_0143947
11.1:0.0486693988)[&Value="97.6/97"]:0.0311057106,(XP_028602039.1:0.0411485868,XP
_025940269.1:0.0366474028)[&Value="0/44"]:0.0034196446)[&Value="78.5/83"]:0.010934
1864,XP_031753959.1:0.2985003976)[&Value="89.3/100"]:0.0313903156)[&Value="96.9/1

00"]:0.0814252407,XP_032819300.1:0.1510354744)[&Value="84.8/97"]:0.0524596799,(((X
P_035676386.1:0.1119709674,(XP_006821224.1:0.1458721869,XP_030827871.1:0.12986
95165)[&Value="74/88"]:0.0538670153)[&Value="68.4/85"]:0.0580954605,PAA85687.1:0.2
694055472)[&Value="36/85"]:0.0409630006,NP_001259946.1:0.2723894755)[&Value="24.
2/74"]:0.0537599282)[&Value="69.2/79"]:0.0382115926)[&Value="98.1/99"]:0.2022563495
,XP_001750431.1:0.5593659166)[&Value="95.5/100"]:0.1346269016,XP_004348308.1:0.2
609758923)[&Value="52.9/85"]:0.0341640779,XP_014148015.1:0.4225073776)[&Value="9
1.3/99"]:0.1227274103,((((((((((((XP_004466363.1:0.1333167023,((NP_002453.2.2:0.00929
76072,NP_001127618.1:0.000003)[&Value="98.5/100"]:0.0809737817,(XP_008569442.1:0
.1070184585,(XP_014388412.1:0.1260099051,XP_005202045.1:0.166548409)[&Value="15
.9/37"]:0.024473723)[&Value="82.6/75"]:0.0362169527)[&Value="79.5/96"]:0.0314595339)
[&Value="89.2/46"]:0.0234931386,(XP_017508130.1:0.0807074602,(NP_001003134.1:0.0
328136991,XP_032211398.1:0.0471297161)[&Value="99.4/100"]:0.0729114942)[&Value="
88.7/68"]:0.0300216821)[&Value="77.6/45"]:0.0150161628,(NP_038634.1:0.0120426309,
NP_034976.1:0.0808355374)[&Value="100/100"]:0.1665822544)[&Value="82/81"]:0.02471
15872,XP_006156437.1:0.0641738895)[&Value="94.1/98"]:0.0554675022,(((((XP_0058857
48.1:0.0253072232,XP_012586448.1:0.3320893938)[&Value="87.4/96"]:0.0232921753,XP
_017508123.1:0.1107387941)[&Value="81.8/97"]:0.0152631719,NP_776366.1:0.15283021
11)[&Value="81.4/84"]:0.0202413158,(((NP_002454.1:0.0053616133,XP_002830747.1:0.0
183071872)[&Value="99.9/100"]:0.1009265089,XP_006156438.1:0.1794631916)[&Value="
77.1/86"]:0.0168008686,(NP_001003133.1:0.1058186122,XP_032211320.1:0.1592266226
)[&Value="95.9/100"]:0.0550999443)[&Value="8.3/31"]:0.0092022905)[&Value="92.2/80"]:
0.0303909377,XP_008569440.1:0.0503541925)[&Value="100/100"]:0.2021615517)[&Value
="5.5/27"]:0.0282226097,XP_004675614.2.2:0.175758699)[&Value="74.1/93"]:0.09178902
03,XP_031752404.1:0.4073266093)[&Value="89.7/92"]:0.0882990621,(((XP_015269256.1:
0.1532002143,XP_028583072.1:0.268723038)[&Value="15.1/74"]:0.0358439795,(XP_0259
33558.1:0.0795543148,XP_009815891.1:0.1201503034)[&Value="100/100"]:0.238320394)
[&Value="56.4/93"]:0.0547421364,((AGU16245.1:0.2431306276,(XP_007904885.1:0.17640
69311,XP_032888405.1:0.2025443887)[&Value="93.9/100"]:0.0908043)[&Value="82.4/91"]
:0.0467634618,(XP_028583068.1:0.2409322461,(NP_001007285.1:0.1192726245,XP_005
167721.2.2:0.0542460417)[&Value="100/100"]:0.3990192287)[&Value="81.6/94"]:0.04411
19097)[&Value="88.3/85"]:0.0495712441)[&Value="70.5/78"]:0.040880328)[&Value="91/98
"]:0.1313111394,(XP_003973512.2.2:0.1552325835,(NP_891987.2.2:0.0498084379,XP_00
9304072.1:0.0198233341)[&Value="99.7/100"]:0.1446974632)[&Value="97.3/100"]:0.1700
42109)[&Value="99.3/100"]:0.2618775182,XP_032804093.1:0.6863467175)[&Value="93.6/
94"]:0.1414790164,((((XP_002608668.1:0.1267559208,XP_019617847.1:0.181026411)[&Va
lue="92.3/100"]:0.1311115785,XP_035690836.1:0.3167343504)[&Value="98.9/100"]:0.211
9622155,(XP_006815062.1:0.6770127506,(CAH1802128.1:0.7352346272,(((PAA92268.1:0
.489617697,PAA69582.1:0.4935674925)[&Value="71.2/100"]:0.1420233503,(PAA74204.1:
0.1944558827,PAA76532.1:0.370680911)[&Value="99.7/100"]:0.3467650496)[&Value="95.
6/100"]:0.198978607,(PAA83069.1:0.1719651195,PAA94353.1:0.2177087863)[&Value="10
0/100"]:0.9149743964)[&Value="94.8/100"]:0.1984586024)[&Value="93.7/93"]:0.15862896
43)[&Value="84.8/83"]:0.1312618768)[&Value="81.9/73"]:0.0845987147,((((((((ABI53802.1:
0.0368030978,(XP_046352531.2:0.009484872,(XP_048248472.1:0.000002,(XP_048248473

.1:0.000002,XP_048248474.1:0.000002)[&Value="0/59"]:0.000002)[&Value="97.9/100"]:0.0316102741)[&Value="86.5/96"]:0.0093563587)[&Value="86.6/100"]:0.0092494171,XP_048258111.1:0.0090782548)[&Value="0/91"]:0.000002,XP_048248476.1:0.0232086794)[&Value="79.4/100"]:0.004863978,XP_046352527.2:0.0279736371)[&Value="99.5/100"]:0.0459420941,(XP_046562919.1:0.0551671186,XP_046563124.1:0.0106361363)[&Value="37.8/72"]:0.0077189103)[&Value="83.5/73"]:0.0121974047,(XP_046563126.1:0.0095092745,XP_046565195.1:0.0134859184)[&Value="97.4/100"]:0.0320274116)[&Value="86.2/94"]:0.0129070789,XP_046563125.1:0.0214982757)[&Value="97.5/100"]:0.1190949205,XP_046565196.1:0.0911729286)[&Value="100/100"]:0.5702044389)[&Value="36.3/58"]:0.020758405)[&Value="85.9/67"]:0.1637404422,((KAI0208044.1:0.086065358,KAI0218869.1:0.1587590282)[&Value="78.8/92"]:0.0261815051,KAI0213370.1:0.000002)[&Value="99/100"]:0.5265685854)[&Value="100/96"]:0.8995576942,((((((XP_006813643.1:0.1391005534,XP_030843280.1:0.2139972818)[&Value="25.5/99"]:0.0308932813,(((((XP_023440724.1:0.0091789899,(XP_005873264.1:0.0093079259,((XP_006163024.2.2:0.0,NP_598513.1:0.0):0.000002,NP_056375.2.2:0.000002)[&Value="0/74"]:0.000002)[&Value="86.3/100"]:0.0094388989)[&Value="96.9/100"]:0.0431864197,(XP_028587646.1:0.0322639945,XP_025913835.1:0.0093458141)[&Value="10.1/87"]:0.0101380062)[&Value="90.9/100"]:0.027276106,(XP_021332524.1:0.0557250032,XP_031757388.1:0.0675382956)[&Value="66.5/100"]:0.0111131663)[&Value="93.4/100"]:0.0393344487,XP_032818114.1:0.0897868837)[&Value="98.5/100"]:0.1081776239,XP_018667792.1:0.1635135977)[&Value="92.4/100"]:0.0581679643)[&Value="93.2/100"]:0.0785984526,(NP_495986.3.3:0.229301677,NP_610941.1:0.2263393818)[&Value="91/83"]:0.0729337305)[&Value="32.5/82"]:0.0284807804,(XP_002602331.1:0.0078070536,XP_019637857.1:0.0105714053)[&Value="98.5/99"]:0.1294450427)[&Value="95.1/89"]:0.3155807743,(PAA68234.1:0.1818284824,PAA87312.1:0.1246319615)[&Value="75.9/94"]:0.1855270416)[&Value="98/98"]:1.942512618,(((((((((((((XP_004479029.1:0.0163296821,NP_284941.2.2:0.0395780108)[&Value="87.1/98"]:0.0107931724,NP_001193437.1:0.0108841906)[&Value="76.4/98"]:0.0055240374,XP_005883071.1:0.027415918)[&Value="72.9/94"]:0.0053751675,(NP_077162.2.2:0.0228684934,XP_006162789.1:0.0103087798)[&Value="0/100"]:0.000983)[&Value="90.2/95"]:0.016158153,XP_025917892.1:0.033977737)[&Value="93.7/100"]:0.0275327407,XP_028587453.1:0.034333563)[&Value="96.4/100"]:0.0418147267,NP_001016189.1:0.0599293853)[&Value="88.6/100"]:0.0328895233,XP_017213868.2.2:0.115773578)[&Value="90.5/99"]:0.0495102426,((((XP_004482574.1:0.0054164485,NP_001272849.1:0.0217974139)[&Value="76.2/95"]:0.0054648442,(XP_006145367.1:0.000002,((XP_014400986.1:0.0108691531,NP_001177198.1:0.0054431175)[&Value="71.5/100"]:0.0054324609,NP_001121132.1:0.021828278)[&Value="0/92"]:0.000002)[&Value="0/78"]:0.000002)[&Value="89.5/94"]:0.0170677372,(XP_025929938.1:0.010967591,(XP_028597443.1:0.0305908478,XP_015268039.1:0.000003)[&Value="93.7/100"]:0.018883645)[&Value="75.5/99"]:0.0050627563)[&Value="97.4/100"]:0.0650866586,NP_001121726.1:0.0810929807)[&Value="97.4/99"]:0.0745599366)[&Value="99.1/99"]:0.1353454032,(((XP_002591612.1:0.0144665077,XP_019628129.1:0.0022146368)[&Value="100/100"]:0.2076786112,NP_996357.1:0.5054739616)[&Value="46.7/63"]:0.0220235808,(XP_006819998.1:0.1752495528,(XP_030846906.1:0.1230185965,XP_030847518.1:0.0349127849)[&Value="100/100"]:0.4174349109)[&Value="80.2/61"]:0.0790351289)[&Value="95.8/46"]:0.1059281886)[&Value="48.8/23"]:0.0332703037,(XP_002126852.1:0.2584904452,NP_495161.1:0.83049798

46)[&Value="83.4/43"]:0.148624264)[&Value="18.3/14"]:0.0924906915,(PAA75551.1:0.015
6703514,PAA75258.1:0.000003)[&Value="100/100"]:0.4859205289)[&Value="35.3/61"]:0.0
622290445,(XP_004365821.1:0.5296052168,(XP_014153836.1:0.7597037004,XP_0017457
40.1:1.3510153826)[&Value="77.6/75"]:0.1398530191)[&Value="85.8/68"]:0.3041890093)[
&Value="100/100"]:6.518150811)[&Value="86.1/97"]:0.6419959325)[&Value="92.4/98"]:0.
465261866)[&Value="98.6/99"]:0.2635679104)[&Value="33.5/81"]:0.0694586346,(XP_0141
53758.1:0.1406937099,XP_014148725.1:0.1548409325)[&Value="92.6/99"]:0.0903285706
)[&Value="85/86"]:0.041833072)[&Value="77.8/39"]:0.0248332738,((PAA65118.1:0.095970
4936,PAA78248.1:0.0351996987)[&Value="20.4/56"]:0.0296905831,(PAA59145.1:0.06761
48537,PAA64382.1:0.1596953076)[&Value="91/100"]:0.0392769458)[&Value="100/100"]:0
.1484050143)[&Value="69.7/35"]:0.0096157375)[&Value="89.1/40"]:0.0342018303)[&Valu
e="63.4/24"]:0.006612426,(XP_005165639.1:0.0438000458,XP_004347890.1:0.237467736
)[&Value="92.4/68"]:0.0397887481)[&Value="75.6/26"]:0.0155875197,XP_026693152.1:0.1
603852464);
end;
