# Supplementary material for: Antiviral Mx proteins have an ancient origin and widespread distribution among eukaryotes
Source: Proc Natl Acad Sci U S A. 2025 Jan 24;122(4):e2416811122. doi: 10.1073/pnas.2416811122 (PMC11789081; doi:10.1073/pnas.2416811122)
Supplement: Supplementary file 13 — Dataset S12 (PDF) [file pnas.2416811122.sd12.pdf]

Dataset S12. Suppl_Figure_2_IQTree

```
#NEXUS
begin taxa;
	dimensions ntax=368;
	taxlabels
	XP_026693152.1
	XP_030853442.1.2
	XP_030853442.1
	XP_032814666.1
	XP_025944940.1
	XP_031757197.1
	EPQ08653.1
	XP_027623811.1
	XP_016856477.1
	XP_012379251.1
	XP_006496668.1
	XP_031753735.1
	XP_025920181.1
	XP_028568434.1
	XP_006510037.1
	NP_001005360.1
	XP_012381548.1
	XP_006161648.2.2
	XP_014389433.1
	XP_021326548.1
	NP_001025299.1
	KMZ10000.1
	NP_001024332.1
	XP_035683496.1
	XP_028570166.1
	XP_025915522.1
	EPQ17174.1
	ELW62001.1
	EAW87759.1
	XP_012378586.1
	BAB27759.1
	KAE8583055.1
	XP_005165639.1
	PAA78248.1
	PAA59145.1
	PAA64382.1
	PAA65118.1
	XP_001749319.1
```

XP_006812840.1
XP_004347890.1
XP_014148725.1
XP_014153758.1
XP_004348308.1
XP_002129967.2
XP_035676386.1
XP_006821224.1
XP_030827871.1
PAA85687.1
XP_032819300.1
NP_957216.1
XP_025940269.1
XP_028602039.1
XP_006168142.1
NP_001317309.1
XP_012382650.2
NP_001392186.1
XP_014394711.1
XP_031753959.1
NP_001259946.1
NP_741403.2
XP_001750431.1
XP_014148015.1
KNE67543.1
KNE61418.1
XP_011392073.1
XP_006461708.1
XP_746923.1
KXN67416.1
NP_013100.1
XP_042914770.1
PWZ09977.1
KAH9304002.1
EFJ15047.1
EFJ37641.1
KAI5070335.1
KAI5070758.1
PTQ35749.1
KAG0555682.1
KAG0554580.1
XP_024362051.1
EFJ35472.1
AAC61784.1

XP_052310486.1
KAH9327796.1
ONM18162.1
XP_042924642.1
NP_001190448.1
XP_002299468.1
XP_002302631.1
AQK88296.1
PTQ45603.1
KAG0556007.1
PTQ29980.1
KAI5072318.1
KAI5058380.1
XP_002987566.1
EFJ15761.1
EFJ23099.1
KAH9306600.1
KAI5602084.1
XP_006375094.1
PWZ36850.1
AAF22292.1
NP_850420.1
XP_002315854.1
NP_001147100.1
AAF79238.1
PSC76263.1
XP_005849062.1
PRW56740.1
GMH43921.1
GMH36208.1
GJP35534.1
CAI5480041.1
KAJ7294545.1
XP_024380180.1
XP_024367947.1
KAG0619429.1
KAG0561847.1
KAH9290598.1
KAH9291961.1
KAH9320939.1
KAH9325151.1
KAH9300179.1
KAH9314974.1
KAF8079489.1

OAP13353.1
OAP19580.1
OAP13972.1
KAF5727250.1
XP_002303204.3
XP_002297993.1
XP_024439231.1
KAK1401877.1
KAH0683503.1
KAF8391993.1
XP_058079501.1
XP_038984915.1
PWZ56863.1
PWZ56864.1
EFJ22917.1
CAG9460856.1
XP_006815062.1
CAH1802128.1
PAA74204.1
PAA76532.1
PAA92268.1
PAA69582.1
PAA83069.1
PAA94353.1
XP_035690836.1
XP_002608668.1
XP_019617847.1
XP_003973512.2.2
NP_891987.2.2
XP_009304072.1
AGU16245.1
XP_007904885.1
XP_032888405.1
XP_028583068.1
NP_001007285.1
XP_005167721.2.2
XP_025933558.1
XP_009815891.1
XP_028583072.1
XP_015269256.1
XP_006156438.1
NP_002454.1
XP_002830747.1
NP_001003133.1

XP_032211320.1
XP_017508123.1
XP_005885748.1
XP_012586448.1
NP_776366.1
XP_008569440.1
XP_004675614.2.2
XP_004466363.1
NP_002453.2.2
NP_001127618.1
XP_008569442.1
XP_014388412.1
XP_005202045.1
XP_017508130.1
NP_001003134.1
XP_032211398.1
NP_038634.1
NP_034976.1
XP_006156437.1
XP_031752404.1
XP_032804093.1
KAI0213370.1
KAI0208044.1
KAI0218869.1
XP_046565196.1
XP_046562919.1
XP_046563124.1
XP_046352527.2
XP_048248476.1
XP_048258111.1
XP_046352531.2
XP_048248472.1
XP_048248473.1
XP_048248474.1
ABI53802.1
XP_046563126.1
XP_046565195.1
XP_046563125.1
KAK3283006.1
GHP04420.1
'KAJ3066410.1'
KAI9324922.1
KAI8836453.1
KAJ3350919.1

KAI8587516.1
XP_047808890.1
KXS17655.1
XP_021869222.1
TVY17522.1
OLL24579.1
KAI9096888.1
RSH87279.1
XP_041144356.1
XP_746402.1
KAJ5704467.1
MCJ1392161.1
KAI9774215.1
XP_002543522.1
XP_751069.1
XP_026607910.1
XP_748757.2
XP_040633937.1
XP_043140374.1
XP_754266.1
KAF9951223.1
GAX85982.1
XP_042923301.1
XP_042924848.1
KAG2488600.1
XP_042924875.1
KAJ9515210.1
KAI3646081.1
OAJ38670.1
XP_006461472.1
XP_006461433.1
XP_006457072.1
XP_750654.1
PAA68234.1
PAA87312.1
XP_002602331.1
XP_019637857.1
XP_006813643.1
XP_030843280.1
XP_018667792.1
XP_032818114.1
XP_021332524.1
XP_031757388.1
XP_028587646.1

XP_025913835.1
XP_023440724.1
XP_005873264.1
XP_006163024.2.2
NP_598513.1
NP_056375.2.2
NP_495986.3.3
NP_610941.1
XP_042918632.1
NP_001130364.1
PWZ11893.1
XP_002317496.2
AAF87857.1
KAI5058044.1
EFJ18064.1
KAG0628798.1
PTQ33908.1
KAH9322298.1
XP_009032466.1
XP_042920073.1
PWZ44616.1
XP_002309632.3
NP_001189935.1
EFJ19523.1
KAI5064281.1
PTQ34556.1
KAG0561482.1
KAG0605142.1
KAH9308354.1
XP_042916771.1
EFJ26018.1
KAG0631008.1
KAH9315399.1
CAD5311589.1
XP_008649599.1
OAE29693.1
KAI5059498.1
OUM66167.1
XP_001481516.1
NP_009738.1
XP_011392385.1
XP_006462464.1
XP_006676761.1
KNE73082.1

KNE65701.1
KXN69997.1
XP_001745740.1
NP_495161.1
PAA75551.1
PAA75258.1
XP_006819998.1
XP_030846906.1
XP_030847518.1
XP_002591612.1
XP_019628129.1
NP_996357.1
NP_001121726.1
XP_025929938.1
XP_015268039.1
XP_028597443.1
XP_004482574.1
NP_001272849.1
XP_006145367.1
XP_014400986.1
NP_001177198.1
NP_001121132.1
XP_017213868.2.2
NP_001016189.1
XP_028587453.1
XP_025917892.1
XP_004479029.1
NP_284941.2.2
NP_001193437.1
XP_005883071.1
NP_077162.2.2
XP_006162789.1
XP_002126852.1
XP_004365821.1
XP_014153836.1
EFJ33653.1
EFJ28901.1
KAG0632288.1
'KAG0555995.1'
XP_024391061.1
XP_024368367.1
OAE31801.1
KAI5073815.1
XP_008646219.1

ACG47836.1
ONM04707.1
'KAG7649995.1'
'NP_172500.1'
XP_006385192.1
KAH9330549.1
KXN72852.1
KNE54706.1
OUM67143.1
XP_011389557.1
XP_006459124.1
XP_752563.1
NP_014854.2
OAJ38404.1
NP_012926.1
KNE68830.1
XP_011389257.1
XP_006458578.1
XP_748106.1
OUM62108.1
OAJ44422.1
KXN66323.1
;
end;

begin trees;
tree tree_1 = [&R]
[&branchAttributeNames={"Value"}](XP_026693152.1:0.1252500521,(((((XP_030853442.1.
2:0.000002,XP_030853442.1:0.000003)[&Value="100/100"]:0.1468334098,((XP_03281466
6.1:0.0664829206,(((((XP_025944940.1:0.052479384,XP_031757197.1:0.1838694875)[&Va
lue="75/100"]:0.0170027768,(EPQ08653.1:0.0094693765,((XP_027623811.1:0.014193334
8,(XP_016856477.1:0.0046850173,XP_012379251.1:0.000002)[&Value="0/98"]:0.000002)[
&Value="0/97"]:0.000002,XP_006496668.1:0.0046480472)[&Value="78.2/98"]:0.01396224
14)[&Value="94.8/100"]:0.0311360779)[&Value="94/100"]:0.0349918374,(XP_031753735.1
:0.045651659,((XP_025920181.1:0.0055512424,XP_028568434.1:0.0040873435)[&Value="
79.5/92"]:0.0086496204,(((XP_006510037.1:0.000002,(NP_001005360.1:0.0046352239,XP
_012381548.1:0.0525900913)[&Value="0/16"]:0.000014)[&Value="0/11"]:0.000003,XP_006
161648.2.2:0.0848346935)[&Value="57.2/97"]:0.0047416116,XP_014389433.1:0.0289353
175)[&Value="88.8/98"]:0.0098928704)[&Value="87.8/94"]:0.0172618323)[&Value="98.1/1
00"]:0.0485228625)[&Value="83.4/98"]:0.0247237884,(XP_021326548.1:0.000002,NP_001
025299.1:0.000002)[&Value="99.5/100"]:0.0613827119)[&Value="39.4/92"]:0.0087468581,
(KMZ10000.1:0.1050634678,NP_001024332.1:0.1791579843)[&Value="96.1/93"]:0.058805
3453)[&Value="87.9/93"]:0.0261572818)[&Value="85.6/89"]:0.0312300424,XP_035683496.
1:0.0956198792)[&Value="82.7/82"]:0.0160573221)[&Value="70/49"]:0.0101983926,((XP_0

28570166.1:0.0192242682,XP_025915522.1:0.000003)[&Value="56/82"]:0.0064167176,(((
EPQ17174.1:0.0093694833,(ELW62001.1:0.000003,(EAW87759.1:0.000002,XP_01237858
6.1:0.0386050393)[&Value="82.5/100"]:0.004693288)[&Value="0/97"]:0.000002)[&Value="
66.8/49"]:0.0046916016,BAB27759.1:0.000002)[&Value="93.3/98"]:0.0223260636,KAE858
3055.1:0.0414609015)[&Value="91.4/89"]:0.0215474298)[&Value="92.6/88"]:0.024854319
3)[&Value="89.8/50"]:0.0220936635,XP_005165639.1:0.0595219324)[&Value="55.5/69"]:0.
0188875863,((((PAA78248.1:0.0198111084,(PAA59145.1:0.0604149352,PAA64382.1:0.143
1731241)[&Value="97.3/100"]:0.0600360371)[&Value="82.6/99"]:0.0273245925,PAA65118
.1:0.0612567871)[&Value="99.6/100"]:0.1202897,XP_001749319.1:0.1681570598)[&Value
="81.8/80"]:0.0293909137,XP_006812840.1:0.3318276981)[&Value="43.4/74"]:0.0225830
794)[&Value="79.4/86"]:0.0322247106,(XP_004347890.1:0.1741948215,((XP_014148725.1
:0.1681506657,XP_014153758.1:0.09053534)[&Value="95.8/100"]:0.071186539,((((XP_004
348308.1:0.2596459167,((((XP_002129967.2:0.360900921,(((XP_035676386.1:0.08921559
33,(XP_006821224.1:0.1152626819,XP_030827871.1:0.122424122)[&Value="79.9/93"]:0.0
587131185)[&Value="64.2/93"]:0.0534331449,PAA85687.1:0.2534641231)[&Value="73.8/9
9"]:0.0576133508,(XP_032819300.1:0.1316754661,(NP_957216.1:0.0323312376,(((XP_02
5940269.1:0.0337387013,XP_028602039.1:0.0380698772)[&Value="8.6/88"]:0.003610034
4,((XP_006168142.1:0.000002,((NP_001317309.1:0.000003,XP_012382650.2:0.065589443
8)[&Value="38.1/87"]:0.0036252023,NP_001392186.1:0.0113636294)[&Value="0/71"]:0.00
0003)[&Value="0/77"]:0.000002,XP_014394711.1:0.044665138)[&Value="97.9/94"]:0.0280
320813)[&Value="77.7/81"]:0.011505172,XP_031753959.1:0.2434076506)[&Value="90.8/1
00"]:0.0284059708)[&Value="97.8/100"]:0.0779835272)[&Value="92/99"]:0.0653574963)[&
Value="85.4/99"]:0.0621243822)[&Value="0/37"]:0.0161202899,(NP_001259946.1:0.1887
482198,NP_741403.2:0.3587511813)[&Value="89.5/80"]:0.0487729331)[&Value="98.9/100
"]:0.1337298843,XP_001750431.1:0.5261873775)[&Value="91.3/97"]:0.0666738641,XP_01
4148015.1:0.4556685749)[&Value="24.6/66"]:0.0166214243)[&Value="96.6/100"]:0.09957
49146,(((KNE67543.1:0.1940700572,KNE61418.1:0.2042482328)[&Value="100/100"]:0.33
18095229,(XP_011392073.1:0.1505179979,XP_006461708.1:0.1589794844)[&Value="95.9
/100"]:0.1120946353)[&Value="64.7/65"]:0.0292115582,((XP_746923.1:0.2121702218,KX
N67416.1:0.3034506662)[&Value="84.9/100"]:0.0564146725,NP_013100.1:0.3687648855)
[&Value="91.2/99"]:0.0824110101)[&Value="98.7/95"]:0.1227060748)[&Value="55.7/67"]:0
.0455412866,((XP_042914770.1:0.4915229577,(PWZ09977.1:0.0508325438,(((KAH930400
2.1:0.1108788944,(((EFJ15047.1:0.000002,EFJ37641.1:0.0042917965)[&Value="96.9/100"]
:0.0432104732,((KAI5070335.1:0.0764187814,KAI5070758.1:0.0715760956)[&Value="93.2
/100"]:0.0329777995,(PTQ35749.1:0.0676896106,(KAG0555682.1:0.0547472176,(KAG055
4580.1:0.0078484109,XP_024362051.1:0.0583183892)[&Value="97.5/100"]:0.0454912446
)[&Value="24.5/98"]:0.0096214065)[&Value="87/100"]:0.0185525799)[&Value="89.6/100"]:
0.0279144542)[&Value="82.8/98"]:0.0235416176,EFJ35472.1:0.5055933925)[&Value="79.
2/93"]:0.0283507204)[&Value="88/86"]:0.0404392251,((AAC61784.1:0.1788795725,XP_05
2310486.1:0.0800867958)[&Value="97.1/100"]:0.0860875736,KAH9327796.1:0.24176128
98)[&Value="86.3/97"]:0.0255111585)[&Value="81.8/83"]:0.0294020815,ONM18162.1:0.2
513497799)[&Value="57.5/74"]:0.0214848723)[&Value="98.9/99"]:0.1774210037)[&Value=
"97.8/100"]:0.1952487402,(((XP_042924642.1:0.4390092719,(((NP_001190448.1:0.09356
57899,XP_002299468.1:0.0536711665)[&Value="27.5/74"]:0.0068986983,(XP_002302631.

1:0.0541191406,AQK88296.1:0.1146575807)[&Value="91.5/97"]:0.039774607)[&Value="8
9.2/93"]:0.0678746526,((((((PTQ45603.1:0.0876251224,(KAG0556007.1:0.0912046042,PT
Q29980.1:0.1514986629)[&Value="37.2/98"]:0.0350933903)[&Value="81.2/10"]:0.0174881
92,KAI5072318.1:0.0635868324)[&Value="0/8"]:0.0164658493,KAI5058380.1:0.099369727
1)[&Value="86.5/86"]:0.0186620476,((XP_002987566.1:0.0690276641,EFJ15761.1:0.12785
82611)[&Value="73.7/99"]:0.0078981893,EFJ23099.1:0.1761772457)[&Value="95.2/99"]:0.
0384797409)[&Value="26/86"]:0.0113156274,KAH9306600.1:0.0880618544)[&Value="69.8
/96"]:0.0127027122,(KAI5602084.1:0.0934049876,(((XP_006375094.1:0.0474678975,PWZ
36850.1:0.1390875765)[&Value="2/25"]:0.0139574293,(AAF22292.1:0.0930503674,NP_85
0420.1:0.1187175105)[&Value="92.8/100"]:0.038589486)[&Value="78.9/97"]:0.013049354
2,((XP_002315854.1:0.0453342219,NP_001147100.1:0.0930903981)[&Value="81.3/100"]:
0.0268566187,AAF79238.1:0.0880204856)[&Value="92.5/99"]:0.0338103703)[&Value="87.
6/100"]:0.0183941859)[&Value="86.1/99"]:0.0219682133)[&Value="92.8/98"]:0.07371557)[
&Value="100/100"]:0.3836136643)[&Value="96.5/99"]:0.1731617444,((((PSC76263.1:0.350
5967573,(XP_005849062.1:0.3471521192,PRW56740.1:0.5465198793)[&Value="87.8/100
"]:0.1357622605)[&Value="100/100"]:0.5167111504,((GMH43921.1:0.5017447274,GMH36
208.1:0.4392374587)[&Value="100/100"]:0.5450813686,(((GJP35534.1:0.1251392588,CAI
5480041.1:0.1181112589)[&Value="98.7/100"]:0.2393621981,((KAJ7294545.1:0.27239559
39,((XP_024380180.1:0.0137894284,XP_024367947.1:0.0080003828)[&Value="100/100"]:
0.229069704,(KAG0619429.1:0.0763357264,KAG0561847.1:0.1271670329)[&Value="92.2/
100"]:0.0652815856)[&Value="96/100"]:0.092293382)[&Value="95.1/100"]:0.1099207268,(
(((KAH9290598.1:0.3010279166,KAH9291961.1:0.3664655836)[&Value="84.8/90"]:0.0617
683629,(KAH9320939.1:0.301930862,(KAH9325151.1:0.1433272134,(KAH9300179.1:0.16
35202696,KAH9314974.1:0.278334753)[&Value="83/100"]:0.1212067015)[&Value="99.3/1
00"]:0.3437275489)[&Value="70.3/75"]:0.0517420903)[&Value="38.5/65"]:0.02549332,((((((
(KAF8079489.1:0.0567209472,(OAP13353.1:0.0948935184,(OAP19580.1:0.108312411,OA
P13972.1:0.0434553227)[&Value="0/68"]:0.000003)[&Value="92.6/100"]:0.1212001719)[&
Value="98.8/100"]:0.2077385443,(KAF5727250.1:0.1171762464,(XP_002303204.3:0.0833
331046,(XP_002297993.1:0.1415599704,XP_024439231.1:0.157553835)[&Value="89.8/96
"]:0.0403848581)[&Value="68/95"]:0.0134816786)[&Value="15.7/89"]:0.0204045654)[&Val
ue="87.4/99"]:0.0213973806,(KAK1401877.1:0.1151601912,KAH0683503.1:0.222076442)[
&Value="48.7/96"]:0.0150527143)[&Value="91.4/96"]:0.035191497,KAF8391993.1:0.0757
236411)[&Value="96.9/99"]:0.0630719967,XP_058079501.1:0.1120758187)[&Value="88.4/
98"]:0.056161018,XP_038984915.1:0.1183519732)[&Value="63.5/98"]:0.0344112014,(PW
Z56863.1:0.0651199656,PWZ56864.1:0.042780384)[&Value="99.5/100"]:0.1105094807)[&
Value="99.7/100"]:0.1477865664)[&Value="95.4/99"]:0.0956952403,EFJ22917.1:0.495208
8491)[&Value="95.2/99"]:0.1019274434)[&Value="98.5/99"]:0.2425218341)[&Value="100/1
00"]:0.4524842524,CAG9460856.1:0.9213809478)[&Value="55.3/98"]:0.0788421308)[&Val
ue="51.6/90"]:0.0978603349)[&Value="98.9/100"]:0.3639435217,(((XP_006815062.1:0.532
2723512,(CAH1802128.1:0.6512580798,(((PAA74204.1:0.1785640119,PAA76532.1:0.3318
694722)[&Value="99.7/100"]:0.3177808106,(PAA92268.1:0.4192876757,PAA69582.1:0.42
95117642)[&Value="83.7/100"]:0.1352353749)[&Value="92.9/100"]:0.1608619545,(PAA83
069.1:0.1294738546,PAA94353.1:0.2196081683)[&Value="100/100"]:0.7525024891)[&Val
ue="95.2/100"]:0.1701129044)[&Value="94.2/100"]:0.1313518207)[&Value="87.8/99"]:0.0

843243802,((XP_035690836.1:0.2593767007,(XP_002608668.1:0.113381334,XP_0196178
47.1:0.1622577328)[&Value="96.3/100"]:0.1394812074)[&Value="99/100"]:0.1890736312,(
(((XP_003973512.2.2:0.138018764,(NP_891987.2.2:0.0474054797,XP_009304072.1:0.017
1284216)[&Value="99.8/100"]:0.1338495438)[&Value="98.9/100"]:0.1365978313,((((AGU1
6245.1:0.2140002388,(XP_007904885.1:0.1560118009,XP_032888405.1:0.1843831691)[&
Value="96.4/100"]:0.0835872822)[&Value="86.3/91"]:0.047562255,(XP_028583068.1:0.21
85008861,(NP_001007285.1:0.1084848292,XP_005167721.2.2:0.0509588351)[&Value="1
00/100"]:0.345356019)[&Value="74.8/92"]:0.0330549494)[&Value="89.9/85"]:0.055272513
8,(((XP_025933558.1:0.0716708891,XP_009815891.1:0.1102493718)[&Value="100/100"]:0
.1872679426,XP_028583072.1:0.2271231086)[&Value="10.3/77"]:0.0419698943,XP_0152
69256.1:0.1479787972)[&Value="48.6/93"]:0.0561185199)[&Value="32.5/79"]:0.03303923
87,((((((XP_006156438.1:0.1634645143,(NP_002454.1:0.0047664216,XP_002830747.1:0.0
170522894)[&Value="100/100"]:0.0963030481)[&Value="78.7/91"]:0.0157617327,(NP_001
003133.1:0.0971825116,XP_032211320.1:0.1423989272)[&Value="95.6/100"]:0.05071152
3)[&Value="9.6/66"]:0.008152409,((XP_017508123.1:0.1006108921,(XP_005885748.1:0.02
43759518,XP_012586448.1:0.3003347372)[&Value="87.2/100"]:0.0207446896)[&Value="8
1/100"]:0.0138314219,NP_776366.1:0.1382834298)[&Value="85.1/85"]:0.0191197336)[&V
alue="92.6/87"]:0.028040418,XP_008569440.1:0.0448283896)[&Value="99.9/100"]:0.1443
353282,(XP_004675614.2.2:0.1717664863,((((XP_004466363.1:0.1218860522,((NP_00245
3.2.2:0.0086576419,NP_001127618.1:0.000003)[&Value="98.9/100"]:0.0766815695,(XP_0
08569442.1:0.0959292522,(XP_014388412.1:0.114993305,XP_005202045.1:0.149372564
6)[&Value="17.8/42"]:0.0224414088)[&Value="89/92"]:0.0345959265)[&Value="76.8/98"]:0
.0288056913)[&Value="88.7/48"]:0.0220837582,(XP_017508130.1:0.076362165,(NP_0010
03134.1:0.0299231368,XP_032211398.1:0.0439022571)[&Value="99.1/100"]:0.064363915
1)[&Value="90.7/76"]:0.0274728565)[&Value="79/46"]:0.0124451517,(NP_038634.1:0.008
5097608,NP_034976.1:0.076374185)[&Value="100/100"]:0.1510143452)[&Value="86.6/85
"]:0.0229157408,XP_006156437.1:0.0623359934)[&Value="94.9/97"]:0.0500006458)[&Val
ue="64.8/98"]:0.056210564)[&Value="90.5/99"]:0.0674149367,XP_031752404.1:0.359115
2644)[&Value="90.1/95"]:0.0705671978)[&Value="95.5/97"]:0.1280442539)[&Value="98.8/
100"]:0.2207103768,XP_032804093.1:0.5885698718)[&Value="94.3/91"]:0.136349111,((K
AI0213370.1:0.003612838,(KAI0208044.1:0.0789960223,KAI0218869.1:0.144596954)[&Va
lue="67.6/96"]:0.0211224685)[&Value="100/100"]:0.5447079004,(XP_046565196.1:0.0774
624807,((((XP_046562919.1:0.0502601438,XP_046563124.1:0.0097381342)[&Value="37.5
/53"]:0.0070741916,(XP_046352527.2:0.0257992893,(XP_048248476.1:0.0213386832,(XP
_048258111.1:0.0083378695,((XP_046352531.2:0.0086921241,(XP_048248472.1:0.00000
2,(XP_048248473.1:0.000002,XP_048248474.1:0.000003)[&Value="0/52"]:0.000003)[&Valu
e="98.8/100"]:0.028870495)[&Value="85.6/96"]:0.0085376745,ABI53802.1:0.0337893394)
[&Value="87.3/100"]:0.0084931812)[&Value="0/97"]:0.000002)[&Value="81/100"]:0.00444
37279)[&Value="99.4/100"]:0.0419106433)[&Value="85.9/53"]:0.0112073563,(XP_0465631
26.1:0.0087215041,XP_046565195.1:0.0123626515)[&Value="98.2/100"]:0.0290764016)[&
Value="82.4/94"]:0.012443243,XP_046563125.1:0.0191560231)[&Value="98.1/99"]:0.1153
154126)[&Value="100/100"]:0.4857880496)[&Value="5.9/49"]:0.0493486252)[&Value="57.
2/55"]:0.044589315)[&Value="57.9/62"]:0.0795537081)[&Value="99.6/99"]:0.3673781415,(
(((KAK3283006.1:0.7505610044,GHP04420.1:0.5738928859)[&Value="96.5/100"]:0.31342

93404,(('KAJ3066410.1':0.1314504037,KAI9324922.1:0.2506646364)[&Value="65.7/95"]:0.
0819950319,(KAI8836453.1:0.2636724504,KAJ3350919.1:0.4951098921)[&Value="77.4/75
"]:0.1552649325)[&Value="100/100"]:0.8101599339)[&Value="52.7/71"]:0.1230928565,((K
AI8587516.1:0.8862582118,(XP_047808890.1:0.7048345972,KXS17655.1:0.6234104246)[
&Value="91.8/100"]:0.2183304059)[&Value="91.9/99"]:0.2293126066,(((XP_021869222.1:0
.5648047058,TVY17522.1:0.4490042025)[&Value="100/100"]:0.5762901399,(OLL24579.1:
0.6524018807,((KAI9096888.1:0.7066470195,(RSH87279.1:0.6574744477,(((XP_04114435
6.1:0.1169620141,XP_746402.1:0.1672402451)[&Value="77.7/100"]:0.0330468219,(KAJ57
04467.1:0.133720857,((MCJ1392161.1:0.092892547,KAI9774215.1:0.0799883374)[&Value
="90.5/100"]:0.0500467412,XP_002543522.1:0.2221196935)[&Value="54.5/94"]:0.045749
1692)[&Value="77.8/94"]:0.0308288212)[&Value="99.8/100"]:0.3797573662,XP_751069.1:
0.8228674516)[&Value="91.9/97"]:0.1612248055)[&Value="8.9/88"]:0.0845805217)[&Valu
e="83.8/99"]:0.1964165528,(XP_026607910.1:0.4687924064,(XP_748757.2:0.4936593742
,(XP_040633937.1:0.2481401808,(XP_043140374.1:0.0655837936,XP_754266.1:0.103823
747)[&Value="99/100"]:0.1639979931)[&Value="98.9/100"]:0.2176240614)[&Value="78.4/1
00"]:0.0815706774)[&Value="100/100"]:0.4981227746)[&Value="74/99"]:0.1551595554)[&
Value="36.7/97"]:0.0604952448)[&Value="92.5/99"]:0.2458845423,KAF9951223.1:1.14791
5779)[&Value="90.7/99"]:0.1730454807)[&Value="96.1/100"]:0.2654948955)[&Value="94.4
/97"]:0.2832469004,((GAX85982.1:0.422847499,(XP_042923301.1:0.264684354,(XP_0429
24848.1:0.3113783749,(KAG2488600.1:0.2992738276,XP_042924875.1:0.3859199841)[&
Value="98.1/95"]:0.1394913241)[&Value="23.4/92"]:0.0792537676)[&Value="99.6/100"]:0.
4557804422)[&Value="99.7/100"]:0.5543909003,(KAJ9515210.1:0.6314879213,((KAI36460
81.1:0.6458649128,OAJ38670.1:0.579373453)[&Value="80.9/99"]:0.1970335064,(((XP_006
461472.1:0.1551524749,XP_006461433.1:0.1600162611)[&Value="100/100"]:0.61487518
39,XP_006457072.1:1.0896252693)[&Value="84/100"]:0.1691782799,XP_750654.1:1.1997
806225)[&Value="72.8/95"]:0.1827495354)[&Value="82/87"]:0.1686376969)[&Value="86/8
7"]:0.1605950755)[&Value="100/100"]:0.5747120205)[&Value="95.7/98"]:0.226942177)[&V
alue="93.8/98"]:0.1807875599)[&Value="85.1/97"]:0.1327123824,(((PAA68234.1:0.062552
0287,(PAA87312.1:0.107762072,((XP_002602331.1:0.0071275111,XP_019637857.1:0.009
2066501)[&Value="98.4/100"]:0.1151024318,(((XP_006813643.1:0.1176921259,XP_03084
3280.1:0.1821838902)[&Value="43.9/99"]:0.0303237678,(XP_018667792.1:0.142278895,(
XP_032818114.1:0.0786415682,((XP_021332524.1:0.0482358903,XP_031757388.1:0.0602
917466)[&Value="8.6/83"]:0.0097147921,((XP_028587646.1:0.0288129856,XP_025913835
.1:0.0082293834)[&Value="16.5/98"]:0.0092408474,(XP_023440724.1:0.0081560548,(XP_
005873264.1:0.0082814694,((XP_006163024.2.2:0.0,NP_598513.1:0.0):0.000002,NP_056
375.2.2:0.000002)[&Value="0/64"]:0.000002)[&Value="88.6/99"]:0.0084426271)[&Value="9
7.8/100"]:0.0380944521)[&Value="89.9/100"]:0.0235962714)[&Value="92.5/100"]:0.03531
14465)[&Value="99.2/100"]:0.0952520354)[&Value="92.3/100"]:0.0506038121)[&Value="9
3.9/100"]:0.0726014817,(NP_495986.3.3:0.1923258209,NP_610941.1:0.1910681196)[&Va
lue="93.9/97"]:0.0662596067)[&Value="40.2/94"]:0.022707103)[&Value="100/100"]:0.4201
237408)[&Value="73.7/94"]:0.0933831173)[&Value="100/100"]:1.371800344,(((XP_042918
632.1:0.1631539294,(((NP_001130364.1:0.0221777855,PWZ11893.1:0.0265022647)[&Val
ue="100/99"]:0.1407364568,(XP_002317496.2:0.1045270248,AAF87857.1:0.0541457454)[
&Value="96.9/99"]:0.0678484559)[&Value="83.5/98"]:0.0481510002,((KAI5058044.1:0.188

9189736,(EFJ18064.1:0.1948028862,(KAG0628798.1:0.2127972395,PTQ33908.1:0.153384
9687)[&Value="67.7/91"]:0.0311762448)[&Value="58.1/86"]:0.03017602)[&Value="38/91"]:
0.0520497057,KAH9322298.1:0.1668057358)[&Value="86.2/96"]:0.0516907758)[&Value="
94.1/94"]:0.1583376032)[&Value="99.4/100"]:0.7017841196,(XP_009032466.1:0.7729321
762,(XP_042920073.1:0.2870495967,((PWZ44616.1:0.3184612676,(XP_002309632.3:0.09
05098851,NP_001189935.1:0.1803919564)[&Value="81.9/100"]:0.0490309549)[&Value="7
8.4/96"]:0.0932095622,((EFJ19523.1:0.1704720089,(KAI5064281.1:0.093776096,(PTQ3455
6.1:0.1200872406,(KAG0561482.1:0.138862253,KAG0605142.1:0.1643639809)[&Value="9
3/95"]:0.0456016801)[&Value="39.7/93"]:0.0423762192)[&Value="78.7/82"]:0.0376822454
)[&Value="33.9/82"]:0.0242521226,KAH9308354.1:0.1441225186)[&Value="90.8/100"]:0.0
804610413)[&Value="91/97"]:0.122373081)[&Value="98.5/100"]:0.4112523768)[&Value="9
9.2/100"]:0.8483102803)[&Value="99.8/100"]:1.376798085,((XP_042916771.1:0.43833507
84,((((EFJ26018.1:0.6447217075,KAG0631008.1:0.1749760729)[&Value="88.9/99"]:0.0867
879353,(KAH9315399.1:0.1792728413,(CAD5311589.1:0.2317330601,XP_008649599.1:0.
3546230218)[&Value="99.8/100"]:0.2703026998)[&Value="99.3/99"]:0.1964010534)[&Valu
e="55.6/36"]:0.0422231597,OAE29693.1:0.2364293738)[&Value="69.5/36"]:0.1118568574
,KAI5059498.1:0.3034216337)[&Value="92.1/99"]:0.3370037717)[&Value="98.9/99"]:1.232
132924,((OUM66167.1:1.5518964497,((XP_001481516.1:0.2151515057,NP_009738.1:0.76
82446065)[&Value="51.9/89"]:0.1330704634,((XP_011392385.1:0.2562196373,XP_006462
464.1:0.1964200354)[&Value="99/100"]:0.2016166675,((XP_006676761.1:0.509106133,(K
NE73082.1:0.2778105845,KNE65701.1:0.2631179712)[&Value="100/100"]:0.5576848946)
[&Value="73.2/98"]:0.1061014325,KXN69997.1:0.5232181378)[&Value="79.9/96"]:0.11080
61943)[&Value="81.2/96"]:0.0734068046)[&Value="97.1/97"]:0.9597662016)[&Value="96/1
00"]:1.257240758,(XP_001745740.1:0.7648589753,(((NP_495161.1:0.5819996557,(((PAA7
5551.1:0.0134579779,PAA75258.1:0.000002)[&Value="100/100"]:0.4697038824,(((XP_006
819998.1:0.1420943096,(XP_030846906.1:0.1003715147,XP_030847518.1:0.0350880358)
[&Value="100/100"]:0.3383455301)[&Value="87.5/91"]:0.0722088037,((XP_002591612.1:0.
0131773964,XP_019628129.1:0.0010131549)[&Value="100/100"]:0.1818504922,NP_9963
57.1:0.4214599113)[&Value="39.6/79"]:0.0199947478)[&Value="91/86"]:0.0855845908,((N
P_001121726.1:0.0681012459,((XP_025929938.1:0.0093634954,(XP_015268039.1:0.0000
02,XP_028597443.1:0.0262092489)[&Value="93.2/100"]:0.0161186409)[&Value="73.9/99"]
:0.004310164,((XP_004482574.1:0.0046314045,NP_001272849.1:0.0185884511)[&Value=
"75.3/95"]:0.0046548336,(XP_006145367.1:0.000002,((XP_014400986.1:0.0092806659,N
P_001177198.1:0.0046448265)[&Value="69.3/100"]:0.0046361176,NP_001121132.1:0.018
6100766)[&Value="0/82"]:0.000002)[&Value="0/68"]:0.000002)[&Value="90.5/86"]:0.01459
71064)[&Value="98.4/99"]:0.0571521762)[&Value="96.9/100"]:0.0635434114,(XP_0172138
68.2.2:0.099919738,(NP_001016189.1:0.0515453491,(XP_028587453.1:0.0296567855,(XP
_025917892.1:0.0290331799,((((XP_004479029.1:0.0140145929,NP_284941.2.2:0.033770
7447)[&Value="85.1/91"]:0.0092817951,NP_001193437.1:0.0093454824)[&Value="77.3/90
"]:0.0047315008,XP_005883071.1:0.023509982)[&Value="73/89"]:0.0046344867,(NP_077
162.2.2:0.0193770382,XP_006162789.1:0.0088116258)[&Value="0/100"]:0.000906)[&Valu
e="91.8/98"]:0.0142025155)[&Value="94.4/100"]:0.02354811)[&Value="95.1/100"]:0.03597
80552)[&Value="88.7/100"]:0.0274012949)[&Value="92.2/100"]:0.0425549617)[&Value="9
8.1/100"]:0.116051194)[&Value="24.1/73"]:0.0296015711)[&Value="82.5/60"]:0.10731784

54,XP_002126852.1:0.2243032437)[&Value="18.4/60"]:0.0826205893)[&Value="91.9/69"]:
0.184420441,XP_004365821.1:0.4722062976)[&Value="83.3/95"]:0.1616493944,XP_0141
53836.1:0.5743593694)[&Value="24.1/77"]:0.2292745342)[&Value="100/100"]:2.24479295
3)[&Value="71.2/97"]:0.7038488834)[&Value="98.5/100"]:1.787199463)[&Value="64/95"]:0
.1290474387)[&Value="83.7/97"]:0.1859625962,(EFJ33653.1:0.1625300825,(EFJ28901.1:0.
267853618,((KAG0632288.1:0.2792739512,('KAG0555995.1':0.0479868302,(XP_02439106
1.1:0.0289596051,XP_024368367.1:0.0254622702)[&Value="100/100"]:0.1128177318)[&V
alue="98.2/100"]:0.0975952111)[&Value="97/100"]:0.1123768886,(OAE31801.1:0.308975
1024,(KAI5073815.1:0.2218658904,(((XP_008646219.1:0.0710686546,ACG47836.1:0.0839
368568)[&Value="90.1/100"]:0.0602386047,ONM04707.1:0.3334981803)[&Value="89.9/10
0"]:0.0648267247,((('KAG7649995.1':0.0190205517,'NP_172500.1':0.0564237848)[&Value
="42.8/96"]:0.0287475284,XP_006385192.1:0.1076091401)[&Value="79.6/100"]:0.051900
0541,KAH9330549.1:0.1535758113)[&Value="89.8/100"]:0.050669477)[&Value="98.4/100"
]:0.1032275709)[&Value="99.2/99"]:0.154714891)[&Value="24.3/95"]:0.0284648772)[&Val
ue="95.5/97"]:0.104409983)[&Value="76.1/96"]:0.1040670273)[&Value="100/100"]:1.8357
45642)[&Value="92.2/95"]:0.3238943329)[&Value="99.1/96"]:0.3822792082)[&Value="32.5
/70"]:0.0773438887,((((KXN72852.1:0.5168844304,KNE54706.1:0.2894232812)[&Value="8
6.3/12"]:0.0670167408,OUM67143.1:0.5064367029)[&Value="68.5/10"]:0.0367031945,((X
P_011389557.1:0.2005705427,XP_006459124.1:0.2321742582)[&Value="95.8/100"]:0.113
5546658,(XP_752563.1:0.239063103,NP_014854.2:0.6015544512)[&Value="95.6/100"]:0.
143436758)[&Value="92.2/99"]:0.0857886687)[&Value="86.6/11"]:0.1001978325,OAJ3840
4.1:0.1503256418)[&Value="100/100"]:1.17078468)[&Value="88.8/92"]:0.0895112331)[&V
alue="88.2/74"]:0.0680298324)[&Value="55.1/88"]:0.0454148905,((((NP_012926.1:0.2274
070465,KNE68830.1:0.1574914041)[&Value="95.4/95"]:0.0672589468,((XP_011389257.1:
0.1134998528,XP_006458578.1:0.2059790504)[&Value="71.9/99"]:0.0310993338,XP_748
106.1:0.1977492612)[&Value="36.3/95"]:0.0287105897)[&Value="75.4/94"]:0.0140851578,
(OUM62108.1:0.1347075269,OAJ44422.1:0.2368813015)[&Value="41.2/91"]:0.043825288
1)[&Value="82/90"]:0.0345376856,KXN66323.1:0.142054723)[&Value="100/100"]:0.25114
95021)[&Value="100/100"]:0.2787699847)[&Value="12.9/86"]:0.0155291834)[&Value="95.
9/94"]:0.0499526994);
end;
