# Supplementary material for: Antiviral Mx proteins have an ancient origin and widespread distribution among eukaryotes
Source: Proc Natl Acad Sci U S A. 2025 Jan 24;122(4):e2416811122. doi: 10.1073/pnas.2416811122 (PMC11789081; doi:10.1073/pnas.2416811122)
Supplement: Supplementary file 15 — Dataset S14 (PDF) [file pnas.2416811122.sd14.pdf]

Dataset S14. Suppl_Figure_3_FastTree

#NEXUS
begin taxa;
dimensions ntax=32;
taxlabels
KAH9290598[&"% Acidic Amino Acids"=14.03%,"% Hydrophobic Amino Acids"=48.21%,Description="hypothetical protein KI387_034715 [Taxus chinensis]","Genetic Code"="Standard",Modified=Mon Aug 12 11:04:35 PDT 2024,Taxonomy="Eukaryota; Viridiplantae; Streptophyta; Embryophyta; Tracheophyta; Spermatophyta; Pinopsida; Pinidae; Conifers II; Cupressales; Taxaceae; Taxus","Extinction Coefficient"=42915.0,dev_stage="mature",Created=Tue Jan 25 00:00:00 PST 2022,isolate="Ta-2019","Isoelectric Point"=6.566585540771484,"% Basic Amino Acids"=15.07%,"% AT-rich Amino Acids"=26.12%,"% Charged Amino Acids"=29.10%,db_xref="taxon:29808",Organism="Taxus chinensis",Accession="KAH9290598.1","% GC-rich Amino Acids"=22.09%,chromosome="10",sex="female","Molecular Weight (kDa)"=75.33005738000003,"# Nucleotide Sequences With Quality"=0,Topology="linear","Charge at pH 7"=-1.8151639582550025,"% Polar Uncharged Amino Acids"=22.99%,tissue_type="Leaf","Molecule Type"="AA"]
XP_002297993[&"% Acidic Amino Acids"=13.50%,"% Hydrophobic Amino Acids"=49.06%,Description="dynamin-related protein 4C [Populus trichocarpa]","Genetic Code"="Standard",Modified=Mon Aug 12 11:04:33 PDT 2024,Taxonomy="Eukaryota; Viridiplantae; Streptophyta; Embryophyta; Tracheophyta; Spermatophyta; Magnoliopsida; eudicotyledons; Gunneridae; Pentapetalae; rosids; fabids; Malpighiales; Salicaceae; Saliceae; Populus","Common Name"="Populus balsamifera subsp. trichocarpa","Extinction Coefficient"=54360.0,Created=Thu Dec 08 00:00:00 PST 2022,isolate="Nisqually-1","Isoelectric Point"=5.398387908935547,"% Basic Amino Acids"=13.35%,"% AT-rich Amino Acids"=22.21%,"% Charged Amino Acids"=26.85%,db_xref="taxon:3694",Organism="Populus trichocarpa",Accession="XP_002297993.1","% GC-rich Amino Acids"=21.34%,chromosome="1","Molecular Weight (kDa)"=77.44427127999998,"# Nucleotide Sequences With Quality"=0,Topology="linear","Charge at pH 7"=-15.136533230290999,"% Polar Uncharged Amino Acids"=24.96%,"Molecule Type"="AA"]
KAF5727250[&"% Acidic Amino Acids"=13.47%,"% Hydrophobic Amino Acids"=49.55%,Description="hypothetical protein HS088_TW22G00939 [Tripterygium wilfordii]","Genetic Code"="Standard",Modified=Mon Aug 12 11:04:35 PDT 2024,Taxonomy="Eukaryota; Viridiplantae; Streptophyta; Embryophyta; Tracheophyta; Spermatophyta; Magnoliopsida; eudicotyledons; Gunneridae; Pentapetalae; rosids; fabids; Celastrales; Celastraceae; Tripterygium","Extinction Coefficient"=37985.0,dev_stage="mature plant",Created=Fri Jul 10 00:00:00 PDT 2020,isolate="XIE 37","Isoelectric Point"=5.683628082275391,"% Basic Amino Acids"=13.92%,"% AT-rich Amino Acids"=22.60%,"% Charged Amino Acids"=27.40%,db_xref="taxon:458696",Organism="Tripterygium

wilfordii",Accession="KAF5727250.1","% GC-rich Amino Acids"=19.91%,chromosome="22","Molecular Weight (kDa)"=74.60867358,"# Nucleotide Sequences With Quality"=0,Topology="linear","Charge at pH 7"=-12.178492403730495,"% Polar Uncharged Amino Acids"=23.50%,tissue_type="leaf","Molecule Type"="AA"]

XP_002303204[&"% Acidic Amino Acids"=13.80%,"% Hydrophobic Amino Acids"=48.02%,Description="dynamin-related protein 4C isoform X2 [Populus trichocarpa]","Genetic Code"="Standard",Modified=Mon Aug 12 11:04:36 PDT 2024,Taxonomy="Eukaryota; Viridiplantae; Streptophyta; Embryophyta; Tracheophyta; Spermatophyta; Magnoliopsida; eudicotyledons; Gunneridae; Pentapetalae; rosids; fabids; Malpighiales; Salicaceae; Saliceae; Populus","Common Name"="Populus balsamifera subsp. trichocarpa","Extinction Coefficient"=43360.0,Created=Thu Dec 08 00:00:00 PST 2022,isolate="Nisqually-1","Isoelectric Point"=5.552570343017578,"% Basic Amino Acids"=14.10%,"% AT-rich Amino Acids"=23.35%,"% Charged Amino Acids"=27.90%,db_xref="taxon:3694",Organism="Populus trichocarpa",Accession="XP_002303204.3","% GC-rich Amino Acids"=20.12%,chromosome="3","Molecular Weight (kDa)"=76.48913967999991,"# Nucleotide Sequences With Quality"=0,Topology="linear","Charge at pH 7"=-16.56672690033052,"% Polar Uncharged Amino Acids"=24.67%,"Molecule Type"="AA"]

XP_024439231[&"% Acidic Amino Acids"=13.46%,"% Hydrophobic Amino Acids"=48.22%,Description="dynamin-related protein 4C [Populus trichocarpa]","Genetic Code"="Standard",Modified=Mon Aug 12 11:04:35 PDT 2024,Taxonomy="Eukaryota; Viridiplantae; Streptophyta; Embryophyta; Tracheophyta; Spermatophyta; Magnoliopsida; eudicotyledons; Gunneridae; Pentapetalae; rosids; fabids; Malpighiales; Salicaceae; Saliceae; Populus","Common Name"="Populus balsamifera subsp. trichocarpa","Extinction Coefficient"=43360.0,Created=Thu Dec 08 00:00:00 PST 2022,isolate="Nisqually-1","Isoelectric Point"=6.389705657958984,"% Basic Amino Acids"=15.68%,"% AT-rich Amino Acids"=24.56%,"% Charged Amino Acids"=29.14%,db_xref="taxon:3694",Organism="Populus trichocarpa",Accession="XP_024439231.1","% GC-rich Amino Acids"=19.82%,chromosome="13","Molecular Weight (kDa)"=75.87577707999995,"# Nucleotide Sequences With Quality"=0,Topology="linear","Charge at pH 7"=-5.450467939091386,"% Polar Uncharged Amino Acids"=23.22%,"Molecule Type"="AA"]

KAF8391993[&"% Acidic Amino Acids"=11.81%,"% Hydrophobic Amino Acids"=48.69%,Description="hypothetical protein HHK36_022333 [Tetracentron sinense]","Genetic Code"="Standard",Modified=Mon Aug 12 11:04:35 PDT 2024,Taxonomy="Eukaryota; Viridiplantae; Streptophyta; Embryophyta; Tracheophyta; Spermatophyta; Magnoliopsida; Trochodendrales; Trochodendraceae; Tetracentron","Extinction Coefficient"=42330.0,Created=Fri Oct 30 00:00:00 PDT 2020,isolate="YNK0","Isoelectric Point"=6.575557708740234,"% Basic Amino Acids"=14.96%,"% AT-rich Amino Acids"=23.23%,"% Charged Amino Acids"=26.77%,db_xref="taxon:13715",Organism="Tetracentron sinense",Accession="KAF8391993.1","% GC-rich Amino Acids"=20.87%,chromosome="16","Molecular Weight (kDa)"=85.30159378000003,"# Nucleotide Sequences With Quality"=0,Topology="linear","Charge at pH 7"=-

4.4926875270876785,"% Polar Uncharged Amino Acids"=24.93%,tissue_type="leaf","Molecule Type"="AA"]
XP_058079501[&"% Acidic Amino Acids"=13.84%,"% Hydrophobic Amino Acids"=47.62%,Description="dynamin-related protein 4C-like [Magnolia sinica]","Genetic Code"="Standard",Modified=Mon Aug 12 11:04:36 PDT 2024,Taxonomy="Eukaryota; Viridiplantae; Streptophyta; Embryophyta; Tracheophyta; Spermatophyta; Magnoliopsida; Magnoliidae; Magnoliales; Magnoliaceae; Magnolia","Extinction Coefficient"=36370.0,Created=Wed Jul 19 00:00:00 PDT 2023,isolate="HGM2019","Isoelectric Point"=5.708515167236328,"% Basic Amino Acids"=14.29%,"% AT-rich Amino Acids"=23.36%,"% Charged Amino Acids"=28.13%,db_xref="taxon:86752",Organism="Magnolia sinica",Accession="XP_058079501.1","% GC-rich Amino Acids"=18.60%,chromosome="15","Molecular Weight (kDa)"=75.59962478000001,"# Nucleotide Sequences With Quality"=0,Topology="linear","Charge at pH 7"=-11.143011250633858,"% Polar Uncharged Amino Acids"=24.70%,tissue_type="leaf","Molecule Type"="AA"]
KAH0683503[&"% Acidic Amino Acids"=13.63%,"% Hydrophobic Amino Acids"=49.19%,Description="hypothetical protein KY289_021255 [Solanum tuberosum]","Genetic Code"="Standard",Modified=Mon Aug 12 11:04:35 PDT 2024,Taxonomy="Eukaryota; Viridiplantae; Streptophyta; Embryophyta; Tracheophyta; Spermatophyta; Magnoliopsida; eudicotyledons; Gunneridae; Pentapetalae; asterids; lamiids; Solanales; Solanaceae; Solanoideae; Solaneae; Solanum","Common Name"="potato","Extinction Coefficient"=42455.0,Created=Tue Sep 28 00:00:00 PDT 2021,isolate="solTubOtavaFocal","Isoelectric Point"=5.458683013916016,"% Basic Amino Acids"=13.63%,"% AT-rich Amino Acids"=25.19%,"% Charged Amino Acids"=27.26%,db_xref="taxon:4113",Organism="Solanum tuberosum",Accession="KAH0683503.1","% GC-rich Amino Acids"=19.85%,chromosome="4","Molecular Weight (kDa)"=75.89402137999996,"# Nucleotide Sequences With Quality"=0,Topology="linear",cultivar="Otava","Charge at pH 7"=-14.213393667195508,"% Polar Uncharged Amino Acids"=24.00%,tissue_type="leaves","Molecule Type"="AA"]
PWZ56864[&"% Acidic Amino Acids"=13.20%,"% Hydrophobic Amino Acids"=49.55%,Description="Dynamin-related protein 4C [Zea mays]","Genetic Code"="Standard",Modified=Mon Aug 12 11:04:35 PDT 2024,Taxonomy="Eukaryota; Viridiplantae; Streptophyta; Embryophyta; Tracheophyta; Spermatophyta; Magnoliopsida; Liliopsida; Poales; Poaceae; PACMAD clade; Panicoideae; Andropogonodae; Andropogoneae; Tripsacinae; Zea","Extinction Coefficient"=37985.0,dev_stage="14-day seedling",Created=Mon Jun 04 00:00:00 PDT 2018,"Isoelectric Point"=6.323802947998047,"% Basic Amino Acids"=14.24%,"% AT-rich Amino Acids"=19.44%,"% Charged Amino Acids"=27.45%,db_xref="taxon:4577",Organism="Zea mays",Accession="PWZ56864.1","% GC-rich Amino Acids"=24.48%,chromosome="1","Molecular Weight (kDa)"=74.55908788,"# Nucleotide Sequences With Quality"=0,Topology="linear",cultivar="inbred line Mo17","Charge at pH

7"=-3.612842663651275,"% Polar Uncharged Amino Acids"=23.44%,tissue_type="seedling","Molecule Type"="AA"]
PWZ56863[&"% Acidic Amino Acids"=13.65%,"% Hydrophobic Amino Acids"=50.00%,Description="Dynamin-related protein 4C [Zea mays]","Genetic Code"="Standard",Modified=Mon Aug 12 11:04:35 PDT 2024,Taxonomy="Eukaryota; Viridiplantae; Streptophyta; Embryophyta; Tracheophyta; Spermatophyta; Magnoliopsida; Liliopsida; Poales; Poaceae; PACMAD clade; Panicoideae; Andropogonodae; Andropogoneae; Tripsacinae; Zea","Extinction Coefficient"=35340.0,dev_stage="14-day seedling",Created=Mon Jun 04 00:00:00 PDT 2018,"Isoelectric Point"=5.948627471923828,"% Basic Amino Acids"=14.24%,"% AT-rich Amino Acids"=21.07%,"% Charged Amino Acids"=27.89%,db_xref="taxon:4577",Organism="Zea mays",Accession="PWZ56863.1","% GC-rich Amino Acids"=24.18%,chromosome="1","Molecular Weight (kDa)"=74.49698797999999,"# Nucleotide Sequences With Quality"=0,Topology="linear",cultivar="inbred line Mo17","Charge at pH 7"=-6.4788212421916676,"% Polar Uncharged Amino Acids"=22.40%,tissue_type="seedling","Molecule Type"="AA"]
XP_038984915[&"% Acidic Amino Acids"=14.39%,"% Hydrophobic Amino Acids"=50.45%,Description="dynamin-related protein 4C-like [Phoenix dactylifera]","Genetic Code"="Standard",Modified=Mon Aug 12 11:04:35 PDT 2024,Taxonomy="Eukaryota; Viridiplantae; Streptophyta; Embryophyta; Tracheophyta; Spermatophyta; Magnoliopsida; Liliopsida; Arecaceae; Coryphoideae; Phoeniceae; Phoenix","Common Name"="date palm","Extinction Coefficient"=37735.0,Created=Wed Jan 27 00:00:00 PST 2021,"Isoelectric Point"=5.626003265380859,"% Basic Amino Acids"=14.70%,"% AT-rich Amino Acids"=20.61%,"% Charged Amino Acids"=29.09%,db_xref="taxon:42345",Organism="Phoenix dactylifera",Accession="XP_038984915.1","% GC-rich Amino Acids"=25.00%,chromosome="8",sex="male","Molecular Weight (kDa)"=73.78827977999997,"# Nucleotide Sequences With Quality"=0,Topology="linear",cultivar="Barhee BC4","Charge at pH 7"=-12.005012141875643,"% Polar Uncharged Amino Acids"=20.91%,tissue_type="young leaves","Molecule Type"="AA"]
KAH9320939[&"% Acidic Amino Acids"=14.55%,"% Hydrophobic Amino Acids"=48.85%,Description="hypothetical protein KI387_015578 [Taxus chinensis]","Genetic Code"="Standard",Modified=Mon Aug 12 11:04:35 PDT 2024,Taxonomy="Eukaryota; Viridiplantae; Streptophyta; Embryophyta; Tracheophyta; Spermatophyta; Pinopsida; Pinidae; Conifers II; Cupressales; Taxaceae; Taxus","Extinction Coefficient"=29630.0,dev_stage="mature",Created=Tue Jan 25 00:00:00 PST 2022,isolate="Ta-2019","Isoelectric Point"=5.030315399169922,"% Basic Amino Acids"=13.40%,"% AT-rich Amino Acids"=21.76%,"% Charged Amino Acids"=27.95%,db_xref="taxon:29808",Organism="Taxus chinensis",Accession="KAH9320939.1","% GC-rich Amino Acids"=21.18%,chromosome="5",sex="female","Molecular Weight (kDa)"=77.18898967999996,"# Nucleotide Sequences With

Quality"=0,Topology="linear","Charge at pH 7"=-19.627328951892576,"% Polar Uncharged Amino Acids"=23.49%,tissue_type="Leaf","Molecule Type"="AA"]

KAH9325151[&"% Acidic Amino Acids"=10.60%,"% Hydrophobic Amino Acids"=49.31%,Description="hypothetical protein KI387_005329, partial [Taxus chinensis]","Genetic Code"="Standard",Modified=Mon Aug 12 11:04:35 PDT 2024,Taxonomy="Eukaryota; Viridiplantae; Streptophyta; Embryophyta; Tracheophyta; Spermatophyta; Pinopsida; Pinidae; Conifers II; Cupressales; Taxaceae; Taxus","Extinction Coefficient"=13200.0,dev_stage="mature",Created=Tue Jan 25 00:00:00 PST 2022,isolate="Ta-2019","Isoelectric Point"=5.268238067626953,"% Basic Amino Acids"=10.14%,"% AT-rich Amino Acids"=29.49%,"% Charged Amino Acids"=20.74%,db_xref="taxon:29808",Organism="Taxus chinensis",Accession="KAH9325151.1","% GC-rich Amino Acids"=16.13%,chromosome="2",sex="female","Molecular Weight (kDa)"=23.75958078000002,"# Nucleotide Sequences With Quality"=0,Topology="linear","Charge at pH 7"=-2.2470332478955557,"% Polar Uncharged Amino Acids"=30.41%,tissue_type="Leaf","Molecule Type"="AA"]

KAK1401877[&"% Acidic Amino Acids"=14.12%,"% Hydrophobic Amino Acids"=48.91%,Description="Dynamin-related protein 4C [Heracleum sosnowskyi]","Genetic Code"="Standard",Modified=Mon Aug 12 11:04:36 PDT 2024,Taxonomy="Eukaryota; Viridiplantae; Streptophyta; Embryophyta; Tracheophyta; Spermatophyta; Magnoliopsida; eudicotyledons; Gunneridae; Pentapetalae; asterids; campanulids; Apiales; Apiaceae; Apioideae; apioid superclade; Tordylieae; Tordyliinae; Heracleum","Extinction Coefficient"=47495.0,dev_stage="plant at anthesis",Created=Mon Aug 21 00:00:00 PDT 2023,isolate="Hsosn_3","Isoelectric Point"=5.616550445556641,"% Basic Amino Acids"=14.41%,"% AT-rich Amino Acids"=23.58%,"% Charged Amino Acids"=28.53%,db_xref="taxon:360622",Organism="Heracleum sosnowskyi",Accession="KAK1401877.1","% GC-rich Amino Acids"=19.07%,chromosome="1","Molecular Weight (kDa)"=77.03545468,"# Nucleotide Sequences With Quality"=0,Topology="linear","Charge at pH 7"=-13.104045396861586,"% Polar Uncharged Amino Acids"=23.29%,tissue_type="leaf","Molecule Type"="AA"]

KAH9300179[&"% Acidic Amino Acids"=11.70%,"% Hydrophobic Amino Acids"=48.54%,Description="hypothetical protein KI387_011762, partial [Taxus chinensis]","Genetic Code"="Standard",Modified=Mon Aug 12 11:04:35 PDT 2024,Taxonomy="Eukaryota; Viridiplantae; Streptophyta; Embryophyta; Tracheophyta; Spermatophyta; Pinopsida; Pinidae; Conifers II; Cupressales; Taxaceae; Taxus","Extinction Coefficient"=13075.0,dev_stage="mature",Created=Tue Jan 25 00:00:00 PST 2022,isolate="Ta-2019","Isoelectric Point"=5.203029632568359,"% Basic Amino Acids"=11.11%,"% AT-rich Amino Acids"=25.15%,"% Charged Amino Acids"=22.81%,db_xref="taxon:29808",Organism="Taxus chinensis",Accession="KAH9300179.1","% GC-rich Amino Acids"=21.05%,chromosome="4",sex="female","Molecular Weight (kDa)"=19.071952580000012,"# Nucleotide Sequences With Quality"=0,Topology="linear","Charge at pH 7"=-2.1811466196014724,"% Polar Uncharged Amino Acids"=29.24%,tissue_type="Leaf","Molecule Type"="AA"]

KAH9314974[&"% Acidic Amino Acids"=10.89%,"% Hydrophobic Amino Acids"=45.97%,Description="hypothetical protein KI387_023601, partial [Taxus chinensis]","Genetic Code"="Standard",Modified=Mon Aug 12 11:04:35 PDT 2024,Taxonomy="Eukaryota; Viridiplantae; Streptophyta; Embryophyta; Tracheophyta; Spermatophyta; Pinopsida; Pinidae; Conifers II; Cupressales; Taxaceae; Taxus","Extinction Coefficient"=30285.0,dev_stage="mature",Created=Tue Jan 25 00:00:00 PST 2022,isolate="Ta-2019","Isoelectric Point"=7.393680572509766,"% Basic Amino Acids"=12.50%,"% AT-rich Amino Acids"=25.81%,"% Charged Amino Acids"=23.39%,db_xref="taxon:29808",Organism="Taxus chinensis",Accession="KAH9314974.1","% GC-rich Amino Acids"=19.76%,chromosome="7",sex="female","Molecular Weight (kDa)"=27.935113980000025,"# Nucleotide Sequences With Quality"=0,Topology="linear","Charge at pH 7"=0.8143130361885939,"% Polar Uncharged Amino Acids"=31.85%,tissue_type="Leaf","Molecule Type"="AA"]

KAF8079489[&"% Acidic Amino Acids"=13.13%,"% Hydrophobic Amino Acids"=48.21%,Description="hypothetical protein N665_1024s0016 [Sinapis alba]","Genetic Code"="Standard",Modified=Thu Oct 22 00:00:00 PDT 2020,Taxonomy="Eukaryota; Viridiplantae; Streptophyta; Embryophyta; Tracheophyta; Spermatophyta; Magnoliopsida; eudicotyledons; Gunneridae; Pentapetalae; rosids; malvids; Brassicales; Brassicaceae; Brassiceae; Sinapis","Common Name"="white mustard","Extinction Coefficient"=43360.0,Created=Thu Oct 22 00:00:00 PDT 2020,"Isoelectric Point"=6.418010711669922,"% Basic Amino Acids"=14.48%,"% AT-rich Amino Acids"=23.58%,"% Charged Amino Acids"=27.61%,strain="S2 GC0560-79",db_xref="taxon:3728",Organism="Sinapis alba",Accession="KAF8079489.1","% GC-rich Amino Acids"=19.85%,chromosome="Unknown","Molecular Weight (kDa)"=74.74961017999996,"# Nucleotide Sequences With Quality"=0,Topology="linear","Charge at pH 7"=-3.279498820243248,"% Polar Uncharged Amino Acids"=24.78%,tissue_type="green leaf","Molecule Type"="AA",isolation_source="green leaf"]

OAP13972[&"% Acidic Amino Acids"=10.76%,"% Hydrophobic Amino Acids"=51.27%,Description="hypothetical protein AXX17_AT1G53540 [Arabidopsis thaliana]","Genetic Code"="Standard",Modified=Mon Aug 12 11:04:34 PDT 2024,Taxonomy="Eukaryota; Viridiplantae; Streptophyta; Embryophyta; Tracheophyta; Spermatophyta; Magnoliopsida; eudicotyledons; Gunneridae; Pentapetalae; rosids; malvids; Brassicales; Brassicaceae; Camelineae; Arabidopsis","Common Name"="thale cress","Extinction Coefficient"=10095.0,dev_stage="adult",Created=Wed May 25 00:00:00 PDT 2016,"Isoelectric Point"=7.011775970458984,"% Basic Amino Acids"=12.66%,"% AT-rich Amino Acids"=18.99%,"% Charged Amino Acids"=23.42%,db_xref="taxon:3702",Organism="Arabidopsis thaliana",Accession="OAP13972.1","% GC-rich Amino Acids"=25.95%,chromosome="1","Molecular Weight (kDa)"=17.169832280000012,"# Nucleotide Sequences With Quality"=0,Topology="linear","Charge at pH 7"=0.015152986826631687,"% Polar Uncharged Amino Acids"=25.95%,tissue_type="leaf","Molecule Type"="AA"]

OAP13353[&"% Acidic Amino Acids"=10.81%,"% Hydrophobic Amino Acids"=50.68%,Description="hypothetical protein AXX17_AT1G53610 [Arabidopsis thaliana]","Genetic Code"="Standard",Modified=Mon Aug 12 11:04:34 PDT 2024,Taxonomy="Eukaryota; Viridiplantae; Streptophyta; Embryophyta; Tracheophyta; Spermatophyta; Magnoliopsida; eudicotyledons; Gunneridae; Pentapetalae; rosids; malvids; Brassicales; Brassicaceae; Camelineae; Arabidopsis","Common Name"="thale cress","Extinction Coefficient"=8605.0,dev_stage="adult",Created=Wed May 25 00:00:00 PDT 2016,"Isoelectric Point"=6.233654022216797,"% Basic Amino Acids"=12.84%,"% AT-rich Amino Acids"=16.89%,"% Charged Amino Acids"=23.65%,db_xref="taxon:3702",Organism="Arabidopsis thaliana",Accession="OAP13353.1","% GC-rich Amino Acids"=25.68%,chromosome="1","Molecular Weight (kDa)"=16.16756978000001,"# Nucleotide Sequences With Quality"=0,Topology="linear","Charge at pH 7"=-1.719159906185574,"% Polar Uncharged Amino Acids"=26.35%,tissue_type="leaf","Molecule Type"="AA"]

OAP19580[&"% Acidic Amino Acids"=12.50%,"% Hydrophobic Amino Acids"=49.38%,Description="DRP4A [Arabidopsis thaliana]","Genetic Code"="Standard",Modified=Mon Aug 12 11:04:34 PDT 2024,Taxonomy="Eukaryota; Viridiplantae; Streptophyta; Embryophyta; Tracheophyta; Spermatophyta; Magnoliopsida; eudicotyledons; Gunneridae; Pentapetalae; rosids; malvids; Brassicales; Brassicaceae; Camelineae; Arabidopsis","Common Name"="thale cress","Extinction Coefficient"=10220.0,dev_stage="adult",Created=Wed May 25 00:00:00 PDT 2016,"Isoelectric Point"=4.586406707763672,"% Basic Amino Acids"=10.00%,"% AT-rich Amino Acids"=17.50%,"% Charged Amino Acids"=22.50%,db_xref="taxon:3702",Organism="Arabidopsis thaliana",Accession="OAP19580.1","% GC-rich Amino Acids"=23.13%,chromosome="1","Molecular Weight (kDa)"=17.32986838000002,"# Nucleotide Sequences With Quality"=0,Topology="linear","Charge at pH 7"=-5.245775862197959,"% Polar Uncharged Amino Acids"=28.75%,tissue_type="leaf","Molecule Type"="AA"]

XP_024380180[&"% Charged Amino Acids"=27.84%,"% Acidic Amino Acids"=13.62%,db_xref="taxon:3218",Organism="Physcomitrium patens","% Hydrophobic Amino Acids"=47.01%,Description="dynamin-related protein 4C-like [Physcomitrium patens]","Genetic Code"="Standard",Modified=Mon Aug 12 11:04:35 PDT 2024,Accession="XP_024380180.1","% GC-rich Amino Acids"=21.86%,Taxonomy="Eukaryota; Viridiplantae; Streptophyta; Embryophyta; Bryophyta; Bryophytina; Bryopsida; Funariidae; Funariales; Funariaceae; Physcomitrium",chromosome="7","Extinction Coefficient"=29380.0,"Molecular Weight (kDa)"=75.34764137999996,"# Nucleotide Sequences With Quality"=0,Topology="linear",Created=Wed Apr 04 00:00:00 PDT 2018,"Charge at pH 7"=-11.043197193814624,"Isoelectric Point"=5.759677886962891,"% Basic Amino Acids"=14.22%,"% AT-rich Amino Acids"=20.66%,"% Polar Uncharged Amino Acids"=25.45%,"Molecule Type"="AA"]

XP_024367947[&"% Charged Amino Acids"=28.82%,"% Acidic Amino Acids"=13.85%,db_xref="taxon:3218",Organism="Physcomitrium patens","% Hydrophobic Amino Acids"=47.45%,Description="dynamin-related protein 4C-like, partial [Physcomitrium patens]","Genetic Code"="Standard",Modified=Mon Aug 12 11:04:35 PDT 2024,Accession="XP_024367947.1","% GC-rich Amino Acids"=22.77%,Taxonomy="Eukaryota; Viridiplantae; Streptophyta; Embryophyta; Bryophyta; Bryophytina; Bryopsida; Funariidae; Funariales; Funariaceae; Physcomitrium",chromosome="Unknown","Extinction Coefficient"=27765.0,"Molecular Weight (kDa)"=70.98869067999993,"# Nucleotide Sequences With Quality"=0,Topology="linear",Created=Wed Apr 04 00:00:00 PDT 2018,"Charge at pH 7"=-8.811216914008607,"Isoelectric Point"=5.974689483642578,"% Basic Amino Acids"=14.97%,"% AT-rich Amino Acids"=20.38%,"% Polar Uncharged Amino Acids"=24.04%,"Molecule Type"="AA"]

KAG0561847[&"% Acidic Amino Acids"=13.88%,"% Hydrophobic Amino Acids"=46.46%,Description="hypothetical protein KC19_9G097200 [Ceratodon purpureus]","Genetic Code"="Standard",Modified=Mon Aug 12 11:04:35 PDT 2024,Taxonomy="Eukaryota; Viridiplantae; Streptophyta; Embryophyta; Bryophyta; Bryophytina; Bryopsida; Dicranidae; Pseudoditrichales; Ditrichaceae; Ceratodon","Extinction Coefficient"=39475.0,Created=Mon Dec 28 00:00:00 PST 2020,"Isoelectric Point"=5.606029510498047,"% Basic Amino Acids"=14.03%,"% AT-rich Amino Acids"=19.76%,"% Charged Amino Acids"=27.90%,strain="R40",db_xref="taxon:3225",Organism="Ceratodon purpureus",Accession="KAG0561847.1","% GC-rich Amino Acids"=22.62%,chromosome="9",sex="male","Molecular Weight (kDa)"=74.42137688,"# Nucleotide Sequences With Quality"=0,Topology="linear","Charge at pH 7"=-10.506836906116629,"% Polar Uncharged Amino Acids"=26.09%,"Molecule Type"="AA"]

KAG0619429[&"% Acidic Amino Acids"=14.56%,"% Hydrophobic Amino Acids"=46.25%,Description="hypothetical protein M758_4G139100 [Ceratodon purpureus]","Genetic Code"="Standard",Modified=Mon Aug 12 11:04:35 PDT 2024,Taxonomy="Eukaryota; Viridiplantae; Streptophyta; Embryophyta; Bryophyta; Bryophytina; Bryopsida; Dicranidae; Pseudoditrichales; Ditrichaceae; Ceratodon","Extinction Coefficient"=35465.0,Created=Mon Dec 28 00:00:00 PST 2020,"Isoelectric Point"=5.657138824462891,"% Basic Amino Acids"=15.02%,"% AT-rich Amino Acids"=21.92%,"% Charged Amino Acids"=29.58%,strain="GG1",db_xref="taxon:3225",Organism="Ceratodon purpureus",Accession="KAG0619429.1","% GC-rich Amino Acids"=20.27%,chromosome="4",sex="female","Molecular Weight (kDa)"=75.43685337999996,"# Nucleotide Sequences With Quality"=0,Topology="linear","Charge at pH 7"=-13.015769556202113,"% Polar Uncharged Amino Acids"=24.47%,"Molecule Type"="AA"]

KAJ7294545[&"% Charged Amino Acids"=29.50%,"% Acidic Amino Acids"=13.92%,db_xref="taxon:34168",Organism="Diphasiastrum complanatum","% Hydrophobic Amino Acids"=46.29%,Description="hypothetical protein O6H91_Y251600 [Diphasiastrum complanatum]","Genetic Code"="Standard",Modified=Mon Mar 20

00:00:00 PDT 2023,Accession="KAJ7294545.1","% GC-rich Amino Acids"=20.88%,Taxonomy="Eukaryota; Viridiplantae; Streptophyta; Embryophyta; Tracheophyta; Lycopodiopsida; Lycopodiales; Lycopodiaceae; Lycopodioideae; Diphasiastrum",chromosome="Unknown","Extinction Coefficient"=42205.0,"Molecular Weight (kDa)"=75.28553777999998,"# Nucleotide Sequences With Quality"=0,Topology="linear",Created=Mon Mar 20 00:00:00 PDT 2023,cultivar="PW_Plant_1","Charge at pH 7"=-5.713675474010753,"Isoelectric Point"=6.275577545166016,"% Basic Amino Acids"=15.58%,"% AT-rich Amino Acids"=23.90%,"% Polar Uncharged Amino Acids"=24.66%,"Molecule Type"="AA"]

KAH9291961[&"% Acidic Amino Acids"=13.11%,"% Hydrophobic Amino Acids"=46.45%,Description="hypothetical protein KI387_042849 [Taxus chinensis]","Genetic Code"="Standard",Modified=Mon Aug 12 11:04:35 PDT 2024,Taxonomy="Eukaryota; Viridiplantae; Streptophyta; Embryophyta; Tracheophyta; Spermatophyta; Pinopsida; Pinidae; Conifers II; Cupressales; Taxaceae; Taxus","Extinction Coefficient"=40505.0,dev_stage="mature",Created=Tue Jan 25 00:00:00 PST 2022,isolate="Ta-2019","Isoelectric Point"=5.592037200927734,"% Basic Amino Acids"=13.11%,"% AT-rich Amino Acids"=22.43%,"% Charged Amino Acids"=26.23%,db_xref="taxon:29808",Organism="Taxus chinensis",Accession="KAH9291961.1","% GC-rich Amino Acids"=19.36%,sex="female","Molecular Weight (kDa)"=91.35399638000017,"# Nucleotide Sequences With Quality"=0,Topology="linear","Charge at pH 7"=-10.58385806878805,"% Polar Uncharged Amino Acids"=27.82%,tissue_type="Leaf","Molecule Type"="AA"]

GJP35534[&"% Charged Amino Acids"=25.64%,strain="NIES-68","% Acidic Amino Acids"=12.97%,db_xref="taxon:2019903",Organism="Closterium sp. NIES-68","% Hydrophobic Amino Acids"=49.77%,Description="hypothetical protein CLOM_g20043 [Closterium sp. NIES-68]","Genetic Code"="Standard",Modified=Mon Aug 12 11:04:36 PDT 2024,Accession="GJP35534.1","% GC-rich Amino Acids"=25.19%,Taxonomy="Eukaryota; Viridiplantae; Streptophyta; Zygnemophyceae; Zygnematophycidae; Desmidiales; Closteriaceae; Closterium; Closterium peracerosum-strigosum-littorale complex","Extinction Coefficient"=27110.0,"Molecular Weight (kDa)"=72.93767118000008,"# Nucleotide Sequences With Quality"=0,Topology="linear",culture_collection="NIES:68",Created=Tue Dec 27 00:00:00 PST 2022,"Charge at pH 7"=-12.75043257997837,"Isoelectric Point"=5.318119049072266,"% Basic Amino Acids"=12.67%,"% AT-rich Amino Acids"=20.21%,"% Polar Uncharged Amino Acids"=24.74%,"Molecule Type"="AA"]

CAI5480041[&"% Charged Amino Acids"=27.08%,strain="NIES-4552=Yama58-4","% Acidic Amino Acids"=14.06%,note="contig: tig00006298",db_xref="taxon:2996821",Organism="Closterium sp. Yama58-4","% Hydrophobic Amino Acids"=47.74%,Description="unnamed protein product [Closterium sp. Yama58-4]","Genetic Code"="Standard",Modified=Sun Jun 18 00:00:00 PDT 2023,Accession="CAI5480041.1","% GC-rich Amino Acids"=23.61%,Taxonomy="Eukaryota; Viridiplantae; Streptophyta; Zygnemophyceae; Zygnematophycidae; Desmidiales; Closteriaceae; Closterium; Closterium peracerosum-strigosum-littorale complex","Extinction Coefficient"=29715.0,"Molecular Weight

(kDa)"=63.69994967999995,"# Nucleotide Sequences With Quality"=0,Topology="linear",Created=Sun Jun 18 00:00:00 PDT 2023,"Charge at pH 7"=-11.91625458415476,"Isoelectric Point"=5.015522003173828,"% Basic Amino Acids"=13.02%,"% AT-rich Amino Acids"=22.40%,"% Polar Uncharged Amino Acids"=25.35%,"Molecule Type"="AA"]

EFJ22917[&"% Charged Amino Acids"=28.89%,"% Acidic Amino Acids"=12.59%,db_xref="taxon:88036",Organism="Selaginella moellendorffii","% Hydrophobic Amino Acids"=44.63%,Description="hypothetical protein SELMODRAFT_104286 [Selaginella moellendorffii]","Genetic Code"="Standard",Modified=Mon Jul 25 00:00:00 PDT 2016,Accession="EFJ22917.1","% GC-rich Amino Acids"=26.67%,Taxonomy="Eukaryota; Viridiplantae; Streptophyta; Embryophyta; Tracheophyta; Lycopodiopsida; Selaginellales; Selaginellaceae; Selaginella",chromosome="Unknown","Extinction Coefficient"=39015.0,"Molecular Weight (kDa)"=60.320899180000005,"# Nucleotide Sequences With Quality"=0,Topology="linear",Created=Mon Jul 25 00:00:00 PDT 2016,"Charge at pH 7"=9.376331029545756,"Isoelectric Point"=8.635631561279297,"% Basic Amino Acids"=16.30%,"% AT-rich Amino Acids"=17.22%,"% Polar Uncharged Amino Acids"=27.22%,"Molecule Type"="AA"]

GMH36208[&"% Charged Amino Acids"=28.68%,strain="KO-2023","% Acidic Amino Acids"=13.70%,collection_date="2019-11-07",db_xref="taxon:3041901",Organism="Bryopsis sp. KO-2023","% Hydrophobic Amino Acids"=48.66%,Description="hypothetical protein BSKO_04076 [Bryopsis sp. KO-2023]","Genetic Code"="Standard",Modified=Mon Aug 12 11:04:36 PDT 2024,Accession="GMH36208.1","% GC-rich Amino Acids"=24.97%,Taxonomy="Eukaryota; Viridiplantae; Chlorophyta; Ulvophyceae; TCBD clade; Bryopsidales; Bryopsidineae; Bryopsidaceae; Bryopsis","Extinction Coefficient"=30995.0,"Molecular Weight (kDa)"=87.66858938000009,"# Nucleotide Sequences With Quality"=0,Topology="linear",Created=Sat May 27 00:00:00 PDT 2023,"Charge at pH 7"=-10.565828367183034,"Isoelectric Point"=6.034984588623047,"% Basic Amino Acids"=14.98%,"% AT-rich Amino Acids"=18.31%,"% Polar Uncharged Amino Acids"=22.92%,"Molecule Type"="AA"]

GMH43921[&"% Charged Amino Acids"=30.06%,strain="KO-2023","% Acidic Amino Acids"=15.18%,collection_date="2019-11-07",db_xref="taxon:3041901",Organism="Bryopsis sp. KO-2023","% Hydrophobic Amino Acids"=48.01%,Description="hypothetical protein BSKO_11855 [Bryopsis sp. KO-2023]","Genetic Code"="Standard",Modified=Mon Aug 12 11:04:36 PDT 2024,Accession="GMH43921.1","% GC-rich Amino Acids"=23.31%,Taxonomy="Eukaryota; Viridiplantae; Chlorophyta; Ulvophyceae; TCBD clade; Bryopsidales; Bryopsidineae; Bryopsidaceae; Bryopsis","Extinction Coefficient"=27555.0,"Molecular Weight (kDa)"=72.50938077999992,"# Nucleotide Sequences With Quality"=0,Topology="linear",Created=Sat May 27 00:00:00 PDT 2023,"Charge at pH 7"=-10.79317009729061,"Isoelectric Point"=5.409976959228516,"% Basic Amino Acids"=14.88%,"% AT-rich Amino Acids"=21.01%,"% Polar Uncharged Amino Acids"=22.39%,"Molecule Type"="AA"]

CAG9460856[&"% Charged Amino Acids"=32.23%,strain="YPF-701","% Acidic Amino Acids"=15.93%,note="contig: scf7180000004436.1247503.F.6849.2197154.F",db_xref="taxon:765719",Organism="Pedinophyceae sp. YPF-701","% Hydrophobic Amino Acids"=49.39%,Description="unnamed protein product [Pedinophyceae sp. YPF-701]","Genetic Code"="Standard",Modified=Mon Jun 27 00:00:00 PDT 2022,Accession="CAG9460856.1","% GC-rich Amino Acids"=28.31%,Taxonomy="Eukaryota; Viridiplantae; Chlorophyta; Pedinophyceae","Extinction Coefficient"=40170.0,"Molecular Weight (kDa)"=90.30091008000021,"# Nucleotide Sequences With Quality"=0,Topology="linear",Created=Mon Jun 27 00:00:00 PDT 2022,"Charge at pH 7"=-14.916549173412331,"Isoelectric Point"=5.661197662353516,"% Basic Amino Acids"=16.30%,"% AT-rich Amino Acids"=16.30%,"% Polar Uncharged Amino Acids"=19.00%,"Molecule Type"="AA"]
;
end;

begin characters;
dimensions nchar=1133;
format datatype=protein missing=? gap=-;
matrix
KAH9290598 M--------------------LSEGGNGRS---
TIERGAGAFEID------------YKNVRKEDGSYSLNACFNREIRPLLDAVDKLRNLGAVKEGI-----
HLPTIVVVGDQSSGKSSVLESLSGIDLPRGQGICTRVPLIMRLQNST--EEYSVIS-VEYKD-RK------
LSINEHQIVDTINLVTEEIAGRNKGISDDPITLHVRKKNVPDLTLVDLPGITRVPVYGQPKDIYEQVYKII
MKYISPRDSIILNVLSATVDFPTCESIRMSQKVDEDGERTLAVVTKVDKAPEGLREKVAED---
AMNIGLGYVCVRNRVE-GE------SIVKARKKENELFKTH-------
PLLSGIDKSIVGIPILAHKLMKIQAAGITNSLPRIMKEIDTKLARRQAELNDLPENLCNPADAMILLTTV
MGSIKDSLNGLLLQGDYQ------EFSEDGGMHCAARLNEMFMGYYRDLCASATDMNDVNGQN-
FLLKEITMLKEA-QGV-GLPNF--LSRQVFLNLVQQRA-
NGVAEISLRVVEKVWDYLDGVMLRVIDRECQT-
YPQLNAATKRAAGHLLIRRNKEDCIEYVKDMIETEKCVDFTVNPLYMDTYTKLHDQKDRFLYALRN--
----------KKKT--
---FSIDGFGE-----VKLEN-
IKE--------------------------------DDQLQAA--
FDMKMSVVAYWKVVIFRLADGIPLHLRFVYRKMVRKE---IGGDLMKEIAGSN--LD-
MIEKIFQESPAVASKRRSLIDSLALLRDAKLVVYNIIDKNNA---

XP_002297993   MSGG----------KRIGRPSKGKNGFHSS----------------------------------
-----------SYEEDSLPLDVRVEENLLAIVG-
GDENQPTPIHSVPIMSSFNDRIRPILDAVDQLRHLMVMKEGI-----
QLPTIVVVGDQSSGKSSVLESLAGISLPRGQGICTRVPLIMRLQHHT--SLIPEMF-LEFNG-KT------
TQTDEANVADDINIATEEIAGSGKGISDAPLTLVIKKNGVPDLTMVDLPGITRVPVHGQPDNIYEQIAG
IVMQYIQPEESIILNVLPASVDFTTCESIRMSRQVDKTGERTLAVVTKADKAPEGLLEKVTAD---
DVNIGLGYVCVRNRIG-DE------SYDNARMEEANLFATH-------

PLLSRIDKSIVGIPVLAKKLMQVQATIMAKCWPEIVRKINEKLNGNVTELNRMPKAMSSVAEFLTAFM
EFIGSVKESLTKILVRGEYD------EYPDDPNMHGVARVVEMFNQYSDELLNCPESEHT---RN-
FLMDEIRVLDDS-KAI-ALPNF--LPRHAFLSLLQRKV-
ERVSHIPFGFVEKAWAYFENVVWSVSRHHTEN-YPQVLLTTKRACQN-
LMVKMREQSTDWVSELVQMEKLTDYTCNPEYLNEWNMLMSHRQTFIDEVQK------------NESSK-
----
---MKIEVFGE-----VEIVN-LRGY-----------------
-------------QPLLSQA--FDLKMRMTAYWKIVSRRLVDCMALHLQLCVRNLVSKE---
LEKEIATELMATN--GG-KLEMMLEEAPSVAAKRKRLNTSIELLREAKDVLSNIMGNVSA---------------
----

KAF5727250 M-------------------------VYSS---
ESEGDSLSMVVN-------------QPRQEVAIDAPIVASCNDRIRPLLDAVDELRHLMVMKEGI-----
QLPTIVVVGDQSSGKSSVLESLAGISLPRGQGICTRVPLVMRLQHHH--IPEPELY-LEFNG-KT------
VQTTESRISEAINLATDEIAGNGKGVSNTPLTLVVKKHGVPDLTMVDLPGITRVPVHDQPENIYEQIA
GIIMEYIKPDESIILNVLSATVDFSTCESIRMSQQVDKTGERTLAVVTKVDKAPEGLLEKVTAD---
DVNVGLGYVCVRNRIG-DE------TYEEARRKEAALFESH-------
PLLCLIDKSIVGVPVLAQKLVQIQAAIIMKCLPEIVKKINDKLNSSLSEFNKLPKTFTSIAEAMTAFMGII
AASKESLRKIILRGEFD------EYPDDQRMHCTARLGEMINQCSQEMQNHRSSDLK---KN-
FLMDEISVLDEAIKGI-RLPNF--LPHSVFIALLCKKV-DSISSMPIEFVEKVWTYIEGVMIEVLTSHSDN-
YHQLQIATRRACLN-
LVAKMKQQSLNWVMEIIQMEKLTDYTCNPEYLLESKKLMAKQDKFMDDVFN------------AVLPSA---
----
--VIYFEGFGG-----VEVVR-LREH------------------
------------KHVVPQA--YDLKARMTAYWKIVLRRLVDSLALHLQLSVHNLVDKE---
LEKEIVNELMMNSLGGG-GVEKLLGESPSVAGKREKLSRTIKLLQESKKVLARIVDEIATA--------------
----

XP_002303204 MGL-----------VLRHCGSVSEEMSNSS--------------------------------
-----------ENEVESLPQSIE------------EKHQELGVSHVPIVSSFNERIRPLLDAVDKLRHLQVMKEGI--
---QLPTIVVVGDQSSGKSSVLESLAGISLPRGQGICTRVPLIMRLQHHT--APEPELS-LEFNG-KT-----
-
VPTSEAKIANAISLATDEIAGNAKGISNTPLTLVVKKNGVPDLTMVDLPGITRVPVHGQPENIYEQIADI
IMEYIRPEESIILNVLSATVDFTTCESIRMSQKVDKNGERTLAVVTKADRAPEGLLEKVTAD---
DVNIGLGYVCVRNRIG-DE------SYKEARKEEADLFENH-------
PLLSKIDKSMVGIPVLAQKLVQIQATIIARCLPEIVRKINEKLNASISELNRMPKTLSSVGEALTTFMSIV
GSAKESLNKIIVRGEYD------EYLEDKNMHCTARLVEMLNQYSGELHNCSENDLT---GN-
FLMDEIQVLEEA-KGI-ELPNF--LPRTTFLSILQKKV-EKISHIPVAFVEKVWTYIEGVVISVLMHHSEN-
YHQLQLSTRRAGHN-
LIARMKEHSRNWVTEIVQMEKLTDYTSNPEYMNDWNKLMAQQHDFTRDVLE------------KVYITT---
----
--FKIEGLGE-----VPIAG-LRGYE-----------------
-----------QHVLLQA--FDLKMRMTAYWKIVLRRLVDFMALHLQFCARNLVNKE---
MEEEIVQELAGRH--DG-AIERMLEESPAVAAKREKLNVSIKLLRESNNVLANIMDKIASNI-------------
----

XP_024439231 MSG----------------GKPSSKRKGVE--------------------------------------
------KYEDASVNTEVELH----------VEHEAIFHDHVPIVSYYNDRITPLLDAVDRLRQLQVMKEGI-----
QLPTIVVVGDQSSGKSSVLESLACINLPRGDGICTRVPLIVRLKHHP--SLVPEIF-LQFNG-KT------
VPTDEAHVADAINLVTDEIAGNGKGISNTELTLVVKKNGVPDLTLVDLPGITRVPVHGQPENIYEQIAY
IIMKYISPDESVILNVLSASVDFSTCESIRMSQKVDKNGQRTLAVVTKVDKSPEGLLEKVTRN---
DVNIGLGYVCVRNRIG-NE------SYEDARKEEAALFATH-------
QLLSKIDKSTVGIQVLAQKLVQIQANIIAKCLPDIVRKIDEKLSASISELNRIPRRLLSVAEVMAAFMGII
GSSKDSLRKILLRGEID------EYRHEKDMHCTARLVEMLNQFSTELHKCSDHT-----KN-
FMINEIEVLEET-KGI-ELPNF--LPHAAILAILQQKV-EEISELQIGFVEKVWAYIRGVVISVLNHHSAN-
YHQLQLFIGRAAHK-LVDKMKDRSIDWVTEILQMEKETDYTCNPEYMKEWNKLIAQQQAVIDNITK--
-----------FGSSR--
--VTIDGSRE-----VVVGD-
LRGH------------------------------KHVLLQA--
FDLKMRLIAYWKIVLMRLVDNMALHLQLSIRNLVNKE---MEKEIVNALLGTG--
GGVAIERMLEEPPSVASKRERLNTSIKLLRESKEVLANIRDKIECGDH----------------------------------
----

KAF8391993 M---------------------VKFGSNFS--
------H----------KEDTKLSAAIAPLVLSYNDQIRPLLDAIDKLRHLQVMKEGI-----
ELPTIVVVGDQSSGKSSVLESLAGISLPRGQGICTRVPLIMRLQHHS--TPSPELH-LEYHN-KI------
IPTNETHVAEAINMATNEIAGNGKGISNTPLTLVVKKKGVPDLTMVDLPGITRVPVHGQPEDIYEQISS
IIMEYIKPKESTILNVLSATVDFPTCESIRMSQRVDKTGERTLAVVTKCDKAPEGLLEKVTAD---
DVNIGLGYVCVRNRIG-EE------LYEEARMEEATLFESH-------
QLLSKIDKSIVGVPILAQKLVQIQASIIAKCLPDIVKNINEKLHANVSELNKMPQNLSSTAEAITVFIRIL
GSAKESLRKILIRGEFD------EYTDENEMHCTARMAEMLNEYSKELQQTSDEKDST--DL-
FLMDEIRILEEA-KGM-TLPNF--LPRAAFLSVLQRKV-NEISSIPVEFVRKGWKYIEDVVIIVLMKHSAN-
YPQLQSSTRRAAHD-LIAKMKEQSIYKVMEIVEMEKLIDYTGNPEYMSTWGRLMVNKEAFMKVIKN--
----------HSNHPQ--
--LVLEGFGQ-----
VEVGH-LIQY------------------------------STVVEQA--
FDMKMRMVAYWKVVHQRLLDNLALHLLFSVHNLVNKE---LEQEIVNELMGSH--GG-
GIKRMLEESSSVSVKRDRINQSIKLLRDSKEVMAEIMGRITANANAPGQVQRLLHLPAASLANRCH
HGHPPHLCALMTATDTNPLLRRDTAIGGNSFR-
HVHDHTPSILFIYSPVTAALPSPQSFHSSPFALPVSTPHLLFYNGHSR

XP_058079501 M-------------------------NSL---
PSSSTSLQEKMA------------MKDHPEKPVLPPLVSSYNDRIRPLLDAVDRLRHLNVMEEGI-----
ELPTIVVVGDQSSGKSSVIESLAGISLPRGQGICTRVPLIMCLQNVP--TDKPQMH-LEYQG-KI------
VLTSENQISDSISMATDEIAGNGKGISNIPLTLVVKKKGVPDLTIVDLPGITRVPVHGQPEDIYEQISNII
MDYIKPKESIILNVLSATVDFPTCESIRMSQHVDKTGERTLAVVTKADKAPEGLLEKVTAD---
DVNIGLGYVCVRNRIG-DE------TYEEARIEEATLFKSH-------
PLLSKIDKSIVGIPVLAQKLFQIQANSLSQCLPDIVKKINDKLNKHVTDLNNMPQNLTNVAEATQAF
MRVVGAAKESLKKVLLRVEFE------DFPDDVKMHCTARMAEMLDGFYKDLQSKSSDNYSTSSCA-
FLMLEIGVLEES-KWI-GLPNF--LQRTAFVALLQRKV-NRIALGPLDFIQSILTYIEEVVIRIVSEHSEE-
YPQLQSMTKRAVHN-LIDKMRDRSVKHVKEIIEMEKIADYTSNSDYVKTWSSLMENRKTFIEVIQD---
---------PNMGTK---

---IMFQDFGE-----VEVGH-
LRQHS----------------------------LSMLEQA--
FDMKMRLTAYWRLVLLRLVDSLALRLLFSVKKLVERE---MEEEIVNELMGSH--TC-
GIERMLEELPSTTQKCERLNKGIKLLRDSKDVVTKIMDGIAAR---
-------------HISD--

KAH0683503 M---------------------AYQNTNDC---
ISDSIEILNAKP------------LAVVASGVVHPPIVASFNDRIRPLLDCIDKLRHLNIMQEGI-----
QLPTIVVVGDQSSGKSSVLESLAGISLPRGQGICTRVPLVMRLKNDPN-ITAPNLQ-LEYNN-KS------
LPVDEIGIADAIILATDEIAGHGKGISNNPLTLVVKKNGVPDLTMVDLPGITRVAVQGQPEDIYEQVYD
IIMKYIVPEESIILNVLSATVDFPTCESIRMSQKVDKTGERTLAVVTKADKAPEGLLEKVTAD---
EVNIGLGYVCVRNRIG-NE------SYEEARSDEQRLFSTH-------
PLLSKIDKSMVSVPILAQKLVRIQASIISKCLPEIVRKINDRLAANLAELNRLPQHLTSVAEALTAFMCIL
SSSKDSLKKILLSGEFD------EYPDEKEMHSAARIVEMLNEYSSELHSKNFEKA----DE-
FLMEEIMVLQET-NGI-RLPNF--LPRAVFLNVLQRKV-
KEIAASPEDFVGKLWNYLERVVIIVLMYHCEN-YPQLQSSTRRAAQN-
LIAKKKNESVDWVREIIGMEKLTDYTCNPDYLTTYSKFMAQQHTFMEIMND------------HGKCSM---
----
--INLEGVGV-----IDVGH-LRKH------------------
------------LDVVQQA--FDLKMRMMAYWKIVLMRLVDSMALHIMFSIRNMINKE---
MENEIIQDLMAPH--GG-GIERMLDESPLVAEKRNRLKKSVKLLKESKEVVANIMDRISLYDD---------
--HESD---

PWZ56864 M---------------------SKKRRTGD---
KLENDVEDTKRA------------ALAIASGVTASAIAASYNDQIRPVLDAVDRLRHLKVTQEGI-----
QLPTIVVVGDQSSGKSSVLESLAGISLPRGQGICTRVPLVMRLQGDPS-TDSPKLQ-LEYSNGRV----
--
VTTTEAKVADAINAATAEIAGSGKGISDAPITLVVRKSGVPDLTLVDLPGITRVPVQGQPEDIYDQIANII
KEYITPKESIILNVLSATVDFPTCESIRMSQQVDRTGERTLAVVTKVDKAPEGLLEKVTMD---
DVHIGLGYVCVRNRVG-EE------TYDQARVAEAQLFKNH-------
PLLSQIDKSMVGIPVLAQRLMQIQASIIAKCLPDIVKQINDRLIRSSTELDRMPPDVINTGDAVRAFLH
IVKKVCTSLENILVRGDFG------CYPDDYYFHGTARVAEMLSRYAKKLPAECPRCSD---EK-
FLAEEMRVLEET-MSI-KLPNF--LPRSAFHSMLKKKV-
EMVSDVPQDLVSEVWEYVEDLVMKVLLQHSEN-FPQVQSSCRRAIQS-
LMEKTRVRSAQHVKEMIEMELVAGYTASPDYMKTWEAIMVGQKKFMGSVEN------------KSGPSK--
----
--VTLECFGE-----VNVGH-LRTH-----------------
-------------PDLAAQA--FDLRARLTAYWKIIVLRLVDGLALHVLRGVKRLVEND---
LEDELANELLGNN--MA-GVERMLSPPPSNGTKRDRLKKSILLLQQSKEVVANIMDRINAADEA------
----

PWZ56863 M---------------------PKKGHMGT---
PKQAYATDGGKG------------DVAAGSTVTASAIASSYDDQIRPLLDAVDRLRHLKVTQEGI-----
QLPTIVVVGDQSSGKSSVLESLAGISLPRGQGICTRVPLVMRLQDDPS-ADSPKLQ-LEYSNGRV----
--
VTTTEADVADAINAATAEIAGSGKGISDAPITLVVRKRGVPDLTLVDLPGITRVPVHGQPEDIYDQVAKI
IKEYIAPKESIILNVLSATVDFPTCESIRMSQQVDRSGERTLAVVTKVDKNPEGLLEKVTMD---

DVNIGLGYVCVRNRIG-DE------TYDQARVEEERLFKYH-------
PLLSKIDKDMVGIPVLANRLMQIQSTIIAKCLPDIVKQINDRLSRSSAELDQMPQDLNNVADAVRVF
FHIVKQVCNSLEKLLVRGDFA------EYPDAREFHGTARIADMLSGYAKELPGQCPINRN---EP-
FLKEEVNILEET-KGI-NLPNF--LPRTAFLVLLKKKV-ETIQEIPQFLANKVSDYVEDLVMKVLLKHSEN-
FPQMQSPCRRAVQT-
LMDKARLRSAHHVKELIAMELVADYTANPDYMKTWTEIMEGYELFMEAVEN-----------TSKPTK-----
----
--ITLKCFGE-----VDVSH-LRVY---------------------
---------ADLAGKA--FDLRARLTAYWKSIVLRQVDGLALHVLLSVKLLVEKD---LEEELGNELLGNK--
LA-GVEKMLSPSPGTGTKRERLKKSIVLLRQSKEVVANIMDRISAAGDI--------------------------------
----

XP_038984915 M-----------------------ANCT---
---------KIA-----------GDMVKTEAKKSTALASSFDDHIRPILDAVDRLRQLKVMQEGI-----
ELPTIVVVGDQSSGKSSVLESLAGISLPRGQGICTRVPLIMRLQDDPS-LSQPQLQ-LEYKD-KA------
IHTSEDGIADAINSATDDIAGSGKGISNAPLTLVVRKRGVPDLTMVDLPGITRVPVHGQPDNIYEQIS
NIIMEYIAPKASIILNVLSATVDFPTCESIRMSQSVDRTGERTLAVVTKADKAPEGLLEKVTAD---
DVNIGLGYVCVRNRIG-DE------SYEEARAEERNLFKRH-------
PLLSRIDKSIVGIPVLAQRLMQIQAASIAKSLPDIVKKINDKLSQHISELDEMPENLRSIGDATRVLLH
MLSTSKESLRRLLIRGEFD------DFPDDASMHATARMAEMLAKFFKELPSDCPSAD----ER-
FLMEEIAVLEEA-KGINGLPNF--LPRHAFLNLLRRKV-
RDISHAPGEFVRKVWAYIEEVVIRVLLHHSEN-YPQIQPMVRRAAQN-
LVGIMRNQSCHFMSEAIEMEMVADYTSSPDYMKKWTELMDGRDGFIAAIEN-----------YYGPNS--
----
---IELKGLGV-----VEVGH-LRQY------------------
------------AAMAEQA--FDMRMRILAYWKIVVLRLVDSLALHIIYSVNCLVERH---
MEKEIVDDLVGPR--MT-GLERMLEESPATAAKRERLRKSIELMKESKEVVAEIMDRVVTTVN----------
----

KAH9320939 MGSS----------CLSERLKMSMLMTNSF--
------------DDE------------AEEVKAEDQGSSLAISYQQQIRPLLDAVDKLRNLDIMKEGI-----
QLPSIVVVGDQSSGKSSVLESLAGIKLPRGQGICTRVPLVMRLQSCAE-ESEEEIS-IEFNG-VE------
KFIQESDITSSIDTATQEIAGNGKGISHTPITLHVTKVGAPDLTMVDLPGITRVPVGGQPGDIFEQICEII
KEYITPKESIILNVLAANVDFPTCESIRMSQKVDELGERTLAVVTKSDRAPDGLKEKVTTD---
AVNIGLGYVCVRNGIG-DE------SNAEAREKEKNLFDFH-------
PLLKDLDKSMVGIPTLAKKLMQIQATTISATLPQIVNKIESMLGKRQAEMRNLPQHLCNPGEADVAF
VKLVHELKESLKKIVILGEFQ------QFPDDPKMHCTARLREKFDMFYRDLSQRGSFSVG---DK-
FLSKESRMLEEA-KGV-GLPNF--LPRSVFLELLQKMV-
EEISEKSLSLAATVWDYLENVISRVIEHYCHC-YPLLESRVRRAVQG-
LVVEKKEECINHVKQMIEMEKGIDFTLSPAYMETYGTLIRSKGQFMDRLGRLVQQHTRLASKGSYNP
SS--
-------------------------------DFDV-------------------------VNKTVVVEDFGE-----MEVGD-LMELP---
-------------------------AERVQEA--YEMQMSLAAYWKVVTLRMGDGIPLHLQFVCRNLVGNE---
LETQILKHVGGPN--FG-AMDKILEESPVVAGKRKSLINSLQLLKDSKTAVANIMDRIAEAA------------
----

KAH9325151 --
--I-----
QLLSIVVVGDQSSGKSSMLRSLAGINLPKGQWICTRVPLIMRLQISK--SQETEIT-IEYSG-VK------
NNIFEYKIIEALNAATDEIAGVGKGISDTPITLNVSKSNIPNITMVDFPCITKMPVHDQPQDIYDQISQV
IKQYITPKESIILNVLFTSVDFPTCESIRMSQLVDVKGEKTLAVVTKVDKAVEGLFEKVTVD-
VVSVNIGLGYVCVRNKIG-NE------SNA--

KAK1401877 MEWI---------------PKQRVEQVNKS--
KFKQIVVKSDQE-HSNMSIIVCEVDESVVPLSSHAPIVSSYNEKIRPLLDAIDKLRNLKVTQEGI-----
QLPTIVVVGDQSSGKSSVLESLAGISLPRGQGICTRVPLIMRLHHHS--KPESELH-LEYCG-KV------
VSTDELKIAESINMATAEIAGDGKGISHTPLTLVVKKNGVPDLTMVDLPGITRVPVHGQPENIYEQISG
IIMEYIKPEESIILNVLSATVDFPTCESIRMSQSVDKTGDRTLAVVTKSDKSPEGLRDKVMAD---
DVNIGLGYVCVRNRIG-DE------SYEEARMAESMLFESH-------
PLLSKIDKSMVSVPVLARKLVQIQARIIAKCLPEIVKTINAKLHANVLELNNLPQKLSTVAEAFTAFMRI
LGLSRESLKKLLVRGEFD------EYPDNKNMHSTARLAEMLDKFSQELKSSSPFITD---EK-
FLIDEIFVLEET-RSV-ALPNF--LPRSAFLHLLQLKV-QAISEIPVSFVGKVWDYIETVVVEILTHHCEN-
YPPLQSSTKRAALN-LIAKMKEQTTCRVLEIIEMEKLADYTCDPEYMALWNKLMKSHANFKEAIDG---
---------DVSWV--
---ADIESFGK-----IDLRH-
LEKI------------------------------SGVRDVA--FDLKMRLTSYWSIVLKRMVDSIALYLLMALKKLVNQG--
-MEEEIVKEVLGPQ--GC-GIERLMDESPSFTIKRERLNQSIGLLEESRDVLAKIIDRIAIADD------------
----

KAH9300179 --
-------------------------SYNERIRPLLDAIDTLRNMGVMK---
AYNCLVLLLWETS--RLEP--------------------------------------
GRSINDTPITLNVGKSNAPDLTMVDFPSITRVPVHGQPQDIYDQISQVIKQYITPKESIIMNVLSASVD
FPTCESIRMSQLVDEKGERTLAVFTKVNKAAEVLFEKVTVD---AVNIGLGYVCVRNRIG-NE------
SNA---

KAH9314974 --

ALNAATNEFAGAGKSISDTPITLNVSKSNAPNLTMVDLPGITWVPVHGQPQDIYDQISQVIKQYITPK

ESIIMNVLSASVDFPTCESIRMSQLVDEKGERTLAVVTKVDNAAEGLFEKVTVD---VVNIGLGYTCV--
-------------YSFAKRSRNLAV--

QACDYLDRIICRVID-------PQLQASSRRAFQA-
LIDRKRDKCIQYVEDAMEMQKSIVYTENPSYSKSLQKMQQWKESFIEVIRQ------------NHKAVK----
----
---IDV--
-------------------------------CLYLVWIAHRIQAQQ--

KAF8079489 M------------------GGSKKRVVSKT--
SPSRSIVKANDP------------NNNNKSVTIESPIVSSYNDQIRPLLDTVDRLRNLNVMKEGI-----
QLPTIVVVGDQSSGKSSVLESLAGISLPRGQGICTRVPLVMRLQGSA--SSEPEIW-LEYSD-NV------
VPTDEEHIAEAISAATDVIAGSHKGVSDAPLTLHVKKAGAPDLTMVDLPGITRVPVKGQPENIYEQIS
GMIMKYIKPQESIILNVLSATVDFTTCESIRMSRQVDKTGERTLAVVTKADMAPEGLLQKVTSD---
DVSIGLGYVCVRNRVG-EE------TYEEARKQEELLFETH-------
PTLSMIDENIVGVPVLAQKLIQIQTTMIARCLPKIVRKINHKMETADLELKKLPMVMASTGEALMKLM
DIISSAKESLLRILIQGDFS------EFPDNHSMHGTARLADMLSQFSDDLQAKPKEVRE------
FLIDEIKVLEEC-KCV-GLPNF--IPRSAFMAILSQHV-DDIHAKPVEFIKNIWDYIEVVLSSVITKYSEN-
FPQIQPSIKRAGRN-
LMGKIKEQSVDRVVQIVEMEKLTDYTCSPEYMKSWTEKIDGQKSFVDAVLND-----------KTKPES----
----
---VFVNGFGN-----VKISH-LREYH-----------------
-----------HAHLLQQA--FDMKMRITCYWKIVLRRVVDNLALYLQLSVKYLVNTQ---
FQKEMVAEMVDPK-GGG-GVERMMEESPLVASKREKLKKSIKVLKESKDAVAAIVDQ----------------
----

OAP13972 M------------------GGSKKHVVTRT---
SSPSLAIVQANP------------HDNREVVPIEAPIISSYNDRIRPLLDTVDRLRNLNVMREGI-----
QLPTIVVVGDQSSGKSSVLDSLAGISLPRGQGICTRVPLVMRLQRSS--SPVPEIW-LEYSD-KI------
VPTDEEHIAEAICAATDVIAGK---FT-------
-------------------------------------LYLGIKCV---

OAP13353 M------------------GRSNKHVVTST---
TPSLAIVQANP------------HPHKDVVPTEAPIVSSYNDHIRPLLDTVDKLRNLNVMQEGI-----
QLPTIVVVGDQSSGKSSVLESLAGISLPRGQGICTRVPLVMRLQRRR--SPEPEIW-LEYGD-KI------
VPTDEEHIAQTICAATDVIAGM---F-------

OAP19580 M------------------GGSK-----MS--
NDYEIDVEAGMS------------SLSIVNTPIEAPIVSSYNDRIRPLLDTVDRLRNLNVMREGI-----
QLPTIVVVGDQSSGKSSVLESLAGINLPRGQGICTRVPLVMRLQRSS--SPEPEIW-LEYSD-KV------
VPTDEEHVAEAICAATDVIAGK---FSLSPSQCSVK--
---CV--
LLQK---

XP_024380180 M------------------SKQSELTMNDS---------------------------------------
------INNMLVVSSVS------------NVNPIKSYDPEALRNSFHSEIRPILDVLDKLRTQGITEENV-----
NIPTIVVVGDQSSGKSSVLESLAGITLPRGQGIATRVPLILRLQSCLS-EQDSKIL-MEYGSVKEM-----
RINSEDDIEAAINAATDDLAGSNKNIRDTPILLHIRKPDAPDLTMVDLPGITRVPVHGQPENIYEQVR
DMIMHYIKPEESIILNVLPAEVDFSTCESIRLSQTVDKKGVRTLAVVTKVDKAPEGLFEKVTSD---
AVSIGLGYVCVRNRTPADD------SIAVARCRELELFNDH-------
PDLRNIDRSMVGIPTLGRRLVKIQSDMVRGCLPRIRDQIHEALQKRRQEMSNVPRSIDSVNEAVAV
FLRLQNEMLNMLTQVVRDGDFS------LVPDDCTLHYTARLHEEFTKFAEDLGKSGLRLSQ---PS-
QTDEIRQMFSEH-QGV-ALPDF--LPHTVLHQLVKKQI-
DSITQTCIFLVDRVFKYAAEVVLHVQSLIFEA-YPHLRDRHHKLAIQ-
VLNETKTTTVEFVERMLAKERTVIFTTNASYLDTIAKINSAVETARRS------------------NQA--------------
----
---YIELDVGRI-REGLILNE-AVPA-----------------------------
-TGWYREA--WEMKVRIAAYSKIMHERLADEIPLEIRNALQRTIVNR---LQELTMMQAFSSP---E-
ELGTLMQQDSKIICRRVRLQQCIDTLEKSLMLVSGMIAA---

XP_024367947 ---
-----------------------------------HSEIRPILDVLDKLRTQGITEENV-----
NIPTIVVVGDQSSGKSSVLESLAGITLPRGQGIATRVPLILRLQSCLS-EQDSKIL-MEYENVKEM-----
RINSEDDIEAAINAATDDLAGSNKNIRNTPISLHIRKPDAPDLTMVDLPGITRVPVHGQPENIYEQVR
DMIMHYIKPEESIILNVLPAEVDFSTCESIRLSQTVDKKGVRTLAVVTKVDKAPEGLFEKVTSD---
AVSIGLGYVCVRNRTPADD------SIAVARCRELELFNDH-------
PDLRNIDRSMVGIPTLARRLVKIQSDMVRGCLPRIRDQIHEALQKRRQEMSNVPRSIDSVNEAVAVF
LRLQNEMLNMLTQVVRDGDFS------LVPDDCTLHYTARLHEEFTKFAEDLGKSGLRLSQ---PS-
QTDEIRQMFSEH-QGV-ALPDF--LPHTVLHQLVKKQI-
DSITQTCIFLVDRVFKYAAEVVLHVQSLIFEA-YPHLRDRHHKLAIQ-
VLNETKTTTVEFVERMLAKERTVIFTTNASYLDTIAKINSAVEKARRS------------------NQA------------

----
--YIELDVGPI-REGLILNE-AVPA----------------------------
--TGWHREA--WEMKVRIAAYSKIMHERLADEIPLEIRYALQRTIVNR---LQELTMMQAFSSP---E-
ELGTLMQQDSKIIGRRVRLQQRIDTLEKSLMLVSGMIAA--

KAG0561847 M-------------------------QSNT---
SPIDIPACDAMS------------LELISRRAALDSLESTFQRDIRPLLDVVDKIRAQGVTEENI-----
QLPTIVVVGDQSSGKSSVLESLAGITLPRGQGIATRVPLVLRLQSCQ--LEESIIK-MDYGNVKDQ-----
EISGEEQIEAAINAATNALAGSGKGVKDSPIQLLIRKPNSPDLTMVDLPGITRVPVHGQPKNIYEQIR
GMIMRYITPEESIILNVLSAQVDFPTCESIRMSQQVDKEGNRTLAVVTKVDKAPEGLLEKVTTD---
AVNIGLGYICVRNRIDVDD------SIAIARQRERELFESH-------
PALKELDGSMVGIPALARKLTKIQSDMVKECLPRIQKQMFEALHKRNQQLSNLPRGIKSDMDARSA
FFQVQNKILTILSQVVRDGNFE------EFPSDAHLHYTARLHQKFQTFADDLHKTGLKFRE---QS-
QTTEIRELLVEH-QGV-GLPDF--LPHSVLHHLMRKQI-
TSVNETCRSLVDEAFEYATEVVLRVNSLCSQG-YPRLEKSYKQLAIE-
TLEEVKTTTMEFVERMLEKESTIIFTTNDYYTATLEKMQTALGEAKRT---------------STYSSR------------
----
--AVELGPGE---DKIALVEILNDP--------------------------
---DRKYQDA--WRLKVSVAAYWKVVQKRLADEIPLEIRYALQCAVVDT---LHQNMMSKPWAGG--
ET-DLRALMEEDSVGAYTRSRLQLRVDALKDCLRLLSGLMC--

KAG0619429 --------------------MDRMLMIGN---
VDTKVVTKMD------------VNQLESTYHLDSLEATFQKEIRPMLDAVDKIRAQGVTEENV-----
QLPTIVVVGDQSSGKSSVLESLAGITLPRGQGIATRVPLILRLQSCDS-TEESLIR-MDYGNVKDR-----
EIDGEEQIEAAINEATNVLAGGNKDVKDTPISLHIRKPHAPDLTMVDLPGITRVPVHGQPQNIYEQIQ
AMIMKHISPEESIILNVLSAQVDFPTCESIRMSQQVDKDGKRTLAVVTKVDKAPEGLLEKVTTD---
AVNIGLGYVCVRNRTDDDD------TISVARIREQRLFESH-------
PALKDLDRSMVGIPALARKLTKIQSDMVKGCLPRIHKQMCDALQKRRQQLNNLPKGIASDNDAILIF
LQIQNRRLDMLTQLVRDGDFE------LFPENLHLHYTARLHEKFMKFADDLHKAGLKLKD---QS-
QAQEIKELLAEH-QGV-GLPDF--LPHSVLHHLVRKQI-
ELIRETCTSLVEEAFEYATDVVSEVNTICSEG-YPNMEKCFKKLATE-
SLEKTKKTTMEFVERMLLKECTLIFTTNDYYLATIAKMNALLDNAKQT---------------QNYNQF---------
----
---VVLEAGGNQ-AEKLGLAE-LQYK--------------------
---------DKEYQDA--WRMKTSLVAYWKVVQKRLADEIPLEIRYALQYAIVDL---LHREMMVKAYSDP---
K-GFQALMQEDSNLSFNRARVQHRVDALKECLLLLNDLMG---

KAJ7294545 M----------------------RFINND--
ELMVPPPEALA------------ERQRSPAFPESALQTHFDQQIRPMLDVVDKLRSLGVTQEGI-----
QLPTIVVVGDQSHGKSSVLESLAEITLPRRQGIATRVPLILRLQSCKV-ASEQSIT-IEYLNVKD------
EIKSEELIEAAIDEATNVLAGPRKDVRDTPISLHVRKLGAPDLTMVDLPGITRVPVHGQPENIYEQIAA
MIQKYINPPESIILNVISATVDFPTCESIRMSQLADKEGKRTLAVVTKVDKAPEGLYEKVTSD---
AVNIGLGYICVRNRTE-KE-----NSNEQARFVEKHLFDTH-------
PSLCKLDKSMVGIPMLAYRLTCIQAQMIQGCLPGLYQQIFDALHKRRRELDSLPTGFQDNAEARVL

FFKIHNEQFKAIDELVREGKLD------KFLEDNHMHYTARLHEMFQNFGDDLRKTGKMFLD---QT-
SLEDIVELLSEH-KGV-SLPNF--LPHAVLHQLVKNEI-
NKISAICYKLVKDSYAYASEVVMAVNRLFFEG-YKHMSAFFRGQAID-
SLRKSEKESTDFVARMLKKEREIIFTTNDYYLDTLDKIRFSLENAKRS---------------SNYNTT-----------
----
--VEIDKEKT-----LRLNE-LHGK----------------------------
---SQDYHEA--WRMKASVAAYWKVLQKRLADEIPLEIRFALQTTITQV---INEQIARKVWSGN--V--
ELKDLMQQDPAIVQKRARLEHSIDTLESSLSLLSSLLVA---

KAH9291961 M---
---------------DKAEEIPSSLSLSYSEGIRPLLDVVDKLQNLNVMNEGI-----
QLPYIVVVGDQSSGKSSVLECLTGISLPRGVGICTRVPLIMRLQNSS--EQDSEIV-VEYND-TV------
EHIIESQITERIDSITKEIAGTNKGISHVPIRLNVKKMNAPDLTLVDLPGIARVSLNGNPDD-
HELISKIVMEYISPADSIILNVLSATVNFRTCESIRMSQRVDVHGERTLGVVTKVDIAPEGLLEKVALD-
--DVNTGLGYVCVRNRVG-DE------CNEEAREAEAELFRSH-------
TQLNKFDEAMVGIPMLARRLMQIQTKRISKCFPDIVKNIEDTLSQRQSELSSLPQQVSNPMEAMVV
FLRLMNGVKDCLNRLLIEGDFS------EFSEETEMHCTARLKEMFDGFYNELVHMSVEDKNA-----
FLVEETKRLEES-KGA-GLSNF--LSRSIF----KKRI-DEVLKTGLSLTANVWDYVEKVVLRVLDLKFRS-
YPRLETATKKDFQL-
LVSKRREQCIHHVNQVAEMEKSLDFTLNPVYMETWTDLLKQKDQFMQELSK------------
TAEEPVTAPQFNIQTSQPVTQQKSPFGVST--
APAKPVTQQKSSSLFKSTSGISTTPAKPVTQQELPSPLESLFHISTMPVKPVTQQKLSSLFESTSGISTT
PAKPVTQQTLPSPFESPFG-
ISTTPAKPVTQQKTPLPFEQTFGLSSFSFSATPAEPITRPKSPKGFTAKPAAVFSGTWMKKTVNIKGFG
E-----VDVEE-VMKMP-----------------------------KEHLEVA--
FEMKARVVSYWKVVVQRVGDGIPMYLQFVYQNLVRND---IDEEIMKKVAGPK--SN-
SMEKLLEENSMISRKRRSLKRSIDSLGEAQCKILEIMDQIAEI---

GJP35534 M----------------------ERAVQQC---
------------------RSVSPARLTMAPLISSFNENVRPMLDAIDKLRLLGLKEEGI-----
ELPTIVVVGDQSSGKSSVLENLSGISLPRGKGIVTRVPLILRLQSCV--KGKDMIT-IEYTP-VTG-
KVSKVLSDEEMIEEEISEATIALAGSRKGVMNCPITLQVQRPDLPDLTLVDLPGITRVPIEDQPKDIYN
QVKNMIMHYITPKESVILNVLAAEVDFSTCESIVMSQEVDSDGDRTLAVVTKVDRAPDGLYEKIQGN
---SVRIGLGYVCVRNKTDADA------SHDDARRAEAAFFNSH-------
PELSQIESHCLGIPALAQRLTEIQAKRVADSIPRIRQAIQKALVGKENELQTIPFAATSNAAALGVISATI
QRRRDVMSGV-ISGKYG------SFQSDDAMHYAARLHEKFNEFEVQMRRVLPDFLG---AD-
YTERCKEALKEV-AGI-SLPNM--FDQAVVKQLVQEVV-
DSIEAPCFLLVNDCFAYAAEVQQAVASQVCGM-YPGLNNAAHLQGLN-
ALRKAMESANRFLKNLLTKEEEVIFTLNHYYMDTVSEIHMKIADYKKAQSG------------NQDQGP----
-----------------------P--
---PVPDVGDFAS---STASLAN-LL--------------------
------------SNDDQPA--RDLQINMFSYAKVMHKRLCDVIPMQIRMCLKNALLDG---
TDGAVWREVHSGD--IS-KIAALKQVD---QQRRARLEESITRLRASESTLLGLAFDGPSLLA------------
----

CAI5480041 ---
---------------------------------MLDAIDRLRILGLKEEGI-----
ELPTIVVVGDQSSGKSSVLENLSGISLPRGNGIVTRVPLILRLQSCT--SKDGEIT-IEYNNPSSG-
KIFKILPDEESIQEEISKATVTLAGSRKGVMDRPITLQVKRSGLPDLTLVDLPGITRVPVDDQPKDIYN
QVKKMIMQYITPEESVILNVLAAEVDFSTCESIVMSQEVDQDGDRTLAVVTKVDRAPDGLYEKIQGN
---SVRIGLGYVCVRNKTDADA------SHAAARLAETDFFDRH-------
PELSRIETDSRGIPALAQRLSEIQAKRVAESIPRIRQSIQKTLIATEEELQKIPLAANSDGAALAMISSTIQ
RRRDFMTGL-IGGRYG------QLQGDISMHYAARLNEKFNEYEALMRKILPDFLG---KK-
YTERCRDALKEV-AGV-SLPNM--LDQAVLSQLVQELI-
DSIEGPSLMLVEDCFSYAADVQKAVITKTCSG-YPNLENAAQLQALM-
ALKKAKDSSIEMTKNLLLKERRVIFTLNHYYMDTVSKIHQSIAKYKADRYG------------N----------------
----
---TPSIDGFSS---NTASLAD-LI-----------------------------
--SNDDQAA--RDLQINVFSYAKVVHKRLCDVIPMEIRMSLEDALVDD---TDAAIWG-------------------
----

EFJ22917 ---
ME------------RYRSRRTPEECALQTPFNENVRSLLDAVDTLRQLSVAEEGI-----
KLPTIVVVGDQSSGKSSVLESLAQVDLPRGQGVVTRVPLVLRLQNTSVTDQSHQVV-IQYGG-KK----
--RVIEEAEISAAVVEATIELAGD-
KHIVNKPISLHITKPGAPDLTMIDLPGITRVPVHGQPEDIEEQIKKIIQEYISPKETIILNVICSTVDFPTC
ESILMSRQVDREGERTMAVVTKVDMSPKDLKEKVMAD---VVGIGLGYICVRNRIG-DE------
THEEGRDREAELFRTD-------
PHLRDLPESMLGIRQLAKRLTEFQADSLRKNLPKLVGNIRSALTAVRRDLDALPQRVADSESALPLA
MDCYQSIRRSLERLLAGDSLP------EFPDDKQMNYAARLHEYFVKLSSQIRSNGDEGSS---
SSGSQRKLEELLHEA-KGV-TLPNI--LQSSVLKQMVAYNV-
KEFHEPSIATVDEVHNYAAQVVMPVISKKTEG-YPKHCSRRTCKA----
CSKNPSRRARSSSRTSSRRRPPSPSLSIQSTWSWS--

RRSSSWRYRRWILAS---------------------------------------CPRTGCTPNPPAPAPR---------------------
----

GMH36208
MERLYHSDRAPAAEARLGLVSHSLQSVSQSDRFKGRHTFEPNGGPVSPGSVSRNSHDWQ
DSRAFLDSGRTFGQGAPNYSPPGPSVG------------
SQSQTTAMGELGKAIASDVRIRPALDAIDFVRPFVKNMPDIAQ---
MLPAIVVVGDQSSGKSSLLEILSGVTLPRGEGICTRVPLELQLRNGT--EVSAQI---EYQTDLDAPRVS-
KHIMVEEVKNEILLATKRIAGMELNIKDLPIVLRMTGPTYQDLTLIDLPGIARMPLRGQPDNIEELTME
MIQKYINGDSKVILCAVPANNEFVTSAALKLASNVDPLGLRTLGVVTKADQFSRGMRRRLEGLDDT
DVKLKLGFVAVRCRTQ-KE-LEEGISLQDVRMREELLFETD-------
PELRDVQPHCRGISTLVDKLVDIQKERLIEQLPRIVKQLDERIADMESSLLEIEDLVESEHHATARTHE
CVRDICDAFGT---LASCQ------NLEPDTTLDIPAETFAMFEDFVQEVERATTGLLN---PE-
LYAEIKTLSERF-RGA-HLPNF--
LPHPVYGQIFKTRILQRLREPAEHLVQDVMGYIEEVVHKLIVRHLKTRFPKLVSLFCEQASE-

FLTAQYESAKFLVGEAVEAQSEFI-TFTPSYMEIMDAFHAIVGH-QASQGG------------KSDPKI--------
------------------P---
--QTPKCFAKIFHERSEAAWFYQQVA-----------------

VSHGDQFGKGCLEMMFSLAAYASTVRVVLTEEIPKQVRRCLVTKVSSREFGLEKFLLEKLTDGR--V--
ELFNLMHDS-QKIQMRHDIVQKLESMRTARQKLRGIIGVKVKTKTRR------------------HSQP--------
------------VPGLSSYRLHEQEL--L

GMH43921 M---
---------------TDNPNGLAEALSKDVKVRAVLDAIDEVRPFLKDMEGLSQ---
MLTAIVVVGDQSSGKSSTLERIAGIDLPRGQGICTRVPLEMQMRKGS--KFSAT---
LEYQQEKGGSKQSVEIKDASKISDAIQAATRDIVGNSKNVEDLPLVLRISSPIYQDLTLIDLPGIARAPL
PGQRSDIEEQTLEMMRRYITGEAKVILCALPATNDFVTSAALKLALQLDPDGERTLGAVTKIDQARKG
IAKKLEGTDASEITLHLGFAGVRCRTE-NE-TDAGITLEQVRQAEEELFRTH-------
DELKHVDDSCKGVSALLQKLVAVQRGRLISHLPKVLHQVDDQLKVQKEIIDKLPDVLENADDAFAE
ARDCVRKICTEFQE---RAKNE------RLDSDVDLRVSIVLSREFEKFYQQIGEICRGLFK---DQ-
VWEHIESLNRDL-KGA-HLPNF--
LPPPVFDILFKEHVLDKLNEPALALLETVSGEVERILGVLTTSATKM-YPNLNGIISSQVSD-
FLGETLEVTEDLVLECTSSQSECM-TNTTMYIDALEEMKA-----IKSRAG------------TKSWML----------
-----------------P---
---AGS-----EELKWFLEKAA-------------------------
GVTTPDKVTT--MEIFCALGAQAKTVGLILAEQIPKFIRRNLVFKLHNNR-ALENYLVKALGRDQQ----
TLFEMMVDE-RLAKKRDKSEQNIKNLKIARRKLRSAIGFGGK---

CAG9460856 M-------------------------SES--
-A------------
TRTASPASATGSFDQRFQSHIKPVLELLDNIRSLLHGCEGLGDVRDKLPTIVVTGDQSAGKSSVLESL
SGIAFPVGDGIVTRLPCQVALREGP--AFRAVCTPPEGHGEAV------
TLTDPKAVTKWIEDTTAAVAGDKKGVLDKPLSIKVEREGSADLTLVDLPGITRVAVDGQADDIEEQVK
RMIQRYISREAAVVLCVLPANVDFSTAECIKMARAVDPGGERTLGVVTKVDRAERGIVTRLNAFGTTG
WALRLGYVAVKNLSQ-
DERAKHGVSTTKVLELEDAFFDDGVGRPAHLAELADLDADMRGLRTLVQKLVQVQGERIEAFMPSL
VDSLREKCRRLEEELSGLSEPVTTEAEALRALSIVVDKFGRIVGDK-
LEGQHKTSALKRKADNSAGISPFALLHDFVSESRAQIRRSMPEFFS---DE-FFDEMRMDLRRR-
RGQ-HLPNFAPLPQDLFEKHFHRHL----EEPISDAVSRSFEMVKGVVVGELGV-FDD-
FPRLKAQVACDVDG-VFERMHIEAKDMMLRLIANERFPD-TLNHYYMDTVSKIMKDIEEHRRDKDK-
-----------QKDKKAVR-----------ELQRGLAGLCSDVAPK--YETYRLADSIKDADLKDGEFTKRCT-------
------VAGAEIEIIVYR------DAERGVPKFDIRNLSSQRLVCRFGANLKRLAPGSSDPEHHSWL------
RHGVDEIEVGA---------------------------RQSVMTVDGANI-----SNVNK-
LRDVDVIMEVFDPGAPWETHVDARSFGDAVLGATSNDEQAA--
VERQVSMAAYTKLVLKALLDCVLKELRTTL---ITER---LTGSLSEEILARR--LEHGTAPLL-----
AAMDNDQLSRKVARLRKEKAAVDEALRQVNNCAW---

;

end;
begin trees;
tree tree_1 = [&R] [&branchAttributeNames={"FastTree support value"}](((((((((KAH0683503[&"% Acidic Amino Acids"=13.63%,"% Hydrophobic Amino Acids"=49.19%,"Genetic Code"="Standard",Modified=Mon Aug 12 11:04:35 PDT 2024,Taxonomy="Eukaryota; Viridiplantae; Streptophyta; Embryophyta; Tracheophyta; Spermatophyta; Magnoliopsida; eudicotyledons; Gunneridae; Pentapetalae; asterids; lamiids; Solanales; Solanaceae; Solanoideae; Solaneae; Solanum","Common Name"="potato","Extinction Coefficient"=42455.0,Created=Tue Sep 28 00:00:00 PDT 2021,isolate="solTubOtavaFocal","Isoelectric Point"=5.458683013916016,"% Basic Amino Acids"=13.63%,"% AT-rich Amino Acids"=25.19%,"% Charged Amino Acids"=27.26%,db_xref="taxon:4113",Organism="Solanum tuberosum",Accession="KAH0683503.1","% GC-rich Amino Acids"=19.85%,chromosome="4","Molecular Weight (kDa)"=75.89402137999996,"# Nucleotide Sequences With Quality"=0,Topology="linear",cultivar="Otava","Charge at pH 7"=-14.213393667195508,"% Polar Uncharged Amino Acids"=24.00%,tissue_type="leaves","Molecule Type"="AA"]:0.27648999999999,KAK1401877[&"% Acidic Amino Acids"=14.12%,"% Hydrophobic Amino Acids"=48.91%,"Genetic Code"="Standard",Modified=Mon Aug 12 11:04:36 PDT 2024,Taxonomy="Eukaryota; Viridiplantae; Streptophyta; Embryophyta; Tracheophyta; Spermatophyta; Magnoliopsida; eudicotyledons; Gunneridae; Pentapetalae; asterids; campanulids; Apiales; Apiaceae; Apioideae; apioid superclade; Tordylieae; Tordyliinae; Heracleum","Extinction Coefficient"=47495.0,dev_stage="plant at anthesis",Created=Mon Aug 21 00:00:00 PDT 2023,isolate="Hsosn_3","Isoelectric Point"=5.616550445556641,"% Basic Amino Acids"=14.41%,"% AT-rich Amino Acids"=23.58%,"% Charged Amino Acids"=28.53%,db_xref="taxon:360622",Organism="Heracleum sosnowskyi",Accession="KAK1401877.1","% GC-rich Amino Acids"=19.07%,chromosome="1","Molecular Weight (kDa)"=77.03545468,"# Nucleotide Sequences With Quality"=0,Topology="linear","Charge at pH 7"=-13.104045396861586,"% Polar Uncharged Amino Acids"=23.29%,tissue_type="leaf","Molecule Type"="AA"]:0.29474)[&"FastTree support value"=0.247]:0.031179999999999986,(KAF8391993[&"% Charged Amino Acids"=26.77%,db_xref="taxon:13715","% Acidic Amino Acids"=11.81%,Organism="Tetracentron sinense","% Hydrophobic Amino Acids"=48.69%,"Genetic Code"="Standard",Modified=Mon Aug 12 11:04:35 PDT 2024,Taxonomy="Eukaryota; Viridiplantae; Streptophyta; Embryophyta; Tracheophyta; Spermatophyta; Magnoliopsida; Trochodendrales; Trochodendraceae; Tetracentron",Accession="KAF8391993.1","% GC-rich Amino Acids"=20.87%,chromosome="16","Molecular Weight (kDa)"=85.30159378000003,"Extinction Coefficient"=42330.0,"# Nucleotide Sequences With Quality"=0,Topology="linear",Created=Fri Oct 30 00:00:00 PDT 2020,isolate="YNK0","Charge at pH 7"=-4.4926875270876785,"Isoelectric Point"=6.575557708740234,"% Basic Amino Acids"=14.96%,"% AT-rich Amino

Acids"=23.23%,"% Polar Uncharged Amino Acids"=24.93%,tissue_type="leaf","Molecule Type"="AA"]:0.16683000000000003,(XP_058079501[&"% Charged Amino Acids"=28.13%,db_xref="taxon:86752","% Acidic Amino Acids"=13.84%,Organism="Magnolia sinica","% Hydrophobic Amino Acids"=47.62%,"Genetic Code"="Standard",Modified=Mon Aug 12 11:04:36 PDT 2024,Taxonomy="Eukaryota; Viridiplantae; Streptophyta; Embryophyta; Tracheophyta; Spermatophyta; Magnoliopsida; Magnoliidae; Magnoliales; Magnoliaceae; Magnolia",Accession="XP_058079501.1","% GC-rich Amino Acids"=18.60%,chromosome="15","Molecular Weight (kDa)"=75.59962478000001,"Extinction Coefficient"=36370.0,"# Nucleotide Sequences With Quality"=0,Topology="linear",Created=Wed Jul 19 00:00:00 PDT 2023,isolate="HGM2019","Charge at pH 7"=-11.143011250633858,"Isoelectric Point"=5.708515167236328,"% Basic Amino Acids"=14.29%,"% AT-rich Amino Acids"=23.36%,"% Polar Uncharged Amino Acids"=24.70%,tissue_type="leaf","Molecule Type"="AA"]:0.21860999999999997,(XP_038984915[&"% Acidic Amino Acids"=14.39%,"% Hydrophobic Amino Acids"=50.45%,"Genetic Code"="Standard",Modified=Mon Aug 12 11:04:35 PDT 2024,Taxonomy="Eukaryota; Viridiplantae; Streptophyta; Embryophyta; Tracheophyta; Spermatophyta; Magnoliopsida; Liliopsida; Arecaceae; Coryphoideae; Phoeniceae; Phoenix","Common Name"="date palm","Extinction Coefficient"=37735.0,Created=Wed Jan 27 00:00:00 PST 2021,"Isoelectric Point"=5.626003265380859,"% Basic Amino Acids"=14.70%,"% AT-rich Amino Acids"=20.61%,"% Charged Amino Acids"=29.09%,db_xref="taxon:42345",Organism="Phoenix dactylifera",Accession="XP_038984915.1","% GC-rich Amino Acids"=25.00%,chromosome="8",sex="male","Molecular Weight (kDa)"=73.78827977999997,"# Nucleotide Sequences With Quality"=0,Topology="linear",cultivar="Barhee BC4","Charge at pH 7"=-12.005012141875643,"% Polar Uncharged Amino Acids"=20.91%,tissue_type="young leaves","Molecule Type"="AA"]:0.20564999999999999,(PWZ56864[&"% Acidic Amino Acids"=13.20%,"% Hydrophobic Amino Acids"=49.55%,"Genetic Code"="Standard",Modified=Mon Aug 12 11:04:35 PDT 2024,Taxonomy="Eukaryota; Viridiplantae; Streptophyta; Embryophyta; Tracheophyta; Spermatophyta; Magnoliopsida; Liliopsida; Poales; Poaceae; PACMAD clade; Panicoideae; Andropogonodae; Andropogoneae; Tripsacinae; Zea","Extinction Coefficient"=37985.0,dev_stage="14-day seedling",Created=Mon Jun 04 00:00:00 PDT 2018,"Isoelectric Point"=6.323802947998047,"% Basic Amino Acids"=14.24%,"% AT-rich Amino Acids"=19.44%,"% Charged Amino Acids"=27.45%,db_xref="taxon:4577",Organism="Zea mays",Accession="PWZ56864.1","% GC-rich Amino Acids"=24.48%,chromosome="1","Molecular Weight (kDa)"=74.55908788,"# Nucleotide Sequences With Quality"=0,Topology="linear",cultivar="inbred line Mo17","Charge at pH 7"=-3.612842663651275,"% Polar Uncharged Amino Acids"=23.44%,tissue_type="seedling","Molecule Type"="AA"]:0.13846000000000025,PWZ56863[&"% Acidic Amino Acids"=13.65%,"% Hydrophobic Amino Acids"=50.00%,"Genetic Code"="Standard",Modified=Mon Aug 12

11:04:35 PDT 2024,Taxonomy="Eukaryota; Viridiplantae; Streptophyta; Embryophyta; Tracheophyta; Spermatophyta; Magnoliopsida; Liliopsida; Poales; Poaceae; PACMAD clade; Panicoideae; Andropogonodae; Andropogoneae; Tripsacinae; Zea","Extinction Coefficient"=35340.0,dev_stage="14-day seedling",Created=Mon Jun 04 00:00:00 PDT 2018,"Isoelectric Point"=5.948627471923828,"% Basic Amino Acids"=14.24%,"% AT-rich Amino Acids"=21.07%,"% Charged Amino Acids"=27.89%,db_xref="taxon:4577",Organism="Zea mays",Accession="PWZ56863.1","% GC-rich Amino Acids"=24.18%,chromosome="1","Molecular Weight (kDa)"=74.49698797999999,"# Nucleotide Sequences With Quality"=0,Topology="linear",cultivar="inbred line Mo17","Charge at pH 7"=-6.4788212421916676,"% Polar Uncharged Amino Acids"=22.40%,tissue_type="seedling","Molecule Type"="AA"]:0.11383999999999972)[&"FastTree support value"=1.0]:0.23421000000000003)[&"FastTree support value"=0.997]:0.10407999999999973)[&"FastTree support value"=0.992]:0.07774000000000036)[&"FastTree support value"=0.948]:0.04428999999999972)[&"FastTree support value"=0.511]:0.026459999999999706,(KAF5727250[&"% Acidic Amino Acids"=13.47%,"% Hydrophobic Amino Acids"=49.55%,"Genetic Code"="Standard",Modified=Mon Aug 12 11:04:35 PDT 2024,Taxonomy="Eukaryota; Viridiplantae; Streptophyta; Embryophyta; Tracheophyta; Spermatophyta; Magnoliopsida; eudicotyledons; Gunneridae; Pentapetalae; rosids; fabids; Celastrales; Celastraceae; Tripterygium","Extinction Coefficient"=37985.0,dev_stage="mature plant",Created=Fri Jul 10 00:00:00 PDT 2020,isolate="XIE 37","Isoelectric Point"=5.683628082275391,"% Basic Amino Acids"=13.92%,"% AT-rich Amino Acids"=22.60%,"% Charged Amino Acids"=27.40%,db_xref="taxon:458696",Organism="Tripterygium wilfordii",Accession="KAF5727250.1","% GC-rich Amino Acids"=19.91%,chromosome="22","Molecular Weight (kDa)"=74.60867358,"# Nucleotide Sequences With Quality"=0,Topology="linear","Charge at pH 7"=-12.178492403730495,"% Polar Uncharged Amino Acids"=23.50%,tissue_type="leaf","Molecule Type"="AA"]:0.21660999999999975,(XP_024439231[&"% Charged Amino Acids"=29.14%,db_xref="taxon:3694","% Acidic Amino Acids"=13.46%,Organism="Populus trichocarpa","% Hydrophobic Amino Acids"=48.22%,"Genetic Code"="Standard",Modified=Mon Aug 12 11:04:35 PDT 2024,Taxonomy="Eukaryota; Viridiplantae; Streptophyta; Embryophyta; Tracheophyta; Spermatophyta; Magnoliopsida; eudicotyledons; Gunneridae; Pentapetalae; rosids; fabids; Malpighiales; Salicaceae; Saliceae; Populus",Accession="XP_024439231.1","% GC-rich Amino Acids"=19.82%,"Common Name"="Populus balsamifera subsp. trichocarpa",chromosome="13","Molecular Weight (kDa)"=75.87577707999995,"Extinction Coefficient"=43360.0,"# Nucleotide Sequences With Quality"=0,Topology="linear",Created=Thu Dec 08 00:00:00 PST 2022,isolate="Nisqually-1","Charge at pH 7"=-5.450467939091386,"Isoelectric Point"=6.389705657958984,"% Basic Amino Acids"=15.68%,"% AT-rich Amino Acids"=24.56%,"% Polar Uncharged Amino Acids"=23.22%,"Molecule Type"="AA"]:0.2300500000000003,(XP_002303204[&"% Charged

Amino Acids"=27.90%,db_xref="taxon:3694","% Acidic Amino Acids"=13.80%,Organism="Populus trichocarpa","% Hydrophobic Amino Acids"=48.02%,"Genetic Code"="Standard",Modified=Mon Aug 12 11:04:36 PDT 2024,Taxonomy="Eukaryota; Viridiplantae; Streptophyta; Embryophyta; Tracheophyta; Spermatophyta; Magnoliopsida; eudicotyledons; Gunneridae; Pentapetalae; rosids; fabids; Malpighiales; Salicaceae; Saliceae; Populus",Accession="XP_002303204.3","% GC-rich Amino Acids"=20.12%,"Common Name"="Populus balsamifera subsp. trichocarpa",chromosome="3","Molecular Weight (kDa)"=76.48913967999991,"Extinction Coefficient"=43360.0,"# Nucleotide Sequences With Quality"=0,Topology="linear",Created=Thu Dec 08 00:00:00 PST 2022,isolate="Nisqually-1","Charge at pH 7"=-16.56672690033052,"Isoelectric Point"=5.552570343017578,"% Basic Amino Acids"=14.10%,"% AT-rich Amino Acids"=23.35%,"% Polar Uncharged Amino Acids"=24.67%,"Molecule Type"="AA"]:0.13331999999999988,XP_002297993[&"% Charged Amino Acids"=26.85%,db_xref="taxon:3694","% Acidic Amino Acids"=13.50%,Organism="Populus trichocarpa","% Hydrophobic Amino Acids"=49.06%,"Genetic Code"="Standard",Modified=Mon Aug 12 11:04:33 PDT 2024,Taxonomy="Eukaryota; Viridiplantae; Streptophyta; Embryophyta; Tracheophyta; Spermatophyta; Magnoliopsida; eudicotyledons; Gunneridae; Pentapetalae; rosids; fabids; Malpighiales; Salicaceae; Saliceae; Populus",Accession="XP_002297993.1","% GC-rich Amino Acids"=21.34%,"Common Name"="Populus balsamifera subsp. trichocarpa",chromosome="1","Molecular Weight (kDa)"=77.44427127999998,"Extinction Coefficient"=54360.0,"# Nucleotide Sequences With Quality"=0,Topology="linear",Created=Thu Dec 08 00:00:00 PST 2022,isolate="Nisqually-1","Charge at pH 7"=-15.136533230290999,"Isoelectric Point"=5.398387908935547,"% Basic Amino Acids"=13.35%,"% AT-rich Amino Acids"=22.21%,"% Polar Uncharged Amino Acids"=24.96%,"Molecule Type"="AA"]:0.24607)[&"FastTree support value"=0.691]:0.024210000000000065)[&"FastTree support value"=0.972]:0.049490000000000034)[&"FastTree support value"=0.993]:0.07020000000000026)[&"FastTree support value"=0.772]:0.05176999999999987,(KAF8079489[&"% Acidic Amino Acids"=13.13%,"% Hydrophobic Amino Acids"=48.21%,"Genetic Code"="Standard",Modified=Thu Oct 22 00:00:00 PDT 2020,Taxonomy="Eukaryota; Viridiplantae; Streptophyta; Embryophyta; Tracheophyta; Spermatophyta; Magnoliopsida; eudicotyledons; Gunneridae; Pentapetalae; rosids; malvids; Brassicales; Brassicaceae; Brassiceae; Sinapis","Common Name"="white mustard","Extinction Coefficient"=43360.0,Created=Thu Oct 22 00:00:00 PDT 2020,"Isoelectric Point"=6.418010711669922,"% Basic Amino Acids"=14.48%,"% AT-rich Amino Acids"=23.58%,strain="S2 GC0560-79","% Charged Amino Acids"=27.61%,db_xref="taxon:3728",Organism="Sinapis alba",Accession="KAF8079489.1","% GC-rich Amino Acids"=19.85%,chromosome="Unknown","Molecular Weight (kDa)"=74.74961017999996,"# Nucleotide Sequences With Quality"=0,Topology="linear","Charge at pH 7"=-3.279498820243248,"% Polar Uncharged Amino Acids"=24.78%,tissue_type="green leaf","Molecule Type"="AA",isolation_source="green leaf"]:0.09432999999999998,(OAP19580[&"% Acidic

Amino Acids"=12.50%,"% Hydrophobic Amino Acids"=49.38%,"Genetic Code"="Standard",Modified=Mon Aug 12 11:04:34 PDT 2024,Taxonomy="Eukaryota; Viridiplantae; Streptophyta; Embryophyta; Tracheophyta; Spermatophyta; Magnoliopsida; eudicotyledons; Gunneridae; Pentapetalae; rosids; malvids; Brassicales; Brassicaceae; Camelineae; Arabidopsis","Common Name"="thale cress","Extinction Coefficient"=10220.0,dev_stage="adult",Created=Wed May 25 00:00:00 PDT 2016,"Isoelectric Point"=4.586406707763672,"% Basic Amino Acids"=10.00%,"% AT-rich Amino Acids"=17.50%,"% Charged Amino Acids"=22.50%,db_xref="taxon:3702",Organism="Arabidopsis thaliana",Accession="OAP19580.1","% GC-rich Amino Acids"=23.13%,chromosome="1","Molecular Weight (kDa)"=17.32986838000002,"# Nucleotide Sequences With Quality"=0,Topology="linear","Charge at pH 7"=-5.245775862197959,"% Polar Uncharged Amino Acids"=28.75%,tissue_type="leaf","Molecule Type"="AA"]:0.1873999999999998,(OAP13972[&"% Acidic Amino Acids"=10.76%,"% Hydrophobic Amino Acids"=51.27%,"Genetic Code"="Standard",Modified=Mon Aug 12 11:04:34 PDT 2024,Taxonomy="Eukaryota; Viridiplantae; Streptophyta; Embryophyta; Tracheophyta; Spermatophyta; Magnoliopsida; eudicotyledons; Gunneridae; Pentapetalae; rosids; malvids; Brassicales; Brassicaceae; Camelineae; Arabidopsis","Common Name"="thale cress","Extinction Coefficient"=10095.0,dev_stage="adult",Created=Wed May 25 00:00:00 PDT 2016,"Isoelectric Point"=7.011775970458984,"% Basic Amino Acids"=12.66%,"% AT-rich Amino Acids"=18.99%,"% Charged Amino Acids"=23.42%,db_xref="taxon:3702",Organism="Arabidopsis thaliana",Accession="OAP13972.1","% GC-rich Amino Acids"=25.95%,chromosome="1","Molecular Weight (kDa)"=17.169832280000012,"# Nucleotide Sequences With Quality"=0,Topology="linear","Charge at pH 7"=0.015152986826631687,"% Polar Uncharged Amino Acids"=25.95%,tissue_type="leaf","Molecule Type"="AA"]:0.02383999999999986,OAP13353[&"% Acidic Amino Acids"=10.81%,"% Hydrophobic Amino Acids"=50.68%,"Genetic Code"="Standard",Modified=Mon Aug 12 11:04:34 PDT 2024,Taxonomy="Eukaryota; Viridiplantae; Streptophyta; Embryophyta; Tracheophyta; Spermatophyta; Magnoliopsida; eudicotyledons; Gunneridae; Pentapetalae; rosids; malvids; Brassicales; Brassicaceae; Camelineae; Arabidopsis","Common Name"="thale cress","Extinction Coefficient"=8605.0,dev_stage="adult",Created=Wed May 25 00:00:00 PDT 2016,"Isoelectric Point"=6.233654022216797,"% Basic Amino Acids"=12.84%,"% AT-rich Amino Acids"=16.89%,"% Charged Amino Acids"=23.65%,db_xref="taxon:3702",Organism="Arabidopsis thaliana",Accession="OAP13353.1","% GC-rich Amino Acids"=25.68%,chromosome="1","Molecular Weight (kDa)"=16.16756978000001,"# Nucleotide Sequences With Quality"=0,Topology="linear","Charge at pH 7"=-1.719159906185574,"% Polar Uncharged Amino Acids"=26.35%,tissue_type="leaf","Molecule

Type"="AA"]:0.11222999999999983)[&"FastTree support value"=0.837]:0.06949000000000005)[&"FastTree support value"=0.868]:0.10060000000000002)[&"FastTree support value"=1.0]:0.28717000000000015)[&"FastTree support value"=1.0]:0.2501599999999997,(((KAH9325151[&"% Acidic Amino Acids"=10.60%,"% Hydrophobic Amino Acids"=49.31%,"Genetic Code"="Standard",Modified=Mon Aug 12 11:04:35 PDT 2024,Taxonomy="Eukaryota; Viridiplantae; Streptophyta; Embryophyta; Tracheophyta; Spermatophyta; Pinopsida; Pinidae; Conifers II; Cupressales; Taxaceae; Taxus","Extinction Coefficient"=13200.0,dev_stage="mature",Created=Tue Jan 25 00:00:00 PST 2022,isolate="Ta-2019","Isoelectric Point"=5.268238067626953,"% Basic Amino Acids"=10.14%,"% AT-rich Amino Acids"=29.49%,"% Charged Amino Acids"=20.74%,db_xref="taxon:29808",Organism="Taxus chinensis",Accession="KAH9325151.1","% GC-rich Amino Acids"=16.13%,chromosome="2",sex="female","Molecular Weight (kDa)"=23.75958078000002,"# Nucleotide Sequences With Quality"=0,Topology="linear","Charge at pH 7"=-2.2470332478955557,"% Polar Uncharged Amino Acids"=30.41%,tissue_type="Leaf","Molecule Type"="AA"]:0.18892999999999982,(KAH9300179[&"% Acidic Amino Acids"=11.70%,"% Hydrophobic Amino Acids"=48.54%,"Genetic Code"="Standard",Modified=Mon Aug 12 11:04:35 PDT 2024,Taxonomy="Eukaryota; Viridiplantae; Streptophyta; Embryophyta; Tracheophyta; Spermatophyta; Pinopsida; Pinidae; Conifers II; Cupressales; Taxaceae; Taxus","Extinction Coefficient"=13075.0,dev_stage="mature",Created=Tue Jan 25 00:00:00 PST 2022,isolate="Ta-2019","Isoelectric Point"=5.203029632568359,"% Basic Amino Acids"=11.11%,"% AT-rich Amino Acids"=25.15%,"% Charged Amino Acids"=22.81%,db_xref="taxon:29808",Organism="Taxus chinensis",Accession="KAH9300179.1","% GC-rich Amino Acids"=21.05%,chromosome="4",sex="female","Molecular Weight (kDa)"=19.071952580000012,"# Nucleotide Sequences With Quality"=0,Topology="linear","Charge at pH 7"=-2.1811466196014724,"% Polar Uncharged Amino Acids"=29.24%,tissue_type="Leaf","Molecule Type"="AA"]:0.16435,KAH9314974[&"% Acidic Amino Acids"=10.89%,"% Hydrophobic Amino Acids"=45.97%,"Genetic Code"="Standard",Modified=Mon Aug 12 11:04:35 PDT 2024,Taxonomy="Eukaryota; Viridiplantae; Streptophyta; Embryophyta; Tracheophyta; Spermatophyta; Pinopsida; Pinidae; Conifers II; Cupressales; Taxaceae; Taxus","Extinction Coefficient"=30285.0,dev_stage="mature",Created=Tue Jan 25 00:00:00 PST 2022,isolate="Ta-2019","Isoelectric Point"=7.393680572509766,"% Basic Amino Acids"=12.50%,"% AT-rich Amino Acids"=25.81%,"% Charged Amino Acids"=23.39%,db_xref="taxon:29808",Organism="Taxus chinensis",Accession="KAH9314974.1","% GC-rich Amino Acids"=19.76%,chromosome="7",sex="female","Molecular Weight (kDa)"=27.935113980000025,"# Nucleotide Sequences With Quality"=0,Topology="linear","Charge at pH 7"=0.8143130361885939,"% Polar Uncharged Amino Acids"=31.85%,tissue_type="Leaf","Molecule Type"="AA"]:0.15616000000000008)[&"FastTree support value"=0.81]:0.10907)[&"FastTree

support value"=0.999]:0.3326300000000002,(KAH9320939[&"% Acidic Amino Acids"=14.55%,"% Hydrophobic Amino Acids"=48.85%,"Genetic Code"="Standard",Modified=Mon Aug 12 11:04:35 PDT 2024,Taxonomy="Eukaryota; Viridiplantae; Streptophyta; Embryophyta; Tracheophyta; Spermatophyta; Pinopsida; Pinidae; Conifers II; Cupressales; Taxaceae; Taxus","Extinction Coefficient"=29630.0,dev_stage="mature",Created=Tue Jan 25 00:00:00 PST 2022,isolate="Ta-2019","Isoelectric Point"=5.030315399169922,"% Basic Amino Acids"=13.40%,"% AT-rich Amino Acids"=21.76%,"% Charged Amino Acids"=27.95%,db_xref="taxon:29808",Organism="Taxus chinensis",Accession="KAH9320939.1","% GC-rich Amino Acids"=21.18%,chromosome="5",sex="female","Molecular Weight (kDa)"=77.18898967999996,"# Nucleotide Sequences With Quality"=0,Topology="linear","Charge at pH 7"=-19.627328951892576,"% Polar Uncharged Amino Acids"=23.49%,tissue_type="Leaf","Molecule Type"="AA"]:0.3275999999999999,KAH9291961[&"% Acidic Amino Acids"=13.11%,"% Hydrophobic Amino Acids"=46.45%,"Genetic Code"="Standard",Modified=Mon Aug 12 11:04:35 PDT 2024,Taxonomy="Eukaryota; Viridiplantae; Streptophyta; Embryophyta; Tracheophyta; Spermatophyta; Pinopsida; Pinidae; Conifers II; Cupressales; Taxaceae; Taxus","Extinction Coefficient"=40505.0,dev_stage="mature",Created=Tue Jan 25 00:00:00 PST 2022,isolate="Ta-2019","Isoelectric Point"=5.592037200927734,"% Basic Amino Acids"=13.11%,"% AT-rich Amino Acids"=22.43%,"% Charged Amino Acids"=26.23%,db_xref="taxon:29808",Organism="Taxus chinensis",Accession="KAH9291961.1","% GC-rich Amino Acids"=19.36%,sex="female","Molecular Weight (kDa)"=91.35399638000017,"# Nucleotide Sequences With Quality"=0,Topology="linear","Charge at pH 7"=-10.58385806878805,"% Polar Uncharged Amino Acids"=27.82%,tissue_type="Leaf","Molecule Type"="AA"]:0.44014)[&"FastTree support value"=0.229]:0.05005000000000015)[&"FastTree support value"=0.338]:0.02275999999999989,KAH9290598[&"% Acidic Amino Acids"=14.03%,"% Hydrophobic Amino Acids"=48.21%,"Genetic Code"="Standard",Modified=Mon Aug 12 11:04:35 PDT 2024,Taxonomy="Eukaryota; Viridiplantae; Streptophyta; Embryophyta; Tracheophyta; Spermatophyta; Pinopsida; Pinidae; Conifers II; Cupressales; Taxaceae; Taxus","Extinction Coefficient"=42915.0,dev_stage="mature",Created=Tue Jan 25 00:00:00 PST 2022,isolate="Ta-2019","Isoelectric Point"=6.566585540771484,"% Basic Amino Acids"=15.07%,"% AT-rich Amino Acids"=26.12%,"% Charged Amino Acids"=29.10%,db_xref="taxon:29808",Organism="Taxus chinensis",Accession="KAH9290598.1","% GC-rich Amino Acids"=22.09%,chromosome="10",sex="female","Molecular Weight (kDa)"=75.33005738000003,"# Nucleotide Sequences With Quality"=0,Topology="linear","Charge at pH 7"=-1.8151639582550025,"% Polar Uncharged Amino Acids"=22.99%,tissue_type="Leaf","Molecule Type"="AA"]:0.40473000000000026)[&"FastTree support value"=0.923]:0.0718399999999999)[&"FastTree support value"=1.0]:0.30382,EFJ22917[&"% Charged Amino

Acids"=28.89%,db_xref="taxon:88036","% Acidic Amino
Acids"=12.59%,Organism="Selaginella moellendorffii","% Hydrophobic Amino
Acids"=44.63%,"Genetic Code"="Standard",Modified=Mon Jul 25 00:00:00 PDT
2016,Taxonomy="Eukaryota; Viridiplantae; Streptophyta; Embryophyta; Tracheophyta;
Lycopodiopsida; Selaginellales; Selaginellaceae; Selaginella",Accession="EFJ22917.1","%
GC-rich Amino Acids"=26.67%,chromosome="Unknown","Molecular Weight
(kDa)"=60.320899180000005,"Extinction Coefficient"=39015.0,"# Nucleotide Sequences
With Quality"=0,Topology="linear",Created=Mon Jul 25 00:00:00 PDT 2016,"Charge at pH
7"=9.376331029545756,"Isoelectric Point"=8.635631561279297,"% Basic Amino
Acids"=16.30%,"% AT-rich Amino Acids"=17.22%,"% Polar Uncharged Amino
Acids"=27.22%,"Molecule Type"="AA"]:0.8690900000000004)[&"FastTree support
value"=0.957]:0.15498000000000012,(KAJ7294545[&"% Charged Amino
Acids"=29.50%,db_xref="taxon:34168","% Acidic Amino
Acids"=13.92%,Organism="Diphasiastrum complanatum","% Hydrophobic Amino
Acids"=46.29%,"Genetic Code"="Standard",Modified=Mon Mar 20 00:00:00 PDT
2023,Taxonomy="Eukaryota; Viridiplantae; Streptophyta; Embryophyta; Tracheophyta;
Lycopodiopsida; Lycopodiales; Lycopodiaceae; Lycopodioideae;
Diphasiastrum",Accession="KAJ7294545.1","% GC-rich Amino
Acids"=20.88%,chromosome="Unknown","Molecular Weight
(kDa)"=75.28553777999998,"Extinction Coefficient"=42205.0,"# Nucleotide Sequences
With Quality"=0,Topology="linear",Created=Mon Mar 20 00:00:00 PDT
2023,cultivar="PW_Plant_1","Charge at pH 7"=-5.713675474010753,"Isoelectric
Point"=6.275577545166016,"% Basic Amino Acids"=15.58%,"% AT-rich Amino
Acids"=23.90%,"% Polar Uncharged Amino Acids"=24.66%,"Molecule
Type"="AA"]:0.3151100000000002,((XP_024380180[&"% Charged Amino
Acids"=27.84%,db_xref="taxon:3218","% Acidic Amino
Acids"=13.62%,Organism="Physcomitrium patens","% Hydrophobic Amino
Acids"=47.01%,"Genetic Code"="Standard",Modified=Mon Aug 12 11:04:35 PDT
2024,Taxonomy="Eukaryota; Viridiplantae; Streptophyta; Embryophyta; Bryophyta;
Bryophytina; Bryopsida; Funariidae; Funariales; Funariaceae;
Physcomitrium",Accession="XP_024380180.1","% GC-rich Amino
Acids"=21.86%,chromosome="7","Molecular Weight
(kDa)"=75.34764137999996,"Extinction Coefficient"=29380.0,"# Nucleotide Sequences
With Quality"=0,Topology="linear",Created=Wed Apr 04 00:00:00 PDT 2018,"Charge at pH
7"=-11.043197193814624,"Isoelectric Point"=5.759677886962891,"% Basic Amino
Acids"=14.22%,"% AT-rich Amino Acids"=20.66%,"% Polar Uncharged Amino
Acids"=25.45%,"Molecule Type"="AA"]:0.01026999999999978,XP_024367947[&"%
Charged Amino Acids"=28.82%,db_xref="taxon:3218","% Acidic Amino
Acids"=13.85%,Organism="Physcomitrium patens","% Hydrophobic Amino
Acids"=47.45%,"Genetic Code"="Standard",Modified=Mon Aug 12 11:04:35 PDT
2024,Taxonomy="Eukaryota; Viridiplantae; Streptophyta; Embryophyta; Bryophyta;
Bryophytina; Bryopsida; Funariidae; Funariales; Funariaceae;
Physcomitrium",Accession="XP_024367947.1","% GC-rich Amino
Acids"=22.77%,chromosome="Unknown","Molecular Weight

(kDa)"=70.98869067999993,"Extinction Coefficient"=27765.0,"# Nucleotide Sequences With Quality"=0,Topology="linear",Created=Wed Apr 04 00:00:00 PDT 2018,"Charge at pH 7"=-8.811216914008607,"Isoelectric Point"=5.974689483642578,"% Basic Amino Acids"=14.97%,"% AT-rich Amino Acids"=20.38%,"% Polar Uncharged Amino Acids"=24.04%,"Molecule Type"="AA"]:0.00586000000000198)[&"FastTree support value"=1.0]:0.34682000000000013,(KAG0561847[&strain="R40","% Charged Amino Acids"=27.90%,db_xref="taxon:3225","% Acidic Amino Acids"=13.88%,Organism="Ceratodon purpureus","% Hydrophobic Amino Acids"=46.46%,"Genetic Code"="Standard",Modified=Mon Aug 12 11:04:35 PDT 2024,Taxonomy="Eukaryota; Viridiplantae; Streptophyta; Embryophyta; Bryophyta; Bryophytina; Bryopsida; Dicranidae; Pseudoditrichales; Ditrichaceae; Ceratodon",Accession="KAG0561847.1","% GC-rich Amino Acids"=22.62%,chromosome="9",sex="male","Molecular Weight (kDa)"=74.42137688,"Extinction Coefficient"=39475.0,"# Nucleotide Sequences With Quality"=0,Topology="linear",Created=Mon Dec 28 00:00:00 PST 2020,"Charge at pH 7"=-10.506836906116629,"Isoelectric Point"=5.606029510498047,"% Basic Amino Acids"=14.03%,"% AT-rich Amino Acids"=19.76%,"% Polar Uncharged Amino Acids"=26.09%,"Molecule Type"="AA"]:0.20638000000000023,KAG0619429[&strain="GG1","% Charged Amino Acids"=29.58%,db_xref="taxon:3225","% Acidic Amino Acids"=14.56%,Organism="Ceratodon purpureus","% Hydrophobic Amino Acids"=46.25%,"Genetic Code"="Standard",Modified=Mon Aug 12 11:04:35 PDT 2024,Taxonomy="Eukaryota; Viridiplantae; Streptophyta; Embryophyta; Bryophyta; Bryophytina; Bryopsida; Dicranidae; Pseudoditrichales; Ditrichaceae; Ceratodon",Accession="KAG0619429.1","% GC-rich Amino Acids"=20.27%,chromosome="4",sex="female","Molecular Weight (kDa)"=75.43685337999996,"Extinction Coefficient"=35465.0,"# Nucleotide Sequences With Quality"=0,Topology="linear",Created=Mon Dec 28 00:00:00 PST 2020,"Charge at pH 7"=-13.015769556202113,"Isoelectric Point"=5.657138824462891,"% Basic Amino Acids"=15.02%,"% AT-rich Amino Acids"=21.92%,"% Polar Uncharged Amino Acids"=24.47%,"Molecule Type"="AA"]:0.1668599999999998)[&"FastTree support value"=0.996]:0.10817999999999994)[&"FastTree support value"=0.998]:0.1659799999999998)[&"FastTree support value"=1.0]:0.21988999999999992)[&"FastTree support value"=0.958]:0.16922999999999977,(GJP35534[&strain="NIES-68","% Charged Amino Acids"=25.64%,db_xref="taxon:2019903","% Acidic Amino Acids"=12.97%,Organism="Closterium sp. NIES-68","% Hydrophobic Amino Acids"=49.77%,"Genetic Code"="Standard",Modified=Mon Aug 12 11:04:36 PDT 2024,Taxonomy="Eukaryota; Viridiplantae; Streptophyta; Zygnemophyceae; Zygnematophycidae; Desmidiales; Closteriaceae; Closterium; Closterium peracerosum-strigosum-littorale complex",Accession="GJP35534.1","% GC-rich Amino Acids"=25.19%,"Molecular Weight (kDa)"=72.93767118000008,"Extinction Coefficient"=27110.0,"# Nucleotide Sequences With Quality"=0,Topology="linear",culture_collection="NIES:68",Created=Tue Dec 27 00:00:00

PST 2022,"Charge at pH 7"=-12.75043257997837,"Isoelectric Point"=5.318119049072266,"% Basic Amino Acids"=12.67%,"% AT-rich Amino Acids"=20.21%,"% Polar Uncharged Amino Acids"=24.74%,"Molecule Type"="AA"]:0.21050000000000013,CAI5480041[&strain="NIES-4552=Yama58-4","% Charged Amino Acids"=27.08%,db_xref="taxon:2996821",note="contig: tig00006298","% Acidic Amino Acids"=14.06%,Organism="Closterium sp. Yama58-4","% Hydrophobic Amino Acids"=47.74%,"Genetic Code"="Standard",Modified=Sun Jun 18 00:00:00 PDT 2023,Taxonomy="Eukaryota; Viridiplantae; Streptophyta; Zygnemophyceae; Zygnematophycidae; Desmidiales; Closteriaceae; Closterium; Closterium peracerosum-strigosum-littorale complex",Accession="CAI5480041.1","% GC-rich Amino Acids"=23.61%,"Molecular Weight (kDa)"=63.69994967999995,"Extinction Coefficient"=29715.0,"# Nucleotide Sequences With Quality"=0,Topology="linear",Created=Sun Jun 18 00:00:00 PDT 2023,"Charge at pH 7"=-11.91625458415476,"Isoelectric Point"=5.015522003173828,"% Basic Amino Acids"=13.02%,"% AT-rich Amino Acids"=22.40%,"% Polar Uncharged Amino Acids"=25.35%,"Molecule Type"="AA"]:0.10796999999999999)[&"FastTree support value"=1.0]:0.46743999999999986):0.20439499999999988,(CAG9460856[&strain="YPF-701","% Charged Amino Acids"=32.23%,db_xref="taxon:765719",note="contig: scf7180000004436.1247503.F.6849.2197154.F","% Acidic Amino Acids"=15.93%,Organism="Pedinophyceae sp. YPF-701","% Hydrophobic Amino Acids"=49.39%,"Genetic Code"="Standard",Modified=Mon Jun 27 00:00:00 PDT 2022,Taxonomy="Eukaryota; Viridiplantae; Chlorophyta; Pedinophyceae",Accession="CAG9460856.1","% GC-rich Amino Acids"=28.31%,"Molecular Weight (kDa)"=90.30091008000021,"Extinction Coefficient"=40170.0,"# Nucleotide Sequences With Quality"=0,Topology="linear",Created=Mon Jun 27 00:00:00 PDT 2022,"Charge at pH 7"=-14.916549173412331,"Isoelectric Point"=5.661197662353516,"% Basic Amino Acids"=16.30%,"% AT-rich Amino Acids"=16.30%,"% Polar Uncharged Amino Acids"=19.00%,"Molecule Type"="AA"]:1.00975,(GMH36208[&strain="KO-2023","% Charged Amino Acids"=28.68%,db_xref="taxon:3041901",collection_date="2019-11-07","% Acidic Amino Acids"=13.70%,Organism="Bryopsis sp. KO-2023","% Hydrophobic Amino Acids"=48.66%,"Genetic Code"="Standard",Modified=Mon Aug 12 11:04:36 PDT 2024,Taxonomy="Eukaryota; Viridiplantae; Chlorophyta; Ulvophyceae; TCBD clade; Bryopsidales; Bryopsidineae; Bryopsidaceae; Bryopsis",Accession="GMH36208.1","% GC-rich Amino Acids"=24.97%,"Molecular Weight (kDa)"=87.66858938000009,"Extinction Coefficient"=30995.0,"# Nucleotide Sequences With Quality"=0,Topology="linear",Created=Sat May 27 00:00:00 PDT 2023,"Charge at pH 7"=-10.565828367183034,"Isoelectric Point"=6.034984588623047,"% Basic Amino Acids"=14.98%,"% AT-rich Amino Acids"=18.31%,"% Polar Uncharged Amino Acids"=22.92%,"Molecule Type"="AA"]:0.5682899999999997,GMH43921[&strain="KO-2023","% Charged Amino Acids"=30.06%,db_xref="taxon:3041901",collection_date="2019-11-07","% Acidic Amino Acids"=15.18%,Organism="Bryopsis sp. KO-2023","% Hydrophobic Amino Acids"=48.01%,"Genetic Code"="Standard",Modified=Mon Aug 12 11:04:36 PDT 2024,Taxonomy="Eukaryota; Viridiplantae; Chlorophyta; Ulvophyceae; TCBD clade; Bryopsidales; Bryopsidineae; Bryopsidaceae; Bryopsis",Accession="GMH43921.1","% GC-

rich Amino Acids"=23.31%,"Molecular Weight (kDa)"=72.50938077999992,"Extinction Coefficient"=27555.0,"# Nucleotide Sequences With Quality"=0,Topology="linear",Created=Sat May 27 00:00:00 PDT 2023,"Charge at pH 7"=-10.79317009729061,"Isoelectric Point"=5.409976959228516,"% Basic Amino Acids"=14.88%,"% AT-rich Amino Acids"=21.01%,"% Polar Uncharged Amino Acids"=22.39%,"Molecule Type"="AA"]:0.6381799999999997)[&"FastTree support value"=1.0]:0.6759200000000001)[&"FastTree support value"=0.998]:0.20439499999999988);
end;
