# Supplementary material for: Antiviral Mx proteins have an ancient origin and widespread distribution among eukaryotes
Source: Proc Natl Acad Sci U S A. 2025 Jan 24;122(4):e2416811122. doi: 10.1073/pnas.2416811122 (PMC11789081; doi:10.1073/pnas.2416811122)
Supplement: Supplementary file 16 — Dataset S15 (PDF) [file pnas.2416811122.sd15.pdf]

Dataset S15. Suppl_Figure_3_MAFFT

>KAH9290598
M--------------------LSEGGNGRS--TIERGAGAFEID------------
YKNVRKEDGSYSLNACFNREIRPLLDAVDKLRNLGAVKEGI-----
HLPTIVVVGDQSSGKSSVLESLSGIDLPRGQGICTRVPLIMRLQNST--EEYSVIS-VEYKD-RK------
LSINEHQIVDTINLVTEEIAGRNKGISDDPITLHVRKKNVPDLTLVDLPGITRVPVYGQPKDIYEQVYKII
MKYISPRDSIILNVLSATVDFPTCESIRMSQKVDEDGERTLAVVTKVDKAPEGLREKVAED---
AMNIGLGYVCVRNRVE-GE------SIVKARKKENELFKTH-------
PLLSGIDKSIVGIPILAHKLMKIQAAGITNSLPRIMKEIDTKLARRQAELNDLPENLCNPADAMILLTTV
MGSIKDSLNGLLLQGDYQ------EFSEDGGMHCAARLNEMFMGYYRDLCASATDMNDVNGQN-
FLLKEITMLKEA-QGV-GLPNF--LSRQVFLNLVQQRA-
NGVAEISLRVVEKVWDYLDGVMLRVIDRECQT-
YPQLNAATKRAAGHLLIRRNKEDCIEYVKDMIETEKCVDFTVNPLYMDTYTKLHDQKDRFLYALRN--
----------KKKT---
---FSIDGFGE-----VKLEN-
IKE-------------------------------DDQLQAA--
FDMKMSVVAYWKVVIFRLADGIPLHLRFVYRKMVRKE---IGGDLMKEIAGSN--LD-
MIEKIFQESPAVASKRRSLIDSLALLRDAKLVVYNIIDKNNA--

>XP_002297993
MSGG----------KRIGRPSKGKNGFHSS--
SYEEDSLPLDVRVEENLLAIVG-GDENQPTPIHSVPIMSSFNDRIRPILDAVDQLRHLMVMKEGI-----
QLPTIVVVGDQSSGKSSVLESLAGISLPRGQGICTRVPLIMRLQHHT--SLIPEMF-LEFNG-KT------
TQTDEANVADDINIATEEIAGSGKGISDAPLTLVIKKNGVPDLTMVDLPGITRVPVHGQPDNIYEQIAG
IVMQYIQPEESIILNVLPASVDFTTCESIRMSRQVDKTGERTLAVVTKADKAPEGLLEKVTAD---
DVNIGLGYVCVRNRIG-DE------SYDNARMEEANLFATH-------
PLLSRIDKSIVGIPVLAKKLMQVQATIMAKCWPEIVRKINEKLNGNVTELNRMPKAMSSVAEFLTAFM
EFIGSVKESLTKILVRGEYD------EYPDDPNMHGVARVVEMFNQYSDELLNCPESEHT---RN-
FLMDEIRVLDDS-KAI-ALPNF--LPRHAFLSLLQRKV-
ERVSHIPFGFVEKAWAYFENVVWSVSRHHTEN-YPQVLLTTKRACQN-
LMVKMREQSTDWVSELVQMEKLTDYTCNPEYLNEWNMLMSHRQTFIDEVQK-------------NESSK-
----
--MKIEVFGE-----VEIVN-LRGY-----------------
-------------QPLLSQA--FDLKMRMTAYWKIVSRRLVDCMALHLQLCVRNLVSKE---
LEKEIATELMATN--GG-KLEMMLEEAPSVAAKRKRLNTSIELLREAKDVLSNIMGNVSA---------------
----
>KAF5727250
M-------------------------VYSS--ESEGDSLSMVVN-------------
QPRQEVAIDAPIVASCNDRIRPLLDAVDELRHLMVMKEGI-----
QLPTIVVVGDQSSGKSSVLESLAGISLPRGQGICTRVPLVMRLQHHH--IPEPELY-LEFNG-KT------
VQTTESRISEAINLATDEIAGNGKGVSNTPLTLVVKKHGVPDLTMVDLPGITRVPVHDQPENIYEQIA
GIIMEYIKPDESIILNVLSATVDFSTCESIRMSQQVDKTGERTLAVVTKVDKAPEGLLEKVTAD---
DVNVGLGYVCVRNRIG-DE------TYEEARRKEAALFESH-------

PLLCLIDKSIVGVPVLAQKLVQIQAAIIMKCLPEIVKKINDKLNSSLSEFNKLPKTFTSIAEAMTAFMGII
AASKESLRKIILRGEFD------EYPDDQRMHCTARLGEMINQCSQEMQNHRSSDLK---KN-
FLMDEISVLDEAIKGI-RLPNF--LPHSVFIALLCKKV-DSISSMPIEFVEKVWTYIEGVMIEVLTSHSDN-
YHQLQIATRRACLN-
LVAKMKQQSLNWVMEIIQMEKLTDYTCNPEYLLESKKLMAKQDKFMDDVFN------------AVLPSA---
----
---VIYFEGFGG-----VEVVR-LREH------------------
------------KHVVPQA--YDLKARMTAYWKIVLRRLVDSLALHLQLSVHNLVDKE---
LEKEIVNELMMNSLGGG-GVEKLLGESPSVAGKREKLSRTIKLLQESKKVLARIVDEIATA-------------
----
>XP_002303204
MGL-----------VLRHCGSVSEEMSNSS--ENEVESLPQSIE-----
-------EKHQELGVSHVPIVSSFNERIRPLLDAVDKLRHLQVMKEGI-----
QLPTIVVVGDQSSGKSSVLESLAGISLPRGQGICTRVPLIMRLQHHT--APEPELS-LEFNG-KT------
VPTSEAKIANAISLATDEIAGNAKGISNTPLTLVVKKNGVPDLTMVDLPGITRVPVHGQPENIYEQIADI
IMEYIRPEESIILNVLSATVDFTTCESIRMSQKVDKNGERTLAVVTKADRAPEGLLEKVTAD---
DVNIGLGYVCVRNRIG-DE------SYKEARKEEADLFENH-------
PLLSKIDKSMVGIPVLAQKLVQIQATIIARCLPEIVRKINEKLNASISELNRMPKTLSSVGEALTTFMSIV
GSAKESLNKIIVRGEYD------EYLEDKNMHCTARLVEMLNQYSGELHNCSENDLT---GN-
FLMDEIQVLEEA-KGI-ELPNF--LPRTTFLSILQKKV-EKISHIPVAFVEKVWTYIEGVVISVLMHHSEN-
YHQLQLSTRRAGHN-
LIARMKEHSRNWVTEIVQMEKLTDYTSNPEYMNDWNKLMAQQHDFTRDVLE------------KVYITT---
----
--FKIEGLGE-----VPIAG-LRGYE------------------
-----------QHVLLQA--FDLKMRMTAYWKIVLRRLVDFMALHLQFCARNLVNKE---
MEEEIVQELAGRH--DG-AIERMLEESPAVAAKREKLNVSIKLLRESNNVLANIMDKIASNI-------------
----
>XP_024439231
MSG----------------GKPSSKRKGVE--KYEDASVNTEVELH-----
-----VEHEAIFHDHVPIVSYYNDRITPLLDAVDRLRQLQVMKEGI-----
QLPTIVVVGDQSSGKSSVLESLACINLPRGDGICTRVPLIVRLKHHP--SLVPEIF-LQFNG-KT------
VPTDEAHVADAINLVTDEIAGNGKGISNTELTLVVKKNGVPDLTLVDLPGITRVPVHGQPENIYEQIAY
IIMKYISPDESVILNVLSASVDFSTCESIRMSQKVDKNGQRTLAVVTKVDKSPEGLLEKVTRN---
DVNIGLGYVCVRNRIG-NE------SYEDARKEEAALFATH-------
QLLSKIDKSTVGIQVLAQKLVQIQANIIAKCLPDIVRKIDEKLSASISELNRIPRRLLSVAEVMAAFMGII
GSSKDSLRKILLRGEID------EYRHEKDMHCTARLVEMLNQFSTELHKCSDHT-----KN-
FMINEIEVLEET-KGI-ELPNF--LPHAAILAILQQKV-EEISELQIGFVEKVWAYIRGVVISVLNHHSAN-
YHQLQLFIGRAAHK-LVDKMKDRSIDWVTEILQMEKETDYTCNPEYMKEWNKLIAQQQAVIDNITK--
-----------FGSSR--
---VTIDGSRE-----VVVGD-
LRGH-----------------------------KHVLLQA--
FDLKMRLIAYWKIVLMRLVDNMALHLQLSIRNLVNKE---MEKEIVNALLGTG--
GGVAIERMLEEPPSVASKRERLNTSIKLLRESKEVLANIRDKIECGDH----------------------------------
----

>KAF8391993
M---------------------VKFGSNFS---H----------
KEDTKLSAAIAPLVLSYNDQIRPLLDAIDKLRHLQVMKEGI-----
ELPTIVVVGDQSSGKSSVLESLAGISLPRGQGICTRVPLIMRLQHHS--TPSPELH-LEYHN-KI------
IPTNETHVAEAINMATNEIAGNGKGISNTPLTLVVKKKGVPDLTMVDLPGITRVPVHGQPEDIYEQISS
IIMEYIKPKESTILNVLSATVDFPTCESIRMSQRVDKTGERTLAVVTKCDKAPEGLLEKVTAD---
DVNIGLGYVCVRNRIG-EE------LYEEARMEEATLFESH-------
QLLSKIDKSIVGVPILAQKLVQIQASIIAKCLPDIVKNINEKLHANVSELNKMPQNLSSTAEAITVFIRIL
GSAKESLRKILIRGEFD------EYTDENEMHCTARMAEMLNEYSKELQQTSDEKDST--DL-
FLMDEIRILEEA-KGM-TLPNF--LPRAAFLSVLQRKV-NEISSIPVEFVRKGWKYIEDVVIIVLMKHSAN-
YPQLQSSTRRAAHD-LIAKMKEQSIYKVMEIVEMEKLIDYTGNPEYMSTWGRLMVNKEAFMKVIKN--
----------HSNHPQ---
--LVLEGFGQ-----
VEVGH-LIQY------------------------------STVVEQA--
FDMKMRMVAYWKVVHQRLLDNLALHLLFSVHNLVNKE---LEQEIVNELMGSH--GG-
GIKRMLEESSSVSVKRDRINQSIKLLRDSKEVMAEIMGRITANANAPGQVQRLLHLPAASLANRCH
HGHPPHLCALMTATDTNPLLRRDTAIGGNSFR-
HVHDHTPSILFIYSPVTAALPSPQSFHSSPFALPVSTPHLLFYNGHSR
>XP_058079501
M--------------------------NSL--PSSSTSLQEKMA------------
MKDHPEKPVLPPLVSSYNDRIRPLLDAVDRLRHLNVMEEGI-----
ELPTIVVVGDQSSGKSSVIESLAGISLPRGQGICTRVPLIMCLQNVP--TDKPQMH-LEYQG-KI------
VLTSENQISDSISMATDEIAGNGKGISNIPLTLVVKKKGVPDLTIVDLPGITRVPVHGQPEDIYEQISNII
MDYIKPKESIILNVLSATVDFPTCESIRMSQHVDKTGERTLAVVTKADKAPEGLLEKVTAD---
DVNIGLGYVCVRNRIG-DE------TYEEARIEEATLFKSH-------
PLLSKIDKSIVGIPVLAQKLFQIQANSLSQCLPDIVKKINDKLNKHVTDLNNMPQNLTNVAEATQAF
MRVVGAAKESLKKVLLRVEFE------DFPDDVKMHCTARMAEMLDGFYKDLQSKSSDNYSTSSCA-
FLMLEIGVLEES-KWI-GLPNF--LQRTAFVALLQRKV-NRIALGPLDFIQSILTYIEEVVIRIVSEHSEE-
YPQLQSMTKRAVHN-LIDKMRDRSVKHVKEIIEMEKIADYTSNSDYVKTWSSLMENRKTFIEVIQD---
---------PNMGTK---
--IMFQDFGE-----VEVGH-
LRQHS-----------------------------LSMLEQA--
FDMKMRLTAYWRLVLLRLVDSLALRLLFSVKKLVERE---MEEEIVNELMGSH--TC-
GIERMLEELPSTTQKCERLNKGIKLLRDSKDVVTKIMDGIAAR---
-------------HISD--
>KAH0683503
M---------------------AYQNTNDC--ISDSIEILNAKP------------
LAVVASGVVHPPIVASFNDRIRPLLDCIDKLRHLNIMQEGI-----
QLPTIVVVGDQSSGKSSVLESLAGISLPRGQGICTRVPLVMRLKNDPN-ITAPNLQ-LEYNN-KS------
LPVDEIGIADAIILATDEIAGHGKGISNNPLTLVVKKNGVPDLTMVDLPGITRVAVQGQPEDIYEQVYD
IIMKYIVPEESIILNVLSATVDFPTCESIRMSQKVDKTGERTLAVVTKADKAPEGLLEKVTAD---
EVNIGLGYVCVRNRIG-NE------SYEEARSDEQRLFSTH-------
PLLSKIDKSMVSVPILAQKLVRIQASIISKCLPEIVRKINDRLAANLAELNRLPQHLTSVAEALTAFMCIL
SSSKDSLKKILLSGEFD------EYPDEKEMHSAARIVEMLNEYSSELHSKNFEKA----DE-

FLMEEIMVLQET-NGI-RLPNF--LPRAVFLNVLQRKV-
KEIAASPEDFVGKLWNYLERVVIIVLMYHCEN-YPQLQSSTRRAAQN-
LIAKKKNESVDWVREIIGMEKLTDYTCNPDYLTTYSKFMAQQHTFMEIMND-----------HGKCSM---
----
---INLEGVGV-----IDVGH-LRKH-----------------
------------LDVVQQA--FDLKMRMMAYWKIVLMRLVDSMALHIMFSIRNMINKE---
MENEIIQDLMAPH--GG-GIERMLDESPLVAEKRNRLKKSVKLLKESKEVVANIMDRISLYDD---------
--HESD--
>PWZ56864
M--------------------SKKRRTGD--KLENDVEDTKRA-----------
ALAIASGVTASAIAASYNDQIRPVLDAVDRLRHLKVTQEGI-----
QLPTIVVVGDQSSGKSSVLESLAGISLPRGQGICTRVPLVMRLQGDPS-TDSPKLQ-LEYSNGRV----
--
VTTTEAKVADAINAATAEIAGSGKGISDAPITLVVRKSGVPDLTLVDLPGITRVPVQGQPEDIYDQIANII
KEYITPKESIILNVLSATVDFPTCESIRMSQQVDRTGERTLAVVTKVDKAPEGLLEKVTMD---
DVHIGLGYVCVRNRVG-EE------TYDQARVAEAQLFKNH-------
PLLSQIDKSMVGIPVLAQRLMQIQASIIAKCLPDIVKQINDRLIRSSTELDRMPPDVINTGDAVRAFLH
IVKKVCTSLENILVRGDFG------CYPDDYYFHGTARVAEMLSRYAKKLPAECPRCSD---EK-
FLAEEMRVLEET-MSI-KLPNF--LPRSAFHSMLKKKV-
EMVSDVPQDLVSEVWEYVEDLVMKVLLQHSEN-FPQVQSSCRRAIQS-
LMEKTRVRSAQHVKEMIEMELVAGYTASPDYMKTWEAIMVGQKKFMGSVEN-----------KSGPSK--
----
--VTLECFGE-----VNVGH-LRTH----------------
------------PDLAAQA--FDLRARLTAYWKIIVLRLVDGLALHVLRGVKRLVEND---
LEDELANELLGNN--MA-GVERMLSPPPSNGTKRDRLKKSILLLQQSKEVVANIMDRINAADEA------
----
>PWZ56863
M--------------------PKKGHMGT--PKQAYATDGGKG----------
-DVAAGSTVTASAIASSYDDQIRPLLDAVDRLRHLKVTQEGI-----
QLPTIVVVGDQSSGKSSVLESLAGISLPRGQGICTRVPLVMRLQDDPS-ADSPKLQ-LEYSNGRV----
--
VTTTEADVADAINAATAEIAGSGKGISDAPITLVVRKRGVPDLTLVDLPGITRVPVHGQPEDIYDQVAKI
IKEYIAPKESIILNVLSATVDFPTCESIRMSQQVDRSGERTLAVVTKVDKNPEGLLEKVTMD---
DVNIGLGYVCVRNRIG-DE------TYDQARVEEERLFKYH-------
PLLSKIDKDMVGIPVLANRLMQIQSTIIAKCLPDIVKQINDRLSRSSAELDQMPQDLNNVADAVRVF
FHIVKQVCNSLEKLLVRGDFA------EYPDAREFHGTARIADMLSGYAKELPGQCPINRN---EP-
FLKEEVNILEET-KGI-NLPNF--LPRTAFLVLLKKKV-ETIQEIPQFLANKVSDYVEDLVMKVLLKHSEN-
FPQMQSPCRRAVQT-
LMDKARLRSAHHVKELIAMELVADYTANPDYMKTWTEIMEGYELFMEAVEN-----------TSKPTK-----
----
--ITLKCFGE-----VDVSH-LRVY--------------------
---------ADLAGKA--FDLRARLTAYWKSIVLRQVDGLALHVLLSVKLLVEKD---LEEELGNELLGNK--
LA-GVEKMLSPSPGTGTKRERLKKSIVLLRQSKEVVANIMDRISAAGDI-------------------------------
----

>XP_038984915
M-------------------------ANCT---KIA-----------
GDMVKTEAKKSTALASSFDDHIRPILDAVDRLRQLKVMQEGI-----
ELPTIVVVGDQSSGKSSVLESLAGISLPRGQGICTRVPLIMRLQDDPS-LSQPQLQ-LEYKD-KA------
IHTSEDGIADAINSATDDIAGSGKGISNAPLTLVVRKRGVPDLTMVDLPGITRVPVHGQPDNIYEQIS
NIIMEYIAPKASIILNVLSATVDFPTCESIRMSQSVDRTGERTLAVVTKADKAPEGLLEKVTAD---
DVNIGLGYVCVRNRIG-DE------SYEEARAEERNLFKRH-------
PLLSRIDKSIVGIPVLAQRLMQIQAASIAKSLPDIVKKINDKLSQHISELDEMPENLRSIGDATRVLLH
MLSTSKESLRRLLIRGEFD------DFPDDASMHATARMAEMLAKFFKELPSDCPSAD----ER-
FLMEEIAVLEEA-KGINGLPNF--LPRHAFLNLLRRKV-
RDISHAPGEFVRKVWAYIEEVVIRVLLHHSEN-YPQIQPMVRRAAQN-
LVGIMRNQSCHFMSEAIEMEMVADYTSSPDYMKKWTELMDGRDGFIAAIEN-----------YYGPNS--
----
---IELKGLGV-----VEVGH-LRQY------------------
------------AAMAEQA--FDMRMRILAYWKIVVLRLVDSLALHIIYSVNCLVERH---
MEKEIVDDLVGPR--MT-GLERMLEESPATAAKRERLRKSIELMKESKEVVAEIMDRVVTTVN----------
----
>KAH9320939
MGSS----------CLSERLKMSMLMTNSF---DDE---------
---AEEVKAEDQGSSLAISYQQQIRPLLDAVDKLRNLDIMKEGI-----
QLPSIVVVGDQSSGKSSVLESLAGIKLPRGQGICTRVPLVMRLQSCAE-ESEEEIS-IEFNG-VE------
KFIQESDITSSIDTATQEIAGNGKGISHTPITLHVTKVGAPDLTMVDLPGITRVPVGGQPGDIFEQICEII
KEYITPKESIILNVLAANVDFPTCESIRMSQKVDELGERTLAVVTKSDRAPDGLKEKVTTD---
AVNIGLGYVCVRNGIG-DE------SNAEAREKEKNLFDFH-------
PLLKDLDKSMVGIPTLAKKLMQIQATTISATLPQIVNKIESMLGKRQAEMRNLPQHLCNPGEADVAF
VKLVHELKESLKKIVILGEFQ------QFPDDPKMHCTARLREKFDMFYRDLSQRGSFSVG---DK-
FLSKESRMLEEA-KGV-GLPNF--LPRSVFLELLQKMV-
EEISEKSLSLAATVWDYLENVISRVIEHYCHC-YPLLESRVRRAVQG-
LVVEKKEECINHVKQMIEMEKGIDFTLSPAYMETYGTLIRSKGQFMDRLGRLVQQHTRLASKGSYNP
SS--
--------------------------------DFDV---------------------------VNKTVVVEDFGE-----MEVGD-LMELP---
--------------------------AERVQEA--YEMQMSLAAYWKVVTLRMGDGIPLHLQFVCRNLVGNE---
LETQILKHVGGPN--FG-AMDKILEESPVVAGKRKSLINSLQLLKDSKTAVANIMDRIAEAA-------------
----
>KAH9325151

------------------------I-----QLLSIVVVGDQSSGKSSMLRSLAGINLPKGQWICTRVPLIMRLQISK--
SQETEIT-IEYSG-VK------
NNIFEYKIIEALNAATDEIAGVGKGISDTPITLNVSKSNIPNITMVDFPCITKMPVHDQPQDIYDQISQV
IKQYITPKESIILNVLFTSVDFPTCESIRMSQLVDVKGEKTLAVVTKVDKAVEGLFEKVTVD-
VVSVNIGLGYVCVRNKIG-NE------SNA--

>KAK1401877
MEWI---------------PKQRVEQVNKS---KFKQIVVKSDQE-
HSNMSIIVCEVDESVVPLSSHAPIVSSYNEKIRPLLDAIDKLRNLKVTQEGI-----
QLPTIVVVGDQSSGKSSVLESLAGISLPRGQGICTRVPLIMRLHHHS--KPESELH-LEYCG-KV------
VSTDELKIAESINMATAEIAGDGKGISHTPLTLVVKKNGVPDLTMVDLPGITRVPVHGQPENIYEQISG
IIMEYIKPEESIILNVLSATVDFPTCESIRMSQSVDKTGDRTLAVVTKSDKSPEGLRDKVMAD---
DVNIGLGYVCVRNRIG-DE------SYEEARMAESMLFESH-------
PLLSKIDKSMVSVPVLARKLVQIQARIIAKCLPEIVKTINAKLHANVLELNNLPQKLSTVAEAFTAFMRI
LGLSRESLKKLLVRGEFD------EYPDNKNMHSTARLAEMLDKFSQELKSSSPFITD---EK-
FLIDEIFVLEET-RSV-ALPNF--LPRSAFLHLLQLKV-QAISEIPVSFVGKVWDYIETVVVEILTHHCEN-
YPPLQSSTKRAALN-LIAKMKEQTTCRVLEIIEMEKLADYTCDPEYMALWNKLMKSHANFKEAIDG---
---------DVSWV---
---ADIESFGK-----IDLRH-
LEKI-----------------------------SGVRDVA--FDLKMRLTSYWSIVLKRMVDSIALYLLMALKKLVNQG--
-MEEEIVKEVLGPQ--GC-GIERLMDESPSFTIKRERLNQSIGLLEESRDVLAKIIDRIAIADD-----------
----
>KAH9300179

SYNERIRPLLDAIDTLRNMGVMK---AYNCLVLLLWETS--RLEP---

GRSINDTPITLNVGKSNAPDLTMVDFPSITRVPVHGQPQDIYDQISQVIKQYITPKESIIMNVLSASVD
FPTCESIRMSQLVDEKGERTLAVFTKVNKAAEVLFEKVTVD---AVNIGLGYVCVRNRIG-NE------
SNA---

>KAH9314974

ALNAATNEFAGAGKSISDTPITLNVSKSNAPNLTMVDLPGITWVPVHGQPQDIYDQISQVIKQYITPK
ESIIMNVLSASVDFPTCESIRMSQLVDEKGERTLAVVTKVDNAAEGLFEKVTVD---VVNIGLGYTCV--
-------------YSFAKRSRNLAV---

QACDYLDRIICRVID-------PQLQASSRRAFQA-
LIDRKRDKCIQYVEDAMEMQKSIVYTENPSYSKSLQKMQQWKESFIEVIRQ------------NHKAVK----
----
--IDV---

```
------------------------------CLYLVWIAHRIQAQQ--------------------------------------------------------
------------------------------------------------------------------------------------------------------------
>KAF8079489
M-------------------GGSKKRVVSKT------------------------------------------SPSRSIVKANDP------------
-NNNNKSVTIESPIVSSYNDQIRPLLDTVDRLRNLNVMKEGI-----
QLPTIVVVGDQSSGKSSVLESLAGISLPRGQGICTRVPLVMRLQGSA--SSEPEIW-LEYSD-NV------
VPTDEEHIAEAISAATDVIAGSHKGVSDAPLTLHVKKAGAPDLTMVDLPGITRVPVKGQPENIYEQIS
GMIMKYIKPQESIILNVLSATVDFTTCESIRMSRQVDKTGERTLAVVTKADMAPEGLLQKVTSD---
DVSIGLGYVCVRNRVG-EE------TYEEARKQEELLFETH-------
PTLSMIDENIVGVPVLAQKLIQIQTTMIARCLPKIVRKINHKMETADLELKKLPMVMASTGEALMKLM
DIISSAKESLLRILIQGDFS------EFPDNHSMHGTARLADMLSQFSDDLQAKPKEVRE------
FLIDEIKVLEEC-KCV-GLPNF--IPRSAFMAILSQHV-DDIHAKPVEFIKNIWDYIEVVLSSVITKYSEN-
FPQIQPSIKRAGRN-
LMGKIKEQSVDRVVQIVEMEKLTDYTCSPEYMKSWTEKIDGQKSFVDAVLND-----------KTKPES----
--------------------------------------------------------------------------------------------------------------
----------------------------------------------------------VFVNGFGN-----VKISH-LREYH------------------
-----------HAHLLQQA--FDMKMRITCYWKIVLRRVVDNLALYLQLSVKYLVNTQ---
FQKEMVAEMVDPK-GGG-GVERMMEESPLVASKREKLKKSIKVLKESKDAVAAIVDQ------------------
----------------------------------------------------------------------------------------
>OAP13972
M-------------------GGSKKHVVTRT------------------------------------------SSPSLAIVQANP-----------
-HDNREVVPIEAPIISSYNDRIRPLLDTVDRLRNLNVMREGI-----
QLPTIVVVGDQSSGKSSVLDSLAGISLPRGQGICTRVPLVMRLQRSS--SPVPEIW-LEYSD-KI------
VPTDEEHIAEAICAATDVIAGK-------------------------------------------------------------------FT-------
-------------------------------------LYLGIKCV-------------------------------------------------------------
--------------------------------------------------------------------------------------------------------------
--------------------------------------------------------------------------------------------------------------
--------------------------------------------------------------------------------------------------------------
--------------------------------------------------------------------------------------------------------------
--------------------------------------------------------------------------------------------------------------
--------------------------------------------------------------------------------------------------------------
-----------------------
>OAP13353
M-------------------GRSNKHVVTST-------------------------------------------TPSLAIVQANP------------
HPHKDVVPTEAPIVSSYNDHIRPLLDTVDKLRNLNVMQEGI-----
QLPTIVVVGDQSSGKSSVLESLAGISLPRGQGICTRVPLVMRLQRRR--SPEPEIW-LEYGD-KI------
VPTDEEHIAQTICAATDVIAGM------------------------------------------------------------------F-------
--------------------------------------------------------------------------------------------------------------
--------------------------------------------------------------------------------------------------------------
--------------------------------------------------------------------------------------------------------------
--------------------------------------------------------------------------------------------------------------
--------------------------------------------------------------------------------------------------------------
--------------------------------------------------------------------------------------------------------------
```


>OAP19580
M----------------GGSK-----MS---------------------------------------NDYEIDVEAGMS-----------
SLSIVNTPIEAPIVSSYNDRIRPLLDTVDRLRNLNVMREGI-----
QLPTIVVVGDQSSGKSSVLESLAGINLPRGQGICTRVPLVMRLQRSS--SPEPEIW-LEYSD-KV------
VPTDEEHVAEAICAATDVIAGK---FSLSPSQCSVK---
--CV--
LLQK---

>XP_024380180
M----------------SKQSELTMNDS--INNMLVVSSVS----------
-NVNPIKSYDPEALRNSFHSEIRPILDVLDKLRTQGITEENV-----
NIPTIVVVGDQSSGKSSVLESLAGITLPRGQGIATRVPLILRLQSCLS-EQDSKIL-MEYGSVKEM-----
RINSEDDIEAAINAATDDLAGSNKNIRDTPILLHIRKPDAPDLTMVDLPGITRVPVHGQPENIYEQVR
DMIMHYIKPEESIILNVLPAEVDFSTCESIRLSQTVDKKGVRTLAVVTKVDKAPEGLFEKVTSD---
AVSIGLGYVCVRNRTPADD------SIAVARCRELELFNDH-------
PDLRNIDRSMVGIPTLGRRLVKIQSDMVRGCLPRIRDQIHEALQKRRQEMSNVPRSIDSVNEAVAV
FLRLQNEMLNMLTQVVRDGDFS------LVPDDCTLHYTARLHEEFTKFAEDLGKSGLRLSQ---PS-
QTDEIRQMFSEH-QGV-ALPDF--LPHTVLHQLVKKQI-
DSITQTCIFLVDRVFKYAAEVVLHVQSLIFEA-YPHLRDRHHKLAIQ-
VLNETKTTTVEFVERMLAKERTVIFTTNASYLDTIAKINSAVETARRS----------------NQA------------
----
--YIELDVGRI-REGLILNE-AVPA--------------------------
-TGWYREA--WEMKVRIAAYSKIMHERLADEIPLEIRNALQRTIVNR---LQELTMMQAFSSP---E-
ELGTLMQQDSKIICRRVRLQQCIDTLEKSLMLVSGMIAA--

>XP_024367947

-HSEIRPILDVLDKLRTQGITEENV-----
NIPTIVVVGDQSSGKSSVLESLAGITLPRGQGIATRVPLILRLQSCLS-EQDSKIL-MEYENVKEM-----
RINSEDDIEAAINAATDDLAGSNKNIRNTPISLHIRKPDAPDLTMVDLPGITRVPVHGQPENIYEQVR
DMIMHYIKPEESIILNVLPAEVDFSTCESIRLSQTVDKKGVRTLAVVTKVDKAPEGLFEKVTSD---
AVSIGLGYVCVRNRTPADD------SIAVARCRELELFNDH-------
PDLRNIDRSMVGIPTLARRLVKIQSDMVRGCLPRIRDQIHEALQKRRQEMSNVPRSIDSVNEAVAVF
LRLQNEMLNMLTQVVRDGDFS------LVPDDCTLHYTARLHEEFTKFAEDLGKSGLRLSQ---PS-
QTDEIRQMFSEH-QGV-ALPDF--LPHTVLHQLVKKQI-
DSITQTCIFLVDRVFKYAAEVVLHVQSLIFEA-YPHLRDRHHKLAIQ-
VLNETKTTTVEFVERMLAKERTVIFTTNASYLDTIAKINSAVEKARRS----------------NQA-----------

----
--YIELDVGPI-REGLILNE-AVPA----------------------------
--TGWHREA--WEMKVRIAAYSKIMHERLADEIPLEIRYALQRTIVNR---LQELTMMQAFSSP---E-
ELGTLMQQDSKIIGRRVRLQQRIDTLEKSLMLVSGMIAA--

>KAG0561847
M-------------------------QSNT--SPIDIPACDAMS------------
LELISRRAALDSLESTFQRDIRPLLDVVDKIRAQGVTEENI-----
QLPTIVVVGDQSSGKSSVLESLAGITLPRGQGIATRVPLVLRLQSCQ--LEESIIK-MDYGNVKDQ-----
EISGEEQIEAAINAATNALAGSGKGVKDSPIQLLIRKPNSPDLTMVDLPGITRVPVHGQPKNIYEQIR
GMIMRYITPEESIILNVLSAQVDFPTCESIRMSQQVDKEGNRTLAVVTKVDKAPEGLLEKVTTD---
AVNIGLGYICVRNRIDVDD------SIAIARQRERELFESH-------
PALKELDGSMVGIPALARKLTKIQSDMVKECLPRIQKQMFEALHKRNQQLSNLPRGIKSDMDARSA
FFQVQNKILTILSQVVRDGNFE------EFPSDAHLHYTARLHQKFQTFADDLHKTGLKFRE---QS-
QTTEIRELLVEH-QGV-GLPDF--LPHSVLHHLMRKQI-
TSVNETCRSLVDEAFEYATEVVLRVNSLCSQG-YPRLEKSYKQLAIE-
TLEEVKTTTMEFVERMLEKESTIIFTTNDYYTATLEKMQTALGEAKRT---------------STYSSR------------
----
---AVELGPGE---DKIALVEILNDP---------------------------
---DRKYQDA--WRLKVSVAAYWKVVQKRLADEIPLEIRYALQCAVVDT---LHQNMMSKPWAGG--
ET-DLRALMEEDSVGAYTRSRLQLRVDALKDCLRLLSGLMC---

>KAG0619429
-------------------MDRMLMIGN--VDTKVVTKMD------------
VNQLESTYHLDSLEATFQKEIRPMLDAVDKIRAQGVTEENV-----
QLPTIVVVGDQSSGKSSVLESLAGITLPRGQGIATRVPLILRLQSCDS-TEESLIR-MDYGNVKDR-----
EIDGEEQIEAAINEATNVLAGGNKDVKDTPISLHIRKPHAPDLTMVDLPGITRVPVHGQPQNIYEQIQ
AMIMKHISPEESIILNVLSAQVDFPTCESIRMSQQVDKDGKRTLAVVTKVDKAPEGLLEKVTTD---
AVNIGLGYVCVRNRTDDDD------TISVARIREQRLFESH-------
PALKDLDRSMVGIPALARKLTKIQSDMVKGCLPRIHKQMCDALQKRRQQLNNLPKGIASDNDAILIF
LQIQNRRLDMLTQLVRDGDFE------LFPENLHLHYTARLHEKFMKFADDLHKAGLKLKD---QS-
QAQEIKELLAEH-QGV-GLPDF--LPHSVLHHLVRKQI-
ELIRETCTSLVEEAFEYATDVVSEVNTICSEG-YPNMEKCFKKLATE-
SLEKTKKTTMEFVERMLLKECTLIFTTNDYYLATIAKMNALLDNAKQT---------------QNYNQF---------
----
---VVLEAGGNQ-AEKLGLAE-LQYK--------------------
---------DKEYQDA--WRMKTSLVAYWKVVQKRLADEIPLEIRYALQYAIVDL---LHREMMVKAYSDP---
K-GFQALMQEDSNLSFNRARVQHRVDALKECLLLLNDLMG--

>KAJ7294545
M----------------------RFINND--ELMVPPPEALA------------
ERQRSPAFPESALQTHFDQQIRPMLDVVDKLRSLGVTQEGI-----
QLPTIVVVGDQSHGKSSVLESLAEITLPRRQGIATRVPLILRLQSCKV-ASEQSIT-IEYLNVKD------
EIKSEELIEAAIDEATNVLAGPRKDVRDTPISLHVRKLGAPDLTMVDLPGITRVPVHGQPENIYEQIAA

MIQKYINPPESIILNVISATVDFPTCESIRMSQLADKEGKRTLAVVTKVDKAPEGLYEKVTSD---
AVNIGLGYICVRNRTE-KE-----NSNEQARFVEKHLFDTH-------
PSLCKLDKSMVGIPMLAYRLTCIQAQMIQGCLPGLYQQIFDALHKRRRELDSLPTGFQDNAEARVL
FFKIHNEQFKAIDELVREGKLD------KFLEDNHMHYTARLHEMFQNFGDDLRKTGKMFLD---QT-
SLEDIVELLSEH-KGV-SLPNF--LPHAVLHQLVKNEI-
NKISAICYKLVKDSYAYASEVVMAVNRLFFEG-YKHMSAFFRGQAID-
SLRKSEKESTDFVARMLKKEREIIFTTNDYYLDTLDKIRFSLENAKRS--------------SNYNTT----------
----
---VEIDKEKT-----LRLNE-LHGK-------------------------
---SQDYHEA--WRMKASVAAYWKVLQKRLADEIPLEIRFALQTTITQV---INEQIARKVWSGN--V--
ELKDLMQQDPAIVQKRARLEHSIDTLESSLSLLSSLLVA--

>KAH9291961
M---
DKAEEIPSSLSLSYSEGIRPLLDVVDKLQNLNVMNEGI-----
QLPYIVVVGDQSSGKSSVLECLTGISLPRGVGICTRVPLIMRLQNSS--EQDSEIV-VEYND-TV------
EHIIESQITERIDSITKEIAGTNKGISHVPIRLNVKKMNAPDLTLVDLPGIARVSLNGNPDD-
HELISKIVMEYISPADSIILNVLSATVNFRTCESIRMSQRVDVHGERTLGVVTKVDIAPEGLLEKVALD-
--DVNTGLGYVCVRNRVG-DE------CNEEAREAEAELFRSH-------
TQLNKFDEAMVGIPMLARRLMQIQTKRISKCFPDIVKNIEDTLSQRQSELSSLPQQVSNPMEAMVV
FLRLMNGVKDCLNRLLIEGDFS------EFSEETEMHCTARLKEMFDGFYNELVHMSVEDKNA-----
FLVEETKRLEES-KGA-GLSNF--LSRSIF----KKRI-DEVLKTGLSLTANVWDYVEKVVLRVLDLKFRS-
YPRLETATKKDFQL-
LVSKRREQCIHHVNQVAEMEKSLDFTLNPVYMETWTDLLKQKDQFMQELSK------------
TAEEPVTAPQFNIQTSQPVTQQKSPFGVST--
APAKPVTQQKSSSLFKSTSGISTTPAKPVTQQELPSPLESLFHISTMPVKPVTQQKLSSLFESTSGISTT
PAKPVTQQTLPSPFESPFG-
ISTTPAKPVTQQKTPLPFEQTFGLSSFSFSATPAEPITRPKSPKGFTAKPAAVFSGTWMKKTVNIKGFG
E-----VDVEE-VMKMP---------------------------KEHLEVA--
FEMKARVVSYWKVVVQRVGDGIPMYLQFVYQNLVRND---IDEEIMKKVAGPK--SN-
SMEKLLEENSMISRKRRSLKRSIDSLGEAQCKILEIMDQIAEI---

>GJP35534
M--------------------ERAVQQC--
RSVSPARLTMAPLISSFNENVRPMLDAIDKLRLLGLKEEGI-----
ELPTIVVVGDQSSGKSSVLENLSGISLPRGKGIVTRVPLILRLQSCV--KGKDMIT-IEYTP-VTG-
KVSKVLSDEEMIEEEISEATIALAGSRKGVMNCPITLQVQRPDLPDLTLVDLPGITRVPIEDQPKDIYN
QVKNMIMHYITPKESVILNVLAAEVDFSTCESIVMSQEVDSDGDRTLAVVTKVDRAPDGLYEKIQGN
---SVRIGLGYVCVRNKTDADA------SHDDARRAEAAFFNSH-------
PELSQIESHCLGIPALAQRLTEIQAKRVADSIPRIRQAIQKALVGKENELQTIPFAATSNAAALGVISATI
QRRRDVMSGV-ISGKYG------SFQSDDAMHYAARLHEKFNEFEVQMRRVLPDFLG---AD-
YTERCKEALKEV-AGI-SLPNM--FDQAVVKQLVQEVV-
DSIEAPCFLLVNDCFAYAAEVQQAVASQVCGM-YPGLNNAAHLQGLN-
ALRKAMESANRFLKNLLTKEEEVIFTLNHYYMDTVSEIHMKIADYKKAQSG-----------NQDQGP----

---------------------P---
---PVPDVGDFAS---STASLAN-LL---------------------
-----------SNDDQPA--RDLQINMFSYAKVMHKRLCDVIPMQIRMCLKNALLDG---
TDGAVWREVHSGD--IS-KIAALKQVD---QQRRARLEESITRLRASESTLLGLAFDGPSLLA------------
----
>CAI5480041

-------MLDAIDRLRILGLKEEGI-----
ELPTIVVVGDQSSGKSSVLENLSGISLPRGNGIVTRVPLILRLQSCT--SKDGEIT-IEYNNPSSG-
KIFKILPDEESIQEEISKATVTLAGSRKGVMDRPITLQVKRSGLPDLTLVDLPGITRVPVDDQPKDIYN
QVKKMIMQYITPEESVILNVLAAEVDFSTCESIVMSQEVDQDGDRTLAVVTKVDRAPDGLYEKIQGN
---SVRIGLGYVCVRNKTDADA------SHAAARLAETDFFDRH-------
PELSRIETDSRGIPALAQRLSEIQAKRVAESIPRIRQSIQKTLIATEEELQKIPLAANSDGAALAMISSTIQ
RRRDFMTGL-IGGRYG------QLQGDISMHYAARLNEKFNEYEALMRKILPDFLG---KK-
YTERCRDALKEV-AGV-SLPNM--LDQAVLSQLVQELI-
DSIEGPSLMLVEDCFSYAADVQKAVITKTCSG-YPNLENAAQLQALM-
ALKKAKDSSIEMTKNLLLKERRVIFTLNHYYMDTVSKIHQSIAKYKADRYG------------N-----------------
----
--TPSIDGFSS---NTASLAD-LI-----------
--SNDDQAA--RDLQINVFSYAKVVHKRLCDVIPMEIRMSLEDALVDD---TDAAIWG--------------------
----

>EFJ22917
--ME------------
RYRSRRTPEECALQTPFNENVRSLLDAVDTLRQLSVAEEGI-----
KLPTIVVVGDQSSGKSSVLESLAQVDLPRGQGVVTRVPLVLRLQNTSVTDQSHQVV-IQYGG-KK----
--RVIEEAEISAAVVEATIELAGD-
KHIVNKPISLHITKPGAPDLTMIDLPGITRVPVHGQPEDIEEQIKKIIQEYISPKETIILNVICSTVDFPTC
ESILMSRQVDREGERTMAVVTKVDMSPKDLKEKVMAD---VVGIGLGYICVRNRIG-DE------
THEEGRDREAELFRTD-------
PHLRDLPESMLGIRQLAKRLTEFQADSLRKNLPKLVGNIRSALTAVRRDLDALPQRVADSESALPLA
MDCYQSIRRSLERLLAGDSLP------EFPDDKQMNYAARLHEYFVKLSSQIRSNGDEGSS---
SSGSQRKLEELLHEA-KGV-TLPNI--LQSSVLKQMVAYNV-
KEFHEPSIATVDEVHNYAAQVVMPVISKKTEG-YPKHCSRRTCKA----
CSKNPSRRARSSSRTSSRRRPPSPSLSIQSTWSWS--

RRSSSWRYRRWILAS--CPRTGCTPNPPAPAPR-----------------------

>GMH36208
MERLYHSDRAPAAEARLGLVSHSLQSVSQSDRFKGRHTFEPNGGPVSPGSVSRNSHDWQDSRAF
LDSGRTFGQGAPNYSPPGPSVG------------
SQSQTTAMGELGKAIASDVRIRPALDAIDFVRPFVKNMPDIAQ---
MLPAIVVVGDQSSGKSSLLEILSGVTLPRGEGICTRVPLELQLRNGT--EVSAQI---EYQTDLDAPRVS-

KHIMVEEVKNEILLATKRIAGMELNIKDLPIVLRMTGPTYQDLTLIDLPGIARMPLRGQPDNIEELTME
MIQKYINGDSKVILCAVPANNEFVTSAALKLASNVDPLGLRTLGVVTKADQFSRGMRRRLEGLDDT
DVKLKLGFVAVRCRTQ-KE-LEEGISLQDVRMREELLFETD-------
PELRDVQPHCRGISTLVDKLVDIQKERLIEQLPRIVKQLDERIADMESSLLEIEDLVESEHHATARTHE
CVRDICDAFGT---LASCQ------NLEPDTTLDIPAETFAMFEDFVQEVERATTGLLN---PE-
LYAEIKTLSERF-RGA-HLPNF--
LPHPVYGQIFKTRILQRLREPAEHLVQDVMGYIEEVVHKLIVRHLKTRFPKLVSLFCEQASE-
FLTAQYESAKFLVGEAVEAQSEFI-TFTPSYMEIMDAFHAIVGH-QASQGG------------KSDPKI--------
-------------------P--
---QTPKCFAKIFHERSEAAWFYQQVA-----------------

VSHGDQFGKGCLEMMFSLAAYASTVRVVLTEEIPKQVRRCLVTKVSSREFGLEKFLLEKLTDGR--V--
ELFNLMHDS-QKIQMRHDIVQKLESMRTARQKLRGIIGVKVKTKTRR-------------------HSQP--------
------------VPGLSSYRLHEQEL---L
>GMH43921
M---
TDNPNGLAEALSKDVKVRAVLDAIDEVRPFLKDMEGLSQ---
MLTAIVVVGDQSSGKSSTLERIAGIDLPRGQGICTRVPLEMQMRKGS--KFSAT---
LEYQQEKGGSKQSVEIKDASKISDAIQAATRDIVGNSKNVEDLPLVLRISSPIYQDLTLIDLPGIARAPL
PGQRSDIEEQTLEMMRRYITGEAKVILCALPATNDFVTSAALKLALQLDPDGERTLGAVTKIDQARKG
IAKKLEGTDASEITLHLGFAGVRCRTE-NE-TDAGITLEQVRQAEEELFRTH-------
DELKHVDDSCKGVSALLQKLVAVQRGRLISHLPKVLHQVDDQLKVQKEIIDKLPDVLENADDAFAE
ARDCVRKICTEFQE---RAKNE------RLDSDVDLRVSIVLSREFEKFYQQIGEICRGLFK---DQ-
VWEHIESLNRDL-KGA-HLPNF--
LPPPVFDILFKEHVLDKLNEPALALLETVSGEVERILGVLTTSATKM-YPNLNGIISSQVSD-
FLGETLEVTEDLVLECTSSQSECM-TNTTMYIDALEEMKA-----IKSRAG------------TKSWML----------
-----------------P--
--AGS-----EELKWFLEKAA--------------------------
GVTTPDKVTT--MEIFCALGAQAKTVGLILAEQIPKFIRRNLVFKLHNNR-ALENYLVKALGRDQQ----
TLFEMMVDE-RLAKKRDKSEQNIKNLKIARRKLRSAIGFGGK---

>CAG9460856
M--------------------------SES--A------------
TRTASPASATGSFDQRFQSHIKPVLELLDNIRSLLHGCEGLGDVRDKLPTIVVTGDQSAGKSSVLESL
SGIAFPVGDGIVTRLPCQVALREGP--AFRAVCTPPEGHGEAV------
TLTDPKAVTKWIEDTTAAVAGDKKGVLDKPLSIKVEREGSADLTLVDLPGITRVAVDGQADDIEEQVK
RMIQRYISREAAVVLCVLPANVDFSTAECIKMARAVDPGGERTLGVVTKVDRAERGIVTRLNAFGTTG
WALRLGYVAVKNLSQ-
DERAKHGVSTTKVLELEDAFFDDGVGRPAHLAELADLDADMRGLRTLVQKLVQVQGERIEAFMPSL
VDSLREKCRRLEEELSGLSEPVTTEAEALRALSIVVDKFGRIVGDK-
LEGQHKTSALKRKADNSAGISPFALLHDFVSESRAQIRRSMPEFFS---DE-FFDEMRMDLRRR-
RGQ-HLPNFAPLPQDLFEKHFHRHL----EEPISDAVSRSFEMVKGVVVGELGV-FDD-
FPRLKAQVACDVDG-VFERMHIEAKDMMLRLIANERFPD-TLNHYYMDTVSKIMKDIEEHRRDKDK-
-----------QKDKKAVR-----------ELQRGLAGLCSDVAPK--YETYRLADSIKDADLKDGEFTKRCT-------

------VAGAEIEIIVYR------DAERGVPKFDIRNLSSQRLVCRFGANLKRLAPGSSDPEHHSWL------
RHGVDEIEVGA----------------------------RQSVMTVDGANI-----SNVNK-
LRDVDVIMEVFDPGAPWETHVDARSFGDAVLGATSNDEQAA--
VERQVSMAAYTKLVLKALLDCVLKELRTTL---ITER---LTGSLSEEILARR--LEHGTAPLL-----
AAMDNDQLSRKVARLRKEKAAVDEALRQVNNCAW--
