# Supplementary material for: Antiviral Mx proteins have an ancient origin and widespread distribution among eukaryotes
Source: Proc Natl Acad Sci U S A. 2025 Jan 24;122(4):e2416811122. doi: 10.1073/pnas.2416811122 (PMC11789081; doi:10.1073/pnas.2416811122)
Supplement: Supplementary file 17 — Dataset S16 (PDF) [file pnas.2416811122.sd16.pdf]

Dataset S16. Suppl_Figure_4_FastTree

```
#NEXUS
begin taxa;
	dimensions ntax=191;
	taxlabels
	XP_014153758.1[&Description="dynamin-3 [Sphaeroforma arctica JP610]"]
	XP_001749319.1[&Description="uncharacterized protein MONBRDRAFT_28892 [Monosiga brevicollis MX1]"]
	XP_004347890.1[&Description="dynamin 1 [Capsaspora owczarzaki ATCC 30864]"]
	PAA64382.1[&Description="hypothetical protein BOX15_Mlig016602g2 [Macrostomum lignano]"]
	PAA59145.1[&Description="hypothetical protein BOX15_Mlig005677g1 [Macrostomum lignano]"]
	PAA65118.1[&Description="hypothetical protein BOX15_Mlig013747g2 [Macrostomum lignano]"]
	PAA78248.1[&Description="hypothetical protein BOX15_Mlig010364g2 [Macrostomum lignano]"]
	NP_001024332.1[&Description="Dynamin [Caenorhabditis elegans]"]
	XP_026693152.1[&Description="dynamin-1 isoform X15 [Ciona intestinalis]"]
	KMZ10000.1[&Description="dynamin [Drosophila melanogaster]"]
	XP_030853442.1.2[&Description="dynamin-1 isoform X1 [Strongylocentrotus purpuratus]"]
	XP_030853442.1[&Description="dynamin-1 isoform X4 [Strongylocentrotus purpuratus]"]
	XP_035683496.1[&Description="hypothetical protein BRAFLDRAFT_121263, partial [Branchiostoma floridae]"]
	XP_031757197.1[&Description="dynamin-3 [Xenopus tropicalis]"]
	XP_032814666.1[&Description="dynamin-1-like isoform X25 [Petromyzon marinus]"]
	XP_005165639.1[&Description="dynamin-1 isoform X3 [Danio rerio]"]
	KAE8583055.1[&Description="dynamin-1 isoform X18 [Xenopus tropicalis]"]
	XP_028570166.1[&Description="dynamin-1 isoform X14 [Podarcis muralis]"]
	XP_025915522.1[&Description="dynamin-1 isoform X1 [Apteryx rowi]"]
	ELW62001.1[&Description="dynamin-1 isoform X3 [Tupaia chinensis]"]
	EAW87759.1[&Description="dynamin-1 isoform 2 [Homo sapiens]"]
	XP_012378586.1[&Description="dynamin-1, partial [Dasypus novemcinctus]"]
	BAB27759.1[&Description="dynamin-1 isoform X5 [Mus musculus]"]
	EPQ17174.1[&Description="PREDICTED: dynamin-1 [Myotis brandtii]"]
	XP_025944940.1[&Description="dynamin-3 isoform X1 [Apteryx rowi]"]
	EPQ08653.1[&Description="PREDICTED: dynamin-3 isoform X1 [Myotis brandtii]"]
	XP_027623811.1[&Description="dynamin-3 isoform X3 [Tupaia chinensis]"]
	XP_016856477.1[&Description="dynamin-3 isoform d [Homo sapiens]"]
	NP_001025299.1[&Description="dynamin-3 [Danio rerio]"]
```

XP_021326548.1[&Description="dynamin-2 isoform X5 [Danio rerio]"]
XP_006161648.2.2[&Description="dynamin-2 [Tupaia chinensis]"]
NP_001005360.1[&Description="dynamin-2 isoform 4 [Homo sapiens]"]
XP_028568434.1[&Description="dynamin-2 isoform X7 [Podarcis muralis]"]
XP_031753735.1[&Description="dynamin-2 isoform X2 [Xenopus tropicalis]"]
XP_025920181.1[&Description="dynamin-2 isoform X1 [Apteryx rowi]"]
XP_014389433.1[&Description="PREDICTED: dynamin-2 isoform X6 [Myotis brandtii]"]
XP_042914770.1[&Description="uncharacterized protein CHLRE_17g724150v5 [Chlamydomonas reinhardtii]"]
ONM18162.1[&Description="Dynamin-related protein 3A [Zea mays]"]
KAH9327796.1[&Description="hypothetical protein KI387_007974, partial [Taxus chinensis]"]
EFJ35472.1[&Description="hypothetical protein SELMODRAFT_404911 [Selaginella moellendorffii]"]
KAH9304002.1[&Description="hypothetical protein KI387_008406 [Taxus chinensis]"]
KAI5070335.1[&Description="hypothetical protein GOP47_0014678 [Adiantum capillus-veneris]"]
KAI5070758.1[&Description="hypothetical protein GOP47_0015101 [Adiantum capillus-veneris]"]
EFJ37641.1[&Description="hypothetical protein SELMODRAFT_437242 [Selaginella moellendorffii]"]
EFJ15047.1[&Description="hypothetical protein SELMODRAFT_119205 [Selaginella moellendorffii]"]
KAG0554580.1[&Description="hypothetical protein KC19_12G102200 [Ceratodon purpureus]"]
XP_024362051.1[&Description="dynamin-related protein 3A-like isoform X1 [Physcomitrium patens]"]
KAG0555682.1[&Description="hypothetical protein KC19_12G188200 [Ceratodon purpureus]"]
PTQ35749.1[&Description="hypothetical protein MARPO_0069s0084 [Marchantia polymorpha]"]
PWZ09977.1[&Description="Dynamin-related protein 3A [Zea mays]"]
XP_052310486.1[&Description="dynamin-related protein 3A [Populus trichocarpa]"]
AAC61784.1[&Description="similar to dynamin-like protein encoded by GenBank Accession Number X99669 [Arabidopsis thaliana]"]
NP_012926.1[&Description="dynamin-like GTPase VPS1 [Saccharomyces cerevisiae S288C]"]
KXN66323.1[&Description="vacuolar dynamin-like GTPase-like protein VpsA [Conidiobolus coronatus NRRL 28638]"]
KNE68830.1[&Description="hypothetical protein AMAG_13468 [Allomyces macrogynus ATCC 38327]"]

OAJ44422.1[&Description="hypothetical protein BDEG_27650 [Batrachochytrium dendrobatidis JEL423]"]
OUM62108.1[&Description="hypothetical protein PIROE2DRAFT_51763 [Piromyces sp. E2]"]
XP_011389257.1[&Description="putative dynamin-like GTPase VPS1 [Ustilago maydis 521]"]
XP_006458578.1[&Description="hypothetical protein AGABI2DRAFT_190843 [Agaricus bisporus var. bisporus H97]"]
XP_748106.1[&Description="vacuolar dynamin-like GTPase VpsA, putative [Aspergillus fumigatus Af293]"]
XP_001750431.1[&Description="uncharacterized protein MONBRDRAFT_34545 [Monosiga brevicollis MX1]"]
KNE61418.1[&Description="hypothetical protein AMAG_06247 [Allomyces macrogynus ATCC 38327]"]
KNE67543.1[&Description="hypothetical protein AMAG_11997 [Allomyces macrogynus ATCC 38327]"]
XP_004348308.1[&Description="dynamin central region family protein [Capsaspora owczarzaki ATCC 30864]"]
NP_741403.2[&Description="Dynamin GTPase [Caenorhabditis elegans]"]
PAA85687.1[&Description="hypothetical protein BOX15_Mlig022202g1 [Macrostomum lignano]"]
XP_002129967.2[&Description="dynamin-1-like protein [Ciona intestinalis]"]
NP_001259946.1[&Description="dynamin related protein 1, isoform B [Drosophila melanogaster]"]
XP_032819300.1[&Description="dynamin-1-like protein isoform X2 [Petromyzon marinus]"]
NP_957216.1[&Description="dynamin-1-like protein [Danio rerio]"]
XP_028602039.1[&Description="dynamin-1-like protein isoform X3 [Podarcis muralis]"]
XP_031753959.1[&Description="dynamin-1-like protein [Xenopus tropicalis]"]
XP_025940269.1[&Description="dynamin-1-like protein isoform X4 [Apteryx rowi]"]
XP_012382650.2[&Description="dynamin-1-like protein, partial [Dasypus novemcinctus]"]
XP_014394711.1[&Description="PREDICTED: dynamin-1-like protein isoform X6 [Myotis brandtii]"]
NP_001392186.1[&Description="dynamin-1-like protein isoform m [Mus musculus]"]
NP_001317309.1[&Description="dynamin-1-like protein isoform 8 [Homo sapiens]"]
XP_006168142.1[&Description="dynamin-1-like protein isoform X1 [Tupaia chinensis]"]
XP_035676386.1[&Description="dynamin-1-like protein isoform X5 [Branchiostoma floridae]"]
XP_006821224.1[&Description="PREDICTED: dynamin-1-like protein-like [Saccoglossus kowalevskii]"]

XP_030827871.1[&Description="dynamin-1-like protein isoform X2 [Strongylocentrotus purpuratus]"]
KXN67416.1[&Description="hypothetical protein CONCODRAFT_19627 [Conidiobolus coronatus NRRL 28638]"]
NP_013100.1[&Description="dynamin-related GTPase DNM1 [Saccharomyces cerevisiae S288C]"]
XP_746923.1[&Description="dynamin-like GTPase Dnm1, putative [Aspergillus fumigatus Af293]"]
XP_006461708.1[&Description="hypothetical protein AGABI2DRAFT_185821 [Agaricus bisporus var. bisporus H97]"]
XP_011392073.1[&Description="dynamin-related GTPase DNM1 [Ustilago maydis 521]"]
EFJ28901.1[&Description="hypothetical protein SELMODRAFT_171046 [Selaginella moellendorffii]"]
EFJ33653.1[&Description="hypothetical protein SELMODRAFT_439053 [Selaginella moellendorffii]"]
ONM04707.1[&Description="Dynamin-2A [Zea mays]"]
KAH9330549.1[&Description="hypothetical protein KI387_002657 [Taxus chinensis]"]
XP_008646219.1[&Description="dynamin-2A [Zea mays]"]
ACG47836.1[&Description="dynamin-2A [Zea mays]"]
XP_006385192.1[&Description="dynamin-2A [Populus trichocarpa]"]
'KAG7649995.1'[&Description="DRP2B [Arabidopsis thaliana]"]
'NP_172500.1'[&Description="unnamed protein product [Arabidopsis thaliana]"]
KAI5073815.1[&Description="hypothetical protein GOP47_0011828 [Adiantum capillus-veneris]"]
OAE31801.1[&Description="hypothetical protein AXG93_1838s1110 [Marchantia polymorpha subsp. ruderalis]"]
KAG0632288.1[&Description="hypothetical protein M758_1G317400 [Ceratodon purpureus]"]
XP_024391061.1[&Description="dynamin-2B-like [Physcomitrium patens]"]
XP_024368367.1[&Description="dynamin-2A-like [Physcomitrium patens]"]
'KAG0555995.1'[&Description="hypothetical protein KC19_11G018700 [Ceratodon purpureus]"]
XP_042924642.1[&Description="uncharacterized protein CHLRE_05g245950v5 [Chlamydomonas reinhardtii]"]
AAF22292.1[&Description="dynamin-like protein 4 [Arabidopsis thaliana]"]
NP_850420.1[&Description="DYNAMIN-like 1D [Arabidopsis thaliana]"]
XP_002302631.1[&Description="phragmoplastin DRP1B isoform X1 [Populus trichocarpa]"]
AQK88296.1[&Description="Dynamin-related protein 1A [Zea mays]"]
NP_001190448.1[&Description="dynamin-like protein [Arabidopsis thaliana]"]
XP_002299468.1[&Description="dynamin-related protein 5A isoform X2 [Populus trichocarpa]"]

PWZ36850.1[&Description="Dynamin-related protein 1E [Zea mays]"]
NP_001147100.1[&Description="dynamin-related protein 1C [Zea mays]"]
AAF79238.1[&Description="F10B6.23 [Arabidopsis thaliana]"]
XP_002315854.1[&Description="phragmoplastin DRP1C [Populus trichocarpa]"]
EFJ15761.1[&Description="hypothetical protein SELMODRAFT_451592 [Selaginella moellendorffii]"]
KAI5072318.1[&Description="hypothetical protein GOP47_0012424 [Adiantum capillus-veneris]"]
EFJ23099.1[&Description="hypothetical protein SELMODRAFT_232702 [Selaginella moellendorffii]"]
KAG0556007.1[&Description="hypothetical protein KC19_11G019300 [Ceratodon purpureus]"]
XP_002987566.1[&Description="dynamin-related protein 1E [Selaginella moellendorffii]"]
KAI5602084.1[&Description="hypothetical protein BDE02_01G133700 [Populus trichocarpa]"]
XP_006375094.1[&Description="phragmoplastin DRP1E [Populus trichocarpa]"]
KAI5058380.1[&Description="hypothetical protein GOP47_0026550 [Adiantum capillus-veneris]"]
PTQ29980.1[&Description="hypothetical protein MARPO_0132s0051 [Marchantia polymorpha]"]
PTQ45603.1[&Description="hypothetical protein MARPO_0014s0125 [Marchantia polymorpha]"]
KAG0561847[&Description="hypothetical protein KC19_9G097200 [Ceratodon purpureus]"]
KAG0619429[&Description="hypothetical protein M758_4G139100 [Ceratodon purpureus]"]
PWZ56863[&Description="Dynamin-related protein 4C [Zea mays]"]
PWZ56864[&Description="Dynamin-related protein 4C [Zea mays]"]
AAB71956[&Description="similar to 'Mx' GTP-binding proteins [Arabidopsis thaliana]"]
NP_176252[&Description="Dynamin related protein 4C [Arabidopsis thaliana]"]
XP_002297993.1[&Description="dynamin-related protein 4C [Populus trichocarpa]"]
XP_002303204[&Description="dynamin-related protein 4C isoform X2 [Populus trichocarpa]"]
XP_024439231.1[&Description="dynamin-related protein 4C [Populus trichocarpa]"]
KAH9291961[&Description="hypothetical protein KI387_042849 [Taxus chinensis]"]
KAH9290598[&Description="hypothetical protein KI387_034715 [Taxus chinensis]"]
KAH9320939[&Description="hypothetical protein KI387_015578 [Taxus chinensis]"]
XP_751402[&Description="dynamin family GTPase, putative [Aspergillus fumigatus Af293]"]

XP_754266[&Description="dynamin GTPase, putative [Aspergillus fumigatus Af293]"]
XP_748757[&Description="dynamin GTPase, putative [Aspergillus fumigatus Af293]"]
XP_751069[&Description="dynamin GTPase, putative [Aspergillus fumigatus Af293]"]
XP_750654[&Description="dynamin GTPase, putative [Aspergillus fumigatus Af293]"]
OAJ38670.1[&Description="hypothetical protein BDEG_22578 [Batrachochytrium dendrobatidis JEL423]"]
XP_006457072.1[&Description="hypothetical protein AGABI2DRAFT_139599 [Agaricus bisporus var. bisporus H97]"]
XP_006461472[&Description="hypothetical protein AGABI2DRAFT_222252 [Agaricus bisporus var. bisporus H97]"]
XP_006461433[&Description="hypothetical protein AGABI2DRAFT_185678 [Agaricus bisporus var. bisporus H97]"]
XP_042924849[&Description="uncharacterized protein CHLRE_05g237200v5 [Chlamydomonas reinhardtii]"]
BAF46281[&Description="dynamin related protein [Chlamydomonas reinhardtii]"]
XP_042924875.1[&Description="uncharacterized protein CHLRE_05g238290v5 [Chlamydomonas reinhardtii]"]
XP_032804093.1[&Description="interferon-induced GTP-binding protein Mx1-like isoform X2 [Petromyzon marinus]"]
XP_006815062.1[&Description="PREDICTED: interferon-induced GTP-binding protein Mx-like [Saccoglossus kowalevskii]"]
XP_035690836.1[&Description="interferon-induced GTP-binding protein Mx3-like [Branchiostoma floridae]"]
XP_019617847.1[&Description="PREDICTED: interferon-induced GTP-binding protein Mx1-like [Branchiostoma belcheri]"]
PAA83069.1[&Description="hypothetical protein BOX15_Mlig009247g1 [Macrostomum lignano]"]
PAA94353.1[&Description="hypothetical protein BOX15_Mlig014920g1 [Macrostomum lignano]"]
PAA74204.1[&Description="hypothetical protein BOX15_Mlig022940g2 [Macrostomum lignano]"]
PAA76532.1[&Description="hypothetical protein BOX15_Mlig002592g2 [Macrostomum lignano]"]
PAA69582.1[&Description="hypothetical protein BOX15_Mlig021727g2 [Macrostomum lignano]"]
PAA92268.1[&Description="hypothetical protein BOX15_Mlig009769g1 [Macrostomum lignano]"]
NP_001007285.1[&Description="interferon-induced GTP-binding protein MxC [Danio rerio]"]

XP_005167721.2.2[&Description="interferon-induced GTP-binding protein MxE isoform X1 [Danio rerio]"]
XP_032888405.1[&Description="interferon-induced GTP-binding protein Mx3-like isoform X1 [Amblyraja radiata]"]
XP_007904885.1[&Description="PREDICTED: interferon-induced GTP-binding protein Mx-like isoform X2 [Callorhinchus milii]"]
XP_003973512.2.2[&Description="interferon-induced GTP-binding protein Mx [Takifugu rubripes]"]
NP_891987.2.2[&Description="interferon-induced GTP-binding protein MxA [Danio rerio]"]
XP_009304072.1[&Description="interferon-induced GTP-binding protein MxB isoform X1 [Danio rerio]"]
XP_028583068.1[&Description="interferon-induced GTP-binding protein Mx1-like isoform X1 [Podarcis muralis]"]
XP_025933558.1[&Description="interferon-induced GTP-binding protein Mx-like isoform X1 [Apteryx rowi]"]
XP_028583072.1[&Description="interferon-induced GTP-binding protein Mx2-like [Podarcis muralis]"]
XP_015269256.1[&Description="PREDICTED: interferon-induced GTP-binding protein Mx1 [Gekko japonicus]"]
XP_031752404.1[&Description="interferon-induced GTP-binding protein Mx2 [Xenopus tropicalis]"]
XP_012586448.1[&Description="PREDICTED: interferon-induced GTP-binding protein Mx2 [Condylura cristata]"]
NP_776366.1[&Description="interferon-induced GTP-binding protein Mx2 [Bos taurus]"]
NP_001003133.1[&Description="interferon-induced GTP-binding protein Mx2 [Canis lupus familiaris]"]
XP_032211320.1[&Description="interferon-induced GTP-binding protein Mx2 isoform X1 [Mustela erminea]"]
XP_006156438.1[&Description="interferon-induced GTP-binding protein Mx2 [Tupaia chinensis]"]
NP_002454.1[&Description="interferon-induced GTP-binding protein Mx2 [Homo sapiens]"]
XP_002830747.1[&Description="interferon-induced GTP-binding protein Mx2 [Pongo abelii]"]
XP_008569440.1[&Description="PREDICTED: interferon-induced GTP-binding protein Mx2 [Galeopterus variegatus]"]
XP_017508123.1[&Description="PREDICTED: interferon-induced GTP-binding protein Mx2-like, partial [Manis javanica]"]
XP_005885748.1[&Description="PREDICTED: interferon-induced GTP-binding protein Mx2 [Myotis brandtii]"]
NP_034976.1[&Description="interferon-induced GTP-binding protein Mx1 [Mus musculus]"]

XP_004675614.2.2[&Description="PREDICTED: interferon-induced GTP-binding protein Mx1 [Condylura cristata]"]
NP_038634.1[&Description="interferon-induced GTP-binding protein Mx2 [Mus musculus]"]
XP_005202045.1[&Description="interferon-induced GTP-binding protein Mx1 isoform X1 [Bos taurus]"]
XP_014388412.1[&Description="PREDICTED: interferon-induced GTP-binding protein Mx1 isoform X1 [Myotis brandtii]"]
NP_001003134.1[&Description="interferon-induced GTP-binding protein Mx1 [Canis lupus familiaris]"]
XP_032211398.1[&Description="interferon-induced GTP-binding protein Mx1 isoform X1 [Mustela erminea]"]
XP_004466363.1[&Description="interferon-induced GTP-binding protein Mx1 [Dasypus novemcinctus]"]
XP_017508130.1[&Description="PREDICTED: interferon-induced GTP-binding protein Mx1 [Manis javanica]"]
NP_002453.2.2[&Description="interferon-induced GTP-binding protein Mx1 isoform a [Homo sapiens]"]
NP_001127618.1[&Description="interferon-induced GTP-binding protein Mx1 [Pongo abelii]"]
XP_008569442.1[&Description="PREDICTED: interferon-induced GTP-binding protein Mx1 [Galeopterus variegatus]"]
XP_006156437.1[&Description="interferon-induced GTP-binding protein Mx1 [Tupaia chinensis]"]
;
end;

begin characters;
dimensions nchar=837;
format datatype=protein missing=? gap=-;
matrix
XP_014153758.1 ---------------------------------------REILENKLLPLRRGYIGVVNR-SQKDITGKKDIRAAQE-----------------AERRFFSTH-----PA---YRHLA-------QNMG-TPKLQKVLNQQLTNHIRDSLPQLRSSLTGQVMQLEKDCSGFKHMMGGRN--------AST--KQLMQLLH--QFSDKWWKS----IDGSADM------------VSFDDLSGGARINRIFFERFPYELSK--HEYDERLLRKEISYAIKNIHGIRSGL-FTPD-QAFETICKKL-IERLRAPS----LFCAEMCMTELMALTDYCG----E--------------------------------ELSRFPRLREEV--NTLVV-Q-YINEA-HAKTEDQID-L-----FIN-------TELSYMNTNHPDFIGFQNASA----------------ERQVETIR--------------------------NLVDSYMAI-----------IGINIRDMIPKC-VARLM----------V---------------------------------QKVKIDIEEELVAGLFNAG--------------------S-PDDLLEESESEKARREDMLNMLSMAKKALKIIGDVNVT---------------------------
XP_001749319.1 ---------------------------------------RAILTNEFLPLRRGYIGVVNR-SQKDIDGRKDIRAALD-----------------AERKFFLMH-----PS---YKDIA-------SKNG-

TPYLQKALNQQLTNHIRECLPGIRNKLQKQLVALESQVAEFKHYDPNDG------T-KNT--
KAMVQMVN--QFANQFEKR----IEGSGDT------------VNVERLSGGARIARVFHERFPFELVK-------
----
-------------------------MDLDERTLRREIGFAIKNIRGIRVGL-FTPD-MAFEAVTKRL-IEKLKMPS----
LKCVDMVCEELVELLNDVT----Q---------------------------------NMARYPRLRDEC--ETLVS-T-
HIRDC-EEKAKVHIN-N-----QIS-------IELSYMNTNHPDFIGFANAAN----------------ERQVEIIR------
---------------------NLVDSYMQI-----------VHKTLRDQVPKV-CMHIM----------V------------------------
----------NNVKEFISDELIAHLYRQS----------------------
PEELMEESPEEVNRRNELLAMYQASKQALDIIKDVNLT---------------------------
XP_004347890.1 --REVLENKLIPLRRGFIGVVNR-
SQKDIDGRKDIKAAMS-----------------AELRFFSTH-----PA---YRDLA-------NKNG-
TMYLQRVLNQQLTNHIRDTLPDLKKKLQNQLNLLEKDVAQMKNMKADDP------A-LRT--
KVMLQMVQ--TFGEDFEKR----IEGSGD--------------VSLSELSGGAKIARIFHERFPFELVK-----------
----
---------------------TEYDEKQLRREISFAILNNHGIRTGL-FTPD-QAFEAIVRKL-IELMRDPS----
LKCVDLVVTELGNVVTQCA----E---------------------------------RLATYPHLRDEM--ENIVR-T-
FLRQA-HDRTNSQIE-M-----LIN-------LELAYMNTNHDDFIGFAGASD----------------ERQVETIR----
-----------------------NLVDSYMAI-----------VSKTVRDLVPKT-IMHLL----------V-------------------------
---------GQVKDVIKSELIAGLYRSG--------------------
ESADQLMEESPEAASRRKEVLQMYNLSKEALDVINNVSIS----------------------------
PAA64382.1 --RDVLENRFLPLRRGYVGVVNR-
SQKDIDGRKDISSAMA-----------------AERKFFLGH-----PA---YRHMA-------ERMG-
TAHLQRCLNQQLVGHIRDTLPTLRAALQSRLTELEAEHRAFGGLDPDDP------N-AKA--KIMATLIN--
QFIENFTKA----IEGFGE--------------VSTKELSGGAKINKIIHDRYMYMLHKSSRYIGEK----------------
----
-------HYTESDEHELRREIKFAIRNIHGIRTGL-FTPD-LAFETVVRQQ-IGRLKEPT----
LRCVELVTAELGSVVHKCA----H---------------------------------EMRSYPRLRETV--EGIVS-A-RIRER-
EVEAKSQLM-L-----TMD-------IQLGYINTNHEDFIGFVNAQK----------------ERQVETIR--------------
-------------SLAKSYMRI-----------VDKAQRDFVPKT-IIHMV----------V----------------------------------
NDLRSFLKTSLPVQLYSCS--------------------D-
QASLMEESEAEATRRVETARAYKATVEALRVLSEVAIG----------------------------
PAA59145.1 --RDVLENRLLPLRRGYIGVVNR-
SQKDIEGKKDIVAAMA-----------------AERKFFLSH-----PA---YRHMA-------ERMG-
TSYLQRCLNQQLTNHIRETLPALRTALQSKLLDMEGDYRMYRNFDPNDP------T-IKT--KAMMTMIN-
-QFTEEFTQA----IEGSGE--------------VNTKELSGGAKINRIFHERFMYLLHK---------------------------
----
------TESDERELRKEISYAIRNIHGIRTGL-FTPD-MAFETIVKQQ-IARMKEPT----
LKCVDMVVEELINVVHHCT----E---------------------------------KMSSYPRLREAT--ESIVT-N-RIREQ-
EGKTKSQLM-L-----LVD-------IQLAYMNTNHEDFIGFANAQQ----------------ERQVETIR-------------
--------------NLVESYMKI-----------VDKTQRDVVPKT-VIHLI----------V----------------------------------
NELKDFLKTSMLAQLYGTQ--------------------D-
QATLMEESEGESVRREETLRIYKATKEALQILSDVSAS----------------------------
PAA65118.1 --REVLENKLLPLRRGYIGVVNR-
SQKDIEGRKDIKAAMA-----------------AERKFFLSH-----PS---YRHMA-------ERMG-

TPYLQRCLNQQLTNHIRETLPSLRSELQTKLLAMEKDYQAYANFSPDDP------S-IKT--KAMMTMIN--
QFTDEFTQS----IEGSGE-------------VSTKELSGGARINRIFHERFMYMLHQ---------------------------
----
-------TESDERELRKEISYTIRNVHGIRTGL-FTPD-MAFERIVKQQ-IMRLKEPT----
LKCVDMVIEELINVVHKCT----Q----------------------------------QMASYPRLREAV--ETTVT-N-RIREQ-
ETRAKTQLM-L-----LVD-------IQLAYMNTNHEDFIGFANAQQ---

PAA78248.1 ---REVLENKLLPLRRGYIGVVNR-
SQKDIEGKKDIAAAMA-----------------AERKFFLSH-----PS---YRHMA-------ERMG-
TPYLQRCLNQQLTNHIRETLPSLRSELQTKLLAMESDYKVYMNFNPDDP------T-IKT--KAMMTFIN--
QFTEDYLQA----IEGTGE-------------VSTKELSGGAKINTIFHERFMYMLHQ----------------------------
----
------TESDERELRKEISYAIRNIHGIRTGL-FTPD-MAFETIVKKQ-IARMKEPT----
LKCVDMVVEELINVVHRCT----T----------------------------------KMSSYPHLQEAV--ESIVT-N-RIREQ-
ESKTKTQLM-L-----LVD-------VQLAYMNTNHEDFIGFANAQQ----------------ERQVETIR-------------
--------------NLVESYMRI-----------VDKTQRDVVPKT-VIHLI----------V-----------------------------------
NELKDFLKTSMLASLYGGY--------------------D-
QNQLMNESPEEAAKREETLRIYKATKDALKILSEVSAG-----------------------------
NP_001024332.1 ---REILENKLFTLRRGYVGVVNR-
GQKDIVGRKDIRAALD-----------------AERKFFISH-----PS---YRHMA-------DRLG-
TSYLQHTLNQQLTNHIRDTLPTLRDSLQKKMFAMEKDVAEYKNYQPNDP------G-RKT--
KALLQMVT--QFNADIERS----IEGSSAKL------------VSTNELSGGARINRLFHERFPFEIVK------------
----
---------------------MEIDEKEMRKEIQYAIRNIHGIRVGL-FTPD-MAFEAIAKKQ-ITRLKEPS----
LKCVDLVVNELANVIRQCA----D----------------------------------TMARYPRLRDEL--ERIVV-S-
HMRER-EQIAKQQIG-L-----IVD-------YELAYMNTNHEDFIGFSNAEA----------------ERQVETIR-----
----------------------NLVDSYMRI-----------ITKTIKDLVPKA-VMHLI----------V-----------------------------
------NQTGEFMKDELLAHLYQCG--------------------D-
TDALMEESQIEAQKREEMLRMYHACKEALRIISEVNMS-----------------------------
XP_026693152.1 ---KHILENKHLPLRRGYVGVVNR-
SQKDIDGNKDIKAALS-----------------AERRFFLSH-----PA---YRHMA-------DKLG-
TPYLQKILNQQLTNHIKETLPALRNKLQKQMMDMEKEVEEFKNFKPDDP------S-RKT--
KAMLQMIQ--GFNNSFEQL----IEGTGAS-------------IDTLELSGGAKINRIFHERFPYELVK------------
----
---------------------MEFDEQTLRKEISVVIQNIHAIRTGL-FTPD-TAFEEIVKSQ-IAKLKDPA----
LKCVELVSTELMNVLRKCS----D----------------------------------KMGRYPMLREET--DRVVS-T-
NVREK-EAMTKEQVA-M-----LID-------FELSYINTNHDDFIGFANASQ----------------ERQVETIR-----
----------------------NLVDSYMAI-----------VSKTIRDLVPKI-IMNIM----------I-----------------------------
------GQTKEFIATEVLAHLYSSG--------------------N-
GTDLMEESHEEAERRDTMLKMYHSLKEALKVMGDINMK-----------------------------
KMZ10000.1 ---RDILENKLLPLRRGYIGVVNR-
SQKDIEGRKDIHQALA-----------------AERKFFLSH-----PS---YRHMA-------DRLG-
TPYLQRVLNQQLTNHIRDTLPGLRDKLQKQMLTLEKEVEEFKHFQPGDA------S-IKT--KAMLQMIQ-

-QLQSDFERT----IEGSGSAL------------VNTNELSGGAKINRIFHERLRFEIVK---------------------------
----
-------MACDEKELRREISFAIRNIHGIRVGL-FTPD-MAFEAIVKRQ-IALLKEPV----
IKCVDLVVQELSVVVRMCT----A-----------------------------------KMSRYPRLREET--ERIIT-T-HVRQR-
EHSCKEQIL-L-----LID-------FELAYMNTNHEDFIGFANAQN--------------ERQVETIR---------------
--------------NLVDSYMKI------------VTKTTRDMVPKA-IMMLI-----------I------------------------------------
NNAKDFINGELLAHLYASG---------------------D-
QAQMMEESAESATRREEMLRMYRACKDALQIIGDVSMA------------------------------
XP_030853442.1.2 --KDILENKLLPLRRGYVGVVNR-
SQRDIEGKKDIKAALA------------------AERKFFLSH-----PS---YRHIA-------DKMG-
TPWLQKILNQQLTNHIRDSLPTLRNRLQAQELSMEKEVAEYKNFSADDP------T-RKT--
KAMLQMVQ--HFGVNFEKR----IEGSGDE--------------INVNELSGGARINRIFHERFPFEVVK---------
----
-------------------------MEYDEKELRREISYAIKNIHGVRVGL-FTPD-MAFEAITKKQ-IGRLKEPS----
IKCVDMVVNELNDVVRHSG----E-----------------------------------GMARYPRLREET--ERIVC-T-
HIRER-EAKTKDQVI-M-----LIN-------IQLAYMNTNHDDFIGFANAAK----------------ERQVETIR------
----------------------NLVDSYMSI-----------ISKQIKDMVPKT-CMALM----------I----------------------------
--------NDTKEFILSEMLAHLYSNQ---------------------D-
QGSLMEESADEAQRRDEMLRMYQATKEALRIIGDINMS------------------------------
XP_030853442.1 --KDILENKLLPLRRGYVGVVNR-
SQRDIEGKKDIKAALA------------------AERKFFLSH-----PS---YRHIA-------DKMG-
TPWLQKILNQQLTNHIRDSLPTLRNRLQAQELSMEKEVAEYKNFSADDP------T-RKT--
KAMLQMVQ--HFGVNFEKR----IEGSGDE--------------INVNELSGGARINRIFHERFPFEVVK---------
----
-------------------------MEYDEKELRREISYAIKNIHGVRVGL-FTPD-MAFEAITKKQ-IGRLKEPS----
IKCVDMVVNELNDVVRHSG----E-----------------------------------GMARYPRLREET--ERIVC-T-
HIRER-EAKTKDQVI-M-----LIN-------IQLAYMNTNHDDFIGFANAAK----------------ERQVETIR------
----------------------NLVDSYMSI-----------ISKQIKDMVPKT-CMALM----------I----------------------------
--------NDTKEFILSEMLAHLYSNQ---------------------D-
QGSLMEESADEAQRRDEMLRMYQATKEALRIIGDINMS------------------------------
XP_035683496.1 --RNILENRTYPLRRGYIGVVNR-
SQADIDGRKDIKAALA------------------AERKFFLSH-----PA---YRHLA-------DRMG-
TPYLQKTLNQQLTNHIRDTLPVLRNKLQGQLLGMEKEVEEYKNFRPDDP------T-RKT--
KAMLQMVN--TFGVDFDKR----IEGSGDQ-------------IDTVELSGGARINRIFHERFPFELVK----------
----
------------------------MEFDEKELRREISYAIKNIHGVRTGL-FTPD-MAFEAICKRQ-IAKLKEPS----
LKCVDMVINELNNVVRQCG----E-----------------------------------KMGRYPQLREET--ERIVT-T-
HIRER-EQRAKDQVV-V-----FVD-------VNLAYINTNHEDFVGFADAQQ----------------ERQVETIR---
-------------------------NLVDSYMGI------------ITKTIRDLVPKT-IMHMI----------V----------------------------
--------NDTKEFIQSEMLAHLYSSG---------------------D-
QASMMEESATEAQRREEMLRMYHSLKEALKIIGDINMT-----------------------------
XP_031757197.1 --QEILENKLLPLRRGYVGVVNR-
SQKDIDGKKNINAALQ------------------AEQMFFLTH-----PA---YRHMA-------DRMG-
TSHLQKMLNQQLTNHIRETLPDLRSKLQDQLLSLQHEVEVYKSINPDDP------S-RKT--

KALLHMVQ--HFAVDFEKR----IAGSGDQ-------------VDTMELSGGAKINRIFHERFPFELVK----------
----
----------------------MVFDEKELRREISYAIKNIHGVRTGL-FTPD-LAFEAIVKKQ-IVKLKGPA----
LKCVDLVMQELINTIKKCT----T--------------------------------KLSNYPRLCVET--ERIVT-F-HIKES-
EGKTKDQVL-L-----MID-------MQLSYINTNHEDFIGFVNAQQ----------------ERQVETIR-------------
--------------NLVESYMTI-----------INKGIRDLIPKT-LMHLM----------I---------------------------------
NSVKEFIHSELLAQLYSYE--------------------D-
QSTLMEESTEQSQKREEKLHMYYCLKEALHIIGDICTS---------------------------

XP_032814666.1 ---------------------------------------RDILENKLLPLRRGYIGVVNR-
SQKDIDGRKDINAAMA-----------------AERKFFLSH-----PS---YRHMA-------DRMG-
TPYLQKTLNQQLTNHIRDTLPALRNKLQGQMLSMEKEVEEYKNFRPDDP------T-RKT--
KALLQMVQ--QFAFDFEKR----IEGSGDQ-------------IDTVELSGGARINRIFHERFPFELVK----------
----
---------------------MEFDEKELRREISYAIRNINGIRTGI-FTPD-LAFETIVKKL-IVKLKEPC----
LKCVDMVIGQLIETVRQCS----E--------------------------------KLGSYPRLRDDT--DRIVT-T-YIRER-
EGRTKDQVL-L-----LID-------IQLAYMNTNHEDFIGFSNAQQ----------------ERQVETIR--------------
-------------NLVDSYMGI-----------VSKNIRDLMPKT-IMHLM----------I---------------------------------
NNTKDFIHSELLAHLYSSS--------------------D-
QTSLMEESAEQAQRRDEVLRMYHTLKDALSIIGDISMS---------------------------

XP_005165639.1 ---------------------------------------REILENKLLPLRRGYIGVVNR-
SQKDIDGKKDITAAMS-----------------AERKFFLTH-----PS---YRHLA-------DRMG-
TPYLQKALNQQLTNHIRDTLPGLRNKLQSQLLSIEKEVEEYKHFRPDDP------S-RKT--KALLQMVQ-
-QFAVDFEKC----IEGSGDQ-------------VDTVELSGGARINRIFHERFPFELVK-------------------------
----
-------MEFDEKELRKEISYAIKNIHGIRTGL-FTPD-MAFETIVKRQ-IAKIKEPC----
QKCVDLVITELVNTVRQCT----K--------------------------------KLAQYPMLREEM--ERIVT-Q-HIRDR-
ESRTKNQVM-L-----LID-------IELAYMNTNHEDFIGFANAQQ----------------ERQVETIR--------------
-------------NLVDSYMAI-----------VNKTVRDLMPKT-IMHLM----------I---------------------------------
NNTKDFIHAELLANLYSCG--------------------D-
QNTLMEESAEQAQHREEMLRMYHALKEALNIIGDISTS---------------------------

KAE8583055.1 ---------------------------------------RDVLENKLLPLRRGYIGVVNR-
SQKDIDGKKDIQAALA-----------------AERKFFLSH-----PS---YRHLA-------DRMG-
TPYLQKALNQQLTNHIRDTLPGLRNKLQSQLLSIEKEVEEYKNFRPDDP------A-RKT--KALLLMVQ--
QFAVDFEKR----IEGSGDQ-------------IDTYELSGGARINRIFHERFPFELVK---------------------------
----
-----MEFDEKELRREISYAIKNIHGIRTGL-FTPD-LAFEAIVKKQ-VQKLKEPS----
LKCVEMVANELTSTIQRCS----E--------------------------------KLSQYPHLREEM--ERIVT-T-HIRER-
EGRTKDQVM-L-----LID-------IELSYMNTNHEDFIGFANAQQ----------------ERQVETIR--------------
-------------NLVDSYMAI-----------VNKTIRDLMPKT-IMHLM----------I--------------------------------
INTKDFIHSDLLANLYSCG--------------------D-
QNTLMEESAEQAQHRDEMLKMYRALKEALCIIGDISTT---------------------------

XP_028570166.1 ---------------------------------------RDVLENKLLPLRRGYIGVVNR-
SQKDIDGKKDIQAALA-----------------AERKFFLTH-----PA---YRHMA-------DRMG-
TPYLQKVLNQQLTNHIRDTLPGLRNKLQSQLLSIEKEVEEYKNFRPDDP------A-RKT--KALLQMVQ-

-QFAVDFEKR----IEGSGDQ-------------IDTYELSGGARINRIFHERFPFELVK---------------------------
----
------MEFDEKELRREISYAIKNIHGIRTGL-FTPD-LAFEAIVKKQ-VQKLNEPC----
LKCVDMVVSELTSTIRKCS----E---------------------------------KLSQYPHLREEM--ERIVT-T-HIRDR-
ESRTKDQVM-L-----LID-------IELAYMNTNHEDFIGFANAQQ----------------ERQVETIR--------------
-------------NLVDSYMAI-----------VNKTIRDLMPKT-IMHLM----------I-----------------------------------
NNTKDFIHSELLANLYSCG--------------------D-
QNTLMEESVEQAQRRDEMLRMYHALKEAHHIIGDINTT----------------------------
XP_025915522.1 --RDVLENKLLPLRRGYIGVVNR-
SQKDIDGKKDIQAALA-----------------AERKFFLSH-----PA---YRHMA-------DRMG-
TPYLQKVLNQQLTNHIRDTLPGLRNKLQSQLLSIEKEVEEYKNFRPDDP------A-RKT--KALLQMVQ-
-QFAVDFEKR----IEGSGDQ-------------IDTYELSGGARINRIFHERFPFELVK---------------------------
----
------MEFDEKELRREISYAIKNIHGIRTGL-FTPD-MAFETIVKKQ-VKKIKEPC----
LKCVDMVISELINTVRQCT----K---------------------------------KLSQYPHLREEM--ERIVT-T-HIRER-
EGRTKDQVM-L-----LID-------IELAYMNTNHEDFIGFANAQQ----------------ERQVETIR--------------
-------------NLVDSYMAI-----------VNKTIRDLMPKT-IMHLM----------I-----------------------------------
NNTKDFIHSELLANLYSCG--------------------D-
QNTLMEESAEQAQRRDEMLRMYHALKEALNIIGDINTS----------------------------
ELW62001.1 --RDVLENKLLPLRRGYIGVVNR-
SQKDIDGKKDITAALA-----------------AERKFFLSH-----PS---YRHLA-------DRMG-
TPYLQKVLNQQLTNHIRDTLPGLRNKLQSQLLSIEKEVDEYKNFRPDDP------A-RKT--KALLQMVQ-
-QFAVDFEKR----IEGSGDQ-------------IDTYELSGGARINRIFHERFPFELVK---------------------------
----
------MEFDEKELRREISYAIKNIHGIRTGL-FTPD-MAFETIVKKQ-VKKIREPC----
LKCVDMVISELISTVRQCT----K---------------------------------KLQQYPRLREEM--ERIVT-T-HIRER-
EGRTKEQVM-L-----LID-------IELAYMNTNHEDFIGFANAQQ----------------ERQVETIR--------------
-------------NLVDSYMAI-----------VNKTVRDLMPKT-IMHLM----------I-----------------------------------
NNTKEFIFSELLANLYSCG--------------------D-
QNTLMEESAEQAQRRDEMLRMYHALKEALNIIGDINTT----------------------------
EAW87759.1 --RDVLENKLLPLRRGYIGVVNR-
SQKDIDGKKDITAALA-----------------AERKFFLSH-----PS---YRHLA-------DRMG-
TPYLQKVLNQQLTNHIRDTLPGLRNKLQSQLLSIEKEVEEYKNFRPDDP------A-RKT--KALLQMVQ-
-QFAVDFEKR----IEGSGDQ-------------IDTYELSGGARINRIFHERFPFELVK---------------------------
----
------MEFDEKELRREISYAIKNIHGIRTGL-FTPD-MAFETIVKKQ-VKKIREPC----
LKCVDMVISELISTVRQCT----K---------------------------------KLQQYPRLREEM--ERIVT-T-HIRER-
EGRTKEQVM-L-----LID-------IELAYMNTNHEDFIGFANAQQ----------------ERQVETIR--------------
-------------NLVDSYMAI-----------VNKTVRDLMPKT-IMHLM----------I-----------------------------------
NNTKEFIFSELLANLYSCG--------------------D-
QNTLMEESAEQAQRRDEMLRMYHALKEALSIIGDINTT----------------------------
XP_012378586.1 --RDVLENKLLPLRRGYIGVVNR-
SQKDIDGKKDITAALA-----------------AERKFFLSH-----PS---YRHLA-------DRMG-
TPYLQKVLNQQLTNHIRDTLPGLRNKLQSQLLSIEKEVDEYKNFRPDDP------A-RKT--KALLQMVQ-

-QFAVDFEKR----IEGSGDQ-------------IDTYELSGGARINRIFHERFPFELVK--------------------------
----
------MEFDEKELRREISYAIKNIHGIRTGL-FTPD-MAFETIVKKQ-VKKIREPC----
LKCVDMVISELISTVRQCT----K--------------------------------KLQQYPRLREEM--ERIVT-T-HIRER-
EGRTKEQVM-L-----LID-------IELAYMNTNHEDFIGFANAQQ----------------ERQVETIR--------------
-------------NLVDSYMAI-----------VNKTVRDLMPKT-IMHLM----------I----------------------------------
NNTKEFIFSELLANLYSCG--------------------D-
QNTLMEESAEQAQRRDEMLRMYHALKEALSIIGDINTT---------------------------

BAB27759.1 ---------------------------------------RDVLENKLLPLRRGYIGVVNR-
SQKDIDGKKDITAALA-----------------AERKFFLSH-----PS---YRHLA-------DRMG-
TPYLQKVLNQQLTNHIRDTLPGLRNKLQSQLLSIEKEVDEYKNFRPDDP------A-RKT--KALLQMVQ-
-QFAVDFEKR----IEGSGDQ-------------IDTYELSGGARINRIFHERFPFELVK--------------------------
----
------MEFDEKELRREISYAIKNIHGIRTGL-FTPD-MAFETIVKKQ-VKKIREPC----
LKCVDMVISELISTVRQCT----K--------------------------------KLQQYPRLREEM--ERIVT-T-HIRER-
EGRTKEQVM-L-----LID-------IELAYMNTNHEDFIGFANAQQ----------------ERQVETIR--------------
-------------NLVDSYMAI-----------VNKTVRDLMPKT-IMHLM----------I----------------------------------
NNTKEFIFSELLANLYSCG--------------------D-
QNTLMEESAEQAQRRDEMLRMYHALKEALSIIGDINTT---------------------------

EPQ17174.1 ---------------------------------------RDVLENKLLPLRRGYIGVVNR-
SQKDIDGKKDITAALA-----------------AERKFFLSH-----PS---YRHLA-------DRMG-
TPYLQKVLNQQLTNHIRDTLPGLRNKLQSQLLSIEKEVDEYKNFRPDDP------A-RKT--KALLQMVQ-
-QFAVDFEKR----IEGSGDQ-------------IDTYELSGGARINRIFHERFPFELVK--------------------------
----
------MEFDEKELRREISYAIKNIHGIRTGL-FTPD-MAFETIVKKQ-VKKIREPC----
LKCVDMVISELINTVRQCT----K--------------------------------KLQQYPRLREEM--ERIVT-T-HIRER-
EGRTKEQVM-L-----LID-------IELAYMNTNHEDFIGFANAQQ----------------ERQVETIR--------------
-------------NLVDSYMAI-----------VNKTVRDLMPKT-IMHLM----------I----------------------------------
NNTKEFIFSELLANLYSCG--------------------D-
QNTLMEESAEQAQRRDEMLRMYHALKEALSIIGDINTT---------------------------

XP_025944940.1 ---------------------------------------REILENKLLPLRRGYIGVVNR-
SQKDIDGKKDIKAALL-----------------AERKFFLSH-----PA---YRHMA-------DRMG-
TPYLQKVLNQQLTNHIRDTLPGFRSKLQSQLLSIEHEVEVYKNFRPEDP------T-RKT--KALLQMVQ--
QFSVDFEKR----IEGSGDQ-------------VDTLELSGGAKINRIFHERFPFELVK--------------------------
----
------MEFNEKELRREISYAIKNIHGIRTGL-FTPD-MAFEAIVKKQ-IVKLKGPC----
LKSVDLVMQELINTVKKCT----K--------------------------------KLATYPRLCEET--ERIVA-G-YIRER-
EEKTKDQVL-L-----LID-------IQVSYINTNHEDFIGFANAQQ----------------ERQVETIR----------------
-----------NLVDSYMSI-----------INKCIRDLIPKT-IMHLM----------I---------------------------------
NNVKEFINAELLAHLYSSE--------------------D-
QNTLMEESAEQAQRRDEMLRMYQALKEALAIIGDISTS---------------------------

EPQ08653.1 ---------------------------------------RDILENKLLPLRRGYVGVVNR-
SQKDIDGKKDIKAAML-----------------AERKFFLSH-----PA---YRHIA-------DRMG-
TPHLQKVLNQQLTNHIRDTLPNFRNKLQAQMLSIEHEVDAYKNFKPEDP------T-RKT--

KALLQMVQ--QFAVDFEKR----IEGSGDQ-------------VDTLELSGGAKINRIFHERFPFEIVK-----------
----
---------------------MEFNEKELRREISYAIKNIHGIRTGL-FTPD-MAFEAIVKKQ-IVKLKGPS----
LKSVDLVMQELINTVKKCT----K--------------------------------KLANFPRLCEET--ERIVA-N-HIRER-
EGKTKDQVL-L-----LID-------IQVSYINTNHEDFIGFANAQQ----------------ERQVETIR---------------
------------NLVDSYMSI-----------INKCIRDLIPKT-IMHLM----------I---------------------------------
NNVKDFINSELLAQLYSSE--------------------D-
QNTLMEESAEQAQRRDEMLHMYQALKEALVIIGDINTA---------------------------

XP_027623811.1 --------------------------------------RDVLENKLLPLRRGYVGVVNR-
SQKDIDGKKDIKAAML-----------------AERKFFLSH-----PA---YRHIA-------DRMG-
TPHLQKVLNQQLTNHIRDTLPNFRNKLQGQLLSIEHEVEAYKNFKPEDP------T-RKT--KALLQMVQ-
-QFAVDFEKR----IEGSGDQ-------------VDTLELSGGAKINRIFHERFPFEIVK--------------------------
----
------MEFNEKELRREISYAIKNIHGIRTGL-FTPD-MAFEAIVKKQ-IVKLKGPS----
LKSVDLVIQELINTVKKCT----K--------------------------------KLANFPRLCEET--ERIVA-N-HIRDR-
EGKTKDQVL-L-----LID-------IQVSYINTNHEDFIGFANAQQ----------------ERQVETIR---------------
------------NLVDSYMSI-----------INKCIRDLIPKT-IMHLM----------I---------------------------------
NNVKEFINSELLAQLYSSE--------------------D-
QNTLMEESAEQAQRRDETLRMYQALKEALVIIGDINTA---------------------------

XP_016856477.1 --------------------------------------RDVLENKLLPLRRGYVGVVNR-
SQKDIDGKKDIKAAML-----------------AERKFFLSH-----PA---YRHIA-------DRMG-
TPHLQKVLNQQLTNHIRDTLPNFRNKLQGQLLSIEHEVEAYKNFKPEDP------T-RKT--KALLQMVQ-
-QFAVDFEKR----IEGSGDQ-------------VDTLELSGGAKINRIFHERFPFEIVK--------------------------
----
------MEFNEKELRREISYAIKNIHGIRTGL-FTPD-MAFEAIVKKQ-IVKLKGPS----
LKSVDLVIQELINTVKKCT----K--------------------------------KLANFPRLCEET--ERIVA-N-HIRER-
EGKTKDQVL-L-----LID-------IQVSYINTNHEDFIGFANAQQ----------------ERQVETIR---------------
------------NLVDSYMSI-----------INKCIRDLIPKT-IMHLM----------I---------------------------------
NNVKDFINSELLAQLYSSE--------------------D-
QNTLMEESAEQAQRRDEMLRMYQALKEALGIIGDISTA---------------------------

NP_001025299.1 --------------------------------------RDILENKLLPLRRGYIGVVNR-
SQKDIDGRKDIRAALA-----------------AERKFFLSH-----PS---YRHMA-------ERMG-
TPHLQKALNQQLTNHIRDTLPGLRSKLQSQLLSLEKEVEEYKNFRPDDP------T-RKT--KALLQMVQ-
-QFGVDFEKC----IEGSGDQ-------------VDTAELSGGARINRIFHERFPFELVK------------------------
----
--------IVFDEKELRREISHAIKNVHGVRTGL-FTPD-LAFEAIVKKQ-IIKLKEPC----
LKCIDLVIQELINTFRQCT----N--------------------------------KLSSYPRLREET--ERIVT-T-YVRER-
EGKTKDQVL-L-----LID-------TELSYINTNHEDFIGFANAQQ----------------ERQVETIR----------------
-----------NLVDSYIGI-----------VNKTIRDLMPKT-IMHLM----------I---------------------------------
NSAKDFIHSELLAYLYSSG--------------------D-
QNSLMEESADQAQRRDEMLRMYHAIKEALSIIGDISTS---------------------------

XP_021326548.1 --------------------------------------RDILENKLLPLRRGYIGVVNR-
SQKDIDGRKDIRAALA-----------------AERKFFLSH-----PS---YRHMA-------ERMG-
TPHLQKALNQQLTNHIRDTLPGLRSKLQSQLLSLEKEVEEYKNFRPDDP------T-RKT--KALLQMVQ-

-QFGVDFEKC----IEGSGDQ-------------VDTAELSGGARINRIFHERFPFELVK-------------------------
----
--------IVFDEKELRREISHAIKNVHGVRTGL-FTPD-LAFEAIVKKQ-IIKLKEPC----
LKCIDLVIQELINTFRQCT----N--------------------------------KLSSYPRLREET--ERIVT-T-YVRER-
EGKTKDQVL-L-----LID-------TELSYINTNHEDFIGFANAQQ----------------ERQVETIR----------------
-----------NLVDSYIGI-----------VNKTIRDLMPKT-IMHLM----------I----------------------------------
NSAKDFIHSELLAYLYSSG--------------------D-
QNSLMEESADQAQRRDEMLRMYHAIKEALSIIGDISTS----------------------------

XP_006161648.2.2 ---------------------------------------RDVLENKLLPLRRGYIGVVNR-
SQKDIEGKKDIRAALA-----------------AERKFFLSH-----PA---YRHMA-------DRMG-
TPHLQKTLNQQLTNHIRESLPALRSKLQSQLLSLEKEVEEYKNFRPDDP------T-RKT--KALLQMVQ--
QFGVDFEKR----IEGSGDQ-------------VDTLELSGGARINRIFHERFPFELVK-------------------------
----
-------MEFDEKDLRREISYAIKNIHGVRTGL-FTPD-MAFEAIVKKQ-IVKLKEPS----
LKCVDLVVSELATVIKKCA----E--------------------------------KLSSYPRLREET--ERIVT-T-YIRER-
EGRTKDQIL-L-----LID-------IEQSYINTNHEDFIGFANAQQ----------------ERQVETIR----------------
-----------NLVDSYVAI-----------INKSIRDLMPKT-IMHLM----------I----------------------------------
NNTKAFIHHELLAYLYSSA--------------------D-
QSSLMEESADQAQRRDDMLRMYHALKEALNIIGDISTS----------------------------

NP_001005360.1 ---------------------------------------RDVLENKLLPLRRGYIGVVNR-
SQKDIEGKKDIRAALA-----------------AERKFFLSH-----PA---YRHMA-------DRMG-
TPHLQKTLNQQLTNHIRESLPALRSKLQSQLLSLEKEVEEYKNFRPDDP------T-RKT--KALLQMVQ--
QFGVDFEKR----IEGSGDQ-------------VDTLELSGGARINRIFHERFPFELVK-------------------------
----
-------MEFDEKDLRREISYAIKNIHGVRTGL-FTPD-MAFEAIVKKQ-IVKLKEPS----
LKCVDLVVSELATVIKKCA----E--------------------------------KLSSYPRLREET--ERIVT-T-YIRER-
EGRTKDQIL-L-----LID-------IEQSYINTNHEDFIGFANAQQ----------------ERQVETIR----------------
-----------NLVDSYVAI-----------INKSIRDLMPKT-IMHLM----------I----------------------------------
NNTKAFIHHELLAYLYSSA--------------------D-
QSSLMEESADQAQRRDDMLRMYHALKEALNIIGDISTS----------------------------

XP_028568434.1 ---------------------------------------RDVLENKLLPLRRGYIGVVNR-
SQKDIDGKKDIRAALA-----------------AERKFFLSH-----PA---YRHMA-------DRMG-
TPHLQKLLNQQLTNHIRETLPSLRSKLQSQLLSLEKEVEEYKNFRPDDP------T-RKT--KALLQMVQ--
QFAVDFEKR----IEGSGDQ-------------VDTLELSGGARINRIFHERFPFELVK--------------------------
----
------MEFDEKDLRREISYAIKNIHGVRTGL-FTPD-MAFEAIVKKQ-IIKLKEPS----
LKCVDLVVSELAMVIKKCA----E--------------------------------KLGSYPRLREET--ERIVT-T-YIRER-
EGKTKDQIL-L-----LID-------IELSYINTNHEDFIGFANAQQ----------------ERQVETIR-----------------
----------NLVDSYVGI-----------INKSIRDLMPKT-IMHLM----------I----------------------------------
NNTKDFIHSELLAYLYSSA--------------------D-
QNSLMEESADQAQRRDDMLRMYHALKEALNIIGDISTS----------------------------

XP_031753735.1 ---------------------------------------KDILENKLLPLRRGYIGVVNR-
SQKDIDGKKDIKAALG-----------------AERKFFLSH-----PG---YRHIA-------ERMG-
TPHLQKTLNQQLTNHIRETLPALRNKLQSQLLSLEKEVEEYKNFRPDDP------T-RKT--KALLQMVQ--

QFGVDFEKR----IEGSGDQ-------------VDTLELSGGARINRIFHERFPFELVK-------------------------
----
-------MEFDEKDLRREISYAIKNIHGVRTGL-FTPD-MAFEAIVKKQ-VVKLKEPC----
LKCVDMVIQELINTVRQCT----A--------------------------------KLLSYPKLREET--ERIVT-T-YIRER-
EGKTKDQIL-L-----LID-------IELSYINTNHEDFIGFANAYY----------------ERQVETIR-----------------
---------NLVDSYICI-----------VNKSIRDLMPKT-IMHLM----------I---------------------------------
NNSKDFIHSELLAYLYSSA-------------------D-
QNSLMEESADQAQRREDMLRMYHALKEALKIIGDISTS---------------------------
XP_025920181.1 --------------------------------------RDVLENKLLPLRRGYIGVVNR-
SQKDIDGKKDIRAALA-----------------AERKFFLSH-----PA---YRHMA-------DRMG-
TPHLQKVLNQQLTNHIRETLPSLRSKLQSQLLSLEKEVEEYKNFRPDDP------T-RKT--KALLQMVQ--
QFGVDFEKR----IEGSGDQ-------------VDTLELSGGARINRIFHERFPFELVK-------------------------
----
-------MEFDEKDLRREISYAIKNIHGVRTGL-FTPD-LAFEAIVKKQ-VVKLKEPC----
LKCVDLVIQELINTVRQCT----S--------------------------------KLGSYPRLREET--ERIVT-T-HIRER-
EGKTKDQIL-L-----LID-------IELSYINTNHEDFIGFANAQQ----------------ERQVETIR----------------
----------NLVDSYVGI-----------INKSIRDLMPKT-IMHLM----------I---------------------------------
NNTKDFIHSELLAYLYSSA-------------------D-
QNSLMEESADQAQRRDDMLRMYHALKEALNIIGDISTS---------------------------
XP_014389433.1 --------------------------------------RDVLENKLLPLRRGYIGVVNR-
SQKDIEGKKDIRAALA-----------------AERKFFLSH-----PA---YRHMA-------DRMG-
TPHLQKTLNQQLTNHIRESLPTLRSKLQSQLLSLEKEVEEYKNFRPDDP------T-RKT--KALLQMVQ--
QFGVDFEKR----IEGSGDQ-------------VDTLELSGGARINRIFHERFPFELVK-------------------------
----
-------MEFDEKDLRREISYAIKNIHGVRTGL-FTPD-LAFEAIVKKQ-VVKLKEPC----
LKCVDLVIQELINTVRQCT----S--------------------------------KLSSYPRLREET--ERIVT-T-YIRER-
EGRTKDQIL-L-----LID-------IEQSYINTNHEDFIGFANAQQ----------------ERQVETIR---------------
-----------NLVDSYVAI-----------INKSIRDLMPKT-IMHLM----------I---------------------------------
NNTKAFIHHELLAYLYSSA-------------------D-
QTSLMEESAEQAQRRDDMLRMYHALKEALNIIGDISTS---------------------------
XP_042914770.1 ---------------------------------
DRGTDAAHILRNAHIPLRLGYIGVVLR-AQADIAAKLPMSECRK----------------REESFFASR-----AE-
--YRDVA-------AHCG-VPTLARRLNVILVEHIRGLLPGLKRRIHEALEVRNAELRALGDPDPVQSK----
-S-AKG--AYLLQLLC--DYAERYAAM----LDGRHLDLH------------
MAQQLSGGARVREVFTEHFLPQ---------LH--

RLDPARDLSDAEVSTVIRNGAGVSGSL-LVAQ-EPFELLTRRA-VQQLMQPA----
LGCKERVHEELVRIAEQAC---PP-------------------------------EAARFPQLQRHL--AHAVV-D-
FIHSG-TLPADSMIR-S-----LVD-------CECDYINCDHPDFIGGRGAIRSVMQDRAQRQA---
GPGAEVEVVR--------------------------KLVDSYFNI-----------SRKNMADMVPKT-IMHFM----------V-
--------------------------------LYTKRGLQQHLIKALYRDE-------------------L-
LDSLLTEAEDVVVRRNAAREAVAVLRAAVTALEEVPHEMATGL----------------------
ONM18162.1 ---------------------------------DRGTDARNFLLGNVIPLKFGYVGVVNR-
SQEDINFNRSVKDALA-----------------FEEKYFLTL-----PA---YHGLA-------HCCG-

VPQLAKKLNMILLKHVTNMLPGLKTRINAQLVAVAKEHAAYGDTVESVPFLPWWKG-KTG--
CLQINFLVEQGYTTFFSQY----LSRVWRCRWG---------
TSPHPAEFFRGTQHLILPAGWISGEVGAAFVVACRVD----QWRSWSCFRCGVGQS--------
FAVMTCCVHFLWSL--------------------GLLVSRWRRVRALA------------DVRCGKSEQLI--------------
----IIT--CDKFRNDDVRWALSVEIDPCKSISDEDIRTTIQNSGGPKGAM-FLPE-VPFEILVRKQ-
IGRLLDPS----LQCAQFIYDELIKISHSCL---IS---------------------------------ELQKFPALKKRL--
SEVVC-S-FLRDG-LRPAETMIT-H-----IIE-------MEASKLRELAEAAS---
RRRINILCVQETKWKGQKVKEVEVAVVK---------------------------LLIKSYYDI-----------
VRKNIEDAIPKA-VMHFL----------V---------------------------------NHTKRDLHNFLIRKLYREN-------
-------------L-LNELMRETDEVLMRRQRIQEMLEILEQAHRTLEEFPFEVDKLE-----------------------
KAH9327796.1 ---------------------------------
DRGTDARNFLLGNVIPLQLGYIGVVNR-SQEDIIANQSIRDALA-----------------YEENFFRGH-----
PV---YCSLA-------DQCG-
IPQLARKLNQILVKHIRVMLPDLKAQIHGQMMSVKKELSSYGEVTESEV--------DQG--NLLLDILR--
KFSDAFRSR----IEGKN----E---------E-TTFELVGGARIHYIIQSIFVKR---------LE------------------------
----
DVDPCEGLSDKDIQTAIQNAMGSRSAL-FVPE-VPFQALLRKK-IAQLLDPS----
LQCVHIIYDELVKLANRCQ---TS--------------------------------DLFRFPLLRRCL--EEVLG-T-CLREG-
LESALTMVS-H-----LIE-------MEMDYINTSHHSFIGGSKAMAA-----------------GIEVTK---------------
------------ILLRSYYDI-----------VRKNIQDRVPKA-IMHFL----------V---------------------------------
NHVKRELYSVLIRNLYREN--------------------L-FAEMLKERDDISARRKQCKDILHILKKAVR----------
----
EFJ35472.1 ---------------------------------DRGTDARSILLGTVIPLRLGYVGVVNR-
SQEVFRSPFFCLKILMKHGCRIFMPTNRSRMLLLRKSIFSEVERCTKAFWIDVASLN-------SRKS-
LIKYELEPVKILVQHIKTVLPELKTRINTQMVALLKELTSYGEATDSKS--------GQG--AMLLNALT--
KYFHVFSSV----IDGKN----Q---------EMSTSELSGGARIHYIFQSIFVKS---------LE--------------------
----
----EVDPCDDLTDEDIRTAIQNATGPKMIL-FVPE-VPFEVLVRRQ-IERLLDPC----
LQCARFIYDELVKMSHRCE---TN---------------------------------ELQRFPVLRRRI--EEAVS-T-
CLREG-LTPTETMIS-H-----LVD-------MEMDYINTSHPRFIGGSKAVET-----------------EVAVTR------
---------------------LLLKSYYEI-----------VRKNIQDAVPKA-IMHFL----------V---------------------------
------GHVKRELLNLLIKKLYREV--------------------L-
FEEMLQERDDIASRRKHCREVLRVLQQAVVTLEDLPVDADAHS-----------------------
KAH9304002.1 ---------------------------------
DRGTDATNFLLGNVIPLRLGYVGVVNR-SQADINGNKSIQDALA-----------------YEERFFRSR-----
PV---YHRLA-------DRCG-IPQLAKKLNYILVQHIRAILPGLKARINAQMVSVVKEISTYGEITESKA-----
---GQG--ALLLNILT--KYSEAFTSV----VEGKN----E---------EMSTAELSGGARIHYIFQSIFVKS--------
-LE--
---------------------------LVDPCDDLTDDDIRMAIQNATGPKNAL-FVPE-IPFEVLVRRQ-ISRLLDPS-
---IQCARFIYDELLKIAHRCK---AL---------------------------------ELERFPVLQRRL--DEVVG-S-
FLREG-LSPAETMIG-H-----LIE-------MEMDYINTSHPAFIGGSKAVEL-----------------EIAVTK--------
-------------------LLLKSYYDI-----------VRKNVQDFVPKA-IMHFL----------V----------------------------
-----NHSKRDLHGILIKKLYREP--------------------L-
FEEVLQEREEIAVRRKRSRELLRVLQQAVWTLDELPLESDMPS----------------------

KAI5070335.1---------------------------------DRGTDARNFLLGSAIPLRLGYIGVVNR-
SQADILGNKSIREALA-----------------YEDNFFRSL-----PV---YHSLA-------DRCG-
IPQLAKKLNNILVQHIKAILPDMKARINTQMVTLSKELASYGELTDSKT--------GQG--ALLLNIIT--
RYSQGFASI----VDGKN----E---------EMSTSELAGGARLHYIFQSIFVKS---------LD------------------
----
-----EVDPCDDLTDEDIRTAVQNATGPKNII-FVPD-VPFEVLVRRQ-IARLLDPS----
LQCARFIYDELVKISHRCE---TY--------------------------------ELQRFPVLRRRI--EEVVA-N-FLREG-
LIPAERMIC-H-----LIE-------MEMDYINTSHPSFIGGSKAVEA-----------------EIAVTR-----------------
---------SLIKSYYDI-----------VRKNVQDAVPKA-IMHFL----------V---------------------------------
NHAKRELHNVFIRKLYREN-------------------L-
FEEMLQEREEVAVKRKRCKEVLRVLQQAAYTLEELPLDYEVPT----------------------
KAI5070758.1---------------------------------DRGTDARNLLLGNVIPLRLGYIGVVNR-
SQEDILGNKSVVDALL-----------------YEENFFRSR-----PV---YHNLA-------DRCG-
IAQLAKKLNTILVQHIKTILPDMKARINTQMVTLAKELASYGELTESKS--------GQG--ALLLNIIT--
RYSQGFAAV----VDGKN----E---------EMSTSELSGGARLHYIFQNIFVKS---------LD-----------------
----
------EVDPCDDLTDEDIRTAIQNATGPKSII-FVPE-VPFEVLVRRQ-IARLLDPS----
LQCARFIYDELVKISHRCE---SH--------------------------------ELQRFPVLRRRI--EEVMS-G-
FLREG-LAPAETMIC-H-----LIE-------MEMDYINTSHPAFIGGNKAVEV-----------------EIAVTR-------
-------------------LLIKSYYDI-----------VRKNIQDAIPKA-VMHFL----------V----------------------------
-----NHTKRELHSVFIRKLYREH-----------------L-
FEEMLQEREDVAVKRKRCKEVLRVLQQAVYTLEELPLDYEAST----------------------
EFJ37641.1 ---------------------------------DRGTDARNFLLGSVIPLRLGYIGVVNR-
SQEDITSNRSIQDALM-----------------YEEQFFRSR-----PV---YHSLS-------DRCG-
IPQLAKKLNQILVQHIRTILPDLKARINTQMVTLQKELATYGELTESKN--------GQG--VLLLGIIT--
KYSQSFSSV----VDGKN----E---------EMSTVELSGGARIHYIFQSIFVKS---------LD-------------------
----
----EVDPCDDLTDEDIRTAIQNATGPKNVL-FVPE-VPFEVLVRRQ-IARLLEPS----
LQCARFIYDELVKISHRCE---SS--------------------------------ELQRFPVLRRNI--EEVIA-S-FLREG-
LSPAETMIG-H-----LIE-------MEMDYINTSHPGFIGGSKAVE-----------------LEIVVTR-----------------
----------LLLKSYYDI-----------VRKNIQDSVPKA-IMHFL----------V---------------------------------
NHAKRELHNVFIRKLYREA-------------------M-
FEELLQEREEIAIKRKRCKEVLKVLQQAAWTIEELPLEYEFPS----------------------
EFJ15047.1 ---------------------------------------RNFLLGSVIPLRLGYIGVVNR-
SQEDITSNRSIQDALM-----------------YEEQFFRSR-----PV---YHSLS-------DRCG-
IPQLAKKLNQILVQHIRTILPDLKARINTQMVTLQKELATYGELTESKN--------GQG--MLLLGIIT--
KYSQSFSSV----VDGKN----E---------EMSTVELSGGARIHYIFQSIFVKS---------LD-------------------
----
----EVDPCDDLTDEDIRTAIQNATGPKNVL-FVPE-VPFEVLVRRQ-IARLLEPS----
LQCARFIYDELVKISHRCE---SS--------------------------------ELQRFPVLRRNI--EEVIA-S-FLREG-
LSPAETMIG-H-----LIE-------MEMDYINTSHPGFIGTSIRLREPPLVLRASDAQTEQESTEIVVTR-------
-------------------LLLKSYYDI-----------VRKNIQDSVPKA-IMHFL----------V---------------------------
------NHAKRELHNVFIRKLYREA-------------------M-FEELLQEREEIAIKRKRCKEVLKVLQQAAWV-
----

KAG0554580.1 ---------------------------------
DRGTDARSFLLGNIIPLRLGYVGVVNR-SQEDISANKSIRDALT-----------------YEENFFRSR-----PV--
-YHNLS-------DRCG-VPQLAKKLNTILVQHIKAILPDLKSRISNQMVALQKELASYGELTDSKS--------
GQG--ALLLGIIT--KYSSDYQAI----VDGKY----E---------EMSTTELSGGARIHYIFQAIFVRS---------
LE--
---------------------------EVDPCDDLTDEDIRTAIQNATGPKNVL-FVPE-VPFEVLVRRQ-IARLLEPS---
-LQCARFIYDELVKISHRCE---SY--------------------------------ELQRFPVLRRRI--EEVVA-N-
FLRDG-LAPAETMIG-H-----LIE-------MEMDYINTSHPAFLGGSKAVEV-----------------EISVTR-------
--------------------LLLKSYYDI-----------VRKNIQDSVPKA-IMHFL----------V---------------------------
------NHSKRELQSIFIRKLYREN--------------------T-
FEEMLQEKEEIAAKRKRCKEILRVLQQAAWSYSDAERGFAECMSCGCFWNCDQTLEELPLDSEGS
S
XP_024362051.1 ---------------------------------
DRGTDARSLLLGNVIPLRLGYVGVVNR-SQEDISRNRSIRDALT-----------------NEENFFRSR-----
PV---YHNLS-------DRCG-VPQLAKKLNTILVQHIKAVLPDLKSRISNQMIFLQKELTSYGELTDSKS----
----GQA--ALLLGIIT--KYSSDYQSI----VEGNY----E---------EMSTTELSGGARIHYIFQEIFVRG--------
-LEVL----V--------------------------------WPL--
NVGGKVTVV------------------FYF--ILNQMEILMELELVQEVDPCDVLTDEDIRTAIQNATGPKNVL-
FVPE-VPFEVLVRRQ-IARLLEPS----LQCARYIYDELVKISQRCE---SY--------------------------------
ELQRFPLLRRRI--DEVVA-N-FLRDG-LAPAETMIG-H-----LIE-------
MEMDYINTSHPAFLGGSKAVEV-----------------EISVTR--------------------------LLLNSYYDI---------
--ARKNIQDSVPKA-IMHFL----------V---------------------------------NHSRRELQSVFIRKLYREN-----
---------------M-
FEEMLQEKEEIAAKRKRCKEILRVLQQAAWSYSDAERGFAECMSCGCFWNSEEMLQELPLDLEAS
S
KAG0555682.1 ---------------------------------
DRGTDARNFLLGNVIPLRLGYIGVVNR-SQEDIIANKSIRDALV-----------------YEEGFFRSK-----PV--
-YHNLA-------DRCG-VPQLAIRLNTILVQHIKAILPDLKSRISTQMITLQKELASYGELTESKS--------
GQG--ALLLNILT--KYSHGFQSV----VDGKN----E---------EMSTTELSGGARIHYIFQAIFVRS---------
LE--
---------------------------EVDPCDDLNDDDIRTAIQNATGPKNVL-FVPE-VPFELLVRRQ-IARLLEPS--
--LQCARFIYDELVKISHRCE---TH--------------------------------ELQRFPQLRRRI--EEVVA-S-
FLREG-LSPAETMIG-H-----LIE-------MEMDYINTSHPGFIGGSKAVEI-----------------EIAVTR--------
-------------------LLLKSYYDI-----------VRKNVQDLVPKA-IMHFL----------V----------------------------
-----NHVKRELHSVFIRKLYREN--------------------L-
FEEMLQEKEEIAVKRKRCKEILRVLQQAAWVKFLLSFDHHC-------------------------
PTQ35749.1 --------------------------------DRGTDARNFLLGNVVPLRLGYIGVVNR-
SQEDIQANKTIREALG-----------------YEENFFRSR-----PV---YHSLS-------ERCG-
IPQLAKKLNSILVQHIRAILPDLKARISTQMVTLQKELAGYGELTDSKS--------GQG--ALLLNIIT--
KYSQGFQSI----VDGKN----E---------EMSTTELSGGARIHYIFQSIFVKS---------LE--------------------
----
----EVDPCDDLTDEDIRTAIQNATGPKNVL-FVPE-VPFEVLVRRQ-IARLLEPS----
LQCARFIYDELVKISHRSE---SY--------------------------------ELQRFPVLRRRI--EEVVA-N-FLREG-
LAPAETMIG-H-----LIE-------MEMDYINTSHPGFIGGSKAVEM-----------------EIAVTR----------------

-----------LLLKSYYDI-----------VRKNIQDSVPKA-IMHFL----------V---------------------------------
NHVKRELHSVFIRKLYRES--------------------L-
FEEMLQEKEEIAAKRKRCKEILRVLQQAAWELDTLSF-----------------------------
PWZ09977.1 ---------------------------------DRGTDARNFLLGNVIPLKLGYVGVVNR-
SQQDINSDLSIKDALA-----------------REEKFFRTQ-----PA---YHGLA-------QYCG-
IPQLAKKLNQILVQHIKTVLPGLKSRISSQLTAVAKELAVYGDPVDSKA--------GQG--AKLLNILA--
KYCEAFSSM----VEGKN----E---------DISTTELSGGARIHYIFQSIFVKS---------LE--------------------
----
---EVDPCEDVTDVDIRMAIQNATGPRSAL-FVPE-VPFEVLVRRQ-ISRLLDPS----
LQCAQFIYDELVKMSHRCL---AT--------------------------------ELQQFPILRRSM--DEVIG-K-
FLRDG-LKPAESMIS-H-----IIE-------MEEDYINTSHPSFIGGSKAVEE-----------------EIAITK----------
-----------------LLLKSYYNI-----------VRKNVEDFIPKA-IMHFL----------V--------------------------------
--NHTKRALHNYLITKLYRDD--------------------L-
LEDLLKEPDELTIKRKQIRENLKVLQQAYKTLDEIPLDAEAVE-----------------------
XP_052310486.1 ---------------------------------
DRGTDARNLLLGKVIPLRLGYVGVVNR-SQEDIILNRSIKDALA-----------------AEEKFFRSR-----PV-
--YNGLA-------DRCG-VPQLAKKLNQILVQHIKSILPGLKSRISSALVSVAKEHASYGEITESKA--------
GQG--TLILNILS--KYSEAFSSM----IEGKN----E---------EMSTSELAGGARIHYIFQSIFVKS---------
LE---
--------------------------EVDPCEDLTDGDIQTIIQNATGPRTPL-FVPE-VPFEVLIRKQ-IARLLDPS----
LQCARFIYNELIKISHHCL---VN--------------------------------ELQRFPVLRKRM--DEVIG-N-
FLRDG-LEPSETMIG-H-----IIE-------MEMDYINTSHPNFVGGSKAVEI-----------------EIAVTK--------
-------------------LLLRSYYDI-----------VRKNIEDSIPKA-IMHFL----------V-------------------------------
---NHTKRELHNVFIRKLYREN--------------------L-
FEEMLQEPDEIAMKRKQTREQLRVLQQAFRTLDELPLEAETVE-----------------------
AAC61784.1 ----------------------------------DKGTDARKLLLGNVVPLRLGYVGVVNR-
CQEDILLNRTVKEALL-----------------AEEKFFRSH-----PV---YHGLA-------DRLG-
VPQLAKKLNQILVQHIKVLLPDLKSRISNALVATAKEHQSYGELTESRA--------GQG--ALLLNFLS--
KYCEAYSSL----LEGKS----E---------EMSTSELSGGARIHYIFQSIFVKS---------LE--------------------
----
---EVDPCEDLTDDDIRTAIQNATGPRSAL-FVPD-VPFEVLVRRQ-ISRLLDPS----
LQCARFIFEELIKISHRCM---MN--------------------------------ELQRFPVLRKRM--DELSGRD-
FLREG-LEPSETMIG-D-----IID-------MEMDYINTSHPNFIGGTKAVEA-----------------EIQITK--------
-------------------LLLRSYYDI-----------VRKNIEDSVPKA-IMHFL----------V------------------------------
----NHTKRELHNVFIKKLYREN-------------------L-
FEEMLQEPDEIAVKRKRTQETLHVLQQAYRTLDELPLEADSVS-----------------------
NP_012926.1 -------------------------------------DVIDILAGRVIPLRYGYIPVINR-
GQKDIEHKKTIREALE-----------------NERKFFENH-----PS---YSSKA-------HYCG-
TPYLAKKLNSILLHHIRQTLPEIKAKIEATLKKYQNELINLGPETMDSA----------S--SVVLSMIT--
DFSNEYAGI----LDGEAKEL-------------SSQELSGGARISYVFHETFKNG---------VD------------------
----
------SLDPFDQIKDSDIRTIMYNSSGSAPSL-FVGT-EAFEVLVKQQ-IRRFEEPS----
LRLVTLVFDELVRMLKQIIS--QP-------------------------------KYSRYPALREAI--SNQFI-Q-FLKDA-
TIPTNEFVV-D-----IIK-------AEQTYINTAHPDLLKGSQAM------------------METEVIK------------------

--------LLISSYFSI-----------VKRTIADIIPKA-LMLKL----------I---------------------------------
VKSKTDIQKVLLEKLYGKQ--------------------D-IEELTKENDITIQRRKECKKMVEILRNASQIVSSV----
----

KXN66323.1 --------------------------------------DVVDILAGRVIPLRLGYVPVINR-
SQRDIESKKNISAALD-----------------HERQFFEKH-----PS---YTSKV-------QYCG-
TPFLARKLSMILMHHIRNTLPEIKAKIQSALTKYQTELAQLGDPIGGDD------A-YSS--NLILNVIT--
EFCNDFRTV----LDGHAKDL-------------TTYELNGGARISFVFFEIYANG---------VS-------------------
----
-----SIDVFDQVKDADIRTILYNSSGSAPSL-FVGT-AAFELIVKQQ-IRRLEEPS----
VKCISFVYDELVRILSQLLQ--KP--------------------------------VFKRFPQLKEQF--YTVVV-N-FFKKS-
TTPTTKLVQ-D-----IIS-------MEANYVNTAHPDFITGHKAM------------------MEIEVIK------------------
---------LLIQSYYNI-----------VKRTVMDMVPKA-IMYNL---------V---------------------------------
FTSKEGLQAELLKELYKSD--------------------F-IEDMLKESEFTRSRRDECKKMIEALQKANEIVASV--
----

KNE68830.1 --------------------------------------DVVDILAGRVIPLRLGYVPVVNR-
GQKDIDKRKSIALALE-----------------HERSYFEEH-----PS---YRSKA-------QYCG-
TPFLARKLNMILLHHIKNTLPEIKNRISAALQKYQAELAQLGDPIDDPS------S-STA--NVVLSVIT--
EFCNDFRNL----LDGTAGDL-------------STVELSGGARISFVFHELFSNG---------IK-------------------
----
-----AIDPFDQIKDTDIRTIMYNSSGSQPAL-FVGA-QAFEVLVKQQ-IKRLEDPS----
LKCVQLVYDELARILAQLLQ--KP--------------------------------VFKRFPDLRDRV--NSNVL-Q-
FLRKA-LIPTNKLVT-D-----LLA-------AEMCYINTGHPDFINGHRAT------------------LETEVIK---------
------------------LLLTSYFNI-----------VKRTIADMVPKS-IMLNL----------V------------------------------
---AYSKENMQRTLLADLYQKN--------------------V-
LDEVLKESDATLARRREVKKMIDALERADEIVSTV------------------------------

OAJ44422.1 --------------------------------------DVIDILAGRVIPLRLGYVPVVNR-
GQRDIENKKKISLALE-----------------AEKNYFENH-----AS---YRSKA-------QYCA-K------------------
-------IQSGLTKFQQELVTLGDPLGEDS------A-NMS--NIILNVIT--EFTSEYRTV----IAGTSNDL-------
------SSDELSGGARISFVFHEIYAAA---------IR--

SMDPFDQVKEVDIRTILYNSSGSSPAL-FVGT-AAFEILVKQQ-IRRLEDPS----
LKCCTMIYDELVRILNRLLQ--RP--------------------------------IFKRFPALKDKF--YNVVI-N-FFQRC-
MNPTNKLVT-D-----LIN-------AEACYINTGHPEFITGHRAM------------------IETEVIK------------------
---------LLLMSYFNI-----------VKRTAADLVPKA-IMLNL---------V--------------------------------
QLSKDELQRELLSELYKRE--------------------E-FDESLKESEFTVQRRVECKKMITALKKADEIIATV---
----

OUM62108.1 --------------------------------------DVIDILAGRVIPLRLGYVPVVNR-
GQKDIETNKSISKALE-----------------AEKQFFEHH-----SS---YKSKA-------QYCG-
TPFLARKLNMILMHHIRNTLPEIKIKIAASLQKYQAELAQLGDPLGDDI------T-SHS--NLVLNIIT--
EFCSEFRTI----IDGNSNDL-------------SSFELSGGARISYVFHDSFKDA---------IT---------------------
----
---SMDPFDQIKDVDIRTIMYNSSGSSPAL-FVGS-TAFEVLVKQQ-IKRLEDPS----
LKCVNMIYDELVRILNQLLQ--RQ--------------------------------VFKRFPSLKEQF--YSTVI-S-FFKAS-
LIPTNKLVQ-S-----IIA-------AEACYINTAHPDFLSGHKAM------------------METEMIK------------------

---------LLLQSYFAI-----------VKRTVADLVPKA-IMLNL---------V---------------------------------
NHAKDELQRHLLSELYQKD-------------------V-FEELLKESDMTLQRRKECKKMIEALTKADEIINSV-
----

XP_011389257.1 -------------------------------------DVVDILAGRVIPLRLGYVPVVNR-
GQRDIDQKKLVSAALT-----------------AEKEFFENH-----PS---YRSKA-------QYCG-
TPFLARKLNTILMHHIRNTLPDIKNKIGSQLAKFQSELASLGGPMGESN---------SA--GVVLQIIT--
EFANEFRTV----IDGNSNDL-------------TVNELAGGARISFVFHELYSNG---------VK------------------
----
-----AIDPFDMVKDTDIRTILYNSSGSSPAL-FVGT-TAFEVIVKQQ-IKRLEDPA----
LRCCSLVYDELVRILAQLLA-KNA---------------------------------SFRRFPALRERF--NTVVI-H-
FFKKC-MAPHHQARL-E-----LCR-------RTSVYLTKK----VNPHAVN------------------LETEVVK--------
-------------------LLIQSYFNV-----------VKVETISMVPKC-IMLNL---------V------------------------------
----TQSKEQMQKELLQEIYRPD-------------------V-
LEELMKESDHVVARRKECVKMISALETASEIIATV------------------------------

XP_006458578.1 -------------------------------------DVVDILAGRIIPLRLGYVPVVNR-
GQRDIESSKPISAALE-----------------YERSFFENH-----AS---YKSKA-------QFCG-
TPFLARKLNMILMHHIKATIPDIKARIAQQLTKYNAELQSLGGFAGESS----------S--NVVLSVIT--
EFTSDFRTT----IDGNTNDL-------------SLNELSGGARISFVFHELFNNG---------IK-------------------
----
----NIDPFDQVKDGDIRTMLYNSSGSTPAL-FVGT-AAFEVIVKQQ-IKRLEEPG----
LKCCQLVYDELIRILGQLLG-KIQ---------------------------------AFRRYPALRERF--NSVVV-N-
FFKKS-MTPTTKLVT-D-----LVS-------MQACYINTTHPDFISGHKAT------------------METEVIK--------
------------------LLIHSYFNI-----------VKREMIDMVPKA-ISLNL---------V------------------------------
---NHSKDNLQRELLQELYKPE-------------------V-
LDDLLKESEYVVSRRKEVVSMVQALNKAEEIVAGV------------------------------

XP_748106.1 -------------------------------------DVVDILAGRIIPLRLGYVPVVNR-
GQRDIENKRPISYALE-----------------HEKNFFESH-----KA---YRNKA-------SYCG-
TPYLARKLNLILMMHIKQTLPDIKARISSSLQKYTAELSQLGDSMLGNS----------A--NIILNIIT--
EFSNEYRTV----LEGNNQEL-------------SSVELSGGARISFVFHELYSNG---------IK------------------
----
-----AVDPFDQVKDIDIRTILYNSSGSSPAL-FVGT-TAFELIVKQQ-IKRLEDPS----
LKCISLVYDELVRILGQLLN--KQ---------------------------------LFRRYPMLKEKF--HAVVI-N-FFKKC-
MEPTNKLVH-D-----LIS-------MEACYINTGHPDFLNGHRAM------------------TEVEVIK---------------
------------LLITSYFNI-----------VKRTMIDMVPKA-IMYTL---------V---------------------------------
QFTKDEMQRELLEQMYRNN-------------------E-
LDELLKESDYTIRRRKECQQMVESLSRASEIVSQVQ-----------------------------

XP_001750431.1 ---LSGEVLPVKLGIIGIVNR-
SQNDINCKTSIQDSLD-----------------NEKRFFRTH----------YPEMA-------DRCG-
CAFLADTLHHLLLQHIRACLPDLKQRIKSLQIQTHKRVQELGEPLKDD-------A-TRG--ATLLTNIM--
RYAEAVKAS----ISGSGAMMRA---------SDEQLPLSTGARIYHIFHYTFGGA---------LN----------------
----
--------KMDAMEGLDSQKILAEIRNAAGPRPSL-FIPE-AAFEALIKKQ-IQRLESPS----
VQCAELIHEELLAVLRQCLK--LR---------------------------------ELARFEALRERL--LDCAR-K-
FLERC-LPRTLDMIR-N-----LIH-------VEMSYINTKHPDFENP---------------------QETQIVT---------

-----------------QLVTSYFTI-----------VRKTITDMVPKT-IMAFM----------V-------------------------------
--SQLQEELHHQLVQELYGAG--------------------A-
DLSLLDEDPRLVKERTAAKEKLRVLDECNTIVSQKYR-----------------------------
KNE61418.1 ------------------------------------GTHALDILAGRVYPLRLGFVGVVNR-
SQRDIDQGKSLEFARK-----------------REQQFFATH-----PV---YAPVA-------SRCG-
TTVLARTLNQVLLAHIRDRLPDLKAKLNALITAKQAELISLGGGDTAHS-----GQ-SPA--STVLRLIT--
KYAAEFQAS----VEGTVPAS-------------ATTSLSGGARLFQVFDQGFGHA---------LD-----------------
----
-------AVDPMSTLTVREIRTAIRNATGARAALFFVPE-AAFDLLVKPQ-IARLEEPA----
LRCVDQVYEELLRLTHECG---SA----------------------------------ELRRYPILRARV--VMPSR-S-
CSRTRQSALPPKFFQ-D-----VEAFAKPKLPASKSPSNDFDPFGPGAGSAAPPLAD-SD---------
EFTIALIR---------------------------MLITSYFGV-----------VRKTLADTVPKA-VMHLL----------V----------
------------------------NETKEALQNRLVQALLQGM--------------------S-W--------------------------------
----
KNE67543.1 -------------------------------------GSHALDILGGRVYPLRLGFVAVVNR-
SQRDIEARRTLEWSRK-----------------REQQFFS-------------------------GRCG-
TAALARTLNSVLLDHIRAQLPDLKARLSALIAAKQNELVTYGESPAVLAATASGSA-SPN--AVVLRLIA-
-LYATEFQAS----IEGTRTAA-------------ALTDLTGGARIFAIFHDGFARA---------LD---------------------
----
---ALDPVSALTVHEIRTAIQNSTGARSALFFVPE-AAFDLLVKPQ-IARLAAPA----
MHCVDHVFEELLRLTHECC---SA----------------------------------ELKRFPTLHARL--VEAVT-A-L-
LKEHLAPTRSFVQ-T-----LVA-------LQSAYINTAHPDFIGGARAVAQLRD-PE---------EFTIALIR-------
--------------------SLITSYFGI-----------VKKTLADAVPKA-VMHLL----------V-----------------------------
-----NDTLAMLQTRLVEALYRDE--------------------M-
LPELLQENPALVKQREACRAQLAVYQRAMEVIAQAY-------------------------------
XP_004348308.1 --LSGRVVPVKLGFIGVVNR-
SQADINTAKPIADSLK-----------------SEEQFFKSH-----PA---YQAIA-------HRCG-
TAYLSKALNKLLMHHIRDCLPDLKTRINAHMAEAQQAYNAYGEPLMDK-------F-NKG--SLMLQIIT--
KFCSNYCGA----IEGTSRDI-------------QTNELTGGARICYIFHETFGRT---------LE---------------------
----
---NVDPLEGLTIGDIRTAIRNATGPRPAL-FVPE-VSFELLVKRQ-IRRMEEPA----
LRCVELVFEELLRITQQCE---TP----------------------------------ELLRFQNLRDHI--SETVT-T-LLRNR-
LPAANVMIE-N-----LVA-------IELAYINTNHPDFTTG----------------------METELIQ--------------------
-------TLIINYFKI-----------VRKNIQDTVPKA-IMHFL----------V----------------------------------
NWIKEHIQSELVSQLYRED--------------------L-FETMLEESVHIAVKRKQAAEMLSALKRAAKVLVEI--
----
NP_741403.2 --LMGKVIPVKLGIIGVVNR-
SQQNILDNKLIVDAVK-----------------DEQSFMQK-------K---YPTLA-------SRNG-
TPYLAKRLNMLLMHHIRNCLPALKARVSIMNAQCQSDLVAFGEPVEDK-----------N--RTLLQIIT--
RFATAYTST----IEGTARNI-------------ETTELCGGARICYIFHDTFGRS---------LE-----------------------
----
-SVNPLENLTQLDILTAIRNATGPRPAL-FVPE-VSFELLVKRQ-IQRLEEPS----
LRCVELVHEEMQRMVQHCGFTTQQ----------------------------------EMIRFPRLYDKI--NEVVS-G-
VLKER-LKPTNELVE-N-----LVA-------IELAYINTKHPEFTEA----------------------RDVAIIE-------------

-------------RLIRNYFII-----------VRKNIQDSVPKA-IMALL----------V---------------------------------
NFVRDNLQSELVRQLYKPD--------------------E-
MDDLLAETEDMAQRRRDTLETMKALQQASVIISEV------------------------------

PAA85687.1 --LLGRVIPVKLGIIGVVNR-
SQADIKNQKQVKEAVR-----------------DESSFLQR-------R---YPSLA-------SRNG-
TPYLARTLNRLLMHHIRDCLPELKTRVNVMAAQHQALLNSFGEEVADK-----------G--HLLLQIIT--
RFASAYCST----IDGVSKNI-------------EIAELCGGARVSYIFHETFYRT---------LS----------------------
----
-KIDPMQGLTTLEILTAIRNSTGPRPAL-FVPE-ISFELLVKRQ-IKRLEEPC----
LRCVELVHEELQRIIQHCSS--QQ--------------------------------EMLRFPKLYERI--MDVVT-S-
LLRTR-LGPTNSMVQ-N-----LVA-------VELAYINTRHPDFTEA----------------------RDCNIIE----------
-----------------RLIKSYFLI-----------VRKNIQDSVPKA-IMHFL----------V--------------------------------
--NYAKDNLQSELVSKLYKSE--------------------Q-
VDSLLQESEHIAQSRQEAADMLAALQRASLIISEI------------------------------

XP_002129967.2 --LCGRIVPVKLGIIGIVNR-
SQLDINKGKSVQDAIK-----------------DEQAFLQK-------K---YPSFA-------NRSG-
SRYLSITLNRLLMHHIRDCLPDLKTRLNVLTSQYQSLLSTYGEEVQEK-----------S--ITLLQIIT--
KFATEYCNT----IDGTSKII-------------ETSEICGGARIAYIFHETYGKT---------LE------------------------
----
SVDPLGGLTDLDILTAIKNANGSRSSW-FVPV-LSFELLVKRQ-IKRLEEPS----
LRCVELVHEELQRIVQNCG---GQ--------------------------------EIVRFMNLRESI--VEVVT-Q-
LLRKR-LPVTNNMVS-H-----LVA-------IELAYINTKHPDFADA----------------------RDVETMR---------
------------------RLIKSYFLI-----------IRKNIQDSVPKA-VMNFL----------V-------------------------------
---NHVQENLQSELVGQLYKHD--------------------Q-
IDHLLTESDTIAQRRAEAVSMLKALHSASLVISEI------------------------------

NP_001259946.1 --LCGRVIPVKLGIIGVMNR-
SQKDIMDQKHIDDQMK-----------------DEAAFLQR-------K---YPTLA-------TRNG-
TPYLAKTLNRLLMHHIRDCLPDLKTRVNIMATQFQSLLNSYGEDVSDK-----------S--QTLLQIIT--
KFSSAYCCT----IEGTARNI-------------ETTELCGGARMGYIFHETFGRT---------LD---------------------
----
---SIHPLAGLSKMDILTAIRNATGPRPAL-FVPE-VSFELLVKRQ-IRRLEEPS----
LRCVELIHEEMQRIVQHCGNEVQQ--------------------------------EMLRFPKLHEKI--VDVVT-Q-
LLRRR-LPHTNVMVE-N-----IVA-------IELAYINTKHPDFHKD----------------------KDCDVIE----------
-----------------HLIKSYFYI-----------VRKSIQDSVPKA-IMHFL----------V-------------------------------
--NYVKDNLQSELVTHLYKSD--------------------K-
AETLLNESDHIAVRRKEAADMLKALTRANHIISEI------------------------------

XP_032819300.1 --LTGRVIPVKLGIIGVVNR-
SQLDINTKKTILDAMQ-----------------DEQSFMQK-------K---YPSLA-------NRNG-
TKFLGKTLNRLLMHHIRDCLPELKTRVNVMAAQYQSLLGGYGNPVDDK-----------N--ATLLQLVT--
KFAAEYCNT----IEGTAKHI-------------ETSELCGGARICYIFHETFGRT---------LE----------------------
----
--SVDPLGGLTTLDVLTAIRNATGPRPAL-FVPE-VSFELLVKRQ-IKRLEEPS----
LRCVELVHEEMQRIIQHCSNYSTQ--------------------------------ELLRFPKLHDAI--VEVVT-C-
LLRRR-LPVSNEMVH-N-----LVA-------IELAYINTKHPDFADA----------------------RDCEVIE----------

-----------------KLIKSYFLI----------VRKNIQDSVPKA-VMHFL---------V------------------------------
--NHVKDNLQSELVGQLYKQQ-------------------L-
LDELLTESEDMAQRRKEASDMLKALQRASQIIAEI------------------------------
NP_957216.1 ---LMGRVIPVKLGLIGVVNR-
SQLDINNKKSVADSIR----------------DEHGFLQK-------K---YPSLA-------NRNG-
TKYLARTLNRLLMHHIRDCLPELKTRINVLSAQYQSLLSSYGEPVEDM----------S--ATLLQLIT--
KFATEYCNT----IEGTAKYI------------ETAELCGGARICYIFHETFGRT---------LE----------------------
----
-SVDPLGGLTTIDVLTAIRNATGPRPAL-FVPE-VSFELLVKRQ-VKRLEEPS----
LRCVELVHEEMQRIIQHCSNYSTQ--------------------------------ELLRFPKLHDAI--VEVVT-S-
LLRKR-LPVTNEMVH-N-----LVA-------IELAYINTKHPDFADA---------------------RDCEVIE---------
-----------------RLIKSYFLI----------VRKNIQDSVPKA-VMHFL---------V------------------------------
--NHVKDSLQSELVGQLYKPA-------------------L-
LDDLLTESEDMAQRRNEAADMLKALQKASQVIAEI------------------------------
XP_028602039.1 ---LMGRVIPVKLGIIGVVNR-
SQLDINNKKSVADSIR----------------DEYGFLQK-------K---YPSLA-------NRNG-
TKYLARTLNRLLMHHIRDCLPELKTRINVLAAQYQSLLNGYGEPVDDK----------S--ATLLQLIT--
KFATEYCNT----IEGTAKYI------------ETSELCGGARICYIFHETFGRT---------LE----------------------
----
-SVDPLGGLNTIDILTAIRNATGPRPAL-FVPE-VSFELLVKRQ-IKRLEEPS----
LRCVELVHEEMQRIIQHCSNYSTQ--------------------------------ELLRFPKLHDAI--VEVVT-S-
LLRRR-LPVTNEMVH-N-----LVA-------IELAYINTKHPDFADA---------------------RDCEVIE---------
-----------------RLIKSYFLI----------VRKNIQDSVPKA-VMHFL---------V------------------------------
--NHVKDSLQSELVGQLYKAL-------------------L-
LDDLLTESEDMAQRRKEAADMLQALQRASHIIAEI------------------------------
XP_031753959.1 ---LLGRVIPVKLGIIGVVNR-
SQLDINNKKSVADSIR----------------DEYGFLQK-------K---YPSLA-------NRNG-
TKYLARTLNRLLMHHIRDCLPELKTRINVLAAQYQSLLNSYGEPVEDK----------S--ATLLQLIT--
KFATEYCNT----IEGTAKYI------------ETSELCGGARICYIFHETFGRT---------LE----------------------
----
-SVDPLGGLTTIDILTAIRNATGPRPAL-FVPE-VSFELLVKRQ-VKRLEEPS----
LRCVELVHEEMQRIIQHCSNYSTQ--------------------------------ELLRFPKLHDAI--VEVVT-S-
LLRKR-LPVTNEMVH-N-----LVA-------IELAYINTKHPDFADA---------------------RDCEVIE---------
-----------------RLIKSYFLI----------VRKNIQDSVPKA-VMHFL---------V------------------------------
--NHVKDTLQSELVGQLYKSM-------------------L-
LEDLLTESEDMAQRRKEAADMLKALQRASQIIAEI------------------------------
XP_025940269.1 ---LMGRVIPVKLGIIGVVNR-
SQLDINNKKSVADSIR----------------DEYGFLQK-------K---YPSLA-------NRNG-
TKYLARTLNRLLMHHIRDCLPELKTRINVLAAQYQSLLNSYGEPVEDK----------S--ATLLQLIT--
KFATEYCNT----IEGTAKYI------------ETSELCGGARICYIFHETFGRT---------LE----------------------
----
-SVDPLGGLNTIDILTAIRNATGPRPAL-FVPE-VSFELLVKRQ-IKRLEEPS----
LRCVELVHEEMQRIIQHCSNYSTQ--------------------------------ELLRFPKLHDAI--VEVVT-C-
LLRRR-LPVTNEMVH-N-----LVA-------IELAYINTKHPDFADA---------------------RDCEVIE---------

-----------------RLIKSYFLI-----------VRKNIQDSVPKA-VMHFL----------V-------------------------------
--NHVKDTLQSELVGQLYKSL--------------------L-
LDDLLTESEDMAQRRKEAADMLKALQRASQIIAEI------------------------------
XP_012382650.2 ---LMGRVIPVKLGIIGIVNR-
SQLDINNKKSVTDSIR-----------------DEYAFLQK-------K---YPSLA-------NRNG-
TKYLARTLNRLLMHHIRDCLPELKTRINVLAAQYQSLLNSYGEPVDDK-----------S--ATLLQLIT--
KFATEYCNT----IEGTAKYI-------------ETSELCGGARICYIFHETFGRT---------LE----------------------
----
-SVDPLGGLNTIDILTAIRNATGPRPAL-FVPE-VSFELLVKRQ-IKRLEEPS----
LRCVELVHEEMQRIIQHCSNYSTQ---------------------------------ELLRFPKLHDAI--VEVVT-C-
LLRKR-LPVTNEMVH-N-----LVA-------IELAYINTKHPDFTDA----------------------RDCEVIE----------
-----------------RLIKSYFLI-----------VRKNIQDSVPKA-VMHFL----------V-------------------------------
--NHVKDTLQSELVGQLYKSS--------------------L-
LDDLLTESEDMAQRRKEAADMLKALQGASQIIAEI------------------------------
XP_014394711.1 ---LMGRVIPVKLGIIGVVNR-
SQLDINNKKSVTDSIR-----------------DEYAFLQK-------K---YPSLA-------NRNG-
TKYLARTLNRLLMHHIRDCLPELKTRINVLAAQYQSLLNSYGEPVDDK-----------S--ATLLQLIT--
KFATEYCNT----IEGTAKYI-------------ETSELCGGARICYIFHETFGRT---------LE----------------------
----
-SVDPLGGLNTIDILTAIRNATGPRPAL-FVPE-VSFELLVKRQ-IKRLEEPS----
LRCVELVHEEMQRIIQHCSNYSTQ---------------------------------ELLRFPKLHDAI--VEVVT-C-
LLRKR-LPVTNEMVH-N-----LVA-------IELAYINTKHPDFADA----------------------RDCEVIE----------
-----------------RLIKSYFLI-----------VRKNIQDSVPKA-VMHFL----------V-------------------------------
--NHVKDTLQSELVGQLYKSS--------------------L-
LDDLLTESEDMAQRRKEAADMLKALQGAGQIIAEI------------------------------
NP_001392186.1 ---LMGRVIPVKLGIIGVVNR-
SQLDINNKKSVTDSIR-----------------DEYAFLQK-------K---YPSLA-------NRNG-
TKYLARTLNRLLMHHIRDCLPELKTRINVLAAQYQSLLNSYGEPVDDK-----------S--ATLLQLIT--
KFATEYCNT----IEGTAKYI-------------ETSELCGGARICYIFHETFGRT---------LE----------------------
----
-SVDPLGGLNTIDILTAIRNATGPRPAL-FVPE-VSFELLVKRQ-IKRLEEPS----
LRCVELVHEEMQRIIQHCSNYSTQ---------------------------------ELLRFPKLHDAI--VEVVT-C-
LLRKR-LPVTNEMVH-N-----LVA-------IELAYINTKHPDFADA----------------------RDCEVIE----------
-----------------RLIKSYFLI-----------VRKNIQDSVPKA-VMHFL----------V-------------------------------
--NHVKDTLQSELVGQLYKSS--------------------L-
LDDLLTESEDMAQRRKEAADMLKALQGASQIIAEI------------------------------
NP_001317309.1 ---LMGRVIPVKLGIIGVVNR-
SQLDINNKKSVTDSIR-----------------DEYAFLQK-------K---YPSLA-------NRNG-
TKYLARTLNRLLMHHIRDCLPELKTRINVLAAQYQSLLNSYGEPVDDK-----------S--ATLLQLIT--
KFATEYCNT----IEGTAKYI-------------ETSELCGGARICYIFHETFGRT---------LE----------------------
----
-SVDPLGGLNTIDILTAIRNATGPRPAL-FVPE-VSFELLVKRQ-IKRLEEPS----
LRCVELVHEEMQRIIQHCSNYSTQ---------------------------------ELLRFPKLHDAI--VEVVT-C-
LLRKR-LPVTNEMVH-N-----LVA-------IELAYINTKHPDFADA----------------------RDCEVIE----------

-----------------RLIKSYFLI-----------VRKNIQDSVPKA-VMHFL----------V-------------------------------
--NHVKDTLQSELVGQLYKSS--------------------L-
LDDLLTESEDMAQRRKEAADMLKALQGASQIIAEI-------------------------------

XP_006168142.1 ---LMGRVIPVKLGIIGVVNR-
SQLDINNKKSVTDSIR-----------------DEYAFLQK-------K---YPSLA-------NRNG-
TKYLARTLNRLLMHHIRDCLPELKTRINVLAAQYQSLLNSYGEPVDDK-----------S--ATLLQLIT--
KFATEYCNT----IEGTAKYI-------------ETSELCGGARICYIFHETFGRT---------LE----------------------
----
-SVDPLGGLNTIDILTAIRNATGPRPAL-FVPE-VSFELLVKRQ-IKRLEEPS----
LRCVELVHEEMQRIIQHCSNYSTQ---------------------------------ELLRFPKLHDAI--VEVVT-C-
LLRKR-LPVTNEMVH-N-----LVA-------IELAYINTKHPDFADA----------------------RDCEVIE----------
-----------------RLIKSYFLI-----------VRKNIQDSVPKA-VMHFL----------V-------------------------------
--NHVKDTLQSELVGQLYKSS--------------------L-
LDDLLTESEDMAQRRKEAADMLKALQGASQIIAEI-------------------------------

XP_035676386.1 ---LMGRVIPVKLGIIGVVNR-
SQMDINKRKPIEEAIK-----------------DEAAFMQR-------K---YPSLA-------SRNG-
TSHLARTLNRLLMHHIRDCLPDLKTRINVMASQFQQLLNSFGEPVDDK-----------G--TTLLQIIT--
KFAAQYCNT----IEGTARNI-------------ETTELCGGARICYIFHETFGRT---------LD----------------------
----
--SIEPLGGLSQLDILTAIRNATGPRPAL-FVPE-VSFELLVKKQ-IRRLEEPS----
LRCVELVHEEMQRIINHCG---TQ---------------------------------ELLRFPRLHDRT--VEVVT-N-
LLRKR-LPVTNSMVE-N-----IVQ-------IELAYVNTKHPDFADA----------------------RDCEVIE---------
------------------RLIKSYFLI-----------VRKNIQDSVPKA-IMHFL----------V------------------------------
---NFVKDNLQSELVSQLYKTD--------------------F-
FSDLLQESDQIGTRRKEAAEMLKALQKAGHIISEI-------------------------------

XP_006821224.1 ---ICGRVIPVKLGIIGVINR-
SQMDINNKKPIQESVK-----------------DEAAFLQR-------K---YPALA-------SRNG-
TPYLAKTLNRLLMHHIRDCLPELKTRVNVMASQFQQLLLSFGEPVENH-------D-DRS--QMLLQIIT-
-RFAAGYYAT----IEGTAKNI-------------ETSELCGGARICYIFHETFGRT---------LD----------------------
----
--SINALGGLTDLDILTAIRNATGPRPAL-FIPE-VSFELLVKRQ-IRRLEEPS----
LRCVELVHEEMQRIVQHCG---TQ---------------------------------ELLRFPKLHDKI--VEVVT-S-
LLRKR-LAPTNAMVE-N-----LVG-------IELAYINTKHPDFADA----------------------RDCDVIQ---------
------------------RLIRSYFLI-----------VRKNIQDSIPKA-VMHFL----------V------------------------------
---NHVKDNLQSELVGQLYKQE-------------------Y-IDELLTESEQMAMRRKEAAEMLRACL----------
----

XP_030827871.1 ---LCGRVIPVKLGIIGVVNR-
SQMDINNKKVIDDAVK-----------------DESAFLQR-------K---YPALA-------SRNG-
TAYLARTLNRLLMHHIRDCLPELKTRVNVMTSQFQQLMASFGEPVEDK-----------S--QTLLQIIT--
KFASEYCAT----IEGTSRNI-------------ETSELCGGARICYIFHETFGRT---------LD----------------------
----
--SIDPLGGLRTIDILTAIRNATGPRPAL-FVPE-VSFELLSKRQ-IRRLEEPS----
MRCIELVHEEMQRIIAHCG---TQ--------------------------------DLLRFPRLHDRI--VEVVT-T-LLRKR-
LPATNAMVE-N-----IVG-------IELAYINTKHPDFADA----------------------RDVDVIK-------------------

-------RLIQSYFLI-----------VRKNIQDSVPKS-IMHFL----------V----------------------------------
NHVQDNLQSELVGQLYRQK--------------------E-
IDTLLNESEHMAQRRKDAQEMLQALQKAGQIISEI-------------------------------
KXN67416.1 ------------------------------------GTNALDILTGRVLNLKLGFIGVINR-
SQQDTVAKKPIRESLE-----------------AELEFFRTH-----PA---YRNIS-------QRCG-
TGHLSKTLNQVLVNHIRDRLPDMKSKLNTLIGQTQQELQYYGDPSIESQ------E-SQG--LLVLRLLT--
DFARNFNSS----IEGSSPQ--------------NGIQLCGGAKIYDIFNSIFGSA---------LK--------------------
----
----SINPNKNLTYQDIRTAIRNSTGPRPSL-FVPE-MAFELLIKPQ-VKLLEQPS----
IRCVELVYEELMKIGHTCS---NK---------------------------------ELLKYPKLNAKI--NEVVS-N-LLRER-
LTPATNYVE-S-----LID-------IQLAYVNTIHPDFIGAKGALQTLHNEHD---------EREIQLIR-------------
--------------SLIDSYFKI-----------TSKSIQDLVPKA-VMHLL----------V----------------------------------
NHTKDNLQNKLVANLYKPD--------------------S-
LNELLMEDETLADERNKCKELLDLYKKAYEIINQAT------------------------------
NP_013100.1 ------------------------------------GTNALDILSGKMYPLKLGFVGVVNR-
SQQDIQLNKTVEESLD-----------------KEEDYFRKH-----PV---YRTIS-------TKCG-
TRYLAKLLNQTLLSHIRDKLPDIKTKLNTLISQTEQELARYGGVGATTN------E-SRA--SLVLQLMN--
KFSTNFISS----IDGTSSDI-------------NTKELCGGARIYYIYNNVFGNS---------LK----------------------
----
--SIDPTSNLSVLDVRTAIRNSTGPRPTL-FVPE-LAFDLLVKPQ-IKLLLEPS----
QRCVELVYEELMKICHKCG---SA---------------------------------ELARYPKLKSML--IEVIS-E-LLRER-
LQPTRSYVE-S-----LID-------IHRAYINTNHPNFLSATEAMDDIMTERE---------ELECELIK------------
---------------RLIVSYFDI-----------IREMIEDQVPKA-VMCLL----------V----------------------------------
NYCKDSVQNRLVTKLYKET--------------------L-FEELLVEDQTLAQDRELCVKSLGVYKKAATLISNIL-
----
XP_746923.1 ------------------------------------GTNAMDILSGRVYPLKLGFIGVVNR-
SQQDIQSGKSLSEALQ-----------------AEAEFFRHH-----PA---YRNMA-------NRCG-
TQFLAKTLNTTLMAHIRDRLPDIKARLNTLMGQTQQELASYGNKQFSGE------E-HRG--SLILQLMT-
-RFASSFISS----IDGTSSEI-------------STKELCGGARIYYIFNSVFGNS---------LE----------------------
----
--TIDPTHNLTVSDIRTAIRNSTGPRPSL-FVPE-LAFDLLVKPQ-IKLLEAPS----
QRCVELVYEELIKICHTCG---SQ---------------------------------ELLRFPRLQAKL--IEVVS-D-LLRER-
LGPCSAYVE-S-----LIS-------IQRAYINTNHPNFLGAAAAMSSVISDRE---------LMETELIR------------
---------------RLISSYFNI-----------VRETIADQVPKA-IMHLL----------V----------------------------------
NHSKDVVQNRLVSELYKEE--------------------L-
FSELLYEDDGIKAEREKCERLLETYKEAARIVGEVL------------------------------
XP_006461708.1 ------------------------------------GTNALDILTGRVYPLKLGFIGIVNR-
SQQDINVEKSLTDAVE-----------------SEAEFFRNH-----AV---YRNIA-------HKNG-
TRYLAKTLNQVLMNHIRDKLPDMKARLNTLMGQAQQELNSFGDAAIFGD-----KN-QQG--
SMILRMMT--QFARDFVSS----IEGTKVDI-------------STKELSGGARIYYIFNDVFGQA---------LA-----
----
-------------------SLDATHNLENHDIRTAIRNSTGPRPSL-FVPE-VAFDLLVKPQ-IKLLEAPS----
LRCVELVYEELVKICHNCT---CS---------------------------------ELERFPRLHAQL--VEVVS-E-LLRER-
LGPTSEYAQ-S-----LIE-------IQAAYINTNHPAFISGTAAASQRASGRE---------EMETNLIR------------

---------------SLITSYFNI-----------VRQSIQDLIPKA-IMHLL----------V---------------------------------
NHTSQHVQNRLVSSLYKPE--------------------L-
FADLLNEDEALVAERTRVKALLDAYKEAFKTLADVSLKSS-------------------------
XP_011392073.1 -----------------------------------GTHALDILTGRVYPLKLGFIGVVNR-
SQQDINGNVSMLAARR-----------------AEEDFFRSH-----AA---YKNIA-------HRCG-
TKYLAKTLNQVLMSHIRDKLPDMKARLNTLMGQTQQELAAFGDTTFLGD------Q-HRG--
SLVLKLMT--QFARDFVAS----IDGTTFDI-------------STKELCGGARIYYIFQDVFGHA---------LT------
----
------------------SINPTHNLTVQDIRTAIRNSTGPRPSL-FVPE-AAFELLIKPQ-IKLLEPPS----
LRCVELVYEELMKICHNCT---SS---------------------------------ELQRFPRLHAQL--IEVVS-E-LLRER-
LGPTSEYVQ-S-----LIQ-------IQAAYINTNHPSFVHDSANIAREQSDRE---------ELETTLIR------------
---------------SLIASYFNI-----------VRLSIQDLVPKA-IMHLL----------V---------------------------------
NFSRESVQNRLVASLYKEN--------------------L-
FEELLYEDEGLTSERKRVKQLLDAYREAFNTLSEVTFKPSGG------------------------
EFJ28901.1 ---LIGQSVSIAG----AHSK--------
DDSLETAWK-----------------AEMESLK-S-----IL---GGASS-------SRLG-
RSSLVEAIAKQIRQRMQQRLPSLLSSLEGRSQDVEEELVRLGEKMVETE------E-GTR--AVALELCR--
EFEDKFLEH----INSGEG----------------------GS--YKVVTS-FEGTLPNRIKQLPLQE--------------------
----
---------LFDLNGLKKVVLEADGYLPYL-LSPE-KGLRELIRRA-LDLAKDPA----
KSCVDEVHRVLVDIVSSAAS---A-------------------------------TPGLGRFPPLKREM--ISVAS-S--
ALDEYRTEAKRMVV-D-----LVD-------MERAYIPPQHFTRLE----LR---------------------IMA------------
---------------QEVRDYVEA-----------VMNSLSANIPKA-AIFCQ----------V-------------------------------
---ERSKDSMLSTLYKSISALP-------------------
TPTIKELLQEDAQVKRRRERCERQASVLSRLVRQLSNNEA-----------------------------
EFJ33653.1 ---LIGQSVSIAAAHAGSVGT--------
DDSLETAWK-----------------AETETLR-S-----IL---TAAPS-------TRLG-
RAALVDVISKQIRKRIRQRLPSLLSGLEGRQQEVEGELVRLGEQMVETE------E-GTR--ALALELCR--
EFEDKYILH----INSGET----------------------GG--WRVISS-FEGALPNKFKNLPLND-------------------
----
----------LFDLNYLKKVVLEADGYQPYL-LSPE-KGLRELVRRA-LELAKDPG----
KHCVDEVHHVLVDIVAASAS---S-------------------------------TPGLGRYPPFKREV--VAIAS-A--
ALDEYRTHAKKMVV-D-----LVD-------MERSYIPPQHFNRLD----LR---------------------FMG---------
------------------QEVRDYVEA-----------VLNSLSANIPKI---
---QELLQEAPDVKRRRDKCQRQSQVLNKLTHQLSMHEA-------

ONM04707.1 ---TIGHSVPTASAQ-SEAGS--------
ETPPEAYWQ-----------------AEVKTLV-S-----TL---GGAPE-------SKLG-
RVALVDSLSKQIKARIKARLPNLLNGLQGKSQVVQDELAKLGEHMVQSS------D-GTK--AIALGLCR-
-EFEDKFLEN----IAGGEG----------------------AG--WKVVAS-FEGKFPTRIKQLPVEK------------------
----
-----------HFDMKNVKKVVLEADGYQPYL-ISPE-KGLRFLIKGL-LELAKEPS----
ILLVDEVHRVLLDIVSSAAN---A-------------------------------TPGLGRYPAFKREV--IAIAS-T--
ALDGFKNEARKMVV-A-----LVD-------MERAFVPPQHFIRLE----LR---------------------WIS-----------

----------------QEVRAYVEA----------VLNNLAGNVPKL-
RPQSLSRLSCVAGCGSLPSRESQRRYAQPVIYFYKSTIAIEFEVFEVFLTKKTKATNSRLSHVVISTQS-
------------------LAKIEELIQEDHNVKRKREKFQLQSSLLSKVTRLLSIHDS----------------------------
KAH9330549.1 ---IASAQSGSVGG--------
ESSLETAWR-----------------AESESLK-A-----IL---TGAPQ-------TKLG-
RIALVETLARQIRKRIKIRLPSILSGLEGRSQVVEEELVRLGEQLVQTA------E-GTR--AIALELCR--
EFEDKFLQH----IATGEG---------------------GG--WKVVAC-FEGNFPNRIKQLPLDR-----------------
----
------------HFDIMNVKRIVLEADGYQPYL-ISPE-KGLRALIKGV-LELAKEPA----
RTCVDEVHRVLIDIVSTAAS---S------------------------------TPGLGRYPPFKREI--VAIAS-A--
ALDNFRGEAKKMVG-S-----LVD-------MERVFVPPQHFIRLE----LR--------------------MMS---------
------------------QEVRGYVEA----------VLNSLAANVPKA-VVLFQ---------V---------------------------
------EKAKEEMLTQLYSSISSQS------------------
TARIEELLQEDQNAKRKRERYQKQASLLSRLTRQLSIHDV----------------------------
XP_008646219.1 --LIGQSVSIASAQSGSVGS------
--DNSLETAWR-----------------AEAETLK-S-----IL---TGAPQ-------SKLG-
RIALVDTIAKQIRKRMKVRLPNLLTGLQGKSQIVQDELARLGEQMVQSA------E-GTR--AVALELCR--
EFEDKFLAH----ITSGEG---------------------SG--WKIVAS-FEGKFPDRIKQLPLDR--------------------
----
---------HFDLNNVKRIVLEADGYQPYL-ISPE-KGLRSLIKGV-LEMAKEPS----
RLCVEEVHRVLLDIVNAAAN---A------------------------------TPGLGRYPPFKREV--IAIAS-N--
ALDAFKSDAKKMVV-A-----LVD-------MERAFVPPQHFIRLE----LR--------------------WMS----------
-----------------QEVRGYVEA----------VLNSLAANVPKA-IVLCQ---------V----------------------------
-----EKSKEDMLNKLYNSISAQS------------------
NAKIEELLQEDHNAKRRREKYQKQSSLLSKLTRQLSIHDN----------------------------
ACG47836.1 --LIGQSVAIASA--QSVGS--------
ENSLETAWR-----------------AEAESLK-N-----IL---TGSPQ-------NKLG-
RIALVDTIAKQIRKRMKVRVPNLLSGLQGKSQMVQDELASLGESMVQSA------E-GTR--
AVALELCR--EFEDKFLAH----ITSGEG---------------------SG--WKIVAS-FEGKFPDRIKQLPLDR----
----
------------------------HFDLNNVKRIVLEADGYQPYL-ISPE-KGLRSLIKIV-LEMAKEPS----
RLCVEEVHRVLLDIVNASAN---A------------------------------TPGLGRYPPFKREV--VAIAS-N--
ALETFKNDAKKMVV-A-----LVD-------MERAFVPPQHFIRLE----LR--------------------WMS----------
-----------------QEVRGYVEA----------VLNSLAANVPKA-IVLCQ---------V----------------------------
-----EKAKEDMLNQLYSSISGQS------------------
NAKIEELLQEDHNAKRRREKYQKQSSLLSKLTRQLSIHDN----------------------------
XP_006385192.1 --LIGQSVSIASVQSGSASS------
--ESSLETAWR-----------------AESESLK-S-----IL---TGAPQ-------SKLG-
RVALVDVLAGQIRSRMKLRLPSLLSGLQGKSQIVQDEMVRLGEQMVSSS------E-GTR--
ALALELCR--EFEDKFLLH----LVGGEG---------------------NG--WKVVAS-FEGNFPNRIKQLPLDR-
----
---------------------------HFDINNVKRIVLEADGYQPYL-ISPE-KGLRSLIKGV-LELAKEPS----
KLCVDEVHRVLLDIVSSAAN---A------------------------------TPGLGRYPPFKREV--VAIAS-S--
VLDGFKNEAKKMVV-A-----LVD-------MERVFVPPQHFIRLE----LR--------------------WMS---------

------------------QEVRGYVEA-----------VLNSLGANVPKA-VVLCQ----------V---------------------------
-------EKAKEDMLNQLYSSISTQS-------------------
TARIEELLQEDQNVKRKRERYQKQSSLLSKLTRQLSIHDN-----------------------------
'KAG7649995.1' --LIGQSVSIASAQSG--GS-------
-ENSLETAWR-----------------AESESLK-S-----IL---TGAPQ-------SKLG-
RIALVDTLASQIRSRMKLRLPNILTGLQGKSQIVQDELARLGEQLVSSA------E-GTR--AIALELCR--
EFEDKFLLH----LAGGEG----------------------SG--WKVVAS-FEGNFPNRIKQLPLDR-----------------
----
------------HFDLNNVKRIVLEADGYQPYL-ISPE-KGLRSLIKTV-LELAKDPA----
RLCVDEVHRVLVDIVSASAN---A-------------------------------TPGLGRYPPFKREV--VAIAS-A--
ALDGFKNEAKKMVV-A-----LVD-------MERAFVPPQHFIRLE----LR---------------------WMS---------
------------------QEVRGYVEA-----------VLNSLAANVPKA-VVLCQ----------V---------------------------
-------EKSKEDMLNQLYSSISAIG-------------------
NERIESLIQEDQNVKRRRDRYQKQSSLLSKLTRQLSIHDN-----------------------------
'NP_172500.1' --VIGQSVSIASAQSG--SG-------
-ENSLETAWR-----------------AESESLK-S-----IL---TGAPQ-------SKLG-
RIALVDTLASQIRSRMKLRLPSVLSGLHGKSQIVQDELARLGEQLVNSA------E-GTR--AIALELCR--
EFEDKFLLH----LAGGEG----------------------SG--WKVVAS-FEGNFPNRIKQLPLDR-----------------
----
------------HFDLNNVKRVVLEADGYQPYL-ISPE-KGLRSLIKIV-LELAKDPA----
RLCVDEVHRVLVDIVSASAN---A-------------------------------TPGLGRYPPFKREV--VAIAS-A--
ALDGFKNEAKKMVV-A-----LVD-------MERAFVPPQHFIRLE----LR---------------------WMS---------
------------------QEVRGYVEA-----------VLNSLAANVPKA-VVLCQ----------V---------------------------
-------EKAKEDMLNQLYSSISAIG-------------------
NERIESLIQEDQNVKRRRERYQKQSSLLSKLTRQLSIHDN-----------------------------
KAI5073815.1--LIGQSASIATAHSSSTGG--------
DNSLETAWR-----------------AEMENLK-S-----VL---GSAPQ-------SKLG-
RIALVDTLSKQIRKRLKLRLPSILSGLEGRTQEVEEELVRLGESLVQSS------E-GTR--AIALELCR--
EFEDKFLQH----INTGEG----------------------GS--WKVVAS-FEGALPGRIKQLPLND------------------
----
-----------LFDLNSVKKIVLEADGYQPYL-ISPE-KGLRALVRKV-LELAKEPA----
KQCVDEVHRILVDIVSSAAS---S-------------------------------TPGLGRYPPFKREV--VALAS-A--
ALDDYRSESRKMVV-A-----LVD-------MERAFIPPQHFIRLD----LK---------------------LMA-----------
----------------QEVRDYVEA-----------VLNSLAANVPKA-VVLCQ----------V-----------------------------
-----ERAKDAMLNQLYSSISAKP-------------------
FEVIQELLQEDQDVKRQRERVRRQSSLLSRLTRQLSVHEA-----------------------------
OAE31801.1 --LIGQSVSIAAAHAG-GGA--------
EDSLETAWR-----------------AEAESLK-Q-----IL---PQASP-------SKLG-
RVALVETLSSQIRKRLKNRLPSLLSGLEGKSQIVEEELVRLGEMRVETS------E-GTR--AIALELCR--
EFDDKFIQT----ISSGET----------------------GG--WKVVSS-FEGTLPNRFKNLPLSE--------------------
----
---------LFDINSIKRLVLEADGYQPYL-LSPE-KGLRALVKKA-LELAKDPA----
KSCVDEVHRILVDIVSAAAS---L------------------------------TPGLGRYPPLKREI--VAIAS-A--
ALEEYRVEARKMVV-A-----LVD-------MERAFIPPQHFIRLD----LR---------------------MMA-----------

----------------QEVRDYVEA-----------VLNSLAANVPKA-VVLCQ----------V-----------------------------
-----ERSKDAMLAQLYSSISAHP-------------------
TPKIEELLKEDGEVKKRRERCQRQANLLSQLTRQLSVHEA-----------------------------
KAG0632288.1 ---MIGQSVSIAAAH-G---S--------
EDSLDTAWK-----------------AEAESLK-S-----LL---TQAAP-------TKLG-
RVALVEAIAKQIRKRLKQRIPTLLSGLHGKSQHVEEELVRLGELRVETS------E-GTR--AIALELCR--
EFEDKFLQH----IQTGEG----------------------GG--WRVVQS-FEGALPKRIKQIPLDN------------------
----
-----------LFELSSIKKLVLEADGYQPYL-LSPE-KGLRALIRKS-LELAKDPA----
KHCVDEVHRILLDIVSASAN---A------------------------------TPGLGKYPPLKREM--VAIAS-A--
ALDEYRSESKKMVV-A-----LVE-------MERVFIPPQHFIRLD----LR--------------------LMA------------
---------------QEVRDYVEA-----------VLNSLAANVPKA-VVLCQ----------V------------------------------
----ERAKDAMLNQLYSSISAHP-------------------
TERIQDLLQEDVEVKRRREKVKRQASMLSRLTRQLSMNEA-----------------------------
XP_024391061.1 ---LIGQSVSIAAAHSS---S--------
EDPLDTAWK-----------------AEMESLN-S-----IL---KGAPP-------AKLG-
RIALLETLASKIQSRLKQRIPNLLSGLEGKSQMVNEELARLGELRVTSS------E-GTV--AVALELCR--
EFEDRFLAH----INTGEG----------------------QG--WKIVAS-FEGVLPKRIKGLPLDQ-------------------
----
----------MFEISSIKKLVLEADGYQPYL-LSPE-KGLRAIIKKA-LELAKEPA----
KSCVDEVHRVLVDIVSASAS---G-------------------------------TPGLGRYPPLKREI--VSIAS-A--
ALEGYRIEAKNMAV-A-----LVD-------MERVFIPPQHFIRLD----LR--------------------LMA------------
---------------QEVRDYVEA-----------VLNSLAANVPKA-VVLCQ----------V------------------------------
----ERAKDAMLNQLYSSISSMA-------------------
TARIQELLMEDQEVKARREKAHKQAAALAKLTKTLGLHEA-----------------------------
XP_024368367.1 ---LIGQSVSIAAAHSS---P--------
EDSLDTAWK-----------------AEMESLK-T-----IL---KGAPS-------AKLG-
RIALLETLASKIQSRLKQRIPNLLSGLEGKSHMVAEELARLGELRVTSS------E-GNV--AVALELCR--
EFEDKFLAH----INTGEG----------------------QG--WKIVAS-FEGVLPKRMKGLPLDQ-----------------
----
------------MFEISSIKKLVLEADGYQPYL-LSPE-KGLRALIKKA-LELAKEPA----
KSCVDEVHRVLVDIVSASAS---G-------------------------------TPGLSRYPPLKREI--VSIAS-A--
ALEEYRVEAKKMAV-A-----LVD-------MERVFIPPQHFIRLD----LR--------------------LMA------------
---------------QEVRDYVEA-----------VLNSLAANVPKA-VVLCQ----------V------------------------------
----ERAKDAMLNQLYSSISSMA-------------------
TARIQELLMEDQEVKTRRERAHKQSAALAKLTKTLGLHEA-----------------------------
'KAG0555995.1' ---LIGQSVSIAAAHSS---G--------
EDSLDTAWK-----------------AEMESLK-S-----IL---NGAPS-------AKLG-
RIALVETLSHQIRTRLKQRLPNLLSGLQGKSQLVEQELARLGEQRVQTS------E-GTR--AIALELCR--
EFEDTFLQH----INTGEG----------------------QG--WKVVSS-FEGVLPKRIKSLPLDQ------------------
----
-----------MFEISSIKKLVLEADGYQPYL-LSPE-KGLRALVRKA-LELAKDPS----
KACVDEVHRILVDIVSAAAN---G------------------------------TAGLGRYPPLKREI--VAIAS-S--
ALDEYRVEAKKMVV-A-----LVD-------MERAFIPPQHFIRLD----LR--------------------LMA-----------

----------------QEVRDYVEA-----------VLNSLSANVPKA-VVLCQ----------V-----------------------------
-----ERAKEAMLNQLYSSISAHA-------------------
TGRIEELLQEDQEVKSRREKCQKQAALSKLTKQLSLQEA-----------------------------

XP_042924642.1 ---------------------------------------RDVLLGKTLKLKHGWVAVVNR-
GQADLNSKVTMKDARA-----------------REQEFFKGK-----PE---YQDL--------QNTG-
TTFLAEKLSNHLINEIMKSLPSIQSYIEGTIAKLQKELTALGGDVSHSR------G-AML--HMTLQLCQ--
KMERAFERI----VDGGKD----------------------GG--EKVLDV-FEIKLKEAINKLPFQK-------------------
----
---------ILTLKNVQMVVNEADGYQPHI-IAPE-NGYRRLIEDG-LSLLRDPA----
LNAIEQVHQILKSIVTLAVN---TPE-----------------------------CRDLARFFNLKSEI--INHAA-S--
TLEKLRKDADGMVR-T-----LVD-------MEASYLSASFFREIVADAHLQ---------------------KIS----------
-----------------DHVSAYLAI-----------VKGQMLATVPKA-IVHTM----------V------------------------------
----VPAKSGLLLDLQEEVAGKE-------------------
EPQLRRLINESEEIAAQRDTIRKRLTLLQRASKEIAAFM------------------------------

AAF22292.1 --
LEVLEGRSYRLQHPWVGISEPFNKQDINKNVDMMLARR-----------------KEREYFDTS-----PD---
YGHLA-------SKMG-SEYLAKLLSKHLESVIRTRIPSILSLINKSIEELERELDRMGRPVAVDA------G-
AQL--YTILEMCR--AFDKIFKEH----LDGGRP----------------------GG--DRIYGV-
FDNQLPAALKKLPFDR---
---HLSLQSVKKIVSEADGYQLTL-IAPE-
QGYRRLIEGA-LGYFRGPA----EASVDAVHYVLKELVRKSIS---E-------------------------------
TEELKRFPSLQVEL--AAAAN-S--SLEKFREESKKSVI-R-----LVD-------
MESAYLTAEFFRKLPGDGHFR---------------------RIA--------------------------SNVSAYVKW----------
-FRTLLRNTIPKA-CVYCQ----------V---------------------------------RQAKLALLNYFYSQISKRE-------
------------GKQLGQLLDEDPALMDRRLECAKRLELYKKARDEIDAVAWV----------------------------

NP_850420.1 ---------------------------------------LDVINGRSYKLKYPWVGIVNR-
SQADINKNVDMMVARR-----------------KEREYFETS-----PD---YGHLA-------TRMG-
SEYLAKLLSKLLESVIRSRIPSILSLINNNIEELERELDQLGRPIAIDA------G-AQL--YTILGMCR--
AFEKIFKEH----LDGGRP----------------------GG--ARIYGI-FDYNLPTAIKKLPFDR---------------------
----
--------HLSLQSVKRIVSESDGYQPHL-IAPE-LGYRRLIEGS-LNHFRGPA----
EASVNAIHLILKELVRKAIA---E-------------------------------TEELKRFPSLQIEL--VAAAN-S--
SLDKFREESMKSVL-R-----LVD-------MESSYLTVDFFRKLHGDGHFR---------------------KIA--------
-------------------SNVAAYIKM-----------VAETLVNTIPKA-VVHCQ----------V----------------------------
-----RQAKLSLLNYFYAQISQSQ-------------------
GKRLGQLLDENPALMERRMQCAKRLELYKKARDEIDAAVWV----------------------------

XP_002302631.1 ---------------------------------------VDILEGKSYKLQFPWIGVVNR-
SQADINKSVDMIAARR-----------------REREYFQSS-----PE---YGHLA-------SRMG-
SEHLGKMLSKHLEQVIKSRIPGLQSLISKTINELETELSRLGRPVATDA------G-GKL--YMIMEICR--
SFDQIFKEH----LDGTRS----------------------GG--DKIYNV-FDNQLPAALKRLQFDK------------------
----
-----------HLSMDNVRKLITEADGYQPHL-IAPE-QGYRRLIEST-LVTIRGPA----
EAAVDAVHVILKDLVHKSIS---E------------------------------TMELKQYPTLRVEV--SAAAV-D--
SLDRMREESKKATL-Q-----LVD-------MESSYLTVEFFRKLPNDSYLR---------------------RIG---------

------------------SNVLAYVNM-----------VCASLRNSIPKS-VVYCQ----------V----------------------------
------REAKRSLLDFFFAELGKKE------------------
TRQLSSLLDEDPAVMQRRTLLGKRLELYRSAQADIDAVTWG---------------------------

AQK88296.1 ---------------------------------------VDILEGRSYRLQTPWVGVVNR-SQQDINKNVDMIAARR-----------------REREYFAST-----PE---YKHMA-------SRMG-SEYLGKMLSKHLEQVIKSRIPGIQSLITKTIAELETELNRLGKPIANDA------G-GKL--YTIMEICR--MFDGIYKEH----LDGVRP----------------------GG--EKVYHV-FDNQFPVAIKRLQFDK-----------------
----
------------QLSMENVRKLITEADGYQPHL-IAPE-QGYRRLIESC-LISIRGPA----EAAVDAVHAILKDLVRKAIN---E------------------------------THELKQFPTLRVEV--GNAAF-E--SLDRMRDESKKNTL-K-----LVD-------MECSYLTVDFFRKLPNDSYLR---------------------RIG--------
-------------------QTVLSYVNM-----------VCSTLRNSIPKS-IVYCQ----------V----------------------------
------REAKRSLLDHFFTELGARE------------------
MKQLSKLLDEDPAVMERRTNLAKRLELYRSAQSEIDAVAWS---------------------------

NP_001190448.1 ---------------------------------------VEILEGRSFKLKYPWVGVVNR-SQADINKNVDMIAARK-----------------REREYFSNT-----TE---YRHLA-------NKMG-SEHLAKMLSKHLERVIKSRIPGIQSLINKTVLELETELSRLGKPIAADA------G-GKL--YSIMEICR--LFDQIFKEH----LDGVRA----------------------GG--EKVYNV-FDNQLPAALKRLQFDK-----------------
----
------------QLAMDNIRKLVTEADGYQPHL-IAPE-QGYRRLIESS-IVSIRGPA----EASVD------TDLVHKSVN---E------------------------------TVELKQYPALRVEV--TNAAI-E--SLDKMREGSKKATL-Q-----LVD-------MECSYLTVDFFRKLPNDSYLR---------------------RIG---------------------------
SNVLSYVNM-----------VCAGLRNSIPKS-IVYCQ----------V----------------------------------
REAKRSLLDHFFAELGTMD------------------
MKRLSSLLNEDPAIMERRSAISKRLELYRAAQSEIDAVAWS---------------------------

XP_002299468.1 ---------------------------------------VDMLEGKSYRLKFPWVGVVNR-SQADINKNVDMIAARR-----------------REREYFSST-----PE---YKHLA-------HRMG-SEHLAKMLSKHLEVVIKSKIPGIQSLVNKTIAELETELSRLGKPIAADA------G-GKM--YSIMEICR--LFDQIYKEH----LDGVRP----------------------GG--DKIYNV-FDNQLPAALKRLQFDK------------------
----
-----------QLSMENIRKLITEADGYQPHL-IAPE-QGYRRLIESS-VVTIRGPA----EAAVDAVHGLLKDLVHKAIN---E------------------------------TIELKQYPALRVEV--SNAAI-E--SLDRMKDTSKKATL-Q-----LVD-------MECSYLTVDFFRKLPNDSYLR---------------------RIG---------
------------------STVLSYVNM-----------VCASLRNSIPKS-IVYCQ----------V----------------------------
-----REAKRSLLDHFFTELGKLE------------------
QKQLSSLLNEDPAVMERRAAIAKRLELYRSAQAEIDAVAWS---------------------------

PWZ36850.1 ---------------------------------------LDVLEGRAYRLQNPWVGIVNR-SQADINRKVDMISARE-----------------KEREYFETS-----PD---YAHLS-------SRMG-SGYLAKLLSQHLESVIKVRIPSITATINKTIDELESELDIIGRAVAADP------G-AQL--YTILDLCR--AFDRVFKEH----LDGGRS----------------------GG--DRIYGV-FDHKLPAAFKKLSFDR-----------------
----
------------YLSVQNVKKVVSEADGYQPHL-MAPE-QGYRRLIEKG-INYFRGPA----EATVDAVHVVLKDLVRKSIG---E------------------------------TEQLRRFPTLQAAI--ATAAN-E--ALERFREDGRSTAL-R-----LVD-------MEAAYVTVEFFRKLPGDGHFR---------------------SIA---------

------------------SNVSQYIRM-----------VGDELLQKIPKA-AVHCQ----------V---------------------------
------REAKRSLLNHFYVQMGKKE------------------
AGEFGHMLDEDPAMMERRQQCFKRLELYKSARDEVDSVAWG---------------------------
NP_001147100.1 ---------------------------------------VDVLEGRQYRLQHPWVGIVNR-
SQADINKNVDMLSARR-----------------KEKEYFESS-----PE---YGHLA-------HKMG-
AEYLAKLLSQHLEAVIRAKIPSIIAMINKTIDEIEAQLDRLGRPIGGDA------G-AQL--YTILDMCR--
AFDRVFKEH----LDGGRP---------------------GG--DRIYGV-FDNQLPAALKKLPFDR-----------------
----
------------HLSMQNVRKVISEADGYQPHL-IAPE-QGYRRLIDSS-LSYFKGPA----
EASVDAVHLVLKELVRRSIA---A------------------------------TEELKRFPTLQSDI--AAAAN-D--
SLERFREDGRKTVL-R-----LVE-------MEASYLTVEFFRKLPQDNHLR--------------------RIG---------
------------------SNVSSYINM-----------VCETLRNTVPKA-IVHCQ----------V---------------------------
------KEAKRNLLNRFYAHVGSKE------------------
KKQLSAMLDEDPALMEKRDALVKRLELYKSARNEIDSVAWK---------------------------
AAF79238.1 ---------------------------------------LDVLEGRSYRLQHPWVGIVNR-
SQADINKRVDMIAARR-----------------KEQEYFETS-----PE---YGHLA-------SRMG-
SEYLAKLLSQHLETVIRQKIPSIVALINKSIDEINAELDRIGRPIAVDS------G-AQL--YTILELCR--
AFDRVFKEH----LDGGRP---------------------GG--DRIYGV-FDHQLPAALKKLPFDR-----------------
----
------------HLSTKNVQKVVSEADGYQPHL-IAPE-QGYRRLIDGS-ISYFKGPA----
EATVDAVHFVLKELVRKSIS---ETEVRTDKMPLVLRTLPSSLRSVLVLTYHCCFFQELKRFPTLASDI--
AAAAN-E--ALERFRDESRKTVL-R-----LVD-------MESSYLTVEFFRKLHSDNHFR--------------------
KIG-TCLSRLPLLRNDTLSSLCLLTLDNSGSNVSAYINM-----------VCDTLRNSLPKA-VVYCQ---------
-V---------------------------------REAKRSLLNFFYAQVGRKE------------------
KEKLGAMLDEDPQLMERRGTLAKRLELYKQARDDIDAVAWK---------------------------
XP_002315854.1 ---------------------------------------LDVIEGRSYRLQHPWVGIVNR-
SQADINKNVDMIAARR-----------------KEREYFETS-----PE---YGHLS-------SKMG-
AEYLAKLLSKHLETVIRQRIPSIIALINKTIDELNAELDRIGRPIAVDS------G-AQL--YTILELCR--
AFDRVFKEH----LDGGRP---------------------GG--DRIYGV-FDHQLPAALKKLPFDR-----------------
----
------------HLSMKNVQKVVSEADGYQPHL-IAPE-QGYRRLIDGS-ISYFKGPA----
EATVDAVHFVLKELVRKSIA---L------------------------------TEELKRFPTLQSDI--AAAAN-E--
ALERFRDESRRTVQ-R-----LVD-------MESSYLTVEFFRKLHNDNHFR--------------------RIG--------
-------------------SNVSAYIGM-----------VCDTLRNSIPKA-VVYCQ----------V---------------------------
------REAKRCLLNNFYAQVGRRE------------------
KERLGAMLDEDPQLMERRTTIAKRLELYKSARDEIDSVAWK---------------------------
EFJ15761.1 ---------------------------------------IDVLEGHSYRLQRPWIGVVNR-
SQADINKSVDMIVARR-----------------REREYFSSS-----PD---YRHLA-------SRMG-
SEYLGRVLSKHLEAVIKARIPSIQSLINKTITELEAELDRLGRPIASDA------G-GQL--YTVLELCR--
AFDHVFKAY----LDGGRP---------------------GG--DRIYNV-FDHQLPAAVKKLPFDR-----------------
----
------------HLSIQNVRKVIAEADGYQPHL-IAPE-QAYRRLIEGS-LGYLRGPA----
EAAVDAVHFILKELVRKAIN---E------------------------------TQELKRFPTFQAEL--SAAAV-E--
ALERFREDSRKFCQ-S-----LVD-------MEAGYLTVEYFRKLPGEAHLR--------------------RIG---------

------------------SNVLQYVHM-----------VCEMLKNAVPKA-VVHCQ----------V--------------------------
---------REAKRSLLHNFYAQIGKRE-------------------
GKQLAQMLDEDPGLMERRSACGKRLDLYKSARDEIDAVVWD----------------------------
KAI5072318.1--LDTLEGRAYRLQHPWVGVVNR-
SQADINKSVDMMAARR-----------------REREYFATS-----PD---YKHLA-------SRMG-
SEYLGQMLSKHLESVIKSRIPSIQMLINKSIDELESELQSLGRPIATDP------G-AQL--YSILELCR--
AFDQVFKEH----LDGVRP---------------------GG--EKIYGV-FDNQFPGALKKLSFDR-----------------
----
------------HLSIQNVRKLINEADGYQPHL-IAPE-QGYRRLIESS-LTLMRGPA----
EAVVDAVHHILKELVRKSVG---E-------------------------------TQELRQFPTLQAEV--GAAAV-S--
ALERFRDESKKTSL-R-----LVD-------MENSYLTVEFFRKLPGDGQLR-------------------RIG---------
------------------ANVLSYVNM-----------ICDTLRYSIPKA-VVHCQ----------V-----------------------------
------REAKRTLLDTFFAELGRKE-------------------
GKQLARLLDEDPAVMERRSACAKRLELYRNARNEIDTVAWA----------------------------
EFJ23099.1 --LEVLEGRAYRLQFQWVGVVNR-
SQADINKSVDMIAARK-----------------KEREFFASS-----PD---YGHLA-------NRMG-
SEYLAKMLSKHLETVIKTRLPSILALINKSIDELEQELNQLGRPISHDA------G-AQL--YTILELCR--
AFDHVFKAH----LDGGRP---------------------GG--ERIYVV-FDNQLPAALKKLPVDK-----------------
----
------------HLSMQNVRKIVTEADGYQPHL-IAPE-QGYRRLIEGT-LGLFRGPA----
EAVVDAVHSVLKELYSFSSA---SL--------------------------SVFLSQELKRFPTLQAEL--AAATT-E--
ALERFRDESRKFVL-R-----LVD-------MEASYLTVEYFRKLPTEAHLR-------------------KIG-RVLF---
--------------AVFSGSHVTSYIMI-----------VCETLRHSIPKA-VVHCQ----------V--------------------------
---------REAKRTLLDTFYTQVGKKE-------------------
EKQLLQMLDEDPALMERRVALAKRLELYKNARDEIDAVMWG----------------------------
KAG0556007.1 --IDVLEGRSYKLIQPWIGVVNR-
SQQDINKNVDMIAARR-----------------REREYFQTS-----PD---YSHLQ-------SKMG-
SEYLGRVLSKHLEAVIRSRIPSIAAMINKTIDDIEAELNQIGRPLANDA------G-AQL--YTILELCR--
AFDRIFKEH----LDGQRA---------------------GG--EKIYNV-FDNQLPAAMKKLPFEK-----------------
----
-----------HLSMQNVRKIVSEADGYQPHL-IAPE-QGYRRLIESS-LVYFRGPA----
EAVVDATHFNLRDLVRKSIG---E-------------------------------CTELRRFPSLQSEI--GQAAI-E--
SLERMRADSKKTAL-R-----LVD-------MEASYLTVDFFRKLPTDGHLR-------------------RIG---------
------------------TNVSSYINM-----------VGDTLRKTLPKA-AVHCQ----------V-----------------------------
------REAKRSLLDHFYTQIGKRE-------------------
GKQLSQMLDEDPALMERRVQLTKRLELFKQARDEIDAVAWA----------------------------
XP_002987566.1 --LDVLEGRSYKLQHPWVGVVNR-
SQADINRSVDMVAARR-----------------REREYFSSS-----AD---YGHLT-------SRMG-
SEYLAKILSKHLEAFIKARIPSILSLINKTIDELEMELNQLGKPVAVDS------G-AQL--YSILELCR--
AFDQVFKSH----LDGGRP---------------------GG--ERIYTV-FDNQLPAALKKLPFDR------------------
----
-----------HLSIQNVRKVVMEADGYQPHL-IAPE-QGYRRLIDGA-LVLFRGPA----
EAVVDAVHFVLKDLVRKAIG---E-------------------------------TMELKRFPTLQAEV--AAAAI-E--
ALERCREESRKFCL-R-----LVD-------MESSYLTVEFFRKLPTDSHLR-------------------RIG----------

-----------------SNVTNYVWM----------VCETIRISIPKA-VVHNQ----------V------------------------------
-----REAKRSLLDNFYTQVGKKE-------------------
GKQLAQLLDEDPALMERRTACAKRLDLYRAARDEIDSVAWG-----------------------------
KAI5602084.1---QDVLEGRAYPLQHPWVGIVNR-
SQADINKNVDMIAARR-----------------REREFFSTS-----PD---YGHLA-------GRMG-
SEYLAKLLSKHLESVIKTRIPGITSLINRSIDDLESELDHLGRPVAIDA------G-AQL--YTILELCR--
AFDRVFKEH----LDGGRP----------------------GG--DRIYGV-FDYQLPTALRKLPFDR------------------
----
-----------HLSLQNVRKVVSEADGYQPHL-IAPE-QGYRRLIDGA-LNYFRGPA----
EASVDAVHFILKEIVRRSIG---E--------------------------------TQELKRFPTLQAEI--ASAAY-D--
ALERFREDSKKTTL-R-----LVE-------MESSYLTVDFFRKLPAEGHFR---------------------RIG------------
----------------SNVSSYAGM----------VSQTLRNSIPKA-VVHCQ----------V------------------------------
-----KEAKRSLLDHFYTQVGKKE-------------------
GKQLAALLDEDPALMERRQKCARRLELYKNARDEIDSVSWA-----------------------------
XP_006375094.1 --LDVIEGRSYRLQHPWVGIVNR-
SQADINKNVDMIVARR-----------------KEREYFATS-----PD---YGHLA-------NKMG-
SEYLAKLLSKHLESAIRARIPSITSLINKTIDELESEMDHLGRPIAVDA------G-AQL--YTILELCR--
AFDKVFKEH----LDGGRP---------------------GG--DRIYGV-FDNQLPAALRKLPFDR-----------------
----
------------HLSLQNVRRVVSEADGYQPHL-IAPE-QGYRRLIESA-LNYFRGPA----
EASADAVHFVLKELVRKSIA---E--------------------------------TQELRRFPSLQAEL--AAAAN-E--
ALERFREDSKKTVL-R-----LVD-------MESSYLTVDFFRRLPSEMHFR---------------------RIG---------
------------------SNVSSYVGM----------VSETLRNTIPKA-VVHCQ----------V-----------------------------
------KEAKQSLLNYFYTQIGKKE-------------------
GKQLSQLLDEDPALMERRQQCAKRLELYKAARDEVDSVSWA----------------------------
KAI5058380.1---IDVLEGRAYHLKNPWIGVVNR-
SQADINKNVDMMAARR-----------------REREYFATS-----SD---YSHLT-------SRMG-
SEYLGKMLSKHLEAVIKARIPSILSLVNKSIDELENEMNQLGRPIAVDA------G-AQL--YTILELCR--
AFDRVFKEH----LDGGRP---------------------GG--DRIYGV-FDNQYPAALKKLPFDR-----------------
----
------------HLSLQNVRKIVSEADGYQPHL-IAPE-QGYRRLIESA-LTYFRGPA----
EAVVDAVHFVLRELVRKSIS---E--------------------------------TKELKRFPTLQAEL--TAAAT-E--
ALEKFRDESRKTSL-R-----MVD-------MEASYLTVDFFRKLPTEGHLR---------------------RIG---------
------------------SNVSAYIGM----------VSESLRVSIPKA-VVHCQ----------V-----------------------------
-----REAKRSLLDHFYTQIGKKE-------------------
GRQLATLLDEDPALMERRAACAKRLELYKQARDEIDAVAWA----------------------------
PTQ29980.1 --LDVLEGRTYRLQLGWVGVVNR-
SQQDINKNTDMLAARR-----------------REREYFQTS-----QD---YGHLA-------SRMG-
SEYLGKLLSKHLEQVIKARIPSILAMINKSIDDLENELNLLGRPIAVDS------G-AQL--YTVLELCR--
AFDHVFKDH----LDGGRP----------------------GG--ERIYSV-FDNQLPAALKRLPFDK-----------------
----
------------HLSMQNVRKVVSEADGYQPHL-IAPE-QGYRRLIESS-LAYFKGPA----
EAVVDAVHFILRDLVRKSVG---E--------------------------------TQELKRFPTLQGEL--AAAAI-E--
SLEKFRDESKKTAI-R-----LVE-------MESSYLTVDFFRKLPTDGHLR---------------------RIG----------

---------------SNVASYVGM----------VCDLLRNSIPKA-VVHCQ---------V---------------------------
-----REAKRALLDHFYIVVGQKE-----------------
SKQLSQMLDEDPALMERRTALAKRLELYKTARNEIDSVAWA--------------------------
PTQ45603.1 --------------------------------------LDVLEGRSYRLQLPWIGVVNR-
SQADINKNVDMIAARR----------------REREYFQSS-----QD---YGHLA-------GKMG-
SEYLAKMLSKHLEAVIKSRIPSILALINKTIDELEGEMSHLGKPIAVDA------G-VQL--YTILELCR--
AFDHVFKDH----LDGGRP--------------------GG--ERIYGV-FDNQLPAALKKLPFDK---------------
----
-----------HLSMQNVRKVVTEADGYQPHL-IAPE-QGYRRLIESS-IAYFKGPA----
EAVVDAVHFILRDLVRKSVS---E----------------------------TQELKRFPTLQAEL--AAAAT-K--
ALEAYREDSRKTAL-K-----LVE-------MEASYLTVDFFRKLPTDAHLR------------------RIG----------
---------------SNVSAYVGM----------VMDILRNSIPKA-AVHCQ---------V---------------------------
-----REAKRSLLDTFYTNIGKKE-----------------
GRQLSVLLDEDPATMERRLACGKRLELYKNARDEIDSVAWA--------------------------
KAG0561847 -------------------------------APEGLLEKVTTDAVNIGLGYICVRNR-IDVD--
--DSIAIARQ----------------RERELFESH-----PA---LKELD------GSMVG-
IPALARKLTKIQSDMVKECLPRIQKQMFEALHKRNQQLSNLPRGIKSDM------D-ARS--
AFFQVQNK--ILTILSQVV----RDGNFEEFPS-----------DAHLHYTARLHQKFQT-FADDLHKTGLK---
F---
---------------------------REQSQTTEIRELLVEHQGVGLPD-FLPH-SVLHHLMRKQ-ITSVNETC----
RSLVDEAFEYATEVVLRVNS---L------------------------------CSQGYPRLEKSY--KQLAI-E--
TLEEVKTTTMEFVE-R-----MLE-------KESTIIFTTNDYYTATLEKMQT------ANDPDRK--YQDAWRLK--
-----------------------VSVAAYWKV----------VQKRLADEIPLE-IRYAL---------Q---------------------
-----------CAVVDTLHQNMMSKPWAGGET-----------------
DLRALMEEDSVGAYTRSRLQLRVDALKDCLRLLSGLMC--------------------------
KAG0619429 -------------------------------APEGLLEKVTTDAVNIGLGYVCVRNR-
TDDD----DTISVARI----------------REQRLFESH-----PA---LKDLD------RSMVG-
IPALARKLTKIQSDMVKGCLPRIHKQMCDALQKRRQQLNNLPKGIASDN------D-AIL--IFLQIQNR--
RLDMLTQLV----RDGDFELFPE-----------NLHLHYTARLHEKFMK-FADDLHKAGLK---L------------
----
--------------KDQSQAQEIKELLAEHQGVGLPD-FLPH-SVLHHLVRKQ-IELIRETC----
TSLVEEAFEYATDVVSEVNT---I------------------------------CSEGYPNMEKCF--KKLAT-E--
SLEKTKKTTMEFVE-R-----MLL-------KECTLIFTTNDYYLATIAKMNA------LQYKDKE--YQDAWRMK-
------------------------TSLVAYWKV----------VQKRLADEIPLE-IRYAL---------Q--------------------
------------YAIVDLLHREMMVKAYSDP-K-----------------
GFQALMQEDSNLSFNRARVQHRVDALKECLLLLNDLMG--------------------------
PWZ56863 -------------------------------NPEGLLEKVTMDDVNIGLGYVCVRNR-IGD-
----ETYDQARV----------------EEERLFKYH-----PL---LSKID------KDMVG-
IPVLANRLMQIQSTIIAKCLPDIVKQINDRLSRSSAELDQMPQDLNNVA------D-AVR--VFFHIVKQ--
VCNSLEKLL----VRGDFAEYPD-----------AREFHGTARIADMLSG-YAKELPGQCPI---N-------------
----
-------------RNEPFLKEEVNILEETKGINLPN-FLPR-TAFLVLLKKK-VETIQEIP----
QFLANKVSDYVEDLVMKVLL---K-----------------------------HSENFPQMQSPC--RRAVQ-T--
LMDKARLRSAHHVK-E-----LIA-------MELVADYTANPDYMKTWTEIME------GVYAD-L--

AGKAFDLR---------------------------ARLTAYWKS-----------IVLRQVDGLALH-VLLSV----------K------
---------------------------LLVEKDLEEELGNELLGNKLA------------------
GVEKMLSPSPGTGTKRERLKKSIVLLRQSKEVVANIMDRIS-------------------------

PWZ56864 ----------------------------------APEGLLEKVTMDDVHIGLGYVCVRNR-VGE-
----ETYDQARV-----------------AEAQLFKNH-----PL---LSQID------KSMVG-
IPVLAQRLMQIQASIIAKCLPDIVKQINDRLIRSSTELDRMPPDVINTG------D-AVR--AFLHIVKK--
VCTSLENIL----VRGDFGCYPD------------DYYFHGTARVAEMLSR-YAKKLPAECPR---C-------------
----
--------------SDEKFLAEEMRVLEETMSIKLPN-FLPR-SAFHSMLKKK-VEMVSDVP----
QDLVSEVWEYVEDLVMKVLL---Q--------------------------------HSENFPQVQSSC--RRAIQ-S--
LMEKTRVRSAQHVK-E-----MIE-------MELVAGYTASPDYMKTWEAIMV------GTHPD-L--
AAQAFDLR---------------------------ARLTAYWKI-----------IVLRLVDGLALH-VLRGV----------K-------
--------------------------RLVENDLEDELANELLGNNMA------------------
GVERMLSPPPSNGTKRDRLKKSILLLQQSKEVVANIMDRIN-------------------------

AAB71956 ---WR-----RAYEQARM-
----------------QEELLFRTH-----PM---LSLID------EDIVG-
IPVIAQKLMLIQATMISRCLPEIVRKINHKMETAVLELNKLPMVMASTG------E-ALM--ALMDIIGS--
AKESLFRIL----VQGDFSEFPD------------DQNMHCTARLADMLSQ-FSDNLQEKPK-----------------
----
---------------EVTEFLMNEIKILDECKCVGLPN-FIPR-SAFLAILSQH-EDAIHVKP----
VEFIKKIWDYIEVVLSSVIA---K--------------------------------YSENFPQIQSAI--KRAGR-N--
LITKIKEHSVERVL-E-----IVE-------MEKLTDYTCNPEYMTSWTQMTA------EKYHAHL--
LQQAFDMK---------------------------MRIASYWTI-----------VLRRIVDSLALY-LQL---------------------
--AG------------------
GGLQRMLEESPSVASKREKLKNSIKLPKESKDAVAAIVDQSS-------------------------

NP_176252 ----------------------------------APEGLLQKVTADDVSIVLGYVCVRNR-IGE---
--ETYEEARM-----------------QEELLFRTH-----PV---LSLID------EDIVG-
IPVLAQKLMLIQSSMIARCLPKIVSKINQKLDTAVLELNKLPMVMASTG------E-ALM--ALMDIIGS--
AKESLLRIL----VQGDFSEYPD------------DQNMHCTARLADMLSQ-FSDSLQAKPK-----------------
----
---------------EVAEFLMDEIKILDECKCVGLPN-FIPR-SAFLAILSQH-VDGIQDKP----
VEFINKIWDYIEDVLSSVTA---K--------------------------------RSDNFPQIQSSI--KRAGR-N--
LISKIKEQSVNRVM-E-----IVE-------MEKLTDYTCNPEYMTSWTQKTS------AKYHAHL--LIPAFDMK-
-------------------------MRITSYWKI-----------VLRRIVDNLALY-LQLSV----------K----------------------
-----------SLVNTRFQKEIVAEMVDPRDG------------------
GGVEKMLEESPLVASKREKLQNSIKLLKESKDAVAAIVDQNC-------------------------

XP_002297993.1 ----------------------------------
APEGLLEKVTADDVNIGLGYVCVRNR-IGD-----ESYDNARM-----------------EEANLFATH-----PL---
LSRID------KSIVG-IPVLAKKLMQVQATIMAKCWPEIVRKINEKLNGNVTELNRMPKAMSSVA------
E-FLT--AFMEFIGS--VKESLTKIL----VRGEYDEYPD------------DPNMHGVARVVEMFNQ-
YSDELLNCPES---E---
---HTRNFLMDEIRVLDDSKAIALPN-FLPR-
HAFLSLLQRK-VERVSHIP----FGFVEKAWAYFENVVWSVSR---H--------------------------------
HTENYPQVLLTT--KRACQ-N--LMVKMREQSTDWVS-E-----LVQ-------

MEKLTDYTCNPEYLNEWNMLMS------HGYQP-L--LSQAFDLK---------------------------
MRMTAYWKI-----------VSRRLVDCMALH-LQLCV----------R----------------------------------
NLVSKELEKEIATELMATNGG-------------------
KLEMMLEEAPSVAAKRKRLNTSIELLREAKDVLSNIMGNVS--------------------------
XP_002303204 -----------------------------------
APEGLLEKVTADDVNIGLGYVCVRNR-IGD-----ESYKEARK-----------------EEADLFENH-----PL---
LSKID------KSMVG-IPVLAQKLVQIQATIIARCLPEIVRKINEKLNASISELNRMPKTLSSVG------E-
ALT--TFMSIVGS--AKESLNKII----VRGEYDEYLE------------DKNMHCTARLVEMLNQ-
YSGELHNCSEN---D--
---LTGNFLMDEIQVLEEAKGIELPN-FLPR-
TTFLSILQKK-VEKISHIP----VAFVEKVWTYIEGVVISVLM---H---------------------------------
HSENYHQLQLST--RRAGH-N--LIARMKEHSRNWVT-E-----IVQ-------
MEKLTDYTSNPEYMNDWNKLMA------QGYEQHV--LLQAFDLK---------------------------
MRMTAYWKI-----------VLRRLVDFMALH-LQFCA----------R----------------------------------
NLVNKEMEEEIVQELAGRHDG-------------------
AIERMLEESPAVAAKREKLNVSIKLLRESNNVLANIMDKIA--------------------------
XP_024439231.1 -----------------------------------
SPEGLLEKVTRNDVNIGLGYVCVRNR-IGN-----ESYEDARK-----------------EEAALFATH-----QL---
LSKID------KSTVG-IQVLAQKLVQIQANIIAKCLPDIVRKIDEKLSASISELNRIPRRLLSVA------E-
VMA--AFMGIIGS--SKDSLRKIL----LRGEIDEYRH------------EKDMHCTARLVEMLNQ-
FSTELHKCSD--
--HTKNFMINEIEVLEETKGIELPN-FLPH-AAILAILQQK-
VEEISELQ----IGFVEKVWAYIRGVVISVLN---H---------------------------------HSANYHQLQLFI--
GRAAH-K--LVDKMKDRSIDWVT-E-----ILQ-------MEKETDYTCNPEYMKEWNKLIA------QGHKH-
V--LLQAFDLK---------------------------MRLIAYWKI-----------VLMRLVDNMALH-LQLSI----------R---
-------------------------------NLVNKEMEKEIVNALLGTGGG------------------
VAIERMLEEPPSVASKRERLNTSIKLLRESKEVLANIRDKIE--------------------------
KAH9291961 -----------------------------------APEGLLEKVALDDVNTGLGYVCVRNR-VGD-
----ECNEEARE-----------------AEAELFRSH-----TQ---LNKFD------EAMVG-
IPMLARRLMQIQTKRISKCFPDIVKNIEDTLSQRQSELSSLPQQVSNPM------E-AMV--VFLRLMNG-
-VKDCLNRLL----IEGDFSEFSE------------ETEMHCTARLKEMFDG-FYNELVHMSV----E-------------
----
---------------DKNAFLVEETKRLEESKGAGLSN-FLSR-SIFKK----R-IDEVLKTG----
LSLTANVWDYVEKVVLRVLD---L---------------------------------KFRSYPRLETAT--KKDFQ-L--
LVSKRREQCIHHVN-Q-----VAE-------MEKSLDFTLNPVYMETWTDLLK------QKMPKEH--
LEVAFEMK---------------------------ARVVSYWKV-----------VVQRVGDGIPMY-LQFVY----------Q----
------------------------------NLVRNDIDEEIMKKVAGPKSN-------------------
SMEKLLEENSMISRKRRSLKRSIDSLGEAQCKILEIMDQIA--------------------------
KAH9290598 -----------------------------------APEGLREKVAEDAMNIGLGYVCVRNR-VEG-
----ESIVKARK-----------------KENELFKTH-----PL---LSGID------KSIVG-
IPILAHKLMKIQAAGITNSLPRIMKEIDTKLARRQAELNDLPENLCNPA------D-AMI--LLTTVMGS--
IKDSLNGLL----LQGDYQEFSE------------DGGMHCAARLNEMFMG-YYRDLCASATDMNDV-------
----
---------------------NGQNFLLKEITMLKEAQGVGLPN-FLSR-QVFLNLVQQR-ANGVAEIS----

LRVVEKVWDYLDGVMLRVID---R--------------------------------ECQTYPQLNAAT--KRAAG-HL-
LIRRNKEDCIEYVK-D-----MIE-------TEKCVDFTVNPLYMDTYTKLHD------Q--EDDQ--
LQAAFDMK---------------------------MSVVAYWKV-----------VIFRLADGIPLH-LRFVY----------R------
----------------------------KMVRKEIGGDLMKEIAGSNLD-------------------
MIEKIFQESPAVASKRRSLIDSLALLRDAKLVVYNIIDKNN-------------------------
KAH9320939 -----------------------------------APDGLKEKVTTDAVNIGLGYVCVRNG-IGD--
---ESNAEARE-----------------KEKNLFDFH-----PL---LKDLD------KSMVG-
IPTLAKKLMQIQATTISATLPQIVNKIESMLGKRQAEMRNLPQHLCNPG------E-ADV--AFVKLVHE--
LKESLKKIV----ILGEFQQFPD-----------DPKMHCTARLREKFDM-FYRDLSQRGSF---S-------------
----
--------------VGDKFLSKESRMLEEAKGVGLPN-FLPR-SVFLELLQKM-VEEISEKS----
LSLAATVWDYLENVISRVIE---H--------------------------------YCHCYPLLESRV--RRAVQ-G--
LVVEKKEECINHVK-Q-----MIE-------MEKGIDFTLSPAYMETYGTLIR------SELPAER--VQEAYEMQ-
--------------------------MSLAAYWKV-----------VTLRMGDGIPLH-LQFVC----------R-------------------
---------------NLVGNELETQILKHVGGPNFG-------------------
AMDKILEESPVVAGKRKSLINSLQLLKDSKTAVANIMDRIA--------------------------
XP_751402 --LDHGWFGLRNR-IPAEAH--
ITDAERDE-----------------REIKEFA-Q-----PA---WEGVG------KDRTG-
IHSLIKYVDKERRAQIQSGLPQIIAEIRRKLHDCESDLGRMGEARDSPK--------AQR--YFIFQFCN--
EMQKMANAS----LIGQYHDIPS-----------EDPRIMLRYRVQRRLDR-FYEEMVDLDNMPI------------
----------LFSSYQHDLE---
ILSSMNPEEW----------------EDKVMNAPGIYSEIYKEAKISEGRSLPG-SIHP-DVEERMFRKL-
TTHWERIA----RSFVEDVKDLVKDCHDVLAR---I---------------------------------AIPNSKVRLEV--
SRVVA-K--TLEEWNRDADAALL-E-----LIK------DNQARPLVTRHPLLISLF--------------------------
VR---------------------------ARLESYYKI-----A------LYRFIDNVAMQVVERHV----------L----------------
------------------GPKCPMLTVSVKTFANLND--------------------
EELNSVAGEDETDIRIRARLERQRSRYVQALEKWERL------------------------------
XP_754266 --LRLGFFLVKNP-
RPIDLEKGMTTAERRK-----------------VEAEFFA-H-----PP---WNKLGLD----PSRVG-
IDNLRIFMQDLLDRHIERELPKVRKDVAQLLHDINKELMDLGDPRTSPA--------QIR--MYLTRIAT--
DFQNLVQAG----VEGIYGNR-DSFFHEIDDE---RDCHRLRAAIHAENGK-FAAYMRRHGQKRKVIS--
--------------AELQ---
EDTETETEA----------------GQILVAKEQMSAWIKKIYDRTRGRELPG-NGNH-ALLSELFHEQ-
SSRWGDIA----RDHVNAITDLVYQFVQSACA---F---------------------------------VIKDTNARQTI--
SSIIT-E--KLGDNAKGALHELS-K-----LLA-------DEAGYPITYNHYYTDNY-------------------------EAE-
--------------------------MDLDAYYKV-----A------RKTFVDNVCRQVIERHI----------L-------------------
--------------TNLPTVFNPMTV--SSFSD-------------------
EDLVCLATESPRVSKRRVEATQLQKALADSLRELR--------------------------------
XP_748757 --LKLGFFLMKNP-
SPEQLKNNISMFEWKQ-----------------KELEFFN-S-----PP---WKDLMLD----HNRVG-
AECLRSFLEKILEEHIERELPKVCDEIQTLLQQTRDSLSDLGDERSSVS--------EQR--KYLLKISM--
DYLNLIQAA----LNGRYQEVEPTFFGNASSK---PSFNRLRARIHELNTN-FATYIRDKGQKRKVAG----
------------DREPGMEDFSADQ--
---TSISCRDAFP----------------EPLRISEQKFKIWVSQIHKNTRGLELPG-NHNH-VFLSELFHEQ-

SSRWPAIA----SQHVRRVNQETADFAHRALD---F----------------------------------IVKDRQVAKEI--
LNIIN-P--ILDINFRAAQEELQ-K-----ICD-------DEKLQPITYNHYYTDTE-------------------------KAM-
--------------------------EELDAYYKV-----S------MKTFVDNICRQVLERHI----------L--------------------
--------------RPLQSTFYPTEV--GAFSD--------------------
RDIRRIASESSKIAQKRQELKNRAEALERSLVLLSES-------------------------------
XP_751069 --LELGWHVLRNR-SFET--
RDISDDARDE-----------------MEKAFFN-Q-----GR---WASLS------RECVG-
IESLRRRLSGVLLRLIRRNLPGLITEIQDKVSDRQQRLSKLGPGRSTLQ--------QQR--GYLLGISS--
KFERITMQA----LNGMYA---DDFFGGLDGTSSTEDFRRLRAVIRQLNEY-FADAMAIRGCRRKIVD---
-------------VHPLFGKN-HHKL--
--AAGNPYTDIW----------------EPEYVERSSLEAEVSKQARNNRGIELPG-NANQ-LLVGSLFRDQ-
SKPWEGLA----KEHLMKAWESARYFTWLVLQ---H----------------------------------ITDDHTSSLLI--
GSIID-P--ELERLKQSLLEKLD-E-----LTA------YAKRGHPLPLGKSFLSQA-------------------------EII--
-------------------------DQMQAYYEV-----RKFFLTAIVTFVDNVAILGIENCL----------L------------------
----------------DPLQRIFTSQVV--NNMDD--------------------
GQVRELSMEPPYIHAERERLAWELDKLQAGLRALRVF-------------------------------
XP_750654 --LGHGYYIVRNN-PNPAIEH----
SRARE-----------------EEAVFFAKS-----PW---ATDLSA----YQNRFG-
TRNLQSALSSLLLEQIQGCLPRIVEQINEKAARIEAELQTLPDPPSANV------P------YILCGKLN--
HLKDQIRAH----IDGGSA------------------QYPLQKIWGNIARD-FKRALLKTRPTVQL-------
LAHSDKMAIPIAREDADS---------DCEMTSIQRSVSVNLKRKTPCDRPTPIP-----------------
ADPKPEQPSTPRPSGYYTEHFNRFERPA-------------------------------------
RMFTWEEIREINEDSYRAGIPD-QTDP-KAVEVMNQLS-VEHWDKPM----
EVFLGATHQLVRDMMMRQLK---D---------------------------------VFRPYYQTSLYRELKRIID-N--
YLQTLRKEHFRHAQ-E-----NYN-------IEHNKPFTMAISALEQL-------------------------KMM----------
-----------------ATTRGYYEI-----------ASSRFIDSICQS-VHTKL----------F--------------------------------
--SKCREELITVIENELRIFDEN----------------
AVERCMELMAEDPERQRRRQYLLKEKEKVLKAQEWLATAKKE----------------------------
OAJ38670.1 --LKLGYFMVRCL-
SKAELAAGNTLQDAQK-----------------LENAFFAQS-----QP---WSTLRRK---S-ARFG-
APALRFELSRLLINLVDMSLPQIKSLTETAIEDAIAELGSIPPALGENA------R------IELFQMIR--
HYCTLVTFN----INAQHD------------------FKLFYQKVRKCFEG-LRDQIVDTRPRFDLEKKG-------
YSAT--
VAPGNFTDSFKPDVNDPPLNVSNISKQDTSSPKKSTPGASFSLASSFSMVIPSMFVSNSNHTVSSD
SIKASTAKANDSGSNPYTQFHPPSLNSTPTSWNTSVAEA----------------
CKLNMRRALTLADLQRVVDAQKGRELQG-YSPY-GAFTFLIAAC-QEEWKRYA----
IECLTNVANELAQLLLDLTE---Q---------------------------------VFGRFANLQAQLRLFTQM-----
FLNELQRVSMEQIH-H-----VIE-------MEMRHPFTLSSRVFVDL-------------------------DLM----------
-----------------ASTLAYMEI-----------AIGRVCDTIPMT-IEHHF----------L--------------------------------
--ARFGDMLEKELVSQLGVLD-G----------------
DAEGIEVLLREDWGVAERRADVEAKKDRLESVWRSLHNFGI-----------------------------
XP_006457072.1 --NQNYLRHGYYCVQLP-
NDQQRQQGLTAH---------------------TLPNYLGVT-----WP---WSEFAGQ---G--RFG-
VTNLVKNVSALLVQMIEANLPALRAAVELALRQCVKRLGQLPPVVSHDN------E---M--ASILLMVN--

AFLRELEAA----SSGES-------------------HKSLAQECRRRYRE-LRDEVEGTCPKFQRNGID-----------
----
--------------DNTYDIPAVRTVIDECTGWELPG-IIPY-DAYKTLIKRF-LAGWKDPV----
HNCFDDVHNTFSAFVTTLAN---K---------------------------------HFSGFSHLNAFV--LNHIR-L--
ELQSSKSTAQISVL-K-----LLD-------IEK-QPPFSQRGDFGEI-------------------------HVM-------------
--------------ATVRTYFKF-----------AFERFADTVLLM-QEQDL----------V---------------------------------
-KSLRPKLEEALYKDVTDLS-G----------------
TPNGLERLLAEDEAIAREREELKSRKERMTDIRNRLNMYGTV----------------------------

XP_006461472 ---LQNNWFCVKQP-
ASSDLKNNWTWQQARQ-----------------KEDEFFTAT-----SP---WNELEAM---YVRYLR-
TKNLVERLSQVLSDLIAKTLPGIQREIEGMISKTRQQINDLPKPPPANA------L------NEVAKLVK--
DFDADIRRN----IEGVAY------------------KEGLIQRIRVPAEK-FRRVIRKTAPPFLPYNNS-------
GIGHNL-ADPEFL--
KQEDDGDGEVYDEEDDEDEDDGATASLD------------------------
ESVVSIYVDEVHRRMTESRSRELPG-NFPF-VVQREYIMDI-LLQWEKPT----
MRLCDRVFQILAAHTKELAH---R----------------------------------HFSMFGQGLLEQRVQLIMY-E--
HLGNCLEAAKKKVA-W-----LLK-------IEG-EPFTVNNHYLADL--------------------------EIM------------
---------------ATVRAYFQV-----------SYKRFVDNVPMA-IDYEL----------V--------------------------------
--RGGGEDVFQLLWGKLELDKPG----------------
AQEMCKDYAQEGAQVADRREELEKKLERLILATRRLINSR------------------------------

XP_006461433 ---LENNWYCVKQP-
SSNDLKNNWTWQEARE-----------------KEQQFFAAT-----AP---WCELEGM---YQKFLR-
TTNLVERLSGVLSDLIAKRLPEIQDELERSVQKTQGLLAQLPREPSKQP------V------NEVATLLH--
EFVADIAQH----ITGIPD------------------ENGLIQSIRPAQER-FRTEVRATAPKFRSYEKK-------
FAGTRSMPRAEFL--DNEDDGYSE--
DECDQFTASGTSTRKRK------------------------RRDSVVYVDEVFERAQRSRTRELPG-NYPF-
VVQQGYIDEC-IKEWRSPA----HILCKAVYNTVSEYFKRMVN---Q----------------------------------
HFATFGQGMLEH--------------------------R-----LLD-------LED-KAFSLNTHYLSDI--------------------
-----EIM----------------------------ADVRAYFQV-----------AYKRFSDNIPMA-IDREL----------V----------
------------------------CGAGRDILPLLWNGLGLNSIE----------------
AHRICKELAQENASVANRREELVKKLQRLDEASQQLLQVSGA----------------------------

XP_042924849 VPEHEAGET---------LKLIRLVGACGAPPAGAGAAGGSAR--
VAHPQHPLGHYVVKNP-SQDGLAMNITFEQARA-----------------DEAAYFAGH-----KH---
WAAALRRQPELQRRMG-
AAALRRGLSGLLVELVIAQLPEMRRSCRELQAAVQEELGAMPQPIQDAP------R-ELD--
RMLTRVAA--ALRCHTR-------ADD--------------------DCTFYQRTQAMYDD-
YCERVMRCLPAFLVGTTLIGTLDASGKGPDAAAAAAAAGGGDSDA-----QLQQVWEG---------------
----GALDVSALAAALGAPAVTTEAE-----AAAFLYSDEV-----------------QQLLT----------------
DHLLPQTFMTLAEVVELRQRHLGRELPG-FSPY-RAMECLLQRF-KGQWRGPA----
LACLGNVAAAAHDLTDQVVA---V---------------------------------EFRDFPAGSRAV--TDALW-R--
RVEGLSDATAEFIE-K-----QLR-------MEDRDVYPGDKAQLQELQLAQP------T-----P--VDDELHMA-
--------------------------ASCLAYFKVSSGGVGGAILVAFGRVRASVPMP-IRDTL----------L-------------
---------------------DRLGDPYEVAAAVQAAGQDIV------------------
AAAPRLLAEHPHLAARRLWCAERQQRLREALEALHSPAAGVP--------------------------

BAF46281 IGPDERTEW---------
GKLCNLVAGARAPTGVPAAAGGSRAAAAPNPHLQLGYYVVKNP-GQEQLAAGISFEQARA----------
-------AEERYFADH-----PL---WASAMKANSLLSQRLG-
TNALRDGLSALLVDKIGEHMPEMRRSARAQLEKRQAELM-----------------------RNLWRISD--
TLAQNALAD----IRGG--------------------SLDFYQELMRHYRE-
YGERSIRSTPAFLVDTSLISALNSSDKGMHSSVDGGATATCELSL-----K----------------------------
ELVS---RIKDGPVVKSAQDA---KAAL--EDERV-----------------KIQLR----------------
QLKFPEGYMTLVEVGQLRKRHLGRELPG-FLPY-SAMEALLSQF-KGKHRDQA----
TACLAAVEAEVHERARRVVG---D--------------------------------QLGRYPRAKGAV--GSALC-S--
HVESLVQETAAELD-K-----LLA-------REDGDVFTLNSYEYEVMRMAQH------T-----A--VDDELYMA-
--------------------------ASCLAYFKV-----------AFKRMQDAVPMA-VRSTL----------L--------------------
----
RRLGDPAALEAAVWRELLGAGAVAAEAVEDSYDSSAAKAAAAVAALQEDPQTASRRQHCVAMAQK
LTEALQVLNTPAAQL---------------------------
XP_042924875.1 ----IAEEA---------HIVCNKLVKLVG---
ARGELGPGGTRSQPDGHLRLGYYVVKNP-SQEQLVEGITFEKARE-----------------IEARYFANH-----
VH---WRPAMATSPGLVQRLG-
ANALRSGLSLLLVERIEEQMPAMRKSAREQLDKLRAELASLPPVCVDPN------H-ELF--
QLLWRVAD--GLDAAAHAR----SDTG--------------------DHAFYQLMDRFYRE-
YGRHVMRSTPAFLVGTTLISALNKSEKMMGAGGGD---DGGDLDL-----Q----------------------------
SVLPVMKQAAGGGAVTSSERV---EAALAAGSEAV-----------------QELLS----------------
SNLLPQRPMTLAEVSRLRQRHLGRELPG-FSPY-SAMEELLRRF-KGQWREHA----
EGCLQEVEAAVRELAGKTVA---E--------------------------------QLRRYPKAERVV--GRALS-S--
HVDTLVAAAEAQLS-E-----LLG-------MEDSDVFTLNDHYLRDTQLAQP------T-----P--VDDELHMA-
--------------------------ASCLAYFKV-----------AFKRIQDEVPMA-IRRTL----------L----------------------
------------RRLGDRQQLEAALRGELPDVA------------------
AEARGLLEEDAAVSERRRQCVDMERRLREALTVLHSPATQL---------------------------
XP_032804093.1 --------------------------------------ALRILQNQVFPLSKGYVLVKCR-
SQRDVEAHQTLAEASR-----------------VEAAFFKKH-----PV---FCHVHN-----
GGKLTTTTVLAAKLTEELVDNIKRTLPKLRKDLAEKLREACAELESQGESIPDDA------R-AKQ--
LFINARVM--RYNDDVRGL----ARGEPSRNL-------------PRNMMLYTLVREHFRT-WLKDLKRAQDA-
----
------------------------------WLEELDNLIREYIDNRSGRELPG-FVSY-HAFENLARVH-VQRLEKPA---
-LNVLAVTCWSVQDVFMRLAD---D--------------------------------VFKFLPEFNRLV--KAKVG-A--
QVQGQEQKARVLIV-T-----LFR-------ME-SMVYTQDMLYQNSLRSLD-----------------EDLDVMN---
------------------------KHLRSYFKI-----------ALMRLMDMVPMV-VRFNM----------L--------------------
--------------DELVVALGAELTALAQE--G--------------------
NLAELLCEDEDAMEKRRMVMERVERLHKACATIENKL------------------------------
XP_006815062.1 --------------------------------------VVRIAENKVINLKKGYTIMKCR-
SQRNLEDAMSLEEAMD-----------------EEERFFREH-----KH---YSVLS-------GQAG-
SRLLAHRLTTELVEQILKSVPQIQHAIKDKLEDTKQELTTLGEEVPMND------V-QRI--DLLVRLLD--
RFPDDFKNA----AEGVYKNATYD------------RSMRLQTIVMKLYDT-FADELESLQP--------------------
----
--------------CDEHLPEIKEELVEQRGLELPP-FVKDRIICENLIRQI-LKQFIPPA----

ERLLQAVNMEVERIGQRLVR---D--------------------------------TYSQFPRLQATV--MSKFC-D--
LQTKAVANSKKAIE-D-----KFK-------ME-DMIHTRDRIYKDTEKDVT------------------------------------
--------------------EPV-----------ATTRLMDDIPLM-ITFYL----------L----------------------------------
QELGNDIKVEMAKFLIE--R-------------------
NIDEFLREDAAVEERRLSLEAKRERLDRASRELAMFSTI---------------------------

XP_035690836.1 --------------------------------------VLQILNNEKYKLRKGYTIIKCR-
GQMDIEKGMSLEEAMD-----------------KEQSYFKSH-----EH---FKSVYK-----EKKAG-
VRTLAGRLSTELVGQIKNSIPFLKEDVKTKLAATRQDLQLMGIGVPQEP------D-KKM--LFLTDLLR--
HFIDDLQKT----AKGELTRVCLKRK---------RTNKRLFGDVRDAFKR-LERAMNKMMP-------V--------
----
----------------STDGSILQEIKNAIVENRGRELPR-FMCY-PVCEAIIREH-LQKFREPA----
SVCLQEVSELVESVSTALAT---A--------------------------------HFQAFQGLDWEI--KGKMN-E--
LRMMHEKKAEESVD-Q-----IFA-------ME-HLVFTQDSIFIRSVTDEG-----------------KEAEKML------
---------------------MYICSYFKV-----------AIRRMVDQVPMA-ISLHL----------L--------------------------
--------KNLTEDITSQVNLMAAY--P------------------
DKTLQLLEEDPDSAERRAMLEEKLKRLMKANQDLARFN-----------------------------

XP_019617847.1 --------------------------------------TVDIVNNRKYALKKGYTIIKCR-
GQVDIENKVSLSDAMD-----------------KEEMFFQKH-----EH---FKILYE-----EKKTG-
TKTLAGKLTTELVEQIKKSIPGLKEDIREKLRDTERQLLLLGDGIPEDP------S-SKM--RFLVELLN--
GFTVDLEKL----TNGEQVRNKMSVKQ--------QKQVWLIGDVRDAYRD-FAAELEGLLP---------------
----
-------------------GEGNLEDIEESIQTNRGRELPG-FLPY-SVCEAFIQKH-MENFQEPA----
SDCLLRVNQKVETVLSLLTT---H--------------------------------WFQAYSQLNMAI--KEKIN-D--
LRLAQERKAQEVIK-Q-----LFE-------ME-NLVFTQDAIFHSALKDQQ-----------------KESEEML------
---------------------QYIKAYFEV-----------SIRRLSDTVPMA-IRLQL----------L----------------------------
------TNFTEEVKYQITLMIAQ--P-------------------
DTLELLHEDPDTTEQRTVLTEKRRRLTKANQKLAKF------------------------------

PAA83069.1 --------------------------------------KLQIARNITFPLSKGYITVKCR-
NQEDIKSRKSLREAKV-----------------DEMRFFSND-----PF---FSQLD------PSQRG-
TDTLAKRLSTELLTLIKKFIPEVIKDVQVVRERLTQQLEELGVGPPEDD------R-EKV--AMIARMYN--
DFVRMFSSE----ADGTAHKFTK--DG--------VKMRNLHQRCRIHWRI-LGEKAATTAKNLGDD--------
----
---------------------ATQLRNSILIFLKEQRGRELPN-FQSAYPIVESLIRDKLIKPLKFLT----
LSCLRDISMEVDITLRDLTD---Q--------------------------------VFSTYPKLNLFI--KEAIQ-D--
TKERMHTLAKEDIE-R-----QFE-------QE-NYIWTNDMLYIGETQEAE-----------------LMIEKSL-------
--------------------NALHAYVKV-----------ATTRIMDNGPLC-MMHYM----------L------------------------
----------YKLADEFHKEVEIYQQD--S------------------
DTLDELLEEKEGVRVHRNESRNRLRALFEAEAELSRISL-----------------------------

PAA94353.1 --------------------------------------KLMIARNITYPLSKGYVTVKCR-
NQEDIKNRKSLKDAKA-----------------DEALFFNTD-----PF---FKQLD------SMYRG-
SDTLARRLSEELLYLVKKFIPELISDVQSVRERLGQELAQMGKGPPEDD------R-SKV--ALIARMFN--
DFTKLFEKE----ASGKSHNFQE--DS--------G-KKNLHQRCRQHWCH-LAEQMEKTVRQIGDD-----
----
-----------------------DAKIRKAVRDFLDDQRGRELPN-FQSAYPVIECIIRQRLVAPLRQLA----

MHCLRDVSVEVDDTLRDLAD---S--------------------------------VFTSYPRLDQYV--KESVQ-G--
IKERMYSRAKDDLD-R-----QFD-------QE-SYVWTSDLTFDDESNEPD-----------------EAIERCL-----
----------------------NSLHAYMKI-----------AFTRMIDNAPLC-MMHFM----------L-----------------------
------------YKLADELCNEVSIFQQE--S-------------------
EKLDQLLEEKEGIRIHRIETKNRLKALAEAENDLCRISL------------------------------

PAA74204.1 --VLALMQNRKIPLKKGYVSVRCR-
TPQQLKDNMSLQQAAR-----------------EEEVFFRTH-----PH---FRALD------KFEYG-
TKTLAVKLSSELYEAIKHNIPELMREVQMKREDICKKLETMGCGPPDSI------E-EMK--NLLVRMIN--
KFTTDFANV----STGTHRHE----------------PDRLFGRCRKHWET-LADEIEQCAFSERSD-------------
----
----------------VTAMWEAAKKDIEEQRGCELPG-FDNVYPVVERLVRNHFLKLVREPC----
SNFLVSVNQEVDETVRTLAD---L--------------------------------VFGDYPTFNQFI--KEVCE-G--
IRTGQVDLAREQIR-N-----IFT-------EE-EFVFTQDRIFLLEKDIVE-----------------RNTQRVL----------
-----------------EGAEAYIRV-----------AVRRLQDTITLA-VMHYM----------L-------------------------------
----HKMSDKLRESLIVLVTE--PQ------------------
PLFDKLREENPLISAKRRHLADRMEKLAAAEQELARF-------------------------------

PAA76532.1 --VLAMMRNERLKLRKGFVTVRCR-
TPQQLKDNMGLREACK-----------------AEEEFFKLH-----PQ---FCALG------DYQRG-
CKTLANKLSVELYQAVKERIPEMLKEIQIKRNDYMTMLEALGTGPPQSE------A-EMK--DLLVKMVE--
EFKKEFSNT----ASGTHWHE----------------ETRLFGPCRLHWEA-LADDIERCAFSESPE-------------
----
----------------NKGLLKILEEDVKRQRGRELVG-FDNTFPVLERLVCSHFLVKLQPHA----
TQFLLSVTQEVDQTLRALSD---R--------------------------------VFGQYPSLNRHI--KQEVE-L--
IKSRKYDDSKKQIT-I-----ILE-------QE-RIIFAQDRIYHSELKLAQ-----------------QNSLTVL------------
---------------EGAEAYVRV-----------SVRRLQDTIPMA-VVYNM----------L--------------------------------
---HGMSSGVSHRLGVLLMD--PG------------------
QLYDLLQEENPETGAKRRQLRQWLDSLMVAEQELSSF------------------------------

PAA69582.1 --IKAIVNNQGRVRLHKGFVMVKCR-
SPKELRNNISLSEVAK-----------------IEEDYFKND-----PH---FSQLP------KDIVG-
TKTLAEKLTNELFKAVAAGIETLEESLKSKLKDYEEELKDLEDSLCETD------S-DKR--AYLMRKLQ--
QFASKVHAA----TASPEDERDA-FAT--------GSSSSLYSSCLTNCSS-FGYSVSRSQPAWIIDY----------
----
------------------SKKVPNGVSDEVQLRRGRELPT-FTFVFPVVQKIVFEEYLPKIEYLA----
KWLLGEVQSQVTKSLENLTE---N--------------------------------CFKAFPRLLLLV--KETVV-K--
AIEAQEAECRADLA-K-----LIA-------HE-RRLFTQDSQFSEKLRSEP-----------------SHEEQIR--------
------------------LGTKAYLLL-----------ACQRISDTVPMS-VLCHM----------L-------------------------
-------DGVATKLVAEITRLFAGGSHQ------------------
VDILDLLREKEGRQRRRRYLIQAACQMRESRKELQSAKQNRML-------------------------

PAA92268.1 --TLKIANNEKIPLKKGYVMVKCR-
SPEELNNGVTLSESVA-----------------NEAAFFKTH-----RH---FSLLP------EQSVG-
IRTLADKLTEELFESVKRNISTLQNDILEMRKKFQEELQRLGQPVAETK------D-EKR--RYLFEMLS--
NFNKDVAIA----TQGEMN-----------------ARFHLYAKCRKHWTE-LGKQIHRAWPGWVTRGHI-------
----
-----------------SHYWQPPEQVTRLITDLRGRELAS-YDHVFPIIERIYKEEFLNKLKQPA----

EQFLEQVSDEVTDVVHLIAQ---K--------------------------------VFGDFQDIFEVV--TETSN-E--
EIAKVLELARADLSGR-----LFL-------QE-ARVFSQDSLFQERIAPIP-----------------DSAERIR---------
------------------RGLDAYLRV-----------ATERLSDTIPQS-VLYFM----------F------------------------------
----DVVSKQLGNRALDLLSESESA------------------
ETFLSLLREPRHKTARRKQLKEMLEQLRLAQRDFNKFRLDVQA-------------------------
NP_001007285.1 --------------------------------------VVRTVNNEVIRLEKGYMIVKCR-
GQQDINDKLNLVEALE-----------------KERRFFDEH-----PQ---FSSLLE-----DGKAT-
IPLLGQRLTEELVEHIAKNVPRLQNQIEMKLQKTFERLKVLGESVPDDD------EIELN--NFLIKKLR--
QFMDALEEV----KRVEEEPV-------------KSDTRVFSKIRQEFVS-WKHILDSKPI----KM---------------
----
------------S-----TDLQEYVRTHRGKELPG-FLNY-GTFAGIIRMH-VEDLEEPA----
LKLLLRNAKDIVHSSVGSIAN---I--------------------------------HFNGYPNLLLAV--KEPIE-K--
CLHEQFQNAEEKIR-S-----QFK-------LE-KTVYCQDDLYTNHLNLGN-----------------SELRETA------
---------------------FHLTSYLTI-----------ACERLANQIPLI-VQYHM----------M---------------------------
-------NEYNSQLQNAMLGLIGTS---------------------
DPGMLLCEDSGVARIRKDLKERLERLKDARRALPKVVHSANS-------------------------
XP_005167721.2.2 --------------------------------------VVRTVNNEVIPLKKGYMIVKCR-
GQQDINDKLGLVEALE-----------------KERRFFDEN-----VH---FRSLLE-----DRKAT-
IPLLAERLTKELVEHIAKNLPQLQNQLEMKLEKTSADLRGLGDGVPLDK------N-EKS--NFLIMKIR--
QFNDVLERV----QMAEEDVE--------------KPNTRVFSKIRSEFVK-WKRILDSKAIKTEETL-------------
----
--------------R-----DEVQEYVKTRRGKELPG-FVNY-RTFENIVKKH-IAELHEPA----
LKLLLKDVTDIVHSSVDHIVN---A--------------------------------HFSSFSPLMRAA--KDPTE-D--
FLHEQFQRAEEKIH-S-----QFR-------ME-KIVYSQDDLYSDQLTNVS-----------------ADVREMA-----
----------------------YHLTSYLTI-----------ACERLANQIPLI-VQYHM----------L---------------------------
-------NQYISQLQNAMLGLIGKN---------------------
SPGMLLCEDSGVARKRKDLKERLERLKSAGRVLSKFVHSA--------------------------
XP_032888405.1 --------------------------------------VVDIVKNLTVELEKGYMIVKCR-
GQNDINENISLVDAIA-----------------KEKEFFEDH-----EQ---FRPLLE-----DGKAG-
IPNLAVRLTKELVNHINKSLPQLRNQIETMLNQSLQTRKKYSHAVPNSY------P-EQI--TFLIEKIN--
SFCSHIKNL----TMGEEPQD-------------KVDGVRYITIVRNEFDE-WNTFLNERISKFTTHM-----------
----
----------------K-----NVVLEYENSYRGRELPG-FVNY-NTFESFIKEE-ISALEDPA----
ITKLKTITDITRDIFMVIAE---Q--------------------------------HFNAFGNLSKAA--KVKIA-I--
MSNKQETEAEKLLQ-A-----QIK-------VE-CVVYSQDSLYSECLSATH-----------------GSIQEMS------
---------------------YHLRAYYKI-----------VSHRLADQIPMV-IRCYI----------L---------------------------
------NEFADKLRTDMLQLIQDKD---------------------
RINVYLMEDEDTQRKRDDLNCKIDRLRKAQKTLLEFG------------------------------
XP_007904885.1 --------------------------------------IVDIVQNLVVELKKGYMIVKCR-
GQKEINDKLTLQDAIA-----------------RENRYFEEH-----EQ---FRTLLD-----EKKAS-
IPHLAERLTNELVYHISKCLPNLRRDVEEKLSKTRKELKLYGNGIPTSE------N-ERL--AFLIDKIT--
EFCSQIISL----VSGEDTGN-------------HTEEMRYFPQVRHEFSA-WKTFLDDKGLNFQDTL----------
----
-----------------R-----DEIEEYDNLHRGQELPG-FVNY-KTFESIMKDE-IFKLEEPA----

IQKLKIIAEITRTSFLNIAE---S---------------------------------HFAAFSNLLRAS--KIKIE-D--
YKQEQEIKAECLLR-T-----HFR-------IE-SVVYSQDTIYSQTLTKLN-----------------ASVEEMS----------
-----------------SHLQTYYKI-----------ASNRMADQVPLI-IRYYI----------L--------------------------------
--KEFSEKLRAGMLELLQERD---------------------
KITVYLYENADTKRKRESLKKQLDRLKRARQELNRFG------------------------------
XP_003973512.2.2 ---------------------------------------VVDIIHNEVIHLKKGYMIVRCR-
GQKEIIDKVSLAEATE-----------------TETAFFRDH-----AH---FQTLYD-----DGQAT-
ILKLAEKLTLELVNHIEKSLPRLEEQIEEKLAHTRAELERYGTGPPSDP------A-EKV--TFLMDKLT--
AFTHDAISL----TTGEDICC--------------GEN--IFSTLRQKFGE-WIRHLDISGVKFNVRL---------------
----
------------E-----REVQEYEVKYRGRELPG-FINY-KTFEYMVKEQ-VKQLEEPA----
VRNLKDIGDAVRKVFIHLAQ---T--------------------------------SFAGLPNLIKTA--KAKIE-T--
IKQRMEKDAEKLLR-T-----QFK-------ME-LLVYTQDRTYSSSLDDNH-----------------ATLTELM-------
--------------------LHLKSYYRI-----------ASQRLSDQIPLV-IRYHM----------L----------------------------
------QEFAIQLQREMLQLLQDRE---------------------
NTELFLKEDLDVGTKRAALQSRQKRLTQAREYLVKF-------------------------------
NP_891987.2.2 ---------------------------------------VVDIVHNEVIHLTKGYMIVRCR-
GQKEIMDQVTLNEATE-----------------TESAFFKDH-----PH---FSKLYE-----EGFAT-
IPKLAEKLTIELVHHIQKSLPRLEEQIETKLAETQKELEAYGNGPPSEP------A-ARL--SFFIDKVT--
AFNQDMLNL----TTGEDVKC--------------TTDLLLFPELRQEFAK-WSHILDRSGDSFNKKI-----------
----
----------------E-----KEVDNYEVKYRGRELPG-FINY-KTFEGLVRDQ-IKLLEEPA----
LKTLKTVSDVVRKKFIQLAQ---C---------------------------------SFIGFPNLLKIA--KTKIE-G--
IKLNKESLAESMLK-T-----QFK-------ME-LIVYSQDGTYSQSLKDNH-----------------ATLREMR-------
--------------------LHLKSYYTI-----------ASKRLADQIPMV-IRYML----------L-----------------------------
-----QEAALELQRNMLQLLQDKD---------------------
GVDNLLKEDCDIGQKRENLLSRQKRLMKARSLLVTF-------------------------------
XP_009304072.1 ---------------------------------------VVDIVHNEVIHLTKGYMIVRCR-
GQKEIMDQVTLNEATE-----------------TESAFFKDH-----PH---FRKLYE-----EGFAT-
IPKLAEKLTIELVHHIQRSLPRLEEQIQTKLAETQKELEAYGDGPPSDP------A-ERL--SFFIDKVT--
AFTCDTLNL----TTGEEVKS--------------ASKLLIFPELRQEFAY-WNSFLDSSGYSFKLKI---------------
----
------------E-----EEVDNYEVKYRGRELPG-FINY-KTFEGLVREQ-MKLLEEPA----
LKMLKNVSDMVKKKFIQLAQ---S---------------------------------SFTGFPILLKIA--KTKIE-A--
IKQDKECLAESMLR-T-----QFK-------ME-LIVYTQDGTYSQSLLSNH-----------------ATLSEMK-------
--------------------LHLESYYMI-----------ASQRLADQIPMV-IRYLL----------L----------------------------
------QEAALELQRSMLQLLQDKD---------------------
GVDDMLKEDFDIGQKRENLLSRQKRLMKAQNLLATY-------------------------------
XP_028583068.1 ---------------------------------------VVDIIRNQRVPLRKGYMIVKCR-
GQSDINDKVTLGDAIE-----------------KEREFFEEH-----DF---FRSLLE-----EGRAT-
IPLLAERLTQELIEHISKTLPTLQKQIKEKLEVTNAELQKCGQDVPGNT------E-EKM--PFLIQKIK--
LFNQDILNK----VQGEDLTD--------------LKETRLFTKVRNIFNQ-WQEEINSNALNVRDTM-----------
----
----------------K-----DEVHSYENKYRGKELPG-FINY-KTFETIVRRQ-IMTLKSPA----

VEMLTDVAELVRENFIQTAY---R--------------------------------HFGAFYNLLRAA--QINIE-D--
IKLKQEAAAVSMIN-T-----QFK-------ME-QIVYSQDSIYSEDLSAKL-----------------CSMEEMT--------
-------------------YHLNAYFKS-----------AGTRLGAQIPMI-IRTYV----------L------------------------------
----QDYADKLQNAMLQLLQNKD--------------------
QFDILLQERYDTSNVRNTLKEKIKRLTKARQHLAKFPM----------------------------
XP_025933558.1 --------------------------------------IVNIIRNLTVPLKKGYMIVKCR-
GQQDIHNNLTLASAIQ-----------------QEKEFFETH-----QH---FSILLN-----EGKAT-
VPLLAEKLTXELVGHIIKTLPTLENQIRSELQKTLQELHKYRRGMPTTE------S-DKL--IFLTDIIK--
LFNEDIAHS----MHGEEHLS--------------GNEIRLFTKIRREFQN-WEQVLQDSSIKVTKIV-------------
----
--------------P-----QKAWKYAAQYRGRELPG-FTNY-RTFENIVKEQ-ITELEEPA----
IAILNAVVGLVEESFLEVTK---K--------------------------------HFADFHNLYRAA--KTRIE-D--
IKERQATEAEKNIR-I-----QFK-------ME-KIVYCQDNSYIASLKQDS-----------------SVVDEIV----------
-----------------AHTQAYFSG-----------ARNRLASQIPLI-ILFSI----------L---------------------------------
-QNFGDNLQITMLHLLQEKE--------------------
KLTFLLQEDGEVAKYRNSLTQRVNRLTKACQYLRNFASLQLS------------------------
XP_028583072.1 --------------------------------------VVNIVRNQVIPLKKGYMIVKCR-
GQQDIQSNMTLASALK-----------------EERAFFEKH-----KC---FSILLQ-----EKKAT-
VPLLAEKLTSELVEHISKSLPTLEEQIRFQLQKTDTEMQRYGKAVPKTD------G-EKQ--YFLAEKIE--
HFIADFGST----VQGEESVS--------------ENEARLFTKVRKEFQK-WGEGVASSGLKIQESL------------
----
---------------L-----PEKWKFENQYRGRELPG-FLNY-HTFEMIIRKY-INNLEMPA----
VDILTRVTEIVRQGFVAIAK---S--------------------------------HFEGFHNLHSEA--KNRIE-S--
IIEKQAKEAEAIVR-I-----QFA-------ME-KTLFCQDNHYQSCLREEG-----------------TAIAETA----------
-----------------FHLEAYFKT-----------VIKRLSSQIPLI-IQYFI----------L---------------------------------
NKYEYRLKNEMMLLLSQRE--------------------
NLSLLVQERTDAAEQRQFLSDRIDRLVQARDHLARLLG----------------------------
XP_015269256.1 --------------------------------------VVDIVRNLIIHLKKGYMIVKCR-
GQQDIQSNLDLASAIQ-----------------KEKAFFEDN-----RH---FRILLA-----EKRAT-
IPLLAEKLTSELVEHINKSLPNLEEQINTQLQKANQDLLKYGKGMPKTE------G-EKL--NFLIEKIK--
LFNEDIINL----TQGEEKLS--------------ENDTRLFTKIRKEFQK-WEKKLNESAVKMQKGS-------------
----
--------------H-----LEVQRFDDQYRGRELPG-FINY-KTFESIIKQL-IMELEAPA----
VEMLKKVTEMVSQDFTKVAK---D--------------------------------HFADFHNLYRAA--KDRIE-D--
IKEKQLEEAECIVR-T-----QFA-------ME-QILYCQDKVYSQDLSEKN-----------------ASVKEMA-------
-------------------YHLEAYFNN-----------AGKRLSCQIPLI-ILFYM----------L----------------------------
------KKNGDKLQNEMLKLLQVKE--------------------
EIGNFLQERKDAAEQRQFLSERIDRLTQARQHLAKFPG----------------------------
XP_031752404.1 --------------------------------------VISVVRNLVYSLNKGYMIVKCR-
GQQEIQENLSLKDALV-----------------NEQNFFKEH-----EH---FSVLLE-----EGYAT-
IACLAGKLTNELVAHIVRNLPTLKTQIRKKLDEAEEKLRNIGSGVPDSE------T-EKL--TFLIDKIR--
RFNDAITHA----TQGEEEMT--------------NGFLKLSTIMRNYFYS-WELTIQETSKGFQNKL-------------
----
--------------K-----EDITVYENQYRGRELPG-FVSF-KVFENIARKQ-IHSLEEPA----

IEKLKQVTDKVKTHFSQIAM---R---------------------------------HFLSFPNLYRCT--KVRIE-D--ICCEQMREAEKTIR-T-----QFK-------ME-KMIYCQDKLYGVLLKPME-----------------ISFDEMR-------
--------------------YHIQAYFRS-----------LTERLSNQIPII-IQYYV----------L-------------------------------
---HEFSNNLQSQIMQLIQERE---------------------
NLDALLAEKNDFSRERKNLKDQIERLSAASQRLAKFHC------------------------------

XP_012586448.1 ---------------------------------------VVNVAQNLTYRLKKGYMVVKCR-GQQDIMDRLSLAQATE-----------------KEVAFFQTH-----PH---FRALLE-----EGKAT-VPRLAEKLTSELILHINKSLPLLENQLRESHQRATEELRQCGPSIPTSD------S-DRM--FFLIEKVK--VFNRDIEKL----IEGEEIVK--------------EKETRLFNKIRKEFKN-WELVLADNIQK------------------------
----
---EIVEHSFIKTAK---D-----------------------
----------NFGEFFNLNQIV--QSKIE-D--IKTKQAAIAENLIQ-L-----QFR-------ME-QLVYCEDHMYSLVLSSPI-----------------SSFTEIG---------------------------VHLNAYFLH-----------
TSSRLTNQIPLI-IHYFI----------L-----------------------------------KESGDILQRVMLQVLQERE----------
-----------HYPWLLQEHSGTAARRAQLKEKLARLGQARQALCDFCSARW--------------------------

NP_776366.1 ---------------------------------------VLKVMQNLTYHLKKGYMIVKCR-GQQDITNKLSLAEATR-----------------KETMFFETH-----PY---FRILLD-----EGKAT-VPLLAERLTTELIWHINKSLPLLENQIKEKHQRATEELQQYGDDIPSDE------G-DKM--FFLIEKIK--VFNEDIGKL----IEGEEIVM--------------ETESRLCNKIREEFTS-WILILTTNIEKVKSIL-------------------
----
--------N-----EEVSKYEKKYRGKELLG-FVNY-KTFETVVKHY-LGQLIDPA----LKMLQKAMEIVWQTFKDTAK---K---------------------------------HFAEFCNLHQTV--QNKIE-D--IKTKQMAEAANLIQ-L-----QFR-------ME-KLVFCQDQIYGVVLNSSI-----------------SSIVEIG---------
------------------VHLNAYFME-----------TSKRLANQIPFI-IQYFM----------L------------------------------
----QENGDKVQKAMMQLLQDTQ---------------------
HYSWLLQEQSDTATKRKFLKEKIFRLTQAQQALYEFPHFKG--------------------------

NP_001003133.1 ---------------------------------------VVNVAQNLTYHLQKGYMIVRCR-GQEEITNQLSLAEATE-----------------KERMFFQTH-----PY---FRALLE-----EGKAT-VPCLAERLTKELILHINKSLPLLEKQIRESHQRATDELHQCGDSIPSNE------A-DKM--FFLIEKIK--LFNQDIDKL----IEGEEIVK--------------KNETRLYNKIREEFEH-WALVLTANTQKVKNIV---------------
----
------------S-----EEVSVYEKQYRGKELLG-FVNY-KTFETIVHQY-IEQLVEPA----LTMLRKTIEIVWQAFTDTAK---K---------------------------------HFSVFSNLSQTI--QNKIE-D--IKTRQAETAENLIR-L-----QFR-------ME-QLVYCQDQIYSVVLRPSM-----------------SSNDEIG-------
--------------------VHLNAYFLE-----------TSKRLANQIPFI-IQYFV----------L------------------------------
----QENGSCLQKAMMQILQERE---------------------
QYSWLLQEHADTSAKRRFLKEKIYRLAQARRALYMFFS-----------------------------

XP_032211320.1 ---------------------------------------
VMKVAQNLTYHLQKGYMMVRCR-GQEEITNRLSLAEATR-----------------KETMFFQKH-----PH---FRALLQ-----EGKAT-VPCLAERLTNELILHINKSLPLLEEQIRESHQRATEELHQCGDDTPSNE------T-DRM--FFLIDKIK--MFNQDIEKL----TEGEEVVK--------------EKETRLFNKIREEFEN-WVLVLTANTQKVKNII--
--H-----EKVSIYEKQYRGKELLG-FVNY-KTFQTIVHQY-LEQLVDPA----LALLQKAVEIIRQTFSDTAK---K---------------------------------HFSGFSNLNQRA--

QNKIE-D--LKTRQAEIAENLIR-L-----QFR-------ME-QLVYCQDEIYSVVLNSSM-----------------
SSITEIG---------------------------VHLNAYFWE-----------TSKRLANQIPFI-IQYFI----------L------------
----------------------QENGSCLQKAMMQVLQERE---------------------
NYSWLLQEQSDIAAKRRFLKDKIYRLSQAQRTLYNFYG-----------------------------
XP_006156438.1 ---------------------------------------VMNVLQNLTFPLKKGYMIVKCR-
GQQEIMNNLSLAEATR-----------------KELMFFQSH-----PH---FRVFLE-----EKKAT-
VPHLAERLTAELIAHIRKSLPSLESEIRERHQGATEELRRCGADIPTQE------T-DKM--FFLIEKIK--
VFNQDVEKL----VEGEEIVR--------------EKETRLYNKIREEFKN-WVLVLAANTQKVKNII--------------
----
-------------H-----EEVSKYEKQYRGKELPG-FVNY-KTFQNIVQQY-IQQLVNPA----
LDMLQKIVELVQEAFIVTAQ---N---------------------------------NFGEFANLNRTA--QSKIE-D--
IKIKQAEKAENMIQ-L-----QFQ-------ME-KLVYCQDQIYSVVLKSPV-----------------SAITEIG---------
------------------VHLNAYFSE-----------TSKRLANQIPFI-IQFFV----------L-------------------------------
---QDNGNWLQKAMMQILQEKE---------------------
RYSWLLQEQGETATKRRFLKERIYRLTQARQALCKFSTEEM--------------------------
NP_002454.1 ---------------------------------------VMNVVRNLTYPLKKGYMIVKCR-
GQQEITNRLSLAEATK-----------------KEITFFQTH-----PY---FRVLLE-----EGSAT-
VPRLAERLTTELIMHIQKSLPLLEGQIRESHQKATEELRRCGADIPSQE------A-DKM--FFLIEKIK--
MFNQDIEKL----VEGEEVVR--------------ENETRLYNKIREDFKN-WVGILATNTQKVKNII-------------
----
--------------H-----EEVEKYEKQYRGKELLG-FVNY-KTFEIIVHQY-IQQLVEPA----
LSMLQKAMEIIQQAFINVAK---K---------------------------------HFGEFFNLNQTV--QSTIE-D--
IKVKHTAKAENMIQ-L-----QFR-------ME-QMVFCQDQIYSVVLKSSV-----------------SSFTEIG-------
--------------------IHLNAYFLE-----------TSKRLANQIPFI-IQYFM----------L------------------------------
----RENGDSLQKAMMQILQEKN---------------------
RYSWLLQEQSETATKRRILKERIYRLTQARHALCQFSSKEIH-------------------------
XP_002830747.1 ---------------------------------------VMNVVRNLTYPLKKGYMIVRCR-
GQQELTNRLSLAEATK-----------------KEITFFQTH-----PY---FRVLLE-----EGSAT-
VPRLAERLTSELIMHIQKSLPLLEEQIRESHQKATEELRRCGADIPSQE------A-DKM--FFLIEKIK--
MFNQDIEKL----VEGEEAVR--------------ENETRLYNKIREDFKN-WVGILATNTQKVKNII-------------
----
--------------H-----EEVEKYEKQYRGKELLG-FVNY-KTFEFIVHQY-IQQLVEPA----
LSMLQKAVEIIRQAFINMAK---K---------------------------------HFGEFFNLNQTV--QSKIE-D--
IKVKHTEKAENMIQ-L-----QFR-------ME-QMVFCQDQIYSVILKSSV-----------------SSFTEIG--------
-------------------IHLNAYFSE-----------TSKRLANQIPFI-IQYFM----------L------------------------------
----RENGDSLQKAMMQILQEKN---------------------
RYSWLLQEQSETTTKRRILKERIYRLTQARHALCQFSGKEIH-------------------------
XP_008569440.1 ---------------------------------------VMNVARNLTYHLKKGYMIVKCR-
GQQDITNKLSLAEATK-----------------KEMAFFQTH-----PY---FRVLLE-----EGKAT-
VPCVAEKLTAELIVHINKSLPLLENQIRENHQRATEELRQCGADIPSQD------S-DKM--FFLIEKVK--
MFNQDIEKL----IEGEEVVK--------------ETETRLYNKIREEFKT-WVLVLNANNKKVKKII---------------
----
------------H-----EEVSKYEKQYRGKELHG-FVSY-KTFEAIVQQY-IQQLVEPA----
LNMLQKAVEIVHQGFTDMAK---K---------------------------------HFTEFSNLNQMA--QNKIE-D--

VKRRQAEKAERMIQ-L-----QFQ-------ME-QLVYCQDQIYSDDLKSSV-----------------SSITEIG------
---------------------VHLNAYFSE-----------TSKRLANQIPFI-IQFFI----------L-----------------------------
-----RENGDWLQKAMMQILQEKD---------------------
HYSWLLQEQSETATKRRFLKERIYRLTQARQALCKFSMEQFR-------------------------
XP_017508123.1 ---------------------------------------IVNVVQNLTYHLKKGYMIVKCR-
GQQEVTNKLSLAEATS-----------------KEMTFFQTH-----PY---FRILLE-----EGKAT-
VPRLAEKLTTELISHINKSLPLLDSQIKESHQKATEELRQCGANVPSSD------T-EKM--FFLIEKIK--
VFNQDIEKL----TEGEEVVR--------------EKETRLYNKIREEFKN-WVLILTANTQKVKNII----------------
----
-----------H-----EEVSKYEKQYRGKELLG-FVNY-KTFQTIVQQY-IEQLVDPA----
LLVLQKAVEIVWQSFTDTAK---K---------------------------------NFGEFSNLNHTA--QSKIE-D--
IKTKQAETAENLIR-L-----QFR-------ME-QLVYCQDQIYSVVLNSSV-----------------SSINEIG---------
------------------VHLDAYFLE-----------TSKRLANQIPFI-IQYFI----------L--------------------------------
--QENSDCLQKAMMQILQEKE---------------------
HYSWLLQEQSGTAAKRNFLKEKIHRLSQARRALCKFSS-----------------------------
XP_005885748.1 ---------------------------------------VVNVAQNLTYRLKKGYMIVKCR-
GQQEITDKLSLAEATK-----------------KEMMFFQTH-----PY---FRVLLE-----EGKAT-
VPRLAERLTTELIWHINKSLPLLENQIRESHLSATEELRQCGEHIPNTD------A-DKM--FFLVEKIK--
VFNRNIERL----VDGEEVVK--------------EKETRLYNKLREEFKN-WVLVLAANSQKVKNII-------------
----
--------------H-----EEVSKYENQYRGKELLG-FVNY-KTFETIVQQY-IQQLVEPA----
LSMLQKTVETVRQTFIDTAK---T---------------------------------HFGEFSNLNQTA--RNKIE-D--
IKTKQAETAANMIH-L-----QFR-------ME-QLVYCQDQIYSEILQSSV-----------------SSITEIG----------
-----------------MHLNAYFLE-----------TSKRLANQIPFI-IQYFM----------L-------------------------------
---QENGDYLQKAMMQILQEKD---------------------
QYSWLLQEQSETALKRKFLKEKIYRLTQARRALYKFSS-----------------------------
NP_034976.1 ---------------------------------------VLDVMRNLVYPLKKGYMIVKCR-
GQQDIQEQLSLTEAFQ-----------------KEQVFFKDH-----SY---FSILLE-----DGKAT-
VPCLAERLTEELTSHICKSLPLLEDQINSSHQSASEELQKYGADIPEDD------R-TRM--SFLVNKIS--
AFNRNIMNL----IQAQETVS--------------EGDSRLFTKLRNEFLA-WDDHIEEYFKKDSPEV-----------
----
----------------Q-----SKMKEFENQYRGRELPG-FVDY-KAFESIIKKR-VKALEESA----
VNMLRRVTKMVQTAFVKILS---N---------------------------------DFGDFLNLCCTA--KSKIK-E--
IRLNQEKEAENLIR-L-----HFQ-------ME-QIVYCQDQVYKETLKQKG-----------------LTTTEMT-------
--------------------QHLKAYYQE-----------CRRNIGRQIPLI-IQYFI----------L-----------------------------
-----KTFGEEIEKMMLQLLQDTS---------------------
KCSWFLEEQSDTREKKKFLKRRLLRLDEARQKLAKFSD-----------------------------
XP_004675614.2.2 ---------------------------------------VVDVIRNFICPLKKGYMIVKCR-
GQKDIQDRLSLAQALQ-----------------KEQAFFEEH-----PH---FRQLLE-----EGRAS-
IPKLADRLTSELIRHISKSLPLLESQVKENHQNVSAELQKCGTDIPEGE------S-EKL--FFLIDKIK--
AFNEDITIL----TEGEETVK--------------SSETRLFTKIRREFHK-WNEEVGKNFLKSFLDI----------------
----
-----------R-----REITKYENQYRGRELPG-FVNY-KTFETLVRRQ-VKMLEEPA----
VQMLHQVADLTRQSFVEVAE---K---------------------------------NFPEFFNFFRTS--KTKIE-D--

IKTEQEQEAEKCIR-L-----HFQ-------ME-QIIYCQDSSYREALQSPE-----------------SSMDEIF--------
-------------------DHLTAYHRE-----------GAAVRS---

NP_038634.1 -------------------------------------VVDVVRNLVYHLKKGYMIVKCR-
GQQDIQEQLSLTEALQ-----------------NEQIFFKEH-----PH---FRVLLE-----DGKAT-
VPCLAERLTAELILHICKSLPLLENQIKESHQSASEELQKYGMDIPEDD------S-EKT--FFLIEKIN--
AFNQDITAL----VQGEENVA--------------EGECRLFTRLRKEFLS-WSKEIEKNFAKGYAVL------------
----
-------------Y-----NEVWAFEKQYRGRELPG-FVNY-KTFENIIRRQ-IKTLEEPA----
IEMLHTVTEIVRAAFTSVSE---K--------------------------------NFSEFYNLHRTT--KSKLE-D--
IRLEQEKEAEMSIR-L-----HFK-------ME-QIIYCQDQIYRGALQLQT-----------------SSMAEIF---------
------------------QHLNAYRQE-----------AHNRISSHVPLI-IQYFI----------L------------------------------
----KMFAERLQKGMLQLLQDKD--------------------
SCSWLLKEQSDTSEKRKFLKERLARLAQARRRLAKFPG----------------------------

XP_005202045.1 -------------------------------------VVDVVRNLVFHLKKGYMIVKCR-
GQQDIKHRMSLDKALQ-----------------RERIFFEDH-----AH---FRDLLE-----EGKAT-
IPCLAERLTSELIMHICKTLPLLENQIKETHQRITEELQKYGKDIPEEE------S-EKM--FCLIEKID--
TFNKEIIST----IEGEEFVE--------------QYDSRLFTKVRAEFSK-WSAVVEKNFEKGYEAI----------------
----
-----------R-----KEIKQFENRYRGRELPG-FVNY-KTFETIIKKQ-VRVLEEPA----
VDMLHTVTDIIRNTFTDVSG---K--------------------------------HFNEFFNLHRTA--KSKIE-D--
IRLEQENEAEKSIR-L-----HFQ-------ME-QLVYCQDQVYRRALQVSE-----------------PSTDEIF-------
--------------------QHLTAYQQE-----------VSTRISGHIPLI-IQFFV----------L------------------------------
----RTYGEQLKKSMLQLLQDKD--------------------
QYDWLLKERTDTRDKRKFLKERLERLTRARQRLAKFPG----------------------------

XP_014388412.1 -------------------------------------VVDVVRNLVYHLKKGYMIVKCR-
GQQDIQYQMSLSKALQ-----------------RERAFFEDH-----PY---FRDLLE-----EGKAT-
IPCLAERLTNELIAHISKSLPLLENQIKENQQSITEQLQKYGMDIPEEE------T-EKM--FFLIDKIN--
TFNQDIQAL----VEGEESVC--------------GDNSRLFTRIRMEFGK-WSIEIEKSFQRGYDDI------------
----
---------------F-----RQIRKFENQYRGRELPG-FVNY-KTFETIIKKQ-VKTLEEPA----
VEMLHKITDMVRLAFTDVSK---K--------------------------------NYEEFFNLFRTC--KSKIE-D--
IKSEQEKEAEKSIR-L-----HFQ-------ME-QIVYCQDQAYRGALQPLT-----------------CSLAEIL--------
-------------------QHLLAYRLE-----------ASNRLSSHIPLI-IQFFV----------L------------------------------
----RSYGQQLQKAMLQLLQDKE--------------------
KYDMLLKEHSDTSDKRKFLKERLARLTKARRRLAQFPG----------------------------

NP_001003134.1 -------------------------------------VVDVAQNLVCHLKKGYMIVKCR-
GQQDIQDQVSLAEALQ-----------------KEKDFFEDH-----PH---FRVLLE-----EGRAT-
VPNLAEKLTSELITHICKTLPLLENQIKENHEKITEELQKYGSDVPEDE------H-EKM--FFLIDKLN--
AFNQDISSL----IQGEESVG--------------EDESRLFTKIRNEFHK-WSAVIEKKFQRGYKAI-------------
----
-------------Y-----KQMEKFENRYRGRELPG-FVNY-KTFEIIIKQQ-IKELEEPA----
VDMLHTITDMVQVAFGDISK---A--------------------------------NFDEFFNLYRTT--KSKIE-D--
IKFELEKEAEKSIR-L-----HFQ-------ME-QIVYCQDHVYQRALQSVN-----------------ISLSEIL---------

------------------EHLLAYRQE-----------ATNRISSHIPLI-IQYFI----------L--------------------------------
-QVYGQKLQNGMLQLLQDKD--------------------
TYSWLLKERSDTSDKRKFLKERLARLAQARRRLAKFPG----------------------------

XP_032211398.1 --------------------------------------VVDVAQNLVCHLKKGYMIVKCR-
GQQDIQDQVTLAEALQ-----------------KERDFFEDH-----PH---FRVLLE-----EGRAT-
VPCLADKLTSELIMHICKTLPLLENQIKENHEKITEELQKYGSDVPEDE------H-EKM--FFLIDKIN--
AFSHDINSL----IEGEESVG--------------ENDSRLFTKIRNEFHK-WNTVIEKAFQKGYKAI--------------
----
-------------Y-----KQIEKFENRYRGRELPG-FVNY-KTFEIIIKQQ-IKELEEPA----
VDMLHTVTDMVQAAFTGISK---A---------------------------------NFDEFFNLYRTT--KSKIE-D--
IKFELEEEAEKSIR-L-----HFQ-------ME-QIVYCQDQVYQRALQLVN-----------------ISLSEIF---------
------------------EHLMAYHQE-----------ATTRISSHIPLI-IQYFI----------L--------------------------------
--QMYGQKLQKAMLQLLQDKD--------------------
TYNWLLKERSDTSDKRKFLKERLARLGQARRRLAKFPG----------------------------

XP_004466363.1 --------------------------------------VVDVVRNLVCHLKKGYMIVRCR-
GQQDIQDRLSLATALQ-----------------KERAFFENH-----EN---FRVLLE-----EGKAT-
VPHLAERLTTELITHISKTLPLLENQIKESHEKTAEALQKCGVDIPEEE------S-EKM--FFLIDKIN--
AFNEAINTL----IQGEELVE--------------EEECRLFTRLRNEFGK-WSNVIEVNFQKSFNAI-------------
----
--------------C-----KKIWKFENQYRGRELPG-FVNF-RTFETIIKEQ-IKALEEPA----
LLMLHVVTDMVRLAFTEVSG---K---------------------------------HFDEFFNLHRTA--KSKIE-D--
IRLEQEKEAEKLIH-L-----HFQ-------ME-QIVYCQDKVYRSALQTTE-----------------SSMAEIF--------
-------------------QHLVAYQQE-----------ASNRLSTHIPLM-VQYFV----------L---------------------------
-------QTYGLQLRKSMLQLLQDKE--------------------
SYDWLLKERSDTSDKRKFLKERLARLNQARRRLAKFPG----------------------------

XP_017508130.1 --------------------------------------VVDVVRNLVCHLKKGYMIVKCR-
GQQDIQDQLSLAEALK-----------------KERAFFEDN-----PY---FRDLLE-----EGRAT-
VPCLADKLTVELITHICKSLPLLENQIKENHQKITEELQKYGMDIPEDE------N-ERM--FFLIDKIN--
TFNQDITSL----IQGEESVG--------------PDETRLFTQIRKEFHK-WSIVIERNFKKGYETI----------------
----
-----------Y-----KQIQKFENQYRGRELPG-FVNY-RTFETIVKQQ-IHALEEPA----
VDMLHTVTDMVRLAFTDVSK---K---------------------------------NFDEFFNLFRTS--KSKIE-E--
IRLEQEKEAERSIR-L-----HFQ-------ME-QIVYCQDKVYQSSLQPED-----------------SSMAEIF-------
--------------------QHLVAYHQE-----------ASNRISSHIPLI-IQFFV----------L----------------------------
-----QTYGQQLQKNMLLLLQDKD--------------------
TYAWLLKERSDTSDKRKFLKERLARLAKARRRLAKFPG----------------------------

NP_002453.2.2 --------------------------------------VVDVVRNLVFHLKKGYMIVKCR-
GQQEIQDQLSLSEALQ-----------------REKIFFENH-----PY---FRDLLE-----EGKAT-
VPCLAEKLTSELITHICKSLPLLENQIKETHQRITEELQKYGVDIPEDE------N-EKM--FFLIDKVN--
AFNQDITAL----MQGEETVG--------------EEDIRLFTRLRHEFHK-WSTIIENNFQEGHKIL-------------
----
--------------S-----RKIQKFENQYRGRELPG-FVNY-RTFETIVKQQ-IKALEEPA----
VDMLHTVTDMVRLAFTDVSI---K---------------------------------NFEEFFNLHRTA--KSKIE-D--
IRAEQEREGEKLIR-L-----HFQ-------ME-QIVYCQDQVYRGALQATD-----------------SSMEEIF------

---------------------QHLMAYHQE-----------ASKRISSHIPLI-IQFFM----------L---------------------------
-------QTYGQQLQKAMLQLLQDKD---------------------
TYSWLLKERSDTSDKRKFLKERLARLTQARRRLAQFPG-----------------------------
NP_001127618.1 ---------------------------------------VVDVVRNLVFHLKKGYMIVKCR-
GQQEIQDQLSLSEALQ-----------------REKIFFEDH-----PY---FRDLLE-----EGKAT-
VPCLAEKLTSELITHICKSLPLLENQIRESHQRITEELQKYGVDVPEDE------N-EKM--FFLIDKIN--
AFNQDITAL----IQGEETVG--------------EEDIRLFTRLRHEFHK-WSIIIENNFQEGHKIL---------------
----
------------S-----RKIQKFENQYRGRGLPG-FVNY-RTFETIVKQQ-IKALEEPA----
VDMLHTVTDMVRLAFTDVSI---K---------------------------------NFEEFFNLHRTA--KSKIE-D--
IRAEQEREGEKLIR-L-----HFQ-------ME-QIVYCQDQVYRGALQATD-----------------SSMEEIF------
---------------------QHLMAYHQE-----------ASKRISSHIPLI-IQFFM----------L---------------------------
-------QTYGQQLQKAMLQLLQDKD---------------------
TYSWLLKERGDTSDKRKFLKERLARLTQARRRLAQFPG-----------------------------
XP_008569442.1 ---------------------------------------VVDVVRNLVYHLKKGYMIVKCR-
GQQDIQDQLSLATALQ-----------------REKDFFEDH-----PQ---FRDLLE-----EGRAT-
IPCLAERLTTELITHICKSLPLLENQIKECHQKITEELQKYGTDIPNDE------N-EKM--LFLIDKIN--
VFNQDINAL----IQGEETVG--------------DEDTRLFTKLRSEFCK-WSDVIEANFKKGYDVI-------------
----
--------------Y-----RKIWTFETQYRGRELPG-FVNY-RTFEAIVKQQ-IKALEEPA----
VDMLHRVTDMVRLAFTDVST---K---------------------------------NFDEFFNLHRTA--KSKIE-D--
IRLEQENEAEKSIR-L-----HFQ-------ME-QIVYCQDHVYSGALQLSE-----------------SSLAEIF--------
-------------------QHLIAYHQE-----------VSKRISSHIPLT-IQFFV----------L-------------------------------
---QTYGQLLQKGMLQLLQDKD---------------------
TYNWLLKERNDTSDKRKFLKERLARLAQARRRLAKFPG-----------------------------
XP_006156437.1 ---------------------------------------VVDVVRNLVCHLKKGYMIVKCR-
GQQDIQDRLSLAEALQ-----------------REKVFFEEH-----PY---FSFLLE-----EGKAT-
IPCLAERLTTELIMHISKSLPLLENQIKESHQKITEELKKYGTDIPEDE------N-EKM--FFLIDKIN--
TFNQNIMVL----IQGEEIVG--------------EDDTRLFTKLRNEFHK-WNIMIEKNFQKGYDLI-------------
----
--------------R-----KKIWKFENQYRGRELPG-FVNY-RTFESIIKEQ-IKALEEPA----
IDMLHTVTDMVRLTFTDASV---K---------------------------------NFDEFFNLHRTA--KSKIE-D--
IRLEQEKEAEKSIR-L-----HFQ-------ME-QIVYCQDHVYRSALQSTE-----------------SSMAEIF--------
-------------------QHLTAYQQE-----------ASNRISSHIPLI-IQFFI----------L-------------------------------
---QTFGQQLQKGMLQLLQDKD---------------------
AYNWLLKERSDTSDKRKFLKERLSRLAQARRRLAKFPG-----------------------------

;
end;
begin trees;
tree tree_1 = [&R] [&branchAttributeNames={"FastTree support value"}]((XP_042924849:0.5572699999999999,(BAF46281:0.4529300000000003,XP_042924875.1:0.3220099999999997)[&"FastTree support value"=0.878]:0.1137100000000002)[&"FastTree support

value"=1.0]:0.45608000000000004,(OAJ38670.1:1.0698799999999995,((XP_750654:1.623
45,(XP_006457072.1:1.1878200000000003,(XP_006461472:0.47588999999999997,XP_006
461433:0.3571900000000001)[&"FastTree support
value"=1.0]:0.8013399999999997)[&"FastTree support
value"=0.808]:0.12636000000000003)[&"FastTree support
value"=0.784]:0.06416999999999984,(((XP_754266:0.5344600000000002,XP_748757:0.5
3179)[&"FastTree support
value"=0.989]:0.36357000000000017,(XP_751069:0.89072,XP_751402:1.45967)[&"FastTre
e support value"=0.9]:0.23665000000000003)[&"FastTree support
value"=1.0]:0.7980600000000004,((((XP_006815062.1:0.8608700000000002,(XP_0356908
36.1:0.4574100000000003,XP_019617847.1:0.31271999999999966)[&"FastTree support
value"=0.998]:0.33844999999999999)[&"FastTree support
value"=0.894]:0.13732999999999995,((PAA69582.1:1.0526999999999997,PAA92268.1:0.5
0908)[&"FastTree support
value"=0.991]:0.41710999999999965,((PAA83069.1:0.31346000000000007,PAA94353.1:0.
19791000000000025)[&"FastTree support
value"=1.0]:0.7703200000000003,(PAA74204.1:0.28476000000000035,PAA76532.1:0.472
3499999999996)[&"FastTree support value"=0.99]:0.29102000000000006)[&"FastTree
support value"=0.803]:0.20582000000000011)[&"FastTree support
value"=0.993]:0.2940100000000001)[&"FastTree support
value"=0.701]:0.05366999999999884,(XP_032804093.1:0.9062499999999996,((NP_0010
07285.1:0.2553599999999996,XP_005167721.2.2:0.10510999999999981)[&"FastTree
support
value"=1.0]:0.4173699999999996,(((XP_032888405.1:0.3998400000000002,XP_00790488
5.1:0.31418999999999997)[&"FastTree support
value"=0.994]:0.1724800000000002,(XP_003973512.2.2:0.2267999999999999,(NP_89198
7.2.2:0.06353000000000009,XP_009304072.1:0.08727999999999998)[&"FastTree support
value"=0.997]:0.19676000000000027)[&"FastTree support
value"=1.0]:0.37371)[&"FastTree support
value"=0.538]:0.08426999999999962,(XP_028583068.1:0.3589399999999996,(XP_031752
404.1:0.5949499999999999,((XP_025933558.1:0.3884800000000004,(XP_028583072.1:0.
3801699999999997,XP_015269256.1:0.19463000000000008)[&"FastTree support
value"=0.518]:0.0740299999999996)[&"FastTree support
value"=0.943]:0.0978399999999997,((XP_012586448.1:0.19507999999999992,(XP_00588
5748.1:0.08253000000000021,((XP_017508123.1:0.0818500000000002,(NP_776366.1:0.2
0167999999999964,(NP_001003133.1:0.09039999999999981,XP_032211320.1:0.097220
00000000008)[&"FastTree support value"=0.982]:0.04969000000000001)[&"FastTree
support value"=0.855]:0.01707999999999984)[&"FastTree support
value"=0.944]:0.02273000000000014,(XP_008569440.1:0.0945999999999998,(XP_006156
438.1:0.12711000000000006,(NP_002454.1:0.01259999999999945,XP_002830747.1:0.0
2193999999999985)[&"FastTree support value"=1.0]:0.09565000000000001)[&"FastTree
support value"=0.549]:0.02519000000000268)[&"FastTree support
value"=0.956]:0.03535000000000215)[&"FastTree support
value"=0.36]:0.00645999999999688)[&"FastTree support

value"=0.934]:0.0578599999999998)[&"FastTree support
value"=1.0]:0.27744000000000035,(XP_004675614.2.2:0.26743000000000006,((NP_038634.1:0.11575999999999986,NP_034976.1:0.39536000000000016)[&"FastTree support
value"=0.932]:0.08265999999999973,((NP_001003134.1:0.04121000000000041,XP_032211398.1:0.04147999999999996)[&"FastTree support
value"=1.0]:0.09720999999999957,((XP_017508130.1:0.08051999999999992,(XP_005202045.1:0.1904300000000001,XP_014388412.1:0.13849)[&"FastTree support
value"=0.939]:0.04443000000000019)[&"FastTree support
value"=0.357]:0.012640000000000207,((NP_002453.2.2:0.011569999999999858,NP_001127618.1:0.009739999999999986)[&"FastTree support
value"=1.0]:0.08596000000000004,(XP_008569442.1:0.08959000000000028,(XP_006156437.1:0.05921000000000021,XP_004466363.1:0.16312000000000015)[&"FastTree support
value"=0.575]:0.022560000000000358)[&"FastTree support
value"=0.74]:0.01578000000000035)[&"FastTree support
value"=0.929]:0.02367000000000008)[&"FastTree support
value"=0.909]:0.02259999999999973)[&"FastTree support
value"=0.842]:0.03420000000000023)[&"FastTree support
value"=0.965]:0.08903000000000016)[&"FastTree support
value"=0.963]:0.11198999999999959)[&"FastTree support
value"=1.0]:0.1548999999999996)[&"FastTree support
value"=0.231]:0.0695800000000002)[&"FastTree support
value"=0.806]:0.08007000000000009)[&"FastTree support
value"=0.923]:0.06680000000000019)[&"FastTree support
value"=0.843]:0.09421999999999997)[&"FastTree support
value"=0.998]:0.3017500000000002)[&"FastTree support
value"=0.961]:0.19616999999999996)[&"FastTree support
value"=0.997]:0.33252999999999977,(((KAG0561847:0.18534999999999968,KAG0619429:0.19228999999999985)[&"FastTree support
value"=0.999]:0.51946,(KAH9291961:0.38612,((KAH9290598:0.4265100000000004,KAH9320939:0.33847000000000005)[&"FastTree support
value"=0.806]:0.07132000000000005,((PWZ56863:0.13614000000000015,PWZ56864:0.16263000000000005)[&"FastTree support
value"=1.0]:0.33061000000000007,((AAB71956:0.08760000000000012,NP_176252:0.0565800000000003)[&"FastTree support
value"=1.0]:0.3312900000000001,(XP_002297993.1:0.21208000000000027,(XP_002303204:0.0950899999999999,XP_024439231.1:0.22184999999999988)[&"FastTree support
value"=0.935]:0.039049999999999585)[&"FastTree support
value"=0.984]:0.0981500000000004)[&"FastTree support
value"=0.97]:0.11765000000000025)[&"FastTree support
value"=1.0]:0.3192300000000001)[&"FastTree support
value"=0.149]:0.07512000000000008)[&"FastTree support
value"=0.999]:0.4563900000000003)[&"FastTree support
value"=1.0]:0.7685200000000001,((((EFJ28901.1:0.1533000000000002,EFJ33653.1:0.1462600000000000028)[&"FastTree support

value"=0.723]:0.03216000000000019,(((OAE31801.1:0.11254000000000008,KAG0632288.1:0.10988000000000042)[&"FastTree support value"=0.21]:0.013679999999999914,('KAG0555995.1':0.0438799999999997,(XP_024391061.1:0.02320000000000011,XP_024368367.1:0.02242000000000033)[&"FastTree support value"=1.0]:0.09776000000000007)[&"FastTree support value"=1.0]:0.07364999999999977)[&"FastTree support value"=0.623]:0.0221600000000004,(KAI5073815.1:0.09428999999999998,(KAH9330549.1:0.07064999999999966,((XP_006385192.1:0.05722999999999967,('KAG7649995.1':0.01034000000000238,'NP_172500.1':0.01949000000000023)[&"FastTree support value"=0.977]:0.03385999999999978)[&"FastTree support value"=0.249]:0.01313999999999993,(ONM04707.1:0.2654899999999998,(XP_008646219.1:0.018819999999999837,ACG47836.1:0.03758999999999979)[&"FastTree support value"=0.969]:0.04023999999999983)[&"FastTree support value"=0.654]:0.022070000000000256)[&"FastTree support value"=0.99]:0.06135999999999964)[&"FastTree support value"=1.0]:0.10073000000000043)[&"FastTree support value"=0.951]:0.04373999999999967)[&"FastTree support value"=0.382]:0.03905000000000003)[&"FastTree support value"=1.0]:0.96848,(XP_042924642.1:0.54013,(EFJ23099.1:0.08311000000000002,((XP_002987566.1:0.09419000000000022,(EFJ15761.1:0.13917000000000002,(KAI5072318.1:0.10263,(XP_002302631.1:0.0719299999999996,(AQK88296.1:0.08442999999999978,(NP_001190448.1:0.09311000000000025,XP_002299468.1:0.041400000000000325)[&"FastTree support value"=0.927]:0.03823999999999961)[&"FastTree support value"=0.513]:0.04086999999999996)[&"FastTree support value"=1.0]:0.1423700000000001)[&"FastTree support value"=0.982]:0.05819999999999981)[&"FastTree support value"=0.938]:0.037599999999999856)[&"FastTree support value"=0.671]:0.011489999999999778,((KAI5058380.1:0.09436,(PTQ45603.1:0.08793000000000006,(PTQ29980.1:0.0809000000000002,KAG0556007.1:0.15458000000000016)[&"FastTree support value"=0.677]:0.026450000000000085)[&"FastTree support value"=0.599]:0.028300000000000214)[&"FastTree support value"=0.418]:0.013129999999999864,((KAI5602084.1:0.09548999999999985,(XP_006375094.1:0.04051999999999989,(AAF22292.1:0.09206000000000003,NP_850420.1:0.1290300000000002)[&"FastTree support value"=1.0]:0.08890999999999982)[&"FastTree support value"=0.626]:0.024630000000000152)[&"FastTree support value"=0.371]:0.01817000000000002,(PWZ36850.1:0.18305000000000016,(NP_001147100.1:0.11617999999999995,(AAF79238.1:0.05282000000000009,XP_002315854.1:0.03460999999999981)[&"FastTree support value"=0.973]:0.05162999999999984)[&"FastTree support value"=0.962]:0.05686999999999976)[&"FastTree support value"=0.574]:0.03534000000000015)[&"FastTree support value"=0.996]:0.04985999999999979)[&"FastTree support value"=0.942]:0.03076999999999964)[&"FastTree support value"=0.944]:0.07057000000000002)[&"FastTree support value"=0.999]:0.47899)[&"FastTree support

value"=0.536]:0.16866000000000003)[&"FastTree support
value"=0.999]:0.5471400000000002,((XP_014153758.1:0.36299000000000037,(XP_00434
7890.1:0.2255200000000004,(XP_001749319.1:0.31578000000000017,(XP_026693152.1:
0.231510000000001,((XP_030853442.1.2:0.0,XP_030853442.1:0.0):0.144620000000000
64,(((NP_001024332.1:0.20892999999999962,KMZ10000.1:0.1586799999999995)[&"Fast
Tree support
value"=0.862]:0.047539999999999694,(PAA65118.1:0.05200999999999224,(PAA78248.1
:0.06203999999999965,(PAA59145.1:0.041669999999999874,PAA64382.1:0.3089599999
999999)[&"FastTree support value"=0.887]:0.03683000000000014)[&"FastTree support
value"=0.665]:0.03420999999999985)[&"FastTree support
value"=1.0]:0.24380999999999986)[&"FastTree support
value"=0.945]:0.04056000000000015,(XP_035683496.1:0.10715999999999948,(XP_03281
4666.1:0.06838999999999995,((XP_005165639.1:0.06280999999999981,((XP_025915522
.1:0.000550,(KAE8583055.1:0.04349000000000025,XP_028570166.1:0.015900000000000
247)[&"FastTree support value"=0.996]:0.030429999999999957)[&"FastTree support
value"=0.825]:0.00714000000000059,(EPQ17174.1:0.000540,(ELW62001.1:0.0023299999
99999721,(EAW87759.1:0.0023599999999994736,(XP_012378586.1:0.0,BAB27759.1:0.0):
0.000550)[&"FastTree support value"=0.52]:0.000550)[&"FastTree support
value"=0.342]:0.002349999999999852)[&"FastTree support
value"=0.994]:0.024169999999999803)[&"FastTree support
value"=0.752]:0.015230000000000743)[&"FastTree support
value"=0.974]:0.027749999999999275,((XP_031757197.1:0.1443499999999993,(XP_0259
44940.1:0.020990000000000286,(EPQ08653.1:0.008760000000000545,(XP_016856477.1:
0.004369999999999763,XP_027623811.1:0.00743000000000027)[&"FastTree support
value"=0.536]:0.006230000000000402)[&"FastTree support
value"=0.994]:0.03732999999999986)[&"FastTree support
value"=0.927]:0.028849999999999376)[&"FastTree support
value"=0.993]:0.05383000000000049,((NP_001025299.1:0.0,XP_021326548.1:0.0):0.0491
9000000000029,(XP_031753735.1:0.04347000000000012,((XP_014389433.1:0.023979999
99999989,XP_025920181.1:0.007229999999999848)[&"FastTree support
value"=0.747]:0.003080000000000638,(XP_028568434.1:0.011519999999999087,(XP_006
161648.2.2:0.0,NP_001005360.1:0.0):0.022149999999999892)[&"FastTree support
value"=0.993]:0.028980000000000672)[&"FastTree support
value"=0.776]:0.009179999999999744)[&"FastTree support
value"=0.958]:0.022520000000000095)[&"FastTree support
value"=0.922]:0.021160000000000068)[&"FastTree support
value"=0.924]:0.02507000000000037)[&"FastTree support
value"=0.895]:0.021730000000000693)[&"FastTree support
value"=0.99]:0.04248999999999992)[&"FastTree support
value"=0.329]:0.01747999999999994)[&"FastTree support
value"=0.311]:0.019529999999999603)[&"FastTree support
value"=0.208]:0.031220000000000248)[&"FastTree support
value"=0.978]:0.07850999999999964)[&"FastTree support
value"=0.821]:0.07812000000000019)[&"FastTree support

value"=0.853]:0.07108999999999988)[&"FastTree support
value"=1.0]:0.5575300000000003,((NP_012926.1:0.34209999999999985,(KNE68830.1:0.1
8889000000000022,((OAJ44422.1:0.17323000000000022,OUM62108.1:0.1384400000000
0012)[&"FastTree support
value"=0.106]:0.05645999999999955,(KXN66323.1:0.20882999999999985,(XP_748106.1
:0.2138,(XP_011389257.1:0.24006999999999978,XP_006458578.1:0.15703000000000022
)[&"FastTree support value"=0.954]:0.05810000000000004)[&"FastTree support
value"=0.214]:0.03526000000000007)[&"FastTree support
value"=0.372]:0.03501000000000021)[&"FastTree support
value"=0.835]:0.05058000000000007)[&"FastTree support
value"=0.637]:0.06985000000000019)[&"FastTree support
value"=1.0]:0.3951199999999999,((XP_042914770.1:0.8702399999999999,(KAH9327796.
1:0.24736000000000002,(((PWZ09977.1:0.11497000000000002,ONM18162.1:0.57389)[&"
FastTree support
value"=0.718]:0.06762999999999986,(XP_052310486.1:0.05896999999999997,AAC61784
.1:0.1171000000000002)[&"FastTree support
value"=0.934]:0.03318999999999983)[&"FastTree support
value"=0.991]:0.07850000000000001,(KAH9304002.1:0.1153900000000001,(EFJ35472.1:
0.26708,((EFJ37641.1:0.000550,EFJ15047.1:0.02188999999999965)[&"FastTree support
value"=0.999]:0.06955,((KAI5070335.1:0.05091000000000001,KAI5070758.1:0.059680000
00000018)[&"FastTree support
value"=0.991]:0.04191000000000114,(PTQ35749.1:0.03739999999999988,(KAG0555682
.1:0.0720200000000002,(KAG0554580.1:0.00764999999999935,XP_024362051.1:0.0689
9999999999995)[&"FastTree support value"=1.0]:0.08282999999999996)[&"FastTree
support value"=0.585]:0.01765999999999787)[&"FastTree support
value"=0.973]:0.03718000000000021)[&"FastTree support
value"=0.581]:0.01827999999999852)[&"FastTree support
value"=0.893]:0.02960999999999914)[&"FastTree support
value"=0.976]:0.05019000000000018)[&"FastTree support
value"=0.72]:0.03026000000000176)[&"FastTree support
value"=0.797]:0.03208000000000011)[&"FastTree support
value"=0.998]:0.24827999999999983)[&"FastTree support
value"=0.987]:0.1961400000000002,(((KNE61418.1:0.30681,KNE67543.1:0.20804)[&"Fast
Tree support
value"=1.0]:0.3894099999999998,(KXN67416.1:0.32962999999999987,((XP_006461708.1:
0.15615999999999985,XP_011392073.1:0.1264400000000001)[&"FastTree support
value"=0.987]:0.09159000000000006,(NP_013100.1:0.29888000000000003,XP_746923.1:
0.11953000000000014)[&"FastTree support
value"=0.918]:0.08707999999999982)[&"FastTree support
value"=0.783]:0.06079999999999965)[&"FastTree support
value"=0.951]:0.10395999999999983)[&"FastTree support
value"=0.991]:0.14464999999999995,(XP_001750431.1:0.7875399999999999,(XP_004348
308.1:0.22115000000000018,(XP_035676386.1:0.08980999999999995,(((XP_006821224.
1:0.0904799999999999,XP_030827871.1:0.09104000000000001)[&"FastTree support

value"=0.711]:0.014720000000000066,(NP_741403.2:0.25044999999999984,(NP_001259
946.1:0.14388999999999985,PAA85687.1:0.16789999999999994)[&"FastTree support
value"=0.747]:0.025110000000000188)[&"FastTree support
value"=0.94]:0.03971999999999998)[&"FastTree support
value"=0.421]:0.023120000000000003,(XP_002129967.2:0.22455999999999987,(XP_03281
9300.1:0.06333000000000011,((XP_025940269.1:0.000550,(XP_028602039.1:0.01489999
9999999913,(XP_031753959.1:0.005539999999999878,NP_957216.1:0.03270000000000
017)[&"FastTree support value"=0.882]:0.009310000000000151)[&"FastTree support
value"=0.063]:0.002489999999999881)[&"FastTree support
value"=0.863]:0.009409999999999918,((XP_014394711.1:0.002460000000001288,XP_01
2382650.2:0.004849999999999799)[&"FastTree support
value"=0.0]:0.000550,(NP_001392186.1:0.0,NP_001317309.1:0.0,XP_006168142.1:0.0):0.
000550)[&"FastTree support value"=0.783]:0.005310000000000148)[&"FastTree support
value"=0.957]:0.028639999999999777)[&"FastTree support
value"=0.926]:0.031120000000000037)[&"FastTree support
value"=0.973]:0.05909999999999993)[&"FastTree support
value"=0.593]:0.01908999999999983)[&"FastTree support
value"=1.0]:0.19187999999999983)[&"FastTree support
value"=0.688]:0.10299000000000014)[&"FastTree support
value"=0.928]:0.07160000000000011)[&"FastTree support
value"=0.916]:0.06679000000000013)[&"FastTree support
value"=0.81]:0.06495000000000006)[&"FastTree support
value"=0.835]:0.13783000000000012)[&"FastTree support
value"=0.992]:0.44665)[&"FastTree support value"=1.0]:0.5211000000000001)[&"FastTree
support value"=0.484]:0.05521000000000002)[&"FastTree support
value"=0.976]:0.24201000000000006)[&"FastTree support
value"=0.882]:0.16798000000000002)[&"FastTree support
value"=0.124]:0.08767000000000014):0.45608000000000004);
end;
