# Supplementary material for: Antiviral Mx proteins have an ancient origin and widespread distribution among eukaryotes
Source: Proc Natl Acad Sci U S A. 2025 Jan 24;122(4):e2416811122. doi: 10.1073/pnas.2416811122 (PMC11789081; doi:10.1073/pnas.2416811122)
Supplement: Supplementary file 18 — Dataset S17 (PDF) [file pnas.2416811122.sd17.pdf]

Dataset S17. Suppl_Figure_5_IQTree

```
#NEXUS
begin taxa;
	dimensions ntax=549;
	taxlabels
	GMI12809.1
	GMI47362.1
	OQR85161.1
	RLO06844.1
	KAF0740912.1
	CAH0521473.1
	KAG2764740.1
	GMF38092.1
	KAH7500220.1
	TMW56688.1
	CCI11042.1
	CCA17876.1
	CEP02405.1
	XP_004348308.1
	NP_741403.2
	XP_002129967.2
	NP_001259946.1
	XP_035676386.1
	XP_030827871.1
	PAA85687.1
	XP_006821224.1
	XP_032819300.1
	NP_957216.1
	XP_025940269.1
	XP_028602039.1
	XP_006168142.1
	NP_001317309.1
	NP_001392186.1
	XP_014394711.1
	XP_012382650.2
	XP_031753959.1
	XP_001750431.1
	XP_014148015.1
	NP_013100.1
	XP_746923.1
	KXN67416.1
	XP_011392073.1
	XP_006461708.1
```

KNE61418.1
KNE67543.1
ABB13595.1
XP_001009829.2
XP_042914770.1
PWZ09977.1
KAH9304002.1
EFJ37641.1
EFJ15047.1
KAI5070758.1
KAI5070335.1
PTQ35749.1
KAG0555682.1
XP_024362051.1
KAG0554580.1
EFJ35472.1
XP_052310486.1
AAC61784.1
KAH9327796.1
ONM18162.1
ETO36135.1
KOO24608.1
KAJ9467322.1
KAJ9466576.1
XP_008860500.1
XP_004185630.1
XP_042924642.1
PTQ29980.1
XP_002302631.1
AQK88296.1
XP_002299468.1
NP_001190448.1
PTQ45603.1
KAI5058380.1
KAI5072318.1
XP_002987566.1
EFJ15761.1
EFJ23099.1
KAH9306600.1
KAI5602084.1
XP_006375094.1
AAF22292.1
NP_850420.1
PWZ36850.1

XP_002315854.1
NP_001147100.1
AAF79238.1
KAG0556007.1
XP_005788601.1
CCW59714.1
KAG5490335.1
XP_003872337.1
KAI5685071.1
XP_028887534.1
EKF32958.1
ESL10883.1
RHW73545.1
CCC89860.1
KAH8605762.1
CAD2212698.1
KNH06820.1
KAF0852279.1
XP_002371703.1
'XP_019914840.1'
'XP_012763851.2_1'
'EUR69800.1_1'
XP_028867889.1
'CAE8701890.1'
XP_001016567.2
'CAD8140652.1_1'
XP_001032891.1
XP_001029982.1
XP_001029985.2
OUM67143.1
KNE54706.1
KXN72852.1
OAJ38404.1
XP_006459124.1
XP_011389557.1
XP_752563.1
NP_014854.2
WGU15254.1
XP_013756556.1
CAH6419740.1
AYV81982.1
CAH6421112.1
ARF12445.1
QKF93607.1

QKU35298.1
AYV83919.1
PSC76263.1
PRW56740.1
XP_005849062.1
GMH36208.1
GMH43921.1
CAG9460856.1
GJP35534.1
CAI5480041.1
KAJ7294545.1
XP_024380180.1
XP_024367947.1
KAG0619429.1
KAG0561847.1
KAH9290598.1
KAH9291961.1
KAH9320939.1
KAH9325151.1
KAH9300179.1
KAH9314974.1
KAF8079489.1
OAP19580.1
OAP13972.1
OAP13353.1
KAF5727250.1
XP_002303204.3
XP_002297993.1
XP_024439231.1
KAK1401877.1
KAH0683503.1
KAF8391993.1
XP_058079501.1
XP_038984915.1
PWZ56863.1
PWZ56864.1
EFJ22917.1
CAD7955538.1
CAD7971727.1
CAE6914669.1
CAI4004018.1
CAI3978736.1
OLP81297.1
CAE7315868.1

CAE8582582.1
KAI0562045.1
KAI0559778.1
KAI0557988.1
XP_005716602.1
CEM15039.1
GBG30247.1
GHP04420.1
OSX73843.1
OSX70108.1
KAK3283006.1
KAJ1474099.1
KAJ1495567.1
KAJ1474882.1
KAJ1487321.1
KAJ1442373.1
CBN78192.1
KAG5184845.1
XP_009039543.1
KAJ8614481.1
XP_009039855.1
XP_009038401.1
KAJ1460532.1
CAH0370685.1
KAH8053135.1
KAJ8603921.1
KAJ1460259.1
KAG5178451.1
CBN76986.1
KAG5185531.1
KAJ1428896.1
KAG5183739.1
XP_005711749.1
KAI0564035.1
PXF49978.1
KAI9324922.1
'KAJ3066410.1'
KAI8836453.1
KAJ3350919.1
TDH66190.1
XP_024574100.1
KAG1692046.1
KAG3062152.1
XP_009533587.1

KAI9918701.1
RMX63821.1
XP_005823288.1
KAJ1441760.1
KAJ1637655.1
KAI8587516.1
KXS17655.1
XP_047808890.1
OLL24579.1
KAI9096888.1
RSH87279.1
XP_041144356.1
XP_746402.1
KAJ5704467.1
KAI9774215.1
MCJ1392161.1
XP_002543522.1
XP_751069.1
XP_026607910.1
XP_748757.2
XP_040633937.1
XP_754266.1
XP_043140374.1
XP_021869222.1
TVY17522.1
KAF9951223.1
ABI33144.1
XP_006815062.1
CAH1802128.1
PAA74204.1
PAA76532.1
PAA92268.1
PAA69582.1
PAA83069.1
PAA94353.1
XP_035690836.1
XP_019617847.1
XP_002608668.1
XP_003973512.2.2
NP_891987.2.2
XP_009304072.1
AGU16245.1
XP_007904885.1
XP_032888405.1

XP_028583068.1
XP_005167721.2.2
NP_001007285.1
XP_009815891.1
XP_025933558.1
XP_028583072.1
XP_015269256.1
XP_006156438.1
XP_002830747.1
NP_002454.1
NP_001003133.1
XP_032211320.1
XP_017508123.1
XP_005885748.1
XP_012586448.1
NP_776366.1
XP_008569440.1
XP_004675614.2.2
XP_004466363.1
NP_002453.2.2
NP_001127618.1
XP_005202045.1
XP_008569442.1
XP_014388412.1
NP_034976.1
NP_038634.1
XP_017508130.1
XP_032211398.1
NP_001003134.1
XP_006156437.1
XP_031752404.1
XP_032804093.1
KAI0213370.1
KAI0208044.1
KAI0218869.1
XP_046565196.1
XP_046563124.1
XP_046562919.1
XP_046352527.2
XP_048248476.1
XP_048258111.1
XP_046352531.2
XP_048248472.1
XP_048248474.1

XP_048248473.1
ABI53802.1
XP_046563126.1
XP_046565195.1
XP_046563125.1
XP_750654.1
XP_006461472.1
XP_006461433.1
XP_006457072.1
KAI3646081.1
KAJ9515210.1
GAX85982.1
KAG2488600.1
XP_042924875.1
XP_042924848.1
XP_042923301.1
ETO25748.1
OAJ38670.1
PAA87312.1
PAA68234.1
NP_495986.3.3
NP_610941.1
XP_006813643.1
XP_030843280.1
XP_018667792.1
XP_032818114.1
XP_021332524.1
XP_028587646.1
XP_025913835.1
XP_023440724.1
XP_005873264.1
NP_598513.1
NP_056375.2.2
XP_006163024.2.2
XP_031757388.1
MEN2496893
XP_002602331.1
XP_019637857.1
KAI5073815.1
XP_008646219.1
ACG47836.1
ONM04707.1
XP_006385192.1
'KAG7649995.1'

'NP_172500.1'
KAH9330549.1
EFJ33653.1
EFJ28901.1
KAG0632288.1
'KAG0555995.1'
XP_024368367.1
XP_024391061.1
OAE31801.1
XP_042918632.1
PWZ11893.1
NP_001130364.1
AAF87857.1
XP_002317496.2
KAI5058044.1
KAH9322298.1
EFJ18064.1
PTQ33908.1
KAG0628798.1
XP_002683545.1
XP_044544418.1
XP_044559198.1
XP_004182822.1
XP_008857507.1
XP_004340186.1
KAH3742895.1
KYQ94066.1
XP_004366192.1
XP_020428321.1
XP_012754836.1
KAF2073270.1
XP_003288319.1
XP_645576.2
PRP82286.1
QYA18543.1
XP_005775651.1
XP_005767412.1
KOO34265.1
EWM28268.1
KAG5184668.1
CBN78455.1
GMI61978.1
GMH67967.1
GMH92561.1

GMI07688.1
EJK67908.1
KAI2494507.1
CAB9516894.1
GKY99394.1
XP_009032466.1.2
XP_009032466.1
XP_042920073.1
PWZ44616.1
NP_001189935.1
XP_002309632.3
EFJ19523.1
KAH9308354.1
KAI5064281.1
PTQ34556.1
KAG0561482.1
KAG0605142.1
XP_004336224.1
KAH3767868.1
XP_002649212.1
XP_003288465.1
KAF2077035.1
XP_020436215.1
XP_012753198.1
XP_004360608.1
KYQ90260.1
CEM26963.1
OII76931.1
KAH8582109.1
POM84969.1
XP_667128.1
XP_001617280.1
XP_028539355.1
UKJ88078.2
XP_004833148.1
GFE54186.1
XP_012766661.1
GIX62800.1
KAH0479249.1
PHJ24853.1
KFG43683.1
ARF10781.1
ARF10780.1
QFG74057.1

KAF5834941.1
KAG8459568.1
KOO23261.1
KAH8061769.1
OUS45493.1
XP_001419538.1
PRP82121.1
XP_004338334.1
TMW65229.1
KAG1689960.1
KAE9027747.1
KAG7385474.1
KAH7489187.1
KAE8986371.1
POM76499.1
KAG7377001.1
XP_008910862.1
KAF1774311.1
KAI9982172.1
'XP_005785253.1'
XP_005792501.1
KAG8471152.1
KOO34643.1
XP_005775544.1
ATZ81043.1
VBB18790.1
ARF10282.1
QKF94243.1
ARF11508.1
ARF08414.1
AYV78912.1
ARF09562.1
AYV76902.1
QFG74079.1
AYV75702.1
ATZ80405.1
PRP80825.1
XP_004355605.1
XP_020436927.1
AER35077.1
KAF2075389.1
XP_003292385.1
XP_044553261.1
XP_044569353.1

XP_044550536.1
XP_002681690.1
XP_004347890.1
NP_001024332.1
KMZ10000.1
XP_006812840.1
XP_032814666.1
XP_025944940.1
XP_031757197.1
EPQ08653.1
XP_006496668.1
XP_016856477.1
XP_012379251.1
XP_027623811.1
XP_031753735.1
XP_028568434.1
XP_025920181.1
XP_006510037.1
NP_001005360.1
XP_012381548.1
XP_006161648.2.2
XP_014389433.1
NP_001025299.1
XP_021326548.1
XP_030853442.1
XP_030853442.1.2
XP_005165639.1
PAA65118.1
PAA78248.1
PAA59145.1
PAA64382.1
XP_028570166.1
XP_025915522.1
EPQ17174.1
BAB27759.1
ELW62001.1
EAW87759.1
XP_012378586.1
KAE8583055.1
XP_035683496.1
XP_026693152.1
XP_014148725.1
XP_014153758.1
XP_001749319.1

GMH85941.1
GMI59178.1
GMI25649.1
GMI62840.1
GMH55978.1
KAH3761456.1
XP_004368323.1
XP_012754660.1
KYR01170.1
XP_003294436.1
PRP81066.1
XP_004184473.1
EMS16943.1
PRP82407.1
NP_012926.1
OUM62108.1
OAJ44422.1
KXN66323.1
XP_011389257.1
XP_006458578.1
KNE68830.1
XP_748106.1
KAJ1432693.1
GAX23670.1
CAB9512103.1
XP_002296064.1
XP_018636213.1
;
end;

begin trees;
tree tree_1 = [&R]
[&branchAttributeNames={"Value"}](GMI12809.1:0.0678889215,GMI47362.1:0.076294607
5,((((OQR85161.1:0.0594980752,(RLO06844.1:0.0366847571,KAF0740912.1:0.011118383
7)[&Value="89.2/100"]:0.0535209203)[&Value="99.6/100"]:0.0855889505,((CAH0521473.1
:0.0713580818,(KAG2764740.1:0.0448025475,(GMF38092.1:0.0269845548,KAH7500220.
1:0.0237912239)[&Value="64.4/95"]:0.0102931702)[&Value="84.8/94"]:0.0214727165)[&V
alue="94.3/99"]:0.052805656,(TMW56688.1:0.0992608621,(CCI11042.1:0.061207702,CC
A17876.1:0.0121541228)[&Value="100/100"]:0.1629882525)[&Value="44.9/97"]:0.0219099
895)[&Value="76.5/97"]:0.0302481607)[&Value="99.7/100"]:0.1341831094,((((CEP02405.1
:0.3409441834,(((XP_004348308.1:0.2700695874,((((NP_741403.2:0.3450338429,XP_0021
29967.2:0.3362773597)[&Value="78.9/85"]:0.0318322525,(NP_001259946.1:0.202352770
9,((((XP_035676386.1:0.1016989887,XP_030827871.1:0.1587361281)[&Value="55.4/75"]:0
.0544808115,PAA85687.1:0.2363162177)[&Value="53.4/74"]:0.0176767818,XP_00682122

4.1:0.1026471898)[&Value="88.7/97"]:0.0516882253,(XP_032819300.1:0.1191829641,(NP
_957216.1:0.0282729937,((XP_025940269.1:0.0320827837,XP_028602039.1:0.037114923
8)[&Value="6.4/87"]:0.0032989501,((XP_006168142.1:0.000003,(NP_001317309.1:0.0034
80187,NP_001392186.1:0.0107548311)[&Value="0/57"]:0.000003)[&Value="0/59"]:0.0000
02,(XP_014394711.1:0.0346853681,(XP_012382650.2:0.0276073795,XP_031753959.1:0.2
06812166)[&Value="96.6/60"]:0.0550549404)[&Value="76.1/59"]:0.0092442861)[&Value="
95.8/59"]:0.0251947444)[&Value="93.4/100"]:0.0400793593)[&Value="97.9/100"]:0.09107
08427)[&Value="88.4/100"]:0.0458854885)[&Value="54.7/96"]:0.0520901892)[&Value="77.
5/88"]:0.0213618732)[&Value="95.7/97"]:0.1182115201,XP_001750431.1:0.5023335495)[
&Value="93.9/99"]:0.0793504976,XP_014148015.1:0.444179774)[&Value="3.2/32"]:0.0108
36689)[&Value="95.9/98"]:0.0761051351,(((NP_013100.1:0.3775674131,(XP_746923.1:0.2
10063585,KXN67416.1:0.2822490943)[&Value="83.4/100"]:0.0479792947)[&Value="82.7/
73"]:0.0598889565,((XP_011392073.1:0.1409203317,XP_006461708.1:0.1449332531)[&Va
lue="96.3/100"]:0.1041142794,(KNE61418.1:0.1850042748,KNE67543.1:0.191054972)[&V
alue="100/100"]:0.3201476412)[&Value="35.2/26"]:0.0244376514)[&Value="93.6/99"]:0.09
62404118,(ABB13595.1:0.2251337632,XP_001009829.2:0.2514640683)[&Value="100/100"
]:0.5801205573)[&Value="88.3/92"]:0.0689634502)[&Value="41.8/44"]:0.0234468415,((((X
P_042914770.1:0.472808632,(PWZ09977.1:0.0503386592,(((KAH9304002.1:0.106521253
2,(((EFJ37641.1:0.0040838504,EFJ15047.1:0.000002)[&Value="95.5/100"]:0.0358186533,((
KAI5070758.1:0.068281104,KAI5070335.1:0.0724232769)[&Value="93.1/100"]:0.03142006
7,(PTQ35749.1:0.0638829103,(KAG0555682.1:0.0517988003,(XP_024362051.1:0.0553492
959,KAG0554580.1:0.0075072803)[&Value="98.9/100"]:0.0432799622)[&Value="31.3/100"
]:0.009273545)[&Value="83.1/100"]:0.0172448598)[&Value="92.6/100"]:0.0310959833)[&V
alue="50.1/99"]:0.0129889764,EFJ35472.1:0.5357792395)[&Value="84.6/99"]:0.03707741
48)[&Value="87.1/98"]:0.0358483643,((XP_052310486.1:0.0760740069,AAC61784.1:0.168
7787488)[&Value="97.5/100"]:0.0816447872,KAH9327796.1:0.227315298)[&Value="85.2/
98"]:0.0231295284)[&Value="77.6/81"]:0.0258768766,ONM18162.1:0.2389906887)[&Valu
e="46.5/75"]:0.0206665662)[&Value="99.3/99"]:0.1789261291)[&Value="94.1/100"]:0.1166
362981,ETO36135.1:0.4848981032)[&Value="94/100"]:0.1143914689,(((KOO24608.1:0.45
1855955,(((KAJ9467322.1:0.3654463911,KAJ9466576.1:0.2074603371)[&Value="99.9/100"
]:0.3540057989,(XP_008860500.1:0.101308856,XP_004185630.1:0.1129685333)[&Value="
100/100"]:0.3659946682)[&Value="30.8/47"]:0.0813778767,((XP_042924642.1:0.4652029
952,(PTQ29980.1:0.0898035689,((((XP_002302631.1:0.0512839365,AQK88296.1:0.113471
5149)[&Value="74.6/98"]:0.0387337426,(XP_002299468.1:0.0506846598,NP_001190448.1
:0.087232764)[&Value="40/89"]:0.0097282556)[&Value="99.7/100"]:0.1182412141,(((PTQ4
5603.1:0.0783386099,KAI5058380.1:0.091997812)[&Value="85.6/76"]:0.0274559273,(KAI
5072318.1:0.0663429563,(((XP_002987566.1:0.0649269803,EFJ15761.1:0.1215099703)[&
Value="73.4/99"]:0.0090796467,EFJ23099.1:0.1857471834)[&Value="93.5/100"]:0.030769
9587,KAH9306600.1:0.0822703807)[&Value="70.3/65"]:0.0082590987)[&Value="81.6/53"]:
0.0115485358)[&Value="87.2/62"]:0.0147459491,(KAI5602084.1:0.0873258282,(((XP_0063
75094.1:0.0483993676,(AAF22292.1:0.0862330605,NP_850420.1:0.1133448567)[&Value=
"90.3/100"]:0.034540745)[&Value="2.2/84"]:0.0141699614,PWZ36850.1:0.1343383867)[&
Value="36.9/94"]:0.0133649131,((XP_002315854.1:0.0423411436,NP_001147100.1:0.088
975161)[&Value="81.4/100"]:0.0244984103,AAF79238.1:0.0894162059)[&Value="91.2/100

"]:0.0360642977)[&Value="80.1/100"]:0.0163211978)[&Value="91.8/100"]:0.0312650154)[
&Value="38.5/50"]:0.0130907812)[&Value="88.3/53"]:0.0351380324,KAG0556007.1:0.096
1757273)[&Value="82.2/76"]:0.0557198225)[&Value="100/100"]:0.3344663752)[&Value="7
5.6/99"]:0.1381079216,XP_005788601.1:0.8194765894)[&Value="31.7/57"]:0.0794667832
)[&Value="78.4/93"]:0.0801133313)[&Value="80.8/84"]:0.052322155,(((((((CCW59714.1:0.
1291165948,((KAG5490335.1:0.0791295733,XP_003872337.1:0.071493893)[&Value="77.5
/100"]:0.0322549416,KAI5685071.1:0.0187514821)[&Value="97.4/100"]:0.0762571324)[&V
alue="99.8/100"]:0.1310994062,(XP_028887534.1:0.0364735252,(EKF32958.1:0.0591036
328,ESL10883.1:0.0692254275)[&Value="97.7/100"]:0.0709667784)[&Value="94.1/100"]:0.
0604465521)[&Value="93.2/97"]:0.0614218278,(RHW73545.1:0.0574039865,CCC89860.1
:0.0832984075)[&Value="11.6/73"]:0.0329535202)[&Value="82.7/96"]:0.0743049232,KAH8
605762.1:0.1529385183)[&Value="95.1/97"]:0.145009126,CAD2212698.1:0.7835689289)[
&Value="92.1/100"]:0.1358324577,KNH06820.1:0.6537337457)[&Value="69.9/97"]:0.0843
186963,KAF0852279.1:0.5814343368)[&Value="37.2/78"]:0.0341492976)[&Value="90.8/92
"]:0.0619787714,(((XP_002371703.1:0.2177170955,(('XP_019914840.1':0.0579147011,('XP
_012763851.2_1':0.000003,'EUR69800.1_1':0.0040698852)[&Value="94.6/100"]:0.0582757
465)[&Value="99.1/100"]:0.1774944468,XP_028867889.1:0.4209922203)[&Value="82/100"
]:0.0925693581)[&Value="96.5/100"]:0.1102485948,('CAE8701890.1':0.3756291271,((XP_
001016567.2:0.3180284459,'CAD8140652.1_1':0.4843167633)[&Value="95.8/100"]:0.1365
646279,(XP_001032891.1:0.269851361,(XP_001029982.1:0.1979905051,XP_001029985.2:
0.1967879344)[&Value="100/100"]:0.6735026991)[&Value="96.8/100"]:0.1463807334)[&V
alue="85.1/100"]:0.0706954997)[&Value="57.1/86"]:0.0341521651)[&Value="100/100"]:0.3
609727243,(((OUM67143.1:0.3844717933,((KNE54706.1:0.277562217,KXN72852.1:0.484
6585706)[&Value="81.1/64"]:0.0592203428,(OAJ38404.1:0.2266820089,((XP_006459124.1
:0.2182602093,XP_011389557.1:0.1955409953)[&Value="96.2/100"]:0.1069677223,(XP_7
52563.1:0.2151193838,NP_014854.2:0.5734161005)[&Value="96/100"]:0.1480756972)[&V
alue="92.8/100"]:0.0830356481)[&Value="83.6/94"]:0.0531254799)[&Value="74.2/63"]:0.0
969240976)[&Value="100/100"]:0.7567204695,((WGU15254.1:0.6078917942,XP_0137565
56.1:0.6131607883)[&Value="95.7/100"]:0.3411148633,(CAH6419740.1:0.8619453833,(A
YV81982.1:0.564187458,(CAH6421112.1:0.5805104977,((ARF12445.1:0.5226349848,(QK
F93607.1:0.3029058743,QKU35298.1:0.650227924)[&Value="60.7/100"]:0.0854235409)[&
Value="81.5/100"]:0.1306109063,AYV83919.1:1.0576063334)[&Value="13.4/93"]:0.060122
1565)[&Value="90.2/97"]:0.1467833054)[&Value="96/99"]:0.2610364128)[&Value="99.8/10
0"]:0.6654749945)[&Value="76.3/97"]:0.2363902283)[&Value="98.5/98"]:0.3701212211,((((
((PSC76263.1:0.337402578,(PRW56740.1:0.506975525,XP_005849062.1:0.3509828587)[
&Value="79.6/100"]:0.1216049769)[&Value="99.3/100"]:0.4185258531,((GMH36208.1:0.4
188446129,GMH43921.1:0.4834908177)[&Value="100/100"]:0.491446675,(CAG9460856.1
:0.8807001665,((GJP35534.1:0.1233315251,CAI5480041.1:0.105549908)[&Value="99/100"
]:0.2225554269,((KAJ7294545.1:0.2593849636,((XP_024380180.1:0.0129864788,XP_0243
67947.1:0.0075502711)[&Value="100/100"]:0.21462094,(KAG0619429.1:0.0743223306,KA
G0561847.1:0.118708845)[&Value="92.4/100"]:0.0607985264)[&Value="94.9/100"]:0.0855
146638)[&Value="94.7/100"]:0.1007742849,((((KAH9290598.1:0.2895876371,KAH9291961
.1:0.3464217377)[&Value="85.1/77"]:0.058231497,(KAH9320939.1:0.2899738946,(KAH93
25151.1:0.133941694,(KAH9300179.1:0.1508616474,KAH9314974.1:0.2545762602)[&Val

ue="84.4/100"]:0.1101435827)[&Value="99/100"]:0.3256811033)[&Value="68.2/90"]:0.047
3347924)[&Value="17.5/66"]:0.0183100934,(((((((KAF8079489.1:0.0549050127,((OAP1958
0.1:0.1072408609,OAP13972.1:0.0425571815)[&Value="0/83"]:0.000002,OAP13353.1:0.0
938770927)[&Value="92.2/100"]:0.1195843723)[&Value="99.3/100"]:0.19276421,(KAF572
7250.1:0.1094871315,(XP_002303204.3:0.0782073061,(XP_002297993.1:0.1341631224,X
P_024439231.1:0.148947406)[&Value="89.9/99"]:0.0379988744)[&Value="71.6/98"]:0.013
4864256)[&Value="23.6/95"]:0.0189004908)[&Value="86.2/100"]:0.0202544769,(KAK1401
877.1:0.1075710055,KAH0683503.1:0.2092781983)[&Value="72.9/99"]:0.0150394933)[&V
alue="92/99"]:0.0327978356,KAF8391993.1:0.0711730438)[&Value="97.3/100"]:0.058439
2933,XP_058079501.1:0.1059222308)[&Value="89.9/100"]:0.0530550149,XP_038984915.
1:0.1113739269)[&Value="73.4/100"]:0.0323933781,(PWZ56863.1:0.0622171174,PWZ568
64.1:0.0404146349)[&Value="99.5/100"]:0.1038899872)[&Value="99.4/100"]:0.139180180
4)[&Value="95.5/96"]:0.0887648625,EFJ22917.1:0.4701774656)[&Value="92.1/99"]:0.1002
50155)[&Value="98.6/99"]:0.2306031036)[&Value="99.8/100"]:0.4226635753)[&Value="72.
6/99"]:0.0914933983)[&Value="92.5/100"]:0.1894386808)[&Value="91.9/100"]:0.21776253
64,((((CAD7955538.1:0.1461869666,CAD7971727.1:0.1368995564)[&Value="100/100"]:0.
5507302078,((CAE6914669.1:0.2463729269,(CAI4004018.1:0.1252296791,(CAI3978736.1
:0.1032719345,(OLP81297.1:0.1531780285,(CAE7315868.1:0.1687141969,CAE8582582.1
:0.2303148868)[&Value="92/100"]:0.0565102743)[&Value="91.6/100"]:0.064704883)[&Val
ue="97.4/100"]:0.0975159008)[&Value="82.1/100"]:0.0947688329)[&Value="100/100"]:0.5
841196802,((KAI0562045.1:0.2783492968,KAI0559778.1:0.7921144636)[&Value="97.4/10
0"]:0.2415071397,(KAI0557988.1:0.2776802767,XP_005716602.1:0.6691841903)[&Value=
"95.3/100"]:0.1788756151)[&Value="99.4/100"]:0.314752997)[&Value="69.3/97"]:0.113069
373)[&Value="97.7/100"]:0.314094101,CEM15039.1:0.9825578105)[&Value="87.7/100"]:0.
1949004931,GBG30247.1:1.3767927305)[&Value="92.3/98"]:0.2782051582)[&Value="94.7
/97"]:0.2466262367,((((GHP04420.1:0.6413938469,(((((OSX73843.1:0.0652651588,OSX70
108.1:0.0981158723)[&Value="100/100"]:0.522377391,(KAK3283006.1:0.3834882835,(((K
AJ1474099.1:0.2228126911,KAJ1495567.1:0.0953319682)[&Value="87.3/93"]:0.05444962
78,KAJ1474882.1:0.1312422672)[&Value="57.3/93"]:0.0575279833,KAJ1487321.1:0.26141
21673)[&Value="100/100"]:0.2871331694)[&Value="99.5/100"]:0.2114856199)[&Value="77
.4/33"]:0.0296916462,((((KAJ1442373.1:0.5972690876,(CBN78192.1:0.1722663367,KAG5
184845.1:0.1958858376)[&Value="87.4/100"]:0.0762957989)[&Value="86.9/100"]:0.09692
44724,(XP_009039543.1:0.1964918558,(KAJ8614481.1:0.1433871723,XP_009039855.1:0.
1887289518)[&Value="88.1/100"]:0.0654894548)[&Value="98.1/100"]:0.1491524329)[&Val
ue="98.7/100"]:0.1617350476,(XP_009038401.1:0.2082676325,KAJ1460532.1:0.19824398
84)[&Value="99.9/100"]:0.2651054221)[&Value="67.9/90"]:0.0894881523,(((CAH0370685.
1:0.4028726959,((KAH8053135.1:0.2444763826,KAJ8603921.1:0.1937016688)[&Value="0/
43"]:0.0581460793,KAJ1460259.1:0.1700468476)[&Value="95/99"]:0.1132229172)[&Value
="99.9/100"]:0.1928951074,(KAG5178451.1:0.2589737427,CBN76986.1:0.2987626737)[&
Value="97.8/100"]:0.1019304255)[&Value="90.1/99"]:0.0641894438,KAG5185531.1:0.441
9923638)[&Value="84.2/98"]:0.0774156004)[&Value="91.4/86"]:0.1227155743)[&Value="6
2.2/32"]:0.0528808951,KAJ1428896.1:0.8682486586)[&Value="81.8/36"]:0.0755300872,(K
AG5183739.1:0.5269326595,(XP_005711749.1:0.1959242092,(KAI0564035.1:0.05971086
53,PXF49978.1:0.0810196392)[&Value="91.5/100"]:0.1264023613)[&Value="100/100"]:0.5

215603037)[&Value="98.2/100"]:0.2189641745)[&Value="93.1/100"]:0.151095912)[&Value
="96.6/99"]:0.246812521,((((((KAI9324922.1:0.2012909966,'KAJ3066410.1':0.1517672977)[
&Value="66.8/100"]:0.1269046204,(KAI8836453.1:0.2541607085,KAJ3350919.1:0.441687
5069)[&Value="72.4/98"]:0.1287833533)[&Value="96.8/99"]:0.211803177,(((TDH66190.1:0
.3959003513,XP_024574100.1:0.1099795572)[&Value="30.9/89"]:0.0196127464,(KAG169
2046.1:0.0518659002,(KAG3062152.1:0.0343713696,(XP_009533587.1:0.0454803162,KAI
9918701.1:0.1204842011)[&Value="62.8/100"]:0.019686678)[&Value="89.9/100"]:0.01817
0445)[&Value="78.6/100"]:0.0209399977)[&Value="92.2/95"]:0.0730843871,RMX63821.1:
0.1503461663)[&Value="99.3/100"]:0.2867082321)[&Value="98.4/100"]:0.3225647356,(XP
_005823288.1:0.6680153066,KAJ1441760.1:0.591453073)[&Value="99.2/100"]:0.4743596
305)[&Value="93.3/100"]:0.2790325972,KAJ1637655.1:1.2460240881)[&Value="83.7/84"]:
0.1283107442,((KAI8587516.1:0.8343732297,(KXS17655.1:0.5091946108,XP_047808890.
1:0.6705254064)[&Value="90.2/100"]:0.1823433104)[&Value="97.8/100"]:0.2702469484,((
OLL24579.1:0.5744929119,(((KAI9096888.1:0.619123927,(RSH87279.1:0.5759778467,(((X
P_041144356.1:0.1029697169,XP_746402.1:0.1469082993)[&Value="79.7/100"]:0.034361
704,(KAJ5704467.1:0.119561174,((KAI9774215.1:0.0711735003,MCJ1392161.1:0.0824261
155)[&Value="90.3/100"]:0.0431010335,XP_002543522.1:0.1980754881)[&Value="26.4/67
"]:0.0399511337)[&Value="78.9/68"]:0.0231985569)[&Value="99.8/100"]:0.3464691209,XP
_751069.1:0.7237329081)[&Value="87.9/100"]:0.1447631441)[&Value="18.6/99"]:0.07719
09618)[&Value="82.1/99"]:0.1611830567,(XP_026607910.1:0.405763386,(XP_748757.2:0.
4276773849,(XP_040633937.1:0.2200778457,(XP_754266.1:0.0917462553,XP_043140374
.1:0.0578032642)[&Value="98/100"]:0.142376929)[&Value="99/100"]:0.1986471685)[&Val
ue="84.2/100"]:0.0759287498)[&Value="100/100"]:0.4107467577)[&Value="87.3/100"]:0.1
242080396,(XP_021869222.1:0.4913254446,TVY17522.1:0.4093553189)[&Value="100/100
"]:0.5561068047)[&Value="14.1/86"]:0.0631791424)[&Value="95.2/100"]:0.22674151,KAF9
951223.1:1.0440497415)[&Value="64.7/95"]:0.1172627954)[&Value="98.5/100"]:0.311326
3709)[&Value="93.1/71"]:0.1446130547)[&Value="0/31"]:0.0354958799,ABI33144.1:1.217
3936431)[&Value="96/98"]:0.2204088696,(((XP_006815062.1:0.5236552709,(CAH1802128
.1:0.6029174185,(((PAA74204.1:0.1623214884,PAA76532.1:0.3122673678)[&Value="99.9/
100"]:0.302621085,(PAA92268.1:0.3854587196,PAA69582.1:0.4043489026)[&Value="85.8
/100"]:0.1271851827)[&Value="92.1/100"]:0.156776478,(PAA83069.1:0.1271378661,PAA9
4353.1:0.1997068772)[&Value="100/100"]:0.6976873742)[&Value="96.8/100"]:0.16892248
41)[&Value="89.1/100"]:0.1061206402)[&Value="86.1/99"]:0.0713439903,((XP_035690836.
1:0.2403457733,(XP_019617847.1:0.1520302576,XP_002608668.1:0.1029818222)[&Value
="97.7/100"]:0.1397331139)[&Value="99.7/100"]:0.1827270704,(((((XP_003973512.2.2:0.1
275923724,(NP_891987.2.2:0.0437315919,XP_009304072.1:0.0167018413)[&Value="99.8
/100"]:0.1252853262)[&Value="97.6/100"]:0.1265646985,((((AGU16245.1:0.2020314456,(X
P_007904885.1:0.1506147899,XP_032888405.1:0.1657619277)[&Value="95.5/100"]:0.082
7441325)[&Value="79.5/87"]:0.0376770354,(XP_028583068.1:0.2039272128,(XP_0051677
21.2.2:0.0471693206,NP_001007285.1:0.1022377707)[&Value="100/100"]:0.326029381)[
&Value="77.9/98"]:0.036879054)[&Value="92/87"]:0.0501998018,(((XP_009815891.1:0.103
7401244,XP_025933558.1:0.0663894233)[&Value="100/100"]:0.1732101492,XP_02858307
2.1:0.2117231273)[&Value="18.6/92"]:0.0396251271,XP_015269256.1:0.1374708883)[&Va
lue="49.3/99"]:0.0521222701)[&Value="44.8/84"]:0.0305757125,((((((XP_006156438.1:0.1

511141712,(XP_002830747.1:0.0141943862,NP_002454.1:0.0062790591)[&Value="100/1
00"]:0.0894418401)[&Value="77.7/83"]:0.0150476329,(NP_001003133.1:0.0904094105,XP
_032211320.1:0.1332102974)[&Value="95.7/100"]:0.0471593239)[&Value="14.2/61"]:0.00
82646003,((XP_017508123.1:0.0943710948,(XP_005885748.1:0.0228524839,XP_0125864
48.1:0.2798024471)[&Value="87.3/100"]:0.0191770341)[&Value="83.3/99"]:0.0132845961,
NP_776366.1:0.1282136949)[&Value="83.1/81"]:0.0179204121)[&Value="92.4/80"]:0.0266
963798,XP_008569440.1:0.0415811146)[&Value="99.7/100"]:0.137725018,(XP_00467561
4.2.2:0.1603841138,((XP_004466363.1:0.1170878536,(((((NP_002453.2.2:0.0042227745,N
P_001127618.1:0.0038911276)[&Value="98.9/100"]:0.0585113325,XP_005202045.1:0.141
137942)[&Value="27.3/51"]:0.0245142429,(XP_008569442.1:0.087662729,XP_014388412.
1:0.1169484939)[&Value="55.4/71"]:0.0315538169)[&Value="88.5/98"]:0.0337722562,(NP
_034976.1:0.0664368415,NP_038634.1:0.013757915)[&Value="100/100"]:0.1380517232)[
&Value="73.9/28"]:0.0088404328,(XP_017508130.1:0.0724696331,(XP_032211398.1:0.03
9323132,NP_001003134.1:0.0296025904)[&Value="98.3/100"]:0.0573935752)[&Value="88
.3/81"]:0.0248853992)[&Value="87.5/43"]:0.0148013746)[&Value="57.5/93"]:0.019020420
1,XP_006156437.1:0.0634401054)[&Value="92.2/98"]:0.0450262573)[&Value="65.4/97"]:0.
05493067)[&Value="89.2/97"]:0.0616470008,XP_031752404.1:0.3342295671)[&Value="92.
1/98"]:0.068889808)[&Value="93.4/99"]:0.1192539777)[&Value="99.3/100"]:0.2018931675
,XP_032804093.1:0.5538217712)[&Value="92.7/97"]:0.107781326,(KAI0213370.1:0.00477
03417,(KAI0208044.1:0.0737949871,KAI0218869.1:0.1353802225)[&Value="38.7/93"]:0.0
186606084)[&Value="100/100"]:0.5135609586)[&Value="24.5/83"]:0.0263481719,(XP_046
565196.1:0.0675638179,((((XP_046563124.1:0.0091485718,XP_046562919.1:0.04742758
23)[&Value="35.1/83"]:0.0066741018,(XP_046352527.2:0.0243705774,(XP_048248476.1:0
.0201751591,(XP_048258111.1:0.0078843316,((XP_046352531.2:0.0082264893,(XP_0482
48472.1:0.000003,(XP_048248474.1:0.000003,XP_048248473.1:0.000003)[&Value="0/53"]
:0.000002)[&Value="98/100"]:0.0273190271)[&Value="87.9/96"]:0.0080674706,ABI53802.
1:0.0319674157)[&Value="86.4/100"]:0.0080356316)[&Value="0/98"]:0.000002)[&Value="8
4/100"]:0.0042046079)[&Value="98.6/100"]:0.0395512819)[&Value="85.2/82"]:0.01057517
85,(XP_046563126.1:0.008225882,XP_046565195.1:0.0116664694)[&Value="97.5/100"]:0.
0274163841)[&Value="85.8/92"]:0.0114560886,XP_046563125.1:0.0182716151)[&Value="
98.2/99"]:0.1132083704)[&Value="100/100"]:0.4758070317)[&Value="75.9/86"]:0.0501811
952)[&Value="88.1/94"]:0.0749466276)[&Value="99.4/100"]:0.3017300207,((XP_750654.1:
0.9410007802,((XP_006461472.1:0.1365880787,XP_006461433.1:0.160886605)[&Value="
100/100"]:0.6214769423,XP_006457072.1:1.2402192736)[&Value="35/93"]:0.1135575773)
[&Value="81.9/99"]:0.1569470487,(KAI3646081.1:0.6752646798,((KAJ9515210.1:0.623995
2797,(GAX85982.1:0.4160077327,(KAG2488600.1:0.2060628009,(XP_042924875.1:0.2349
54412,(XP_042924848.1:0.2893311056,XP_042923301.1:0.2761286055)[&Value="97.9/80
"]:0.1283429749)[&Value="56.7/64"]:0.105865159)[&Value="99.9/100"]:0.3955156434)[&V
alue="99.9/100"]:0.4129556251)[&Value="92.2/100"]:0.1988646361,(ETO25748.1:0.77039
18293,OAJ38670.1:0.6292877766)[&Value="74.2/72"]:0.1075769062)[&Value="54.9/47"]:0.
0735672596)[&Value="83.9/72"]:0.1133668864)[&Value="100/100"]:0.5997658494)[&Valu
e="81/58"]:0.1275087907)[&Value="96.7/100"]:0.1775933757)[&Value="58.4/93"]:0.12148
17297,((((PAA87312.1:0.1084999222,PAA68234.1:0.150890826)[&Value="61.2/93"]:0.1385
37599,(((NP_495986.3.3:0.1936896987,NP_610941.1:0.180965887)[&Value="92/87"]:0.06

24911143,(((XP_006813643.1:0.1171120407,XP_030843280.1:0.1770182371)[&Value="28/
97"]:0.0278253048,(XP_018667792.1:0.1424452503,(XP_032818114.1:0.0738589718,(XP_
021332524.1:0.0474293018,(((XP_028587646.1:0.0280581565,XP_025913835.1:0.007750
2898)[&Value="29.9/99"]:0.008641412,(XP_023440724.1:0.0078195679,(XP_005873264.1:
0.0080185393,((NP_598513.1:0.0,NP_056375.2.2:0.0):0.000003,XP_006163024.2.2:0.000
003)[&Value="0/89"]:0.000002)[&Value="85.5/100"]:0.0082794424)[&Value="97.5/100"]:0.
0374670905)[&Value="88.4/100"]:0.0254523507,XP_031757388.1:0.0605108485)[&Value=
"0/46"]:0.006224448)[&Value="90.5/100"]:0.0331834761)[&Value="98.8/100"]:0.09035844
18)[&Value="90.3/94"]:0.0475916768)[&Value="80.8/94"]:0.0287655227,MEN2496893:0.7
676528316)[&Value="1.4/25"]:0.0427519502)[&Value="26.8/64"]:0.0242712955,(XP_00260
2331.1:0.0069256779,XP_019637857.1:0.0089022887)[&Value="98.6/100"]:0.1095874429
)[&Value="98/99"]:0.2738440886)[&Value="100/100"]:1.509998376,(((((KAI5073815.1:0.22
22194493,(((XP_008646219.1:0.06562661,ACG47836.1:0.0862066354)[&Value="94.5/100"
]:0.0585270869,ONM04707.1:0.3150209884)[&Value="88.6/99"]:0.0572438153,((XP_0063
85192.1:0.1082745123,('KAG7649995.1':0.0184936274,'NP_172500.1':0.0548152728)[&Va
lue="16.8/87"]:0.0252152973)[&Value="70/100"]:0.0478422416,KAH9330549.1:0.1515200
465)[&Value="88.2/100"]:0.045108374)[&Value="96.2/99"]:0.0993522274)[&Value="99.7/8
2"]:0.1574038856,(EFJ33653.1:0.2487410688,EFJ28901.1:0.2569398172)[&Value="96.7/78
"]:0.1032788585)[&Value="41.1/60"]:0.047359979,(KAG0632288.1:0.2706520959,('KAG05
55995.1':0.0474343702,(XP_024368367.1:0.0251492103,XP_024391061.1:0.02758063)[&
Value="99.8/100"]:0.1080097308)[&Value="97.2/100"]:0.0966429062)[&Value="95.6/100"]:
0.0974703945)[&Value="87.1/59"]:0.1118500392,OAE31801.1:0.1571648335)[&Value="10
0/100"]:1.595630434,((((XP_042918632.1:0.1748685627,(((PWZ11893.1:0.0248132618,NP
_001130364.1:0.021484377)[&Value="99.9/100"]:0.1347939923,(AAF87857.1:0.05092611
44,XP_002317496.2:0.1001057962)[&Value="95.8/100"]:0.0599821016)[&Value="90.8/68"]
:0.0536342415,((KAI5058044.1:0.1600356301,KAH9322298.1:0.1451773669)[&Value="9.3
/92"]:0.04669328,(EFJ18064.1:0.1879633972,(PTQ33908.1:0.1385464236,KAG0628798.1:
0.2067786071)[&Value="77.3/95"]:0.0308766832)[&Value="51.5/93"]:0.0327240221)[&Val
ue="90.3/96"]:0.0653242665)[&Value="93.3/64"]:0.1276230833)[&Value="100/100"]:0.712
4370393,((XP_002683545.1:0.0704981966,(XP_044544418.1:0.0118148293,XP_04455919
8.1:0.000002)[&Value="96.2/100"]:0.1318953089)[&Value="100/100"]:0.5687283098,(((XP
_004182822.1:0.058741219,XP_008857507.1:0.164600245)[&Value="100/100"]:0.4280929
436,(XP_004340186.1:0.2860500747,((KAH3742895.1:0.2838339561,(KYQ94066.1:0.1989
41644,((((XP_004366192.1:0.1549436987,(XP_020428321.1:0.0924896746,XP_012754836
.1:0.06783129)[&Value="89.6/100"]:0.0384230351)[&Value="87.4/98"]:0.0500249696,KAF
2073270.1:0.1034183386)[&Value="90.4/95"]:0.0443640872,XP_003288319.1:0.06976698
92)[&Value="70.8/95"]:0.0396510199,XP_645576.2:0.0700769594)[&Value="90/98"]:0.049
5079478)[&Value="99.8/100"]:0.2037741646)[&Value="10/85"]:0.0665566033,PRP82286.1
:0.3120668973)[&Value="49.1/63"]:0.0637298904)[&Value="86.3/63"]:0.0778799555)[&Val
ue="37.4/32"]:0.1096837722,QYA18543.1:0.4069108215)[&Value="95.7/99"]:0.186970402
7)[&Value="97.6/100"]:0.2679824322)[&Value="95/100"]:0.3239363991,((XP_005775651.1:
1.4120768172,((XP_005767412.1:0.5057868841,KOO34265.1:0.4531211018)[&Value="90.
5/100"]:0.1761676101,((((EWM28268.1:0.3044278202,(KAG5184668.1:0.1339854562,CBN
78455.1:0.115066141)[&Value="80.1/100"]:0.0646023746)[&Value="95.6/100"]:0.1229914

038,((GMI61978.1:0.2451884928,(GMH67967.1:0.0802310472,(GMH92561.1:0.02027592
63,GMI07688.1:0.0477388255)[&Value="92.9/100"]:0.0483533938)[&Value="95.1/100"]:0.
0705297911)[&Value="41.4/99"]:0.0786932743,(EJK67908.1:0.1846497083,(KAI2494507.1
:0.2054144421,(CAB9516894.1:0.1436481308,GKY99394.1:0.1989448319)[&Value="86.3/
100"]:0.0616182149)[&Value="98.9/100"]:0.1221178461)[&Value="25.1/99"]:0.050306272
5)[&Value="98.9/100"]:0.1969631289)[&Value="83.1/99"]:0.1389785371,(XP_009032466.1.
2:0.000003,XP_009032466.1:0.000003)[&Value="100/100"]:0.7575855435)[&Value="98.1/
100"]:0.2474016755,(XP_042920073.1:0.3257802153,((PWZ44616.1:0.3066469621,(NP_0
01189935.1:0.1686872724,XP_002309632.3:0.0905890497)[&Value="87.1/100"]:0.043633
6924)[&Value="73.4/98"]:0.0767520053,(EFJ19523.1:0.1653340998,(KAH9308354.1:0.140
5675838,(KAI5064281.1:0.0860341394,(PTQ34556.1:0.1228526809,(KAG0561482.1:0.130
0490175,KAG0605142.1:0.1616035435)[&Value="91.3/100"]:0.0409277727)[&Value="62/9
6"]:0.0411955176)[&Value="58.1/95"]:0.0285001479)[&Value="26.7/93"]:0.0228128233)[&
Value="95.4/100"]:0.0996803352)[&Value="93.5/99"]:0.1009977309)[&Value="99.5/100"]:0
.2640893806)[&Value="86/99"]:0.1103069527)[&Value="89.9/100"]:0.219802975)[&Value=
"99/100"]:0.6027746003,((XP_004336224.1:0.4311701785,KAH3767868.1:1.2291058348)[
&Value="61.9/86"]:0.112599884,((XP_002649212.1:0.1127662343,XP_003288465.1:0.080
9362425)[&Value="96.1/100"]:0.1498229837,(KAF2077035.1:0.1247993023,(((XP_0204362
15.1:0.0545934748,XP_012753198.1:0.1767513863)[&Value="96.6/100"]:0.0705509416,X
P_004360608.1:0.199045807)[&Value="95.5/100"]:0.0599763111,KYQ90260.1:0.18571989
5)[&Value="14.2/77"]:0.0343102496)[&Value="95.6/100"]:0.1131487352)[&Value="100/100
"]:0.5282532326)[&Value="99.9/100"]:0.6524531376)[&Value="20.8/89"]:0.1268317371)[&
Value="99.4/100"]:1.055177612,(CEM26963.1:0.9225535358,((OII76931.1:0.630046708,(K
AH8582109.1:0.2018665668,(POM84969.1:0.1410397951,XP_667128.1:0.000003)[&Value
="90.7/100"]:0.0639478892)[&Value="99.4/100"]:0.2992168031)[&Value="99.9/100"]:0.513
2904893,((((XP_001617280.1:0.078784318,XP_028539355.1:0.000150)[&Value="100/100"]
:0.5703816858,((UKJ88078.2:0.1383026657,XP_004833148.1:0.0422322372)[&Value="97.
8/100"]:0.1469615664,(GFE54186.1:0.0789611628,(XP_012766661.1:0.0842089813,GIX62
800.1:0.0596363335)[&Value="92.3/100"]:0.0488024978)[&Value="99.8/100"]:0.22111006
67)[&Value="99.2/100"]:0.2278979323)[&Value="0/2"]:0.0758665297,KAH0479249.1:0.430
3433884)[&Value="77.6/6"]:0.067992176,(PHJ24853.1:0.0762841345,KFG43683.1:0.1022
820849)[&Value="97.6/100"]:0.1665176597)[&Value="99.5/98"]:0.3928667805)[&Value="9
4.3/99"]:0.4112790734)[&Value="100/100"]:1.980370215)[&Value="91.4/99"]:0.532774849
7)[&Value="66.5/64"]:0.2611456799)[&Value="35.4/44"]:0.0256850414,(((((ARF10781.1:0.2
833863997,ARF10780.1:0.1396473954)[&Value="99.8/100"]:0.9846518493,QFG74057.1:1
.95289125)[&Value="65.5/58"]:0.2700157412,KAF5834941.1:1.1200492916)[&Value="95.5
/99"]:0.4459620222,(KAG8459568.1:1.8396388237,KOO23261.1:1.3570289765)[&Value="
85.6/99"]:0.3010259609)[&Value="85/57"]:0.1509997228,((KAH8061769.1:0.7050690551,(
(OUS45493.1:0.1930389964,XP_001419538.1:0.1899342266)[&Value="100/100"]:0.72786
1926,((PRP82121.1:0.6024428096,XP_004338334.1:0.755400475)[&Value="66/96"]:0.170
3032301,(TMW65229.1:0.2496010185,((KAG1689960.1:0.1266236759,(KAE9027747.1:0.1
317590401,KAG7385474.1:0.1036767445)[&Value="23.7/96"]:0.0337511888)[&Value="28.
4/84"]:0.0393794174,((KAH7489187.1:0.1588412868,KAE8986371.1:0.245254747)[&Value
="81.7/95"]:0.052124327,(POM76499.1:0.1406810471,(KAG7377001.1:0.0836456259,(XP_

008910862.1:0.0367547544,(KAF1774311.1:0.0500891053,KAI9982172.1:0.1740401536)[
&Value="38.7/100"]:0.0109362979)[&Value="99.7/100"]:0.0961997265)[&Value="98/100"]:
0.0747918079)[&Value="77.9/100"]:0.0395355185)[&Value="93.2/95"]:0.0650643185)[&Va
lue="98.5/95"]:0.1782649433)[&Value="100/100"]:0.5319641215)[&Value="25.2/78"]:0.056
7644905)[&Value="87.1/92"]:0.2244839139)[&Value="90.9/66"]:0.2342217496,('XP_00578
5253.1':0.0110066634,XP_005792501.1:0.0506107582)[&Value="100/100"]:1.577502005)[
&Value="37.5/56"]:0.1777677726)[&Value="99.4/100"]:0.4942881482)[&Value="89.2/79"]:
0.1649860242)[&Value="89.6/78"]:0.1586942038,(((KAG8471152.1:0.4045423395,(KOO34
643.1:0.2653686054,XP_005775544.1:0.2931761452)[&Value="99.5/100"]:0.3567625231)[
&Value="97.4/100"]:0.3105249733,(((ATZ81043.1:0.6053058605,VBB18790.1:0.805483814
6)[&Value="78.8/84"]:0.0951305087,(ARF10282.1:0.2797844226,(((QKF94243.1:0.131752
0017,ARF11508.1:0.131706945)[&Value="97.5/100"]:0.0769205483,(ARF08414.1:0.35520
09545,AYV78912.1:0.40927585)[&Value="99.6/83"]:0.2384828407)[&Value="49.5/58"]:0.0
406946471,(ARF09562.1:0.1768648775,AYV76902.1:0.2314819042)[&Value="89.9/100"]:0
.0735522621)[&Value="92.7/80"]:0.1105140369)[&Value="100/81"]:0.3864086822)[&Value
="33.8/58"]:0.0999635909,QFG74079.1:1.0590763286)[&Value="37.7/53"]:0.052973179)[
&Value="93.4/55"]:0.2679771141,(AYV75702.1:1.5010350832,ATZ80405.1:1.6386124498)[
&Value="84.8/84"]:0.2457079595)[&Value="97.7/100"]:0.3106857897)[&Value="79.2/89"]:
0.072572618)[&Value="93.3/97"]:0.1245432708)[&Value="99.6/99"]:0.2013890414)[&Valu
e="82.4/89"]:0.0288391957)[&Value="85.1/84"]:0.0453981102,(PRP80825.1:0.306859036
4,((XP_004355605.1:0.140690195,XP_020436927.1:0.1250734698)[&Value="99/100"]:0.11
21833405,(AER35077.1:0.1579354789,(KAF2075389.1:0.188927348,XP_003292385.1:0.22
01915438)[&Value="80.2/89"]:0.044942993)[&Value="13.4/79"]:0.0200462686)[&Value="9
9.6/100"]:0.1887690215)[&Value="99.7/100"]:0.1763333944)[&Value="86.1/40"]:0.034534
1718)[&Value="78.5/42"]:0.0119122865)[&Value="75.7/69"]:0.0415524035,(((XP_0445532
61.1:0.0203509898,XP_044569353.1:0.0128544599)[&Value="99.7/100"]:0.1547971323,(X
P_044550536.1:0.0727934498,XP_002681690.1:0.1088663493)[&Value="98.7/100"]:0.105
4428941)[&Value="98.9/100"]:0.111106855,((((XP_004347890.1:0.1830637649,((((((NP_00
1024332.1:0.1843642236,KMZ10000.1:0.0865975164)[&Value="84.5/97"]:0.0361496887,X
P_006812840.1:0.3127976563)[&Value="80/58"]:0.0166975237,(XP_032814666.1:0.07569
8636,((((XP_025944940.1:0.0492894804,XP_031757197.1:0.1731803561)[&Value="74.4/1
00"]:0.0162018325,(EPQ08653.1:0.0091255023,(XP_006496668.1:0.0044032333,((XP_016
856477.1:0.0044373063,XP_012379251.1:0.000003)[&Value="0/94"]:0.000003,XP_027623
811.1:0.0134253352)[&Value="0/95"]:0.000003)[&Value="69.4/94"]:0.0130644641)[&Value
="96/100"]:0.0294393373)[&Value="90.9/100"]:0.0275638698,(XP_031753735.1:0.043859
7186,((XP_028568434.1:0.0043264938,XP_025920181.1:0.0047033197)[&Value="44.8/97"
]:0.0066147902,(((XP_006510037.1:0.000003,(NP_001005360.1:0.000032,XP_012381548.
1:0.0496875541)[&Value="0/21"]:0.0043907719)[&Value="0/19"]:0.000003,XP_006161648.
2.2:0.0806536202)[&Value="46.1/95"]:0.0044650559,XP_014389433.1:0.0275564082)[&Va
lue="87.8/98"]:0.0112545986)[&Value="83.6/97"]:0.017888413)[&Value="97.6/100"]:0.048
341189)[&Value="81.8/96"]:0.0272234614,(NP_001025299.1:0.000003,XP_021326548.1:0.
000003)[&Value="99.3/100"]:0.0584675084)[&Value="63.7/95"]:0.018019741)[&Value="62.
3/71"]:0.0215989941)[&Value="83.5/36"]:0.019293298,((XP_030853442.1:0.000003,XP_03
0853442.1.2:0.000003)[&Value="100/100"]:0.1377786503,((XP_005165639.1:0.037945531

6,(PAA65118.1:0.0584782078,(PAA78248.1:0.0199545404,(PAA59145.1:0.0576624242,PA
A64382.1:0.1372408252)[&Value="97.8/100"]:0.0548678254)[&Value="45.1/89"]:0.028573
168)[&Value="100/100"]:0.1420150078)[&Value="93.2/74"]:0.0331512707,((XP_028570166
.1:0.0133674639,XP_025915522.1:0.0071910833)[&Value="74.6/72"]:0.0065417091,((EPQ
17174.1:0.000003,(BAB27759.1:0.0044254079,(ELW62001.1:0.000003,(EAW87759.1:0.00
0003,XP_012378586.1:0.0366721388)[&Value="82.9/100"]:0.004460293)[&Value="0/33"]:0
.000003)[&Value="65.5/31"]:0.0089238859)[&Value="95.8/98"]:0.0229664647,KAE858305
5.1:0.0363149186)[&Value="90.5/75"]:0.0204150384)[&Value="88.6/73"]:0.0217411266)[&
Value="80.9/64"]:0.0122886042)[&Value="82.3/38"]:0.015193275)[&Value="16/13"]:0.0162
915721,XP_035683496.1:0.0939593854)[&Value="84.9/54"]:0.0255292842,XP_026693152
.1:0.1146631)[&Value="74.5/49"]:0.0278052473)[&Value="84.7/82"]:0.0482151934,(XP_01
4148725.1:0.153569162,XP_014153758.1:0.0861729276)[&Value="90.6/100"]:0.04902716
9)[&Value="55.3/55"]:0.0451462982,XP_001749319.1:0.1254914667)[&Value="99.6/100"]:
0.154021455,((GMH85941.1:0.085040734,GMI59178.1:0.0542074536)[&Value="99/100"]:
0.1306762654,((GMI25649.1:0.0720309159,GMI62840.1:0.0515792354)[&Value="22.3/82"
]:0.0223930335,GMH55978.1:0.0456499496)[&Value="97.4/100"]:0.0897399415)[&Value=
"100/100"]:0.2607023342)[&Value="95.6/100"]:0.0939577698)[&Value="94.1/99"]:0.05885
30509)[&Value="50.3/14"]:0.013041936,((((KAH3761456.1:0.3488138014,XP_004368323.1
:0.171005669)[&Value="86/80"]:0.0423998558,((XP_012754660.1:0.0549079241,(KYR0117
0.1:0.0443879976,XP_003294436.1:0.0328815995)[&Value="94.1/100"]:0.039603805)[&Va
lue="99.9/100"]:0.1392397586,PRP81066.1:0.2890944489)[&Value="90.5/99"]:0.0492177
396)[&Value="11.2/65"]:0.0161490579,(XP_004184473.1:0.0738682396,EMS16943.1:0.09
27057944)[&Value="100/100"]:0.4467334394)[&Value="47.5/78"]:0.0189426768,PRP8240
7.1:0.2482816802)[&Value="93.6/92"]:0.0541932282)[&Value="40.4/26"]:0.0338531824,(N
P_012926.1:0.2132518394,((OUM62108.1:0.126424544,OAJ44422.1:0.2095575064)[&Valu
e="47.6/99"]:0.0309389721,(KXN66323.1:0.1571062263,(((XP_011389257.1:0.108615400
6,XP_006458578.1:0.2018674905)[&Value="62.3/90"]:0.0203584194,KNE68830.1:0.18253
22352)[&Value="61.3/23"]:0.0227322799,XP_748106.1:0.1925152322)[&Value="77.9/29"]:
0.0190925104)[&Value="85/29"]:0.0293239155)[&Value="92.6/29"]:0.0630779202)[&Value
="99.9/100"]:0.1815359702)[&Value="95.5/99"]:0.0743027703)[&Value="43.5/95"]:0.03648
60477,((KAJ1432693.1:0.3664146671,((GAX23670.1:0.1976944796,CAB9512103.1:0.2023
211596)[&Value="15.1/86"]:0.0708055153,XP_002296064.1:0.3259912241)[&Value="96.9/
99"]:0.1183182279)[&Value="30.2/83"]:0.0444846211,XP_018636213.1:0.4595815274)[&V
alue="79.3/88"]:0.0573682239)[&Value="100/100"]:0.2260433357);
end;
